# Data on statistical experimental design to formulate amphotericin B-loaded Eudragit RL100 nanoparticles coated with hyaluronic acid for the treatment of vulvovaginal candidiasis

**DOI:** 10.1016/j.dib.2020.105311

**Published:** 2020-03-05

**Authors:** Carolina M. Melo, Jéssica F. Cardoso, Fernanda B. Perasoli, Maria Betânia F. Marques, Wagner N. Mussel, Sandra A.L. Moura, Gisele R. Da Silva

**Affiliations:** a School of Pharmacy, Federal University of São João del-Rei, Divinópolis, Minas Gerais, Brazil; b School of Pharmacy, Federal University of Ouro Preto, Ouro Preto, Minas Gerais, Brazil; c Chemistry Department, Federal University of Minas Gerais, Belo Horizonte, Minas Gerais, Brazil; d Department of Biological Sciences, Federal University of Ouro Preto, Ouro Preto, Minas Gerais, Brazil

**Keywords:** Eudragit RL100, Amphotericin B, Hyaluronic acid, Amphotericin B-loaded Eudragit RL100 nanoparticles coated with hyaluronic acid, Vulvovaginal candidiasis

## Abstract

Data described in this article are related to the research article entitled “Amphotericin B-loaded Eudragit RL100 nanoparticles coated with hyaluronic acid (AMP EUD nanoparticles/HA) for the treatment of vulvovaginal candidiasis” [1]. In this work, we report original data on the statistical experimental design to formulate uncoated AMP EUD nanoparticles, data on the validation of spectrophotometric method to quantify the AMP released from uncoated EUD nanoparticles and coated with HA to obtain the *in vitro* drug release profiles as well as the drug encapsulation efficiency. In addition, we describe original data on characterization, including diameter size, polydispersity index, zeta potential, FTIR, DSC/TG, and XRD; data on diameter of *in vitro* inhibition halos of *Candida albicans*; and on the vaginal burden of infected animals treated with uncoated AMP EUD nanoparticles and AMP EUD nanoparticles/HA. Finally, different histological sections of endocervix collected from treated and untreated animals were inserted into this manuscript.

**Table:** Specifications Table

Subject	Pharmaceutical Technology
Specific subject area	Drug Delivery Systems
Type of data	Tables, graphics, and figures
How data was acquired	Diameter and polydispersity index were determined by Photon Correlation Spectroscopy (Malvern S4700 PCS System, Malvern Instruments, UK). Zeta potential was measured by electrophoretic mobility by Laser Doppler Anemometry (Malvern S4700 PCS System, Malvern Instruments, UK). AMP efficiency of encapsulation and AMP released from EUD nanoparticles were determined by spectrophotometer at ultraviolet region (Shimadzu IRAffiniZy-1, Kyoto, Japan). Statistical experimental design was calculated by Statistica v7.0.61.0 EN software. Infrared spectra were collected in a Fourier Transform Infrared (FTIR) spectrophotometer (Perkin Elmer, Spectrum 1000). Powder X-Ray Diffraction (XRD) was recorded using an X-Ray Diffractometer (Shimadzu, XRD-7000, Kyoto, Japan). The thermal behavior was evaluated by Differential Scanning Calorimetry (DSC) (DSC60 Shimadzu, Kyoto, Japan) Thermogravimetry (TG)/Differential Thermal Analysis (DTA) (Shimadzu DTG60 thermobalance, Kyoto, Japan). Inhibition halos were measured using a caliper. Histological sections were obtained by using a Semi automatic Microtome (CUT 5062, Slee Mainz, Germany). Photomicrographs of the histological sections were obtained from Light Microscope (Kasvi ECO K112L, São João dos Pinhais, Paraná, Brazil), and images were digitized through a JVC TK–1270/JGB microcamera (Kontron Eletronics KS300, Carl Zeiss, Germany).
Data format	Raw and analyzed data
Parameters for data collection	Calibration curves were obtained using six AMP concentrations, and each concentration was analyzed in triplicate. Precision and accuracy were obtained by using three AMP concentrations, and each concentration was analyzed in triplicate. The AMP release study was investigated by using three bachtes of nanoparticles; and the drug assay was performed in triplicate. FTIR spectra were a result of 32 scans with a resolution of 4 cm^−1^. XRD diffractograms were analyzed at the angle range of 5 up to 35° of 2Ɵ with a step size of 0.02^o^, at a rate of 1.2 s.step^−1^. DSC curves were obtained in a heating rate of 10 °C min^−1^, from 30 to 400 °C. TG/DTA curves were obtained in a heating rate of 10 °C min-1, from 30 to 600 °C. Inhibition halos of *Candida albicans* were obtained by inoculating nanoparticles and pure AMP in solid Muller-Hinton agar, and measuring the diameter of the inhibition of the microorganism, in sextuplicate. Female Wistar rats were infected with 1 × 10^7^ yeast *Candida albicans* cells mL^-1^. They were separated into 6 groups: Group 1: infected animals, which received 0.1 mL of sterile saline solution (infected control); Group 2: infected animals, which received 0.1 mL of unloaded EUD nanoparticles/HA; Group 3: infected animals, which received 0.1 mL of unloaded and uncoated EUD nanoparticles; Group 4: infected and treated animals, which received 0.1 mL of AMP EUD nanoparticles/HA; Group 5: infected and treated animals, which received 0.1 mL of uncoated AMP EUD nanoparticles; Group 6: infected and treated animals, which received 0.1 mL of pure AMP in solution (2 mg) (n = 6 for each group). The number of CFU mL^−1^ of the vaginal liquid was counted on each animal before the treatment (zero time) at 24 and 48 hours post-treatment. Histopathological analyses were determined for animals of Groups 1, 2, and 4 after cutting the endocervix sections (4 sections for group). Photomicrographs were obtained for each endocervix section (4 photomicrographs for section).
Data collection description	Diameter and zeta potential were described as nanometer and milliVolt, respectively. Statistical experimental design was evaluated using the analysis of variance (ANOVA) at the 5% significance level. A model was considered significant if the p value was lower than 0.05. Linearity and matrix effect were described as calibration curve for AMP and calibration curve for AMP associated with the matrix, being represented by the components of the nanoparticles (EUD, Tween 80, and HA). Linear regression analysis was done by the ordinal least squares method. Residue analysis was performed. Normality, homoscedasticity, and independency were calculated. Lack-of-fit test (ANOVA) (p > 0.05) and the significance of regression (p > 0.05) were evaluated. As the linear model was suitable, slope and intercept were calculated to establish the equation that describes each calibration curve. Finally, these calibration curves were compared by t-Student test assuming combined or distinct variances [2]. Precision was described as AMP content ± relative standard deviation (RSD). RSD lower than 5% represents precision. Accuracy was described as percentage of AMP recovery in a matrix. Recovery between 98 and 102% represents accuracy. Limit of Quantitation was calculated using Equation (1) (section 2). AMP encapsulation efficiency was described as percentage. FTIR spectra were expressed as transmittance versus wavelength (nm). XRD diffractograms were expressed as intensity versus angle (2Ө). DSC thermograms were expressed as heat flow versus temperature (^°^C). TG/DTA thermograms were expressed as uV versus temperature (^°^C). AMP released from nanoparticles was described as accumulated percentage over time in hours. Inhibition halos were expressed as the diameter average of inhibition halos (mm) ± RSD. The number of colonies was expressed as average of CFU mL^−1^ ± RSD. Analyses of the vaginal endocervix and vaginal epithelium were performed to identify the presence of *Candida albicans* contamination and inflammatory infiltrate, respectively, in each tissue.
Data Source Location	School of Pharmacy, Federal University of São João del-Rei, Divinópolis, Minas Gerais, Brazil. School of Pharmacy, Federal University of Ouro Preto, Ouro Preto, Minas Gerais, Brazil. Chemistry Department, Federal University of Minas Gerais, Belo Horizonte, Minas Gerais, Brazil. Department of Biological Sciences, Federal University of Ouro Preto, Ouro Preto, Minas Gerais, Brazil
Data accessibility	Data are available in this article.
Related Research Article	Amphotericin B-loaded Eudragit RL100 nanoparticles coated with hyaluronic acid for the treatment of vulvovaginal candidiasis. Carolina M. Melo, Jéssica F. Cardoso, Fernanda B. Perassoli, Luccas M. Pinto, Ari S.O. Neto, Juliana T. Magalhães, Maria Betânia F. Marques, Wagner N. Mussel, Marcelo G.F. Araújo, Sandra A.L. Moura, Gisele R. Da Silva. Carbohydrate Polymers, 15;230, 2020, 115608 https://doi.org/10.1016/j.carbpol.2019.115608

**Table:** 

**Value of the Data** • Data on statistical experimental design are valuable to rationally formulate polymeric nanoparticles. • A rational formulation of nanoparticles can be used for researchers and veterinary/pharmaceutical industries to other studies on development of polymeric nanoparticles. • Our polymeric nanoparticles may be a precursor formulation to incorporate other drugs or active compounds to treat or add in the treatment of different diseases.

## 1 Data

Data described in this article are related to the research article entitled “Amphotericin B-loaded Eudragit RL100 nanoparticles coated with hyaluronic acid for the treatment of vulvovaginal candidiasis” [1].

In section 1.1, data on diameter, polydispersity index, and zeta potential of AMP EUD nanoparticles from formulations 1 to 8, including the formulation 9 (central point) are presented.

In section 1.2, data on statistical experimental design are presented.

In section 1.3, data on linearity, matrix effect, precision, accuracy, and limit of quantitation are presented.

In section 1.4, data on AMP EUD nanoparticles/HA are presented.

In section 1.5, data on AMP encapsulation efficiency are presented.

In section 1.6, data on characterization of uncoated AMP EUD nanoparticles and AMP EUD nanoparticles/HA by FTIR, DSC/TG, and XRD are presented.

In section 1.7, data on AMP released from uncoated EUD nanoparticles and EUD nanoparticles/HA are presented.

In section 1.8, data on *in* *vitro* fungicidal activity of uncoated AMP EUD nanoparticles and AMP EUD nanoparticles/HA by agar diffusion method are presented.

In section 1.9, quantitative data on *in* *vivo* fungicidal activity of AMP EUD nanoparticles/HA in the vulvovaginal candidiasis murine model are presented.

In section 1.10, qualitative data on *in vivo Candida albicans* contamination after AMP EUD nanoparticles/HA treatment are presented.

### 1.1 Diameter, polydispersity index, and zeta potential of AMP EUD nanoparticles from formulations 1 to 8, including the formulation 9 (central point) (Table 1)

Diameter, polydispersity index, and zeta potential of uncoated AMP EUD nanoparticles from formulations 1 to 8, including the formulation 9 (central point) are described in Table 1.

**Table 1:** Diameter, polydispersity index, and zeta potential of uncoated AMP EUD nanoparticles from formulations 1 to 8, including the formulation 9 (central point).

Formulation	Diameter (nm)	Polydispersity index	Zeta potential (mV)
1	104.80	0.47	2.55
	99.80	0.47	14.9
	99.44	0.69	4.71
Average ± RSD	101.3 ± 2.9	0.541 ± 0.13	7.39 ± 6.6
2	163.10	0.34	8.39
	185.50	0.51	24
	272.50	0.38	9.33
Average ± RSD	207 ± 27.9	0.412 ± 0.09	13.9 ± 8.75
3	139.20	0.45	12.4
	109.20	0.39	10.8
	130.50	0.40	4.68
Average ± RSD	126.3 ± 12.2	0.415 ± 0.03	9.29 ± 4.07
4	118.40	0.63	17.6
	135.60	0.42	7.32
	127.10	0.45	4.08
Average ± RSD	127 ± 6.8	0.497 ± 0.11	9.67 ± 7.06
5	279.40	0.38	9.49
	156.50	0.45	12.6
	150.10	0.47	10.4
Average ± RSD	195.3 ± 37.3	0.436 ± 0.04	10.8 ± 1.60
6	328.60	0.32	5.83
	178.50	0.28	2.94
	174.60	0.27	2.66
Average ± RSD	227.2 ± 38.6	0.292 ± 0.03	3.81 ± 1.75
7	188.60	0.27	15
	161.30	0.38	10.9
	147.30	0.32	21.9
Average ± RSD	165.7 ± 12.7	0.32 ± 0.06	15.9 ± 5.56
8	120.50	0.36	8.64
	110.40	0.30	17
	130.40	0.24	12.7
Average ± RSD	120.4 ± 8.3	0.3 ± 0.06	12.8 ± 4.18
9	245.00	0.92	22.30
	255.20	0.33	25.70
	196.50	0.34	5.54
	247.80	0.43	26.30
	226.40	0.52	11.50
Average ± RSD	234.18 ± 19.3	0.50 ± 0.11	18.26 ± 2.95

### 1.2 Statistical experimental design (Table 2)

Statistical parameters derived from regression analysis and ANOVA related to the statistical experimental design are described in Table 2.

**Table 2:** Statistical parameters derived from regression analysis and ANOVA of 3 independent variables, 13 runs, and 4 factors: particle size, polydispersity index, zeta potential, and encapsulation efficiency.

Independent variables	Particle size		Polydispersity index		Zeta potential		Encapsulation efficiency	
	Coefficient	*p*-Value	Coefficient	*p*-Value	Coefficient	*p*-Value	Coefficient	*p*-Value
EUD mass (mg)	−106.98	**0.0016**	0.169	0.7438	−7.51	0.9075	2.43	0.1589
Tween 80 concentration [% (w/v)]	−43.51	**0.0256**	0.177	0.8169	−6.41	0.6521	−2.12	0.0527
Flow time of the organic phase (min)	−1.20	0.0607	0.131	0.4359	−7.50	0.9056	1.77	0.0848
EUD mass (mg) × Tween 80 concentration [% (w/v)]	−42.36	**0.0304**	0.238	0.6069	−7.39	0.8781	−1.18	0.6342
EUD mass (mg) × Flow time of the organic phase (min)	−34.56	0.1065	0.181	0.8556	−9.23	0.6789	−2.38	0.5826
Tween 80 concentration [% (w/v)] × Flow time of the organic phase (min)	−29.75	0.2392	0.187	0.9169	−5.83	0.5332	2.20	0.2660
EUD mass (mg) × Tween 80 concentration [% (w/v)] × Flow time of the organic phase (min)	−37.589	0.1087	0.169	0.7438	−7.51	0.9075	1.83	0.0962
Determination coefficient for model (R^2^)	0.996		0.989		0.991		0.992	
Model *p*-Value	**0.028**		0.955		0.845		0.871	
F- ratio	8.38		0.23		0.42		0.50	

Significant effect of factors was shown in bold type. F-ratios are lower than the theoretical values.

Determination coefficient (R^2^) higher than 0.99 indicates that at least 99% of the variation in response might be explained by the model and confirms the goodness of fit to the model. p-values lower than 0.05 indicate the significance of the regression model with a confidence of 95%. F-ratio higher than the theoretical value (Fisher test critical value) indicates the significance of the regression model with a confidence of 95% [3]. Therefore, the individual modification of EUD mass (mg) and Tween 80 concentration [% (w/v)] at higher (+1) levels produced significant effects on amphotericin B EUD nanoparticle diameter (p < 0.05). The synergistic influence of these independent variables at higher (+1) values on nanoparticles diameter was also significant (p < 0.05).

**Fig. 1:**
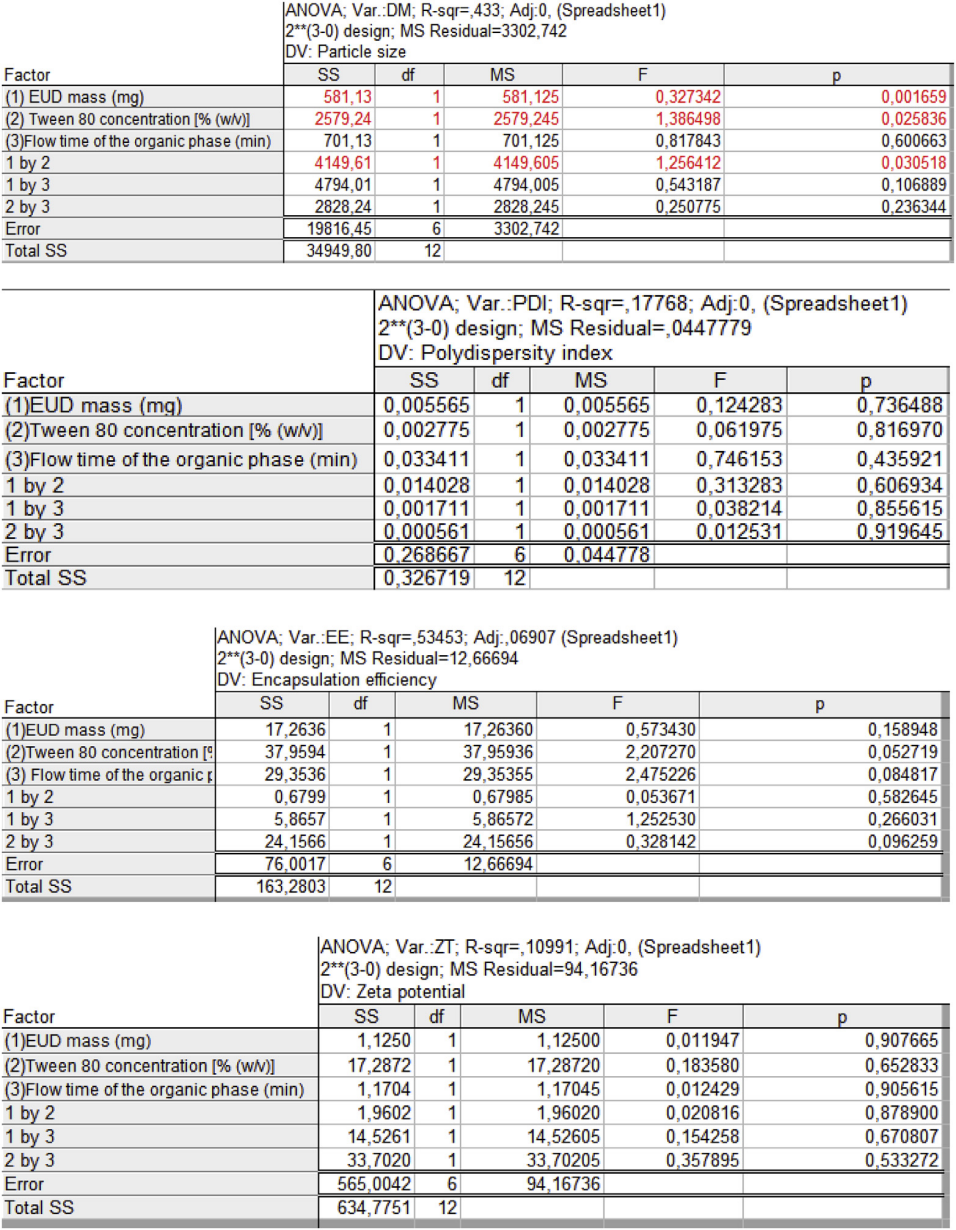
Original data exported from the Statistica v7.0.61.0 EN software to obtain data described in Table 2.

Fig. 1 indicates the original data exported from the Statistica v7.0.61.0 EN software for generating the results described in Table 2 (statistical experimental design).

### 1.3 Validation: linearity, matrix effect, precision, accuracy, and limit of quantitation

#### 1.3.1 Linearity and matrix effect (Table 3, Table 4, Table 5, Table 6, Table 7, Table 8, Table 9, Table 10 - Fig. 2, Fig. 3, Fig. 4)

Calibration curves of AMP and AMP in contact with matrix (compounds of the nanoparticles) were obtained from six drug concentrations (5; 10; 15; 20; 30 and 35 μg mL^−1^) in 3 independent replicates, performed in random order. The absorvance values obtained for each AMP concentration are described in Table 3.

**Fig. 2:**
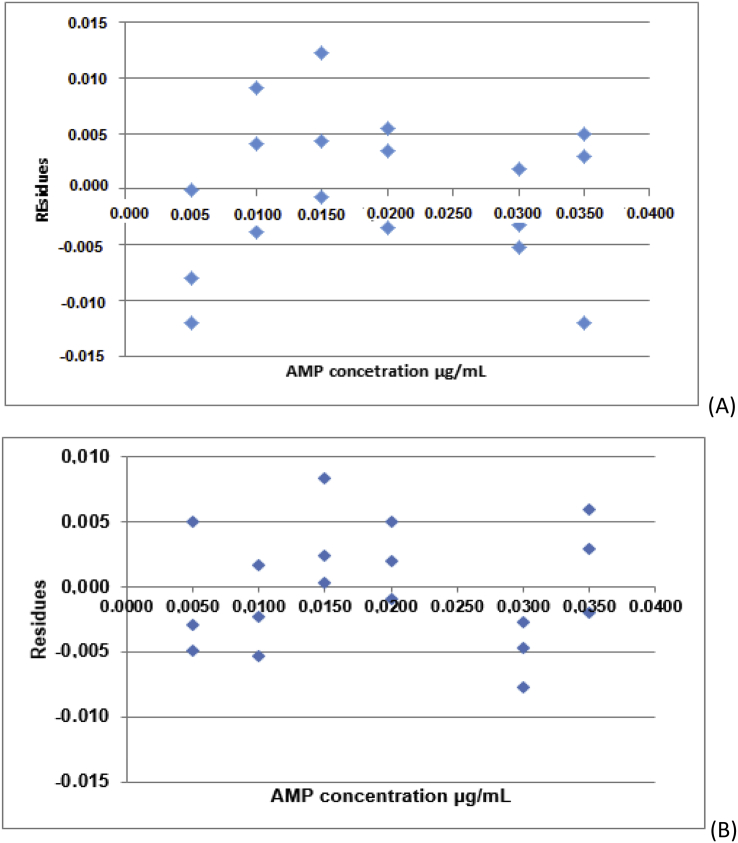
Graphics of residues (regression of residues versus AMP concentration levels) by Jacknife standardized residuals test. (A) AMP in the absence of the matrix and (B) AMP in the presence of the matrix (compounds of the nanoparticles).

**Fig. 3:**
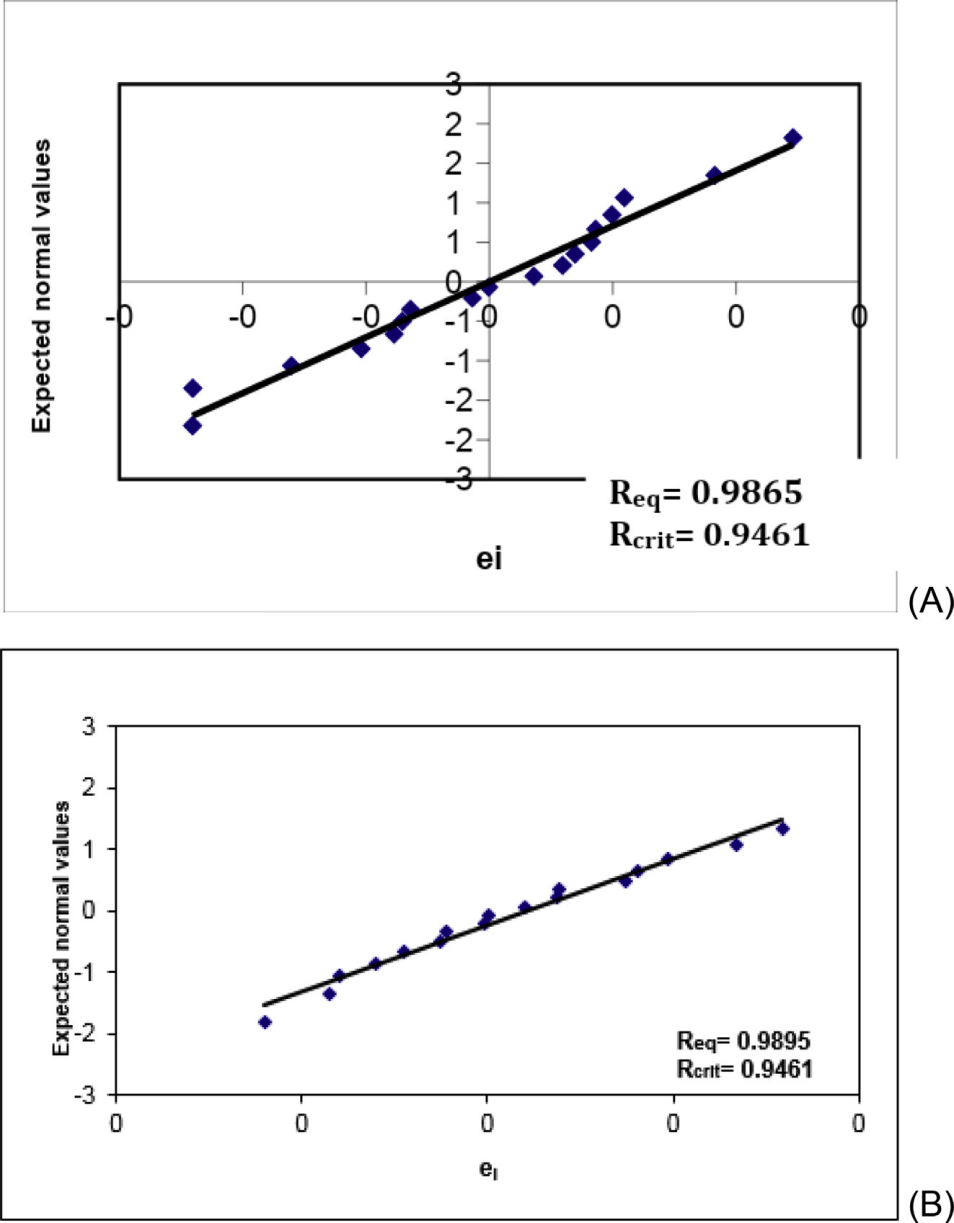
Normal QQ plots of residues for (A) AMP in the absence of the matrix and (B) AMP in the presence of the matrix (compounds of the nanoparticles). ei: residues. R: correlation coefficient of Ryan-Joiner test.

**Fig. 4:**
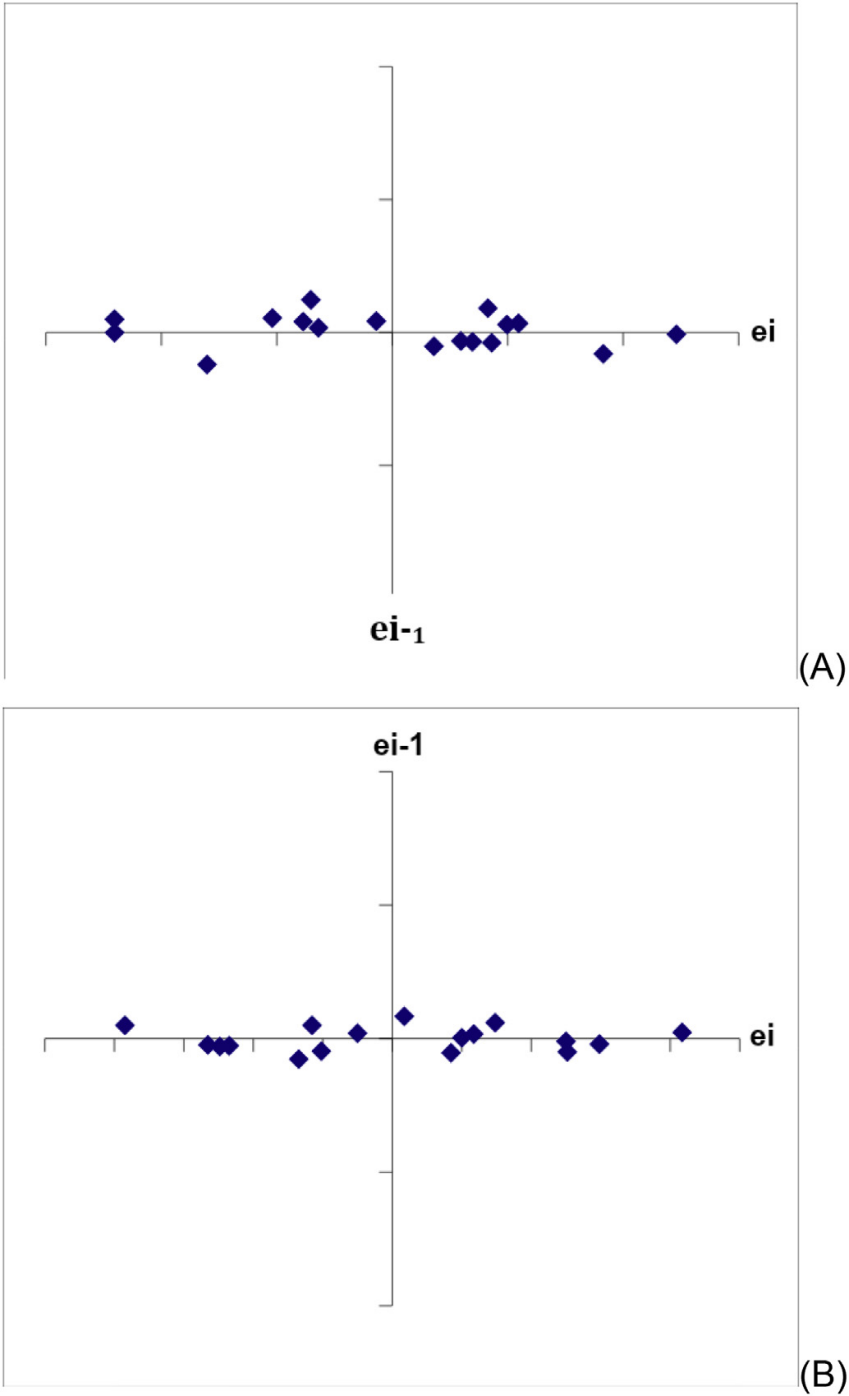
Independence of the residues by the Durbin-Watson test for (A) AMP in the absence of the matrix and (B) AMP in the presence of the matrix (compounds of the nanoparticles). ei: residues.

**Table 3:** Theoretical AMP concentration and absorvance equivalent to each AMP concentration in the absence of the matrix and in the presence of the matrix (compounds of the nanoparticles).

Replicates	AMP Concentration (μg mL^−1^)	Absorvance (nm) in the absence of the matrix	Absorvance (nm) in the presence of the matrix
1	5	0.143	0.145
2	5	0.131	0.149
3	5	0.135	0.153
4	10	0.222	0.214
5	10	0.217	0.211
6	10	0.209	0.218
7	15	0.287	0.287
8	15	0.282	0.293
9	15	0.295	0.285
10	20	0.349	0.355
11	20	0.356	0.352
12	20	0.358	0.358
13	30	0.487	0.479
14	30	0.494	0.487
15	30	0.489	0.485
16	35	0.565	0.556
17	35	0.567	0.564
18	35	0.55	0.561

Table 4 represents the original data for calculating the residues for AMP in the absence of the matrix and in the presence of the matrix (compounds of the nanoparticles) by the Jacknife test. Fig. 2 represents the graphics of residues (regression of residues versus AMP concentration levels) for AMP in the absence of the matrix (Fig. 1A) and in the presence of the matrix (compounds of the nanoparticles) (Fig. 1B). Lines correspond to ± t(1-α/2; n-2)Sres, which is the acceptable variation range for regression residues.

**Table 4:** Original data to calculate the residues for AMP in the absence and in the presence of the matrix (compounds of the nanoparticles) by the Jacknife test.

Replicates	xi	yi	ei	Jei	ri	hi
**Residues for AMP in the absence of the matrix**						
1	5	0.143	0.000	−0.004	−0.004	0.155
2	5	0.131	−0.012	−2.074	−1.888	0.155
3	5	0.135	−0.008	−1.286	−1.260	0.155
4	10	0.222	0.009	1.433	1.388	0.097
5	10	0.217	0.004	0.617	0.629	0.097
6	10	0.209	−0.004	−0.574	−0.586	0.097
7	15	0.287	0.004	0.630	0.642	0.064
8	15	0.282	−0.001	−0.100	−0.103	0.064
9	15	0.295	0.012	2.001	1.836	0.064
10	20	0.349	−0.004	−0.512	−0.524	0.056
11	20	0.356	0.,003	0.504	0.516	0.056
12	20	0.358	0.005	0.804	0.813	0.056
13	30	0.487	−0.005	−0.787	−0.797	0.114
14	30	0.494	0.002	0.268	0.276	0.114
15	30	0.489	−0.003	−0.478	−0.490	0.114
16	35	0.565	0.003	0.461	0.473	0.180
17	35	0.567	0.005	0.782	0.792	0.180
18	35	0.55	−0.012	−2.116	−1.918	0.180
**Residues for AMP in the presence of the matrix**						
1	5	0.145	−0.003	−0.691	−0.703	0.155
2	5	0.149	−0.005	−1.192	−1.176	0.155
3	5	0.153	0.005	1.210	1.193	0.155
4	10	0.214	−0.002	−0.517	−0.530	0.097
5	10	0.211	−0.005	−1.237	−1.217	0.097
6	10	0.218	0.002	0.377	0.387	0.097
7	15	0.287	0.002	0.515	0.528	0.064
8	15	0.293	0.008	2.060	1.878	0.064
9	15	0.285	0.000	0.075	0.077	0.064
10	20	0.355	0.002	0.436	0.448	0.056
11	20	0.352	−0.001	−0.218	−0.225	0.056
12	20	0.358	0.005	1.130	1.120	0.056
13	30	0.479	−0.008	−1.925	−1.780	0.114
14	30	0.487	−0.003	−0.611	−0.623	0.114
15	30	0.485	−0.005	−1.092	−1.086	0.114
16	35	0.556	−0.002	−0.479	−0.491	0.180
17	35	0.564	0.006	1.487	1.434	0.180
18	35	0.561	0.003	0.701	0.712	0.180

The assumption that residues followed the normal distribution was evaluated by the Ryan-Joiner test. The original data to indicate the normality of the residues for AMP in the absence and in the presence of the matrix (compounds of the nanoparticles) are described in Table 5. Fig. 3 depicts the QQ plots, and their Ryan-Joiner correlation coefficients, showing a significant correlation between the two components (Req > Rcrit), indicating that there was no deviation from normality for (A) AMP in the absence of the matrix and (B) AMP in the presence of the matrix (compounds of the nanoparticles) when α = 0.10.

**Table 5:** Original data to calculate the normality of the residues for AMP in the absence and in the presence of the matrix (compounds of the nanoparticles) by the Ryan-Joiner test.

Replicates	pi	qi	ei
**Normality of the residues for AMP in the absence of the matrix**			
1	0.0342	−1.8217	−0.012
2	0.0890	−1.3467	−0.012
3	0.1438	−1.0632	−0.008
4	0.1986	−0.8465	−0.005
5	0.2534	−0.6638	−0.004
6	0.3082	−0.5009	−0.004
7	0.3630	−0.3504	−0.003
8	0.4178	−0.2075	−0.001
9	0.4726	−0.0687	0.000
10	0.5274	0.0687	0.002
11	0.5822	0.2075	0.003
12	0.6370	0.3504	0.003
13	0.6918	0.5009	0.004
14	0.7466	0.6638	0.004
15	0.8014	0.8465	0.005
16	0.8562	1.0632	0.005
17	0.9110	1.3467	0.009
18	0.9658	1.8217	0.012
**Normality of the residues for AMP in the presence of the matrix**			
1	0.0342	−1.8217	−0.006
2	0.0890	−1.3467	−0.004
3	0.1438	−1.0632	−0.004
4	0.1986	−0.8465	−0.003
5	0.2534	−0.6638	−0.002
6	0.3082	−0.5009	−0.001
7	0.3630	−0.3504	−0.001
8	0.4178	−0.2075	0.000
9	0.4726	−0.0687	0.000
10	0.5274	0.0687	0.001
11	0.5822	0.2075	0.002
12	0.6370	0.3504	0.002
13	0.6918	0.5009	0.004
14	0.7466	0.6638	0.004
15	0.8014	0.8465	0.005
16	0.8562	1.0632	0.007
17	0.9110	1.3467	0.008
18	0.9658	1.8217	0.008

The original data to indicate the independence of the residues for AMP in the absence and in the presence of the matrix (compounds of the nanoparticles) are described in Table 6. The correlation among the residues was not confirmed since d = 2.12 and d = 1.88 were in the range of 1.39 and 2.61 for AMP in the absence and in the presence of the matrix, respectively, which indicated the existence of the independence of the residues. Fig. 4 depicts the graphics of autocorrelation of the residues (independence) by the Durbin-Watson test for (A) AMP in the absence of the matrix and (B) AMP in the presence of the matrix (compounds of the nanoparticles).

**Table 6:** Original data to calculate the independence of the residues for AMP in the absence and in the presence of the matrix (compounds of the nanoparticles) by the Durbin-Watson test.

Replicates	ei	ei-1	e − ei-1
**Independence of the residues for AMP in the absence of the matrix**			
1	0.000		
2	−0.012	0.00	−0.012
3	−0.008	−0.01	0.004
4	0.009	−0.01	0.017
5	0.004	0.01	−0.005
6	−0.004	0.00	−0.008
7	0.004	0.00	0.008
8	−0.001	0.00	−0.005
9	0.012	0.00	0.013
10	−0.004	0.01	−0.016
11	0.003	0.00	0.007
12	0.005	0.00	0.002
13	−0.005	0.01	−0.011
14	0.002	−0.01	0.007
15	−0.003	0.00	−0.005
16	0.003	0.00	0.006
17	0.005	0.00	0.002
18	−0.012	0.00	−0.017
**Independence of the residues for AMP in the presence of the matrix**			
1	−0.003		
2	−0.005	0.00	−0.002
3	0.005	0.00	0.010
4	−0.002	0.01	−0.007
5	−0.005	0.00	−0.003
6	0.002	−0.01	0.007
7	0.002	0.00	0.001
8	0.008	0.00	0.006
9	0.000	0.01	−0.008
10	0.002	0.00	0.002
11	−0.001	0.00	−0.003
12	0.005	0.00	0.006
13	−0.008	0.00	−0.013
14	−0.003	−0.01	0.005
15	−0.005	0.00	−0.002
16	−0.002	0.00	0.003
17	0.006	0.00	0.008
18	0.003	0.01	−0.003

The homoscedasticity of data was evaluated by Levene test, adapted by Brown-Forsythe. As t_calculated_ (tL) value was higher than the t_critical_ value (α = 0.05), the homoscedasticity was determined for AMP in the absence and in the presence of the matrix (compounds of the nanoparticles). Table 7 shows the original data to calculate the homoscedasticity for AMP in the absence and in the presence of the matrix. Table 8 depicts the statistical data for determining the homoscedasticity.

**Table 7:** Homoscedasticity of the residues by modified Levene test for (A) AMP in the absence of the matrix and (B) AMP in the presence of the matrix (compounds of the nanoparticles).

Group K1	Group K2		
e1j	e2j	|d1|	|d2|
**Homoscedasticity of the residues for AMP in the absence of the matrix**			
0.000	−0.004	0.0000	0.0053
−0.012	0.003	0.0120	0.0017
−0.008	0.005	0.0080	0.0037
0.009	−0.005	0.0092	0.0070
0.004	0.002	0.0042	0.0000
−0.004	−0.003	0.0038	0.0050
0.004	0.003	0.0043	0.0012
−0.001	0.005	0.0007	0.0032
0.012	−0.012	0.0123	0.0138
**Homoscedasticity of the residues for AMP in the presence of the matrix**			
−0.003	0.002	0.0033	0.0030
−0.005	−0.001	0.0053	0.0000
0.005	0.005	0.0047	0.0060
−0.002	−0.008	0.0027	0.0067
−0,005	−0.003	0.0057	0.0017
0.002	−0.005	0.0013	0.0037
0.002	−0.002	0.0020	0.0010
0.008	0.006	0.0080	0.0070
0.000	0.003	0.0000	0.0040

**Table 8:** Homoscedasticity of the residues by modified Levene test for (A) AMP in the absence of the matrix and (B) AMP in the presence of the matrix (compounds of the nanoparticles).

Statistic	(A)		(B)	
	Group K1	Group K2	Group K1	Group K2
n_k_	9	0.007	9	9
e_k_ (mediana)	−2.5 E-05	0.994	3.4 E-04	−1.0 E-03
d_k_ (average)	6.06 E-03		3.66 E-03	3.67 E-03
SQD_k_	1.65 E-04		4.92 E−05	5.03 E-05
s^2^_p_	1.88 E-05		6.22 E-06	
**t**_**L**_	0.742		0.007	
p	0.468938		0.994466	

Considering that the ordinal least squares method (OLSM) can be applied to define the regression equations for AMP in the absence and in the presence of the matrix, the linear regression analyzes were performed. Then, the calculation of regression parameters and their deviations, significance, and confidence intervals was obtained. Table 9 shows the data for defining the regression parameters, and finally the equations (model Y = ax + b) to describe the linearity curves for AMP in the absence and in the presence of the matrix (matrix effect). According to the obtained data, the regression was significative and there was no linearity deviation.

**Table 9:** Regression parameters to define the regression statistics, linearity deviation, significance of the regression, and confidence intervals to define the linearity equations for (A) AMP in the absence of the matrix and (B) AMP in the presence of the matrix (compounds of the nanoparticles).

	(A)	(B)
**Regression statistics**		
Coefficient	R2 = 0.9980 (n = 18)	R2 = 0.9990 (n = 18)
	Linear (intercept) - 0.0732 Angular (slope) - 13.96687 s(EP) (intercept) - 0.0034 s(EP) (slope) - 0.15446	Linear (intercept) - 0.0796 Angular (slope) - 13.66915 s(EP) (intercept) - 0.0022 s(EP) (slope) - 0.10235

**ANOVA (linearity deviation and significance of the regression)**							
FV	GL	SQ	QM	F	p	Sign	F critical
Regression	(A) 1 (B) 1	(A) 3.93E-01 (B) 3.76E-01	(A) 3.93E-01 (B) 3.76E-01	(A) 8176.35 (B) 17,835.93	(A) 0.00E+00 (B) 8.18E-26		(A) 4.493998478 (B) 4.493998478
Residue	(A) 16 (B) 16	(A) 7.68E-04 (B) 3.37E-04	(A) 4.80E-05 (B) 2.11E-05				
Linearity deviation	(A) 4 (B) 4	(A) 2.78E-04 (B) 1.59E-04	(A) 6.96E-05 (B) 3.97E-05	(A) 1.703 (B) 2.664	(A) 2.14E-01 (B) 0.084		(A) 3.259166727 (B) 3.259166727
Between levels	(A) 5 (B) 5	(A) 3.93E-01 (B) 3.76E-01					
Error	(A) 12 (B) 12	(A) 4.90E-04 (B) 1.79E-04	(A) 4.08E-05 (B) 1.49E-05				
Total	(A) 17 (B) 17	(A) 3.93E-01 (B) 3.76E-01					

After verifying the premises required by ordinal least squares method (OLSM), the following regression equations were retrieved: Abs = 13.967 [AMP] + 0.0732 (R^2^ = 0.9980) to the AMP in the absence of the matrix and Abs = 13.611 [AMP] – 0.0809 (R^2^ = 0.9990) to the AMP in the presence of the matrix (compounds of the nanoparticles). The regression parameters for the analytical curves obtained for AMP concentration in the absence and in the presence of the matrix are indicated in Table 10. The linearity of the method ranged from 5 to 35 μg mL^−1^, and the matrix did not interfere with analyte quantitation.

**Table 10:** Linearity and matrix effect: regression parameters for calibration curves for AMP and AMP associated with the matrix in the range of 5–35 μg mL^−1^, including the lack-of-fit evaluation.

Regression parameters	AMP in the absence of matrix	AMP in the presence of matrix
Slope ± SD	13.967 ± 0.113	13.611 ± 0.102
Intercept ± SD	0.0732 ± 0.0025	0.0809 ± 0.0022
Determination coefficient (R^2^)	0.9980	0.9990
Correlation coefficient (r)	0.99938	0.99951
Normality of residues	0.9865 (Rcritical = 0.9461)	0.9895 (Rcritical = 0.9461)
Independency of residues	2.117 (1.160–2.840)	1.880 (1.160–2.840)
Homoscedasticity	0.4689 (TL = 0,742)	0.9945 (TL = 0,007)
Lack-of-fit (p)	0.214	0.084

#### 1.3.2 Precision and accuracy (Table 11)

Data on precision and accuracy are described in Table 11.

**Table 11:** Assayed (A) AMP concentrations in the absence of the matrix and (B) in the presence of the matrix (compounds of the nanoparticles) to determine intra-day and inter-days precision. Recovered percentage of AMP to determine intra-day and inter-days accuracy. Replicates 1, 2, and 3 for each day.

Intra-day precision and accuracy	Replicates Absorbance (nm)	Theoretical AMP concentration					
		5.0 μg mL^−1^		20.0 μg mL^−1^		35 μg mL^−1^	
		(A)	(B)	(A)	(B)	(A)	(B)
Day 1	1	0.143	0.145	0.354	0.355	0.566	0.556
	2	0.147	0.149	0.352	0.352	0.559	0.564
	3	0.148	0.154	0.361	0.358	0.564	0.561
Average ± RSD		0.146 ± 1.027	0.149 ± 1.563	0.356 ± 0.750	0.355 ± 0.423	0.563 ± 0.355	0.560 ± 0.387
AMP concentration (μg mL^−1^)		4.9	5.0	20.1	20.1	35.5	35.2
Recovery (%)		98.91	100.56	100.75	100.69	100.53	100.64
Day 2	1	0.146	0.144	0.357	0.349	0.563	0.553
	2	0.148	0.152	0.349	0.356	0.559	0.562
	3	0.142	0.147	0.356	0.354	0.560	0.564
Average ± RSD		0.145 ± 1.147	0.148 ± 1.467	0.354 ± 0.706	0.353 ± 0.567	0.561 ± 0.208	0.560 ± 0.596
AMP concentration (μg mL^−1^)		4.9	4.9	20.0	20.0	35.0	35.2
Recovery (%)		97.95	98.11	100.15	99.96	100.04	100.50
Inter-day precision		0.146 ± 1.78	0.149 ± 2.65	0.355 ± 1.17	0.354 ± 0.89	0.562 ± 0.52	0.560 ± 0.81
Inter-day accuracy (Recovery %)		98.43 ± 0.69	99.34 ± 1.73	100.45 ± 0.42	100.33 ± 0.52	100.29 ± 0.35	100.57 ± 0.09

#### 1.3.3 Limit of quantitation

The Limit of Quantification (LOQ) was 2.42 μg mL^−1^.

### 1.4 Diameter, polydispersity index, and zeta potential of AMP EUD nanoparticles (selected formulation to be coated with HA) (Table 12)

Diameter, polydispersity index, and zeta potential of AMP EUD nanoparticles/HA in different concentrations are described in Table 12.

**Table 12:** Diameter, polydispersity index, and zeta potential of AMP EUD nanoparticles/HA in different concentrations.

HA concentration %(w/v)	Diameter (nm)	Polydispersity index	Zeta potential (mV)
0.25	133.4	0.640	−22.48
	105.7	0.630	−20.04
	235.9	0.621	−19.04
	158.3 ± 13.8	0.630 ± 0.19	−20.52 ± 1.77
0.50	145.7	0.571	−23.95
	147.54	0.567	−23.65
	140.00	0.574	−23.66
	144.4 ± 12.6	0.571 ± 0.25	−23.78 ± 0.15
1.5	129.8	0.547	−24.12
	133	0.541	−24.98
	130.3	0.536	−28.38
	131.4 ± 7.6	0.541 ± 0.10	−25.83 ± 2.26
3.0	147.2	0.303	−32.01
	147.8	0.300	−28.80
	147.9	0.301	−29.01
	147.6 ± 16.7	0.301 ± 0.09	−29.94 ± 1.76

### 1.5 Amphotericin B encapsulation efficiency (Table 13)

The AMP encapsulation efficiency (EE%) in uncoated EUD nanoparticles from formulations 1 to 8, including the formulation 9 (central point), is described in Table 13.

**Table 13:** AMP mass (mg) in the supernatant after ultracentrifugation of uncoated EUD nanoparticles from formulations 1 to 8, including the formulation 9 (central point), and AMP encapsulation efficiency (EE%).

Formulation	AMP mass (mg) in the supernatant	EE%
1	0.553	77.88
2	0.455	81.80
3	0.516	79.38
4	0.377	84.90
5	0.425	83.00
6	0.457	81.72
7	0.305	87.81
8	0.318	87.27
9	*0.313*	*87.49*

### 1.6 Characterization of uncoated AMP EUD nanoparticles and AMP EUD nanoparticles/HA

#### 1.6.1 Fourier Transform Infrared Spectroscopy (FTIR), Powder X-Ray Diffraction (XRD), and Thermal analysis (DSC)

Data on FTIR, XRD, and DSC of pure AMP, pure EUD, pure HA, EUD nanoparticles, AMP EUD nanoparticles, EUD nanoparticles/HA, and AMP EUD nanoparticles/HA were shown in the supplementary files.

### 1.7 AMP released from uncoated EUD nanoparticles and EUD nanoparticles/HA – AMP release profiles (Table 14, Table 15)

Data on the AMP released from uncoated EUD nanoparticles (formulation 8) and EUD nanoparticles/HA are described in Table 14, Table 15, respectively.

**Table 14:** AMP released from selected uncoated EUD nanoparticles (formulation 8). 3 batches of uncoated AMP EUD nanoparticles (1, 2 and 3). Data were expressed as accumulated percentage of AMP released over time for each batch, average percentages ± standard deviation (SD).

Time (hours)	Percentage of AMP released over time			Average percentages ± SD
	1	2	3	
0	0.00	0.00	0.00	0.00
0.5	0.00	0.00	0.00	0.00
1	0.00	0.00	0.00	0.00
2	0.00	0.00	0.00	0.00
4	3.20	3.75	1.23	2.73 ± 0.75
8	5.96	5.41	4.03	5.13 ± 0.55
12	13.69	15.90	14.09	14.56 ± 0.67
24	26.39	29.71	27.52	27.87 ± 0.92
48	32.47	34.68	28.63	31.93 ± 1.65
72	57.87	59.52	62.75	60.05 ± 1.35
96	84.37	82.71	78.41	81.83 ± 1.71

**Table 15:** AMP released from selected EUD nanoparticles/HA (formulation 8). 3 batches of AMP EUD nanoparticles/HA (1, 2 and 3). Data were expressed as accumulated percentage of AMP released over time for each batch, average percentages ± standard deviation (SD).

Time (hours)	Percentage of AMP released over time			Average percentages ± SD
	1	2	3	
0	0.00	0.00	0.00	0.00
0.5	0.00	0.00	0.00	0.00
1	0.00	0.00	0.00	0.00
2	0.00	0.00	0.00	0.00
4	3.75	4.31	3.75	3.94 ± 0.26
8	8.72	8.72	8.17	8.54 ± 0.26
12	17.01	17.01	16.45	16.82 ± 0.25
24	29.15	28.60	29.71	29.15 ± 0.45
48	35.78	37.99	35.78	36.52 ± 1.04
72	66.15	67.25	67.25	66.89 ± 0.52
96	82.71	83.82	82.33	82.95 ± 0.63

### 1.8 In vitro antifungal activity of uncoated AMP EUD nanoparticles and EUD nanoparticles/HA (Table 16)

Data on *in vitro* antifungal activity of uncoated AMP EUD nanoparticles and EUD nanoparticles/HA are described in Table 16.

**Table 16:** Diameter of inhibiton halos (mm) induced by AMP released from 6 batches of unloaded EUD nanoparticles/HA, AMP EUD nanoparticles/HA, unloaded and uncoated EUD nanoparticles, uncoated AMP EUD nanoparticles (1, 2, 3, 4, 5, 6), and pure AMP.

Formulation	Inhibition halos – diameter (mm)						
	1	2	3	4	5	6	Average ± RSD
Pure AMP	22	19	19	19	14	17	18.33 ± 2.66
EUD nanoparticles/HA	0	0	0	0	0	0	0
AMP EUD nanoparticles/HA	11	12	13	14	14	11	12.50 ± 1.37
EUD nanoparticles	0	0	0	0	0	0	0
Uncoated AMP EUD nanoparticles	12	14	15	17	17	14	14.83 ± 1.94

### 1.9 In vivo antifungal activity of uncoated AMP EUD nanoparticles and EUD nanoparticles/HA (Table 17)

The vaginal fungal burden (CFU mL^-1^) in animals treated with uncoated AMP EUD nanoparticles and EUD nanoparticles/HA is described in Table 17.

**Table 17:** Vaginal fungal burden (CFU mL^−1^) in each animal of infected control; infected groups receiving unloaded EUD nanoparticles/HA and unloaded and uncoated EUD nanoparticles, respectively; infected groups receiving AMP EUD nanoparticles/HA and uncoated AMP EUD nanoparticles, respectively; infected animals receiving pure AMP in solution. Animals were numbered as 1, 2, 3, 4, 5, and 6. The vaginal fungal burden was evaluated at 0, 24 and 48 hours post-treatment.

Formulation	1	2	3	4	5	6	Average ± RSD
Infected control	2.48	2.65	3.73	3.41	2.00	2.96	2.87 ± 0.57
**CFU mL**^**−1**^**(time zero)**							
EUD nanoparticles/HA	2.00	3.20	3.43	2.87	2.76	3.81	3.01 ± 0.58
EUD nanoparticles	3.25	3.38	3.60	3.09	2.39	3.11	3.14 ± 0.41
AMP EUD nanoparticles/HA	2.87	3.43	3.81	3.41	2.76	2.00	3.05 ± 0.59
Uncoated AMP EUD nanoparticles	3.17	3.47	3.40	3.00	2.50	2.39	2.98 ± 0.45
AMP solution	2.7	3.26	3.18	3.36	2.92	2.94	3.07 ± 0.25
**CFU mL**^**−1**^**(24 hours)**							
Infected control	2.76	2.67	3.17	3.65	2.15	3.14	2.92 ± 0.83
EUD nanoparticles/HA	2.23	2.85	2.00	1.19	3.42	3.55	2.34 ± 0.47
EUD nanoparticles	3.25	3.32	3.20	2.93	2.80	3.02	3.08 ± 0.20
AMP EUD nanoparticles/HA	0.0	0.0	0.0	0.0	0.0	0.0	0.0
Uncoated AMP EUD nanoparticles	1.44	1.25	1.30	1.17	0.55	0.18	0.98 ± 0.50
AMP solution	1.18	1.18	1.31	1.32	1.34	1.14	1.25 ± 0.09
**CFU mL**^**−1**^**(48 hours)**							
Infected control	2.59	3.22	3.21	4.05	2.08	2.74	2.98 ± 0.67
EUD nanoparticles/HA	3.41	3.28	3.08	2.13	3.04	3.15	3.02 ± 0.45
EUD nanoparticles	3.73	3.84	2.97	2.52	3.63	3.70	3.39 ± 0.53
AMP EUD nanoparticles/HA	0.0	0.0	0.0	0.0	0.0	0.0	0.0
Uncoated AMP EUD nanoparticles	0.0	0.0	0.0	0.0	0.0	0.0	0.0
AMP solution	0.6	0.8	1.45	0.97	1.25	0.77	0.97 ± 0.32

### 1.10 In vivo antifungal activity of uncoated AMP EUD nanoparticles and EUD nanoparticles/HA (Fig. 5)

**Fig. 5:**
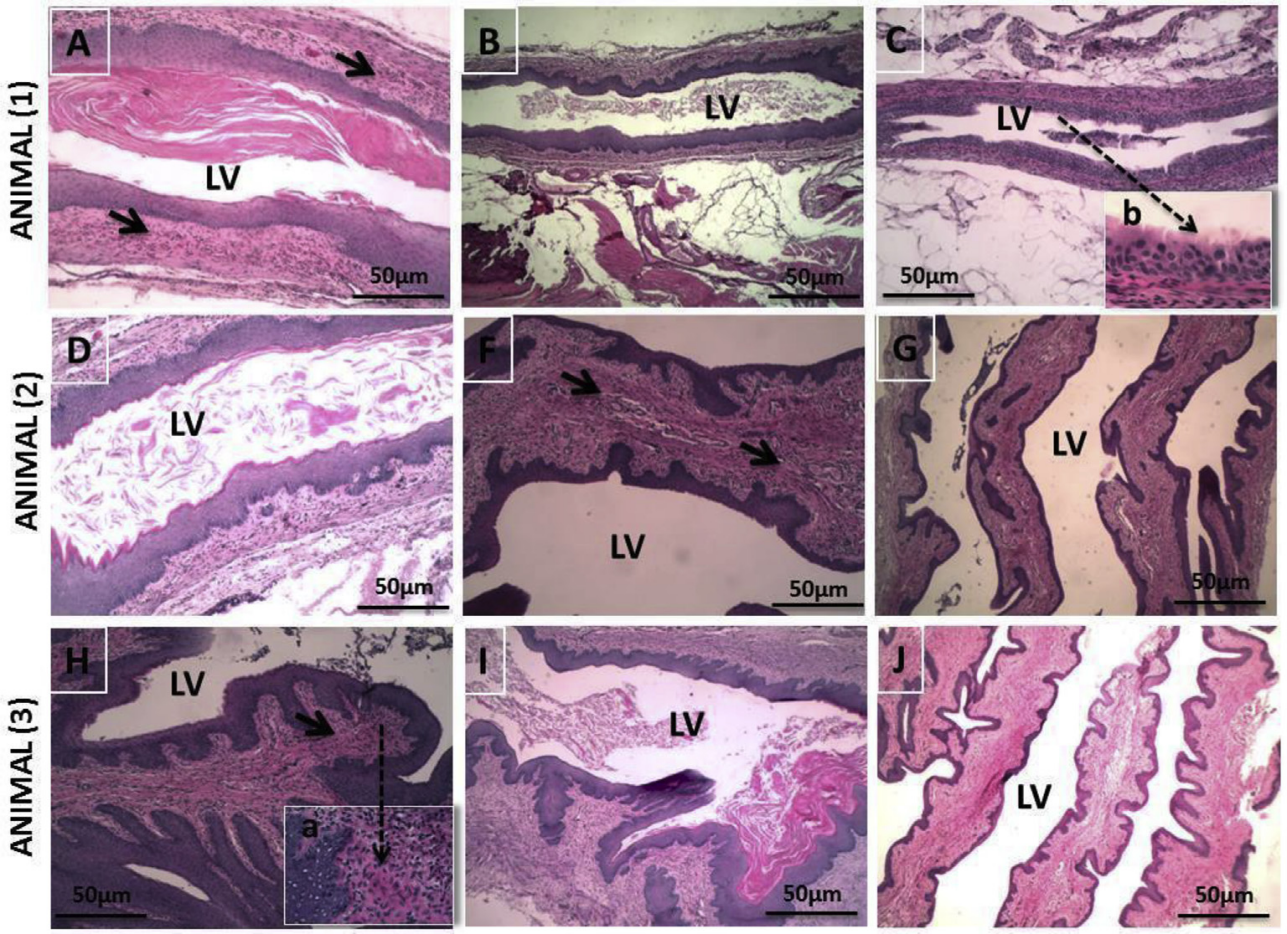
Histological sections of the endocervix collected 24 hours post-infection from 3 animals of each group (1, 2, and 3). (A) Infected control receiving no treatment (Group 1) (A, D, and H). The Vaginal Lumen (LV) showed Candida albicans hyphae. The vaginal epithelium showed inflammatory infiltrate (Head Arrow). The high resolution (a) (Dotted Arrow) showed, in detail, the inflammatory infiltrate. (B) Infected animals receiving unloaded EUD nanoparticles/HA (Group 2) (B, F, and I). The LV showed Candida albicans hyphae and the vaginal epithelium showed intense inflammatory cells (Head Arrow). (C) Infected animals receiving AMP EUD nanoparticles/HA (Group 3) (C, G, J). The LV did not contain fungal contamination. The high resolution (b) (Dotted Arrow) showed, in the detail, the integrity of the vaginal epithelium. Scale bar: 50 μm.

Histological sections of the endocervix collected 24 hours post-infection from animals receveing no treatment (Group 1), unloaded EUD nanoparticles/HA (Group 2), and AMP EUD nanoparticles/HA (Group 3) are presented in Fig. 5.

## 2 Experimental design, materials and methods

In section 1.1, data on diameter, polydispersity index, and zeta potential of AMP EUD nanoparticles from formulations 1 to 8, including the formulation 9 (central point) were obtained in triplicate, and the average values were calculated.

In section 1.2, the statistical experimental design (2^3^ full factorial design) was performed to evaluate the influence of independent variables: (A) EUD mass (mg), (B) Tween 80 concentration [% (w/v)], (C) Flow time of the organic phase (minutes), on diameter, polydispersity index, zeta potential, and AMP encapsulation efficiency for uncoated amphotericin B EUD nanoparticles. This influence was calculated by using the analysis of variance (ANOVA) in an individual analysis (A, B, C) as well as in a combination of analyzes (AB, AC, BC, ABC) (p < 0.05). The statistical parameters derived from ANOVA and regression analysis namely, model determination coefficient, F-ratio, model p value, coefficient estimates of all risk independent variables, and their respective p values [3] are tabulated in Table 2.

In section 1.3, data on linearity, matrix effect, precision, accuracy, and limit of quantitation were obtained as described below:

*Linearity and matrix effect* - Calibration curves were obtained using six AMP reference standard concentrations (5.0; 10.0; 15.0; 20.0; 30.0; and 35.0 μg mL^−1^) in 3 independent replicates run in random order. To verify the matrix effect, calibration curves were plotted using six amphotericin B reference standard concentrations (5.0; 10.0; 15.0; 20.0; 30.0; and 35.0 μg mL^−1^) associated with EUD, Tween 80 and HA at the concentration of 35 μg mL^−1^ in 3 independent replicates run in random order. Linear regression analysis was done by the ordinal least squares method. Residue analysis was performed [4], and outliers were deleted by using the Jacknife standardized residual test [5]. Maximum exclusion of 22.2% of original points was considered [6]. Then, normality by Ryan-Joiner test [7], homoscedasticity by Brown-Forsythe test [8,9], and independency by Durbin-Watson test [10] were achieved. For this model assumption, the lack-of-fit test (ANOVA) (p > 0.05), and the significance of regression (p > 0.05) were considered. Finally, as the linear model was suitable, slope and intercept were calculated to establish the equation that describes each calibration curve (calibration curve for AMP and calibration curve for AMP associated with EUD, Tween 80 and HA – matrix effect). Then, these calibration curves were compared by t-Student test assuming combined or distinct variances [2,11].

*Precision -* Precision was determined based on repeatability and intermediate precision. Repeatability was assessed through the assay of solutions at concentrations of 5.0; 20.0; and 35.0 μg mL^−1^ on the same day. Solutions were prepared in triplicate with AMP associated with EUD, Tween 80 and HA at the concentration of 35 μg mL^−1^. Intermediate precision was verified by evaluating the results on 2 different days (n = 6 for each concentration). Precision was expressed as mean content of AMP ± RSD.

*Accuracy -* To determine accuracy, standard solutions at concentrations of 5.0; 20.0; and 35.0 μg mL^−1^ were prepared in triplicate with AMP associated with EUD, Tween 80 and HA at the concentration of 35 μg mL^−1^. Solutions were assayed on 2 different days (n = 6 for each concentration). The percent recovery of added AMP was calculated comparing absorvances of resultant solutions with AMP standard solutions at the same concentration. The RSD was also calculated.

*Limit of Quantitation -* The limit of quantitation value (LOQ) is defined as the lowest concentration that can be quantitatively determined with suitable precision and accuracy. The LOQ was calculated directly from the calibration curve and can be expressed as:

(1) $$LOQ=\frac{10\sigma }{b}$$

where, σ is the standard deviation of the response and b is the slope of the calibration curve.

In section 1.4, data on diameter, polydispersity index, and zeta potential of AMP EUD nanoparticles/HA were obtained in triplicate, and the average values were calculated.

In section 1.5, to calculate the AMP of uncoated EUD nanoparticles from formulations 1 to 8, including the formulation 9 (central point), the nanoparticles were ultracentrifuged at 14.000 *g* for 30 minutes at 8 °C. The supernatant (1 mL) was collected and diluted in 1 mL of methanol and phosphate buffer solution (PBS, pH 7.4) (1:2) to quantify the non-encapsulated drug. Then, the theoretical AMP mass to formulate the uncoated EUD nanoparticles from formulations was 2.5 mg, which is equivalent to 100% of the drug. This value was subtracted from the AMP mass in the supernatant, resulting in the amount (mg) of AMP encapsulated into the uncoated EUD nanoparticles. These values were expressed as percentages, representing the AMP encapsulation efficiency of uncoated EUD nanoparticles from formulations 1 to 8, including the formulation 9 (central point).

In section 1.6, FTIR spectra, XRD diffractograms, and DSC/TG/DTA thermograms were directly obtained for pure AMP, pure EUD, and pure HA, since these substances are solids in normal temperature and pressure conditions. To analyze the unloaded EUD nanoparticles, AMP EUD nanoparticles, unloaded and uncoated EUD nanoparticles, and AMP EUD nanoparticles/HA, three batches of each sample were ultracentrifuged at 14.000 *g* for 30 minutes at 8 °C after recent preparation. Then, the supernatant was discarded, and the resulting pellet was collected and stored in plastic microtubes (2 mL). The microtubes were kept in desiccator for 15 days for complete pellet drying. Finally, the solid nanoparticles were gathered, and analyzed by using the different analytical techniques previously described.

In section 1.7, AMP released from uncoated EUD nanoparticles and EUD nanoparticles/HA was measured in 3 batches for each formulation, and the percentage of AMP released from nanoparticles was expressed to show the existence of a prolonged and controlled drug delivery systems. Uncoated and coated AMP EUD nanoparticles were placed in dialysis bags composed of cellulose. They were immersed in tubes containing the phosphate buffer (pH 5.5), and the tubes were sealed to perform the *in vitro* drug release study. The sink conditions were attained. The phosphate buffer (pH 5.5) was applied to simulate the pH of the infected vaginal cavity on a condition of vulvovaginal candidiasis.

In section 1.8, the solution of pure AMP and nanoparticles in suspension were transferred into metal tubes, which were placed on the Muller-Hinton agar previously inoculated with *C. albicans*. The diffusion of drug and formulation into the agar induced the inhibition of *C. albicans* growth, creating inhibition halos; and their diameters were measured by using the caliper.

In section 1.9, vaginal *Candida* burden of rats of all groups was determined after vaginal lavage, collection of the lavage liquid, and incubation in plates containing Sabouraud Dextrose agar supplemented with chloramphenicol at 24 and 48 hours post-treatment.

In section 1.10, the C*andida* contamination in the vaginal lumen was qualitatively evaluated using the histological sections. After 24 hours post-infection, the animals were euthanized and the vaginas were fixed in 10% formalin in isotonic saline solution, embedded in paraffin, and sectioned to obtain histopathological data. In addition, the vaginal epithelium was evaluated to determine the existence of inflammatory infiltrate.
