# Supplementary material for: Data on statistical experimental design to formulate amphotericin B-loaded Eudragit RL100 nanoparticles coated with hyaluronic acid for the treatment of vulvovaginal candidiasis
Source: Data Brief. 2020 Mar 5;29:105311. doi: 10.1016/j.dib.2020.105311 (PMC7082528; doi:10.1016/j.dib.2020.105311)
Supplement: Multimedia component 1 [file mmc1.pdf]

File Name:	**pure EUD**		
[Data]			
Time	Temp	**DTA**	**TGA**
sec	C	uV	mg
0	241.857	-296	11.7185
1.2000	241.746	-322	11.7186
2.2000	241.886	-337	11.7183
3.2000	241.705	-349	11.7181
4.2000	241.882	-456	11.7180
5.2000	241.786	-366	11.7181
6.2000	241.887	-527	11.7181
7.2000	242.003	-510	11.7181
8.2000	241.967	-631	11.7184
9.2000	242.185	-661	11.7187
10.2000	242.224	-862	11.7187
11.2000	242.490	-930	11.7187
12.2000	242.492	-1.194	11.7187
130.000	242.932	-1.368	11.7186
140.000	242.773	-1.612	11.7184
150.000	243.320	-1.991	11.7183
160.000	243.356	-2.249	11.7184
170.000	243.819	-2.661	11.7183
180.000	243.982	-3.108	11.7183
190.000	244.484	-3.557	11.7182
200.000	244.674	-4.049	11.7182
210.000	245.235	-4.735	11.7183
220.000	245.475	-5.205	11.7181
230.000	245.979	-5.986	11.7182
240.000	246.453	-6.668	11.7183

250.000	246.804	-7.432	11.7182
260.000	247.460	-8.197	11.7182
270.000	247.803	-9.156	11.7182
280.000	248.479	-9.948	11.7182
290.000	248.806	-10.968	11.7181
300.000	249.638	-11.968	11.7181
310.000	249.938	-12.948	11.7181
320.000	250.834	-14.107	11.7181
330.000	251.261	-15.140	11.7183
340.000	252.119	-16.311	11.7188
350.000	252.580	-17.481	11.7190
360.000	253.440	-18.682	11.7191
370.000	253.968	-19.861	11.7195
380.000	254.763	-21.227	11.7199
390.000	255.461	-22.399	11.7200
400.000	256.181	-23.789	11.7199
410.000	257.033	-25.075	11.7201
420.000	257.726	-26.432	11.7205
430.000	258.583	-27.782	11.7205
440.000	259.357	-29.265	11.7202
450.000	260.395	-30.561	11.7204
460.000	260.891	-32.064	11.7206
470.000	262.130	-33.536	11.7206
480.000	262.606	-34.936	11.7204
490.000	263.782	-36.484	11.7204
500.000	264.524	-37.948	11.7205
510.000	265.558	-39.456	11.7205
520.000	266.319	-40.946	11.7205
530.000	267.510	-42.516	11.7207
540.000	268.197	-43.941	11.7207

550.000	269.353	-45.609	11.7207
560.000	270.326	-47.034	11.7210
570.000	271.282	-48.668	11.7214
580.000	272.386	-50.175	11.7215
590.000	273.314	-51.786	11.7215
600.000	274.450	-53.254	11.7215
610.000	275.400	-54.954	11.7217
620.000	276.626	-56.421	11.7219
630.000	277.417	-58.044	11.7218
640.000	278.831	-59.695	11.7218
650.000	279.596	-61.187	11.7221
660.000	280.994	-62.855	11.7224
670.000	281.982	-64.467	11.7224
680.000	283.257	-66.009	11.7223
690.000	284.202	-67.595	11.7224
700.000	285.591	-69.262	11.7225
710.000	286.581	-70.709	11.7224
720.000	287.853	-72.430	11.7221
730.000	289.017	-73.932	11.7221
740.000	290.168	-75.549	11.7224
750.000	291.459	-77.114	11.7223
760.000	292.594	-78.715	11.7221
770.000	293.961	-80.209	11.7219
780.000	295.061	-81.877	11.7220
790.000	296.567	-83.368	11.7219
800.000	297.463	-84.941	11.7218
810.000	299.057	-86.563	11.7218
820.000	300.102	-88.052	11.7221
830.000	301.556	-89.645	11.7223
840.000	302.656	-91.186	11.7222

850.000	304.149	-92.697	11.7223
860.000	305.211	-94.187	11.7225
870.000	306.665	-95.779	11.7228
880.000	307.826	-97.174	11.7228
890.000	309.244	-98.764	11.7226
900.000	310.616	-100.222	11.7228
910.000	311.827	-101.723	11.7232
920.000	313.240	-103.148	11.7233
930.000	314.471	-104.721	11.7231
940.000	315.908	-106.041	11.7230
950.000	317.057	-107.591	11.7231
960.000	318.718	-109.043	11.7233
970.000	319.712	-110.447	11.7232
980.000	321.404	-111.944	11.7230
990.000	322.526	-113.302	11.7230
1.000.000	324.099	-114.751	11.7231
1.010.000	325.391	-116.186	11.7233
1.020.000	326.984	-117.577	11.7230
1.030.000	328.136	-118.898	11.7230
1.040.000	329.714	-120.401	11.7232
1.050.000	330.986	-121.633	11.7232
1.060.000	332.412	-123.080	11.7233
1.070.000	333.899	-124.420	11.7232
1.080.000	335.270	-125.738	11.7229
1.090.000	336.729	-127.019	11.7226
1.100.000	338.105	-128.474	11.7224
1.110.000	339.723	-129.662	11.7224
1.120.000	340.983	-131.059	11.7220
1.130.000	342.742	-132.407	11.7217
1.140.000	343.854	-133.621	11.7215

1.150.000	345.587	-134.969	11.7216
1.160.000	346.857	-136.229	11.7216
1.170.000	348.510	-137.484	11.7216
1.180.000	349.759	-138.762	11.7215
1.190.000	351.468	-140.033	11.7215
1.200.000	352.760	-141.211	11.7215
1.210.000	354.382	-142.537	11.7217
1.220.000	355.876	-143.676	11.7216
1.230.000	357.397	-144.953	11.7215
1.240.000	358.939	-146.148	11.7215
1.250.000	360.428	-147.370	11.7216
1.260.000	361.995	-148.500	11.7219
1.270.000	363.383	-149.793	11.7222
1.280.000	365.118	-150.851	11.7222
1.290.000	366.355	-152.080	11.7221
1.300.000	368.205	-153.292	11.7222
1.310.000	369.477	-154.367	11.7223
1.320.000	371.223	-155.582	11.7225
1.330.000	372.602	-156.738	11.7223
1.340.000	374.388	-157.858	11.7221
1.350.000	375.728	-158.957	11.7220
1.360.000	377.514	-160.151	11.7221
1.370.000	378.855	-161.148	11.7220
1.380.000	380.543	-162.361	11.7217
1.390.000	382.096	-163.389	11.7215
1.400.000	383.633	-164.517	11.7215
1.410.000	385.264	-165.566	11.7217
1.420.000	386.780	-166.714	11.7217
1.430.000	388.430	-167.651	11.7214
1.440.000	389.899	-168.833	11.7213

1.450.000	391.760	-169.820	11.7214
1.460.000	392.999	-170.854	11.7214
1.470.000	394.944	-171.996	11.7212
1.480.000	396.237	-172.936	11.7209
1.490.000	398.032	-173.987	11.7209
1.500.000	399.468	-175.041	11.7211
1.510.000	401.253	-175.999	11.7212
1.520.000	402.587	-176.961	11.7211
1.530.000	404.415	-178.061	11.7207
1.540.000	405.832	-178.895	11.7206
1.550.000	407.514	-179.961	11.7207
1.560.000	409.140	-180.923	11.7207
1.570.000	410.693	-181.877	11.7206
1.580.000	412.321	-182.825	11.7203
1.590.000	413.850	-183.864	11.7201
1.600.000	415.562	-184.667	11.7202
1.610.000	416.933	-185.699	11.7203
1.620.000	418.837	-186.626	11.7201
1.630.000	420.071	-187.526	11.7198
1.640.000	421.938	-188.505	11.7196
1.650.000	423.360	-189.390	11.7196
1.660.000	425.098	-190.319	11.7196
1.670.000	426.544	-191.213	11.7193
1.680.000	428.411	-192.134	11.7189
1.690.000	429.752	-192.943	11.7188
1.700.000	431.642	-193.962	11.7186
1.710.000	433.140	-194.715	11.7184
1.720.000	434.739	-195.627	11.7181
1.730.000	436.400	-196.488	11.7180
1.740.000	437.994	-197.366	11.7180

1.750.000	439.570	-198.131	11.7178
1.760.000	441.164	-199.107	11.7175
1.770.000	443.019	-199.812	11.7175
1.780.000	444.389	-200.708	11.7176
1.790.000	446.353	-201.590	11.7177
1.800.000	447.717	-202.345	11.7175
1.810.000	449.553	-203.232	11.7176
1.820.000	451.002	-204.047	11.7177
1.830.000	452.828	-204.823	11.7177
1.840.000	454.264	-205.655	11.7174
1.850.000	456.112	-206.509	11.7169
1.860.000	457.537	-207.184	11.7168
1.870.000	459.352	-208.110	11.7166
1.880.000	460.930	-208.825	11.7164
1.890.000	462.620	-209.639	11.7162
1.900.000	464.337	-210.408	11.7159
1.910.000	465.978	-211.199	11.7160
1.920.000	467.616	-211.900	11.7162
1.930.000	469.202	-212.793	11.7163
1.940.000	471.091	-213.445	11.7160
1.950.000	472.444	-214.235	11.7158
1.960.000	474.436	-215.075	11.7157
1.970.000	475.810	-215.722	11.7157
1.980.000	477.679	-216.518	11.7153
1.990.000	479.140	-217.301	11.7148
2.000.000	481.053	-218.004	11.7144
2.010.000	482.498	-218.772	11.7144
2.020.000	484.408	-219.581	11.7140
2.030.000	485.901	-220.179	11.7136
2.040.000	487.685	-221.015	11.7133

2.050.000	489.366	-221.707	11.7133
2.060.000	490.968	-222.432	11.7133
2.070.000	492.684	-223.119	11.7131
2.080.000	494.346	-223.926	11.7132
2.090.000	496.033	-224.533	11.7132
2.100.000	497.588	-225.351	11.7135
2.110.000	499.552	-226.035	11.7136
2.120.000	500.905	-226.768	11.7135
2.130.000	502.943	-227.538	11.7135
2.140.000	504.355	-228.184	11.7133
2.150.000	506.202	-228.919	11.7132
2.160.000	507.639	-229.657	11.7126
2.170.000	509.510	-230.342	11.7122
2.180.000	510.932	-230.999	11.7119
2.190.000	512.775	-231.816	11.7117
2.200.000	514.277	-232.387	11.7114
2.210.000	515.974	-233.171	11.7110
2.220.000	517.669	-233.849	11.7110
2.230.000	519.273	-234.549	11.7111
2.240.000	520.965	-235.214	11.7110
2.250.000	522.636	-236.034	11.7107
2.260.000	524.368	-236.565	11.7109
2.270.000	525.814	-237.363	11.7110
2.280.000	527.766	-238.060	11.7107
2.290.000	529.099	-238.701	11.7105
2.300.000	531.019	-239.460	11.7103
2.310.000	532.454	-240.138	11.7104
2.320.000	534.294	-240.813	11.7102
2.330.000	535.810	-241.535	11.7098
2.340.000	537.659	-242.253	11.7095

2.350.000	539.162	-242.871	11.7093
2.360.000	540.913	-243.663	11.7091
2.370.000	542.511	-244.261	11.7085
2.380.000	544.218	-245.026	11.7079
2.390.000	545.892	-245.701	11.7079
2.400.000	547.512	-246.424	11.7079
2.410.000	549.208	-247.058	11.7076
2.420.000	550.807	-247.895	11.7073
2.430.000	552.608	-248.476	11.7073
2.440.000	554.009	-249.241	11.7075
2.450.000	556.026	-250.015	11.7070
2.460.000	557.384	-250.641	11.7068
2.470.000	559.313	-251.434	11.7068
2.480.000	560.850	-252.144	11.7068
2.490.000	562.666	-252.830	11.7063
2.500.000	564.124	-253.521	11.7060
2.510.000	566.023	-254.298	11.7058
2.520.000	567.421	-254.880	11.7056
2.530.000	569.259	-255.665	11.7049
2.540.000	570.903	-256.305	11.7046
2.550.000	572.560	-257.011	11.7045
2.560.000	574.314	-257.657	11.7045
2.570.000	575.966	-258.406	11.7040
2.580.000	577.738	-258.962	11.7038
2.590.000	579.281	-259.720	11.7038
2.600.000	581.160	-260.301	11.7038
2.610.000	582.570	-260.958	11.7037
2.620.000	584.564	-261.650	11.7033
2.630.000	585.944	-262.221	11.7030
2.640.000	587.902	-262.851	11.7031

2.650.000	589.367	-263.498	11.7030
2.660.000	591.286	-264.054	11.7026
2.670.000	592.767	-264.573	11.7024
2.680.000	594.667	-265.267	11.7026
2.690.000	596.269	-265.668	11.7031
2.700.000	598.048	-266.307	11.7031
2.710.000	599.704	-266.791	11.7028
2.720.000	601.405	-267.306	11.7028
2.730.000	603.106	-267.767	11.7028
2.740.000	604.759	-268.350	11.7023
2.750.000	606.586	-268.696	11.7018
2.760.000	608.034	-269.260	11.7015
2.770.000	610.043	-269.702	11.7015
2.780.000	611.429	-270.110	11.7013
2.790.000	613.347	-270.612	11.7009
2.800.000	614.862	-271.000	11.7009
2.810.000	616.688	-271.412	11.7010
2.820.000	618.213	-271.824	11.7010
2.830.000	620.149	-272.242	11.7006
2.840.000	621.587	-272.598	11.7002
2.850.000	623.492	-273.065	11.7001
2.860.000	625.073	-273.331	11.7003
2.870.000	626.750	-273.778	11.7000
2.880.000	628.545	-274.123	11.6997
2.890.000	630.168	-274.508	11.6995
2.900.000	631.904	-274.800	11.6994
2.910.000	633.564	-275.281	11.6992
2.920.000	635.385	-275.519	11.6988
2.930.000	636.827	-275.937	11.6983
2.940.000	638.821	-276.337	11.6978

2.950.000	640.193	-276.612	11.6976
2.960.000	642.195	-277.050	11.6976
2.970.000	643.653	-277.402	11.6975
2.980.000	645.527	-277.722	11.6974
2.990.000	647.066	-278.093	11.6972
3.000.000	649.022	-278.518	11.6971
3.010.000	650.481	-278.754	11.6969
3.020.000	652.350	-279.227	11.6964
3.030.000	653.990	-279.496	11.6959
3.040.000	655.651	-279.869	11.6956
3.050.000	657.402	-280.201	11.6957
3.060.000	659.102	-280.575	11.6957
3.070.000	660.863	-280.854	11.6957
3.080.000	662.405	-281.310	11.6955
3.090.000	664.328	-281.577	11.6953
3.100.000	665.740	-281.951	11.6953
3.110.000	667.779	-282.388	11.6952
3.120.000	669.196	-282.648	11.6949
3.130.000	671.109	-283.065	11.6945
3.140.000	672.682	-283.442	11.6941
3.150.000	674.589	-283.766	11.6940
3.160.000	676.083	-284.128	11.6938
3.170.000	677.934	-284.563	11.6934
3.180.000	679.500	-284.803	11.6928
3.190.000	681.253	-285.287	11.6925
3.200.000	682.964	-285.576	11.6925
3.210.000	684.630	-285.978	11.6927
3.220.000	686.385	-286.295	11.6927
3.230.000	688.020	-286.760	11.6923
3.240.000	689.859	-287.003	11.6920

3.250.000	691.424	-287.475	11.6918
3.260.000	693.426	-287.817	11.6917
3.270.000	694.758	-288.160	11.6912
3.280.000	696.763	-288.593	11.6905
3.290.000	698.246	-288.922	11.6903
3.300.000	700.091	-289.334	11.6903
3.310.000	701.640	-289.698	11.6905
3.320.000	703.565	-290.083	11.6904
3.330.000	705.008	-290.380	11.6901
3.340.000	706.907	-290.873	11.6898
3.350.000	708.453	-291.116	11.6896
3.360.000	710.176	-291.564	11.6894
3.370.000	711.948	-291.907	11.6892
3.380.000	713.556	-292.265	11.6889
3.390.000	715.267	-292.578	11.6882
3.400.000	716.890	-293.056	11.6880
3.410.000	718.656	-293.275	11.6880
3.420.000	720.152	-293.736	11.6876
3.430.000	722.136	-294.156	11.6874
3.440.000	723.495	-294.436	11.6868
3.450.000	725.451	-294.875	11.6867
3.460.000	726.911	-295.241	11.6866
3.470.000	728.764	-295.610	11.6865
3.480.000	730.358	-295.961	11.6865
3.490.000	732.214	-296.395	11.6859
3.500.000	733.712	-296.651	11.6858
3.510.000	735.469	-297.128	11.6858
3.520.000	737.104	-297.383	11.6856
3.530.000	738.803	-297.815	11.6855
3.540.000	740.540	-298.158	11.6850

3.550.000	742.184	-298.549	11.6846
3.560.000	743.972	-298.840	11.6844
3.570.000	745.493	-299.312	11.6842
3.580.000	747.422	-299.590	11.6840
3.590.000	748.844	-300.016	11.6839
3.600.000	750.837	-300.421	11.6837
3.610.000	752.260	-300.724	11.6834
3.620.000	754.167	-301.149	11.6832
3.630.000	755.670	-301.528	11.6829
3.640.000	757.558	-301.873	11.6826
3.650.000	759.029	-302.217	11.6823
3.660.000	760.965	-302.676	11.6817
3.670.000	762.475	-302.949	11.6814
3.680.000	764.234	-303.393	11.6813
3.690.000	765.982	-303.728	11.6811
3.700.000	767.564	-304.107	11.6810
3.710.000	769.360	-304.437	11.6806
3.720.000	771.017	-304.892	11.6803
3.730.000	772.780	-305.140	11.6801
3.740.000	774.309	-305.610	11.6798
3.750.000	776.305	-305.994	11.6796
3.760.000	777.628	-306.313	11.6791
3.770.000	779.661	-306.778	11.6785
3.780.000	781.095	-307.082	11.6782
3.790.000	783.000	-307.486	11.6780
3.800.000	784.509	-307.870	11.6777
3.810.000	786.371	-308.241	11.6773
3.820.000	787.900	-308.577	11.6771
3.830.000	789.799	-309.074	11.6770
3.840.000	791.398	-309.305	11.6770

3.850.000	793.133	-309.758	11.6767
3.860.000	794.859	-310.108	11.6762
3.870.000	796.500	-310.465	11.6756
3.880.000	798.302	-310.804	11.6751
3.890.000	799.898	-311.273	11.6745
3.900.000	801.743	-311.507	11.6738
3.910.000	803.232	-311.980	11.6731
3.920.000	805.200	-312.347	11.6728
3.930.000	806.557	-312.676	11.6726
3.940.000	808.552	-313.107	11.6727
3.950.000	810.018	-313.429	11.6725
3.960.000	811.834	-313.818	11.6725
3.970.000	813.369	-314.176	11.6725
3.980.000	815.249	-314.595	11.6725
3.990.000	816.740	-314.897	11.6724
4.000.000	818.544	-315.351	11.6722
4.010.000	820.167	-315.638	11.6717
4.020.000	821.778	-316.052	11.6712
4.030.000	823.584	-316.383	11.6710
4.040.000	825.183	-316.781	11.6708
4.050.000	826.980	-317.077	11.6705
4.060.000	828.548	-317.559	11.6698
4.070.000	830.419	-317.817	11.6694
4.080.000	831.814	-318.203	11.6692
4.090.000	833.799	-318.638	11.6689
4.100.000	835.224	-318.915	11.6685
4.110.000	837.124	-319.324	11.6681
4.120.000	838.601	-319.677	11.6679
4.130.000	840.476	-320.020	11.6677
4.140.000	842.026	-320.359	11.6676

4.150.000	843.870	-320.794	11.6674
4.160.000	845.406	-321.034	11.6669
4.170.000	847.164	-321.480	11.6667
4.180.000	848.871	-321.775	11.6664
4.190.000	850.467	-322.128	11.6662
4.200.000	852.232	-322.454	11.6660
4.210.000	853.880	-322.876	11.6655
4.220.000	855.665	-323.119	11.6652
4.230.000	857.189	-323.580	11.6651
4.240.000	859.129	-323.865	11.6650
4.250.000	860.510	-324.212	11.6648
4.260.000	862.479	-324.653	11.6642
4.270.000	864.014	-324.921	11.6639
4.280.000	865.827	-325.302	11.6637
4.290.000	867.366	-325.675	11.6635
4.300.000	869.266	-326.014	11.6630
4.310.000	870.747	-326.318	11.6625
4.320.000	872.626	-326.777	11.6623
4.330.000	874.238	-327.015	11.6620
4.340.000	875.923	-327.458	11.6615
4.350.000	877.685	-327.749	11.6610
4.360.000	879.279	-328.119	11.6607
4.370.000	881.070	-328.434	11.6605
4.380.000	882.726	-328.868	11.6601
4.390.000	884.521	-329.109	11.6597
4.400.000	885.985	-329.524	11.6593
4.410.000	887.964	-329.887	11.6590
4.420.000	889.374	-330.199	11.6585
4.430.000	891.359	-330.598	11.6579
4.440.000	892.835	-330.896	11.6575

4.450.000	894.659	-331.268	11.6573
4.460.000	896.263	-331.612	11.6569
4.470.000	898.118	-331.969	11.6564
4.480.000	899.602	-332.255	11.6559
4.490.000	901.475	-332.705	11.6557
4.500.000	903.128	-332.947	11.6555
4.510.000	904.750	-333.348	11.6550
4.520.000	906.532	-333.639	11.6547
4.530.000	908.096	-333.995	11.6546
4.540.000	909.937	-334.290	11.6543
4.550.000	911.469	-334.702	11.6539
4.560.000	913.317	-334.967	11.6534
4.570.000	914.734	-335.342	11.6531
4.580.000	916.743	-335.754	11.6528
4.590.000	918.117	-335.982	11.6523
4.600.000	920.052	-336.385	11.6517
4.610.000	921.542	-336.706	11.6513
4.620.000	923.345	-337.023	11.6510
4.630.000	924.869	-337.358	11.6505
4.640.000	926.736	-337.766	11.6499
4.650.000	928.268	-337.981	11.6494
4.660.000	930.041	-338.433	11.6490
4.670.000	931.741	-338.672	11.6487
4.680.000	933.318	-339.065	11.6481
4.690.000	935.073	-339.336	11.6476
4.700.000	936.699	-339.723	11.6473
4.710.000	938.492	-339.989	11.6469
4.720.000	940.024	-340.412	11.6463
4.730.000	941.971	-340.674	11.6457
4.740.000	943.314	-341.028	11.6454

4.750.000	945.274	-341.409	11.6451
4.760.000	946.730	-341.684	11.6445
4.770.000	948.588	-342.055	11.6439
4.780.000	950.144	-342.373	11.6434
4.790.000	951.965	-342.704	11.6429
4.800.000	953.506	-343.012	11.6422
4.810.000	955.339	-343.440	11.6416
4.820.000	956.934	-343.661	11.6412
4.830.000	958.650	-344.076	11.6407
4.840.000	960.441	-344.377	11.6401
4.850.000	961.980	-344.714	11.6398
4.860.000	963.820	-345.015	11.6397
4.870.000	965.424	-345.441	11.6394
4.880.000	967.226	-345.686	11.6388
4.890.000	968.737	-346.087	11.6385
4.900.000	970.688	-346.465	11.6384
4.910.000	972.052	-346.740	11.6380
4.920.000	974.030	-347.160	11.6374
4.930.000	975.504	-347.462	11.6370
4.940.000	977.337	-347.824	11.6368
4.950.000	978.969	-348.180	11.6364
4.960.000	980.802	-348.538	11.6357
4.970.000	982.356	-348.834	11.6354
4.980.000	984.176	-349.282	11.6352
4.990.000	985.814	-349.489	11.6348
5.000.000	987.514	-349.916	11.6344
5.010.000	989.271	-350.235	11.6342
5.020.000	990.853	-350.572	11.6340
5.030.000	992.704	-350.877	11.6335
5.040.000	994.262	-351.305	11.6329

5.050.000	996.158	-351.577	11.6326
5.060.000	997.594	-351.955	11.6324
5.070.000	999.571	-352.351	11.6320
5.080.000	1.000.998	-352.613	11.6315
5.090.000	1.002.932	-353.031	11.6313
5.100.000	1.004.493	-353.361	11.6311
5.110.000	1.006.301	-353.690	11.6306
5.120.000	1.007.838	-354.021	11.6299
5.130.000	1.009.674	-354.417	11.6296
5.140.000	1.011.201	-354.646	11.6294
5.150.000	1.012.950	-355.080	11.6288
5.160.000	1.014.629	-355.338	11.6281
5.170.000	1.016.263	-355.722	11.6278
5.180.000	1.018.028	-356.000	11.6276
5.190.000	1.019.593	-356.381	11.6272
5.200.000	1.021.382	-356.653	11.6267
5.210.000	1.022.935	-357.064	11.6264
5.220.000	1.024.838	-357.356	11.6264
5.230.000	1.026.210	-357.686	11.6263
5.240.000	1.028.177	-358.084	11.6258
5.250.000	1.029.647	-358.344	11.6252
5.260.000	1.031.492	-358.719	11.6249
5.270.000	1.033.030	-359.049	11.6245
5.280.000	1.034.852	-359.373	11.6241
5.290.000	1.036.373	-359.662	11.6234
5.300.000	1.038.198	-360.075	11.6229
5.310.000	1.039.775	-360.292	11.6227
5.320.000	1.041.495	-360.714	11.6223
5.330.000	1.043.271	-361.011	11.6218
5.340.000	1.044.808	-361.331	11.6212

5.350.000	1.046.629	-361.619	11.6208
5.360.000	1.048.152	-362.006	11.6205
5.370.000	1.049.970	-362.257	11.6200
5.380.000	1.051.460	-362.646	11.6195
5.390.000	1.053.483	-362.985	11.6191
5.400.000	1.054.885	-363.258	11.6189
5.410.000	1.056.765	-363.663	11.6186
5.420.000	1.058.276	-363.912	11.6181
5.430.000	1.060.108	-364.270	11.6177
5.440.000	1.061.691	-364.600	11.6175
5.450.000	1.063.508	-364.939	11.6173
5.460.000	1.065.071	-365.197	11.6168
5.470.000	1.066.816	-365.638	11.6163
5.480.000	1.068.515	-365.845	11.6158
5.490.000	1.070.167	-366.270	11.6156
5.500.000	1.071.924	-366.566	11.6152
5.510.000	1.073.532	-366.889	11.6145
5.520.000	1.075.318	-367.189	11.6139
5.530.000	1.076.891	-367.603	11.6136
5.540.000	1.078.798	-367.890	11.6134
5.550.000	1.080.264	-368.248	11.6131
5.560.000	1.082.214	-368.647	11.6126
5.570.000	1.083.651	-368.903	11.6122
5.580.000	1.085.543	-369.309	11.6120
5.590.000	1.087.096	-369.622	11.6118
5.600.000	1.088.874	-369.971	11.6114
5.610.000	1.090.468	-370.295	11.6107
5.620.000	1.092.357	-370.686	11.6103
5.630.000	1.093.896	-370.942	11.6100
5.640.000	1.095.678	-371.383	11.6096

5.650.000	1.097.438	-371.642	11.6089
5.660.000	1.099.023	-372.030	11.6084
5.670.000	1.100.857	-372.322	11.6081
5.680.000	1.102.429	-372.693	11.6078
5.690.000	1.104.258	-372.983	11.6073
5.700.000	1.105.736	-373.387	11.6066
5.710.000	1.107.633	-373.705	11.6060
5.720.000	1.109.016	-374.027	11.6056
5.730.000	1.111.001	-374.441	11.6052
5.740.000	1.112.463	-374.698	11.6048
5.750.000	1.114.314	-375.090	11.6041
5.760.000	1.115.888	-375.416	11.6036
5.770.000	1.117.655	-375.740	11.6033
5.780.000	1.119.194	-376.040	11.6029
5.790.000	1.120.998	-376.466	11.6022
5.800.000	1.122.620	-376.677	11.6017
5.810.000	1.124.291	-377.123	11.6013
5.820.000	1.126.051	-377.372	11.6011
5.830.000	1.127.574	-377.716	11.6009
5.840.000	1.129.402	-378.026	11.6003
5.850.000	1.130.984	-378.398	11.5999
5.860.000	1.132.806	-378.673	11.5995
5.870.000	1.134.275	-379.052	11.5992
5.880.000	1.136.281	-379.368	11.5989
5.890.000	1.137.626	-379.667	11.5981
5.900.000	1.139.572	-380.049	11.5976
5.910.000	1.141.061	-380.278	11.5974
5.920.000	1.142.857	-380.686	11.5970
5.930.000	1.144.463	-380.975	11.5966
5.940.000	1.146.230	-381.302	11.5960

5.950.000	1.147.795	-381.596	11.5953
5.960.000	1.149.643	-382.040	11.5948
5.970.000	1.151.265	-382.226	11.5945
5.980.000	1.152.925	-382.635	11.5943
5.990.000	1.154.745	-382.940	11.5938
6.000.000	1.156.279	-383.279	11.5930
6.010.000	1.158.122	-383.589	11.5926
6.020.000	1.159.689	-383.999	11.5922
6.030.000	1.161.510	-384.279	11.5917
6.040.000	1.162.975	-384.636	11.5909
6.050.000	1.164.933	-385.038	11.5900
6.060.000	1.166.363	-385.292	11.5895
6.070.000	1.168.279	-385.706	11.5892
6.080.000	1.169.802	-386.015	11.5887
6.090.000	1.171.640	-386.361	11.5883
6.100.000	1.173.234	-386.699	11.5880
6.110.000	1.175.051	-387.079	11.5874
6.120.000	1.176.635	-387.335	11.5870
6.130.000	1.178.345	-387.770	11.5865
6.140.000	1.180.088	-388.002	11.5861
6.150.000	1.181.698	-388.405	11.5857
6.160.000	1.183.523	-388.708	11.5852
6.170.000	1.185.061	-389.047	11.5847
6.180.000	1.186.953	-389.377	11.5844
6.190.000	1.188.456	-389.772	11.5842
6.200.000	1.190.413	-390.071	11.5838
6.210.000	1.191.826	-390.431	11.5831
6.220.000	1.193.803	-390.816	11.5823
6.230.000	1.195.236	-391.062	11.5819
6.240.000	1.197.067	-391.458	11.5815

6.250.000	1.198.709	-391.788	11.5810
6.260.000	1.200.472	-392.120	11.5803
6.270.000	1.202.073	-392.443	11.5797
6.280.000	1.203.892	-392.845	11.5793
6.290.000	1.205.502	-393.069	11.5788
6.300.000	1.207.201	-393.494	11.5783
6.310.000	1.209.014	-393.769	11.5776
6.320.000	1.210.547	-394.117	11.5772
6.330.000	1.212.357	-394.429	11.5770
6.340.000	1.213.918	-394.793	11.5768
6.350.000	1.215.714	-395.104	11.5766
6.360.000	1.217.221	-395.468	11.5761
6.370.000	1.219.132	-395.801	11.5758
6.380.000	1.220.499	-396.117	11.5755
6.390.000	1.222.491	-396.515	11.5751
6.400.000	1.223.989	-396.792	11.5746
6.410.000	1.225.800	-397.177	11.5740
6.420.000	1.227.407	-397.494	11.5732
6.430.000	1.229.180	-397.828	11.5726
6.440.000	1.230.771	-398.120	11.5721
6.450.000	1.232.522	-398.538	11.5716
6.460.000	1.234.190	-398.728	11.5709
6.470.000	1.235.810	-399.165	11.5702
6.480.000	1.237.638	-399.457	11.5695
6.490.000	1.239.085	-399.772	11.5691
6.500.000	1.240.988	-400.103	11.5686
6.510.000	1.242.526	-400.477	11.5676
6.520.000	1.244.358	-400.762	11.5666
6.530.000	1.245.881	-401.122	11.5659
6.540.000	1.247.824	-401.483	11.5655

6.550.000	1.249.245	-401.745	11.5650
6.560.000	1.251.151	-402.159	11.5642
6.570.000	1.252.704	-402.428	11.5637
6.580.000	1.254.467	-402.762	11.5635
6.590.000	1.256.035	-403.084	11.5631
6.600.000	1.257.826	-403.440	11.5625
6.610.000	1.259.431	-403.679	11.5617
6.620.000	1.261.112	-404.097	11.5612
6.630.000	1.262.935	-404.333	11.5609
6.640.000	1.264.511	-404.723	11.5603
6.650.000	1.266.379	-405.025	11.5596
6.660.000	1.267.909	-405.352	11.5592
6.670.000	1.269.752	-405.686	11.5589
6.680.000	1.271.263	-406.069	11.5585
6.690.000	1.273.202	-406.359	11.5579
6.700.000	1.274.572	-406.670	11.5571
6.710.000	1.276.553	-407.090	11.5567
6.720.000	1.278.051	-407.308	11.5561
6.730.000	1.279.828	-407.717	11.5553
6.740.000	1.281.490	-408.035	11.5544
6.750.000	1.283.266	-408.376	11.5539
6.760.000	1.284.925	-408.690	11.5537
6.770.000	1.286.705	-409.079	11.5531
6.780.000	1.288.326	-409.294	11.5524
6.790.000	1.289.995	-409.721	11.5521
6.800.000	1.291.834	-409.977	11.5518
6.810.000	1.293.296	-410.329	11.5513
6.820.000	1.295.229	-410.661	11.5505
6.830.000	1.296.729	-411.036	11.5499
6.840.000	1.298.601	-411.334	11.5495

6.850.000	1.300.135	-411.706	11.5491
6.860.000	1.302.046	-412.032	11.5484
6.870.000	1.303.523	-412.343	11.5477
6.880.000	1.305.436	-412.738	11.5472
6.890.000	1.306.939	-412.975	11.5468
6.900.000	1.308.728	-413.363	11.5462
6.910.000	1.310.340	-413.696	11.5454
6.920.000	1.312.067	-414.001	11.5447
6.930.000	1.313.683	-414.295	11.5441
6.940.000	1.315.427	-414.716	11.5437
6.950.000	1.317.112	-414.909	11.5429
6.960.000	1.318.701	-415.330	11.5421
6.970.000	1.320.567	-415.638	11.5413
6.980.000	1.322.104	-415.945	11.5410
6.990.000	1.323.978	-416.312	11.5405
7.000.000	1.325.426	-416.655	11.5398
7.010.000	1.327.311	-416.962	11.5393
7.020.000	1.328.764	-417.302	11.5389
7.030.000	1.330.679	-417.650	11.5386
7.040.000	1.332.124	-417.903	11.5380
7.050.000	1.333.971	-418.297	11.5374
7.060.000	1.335.585	-418.579	11.5370
7.070.000	1.337.334	-418.929	11.5365
7.080.000	1.338.942	-419.227	11.5358
7.090.000	1.340.781	-419.567	11.5350
7.100.000	1.342.363	-419.803	11.5346
7.110.000	1.344.058	-420.206	11.5341
7.120.000	1.345.807	-420.410	11.5334
7.130.000	1.347.318	-420.773	11.5326
7.140.000	1.349.265	-421.064	11.5322

7.150.000	1.350.730	-421.365	11.5320
7.160.000	1.352.615	-421.679	11.5315
7.170.000	1.354.092	-422.030	11.5308
7.180.000	1.356.001	-422.308	11.5302
7.190.000	1.357.454	-422.594	11.5296
7.200.000	1.359.418	-422.970	11.5289
7.210.000	1.360.894	-423.182	11.5280
7.220.000	1.362.724	-423.555	11.5274
7.230.000	1.364.318	-423.820	11.5270
7.240.000	1.366.068	-424.120	11.5265
7.250.000	1.367.700	-424.402	11.5258
7.260.000	1.369.448	-424.774	11.5252
7.270.000	1.371.122	-424.953	11.5247
7.280.000	1.372.759	-425.376	11.5238
7.290.000	1.374.629	-425.602	11.5232
7.300.000	1.376.136	-425.936	11.5227
7.310.000	1.378.055	-426.251	11.5224
7.320.000	1.379.526	-426.536	11.5218
7.330.000	1.381.406	-426.848	11.5212
7.340.000	1.382.893	-427.181	11.5206
7.350.000	1.384.821	-427.458	11.5201
7.360.000	1.386.269	-427.735	11.5192
7.370.000	1.388.182	-428.108	11.5184
7.380.000	1.389.738	-428.321	11.5178
7.390.000	1.391.473	-428.689	11.5173
7.400.000	1.393.130	-428.960	11.5168
7.410.000	1.394.874	-429.247	11.5162
7.420.000	1.396.552	-429.525	11.5156
7.430.000	1.398.339	-429.900	11.5150
7.440.000	1.400.031	-430.057	11.5143

7.450.000	1.401.613	-430.459	11.5134
7.460.000	1.403.522	-430.695	11.5129
7.470.000	1.404.972	-430.956	11.5124
7.480.000	1.406.858	-431.267	11.5118
7.490.000	1.408.367	-431.583	11.5112
7.500.000	1.410.219	-431.849	11.5108
7.510.000	1.411.691	-432.136	11.5106
7.520.000	1.413.633	-432.476	11.5101
7.530.000	1.415.111	-432.678	11.5096
7.540.000	1.417.016	-433.041	11.5093
7.550.000	1.418.582	-433.240	11.5091
7.560.000	1.420.279	-433.540	11.5090
7.570.000	1.421.935	-433.784	11.5085
7.580.000	1.423.674	-434.063	11.5078
7.590.000	1.425.324	-434.229	11.5072
7.600.000	1.427.032	-434.593	11.5066
7.610.000	1.428.798	-434.695	11.5059
7.620.000	1.430.323	-434.987	11.5051
7.630.000	1.432.233	-435.201	11.5044
7.640.000	1.433.737	-435.380	11.5039
7.650.000	1.435.698	-435.590	11.5032
7.660.000	1.437.196	-435.793	11.5023
7.670.000	1.439.081	-435.897	11.5013
7.680.000	1.440.570	-436.035	11.5006
7.690.000	1.442.442	-436.222	11.4998
7.700.000	1.443.932	-436.240	11.4990
7.710.000	1.445.730	-436.457	11.4980
7.720.000	1.447.310	-436.574	11.4970
7.730.000	1.449.012	-436.755	11.4961
7.740.000	1.450.662	-436.923	11.4951

7.750.000	1.452.395	-437.217	11.4937
7.760.000	1.454.078	-437.379	11.4925
7.770.000	1.455.680	-437.770	11.4917
7.780.000	1.457.517	-438.020	11.4910
7.790.000	1.458.950	-438.396	11.4903
7.800.000	1.460.838	-438.796	11.4895
7.810.000	1.462.340	-439.175	11.4888
7.820.000	1.464.148	-439.598	11.4882
7.830.000	1.465.661	-440.035	11.4872
7.840.000	1.467.563	-440.452	11.4864
7.850.000	1.468.999	-440.847	11.4853
7.860.000	1.470.926	-441.374	11.4848
7.870.000	1.472.483	-441.700	11.4843
7.880.000	1.474.229	-442.196	11.4837
7.890.000	1.475.893	-442.598	11.4829
7.900.000	1.477.582	-443.021	11.4823
7.910.000	1.479.223	-443.403	11.4817
7.920.000	1.480.978	-443.893	11.4811
7.930.000	1.482.674	-444.164	11.4802
7.940.000	1.484.280	-444.656	11.4791
7.950.000	1.486.156	-445.009	11.4785
7.960.000	1.487.589	-445.358	11.4779
7.970.000	1.489.541	-445.765	11.4770
7.980.000	1.491.084	-446.146	11.4760
7.990.000	1.492.890	-446.445	11.4752
8.000.000	1.494.510	-446.808	11.4747
8.010.000	1.496.361	-447.212	11.4743
8.020.000	1.497.832	-447.484	11.4737
8.030.000	1.499.753	-447.917	11.4731
8.040.000	1.501.301	-448.145	11.4725

8.050.000	1.503.088	-448.535	11.4718
8.060.000	1.504.696	-448.837	11.4711
8.070.000	1.506.432	-449.151	11.4702
8.080.000	1.508.149	-449.394	11.4691
8.090.000	1.509.767	-449.795	11.4680
8.100.000	1.511.671	-449.960	11.4672
8.110.000	1.513.111	-450.348	11.4665
8.120.000	1.514.986	-450.618	11.4658
8.130.000	1.516.455	-450.863	11.4648
8.140.000	1.518.337	-451.193	11.4641
8.150.000	1.519.818	-451.481	11.4636
8.160.000	1.521.719	-451.722	11.4628
8.170.000	1.523.169	-451.999	11.4618
8.180.000	1.525.095	-452.322	11.4608
8.190.000	1.526.620	-452.505	11.4600
8.200.000	1.528.441	-452.836	11.4594
8.210.000	1.530.080	-453.077	11.4585
8.220.000	1.531.778	-453.319	11.4578
8.230.000	1.533.424	-453.539	11.4569
8.240.000	1.535.155	-453.860	11.4564
8.250.000	1.536.822	-453.993	11.4557
8.260.000	1.538.435	-454.318	11.4549
8.270.000	1.540.312	-454.484	11.4539
8.280.000	1.541.721	-454.732	11.4531
8.290.000	1.543.654	-454.999	11.4523
8.300.000	1.545.155	-455.197	11.4520
8.310.000	1.547.009	-455.438	11.4511
8.320.000	1.548.538	-455.708	11.4504
8.330.000	1.550.462	-455.915	11.4498
8.340.000	1.551.916	-456.095	11.4498

8.350.000	1.553.791	-456.430	11.4496
8.360.000	1.555.334	-456.551	11.4488
8.370.000	1.557.180	-456.854	11.4477
8.380.000	1.558.756	-457.043	11.4469
8.390.000	1.560.484	-457.246	11.4467
8.400.000	1.562.161	-457.453	11.4457
8.410.000	1.563.896	-457.763	11.4443
8.420.000	1.565.603	-457.836	11.4433
8.430.000	1.567.198	-458.178	11.4426
8.440.000	1.569.122	-458.358	11.4420
8.450.000	1.570.594	-458.563	11.4411
8.460.000	1.572.472	-458.813	11.4399
8.470.000	1.573.999	-459.053	11.4391
8.480.000	1.575.866	-459.249	11.4386
8.490.000	1.577.368	-459.487	11.4381
8.500.000	1.579.268	-459.722	11.4374
8.510.000	1.580.739	-459.891	11.4367
8.520.000	1.582.647	-460.198	11.4358
8.530.000	1.584.227	-460.351	11.4351
8.540.000	1.585.976	-460.626	11.4345
8.550.000	1.587.716	-460.828	11.4340
8.560.000	1.589.455	-461.059	11.4334
8.570.000	1.591.115	-461.219	11.4327
8.580.000	1.592.793	-461.536	11.4321
8.590.000	1.594.610	-461.636	11.4317
8.600.000	1.596.095	-461.921	11.4310
8.610.000	1.598.051	-462.142	11.4301
8.620.000	1.599.524	-462.337	11.4289
8.630.000	1.601.421	-462.599	11.4280
8.640.000	1.602.997	-462.858	11.4273

8.650.000	1.604.882	-463.042	11.4266
8.660.000	1.606.373	-463.265	11.4258
8.670.000	1.608.262	-463.555	11.4249
8.680.000	1.609.727	-463.677	11.4243
8.690.000	1.611.584	-463.978	11.4238
8.700.000	1.613.179	-464.169	11.4230
8.710.000	1.614.883	-464.382	11.4219
8.720.000	1.616.544	-464.571	11.4210
8.730.000	1.618.260	-464.850	11.4204
8.740.000	1.619.945	-464.962	11.4198
8.750.000	1.621.598	-465.265	11.4190
8.760.000	1.623.435	-465.405	11.4181
8.770.000	1.624.924	-465.627	11.4176
8.780.000	1.626.853	-465.877	11.4171
8.790.000	1.628.314	-466.049	11.4164
8.800.000	1.630.186	-466.277	11.4156
8.810.000	1.631.673	-466.502	11.4147
8.820.000	1.633.570	-466.687	11.4142
8.830.000	1.635.057	-466.876	11.4137
8.840.000	1.636.917	-467.159	11.4129
8.850.000	1.638.458	-467.240	11.4121
8.860.000	1.640.211	-467.516	11.4113
8.870.000	1.641.917	-467.714	11.4109
8.880.000	1.643.642	-467.912	11.4105
8.890.000	1.645.290	-468.086	11.4098
8.900.000	1.646.982	-468.364	11.4089
8.910.000	1.648.716	-468.431	11.4082
8.920.000	1.650.281	-468.746	11.4079
8.930.000	1.652.209	-468.911	11.4074
8.940.000	1.653.649	-469.089	11.4067

8.950.000	1.655.576	-469.356	11.4057
8.960.000	1.657.112	-469.572	11.4050
8.970.000	1.658.953	-469.763	11.4046
8.980.000	1.660.461	-469.986	11.4041
8.990.000	1.662.381	-470.238	11.4030
9.000.000	1.663.910	-470.427	11.4022
9.010.000	1.665.775	-470.711	11.4018
9.020.000	1.667.390	-470.873	11.4016
9.030.000	1.669.065	-471.141	11.4010
9.040.000	1.670.799	-471.341	11.4001
9.050.000	1.672.529	-471.570	11.3995
9.060.000	1.674.167	-471.754	11.3991
9.070.000	1.675.840	-472.089	11.3985
9.080.000	1.677.660	-472.214	11.3976
9.090.000	1.679.194	-472.504	11.3968
9.100.000	1.681.151	-472.775	11.3961
9.110.000	1.682.586	-472.965	11.3954
9.120.000	1.684.526	-473.247	11.3945
9.130.000	1.686.022	-473.505	11.3936
9.140.000	1.687.883	-473.724	11.3929
9.150.000	1.689.425	-473.947	11.3921
9.160.000	1.691.315	-474.277	11.3914
9.170.000	1.692.834	-474.391	11.3906
9.180.000	1.694.638	-474.726	11.3900
9.190.000	1.696.288	-474.939	11.3894
9.200.000	1.698.029	-475.196	11.3885
9.210.000	1.699.733	-475.417	11.3878
9.220.000	1.701.437	-475.703	11.3872
9.230.000	1.703.172	-475.860	11.3865
9.240.000	1.704.797	-476.209	11.3857

9.250.000	1.706.613	-476.360	11.3851
9.260.000	1.708.102	-476.627	11.3848
9.270.000	1.709.978	-476.909	11.3842
9.280.000	1.711.481	-477.114	11.3833
9.290.000	1.713.344	-477.363	11.3827
9.300.000	1.714.847	-477.635	11.3822
9.310.000	1.716.747	-477.866	11.3816
9.320.000	1.718.205	-478.109	11.3807
9.330.000	1.720.138	-478.425	11.3800
9.340.000	1.721.659	-478.573	11.3796
9.350.000	1.723.370	-478.872	11.3792
9.360.000	1.725.074	-479.105	11.3784
9.370.000	1.726.713	-479.332	11.3776
9.380.000	1.728.364	-479.550	11.3771
9.390.000	1.730.064	-479.878	11.3767
9.400.000	1.731.817	-480.013	11.3762
9.410.000	1.733.381	-480.331	11.3755
9.420.000	1.735.313	-480.586	11.3747
9.430.000	1.736.687	-480.779	11.3741
9.440.000	1.738.624	-481.091	11.3736
9.450.000	1.740.138	-481.343	11.3728
9.460.000	1.741.993	-481.569	11.3718
9.470.000	1.743.490	-481.839	11.3709
9.480.000	1.745.346	-482.115	11.3704
9.490.000	1.746.821	-482.298	11.3697
9.500.000	1.748.706	-482.660	11.3687
9.510.000	1.750.280	-482.829	11.3678
9.520.000	1.752.003	-483.116	11.3672
9.530.000	1.753.678	-483.376	11.3667
9.540.000	1.755.381	-483.637	11.3657

9.550.000	1.757.068	-483.841	11.3649
9.560.000	1.758.767	-484.220	11.3644
9.570.000	1.760.536	-484.342	11.3639
9.580.000	1.762.038	-484.687	11.3632
9.590.000	1.763.976	-484.975	11.3624
9.600.000	1.765.375	-485.181	11.3618
9.610.000	1.767.262	-485.517	11.3612
9.620.000	1.768.780	-485.792	11.3604
9.630.000	1.770.673	-486.037	11.3595
9.640.000	1.772.184	-486.301	11.3586
9.650.000	1.774.066	-486.657	11.3580
9.660.000	1.775.548	-486.821	11.3571
9.670.000	1.777.365	-487.230	11.3561
9.680.000	1.779.018	-487.442	11.3551
9.690.000	1.780.717	-487.726	11.3545
9.700.000	1.782.395	-487.991	11.3539
9.710.000	1.784.115	-488.337	11.3529
9.720.000	1.785.783	-488.509	11.3521
9.730.000	1.787.373	-488.899	11.3514
9.740.000	1.789.310	-489.135	11.3507
9.750.000	1.790.741	-489.460	11.3498
9.760.000	1.792.642	-489.767	11.3489
9.770.000	1.794.129	-490.038	11.3484
9.780.000	1.796.008	-490.356	11.3477
9.790.000	1.797.550	-490.672	11.3469
9.800.000	1.799.451	-490.948	11.3460
9.810.000	1.800.929	-491.221	11.3454
9.820.000	1.802.816	-491.600	11.3446
9.830.000	1.804.335	-491.806	11.3436
9.840.000	1.806.050	-492.148	11.3427

9.850.000	1.807.740	-492.439	11.3422
9.860.000	1.809.407	-492.742	11.3417
9.870.000	1.811.123	-493.015	11.3409
9.880.000	1.812.762	-493.400	11.3401
9.890.000	1.814.503	-493.607	11.3396
9.900.000	1.816.055	-494.000	11.3391
9.910.000	1.817.946	-494.301	11.3383
9.920.000	1.819.383	-494.564	11.3375
9.930.000	1.821.236	-494.932	11.3367
9.940.000	1.822.725	-495.269	11.3358
9.950.000	1.824.556	-495.595	11.3348
9.960.000	1.826.012	-495.954	11.3337
9.970.000	1.827.905	-496.373	11.3326
9.980.000	1.829.336	-496.709	11.3315
9.990.000	1.831.154	-497.237	11.3302
10.000.000	1.832.752	-497.585	11.3293
10.010.000	1.834.453	-498.100	11.3285
10.020.000	1.836.170	-498.540	11.3275
10.030.000	1.837.787	-499.010	11.3266
10.040.000	1.839.484	-499.409	11.3259
10.050.000	1.841.048	-499.968	11.3252
10.060.000	1.842.889	-500.301	11.3245
10.070.000	1.844.362	-500.841	11.3235
10.080.000	1.846.293	-501.315	11.3230
10.090.000	1.847.713	-501.708	11.3226
10.100.000	1.849.566	-502.197	11.3219
10.110.000	1.851.114	-502.645	11.3210
10.120.000	1.852.978	-503.051	11.3202
10.130.000	1.854.503	-503.470	11.3197
10.140.000	1.856.367	-503.931	11.3190

10.150.000	1.857.860	-504.251	11.3179
10.160.000	1.859.690	-504.725	11.3171
10.170.000	1.861.336	-505.056	11.3165
10.180.000	1.862.953	-505.424	11.3160
10.190.000	1.864.723	-505.750	11.3152
10.200.000	1.866.349	-506.174	11.3142
10.210.000	1.868.092	-506.410	11.3133
10.220.000	1.869.679	-506.851	11.3125
10.230.000	1.871.572	-507.145	11.3116
10.240.000	1.873.004	-507.467	11.3105
10.250.000	1.874.940	-507.840	11.3097
10.260.000	1.876.385	-508.117	11.3091
10.270.000	1.878.303	-508.447	11.3085
10.280.000	1.879.799	-508.770	11.3074
10.290.000	1.881.680	-509.062	11.3065
10.300.000	1.883.201	-509.309	11.3061
10.310.000	1.885.050	-509.703	11.3053
10.320.000	1.886.638	-509.882	11.3044
10.330.000	1.888.369	-510.227	11.3035
10.340.000	1.890.040	-510.469	11.3029
10.350.000	1.891.751	-510.737	11.3025
10.360.000	1.893.467	-510.954	11.3015
10.370.000	1.895.106	-511.313	11.3004
10.380.000	1.896.908	-511.448	11.2997
10.390.000	1.898.417	-511.794	11.2992
10.400.000	1.900.370	-512.027	11.2984
10.410.000	1.901.844	-512.236	11.2974
10.420.000	1.903.720	-512.538	11.2967
10.430.000	1.905.262	-512.754	11.2962
10.440.000	1.907.094	-512.982	11.2958

10.450.000	1.908.628	-513.230	11.2951
10.460.000	1.910.531	-513.486	11.2942
10.470.000	1.912.007	-513.633	11.2934
10.480.000	1.913.833	-513.971	11.2928
10.490.000	1.915.463	-514.112	11.2922
10.500.000	1.917.095	-514.357	11.2912
10.510.000	1.918.817	-514.552	11.2900
10.520.000	1.920.506	-514.775	11.2891
10.530.000	1.922.207	-514.925	11.2885
10.540.000	1.923.815	-515.222	11.2878
10.550.000	1.925.667	-515.303	11.2869
10.560.000	1.927.096	-515.547	11.2861
10.570.000	1.929.100	-515.802	11.2855
10.580.000	1.930.508	-515.932	11.2850
10.590.000	1.932.401	-516.142	11.2846
10.600.000	1.933.922	-516.347	11.2837
10.610.000	1.935.757	-516.493	11.2826
10.620.000	1.937.288	-516.668	11.2816
10.630.000	1.939.181	-516.915	11.2810
10.640.000	1.940.667	-516.979	11.2804
10.650.000	1.942.461	-517.243	11.2795
10.660.000	1.944.138	-517.383	11.2787
10.670.000	1.945.764	-517.539	11.2779
10.680.000	1.947.538	-517.699	11.2774
10.690.000	1.949.168	-517.909	11.2767
10.700.000	1.950.910	-517.977	11.2757
10.710.000	1.952.492	-518.230	11.2745
10.720.000	1.954.382	-518.333	11.2735
10.730.000	1.955.851	-518.522	11.2728
10.740.000	1.957.782	-518.732	11.2722

10.750.000	1.959.261	-518.842	11.2712
10.760.000	1.961.156	-519.033	11.2700
10.770.000	1.962.661	-519.195	11.2691
10.780.000	1.964.500	-519.365	11.2686
10.790.000	1.966.061	-519.504	11.2681
10.800.000	1.967.929	-519.784	11.2673
10.810.000	1.969.562	-519.843	11.2666
10.820.000	1.971.247	-520.083	11.2661
10.830.000	1.972.977	-520.242	11.2656
10.840.000	1.974.672	-520.408	11.2651
10.850.000	1.976.427	-520.530	11.2643
10.860.000	1.978.062	-520.791	11.2636
10.870.000	1.979.911	-520.849	11.2628
10.880.000	1.981.377	-521.057	11.2621
10.890.000	1.983.349	-521.246	11.2614
10.900.000	1.984.781	-521.343	11.2606
10.910.000	1.986.739	-521.549	11.2595
10.920.000	1.988.258	-521.659	11.2583
10.930.000	1.990.121	-521.787	11.2573
10.940.000	1.991.662	-521.914	11.2565
10.950.000	1.993.535	-522.099	11.2558
10.960.000	1.995.074	-522.157	11.2545
10.970.000	1.996.893	-522.384	11.2537
10.980.000	1.998.543	-522.445	11.2527
10.990.000	2.000.198	-522.627	11.2522
11.000.000	2.001.966	-522.744	11.2517
11.010.000	2.003.604	-522.932	11.2508
11.020.000	2.005.361	-523.027	11.2501
11.030.000	2.006.949	-523.301	11.2492
11.040.000	2.008.792	-523.401	11.2486

11.050.000	2.010.263	-523.620	11.2480
11.060.000	2.012.162	-523.863	11.2469
11.070.000	2.013.598	-524.018	11.2457
11.080.000	2.015.499	-524.283	11.2444
11.090.000	2.017.015	-524.504	11.2434
11.100.000	2.018.805	-524.682	11.2426
11.110.000	2.020.343	-524.896	11.2415
11.120.000	2.022.199	-525.195	11.2406
11.130.000	2.023.764	-525.313	11.2397
11.140.000	2.025.502	-525.618	11.2387
11.150.000	2.027.154	-525.802	11.2378
11.160.000	2.028.740	-526.037	11.2368
11.170.000	2.030.468	-526.301	11.2361
11.180.000	2.032.078	-526.656	11.2347
11.190.000	2.033.812	-526.910	11.2332
11.200.000	2.035.313	-527.350	11.2319
11.210.000	2.037.226	-527.735	11.2310
11.220.000	2.038.615	-528.140	11.2305
11.230.000	2.040.545	-528.594	11.2290
11.240.000	2.042.053	-528.982	11.2277
11.250.000	2.043.897	-529.400	11.2268
11.260.000	2.045.437	-529.765	11.2259
11.270.000	2.047.305	-530.114	11.2252
11.280.000	2.048.822	-530.382	11.2239
11.290.000	2.050.693	-530.752	11.2227
11.300.000	2.052.319	-530.906	11.2218
11.310.000	2.053.991	-531.164	11.2208
11.320.000	2.055.756	-531.347	11.2200
11.330.000	2.057.341	-531.545	11.2190
11.340.000	2.059.126	-531.642	11.2180

11.350.000	2.060.774	-531.896	11.2167
11.360.000	2.062.535	-531.928	11.2158
11.370.000	2.064.041	-532.127	11.2148
11.380.000	2.065.965	-532.306	11.2139
11.390.000	2.067.401	-532.374	11.2128
11.400.000	2.069.318	-532.605	11.2115
11.410.000	2.070.807	-532.751	11.2106
11.420.000	2.072.646	-532.906	11.2098
11.430.000	2.074.172	-533.062	11.2090
11.440.000	2.076.020	-533.311	11.2078
11.450.000	2.077.560	-533.404	11.2066
11.460.000	2.079.326	-533.718	11.2053
11.470.000	2.080.992	-533.861	11.2042
11.480.000	2.082.648	-534.142	11.2028
11.490.000	2.084.372	-534.344	11.2013
11.500.000	2.085.987	-534.615	11.1999
11.510.000	2.087.743	-534.787	11.1987
11.520.000	2.089.320	-535.121	11.1977
11.530.000	2.091.234	-535.310	11.1964
11.540.000	2.092.619	-535.602	11.1954
11.550.000	2.094.542	-535.919	11.1941
11.560.000	2.095.993	-536.136	11.1928
11.570.000	2.097.835	-536.472	11.1915
11.580.000	2.099.360	-536.744	11.1901
11.590.000	2.101.210	-537.040	11.1887
11.600.000	2.102.710	-537.312	11.1871
11.610.000	2.104.568	-537.690	11.1854
11.620.000	2.106.129	-537.889	11.1841
11.630.000	2.107.849	-538.286	11.1828
11.640.000	2.109.563	-538.556	11.1813

11.650.000	2.111.140	-538.864	11.1796
11.660.000	2.112.874	-539.134	11.1782
11.670.000	2.114.484	-539.514	11.1770
11.680.000	2.116.258	-539.720	11.1756
11.690.000	2.117.762	-540.054	11.1740
11.700.000	2.119.734	-540.359	11.1727
11.710.000	2.121.098	-540.581	11.1716
11.720.000	2.123.053	-540.918	11.1704
11.730.000	2.124.562	-541.136	11.1690
11.740.000	2.126.367	-541.387	11.1675
11.750.000	2.127.941	-541.627	11.1663
11.760.000	2.129.752	-541.847	11.1650
11.770.000	2.131.264	-542.012	11.1635
11.780.000	2.133.065	-542.318	11.1619
11.790.000	2.134.709	-542.422	11.1605
11.800.000	2.136.400	-542.681	11.1595
11.810.000	2.138.138	-542.843	11.1585
11.820.000	2.139.790	-543.016	11.1573
11.830.000	2.141.566	-543.156	11.1559
11.840.000	2.143.171	-543.409	11.1547
11.850.000	2.144.994	-543.440	11.1533
11.860.000	2.146.478	-543.660	11.1519
11.870.000	2.148.421	-543.839	11.1502
11.880.000	2.149.859	-543.907	11.1487
11.890.000	2.151.744	-544.097	11.1475
11.900.000	2.153.287	-544.211	11.1464
11.910.000	2.155.097	-544.304	11.1453
11.920.000	2.156.692	-544.436	11.1436
11.930.000	2.158.566	-544.581	11.1422
11.940.000	2.160.091	-544.623	11.1408

11.950.000	2.161.877	-544.815	11.1394
11.960.000	2.163.604	-544.866	11.1379
11.970.000	2.165.193	-544.979	11.1363
11.980.000	2.166.964	-545.056	11.1350
11.990.000	2.168.604	-545.188	11.1336
12.000.000	2.170.391	-545.213	11.1319
12.010.000	2.171.973	-545.356	11.1301
12.020.000	2.173.879	-545.394	11.1285
12.030.000	2.175.322	-545.464	11.1270
12.040.000	2.177.308	-545.586	11.1255
12.050.000	2.178.771	-545.569	11.1237
12.060.000	2.180.632	-545.654	11.1222
12.070.000	2.182.254	-545.711	11.1209
12.080.000	2.184.056	-545.741	11.1194
12.090.000	2.185.611	-545.740	11.1176
12.100.000	2.187.448	-545.846	11.1162
12.110.000	2.189.076	-545.775	11.1148
12.120.000	2.190.801	-545.880	11.1137
12.130.000	2.192.556	-545.871	11.1122
12.140.000	2.194.179	-545.877	11.1107
12.150.000	2.196.005	-545.881	11.1095
12.160.000	2.197.630	-545.960	11.1082
12.170.000	2.199.476	-545.890	11.1066
12.180.000	2.201.027	-545.933	11.1051
12.190.000	2.202.977	-545.958	11.1037
12.200.000	2.204.426	-545.909	11.1025
12.210.000	2.206.328	-545.954	11.1010
12.220.000	2.207.854	-545.907	11.0997
12.230.000	2.209.686	-545.911	11.0984
12.240.000	2.211.261	-545.907	11.0973

12.250.000	2.213.108	-545.892	11.0958
12.260.000	2.214.691	-545.815	11.0941
12.270.000	2.216.510	-545.896	11.0928
12.280.000	2.218.222	-545.758	11.0916
12.290.000	2.219.828	-545.753	11.0903
12.300.000	2.221.634	-545.686	11.0888
12.310.000	2.223.259	-545.649	11.0875
12.320.000	2.225.031	-545.548	11.0867
12.330.000	2.226.643	-545.578	11.0858
12.340.000	2.228.509	-545.425	11.0843
12.350.000	2.229.966	-545.400	11.0828
12.360.000	2.231.958	-545.348	11.0816
12.370.000	2.233.386	-545.202	11.0806
12.380.000	2.235.355	-545.169	11.0794
12.390.000	2.236.900	-545.044	11.0780
12.400.000	2.238.726	-544.933	11.0767
12.410.000	2.240.303	-544.775	11.0757
12.420.000	2.242.192	-544.733	11.0746
12.430.000	2.243.748	-544.522	11.0733
12.440.000	2.245.577	-544.476	11.0717
12.450.000	2.247.243	-544.275	11.0703
12.460.000	2.248.875	-544.160	11.0691
12.470.000	2.250.671	-543.981	11.0676
12.480.000	2.252.290	-543.875	11.0661
12.490.000	2.254.125	-543.666	11.0645
12.500.000	2.255.695	-543.570	11.0633
12.510.000	2.257.643	-543.403	11.0623
12.520.000	2.259.112	-543.234	11.0612
12.530.000	2.261.056	-543.130	11.0601
12.540.000	2.262.564	-542.909	11.0590

12.550.000	2.264.452	-542.798	11.0580
12.560.000	2.266.040	-542.657	11.0573
12.570.000	2.267.874	-542.485	11.0562
12.580.000	2.269.443	-542.295	11.0549
12.590.000	2.271.257	-542.228	11.0536
12.600.000	2.272.925	-541.971	11.0527
12.610.000	2.274.615	-541.911	11.0519
12.620.000	2.276.437	-541.750	11.0506
12.630.000	2.278.080	-541.627	11.0492
12.640.000	2.279.905	-541.455	11.0483
12.650.000	2.281.469	-541.395	11.0476
12.660.000	2.283.315	-541.178	11.0469
12.670.000	2.284.818	-541.106	11.0460
12.680.000	2.286.799	-541.006	11.0450
12.690.000	2.288.215	-540.787	11.0441
12.700.000	2.290.243	-540.745	11.0435
12.710.000	2.291.780	-540.640	11.0431
12.720.000	2.293.571	-540.512	11.0422
12.730.000	2.295.162	-540.396	11.0412
12.740.000	2.297.043	-540.343	11.0404
12.750.000	2.298.570	-540.199	11.0398
12.760.000	2.300.374	-540.227	11.0391
12.770.000	2.302.074	-540.078	11.0377
12.780.000	2.303.715	-540.058	11.0365
12.790.000	2.305.499	-539.979	11.0354
12.800.000	2.307.073	-539.933	11.0343
12.810.000	2.308.877	-539.841	11.0332
12.820.000	2.310.448	-539.886	11.0323
12.830.000	2.312.404	-539.818	11.0313
12.840.000	2.313.853	-539.813	11.0303

12.850.000	2.315.777	-539.841	11.0291
12.860.000	2.317.207	-539.763	11.0281
12.870.000	2.319.110	-539.823	11.0272
12.880.000	2.320.686	-539.817	11.0264
12.890.000	2.322.432	-539.850	11.0251
12.900.000	2.324.026	-539.855	11.0240
12.910.000	2.325.828	-539.991	11.0231
12.920.000	2.327.405	-539.939	11.0222
12.930.000	2.329.152	-540.115	11.0212
12.940.000	2.330.890	-540.142	11.0200
12.950.000	2.332.539	-540.246	11.0188
12.960.000	2.334.364	-540.301	11.0179
12.970.000	2.335.863	-540.432	11.0169
12.980.000	2.337.630	-540.484	11.0160
12.990.000	2.339.210	-540.695	11.0151
13.000.000	2.341.056	-540.762	11.0140
13.010.000	2.342.509	-540.899	11.0129
13.020.000	2.344.417	-541.087	11.0118
13.030.000	2.345.884	-541.164	11.0110
13.040.000	2.347.762	-541.347	11.0102
13.050.000	2.349.340	-541.527	11.1986
13.060.000	2.351.128	-541.686	11.1973
13.070.000	2.352.696	-541.848	11.1965
13.080.000	2.354.426	-542.148	11.1960
13.090.000	2.356.101	-542.212	11.1950
13.100.000	2.357.662	-542.372	11.1935
13.110.000	2.359.568	-542.672	11.2018
13.120.000	2.361.126	-542.723	11.2003
13.130.000	2.362.047	-542.865	10.9997
13.140.000	2.364.822	-543.447	10.9987

13.150.000	2.366.187	-543.449	10.9974
13.160.000	2.367.742	-543.840	10.9968
13.170.000	2.369.692	-544.125	10.9965
13.180.000	2.371.247	-544.471	10.9954
13.190.000	2.373.050	-544.573	10.9947
13.200.000	2.374.533	-544.726	10.9872
13.210.000	2.376.571	-544.956	10.9838
13.220.000	2.377.836	-545.083	10.9822
13.230.000	2.379.687	-545.374	10.9817
13.240.000	2.381.186	-545.547	10.9760
13.250.000	2.382.942	-545.929	10.9610
13.260.000	2.384.593	-546.286	10.9584
13.270.000	2.386.241	-546.725	10.9594
13.280.000	2.388.121	-546.919	10.9562
13.290.000	2.389.632	-547.226	10.9543
13.300.000	2.391.496	-547.482	10.9501
13.310.000	2.393.022	-547.893	10.9583
13.320.000	2.394.863	-548.081	10.9691
13.330.000	2.396.297	-548.361	10.9676
13.340.000	2.398.243	-548.699	10.9698
13.350.000	2.399.681	-548.915	10.9685
13.360.000	2.401.544	-549.277	10.9726
13.370.000	2.403.073	-549.551	10.9721
13.380.000	2.404.850	-549.817	10.9713
13.390.000	2.406.415	-550.117	10.9706
13.400.000	2.408.269	-550.430	10.9692
13.410.000	2.409.817	-550.606	10.9679
13.420.000	2.411.494	-550.966	10.9666
13.430.000	2.413.216	-551.174	10.9655
13.440.000	2.414.792	-551.459	10.9639

13.450.000	2.416.515	-551.667	10.9620
13.460.000	2.418.116	-551.986	10.9604
13.470.000	2.419.900	-552.196	10.9590
13.480.000	2.421.408	-552.491	10.9574
13.490.000	2.423.308	-552.728	10.9557
13.500.000	2.424.743	-552.953	10.9538
13.510.000	2.426.692	-553.245	10.9521
13.520.000	2.428.184	-553.412	10.9508
13.530.000	2.429.986	-553.676	10.9492
13.540.000	2.431.586	-553.889	10.9478
13.550.000	2.433.328	-554.110	10.9456
13.560.000	2.434.869	-554.276	10.9438
13.570.000	2.436.686	-554.565	10.9429
13.580.000	2.438.302	-554.640	10.9415
13.590.000	2.439.970	-554.883	10.9404
13.600.000	2.441.733	-555.026	10.9391
13.610.000	2.443.296	-555.190	10.9379
13.620.000	2.445.150	-555.293	10.9373
13.630.000	2.446.701	-555.499	10.9357
13.640.000	2.448.516	-555.563	10.9346
13.650.000	2.450.029	-555.715	10.9333
13.660.000	2.451.942	-555.875	10.9318
13.670.000	2.453.373	-555.897	10.9303
13.680.000	2.455.271	-556.062	10.9292
13.690.000	2.456.833	-556.138	10.9278
13.700.000	2.458.648	-556.220	10.9263
13.710.000	2.460.231	-556.264	10.9244
13.720.000	2.462.047	-556.384	10.9229
13.730.000	2.463.644	-556.381	10.9218
13.740.000	2.465.385	-556.492	10.9204

13.750.000	2.467.086	-556.437	10.9192
13.760.000	2.468.729	-556.508	10.9179
13.770.000	2.470.506	-556.468	10.9165
13.780.000	2.472.121	-556.489	10.9152
13.790.000	2.473.923	-556.421	10.9136
13.800.000	2.475.478	-556.445	10.9120
13.810.000	2.477.418	-556.367	10.9101
13.820.000	2.478.802	-556.301	10.9084
13.830.000	2.480.803	-556.296	10.9071
13.840.000	2.482.372	-556.160	10.9060
13.850.000	2.484.218	-556.117	10.9048
13.860.000	2.485.828	-556.029	10.9034
13.870.000	2.487.615	-555.923	10.9023
13.880.000	2.489.195	-555.785	10.9012
13.890.000	2.491.053	-555.770	10.9001
13.900.000	2.492.691	-555.539	10.8986
13.910.000	2.494.384	-555.481	10.8969
13.920.000	2.496.199	-555.290	10.8955
13.930.000	2.497.757	-555.150	10.8943
13.940.000	2.499.609	-554.969	10.8928
13.950.000	2.501.272	-554.874	10.8913
13.960.000	2.503.110	-554.659	10.8898
13.970.000	2.504.671	-554.537	10.8886
13.980.000	2.506.587	-554.391	10.8875
13.990.000	2.508.020	-554.154	10.8861
14.000.000	2.509.967	-554.040	10.8849
14.010.000	2.511.517	-553.802	10.8837
14.020.000	2.513.351	-553.624	10.8826
14.030.000	2.514.944	-553.427	10.8817
14.040.000	2.516.788	-553.252	10.8807

14.050.000	2.518.422	-553.023	10.8795
14.060.000	2.520.227	-552.908	10.8783
14.070.000	2.521.902	-552.593	10.8773
14.080.000	2.523.576	-552.476	10.8764
14.090.000	2.525.385	-552.262	10.8751
14.100.000	2.526.986	-552.052	10.8737
14.110.000	2.528.825	-551.820	10.8726
14.120.000	2.530.379	-551.707	10.8721
14.130.000	2.532.284	-551.446	10.8711
14.140.000	2.533.741	-551.272	10.8698
14.150.000	2.535.709	-551.140	10.8685
14.160.000	2.537.170	-550.882	10.8674
14.170.000	2.539.059	-550.756	10.8663
14.180.000	2.540.682	-550.558	10.8648
14.190.000	2.542.488	-550.374	10.8633
14.200.000	2.544.091	-550.182	10.8620
14.210.000	2.545.918	-550.071	10.8609
14.220.000	2.547.508	-549.810	10.8599
14.230.000	2.549.247	-549.717	10.8586
14.240.000	2.550.984	-549.491	10.8570
14.250.000	2.552.609	-549.383	10.8560
14.260.000	2.554.417	-549.189	10.8550
14.270.000	2.555.999	-549.079	10.8538
14.280.000	2.557.842	-548.914	10.8526
14.290.000	2.559.408	-548.839	10.8518
14.300.000	2.561.328	-548.717	10.8511
14.310.000	2.562.752	-548.560	10.8499
14.320.000	2.564.703	-548.505	10.8487
14.330.000	2.566.213	-548.323	10.8477
14.340.000	2.568.055	-548.259	10.8468

14.350.000	2.569.657	-548.155	10.8457
14.360.000	2.571.442	-548.059	10.8444
14.370.000	2.573.060	-547.939	10.8432
14.380.000	2.574.806	-547.941	10.8421
14.390.000	2.576.484	-547.739	10.8411
14.400.000	2.578.199	-547.755	10.8398
14.410.000	2.580.010	-547.656	10.8386
14.420.000	2.581.620	-547.580	10.8377
14.430.000	2.583.413	-547.495	10.8370
14.440.000	2.584.966	-547.495	10.8361
14.450.000	2.586.817	-547.388	10.8352
14.460.000	2.588.314	-547.368	10.8344
14.470.000	2.590.272	-547.355	10.8336
14.480.000	2.591.698	-547.264	10.8328
14.490.000	2.593.594	-547.313	10.8318
14.500.000	2.595.199	-547.225	10.8309
14.510.000	2.596.975	-547.221	10.8300
14.520.000	2.598.573	-547.192	10.8291
14.530.000	2.600.397	-547.186	10.8282
14.540.000	2.601.985	-547.112	10.8269
14.550.000	2.603.743	-547.180	10.8259
14.560.000	2.605.461	-547.054	10.8246
14.570.000	2.607.039	-547.110	10.8234
14.580.000	2.608.846	-547.039	10.8222
14.590.000	2.610.437	-547.050	10.8207
14.600.000	2.612.220	-547.023	10.8196
14.610.000	2.613.799	-547.064	10.8187
14.620.000	2.615.663	-547.033	10.8177
14.630.000	2.617.134	-547.036	10.8165
14.640.000	2.619.066	-547.099	10.8152

14.650.000	2.620.519	-547.031	10.8145
14.660.000	2.622.397	-547.096	10.8136
14.670.000	2.623.965	-547.082	10.8124
14.680.000	2.625.711	-547.079	10.8110
14.690.000	2.627.355	-547.071	10.8100
14.700.000	2.629.109	-547.133	10.8091
14.710.000	2.630.697	-547.042	10.8082
14.720.000	2.632.432	-547.133	10.8069
14.730.000	2.634.195	-547.094	10.8058
14.740.000	2.635.783	-547.110	10.8048
14.750.000	2.637.612	-547.112	10.8035
14.760.000	2.639.158	-547.153	10.8021
14.770.000	2.641.004	-547.119	10.8009
14.780.000	2.642.494	-547.185	10.7995
14.790.000	2.644.406	-547.188	10.7981
14.800.000	2.645.849	-547.185	10.7967
14.810.000	2.647.749	-547.256	10.7956
14.820.000	2.649.259	-547.184	10.7948
14.830.000	2.651.099	-547.246	10.7936
14.840.000	2.652.645	-547.260	10.7925
14.850.000	2.654.485	-547.267	10.7914
14.860.000	2.656.093	-547.257	10.7906
14.870.000	2.657.843	-547.360	10.7894
14.880.000	2.659.523	-547.270	10.7881
14.890.000	2.661.159	-547.357	10.7868
14.900.000	2.662.942	-547.337	10.7858
14.910.000	2.664.517	-547.362	10.7848
14.920.000	2.666.325	-547.349	10.7833
14.930.000	2.667.880	-547.403	10.7819
14.940.000	2.669.728	-547.389	10.7808

14.950.000	2.671.227	-547.411	10.7799
14.960.000	2.673.168	-547.472	10.7787
14.970.000	2.674.638	-547.414	10.7774
14.980.000	2.676.527	-547.479	10.7767
14.990.000	2.678.068	-547.474	10.7758
15.000.000	2.679.836	-547.486	10.7749
15.010.000	2.681.451	-547.484	10.7733
15.020.000	2.683.274	-547.545	10.7719
15.030.000	2.684.866	-547.495	10.7708
15.040.000	2.686.592	-547.587	10.7697
15.050.000	2.688.331	-547.529	10.7683
15.060.000	2.689.881	-547.587	10.7669
15.070.000	2.691.761	-547.594	10.7656
15.080.000	2.693.324	-547.622	10.7646
15.090.000	2.695.150	-547.607	10.7636
15.100.000	2.696.654	-547.687	10.7624
15.110.000	2.698.559	-547.694	10.7609
15.120.000	2.700.054	-547.717	10.7596
15.130.000	2.701.939	-547.787	10.7586
15.140.000	2.703.406	-547.723	10.7576
15.150.000	2.705.243	-547.792	10.7563
15.160.000	2.706.871	-547.818	10.7550
15.170.000	2.708.594	-547.809	10.7539
15.180.000	2.710.190	-547.814	10.7529
15.190.000	2.711.993	-547.917	10.7514
15.200.000	2.713.671	-547.835	10.7497
15.210.000	2.715.306	-547.938	10.7481
15.220.000	2.717.076	-547.913	10.7468
15.230.000	2.718.609	-547.944	10.7452
15.240.000	2.720.452	-547.965	10.7435

15.250.000	2.722.008	-548.006	10.7421
15.260.000	2.723.776	-547.992	10.7410
15.270.000	2.725.308	-548.067	10.7398
15.280.000	2.727.249	-548.102	10.7384
15.290.000	2.728.661	-548.075	10.7370
15.300.000	2.730.551	-548.163	10.7360
15.310.000	2.732.125	-548.127	10.7351
15.320.000	2.733.872	-548.196	10.7339
15.330.000	2.735.477	-548.214	10.7325
15.340.000	2.737.219	-548.235	10.7311
15.350.000	2.738.768	-548.199	10.7300
15.360.000	2.740.561	-548.297	10.7287
15.370.000	2.742.206	-548.203	10.7273
15.380.000	2.743.877	-548.289	10.7259
15.390.000	2.745.657	-548.306	10.7244
15.400.000	2.747.187	-548.305	10.7231
15.410.000	2.749.037	-548.347	10.7217
15.420.000	2.750.605	-548.446	10.7204
15.430.000	2.752.455	-548.429	10.7189
15.440.000	2.753.952	-548.467	10.7171
15.450.000	2.755.868	-548.583	10.7155
15.460.000	2.757.301	-548.532	10.7141
15.470.000	2.759.174	-548.618	10.7127
15.480.000	2.760.705	-548.642	10.7109
15.490.000	2.762.505	-548.671	10.7091
15.500.000	2.764.086	-548.685	10.7074
15.510.000	2.765.823	-548.779	10.7059
15.520.000	2.767.456	-548.733	10.7044
15.530.000	2.769.212	-548.840	10.7029
15.540.000	2.770.930	-548.815	10.7012

15.550.000	2.772.471	-548.854	10.6996
15.560.000	2.774.289	-548.884	10.6983
15.570.000	2.775.810	-548.947	10.6969
15.580.000	2.777.656	-548.961	10.6952
15.590.000	2.779.122	-549.042	10.6933
15.600.000	2.781.050	-549.069	10.6916
15.610.000	2.782.497	-549.087	10.6901
15.620.000	2.784.347	-549.161	10.6886
15.630.000	2.785.923	-549.148	10.6873
15.640.000	2.787.701	-549.244	10.6855
15.650.000	2.789.289	-549.261	10.6841
15.660.000	2.791.084	-549.295	10.6826
15.670.000	2.792.612	-549.339	10.6811
15.680.000	2.794.433	-549.419	10.6797
15.690.000	2.796.061	-549.398	10.6780
15.700.000	2.797.710	-549.541	10.6761
15.710.000	2.799.579	-549.542	10.6745
15.720.000	2.801.079	-549.622	10.6730
15.730.000	2.802.919	-549.676	10.6717
15.740.000	2.804.423	-549.752	10.6701
15.750.000	2.806.248	-549.800	10.6685
15.760.000	2.807.749	-549.876	10.6669
15.770.000	2.809.652	-550.009	10.6654
15.780.000	2.811.151	-549.995	10.6637
15.790.000	2.813.006	-550.150	10.6618
15.800.000	2.814.548	-550.197	10.6602
15.810.000	2.816.310	-550.279	10.6586
15.820.000	2.817.934	-550.346	10.6570
15.830.000	2.819.686	-550.466	10.6557
15.840.000	2.821.246	-550.444	10.6546

15.850.000	2.822.973	-550.637	10.6531
15.860.000	2.824.711	-550.623	10.6510
15.870.000	2.826.291	-550.767	10.6490
15.880.000	2.828.115	-550.801	10.6473
15.890.000	2.829.652	-550.867	10.6456
15.900.000	2.831.460	-550.957	10.6435
15.910.000	2.833.012	-551.068	10.6412
15.920.000	2.834.847	-551.103	10.6397
15.930.000	2.836.335	-551.199	10.6384
15.940.000	2.838.205	-551.329	10.6368
15.950.000	2.839.697	-551.316	10.6351
15.960.000	2.841.510	-551.456	10.6333
15.970.000	2.843.090	-551.539	10.6318
15.980.000	2.844.880	-551.613	10.6303
15.990.000	2.846.469	-551.689	10.6285
16.000.000	2.848.225	-551.822	10.6265
16.010.000	2.849.829	-551.812	10.6247
16.020.000	2.851.546	-551.962	10.6231
16.030.000	2.853.287	-551.974	10.6215
16.040.000	2.854.793	-552.113	10.6202
16.050.000	2.856.665	-552.149	10.6186
16.060.000	2.858.188	-552.243	10.6170
16.070.000	2.859.968	-552.293	10.6154
16.080.000	2.861.483	-552.405	10.6136
16.090.000	2.863.406	-552.493	10.6120
16.100.000	2.864.832	-552.558	10.6102
16.110.000	2.866.719	-552.704	10.6080
16.120.000	2.868.236	-552.719	10.6061
16.130.000	2.870.003	-552.845	10.6043
16.140.000	2.871.624	-552.929	10.6028

16.150.000	2.873.362	-553.017	10.6008
16.160.000	2.874.964	-553.046	10.5986
16.170.000	2.876.713	-553.235	10.5966
16.180.000	2.878.355	-553.199	10.5949
16.190.000	2.879.996	-553.347	10.5931
16.200.000	2.881.810	-553.401	10.5915
16.210.000	2.883.339	-553.480	10.5898
16.220.000	2.885.188	-553.568	10.5882
16.230.000	2.886.703	-553.719	10.5866
16.240.000	2.888.532	-553.766	10.5849
16.250.000	2.890.028	-553.885	10.5831
16.260.000	2.891.903	-554.025	10.5811
16.270.000	2.893.393	-554.055	10.5790
16.280.000	2.895.202	-554.221	10.5776
16.290.000	2.896.783	-554.303	10.5756
16.300.000	2.898.534	-554.417	10.5739
16.310.000	2.900.139	-554.531	10.5718
16.320.000	2.901.947	-554.688	10.5701
16.330.000	2.903.591	-554.714	10.5686
16.340.000	2.905.280	-554.904	10.5664
16.350.000	2.906.930	-554.930	10.5645
16.360.000	2.908.534	-555.102	10.5626
16.370.000	2.910.344	-555.170	10.5606
16.380.000	2.911.897	-555.308	10.5588
16.390.000	2.913.776	-555.416	10.5567
16.400.000	2.915.169	-555.566	10.5549
16.410.000	2.917.095	-555.661	10.5529
16.420.000	2.918.498	-555.770	10.5510
16.430.000	2.920.436	-555.935	10.5487
16.440.000	2.921.890	-555.995	10.5467

16.450.000	2.923.686	-556.153	10.5440
16.460.000	2.925.258	-556.277	10.5416
16.470.000	2.927.026	-556.388	10.5398
16.480.000	2.928.592	-556.468	10.5386
16.490.000	2.930.361	-556.679	10.5378
16.500.000	2.932.023	-556.683	10.5360
16.510.000	2.933.635	-556.895	10.5347
16.520.000	2.935.421	-556.979	10.5333
16.530.000	2.936.921	-557.108	10.5316
16.540.000	2.938.806	-557.242	10.5292
16.550.000	2.940.319	-557.396	10.5266
16.560.000	2.942.111	-557.481	10.5246
16.570.000	2.943.617	-557.658	10.5228
16.580.000	2.945.509	-557.829	10.5208
16.590.000	2.946.896	-557.865	10.5189
16.600.000	2.948.814	-558.117	10.5169
16.610.000	2.950.386	-558.178	10.5151
16.620.000	2.952.086	-558.356	10.5134
16.630.000	2.953.691	-558.457	10.5118
16.640.000	2.955.426	-558.600	10.5101
16.650.000	2.957.047	-558.691	10.5085
16.660.000	2.958.751	-558.931	10.5072
16.670.000	2.960.510	-558.939	10.5054
16.680.000	2.961.984	-559.129	10.5035
16.690.000	2.963.826	-559.249	10.5017
16.700.000	2.965.355	-559.386	10.4999
16.710.000	2.967.160	-559.493	10.4981
16.720.000	2.968.632	-559.678	10.4958
16.730.000	2.970.530	-559.776	10.4936
16.740.000	2.971.954	-559.914	10.4920

16.750.000	2.973.858	-560.082	10.4903
16.760.000	2.975.304	-560.134	10.4885
16.770.000	2.977.183	-560.330	10.4865
16.780.000	2.978.752	-560.450	10.4847
16.790.000	2.980.464	-560.559	10.4830
16.800.000	2.982.046	-560.705	10.4811
16.810.000	2.983.844	-560.929	10.4791
16.820.000	2.985.445	-560.978	10.4775
16.830.000	2.987.116	-561.202	10.4761
16.840.000	2.988.875	-561.305	10.4744
16.850.000	2.990.400	-561.487	10.4731
16.860.000	2.992.251	-561.621	10.4718
16.870.000	2.993.814	-561.826	10.4704
16.880.000	2.995.600	-561.953	10.4687
16.890.000	2.997.026	-562.175	10.4667
16.900.000	2.998.969	-562.336	10.4650
16.910.000	3.000.406	-562.463	10.4634
16.920.000	3.002.307	-562.702	10.4616
16.930.000	3.003.840	-562.811	10.4597
16.940.000	3.005.618	-563.005	10.4582
16.950.000	3.007.173	-563.166	10.4567
16.960.000	3.008.925	-563.326	10.4548
16.970.000	3.010.509	-563.463	10.4530
16.980.000	3.012.221	-563.719	10.4510
16.990.000	3.013.899	-563.765	10.4492
17.000.000	3.015.478	-564.035	10.4475
17.010.000	3.017.264	-564.175	10.4453
17.020.000	3.018.787	-564.353	10.4435
17.030.000	3.020.599	-564.530	10.4421
17.040.000	3.022.138	-564.758	10.4405

17.050.000	3.023.983	-564.919	10.4387
17.060.000	3.025.399	-565.132	10.4368
17.070.000	3.027.352	-565.365	10.4353
17.080.000	3.028.758	-565.479	10.4339
17.090.000	3.030.626	-565.749	10.4319
17.100.000	3.032.182	-565.907	10.4303
17.110.000	3.033.936	-566.145	10.4291
17.120.000	3.035.520	-566.370	10.4279
17.130.000	3.037.248	-566.615	10.4263
17.140.000	3.038.796	-566.774	10.4250
17.150.000	3.040.435	-567.094	10.4237
17.160.000	3.042.235	-567.307	10.4223
17.170.000	3.043.736	-567.587	10.4207
17.180.000	3.045.546	-567.787	10.4191
17.190.000	3.047.073	-568.106	10.4179
17.200.000	3.048.870	-568.381	10.4166
17.210.000	3.050.355	-568.694	10.4151
17.220.000	3.052.230	-568.900	10.4136
17.230.000	3.053.640	-569.191	10.4123
17.240.000	3.055.521	-569.499	10.4109
17.250.000	3.057.023	-569.685	10.4095
17.260.000	3.058.805	-569.968	10.4077
17.270.000	3.060.370	-570.248	10.4061
17.280.000	3.062.126	-570.489	10.4046
17.290.000	3.063.724	-570.713	10.4030
17.300.000	3.065.431	-571.050	10.4013
17.310.000	3.067.017	-571.131	10.3995
17.320.000	3.068.717	-571.492	10.3982
17.330.000	3.070.460	-571.723	10.3968
17.340.000	3.071.962	-571.961	10.3952

17.350.000	3.073.809	-572.196	10.3936
17.360.000	3.075.310	-572.469	10.3925
17.370.000	3.077.144	-572.685	10.3915
17.380.000	3.078.619	-572.933	10.3898
17.390.000	3.080.481	-573.188	10.3884
17.400.000	3.081.942	-573.389	10.3873
17.410.000	3.083.755	-573.684	10.3862
17.420.000	3.085.308	-573.846	10.3850
17.430.000	3.087.041	-574.135	10.3831
17.440.000	3.088.635	-574.338	10.3821
17.450.000	3.090.384	-574.606	10.3809
17.460.000	3.091.979	-574.780	10.3796
17.470.000	3.093.660	-575.119	10.3783
17.480.000	3.095.415	-575.283	10.3767
17.490.000	3.096.953	-575.608	10.3753
17.500.000	3.098.737	-575.818	10.3740
17.510.000	3.100.275	-576.083	10.3727
17.520.000	3.102.047	-576.332	10.3714
17.530.000	3.103.551	-576.643	10.3700
17.540.000	3.105.399	-576.870	10.3687
17.550.000	3.106.832	-577.151	10.3672
17.560.000	3.108.738	-577.465	10.3658
17.570.000	3.110.176	-577.654	10.3645
17.580.000	3.111.980	-577.968	10.3632
17.590.000	3.113.541	-578.208	10.3616
17.600.000	3.115.253	-578.472	10.3599
17.610.000	3.116.874	-578.696	10.3585
17.620.000	3.118.604	-579.002	10.3573
17.630.000	3.120.205	-579.155	10.3561
17.640.000	3.121.836	-579.505	10.3547

17.650.000	3.123.603	-579.708	10.3533
17.660.000	3.125.111	-579.991	10.3521
17.670.000	3.126.957	-580.233	10.3510
17.680.000	3.128.487	-580.521	10.3499
17.690.000	3.130.296	-580.771	10.3486
17.700.000	3.131.787	-581.065	10.3471
17.710.000	3.133.673	-581.322	10.3457
17.720.000	3.135.103	-581.552	10.3445
17.730.000	3.136.933	-581.879	10.3431
17.740.000	3.138.432	-582.069	10.3416
17.750.000	3.140.187	-582.368	10.3398
17.760.000	3.141.760	-582.624	10.3383
17.770.000	3.143.486	-582.878	10.3369
17.780.000	3.145.105	-583.100	10.3357
17.790.000	3.146.823	-583.425	10.3342
17.800.000	3.148.482	-583.568	10.3326
17.810.000	3.150.078	-583.891	10.3314
17.820.000	3.151.843	-584.070	10.3303
17.830.000	3.153.328	-584.298	10.3291
17.840.000	3.155.135	-584.541	10.3278
17.850.000	3.156.645	-584.824	10.3265
17.860.000	3.158.480	-585.008	10.3256
17.870.000	3.159.921	-585.273	10.3247
17.880.000	3.161.826	-585.553	10.3236
17.890.000	3.163.233	-585.720	10.3222
17.900.000	3.165.083	-586.004	10.3209
17.910.000	3.166.625	-586.212	10.3197
17.920.000	3.168.352	-586.456	10.3184
17.930.000	3.169.975	-586.662	10.3172
17.940.000	3.171.710	-586.944	10.3161

17.950.000	3.173.265	-587.117	10.3150
17.960.000	3.174.996	-587.423	10.3140
17.970.000	3.176.682	-587.602	10.3129
17.980.000	3.178.228	-587.879	10.3114
17.990.000	3.180.027	-588.098	10.3100
18.000.000	3.181.539	-588.357	10.3085
18.010.000	3.183.368	-588.579	10.3071
18.020.000	3.184.858	-588.879	10.3058
18.030.000	3.186.705	-589.086	10.3047
18.040.000	3.188.173	-589.333	10.3035
18.050.000	3.190.039	-589.645	10.3023
18.060.000	3.191.515	-589.798	10.3008
18.070.000	3.193.312	-590.107	10.2990
18.080.000	3.194.866	-590.350	10.2973
18.090.000	3.196.622	-590.589	10.2955
18.100.000	3.198.209	-590.803	10.2938
18.110.000	3.199.924	-591.149	10.2924
18.120.000	3.201.577	-591.302	10.2913
18.130.000	3.203.205	-591.634	10.2904
18.140.000	3.204.977	-591.880	10.2895
18.150.000	3.206.493	-592.126	10.2883
18.160.000	3.208.283	-592.382	10.2871
18.170.000	3.209.764	-592.671	10.2860
18.180.000	3.211.595	-592.904	10.2850
18.190.000	3.213.034	-593.186	10.2838
18.200.000	3.214.959	-593.471	10.2824
18.210.000	3.216.372	-593.678	10.2810
18.220.000	3.218.194	-594.012	10.2796
18.230.000	3.219.791	-594.244	10.2783
18.240.000	3.221.501	-594.551	10.2770

18.250.000	3.223.110	-594.804	10.2755
18.260.000	3.224.834	-595.112	10.2741
18.270.000	3.226.428	-595.350	10.2725
18.280.000	3.228.158	-595.705	10.2711
18.290.000	3.229.833	-595.899	10.2697
18.300.000	3.231.372	-596.237	10.2682
18.310.000	3.233.194	-596.504	10.2668
18.320.000	3.234.686	-596.792	10.2653
18.330.000	3.236.494	-597.061	10.2637
18.340.000	3.238.005	-597.394	10.2623
18.350.000	3.239.870	-597.650	10.2609
18.360.000	3.241.311	-597.922	10.2595
18.370.000	3.243.190	-598.259	10.2581
18.380.000	3.244.623	-598.449	10.2567
18.390.000	3.246.445	-598.796	10.2552
18.400.000	3.247.980	-599.053	10.2538
18.410.000	3.249.739	-599.324	10.2524
18.420.000	3.251.302	-599.597	10.2510
18.430.000	3.253.034	-599.939	10.2493
18.440.000	3.254.625	-600.110	10.2475
18.450.000	3.256.263	-600.496	10.2459
18.460.000	3.258.054	-600.718	10.2443
18.470.000	3.259.568	-601.018	10.2429
18.480.000	3.261.368	-601.281	10.2414
18.490.000	3.262.861	-601.579	10.2398
18.500.000	3.264.669	-601.847	10.2381
18.510.000	3.266.117	-602.161	10.2366
18.520.000	3.267.993	-602.431	10.2351
18.530.000	3.269.381	-602.682	10.2336
18.540.000	3.271.253	-603.040	10.2321

18.550.000	3.272.778	-603.269	10.2305
18.560.000	3.274.516	-603.600	10.2290
18.570.000	3.276.094	-603.880	10.2276
18.580.000	3.277.866	-604.188	10.2262
18.590.000	3.279.432	-604.446	10.2246
18.600.000	3.281.186	-604.820	10.2230
18.610.000	3.282.822	-605.001	10.2215
18.620.000	3.284.381	-605.367	10.2198
18.630.000	3.286.153	-605.635	10.2181
18.640.000	3.287.664	-605.927	10.2163
18.650.000	3.289.497	-606.222	10.2145
18.660.000	3.290.980	-606.571	10.2128
18.670.000	3.292.786	-606.840	10.2107
18.680.000	3.294.274	-607.151	10.2086
18.690.000	3.296.176	-607.514	10.2066
18.700.000	3.297.609	-607.743	10.2050
18.710.000	3.299.436	-608.116	10.2031
18.720.000	3.300.988	-608.396	10.2011
18.730.000	3.302.698	-608.709	10.1991
18.740.000	3.304.271	-609.006	10.1973
18.750.000	3.306.006	-609.377	10.1956
18.760.000	3.307.573	-609.607	10.1938
18.770.000	3.309.247	-610.018	10.1917
18.780.000	3.310.956	-610.253	10.1894
18.790.000	3.312.506	-610.645	10.1875
18.800.000	3.314.336	-610.943	10.1858
18.810.000	3.315.857	-611.284	10.1839
18.820.000	3.317.676	-611.613	10.1819
18.830.000	3.319.137	-612.010	10.1797
18.840.000	3.321.005	-612.330	10.1780

18.850.000	3.322.412	-612.673	10.1764
18.860.000	3.324.315	-613.093	10.1745
18.870.000	3.325.765	-613.381	10.1726
18.880.000	3.327.554	-613.798	10.1707
18.890.000	3.329.092	-614.165	10.1689
18.900.000	3.330.870	-614.544	10.1672
18.910.000	3.332.435	-614.911	10.1650
18.920.000	3.334.185	-615.367	10.1627
18.930.000	3.335.800	-615.662	10.1604
18.940.000	3.337.428	-616.142	10.1582
18.950.000	3.339.147	-616.474	10.1562
18.960.000	3.340.639	-616.891	10.1539
18.970.000	3.342.450	-617.264	10.1516
18.980.000	3.343.965	-617.733	10.1494
18.990.000	3.345.767	-618.115	10.1475
19.000.000	3.347.199	-618.557	10.1453
19.010.000	3.349.094	-618.993	10.1429
19.020.000	3.350.501	-619.357	10.1407
19.030.000	3.352.395	-619.852	10.1388
19.040.000	3.353.880	-620.218	10.1368
19.050.000	3.355.552	-620.673	10.1347
19.060.000	3.357.169	-621.111	10.1323
19.070.000	3.358.903	-621.582	10.1303
19.080.000	3.360.444	-621.986	10.1282
19.090.000	3.362.179	-622.512	10.1259
19.100.000	3.363.820	-622.874	10.1234
19.110.000	3.365.356	-623.394	10.1208
19.120.000	3.367.135	-623.841	10.1183
19.130.000	3.368.650	-624.312	10.1160
19.140.000	3.370.457	-624.772	10.1135

19.150.000	3.371.947	-625.350	10.1109
19.160.000	3.373.770	-625.800	10.1083
19.170.000	3.375.161	-626.331	10.1060
19.180.000	3.377.039	-626.919	10.1036
19.190.000	3.378.502	-627.376	10.1010
19.200.000	3.380.267	-627.991	10.0983
19.210.000	3.381.774	-628.532	10.0958
19.220.000	3.383.497	-629.085	10.0934
19.230.000	3.385.075	-629.659	10.0908
19.240.000	3.386.792	-630.291	10.0879
19.250.000	3.388.406	-630.798	10.0851
19.260.000	3.390.063	-631.489	10.0827
19.270.000	3.391.817	-632.033	10.0800
19.280.000	3.393.294	-632.673	10.0771
19.290.000	3.395.079	-633.268	10.0743
19.300.000	3.396.617	-633.942	10.0718
19.310.000	3.398.375	-634.555	10.0691
19.320.000	3.399.803	-635.222	10.0662
19.330.000	3.401.670	-635.871	10.0633
19.340.000	3.403.098	-636.528	10.0605
19.350.000	3.404.973	-637.258	10.0576
19.360.000	3.406.484	-637.871	10.0548
19.370.000	3.408.237	-638.604	10.0518
19.380.000	3.409.789	-639.288	10.0490
19.390.000	3.411.515	-639.992	10.0462
19.400.000	3.413.011	-640.659	10.0435
19.410.000	3.414.763	-641.475	10.0406
19.420.000	3.416.343	-642.112	10.0373
19.430.000	3.417.945	-642.919	10.0343
19.440.000	3.419.702	-643.638	10.0315

19.450.000	3.421.193	-644.429	10.0283
19.460.000	3.422.995	-645.166	10.0251
19.470.000	3.424.512	-646.022	10.0221
19.480.000	3.426.301	-646.777	10.0194
19.490.000	3.427.727	-647.610	10.0164
19.500.000	3.429.589	-648.476	10.0130
19.510.000	3.430.995	-649.224	10.1998
19.520.000	3.432.826	-650.124	10.1968
19.530.000	3.434.328	-650.951	10.1938
19.540.000	3.436.114	-651.810	10.2008
19.550.000	3.437.628	-652.665	9.9977
19.560.000	3.439.389	-653.584	9.9945
19.570.000	3.440.954	-654.398	9.9913
19.580.000	3.442.631	-655.383	9.9878
19.590.000	3.444.319	-656.208	9.9845
19.600.000	3.445.857	-657.176	9.9812
19.610.000	3.447.616	-658.070	9.9777
19.620.000	3.449.148	-659.025	9.9742
19.630.000	3.450.901	-659.941	9.9708
19.640.000	3.452.377	-660.943	9.9675
19.650.000	3.454.231	-661.872	9.9641
19.660.000	3.455.610	-662.850	9.9605
19.670.000	3.457.456	-663.865	9.9568
19.680.000	3.458.933	-664.790	9.9530
19.690.000	3.460.712	-665.830	9.9493
19.700.000	3.462.263	-666.825	9.9456
19.710.000	3.464.007	-667.840	9.9419
19.720.000	3.465.513	-668.822	9.9380
19.730.000	3.467.239	-669.934	9.9341
19.740.000	3.468.814	-670.873	9.9304

19.750.000	3.470.461	-672.017	9.9266
19.760.000	3.472.187	-673.053	9.9226
19.770.000	3.473.656	-674.125	9.9187
19.780.000	3.475.446	-675.200	9.9147
19.790.000	3.476.951	-676.336	9.9106
19.800.000	3.478.698	-677.397	9.9064
19.810.000	3.480.203	-678.574	9.9021
19.820.000	3.482.103	-679.733	9.8979
19.830.000	3.483.461	-680.810	9.8938
19.840.000	3.485.352	-682.033	9.8896
19.850.000	3.486.798	-683.152	9.8854
19.860.000	3.488.570	-684.352	9.8811
19.870.000	3.490.083	-685.533	9.8768
19.880.000	3.491.828	-686.746	9.8726
19.890.000	3.493.371	-687.906	9.8685
19.900.000	3.495.067	-689.197	9.8640
19.910.000	3.496.704	-690.339	9.8596
19.920.000	3.498.347	-691.648	9.8550
19.930.000	3.500.067	-692.871	9.8509
19.940.000	3.501.592	-694.106	9.8466
19.950.000	3.503.297	-695.340	9.8421
19.960.000	3.504.799	-696.667	9.8377
19.970.000	3.506.627	-697.878	9.8329
19.980.000	3.508.011	-699.164	9.8283
19.990.000	3.509.853	-700.480	9.8236
20.000.000	3.511.276	-701.679	9.8190
20.010.000	3.513.063	-703.025	9.8144
20.020.000	3.514.608	-704.290	9.8096
20.030.000	3.516.317	-705.584	9.8046
20.040.000	3.517.899	-706.884	9.7998

20.050.000	3.519.657	-708.261	9.7952
20.060.000	3.521.173	-709.482	9.7905
20.070.000	3.522.807	-710.896	9.7858
20.080.000	3.524.483	-712.169	9.7809
20.090.000	3.526.002	-713.571	9.7761
20.100.000	3.527.744	-714.888	9.7711
20.110.000	3.529.296	-716.297	9.7658
20.120.000	3.531.033	-717.620	9.7605
20.130.000	3.532.517	-719.051	9.7551
20.140.000	3.534.363	-720.427	9.7495
20.150.000	3.535.769	-721.800	9.7441
20.160.000	3.537.642	-723.249	9.7387
20.170.000	3.539.073	-724.597	9.7335
20.180.000	3.540.850	-726.005	9.7284
20.190.000	3.542.353	-727.398	9.7232
20.200.000	3.544.041	-728.817	9.7178
20.210.000	3.545.560	-730.211	9.7123
20.220.000	3.547.243	-731.725	9.7070
20.230.000	3.548.775	-733.107	9.7019
20.240.000	3.550.393	-734.679	9.6967
20.250.000	3.552.089	-736.224	9.6914
20.260.000	3.553.621	-737.814	9.6861
20.270.000	3.555.323	-739.390	9.6809
20.280.000	3.556.765	-741.136	9.6757
20.290.000	3.558.521	-742.802	9.6705
20.300.000	3.559.904	-744.604	9.6651
20.310.000	3.561.722	-746.478	9.6598
20.320.000	3.563.085	-748.266	9.6546
20.330.000	3.564.863	-750.251	9.6492
20.340.000	3.566.304	-752.141	9.6437

20.350.000	3.568.019	-754.111	9.6383
20.360.000	3.569.489	-756.095	9.6327
20.370.000	3.571.241	-758.147	9.6272
20.380.000	3.572.711	-760.094	9.6216
20.390.000	3.574.369	-762.215	9.6158
20.400.000	3.576.000	-764.168	9.6101
20.410.000	3.577.505	-766.256	9.6042
20.420.000	3.579.176	-768.242	9.5983
20.430.000	3.580.692	-770.285	9.5923
20.440.000	3.582.348	-772.240	9.5862
20.450.000	3.583.832	-774.321	9.5800
20.460.000	3.585.615	-776.272	9.5739
20.470.000	3.586.980	-778.286	9.5678
20.480.000	3.588.861	-780.324	9.5617
20.490.000	3.590.240	-782.242	9.5554
20.500.000	3.591.988	-784.240	9.5494
20.510.000	3.593.512	-786.202	9.5434
20.520.000	3.595.219	-788.128	9.5374
20.530.000	3.596.678	-790.035	9.5315
20.540.000	3.598.396	-792.024	9.5255
20.550.000	3.599.898	-793.821	9.5194
20.560.000	3.601.524	-795.773	9.5129
20.570.000	3.603.187	-797.614	9.5062
20.580.000	3.604.665	-799.454	9.4995
20.590.000	3.606.393	-801.254	9.4929
20.600.000	3.607.911	-803.128	9.4863
20.610.000	3.609.621	-804.834	9.4799
20.620.000	3.611.004	-806.650	9.4734
20.630.000	3.612.834	-808.406	9.4666
20.640.000	3.614.182	-810.108	9.4598

20.650.000	3.615.951	-811.887	9.4530
20.660.000	3.617.396	-813.525	9.4462
20.670.000	3.619.114	-815.240	9.4393
20.680.000	3.620.569	-816.917	9.4323
20.690.000	3.622.330	-818.589	9.4255
20.700.000	3.623.806	-820.170	9.4185
20.710.000	3.625.497	-821.873	9.4116
20.720.000	3.627.105	-823.344	9.4048
20.730.000	3.628.643	-824.952	9.3978
20.740.000	3.630.289	-826.435	9.3909
20.750.000	3.631.829	-827.943	9.3839
20.760.000	3.633.485	-829.386	9.3771
20.770.000	3.634.964	-830.969	9.3702
20.780.000	3.636.712	-832.426	9.3633
20.790.000	3.638.040	-833.975	9.3562
20.800.000	3.639.882	-835.589	9.3492
20.810.000	3.641.249	-837.094	9.3419
20.820.000	3.642.984	-838.730	9.3348
20.830.000	3.644.470	-840.317	9.3276
20.840.000	3.646.114	-841.901	9.3204
20.850.000	3.647.557	-843.462	9.3130
20.860.000	3.649.244	-845.107	9.3055
20.870.000	3.650.696	-846.576	9.2980
20.880.000	3.652.370	-848.187	9.2907
20.890.000	3.653.939	-849.663	9.2833
20.900.000	3.655.424	-851.161	9.2757
20.910.000	3.657.094	-852.595	9.2680
20.920.000	3.658.629	-854.088	9.2601
20.930.000	3.660.314	-855.388	9.2524
20.940.000	3.661.773	-856.784	9.2446

20.950.000	3.663.561	-858.036	9.2365
20.960.000	3.664.894	-859.227	9.2282
20.970.000	3.666.690	-860.439	9.2199
20.980.000	3.668.108	-861.463	9.2120
20.990.000	3.669.823	-862.517	9.2041
21.000.000	3.671.253	-863.467	9.1962
21.010.000	3.673.003	-864.360	9.1882
21.020.000	3.674.456	-865.180	9.1800
21.030.000	3.676.135	-866.048	9.1716
21.040.000	3.677.709	-866.682	9.1630
21.050.000	3.679.283	-867.453	9.1541
21.060.000	3.680.942	-868.083	9.1452
21.070.000	3.682.428	-868.718	9.1363
21.080.000	3.684.078	-869.292	9.1277
21.090.000	3.685.589	-869.940	9.1190
21.100.000	3.687.293	-870.434	9.1103
21.110.000	3.688.656	-871.012	9.1016
21.120.000	3.690.491	-871.573	9.0929
21.130.000	3.691.859	-872.019	9.0840
21.140.000	3.693.666	-872.569	9.0750
21.150.000	3.695.184	-873.009	9.0658
21.160.000	3.696.919	-873.419	9.0569
21.170.000	3.698.418	-873.777	9.0480
21.180.000	3.700.190	-874.141	9.0389
21.190.000	3.701.649	-874.313	9.0296
21.200.000	3.703.374	-874.591	9.0200
21.210.000	3.705.018	-874.649	9.0105
21.220.000	3.706.595	-874.735	9.2008
21.230.000	3.708.367	-874.693	8.9911
21.240.000	3.709.939	-874.605	8.9814

21.250.000	3.711.672	-874.359	8.9716
21.260.000	3.713.220	-874.160	8.9616
21.270.000	3.715.115	-873.743	8.9515
21.280.000	3.716.522	-873.264	8.9411
21.290.000	3.718.431	-872.745	8.9303
21.300.000	3.719.886	-872.010	8.9193
21.310.000	3.721.713	-871.300	8.9079
21.320.000	3.723.309	-870.460	8.8963
21.330.000	3.725.078	-869.479	8.8844
21.340.000	3.726.659	-868.455	8.8723
21.350.000	3.728.450	-867.455	8.8602
21.360.000	3.730.074	-866.203	8.8477
21.370.000	3.731.796	-865.118	8.8350
21.380.000	3.733.515	-863.901	8.8221
21.390.000	3.735.169	-862.692	8.8092
21.400.000	3.736.945	-861.446	8.7964
21.410.000	3.738.540	-860.311	8.7833
21.420.000	3.740.318	-859.038	8.7698
21.430.000	3.741.853	-857.958	8.7563
21.440.000	3.743.783	-856.828	8.7429
21.450.000	3.745.223	-855.671	8.7296
21.460.000	3.747.071	-854.644	8.7163
21.470.000	3.748.613	-853.605	8.7028
21.480.000	3.750.371	-852.679	8.6895
21.490.000	3.751.882	-851.864	8.6765
21.500.000	3.753.665	-851.173	8.6634
21.510.000	3.755.134	-850.587	8.6503
21.520.000	3.756.861	-850.214	8.6367
21.530.000	3.758.479	-849.841	8.6230
21.540.000	3.760.063	-849.667	8.6092

21.550.000	3.761.765	-849.504	8.5952
21.560.000	3.763.321	-849.410	8.5810
21.570.000	3.765.051	-849.294	8.5663
21.580.000	3.766.653	-849.319	8.5514
21.590.000	3.768.511	-849.207	8.5365
21.600.000	3.769.970	-849.131	8.5214
21.610.000	3.771.913	-849.093	8.5060
21.620.000	3.773.381	-848.880	8.4900
21.630.000	3.775.257	-848.748	8.4737
21.640.000	3.776.809	-848.516	8.4573
21.650.000	3.778.605	-848.199	8.4406
21.660.000	3.780.144	-847.863	8.4235
21.670.000	3.781.999	-847.560	8.4058
21.680.000	3.783.593	-847.010	8.3880
21.690.000	3.785.316	-846.593	8.3702
21.700.000	3.787.059	-846.011	8.3522
21.710.000	3.788.681	-845.436	8.3339
21.720.000	3.790.471	-844.798	8.3151
21.730.000	3.792.151	-844.201	8.2962
21.740.000	3.793.911	-843.437	8.2771
21.750.000	3.795.453	-842.807	8.2580
21.760.000	3.797.383	-842.064	8.2385
21.770.000	3.798.826	-841.327	8.2185
21.780.000	3.800.755	-840.663	8.1984
21.790.000	3.802.268	-839.863	8.1783
21.800.000	3.804.102	-839.145	8.1580
21.810.000	3.805.647	-838.431	8.1374
21.820.000	3.807.501	-837.707	8.1163
21.830.000	3.809.039	-836.972	8.0949
21.840.000	3.810.875	-836.352	8.0733

21.850.000	3.812.471	-835.577	8.0515
21.860.000	3.814.086	-834.966	8.0294
21.870.000	3.815.812	-834.308	8.1970
21.880.000	3.817.404	-833.703	7.9842
21.890.000	3.819.053	-833.048	7.9614
21.900.000	3.820.664	-832.553	7.9388
21.910.000	3.822.444	-831.944	7.9157
21.920.000	3.823.898	-831.473	7.8921
21.930.000	3.825.809	-831.031	7.8682
21.940.000	3.827.164	-830.533	7.8442
21.950.000	3.829.063	-830.178	7.8201
21.960.000	3.830.537	-829.795	7.7958
21.970.000	3.832.274	-829.446	7.7709
21.980.000	3.833.793	-829.131	7.7457
21.990.000	3.835.572	-828.923	7.7206
22.000.000	3.837.050	-828.597	7.6955
22.010.000	3.838.792	-828.463	7.6702
22.020.000	3.840.433	-828.212	7.6447
22.030.000	3.842.009	-828.059	7.6189
22.040.000	3.843.749	-827.890	7.5929
22.050.000	3.845.319	-827.783	7.5668
22.060.000	3.846.983	-827.590	7.5406
22.070.000	3.848.525	-827.566	7.5139
22.080.000	3.850.260	-827.403	7.4868
22.090.000	3.851.675	-827.373	7.4594
22.100.000	3.853.511	-827.401	7.4321
22.110.000	3.854.921	-827.353	7.4046
22.120.000	3.856.719	-827.468	7.3768
22.130.000	3.858.233	-827.588	7.3485
22.140.000	3.860.011	-827.726	7.3201

22.150.000	3.861.465	-827.878	7.2916
22.160.000	3.863.230	-828.183	7.2633
22.170.000	3.864.726	-828.334	7.2349
22.180.000	3.866.376	-828.680	7.2063
22.190.000	3.867.966	-828.940	7.1775
22.200.000	3.869.519	-829.271	7.1487
22.210.000	3.871.123	-829.614	7.1199
22.220.000	3.872.654	-830.125	7.0908
22.230.000	3.874.317	-830.489	7.0616
22.240.000	3.875.662	-831.093	7.0324
22.250.000	3.877.420	-831.730	7.1932
22.260.000	3.878.667	-832.435	6.9743
22.270.000	3.880.391	-833.320	6.9454
22.280.000	3.881.765	-834.272	6.9164
22.290.000	3.883.440	-835.385	6.8873
22.300.000	3.884.832	-836.585	6.8580
22.310.000	3.886.558	-837.913	6.8284
22.320.000	3.887.941	-839.204	6.7989
22.330.000	3.889.635	-840.671	6.7691
22.340.000	3.891.109	-842.000	6.7390
22.350.000	3.892.644	-843.421	6.7084
22.360.000	3.894.196	-844.810	6.6778
22.370.000	3.895.663	-846.233	6.6473
22.380.000	3.897.211	-847.601	6.6165
22.390.000	3.898.648	-849.145	6.5853
22.400.000	3.900.224	-850.529	6.5540
22.410.000	3.901.381	-852.114	6.5232
22.420.000	3.902.867	-853.847	6.4925
22.430.000	3.903.866	-855.715	6.4618
22.440.000	3.905.248	-858.021	6.4309

22.450.000	3.906.374	-860.674	6.3999
22.460.000	3.907.921	-863.753	6.3691
22.470.000	3.909.255	-867.187	6.3385
22.480.000	3.910.851	-870.925	6.3076
22.490.000	3.912.171	-874.640	6.2767
22.500.000	3.913.672	-878.544	6.2455
22.510.000	3.915.089	-882.241	6.2144
22.520.000	3.916.466	-885.872	6.1832
22.530.000	3.917.917	-889.347	6.1518
22.540.000	3.919.334	-892.801	6.1202
22.550.000	3.920.862	-896.072	6.0886
22.560.000	3.922.249	-899.432	6.0570
22.570.000	3.923.999	-902.563	6.0257
22.580.000	3.925.219	-905.570	5.9941
22.590.000	3.926.780	-908.534	5.9624
22.600.000	3.928.025	-911.328	5.9305
22.610.000	3.929.507	-914.119	5.8983
22.620.000	3.930.824	-916.909	5.8662
22.630.000	3.932.352	-919.711	5.8340
22.640.000	3.933.457	-922.578	5.8019
22.650.000	3.934.950	-925.707	5.7698
22.660.000	3.936.195	-928.813	5.7377
22.670.000	3.937.579	-932.206	5.7056
22.680.000	3.939.037	-935.638	5.6733
22.690.000	3.940.474	-939.160	5.6408
22.700.000	3.942.006	-942.703	5.6082
22.710.000	3.943.517	-946.342	5.5757
22.720.000	3.945.152	-949.679	5.5434
22.730.000	3.946.570	-952.986	5.5111
22.740.000	3.948.156	-956.068	5.4787

22.750.000	3.949.349	-958.933	5.4461
22.760.000	3.951.011	-961.784	5.4135
22.770.000	3.952.300	-964.496	5.3806
22.780.000	3.953.938	-967.122	5.3473
22.790.000	3.955.328	-969.781	5.3140
22.800.000	3.957.018	-972.480	5.2806
22.810.000	3.958.428	-975.004	5.2472
22.820.000	3.960.077	-977.589	5.2138
22.830.000	3.961.617	-979.937	5.1802
22.840.000	3.963.101	-982.233	5.1467
22.850.000	3.964.773	-984.406	5.1131
22.860.000	3.966.334	-986.434	5.0794
22.870.000	3.967.895	-988.287	5.0453
22.880.000	3.969.416	-990.189	5.0112
22.890.000	3.971.131	-991.874	4.9769
22.900.000	3.972.531	-993.583	4.9425
22.910.000	3.974.379	-995.258	4.9081
22.920.000	3.975.808	-996.779	4.8735
22.930.000	3.977.625	-998.327	4.8391
22.940.000	3.979.074	-999.743	4.8046
22.950.000	3.980.855	-1.001.059	4.7699
22.960.000	3.982.272	-1.002.277	4.7351
22.970.000	3.984.121	-1.003.522	4.7001
22.980.000	3.985.631	-1.004.499	4.6649
22.990.000	3.987.364	-1.005.552	4.6298
23.000.000	3.988.971	-1.006.434	4.5946
23.010.000	3.990.620	-1.007.306	4.5591
23.020.000	3.992.312	-1.008.044	4.5236
23.030.000	3.993.960	-1.008.815	4.4881
23.040.000	3.995.694	-1.009.354	4.4523

23.050.000	3.997.247	-1.009.989	4.4165
23.060.000	3.999.152	-1.010.478	4.3805
23.070.000	4.000.563	-1.010.876	4.3449
23.080.000	4.002.503	-1.011.279	4.3097
23.090.000	4.004.044	-1.011.574	4.2744
23.100.000	4.005.791	-1.011.731	4.2387
23.110.000	4.007.302	-1.011.876	4.2030
23.120.000	4.009.183	-1.012.013	4.1671
23.130.000	4.010.698	-1.011.975	4.1311
23.140.000	4.012.473	-1.012.065	4.0949
23.150.000	4.014.076	-1.012.004	4.0588
23.160.000	4.015.805	-1.012.005	4.0227
23.170.000	4.017.509	-1.011.991	3.9865
23.180.000	4.019.272	-1.011.950	3.9505
23.190.000	4.021.014	-1.011.767	3.9144
23.200.000	4.022.737	-1.011.677	3.8787
23.210.000	4.024.594	-1.011.247	3.8430
23.220.000	4.026.024	-1.010.837	3.8071
23.230.000	4.027.949	-1.010.403	3.7716
23.240.000	4.029.484	-1.009.817	3.7365
23.250.000	4.031.425	-1.009.278	3.7015
23.260.000	4.032.960	-1.008.635	3.6666
23.270.000	4.034.795	-1.007.935	3.6316
23.280.000	4.036.291	-1.007.307	3.5969
23.290.000	4.038.161	-1.006.720	3.5624
23.300.000	4.039.701	-1.005.971	3.5282
23.310.000	4.041.401	-1.005.416	3.4940
23.320.000	4.043.071	-1.004.867	3.4599
23.330.000	4.044.756	-1.004.389	3.4259
23.340.000	4.046.521	-1.003.924	3.3918

23.350.000	4.048.317	-1.003.592	3.3581
23.360.000	4.050.031	-1.003.062	3.3244
23.370.000	4.051.727	-1.002.698	3.2910
23.380.000	4.053.652	-1.002.159	3.2577
23.390.000	4.055.158	-1.001.590	3.2245
23.400.000	4.057.137	-1.000.988	3.1916
23.410.000	4.058.766	-1.000.277	3.1589
23.420.000	4.060.661	-999.502	3.1263
23.430.000	4.062.267	-998.680	3.0938
23.440.000	4.064.195	-997.763	3.0613
23.450.000	4.065.739	-996.768	3.0290
23.460.000	4.067.671	-995.840	2.9968
23.470.000	4.069.277	-994.730	2.9648
23.480.000	4.071.094	-993.740	2.9327
23.490.000	4.072.824	-992.697	2.9009
23.500.000	4.074.597	-991.650	2.8693
23.510.000	4.076.367	-990.576	2.8377
23.520.000	4.078.118	-989.647	2.8063
23.530.000	4.079.962	-988.455	2.7749
23.540.000	4.081.617	-987.458	2.7438
23.550.000	4.083.545	-986.345	2.7129
23.560.000	4.085.105	-985.174	2.6822
23.570.000	4.087.099	-984.064	2.6515
23.580.000	4.088.669	-982.866	2.6209
23.590.000	4.090.564	-981.594	2.5906
23.600.000	4.092.139	-980.351	2.5607
23.610.000	4.094.082	-979.128	2.5309
23.620.000	4.095.669	-977.755	2.5009
23.630.000	4.097.570	-976.566	2.4711
23.640.000	4.099.316	-975.255	2.4415

23.650.000	4.101.089	-973.975	2.4121
23.660.000	4.102.851	-972.656	2.3829
23.670.000	4.104.676	-971.384	2.3538
23.680.000	4.106.471	-969.979	2.3248
23.690.000	4.108.191	-968.677	2.2959
23.700.000	4.110.078	-967.209	2.2670
23.710.000	4.111.635	-965.828	2.2386
23.720.000	4.113.634	-964.413	2.2103
23.730.000	4.115.231	-962.985	2.1822
23.740.000	4.117.164	-961.556	2.1541
23.750.000	4.118.789	-960.140	2.1264
23.760.000	4.120.761	-958.693	2.0990
23.770.000	4.122.302	-957.209	2.0719
23.780.000	4.124.261	-955.829	2.0448
23.790.000	4.125.919	-954.282	2.0181
23.800.000	4.127.827	-952.890	1.9915
23.810.000	4.129.550	-951.416	1.9651
23.820.000	4.131.378	-949.928	1.9388
23.830.000	4.133.098	-948.435	1.9126
23.840.000	4.134.885	-947.003	1.8867
23.850.000	4.136.674	-945.395	1.8610
23.860.000	4.138.398	-943.992	1.8355
23.870.000	4.140.351	-942.436	1.8103
23.880.000	4.141.917	-940.919	1.7852
23.890.000	4.143.884	-939.451	1.7600
23.900.000	4.145.528	-937.983	1.7351
23.910.000	4.147.459	-936.468	1.7103
23.920.000	4.149.028	-934.991	1.6857
23.930.000	4.151.020	-933.513	1.6614
23.940.000	4.152.550	-931.979	1.6372

23.950.000	4.154.480	-930.568	1.6134
23.960.000	4.156.169	-929.050	1.5898
23.970.000	4.157.997	-927.629	1.5665
23.980.000	4.159.730	-926.166	1.5432
23.990.000	4.161.553	-924.736	1.5197
24.000.000	4.163.278	-923.258	1.4967
24.010.000	4.165.047	-921.910	1.4741
24.020.000	4.166.929	-920.390	1.4518
24.030.000	4.168.539	-919.028	1.4295
24.040.000	4.170.488	-917.602	1.4073
24.050.000	4.172.049	-916.180	1.3857
24.060.000	4.173.987	-914.823	1.3643
24.070.000	4.175.605	-913.482	1.3430
24.080.000	4.177.529	-912.086	1.3217
24.090.000	4.179.128	-910.737	1.3005
24.100.000	4.181.100	-909.448	1.2798
24.110.000	4.182.631	-908.033	1.2592
24.120.000	4.184.557	-906.773	1.2387
24.130.000	4.186.241	-905.449	1.2181
24.140.000	4.188.029	-904.136	1.1980
24.150.000	4.189.749	-902.818	1.1778
24.160.000	4.191.586	-901.606	1.1579
24.170.000	4.193.284	-900.213	1.1383
24.180.000	4.195.048	-899.050	1.1189
24.190.000	4.196.922	-897.748	1.0996
24.200.000	4.198.521	-896.511	1.0805
24.210.000	4.200.479	-895.266	1.0618
24.220.000	4.202.050	-894.061	1.0432
24.230.000	4.203.971	-892.852	1.0249
24.240.000	4.205.595	-891.658	1.1964

24.250.000	4.207.498	-890.466	9.882
24.260.000	4.209.076	-889.253	9.705
24.270.000	4.210.989	-888.134	9.530
24.280.000	4.212.586	-886.875	9.357
24.290.000	4.214.387	-885.740	9.184
24.300.000	4.216.085	-884.548	9.013
24.310.000	4.217.954	-883.398	8.845
24.320.000	4.219.646	-882.200	8.680
24.330.000	4.221.438	-881.119	8.516
24.340.000	4.223.194	-879.885	8.351
24.350.000	4.224.861	-878.816	8.188
24.360.000	4.226.755	-877.652	8.031
24.370.000	4.228.400	-876.525	7.876
24.380.000	4.230.265	-875.400	7.723
24.390.000	4.231.849	-874.324	7.571
24.400.000	4.233.769	-873.203	7.420
24.410.000	4.235.305	-872.124	7.273
24.420.000	4.237.287	-871.094	7.128
24.430.000	4.238.845	-869.956	6.983
24.440.000	4.240.721	-868.936	6.838
24.450.000	4.242.399	-867.871	6.699
24.460.000	4.244.206	-866.805	6.564
24.470.000	4.245.871	-865.716	6.433
24.480.000	4.247.669	-864.698	6.301
24.490.000	4.249.348	-863.593	6.170
24.500.000	4.251.113	-862.619	6.045
24.510.000	4.252.946	-861.533	5.921
24.520.000	4.254.557	-860.536	5.800
24.530.000	4.256.423	-859.495	5.677
24.540.000	4.258.041	-858.523	5.557

24.550.000	4.259.927	-857.501	5.442
24.560.000	4.261.504	-856.554	5.331
24.570.000	4.263.417	-855.563	5.219
24.580.000	4.264.939	-854.567	5.108
24.590.000	4.266.883	-853.673	5.001
24.600.000	4.268.422	-852.637	4.899
24.610.000	4.270.293	-851.746	4.800
24.620.000	4.271.944	-850.816	4.701
24.630.000	4.273.755	-849.891	4.604
24.640.000	4.275.405	-848.971	4.510
24.650.000	4.277.233	-848.152	4.420
24.660.000	4.278.971	-847.185	4.332
24.670.000	4.280.654	-846.423	4.242
24.680.000	4.282.483	-845.525	4.154
24.690.000	4.284.058	-844.724	4.070
24.700.000	4.285.900	-843.908	3.990
24.710.000	4.287.501	-843.164	3.912
24.720.000	4.289.353	-842.341	3.834
24.730.000	4.290.850	-841.616	3.755
24.740.000	4.292.816	-840.917	3.679
24.750.000	4.294.254	-840.147	3.607
24.760.000	4.296.177	-839.499	3.538
24.770.000	4.297.786	-838.793	3.469
24.780.000	4.299.603	-838.172	3.402
24.790.000	4.301.228	-837.486	3.339
24.800.000	4.303.010	-836.919	3.280
24.810.000	4.304.583	-836.232	3.221
24.820.000	4.306.348	-835.744	3.163
24.830.000	4.308.069	-835.090	3.105
24.840.000	4.309.626	-834.603	3.049

24.850.000	4.311.482	-834.064	2.994
24.860.000	4.313.043	-833.607	2.942
24.870.000	4.314.855	-833.108	2.892
24.880.000	4.316.433	-832.733	2.843
24.890.000	4.318.313	-832.307	2.796
24.900.000	4.319.808	-831.934	2.749
24.910.000	4.321.737	-831.633	2.706
24.920.000	4.323.202	-831.227	2.664
24.930.000	4.325.076	-830.974	2.624
24.940.000	4.326.623	-830.732	2.585
24.950.000	4.328.395	-830.460	2.546
24.960.000	4.330.020	-830.229	2.508
24.970.000	4.331.781	-830.113	2.471
24.980.000	4.333.382	-829.840	2.436
24.990.000	4.335.073	-829.766	2.402
25.000.000	4.336.808	-829.613	2.370
25.010.000	4.338.393	-829.522	2.339
25.020.000	4.340.154	-829.424	2.308
25.030.000	4.341.760	-829.405	2.279
25.040.000	4.343.568	-829.333	2.251
25.050.000	4.345.039	-829.359	2.222
25.060.000	4.346.952	-829.373	2.195
25.070.000	4.348.385	-829.370	2.167
25.080.000	4.350.231	-829.490	2.139
25.090.000	4.351.796	-829.541	2.113
25.100.000	4.353.593	-829.660	2.088
25.110.000	4.355.149	-829.794	2.065
25.120.000	4.356.937	-829.955	2.039
25.130.000	4.358.467	-830.076	2.017
25.140.000	4.360.228	-830.328	1.996

25.150.000	4.361.855	-830.443	1.976
25.160.000	4.363.482	-830.712	1.957
25.170.000	4.365.257	-830.901	1.939
25.180.000	4.366.781	-831.151	1.920
25.190.000	4.368.528	-831.351	1.899
25.200.000	4.370.083	-831.695	1.877
25.210.000	4.371.886	-831.901	1.858
25.220.000	4.373.362	-832.202	1.840
25.230.000	4.375.236	-832.555	1.823
25.240.000	4.376.694	-832.790	1.805
25.250.000	4.378.539	-833.157	1.788
25.260.000	4.380.055	-833.470	1.772
25.270.000	4.381.798	-833.807	1.756
25.280.000	4.383.330	-834.145	1.741
25.290.000	4.385.103	-834.565	1.726
25.300.000	4.386.649	-834.826	1.712
25.310.000	4.388.388	-835.281	1.698
25.320.000	4.390.090	-835.630	1.686
25.330.000	4.391.704	-836.046	1.674
25.340.000	4.393.424	-836.410	1.664
25.350.000	4.394.996	-836.857	1.654
25.360.000	4.396.725	-837.201	1.644
25.370.000	4.398.230	-837.700	1.633
25.380.000	4.400.107	-838.082	1.624
25.390.000	4.401.519	-838.529	1.617
25.400.000	4.403.374	-839.013	1.609
25.410.000	4.404.852	-839.401	1.600
25.420.000	4.406.632	-839.869	1.589
25.430.000	4.408.169	-840.340	1.580
25.440.000	4.409.979	-840.812	1.571

25.450.000	4.411.511	-841.277	1.564
25.460.000	4.413.266	-841.834	1.557
25.470.000	4.414.842	-842.248	1.549
25.480.000	4.416.515	-842.862	1.541
25.490.000	4.418.215	-843.383	1.536
25.500.000	4.419.771	-843.947	1.532
25.510.000	4.421.514	-844.531	1.527
25.520.000	4.423.121	-845.228	1.521
25.530.000	4.424.851	-845.798	1.515
25.540.000	4.426.318	-846.523	1.511
25.550.000	4.428.248	-847.285	1.508
25.560.000	4.429.626	-847.991	1.506
25.570.000	4.431.511	-848.853	1.504
25.580.000	4.432.999	-849.683	1.503
25.590.000	4.434.753	-850.560	1.499
25.600.000	4.436.257	-851.505	1.496
25.610.000	4.438.027	-852.537	1.495
25.620.000	4.439.522	-853.491	1.492
25.630.000	4.441.262	-854.656	1.487
25.640.000	4.442.858	-855.716	1.480
25.650.000	4.444.523	-856.931	1.473
25.660.000	4.446.188	-858.131	1.468
25.670.000	4.447.832	-859.414	1.463
25.680.000	4.449.518	-860.662	1.460
25.690.000	4.451.072	-862.102	1.456
25.700.000	4.452.850	-863.396	1.452
25.710.000	4.454.295	-864.823	1.450
25.720.000	4.456.169	-866.323	1.448
25.730.000	4.457.609	-867.713	1.447
25.740.000	4.459.414	-869.219	1.444

25.750.000	4.460.895	-870.706	1.439
25.760.000	4.462.693	-872.160	1.434
25.770.000	4.464.185	-873.632	1.432
25.780.000	4.466.042	-875.173	1.431
25.790.000	4.467.587	-876.528	1.429
25.800.000	4.469.270	-878.043	1.425
25.810.000	4.470.929	-879.423	1.423
25.820.000	4.472.555	-880.805	1.422
25.830.000	4.474.175	-882.149	1.422
25.840.000	4.475.826	-883.529	1.420
25.850.000	4.477.528	-884.724	1.417
25.860.000	4.478.996	-886.021	1.415
25.870.000	4.480.842	-887.205	1.413
25.880.000	4.482.252	-888.330	1.413
25.890.000	4.484.134	-889.468	1.413
25.900.000	4.485.592	-890.471	1.413
25.910.000	4.487.384	-891.454	1.414
25.920.000	4.488.880	-892.368	1.413
25.930.000	4.490.717	-893.206	1.415
25.940.000	4.492.163	-893.907	1.417
25.950.000	4.493.946	-894.658	1.418
25.960.000	4.495.509	-895.167	1.417
25.970.000	4.497.166	-895.706	1.414
25.980.000	4.498.810	-896.072	1.411
25.990.000	4.500.429	-896.387	1.409
26.000.000	4.502.086	-896.544	1.407
26.010.000	4.503.732	-896.709	1.405
26.020.000	4.505.440	-896.579	1.402
26.030.000	4.506.895	-896.476	1.399
26.040.000	4.508.727	-896.244	1.399

26.050.000	4.510.155	-895.830	1.400
26.060.000	4.511.961	-895.407	1.399
26.070.000	4.513.453	-894.844	1.395
26.080.000	4.515.216	-894.160	1.391
26.090.000	4.516.673	-893.383	1.392
26.100.000	4.518.497	-892.614	1.392
26.110.000	4.520.003	-891.627	1.391
26.120.000	4.521.776	-890.730	1.389
26.130.000	4.523.363	-889.616	1.387
26.140.000	4.525.004	-888.521	1.389
26.150.000	4.526.636	-887.316	1.392
26.160.000	4.528.325	-886.136	1.391
26.170.000	4.529.928	-884.748	1.391
26.180.000	4.531.489	-883.504	1.391
26.190.000	4.533.309	-882.055	1.395
26.200.000	4.534.725	-880.673	1.397
26.210.000	4.536.596	-879.214	1.396
26.220.000	4.538.049	-877.693	1.394
26.230.000	4.539.871	-876.169	1.392
26.240.000	4.541.366	-874.627	1.391
26.250.000	4.543.208	-872.997	1.390
26.260.000	4.544.641	-871.359	1.387
26.270.000	4.546.488	-869.767	1.383
26.280.000	4.547.975	-867.984	1.380
26.290.000	4.549.692	-866.330	1.380
26.300.000	4.551.343	-864.561	1.382
26.310.000	4.552.951	-862.788	1.382
26.320.000	4.554.589	-860.958	1.379
26.330.000	4.556.256	-859.237	1.379
26.340.000	4.557.936	-857.276	1.381

26.350.000	4.559.518	-855.511	1.383
26.360.000	4.561.315	-853.631	1.384
26.370.000	4.562.720	-851.717	1.383
26.380.000	4.564.598	-849.877	1.383
26.390.000	4.566.021	-847.987	1.384
26.400.000	4.567.792	-846.042	1.386
26.410.000	4.569.256	-844.166	1.387
26.420.000	4.571.097	-842.293	1.385
26.430.000	4.572.539	-840.328	1.383
26.440.000	4.574.348	-838.510	1.385
26.450.000	4.575.905	-836.549	1.386
26.460.000	4.577.603	-834.693	1.383
26.470.000	4.579.206	-832.796	1.380
26.480.000	4.580.896	-830.942	1.377
26.490.000	4.582.468	-829.024	1.377
26.500.000	4.584.101	-827.277	1.376
26.510.000	4.585.835	-825.352	1.371
26.520.000	4.587.321	-823.611	1.370
26.530.000	4.589.131	-821.819	1.369
26.540.000	4.590.582	-820.019	1.368
26.550.000	4.592.382	-818.301	1.369
26.560.000	4.593.897	-816.604	1.367
26.570.000	4.595.713	-814.882	1.368
26.580.000	4.597.124	-813.217	1.371
26.590.000	4.598.995	-811.649	1.371
26.600.000	4.600.440	-809.979	1.371
26.610.000	4.602.195	-808.453	1.369
26.620.000	4.603.780	-806.906	1.369
26.630.000	4.605.460	-805.398	1.367
26.640.000	4.607.043	-803.885	1.362

26.650.000	4.608.707	-802.521	1.360
26.660.000	4.610.333	-801.015	1.362
26.670.000	4.611.979	-799.742	1.365
26.680.000	4.613.715	-798.343	1.368
26.690.000	4.615.188	-797.036	1.369
26.700.000	4.616.976	-795.765	1.371
26.710.000	4.618.478	-794.566	1.373
26.720.000	4.620.242	-793.335	1.373
26.730.000	4.621.712	-792.177	1.375
26.740.000	4.623.548	-791.056	1.375
26.750.000	4.624.978	-789.902	1.373
26.760.000	4.626.777	-788.883	1.373
26.770.000	4.628.287	-787.771	1.372
26.780.000	4.630.009	-786.772	1.372
26.790.000	4.631.601	-785.767	1.372
26.800.000	4.633.281	-784.792	1.371
26.810.000	4.634.839	-783.796	1.372
26.820.000	4.636.508	-782.949	1.373
26.830.000	4.638.189	-781.935	1.374
26.840.000	4.639.684	-781.118	1.375
26.850.000	4.641.450	-780.228	1.376
26.860.000	4.642.938	-779.397	1.377
26.870.000	4.644.746	-778.606	1.376
26.880.000	4.646.211	-777.850	1.376
26.890.000	4.648.053	-777.059	1.375
26.900.000	4.649.484	-776.339	1.376
26.910.000	4.651.338	-775.681	1.375
26.920.000	4.652.735	-774.918	1.374
26.930.000	4.654.525	-774.303	1.370
26.940.000	4.656.079	-773.629	1.368

26.950.000	4.657.755	-772.998	1.368
26.960.000	4.659.311	-772.354	1.369
26.970.000	4.661.032	-771.833	1.367
26.980.000	4.662.604	-771.163	1.366
26.990.000	4.664.241	-770.676	1.365
27.000.000	4.665.939	-770.085	1.365
27.010.000	4.667.448	-769.597	1.366
27.020.000	4.669.269	-769.064	1.367
27.030.000	4.670.733	-768.600	1.369
27.040.000	4.672.465	-768.099	1.369
27.050.000	4.673.947	-767.684	1.371
27.060.000	4.675.782	-767.238	1.373
27.070.000	4.677.181	-766.773	1.373
27.080.000	4.679.015	-766.415	1.373
27.090.000	4.680.461	-765.950	1.372
27.100.000	4.682.239	-765.625	1.371
27.110.000	4.683.785	-765.246	1.371
27.120.000	4.685.524	-764.898	1.373
27.130.000	4.687.045	-764.518	1.371
27.140.000	4.688.767	-764.277	1.368
27.150.000	4.690.369	-763.848	1.367
27.160.000	4.691.973	-763.608	1.366
27.170.000	4.693.680	-763.296	1.364
27.180.000	4.695.197	-763.007	1.363
27.190.000	4.696.950	-762.722	1.363
27.200.000	4.698.433	-762.519	1.362
27.210.000	4.700.259	-762.236	1.362
27.220.000	4.701.703	-762.021	1.361
27.230.000	4.703.541	-761.822	1.360
27.240.000	4.705.001	-761.556	1.359

27.250.000	4.706.794	-761.395	1.359
27.260.000	4.708.312	-761.163	1.359
27.270.000	4.709.992	-760.975	1.360
27.280.000	4.711.520	-760.776	1.360
27.290.000	4.713.267	-760.645	1.361
27.300.000	4.714.788	-760.408	1.363
27.310.000	4.716.462	-760.297	1.365
27.320.000	4.718.142	-760.086	1.367
27.330.000	4.719.705	-759.971	1.367
27.340.000	4.721.434	-759.789	1.364
27.350.000	4.722.991	-759.658	1.362
27.360.000	4.724.726	-759.501	1.360
27.370.000	4.726.246	-759.434	1.359
27.380.000	4.728.054	-759.297	1.360
27.390.000	4.729.464	-759.172	1.360
27.400.000	4.731.307	-759.110	1.361
27.410.000	4.732.734	-758.950	1.362
27.420.000	4.734.522	-758.882	1.361
27.430.000	4.736.050	-758.776	1.358
27.440.000	4.737.763	-758.690	1.354
27.450.000	4.739.293	-758.568	1.353
27.460.000	4.741.010	-758.575	1.352
27.470.000	4.742.625	-758.391	1.352
27.480.000	4.744.246	-758.377	1.351
27.490.000	4.745.921	-758.279	1.352
27.500.000	4.747.474	-758.213	1.352
27.510.000	4.749.198	-758.108	1.353
27.520.000	4.750.701	-758.105	1.351
27.530.000	4.752.440	-757.979	1.350
27.540.000	4.753.895	-757.989	1.349

27.550.000	4.755.767	-757.948	1.346
27.560.000	4.757.149	-757.847	1.345
27.570.000	4.758.965	-757.852	1.344
27.580.000	4.760.474	-757.775	1.345
27.590.000	4.762.171	-757.733	1.346
27.600.000	4.763.669	-757.682	1.346
27.610.000	4.765.465	-757.690	1.346
27.620.000	4.766.966	-757.596	1.346
27.630.000	4.768.641	-757.636	1.347
27.640.000	4.770.269	-757.529	1.347
27.650.000	4.771.807	-757.554	1.346
27.660.000	4.773.554	-757.505	1.343
27.670.000	4.775.113	-757.503	1.342
27.680.000	4.776.794	-757.448	1.339
27.690.000	4.778.315	-757.518	1.336
27.700.000	4.780.121	-757.479	1.336
27.710.000	4.781.479	-757.491	1.336
27.720.000	4.783.363	-757.555	1.336
27.730.000	4.784.785	-757.516	1.338
27.740.000	4.786.558	-757.578	1.339
27.750.000	4.788.053	-757.609	1.338
27.760.000	4.789.778	-757.630	1.334
27.770.000	4.791.312	-757.661	1.333
27.780.000	4.793.056	-757.781	1.334
27.790.000	4.794.579	-757.717	1.335
27.800.000	4.796.258	-757.850	1.335
27.810.000	4.797.921	-757.869	1.336
27.820.000	4.799.498	-757.907	1.338
27.830.000	4.801.146	-757.945	1.340
27.840.000	4.802.729	-758.061	1.339

27.850.000	4.804.456	-758.041	1.336
27.860.000	4.805.938	-758.175	1.334
27.870.000	4.807.770	-758.247	1.334
27.880.000	4.809.175	-758.281	1.333
27.890.000	4.811.008	-758.421	1.332
27.900.000	4.812.462	-758.450	1.329
27.910.000	4.814.176	-758.541	1.326
27.920.000	4.815.716	-758.626	1.324
27.930.000	4.817.513	-758.720	1.324
27.940.000	4.818.952	-758.763	1.323
27.950.000	4.820.684	-758.925	1.323
27.960.000	4.822.284	-758.927	1.321
27.970.000	4.823.870	-759.081	1.319
27.980.000	4.825.549	-759.132	1.317
27.990.000	4.827.135	-759.242	1.315
28.000.000	4.828.811	-759.286	1.315
28.010.000	4.830.366	-759.464	1.312
28.020.000	4.832.111	-759.466	1.311
28.030.000	4.833.541	-759.604	1.310
28.040.000	4.835.408	-759.756	1.312
28.050.000	4.836.823	-759.799	1.314
28.060.000	4.838.618	-759.934	1.313
28.070.000	4.840.117	-760.038	1.313
28.080.000	4.841.859	-760.125	1.311
28.090.000	4.843.377	-760.238	1.311
28.100.000	4.845.171	-760.420	1.312
28.110.000	4.846.628	-760.435	1.310
28.120.000	4.848.361	-760.634	1.306
28.130.000	4.849.985	-760.735	1.305
28.140.000	4.851.581	-760.845	1.306

28.150.000	4.853.283	-760.951	1.307
28.160.000	4.854.871	-761.133	1.306
28.170.000	4.856.549	-761.164	1.302
28.180.000	4.858.029	-761.364	1.302
28.190.000	4.859.828	-761.461	1.303
28.200.000	4.861.252	-761.585	1.302
28.210.000	4.863.096	-761.785	1.300
28.220.000	4.864.543	-761.858	1.297
28.230.000	4.866.356	-762.018	1.296
28.240.000	4.867.800	-762.133	1.294
28.250.000	4.869.638	-762.253	1.294
28.260.000	4.871.084	-762.338	1.294
28.270.000	4.872.829	-762.536	1.294
28.280.000	4.874.364	-762.563	1.294
28.290.000	4.875.987	-762.747	1.295
28.300.000	4.877.643	-762.834	1.297
28.310.000	4.879.275	-762.970	1.296
28.320.000	4.880.892	-763.041	1.293
28.330.000	4.882.499	-763.252	1.288
28.340.000	4.884.222	-763.270	1.289
28.350.000	4.885.657	-763.463	1.290
28.360.000	4.887.534	-763.594	1.291
28.370.000	4.888.919	-763.700	1.291
28.380.000	4.890.730	-763.864	1.293
28.390.000	4.892.187	-763.996	1.295
28.400.000	4.893.945	-764.098	1.295
28.410.000	4.895.412	-764.223	1.295
28.420.000	4.897.244	-764.399	1.295
28.430.000	4.898.687	-764.451	1.294
28.440.000	4.900.447	-764.646	1.293

28.450.000	4.902.041	-764.743	1.291
28.460.000	4.903.616	-764.862	1.288
28.470.000	4.905.297	-764.987	1.285
28.480.000	4.906.947	-765.169	1.282
28.490.000	4.908.544	-765.195	1.280
28.500.000	4.910.133	-765.427	1.279
28.510.000	4.911.913	-765.489	1.277
28.520.000	4.913.305	-765.634	1.277
28.530.000	4.915.141	-765.800	1.279
28.540.000	4.916.572	-765.888	1.279
28.550.000	4.918.359	-766.040	1.278
28.560.000	4.919.831	-766.194	1.274
28.570.000	4.921.584	-766.276	1.273
28.580.000	4.923.021	-766.381	1.274
28.590.000	4.924.819	-766.567	1.276
28.600.000	4.926.333	-766.584	1.278
28.610.000	4.928.038	-766.765	1.280
28.620.000	4.929.618	-766.864	1.281
28.630.000	4.931.258	-766.970	1.283
28.640.000	4.932.867	-767.055	1.284
28.650.000	4.934.487	-767.254	1.284
28.660.000	4.936.193	-767.271	1.283
28.670.000	4.937.711	-767.469	1.282
28.680.000	4.939.553	-767.602	1.282
28.690.000	4.940.932	-767.680	1.281
28.700.000	4.942.755	-767.867	1.279
28.710.000	4.944.241	-767.990	1.277
28.720.000	4.946.014	-768.080	1.274
28.730.000	4.947.473	-768.203	1.274
28.740.000	4.949.279	-768.348	1.273

28.750.000	4.950.672	-768.373	1.270
28.760.000	4.952.453	-768.538	1.268
28.770.000	4.954.002	-768.597	1.266
28.780.000	4.955.640	-768.712	1.266
28.790.000	4.957.277	-768.786	1.265
28.800.000	4.958.916	-768.908	1.263
28.810.000	4.960.537	-768.929	1.259
28.820.000	4.962.169	-769.125	1.260
28.830.000	4.963.908	-769.132	1.262
28.840.000	4.965.329	-769.251	1.260
28.850.000	4.967.181	-769.357	1.256
28.860.000	4.968.620	-769.435	1.254
28.870.000	4.970.412	-769.557	1.255
28.880.000	4.971.867	-769.657	1.254
28.890.000	4.973.682	-769.712	1.249
28.900.000	4.975.093	-769.792	1.247
28.910.000	4.976.908	-769.940	1.249
28.920.000	4.978.382	-769.924	1.252
28.930.000	4.980.114	-770.078	1.251
28.940.000	4.981.672	-770.135	1.251
28.950.000	4.983.350	-770.206	1.253
28.960.000	4.984.921	-770.233	1.255
28.970.000	4.986.573	-770.379	1.254
28.980.000	4.988.212	-770.319	1.251
28.990.000	4.989.730	-770.481	1.249
29.000.000	4.991.560	-770.521	1.250
29.010.000	4.992.951	-770.573	1.251
29.020.000	4.994.752	-770.690	1.248
29.030.000	4.996.260	-770.792	1.245
29.040.000	4.998.061	-770.848	1.244

29.050.000	4.999.519	-770.942	1.244
29.060.000	5.001.361	-771.052	1.244
29.070.000	5.002.764	-771.083	1.240
29.080.000	5.004.538	-771.204	1.239
29.090.000	5.006.074	-771.237	1.239
29.100.000	5.007.748	-771.332	1.239
29.110.000	5.009.365	-771.381	1.236
29.120.000	5.011.004	-771.465	1.232
29.130.000	5.012.593	-771.463	1.229
29.140.000	5.014.200	-771.617	1.229
29.150.000	5.015.909	-771.589	1.231
29.160.000	5.017.419	-771.699	1.232
29.170.000	5.019.265	-771.757	1.232
29.180.000	5.020.682	-771.796	1.233
29.190.000	5.022.501	-771.866	1.235
29.200.000	5.023.914	-771.954	1.237
29.210.000	5.025.743	-771.987	1.235
29.220.000	5.027.181	-772.036	1.232
29.230.000	5.028.973	-772.168	1.229
29.240.000	5.030.453	-772.121	1.230
29.250.000	5.032.153	-772.242	1.230
29.260.000	5.033.736	-772.296	1.231
29.270.000	5.035.429	-772.350	1.229
29.280.000	5.037.005	-772.390	1.228
29.290.000	5.038.675	-772.517	1.228
29.300.000	5.040.325	-772.472	1.228
29.310.000	5.041.875	-772.622	1.227
29.320.000	5.043.679	-772.651	1.223
29.330.000	5.045.131	-772.707	1.219
29.340.000	5.046.927	-772.791	1.217

29.350.000	5.048.404	-772.871	1.216
29.360.000	5.050.157	-772.934	1.215
29.370.000	5.051.595	-773.021	1.212
29.380.000	5.053.491	-773.134	1.212
29.390.000	5.054.891	-773.146	1.212
29.400.000	5.056.678	-773.286	1.213
29.410.000	5.058.250	-773.316	1.214
29.420.000	5.059.913	-773.421	1.214
29.430.000	5.061.504	-773.492	1.213
29.440.000	5.063.216	-773.595	1.212
29.450.000	5.064.762	-773.608	1.212
29.460.000	5.066.417	-773.764	1.212
29.470.000	5.068.097	-773.763	1.210
29.480.000	5.069.573	-773.875	1.207
29.490.000	5.071.433	-773.949	1.205
29.500.000	5.072.849	-774.018	1.206
29.510.000	5.074.659	-774.086	1.208
29.520.000	5.076.127	-774.201	1.208
29.530.000	5.077.934	-774.261	1.207
29.540.000	5.079.347	-774.329	1.206
29.550.000	5.081.193	-774.476	1.206
29.560.000	5.082.610	-774.450	1.205
29.570.000	5.084.359	-774.596	1.205
29.580.000	5.085.925	-774.651	1.203
29.590.000	5.087.575	-774.716	1.200
29.600.000	5.089.169	-774.791	1.198
29.610.000	5.090.862	-774.927	1.198
29.620.000	5.092.485	-774.891	1.197
29.630.000	5.094.044	-775.042	1.197
29.640.000	5.095.781	-775.075	1.195

29.650.000	5.097.286	-775.168	1.193
29.660.000	5.099.048	-775.250	1.194
29.670.000	5.100.536	-775.361	1.195
29.680.000	5.102.297	-775.424	1.197
29.690.000	5.103.725	-775.540	1.197
29.700.000	5.105.600	-775.657	1.195
29.710.000	5.106.979	-775.692	1.195
29.720.000	5.108.835	-775.854	1.195
29.730.000	5.110.328	-775.904	1.195
29.740.000	5.112.019	-776.041	1.193
29.750.000	5.113.604	-776.121	1.189
29.760.000	5.115.344	-776.246	1.185
29.770.000	5.116.844	-776.298	1.185
29.780.000	5.118.538	-776.488	1.185
29.790.000	5.120.174	-776.500	1.185
29.800.000	5.121.718	-776.682	1.183
29.810.000	5.123.446	-776.768	1.180
29.820.000	5.124.940	-776.896	1.179
29.830.000	5.126.749	-776.996	1.176
29.840.000	5.128.228	-777.173	1.174
29.850.000	5.129.996	-777.236	1.171
29.860.000	5.131.414	-777.345	1.169
29.870.000	5.133.323	-777.530	1.167
29.880.000	5.134.717	-777.566	1.165
29.890.000	5.136.522	-777.734	1.164
29.900.000	5.138.036	-777.866	1.164
29.910.000	5.139.729	-777.964	1.163
29.920.000	5.141.303	-778.088	1.160
29.930.000	5.142.972	-778.271	1.156
29.940.000	5.144.539	-778.302	1.154

29.950.000	5.146.189	-778.517	1.155
29.960.000	5.147.879	-778.608	1.156
29.970.000	5.149.404	-778.754	1.155
29.980.000	5.151.130	-778.871	1.154
29.990.000	5.152.647	-779.038	1.154
30.000.000	5.154.412	-779.138	1.153
30.010.000	5.155.847	-779.315	1.152
30.020.000	5.157.705	-779.470	1.150
30.030.000	5.159.090	-779.568	1.148
30.040.000	5.160.889	-779.778	1.146
30.050.000	5.162.400	-779.889	1.145
30.060.000	5.164.129	-780.089	1.144
30.070.000	5.165.644	-780.216	1.144
30.080.000	5.167.416	-780.414	1.143
30.090.000	5.168.921	-780.526	1.142
30.100.000	5.170.648	-780.776	1.138
30.110.000	5.172.261	-780.859	1.134
30.120.000	5.173.835	-781.094	1.132
30.130.000	5.175.565	-781.247	1.132
30.140.000	5.177.085	-781.451	1.129
30.150.000	5.178.832	-781.583	1.127
30.160.000	5.180.324	-781.843	1.123
30.170.000	5.182.130	-781.992	1.122
30.180.000	5.183.566	-782.188	1.124
30.190.000	5.185.405	-782.439	1.124
30.200.000	5.186.842	-782.581	1.122
30.210.000	5.188.652	-782.803	1.119
30.220.000	5.190.143	-783.004	1.120
30.230.000	5.191.836	-783.185	1.122
30.240.000	5.193.376	-783.354	1.120

30.250.000	5.195.060	-783.625	1.115
30.260.000	5.196.623	-783.715	1.112
30.270.000	5.198.264	-784.001	1.111
30.280.000	5.199.953	-784.179	1.112
30.290.000	5.201.521	-784.389	1.112
30.300.000	5.203.271	-784.576	1.113
30.310.000	5.204.786	-784.849	1.113
30.320.000	5.206.543	-785.010	1.114
30.330.000	5.208.013	-785.320	1.113
30.340.000	5.209.867	-785.541	1.111
30.350.000	5.211.270	-785.742	1.110
30.360.000	5.213.068	-786.043	1.108
30.370.000	5.214.552	-786.252	1.105
30.380.000	5.216.268	-786.498	1.103
30.390.000	5.217.780	-786.748	1.102
30.400.000	5.219.579	-787.024	1.103
30.410.000	5.221.047	-787.229	1.103
30.420.000	5.222.763	-787.552	1.102
30.430.000	5.224.403	-787.717	1.100
30.440.000	5.225.942	-788.017	1.100
30.450.000	5.227.676	-788.244	1.102
30.460.000	5.229.213	-788.508	1.103
30.470.000	5.230.883	-788.705	1.101
30.480.000	5.232.434	-789.034	1.099
30.490.000	5.234.221	-789.256	1.098
30.500.000	5.235.602	-789.513	1.099
30.510.000	5.237.460	-789.832	1.100
30.520.000	5.238.871	-790.028	1.098
30.530.000	5.240.675	-790.317	1.095
30.540.000	5.242.154	-790.588	1.094

30.550.000	5.243.906	-790.833	1.095
30.560.000	5.245.386	-791.089	1.095
30.570.000	5.247.132	-791.425	1.095
30.580.000	5.248.665	-791.574	1.095
30.590.000	5.250.339	-791.928	1.093
30.600.000	5.251.974	-792.148	1.092
30.610.000	5.253.517	-792.421	1.092
30.620.000	5.255.237	-792.677	1.090
30.630.000	5.256.774	-793.003	1.087
30.640.000	5.258.504	-793.191	1.082
30.650.000	5.259.965	-793.524	1.080
30.660.000	5.261.805	-793.775	1.079
30.670.000	5.263.173	-794.030	1.078
30.680.000	5.265.020	-794.356	1.075
30.690.000	5.266.479	-794.588	1.072
30.700.000	5.268.221	-794.864	1.072
30.710.000	5.269.690	-795.119	1.072
30.720.000	5.271.468	-795.409	1.071
30.730.000	5.272.920	-795.621	1.067
30.740.000	5.274.660	-795.957	1.064
30.750.000	5.276.249	-796.148	1.063
30.760.000	5.277.858	-796.440	1.063
30.770.000	5.279.501	-796.660	1.062
30.780.000	5.281.058	-796.937	1.060
30.790.000	5.282.725	-797.121	1.058
30.800.000	5.284.258	-797.469	1.058
30.810.000	5.286.016	-797.630	1.059
30.820.000	5.287.427	-797.918	1.059
30.830.000	5.289.293	-798.237	1.056
30.840.000	5.290.705	-798.431	1.053

30.850.000	5.292.494	-798.723	1.054
30.860.000	5.293.996	-798.982	1.054
30.870.000	5.295.739	-799.214	1.052
30.880.000	5.297.202	-799.462	1.049
30.890.000	5.298.987	-799.788	1.046
30.900.000	5.300.460	-799.937	1.046
30.910.000	5.302.156	-800.291	1.043
30.920.000	5.303.777	-800.518	1.039
30.930.000	5.305.342	-800.768	1.037
30.940.000	5.307.015	-800.996	1.036
30.950.000	5.308.573	-801.334	1.038
30.960.000	5.310.309	-801.490	1.039
30.970.000	5.311.798	-801.825	1.040
30.980.000	5.313.619	-802.071	1.039
30.990.000	5.315.002	-802.318	1.040
31.000.000	5.316.833	-802.621	1.041
31.010.000	5.318.256	-802.844	1.037
31.020.000	5.319.982	-803.110	1.031
31.030.000	5.321.487	-803.379	1.030
31.040.000	5.323.250	-803.646	1.030
31.050.000	5.324.682	-803.851	1.030
31.060.000	5.326.449	-804.196	1.026
31.070.000	5.328.063	-804.379	1.024
31.080.000	5.329.669	-804.704	1.025
31.090.000	5.331.329	-804.949	1.026
31.100.000	5.332.925	-805.218	1.026
31.110.000	5.334.545	-805.435	1.023
31.120.000	5.336.113	-805.782	1.021
31.130.000	5.337.824	-805.948	1.023
31.140.000	5.339.252	-806.251	1.023

31.150.000	5.341.078	-806.545	1.024
31.160.000	5.342.464	-806.738	1.024
31.170.000	5.344.244	-807.025	1.024
31.180.000	5.345.747	-807.290	1.025
31.190.000	5.347.497	-807.507	1.026
31.200.000	5.348.994	-807.744	1.027
31.210.000	5.350.756	-808.056	1.026
31.220.000	5.352.205	-808.209	1.024
31.230.000	5.353.926	-808.526	1.023
31.240.000	5.355.521	-808.740	1.025
31.250.000	5.357.074	-808.959	1.027
31.260.000	5.358.737	-809.174	1.026
31.270.000	5.360.322	-809.485	1.023
31.280.000	5.362.005	-809.600	1.020
31.290.000	5.363.507	-809.917	1.018
31.300.000	5.365.321	-810.140	1.017
31.310.000	5.366.713	-810.354	1.015
31.320.000	5.368.538	-810.636	1.013
31.330.000	5.369.943	-810.841	1.012
31.340.000	5.371.751	-811.090	1.013
31.350.000	5.373.220	-811.340	1.015
31.360.000	5.374.987	-811.574	1.017
31.370.000	5.376.432	-811.771	1.017
31.380.000	5.378.173	-812.074	1.015
31.390.000	5.379.728	-812.244	1.013
31.400.000	5.381.354	-812.504	1.014
31.410.000	5.382.983	-812.714	1.014
31.420.000	5.384.595	-812.955	1.013
31.430.000	5.386.233	-813.138	1.011
31.440.000	5.387.788	-813.430	1.010

31.450.000	5.389.506	-813.545	1.010
31.460.000	5.390.909	-813.801	1.010
31.470.000	5.392.747	-814.054	1.008
31.480.000	5.394.135	-814.198	1.007
31.490.000	5.395.940	-814.434	1.007
31.500.000	5.397.422	-814.655	1.005
31.510.000	5.399.175	-814.832	1.003
31.520.000	5.400.639	-815.021	1.002
31.530.000	5.402.443	-815.286	1.002
31.540.000	5.403.872	-815.389	1.001
31.550.000	5.405.579	-815.649	998
31.560.000	5.407.164	-815.824	996
31.570.000	5.408.729	-815.999	996
31.580.000	5.410.354	-816.155	996
31.590.000	5.411.952	-816.434	996
31.600.000	5.413.617	-816.488	995
31.610.000	5.415.157	-816.759	993
31.620.000	5.416.934	-816.924	992
31.630.000	5.418.366	-817.083	992
31.640.000	5.420.204	-817.311	992
31.650.000	5.421.643	-817.490	992
31.660.000	5.423.387	-817.655	992
31.670.000	5.424.797	-817.861	992
31.680.000	5.426.624	-818.047	992
31.690.000	5.428.044	-818.166	992
31.700.000	5.429.792	-818.425	990
31.710.000	5.431.348	-818.535	989
31.720.000	5.432.958	-818.719	988
31.730.000	5.434.581	-818.884	986
31.740.000	5.436.229	-819.069	984

31.750.000	5.437.855	-819.186	983
31.760.000	5.439.430	-819.418	982
31.770.000	5.441.175	-819.483	981
31.780.000	5.442.584	-819.691	978
31.790.000	5.444.417	-819.868	977
31.800.000	5.445.863	-820.007	977
31.810.000	5.447.620	-820.194	977
31.820.000	5.449.115	-820.359	978
31.830.000	5.450.881	-820.495	978
31.840.000	5.452.311	-820.623	979
31.850.000	5.454.109	-820.870	979
31.860.000	5.455.631	-820.927	978
31.870.000	5.457.355	-821.149	976
31.880.000	5.458.935	-821.284	975
31.890.000	5.460.523	-821.413	975
31.900.000	5.462.126	-821.545	976
31.910.000	5.463.776	-821.747	978
31.920.000	5.465.403	-821.782	977
31.930.000	5.466.940	-822.001	977
31.940.000	5.468.739	-822.126	977
31.950.000	5.470.128	-822.221	979
31.960.000	5.471.922	-822.396	978
31.970.000	5.473.412	-822.542	976
31.980.000	5.475.166	-822.653	975
31.990.000	5.476.628	-822.774	975
32.000.000	5.478.412	-822.920	974
32.010.000	5.479.828	-822.978	974
32.020.000	5.481.603	-823.162	974
32.030.000	5.483.143	-823.194	974
32.040.000	5.484.778	-823.324	974

32.050.000	5.486.371	-823.416	973
32.060.000	5.488.054	-823.535	972
32.070.000	5.489.637	-823.578	970
32.080.000	5.491.228	-823.759	967
32.090.000	5.492.968	-823.745	965
32.100.000	5.494.422	-823.901	964
32.110.000	5.496.214	-823.995	965
32.120.000	5.497.702	-824.061	966
32.130.000	5.499.403	-824.174	967
32.140.000	5.500.918	-824.287	968
32.150.000	5.502.710	-824.349	968
32.160.000	5.504.106	-824.427	968
32.170.000	5.505.912	-824.577	967
32.180.000	5.507.359	-824.601	966
32.190.000	5.509.056	-824.751	966
32.200.000	5.510.702	-824.823	968
32.210.000	5.512.294	-824.900	968
32.220.000	5.513.898	-824.946	967
32.230.000	5.515.526	-825.108	965
32.240.000	5.517.144	-825.064	964
32.250.000	5.518.672	-825.227	964
32.260.000	5.520.440	-825.271	962
32.270.000	5.521.832	-825.312	959
32.280.000	5.523.656	-825.414	958
32.290.000	5.525.102	-825.508	957
32.300.000	5.526.889	-825.551	958
32.310.000	5.528.373	-825.639	959
32.320.000	5.530.184	-825.740	960
32.330.000	5.531.579	-825.725	960
32.340.000	5.533.322	-825.883	959

32.350.000	5.534.883	-825.902	957
32.360.000	5.536.511	-825.980	956
32.370.000	5.538.067	-826.023	954
32.380.000	5.539.737	-826.127	953
32.390.000	5.541.307	-826.118	951
32.400.000	5.542.898	-826.248	950
32.410.000	5.544.651	-826.235	950
32.420.000	5.546.124	-826.353	949
32.430.000	5.547.944	-826.385	948
32.440.000	5.549.401	-826.444	947
32.450.000	5.551.128	-826.525	947
32.460.000	5.552.582	-826.595	946
32.470.000	5.554.383	-826.633	946
32.480.000	5.555.744	-826.678	946
32.490.000	5.557.579	-826.802	947
32.500.000	5.559.000	-826.768	948
32.510.000	5.560.687	-826.862	947
32.520.000	5.562.265	-826.893	948
32.530.000	5.563.907	-826.950	949
32.540.000	5.565.455	-826.940	949
32.550.000	5.567.117	-827.063	948
32.560.000	5.568.694	-826.992	947
32.570.000	5.570.271	-827.102	946
32.580.000	5.571.990	-827.109	946
32.590.000	5.573.404	-827.119	946
32.600.000	5.575.193	-827.175	945
32.610.000	5.576.594	-827.260	946
32.620.000	5.578.366	-827.238	945
32.630.000	5.579.793	-827.299	944
32.640.000	5.581.621	-827.394	942

32.650.000	5.583.043	-827.357	941
32.660.000	5.584.778	-827.478	941
32.670.000	5.586.307	-827.507	941
32.680.000	5.587.977	-827.566	941
32.690.000	5.589.534	-827.617	942
32.700.000	5.591.174	-827.709	944
32.710.000	5.592.725	-827.697	945
32.720.000	5.594.301	-827.844	946
32.730.000	5.595.983	-827.838	944
32.740.000	5.597.446	-827.934	942
32.750.000	5.599.212	-827.994	940
32.760.000	5.600.677	-828.055	938
32.770.000	5.602.403	-828.126	938
32.780.000	5.603.824	-828.200	938
32.790.000	5.605.652	-828.267	939
32.800.000	5.607.017	-828.295	939
32.810.000	5.608.821	-828.411	939
32.820.000	5.610.256	-828.409	937
32.830.000	5.611.940	-828.540	934
32.840.000	5.613.496	-828.596	932
32.850.000	5.615.160	-828.711	927
32.860.000	5.616.692	-828.741	924
32.870.000	5.618.379	-828.923	922
32.880.000	5.619.987	-828.916	922
32.890.000	5.621.517	-829.073	922
32.900.000	5.623.235	-829.157	921
32.910.000	5.624.679	-829.238	920
32.920.000	5.626.439	-829.332	920
32.930.000	5.627.869	-829.474	921
32.940.000	5.629.612	-829.533	922

32.950.000	5.631.041	-829.643	922
32.960.000	5.632.846	-829.801	921
32.970.000	5.634.263	-829.823	921
32.980.000	5.636.033	-830.012	923
32.990.000	5.637.521	-830.102	924
33.000.000	5.639.186	-830.205	923
33.010.000	5.640.737	-830.307	919
33.020.000	5.642.394	-830.484	916
33.030.000	5.643.948	-830.509	915
33.040.000	5.645.512	-830.723	915
33.050.000	5.647.214	-830.774	914
33.060.000	5.648.650	-830.904	912
33.070.000	5.650.371	-831.001	909
33.080.000	5.651.897	-831.163	908
33.090.000	5.653.648	-831.256	907
33.100.000	5.655.060	-831.404	907
33.110.000	5.656.904	-831.536	908
33.120.000	5.658.263	-831.630	909
33.130.000	5.660.048	-831.823	909
33.140.000	5.661.497	-831.903	912
33.150.000	5.663.205	-832.073	915
33.160.000	5.664.753	-832.193	916
33.170.000	5.666.455	-832.361	913
33.180.000	5.667.946	-832.459	910
33.190.000	5.669.620	-832.686	911
33.200.000	5.671.234	-832.760	911
33.210.000	5.672.789	-832.971	908
33.220.000	5.674.494	-833.108	905
33.230.000	5.675.974	-833.279	906
33.240.000	5.677.709	-833.437	906

33.250.000	5.679.164	-833.651	905
33.260.000	5.680.936	-833.776	902
33.270.000	5.682.327	-833.938	903
33.280.000	5.684.127	-834.152	906
33.290.000	5.685.548	-834.239	906
33.300.000	5.687.289	-834.452	905
33.310.000	5.688.804	-834.610	905
33.320.000	5.690.517	-834.778	908
33.330.000	5.692.016	-834.925	910
33.340.000	5.693.706	-835.172	909
33.350.000	5.695.261	-835.246	908
33.360.000	5.696.812	-835.499	907
33.370.000	5.698.521	-835.650	909
33.380.000	5.699.981	-835.837	908
33.390.000	5.701.689	-835.993	907
33.400.000	5.703.166	-836.229	903
33.410.000	5.704.903	-836.386	901
33.420.000	5.706.325	-836.612	901
33.430.000	5.708.163	-836.834	900
33.440.000	5.709.508	-836.972	897
33.450.000	5.711.269	-837.212	895
33.460.000	5.712.735	-837.375	895
33.470.000	5.714.390	-837.571	894
33.480.000	5.715.892	-837.734	892
33.490.000	5.717.614	-837.983	890
33.500.000	5.719.084	-838.095	891
33.510.000	5.720.758	-838.366	891
33.520.000	5.722.401	-838.497	891
33.530.000	5.723.873	-838.742	893
33.540.000	5.725.602	-838.931	897

33.550.000	5.727.078	-839.161	900
33.560.000	5.728.794	-839.327	901
33.570.000	5.730.196	-839.594	901
33.580.000	5.732.036	-839.775	905
33.590.000	5.733.372	-839.968	906
33.600.000	5.735.179	-840.232	905
33.610.000	5.736.613	-840.369	905
33.620.000	5.738.315	-840.620	906
33.630.000	5.739.821	-840.825	908
33.640.000	5.741.544	-841.023	906
33.650.000	5.743.004	-841.184	903
33.660.000	5.744.711	-841.474	902
33.670.000	5.746.284	-841.560	903
33.680.000	5.747.823	-841.819	903
33.690.000	5.749.514	-841.973	903
33.700.000	5.751.006	-842.173	903
33.710.000	5.752.697	-842.333	905
33.720.000	5.754.181	-842.577	906
33.730.000	5.755.945	-842.719	905
33.740.000	5.757.338	-842.928	901
33.750.000	5.759.232	-843.152	899
33.760.000	5.760.580	-843.285	900
33.770.000	5.762.387	-843.528	900
33.780.000	5.763.868	-843.690	899
33.790.000	5.765.537	-843.886	897
33.800.000	5.767.066	-844.044	899
33.810.000	5.768.784	-844.290	903
33.820.000	5.770.296	-844.389	906
33.830.000	5.771.950	-844.630	905
33.840.000	5.773.625	-844.779	907

33.850.000	5.775.102	-844.983	910
33.860.000	5.776.895	-845.135	912
33.870.000	5.778.372	-845.343	914
33.880.000	5.780.106	-845.472	913
33.890.000	5.781.590	-845.714	912
33.900.000	5.783.419	-845.907	913
33.910.000	5.784.798	-846.052	914
33.920.000	5.786.619	-846.290	914
33.930.000	5.788.030	-846.415	912
33.940.000	5.789.779	-846.627	911
33.950.000	5.791.265	-846.788	913
33.960.000	5.792.985	-846.963	916
33.970.000	5.794.530	-847.090	920
33.980.000	5.796.213	-847.330	923
33.990.000	5.797.819	-847.391	925
34.000.000	5.799.369	-847.604	927
34.010.000	5.801.069	-847.735	931
34.020.000	5.802.570	-847.893	934
34.030.000	5.804.275	-848.011	935
34.040.000	5.805.756	-848.219	934
34.050.000	5.807.530	-848.302	932
34.060.000	5.808.933	-848.483	933
34.070.000	5.810.816	-848.671	931
34.080.000	5.812.237	-848.749	929
34.090.000	5.813.993	-848.937	926
34.100.000	5.815.492	-849.083	924
34.110.000	5.817.238	-849.221	923
34.120.000	5.818.729	-849.372	925
34.130.000	5.820.496	-849.614	927
34.140.000	5.821.981	-849.683	928

34.150.000	5.823.608	-849.912	928
34.160.000	5.825.301	-850.047	929
34.170.000	5.826.802	-850.215	933
34.180.000	5.828.514	-850.337	935
34.190.000	5.830.028	-850.565	935
34.200.000	5.831.738	-850.676	934
34.210.000	5.833.183	-850.900	934
34.220.000	5.835.043	-851.125	934
34.230.000	5.836.419	-851.261	933
34.240.000	5.838.218	-851.511	932
34.250.000	5.839.691	-851.676	931
34.260.000	5.841.403	-851.876	931
34.270.000	5.842.883	-852.058	933
34.280.000	5.844.624	-852.252	935
34.290.000	5.846.144	-852.371	937
34.300.000	5.847.835	-852.603	940
34.310.000	5.849.421	-852.688	941
34.320.000	5.850.966	-852.891	943
34.330.000	5.852.680	-853.035	944
34.340.000	5.854.241	-853.198	946
34.350.000	5.855.906	-853.307	949
34.360.000	5.857.403	-853.536	949
34.370.000	5.859.195	-853.652	949
34.380.000	5.860.534	-853.827	949
34.390.000	5.862.395	-854.049	948
34.400.000	5.863.811	-854.144	949
34.410.000	5.865.580	-854.355	949
34.420.000	5.867.088	-854.518	950
34.430.000	5.868.809	-854.679	951
34.440.000	5.870.277	-854.830	952

34.450.000	5.872.042	-855.054	952
34.460.000	5.873.559	-855.114	952
34.470.000	5.875.172	-855.356	950
34.480.000	5.876.843	-855.488	950
34.490.000	5.878.356	-855.671	949
34.500.000	5.880.074	-855.791	949
34.510.000	5.881.603	-856.044	949
34.520.000	5.883.363	-856.150	950
34.530.000	5.884.785	-856.363	951
34.540.000	5.886.607	-856.568	952
34.550.000	5.887.978	-856.680	954
34.560.000	5.889.840	-856.923	954
34.570.000	5.891.292	-857.107	954
34.580.000	5.893.038	-857.273	954
34.590.000	5.894.514	-857.438	953
34.600.000	5.896.280	-857.679	950
34.610.000	5.897.760	-857.775	946
34.620.000	5.899.438	-858.036	942
34.630.000	5.901.071	-858.171	938
34.640.000	5.902.635	-858.386	935
34.650.000	5.904.349	-858.559	933
34.660.000	5.905.878	-858.782	933
34.670.000	5.907.594	-858.905	934
34.680.000	5.909.074	-859.129	936
34.690.000	5.910.911	-859.283	940
34.700.000	5.912.234	-859.420	942
34.710.000	5.914.080	-859.634	941
34.720.000	5.915.528	-859.726	939
34.730.000	5.917.256	-859.902	939
34.740.000	5.918.764	-860.030	939

34.750.000	5.920.509	-860.167	941
34.760.000	5.921.955	-860.259	944
34.770.000	5.923.699	-860.480	949
34.780.000	5.925.305	-860.531	953
34.790.000	5.926.861	-860.709	955
34.800.000	5.928.564	-860.826	952
34.810.000	5.930.097	-861.003	948
34.820.000	5.931.753	-861.086	943
34.830.000	5.933.296	-861.320	939
34.840.000	5.935.036	-861.421	936
34.850.000	5.936.469	-861.628	938
34.860.000	5.938.310	-861.844	944
34.870.000	5.939.676	-861.949	951
34.880.000	5.941.477	-862.175	957
34.890.000	5.942.968	-862.372	962
34.900.000	5.944.720	-862.525	965
34.910.000	5.946.236	-862.700	964
34.920.000	5.947.966	-862.946	961
34.930.000	5.949.442	-863.019	956
34.940.000	5.951.111	-863.282	951
34.950.000	5.952.740	-863.429	950
34.960.000	5.954.268	-863.618	951
34.970.000	5.955.963	-863.766	956
34.980.000	5.957.479	-864.001	957
34.990.000	5.959.195	-864.116	960
35.000.000	5.960.638	-864.367	961
35.010.000	5.962.490	-864.564	960
35.020.000	5.963.860	-864.712	958
35.030.000	5.965.674	-864.941	956
35.040.000	5.967.135	-865.104	956

35.050.000	5.968.822	-865.293	957
35.060.000	5.970.311	-865.471	961
35.070.000	5.972.058	-865.687	963
35.080.000	5.973.567	-865.829	965
35.090.000	5.975.262	-866.057	967
35.100.000	5.976.872	-866.166	969
35.110.000	5.978.408	-866.399	969
35.120.000	5.980.161	-866.553	966
35.130.000	5.981.715	-866.751	965
35.140.000	5.983.380	-866.893	966
35.150.000	5.984.893	-867.151	967
35.160.000	5.986.705	-867.327	968
35.170.000	5.988.050	-867.497	969
35.180.000	5.989.921	-867.758	969
35.190.000	5.991.375	-867.874	970
35.200.000	5.993.128	-868.104	973
35.210.000	5.994.664	-868.294	975
35.220.000	5.996.386	-868.485	976
35.230.000	5.997.859	-868.637	976
35.240.000	5.999.597	-868.918	976
35.250.000	6.001.165	-869.003	976
35.260.000	6.002.737	-869.256	977
35.270.000	6.004.418	-869.410	978
35.280.000	6.005.929	-869.600	977
35.290.000	6.007.644	-869.741	977
35.300.000	6.009.119	-869.987	977
35.310.000	6.010.900	-870.106	978
35.320.000	6.012.314	-870.303	981
35.330.000	6.014.117	-870.518	982
35.340.000	6.015.503	-870.615	981

35.350.000	6.017.346	-870.859	980
