# Supplementary material for: Data on statistical experimental design to formulate amphotericin B-loaded Eudragit RL100 nanoparticles coated with hyaluronic acid for the treatment of vulvovaginal candidiasis
Source: Data Brief. 2020 Mar 5;29:105311. doi: 10.1016/j.dib.2020.105311 (PMC7082528; doi:10.1016/j.dib.2020.105311)
Supplement: Multimedia component 2 [file mmc2.pdf]

File Name:	**pure AMP**		
[Data]			
Time	Temp	**DTA**	**TGA**
sec	C	uV	mg
0	311.939	-3.640	7.861
1.2000	312.302	-3.715	7.859
2.2000	313.027	-4.034	7.857
3.2000	313.532	-4.139	7.855
4.2000	314.015	-4.265	7.852
5.2000	314.620	-4.396	7.850
6.2000	315.169	-4.624	7.847
7.2000	315.852	-4.598	7.844
8.2000	316.223	-4.839	7.840
9.2000	317.134	-4.984	7.837
10.2000	317.392	-5.031	7.836
11.2000	318.288	-5.269	7.837
12.2000	318.760	-5.395	7.838
130.000	319.590	-5.557	7.838
140.000	320.106	-5.745	7.841
150.000	321.044	-6.042	7.845
160.000	321.567	-6.156	7.847
170.000	322.386	-6.522	7.847
180.000	323.249	-6.777	7.847
190.000	323.918	-7.132	7.849
200.000	324.872	-7.433	7.847
210.000	325.590	-7.914	7.845
220.000	326.633	-8.269	7.842
230.000	327.301	-8.798	7.843
240.000	328.530	-9.354	7.844

250.000	329.113	-9.796	7.842
260.000	330.319	-10.448	7.841
270.000	331.119	-11.041	7.842
280.000	332.226	-11.656	7.843
290.000	333.052	-12.316	7.841
300.000	334.229	-13.092	7.838
310.000	335.169	-13.689	7.838
320.000	336.198	-14.536	7.837
330.000	337.377	-15.268	7.834
340.000	338.310	-16.103	7.832
350.000	339.628	-16.916	7.832
360.000	340.524	-17.817	7.832
370.000	341.834	-18.674	7.831
380.000	342.720	-19.570	7.829
390.000	344.170	-20.556	7.829
400.000	344.983	-21.405	7.828
410.000	346.424	-22.459	7.825
420.000	347.476	-23.389	7.822
430.000	348.720	-24.399	7.821
440.000	349.920	-25.380	7.822
450.000	351.255	-26.489	7.821
460.000	352.457	-27.402	7.818
470.000	353.701	-28.556	7.820
480.000	355.127	-29.535	7.822
490.000	356.094	-30.616	7.821
500.000	357.662	-31.729	7.819
510.000	358.699	-32.839	7.819
520.000	360.205	-33.907	7.820
530.000	361.300	-35.050	7.817
540.000	362.893	-36.230	7.816

550.000	363.996	-37.265	7.813
560.000	365.556	-38.513	7.813
570.000	366.838	-39.591	7.808
580.000	368.228	-40.741	7.803
590.000	369.594	-41.862	7.801
600.000	370.984	-43.087	7.797
610.000	372.386	-44.121	7.793
620.000	373.669	-45.414	7.790
630.000	375.352	-46.528	7.788
640.000	376.394	-47.678	7.785
650.000	378.140	-48.924	7.780
660.000	379.368	-50.063	7.778
670.000	380.967	-51.238	7.776
680.000	382.290	-52.417	7.772
690.000	384.015	-53.635	7.767
700.000	385.213	-54.705	7.765
710.000	386.797	-55.962	7.765
720.000	388.309	-57.052	7.764
730.000	389.684	-58.240	7.760
740.000	391.287	-59.355	7.760
750.000	392.702	-60.566	7.762
760.000	394.339	-61.622	7.760
770.000	395.657	-62.844	7.758
780.000	397.441	-64.016	7.756
790.000	398.657	-65.071	7.755
800.000	400.486	-66.278	7.752
810.000	401.843	-67.365	7.749
820.000	403.521	-68.481	7.747
830.000	404.908	-69.561	7.746
840.000	406.567	-70.701	7.742

850.000	407.982	-71.690	7.737
860.000	409.529	-72.853	7.735
870.000	411.165	-73.842	7.733
880.000	412.570	-74.956	7.728
890.000	414.274	-75.945	7.724
900.000	415.721	-77.031	7.720
910.000	417.466	-78.043	7.717
920.000	418.807	-79.081	7.713
930.000	420.687	-80.118	7.710
940.000	421.891	-81.039	7.707
950.000	423.770	-82.108	7.704
960.000	425.167	-83.035	7.703
970.000	426.834	-84.028	7.701
980.000	428.396	-84.936	7.698
990.000	430.056	-85.944	7.692
1.000.000	431.592	-86.778	7.689
1.010.000	433.229	-87.819	7.686
1.020.000	434.989	-88.626	7.683
1.030.000	436.351	-89.539	7.680
1.040.000	438.237	-90.469	7.679
1.050.000	439.661	-91.346	7.677
1.060.000	441.447	-92.185	7.675
1.070.000	442.842	-93.068	7.672
1.080.000	444.758	-93.946	7.667
1.090.000	446.143	-94.699	7.662
1.100.000	447.987	-95.634	7.658
1.110.000	449.524	-96.354	7.654
1.120.000	451.269	-97.205	7.650
1.130.000	452.932	-98.001	7.646
1.140.000	454.645	-98.793	7.643

1.150.000	456.271	-99.479	7.641
1.160.000	457.836	-100.338	7.638
1.170.000	459.770	-101.015	7.636
1.180.000	461.100	-101.755	7.633
1.190.000	463.100	-102.563	7.631
1.200.000	464.579	-103.240	7.629
1.210.000	466.424	-103.986	7.627
1.220.000	467.899	-104.675	7.623
1.230.000	469.834	-105.408	7.619
1.240.000	471.327	-106.023	7.618
1.250.000	473.192	-106.801	7.618
1.260.000	474.831	-107.382	7.615
1.270.000	476.470	-108.064	7.613
1.280.000	478.246	-108.685	7.613
1.290.000	479.895	-109.375	7.613
1.300.000	481.687	-109.893	7.609
1.310.000	483.208	-110.630	7.604
1.320.000	485.217	-111.239	7.601
1.330.000	486.624	-111.823	7.599
1.340.000	488.636	-112.491	7.597
1.350.000	490.150	-113.018	7.594
1.360.000	492.015	-113.649	7.592
1.370.000	493.600	-114.213	7.592
1.380.000	495.534	-114.818	7.592
1.390.000	497.097	-115.327	7.591
1.400.000	498.925	-115.988	7.589
1.410.000	500.669	-116.426	7.587
1.420.000	502.283	-117.050	7.587
1.430.000	504.136	-117.542	7.583
1.440.000	505.682	-118.085	7.579

1.450.000	507.584	-118.611	7.575
1.460.000	509.082	-119.181	7.573
1.470.000	511.108	-119.697	7.569
1.480.000	512.460	-120.165	7.563
1.490.000	514.474	-120.750	7.559
1.500.000	516.009	-121.176	7.557
1.510.000	517.813	-121.725	7.554
1.520.000	519.482	-122.189	7.549
1.530.000	521.301	-122.684	7.547
1.540.000	522.907	-123.087	7.545
1.550.000	524.647	-123.680	7.543
1.560.000	526.480	-124.036	7.541
1.570.000	528.019	-124.539	7.539
1.580.000	530.029	-125.031	7.538
1.590.000	531.534	-125.449	7.535
1.600.000	533.444	-125.939	7.534
1.610.000	534.972	-126.393	7.531
1.620.000	536.954	-126.834	7.528
1.630.000	538.430	-127.232	7.526
1.640.000	540.343	-127.782	7.526
1.650.000	541.961	-128.088	7.525
1.660.000	543.726	-128.583	7.523
1.670.000	545.459	-128.981	7.523
1.680.000	547.230	-129.422	7.523
1.690.000	548.993	-129.751	7.523
1.700.000	550.643	-130.273	7.522
1.710.000	552.568	-130.595	7.521
1.720.000	554.063	-131.042	7.521
1.730.000	556.068	-131.487	7.519
1.740.000	557.547	-131.844	7.517

1.750.000	559.484	-132.268	7.514
1.760.000	561.049	-132.680	7.514
1.770.000	562.995	-133.075	7.514
1.780.000	564.502	-133.415	7.511
1.790.000	566.457	-133.908	7.509
1.800.000	568.141	-134.197	7.509
1.810.000	569.887	-134.645	7.507
1.820.000	571.760	-135.017	7.505
1.830.000	573.420	-135.400	7.502
1.840.000	575.231	-135.702	7.500
1.850.000	576.842	-136.171	7.499
1.860.000	578.820	-136.484	7.497
1.870.000	580.287	-136.858	7.497
1.880.000	582.323	-137.310	7.497
1.890.000	583.812	-137.579	7.495
1.900.000	585.776	-137.996	7.493
1.910.000	587.370	-138.353	7.494
1.920.000	589.304	-138.704	7.493
1.930.000	590.908	-139.019	7.488
1.940.000	592.768	-139.476	7.486
1.950.000	594.504	-139.691	7.486
1.960.000	596.192	-140.140	7.486
1.970.000	598.087	-140.424	7.483
1.980.000	599.727	-140.773	7.479
1.990.000	601.597	-141.112	7.477
2.000.000	603.185	-141.502	7.477
2.010.000	605.190	-141.830	7.476
2.020.000	606.598	-142.158	7.472
2.030.000	608.653	-142.565	7.471
2.040.000	610.180	-142.788	7.473

2.050.000	612.027	-143.208	7.471
2.060.000	613.693	-143.513	7.469
2.070.000	615.499	-143.859	7.466
2.080.000	617.172	-144.118	7.466
2.090.000	618.930	-144.539	7.467
2.100.000	620.713	-144.730	7.464
2.110.000	622.306	-145.130	7.463
2.120.000	624.297	-145.449	7.466
2.130.000	625.779	-145.719	7.467
2.140.000	627.744	-146.081	7.465
2.150.000	629.265	-146.436	7.463
2.160.000	631.201	-146.705	7.464
2.170.000	632.744	-147.007	7.463
2.180.000	634.668	-147.400	7.461
2.190.000	636.252	-147.580	7.458
2.200.000	638.065	-147.981	7.458
2.210.000	639.769	-148.248	7.458
2.220.000	641.525	-148.575	7.457
2.230.000	643.282	-148.834	7.454
2.240.000	644.961	-149.225	7.452
2.250.000	646.897	-149.410	7.452
2.260.000	648.371	-149.761	7.450
2.270.000	650.439	-150.105	7.445
2.280.000	651.889	-150.358	7.442
2.290.000	653.841	-150.687	7.442
2.300.000	655.406	-150.995	7.444
2.310.000	657.316	-151.290	7.443
2.320.000	658.825	-151.535	7.440
2.330.000	660.786	-151.947	7.439
2.340.000	662.424	-152.130	7.441

2.350.000	664.173	-152.493	7.440
2.360.000	665.978	-152.775	7.437
2.370.000	667.651	-153.062	7.436
2.380.000	669.506	-153.298	7.437
2.390.000	671.141	-153.716	7.439
2.400.000	673.072	-153.916	7.437
2.410.000	674.533	-154.233	7.437
2.420.000	676.574	-154.616	7.438
2.430.000	678.030	-154.791	7.439
2.440.000	680.008	-155.149	7.437
2.450.000	681.609	-155.447	7.434
2.460.000	683.522	-155.724	7.434
2.470.000	685.085	-155.975	7.435
2.480.000	686.973	-156.372	7.435
2.490.000	688.678	-156.529	7.433
2.500.000	690.371	-156.935	7.432
2.510.000	692.270	-157.173	7.432
2.520.000	693.823	-157.448	7.432
2.530.000	695.742	-157.735	7.431
2.540.000	697.348	-158.085	7.429
2.550.000	699.280	-158.314	7.426
2.560.000	700.725	-158.623	7.425
2.570.000	702.815	-158.966	7.425
2.580.000	704.276	-159.141	7.424
2.590.000	706.201	-159.522	7.421
2.600.000	707.829	-159.770	7.418
2.610.000	709.596	-160.038	7.418
2.620.000	711.292	-160.280	7.419
2.630.000	713.056	-160.650	7.418
2.640.000	714.796	-160.776	7.416

2.650.000	716.365	-161.148	7.416
2.660.000	718.329	-161.430	7.416
2.670.000	719.801	-161.661	7.416
2.680.000	721.782	-161.976	7.414
2.690.000	723.269	-162.276	7.412
2.700.000	725.227	-162.508	7.411
2.710.000	726.713	-162.780	7.409
2.720.000	728.652	-163.128	7.406
2.730.000	730.194	-163.273	7.402
2.740.000	732.030	-163.683	7.399
2.750.000	733.739	-163.898	7.396
2.760.000	735.453	-164.172	7.394
2.770.000	737.190	-164.390	7.392
2.780.000	738.914	-164.752	7.392
2.790.000	740.720	-164.901	7.393
2.800.000	742.238	-165.243	7.393
2.810.000	744.265	-165.544	7.393
2.820.000	745.688	-165.752	7.396
2.830.000	747.641	-166.087	7.397
2.840.000	749.226	-166.344	7.395
2.850.000	751.080	-166.609	7.395
2.860.000	752.648	-166.855	7.395
2.870.000	754.561	-167.211	7.396
2.880.000	756.145	-167.355	7.393
2.890.000	757.933	-167.727	7.392
2.900.000	759.714	-167.950	7.393
2.910.000	761.363	-168.217	7.396
2.920.000	763.212	-168.452	7.397
2.930.000	764.821	-168.821	7.397
2.940.000	766.731	-168.986	7.399

2.950.000	768.212	-169.310	7.400
2.960.000	770.193	-169.635	7.401
2.970.000	771.669	-169.808	7.402
2.980.000	773.592	-170.152	7.403
2.990.000	775.165	-170.400	7.405
3.000.000	777.040	-170.684	7.405
3.010.000	778.650	-170.945	7.405
3.020.000	780.508	-171.297	7.406
3.030.000	782.204	-171.458	7.404
3.040.000	783.943	-171.840	7.400
3.050.000	785.834	-172.046	7.399
3.060.000	787.346	-172.342	7.398
3.070.000	789.296	-172.614	7.397
3.080.000	790.817	-172.913	7.394
3.090.000	792.721	-173.200	7.393
3.100.000	794.236	-173.468	7.396
3.110.000	796.258	-173.809	7.393
3.120.000	797.705	-174.004	7.390
3.130.000	799.666	-174.360	7.390
3.140.000	801.274	-174.589	7.391
3.150.000	803.031	-174.886	7.391
3.160.000	804.789	-175.139	7.389
3.170.000	806.529	-175.494	7.388
3.180.000	808.231	-175.635	7.391
3.190.000	809.913	-176.024	7.389
3.200.000	811.777	-176.238	7.387
3.210.000	813.213	-176.498	7.387
3.220.000	815.194	-176.825	7.387
3.230.000	816.703	-177.088	7.387
3.240.000	818.622	-177.352	7.386

3.250.000	820.128	-177.631	7.386
3.260.000	822.073	-177.951	7.386
3.270.000	823.570	-178.135	7.382
3.280.000	825.449	-178.521	7.381
3.290.000	827.110	-178.699	7.381
3.300.000	828.817	-179.006	7.380
3.310.000	830.516	-179.241	7.376
3.320.000	832.223	-179.569	7.376
3.330.000	833.970	-179.711	7.379
3.340.000	835.549	-180.088	7.380
3.350.000	837.550	-180.318	7.380
3.360.000	838.914	-180.568	7.382
3.370.000	840.913	-180.897	7.384
3.380.000	842.476	-181.131	7.385
3.390.000	844.283	-181.402	7.384
3.400.000	845.872	-181.656	7.384
3.410.000	847.756	-181.980	7.383
3.420.000	849.290	-182.138	7.379
3.430.000	851.129	-182.505	7.379
3.440.000	852.828	-182.698	7.380
3.450.000	854.444	-182.994	7.381
3.460.000	856.298	-183.225	7.380
3.470.000	857.917	-183.540	7.380
3.480.000	859.749	-183.733	7.382
3.490.000	861.299	-184.084	7.379
3.500.000	863.271	-184.351	7.377
3.510.000	864.719	-184.572	7.377
3.520.000	866.664	-184.944	7.378
3.530.000	868.215	-185.141	7.377
3.540.000	870.083	-185.441	7.375

3.550.000	871.648	-185.705	7.375
3.560.000	873.543	-186.006	7.377
3.570.000	875.135	-186.199	7.377
3.580.000	876.842	-186.568	7.376
3.590.000	878.687	-186.748	7.376
3.600.000	880.271	-187.100	7.378
3.610.000	882.173	-187.327	7.378
3.620.000	883.735	-187.617	7.377
3.630.000	885.627	-187.887	7.377
3.640.000	887.101	-188.171	7.379
3.650.000	889.097	-188.449	7.377
3.660.000	890.519	-188.683	7.374
3.670.000	892.494	-189.032	7.374
3.680.000	894.019	-189.230	7.375
3.690.000	895.838	-189.549	7.375
3.700.000	897.494	-189.801	7.373
3.710.000	899.295	-190.126	7.373
3.720.000	901.053	-190.304	7.375
3.730.000	902.711	-190.685	7.376
3.740.000	904.544	-190.842	7.375
3.750.000	906.052	-191.177	7.375
3.760.000	908.008	-191.481	7.375
3.770.000	909.439	-191.694	7.374
3.780.000	911.337	-192.000	7.372
3.790.000	912.829	-192.304	7.370
3.800.000	914.753	-192.558	7.368
3.810.000	916.251	-192.779	7.367
3.820.000	918.108	-193.158	7.367
3.830.000	919.737	-193.306	7.369
3.840.000	921.479	-193.662	7.370

3.850.000	923.197	-193.902	7.371
3.860.000	924.870	-194.165	7.372
3.870.000	926.590	-194.392	7.375
3.880.000	928.188	-194.747	7.376
3.890.000	930.084	-194.938	7.376
3.900.000	931.485	-195.232	7.376
3.910.000	933.481	-195.561	7.377
3.920.000	934.912	-195.751	7.378
3.930.000	936.800	-196.057	7.377
3.940.000	938.374	-196.332	7.375
3.950.000	940.216	-196.614	7.374
3.960.000	941.766	-196.818	7.374
3.970.000	943.586	-197.180	7.373
3.980.000	945.208	-197.328	7.371
3.990.000	946.917	-197.664	7.370
4.000.000	948.751	-197.896	7.372
4.010.000	950.292	-198.166	7.372
4.020.000	952.160	-198.405	7.368
4.030.000	953.673	-198.736	7.365
4.040.000	955.585	-198.949	7.366
4.050.000	957.011	-199.232	7.365
4.060.000	959.009	-199.576	7.362
4.070.000	960.486	-199.769	7.361
4.080.000	962.361	-200.088	7.362
4.090.000	963.907	-200.340	7.360
4.100.000	965.775	-200.633	7.359
4.110.000	967.375	-200.866	7.358
4.120.000	969.143	-201.230	7.360
4.130.000	970.881	-201.379	7.360
4.140.000	972.466	-201.763	7.358

4.150.000	974.347	-201.982	7.360
4.160.000	975.851	-202.269	7.361
4.170.000	977.809	-202.552	7.361
4.180.000	979.303	-202.876	7.358
4.190.000	981.238	-203.107	7.359
4.200.000	982.681	-203.381	7.362
4.210.000	984.647	-203.739	7.363
4.220.000	986.157	-203.950	7.363
4.230.000	987.988	-204.279	7.363
4.240.000	989.637	-204.537	7.366
4.250.000	991.387	-204.828	7.366
4.260.000	993.097	-205.064	7.364
4.270.000	994.825	-205.452	7.362
4.280.000	996.625	-205.596	7.363
4.290.000	998.191	-205.958	7.363
4.300.000	1.000.168	-206.270	7.360
4.310.000	1.001.578	-206.485	7.359
4.320.000	1.003.531	-206.817	7.360
4.330.000	1.005.005	-207.118	7.361
4.340.000	1.006.909	-207.360	7.361
4.350.000	1.008.378	-207.631	7.360
4.360.000	1.010.267	-207.995	7.361
4.370.000	1.011.783	-208.151	7.362
4.380.000	1.013.555	-208.523	7.361
4.390.000	1.015.269	-208.787	7.359
4.400.000	1.016.944	-209.056	7.359
4.410.000	1.018.665	-209.307	7.360
4.420.000	1.020.319	-209.656	7.359
4.430.000	1.022.112	-209.832	7.358
4.440.000	1.023.588	-210.186	7.358

4.450.000	1.025.596	-210.520	7.361
4.460.000	1.026.960	-210.711	7.360
4.470.000	1.028.879	-211.041	7.357
4.480.000	1.030.401	-211.320	7.357
4.490.000	1.032.206	-211.563	7.357
4.500.000	1.033.790	-211.847	7.356
4.510.000	1.035.608	-212.200	7.353
4.520.000	1.037.197	-212.344	7.352
4.530.000	1.038.928	-212.736	7.355
4.540.000	1.040.724	-212.964	7.357
4.550.000	1.042.248	-213.230	7.355
4.560.000	1.044.128	-213.489	7.355
4.570.000	1.045.678	-213.850	7.358
4.580.000	1.047.502	-214.038	7.359
4.590.000	1.048.926	-214.354	7.358
4.600.000	1.050.917	-214.682	7.356
4.610.000	1.052.357	-214.876	7.358
4.620.000	1.054.296	-215.222	7.359
4.630.000	1.055.869	-215.496	7.358
4.640.000	1.057.673	-215.765	7.357
4.650.000	1.059.214	-216.038	7.357
4.660.000	1.061.058	-216.397	7.360
4.670.000	1.062.710	-216.560	7.363
4.680.000	1.064.342	-216.946	7.364
4.690.000	1.066.222	-217.173	7.364
4.700.000	1.067.720	-217.439	7.367
4.710.000	1.069.590	-217.764	7.369
4.720.000	1.071.107	-218.068	7.370
4.730.000	1.073.060	-218.365	7.369
4.740.000	1.074.528	-218.631	7.367

4.750.000	1.076.486	-218.985	7.367
4.760.000	1.077.923	-219.167	7.368
4.770.000	1.079.817	-219.546	7.369
4.780.000	1.081.428	-219.789	7.368
4.790.000	1.083.129	-220.094	7.366
4.800.000	1.084.822	-220.346	7.365
4.810.000	1.086.548	-220.710	7.366
4.820.000	1.088.260	-220.882	7.367
4.830.000	1.089.846	-221.271	7.365
4.840.000	1.091.796	-221.529	7.363
4.850.000	1.093.271	-221.805	7.363
4.860.000	1.095.247	-222.153	7.363
4.870.000	1.096.696	-222.422	7.363
4.880.000	1.098.607	-222.717	7.360
4.890.000	1.100.134	-223.016	7.358
4.900.000	1.102.052	-223.336	7.356
4.910.000	1.103.585	-223.549	7.356
4.920.000	1.105.360	-223.920	7.357
4.930.000	1.107.010	-224.136	7.357
4.940.000	1.108.695	-224.455	7.355
4.950.000	1.110.426	-224.711	7.353
4.960.000	1.112.106	-225.055	7.353
4.970.000	1.113.824	-225.232	7.355
4.980.000	1.115.383	-225.593	7.356
4.990.000	1.117.284	-225.859	7.355
5.000.000	1.118.660	-226.125	7.354
5.010.000	1.120.629	-226.463	7.358
5.020.000	1.122.099	-226.699	7.362
5.030.000	1.123.899	-226.988	7.363
5.040.000	1.125.472	-227.252	7.361

5.050.000	1.127.323	-227.572	7.360
5.060.000	1.128.835	-227.766	7.360
5.070.000	1.130.632	-228.162	7.359
5.080.000	1.132.343	-228.346	7.359
5.090.000	1.133.917	-228.655	7.357
5.100.000	1.135.780	-228.921	7.357
5.110.000	1.137.338	-229.213	7.357
5.120.000	1.139.163	-229.451	7.360
5.130.000	1.140.635	-229.767	7.359
5.140.000	1.142.591	-230.041	7.356
5.150.000	1.143.964	-230.295	7.356
5.160.000	1.145.909	-230.663	7.356
5.170.000	1.147.440	-230.852	7.357
5.180.000	1.149.258	-231.176	7.354
5.190.000	1.150.823	-231.433	7.352
5.200.000	1.152.637	-231.730	7.353
5.210.000	1.154.213	-231.953	7.355
5.220.000	1.155.965	-232.326	7.355
5.230.000	1.157.724	-232.493	7.355
5.240.000	1.159.248	-232.851	7.354
5.250.000	1.161.161	-233.110	7.355
5.260.000	1.162.679	-233.389	7.354
5.270.000	1.164.522	-233.696	7.354
5.280.000	1.166.051	-234.013	7.353
5.290.000	1.167.975	-234.287	7.351
5.300.000	1.169.415	-234.557	7.351
5.310.000	1.171.337	-234.945	7.352
5.320.000	1.172.959	-235.145	7.354
5.330.000	1.174.653	-235.490	7.352
5.340.000	1.176.342	-235.765	7.350

5.350.000	1.178.061	-236.073	7.349
5.360.000	1.179.775	-236.298	7.348
5.370.000	1.181.429	-236.719	7.348
5.380.000	1.183.291	-236.893	7.345
5.390.000	1.184.790	-237.253	7.343
5.400.000	1.186.738	-237.580	7.344
5.410.000	1.188.187	-237.832	7.344
5.420.000	1.190.135	-238.172	7.343
5.430.000	1.191.633	-238.501	7.341
5.440.000	1.193.578	-238.777	7.342
5.450.000	1.195.083	-239.045	7.345
5.460.000	1.196.925	-239.432	7.347
5.470.000	1.198.520	-239.618	7.349
5.480.000	1.200.259	-239.982	7.349
5.490.000	1.201.983	-240.262	7.352
5.500.000	1.203.647	-240.563	7.352
5.510.000	1.205.361	-240.810	7.351
5.520.000	1.207.036	-241.211	7.348
5.530.000	1.208.877	-241.402	7.344
5.540.000	1.210.290	-241.727	7.344
5.550.000	1.212.293	-242.093	7.344
5.560.000	1.213.674	-242.312	7.343
5.570.000	1.215.607	-242.655	7.342
5.580.000	1.217.133	-242.948	7.344
5.590.000	1.218.925	-243.248	7.347
5.600.000	1.220.461	-243.488	7.350
5.610.000	1.222.279	-243.873	7.350
5.620.000	1.223.895	-244.053	7.351
5.630.000	1.225.619	-244.433	7.352
5.640.000	1.227.383	-244.689	7.353

5.650.000	1.228.906	-244.967	7.354
5.660.000	1.230.798	-245.235	7.353
5.670.000	1.232.318	-245.604	7.351
5.680.000	1.234.179	-245.833	7.351
5.690.000	1.235.616	-246.130	7.351
5.700.000	1.237.559	-246.502	7.352
5.710.000	1.239.040	-246.678	7.350
5.720.000	1.240.885	-247.054	7.349
5.730.000	1.242.463	-247.315	7.352
5.740.000	1.244.211	-247.611	7.353
5.750.000	1.245.868	-247.891	7.352
5.760.000	1.247.635	-248.263	7.350
5.770.000	1.249.300	-248.406	7.351
5.780.000	1.250.913	-248.809	7.352
5.790.000	1.252.812	-249.046	7.350
5.800.000	1.254.266	-249.338	7.348
5.810.000	1.256.157	-249.667	7.349
5.820.000	1.257.685	-249.976	7.351
5.830.000	1.259.549	-250.253	7.348
5.840.000	1.261.007	-250.536	7.345
5.850.000	1.262.987	-250.914	7.344
5.860.000	1.264.469	-251.110	7.344
5.870.000	1.266.321	-251.489	7.343
5.880.000	1.267.958	-251.761	7.341
5.890.000	1.269.676	-252.062	7.342
5.900.000	1.271.388	-252.324	7.343
5.910.000	1.273.108	-252.702	7.341
5.920.000	1.274.790	-252.865	7.342
5.930.000	1.276.375	-253.269	7.343
5.940.000	1.278.317	-253.570	7.342

5.950.000	1.279.722	-253.813	7.340
5.960.000	1.281.672	-254.173	7.338
5.970.000	1.283.220	-254.478	7.338
5.980.000	1.285.111	-254.766	7.338
5.990.000	1.286.619	-255.072	7.335
6.000.000	1.288.544	-255.426	7.334
6.010.000	1.290.034	-255.647	7.336
6.020.000	1.291.857	-256.046	7.337
6.030.000	1.293.538	-256.286	7.337
6.040.000	1.295.210	-256.598	7.338
6.050.000	1.296.934	-256.872	7.341
6.060.000	1.298.636	-257.250	7.345
6.070.000	1.300.439	-257.428	7.345
6.080.000	1.301.974	-257.816	7.345
6.090.000	1.303.952	-258.125	7.346
6.100.000	1.305.321	-258.395	7.348
6.110.000	1.307.251	-258.765	7.349
6.120.000	1.308.765	-259.016	7.348
6.130.000	1.310.572	-259.327	7.347
6.140.000	1.312.104	-259.605	7.349
6.150.000	1.313.970	-259.956	7.350
6.160.000	1.315.489	-260.169	7.350
6.170.000	1.317.226	-260.536	7.346
6.180.000	1.318.935	-260.775	7.343
6.190.000	1.320.533	-261.095	7.343
6.200.000	1.322.402	-261.367	7.344
6.210.000	1.323.950	-261.687	7.342
6.220.000	1.325.805	-261.914	7.340
6.230.000	1.327.281	-262.264	7.342
6.240.000	1.329.191	-262.556	7.343

6.250.000	1.330.608	-262.807	7.343
6.260.000	1.332.562	-263.182	7.340
6.270.000	1.334.052	-263.407	7.340
6.280.000	1.335.896	-263.733	7.340
6.290.000	1.337.450	-264.003	7.339
6.300.000	1.339.282	-264.317	7.336
6.310.000	1.340.913	-264.536	7.337
6.320.000	1.342.602	-264.919	7.338
6.330.000	1.344.361	-265.102	7.338
6.340.000	1.345.916	-265.454	7.336
6.350.000	1.347.783	-265.706	7.334
6.360.000	1.349.286	-265.992	7.337
6.370.000	1.351.171	-266.289	7.339
6.380.000	1.352.643	-266.586	7.337
6.390.000	1.354.568	-266.852	7.337
6.400.000	1.355.977	-267.094	7.339
6.410.000	1.357.907	-267.443	7.340
6.420.000	1.359.457	-267.634	7.337
6.430.000	1.361.250	-267.987	7.334
6.440.000	1.362.922	-268.232	7.334
6.450.000	1.364.640	-268.531	7.335
6.460.000	1.366.300	-268.727	7.337
6.470.000	1.367.982	-269.120	7.337
6.480.000	1.369.771	-269.262	7.336
6.490.000	1.371.275	-269.605	7.337
6.500.000	1.373.221	-269.896	7.340
6.510.000	1.374.676	-270.138	7.342
6.520.000	1.376.607	-270.442	7.339
6.530.000	1.378.149	-270.747	7.338
6.540.000	1.380.023	-270.991	7.339

6.550.000	1.381.536	-271.248	7.340
6.560.000	1.383.394	-271.601	7.339
6.570.000	1.384.973	-271.758	7.336
6.580.000	1.386.717	-272.118	7.334
6.590.000	1.388.456	-272.368	7.337
6.600.000	1.390.091	-272.648	7.340
6.610.000	1.391.850	-272.878	7.340
6.620.000	1.393.454	-273.250	7.339
6.630.000	1.395.328	-273.413	7.339
6.640.000	1.396.751	-273.756	7.340
6.650.000	1.398.814	-274.074	7.341
6.660.000	1.400.252	-274.283	7.340
6.670.000	1.402.144	-274.618	7.337
6.680.000	1.403.674	-274.882	7.337
6.690.000	1.405.469	-275.160	7.338
6.700.000	1.406.977	-275.371	7.340
6.710.000	1.408.862	-275.737	7.341
6.720.000	1.410.427	-275.889	7.340
6.730.000	1.412.119	-276.224	7.339
6.740.000	1.413.857	-276.471	7.340
6.750.000	1.415.461	-276.760	7.343
6.760.000	1.417.302	-276.990	7.345
6.770.000	1.418.892	-277.342	7.345
6.780.000	1.420.748	-277.537	7.346
6.790.000	1.422.162	-277.820	7.345
6.800.000	1.424.112	-278.165	7.345
6.810.000	1.425.567	-278.339	7.345
6.820.000	1.427.431	-278.666	7.346
6.830.000	1.428.971	-278.909	7.345
6.840.000	1.430.752	-279.174	7.343

6.850.000	1.432.337	-279.423	7.341
6.860.000	1.434.100	-279.744	7.342
6.870.000	1.435.808	-279.920	7.342
6.880.000	1.437.480	-280.289	7.339
6.890.000	1.439.269	-280.475	7.334
6.900.000	1.440.799	-280.753	7.335
6.910.000	1.442.661	-281.012	7.337
6.920.000	1.444.144	-281.311	7.336
6.930.000	1.446.032	-281.547	7.333
6.940.000	1.447.477	-281.823	7.331
6.950.000	1.449.440	-282.132	7.333
6.960.000	1.450.878	-282.315	7.333
6.970.000	1.452.741	-282.640	7.332
6.980.000	1.454.370	-282.874	7.332
6.990.000	1.456.096	-283.149	7.334
7.000.000	1.457.752	-283.374	7.338
7.010.000	1.459.456	-283.703	7.340
7.020.000	1.461.154	-283.851	7.342
7.030.000	1.462.775	-284.222	7.343
7.040.000	1.464.643	-284.446	7.343
7.050.000	1.466.089	-284.697	7.343
7.060.000	1.468.013	-285.019	7.343
7.070.000	1.469.502	-285.278	7.343
7.080.000	1.471.383	-285.513	7.341
7.090.000	1.472.900	-285.809	7.338
7.100.000	1.474.810	-286.109	7.337
7.110.000	1.476.333	-286.298	7.336
7.120.000	1.478.157	-286.676	7.336
7.130.000	1.479.786	-286.850	7.335
7.140.000	1.481.476	-287.158	7.333

7.150.000	1.483.180	-287.404	7.335
7.160.000	1.484.871	-287.728	7.338
7.170.000	1.486.629	-287.900	7.340
7.180.000	1.488.181	-288.274	7.339
7.190.000	1.490.128	-288.520	7.337
7.200.000	1.491.539	-288.816	7.337
7.210.000	1.493.554	-289.137	7.340
7.220.000	1.495.030	-289.382	7.342
7.230.000	1.496.880	-289.681	7.342
7.240.000	1.498.487	-289.957	7.341
7.250.000	1.500.303	-290.256	7.342
7.260.000	1.501.844	-290.477	7.344
7.270.000	1.503.620	-290.838	7.343
7.280.000	1.505.294	-291.052	7.342
7.290.000	1.506.913	-291.354	7.339
7.300.000	1.508.691	-291.606	7.337
7.310.000	1.510.255	-291.934	7.336
7.320.000	1.512.110	-292.123	7.336
7.330.000	1.513.653	-292.483	7.335
7.340.000	1.515.562	-292.742	7.333
7.350.000	1.516.966	-293.003	7.333
7.360.000	1.518.920	-293.348	7.334
7.370.000	1.520.363	-293.549	7.333
7.380.000	1.522.217	-293.856	7.334
7.390.000	1.523.737	-294.149	7.334
7.400.000	1.525.561	-294.408	7.335
7.410.000	1.527.084	-294.639	7.335
7.420.000	1.528.873	-295.004	7.334
7.430.000	1.530.631	-295.159	7.334
7.440.000	1.532.230	-295.516	7.336

7.450.000	1.534.010	-295.747	7.338
7.460.000	1.535.586	-296.013	7.338
7.470.000	1.537.396	-296.294	7.335
7.480.000	1.538.891	-296.607	7.333
7.490.000	1.540.814	-296.852	7.332
7.500.000	1.542.207	-297.099	7.331
7.510.000	1.544.151	-297.438	7.330
7.520.000	1.545.655	-297.626	7.329
7.530.000	1.547.430	-297.943	7.327
7.540.000	1.549.092	-298.212	7.327
7.550.000	1.550.862	-298.501	7.328
7.560.000	1.552.452	-298.720	7.329
7.570.000	1.554.187	-299.093	7.328
7.580.000	1.555.940	-299.248	7.327
7.590.000	1.557.481	-299.586	7.325
7.600.000	1.559.379	-299.848	7.325
7.610.000	1.560.814	-300.102	7.325
7.620.000	1.562.714	-300.412	7.324
7.630.000	1.564.172	-300.730	7.321
7.640.000	1.566.073	-300.956	7.319
7.650.000	1.567.567	-301.227	7.320
7.660.000	1.569.501	-301.587	7.320
7.670.000	1.571.029	-301.754	7.321
7.680.000	1.572.785	-302.112	7.321
7.690.000	1.574.416	-302.357	7.323
7.700.000	1.576.169	-302.630	7.324
7.710.000	1.577.856	-302.899	7.324
7.720.000	1.579.507	-303.255	7.323
7.730.000	1.581.311	-303.393	7.321
7.740.000	1.582.796	-303.762	7.320

7.750.000	1.584.738	-304.062	7.321
7.760.000	1.586.175	-304.300	7.322
7.770.000	1.588.099	-304.649	7.321
7.780.000	1.589.620	-304.931	7.321
7.790.000	1.591.495	-305.220	7.322
7.800.000	1.593.000	-305.486	7.324
7.810.000	1.594.914	-305.854	7.321
7.820.000	1.596.438	-306.049	7.319
7.830.000	1.598.196	-306.412	7.319
7.840.000	1.599.950	-306.685	7.319
7.850.000	1.601.547	-306.958	7.318
7.860.000	1.603.290	-307.210	7.314
7.870.000	1.604.946	-307.603	7.313
7.880.000	1.606.727	-307.791	7.315
7.890.000	1.608.267	-308.142	7.314
7.900.000	1.610.147	-308.473	7.313
7.910.000	1.611.582	-308.679	7.311
7.920.000	1.613.465	-309.035	7.310
7.930.000	1.614.980	-309.316	7.310
7.940.000	1.616.847	-309.595	7.309
7.950.000	1.618.348	-309.868	7.307
7.960.000	1.620.152	-310.221	7.303
7.970.000	1.621.725	-310.414	7.302
7.980.000	1.623.464	-310.794	7.302
7.990.000	1.625.190	-311.011	7.302
8.000.000	1.626.806	-311.336	7.301
8.010.000	1.628.555	-311.590	7.298
8.020.000	1.630.135	-311.917	7.299
8.030.000	1.631.975	-312.185	7.300
8.040.000	1.633.424	-312.502	7.299

8.050.000	1.635.389	-312.809	7.296
8.060.000	1.636.809	-313.066	7.294
8.070.000	1.638.694	-313.412	7.293
8.080.000	1.640.206	-313.653	7.292
8.090.000	1.641.987	-313.977	7.288
8.100.000	1.643.600	-314.251	7.286
8.110.000	1.645.396	-314.568	7.287
8.120.000	1.646.959	-314.800	7.289
8.130.000	1.648.697	-315.177	7.288
8.140.000	1.650.481	-315.385	7.287
8.150.000	1.651.960	-315.714	7.288
8.160.000	1.653.862	-316.014	7.288
8.170.000	1.655.343	-316.306	7.289
8.180.000	1.657.188	-316.598	7.288
8.190.000	1.658.664	-316.928	7.284
8.200.000	1.660.570	-317.226	7.282
8.210.000	1.662.067	-317.507	7.281
8.220.000	1.663.940	-317.906	7.281
8.230.000	1.665.506	-318.104	7.279
8.240.000	1.667.289	-318.478	7.276
8.250.000	1.668.929	-318.766	7.274
8.260.000	1.670.673	-319.063	7.274
8.270.000	1.672.309	-319.329	7.272
8.280.000	1.673.960	-319.753	7.269
8.290.000	1.675.786	-319.938	7.267
8.300.000	1.677.255	-320.321	7.266
8.310.000	1.679.201	-320.647	7.266
8.320.000	1.680.695	-320.924	7.266
8.330.000	1.682.562	-321.305	7.264
8.340.000	1.684.107	-321.623	7.262

8.350.000	1.685.971	-321.908	7.263
8.360.000	1.687.416	-322.197	7.262
8.370.000	1.689.315	-322.614	7.258
8.380.000	1.690.874	-322.815	7.255
8.390.000	1.692.594	-323.204	7.254
8.400.000	1.694.323	-323.504	7.254
8.410.000	1.695.982	-323.820	7.251
8.420.000	1.697.706	-324.095	7.246
8.430.000	1.699.364	-324.508	7.245
8.440.000	1.701.148	-324.733	7.246
8.450.000	1.702.682	-325.107	7.244
8.460.000	1.704.608	-325.470	7.241
8.470.000	1.706.027	-325.715	7.240
8.480.000	1.707.920	-326.098	7.241
8.490.000	1.709.416	-326.435	7.241
8.500.000	1.711.256	-326.725	7.240
8.510.000	1.712.764	-327.021	7.235
8.520.000	1.714.570	-327.431	7.234
8.530.000	1.716.164	-327.630	7.234
8.540.000	1.717.902	-328.044	7.233
8.550.000	1.719.613	-328.333	7.229
8.560.000	1.721.215	-328.670	7.228
8.570.000	1.722.973	-328.950	7.230
8.580.000	1.724.571	-329.347	7.231
8.590.000	1.726.378	-329.607	7.230
8.600.000	1.727.814	-329.968	7.226
8.610.000	1.729.723	-330.325	7.225
8.620.000	1.731.117	-330.581	7.224
8.630.000	1.733.024	-330.971	7.222
8.640.000	1.734.608	-331.269	7.218

8.650.000	1.736.349	-331.599	7.213
8.660.000	1.737.936	-331.900	7.208
8.670.000	1.739.727	-332.306	7.208
8.680.000	1.741.322	-332.517	7.205
8.690.000	1.743.023	-332.919	7.202
8.700.000	1.744.786	-333.203	7.199
8.710.000	1.746.307	-333.543	7.196
8.720.000	1.748.192	-333.864	7.195
8.730.000	1.749.668	-334.229	7.194
8.740.000	1.751.522	-334.512	7.189
8.750.000	1.752.971	-334.857	7.183
8.760.000	1.754.929	-335.228	7.182
8.770.000	1.756.362	-335.469	7.181
8.780.000	1.758.224	-335.874	7.179
8.790.000	1.759.795	-336.149	7.174
8.800.000	1.761.575	-336.485	7.172
8.810.000	1.763.139	-336.772	7.171
8.820.000	1.764.898	-337.137	7.169
8.830.000	1.766.555	-337.366	7.163
8.840.000	1.768.183	-337.798	7.159
8.850.000	1.770.031	-338.024	7.156
8.860.000	1.771.498	-338.371	7.153
8.870.000	1.773.386	-338.716	7.149
8.880.000	1.774.914	-339.033	7.144
8.890.000	1.776.750	-339.344	7.140
8.900.000	1.778.296	-339.672	7.138
8.910.000	1.780.180	-339.997	7.136
8.920.000	1.781.662	-340.243	7.132
8.930.000	1.783.525	-340.637	7.128
8.940.000	1.785.132	-340.883	7.126

8.950.000	1.786.817	-341.225	7.126
8.960.000	1.788.563	-341.509	7.124
8.970.000	1.790.214	-341.828	7.121
8.980.000	1.791.944	-342.037	7.117
8.990.000	1.793.588	-342.441	7.115
9.000.000	1.795.443	-342.656	7.113
9.010.000	1.796.916	-342.966	7.111
9.020.000	1.798.872	-343.305	7.107
9.030.000	1.800.348	-343.532	7.103
9.040.000	1.802.198	-343.830	7.100
9.050.000	1.803.720	-344.119	7.097
9.060.000	1.805.610	-344.383	7.096
9.070.000	1.807.109	-344.589	7.091
9.080.000	1.808.945	-344.946	7.085
9.090.000	1.810.570	-345.090	7.081
9.100.000	1.812.251	-345.384	7.080
9.110.000	1.813.953	-345.621	7.079
9.120.000	1.815.631	-345.863	7.078
9.130.000	1.817.400	-346.042	7.072
9.140.000	1.818.971	-346.368	7.069
9.150.000	1.820.834	-346.494	7.068
9.160.000	1.822.265	-346.751	7.066
9.170.000	1.824.209	-347.012	7.062
9.180.000	1.825.659	-347.150	7.056
9.190.000	1.827.548	-347.398	7.051
9.200.000	1.829.089	-347.581	7.049
9.210.000	1.830.919	-347.769	7.047
9.220.000	1.832.496	-347.920	7.043
9.230.000	1.834.310	-348.196	7.038
9.240.000	1.835.947	-348.258	7.034

9.250.000	1.837.622	-348.495	7.033
9.260.000	1.839.403	-348.645	7.033
9.270.000	1.840.955	-348.807	7.032
9.280.000	1.842.781	-348.924	7.030
9.290.000	1.844.346	-349.177	7.027
9.300.000	1.846.223	-349.268	7.025
9.310.000	1.847.725	-349.458	7.024
9.320.000	1.849.643	-349.668	7.022
9.330.000	1.851.091	-349.727	7.019
9.340.000	1.853.019	-349.973	7.015
9.350.000	1.854.616	-350.119	7.012
9.360.000	1.856.359	-350.244	7.009
9.370.000	1.858.019	-350.395	7.006
9.380.000	1.859.804	-350.605	7.001
9.390.000	1.861.396	-350.635	6.995
9.400.000	1.863.091	-350.857	6.991
9.410.000	1.864.882	-350.959	6.988
9.420.000	1.866.410	-351.107	6.987
9.430.000	1.868.307	-351.269	6.983
9.440.000	1.869.828	-351.436	6.981
9.450.000	1.871.700	-351.562	6.980
9.460.000	1.873.206	-351.747	6.978
9.470.000	1.875.172	-351.932	6.977
9.480.000	1.876.619	-352.008	6.976
9.490.000	1.878.509	-352.246	6.974
9.500.000	1.880.097	-352.345	6.972
9.510.000	1.881.832	-352.519	6.969
9.520.000	1.883.480	-352.640	6.969
9.530.000	1.885.230	-352.852	6.970
9.540.000	1.886.892	-352.904	6.971

9.550.000	1.888.574	-353.154	6.971
9.560.000	1.890.406	-353.254	6.969
9.570.000	1.891.927	-353.429	6.966
9.580.000	1.893.858	-353.611	6.964
9.590.000	1.895.343	-353.779	6.961
9.600.000	1.897.204	-353.943	6.956
9.610.000	1.898.759	-354.117	6.954
9.620.000	1.900.646	-354.313	6.953
9.630.000	1.902.126	-354.414	6.954
9.640.000	1.904.039	-354.687	6.954
9.650.000	1.905.622	-354.786	6.952
9.660.000	1.907.316	-354.983	6.952
9.670.000	1.908.982	-355.143	6.951
9.680.000	1.910.663	-355.349	6.947
9.690.000	1.912.421	-355.480	6.944
9.700.000	1.914.006	-355.749	6.943
9.710.000	1.915.855	-355.842	6.942
9.720.000	1.917.304	-356.063	6.940
9.730.000	1.919.209	-356.291	6.936
9.740.000	1.920.684	-356.425	6.932
9.750.000	1.922.551	-356.660	6.930
9.760.000	1.924.081	-356.853	6.931
9.770.000	1.925.929	-357.025	6.932
9.780.000	1.927.421	-357.177	6.932
9.790.000	1.929.261	-357.468	6.931
9.800.000	1.930.897	-357.542	6.930
9.810.000	1.932.543	-357.799	6.931
9.820.000	1.934.319	-357.967	6.931
9.830.000	1.935.924	-358.181	6.928
9.840.000	1.937.673	-358.318	6.925

9.850.000	1.939.277	-358.608	6.923
9.860.000	1.941.120	-358.758	6.922
9.870.000	1.942.575	-358.976	6.922
9.880.000	1.944.491	-359.238	6.921
9.890.000	1.945.933	-359.378	6.918
9.900.000	1.947.800	-359.639	6.914
9.910.000	1.949.358	-359.847	6.913
9.920.000	1.951.182	-360.053	6.913
9.930.000	1.952.712	-360.231	6.913
9.940.000	1.954.530	-360.548	6.912
9.950.000	1.956.156	-360.647	6.910
9.960.000	1.957.830	-360.925	6.910
9.970.000	1.959.602	-361.114	6.909
9.980.000	1.961.181	-361.342	6.908
9.990.000	1.962.995	-361.519	6.907
10.000.000	1.964.503	-361.812	6.904
10.010.000	1.966.424	-361.992	6.900
10.020.000	1.967.878	-362.260	6.898
10.030.000	1.969.826	-362.532	6.897
10.040.000	1.971.285	-362.681	6.894
10.050.000	1.973.126	-362.984	6.889
10.060.000	1.974.692	-363.194	6.884
10.070.000	1.976.499	-363.424	6.883
10.080.000	1.978.061	-363.638	6.883
10.090.000	1.979.879	-363.958	6.884
10.100.000	1.981.489	-364.082	6.883
10.110.000	1.983.144	-364.404	6.882
10.120.000	1.984.975	-364.594	6.881
10.130.000	1.986.486	-364.845	6.882
10.140.000	1.988.408	-365.076	6.881

10.150.000	1.989.920	-365.352	6.880
10.160.000	1.991.779	-365.556	6.879
10.170.000	1.993.255	-365.813	6.878
10.180.000	1.995.196	-366.107	6.877
10.190.000	1.996.658	-366.263	6.877
10.200.000	1.998.499	-366.591	6.878
10.210.000	2.000.113	-366.782	6.877
10.220.000	2.001.852	-367.026	6.874
10.230.000	2.003.490	-367.250	6.873
10.240.000	2.005.227	-367.510	6.871
10.250.000	2.006.879	-367.672	6.871
10.260.000	2.008.562	-368.014	6.868
10.270.000	2.010.349	-368.135	6.865
10.280.000	2.011.833	-368.407	6.862
10.290.000	2.013.712	-368.641	6.863
10.300.000	2.015.225	-368.858	6.865
10.310.000	2.017.025	-369.082	6.866
10.320.000	2.018.539	-369.325	6.864
10.330.000	2.020.485	-369.542	6.865
10.340.000	2.021.899	-369.720	6.867
10.350.000	2.023.761	-370.008	6.866
10.360.000	2.025.412	-370.160	6.864
10.370.000	2.027.110	-370.425	6.862
10.380.000	2.028.769	-370.598	6.863
10.390.000	2.030.499	-370.832	6.864
10.400.000	2.032.166	-370.978	6.861
10.410.000	2.033.819	-371.291	6.858
10.420.000	2.035.626	-371.378	6.858
10.430.000	2.037.140	-371.627	6.857
10.440.000	2.039.054	-371.865	6.857

10.450.000	2.040.497	-372.018	6.855
10.460.000	2.042.365	-372.228	6.854
10.470.000	2.043.933	-372.440	6.854
10.480.000	2.045.798	-372.600	6.854
10.490.000	2.047.292	-372.742	6.854
10.500.000	2.049.142	-373.000	6.852
10.510.000	2.050.693	-373.059	6.848
10.520.000	2.052.457	-373.310	6.844
10.530.000	2.054.200	-373.461	6.842
10.540.000	2.055.778	-373.598	6.841
10.550.000	2.057.552	-373.692	6.838
10.560.000	2.059.138	-373.935	6.834
10.570.000	2.061.009	-373.981	6.833
10.580.000	2.062.473	-374.175	6.833
10.590.000	2.064.458	-374.378	6.833
10.600.000	2.065.922	-374.441	6.829
10.610.000	2.067.791	-374.643	6.827
10.620.000	2.069.342	-374.781	6.827
10.630.000	2.071.177	-374.900	6.827
10.640.000	2.072.740	-375.029	6.825
10.650.000	2.074.565	-375.268	6.823
10.660.000	2.076.148	-375.269	6.821
10.670.000	2.077.839	-375.495	6.822
10.680.000	2.079.587	-375.619	6.822
10.690.000	2.081.192	-375.783	6.820
10.700.000	2.083.015	-375.903	6.819
10.710.000	2.084.577	-376.136	6.819
10.720.000	2.086.397	-376.229	6.820
10.730.000	2.087.903	-376.444	6.819
10.740.000	2.089.888	-376.691	6.819

10.750.000	2.091.277	-376.821	6.817
10.760.000	2.093.209	-377.075	6.818
10.770.000	2.094.764	-377.279	6.818
10.780.000	2.096.516	-377.490	6.817
10.790.000	2.098.114	-377.701	6.814
10.800.000	2.099.930	-378.009	6.814
10.810.000	2.101.559	-378.149	6.816
10.820.000	2.103.266	-378.499	6.815
10.830.000	2.105.027	-378.706	6.814
10.840.000	2.106.566	-378.984	6.815
10.850.000	2.108.385	-379.243	6.818
10.860.000	2.109.928	-379.557	6.819
10.870.000	2.111.753	-379.830	6.819
10.880.000	2.113.248	-380.161	6.819
10.890.000	2.115.165	-380.469	6.822
10.900.000	2.116.548	-380.724	6.822
10.910.000	2.118.428	-381.106	6.821
10.920.000	2.120.003	-381.360	6.818
10.930.000	2.121.776	-381.718	6.816
10.940.000	2.123.367	-382.021	6.815
10.950.000	2.125.117	-382.358	6.813
10.960.000	2.126.702	-382.645	6.810
10.970.000	2.128.429	-383.026	6.807
10.980.000	2.130.184	-383.243	6.805
10.990.000	2.131.654	-383.625	6.805
11.000.000	2.133.572	-383.941	6.804
11.010.000	2.135.068	-384.231	6.802
11.020.000	2.136.882	-384.589	6.800
11.030.000	2.138.392	-384.941	6.800
11.040.000	2.140.303	-385.247	6.801

11.050.000	2.141.734	-385.530	6.800
11.060.000	2.143.639	-385.910	6.796
11.070.000	2.145.138	-386.126	6.794
11.080.000	2.146.906	-386.506	6.793
11.090.000	2.148.547	-386.789	6.794
11.100.000	2.150.251	-387.090	6.793
11.110.000	2.151.908	-387.349	6.790
11.120.000	2.153.573	-387.759	6.789
11.130.000	2.155.303	-387.900	6.789
11.140.000	2.156.816	-388.292	6.789
11.150.000	2.158.758	-388.591	6.786
11.160.000	2.160.224	-388.835	6.781
11.170.000	2.162.045	-389.155	6.779
11.180.000	2.163.553	-389.455	6.779
11.190.000	2.165.464	-389.704	6.778
11.200.000	2.166.902	-389.985	6.774
11.210.000	2.168.793	-390.345	6.771
11.220.000	2.170.350	-390.511	6.770
11.230.000	2.172.062	-390.875	6.771
11.240.000	2.173.739	-391.114	6.770
11.250.000	2.175.409	-391.393	6.766
11.260.000	2.177.111	-391.657	6.764
11.270.000	2.178.839	-392.022	6.765
11.280.000	2.180.589	-392.206	6.764
11.290.000	2.182.060	-392.542	6.760
11.300.000	2.184.027	-392.839	6.756
11.310.000	2.185.426	-393.066	6.756
11.320.000	2.187.352	-393.396	6.757
11.330.000	2.188.904	-393.678	6.756
11.340.000	2.190.703	-393.932	6.753

11.350.000	2.192.242	-394.191	6.751
11.360.000	2.194.088	-394.549	6.753
11.370.000	2.195.609	-394.712	6.754
11.380.000	2.197.430	-395.061	6.753
11.390.000	2.199.087	-395.289	6.752
11.400.000	2.200.727	-395.555	6.751
11.410.000	2.202.500	-395.801	6.752
11.420.000	2.204.097	-396.120	6.753
11.430.000	2.205.892	-396.284	6.754
11.440.000	2.207.361	-396.617	6.751
11.450.000	2.209.270	-396.861	6.749
11.460.000	2.210.660	-397.079	6.749
11.470.000	2.212.578	-397.410	6.750
11.480.000	2.214.120	-397.626	6.750
11.490.000	2.215.910	-397.882	6.747
11.500.000	2.217.453	-398.123	6.746
11.510.000	2.219.286	-398.407	6.746
11.520.000	2.220.838	-398.582	6.747
11.530.000	2.222.566	-398.890	6.748
11.540.000	2.224.268	-399.054	6.746
11.550.000	2.225.859	-399.367	6.744
11.560.000	2.227.638	-399.537	6.744
11.570.000	2.229.211	-399.814	6.744
11.580.000	2.230.982	-400.010	6.744
11.590.000	2.232.531	-400.302	6.744
11.600.000	2.234.430	-400.521	6.745
11.610.000	2.235.824	-400.747	6.745
11.620.000	2.237.768	-401.048	6.744
11.630.000	2.239.248	-401.244	6.745
11.640.000	2.241.065	-401.494	6.744

11.650.000	2.242.639	-401.744	6.744
11.660.000	2.244.387	-401.986	6.742
11.670.000	2.245.977	-402.173	6.740
11.680.000	2.247.737	-402.503	6.739
11.690.000	2.249.428	-402.638	6.738
11.700.000	2.251.021	-402.934	6.739
11.710.000	2.252.815	-403.142	6.738
11.720.000	2.254.330	-403.377	6.737
11.730.000	2.256.165	-403.608	6.735
11.740.000	2.257.661	-403.896	6.732
11.750.000	2.259.569	-404.089	6.730
11.760.000	2.261.026	-404.332	6.727
11.770.000	2.262.919	-404.635	6.724
11.780.000	2.264.428	-404.780	6.723
11.790.000	2.266.218	-405.094	6.725
11.800.000	2.267.815	-405.304	6.727
11.810.000	2.269.557	-405.533	6.728
11.820.000	2.271.185	-405.749	6.727
11.830.000	2.272.901	-406.053	6.723
11.840.000	2.274.589	-406.169	6.719
11.850.000	2.276.177	-406.480	6.715
11.860.000	2.278.033	-406.678	6.712
11.870.000	2.279.509	-406.906	6.708
11.880.000	2.281.408	-407.165	6.705
11.890.000	2.282.888	-407.423	6.704
11.900.000	2.284.734	-407.627	6.702
11.910.000	2.286.210	-407.858	6.700
11.920.000	2.288.126	-408.155	6.698
11.930.000	2.289.619	-408.298	6.695
11.940.000	2.291.454	-408.626	6.692

11.950.000	2.293.058	-408.821	6.691
11.960.000	2.294.785	-409.066	6.690
11.970.000	2.296.486	-409.298	6.691
11.980.000	2.298.159	-409.602	6.692
11.990.000	2.299.880	-409.722	6.691
12.000.000	2.301.467	-410.051	6.689
12.010.000	2.303.318	-410.245	6.689
12.020.000	2.304.746	-410.464	6.687
12.030.000	2.306.634	-410.751	6.686
12.040.000	2.308.101	-410.970	6.683
12.050.000	2.309.980	-411.205	6.682
12.060.000	2.311.442	-411.446	6.681
12.070.000	2.313.325	-411.703	6.680
12.080.000	2.314.854	-411.866	6.678
12.090.000	2.316.609	-412.191	6.676
12.100.000	2.318.236	-412.344	6.676
12.110.000	2.319.894	-412.606	6.675
12.120.000	2.321.571	-412.808	6.674
12.130.000	2.323.228	-413.095	6.674
12.140.000	2.324.936	-413.244	6.673
12.150.000	2.326.518	-413.573	6.671
12.160.000	2.328.409	-413.757	6.667
12.170.000	2.329.827	-414.004	6.663
12.180.000	2.331.760	-414.286	6.661
12.190.000	2.333.183	-414.478	6.660
12.200.000	2.335.083	-414.737	6.659
12.210.000	2.336.573	-414.985	6.659
12.220.000	2.338.398	-415.223	6.659
12.230.000	2.339.903	-415.411	6.658
12.240.000	2.341.704	-415.748	6.655

12.250.000	2.343.275	-415.877	6.653
12.260.000	2.344.975	-416.169	6.653
12.270.000	2.346.725	-416.391	6.654
12.280.000	2.348.323	-416.628	6.654
12.290.000	2.350.094	-416.814	6.652
12.300.000	2.351.665	-417.137	6.649
12.310.000	2.353.512	-417.301	6.646
12.320.000	2.354.964	-417.553	6.645
12.330.000	2.356.857	-417.848	6.644
12.340.000	2.358.306	-418.005	6.644
12.350.000	2.360.155	-418.285	6.643
12.360.000	2.361.702	-418.518	6.643
12.370.000	2.363.508	-418.753	6.642
12.380.000	2.365.077	-418.964	6.641
12.390.000	2.366.876	-419.265	6.639
12.400.000	2.368.499	-419.402	6.638
12.410.000	2.370.161	-419.696	6.637
12.420.000	2.371.947	-419.900	6.637
12.430.000	2.373.510	-420.124	6.636
12.440.000	2.375.310	-420.324	6.636
12.450.000	2.376.839	-420.618	6.636
12.460.000	2.378.695	-420.808	6.633
12.470.000	2.380.135	-421.050	6.629
12.480.000	2.382.096	-421.344	6.624
12.490.000	2.383.534	-421.488	6.623
12.500.000	2.385.431	-421.768	6.623
12.510.000	2.387.009	-421.990	6.622
12.520.000	2.388.746	-422.215	6.619
12.530.000	2.390.386	-422.417	6.617
12.540.000	2.392.148	-422.709	6.617

12.550.000	2.393.776	-422.825	6.616
12.560.000	2.395.435	-423.143	6.614
12.570.000	2.397.243	-423.319	6.611
12.580.000	2.398.733	-423.540	6.608
12.590.000	2.400.583	-423.761	6.607
12.600.000	2.402.091	-424.014	6.606
12.610.000	2.403.990	-424.225	6.605
12.620.000	2.405.484	-424.474	6.602
12.630.000	2.407.368	-424.707	6.599
12.640.000	2.408.813	-424.889	6.598
12.650.000	2.410.691	-425.200	6.598
12.660.000	2.412.221	-425.335	6.597
12.670.000	2.413.946	-425.609	6.594
12.680.000	2.415.587	-425.801	6.591
12.690.000	2.417.318	-426.054	6.590
12.700.000	2.418.953	-426.217	6.590
12.710.000	2.420.603	-426.516	6.588
12.720.000	2.422.403	-426.669	6.584
12.730.000	2.423.903	-426.941	6.583
12.740.000	2.425.827	-427.185	6.583
12.750.000	2.427.285	-427.368	6.582
12.760.000	2.429.112	-427.626	6.580
12.770.000	2.430.604	-427.844	6.578
12.780.000	2.432.482	-428.066	6.577
12.790.000	2.433.918	-428.257	6.579
12.800.000	2.435.844	-428.562	6.578
12.810.000	2.437.394	-428.691	6.577
12.820.000	2.439.106	-428.979	6.577
12.830.000	2.440.817	-429.191	6.578
12.840.000	2.442.512	-429.413	6.578

12.850.000	2.444.188	-429.578	6.576
12.860.000	2.445.861	-429.902	6.572
12.870.000	2.447.606	-430.020	6.569
12.880.000	2.449.103	-430.306	6.568
12.890.000	2.451.073	-430.572	6.567
12.900.000	2.452.457	-430.718	6.564
12.910.000	2.454.336	-430.990	6.559
12.920.000	2.455.823	-431.231	6.558
12.930.000	2.457.661	-431.404	6.557
12.940.000	2.459.183	-431.623	6.556
12.950.000	2.461.032	-431.919	6.552
12.960.000	2.462.597	-432.043	6.548
12.970.000	2.464.331	-432.330	6.549
12.980.000	2.466.042	-432.506	6.550
12.990.000	2.467.636	-432.704	6.548
13.000.000	2.469.371	-432.901	6.544
13.010.000	2.471.020	-433.190	6.541
13.020.000	2.472.753	-433.300	6.543
13.030.000	2.474.305	-433.583	6.541
13.040.000	2.476.205	-433.829	6.538
13.050.000	2.477.606	-433.962	6.534
13.060.000	2.479.506	-434.250	6.534
13.070.000	2.481.042	-434.459	6.537
13.080.000	2.482.877	-434.661	6.535
13.090.000	2.484.396	-434.856	6.532
13.100.000	2.486.256	-435.134	6.531
13.110.000	2.487.766	-435.256	6.532
13.120.000	2.489.525	-435.543	6.530
13.130.000	2.491.196	-435.706	6.526
13.140.000	2.492.776	-435.925	6.523

13.150.000	2.494.570	-436.114	6.523
13.160.000	2.496.159	-436.396	6.523
13.170.000	2.497.952	-436.516	6.519
13.180.000	2.499.499	-436.791	6.516
13.190.000	2.501.406	-437.011	6.515
13.200.000	2.502.833	-437.180	6.515
13.210.000	2.504.738	-437.430	6.511
13.220.000	2.506.223	-437.589	6.508
13.230.000	2.508.016	-437.822	6.508
13.240.000	2.509.567	-438.030	6.510
13.250.000	2.511.375	-438.247	6.508
13.260.000	2.512.881	-438.391	6.505
13.270.000	2.514.604	-438.675	6.504
13.280.000	2.516.324	-438.792	6.504
13.290.000	2.517.952	-439.062	6.503
13.300.000	2.519.720	-439.222	6.499
13.310.000	2.521.281	-439.453	6.497
13.320.000	2.523.066	-439.633	6.499
13.330.000	2.524.563	-439.874	6.500
13.340.000	2.526.482	-440.054	6.499
13.350.000	2.527.861	-440.236	6.497
13.360.000	2.529.785	-440.506	6.497
13.370.000	2.531.263	-440.628	6.498
13.380.000	2.533.054	-440.892	6.495
13.390.000	2.534.633	-441.074	6.492
13.400.000	2.536.417	-441.300	6.490
13.410.000	2.537.989	-441.456	6.491
13.420.000	2.539.750	-441.740	6.490
13.430.000	2.541.420	-441.844	6.488
13.440.000	2.542.990	-442.099	6.487

13.450.000	2.544.815	-442.287	6.488
13.460.000	2.546.293	-442.487	6.489
13.470.000	2.548.124	-442.680	6.488
13.480.000	2.549.630	-442.937	6.485
13.490.000	2.551.478	-443.096	6.483
13.500.000	2.552.918	-443.305	6.483
13.510.000	2.554.848	-443.581	6.482
13.520.000	2.556.367	-443.690	6.478
13.530.000	2.558.169	-443.958	6.476
13.540.000	2.559.735	-444.151	6.475
13.550.000	2.561.497	-444.346	6.473
13.560.000	2.563.083	-444.536	6.469
13.570.000	2.564.804	-444.809	6.465
13.580.000	2.566.471	-444.901	6.463
13.590.000	2.568.050	-445.202	6.463
13.600.000	2.569.910	-445.365	6.463
13.610.000	2.571.349	-445.574	6.460
13.620.000	2.573.250	-445.815	6.457
13.630.000	2.574.794	-446.032	6.457
13.640.000	2.576.587	-446.232	6.456
13.650.000	2.578.109	-446.449	6.453
13.660.000	2.579.994	-446.686	6.450
13.670.000	2.581.439	-446.818	6.449
13.680.000	2.583.255	-447.103	6.450
13.690.000	2.584.880	-447.276	6.450
13.700.000	2.586.549	-447.483	6.448
13.710.000	2.588.241	-447.663	6.446
13.720.000	2.589.905	-447.925	6.445
13.730.000	2.591.596	-448.046	6.446
13.740.000	2.593.241	-448.342	6.447

13.750.000	2.595.067	-448.524	6.445
13.760.000	2.596.531	-448.725	6.445
13.770.000	2.598.461	-448.992	6.445
13.780.000	2.599.885	-449.166	6.445
13.790.000	2.601.713	-449.380	6.443
13.800.000	2.603.209	-449.620	6.439
13.810.000	2.605.071	-449.835	6.436
13.820.000	2.606.563	-450.007	6.434
13.830.000	2.608.375	-450.327	6.432
13.840.000	2.609.992	-450.441	6.432
13.850.000	2.611.690	-450.719	6.431
13.860.000	2.613.381	-450.916	6.431
13.870.000	2.615.031	-451.165	6.429
13.880.000	2.616.733	-451.312	6.431
13.890.000	2.618.304	-451.630	6.429
13.900.000	2.620.180	-451.785	6.428
13.910.000	2.621.582	-452.052	6.425
13.920.000	2.623.500	-452.311	6.423
13.930.000	2.624.997	-452.492	6.423
13.940.000	2.626.765	-452.738	6.423
13.950.000	2.628.300	-452.994	6.423
13.960.000	2.630.158	-453.215	6.420
13.970.000	2.631.651	-453.399	6.418
13.980.000	2.633.496	-453.720	6.417
13.990.000	2.635.072	-453.859	6.416
14.000.000	2.636.741	-454.132	6.414
14.010.000	2.638.457	-454.338	6.413
14.020.000	2.640.018	-454.583	6.410
14.030.000	2.641.754	-454.745	6.407
14.040.000	2.643.367	-455.060	6.406

14.050.000	2.645.170	-455.209	6.404
14.060.000	2.646.648	-455.475	6.402
14.070.000	2.648.580	-455.747	6.400
14.080.000	2.650.023	-455.916	6.398
14.090.000	2.651.902	-456.191	6.398
14.100.000	2.653.422	-456.425	6.399
14.110.000	2.655.250	-456.643	6.397
14.120.000	2.656.771	-456.843	6.394
14.130.000	2.658.532	-457.140	6.390
14.140.000	2.660.096	-457.258	6.387
14.150.000	2.661.805	-457.578	6.387
14.160.000	2.663.528	-457.738	6.385
14.170.000	2.665.117	-457.977	6.383
14.180.000	2.666.897	-458.161	6.380
14.190.000	2.668.492	-458.424	6.380
14.200.000	2.670.292	-458.601	6.381
14.210.000	2.671.748	-458.857	6.381
14.220.000	2.673.707	-459.100	6.379
14.230.000	2.675.090	-459.254	6.375
14.240.000	2.676.930	-459.546	6.374
14.250.000	2.678.507	-459.750	6.373
14.260.000	2.680.245	-459.981	6.372
14.270.000	2.681.842	-460.179	6.368
14.280.000	2.683.605	-460.458	6.365
14.290.000	2.685.188	-460.595	6.363
14.300.000	2.686.908	-460.910	6.363
14.310.000	2.688.670	-461.063	6.363
14.320.000	2.690.218	-461.320	6.361
14.330.000	2.692.007	-461.515	6.359
14.340.000	2.693.546	-461.762	6.357

14.350.000	2.695.346	-461.966	6.354
14.360.000	2.696.815	-462.224	6.353
14.370.000	2.698.737	-462.451	6.351
14.380.000	2.700.177	-462.631	6.349
14.390.000	2.702.022	-462.931	6.347
14.400.000	2.703.553	-463.072	6.346
14.410.000	2.705.382	-463.366	6.344
14.420.000	2.706.942	-463.568	6.341
14.430.000	2.708.681	-463.797	6.339
14.440.000	2.710.266	-463.971	6.336
14.450.000	2.711.949	-464.276	6.334
14.460.000	2.713.683	-464.390	6.332
14.470.000	2.715.188	-464.681	6.333
14.480.000	2.717.028	-464.877	6.334
14.490.000	2.718.523	-465.094	6.335
14.500.000	2.720.329	-465.321	6.335
14.510.000	2.721.833	-465.576	6.334
14.520.000	2.723.722	-465.778	6.332
14.530.000	2.725.143	-465.982	6.331
14.540.000	2.727.032	-466.257	6.330
14.550.000	2.728.505	-466.397	6.329
14.560.000	2.730.292	-466.687	6.327
14.570.000	2.731.905	-466.887	6.324
14.580.000	2.733.577	-467.083	6.321
14.590.000	2.735.237	-467.277	6.319
14.600.000	2.736.938	-467.581	6.318
14.610.000	2.738.620	-467.691	6.317
14.620.000	2.740.192	-467.986	6.315
14.630.000	2.742.037	-468.202	6.313
14.640.000	2.743.496	-468.400	6.312

14.650.000	2.745.373	-468.666	6.313
14.660.000	2.746.865	-468.906	6.311
14.670.000	2.748.677	-469.095	6.308
14.680.000	2.750.206	-469.321	6.305
14.690.000	2.752.016	-469.593	6.302
14.700.000	2.753.498	-469.708	6.303
14.710.000	2.755.251	-470.018	6.302
14.720.000	2.756.863	-470.198	6.299
14.730.000	2.758.591	-470.417	6.297
14.740.000	2.760.207	-470.585	6.297
14.750.000	2.761.887	-470.850	6.298
14.760.000	2.763.634	-470.983	6.295
14.770.000	2.765.185	-471.286	6.291
14.780.000	2.767.053	-471.465	6.288
14.790.000	2.768.480	-471.669	6.289
14.800.000	2.770.363	-471.951	6.288
14.810.000	2.771.852	-472.153	6.285
14.820.000	2.773.670	-472.385	6.282
14.830.000	2.775.158	-472.620	6.282
14.840.000	2.777.024	-472.876	6.283
14.850.000	2.778.527	-473.070	6.280
14.860.000	2.780.350	-473.387	6.277
14.870.000	2.781.967	-473.538	6.273
14.880.000	2.783.628	-473.816	6.271
14.890.000	2.785.342	-474.041	6.268
14.900.000	2.786.955	-474.272	6.267
14.910.000	2.788.669	-474.445	6.264
14.920.000	2.790.292	-474.760	6.261
14.930.000	2.792.082	-474.914	6.258
14.940.000	2.793.520	-475.161	6.258

14.950.000	2.795.493	-475.465	6.257
14.960.000	2.796.965	-475.628	6.254
14.970.000	2.798.803	-475.895	6.249
14.980.000	2.800.381	-476.143	6.248
14.990.000	2.802.152	-476.366	6.248
15.000.000	2.803.702	-476.563	6.249
15.010.000	2.805.466	-476.902	6.247
15.020.000	2.807.061	-477.018	6.245
15.030.000	2.808.724	-477.316	6.246
15.040.000	2.810.432	-477.513	6.246
15.050.000	2.812.009	-477.737	6.248
15.060.000	2.813.762	-477.917	6.247
15.070.000	2.815.305	-478.210	6.246
15.080.000	2.817.127	-478.355	6.245
15.090.000	2.818.602	-478.603	6.246
15.100.000	2.820.459	-478.847	6.246
15.110.000	2.821.886	-478.988	6.244
15.120.000	2.823.765	-479.246	6.244
15.130.000	2.825.302	-479.455	6.241
15.140.000	2.827.049	-479.650	6.240
15.150.000	2.828.574	-479.845	6.240
15.160.000	2.830.405	-480.143	6.239
15.170.000	2.831.962	-480.260	6.238
15.180.000	2.833.620	-480.531	6.234
15.190.000	2.835.350	-480.714	6.232
15.200.000	2.836.917	-480.940	6.232
15.210.000	2.838.713	-481.103	6.231
15.220.000	2.840.222	-481.366	6.230
15.230.000	2.842.014	-481.521	6.228
15.240.000	2.843.492	-481.764	6.227

15.250.000	2.845.416	-482.004	6.228
15.260.000	2.846.809	-482.141	6.229
15.270.000	2.848.672	-482.406	6.228
15.280.000	2.850.175	-482.582	6.226
15.290.000	2.851.945	-482.804	6.223
15.300.000	2.853.514	-482.999	6.222
15.310.000	2.855.297	-483.258	6.220
15.320.000	2.856.886	-483.397	6.218
15.330.000	2.858.574	-483.683	6.215
15.340.000	2.860.280	-483.811	6.212
15.350.000	2.861.831	-484.070	6.211
15.360.000	2.863.625	-484.269	6.210
15.370.000	2.865.127	-484.474	6.207
15.380.000	2.866.949	-484.682	6.206
15.390.000	2.868.426	-484.933	6.204
15.400.000	2.870.319	-485.129	6.201
15.410.000	2.871.729	-485.308	6.199
15.420.000	2.873.585	-485.587	6.198
15.430.000	2.875.137	-485.727	6.196
15.440.000	2.876.885	-486.002	6.192
15.450.000	2.878.464	-486.176	6.186
15.460.000	2.880.205	-486.391	6.183
15.470.000	2.881.794	-486.557	6.183
15.480.000	2.883.520	-486.859	6.181
15.490.000	2.885.214	-486.958	6.176
15.500.000	2.886.783	-487.242	6.173
15.510.000	2.888.640	-487.453	6.173
15.520.000	2.890.117	-487.669	6.171
15.530.000	2.891.932	-487.893	6.166
15.540.000	2.893.447	-488.155	6.162

15.550.000	2.895.268	-488.339	6.162
15.560.000	2.896.739	-488.564	6.162
15.570.000	2.898.610	-488.833	6.161
15.580.000	2.900.060	-488.949	6.159
15.590.000	2.901.896	-489.238	6.160
15.600.000	2.903.456	-489.448	6.160
15.610.000	2.905.159	-489.635	6.157
15.620.000	2.906.836	-489.838	6.154
15.630.000	2.908.515	-490.116	6.152
15.640.000	2.910.164	-490.246	6.150
15.650.000	2.911.784	-490.550	6.145
15.660.000	2.913.599	-490.756	6.141
15.670.000	2.915.065	-490.964	6.139
15.680.000	2.916.910	-491.238	6.137
15.690.000	2.918.383	-491.451	6.136
15.700.000	2.920.166	-491.657	6.134
15.710.000	2.921.645	-491.876	6.134
15.720.000	2.923.503	-492.117	6.136
15.730.000	2.924.977	-492.263	6.135
15.740.000	2.926.782	-492.553	6.132
15.750.000	2.928.389	-492.705	6.131
15.760.000	2.930.075	-492.961	6.131
15.770.000	2.931.759	-493.126	6.131
15.780.000	2.933.449	-493.408	6.129
15.790.000	2.935.088	-493.543	6.127
15.800.000	2.936.699	-493.839	6.127
15.810.000	2.938.497	-494.012	6.128
15.820.000	2.939.949	-494.263	6.126
15.830.000	2.941.840	-494.532	6.121
15.840.000	2.943.292	-494.723	6.117

15.850.000	2.945.079	-494.967	6.117
15.860.000	2.946.599	-495.173	6.114
15.870.000	2.948.425	-495.420	6.109
15.880.000	2.949.956	-495.586	6.107
15.890.000	2.951.766	-495.886	6.107
15.900.000	2.953.326	-495.999	6.107
15.910.000	2.955.054	-496.271	6.104
15.920.000	2.956.742	-496.481	6.102
15.930.000	2.958.364	-496.692	6.103
15.940.000	2.960.076	-496.873	6.105
15.950.000	2.961.694	-497.200	6.103
15.960.000	2.963.519	-497.327	6.101
15.970.000	2.964.958	-497.604	6.101
15.980.000	2.966.850	-497.883	6.102
15.990.000	2.968.326	-498.048	6.102
16.000.000	2.970.153	-498.309	6.100
16.010.000	2.971.680	-498.528	6.099
16.020.000	2.973.499	-498.722	6.098
16.030.000	2.974.991	-498.940	6.098
16.040.000	2.976.821	-499.215	6.097
16.050.000	2.978.388	-499.320	6.094
16.060.000	2.980.133	-499.611	6.090
16.070.000	2.981.853	-499.792	6.086
16.080.000	2.983.460	-499.994	6.082
16.090.000	2.985.169	-500.162	6.080
16.100.000	2.986.801	-500.445	6.076
16.110.000	2.988.560	-500.553	6.071
16.120.000	2.990.029	-500.819	6.067
16.130.000	2.991.977	-501.047	6.065
16.140.000	2.993.346	-501.189	6.064

16.150.000	2.995.248	-501.431	6.062
16.160.000	2.996.773	-501.615	6.057
16.170.000	2.998.551	-501.784	6.054
16.180.000	3.000.146	-501.965	6.054
16.190.000	3.001.927	-502.236	6.052
16.200.000	3.003.484	-502.297	6.049
16.210.000	3.005.232	-502.584	6.044
16.220.000	3.006.925	-502.716	6.040
16.230.000	3.008.512	-502.916	6.039
16.240.000	3.010.256	-503.075	6.037
16.250.000	3.011.850	-503.312	6.034
16.260.000	3.013.641	-503.446	6.029
16.270.000	3.015.121	-503.679	6.028
16.280.000	3.017.054	-503.870	6.026
16.290.000	3.018.461	-504.042	6.022
16.300.000	3.020.323	-504.273	6.017
16.310.000	3.021.824	-504.411	6.012
16.320.000	3.023.646	-504.629	6.009
16.330.000	3.025.205	-504.807	6.006
16.340.000	3.027.011	-505.023	6.003
16.350.000	3.028.536	-505.149	5.999
16.360.000	3.030.308	-505.433	5.994
16.370.000	3.031.978	-505.538	5.991
16.380.000	3.033.572	-505.782	5.988
16.390.000	3.035.364	-505.951	5.984
16.400.000	3.036.948	-506.166	5.982
16.410.000	3.038.727	-506.319	5.978
16.420.000	3.040.234	-506.561	5.976
16.430.000	3.042.097	-506.717	5.973
16.440.000	3.043.534	-506.897	5.970

16.450.000	3.045.458	-507.144	5.967
16.460.000	3.046.917	-507.246	5.964
16.470.000	3.048.740	-507.487	5.959
16.480.000	3.050.286	-507.660	5.957
16.490.000	3.052.018	-507.835	5.957
16.500.000	3.053.642	-507.989	5.956
16.510.000	3.055.378	-508.255	5.955
16.520.000	3.057.027	-508.320	5.951
16.530.000	3.058.655	-508.590	5.948
16.540.000	3.060.413	-508.745	5.945
16.550.000	3.061.955	-508.916	5.940
16.560.000	3.063.819	-509.101	5.937
16.570.000	3.065.330	-509.332	5.934
16.580.000	3.067.173	-509.479	5.929
16.590.000	3.068.612	-509.671	5.923
16.600.000	3.070.507	-509.911	5.920
16.610.000	3.071.968	-510.026	5.918
16.620.000	3.073.843	-510.281	5.915
16.630.000	3.075.415	-510.445	5.913
16.640.000	3.077.132	-510.626	5.912
16.650.000	3.078.749	-510.797	5.911
16.660.000	3.080.477	-511.053	5.909
16.670.000	3.082.140	-511.158	5.908
16.680.000	3.083.803	-511.439	5.905
16.690.000	3.085.572	-511.567	5.901
16.700.000	3.087.057	-511.778	5.896
16.710.000	3.088.916	-511.957	5.890
16.720.000	3.090.432	-512.173	5.888
16.730.000	3.092.224	-512.340	5.886
16.740.000	3.093.704	-512.542	5.884

16.750.000	3.095.599	-512.746	5.882
16.760.000	3.097.021	-512.881	5.880
16.770.000	3.098.887	-513.145	5.877
16.780.000	3.100.443	-513.289	5.872
16.790.000	3.102.171	-513.508	5.868
16.800.000	3.103.850	-513.698	5.865
16.810.000	3.105.542	-513.915	5.860
16.820.000	3.107.159	-514.042	5.853
16.830.000	3.108.858	-514.328	5.849
16.840.000	3.110.548	-514.429	5.847
16.850.000	3.112.120	-514.688	5.844
16.860.000	3.113.962	-514.891	5.840
16.870.000	3.115.420	-515.068	5.836
16.880.000	3.117.245	-515.299	5.834
16.890.000	3.118.744	-515.527	5.832
16.900.000	3.120.619	-515.700	5.830
16.910.000	3.122.046	-515.891	5.827
16.920.000	3.123.945	-516.169	5.822
16.930.000	3.125.444	-516.258	5.816
16.940.000	3.127.186	-516.534	5.813
16.950.000	3.128.821	-516.710	5.812
16.960.000	3.130.520	-516.904	5.810
16.970.000	3.132.146	-517.063	5.804
16.980.000	3.133.815	-517.340	5.799
16.990.000	3.135.539	-517.424	5.796
17.000.000	3.137.054	-517.700	5.793
17.010.000	3.138.967	-517.897	5.791
17.020.000	3.140.393	-518.064	5.786
17.030.000	3.142.235	-518.308	5.782
17.040.000	3.143.756	-518.522	5.780

17.050.000	3.145.585	-518.712	5.778
17.060.000	3.147.077	-518.901	5.775
17.070.000	3.148.936	-519.187	5.773
17.080.000	3.150.417	-519.297	5.769
17.090.000	3.152.200	-519.581	5.763
17.100.000	3.153.818	-519.750	5.761
17.110.000	3.155.433	-519.955	5.758
17.120.000	3.157.145	-520.121	5.755
17.130.000	3.158.786	-520.404	5.749
17.140.000	3.160.453	-520.469	5.744
17.150.000	3.162.032	-520.765	5.742
17.160.000	3.163.876	-520.930	5.740
17.170.000	3.165.297	-521.096	5.737
17.180.000	3.167.206	-521.340	5.732
17.190.000	3.168.680	-521.507	5.728
17.200.000	3.170.481	-521.706	5.727
17.210.000	3.171.970	-521.914	5.727
17.220.000	3.173.814	-522.128	5.724
17.230.000	3.175.317	-522.271	5.720
17.240.000	3.177.085	-522.541	5.716
17.250.000	3.178.721	-522.676	5.715
17.260.000	3.180.344	-522.905	5.713
17.270.000	3.182.070	-523.098	5.710
17.280.000	3.183.738	-523.322	5.704
17.290.000	3.185.400	-523.473	5.701
17.300.000	3.186.969	-523.747	5.700
17.310.000	3.188.810	-523.906	5.699
17.320.000	3.190.220	-524.126	5.697
17.330.000	3.192.162	-524.402	5.691
17.340.000	3.193.608	-524.553	5.689

17.350.000	3.195.392	-524.781	5.688
17.360.000	3.196.938	-524.997	5.685
17.370.000	3.198.754	-525.207	5.682
17.380.000	3.200.234	-525.367	5.678
17.390.000	3.202.060	-525.660	5.677
17.400.000	3.203.625	-525.750	5.676
17.410.000	3.205.258	-526.006	5.674
17.420.000	3.206.959	-526.188	5.669
17.430.000	3.208.567	-526.394	5.667
17.440.000	3.210.284	-526.544	5.665
17.450.000	3.211.865	-526.865	5.662
17.460.000	3.213.683	-526.972	5.658
17.470.000	3.215.126	-527.229	5.654
17.480.000	3.217.006	-527.487	5.653
17.490.000	3.218.463	-527.637	5.652
17.500.000	3.220.275	-527.911	5.650
17.510.000	3.221.778	-528.116	5.646
17.520.000	3.223.562	-528.314	5.644
17.530.000	3.225.076	-528.533	5.643
17.540.000	3.226.842	-528.816	5.642
17.550.000	3.228.400	-528.927	5.641
17.560.000	3.230.107	-529.232	5.639
17.570.000	3.231.842	-529.403	5.638
17.580.000	3.233.402	-529.628	5.637
17.590.000	3.235.132	-529.829	5.634
17.600.000	3.236.747	-530.117	5.628
17.610.000	3.238.511	-530.277	5.619
17.620.000	3.239.967	-530.543	5.610
17.630.000	3.241.858	-530.791	5.603
17.640.000	3.243.229	-530.968	5.597

17.650.000	3.245.125	-531.222	5.590
17.660.000	3.246.595	-531.421	5.584
17.670.000	3.248.334	-531.637	5.580
17.680.000	3.249.907	-531.836	5.579
17.690.000	3.251.679	-532.106	5.576
17.700.000	3.253.197	-532.226	5.570
17.710.000	3.254.953	-532.511	5.562
17.720.000	3.256.642	-532.670	5.556
17.730.000	3.258.207	-532.905	5.552
17.740.000	3.259.953	-533.082	5.547
17.750.000	3.261.479	-533.318	5.540
17.760.000	3.263.275	-533.499	5.535
17.770.000	3.264.734	-533.754	5.534
17.780.000	3.266.647	-533.963	5.531
17.790.000	3.268.053	-534.160	5.526
17.800.000	3.269.928	-534.434	5.520
17.810.000	3.271.427	-534.576	5.515
17.820.000	3.273.185	-534.831	5.511
17.830.000	3.274.774	-535.023	5.506
17.840.000	3.276.526	-535.236	5.499
17.850.000	3.278.071	-535.402	5.493
17.860.000	3.279.812	-535.684	5.489
17.870.000	3.281.468	-535.793	5.486
17.880.000	3.283.021	-536.079	5.480
17.890.000	3.284.817	-536.237	5.474
17.900.000	3.286.369	-536.451	5.469
17.910.000	3.288.161	-536.654	5.467
17.920.000	3.289.720	-536.907	5.464
17.930.000	3.291.569	-537.075	5.461
17.940.000	3.292.976	-537.292	5.458

17.950.000	3.294.913	-537.547	5.455
17.960.000	3.296.346	-537.667	5.453
17.970.000	3.298.146	-537.915	5.449
17.980.000	3.299.717	-538.091	5.444
17.990.000	3.301.415	-538.264	5.443
18.000.000	3.303.051	-538.424	5.441
18.010.000	3.304.784	-538.688	5.438
18.020.000	3.306.405	-538.743	5.433
18.030.000	3.308.035	-539.014	5.431
18.040.000	3.309.843	-539.142	5.429
18.050.000	3.311.328	-539.308	5.425
18.060.000	3.313.147	-539.481	5.420
18.070.000	3.314.659	-539.687	5.420
18.080.000	3.316.476	-539.806	5.420
18.090.000	3.317.934	-539.983	5.418
18.100.000	3.319.822	-540.175	5.413
18.110.000	3.321.263	-540.249	5.410
18.120.000	3.323.089	-540.481	5.409
18.130.000	3.324.662	-540.576	5.406
18.140.000	3.326.358	-540.756	5.400
18.150.000	3.327.978	-540.886	5.392
18.160.000	3.329.731	-541.081	5.386
18.170.000	3.331.351	-541.156	5.383
18.180.000	3.332.971	-541.391	5.378
18.190.000	3.334.751	-541.482	5.370
18.200.000	3.336.223	-541.661	5.361
18.210.000	3.338.047	-541.814	5.354
18.220.000	3.339.548	-541.965	5.350
18.230.000	3.341.336	-542.118	5.345
18.240.000	3.342.844	-542.293	5.341

18.250.000	3.344.688	-542.448	5.337
18.260.000	3.346.112	-542.586	5.333
18.270.000	3.347.978	-542.809	5.332
18.280.000	3.349.527	-542.879	5.333
18.290.000	3.351.188	-543.083	5.332
18.300.000	3.352.836	-543.230	5.326
18.310.000	3.354.473	-543.373	5.320
18.320.000	3.356.079	-543.481	5.315
18.330.000	3.357.769	-543.746	5.313
18.340.000	3.359.474	-543.773	5.309
18.350.000	3.360.951	-543.982	5.303
18.360.000	3.362.831	-544.155	5.299
18.370.000	3.364.262	-544.281	5.296
18.380.000	3.366.103	-544.481	5.296
18.390.000	3.367.564	-544.651	5.296
18.400.000	3.369.386	-544.779	5.293
18.410.000	3.370.835	-544.943	5.288
18.420.000	3.372.653	-545.178	5.287
18.430.000	3.374.165	-545.262	5.289
18.440.000	3.375.876	-545.499	5.286
18.450.000	3.377.482	-545.665	5.279
18.460.000	3.379.145	-545.841	5.273
18.470.000	3.380.790	-546.023	5.270
18.480.000	3.382.467	-546.279	5.268
18.490.000	3.384.154	-546.361	5.265
18.500.000	3.385.692	-546.665	5.257
18.510.000	3.387.561	-546.849	5.252
18.520.000	3.388.933	-547.018	5.246
18.530.000	3.390.788	-547.288	5.242
18.540.000	3.392.295	-547.505	5.238

18.550.000	3.394.039	-547.685	5.233
18.560.000	3.395.524	-547.901	5.230
18.570.000	3.397.391	-548.175	5.228
18.580.000	3.398.851	-548.321	5.226
18.590.000	3.400.651	-548.619	5.224
18.600.000	3.402.254	-548.812	5.221
18.610.000	3.403.894	-549.038	5.218
18.620.000	3.405.593	-549.255	5.214
18.630.000	3.407.196	-549.539	5.207
18.640.000	3.408.885	-549.681	5.203
18.650.000	3.410.435	-549.994	5.201
18.660.000	3.412.256	-550.196	5.198
18.670.000	3.413.639	-550.418	5.194
18.680.000	3.415.503	-550.713	5.191
18.690.000	3.416.966	-550.928	5.191
18.700.000	3.418.798	-551.176	5.188
18.710.000	3.420.297	-551.443	5.181
18.720.000	3.422.085	-551.670	5.175
18.730.000	3.423.542	-551.871	5.168
18.740.000	3.425.347	-552.191	5.163
18.750.000	3.426.921	-552.334	5.155
18.760.000	3.428.543	-552.610	5.150
18.770.000	3.430.251	-552.833	5.148
18.780.000	3.431.816	-553.048	5.146
18.790.000	3.433.530	-553.233	5.144
18.800.000	3.435.092	-553.529	5.138
18.810.000	3.436.921	-553.683	5.131
18.820.000	3.438.332	-553.950	5.123
18.830.000	3.440.239	-554.226	5.116
18.840.000	3.441.689	-554.402	5.107

18.850.000	3.443.475	-554.679	5.098
18.860.000	3.444.991	-554.906	5.090
18.870.000	3.446.765	-555.130	5.085
18.880.000	3.448.221	-555.326	5.079
18.890.000	3.450.020	-555.646	5.075
18.900.000	3.451.586	-555.793	5.072
18.910.000	3.453.254	-556.073	5.068
18.920.000	3.454.970	-556.269	5.065
18.930.000	3.456.520	-556.489	5.063
18.940.000	3.458.277	-556.696	5.061
18.950.000	3.459.860	-556.985	5.061
18.960.000	3.461.607	-557.115	5.061
18.970.000	3.463.075	-557.389	5.060
18.980.000	3.464.942	-557.656	5.057
18.990.000	3.466.340	-557.802	5.053
19.000.000	3.468.183	-558.071	5.050
19.010.000	3.469.671	-558.273	5.048
19.020.000	3.471.430	-558.476	5.047
19.030.000	3.472.979	-558.689	5.043
19.040.000	3.474.811	-558.956	5.037
19.050.000	3.476.333	-559.077	5.031
19.060.000	3.478.034	-559.394	5.028
19.070.000	3.479.723	-559.530	5.024
19.080.000	3.481.265	-559.773	5.019
19.090.000	3.483.015	-559.958	5.012
19.100.000	3.484.596	-560.226	5.006
19.110.000	3.486.359	-560.395	5.001
19.120.000	3.487.809	-560.662	4.998
19.130.000	3.489.714	-560.889	4.994
19.140.000	3.491.121	-561.124	4.989

19.150.000	3.493.005	-561.392	4.984
19.160.000	3.494.517	-561.568	4.984
19.170.000	3.496.272	-561.842	4.985
19.180.000	3.497.828	-562.066	4.986
19.190.000	3.499.596	-562.302	4.986
19.200.000	3.501.108	-562.469	4.986
19.210.000	3.502.836	-562.785	4.986
19.220.000	3.504.502	-562.917	4.985
19.230.000	3.506.058	-563.181	4.982
19.240.000	3.507.846	-563.379	4.980
19.250.000	3.509.352	-563.620	4.976
19.260.000	3.511.146	-563.830	4.972
19.270.000	3.512.671	-564.125	4.969
19.280.000	3.514.531	-564.329	4.966
19.290.000	3.515.933	-564.571	4.962
19.300.000	3.517.813	-564.898	4.960
19.310.000	3.519.268	-565.049	4.955
19.320.000	3.521.050	-565.352	4.952
19.330.000	3.522.617	-565.595	4.947
19.340.000	3.524.352	-565.821	4.943
19.350.000	3.525.874	-566.027	4.940
19.360.000	3.527.634	-566.346	4.938
19.370.000	3.529.301	-566.461	4.937
19.380.000	3.530.904	-566.777	4.935
19.390.000	3.532.675	-566.951	4.934
19.400.000	3.534.209	-567.182	4.933
19.410.000	3.535.982	-567.406	4.933
19.420.000	3.537.484	-567.673	4.932
19.430.000	3.539.251	-567.863	4.931
19.440.000	3.540.712	-568.131	4.928

19.450.000	3.542.593	-568.398	4.924
19.460.000	3.544.018	-568.552	4.922
19.470.000	3.545.854	-568.840	4.920
19.480.000	3.547.429	-569.049	4.920
19.490.000	3.549.163	-569.273	4.918
19.500.000	3.550.722	-569.460	4.917
19.510.000	3.552.472	-569.747	4.918
19.520.000	3.554.008	-569.860	4.918
19.530.000	3.555.682	-570.161	4.918
19.540.000	3.557.440	-570.332	4.917
19.550.000	3.558.911	-570.570	4.915
19.560.000	3.560.748	-570.777	4.912
19.570.000	3.562.226	-571.027	4.911
19.580.000	3.564.032	-571.218	4.912
19.590.000	3.565.521	-571.473	4.913
19.600.000	3.567.406	-571.703	4.914
19.610.000	3.568.855	-571.877	4.914
19.620.000	3.570.659	-572.162	4.915
19.630.000	3.572.212	-572.332	4.916
19.640.000	3.573.936	-572.607	4.917
19.650.000	3.575.516	-572.825	4.916
19.660.000	3.577.228	-573.067	4.915
19.670.000	3.578.831	-573.245	4.913
19.680.000	3.580.481	-573.563	4.911
19.690.000	3.582.219	-573.681	4.909
19.700.000	3.583.740	-573.986	4.908
19.710.000	3.585.603	-574.208	4.909
19.720.000	3.587.089	-574.418	4.908
19.730.000	3.588.842	-574.668	4.906
19.740.000	3.590.365	-574.926	4.905

19.750.000	3.592.191	-575.142	4.905
19.760.000	3.593.659	-575.372	4.905
19.770.000	3.595.511	-575.657	4.902
19.780.000	3.596.984	-575.803	4.899
19.790.000	3.598.726	-576.100	4.895
19.800.000	3.600.353	-576.306	4.894
19.810.000	3.602.031	-576.548	4.893
19.820.000	3.603.677	-576.743	4.892
19.830.000	3.605.382	-577.052	4.890
19.840.000	3.607.032	-577.162	4.888
19.850.000	3.608.579	-577.453	4.888
19.860.000	3.610.402	-577.669	4.890
19.870.000	3.611.842	-577.861	4.891
19.880.000	3.613.653	-578.107	4.892
19.890.000	3.615.153	-578.357	4.891
19.900.000	3.616.931	-578.527	4.888
19.910.000	3.618.403	-578.767	4.887
19.920.000	3.620.244	-579.010	4.888
19.930.000	3.621.716	-579.136	4.888
19.940.000	3.623.483	-579.409	4.886
19.950.000	3.625.083	-579.595	4.883
19.960.000	3.626.747	-579.810	4.883
19.970.000	3.628.383	-579.994	4.886
19.980.000	3.630.043	-580.242	4.889
19.990.000	3.631.701	-580.357	4.889
20.000.000	3.633.256	-580.658	4.886
20.010.000	3.635.074	-580.794	4.884
20.020.000	3.636.485	-581.009	4.886
20.030.000	3.638.352	-581.263	4.889
20.040.000	3.639.868	-581.462	4.891

20.050.000	3.641.654	-581.667	4.891
20.060.000	3.643.138	-581.885	4.891
20.070.000	3.645.018	-582.123	4.896
20.080.000	3.646.458	-582.267	4.904
20.090.000	3.648.251	-582.563	4.908
20.100.000	3.649.839	-582.696	4.905
20.110.000	3.651.486	-582.953	4.905
20.120.000	3.653.121	-583.128	4.910
20.130.000	3.654.772	-583.352	4.917
20.140.000	3.656.433	-583.503	4.916
20.150.000	3.658.077	-583.822	4.910
20.160.000	3.659.870	-583.948	4.912
20.170.000	3.661.308	-584.195	4.917
20.180.000	3.663.213	-584.445	4.913
20.190.000	3.664.643	-584.601	4.904
20.200.000	3.666.469	-584.828	4.899
20.210.000	3.667.962	-585.028	4.901
20.220.000	3.669.753	-585.204	4.902
20.230.000	3.671.235	-585.389	4.896
20.240.000	3.673.056	-585.669	4.889
20.250.000	3.674.598	-585.767	4.889
20.260.000	3.676.289	-586.060	4.893
20.270.000	3.678.013	-586.257	4.893
20.280.000	3.679.660	-586.467	4.888
20.290.000	3.681.304	-586.645	4.883
20.300.000	3.682.929	-586.955	4.886
20.310.000	3.684.666	-587.078	4.892
20.320.000	3.686.135	-587.368	4.889
20.330.000	3.688.021	-587.608	4.883
20.340.000	3.689.455	-587.769	4.880

20.350.000	3.691.282	-588.038	4.888
20.360.000	3.692.775	-588.248	4.894
20.370.000	3.694.577	-588.440	4.892
20.380.000	3.696.120	-588.649	4.887
20.390.000	3.697.939	-588.911	4.890
20.400.000	3.699.469	-589.032	4.897
20.410.000	3.701.165	-589.287	4.897
20.420.000	3.702.830	-589.456	4.888
20.430.000	3.704.386	-589.641	4.884
20.440.000	3.706.103	-589.800	4.887
20.450.000	3.707.708	-590.066	4.891
20.460.000	3.709.410	-590.159	4.884
20.470.000	3.710.927	-590.387	4.874
20.480.000	3.712.807	-590.600	4.869
20.490.000	3.714.240	-590.749	4.870
20.500.000	3.716.104	-590.990	4.868
20.510.000	3.717.578	-591.121	4.861
20.520.000	3.719.358	-591.286	4.854
20.530.000	3.720.866	-591.437	4.853
20.540.000	3.722.652	-591.637	4.856
20.550.000	3.724.168	-591.748	4.855
20.560.000	3.725.882	-592.006	4.849
20.570.000	3.727.529	-592.097	4.841
20.580.000	3.729.129	-592.321	4.838
20.590.000	3.730.849	-592.491	4.842
20.600.000	3.732.445	-592.698	4.842
20.610.000	3.734.195	-592.860	4.838
20.620.000	3.735.726	-593.130	4.833
20.630.000	3.737.573	-593.288	4.833
20.640.000	3.738.928	-593.470	4.837

20.650.000	3.740.803	-593.738	4.836
20.660.000	3.742.248	-593.859	4.829
20.670.000	3.744.029	-594.101	4.826
20.680.000	3.745.557	-594.260	4.828
20.690.000	3.747.336	-594.454	4.832
20.700.000	3.748.836	-594.604	4.831
20.710.000	3.750.610	-594.872	4.825
20.720.000	3.752.215	-594.945	4.822
20.730.000	3.753.842	-595.177	4.825
20.740.000	3.755.578	-595.303	4.824
20.750.000	3.757.130	-595.493	4.820
20.760.000	3.758.853	-595.590	4.814
20.770.000	3.760.407	-595.811	4.815
20.780.000	3.762.184	-595.917	4.820
20.790.000	3.763.642	-596.095	4.817
20.800.000	3.765.527	-596.308	4.812
20.810.000	3.766.969	-596.428	4.811
20.820.000	3.768.783	-596.666	4.818
20.830.000	3.770.328	-596.859	4.822
20.840.000	3.772.061	-597.021	4.821
20.850.000	3.773.599	-597.177	4.819
20.860.000	3.775.367	-597.434	4.820
20.870.000	3.776.951	-597.516	4.824
20.880.000	3.778.590	-597.783	4.823
20.890.000	3.780.307	-597.914	4.816
20.900.000	3.781.844	-598.116	4.810
20.910.000	3.783.577	-598.276	4.812
20.920.000	3.785.145	-598.498	4.812
20.930.000	3.786.939	-598.639	4.807
20.940.000	3.788.394	-598.845	4.798

20.950.000	3.790.302	-599.045	4.797
20.960.000	3.791.699	-599.156	4.803
20.970.000	3.793.546	-599.371	4.805
20.980.000	3.795.074	-599.498	4.803
20.990.000	3.796.781	-599.666	4.799
21.000.000	3.798.342	-599.789	4.797
21.010.000	3.800.119	-599.964	4.798
21.020.000	3.801.614	-600.014	4.794
21.030.000	3.803.311	-600.241	4.788
21.040.000	3.805.011	-600.316	4.782
21.050.000	3.806.537	-600.528	4.779
21.060.000	3.808.353	-600.677	4.782
21.070.000	3.809.854	-600.888	4.787
21.080.000	3.811.614	-601.044	4.791
21.090.000	3.813.119	-601.285	4.790
21.100.000	3.814.935	-601.453	4.789
21.110.000	3.816.357	-601.626	4.787
21.120.000	3.818.204	-601.885	4.784
21.130.000	3.819.670	-602.001	4.782
21.140.000	3.821.430	-602.240	4.779
21.150.000	3.823.018	-602.406	4.777
21.160.000	3.824.715	-602.592	4.777
21.170.000	3.826.289	-602.729	4.777
21.180.000	3.827.988	-602.982	4.775
21.190.000	3.829.640	-603.037	4.771
21.200.000	3.831.224	-603.273	4.767
21.210.000	3.832.967	-603.396	4.765
21.220.000	3.834.464	-603.544	4.762
21.230.000	3.836.244	-603.713	4.761
21.240.000	3.837.721	-603.891	4.760

21.250.000	3.839.563	-604.004	4.761
21.260.000	3.840.956	-604.139	4.762
21.270.000	3.842.850	-604.309	4.763
21.280.000	3.844.306	-604.345	4.760
21.290.000	3.846.108	-604.521	4.756
21.300.000	3.847.646	-604.597	4.755
21.310.000	3.849.359	-604.712	4.752
21.320.000	3.850.923	-604.805	4.749
21.330.000	3.852.635	-604.993	4.747
21.340.000	3.854.235	-605.037	4.749
21.350.000	3.855.808	-605.244	4.753
21.360.000	3.857.584	-605.344	4.756
21.370.000	3.859.059	-605.497	4.758
21.380.000	3.860.898	-605.661	4.757
21.390.000	3.862.388	-605.824	4.757
21.400.000	3.864.177	-605.947	4.754
21.410.000	3.865.647	-606.111	4.749
21.420.000	3.867.475	-606.303	4.744
21.430.000	3.868.920	-606.374	4.739
21.440.000	3.870.728	-606.601	4.738
21.450.000	3.872.273	-606.700	4.738
21.460.000	3.874.000	-606.892	4.737
21.470.000	3.875.601	-607.048	4.736
21.480.000	3.877.299	-607.232	4.734
21.490.000	3.878.901	-607.329	4.732
21.500.000	3.880.498	-607.612	4.731
21.510.000	3.882.281	-607.693	4.732
21.520.000	3.883.772	-607.910	4.732
21.530.000	3.885.574	-608.091	4.728
21.540.000	3.887.095	-608.244	4.726

21.550.000	3.888.878	-608.412	4.723
21.560.000	3.890.343	-608.579	4.718
21.570.000	3.892.202	-608.710	4.707
21.580.000	3.893.611	-608.825	4.696
21.590.000	3.895.455	-609.033	4.688
21.600.000	3.897.007	-609.090	4.680
21.610.000	3.898.672	-609.285	4.675
21.620.000	3.900.304	-609.419	4.674
21.630.000	3.902.003	-609.600	4.680
21.640.000	3.903.621	-609.716	4.683
21.650.000	3.905.266	-609.967	4.677
21.660.000	3.906.953	-610.048	4.670
21.670.000	3.908.479	-610.274	4.665
21.680.000	3.910.332	-610.460	4.661
21.690.000	3.911.751	-610.627	4.657
21.700.000	3.913.595	-610.840	4.653
21.710.000	3.915.084	-611.057	4.649
21.720.000	3.916.885	-611.214	4.644
21.730.000	3.918.377	-611.408	4.640
21.740.000	3.920.201	-611.666	4.635
21.750.000	3.921.685	-611.762	4.629
21.760.000	3.923.457	-612.046	4.626
21.770.000	3.925.057	-612.223	4.627
21.780.000	3.926.703	-612.405	4.628
21.790.000	3.928.364	-612.577	4.628
21.800.000	3.930.009	-612.841	4.631
21.810.000	3.931.719	-612.927	4.638
21.820.000	3.933.264	-613.218	4.643
21.830.000	3.935.110	-613.371	4.648
21.840.000	3.936.532	-613.547	4.652

21.850.000	3.938.383	-613.751	4.657
21.860.000	3.939.844	-613.891	4.659
21.870.000	3.941.625	-614.044	4.659
21.880.000	3.943.096	-614.197	4.658
21.890.000	3.944.955	-614.364	4.657
21.900.000	3.946.402	-614.444	4.658
21.910.000	3.948.169	-614.671	4.659
21.920.000	3.949.775	-614.772	4.660
21.930.000	3.951.409	-614.972	4.662
21.940.000	3.953.078	-615.096	4.664
21.950.000	3.954.714	-615.314	4.664
21.960.000	3.956.391	-615.408	4.658
21.970.000	3.957.972	-615.664	4.650
21.980.000	3.959.758	-615.768	4.646
21.990.000	3.961.195	-615.972	4.645
22.000.000	3.963.044	-616.207	4.646
22.010.000	3.964.508	-616.337	4.642
22.020.000	3.966.304	-616.536	4.635
22.030.000	3.967.840	-616.747	4.631
22.040.000	3.969.647	-616.944	4.632
22.050.000	3.971.135	-617.100	4.634
22.060.000	3.972.930	-617.378	4.628
22.070.000	3.974.524	-617.496	4.619
22.080.000	3.976.184	-617.759	4.615
22.090.000	3.977.864	-617.944	4.617
22.100.000	3.979.467	-618.153	4.614
22.110.000	3.981.168	-618.307	4.604
22.120.000	3.982.743	-618.612	4.596
22.130.000	3.984.539	-618.727	4.597
22.140.000	3.986.020	-618.992	4.600

22.150.000	3.987.899	-619.250	4.594
22.160.000	3.989.333	-619.410	4.586
22.170.000	3.991.128	-619.652	4.582
22.180.000	3.992.627	-619.874	4.587
22.190.000	3.994.452	-620.073	4.589
22.200.000	3.995.923	-620.267	4.582
22.210.000	3.997.732	-620.539	4.574
22.220.000	3.999.235	-620.653	4.569
22.230.000	4.000.939	-620.935	4.568
22.240.000	4.002.639	-621.116	4.566
22.250.000	4.004.267	-621.341	4.554
22.260.000	4.005.967	-621.513	4.544
22.270.000	4.007.535	-621.816	4.537
22.280.000	4.009.276	-621.938	4.535
22.290.000	4.010.780	-622.208	4.537
22.300.000	4.012.640	-622.434	4.533
22.310.000	4.014.060	-622.580	4.527
22.320.000	4.015.934	-622.811	4.523
22.330.000	4.017.401	-622.994	4.523
22.340.000	4.019.147	-623.152	4.526
22.350.000	4.020.673	-623.344	4.521
22.360.000	4.022.448	-623.580	4.511
22.370.000	4.023.985	-623.702	4.504
22.380.000	4.025.701	-624.015	4.499
22.390.000	4.027.354	-624.165	4.499
22.400.000	4.028.941	-624.404	4.492
22.410.000	4.030.672	-624.605	4.483
22.420.000	4.032.255	-624.891	4.479
22.430.000	4.034.001	-625.067	4.478
22.440.000	4.035.480	-625.383	4.479

22.450.000	4.037.324	-625.599	4.473
22.460.000	4.038.728	-625.825	4.464
22.470.000	4.040.562	-626.130	4.458
22.480.000	4.042.107	-626.321	4.458
22.490.000	4.043.831	-626.595	4.458
22.500.000	4.045.353	-626.839	4.453
22.510.000	4.047.134	-627.117	4.448
22.520.000	4.048.693	-627.346	4.447
22.530.000	4.050.412	-627.685	4.449
22.540.000	4.052.055	-627.843	4.450
22.550.000	4.053.628	-628.152	4.445
22.560.000	4.055.373	-628.397	4.438
22.570.000	4.056.934	-628.672	4.436
22.580.000	4.058.678	-628.899	4.437
22.590.000	4.060.211	-629.233	4.434
22.600.000	4.062.050	-629.486	4.425
22.610.000	4.063.456	-629.737	4.417
22.620.000	4.065.328	-630.062	4.414
22.630.000	4.066.793	-630.281	4.413
22.640.000	4.068.628	-630.600	4.405
22.650.000	4.070.146	-630.865	4.396
22.660.000	4.071.901	-631.143	4.389
22.670.000	4.073.453	-631.377	4.387
22.680.000	4.075.172	-631.733	4.384
22.690.000	4.076.808	-631.907	4.375
22.700.000	4.078.467	-632.239	4.366
22.710.000	4.080.182	-632.492	4.359
22.720.000	4.081.731	-632.790	4.355
22.730.000	4.083.520	-633.055	4.350
22.740.000	4.085.044	-633.410	4.343

22.750.000	4.086.903	-633.686	4.335
22.760.000	4.088.328	-634.029	4.332
22.770.000	4.090.195	-634.364	4.330
22.780.000	4.091.635	-634.622	4.324
22.790.000	4.093.446	-634.994	4.315
22.800.000	4.095.024	-635.290	4.307
22.810.000	4.096.762	-635.630	4.301
22.820.000	4.098.318	-635.924	4.294
22.830.000	4.100.051	-636.287	4.284
22.840.000	4.101.644	-636.524	4.277
22.850.000	4.103.274	-636.905	4.271
22.860.000	4.105.044	-637.146	4.265
22.870.000	4.106.585	-637.494	4.258
22.880.000	4.108.381	-637.758	4.250
22.890.000	4.109.931	-638.082	4.243
22.900.000	4.111.699	-638.367	4.238
22.910.000	4.113.189	-638.679	4.230
22.920.000	4.115.055	-638.998	4.224
22.930.000	4.116.475	-639.233	4.218
22.940.000	4.118.312	-639.583	4.214
22.950.000	4.119.874	-639.816	4.209
22.960.000	4.121.618	-640.124	4.202
22.970.000	4.123.220	-640.416	4.196
22.980.000	4.124.952	-640.699	4.190
22.990.000	4.126.547	-640.927	4.184
23.000.000	4.128.266	-641.284	4.178
23.010.000	4.129.939	-641.446	4.171
23.020.000	4.131.516	-641.772	4.164
23.030.000	4.133.285	-642.019	4.158
23.040.000	4.134.807	-642.280	4.154

23.050.000	4.136.569	-642.526	4.149
23.060.000	4.138.039	-642.777	4.142
23.070.000	4.139.864	-642.945	4.134
23.080.000	4.141.290	-643.131	4.128
23.090.000	4.143.157	-643.367	4.122
23.100.000	4.144.634	-643.457	4.116
23.110.000	4.146.386	-643.747	4.108
23.120.000	4.147.944	-644.013	4.102
23.130.000	4.149.652	-644.392	4.098
23.140.000	4.151.194	-644.867	4.095
23.150.000	4.152.915	-645.574	4.092
23.160.000	4.154.545	-646.214	4.090
23.170.000	4.156.082	-647.127	4.089
23.180.000	4.157.879	-647.990	4.086
23.190.000	4.159.322	-648.928	4.084
23.200.000	4.161.186	-649.873	4.080
23.210.000	4.162.664	-650.798	4.078
23.220.000	4.164.413	-651.628	4.074
23.230.000	4.165.902	-652.436	4.069
23.240.000	4.167.730	-653.219	4.067
23.250.000	4.169.151	-653.810	4.065
23.260.000	4.170.965	-654.498	4.066
23.270.000	4.172.508	-655.042	4.066
23.280.000	4.174.201	-655.522	4.066
23.290.000	4.175.792	-655.942	4.066
23.300.000	4.177.476	-656.391	4.065
23.310.000	4.179.118	-656.647	4.065
23.320.000	4.180.714	-657.045	4.065
23.330.000	4.182.467	-657.258	4.062
23.340.000	4.183.935	-657.528	4.059

23.350.000	4.185.776	-657.794	4.057
23.360.000	4.187.240	-657.982	4.055
23.370.000	4.189.044	-658.191	4.054
23.380.000	4.190.483	-658.389	4.054
23.390.000	4.192.326	-658.588	4.053
23.400.000	4.193.742	-658.713	4.052
23.410.000	4.195.542	-658.957	4.050
23.420.000	4.197.099	-659.067	4.049
23.430.000	4.198.798	-659.305	4.048
23.440.000	4.200.377	-659.436	4.045
23.450.000	4.202.082	-659.647	4.042
23.460.000	4.203.682	-659.776	4.040
23.470.000	4.205.324	-660.048	4.036
23.480.000	4.207.048	-660.148	4.033
23.490.000	4.208.520	-660.406	4.031
23.500.000	4.210.350	-660.639	4.028
23.510.000	4.211.783	-660.857	4.025
23.520.000	4.213.562	-661.137	4.020
23.530.000	4.215.044	-661.474	4.017
23.540.000	4.216.831	-661.830	4.016
23.550.000	4.218.295	-662.232	4.012
23.560.000	4.220.085	-662.798	4.008
23.570.000	4.221.612	-663.249	4.004
23.580.000	4.223.319	-663.895	4.001
23.590.000	4.224.923	-664.497	3.996
23.600.000	4.226.562	-665.099	3.993
23.610.000	4.228.207	-665.662	3.990
23.620.000	4.229.823	-666.315	3.988
23.630.000	4.231.523	-666.737	3.985
23.640.000	4.232.987	-667.290	3.982

23.650.000	4.234.806	-667.753	3.980
23.660.000	4.236.263	-668.111	3.980
23.670.000	4.238.080	-668.506	3.979
23.680.000	4.239.535	-668.815	3.976
23.690.000	4.241.350	-669.062	3.975
23.700.000	4.242.807	-669.301	3.973
23.710.000	4.244.644	-669.559	3.971
23.720.000	4.246.115	-669.643	3.970
23.730.000	4.247.828	-669.864	3.970
23.740.000	4.249.450	-669.970	3.970
23.750.000	4.251.051	-670.070	3.970
23.760.000	4.252.710	-670.153	3.969
23.770.000	4.254.353	-670.303	3.970
23.780.000	4.256.014	-670.298	3.972
23.790.000	4.257.563	-670.449	3.972
23.800.000	4.259.402	-670.535	3.971
23.810.000	4.260.818	-670.616	3.968
23.820.000	4.262.660	-670.787	3.964
23.830.000	4.264.142	-670.923	3.960
23.840.000	4.265.903	-671.111	3.954
23.850.000	4.267.405	-671.346	3.948
23.860.000	4.269.225	-671.658	3.941
23.870.000	4.270.715	-671.896	3.933
23.880.000	4.272.493	-672.315	3.925
23.890.000	4.274.102	-672.595	3.921
23.900.000	4.275.719	-673.020	3.919
23.910.000	4.277.406	-673.369	3.916
23.920.000	4.278.992	-673.762	3.909
23.930.000	4.280.702	-674.056	3.904
23.940.000	4.282.232	-674.487	3.900

23.950.000	4.284.026	-674.754	3.897
23.960.000	4.285.440	-675.089	3.893
23.970.000	4.287.302	-675.446	3.882
23.980.000	4.288.771	-675.675	3.873
23.990.000	4.290.579	-675.982	3.866
24.000.000	4.292.106	-676.248	3.858
24.010.000	4.293.873	-676.473	3.852
24.020.000	4.295.390	-676.671	3.844
24.030.000	4.297.173	-676.967	3.839
24.040.000	4.298.744	-677.045	3.834
24.050.000	4.300.407	-677.242	3.827
24.060.000	4.302.073	-677.324	3.821
24.070.000	4.303.630	-677.389	3.811
24.080.000	4.305.358	-677.393	3.802
24.090.000	4.306.916	-677.496	3.788
24.100.000	4.308.710	-677.388	3.775
24.110.000	4.310.117	-677.405	3.764
24.120.000	4.312.012	-677.415	3.755
24.130.000	4.313.391	-677.287	3.749
24.140.000	4.315.240	-677.265	3.742
24.150.000	4.316.729	-677.167	3.732
24.160.000	4.318.467	-677.024	3.725
24.170.000	4.320.005	-676.846	3.719
24.180.000	4.321.781	-676.721	3.714
24.190.000	4.323.291	-676.344	3.706
24.200.000	4.324.962	-676.124	3.698
24.210.000	4.326.661	-675.778	3.691
24.220.000	4.328.221	-675.396	3.687
24.230.000	4.329.951	-675.027	3.685
24.240.000	4.331.517	-674.768	3.683

24.250.000	4.333.246	-674.451	3.682
24.260.000	4.334.718	-674.333	3.681
24.270.000	4.336.585	-674.313	3.680
24.280.000	4.337.934	-674.321	3.681
24.290.000	4.339.762	-674.572	3.682
24.300.000	4.341.283	-674.825	3.682
24.310.000	4.342.995	-675.208	3.683
24.320.000	4.344.541	-675.658	3.681
24.330.000	4.346.337	-676.206	3.682
24.340.000	4.347.814	-676.674	3.685
24.350.000	4.349.506	-677.323	3.688
24.360.000	4.351.176	-677.847	3.692
24.370.000	4.352.697	-678.486	3.694
24.380.000	4.354.407	-679.021	3.696
24.390.000	4.355.911	-679.612	3.699
24.400.000	4.357.661	-680.113	3.701
24.410.000	4.359.166	-680.677	3.703
24.420.000	4.360.979	-681.142	3.706
24.430.000	4.362.378	-681.571	3.706
24.440.000	4.364.298	-682.025	3.705
24.450.000	4.365.744	-682.315	3.703
24.460.000	4.367.529	-682.656	3.702
24.470.000	4.369.066	-682.900	3.699
24.480.000	4.370.783	-683.091	3.695
24.490.000	4.372.306	-683.183	3.688
24.500.000	4.373.986	-683.348	3.680
24.510.000	4.375.598	-683.265	3.672
24.520.000	4.377.183	-683.264	3.663
24.530.000	4.378.936	-683.099	3.652
24.540.000	4.380.464	-682.882	3.640

24.550.000	4.382.229	-682.568	3.628
24.560.000	4.383.733	-682.292	3.616
24.570.000	4.385.582	-681.858	3.606
24.580.000	4.386.990	-681.492	3.596
24.590.000	4.388.854	-681.149	3.588
24.600.000	4.390.264	-680.707	3.580
24.610.000	4.392.075	-680.468	3.570
24.620.000	4.393.609	-680.188	3.561
24.630.000	4.395.324	-679.963	3.553
24.640.000	4.396.866	-679.803	3.549
24.650.000	4.398.633	-679.812	3.541
24.660.000	4.400.223	-679.701	3.532
24.670.000	4.401.851	-679.844	3.524
24.680.000	4.403.624	-679.929	3.519
24.690.000	4.405.086	-680.105	3.513
24.700.000	4.406.866	-680.336	3.507
24.710.000	4.408.338	-680.654	3.499
24.720.000	4.410.120	-680.941	3.492
24.730.000	4.411.552	-681.287	3.485
24.740.000	4.413.406	-681.691	3.478
24.750.000	4.414.814	-681.968	3.470
24.760.000	4.416.604	-682.366	3.459
24.770.000	4.418.145	-682.674	3.448
24.780.000	4.419.846	-682.942	3.437
24.790.000	4.421.470	-683.165	3.428
24.800.000	4.423.163	-683.411	3.418
24.810.000	4.424.720	-683.449	3.408
24.820.000	4.426.349	-683.611	3.399
24.830.000	4.428.060	-683.616	3.390
24.840.000	4.429.554	-683.691	3.380

24.850.000	4.431.339	-683.720	3.370
24.860.000	4.432.828	-683.771	3.361
24.870.000	4.434.597	-683.804	3.349
24.880.000	4.436.082	-683.896	3.337
24.890.000	4.437.953	-683.954	3.327
24.900.000	4.439.332	-683.987	3.318
24.910.000	4.441.147	-684.122	3.310
24.920.000	4.442.703	-684.140	3.300
24.930.000	4.444.375	-684.230	3.290
24.940.000	4.445.949	-684.260	3.281
24.950.000	4.447.659	-684.324	3.271
24.960.000	4.449.207	-684.317	3.262
24.970.000	4.450.867	-684.411	3.252
24.980.000	4.452.588	-684.328	3.240
24.990.000	4.454.068	-684.408	3.229
25.000.000	4.455.938	-684.420	3.220
25.010.000	4.457.432	-684.419	3.211
25.020.000	4.459.195	-684.447	3.201
25.030.000	4.460.664	-684.498	3.190
25.040.000	4.462.470	-684.509	3.177
25.050.000	4.463.878	-684.516	3.169
25.060.000	4.465.697	-684.618	3.159
25.070.000	4.467.155	-684.561	3.149
25.080.000	4.468.904	-684.658	3.136
25.090.000	4.470.502	-684.672	3.124
25.100.000	4.472.182	-684.687	3.115
25.110.000	4.473.767	-684.695	3.105
25.120.000	4.475.431	-684.815	3.095
25.130.000	4.477.139	-684.723	3.085
25.140.000	4.478.646	-684.823	3.074

25.150.000	4.480.447	-684.847	3.063
25.160.000	4.481.887	-684.857	3.053
25.170.000	4.483.654	-684.937	3.042
25.180.000	4.485.104	-685.008	3.031
25.190.000	4.486.924	-685.038	3.017
25.200.000	4.488.387	-685.125	3.003
25.210.000	4.490.205	-685.259	2.994
25.220.000	4.491.680	-685.248	2.984
25.230.000	4.493.454	-685.422	2.974
25.240.000	4.495.079	-685.481	2.963
25.250.000	4.496.700	-685.571	2.951
25.260.000	4.498.289	-685.655	2.941
25.270.000	4.499.976	-685.840	2.929
25.280.000	4.501.627	-685.873	2.919
25.290.000	4.503.178	-686.076	2.908
25.300.000	4.504.977	-686.189	2.896
25.310.000	4.506.394	-686.315	2.886
25.320.000	4.508.253	-686.499	2.877
25.330.000	4.509.695	-686.661	2.868
25.340.000	4.511.500	-686.819	2.858
25.350.000	4.513.008	-686.993	2.845
25.360.000	4.514.831	-687.202	2.833
25.370.000	4.516.246	-687.303	2.823
25.380.000	4.518.037	-687.565	2.814
25.390.000	4.519.637	-687.692	2.803
25.400.000	4.521.256	-687.907	2.790
25.410.000	4.522.911	-688.062	2.781
25.420.000	4.524.576	-688.284	2.772
25.430.000	4.526.165	-688.380	2.763
25.440.000	4.527.755	-688.658	2.752

25.450.000	4.529.558	-688.772	2.742
25.460.000	4.530.989	-688.978	2.733
25.470.000	4.532.835	-689.245	2.725
25.480.000	4.534.300	-689.374	2.718
25.490.000	4.536.096	-689.619	2.711
25.500.000	4.537.548	-689.812	2.701
25.510.000	4.539.399	-690.015	2.690
25.520.000	4.540.831	-690.187	2.679
25.530.000	4.542.619	-690.467	2.671
25.540.000	4.544.178	-690.593	2.662
25.550.000	4.545.816	-690.860	2.653
25.560.000	4.547.484	-691.050	2.643
25.570.000	4.549.113	-691.277	2.635
25.580.000	4.550.756	-691.428	2.627
25.590.000	4.552.339	-691.718	2.617
25.600.000	4.554.075	-691.847	2.609
25.610.000	4.555.499	-692.085	2.601
25.620.000	4.557.374	-692.336	2.594
25.630.000	4.558.771	-692.505	2.588
25.640.000	4.560.565	-692.738	2.581
25.650.000	4.562.099	-692.967	2.572
25.660.000	4.563.900	-693.163	2.562
25.670.000	4.565.371	-693.365	2.554
25.680.000	4.567.218	-693.667	2.544
25.690.000	4.568.712	-693.790	2.536
25.700.000	4.570.399	-694.097	2.529
25.710.000	4.572.030	-694.287	2.521
25.720.000	4.573.634	-694.525	2.513
25.730.000	4.575.323	-694.730	2.504
25.740.000	4.576.915	-695.058	2.495

25.750.000	4.578.666	-695.201	2.484
25.760.000	4.580.101	-695.484	2.475
25.770.000	4.581.962	-695.769	2.466
25.780.000	4.583.373	-695.960	2.456
25.790.000	4.585.213	-696.275	2.446
25.800.000	4.586.701	-696.511	2.435
25.810.000	4.588.435	-696.736	2.424
25.820.000	4.589.915	-696.989	2.414
25.830.000	4.591.713	-697.257	2.404
25.840.000	4.593.188	-697.420	2.393
25.850.000	4.594.887	-697.716	2.383
25.860.000	4.596.561	-697.894	2.373
25.870.000	4.598.115	-698.131	2.362
25.880.000	4.599.775	-698.312	2.351
25.890.000	4.601.402	-698.611	2.340
25.900.000	4.603.129	-698.751	2.330
25.910.000	4.604.610	-699.027	2.319
25.920.000	4.606.436	-699.263	2.309
25.930.000	4.607.802	-699.440	2.299
25.940.000	4.609.652	-699.718	2.289
25.950.000	4.611.124	-699.903	2.280
25.960.000	4.612.832	-700.134	2.271
25.970.000	4.614.354	-700.342	2.263
25.980.000	4.616.100	-700.595	2.254
25.990.000	4.617.585	-700.741	2.244
26.000.000	4.619.312	-701.067	2.232
26.010.000	4.620.969	-701.212	2.219
26.020.000	4.622.550	-701.473	2.207
26.030.000	4.624.231	-701.666	2.198
26.040.000	4.625.791	-701.919	2.191

26.050.000	4.627.508	-702.102	2.185
26.060.000	4.629.008	-702.418	2.181
26.070.000	4.630.830	-702.604	2.175
26.080.000	4.632.176	-702.840	2.167
26.090.000	4.634.041	-703.118	2.159
26.100.000	4.635.515	-703.296	2.150
26.110.000	4.637.240	-703.559	2.141
26.120.000	4.638.821	-703.788	2.132
26.130.000	4.640.578	-704.031	2.123
26.140.000	4.642.081	-704.230	2.114
26.150.000	4.643.809	-704.551	2.103
26.160.000	4.645.370	-704.691	2.095
26.170.000	4.646.943	-704.988	2.087
26.180.000	4.648.680	-705.203	2.080
26.190.000	4.650.158	-705.452	2.075
26.200.000	4.651.919	-705.663	2.070
26.210.000	4.653.426	-705.976	2.063
26.220.000	4.655.206	-706.184	2.054
26.230.000	4.656.616	-706.449	2.045
26.240.000	4.658.517	-706.780	2.034
26.250.000	4.659.929	-706.966	2.025
26.260.000	4.661.716	-707.267	2.017
26.270.000	4.663.237	-707.527	2.010
26.280.000	4.664.922	-707.783	2.004
26.290.000	4.666.464	-708.047	1.996
26.300.000	4.668.196	-708.374	1.989
26.310.000	4.669.767	-708.545	1.981
26.320.000	4.671.372	-708.888	1.973
26.330.000	4.673.060	-709.128	1.965
26.340.000	4.674.611	-709.422	1.956

26.350.000	4.676.403	-709.699	1.948
26.360.000	4.677.880	-710.043	1.941
26.370.000	4.679.698	-710.330	1.935
26.380.000	4.681.118	-710.649	1.928
26.390.000	4.682.953	-710.999	1.921
26.400.000	4.684.370	-711.246	1.913
26.410.000	4.686.202	-711.642	1.907
26.420.000	4.687.716	-711.932	1.900
26.430.000	4.689.402	-712.275	1.894
26.440.000	4.690.935	-712.593	1.887
26.450.000	4.692.713	-712.989	1.879
26.460.000	4.694.270	-713.265	1.872
26.470.000	4.695.917	-713.682	1.866
26.480.000	4.697.644	-713.990	1.859
26.490.000	4.699.126	-714.391	1.854
26.500.000	4.700.918	-714.751	1.849
26.510.000	4.702.421	-715.146	1.843
26.520.000	4.704.165	-715.520	1.838
26.530.000	4.705.623	-715.932	1.832
26.540.000	4.707.424	-716.311	1.826
26.550.000	4.708.859	-716.669	1.819
26.560.000	4.710.656	-717.109	1.811
26.570.000	4.712.151	-717.430	1.806
26.580.000	4.713.888	-717.843	1.798
26.590.000	4.715.475	-718.198	1.791
26.600.000	4.717.152	-718.577	1.785
26.610.000	4.718.717	-718.898	1.780
26.620.000	4.720.356	-719.347	1.774
26.630.000	4.722.059	-719.624	1.767
26.640.000	4.723.536	-720.055	1.760

26.650.000	4.725.350	-720.427	1.753
26.660.000	4.726.789	-720.753	1.745
26.670.000	4.728.564	-721.135	1.738
26.680.000	4.730.036	-721.522	1.731
26.690.000	4.731.876	-721.861	1.725
26.700.000	4.733.289	-722.201	1.718
26.710.000	4.735.116	-722.624	1.712
26.720.000	4.736.612	-722.898	1.705
26.730.000	4.738.346	-723.337	1.700
26.740.000	4.739.886	-723.660	1.694
26.750.000	4.741.570	-724.026	1.686
26.760.000	4.743.168	-724.362	1.676
26.770.000	4.744.781	-724.800	1.669
26.780.000	4.746.476	-725.056	1.665
26.790.000	4.748.033	-725.503	1.662
26.800.000	4.749.832	-725.860	1.658
26.810.000	4.751.280	-726.207	1.653
26.820.000	4.753.082	-726.627	1.649
26.830.000	4.754.575	-727.014	1.647
26.840.000	4.756.304	-727.356	1.644
26.850.000	4.757.774	-727.751	1.641
26.860.000	4.759.612	-728.190	1.636
26.870.000	4.761.003	-728.480	1.631
26.880.000	4.762.785	-728.929	1.628
26.890.000	4.764.323	-729.280	1.623
26.900.000	4.765.990	-729.635	1.619
26.910.000	4.767.617	-729.998	1.614
26.920.000	4.769.242	-730.421	1.608
26.930.000	4.770.892	-730.684	1.602
26.940.000	4.772.452	-731.132	1.596

26.950.000	4.774.192	-731.470	1.591
26.960.000	4.775.644	-731.841	1.586
26.970.000	4.777.481	-732.257	1.582
26.980.000	4.778.909	-732.649	1.578
26.990.000	4.780.669	-733.010	1.574
27.000.000	4.782.132	-733.403	1.571
27.010.000	4.783.969	-733.820	1.569
27.020.000	4.785.382	-734.146	1.565
27.030.000	4.787.141	-734.593	1.560
27.040.000	4.788.734	-734.918	1.553
27.050.000	4.790.357	-735.311	1.547
27.060.000	4.791.959	-735.651	1.543
27.070.000	4.793.564	-736.057	1.540
27.080.000	4.795.190	-736.359	1.537
27.090.000	4.796.766	-736.789	1.533
27.100.000	4.798.550	-737.082	1.529
27.110.000	4.799.917	-737.475	1.525
27.120.000	4.801.773	-737.861	1.522
27.130.000	4.803.202	-738.176	1.516
27.140.000	4.804.968	-738.565	1.510
27.150.000	4.806.483	-738.932	1.503
27.160.000	4.808.249	-739.277	1.498
27.170.000	4.809.680	-739.597	1.494
27.180.000	4.811.458	-740.022	1.491
27.190.000	4.812.951	-740.283	1.488
27.200.000	4.814.621	-740.688	1.483
27.210.000	4.816.268	-741.007	1.479
27.220.000	4.817.820	-741.337	1.477
27.230.000	4.819.518	-741.636	1.474
27.240.000	4.821.074	-742.081	1.471

27.250.000	4.822.855	-742.299	1.468
27.260.000	4.824.254	-742.667	1.465
27.270.000	4.826.064	-743.045	1.462
27.280.000	4.827.491	-743.288	1.459
27.290.000	4.829.284	-743.658	1.457
27.300.000	4.830.747	-743.964	1.455
27.310.000	4.832.495	-744.244	1.451
27.320.000	4.833.975	-744.525	1.446
27.330.000	4.835.744	-744.899	1.441
27.340.000	4.837.270	-745.079	1.439
27.350.000	4.838.941	-745.429	1.437
27.360.000	4.840.598	-745.678	1.432
27.370.000	4.842.192	-745.952	1.426
27.380.000	4.843.876	-746.190	1.423
27.390.000	4.845.462	-746.547	1.421
27.400.000	4.847.156	-746.724	1.417
27.410.000	4.848.589	-747.030	1.413
27.420.000	4.850.489	-747.341	1.409
27.430.000	4.851.839	-747.527	1.407
27.440.000	4.853.686	-747.837	1.404
27.450.000	4.855.175	-748.090	1.398
27.460.000	4.856.874	-748.303	1.394
27.470.000	4.858.380	-748.540	1.391
27.480.000	4.860.198	-748.854	1.389
27.490.000	4.861.670	-748.975	1.386
27.500.000	4.863.394	-749.269	1.383
27.510.000	4.864.989	-749.438	1.381
27.520.000	4.866.568	-749.638	1.380
27.530.000	4.868.273	-749.814	1.378
27.540.000	4.869.822	-750.077	1.374

27.550.000	4.871.576	-750.191	1.370
27.560.000	4.873.064	-750.428	1.369
27.570.000	4.874.850	-750.602	1.369
27.580.000	4.876.272	-750.740	1.366
27.590.000	4.878.090	-750.981	1.363
27.600.000	4.879.608	-751.091	1.363
27.610.000	4.881.335	-751.258	1.364
27.620.000	4.882.814	-751.415	1.362
27.630.000	4.884.601	-751.591	1.359
27.640.000	4.886.095	-751.662	1.357
27.650.000	4.887.771	-751.865	1.357
27.660.000	4.889.413	-751.931	1.355
27.670.000	4.890.937	-752.106	1.352
27.680.000	4.892.682	-752.193	1.348
27.690.000	4.894.251	-752.346	1.348
27.700.000	4.895.933	-752.426	1.349
27.710.000	4.897.446	-752.613	1.349
27.720.000	4.899.263	-752.705	1.347
27.730.000	4.900.623	-752.798	1.344
27.740.000	4.902.482	-752.973	1.345
27.750.000	4.903.950	-753.026	1.345
27.760.000	4.905.652	-753.131	1.344
27.770.000	4.907.167	-753.219	1.342
27.780.000	4.908.946	-753.305	1.340
27.790.000	4.910.476	-753.336	1.340
27.800.000	4.912.147	-753.496	1.341
27.810.000	4.913.730	-753.471	1.340
27.820.000	4.915.360	-753.596	1.340
27.830.000	4.917.059	-753.627	1.338
27.840.000	4.918.578	-753.686	1.335

27.850.000	4.920.334	-753.725	1.332
27.860.000	4.921.801	-753.812	1.331
27.870.000	4.923.612	-753.799	1.332
27.880.000	4.924.976	-753.840	1.329
27.890.000	4.926.827	-753.913	1.328
27.900.000	4.928.298	-753.854	1.328
27.910.000	4.930.027	-753.924	1.328
27.920.000	4.931.602	-753.914	1.328
27.930.000	4.933.287	-753.907	1.326
27.940.000	4.934.805	-753.870	1.325
27.950.000	4.936.533	-753.917	1.325
27.960.000	4.938.092	-753.786	1.324
27.970.000	4.939.694	-753.830	1.324
27.980.000	4.941.459	-753.740	1.325
27.990.000	4.942.912	-753.694	1.324
28.000.000	4.944.698	-753.609	1.322
28.010.000	4.946.181	-753.573	1.320
28.020.000	4.947.948	-753.457	1.318
28.030.000	4.949.393	-753.388	1.316
28.040.000	4.951.240	-753.342	1.314
28.050.000	4.952.689	-753.169	1.313
28.060.000	4.954.459	-753.113	1.312
28.070.000	4.955.963	-752.973	1.311
28.080.000	4.957.658	-752.842	1.308
28.090.000	4.959.209	-752.688	1.306
28.100.000	4.960.921	-752.610	1.304
28.110.000	4.962.530	-752.371	1.304
28.120.000	4.964.120	-752.291	1.305
28.130.000	4.965.833	-752.082	1.307
28.140.000	4.967.340	-751.943	1.309

28.150.000	4.969.092	-751.763	1.310
28.160.000	4.970.559	-751.626	1.313
28.170.000	4.972.349	-751.434	1.311
28.180.000	4.973.767	-751.292	1.308
28.190.000	4.975.626	-751.122	1.305
28.200.000	4.977.015	-750.893	1.303
28.210.000	4.978.801	-750.779	1.303
28.220.000	4.980.335	-750.541	1.303
28.230.000	4.982.005	-750.381	1.303
28.240.000	4.983.567	-750.177	1.302
28.250.000	4.985.261	-750.021	1.299
28.260.000	4.986.822	-749.771	1.299
28.270.000	4.988.453	-749.667	1.298
28.280.000	4.990.142	-749.403	1.297
28.290.000	4.991.620	-749.249	1.297
28.300.000	4.993.402	-749.048	1.296
28.310.000	4.994.856	-748.863	1.293
28.320.000	4.996.655	-748.674	1.291
28.330.000	4.998.139	-748.509	1.288
28.340.000	4.999.952	-748.313	1.286
28.350.000	5.001.345	-748.081	1.285
28.360.000	5.003.161	-747.961	1.285
28.370.000	5.004.641	-747.706	1.284
28.380.000	5.006.339	-747.574	1.281
28.390.000	5.007.928	-747.397	1.279
28.400.000	5.009.548	-747.216	1.277
28.410.000	5.011.168	-747.024	1.277
28.420.000	5.012.763	-746.934	1.275
28.430.000	5.014.472	-746.682	1.275
28.440.000	5.015.952	-746.589	1.275

28.450.000	5.017.777	-746.454	1.274
28.460.000	5.019.222	-746.281	1.271
28.470.000	5.021.000	-746.188	1.267
28.480.000	5.022.430	-746.077	1.265
28.490.000	5.024.274	-745.949	1.265
28.500.000	5.025.699	-745.813	1.267
28.510.000	5.027.513	-745.787	1.270
28.520.000	5.028.994	-745.589	1.272
28.530.000	5.030.692	-745.564	1.272
28.540.000	5.032.237	-745.455	1.270
28.550.000	5.033.883	-745.393	1.269
28.560.000	5.035.502	-745.298	1.266
28.570.000	5.037.143	-745.318	1.264
28.580.000	5.038.797	-745.158	1.261
28.590.000	5.040.308	-745.189	1.259
28.600.000	5.042.156	-745.172	1.258
28.610.000	5.043.513	-745.109	1.256
28.620.000	5.045.383	-745.156	1.253
28.630.000	5.046.831	-745.166	1.250
28.640.000	5.048.584	-745.141	1.248
28.650.000	5.050.063	-745.167	1.245
28.660.000	5.051.855	-745.254	1.243
28.670.000	5.053.307	-745.202	1.240
28.680.000	5.055.075	-745.331	1.238
28.690.000	5.056.646	-745.356	1.235
28.700.000	5.058.255	-745.411	1.235
28.710.000	5.059.873	-745.480	1.235
28.720.000	5.061.514	-745.606	1.234
28.730.000	5.063.190	-745.609	1.233
28.740.000	5.064.691	-745.761	1.230

28.750.000	5.066.514	-745.851	1.228
28.760.000	5.067.907	-745.951	1.227
28.770.000	5.069.749	-746.122	1.227
28.780.000	5.071.210	-746.220	1.229
28.790.000	5.072.967	-746.361	1.230
28.800.000	5.074.436	-746.518	1.230
28.810.000	5.076.212	-746.698	1.228
28.820.000	5.077.665	-746.801	1.227
28.830.000	5.079.435	-747.054	1.226
28.840.000	5.081.028	-747.160	1.225
28.850.000	5.082.579	-747.370	1.225
28.860.000	5.084.246	-747.518	1.225
28.870.000	5.085.822	-747.732	1.225
28.880.000	5.087.494	-747.845	1.223
28.890.000	5.089.028	-748.111	1.220
28.900.000	5.090.805	-748.271	1.217
28.910.000	5.092.185	-748.456	1.213
28.920.000	5.094.024	-748.741	1.210
28.930.000	5.095.468	-748.897	1.207
28.940.000	5.097.229	-749.133	1.206
28.950.000	5.098.755	-749.373	1.205
28.960.000	5.100.493	-749.606	1.205
28.970.000	5.101.956	-749.799	1.203
28.980.000	5.103.702	-750.110	1.202
28.990.000	5.105.233	-750.256	1.201
29.000.000	5.106.844	-750.545	1.200
29.010.000	5.108.509	-750.779	1.201
29.020.000	5.110.064	-751.020	1.201
29.030.000	5.111.750	-751.218	1.201
29.040.000	5.113.266	-751.556	1.199

29.050.000	5.115.040	-751.730	1.199
29.060.000	5.116.432	-752.004	1.198
29.070.000	5.118.313	-752.315	1.198
29.080.000	5.119.726	-752.490	1.198
29.090.000	5.121.490	-752.811	1.195
29.100.000	5.122.976	-753.053	1.192
29.110.000	5.124.747	-753.302	1.192
29.120.000	5.126.202	-753.538	1.193
29.130.000	5.127.965	-753.868	1.192
29.140.000	5.129.528	-754.031	1.188
29.150.000	5.131.150	-754.354	1.186
29.160.000	5.132.837	-754.602	1.186
29.170.000	5.134.387	-754.853	1.187
29.180.000	5.136.064	-755.072	1.188
29.190.000	5.137.643	-755.387	1.187
29.200.000	5.139.360	-755.596	1.186
29.210.000	5.140.825	-755.856	1.186
29.220.000	5.142.689	-756.160	1.187
29.230.000	5.144.033	-756.308	1.187
29.240.000	5.145.844	-756.593	1.187
29.250.000	5.147.341	-756.803	1.186
29.260.000	5.149.043	-757.013	1.184
29.270.000	5.150.566	-757.215	1.183
29.280.000	5.152.319	-757.523	1.183
29.290.000	5.153.855	-757.613	1.181
29.300.000	5.155.517	-757.900	1.180
29.310.000	5.157.210	-758.067	1.178
29.320.000	5.158.750	-758.291	1.178
29.330.000	5.160.477	-758.477	1.178
29.340.000	5.161.993	-758.733	1.181

29.350.000	5.163.721	-758.881	1.182
29.360.000	5.165.154	-759.132	1.181
29.370.000	5.166.998	-759.362	1.180
29.380.000	5.168.404	-759.501	1.180
29.390.000	5.170.200	-759.779	1.179
29.400.000	5.171.733	-759.942	1.178
29.410.000	5.173.438	-760.130	1.177
29.420.000	5.174.956	-760.300	1.176
29.430.000	5.176.713	-760.538	1.175
29.440.000	5.178.241	-760.650	1.173
29.450.000	5.179.896	-760.909	1.172
29.460.000	5.181.604	-761.035	1.172
29.470.000	5.183.082	-761.258	1.172
29.480.000	5.184.866	-761.407	1.171
29.490.000	5.186.365	-761.645	1.170
29.500.000	5.188.139	-761.823	1.170
29.510.000	5.189.596	-762.048	1.170
29.520.000	5.191.445	-762.254	1.170
29.530.000	5.192.817	-762.404	1.169
29.540.000	5.194.653	-762.663	1.167
29.550.000	5.196.167	-762.813	1.165
29.560.000	5.197.889	-763.022	1.164
29.570.000	5.199.430	-763.222	1.163
29.580.000	5.201.151	-763.432	1.164
29.590.000	5.202.682	-763.560	1.164
29.600.000	5.204.323	-763.848	1.162
29.610.000	5.206.006	-763.949	1.160
29.620.000	5.207.555	-764.190	1.160
29.630.000	5.209.321	-764.339	1.161
29.640.000	5.210.818	-764.519	1.162

29.650.000	5.212.589	-764.685	1.160
29.660.000	5.214.054	-764.893	1.158
29.670.000	5.215.891	-765.045	1.158
29.680.000	5.217.299	-765.192	1.159
29.690.000	5.219.083	-765.437	1.160
29.700.000	5.220.563	-765.535	1.158
29.710.000	5.222.280	-765.783	1.157
29.720.000	5.223.860	-765.935	1.157
29.730.000	5.225.576	-766.125	1.158
29.740.000	5.227.143	-766.270	1.158
29.750.000	5.228.840	-766.539	1.157
29.760.000	5.230.491	-766.606	1.155
29.770.000	5.232.008	-766.841	1.153
29.780.000	5.233.789	-767.010	1.151
29.790.000	5.235.235	-767.186	1.152
29.800.000	5.237.014	-767.352	1.151
29.810.000	5.238.480	-767.557	1.148
29.820.000	5.240.280	-767.710	1.146
29.830.000	5.241.685	-767.873	1.144
29.840.000	5.243.564	-768.105	1.144
29.850.000	5.245.012	-768.191	1.142
29.860.000	5.246.778	-768.418	1.138
29.870.000	5.248.327	-768.563	1.136
29.880.000	5.249.959	-768.735	1.136
29.890.000	5.251.581	-768.884	1.138
29.900.000	5.253.233	-769.120	1.141
29.910.000	5.254.867	-769.189	1.140
29.920.000	5.256.393	-769.444	1.141
29.930.000	5.258.180	-769.596	1.143
29.940.000	5.259.617	-769.746	1.145

29.950.000	5.261.430	-769.970	1.145
29.960.000	5.262.920	-770.147	1.143
29.970.000	5.264.713	-770.301	1.140
29.980.000	5.266.157	-770.491	1.139
29.990.000	5.267.969	-770.719	1.140
30.000.000	5.269.438	-770.827	1.141
30.010.000	5.271.177	-771.081	1.141
30.020.000	5.272.752	-771.234	1.141
30.030.000	5.274.365	-771.424	1.141
30.040.000	5.275.984	-771.580	1.142
30.050.000	5.277.635	-771.808	1.144
30.060.000	5.279.286	-771.896	1.142
30.070.000	5.280.865	-772.146	1.140
30.080.000	5.282.669	-772.275	1.137
30.090.000	5.284.084	-772.448	1.137
30.100.000	5.285.920	-772.666	1.138
30.110.000	5.287.372	-772.836	1.139
30.120.000	5.289.134	-772.997	1.139
30.130.000	5.290.585	-773.188	1.139
30.140.000	5.292.397	-773.392	1.140
30.150.000	5.293.823	-773.500	1.141
30.160.000	5.295.603	-773.763	1.143
30.170.000	5.297.154	-773.874	1.142
30.180.000	5.298.827	-774.080	1.139
30.190.000	5.300.446	-774.223	1.139
30.200.000	5.302.069	-774.422	1.139
30.210.000	5.303.725	-774.532	1.140
30.220.000	5.305.285	-774.809	1.137
30.230.000	5.307.054	-774.900	1.133
30.240.000	5.308.450	-775.097	1.132

30.250.000	5.310.286	-775.318	1.131
30.260.000	5.311.721	-775.461	1.131
30.270.000	5.313.491	-775.637	1.131
30.280.000	5.314.953	-775.838	1.129
30.290.000	5.316.800	-776.017	1.130
30.300.000	5.318.230	-776.171	1.130
30.310.000	5.320.011	-776.412	1.131
30.320.000	5.321.561	-776.515	1.133
30.330.000	5.323.197	-776.751	1.135
30.340.000	5.324.846	-776.904	1.137
30.350.000	5.326.437	-777.098	1.136
30.360.000	5.328.084	-777.221	1.137
30.370.000	5.329.629	-777.487	1.139
30.380.000	5.331.428	-777.592	1.139
30.390.000	5.332.847	-777.774	1.138
30.400.000	5.334.679	-778.018	1.137
30.410.000	5.336.102	-778.130	1.137
30.420.000	5.337.906	-778.351	1.136
30.430.000	5.339.396	-778.551	1.134
30.440.000	5.341.183	-778.698	1.133
30.450.000	5.342.623	-778.873	1.133
30.460.000	5.344.389	-779.139	1.134
30.470.000	5.345.890	-779.201	1.136
30.480.000	5.347.556	-779.454	1.137
30.490.000	5.349.198	-779.607	1.136
30.500.000	5.350.780	-779.776	1.135
30.510.000	5.352.459	-779.930	1.133
30.520.000	5.354.036	-780.180	1.131
30.530.000	5.355.795	-780.289	1.130
30.540.000	5.357.227	-780.510	1.130

30.550.000	5.359.069	-780.729	1.131
30.560.000	5.360.485	-780.853	1.131
30.570.000	5.362.276	-781.079	1.130
30.580.000	5.363.758	-781.242	1.129
30.590.000	5.365.479	-781.415	1.128
30.600.000	5.366.983	-781.588	1.127
30.610.000	5.368.739	-781.842	1.127
30.620.000	5.370.256	-781.917	1.127
30.630.000	5.371.897	-782.165	1.128
30.640.000	5.373.612	-782.298	1.131
30.650.000	5.375.135	-782.484	1.133
30.660.000	5.376.868	-782.621	1.134
30.670.000	5.378.441	-782.864	1.136
30.680.000	5.380.177	-782.958	1.140
30.690.000	5.381.607	-783.175	1.142
30.700.000	5.383.458	-783.386	1.142
30.710.000	5.384.810	-783.504	1.140
30.720.000	5.386.639	-783.738	1.136
30.730.000	5.388.134	-783.916	1.133
30.740.000	5.389.865	-784.075	1.130
30.750.000	5.391.396	-784.260	1.128
30.760.000	5.393.155	-784.502	1.130
30.770.000	5.394.675	-784.607	1.132
30.780.000	5.396.359	-784.860	1.134
30.790.000	5.398.022	-784.988	1.135
30.800.000	5.399.539	-785.188	1.133
30.810.000	5.401.281	-785.346	1.131
30.820.000	5.402.792	-785.545	1.129
30.830.000	5.404.516	-785.678	1.126
30.840.000	5.405.989	-785.896	1.126

30.850.000	5.407.860	-786.082	1.127
30.860.000	5.409.232	-786.213	1.128
30.870.000	5.411.051	-786.428	1.129
30.880.000	5.412.585	-786.563	1.128
30.890.000	5.414.269	-786.770	1.126
30.900.000	5.415.824	-786.900	1.124
30.910.000	5.417.547	-787.117	1.123
30.920.000	5.419.062	-787.223	1.123
30.930.000	5.420.734	-787.466	1.123
30.940.000	5.422.382	-787.557	1.123
30.950.000	5.423.875	-787.780	1.124
30.960.000	5.425.661	-787.927	1.124
30.970.000	5.427.192	-788.131	1.124
30.980.000	5.428.957	-788.278	1.124
30.990.000	5.430.434	-788.492	1.124
31.000.000	5.432.237	-788.655	1.126
31.010.000	5.433.629	-788.790	1.126
31.020.000	5.435.430	-789.038	1.124
31.030.000	5.436.898	-789.156	1.122
31.040.000	5.438.609	-789.361	1.122
31.050.000	5.440.161	-789.536	1.121
31.060.000	5.441.849	-789.717	1.118
31.070.000	5.443.411	-789.824	1.117
31.080.000	5.445.069	-790.091	1.116
31.090.000	5.446.732	-790.147	1.116
31.100.000	5.448.266	-790.380	1.114
31.110.000	5.450.026	-790.532	1.112
31.120.000	5.451.526	-790.703	1.111
31.130.000	5.453.265	-790.885	1.111
31.140.000	5.454.712	-791.068	1.113

31.150.000	5.456.527	-791.217	1.115
31.160.000	5.457.912	-791.382	1.115
31.170.000	5.459.754	-791.608	1.114
31.180.000	5.461.236	-791.714	1.112
31.190.000	5.462.942	-791.936	1.110
31.200.000	5.464.547	-792.100	1.109
31.210.000	5.466.217	-792.271	1.108
31.220.000	5.467.797	-792.412	1.109
31.230.000	5.469.444	-792.666	1.110
31.240.000	5.471.076	-792.756	1.112
31.250.000	5.472.571	-792.985	1.110
31.260.000	5.474.365	-793.148	1.106
31.270.000	5.475.760	-793.305	1.103
31.280.000	5.477.565	-793.504	1.102
31.290.000	5.479.020	-793.700	1.102
31.300.000	5.480.817	-793.849	1.104
31.310.000	5.482.280	-794.024	1.109
31.320.000	5.484.135	-794.285	1.115
31.330.000	5.485.567	-794.377	1.120
31.340.000	5.487.312	-794.625	1.123
31.350.000	5.488.849	-794.785	1.122
31.360.000	5.490.482	-794.969	1.121
31.370.000	5.492.086	-795.129	1.119
31.380.000	5.493.710	-795.388	1.117
31.390.000	5.495.365	-795.465	1.113
31.400.000	5.496.898	-795.742	1.109
31.410.000	5.498.677	-795.879	1.107
31.420.000	5.500.119	-796.070	1.107
31.430.000	5.501.969	-796.299	1.107
31.440.000	5.503.416	-796.503	1.107

31.450.000	5.505.170	-796.676	1.107
31.460.000	5.506.622	-796.871	1.107
31.470.000	5.508.465	-797.122	1.108
31.480.000	5.509.893	-797.257	1.107
31.490.000	5.511.632	-797.509	1.106
31.500.000	5.513.198	-797.681	1.105
31.510.000	5.514.824	-797.889	1.106
31.520.000	5.516.438	-798.058	1.105
31.530.000	5.518.134	-798.317	1.105
31.540.000	5.519.758	-798.419	1.105
31.550.000	5.521.333	-798.703	1.104
31.560.000	5.523.111	-798.881	1.105
31.570.000	5.524.511	-799.055	1.104
31.580.000	5.526.381	-799.293	1.104
31.590.000	5.527.780	-799.479	1.103
31.600.000	5.529.554	-799.675	1.103
31.610.000	5.531.010	-799.883	1.102
31.620.000	5.532.814	-800.123	1.101
31.630.000	5.534.248	-800.260	1.102
31.640.000	5.536.012	-800.566	1.101
31.650.000	5.537.593	-800.706	1.103
31.660.000	5.539.256	-800.947	1.104
31.670.000	5.540.889	-801.131	1.105
31.680.000	5.542.512	-801.358	1.104
31.690.000	5.544.134	-801.474	1.103
31.700.000	5.545.668	-801.745	1.101
31.710.000	5.547.441	-801.874	1.098
31.720.000	5.548.825	-802.073	1.096
31.730.000	5.550.701	-802.304	1.095
31.740.000	5.552.161	-802.450	1.095

31.750.000	5.553.896	-802.667	1.096
31.760.000	5.555.401	-802.846	1.095
31.770.000	5.557.198	-803.033	1.094
31.780.000	5.558.649	-803.198	1.095
31.790.000	5.560.398	-803.457	1.094
31.800.000	5.561.935	-803.564	1.095
31.810.000	5.563.513	-803.796	1.094
31.820.000	5.565.232	-803.968	1.092
31.830.000	5.566.820	-804.184	1.092
31.840.000	5.568.464	-804.295	1.093
31.850.000	5.570.003	-804.547	1.093
31.860.000	5.571.734	-804.680	1.091
31.870.000	5.573.181	-804.872	1.089
31.880.000	5.575.033	-805.114	1.087
31.890.000	5.576.440	-805.241	1.088
31.900.000	5.578.233	-805.453	1.089
31.910.000	5.579.687	-805.649	1.088
31.920.000	5.581.408	-805.813	1.087
31.930.000	5.582.911	-805.981	1.086
31.940.000	5.584.615	-806.239	1.085
31.950.000	5.586.209	-806.323	1.086
31.960.000	5.587.781	-806.576	1.085
31.970.000	5.589.481	-806.734	1.085
31.980.000	5.591.063	-806.952	1.086
31.990.000	5.592.743	-807.101	1.086
32.000.000	5.594.265	-807.358	1.089
32.010.000	5.596.043	-807.521	1.089
32.020.000	5.597.436	-807.741	1.090
32.030.000	5.599.294	-807.989	1.091
32.040.000	5.600.720	-808.131	1.091

32.050.000	5.602.453	-808.382	1.091
32.060.000	5.603.972	-808.579	1.093
32.070.000	5.605.712	-808.781	1.093
32.080.000	5.607.201	-808.934	1.091
32.090.000	5.608.964	-809.212	1.090
32.100.000	5.610.519	-809.311	1.088
32.110.000	5.612.169	-809.567	1.087
32.120.000	5.613.853	-809.726	1.086
32.130.000	5.615.360	-809.931	1.086
32.140.000	5.617.116	-810.098	1.085
32.150.000	5.618.611	-810.356	1.084
32.160.000	5.620.372	-810.508	1.083
32.170.000	5.621.765	-810.723	1.083
32.180.000	5.623.598	-810.977	1.084
32.190.000	5.624.975	-811.088	1.082
32.200.000	5.626.796	-811.341	1.080
32.210.000	5.628.281	-811.507	1.079
32.220.000	5.629.980	-811.673	1.080
32.230.000	5.631.523	-811.834	1.081
32.240.000	5.633.216	-812.082	1.083
32.250.000	5.634.778	-812.147	1.084
32.260.000	5.636.398	-812.416	1.084
32.270.000	5.638.097	-812.528	1.085
32.280.000	5.639.581	-812.726	1.086
32.290.000	5.641.335	-812.890	1.087
32.300.000	5.642.811	-813.119	1.087
32.310.000	5.644.587	-813.278	1.086
32.320.000	5.646.050	-813.498	1.086
32.330.000	5.647.920	-813.723	1.086
32.340.000	5.649.295	-813.869	1.087

32.350.000	5.651.093	-814.129	1.087
32.360.000	5.652.602	-814.300	1.085
32.370.000	5.654.240	-814.507	1.083
32.380.000	5.655.791	-814.675	1.081
32.390.000	5.657.488	-814.922	1.080
32.400.000	5.659.036	-815.041	1.080
32.410.000	5.660.684	-815.315	1.079
32.420.000	5.662.365	-815.457	1.078
32.430.000	5.663.863	-815.672	1.077
32.440.000	5.665.675	-815.865	1.078
32.450.000	5.667.083	-816.076	1.080
32.460.000	5.668.852	-816.262	1.081
32.470.000	5.670.335	-816.493	1.081
32.480.000	5.672.119	-816.693	1.078
32.490.000	5.673.543	-816.860	1.077
32.500.000	5.675.317	-817.135	1.078
32.510.000	5.676.816	-817.259	1.076
32.520.000	5.678.492	-817.511	1.072
32.530.000	5.680.069	-817.675	1.069
32.540.000	5.681.791	-817.897	1.068
32.550.000	5.683.366	-818.057	1.069
32.560.000	5.684.986	-818.342	1.069
32.570.000	5.686.677	-818.448	1.068
32.580.000	5.688.155	-818.725	1.068
32.590.000	5.689.968	-818.911	1.069
32.600.000	5.691.392	-819.119	1.069
32.610.000	5.693.157	-819.353	1.070
32.620.000	5.694.614	-819.583	1.071
32.630.000	5.696.454	-819.778	1.071
32.640.000	5.697.856	-819.980	1.068

32.650.000	5.699.709	-820.268	1.068
32.660.000	5.701.193	-820.392	1.069
32.670.000	5.702.857	-820.676	1.070
32.680.000	5.704.474	-820.867	1.069
32.690.000	5.706.076	-821.094	1.068
32.700.000	5.707.674	-821.245	1.068
32.710.000	5.709.309	-821.543	1.067
32.720.000	5.710.994	-821.626	1.067
32.730.000	5.712.463	-821.888	1.068
32.740.000	5.714.289	-822.094	1.068
32.750.000	5.715.677	-822.245	1.067
32.760.000	5.717.484	-822.476	1.066
32.770.000	5.718.964	-822.704	1.066
32.780.000	5.720.767	-822.856	1.066
32.790.000	5.722.171	-823.043	1.066
32.800.000	5.723.945	-823.297	1.065
32.810.000	5.725.463	-823.396	1.063
32.820.000	5.727.115	-823.659	1.064
32.830.000	5.728.715	-823.834	1.064
32.840.000	5.730.336	-824.025	1.064
32.850.000	5.731.935	-824.199	1.064
32.860.000	5.733.539	-824.466	1.063
32.870.000	5.735.250	-824.561	1.063
32.880.000	5.736.702	-824.828	1.063
32.890.000	5.738.560	-825.023	1.063
32.900.000	5.739.944	-825.187	1.064
32.910.000	5.741.713	-825.416	1.065
32.920.000	5.743.188	-825.624	1.067
32.930.000	5.744.935	-825.799	1.066
32.940.000	5.746.375	-825.985	1.066

32.950.000	5.748.215	-826.241	1.066
32.960.000	5.749.659	-826.330	1.065
32.970.000	5.751.313	-826.582	1.065
32.980.000	5.752.958	-826.769	1.064
32.990.000	5.754.565	-826.959	1.063
33.000.000	5.756.212	-827.137	1.062
33.010.000	5.757.784	-827.407	1.060
33.020.000	5.759.469	-827.493	1.060
33.030.000	5.760.956	-827.759	1.060
33.040.000	5.762.750	-827.985	1.060
33.050.000	5.764.138	-828.108	1.062
33.060.000	5.765.949	-828.373	1.065
33.070.000	5.767.387	-828.561	1.070
33.080.000	5.769.118	-828.732	1.075
33.090.000	5.770.579	-828.931	1.075
33.100.000	5.772.349	-829.170	1.074
33.110.000	5.773.853	-829.271	1.072
33.120.000	5.775.554	-829.558	1.070
33.130.000	5.777.130	-829.693	1.068
33.140.000	5.778.724	-829.871	1.067
33.150.000	5.780.357	-830.028	1.070
33.160.000	5.781.959	-830.296	1.074
33.170.000	5.783.650	-830.411	1.078
33.180.000	5.785.116	-830.676	1.083
33.190.000	5.786.993	-830.907	1.087
33.200.000	5.788.321	-831.102	1.088
33.210.000	5.790.132	-831.357	1.087
33.220.000	5.791.610	-831.568	1.086
33.230.000	5.793.339	-831.792	1.086
33.240.000	5.794.829	-831.997	1.087

33.250.000	5.796.595	-832.258	1.088
33.260.000	5.798.060	-832.403	1.092
33.270.000	5.799.758	-832.692	1.095
33.280.000	5.801.366	-832.862	1.097
33.290.000	5.802.905	-833.077	1.095
33.300.000	5.804.648	-833.271	1.091
33.310.000	5.806.160	-833.529	1.088
33.320.000	5.807.886	-833.679	1.085
33.330.000	5.809.387	-833.968	1.082
33.340.000	5.811.229	-834.191	1.082
33.350.000	5.812.603	-834.392	1.081
33.360.000	5.814.460	-834.694	1.081
33.370.000	5.815.908	-834.868	1.079
33.380.000	5.817.662	-835.133	1.075
33.390.000	5.819.166	-835.370	1.072
33.400.000	5.820.917	-835.612	1.070
33.410.000	5.822.403	-835.812	1.069
33.420.000	5.824.105	-836.124	1.069
33.430.000	5.825.765	-836.298	1.071
33.440.000	5.827.340	-836.587	1.072
33.450.000	5.829.036	-836.790	1.072
33.460.000	5.830.605	-837.053	1.071
33.470.000	5.832.277	-837.234	1.069
33.480.000	5.833.762	-837.508	1.069
33.490.000	5.835.583	-837.721	1.069
33.500.000	5.836.928	-837.938	1.071
33.510.000	5.838.787	-838.201	1.076
33.520.000	5.840.229	-838.360	1.080
33.530.000	5.841.983	-838.596	1.083
33.540.000	5.843.541	-838.811	1.084

33.550.000	5.845.227	-839.010	1.083
33.560.000	5.846.756	-839.166	1.081
33.570.000	5.848.460	-839.459	1.078
33.580.000	5.850.072	-839.549	1.078
33.590.000	5.851.608	-839.810	1.078
33.600.000	5.853.333	-839.983	1.081
33.610.000	5.854.787	-840.179	1.084
33.620.000	5.856.538	-840.366	1.088
33.630.000	5.858.015	-840.631	1.087
33.640.000	5.859.797	-840.783	1.085
33.650.000	5.861.184	-840.972	1.080
33.660.000	5.862.983	-841.226	1.077
33.670.000	5.864.466	-841.350	1.074
33.680.000	5.866.164	-841.586	1.073
33.690.000	5.867.701	-841.765	1.074
33.700.000	5.869.415	-841.962	1.075
33.710.000	5.870.897	-842.116	1.075
33.720.000	5.872.588	-842.379	1.073
33.730.000	5.874.222	-842.486	1.070
33.740.000	5.875.726	-842.761	1.066
33.750.000	5.877.542	-842.933	1.063
33.760.000	5.878.938	-843.127	1.060
33.770.000	5.880.702	-843.341	1.057
33.780.000	5.882.214	-843.588	1.058
33.790.000	5.883.969	-843.768	1.059
33.800.000	5.885.390	-843.978	1.060
33.810.000	5.887.239	-844.249	1.059
33.820.000	5.888.633	-844.385	1.059
33.830.000	5.890.391	-844.647	1.060
33.840.000	5.891.921	-844.851	1.062

33.850.000	5.893.567	-845.064	1.063
33.860.000	5.895.126	-845.252	1.064
33.870.000	5.896.804	-845.549	1.065
33.880.000	5.898.391	-845.637	1.066
33.890.000	5.899.961	-845.940	1.066
33.900.000	5.901.716	-846.118	1.068
33.910.000	5.903.183	-846.309	1.068
33.920.000	5.904.955	-846.555	1.070
33.930.000	5.906.394	-846.794	1.070
33.940.000	5.908.144	-846.969	1.071
33.950.000	5.909.557	-847.206	1.073
33.960.000	5.911.373	-847.458	1.073
33.970.000	5.912.812	-847.598	1.072
33.980.000	5.914.530	-847.895	1.071
33.990.000	5.916.116	-848.071	1.070
34.000.000	5.917.767	-848.292	1.068
34.010.000	5.919.375	-848.499	1.066
34.020.000	5.921.031	-848.776	1.067
34.030.000	5.922.624	-848.881	1.068
34.040.000	5.924.158	-849.198	1.068
34.050.000	5.925.942	-849.383	1.068
34.060.000	5.927.297	-849.605	1.068
34.070.000	5.929.122	-849.843	1.069
34.080.000	5.930.557	-850.086	1.070
34.090.000	5.932.328	-850.287	1.070
34.100.000	5.933.805	-850.499	1.071
34.110.000	5.935.577	-850.747	1.072
34.120.000	5.937.044	-850.876	1.073
34.130.000	5.938.817	-851.163	1.074
34.140.000	5.940.343	-851.329	1.074

34.150.000	5.941.937	-851.546	1.076
34.160.000	5.943.588	-851.750	1.076
34.170.000	5.945.215	-852.025	1.074
34.180.000	5.946.856	-852.148	1.074
34.190.000	5.948.384	-852.456	1.073
34.200.000	5.950.176	-852.657	1.072
34.210.000	5.951.617	-852.872	1.070
34.220.000	5.953.402	-853.165	1.071
34.230.000	5.954.853	-853.363	1.070
34.240.000	5.956.608	-853.577	1.069
34.250.000	5.958.057	-853.810	1.068
34.260.000	5.959.878	-854.048	1.069
34.270.000	5.961.285	-854.210	1.069
34.280.000	5.963.035	-854.496	1.068
34.290.000	5.964.632	-854.653	1.067
34.300.000	5.966.209	-854.911	1.068
34.310.000	5.967.863	-855.085	1.068
34.320.000	5.969.473	-855.334	1.066
34.330.000	5.971.078	-855.474	1.065
34.340.000	5.972.623	-855.772	1.064
34.350.000	5.974.430	-855.949	1.063
34.360.000	5.975.802	-856.177	1.062
34.370.000	5.977.645	-856.439	1.060
34.380.000	5.979.101	-856.639	1.058
34.390.000	5.980.834	-856.862	1.056
34.400.000	5.982.370	-857.092	1.057
34.410.000	5.984.112	-857.331	1.057
34.420.000	5.985.608	-857.503	1.053
34.430.000	5.987.359	-857.819	1.051
34.440.000	5.988.891	-857.957	1.050

34.450.000	5.990.497	-858.233	1.051
34.460.000	5.992.173	-858.442	1.050
34.470.000	5.993.717	-858.690	1.049
34.480.000	5.995.436	-858.858	1.048
34.490.000	5.996.884	-859.169	1.050
34.500.000	5.998.735	-859.329	1.052
34.510.000	6.000.114	-859.570	1.053
34.520.000	6.001.939	-859.843	1.053
34.530.000	6.003.419	-860.015	1.051
34.540.000	6.005.133	-860.265	1.049
34.550.000	6.006.639	-860.483	1.048
34.560.000	6.008.397	-860.710	1.048
34.570.000	6.009.880	-860.903	1.047
34.580.000	6.011.602	-861.201	1.045
34.590.000	6.013.215	-861.335	1.044
34.600.000	6.014.751	-861.610	1.046
34.610.000	6.016.454	-861.790	1.049
34.620.000	6.017.971	-862.032	1.049
