# Supplementary material for: Data on statistical experimental design to formulate amphotericin B-loaded Eudragit RL100 nanoparticles coated with hyaluronic acid for the treatment of vulvovaginal candidiasis
Source: Data Brief. 2020 Mar 5;29:105311. doi: 10.1016/j.dib.2020.105311 (PMC7082528; doi:10.1016/j.dib.2020.105311)
Supplement: Multimedia component 3 [file mmc3.pdf]

| | | | |
|---|---|---|---|
| File Name: | **Pure HA** | | |
| | | | |
| [Data] | | | |
| Time | Temp | **DTA** | **TGA** |
| sec | C | uV | mg |
| 0 | 256.676 | 38 | 2.0263 |
| 1.2000 | 256.574 | 80 | 2.0262 |
| 2.2000 | 256.764 | 57 | 2.0261 |
| 3.2000 | 256.539 | 194 | 2.0261 |
| 4.2000 | 256.706 | 100 | 2.0264 |
| 5.2000 | 256.676 | 262 | 2.0262 |
| 6.2000 | 256.632 | 219 | 2.0263 |
| 7.2000 | 256.864 | 238 | 2.0266 |
| 8.2000 | 256.711 | 225 | 2.0267 |
| 9.2000 | 257.015 | 256 | 2.0268 |
| 10.2000 | 256.890 | 119 | 2.0269 |
| 11.2000 | 257.285 | 112 | 2.0272 |
| 12.2000 | 257.137 | 54 | 2.0277 |
| 130.000 | 257.523 | -127 | 2.0278 |
| 140.000 | 257.471 | -153 | 2.0278 |
| 150.000 | 257.827 | -429 | 2.0280 |
| 160.000 | 257.938 | -605 | 2.0280 |
| 170.000 | 258.377 | -869 | 2.0278 |
| 180.000 | 258.520 | -1.109 | 2.0276 |
| 190.000 | 258.973 | -1.549 | 2.0277 |
| 200.000 | 259.213 | -1.749 | 2.0278 |
| 210.000 | 259.466 | -2.330 | 2.0279 |
| 220.000 | 260.200 | -2.745 | 2.0280 |
| 230.000 | 260.360 | -3.257 | 2.0281 |
| 240.000 | 260.923 | -3.766 | 2.0282 |

| | | | |
|---|---|---|---|
| 250.000 | 261.243 | -4.437 | 2.0279 |
| 260.000 | 261.898 | -5.023 | 2.0278 |
| 270.000 | 262.143 | -5.738 | 2.0276 |
| 280.000 | 262.919 | -6.463 | 2.0277 |
| 290.000 | 263.243 | -7.140 | 2.0280 |
| 300.000 | 264.054 | -8.036 | 2.0281 |
| 310.000 | 264.466 | -8.806 | 2.0285 |
| 320.000 | 265.173 | -9.677 | 2.0288 |
| 330.000 | 265.721 | -10.567 | 2.0289 |
| 340.000 | 266.459 | -11.540 | 2.0290 |
| 350.000 | 267.069 | -12.327 | 2.0289 |
| 360.000 | 267.687 | -13.444 | 2.0288 |
| 370.000 | 268.505 | -14.338 | 2.0287 |
| 380.000 | 269.006 | -15.407 | 2.0286 |
| 390.000 | 270.011 | -16.459 | 2.0286 |
| 400.000 | 270.585 | -17.557 | 2.0287 |
| 410.000 | 271.539 | -18.647 | 2.0286 |
| 420.000 | 272.133 | -19.779 | 2.0284 |
| 430.000 | 273.290 | -20.946 | 2.0285 |
| 440.000 | 273.722 | -22.065 | 2.0287 |
| 450.000 | 274.756 | -23.361 | 2.0285 |
| 460.000 | 275.526 | -24.462 | 2.0285 |
| 470.000 | 276.465 | -25.730 | 2.0285 |
| 480.000 | 277.305 | -26.921 | 2.0287 |
| 490.000 | 278.221 | -28.184 | 2.0288 |
| 500.000 | 279.102 | -29.393 | 2.0285 |
| 510.000 | 280.087 | -30.817 | 2.0285 |
| 520.000 | 281.146 | -31.954 | 2.0286 |
| 530.000 | 281.927 | -33.352 | 2.0285 |
| 540.000 | 283.175 | -34.647 | 2.0280 |

| | | | |
|---|---|---|---|
| 550.000 | 283.873 | -35.957 | 2.0277 |
| 560.000 | 285.145 | -37.335 | 2.0276 |
| 570.000 | 285.940 | -38.714 | 2.0276 |
| 580.000 | 287.125 | -40.019 | 2.0275 |
| 590.000 | 287.926 | -41.390 | 2.0270 |
| 600.000 | 289.306 | -42.881 | 2.0270 |
| 610.000 | 290.158 | -44.183 | 2.0270 |
| 620.000 | 291.379 | -45.640 | 2.0272 |
| 630.000 | 292.520 | -47.006 | 2.0271 |
| 640.000 | 293.590 | -48.435 | 2.0270 |
| 650.000 | 294.787 | -49.790 | 2.0269 |
| 660.000 | 295.742 | -51.288 | 2.0269 |
| 670.000 | 297.086 | -52.578 | 2.0267 |
| 680.000 | 298.026 | -54.085 | 2.0263 |
| 690.000 | 299.427 | -55.536 | 2.0260 |
| 700.000 | 300.308 | -56.872 | 2.0258 |
| 710.000 | 301.728 | -58.377 | 2.0258 |
| 720.000 | 302.701 | -59.810 | 2.0256 |
| 730.000 | 304.134 | -61.232 | 2.0255 |
| 740.000 | 305.103 | -62.650 | 2.0254 |
| 750.000 | 306.575 | -64.172 | 2.0255 |
| 760.000 | 307.569 | -65.489 | 2.0255 |
| 770.000 | 308.890 | -67.043 | 2.0252 |
| 780.000 | 310.090 | -68.395 | 2.0249 |
| 790.000 | 311.296 | -69.850 | 2.0247 |
| 800.000 | 312.525 | -71.239 | 2.0245 |
| 810.000 | 313.762 | -72.756 | 2.0240 |
| 820.000 | 315.171 | -74.037 | 2.0237 |
| 830.000 | 316.183 | -75.515 | 2.0234 |
| 840.000 | 317.627 | -76.916 | 2.0235 |

| | | | |
|---|---|---|---|
| 850.000 | 318.658 | -78.357 | 2.0236 |
| 860.000 | 320.329 | -79.824 | 2.0237 |
| 870.000 | 321.410 | -81.183 | 2.0237 |
| 880.000 | 322.874 | -82.617 | 2.0238 |
| 890.000 | 324.015 | -84.022 | 2.0238 |
| 900.000 | 325.556 | -85.439 | 2.0235 |
| 910.000 | 326.648 | -86.765 | 2.0231 |
| 920.000 | 328.117 | -88.274 | 2.0223 |
| 930.000 | 329.406 | -89.557 | 2.0218 |
| 940.000 | 330.713 | -91.001 | 2.0213 |
| 950.000 | 332.162 | -92.359 | 2.0208 |
| 960.000 | 333.514 | -93.732 | 2.0203 |
| 970.000 | 334.900 | -95.056 | 2.0199 |
| 980.000 | 336.145 | -96.526 | 2.0196 |
| 990.000 | 337.778 | -97.812 | 2.0195 |
| 1.000.000 | 338.804 | -99.198 | 2.0191 |
| 1.010.000 | 340.560 | -100.614 | 2.0186 |
| 1.020.000 | 341.629 | -101.846 | 2.0182 |
| 1.030.000 | 343.230 | -103.265 | 2.0180 |
| 1.040.000 | 344.593 | -104.627 | 2.0177 |
| 1.050.000 | 346.130 | -105.951 | 2.0171 |
| 1.060.000 | 347.329 | -107.236 | 2.0166 |
| 1.070.000 | 348.932 | -108.660 | 2.0163 |
| 1.080.000 | 350.265 | -109.874 | 2.0162 |
| 1.090.000 | 351.740 | -111.274 | 2.0159 |
| 1.100.000 | 353.240 | -112.511 | 2.0155 |
| 1.110.000 | 354.568 | -113.850 | 2.0150 |
| 1.120.000 | 356.203 | -115.098 | 2.0149 |
| 1.130.000 | 357.474 | -116.468 | 2.0146 |
| 1.140.000 | 359.129 | -117.704 | 2.0141 |

| | | | |
|---|---|---|---|
| 1.150.000 | 360.314 | -119.022 | 2.0135 |
| 1.160.000 | 362.112 | -120.356 | 2.0130 |
| 1.170.000 | 363.296 | -121.561 | 2.0125 |
| 1.180.000 | 365.020 | -122.900 | 2.0121 |
| 1.190.000 | 366.394 | -124.181 | 2.0116 |
| 1.200.000 | 368.001 | -125.421 | 2.0112 |
| 1.210.000 | 369.376 | -126.673 | 2.0108 |
| 1.220.000 | 371.014 | -128.018 | 2.0104 |
| 1.230.000 | 372.396 | -129.154 | 2.1999 |
| 1.240.000 | 373.919 | -130.512 | 2.1995 |
| 1.250.000 | 375.551 | -131.698 | 2.1988 |
| 1.260.000 | 376.893 | -132.970 | 2.1981 |
| 1.270.000 | 378.626 | -134.178 | 2.1974 |
| 1.280.000 | 379.925 | -135.480 | 2.1969 |
| 1.290.000 | 381.638 | -136.669 | 2.1965 |
| 1.300.000 | 382.990 | -137.957 | 2.1959 |
| 1.310.000 | 384.791 | -139.212 | 2.1953 |
| 1.320.000 | 386.089 | -140.384 | 2.1950 |
| 1.330.000 | 387.826 | -141.703 | 2.1947 |
| 1.340.000 | 389.190 | -142.825 | 2.1942 |
| 1.350.000 | 390.833 | -144.077 | 2.1935 |
| 1.360.000 | 392.401 | -145.303 | 2.2027 |
| 1.370.000 | 393.961 | -146.523 | 2.2021 |
| 1.380.000 | 395.482 | -147.633 | 2.2015 |
| 1.390.000 | 396.966 | -148.969 | 2.2006 |
| 1.400.000 | 398.720 | -150.017 | 1.9998 |
| 1.410.000 | 400.053 | -151.284 | 1.9993 |
| 1.420.000 | 401.851 | -152.453 | 1.9989 |
| 1.430.000 | 403.224 | -153.614 | 1.9984 |
| 1.440.000 | 404.917 | -154.810 | 1.9976 |

| | | | |
|---|---|---|---|
| 1.450.000 | 406.228 | -156.040 | 1.9970 |
| 1.460.000 | 408.034 | -157.180 | 1.9965 |
| 1.470.000 | 409.311 | -158.313 | 1.9957 |
| 1.480.000 | 411.210 | -159.554 | 1.9947 |
| 1.490.000 | 412.412 | -160.611 | 1.9938 |
| 1.500.000 | 414.099 | -161.875 | 1.9931 |
| 1.510.000 | 415.676 | -162.963 | 1.9926 |
| 1.520.000 | 417.270 | -164.134 | 1.9919 |
| 1.530.000 | 418.812 | -165.201 | 1.9911 |
| 1.540.000 | 420.371 | -166.460 | 1.9903 |
| 1.550.000 | 421.988 | -167.442 | 1.9898 |
| 1.560.000 | 423.402 | -168.659 | 1.9892 |
| 1.570.000 | 425.212 | -169.775 | 1.9884 |
| 1.580.000 | 426.504 | -170.857 | 1.9874 |
| 1.590.000 | 428.345 | -172.023 | 1.9865 |
| 1.600.000 | 429.679 | -173.173 | 1.9859 |
| 1.610.000 | 431.467 | -174.216 | 1.9853 |
| 1.620.000 | 432.806 | -175.329 | 1.9843 |
| 1.630.000 | 434.681 | -176.523 | 1.9835 |
| 1.640.000 | 436.077 | -177.490 | 1.9830 |
| 1.650.000 | 437.813 | -178.666 | 1.9825 |
| 1.660.000 | 439.339 | -179.715 | 1.9816 |
| 1.670.000 | 440.906 | -180.811 | 1.9803 |
| 1.680.000 | 442.548 | -181.863 | 1.9793 |
| 1.690.000 | 444.162 | -183.021 | 1.9783 |
| 1.700.000 | 445.866 | -183.940 | 1.9772 |
| 1.710.000 | 447.269 | -185.110 | 1.9757 |
| 1.720.000 | 449.184 | -186.199 | 1.9746 |
| 1.730.000 | 450.444 | -187.155 | 1.9737 |
| 1.740.000 | 452.301 | -188.269 | 1.9729 |

| | | | |
|---|---|---|---|
| 1.750.000 | 453.721 | -189.306 | 1.9721 |
| 1.760.000 | 455.505 | -190.324 | 1.9711 |
| 1.770.000 | 456.938 | -191.345 | 1.9702 |
| 1.780.000 | 458.825 | -192.422 | 1.9693 |
| 1.790.000 | 460.221 | -193.352 | 1.9685 |
| 1.800.000 | 461.983 | -194.464 | 1.9674 |
| 1.810.000 | 463.575 | -195.377 | 1.9662 |
| 1.820.000 | 465.154 | -196.396 | 1.9652 |
| 1.830.000 | 466.839 | -197.365 | 1.9643 |
| 1.840.000 | 468.519 | -198.401 | 1.9632 |
| 1.850.000 | 470.146 | -199.266 | 1.9619 |
| 1.860.000 | 471.649 | -200.340 | 1.9609 |
| 1.870.000 | 473.557 | -201.248 | 1.9603 |
| 1.880.000 | 474.860 | -202.192 | 1.9595 |
| 1.890.000 | 476.796 | -203.200 | 1.9585 |
| 1.900.000 | 478.160 | -204.061 | 1.9578 |
| 1.910.000 | 480.005 | -205.020 | 1.9571 |
| 1.920.000 | 481.502 | -205.954 | 1.9562 |
| 1.930.000 | 483.275 | -206.852 | 1.9552 |
| 1.940.000 | 484.790 | -207.716 | 1.9543 |
| 1.950.000 | 486.566 | -208.713 | 1.9536 |
| 1.960.000 | 488.199 | -209.470 | 1.9529 |
| 1.970.000 | 489.825 | -210.423 | 1.9522 |
| 1.980.000 | 491.607 | -211.266 | 1.9516 |
| 1.990.000 | 493.116 | -212.131 | 1.9509 |
| 2.000.000 | 494.961 | -212.946 | 1.9501 |
| 2.010.000 | 496.401 | -213.868 | 1.9493 |
| 2.020.000 | 498.289 | -214.665 | 1.9483 |
| 2.030.000 | 499.663 | -215.497 | 1.9473 |
| 2.040.000 | 501.668 | -216.394 | 1.9460 |

| | | | |
|---|---|---|---|
| 2.050.000 | 503.031 | -217.113 | 1.9449 |
| 2.060.000 | 504.859 | -217.978 | 1.9439 |
| 2.070.000 | 506.401 | -218.758 | 1.9429 |
| 2.080.000 | 508.166 | -219.557 | 1.9420 |
| 2.090.000 | 509.724 | -220.283 | 1.9409 |
| 2.100.000 | 511.487 | -221.155 | 1.9400 |
| 2.110.000 | 513.076 | -221.780 | 1.9393 |
| 2.120.000 | 514.673 | -222.625 | 1.9383 |
| 2.130.000 | 516.445 | -223.333 | 1.9372 |
| 2.140.000 | 517.893 | -224.057 | 1.9362 |
| 2.150.000 | 519.814 | -224.800 | 1.9350 |
| 2.160.000 | 521.232 | -225.582 | 1.9340 |
| 2.170.000 | 523.065 | -226.243 | 1.9329 |
| 2.180.000 | 524.490 | -226.960 | 1.9317 |
| 2.190.000 | 526.427 | -227.717 | 1.9309 |
| 2.200.000 | 527.828 | -228.307 | 1.9301 |
| 2.210.000 | 529.766 | -229.105 | 1.9292 |
| 2.220.000 | 531.273 | -229.714 | 1.9283 |
| 2.230.000 | 532.975 | -230.398 | 1.9274 |
| 2.240.000 | 534.564 | -231.026 | 1.9265 |
| 2.250.000 | 536.270 | -231.736 | 1.9254 |
| 2.260.000 | 537.935 | -232.283 | 1.9243 |
| 2.270.000 | 539.485 | -233.015 | 1.9231 |
| 2.280.000 | 541.445 | -233.576 | 1.9222 |
| 2.290.000 | 542.810 | -234.235 | 1.9212 |
| 2.300.000 | 544.764 | -234.892 | 1.9201 |
| 2.310.000 | 546.293 | -235.501 | 1.9190 |
| 2.320.000 | 548.124 | -236.120 | 1.9180 |
| 2.330.000 | 549.563 | -236.717 | 1.9169 |
| 2.340.000 | 551.537 | -237.314 | 1.9159 |

| | | | |
|---|---|---|---|
| 2.350.000 | 552.903 | -237.867 | 1.9146 |
| 2.360.000 | 554.854 | -238.541 | 1.9136 |
| 2.370.000 | 556.394 | -239.021 | 1.9126 |
| 2.380.000 | 558.105 | -239.666 | 1.9114 |
| 2.390.000 | 559.814 | -240.187 | 1.9100 |
| 2.400.000 | 561.532 | -240.756 | 1.9089 |
| 2.410.000 | 563.189 | -241.233 | 1.9078 |
| 2.420.000 | 564.845 | -241.897 | 1.9065 |
| 2.430.000 | 566.705 | -242.314 | 1.9053 |
| 2.440.000 | 568.137 | -242.887 | 1.9042 |
| 2.450.000 | 570.118 | -243.467 | 1.9031 |
| 2.460.000 | 571.550 | -243.929 | 1.9018 |
| 2.470.000 | 573.462 | -244.481 | 1.9006 |
| 2.480.000 | 574.954 | -245.003 | 1.8995 |
| 2.490.000 | 576.855 | -245.485 | 1.8985 |
| 2.500.000 | 578.358 | -245.925 | 1.8973 |
| 2.510.000 | 580.196 | -246.538 | 1.8966 |
| 2.520.000 | 581.839 | -246.883 | 1.8960 |
| 2.530.000 | 583.628 | -247.457 | 1.8954 |
| 2.540.000 | 585.317 | -247.908 | 1.8946 |
| 2.550.000 | 586.982 | -248.342 | 1.8938 |
| 2.560.000 | 588.752 | -248.745 | 1.8930 |
| 2.570.000 | 590.329 | -249.308 | 1.8918 |
| 2.580.000 | 592.213 | -249.587 | 1.8906 |
| 2.590.000 | 593.601 | -250.132 | 1.8896 |
| 2.600.000 | 595.719 | -250.604 | 1.8886 |
| 2.610.000 | 597.045 | -250.958 | 1.8877 |
| 2.620.000 | 599.017 | -251.458 | 1.8867 |
| 2.630.000 | 600.559 | -251.867 | 1.8860 |
| 2.640.000 | 602.478 | -252.249 | 1.8853 |

| | | | |
|---|---|---|---|
| 2.650.000 | 603.954 | -252.648 | 1.8848 |
| 2.660.000 | 605.881 | -253.131 | 1.8839 |
| 2.670.000 | 607.406 | -253.388 | 1.8831 |
| 2.680.000 | 609.155 | -253.874 | 1.8824 |
| 2.690.000 | 610.922 | -254.213 | 1.8817 |
| 2.700.000 | 612.468 | -254.579 | 1.8808 |
| 2.710.000 | 614.318 | -254.895 | 1.8797 |
| 2.720.000 | 615.862 | -255.333 | 1.8785 |
| 2.730.000 | 617.655 | -255.610 | 1.8778 |
| 2.740.000 | 619.199 | -256.007 | 1.8769 |
| 2.750.000 | 621.154 | -256.405 | 1.8758 |
| 2.760.000 | 622.551 | -256.665 | 1.8747 |
| 2.770.000 | 624.559 | -257.085 | 1.8739 |
| 2.780.000 | 626.025 | -257.364 | 1.8732 |
| 2.790.000 | 627.885 | -257.708 | 1.8723 |
| 2.800.000 | 629.436 | -257.996 | 1.8712 |
| 2.810.000 | 631.277 | -258.361 | 1.8702 |
| 2.820.000 | 632.860 | -258.579 | 1.8695 |
| 2.830.000 | 634.615 | -258.974 | 1.8686 |
| 2.840.000 | 636.387 | -259.185 | 1.8676 |
| 2.850.000 | 637.950 | -259.530 | 1.8665 |
| 2.860.000 | 639.865 | -259.794 | 1.8656 |
| 2.870.000 | 641.369 | -260.092 | 1.8647 |
| 2.880.000 | 643.264 | -260.381 | 1.8637 |
| 2.890.000 | 644.757 | -260.682 | 1.8626 |
| 2.900.000 | 646.734 | -260.956 | 1.8616 |
| 2.910.000 | 648.120 | -261.208 | 1.8607 |
| 2.920.000 | 650.103 | -261.536 | 1.8601 |
| 2.930.000 | 651.614 | -261.707 | 1.8592 |
| 2.940.000 | 653.462 | -262.039 | 1.8582 |

| | | | |
|---|---|---|---|
| 2.950.000 | 655.122 | -262.267 | 1.8571 |
| 2.960.000 | 656.910 | -262.535 | 1.8564 |
| 2.970.000 | 658.545 | -262.708 | 1.8556 |
| 2.980.000 | 660.309 | -263.101 | 1.8547 |
| 2.990.000 | 662.050 | -263.131 | 1.8538 |
| 3.000.000 | 663.601 | -263.477 | 1.8531 |
| 3.010.000 | 665.614 | -263.688 | 1.8526 |
| 3.020.000 | 667.083 | -263.890 | 1.8520 |
| 3.030.000 | 669.029 | -264.163 | 1.8508 |
| 3.040.000 | 670.496 | -264.391 | 1.8499 |
| 3.050.000 | 672.415 | -264.613 | 1.8492 |
| 3.060.000 | 673.891 | -264.816 | 1.8485 |
| 3.070.000 | 675.914 | -265.105 | 1.8476 |
| 3.080.000 | 677.407 | -265.172 | 1.8467 |
| 3.090.000 | 679.263 | -265.463 | 1.8462 |
| 3.100.000 | 680.973 | -265.688 | 1.8455 |
| 3.110.000 | 682.769 | -265.846 | 1.8446 |
| 3.120.000 | 684.444 | -265.999 | 1.8436 |
| 3.130.000 | 686.148 | -266.287 | 1.8428 |
| 3.140.000 | 687.953 | -266.324 | 1.8421 |
| 3.150.000 | 689.466 | -266.607 | 1.8414 |
| 3.160.000 | 691.473 | -266.784 | 1.8406 |
| 3.170.000 | 692.851 | -266.900 | 1.8398 |
| 3.180.000 | 694.862 | -267.138 | 1.8391 |
| 3.190.000 | 696.422 | -267.316 | 1.8384 |
| 3.200.000 | 698.311 | -267.453 | 1.8377 |
| 3.210.000 | 699.846 | -267.628 | 1.8367 |
| 3.220.000 | 701.867 | -267.851 | 1.8358 |
| 3.230.000 | 703.351 | -267.910 | 1.8351 |
| 3.240.000 | 705.156 | -268.145 | 1.8343 |

| | | | |
|---|---|---|---|
| 3.250.000 | 706.902 | -268.234 | 1.8336 |
| 3.260.000 | 708.508 | -268.397 | 1.8330 |
| 3.270.000 | 710.272 | -268.507 | 1.8322 |
| 3.280.000 | 711.942 | -268.727 | 1.8315 |
| 3.290.000 | 713.729 | -268.755 | 1.8308 |
| 3.300.000 | 715.302 | -268.987 | 1.8301 |
| 3.310.000 | 717.298 | -269.103 | 1.8294 |
| 3.320.000 | 718.623 | -269.211 | 1.8284 |
| 3.330.000 | 720.719 | -269.404 | 1.8273 |
| 3.340.000 | 722.139 | -269.498 | 1.8266 |
| 3.350.000 | 724.032 | -269.629 | 1.8259 |
| 3.360.000 | 725.605 | -269.766 | 1.8252 |
| 3.370.000 | 727.496 | -269.905 | 1.8241 |
| 3.380.000 | 728.998 | -269.954 | 1.8233 |
| 3.390.000 | 730.836 | -270.184 | 1.8227 |
| 3.400.000 | 732.532 | -270.197 | 1.8222 |
| 3.410.000 | 734.169 | -270.358 | 1.8217 |
| 3.420.000 | 736.014 | -270.453 | 1.8213 |
| 3.430.000 | 737.612 | -270.582 | 1.8209 |
| 3.440.000 | 739.440 | -270.649 | 1.8203 |
| 3.450.000 | 741.008 | -270.838 | 1.8198 |
| 3.460.000 | 742.984 | -270.883 | 1.8191 |
| 3.470.000 | 744.340 | -270.980 | 1.8184 |
| 3.480.000 | 746.349 | -271.155 | 1.8174 |
| 3.490.000 | 747.888 | -271.161 | 1.8161 |
| 3.500.000 | 749.707 | -271.345 | 1.8152 |
| 3.510.000 | 751.304 | -271.418 | 1.8145 |
| 3.520.000 | 753.160 | -271.504 | 1.8138 |
| 3.530.000 | 754.740 | -271.561 | 1.8132 |
| 3.540.000 | 756.545 | -271.758 | 1.8123 |

| | | | |
|---|---|---|---|
| 3.550.000 | 758.268 | -271.730 | 1.8116 |
| 3.560.000 | 759.931 | -271.914 | 1.8112 |
| 3.570.000 | 761.811 | -271.960 | 1.8107 |
| 3.580.000 | 763.289 | -272.053 | 1.8103 |
| 3.590.000 | 765.252 | -272.121 | 1.8097 |
| 3.600.000 | 766.740 | -272.282 | 1.8089 |
| 3.610.000 | 768.705 | -272.308 | 1.8084 |
| 3.620.000 | 770.135 | -272.421 | 1.8078 |
| 3.630.000 | 772.217 | -272.582 | 1.8072 |
| 3.640.000 | 773.632 | -272.555 | 1.8064 |
| 3.650.000 | 775.584 | -272.748 | 1.8056 |
| 3.660.000 | 777.212 | -272.829 | 1.8048 |
| 3.670.000 | 779.004 | -272.899 | 1.8043 |
| 3.680.000 | 780.684 | -272.967 | 1.8039 |
| 3.690.000 | 782.453 | -273.134 | 1.8034 |
| 3.700.000 | 784.168 | -273.101 | 1.8028 |
| 3.710.000 | 785.823 | -273.285 | 1.8020 |
| 3.720.000 | 787.715 | -273.297 | 1.8016 |
| 3.730.000 | 789.242 | -273.382 | 1.8010 |
| 3.740.000 | 791.235 | -273.542 | 1.8005 |
| 3.750.000 | 792.746 | -273.598 | 1.8001 |
| 3.760.000 | 794.630 | -273.700 | 1.7994 |
| 3.770.000 | 796.147 | -273.812 | 1.7986 |
| 3.780.000 | 798.184 | -273.878 | 1.7982 |
| 3.790.000 | 799.591 | -273.915 | 1.7978 |
| 3.800.000 | 801.542 | -274.099 | 1.7976 |
| 3.810.000 | 803.176 | -274.102 | 1.7969 |
| 3.820.000 | 804.893 | -274.223 | 1.7963 |
| 3.830.000 | 806.563 | -274.261 | 1.7962 |
| 3.840.000 | 808.312 | -274.383 | 1.7957 |

| | | | |
|---|---|---|---|
| 3.850.000 | 810.026 | -274.349 | 1.7950 |
| 3.860.000 | 811.644 | -274.528 | 1.7943 |
| 3.870.000 | 813.550 | -274.532 | 1.7936 |
| 3.880.000 | 814.975 | -274.649 | 1.7931 |
| 3.890.000 | 817.032 | -274.766 | 1.7926 |
| 3.900.000 | 818.321 | -274.758 | 1.7922 |
| 3.910.000 | 820.254 | -274.898 | 1.7917 |
| 3.920.000 | 821.909 | -274.963 | 1.7910 |
| 3.930.000 | 823.795 | -275.002 | 1.7904 |
| 3.940.000 | 825.296 | -275.036 | 1.7898 |
| 3.950.000 | 827.161 | -275.214 | 1.7893 |
| 3.960.000 | 828.785 | -275.162 | 1.7887 |
| 3.970.000 | 830.516 | -275.302 | 1.7882 |
| 3.980.000 | 832.296 | -275.366 | 1.7876 |
| 3.990.000 | 833.868 | -275.433 | 1.7869 |
| 4.000.000 | 835.686 | -275.463 | 1.7868 |
| 4.010.000 | 837.288 | -275.610 | 1.7863 |
| 4.020.000 | 839.205 | -275.594 | 1.7858 |
| 4.030.000 | 840.621 | -275.719 | 1.7849 |
| 4.040.000 | 842.641 | -275.853 | 1.7839 |
| 4.050.000 | 844.048 | -275.822 | 1.7834 |
| 4.060.000 | 845.966 | -275.984 | 1.7828 |
| 4.070.000 | 847.561 | -276.046 | 1.7819 |
| 4.080.000 | 849.394 | -276.096 | 1.7813 |
| 4.090.000 | 850.934 | -276.153 | 1.7809 |
| 4.100.000 | 852.822 | -276.322 | 1.7805 |
| 4.110.000 | 854.412 | -276.273 | 1.7810 |
| 4.120.000 | 856.145 | -276.483 | 1.7807 |
| 4.130.000 | 858.007 | -276.503 | 1.7805 |
| 4.140.000 | 859.567 | -276.600 | 1.7800 |

| | | | |
|---|---|---|---|
| 4.150.000 | 861.441 | -276.641 | 1.7795 |
| 4.160.000 | 863.059 | -276.789 | 1.7791 |
| 4.170.000 | 864.905 | -276.803 | 1.7785 |
| 4.180.000 | 866.364 | -276.909 | 1.7780 |
| 4.190.000 | 868.397 | -277.055 | 1.7774 |
| 4.200.000 | 869.750 | -277.035 | 1.7768 |
| 4.210.000 | 871.724 | -277.212 | 1.7766 |
| 4.220.000 | 873.312 | -277.295 | 1.7762 |
| 4.230.000 | 875.124 | -277.371 | 1.7758 |
| 4.240.000 | 876.768 | -277.448 | 1.7754 |
| 4.250.000 | 878.602 | -277.614 | 1.7747 |
| 4.260.000 | 880.263 | -277.589 | 1.7742 |
| 4.270.000 | 881.956 | -277.778 | 1.7737 |
| 4.280.000 | 883.757 | -277.800 | 1.7734 |
| 4.290.000 | 885.325 | -277.924 | 1.7729 |
| 4.300.000 | 887.263 | -278.023 | 1.7724 |
| 4.310.000 | 888.820 | -278.115 | 1.7718 |
| 4.320.000 | 890.713 | -278.217 | 1.7713 |
| 4.330.000 | 892.216 | -278.348 | 1.7708 |
| 4.340.000 | 894.226 | -278.453 | 1.7705 |
| 4.350.000 | 895.647 | -278.493 | 1.7701 |
| 4.360.000 | 897.601 | -278.670 | 1.7697 |
| 4.370.000 | 899.168 | -278.706 | 1.7693 |
| 4.380.000 | 900.961 | -278.842 | 1.7692 |
| 4.390.000 | 902.613 | -278.923 | 1.7689 |
| 4.400.000 | 904.415 | -279.049 | 1.7684 |
| 4.410.000 | 906.091 | -279.094 | 1.7679 |
| 4.420.000 | 907.783 | -279.307 | 1.7676 |
| 4.430.000 | 909.594 | -279.259 | 1.7674 |
| 4.440.000 | 911.016 | -279.420 | 1.7671 |

| | | | |
|---|---|---|---|
| 4.450.000 | 913.033 | -279.552 | 1.7666 |
| 4.460.000 | 914.501 | -279.615 | 1.7663 |
| 4.470.000 | 916.395 | -279.740 | 1.7660 |
| 4.480.000 | 917.888 | -279.881 | 1.7656 |
| 4.490.000 | 919.804 | -279.946 | 1.7653 |
| 4.500.000 | 921.265 | -280.022 | 1.7647 |
| 4.510.000 | 923.209 | -280.227 | 1.7644 |
| 4.520.000 | 924.722 | -280.219 | 1.7642 |
| 4.530.000 | 926.522 | -280.396 | 1.7639 |
| 4.540.000 | 928.202 | -280.485 | 1.7634 |
| 4.550.000 | 929.875 | -280.576 | 1.7627 |
| 4.560.000 | 931.611 | -280.645 | 1.7623 |
| 4.570.000 | 933.280 | -280.877 | 1.7619 |
| 4.580.000 | 935.114 | -280.840 | 1.7615 |
| 4.590.000 | 936.594 | -281.034 | 1.7609 |
| 4.600.000 | 938.576 | -281.176 | 1.7606 |
| 4.610.000 | 939.986 | -281.214 | 1.7604 |
| 4.620.000 | 941.906 | -281.396 | 1.7604 |
| 4.630.000 | 943.419 | -281.493 | 1.7600 |
| 4.640.000 | 945.327 | -281.592 | 1.7596 |
| 4.650.000 | 946.827 | -281.694 | 1.7593 |
| 4.660.000 | 948.733 | -281.880 | 1.7592 |
| 4.670.000 | 950.278 | -281.897 | 1.7590 |
| 4.680.000 | 952.096 | -282.114 | 1.7588 |
| 4.690.000 | 953.812 | -282.177 | 1.7585 |
| 4.700.000 | 955.477 | -282.285 | 1.7581 |
| 4.710.000 | 957.214 | -282.371 | 1.7581 |
| 4.720.000 | 958.872 | -282.600 | 1.7579 |
| 4.730.000 | 960.673 | -282.606 | 1.7574 |
| 4.740.000 | 962.189 | -282.810 | 1.7568 |

| | | | |
|---|---|---|---|
| 4.750.000 | 964.204 | -282.952 | 1.7563 |
| 4.760.000 | 965.584 | -283.021 | 1.7561 |
| 4.770.000 | 967.553 | -283.221 | 1.7557 |
| 4.780.000 | 969.092 | -283.337 | 1.7550 |
| 4.790.000 | 970.965 | -283.464 | 1.7543 |
| 4.800.000 | 972.515 | -283.593 | 1.7539 |
| 4.810.000 | 974.398 | -283.787 | 1.7537 |
| 4.820.000 | 975.984 | -283.803 | 1.7533 |
| 4.830.000 | 977.767 | -284.027 | 1.7528 |
| 4.840.000 | 979.506 | -284.086 | 1.7525 |
| 4.850.000 | 981.123 | -284.276 | 1.7523 |
| 4.860.000 | 983.004 | -284.374 | 1.7522 |
| 4.870.000 | 984.522 | -284.534 | 1.7518 |
| 4.880.000 | 986.413 | -284.637 | 1.7514 |
| 4.890.000 | 987.919 | -284.845 | 1.7511 |
| 4.900.000 | 989.906 | -284.980 | 1.7509 |
| 4.910.000 | 991.335 | -285.089 | 1.7507 |
| 4.920.000 | 993.307 | -285.316 | 1.7504 |
| 4.930.000 | 994.816 | -285.377 | 1.7500 |
| 4.940.000 | 996.672 | -285.567 | 1.7497 |
| 4.950.000 | 998.293 | -285.717 | 1.7496 |
| 4.960.000 | 1.000.160 | -285.891 | 1.7493 |
| 4.970.000 | 1.001.760 | -285.978 | 1.7489 |
| 4.980.000 | 1.003.531 | -286.223 | 1.7483 |
| 4.990.000 | 1.005.249 | -286.234 | 1.7480 |
| 5.000.000 | 1.006.776 | -286.459 | 1.7478 |
| 5.010.000 | 1.008.704 | -286.595 | 1.7475 |
| 5.020.000 | 1.010.226 | -286.727 | 1.7470 |
| 5.030.000 | 1.012.074 | -286.883 | 1.7468 |
| 5.040.000 | 1.013.579 | -287.079 | 1.7469 |

| | | | |
|---|---|---|---|
| 5.050.000 | 1.015.518 | -287.206 | 1.7469 |
| 5.060.000 | 1.016.919 | -287.342 | 1.7467 |
| 5.070.000 | 1.018.901 | -287.560 | 1.7465 |
| 5.080.000 | 1.020.377 | -287.614 | 1.7464 |
| 5.090.000 | 1.022.224 | -287.823 | 1.7462 |
| 5.100.000 | 1.023.863 | -287.958 | 1.7459 |
| 5.110.000 | 1.025.509 | -288.095 | 1.7455 |
| 5.120.000 | 1.027.175 | -288.227 | 1.7450 |
| 5.130.000 | 1.028.909 | -288.472 | 1.7448 |
| 5.140.000 | 1.030.644 | -288.491 | 1.7444 |
| 5.150.000 | 1.032.227 | -288.743 | 1.7440 |
| 5.160.000 | 1.034.125 | -288.867 | 1.7436 |
| 5.170.000 | 1.035.583 | -288.998 | 1.7431 |
| 5.180.000 | 1.037.530 | -289.200 | 1.7429 |
| 5.190.000 | 1.039.011 | -289.373 | 1.7428 |
| 5.200.000 | 1.040.892 | -289.487 | 1.7428 |
| 5.210.000 | 1.042.347 | -289.650 | 1.7427 |
| 5.220.000 | 1.044.270 | -289.855 | 1.7425 |
| 5.230.000 | 1.045.766 | -289.927 | 1.7423 |
| 5.240.000 | 1.047.658 | -290.198 | 1.7421 |
| 5.250.000 | 1.049.292 | -290.277 | 1.7420 |
| 5.260.000 | 1.050.955 | -290.460 | 1.7416 |
| 5.270.000 | 1.052.689 | -290.603 | 1.7411 |
| 5.280.000 | 1.054.410 | -290.826 | 1.7407 |
| 5.290.000 | 1.056.116 | -290.869 | 1.7407 |
| 5.300.000 | 1.057.702 | -291.127 | 1.7406 |
| 5.310.000 | 1.059.661 | -291.254 | 1.7404 |
| 5.320.000 | 1.061.064 | -291.422 | 1.7401 |
| 5.330.000 | 1.063.047 | -291.639 | 1.7399 |
| 5.340.000 | 1.064.501 | -291.771 | 1.7399 |

| | | | |
|---|---|---|---|
| 5.350.000 | 1.066.397 | -291.968 | 1.7398 |
| 5.360.000 | 1.067.967 | -292.156 | 1.7397 |
| 5.370.000 | 1.069.834 | -292.325 | 1.7395 |
| 5.380.000 | 1.071.325 | -292.434 | 1.7392 |
| 5.390.000 | 1.073.204 | -292.725 | 1.7390 |
| 5.400.000 | 1.074.868 | -292.808 | 1.7388 |
| 5.410.000 | 1.076.509 | -293.020 | 1.7385 |
| 5.420.000 | 1.078.329 | -293.189 | 1.7382 |
| 5.430.000 | 1.079.946 | -293.392 | 1.7379 |
| 5.440.000 | 1.081.731 | -293.490 | 1.7374 |
| 5.450.000 | 1.083.295 | -293.767 | 1.7370 |
| 5.460.000 | 1.085.270 | -293.901 | 1.7368 |
| 5.470.000 | 1.086.715 | -294.090 | 1.7365 |
| 5.480.000 | 1.088.677 | -294.357 | 1.7364 |
| 5.490.000 | 1.090.156 | -294.451 | 1.7360 |
| 5.500.000 | 1.092.021 | -294.681 | 1.7356 |
| 5.510.000 | 1.093.617 | -294.872 | 1.7355 |
| 5.520.000 | 1.095.476 | -295.064 | 1.7355 |
| 5.530.000 | 1.096.983 | -295.200 | 1.7354 |
| 5.540.000 | 1.098.819 | -295.489 | 1.7352 |
| 5.550.000 | 1.100.478 | -295.561 | 1.7350 |
| 5.560.000 | 1.102.131 | -295.834 | 1.7350 |
| 5.570.000 | 1.103.968 | -295.967 | 1.7350 |
| 5.580.000 | 1.105.534 | -296.168 | 1.7350 |
| 5.590.000 | 1.107.385 | -296.331 | 1.7349 |
| 5.600.000 | 1.108.926 | -296.577 | 1.7349 |
| 5.610.000 | 1.110.795 | -296.726 | 1.7348 |
| 5.620.000 | 1.112.255 | -296.920 | 1.7347 |
| 5.630.000 | 1.114.211 | -297.177 | 1.7346 |
| 5.640.000 | 1.115.675 | -297.303 | 1.7345 |

| | | | |
|---|---|---|---|
| 5.650.000 | 1.117.492 | -297.541 | 1.7344 |
| 5.660.000 | 1.119.114 | -297.713 | 1.7340 |
| 5.670.000 | 1.120.852 | -297.895 | 1.7336 |
| 5.680.000 | 1.122.482 | -298.069 | 1.7334 |
| 5.690.000 | 1.124.267 | -298.341 | 1.7333 |
| 5.700.000 | 1.125.896 | -298.395 | 1.7330 |
| 5.710.000 | 1.127.587 | -298.698 | 1.7329 |
| 5.720.000 | 1.129.425 | -298.854 | 1.7328 |
| 5.730.000 | 1.130.917 | -299.038 | 1.7328 |
| 5.740.000 | 1.132.822 | -299.233 | 1.7329 |
| 5.750.000 | 1.134.322 | -299.456 | 1.7327 |
| 5.760.000 | 1.136.199 | -299.605 | 1.7325 |
| 5.770.000 | 1.137.675 | -299.834 | 1.7324 |
| 5.780.000 | 1.139.594 | -300.055 | 1.7320 |
| 5.790.000 | 1.141.065 | -300.164 | 1.7317 |
| 5.800.000 | 1.142.945 | -300.466 | 1.7315 |
| 5.810.000 | 1.144.522 | -300.565 | 1.7315 |
| 5.820.000 | 1.146.249 | -300.784 | 1.7314 |
| 5.830.000 | 1.147.917 | -300.963 | 1.7314 |
| 5.840.000 | 1.149.675 | -301.179 | 1.7311 |
| 5.850.000 | 1.151.339 | -301.280 | 1.7309 |
| 5.860.000 | 1.152.989 | -301.590 | 1.7307 |
| 5.870.000 | 1.154.838 | -301.664 | 1.7306 |
| 5.880.000 | 1.156.305 | -301.913 | 1.7304 |
| 5.890.000 | 1.158.218 | -302.142 | 1.7303 |
| 5.900.000 | 1.159.697 | -302.325 | 1.7303 |
| 5.910.000 | 1.161.589 | -302.544 | 1.7302 |
| 5.920.000 | 1.163.135 | -302.768 | 1.7300 |
| 5.930.000 | 1.165.088 | -302.977 | 1.7298 |
| 5.940.000 | 1.166.527 | -303.138 | 1.7294 |

| | | | |
|---|---|---|---|
| 5.950.000 | 1.168.446 | -303.438 | 1.7292 |
| 5.960.000 | 1.170.000 | -303.551 | 1.7290 |
| 5.970.000 | 1.171.727 | -303.802 | 1.7288 |
| 5.980.000 | 1.173.450 | -303.995 | 1.7287 |
| 5.990.000 | 1.175.124 | -304.213 | 1.7287 |
| 6.000.000 | 1.176.875 | -304.353 | 1.7286 |
| 6.010.000 | 1.178.514 | -304.676 | 1.7284 |
| 6.020.000 | 1.180.370 | -304.787 | 1.7283 |
| 6.030.000 | 1.181.862 | -305.049 | 1.7282 |
| 6.040.000 | 1.183.829 | -305.325 | 1.7284 |
| 6.050.000 | 1.185.277 | -305.464 | 1.7284 |
| 6.060.000 | 1.187.204 | -305.728 | 1.7283 |
| 6.070.000 | 1.188.734 | -305.957 | 1.7281 |
| 6.080.000 | 1.190.597 | -306.140 | 1.7279 |
| 6.090.000 | 1.192.122 | -306.346 | 1.7276 |
| 6.100.000 | 1.194.007 | -306.661 | 1.7273 |
| 6.110.000 | 1.195.601 | -306.758 | 1.7273 |
| 6.120.000 | 1.197.342 | -307.063 | 1.7272 |
| 6.130.000 | 1.199.066 | -307.224 | 1.7271 |
| 6.140.000 | 1.200.707 | -307.471 | 1.7270 |
| 6.150.000 | 1.202.545 | -307.661 | 1.7269 |
| 6.160.000 | 1.204.155 | -307.964 | 1.7268 |
| 6.170.000 | 1.206.013 | -308.083 | 1.7264 |
| 6.180.000 | 1.207.457 | -308.350 | 1.7260 |
| 6.190.000 | 1.209.432 | -308.611 | 1.7258 |
| 6.200.000 | 1.210.826 | -308.740 | 1.7257 |
| 6.210.000 | 1.212.720 | -309.031 | 1.7257 |
| 6.220.000 | 1.214.274 | -309.240 | 1.7254 |
| 6.230.000 | 1.216.075 | -309.454 | 1.7250 |
| 6.240.000 | 1.217.593 | -309.633 | 1.7247 |

| | | | |
|---|---|---|---|
| 6.250.000 | 1.219.460 | -309.939 | 1.7245 |
| 6.260.000 | 1.221.058 | -310.051 | 1.7245 |
| 6.270.000 | 1.222.793 | -310.358 | 1.7244 |
| 6.280.000 | 1.224.525 | -310.501 | 1.7244 |
| 6.290.000 | 1.226.110 | -310.746 | 1.7244 |
| 6.300.000 | 1.227.935 | -310.935 | 1.7244 |
| 6.310.000 | 1.229.486 | -311.195 | 1.7243 |
| 6.320.000 | 1.231.339 | -311.374 | 1.7240 |
| 6.330.000 | 1.232.795 | -311.628 | 1.7238 |
| 6.340.000 | 1.234.717 | -311.853 | 1.7235 |
| 6.350.000 | 1.236.147 | -312.028 | 1.7232 |
| 6.360.000 | 1.238.093 | -312.318 | 1.7229 |
| 6.370.000 | 1.239.620 | -312.475 | 1.7227 |
| 6.380.000 | 1.241.465 | -312.743 | 1.7227 |
| 6.390.000 | 1.243.054 | -312.964 | 1.7227 |
| 6.400.000 | 1.244.830 | -313.180 | 1.7224 |
| 6.410.000 | 1.246.433 | -313.331 | 1.7221 |
| 6.420.000 | 1.248.137 | -313.655 | 1.7221 |
| 6.430.000 | 1.249.902 | -313.750 | 1.7221 |
| 6.440.000 | 1.251.434 | -314.033 | 1.7221 |
| 6.450.000 | 1.253.344 | -314.245 | 1.7220 |
| 6.460.000 | 1.254.845 | -314.449 | 1.7220 |
| 6.470.000 | 1.256.701 | -314.706 | 1.7220 |
| 6.480.000 | 1.258.260 | -314.963 | 1.7221 |
| 6.490.000 | 1.260.160 | -315.141 | 1.7220 |
| 6.500.000 | 1.261.616 | -315.362 | 1.7219 |
| 6.510.000 | 1.263.600 | -315.684 | 1.7218 |
| 6.520.000 | 1.265.071 | -315.786 | 1.7216 |
| 6.530.000 | 1.266.875 | -316.084 | 1.7215 |
| 6.540.000 | 1.268.517 | -316.290 | 1.7211 |

| | | | |
|---|---|---|---|
| 6.550.000 | 1.270.247 | -316.531 | 1.7209 |
| 6.560.000 | 1.271.924 | -316.722 | 1.7205 |
| 6.570.000 | 1.273.677 | -317.043 | 1.7204 |
| 6.580.000 | 1.275.404 | -317.146 | 1.7201 |
| 6.590.000 | 1.277.015 | -317.461 | 1.7197 |
| 6.600.000 | 1.278.909 | -317.682 | 1.7195 |
| 6.610.000 | 1.280.359 | -317.879 | 1.7195 |
| 6.620.000 | 1.282.320 | -318.174 | 1.7196 |
| 6.630.000 | 1.283.803 | -318.433 | 1.7195 |
| 6.640.000 | 1.285.675 | -318.616 | 1.7193 |
| 6.650.000 | 1.287.193 | -318.873 | 1.7190 |
| 6.660.000 | 1.289.094 | -319.176 | 1.7189 |
| 6.670.000 | 1.290.608 | -319.290 | 1.7189 |
| 6.680.000 | 1.292.452 | -319.633 | 1.7188 |
| 6.690.000 | 1.294.089 | -319.818 | 1.7188 |
| 6.700.000 | 1.295.753 | -320.055 | 1.7188 |
| 6.710.000 | 1.297.542 | -320.285 | 1.7188 |
| 6.720.000 | 1.299.222 | -320.584 | 1.7189 |
| 6.730.000 | 1.301.012 | -320.714 | 1.7189 |
| 6.740.000 | 1.302.551 | -321.051 | 1.7189 |
| 6.750.000 | 1.304.524 | -321.278 | 1.7188 |
| 6.760.000 | 1.305.885 | -321.487 | 1.7187 |
| 6.770.000 | 1.307.821 | -321.781 | 1.7185 |
| 6.780.000 | 1.309.346 | -321.984 | 1.7184 |
| 6.790.000 | 1.311.159 | -322.253 | 1.7184 |
| 6.800.000 | 1.312.653 | -322.482 | 1.7183 |
| 6.810.000 | 1.314.577 | -322.756 | 1.7182 |
| 6.820.000 | 1.316.060 | -322.934 | 1.7179 |
| 6.830.000 | 1.317.901 | -323.266 | 1.7176 |
| 6.840.000 | 1.319.586 | -323.416 | 1.7176 |

| | | | |
|---|---|---|---|
| 6.850.000 | 1.321.211 | -323.691 | 1.7173 |
| 6.860.000 | 1.323.005 | -323.892 | 1.7170 |
| 6.870.000 | 1.324.599 | -324.154 | 1.7166 |
| 6.880.000 | 1.326.355 | -324.326 | 1.7163 |
| 6.890.000 | 1.327.937 | -324.634 | 1.7164 |
| 6.900.000 | 1.329.827 | -324.848 | 1.7164 |
| 6.910.000 | 1.331.231 | -325.066 | 1.7163 |
| 6.920.000 | 1.333.183 | -325.360 | 1.7163 |
| 6.930.000 | 1.334.632 | -325.511 | 1.7162 |
| 6.940.000 | 1.336.525 | -325.821 | 1.7162 |
| 6.950.000 | 1.338.096 | -326.032 | 1.7162 |
| 6.960.000 | 1.339.904 | -326.262 | 1.7161 |
| 6.970.000 | 1.341.489 | -326.460 | 1.7162 |
| 6.980.000 | 1.343.237 | -326.769 | 1.7159 |
| 6.990.000 | 1.344.894 | -326.861 | 1.7157 |
| 7.000.000 | 1.346.505 | -327.169 | 1.7157 |
| 7.010.000 | 1.348.304 | -327.366 | 1.7155 |
| 7.020.000 | 1.349.875 | -327.594 | 1.7153 |
| 7.030.000 | 1.351.718 | -327.809 | 1.7149 |
| 7.040.000 | 1.353.250 | -328.083 | 1.7146 |
| 7.050.000 | 1.355.150 | -328.249 | 1.7147 |
| 7.060.000 | 1.356.642 | -328.499 | 1.7150 |
| 7.070.000 | 1.358.588 | -328.770 | 1.7151 |
| 7.080.000 | 1.360.025 | -328.899 | 1.7151 |
| 7.090.000 | 1.361.867 | -329.200 | 1.7151 |
| 7.100.000 | 1.363.485 | -329.422 | 1.7150 |
| 7.110.000 | 1.365.229 | -329.617 | 1.7150 |
| 7.120.000 | 1.366.847 | -329.821 | 1.7146 |
| 7.130.000 | 1.368.602 | -330.139 | 1.7143 |
| 7.140.000 | 1.370.280 | -330.233 | 1.7140 |

| | | | |
|---|---|---|---|
| 7.150.000 | 1.371.952 | -330.558 | 1.7135 |
| 7.160.000 | 1.373.833 | -330.738 | 1.7134 |
| 7.170.000 | 1.375.300 | -330.956 | 1.7132 |
| 7.180.000 | 1.377.240 | -331.187 | 1.7133 |
| 7.190.000 | 1.378.697 | -331.425 | 1.7132 |
| 7.200.000 | 1.380.563 | -331.624 | 1.7130 |
| 7.210.000 | 1.382.061 | -331.867 | 1.7131 |
| 7.220.000 | 1.383.998 | -332.156 | 1.7132 |
| 7.230.000 | 1.385.460 | -332.290 | 1.7134 |
| 7.240.000 | 1.387.359 | -332.601 | 1.7133 |
| 7.250.000 | 1.388.940 | -332.772 | 1.7132 |
| 7.260.000 | 1.390.705 | -333.038 | 1.7132 |
| 7.270.000 | 1.392.420 | -333.234 | 1.7133 |
| 7.280.000 | 1.394.160 | -333.507 | 1.7133 |
| 7.290.000 | 1.395.844 | -333.644 | 1.7132 |
| 7.300.000 | 1.397.450 | -333.987 | 1.7129 |
| 7.310.000 | 1.399.342 | -334.132 | 1.7128 |
| 7.320.000 | 1.400.794 | -334.389 | 1.7128 |
| 7.330.000 | 1.402.747 | -334.656 | 1.7126 |
| 7.340.000 | 1.404.232 | -334.847 | 1.7125 |
| 7.350.000 | 1.406.052 | -335.082 | 1.7122 |
| 7.360.000 | 1.407.572 | -335.331 | 1.7119 |
| 7.370.000 | 1.409.445 | -335.559 | 1.7119 |
| 7.380.000 | 1.410.933 | -335.768 | 1.7119 |
| 7.390.000 | 1.412.855 | -336.080 | 1.7119 |
| 7.400.000 | 1.414.427 | -336.212 | 1.7116 |
| 7.410.000 | 1.416.125 | -336.508 | 1.7116 |
| 7.420.000 | 1.417.845 | -336.714 | 1.7118 |
| 7.430.000 | 1.419.522 | -336.935 | 1.7120 |
| 7.440.000 | 1.421.206 | -337.109 | 1.7122 |

| | | | |
|---|---|---|---|
| 7.450.000 | 1.422.830 | -337.436 | 1.7121 |
| 7.460.000 | 1.424.627 | -337.578 | 1.7121 |
| 7.470.000 | 1.426.096 | -337.834 | 1.7122 |
| 7.480.000 | 1.428.075 | -338.134 | 1.7121 |
| 7.490.000 | 1.429.473 | -338.275 | 1.7122 |
| 7.500.000 | 1.431.425 | -338.562 | 1.7121 |
| 7.510.000 | 1.432.945 | -338.800 | 1.7119 |
| 7.520.000 | 1.434.792 | -338.991 | 1.7118 |
| 7.530.000 | 1.436.318 | -339.192 | 1.7119 |
| 7.540.000 | 1.438.135 | -339.526 | 1.7120 |
| 7.550.000 | 1.439.725 | -339.607 | 1.7120 |
| 7.560.000 | 1.441.430 | -339.918 | 1.7117 |
| 7.570.000 | 1.443.140 | -340.113 | 1.7115 |
| 7.580.000 | 1.444.727 | -340.319 | 1.7115 |
| 7.590.000 | 1.446.535 | -340.509 | 1.7116 |
| 7.600.000 | 1.448.101 | -340.830 | 1.7116 |
| 7.610.000 | 1.450.050 | -340.964 | 1.7116 |
| 7.620.000 | 1.451.461 | -341.255 | 1.7115 |
| 7.630.000 | 1.453.432 | -341.512 | 1.7115 |
| 7.640.000 | 1.454.811 | -341.653 | 1.7115 |
| 7.650.000 | 1.456.732 | -341.941 | 1.7114 |
| 7.660.000 | 1.458.267 | -342.162 | 1.7113 |
| 7.670.000 | 1.460.095 | -342.377 | 1.7113 |
| 7.680.000 | 1.461.644 | -342.575 | 1.7113 |
| 7.690.000 | 1.463.473 | -342.894 | 1.7113 |
| 7.700.000 | 1.465.058 | -343.011 | 1.7113 |
| 7.710.000 | 1.466.767 | -343.339 | 1.7113 |
| 7.720.000 | 1.468.614 | -343.510 | 1.7114 |
| 7.730.000 | 1.470.101 | -343.749 | 1.7113 |
| 7.740.000 | 1.471.996 | -343.958 | 1.7110 |

| | | | |
|---|---|---|---|
| 7.750.000 | 1.473.539 | -344.234 | 1.7107 |
| 7.760.000 | 1.475.368 | -344.444 | 1.7107 |
| 7.770.000 | 1.476.867 | -344.694 | 1.7108 |
| 7.780.000 | 1.478.798 | -344.948 | 1.7108 |
| 7.790.000 | 1.480.202 | -345.119 | 1.7106 |
| 7.800.000 | 1.482.170 | -345.434 | 1.7105 |
| 7.810.000 | 1.483.723 | -345.619 | 1.7104 |
| 7.820.000 | 1.485.494 | -345.876 | 1.7105 |
| 7.830.000 | 1.487.171 | -346.109 | 1.7105 |
| 7.840.000 | 1.488.940 | -346.377 | 1.7105 |
| 7.850.000 | 1.490.571 | -346.538 | 1.7105 |
| 7.860.000 | 1.492.276 | -346.874 | 1.7105 |
| 7.870.000 | 1.494.042 | -347.013 | 1.7105 |
| 7.880.000 | 1.495.569 | -347.317 | 1.7105 |
| 7.890.000 | 1.497.495 | -347.545 | 1.7105 |
| 7.900.000 | 1.498.995 | -347.763 | 1.7105 |
| 7.910.000 | 1.500.856 | -348.049 | 1.7105 |
| 7.920.000 | 1.502.362 | -348.306 | 1.7105 |
| 7.930.000 | 1.504.280 | -348.538 | 1.7104 |
| 7.940.000 | 1.505.775 | -348.778 | 1.7104 |
| 7.950.000 | 1.507.679 | -349.067 | 1.7102 |
| 7.960.000 | 1.509.232 | -349.228 | 1.7102 |
| 7.970.000 | 1.510.971 | -349.555 | 1.7102 |
| 7.980.000 | 1.512.650 | -349.770 | 1.7102 |
| 7.990.000 | 1.514.363 | -350.011 | 1.7103 |
| 8.000.000 | 1.515.997 | -350.216 | 1.7105 |
| 8.010.000 | 1.517.709 | -350.554 | 1.7106 |
| 8.020.000 | 1.519.435 | -350.655 | 1.7106 |
| 8.030.000 | 1.520.935 | -350.983 | 1.7104 |
| 8.040.000 | 1.522.878 | -351.240 | 1.7101 |

| | | | |
|---|---|---|---|
| 8.050.000 | 1.524.304 | -351.434 | 1.7099 |
| 8.060.000 | 1.526.254 | -351.724 | 1.7098 |
| 8.070.000 | 1.527.761 | -351.984 | 1.7099 |
| 8.080.000 | 1.529.612 | -352.184 | 1.7098 |
| 8.090.000 | 1.531.099 | -352.429 | 1.7095 |
| 8.100.000 | 1.533.021 | -352.733 | 1.7094 |
| 8.110.000 | 1.534.510 | -352.866 | 1.7094 |
| 8.120.000 | 1.536.294 | -353.191 | 1.7095 |
| 8.130.000 | 1.537.961 | -353.411 | 1.7096 |
| 8.140.000 | 1.539.617 | -353.632 | 1.7096 |
| 8.150.000 | 1.541.373 | -353.839 | 1.7098 |
| 8.160.000 | 1.543.028 | -354.170 | 1.7099 |
| 8.170.000 | 1.544.827 | -354.276 | 1.7099 |
| 8.180.000 | 1.546.330 | -354.604 | 1.7099 |
| 8.190.000 | 1.548.250 | -354.823 | 1.7096 |
| 8.200.000 | 1.549.644 | -355.004 | 1.7095 |
| 8.210.000 | 1.551.547 | -355.319 | 1.7094 |
| 8.220.000 | 1.553.065 | -355.513 | 1.7097 |
| 8.230.000 | 1.554.899 | -355.738 | 1.7100 |
| 8.240.000 | 1.556.415 | -355.962 | 1.7103 |
| 8.250.000 | 1.558.313 | -356.255 | 1.7105 |
| 8.260.000 | 1.559.805 | -356.385 | 1.7106 |
| 8.270.000 | 1.561.570 | -356.716 | 1.7104 |
| 8.280.000 | 1.563.388 | -356.902 | 1.7104 |
| 8.290.000 | 1.564.976 | -357.162 | 1.7104 |
| 8.300.000 | 1.566.736 | -357.358 | 1.7103 |
| 8.310.000 | 1.568.373 | -357.654 | 1.7104 |
| 8.320.000 | 1.570.130 | -357.818 | 1.7104 |
| 8.330.000 | 1.571.700 | -358.118 | 1.7103 |
| 8.340.000 | 1.573.612 | -358.354 | 1.7101 |

| | | | |
|---|---|---|---|
| 8.350.000 | 1.574.988 | -358.553 | 1.7097 |
| 8.360.000 | 1.576.961 | -358.861 | 1.7095 |
| 8.370.000 | 1.578.478 | -359.063 | 1.7092 |
| 8.380.000 | 1.580.290 | -359.327 | 1.7089 |
| 8.390.000 | 1.581.912 | -359.562 | 1.7088 |
| 8.400.000 | 1.583.716 | -359.809 | 1.7087 |
| 8.410.000 | 1.585.302 | -359.996 | 1.7085 |
| 8.420.000 | 1.587.078 | -360.324 | 1.7085 |
| 8.430.000 | 1.588.730 | -360.460 | 1.7085 |
| 8.440.000 | 1.590.380 | -360.780 | 1.7087 |
| 8.450.000 | 1.592.181 | -361.000 | 1.7091 |
| 8.460.000 | 1.593.750 | -361.227 | 1.7095 |
| 8.470.000 | 1.595.595 | -361.449 | 1.7100 |
| 8.480.000 | 1.597.078 | -361.744 | 1.7101 |
| 8.490.000 | 1.599.043 | -361.951 | 1.7100 |
| 8.500.000 | 1.600.446 | -362.205 | 1.7098 |
| 8.510.000 | 1.602.428 | -362.516 | 1.7095 |
| 8.520.000 | 1.603.923 | -362.663 | 1.7093 |
| 8.530.000 | 1.605.737 | -362.990 | 1.7092 |
| 8.540.000 | 1.607.368 | -363.202 | 1.7091 |
| 8.550.000 | 1.609.084 | -363.433 | 1.7090 |
| 8.560.000 | 1.610.674 | -363.652 | 1.7089 |
| 8.570.000 | 1.612.451 | -363.977 | 1.7089 |
| 8.580.000 | 1.614.141 | -364.087 | 1.7088 |
| 8.590.000 | 1.615.725 | -364.424 | 1.7087 |
| 8.600.000 | 1.617.620 | -364.634 | 1.7085 |
| 8.610.000 | 1.619.065 | -364.854 | 1.7086 |
| 8.620.000 | 1.620.976 | -365.128 | 1.7088 |
| 8.630.000 | 1.622.478 | -365.402 | 1.7086 |
| 8.640.000 | 1.624.309 | -365.605 | 1.7084 |

| | | | |
|---|---|---|---|
| 8.650.000 | 1.625.775 | -365.874 | 1.7081 |
| 8.660.000 | 1.627.707 | -366.149 | 1.7081 |
| 8.670.000 | 1.629.164 | -366.302 | 1.7080 |
| 8.680.000 | 1.631.010 | -366.635 | 1.7079 |
| 8.690.000 | 1.632.614 | -366.838 | 1.7080 |
| 8.700.000 | 1.634.287 | -367.076 | 1.7083 |
| 8.710.000 | 1.635.971 | -367.300 | 1.7084 |
| 8.720.000 | 1.637.681 | -367.611 | 1.7082 |
| 8.730.000 | 1.639.396 | -367.746 | 1.7080 |
| 8.740.000 | 1.641.034 | -368.091 | 1.7080 |
| 8.750.000 | 1.642.912 | -368.278 | 1.7078 |
| 8.760.000 | 1.644.316 | -368.510 | 1.7078 |
| 8.770.000 | 1.646.239 | -368.794 | 1.7081 |
| 8.780.000 | 1.647.715 | -369.023 | 1.7083 |
| 8.790.000 | 1.649.547 | -369.259 | 1.7085 |
| 8.800.000 | 1.651.061 | -369.530 | 1.7084 |
| 8.810.000 | 1.652.983 | -369.784 | 1.7084 |
| 8.820.000 | 1.654.439 | -369.946 | 1.7082 |
| 8.830.000 | 1.656.320 | -370.308 | 1.7081 |
| 8.840.000 | 1.657.968 | -370.463 | 1.7080 |
| 8.850.000 | 1.659.628 | -370.740 | 1.7077 |
| 8.860.000 | 1.661.337 | -370.939 | 1.7076 |
| 8.870.000 | 1.663.061 | -371.212 | 1.7074 |
| 8.880.000 | 1.664.706 | -371.373 | 1.7073 |
| 8.890.000 | 1.666.313 | -371.703 | 1.7073 |
| 8.900.000 | 1.668.179 | -371.871 | 1.7072 |
| 8.910.000 | 1.669.631 | -372.149 | 1.7074 |
| 8.920.000 | 1.671.587 | -372.467 | 1.7075 |
| 8.930.000 | 1.673.077 | -372.652 | 1.7076 |
| 8.940.000 | 1.674.910 | -372.914 | 1.7075 |

| | | | |
|---|---|---|---|
| 8.950.000 | 1.676.475 | -373.174 | 1.7071 |
| 8.960.000 | 1.678.353 | -373.435 | 1.7071 |
| 8.970.000 | 1.679.864 | -373.637 | 1.7071 |
| 8.980.000 | 1.681.708 | -373.985 | 1.7073 |
| 8.990.000 | 1.683.328 | -374.145 | 1.7074 |
| 9.000.000 | 1.685.013 | -374.462 | 1.7077 |
| 9.010.000 | 1.686.736 | -374.684 | 1.7079 |
| 9.020.000 | 1.688.380 | -374.958 | 1.7079 |
| 9.030.000 | 1.690.164 | -375.159 | 1.7076 |
| 9.040.000 | 1.691.760 | -375.507 | 1.7074 |
| 9.050.000 | 1.693.646 | -375.692 | 1.7073 |
| 9.060.000 | 1.695.088 | -375.985 | 1.7073 |
| 9.070.000 | 1.697.073 | -376.287 | 1.7072 |
| 9.080.000 | 1.698.535 | -376.467 | 1.7071 |
| 9.090.000 | 1.700.430 | -376.784 | 1.7071 |
| 9.100.000 | 1.701.981 | -377.024 | 1.7070 |
| 9.110.000 | 1.703.805 | -377.267 | 1.7069 |
| 9.120.000 | 1.705.335 | -377.514 | 1.7068 |
| 9.130.000 | 1.707.189 | -377.840 | 1.7066 |
| 9.140.000 | 1.708.760 | -377.977 | 1.7064 |
| 9.150.000 | 1.710.419 | -378.317 | 1.7062 |
| 9.160.000 | 1.712.218 | -378.514 | 1.7062 |
| 9.170.000 | 1.713.740 | -378.761 | 1.7063 |
| 9.180.000 | 1.715.612 | -379.006 | 1.7061 |
| 9.190.000 | 1.717.182 | -379.297 | 1.7060 |
| 9.200.000 | 1.718.992 | -379.508 | 1.7060 |
| 9.210.000 | 1.720.461 | -379.783 | 1.7061 |
| 9.220.000 | 1.722.413 | -380.085 | 1.7060 |
| 9.230.000 | 1.723.841 | -380.258 | 1.7060 |
| 9.240.000 | 1.725.705 | -380.582 | 1.7058 |

| | | | |
|---|---|---|---|
| 9.250.000 | 1.727.267 | -380.797 | 1.7058 |
| 9.260.000 | 1.729.026 | -381.059 | 1.7055 |
| 9.270.000 | 1.730.668 | -381.295 | 1.7056 |
| 9.280.000 | 1.732.415 | -381.611 | 1.7057 |
| 9.290.000 | 1.734.047 | -381.742 | 1.7056 |
| 9.300.000 | 1.735.780 | -382.099 | 1.7055 |
| 9.310.000 | 1.737.524 | -382.255 | 1.7054 |
| 9.320.000 | 1.739.079 | -382.537 | 1.7056 |
| 9.330.000 | 1.740.924 | -382.763 | 1.7056 |
| 9.340.000 | 1.742.433 | -383.022 | 1.7056 |
| 9.350.000 | 1.744.300 | -383.264 | 1.7054 |
| 9.360.000 | 1.745.763 | -383.557 | 1.7055 |
| 9.370.000 | 1.747.684 | -383.783 | 1.7054 |
| 9.380.000 | 1.749.121 | -383.998 | 1.7053 |
| 9.390.000 | 1.750.995 | -384.304 | 1.7054 |
| 9.400.000 | 1.752.572 | -384.487 | 1.7053 |
| 9.410.000 | 1.754.348 | -384.778 | 1.7051 |
| 9.420.000 | 1.756.039 | -385.016 | 1.7049 |
| 9.430.000 | 1.757.763 | -385.260 | 1.7048 |
| 9.440.000 | 1.759.373 | -385.426 | 1.7049 |
| 9.450.000 | 1.761.016 | -385.769 | 1.7049 |
| 9.460.000 | 1.762.840 | -385.895 | 1.7047 |
| 9.470.000 | 1.764.295 | -386.198 | 1.7045 |
| 9.480.000 | 1.766.280 | -386.471 | 1.7045 |
| 9.490.000 | 1.767.719 | -386.667 | 1.7046 |
| 9.500.000 | 1.769.589 | -386.942 | 1.7047 |
| 9.510.000 | 1.771.146 | -387.226 | 1.7044 |
| 9.520.000 | 1.773.021 | -387.446 | 1.7043 |
| 9.530.000 | 1.774.534 | -387.663 | 1.7044 |
| 9.540.000 | 1.776.428 | -388.004 | 1.7044 |

| | | | |
|---|---|---|---|
| 9.550.000 | 1.777.963 | -388.152 | 1.7046 |
| 9.560.000 | 1.779.710 | -388.468 | 1.7043 |
| 9.570.000 | 1.781.411 | -388.702 | 1.7043 |
| 9.580.000 | 1.783.082 | -388.934 | 1.7043 |
| 9.590.000 | 1.784.754 | -389.148 | 1.7045 |
| 9.600.000 | 1.786.427 | -389.501 | 1.7046 |
| 9.610.000 | 1.788.267 | -389.621 | 1.7044 |
| 9.620.000 | 1.789.746 | -389.949 | 1.7041 |
| 9.630.000 | 1.791.750 | -390.246 | 1.7039 |
| 9.640.000 | 1.793.218 | -390.442 | 1.7038 |
| 9.650.000 | 1.795.075 | -390.732 | 1.7037 |
| 9.660.000 | 1.796.604 | -390.989 | 1.7035 |
| 9.670.000 | 1.798.476 | -391.237 | 1.7034 |
| 9.680.000 | 1.799.970 | -391.473 | 1.7035 |
| 9.690.000 | 1.801.846 | -391.795 | 1.7039 |
| 9.700.000 | 1.803.422 | -391.953 | 1.7040 |
| 9.710.000 | 1.805.187 | -392.297 | 1.7040 |
| 9.720.000 | 1.806.866 | -392.483 | 1.7038 |
| 9.730.000 | 1.808.508 | -392.758 | 1.7037 |
| 9.740.000 | 1.810.294 | -392.990 | 1.7037 |
| 9.750.000 | 1.811.910 | -393.302 | 1.7037 |
| 9.760.000 | 1.813.675 | -393.458 | 1.7034 |
| 9.770.000 | 1.815.165 | -393.776 | 1.7033 |
| 9.780.000 | 1.817.109 | -394.041 | 1.7032 |
| 9.790.000 | 1.818.499 | -394.237 | 1.7033 |
| 9.800.000 | 1.820.427 | -394.535 | 1.7033 |
| 9.810.000 | 1.821.958 | -394.768 | 1.7032 |
| 9.820.000 | 1.823.749 | -395.010 | 1.7031 |
| 9.830.000 | 1.825.310 | -395.255 | 1.7032 |
| 9.840.000 | 1.827.136 | -395.555 | 1.7034 |

| | | | |
|---|---|---|---|
| 9.850.000 | 1.828.708 | -395.715 | 1.7035 |
| 9.860.000 | 1.830.494 | -396.051 | 1.7034 |
| 9.870.000 | 1.832.169 | -396.220 | 1.7032 |
| 9.880.000 | 1.833.816 | -396.528 | 1.7032 |
| 9.890.000 | 1.835.622 | -396.749 | 1.7035 |
| 9.900.000 | 1.837.179 | -397.020 | 1.7036 |
| 9.910.000 | 1.839.027 | -397.255 | 1.7035 |
| 9.920.000 | 1.840.514 | -397.564 | 1.7036 |
| 9.930.000 | 1.842.432 | -397.792 | 1.7039 |
| 9.940.000 | 1.843.855 | -398.018 | 1.7042 |
| 9.950.000 | 1.845.746 | -398.346 | 1.7043 |
| 9.960.000 | 1.847.244 | -398.510 | 1.7045 |
| 9.970.000 | 1.849.095 | -398.828 | 1.7046 |
| 9.980.000 | 1.850.675 | -399.063 | 1.7045 |
| 9.990.000 | 1.852.460 | -399.307 | 1.7045 |
| 10.000.000 | 1.854.069 | -399.520 | 1.7044 |
| 10.010.000 | 1.855.792 | -399.846 | 1.7041 |
| 10.020.000 | 1.857.508 | -399.970 | 1.7039 |
| 10.030.000 | 1.859.071 | -400.305 | 1.7036 |
| 10.040.000 | 1.860.939 | -400.527 | 1.7035 |
| 10.050.000 | 1.862.480 | -400.764 | 1.7037 |
| 10.060.000 | 1.864.314 | -401.034 | 1.7038 |
| 10.070.000 | 1.865.869 | -401.317 | 1.7038 |
| 10.080.000 | 1.867.771 | -401.555 | 1.7037 |
| 10.090.000 | 1.869.206 | -401.785 | 1.7036 |
| 10.100.000 | 1.871.169 | -402.099 | 1.7037 |
| 10.110.000 | 1.872.598 | -402.260 | 1.7038 |
| 10.120.000 | 1.874.444 | -402.573 | 1.7036 |
| 10.130.000 | 1.876.059 | -402.807 | 1.7035 |
| 10.140.000 | 1.877.768 | -403.037 | 1.7035 |

| | | | |
|---|---|---|---|
| 10.150.000 | 1.879.475 | -403.262 | 1.7037 |
| 10.160.000 | 1.881.179 | -403.591 | 1.7038 |
| 10.170.000 | 1.882.864 | -403.707 | 1.7040 |
| 10.180.000 | 1.884.488 | -404.056 | 1.7039 |
| 10.190.000 | 1.886.368 | -404.286 | 1.7039 |
| 10.200.000 | 1.887.824 | -404.474 | 1.7040 |
| 10.210.000 | 1.889.772 | -404.776 | 1.7040 |
| 10.220.000 | 1.891.226 | -405.021 | 1.7040 |
| 10.230.000 | 1.893.096 | -405.245 | 1.7041 |
| 10.240.000 | 1.894.579 | -405.496 | 1.7041 |
| 10.250.000 | 1.896.506 | -405.796 | 1.7039 |
| 10.260.000 | 1.898.023 | -405.945 | 1.7038 |
| 10.270.000 | 1.899.830 | -406.299 | 1.7040 |
| 10.280.000 | 1.901.498 | -406.477 | 1.7039 |
| 10.290.000 | 1.903.185 | -406.741 | 1.7039 |
| 10.300.000 | 1.904.849 | -406.966 | 1.7037 |
| 10.310.000 | 1.906.575 | -407.277 | 1.7035 |
| 10.320.000 | 1.908.275 | -407.425 | 1.7037 |
| 10.330.000 | 1.909.845 | -407.743 | 1.7038 |
| 10.340.000 | 1.911.734 | -407.977 | 1.7036 |
| 10.350.000 | 1.913.145 | -408.214 | 1.7035 |
| 10.360.000 | 1.915.089 | -408.524 | 1.7033 |
| 10.370.000 | 1.916.590 | -408.729 | 1.7035 |
| 10.380.000 | 1.918.378 | -408.986 | 1.7036 |
| 10.390.000 | 1.919.934 | -409.246 | 1.7036 |
| 10.400.000 | 1.921.796 | -409.519 | 1.7036 |
| 10.410.000 | 1.923.276 | -409.698 | 1.7035 |
| 10.420.000 | 1.925.135 | -410.047 | 1.7036 |
| 10.430.000 | 1.926.743 | -410.204 | 1.7035 |
| 10.440.000 | 1.928.435 | -410.481 | 1.7035 |

| | | | |
|---|---|---|---|
| 10.450.000 | 1.930.183 | -410.716 | 1.7034 |
| 10.460.000 | 1.931.767 | -410.981 | 1.7034 |
| 10.470.000 | 1.933.527 | -411.145 | 1.7034 |
| 10.480.000 | 1.935.078 | -411.482 | 1.7034 |
| 10.490.000 | 1.936.967 | -411.658 | 1.7034 |
| 10.500.000 | 1.938.383 | -411.919 | 1.7033 |
| 10.510.000 | 1.940.323 | -412.231 | 1.7034 |
| 10.520.000 | 1.941.805 | -412.405 | 1.7034 |
| 10.530.000 | 1.943.653 | -412.708 | 1.7033 |
| 10.540.000 | 1.945.237 | -412.967 | 1.7033 |
| 10.550.000 | 1.947.082 | -413.179 | 1.7034 |
| 10.560.000 | 1.948.556 | -413.391 | 1.7035 |
| 10.570.000 | 1.950.351 | -413.731 | 1.7036 |
| 10.580.000 | 1.952.019 | -413.863 | 1.7037 |
| 10.590.000 | 1.953.664 | -414.197 | 1.7041 |
| 10.600.000 | 1.955.440 | -414.391 | 1.7044 |
| 10.610.000 | 1.957.036 | -414.653 | 1.7044 |
| 10.620.000 | 1.958.813 | -414.853 | 1.7044 |
| 10.630.000 | 1.960.374 | -415.181 | 1.7045 |
| 10.640.000 | 1.962.255 | -415.374 | 1.7044 |
| 10.650.000 | 1.963.708 | -415.639 | 1.7044 |
| 10.660.000 | 1.965.630 | -415.923 | 1.7046 |
| 10.670.000 | 1.967.093 | -416.091 | 1.7049 |
| 10.680.000 | 1.968.962 | -416.393 | 1.7050 |
| 10.690.000 | 1.970.552 | -416.617 | 1.7051 |
| 10.700.000 | 1.972.270 | -416.852 | 1.7051 |
| 10.710.000 | 1.973.917 | -417.072 | 1.7049 |
| 10.720.000 | 1.975.704 | -417.383 | 1.7047 |
| 10.730.000 | 1.977.306 | -417.498 | 1.7045 |
| 10.740.000 | 1.978.978 | -417.849 | 1.7046 |

| | | | |
|---|---|---|---|
| 10.750.000 | 1.980.775 | -418.041 | 1.7047 |
| 10.760.000 | 1.982.325 | -418.284 | 1.7045 |
| 10.770.000 | 1.984.219 | -418.537 | 1.7042 |
| 10.780.000 | 1.985.716 | -418.795 | 1.7040 |
| 10.790.000 | 1.987.569 | -419.019 | 1.7037 |
| 10.800.000 | 1.989.076 | -419.295 | 1.7036 |
| 10.810.000 | 1.991.013 | -419.563 | 1.7032 |
| 10.820.000 | 1.992.460 | -419.756 | 1.7031 |
| 10.830.000 | 1.994.342 | -420.087 | 1.7032 |
| 10.840.000 | 1.995.894 | -420.279 | 1.7032 |
| 10.850.000 | 1.997.664 | -420.570 | 1.7032 |
| 10.860.000 | 1.999.332 | -420.805 | 1.7030 |
| 10.870.000 | 2.001.089 | -421.064 | 1.7026 |
| 10.880.000 | 2.002.768 | -421.240 | 1.7023 |
| 10.890.000 | 2.004.398 | -421.587 | 1.7022 |
| 10.900.000 | 2.006.213 | -421.716 | 1.7023 |
| 10.910.000 | 2.007.678 | -422.010 | 1.7024 |
| 10.920.000 | 2.009.557 | -422.268 | 1.7025 |
| 10.930.000 | 2.011.056 | -422.483 | 1.7027 |
| 10.940.000 | 2.012.909 | -422.743 | 1.7029 |
| 10.950.000 | 2.014.431 | -422.998 | 1.7030 |
| 10.960.000 | 2.016.331 | -423.227 | 1.7029 |
| 10.970.000 | 2.017.737 | -423.446 | 1.7026 |
| 10.980.000 | 2.019.661 | -423.761 | 1.7025 |
| 10.990.000 | 2.021.210 | -423.910 | 1.7024 |
| 11.000.000 | 2.022.946 | -424.194 | 1.7025 |
| 11.010.000 | 2.024.635 | -424.416 | 1.7026 |
| 11.020.000 | 2.026.270 | -424.656 | 1.7027 |
| 11.030.000 | 2.028.004 | -424.835 | 1.7027 |
| 11.040.000 | 2.029.622 | -425.179 | 1.7025 |

| | | | |
|---|---|---|---|
| 11.050.000 | 2.031.425 | -425.315 | 1.7027 |
| 11.060.000 | 2.032.943 | -425.590 | 1.7027 |
| 11.070.000 | 2.034.828 | -425.882 | 1.7029 |
| 11.080.000 | 2.036.259 | -426.043 | 1.7032 |
| 11.090.000 | 2.038.176 | -426.329 | 1.7034 |
| 11.100.000 | 2.039.695 | -426.586 | 1.7035 |
| 11.110.000 | 2.041.507 | -426.768 | 1.7036 |
| 11.120.000 | 2.043.017 | -427.009 | 1.7036 |
| 11.130.000 | 2.044.854 | -427.322 | 1.7035 |
| 11.140.000 | 2.046.409 | -427.429 | 1.7034 |
| 11.150.000 | 2.048.195 | -427.739 | 1.7033 |
| 11.160.000 | 2.049.892 | -427.952 | 1.7033 |
| 11.170.000 | 2.051.514 | -428.186 | 1.7031 |
| 11.180.000 | 2.053.257 | -428.380 | 1.7030 |
| 11.190.000 | 2.054.850 | -428.682 | 1.7029 |
| 11.200.000 | 2.056.664 | -428.800 | 1.7026 |
| 11.210.000 | 2.058.173 | -429.110 | 1.7027 |
| 11.220.000 | 2.060.100 | -429.363 | 1.7029 |
| 11.230.000 | 2.061.463 | -429.510 | 1.7030 |
| 11.240.000 | 2.063.402 | -429.805 | 1.7029 |
| 11.250.000 | 2.064.920 | -430.030 | 1.7026 |
| 11.260.000 | 2.066.769 | -430.250 | 1.7024 |
| 11.270.000 | 2.068.314 | -430.453 | 1.7021 |
| 11.280.000 | 2.070.149 | -430.739 | 1.7019 |
| 11.290.000 | 2.071.674 | -430.877 | 1.7019 |
| 11.300.000 | 2.073.406 | -431.167 | 1.7018 |
| 11.310.000 | 2.075.128 | -431.335 | 1.7017 |
| 11.320.000 | 2.076.706 | -431.592 | 1.7018 |
| 11.330.000 | 2.078.537 | -431.772 | 1.7018 |
| 11.340.000 | 2.080.113 | -432.039 | 1.7018 |

| | | | |
|---|---|---|---|
| 11.350.000 | 2.081.911 | -432.198 | 1.7015 |
| 11.360.000 | 2.083.419 | -432.477 | 1.7013 |
| 11.370.000 | 2.085.378 | -432.695 | 1.7012 |
| 11.380.000 | 2.086.786 | -432.891 | 1.7012 |
| 11.390.000 | 2.088.690 | -433.175 | 1.7011 |
| 11.400.000 | 2.090.205 | -433.345 | 1.7012 |
| 11.410.000 | 2.091.987 | -433.587 | 1.7013 |
| 11.420.000 | 2.093.599 | -433.810 | 1.7014 |
| 11.430.000 | 2.095.381 | -434.060 | 1.7013 |
| 11.440.000 | 2.096.999 | -434.233 | 1.7011 |
| 11.450.000 | 2.098.720 | -434.549 | 1.7011 |
| 11.460.000 | 2.100.452 | -434.667 | 1.7010 |
| 11.470.000 | 2.102.070 | -434.974 | 1.7008 |
| 11.480.000 | 2.103.886 | -435.179 | 1.7007 |
| 11.490.000 | 2.105.430 | -435.397 | 1.7008 |
| 11.500.000 | 2.107.245 | -435.645 | 1.7009 |
| 11.510.000 | 2.108.734 | -435.921 | 1.7007 |
| 11.520.000 | 2.110.621 | -436.103 | 1.7006 |
| 11.530.000 | 2.112.030 | -436.332 | 1.7005 |
| 11.540.000 | 2.113.987 | -436.622 | 1.7005 |
| 11.550.000 | 2.115.453 | -436.751 | 1.7005 |
| 11.560.000 | 2.117.250 | -437.045 | 1.7003 |
| 11.570.000 | 2.118.901 | -437.270 | 1.7003 |
| 11.580.000 | 2.120.600 | -437.482 | 1.7005 |
| 11.590.000 | 2.122.210 | -437.659 | 1.7005 |
| 11.600.000 | 2.123.961 | -437.982 | 1.7004 |
| 11.610.000 | 2.125.637 | -438.077 | 1.7002 |
| 11.620.000 | 2.127.193 | -438.386 | 1.7000 |
| 11.630.000 | 2.129.078 | -438.585 | 1.6999 |
| 11.640.000 | 2.130.500 | -438.780 | 1.6998 |

| | | | |
|---|---|---|---|
| 11.650.000 | 2.132.390 | -439.032 | 1.6997 |
| 11.660.000 | 2.133.948 | -439.308 | 1.6992 |
| 11.670.000 | 2.135.745 | -439.471 | 1.6989 |
| 11.680.000 | 2.137.248 | -439.693 | 1.6989 |
| 11.690.000 | 2.139.162 | -439.979 | 1.6990 |
| 11.700.000 | 2.140.619 | -440.088 | 1.6991 |
| 11.710.000 | 2.142.465 | -440.416 | 1.6991 |
| 11.720.000 | 2.144.100 | -440.594 | 1.6990 |
| 11.730.000 | 2.145.766 | -440.798 | 1.6991 |
| 11.740.000 | 2.147.448 | -441.011 | 1.6989 |
| 11.750.000 | 2.149.127 | -441.288 | 1.6989 |
| 11.760.000 | 2.150.833 | -441.394 | 1.6987 |
| 11.770.000 | 2.152.396 | -441.712 | 1.6985 |
| 11.780.000 | 2.154.313 | -441.890 | 1.6984 |
| 11.790.000 | 2.155.731 | -442.083 | 1.6983 |
| 11.800.000 | 2.157.645 | -442.357 | 1.6984 |
| 11.810.000 | 2.159.180 | -442.572 | 1.6984 |
| 11.820.000 | 2.160.982 | -442.778 | 1.6985 |
| 11.830.000 | 2.162.484 | -442.967 | 1.6986 |
| 11.840.000 | 2.164.388 | -443.238 | 1.6985 |
| 11.850.000 | 2.165.841 | -443.370 | 1.6986 |
| 11.860.000 | 2.167.665 | -443.664 | 1.6985 |
| 11.870.000 | 2.169.294 | -443.811 | 1.6983 |
| 11.880.000 | 2.170.936 | -444.052 | 1.6982 |
| 11.890.000 | 2.172.693 | -444.225 | 1.6979 |
| 11.900.000 | 2.174.347 | -444.474 | 1.6976 |
| 11.910.000 | 2.176.076 | -444.598 | 1.6973 |
| 11.920.000 | 2.177.677 | -444.887 | 1.6972 |
| 11.930.000 | 2.179.565 | -445.040 | 1.6972 |
| 11.940.000 | 2.180.981 | -445.231 | 1.6969 |

| | | | |
|---|---|---|---|
| 11.950.000 | 2.182.917 | -445.502 | 1.6966 |
| 11.960.000 | 2.184.397 | -445.653 | 1.6964 |
| 11.970.000 | 2.186.244 | -445.892 | 1.6964 |
| 11.980.000 | 2.187.799 | -446.123 | 1.6962 |
| 11.990.000 | 2.189.657 | -446.334 | 1.6962 |
| 12.000.000 | 2.191.177 | -446.512 | 1.6961 |
| 12.010.000 | 2.192.980 | -446.824 | 1.6960 |
| 12.020.000 | 2.194.633 | -446.919 | 1.6960 |
| 12.030.000 | 2.196.264 | -447.195 | 1.6960 |
| 12.040.000 | 2.198.030 | -447.372 | 1.6961 |
| 12.050.000 | 2.199.645 | -447.576 | 1.6958 |
| 12.060.000 | 2.201.423 | -447.716 | 1.6954 |
| 12.070.000 | 2.202.941 | -447.987 | 1.6953 |
| 12.080.000 | 2.204.840 | -448.131 | 1.6953 |
| 12.090.000 | 2.206.236 | -448.334 | 1.6953 |
| 12.100.000 | 2.208.187 | -448.571 | 1.6951 |
| 12.110.000 | 2.209.662 | -448.690 | 1.6949 |
| 12.120.000 | 2.211.505 | -448.925 | 1.6947 |
| 12.130.000 | 2.213.084 | -449.110 | 1.6948 |
| 12.140.000 | 2.214.824 | -449.262 | 1.6947 |
| 12.150.000 | 2.216.395 | -449.440 | 1.6945 |
| 12.160.000 | 2.218.166 | -449.690 | 1.6942 |
| 12.170.000 | 2.219.804 | -449.756 | 1.6939 |
| 12.180.000 | 2.221.406 | -450.007 | 1.6937 |
| 12.190.000 | 2.223.209 | -450.131 | 1.6934 |
| 12.200.000 | 2.224.696 | -450.302 | 1.6930 |
| 12.210.000 | 2.226.545 | -450.473 | 1.6926 |
| 12.220.000 | 2.228.085 | -450.684 | 1.6921 |
| 12.230.000 | 2.229.922 | -450.824 | 1.6919 |
| 12.240.000 | 2.231.412 | -451.012 | 1.6917 |

| | | | |
|---|---|---|---|
| 12.250.000 | 2.233.305 | -451.231 | 1.6913 |
| 12.260.000 | 2.234.731 | -451.295 | 1.6911 |
| 12.270.000 | 2.236.596 | -451.529 | 1.6909 |
| 12.280.000 | 2.238.140 | -451.654 | 1.6909 |
| 12.290.000 | 2.239.885 | -451.830 | 1.6909 |
| 12.300.000 | 2.241.535 | -451.952 | 1.6909 |
| 12.310.000 | 2.243.243 | -452.165 | 1.6908 |
| 12.320.000 | 2.244.906 | -452.232 | 1.6908 |
| 12.330.000 | 2.246.585 | -452.513 | 1.6908 |
| 12.340.000 | 2.248.430 | -452.592 | 1.6908 |
| 12.350.000 | 2.249.889 | -452.768 | 1.6906 |
| 12.360.000 | 2.251.771 | -452.953 | 1.6903 |
| 12.370.000 | 2.253.273 | -453.105 | 1.6901 |
| 12.380.000 | 2.255.067 | -453.256 | 1.6899 |
| 12.390.000 | 2.256.563 | -453.433 | 1.6897 |
| 12.400.000 | 2.258.477 | -453.595 | 1.6895 |
| 12.410.000 | 2.259.872 | -453.679 | 1.6893 |
| 12.420.000 | 2.261.721 | -453.874 | 1.6890 |
| 12.430.000 | 2.263.372 | -453.960 | 1.6887 |
| 12.440.000 | 2.265.073 | -454.127 | 1.6883 |
| 12.450.000 | 2.266.749 | -454.229 | 1.6881 |
| 12.460.000 | 2.268.465 | -454.377 | 1.6879 |
| 12.470.000 | 2.270.125 | -454.445 | 1.6878 |
| 12.480.000 | 2.271.803 | -454.676 | 1.6877 |
| 12.490.000 | 2.273.584 | -454.718 | 1.6877 |
| 12.500.000 | 2.275.052 | -454.895 | 1.6877 |
| 12.510.000 | 2.277.012 | -455.096 | 1.6875 |
| 12.520.000 | 2.278.432 | -455.159 | 1.6870 |
| 12.530.000 | 2.280.289 | -455.326 | 1.6866 |
| 12.540.000 | 2.281.818 | -455.482 | 1.6862 |

| | | | |
|---|---|---|---|
| 12.550.000 | 2.283.663 | -455.576 | 1.6857 |
| 12.560.000 | 2.285.188 | -455.685 | 1.6850 |
| 12.570.000 | 2.287.070 | -455.900 | 1.6847 |
| 12.580.000 | 2.288.637 | -455.897 | 1.6844 |
| 12.590.000 | 2.290.390 | -456.081 | 1.6841 |
| 12.600.000 | 2.292.049 | -456.176 | 1.6836 |
| 12.610.000 | 2.293.737 | -456.300 | 1.6831 |
| 12.620.000 | 2.295.481 | -456.384 | 1.6826 |
| 12.630.000 | 2.297.090 | -456.594 | 1.6824 |
| 12.640.000 | 2.298.981 | -456.621 | 1.6821 |
| 12.650.000 | 2.300.415 | -456.801 | 1.6817 |
| 12.660.000 | 2.302.401 | -456.959 | 1.6812 |
| 12.670.000 | 2.303.861 | -457.012 | 1.6806 |
| 12.680.000 | 2.305.734 | -457.176 | 1.6803 |
| 12.690.000 | 2.307.278 | -457.286 | 1.6799 |
| 12.700.000 | 2.309.134 | -457.399 | 1.6795 |
| 12.710.000 | 2.310.662 | -457.487 | 1.6789 |
| 12.720.000 | 2.312.536 | -457.672 | 1.6783 |
| 12.730.000 | 2.314.094 | -457.689 | 1.6778 |
| 12.740.000 | 2.315.801 | -457.878 | 1.6774 |
| 12.750.000 | 2.317.594 | -457.954 | 1.6768 |
| 12.760.000 | 2.319.189 | -458.062 | 1.6763 |
| 12.770.000 | 2.320.990 | -458.134 | 1.6761 |
| 12.780.000 | 2.322.586 | -458.331 | 1.6754 |
| 12.790.000 | 2.324.448 | -458.354 | 1.6749 |
| 12.800.000 | 2.325.952 | -458.499 | 1.6741 |
| 12.810.000 | 2.327.915 | -458.627 | 1.6733 |
| 12.820.000 | 2.329.324 | -458.634 | 1.6727 |
| 12.830.000 | 2.331.211 | -458.773 | 1.6719 |
| 12.840.000 | 2.332.812 | -458.809 | 1.6712 |

| | | | |
|---|---|---|---|
| 12.850.000 | 2.334.595 | -458.852 | 1.6705 |
| 12.860.000 | 2.336.190 | -458.900 | 1.6699 |
| 12.870.000 | 2.337.999 | -459.007 | 1.6692 |
| 12.880.000 | 2.339.638 | -458.944 | 1.6683 |
| 12.890.000 | 2.341.332 | -459.055 | 1.6675 |
| 12.900.000 | 2.343.137 | -459.008 | 1.6668 |
| 12.910.000 | 2.344.699 | -459.082 | 1.6656 |
| 12.920.000 | 2.346.572 | -459.065 | 1.6648 |
| 12.930.000 | 2.348.127 | -459.099 | 1.6640 |
| 12.940.000 | 2.349.984 | -459.085 | 1.6633 |
| 12.950.000 | 2.351.469 | -459.130 | 1.6625 |
| 12.960.000 | 2.353.442 | -459.118 | 1.6615 |
| 12.970.000 | 2.354.875 | -459.065 | 1.6607 |
| 12.980.000 | 2.356.799 | -459.099 | 1.6599 |
| 12.990.000 | 2.358.364 | -459.001 | 1.6590 |
| 13.000.000 | 2.360.143 | -459.010 | 1.6581 |
| 13.010.000 | 2.361.826 | -458.954 | 1.6571 |
| 13.020.000 | 2.363.670 | -458.925 | 1.6565 |
| 13.030.000 | 2.365.274 | -458.804 | 1.6560 |
| 13.040.000 | 2.367.009 | -458.812 | 1.6552 |
| 13.050.000 | 2.368.794 | -458.644 | 1.6541 |
| 13.060.000 | 2.370.371 | -458.610 | 1.6529 |
| 13.070.000 | 2.372.270 | -458.516 | 1.6516 |
| 13.080.000 | 2.373.833 | -458.409 | 1.6502 |
| 13.090.000 | 2.375.689 | -458.293 | 1.6486 |
| 13.100.000 | 2.377.210 | -458.203 | 1.6472 |
| 13.110.000 | 2.379.140 | -458.059 | 1.6459 |
| 13.120.000 | 2.380.712 | -457.919 | 1.6444 |
| 13.130.000 | 2.382.644 | -457.827 | 1.6428 |
| 13.140.000 | 2.384.247 | -457.604 | 1.6409 |

| | | | |
|---|---|---|---|
| 13.150.000 | 2.386.076 | -457.505 | 1.6392 |
| 13.160.000 | 2.387.767 | -457.337 | 1.6375 |
| 13.170.000 | 2.389.523 | -457.142 | 1.6356 |
| 13.180.000 | 2.391.231 | -456.919 | 1.6337 |
| 13.190.000 | 2.392.958 | -456.814 | 1.6317 |
| 13.200.000 | 2.394.759 | -456.489 | 1.6299 |
| 13.210.000 | 2.396.327 | -456.342 | 1.6279 |
| 13.220.000 | 2.398.295 | -456.115 | 1.6258 |
| 13.230.000 | 2.399.795 | -455.852 | 1.6237 |
| 13.240.000 | 2.401.794 | -455.688 | 1.6215 |
| 13.250.000 | 2.403.378 | -455.424 | 1.6194 |
| 13.260.000 | 2.405.257 | -455.144 | 1.6172 |
| 13.270.000 | 2.406.775 | -454.891 | 1.6146 |
| 13.280.000 | 2.408.723 | -454.685 | 1.6118 |
| 13.290.000 | 2.410.268 | -454.313 | 1.6091 |
| 13.300.000 | 2.412.087 | -454.135 | 1.6063 |
| 13.310.000 | 2.413.778 | -453.844 | 1.6035 |
| 13.320.000 | 2.415.461 | -453.577 | 1.6006 |
| 13.330.000 | 2.417.227 | -453.289 | 1.5977 |
| 13.340.000 | 2.418.949 | -453.137 | 1.5947 |
| 13.350.000 | 2.420.742 | -452.786 | 1.5913 |
| 13.360.000 | 2.422.329 | -452.648 | 1.5878 |
| 13.370.000 | 2.424.246 | -452.439 | 1.5844 |
| 13.380.000 | 2.425.685 | -452.226 | 1.5809 |
| 13.390.000 | 2.427.633 | -452.128 | 1.5775 |
| 13.400.000 | 2.429.143 | -451.982 | 1.5738 |
| 13.410.000 | 2.430.949 | -451.852 | 1.5700 |
| 13.420.000 | 2.432.480 | -451.792 | 1.5661 |
| 13.430.000 | 2.434.342 | -451.779 | 1.5618 |
| 13.440.000 | 2.435.879 | -451.713 | 1.5575 |

| | | | |
|---|---|---|---|
| 13.450.000 | 2.437.645 | -451.827 | 1.5531 |
| 13.460.000 | 2.439.356 | -451.854 | 1.5489 |
| 13.470.000 | 2.441.042 | -452.002 | 1.5447 |
| 13.480.000 | 2.442.737 | -452.134 | 1.5404 |
| 13.490.000 | 2.444.350 | -452.338 | 1.5362 |
| 13.500.000 | 2.446.118 | -452.509 | 1.5320 |
| 13.510.000 | 2.447.611 | -452.858 | 1.5276 |
| 13.520.000 | 2.449.547 | -453.123 | 1.5232 |
| 13.530.000 | 2.450.898 | -453.449 | 1.5188 |
| 13.540.000 | 2.452.771 | -453.880 | 1.5145 |
| 13.550.000 | 2.454.265 | -454.222 | 1.5102 |
| 13.560.000 | 2.456.074 | -454.704 | 1.5060 |
| 13.570.000 | 2.457.638 | -455.138 | 1.5017 |
| 13.580.000 | 2.459.430 | -455.631 | 1.4975 |
| 13.590.000 | 2.460.931 | -456.075 | 1.4933 |
| 13.600.000 | 2.462.724 | -456.685 | 1.4891 |
| 13.610.000 | 2.464.332 | -457.124 | 1.4848 |
| 13.620.000 | 2.465.916 | -457.727 | 1.4808 |
| 13.630.000 | 2.467.705 | -458.260 | 1.4768 |
| 13.640.000 | 2.469.210 | -458.850 | 1.4729 |
| 13.650.000 | 2.470.987 | -459.380 | 1.4689 |
| 13.660.000 | 2.472.501 | -460.040 | 1.4649 |
| 13.670.000 | 2.474.345 | -460.605 | 1.4608 |
| 13.680.000 | 2.475.778 | -461.195 | 1.4569 |
| 13.690.000 | 2.477.661 | -461.847 | 1.4531 |
| 13.700.000 | 2.479.109 | -462.368 | 1.4493 |
| 13.710.000 | 2.480.963 | -463.024 | 1.4454 |
| 13.720.000 | 2.482.499 | -463.604 | 1.4416 |
| 13.730.000 | 2.484.200 | -464.200 | 1.4379 |
| 13.740.000 | 2.485.815 | -464.764 | 1.4344 |

| | | | |
|---|---|---|---|
| 13.750.000 | 2.487.523 | -465.424 | 1.4307 |
| 13.760.000 | 2.489.171 | -465.879 | 1.4271 |
| 13.770.000 | 2.490.763 | -466.546 | 1.4235 |
| 13.780.000 | 2.492.550 | -467.073 | 1.4199 |
| 13.790.000 | 2.494.057 | -467.621 | 1.4165 |
| 13.800.000 | 2.495.932 | -468.196 | 1.4131 |
| 13.810.000 | 2.497.432 | -468.761 | 1.4098 |
| 13.820.000 | 2.499.244 | -469.262 | 1.4064 |
| 13.830.000 | 2.500.673 | -469.794 | 1.4032 |
| 13.840.000 | 2.502.565 | -470.357 | 1.4000 |
| 13.850.000 | 2.504.024 | -470.789 | 1.3967 |
| 13.860.000 | 2.505.822 | -471.357 | 1.3932 |
| 13.870.000 | 2.507.390 | -471.815 | 1.3897 |
| 13.880.000 | 2.509.072 | -472.303 | 1.3862 |
| 13.890.000 | 2.510.682 | -472.747 | 1.3827 |
| 13.900.000 | 2.512.400 | -473.271 | 1.3791 |
| 13.910.000 | 2.514.083 | -473.661 | 1.3755 |
| 13.920.000 | 2.515.701 | -474.162 | 1.3722 |
| 13.930.000 | 2.517.498 | -474.549 | 1.3689 |
| 13.940.000 | 2.518.951 | -474.985 | 1.3657 |
| 13.950.000 | 2.520.831 | -475.429 | 1.3625 |
| 13.960.000 | 2.522.291 | -475.821 | 1.3594 |
| 13.970.000 | 2.524.086 | -476.224 | 1.3561 |
| 13.980.000 | 2.525.589 | -476.630 | 1.3529 |
| 13.990.000 | 2.527.435 | -477.043 | 1.3498 |
| 14.000.000 | 2.528.927 | -477.352 | 1.3466 |
| 14.010.000 | 2.530.736 | -477.807 | 1.3432 |
| 14.020.000 | 2.532.313 | -478.091 | 1.3399 |
| 14.030.000 | 2.534.071 | -478.497 | 1.3366 |
| 14.040.000 | 2.535.721 | -478.824 | 1.3333 |

| | | | |
|---|---|---|---|
| 14.050.000 | 2.537.395 | -479.194 | 1.3301 |
| 14.060.000 | 2.539.044 | -479.461 | 1.3270 |
| 14.070.000 | 2.540.699 | -479.899 | 1.3237 |
| 14.080.000 | 2.542.462 | -480.111 | 1.3204 |
| 14.090.000 | 2.543.917 | -480.465 | 1.3171 |
| 14.100.000 | 2.545.804 | -480.826 | 1.3141 |
| 14.110.000 | 2.547.270 | -481.073 | 1.3109 |
| 14.120.000 | 2.549.113 | -481.400 | 1.3077 |
| 14.130.000 | 2.550.638 | -481.704 | 1.3045 |
| 14.140.000 | 2.552.563 | -481.982 | 1.3014 |
| 14.150.000 | 2.554.022 | -482.218 | 1.2983 |
| 14.160.000 | 2.555.820 | -482.564 | 1.2952 |
| 14.170.000 | 2.557.433 | -482.723 | 1.2920 |
| 14.180.000 | 2.559.075 | -483.037 | 1.2889 |
| 14.190.000 | 2.560.814 | -483.260 | 1.2858 |
| 14.200.000 | 2.562.435 | -483.508 | 1.2826 |
| 14.210.000 | 2.564.137 | -483.707 | 1.2793 |
| 14.220.000 | 2.565.757 | -484.021 | 1.2760 |
| 14.230.000 | 2.567.575 | -484.195 | 1.2728 |
| 14.240.000 | 2.569.071 | -484.444 | 1.2696 |
| 14.250.000 | 2.571.015 | -484.722 | 1.2665 |
| 14.260.000 | 2.572.417 | -484.848 | 1.2633 |
| 14.270.000 | 2.574.315 | -485.109 | 1.2603 |
| 14.280.000 | 2.575.815 | -485.326 | 1.2572 |
| 14.290.000 | 2.577.573 | -485.492 | 1.2541 |
| 14.300.000 | 2.579.140 | -485.671 | 1.2508 |
| 14.310.000 | 2.580.952 | -485.962 | 1.2476 |
| 14.320.000 | 2.582.503 | -486.038 | 1.2444 |
| 14.330.000 | 2.584.227 | -486.310 | 1.2412 |
| 14.340.000 | 2.585.971 | -486.461 | 1.2381 |

| | | | |
|---|---|---|---|
| 14.350.000 | 2.587.532 | -486.643 | 1.2350 |
| 14.360.000 | 2.589.365 | -486.802 | 1.2320 |
| 14.370.000 | 2.590.928 | -487.058 | 1.2290 |
| 14.380.000 | 2.592.742 | -487.185 | 1.2258 |
| 14.390.000 | 2.594.198 | -487.440 | 1.2227 |
| 14.400.000 | 2.596.137 | -487.646 | 1.2196 |
| 14.410.000 | 2.597.548 | -487.774 | 1.2166 |
| 14.420.000 | 2.599.413 | -488.037 | 1.2137 |
| 14.430.000 | 2.600.940 | -488.195 | 1.2107 |
| 14.440.000 | 2.602.695 | -488.393 | 1.2077 |
| 14.450.000 | 2.604.268 | -488.561 | 1.2047 |
| 14.460.000 | 2.606.066 | -488.824 | 1.2018 |
| 14.470.000 | 2.607.642 | -488.943 | 1.1990 |
| 14.480.000 | 2.609.354 | -489.217 | 1.1963 |
| 14.490.000 | 2.611.120 | -489.373 | 1.1935 |
| 14.500.000 | 2.612.618 | -489.612 | 1.1906 |
| 14.510.000 | 2.614.449 | -489.798 | 1.1877 |
| 14.520.000 | 2.615.959 | -490.043 | 1.1850 |
| 14.530.000 | 2.617.776 | -490.266 | 1.1824 |
| 14.540.000 | 2.619.249 | -490.505 | 1.1795 |
| 14.550.000 | 2.621.113 | -490.760 | 1.1767 |
| 14.560.000 | 2.622.525 | -490.940 | 1.1740 |
| 14.570.000 | 2.624.418 | -491.254 | 1.1713 |
| 14.580.000 | 2.625.943 | -491.415 | 1.1687 |
| 14.590.000 | 2.627.697 | -491.691 | 1.1660 |
| 14.600.000 | 2.629.306 | -491.923 | 1.1633 |
| 14.610.000 | 2.631.025 | -492.177 | 1.1604 |
| 14.620.000 | 2.632.643 | -492.370 | 1.1577 |
| 14.630.000 | 2.634.320 | -492.715 | 1.1553 |
| 14.640.000 | 2.636.053 | -492.843 | 1.1528 |

| | | | |
|---|---|---|---|
| 14.650.000 | 2.637.523 | -493.172 | 1.1501 |
| 14.660.000 | 2.639.396 | -493.430 | 1.1475 |
| 14.670.000 | 2.640.858 | -493.676 | 1.1450 |
| 14.680.000 | 2.642.679 | -493.974 | 1.1425 |
| 14.690.000 | 2.644.181 | -494.278 | 1.1402 |
| 14.700.000 | 2.646.055 | -494.514 | 1.1376 |
| 14.710.000 | 2.647.472 | -494.780 | 1.1352 |
| 14.720.000 | 2.649.339 | -495.128 | 1.1328 |
| 14.730.000 | 2.650.861 | -495.317 | 1.1306 |
| 14.740.000 | 2.652.602 | -495.670 | 1.1284 |
| 14.750.000 | 2.654.217 | -495.922 | 1.1262 |
| 14.760.000 | 2.655.902 | -496.208 | 1.1238 |
| 14.770.000 | 2.657.522 | -496.468 | 1.1214 |
| 14.780.000 | 2.659.230 | -496.866 | 1.1190 |
| 14.790.000 | 2.660.948 | -497.028 | 1.1169 |
| 14.800.000 | 2.662.477 | -497.395 | 1.1146 |
| 14.810.000 | 2.664.369 | -497.723 | 1.1122 |
| 14.820.000 | 2.665.803 | -497.978 | 1.1099 |
| 14.830.000 | 2.667.641 | -498.332 | 1.1078 |
| 14.840.000 | 2.669.145 | -498.651 | 1.1057 |
| 14.850.000 | 2.670.908 | -498.921 | 1.1033 |
| 14.860.000 | 2.672.457 | -499.242 | 1.1012 |
| 14.870.000 | 2.674.289 | -499.622 | 1.0991 |
| 14.880.000 | 2.675.792 | -499.824 | 1.0970 |
| 14.890.000 | 2.677.506 | -500.212 | 1.0951 |
| 14.900.000 | 2.679.148 | -500.513 | 1.0930 |
| 14.910.000 | 2.680.779 | -500.811 | 1.0911 |
| 14.920.000 | 2.682.528 | -501.115 | 1.0893 |
| 14.930.000 | 2.684.179 | -501.512 | 1.0874 |
| 14.940.000 | 2.685.895 | -501.707 | 1.0856 |

| | | | |
|---|---|---|---|
| 14.950.000 | 2.687.400 | -502.092 | 1.0837 |
| 14.960.000 | 2.689.282 | -502.422 | 1.0819 |
| 14.970.000 | 2.690.687 | -502.709 | 1.0801 |
| 14.980.000 | 2.692.559 | -503.091 | 1.0782 |
| 14.990.000 | 2.694.058 | -503.368 | 1.0763 |
| 15.000.000 | 2.695.810 | -503.671 | 1.0745 |
| 15.010.000 | 2.697.327 | -503.981 | 1.0727 |
| 15.020.000 | 2.699.195 | -504.352 | 1.0710 |
| 15.030.000 | 2.700.657 | -504.554 | 1.0693 |
| 15.040.000 | 2.702.473 | -504.957 | 1.0677 |
| 15.050.000 | 2.704.160 | -505.203 | 1.0660 |
| 15.060.000 | 2.705.716 | -505.542 | 1.0644 |
| 15.070.000 | 2.707.448 | -505.814 | 1.0626 |
| 15.080.000 | 2.709.016 | -506.174 | 1.0607 |
| 15.090.000 | 2.710.759 | -506.415 | 1.0592 |
| 15.100.000 | 2.712.254 | -506.798 | 1.0575 |
| 15.110.000 | 2.714.111 | -507.093 | 1.0559 |
| 15.120.000 | 2.715.496 | -507.380 | 1.0543 |
| 15.130.000 | 2.717.390 | -507.737 | 1.0529 |
| 15.140.000 | 2.718.863 | -507.995 | 1.0515 |
| 15.150.000 | 2.720.630 | -508.314 | 1.0500 |
| 15.160.000 | 2.722.206 | -508.601 | 1.0485 |
| 15.170.000 | 2.723.962 | -508.914 | 1.0470 |
| 15.180.000 | 2.725.496 | -509.165 | 1.0454 |
| 15.190.000 | 2.727.229 | -509.552 | 1.0438 |
| 15.200.000 | 2.728.895 | -509.749 | 1.0423 |
| 15.210.000 | 2.730.427 | -510.105 | 1.0409 |
| 15.220.000 | 2.732.234 | -510.389 | 1.0395 |
| 15.230.000 | 2.733.753 | -510.687 | 1.0381 |
| 15.240.000 | 2.735.543 | -510.963 | 1.0367 |

| | | | |
|---|---|---|---|
| 15.250.000 | 2.737.002 | -511.312 | 1.0353 |
| 15.260.000 | 2.738.913 | -511.581 | 1.0340 |
| 15.270.000 | 2.740.317 | -511.868 | 1.0328 |
| 15.280.000 | 2.742.205 | -512.227 | 1.0316 |
| 15.290.000 | 2.743.684 | -512.424 | 1.0303 |
| 15.300.000 | 2.745.444 | -512.790 | 1.0290 |
| 15.310.000 | 2.747.002 | -513.053 | 1.0277 |
| 15.320.000 | 2.748.754 | -513.355 | 1.0264 |
| 15.330.000 | 2.750.276 | -513.600 | 1.0253 |
| 15.340.000 | 2.752.030 | -513.968 | 1.0239 |
| 15.350.000 | 2.753.688 | -514.158 | 1.0226 |
| 15.360.000 | 2.755.245 | -514.528 | 1.0214 |
| 15.370.000 | 2.757.091 | -514.782 | 1.0203 |
| 15.380.000 | 2.758.562 | -515.069 | 1.0191 |
| 15.390.000 | 2.760.351 | -515.343 | 1.0178 |
| 15.400.000 | 2.761.885 | -515.665 | 1.0166 |
| 15.410.000 | 2.763.704 | -515.919 | 1.0156 |
| 15.420.000 | 2.765.147 | -516.205 | 1.0147 |
| 15.430.000 | 2.767.046 | -516.535 | 1.0136 |
| 15.440.000 | 2.768.469 | -516.732 | 1.0126 |
| 15.450.000 | 2.770.293 | -517.090 | 1.0116 |
| 15.460.000 | 2.771.873 | -517.349 | 1.0105 |
| 15.470.000 | 2.773.545 | -517.615 | 1.1993 |
| 15.480.000 | 2.775.182 | -517.874 | 1.1981 |
| 15.490.000 | 2.776.869 | -518.225 | 1.1971 |
| 15.500.000 | 2.778.541 | -518.380 | 1.1961 |
| 15.510.000 | 2.780.089 | -518.751 | 1.1951 |
| 15.520.000 | 2.781.935 | -518.974 | 1.1943 |
| 15.530.000 | 2.783.375 | -519.246 | 1.1934 |
| 15.540.000 | 2.785.231 | -519.547 | 1.2024 |

| | | | |
|---|---|---|---|
| 15.550.000 | 2.786.736 | -519.821 | 1.2013 |
| 15.560.000 | 2.788.529 | -520.062 | 1.2000 |
| 15.570.000 | 2.790.037 | -520.350 | 9.989 |
| 15.580.000 | 2.791.917 | -520.640 | 9.978 |
| 15.590.000 | 2.793.387 | -520.847 | 9.969 |
| 15.600.000 | 2.795.189 | -521.182 | 9.962 |
| 15.610.000 | 2.796.762 | -521.397 | 9.954 |
| 15.620.000 | 2.798.445 | -521.686 | 9.945 |
| 15.630.000 | 2.800.129 | -521.938 | 9.935 |
| 15.640.000 | 2.801.786 | -522.233 | 9.926 |
| 15.650.000 | 2.803.439 | -522.427 | 9.916 |
| 15.660.000 | 2.805.046 | -522.768 | 9.908 |
| 15.670.000 | 2.806.890 | -522.996 | 9.901 |
| 15.680.000 | 2.808.295 | -523.259 | 9.894 |
| 15.690.000 | 2.810.188 | -523.587 | 9.886 |
| 15.700.000 | 2.811.647 | -523.809 | 9.877 |
| 15.710.000 | 2.813.475 | -524.100 | 9.866 |
| 15.720.000 | 2.814.976 | -524.353 | 9.854 |
| 15.730.000 | 2.816.809 | -524.632 | 9.844 |
| 15.740.000 | 2.818.263 | -524.827 | 9.836 |
| 15.750.000 | 2.820.055 | -525.169 | 9.829 |
| 15.760.000 | 2.821.640 | -525.332 | 9.822 |
| 15.770.000 | 2.823.302 | -525.622 | 9.816 |
| 15.780.000 | 2.824.987 | -525.848 | 9.809 |
| 15.790.000 | 2.826.624 | -526.124 | 9.801 |
| 15.800.000 | 2.828.333 | -526.318 | 9.794 |
| 15.810.000 | 2.829.896 | -526.661 | 9.786 |
| 15.820.000 | 2.831.756 | -526.852 | 9.780 |
| 15.830.000 | 2.833.174 | -527.118 | 9.774 |
| 15.840.000 | 2.835.079 | -527.425 | 9.768 |

| | | | |
|---|---|---|---|
| 15.850.000 | 2.836.524 | -527.605 | 9.761 |
| 15.860.000 | 2.838.312 | -527.892 | 9.752 |
| 15.870.000 | 2.839.869 | -528.139 | 9.745 |
| 15.880.000 | 2.841.653 | -528.384 | 9.736 |
| 15.890.000 | 2.843.166 | -528.619 | 9.729 |
| 15.900.000 | 2.844.937 | -528.938 | 9.723 |
| 15.910.000 | 2.846.568 | -529.071 | 9.716 |
| 15.920.000 | 2.848.160 | -529.378 | 9.711 |
| 15.930.000 | 2.849.936 | -529.585 | 9.703 |
| 15.940.000 | 2.851.491 | -529.819 | 9.696 |
| 15.950.000 | 2.853.248 | -530.021 | 9.689 |
| 15.960.000 | 2.854.791 | -530.336 | 9.682 |
| 15.970.000 | 2.856.602 | -530.531 | 9.677 |
| 15.980.000 | 2.858.026 | -530.765 | 9.671 |
| 15.990.000 | 2.859.950 | -531.069 | 9.664 |
| 16.000.000 | 2.861.365 | -531.216 | 9.656 |
| 16.010.000 | 2.863.199 | -531.521 | 9.648 |
| 16.020.000 | 2.864.766 | -531.728 | 9.640 |
| 16.030.000 | 2.866.506 | -531.961 | 9.632 |
| 16.040.000 | 2.868.086 | -532.180 | 9.625 |
| 16.050.000 | 2.869.876 | -532.498 | 9.618 |
| 16.060.000 | 2.871.469 | -532.606 | 9.612 |
| 16.070.000 | 2.873.118 | -532.933 | 9.605 |
| 16.080.000 | 2.874.886 | -533.120 | 9.599 |
| 16.090.000 | 2.876.395 | -533.362 | 9.592 |
| 16.100.000 | 2.878.249 | -533.605 | 9.586 |
| 16.110.000 | 2.879.760 | -533.877 | 9.580 |
| 16.120.000 | 2.881.591 | -534.096 | 9.572 |
| 16.130.000 | 2.883.040 | -534.359 | 9.566 |
| 16.140.000 | 2.884.935 | -534.637 | 9.560 |

| | | | |
|---|---|---|---|
| 16.150.000 | 2.886.382 | -534.815 | 9.555 |
| 16.160.000 | 2.888.200 | -535.140 | 9.549 |
| 16.170.000 | 2.889.820 | -535.327 | 9.544 |
| 16.180.000 | 2.891.531 | -535.600 | 9.539 |
| 16.190.000 | 2.893.126 | -535.827 | 9.534 |
| 16.200.000 | 2.894.897 | -536.102 | 9.527 |
| 16.210.000 | 2.896.483 | -536.313 | 9.521 |
| 16.220.000 | 2.898.145 | -536.625 | 9.517 |
| 16.230.000 | 2.899.958 | -536.801 | 9.513 |
| 16.240.000 | 2.901.424 | -537.084 | 9.507 |
| 16.250.000 | 2.903.341 | -537.342 | 9.502 |
| 16.260.000 | 2.904.796 | -537.574 | 9.497 |
| 16.270.000 | 2.906.653 | -537.839 | 9.491 |
| 16.280.000 | 2.908.141 | -538.086 | 9.486 |
| 16.290.000 | 2.909.981 | -538.347 | 9.479 |
| 16.300.000 | 2.911.466 | -538.545 | 9.473 |
| 16.310.000 | 2.913.314 | -538.863 | 9.467 |
| 16.320.000 | 2.914.835 | -539.028 | 9.463 |
| 16.330.000 | 2.916.584 | -539.329 | 9.459 |
| 16.340.000 | 2.918.240 | -539.566 | 9.454 |
| 16.350.000 | 2.919.891 | -539.815 | 9.449 |
| 16.360.000 | 2.921.597 | -540.010 | 9.442 |
| 16.370.000 | 2.923.202 | -540.358 | 9.437 |
| 16.380.000 | 2.924.972 | -540.488 | 9.433 |
| 16.390.000 | 2.926.455 | -540.776 | 9.428 |
| 16.400.000 | 2.928.371 | -541.055 | 9.423 |
| 16.410.000 | 2.929.788 | -541.240 | 9.418 |
| 16.420.000 | 2.931.616 | -541.503 | 9.414 |
| 16.430.000 | 2.933.093 | -541.758 | 9.409 |
| 16.440.000 | 2.934.938 | -541.974 | 9.404 |

| | | | |
|---|---|---|---|
| 16.450.000 | 2.936.448 | -542.186 | 9.401 |
| 16.460.000 | 2.938.268 | -542.489 | 9.395 |
| 16.470.000 | 2.939.827 | -542.607 | 9.389 |
| 16.480.000 | 2.941.528 | -542.904 | 9.384 |
| 16.490.000 | 2.943.226 | -543.113 | 9.379 |
| 16.500.000 | 2.944.860 | -543.327 | 9.373 |
| 16.510.000 | 2.946.559 | -543.520 | 9.367 |
| 16.520.000 | 2.948.184 | -543.851 | 9.361 |
| 16.530.000 | 2.949.947 | -543.953 | 9.357 |
| 16.540.000 | 2.951.438 | -544.237 | 9.352 |
| 16.550.000 | 2.953.354 | -544.517 | 9.348 |
| 16.560.000 | 2.954.749 | -544.663 | 9.345 |
| 16.570.000 | 2.956.623 | -544.931 | 9.340 |
| 16.580.000 | 2.958.138 | -545.173 | 9.335 |
| 16.590.000 | 2.959.961 | -545.373 | 9.332 |
| 16.600.000 | 2.961.482 | -545.587 | 9.329 |
| 16.610.000 | 2.963.273 | -545.878 | 9.327 |
| 16.620.000 | 2.964.852 | -546.017 | 9.323 |
| 16.630.000 | 2.966.580 | -546.323 | 9.320 |
| 16.640.000 | 2.968.291 | -546.505 | 9.316 |
| 16.650.000 | 2.969.885 | -546.723 | 9.312 |
| 16.660.000 | 2.971.643 | -546.908 | 9.306 |
| 16.670.000 | 2.973.200 | -547.206 | 9.301 |
| 16.680.000 | 2.975.038 | -547.354 | 9.296 |
| 16.690.000 | 2.976.532 | -547.613 | 9.293 |
| 16.700.000 | 2.978.408 | -547.864 | 9.288 |
| 16.710.000 | 2.979.821 | -548.041 | 9.281 |
| 16.720.000 | 2.981.708 | -548.326 | 9.276 |
| 16.730.000 | 2.983.248 | -548.509 | 9.270 |
| 16.740.000 | 2.985.017 | -548.719 | 9.262 |

| | | | |
|---|---|---|---|
| 16.750.000 | 2.986.569 | -548.918 | 9.255 |
| 16.760.000 | 2.988.372 | -549.182 | 9.249 |
| 16.770.000 | 2.989.912 | -549.296 | 9.245 |
| 16.780.000 | 2.991.609 | -549.595 | 9.242 |
| 16.790.000 | 2.993.360 | -549.734 | 9.238 |
| 16.800.000 | 2.994.905 | -549.995 | 9.237 |
| 16.810.000 | 2.996.721 | -550.157 | 9.234 |
| 16.820.000 | 2.998.245 | -550.397 | 9.231 |
| 16.830.000 | 3.000.045 | -550.591 | 9.229 |
| 16.840.000 | 3.001.574 | -550.839 | 9.227 |
| 16.850.000 | 3.003.478 | -551.053 | 9.223 |
| 16.860.000 | 3.004.843 | -551.221 | 9.219 |
| 16.870.000 | 3.006.735 | -551.500 | 9.215 |
| 16.880.000 | 3.008.245 | -551.661 | 9.213 |
| 16.890.000 | 3.009.997 | -551.905 | 9.211 |
| 16.900.000 | 3.011.623 | -552.098 | 9.209 |
| 16.910.000 | 3.013.336 | -552.319 | 9.205 |
| 16.920.000 | 3.014.916 | -552.464 | 9.203 |
| 16.930.000 | 3.016.639 | -552.755 | 9.199 |
| 16.940.000 | 3.018.326 | -552.865 | 9.194 |
| 16.950.000 | 3.019.907 | -553.146 | 9.191 |
| 16.960.000 | 3.021.728 | -553.328 | 9.188 |
| 16.970.000 | 3.023.226 | -553.505 | 9.185 |
| 16.980.000 | 3.025.037 | -553.744 | 9.180 |
| 16.990.000 | 3.026.526 | -553.982 | 9.175 |
| 17.000.000 | 3.028.401 | -554.141 | 9.172 |
| 17.010.000 | 3.029.866 | -554.334 | 9.169 |
| 17.020.000 | 3.031.724 | -554.611 | 9.164 |
| 17.030.000 | 3.033.194 | -554.720 | 9.158 |
| 17.040.000 | 3.034.935 | -555.007 | 9.153 |

| | | | |
|---|---|---|---|
| 17.050.000 | 3.036.534 | -555.160 | 9.149 |
| 17.060.000 | 3.038.239 | -555.378 | 9.147 |
| 17.070.000 | 3.039.895 | -555.534 | 9.143 |
| 17.080.000 | 3.041.579 | -555.812 | 9.138 |
| 17.090.000 | 3.043.260 | -555.890 | 9.133 |
| 17.100.000 | 3.044.803 | -556.168 | 9.130 |
| 17.110.000 | 3.046.689 | -556.360 | 9.127 |
| 17.120.000 | 3.048.137 | -556.536 | 9.124 |
| 17.130.000 | 3.049.964 | -556.773 | 9.120 |
| 17.140.000 | 3.051.483 | -556.994 | 9.115 |
| 17.150.000 | 3.053.273 | -557.176 | 9.112 |
| 17.160.000 | 3.054.738 | -557.371 | 9.110 |
| 17.170.000 | 3.056.604 | -557.652 | 9.108 |
| 17.180.000 | 3.058.091 | -557.751 | 9.107 |
| 17.190.000 | 3.059.869 | -558.034 | 9.103 |
| 17.200.000 | 3.061.509 | -558.224 | 9.101 |
| 17.210.000 | 3.063.119 | -558.388 | 9.100 |
| 17.220.000 | 3.064.835 | -558.576 | 9.097 |
| 17.230.000 | 3.066.480 | -558.841 | 9.093 |
| 17.240.000 | 3.068.211 | -558.920 | 9.087 |
| 17.250.000 | 3.069.715 | -559.200 | 9.083 |
| 17.260.000 | 3.071.621 | -559.401 | 9.082 |
| 17.270.000 | 3.073.021 | -559.553 | 9.080 |
| 17.280.000 | 3.074.889 | -559.823 | 9.078 |
| 17.290.000 | 3.076.400 | -560.010 | 9.074 |
| 17.300.000 | 3.078.220 | -560.198 | 9.071 |
| 17.310.000 | 3.079.711 | -560.414 | 9.068 |
| 17.320.000 | 3.081.546 | -560.653 | 9.066 |
| 17.330.000 | 3.083.062 | -560.787 | 9.064 |
| 17.340.000 | 3.084.830 | -561.062 | 9.061 |

| | | | |
|---|---|---|---|
| 17.350.000 | 3.086.485 | -561.199 | 9.058 |
| 17.360.000 | 3.088.088 | -561.411 | 9.055 |
| 17.370.000 | 3.089.772 | -561.584 | 9.052 |
| 17.380.000 | 3.091.434 | -561.837 | 9.048 |
| 17.390.000 | 3.093.176 | -561.954 | 9.045 |
| 17.400.000 | 3.094.717 | -562.220 | 9.041 |
| 17.410.000 | 3.096.606 | -562.426 | 9.038 |
| 17.420.000 | 3.098.006 | -562.609 | 9.035 |
| 17.430.000 | 3.099.930 | -562.874 | 9.032 |
| 17.440.000 | 3.101.403 | -563.022 | 9.029 |
| 17.450.000 | 3.103.205 | -563.254 | 9.026 |
| 17.460.000 | 3.104.738 | -563.467 | 9.024 |
| 17.470.000 | 3.106.534 | -563.713 | 9.022 |
| 17.480.000 | 3.108.050 | -563.861 | 9.019 |
| 17.490.000 | 3.109.787 | -564.165 | 9.017 |
| 17.500.000 | 3.111.439 | -564.291 | 9.015 |
| 17.510.000 | 3.113.083 | -564.552 | 9.013 |
| 17.520.000 | 3.114.836 | -564.732 | 9.008 |
| 17.530.000 | 3.116.420 | -564.968 | 9.004 |
| 17.540.000 | 3.118.201 | -565.135 | 9.001 |
| 17.550.000 | 3.119.699 | -565.406 | 8.997 |
| 17.560.000 | 3.121.586 | -565.583 | 8.994 |
| 17.570.000 | 3.122.991 | -565.786 | 8.991 |
| 17.580.000 | 3.124.901 | -566.057 | 8.988 |
| 17.590.000 | 3.126.369 | -566.198 | 8.986 |
| 17.600.000 | 3.128.168 | -566.429 | 8.985 |
| 17.610.000 | 3.129.690 | -566.627 | 8.983 |
| 17.620.000 | 3.131.471 | -566.845 | 8.979 |
| 17.630.000 | 3.133.056 | -567.020 | 8.977 |
| 17.640.000 | 3.134.763 | -567.293 | 8.976 |

| | | | |
|---|---|---|---|
| 17.650.000 | 3.136.464 | -567.395 | 8.975 |
| 17.660.000 | 3.138.052 | -567.659 | 8.976 |
| 17.670.000 | 3.139.861 | -567.838 | 8.977 |
| 17.680.000 | 3.141.390 | -568.037 | 8.979 |
| 17.690.000 | 3.143.169 | -568.231 | 8.979 |
| 17.700.000 | 3.144.674 | -568.482 | 8.977 |
| 17.710.000 | 3.146.509 | -568.660 | 8.972 |
| 17.720.000 | 3.147.920 | -568.845 | 8.966 |
| 17.730.000 | 3.149.841 | -569.109 | 8.962 |
| 17.740.000 | 3.151.327 | -569.222 | 8.958 |
| 17.750.000 | 3.153.119 | -569.485 | 8.954 |
| 17.760.000 | 3.154.748 | -569.652 | 8.952 |
| 17.770.000 | 3.156.453 | -569.855 | 8.950 |
| 17.780.000 | 3.158.050 | -570.013 | 8.948 |
| 17.790.000 | 3.159.754 | -570.285 | 8.944 |
| 17.800.000 | 3.161.414 | -570.353 | 8.938 |
| 17.810.000 | 3.162.987 | -570.622 | 8.933 |
| 17.820.000 | 3.164.797 | -570.800 | 8.928 |
| 17.830.000 | 3.166.283 | -570.978 | 8.926 |
| 17.840.000 | 3.168.145 | -571.211 | 8.923 |
| 17.850.000 | 3.169.648 | -571.426 | 8.924 |
| 17.860.000 | 3.171.489 | -571.588 | 8.924 |
| 17.870.000 | 3.172.936 | -571.798 | 8.921 |
| 17.880.000 | 3.174.837 | -572.024 | 8.917 |
| 17.890.000 | 3.176.290 | -572.142 | 8.913 |
| 17.900.000 | 3.178.115 | -572.421 | 8.911 |
| 17.910.000 | 3.179.710 | -572.579 | 8.906 |
| 17.920.000 | 3.181.411 | -572.786 | 8.902 |
| 17.930.000 | 3.183.044 | -572.952 | 8.900 |
| 17.940.000 | 3.184.707 | -573.210 | 8.896 |

| | | | |
|---|---|---|---|
| 17.950.000 | 3.186.379 | -573.311 | 8.893 |
| 17.960.000 | 3.187.945 | -573.573 | 8.891 |
| 17.970.000 | 3.189.838 | -573.737 | 8.889 |
| 17.980.000 | 3.191.239 | -573.918 | 8.888 |
| 17.990.000 | 3.193.116 | -574.145 | 8.889 |
| 18.000.000 | 3.194.597 | -574.315 | 8.888 |
| 18.010.000 | 3.196.425 | -574.511 | 8.885 |
| 18.020.000 | 3.197.920 | -574.713 | 8.881 |
| 18.030.000 | 3.199.744 | -574.912 | 8.877 |
| 18.040.000 | 3.201.255 | -575.042 | 8.873 |
| 18.050.000 | 3.203.032 | -575.314 | 8.869 |
| 18.060.000 | 3.204.597 | -575.432 | 8.866 |
| 18.070.000 | 3.206.279 | -575.655 | 8.862 |
| 18.080.000 | 3.208.008 | -575.826 | 8.865 |
| 18.090.000 | 3.209.621 | -576.034 | 8.864 |
| 18.100.000 | 3.211.351 | -576.156 | 8.860 |
| 18.110.000 | 3.212.895 | -576.441 | 8.854 |
| 18.120.000 | 3.214.717 | -576.546 | 8.850 |
| 18.130.000 | 3.216.126 | -576.736 | 8.847 |
| 18.140.000 | 3.217.992 | -576.978 | 8.843 |
| 18.150.000 | 3.219.474 | -577.089 | 8.837 |
| 18.160.000 | 3.221.259 | -577.313 | 8.832 |
| 18.170.000 | 3.222.758 | -577.493 | 8.829 |
| 18.180.000 | 3.224.573 | -577.667 | 8.826 |
| 18.190.000 | 3.226.113 | -577.828 | 8.822 |
| 18.200.000 | 3.227.878 | -578.077 | 8.820 |
| 18.210.000 | 3.229.489 | -578.147 | 8.818 |
| 18.220.000 | 3.231.098 | -578.394 | 8.817 |
| 18.230.000 | 3.232.871 | -578.540 | 8.817 |
| 18.240.000 | 3.234.444 | -578.748 | 8.815 |

| | | | |
|---|---|---|---|
| 18.250.000 | 3.236.194 | -578.887 | 8.812 |
| 18.260.000 | 3.237.736 | -579.136 | 8.806 |
| 18.270.000 | 3.239.554 | -579.286 | 8.803 |
| 18.280.000 | 3.240.957 | -579.474 | 8.800 |
| 18.290.000 | 3.242.872 | -579.722 | 8.799 |
| 18.300.000 | 3.244.308 | -579.851 | 8.798 |
| 18.310.000 | 3.246.130 | -580.066 | 8.799 |
| 18.320.000 | 3.247.644 | -580.269 | 8.799 |
| 18.330.000 | 3.249.425 | -580.453 | 8.798 |
| 18.340.000 | 3.250.986 | -580.600 | 8.795 |
| 18.350.000 | 3.252.742 | -580.877 | 8.792 |
| 18.360.000 | 3.254.323 | -580.941 | 8.788 |
| 18.370.000 | 3.255.970 | -581.206 | 8.785 |
| 18.380.000 | 3.257.696 | -581.372 | 8.785 |
| 18.390.000 | 3.259.228 | -581.548 | 8.786 |
| 18.400.000 | 3.261.031 | -581.710 | 8.785 |
| 18.410.000 | 3.262.551 | -581.964 | 8.782 |
| 18.420.000 | 3.264.420 | -582.105 | 8.778 |
| 18.430.000 | 3.265.843 | -582.312 | 8.773 |
| 18.440.000 | 3.267.728 | -582.544 | 8.768 |
| 18.450.000 | 3.269.178 | -582.662 | 8.761 |
| 18.460.000 | 3.271.004 | -582.933 | 8.755 |
| 18.470.000 | 3.272.568 | -583.101 | 8.752 |
| 18.480.000 | 3.274.278 | -583.307 | 8.747 |
| 18.490.000 | 3.275.873 | -583.498 | 8.742 |
| 18.500.000 | 3.277.628 | -583.756 | 8.738 |
| 18.510.000 | 3.279.208 | -583.857 | 8.736 |
| 18.520.000 | 3.280.894 | -584.152 | 8.734 |
| 18.530.000 | 3.282.684 | -584.289 | 8.732 |
| 18.540.000 | 3.284.150 | -584.510 | 8.730 |

| | | | |
|---|---|---|---|
| 18.550.000 | 3.285.998 | -584.701 | 8.725 |
| 18.560.000 | 3.287.531 | -584.914 | 8.721 |
| 18.570.000 | 3.289.326 | -585.103 | 8.716 |
| 18.580.000 | 3.290.803 | -585.309 | 8.711 |
| 18.590.000 | 3.292.680 | -585.523 | 8.708 |
| 18.600.000 | 3.294.083 | -585.651 | 8.706 |
| 18.610.000 | 3.295.963 | -585.926 | 8.706 |
| 18.620.000 | 3.297.493 | -586.078 | 8.706 |
| 18.630.000 | 3.299.195 | -586.292 | 8.703 |
| 18.640.000 | 3.300.867 | -586.479 | 8.700 |
| 18.650.000 | 3.302.548 | -586.688 | 8.698 |
| 18.660.000 | 3.304.189 | -586.805 | 8.694 |
| 18.670.000 | 3.305.823 | -587.080 | 8.691 |
| 18.680.000 | 3.307.586 | -587.186 | 8.689 |
| 18.690.000 | 3.309.027 | -587.419 | 8.689 |
| 18.700.000 | 3.310.946 | -587.643 | 8.687 |
| 18.710.000 | 3.312.393 | -587.801 | 8.684 |
| 18.720.000 | 3.314.221 | -588.015 | 8.683 |
| 18.730.000 | 3.315.693 | -588.226 | 8.680 |
| 18.740.000 | 3.317.572 | -588.415 | 8.679 |
| 18.750.000 | 3.319.046 | -588.588 | 8.677 |
| 18.760.000 | 3.320.876 | -588.858 | 8.676 |
| 18.770.000 | 3.322.453 | -588.956 | 8.674 |
| 18.780.000 | 3.324.106 | -589.217 | 8.671 |
| 18.790.000 | 3.325.777 | -589.391 | 8.667 |
| 18.800.000 | 3.327.408 | -589.590 | 8.662 |
| 18.810.000 | 3.329.071 | -589.721 | 8.656 |
| 18.820.000 | 3.330.684 | -589.999 | 8.650 |
| 18.830.000 | 3.332.473 | -590.092 | 8.645 |
| 18.840.000 | 3.333.873 | -590.304 | 8.639 |

| | | | |
|---|---|---|---|
| 18.850.000 | 3.335.798 | -590.543 | 8.632 |
| 18.860.000 | 3.337.221 | -590.668 | 8.627 |
| 18.870.000 | 3.339.060 | -590.876 | 8.622 |
| 18.880.000 | 3.340.573 | -591.055 | 8.615 |
| 18.890.000 | 3.342.375 | -591.209 | 8.610 |
| 18.900.000 | 3.343.814 | -591.328 | 8.607 |
| 18.910.000 | 3.345.654 | -591.586 | 8.606 |
| 18.920.000 | 3.347.180 | -591.634 | 8.604 |
| 18.930.000 | 3.348.859 | -591.870 | 8.601 |
| 18.940.000 | 3.350.569 | -592.015 | 8.594 |
| 18.950.000 | 3.352.125 | -592.173 | 8.586 |
| 18.960.000 | 3.353.844 | -592.308 | 8.578 |
| 18.970.000 | 3.355.417 | -592.563 | 8.571 |
| 18.980.000 | 3.357.227 | -592.649 | 8.565 |
| 18.990.000 | 3.358.662 | -592.861 | 8.557 |
| 19.000.000 | 3.360.562 | -593.086 | 8.551 |
| 19.010.000 | 3.361.969 | -593.185 | 8.546 |
| 19.020.000 | 3.363.811 | -593.427 | 8.540 |
| 19.030.000 | 3.365.352 | -593.585 | 8.535 |
| 19.040.000 | 3.367.108 | -593.748 | 8.529 |
| 19.050.000 | 3.368.629 | -593.914 | 8.522 |
| 19.060.000 | 3.370.394 | -594.158 | 8.516 |
| 19.070.000 | 3.371.970 | -594.236 | 8.509 |
| 19.080.000 | 3.373.626 | -594.506 | 8.505 |
| 19.090.000 | 3.375.401 | -594.658 | 8.501 |
| 19.100.000 | 3.376.959 | -594.856 | 8.495 |
| 19.110.000 | 3.378.738 | -595.017 | 8.488 |
| 19.120.000 | 3.380.293 | -595.263 | 8.482 |
| 19.130.000 | 3.382.079 | -595.395 | 8.477 |
| 19.140.000 | 3.383.523 | -595.608 | 8.474 |

| | | | |
|---|---|---|---|
| 19.150.000 | 3.385.445 | -595.844 | 8.470 |
| 19.160.000 | 3.386.838 | -595.966 | 8.468 |
| 19.170.000 | 3.388.689 | -596.213 | 8.466 |
| 19.180.000 | 3.390.228 | -596.394 | 8.463 |
| 19.190.000 | 3.391.962 | -596.588 | 8.463 |
| 19.200.000 | 3.393.555 | -596.777 | 8.466 |
| 19.210.000 | 3.395.332 | -597.029 | 8.471 |
| 19.220.000 | 3.396.928 | -597.129 | 8.474 |
| 19.230.000 | 3.398.593 | -597.410 | 8.476 |
| 19.240.000 | 3.400.322 | -597.546 | 8.479 |
| 19.250.000 | 3.401.880 | -597.781 | 8.489 |
| 19.260.000 | 3.403.670 | -597.952 | 8.497 |
| 19.270.000 | 3.405.136 | -598.167 | 8.505 |
| 19.280.000 | 3.406.973 | -598.342 | 8.509 |
| 19.290.000 | 3.408.377 | -598.578 | 8.514 |
| 19.300.000 | 3.410.256 | -598.767 | 8.522 |
| 19.310.000 | 3.411.737 | -598.926 | 8.531 |
| 19.320.000 | 3.413.594 | -599.202 | 8.536 |
| 19.330.000 | 3.415.140 | -599.333 | 8.540 |
| 19.340.000 | 3.416.859 | -599.569 | 8.540 |
| 19.350.000 | 3.418.475 | -599.755 | 8.542 |
| 19.360.000 | 3.420.162 | -599.985 | 8.547 |
| 19.370.000 | 3.421.778 | -600.118 | 8.551 |
| 19.380.000 | 3.423.444 | -600.401 | 8.551 |
| 19.390.000 | 3.425.182 | -600.529 | 8.548 |
| 19.400.000 | 3.426.655 | -600.762 | 8.544 |
| 19.410.000 | 3.428.514 | -600.973 | 8.542 |
| 19.420.000 | 3.429.978 | -601.156 | 8.540 |
| 19.430.000 | 3.431.819 | -601.364 | 8.535 |
| 19.440.000 | 3.433.351 | -601.590 | 8.527 |

| | | | |
|---|---|---|---|
| 19.450.000 | 3.435.166 | -601.772 | 8.519 |
| 19.460.000 | 3.436.621 | -601.944 | 8.513 |
| 19.470.000 | 3.438.445 | -602.219 | 8.510 |
| 19.480.000 | 3.439.980 | -602.330 | 8.506 |
| 19.490.000 | 3.441.721 | -602.592 | 8.499 |
| 19.500.000 | 3.443.334 | -602.767 | 8.490 |
| 19.510.000 | 3.444.998 | -602.973 | 8.484 |
| 19.520.000 | 3.446.637 | -603.119 | 8.480 |
| 19.530.000 | 3.448.252 | -603.389 | 8.476 |
| 19.540.000 | 3.450.025 | -603.487 | 8.469 |
| 19.550.000 | 3.451.566 | -603.738 | 8.461 |
| 19.560.000 | 3.453.414 | -603.966 | 8.458 |
| 19.570.000 | 3.454.866 | -604.097 | 8.460 |
| 19.580.000 | 3.456.696 | -604.320 | 8.462 |
| 19.590.000 | 3.458.155 | -604.517 | 8.463 |
| 19.600.000 | 3.459.987 | -604.694 | 8.460 |
| 19.610.000 | 3.461.485 | -604.869 | 8.457 |
| 19.620.000 | 3.463.275 | -605.136 | 8.458 |
| 19.630.000 | 3.464.838 | -605.224 | 8.459 |
| 19.640.000 | 3.466.531 | -605.485 | 8.457 |
| 19.650.000 | 3.468.254 | -605.654 | 8.454 |
| 19.660.000 | 3.469.887 | -605.829 | 8.452 |
| 19.670.000 | 3.471.583 | -605.988 | 8.454 |
| 19.680.000 | 3.473.199 | -606.269 | 8.457 |
| 19.690.000 | 3.474.957 | -606.351 | 8.459 |
| 19.700.000 | 3.476.410 | -606.607 | 8.457 |
| 19.710.000 | 3.478.324 | -606.848 | 8.453 |
| 19.720.000 | 3.479.713 | -606.981 | 8.451 |
| 19.730.000 | 3.481.594 | -607.223 | 8.448 |
| 19.740.000 | 3.483.048 | -607.424 | 8.449 |

| | | | |
|---|---|---|---|
| 19.750.000 | 3.484.858 | -607.599 | 8.448 |
| 19.760.000 | 3.486.362 | -607.793 | 8.447 |
| 19.770.000 | 3.488.210 | -608.053 | 8.445 |
| 19.780.000 | 3.489.729 | -608.133 | 8.441 |
| 19.790.000 | 3.491.482 | -608.400 | 8.438 |
| 19.800.000 | 3.493.140 | -608.524 | 8.434 |
| 19.810.000 | 3.494.708 | -608.703 | 8.428 |
| 19.820.000 | 3.496.454 | -608.874 | 8.422 |
| 19.830.000 | 3.497.995 | -609.099 | 8.415 |
| 19.840.000 | 3.499.783 | -609.227 | 8.411 |
| 19.850.000 | 3.501.236 | -609.451 | 8.407 |
| 19.860.000 | 3.503.114 | -609.649 | 8.403 |
| 19.870.000 | 3.504.498 | -609.777 | 8.397 |
| 19.880.000 | 3.506.391 | -610.050 | 8.387 |
| 19.890.000 | 3.507.933 | -610.184 | 8.380 |
| 19.900.000 | 3.509.681 | -610.380 | 8.371 |
| 19.910.000 | 3.511.255 | -610.551 | 8.363 |
| 19.920.000 | 3.513.000 | -610.775 | 8.355 |
| 19.930.000 | 3.514.571 | -610.881 | 8.348 |
| 19.940.000 | 3.516.235 | -611.127 | 8.341 |
| 19.950.000 | 3.517.979 | -611.256 | 8.334 |
| 19.960.000 | 3.519.477 | -611.486 | 8.328 |
| 19.970.000 | 3.521.286 | -611.637 | 8.321 |
| 19.980.000 | 3.522.802 | -611.867 | 8.315 |
| 19.990.000 | 3.524.593 | -612.040 | 8.310 |
| 20.000.000 | 3.526.130 | -612.282 | 8.305 |
| 20.010.000 | 3.527.990 | -612.469 | 8.301 |
| 20.020.000 | 3.529.355 | -612.641 | 8.297 |
| 20.030.000 | 3.531.274 | -612.907 | 8.296 |
| 20.040.000 | 3.532.769 | -613.048 | 8.297 |

| | | | |
|---|---|---|---|
| 20.050.000 | 3.534.491 | -613.258 | 8.295 |
| 20.060.000 | 3.536.090 | -613.437 | 8.291 |
| 20.070.000 | 3.537.791 | -613.627 | 8.285 |
| 20.080.000 | 3.539.379 | -613.768 | 8.279 |
| 20.090.000 | 3.541.114 | -614.040 | 8.274 |
| 20.100.000 | 3.542.778 | -614.112 | 8.270 |
| 20.110.000 | 3.544.308 | -614.354 | 8.264 |
| 20.120.000 | 3.546.139 | -614.534 | 8.258 |
| 20.130.000 | 3.547.624 | -614.691 | 8.252 |
| 20.140.000 | 3.549.421 | -614.901 | 8.246 |
| 20.150.000 | 3.550.897 | -615.122 | 8.239 |
| 20.160.000 | 3.552.738 | -615.257 | 8.231 |
| 20.170.000 | 3.554.152 | -615.443 | 8.224 |
| 20.180.000 | 3.556.015 | -615.699 | 8.220 |
| 20.190.000 | 3.557.535 | -615.793 | 8.214 |
| 20.200.000 | 3.559.250 | -616.046 | 8.208 |
| 20.210.000 | 3.560.856 | -616.204 | 8.204 |
| 20.220.000 | 3.562.628 | -616.389 | 8.201 |
| 20.230.000 | 3.564.216 | -616.522 | 8.198 |
| 20.240.000 | 3.565.865 | -616.790 | 8.192 |
| 20.250.000 | 3.567.570 | -616.859 | 8.188 |
| 20.260.000 | 3.569.093 | -617.118 | 8.183 |
| 20.270.000 | 3.570.957 | -617.324 | 8.178 |
| 20.280.000 | 3.572.395 | -617.478 | 8.172 |
| 20.290.000 | 3.574.241 | -617.710 | 8.162 |
| 20.300.000 | 3.575.738 | -617.929 | 8.155 |
| 20.310.000 | 3.577.547 | -618.102 | 8.149 |
| 20.320.000 | 3.579.049 | -618.304 | 8.145 |
| 20.330.000 | 3.580.889 | -618.587 | 8.143 |
| 20.340.000 | 3.582.406 | -618.703 | 8.140 |

| | | | |
|---|---|---|---|
| 20.350.000 | 3.584.158 | -618.987 | 8.135 |
| 20.360.000 | 3.585.791 | -619.161 | 8.127 |
| 20.370.000 | 3.587.419 | -619.360 | 8.117 |
| 20.380.000 | 3.589.105 | -619.516 | 8.107 |
| 20.390.000 | 3.590.688 | -619.783 | 8.099 |
| 20.400.000 | 3.592.400 | -619.852 | 8.094 |
| 20.410.000 | 3.593.929 | -620.117 | 8.091 |
| 20.420.000 | 3.595.799 | -620.320 | 8.086 |
| 20.430.000 | 3.597.202 | -620.467 | 8.079 |
| 20.440.000 | 3.599.093 | -620.717 | 8.073 |
| 20.450.000 | 3.600.595 | -620.914 | 8.067 |
| 20.460.000 | 3.602.385 | -621.105 | 8.061 |
| 20.470.000 | 3.603.893 | -621.291 | 8.056 |
| 20.480.000 | 3.605.714 | -621.546 | 8.050 |
| 20.490.000 | 3.607.172 | -621.656 | 8.047 |
| 20.500.000 | 3.608.910 | -621.928 | 8.046 |
| 20.510.000 | 3.610.595 | -622.078 | 8.044 |
| 20.520.000 | 3.612.156 | -622.283 | 8.042 |
| 20.530.000 | 3.613.888 | -622.422 | 8.040 |
| 20.540.000 | 3.615.484 | -622.687 | 8.039 |
| 20.550.000 | 3.617.241 | -622.783 | 8.037 |
| 20.560.000 | 3.618.730 | -623.019 | 8.034 |
| 20.570.000 | 3.620.601 | -623.244 | 8.032 |
| 20.580.000 | 3.622.008 | -623.398 | 8.031 |
| 20.590.000 | 3.623.900 | -623.664 | 8.029 |
| 20.600.000 | 3.625.390 | -623.854 | 8.031 |
| 20.610.000 | 3.627.118 | -624.062 | 8.035 |
| 20.620.000 | 3.628.677 | -624.265 | 8.037 |
| 20.630.000 | 3.630.449 | -624.517 | 8.035 |
| 20.640.000 | 3.631.963 | -624.658 | 8.031 |

| | | | |
|---|---|---|---|
| 20.650.000 | 3.633.664 | -624.954 | 8.027 |
| 20.660.000 | 3.635.361 | -625.106 | 8.021 |
| 20.670.000 | 3.636.955 | -625.340 | 8.016 |
| 20.680.000 | 3.638.731 | -625.530 | 8.013 |
| 20.690.000 | 3.640.261 | -625.750 | 8.008 |
| 20.700.000 | 3.642.045 | -625.918 | 8.005 |
| 20.710.000 | 3.643.520 | -626.173 | 8.002 |
| 20.720.000 | 3.645.360 | -626.332 | 7.998 |
| 20.730.000 | 3.646.758 | -626.506 | 7.993 |
| 20.740.000 | 3.648.599 | -626.757 | 7.987 |
| 20.750.000 | 3.650.088 | -626.894 | 7.982 |
| 20.760.000 | 3.651.864 | -627.121 | 7.979 |
| 20.770.000 | 3.653.425 | -627.325 | 7.974 |
| 20.780.000 | 3.655.198 | -627.529 | 7.971 |
| 20.790.000 | 3.656.735 | -627.689 | 7.967 |
| 20.800.000 | 3.658.419 | -627.954 | 7.964 |
| 20.810.000 | 3.660.120 | -628.067 | 7.964 |
| 20.820.000 | 3.661.653 | -628.326 | 7.965 |
| 20.830.000 | 3.663.462 | -628.519 | 7.964 |
| 20.840.000 | 3.664.959 | -628.713 | 7.960 |
| 20.850.000 | 3.666.741 | -628.919 | 7.952 |
| 20.860.000 | 3.668.241 | -629.162 | 7.945 |
| 20.870.000 | 3.670.078 | -629.356 | 7.938 |
| 20.880.000 | 3.671.539 | -629.533 | 7.933 |
| 20.890.000 | 3.673.413 | -629.798 | 7.926 |
| 20.900.000 | 3.674.900 | -629.909 | 7.920 |
| 20.910.000 | 3.676.689 | -630.174 | 7.914 |
| 20.920.000 | 3.678.248 | -630.373 | 7.908 |
| 20.930.000 | 3.679.930 | -630.587 | 7.905 |
| 20.940.000 | 3.681.555 | -630.755 | 7.900 |

| | | | |
|---|---|---|---|
| 20.950.000 | 3.683.223 | -631.068 | 7.897 |
| 20.960.000 | 3.684.902 | -631.170 | 7.895 |
| 20.970.000 | 3.686.450 | -631.445 | 7.893 |
| 20.980.000 | 3.688.269 | -631.662 | 7.891 |
| 20.990.000 | 3.689.751 | -631.836 | 7.888 |
| 21.000.000 | 3.691.593 | -632.102 | 7.887 |
| 21.010.000 | 3.693.065 | -632.336 | 7.884 |
| 21.020.000 | 3.694.913 | -632.527 | 7.883 |
| 21.030.000 | 3.696.380 | -632.751 | 7.879 |
| 21.040.000 | 3.698.197 | -633.023 | 7.874 |
| 21.050.000 | 3.699.686 | -633.123 | 7.868 |
| 21.060.000 | 3.701.431 | -633.409 | 7.861 |
| 21.070.000 | 3.703.040 | -633.579 | 7.854 |
| 21.080.000 | 3.704.698 | -633.760 | 7.847 |
| 21.090.000 | 3.706.352 | -633.906 | 7.841 |
| 21.100.000 | 3.707.981 | -634.188 | 7.837 |
| 21.110.000 | 3.709.682 | -634.285 | 7.834 |
| 21.120.000 | 3.711.263 | -634.557 | 7.827 |
| 21.130.000 | 3.713.116 | -634.761 | 7.819 |
| 21.140.000 | 3.714.479 | -634.937 | 7.812 |
| 21.150.000 | 3.716.385 | -635.201 | 7.807 |
| 21.160.000 | 3.717.852 | -635.418 | 7.802 |
| 21.170.000 | 3.719.606 | -635.618 | 7.797 |
| 21.180.000 | 3.721.142 | -635.841 | 7.793 |
| 21.190.000 | 3.722.922 | -636.121 | 7.789 |
| 21.200.000 | 3.724.409 | -636.233 | 7.784 |
| 21.210.000 | 3.726.147 | -636.525 | 7.780 |
| 21.220.000 | 3.727.774 | -636.706 | 7.776 |
| 21.230.000 | 3.729.389 | -636.923 | 7.775 |
| 21.240.000 | 3.731.099 | -637.112 | 7.774 |

| | | | |
|---|---|---|---|
| 21.250.000 | 3.732.688 | -637.378 | 7.773 |
| 21.260.000 | 3.734.429 | -637.481 | 7.771 |
| 21.270.000 | 3.735.936 | -637.769 | 7.767 |
| 21.280.000 | 3.737.795 | -637.948 | 7.762 |
| 21.290.000 | 3.739.179 | -638.089 | 7.758 |
| 21.300.000 | 3.741.018 | -638.338 | 7.754 |
| 21.310.000 | 3.742.542 | -638.490 | 7.750 |
| 21.320.000 | 3.744.287 | -638.684 | 7.746 |
| 21.330.000 | 3.745.842 | -638.884 | 7.743 |
| 21.340.000 | 3.747.650 | -639.128 | 7.741 |
| 21.350.000 | 3.749.174 | -639.249 | 7.738 |
| 21.360.000 | 3.750.887 | -639.536 | 7.733 |
| 21.370.000 | 3.752.536 | -639.682 | 7.729 |
| 21.380.000 | 3.754.106 | -639.927 | 7.726 |
| 21.390.000 | 3.755.886 | -640.115 | 7.725 |
| 21.400.000 | 3.757.407 | -640.363 | 7.721 |
| 21.410.000 | 3.759.153 | -640.528 | 7.715 |
| 21.420.000 | 3.760.678 | -640.782 | 7.709 |
| 21.430.000 | 3.762.513 | -641.012 | 7.704 |
| 21.440.000 | 3.763.911 | -641.193 | 7.700 |
| 21.450.000 | 3.765.797 | -641.468 | 7.696 |
| 21.460.000 | 3.767.293 | -641.627 | 7.691 |
| 21.470.000 | 3.769.031 | -641.850 | 7.688 |
| 21.480.000 | 3.770.595 | -642.058 | 7.688 |
| 21.490.000 | 3.772.349 | -642.269 | 7.686 |
| 21.500.000 | 3.773.896 | -642.412 | 7.684 |
| 21.510.000 | 3.775.584 | -642.657 | 7.682 |
| 21.520.000 | 3.777.240 | -642.751 | 7.680 |
| 21.530.000 | 3.778.818 | -642.980 | 7.678 |
| 21.540.000 | 3.780.581 | -643.141 | 7.675 |

| | | | |
|---|---|---|---|
| 21.550.000 | 3.782.095 | -643.310 | 7.672 |
| 21.560.000 | 3.783.897 | -643.497 | 7.669 |
| 21.570.000 | 3.785.413 | -643.721 | 7.667 |
| 21.580.000 | 3.787.260 | -643.895 | 7.665 |
| 21.590.000 | 3.788.645 | -644.078 | 7.664 |
| 21.600.000 | 3.790.553 | -644.349 | 7.663 |
| 21.610.000 | 3.792.015 | -644.475 | 7.663 |
| 21.620.000 | 3.793.775 | -644.736 | 7.661 |
| 21.630.000 | 3.795.329 | -644.926 | 7.657 |
| 21.640.000 | 3.797.042 | -645.143 | 7.656 |
| 21.650.000 | 3.798.638 | -645.314 | 7.653 |
| 21.660.000 | 3.800.337 | -645.614 | 7.652 |
| 21.670.000 | 3.802.011 | -645.711 | 7.651 |
| 21.680.000 | 3.803.578 | -645.994 | 7.650 |
| 21.690.000 | 3.805.398 | -646.180 | 7.649 |
| 21.700.000 | 3.806.834 | -646.374 | 7.647 |
| 21.710.000 | 3.808.643 | -646.591 | 7.646 |
| 21.720.000 | 3.810.104 | -646.808 | 7.645 |
| 21.730.000 | 3.811.953 | -646.981 | 7.643 |
| 21.740.000 | 3.813.372 | -647.149 | 7.641 |
| 21.750.000 | 3.815.210 | -647.375 | 7.639 |
| 21.760.000 | 3.816.694 | -647.471 | 7.637 |
| 21.770.000 | 3.818.435 | -647.697 | 7.636 |
| 21.780.000 | 3.820.069 | -647.857 | 7.633 |
| 21.790.000 | 3.821.711 | -648.034 | 7.630 |
| 21.800.000 | 3.823.383 | -648.188 | 7.626 |
| 21.810.000 | 3.825.002 | -648.439 | 7.624 |
| 21.820.000 | 3.826.717 | -648.531 | 7.622 |
| 21.830.000 | 3.828.225 | -648.783 | 7.622 |
| 21.840.000 | 3.830.060 | -648.968 | 7.621 |

| | | | |
|---|---|---|---|
| 21.850.000 | 3.831.480 | -649.123 | 7.620 |
| 21.860.000 | 3.833.318 | -649.373 | 7.618 |
| 21.870.000 | 3.834.800 | -649.551 | 7.616 |
| 21.880.000 | 3.836.599 | -649.727 | 7.616 |
| 21.890.000 | 3.838.085 | -649.920 | 7.614 |
| 21.900.000 | 3.839.931 | -650.192 | 7.613 |
| 21.910.000 | 3.841.451 | -650.308 | 7.612 |
| 21.920.000 | 3.843.175 | -650.599 | 7.611 |
| 21.930.000 | 3.844.796 | -650.764 | 7.611 |
| 21.940.000 | 3.846.391 | -650.976 | 7.611 |
| 21.950.000 | 3.848.074 | -651.152 | 7.610 |
| 21.960.000 | 3.849.711 | -651.434 | 7.606 |
| 21.970.000 | 3.851.392 | -651.522 | 7.603 |
| 21.980.000 | 3.852.886 | -651.789 | 7.601 |
| 21.990.000 | 3.854.767 | -651.985 | 7.600 |
| 22.000.000 | 3.856.154 | -652.117 | 7.598 |
| 22.010.000 | 3.858.029 | -652.342 | 7.596 |
| 22.020.000 | 3.859.544 | -652.509 | 7.595 |
| 22.030.000 | 3.861.301 | -652.665 | 7.592 |
| 22.040.000 | 3.862.839 | -652.840 | 7.590 |
| 22.050.000 | 3.864.608 | -653.077 | 7.589 |
| 22.060.000 | 3.866.102 | -653.183 | 7.588 |
| 22.070.000 | 3.867.838 | -653.446 | 7.587 |
| 22.080.000 | 3.869.485 | -653.589 | 7.584 |
| 22.090.000 | 3.871.057 | -653.805 | 7.582 |
| 22.100.000 | 3.872.813 | -653.978 | 7.580 |
| 22.110.000 | 3.874.343 | -654.208 | 7.578 |
| 22.120.000 | 3.876.148 | -654.362 | 7.574 |
| 22.130.000 | 3.877.630 | -654.610 | 7.570 |
| 22.140.000 | 3.879.505 | -654.813 | 7.568 |

| | | | |
|---|---|---|---|
| 22.150.000 | 3.880.893 | -654.981 | 7.564 |
| 22.160.000 | 3.882.754 | -655.236 | 7.562 |
| 22.170.000 | 3.884.236 | -655.387 | 7.561 |
| 22.180.000 | 3.885.979 | -655.626 | 7.560 |
| 22.190.000 | 3.887.530 | -655.797 | 7.558 |
| 22.200.000 | 3.889.290 | -656.035 | 7.557 |
| 22.210.000 | 3.890.825 | -656.186 | 7.556 |
| 22.220.000 | 3.892.536 | -656.435 | 7.554 |
| 22.230.000 | 3.894.226 | -656.558 | 7.552 |
| 22.240.000 | 3.895.724 | -656.809 | 7.550 |
| 22.250.000 | 3.897.563 | -656.965 | 7.549 |
| 22.260.000 | 3.899.103 | -657.169 | 7.548 |
| 22.270.000 | 3.900.844 | -657.310 | 7.547 |
| 22.280.000 | 3.902.321 | -657.522 | 7.545 |
| 22.290.000 | 3.904.191 | -657.683 | 7.544 |
| 22.300.000 | 3.905.598 | -657.807 | 7.543 |
| 22.310.000 | 3.907.461 | -658.023 | 7.543 |
| 22.320.000 | 3.908.909 | -658.090 | 7.542 |
| 22.330.000 | 3.910.694 | -658.291 | 7.540 |
| 22.340.000 | 3.912.253 | -658.440 | 7.538 |
| 22.350.000 | 3.913.967 | -658.611 | 7.538 |
| 22.360.000 | 3.915.573 | -658.729 | 7.536 |
| 22.370.000 | 3.917.213 | -658.983 | 7.535 |
| 22.380.000 | 3.918.902 | -659.017 | 7.533 |
| 22.390.000 | 3.920.426 | -659.266 | 7.532 |
| 22.400.000 | 3.922.208 | -659.415 | 7.531 |
| 22.410.000 | 3.923.680 | -659.579 | 7.530 |
| 22.420.000 | 3.925.472 | -659.762 | 7.531 |
| 22.430.000 | 3.926.978 | -659.971 | 7.531 |
| 22.440.000 | 3.928.828 | -660.111 | 7.529 |

| | | | |
|---|---|---|---|
| 22.450.000 | 3.930.198 | -660.266 | 7.527 |
| 22.460.000 | 3.932.039 | -660.479 | 7.526 |
| 22.470.000 | 3.933.561 | -660.584 | 7.526 |
| 22.480.000 | 3.935.267 | -660.804 | 7.526 |
| 22.490.000 | 3.936.890 | -660.950 | 7.527 |
| 22.500.000 | 3.938.550 | -661.109 | 7.527 |
| 22.510.000 | 3.940.164 | -661.241 | 7.525 |
| 22.520.000 | 3.941.845 | -661.490 | 7.525 |
| 22.530.000 | 3.943.517 | -661.542 | 7.527 |
| 22.540.000 | 3.945.011 | -661.761 | 7.528 |
| 22.550.000 | 3.946.873 | -661.915 | 7.530 |
| 22.560.000 | 3.948.284 | -662.032 | 7.533 |
| 22.570.000 | 3.950.126 | -662.199 | 7.536 |
| 22.580.000 | 3.951.604 | -662.351 | 7.539 |
| 22.590.000 | 3.953.408 | -662.447 | 7.540 |
| 22.600.000 | 3.954.868 | -662.590 | 7.541 |
| 22.610.000 | 3.956.656 | -662.784 | 7.542 |
| 22.620.000 | 3.958.170 | -662.847 | 7.543 |
| 22.630.000 | 3.959.898 | -663.089 | 7.545 |
| 22.640.000 | 3.961.541 | -663.223 | 7.548 |
| 22.650.000 | 3.963.151 | -663.382 | 7.550 |
| 22.660.000 | 3.964.825 | -663.534 | 7.548 |
| 22.670.000 | 3.966.428 | -663.777 | 7.547 |
| 22.680.000 | 3.968.168 | -663.847 | 7.546 |
| 22.690.000 | 3.969.635 | -664.094 | 7.547 |
| 22.700.000 | 3.971.545 | -664.282 | 7.547 |
| 22.710.000 | 3.972.940 | -664.416 | 7.546 |
| 22.720.000 | 3.974.781 | -664.641 | 7.545 |
| 22.730.000 | 3.976.258 | -664.811 | 7.543 |
| 22.740.000 | 3.978.015 | -664.973 | 7.542 |

| | | | |
|---|---|---|---|
| 22.750.000 | 3.979.501 | -665.155 | 7.540 |
| 22.760.000 | 3.981.317 | -665.410 | 7.537 |
| 22.770.000 | 3.982.839 | -665.508 | 7.537 |
| 22.780.000 | 3.984.529 | -665.762 | 7.538 |
| 22.790.000 | 3.986.224 | -665.935 | 7.536 |
| 22.800.000 | 3.987.797 | -666.144 | 7.533 |
| 22.810.000 | 3.989.522 | -666.308 | 7.530 |
| 22.820.000 | 3.991.116 | -666.570 | 7.528 |
| 22.830.000 | 3.992.839 | -666.691 | 7.527 |
| 22.840.000 | 3.994.315 | -666.944 | 7.527 |
| 22.850.000 | 3.996.184 | -667.154 | 7.528 |
| 22.860.000 | 3.997.565 | -667.277 | 7.528 |
| 22.870.000 | 3.999.428 | -667.514 | 7.526 |
| 22.880.000 | 4.000.933 | -667.651 | 7.525 |
| 22.890.000 | 4.002.680 | -667.813 | 7.525 |
| 22.900.000 | 4.004.250 | -667.964 | 7.526 |
| 22.910.000 | 4.006.008 | -668.184 | 7.529 |
| 22.920.000 | 4.007.568 | -668.278 | 7.533 |
| 22.930.000 | 4.009.262 | -668.521 | 7.538 |
| 22.940.000 | 4.010.916 | -668.632 | 7.539 |
| 22.950.000 | 4.012.478 | -668.868 | 7.536 |
| 22.960.000 | 4.014.244 | -669.030 | 7.534 |
| 22.970.000 | 4.015.794 | -669.248 | 7.533 |
| 22.980.000 | 4.017.560 | -669.419 | 7.532 |
| 22.990.000 | 4.018.993 | -669.639 | 7.531 |
| 23.000.000 | 4.020.879 | -669.853 | 7.531 |
| 23.010.000 | 4.022.286 | -670.001 | 7.528 |
| 23.020.000 | 4.024.115 | -670.255 | 7.521 |
| 23.030.000 | 4.025.689 | -670.400 | 7.512 |
| 23.040.000 | 4.027.422 | -670.658 | 7.503 |

| | | | |
|---|---|---|---|
| 23.050.000 | 4.028.975 | -670.828 | 7.498 |
| 23.060.000 | 4.030.706 | -671.042 | 7.498 |
| 23.070.000 | 4.032.262 | -671.190 | 7.504 |
| 23.080.000 | 4.033.962 | -671.468 | 7.516 |
| 23.090.000 | 4.035.655 | -671.577 | 7.526 |
| 23.100.000 | 4.037.166 | -671.827 | 7.533 |
| 23.110.000 | 4.038.991 | -672.009 | 7.535 |
| 23.120.000 | 4.040.464 | -672.206 | 7.531 |
| 23.130.000 | 4.042.249 | -672.400 | 7.521 |
| 23.140.000 | 4.043.706 | -672.609 | 7.509 |
| 23.150.000 | 4.045.561 | -672.773 | 7.500 |
| 23.160.000 | 4.046.994 | -672.948 | 7.497 |
| 23.170.000 | 4.048.853 | -673.178 | 7.499 |
| 23.180.000 | 4.050.342 | -673.282 | 7.507 |
| 23.190.000 | 4.052.055 | -673.509 | 7.516 |
| 23.200.000 | 4.053.679 | -673.683 | 7.523 |
| 23.210.000 | 4.055.356 | -673.848 | 7.525 |
| 23.220.000 | 4.056.967 | -673.981 | 7.520 |
| 23.230.000 | 4.058.565 | -674.244 | 7.511 |
| 23.240.000 | 4.060.305 | -674.318 | 7.504 |
| 23.250.000 | 4.061.779 | -674.549 | 7.501 |
| 23.260.000 | 4.063.625 | -674.739 | 7.502 |
| 23.270.000 | 4.065.080 | -674.902 | 7.503 |
| 23.280.000 | 4.066.885 | -675.127 | 7.505 |
| 23.290.000 | 4.068.380 | -675.335 | 7.505 |
| 23.300.000 | 4.070.188 | -675.498 | 7.501 |
| 23.310.000 | 4.071.604 | -675.675 | 7.493 |
| 23.320.000 | 4.073.458 | -675.951 | 7.483 |
| 23.330.000 | 4.074.953 | -676.048 | 7.480 |
| 23.340.000 | 4.076.678 | -676.319 | 7.485 |

| | | | |
|---|---|---|---|
| 23.350.000 | 4.078.286 | -676.469 | 7.493 |
| 23.360.000 | 4.079.909 | -676.661 | 7.506 |
| 23.370.000 | 4.081.544 | -676.794 | 7.518 |
| 23.380.000 | 4.083.189 | -677.077 | 7.525 |
| 23.390.000 | 4.084.900 | -677.135 | 7.523 |
| 23.400.000 | 4.086.376 | -677.360 | 7.515 |
| 23.410.000 | 4.088.268 | -677.571 | 7.505 |
| 23.420.000 | 4.089.658 | -677.696 | 7.503 |
| 23.430.000 | 4.091.481 | -677.914 | 7.512 |
| 23.440.000 | 4.092.991 | -678.112 | 7.533 |
| 23.450.000 | 4.094.773 | -678.252 | 7.555 |
| 23.460.000 | 4.096.234 | -678.425 | 7.573 |
| 23.470.000 | 4.098.034 | -678.676 | 7.587 |
| 23.480.000 | 4.099.578 | -678.750 | 7.592 |
| 23.490.000 | 4.101.268 | -679.001 | 7.593 |
| 23.500.000 | 4.102.928 | -679.155 | 7.590 |
| 23.510.000 | 4.104.540 | -679.326 | 7.587 |
| 23.520.000 | 4.106.224 | -679.481 | 7.584 |
| 23.530.000 | 4.107.802 | -679.725 | 7.583 |
| 23.540.000 | 4.109.593 | -679.809 | 7.581 |
| 23.550.000 | 4.111.016 | -680.030 | 7.584 |
| 23.560.000 | 4.112.907 | -680.229 | 7.588 |
| 23.570.000 | 4.114.308 | -680.343 | 7.595 |
| 23.580.000 | 4.116.097 | -680.576 | 7.603 |
| 23.590.000 | 4.117.651 | -680.749 | 7.609 |
| 23.600.000 | 4.119.406 | -680.909 | 7.615 |
| 23.610.000 | 4.120.899 | -681.065 | 7.611 |
| 23.620.000 | 4.122.699 | -681.309 | 7.592 |
| 23.630.000 | 4.124.244 | -681.384 | 7.569 |
| 23.640.000 | 4.125.924 | -681.625 | 7.558 |

| | | | |
|---|---|---|---|
| 23.650.000 | 4.127.597 | -681.763 | 7.558 |
| 23.660.000 | 4.129.152 | -681.930 | 7.559 |
| 23.670.000 | 4.130.941 | -682.050 | 7.561 |
| 23.680.000 | 4.132.461 | -682.240 | 7.563 |
| 23.690.000 | 4.134.219 | -682.311 | 7.564 |
| 23.700.000 | 4.135.699 | -682.474 | 7.563 |
| 23.710.000 | 4.137.555 | -682.642 | 7.563 |
| 23.720.000 | 4.138.950 | -682.707 | 7.563 |
| 23.730.000 | 4.140.794 | -682.912 | 7.563 |
| 23.740.000 | 4.142.297 | -683.045 | 7.564 |
| 23.750.000 | 4.144.019 | -683.218 | 7.562 |
| 23.760.000 | 4.145.590 | -683.342 | 7.551 |
| 23.770.000 | 4.147.306 | -683.565 | 7.531 |
| 23.780.000 | 4.148.889 | -683.643 | 7.514 |
| 23.790.000 | 4.150.527 | -683.887 | 7.501 |
| 23.800.000 | 4.152.239 | -683.986 | 7.490 |
| 23.810.000 | 4.153.724 | -684.180 | 7.481 |
| 23.820.000 | 4.155.512 | -684.338 | 7.476 |
| 23.830.000 | 4.157.052 | -684.529 | 7.473 |
| 23.840.000 | 4.158.784 | -684.688 | 7.463 |
| 23.850.000 | 4.160.267 | -684.878 | 7.450 |
| 23.860.000 | 4.162.153 | -685.070 | 7.439 |
| 23.870.000 | 4.163.538 | -685.211 | 7.430 |
| 23.880.000 | 4.165.353 | -685.432 | 7.421 |
| 23.890.000 | 4.166.882 | -685.560 | 7.411 |
| 23.900.000 | 4.168.595 | -685.767 | 7.402 |
| 23.910.000 | 4.170.169 | -685.920 | 7.395 |
| 23.920.000 | 4.171.874 | -686.139 | 7.388 |
| 23.930.000 | 4.173.457 | -686.235 | 7.382 |
| 23.940.000 | 4.175.129 | -686.509 | 7.381 |

| | | | |
|---|---|---|---|
| 23.950.000 | 4.176.839 | -686.601 | 7.380 |
| 23.960.000 | 4.178.308 | -686.798 | 7.379 |
| 23.970.000 | 4.180.159 | -687.011 | 7.377 |
| 23.980.000 | 4.181.663 | -687.175 | 7.375 |
| 23.990.000 | 4.183.463 | -687.383 | 7.373 |
| 24.000.000 | 4.184.903 | -687.576 | 7.372 |
| 24.010.000 | 4.186.754 | -687.766 | 7.367 |
| 24.020.000 | 4.188.213 | -687.930 | 7.363 |
| 24.030.000 | 4.189.989 | -688.176 | 7.361 |
| 24.040.000 | 4.191.561 | -688.305 | 7.359 |
| 24.050.000 | 4.193.272 | -688.539 | 7.357 |
| 24.060.000 | 4.194.882 | -688.714 | 7.353 |
| 24.070.000 | 4.196.572 | -688.912 | 7.351 |
| 24.080.000 | 4.198.210 | -689.047 | 7.349 |
| 24.090.000 | 4.199.798 | -689.322 | 7.346 |
| 24.100.000 | 4.201.576 | -689.417 | 7.343 |
| 24.110.000 | 4.202.998 | -689.652 | 7.341 |
| 24.120.000 | 4.204.872 | -689.868 | 7.340 |
| 24.130.000 | 4.206.282 | -689.991 | 7.342 |
| 24.140.000 | 4.208.075 | -690.211 | 7.352 |
| 24.150.000 | 4.209.627 | -690.416 | 7.364 |
| 24.160.000 | 4.211.416 | -690.586 | 7.375 |
| 24.170.000 | 4.212.891 | -690.735 | 7.384 |
| 24.180.000 | 4.214.684 | -691.007 | 7.392 |
| 24.190.000 | 4.216.246 | -691.092 | 7.397 |
| 24.200.000 | 4.217.901 | -691.321 | 7.405 |
| 24.210.000 | 4.219.592 | -691.489 | 7.410 |
| 24.220.000 | 4.221.156 | -691.659 | 7.414 |
| 24.230.000 | 4.222.852 | -691.784 | 7.415 |
| 24.240.000 | 4.224.420 | -692.052 | 7.416 |

| | | | |
|---|---|---|---|
| 24.250.000 | 4.226.170 | -692.160 | 7.424 |
| 24.260.000 | 4.227.620 | -692.366 | 7.433 |
| 24.270.000 | 4.229.481 | -692.618 | 7.434 |
| 24.280.000 | 4.230.893 | -692.711 | 7.433 |
| 24.290.000 | 4.232.705 | -692.943 | 7.431 |
| 24.300.000 | 4.234.209 | -693.122 | 7.432 |
| 24.310.000 | 4.235.977 | -693.266 | 7.438 |
| 24.320.000 | 4.237.478 | -693.421 | 7.438 |
| 24.330.000 | 4.239.238 | -693.664 | 7.432 |
| 24.340.000 | 4.240.796 | -693.727 | 7.424 |
| 24.350.000 | 4.242.424 | -693.963 | 7.418 |
| 24.360.000 | 4.244.130 | -694.109 | 7.417 |
| 24.370.000 | 4.245.693 | -694.278 | 7.420 |
| 24.380.000 | 4.247.441 | -694.448 | 7.418 |
| 24.390.000 | 4.248.958 | -694.660 | 7.413 |
| 24.400.000 | 4.250.789 | -694.794 | 7.408 |
| 24.410.000 | 4.252.191 | -694.982 | 7.406 |
| 24.420.000 | 4.254.110 | -695.211 | 7.411 |
| 24.430.000 | 4.255.502 | -695.314 | 7.419 |
| 24.440.000 | 4.257.288 | -695.544 | 7.424 |
| 24.450.000 | 4.258.833 | -695.695 | 7.428 |
| 24.460.000 | 4.260.529 | -695.888 | 7.431 |
| 24.470.000 | 4.262.060 | -696.010 | 7.436 |
| 24.480.000 | 4.263.775 | -696.252 | 7.443 |
| 24.490.000 | 4.265.397 | -696.332 | 7.446 |
| 24.500.000 | 4.267.051 | -696.579 | 7.440 |
| 24.510.000 | 4.268.791 | -696.701 | 7.431 |
| 24.520.000 | 4.270.295 | -696.886 | 7.424 |
| 24.530.000 | 4.272.087 | -697.068 | 7.422 |
| 24.540.000 | 4.273.593 | -697.303 | 7.414 |

| | | | |
|---|---|---|---|
| 24.550.000 | 4.275.380 | -697.420 | 7.396 |
| 24.560.000 | 4.276.816 | -697.608 | 7.375 |
| 24.570.000 | 4.278.645 | -697.829 | 7.362 |
| 24.580.000 | 4.280.080 | -697.934 | 7.363 |
| 24.590.000 | 4.281.835 | -698.169 | 7.368 |
| 24.600.000 | 4.283.404 | -698.316 | 7.373 |
| 24.610.000 | 4.285.089 | -698.504 | 7.374 |
| 24.620.000 | 4.286.680 | -698.669 | 7.377 |
| 24.630.000 | 4.288.379 | -698.884 | 7.384 |
| 24.640.000 | 4.290.018 | -698.968 | 7.391 |
| 24.650.000 | 4.291.569 | -699.222 | 7.389 |
| 24.660.000 | 4.293.361 | -699.356 | 7.384 |
| 24.670.000 | 4.294.821 | -699.527 | 7.378 |
| 24.680.000 | 4.296.622 | -699.722 | 7.374 |
| 24.690.000 | 4.298.099 | -699.914 | 7.367 |
| 24.700.000 | 4.299.869 | -700.065 | 7.354 |
| 24.710.000 | 4.301.318 | -700.249 | 7.344 |
| 24.720.000 | 4.303.224 | -700.471 | 7.335 |
| 24.730.000 | 4.304.649 | -700.573 | 7.329 |
| 24.740.000 | 4.306.445 | -700.828 | 7.322 |
| 24.750.000 | 4.308.043 | -700.964 | 7.319 |
| 24.760.000 | 4.309.664 | -701.171 | 7.324 |
| 24.770.000 | 4.311.328 | -701.314 | 7.325 |
| 24.780.000 | 4.312.941 | -701.565 | 7.321 |
| 24.790.000 | 4.314.582 | -701.652 | 7.316 |
| 24.800.000 | 4.316.159 | -701.907 | 7.312 |
| 24.810.000 | 4.317.949 | -702.075 | 7.311 |
| 24.820.000 | 4.319.384 | -702.254 | 7.310 |
| 24.830.000 | 4.321.242 | -702.501 | 7.313 |
| 24.840.000 | 4.322.706 | -702.674 | 7.323 |

| | | | |
|---|---|---|---|
| 24.850.000 | 4.324.487 | -702.854 | 7.332 |
| 24.860.000 | 4.325.984 | -703.087 | 7.339 |
| 24.870.000 | 4.327.786 | -703.280 | 7.347 |
| 24.880.000 | 4.329.267 | -703.416 | 7.355 |
| 24.890.000 | 4.331.008 | -703.679 | 7.361 |
| 24.900.000 | 4.332.605 | -703.789 | 7.363 |
| 24.910.000 | 4.334.229 | -704.021 | 7.362 |
| 24.920.000 | 4.335.891 | -704.157 | 7.363 |
| 24.930.000 | 4.337.494 | -704.370 | 7.365 |
| 24.940.000 | 4.339.184 | -704.484 | 7.369 |
| 24.950.000 | 4.340.755 | -704.727 | 7.371 |
| 24.960.000 | 4.342.596 | -704.876 | 7.374 |
| 24.970.000 | 4.343.956 | -705.046 | 7.379 |
| 24.980.000 | 4.345.857 | -705.287 | 7.384 |
| 24.990.000 | 4.347.321 | -705.438 | 7.387 |
| 25.000.000 | 4.349.068 | -705.646 | 7.383 |
| 25.010.000 | 4.350.584 | -705.818 | 7.369 |
| 25.020.000 | 4.352.338 | -706.017 | 7.354 |
| 25.030.000 | 4.353.811 | -706.125 | 7.343 |
| 25.040.000 | 4.355.571 | -706.385 | 7.338 |
| 25.050.000 | 4.357.220 | -706.496 | 7.342 |
| 25.060.000 | 4.358.783 | -706.692 | 7.350 |
| 25.070.000 | 4.360.528 | -706.850 | 7.357 |
| 25.080.000 | 4.362.101 | -707.049 | 7.359 |
| 25.090.000 | 4.363.837 | -707.175 | 7.353 |
| 25.100.000 | 4.365.297 | -707.401 | 7.339 |
| 25.110.000 | 4.367.134 | -707.562 | 7.326 |
| 25.120.000 | 4.368.533 | -707.727 | 7.318 |
| 25.130.000 | 4.370.384 | -707.993 | 7.316 |
| 25.140.000 | 4.371.855 | -708.087 | 7.319 |

| | | | |
|---|---|---|---|
| 25.150.000 | 4.373.569 | -708.305 | 7.320 |
| 25.160.000 | 4.375.096 | -708.482 | 7.321 |
| 25.170.000 | 4.376.883 | -708.680 | 7.318 |
| 25.180.000 | 4.378.433 | -708.817 | 7.314 |
| 25.190.000 | 4.380.141 | -709.081 | 7.308 |
| 25.200.000 | 4.381.791 | -709.177 | 7.302 |
| 25.210.000 | 4.383.361 | -709.427 | 7.296 |
| 25.220.000 | 4.385.120 | -709.582 | 7.289 |
| 25.230.000 | 4.386.561 | -709.767 | 7.286 |
| 25.240.000 | 4.388.333 | -709.953 | 7.284 |
| 25.250.000 | 4.389.809 | -710.170 | 7.282 |
| 25.260.000 | 4.391.599 | -710.328 | 7.280 |
| 25.270.000 | 4.393.040 | -710.492 | 7.278 |
| 25.280.000 | 4.394.914 | -710.731 | 7.276 |
| 25.290.000 | 4.396.383 | -710.825 | 7.273 |
| 25.300.000 | 4.398.153 | -711.035 | 7.266 |
| 25.310.000 | 4.399.746 | -711.184 | 7.254 |
| 25.320.000 | 4.401.382 | -711.351 | 7.238 |
| 25.330.000 | 4.402.953 | -711.475 | 7.225 |
| 25.340.000 | 4.404.630 | -711.739 | 7.220 |
| 25.350.000 | 4.406.306 | -711.795 | 7.222 |
| 25.360.000 | 4.407.794 | -712.051 | 7.232 |
| 25.370.000 | 4.409.609 | -712.227 | 7.242 |
| 25.380.000 | 4.411.030 | -712.385 | 7.251 |
| 25.390.000 | 4.412.856 | -712.620 | 7.254 |
| 25.400.000 | 4.414.356 | -712.833 | 7.253 |
| 25.410.000 | 4.416.147 | -712.987 | 7.248 |
| 25.420.000 | 4.417.581 | -713.176 | 7.240 |
| 25.430.000 | 4.419.416 | -713.434 | 7.235 |
| 25.440.000 | 4.420.906 | -713.529 | 7.235 |

| | | | |
|---|---|---|---|
| 25.450.000 | 4.422.576 | -713.786 | 7.237 |
| 25.460.000 | 4.424.186 | -713.956 | 7.238 |
| 25.470.000 | 4.425.833 | -714.147 | 7.237 |
| 25.480.000 | 4.427.478 | -714.308 | 7.233 |
| 25.490.000 | 4.429.096 | -714.587 | 7.228 |
| 25.500.000 | 4.430.824 | -714.675 | 7.223 |
| 25.510.000 | 4.432.320 | -714.938 | 7.219 |
| 25.520.000 | 4.434.187 | -715.153 | 7.218 |
| 25.530.000 | 4.435.582 | -715.310 | 7.216 |
| 25.540.000 | 4.437.385 | -715.531 | 7.214 |
| 25.550.000 | 4.438.883 | -715.735 | 7.210 |
| 25.560.000 | 4.440.619 | -715.881 | 7.201 |
| 25.570.000 | 4.442.098 | -716.045 | 7.188 |
| 25.580.000 | 4.443.882 | -716.294 | 7.175 |
| 25.590.000 | 4.445.356 | -716.360 | 7.167 |
| 25.600.000 | 4.447.053 | -716.617 | 7.166 |
| 25.610.000 | 4.448.703 | -716.760 | 7.171 |
| 25.620.000 | 4.450.277 | -716.939 | 7.178 |
| 25.630.000 | 4.452.010 | -717.099 | 7.186 |
| 25.640.000 | 4.453.615 | -717.366 | 7.188 |
| 25.650.000 | 4.455.313 | -717.453 | 7.189 |
| 25.660.000 | 4.456.786 | -717.693 | 7.185 |
| 25.670.000 | 4.458.619 | -717.927 | 7.178 |
| 25.680.000 | 4.460.015 | -718.048 | 7.170 |
| 25.690.000 | 4.461.847 | -718.290 | 7.167 |
| 25.700.000 | 4.463.341 | -718.486 | 7.169 |
| 25.710.000 | 4.465.068 | -718.667 | 7.170 |
| 25.720.000 | 4.466.588 | -718.867 | 7.174 |
| 25.730.000 | 4.468.391 | -719.116 | 7.175 |
| 25.740.000 | 4.469.926 | -719.222 | 7.172 |

| | | | |
|---|---|---|---|
| 25.750.000 | 4.471.586 | -719.486 | 7.166 |
| 25.760.000 | 4.473.281 | -719.634 | 7.158 |
| 25.770.000 | 4.474.806 | -719.809 | 7.152 |
| 25.780.000 | 4.476.533 | -719.980 | 7.148 |
| 25.790.000 | 4.478.089 | -720.238 | 7.148 |
| 25.800.000 | 4.479.829 | -720.388 | 7.148 |
| 25.810.000 | 4.481.264 | -720.598 | 7.147 |
| 25.820.000 | 4.483.138 | -720.829 | 7.146 |
| 25.830.000 | 4.484.514 | -720.949 | 7.143 |
| 25.840.000 | 4.486.366 | -721.189 | 7.138 |
| 25.850.000 | 4.487.906 | -721.324 | 7.132 |
| 25.860.000 | 4.489.598 | -721.482 | 7.127 |
| 25.870.000 | 4.491.155 | -721.592 | 7.121 |
| 25.880.000 | 4.492.902 | -721.790 | 7.117 |
| 25.890.000 | 4.494.455 | -721.805 | 7.113 |
| 25.900.000 | 4.496.080 | -721.991 | 7.108 |
| 25.910.000 | 4.497.764 | -722.043 | 7.104 |
| 25.920.000 | 4.499.249 | -722.202 | 7.101 |
| 25.930.000 | 4.501.032 | -722.327 | 7.099 |
| 25.940.000 | 4.502.518 | -722.510 | 7.097 |
| 25.950.000 | 4.504.309 | -722.658 | 7.095 |
| 25.960.000 | 4.505.756 | -722.857 | 7.093 |
| 25.970.000 | 4.507.624 | -723.037 | 7.090 |
| 25.980.000 | 4.509.031 | -723.168 | 7.088 |
| 25.990.000 | 4.510.814 | -723.421 | 7.087 |
| 26.000.000 | 4.512.385 | -723.559 | 7.086 |
| 26.010.000 | 4.514.059 | -723.783 | 7.084 |
| 26.020.000 | 4.515.611 | -723.961 | 7.082 |
| 26.030.000 | 4.517.290 | -724.196 | 7.081 |
| 26.040.000 | 4.518.889 | -724.349 | 7.079 |

| | | | |
|---|---|---|---|
| 26.050.000 | 4.520.505 | -724.622 | 7.078 |
| 26.060.000 | 4.522.259 | -724.766 | 7.075 |
| 26.070.000 | 4.523.685 | -725.001 | 7.072 |
| 26.080.000 | 4.525.564 | -725.229 | 7.071 |
| 26.090.000 | 4.527.030 | -725.442 | 7.068 |
| 26.100.000 | 4.528.803 | -725.666 | 7.066 |
| 26.110.000 | 4.530.286 | -725.894 | 7.061 |
| 26.120.000 | 4.532.114 | -726.137 | 7.058 |
| 26.130.000 | 4.533.543 | -726.306 | 7.056 |
| 26.140.000 | 4.535.356 | -726.601 | 7.055 |
| 26.150.000 | 4.536.858 | -726.760 | 7.054 |
| 26.160.000 | 4.538.509 | -727.008 | 7.053 |
| 26.170.000 | 4.540.155 | -727.218 | 7.050 |
| 26.180.000 | 4.541.769 | -727.467 | 7.049 |
| 26.190.000 | 4.543.488 | -727.622 | 7.047 |
| 26.200.000 | 4.545.049 | -727.950 | 7.045 |
| 26.210.000 | 4.546.820 | -728.084 | 7.044 |
| 26.220.000 | 4.548.263 | -728.343 | 7.042 |
| 26.230.000 | 4.550.063 | -728.596 | 7.041 |
| 26.240.000 | 4.551.518 | -728.770 | 7.041 |
| 26.250.000 | 4.553.313 | -729.023 | 7.041 |
| 26.260.000 | 4.554.765 | -729.248 | 7.041 |
| 26.270.000 | 4.556.572 | -729.468 | 7.039 |
| 26.280.000 | 4.558.060 | -729.658 | 7.038 |
| 26.290.000 | 4.559.820 | -729.943 | 7.039 |
| 26.300.000 | 4.561.436 | -730.093 | 7.039 |
| 26.310.000 | 4.563.040 | -730.368 | 7.039 |
| 26.320.000 | 4.564.775 | -730.566 | 7.039 |
| 26.330.000 | 4.566.330 | -730.799 | 7.039 |
| 26.340.000 | 4.568.023 | -730.970 | 7.040 |

| | | | |
|---|---|---|---|
| 26.350.000 | 4.569.572 | -731.269 | 7.039 |
| 26.360.000 | 4.571.366 | -731.461 | 7.040 |
| 26.370.000 | 4.572.775 | -731.662 | 7.041 |
| 26.380.000 | 4.574.632 | -731.939 | 7.042 |
| 26.390.000 | 4.576.056 | -732.099 | 7.042 |
| 26.400.000 | 4.577.808 | -732.336 | 7.042 |
| 26.410.000 | 4.579.366 | -732.558 | 7.043 |
| 26.420.000 | 4.581.114 | -732.758 | 7.046 |
| 26.430.000 | 4.582.653 | -732.939 | 7.048 |
| 26.440.000 | 4.584.385 | -733.243 | 7.050 |
| 26.450.000 | 4.585.960 | -733.319 | 7.051 |
| 26.460.000 | 4.587.572 | -733.595 | 7.052 |
| 26.470.000 | 4.589.263 | -733.790 | 7.056 |
| 26.480.000 | 4.590.801 | -733.971 | 7.058 |
| 26.490.000 | 4.592.545 | -734.150 | 7.062 |
| 26.500.000 | 4.594.027 | -734.407 | 7.066 |
| 26.510.000 | 4.595.862 | -734.589 | 7.072 |
| 26.520.000 | 4.597.270 | -734.813 | 7.077 |
| 26.530.000 | 4.599.167 | -735.068 | 7.084 |
| 26.540.000 | 4.600.600 | -735.217 | 7.091 |
| 26.550.000 | 4.602.349 | -735.489 | 7.098 |
| 26.560.000 | 4.603.888 | -735.669 | 7.104 |
| 26.570.000 | 4.605.617 | -735.903 | 7.111 |
| 26.580.000 | 4.607.121 | -736.087 | 7.118 |
| 26.590.000 | 4.608.834 | -736.361 | 7.124 |
| 26.600.000 | 4.610.466 | -736.476 | 7.129 |
| 26.610.000 | 4.611.990 | -736.749 | 7.135 |
| 26.620.000 | 4.613.766 | -736.941 | 7.140 |
| 26.630.000 | 4.615.256 | -737.152 | 7.145 |
| 26.640.000 | 4.617.081 | -737.361 | 7.150 |

| | | | |
|---|---|---|---|
| 26.650.000 | 4.618.547 | -737.605 | 7.157 |
| 26.660.000 | 4.620.327 | -737.789 | 7.166 |
| 26.670.000 | 4.621.745 | -738.000 | 7.176 |
| 26.680.000 | 4.623.607 | -738.270 | 7.186 |
| 26.690.000 | 4.625.025 | -738.410 | 7.194 |
| 26.700.000 | 4.626.787 | -738.683 | 7.204 |
| 26.710.000 | 4.628.317 | -738.895 | 7.216 |
| 26.720.000 | 4.630.013 | -739.120 | 7.227 |
| 26.730.000 | 4.631.622 | -739.305 | 7.238 |
| 26.740.000 | 4.633.246 | -739.592 | 7.248 |
| 26.750.000 | 4.634.956 | -739.716 | 7.257 |
| 26.760.000 | 4.636.493 | -740.014 | 7.268 |
| 26.770.000 | 4.638.288 | -740.218 | 7.279 |
| 26.780.000 | 4.639.710 | -740.413 | 7.289 |
| 26.790.000 | 4.641.508 | -740.670 | 7.299 |
| 26.800.000 | 4.643.028 | -740.914 | 7.309 |
| 26.810.000 | 4.644.797 | -741.119 | 7.317 |
| 26.820.000 | 4.646.232 | -741.335 | 7.325 |
| 26.830.000 | 4.648.069 | -741.621 | 7.331 |
| 26.840.000 | 4.649.531 | -741.763 | 7.338 |
| 26.850.000 | 4.651.269 | -742.061 | 7.344 |
| 26.860.000 | 4.652.939 | -742.279 | 7.351 |
| 26.870.000 | 4.654.514 | -742.477 | 7.358 |
| 26.880.000 | 4.656.179 | -742.682 | 7.364 |
| 26.890.000 | 4.657.809 | -742.997 | 7.367 |
| 26.900.000 | 4.659.458 | -743.098 | 7.369 |
| 26.910.000 | 4.660.987 | -743.394 | 7.367 |
| 26.920.000 | 4.662.809 | -743.636 | 7.365 |
| 26.930.000 | 4.664.212 | -743.819 | 7.361 |
| 26.940.000 | 4.666.053 | -744.091 | 7.355 |

| | | | |
|---|---|---|---|
| 26.950.000 | 4.667.510 | -744.308 | 7.352 |
| 26.960.000 | 4.669.292 | -744.522 | 7.350 |
| 26.970.000 | 4.670.821 | -744.740 | 7.348 |
| 26.980.000 | 4.672.611 | -745.028 | 7.346 |
| 26.990.000 | 4.674.098 | -745.141 | 7.342 |
| 27.000.000 | 4.675.796 | -745.431 | 7.340 |
| 27.010.000 | 4.677.430 | -745.626 | 7.340 |
| 27.020.000 | 4.679.014 | -745.855 | 7.341 |
| 27.030.000 | 4.680.710 | -746.031 | 7.342 |
| 27.040.000 | 4.682.265 | -746.327 | 7.342 |
| 27.050.000 | 4.683.994 | -746.462 | 7.342 |
| 27.060.000 | 4.685.435 | -746.722 | 7.344 |
| 27.070.000 | 4.687.316 | -746.976 | 7.343 |
| 27.080.000 | 4.688.697 | -747.128 | 7.342 |
| 27.090.000 | 4.690.546 | -747.415 | 7.341 |
| 27.100.000 | 4.692.069 | -747.622 | 7.341 |
| 27.110.000 | 4.693.808 | -747.831 | 7.341 |
| 27.120.000 | 4.695.317 | -748.034 | 7.342 |
| 27.130.000 | 4.697.086 | -748.321 | 7.342 |
| 27.140.000 | 4.698.601 | -748.460 | 7.343 |
| 27.150.000 | 4.700.264 | -748.752 | 7.344 |
| 27.160.000 | 4.701.960 | -748.943 | 7.346 |
| 27.170.000 | 4.703.457 | -749.198 | 7.346 |
| 27.180.000 | 4.705.253 | -749.394 | 7.347 |
| 27.190.000 | 4.706.742 | -749.674 | 7.348 |
| 27.200.000 | 4.708.506 | -749.854 | 7.348 |
| 27.210.000 | 4.709.969 | -750.113 | 7.347 |
| 27.220.000 | 4.711.824 | -750.385 | 7.345 |
| 27.230.000 | 4.713.193 | -750.539 | 7.345 |
| 27.240.000 | 4.715.011 | -750.822 | 7.342 |

| | | | |
|---|---|---|---|
| 27.250.000 | 4.716.474 | -751.015 | 7.342 |
| 27.260.000 | 4.718.223 | -751.241 | 7.341 |
| 27.270.000 | 4.719.748 | -751.437 | 7.340 |
| 27.280.000 | 4.721.427 | -751.706 | 7.339 |
| 27.290.000 | 4.722.995 | -751.839 | 7.338 |
| 27.300.000 | 4.724.660 | -752.143 | 7.337 |
| 27.310.000 | 4.726.374 | -752.285 | 7.338 |
| 27.320.000 | 4.727.891 | -752.518 | 7.339 |
| 27.330.000 | 4.729.620 | -752.718 | 7.340 |
| 27.340.000 | 4.731.121 | -752.952 | 7.342 |
| 27.350.000 | 4.732.868 | -753.166 | 7.344 |
| 27.360.000 | 4.734.302 | -753.405 | 7.346 |
| 27.370.000 | 4.736.149 | -753.655 | 7.348 |
| 27.380.000 | 4.737.561 | -753.810 | 7.351 |
| 27.390.000 | 4.739.340 | -754.091 | 7.354 |
| 27.400.000 | 4.740.866 | -754.295 | 7.356 |
| 27.410.000 | 4.742.539 | -754.531 | 7.358 |
| 27.420.000 | 4.744.167 | -754.738 | 7.361 |
| 27.430.000 | 4.745.823 | -754.990 | 7.363 |
| 27.440.000 | 4.747.420 | -755.148 | 7.366 |
| 27.450.000 | 4.749.021 | -755.445 | 7.369 |
| 27.460.000 | 4.750.782 | -755.590 | 7.372 |
| 27.470.000 | 4.752.193 | -755.818 | 7.373 |
| 27.480.000 | 4.754.015 | -756.072 | 7.372 |
| 27.490.000 | 4.755.454 | -756.278 | 7.370 |
| 27.500.000 | 4.757.264 | -756.501 | 7.367 |
| 27.510.000 | 4.758.711 | -756.732 | 7.364 |
| 27.520.000 | 4.760.525 | -756.979 | 7.363 |
| 27.530.000 | 4.761.962 | -757.158 | 7.361 |
| 27.540.000 | 4.763.775 | -757.472 | 7.363 |

| | | | |
|---|---|---|---|
| 27.550.000 | 4.765.293 | -757.637 | 7.365 |
| 27.560.000 | 4.766.934 | -757.907 | 7.365 |
| 27.570.000 | 4.768.578 | -758.133 | 7.365 |
| 27.580.000 | 4.770.185 | -758.383 | 7.366 |
| 27.590.000 | 4.771.831 | -758.542 | 7.366 |
| 27.600.000 | 4.773.390 | -758.862 | 7.367 |
| 27.610.000 | 4.775.213 | -759.058 | 7.367 |
| 27.620.000 | 4.776.596 | -759.304 | 7.369 |
| 27.630.000 | 4.778.449 | -759.577 | 7.371 |
| 27.640.000 | 4.779.890 | -759.771 | 7.373 |
| 27.650.000 | 4.781.680 | -760.060 | 7.373 |
| 27.660.000 | 4.783.154 | -760.282 | 7.371 |
| 27.670.000 | 4.784.944 | -760.538 | 7.369 |
| 27.680.000 | 4.786.387 | -760.733 | 7.366 |
| 27.690.000 | 4.788.154 | -761.038 | 7.366 |
| 27.700.000 | 4.789.712 | -761.188 | 7.366 |
| 27.710.000 | 4.791.301 | -761.471 | 7.366 |
| 27.720.000 | 4.793.048 | -761.679 | 7.368 |
| 27.730.000 | 4.794.595 | -761.963 | 7.370 |
| 27.740.000 | 4.796.321 | -762.135 | 7.369 |
| 27.750.000 | 4.797.834 | -762.456 | 7.367 |
| 27.760.000 | 4.799.676 | -762.670 | 7.363 |
| 27.770.000 | 4.801.068 | -762.901 | 7.361 |
| 27.780.000 | 4.802.880 | -763.196 | 7.360 |
| 27.790.000 | 4.804.330 | -763.371 | 7.360 |
| 27.800.000 | 4.806.077 | -763.647 | 7.362 |
| 27.810.000 | 4.807.620 | -763.884 | 7.365 |
| 27.820.000 | 4.809.333 | -764.123 | 7.366 |
| 27.830.000 | 4.810.830 | -764.308 | 7.365 |
| 27.840.000 | 4.812.537 | -764.618 | 7.362 |

| | | | |
|---|---|---|---|
| 27.850.000 | 4.814.209 | -764.767 | 7.359 |
| 27.860.000 | 4.815.727 | -765.044 | 7.355 |
| 27.870.000 | 4.817.455 | -765.252 | 7.355 |
| 27.880.000 | 4.818.999 | -765.506 | 7.357 |
| 27.890.000 | 4.820.747 | -765.731 | 7.361 |
| 27.900.000 | 4.822.186 | -765.994 | 7.367 |
| 27.910.000 | 4.824.030 | -766.224 | 7.372 |
| 27.920.000 | 4.825.409 | -766.430 | 7.376 |
| 27.930.000 | 4.827.246 | -766.716 | 7.378 |
| 27.940.000 | 4.828.708 | -766.887 | 7.377 |
| 27.950.000 | 4.830.390 | -767.153 | 7.372 |
| 27.960.000 | 4.831.975 | -767.373 | 7.368 |
| 27.970.000 | 4.833.722 | -767.641 | 7.364 |
| 27.980.000 | 4.835.253 | -767.800 | 7.362 |
| 27.990.000 | 4.836.937 | -768.132 | 7.360 |
| 28.000.000 | 4.838.592 | -768.281 | 7.359 |
| 28.010.000 | 4.840.123 | -768.563 | 7.359 |
| 28.020.000 | 4.841.930 | -768.817 | 7.357 |
| 28.030.000 | 4.843.371 | -769.047 | 7.355 |
| 28.040.000 | 4.845.168 | -769.322 | 7.352 |
| 28.050.000 | 4.846.650 | -769.610 | 7.349 |
| 28.060.000 | 4.848.479 | -769.857 | 7.346 |
| 28.070.000 | 4.849.907 | -770.082 | 7.345 |
| 28.080.000 | 4.851.704 | -770.432 | 7.346 |
| 28.090.000 | 4.853.212 | -770.607 | 7.347 |
| 28.100.000 | 4.854.940 | -770.920 | 7.349 |
| 28.110.000 | 4.856.518 | -771.176 | 7.348 |
| 28.120.000 | 4.858.176 | -771.432 | 7.348 |
| 28.130.000 | 4.859.790 | -771.657 | 7.347 |
| 28.140.000 | 4.861.385 | -771.989 | 7.346 |

| | | | |
|---|---|---|---|
| 28.150.000 | 4.863.109 | -772.131 | 7.347 |
| 28.160.000 | 4.864.538 | -772.457 | 7.348 |
| 28.170.000 | 4.866.369 | -772.702 | 7.351 |
| 28.180.000 | 4.867.789 | -772.911 | 7.354 |
| 28.190.000 | 4.869.577 | -773.170 | 7.354 |
| 28.200.000 | 4.871.045 | -773.430 | 7.353 |
| 28.210.000 | 4.872.868 | -773.630 | 7.351 |
| 28.220.000 | 4.874.264 | -773.865 | 7.348 |
| 28.230.000 | 4.876.099 | -774.171 | 7.345 |
| 28.240.000 | 4.877.581 | -774.320 | 7.341 |
| 28.250.000 | 4.879.220 | -774.613 | 7.336 |
| 28.260.000 | 4.880.901 | -774.831 | 7.333 |
| 28.270.000 | 4.882.455 | -775.080 | 7.330 |
| 28.280.000 | 4.884.159 | -775.282 | 7.330 |
| 28.290.000 | 4.885.722 | -775.612 | 7.332 |
| 28.300.000 | 4.887.462 | -775.763 | 7.334 |
| 28.310.000 | 4.888.921 | -776.040 | 7.336 |
| 28.320.000 | 4.890.776 | -776.363 | 7.336 |
| 28.330.000 | 4.892.182 | -776.528 | 7.337 |
| 28.340.000 | 4.893.994 | -776.821 | 7.337 |
| 28.350.000 | 4.895.466 | -777.072 | 7.335 |
| 28.360.000 | 4.897.230 | -777.309 | 7.332 |
| 28.370.000 | 4.898.687 | -777.531 | 7.330 |
| 28.380.000 | 4.900.434 | -777.859 | 7.328 |
| 28.390.000 | 4.901.993 | -777.992 | 7.328 |
| 28.400.000 | 4.903.599 | -778.331 | 7.328 |
| 28.410.000 | 4.905.295 | -778.519 | 7.328 |
| 28.420.000 | 4.906.855 | -778.781 | 7.330 |
| 28.430.000 | 4.908.566 | -779.003 | 7.332 |
| 28.440.000 | 4.910.110 | -779.305 | 7.333 |

| | | | |
|---|---|---|---|
| 28.450.000 | 4.911.900 | -779.508 | 7.335 |
| 28.460.000 | 4.913.282 | -779.782 | 7.334 |
| 28.470.000 | 4.915.162 | -780.089 | 7.333 |
| 28.480.000 | 4.916.564 | -780.290 | 7.332 |
| 28.490.000 | 4.918.357 | -780.589 | 7.332 |
| 28.500.000 | 4.919.894 | -780.853 | 7.330 |
| 28.510.000 | 4.921.551 | -781.108 | 7.327 |
| 28.520.000 | 4.923.113 | -781.341 | 7.327 |
| 28.530.000 | 4.924.817 | -781.671 | 7.324 |
| 28.540.000 | 4.926.370 | -781.821 | 7.323 |
| 28.550.000 | 4.927.997 | -782.146 | 7.320 |
| 28.560.000 | 4.929.739 | -782.391 | 7.317 |
| 28.570.000 | 4.931.220 | -782.611 | 7.315 |
| 28.580.000 | 4.932.991 | -782.875 | 7.315 |
| 28.590.000 | 4.934.458 | -783.162 | 7.314 |
| 28.600.000 | 4.936.233 | -783.390 | 7.315 |
| 28.610.000 | 4.937.644 | -783.639 | 7.317 |
| 28.620.000 | 4.939.469 | -783.964 | 7.321 |
| 28.630.000 | 4.940.927 | -784.133 | 7.325 |
| 28.640.000 | 4.942.656 | -784.473 | 7.330 |
| 28.650.000 | 4.944.197 | -784.703 | 7.334 |
| 28.660.000 | 4.945.872 | -784.962 | 7.335 |
| 28.670.000 | 4.947.454 | -785.205 | 7.334 |
| 28.680.000 | 4.949.145 | -785.530 | 7.333 |
| 28.690.000 | 4.950.746 | -785.669 | 7.333 |
| 28.700.000 | 4.952.280 | -786.009 | 7.331 |
| 28.710.000 | 4.954.057 | -786.243 | 7.328 |
| 28.720.000 | 4.955.476 | -786.459 | 7.326 |
| 28.730.000 | 4.957.264 | -786.759 | 7.324 |
| 28.740.000 | 4.958.735 | -787.026 | 7.325 |

| | | | |
|---|---|---|---|
| 28.750.000 | 4.960.501 | -787.269 | 7.325 |
| 28.760.000 | 4.961.988 | -787.518 | 7.323 |
| 28.770.000 | 4.963.778 | -787.835 | 7.321 |
| 28.780.000 | 4.965.218 | -788.017 | 7.321 |
| 28.790.000 | 4.966.984 | -788.345 | 7.322 |
| 28.800.000 | 4.968.550 | -788.587 | 7.321 |
| 28.810.000 | 4.970.147 | -788.843 | 7.320 |
| 28.820.000 | 4.971.804 | -789.079 | 7.319 |
| 28.830.000 | 4.973.415 | -789.432 | 7.318 |
| 28.840.000 | 4.975.084 | -789.587 | 7.318 |
| 28.850.000 | 4.976.621 | -789.930 | 7.318 |
| 28.860.000 | 4.978.424 | -790.209 | 7.318 |
| 28.870.000 | 4.979.829 | -790.414 | 7.319 |
| 28.880.000 | 4.981.630 | -790.725 | 7.319 |
| 28.890.000 | 4.983.090 | -790.994 | 7.318 |
| 28.900.000 | 4.984.851 | -791.229 | 7.317 |
| 28.910.000 | 4.986.292 | -791.473 | 7.318 |
| 28.920.000 | 4.988.081 | -791.788 | 7.318 |
| 28.930.000 | 4.989.576 | -791.950 | 7.318 |
| 28.940.000 | 4.991.277 | -792.288 | 7.317 |
| 28.950.000 | 4.992.929 | -792.506 | 7.316 |
| 28.960.000 | 4.994.504 | -792.772 | 7.315 |
| 28.970.000 | 4.996.181 | -792.999 | 7.316 |
| 28.980.000 | 4.997.767 | -793.343 | 7.315 |
| 28.990.000 | 4.999.507 | -793.540 | 7.312 |
| 29.000.000 | 5.000.953 | -793.864 | 7.309 |
| 29.010.000 | 5.002.830 | -794.165 | 7.307 |
| 29.020.000 | 5.004.200 | -794.397 | 7.307 |
| 29.030.000 | 5.006.035 | -794.716 | 7.309 |
| 29.040.000 | 5.007.516 | -794.985 | 7.309 |

| | | | |
|---|---|---|---|
| 29.050.000 | 5.009.248 | -795.244 | 7.306 |
| 29.060.000 | 5.010.783 | -795.501 | 7.304 |
| 29.070.000 | 5.012.526 | -795.847 | 7.306 |
| 29.080.000 | 5.014.048 | -796.001 | 7.306 |
| 29.090.000 | 5.015.692 | -796.353 | 7.307 |
| 29.100.000 | 5.017.367 | -796.582 | 7.304 |
| 29.110.000 | 5.018.902 | -796.849 | 7.302 |
| 29.120.000 | 5.020.654 | -797.094 | 7.304 |
| 29.130.000 | 5.022.169 | -797.416 | 7.304 |
| 29.140.000 | 5.023.919 | -797.619 | 7.303 |
| 29.150.000 | 5.025.338 | -797.897 | 7.303 |
| 29.160.000 | 5.027.185 | -798.204 | 7.302 |
| 29.170.000 | 5.028.624 | -798.410 | 7.303 |
| 29.180.000 | 5.030.397 | -798.721 | 7.305 |
| 29.190.000 | 5.031.919 | -798.956 | 7.306 |
| 29.200.000 | 5.033.636 | -799.226 | 7.308 |
| 29.210.000 | 5.035.149 | -799.443 | 7.310 |
| 29.220.000 | 5.036.896 | -799.769 | 7.311 |
| 29.230.000 | 5.038.462 | -799.931 | 7.312 |
| 29.240.000 | 5.040.031 | -800.273 | 7.310 |
| 29.250.000 | 5.041.769 | -800.490 | 7.309 |
| 29.260.000 | 5.043.207 | -800.743 | 7.309 |
| 29.270.000 | 5.045.036 | -801.000 | 7.309 |
| 29.280.000 | 5.046.486 | -801.282 | 7.309 |
| 29.290.000 | 5.048.276 | -801.516 | 7.309 |
| 29.300.000 | 5.049.717 | -801.786 | 7.310 |
| 29.310.000 | 5.051.535 | -802.072 | 7.310 |
| 29.320.000 | 5.052.912 | -802.252 | 7.312 |
| 29.330.000 | 5.054.717 | -802.590 | 7.310 |
| 29.340.000 | 5.056.241 | -802.802 | 7.309 |

| | | | |
|---|---|---|---|
| 29.350.000 | 5.057.880 | -803.065 | 7.310 |
| 29.360.000 | 5.059.493 | -803.291 | 7.311 |
| 29.370.000 | 5.061.120 | -803.591 | 7.313 |
| 29.380.000 | 5.062.747 | -803.723 | 7.314 |
| 29.390.000 | 5.064.310 | -804.058 | 7.314 |
| 29.400.000 | 5.066.097 | -804.244 | 7.313 |
| 29.410.000 | 5.067.505 | -804.494 | 7.313 |
| 29.420.000 | 5.069.322 | -804.772 | 7.313 |
| 29.430.000 | 5.070.784 | -805.021 | 7.314 |
| 29.440.000 | 5.072.523 | -805.259 | 7.314 |
| 29.450.000 | 5.074.013 | -805.529 | 7.311 |
| 29.460.000 | 5.075.828 | -805.819 | 7.308 |
| 29.470.000 | 5.077.257 | -806.006 | 7.305 |
| 29.480.000 | 5.078.995 | -806.347 | 7.303 |
| 29.490.000 | 5.080.584 | -806.558 | 7.302 |
| 29.500.000 | 5.082.163 | -806.825 | 7.303 |
| 29.510.000 | 5.083.847 | -807.053 | 7.304 |
| 29.520.000 | 5.085.450 | -807.378 | 7.305 |
| 29.530.000 | 5.087.119 | -807.551 | 7.305 |
| 29.540.000 | 5.088.619 | -807.880 | 7.303 |
| 29.550.000 | 5.090.444 | -808.144 | 7.303 |
| 29.560.000 | 5.091.847 | -808.380 | 7.303 |
| 29.570.000 | 5.093.710 | -808.706 | 7.303 |
| 29.580.000 | 5.095.168 | -808.962 | 7.303 |
| 29.590.000 | 5.096.891 | -809.225 | 7.305 |
| 29.600.000 | 5.098.413 | -809.499 | 7.306 |
| 29.610.000 | 5.100.191 | -809.824 | 7.308 |
| 29.620.000 | 5.101.686 | -810.023 | 7.307 |
| 29.630.000 | 5.103.413 | -810.390 | 7.307 |
| 29.640.000 | 5.105.044 | -810.607 | 7.307 |

| | | | |
|---|---|---|---|
| 29.650.000 | 5.106.593 | -810.910 | 7.306 |
| 29.660.000 | 5.108.296 | -811.153 | 7.305 |
| 29.670.000 | 5.109.854 | -811.486 | 7.303 |
| 29.680.000 | 5.111.609 | -811.704 | 7.302 |
| 29.690.000 | 5.113.097 | -812.050 | 7.302 |
| 29.700.000 | 5.114.949 | -812.318 | 7.302 |
| 29.710.000 | 5.116.324 | -812.558 | 7.304 |
| 29.720.000 | 5.118.127 | -812.876 | 7.309 |
| 29.730.000 | 5.119.648 | -813.127 | 7.312 |
| 29.740.000 | 5.121.383 | -813.418 | 7.317 |
| 29.750.000 | 5.122.920 | -813.679 | 7.318 |
| 29.760.000 | 5.124.636 | -813.992 | 7.318 |
| 29.770.000 | 5.126.162 | -814.194 | 7.315 |
| 29.780.000 | 5.127.855 | -814.547 | 7.313 |
| 29.790.000 | 5.129.587 | -814.754 | 7.313 |
| 29.800.000 | 5.131.049 | -815.058 | 7.314 |
| 29.810.000 | 5.132.833 | -815.295 | 7.317 |
| 29.820.000 | 5.134.326 | -815.594 | 7.322 |
| 29.830.000 | 5.136.079 | -815.852 | 7.327 |
| 29.840.000 | 5.137.561 | -816.150 | 7.329 |
| 29.850.000 | 5.139.380 | -816.426 | 7.330 |
| 29.860.000 | 5.140.760 | -816.639 | 7.327 |
| 29.870.000 | 5.142.591 | -816.952 | 7.324 |
| 29.880.000 | 5.144.065 | -817.178 | 7.323 |
| 29.890.000 | 5.145.779 | -817.444 | 7.321 |
| 29.900.000 | 5.147.349 | -817.691 | 7.322 |
| 29.910.000 | 5.149.047 | -817.965 | 7.325 |
| 29.920.000 | 5.150.656 | -818.162 | 7.329 |
| 29.930.000 | 5.152.275 | -818.487 | 7.331 |
| 29.940.000 | 5.153.965 | -818.651 | 7.329 |

| | | | |
|---|---|---|---|
| 29.950.000 | 5.155.422 | -818.924 | 7.328 |
| 29.960.000 | 5.157.231 | -819.193 | 7.327 |
| 29.970.000 | 5.158.697 | -819.430 | 7.325 |
| 29.980.000 | 5.160.438 | -819.671 | 7.324 |
| 29.990.000 | 5.161.880 | -819.943 | 7.325 |
| 30.000.000 | 5.163.731 | -820.193 | 7.326 |
| 30.010.000 | 5.165.198 | -820.401 | 7.329 |
| 30.020.000 | 5.166.930 | -820.710 | 7.329 |
| 30.030.000 | 5.168.517 | -820.901 | 7.328 |
| 30.040.000 | 5.170.116 | -821.177 | 7.326 |
| 30.050.000 | 5.171.760 | -821.392 | 7.323 |
| 30.060.000 | 5.173.421 | -821.687 | 7.322 |
| 30.070.000 | 5.175.032 | -821.877 | 7.323 |
| 30.080.000 | 5.176.613 | -822.205 | 7.325 |
| 30.090.000 | 5.178.357 | -822.410 | 7.328 |
| 30.100.000 | 5.179.757 | -822.670 | 7.331 |
| 30.110.000 | 5.181.612 | -822.980 | 7.335 |
| 30.120.000 | 5.183.055 | -823.220 | 7.334 |
| 30.130.000 | 5.184.845 | -823.476 | 7.330 |
| 30.140.000 | 5.186.344 | -823.737 | 7.325 |
| 30.150.000 | 5.188.133 | -824.019 | 7.319 |
| 30.160.000 | 5.189.605 | -824.222 | 7.315 |
| 30.170.000 | 5.191.353 | -824.552 | 7.311 |
| 30.180.000 | 5.192.951 | -824.723 | 7.310 |
| 30.190.000 | 5.194.545 | -825.009 | 7.311 |
| 30.200.000 | 5.196.188 | -825.250 | 7.315 |
| 30.210.000 | 5.197.761 | -825.518 | 7.321 |
| 30.220.000 | 5.199.441 | -825.701 | 7.327 |
| 30.230.000 | 5.200.939 | -826.027 | 7.329 |
| 30.240.000 | 5.202.812 | -826.276 | 7.332 |

| | | | |
|---|---|---|---|
| 30.250.000 | 5.204.183 | -826.510 | 7.333 |
| 30.260.000 | 5.206.057 | -826.819 | 7.331 |
| 30.270.000 | 5.207.507 | -827.023 | 7.326 |
| 30.280.000 | 5.209.261 | -827.311 | 7.321 |
| 30.290.000 | 5.210.800 | -827.543 | 7.318 |
| 30.300.000 | 5.212.532 | -827.821 | 7.316 |
| 30.310.000 | 5.214.018 | -828.014 | 7.315 |
| 30.320.000 | 5.215.696 | -828.364 | 7.318 |
| 30.330.000 | 5.217.353 | -828.532 | 7.321 |
| 30.340.000 | 5.218.849 | -828.827 | 7.322 |
| 30.350.000 | 5.220.628 | -829.057 | 7.323 |
| 30.360.000 | 5.222.138 | -829.330 | 7.322 |
| 30.370.000 | 5.223.877 | -829.530 | 7.319 |
| 30.380.000 | 5.225.341 | -829.804 | 7.314 |
| 30.390.000 | 5.227.182 | -830.063 | 7.312 |
| 30.400.000 | 5.228.561 | -830.255 | 7.311 |
| 30.410.000 | 5.230.386 | -830.542 | 7.313 |
| 30.420.000 | 5.231.870 | -830.721 | 7.318 |
| 30.430.000 | 5.233.587 | -830.997 | 7.323 |
| 30.440.000 | 5.235.133 | -831.219 | 7.327 |
| 30.450.000 | 5.236.841 | -831.484 | 7.330 |
| 30.460.000 | 5.238.417 | -831.671 | 7.330 |
| 30.470.000 | 5.240.053 | -831.988 | 7.328 |
| 30.480.000 | 5.241.745 | -832.140 | 7.324 |
| 30.490.000 | 5.243.213 | -832.420 | 7.319 |
| 30.500.000 | 5.244.993 | -832.644 | 7.317 |
| 30.510.000 | 5.246.460 | -832.878 | 7.317 |
| 30.520.000 | 5.248.241 | -833.122 | 7.319 |
| 30.530.000 | 5.249.661 | -833.367 | 7.320 |
| 30.540.000 | 5.251.507 | -833.613 | 7.321 |

| | | | |
|---|---|---|---|
| 30.550.000 | 5.252.889 | -833.840 | 7.321 |
| 30.560.000 | 5.254.699 | -834.130 | 7.321 |
| 30.570.000 | 5.256.233 | -834.332 | 7.321 |
| 30.580.000 | 5.257.913 | -834.616 | 7.320 |
| 30.590.000 | 5.259.528 | -834.849 | 7.321 |
| 30.600.000 | 5.261.155 | -835.137 | 7.323 |
| 30.610.000 | 5.262.766 | -835.311 | 7.328 |
| 30.620.000 | 5.264.379 | -835.657 | 7.334 |
| 30.630.000 | 5.266.118 | -835.858 | 7.337 |
| 30.640.000 | 5.267.540 | -836.118 | 7.338 |
| 30.650.000 | 5.269.409 | -836.430 | 7.336 |
| 30.660.000 | 5.270.837 | -836.655 | 7.335 |
| 30.670.000 | 5.272.600 | -836.925 | 7.331 |
| 30.680.000 | 5.274.065 | -837.186 | 7.329 |
| 30.690.000 | 5.275.862 | -837.433 | 7.328 |
| 30.700.000 | 5.277.337 | -837.633 | 7.329 |
| 30.710.000 | 5.279.083 | -837.982 | 7.332 |
| 30.720.000 | 5.280.658 | -838.131 | 7.336 |
| 30.730.000 | 5.282.301 | -838.422 | 7.338 |
| 30.740.000 | 5.283.949 | -838.635 | 7.340 |
| 30.750.000 | 5.285.546 | -838.917 | 7.338 |
| 30.760.000 | 5.287.212 | -839.082 | 7.337 |
| 30.770.000 | 5.288.718 | -839.403 | 7.333 |
| 30.780.000 | 5.290.558 | -839.622 | 7.331 |
| 30.790.000 | 5.291.903 | -839.867 | 7.330 |
| 30.800.000 | 5.293.794 | -840.173 | 7.330 |
| 30.810.000 | 5.295.250 | -840.366 | 7.332 |
| 30.820.000 | 5.296.988 | -840.642 | 7.332 |
| 30.830.000 | 5.298.530 | -840.896 | 7.331 |
| 30.840.000 | 5.300.318 | -841.169 | 7.330 |

| | | | |
|---|---|---|---|
| 30.850.000 | 5.301.759 | -841.354 | 7.327 |
| 30.860.000 | 5.303.476 | -841.678 | 7.325 |
| 30.870.000 | 5.305.134 | -841.869 | 7.326 |
| 30.880.000 | 5.306.677 | -842.150 | 7.327 |
| 30.890.000 | 5.308.380 | -842.359 | 7.328 |
| 30.900.000 | 5.309.887 | -842.643 | 7.328 |
| 30.910.000 | 5.311.652 | -842.854 | 7.327 |
| 30.920.000 | 5.313.140 | -843.141 | 7.325 |
| 30.930.000 | 5.314.967 | -843.376 | 7.321 |
| 30.940.000 | 5.316.355 | -843.611 | 7.319 |
| 30.950.000 | 5.318.214 | -843.923 | 7.318 |
| 30.960.000 | 5.319.687 | -844.098 | 7.317 |
| 30.970.000 | 5.321.432 | -844.365 | 7.318 |
| 30.980.000 | 5.322.941 | -844.593 | 7.319 |
| 30.990.000 | 5.324.653 | -844.864 | 7.319 |
| 31.000.000 | 5.326.191 | -845.036 | 7.317 |
| 31.010.000 | 5.327.802 | -845.340 | 7.316 |
| 31.020.000 | 5.329.514 | -845.505 | 7.316 |
| 31.030.000 | 5.331.033 | -845.797 | 7.319 |
| 31.040.000 | 5.332.830 | -846.002 | 7.322 |
| 31.050.000 | 5.334.299 | -846.249 | 7.324 |
| 31.060.000 | 5.336.093 | -846.480 | 7.325 |
| 31.070.000 | 5.337.552 | -846.732 | 7.326 |
| 31.080.000 | 5.339.396 | -846.957 | 7.325 |
| 31.090.000 | 5.340.735 | -847.145 | 7.325 |
| 31.100.000 | 5.342.554 | -847.452 | 7.323 |
| 31.110.000 | 5.344.057 | -847.617 | 7.324 |
| 31.120.000 | 5.345.694 | -847.872 | 7.324 |
| 31.130.000 | 5.347.291 | -848.073 | 7.326 |
| 31.140.000 | 5.348.993 | -848.330 | 7.327 |

| | | | |
|---|---|---|---|
| 31.150.000 | 5.350.587 | -848.481 | 7.326 |
| 31.160.000 | 5.352.164 | -848.779 | 7.326 |
| 31.170.000 | 5.353.903 | -848.906 | 7.327 |
| 31.180.000 | 5.355.355 | -849.163 | 7.326 |
| 31.190.000 | 5.357.164 | -849.389 | 7.326 |
| 31.200.000 | 5.358.571 | -849.594 | 7.325 |
| 31.210.000 | 5.360.360 | -849.832 | 7.324 |
| 31.220.000 | 5.361.817 | -850.074 | 7.322 |
| 31.230.000 | 5.363.609 | -850.293 | 7.321 |
| 31.240.000 | 5.365.063 | -850.467 | 7.321 |
| 31.250.000 | 5.366.817 | -850.768 | 7.318 |
| 31.260.000 | 5.368.379 | -850.922 | 7.319 |
| 31.270.000 | 5.370.020 | -851.173 | 7.321 |
| 31.280.000 | 5.371.666 | -851.388 | 7.322 |
| 31.290.000 | 5.373.291 | -851.632 | 7.322 |
| 31.300.000 | 5.374.929 | -851.813 | 7.323 |
| 31.310.000 | 5.376.471 | -852.099 | 7.324 |
| 31.320.000 | 5.378.271 | -852.291 | 7.325 |
| 31.330.000 | 5.379.621 | -852.525 | 7.323 |
| 31.340.000 | 5.381.506 | -852.812 | 7.321 |
| 31.350.000 | 5.382.947 | -853.012 | 7.322 |
| 31.360.000 | 5.384.708 | -853.266 | 7.322 |
| 31.370.000 | 5.386.241 | -853.506 | 7.320 |
| 31.380.000 | 5.387.995 | -853.775 | 7.319 |
| 31.390.000 | 5.389.487 | -853.964 | 7.319 |
| 31.400.000 | 5.391.228 | -854.291 | 7.320 |
| 31.410.000 | 5.392.815 | -854.466 | 7.319 |
| 31.420.000 | 5.394.402 | -854.739 | 7.318 |
| 31.430.000 | 5.396.092 | -854.970 | 7.318 |
| 31.440.000 | 5.397.646 | -855.241 | 7.319 |

| | | | |
|---|---|---|---|
| 31.450.000 | 5.399.360 | -855.430 | 7.321 |
| 31.460.000 | 5.400.818 | -855.746 | 7.323 |
| 31.470.000 | 5.402.678 | -855.970 | 7.327 |
| 31.480.000 | 5.404.086 | -856.220 | 7.330 |
| 31.490.000 | 5.405.932 | -856.525 | 7.332 |
| 31.500.000 | 5.407.403 | -856.712 | 7.331 |
| 31.510.000 | 5.409.127 | -856.990 | 7.333 |
| 31.520.000 | 5.410.610 | -857.207 | 7.335 |
| 31.530.000 | 5.412.320 | -857.478 | 7.336 |
| 31.540.000 | 5.413.859 | -857.656 | 7.339 |
| 31.550.000 | 5.415.503 | -857.955 | 7.343 |
| 31.560.000 | 5.417.181 | -858.094 | 7.347 |
| 31.570.000 | 5.418.681 | -858.356 | 7.348 |
| 31.580.000 | 5.420.444 | -858.553 | 7.349 |
| 31.590.000 | 5.421.958 | -858.782 | 7.349 |
| 31.600.000 | 5.423.710 | -858.996 | 7.349 |
| 31.610.000 | 5.425.195 | -859.243 | 7.348 |
| 31.620.000 | 5.427.000 | -859.461 | 7.348 |
| 31.630.000 | 5.428.388 | -859.639 | 7.351 |
| 31.640.000 | 5.430.194 | -859.919 | 7.355 |
| 31.650.000 | 5.431.686 | -860.106 | 7.355 |
| 31.660.000 | 5.433.351 | -860.347 | 7.356 |
| 31.670.000 | 5.434.919 | -860.555 | 7.355 |
| 31.680.000 | 5.436.567 | -860.802 | 7.353 |
| 31.690.000 | 5.438.145 | -860.958 | 7.350 |
| 31.700.000 | 5.439.791 | -861.267 | 7.348 |
| 31.710.000 | 5.441.519 | -861.403 | 7.348 |
| 31.720.000 | 5.442.975 | -861.658 | 7.348 |
| 31.730.000 | 5.444.787 | -861.908 | 7.347 |
| 31.740.000 | 5.446.250 | -862.100 | 7.348 |

| | | | |
|---|---|---|---|
| 31.750.000 | 5.447.981 | -862.346 | 7.349 |
| 31.760.000 | 5.449.427 | -862.579 | 7.350 |
| 31.770.000 | 5.451.276 | -862.816 | 7.349 |
| 31.780.000 | 5.452.669 | -863.002 | 7.349 |
| 31.790.000 | 5.454.446 | -863.314 | 7.349 |
| 31.800.000 | 5.456.049 | -863.497 | 7.348 |
| 31.810.000 | 5.457.657 | -863.775 | 7.348 |
| 31.820.000 | 5.459.315 | -863.982 | 7.348 |
| 31.830.000 | 5.460.945 | -864.261 | 7.349 |
| 31.840.000 | 5.462.541 | -864.404 | 7.350 |
| 31.850.000 | 5.464.122 | -864.738 | 7.352 |
| 31.860.000 | 5.465.904 | -864.931 | 7.356 |
| 31.870.000 | 5.467.277 | -865.156 | 7.358 |
| 31.880.000 | 5.469.138 | -865.456 | 7.359 |
| 31.890.000 | 5.470.574 | -865.661 | 7.359 |
| 31.900.000 | 5.472.313 | -865.899 | 7.359 |
| 31.910.000 | 5.473.815 | -866.135 | 7.361 |
| 31.920.000 | 5.475.541 | -866.388 | 7.363 |
| 31.930.000 | 5.477.041 | -866.559 | 7.366 |
| 31.940.000 | 5.478.773 | -866.885 | 7.368 |
| 31.950.000 | 5.480.362 | -867.042 | 7.369 |
| 31.960.000 | 5.481.946 | -867.292 | 7.370 |
| 31.970.000 | 5.483.614 | -867.506 | 7.370 |
| 31.980.000 | 5.485.172 | -867.765 | 7.368 |
| 31.990.000 | 5.486.885 | -867.934 | 7.368 |
| 32.000.000 | 5.488.355 | -868.228 | 7.369 |
| 32.010.000 | 5.490.177 | -868.425 | 7.371 |
| 32.020.000 | 5.491.531 | -868.631 | 7.374 |
| 32.030.000 | 5.493.363 | -868.908 | 7.379 |
| 32.040.000 | 5.494.879 | -869.119 | 7.381 |

| | | | |
|---|---|---|---|
| 32.050.000 | 5.496.609 | -869.379 | 7.381 |
| 32.060.000 | 5.498.132 | -869.581 | 7.381 |
| 32.070.000 | 5.499.875 | -869.844 | 7.377 |
| 32.080.000 | 5.501.335 | -870.013 | 7.374 |
| 32.090.000 | 5.503.016 | -870.308 | 7.370 |
| 32.100.000 | 5.504.661 | -870.445 | 7.369 |
| 32.110.000 | 5.506.183 | -870.721 | 7.368 |
| 32.120.000 | 5.507.969 | -870.929 | 7.369 |
| 32.130.000 | 5.509.450 | -871.156 | 7.370 |
| 32.140.000 | 5.511.195 | -871.386 | 7.369 |
| 32.150.000 | 5.512.667 | -871.652 | 7.368 |
| 32.160.000 | 5.514.520 | -871.919 | 7.365 |
| 32.170.000 | 5.515.932 | -872.110 | 7.363 |
| 32.180.000 | 5.517.722 | -872.412 | 7.361 |
| 32.190.000 | 5.519.180 | -872.593 | 7.361 |
| 32.200.000 | 5.520.882 | -872.860 | 7.364 |
| 32.210.000 | 5.522.475 | -873.087 | 7.369 |
| 32.220.000 | 5.524.149 | -873.351 | 7.373 |
| 32.230.000 | 5.525.689 | -873.512 | 7.375 |
| 32.240.000 | 5.527.297 | -873.840 | 7.375 |
| 32.250.000 | 5.529.048 | -873.972 | 7.375 |
| 32.260.000 | 5.530.497 | -874.237 | 7.371 |
| 32.270.000 | 5.532.335 | -874.480 | 7.368 |
| 32.280.000 | 5.533.797 | -874.703 | 7.365 |
| 32.290.000 | 5.535.542 | -874.927 | 7.366 |
| 32.300.000 | 5.536.991 | -875.165 | 7.366 |
| 32.310.000 | 5.538.817 | -875.391 | 7.369 |
| 32.320.000 | 5.540.188 | -875.573 | 7.372 |
| 32.330.000 | 5.541.948 | -875.857 | 7.373 |
| 32.340.000 | 5.543.507 | -876.017 | 7.373 |

| | | | |
|---|---|---|---|
| 32.350.000 | 5.545.142 | -876.257 | 7.372 |
| 32.360.000 | 5.546.743 | -876.460 | 7.370 |
| 32.370.000 | 5.548.393 | -876.713 | 7.369 |
| 32.380.000 | 5.550.054 | -876.867 | 7.368 |
| 32.390.000 | 5.551.609 | -877.167 | 7.370 |
| 32.400.000 | 5.553.383 | -877.355 | 7.374 |
| 32.410.000 | 5.554.769 | -877.567 | 7.379 |
| 32.420.000 | 5.556.590 | -877.833 | 7.383 |
| 32.430.000 | 5.558.025 | -878.034 | 7.387 |
| 32.440.000 | 5.559.770 | -878.257 | 7.388 |
| 32.450.000 | 5.561.229 | -878.489 | 7.387 |
| 32.460.000 | 5.562.998 | -878.742 | 7.384 |
| 32.470.000 | 5.564.481 | -878.892 | 7.383 |
| 32.480.000 | 5.566.205 | -879.187 | 7.383 |
| 32.490.000 | 5.567.824 | -879.342 | 7.385 |
| 32.500.000 | 5.569.408 | -879.581 | 7.386 |
| 32.510.000 | 5.571.104 | -879.769 | 7.388 |
| 32.520.000 | 5.572.654 | -880.036 | 7.388 |
| 32.530.000 | 5.574.339 | -880.201 | 7.386 |
| 32.540.000 | 5.575.845 | -880.489 | 7.384 |
| 32.550.000 | 5.577.655 | -880.736 | 7.380 |
| 32.560.000 | 5.579.008 | -880.932 | 7.376 |
| 32.570.000 | 5.580.816 | -881.209 | 7.375 |
| 32.580.000 | 5.582.277 | -881.413 | 7.376 |
| 32.590.000 | 5.584.003 | -881.676 | 7.379 |
| 32.600.000 | 5.585.509 | -881.897 | 7.383 |
| 32.610.000 | 5.587.302 | -882.163 | 7.384 |
| 32.620.000 | 5.588.785 | -882.326 | 7.383 |
| 32.630.000 | 5.590.491 | -882.627 | 7.382 |
| 32.640.000 | 5.592.135 | -882.768 | 7.382 |

| | | | |
|---|---|---|---|
| 32.650.000 | 5.593.600 | -883.032 | 7.382 |
| 32.660.000 | 5.595.356 | -883.233 | 7.384 |
| 32.670.000 | 5.596.839 | -883.505 | 7.387 |
| 32.680.000 | 5.598.576 | -883.683 | 7.391 |
| 32.690.000 | 5.600.003 | -883.938 | 7.395 |
| 32.700.000 | 5.601.842 | -884.197 | 7.400 |
| 32.710.000 | 5.603.241 | -884.379 | 7.399 |
| 32.720.000 | 5.605.062 | -884.656 | 7.398 |
| 32.730.000 | 5.606.551 | -884.841 | 7.398 |
| 32.740.000 | 5.608.236 | -885.090 | 7.398 |
| 32.750.000 | 5.609.783 | -885.295 | 7.399 |
| 32.760.000 | 5.611.496 | -885.562 | 7.400 |
| 32.770.000 | 5.613.098 | -885.703 | 7.404 |
| 32.780.000 | 5.614.660 | -886.009 | 7.408 |
| 32.790.000 | 5.616.413 | -886.175 | 7.410 |
| 32.800.000 | 5.617.850 | -886.426 | 7.410 |
| 32.810.000 | 5.619.626 | -886.656 | 7.410 |
| 32.820.000 | 5.621.101 | -886.896 | 7.409 |
| 32.830.000 | 5.622.888 | -887.134 | 7.406 |
| 32.840.000 | 5.624.306 | -887.370 | 7.404 |
| 32.850.000 | 5.626.169 | -887.642 | 7.405 |
| 32.860.000 | 5.627.576 | -887.809 | 7.407 |
| 32.870.000 | 5.629.320 | -888.091 | 7.408 |
| 32.880.000 | 5.630.873 | -888.288 | 7.407 |
| 32.890.000 | 5.632.493 | -888.543 | 7.407 |
| 32.900.000 | 5.634.126 | -888.752 | 7.405 |
| 32.910.000 | 5.635.735 | -889.038 | 7.402 |
| 32.920.000 | 5.637.391 | -889.178 | 7.401 |
| 32.930.000 | 5.638.944 | -889.500 | 7.401 |
| 32.940.000 | 5.640.735 | -889.718 | 7.402 |

| | | | |
|---|---|---|---|
| 32.950.000 | 5.642.141 | -889.941 | 7.404 |
| 32.960.000 | 5.643.974 | -890.232 | 7.405 |
| 32.970.000 | 5.645.405 | -890.453 | 7.407 |
| 32.980.000 | 5.647.141 | -890.676 | 7.406 |
| 32.990.000 | 5.648.622 | -890.914 | 7.405 |
| 33.000.000 | 5.650.404 | -891.189 | 7.404 |
| 33.010.000 | 5.651.889 | -891.342 | 7.404 |
| 33.020.000 | 5.653.596 | -891.667 | 7.406 |
| 33.030.000 | 5.655.178 | -891.828 | 7.406 |
| 33.040.000 | 5.656.754 | -892.065 | 7.407 |
| 33.050.000 | 5.658.444 | -892.279 | 7.408 |
| 33.060.000 | 5.660.074 | -892.555 | 7.408 |
| 33.070.000 | 5.661.718 | -892.682 | 7.407 |
| 33.080.000 | 5.663.163 | -892.962 | 7.406 |
| 33.090.000 | 5.665.026 | -893.200 | 7.407 |
| 33.100.000 | 5.666.361 | -893.379 | 7.410 |
| 33.110.000 | 5.668.197 | -893.625 | 7.415 |
| 33.120.000 | 5.669.667 | -893.846 | 7.419 |
| 33.130.000 | 5.671.374 | -894.038 | 7.423 |
| 33.140.000 | 5.672.895 | -894.266 | 7.426 |
| 33.150.000 | 5.674.671 | -894.550 | 7.426 |
| 33.160.000 | 5.676.198 | -894.692 | 7.425 |
| 33.170.000 | 5.677.856 | -894.991 | 7.423 |
| 33.180.000 | 5.679.525 | -895.209 | 7.423 |
| 33.190.000 | 5.681.024 | -895.437 | 7.422 |
| 33.200.000 | 5.682.743 | -895.646 | 7.422 |
| 33.210.000 | 5.684.257 | -895.932 | 7.422 |
| 33.220.000 | 5.685.997 | -896.102 | 7.422 |
| 33.230.000 | 5.687.406 | -896.353 | 7.420 |
| 33.240.000 | 5.689.238 | -896.614 | 7.417 |

| | | | |
|---|---|---|---|
| 33.250.000 | 5.690.640 | -896.772 | 7.414 |
| 33.260.000 | 5.692.465 | -897.077 | 7.411 |
| 33.270.000 | 5.693.975 | -897.262 | 7.411 |
| 33.280.000 | 5.695.677 | -897.503 | 7.413 |
| 33.290.000 | 5.697.213 | -897.710 | 7.416 |
| 33.300.000 | 5.698.920 | -898.002 | 7.417 |
| 33.310.000 | 5.700.468 | -898.145 | 7.416 |
| 33.320.000 | 5.702.070 | -898.460 | 7.415 |
| 33.330.000 | 5.703.805 | -898.643 | 7.415 |
| 33.340.000 | 5.705.208 | -898.874 | 7.413 |
| 33.350.000 | 5.707.027 | -899.127 | 7.410 |
| 33.360.000 | 5.708.521 | -899.378 | 7.410 |
| 33.370.000 | 5.710.279 | -899.596 | 7.412 |
| 33.380.000 | 5.711.751 | -899.826 | 7.412 |
| 33.390.000 | 5.713.628 | -900.122 | 7.412 |
| 33.400.000 | 5.715.024 | -900.280 | 7.412 |
| 33.410.000 | 5.716.795 | -900.592 | 7.414 |
| 33.420.000 | 5.718.330 | -900.797 | 7.416 |
| 33.430.000 | 5.719.932 | -901.028 | 7.418 |
| 33.440.000 | 5.721.514 | -901.252 | 7.421 |
| 33.450.000 | 5.723.183 | -901.564 | 7.424 |
| 33.460.000 | 5.724.800 | -901.658 | 7.425 |
| 33.470.000 | 5.726.337 | -901.985 | 7.425 |
| 33.480.000 | 5.728.149 | -902.194 | 7.426 |
| 33.490.000 | 5.729.566 | -902.389 | 7.425 |
| 33.500.000 | 5.731.374 | -902.664 | 7.425 |
| 33.510.000 | 5.732.857 | -902.909 | 7.426 |
| 33.520.000 | 5.734.665 | -903.112 | 7.427 |
| 33.530.000 | 5.736.089 | -903.363 | 7.428 |
| 33.540.000 | 5.737.895 | -903.641 | 7.427 |

| | | | |
|---|---|---|---|
| 33.550.000 | 5.739.348 | -903.784 | 7.426 |
| 33.560.000 | 5.741.023 | -904.080 | 7.425 |
| 33.570.000 | 5.742.656 | -904.280 | 7.424 |
| 33.580.000 | 5.744.209 | -904.515 | 7.424 |
| 33.590.000 | 5.745.879 | -904.715 | 7.426 |
| 33.600.000 | 5.747.479 | -905.025 | 7.430 |
| 33.610.000 | 5.749.190 | -905.160 | 7.435 |
| 33.620.000 | 5.750.626 | -905.430 | 7.439 |
| 33.630.000 | 5.752.477 | -905.700 | 7.440 |
| 33.640.000 | 5.753.825 | -905.863 | 7.440 |
| 33.650.000 | 5.755.653 | -906.125 | 7.441 |
| 33.660.000 | 5.757.119 | -906.373 | 7.441 |
| 33.670.000 | 5.758.888 | -906.585 | 7.440 |
| 33.680.000 | 5.760.412 | -906.813 | 7.439 |
| 33.690.000 | 5.762.157 | -907.119 | 7.441 |
| 33.700.000 | 5.763.655 | -907.240 | 7.442 |
| 33.710.000 | 5.765.256 | -907.539 | 7.442 |
| 33.720.000 | 5.766.933 | -907.749 | 7.441 |
| 33.730.000 | 5.768.503 | -907.997 | 7.439 |
| 33.740.000 | 5.770.230 | -908.211 | 7.438 |
| 33.750.000 | 5.771.726 | -908.521 | 7.436 |
| 33.760.000 | 5.773.482 | -908.697 | 7.432 |
| 33.770.000 | 5.774.890 | -908.957 | 7.430 |
| 33.780.000 | 5.776.716 | -909.225 | 7.428 |
| 33.790.000 | 5.778.105 | -909.377 | 7.428 |
| 33.800.000 | 5.779.913 | -909.683 | 7.428 |
| 33.810.000 | 5.781.427 | -909.891 | 7.428 |
| 33.820.000 | 5.783.109 | -910.109 | 7.430 |
| 33.830.000 | 5.784.629 | -910.307 | 7.433 |
| 33.840.000 | 5.786.330 | -910.626 | 7.436 |

| | | | |
|---|---|---|---|
| 33.850.000 | 5.787.937 | -910.734 | 7.437 |
| 33.860.000 | 5.789.459 | -911.041 | 7.436 |
| 33.870.000 | 5.791.239 | -911.236 | 7.436 |
| 33.880.000 | 5.792.664 | -911.466 | 7.435 |
| 33.890.000 | 5.794.416 | -911.692 | 7.435 |
| 33.900.000 | 5.795.881 | -911.956 | 7.437 |
| 33.910.000 | 5.797.644 | -912.144 | 7.439 |
| 33.920.000 | 5.799.079 | -912.373 | 7.440 |
| 33.930.000 | 5.800.913 | -912.664 | 7.440 |
| 33.940.000 | 5.802.337 | -912.810 | 7.441 |
| 33.950.000 | 5.804.061 | -913.114 | 7.442 |
| 33.960.000 | 5.805.616 | -913.331 | 7.442 |
| 33.970.000 | 5.807.275 | -913.564 | 7.443 |
| 33.980.000 | 5.808.898 | -913.755 | 7.443 |
| 33.990.000 | 5.810.494 | -914.082 | 7.445 |
| 34.000.000 | 5.812.189 | -914.212 | 7.446 |
| 34.010.000 | 5.813.679 | -914.511 | 7.444 |
| 34.020.000 | 5.815.469 | -914.750 | 7.443 |
| 34.030.000 | 5.816.876 | -914.938 | 7.442 |
| 34.040.000 | 5.818.677 | -915.215 | 7.442 |
| 34.050.000 | 5.820.138 | -915.457 | 7.442 |
| 34.060.000 | 5.821.922 | -915.668 | 7.444 |
| 34.070.000 | 5.823.397 | -915.904 | 7.445 |
| 34.080.000 | 5.825.205 | -916.206 | 7.446 |
| 34.090.000 | 5.826.676 | -916.341 | 7.446 |
| 34.100.000 | 5.828.333 | -916.647 | 7.449 |
| 34.110.000 | 5.829.977 | -916.881 | 7.454 |
| 34.120.000 | 5.831.530 | -917.123 | 7.456 |
| 34.130.000 | 5.833.217 | -917.310 | 7.459 |
| 34.140.000 | 5.834.752 | -917.625 | 7.460 |

| | | | |
|---|---|---|---|
| 34.150.000 | 5.836.477 | -917.785 | 7.460 |
| 34.160.000 | 5.837.905 | -918.055 | 7.460 |
| 34.170.000 | 5.839.756 | -918.353 | 7.456 |
| 34.180.000 | 5.841.177 | -918.514 | 7.454 |
| 34.190.000 | 5.842.955 | -918.793 | 7.454 |
| 34.200.000 | 5.844.410 | -919.039 | 7.455 |
| 34.210.000 | 5.846.156 | -919.251 | 7.456 |
| 34.220.000 | 5.847.653 | -919.451 | 7.455 |
| 34.230.000 | 5.849.393 | -919.773 | 7.454 |
| 34.240.000 | 5.850.983 | -919.903 | 7.453 |
| 34.250.000 | 5.852.556 | -920.191 | 7.453 |
| 34.260.000 | 5.854.263 | -920.402 | 7.455 |
| 34.270.000 | 5.855.806 | -920.654 | 7.458 |
| 34.280.000 | 5.857.524 | -920.865 | 7.461 |
| 34.290.000 | 5.858.992 | -921.175 | 7.464 |
| 34.300.000 | 5.860.841 | -921.390 | 7.467 |
| 34.310.000 | 5.862.210 | -921.645 | 7.468 |
| 34.320.000 | 5.863.996 | -921.923 | 7.464 |
| 34.330.000 | 5.865.429 | -922.096 | 7.462 |
| 34.340.000 | 5.867.196 | -922.394 | 7.460 |
| 34.350.000 | 5.868.731 | -922.615 | 7.459 |
| 34.360.000 | 5.870.444 | -922.860 | 7.461 |
| 34.370.000 | 5.871.986 | -923.054 | 7.463 |
| 34.380.000 | 5.873.681 | -923.383 | 7.467 |
| 34.390.000 | 5.875.347 | -923.536 | 7.469 |
| 34.400.000 | 5.876.865 | -923.844 | 7.469 |
| 34.410.000 | 5.878.687 | -924.081 | 7.470 |
| 34.420.000 | 5.880.106 | -924.313 | 7.468 |
| 34.430.000 | 5.881.889 | -924.573 | 7.465 |
| 34.440.000 | 5.883.325 | -924.853 | 7.461 |

| | | | |
|---|---|---|---|
| 34.450.000 | 5.885.113 | -925.082 | 7.458 |
| 34.460.000 | 5.886.521 | -925.323 | 7.459 |
| 34.470.000 | 5.888.319 | -925.644 | 7.463 |
| 34.480.000 | 5.889.856 | -925.808 | 7.466 |
| 34.490.000 | 5.891.571 | -926.116 | 7.467 |
| 34.500.000 | 5.893.138 | -926.333 | 7.469 |
| 34.510.000 | 5.894.753 | -926.581 | 7.471 |
| 34.520.000 | 5.896.396 | -926.789 | 7.473 |
| 34.530.000 | 5.897.976 | -927.120 | 7.473 |
| 34.540.000 | 5.899.706 | -927.235 | 7.473 |
| 34.550.000 | 5.901.103 | -927.535 | 7.474 |
| 34.560.000 | 5.902.935 | -927.813 | 7.476 |
| 34.570.000 | 5.904.385 | -928.003 | 7.476 |
| 34.580.000 | 5.906.128 | -928.267 | 7.477 |
| 34.590.000 | 5.907.611 | -928.506 | 7.476 |
| 34.600.000 | 5.909.411 | -928.751 | 7.474 |
| 34.610.000 | 5.910.832 | -928.937 | 7.473 |
| 34.620.000 | 5.912.636 | -929.253 | 7.474 |
| 34.630.000 | 5.914.217 | -929.429 | 7.475 |
| 34.640.000 | 5.915.805 | -929.701 | 7.477 |
| 34.650.000 | 5.917.476 | -929.935 | 7.479 |
| 34.660.000 | 5.919.025 | -930.202 | 7.483 |
| 34.670.000 | 5.920.694 | -930.373 | 7.485 |
| 34.680.000 | 5.922.184 | -930.691 | 7.485 |
| 34.690.000 | 5.923.978 | -930.899 | 7.482 |
| 34.700.000 | 5.925.354 | -931.127 | 7.479 |
| 34.710.000 | 5.927.196 | -931.410 | 7.476 |
| 34.720.000 | 5.928.635 | -931.595 | 7.476 |
| 34.730.000 | 5.930.375 | -931.849 | 7.476 |
| 34.740.000 | 5.931.892 | -932.087 | 7.478 |

| | | | |
|---|---|---|---|
| 34.750.000 | 5.933.653 | -932.327 | 7.482 |
| 34.760.000 | 5.935.146 | -932.494 | 7.486 |
| 34.770.000 | 5.936.821 | -932.803 | 7.489 |
| 34.780.000 | 5.938.449 | -932.955 | 7.489 |
| 34.790.000 | 5.939.997 | -933.255 | 7.486 |
| 34.800.000 | 5.941.729 | -933.466 | 7.483 |
| 34.810.000 | 5.943.241 | -933.726 | 7.479 |
| 34.820.000 | 5.945.033 | -933.968 | 7.477 |
| 34.830.000 | 5.946.429 | -934.229 | 7.478 |
| 34.840.000 | 5.948.262 | -934.488 | 7.481 |
| 34.850.000 | 5.949.621 | -934.693 | 7.486 |
| 34.860.000 | 5.951.513 | -935.007 | 7.492 |
| 34.870.000 | 5.952.978 | -935.198 | 7.496 |
| 34.880.000 | 5.954.629 | -935.460 | 7.499 |
| 34.890.000 | 5.956.192 | -935.682 | 7.498 |
| 34.900.000 | 5.957.876 | -935.964 | 7.497 |
| 34.910.000 | 5.959.401 | -936.110 | 7.496 |
| 34.920.000 | 5.961.058 | -936.443 | 7.494 |
| 34.930.000 | 5.962.777 | -936.614 | 7.495 |
| 34.940.000 | 5.964.232 | -936.885 | 7.497 |
| 34.950.000 | 5.966.057 | -937.142 | 7.500 |
| 34.960.000 | 5.967.522 | -937.404 | 7.503 |
| 34.970.000 | 5.969.302 | -937.676 | 7.504 |
| 34.980.000 | 5.970.757 | -937.953 | 7.506 |
| 34.990.000 | 5.972.599 | -938.239 | 7.505 |
| 35.000.000 | 5.974.021 | -938.447 | 7.503 |
| 35.010.000 | 5.975.778 | -938.785 | 7.499 |
| 35.020.000 | 5.977.359 | -939.007 | 7.498 |
| 35.030.000 | 5.978.991 | -939.291 | 7.499 |
| 35.040.000 | 5.980.623 | -939.530 | 7.500 |

| | | | |
|---|---|---|---|
| 35.050.000 | 5.982.232 | -939.856 | 7.501 |
| 35.060.000 | 5.983.887 | -940.021 | 7.502 |
| 35.070.000 | 5.985.403 | -940.348 | 7.503 |
| 35.080.000 | 5.987.228 | -940.582 | 7.503 |
| 35.090.000 | 5.988.637 | -940.848 | 7.501 |
| 35.100.000 | 5.990.460 | -941.116 | 7.500 |
| 35.110.000 | 5.991.909 | -941.354 | 7.498 |
| 35.120.000 | 5.993.639 | -941.596 | 7.497 |
| 35.130.000 | 5.995.139 | -941.839 | 7.496 |
| 35.140.000 | 5.996.913 | -942.127 | 7.497 |
| 35.150.000 | 5.998.376 | -942.284 | 7.497 |
| 35.160.000 | 6.000.107 | -942.609 | 7.497 |
| 35.170.000 | 6.001.738 | -942.823 | 7.496 |
| 35.180.000 | 6.003.278 | -943.050 | 7.497 |
| 35.190.000 | 6.004.960 | -943.277 | 7.499 |
| 35.200.000 | 6.006.537 | -943.582 | 7.501 |
| 35.210.000 | 6.008.285 | -943.734 | 7.504 |
| 35.220.000 | 6.009.745 | -944.026 | 7.507 |
| 35.230.000 | 6.011.557 | -944.293 | 7.508 |
| 35.240.000 | 6.012.976 | -944.491 | 7.509 |
| 35.250.000 | 6.014.767 | -944.782 | 7.509 |
| 35.260.000 | 6.016.248 | -945.015 | 7.507 |
| 35.270.000 | 6.017.980 | -945.273 | 7.505 |
