# Supplementary material for: Data on statistical experimental design to formulate amphotericin B-loaded Eudragit RL100 nanoparticles coated with hyaluronic acid for the treatment of vulvovaginal candidiasis
Source: Data Brief. 2020 Mar 5;29:105311. doi: 10.1016/j.dib.2020.105311 (PMC7082528; doi:10.1016/j.dib.2020.105311)
Supplement: Multimedia component 4 [file mmc4.pdf]

File Name:	**EUD nanoparticles**		
[Data]			
Time	Temp	**DTA**	**TGA**
sec	C	uV	mg
0	272.176	-567	1.7750
1.2000	272.603	-631	1.7749
2.2000	272.350	-519	1.7748
3.2000	272.631	-591	1.7749
4.2000	272.570	-568	1.7749
5.2000	272.770	-534	1.7747
6.2000	272.748	-521	1.7745
7.2000	273.001	-608	1.7745
8.2000	273.066	-474	1.7745
9.2000	273.222	-611	1.7743
10.2000	273.524	-602	1.7740
11.2000	273.543	-647	1.7739
12.2000	273.935	-726	1.7739
130.000	273.906	-892	1.7738
140.000	274.467	-984	1.7735
150.000	274.439	-1.184	1.7733
160.000	275.074	-1.440	1.7733
170.000	275.150	-1.584	1.7734
180.000	275.715	-1.974	1.7732
190.000	276.013	-2.239	1.7730
200.000	276.491	-2.605	1.7728
210.000	276.865	-2.976	1.7728
220.000	277.411	-3.500	1.7729
230.000	277.881	-3.822	1.7727
240.000	278.307	-4.452	1.7724

250.000	279.041	-4.973	1.7722
260.000	279.328	-5.552	1.7721
270.000	280.185	-6.209	1.7722
280.000	280.586	-6.917	1.7721
290.000	281.405	-7.583	1.7718
300.000	281.854	-8.352	1.7715
310.000	282.830	-9.161	1.7714
320.000	283.238	-9.852	1.7715
330.000	284.119	-10.768	1.7716
340.000	284.753	-11.583	1.7715
350.000	285.519	-12.449	1.7714
360.000	286.294	-13.328	1.7715
370.000	287.061	-14.340	1.7716
380.000	287.926	-15.140	1.7716
390.000	288.601	-16.220	1.7713
400.000	289.733	-17.169	1.7710
410.000	290.299	-18.160	1.7708
420.000	291.446	-19.281	1.7708
430.000	292.145	-20.308	1.7707
440.000	293.203	-21.359	1.7707
450.000	293.969	-22.442	1.7705
460.000	295.187	-23.595	1.7703
470.000	295.855	-24.586	1.7704
480.000	296.990	-25.837	1.7706
490.000	297.979	-26.901	1.7704
500.000	298.880	-28.082	1.7701
510.000	300.002	-29.189	1.7697
520.000	300.986	-30.429	1.7697
530.000	302.130	-31.498	1.7695
540.000	303.021	-32.781	1.7691

550.000	304.424	-33.971	1.7686
560.000	305.093	-35.130	1.7684
570.000	306.499	-36.404	1.7686
580.000	307.381	-37.603	1.7687
590.000	308.590	-38.805	1.7684
600.000	309.578	-40.030	1.7682
610.000	310.870	-41.307	1.7683
620.000	311.845	-42.428	1.7684
630.000	313.099	-43.777	1.7681
640.000	314.268	-44.920	1.7678
650.000	315.381	-46.218	1.7676
660.000	316.719	-47.411	1.7677
670.000	317.717	-48.654	1.7675
680.000	319.072	-49.870	1.7671
690.000	320.047	-51.150	1.7668
700.000	321.582	-52.405	1.7665
710.000	322.491	-53.575	1.7665
720.000	323.968	-54.908	1.7665
730.000	325.042	-56.077	1.7663
740.000	326.490	-57.373	1.7660
750.000	327.628	-58.572	1.7660
760.000	329.065	-59.839	1.7661
770.000	330.217	-60.970	1.7659
780.000	331.526	-62.304	1.7655
790.000	332.925	-63.426	1.7652
800.000	334.024	-64.686	1.7651
810.000	335.595	-65.900	1.7648
820.000	336.684	-67.114	1.7643
830.000	338.211	-68.318	1.7638
840.000	339.325	-69.550	1.7635

850.000	340.978	-70.747	1.7632
860.000	342.057	-71.890	1.7628
870.000	343.667	-73.174	1.7623
880.000	344.881	-74.238	1.7622
890.000	346.326	-75.489	1.7622
900.000	347.686	-76.626	1.7622
910.000	349.093	-77.815	1.7619
920.000	350.498	-78.874	1.7617
930.000	351.851	-80.173	1.7616
940.000	353.409	-81.185	1.7611
950.000	354.611	-82.364	1.7605
960.000	356.331	-83.559	1.7599
970.000	357.529	-84.636	1.7594
980.000	359.213	-85.777	1.7590
990.000	360.468	-86.910	1.7585
1.000.000	362.122	-88.006	1.7579
1.010.000	363.324	-89.077	1.7576
1.020.000	365.006	-90.249	1.7574
1.030.000	366.404	-91.237	1.7569
1.040.000	367.879	-92.371	1.7564
1.050.000	369.441	-93.414	1.7559
1.060.000	370.895	-94.530	1.7555
1.070.000	372.463	-95.493	1.7551
1.080.000	373.878	-96.655	1.7544
1.090.000	375.629	-97.635	1.7539
1.100.000	376.834	-98.669	1.7535
1.110.000	378.658	-99.769	1.7532
1.120.000	379.927	-100.711	1.7527
1.130.000	381.624	-101.743	1.7523
1.140.000	382.997	-102.764	1.7521

1.150.000	384.723	-103.762	1.7515
1.160.000	386.105	-104.681	1.7511
1.170.000	387.734	-105.775	1.7509
1.180.000	389.286	-106.620	1.7507
1.190.000	390.792	-107.661	1.7505
1.200.000	392.517	-108.564	1.7502
1.210.000	393.985	-109.526	1.7500
1.220.000	395.716	-110.426	1.7500
1.230.000	397.036	-111.410	1.7496
1.240.000	398.905	-112.314	1.7491
1.250.000	400.185	-113.230	1.7488
1.260.000	402.074	-114.196	1.7486
1.270.000	403.425	-114.970	1.7482
1.280.000	405.110	-115.915	1.7476
1.290.000	406.595	-116.754	1.7471
1.300.000	408.317	-117.640	1.7467
1.310.000	409.792	-118.427	1.7464
1.320.000	411.424	-119.373	1.7457
1.330.000	413.057	-120.085	1.7453
1.340.000	414.450	-120.977	1.7450
1.350.000	416.328	-121.780	1.7444
1.360.000	417.643	-122.570	1.7439
1.370.000	419.458	-123.404	1.7434
1.380.000	420.841	-124.220	1.7427
1.390.000	422.638	-124.964	1.7419
1.400.000	424.039	-125.735	1.7411
1.410.000	425.884	-126.611	1.7407
1.420.000	427.320	-127.225	1.7402
1.430.000	429.042	-128.045	1.7397
1.440.000	430.611	-128.771	1.7394

1.450.000	432.251	-129.492	1.7393
1.460.000	433.856	-130.144	1.7389
1.470.000	435.433	-130.998	1.7386
1.480.000	437.240	-131.553	1.7385
1.490.000	438.598	-132.325	1.7383
1.500.000	440.533	-133.058	1.7381
1.510.000	441.908	-133.676	1.7377
1.520.000	443.749	-134.423	1.7375
1.530.000	445.225	-135.089	1.7372
1.540.000	447.096	-135.711	1.7366
1.550.000	448.477	-136.350	1.7363
1.560.000	450.326	-137.119	1.7358
1.570.000	451.928	-137.610	1.7354
1.580.000	453.552	-138.321	1.7351
1.590.000	455.299	-138.926	1.7351
1.600.000	456.837	-139.577	1.7350
1.610.000	458.613	-140.103	1.7349
1.620.000	460.159	-140.830	1.7347
1.630.000	462.036	-141.355	1.7346
1.640.000	463.471	-141.992	1.7344
1.650.000	465.432	-142.648	1.7341
1.660.000	466.812	-143.125	1.7339
1.670.000	468.716	-143.783	1.7337
1.680.000	470.271	-144.351	1.7334
1.690.000	472.004	-144.879	1.7331
1.700.000	473.632	-145.437	1.7328
1.710.000	475.402	-146.081	1.7325
1.720.000	477.064	-146.459	1.7320
1.730.000	478.660	-147.112	1.7316
1.740.000	480.522	-147.608	1.7312

1.750.000	482.056	-148.127	1.7308
1.760.000	483.967	-148.672	1.7302
1.770.000	485.416	-149.223	1.7296
1.780.000	487.352	-149.697	1.7292
1.790.000	488.734	-150.217	1.7286
1.800.000	490.777	-150.784	1.7279
1.810.000	492.193	-151.158	1.7275
1.820.000	494.043	-151.760	1.7271
1.830.000	495.722	-152.210	1.7266
1.840.000	497.432	-152.697	1.7262
1.850.000	499.067	-153.099	1.7259
1.860.000	500.855	-153.712	1.7258
1.870.000	502.647	-154.016	1.7256
1.880.000	504.177	-154.557	1.7253
1.890.000	506.104	-155.016	1.7254
1.900.000	507.508	-155.413	1.7254
1.910.000	509.416	-155.927	1.7253
1.920.000	510.925	-156.376	1.7252
1.930.000	512.768	-156.747	1.7251
1.940.000	514.263	-157.195	1.7249
1.950.000	516.156	-157.685	1.7248
1.960.000	517.641	-157.980	1.7245
1.970.000	519.477	-158.496	1.7242
1.980.000	521.155	-158.870	1.7240
1.990.000	522.796	-159.270	1.7238
2.000.000	524.565	-159.619	1.7234
2.010.000	526.167	-160.141	1.7231
2.020.000	528.059	-160.403	1.7229
2.030.000	529.505	-160.874	1.7226
2.040.000	531.495	-161.305	1.7223

2.050.000	532.853	-161.594	1.7220
2.060.000	534.784	-162.049	1.7219
2.070.000	536.324	-162.422	1.7217
2.080.000	538.181	-162.796	1.7212
2.090.000	539.766	-163.128	1.7206
2.100.000	541.646	-163.601	1.7204
2.110.000	543.223	-163.826	1.7202
2.120.000	544.936	-164.287	1.7197
2.130.000	546.760	-164.611	1.7193
2.140.000	548.303	-164.992	1.7193
2.150.000	550.209	-165.308	1.7193
2.160.000	551.695	-165.725	1.7192
2.170.000	553.626	-166.017	1.7189
2.180.000	555.032	-166.384	1.7189
2.190.000	557.077	-166.800	1.7189
2.200.000	558.555	-167.045	1.7188
2.210.000	560.486	-167.484	1.7187
2.220.000	562.079	-167.796	1.7185
2.230.000	563.905	-168.129	1.7186
2.240.000	565.501	-168.450	1.7189
2.250.000	567.304	-168.859	1.7189
2.260.000	568.995	-169.053	1.7189
2.270.000	570.647	-169.492	1.7190
2.280.000	572.589	-169.781	1.7192
2.290.000	574.008	-170.072	1.7192
2.300.000	575.944	-170.449	1.7189
2.310.000	577.528	-170.790	1.7185
2.320.000	579.464	-171.098	1.7182
2.330.000	580.916	-171.426	1.7180
2.340.000	582.932	-171.801	1.7178

2.350.000	584.386	-172.001	1.7175
2.360.000	586.255	-172.425	1.7173
2.370.000	587.974	-172.694	1.7171
2.380.000	589.665	-173.004	1.7173
2.390.000	591.403	-173.283	1.7173
2.400.000	593.107	-173.682	1.7172
2.410.000	594.916	-173.843	1.7172
2.420.000	596.511	-174.259	1.7170
2.430.000	598.548	-174.578	1.7170
2.440.000	599.924	-174.812	1.7169
2.450.000	601.955	-175.200	1.7169
2.460.000	603.468	-175.469	1.7168
2.470.000	605.324	-175.744	1.7167
2.480.000	606.826	-176.028	1.7165
2.490.000	608.701	-176.402	1.7162
2.500.000	610.241	-176.566	1.7159
2.510.000	612.012	-176.950	1.7155
2.520.000	613.793	-177.195	1.7150
2.530.000	615.414	-177.505	1.7146
2.540.000	617.264	-177.763	1.7143
2.550.000	618.833	-178.094	1.7142
2.560.000	620.714	-178.330	1.7138
2.570.000	622.162	-178.655	1.7135
2.580.000	624.192	-178.966	1.7132
2.590.000	625.567	-179.177	1.7127
2.600.000	627.531	-179.530	1.7124
2.610.000	629.091	-179.766	1.7122
2.620.000	630.841	-180.054	1.7122
2.630.000	632.463	-180.293	1.7122
2.640.000	634.269	-180.633	1.7121

2.650.000	635.914	-180.804	1.7121
2.660.000	637.655	-181.189	1.7123
2.670.000	639.528	-181.379	1.7124
2.680.000	640.994	-181.681	1.7124
2.690.000	642.973	-181.964	1.7123
2.700.000	644.424	-182.261	1.7122
2.710.000	646.334	-182.512	1.7123
2.720.000	647.844	-182.808	1.7122
2.730.000	649.819	-183.098	1.7120
2.740.000	651.262	-183.299	1.7118
2.750.000	653.182	-183.680	1.7117
2.760.000	654.791	-183.886	1.7115
2.770.000	656.566	-184.195	1.7114
2.780.000	658.265	-184.453	1.7113
2.790.000	660.011	-184.754	1.7112
2.800.000	661.745	-184.936	1.7112
2.810.000	663.320	-185.320	1.7113
2.820.000	665.286	-185.524	1.7115
2.830.000	666.683	-185.788	1.7115
2.840.000	668.670	-186.152	1.7114
2.850.000	670.194	-186.371	1.7113
2.860.000	672.086	-186.675	1.7112
2.870.000	673.643	-186.947	1.7109
2.880.000	675.635	-187.260	1.7106
2.890.000	677.106	-187.446	1.7103
2.900.000	678.934	-187.818	1.7101
2.910.000	680.646	-188.003	1.7098
2.920.000	682.318	-188.309	1.7094
2.930.000	684.107	-188.551	1.7092
2.940.000	685.752	-188.891	1.7091

2.950.000	687.566	-189.056	1.7089
2.960.000	689.085	-189.401	1.7086
2.970.000	691.117	-189.699	1.7085
2.980.000	692.544	-189.928	1.7084
2.990.000	694.591	-190.271	1.7083
3.000.000	696.122	-190.497	1.7081
3.010.000	697.995	-190.811	1.7077
3.020.000	699.550	-191.068	1.7076
3.030.000	701.403	-191.364	1.7075
3.040.000	703.037	-191.554	1.7076
3.050.000	704.708	-191.925	1.7076
3.060.000	706.545	-192.078	1.7075
3.070.000	708.058	-192.418	1.7073
3.080.000	709.947	-192.639	1.7072
3.090.000	711.484	-192.923	1.7070
3.100.000	713.386	-193.197	1.7070
3.110.000	714.859	-193.483	1.7067
3.120.000	716.825	-193.747	1.7063
3.130.000	718.263	-193.987	1.7062
3.140.000	720.168	-194.323	1.7064
3.150.000	721.711	-194.489	1.7064
3.160.000	723.469	-194.813	1.7061
3.170.000	725.138	-195.060	1.7059
3.180.000	726.897	-195.338	1.7060
3.190.000	728.587	-195.529	1.7061
3.200.000	730.233	-195.895	1.7060
3.210.000	732.090	-196.071	1.7058
3.220.000	733.546	-196.368	1.7056
3.230.000	735.560	-196.671	1.7057
3.240.000	736.970	-196.901	1.7057

3.250.000	738.908	-197.192	1.7054
3.260.000	740.429	-197.481	1.7051
3.270.000	742.337	-197.738	1.7048
3.280.000	743.828	-197.955	1.7048
3.290.000	745.702	-198.344	1.7047
3.300.000	747.336	-198.485	1.7045
3.310.000	749.068	-198.815	1.7043
3.320.000	750.818	-199.066	1.7041
3.330.000	752.499	-199.343	1.7042
3.340.000	754.257	-199.553	1.7042
3.350.000	755.798	-199.917	1.7041
3.360.000	757.789	-200.132	1.7038
3.370.000	759.144	-200.415	1.7036
3.380.000	761.126	-200.748	1.7035
3.390.000	762.616	-200.943	1.7032
3.400.000	764.467	-201.268	1.7031
3.410.000	766.084	-201.536	1.7029
3.420.000	767.930	-201.824	1.7027
3.430.000	769.496	-202.029	1.7028
3.440.000	771.342	-202.420	1.7031
3.450.000	773.062	-202.598	1.7032
3.460.000	774.667	-202.933	1.7033
3.470.000	776.527	-203.170	1.7031
3.480.000	778.060	-203.442	1.7032
3.490.000	779.981	-203.707	1.7032
3.500.000	781.442	-204.049	1.7032
3.510.000	783.383	-204.300	1.7030
3.520.000	784.844	-204.540	1.7026
3.530.000	786.829	-204.927	1.7025
3.540.000	788.338	-205.098	1.7024

3.550.000	790.226	-205.447	1.7024
3.560.000	791.824	-205.710	1.7025
3.570.000	793.619	-205.987	1.7026
3.580.000	795.290	-206.200	1.7026
3.590.000	796.974	-206.582	1.7027
3.600.000	798.797	-206.723	1.7028
3.610.000	800.316	-207.082	1.7027
3.620.000	802.283	-207.370	1.7025
3.630.000	803.742	-207.606	1.7021
3.640.000	805.661	-207.933	1.7018
3.650.000	807.174	-208.213	1.7018
3.660.000	809.060	-208.455	1.7018
3.670.000	810.490	-208.706	1.7018
3.680.000	812.415	-209.085	1.7016
3.690.000	814.011	-209.251	1.7015
3.700.000	815.687	-209.596	1.7015
3.710.000	817.465	-209.836	1.7016
3.720.000	819.012	-210.121	1.7015
3.730.000	820.830	-210.324	1.7014
3.740.000	822.445	-210.721	1.7012
3.750.000	824.305	-210.896	1.7012
3.760.000	825.749	-211.206	1.7014
3.770.000	827.765	-211.566	1.7012
3.780.000	829.187	-211.747	1.7010
3.790.000	831.079	-212.084	1.7008
3.800.000	832.613	-212.351	1.7010
3.810.000	834.447	-212.609	1.7010
3.820.000	836.008	-212.851	1.7008
3.830.000	837.799	-213.212	1.7006
3.840.000	839.435	-213.348	1.7005

3.850.000	841.051	-213.717	1.7004
3.860.000	842.905	-213.934	1.7003
3.870.000	844.465	-214.223	1.7001
3.880.000	846.345	-214.483	1.7000
3.890.000	847.845	-214.801	1.7000
3.900.000	849.774	-215.053	1.7002
3.910.000	851.161	-215.331	1.7000
3.920.000	853.158	-215.672	1.6998
3.930.000	854.655	-215.854	1.6996
3.940.000	856.457	-216.210	1.6998
3.950.000	858.118	-216.458	1.6999
3.960.000	859.825	-216.753	1.6998
3.970.000	861.488	-216.962	1.6997
3.980.000	863.193	-217.352	1.6999
3.990.000	865.014	-217.503	1.7003
4.000.000	866.556	-217.872	1.7004
4.010.000	868.533	-218.151	1.7002
4.020.000	869.927	-218.386	1.7003
4.030.000	871.872	-218.727	1.7003
4.040.000	873.372	-219.031	1.7002
4.050.000	875.236	-219.267	1.6998
4.060.000	876.732	-219.550	1.6994
4.070.000	878.654	-219.927	1.6992
4.080.000	880.247	-220.089	1.6988
4.090.000	882.013	-220.450	1.6983
4.100.000	883.708	-220.703	1.6981
4.110.000	885.371	-220.987	1.6980
4.120.000	887.095	-221.211	1.6981
4.130.000	888.754	-221.598	1.6982
4.140.000	890.641	-221.790	1.6983

4.150.000	892.092	-222.113	1.6984
4.160.000	894.072	-222.453	1.6987
4.170.000	895.533	-222.663	1.6989
4.180.000	897.433	-222.996	1.6989
4.190.000	898.991	-223.293	1.6988
4.200.000	900.867	-223.574	1.6989
4.210.000	902.355	-223.796	1.6990
4.220.000	904.227	-224.187	1.6988
4.230.000	905.844	-224.339	1.6983
4.240.000	907.531	-224.699	1.6980
4.250.000	909.315	-224.946	1.6981
4.260.000	910.834	-225.212	1.6980
4.270.000	912.695	-225.466	1.6978
4.280.000	914.186	-225.825	1.6977
4.290.000	916.079	-226.054	1.6979
4.300.000	917.546	-226.352	1.6980
4.310.000	919.488	-226.713	1.6980
4.320.000	920.925	-226.884	1.6978
4.330.000	922.821	-227.245	1.6977
4.340.000	924.398	-227.514	1.6977
4.350.000	926.166	-227.778	1.6979
4.360.000	927.765	-228.030	1.6982
4.370.000	929.511	-228.400	1.6983
4.380.000	931.187	-228.550	1.6982
4.390.000	932.750	-228.916	1.6982
4.400.000	934.680	-229.187	1.6983
4.410.000	936.126	-229.435	1.6982
4.420.000	938.062	-229.776	1.6979
4.430.000	939.522	-230.057	1.6976
4.440.000	941.381	-230.314	1.6976

4.450.000	942.889	-230.591	1.6977
4.460.000	944.831	-230.964	1.6974
4.470.000	946.300	-231.111	1.6974
4.480.000	948.081	-231.493	1.6972
4.490.000	949.788	-231.742	1.6973
4.500.000	951.458	-232.033	1.6975
4.510.000	953.200	-232.276	1.6974
4.520.000	954.853	-232.655	1.6975
4.530.000	956.657	-232.806	1.6976
4.540.000	958.150	-233.173	1.6976
4.550.000	960.079	-233.470	1.6977
4.560.000	961.509	-233.706	1.6977
4.570.000	963.456	-234.043	1.6979
4.580.000	964.984	-234.332	1.6981
4.590.000	966.830	-234.594	1.6983
4.600.000	968.335	-234.865	1.6984
4.610.000	970.195	-235.234	1.6983
4.620.000	971.802	-235.401	1.6982
4.630.000	973.520	-235.778	1.6980
4.640.000	975.306	-236.021	1.6980
4.650.000	976.864	-236.313	1.6977
4.660.000	978.725	-236.585	1.6976
4.670.000	980.304	-236.937	1.6976
4.680.000	982.156	-237.185	1.6976
4.690.000	983.617	-237.482	1.6975
4.700.000	985.627	-237.841	1.6971
4.710.000	987.047	-238.038	1.6969
4.720.000	988.914	-238.405	1.6966
4.730.000	990.538	-238.677	1.6964
4.740.000	992.318	-238.970	1.6962

4.750.000	993.932	-239.217	1.6961
4.760.000	995.716	-239.622	1.6961
4.770.000	997.396	-239.777	1.6963
4.780.000	999.020	-240.170	1.6962
4.790.000	1.000.955	-240.425	1.6962
4.800.000	1.002.391	-240.701	1.6961
4.810.000	1.004.361	-241.025	1.6959
4.820.000	1.005.793	-241.341	1.6958
4.830.000	1.007.671	-241.587	1.6954
4.840.000	1.009.143	-241.893	1.6950
4.850.000	1.011.074	-242.240	1.6947
4.860.000	1.012.487	-242.419	1.6946
4.870.000	1.014.326	-242.789	1.6947
4.880.000	1.015.973	-243.049	1.6947
4.890.000	1.017.662	-243.351	1.6947
4.900.000	1.019.383	-243.633	1.6948
4.910.000	1.021.060	-243.970	1.6948
4.920.000	1.022.765	-244.132	1.6947
4.930.000	1.024.295	-244.516	1.6945
4.940.000	1.026.255	-244.807	1.6943
4.950.000	1.027.623	-245.048	1.6940
4.960.000	1.029.571	-245.408	1.6937
4.970.000	1.031.085	-245.680	1.6936
4.980.000	1.032.929	-245.972	1.6936
4.990.000	1.034.424	-246.242	1.6938
5.000.000	1.036.273	-246.590	1.6941
5.010.000	1.037.814	-246.779	1.6942
5.020.000	1.039.598	-247.164	1.6945
5.030.000	1.041.285	-247.376	1.6945
5.040.000	1.042.871	-247.692	1.6944

5.050.000	1.044.711	-247.938	1.6941
5.060.000	1.046.284	-248.309	1.6939
5.070.000	1.048.149	-248.519	1.6936
5.080.000	1.049.577	-248.849	1.6934
5.090.000	1.051.525	-249.161	1.6931
5.100.000	1.052.925	-249.376	1.6931
5.110.000	1.054.882	-249.748	1.6932
5.120.000	1.056.444	-250.006	1.6931
5.130.000	1.058.215	-250.293	1.6931
5.140.000	1.059.829	-250.587	1.6930
5.150.000	1.061.602	-250.921	1.6929
5.160.000	1.063.234	-251.111	1.6927
5.170.000	1.064.857	-251.497	1.6925
5.180.000	1.066.753	-251.733	1.6923
5.190.000	1.068.222	-252.046	1.6920
5.200.000	1.070.127	-252.350	1.6916
5.210.000	1.071.631	-252.676	1.6914
5.220.000	1.073.508	-252.956	1.6913
5.230.000	1.074.943	-253.264	1.6913
5.240.000	1.076.970	-253.611	1.6911
5.250.000	1.078.406	-253.835	1.6912
5.260.000	1.080.280	-254.219	1.6912
5.270.000	1.081.871	-254.448	1.6913
5.280.000	1.083.569	-254.776	1.6912
5.290.000	1.085.328	-255.049	1.6911
5.300.000	1.087.015	-255.407	1.6911
5.310.000	1.088.733	-255.579	1.6912
5.320.000	1.090.275	-255.983	1.6913
5.330.000	1.092.263	-256.247	1.6913
5.340.000	1.093.662	-256.520	1.6914

5.350.000	1.095.642	-256.879	1.6915
5.360.000	1.097.144	-257.146	1.6916
5.370.000	1.099.007	-257.473	1.6917
5.380.000	1.100.561	-257.766	1.6918
5.390.000	1.102.400	-258.093	1.6917
5.400.000	1.103.959	-258.308	1.6917
5.410.000	1.105.741	-258.703	1.6917
5.420.000	1.107.416	-258.903	1.6919
5.430.000	1.109.010	-259.244	1.6920
5.440.000	1.110.777	-259.497	1.6920
5.450.000	1.112.369	-259.830	1.6920
5.460.000	1.114.164	-260.058	1.6920
5.470.000	1.115.700	-260.428	1.6918
5.480.000	1.117.649	-260.708	1.6916
5.490.000	1.118.998	-260.978	1.6914
5.500.000	1.120.977	-261.318	1.6911
5.510.000	1.122.475	-261.553	1.6908
5.520.000	1.124.221	-261.876	1.6907
5.530.000	1.125.834	-262.138	1.6906
5.540.000	1.127.600	-262.460	1.6906
5.550.000	1.129.178	-262.667	1.6906
5.560.000	1.130.891	-263.046	1.6904
5.570.000	1.132.686	-263.260	1.6903
5.580.000	1.134.166	-263.592	1.6904
5.590.000	1.136.099	-263.868	1.6906
5.600.000	1.137.542	-264.144	1.6906
5.610.000	1.139.446	-264.444	1.6904
5.620.000	1.140.889	-264.757	1.6903
5.630.000	1.142.867	-265.048	1.6903
5.640.000	1.144.260	-265.270	1.6905

5.650.000	1.146.166	-265.665	1.6904
5.660.000	1.147.730	-265.862	1.6905
5.670.000	1.149.472	-266.193	1.6906
5.680.000	1.151.104	-266.456	1.6905
5.690.000	1.152.874	-266.775	1.6903
5.700.000	1.154.584	-266.976	1.6900
5.710.000	1.156.157	-267.372	1.6898
5.720.000	1.158.060	-267.567	1.6896
5.730.000	1.159.454	-267.893	1.6897
5.740.000	1.161.428	-268.238	1.6899
5.750.000	1.162.895	-268.479	1.6901
5.760.000	1.164.730	-268.784	1.6900
5.770.000	1.166.282	-269.082	1.6900
5.780.000	1.168.171	-269.371	1.6897
5.790.000	1.169.623	-269.622	1.6893
5.800.000	1.171.551	-270.034	1.6890
5.810.000	1.173.188	-270.224	1.6887
5.820.000	1.174.819	-270.547	1.6886
5.830.000	1.176.602	-270.820	1.6883
5.840.000	1.178.167	-271.135	1.6880
5.850.000	1.180.054	-271.368	1.6879
5.860.000	1.181.562	-271.740	1.6879
5.870.000	1.183.495	-272.009	1.6876
5.880.000	1.184.922	-272.282	1.6875
5.890.000	1.186.877	-272.672	1.6876
5.900.000	1.188.323	-272.881	1.6878
5.910.000	1.190.246	-273.220	1.6877
5.920.000	1.191.811	-273.515	1.6877
5.930.000	1.193.630	-273.806	1.6875
5.940.000	1.195.209	-274.038	1.6872

5.950.000	1.196.962	-274.429	1.6871
5.960.000	1.198.716	-274.624	1.6869
5.970.000	1.200.337	-274.996	1.6869
5.980.000	1.202.203	-275.247	1.6868
5.990.000	1.203.694	-275.522	1.6864
6.000.000	1.205.530	-275.826	1.6862
6.010.000	1.207.035	-276.150	1.6859
6.020.000	1.208.944	-276.434	1.6855
6.030.000	1.210.384	-276.690	1.6851
6.040.000	1.212.291	-277.056	1.6843
6.050.000	1.213.795	-277.254	1.6839
6.060.000	1.215.541	-277.580	1.6838
6.070.000	1.217.223	-277.869	1.6837
6.080.000	1.218.908	-278.149	1.6839
6.090.000	1.220.556	-278.375	1.6841
6.100.000	1.222.256	-278.778	1.6841
6.110.000	1.224.042	-278.932	1.6842
6.120.000	1.225.481	-279.261	1.6846
6.130.000	1.227.460	-279.605	1.6846
6.140.000	1.228.889	-279.836	1.6844
6.150.000	1.230.807	-280.178	1.6841
6.160.000	1.232.255	-280.459	1.6842
6.170.000	1.234.166	-280.740	1.6842
6.180.000	1.235.651	-281.001	1.6843
6.190.000	1.237.504	-281.388	1.6842
6.200.000	1.239.118	-281.544	1.6841
6.210.000	1.240.790	-281.901	1.6841
6.220.000	1.242.527	-282.151	1.6841
6.230.000	1.244.175	-282.465	1.6843
6.240.000	1.245.918	-282.676	1.6844

6.250.000	1.247.450	-283.047	1.6843
6.260.000	1.249.365	-283.267	1.6845
6.270.000	1.250.747	-283.562	1.6849
6.280.000	1.252.764	-283.915	1.6852
6.290.000	1.254.227	-284.123	1.6853
6.300.000	1.256.089	-284.453	1.6850
6.310.000	1.257.649	-284.728	1.6849
6.320.000	1.259.458	-285.025	1.6849
6.330.000	1.260.975	-285.240	1.6847
6.340.000	1.262.772	-285.633	1.6846
6.350.000	1.264.462	-285.799	1.6843
6.360.000	1.266.060	-286.143	1.6841
6.370.000	1.267.924	-286.415	1.6841
6.380.000	1.269.446	-286.691	1.6840
6.390.000	1.271.349	-286.975	1.6838
6.400.000	1.272.850	-287.329	1.6835
6.410.000	1.274.784	-287.575	1.6833
6.420.000	1.276.183	-287.874	1.6833
6.430.000	1.278.128	-288.237	1.6833
6.440.000	1.279.632	-288.430	1.6833
6.450.000	1.281.448	-288.803	1.6833
6.460.000	1.283.053	-289.075	1.6831
6.470.000	1.284.798	-289.366	1.6832
6.480.000	1.286.522	-289.629	1.6833
6.490.000	1.288.231	-290.001	1.6830
6.500.000	1.289.987	-290.167	1.6827
6.510.000	1.291.553	-290.561	1.6827
6.520.000	1.293.507	-290.845	1.6829
6.530.000	1.294.911	-291.074	1.6831
6.540.000	1.296.808	-291.441	1.6830

6.550.000	1.298.322	-291.757	1.6830
6.560.000	1.300.217	-292.000	1.6833
6.570.000	1.301.706	-292.287	1.6834
6.580.000	1.303.629	-292.670	1.6833
6.590.000	1.305.169	-292.845	1.6829
6.600.000	1.306.930	-293.220	1.6828
6.610.000	1.308.629	-293.460	1.6827
6.620.000	1.310.264	-293.762	1.6827
6.630.000	1.312.028	-294.004	1.6826
6.640.000	1.313.609	-294.377	1.6822
6.650.000	1.315.434	-294.561	1.6821
6.660.000	1.316.831	-294.894	1.6821
6.670.000	1.318.807	-295.250	1.6823
6.680.000	1.320.196	-295.424	1.6823
6.690.000	1.322.105	-295.790	1.6827
6.700.000	1.323.659	-296.062	1.6831
6.710.000	1.325.484	-296.327	1.6835
6.720.000	1.327.041	-296.577	1.6840
6.730.000	1.328.863	-296.942	1.6842
6.740.000	1.330.476	-297.093	1.6842
6.750.000	1.332.123	-297.474	1.6839
6.760.000	1.333.910	-297.680	1.6837
6.770.000	1.335.459	-297.958	1.6836
6.780.000	1.337.287	-298.214	1.6832
6.790.000	1.338.819	-298.534	1.6827
6.800.000	1.340.661	-298.761	1.6824
6.810.000	1.342.081	-299.051	1.6825
6.820.000	1.344.089	-299.369	1.6826
6.830.000	1.345.552	-299.571	1.6827
6.840.000	1.347.411	-299.908	1.6826

6.850.000	1.349.001	-300.153	1.6824
6.860.000	1.350.719	-300.422	1.6824
6.870.000	1.352.373	-300.657	1.6824
6.880.000	1.354.076	-301.006	1.6821
6.890.000	1.355.772	-301.149	1.6817
6.900.000	1.357.365	-301.521	1.6813
6.910.000	1.359.268	-301.759	1.6813
6.920.000	1.360.703	-301.984	1.6815
6.930.000	1.362.649	-302.299	1.6815
6.940.000	1.364.128	-302.584	1.6814
6.950.000	1.366.001	-302.817	1.6812
6.960.000	1.367.524	-303.087	1.6811
6.970.000	1.369.417	-303.427	1.6810
6.980.000	1.370.944	-303.593	1.6810
6.990.000	1.372.730	-303.952	1.6808
7.000.000	1.374.387	-304.175	1.6807
7.010.000	1.376.083	-304.431	1.6808
7.020.000	1.377.806	-304.675	1.6808
7.030.000	1.379.456	-305.041	1.6808
7.040.000	1.381.249	-305.193	1.6809
7.050.000	1.382.812	-305.546	1.6810
7.060.000	1.384.753	-305.853	1.6810
7.070.000	1.386.170	-306.076	1.6809
7.080.000	1.388.104	-306.392	1.6809
7.090.000	1.389.642	-306.647	1.6810
7.100.000	1.391.418	-306.905	1.6810
7.110.000	1.392.982	-307.150	1.6810
7.120.000	1.394.848	-307.507	1.6809
7.130.000	1.396.399	-307.657	1.6811
7.140.000	1.398.160	-308.018	1.6812

7.150.000	1.399.905	-308.272	1.6811
7.160.000	1.401.507	-308.543	1.6810
7.170.000	1.403.374	-308.790	1.6810
7.180.000	1.404.925	-309.121	1.6811
7.190.000	1.406.764	-309.325	1.6812
7.200.000	1.408.232	-309.629	1.6811
7.210.000	1.410.126	-309.937	1.6810
7.220.000	1.411.566	-310.122	1.6808
7.230.000	1.413.463	-310.476	1.6806
7.240.000	1.415.009	-310.712	1.6803
7.250.000	1.416.798	-310.983	1.6799
7.260.000	1.418.374	-311.248	1.6795
7.270.000	1.420.205	-311.581	1.6793
7.280.000	1.421.844	-311.724	1.6792
7.290.000	1.423.499	-312.097	1.6789
7.300.000	1.425.335	-312.309	1.6785
7.310.000	1.426.833	-312.574	1.6781
7.320.000	1.428.719	-312.878	1.6780
7.330.000	1.430.209	-313.155	1.6780
7.340.000	1.432.020	-313.408	1.6783
7.350.000	1.433.519	-313.677	1.6785
7.360.000	1.435.446	-313.991	1.6788
7.370.000	1.436.904	-314.160	1.6790
7.380.000	1.438.758	-314.515	1.6791
7.390.000	1.440.391	-314.733	1.6789
7.400.000	1.442.083	-314.998	1.6788
7.410.000	1.443.810	-315.238	1.6785
7.420.000	1.445.452	-315.580	1.6783
7.430.000	1.447.205	-315.707	1.6784
7.440.000	1.448.771	-316.065	1.6781

7.450.000	1.450.652	-316.299	1.6780
7.460.000	1.452.088	-316.518	1.6779
7.470.000	1.453.970	-316.825	1.6779
7.480.000	1.455.480	-317.089	1.6779
7.490.000	1.457.321	-317.342	1.6778
7.500.000	1.458.908	-317.610	1.6779
7.510.000	1.460.801	-317.909	1.6780
7.520.000	1.462.319	-318.087	1.6781
7.530.000	1.464.071	-318.419	1.6780
7.540.000	1.465.793	-318.604	1.6780
7.550.000	1.467.408	-318.882	1.6781
7.560.000	1.469.168	-319.109	1.6781
7.570.000	1.470.753	-319.417	1.6780
7.580.000	1.472.613	-319.645	1.6778
7.590.000	1.474.100	-319.936	1.6777
7.600.000	1.476.080	-320.224	1.6777
7.610.000	1.477.476	-320.440	1.6777
7.620.000	1.479.425	-320.747	1.6779
7.630.000	1.480.965	-320.965	1.6782
7.640.000	1.482.729	-321.259	1.6783
7.650.000	1.484.376	-321.488	1.6783
7.660.000	1.486.167	-321.814	1.6783
7.670.000	1.487.727	-321.969	1.6781
7.680.000	1.489.482	-322.333	1.6778
7.690.000	1.491.238	-322.519	1.6774
7.700.000	1.492.781	-322.812	1.6770
7.710.000	1.494.699	-323.084	1.6767
7.720.000	1.496.240	-323.352	1.6764
7.730.000	1.498.090	-323.612	1.6762
7.740.000	1.499.602	-323.905	1.6760

7.750.000	1.501.523	-324.156	1.6760
7.760.000	1.502.988	-324.368	1.6762
7.770.000	1.504.841	-324.717	1.6765
7.780.000	1.506.461	-324.899	1.6768
7.790.000	1.508.115	-325.191	1.6767
7.800.000	1.509.764	-325.410	1.6767
7.810.000	1.511.516	-325.726	1.6766
7.820.000	1.513.175	-325.892	1.6765
7.830.000	1.514.804	-326.261	1.6765
7.840.000	1.516.711	-326.446	1.6764
7.850.000	1.518.132	-326.720	1.6762
7.860.000	1.520.088	-327.044	1.6761
7.870.000	1.521.544	-327.271	1.6759
7.880.000	1.523.366	-327.545	1.6757
7.890.000	1.524.934	-327.833	1.6754
7.900.000	1.526.774	-328.087	1.6755
7.910.000	1.528.246	-328.289	1.6755
7.920.000	1.530.050	-328.641	1.6754
7.930.000	1.531.729	-328.850	1.6749
7.940.000	1.533.373	-329.137	1.6752
7.950.000	1.535.176	-329.380	1.6758
7.960.000	1.536.774	-329.674	1.6747
7.970.000	1.538.568	-329.871	1.6742
7.980.000	1.540.076	-330.207	1.6733
7.990.000	1.541.998	-330.435	1.6729
8.000.000	1.543.410	-330.686	1.6723
8.010.000	1.545.328	-331.020	1.6704
8.020.000	1.546.824	-331.212	1.6701
8.030.000	1.548.624	-331.500	1.6699
8.040.000	1.550.213	-331.748	1.6699

8.050.000	1.552.027	-332.040	1.6705
8.060.000	1.553.654	-332.243	1.6709
8.070.000	1.555.407	-332.608	1.6712
8.080.000	1.557.168	-332.742	1.6714
8.090.000	1.558.707	-333.062	1.6716
8.100.000	1.560.556	-333.288	1.6716
8.110.000	1.562.063	-333.551	1.6716
8.120.000	1.563.919	-333.804	1.6714
8.130.000	1.565.367	-334.102	1.6713
8.140.000	1.567.312	-334.345	1.6712
8.150.000	1.568.767	-334.571	1.6710
8.160.000	1.570.688	-334.927	1.6708
8.170.000	1.572.234	-335.083	1.6707
8.180.000	1.574.023	-335.412	1.6705
8.190.000	1.575.679	-335.652	1.6706
8.200.000	1.577.384	-335.923	1.6705
8.210.000	1.579.052	-336.100	1.6706
8.220.000	1.580.735	-336.483	1.6707
8.230.000	1.582.519	-336.618	1.6706
8.240.000	1.584.012	-336.930	1.6705
8.250.000	1.585.966	-337.221	1.6703
8.260.000	1.587.389	-337.425	1.6701
8.270.000	1.589.322	-337.713	1.6698
8.280.000	1.590.811	-337.983	1.6695
8.290.000	1.592.713	-338.201	1.6694
8.300.000	1.594.187	-338.438	1.6694
8.310.000	1.596.077	-338.777	1.6694
8.320.000	1.597.680	-338.928	1.6693
8.330.000	1.599.352	-339.248	1.6692
8.340.000	1.601.077	-339.459	1.6689

8.350.000	1.602.719	-339.734	1.6688
8.360.000	1.604.468	-339.930	1.6685
8.370.000	1.606.009	-340.272	1.6683
8.380.000	1.607.907	-340.471	1.6682
8.390.000	1.609.314	-340.753	1.6681
8.400.000	1.611.311	-341.077	1.6681
8.410.000	1.612.758	-341.250	1.6681
8.420.000	1.614.613	-341.558	1.6680
8.430.000	1.616.186	-341.825	1.6681
8.440.000	1.617.967	-342.091	1.6680
8.450.000	1.619.518	-342.288	1.6679
8.460.000	1.621.307	-342.637	1.6678
8.470.000	1.622.940	-342.777	1.6677
8.480.000	1.624.583	-343.113	1.6677
8.490.000	1.626.384	-343.328	1.6675
8.500.000	1.627.963	-343.601	1.6675
8.510.000	1.629.829	-343.851	1.6676
8.520.000	1.631.330	-344.180	1.6675
8.530.000	1.633.239	-344.381	1.6675
8.540.000	1.634.649	-344.631	1.6675
8.550.000	1.636.589	-344.974	1.6674
8.560.000	1.638.053	-345.131	1.6674
8.570.000	1.639.853	-345.442	1.6674
8.580.000	1.641.458	-345.695	1.6671
8.590.000	1.643.245	-345.964	1.6669
8.600.000	1.644.872	-346.183	1.6667
8.610.000	1.646.563	-346.548	1.6665
8.620.000	1.648.376	-346.688	1.6662
8.630.000	1.649.873	-347.010	1.6657
8.640.000	1.651.796	-347.281	1.6654

8.650.000	1.653.248	-347.506	1.6651
8.660.000	1.655.109	-347.802	1.6649
8.670.000	1.656.641	-348.082	1.6648
8.680.000	1.658.478	-348.321	1.6648
8.690.000	1.659.958	-348.550	1.6649
8.700.000	1.661.839	-348.906	1.6650
8.710.000	1.663.381	-349.067	1.6652
8.720.000	1.665.193	-349.410	1.6652
8.730.000	1.666.916	-349.663	1.6651
8.740.000	1.668.554	-349.917	1.6649
8.750.000	1.670.300	-350.154	1.6646
8.760.000	1.671.942	-350.533	1.6642
8.770.000	1.673.722	-350.679	1.6639
8.780.000	1.675.192	-350.990	1.6636
8.790.000	1.677.168	-351.325	1.6635
8.800.000	1.678.600	-351.506	1.6633
8.810.000	1.680.470	-351.832	1.6629
8.820.000	1.682.001	-352.107	1.6626
8.830.000	1.683.856	-352.367	1.6625
8.840.000	1.685.436	-352.630	1.6626
8.850.000	1.687.243	-352.973	1.6628
8.860.000	1.688.885	-353.118	1.6628
8.870.000	1.690.555	-353.476	1.6627
8.880.000	1.692.378	-353.697	1.6625
8.890.000	1.693.887	-353.973	1.6624
8.900.000	1.695.732	-354.217	1.6621
8.910.000	1.697.285	-354.553	1.6616
8.920.000	1.699.163	-354.772	1.6613
8.930.000	1.700.584	-355.060	1.6612
8.940.000	1.702.599	-355.388	1.6612

8.950.000	1.704.012	-355.565	1.6611
8.960.000	1.705.902	-355.915	1.6611
8.970.000	1.707.492	-356.159	1.6611
8.980.000	1.709.207	-356.408	1.6610
8.990.000	1.710.836	-356.666	1.6608
9.000.000	1.712.608	-357.013	1.6605
9.010.000	1.714.262	-357.145	1.6604
9.020.000	1.715.815	-357.498	1.6603
9.030.000	1.717.682	-357.761	1.6602
9.040.000	1.719.168	-357.978	1.6600
9.050.000	1.721.039	-358.285	1.6599
9.060.000	1.722.547	-358.595	1.6598
9.070.000	1.724.482	-358.818	1.6598
9.080.000	1.725.897	-359.072	1.6598
9.090.000	1.727.794	-359.409	1.6596
9.100.000	1.729.316	-359.576	1.6593
9.110.000	1.731.077	-359.943	1.6591
9.120.000	1.732.729	-360.153	1.6588
9.130.000	1.734.384	-360.421	1.6587
9.140.000	1.736.112	-360.658	1.6584
9.150.000	1.737.764	-361.014	1.6581
9.160.000	1.739.565	-361.169	1.6577
9.170.000	1.741.050	-361.502	1.6576
9.180.000	1.743.054	-361.804	1.6577
9.190.000	1.744.463	-362.023	1.6575
9.200.000	1.746.324	-362.320	1.6573
9.210.000	1.747.860	-362.599	1.6572
9.220.000	1.749.661	-362.848	1.6572
9.230.000	1.751.230	-363.107	1.6572
9.240.000	1.753.047	-363.462	1.6572

9.250.000	1.754.612	-363.619	1.6570
9.260.000	1.756.339	-363.962	1.6566
9.270.000	1.758.090	-364.213	1.6564
9.280.000	1.759.672	-364.466	1.6563
9.290.000	1.761.497	-364.705	1.6562
9.300.000	1.763.046	-365.034	1.6560
9.310.000	1.764.906	-365.236	1.6558
9.320.000	1.766.370	-365.540	1.6556
9.330.000	1.768.313	-365.840	1.6555
9.340.000	1.769.754	-366.028	1.6555
9.350.000	1.771.604	-366.391	1.6552
9.360.000	1.773.179	-366.601	1.6551
9.370.000	1.774.924	-366.874	1.6552
9.380.000	1.776.549	-367.135	1.6552
9.390.000	1.778.363	-367.459	1.6552
9.400.000	1.780.023	-367.626	1.6553
9.410.000	1.781.674	-367.999	1.6553
9.420.000	1.783.545	-368.213	1.6555
9.430.000	1.784.992	-368.489	1.6553
9.440.000	1.786.920	-368.777	1.6553
9.450.000	1.788.437	-369.078	1.6552
9.460.000	1.790.271	-369.326	1.6551
9.470.000	1.791.796	-369.622	1.6549
9.480.000	1.793.730	-369.941	1.6548
9.490.000	1.795.181	-370.142	1.6547
9.500.000	1.797.053	-370.498	1.6546
9.510.000	1.798.707	-370.760	1.6544
9.520.000	1.800.407	-371.013	1.6543
9.530.000	1.802.108	-371.271	1.6543
9.540.000	1.803.799	-371.625	1.6542

9.550.000	1.805.567	-371.765	1.6540
9.560.000	1.807.067	-372.129	1.6538
9.570.000	1.808.960	-372.387	1.6537
9.580.000	1.810.411	-372.615	1.6536
9.590.000	1.812.297	-372.962	1.6536
9.600.000	1.813.782	-373.213	1.6535
9.610.000	1.815.649	-373.465	1.6533
9.620.000	1.817.151	-373.738	1.6532
9.630.000	1.819.025	-374.050	1.6533
9.640.000	1.820.545	-374.245	1.6533
9.650.000	1.822.278	-374.601	1.6530
9.660.000	1.823.994	-374.812	1.6528
9.670.000	1.825.592	-375.103	1.6525
9.680.000	1.827.412	-375.338	1.6523
9.690.000	1.828.997	-375.674	1.6520
9.700.000	1.830.777	-375.884	1.6518
9.710.000	1.832.265	-376.186	1.6517
9.720.000	1.834.233	-376.495	1.6515
9.730.000	1.835.605	-376.699	1.6515
9.740.000	1.837.539	-377.054	1.6514
9.750.000	1.839.093	-377.269	1.6513
9.760.000	1.840.842	-377.560	1.6511
9.770.000	1.842.440	-377.814	1.6509
9.780.000	1.844.220	-378.118	1.6507
9.790.000	1.845.823	-378.306	1.6505
9.800.000	1.847.479	-378.649	1.6504
9.810.000	1.849.276	-378.834	1.6501
9.820.000	1.850.784	-379.137	1.6497
9.830.000	1.852.706	-379.406	1.6495
9.840.000	1.854.200	-379.678	1.6494

9.850.000	1.856.065	-379.953	1.6492
9.860.000	1.857.571	-380.239	1.6491
9.870.000	1.859.500	-380.524	1.6490
9.880.000	1.860.954	-380.745	1.6490
9.890.000	1.862.798	-381.097	1.6489
9.900.000	1.864.415	-381.304	1.6487
9.910.000	1.866.128	-381.623	1.6485
9.920.000	1.867.840	-381.871	1.6483
9.930.000	1.869.542	-382.190	1.6483
9.940.000	1.871.215	-382.370	1.6483
9.950.000	1.872.862	-382.741	1.6483
9.960.000	1.874.704	-382.961	1.6481
9.970.000	1.876.113	-383.247	1.6480
9.980.000	1.878.128	-383.571	1.6477
9.990.000	1.879.605	-383.819	1.6475
10.000.000	1.881.429	-384.103	1.6473
10.010.000	1.882.980	-384.375	1.6471
10.020.000	1.884.854	-384.683	1.6468
10.030.000	1.886.369	-384.882	1.6464
10.040.000	1.888.155	-385.262	1.6461
10.050.000	1.889.844	-385.466	1.6461
10.060.000	1.891.514	-385.757	1.6457
10.070.000	1.893.285	-386.020	1.6453
10.080.000	1.894.902	-386.337	1.6455
10.090.000	1.896.706	-386.540	1.6457
10.100.000	1.898.263	-386.907	1.6455
10.110.000	1.900.141	-387.177	1.6451
10.120.000	1.901.558	-387.413	1.6446
10.130.000	1.903.524	-387.760	1.6443
10.140.000	1.905.013	-387.962	1.6438

10.150.000	1.906.800	-388.265	1.6435
10.160.000	1.908.339	-388.513	1.6435
10.170.000	1.910.199	-388.829	1.6437
10.180.000	1.911.778	-389.023	1.6438
10.190.000	1.913.498	-389.383	1.6434
10.200.000	1.915.252	-389.559	1.6432
10.210.000	1.916.796	-389.878	1.6430
10.220.000	1.918.654	-390.122	1.6425
10.230.000	1.920.160	-390.395	1.6422
10.240.000	1.921.967	-390.654	1.6418
10.250.000	1.923.469	-390.950	1.6416
10.260.000	1.925.377	-391.214	1.6412
10.270.000	1.926.793	-391.430	1.6408
10.280.000	1.928.697	-391.772	1.6404
10.290.000	1.930.251	-391.979	1.6402
10.300.000	1.931.987	-392.260	1.6400
10.310.000	1.933.656	-392.524	1.6400
10.320.000	1.935.357	-392.792	1.6400
10.330.000	1.937.030	-393.000	1.6399
10.340.000	1.938.704	-393.362	1.6395
10.350.000	1.940.502	-393.539	1.6389
10.360.000	1.942.021	-393.834	1.6384
10.370.000	1.943.903	-394.145	1.6379
10.380.000	1.945.325	-394.361	1.6377
10.390.000	1.947.180	-394.644	1.6374
10.400.000	1.948.687	-394.920	1.6373
10.410.000	1.950.569	-395.191	1.6371
10.420.000	1.952.063	-395.402	1.6369
10.430.000	1.953.924	-395.758	1.6363
10.440.000	1.955.567	-395.927	1.6360

10.450.000	1.957.171	-396.245	1.6356
10.460.000	1.958.929	-396.457	1.6353
10.470.000	1.960.546	-396.732	1.6353
10.480.000	1.962.281	-396.930	1.6353
10.490.000	1.963.858	-397.259	1.6352
10.500.000	1.965.771	-397.482	1.6353
10.510.000	1.967.156	-397.754	1.6348
10.520.000	1.969.121	-398.054	1.6345
10.530.000	1.970.612	-398.271	1.6341
10.540.000	1.972.414	-398.549	1.6340
10.550.000	1.974.002	-398.807	1.6338
10.560.000	1.975.786	-399.072	1.6333
10.570.000	1.977.363	-399.273	1.6331
10.580.000	1.979.114	-399.612	1.6328
10.590.000	1.980.804	-399.757	1.6326
10.600.000	1.982.410	-400.099	1.6320
10.610.000	1.984.265	-400.337	1.6315
10.620.000	1.985.766	-400.573	1.6312
10.630.000	1.987.625	-400.839	1.6310
10.640.000	1.989.128	-401.158	1.6307
10.650.000	1.991.086	-401.396	1.6303
10.660.000	1.992.492	-401.627	1.6298
10.670.000	1.994.367	-401.960	1.6294
10.680.000	1.995.935	-402.141	1.6291
10.690.000	1.997.695	-402.460	1.6288
10.700.000	1.999.298	-402.688	1.6286
10.710.000	2.001.059	-402.976	1.6285
10.720.000	2.002.714	-403.177	1.6283
10.730.000	2.004.408	-403.534	1.6282
10.740.000	2.006.189	-403.682	1.6281

10.750.000	2.007.677	-403.986	1.6278
10.760.000	2.009.618	-404.275	1.6274
10.770.000	2.011.062	-404.500	1.6271
10.780.000	2.012.881	-404.771	1.6268
10.790.000	2.014.436	-405.056	1.6263
10.800.000	2.016.252	-405.290	1.6259
10.810.000	2.017.761	-405.521	1.6255
10.820.000	2.019.645	-405.862	1.6253
10.830.000	2.021.147	-406.019	1.6251
10.840.000	2.022.887	-406.324	1.6248
10.850.000	2.024.587	-406.574	1.6245
10.860.000	2.026.232	-406.826	1.6242
10.870.000	2.027.995	-407.028	1.6239
10.880.000	2.029.552	-407.374	1.6237
10.890.000	2.031.430	-407.534	1.6235
10.900.000	2.032.841	-407.825	1.6232
10.910.000	2.034.769	-408.132	1.6229
10.920.000	2.036.218	-408.306	1.6229
10.930.000	2.038.070	-408.622	1.6228
10.940.000	2.039.641	-408.877	1.6225
10.950.000	2.041.457	-409.105	1.6220
10.960.000	2.043.006	-409.342	1.6215
10.970.000	2.044.753	-409.669	1.6210
10.980.000	2.046.459	-409.800	1.6204
10.990.000	2.048.065	-410.143	1.6199
11.000.000	2.049.900	-410.361	1.6195
11.010.000	2.051.426	-410.589	1.6191
11.020.000	2.053.211	-410.832	1.6186
11.030.000	2.054.791	-411.135	1.6181
11.040.000	2.056.608	-411.341	1.6178

11.050.000	2.058.067	-411.588	1.6174
11.060.000	2.060.020	-411.908	1.6170
11.070.000	2.061.473	-412.057	1.6167
11.080.000	2.063.292	-412.369	1.6166
11.090.000	2.064.906	-412.620	1.6166
11.100.000	2.066.658	-412.855	1.6163
11.110.000	2.068.299	-413.070	1.6161
11.120.000	2.070.004	-413.420	1.6159
11.130.000	2.071.766	-413.534	1.6154
11.140.000	2.073.292	-413.844	1.6150
11.150.000	2.075.185	-414.083	1.6146
11.160.000	2.076.642	-414.283	1.6142
11.170.000	2.078.525	-414.552	1.6138
11.180.000	2.080.026	-414.822	1.6134
11.190.000	2.081.916	-415.014	1.6130
11.200.000	2.083.363	-415.246	1.6124
11.210.000	2.085.281	-415.572	1.6118
11.220.000	2.086.827	-415.725	1.6115
11.230.000	2.088.592	-416.029	1.6113
11.240.000	2.090.260	-416.247	1.6111
11.250.000	2.091.936	-416.500	1.6109
11.260.000	2.093.669	-416.709	1.6108
11.270.000	2.095.326	-417.038	1.6106
11.280.000	2.097.148	-417.203	1.6103
11.290.000	2.098.592	-417.484	1.6099
11.300.000	2.100.573	-417.782	1.6096
11.310.000	2.101.990	-417.943	1.6092
11.320.000	2.103.888	-418.232	1.6087
11.330.000	2.105.432	-418.494	1.6084
11.340.000	2.107.238	-418.708	1.6080

11.350.000	2.108.773	-418.912	1.6077
11.360.000	2.110.576	-419.241	1.6074
11.370.000	2.112.176	-419.371	1.6068
11.380.000	2.113.849	-419.678	1.6063
11.390.000	2.115.605	-419.888	1.6059
11.400.000	2.117.140	-420.121	1.6056
11.410.000	2.118.953	-420.337	1.6053
11.420.000	2.120.480	-420.641	1.6051
11.430.000	2.122.364	-420.835	1.6047
11.440.000	2.123.796	-421.075	1.6042
11.450.000	2.125.735	-421.360	1.6036
11.460.000	2.127.198	-421.495	1.6032
11.470.000	2.129.023	-421.812	1.6030
11.480.000	2.130.609	-422.017	1.6026
11.490.000	2.132.353	-422.245	1.6020
11.500.000	2.133.988	-422.482	1.6019
11.510.000	2.135.730	-422.776	1.6022
11.520.000	2.137.367	-422.870	1.6019
11.530.000	2.138.962	-423.198	1.6014
11.540.000	2.140.807	-423.414	1.6011
11.550.000	2.142.339	-423.644	1.6009
11.560.000	2.144.193	-423.893	1.6006
11.570.000	2.145.675	-424.152	1.6001
11.580.000	2.147.526	-424.327	1.5998
11.590.000	2.149.004	-424.561	1.5995
11.600.000	2.150.909	-424.842	1.5990
11.610.000	2.152.409	-424.956	1.5986
11.620.000	2.154.192	-425.258	1.5984
11.630.000	2.155.849	-425.448	1.5982
11.640.000	2.157.511	-425.630	1.5978

11.650.000	2.159.210	-425.833	1.5975
11.660.000	2.160.884	-426.157	1.5974
11.670.000	2.162.648	-426.248	1.5973
11.680.000	2.164.173	-426.546	1.5972
11.690.000	2.166.073	-426.787	1.5970
11.700.000	2.167.521	-426.953	1.5968
11.710.000	2.169.419	-427.250	1.5966
11.720.000	2.170.909	-427.454	1.5962
11.730.000	2.172.763	-427.650	1.5958
11.740.000	2.174.261	-427.850	1.5955
11.750.000	2.176.140	-428.156	1.5952
11.760.000	2.177.696	-428.262	1.5948
11.770.000	2.179.391	-428.587	1.5945
11.780.000	2.181.176	-428.780	1.5943
11.790.000	2.182.762	-429.018	1.5942
11.800.000	2.184.540	-429.199	1.5939
11.810.000	2.186.082	-429.499	1.5935
11.820.000	2.187.938	-429.666	1.5933
11.830.000	2.189.395	-429.895	1.5929
11.840.000	2.191.313	-430.163	1.5928
11.850.000	2.192.739	-430.292	1.5926
11.860.000	2.194.628	-430.575	1.5926
11.870.000	2.196.196	-430.774	1.5925
11.880.000	2.197.958	-430.958	1.5922
11.890.000	2.199.558	-431.149	1.5919
11.900.000	2.201.305	-431.429	1.5912
11.910.000	2.202.964	-431.509	1.5908
11.920.000	2.204.569	-431.809	1.5901
11.930.000	2.206.412	-431.982	1.5896
11.940.000	2.207.900	-432.181	1.5893

11.950.000	2.209.774	-432.423	1.5890
11.960.000	2.211.243	-432.637	1.5890
11.970.000	2.213.091	-432.820	1.5887
11.980.000	2.214.534	-433.029	1.5883
11.990.000	2.216.452	-433.292	1.5878
12.000.000	2.217.935	-433.414	1.5874
12.010.000	2.219.757	-433.709	1.5871
12.020.000	2.221.370	-433.893	1.5868
12.030.000	2.223.032	-434.102	1.5866
12.040.000	2.224.726	-434.287	1.5864
12.050.000	2.226.385	-434.575	1.5862
12.060.000	2.228.073	-434.667	1.5858
12.070.000	2.229.644	-434.957	1.5855
12.080.000	2.231.549	-435.178	1.5851
12.090.000	2.232.944	-435.352	1.5848
12.100.000	2.234.880	-435.637	1.5845
12.110.000	2.236.400	-435.852	1.5842
12.120.000	2.238.217	-436.072	1.5840
12.130.000	2.239.742	-436.276	1.5837
12.140.000	2.241.608	-436.579	1.5835
12.150.000	2.243.089	-436.688	1.5833
12.160.000	2.244.880	-436.991	1.5832
12.170.000	2.246.546	-437.157	1.5832
12.180.000	2.248.139	-437.380	1.5830
12.190.000	2.249.891	-437.550	1.5827
12.200.000	2.251.452	-437.823	1.5824
12.210.000	2.253.246	-437.959	1.5820
12.220.000	2.254.758	-438.227	1.5818
12.230.000	2.256.669	-438.446	1.5817
12.240.000	2.258.107	-438.594	1.5817

12.250.000	2.259.965	-438.846	1.5815
12.260.000	2.261.490	-439.041	1.5812
12.270.000	2.263.286	-439.244	1.5808
12.280.000	2.264.853	-439.432	1.5805
12.290.000	2.266.633	-439.717	1.5802
12.300.000	2.268.226	-439.834	1.5797
12.310.000	2.269.902	-440.129	1.5794
12.320.000	2.271.706	-440.274	1.5795
12.330.000	2.273.240	-440.510	1.5794
12.340.000	2.275.108	-440.706	1.5791
12.350.000	2.276.642	-440.973	1.5786
12.360.000	2.278.494	-441.174	1.5779
12.370.000	2.279.974	-441.424	1.5775
12.380.000	2.281.891	-441.703	1.5770
12.390.000	2.283.333	-441.848	1.5768
12.400.000	2.285.213	-442.170	1.5767
12.410.000	2.286.785	-442.374	1.5767
12.420.000	2.288.516	-442.622	1.5766
12.430.000	2.290.148	-442.830	1.5761
12.440.000	2.291.867	-443.117	1.5755
12.450.000	2.293.587	-443.250	1.5746
12.460.000	2.295.220	-443.594	1.5736
12.470.000	2.297.075	-443.776	1.5730
12.480.000	2.298.562	-444.018	1.5726
12.490.000	2.300.457	-444.290	1.5724
12.500.000	2.301.961	-444.516	1.5723
12.510.000	2.303.802	-444.763	1.5723
12.520.000	2.305.317	-444.994	1.5721
12.530.000	2.307.189	-445.279	1.5716
12.540.000	2.308.665	-445.455	1.5710

12.550.000	2.310.473	-445.770	1.5703
12.560.000	2.312.180	-445.977	1.5697
12.570.000	2.313.759	-446.219	1.5693
12.580.000	2.315.547	-446.411	1.5691
12.590.000	2.317.174	-446.726	1.5692
12.600.000	2.318.908	-446.866	1.5691
12.610.000	2.320.468	-447.164	1.5690
12.620.000	2.322.371	-447.423	1.5687
12.630.000	2.323.753	-447.598	1.5682
12.640.000	2.325.698	-447.892	1.5676
12.650.000	2.327.180	-448.110	1.5670
12.660.000	2.328.980	-448.338	1.5665
12.670.000	2.330.572	-448.566	1.5663
12.680.000	2.332.382	-448.839	1.5662
12.690.000	2.333.970	-448.983	1.5663
12.700.000	2.335.675	-449.329	1.5664
12.710.000	2.337.451	-449.467	1.5662
12.720.000	2.338.983	-449.729	1.5660
12.730.000	2.340.840	-449.948	1.5655
12.740.000	2.342.366	-450.207	1.5649
12.750.000	2.344.190	-450.431	1.5642
12.760.000	2.345.678	-450.697	1.5637
12.770.000	2.347.635	-450.943	1.5633
12.780.000	2.349.055	-451.134	1.5632
12.790.000	2.350.890	-451.431	1.5633
12.800.000	2.352.510	-451.628	1.5635
12.810.000	2.354.228	-451.896	1.5634
12.820.000	2.355.891	-452.077	1.5632
12.830.000	2.357.608	-452.353	1.5630
12.840.000	2.359.263	-452.495	1.5627

12.850.000	2.360.902	-452.815	1.5623
12.860.000	2.362.748	-452.980	1.5620
12.870.000	2.364.179	-453.200	1.5618
12.880.000	2.366.107	-453.477	1.5616
12.890.000	2.367.568	-453.699	1.5612
12.900.000	2.369.440	-453.888	1.5606
12.910.000	2.370.940	-454.128	1.5602
12.920.000	2.372.812	-454.376	1.5598
12.930.000	2.374.332	-454.524	1.5595
12.940.000	2.376.128	-454.830	1.5594
12.950.000	2.377.765	-454.969	1.5596
12.960.000	2.379.423	-455.235	1.5598
12.970.000	2.381.150	-455.426	1.5599
12.980.000	2.382.791	-455.683	1.5598
12.990.000	2.384.528	-455.826	1.5596
13.000.000	2.386.068	-456.137	1.5591
13.010.000	2.388.020	-456.327	1.5589
13.020.000	2.389.452	-456.554	1.5588
13.030.000	2.391.365	-456.824	1.5587
13.040.000	2.392.904	-457.005	1.5585
13.050.000	2.394.695	-457.264	1.5582
13.060.000	2.396.286	-457.481	1.5579
13.070.000	2.398.082	-457.737	1.5577
13.080.000	2.399.649	-457.890	1.5573
13.090.000	2.401.389	-458.183	1.5570
13.100.000	2.403.099	-458.345	1.5568
13.110.000	2.404.688	-458.603	1.5566
13.120.000	2.406.508	-458.801	1.5562
13.130.000	2.408.060	-459.056	1.5557
13.140.000	2.409.890	-459.254	1.5551

13.150.000	2.411.415	-459.519	1.5546
13.160.000	2.413.305	-459.744	1.5540
13.170.000	2.414.711	-459.937	1.5534
13.180.000	2.416.574	-460.239	1.5528
13.190.000	2.418.127	-460.400	1.5524
13.200.000	2.419.888	-460.675	1.5521
13.210.000	2.421.490	-460.879	1.5516
13.220.000	2.423.271	-461.131	1.5511
13.230.000	2.424.879	-461.305	1.5506
13.240.000	2.426.558	-461.610	1.5504
13.250.000	2.428.368	-461.737	1.5502
13.260.000	2.429.854	-462.009	1.5500
13.270.000	2.431.735	-462.267	1.5497
13.280.000	2.433.246	-462.466	1.5494
13.290.000	2.435.060	-462.711	1.5493
13.300.000	2.436.597	-462.941	1.5492
13.310.000	2.438.478	-463.166	1.5491
13.320.000	2.439.898	-463.354	1.5490
13.330.000	2.441.762	-463.653	1.5489
13.340.000	2.443.338	-463.813	1.5488
13.350.000	2.445.060	-464.078	1.5486
13.360.000	2.446.794	-464.281	1.5482
13.370.000	2.448.475	-464.550	1.5479
13.380.000	2.450.160	-464.688	1.5475
13.390.000	2.451.743	-465.012	1.5471
13.400.000	2.453.622	-465.193	1.5467
13.410.000	2.455.048	-465.401	1.5464
13.420.000	2.456.948	-465.689	1.5460
13.430.000	2.458.411	-465.853	1.5455
13.440.000	2.460.288	-466.115	1.5452

13.450.000	2.461.808	-466.320	1.5448
13.460.000	2.463.649	-466.549	1.5444
13.470.000	2.465.199	-466.720	1.5441
13.480.000	2.466.967	-467.047	1.5439
13.490.000	2.468.619	-467.168	1.5438
13.500.000	2.470.269	-467.440	1.5437
13.510.000	2.472.015	-467.630	1.5436
13.520.000	2.473.610	-467.878	1.5434
13.530.000	2.475.398	-468.071	1.5432
13.540.000	2.476.863	-468.336	1.5430
13.550.000	2.478.789	-468.555	1.5427
13.560.000	2.480.217	-468.778	1.5425
13.570.000	2.482.118	-469.042	1.5421
13.580.000	2.483.662	-469.212	1.5417
13.590.000	2.485.464	-469.483	1.5414
13.600.000	2.487.099	-469.685	1.5414
13.610.000	2.488.818	-469.920	1.5414
13.620.000	2.490.440	-470.088	1.5412
13.630.000	2.492.136	-470.409	1.5408
13.640.000	2.493.896	-470.541	1.5404
13.650.000	2.495.387	-470.801	1.5400
13.660.000	2.497.264	-471.030	1.5397
13.670.000	2.498.778	-471.232	1.5393
13.680.000	2.500.650	-471.479	1.5388
13.690.000	2.502.162	-471.719	1.5382
13.700.000	2.504.043	-471.923	1.5376
13.710.000	2.505.494	-472.117	1.5374
13.720.000	2.507.356	-472.427	1.5371
13.730.000	2.508.913	-472.545	1.5368
13.740.000	2.510.631	-472.846	1.5365

13.750.000	2.512.336	-473.052	1.5363
13.760.000	2.513.981	-473.287	1.5362
13.770.000	2.515.661	-473.456	1.5362
13.780.000	2.517.273	-473.776	1.5358
13.790.000	2.519.110	-473.922	1.5353
13.800.000	2.520.575	-474.191	1.5348
13.810.000	2.522.472	-474.463	1.5346
13.820.000	2.523.965	-474.635	1.5343
13.830.000	2.525.789	-474.896	1.5340
13.840.000	2.527.330	-475.124	1.5334
13.850.000	2.529.155	-475.349	1.5331
13.860.000	2.530.640	-475.517	1.5329
13.870.000	2.532.448	-475.832	1.5327
13.880.000	2.534.091	-475.973	1.5325
13.890.000	2.535.729	-476.239	1.5324
13.900.000	2.537.483	-476.440	1.5322
13.910.000	2.539.123	-476.690	1.5320
13.920.000	2.540.909	-476.848	1.5318
13.930.000	2.542.423	-477.158	1.5315
13.940.000	2.544.294	-477.355	1.5311
13.950.000	2.545.705	-477.558	1.5308
13.960.000	2.547.625	-477.858	1.5305
13.970.000	2.549.122	-478.008	1.5303
13.980.000	2.550.895	-478.265	1.5301
13.990.000	2.552.460	-478.485	1.5300
14.000.000	2.554.226	-478.705	1.5298
14.010.000	2.555.833	-478.862	1.5294
14.020.000	2.557.551	-479.173	1.5292
14.030.000	2.559.301	-479.279	1.5293
14.040.000	2.560.847	-479.554	1.5293

14.050.000	2.562.705	-479.755	1.5293
14.060.000	2.564.212	-479.959	1.5292
14.070.000	2.566.023	-480.204	1.5290
14.080.000	2.567.526	-480.443	1.5287
14.090.000	2.569.393	-480.626	1.5282
14.100.000	2.570.826	-480.829	1.5280
14.110.000	2.572.731	-481.130	1.5277
14.120.000	2.574.232	-481.237	1.5276
14.130.000	2.576.015	-481.527	1.5275
14.140.000	2.577.721	-481.734	1.5273
14.150.000	2.579.364	-481.953	1.5273
14.160.000	2.581.056	-482.120	1.5271
14.170.000	2.582.712	-482.434	1.5269
14.180.000	2.584.485	-482.535	1.5267
14.190.000	2.585.959	-482.822	1.5264
14.200.000	2.587.892	-483.097	1.5262
14.210.000	2.589.300	-483.251	1.5260
14.220.000	2.591.163	-483.523	1.5256
14.230.000	2.592.694	-483.779	1.5253
14.240.000	2.594.545	-483.964	1.5251
14.250.000	2.596.089	-484.184	1.5247
14.260.000	2.597.932	-484.488	1.5246
14.270.000	2.599.523	-484.601	1.5244
14.280.000	2.601.207	-484.889	1.5242
14.290.000	2.602.946	-485.101	1.5240
14.300.000	2.604.570	-485.315	1.5239
14.310.000	2.606.298	-485.510	1.5236
14.320.000	2.607.859	-485.807	1.5233
14.330.000	2.609.714	-485.975	1.5229
14.340.000	2.611.118	-486.226	1.5225

14.350.000	2.613.086	-486.509	1.5223
14.360.000	2.614.558	-486.669	1.5220
14.370.000	2.616.343	-486.943	1.5219
14.380.000	2.617.922	-487.172	1.5218
14.390.000	2.619.672	-487.428	1.5217
14.400.000	2.621.203	-487.576	1.5215
14.410.000	2.622.983	-487.896	1.5210
14.420.000	2.624.625	-488.008	1.5206
14.430.000	2.626.197	-488.282	1.5202
14.440.000	2.628.036	-488.495	1.5197
14.450.000	2.629.508	-488.718	1.5194
14.460.000	2.631.372	-488.941	1.5190
14.470.000	2.632.860	-489.201	1.5186
14.480.000	2.634.723	-489.383	1.5182
14.490.000	2.636.173	-489.589	1.5180
14.500.000	2.638.033	-489.876	1.5178
14.510.000	2.639.539	-489.977	1.5178
14.520.000	2.641.301	-490.265	1.5178
14.530.000	2.642.900	-490.439	1.5178
14.540.000	2.644.608	-490.631	1.5177
14.550.000	2.646.263	-490.803	1.5173
14.560.000	2.647.914	-491.091	1.5170
14.570.000	2.649.668	-491.173	1.5165
14.580.000	2.651.197	-491.461	1.5161
14.590.000	2.653.087	-491.693	1.5160
14.600.000	2.654.534	-491.847	1.5158
14.610.000	2.656.401	-492.115	1.5155
14.620.000	2.657.925	-492.348	1.5152
14.630.000	2.659.706	-492.509	1.5149
14.640.000	2.661.186	-492.720	1.5145

14.650.000	2.663.047	-493.015	1.5142
14.660.000	2.664.561	-493.126	1.5141
14.670.000	2.666.246	-493.404	1.5143
14.680.000	2.667.983	-493.594	1.5145
14.690.000	2.669.580	-493.804	1.5146
14.700.000	2.671.378	-493.977	1.5148
14.710.000	2.672.957	-494.270	1.5148
14.720.000	2.674.798	-494.415	1.5146
14.730.000	2.676.218	-494.660	1.5141
14.740.000	2.678.146	-494.929	1.5139
14.750.000	2.679.582	-495.057	1.5137
14.760.000	2.681.406	-495.336	1.5133
14.770.000	2.682.924	-495.541	1.5131
14.780.000	2.684.735	-495.752	1.5131
14.790.000	2.686.262	-495.923	1.5132
14.800.000	2.688.036	-496.217	1.5131
14.810.000	2.689.716	-496.331	1.5126
14.820.000	2.691.325	-496.613	1.5121
14.830.000	2.693.092	-496.779	1.5117
14.840.000	2.694.608	-496.997	1.5111
14.850.000	2.696.420	-497.214	1.5105
14.860.000	2.697.946	-497.456	1.5103
14.870.000	2.699.801	-497.658	1.5101
14.880.000	2.701.232	-497.861	1.5100
14.890.000	2.703.141	-498.136	1.5097
14.900.000	2.704.595	-498.247	1.5094
14.910.000	2.706.348	-498.533	1.5092
14.920.000	2.708.005	-498.736	1.5089
14.930.000	2.709.723	-498.946	1.5086
14.940.000	2.711.364	-499.117	1.5085

14.950.000	2.713.026	-499.409	1.5085
14.960.000	2.714.703	-499.502	1.5087
14.970.000	2.716.252	-499.789	1.5086
14.980.000	2.718.111	-500.006	1.5083
14.990.000	2.719.523	-500.178	1.5080
15.000.000	2.721.370	-500.435	1.5076
15.010.000	2.722.846	-500.663	1.5072
15.020.000	2.724.719	-500.844	1.5070
15.030.000	2.726.195	-501.066	1.5068
15.040.000	2.728.042	-501.342	1.5065
15.050.000	2.729.562	-501.441	1.5061
15.060.000	2.731.291	-501.723	1.5057
15.070.000	2.732.897	-501.909	1.5053
15.080.000	2.734.529	-502.103	1.5048
15.090.000	2.736.250	-502.289	1.5042
15.100.000	2.737.838	-502.590	1.5038
15.110.000	2.739.604	-502.701	1.5031
15.120.000	2.741.064	-502.946	1.5025
15.130.000	2.742.922	-503.198	1.5020
15.140.000	2.744.357	-503.351	1.5016
15.150.000	2.746.221	-503.608	1.5012
15.160.000	2.747.747	-503.805	1.5009
15.170.000	2.749.524	-504.012	1.5007
15.180.000	2.751.052	-504.179	1.5005
15.190.000	2.752.827	-504.470	1.5000
15.200.000	2.754.408	-504.567	1.4995
15.210.000	2.756.069	-504.862	1.4993
15.220.000	2.757.825	-505.030	1.4992
15.230.000	2.759.335	-505.235	1.4993
15.240.000	2.761.146	-505.444	1.4994

15.250.000	2.762.665	-505.725	1.4996
15.260.000	2.764.527	-505.885	1.4997
15.270.000	2.765.995	-506.121	1.4995
15.280.000	2.767.870	-506.382	1.4992
15.290.000	2.769.356	-506.531	1.4989
15.300.000	2.771.171	-506.810	1.4987
15.310.000	2.772.727	-507.010	1.4986
15.320.000	2.774.438	-507.243	1.4985
15.330.000	2.776.045	-507.436	1.4982
15.340.000	2.777.752	-507.729	1.4978
15.350.000	2.779.458	-507.844	1.4972
15.360.000	2.780.987	-508.145	1.4968
15.370.000	2.782.883	-508.362	1.4964
15.380.000	2.784.353	-508.559	1.4962
15.390.000	2.786.232	-508.833	1.4959
15.400.000	2.787.694	-509.066	1.4957
15.410.000	2.789.532	-509.277	1.4954
15.420.000	2.791.005	-509.495	1.4948
15.430.000	2.792.882	-509.797	1.4943
15.440.000	2.794.382	-509.926	1.4939
15.450.000	2.796.142	-510.223	1.4936
15.460.000	2.797.826	-510.432	1.4933
15.470.000	2.799.468	-510.653	1.4932
15.480.000	2.801.209	-510.842	1.4931
15.490.000	2.802.832	-511.175	1.4932
15.500.000	2.804.580	-511.270	1.4931
15.510.000	2.806.042	-511.558	1.4928
15.520.000	2.807.956	-511.833	1.4927
15.530.000	2.809.358	-511.985	1.4926
15.540.000	2.811.236	-512.268	1.4924

15.550.000	2.812.718	-512.493	1.4921
15.560.000	2.814.529	-512.691	1.4919
15.570.000	2.816.063	-512.914	1.4915
15.580.000	2.817.862	-513.229	1.4913
15.590.000	2.819.457	-513.338	1.4914
15.600.000	2.821.156	-513.646	1.4914
15.610.000	2.822.905	-513.837	1.4912
15.620.000	2.824.455	-514.073	1.4909
15.630.000	2.826.241	-514.297	1.4905
15.640.000	2.827.785	-514.576	1.4901
15.650.000	2.829.584	-514.752	1.4896
15.660.000	2.831.053	-515.005	1.4890
15.670.000	2.832.975	-515.278	1.4884
15.680.000	2.834.395	-515.434	1.4880
15.690.000	2.836.242	-515.717	1.4877
15.700.000	2.837.767	-515.920	1.4874
15.710.000	2.839.541	-516.156	1.4871
15.720.000	2.841.165	-516.352	1.4868
15.730.000	2.842.879	-516.656	1.4866
15.740.000	2.844.540	-516.746	1.4863
15.750.000	2.846.144	-517.064	1.4858
15.760.000	2.847.949	-517.253	1.4853
15.770.000	2.849.414	-517.448	1.4848
15.780.000	2.851.258	-517.700	1.4844
15.790.000	2.852.743	-517.938	1.4837
15.800.000	2.854.592	-518.135	1.4832
15.810.000	2.856.076	-518.362	1.4828
15.820.000	2.857.953	-518.629	1.4825
15.830.000	2.859.479	-518.750	1.4821
15.840.000	2.861.227	-519.053	1.4820

15.850.000	2.862.871	-519.229	1.4818
15.860.000	2.864.550	-519.441	1.4817
15.870.000	2.866.184	-519.624	1.4816
15.880.000	2.867.845	-519.925	1.4816
15.890.000	2.869.582	-520.022	1.4818
15.900.000	2.871.090	-520.300	1.4817
15.910.000	2.873.007	-520.542	1.4817
15.920.000	2.874.416	-520.721	1.4817
15.930.000	2.876.308	-520.975	1.4815
15.940.000	2.877.828	-521.192	1.4813
15.950.000	2.879.619	-521.393	1.4809
15.960.000	2.881.169	-521.605	1.4805
15.970.000	2.882.978	-521.901	1.4800
15.980.000	2.884.529	-522.015	1.4797
15.990.000	2.886.256	-522.304	1.4794
16.000.000	2.887.928	-522.513	1.4789
16.010.000	2.889.502	-522.738	1.4785
16.020.000	2.891.326	-522.931	1.4780
16.030.000	2.892.875	-523.217	1.4774
16.040.000	2.894.706	-523.390	1.4769
16.050.000	2.896.197	-523.652	1.4766
16.060.000	2.898.112	-523.912	1.4764
16.070.000	2.899.544	-524.053	1.4763
16.080.000	2.901.399	-524.361	1.4761
16.090.000	2.902.934	-524.554	1.4760
16.100.000	2.904.669	-524.777	1.4758
16.110.000	2.906.233	-524.982	1.4755
16.120.000	2.908.010	-525.270	1.4754
16.130.000	2.909.606	-525.390	1.4754
16.140.000	2.911.237	-525.698	1.4752

16.150.000	2.913.034	-525.878	1.4750
16.160.000	2.914.494	-526.090	1.4746
16.170.000	2.916.373	-526.328	1.4742
16.180.000	2.917.860	-526.585	1.4737
16.190.000	2.919.643	-526.771	1.4732
16.200.000	2.921.125	-527.006	1.4728
16.210.000	2.923.047	-527.294	1.4724
16.220.000	2.924.478	-527.423	1.4721
16.230.000	2.926.270	-527.744	1.4718
16.240.000	2.927.877	-527.944	1.4716
16.250.000	2.929.533	-528.164	1.4714
16.260.000	2.931.217	-528.365	1.4711
16.270.000	2.932.842	-528.656	1.4708
16.280.000	2.934.585	-528.763	1.4704
16.290.000	2.936.142	-529.075	1.4700
16.300.000	2.938.025	-529.310	1.4696
16.310.000	2.939.445	-529.484	1.4693
16.320.000	2.941.285	-529.767	1.4691
16.330.000	2.942.766	-529.999	1.4687
16.340.000	2.944.616	-530.217	1.4684
16.350.000	2.946.097	-530.437	1.4681
16.360.000	2.947.928	-530.730	1.4679
16.370.000	2.949.466	-530.865	1.4678
16.380.000	2.951.211	-531.188	1.4675
16.390.000	2.952.895	-531.366	1.4670
16.400.000	2.954.449	-531.596	1.4666
16.410.000	2.956.202	-531.801	1.4662
16.420.000	2.957.791	-532.092	1.4657
16.430.000	2.959.595	-532.250	1.4653
16.440.000	2.961.058	-532.506	1.4649

16.450.000	2.962.931	-532.790	1.4644
16.460.000	2.964.364	-532.930	1.4641
16.470.000	2.966.248	-533.221	1.4639
16.480.000	2.967.759	-533.433	1.4638
16.490.000	2.969.500	-533.662	1.4636
16.500.000	2.971.130	-533.879	1.4634
16.510.000	2.972.884	-534.178	1.4630
16.520.000	2.974.479	-534.292	1.4626
16.530.000	2.976.140	-534.613	1.4621
16.540.000	2.977.915	-534.797	1.4615
16.550.000	2.979.414	-535.035	1.4612
16.560.000	2.981.264	-535.273	1.4610
16.570.000	2.982.767	-535.530	1.4607
16.580.000	2.984.604	-535.740	1.4604
16.590.000	2.986.067	-535.982	1.4602
16.600.000	2.987.965	-536.257	1.4602
16.610.000	2.989.395	-536.415	1.4601
16.620.000	2.991.244	-536.733	1.4599
16.630.000	2.992.857	-536.929	1.4596
16.640.000	2.994.542	-537.160	1.4594
16.650.000	2.996.176	-537.359	1.4593
16.660.000	2.997.891	-537.672	1.4590
16.670.000	2.999.569	-537.776	1.4587
16.680.000	3.001.114	-538.096	1.4583
16.690.000	3.003.027	-538.311	1.4580
16.700.000	3.004.437	-538.512	1.4579
16.710.000	3.006.301	-538.780	1.4576
16.720.000	3.007.801	-539.016	1.4571
16.730.000	3.009.599	-539.219	1.4568
16.740.000	3.011.113	-539.450	1.4564

16.750.000	3.012.969	-539.737	1.4560
16.760.000	3.014.470	-539.854	1.4555
16.770.000	3.016.210	-540.159	1.4554
16.780.000	3.017.873	-540.326	1.4551
16.790.000	3.019.459	-540.555	1.4548
16.800.000	3.021.195	-540.734	1.4545
16.810.000	3.022.791	-541.030	1.4540
16.820.000	3.024.555	-541.141	1.4536
16.830.000	3.026.044	-541.423	1.4530
16.840.000	3.027.929	-541.652	1.4525
16.850.000	3.029.342	-541.807	1.4523
16.860.000	3.031.199	-542.087	1.4520
16.870.000	3.032.741	-542.257	1.4516
16.880.000	3.034.510	-542.473	1.4512
16.890.000	3.036.027	-542.658	1.4507
16.900.000	3.037.822	-542.936	1.4503
16.910.000	3.039.388	-543.055	1.4499
16.920.000	3.041.079	-543.336	1.4492
16.930.000	3.042.810	-543.496	1.4488
16.940.000	3.044.304	-543.720	1.4484
16.950.000	3.046.163	-543.941	1.4481
16.960.000	3.047.675	-544.193	1.4479
16.970.000	3.049.476	-544.392	1.4476
16.980.000	3.050.956	-544.627	1.4474
16.990.000	3.052.881	-544.885	1.4472
17.000.000	3.054.269	-545.025	1.4470
17.010.000	3.056.126	-545.300	1.4466
17.020.000	3.057.677	-545.510	1.4461
17.030.000	3.059.362	-545.706	1.4457
17.040.000	3.061.023	-545.907	1.4454

17.050.000	3.062.687	-546.161	1.4451
17.060.000	3.064.371	-546.279	1.4450
17.070.000	3.065.988	-546.563	1.4450
17.080.000	3.067.802	-546.754	1.4451
17.090.000	3.069.242	-546.949	1.4451
17.100.000	3.071.133	-547.217	1.4448
17.110.000	3.072.590	-547.423	1.4444
17.120.000	3.074.403	-547.620	1.4438
17.130.000	3.075.900	-547.851	1.4432
17.140.000	3.077.776	-548.104	1.4426
17.150.000	3.079.226	-548.207	1.4420
17.160.000	3.080.972	-548.520	1.4414
17.170.000	3.082.662	-548.671	1.4411
17.180.000	3.084.277	-548.912	1.4409
17.190.000	3.085.992	-549.078	1.4404
17.200.000	3.087.641	-549.365	1.4397
17.210.000	3.089.342	-549.477	1.4391
17.220.000	3.090.837	-549.753	1.4385
17.230.000	3.092.730	-549.990	1.4379
17.240.000	3.094.153	-550.149	1.4375
17.250.000	3.096.029	-550.424	1.4372
17.260.000	3.097.561	-550.653	1.4368
17.270.000	3.099.303	-550.843	1.4365
17.280.000	3.100.862	-551.049	1.4361
17.290.000	3.102.662	-551.340	1.4356
17.300.000	3.104.227	-551.455	1.4353
17.310.000	3.105.925	-551.766	1.4348
17.320.000	3.107.643	-551.927	1.4347
17.330.000	3.109.185	-552.167	1.4347
17.340.000	3.110.956	-552.366	1.4346

17.350.000	3.112.479	-552.630	1.4344
17.360.000	3.114.321	-552.824	1.4339
17.370.000	3.115.752	-553.058	1.4333
17.380.000	3.117.636	-553.318	1.4328
17.390.000	3.119.075	-553.467	1.4323
17.400.000	3.120.922	-553.754	1.4319
17.410.000	3.122.497	-553.941	1.4316
17.420.000	3.124.223	-554.182	1.4314
17.430.000	3.125.814	-554.375	1.4311
17.440.000	3.127.559	-554.640	1.4308
17.450.000	3.129.137	-554.752	1.4304
17.460.000	3.130.822	-555.050	1.4299
17.470.000	3.132.603	-555.226	1.4295
17.480.000	3.134.032	-555.439	1.4293
17.490.000	3.135.902	-555.675	1.4292
17.500.000	3.137.411	-555.921	1.4291
17.510.000	3.139.233	-556.135	1.4287
17.520.000	3.140.722	-556.364	1.4283
17.530.000	3.142.568	-556.624	1.4278
17.540.000	3.144.047	-556.761	1.4270
17.550.000	3.145.822	-557.058	1.4264
17.560.000	3.147.441	-557.240	1.4259
17.570.000	3.149.093	-557.458	1.4255
17.580.000	3.150.783	-557.654	1.4253
17.590.000	3.152.400	-557.913	1.4251
17.600.000	3.154.106	-558.018	1.4248
17.610.000	3.155.582	-558.301	1.4243
17.620.000	3.157.473	-558.507	1.4236
17.630.000	3.158.913	-558.681	1.4229
17.640.000	3.160.779	-558.933	1.4222

17.650.000	3.162.258	-559.106	1.4217
17.660.000	3.164.032	-559.303	1.4213
17.670.000	3.165.533	-559.488	1.4208
17.680.000	3.167.358	-559.745	1.4206
17.690.000	3.168.895	-559.860	1.4202
17.700.000	3.170.603	-560.134	1.4198
17.710.000	3.172.319	-560.277	1.4192
17.720.000	3.173.870	-560.505	1.4185
17.730.000	3.175.642	-560.656	1.4180
17.740.000	3.177.202	-560.922	1.4176
17.750.000	3.178.977	-561.079	1.4174
17.760.000	3.180.478	-561.326	1.4171
17.770.000	3.182.342	-561.578	1.4168
17.780.000	3.183.736	-561.699	1.4166
17.790.000	3.185.617	-561.980	1.4161
17.800.000	3.187.117	-562.162	1.4155
17.810.000	3.188.864	-562.362	1.4147
17.820.000	3.190.453	-562.558	1.4140
17.830.000	3.192.154	-562.817	1.4135
17.840.000	3.193.782	-562.919	1.4129
17.850.000	3.195.415	-563.244	1.4126
17.860.000	3.197.234	-563.375	1.4124
17.870.000	3.198.728	-563.604	1.4120
17.880.000	3.200.542	-563.820	1.4115
17.890.000	3.202.062	-564.038	1.4107
17.900.000	3.203.881	-564.247	1.4099
17.910.000	3.205.330	-564.451	1.4091
17.920.000	3.207.202	-564.703	1.4085
17.930.000	3.208.638	-564.851	1.4080
17.940.000	3.210.403	-565.116	1.4074

17.950.000	3.212.010	-565.291	1.4072
17.960.000	3.213.657	-565.509	1.4068
17.970.000	3.215.353	-565.679	1.4064
17.980.000	3.217.000	-565.959	1.4059
17.990.000	3.218.680	-566.062	1.4053
18.000.000	3.220.244	-566.358	1.4048
18.010.000	3.222.113	-566.577	1.4042
18.020.000	3.223.511	-566.745	1.4037
18.030.000	3.225.381	-567.018	1.4033
18.040.000	3.226.848	-567.217	1.4030
18.050.000	3.228.625	-567.418	1.4028
18.060.000	3.230.123	-567.630	1.4024
18.070.000	3.231.949	-567.896	1.4021
18.080.000	3.233.505	-568.037	1.4018
18.090.000	3.235.216	-568.335	1.4014
18.100.000	3.236.859	-568.485	1.4010
18.110.000	3.238.473	-568.716	1.4007
18.120.000	3.240.227	-568.911	1.4003
18.130.000	3.241.811	-569.184	1.3999
18.140.000	3.243.605	-569.347	1.3994
18.150.000	3.245.058	-569.606	1.3988
18.160.000	3.246.969	-569.856	1.3982
18.170.000	3.248.313	-570.023	1.3975
18.180.000	3.250.175	-570.292	1.3967
18.190.000	3.251.755	-570.479	1.3961
18.200.000	3.253.494	-570.733	1.3955
18.210.000	3.255.034	-570.914	1.3950
18.220.000	3.256.799	-571.197	1.3945
18.230.000	3.258.365	-571.334	1.3943
18.240.000	3.260.057	-571.624	1.3942

18.250.000	3.261.818	-571.787	1.3939
18.260.000	3.263.333	-572.020	1.3936
18.270.000	3.265.152	-572.252	1.3933
18.280.000	3.266.623	-572.506	1.3928
18.290.000	3.268.446	-572.716	1.3923
18.300.000	3.269.917	-572.962	1.3917
18.310.000	3.271.800	-573.216	1.3911
18.320.000	3.273.230	-573.360	1.3906
18.330.000	3.275.071	-573.671	1.3900
18.340.000	3.276.632	-573.844	1.3895
18.350.000	3.278.339	-574.090	1.3890
18.360.000	3.279.969	-574.295	1.3885
18.370.000	3.281.661	-574.564	1.3879
18.380.000	3.283.320	-574.680	1.3875
18.390.000	3.284.905	-574.985	1.3871
18.400.000	3.286.727	-575.177	1.3868
18.410.000	3.288.143	-575.372	1.3864
18.420.000	3.290.062	-575.639	1.3857
18.430.000	3.291.545	-575.840	1.3853
18.440.000	3.293.305	-576.036	1.3847
18.450.000	3.294.824	-576.254	1.3842
18.460.000	3.296.694	-576.521	1.3838
18.470.000	3.298.158	-576.649	1.3833
18.480.000	3.299.931	-576.939	1.3827
18.490.000	3.301.586	-577.113	1.3821
18.500.000	3.303.198	-577.332	1.3815
18.510.000	3.304.929	-577.522	1.3810
18.520.000	3.306.491	-577.804	1.3802
18.530.000	3.308.277	-577.939	1.3794
18.540.000	3.309.776	-578.218	1.3788

18.550.000	3.311.680	-578.454	1.3782
18.560.000	3.313.056	-578.621	1.3776
18.570.000	3.314.919	-578.890	1.3768
18.580.000	3.316.450	-579.083	1.3761
18.590.000	3.318.191	-579.286	1.3754
18.600.000	3.319.691	-579.489	1.3748
18.610.000	3.321.496	-579.752	1.3743
18.620.000	3.323.070	-579.861	1.3736
18.630.000	3.324.735	-580.153	1.3728
18.640.000	3.326.500	-580.298	1.3722
18.650.000	3.327.999	-580.520	1.3717
18.660.000	3.329.791	-580.707	1.3713
18.670.000	3.331.317	-580.943	1.3708
18.680.000	3.333.119	-581.123	1.3702
18.690.000	3.334.569	-581.347	1.3697
18.700.000	3.336.454	-581.592	1.3693
18.710.000	3.337.910	-581.714	1.3689
18.720.000	3.339.656	-581.983	1.3683
18.730.000	3.341.239	-582.164	1.3679
18.740.000	3.342.933	-582.376	1.3673
18.750.000	3.344.572	-582.577	1.3667
18.760.000	3.346.283	-582.818	1.3663
18.770.000	3.347.930	-582.931	1.3658
18.780.000	3.349.522	-583.242	1.3654
18.790.000	3.351.333	-583.395	1.3650
18.800.000	3.352.748	-583.591	1.3647
18.810.000	3.354.616	-583.845	1.3642
18.820.000	3.356.087	-584.057	1.3636
18.830.000	3.357.912	-584.263	1.3629
18.840.000	3.359.397	-584.467	1.3622

18.850.000	3.361.215	-584.731	1.3615
18.860.000	3.362.709	-584.869	1.3607
18.870.000	3.364.508	-585.171	1.3600
18.880.000	3.366.125	-585.321	1.3594
18.890.000	3.367.764	-585.546	1.3587
18.900.000	3.369.450	-585.748	1.3581
18.910.000	3.371.090	-586.020	1.3574
18.920.000	3.372.812	-586.152	1.3565
18.930.000	3.374.332	-586.433	1.3556
18.940.000	3.376.226	-586.659	1.3549
18.950.000	3.377.615	-586.859	1.3542
18.960.000	3.379.448	-587.091	1.3536
18.970.000	3.380.948	-587.304	1.3530
18.980.000	3.382.743	-587.532	1.3526
18.990.000	3.384.311	-587.745	1.3523
19.000.000	3.386.107	-588.029	1.3521
19.010.000	3.387.630	-588.138	1.3515
19.020.000	3.389.307	-588.440	1.3508
19.030.000	3.391.037	-588.617	1.3501
19.040.000	3.392.586	-588.844	1.3493
19.050.000	3.394.366	-589.032	1.3484
19.060.000	3.395.904	-589.292	1.3477
19.070.000	3.397.701	-589.477	1.3472
19.080.000	3.399.167	-589.721	1.3465
19.090.000	3.401.067	-589.972	1.3456
19.100.000	3.402.511	-590.131	1.3448
19.110.000	3.404.304	-590.418	1.3438
19.120.000	3.405.879	-590.605	1.3428
19.130.000	3.407.590	-590.833	1.3421
19.140.000	3.409.172	-591.030	1.3417

19.150.000	3.410.902	-591.301	1.3415
19.160.000	3.412.496	-591.415	1.3414
19.170.000	3.414.082	-591.736	1.3412
19.180.000	3.415.898	-591.897	1.3409
19.190.000	3.417.352	-592.124	1.3405
19.200.000	3.419.173	-592.374	1.3399
19.210.000	3.420.697	-592.601	1.3392
19.220.000	3.422.487	-592.810	1.3387
19.230.000	3.423.974	-593.030	1.3382
19.240.000	3.425.835	-593.291	1.3378
19.250.000	3.427.266	-593.430	1.3374
19.260.000	3.429.078	-593.731	1.3368
19.270.000	3.430.658	-593.916	1.3360
19.280.000	3.432.281	-594.143	1.3352
19.290.000	3.433.998	-594.343	1.3342
19.300.000	3.435.603	-594.644	1.3333
19.310.000	3.437.305	-594.762	1.3325
19.320.000	3.438.868	-595.070	1.3318
19.330.000	3.440.725	-595.273	1.3312
19.340.000	3.442.144	-595.470	1.3307
19.350.000	3.444.025	-595.745	1.3300
19.360.000	3.445.497	-595.932	1.3291
19.370.000	3.447.259	-596.146	1.3282
19.380.000	3.448.778	-596.374	1.3273
19.390.000	3.450.542	-596.629	1.3265
19.400.000	3.452.090	-596.760	1.3257
19.410.000	3.453.777	-597.055	1.3251
19.420.000	3.455.478	-597.221	1.3244
19.430.000	3.457.025	-597.444	1.3238
19.440.000	3.458.780	-597.622	1.3232

19.450.000	3.460.347	-597.897	1.3224
19.460.000	3.462.112	-598.064	1.3217
19.470.000	3.463.574	-598.291	1.3209
19.480.000	3.465.450	-598.527	1.3202
19.490.000	3.466.859	-598.679	1.3197
19.500.000	3.468.710	-598.951	1.3191
19.510.000	3.470.236	-599.145	1.3187
19.520.000	3.471.973	-599.354	1.3181
19.530.000	3.473.568	-599.544	1.3176
19.540.000	3.475.304	-599.820	1.3170
19.550.000	3.476.903	-599.920	1.3163
19.560.000	3.478.500	-600.221	1.3155
19.570.000	3.480.295	-600.388	1.3149
19.580.000	3.481.777	-600.593	1.3142
19.590.000	3.483.606	-600.835	1.3136
19.600.000	3.485.070	-601.063	1.3130
19.610.000	3.486.871	-601.280	1.3126
19.620.000	3.488.350	-601.515	1.3122
19.630.000	3.490.211	-601.778	1.3117
19.640.000	3.491.663	-601.918	1.3112
19.650.000	3.493.439	-602.217	1.3107
19.660.000	3.495.056	-602.393	1.3104
19.670.000	3.496.747	-602.640	1.3103
19.680.000	3.498.374	-602.816	1.3102
19.690.000	3.500.037	-603.095	1.3102
19.700.000	3.501.690	-603.219	1.3101
19.710.000	3.503.230	-603.510	1.3098
19.720.000	3.505.081	-603.713	1.3092
19.730.000	3.506.463	-603.901	1.3083
19.740.000	3.508.324	-604.150	1.3072

19.750.000	3.509.791	-604.367	1.3063
19.760.000	3.511.536	-604.564	1.3055
19.770.000	3.513.103	-604.788	1.3052
19.780.000	3.514.947	-605.067	1.3051
19.790.000	3.516.400	-605.188	1.3048
19.800.000	3.518.150	-605.484	1.3046
19.810.000	3.519.793	-605.661	1.3041
19.820.000	3.521.390	-605.881	1.3035
19.830.000	3.523.141	-606.081	1.3028
19.840.000	3.524.664	-606.355	1.3020
19.850.000	3.526.428	-606.495	1.3015
19.860.000	3.527.875	-606.762	1.3011
19.870.000	3.529.769	-607.012	1.3008
19.880.000	3.531.202	-607.175	1.3005
19.890.000	3.533.053	-607.476	1.3001
19.900.000	3.534.587	-607.653	1.2994
19.910.000	3.536.314	-607.889	1.2985
19.920.000	3.537.854	-608.091	1.2973
19.930.000	3.539.615	-608.382	1.2961
19.940.000	3.541.152	-608.494	1.2949
19.950.000	3.542.797	-608.809	1.2939
19.960.000	3.544.573	-608.978	1.2932
19.970.000	3.546.034	-609.207	1.2928
19.980.000	3.547.884	-609.418	1.2925
19.990.000	3.549.384	-609.682	1.2922
20.000.000	3.551.171	-609.888	1.2917
20.010.000	3.552.691	-610.119	1.2911
20.020.000	3.554.548	-610.397	1.2903
20.030.000	3.555.996	-610.546	1.2893
20.040.000	3.557.778	-610.860	1.2883

20.050.000	3.559.338	-611.065	1.2875
20.060.000	3.561.015	-611.287	1.2869
20.070.000	3.562.652	-611.498	1.2864
20.080.000	3.564.292	-611.792	1.2858
20.090.000	3.566.018	-611.913	1.2851
20.100.000	3.567.532	-612.223	1.2840
20.110.000	3.569.392	-612.438	1.2828
20.120.000	3.570.826	-612.644	1.2815
20.130.000	3.572.692	-612.922	1.2804
20.140.000	3.574.175	-613.140	1.2794
20.150.000	3.575.937	-613.350	1.2785
20.160.000	3.577.428	-613.566	1.2780
20.170.000	3.579.246	-613.841	1.2772
20.180.000	3.580.718	-613.976	1.2760
20.190.000	3.582.454	-614.286	1.2743
20.200.000	3.584.156	-614.486	1.2726
20.210.000	3.585.717	-614.724	1.2708
20.220.000	3.587.438	-614.919	1.2693
20.230.000	3.589.073	-615.213	1.2681
20.240.000	3.590.796	-615.370	1.2672
20.250.000	3.592.299	-615.644	1.2664
20.260.000	3.594.175	-615.914	1.2655
20.270.000	3.595.530	-616.074	1.2643
20.280.000	3.597.404	-616.367	1.2625
20.290.000	3.598.931	-616.591	1.2604
20.300.000	3.600.645	-616.802	1.2581
20.310.000	3.602.222	-617.025	1.2558
20.320.000	3.603.983	-617.335	1.2538
20.330.000	3.605.558	-617.457	1.2523
20.340.000	3.607.231	-617.771	1.2516

20.350.000	3.608.916	-617.974	1.2513
20.360.000	3.610.469	-618.223	1.2511
20.370.000	3.612.261	-618.456	1.2510
20.380.000	3.613.744	-618.726	1.2506
20.390.000	3.615.564	-618.928	1.2498
20.400.000	3.616.983	-619.193	1.2484
20.410.000	3.618.870	-619.473	1.2465
20.420.000	3.620.280	-619.636	1.2445
20.430.000	3.622.080	-619.931	1.2429
20.440.000	3.623.682	-620.148	1.2417
20.450.000	3.625.345	-620.384	1.2406
20.460.000	3.626.967	-620.590	1.2395
20.470.000	3.628.658	-620.896	1.2384
20.480.000	3.630.294	-621.038	1.2371
20.490.000	3.631.879	-621.348	1.2357
20.500.000	3.633.694	-621.566	1.2342
20.510.000	3.635.074	-621.781	1.2331
20.520.000	3.636.961	-622.071	1.2320
20.530.000	3.638.430	-622.320	1.2311
20.540.000	3.640.204	-622.516	1.2302
20.550.000	3.641.709	-622.779	1.2290
20.560.000	3.643.542	-623.064	1.2275
20.570.000	3.645.001	-623.199	1.2259
20.580.000	3.646.764	-623.512	1.2243
20.590.000	3.648.372	-623.696	1.2228
20.600.000	3.649.991	-623.928	1.2217
20.610.000	3.651.689	-624.111	1.2207
20.620.000	3.653.261	-624.410	1.2196
20.630.000	3.655.016	-624.554	1.2182
20.640.000	3.656.480	-624.860	1.2165

20.650.000	3.658.383	-625.113	1.2149
20.660.000	3.659.790	-625.295	1.2132
20.670.000	3.661.636	-625.601	1.2117
20.680.000	3.663.141	-625.822	1.2102
20.690.000	3.664.920	-626.047	1.2089
20.700.000	3.666.426	-626.295	1.2076
20.710.000	3.668.254	-626.607	1.2061
20.720.000	3.669.765	-626.753	1.2045
20.730.000	3.671.435	-627.079	1.2028
20.740.000	3.673.152	-627.303	1.2012
20.750.000	3.674.689	-627.544	1.1998
20.760.000	3.676.457	-627.782	1.1985
20.770.000	3.678.012	-628.097	1.1972
20.780.000	3.679.807	-628.294	1.1958
20.790.000	3.681.262	-628.583	1.1942
20.800.000	3.683.127	-628.859	1.1926
20.810.000	3.684.556	-629.033	1.1909
20.820.000	3.686.374	-629.343	1.1894
20.830.000	3.687.933	-629.561	1.1880
20.840.000	3.689.619	-629.794	1.1867
20.850.000	3.691.192	-630.024	1.1855
20.860.000	3.692.888	-630.321	1.1842
20.870.000	3.694.521	-630.442	1.1828
20.880.000	3.696.125	-630.785	1.1813
20.890.000	3.697.935	-630.972	1.1800
20.900.000	3.699.396	-631.200	1.1789
20.910.000	3.701.236	-631.489	1.1778
20.920.000	3.702.700	-631.740	1.1769
20.930.000	3.704.495	-631.950	1.1759
20.940.000	3.705.929	-632.197	1.1748

20.950.000	3.707.782	-632.500	1.1736
20.960.000	3.709.250	-632.636	1.1723
20.970.000	3.710.957	-632.949	1.1712
20.980.000	3.712.614	-633.185	1.1700
20.990.000	3.714.236	-633.428	1.1689
21.000.000	3.715.897	-633.638	1.1678
21.010.000	3.717.555	-633.961	1.1666
21.020.000	3.719.227	-634.101	1.1652
21.030.000	3.720.750	-634.418	1.1638
21.040.000	3.722.596	-634.687	1.1625
21.050.000	3.723.998	-634.877	1.1614
21.060.000	3.725.857	-635.184	1.1601
21.070.000	3.727.297	-635.410	1.1588
21.080.000	3.729.091	-635.661	1.1577
21.090.000	3.730.599	-635.889	1.1564
21.100.000	3.732.369	-636.212	1.1551
21.110.000	3.733.960	-636.349	1.1537
21.120.000	3.735.643	-636.682	1.1523
21.130.000	3.737.349	-636.889	1.1510
21.140.000	3.738.902	-637.156	1.1497
21.150.000	3.740.620	-637.387	1.1484
21.160.000	3.742.179	-637.695	1.1471
21.170.000	3.743.943	-637.898	1.1458
21.180.000	3.745.371	-638.192	1.1443
21.190.000	3.747.264	-638.500	1.1427
21.200.000	3.748.665	-638.682	1.1412
21.210.000	3.750.495	-639.001	1.1396
21.220.000	3.752.053	-639.246	1.1381
21.230.000	3.753.789	-639.516	1.1364
21.240.000	3.755.351	-639.748	1.1347

21.250.000	3.757.096	-640.080	1.1332
21.260.000	3.758.705	-640.229	1.1316
21.270.000	3.760.292	-640.545	1.1299
21.280.000	3.762.047	-640.771	1.1281
21.290.000	3.763.512	-641.018	1.1264
21.300.000	3.765.345	-641.294	1.1248
21.310.000	3.766.777	-641.558	1.1230
21.320.000	3.768.584	-641.773	1.1216
21.330.000	3.770.061	-642.023	1.1201
21.340.000	3.771.921	-642.335	1.1186
21.350.000	3.773.397	-642.485	1.1168
21.360.000	3.775.155	-642.804	1.1151
21.370.000	3.776.732	-643.034	1.1135
21.380.000	3.778.402	-643.270	1.1118
21.390.000	3.780.022	-643.495	1.1102
21.400.000	3.781.627	-643.798	1.1087
21.410.000	3.783.338	-643.934	1.1071
21.420.000	3.784.863	-644.252	1.1055
21.430.000	3.786.714	-644.519	1.1037
21.440.000	3.788.139	-644.699	1.1019
21.450.000	3.789.952	-645.009	1.1002
21.460.000	3.791.483	-645.243	1.0987
21.470.000	3.793.239	-645.461	1.0970
21.480.000	3.794.738	-645.691	1.0953
21.490.000	3.796.539	-646.006	1.0935
21.500.000	3.798.049	-646.168	1.0919
21.510.000	3.799.749	-646.478	1.0900
21.520.000	3.801.417	-646.687	1.0881
21.530.000	3.802.975	-646.934	1.0862
21.540.000	3.804.720	-647.142	1.0844

21.550.000	3.806.290	-647.448	1.0825
21.560.000	3.808.045	-647.638	1.0806
21.570.000	3.809.533	-647.929	1.0784
21.580.000	3.811.383	-648.221	1.0762
21.590.000	3.812.807	-648.380	1.0741
21.600.000	3.814.595	-648.692	1.0719
21.610.000	3.816.156	-648.919	1.0699
21.620.000	3.817.886	-649.151	1.0680
21.630.000	3.819.402	-649.371	1.0660
21.640.000	3.821.122	-649.674	1.0640
21.650.000	3.822.738	-649.813	1.0618
21.660.000	3.824.307	-650.122	1.0596
21.670.000	3.826.077	-650.325	1.0571
21.680.000	3.827.581	-650.580	1.0549
21.690.000	3.829.379	-650.815	1.0528
21.700.000	3.830.867	-651.085	1.0508
21.710.000	3.832.666	-651.300	1.0488
21.720.000	3.834.096	-651.534	1.0468
21.730.000	3.835.976	-651.840	1.0446
21.740.000	3.837.425	-652.000	1.0422
21.750.000	3.839.172	-652.276	1.0396
21.760.000	3.840.734	-652.484	1.0372
21.770.000	3.842.427	-652.736	1.0348
21.780.000	3.844.079	-652.918	1.0324
21.790.000	3.845.704	-653.243	1.0302
21.800.000	3.847.375	-653.342	1.0280
21.810.000	3.848.906	-653.654	1.0256
21.820.000	3.850.740	-653.906	1.0231
21.830.000	3.852.126	-654.076	1.0204
21.840.000	3.854.013	-654.379	1.0179

21.850.000	3.855.472	-654.614	1.0153
21.860.000	3.857.244	-654.819	1.0129
21.870.000	3.858.728	-655.033	1.0106
21.880.000	3.860.560	-655.356	1.1984
21.890.000	3.862.088	-655.482	1.1962
21.900.000	3.863.747	-655.796	1.1939
21.910.000	3.865.441	-656.004	1.2013
21.920.000	3.867.027	-656.239	9.986
21.930.000	3.868.718	-656.457	9.958
21.940.000	3.870.284	-656.745	9.928
21.950.000	3.872.073	-656.924	9.897
21.960.000	3.873.455	-657.198	9.868
21.970.000	3.875.359	-657.506	9.838
21.980.000	3.876.768	-657.665	9.810
21.990.000	3.878.557	-657.961	9.783
22.000.000	3.880.131	-658.214	9.756
22.010.000	3.881.858	-658.453	9.730
22.020.000	3.883.382	-658.661	9.702
22.030.000	3.885.148	-658.978	9.674
22.040.000	3.886.732	-659.119	9.645
22.050.000	3.888.360	-659.433	9.615
22.060.000	3.890.090	-659.633	9.583
22.070.000	3.891.601	-659.863	9.550
22.080.000	3.893.384	-660.089	9.521
22.090.000	3.894.861	-660.381	9.491
22.100.000	3.896.680	-660.580	9.461
22.110.000	3.898.107	-660.808	9.430
22.120.000	3.899.974	-661.105	9.401
22.130.000	3.901.480	-661.257	9.371
22.140.000	3.903.245	-661.546	9.339

22.150.000	3.904.797	-661.764	9.307
22.160.000	3.906.513	-662.008	9.277
22.170.000	3.908.112	-662.215	9.246
22.180.000	3.909.751	-662.517	9.216
22.190.000	3.911.424	-662.654	9.187
22.200.000	3.912.947	-662.946	9.157
22.210.000	3.914.774	-663.186	9.126
22.220.000	3.916.205	-663.372	9.093
22.230.000	3.918.040	-663.634	9.059
22.240.000	3.919.523	-663.888	9.025
22.250.000	3.921.311	-664.075	8.991
22.260.000	3.922.766	-664.273	8.958
22.270.000	3.924.595	-664.569	8.926
22.280.000	3.926.079	-664.677	8.893
22.290.000	3.927.746	-664.946	8.860
22.300.000	3.929.432	-665.151	8.826
22.310.000	3.931.018	-665.356	8.790
22.320.000	3.932.718	-665.527	8.756
22.330.000	3.934.295	-665.833	8.720
22.340.000	3.936.071	-665.954	8.685
22.350.000	3.937.480	-666.200	8.649
22.360.000	3.939.377	-666.468	8.613
22.370.000	3.940.837	-666.616	8.576
22.380.000	3.942.600	-666.863	8.538
22.390.000	3.944.100	-667.087	8.501
22.400.000	3.945.908	-667.295	8.463
22.410.000	3.947.374	-667.477	8.427
22.420.000	3.949.145	-667.782	8.391
22.430.000	3.950.761	-667.902	8.355
22.440.000	3.952.307	-668.179	8.319

22.450.000	3.954.079	-668.359	8.281
22.460.000	3.955.602	-668.581	8.240
22.470.000	3.957.378	-668.771	8.198
22.480.000	3.958.887	-669.041	8.157
22.490.000	3.960.750	-669.221	8.115
22.500.000	3.962.120	-669.430	8.077
22.510.000	3.963.985	-669.695	8.039
22.520.000	3.965.461	-669.857	8.004
22.530.000	3.967.210	-670.095	7.968
22.540.000	3.968.785	-670.299	7.932
22.550.000	3.970.476	-670.518	7.896
22.560.000	3.972.039	-670.677	7.856
22.570.000	3.973.743	-670.984	7.816
22.580.000	3.975.408	-671.101	7.775
22.590.000	3.976.988	-671.398	7.736
22.600.000	3.978.812	-671.626	7.697
22.610.000	3.980.245	-671.789	7.656
22.620.000	3.982.044	-672.035	7.616
22.630.000	3.983.496	-672.271	7.574
22.640.000	3.985.342	-672.450	7.532
22.650.000	3.986.760	-672.648	7.489
22.660.000	3.988.579	-672.932	7.446
22.670.000	3.990.153	-673.061	7.403
22.680.000	3.991.819	-673.322	7.363
22.690.000	3.993.474	-673.510	7.325
22.700.000	3.995.126	-673.706	7.287
22.710.000	3.996.785	-673.862	7.247
22.720.000	3.998.379	-674.148	7.205
22.730.000	4.000.153	-674.254	7.164
22.740.000	4.001.542	-674.477	7.122

22.750.000	4.003.409	-674.720	7.080
22.760.000	4.004.859	-674.864	7.039
22.770.000	4.006.635	-675.070	6.999
22.780.000	4.008.126	-675.252	6.962
22.790.000	4.009.907	-675.433	6.923
22.800.000	4.011.429	-675.571	6.882
22.810.000	4.013.210	-675.830	6.837
22.820.000	4.014.806	-675.923	6.791
22.830.000	4.016.397	-676.139	6.747
22.840.000	4.018.111	-676.301	6.702
22.850.000	4.019.668	-676.496	6.662
22.860.000	4.021.404	-676.628	6.624
22.870.000	4.022.870	-676.869	6.585
22.880.000	4.024.733	-677.040	6.548
22.890.000	4.026.119	-677.186	6.508
22.900.000	4.027.923	-677.421	6.464
22.910.000	4.029.448	-677.535	6.416
22.920.000	4.031.218	-677.750	6.368
22.930.000	4.032.736	-677.908	6.321
22.940.000	4.034.464	-678.083	6.278
22.950.000	4.036.015	-678.208	6.238
22.960.000	4.037.686	-678.455	6.200
22.970.000	4.039.367	-678.524	6.159
22.980.000	4.040.880	-678.751	6.118
22.990.000	4.042.687	-678.905	6.077
23.000.000	4.044.140	-679.063	6.036
23.010.000	4.045.910	-679.236	5.991
23.020.000	4.047.386	-679.426	5.944
23.030.000	4.049.204	-679.589	5.894
23.040.000	4.050.619	-679.724	5.846

23.050.000	4.052.443	-679.988	5.797
23.060.000	4.053.966	-680.101	5.748
23.070.000	4.055.671	-680.340	5.700
23.080.000	4.057.269	-680.503	5.655
23.090.000	4.058.951	-680.727	5.613
23.100.000	4.060.624	-680.862	5.573
23.110.000	4.062.191	-681.147	5.529
23.120.000	4.063.976	-681.260	5.485
23.130.000	4.065.405	-681.465	5.439
23.140.000	4.067.291	-681.712	5.394
23.150.000	4.068.750	-681.844	5.350
23.160.000	4.070.528	-682.036	5.308
23.170.000	4.072.042	-682.235	5.265
23.180.000	4.073.837	-682.414	5.220
23.190.000	4.075.311	-682.551	5.174
23.200.000	4.077.054	-682.787	5.126
23.210.000	4.078.697	-682.893	5.080
23.220.000	4.080.289	-683.088	5.036
23.230.000	4.082.009	-683.215	4.998
23.240.000	4.083.582	-683.422	4.966
23.250.000	4.085.301	-683.504	4.934
23.260.000	4.086.848	-683.724	4.899
23.270.000	4.088.672	-683.880	4.857
23.280.000	4.090.093	-683.987	4.818
23.290.000	4.091.951	-684.184	4.779
23.300.000	4.093.438	-684.280	4.736
23.310.000	4.095.194	-684.434	4.689
23.320.000	4.096.747	-684.568	4.642
23.330.000	4.098.502	-684.734	4.599
23.340.000	4.100.073	-684.799	4.561

23.350.000	4.101.741	-685.035	4.524
23.360.000	4.103.413	-685.086	4.490
23.370.000	4.105.002	-685.267	4.454
23.380.000	4.106.804	-685.403	4.415
23.390.000	4.108.305	-685.535	4.372
23.400.000	4.110.105	-685.674	4.323
23.410.000	4.111.555	-685.834	4.270
23.420.000	4.113.408	-685.962	4.220
23.430.000	4.114.858	-686.058	4.180
23.440.000	4.116.660	-686.244	4.152
23.450.000	4.118.222	-686.306	4.126
23.460.000	4.119.883	-686.467	4.097
23.470.000	4.121.531	-686.563	4.066
23.480.000	4.123.213	-686.730	4.034
23.490.000	4.124.831	-686.782	4.001
23.500.000	4.126.464	-686.992	3.965
23.510.000	4.128.278	-687.061	3.930
23.520.000	4.129.694	-687.186	3.895
23.530.000	4.131.551	-687.365	3.860
23.540.000	4.133.028	-687.492	3.819
23.550.000	4.134.802	-687.596	3.773
23.560.000	4.136.300	-687.722	3.726
23.570.000	4.138.129	-687.890	3.680
23.580.000	4.139.632	-687.935	3.642
23.590.000	4.141.369	-688.123	3.607
23.600.000	4.142.998	-688.178	3.572
23.610.000	4.144.652	-688.308	3.535
23.620.000	4.146.331	-688.400	3.494
23.630.000	4.147.916	-688.536	3.452
23.640.000	4.149.673	-688.558	3.408

23.650.000	4.151.112	-688.715	3.364
23.660.000	4.153.013	-688.828	3.322
23.670.000	4.154.381	-688.861	3.286
23.680.000	4.156.224	-689.003	3.250
23.690.000	4.157.754	-689.071	3.212
23.700.000	4.159.491	-689.172	3.175
23.710.000	4.161.054	-689.233	3.135
23.720.000	4.162.834	-689.371	3.096
23.730.000	4.164.355	-689.365	3.054
23.740.000	4.166.063	-689.518	3.011
23.750.000	4.167.752	-689.559	2.966
23.760.000	4.169.265	-689.657	2.923
23.770.000	4.171.075	-689.720	2.881
23.780.000	4.172.566	-689.849	2.836
23.790.000	4.174.358	-689.891	2.789
23.800.000	4.175.840	-690.007	2.745
23.810.000	4.177.661	-690.106	2.706
23.820.000	4.179.138	-690.101	2.670
23.830.000	4.180.950	-690.252	2.633
23.840.000	4.182.499	-690.279	2.602
23.850.000	4.184.154	-690.349	2.572
23.860.000	4.185.764	-690.410	2.539
23.870.000	4.187.436	-690.508	2.504
23.880.000	4.189.092	-690.474	2.468
23.890.000	4.190.684	-690.625	2.433
23.900.000	4.192.520	-690.647	2.395
23.910.000	4.193.942	-690.684	2.359
23.920.000	4.195.780	-690.789	2.323
23.930.000	4.197.283	-690.861	2.285
23.940.000	4.199.084	-690.908	2.246

23.950.000	4.200.567	-690.975	2.209
23.960.000	4.202.445	-691.092	2.172
23.970.000	4.203.869	-691.074	2.136
23.980.000	4.205.634	-691.207	2.106
23.990.000	4.207.278	-691.243	2.080
24.000.000	4.208.860	-691.297	2.047
24.010.000	4.210.564	-691.333	2.012
24.020.000	4.212.170	-691.468	1.979
24.030.000	4.213.875	-691.419	1.945
24.040.000	4.215.381	-691.553	1.914
24.050.000	4.217.307	-691.660	1.884
24.060.000	4.218.703	-691.629	1.855
24.070.000	4.220.543	-691.740	1.823
24.080.000	4.222.022	-691.791	1.779
24.090.000	4.223.823	-691.829	1.729
24.100.000	4.225.344	-691.886	1.682
24.110.000	4.227.102	-692.021	1.639
24.120.000	4.228.679	-691.986	1.600
24.130.000	4.230.325	-692.125	1.568
24.140.000	4.232.051	-692.129	1.537
24.150.000	4.233.585	-692.189	1.508
24.160.000	4.235.389	-692.225	1.482
24.170.000	4.236.918	-692.337	1.456
24.180.000	4.238.696	-692.346	1.428
24.190.000	4.240.131	-692.419	1.400
24.200.000	4.242.039	-692.510	1.371
24.210.000	4.243.457	-692.500	1.341
24.220.000	4.245.252	-692.595	1.303
24.230.000	4.246.830	-692.644	1.262
24.240.000	4.248.494	-692.670	1.222

24.250.000	4.250.122	-692.712	1.188
24.260.000	4.251.825	-692.838	1.165
24.270.000	4.253.474	-692.781	1.155
24.280.000	4.255.092	-692.906	1.154
24.290.000	4.256.867	-692.952	1.156
24.300.000	4.258.311	-692.966	1.152
24.310.000	4.260.161	-693.040	1.140
24.320.000	4.261.605	-693.114	1.111
24.330.000	4.263.430	-693.119	1.067
24.340.000	4.264.917	-693.176	1.016
24.350.000	4.266.723	-693.285	969
24.360.000	4.268.229	-693.233	929
24.370.000	4.269.926	-693.368	898
24.380.000	4.271.576	-693.389	880
24.390.000	4.273.228	-693.447	873
24.400.000	4.274.885	-693.464	874
24.410.000	4.276.519	-693.600	870
24.420.000	4.278.275	-693.552	852
24.430.000	4.279.737	-693.669	819
24.440.000	4.281.644	-693.752	772
24.450.000	4.283.034	-693.765	723
24.460.000	4.284.841	-693.856	678
24.470.000	4.286.388	-693.933	644
24.480.000	4.288.120	-693.995	626
24.490.000	4.289.674	-694.040	623
24.500.000	4.291.450	-694.206	632
24.510.000	4.292.999	-694.168	645
24.520.000	4.294.697	-694.307	647
24.530.000	4.296.403	-694.344	630
24.540.000	4.297.922	-694.401	604

24.550.000	4.299.690	-694.444	578
24.560.000	4.301.208	-694.604	553
24.570.000	4.303.023	-694.602	533
24.580.000	4.304.445	-694.704	520
24.590.000	4.306.315	-694.816	515
24.600.000	4.307.776	-694.811	513
24.610.000	4.309.545	-694.946	500
24.620.000	4.311.107	-694.979	474
24.630.000	4.312.812	-695.058	433
24.640.000	4.314.383	-695.096	381
24.650.000	4.316.089	-695.220	328
24.660.000	4.317.751	-695.180	284
24.670.000	4.319.255	-695.314	249
24.680.000	4.321.075	-695.371	226
24.690.000	4.322.543	-695.424	219
24.700.000	4.324.341	-695.532	228
24.710.000	4.325.831	-695.621	246
24.720.000	4.327.655	-695.693	262
24.730.000	4.329.105	-695.753	270
24.740.000	4.330.925	-695.892	266
24.750.000	4.332.417	-695.889	248
24.760.000	4.334.170	-696.015	218
24.770.000	4.335.782	-696.080	186
24.780.000	4.337.397	-696.171	156
24.790.000	4.339.058	-696.206	136
24.800.000	4.340.656	-696.391	127
24.810.000	4.342.420	-696.379	124
24.820.000	4.343.838	-696.494	121
24.830.000	4.345.679	-696.638	109
24.840.000	4.347.201	-696.678	86

24.850.000	4.348.991	-696.805	54
24.860.000	4.350.484	-696.918	21
24.870.000	4.352.290	-696.989	-9
24.880.000	4.353.767	-697.078	-25
24.890.000	4.355.514	-697.255	-28
24.900.000	4.357.143	-697.262	-23
24.910.000	4.358.731	-697.426	-20
24.920.000	4.360.448	-697.501	-25
24.930.000	4.362.018	-697.626	-40
24.940.000	4.363.743	-697.702	-66
24.950.000	4.365.297	-697.869	-96
24.960.000	4.367.099	-697.935	-123
24.970.000	4.368.483	-698.055	-141
24.980.000	4.370.393	-698.234	-144
24.990.000	4.371.817	-698.287	-140
25.000.000	4.373.598	-698.428	-135
25.010.000	4.375.128	-698.542	-137
25.020.000	4.376.817	-698.664	-148
25.030.000	4.378.394	-698.730	-165
25.040.000	4.380.093	-698.936	-187
25.050.000	4.381.796	-698.957	-208
25.060.000	4.383.326	-699.146	-218
25.070.000	4.385.090	-699.257	-214
25.080.000	4.386.601	-699.372	-203
25.090.000	4.388.370	-699.524	-191
25.100.000	4.389.831	-699.676	-186
25.110.000	4.391.664	-699.802	-191
25.120.000	4.393.104	-699.930	-203
25.130.000	4.394.944	-700.134	-221
25.140.000	4.396.485	-700.201	-240

25.150.000	4.398.168	-700.418	-253
25.160.000	4.399.760	-700.525	-259
25.170.000	4.401.438	-700.688	-260
25.180.000	4.403.083	-700.790	-260
25.190.000	4.404.672	-701.017	-267
25.200.000	4.406.446	-701.097	-276
25.210.000	4.407.918	-701.297	-287
25.220.000	4.409.764	-701.487	-299
25.230.000	4.411.195	-701.611	-306
25.240.000	4.412.976	-701.788	-308
25.250.000	4.414.476	-701.948	-313
25.260.000	4.416.246	-702.118	-319
25.270.000	4.417.708	-702.214	-329
25.280.000	4.419.480	-702.462	-338
25.290.000	4.421.101	-702.546	-351
25.300.000	4.422.702	-702.735	-364
25.310.000	4.424.418	-702.894	-367
25.320.000	4.426.020	-703.067	-365
25.330.000	4.427.740	-703.180	-362
25.340.000	4.429.249	-703.397	-368
25.350.000	4.431.051	-703.532	-379
25.360.000	4.432.456	-703.684	-394
25.370.000	4.434.288	-703.908	-414
25.380.000	4.435.782	-704.035	-436
25.390.000	4.437.496	-704.213	-452
25.400.000	4.439.024	-704.375	-457
25.410.000	4.440.794	-704.586	-455
25.420.000	4.442.305	-704.689	-456
25.430.000	4.443.983	-704.924	-457
25.440.000	4.445.679	-705.033	-460

25.450.000	4.447.181	-705.234	-467
25.460.000	4.448.983	-705.392	-477
25.470.000	4.450.463	-705.584	-487
25.480.000	4.452.186	-705.746	-490
25.490.000	4.453.638	-705.943	-490
25.500.000	4.455.488	-706.164	-491
25.510.000	4.456.909	-706.272	-493
25.520.000	4.458.702	-706.511	-495
25.530.000	4.460.242	-706.648	-504
25.540.000	4.461.943	-706.834	-516
25.550.000	4.463.540	-706.983	-530
25.560.000	4.465.178	-707.185	-539
25.570.000	4.466.798	-707.279	-540
25.580.000	4.468.356	-707.548	-537
25.590.000	4.470.122	-707.662	-536
25.600.000	4.471.564	-707.845	-537
25.610.000	4.473.381	-708.076	-539
25.620.000	4.474.865	-708.248	-545
25.630.000	4.476.632	-708.428	-554
25.640.000	4.478.126	-708.629	-564
25.650.000	4.479.981	-708.859	-570
25.660.000	4.481.390	-709.007	-572
25.670.000	4.483.134	-709.260	-576
25.680.000	4.484.776	-709.440	-578
25.690.000	4.486.344	-709.652	-581
25.700.000	4.488.077	-709.821	-585
25.710.000	4.489.637	-710.093	-590
25.720.000	4.491.317	-710.208	-594
25.730.000	4.492.844	-710.490	-594
25.740.000	4.494.699	-710.721	-594

25.750.000	4.496.118	-710.875	-594
25.760.000	4.497.937	-711.153	-595
25.770.000	4.499.416	-711.370	-598
25.780.000	4.501.180	-711.558	-604
25.790.000	4.502.700	-711.767	-611
25.800.000	4.504.436	-712.030	-615
25.810.000	4.505.969	-712.149	-618
25.820.000	4.507.624	-712.443	-620
25.830.000	4.509.305	-712.603	-622
25.840.000	4.510.844	-712.819	-622
25.850.000	4.512.586	-712.999	-623
25.860.000	4.514.103	-713.255	-628
25.870.000	4.515.891	-713.424	-632
25.880.000	4.517.292	-713.649	-634
25.890.000	4.519.188	-713.897	-636
25.900.000	4.520.589	-714.032	-638
25.910.000	4.522.347	-714.306	-643
25.920.000	4.523.892	-714.486	-646
25.930.000	4.525.558	-714.721	-647
25.940.000	4.527.151	-714.887	-648
25.950.000	4.528.841	-715.164	-649
25.960.000	4.530.409	-715.253	-650
25.970.000	4.532.042	-715.550	-652
25.980.000	4.533.848	-715.721	-650
25.990.000	4.535.229	-715.902	-652
26.000.000	4.537.100	-716.154	-654
26.010.000	4.538.544	-716.386	-656
26.020.000	4.540.326	-716.558	-658
26.030.000	4.541.781	-716.776	-659
26.040.000	4.543.585	-717.059	-661

26.050.000	4.545.092	-717.186	-663
26.060.000	4.546.801	-717.470	-666
26.070.000	4.548.394	-717.673	-668
26.080.000	4.550.047	-717.906	-669
26.090.000	4.551.713	-718.117	-670
26.100.000	4.553.324	-718.398	-670
26.110.000	4.555.033	-718.522	-669
26.120.000	4.556.484	-718.814	-668
26.130.000	4.558.339	-719.062	-668
26.140.000	4.559.780	-719.245	-667
26.150.000	4.561.561	-719.512	-668
26.160.000	4.563.084	-719.751	-669
26.170.000	4.564.848	-719.976	-669
26.180.000	4.566.317	-720.183	-670
26.190.000	4.568.095	-720.498	-670
26.200.000	4.569.677	-720.637	-671
26.210.000	4.571.322	-720.930	-671
26.220.000	4.573.025	-721.143	-672
26.230.000	4.574.534	-721.374	-672
26.240.000	4.576.284	-721.576	-673
26.250.000	4.577.772	-721.871	-672
26.260.000	4.579.570	-722.058	-672
26.270.000	4.581.006	-722.275	-672
26.280.000	4.582.807	-722.560	-672
26.290.000	4.584.263	-722.696	-672
26.300.000	4.586.062	-723.003	-670
26.310.000	4.587.606	-723.214	-671
26.320.000	4.589.283	-723.418	-672
26.330.000	4.590.871	-723.617	-672
26.340.000	4.592.569	-723.904	-673

26.350.000	4.594.218	-724.015	-673
26.360.000	4.595.722	-724.315	-675
26.370.000	4.597.532	-724.508	-675
26.380.000	4.598.983	-724.702	-678
26.390.000	4.600.763	-724.959	-681
26.400.000	4.602.254	-725.211	-682
26.410.000	4.604.052	-725.413	-683
26.420.000	4.605.520	-725.623	-683
26.430.000	4.607.362	-725.928	-684
26.440.000	4.608.860	-726.075	-685
26.450.000	4.610.528	-726.340	-683
26.460.000	4.612.158	-726.552	-685
26.470.000	4.613.756	-726.775	-686
26.480.000	4.615.412	-726.953	-686
26.490.000	4.616.999	-727.264	-686
26.500.000	4.618.732	-727.405	-686
26.510.000	4.620.157	-727.644	-686
26.520.000	4.622.023	-727.926	-685
26.530.000	4.623.436	-728.070	-683
26.540.000	4.625.253	-728.329	-682
26.550.000	4.626.716	-728.542	-680
26.560.000	4.628.480	-728.748	-680
26.570.000	4.629.960	-728.916	-681
26.580.000	4.631.700	-729.213	-682
26.590.000	4.633.307	-729.339	-681
26.600.000	4.634.933	-729.616	-676
26.610.000	4.636.581	-729.798	-670
26.620.000	4.638.162	-730.033	-663
26.630.000	4.639.888	-730.210	-655
26.640.000	4.641.444	-730.501	-648

26.650.000	4.643.271	-730.705	-644
26.660.000	4.644.640	-730.915	-645
26.670.000	4.646.515	-731.216	-647
26.680.000	4.647.970	-731.385	-646
26.690.000	4.649.695	-731.644	-642
26.700.000	4.651.269	-731.884	-636
26.710.000	4.652.966	-732.138	-627
26.720.000	4.654.534	-732.317	-616
26.730.000	4.656.194	-732.645	-604
26.740.000	4.657.876	-732.797	-595
26.750.000	4.659.432	-733.094	-585
26.760.000	4.661.225	-733.300	-574
26.770.000	4.662.685	-733.537	-562
26.780.000	4.664.460	-733.761	-550
26.790.000	4.665.902	-734.011	-536
26.800.000	4.667.708	-734.208	-521
26.810.000	4.669.140	-734.398	-508
26.820.000	4.670.909	-734.679	-499
26.830.000	4.672.459	-734.822	-491
26.840.000	4.674.098	-735.070	-483
26.850.000	4.675.704	-735.260	-471
26.860.000	4.677.391	-735.513	-460
26.870.000	4.679.044	-735.658	-444
26.880.000	4.680.610	-735.941	-427
26.890.000	4.682.412	-736.107	-411
26.900.000	4.683.768	-736.328	-399
26.910.000	4.685.644	-736.594	-391
26.920.000	4.687.121	-736.797	-384
26.930.000	4.688.839	-737.016	-379
26.940.000	4.690.340	-737.256	-373

26.950.000	4.692.153	-737.521	-363
26.960.000	4.693.604	-737.657	-351
26.970.000	4.695.344	-737.961	-335
26.980.000	4.696.937	-738.136	-319
26.990.000	4.698.536	-738.363	-306
27.000.000	4.700.233	-738.545	-297
27.010.000	4.701.768	-738.811	-293
27.020.000	4.703.514	-738.962	-289
27.030.000	4.704.969	-739.235	-285
27.040.000	4.706.803	-739.457	-280
27.050.000	4.708.185	-739.618	-275
27.060.000	4.709.979	-739.891	-266
27.070.000	4.711.533	-740.074	-259
27.080.000	4.713.217	-740.314	-254
27.090.000	4.714.794	-740.517	-252
27.100.000	4.716.540	-740.783	-255
27.110.000	4.718.095	-740.931	-260
27.120.000	4.719.718	-741.238	-268
27.130.000	4.721.439	-741.401	-274
27.140.000	4.722.881	-741.651	-278
27.150.000	4.724.692	-741.860	-280
27.160.000	4.726.179	-742.114	-280
27.170.000	4.727.926	-742.314	-280
27.180.000	4.729.398	-742.551	-278
27.190.000	4.731.263	-742.814	-278
27.200.000	4.732.673	-742.960	-282
27.210.000	4.734.483	-743.238	-291
27.220.000	4.736.009	-743.448	-302
27.230.000	4.737.673	-743.641	-312
27.240.000	4.739.289	-743.836	-321

27.250.000	4.740.911	-744.123	-328
27.260.000	4.742.563	-744.212	-332
27.270.000	4.744.093	-744.519	-332
27.280.000	4.745.918	-744.721	-329
27.290.000	4.747.313	-744.914	-326
27.300.000	4.749.110	-745.190	-326
27.310.000	4.750.616	-745.400	-327
27.320.000	4.752.391	-745.592	-330
27.330.000	4.753.840	-745.810	-336
27.340.000	4.755.612	-746.062	-341
27.350.000	4.757.061	-746.180	-345
27.360.000	4.758.717	-746.450	-345
27.370.000	4.760.388	-746.615	-345
27.380.000	4.761.919	-746.823	-341
27.390.000	4.763.613	-746.964	-337
27.400.000	4.765.170	-747.231	-333
27.410.000	4.766.921	-747.352	-328
27.420.000	4.768.369	-747.586	-327
27.430.000	4.770.244	-747.840	-325
27.440.000	4.771.622	-747.975	-326
27.450.000	4.773.398	-748.218	-328
27.460.000	4.774.896	-748.434	-331
27.470.000	4.776.640	-748.643	-333
27.480.000	4.778.150	-748.820	-335
27.490.000	4.779.859	-749.096	-336
27.500.000	4.781.482	-749.204	-336
27.510.000	4.783.054	-749.496	-335
27.520.000	4.784.805	-749.682	-331
27.530.000	4.786.299	-749.906	-329
27.540.000	4.788.110	-750.136	-327

27.550.000	4.789.566	-750.394	-326
27.560.000	4.791.378	-750.594	-328
27.570.000	4.792.765	-750.831	-330
27.580.000	4.794.598	-751.111	-332
27.590.000	4.796.089	-751.253	-335
27.600.000	4.797.751	-751.537	-335
27.610.000	4.799.356	-751.764	-334
27.620.000	4.800.998	-751.961	-333
27.630.000	4.802.630	-752.157	-330
27.640.000	4.804.275	-752.456	-329
27.650.000	4.805.956	-752.587	-329
27.660.000	4.807.433	-752.853	-331
27.670.000	4.809.264	-753.095	-331
27.680.000	4.810.684	-753.285	-330
27.690.000	4.812.522	-753.548	-330
27.700.000	4.813.968	-753.786	-329
27.710.000	4.815.746	-753.996	-329
27.720.000	4.817.216	-754.203	-327
27.730.000	4.819.006	-754.509	-326
27.740.000	4.820.539	-754.658	-323
27.750.000	4.822.169	-754.955	-322
27.760.000	4.823.848	-755.159	-319
27.770.000	4.825.462	-755.391	-316
27.780.000	4.827.132	-755.573	-314
27.790.000	4.828.649	-755.878	-314
27.800.000	4.830.470	-756.067	-316
27.810.000	4.831.860	-756.310	-318
27.820.000	4.833.712	-756.600	-320
27.830.000	4.835.198	-756.779	-320
27.840.000	4.836.942	-757.039	-319

27.850.000	4.838.473	-757.286	-317
27.860.000	4.840.218	-757.543	-315
27.870.000	4.841.751	-757.735	-315
27.880.000	4.843.483	-758.064	-315
27.890.000	4.845.126	-758.229	-315
27.900.000	4.846.647	-758.503	-314
27.910.000	4.848.441	-758.715	-313
27.920.000	4.849.909	-758.969	-312
27.930.000	4.851.719	-759.196	-311
27.940.000	4.853.162	-759.456	-311
27.950.000	4.854.967	-759.699	-311
27.960.000	4.856.411	-759.894	-313
27.970.000	4.858.234	-760.200	-315
27.980.000	4.859.747	-760.377	-316
27.990.000	4.861.482	-760.662	-315
28.000.000	4.863.053	-760.886	-314
28.010.000	4.864.748	-761.141	-314
28.020.000	4.866.316	-761.301	-314
28.030.000	4.867.900	-761.624	-315
28.040.000	4.869.696	-761.795	-315
28.050.000	4.871.093	-762.025	-315
28.060.000	4.872.934	-762.286	-314
28.070.000	4.874.418	-762.499	-315
28.080.000	4.876.179	-762.717	-314
28.090.000	4.877.670	-762.927	-314
28.100.000	4.879.489	-763.183	-312
28.110.000	4.880.929	-763.333	-310
28.120.000	4.882.690	-763.640	-309
28.130.000	4.884.259	-763.791	-307
28.140.000	4.885.830	-764.020	-307

28.150.000	4.887.516	-764.208	-305
28.160.000	4.889.084	-764.469	-306
28.170.000	4.890.775	-764.583	-307
28.180.000	4.892.260	-764.855	-307
28.190.000	4.894.129	-765.088	-308
28.200.000	4.895.490	-765.244	-308
28.210.000	4.897.348	-765.514	-309
28.220.000	4.898.830	-765.702	-307
28.230.000	4.900.598	-765.923	-308
28.240.000	4.902.088	-766.128	-308
28.250.000	4.903.836	-766.386	-310
28.260.000	4.905.371	-766.519	-309
28.270.000	4.906.999	-766.799	-309
28.280.000	4.908.712	-766.975	-307
28.290.000	4.910.195	-767.213	-306
28.300.000	4.911.970	-767.408	-305
28.310.000	4.913.521	-767.681	-302
28.320.000	4.915.245	-767.865	-301
28.330.000	4.916.715	-768.094	-300
28.340.000	4.918.562	-768.356	-298
28.350.000	4.919.943	-768.500	-296
28.360.000	4.921.729	-768.761	-294
28.370.000	4.923.244	-768.963	-292
28.380.000	4.924.895	-769.174	-291
28.390.000	4.926.487	-769.365	-289
28.400.000	4.928.138	-769.638	-287
28.410.000	4.929.779	-769.739	-286
28.420.000	4.931.307	-770.023	-286
28.430.000	4.933.088	-770.225	-284
28.440.000	4.934.564	-770.398	-283

28.450.000	4.936.348	-770.656	-281
28.460.000	4.937.755	-770.873	-278
28.470.000	4.939.567	-771.044	-279
28.480.000	4.940.975	-771.253	-279
28.490.000	4.942.793	-771.526	-281
28.500.000	4.944.312	-771.669	-282
28.510.000	4.946.017	-771.968	-281
28.520.000	4.947.653	-772.153	-278
28.530.000	4.949.262	-772.367	-273
28.540.000	4.950.893	-772.566	-271
28.550.000	4.952.543	-772.879	-270
28.560.000	4.954.238	-772.992	-269
28.570.000	4.955.710	-773.287	-269
28.580.000	4.957.551	-773.560	-269
28.590.000	4.958.927	-773.727	-271
28.600.000	4.960.727	-774.005	-272
28.610.000	4.962.220	-774.226	-270
28.620.000	4.963.944	-774.446	-269
28.630.000	4.965.477	-774.651	-269
28.640.000	4.967.207	-774.970	-267
28.650.000	4.968.762	-775.080	-266
28.660.000	4.970.387	-775.399	-264
28.670.000	4.972.117	-775.600	-260
28.680.000	4.973.631	-775.815	-258
28.690.000	4.975.349	-776.016	-257
28.700.000	4.976.844	-776.300	-257
28.710.000	4.978.617	-776.488	-258
28.720.000	4.980.009	-776.710	-259
28.730.000	4.981.851	-776.985	-258
28.740.000	4.983.325	-777.142	-256

28.750.000	4.985.054	-777.407	-252
28.760.000	4.986.608	-777.603	-250
28.770.000	4.988.293	-777.839	-250
28.780.000	4.989.858	-778.006	-251
28.790.000	4.991.537	-778.304	-254
28.800.000	4.993.210	-778.424	-256
28.810.000	4.994.696	-778.692	-257
28.820.000	4.996.521	-778.917	-257
28.830.000	4.997.922	-779.105	-256
28.840.000	4.999.709	-779.352	-254
28.850.000	5.001.163	-779.611	-253
28.860.000	5.002.980	-779.826	-253
28.870.000	5.004.403	-780.015	-254
28.880.000	5.006.189	-780.306	-254
28.890.000	5.007.720	-780.451	-251
28.900.000	5.009.395	-780.718	-248
28.910.000	5.011.033	-780.919	-246
28.920.000	5.012.640	-781.167	-244
28.930.000	5.014.298	-781.323	-243
28.940.000	5.015.835	-781.643	-244
28.950.000	5.017.649	-781.807	-244
28.960.000	5.019.049	-782.049	-247
28.970.000	5.020.895	-782.331	-247
28.980.000	5.022.357	-782.523	-245
28.990.000	5.024.109	-782.763	-241
29.000.000	5.025.620	-783.006	-236
29.010.000	5.027.400	-783.247	-233
29.020.000	5.028.858	-783.415	-229
29.030.000	5.030.583	-783.726	-226
29.040.000	5.032.212	-783.895	-225

29.050.000	5.033.762	-784.135	-225
29.060.000	5.035.507	-784.329	-228
29.070.000	5.037.041	-784.609	-229
29.080.000	5.038.745	-784.793	-228
29.090.000	5.040.218	-785.074	-228
29.100.000	5.042.053	-785.338	-226
29.110.000	5.043.441	-785.518	-223
29.120.000	5.045.278	-785.809	-220
29.130.000	5.046.755	-786.007	-217
29.140.000	5.048.464	-786.253	-216
29.150.000	5.050.009	-786.455	-215
29.160.000	5.051.703	-786.724	-216
29.170.000	5.053.276	-786.875	-218
29.180.000	5.054.932	-787.176	-221
29.190.000	5.056.624	-787.331	-222
29.200.000	5.058.127	-787.587	-222
29.210.000	5.059.934	-787.824	-224
29.220.000	5.061.391	-788.067	-223
29.230.000	5.063.153	-788.285	-221
29.240.000	5.064.620	-788.546	-216
29.250.000	5.066.455	-788.808	-213
29.260.000	5.067.882	-788.949	-210
29.270.000	5.069.626	-789.257	-209
29.280.000	5.071.219	-789.458	-207
29.290.000	5.072.854	-789.706	-207
29.300.000	5.074.493	-789.912	-208
29.310.000	5.076.109	-790.213	-211
29.320.000	5.077.756	-790.349	-213
29.330.000	5.079.291	-790.669	-215
29.340.000	5.081.127	-790.910	-217

29.350.000	5.082.533	-791.106	-217
29.360.000	5.084.379	-791.395	-215
29.370.000	5.085.809	-791.627	-211
29.380.000	5.087.572	-791.860	-209
29.390.000	5.089.061	-792.081	-208
29.400.000	5.090.842	-792.400	-208
29.410.000	5.092.405	-792.539	-210
29.420.000	5.094.026	-792.834	-213
29.430.000	5.095.675	-793.046	-218
29.440.000	5.097.243	-793.276	-221
29.450.000	5.098.971	-793.490	-222
29.460.000	5.100.484	-793.794	-221
29.470.000	5.102.250	-793.956	-219
29.480.000	5.103.675	-794.222	-215
29.490.000	5.105.539	-794.491	-211
29.500.000	5.106.944	-794.651	-209
29.510.000	5.108.729	-794.932	-206
29.520.000	5.110.269	-795.159	-206
29.530.000	5.111.975	-795.373	-207
29.540.000	5.113.498	-795.589	-209
29.550.000	5.115.197	-795.885	-211
29.560.000	5.116.824	-796.023	-212
29.570.000	5.118.392	-796.320	-210
29.580.000	5.120.161	-796.525	-208
29.590.000	5.121.630	-796.747	-207
29.600.000	5.123.437	-796.996	-205
29.610.000	5.124.855	-797.252	-203
29.620.000	5.126.663	-797.466	-203
29.630.000	5.128.118	-797.695	-204
29.640.000	5.129.924	-798.002	-206

29.650.000	5.131.425	-798.152	-208
29.660.000	5.133.103	-798.439	-208
29.670.000	5.134.695	-798.651	-208
29.680.000	5.136.397	-798.893	-206
29.690.000	5.138.015	-799.081	-203
29.700.000	5.139.608	-799.402	-199
29.710.000	5.141.342	-799.548	-197
29.720.000	5.142.757	-799.814	-194
29.730.000	5.144.619	-800.100	-194
29.740.000	5.146.069	-800.303	-196
29.750.000	5.147.868	-800.561	-198
29.760.000	5.149.341	-800.809	-198
29.770.000	5.151.130	-801.043	-198
29.780.000	5.152.612	-801.256	-197
29.790.000	5.154.429	-801.576	-197
29.800.000	5.155.966	-801.737	-197
29.810.000	5.157.594	-802.024	-199
29.820.000	5.159.286	-802.261	-199
29.830.000	5.160.838	-802.524	-201
29.840.000	5.162.575	-802.715	-203
29.850.000	5.164.087	-803.036	-202
29.860.000	5.165.952	-803.263	-199
29.870.000	5.167.307	-803.503	-195
29.880.000	5.169.153	-803.793	-192
29.890.000	5.170.624	-803.986	-192
29.900.000	5.172.360	-804.267	-192
29.910.000	5.173.878	-804.507	-193
29.920.000	5.175.629	-804.770	-194
29.930.000	5.177.145	-804.954	-193
29.940.000	5.178.820	-805.272	-190

29.950.000	5.180.490	-805.443	-189
29.960.000	5.181.991	-805.721	-189
29.970.000	5.183.771	-805.950	-190
29.980.000	5.185.240	-806.207	-191
29.990.000	5.187.022	-806.451	-193
30.000.000	5.188.472	-806.701	-196
30.010.000	5.190.312	-806.963	-196
30.020.000	5.191.753	-807.162	-195
30.030.000	5.193.522	-807.461	-192
30.040.000	5.195.070	-807.644	-188
30.050.000	5.196.723	-807.902	-187
30.060.000	5.198.311	-808.106	-187
30.070.000	5.199.945	-808.395	-190
30.080.000	5.201.584	-808.533	-191
30.090.000	5.203.153	-808.857	-192
30.100.000	5.204.935	-809.065	-193
30.110.000	5.206.333	-809.281	-193
30.120.000	5.208.165	-809.568	-191
30.130.000	5.209.654	-809.797	-189
30.140.000	5.211.412	-810.021	-188
30.150.000	5.212.860	-810.245	-189
30.160.000	5.214.656	-810.533	-189
30.170.000	5.216.135	-810.701	-188
30.180.000	5.217.868	-811.007	-186
30.190.000	5.219.500	-811.201	-183
30.200.000	5.221.055	-811.445	-180
30.210.000	5.222.749	-811.644	-176
30.220.000	5.224.310	-811.945	-174
30.230.000	5.226.039	-812.111	-175
30.240.000	5.227.482	-812.392	-177

30.250.000	5.229.350	-812.671	-179
30.260.000	5.230.699	-812.848	-180
30.270.000	5.232.541	-813.132	-180
30.280.000	5.234.036	-813.368	-177
30.290.000	5.235.756	-813.610	-173
30.300.000	5.237.308	-813.824	-170
30.310.000	5.239.030	-814.146	-168
30.320.000	5.240.614	-814.251	-166
30.330.000	5.242.177	-814.571	-167
30.340.000	5.243.899	-814.769	-170
30.350.000	5.245.423	-815.006	-173
30.360.000	5.247.198	-815.257	-175
30.370.000	5.248.650	-815.535	-175
30.380.000	5.250.427	-815.753	-173
30.390.000	5.251.856	-815.995	-170
30.400.000	5.253.684	-816.314	-167
30.410.000	5.255.169	-816.472	-163
30.420.000	5.256.896	-816.805	-160
30.430.000	5.258.453	-817.029	-160
30.440.000	5.260.134	-817.269	-162
30.450.000	5.261.724	-817.478	-163
30.460.000	5.263.371	-817.815	-163
30.470.000	5.265.077	-817.976	-161
30.480.000	5.266.568	-818.279	-160
30.490.000	5.268.418	-818.559	-159
30.500.000	5.269.834	-818.787	-158
30.510.000	5.271.599	-819.057	-159
30.520.000	5.273.095	-819.329	-159
30.530.000	5.274.838	-819.570	-161
30.540.000	5.276.325	-819.791	-163

30.550.000	5.278.087	-820.144	-163
30.560.000	5.279.657	-820.291	-163
30.570.000	5.281.292	-820.602	-161
30.580.000	5.282.950	-820.837	-159
30.590.000	5.284.553	-821.099	-160
30.600.000	5.286.239	-821.288	-160
30.610.000	5.287.752	-821.616	-160
30.620.000	5.289.584	-821.825	-160
30.630.000	5.290.989	-822.081	-161
30.640.000	5.292.820	-822.380	-160
30.650.000	5.294.278	-822.566	-159
30.660.000	5.296.029	-822.853	-158
30.670.000	5.297.552	-823.082	-159
30.680.000	5.299.297	-823.348	-160
30.690.000	5.300.829	-823.542	-160
30.700.000	5.302.531	-823.848	-159
30.710.000	5.304.205	-824.018	-159
30.720.000	5.305.676	-824.303	-157
30.730.000	5.307.471	-824.532	-155
30.740.000	5.308.968	-824.783	-153
30.750.000	5.310.678	-825.017	-152
30.760.000	5.312.141	-825.273	-152
30.770.000	5.313.946	-825.535	-153
30.780.000	5.315.338	-825.709	-154
30.790.000	5.317.184	-826.024	-155
30.800.000	5.318.710	-826.215	-157
30.810.000	5.320.376	-826.460	-159
30.820.000	5.321.949	-826.666	-160
30.830.000	5.323.582	-826.928	-161
30.840.000	5.325.213	-827.082	-159

30.850.000	5.326.758	-827.401	-158
30.860.000	5.328.516	-827.562	-159
30.870.000	5.329.939	-827.789	-158
30.880.000	5.331.754	-828.060	-159
30.890.000	5.333.214	-828.274	-159
30.900.000	5.334.975	-828.486	-159
30.910.000	5.336.421	-828.713	-160
30.920.000	5.338.293	-828.987	-159
30.930.000	5.339.717	-829.153	-157
30.940.000	5.341.437	-829.442	-155
30.950.000	5.343.077	-829.646	-155
30.960.000	5.344.636	-829.878	-155
30.970.000	5.346.326	-830.071	-154
30.980.000	5.347.873	-830.386	-151
30.990.000	5.349.600	-830.552	-148
31.000.000	5.351.072	-830.828	-144
31.010.000	5.352.918	-831.129	-139
31.020.000	5.354.294	-831.288	-136
31.030.000	5.356.130	-831.579	-135
31.040.000	5.357.630	-831.818	-137
31.050.000	5.359.356	-832.051	-138
31.060.000	5.360.904	-832.279	-139
31.070.000	5.362.605	-832.578	-138
31.080.000	5.364.142	-832.708	-136
31.090.000	5.365.719	-833.040	-133
31.100.000	5.367.477	-833.230	-130
31.110.000	5.368.958	-833.465	-126
31.120.000	5.370.715	-833.708	-125
31.130.000	5.372.193	-833.984	-124
31.140.000	5.374.032	-834.204	-124

31.150.000	5.375.421	-834.432	-125
31.160.000	5.377.290	-834.738	-124
31.170.000	5.378.756	-834.903	-122
31.180.000	5.380.470	-835.212	-120
31.190.000	5.382.053	-835.427	-119
31.200.000	5.383.665	-835.696	-117
31.210.000	5.385.287	-835.893	-116
31.220.000	5.386.908	-836.223	-116
31.230.000	5.388.589	-836.358	-116
31.240.000	5.390.071	-836.662	-117
31.250.000	5.391.897	-836.929	-117
31.260.000	5.393.315	-837.119	-117
31.270.000	5.395.089	-837.384	-116
31.280.000	5.396.569	-837.647	-116
31.290.000	5.398.355	-837.858	-115
31.300.000	5.399.812	-838.079	-116
31.310.000	5.401.545	-838.388	-115
31.320.000	5.403.096	-838.533	-115
31.330.000	5.404.699	-838.815	-114
31.340.000	5.406.366	-839.024	-111
31.350.000	5.407.941	-839.274	-110
31.360.000	5.409.630	-839.464	-108
31.370.000	5.411.154	-839.759	-107
31.380.000	5.412.976	-839.966	-106
31.390.000	5.414.349	-840.197	-107
31.400.000	5.416.222	-840.501	-108
31.410.000	5.417.683	-840.683	-109
31.420.000	5.419.378	-840.952	-108
31.430.000	5.420.908	-841.172	-107
31.440.000	5.422.638	-841.452	-105

31.450.000	5.424.155	-841.630	-102
31.460.000	5.425.813	-841.932	-99
31.470.000	5.427.490	-842.128	-97
31.480.000	5.429.023	-842.403	-97
31.490.000	5.430.814	-842.628	-98
31.500.000	5.432.272	-842.881	-98
31.510.000	5.434.057	-843.136	-97
31.520.000	5.435.495	-843.385	-96
31.530.000	5.437.309	-843.650	-95
31.540.000	5.438.720	-843.854	-92
31.550.000	5.440.509	-844.170	-89
31.560.000	5.442.032	-844.347	-89
31.570.000	5.443.662	-844.604	-90
31.580.000	5.445.263	-844.819	-90
31.590.000	5.446.947	-845.098	-91
31.600.000	5.448.550	-845.247	-90
31.610.000	5.450.112	-845.559	-90
31.620.000	5.451.889	-845.748	-86
31.630.000	5.453.304	-845.980	-81
31.640.000	5.455.102	-846.240	-77
31.650.000	5.456.553	-846.470	-72
31.660.000	5.458.253	-846.677	-71
31.670.000	5.459.751	-846.897	-72
31.680.000	5.461.520	-847.188	-74
31.690.000	5.462.982	-847.325	-76
31.700.000	5.464.742	-847.658	-77
31.710.000	5.466.369	-847.858	-78
31.720.000	5.467.938	-848.090	-77
31.730.000	5.469.648	-848.290	-75
31.740.000	5.471.195	-848.606	-75

31.750.000	5.472.906	-848.747	-74
31.760.000	5.474.374	-849.033	-75
31.770.000	5.476.205	-849.329	-79
31.780.000	5.477.566	-849.494	-83
31.790.000	5.479.365	-849.791	-85
31.800.000	5.480.845	-850.015	-86
31.810.000	5.482.559	-850.255	-86
31.820.000	5.484.107	-850.467	-85
31.830.000	5.485.802	-850.760	-81
31.840.000	5.487.377	-850.887	-78
31.850.000	5.488.981	-851.197	-76
31.860.000	5.490.713	-851.389	-73
31.870.000	5.492.173	-851.607	-72
31.880.000	5.493.937	-851.839	-72
31.890.000	5.495.405	-852.113	-74
31.900.000	5.497.160	-852.328	-75
31.910.000	5.498.559	-852.543	-76
31.920.000	5.500.423	-852.845	-76
31.930.000	5.501.884	-853.005	-75
31.940.000	5.503.590	-853.300	-73
31.950.000	5.505.182	-853.521	-71
31.960.000	5.506.827	-853.766	-69
31.970.000	5.508.444	-853.964	-68
31.980.000	5.510.045	-854.288	-69
31.990.000	5.511.723	-854.420	-71
32.000.000	5.513.165	-854.691	-72
32.010.000	5.514.976	-854.964	-75
32.020.000	5.516.402	-855.179	-74
32.030.000	5.518.199	-855.445	-70
32.040.000	5.519.686	-855.698	-66

32.050.000	5.521.466	-855.927	-61
32.060.000	5.522.918	-856.138	-58
32.070.000	5.524.680	-856.452	-56
32.080.000	5.526.216	-856.593	-54
32.090.000	5.527.796	-856.884	-57
32.100.000	5.529.487	-857.097	-58
32.110.000	5.531.036	-857.377	-60
32.120.000	5.532.752	-857.551	-62
32.130.000	5.534.243	-857.853	-63
32.140.000	5.536.057	-858.086	-62
32.150.000	5.537.433	-858.287	-60
32.160.000	5.539.309	-858.590	-60
32.170.000	5.540.753	-858.774	-60
32.180.000	5.542.485	-859.014	-60
32.190.000	5.544.027	-859.254	-61
32.200.000	5.545.735	-859.522	-63
32.210.000	5.547.253	-859.673	-65
32.220.000	5.548.880	-859.967	-66
32.230.000	5.550.567	-860.157	-64
32.240.000	5.552.086	-860.418	-61
32.250.000	5.553.828	-860.640	-60
32.260.000	5.555.301	-860.895	-57
32.270.000	5.557.091	-861.117	-56
32.280.000	5.558.531	-861.380	-56
32.290.000	5.560.385	-861.644	-57
32.300.000	5.561.761	-861.817	-58
32.310.000	5.563.540	-862.110	-55
32.320.000	5.565.083	-862.319	-53
32.330.000	5.566.710	-862.571	-51
32.340.000	5.568.339	-862.770	-49

32.350.000	5.569.983	-863.063	-47
32.360.000	5.571.620	-863.198	-48
32.370.000	5.573.163	-863.509	-49
32.380.000	5.574.977	-863.722	-51
32.390.000	5.576.357	-863.935	-50
32.400.000	5.578.198	-864.208	-47
32.410.000	5.579.641	-864.435	-45
32.420.000	5.581.376	-864.628	-44
32.430.000	5.582.838	-864.840	-43
32.440.000	5.584.596	-865.136	-45
32.450.000	5.586.110	-865.242	-44
32.460.000	5.587.766	-865.533	-45
32.470.000	5.589.396	-865.724	-44
32.480.000	5.591.002	-865.933	-42
32.490.000	5.592.625	-866.111	-42
32.500.000	5.594.198	-866.409	-42
32.510.000	5.595.959	-866.561	-43
32.520.000	5.597.344	-866.815	-45
32.530.000	5.599.230	-867.085	-46
32.540.000	5.600.604	-867.243	-47
32.550.000	5.602.357	-867.496	-47
32.560.000	5.603.860	-867.714	-45
32.570.000	5.605.560	-867.934	-43
32.580.000	5.607.074	-868.104	-42
32.590.000	5.608.776	-868.412	-41
32.600.000	5.610.379	-868.540	-41
32.610.000	5.611.965	-868.814	-41
32.620.000	5.613.740	-869.007	-40
32.630.000	5.615.179	-869.220	-37
32.640.000	5.617.010	-869.458	-34

32.650.000	5.618.429	-869.719	-30
32.660.000	5.620.227	-869.925	-25
32.670.000	5.621.631	-870.151	-24
32.680.000	5.623.477	-870.477	-24
32.690.000	5.624.957	-870.616	-24
32.700.000	5.626.630	-870.910	-24
32.710.000	5.628.214	-871.112	-24
32.720.000	5.629.916	-871.348	-25
32.730.000	5.631.494	-871.527	-26
32.740.000	5.633.058	-871.825	-28
32.750.000	5.634.825	-871.953	-28
32.760.000	5.636.205	-872.225	-29
32.770.000	5.638.037	-872.459	-27
32.780.000	5.639.488	-872.659	-25
32.790.000	5.641.219	-872.877	-24
32.800.000	5.642.700	-873.097	-24
32.810.000	5.644.478	-873.331	-25
32.820.000	5.645.900	-873.484	-25
32.830.000	5.647.668	-873.765	-24
32.840.000	5.649.240	-873.944	-25
32.850.000	5.650.807	-874.164	-26
32.860.000	5.652.494	-874.342	-27
32.870.000	5.654.022	-874.613	-25
32.880.000	5.655.755	-874.745	-23
32.890.000	5.657.255	-875.035	-24
32.900.000	5.659.064	-875.257	-26
32.910.000	5.660.443	-875.424	-25
32.920.000	5.662.264	-875.705	-23
32.930.000	5.663.735	-875.911	-21
32.940.000	5.665.479	-876.139	-19

32.950.000	5.667.003	-876.363	-18
32.960.000	5.668.725	-876.645	-17
32.970.000	5.670.269	-876.771	-16
32.980.000	5.671.877	-877.089	-16
32.990.000	5.673.648	-877.288	-15
33.000.000	5.675.117	-877.515	-15
33.010.000	5.676.888	-877.740	-14
33.020.000	5.678.349	-878.014	-14
33.030.000	5.680.121	-878.221	-14
33.040.000	5.681.559	-878.456	-14
33.050.000	5.683.382	-878.734	-13
33.060.000	5.684.819	-878.868	-13
33.070.000	5.686.612	-879.193	-12
33.080.000	5.688.141	-879.392	-12
33.090.000	5.689.770	-879.604	-11
33.100.000	5.691.420	-879.800	-11
33.110.000	5.693.006	-880.123	-9
33.120.000	5.694.668	-880.220	-7
33.130.000	5.696.153	-880.504	-7
33.140.000	5.697.950	-880.745	-7
33.150.000	5.699.346	-880.902	-9
33.160.000	5.701.168	-881.160	-7
33.170.000	5.702.628	-881.357	-7
33.180.000	5.704.381	-881.551	-7
33.190.000	5.705.807	-881.760	-6
33.200.000	5.707.561	-882.023	-6
33.210.000	5.709.115	-882.134	-6
33.220.000	5.710.692	-882.390	-6
33.230.000	5.712.400	-882.568	-5
33.240.000	5.713.927	-882.789	-3

33.250.000	5.715.615	-882.946	2
33.260.000	5.717.125	-883.227	5
33.270.000	5.718.961	-883.412	9
33.280.000	5.720.340	-883.619	11
33.290.000	5.722.190	-883.899	13
33.300.000	5.723.651	-884.071	14
33.310.000	5.725.363	-884.309	13
33.320.000	5.726.849	-884.535	13
33.330.000	5.728.542	-884.754	15
33.340.000	5.730.091	-884.930	18
33.350.000	5.731.712	-885.229	21
33.360.000	5.733.378	-885.370	22
33.370.000	5.734.903	-885.655	21
33.380.000	5.736.683	-885.866	18
33.390.000	5.738.122	-886.078	17
33.400.000	5.739.880	-886.294	17
33.410.000	5.741.320	-886.547	16
33.420.000	5.743.177	-886.775	16
33.430.000	5.744.537	-886.947	17
33.440.000	5.746.325	-887.226	21
33.450.000	5.747.860	-887.399	22
33.460.000	5.749.474	-887.642	21
33.470.000	5.751.098	-887.833	17
33.480.000	5.752.749	-888.105	16
33.490.000	5.754.376	-888.240	14
33.500.000	5.755.934	-888.547	12
33.510.000	5.757.758	-888.750	11
33.520.000	5.759.117	-888.955	10
33.530.000	5.760.947	-889.242	11
33.540.000	5.762.367	-889.480	11

33.550.000	5.764.158	-889.680	11
33.560.000	5.765.595	-889.903	14
33.570.000	5.767.370	-890.206	17
33.580.000	5.768.858	-890.321	20
33.590.000	5.770.518	-890.617	24
33.600.000	5.772.186	-890.814	26
33.610.000	5.773.737	-891.032	29
33.620.000	5.775.440	-891.214	30
33.630.000	5.776.959	-891.515	29
33.640.000	5.778.703	-891.677	30
33.650.000	5.780.091	-891.935	32
33.660.000	5.781.921	-892.194	36
33.670.000	5.783.336	-892.336	40
33.680.000	5.785.074	-892.618	42
33.690.000	5.786.641	-892.817	46
33.700.000	5.788.289	-893.028	49
33.710.000	5.789.821	-893.226	49
33.720.000	5.791.532	-893.542	49
33.730.000	5.793.157	-893.665	46
33.740.000	5.794.683	-893.944	45
33.750.000	5.796.421	-894.133	41
33.760.000	5.797.842	-894.354	39
33.770.000	5.799.638	-894.571	36
33.780.000	5.801.059	-894.820	34
33.790.000	5.802.854	-895.005	33
33.800.000	5.804.239	-895.201	34
33.810.000	5.806.042	-895.484	41
33.820.000	5.807.523	-895.610	48
33.830.000	5.809.214	-895.878	54
33.840.000	5.810.835	-896.096	58

33.850.000	5.812.463	-896.297	62
33.860.000	5.814.094	-896.480	64
33.870.000	5.815.667	-896.792	63
33.880.000	5.817.402	-896.929	57
33.890.000	5.818.796	-897.183	54
33.900.000	5.820.621	-897.449	51
33.910.000	5.822.061	-897.636	50
33.920.000	5.823.765	-897.881	49
33.930.000	5.825.281	-898.100	49
33.940.000	5.827.066	-898.350	50
33.950.000	5.828.532	-898.526	53
33.960.000	5.830.252	-898.828	57
33.970.000	5.831.833	-898.966	58
33.980.000	5.833.384	-899.219	59
33.990.000	5.835.075	-899.405	60
34.000.000	5.836.613	-899.673	58
34.010.000	5.838.320	-899.818	53
34.020.000	5.839.741	-900.082	49
34.030.000	5.841.576	-900.314	47
34.040.000	5.842.949	-900.478	46
34.050.000	5.844.766	-900.755	48
34.060.000	5.846.241	-900.929	51
34.070.000	5.847.960	-901.165	55
34.080.000	5.849.513	-901.373	62
34.090.000	5.851.220	-901.640	67
34.100.000	5.852.783	-901.787	72
34.110.000	5.854.393	-902.103	75
34.120.000	5.856.132	-902.279	78
34.130.000	5.857.544	-902.517	81
34.140.000	5.859.383	-902.776	80

34.150.000	5.860.851	-903.037	78
34.160.000	5.862.587	-903.241	77
34.170.000	5.864.070	-903.494	76
34.180.000	5.865.868	-903.777	75
34.190.000	5.867.289	-903.914	76
34.200.000	5.869.011	-904.222	78
34.210.000	5.870.664	-904.431	82
34.220.000	5.872.210	-904.660	84
34.230.000	5.873.865	-904.860	86
34.240.000	5.875.453	-905.182	85
34.250.000	5.877.175	-905.309	83
34.260.000	5.878.614	-905.590	82
34.270.000	5.880.440	-905.872	79
34.280.000	5.881.863	-906.047	79
34.290.000	5.883.650	-906.323	80
34.300.000	5.885.145	-906.559	80
34.310.000	5.886.896	-906.764	83
34.320.000	5.888.354	-906.968	82
34.330.000	5.890.090	-907.283	80
34.340.000	5.891.676	-907.406	78
34.350.000	5.893.252	-907.687	72
34.360.000	5.894.921	-907.862	66
34.370.000	5.896.434	-908.101	61
34.380.000	5.898.165	-908.289	59
34.390.000	5.899.652	-908.562	59
34.400.000	5.901.478	-908.770	62
34.410.000	5.902.856	-908.950	66
34.420.000	5.904.677	-909.236	74
34.430.000	5.906.118	-909.405	83
34.440.000	5.907.854	-909.647	92

34.450.000	5.909.394	-909.867	98
34.460.000	5.911.101	-910.114	101
34.470.000	5.912.671	-910.275	103
34.480.000	5.914.277	-910.575	101
34.490.000	5.916.020	-910.735	98
34.500.000	5.917.479	-910.998	95
34.510.000	5.919.296	-911.253	93
34.520.000	5.920.759	-911.478	92
34.530.000	5.922.557	-911.704	95
34.540.000	5.923.985	-911.944	98
34.550.000	5.925.831	-912.208	100
34.560.000	5.927.238	-912.368	102
34.570.000	5.928.976	-912.687	103
34.580.000	5.930.595	-912.876	102
34.590.000	5.932.176	-913.120	101
34.600.000	5.933.860	-913.325	98
34.610.000	5.935.445	-913.622	98
34.620.000	5.937.087	-913.756	100
34.630.000	5.938.623	-914.039	101
34.640.000	5.940.439	-914.296	101
34.650.000	5.941.792	-914.473	98
34.660.000	5.943.651	-914.746	96
34.670.000	5.945.128	-914.946	94
34.680.000	5.946.819	-915.145	91
34.690.000	5.948.356	-915.350	92
34.700.000	5.950.080	-915.647	93
34.710.000	5.951.624	-915.746	96
34.720.000	5.953.248	-916.029	102
34.730.000	5.954.920	-916.206	107
34.740.000	5.956.426	-916.420	110

34.750.000	5.958.205	-916.622	108
34.760.000	5.959.661	-916.873	108
34.770.000	5.961.445	-917.051	107
34.780.000	5.962.840	-917.265	104
34.790.000	5.964.626	-917.506	100
34.800.000	5.966.052	-917.629	98
34.810.000	5.967.797	-917.893	100
34.820.000	5.969.363	-918.088	102
34.830.000	5.971.045	-918.288	104
34.840.000	5.972.612	-918.447	108
34.850.000	5.974.255	-918.759	114
34.860.000	5.975.920	-918.846	119
34.870.000	5.977.374	-919.098	122
34.880.000	5.979.205	-919.344	123
34.890.000	5.980.609	-919.526	124
34.900.000	5.982.374	-919.765	125
34.910.000	5.983.857	-919.987	124
34.920.000	5.985.656	-920.212	121
34.930.000	5.987.097	-920.389	116
34.940.000	5.988.871	-920.694	117
34.950.000	5.990.415	-920.839	121
34.960.000	5.992.038	-921.101	121
34.970.000	5.993.688	-921.318	122
34.980.000	5.995.316	-921.559	123
34.990.000	5.996.971	-921.720	126
35.000.000	5.998.454	-922.007	125
35.010.000	6.000.283	-922.240	123
35.020.000	6.001.642	-922.434	121
35.030.000	6.003.477	-922.726	121
35.040.000	6.004.942	-922.920	123

35.050.000	6.006.684	-923.181	125
35.060.000	6.008.204	-923.368	125
35.070.000	6.009.905	-923.671	126
35.080.000	6.011.477	-923.828	128
35.090.000	6.013.140	-924.125	127
35.100.000	6.014.827	-924.298	126
35.110.000	6.016.288	-924.532	127
