# Supplementary material for: Data on statistical experimental design to formulate amphotericin B-loaded Eudragit RL100 nanoparticles coated with hyaluronic acid for the treatment of vulvovaginal candidiasis
Source: Data Brief. 2020 Mar 5;29:105311. doi: 10.1016/j.dib.2020.105311 (PMC7082528; doi:10.1016/j.dib.2020.105311)
Supplement: Multimedia component 5 [file mmc5.pdf]

File Name:	**EUD nanoparticles HA**		
[Data]			
Time	Temp	**DTA**	**TGA**
sec	C	uV	mg
0	234.873	-1.625	1.5653
1.2000	235.155	-1.723	1.5650
2.2000	234.986	-1.687	1.5648
3.2000	235.136	-1.726	1.5646
4.2000	234.990	-1.741	1.5643
5.2000	235.234	-1.776	1.5639
6.2000	235.066	-1.752	1.5636
7.2000	235.328	-1.893	1.5633
8.2000	235.267	-1.797	1.5630
9.2000	235.366	-1.966	1.5632
10.2000	235.527	-2.009	1.5634
11.2000	235.575	-2.077	1.5635
12.2000	235.787	-2.161	1.5638
130.000	235.951	-2.416	1.5638
140.000	236.218	-2.465	1.5640
150.000	236.335	-2.799	1.5640
160.000	236.886	-2.979	1.5639
170.000	236.764	-3.249	1.5638
180.000	237.423	-3.649	1.5640
190.000	237.533	-3.955	1.5641
200.000	238.076	-4.356	1.5641
210.000	238.253	-4.821	1.5641
220.000	238.868	-5.270	1.5639
230.000	239.060	-5.750	1.5639
240.000	239.787	-6.415	1.5638

250.000	240.080	-6.868	1.5635
260.000	240.696	-7.607	1.5635
270.000	241.217	-8.258	1.5636
280.000	241.759	-8.933	1.5636
290.000	242.324	-9.647	1.5637
300.000	242.973	-10.485	1.5637
310.000	243.586	-11.126	1.5637
320.000	244.114	-12.069	1.5639
330.000	244.953	-12.824	1.5641
340.000	245.386	-13.741	1.5643
350.000	246.369	-14.666	1.5645
360.000	246.866	-15.551	1.5645
370.000	247.824	-16.536	1.5647
380.000	248.354	-17.536	1.5651
390.000	249.426	-18.464	1.5650
400.000	249.928	-19.466	1.5651
410.000	251.005	-20.561	1.5651
420.000	251.573	-21.470	1.5651
430.000	252.609	-22.627	1.5651
440.000	253.341	-23.659	1.5649
450.000	254.304	-24.717	1.5647
460.000	255.054	-25.798	1.5648
470.000	256.187	-26.982	1.5649
480.000	256.976	-27.975	1.5652
490.000	257.919	-29.215	1.5657
500.000	258.996	-30.232	1.5662
510.000	259.767	-31.449	1.5666
520.000	260.918	-32.543	1.5671
530.000	261.788	-33.710	1.5675
540.000	262.923	-34.836	1.5677

550.000	263.758	-36.073	1.5679
560.000	265.059	-37.213	1.5681
570.000	265.733	-38.392	1.5682
580.000	267.242	-39.659	1.5683
590.000	268.000	-40.747	1.5684
600.000	269.319	-42.007	1.5684
610.000	270.291	-43.203	1.5685
620.000	271.522	-44.377	1.5683
630.000	272.486	-45.535	1.5680
640.000	273.810	-46.832	1.5677
650.000	274.784	-47.915	1.5674
660.000	276.080	-49.234	1.5669
670.000	277.252	-50.363	1.5666
680.000	278.324	-51.597	1.5663
690.000	279.671	-52.791	1.5662
700.000	280.757	-54.029	1.5659
710.000	282.127	-55.141	1.5658
720.000	283.209	-56.467	1.5656
730.000	284.656	-57.564	1.5656
740.000	285.634	-58.824	1.5655
750.000	287.176	-60.061	1.5656
760.000	288.130	-61.183	1.5658
770.000	289.651	-62.453	1.5660
780.000	290.733	-63.645	1.5664
790.000	292.236	-64.810	1.5667
800.000	293.336	-65.996	1.5671
810.000	294.911	-67.260	1.5674
820.000	296.022	-68.321	1.5679
830.000	297.533	-69.628	1.5682
840.000	298.796	-70.692	1.5684

850.000	300.103	-71.900	1.5687
860.000	301.489	-73.050	1.5687
870.000	302.892	-74.208	1.5688
880.000	304.257	-75.292	1.5689
890.000	305.516	-76.569	1.5688
900.000	307.038	-77.530	1.5688
910.000	308.136	-78.779	1.5689
920.000	309.829	-79.930	1.5690
930.000	310.968	-80.988	1.5690
940.000	312.600	-82.177	1.5687
950.000	313.807	-83.288	1.5684
960.000	315.347	-84.355	1.5679
970.000	316.527	-85.464	1.5673
980.000	318.195	-86.606	1.5667
990.000	319.331	-87.608	1.5662
1.000.000	320.954	-88.786	1.5658
1.010.000	322.304	-89.788	1.5654
1.020.000	323.757	-90.899	1.5652
1.030.000	325.222	-91.950	1.5651
1.040.000	326.739	-93.017	1.5649
1.050.000	328.084	-93.984	1.5647
1.060.000	329.567	-95.157	1.5646
1.070.000	331.128	-96.046	1.5644
1.080.000	332.375	-97.165	1.5641
1.090.000	334.147	-98.168	1.5641
1.100.000	335.346	-99.159	1.5639
1.110.000	337.044	-100.190	1.5637
1.120.000	338.315	-101.210	1.5637
1.130.000	340.000	-102.156	1.5637
1.140.000	341.279	-103.184	1.5637

1.150.000	343.074	-104.197	1.5638
1.160.000	344.284	-105.093	1.5643
1.170.000	346.127	-106.159	1.5648
1.180.000	347.443	-107.044	1.5652
1.190.000	349.033	-108.038	1.5657
1.200.000	350.512	-108.954	1.5663
1.210.000	352.119	-109.920	1.5668
1.220.000	353.548	-110.788	1.5668
1.230.000	355.143	-111.838	1.5669
1.240.000	356.665	-112.604	1.5669
1.250.000	358.167	-113.622	1.5667
1.260.000	359.899	-114.517	1.5663
1.270.000	361.298	-115.406	1.5660
1.280.000	363.064	-116.288	1.5659
1.290.000	364.411	-117.251	1.5658
1.300.000	366.191	-118.084	1.5659
1.310.000	367.515	-118.978	1.5662
1.320.000	369.373	-119.896	1.5664
1.330.000	370.673	-120.708	1.5665
1.340.000	372.459	-121.636	1.5667
1.350.000	373.855	-122.413	1.5666
1.360.000	375.624	-123.317	1.5664
1.370.000	377.077	-124.146	1.5660
1.380.000	378.874	-125.014	1.5657
1.390.000	380.309	-125.769	1.5657
1.400.000	382.009	-126.710	1.5656
1.410.000	383.613	-127.417	1.5653
1.420.000	385.154	-128.308	1.5651
1.430.000	386.909	-129.046	1.5650
1.440.000	388.343	-129.858	1.5650

1.450.000	390.133	-130.621	1.5647
1.460.000	391.620	-131.498	1.5645
1.470.000	393.404	-132.171	1.5645
1.480.000	394.782	-133.031	1.5645
1.490.000	396.773	-133.824	1.5641
1.500.000	398.078	-134.559	1.5638
1.510.000	400.011	-135.352	1.5636
1.520.000	401.429	-136.055	1.5631
1.530.000	403.203	-136.831	1.5626
1.540.000	404.680	-137.549	1.5623
1.550.000	406.412	-138.290	1.5620
1.560.000	407.860	-138.939	1.5618
1.570.000	409.605	-139.775	1.5619
1.580.000	411.146	-140.379	1.5622
1.590.000	412.795	-141.144	1.5622
1.600.000	414.532	-141.833	1.5622
1.610.000	416.104	-142.549	1.5624
1.620.000	417.831	-143.172	1.5625
1.630.000	419.330	-143.970	1.5625
1.640.000	421.124	-144.516	1.5623
1.650.000	422.536	-145.280	1.5621
1.660.000	424.430	-145.953	1.5621
1.670.000	425.754	-146.566	1.5620
1.680.000	427.698	-147.299	1.5618
1.690.000	429.057	-147.906	1.5616
1.700.000	430.977	-148.582	1.5615
1.710.000	432.415	-149.223	1.5614
1.720.000	434.300	-149.866	1.5612
1.730.000	435.657	-150.435	1.5608
1.740.000	437.586	-151.192	1.5603

1.750.000	439.062	-151.672	1.5598
1.760.000	440.773	-152.379	1.5591
1.770.000	442.418	-152.961	1.5586
1.780.000	444.027	-153.550	1.5582
1.790.000	445.754	-154.119	1.5577
1.800.000	447.375	-154.831	1.5574
1.810.000	449.129	-155.267	1.5574
1.820.000	450.664	-156.000	1.5576
1.830.000	452.621	-156.533	1.5575
1.840.000	453.967	-157.109	1.5577
1.850.000	455.910	-157.736	1.5580
1.860.000	457.369	-158.299	1.5581
1.870.000	459.229	-158.846	1.5582
1.880.000	460.655	-159.450	1.5583
1.890.000	462.611	-160.003	1.5582
1.900.000	463.986	-160.538	1.5580
1.910.000	465.886	-161.170	1.5578
1.920.000	467.453	-161.624	1.5575
1.930.000	469.227	-162.253	1.5570
1.940.000	470.892	-162.762	1.5565
1.950.000	472.577	-163.307	1.5560
1.960.000	474.215	-163.811	1.5554
1.970.000	475.993	-164.449	1.5550
1.980.000	477.660	-164.847	1.5547
1.990.000	479.220	-165.474	1.5544
2.000.000	481.152	-165.940	1.5542
2.010.000	482.522	-166.457	1.5542
2.020.000	484.528	-166.984	1.5541
2.030.000	486.001	-167.487	1.5542
2.040.000	487.872	-168.004	1.5540

2.050.000	489.452	-168.538	1.5542
2.060.000	491.394	-169.009	1.5542
2.070.000	492.763	-169.443	1.5543
2.080.000	494.755	-170.015	1.5545
2.090.000	496.255	-170.360	1.5546
2.100.000	498.049	-170.899	1.5545
2.110.000	499.675	-171.341	1.5544
2.120.000	501.423	-171.802	1.5541
2.130.000	503.111	-172.200	1.5538
2.140.000	504.832	-172.744	1.5531
2.150.000	506.498	-173.039	1.5528
2.160.000	508.165	-173.590	1.5525
2.170.000	510.003	-173.949	1.5523
2.180.000	511.509	-174.399	1.5520
2.190.000	513.385	-174.815	1.5520
2.200.000	514.849	-175.265	1.5520
2.210.000	516.762	-175.665	1.5518
2.220.000	518.168	-176.094	1.5516
2.230.000	520.134	-176.513	1.5516
2.240.000	521.511	-176.893	1.5517
2.250.000	523.491	-177.400	1.5514
2.260.000	524.952	-177.686	1.5511
2.270.000	526.832	-178.175	1.5508
2.280.000	528.414	-178.578	1.5505
2.290.000	530.219	-178.980	1.5502
2.300.000	531.818	-179.353	1.5498
2.310.000	533.594	-179.840	1.5496
2.320.000	535.236	-180.095	1.5496
2.330.000	536.869	-180.632	1.5497
2.340.000	538.669	-180.953	1.5500

2.350.000	540.205	-181.352	1.5501
2.360.000	542.080	-181.732	1.5499
2.370.000	543.626	-182.177	1.5498
2.380.000	545.502	-182.528	1.5498
2.390.000	546.979	-182.984	1.5494
2.400.000	549.016	-183.355	1.5495
2.410.000	550.386	-183.761	1.5495
2.420.000	552.368	-184.201	1.5493
2.430.000	553.814	-184.494	1.5496
2.440.000	555.672	-184.936	1.5494
2.450.000	557.288	-185.340	1.5494
2.460.000	559.104	-185.724	1.5495
2.470.000	560.660	-186.070	1.5494
2.480.000	562.498	-186.549	1.5492
2.490.000	564.122	-186.848	1.5490
2.500.000	565.867	-187.297	1.5486
2.510.000	567.677	-187.638	1.5484
2.520.000	569.223	-188.035	1.5480
2.530.000	571.047	-188.373	1.5474
2.540.000	572.679	-188.821	1.5469
2.550.000	574.456	-189.115	1.5467
2.560.000	576.021	-189.573	1.5465
2.570.000	577.989	-189.971	1.5464
2.580.000	579.362	-190.307	1.5463
2.590.000	581.387	-190.735	1.5462
2.600.000	582.857	-191.069	1.5462
2.610.000	584.793	-191.471	1.5464
2.620.000	586.351	-191.858	1.5466
2.630.000	588.203	-192.211	1.5466
2.640.000	589.757	-192.540	1.5467

2.650.000	591.619	-193.052	1.5469
2.660.000	593.234	-193.257	1.5470
2.670.000	594.978	-193.722	1.5471
2.680.000	596.687	-194.040	1.5472
2.690.000	598.350	-194.396	1.5474
2.700.000	600.173	-194.741	1.5476
2.710.000	601.779	-195.180	1.5477
2.720.000	603.630	-195.419	1.5480
2.730.000	605.145	-195.888	1.5483
2.740.000	607.095	-196.185	1.5482
2.750.000	608.439	-196.530	1.5480
2.760.000	610.432	-196.955	1.5481
2.770.000	611.910	-197.240	1.5481
2.780.000	613.748	-197.614	1.5481
2.790.000	615.254	-197.967	1.5480
2.800.000	617.184	-198.319	1.5479
2.810.000	618.642	-198.639	1.5476
2.820.000	620.531	-199.061	1.5472
2.830.000	622.138	-199.292	1.5473
2.840.000	623.872	-199.733	1.5472
2.850.000	625.570	-200.039	1.5470
2.860.000	627.240	-200.366	1.5470
2.870.000	628.944	-200.668	1.5472
2.880.000	630.615	-201.095	1.5472
2.890.000	632.346	-201.302	1.5469
2.900.000	633.922	-201.745	1.5466
2.910.000	635.862	-202.023	1.5463
2.920.000	637.234	-202.363	1.5462
2.930.000	639.254	-202.745	1.5460
2.940.000	640.705	-203.044	1.5460

2.950.000	642.621	-203.404	1.5460
2.960.000	644.158	-203.758	1.5458
2.970.000	646.010	-204.069	1.5457
2.980.000	647.497	-204.365	1.5453
2.990.000	649.413	-204.788	1.5449
3.000.000	650.924	-204.979	1.5444
3.010.000	652.732	-205.406	1.5441
3.020.000	654.340	-205.694	1.5437
3.030.000	656.078	-206.013	1.5433
3.040.000	657.803	-206.330	1.5430
3.050.000	659.519	-206.764	1.5430
3.060.000	661.290	-206.935	1.5428
3.070.000	662.918	-207.387	1.5426
3.080.000	664.780	-207.634	1.5426
3.090.000	666.233	-207.979	1.5424
3.100.000	668.179	-208.342	1.5424
3.110.000	669.666	-208.635	1.5424
3.120.000	671.589	-208.995	1.5424
3.130.000	673.068	-209.341	1.5423
3.140.000	675.033	-209.641	1.5424
3.150.000	676.467	-209.951	1.5428
3.160.000	678.423	-210.368	1.5430
3.170.000	679.943	-210.558	1.5431
3.180.000	681.778	-210.977	1.5434
3.190.000	683.422	-211.278	1.5435
3.200.000	685.158	-211.572	1.5436
3.210.000	686.820	-211.878	1.5435
3.220.000	688.566	-212.273	1.5435
3.230.000	690.295	-212.460	1.5433
3.240.000	691.941	-212.895	1.5431

3.250.000	693.802	-213.109	1.5430
3.260.000	695.268	-213.463	1.5426
3.270.000	697.240	-213.776	1.5421
3.280.000	698.737	-214.085	1.5418
3.290.000	700.670	-214.427	1.5415
3.300.000	702.148	-214.786	1.5412
3.310.000	704.136	-215.054	1.5408
3.320.000	705.486	-215.345	1.5408
3.330.000	707.505	-215.728	1.5407
3.340.000	708.950	-215.937	1.5404
3.350.000	710.743	-216.334	1.5401
3.360.000	712.394	-216.607	1.5401
3.370.000	714.135	-216.924	1.5398
3.380.000	715.725	-217.189	1.5396
3.390.000	717.542	-217.576	1.5395
3.400.000	719.149	-217.768	1.5395
3.410.000	720.859	-218.186	1.5394
3.420.000	722.709	-218.420	1.5392
3.430.000	724.158	-218.749	1.5392
3.440.000	726.077	-219.016	1.5392
3.450.000	727.568	-219.354	1.5389
3.460.000	729.419	-219.613	1.5387
3.470.000	730.898	-219.958	1.5388
3.480.000	732.841	-220.257	1.5387
3.490.000	734.271	-220.521	1.5386
3.500.000	736.255	-220.921	1.5386
3.510.000	737.721	-221.132	1.5386
3.520.000	739.577	-221.480	1.5383
3.530.000	741.125	-221.760	1.5382
3.540.000	742.916	-222.058	1.5381

3.550.000	744.497	-222.325	1.5379
3.560.000	746.323	-222.730	1.5376
3.570.000	747.935	-222.871	1.5374
3.580.000	749.609	-223.277	1.5374
3.590.000	751.382	-223.506	1.5372
3.600.000	752.987	-223.843	1.5370
3.610.000	754.832	-224.107	1.5370
3.620.000	756.431	-224.462	1.5370
3.630.000	758.316	-224.706	1.5369
3.640.000	759.791	-225.081	1.5370
3.650.000	761.730	-225.341	1.5373
3.660.000	763.143	-225.634	1.5374
3.670.000	765.128	-226.011	1.5373
3.680.000	766.559	-226.212	1.5375
3.690.000	768.401	-226.567	1.5378
3.700.000	769.978	-226.865	1.5377
3.710.000	771.822	-227.154	1.5375
3.720.000	773.370	-227.454	1.5380
3.730.000	775.265	-227.841	1.5383
3.740.000	776.866	-228.016	1.5383
3.750.000	778.615	-228.424	1.5383
3.760.000	780.403	-228.658	1.5386
3.770.000	781.967	-228.990	1.5388
3.780.000	783.724	-229.228	1.5385
3.790.000	785.378	-229.584	1.5382
3.800.000	787.175	-229.835	1.5383
3.810.000	788.735	-230.209	1.5383
3.820.000	790.685	-230.465	1.5378
3.830.000	792.090	-230.767	1.5374
3.840.000	794.106	-231.152	1.5375

3.850.000	795.582	-231.371	1.5374
3.860.000	797.490	-231.705	1.5371
3.870.000	798.994	-232.014	1.5369
3.880.000	800.850	-232.309	1.5370
3.890.000	802.360	-232.566	1.5369
3.900.000	804.272	-232.976	1.5368
3.910.000	805.800	-233.136	1.5368
3.920.000	807.601	-233.525	1.5369
3.930.000	809.284	-233.788	1.5368
3.940.000	810.902	-234.084	1.5367
3.950.000	812.664	-234.358	1.5367
3.960.000	814.338	-234.718	1.5366
3.970.000	816.054	-234.886	1.5363
3.980.000	817.609	-235.300	1.5361
3.990.000	819.506	-235.497	1.5360
4.000.000	820.916	-235.820	1.5357
4.010.000	822.901	-236.185	1.5352
4.020.000	824.325	-236.402	1.5349
4.030.000	826.226	-236.748	1.5344
4.040.000	827.740	-237.071	1.5340
4.050.000	829.619	-237.315	1.5336
4.060.000	831.102	-237.602	1.5333
4.070.000	833.016	-237.991	1.5330
4.080.000	834.475	-238.148	1.5329
4.090.000	836.303	-238.544	1.5327
4.100.000	837.951	-238.794	1.5325
4.110.000	839.632	-239.081	1.5324
4.120.000	841.366	-239.365	1.5320
4.130.000	842.996	-239.695	1.5320
4.140.000	844.723	-239.877	1.5320

4.150.000	846.300	-240.272	1.5320
4.160.000	848.233	-240.463	1.5323
4.170.000	849.604	-240.761	1.5324
4.180.000	851.570	-241.091	1.5328
4.190.000	853.013	-241.337	1.5331
4.200.000	854.911	-241.650	1.5331
4.210.000	856.448	-241.954	1.5334
4.220.000	858.340	-242.202	1.5335
4.230.000	859.794	-242.496	1.5335
4.240.000	861.718	-242.839	1.5334
4.250.000	863.194	-243.008	1.5337
4.260.000	865.026	-243.407	1.5339
4.270.000	866.669	-243.648	1.5338
4.280.000	868.365	-243.925	1.5338
4.290.000	870.065	-244.193	1.5340
4.300.000	871.840	-244.552	1.5339
4.310.000	873.463	-244.729	1.5339
4.320.000	875.152	-245.117	1.5339
4.330.000	876.984	-245.293	1.5340
4.340.000	878.432	-245.638	1.5339
4.350.000	880.408	-245.934	1.5338
4.360.000	881.879	-246.188	1.5339
4.370.000	883.791	-246.510	1.5337
4.380.000	885.276	-246.829	1.5336
4.390.000	887.170	-247.091	1.5337
4.400.000	888.648	-247.360	1.5337
4.410.000	890.680	-247.731	1.5336
4.420.000	892.080	-247.897	1.5336
4.430.000	893.973	-248.282	1.5337
4.440.000	895.602	-248.551	1.5338

4.450.000	897.335	-248.842	1.5338
4.460.000	898.991	-249.111	1.5340
4.470.000	900.741	-249.457	1.5340
4.480.000	902.420	-249.645	1.5339
4.490.000	904.099	-250.036	1.5340
4.500.000	905.882	-250.222	1.5340
4.510.000	907.351	-250.547	1.5337
4.520.000	909.291	-250.846	1.5333
4.530.000	910.795	-251.112	1.5332
4.540.000	912.685	-251.390	1.5330
4.550.000	914.126	-251.720	1.5327
4.560.000	916.050	-251.945	1.5324
4.570.000	917.420	-252.237	1.5324
4.580.000	919.381	-252.571	1.5322
4.590.000	920.834	-252.760	1.5319
4.600.000	922.700	-253.122	1.5318
4.610.000	924.251	-253.360	1.5316
4.620.000	926.000	-253.648	1.5314
4.630.000	927.628	-253.924	1.5313
4.640.000	929.455	-254.248	1.5316
4.650.000	931.054	-254.439	1.5317
4.660.000	932.740	-254.818	1.5318
4.670.000	934.457	-254.989	1.5321
4.680.000	935.998	-255.339	1.5323
4.690.000	937.865	-255.570	1.5323
4.700.000	939.414	-255.870	1.5323
4.710.000	941.205	-256.120	1.5323
4.720.000	942.717	-256.435	1.5323
4.730.000	944.637	-256.692	1.5321
4.740.000	946.060	-256.992	1.5320

4.750.000	948.095	-257.335	1.5319
4.760.000	949.491	-257.540	1.5314
4.770.000	951.359	-257.859	1.5310
4.780.000	952.945	-258.137	1.5305
4.790.000	954.717	-258.421	1.5299
4.800.000	956.288	-258.663	1.5293
4.810.000	958.161	-259.036	1.5288
4.820.000	959.705	-259.192	1.5284
4.830.000	961.484	-259.584	1.5282
4.840.000	963.190	-259.780	1.5281
4.850.000	964.769	-260.094	1.5281
4.860.000	966.637	-260.368	1.5284
4.870.000	968.209	-260.676	1.5287
4.880.000	970.020	-260.898	1.5289
4.890.000	971.564	-261.271	1.5291
4.900.000	973.460	-261.501	1.5293
4.910.000	974.928	-261.811	1.5296
4.920.000	976.930	-262.161	1.5298
4.930.000	978.328	-262.342	1.5300
4.940.000	980.236	-262.705	1.5302
4.950.000	981.759	-262.992	1.5303
4.960.000	983.626	-263.246	1.5301
4.970.000	985.188	-263.540	1.5299
4.980.000	987.067	-263.899	1.5298
4.990.000	988.604	-264.068	1.5296
5.000.000	990.402	-264.453	1.5292
5.010.000	992.087	-264.669	1.5291
5.020.000	993.730	-265.016	1.5290
5.030.000	995.507	-265.273	1.5289
5.040.000	997.167	-265.600	1.5288

5.050.000	998.939	-265.821	1.5287
5.060.000	1.000.543	-266.210	1.5286
5.070.000	1.002.479	-266.422	1.5287
5.080.000	1.003.859	-266.739	1.5286
5.090.000	1.005.864	-267.084	1.5286
5.100.000	1.007.311	-267.289	1.5288
5.110.000	1.009.182	-267.653	1.5290
5.120.000	1.010.684	-267.908	1.5292
5.130.000	1.012.507	-268.166	1.5290
5.140.000	1.013.998	-268.449	1.5289
5.150.000	1.015.896	-268.798	1.5291
5.160.000	1.017.338	-268.955	1.5291
5.170.000	1.019.146	-269.333	1.5288
5.180.000	1.020.822	-269.546	1.5287
5.190.000	1.022.430	-269.863	1.5285
5.200.000	1.024.211	-270.108	1.5285
5.210.000	1.025.849	-270.409	1.5285
5.220.000	1.027.558	-270.606	1.5285
5.230.000	1.029.170	-270.993	1.5286
5.240.000	1.031.026	-271.171	1.5286
5.250.000	1.032.439	-271.484	1.5287
5.260.000	1.034.435	-271.818	1.5287
5.270.000	1.035.845	-272.027	1.5285
5.280.000	1.037.735	-272.355	1.5283
5.290.000	1.039.217	-272.636	1.5280
5.300.000	1.041.122	-272.897	1.5277
5.310.000	1.042.640	-273.152	1.5278
5.320.000	1.044.542	-273.508	1.5279
5.330.000	1.045.999	-273.666	1.5279
5.340.000	1.047.810	-274.038	1.5280

5.350.000	1.049.450	-274.272	1.5282
5.360.000	1.051.131	-274.565	1.5283
5.370.000	1.052.843	-274.813	1.5280
5.380.000	1.054.511	-275.116	1.5280
5.390.000	1.056.219	-275.309	1.5282
5.400.000	1.057.857	-275.707	1.5281
5.410.000	1.059.686	-275.855	1.5279
5.420.000	1.061.170	-276.206	1.5279
5.430.000	1.063.151	-276.531	1.5283
5.440.000	1.064.568	-276.728	1.5285
5.450.000	1.066.510	-277.068	1.5285
5.460.000	1.067.984	-277.360	1.5286
5.470.000	1.069.894	-277.620	1.5288
5.480.000	1.071.372	-277.918	1.5285
5.490.000	1.073.319	-278.263	1.5283
5.500.000	1.074.788	-278.419	1.5282
5.510.000	1.076.649	-278.829	1.5279
5.520.000	1.078.241	-279.041	1.5274
5.530.000	1.079.959	-279.359	1.5271
5.540.000	1.081.658	-279.627	1.5271
5.550.000	1.083.409	-279.939	1.5270
5.560.000	1.085.098	-280.145	1.5266
5.570.000	1.086.720	-280.543	1.5266
5.580.000	1.088.570	-280.709	1.5270
5.590.000	1.090.050	-281.069	1.5272
5.600.000	1.091.995	-281.360	1.5272
5.610.000	1.093.488	-281.620	1.5274
5.620.000	1.095.379	-281.920	1.5276
5.630.000	1.096.881	-282.206	1.5276
5.640.000	1.098.764	-282.484	1.5274

5.650.000	1.100.264	-282.782	1.5275
5.660.000	1.102.221	-283.116	1.5276
5.670.000	1.103.706	-283.300	1.5273
5.680.000	1.105.512	-283.668	1.5271
5.690.000	1.107.093	-283.897	1.5271
5.700.000	1.108.845	-284.202	1.5270
5.710.000	1.110.463	-284.462	1.5266
5.720.000	1.112.233	-284.783	1.5263
5.730.000	1.113.866	-284.976	1.5263
5.740.000	1.115.558	-285.360	1.5262
5.750.000	1.117.299	-285.509	1.5262
5.760.000	1.118.834	-285.866	1.5262
5.770.000	1.120.763	-286.131	1.5263
5.780.000	1.122.233	-286.391	1.5262
5.790.000	1.124.088	-286.671	1.5261
5.800.000	1.125.618	-286.996	1.5263
5.810.000	1.127.481	-287.232	1.5263
5.820.000	1.128.938	-287.523	1.5259
5.830.000	1.130.891	-287.868	1.5257
5.840.000	1.132.312	-288.027	1.5258
5.850.000	1.134.187	-288.383	1.5257
5.860.000	1.135.724	-288.639	1.5253
5.870.000	1.137.504	-288.926	1.5252
5.880.000	1.139.169	-289.184	1.5255
5.890.000	1.140.908	-289.493	1.5257
5.900.000	1.142.548	-289.703	1.5254
5.910.000	1.144.212	-290.058	1.5251
5.920.000	1.145.994	-290.232	1.5252
5.930.000	1.147.578	-290.562	1.5252
5.940.000	1.149.392	-290.800	1.5248

5.950.000	1.150.919	-291.077	1.5242
5.960.000	1.152.777	-291.336	1.5237
5.970.000	1.154.300	-291.654	1.5236
5.980.000	1.156.234	-291.910	1.5234
5.990.000	1.157.675	-292.207	1.5231
6.000.000	1.159.629	-292.526	1.5227
6.010.000	1.161.091	-292.720	1.5227
6.020.000	1.162.969	-293.072	1.5227
6.030.000	1.164.545	-293.312	1.5226
6.040.000	1.166.348	-293.608	1.5223
6.050.000	1.167.916	-293.872	1.5223
6.060.000	1.169.760	-294.199	1.5224
6.070.000	1.171.309	-294.404	1.5226
6.080.000	1.173.037	-294.761	1.5224
6.090.000	1.174.778	-294.942	1.5223
6.100.000	1.176.417	-295.325	1.5224
6.110.000	1.178.265	-295.538	1.5224
6.120.000	1.179.809	-295.842	1.5223
6.130.000	1.181.600	-296.072	1.5220
6.140.000	1.183.172	-296.432	1.5216
6.150.000	1.185.116	-296.669	1.5215
6.160.000	1.186.522	-296.958	1.5214
6.170.000	1.188.516	-297.288	1.5211
6.180.000	1.189.911	-297.504	1.5210
6.190.000	1.191.840	-297.825	1.5210
6.200.000	1.193.387	-298.083	1.5211
6.210.000	1.195.216	-298.371	1.5213
6.220.000	1.196.833	-298.624	1.5213
6.230.000	1.198.659	-298.967	1.5219
6.240.000	1.200.212	-299.140	1.5224

6.250.000	1.202.024	-299.520	1.5225
6.260.000	1.203.698	-299.716	1.5228
6.270.000	1.205.319	-300.012	1.5232
6.280.000	1.207.090	-300.252	1.5233
6.290.000	1.208.640	-300.527	1.5229
6.300.000	1.210.424	-300.731	1.5229
6.310.000	1.211.988	-301.095	1.5228
6.320.000	1.213.853	-301.271	1.5228
6.330.000	1.215.283	-301.583	1.5226
6.340.000	1.217.277	-301.895	1.5228
6.350.000	1.218.648	-302.069	1.5232
6.360.000	1.220.597	-302.420	1.5233
6.370.000	1.222.083	-302.669	1.5236
6.380.000	1.223.910	-302.909	1.5240
6.390.000	1.225.402	-303.171	1.5243
6.400.000	1.227.277	-303.492	1.5242
6.410.000	1.228.829	-303.663	1.5240
6.420.000	1.230.600	-304.031	1.5240
6.430.000	1.232.249	-304.205	1.5238
6.440.000	1.233.955	-304.537	1.5232
6.450.000	1.235.675	-304.766	1.5226
6.460.000	1.237.353	-305.068	1.5222
6.470.000	1.239.063	-305.255	1.5221
6.480.000	1.240.628	-305.631	1.5217
6.490.000	1.242.535	-305.794	1.5212
6.500.000	1.243.955	-306.116	1.5210
6.510.000	1.245.906	-306.404	1.5210
6.520.000	1.247.359	-306.623	1.5208
6.530.000	1.249.221	-306.926	1.5204
6.540.000	1.250.755	-307.184	1.5202

6.550.000	1.252.624	-307.431	1.5204
6.560.000	1.254.122	-307.711	1.5203
6.570.000	1.256.043	-308.018	1.5200
6.580.000	1.257.542	-308.194	1.5199
6.590.000	1.259.353	-308.547	1.5201
6.600.000	1.261.005	-308.737	1.5201
6.610.000	1.262.676	-309.048	1.5197
6.620.000	1.264.413	-309.289	1.5197
6.630.000	1.266.093	-309.555	1.5198
6.640.000	1.267.760	-309.757	1.5199
6.650.000	1.269.411	-310.139	1.5196
6.660.000	1.271.269	-310.307	1.5195
6.670.000	1.272.766	-310.649	1.5195
6.680.000	1.274.720	-310.911	1.5193
6.690.000	1.276.165	-311.141	1.5191
6.700.000	1.278.078	-311.451	1.5191
6.710.000	1.279.614	-311.738	1.5192
6.720.000	1.281.436	-311.971	1.5192
6.730.000	1.282.950	-312.254	1.5189
6.740.000	1.284.876	-312.578	1.5188
6.750.000	1.286.356	-312.744	1.5190
6.760.000	1.288.211	-313.108	1.5191
6.770.000	1.289.816	-313.323	1.5188
6.780.000	1.291.610	-313.625	1.5185
6.790.000	1.293.278	-313.876	1.5188
6.800.000	1.294.986	-314.162	1.5193
6.810.000	1.296.693	-314.367	1.5194
6.820.000	1.298.362	-314.768	1.5192
6.830.000	1.300.179	-314.915	1.5194
6.840.000	1.301.686	-315.251	1.5199

6.850.000	1.303.627	-315.528	1.5199
6.860.000	1.305.077	-315.762	1.5196
6.870.000	1.306.949	-316.084	1.5195
6.880.000	1.308.438	-316.376	1.5198
6.890.000	1.310.360	-316.608	1.5202
6.900.000	1.311.807	-316.907	1.5203
6.910.000	1.313.745	-317.231	1.5205
6.920.000	1.315.175	-317.398	1.5210
6.930.000	1.317.023	-317.763	1.5217
6.940.000	1.318.627	-317.981	1.5218
6.950.000	1.320.326	-318.277	1.5218
6.960.000	1.322.011	-318.531	1.5221
6.970.000	1.323.776	-318.831	1.5224
6.980.000	1.325.375	-319.053	1.5222
6.990.000	1.327.087	-319.396	1.5219
7.000.000	1.328.860	-319.548	1.5218
7.010.000	1.330.375	-319.894	1.5220
7.020.000	1.332.260	-320.133	1.5218
7.030.000	1.333.772	-320.410	1.5215
7.040.000	1.335.637	-320.673	1.5215
7.050.000	1.337.172	-320.994	1.5218
7.060.000	1.339.032	-321.231	1.5218
7.070.000	1.340.441	-321.499	1.5215
7.080.000	1.342.404	-321.844	1.5216
7.090.000	1.343.825	-322.011	1.5219
7.100.000	1.345.705	-322.343	1.5219
7.110.000	1.347.292	-322.578	1.5218
7.120.000	1.349.035	-322.849	1.5219
7.130.000	1.350.629	-323.104	1.5221
7.140.000	1.352.463	-323.392	1.5220

7.150.000	1.354.046	-323.565	1.5217
7.160.000	1.355.785	-323.912	1.5217
7.170.000	1.357.490	-324.072	1.5218
7.180.000	1.359.094	-324.393	1.5219
7.190.000	1.360.939	-324.619	1.5217
7.200.000	1.362.425	-324.862	1.5217
7.210.000	1.364.336	-325.126	1.5218
7.220.000	1.365.853	-325.467	1.5217
7.230.000	1.367.787	-325.684	1.5215
7.240.000	1.369.240	-325.964	1.5213
7.250.000	1.371.169	-326.288	1.5210
7.260.000	1.372.615	-326.468	1.5210
7.270.000	1.374.530	-326.815	1.5207
7.280.000	1.376.061	-327.047	1.5207
7.290.000	1.377.833	-327.315	1.5205
7.300.000	1.379.454	-327.582	1.5203
7.310.000	1.381.267	-327.884	1.5203
7.320.000	1.382.828	-328.060	1.5203
7.330.000	1.384.577	-328.452	1.5203
7.340.000	1.386.343	-328.604	1.5200
7.350.000	1.387.992	-328.940	1.5202
7.360.000	1.389.758	-329.182	1.5205
7.370.000	1.391.365	-329.451	1.5204
7.380.000	1.393.206	-329.692	1.5201
7.390.000	1.394.728	-330.025	1.5201
7.400.000	1.396.619	-330.235	1.5203
7.410.000	1.398.022	-330.536	1.5203
7.420.000	1.400.008	-330.848	1.5199
7.430.000	1.401.466	-331.041	1.5197
7.440.000	1.403.338	-331.367	1.5199

7.450.000	1.404.942	-331.634	1.5201
7.460.000	1.406.754	-331.899	1.5199
7.470.000	1.408.287	-332.150	1.5201
7.480.000	1.410.169	-332.452	1.5205
7.490.000	1.411.667	-332.654	1.5207
7.500.000	1.413.446	-332.988	1.5205
7.510.000	1.415.132	-333.166	1.5204
7.520.000	1.416.726	-333.499	1.5205
7.530.000	1.418.560	-333.721	1.5204
7.540.000	1.420.134	-334.006	1.5202
7.550.000	1.421.859	-334.201	1.5201
7.560.000	1.423.431	-334.574	1.5203
7.570.000	1.425.332	-334.746	1.5204
7.580.000	1.426.760	-335.050	1.5205
7.590.000	1.428.761	-335.384	1.5208
7.600.000	1.430.141	-335.565	1.5211
7.610.000	1.432.095	-335.905	1.5211
7.620.000	1.433.585	-336.146	1.5212
7.630.000	1.435.403	-336.393	1.5213
7.640.000	1.436.944	-336.664	1.5211
7.650.000	1.438.792	-336.995	1.5204
7.660.000	1.440.308	-337.141	1.5201
7.670.000	1.442.121	-337.526	1.5200
7.680.000	1.443.789	-337.709	1.5197
7.690.000	1.445.490	-338.002	1.5196
7.700.000	1.447.189	-338.235	1.5195
7.710.000	1.448.858	-338.512	1.5195
7.720.000	1.450.587	-338.696	1.5195
7.730.000	1.452.168	-339.054	1.5193
7.740.000	1.453.998	-339.196	1.5189

7.750.000	1.455.461	-339.530	1.5185
7.760.000	1.457.430	-339.821	1.5182
7.770.000	1.458.799	-340.002	1.5179
7.780.000	1.460.754	-340.319	1.5176
7.790.000	1.462.265	-340.583	1.5172
7.800.000	1.464.134	-340.818	1.5171
7.810.000	1.465.634	-341.079	1.5170
7.820.000	1.467.546	-341.368	1.5167
7.830.000	1.469.016	-341.540	1.5165
7.840.000	1.470.855	-341.890	1.5165
7.850.000	1.472.492	-342.066	1.5166
7.860.000	1.474.150	-342.364	1.5164
7.870.000	1.475.857	-342.594	1.5163
7.880.000	1.477.562	-342.874	1.5166
7.890.000	1.479.247	-343.066	1.5167
7.900.000	1.480.910	-343.434	1.5167
7.910.000	1.482.782	-343.572	1.5169
7.920.000	1.484.288	-343.900	1.5173
7.930.000	1.486.234	-344.185	1.5177
7.940.000	1.487.669	-344.399	1.5178
7.950.000	1.489.575	-344.700	1.5181
7.960.000	1.491.070	-344.978	1.5185
7.970.000	1.492.968	-345.241	1.5185
7.980.000	1.494.448	-345.489	1.5182
7.990.000	1.496.357	-345.815	1.5180
8.000.000	1.497.820	-346.001	1.5181
8.010.000	1.499.735	-346.358	1.5176
8.020.000	1.501.373	-346.582	1.5171
8.030.000	1.503.084	-346.861	1.5169
8.040.000	1.504.763	-347.115	1.5169

8.050.000	1.506.474	-347.408	1.5166
8.060.000	1.508.107	-347.609	1.5162
8.070.000	1.509.806	-347.991	1.5161
8.080.000	1.511.550	-348.117	1.5159
8.090.000	1.513.084	-348.451	1.5156
8.100.000	1.514.955	-348.710	1.5153
8.110.000	1.516.400	-348.933	1.5152
8.120.000	1.518.309	-349.259	1.5153
8.130.000	1.519.823	-349.566	1.5152
8.140.000	1.521.675	-349.773	1.5152
8.150.000	1.523.206	-350.059	1.5155
8.160.000	1.525.088	-350.366	1.5158
8.170.000	1.526.543	-350.529	1.5160
8.180.000	1.528.365	-350.875	1.5161
8.190.000	1.529.949	-351.075	1.5163
8.200.000	1.531.697	-351.346	1.5166
8.210.000	1.533.319	-351.609	1.5165
8.220.000	1.535.024	-351.860	1.5162
8.230.000	1.536.684	-352.065	1.5160
8.240.000	1.538.387	-352.425	1.5159
8.250.000	1.540.124	-352.549	1.5159
8.260.000	1.541.700	-352.887	1.5156
8.270.000	1.543.571	-353.121	1.5155
8.280.000	1.545.049	-353.359	1.5156
8.290.000	1.546.937	-353.642	1.5156
8.300.000	1.548.414	-353.923	1.5154
8.310.000	1.550.281	-354.150	1.5150
8.320.000	1.551.730	-354.424	1.5152
8.330.000	1.553.677	-354.727	1.5155
8.340.000	1.555.118	-354.889	1.5156

8.350.000	1.556.975	-355.232	1.5154
8.360.000	1.558.635	-355.458	1.5154
8.370.000	1.560.334	-355.724	1.5158
8.380.000	1.561.967	-355.959	1.5159
8.390.000	1.563.765	-356.256	1.5157
8.400.000	1.565.312	-356.443	1.5153
8.410.000	1.567.087	-356.792	1.5156
8.420.000	1.568.804	-356.947	1.5158
8.430.000	1.570.327	-357.289	1.5157
8.440.000	1.572.213	-357.509	1.5156
8.450.000	1.573.750	-357.783	1.5156
8.460.000	1.575.628	-358.017	1.5159
8.470.000	1.577.211	-358.364	1.5161
8.480.000	1.579.070	-358.564	1.5160
8.490.000	1.580.509	-358.851	1.5160
8.500.000	1.582.507	-359.181	1.5163
8.510.000	1.583.907	-359.367	1.5165
8.520.000	1.585.804	-359.712	1.5164
8.530.000	1.587.346	-359.955	1.5161
8.540.000	1.589.150	-360.239	1.5161
8.550.000	1.590.738	-360.493	1.5162
8.560.000	1.592.550	-360.831	1.5162
8.570.000	1.594.150	-361.022	1.5160
8.580.000	1.595.963	-361.397	1.5159
8.590.000	1.597.655	-361.582	1.5161
8.600.000	1.599.283	-361.920	1.5162
8.610.000	1.601.102	-362.174	1.5160
8.620.000	1.602.665	-362.453	1.5157
8.630.000	1.604.432	-362.664	1.5158
8.640.000	1.605.978	-363.014	1.5160

8.650.000	1.607.855	-363.222	1.5161
8.660.000	1.609.310	-363.529	1.5158
8.670.000	1.611.244	-363.824	1.5158
8.680.000	1.612.687	-364.025	1.5161
8.690.000	1.614.600	-364.362	1.5164
8.700.000	1.616.099	-364.597	1.5163
8.710.000	1.617.976	-364.863	1.5161
8.720.000	1.619.498	-365.115	1.5162
8.730.000	1.621.341	-365.426	1.5165
8.740.000	1.622.843	-365.605	1.5164
8.750.000	1.624.595	-365.962	1.5161
8.760.000	1.626.310	-366.152	1.5158
8.770.000	1.627.900	-366.460	1.5157
8.780.000	1.629.659	-366.687	1.5155
8.790.000	1.631.268	-366.963	1.5151
8.800.000	1.633.047	-367.187	1.5148
8.810.000	1.634.694	-367.530	1.5150
8.820.000	1.636.506	-367.700	1.5155
8.830.000	1.637.924	-368.003	1.5159
8.840.000	1.639.891	-368.294	1.5161
8.850.000	1.641.291	-368.502	1.5165
8.860.000	1.643.150	-368.803	1.5170
8.870.000	1.644.723	-369.072	1.5172
8.880.000	1.646.535	-369.310	1.5171
8.890.000	1.648.045	-369.560	1.5168
8.900.000	1.649.939	-369.880	1.5169
8.910.000	1.651.434	-370.064	1.5172
8.920.000	1.653.278	-370.418	1.5172
8.930.000	1.654.929	-370.593	1.5170
8.940.000	1.656.549	-370.893	1.5170

8.950.000	1.658.293	-371.142	1.5174
8.960.000	1.659.950	-371.417	1.5174
8.970.000	1.661.632	-371.605	1.5171
8.980.000	1.663.285	-371.995	1.5169
8.990.000	1.665.129	-372.128	1.5170
9.000.000	1.666.578	-372.467	1.5168
9.010.000	1.668.560	-372.774	1.5164
9.020.000	1.670.005	-372.970	1.5163
9.030.000	1.671.948	-373.303	1.5166
9.040.000	1.673.430	-373.589	1.5169
9.050.000	1.675.294	-373.821	1.5168
9.060.000	1.676.807	-374.077	1.5168
9.070.000	1.678.663	-374.398	1.5170
9.080.000	1.680.165	-374.600	1.5170
9.090.000	1.682.027	-374.943	1.5165
9.100.000	1.683.638	-375.138	1.5160
9.110.000	1.685.367	-375.453	1.5159
9.120.000	1.687.035	-375.742	1.5156
9.130.000	1.688.769	-376.011	1.5151
9.140.000	1.690.515	-376.219	1.5147
9.150.000	1.692.163	-376.610	1.5146
9.160.000	1.693.992	-376.758	1.5146
9.170.000	1.695.466	-377.083	1.5141
9.180.000	1.697.394	-377.386	1.5140
9.190.000	1.698.857	-377.617	1.5141
9.200.000	1.700.760	-377.944	1.5140
9.210.000	1.702.286	-378.221	1.5137
9.220.000	1.704.150	-378.454	1.5136
9.230.000	1.705.608	-378.726	1.5136
9.240.000	1.707.553	-379.047	1.5135

9.250.000	1.709.008	-379.231	1.5132
9.260.000	1.710.833	-379.565	1.5132
9.270.000	1.712.416	-379.778	1.5133
9.280.000	1.714.127	-380.088	1.5132
9.290.000	1.715.792	-380.326	1.5128
9.300.000	1.717.507	-380.599	1.5127
9.310.000	1.719.162	-380.807	1.5128
9.320.000	1.720.838	-381.171	1.5126
9.330.000	1.722.597	-381.303	1.5123
9.340.000	1.724.112	-381.660	1.5122
9.350.000	1.726.024	-381.920	1.5124
9.360.000	1.727.505	-382.178	1.5124
9.370.000	1.729.416	-382.439	1.5121
9.380.000	1.730.864	-382.752	1.5121
9.390.000	1.732.723	-382.976	1.5122
9.400.000	1.734.242	-383.248	1.5122
9.410.000	1.736.099	-383.554	1.5120
9.420.000	1.737.562	-383.731	1.5119
9.430.000	1.739.438	-384.085	1.5122
9.440.000	1.741.025	-384.314	1.5121
9.450.000	1.742.730	-384.575	1.5118
9.460.000	1.744.385	-384.824	1.5115
9.470.000	1.746.114	-385.111	1.5116
9.480.000	1.747.787	-385.307	1.5114
9.490.000	1.749.473	-385.660	1.5111
9.500.000	1.751.225	-385.804	1.5109
9.510.000	1.752.721	-386.127	1.5112
9.520.000	1.754.592	-386.361	1.5112
9.530.000	1.756.065	-386.584	1.5109
9.540.000	1.757.925	-386.864	1.5110

9.550.000	1.759.430	-387.168	1.5113
9.560.000	1.761.326	-387.376	1.5114
9.570.000	1.762.751	-387.648	1.5112
9.580.000	1.764.727	-387.969	1.5112
9.590.000	1.766.171	-388.144	1.5113
9.600.000	1.768.051	-388.478	1.5111
9.610.000	1.769.618	-388.695	1.5109
9.620.000	1.771.358	-388.977	1.5109
9.630.000	1.772.965	-389.199	1.5110
9.640.000	1.774.756	-389.494	1.5110
9.650.000	1.776.312	-389.689	1.5110
9.660.000	1.778.039	-390.033	1.5113
9.670.000	1.779.766	-390.191	1.5117
9.680.000	1.781.356	-390.514	1.5119
9.690.000	1.783.166	-390.725	1.5120
9.700.000	1.784.750	-390.997	1.5124
9.710.000	1.786.595	-391.231	1.5128
9.720.000	1.788.099	-391.554	1.5129
9.730.000	1.790.017	-391.776	1.5128
9.740.000	1.791.436	-392.049	1.5130
9.750.000	1.793.411	-392.376	1.5130
9.760.000	1.794.821	-392.559	1.5125
9.770.000	1.796.741	-392.883	1.5120
9.780.000	1.798.310	-393.134	1.5117
9.790.000	1.800.108	-393.398	1.5114
9.800.000	1.801.718	-393.652	1.5109
9.810.000	1.803.522	-393.977	1.5105
9.820.000	1.805.102	-394.163	1.5102
9.830.000	1.806.840	-394.520	1.5098
9.840.000	1.808.532	-394.719	1.5094

9.850.000	1.810.141	-395.041	1.5092
9.860.000	1.811.937	-395.279	1.5092
9.870.000	1.813.469	-395.539	1.5089
9.880.000	1.815.260	-395.753	1.5086
9.890.000	1.816.800	-396.107	1.5085
9.900.000	1.818.669	-396.318	1.5084
9.910.000	1.820.136	-396.619	1.5080
9.920.000	1.822.079	-396.945	1.5077
9.930.000	1.823.496	-397.116	1.5077
9.940.000	1.825.398	-397.434	1.5075
9.950.000	1.826.874	-397.697	1.5072
9.960.000	1.828.681	-397.938	1.5071
9.970.000	1.830.222	-398.200	1.5069
9.980.000	1.832.062	-398.520	1.5069
9.990.000	1.833.589	-398.685	1.5066
10.000.000	1.835.356	-399.060	1.5064
10.010.000	1.837.028	-399.218	1.5063
10.020.000	1.838.686	-399.531	1.5061
10.030.000	1.840.430	-399.772	1.5062
10.040.000	1.842.072	-400.034	1.5062
10.050.000	1.843.819	-400.245	1.5061
10.060.000	1.845.373	-400.601	1.5061
10.070.000	1.847.276	-400.776	1.5062
10.080.000	1.848.670	-401.079	1.5061
10.090.000	1.850.643	-401.369	1.5060
10.100.000	1.852.031	-401.555	1.5059
10.110.000	1.853.905	-401.883	1.5059
10.120.000	1.855.484	-402.139	1.5059
10.130.000	1.857.282	-402.376	1.5060
10.140.000	1.858.838	-402.634	1.5061

10.150.000	1.860.726	-402.947	1.5062
10.160.000	1.862.202	-403.128	1.5062
10.170.000	1.864.014	-403.472	1.5063
10.180.000	1.865.651	-403.665	1.5065
10.190.000	1.867.330	-403.969	1.5065
10.200.000	1.869.055	-404.197	1.5067
10.210.000	1.870.728	-404.482	1.5067
10.220.000	1.872.421	-404.674	1.5067
10.230.000	1.874.044	-405.045	1.5068
10.240.000	1.875.870	-405.207	1.5068
10.250.000	1.877.360	-405.514	1.5067
10.260.000	1.879.333	-405.822	1.5066
10.270.000	1.880.751	-406.020	1.5066
10.280.000	1.882.680	-406.333	1.5065
10.290.000	1.884.186	-406.629	1.5064
10.300.000	1.886.042	-406.870	1.5065
10.310.000	1.887.570	-407.123	1.5064
10.320.000	1.889.436	-407.453	1.5061
10.330.000	1.890.909	-407.637	1.5061
10.340.000	1.892.756	-407.992	1.5059
10.350.000	1.894.362	-408.195	1.5060
10.360.000	1.896.111	-408.492	1.5060
10.370.000	1.897.798	-408.734	1.5061
10.380.000	1.899.496	-409.024	1.5061
10.390.000	1.901.180	-409.223	1.5058
10.400.000	1.902.844	-409.604	1.5061
10.410.000	1.904.639	-409.740	1.5064
10.420.000	1.906.126	-410.065	1.5064
10.430.000	1.908.057	-410.346	1.5062
10.440.000	1.909.492	-410.562	1.5061

10.450.000	1.911.356	-410.866	1.5062
10.460.000	1.912.876	-411.150	1.5062
10.470.000	1.914.717	-411.368	1.5057
10.480.000	1.916.210	-411.654	1.5057
10.490.000	1.918.119	-411.956	1.5057
10.500.000	1.919.593	-412.137	1.5058
10.510.000	1.921.420	-412.488	1.5056
10.520.000	1.923.021	-412.698	1.5057
10.530.000	1.924.723	-412.991	1.5058
10.540.000	1.926.364	-413.224	1.5056
10.550.000	1.928.129	-413.515	1.5053
10.560.000	1.929.788	-413.721	1.5051
10.570.000	1.931.408	-414.075	1.5051
10.580.000	1.933.222	-414.218	1.5047
10.590.000	1.934.732	-414.553	1.5045
10.600.000	1.936.606	-414.797	1.5045
10.610.000	1.938.127	-415.028	1.5044
10.620.000	1.939.940	-415.299	1.5039
10.630.000	1.941.468	-415.595	1.5035
10.640.000	1.943.325	-415.824	1.5034
10.650.000	1.944.775	-416.079	1.5032
10.660.000	1.946.721	-416.379	1.5028
10.670.000	1.948.171	-416.554	1.5029
10.680.000	1.950.032	-416.884	1.5031
10.690.000	1.951.629	-417.102	1.5031
10.700.000	1.953.340	-417.371	1.5027
10.710.000	1.954.966	-417.598	1.5026
10.720.000	1.956.768	-417.891	1.5025
10.730.000	1.958.382	-418.060	1.5019
10.740.000	1.960.118	-418.416	1.5012

10.750.000	1.961.842	-418.566	1.5008
10.760.000	1.963.388	-418.881	1.5007
10.770.000	1.965.231	-419.102	1.5004
10.780.000	1.966.719	-419.355	1.5001
10.790.000	1.968.569	-419.596	1.5001
10.800.000	1.970.114	-419.904	1.5001
10.810.000	1.971.965	-420.105	1.4999
10.820.000	1.973.457	-420.377	1.4998
10.830.000	1.975.363	-420.697	1.4999
10.840.000	1.976.835	-420.831	1.4998
10.850.000	1.978.695	-421.164	1.4993
10.860.000	1.980.258	-421.384	1.4989
10.870.000	1.982.000	-421.628	1.4989
10.880.000	1.983.621	-421.872	1.4987
10.890.000	1.985.404	-422.154	1.4981
10.900.000	1.987.029	-422.340	1.4978
10.910.000	1.988.761	-422.680	1.4980
10.920.000	1.990.495	-422.819	1.4979
10.930.000	1.992.046	-423.130	1.4975
10.940.000	1.993.908	-423.348	1.4973
10.950.000	1.995.483	-423.608	1.4971
10.960.000	1.997.297	-423.816	1.4970
10.970.000	1.998.818	-424.138	1.4964
10.980.000	2.000.732	-424.340	1.4961
10.990.000	2.002.154	-424.602	1.4962
11.000.000	2.004.073	-424.887	1.4959
11.010.000	2.005.537	-425.043	1.4955
11.020.000	2.007.390	-425.358	1.4956
11.030.000	2.008.944	-425.567	1.4958
11.040.000	2.010.731	-425.803	1.4957

11.050.000	2.012.297	-426.042	1.4952
11.060.000	2.014.177	-426.345	1.4950
11.070.000	2.015.708	-426.492	1.4950
11.080.000	2.017.434	-426.808	1.4947
11.090.000	2.019.144	-426.961	1.4939
11.100.000	2.020.742	-427.244	1.4935
11.110.000	2.022.515	-427.449	1.4934
11.120.000	2.024.115	-427.718	1.4934
11.130.000	2.025.900	-427.896	1.4931
11.140.000	2.027.425	-428.221	1.4928
11.150.000	2.029.283	-428.413	1.4929
11.160.000	2.030.743	-428.653	1.4930
11.170.000	2.032.683	-428.945	1.4927
11.180.000	2.034.100	-429.096	1.4922
11.190.000	2.036.007	-429.384	1.4919
11.200.000	2.037.532	-429.619	1.4920
11.210.000	2.039.340	-429.830	1.4918
11.220.000	2.040.873	-430.053	1.4914
11.230.000	2.042.727	-430.375	1.4912
11.240.000	2.044.233	-430.498	1.4914
11.250.000	2.046.043	-430.833	1.4913
11.260.000	2.047.701	-431.010	1.4910
11.270.000	2.049.339	-431.265	1.4908
11.280.000	2.051.091	-431.484	1.4909
11.290.000	2.052.726	-431.748	1.4909
11.300.000	2.054.474	-431.897	1.4905
11.310.000	2.056.028	-432.245	1.4902
11.320.000	2.057.947	-432.380	1.4902
11.330.000	2.059.346	-432.660	1.4899
11.340.000	2.061.291	-432.928	1.4893

11.350.000	2.062.767	-433.092	1.4888
11.360.000	2.064.629	-433.398	1.4885
11.370.000	2.066.200	-433.639	1.4882
11.380.000	2.068.027	-433.840	1.4874
11.390.000	2.069.513	-434.067	1.4868
11.400.000	2.071.395	-434.368	1.4865
11.410.000	2.072.934	-434.518	1.4860
11.420.000	2.074.702	-434.841	1.4854
11.430.000	2.076.402	-435.027	1.4849
11.440.000	2.078.045	-435.273	1.4848
11.450.000	2.079.786	-435.499	1.4845
11.460.000	2.081.458	-435.762	1.4840
11.470.000	2.083.151	-435.918	1.4836
11.480.000	2.084.790	-436.256	1.4833
11.490.000	2.086.615	-436.392	1.4831
11.500.000	2.088.104	-436.687	1.4824
11.510.000	2.090.092	-436.952	1.4819
11.520.000	2.091.512	-437.122	1.4818
11.530.000	2.093.427	-437.409	1.4816
11.540.000	2.094.933	-437.647	1.4811
11.550.000	2.096.797	-437.869	1.4806
11.560.000	2.098.289	-438.093	1.4804
11.570.000	2.100.210	-438.391	1.4803
11.580.000	2.101.704	-438.531	1.4798
11.590.000	2.103.525	-438.835	1.4794
11.600.000	2.105.139	-439.035	1.4793
11.610.000	2.106.809	-439.271	1.4793
11.620.000	2.108.512	-439.482	1.4789
11.630.000	2.110.229	-439.751	1.4786
11.640.000	2.111.876	-439.892	1.4786

11.650.000	2.113.573	-440.213	1.4786
11.660.000	2.115.300	-440.337	1.4785
11.670.000	2.116.839	-440.619	1.4783
11.680.000	2.118.753	-440.853	1.4787
11.690.000	2.120.171	-441.041	1.4789
11.700.000	2.122.065	-441.315	1.4787
11.710.000	2.123.593	-441.563	1.4784
11.720.000	2.125.411	-441.750	1.4781
11.730.000	2.126.902	-441.992	1.4780
11.740.000	2.128.808	-442.270	1.4772
11.750.000	2.130.279	-442.390	1.4766
11.760.000	2.132.131	-442.708	1.4763
11.770.000	2.133.697	-442.880	1.4759
11.780.000	2.135.464	-443.136	1.4754
11.790.000	2.137.096	-443.348	1.4750
11.800.000	2.138.831	-443.609	1.4748
11.810.000	2.140.515	-443.759	1.4743
11.820.000	2.142.140	-444.071	1.4737
11.830.000	2.143.904	-444.196	1.4733
11.840.000	2.145.467	-444.492	1.4731
11.850.000	2.147.331	-444.702	1.4728
11.860.000	2.148.863	-444.914	1.4724
11.870.000	2.150.674	-445.142	1.4722
11.880.000	2.152.157	-445.385	1.4720
11.890.000	2.154.051	-445.576	1.4714
11.900.000	2.155.494	-445.801	1.4711
11.910.000	2.157.417	-446.067	1.4707
11.920.000	2.158.891	-446.195	1.4701
11.930.000	2.160.724	-446.483	1.4697
11.940.000	2.162.321	-446.667	1.4695

11.950.000	2.164.058	-446.882	1.4694
11.960.000	2.165.632	-447.075	1.4691
11.970.000	2.167.480	-447.345	1.4690
11.980.000	2.169.061	-447.481	1.4691
11.990.000	2.170.776	-447.766	1.4689
12.000.000	2.172.503	-447.902	1.4686
12.010.000	2.174.072	-448.189	1.4684
12.020.000	2.175.943	-448.364	1.4683
12.030.000	2.177.460	-448.593	1.4678
12.040.000	2.179.277	-448.789	1.4673
12.050.000	2.180.775	-449.074	1.4670
12.060.000	2.182.665	-449.246	1.4666
12.070.000	2.184.124	-449.492	1.4661
12.080.000	2.186.070	-449.775	1.4658
12.090.000	2.187.513	-449.915	1.4659
12.100.000	2.189.405	-450.212	1.4658
12.110.000	2.190.951	-450.424	1.4655
12.120.000	2.192.734	-450.644	1.4653
12.130.000	2.194.340	-450.851	1.4652
12.140.000	2.196.125	-451.147	1.4649
12.150.000	2.197.700	-451.278	1.4644
12.160.000	2.199.459	-451.602	1.4640
12.170.000	2.201.158	-451.755	1.4639
12.180.000	2.202.782	-452.037	1.4636
12.190.000	2.204.595	-452.223	1.4633
12.200.000	2.206.153	-452.489	1.4632
12.210.000	2.207.934	-452.646	1.4633
12.220.000	2.209.463	-452.968	1.4631
12.230.000	2.211.351	-453.125	1.4629
12.240.000	2.212.783	-453.388	1.4631

12.250.000	2.214.712	-453.651	1.4633
12.260.000	2.216.173	-453.809	1.4630
12.270.000	2.217.995	-454.090	1.4628
12.280.000	2.219.527	-454.305	1.4625
12.290.000	2.221.355	-454.519	1.4622
12.300.000	2.222.894	-454.752	1.4617
12.310.000	2.224.725	-455.030	1.4613
12.320.000	2.226.261	-455.159	1.4610
12.330.000	2.228.028	-455.478	1.4607
12.340.000	2.229.737	-455.642	1.4605
12.350.000	2.231.347	-455.890	1.4605
12.360.000	2.233.078	-456.093	1.4605
12.370.000	2.234.748	-456.365	1.4604
12.380.000	2.236.467	-456.537	1.4606
12.390.000	2.238.038	-456.849	1.4607
12.400.000	2.239.872	-457.002	1.4605
12.410.000	2.241.385	-457.270	1.4601
12.420.000	2.243.291	-457.553	1.4599
12.430.000	2.244.750	-457.706	1.4599
12.440.000	2.246.598	-457.993	1.4595
12.450.000	2.248.144	-458.211	1.4593
12.460.000	2.249.957	-458.431	1.4593
12.470.000	2.251.478	-458.644	1.4592
12.480.000	2.253.361	-458.931	1.4589
12.490.000	2.254.838	-459.052	1.4586
12.500.000	2.256.594	-459.360	1.4584
12.510.000	2.258.284	-459.540	1.4579
12.520.000	2.259.939	-459.789	1.4575
12.530.000	2.261.676	-459.994	1.4571
12.540.000	2.263.344	-460.285	1.4567

12.550.000	2.265.041	-460.417	1.4562
12.560.000	2.266.682	-460.744	1.4557
12.570.000	2.268.471	-460.893	1.4553
12.580.000	2.269.985	-461.168	1.4547
12.590.000	2.271.910	-461.416	1.4542
12.600.000	2.273.325	-461.593	1.4540
12.610.000	2.275.235	-461.861	1.4539
12.620.000	2.276.724	-462.123	1.4535
12.630.000	2.278.580	-462.303	1.4535
12.640.000	2.280.085	-462.538	1.4537
12.650.000	2.281.972	-462.831	1.4534
12.660.000	2.283.470	-462.953	1.4529
12.670.000	2.285.293	-463.278	1.4527
12.680.000	2.286.934	-463.461	1.4525
12.690.000	2.288.650	-463.703	1.4520
12.700.000	2.290.305	-463.929	1.4515
12.710.000	2.292.017	-464.186	1.4514
12.720.000	2.293.692	-464.321	1.4514
12.730.000	2.295.295	-464.645	1.4510
12.740.000	2.297.125	-464.768	1.4508
12.750.000	2.298.621	-465.046	1.4505
12.760.000	2.300.541	-465.288	1.4502
12.770.000	2.302.036	-465.491	1.4497
12.780.000	2.303.874	-465.741	1.4493
12.790.000	2.305.377	-465.976	1.4489
12.800.000	2.307.238	-466.182	1.4484
12.810.000	2.308.694	-466.410	1.4481
12.820.000	2.310.614	-466.682	1.4478
12.830.000	2.312.067	-466.818	1.4475
12.840.000	2.313.865	-467.113	1.4471

12.850.000	2.315.531	-467.323	1.4469
12.860.000	2.317.261	-467.569	1.4467
12.870.000	2.318.861	-467.778	1.4463
12.880.000	2.320.634	-468.048	1.4457
12.890.000	2.322.259	-468.209	1.4453
12.900.000	2.323.922	-468.531	1.4445
12.910.000	2.325.722	-468.667	1.4438
12.920.000	2.327.168	-468.940	1.4435
12.930.000	2.329.087	-469.173	1.4431
12.940.000	2.330.576	-469.384	1.4428
12.950.000	2.332.376	-469.620	1.4427
12.960.000	2.333.877	-469.873	1.4428
12.970.000	2.335.752	-470.054	1.4429
12.980.000	2.337.240	-470.295	1.4427
12.990.000	2.339.154	-470.579	1.4427
13.000.000	2.340.592	-470.704	1.4429
13.010.000	2.342.448	-471.002	1.4428
13.020.000	2.344.024	-471.198	1.4425
13.030.000	2.345.746	-471.428	1.4424
13.040.000	2.347.366	-471.637	1.4425
13.050.000	2.349.126	-471.913	1.4424
13.060.000	2.350.724	-472.039	1.4417
13.070.000	2.352.406	-472.339	1.4412
13.080.000	2.354.154	-472.499	1.4411
13.090.000	2.355.764	-472.760	1.4406
13.100.000	2.357.551	-472.964	1.4398
13.110.000	2.359.079	-473.172	1.4393
13.120.000	2.360.931	-473.395	1.4392
13.130.000	2.362.423	-473.666	1.4388
13.140.000	2.364.315	-473.838	1.4383

13.150.000	2.365.731	-474.069	1.4380
13.160.000	2.367.660	-474.347	1.4381
13.170.000	2.369.133	-474.464	1.4377
13.180.000	2.370.948	-474.773	1.4373
13.190.000	2.372.528	-474.975	1.4372
13.200.000	2.374.334	-475.194	1.4372
13.210.000	2.375.895	-475.409	1.4368
13.220.000	2.377.725	-475.699	1.4364
13.230.000	2.379.314	-475.828	1.4367
13.240.000	2.381.018	-476.142	1.4367
13.250.000	2.382.761	-476.308	1.4366
13.260.000	2.384.307	-476.575	1.4365
13.270.000	2.386.133	-476.761	1.4367
13.280.000	2.387.719	-477.029	1.4368
13.290.000	2.389.532	-477.217	1.4363
13.300.000	2.391.047	-477.503	1.4360
13.310.000	2.392.915	-477.698	1.4361
13.320.000	2.394.378	-477.952	1.4359
13.330.000	2.396.341	-478.255	1.4353
13.340.000	2.397.800	-478.402	1.4350
13.350.000	2.399.629	-478.692	1.4351
13.360.000	2.401.188	-478.931	1.4350
13.370.000	2.402.992	-479.165	1.4344
13.380.000	2.404.555	-479.365	1.4339
13.390.000	2.406.358	-479.684	1.4338
13.400.000	2.407.894	-479.796	1.4335
13.410.000	2.409.622	-480.118	1.4326
13.420.000	2.411.370	-480.303	1.4319
13.430.000	2.412.973	-480.552	1.4314
13.440.000	2.414.733	-480.743	1.4310

13.450.000	2.416.339	-481.017	1.4303
13.460.000	2.418.084	-481.182	1.4298
13.470.000	2.419.654	-481.487	1.4297
13.480.000	2.421.504	-481.685	1.4295
13.490.000	2.422.942	-481.925	1.4289
13.500.000	2.424.872	-482.213	1.4285
13.510.000	2.426.281	-482.352	1.4284
13.520.000	2.428.154	-482.645	1.4281
13.530.000	2.429.707	-482.894	1.4277
13.540.000	2.431.525	-483.070	1.4271
13.550.000	2.433.033	-483.290	1.4269
13.560.000	2.434.886	-483.578	1.4266
13.570.000	2.436.410	-483.697	1.4262
13.580.000	2.438.225	-484.014	1.4255
13.590.000	2.439.858	-484.185	1.4249
13.600.000	2.441.487	-484.415	1.4247
13.610.000	2.443.195	-484.615	1.4244
13.620.000	2.444.848	-484.890	1.4240
13.630.000	2.446.579	-485.036	1.4235
13.640.000	2.448.167	-485.348	1.4234
13.650.000	2.450.045	-485.496	1.4236
13.660.000	2.451.477	-485.754	1.4234
13.670.000	2.453.386	-485.993	1.4229
13.680.000	2.454.890	-486.181	1.4226
13.690.000	2.456.704	-486.425	1.4227
13.700.000	2.458.207	-486.644	1.4226
13.710.000	2.460.081	-486.842	1.4221
13.720.000	2.461.544	-487.039	1.4216
13.730.000	2.463.417	-487.329	1.4215
13.740.000	2.464.931	-487.440	1.4214

13.750.000	2.466.719	-487.740	1.4210
13.760.000	2.468.384	-487.915	1.4203
13.770.000	2.470.062	-488.149	1.4200
13.780.000	2.471.735	-488.322	1.4199
13.790.000	2.473.473	-488.591	1.4198
13.800.000	2.475.110	-488.710	1.4193
13.810.000	2.476.722	-489.024	1.4188
13.820.000	2.478.591	-489.160	1.4186
13.830.000	2.480.026	-489.379	1.4183
13.840.000	2.481.926	-489.605	1.4175
13.850.000	2.483.376	-489.781	1.4168
13.860.000	2.485.215	-490.001	1.4166
13.870.000	2.486.757	-490.259	1.4165
13.880.000	2.488.641	-490.435	1.4162
13.890.000	2.490.140	-490.637	1.4157
13.900.000	2.492.053	-490.907	1.4154
13.910.000	2.493.490	-491.002	1.4157
13.920.000	2.495.325	-491.301	1.4158
13.930.000	2.496.919	-491.505	1.4155
13.940.000	2.498.653	-491.698	1.4154
13.950.000	2.500.293	-491.878	1.4152
13.960.000	2.502.014	-492.166	1.4151
13.970.000	2.503.674	-492.248	1.4147
13.980.000	2.505.280	-492.555	1.4138
13.990.000	2.507.125	-492.691	1.4135
14.000.000	2.508.634	-492.919	1.4133
14.010.000	2.510.484	-493.136	1.4131
14.020.000	2.512.004	-493.323	1.4130
14.030.000	2.513.819	-493.549	1.4133
14.040.000	2.515.296	-493.771	1.4136

14.050.000	2.517.202	-493.942	1.4135
14.060.000	2.518.580	-494.127	1.4135
14.070.000	2.520.520	-494.388	1.4137
14.080.000	2.522.015	-494.518	1.4135
14.090.000	2.523.808	-494.802	1.4131
14.100.000	2.525.442	-494.962	1.4129
14.110.000	2.527.255	-495.145	1.4131
14.120.000	2.528.803	-495.292	1.4129
14.130.000	2.530.597	-495.557	1.4127
14.140.000	2.532.239	-495.576	1.4128
14.150.000	2.533.909	-495.807	1.4129
14.160.000	2.535.729	-495.852	1.4128
14.170.000	2.537.251	-496.016	1.4125
14.180.000	2.539.106	-496.073	1.4128
14.190.000	2.540.683	-496.157	1.4128
14.200.000	2.542.490	-496.223	1.4122
14.210.000	2.544.085	-496.319	1.4117
14.220.000	2.545.984	-496.312	1.4115
14.230.000	2.547.420	-496.325	1.4110
14.240.000	2.549.368	-496.385	1.4104
14.250.000	2.550.833	-496.299	1.4097
14.260.000	2.552.681	-496.308	1.4097
14.270.000	2.554.304	-496.259	1.4094
14.280.000	2.556.102	-496.194	1.4086
14.290.000	2.557.689	-496.112	1.4082
14.300.000	2.559.493	-496.100	1.4077
14.310.000	2.561.096	-495.929	1.4073
14.320.000	2.562.882	-495.936	1.4065
14.330.000	2.564.635	-495.795	1.4060
14.340.000	2.566.219	-495.701	1.4058

14.350.000	2.568.056	-495.566	1.4054
14.360.000	2.569.640	-495.501	1.4048
14.370.000	2.571.487	-495.343	1.4046
14.380.000	2.573.042	-495.292	1.4043
14.390.000	2.574.950	-495.148	1.4040
14.400.000	2.576.418	-495.034	1.4035
14.410.000	2.578.362	-494.978	1.4033
14.420.000	2.579.840	-494.796	1.4031
14.430.000	2.581.729	-494.723	1.4026
14.440.000	2.583.348	-494.598	1.4022
14.450.000	2.585.172	-494.472	1.4018
14.460.000	2.586.757	-494.346	1.4013
14.470.000	2.588.605	-494.293	1.4007
14.480.000	2.590.169	-494.068	1.4003
14.490.000	2.591.941	-494.050	1.4000
14.500.000	2.593.670	-493.882	1.3994
14.510.000	2.595.328	-493.808	1.3988
14.520.000	2.597.063	-493.682	1.3985
14.530.000	2.598.735	-493.643	1.3980
14.540.000	2.600.525	-493.512	1.3976
14.550.000	2.602.087	-493.514	1.3971
14.560.000	2.604.011	-493.428	1.3968
14.570.000	2.605.424	-493.375	1.3963
14.580.000	2.607.376	-493.406	1.3959
14.590.000	2.608.856	-493.324	1.3954
14.600.000	2.610.683	-493.333	1.3949
14.610.000	2.612.206	-493.317	1.3947
14.620.000	2.614.043	-493.312	1.3943
14.630.000	2.615.569	-493.296	1.3939
14.640.000	2.617.404	-493.398	1.3937

14.650.000	2.618.959	-493.325	1.3934
14.660.000	2.620.768	-493.452	1.3930
14.670.000	2.622.451	-493.487	1.3925
14.680.000	2.624.088	-493.547	1.3920
14.690.000	2.625.821	-493.594	1.3917
14.700.000	2.627.473	-493.748	1.3912
14.710.000	2.629.175	-493.745	1.3908
14.720.000	2.630.743	-493.930	1.3906
14.730.000	2.632.606	-493.966	1.3903
14.740.000	2.634.016	-494.089	1.3900
14.750.000	2.635.949	-494.260	1.3896
14.760.000	2.637.427	-494.322	1.3891
14.770.000	2.639.307	-494.481	1.3887
14.780.000	2.640.835	-494.640	1.3882
14.790.000	2.642.662	-494.728	1.3877
14.800.000	2.644.155	-494.860	1.3873
14.810.000	2.646.017	-495.087	1.3869
14.820.000	2.647.531	-495.125	1.3866
14.830.000	2.649.291	-495.353	1.3862
14.840.000	2.650.921	-495.498	1.3854
14.850.000	2.652.610	-495.650	1.3851
14.860.000	2.654.283	-495.811	1.3845
14.870.000	2.655.970	-496.058	1.3836
14.880.000	2.657.686	-496.134	1.3829
14.890.000	2.659.309	-496.440	1.3822
14.900.000	2.661.103	-496.558	1.3816
14.910.000	2.662.622	-496.780	1.3811
14.920.000	2.664.467	-497.009	1.3808
14.930.000	2.665.963	-497.185	1.3804
14.940.000	2.667.770	-497.418	1.3801

14.950.000	2.669.263	-497.655	1.3799
14.960.000	2.671.118	-497.864	1.3796
14.970.000	2.672.578	-498.095	1.3792
14.980.000	2.674.462	-498.378	1.3787
14.990.000	2.676.009	-498.541	1.3782
15.000.000	2.677.782	-498.841	1.3776
15.010.000	2.679.402	-499.054	1.3773
15.020.000	2.681.131	-499.313	1.3769
15.030.000	2.682.712	-499.540	1.3765
15.040.000	2.684.462	-499.864	1.3762
15.050.000	2.686.101	-500.015	1.3762
15.060.000	2.687.730	-500.367	1.3760
15.070.000	2.689.520	-500.561	1.3755
15.080.000	2.690.998	-500.845	1.3752
15.090.000	2.692.878	-501.102	1.3751
15.100.000	2.694.393	-501.388	1.3748
15.110.000	2.696.217	-501.656	1.3742
15.120.000	2.697.746	-501.960	1.3740
15.130.000	2.699.625	-502.225	1.3737
15.140.000	2.701.041	-502.477	1.3733
15.150.000	2.702.947	-502.834	1.3725
15.160.000	2.704.408	-503.031	1.3719
15.170.000	2.706.206	-503.353	1.3714
15.180.000	2.707.782	-503.638	1.3706
15.190.000	2.709.518	-503.913	1.3698
15.200.000	2.711.082	-504.167	1.3693
15.210.000	2.712.831	-504.553	1.3688
15.220.000	2.714.485	-504.729	1.3684
15.230.000	2.716.133	-505.096	1.3681
15.240.000	2.717.849	-505.341	1.3678

15.250.000	2.719.391	-505.645	1.3675
15.260.000	2.721.150	-505.914	1.3670
15.270.000	2.722.703	-506.255	1.3665
15.280.000	2.724.464	-506.508	1.3660
15.290.000	2.725.947	-506.845	1.3653
15.300.000	2.727.840	-507.138	1.3644
15.310.000	2.729.213	-507.418	1.3637
15.320.000	2.731.147	-507.806	1.3633
15.330.000	2.732.648	-508.026	1.3629
15.340.000	2.734.446	-508.408	1.3625
15.350.000	2.735.987	-508.709	1.3622
15.360.000	2.737.762	-509.006	1.3619
15.370.000	2.739.293	-509.285	1.3616
15.380.000	2.741.057	-509.701	1.3613
15.390.000	2.742.663	-509.905	1.3608
15.400.000	2.744.327	-510.311	1.3604
15.410.000	2.746.030	-510.577	1.3599
15.420.000	2.747.579	-510.915	1.3594
15.430.000	2.749.310	-511.196	1.3589
15.440.000	2.750.933	-511.582	1.3584
15.450.000	2.752.692	-511.833	1.3580
15.460.000	2.754.185	-512.211	1.3573
15.470.000	2.756.052	-512.515	1.3569
15.480.000	2.757.467	-512.836	1.3567
15.490.000	2.759.360	-513.207	1.3564
15.500.000	2.760.842	-513.476	1.3562
15.510.000	2.762.614	-513.826	1.3558
15.520.000	2.764.150	-514.145	1.3557
15.530.000	2.765.939	-514.453	1.3553
15.540.000	2.767.435	-514.747	1.3548

15.550.000	2.769.276	-515.156	1.3544
15.560.000	2.770.844	-515.397	1.3539
15.570.000	2.772.536	-515.784	1.3534
15.580.000	2.774.225	-516.082	1.3529
15.590.000	2.775.818	-516.418	1.3525
15.600.000	2.777.505	-516.728	1.3519
15.610.000	2.779.140	-517.143	1.3511
15.620.000	2.780.845	-517.385	1.3503
15.630.000	2.782.410	-517.795	1.3492
15.640.000	2.784.260	-518.133	1.3484
15.650.000	2.785.651	-518.447	1.3478
15.660.000	2.787.564	-518.854	1.3472
15.670.000	2.789.045	-519.149	1.3465
15.680.000	2.790.879	-519.517	1.3461
15.690.000	2.792.391	-519.873	1.3457
15.700.000	2.794.218	-520.178	1.3453
15.710.000	2.795.669	-520.516	1.3446
15.720.000	2.797.520	-520.942	1.3443
15.730.000	2.799.033	-521.162	1.3440
15.740.000	2.800.752	-521.577	1.3435
15.750.000	2.802.418	-521.898	1.3430
15.760.000	2.804.073	-522.231	1.3426
15.770.000	2.805.752	-522.547	1.3421
15.780.000	2.807.380	-522.961	1.3416
15.790.000	2.809.108	-523.195	1.3412
15.800.000	2.810.652	-523.615	1.3408
15.810.000	2.812.495	-523.926	1.3406
15.820.000	2.813.949	-524.273	1.3403
15.830.000	2.815.810	-524.643	1.3402
15.840.000	2.817.265	-524.954	1.3399

15.850.000	2.819.065	-525.300	1.3393
15.860.000	2.820.537	-525.637	1.3388
15.870.000	2.822.425	-525.972	1.3385
15.880.000	2.823.874	-526.275	1.3380
15.890.000	2.825.734	-526.670	1.3373
15.900.000	2.827.284	-526.923	1.3367
15.910.000	2.829.015	-527.299	1.3364
15.920.000	2.830.641	-527.610	1.3360
15.930.000	2.832.311	-527.934	1.3354
15.940.000	2.833.963	-528.243	1.3349
15.950.000	2.835.622	-528.634	1.3348
15.960.000	2.837.282	-528.843	1.3345
15.970.000	2.838.854	-529.258	1.3340
15.980.000	2.840.695	-529.537	1.3339
15.990.000	2.842.143	-529.866	1.3341
16.000.000	2.843.996	-530.182	1.3339
16.010.000	2.845.518	-530.506	1.3335
16.020.000	2.847.332	-530.817	1.3333
16.030.000	2.848.816	-531.125	1.3334
16.040.000	2.850.673	-531.429	1.3334
16.050.000	2.852.084	-531.695	1.3329
16.060.000	2.853.963	-532.065	1.3326
16.070.000	2.855.453	-532.287	1.3325
16.080.000	2.857.230	-532.643	1.3322
16.090.000	2.858.806	-532.930	1.3316
16.100.000	2.860.542	-533.228	1.3312
16.110.000	2.862.161	-533.487	1.3310
16.120.000	2.863.888	-533.876	1.3305
16.130.000	2.865.526	-534.056	1.3299
16.140.000	2.867.151	-534.433	1.3296

16.150.000	2.868.930	-534.691	1.3290
16.160.000	2.870.439	-534.985	1.3282
16.170.000	2.872.262	-535.274	1.3273
16.180.000	2.873.730	-535.590	1.3268
16.190.000	2.875.583	-535.864	1.3263
16.200.000	2.877.044	-536.184	1.3256
16.210.000	2.878.948	-536.478	1.3252
16.220.000	2.880.388	-536.721	1.3251
16.230.000	2.882.259	-537.090	1.3248
16.240.000	2.883.799	-537.296	1.3242
16.250.000	2.885.583	-537.618	1.3235
16.260.000	2.887.117	-537.890	1.3231
16.270.000	2.888.903	-538.181	1.3222
16.280.000	2.890.461	-538.436	1.3214
16.290.000	2.892.186	-538.783	1.3209
16.300.000	2.893.854	-538.951	1.3205
16.310.000	2.895.440	-539.308	1.3199
16.320.000	2.897.247	-539.544	1.3192
16.330.000	2.898.781	-539.832	1.3186
16.340.000	2.900.601	-540.093	1.3184
16.350.000	2.902.168	-540.417	1.3177
16.360.000	2.903.944	-540.656	1.3171
16.370.000	2.905.418	-540.970	1.3166
16.380.000	2.907.311	-541.264	1.3163
16.390.000	2.908.701	-541.514	1.3158
16.400.000	2.910.587	-541.835	1.3150
16.410.000	2.912.080	-542.085	1.3146
16.420.000	2.913.865	-542.400	1.3142
16.430.000	2.915.441	-542.659	1.3135
16.440.000	2.917.206	-542.969	1.3129

16.450.000	2.918.707	-543.178	1.3125
16.460.000	2.920.526	-543.549	1.3120
16.470.000	2.922.093	-543.717	1.3113
16.480.000	2.923.757	-544.036	1.3109
16.490.000	2.925.432	-544.291	1.3105
16.500.000	2.927.040	-544.569	1.3103
16.510.000	2.928.780	-544.787	1.3097
16.520.000	2.930.341	-545.136	1.3093
16.530.000	2.932.142	-545.322	1.3092
16.540.000	2.933.634	-545.657	1.3088
16.550.000	2.935.551	-545.927	1.3081
16.560.000	2.936.904	-546.138	1.3075
16.570.000	2.938.821	-546.468	1.3072
16.580.000	2.940.290	-546.702	1.3069
16.590.000	2.942.083	-546.976	1.3062
16.600.000	2.943.576	-547.234	1.3057
16.610.000	2.945.414	-547.498	1.3055
16.620.000	2.946.893	-547.709	1.3054
16.630.000	2.948.674	-548.057	1.3049
16.640.000	2.950.261	-548.188	1.3044
16.650.000	2.951.938	-548.533	1.3042
16.660.000	2.953.619	-548.773	1.3041
16.670.000	2.955.258	-549.016	1.3038
16.680.000	2.956.993	-549.258	1.3032
16.690.000	2.958.588	-549.605	1.3031
16.700.000	2.960.363	-549.752	1.3029
16.710.000	2.961.878	-550.111	1.3025
16.720.000	2.963.766	-550.365	1.3020
16.730.000	2.965.171	-550.610	1.3017
16.740.000	2.967.053	-550.927	1.3017

16.750.000	2.968.547	-551.186	1.3013
16.760.000	2.970.355	-551.462	1.3006
16.770.000	2.971.874	-551.744	1.3000
16.780.000	2.973.743	-552.043	1.2995
16.790.000	2.975.205	-552.291	1.2990
16.800.000	2.977.043	-552.655	1.2979
16.810.000	2.978.624	-552.844	1.2967
16.820.000	2.980.305	-553.168	1.2961
16.830.000	2.981.949	-553.432	1.2954
16.840.000	2.983.624	-553.697	1.2945
16.850.000	2.985.273	-553.946	1.2935
16.860.000	2.986.956	-554.291	1.2929
16.870.000	2.988.663	-554.468	1.2924
16.880.000	2.990.216	-554.812	1.2916
16.890.000	2.992.086	-555.064	1.2912
16.900.000	2.993.502	-555.295	1.2909
16.910.000	2.995.374	-555.610	1.2906
16.920.000	2.996.897	-555.886	1.2902
16.930.000	2.998.707	-556.133	1.2900
16.940.000	3.000.197	-556.421	1.2899
16.950.000	3.002.094	-556.716	1.2895
16.960.000	3.003.530	-556.931	1.2890
16.970.000	3.005.408	-557.270	1.2886
16.980.000	3.006.893	-557.465	1.2883
16.990.000	3.008.657	-557.792	1.2878
17.000.000	3.010.291	-558.032	1.2873
17.010.000	3.011.962	-558.304	1.2869
17.020.000	3.013.644	-558.570	1.2866
17.030.000	3.015.325	-558.941	1.2861
17.040.000	3.016.979	-559.082	1.2856

17.050.000	3.018.555	-559.451	1.2854
17.060.000	3.020.376	-559.702	1.2851
17.070.000	3.021.864	-559.976	1.2846
17.080.000	3.023.700	-560.262	1.2843
17.090.000	3.025.207	-560.579	1.2842
17.100.000	3.027.048	-560.833	1.2837
17.110.000	3.028.492	-561.145	1.2830
17.120.000	3.030.387	-561.424	1.2825
17.130.000	3.031.832	-561.660	1.2823
17.140.000	3.033.664	-562.009	1.2817
17.150.000	3.035.185	-562.201	1.2810
17.160.000	3.036.932	-562.531	1.2803
17.170.000	3.038.537	-562.771	1.2797
17.180.000	3.040.274	-563.081	1.2787
17.190.000	3.041.868	-563.313	1.2775
17.200.000	3.043.591	-563.664	1.2766
17.210.000	3.045.291	-563.850	1.2759
17.220.000	3.046.862	-564.196	1.2750
17.230.000	3.048.669	-564.437	1.2742
17.240.000	3.050.169	-564.729	1.2736
17.250.000	3.051.992	-565.012	1.2730
17.260.000	3.053.510	-565.349	1.2721
17.270.000	3.055.319	-565.593	1.2713
17.280.000	3.056.773	-565.886	1.2705
17.290.000	3.058.660	-566.200	1.2698
17.300.000	3.060.048	-566.406	1.2692
17.310.000	3.061.955	-566.738	1.2688
17.320.000	3.063.483	-566.978	1.2684
17.330.000	3.065.213	-567.269	1.2680
17.340.000	3.066.790	-567.524	1.2678

17.350.000	3.068.549	-567.806	1.2675
17.360.000	3.070.116	-568.040	1.2672
17.370.000	3.071.874	-568.396	1.2668
17.380.000	3.073.517	-568.562	1.2665
17.390.000	3.075.120	-568.885	1.2661
17.400.000	3.076.871	-569.143	1.2655
17.410.000	3.078.423	-569.432	1.2650
17.420.000	3.080.202	-569.665	1.2647
17.430.000	3.081.784	-570.024	1.2640
17.440.000	3.083.601	-570.232	1.2633
17.450.000	3.085.044	-570.538	1.2626
17.460.000	3.086.932	-570.870	1.2620
17.470.000	3.088.419	-571.104	1.2610
17.480.000	3.090.271	-571.443	1.2598
17.490.000	3.091.768	-571.731	1.2587
17.500.000	3.093.573	-572.020	1.2578
17.510.000	3.095.089	-572.310	1.2569
17.520.000	3.096.900	-572.638	1.2557
17.530.000	3.098.427	-572.871	1.2549
17.540.000	3.100.192	-573.243	1.2542
17.550.000	3.101.847	-573.460	1.2534
17.560.000	3.103.487	-573.791	1.2527
17.570.000	3.105.190	-574.061	1.2520
17.580.000	3.106.800	-574.362	1.2515
17.590.000	3.108.555	-574.612	1.2506
17.600.000	3.110.139	-574.965	1.2499
17.610.000	3.111.924	-575.167	1.2494
17.620.000	3.113.379	-575.518	1.2488
17.630.000	3.115.303	-575.818	1.2479
17.640.000	3.116.673	-576.058	1.2472

17.650.000	3.118.560	-576.392	1.2466
17.660.000	3.120.082	-576.676	1.2458
17.670.000	3.121.859	-576.965	1.2453
17.680.000	3.123.372	-577.235	1.2448
17.690.000	3.125.228	-577.583	1.2445
17.700.000	3.126.717	-577.805	1.2441
17.710.000	3.128.518	-578.160	1.2438
17.720.000	3.130.093	-578.370	1.2436
17.730.000	3.131.768	-578.731	1.2432
17.740.000	3.133.458	-578.995	1.2427
17.750.000	3.135.095	-579.331	1.2421
17.760.000	3.136.778	-579.539	1.2417
17.770.000	3.138.398	-579.935	1.2410
17.780.000	3.140.153	-580.107	1.2404
17.790.000	3.141.641	-580.447	1.2401
17.800.000	3.143.547	-580.748	1.2395
17.810.000	3.144.977	-580.998	1.2389
17.820.000	3.146.817	-581.323	1.2383
17.830.000	3.148.331	-581.637	1.2377
17.840.000	3.150.122	-581.865	1.2371
17.850.000	3.151.599	-582.144	1.2362
17.860.000	3.153.503	-582.501	1.2356
17.870.000	3.154.945	-582.707	1.2348
17.880.000	3.156.714	-583.065	1.2336
17.890.000	3.158.321	-583.308	1.2326
17.900.000	3.160.005	-583.620	1.2321
17.910.000	3.161.684	-583.898	1.2316
17.920.000	3.163.375	-584.227	1.2303
17.930.000	3.165.009	-584.435	1.2301
17.940.000	3.166.678	-584.811	1.2299

17.950.000	3.168.431	-584.988	1.2294
17.960.000	3.169.912	-585.341	1.2287
17.970.000	3.171.804	-585.630	1.2279
17.980.000	3.173.267	-585.873	1.2276
17.990.000	3.175.092	-586.221	1.2266
18.000.000	3.176.593	-586.526	1.2254
18.010.000	3.178.405	-586.781	1.2246
18.020.000	3.179.887	-587.100	1.2243
18.030.000	3.181.741	-587.431	1.2237
18.040.000	3.183.221	-587.624	1.2227
18.050.000	3.185.023	-588.008	1.2221
18.060.000	3.186.612	-588.239	1.2213
18.070.000	3.188.356	-588.575	1.2203
18.080.000	3.189.956	-588.829	1.2189
18.090.000	3.191.678	-589.147	1.2179
18.100.000	3.193.300	-589.361	1.2172
18.110.000	3.194.972	-589.735	1.2163
18.120.000	3.196.710	-589.918	1.2151
18.130.000	3.198.206	-590.264	1.2144
18.140.000	3.200.053	-590.546	1.2138
18.150.000	3.201.575	-590.820	1.2129
18.160.000	3.203.399	-591.126	1.2119
18.170.000	3.204.888	-591.450	1.2108
18.180.000	3.206.736	-591.730	1.2100
18.190.000	3.208.168	-592.034	1.2093
18.200.000	3.210.099	-592.378	1.2085
18.210.000	3.211.538	-592.600	1.2075
18.220.000	3.213.368	-592.967	1.2068
18.230.000	3.214.882	-593.231	1.2061
18.240.000	3.216.594	-593.529	1.2056

18.250.000	3.218.166	-593.800	1.2048
18.260.000	3.219.960	-594.164	1.2041
18.270.000	3.221.540	-594.365	1.2034
18.280.000	3.223.253	-594.748	1.2025
18.290.000	3.224.913	-594.959	1.2014
18.300.000	3.226.483	-595.304	1.2006
18.310.000	3.228.291	-595.560	1.1998
18.320.000	3.229.813	-595.876	1.1989
18.330.000	3.231.610	-596.152	1.1979
18.340.000	3.233.088	-596.525	1.1973
18.350.000	3.234.985	-596.776	1.1968
18.360.000	3.236.438	-597.092	1.1961
18.370.000	3.238.318	-597.455	1.1953
18.380.000	3.239.791	-597.676	1.1947
18.390.000	3.241.584	-598.050	1.1944
18.400.000	3.243.126	-598.333	1.1941
18.410.000	3.244.923	-598.637	1.1933
18.420.000	3.246.464	-598.950	1.1927
18.430.000	3.248.252	-599.296	1.1922
18.440.000	3.249.820	-599.521	1.1916
18.450.000	3.251.460	-599.897	1.1906
18.460.000	3.253.197	-600.161	1.1897
18.470.000	3.254.788	-600.489	1.1891
18.480.000	3.256.504	-600.757	1.1887
18.490.000	3.258.104	-601.088	1.1879
18.500.000	3.259.858	-601.334	1.1870
18.510.000	3.261.371	-601.703	1.1864
18.520.000	3.263.244	-601.957	1.1857
18.530.000	3.264.648	-602.261	1.1848
18.540.000	3.266.559	-602.595	1.1837

18.550.000	3.268.010	-602.830	1.1828
18.560.000	3.269.789	-603.158	1.1820
18.570.000	3.271.331	-603.470	1.1812
18.580.000	3.273.106	-603.729	1.1800
18.590.000	3.274.620	-604.015	1.1790
18.600.000	3.276.475	-604.396	1.1780
18.610.000	3.277.975	-604.593	1.1772
18.620.000	3.279.755	-604.967	1.1763
18.630.000	3.281.405	-605.214	1.1750
18.640.000	3.282.991	-605.518	1.1743
18.650.000	3.284.746	-605.794	1.1735
18.660.000	3.286.333	-606.131	1.1725
18.670.000	3.288.069	-606.349	1.1711
18.680.000	3.289.634	-606.735	1.1697
18.690.000	3.291.473	-606.959	1.1689
18.700.000	3.292.844	-607.290	1.1680
18.710.000	3.294.821	-607.652	1.1670
18.720.000	3.296.252	-607.912	1.1659
18.730.000	3.298.109	-608.260	1.1651
18.740.000	3.299.606	-608.596	1.1644
18.750.000	3.301.395	-608.874	1.1634
18.760.000	3.302.926	-609.159	1.1621
18.770.000	3.304.729	-609.541	1.1608
18.780.000	3.306.273	-609.762	1.1596
18.790.000	3.307.978	-610.136	1.1587
18.800.000	3.309.598	-610.405	1.1575
18.810.000	3.311.264	-610.688	1.1562
18.820.000	3.312.912	-610.980	1.1555
18.830.000	3.314.549	-611.317	1.1548
18.840.000	3.316.234	-611.521	1.1539

18.850.000	3.317.807	-611.874	1.1527
18.860.000	3.319.634	-612.094	1.1517
18.870.000	3.321.062	-612.401	1.1510
18.880.000	3.322.926	-612.717	1.1500
18.890.000	3.324.378	-612.962	1.1488
18.900.000	3.326.180	-613.277	1.1477
18.910.000	3.327.681	-613.561	1.1468
18.920.000	3.329.523	-613.837	1.1460
18.930.000	3.330.932	-614.107	1.1451
18.940.000	3.332.818	-614.435	1.1440
18.950.000	3.334.299	-614.659	1.1433
18.960.000	3.336.055	-615.020	1.1426
18.970.000	3.337.684	-615.301	1.1419
18.980.000	3.339.373	-615.592	1.1413
18.990.000	3.341.003	-615.856	1.1404
19.000.000	3.342.710	-616.230	1.1397
19.010.000	3.344.344	-616.438	1.1390
19.020.000	3.345.957	-616.817	1.1382
19.030.000	3.347.715	-617.073	1.1372
19.040.000	3.349.181	-617.384	1.1361
19.050.000	3.351.072	-617.731	1.1350
19.060.000	3.352.540	-618.027	1.1342
19.070.000	3.354.337	-618.339	1.1330
19.080.000	3.355.844	-618.680	1.1317
19.090.000	3.357.700	-618.954	1.1304
19.100.000	3.359.157	-619.234	1.1292
19.110.000	3.361.009	-619.623	1.1283
19.120.000	3.362.503	-619.845	1.1270
19.130.000	3.364.269	-620.207	1.1259
19.140.000	3.365.866	-620.517	1.1250

19.150.000	3.367.561	-620.807	1.1242
19.160.000	3.369.183	-621.100	1.1232
19.170.000	3.370.872	-621.496	1.1221
19.180.000	3.372.506	-621.697	1.1211
19.190.000	3.374.094	-622.107	1.1203
19.200.000	3.375.912	-622.355	1.1193
19.210.000	3.377.403	-622.674	1.1180
19.220.000	3.379.218	-622.972	1.1168
19.230.000	3.380.717	-623.288	1.1159
19.240.000	3.382.553	-623.580	1.1148
19.250.000	3.384.026	-623.924	1.1135
19.260.000	3.385.930	-624.237	1.1123
19.270.000	3.387.333	-624.537	1.1113
19.280.000	3.389.243	-624.920	1.1105
19.290.000	3.390.712	-625.158	1.1092
19.300.000	3.392.462	-625.541	1.1080
19.310.000	3.394.049	-625.857	1.1071
19.320.000	3.395.828	-626.167	1.1059
19.330.000	3.397.359	-626.446	1.1046
19.340.000	3.399.086	-626.841	1.1032
19.350.000	3.400.714	-627.038	1.1021
19.360.000	3.402.349	-627.436	1.1010
19.370.000	3.404.077	-627.698	1.0998
19.380.000	3.405.655	-628.041	1.0985
19.390.000	3.407.428	-628.312	1.0976
19.400.000	3.408.986	-628.685	1.0968
19.410.000	3.410.714	-628.945	1.0956
19.420.000	3.412.243	-629.311	1.0946
19.430.000	3.414.147	-629.634	1.0939
19.440.000	3.415.517	-629.931	1.0933

19.450.000	3.417.408	-630.325	1.0924
19.460.000	3.418.916	-630.623	1.0912
19.470.000	3.420.673	-630.982	1.0902
19.480.000	3.422.229	-631.308	1.0892
19.490.000	3.424.022	-631.675	1.0877
19.500.000	3.425.566	-631.961	1.0863
19.510.000	3.427.367	-632.382	1.0852
19.520.000	3.428.933	-632.617	1.0842
19.530.000	3.430.618	-633.037	1.0832
19.540.000	3.432.363	-633.351	1.0822
19.550.000	3.433.935	-633.696	1.0814
19.560.000	3.435.647	-634.006	1.0805
19.570.000	3.437.223	-634.430	1.0792
19.580.000	3.438.966	-634.688	1.0780
19.590.000	3.440.464	-635.088	1.0769
19.600.000	3.442.339	-635.463	1.0755
19.610.000	3.443.814	-635.757	1.0742
19.620.000	3.445.668	-636.155	1.0727
19.630.000	3.447.167	-636.449	1.0714
19.640.000	3.448.997	-636.804	1.0699
19.650.000	3.450.517	-637.152	1.0686
19.660.000	3.452.326	-637.480	1.0675
19.670.000	3.453.849	-637.785	1.0659
19.680.000	3.455.617	-638.208	1.0643
19.690.000	3.457.220	-638.451	1.0630
19.700.000	3.458.911	-638.859	1.0620
19.710.000	3.460.569	-639.175	1.0607
19.720.000	3.462.214	-639.530	1.0591
19.730.000	3.463.900	-639.822	1.0579
19.740.000	3.465.530	-640.247	1.0574

19.750.000	3.467.314	-640.487	1.0564
19.760.000	3.468.799	-640.885	1.0554
19.770.000	3.470.697	-641.251	1.0544
19.780.000	3.472.151	-641.557	1.0540
19.790.000	3.473.993	-641.961	1.0533
19.800.000	3.475.511	-642.295	1.0521
19.810.000	3.477.295	-642.638	1.0511
19.820.000	3.478.773	-642.997	1.0500
19.830.000	3.480.644	-643.360	1.0487
19.840.000	3.482.156	-643.669	1.0472
19.850.000	3.483.959	-644.098	1.0456
19.860.000	3.485.570	-644.383	1.0440
19.870.000	3.487.253	-644.775	1.0426
19.880.000	3.488.936	-645.112	1.0412
19.890.000	3.490.574	-645.458	1.0397
19.900.000	3.492.223	-645.760	1.0382
19.910.000	3.493.899	-646.218	1.0369
19.920.000	3.495.619	-646.428	1.0358
19.930.000	3.497.138	-646.863	1.0345
19.940.000	3.499.018	-647.204	1.0330
19.950.000	3.500.467	-647.524	1.0318
19.960.000	3.502.362	-647.933	1.0309
19.970.000	3.503.867	-648.292	1.0298
19.980.000	3.505.683	-648.593	1.0287
19.990.000	3.507.186	-648.966	1.0277
20.000.000	3.509.046	-649.342	1.0273
20.010.000	3.510.468	-649.627	1.0264
20.020.000	3.512.307	-650.044	1.0252
20.030.000	3.513.872	-650.338	1.0242
20.040.000	3.515.557	-650.726	1.0232

20.050.000	3.517.184	-651.071	1.0219
20.060.000	3.518.915	-651.431	1.0203
20.070.000	3.520.563	-651.741	1.0189
20.080.000	3.522.233	-652.181	1.0176
20.090.000	3.523.972	-652.411	1.0160
20.100.000	3.525.523	-652.831	1.0143
20.110.000	3.527.339	-653.146	1.0129
20.120.000	3.528.777	-653.483	1.0111
20.130.000	3.530.662	-653.861	1.1993
20.140.000	3.532.127	-654.246	1.1978
20.150.000	3.533.994	-654.567	1.1966
20.160.000	3.535.444	-654.937	1.1950
20.170.000	3.537.311	-655.319	1.1934
20.180.000	3.538.817	-655.640	1.2023
20.190.000	3.540.648	-656.056	1.2013
20.200.000	3.542.216	-656.367	9.998
20.210.000	3.543.930	-656.759	9.981
20.220.000	3.545.550	-657.097	9.969
20.230.000	3.547.327	-657.496	9.958
20.240.000	3.548.751	-657.761	9.943
20.250.000	3.550.518	-658.171	9.927
20.260.000	3.552.389	-658.427	9.908
20.270.000	3.553.865	-658.755	9.879
20.280.000	3.555.499	-659.308	9.864
20.290.000	3.557.149	-659.550	9.847
20.300.000	3.558.963	-659.887	9.834
20.310.000	3.560.451	-660.300	9.812
20.320.000	3.562.273	-660.650	9.789
20.330.000	3.563.679	-660.931	9.777
20.340.000	3.565.651	-661.242	9.756

20.350.000	3.567.030	-661.552	9.734
20.360.000	3.568.869	-661.983	9.709
20.370.000	3.570.563	-662.490	9.690
20.380.000	3.572.115	-662.725	9.670
20.390.000	3.573.771	-663.189	9.657
20.400.000	3.575.523	-663.615	9.650
20.410.000	3.577.126	-663.883	9.640
20.420.000	3.578.868	-664.342	9.628
20.430.000	3.580.499	-664.581	9.609
20.440.000	3.582.132	-664.986	9.603
20.450.000	3.583.876	-665.332	9.592
20.460.000	3.585.440	-665.768	9.580
20.470.000	3.587.202	-666.031	9.566
20.480.000	3.588.716	-666.461	9.556
20.490.000	3.590.603	-666.781	9.542
20.500.000	3.591.966	-667.153	9.530
20.510.000	3.593.848	-667.564	9.517
20.520.000	3.595.335	-667.844	9.505
20.530.000	3.597.181	-668.277	9.490
20.540.000	3.598.646	-668.629	9.476
20.550.000	3.600.447	-668.985	9.464
20.560.000	3.602.030	-669.399	9.453
20.570.000	3.603.761	-669.878	9.433
20.580.000	3.605.349	-670.143	9.419
20.590.000	3.607.085	-670.625	9.406
20.600.000	3.608.702	-670.889	9.400
20.610.000	3.610.322	-671.227	9.390
20.620.000	3.612.227	-671.630	9.373
20.630.000	3.613.739	-671.989	9.363
20.640.000	3.615.422	-672.242	9.351

20.650.000	3.616.979	-672.694	9.336
20.660.000	3.618.870	-672.988	9.315
20.670.000	3.620.126	-673.344	9.287
20.680.000	3.622.094	-673.878	9.267
20.690.000	3.623.707	-674.072	9.247
20.700.000	3.625.469	-674.493	9.225
20.710.000	3.627.001	-674.950	9.204
20.720.000	3.628.587	-675.192	9.184
20.730.000	3.630.367	-675.539	9.159
20.740.000	3.632.038	-676.055	9.144
20.750.000	3.633.476	-676.055	9.129
20.760.000	3.634.976	-676.562	9.116
20.770.000	3.637.133	-676.744	9.108
20.780.000	3.638.592	-677.135	9.091
20.790.000	3.640.211	-677.304	9.094
20.800.000	3.641.906	-677.743	9.150
20.810.000	3.643.457	-678.118	9.163
20.820.000	3.645.269	-678.414	9.142
20.830.000	3.647.532	-678.686	9.111
20.840.000	3.648.718	-679.476	9.086
20.850.000	3.650.117	-679.880	9.060
20.860.000	3.651.913	-680.191	8.995
20.870.000	3.653.562	-680.631	8.933
20.880.000	3.654.759	-681.086	8.905
20.890.000	3.656.627	-681.609	8.880
20.900.000	3.658.400	-681.895	8.891
20.910.000	3.660.126	-682.331	8.826
20.920.000	3.661.670	-682.589	8.757
20.930.000	3.663.459	-683.085	8.712
20.940.000	3.665.140	-683.453	8.704

20.950.000	3.666.663	-684.008	8.665
20.960.000	3.668.379	-684.258	8.648
20.970.000	3.670.169	-684.575	8.645
20.980.000	3.671.603	-684.816	8.686
20.990.000	3.673.015	-685.487	8.704
21.000.000	3.675.074	-685.768	8.654
21.010.000	3.676.548	-686.039	8.628
21.020.000	3.678.310	-686.350	8.660
21.030.000	3.679.931	-686.871	8.614
21.040.000	3.681.642	-687.142	8.635
21.050.000	3.683.116	-687.331	8.555
21.060.000	3.685.025	-687.647	8.549
21.070.000	3.686.465	-687.998	8.525
21.080.000	3.688.374	-688.435	8.507
21.090.000	3.689.861	-688.699	8.492
21.100.000	3.691.673	-689.069	8.516
21.110.000	3.693.220	-689.543	8.478
21.120.000	3.694.873	-689.893	8.463
21.130.000	3.696.471	-690.259	8.460
21.140.000	3.698.239	-690.702	8.456
21.150.000	3.699.864	-690.954	8.397
21.160.000	3.701.475	-691.410	8.342
21.170.000	3.703.179	-691.684	8.379
21.180.000	3.704.622	-692.048	8.416
21.190.000	3.706.529	-692.408	8.402
21.200.000	3.708.033	-692.809	8.366
21.210.000	3.709.844	-693.167	8.378
21.220.000	3.711.332	-693.509	8.350
21.230.000	3.713.119	-693.850	8.312
21.240.000	3.714.564	-694.147	8.205

21.250.000	3.716.401	-694.544	8.211
21.260.000	3.717.888	-694.829	8.187
21.270.000	3.719.655	-695.198	8.158
21.280.000	3.721.238	-695.570	8.132
21.290.000	3.722.953	-695.935	8.110
21.300.000	3.724.550	-696.281	8.106
21.310.000	3.726.284	-696.735	8.067
21.320.000	3.727.938	-697.010	8.060
21.330.000	3.729.543	-697.453	8.047
21.340.000	3.731.278	-697.767	8.036
21.350.000	3.732.791	-698.151	8.025
21.360.000	3.734.595	-698.491	8.005
21.370.000	3.736.129	-698.889	8.011
21.380.000	3.737.873	-699.159	7.994
21.390.000	3.739.366	-699.568	7.967
21.400.000	3.741.266	-699.922	7.945
21.410.000	3.742.631	-700.231	7.924
21.420.000	3.744.528	-700.627	7.906
21.430.000	3.746.032	-700.913	7.886
21.440.000	3.747.769	-701.272	7.862
21.450.000	3.749.292	-701.575	7.847
21.460.000	3.751.104	-701.892	7.831
21.470.000	3.752.570	-702.166	7.812
21.480.000	3.754.320	-702.545	7.794
21.490.000	3.755.899	-702.757	7.777
21.500.000	3.757.510	-703.107	7.761
21.510.000	3.759.225	-703.367	7.744
21.520.000	3.760.821	-703.678	7.726
21.530.000	3.762.570	-703.914	7.708
21.540.000	3.764.127	-704.268	7.692

21.550.000	3.765.927	-704.472	7.676
21.560.000	3.767.399	-704.796	7.658
21.570.000	3.769.247	-705.087	7.638
21.580.000	3.770.623	-705.294	7.623
21.590.000	3.772.486	-705.615	7.610
21.600.000	3.774.003	-705.837	7.596
21.610.000	3.775.730	-706.102	7.578
21.620.000	3.777.264	-706.345	7.563
21.630.000	3.779.076	-706.583	7.550
21.640.000	3.780.551	-706.769	7.533
21.650.000	3.782.317	-707.054	7.514
21.660.000	3.783.919	-707.174	7.496
21.670.000	3.785.540	-707.413	7.480
21.680.000	3.787.296	-707.604	7.461
21.690.000	3.788.888	-707.758	7.437
21.700.000	3.790.574	-707.892	7.415
21.710.000	3.792.159	-708.132	7.398
21.720.000	3.793.906	-708.196	7.378
21.730.000	3.795.372	-708.418	7.355
21.740.000	3.797.249	-708.597	7.333
21.750.000	3.798.618	-708.683	7.319
21.760.000	3.800.491	-708.896	7.308
21.770.000	3.802.002	-708.989	7.292
21.780.000	3.803.768	-709.077	7.275
21.790.000	3.805.232	-709.179	7.262
21.800.000	3.807.089	-709.274	7.249
21.810.000	3.808.519	-709.281	7.232
21.820.000	3.810.289	-709.415	7.214
21.830.000	3.811.871	-709.390	7.201
21.840.000	3.813.540	-709.492	7.183

21.850.000	3.815.206	-709.495	7.165
21.860.000	3.816.817	-709.512	7.150
21.870.000	3.818.465	-709.481	7.133
21.880.000	3.820.109	-709.571	7.118
21.890.000	3.821.849	-709.440	7.100
21.900.000	3.823.305	-709.482	7.085
21.910.000	3.825.166	-709.436	7.071
21.920.000	3.826.580	-709.359	7.058
21.930.000	3.828.439	-709.335	7.045
21.940.000	3.829.919	-709.265	7.030
21.950.000	3.831.687	-709.175	7.013
21.960.000	3.833.171	-709.100	6.995
21.970.000	3.834.998	-709.045	6.980
21.980.000	3.836.455	-708.859	6.962
21.990.000	3.838.249	-708.822	6.942
22.000.000	3.839.780	-708.622	6.924
22.010.000	3.841.411	-708.496	6.910
22.020.000	3.843.062	-708.323	6.894
22.030.000	3.844.729	-708.164	6.875
22.040.000	3.846.296	-707.903	6.859
22.050.000	3.847.939	-707.814	6.844
22.060.000	3.849.651	-707.506	6.826
22.070.000	3.851.125	-707.349	6.808
22.080.000	3.852.963	-707.128	6.790
22.090.000	3.854.402	-706.890	6.775
22.100.000	3.856.239	-706.687	6.758
22.110.000	3.857.690	-706.512	6.744
22.120.000	3.859.492	-706.217	6.734
22.130.000	3.860.952	-706.006	6.724
22.140.000	3.862.760	-705.819	6.711

22.150.000	3.864.206	-705.490	6.700
22.160.000	3.866.002	-705.310	6.690
22.170.000	3.867.539	-705.042	6.677
22.180.000	3.869.228	-704.799	6.662
22.190.000	3.870.833	-704.550	6.648
22.200.000	3.872.546	-704.338	6.633
22.210.000	3.874.114	-704.009	6.618
22.220.000	3.875.750	-703.838	6.603
22.230.000	3.877.463	-703.511	6.591
22.240.000	3.878.949	-703.299	6.578
22.250.000	3.880.733	-703.037	6.564
22.260.000	3.882.199	-702.778	6.552
22.270.000	3.883.970	-702.538	6.544
22.280.000	3.885.442	-702.319	6.534
22.290.000	3.887.266	-702.046	6.522
22.300.000	3.888.708	-701.800	6.512
22.310.000	3.890.579	-701.622	6.505
22.320.000	3.892.065	-701.291	6.494
22.330.000	3.893.812	-701.109	6.481
22.340.000	3.895.362	-700.861	6.471
22.350.000	3.897.083	-700.631	6.463
22.360.000	3.898.594	-700.364	6.454
22.370.000	3.900.374	-700.193	6.444
22.380.000	3.901.918	-699.878	6.437
22.390.000	3.903.538	-699.724	6.430
22.400.000	3.905.241	-699.459	6.421
22.410.000	3.906.750	-699.351	6.409
22.420.000	3.908.536	-699.278	6.399
22.430.000	3.910.078	-699.359	6.387
22.440.000	3.911.878	-699.474	6.369

22.450.000	3.913.349	-699.814	6.351
22.460.000	3.915.158	-700.211	6.335
22.470.000	3.916.570	-700.726	6.317
22.480.000	3.918.465	-701.383	6.295
22.490.000	3.919.882	-701.992	6.273
22.500.000	3.921.697	-702.784	6.254
22.510.000	3.923.251	-703.565	6.232
22.520.000	3.924.984	-704.280	6.208
22.530.000	3.926.502	-705.076	6.184
22.540.000	3.928.289	-705.987	6.163
22.550.000	3.929.885	-706.661	6.140
22.560.000	3.931.597	-707.539	6.116
22.570.000	3.933.217	-708.274	6.095
22.580.000	3.934.808	-709.053	6.071
22.590.000	3.936.518	-709.739	6.050
22.600.000	3.938.107	-710.479	6.030
22.610.000	3.939.875	-711.068	6.011
22.620.000	3.941.354	-711.756	5.994
22.630.000	3.943.234	-712.328	5.975
22.640.000	3.944.629	-712.854	5.959
22.650.000	3.946.530	-713.419	5.946
22.660.000	3.948.015	-713.841	5.928
22.670.000	3.949.799	-714.281	5.906
22.680.000	3.951.291	-714.689	5.885
22.690.000	3.953.081	-715.064	5.870
22.700.000	3.954.583	-715.405	5.850
22.710.000	3.956.262	-715.833	5.830
22.720.000	3.957.906	-716.144	5.811
22.730.000	3.959.564	-716.656	5.793
22.740.000	3.961.270	-717.151	5.778

22.750.000	3.962.870	-717.737	5.756
22.760.000	3.964.592	-718.360	5.737
22.770.000	3.966.190	-719.154	5.714
22.780.000	3.967.935	-719.839	5.691
22.790.000	3.969.441	-720.737	5.669
22.800.000	3.971.296	-721.604	5.648
22.810.000	3.972.699	-722.465	5.627
22.820.000	3.974.572	-723.425	5.603
22.830.000	3.976.051	-724.298	5.584
22.840.000	3.977.814	-725.210	5.564
22.850.000	3.979.343	-726.123	5.543
22.860.000	3.981.181	-726.999	5.524
22.870.000	3.982.610	-727.823	5.505
22.880.000	3.984.477	-728.771	5.486
22.890.000	3.986.069	-729.505	5.468
22.900.000	3.987.718	-730.353	5.452
22.910.000	3.989.388	-731.117	5.431
22.920.000	3.991.024	-731.852	5.410
22.930.000	3.992.702	-732.523	5.390
22.940.000	3.994.332	-733.296	5.369
22.950.000	3.996.051	-733.812	5.349
22.960.000	3.997.565	-734.528	5.326
22.970.000	3.999.422	-735.111	5.306
22.980.000	4.000.858	-735.654	5.291
22.990.000	4.002.740	-736.246	5.273
23.000.000	4.004.214	-736.726	5.254
23.010.000	4.005.980	-737.193	5.235
23.020.000	4.007.503	-737.644	5.216
23.030.000	4.009.324	-738.085	5.197
23.040.000	4.010.786	-738.407	5.179

23.050.000	4.012.561	-738.831	5.156
23.060.000	4.014.139	-739.060	5.137
23.070.000	4.015.806	-739.374	5.117
23.080.000	4.017.397	-739.590	5.100
23.090.000	4.019.097	-739.789	5.081
23.100.000	4.020.736	-739.921	5.061
23.110.000	4.022.369	-740.109	5.044
23.120.000	4.024.045	-740.065	5.028
23.130.000	4.025.557	-740.205	5.011
23.140.000	4.027.409	-740.234	4.993
23.150.000	4.028.854	-740.195	4.977
23.160.000	4.030.624	-740.194	4.964
23.170.000	4.032.143	-740.165	4.949
23.180.000	4.033.935	-740.072	4.934
23.190.000	4.035.378	-739.977	4.918
23.200.000	4.037.251	-739.909	4.901
23.210.000	4.038.673	-739.713	4.887
23.220.000	4.040.523	-739.648	4.869
23.230.000	4.042.057	-739.463	4.853
23.240.000	4.043.726	-739.300	4.838
23.250.000	4.045.357	-739.105	4.823
23.260.000	4.047.007	-738.963	4.808
23.270.000	4.048.583	-738.705	4.791
23.280.000	4.050.260	-738.592	4.776
23.290.000	4.051.914	-738.297	4.760
23.300.000	4.053.491	-738.157	4.742
23.310.000	4.055.270	-737.939	4.726
23.320.000	4.056.756	-737.707	4.711
23.330.000	4.058.582	-737.510	4.697
23.340.000	4.060.048	-737.343	4.682

23.350.000	4.061.863	-737.076	4.668
23.360.000	4.063.301	-736.892	4.653
23.370.000	4.065.130	-736.718	4.640
23.380.000	4.066.591	-736.449	4.625
23.390.000	4.068.373	-736.313	4.610
23.400.000	4.069.906	-736.115	4.596
23.410.000	4.071.640	-735.946	4.582
23.420.000	4.073.186	-735.769	4.567
23.430.000	4.074.912	-735.632	4.552
23.440.000	4.076.478	-735.407	4.538
23.450.000	4.078.181	-735.342	4.524
23.460.000	4.079.870	-735.136	4.509
23.470.000	4.081.385	-735.052	4.495
23.480.000	4.083.171	-734.903	4.481
23.490.000	4.084.654	-734.821	4.468
23.500.000	4.086.404	-734.680	4.453
23.510.000	4.087.931	-734.657	4.440
23.520.000	4.089.769	-734.558	4.426
23.530.000	4.091.155	-734.497	4.411
23.540.000	4.093.031	-734.499	4.395
23.550.000	4.094.490	-734.419	4.381
23.560.000	4.096.289	-734.471	4.367
23.570.000	4.097.817	-734.503	4.350
23.580.000	4.099.592	-734.573	4.333
23.590.000	4.101.088	-734.645	4.316
23.600.000	4.102.864	-734.859	4.299
23.610.000	4.104.424	-734.923	4.285
23.620.000	4.106.082	-735.215	4.266
23.630.000	4.107.792	-735.397	4.250
23.640.000	4.109.340	-735.697	4.235

23.650.000	4.111.093	-735.979	4.218
23.660.000	4.112.639	-736.406	4.201
23.670.000	4.114.422	-736.744	4.184
23.680.000	4.115.942	-737.255	4.167
23.690.000	4.117.800	-737.710	4.151
23.700.000	4.119.223	-738.219	4.132
23.710.000	4.121.087	-738.800	4.115
23.720.000	4.122.544	-739.279	4.099
23.730.000	4.124.359	-739.893	4.083
23.740.000	4.125.907	-740.490	4.065
23.750.000	4.127.671	-741.064	4.045
23.760.000	4.129.196	-741.653	4.027
23.770.000	4.130.963	-742.388	4.012
23.780.000	4.132.550	-742.934	3.995
23.790.000	4.134.263	-743.695	3.976
23.800.000	4.135.948	-744.401	3.956
23.810.000	4.137.546	-745.138	3.940
23.820.000	4.139.260	-745.882	3.924
23.830.000	4.140.888	-746.751	3.903
23.840.000	4.142.644	-747.460	3.881
23.850.000	4.144.188	-748.355	3.862
23.860.000	4.146.060	-749.172	3.845
23.870.000	4.147.447	-749.932	3.828
23.880.000	4.149.324	-750.781	3.807
23.890.000	4.150.847	-751.523	3.785
23.900.000	4.152.632	-752.318	3.766
23.910.000	4.154.163	-753.057	3.747
23.920.000	4.155.981	-753.800	3.725
23.930.000	4.157.447	-754.476	3.702
23.940.000	4.159.281	-755.291	3.678

23.950.000	4.160.830	-755.915	3.658
23.960.000	4.162.501	-756.700	3.639
23.970.000	4.164.198	-757.408	3.616
23.980.000	4.165.822	-758.131	3.591
23.990.000	4.167.472	-758.814	3.571
24.000.000	4.169.139	-759.614	3.551
24.010.000	4.170.859	-760.205	3.529
24.020.000	4.172.414	-760.962	3.505
24.030.000	4.174.289	-761.611	3.484
24.040.000	4.175.690	-762.229	3.466
24.050.000	4.177.589	-762.887	3.444
24.060.000	4.179.071	-763.491	3.420
24.070.000	4.180.841	-764.042	3.401
24.080.000	4.182.373	-764.605	3.384
24.090.000	4.184.210	-765.136	3.363
24.100.000	4.185.682	-765.565	3.340
24.110.000	4.187.492	-766.080	3.321
24.120.000	4.189.050	-766.418	3.306
24.130.000	4.190.766	-766.826	3.285
24.140.000	4.192.370	-767.150	3.260
24.150.000	4.194.047	-767.418	3.240
24.160.000	4.195.688	-767.610	3.225
24.170.000	4.197.282	-767.910	3.208
24.180.000	4.199.043	-767.960	3.186
24.190.000	4.200.552	-768.173	3.166
24.200.000	4.202.391	-768.260	3.152
24.210.000	4.203.867	-768.281	3.137
24.220.000	4.205.679	-768.340	3.117
24.230.000	4.207.202	-768.346	3.099
24.240.000	4.208.987	-768.279	3.086

24.250.000	4.210.449	-768.217	3.071
24.260.000	4.212.297	-768.157	3.052
24.270.000	4.213.715	-767.955	3.033
24.280.000	4.215.518	-767.870	3.018
24.290.000	4.217.099	-767.665	3.000
24.300.000	4.218.775	-767.487	2.976
24.310.000	4.220.361	-767.243	2.957
24.320.000	4.222.093	-767.032	2.943
24.330.000	4.223.665	-766.688	2.926
24.340.000	4.225.319	-766.481	2.906
24.350.000	4.227.064	-766.103	2.891
24.360.000	4.228.541	-765.807	2.879
24.370.000	4.230.339	-765.438	2.862
24.380.000	4.231.826	-765.063	2.841
24.390.000	4.233.629	-764.692	2.825
24.400.000	4.235.133	-764.324	2.812
24.410.000	4.236.952	-763.888	2.795
24.420.000	4.238.370	-763.454	2.776
24.430.000	4.240.239	-763.080	2.762
24.440.000	4.241.674	-762.557	2.751
24.450.000	4.243.495	-762.186	2.737
24.460.000	4.245.058	-761.695	2.717
24.470.000	4.246.744	-761.234	2.699
24.480.000	4.248.354	-760.755	2.688
24.490.000	4.250.051	-760.340	2.671
24.500.000	4.251.616	-759.767	2.653
24.510.000	4.253.279	-759.357	2.637
24.520.000	4.254.956	-758.792	2.623
24.530.000	4.256.503	-758.326	2.608
24.540.000	4.258.280	-757.774	2.594

24.550.000	4.259.770	-757.296	2.579
24.560.000	4.261.528	-756.738	2.566
24.570.000	4.263.045	-756.266	2.550
24.580.000	4.264.877	-755.670	2.537
24.590.000	4.266.252	-755.128	2.527
24.600.000	4.268.105	-754.620	2.512
24.610.000	4.269.518	-753.980	2.495
24.620.000	4.271.338	-753.462	2.481
24.630.000	4.272.812	-752.877	2.469
24.640.000	4.274.572	-752.306	2.453
24.650.000	4.276.091	-751.701	2.436
24.660.000	4.277.827	-751.181	2.425
24.670.000	4.279.421	-750.501	2.416
24.680.000	4.281.073	-749.997	2.401
24.690.000	4.282.812	-749.354	2.387
24.700.000	4.284.338	-748.773	2.379
24.710.000	4.286.054	-748.159	2.367
24.720.000	4.287.619	-747.611	2.348
24.730.000	4.289.332	-746.974	2.333
24.740.000	4.290.816	-746.427	2.324
24.750.000	4.292.685	-745.833	2.312
24.760.000	4.294.034	-745.228	2.297
24.770.000	4.295.926	-744.701	2.283
24.780.000	4.297.411	-744.057	2.276
24.790.000	4.299.112	-743.486	2.263
24.800.000	4.300.689	-742.902	2.246
24.810.000	4.302.441	-742.322	2.233
24.820.000	4.303.946	-741.691	2.220
24.830.000	4.305.728	-741.211	2.200
24.840.000	4.307.275	-740.540	2.184

24.850.000	4.308.902	-740.015	2.175
24.860.000	4.310.595	-739.433	2.165
24.870.000	4.312.119	-738.863	2.152
24.880.000	4.313.856	-738.265	2.137
24.890.000	4.315.406	-737.787	2.129
24.900.000	4.317.129	-737.135	2.123
24.910.000	4.318.654	-736.651	2.112
24.920.000	4.320.519	-736.097	2.098
24.930.000	4.321.898	-735.506	2.088
24.940.000	4.323.728	-735.025	2.079
24.950.000	4.325.214	-734.466	2.073
24.960.000	4.326.986	-733.940	2.058
24.970.000	4.328.458	-733.413	2.046
24.980.000	4.330.227	-732.898	2.037
24.990.000	4.331.671	-732.326	2.025
25.000.000	4.333.427	-731.887	2.011
25.010.000	4.335.019	-731.248	1.996
25.020.000	4.336.622	-730.774	1.986
25.030.000	4.338.281	-730.223	1.973
25.040.000	4.339.856	-729.672	1.957
25.050.000	4.341.496	-729.083	1.944
25.060.000	4.343.078	-728.643	1.939
25.070.000	4.344.813	-727.991	1.932
25.080.000	4.346.222	-727.533	1.923
25.090.000	4.348.091	-727.010	1.919
25.100.000	4.349.478	-726.435	1.912
25.110.000	4.351.261	-725.959	1.905
25.120.000	4.352.738	-725.439	1.898
25.130.000	4.354.522	-724.914	1.889
25.140.000	4.356.006	-724.409	1.880

25.150.000	4.357.810	-723.945	1.872
25.160.000	4.359.261	-723.397	1.866
25.170.000	4.361.042	-722.985	1.861
25.180.000	4.362.629	-722.491	1.855
25.190.000	4.364.239	-722.060	1.848
25.200.000	4.365.872	-721.613	1.844
25.210.000	4.367.500	-721.208	1.835
25.220.000	4.369.075	-720.707	1.826
25.230.000	4.370.685	-720.396	1.822
25.240.000	4.372.415	-719.881	1.816
25.250.000	4.373.871	-719.513	1.809
25.260.000	4.375.733	-719.119	1.803
25.270.000	4.377.137	-718.669	1.800
25.280.000	4.378.963	-718.302	1.796
25.290.000	4.380.429	-717.919	1.790
25.300.000	4.382.207	-717.485	1.786
25.310.000	4.383.643	-717.117	1.782
25.320.000	4.385.449	-716.799	1.774
25.330.000	4.386.900	-716.326	1.767
25.340.000	4.388.647	-716.043	1.760
25.350.000	4.390.208	-715.638	1.752
25.360.000	4.391.876	-715.297	1.743
25.370.000	4.393.518	-714.931	1.736
25.380.000	4.395.158	-714.619	1.731
25.390.000	4.396.779	-714.209	1.722
25.400.000	4.398.390	-713.983	1.713
25.410.000	4.400.102	-713.568	1.707
25.420.000	4.401.569	-713.307	1.699
25.430.000	4.403.330	-712.996	1.689
25.440.000	4.404.805	-712.691	1.679

25.450.000	4.406.572	-712.410	1.674
25.460.000	4.408.024	-712.170	1.667
25.470.000	4.409.878	-711.866	1.660
25.480.000	4.411.255	-711.603	1.655
25.490.000	4.413.095	-711.395	1.650
25.500.000	4.414.558	-711.056	1.643
25.510.000	4.416.285	-710.865	1.638
25.520.000	4.417.864	-710.610	1.635
25.530.000	4.419.521	-710.366	1.630
25.540.000	4.421.073	-710.123	1.626
25.550.000	4.422.780	-709.965	1.622
25.560.000	4.424.325	-709.665	1.618
25.570.000	4.425.938	-709.522	1.611
25.580.000	4.427.702	-709.266	1.606
25.590.000	4.429.160	-709.080	1.601
25.600.000	4.430.912	-708.852	1.593
25.610.000	4.432.429	-708.678	1.585
25.620.000	4.434.148	-708.453	1.581
25.630.000	4.435.638	-708.305	1.577
25.640.000	4.437.460	-708.136	1.570
25.650.000	4.438.844	-707.909	1.563
25.660.000	4.440.650	-707.808	1.560
25.670.000	4.442.095	-707.569	1.559
25.680.000	4.443.849	-707.452	1.553
25.690.000	4.445.393	-707.310	1.546
25.700.000	4.447.110	-707.150	1.543
25.710.000	4.448.601	-706.973	1.543
25.720.000	4.450.341	-706.916	1.540
25.730.000	4.451.897	-706.656	1.535
25.740.000	4.453.501	-706.623	1.533

25.750.000	4.455.221	-706.465	1.532
25.760.000	4.456.706	-706.357	1.528
25.770.000	4.458.439	-706.212	1.522
25.780.000	4.459.937	-706.178	1.517
25.790.000	4.461.717	-706.023	1.515
25.800.000	4.463.142	-705.979	1.510
25.810.000	4.464.975	-705.901	1.505
25.820.000	4.466.386	-705.791	1.501
25.830.000	4.468.239	-705.771	1.499
25.840.000	4.469.720	-705.630	1.497
25.850.000	4.471.435	-705.581	1.489
25.860.000	4.472.911	-705.513	1.485
25.870.000	4.474.675	-705.459	1.484
25.880.000	4.476.137	-705.339	1.480
25.890.000	4.477.843	-705.369	1.473
25.900.000	4.479.443	-705.233	1.469
25.910.000	4.481.016	-705.235	1.468
25.920.000	4.482.749	-705.190	1.465
25.930.000	4.484.318	-705.168	1.459
25.940.000	4.485.998	-705.093	1.457
25.950.000	4.487.569	-705.147	1.458
25.960.000	4.489.289	-705.032	1.459
25.970.000	4.490.735	-705.067	1.456
25.980.000	4.492.590	-705.078	1.454
25.990.000	4.493.946	-705.016	1.455
26.000.000	4.495.773	-705.067	1.456
26.010.000	4.497.228	-705.063	1.452
26.020.000	4.498.977	-705.058	1.447
26.030.000	4.500.495	-705.088	1.446
26.040.000	4.502.310	-705.137	1.446

26.050.000	4.503.756	-705.110	1.442
26.060.000	4.505.502	-705.203	1.437
26.070.000	4.507.046	-705.156	1.435
26.080.000	4.508.692	-705.238	1.434
26.090.000	4.510.372	-705.260	1.433
26.100.000	4.511.947	-705.305	1.427
26.110.000	4.513.591	-705.293	1.424
26.120.000	4.515.182	-705.431	1.423
26.130.000	4.516.904	-705.354	1.424
26.140.000	4.518.417	-705.470	1.424
26.150.000	4.520.248	-705.526	1.421
26.160.000	4.521.672	-705.521	1.417
26.170.000	4.523.494	-705.609	1.415
26.180.000	4.524.920	-705.657	1.414
26.190.000	4.526.692	-705.679	1.413
26.200.000	4.528.186	-705.738	1.408
26.210.000	4.529.962	-705.807	1.403
26.220.000	4.531.430	-705.760	1.402
26.230.000	4.533.160	-705.884	1.402
26.240.000	4.534.743	-705.844	1.402
26.250.000	4.536.405	-705.923	1.398
26.260.000	4.538.009	-705.930	1.392
26.270.000	4.539.673	-705.982	1.390
26.280.000	4.541.271	-705.990	1.391
26.290.000	4.542.871	-706.108	1.390
26.300.000	4.544.582	-706.069	1.386
26.310.000	4.546.056	-706.200	1.383
26.320.000	4.547.864	-706.238	1.383
26.330.000	4.549.305	-706.293	1.385
26.340.000	4.551.120	-706.370	1.383

26.350.000	4.552.588	-706.452	1.378
26.360.000	4.554.379	-706.483	1.373
26.370.000	4.555.852	-706.530	1.370
26.380.000	4.557.717	-706.626	1.369
26.390.000	4.559.146	-706.615	1.365
26.400.000	4.560.914	-706.722	1.360
26.410.000	4.562.464	-706.740	1.354
26.420.000	4.564.129	-706.802	1.352
26.430.000	4.565.740	-706.817	1.351
26.440.000	4.567.423	-706.918	1.347
26.450.000	4.568.989	-706.850	1.341
26.460.000	4.570.635	-706.949	1.338
26.470.000	4.572.344	-706.919	1.338
26.480.000	4.573.875	-706.956	1.337
26.490.000	4.575.644	-706.951	1.332
26.500.000	4.577.138	-706.963	1.329
26.510.000	4.578.923	-706.974	1.327
26.520.000	4.580.397	-707.022	1.327
26.530.000	4.582.233	-707.014	1.325
26.540.000	4.583.690	-707.016	1.321
26.550.000	4.585.502	-707.116	1.317
26.560.000	4.586.976	-707.037	1.315
26.570.000	4.588.712	-707.116	1.311
26.580.000	4.590.248	-707.153	1.306
26.590.000	4.591.981	-707.170	1.300
26.600.000	4.593.527	-707.194	1.296
26.610.000	4.595.283	-707.303	1.293
26.620.000	4.596.860	-707.250	1.290
26.630.000	4.598.484	-707.364	1.286
26.640.000	4.600.176	-707.354	1.281

26.650.000	4.601.725	-707.417	1.281
26.660.000	4.603.491	-707.426	1.278
26.670.000	4.605.003	-707.481	1.273
26.680.000	4.606.756	-707.474	1.268
26.690.000	4.608.221	-707.531	1.266
26.700.000	4.610.078	-707.549	1.264
26.710.000	4.611.520	-707.540	1.262
26.720.000	4.613.382	-707.602	1.257
26.730.000	4.614.820	-707.536	1.254
26.740.000	4.616.585	-707.573	1.253
26.750.000	4.618.088	-707.572	1.252
26.760.000	4.619.846	-707.569	1.248
26.770.000	4.621.369	-707.531	1.245
26.780.000	4.623.107	-707.624	1.244
26.790.000	4.624.701	-707.528	1.244
26.800.000	4.626.307	-707.584	1.241
26.810.000	4.628.031	-707.555	1.237
26.820.000	4.629.614	-707.580	1.235
26.830.000	4.631.346	-707.561	1.235
26.840.000	4.632.924	-707.656	1.232
26.850.000	4.634.646	-707.577	1.229
26.860.000	4.636.128	-707.646	1.227
26.870.000	4.637.997	-707.679	1.226
26.880.000	4.639.365	-707.647	1.226
26.890.000	4.641.216	-707.718	1.223
26.900.000	4.642.689	-707.693	1.221
26.910.000	4.644.460	-707.718	1.220
26.920.000	4.645.982	-707.740	1.218
26.930.000	4.647.797	-707.772	1.216
26.940.000	4.649.285	-707.736	1.214

26.950.000	4.651.083	-707.840	1.212
26.960.000	4.652.620	-707.738	1.210
26.970.000	4.654.273	-707.818	1.209
26.980.000	4.655.955	-707.803	1.207
26.990.000	4.657.535	-707.796	1.207
27.000.000	4.659.199	-707.755	1.205
27.010.000	4.660.785	-707.839	1.203
27.020.000	4.662.546	-707.748	1.202
27.030.000	4.664.037	-707.796	1.201
27.040.000	4.665.880	-707.800	1.199
27.050.000	4.667.324	-707.753	1.198
27.060.000	4.669.201	-707.792	1.198
27.070.000	4.670.679	-707.787	1.197
27.080.000	4.672.478	-707.756	1.196
27.090.000	4.673.939	-707.760	1.195
27.100.000	4.675.764	-707.795	1.194
27.110.000	4.677.220	-707.728	1.193
27.120.000	4.678.980	-707.797	1.192
27.130.000	4.680.559	-707.737	1.190
27.140.000	4.682.197	-707.761	1.189
27.150.000	4.683.868	-707.729	1.188
27.160.000	4.685.543	-707.744	1.187
27.170.000	4.687.164	-707.669	1.186
27.180.000	4.688.777	-707.750	1.184
27.190.000	4.690.495	-707.651	1.182
27.200.000	4.691.991	-707.688	1.180
27.210.000	4.693.862	-707.684	1.180
27.220.000	4.695.274	-707.664	1.181
27.230.000	4.697.117	-707.681	1.183
27.240.000	4.698.604	-707.694	1.184

27.250.000	4.700.376	-707.665	1.184
27.260.000	4.701.847	-707.654	1.185
27.270.000	4.703.731	-707.720	1.192
27.280.000	4.705.175	-707.631	1.199
27.290.000	4.706.995	-707.684	1.203
27.300.000	4.708.559	-707.641	1.210
27.310.000	4.710.276	-707.634	1.223
27.320.000	4.711.874	-707.598	1.235
27.330.000	4.713.550	-707.616	1.244
27.340.000	4.715.181	-707.526	1.254
27.350.000	4.716.828	-707.596	1.269
27.360.000	4.718.520	-707.479	1.284
27.370.000	4.720.063	-707.527	1.297
27.380.000	4.721.880	-707.503	1.313
27.390.000	4.723.405	-707.470	1.334
27.400.000	4.725.197	-707.483	1.349
27.410.000	4.726.656	-707.493	1.361
27.420.000	4.728.481	-707.446	1.375
27.430.000	4.729.935	-707.443	1.390
27.440.000	4.731.805	-707.492	1.401
27.450.000	4.733.234	-707.403	1.410
27.460.000	4.735.017	-707.479	1.421
27.470.000	4.736.539	-707.460	1.433
27.480.000	4.738.258	-707.472	1.441
27.490.000	4.739.846	-707.474	1.448
27.500.000	4.741.623	-707.545	1.457
27.510.000	4.743.169	-707.457	1.465
27.520.000	4.744.805	-707.555	1.470
27.530.000	4.746.522	-707.512	1.477
27.540.000	4.748.042	-707.560	1.484

27.550.000	4.749.816	-707.544	1.489
27.560.000	4.751.316	-707.587	1.494
27.570.000	4.753.033	-707.570	1.498
27.580.000	4.754.523	-707.617	1.501
27.590.000	4.756.359	-707.599	1.502
27.600.000	4.757.762	-707.586	1.505
27.610.000	4.759.649	-707.653	1.505
27.620.000	4.761.124	-707.600	1.501
27.630.000	4.762.890	-707.631	1.498
27.640.000	4.764.411	-707.645	1.494
27.650.000	4.766.151	-707.662	1.489
27.660.000	4.767.656	-707.646	1.482
27.670.000	4.769.427	-707.761	1.476
27.680.000	4.770.973	-707.687	1.472
27.690.000	4.772.618	-707.787	1.468
27.700.000	4.774.290	-707.781	1.465
27.710.000	4.775.881	-707.807	1.463
27.720.000	4.777.618	-707.802	1.461
27.730.000	4.779.222	-707.878	1.460
27.740.000	4.780.945	-707.809	1.461
27.750.000	4.782.447	-707.896	1.460
27.760.000	4.784.300	-707.903	1.460
27.770.000	4.785.724	-707.899	1.462
27.780.000	4.787.587	-707.960	1.464
27.790.000	4.789.025	-707.892	1.463
27.800.000	4.790.792	-707.907	1.463
27.810.000	4.792.318	-707.907	1.463
27.820.000	4.794.120	-707.873	1.460
27.830.000	4.795.634	-707.837	1.456
27.840.000	4.797.423	-707.898	1.456

27.850.000	4.798.946	-707.789	1.456
27.860.000	4.800.644	-707.871	1.455
27.870.000	4.802.285	-707.843	1.456
27.880.000	4.803.884	-707.841	1.459
27.890.000	4.805.562	-707.810	1.460
27.900.000	4.807.145	-707.904	1.460
27.910.000	4.808.842	-707.815	1.459
27.920.000	4.810.355	-707.912	1.460
27.930.000	4.812.214	-707.921	1.459
27.940.000	4.813.621	-707.933	1.456
27.950.000	4.815.438	-707.987	1.456
27.960.000	4.816.957	-708.004	1.459
27.970.000	4.818.691	-708.043	1.459
27.980.000	4.820.175	-708.080	1.458
27.990.000	4.821.983	-708.143	1.460
28.000.000	4.823.381	-708.136	1.463
28.010.000	4.825.202	-708.264	1.464
28.020.000	4.826.726	-708.251	1.463
28.030.000	4.828.416	-708.344	1.463
28.040.000	4.830.053	-708.383	1.466
28.050.000	4.831.698	-708.441	1.467
28.060.000	4.833.337	-708.442	1.465
28.070.000	4.834.984	-708.600	1.465
28.080.000	4.836.637	-708.548	1.465
28.090.000	4.838.184	-708.673	1.464
28.100.000	4.839.968	-708.727	1.462
28.110.000	4.841.370	-708.756	1.462
28.120.000	4.843.195	-708.844	1.463
28.130.000	4.844.649	-708.931	1.462
28.140.000	4.846.441	-708.943	1.460

28.150.000	4.847.892	-709.036	1.460
28.160.000	4.849.739	-709.113	1.460
28.170.000	4.851.186	-709.133	1.458
28.180.000	4.852.988	-709.260	1.456
28.190.000	4.854.468	-709.251	1.456
28.200.000	4.856.201	-709.331	1.458
28.210.000	4.857.734	-709.368	1.458
28.220.000	4.859.424	-709.425	1.456
28.230.000	4.861.000	-709.414	1.457
28.240.000	4.862.629	-709.526	1.460
28.250.000	4.864.320	-709.471	1.458
28.260.000	4.865.841	-709.576	1.455
28.270.000	4.867.590	-709.593	1.453
28.280.000	4.869.081	-709.637	1.453
28.290.000	4.870.903	-709.709	1.452
28.300.000	4.872.366	-709.778	1.449
28.310.000	4.874.144	-709.807	1.448
28.320.000	4.875.593	-709.855	1.447
28.330.000	4.877.438	-709.936	1.448
28.340.000	4.878.875	-709.898	1.446
28.350.000	4.880.674	-709.989	1.444
28.360.000	4.882.195	-709.982	1.443
28.370.000	4.883.884	-710.017	1.441
28.380.000	4.885.480	-710.006	1.438
28.390.000	4.887.219	-710.076	1.434
28.400.000	4.888.729	-709.984	1.431
28.410.000	4.890.407	-710.081	1.431
28.420.000	4.892.061	-710.011	1.432
28.430.000	4.893.597	-710.062	1.434
28.440.000	4.895.359	-710.050	1.434

28.450.000	4.896.899	-710.096	1.435
28.460.000	4.898.651	-710.070	1.438
28.470.000	4.900.148	-710.151	1.440
28.480.000	4.901.959	-710.156	1.437
28.490.000	4.903.378	-710.194	1.433
28.500.000	4.905.222	-710.291	1.431
28.510.000	4.906.673	-710.261	1.431
28.520.000	4.908.473	-710.359	1.431
28.530.000	4.909.960	-710.410	1.430
28.540.000	4.911.721	-710.440	1.427
28.550.000	4.913.226	-710.495	1.428
28.560.000	4.914.986	-710.617	1.431
28.570.000	4.916.511	-710.583	1.431
28.580.000	4.918.181	-710.735	1.429
28.590.000	4.919.866	-710.747	1.426
28.600.000	4.921.431	-710.836	1.428
28.610.000	4.923.128	-710.869	1.431
28.620.000	4.924.727	-710.992	1.429
28.630.000	4.926.446	-710.988	1.426
28.640.000	4.927.927	-711.129	1.425
28.650.000	4.929.767	-711.163	1.428
28.660.000	4.931.146	-711.251	1.426
28.670.000	4.932.970	-711.394	1.422
28.680.000	4.934.437	-711.420	1.420
28.690.000	4.936.198	-711.541	1.421
28.700.000	4.937.675	-711.626	1.420
28.710.000	4.939.465	-711.707	1.414
28.720.000	4.940.907	-711.769	1.412
28.730.000	4.942.712	-711.936	1.412
28.740.000	4.944.251	-711.930	1.411

28.750.000	4.945.924	-712.112	1.407
28.760.000	4.947.560	-712.190	1.404
28.770.000	4.949.152	-712.289	1.404
28.780.000	4.950.787	-712.352	1.405
28.790.000	4.952.371	-712.548	1.403
28.800.000	4.954.038	-712.562	1.398
28.810.000	4.955.571	-712.748	1.396
28.820.000	4.957.394	-712.832	1.396
28.830.000	4.958.758	-712.942	1.395
28.840.000	4.960.626	-713.109	1.391
28.850.000	4.962.088	-713.205	1.384
28.860.000	4.963.816	-713.316	1.382
28.870.000	4.965.335	-713.449	1.381
28.880.000	4.967.145	-713.556	1.380
28.890.000	4.968.537	-713.626	1.374
28.900.000	4.970.336	-713.827	1.372
28.910.000	4.971.852	-713.850	1.372
28.920.000	4.973.534	-714.009	1.371
28.930.000	4.975.141	-714.108	1.369
28.940.000	4.976.774	-714.194	1.364
28.950.000	4.978.383	-714.225	1.361
28.960.000	4.980.046	-714.370	1.360
28.970.000	4.981.713	-714.306	1.357
28.980.000	4.983.229	-714.427	1.354
28.990.000	4.984.989	-714.435	1.351
29.000.000	4.986.457	-714.466	1.349
29.010.000	4.988.219	-714.535	1.347
29.020.000	4.989.669	-714.582	1.344
29.030.000	4.991.446	-714.599	1.341
29.040.000	4.992.900	-714.663	1.342

29.050.000	4.994.751	-714.732	1.341
29.060.000	4.996.164	-714.733	1.339
29.070.000	4.997.944	-714.830	1.339
29.080.000	4.999.460	-714.831	1.341
29.090.000	5.001.152	-714.923	1.341
29.100.000	5.002.707	-714.958	1.337
29.110.000	5.004.383	-715.030	1.333
29.120.000	5.005.920	-715.066	1.333
29.130.000	5.007.579	-715.200	1.332
29.140.000	5.009.232	-715.176	1.328
29.150.000	5.010.770	-715.314	1.324
29.160.000	5.012.530	-715.362	1.322
29.170.000	5.014.008	-715.437	1.323
29.180.000	5.015.758	-715.500	1.322
29.190.000	5.017.251	-715.636	1.319
29.200.000	5.019.006	-715.690	1.319
29.210.000	5.020.422	-715.766	1.321
29.220.000	5.022.264	-715.893	1.321
29.230.000	5.023.657	-715.919	1.318
29.240.000	5.025.462	-716.068	1.318
29.250.000	5.026.955	-716.141	1.318
29.260.000	5.028.666	-716.250	1.317
29.270.000	5.030.224	-716.326	1.312
29.280.000	5.031.954	-716.499	1.308
29.290.000	5.033.477	-716.515	1.309
29.300.000	5.035.142	-716.701	1.308
29.310.000	5.036.782	-716.727	1.304
29.320.000	5.038.345	-716.875	1.301
29.330.000	5.040.023	-716.943	1.301
29.340.000	5.041.549	-717.066	1.302

29.350.000	5.043.272	-717.125	1.298
29.360.000	5.044.790	-717.302	1.293
29.370.000	5.046.590	-717.365	1.289
29.380.000	5.047.992	-717.491	1.287
29.390.000	5.049.850	-717.655	1.284
29.400.000	5.051.278	-717.690	1.279
29.410.000	5.053.055	-717.854	1.276
29.420.000	5.054.547	-717.955	1.276
29.430.000	5.056.276	-718.050	1.275
29.440.000	5.057.741	-718.160	1.272
29.450.000	5.059.544	-718.318	1.268
29.460.000	5.061.016	-718.337	1.267
29.470.000	5.062.696	-718.510	1.266
29.480.000	5.064.333	-718.570	1.264
29.490.000	5.065.888	-718.678	1.261
29.500.000	5.067.570	-718.750	1.262
29.510.000	5.069.136	-718.906	1.262
29.520.000	5.070.828	-718.936	1.258
29.530.000	5.072.375	-719.100	1.254
29.540.000	5.074.180	-719.159	1.253
29.550.000	5.075.537	-719.291	1.250
29.560.000	5.077.402	-719.432	1.245
29.570.000	5.078.820	-719.477	1.244
29.580.000	5.080.584	-719.616	1.244
29.590.000	5.082.102	-719.743	1.242
29.600.000	5.083.845	-719.833	1.237
29.610.000	5.085.293	-719.894	1.236
29.620.000	5.087.057	-720.082	1.239
29.630.000	5.088.564	-720.096	1.238
29.640.000	5.090.293	-720.296	1.235

29.650.000	5.091.901	-720.398	1.236
29.660.000	5.093.501	-720.501	1.238
29.670.000	5.095.111	-720.600	1.237
29.680.000	5.096.729	-720.812	1.235
29.690.000	5.098.343	-720.816	1.232
29.700.000	5.099.892	-721.033	1.229
29.710.000	5.101.672	-721.109	1.225
29.720.000	5.103.076	-721.234	1.223
29.730.000	5.104.905	-721.395	1.224
29.740.000	5.106.320	-721.516	1.220
29.750.000	5.108.096	-721.653	1.215
29.760.000	5.109.556	-721.812	1.214
29.770.000	5.111.351	-721.941	1.213
29.780.000	5.112.754	-722.051	1.212
29.790.000	5.114.554	-722.280	1.206
29.800.000	5.116.059	-722.343	1.205
29.810.000	5.117.710	-722.577	1.208
29.820.000	5.119.289	-722.710	1.208
29.830.000	5.120.941	-722.886	1.206
29.840.000	5.122.513	-723.050	1.206
29.850.000	5.124.152	-723.310	1.207
29.860.000	5.125.781	-723.393	1.208
29.870.000	5.127.343	-723.701	1.207
29.880.000	5.129.100	-723.857	1.207
29.890.000	5.130.527	-724.076	1.210
29.900.000	5.132.322	-724.310	1.211
29.910.000	5.133.794	-724.561	1.209
29.920.000	5.135.549	-724.766	1.205
29.930.000	5.137.005	-725.024	1.202
29.940.000	5.138.829	-725.285	1.200

29.950.000	5.140.204	-725.488	1.194
29.960.000	5.141.984	-725.775	1.190
29.970.000	5.143.485	-725.970	1.190
29.980.000	5.145.180	-726.257	1.189
29.990.000	5.146.751	-726.482	1.186
30.000.000	5.148.452	-726.730	1.184
30.010.000	5.149.945	-726.925	1.185
30.020.000	5.151.630	-727.217	1.185
30.030.000	5.153.233	-727.359	1.181
30.040.000	5.154.792	-727.639	1.178
30.050.000	5.156.526	-727.820	1.178
30.060.000	5.157.961	-728.042	1.180
30.070.000	5.159.726	-728.220	1.177
30.080.000	5.161.207	-728.495	1.174
30.090.000	5.162.963	-728.629	1.175
30.100.000	5.164.386	-728.843	1.175
30.110.000	5.166.196	-729.081	1.173
30.120.000	5.167.605	-729.186	1.169
30.130.000	5.169.399	-729.422	1.168
30.140.000	5.170.869	-729.588	1.168
30.150.000	5.172.597	-729.750	1.166
30.160.000	5.174.090	-729.915	1.161
30.170.000	5.175.821	-730.146	1.158
30.180.000	5.177.330	-730.254	1.158
30.190.000	5.178.957	-730.529	1.154
30.200.000	5.180.618	-730.628	1.151
30.210.000	5.182.131	-730.850	1.150
30.220.000	5.183.831	-731.020	1.152
30.230.000	5.185.361	-731.206	1.152
30.240.000	5.187.053	-731.359	1.150

30.250.000	5.188.555	-731.616	1.151
30.260.000	5.190.350	-731.756	1.153
30.270.000	5.191.725	-731.975	1.151
30.280.000	5.193.572	-732.226	1.150
30.290.000	5.195.008	-732.365	1.153
30.300.000	5.196.712	-732.600	1.155
30.310.000	5.198.276	-732.805	1.154
30.320.000	5.199.965	-732.966	1.151
30.330.000	5.201.409	-733.155	1.150
30.340.000	5.203.180	-733.406	1.149
30.350.000	5.204.619	-733.491	1.144
30.360.000	5.206.307	-733.750	1.138
30.370.000	5.207.919	-733.890	1.134
30.380.000	5.209.469	-734.081	1.133
30.390.000	5.211.143	-734.218	1.131
30.400.000	5.212.725	-734.439	1.125
30.410.000	5.214.374	-734.526	1.122
30.420.000	5.215.921	-734.783	1.122
30.430.000	5.217.688	-734.881	1.119
30.440.000	5.219.059	-735.076	1.115
30.450.000	5.220.884	-735.299	1.111
30.460.000	5.222.291	-735.430	1.111
30.470.000	5.224.039	-735.641	1.109
30.480.000	5.225.537	-735.862	1.106
30.490.000	5.227.299	-736.019	1.104
30.500.000	5.228.736	-736.201	1.105
30.510.000	5.230.483	-736.457	1.103
30.520.000	5.231.965	-736.555	1.100
30.530.000	5.233.666	-736.814	1.099
30.540.000	5.235.262	-736.970	1.100

30.550.000	5.236.868	-737.149	1.099
30.560.000	5.238.498	-737.309	1.097
30.570.000	5.240.064	-737.551	1.096
30.580.000	5.241.727	-737.631	1.100
30.590.000	5.243.255	-737.909	1.102
30.600.000	5.245.002	-738.024	1.102
30.610.000	5.246.425	-738.198	1.100
30.620.000	5.248.222	-738.407	1.101
30.630.000	5.249.694	-738.555	1.102
30.640.000	5.251.407	-738.728	1.101
30.650.000	5.252.840	-738.905	1.098
30.660.000	5.254.704	-739.066	1.099
30.670.000	5.256.075	-739.193	1.101
30.680.000	5.257.852	-739.410	1.102
30.690.000	5.259.357	-739.485	1.100
30.700.000	5.261.022	-739.694	1.099
30.710.000	5.262.583	-739.829	1.101
30.720.000	5.264.245	-739.980	1.101
30.730.000	5.265.801	-740.098	1.098
30.740.000	5.267.440	-740.322	1.096
30.750.000	5.269.054	-740.372	1.098
30.760.000	5.270.590	-740.614	1.097
30.770.000	5.272.373	-740.743	1.093
30.780.000	5.273.771	-740.892	1.090
30.790.000	5.275.516	-741.047	1.089
30.800.000	5.277.004	-741.235	1.087
30.810.000	5.278.730	-741.369	1.082
30.820.000	5.280.176	-741.550	1.079
30.830.000	5.281.982	-741.725	1.080
30.840.000	5.283.321	-741.842	1.082

30.850.000	5.285.147	-742.066	1.081
30.860.000	5.286.601	-742.186	1.078
30.870.000	5.288.291	-742.375	1.080
30.880.000	5.289.848	-742.524	1.083
30.890.000	5.291.533	-742.706	1.082
30.900.000	5.293.058	-742.811	1.077
30.910.000	5.294.719	-743.064	1.074
30.920.000	5.296.310	-743.138	1.077
30.930.000	5.297.888	-743.368	1.079
30.940.000	5.299.575	-743.539	1.077
30.950.000	5.301.056	-743.710	1.075
30.960.000	5.302.782	-743.868	1.074
30.970.000	5.304.225	-744.114	1.074
30.980.000	5.305.999	-744.258	1.073
30.990.000	5.307.414	-744.469	1.069
31.000.000	5.309.233	-744.695	1.066
31.010.000	5.310.598	-744.819	1.064
31.020.000	5.312.373	-745.091	1.064
31.030.000	5.313.870	-745.259	1.066
31.040.000	5.315.576	-745.457	1.064
31.050.000	5.317.043	-745.650	1.061
31.060.000	5.318.769	-745.924	1.060
31.070.000	5.320.248	-746.042	1.060
31.080.000	5.321.869	-746.327	1.059
31.090.000	5.323.487	-746.486	1.055
31.100.000	5.325.043	-746.747	1.051
31.110.000	5.326.707	-746.938	1.051
31.120.000	5.328.282	-747.200	1.051
31.130.000	5.329.897	-747.348	1.048
31.140.000	5.331.429	-747.658	1.045

31.150.000	5.333.198	-747.830	1.048
31.160.000	5.334.564	-748.098	1.050
31.170.000	5.336.396	-748.371	1.049
31.180.000	5.337.751	-748.554	1.045
31.190.000	5.339.501	-748.846	1.045
31.200.000	5.340.963	-749.103	1.046
31.210.000	5.342.665	-749.343	1.045
31.220.000	5.344.114	-749.591	1.041
31.230.000	5.345.838	-749.926	1.037
31.240.000	5.347.322	-750.106	1.034
31.250.000	5.349.028	-750.462	1.034
31.260.000	5.350.601	-750.685	1.034
31.270.000	5.352.128	-750.967	1.031
31.280.000	5.353.778	-751.227	1.028
31.290.000	5.355.364	-751.569	1.026
31.300.000	5.356.974	-751.752	1.027
31.310.000	5.358.442	-752.137	1.027
31.320.000	5.360.209	-752.372	1.024
31.330.000	5.361.596	-752.677	1.023
31.340.000	5.363.393	-752.998	1.024
31.350.000	5.364.803	-753.254	1.026
31.360.000	5.366.554	-753.570	1.027
31.370.000	5.368.002	-753.877	1.026
31.380.000	5.369.759	-754.143	1.024
31.390.000	5.371.149	-754.419	1.023
31.400.000	5.372.896	-754.792	1.021
31.410.000	5.374.380	-754.994	1.019
31.420.000	5.376.004	-755.350	1.017
31.430.000	5.377.601	-755.614	1.015
31.440.000	5.379.222	-755.901	1.017

31.450.000	5.380.820	-756.174	1.020
31.460.000	5.382.403	-756.517	1.020
31.470.000	5.384.020	-756.696	1.017
31.480.000	5.385.542	-757.074	1.017
31.490.000	5.387.288	-757.325	1.018
31.500.000	5.388.690	-757.607	1.019
31.510.000	5.390.444	-757.921	1.015
31.520.000	5.391.900	-758.224	1.012
31.530.000	5.393.589	-758.485	1.012
31.540.000	5.395.017	-758.782	1.012
31.550.000	5.396.842	-759.087	1.008
31.560.000	5.398.211	-759.319	1.005
31.570.000	5.399.977	-759.674	1.004
31.580.000	5.401.467	-759.898	1.008
31.590.000	5.403.116	-760.204	1.010
31.600.000	5.404.664	-760.480	1.010
31.610.000	5.406.312	-760.787	1.011
31.620.000	5.407.852	-761.039	1.013
31.630.000	5.409.482	-761.415	1.015
31.640.000	5.411.080	-761.625	1.015
31.650.000	5.412.586	-761.997	1.013
31.660.000	5.414.294	-762.283	1.011
31.670.000	5.415.751	-762.609	1.012
31.680.000	5.417.492	-762.917	1.014
31.690.000	5.418.932	-763.289	1.013
31.700.000	5.420.667	-763.556	1.008
31.710.000	5.422.061	-763.896	1.006
31.720.000	5.423.872	-764.261	1.005
31.730.000	5.425.241	-764.507	1.003
31.740.000	5.427.026	-764.883	996

31.750.000	5.428.502	-765.201	990
31.760.000	5.430.144	-765.525	989
31.770.000	5.431.659	-765.878	988
31.780.000	5.433.398	-766.234	987
31.790.000	5.434.884	-766.511	986
31.800.000	5.436.518	-766.921	988
31.810.000	5.438.143	-767.189	992
31.820.000	5.439.667	-767.587	993
31.830.000	5.441.328	-767.906	991
31.840.000	5.442.856	-768.264	993
31.850.000	5.444.543	-768.600	994
31.860.000	5.445.976	-768.990	992
31.870.000	5.447.749	-769.298	990
31.880.000	5.449.129	-769.663	991
31.890.000	5.451.025	-770.067	992
31.900.000	5.452.377	-770.333	992
31.910.000	5.454.111	-770.752	990
31.920.000	5.455.601	-771.098	991
31.930.000	5.457.269	-771.439	994
31.940.000	5.458.712	-771.761	994
31.950.000	5.460.430	-772.182	991
31.960.000	5.461.921	-772.437	991
31.970.000	5.463.557	-772.852	993
31.980.000	5.465.157	-773.160	992
31.990.000	5.466.685	-773.516	990
32.000.000	5.468.372	-773.836	989
32.010.000	5.469.897	-774.250	989
32.020.000	5.471.559	-774.524	988
32.030.000	5.473.032	-774.947	985
32.040.000	5.474.786	-775.277	986

32.050.000	5.476.157	-775.623	988
32.060.000	5.477.958	-776.050	990
32.070.000	5.479.358	-776.354	987
32.080.000	5.481.111	-776.740	985
32.090.000	5.482.566	-777.118	986
32.100.000	5.484.308	-777.456	987
32.110.000	5.485.753	-777.798	984
32.120.000	5.487.475	-778.241	981
32.130.000	5.489.002	-778.511	979
32.140.000	5.490.632	-778.958	980
32.150.000	5.492.222	-779.308	980
32.160.000	5.493.787	-779.672	977
32.170.000	5.495.406	-780.017	976
32.180.000	5.496.969	-780.447	976
32.190.000	5.498.611	-780.711	977
32.200.000	5.500.105	-781.163	976
32.210.000	5.502.118	-781.498	973
32.220.000	5.503.538	-781.728	974
32.230.000	5.505.387	-781.906	975
32.240.000	5.506.813	-781.934	975
32.250.000	5.508.510	-781.896	971
32.260.000	5.509.981	-781.840	972
32.270.000	5.511.739	-781.734	975
32.280.000	5.513.117	-781.627	976
32.290.000	5.514.872	-781.690	975
32.300.000	5.516.342	-781.700	975
32.310.000	5.517.970	-781.928	979
32.320.000	5.519.560	-782.128	981
32.330.000	5.521.180	-782.408	980
32.340.000	5.522.769	-782.674	979

32.350.000	5.524.368	-783.090	983
32.360.000	5.525.975	-783.336	985
32.370.000	5.527.469	-783.765	984
32.380.000	5.529.198	-784.119	981
32.390.000	5.530.582	-784.480	984
32.400.000	5.532.388	-784.890	987
32.410.000	5.533.808	-785.289	986
32.420.000	5.535.543	-785.633	984
32.430.000	5.536.962	-786.037	987
32.440.000	5.538.729	-786.456	990
32.450.000	5.540.110	-786.770	989
32.460.000	5.541.894	-787.223	986
32.470.000	5.543.394	-787.565	986
32.480.000	5.545.036	-787.962	985
32.490.000	5.546.557	-788.323	983
32.500.000	5.548.217	-788.707	981
32.510.000	5.549.759	-789.024	982
32.520.000	5.551.360	-789.480	984
32.530.000	5.553.015	-789.734	984
32.540.000	5.554.516	-790.146	984
32.550.000	5.556.224	-790.491	988
32.560.000	5.557.740	-790.868	988
32.570.000	5.559.434	-791.222	986
32.580.000	5.560.892	-791.630	986
32.590.000	5.562.657	-791.974	987
32.600.000	5.564.052	-792.367	984
32.610.000	5.565.868	-792.753	980
32.620.000	5.567.246	-793.040	979
32.630.000	5.568.979	-793.475	981
32.640.000	5.570.481	-793.802	982

32.650.000	5.572.170	-794.146	980
32.660.000	5.573.661	-794.472	981
32.670.000	5.575.344	-794.880	984
32.680.000	5.576.898	-795.147	987
32.690.000	5.578.489	-795.554	985
32.700.000	5.580.177	-795.846	985
32.710.000	5.581.633	-796.198	986
32.720.000	5.583.335	-796.512	984
32.730.000	5.584.847	-796.888	981
32.740.000	5.586.504	-797.186	980
32.750.000	5.587.974	-797.584	980
32.760.000	5.589.761	-797.911	978
32.770.000	5.591.092	-798.221	976
32.780.000	5.592.935	-798.609	978
32.790.000	5.594.385	-798.889	981
32.800.000	5.596.138	-799.257	981
32.810.000	5.597.603	-799.576	981
32.820.000	5.599.282	-799.878	985
32.830.000	5.600.746	-800.197	989
32.840.000	5.602.429	-800.618	988
32.850.000	5.603.955	-800.830	987
32.860.000	5.605.561	-801.226	988
32.870.000	5.607.196	-801.539	991
32.880.000	5.608.722	-801.856	993
32.890.000	5.610.402	-802.139	993
32.900.000	5.611.949	-802.544	995
32.910.000	5.613.691	-802.781	999
32.920.000	5.615.107	-803.167	1.003
32.930.000	5.616.884	-803.469	1.004
32.940.000	5.618.245	-803.762	1.002

32.950.000	5.620.038	-804.133	1.000
32.960.000	5.621.504	-804.431	1.001
32.970.000	5.623.214	-804.749	1.002
32.980.000	5.624.661	-805.068	1.000
32.990.000	5.626.429	-805.401	998
33.000.000	5.627.857	-805.679	999
33.010.000	5.629.581	-806.076	1.003
33.020.000	5.631.135	-806.308	1.003
33.030.000	5.632.740	-806.672	1.001
33.040.000	5.634.358	-806.961	1.001
33.050.000	5.635.942	-807.291	1.003
33.060.000	5.637.555	-807.550	1.004
33.070.000	5.639.146	-807.965	1.001
33.080.000	5.640.846	-808.198	999
33.090.000	5.642.237	-808.549	999
33.100.000	5.644.076	-808.892	1.002
33.110.000	5.645.447	-809.169	1.004
33.120.000	5.647.204	-809.519	1.002
33.130.000	5.648.695	-809.857	1.003
33.140.000	5.650.445	-810.150	1.008
33.150.000	5.651.899	-810.463	1.014
33.160.000	5.653.685	-810.835	1.015
33.170.000	5.655.118	-811.059	1.013
33.180.000	5.656.834	-811.447	1.015
33.190.000	5.658.359	-811.708	1.017
33.200.000	5.659.961	-812.025	1.014
33.210.000	5.661.557	-812.323	1.009
33.220.000	5.663.193	-812.681	1.006
33.230.000	5.664.808	-812.912	1.006
33.240.000	5.666.357	-813.320	1.008

33.250.000	5.668.077	-813.539	1.009
33.260.000	5.669.509	-813.883	1.010
33.270.000	5.671.296	-814.196	1.011
33.280.000	5.672.719	-814.461	1.013
33.290.000	5.674.454	-814.791	1.016
33.300.000	5.675.924	-815.105	1.015
33.310.000	5.677.741	-815.363	1.012
33.320.000	5.679.122	-815.645	1.009
33.330.000	5.680.900	-816.003	1.009
33.340.000	5.682.342	-816.187	1.009
33.350.000	5.684.059	-816.560	1.005
33.360.000	5.685.639	-816.819	1.001
33.370.000	5.687.280	-817.097	999
33.380.000	5.688.820	-817.369	999
33.390.000	5.690.498	-817.710	998
33.400.000	5.692.046	-817.889	994
33.410.000	5.693.629	-818.253	992
33.420.000	5.695.340	-818.469	993
33.430.000	5.696.766	-818.755	993
33.440.000	5.698.504	-819.016	992
33.450.000	5.700.012	-819.309	990
33.460.000	5.701.743	-819.572	989
33.470.000	5.703.210	-819.856	990
33.480.000	5.704.998	-820.140	989
33.490.000	5.706.398	-820.377	990
33.500.000	5.708.202	-820.709	990
33.510.000	5.709.679	-820.913	991
33.520.000	5.711.347	-821.215	995
33.530.000	5.712.874	-821.484	999
33.540.000	5.714.523	-821.759	1.001

33.550.000	5.716.064	-821.980	1.002
33.560.000	5.717.726	-822.320	1.001
33.570.000	5.719.310	-822.488	1.004
33.580.000	5.720.933	-822.824	1.008
33.590.000	5.722.597	-823.066	1.010
33.600.000	5.724.088	-823.320	1.010
33.610.000	5.725.791	-823.539	1.011
33.620.000	5.727.256	-823.851	1.016
33.630.000	5.729.023	-824.040	1.020
33.640.000	5.730.409	-824.314	1.017
33.650.000	5.732.225	-824.598	1.014
33.660.000	5.733.590	-824.764	1.014
33.670.000	5.735.347	-825.065	1.017
33.680.000	5.736.832	-825.285	1.019
33.690.000	5.738.580	-825.534	1.017
33.700.000	5.740.064	-825.769	1.017
33.710.000	5.741.819	-826.029	1.019
33.720.000	5.743.284	-826.189	1.023
33.730.000	5.744.964	-826.516	1.027
33.740.000	5.746.589	-826.686	1.028
33.750.000	5.748.133	-826.954	1.029
33.760.000	5.749.785	-827.180	1.032
33.770.000	5.751.343	-827.450	1.036
33.780.000	5.752.988	-827.647	1.038
33.790.000	5.754.497	-827.969	1.038
33.800.000	5.756.318	-828.182	1.037
33.810.000	5.757.717	-828.466	1.037
33.820.000	5.759.567	-828.775	1.039
33.830.000	5.760.933	-828.966	1.040
33.840.000	5.762.717	-829.263	1.039

33.850.000	5.764.198	-829.524	1.035
33.860.000	5.765.916	-829.762	1.035
33.870.000	5.767.387	-830.010	1.036
33.880.000	5.769.159	-830.335	1.038
33.890.000	5.770.612	-830.493	1.037
33.900.000	5.772.329	-830.826	1.037
33.910.000	5.773.923	-831.059	1.040
33.920.000	5.775.528	-831.313	1.042
33.930.000	5.777.196	-831.523	1.042
33.940.000	5.778.744	-831.825	1.039
33.950.000	5.780.375	-831.981	1.039
33.960.000	5.781.885	-832.316	1.040
33.970.000	5.783.672	-832.503	1.040
33.980.000	5.785.068	-832.756	1.037
33.990.000	5.786.903	-833.034	1.038
34.000.000	5.788.298	-833.218	1.040
34.010.000	5.790.048	-833.471	1.042
34.020.000	5.791.528	-833.737	1.040
34.030.000	5.793.322	-833.942	1.037
34.040.000	5.794.750	-834.147	1.038
34.050.000	5.796.491	-834.441	1.039
34.060.000	5.798.000	-834.568	1.037
34.070.000	5.799.640	-834.846	1.037
34.080.000	5.801.233	-835.045	1.039
34.090.000	5.802.868	-835.245	1.039
34.100.000	5.804.445	-835.441	1.037
34.110.000	5.806.071	-835.711	1.036
34.120.000	5.807.706	-835.819	1.039
34.130.000	5.809.257	-836.118	1.039
34.140.000	5.811.040	-836.304	1.038

34.150.000	5.812.427	-836.501	1.040
34.160.000	5.814.226	-836.748	1.044
34.170.000	5.815.689	-836.970	1.046
34.180.000	5.817.404	-837.165	1.045
34.190.000	5.818.830	-837.377	1.043
34.200.000	5.820.658	-837.614	1.045
34.210.000	5.822.024	-837.755	1.046
34.220.000	5.823.818	-838.034	1.045
34.230.000	5.825.337	-838.184	1.046
34.240.000	5.827.014	-838.425	1.048
34.250.000	5.828.616	-838.618	1.051
34.260.000	5.830.252	-838.850	1.049
34.270.000	5.831.792	-838.995	1.049
34.280.000	5.833.450	-839.301	1.051
34.290.000	5.835.082	-839.408	1.052
34.300.000	5.836.590	-839.678	1.051
34.310.000	5.838.338	-839.873	1.052
34.320.000	5.839.791	-840.065	1.053
34.330.000	5.841.535	-840.279	1.053
34.340.000	5.842.975	-840.542	1.051
34.350.000	5.844.767	-840.696	1.051
34.360.000	5.846.163	-840.923	1.051
34.370.000	5.848.027	-841.182	1.048
34.380.000	5.849.403	-841.310	1.048
34.390.000	5.851.150	-841.590	1.052
34.400.000	5.852.653	-841.765	1.055
34.410.000	5.854.331	-841.976	1.056
34.420.000	5.855.888	-842.176	1.057
34.430.000	5.857.553	-842.428	1.059
34.440.000	5.859.093	-842.547	1.063

34.450.000	5.860.701	-842.834	1.061
34.460.000	5.862.422	-842.988	1.059
34.470.000	5.863.883	-843.236	1.060
34.480.000	5.865.648	-843.430	1.062
34.490.000	5.867.130	-843.664	1.061
34.500.000	5.868.822	-843.842	1.060
34.510.000	5.870.300	-844.090	1.060
34.520.000	5.872.084	-844.283	1.062
34.530.000	5.873.453	-844.487	1.062
34.540.000	5.875.272	-844.741	1.060
34.550.000	5.876.708	-844.893	1.062
34.560.000	5.878.426	-845.131	1.063
34.570.000	5.879.936	-845.346	1.063
34.580.000	5.881.635	-845.555	1.060
34.590.000	5.883.179	-845.734	1.062
34.600.000	5.884.862	-846.024	1.065
34.610.000	5.886.401	-846.111	1.063
34.620.000	5.887.987	-846.376	1.061
34.630.000	5.889.650	-846.558	1.062
34.640.000	5.891.138	-846.744	1.062
34.650.000	5.892.850	-846.900	1.062
34.660.000	5.894.348	-847.156	1.059
34.670.000	5.896.084	-847.260	1.062
34.680.000	5.897.514	-847.484	1.064
34.690.000	5.899.325	-847.688	1.064
34.700.000	5.900.683	-847.821	1.064
34.710.000	5.902.516	-848.058	1.066
34.720.000	5.903.922	-848.200	1.067
34.730.000	5.905.667	-848.401	1.067
34.740.000	5.907.130	-848.578	1.066

34.750.000	5.908.893	-848.786	1.067
34.760.000	5.910.336	-848.929	1.068
34.770.000	5.912.045	-849.197	1.066
34.780.000	5.913.634	-849.291	1.063
34.790.000	5.915.202	-849.538	1.063
34.800.000	5.916.844	-849.720	1.064
34.810.000	5.918.457	-849.941	1.063
34.820.000	5.920.073	-850.084	1.063
34.830.000	5.921.605	-850.400	1.067
34.840.000	5.923.379	-850.518	1.070
34.850.000	5.924.772	-850.792	1.070
34.860.000	5.926.644	-851.022	1.070
34.870.000	5.928.020	-851.189	1.072
34.880.000	5.929.805	-851.441	1.072
34.890.000	5.931.285	-851.664	1.068
34.900.000	5.933.068	-851.868	1.066
34.910.000	5.934.504	-852.081	1.068
34.920.000	5.936.316	-852.376	1.067
34.930.000	5.937.778	-852.538	1.064
34.940.000	5.939.499	-852.841	1.065
34.950.000	5.941.085	-853.034	1.068
34.960.000	5.942.666	-853.268	1.068
34.970.000	5.944.307	-853.478	1.068
34.980.000	5.945.929	-853.745	1.071
34.990.000	5.947.540	-853.892	1.074
35.000.000	5.949.086	-854.205	1.073
35.010.000	5.950.865	-854.371	1.073
35.020.000	5.952.301	-854.610	1.077
35.030.000	5.954.119	-854.854	1.082
35.040.000	5.955.544	-855.042	1.085

35.050.000	5.957.326	-855.281	1.087
35.060.000	5.958.767	-855.515	1.093
35.070.000	5.960.520	-855.709	1.096
35.080.000	5.961.947	-855.901	1.096
35.090.000	5.963.757	-856.202	1.094
35.100.000	5.965.236	-856.301	1.096
35.110.000	5.966.928	-856.587	1.096
35.120.000	5.968.502	-856.769	1.093
35.130.000	5.970.169	-856.968	1.092
35.140.000	5.971.764	-857.168	1.095
35.150.000	5.973.400	-857.456	1.096
35.160.000	5.975.039	-857.565	1.093
35.170.000	5.976.560	-857.894	1.091
35.180.000	5.978.307	-858.057	1.096
35.190.000	5.979.719	-858.282	1.100
35.200.000	5.981.525	-858.514	1.100
35.210.000	5.982.939	-858.747	1.099
35.220.000	5.984.685	-858.948	1.100
35.230.000	5.986.095	-859.162	1.102
35.240.000	5.987.958	-859.386	1.101
35.250.000	5.989.345	-859.549	1.098
35.260.000	5.991.150	-859.815	1.097
35.270.000	5.992.652	-859.942	1.099
35.280.000	5.994.309	-860.197	1.102
35.290.000	5.995.892	-860.387	1.103
35.300.000	5.997.552	-860.618	1.102
35.310.000	5.999.108	-860.791	1.104
35.320.000	6.000.760	-861.090	1.109
35.330.000	6.002.402	-861.234	1.111
35.340.000	6.003.870	-861.499	1.109

35.350.000	6.005.626	-861.698	1.110
35.360.000	6.007.123	-861.943	1.112
35.370.000	6.008.885	-862.147	1.114
35.380.000	6.010.344	-862.402	1.111
35.390.000	6.012.104	-862.591	1.109
35.400.000	6.013.554	-862.824	1.111
35.410.000	6.015.366	-863.086	1.115
35.420.000	6.016.755	-863.237	1.114
