# Supplementary material for: Data on statistical experimental design to formulate amphotericin B-loaded Eudragit RL100 nanoparticles coated with hyaluronic acid for the treatment of vulvovaginal candidiasis
Source: Data Brief. 2020 Mar 5;29:105311. doi: 10.1016/j.dib.2020.105311 (PMC7082528; doi:10.1016/j.dib.2020.105311)
Supplement: Multimedia component 6 [file mmc6.pdf]

File Name:	**AMP EUD nanoparticles**		
[Data]			
Time	Temp	**DTA**	**TGA**
sec	C	uV	mg
0	319.096	-10.572	1.9624
1.2000	319.297	-10.524	1.9626
2.2000	319.898	-10.676	1.9626
3.2000	320.338	-10.664	1.9626
4.2000	320.734	-10.685	1.9627
5.2000	321.217	-10.679	1.9627
6.2000	321.627	-10.775	1.9626
7.2000	322.205	-10.657	1.9623
8.2000	322.453	-10.771	1.9621
9.2000	323.308	-10.837	1.9622
10.2000	323.478	-10.788	1.9621
11.2000	324.247	-10.912	1.9621
12.2000	324.655	-10.988	1.9622
130.000	325.350	-11.080	1.9625
140.000	325.813	-11.144	1.9625
150.000	326.598	-11.394	1.9623
160.000	327.186	-11.432	1.9623
170.000	327.802	-11.723	1.9623
180.000	328.635	-11.922	1.9621
190.000	329.165	-12.181	1.9617
200.000	330.119	-12.510	1.9617
210.000	330.613	-12.904	1.9620
220.000	331.700	-13.252	1.9619
230.000	332.191	-13.665	1.9616
240.000	333.263	-14.222	1.9615

250.000	333.928	-14.589	1.9616
260.000	334.896	-15.200	1.9615
270.000	335.762	-15.728	1.9612
280.000	336.702	-16.347	1.9609
290.000	337.581	-16.891	1.9607
300.000	338.447	-17.642	1.9606
310.000	339.643	-18.245	1.9606
320.000	340.268	-18.970	1.9607
330.000	341.560	-19.756	1.9609
340.000	342.285	-20.488	1.9611
350.000	343.486	-21.270	1.9615
360.000	344.321	-22.077	1.9618
370.000	345.608	-22.944	1.9619
380.000	346.514	-23.692	1.9619
390.000	347.713	-24.656	1.9620
400.000	348.789	-25.452	1.9621
410.000	349.788	-26.361	1.9621
420.000	351.061	-27.224	1.9620
430.000	352.011	-28.217	1.9620
440.000	353.434	-29.100	1.9619
450.000	354.302	-30.084	1.9617
460.000	355.810	-31.106	1.9615
470.000	356.661	-31.987	1.9613
480.000	358.091	-33.083	1.9610
490.000	359.247	-34.032	1.9608
500.000	360.473	-35.039	1.9606
510.000	361.640	-36.028	1.9603
520.000	362.978	-37.125	1.9601
530.000	364.239	-38.019	1.9601
540.000	365.352	-39.152	1.9600

550.000	366.928	-40.175	1.9599
560.000	367.933	-41.204	1.9598
570.000	369.475	-42.292	1.9600
580.000	370.575	-43.368	1.9599
590.000	372.110	-44.404	1.9596
600.000	373.217	-45.424	1.9596
610.000	374.764	-46.598	1.9595
620.000	375.926	-47.538	1.9596
630.000	377.399	-48.697	1.9595
640.000	378.791	-49.749	1.9596
650.000	380.081	-50.812	1.9596
660.000	381.603	-51.817	1.9595
670.000	382.825	-52.992	1.9594
680.000	384.511	-53.991	1.9592
690.000	385.560	-55.058	1.9590
700.000	387.283	-56.214	1.9588
710.000	388.522	-57.207	1.9587
720.000	390.083	-58.292	1.9587
730.000	391.412	-59.343	1.9588
740.000	393.011	-60.431	1.9588
750.000	394.345	-61.416	1.9588
760.000	395.852	-62.533	1.9587
770.000	397.481	-63.488	1.9585
780.000	398.722	-64.546	1.9583
790.000	400.465	-65.581	1.9580
800.000	401.712	-66.619	1.9577
810.000	403.403	-67.611	1.9575
820.000	404.693	-68.646	1.9574
830.000	406.422	-69.695	1.9572
840.000	407.614	-70.581	1.9568

850.000	409.279	-71.649	1.9567
860.000	410.715	-72.588	1.9566
870.000	412.209	-73.539	1.9563
880.000	413.737	-74.473	1.9561
890.000	415.221	-75.543	1.9560
900.000	416.824	-76.345	1.9561
910.000	418.170	-77.397	1.9562
920.000	419.994	-78.367	1.9563
930.000	421.238	-79.240	1.9566
940.000	423.023	-80.243	1.9569
950.000	424.374	-81.165	1.9569
960.000	426.091	-82.063	1.9567
970.000	427.442	-82.958	1.9567
980.000	429.162	-83.975	1.9566
990.000	430.666	-84.748	1.9562
1.000.000	432.168	-85.710	1.9560
1.010.000	433.881	-86.580	1.9558
1.020.000	435.275	-87.476	1.9558
1.030.000	437.034	-88.333	1.9557
1.040.000	438.396	-89.295	1.9555
1.050.000	440.292	-90.130	1.9554
1.060.000	441.563	-90.966	1.9553
1.070.000	443.428	-91.891	1.9551
1.080.000	444.806	-92.644	1.9548
1.090.000	446.534	-93.551	1.9545
1.100.000	448.075	-94.383	1.9543
1.110.000	449.761	-95.224	1.9539
1.120.000	451.346	-95.966	1.9536
1.130.000	452.931	-96.909	1.9536
1.140.000	454.739	-97.609	1.9534

1.150.000	456.105	-98.457	1.9531
1.160.000	458.055	-99.323	1.9529
1.170.000	459.438	-100.079	1.9527
1.180.000	461.259	-100.921	1.9526
1.190.000	462.708	-101.685	1.9525
1.200.000	464.550	-102.508	1.9522
1.210.000	466.008	-103.206	1.9522
1.220.000	467.814	-104.076	1.9523
1.230.000	469.474	-104.785	1.9524
1.240.000	470.970	-105.557	1.9525
1.250.000	472.783	-106.283	1.9524
1.260.000	474.302	-107.108	1.9522
1.270.000	476.160	-107.796	1.9521
1.280.000	477.644	-108.579	1.9520
1.290.000	479.565	-109.375	1.9517
1.300.000	480.947	-110.010	1.9512
1.310.000	482.863	-110.831	1.9510
1.320.000	484.361	-111.527	1.9510
1.330.000	486.167	-112.231	1.9511
1.340.000	487.757	-112.926	1.9510
1.350.000	489.474	-113.715	1.9509
1.360.000	491.208	-114.269	1.9510
1.370.000	492.723	-115.088	1.9512
1.380.000	494.728	-115.763	1.9510
1.390.000	496.115	-116.409	1.9508
1.400.000	498.042	-117.149	1.9504
1.410.000	499.528	-117.856	1.9503
1.420.000	501.424	-118.481	1.9503
1.430.000	502.842	-119.133	1.9498
1.440.000	504.791	-119.909	1.9494

1.450.000	506.343	-120.458	1.9494
1.460.000	508.026	-121.156	1.9493
1.470.000	509.752	-121.777	1.9491
1.480.000	511.351	-122.440	1.9489
1.490.000	513.139	-123.001	1.9488
1.500.000	514.718	-123.744	1.9490
1.510.000	516.600	-124.315	1.9490
1.520.000	517.970	-124.932	1.9489
1.530.000	519.957	-125.641	1.9488
1.540.000	521.393	-126.184	1.9488
1.550.000	523.295	-126.855	1.9488
1.560.000	524.837	-127.425	1.9486
1.570.000	526.660	-128.080	1.9484
1.580.000	528.218	-128.591	1.9483
1.590.000	529.951	-129.285	1.9482
1.600.000	531.789	-129.796	1.9483
1.610.000	533.292	-130.416	1.9482
1.620.000	535.193	-131.004	1.9483
1.630.000	536.689	-131.595	1.9486
1.640.000	538.570	-132.166	1.9487
1.650.000	540.098	-132.746	1.9486
1.660.000	542.056	-133.374	1.9483
1.670.000	543.511	-133.838	1.9480
1.680.000	545.370	-134.493	1.9478
1.690.000	547.013	-135.022	1.9475
1.700.000	548.691	-135.559	1.9470
1.710.000	550.503	-136.085	1.9467
1.720.000	552.152	-136.754	1.9464
1.730.000	554.009	-137.153	1.9462
1.740.000	555.500	-137.779	1.9459

1.750.000	557.532	-138.382	1.9454
1.760.000	558.937	-138.817	1.9449
1.770.000	560.885	-139.440	1.9447
1.780.000	562.460	-139.937	1.9446
1.790.000	564.310	-140.476	1.9444
1.800.000	565.890	-140.977	1.9441
1.810.000	567.717	-141.595	1.9438
1.820.000	569.458	-141.982	1.9435
1.830.000	571.068	-142.588	1.9434
1.840.000	572.965	-143.063	1.9432
1.850.000	574.503	-143.570	1.9431
1.860.000	576.391	-144.070	1.9429
1.870.000	577.950	-144.628	1.9428
1.880.000	579.929	-145.105	1.9429
1.890.000	581.359	-145.559	1.9429
1.900.000	583.366	-146.156	1.9428
1.910.000	584.896	-146.526	1.9427
1.920.000	586.729	-147.069	1.9424
1.930.000	588.451	-147.524	1.9423
1.940.000	590.155	-148.025	1.9422
1.950.000	591.940	-148.421	1.9421
1.960.000	593.596	-149.010	1.9420
1.970.000	595.539	-149.400	1.9417
1.980.000	596.961	-149.869	1.9416
1.990.000	599.008	-150.422	1.9416
2.000.000	600.581	-150.842	1.9416
2.010.000	602.447	-151.324	1.9414
2.020.000	603.987	-151.767	1.9412
2.030.000	605.894	-152.299	1.9409
2.040.000	607.460	-152.640	1.9409

2.050.000	609.213	-153.173	1.9408
2.060.000	611.063	-153.561	1.9408
2.070.000	612.568	-154.022	1.9406
2.080.000	614.486	-154.421	1.9405
2.090.000	616.082	-154.934	1.9405
2.100.000	617.932	-155.322	1.9405
2.110.000	619.421	-155.769	1.9404
2.120.000	621.463	-156.266	1.9401
2.130.000	622.867	-156.585	1.9397
2.140.000	624.787	-157.100	1.9396
2.150.000	626.409	-157.519	1.9395
2.160.000	628.171	-157.911	1.9392
2.170.000	629.845	-158.302	1.9389
2.180.000	631.603	-158.812	1.9386
2.190.000	633.424	-159.072	1.9384
2.200.000	634.932	-159.569	1.9384
2.210.000	636.940	-160.012	1.9384
2.220.000	638.387	-160.336	1.9383
2.230.000	640.337	-160.810	1.9382
2.240.000	641.848	-161.198	1.9381
2.250.000	643.760	-161.581	1.9379
2.260.000	645.233	-161.919	1.9375
2.270.000	647.159	-162.403	1.9372
2.280.000	648.821	-162.692	1.9368
2.290.000	650.529	-163.156	1.9365
2.300.000	652.394	-163.499	1.9365
2.310.000	654.023	-163.913	1.9365
2.320.000	655.879	-164.246	1.9366
2.330.000	657.412	-164.683	1.9367
2.340.000	659.372	-165.057	1.9367

2.350.000	660.819	-165.387	1.9370
2.360.000	662.812	-165.850	1.9370
2.370.000	664.343	-166.134	1.9369
2.380.000	666.180	-166.549	1.9367
2.390.000	667.870	-166.867	1.9366
2.400.000	669.674	-167.307	1.9365
2.410.000	671.394	-167.570	1.9361
2.420.000	673.064	-168.049	1.9358
2.430.000	674.997	-168.326	1.9358
2.440.000	676.442	-168.686	1.9358
2.450.000	678.459	-169.112	1.9355
2.460.000	679.979	-169.467	1.9353
2.470.000	681.915	-169.777	1.9353
2.480.000	683.399	-170.129	1.9351
2.490.000	685.363	-170.577	1.9346
2.500.000	686.920	-170.803	1.9344
2.510.000	688.752	-171.243	1.9342
2.520.000	690.536	-171.538	1.9341
2.530.000	692.191	-171.888	1.9337
2.540.000	694.072	-172.181	1.9335
2.550.000	695.679	-172.615	1.9335
2.560.000	697.609	-172.872	1.9335
2.570.000	699.046	-173.241	1.9333
2.580.000	701.064	-173.648	1.9331
2.590.000	702.541	-173.901	1.9330
2.600.000	704.465	-174.301	1.9330
2.610.000	706.042	-174.612	1.9328
2.620.000	707.877	-174.947	1.9324
2.630.000	709.562	-175.234	1.9322
2.640.000	711.286	-175.670	1.9321

2.650.000	713.106	-175.866	1.9321
2.660.000	714.627	-176.258	1.9322
2.670.000	716.594	-176.603	1.9322
2.680.000	718.049	-176.889	1.9323
2.690.000	719.969	-177.269	1.9325
2.700.000	721.430	-177.596	1.9326
2.710.000	723.445	-177.924	1.9326
2.720.000	724.846	-178.166	1.9324
2.730.000	726.783	-178.612	1.9323
2.740.000	728.462	-178.834	1.9323
2.750.000	730.140	-179.216	1.9322
2.760.000	731.955	-179.504	1.9318
2.770.000	733.584	-179.881	1.9315
2.780.000	735.374	-180.108	1.9313
2.790.000	736.948	-180.504	1.9311
2.800.000	738.901	-180.840	1.9306
2.810.000	740.300	-181.098	1.9303
2.820.000	742.253	-181.491	1.9302
2.830.000	743.796	-181.768	1.9302
2.840.000	745.657	-182.110	1.9302
2.850.000	747.291	-182.408	1.9302
2.860.000	749.131	-182.778	1.9301
2.870.000	750.763	-182.992	1.9301
2.880.000	752.461	-183.409	1.9300
2.890.000	754.369	-183.654	1.9296
2.900.000	755.848	-183.995	1.9292
2.910.000	757.810	-184.338	1.9289
2.920.000	759.323	-184.684	1.9288
2.930.000	761.228	-184.958	1.9286
2.940.000	762.687	-185.263	1.9283

2.950.000	764.669	-185.677	1.9279
2.960.000	766.224	-185.869	1.9279
2.970.000	768.067	-186.281	1.9280
2.980.000	769.772	-186.582	1.9279
2.990.000	771.467	-186.884	1.9278
3.000.000	773.286	-187.163	1.9276
3.010.000	774.900	-187.573	1.9276
3.020.000	776.808	-187.789	1.9275
3.030.000	778.282	-188.158	1.9272
3.040.000	780.279	-188.558	1.9270
3.050.000	781.755	-188.771	1.9268
3.060.000	783.664	-189.176	1.9269
3.070.000	785.232	-189.485	1.9271
3.080.000	787.170	-189.811	1.9272
3.090.000	788.710	-190.060	1.9271
3.100.000	790.545	-190.496	1.9270
3.110.000	792.326	-190.686	1.9268
3.120.000	793.887	-191.071	1.9266
3.130.000	795.824	-191.355	1.9263
3.140.000	797.338	-191.687	1.9258
3.150.000	799.246	-191.997	1.9255
3.160.000	800.767	-192.325	1.9257
3.170.000	802.760	-192.673	1.9257
3.180.000	804.198	-192.916	1.9252
3.190.000	806.148	-193.317	1.9249
3.200.000	807.768	-193.547	1.9249
3.210.000	809.462	-193.882	1.9252
3.220.000	811.235	-194.157	1.9251
3.230.000	812.883	-194.508	1.9248
3.240.000	814.647	-194.693	1.9247

3.250.000	816.214	-195.086	1.9247
3.260.000	818.139	-195.391	1.9246
3.270.000	819.549	-195.632	1.9243
3.280.000	821.502	-196.021	1.9238
3.290.000	823.045	-196.277	1.9237
3.300.000	824.920	-196.573	1.9237
3.310.000	826.465	-196.849	1.9237
3.320.000	828.331	-197.237	1.9235
3.330.000	829.966	-197.399	1.9232
3.340.000	831.608	-197.782	1.9232
3.350.000	833.491	-198.045	1.9234
3.360.000	835.012	-198.344	1.9234
3.370.000	836.865	-198.629	1.9234
3.380.000	838.408	-198.963	1.9233
3.390.000	840.314	-199.247	1.9233
3.400.000	841.766	-199.512	1.9233
3.410.000	843.758	-199.908	1.9231
3.420.000	845.254	-200.076	1.9229
3.430.000	847.093	-200.449	1.9226
3.440.000	848.786	-200.703	1.9225
3.450.000	850.444	-200.983	1.9226
3.460.000	852.167	-201.214	1.9226
3.470.000	853.843	-201.645	1.9223
3.480.000	855.721	-201.807	1.9221
3.490.000	857.150	-202.138	1.9222
3.500.000	859.128	-202.496	1.9224
3.510.000	860.615	-202.724	1.9225
3.520.000	862.525	-203.052	1.9224
3.530.000	864.049	-203.345	1.9223
3.540.000	866.025	-203.674	1.9224

3.550.000	867.552	-203.906	1.9222
3.560.000	869.330	-204.273	1.9217
3.570.000	871.085	-204.486	1.9212
3.580.000	872.675	-204.816	1.9210
3.590.000	874.553	-205.074	1.9210
3.600.000	876.121	-205.405	1.9209
3.610.000	877.984	-205.688	1.9206
3.620.000	879.484	-205.995	1.9203
3.630.000	881.488	-206.344	1.9200
3.640.000	882.918	-206.549	1.9200
3.650.000	884.909	-206.933	1.9198
3.660.000	886.475	-207.163	1.9194
3.670.000	888.209	-207.461	1.9190
3.680.000	889.913	-207.730	1.9189
3.690.000	891.639	-208.103	1.9189
3.700.000	893.420	-208.256	1.9186
3.710.000	894.951	-208.650	1.9184
3.720.000	896.971	-208.948	1.9180
3.730.000	898.404	-209.191	1.9180
3.740.000	900.339	-209.551	1.9180
3.750.000	901.910	-209.828	1.9178
3.760.000	903.832	-210.105	1.9176
3.770.000	905.321	-210.375	1.9175
3.780.000	907.208	-210.759	1.9174
3.790.000	908.755	-210.901	1.9174
3.800.000	910.436	-211.275	1.9173
3.810.000	912.260	-211.534	1.9172
3.820.000	913.788	-211.814	1.9169
3.830.000	915.630	-212.064	1.9168
3.840.000	917.132	-212.417	1.9167

3.850.000	919.066	-212.661	1.9167
3.860.000	920.534	-212.939	1.9165
3.870.000	922.527	-213.303	1.9162
3.880.000	924.043	-213.483	1.9159
3.890.000	925.812	-213.813	1.9159
3.900.000	927.437	-214.110	1.9160
3.910.000	929.227	-214.387	1.9159
3.920.000	930.859	-214.587	1.9159
3.930.000	932.488	-214.962	1.9160
3.940.000	934.372	-215.146	1.9161
3.950.000	935.796	-215.468	1.9160
3.960.000	937.755	-215.790	1.9160
3.970.000	939.248	-216.019	1.9159
3.980.000	941.174	-216.311	1.9159
3.990.000	942.650	-216.575	1.9158
4.000.000	944.594	-216.900	1.9159
4.010.000	946.080	-217.089	1.9159
4.020.000	947.906	-217.445	1.9160
4.030.000	949.610	-217.641	1.9159
4.040.000	951.190	-217.936	1.9157
4.050.000	953.043	-218.168	1.9154
4.060.000	954.588	-218.498	1.9152
4.070.000	956.457	-218.707	1.9151
4.080.000	957.953	-219.035	1.9149
4.090.000	959.981	-219.386	1.9147
4.100.000	961.378	-219.545	1.9145
4.110.000	963.315	-219.925	1.9144
4.120.000	964.883	-220.151	1.9144
4.130.000	966.661	-220.431	1.9144
4.140.000	968.315	-220.692	1.9143

4.150.000	970.049	-221.055	1.9142
4.160.000	971.812	-221.189	1.9141
4.170.000	973.330	-221.566	1.9140
4.180.000	975.309	-221.849	1.9137
4.190.000	976.778	-222.093	1.9136
4.200.000	978.728	-222.424	1.9135
4.210.000	980.280	-222.740	1.9135
4.220.000	982.177	-223.007	1.9135
4.230.000	983.669	-223.254	1.9134
4.240.000	985.587	-223.660	1.9134
4.250.000	987.191	-223.817	1.9133
4.260.000	988.901	-224.174	1.9132
4.270.000	990.693	-224.450	1.9131
4.280.000	992.289	-224.736	1.9129
4.290.000	994.139	-224.954	1.9129
4.300.000	995.671	-225.331	1.9126
4.310.000	997.700	-225.611	1.9125
4.320.000	999.122	-225.879	1.9124
4.330.000	1.001.099	-226.230	1.9122
4.340.000	1.002.627	-226.445	1.9121
4.350.000	1.004.470	-226.779	1.9119
4.360.000	1.006.014	-227.037	1.9117
4.370.000	1.007.817	-227.343	1.9114
4.380.000	1.009.406	-227.549	1.9112
4.390.000	1.011.094	-227.920	1.9109
4.400.000	1.012.953	-228.115	1.9107
4.410.000	1.014.391	-228.404	1.9103
4.420.000	1.016.333	-228.728	1.9101
4.430.000	1.017.890	-229.010	1.9101
4.440.000	1.019.727	-229.282	1.9101

4.450.000	1.021.224	-229.550	1.9102
4.460.000	1.023.155	-229.891	1.9102
4.470.000	1.024.599	-230.064	1.9101
4.480.000	1.026.433	-230.421	1.9100
4.490.000	1.028.105	-230.659	1.9100
4.500.000	1.029.689	-230.927	1.9099
4.510.000	1.031.540	-231.165	1.9097
4.520.000	1.033.127	-231.531	1.9095
4.530.000	1.034.939	-231.690	1.9094
4.540.000	1.036.450	-232.050	1.9092
4.550.000	1.038.423	-232.385	1.9093
4.560.000	1.039.839	-232.544	1.9094
4.570.000	1.041.712	-232.898	1.9096
4.580.000	1.043.304	-233.161	1.9096
4.590.000	1.045.103	-233.415	1.9096
4.600.000	1.046.662	-233.666	1.9096
4.610.000	1.048.459	-234.013	1.9094
4.620.000	1.050.154	-234.155	1.9093
4.630.000	1.051.707	-234.511	1.9091
4.640.000	1.053.638	-234.755	1.9089
4.650.000	1.055.136	-235.014	1.9089
4.660.000	1.057.014	-235.316	1.9087
4.670.000	1.058.503	-235.619	1.9086
4.680.000	1.060.446	-235.888	1.9086
4.690.000	1.061.926	-236.119	1.9085
4.700.000	1.063.827	-236.491	1.9082
4.710.000	1.065.369	-236.655	1.9079
4.720.000	1.067.135	-237.004	1.9076
4.730.000	1.068.846	-237.243	1.9076
4.740.000	1.070.472	-237.537	1.9077

4.750.000	1.072.308	-237.751	1.9075
4.760.000	1.073.867	-238.121	1.9072
4.770.000	1.075.797	-238.375	1.9069
4.780.000	1.077.194	-238.623	1.9068
4.790.000	1.079.192	-239.013	1.9067
4.800.000	1.080.707	-239.214	1.9065
4.810.000	1.082.527	-239.534	1.9063
4.820.000	1.084.106	-239.790	1.9061
4.830.000	1.085.947	-240.139	1.9060
4.840.000	1.087.539	-240.308	1.9061
4.850.000	1.089.246	-240.673	1.9060
4.860.000	1.091.072	-240.901	1.9059
4.870.000	1.092.620	-241.234	1.9056
4.880.000	1.094.565	-241.485	1.9055
4.890.000	1.096.070	-241.805	1.9055
4.900.000	1.097.927	-242.058	1.9055
4.910.000	1.099.460	-242.342	1.9053
4.920.000	1.101.409	-242.699	1.9050
4.930.000	1.102.874	-242.865	1.9048
4.940.000	1.104.724	-243.234	1.9044
4.950.000	1.106.350	-243.492	1.9041
4.960.000	1.108.008	-243.738	1.9041
4.970.000	1.109.733	-243.974	1.9040
4.980.000	1.111.380	-244.342	1.9039
4.990.000	1.113.224	-244.510	1.9039
5.000.000	1.114.736	-244.843	1.9038
5.010.000	1.116.656	-245.168	1.9037
5.020.000	1.118.084	-245.360	1.9035
5.030.000	1.119.956	-245.704	1.9034
5.040.000	1.121.477	-245.953	1.9033

5.050.000	1.123.323	-246.226	1.9031
5.060.000	1.124.834	-246.445	1.9029
5.070.000	1.126.605	-246.805	1.9029
5.080.000	1.128.273	-246.964	1.9029
5.090.000	1.129.893	-247.303	1.9028
5.100.000	1.131.792	-247.544	1.9025
5.110.000	1.133.284	-247.811	1.9024
5.120.000	1.135.164	-248.092	1.9024
5.130.000	1.136.658	-248.400	1.9024
5.140.000	1.138.567	-248.660	1.9023
5.150.000	1.139.963	-248.865	1.9021
5.160.000	1.141.908	-249.243	1.9019
5.170.000	1.143.424	-249.414	1.9017
5.180.000	1.145.174	-249.713	1.9016
5.190.000	1.146.844	-249.961	1.9015
5.200.000	1.148.607	-250.268	1.9012
5.210.000	1.150.297	-250.433	1.9010
5.220.000	1.151.907	-250.801	1.9009
5.230.000	1.153.840	-251.020	1.9009
5.240.000	1.155.225	-251.264	1.9009
5.250.000	1.157.171	-251.608	1.9007
5.260.000	1.158.669	-251.838	1.9006
5.270.000	1.160.491	-252.110	1.9007
5.280.000	1.161.992	-252.357	1.9007
5.290.000	1.163.899	-252.702	1.9004
5.300.000	1.165.480	-252.873	1.9001
5.310.000	1.167.205	-253.230	1.9000
5.320.000	1.169.004	-253.457	1.8999
5.330.000	1.170.575	-253.744	1.8999
5.340.000	1.172.478	-253.998	1.8998

5.350.000	1.174.001	-254.343	1.8997
5.360.000	1.175.840	-254.568	1.8998
5.370.000	1.177.324	-254.868	1.8999
5.380.000	1.179.272	-255.204	1.8998
5.390.000	1.180.693	-255.390	1.8995
5.400.000	1.182.622	-255.749	1.8994
5.410.000	1.184.229	-256.036	1.8991
5.420.000	1.185.981	-256.295	1.8989
5.430.000	1.187.666	-256.529	1.8989
5.440.000	1.189.381	-256.918	1.8990
5.450.000	1.191.178	-257.077	1.8990
5.460.000	1.192.702	-257.425	1.8990
5.470.000	1.194.671	-257.743	1.8992
5.480.000	1.196.129	-257.976	1.8994
5.490.000	1.198.005	-258.320	1.8994
5.500.000	1.199.530	-258.601	1.8992
5.510.000	1.201.442	-258.892	1.8990
5.520.000	1.202.908	-259.122	1.8988
5.530.000	1.204.772	-259.515	1.8986
5.540.000	1.206.418	-259.694	1.8984
5.550.000	1.208.080	-260.031	1.8981
5.560.000	1.209.898	-260.278	1.8979
5.570.000	1.211.437	-260.597	1.8977
5.580.000	1.213.271	-260.829	1.8974
5.590.000	1.214.805	-261.156	1.8972
5.600.000	1.216.708	-261.434	1.8971
5.610.000	1.218.077	-261.660	1.8969
5.620.000	1.220.014	-262.028	1.8967
5.630.000	1.221.577	-262.247	1.8965
5.640.000	1.223.332	-262.570	1.8965

5.650.000	1.224.982	-262.808	1.8966
5.660.000	1.226.735	-263.139	1.8967
5.670.000	1.228.426	-263.314	1.8966
5.680.000	1.230.007	-263.695	1.8965
5.690.000	1.231.944	-263.913	1.8965
5.700.000	1.233.323	-264.178	1.8965
5.710.000	1.235.240	-264.494	1.8963
5.720.000	1.236.730	-264.778	1.8963
5.730.000	1.238.608	-265.017	1.8962
5.740.000	1.240.078	-265.281	1.8961
5.750.000	1.242.004	-265.648	1.8960
5.760.000	1.243.535	-265.799	1.8960
5.770.000	1.245.301	-266.155	1.8958
5.780.000	1.247.088	-266.392	1.8955
5.790.000	1.248.650	-266.655	1.8953
5.800.000	1.250.464	-266.879	1.8954
5.810.000	1.252.033	-267.214	1.8955
5.820.000	1.253.904	-267.416	1.8955
5.830.000	1.255.309	-267.695	1.8954
5.840.000	1.257.260	-268.018	1.8955
5.850.000	1.258.704	-268.191	1.8955
5.860.000	1.260.580	-268.531	1.8954
5.870.000	1.262.182	-268.779	1.8954
5.880.000	1.263.957	-269.043	1.8953
5.890.000	1.265.629	-269.278	1.8953
5.900.000	1.267.332	-269.629	1.8954
5.910.000	1.269.106	-269.778	1.8953
5.920.000	1.270.627	-270.110	1.8953
5.930.000	1.272.570	-270.392	1.8954
5.940.000	1.274.009	-270.626	1.8955

5.950.000	1.275.917	-270.922	1.8955
5.960.000	1.277.438	-271.193	1.8954
5.970.000	1.279.370	-271.468	1.8952
5.980.000	1.280.835	-271.685	1.8951
5.990.000	1.282.722	-272.040	1.8949
6.000.000	1.284.357	-272.230	1.8948
6.010.000	1.286.033	-272.552	1.8947
6.020.000	1.287.803	-272.785	1.8945
6.030.000	1.289.425	-273.101	1.8944
6.040.000	1.291.226	-273.275	1.8944
6.050.000	1.292.765	-273.627	1.8943
6.060.000	1.294.733	-273.904	1.8943
6.070.000	1.296.152	-274.124	1.8941
6.080.000	1.298.117	-274.481	1.8940
6.090.000	1.299.651	-274.692	1.8939
6.100.000	1.301.468	-274.977	1.8938
6.110.000	1.303.087	-275.258	1.8936
6.120.000	1.304.884	-275.566	1.8935
6.130.000	1.306.523	-275.718	1.8933
6.140.000	1.308.160	-276.074	1.8931
6.150.000	1.310.003	-276.301	1.8930
6.160.000	1.311.462	-276.533	1.8929
6.170.000	1.313.370	-276.836	1.8929
6.180.000	1.314.857	-277.123	1.8927
6.190.000	1.316.705	-277.371	1.8926
6.200.000	1.318.203	-277.614	1.8927
6.210.000	1.320.114	-277.961	1.8928
6.220.000	1.321.611	-278.108	1.8928
6.230.000	1.323.395	-278.463	1.8927
6.240.000	1.325.085	-278.693	1.8926

6.250.000	1.326.722	-278.940	1.8925
6.260.000	1.328.483	-279.172	1.8925
6.270.000	1.330.123	-279.531	1.8923
6.280.000	1.331.922	-279.677	1.8924
6.290.000	1.333.338	-279.977	1.8923
6.300.000	1.335.321	-280.313	1.8924
6.310.000	1.336.791	-280.484	1.8924
6.320.000	1.338.645	-280.802	1.8923
6.330.000	1.340.174	-281.052	1.8923
6.340.000	1.342.036	-281.342	1.8924
6.350.000	1.343.600	-281.564	1.8926
6.360.000	1.345.333	-281.904	1.8927
6.370.000	1.347.080	-282.048	1.8926
6.380.000	1.348.630	-282.388	1.8925
6.390.000	1.350.502	-282.613	1.8925
6.400.000	1.351.999	-282.870	1.8925
6.410.000	1.353.876	-283.150	1.8925
6.420.000	1.355.403	-283.429	1.8923
6.430.000	1.357.332	-283.699	1.8923
6.440.000	1.358.737	-283.905	1.8923
6.450.000	1.360.647	-284.271	1.8922
6.460.000	1.362.276	-284.463	1.8921
6.470.000	1.363.978	-284.739	1.8920
6.480.000	1.365.700	-284.989	1.8918
6.490.000	1.367.357	-285.293	1.8917
6.500.000	1.369.143	-285.451	1.8916
6.510.000	1.370.657	-285.803	1.8915
6.520.000	1.372.615	-286.070	1.8915
6.530.000	1.374.048	-286.288	1.8914
6.540.000	1.375.962	-286.625	1.8915

6.550.000	1.377.517	-286.873	1.8914
6.560.000	1.379.355	-287.143	1.8913
6.570.000	1.380.914	-287.382	1.8911
6.580.000	1.382.749	-287.724	1.8910
6.590.000	1.384.367	-287.875	1.8908
6.600.000	1.386.024	-288.229	1.8908
6.610.000	1.387.842	-288.437	1.8908
6.620.000	1.389.375	-288.695	1.8908
6.630.000	1.391.269	-288.969	1.8908
6.640.000	1.392.789	-289.276	1.8905
6.650.000	1.394.679	-289.511	1.8902
6.660.000	1.396.177	-289.778	1.8898
6.670.000	1.398.053	-290.121	1.8894
6.680.000	1.399.592	-290.277	1.8892
6.690.000	1.401.459	-290.634	1.8890
6.700.000	1.403.113	-290.877	1.8889
6.710.000	1.404.791	-291.121	1.8887
6.720.000	1.406.472	-291.334	1.8889
6.730.000	1.408.120	-291.705	1.8889
6.740.000	1.409.967	-291.847	1.8890
6.750.000	1.411.385	-292.154	1.8893
6.760.000	1.413.327	-292.490	1.8896
6.770.000	1.414.789	-292.693	1.8898
6.780.000	1.416.661	-292.996	1.8900
6.790.000	1.418.219	-293.268	1.8900
6.800.000	1.420.049	-293.531	1.8897
6.810.000	1.421.581	-293.753	1.8895
6.820.000	1.423.344	-294.092	1.8894
6.830.000	1.425.043	-294.253	1.8894
6.840.000	1.426.641	-294.565	1.8895

6.850.000	1.428.480	-294.817	1.8897
6.860.000	1.429.989	-295.065	1.8896
6.870.000	1.431.825	-295.336	1.8894
6.880.000	1.433.351	-295.630	1.8889
6.890.000	1.435.246	-295.913	1.8886
6.900.000	1.436.668	-296.113	1.8882
6.910.000	1.438.602	-296.461	1.8878
6.920.000	1.440.188	-296.660	1.8877
6.930.000	1.441.909	-296.935	1.8877
6.940.000	1.443.580	-297.174	1.8876
6.950.000	1.445.276	-297.493	1.8873
6.960.000	1.446.982	-297.641	1.8871
6.970.000	1.448.587	-298.010	1.8870
6.980.000	1.450.512	-298.247	1.8867
6.990.000	1.451.891	-298.462	1.8865
7.000.000	1.453.818	-298.792	1.8866
7.010.000	1.455.375	-299.045	1.8868
7.020.000	1.457.236	-299.287	1.8869
7.030.000	1.458.713	-299.530	1.8867
7.040.000	1.460.603	-299.880	1.8866
7.050.000	1.462.154	-300.035	1.8865
7.060.000	1.463.893	-300.366	1.8864
7.070.000	1.465.649	-300.595	1.8864
7.080.000	1.467.175	-300.847	1.8865
7.090.000	1.469.049	-301.103	1.8865
7.100.000	1.470.603	-301.447	1.8862
7.110.000	1.472.464	-301.679	1.8861
7.120.000	1.473.932	-301.956	1.8860
7.130.000	1.475.911	-302.295	1.8858
7.140.000	1.477.374	-302.466	1.8857

7.150.000	1.479.196	-302.818	1.8855
7.160.000	1.480.831	-303.093	1.8853
7.170.000	1.482.578	-303.356	1.8851
7.180.000	1.484.242	-303.584	1.8847
7.190.000	1.485.927	-303.970	1.8844
7.200.000	1.487.739	-304.129	1.8842
7.210.000	1.489.219	-304.452	1.8842
7.220.000	1.491.159	-304.782	1.8842
7.230.000	1.492.662	-305.022	1.8844
7.240.000	1.494.532	-305.335	1.8846
7.250.000	1.496.067	-305.621	1.8846
7.260.000	1.497.946	-305.916	1.8842
7.270.000	1.499.448	-306.143	1.8839
7.280.000	1.501.293	-306.512	1.8838
7.290.000	1.502.943	-306.712	1.8837
7.300.000	1.504.586	-307.023	1.8836
7.310.000	1.506.401	-307.283	1.8834
7.320.000	1.507.959	-307.592	1.8834
7.330.000	1.509.741	-307.826	1.8832
7.340.000	1.511.231	-308.167	1.8829
7.350.000	1.513.192	-308.488	1.8827
7.360.000	1.514.585	-308.699	1.8826
7.370.000	1.516.512	-309.079	1.8826
7.380.000	1.518.036	-309.303	1.8827
7.390.000	1.519.812	-309.629	1.8826
7.400.000	1.521.406	-309.887	1.8825
7.410.000	1.523.180	-310.232	1.8824
7.420.000	1.524.815	-310.400	1.8822
7.430.000	1.526.443	-310.796	1.8820
7.440.000	1.528.334	-311.060	1.8818

7.450.000	1.529.737	-311.314	1.8817
7.460.000	1.531.636	-311.661	1.8817
7.470.000	1.533.168	-311.979	1.8816
7.480.000	1.534.999	-312.222	1.8814
7.490.000	1.536.470	-312.506	1.8813
7.500.000	1.538.359	-312.896	1.8814
7.510.000	1.539.897	-313.073	1.8814
7.520.000	1.541.659	-313.439	1.8815
7.530.000	1.543.356	-313.730	1.8815
7.540.000	1.544.970	-314.012	1.8814
7.550.000	1.546.774	-314.270	1.8812
7.560.000	1.548.329	-314.675	1.8810
7.570.000	1.550.182	-314.904	1.8808
7.580.000	1.551.641	-315.246	1.8805
7.590.000	1.553.590	-315.619	1.8802
7.600.000	1.555.045	-315.828	1.8799
7.610.000	1.556.886	-316.189	1.8795
7.620.000	1.558.438	-316.507	1.8791
7.630.000	1.560.271	-316.814	1.8788
7.640.000	1.561.892	-317.092	1.8785
7.650.000	1.563.590	-317.480	1.8783
7.660.000	1.565.370	-317.687	1.8779
7.670.000	1.566.843	-318.069	1.8778
7.680.000	1.568.760	-318.373	1.8778
7.690.000	1.570.238	-318.678	1.8778
7.700.000	1.572.126	-319.006	1.8776
7.710.000	1.573.658	-319.348	1.8774
7.720.000	1.575.540	-319.662	1.8773
7.730.000	1.576.982	-319.922	1.8772
7.740.000	1.578.869	-320.336	1.8771

7.750.000	1.580.494	-320.586	1.8769
7.760.000	1.582.144	-320.922	1.8767
7.770.000	1.583.921	-321.200	1.8764
7.780.000	1.585.510	-321.561	1.8763
7.790.000	1.587.303	-321.789	1.8763
7.800.000	1.588.861	-322.194	1.8763
7.810.000	1.590.796	-322.514	1.8764
7.820.000	1.592.226	-322.768	1.8763
7.830.000	1.594.152	-323.150	1.8763
7.840.000	1.595.680	-323.423	1.8764
7.850.000	1.597.483	-323.729	1.8765
7.860.000	1.599.056	-324.033	1.8764
7.870.000	1.600.887	-324.404	1.8761
7.880.000	1.602.528	-324.594	1.8757
7.890.000	1.604.153	-324.975	1.8756
7.900.000	1.606.024	-325.237	1.8755
7.910.000	1.607.471	-325.525	1.8755
7.920.000	1.609.372	-325.858	1.8752
7.930.000	1.610.890	-326.184	1.8751
7.940.000	1.612.679	-326.457	1.8749
7.950.000	1.614.173	-326.748	1.8746
7.960.000	1.616.108	-327.125	1.8743
7.970.000	1.617.593	-327.294	1.8740
7.980.000	1.619.373	-327.669	1.8739
7.990.000	1.621.026	-327.940	1.8737
8.000.000	1.622.649	-328.218	1.8735
8.010.000	1.624.405	-328.469	1.8734
8.020.000	1.625.967	-328.857	1.8734
8.030.000	1.627.871	-329.060	1.8734
8.040.000	1.629.317	-329.386	1.8733

8.050.000	1.631.243	-329.730	1.8732
8.060.000	1.632.712	-329.942	1.8732
8.070.000	1.634.576	-330.278	1.8731
8.080.000	1.636.097	-330.565	1.8730
8.090.000	1.637.954	-330.881	1.8729
8.100.000	1.639.510	-331.109	1.8727
8.110.000	1.641.219	-331.462	1.8723
8.120.000	1.642.983	-331.663	1.8721
8.130.000	1.644.509	-332.016	1.8721
8.140.000	1.646.368	-332.280	1.8722
8.150.000	1.647.920	-332.573	1.8722
8.160.000	1.649.765	-332.868	1.8721
8.170.000	1.651.264	-333.170	1.8722
8.180.000	1.653.171	-333.469	1.8722
8.190.000	1.654.603	-333.690	1.8720
8.200.000	1.656.483	-334.055	1.8719
8.210.000	1.658.080	-334.281	1.8717
8.220.000	1.659.752	-334.587	1.8715
8.230.000	1.661.474	-334.834	1.8711
8.240.000	1.663.141	-335.175	1.8708
8.250.000	1.664.886	-335.371	1.8706
8.260.000	1.666.482	-335.725	1.8704
8.270.000	1.668.392	-336.010	1.8701
8.280.000	1.669.810	-336.247	1.8700
8.290.000	1.671.712	-336.607	1.8700
8.300.000	1.673.260	-336.863	1.8698
8.310.000	1.675.055	-337.130	1.8697
8.320.000	1.676.598	-337.398	1.8694
8.330.000	1.678.468	-337.756	1.8690
8.340.000	1.680.055	-337.937	1.8687

8.350.000	1.681.735	-338.304	1.8683
8.360.000	1.683.551	-338.547	1.8681
8.370.000	1.685.111	-338.849	1.8679
8.380.000	1.686.962	-339.108	1.8676
8.390.000	1.688.505	-339.437	1.8674
8.400.000	1.690.357	-339.681	1.8673
8.410.000	1.691.859	-339.971	1.8672
8.420.000	1.693.787	-340.316	1.8668
8.430.000	1.695.269	-340.493	1.8665
8.440.000	1.697.116	-340.858	1.8665
8.450.000	1.698.762	-341.130	1.8664
8.460.000	1.700.453	-341.382	1.8661
8.470.000	1.702.157	-341.636	1.8659
8.480.000	1.703.817	-342.026	1.8656
8.490.000	1.705.634	-342.169	1.8655
8.500.000	1.707.099	-342.501	1.8654
8.510.000	1.709.059	-342.834	1.8652
8.520.000	1.710.502	-343.061	1.8653
8.530.000	1.712.349	-343.360	1.8652
8.540.000	1.713.853	-343.626	1.8652
8.550.000	1.715.704	-343.922	1.8651
8.560.000	1.717.214	-344.160	1.8648
8.570.000	1.719.026	-344.500	1.8645
8.580.000	1.720.687	-344.679	1.8642
8.590.000	1.722.310	-345.012	1.8639
8.600.000	1.724.141	-345.243	1.8636
8.610.000	1.725.680	-345.532	1.8632
8.620.000	1.727.512	-345.774	1.8631
8.630.000	1.729.050	-346.096	1.8630
8.640.000	1.730.951	-346.392	1.8629

8.650.000	1.732.366	-346.591	1.8627
8.660.000	1.734.281	-346.956	1.8628
8.670.000	1.735.847	-347.179	1.8629
8.680.000	1.737.595	-347.453	1.8629
8.690.000	1.739.222	-347.695	1.8626
8.700.000	1.740.981	-348.026	1.8623
8.710.000	1.742.683	-348.184	1.8621
8.720.000	1.744.278	-348.551	1.8618
8.730.000	1.746.158	-348.763	1.8615
8.740.000	1.747.616	-349.014	1.8615
8.750.000	1.749.530	-349.332	1.8614
8.760.000	1.751.016	-349.575	1.8612
8.770.000	1.752.882	-349.823	1.8610
8.780.000	1.754.385	-350.074	1.8606
8.790.000	1.756.254	-350.395	1.8601
8.800.000	1.757.768	-350.531	1.8598
8.810.000	1.759.549	-350.863	1.8595
8.820.000	1.761.286	-351.080	1.8592
8.830.000	1.762.899	-351.335	1.8590
8.840.000	1.764.697	-351.516	1.8588
8.850.000	1.766.264	-351.855	1.8586
8.860.000	1.768.129	-352.032	1.8583
8.870.000	1.769.597	-352.295	1.8581
8.880.000	1.771.535	-352.588	1.8579
8.890.000	1.772.988	-352.734	1.8578
8.900.000	1.774.841	-353.040	1.8578
8.910.000	1.776.434	-353.278	1.8576
8.920.000	1.778.223	-353.492	1.8572
8.930.000	1.779.868	-353.694	1.8568
8.940.000	1.781.585	-354.025	1.8564

8.950.000	1.783.373	-354.115	1.8562
8.960.000	1.784.888	-354.415	1.8560
8.970.000	1.786.816	-354.653	1.8558
8.980.000	1.788.310	-354.864	1.8559
8.990.000	1.790.170	-355.123	1.8560
9.000.000	1.791.691	-355.357	1.8559
9.010.000	1.793.586	-355.593	1.8556
9.020.000	1.795.089	-355.778	1.8551
9.030.000	1.796.938	-356.091	1.8547
9.040.000	1.798.593	-356.246	1.8543
9.050.000	1.800.278	-356.506	1.8541
9.060.000	1.802.049	-356.701	1.8541
9.070.000	1.803.698	-356.966	1.8540
9.080.000	1.805.452	-357.111	1.8538
9.090.000	1.806.968	-357.409	1.8535
9.100.000	1.808.891	-357.639	1.8532
9.110.000	1.810.292	-357.809	1.8530
9.120.000	1.812.168	-358.089	1.8526
9.130.000	1.813.696	-358.279	1.8526
9.140.000	1.815.499	-358.525	1.8527
9.150.000	1.817.126	-358.735	1.8529
9.160.000	1.818.896	-359.022	1.8528
9.170.000	1.820.515	-359.146	1.8527
9.180.000	1.822.165	-359.463	1.8524
9.190.000	1.824.026	-359.626	1.8521
9.200.000	1.825.488	-359.854	1.8517
9.210.000	1.827.381	-360.121	1.8515
9.220.000	1.828.910	-360.362	1.8514
9.230.000	1.830.727	-360.579	1.8514
9.240.000	1.832.184	-360.784	1.8514

9.250.000	1.834.132	-361.102	1.8512
9.260.000	1.835.614	-361.229	1.8508
9.270.000	1.837.403	-361.542	1.8505
9.280.000	1.839.079	-361.753	1.8499
9.290.000	1.840.738	-361.980	1.8494
9.300.000	1.842.502	-362.182	1.8491
9.310.000	1.844.132	-362.533	1.8488
9.320.000	1.845.941	-362.667	1.8488
9.330.000	1.847.422	-362.967	1.8487
9.340.000	1.849.337	-363.283	1.8485
9.350.000	1.850.774	-363.431	1.8483
9.360.000	1.852.655	-363.735	1.8479
9.370.000	1.854.206	-363.972	1.8473
9.380.000	1.856.041	-364.238	1.8470
9.390.000	1.857.610	-364.451	1.8467
9.400.000	1.859.391	-364.799	1.8465
9.410.000	1.861.102	-364.926	1.8462
9.420.000	1.862.687	-365.264	1.8462
9.430.000	1.864.539	-365.468	1.8460
9.440.000	1.866.051	-365.733	1.8456
9.450.000	1.867.883	-365.986	1.8451
9.460.000	1.869.354	-366.264	1.8446
9.470.000	1.871.283	-366.528	1.8442
9.480.000	1.872.709	-366.757	1.8440
9.490.000	1.874.661	-367.110	1.8436
9.500.000	1.876.212	-367.297	1.8434
9.510.000	1.877.947	-367.598	1.8431
9.520.000	1.879.688	-367.843	1.8428
9.530.000	1.881.351	-368.136	1.8424
9.540.000	1.883.077	-368.304	1.8420

9.550.000	1.884.657	-368.663	1.8418
9.560.000	1.886.554	-368.894	1.8418
9.570.000	1.887.970	-369.154	1.8418
9.580.000	1.889.929	-369.474	1.8419
9.590.000	1.891.420	-369.713	1.8420
9.600.000	1.893.252	-369.984	1.8420
9.610.000	1.894.838	-370.265	1.8416
9.620.000	1.896.726	-370.589	1.8411
9.630.000	1.898.275	-370.762	1.8405
9.640.000	1.899.997	-371.119	1.8402
9.650.000	1.901.766	-371.343	1.8397
9.660.000	1.903.306	-371.613	1.8392
9.670.000	1.905.121	-371.855	1.8387
9.680.000	1.906.681	-372.184	1.8382
9.690.000	1.908.517	-372.421	1.8377
9.700.000	1.909.985	-372.683	1.8371
9.710.000	1.911.902	-373.019	1.8366
9.720.000	1.913.365	-373.199	1.8363
9.730.000	1.915.231	-373.556	1.8361
9.740.000	1.916.822	-373.805	1.8360
9.750.000	1.918.508	-374.056	1.8360
9.760.000	1.920.212	-374.305	1.8357
9.770.000	1.921.871	-374.680	1.8353
9.780.000	1.923.615	-374.810	1.8347
9.790.000	1.925.137	-375.156	1.8342
9.800.000	1.927.052	-375.458	1.8336
9.810.000	1.928.480	-375.658	1.8330
9.820.000	1.930.346	-375.980	1.8323
9.830.000	1.931.889	-376.266	1.8318
9.840.000	1.933.779	-376.521	1.8314

9.850.000	1.935.253	-376.766	1.8310
9.860.000	1.937.059	-377.111	1.8307
9.870.000	1.938.720	-377.267	1.8306
9.880.000	1.940.336	-377.589	1.8304
9.890.000	1.942.131	-377.829	1.8302
9.900.000	1.943.703	-378.109	1.8301
9.910.000	1.945.483	-378.343	1.8299
9.920.000	1.947.002	-378.670	1.8293
9.930.000	1.948.926	-378.958	1.8289
9.940.000	1.950.376	-379.200	1.8285
9.950.000	1.952.283	-379.536	1.8282
9.960.000	1.953.817	-379.742	1.8280
9.970.000	1.955.594	-380.051	1.8275
9.980.000	1.957.201	-380.305	1.8272
9.990.000	1.958.902	-380.615	1.8268
10.000.000	1.960.585	-380.790	1.8264
10.010.000	1.962.218	-381.152	1.8259
10.020.000	1.964.057	-381.349	1.8250
10.030.000	1.965.511	-381.639	1.8247
10.040.000	1.967.408	-381.956	1.8243
10.050.000	1.968.922	-382.223	1.8237
10.060.000	1.970.811	-382.481	1.8230
10.070.000	1.972.340	-382.765	1.8225
10.080.000	1.974.237	-383.064	1.8224
10.090.000	1.975.704	-383.228	1.8219
10.100.000	1.977.480	-383.583	1.8214
10.110.000	1.979.181	-383.815	1.8211
10.120.000	1.980.804	-384.078	1.8207
10.130.000	1.982.546	-384.289	1.8204
10.140.000	1.984.159	-384.635	1.8201

10.150.000	1.985.979	-384.838	1.8199
10.160.000	1.987.447	-385.144	1.8197
10.170.000	1.989.456	-385.463	1.8194
10.180.000	1.990.893	-385.649	1.8192
10.190.000	1.992.778	-385.990	1.8187
10.200.000	1.994.330	-386.247	1.8181
10.210.000	1.996.095	-386.495	1.8177
10.220.000	1.997.723	-386.741	1.8171
10.230.000	1.999.463	-387.084	1.8168
10.240.000	2.001.140	-387.212	1.8164
10.250.000	2.002.744	-387.559	1.8159
10.260.000	2.004.595	-387.813	1.8154
10.270.000	2.006.052	-388.033	1.8149
10.280.000	2.007.942	-388.357	1.8144
10.290.000	2.009.482	-388.626	1.8140
10.300.000	2.011.334	-388.865	1.8134
10.310.000	2.012.807	-389.117	1.8128
10.320.000	2.014.676	-389.449	1.8123
10.330.000	2.016.256	-389.617	1.8119
10.340.000	2.017.944	-389.920	1.8113
10.350.000	2.019.668	-390.165	1.8103
10.360.000	2.021.311	-390.433	1.8096
10.370.000	2.023.012	-390.624	1.8090
10.380.000	2.024.590	-390.978	1.8085
10.390.000	2.026.502	-391.220	1.8080
10.400.000	2.027.954	-391.468	1.8075
10.410.000	2.029.870	-391.781	1.8070
10.420.000	2.031.386	-391.980	1.8067
10.430.000	2.033.190	-392.269	1.8062
10.440.000	2.034.727	-392.501	1.8057

10.450.000	2.036.520	-392.779	1.8053
10.460.000	2.038.107	-392.961	1.8050
10.470.000	2.039.824	-393.296	1.8045
10.480.000	2.041.572	-393.452	1.8040
10.490.000	2.043.065	-393.731	1.8035
10.500.000	2.044.984	-393.983	1.8030
10.510.000	2.046.532	-394.235	1.8026
10.520.000	2.048.323	-394.469	1.8021
10.530.000	2.049.798	-394.721	1.8016
10.540.000	2.051.738	-395.011	1.8012
10.550.000	2.053.175	-395.175	1.8008
10.560.000	2.055.015	-395.494	1.8003
10.570.000	2.056.660	-395.700	1.7996
10.580.000	2.058.323	-395.943	1.7991
10.590.000	2.060.005	-396.131	1.7984
10.600.000	2.061.654	-396.465	1.7977
10.610.000	2.063.452	-396.602	1.7972
10.620.000	2.065.043	-396.918	1.7967
10.630.000	2.066.957	-397.220	1.7965
10.640.000	2.068.355	-397.373	1.7961
10.650.000	2.070.286	-397.680	1.7958
10.660.000	2.071.807	-397.943	1.7955
10.670.000	2.073.620	-398.147	1.7951
10.680.000	2.075.144	-398.370	1.7947
10.690.000	2.076.959	-398.690	1.7943
10.700.000	2.078.584	-398.814	1.7937
10.710.000	2.080.252	-399.128	1.7931
10.720.000	2.082.038	-399.337	1.7925
10.730.000	2.083.643	-399.590	1.7918
10.740.000	2.085.467	-399.826	1.7912

10.750.000	2.086.964	-400.096	1.7905
10.760.000	2.088.893	-400.304	1.7898
10.770.000	2.090.279	-400.532	1.7893
10.780.000	2.092.243	-400.840	1.7888
10.790.000	2.093.758	-400.974	1.7883
10.800.000	2.095.514	-401.274	1.7878
10.810.000	2.097.203	-401.484	1.7874
10.820.000	2.098.890	-401.733	1.7871
10.830.000	2.100.583	-401.894	1.7865
10.840.000	2.102.241	-402.247	1.7860
10.850.000	2.104.119	-402.404	1.7855
10.860.000	2.105.563	-402.658	1.7850
10.870.000	2.107.469	-402.953	1.7845
10.880.000	2.108.903	-403.134	1.7841
10.890.000	2.110.762	-403.410	1.7836
10.900.000	2.112.301	-403.648	1.7832
10.910.000	2.114.130	-403.891	1.7829
10.920.000	2.115.644	-404.097	1.7825
10.930.000	2.117.419	-404.401	1.7820
10.940.000	2.119.113	-404.548	1.7814
10.950.000	2.120.685	-404.830	1.7809
10.960.000	2.122.517	-405.055	1.7804
10.970.000	2.124.127	-405.324	1.7800
10.980.000	2.125.913	-405.524	1.7794
10.990.000	2.127.413	-405.791	1.7790
11.000.000	2.129.330	-406.057	1.7786
11.010.000	2.130.731	-406.213	1.7780
11.020.000	2.132.596	-406.520	1.7774
11.030.000	2.134.173	-406.729	1.7769
11.040.000	2.135.867	-406.942	1.7764

11.050.000	2.137.548	-407.157	1.7758
11.060.000	2.139.329	-407.478	1.7755
11.070.000	2.140.991	-407.559	1.7752
11.080.000	2.142.585	-407.888	1.7748
11.090.000	2.144.462	-408.106	1.7741
11.100.000	2.145.895	-408.300	1.7736
11.110.000	2.147.795	-408.577	1.7730
11.120.000	2.149.276	-408.805	1.7724
11.130.000	2.151.105	-408.993	1.7720
11.140.000	2.152.627	-409.207	1.7714
11.150.000	2.154.458	-409.511	1.7710
11.160.000	2.156.051	-409.629	1.7706
11.170.000	2.157.792	-409.931	1.7701
11.180.000	2.159.520	-410.118	1.7694
11.190.000	2.161.107	-410.348	1.7687
11.200.000	2.162.883	-410.531	1.7681
11.210.000	2.164.474	-410.812	1.7675
11.220.000	2.166.379	-411.001	1.7671
11.230.000	2.167.754	-411.236	1.7668
11.240.000	2.169.721	-411.511	1.7664
11.250.000	2.171.193	-411.682	1.7660
11.260.000	2.172.995	-411.939	1.7655
11.270.000	2.174.612	-412.136	1.7649
11.280.000	2.176.344	-412.373	1.7645
11.290.000	2.178.029	-412.534	1.7639
11.300.000	2.179.744	-412.867	1.7635
11.310.000	2.181.484	-412.992	1.7632
11.320.000	2.183.042	-413.268	1.7630
11.330.000	2.184.939	-413.520	1.7628
11.340.000	2.186.383	-413.704	1.7623

11.350.000	2.188.271	-413.960	1.7616
11.360.000	2.189.732	-414.190	1.7608
11.370.000	2.191.622	-414.417	1.7602
11.380.000	2.193.116	-414.585	1.7596
11.390.000	2.194.935	-414.893	1.7588
11.400.000	2.196.636	-415.057	1.7579
11.410.000	2.198.303	-415.318	1.7572
11.420.000	2.200.037	-415.491	1.7565
11.430.000	2.201.702	-415.760	1.7558
11.440.000	2.203.445	-415.908	1.7552
11.450.000	2.204.930	-416.160	1.7547
11.460.000	2.206.904	-416.421	1.7545
11.470.000	2.208.242	-416.571	1.7542
11.480.000	2.210.160	-416.854	1.7539
11.490.000	2.211.693	-417.038	1.7536
11.500.000	2.213.472	-417.249	1.7531
11.510.000	2.215.061	-417.450	1.7525
11.520.000	2.216.874	-417.770	1.7519
11.530.000	2.218.481	-417.840	1.7515
11.540.000	2.220.130	-418.159	1.7512
11.550.000	2.221.924	-418.323	1.7510
11.560.000	2.223.441	-418.526	1.7507
11.570.000	2.225.311	-418.767	1.7504
11.580.000	2.226.763	-419.006	1.7498
11.590.000	2.228.649	-419.179	1.7492
11.600.000	2.230.123	-419.413	1.7486
11.610.000	2.232.000	-419.689	1.7478
11.620.000	2.233.522	-419.797	1.7473
11.630.000	2.235.315	-420.118	1.7470
11.640.000	2.236.979	-420.286	1.7467

11.650.000	2.238.658	-420.510	1.7464
11.660.000	2.240.319	-420.679	1.7460
11.670.000	2.241.980	-421.005	1.7455
11.680.000	2.243.786	-421.125	1.7448
11.690.000	2.245.218	-421.367	1.7438
11.700.000	2.247.170	-421.648	1.7428
11.710.000	2.248.630	-421.803	1.7419
11.720.000	2.250.423	-422.055	1.7413
11.730.000	2.252.036	-422.277	1.7408
11.740.000	2.253.852	-422.508	1.7403
11.750.000	2.255.440	-422.682	1.7400
11.760.000	2.257.137	-422.997	1.7396
11.770.000	2.258.863	-423.102	1.7390
11.780.000	2.260.471	-423.387	1.7384
11.790.000	2.262.242	-423.583	1.7377
11.800.000	2.263.809	-423.808	1.7372
11.810.000	2.265.660	-424.031	1.7367
11.820.000	2.267.111	-424.283	1.7363
11.830.000	2.269.040	-424.514	1.7358
11.840.000	2.270.500	-424.689	1.7352
11.850.000	2.272.379	-425.004	1.7346
11.860.000	2.273.980	-425.147	1.7340
11.870.000	2.275.698	-425.445	1.7334
11.880.000	2.277.366	-425.629	1.7327
11.890.000	2.279.092	-425.905	1.7322
11.900.000	2.280.766	-426.019	1.7320
11.910.000	2.282.369	-426.338	1.7319
11.920.000	2.284.263	-426.528	1.7318
11.930.000	2.285.687	-426.740	1.7315
11.940.000	2.287.632	-427.021	1.7311

11.950.000	2.289.152	-427.241	1.7306
11.960.000	2.290.960	-427.450	1.7301
11.970.000	2.292.521	-427.662	1.7294
11.980.000	2.294.358	-427.944	1.7289
11.990.000	2.295.928	-428.071	1.7283
12.000.000	2.297.664	-428.359	1.7279
12.010.000	2.299.373	-428.539	1.7277
12.020.000	2.300.951	-428.759	1.7273
12.030.000	2.302.743	-428.951	1.7268
12.040.000	2.304.309	-429.231	1.7262
12.050.000	2.306.165	-429.398	1.7254
12.060.000	2.307.606	-429.630	1.7246
12.070.000	2.309.494	-429.890	1.7236
12.080.000	2.310.985	-430.040	1.7229
12.090.000	2.312.810	-430.327	1.7226
12.100.000	2.314.432	-430.517	1.7221
12.110.000	2.316.157	-430.733	1.7217
12.120.000	2.317.747	-430.904	1.7212
12.130.000	2.319.476	-431.206	1.7207
12.140.000	2.321.210	-431.308	1.7201
12.150.000	2.322.714	-431.576	1.7193
12.160.000	2.324.610	-431.817	1.7186
12.170.000	2.326.057	-431.983	1.7181
12.180.000	2.327.915	-432.236	1.7176
12.190.000	2.329.452	-432.463	1.7171
12.200.000	2.331.304	-432.668	1.7166
12.210.000	2.332.775	-432.856	1.7161
12.220.000	2.334.597	-433.168	1.7154
12.230.000	2.336.207	-433.243	1.7149
12.240.000	2.337.851	-433.523	1.7144

12.250.000	2.339.600	-433.707	1.7139
12.260.000	2.341.227	-433.950	1.7134
12.270.000	2.342.995	-434.111	1.7130
12.280.000	2.344.502	-434.396	1.7124
12.290.000	2.346.418	-434.601	1.7117
12.300.000	2.347.815	-434.799	1.7110
12.310.000	2.349.754	-435.071	1.7104
12.320.000	2.351.304	-435.241	1.7099
12.330.000	2.353.025	-435.476	1.7095
12.340.000	2.354.655	-435.676	1.7090
12.350.000	2.356.404	-435.934	1.7085
12.360.000	2.358.000	-436.068	1.7080
12.370.000	2.359.646	-436.367	1.7076
12.380.000	2.361.457	-436.523	1.7070
12.390.000	2.362.942	-436.736	1.7066
12.400.000	2.364.820	-436.978	1.7061
12.410.000	2.366.309	-437.209	1.7057
12.420.000	2.368.179	-437.401	1.7051
12.430.000	2.369.679	-437.618	1.7046
12.440.000	2.371.550	-437.892	1.7041
12.450.000	2.373.095	-438.021	1.7038
12.460.000	2.374.868	-438.316	1.7033
12.470.000	2.376.492	-438.483	1.7031
12.480.000	2.378.118	-438.696	1.7028
12.490.000	2.379.853	-438.874	1.7025
12.500.000	2.381.506	-439.183	1.7019
12.510.000	2.383.324	-439.330	1.7014
12.520.000	2.384.804	-439.576	1.7008
12.530.000	2.386.734	-439.857	1.7001
12.540.000	2.388.197	-440.007	1.6995

12.550.000	2.390.068	-440.292	1.6991
12.560.000	2.391.633	-440.514	1.6986
12.570.000	2.393.408	-440.740	1.6980
12.580.000	2.394.973	-440.922	1.6975
12.590.000	2.396.729	-441.220	1.6970
12.600.000	2.398.404	-441.338	1.6965
12.610.000	2.400.015	-441.639	1.6961
12.620.000	2.401.877	-441.827	1.6958
12.630.000	2.403.341	-442.025	1.6954
12.640.000	2.405.201	-442.281	1.6949
12.650.000	2.406.725	-442.531	1.6945
12.660.000	2.408.568	-442.720	1.6942
12.670.000	2.410.032	-442.929	1.6939
12.680.000	2.411.874	-443.224	1.6935
12.690.000	2.413.446	-443.347	1.6931
12.700.000	2.415.134	-443.621	1.6928
12.710.000	2.416.808	-443.810	1.6924
12.720.000	2.418.494	-444.055	1.6919
12.730.000	2.420.203	-444.200	1.6914
12.740.000	2.421.740	-444.496	1.6909
12.750.000	2.423.637	-444.663	1.6904
12.760.000	2.425.054	-444.896	1.6898
12.770.000	2.426.991	-445.174	1.6894
12.780.000	2.428.476	-445.339	1.6890
12.790.000	2.430.265	-445.591	1.6885
12.800.000	2.431.835	-445.804	1.6882
12.810.000	2.433.653	-446.055	1.6878
12.820.000	2.435.196	-446.204	1.6875
12.830.000	2.436.935	-446.511	1.6873
12.840.000	2.438.678	-446.666	1.6868

12.850.000	2.440.251	-446.912	1.6862
12.860.000	2.442.094	-447.119	1.6858
12.870.000	2.443.599	-447.380	1.6852
12.880.000	2.445.441	-447.574	1.6846
12.890.000	2.446.897	-447.811	1.6840
12.900.000	2.448.801	-448.085	1.6834
12.910.000	2.450.261	-448.231	1.6829
12.920.000	2.452.124	-448.540	1.6824
12.930.000	2.453.718	-448.725	1.6819
12.940.000	2.455.455	-448.942	1.6814
12.950.000	2.457.090	-449.136	1.6811
12.960.000	2.458.812	-449.442	1.6806
12.970.000	2.460.523	-449.540	1.6803
12.980.000	2.462.056	-449.837	1.6800
12.990.000	2.463.947	-450.072	1.6797
13.000.000	2.465.359	-450.232	1.6794
13.010.000	2.467.273	-450.506	1.6788
13.020.000	2.468.828	-450.733	1.6785
13.030.000	2.470.589	-450.911	1.6782
13.040.000	2.472.116	-451.103	1.6780
13.050.000	2.473.977	-451.413	1.6775
13.060.000	2.475.549	-451.501	1.6771
13.070.000	2.477.232	-451.792	1.6767
13.080.000	2.478.962	-451.980	1.6762
13.090.000	2.480.542	-452.198	1.6758
13.100.000	2.482.296	-452.383	1.6755
13.110.000	2.483.809	-452.643	1.6750
13.120.000	2.485.703	-452.810	1.6746
13.130.000	2.487.122	-453.016	1.6742
13.140.000	2.489.027	-453.274	1.6738

13.150.000	2.490.537	-453.405	1.6734
13.160.000	2.492.363	-453.674	1.6729
13.170.000	2.493.945	-453.833	1.6723
13.180.000	2.495.710	-454.051	1.6720
13.190.000	2.497.322	-454.212	1.6715
13.200.000	2.499.010	-454.513	1.6712
13.210.000	2.500.830	-454.627	1.6708
13.220.000	2.502.274	-454.884	1.6705
13.230.000	2.504.217	-455.140	1.6703
13.240.000	2.505.647	-455.318	1.6702
13.250.000	2.507.425	-455.538	1.6698
13.260.000	2.508.948	-455.756	1.6696
13.270.000	2.510.812	-455.994	1.6694
13.280.000	2.512.262	-456.123	1.6690
13.290.000	2.514.098	-456.410	1.6684
13.300.000	2.515.731	-456.546	1.6678
13.310.000	2.517.373	-456.773	1.6672
13.320.000	2.519.092	-456.934	1.6666
13.330.000	2.520.667	-457.172	1.6662
13.340.000	2.522.491	-457.311	1.6660
13.350.000	2.523.958	-457.584	1.6658
13.360.000	2.525.864	-457.793	1.6655
13.370.000	2.527.285	-457.937	1.6651
13.380.000	2.529.137	-458.196	1.6647
13.390.000	2.530.645	-458.368	1.6646
13.400.000	2.532.417	-458.574	1.6644
13.410.000	2.533.987	-458.746	1.6641
13.420.000	2.535.741	-459.023	1.6640
13.430.000	2.537.366	-459.130	1.6637
13.440.000	2.539.003	-459.386	1.6633

13.450.000	2.540.807	-459.549	1.6628
13.460.000	2.542.235	-459.735	1.6623
13.470.000	2.544.156	-459.968	1.6618
13.480.000	2.545.626	-460.194	1.6614
13.490.000	2.547.417	-460.353	1.6611
13.500.000	2.548.945	-460.571	1.6609
13.510.000	2.550.812	-460.835	1.6606
13.520.000	2.552.313	-460.929	1.6601
13.530.000	2.554.030	-461.198	1.6596
13.540.000	2.555.701	-461.370	1.6591
13.550.000	2.557.380	-461.579	1.6586
13.560.000	2.559.058	-461.726	1.6583
13.570.000	2.560.612	-461.994	1.6581
13.580.000	2.562.436	-462.129	1.6580
13.590.000	2.563.857	-462.334	1.6578
13.600.000	2.565.783	-462.581	1.6574
13.610.000	2.567.247	-462.705	1.6572
13.620.000	2.569.049	-462.946	1.6568
13.630.000	2.570.610	-463.116	1.6565
13.640.000	2.572.408	-463.346	1.6562
13.650.000	2.573.973	-463.497	1.6559
13.660.000	2.575.755	-463.795	1.6559
13.670.000	2.577.440	-463.909	1.6557
13.680.000	2.578.997	-464.190	1.6553
13.690.000	2.580.827	-464.376	1.6547
13.700.000	2.582.278	-464.610	1.6541
13.710.000	2.584.139	-464.833	1.6537
13.720.000	2.585.634	-465.064	1.6531
13.730.000	2.587.518	-465.289	1.6528
13.740.000	2.588.963	-465.463	1.6526

13.750.000	2.590.819	-465.749	1.6525
13.760.000	2.592.402	-465.902	1.6524
13.770.000	2.594.112	-466.161	1.6522
13.780.000	2.595.829	-466.361	1.6519
13.790.000	2.597.493	-466.615	1.6514
13.800.000	2.599.193	-466.753	1.6508
13.810.000	2.600.762	-467.054	1.6503
13.820.000	2.602.683	-467.272	1.6500
13.830.000	2.604.076	-467.458	1.6498
13.840.000	2.605.937	-467.742	1.6497
13.850.000	2.607.458	-467.960	1.6498
13.860.000	2.609.245	-468.181	1.6499
13.870.000	2.610.759	-468.390	1.6499
13.880.000	2.612.606	-468.685	1.6498
13.890.000	2.614.170	-468.827	1.6496
13.900.000	2.615.896	-469.120	1.6493
13.910.000	2.617.648	-469.326	1.6488
13.920.000	2.619.176	-469.572	1.6484
13.930.000	2.621.034	-469.781	1.6479
13.940.000	2.622.543	-470.075	1.6474
13.950.000	2.624.352	-470.266	1.6470
13.960.000	2.625.797	-470.535	1.6467
13.970.000	2.627.679	-470.820	1.6464
13.980.000	2.629.176	-470.956	1.6463
13.990.000	2.630.979	-471.260	1.6461
14.000.000	2.632.593	-471.473	1.6456
14.010.000	2.634.326	-471.706	1.6450
14.020.000	2.635.943	-471.884	1.6443
14.030.000	2.637.620	-472.211	1.6436
14.040.000	2.639.394	-472.317	1.6432

14.050.000	2.640.893	-472.630	1.6427
14.060.000	2.642.804	-472.870	1.6426
14.070.000	2.644.237	-473.055	1.6426
14.080.000	2.646.081	-473.336	1.6424
14.090.000	2.647.574	-473.564	1.6421
14.100.000	2.649.446	-473.786	1.6415
14.110.000	2.650.961	-473.979	1.6408
14.120.000	2.652.782	-474.299	1.6404
14.130.000	2.654.381	-474.437	1.6399
14.140.000	2.656.041	-474.704	1.6397
14.150.000	2.657.787	-474.907	1.6395
14.160.000	2.659.375	-475.155	1.6394
14.170.000	2.661.173	-475.343	1.6393
14.180.000	2.662.698	-475.627	1.6387
14.190.000	2.664.570	-475.843	1.6383
14.200.000	2.665.982	-476.055	1.6380
14.210.000	2.667.886	-476.356	1.6378
14.220.000	2.669.404	-476.527	1.6377
14.230.000	2.671.239	-476.797	1.6376
14.240.000	2.672.791	-477.010	1.6377
14.250.000	2.674.585	-477.271	1.6376
14.260.000	2.676.201	-477.424	1.6373
14.270.000	2.677.844	-477.751	1.6366
14.280.000	2.679.667	-477.906	1.6360
14.290.000	2.681.158	-478.156	1.6355
14.300.000	2.683.018	-478.425	1.6353
14.310.000	2.684.525	-478.665	1.6350
14.320.000	2.686.353	-478.883	1.6350
14.330.000	2.687.857	-479.110	1.6352
14.340.000	2.689.775	-479.394	1.6352

14.350.000	2.691.240	-479.540	1.6353
14.360.000	2.693.067	-479.860	1.6351
14.370.000	2.694.745	-480.037	1.6346
14.380.000	2.696.351	-480.267	1.6339
14.390.000	2.698.100	-480.470	1.6334
14.400.000	2.699.735	-480.785	1.6329
14.410.000	2.701.510	-480.901	1.6324
14.420.000	2.703.008	-481.187	1.6318
14.430.000	2.704.916	-481.451	1.6316
14.440.000	2.706.356	-481.614	1.6314
14.450.000	2.708.221	-481.897	1.6312
14.460.000	2.709.750	-482.101	1.6308
14.470.000	2.711.565	-482.320	1.6304
14.480.000	2.713.115	-482.530	1.6302
14.490.000	2.714.897	-482.837	1.6299
14.500.000	2.716.537	-482.954	1.6297
14.510.000	2.718.147	-483.272	1.6297
14.520.000	2.719.970	-483.462	1.6294
14.530.000	2.721.467	-483.701	1.6292
14.540.000	2.723.314	-483.946	1.6287
14.550.000	2.724.814	-484.229	1.6280
14.560.000	2.726.687	-484.430	1.6273
14.570.000	2.728.146	-484.637	1.6268
14.580.000	2.730.021	-484.966	1.6263
14.590.000	2.731.548	-485.099	1.6259
14.600.000	2.733.299	-485.387	1.6256
14.610.000	2.734.959	-485.611	1.6254
14.620.000	2.736.607	-485.837	1.6251
14.630.000	2.738.313	-486.012	1.6247
14.640.000	2.739.897	-486.321	1.6242

14.650.000	2.741.749	-486.486	1.6236
14.660.000	2.743.185	-486.731	1.6233
14.670.000	2.745.125	-487.026	1.6231
14.680.000	2.746.590	-487.176	1.6231
14.690.000	2.748.404	-487.431	1.6229
14.700.000	2.749.976	-487.666	1.6227
14.710.000	2.751.822	-487.908	1.6224
14.720.000	2.753.315	-488.064	1.6219
14.730.000	2.755.022	-488.354	1.6218
14.740.000	2.756.766	-488.527	1.6216
14.750.000	2.758.313	-488.765	1.6214
14.760.000	2.760.101	-488.936	1.6213
14.770.000	2.761.636	-489.194	1.6212
14.780.000	2.763.448	-489.387	1.6209
14.790.000	2.764.950	-489.626	1.6205
14.800.000	2.766.853	-489.854	1.6200
14.810.000	2.768.294	-490.010	1.6198
14.820.000	2.770.186	-490.295	1.6194
14.830.000	2.771.762	-490.488	1.6191
14.840.000	2.773.441	-490.696	1.6188
14.850.000	2.775.104	-490.893	1.6186
14.860.000	2.776.790	-491.187	1.6182
14.870.000	2.778.517	-491.282	1.6179
14.880.000	2.780.068	-491.591	1.6176
14.890.000	2.781.944	-491.824	1.6172
14.900.000	2.783.387	-492.000	1.6170
14.910.000	2.785.260	-492.269	1.6169
14.920.000	2.786.740	-492.487	1.6165
14.930.000	2.788.605	-492.695	1.6162
14.940.000	2.790.103	-492.904	1.6157

14.950.000	2.791.957	-493.204	1.6153
14.960.000	2.793.551	-493.325	1.6147
14.970.000	2.795.197	-493.598	1.6142
14.980.000	2.796.939	-493.788	1.6137
14.990.000	2.798.550	-494.055	1.6133
15.000.000	2.800.315	-494.216	1.6129
15.010.000	2.801.854	-494.497	1.6124
15.020.000	2.803.704	-494.681	1.6122
15.030.000	2.805.181	-494.901	1.6121
15.040.000	2.807.102	-495.199	1.6119
15.050.000	2.808.551	-495.333	1.6119
15.060.000	2.810.349	-495.601	1.6118
15.070.000	2.811.961	-495.823	1.6116
15.080.000	2.813.636	-496.023	1.6112
15.090.000	2.815.282	-496.198	1.6107
15.100.000	2.816.943	-496.505	1.6103
15.110.000	2.818.716	-496.609	1.6100
15.120.000	2.820.197	-496.880	1.6095
15.130.000	2.822.086	-497.123	1.6092
15.140.000	2.823.579	-497.302	1.6090
15.150.000	2.825.382	-497.527	1.6086
15.160.000	2.826.890	-497.755	1.6082
15.170.000	2.828.755	-497.974	1.6080
15.180.000	2.830.198	-498.149	1.6077
15.190.000	2.831.992	-498.423	1.6073
15.200.000	2.833.648	-498.583	1.6070
15.210.000	2.835.270	-498.820	1.6068
15.220.000	2.837.009	-499.002	1.6063
15.230.000	2.838.599	-499.273	1.6058
15.240.000	2.840.373	-499.414	1.6054

15.250.000	2.841.917	-499.693	1.6051
15.260.000	2.843.783	-499.925	1.6047
15.270.000	2.845.186	-500.081	1.6042
15.280.000	2.847.074	-500.370	1.6038
15.290.000	2.848.569	-500.557	1.6034
15.300.000	2.850.349	-500.765	1.6030
15.310.000	2.851.935	-500.959	1.6026
15.320.000	2.853.671	-501.238	1.6024
15.330.000	2.855.294	-501.351	1.6022
15.340.000	2.856.923	-501.652	1.6019
15.350.000	2.858.754	-501.810	1.6015
15.360.000	2.860.230	-502.027	1.6014
15.370.000	2.862.088	-502.272	1.6011
15.380.000	2.863.602	-502.505	1.6009
15.390.000	2.865.437	-502.705	1.6006
15.400.000	2.866.893	-502.919	1.6004
15.410.000	2.868.785	-503.199	1.6002
15.420.000	2.870.263	-503.335	1.5998
15.430.000	2.872.024	-503.613	1.5995
15.440.000	2.873.681	-503.803	1.5990
15.450.000	2.875.305	-504.031	1.5986
15.460.000	2.877.036	-504.204	1.5983
15.470.000	2.878.692	-504.530	1.5981
15.480.000	2.880.471	-504.660	1.5979
15.490.000	2.881.930	-504.910	1.5976
15.500.000	2.883.867	-505.190	1.5973
15.510.000	2.885.254	-505.335	1.5970
15.520.000	2.887.101	-505.594	1.5970
15.530.000	2.888.625	-505.830	1.5969
15.540.000	2.890.434	-506.022	1.5968

15.550.000	2.891.969	-506.209	1.5965
15.560.000	2.893.733	-506.514	1.5962
15.570.000	2.895.401	-506.637	1.5956
15.580.000	2.897.032	-506.909	1.5950
15.590.000	2.898.807	-507.090	1.5943
15.600.000	2.900.340	-507.298	1.5940
15.610.000	2.902.145	-507.545	1.5938
15.620.000	2.903.647	-507.770	1.5937
15.630.000	2.905.515	-507.970	1.5935
15.640.000	2.906.911	-508.158	1.5932
15.650.000	2.908.764	-508.431	1.5928
15.660.000	2.910.281	-508.569	1.5920
15.670.000	2.911.992	-508.825	1.5916
15.680.000	2.913.654	-509.030	1.5911
15.690.000	2.915.390	-509.290	1.5908
15.700.000	2.917.026	-509.419	1.5905
15.710.000	2.918.640	-509.725	1.5901
15.720.000	2.920.494	-509.900	1.5899
15.730.000	2.921.895	-510.117	1.5897
15.740.000	2.923.774	-510.398	1.5894
15.750.000	2.925.239	-510.587	1.5889
15.760.000	2.927.022	-510.813	1.5886
15.770.000	2.928.540	-511.039	1.5884
15.780.000	2.930.339	-511.317	1.5880
15.790.000	2.931.884	-511.433	1.5875
15.800.000	2.933.613	-511.752	1.5870
15.810.000	2.935.332	-511.922	1.5865
15.820.000	2.936.907	-512.144	1.5860
15.830.000	2.938.650	-512.339	1.5855
15.840.000	2.940.210	-512.602	1.5852

15.850.000	2.942.028	-512.759	1.5848
15.860.000	2.943.455	-513.023	1.5845
15.870.000	2.945.385	-513.295	1.5842
15.880.000	2.946.800	-513.448	1.5840
15.890.000	2.948.616	-513.705	1.5839
15.900.000	2.950.179	-513.913	1.5836
15.910.000	2.951.922	-514.133	1.5834
15.920.000	2.953.540	-514.301	1.5835
15.930.000	2.955.263	-514.615	1.5834
15.940.000	2.956.928	-514.711	1.5830
15.950.000	2.958.486	-515.009	1.5826
15.960.000	2.960.349	-515.251	1.5823
15.970.000	2.961.796	-515.428	1.5821
15.980.000	2.963.678	-515.702	1.5818
15.990.000	2.965.129	-515.943	1.5816
16.000.000	2.966.948	-516.133	1.5815
16.010.000	2.968.424	-516.345	1.5812
16.020.000	2.970.287	-516.655	1.5809
16.030.000	2.971.875	-516.787	1.5804
16.040.000	2.973.571	-517.086	1.5796
16.050.000	2.975.252	-517.275	1.5790
16.060.000	2.976.880	-517.514	1.5784
16.070.000	2.978.589	-517.696	1.5780
16.080.000	2.980.157	-517.993	1.5778
16.090.000	2.982.031	-518.176	1.5777
16.100.000	2.983.402	-518.411	1.5777
16.110.000	2.985.325	-518.712	1.5776
16.120.000	2.986.817	-518.897	1.5773
16.130.000	2.988.571	-519.154	1.5768
16.140.000	2.990.196	-519.379	1.5760

16.150.000	2.991.972	-519.654	1.5754
16.160.000	2.993.561	-519.796	1.5749
16.170.000	2.995.247	-520.114	1.5744
16.180.000	2.996.963	-520.262	1.5740
16.190.000	2.998.483	-520.511	1.5737
16.200.000	3.000.331	-520.741	1.5733
16.210.000	3.001.808	-520.953	1.5729
16.220.000	3.003.624	-521.194	1.5723
16.230.000	3.005.117	-521.432	1.5718
16.240.000	3.006.953	-521.661	1.5715
16.250.000	3.008.453	-521.815	1.5712
16.260.000	3.010.249	-522.109	1.5711
16.270.000	3.011.887	-522.296	1.5711
16.280.000	3.013.492	-522.523	1.5709
16.290.000	3.015.195	-522.709	1.5706
16.300.000	3.016.827	-523.006	1.5702
16.310.000	3.018.541	-523.117	1.5696
16.320.000	3.020.047	-523.400	1.5688
16.330.000	3.021.918	-523.661	1.5678
16.340.000	3.023.329	-523.817	1.5670
16.350.000	3.025.213	-524.094	1.5664
16.360.000	3.026.756	-524.330	1.5660
16.370.000	3.028.566	-524.551	1.5656
16.380.000	3.030.091	-524.763	1.5656
16.390.000	3.031.851	-525.059	1.5656
16.400.000	3.033.446	-525.178	1.5655
16.410.000	3.035.090	-525.485	1.5650
16.420.000	3.036.823	-525.664	1.5644
16.430.000	3.038.328	-525.892	1.5635
16.440.000	3.040.131	-526.099	1.5627

16.450.000	3.041.620	-526.352	1.5619
16.460.000	3.043.457	-526.544	1.5612
16.470.000	3.044.944	-526.762	1.5609
16.480.000	3.046.789	-527.025	1.5606
16.490.000	3.048.292	-527.138	1.5606
16.500.000	3.050.021	-527.414	1.5606
16.510.000	3.051.629	-527.585	1.5602
16.520.000	3.053.313	-527.798	1.5598
16.530.000	3.054.908	-527.943	1.5589
16.540.000	3.056.570	-528.244	1.5581
16.550.000	3.058.327	-528.330	1.5573
16.560.000	3.059.771	-528.569	1.5568
16.570.000	3.061.656	-528.815	1.5564
16.580.000	3.063.070	-528.983	1.5560
16.590.000	3.064.919	-529.200	1.5558
16.600.000	3.066.425	-529.404	1.5556
16.610.000	3.068.239	-529.621	1.5550
16.620.000	3.069.729	-529.761	1.5544
16.630.000	3.071.466	-530.033	1.5536
16.640.000	3.073.103	-530.154	1.5529
16.650.000	3.074.732	-530.391	1.5524
16.660.000	3.076.457	-530.565	1.5518
16.670.000	3.077.981	-530.788	1.5513
16.680.000	3.079.830	-530.970	1.5507
16.690.000	3.081.241	-531.205	1.5499
16.700.000	3.083.203	-531.437	1.5492
16.710.000	3.084.623	-531.602	1.5486
16.720.000	3.086.465	-531.881	1.5480
16.730.000	3.088.008	-532.056	1.5476
16.740.000	3.089.716	-532.303	1.5472

16.750.000	3.091.334	-532.479	1.5470
16.760.000	3.093.029	-532.755	1.5467
16.770.000	3.094.636	-532.882	1.5461
16.780.000	3.096.250	-533.182	1.5453
16.790.000	3.098.084	-533.381	1.5444
16.800.000	3.099.470	-533.576	1.5438
16.810.000	3.101.434	-533.859	1.5433
16.820.000	3.102.897	-534.090	1.5428
16.830.000	3.104.721	-534.287	1.5424
16.840.000	3.106.204	-534.518	1.5419
16.850.000	3.108.031	-534.818	1.5414
16.860.000	3.109.560	-534.941	1.5408
16.870.000	3.111.256	-535.250	1.5402
16.880.000	3.112.920	-535.445	1.5397
16.890.000	3.114.528	-535.681	1.5390
16.900.000	3.116.247	-535.871	1.5383
16.910.000	3.117.788	-536.165	1.5376
16.920.000	3.119.614	-536.336	1.5371
16.930.000	3.121.062	-536.616	1.5363
16.940.000	3.122.968	-536.881	1.5357
16.950.000	3.124.415	-537.050	1.5351
16.960.000	3.126.203	-537.327	1.5346
16.970.000	3.127.758	-537.539	1.5341
16.980.000	3.129.505	-537.777	1.5333
16.990.000	3.131.064	-537.973	1.5326
17.000.000	3.132.801	-538.295	1.5318
17.010.000	3.134.483	-538.434	1.5309
17.020.000	3.135.998	-538.709	1.5301
17.030.000	3.137.874	-538.948	1.5294
17.040.000	3.139.331	-539.165	1.5290

17.050.000	3.141.159	-539.420	1.5285
17.060.000	3.142.657	-539.676	1.5282
17.070.000	3.144.474	-539.879	1.5278
17.080.000	3.145.940	-540.074	1.5273
17.090.000	3.147.743	-540.400	1.5269
17.100.000	3.149.302	-540.539	1.5263
17.110.000	3.150.981	-540.797	1.5254
17.120.000	3.152.620	-540.999	1.5247
17.130.000	3.154.280	-541.247	1.5240
17.140.000	3.155.976	-541.380	1.5233
17.150.000	3.157.535	-541.682	1.5226
17.160.000	3.159.410	-541.864	1.5220
17.170.000	3.160.784	-542.076	1.5213
17.180.000	3.162.676	-542.347	1.5207
17.190.000	3.164.173	-542.534	1.5201
17.200.000	3.165.908	-542.757	1.5194
17.210.000	3.167.484	-542.959	1.5188
17.220.000	3.169.232	-543.228	1.5183
17.230.000	3.170.758	-543.355	1.5178
17.240.000	3.172.451	-543.654	1.5173
17.250.000	3.174.198	-543.825	1.5168
17.260.000	3.175.703	-544.059	1.5160
17.270.000	3.177.573	-544.253	1.5152
17.280.000	3.179.053	-544.531	1.5144
17.290.000	3.180.905	-544.724	1.5135
17.300.000	3.182.314	-544.959	1.5126
17.310.000	3.184.171	-545.225	1.5117
17.320.000	3.185.697	-545.377	1.5110
17.330.000	3.187.440	-545.652	1.5104
17.340.000	3.189.012	-545.870	1.5099

17.350.000	3.190.773	-546.118	1.5093
17.360.000	3.192.397	-546.311	1.5088
17.370.000	3.194.096	-546.629	1.5082
17.380.000	3.195.783	-546.740	1.5075
17.390.000	3.197.302	-547.047	1.5068
17.400.000	3.199.188	-547.295	1.5060
17.410.000	3.200.602	-547.477	1.5052
17.420.000	3.202.467	-547.771	1.5044
17.430.000	3.204.001	-548.032	1.5037
17.440.000	3.205.787	-548.254	1.5029
17.450.000	3.207.268	-548.464	1.5020
17.460.000	3.209.076	-548.787	1.5011
17.470.000	3.210.635	-548.908	1.5003
17.480.000	3.212.292	-549.229	1.4993
17.490.000	3.214.066	-549.454	1.4986
17.500.000	3.215.651	-549.703	1.4979
17.510.000	3.217.392	-549.896	1.4972
17.520.000	3.218.915	-550.211	1.4966
17.530.000	3.220.739	-550.422	1.4958
17.540.000	3.222.131	-550.646	1.4951
17.550.000	3.224.021	-550.966	1.4944
17.560.000	3.225.529	-551.151	1.4935
17.570.000	3.227.296	-551.429	1.4928
17.580.000	3.228.859	-551.653	1.4922
17.590.000	3.230.642	-551.923	1.4916
17.600.000	3.232.277	-552.097	1.4910
17.610.000	3.233.942	-552.436	1.4905
17.620.000	3.235.701	-552.586	1.4899
17.630.000	3.237.169	-552.872	1.4891
17.640.000	3.239.029	-553.138	1.4883

17.650.000	3.240.514	-553.389	1.4873
17.660.000	3.242.298	-553.640	1.4865
17.670.000	3.243.832	-553.885	1.4856
17.680.000	3.245.660	-554.157	1.4846
17.690.000	3.247.115	-554.328	1.4839
17.700.000	3.248.913	-554.651	1.4830
17.710.000	3.250.558	-554.876	1.4821
17.720.000	3.252.197	-555.091	1.4814
17.730.000	3.253.903	-555.293	1.4807
17.740.000	3.255.499	-555.601	1.4799
17.750.000	3.257.285	-555.739	1.4790
17.760.000	3.258.758	-556.023	1.4782
17.770.000	3.260.625	-556.285	1.4774
17.780.000	3.262.045	-556.450	1.4767
17.790.000	3.263.872	-556.739	1.4758
17.800.000	3.265.389	-556.950	1.4749
17.810.000	3.267.177	-557.167	1.4741
17.820.000	3.268.747	-557.377	1.4734
17.830.000	3.270.521	-557.678	1.4727
17.840.000	3.272.119	-557.788	1.4721
17.850.000	3.273.721	-558.108	1.4713
17.860.000	3.275.519	-558.292	1.4705
17.870.000	3.276.991	-558.500	1.4697
17.880.000	3.278.812	-558.748	1.4690
17.890.000	3.280.292	-559.009	1.4681
17.900.000	3.282.114	-559.192	1.4671
17.910.000	3.283.565	-559.407	1.4663
17.920.000	3.285.413	-559.717	1.4654
17.930.000	3.286.934	-559.840	1.4644
17.940.000	3.288.704	-560.112	1.4635

17.950.000	3.290.332	-560.313	1.4626
17.960.000	3.291.984	-560.530	1.4616
17.970.000	3.293.665	-560.703	1.4607
17.980.000	3.295.255	-561.014	1.4599
17.990.000	3.297.071	-561.152	1.4592
18.000.000	3.298.515	-561.395	1.4585
18.010.000	3.300.378	-561.675	1.4577
18.020.000	3.301.840	-561.839	1.4568
18.030.000	3.303.630	-562.090	1.4558
18.040.000	3.305.188	-562.311	1.4550
18.050.000	3.306.993	-562.550	1.4543
18.060.000	3.308.526	-562.718	1.4535
18.070.000	3.310.255	-563.015	1.4528
18.080.000	3.311.930	-563.158	1.4521
18.090.000	3.313.491	-563.423	1.4514
18.100.000	3.315.267	-563.626	1.4504
18.110.000	3.316.800	-563.875	1.4492
18.120.000	3.318.578	-564.092	1.4482
18.130.000	3.320.055	-564.345	1.4473
18.140.000	3.321.921	-564.590	1.4464
18.150.000	3.323.342	-564.741	1.4456
18.160.000	3.325.180	-565.050	1.4449
18.170.000	3.326.750	-565.233	1.4440
18.180.000	3.328.449	-565.467	1.4429
18.190.000	3.330.063	-565.662	1.4417
18.200.000	3.331.739	-565.930	1.4405
18.210.000	3.333.412	-566.052	1.4394
18.220.000	3.334.963	-566.345	1.4383
18.230.000	3.336.768	-566.555	1.4373
18.240.000	3.338.230	-566.746	1.4365

18.250.000	3.340.088	-567.014	1.4357
18.260.000	3.341.576	-567.221	1.4346
18.270.000	3.343.361	-567.419	1.4335
18.280.000	3.344.891	-567.651	1.4323
18.290.000	3.346.715	-567.935	1.4310
18.300.000	3.348.205	-568.037	1.4298
18.310.000	3.349.898	-568.343	1.4286
18.320.000	3.351.589	-568.538	1.4276
18.330.000	3.353.142	-568.764	1.4266
18.340.000	3.354.920	-568.939	1.4257
18.350.000	3.356.399	-569.229	1.4245
18.360.000	3.358.273	-569.416	1.4233
18.370.000	3.359.710	-569.646	1.4220
18.380.000	3.361.580	-569.917	1.4207
18.390.000	3.363.031	-570.058	1.4196
18.400.000	3.364.820	-570.334	1.4187
18.410.000	3.366.399	-570.533	1.4179
18.420.000	3.368.096	-570.747	1.4169
18.430.000	3.369.692	-570.943	1.4160
18.440.000	3.371.358	-571.242	1.4150
18.450.000	3.373.083	-571.358	1.4138
18.460.000	3.374.594	-571.625	1.4125
18.470.000	3.376.461	-571.863	1.4114
18.480.000	3.377.932	-572.062	1.4103
18.490.000	3.379.718	-572.325	1.4094
18.500.000	3.381.238	-572.565	1.4088
18.510.000	3.383.072	-572.783	1.4081
18.520.000	3.384.536	-572.987	1.4072
18.530.000	3.386.341	-573.292	1.4062
18.540.000	3.387.885	-573.445	1.4050

18.550.000	3.389.558	-573.730	1.4037
18.560.000	3.391.289	-573.929	1.4024
18.570.000	3.392.874	-574.211	1.4012
18.580.000	3.394.631	-574.377	1.4003
18.590.000	3.396.168	-574.676	1.3993
18.600.000	3.397.996	-574.914	1.3985
18.610.000	3.399.420	-575.123	1.3975
18.620.000	3.401.323	-575.428	1.3965
18.630.000	3.402.791	-575.636	1.3952
18.640.000	3.404.569	-575.882	1.3937
18.650.000	3.406.135	-576.130	1.3921
18.660.000	3.407.886	-576.403	1.3907
18.670.000	3.409.451	-576.558	1.3893
18.680.000	3.411.105	-576.899	1.3881
18.690.000	3.412.851	-577.074	1.3871
18.700.000	3.414.371	-577.334	1.3860
18.710.000	3.416.187	-577.579	1.3848
18.720.000	3.417.716	-577.876	1.3835
18.730.000	3.419.513	-578.086	1.3821
18.740.000	3.420.959	-578.346	1.3807
18.750.000	3.422.845	-578.642	1.3793
18.760.000	3.424.289	-578.795	1.3782
18.770.000	3.426.053	-579.100	1.3772
18.780.000	3.427.647	-579.313	1.3764
18.790.000	3.429.279	-579.534	1.3757
18.800.000	3.430.964	-579.722	1.3749
18.810.000	3.432.581	-580.044	1.3740
18.820.000	3.434.311	-580.159	1.3728
18.830.000	3.435.776	-580.433	1.3716
18.840.000	3.437.697	-580.732	1.3704

18.850.000	3.439.094	-580.882	1.3692
18.860.000	3.440.923	-581.164	1.3679
18.870.000	3.442.433	-581.392	1.3668
18.880.000	3.444.247	-581.610	1.3659
18.890.000	3.445.711	-581.793	1.3651
18.900.000	3.447.457	-582.106	1.3641
18.910.000	3.449.114	-582.239	1.3631
18.920.000	3.450.698	-582.538	1.3618
18.930.000	3.452.426	-582.729	1.3604
18.940.000	3.453.988	-582.951	1.3590
18.950.000	3.455.774	-583.174	1.3577
18.960.000	3.457.288	-583.441	1.3568
18.970.000	3.459.141	-583.661	1.3561
18.980.000	3.460.530	-583.852	1.3555
18.990.000	3.462.419	-584.166	1.3546
19.000.000	3.463.956	-584.344	1.3538
19.010.000	3.465.667	-584.608	1.3526
19.020.000	3.467.292	-584.821	1.3514
19.030.000	3.468.975	-585.102	1.3501
19.040.000	3.470.595	-585.251	1.3489
19.050.000	3.472.209	-585.572	1.3482
19.060.000	3.474.006	-585.753	1.3475
19.070.000	3.475.455	-586.002	1.3469
19.080.000	3.477.317	-586.287	1.3461
19.090.000	3.478.801	-586.501	1.3450
19.100.000	3.480.537	-586.719	1.3436
19.110.000	3.482.035	-586.968	1.3418
19.120.000	3.483.900	-587.247	1.3400
19.130.000	3.485.396	-587.380	1.3385
19.140.000	3.487.095	-587.691	1.3372

19.150.000	3.488.796	-587.870	1.3360
19.160.000	3.490.348	-588.109	1.3349
19.170.000	3.492.094	-588.304	1.3339
19.180.000	3.493.692	-588.600	1.3326
19.190.000	3.495.465	-588.774	1.3310
19.200.000	3.496.903	-589.015	1.3292
19.210.000	3.498.780	-589.281	1.3273
19.220.000	3.500.194	-589.437	1.3255
19.230.000	3.502.032	-589.704	1.3242
19.240.000	3.503.534	-589.909	1.3230
19.250.000	3.505.275	-590.134	1.3220
19.260.000	3.506.886	-590.320	1.3208
19.270.000	3.508.557	-590.633	1.3195
19.280.000	3.510.191	-590.726	1.3181
19.290.000	3.511.822	-591.048	1.3165
19.300.000	3.513.634	-591.257	1.3148
19.310.000	3.515.056	-591.461	1.3135
19.320.000	3.516.889	-591.728	1.3123
19.330.000	3.518.360	-591.970	1.3114
19.340.000	3.520.200	-592.172	1.3104
19.350.000	3.521.660	-592.380	1.3092
19.360.000	3.523.432	-592.681	1.3078
19.370.000	3.525.030	-592.831	1.3061
19.380.000	3.526.682	-593.131	1.3044
19.390.000	3.528.346	-593.310	1.3027
19.400.000	3.530.001	-593.566	1.3013
19.410.000	3.531.685	-593.727	1.2997
19.420.000	3.533.216	-594.035	1.2981
19.430.000	3.535.064	-594.213	1.2965
19.440.000	3.536.473	-594.445	1.2948

19.450.000	3.538.315	-594.722	1.2932
19.460.000	3.539.798	-594.916	1.2914
19.470.000	3.541.575	-595.146	1.2899
19.480.000	3.543.101	-595.370	1.2884
19.490.000	3.544.852	-595.612	1.2869
19.500.000	3.546.384	-595.767	1.2852
19.510.000	3.548.089	-596.060	1.2832
19.520.000	3.549.809	-596.200	1.2812
19.530.000	3.551.318	-596.455	1.2791
19.540.000	3.553.143	-596.654	1.2773
19.550.000	3.554.627	-596.887	1.2758
19.560.000	3.556.395	-597.092	1.2741
19.570.000	3.557.874	-597.324	1.2723
19.580.000	3.559.778	-597.576	1.2703
19.590.000	3.561.176	-597.721	1.2682
19.600.000	3.562.945	-598.003	1.2661
19.610.000	3.564.528	-598.178	1.2642
19.620.000	3.566.197	-598.405	1.2624
19.630.000	3.567.862	-598.599	1.2608
19.640.000	3.569.506	-598.881	1.2592
19.650.000	3.571.178	-598.984	1.2573
19.660.000	3.572.703	-599.260	1.2551
19.670.000	3.574.557	-599.500	1.2526
19.680.000	3.575.968	-599.678	1.2500
19.690.000	3.577.827	-599.936	1.2475
19.700.000	3.579.283	-600.152	1.2454
19.710.000	3.581.073	-600.335	1.2437
19.720.000	3.582.602	-600.555	1.2419
19.730.000	3.584.374	-600.851	1.2401
19.740.000	3.585.943	-600.962	1.2381

19.750.000	3.587.602	-601.254	1.2359
19.760.000	3.589.331	-601.456	1.2338
19.770.000	3.590.856	-601.680	1.2317
19.780.000	3.592.640	-601.902	1.2294
19.790.000	3.594.152	-602.183	1.2273
19.800.000	3.596.016	-602.381	1.2251
19.810.000	3.597.429	-602.604	1.2230
19.820.000	3.599.278	-602.878	1.2208
19.830.000	3.600.774	-603.037	1.2187
19.840.000	3.602.514	-603.328	1.2168
19.850.000	3.604.155	-603.545	1.2151
19.860.000	3.605.804	-603.778	1.2135
19.870.000	3.607.421	-603.952	1.2117
19.880.000	3.609.076	-604.300	1.2098
19.890.000	3.610.822	-604.427	1.2077
19.900.000	3.612.269	-604.698	1.2056
19.910.000	3.614.157	-604.985	1.2034
19.920.000	3.615.613	-605.169	1.2012
19.930.000	3.617.407	-605.420	1.1989
19.940.000	3.618.876	-605.647	1.1967
19.950.000	3.620.682	-605.869	1.1943
19.960.000	3.622.164	-606.063	1.1919
19.970.000	3.623.954	-606.351	1.1895
19.980.000	3.625.557	-606.496	1.1872
19.990.000	3.627.158	-606.764	1.1851
20.000.000	3.628.867	-606.963	1.1830
20.010.000	3.630.495	-607.226	1.1808
20.020.000	3.632.221	-607.376	1.1786
20.030.000	3.633.678	-607.660	1.1763
20.040.000	3.635.564	-607.889	1.1740

20.050.000	3.636.953	-608.067	1.1718
20.060.000	3.638.767	-608.333	1.1697
20.070.000	3.640.336	-608.521	1.1674
20.080.000	3.642.041	-608.735	1.1651
20.090.000	3.643.603	-608.911	1.1628
20.100.000	3.645.341	-609.185	1.1603
20.110.000	3.646.934	-609.289	1.1579
20.120.000	3.648.569	-609.578	1.1554
20.130.000	3.650.367	-609.753	1.1530
20.140.000	3.651.791	-609.930	1.1508
20.150.000	3.653.668	-610.185	1.1486
20.160.000	3.655.108	-610.432	1.1464
20.170.000	3.656.917	-610.591	1.1441
20.180.000	3.658.402	-610.805	1.1418
20.190.000	3.660.236	-611.081	1.1396
20.200.000	3.661.714	-611.183	1.1373
20.210.000	3.663.442	-611.477	1.1351
20.220.000	3.665.107	-611.668	1.1328
20.230.000	3.666.766	-611.879	1.1305
20.240.000	3.668.409	-612.090	1.1282
20.250.000	3.670.023	-612.370	1.1259
20.260.000	3.671.812	-612.499	1.1236
20.270.000	3.673.226	-612.752	1.1214
20.280.000	3.675.112	-613.014	1.1192
20.290.000	3.676.570	-613.154	1.1170
20.300.000	3.678.345	-613.400	1.1149
20.310.000	3.679.907	-613.603	1.1128
20.320.000	3.681.652	-613.840	1.1107
20.330.000	3.683.198	-613.983	1.1083
20.340.000	3.684.924	-614.264	1.1059

20.350.000	3.686.571	-614.370	1.1034
20.360.000	3.688.173	-614.621	1.1011
20.370.000	3.689.954	-614.799	1.0988
20.380.000	3.691.435	-615.009	1.0968
20.390.000	3.693.244	-615.222	1.0949
20.400.000	3.694.700	-615.443	1.0931
20.410.000	3.696.569	-615.651	1.0912
20.420.000	3.698.004	-615.821	1.0891
20.430.000	3.699.852	-616.108	1.0866
20.440.000	3.701.406	-616.232	1.0839
20.450.000	3.703.059	-616.462	1.0809
20.460.000	3.704.722	-616.656	1.0777
20.470.000	3.706.378	-616.884	1.0747
20.480.000	3.708.030	-616.984	1.0717
20.490.000	3.709.577	-617.265	1.0687
20.500.000	3.711.446	-617.440	1.0659
20.510.000	3.712.797	-617.619	1.0630
20.520.000	3.714.689	-617.871	1.0601
20.530.000	3.716.196	-618.054	1.0572
20.540.000	3.717.971	-618.237	1.0543
20.550.000	3.719.467	-618.442	1.0514
20.560.000	3.721.252	-618.686	1.0484
20.570.000	3.722.777	-618.792	1.0457
20.580.000	3.724.489	-619.056	1.0429
20.590.000	3.726.154	-619.228	1.0400
20.600.000	3.727.685	-619.416	1.0371
20.610.000	3.729.470	-619.582	1.0340
20.620.000	3.730.987	-619.847	1.0310
20.630.000	3.732.802	-619.988	1.0281
20.640.000	3.734.256	-620.201	1.0253

20.650.000	3.736.127	-620.439	1.0228
20.660.000	3.737.558	-620.538	1.0204
20.670.000	3.739.359	-620.823	1.0180
20.680.000	3.740.937	-620.987	1.0154
20.690.000	3.742.686	-621.194	1.0128
20.700.000	3.744.219	-621.334	1.0101
20.710.000	3.745.911	-621.614	1.1972
20.720.000	3.747.614	-621.688	1.1943
20.730.000	3.749.114	-621.952	1.2014
20.740.000	3.751.000	-622.144	9.988
20.750.000	3.752.448	-622.293	9.963
20.760.000	3.754.245	-622.519	9.939
20.770.000	3.755.759	-622.714	9.914
20.780.000	3.757.591	-622.916	9.887
20.790.000	3.759.040	-623.056	9.858
20.800.000	3.760.846	-623.313	9.829
20.810.000	3.762.400	-623.409	9.803
20.820.000	3.764.050	-623.649	9.777
20.830.000	3.765.758	-623.796	9.751
20.840.000	3.767.338	-624.005	9.726
20.850.000	3.769.052	-624.125	9.699
20.860.000	3.770.574	-624.391	9.671
20.870.000	3.772.462	-624.549	9.640
20.880.000	3.773.854	-624.704	9.609
20.890.000	3.775.702	-624.949	9.577
20.900.000	3.777.250	-625.077	9.549
20.910.000	3.778.963	-625.265	9.523
20.920.000	3.780.521	-625.435	9.499
20.930.000	3.782.293	-625.659	9.473
20.940.000	3.783.852	-625.767	9.446

20.950.000	3.785.502	-626.003	9.415
20.960.000	3.787.246	-626.135	9.382
20.970.000	3.788.725	-626.322	9.350
20.980.000	3.790.549	-626.507	9.318
20.990.000	3.792.033	-626.711	9.289
21.000.000	3.793.846	-626.862	9.261
21.010.000	3.795.312	-627.043	9.234
21.020.000	3.797.198	-627.274	9.207
21.030.000	3.798.632	-627.363	9.179
21.040.000	3.800.438	-627.606	9.147
21.050.000	3.802.025	-627.761	9.112
21.060.000	3.803.697	-627.896	9.077
21.070.000	3.805.302	-628.044	9.045
21.080.000	3.806.884	-628.299	9.016
21.090.000	3.808.659	-628.379	8.986
21.100.000	3.810.173	-628.610	8.956
21.110.000	3.812.035	-628.826	8.927
21.120.000	3.813.464	-628.933	8.895
21.130.000	3.815.286	-629.175	8.861
21.140.000	3.816.825	-629.342	8.827
21.150.000	3.818.582	-629.517	8.794
21.160.000	3.820.069	-629.654	8.763
21.170.000	3.821.846	-629.917	8.733
21.180.000	3.823.446	-629.996	8.704
21.190.000	3.825.025	-630.247	8.675
21.200.000	3.826.799	-630.395	8.643
21.210.000	3.828.353	-630.589	8.607
21.220.000	3.830.132	-630.749	8.568
21.230.000	3.831.611	-630.968	8.529
21.240.000	3.833.450	-631.137	8.492

21.250.000	3.834.884	-631.288	8.456
21.260.000	3.836.709	-631.530	8.423
21.270.000	3.838.225	-631.628	8.389
21.280.000	3.839.935	-631.851	8.356
21.290.000	3.841.550	-631.995	8.321
21.300.000	3.843.230	-632.182	8.285
21.310.000	3.844.872	-632.308	8.247
21.320.000	3.846.509	-632.555	8.209
21.330.000	3.848.304	-632.660	8.172
21.340.000	3.849.725	-632.848	8.138
21.350.000	3.851.617	-633.071	8.105
21.360.000	3.853.043	-633.212	8.071
21.370.000	3.854.836	-633.396	8.037
21.380.000	3.856.343	-633.569	8.002
21.390.000	3.858.181	-633.789	7.966
21.400.000	3.859.656	-633.885	7.930
21.410.000	3.861.405	-634.137	7.893
21.420.000	3.863.073	-634.270	7.858
21.430.000	3.864.651	-634.459	7.823
21.440.000	3.866.384	-634.596	7.787
21.450.000	3.867.913	-634.823	7.752
21.460.000	3.869.756	-634.939	7.716
21.470.000	3.871.151	-635.134	7.678
21.480.000	3.873.031	-635.349	7.639
21.490.000	3.874.450	-635.428	7.601
21.500.000	3.876.254	-635.659	7.564
21.510.000	3.877.795	-635.790	7.530
21.520.000	3.879.517	-635.932	7.495
21.530.000	3.881.105	-636.065	7.460
21.540.000	3.882.803	-636.304	7.425

21.550.000	3.884.465	-636.328	7.388
21.560.000	3.886.022	-636.581	7.351
21.570.000	3.887.871	-636.709	7.313
21.580.000	3.889.293	-636.838	7.278
21.590.000	3.891.111	-637.040	7.243
21.600.000	3.892.608	-637.212	7.207
21.610.000	3.894.429	-637.357	7.173
21.620.000	3.895.852	-637.497	7.137
21.630.000	3.897.703	-637.740	7.102
21.640.000	3.899.235	-637.828	7.064
21.650.000	3.900.909	-638.043	7.027
21.660.000	3.902.636	-638.193	6.989
21.670.000	3.904.196	-638.370	6.952
21.680.000	3.905.919	-638.453	6.915
21.690.000	3.907.433	-638.700	6.877
21.700.000	3.909.261	-638.824	6.841
21.710.000	3.910.703	-638.970	6.805
21.720.000	3.912.551	-639.189	6.768
21.730.000	3.914.006	-639.271	6.732
21.740.000	3.915.782	-639.456	6.697
21.750.000	3.917.267	-639.588	6.659
21.760.000	3.919.059	-639.776	6.620
21.770.000	3.920.605	-639.870	6.583
21.780.000	3.922.321	-640.102	6.547
21.790.000	3.924.051	-640.189	6.510
21.800.000	3.925.528	-640.388	6.474
21.810.000	3.927.327	-640.521	6.437
21.820.000	3.928.863	-640.712	6.400
21.830.000	3.930.613	-640.877	6.363
21.840.000	3.932.054	-641.022	6.324

21.850.000	3.933.914	-641.235	6.286
21.860.000	3.935.376	-641.320	6.247
21.870.000	3.937.153	-641.554	6.210
21.880.000	3.938.726	-641.678	6.174
21.890.000	3.940.378	-641.839	6.137
21.900.000	3.942.041	-641.964	6.100
21.910.000	3.943.693	-642.218	6.063
21.920.000	3.945.358	-642.239	6.024
21.930.000	3.946.869	-642.468	5.985
21.940.000	3.948.726	-642.648	5.948
21.950.000	3.950.118	-642.757	5.911
21.960.000	3.951.958	-642.964	5.874
21.970.000	3.953.447	-643.130	5.837
21.980.000	3.955.224	-643.257	5.799
21.990.000	3.956.741	-643.428	5.762
22.000.000	3.958.545	-643.645	5.723
22.010.000	3.960.127	-643.711	5.686
22.020.000	3.961.730	-643.934	5.649
22.030.000	3.963.480	-644.062	5.611
22.040.000	3.965.001	-644.218	5.574
22.050.000	3.966.758	-644.362	5.538
22.060.000	3.968.242	-644.562	5.501
22.070.000	3.970.053	-644.713	5.465
22.080.000	3.971.464	-644.855	5.427
22.090.000	3.973.338	-645.077	5.390
22.100.000	3.974.835	-645.173	5.352
22.110.000	3.976.550	-645.364	5.315
22.120.000	3.978.173	-645.525	5.278
22.130.000	3.979.864	-645.686	5.241
22.140.000	3.981.466	-645.793	5.203

22.150.000	3.983.104	-646.038	5.164
22.160.000	3.984.806	-646.112	5.126
22.170.000	3.986.294	-646.302	5.090
22.180.000	3.988.143	-646.512	5.055
22.190.000	3.989.617	-646.617	5.019
22.200.000	3.991.390	-646.793	4.983
22.210.000	3.992.884	-646.960	4.947
22.220.000	3.994.776	-647.144	4.911
22.230.000	3.996.202	-647.235	4.877
22.240.000	3.997.953	-647.464	4.843
22.250.000	3.999.597	-647.557	4.808
22.260.000	4.001.188	-647.742	4.774
22.270.000	4.002.923	-647.857	4.739
22.280.000	4.004.493	-648.066	4.704
22.290.000	4.006.235	-648.147	4.670
22.300.000	4.007.683	-648.353	4.636
22.310.000	4.009.577	-648.536	4.602
22.320.000	4.010.959	-648.633	4.566
22.330.000	4.012.809	-648.858	4.532
22.340.000	4.014.336	-649.012	4.500
22.350.000	4.016.057	-649.169	4.467
22.360.000	4.017.646	-649.329	4.433
22.370.000	4.019.350	-649.571	4.398
22.380.000	4.020.951	-649.642	4.363
22.390.000	4.022.530	-649.906	4.328
22.400.000	4.024.292	-650.051	4.292
22.410.000	4.025.774	-650.225	4.256
22.420.000	4.027.575	-650.457	4.220
22.430.000	4.029.052	-650.669	4.185
22.440.000	4.030.854	-650.823	4.150

22.450.000	4.032.382	-651.043	4.115
22.460.000	4.034.192	-651.264	4.082
22.470.000	4.035.706	-651.372	4.047
22.480.000	4.037.377	-651.610	4.012
22.490.000	4.039.058	-651.774	3.980
22.500.000	4.040.675	-651.945	3.950
22.510.000	4.042.332	-652.051	3.922
22.520.000	4.043.908	-652.300	3.894
22.530.000	4.045.675	-652.404	3.867
22.540.000	4.047.101	-652.564	3.841
22.550.000	4.048.998	-652.793	3.815
22.560.000	4.050.470	-652.887	3.790
22.570.000	4.052.298	-653.071	3.763
22.580.000	4.053.801	-653.220	3.735
22.590.000	4.055.556	-653.388	3.708
22.600.000	4.057.101	-653.486	3.681
22.610.000	4.058.766	-653.713	3.654
22.620.000	4.060.444	-653.771	3.626
22.630.000	4.062.007	-653.984	3.599
22.640.000	4.063.790	-654.113	3.571
22.650.000	4.065.277	-654.271	3.545
22.660.000	4.067.054	-654.432	3.519
22.670.000	4.068.562	-654.593	3.491
22.680.000	4.070.450	-654.762	3.463
22.690.000	4.071.842	-654.851	3.436
22.700.000	4.073.666	-655.062	3.410
22.710.000	4.075.237	-655.170	3.385
22.720.000	4.076.908	-655.332	3.359
22.730.000	4.078.536	-655.443	3.334
22.740.000	4.080.186	-655.673	3.306

22.750.000	4.081.882	-655.711	3.275
22.760.000	4.083.450	-655.947	3.241
22.770.000	4.085.251	-656.097	3.209
22.780.000	4.086.663	-656.214	3.180
22.790.000	4.088.542	-656.421	3.156
22.800.000	4.090.040	-656.589	3.134
22.810.000	4.091.802	-656.713	3.115
22.820.000	4.093.301	-656.868	3.093
22.830.000	4.095.083	-657.095	3.072
22.840.000	4.096.626	-657.135	3.053
22.850.000	4.098.302	-657.350	3.034
22.860.000	4.099.983	-657.472	3.014
22.870.000	4.101.534	-657.607	2.993
22.880.000	4.103.290	-657.739	2.972
22.890.000	4.104.846	-657.946	2.951
22.900.000	4.106.679	-658.047	2.929
22.910.000	4.108.082	-658.204	2.907
22.920.000	4.109.954	-658.386	2.884
22.930.000	4.111.385	-658.449	2.862
22.940.000	4.113.159	-658.652	2.844
22.950.000	4.114.728	-658.780	2.829
22.960.000	4.116.441	-658.936	2.810
22.970.000	4.117.994	-659.011	2.784
22.980.000	4.119.675	-659.251	2.754
22.990.000	4.121.382	-659.306	2.726
23.000.000	4.122.906	-659.490	2.702
23.010.000	4.124.754	-659.656	2.681
23.020.000	4.126.199	-659.766	2.664
23.030.000	4.128.004	-659.935	2.650
23.040.000	4.129.465	-660.092	2.635

23.050.000	4.131.267	-660.229	2.617
23.060.000	4.132.751	-660.334	2.598
23.070.000	4.134.504	-660.561	2.581
23.080.000	4.136.114	-660.644	2.568
23.090.000	4.137.704	-660.814	2.560
23.100.000	4.139.405	-660.919	2.560
23.110.000	4.141.011	-661.130	2.561
23.120.000	4.142.751	-661.210	2.558
23.130.000	4.144.239	-661.415	2.544
23.140.000	4.146.142	-661.576	2.522
23.150.000	4.147.489	-661.673	2.498
23.160.000	4.149.318	-661.867	2.476
23.170.000	4.150.856	-662.007	2.462
23.180.000	4.152.513	-662.135	2.462
23.190.000	4.154.097	-662.285	2.475
23.200.000	4.155.833	-662.503	2.491
23.210.000	4.157.407	-662.550	2.505
23.220.000	4.159.042	-662.799	2.507
23.230.000	4.160.839	-662.939	2.498
23.240.000	4.162.303	-663.075	2.485
23.250.000	4.164.122	-663.245	2.477
23.260.000	4.165.567	-663.427	2.473
23.270.000	4.167.377	-663.557	2.471
23.280.000	4.168.816	-663.710	2.464
23.290.000	4.170.645	-663.920	2.460
23.300.000	4.172.133	-664.003	2.449
23.310.000	4.173.826	-664.221	2.431
23.320.000	4.175.464	-664.359	2.410
23.330.000	4.177.127	-664.507	2.397
23.340.000	4.178.764	-664.646	2.394

23.350.000	4.180.424	-664.895	2.397
23.360.000	4.182.150	-664.967	2.406
23.370.000	4.183.598	-665.168	2.411
23.380.000	4.185.475	-665.372	2.401
23.390.000	4.186.907	-665.485	2.375
23.400.000	4.188.658	-665.680	2.339
23.410.000	4.190.206	-665.847	2.296
23.420.000	4.191.980	-666.026	2.256
23.430.000	4.193.495	-666.141	2.224
23.440.000	4.195.200	-666.397	2.207
23.450.000	4.196.856	-666.495	2.204
23.460.000	4.198.484	-666.731	2.205
23.470.000	4.200.230	-666.883	2.204
23.480.000	4.201.762	-667.072	2.195
23.490.000	4.203.501	-667.228	2.176
23.500.000	4.204.953	-667.455	2.153
23.510.000	4.206.801	-667.614	2.130
23.520.000	4.208.207	-667.748	2.110
23.530.000	4.210.023	-667.972	2.090
23.540.000	4.211.532	-668.098	2.070
23.550.000	4.213.236	-668.281	2.048
23.560.000	4.214.831	-668.409	2.025
23.570.000	4.216.551	-668.634	2.001
23.580.000	4.218.156	-668.708	1.977
23.590.000	4.219.706	-668.964	1.958
23.600.000	4.221.554	-669.095	1.949
23.610.000	4.222.946	-669.248	1.946
23.620.000	4.224.786	-669.461	1.943
23.630.000	4.226.254	-669.623	1.927
23.640.000	4.228.020	-669.772	1.902

23.650.000	4.229.484	-669.943	1.874
23.660.000	4.231.266	-670.188	1.847
23.670.000	4.232.808	-670.258	1.827
23.680.000	4.234.559	-670.519	1.819
23.690.000	4.236.214	-670.662	1.822
23.700.000	4.237.796	-670.835	1.836
23.710.000	4.239.521	-671.000	1.846
23.720.000	4.241.040	-671.259	1.849
23.730.000	4.242.838	-671.374	1.843
23.740.000	4.244.225	-671.574	1.824
23.750.000	4.246.031	-671.803	1.791
23.760.000	4.247.486	-671.910	1.757
23.770.000	4.249.272	-672.139	1.724
23.780.000	4.250.828	-672.315	1.697
23.790.000	4.252.558	-672.501	1.679
23.800.000	4.254.140	-672.654	1.670
23.810.000	4.255.854	-672.910	1.672
23.820.000	4.257.482	-672.976	1.675
23.830.000	4.258.961	-673.207	1.673
23.840.000	4.260.839	-673.387	1.663
23.850.000	4.262.252	-673.555	1.641
23.860.000	4.264.025	-673.731	1.613
23.870.000	4.265.481	-673.937	1.588
23.880.000	4.267.304	-674.116	1.571
23.890.000	4.268.770	-674.254	1.562
23.900.000	4.270.551	-674.517	1.559
23.910.000	4.272.126	-674.625	1.562
23.920.000	4.273.770	-674.836	1.565
23.930.000	4.275.392	-674.996	1.556
23.940.000	4.277.016	-675.208	1.543

23.950.000	4.278.690	-675.327	1.526
23.960.000	4.280.196	-675.564	1.512
23.970.000	4.282.030	-675.737	1.503
23.980.000	4.283.393	-675.898	1.495
23.990.000	4.285.277	-676.136	1.494
24.000.000	4.286.752	-676.309	1.490
24.010.000	4.288.476	-676.489	1.477
24.020.000	4.290.038	-676.655	1.457
24.030.000	4.291.769	-676.901	1.435
24.040.000	4.293.278	-677.005	1.416
24.050.000	4.294.954	-677.245	1.407
24.060.000	4.296.634	-677.398	1.404
24.070.000	4.298.162	-677.600	1.411
24.080.000	4.299.937	-677.772	1.418
24.090.000	4.301.415	-678.016	1.415
24.100.000	4.303.176	-678.161	1.410
24.110.000	4.304.636	-678.355	1.404
24.120.000	4.306.471	-678.603	1.398
24.130.000	4.307.932	-678.706	1.394
24.140.000	4.309.683	-678.970	1.392
24.150.000	4.311.268	-679.156	1.394
24.160.000	4.312.916	-679.352	1.392
24.170.000	4.314.545	-679.506	1.379
24.180.000	4.316.151	-679.798	1.362
24.190.000	4.317.897	-679.879	1.345
24.200.000	4.319.331	-680.162	1.330
24.210.000	4.321.200	-680.392	1.320
24.220.000	4.322.670	-680.556	1.316
24.230.000	4.324.429	-680.805	1.313
24.240.000	4.325.947	-681.012	1.305

24.250.000	4.327.724	-681.214	1.290
24.260.000	4.329.177	-681.372	1.276
24.270.000	4.330.973	-681.665	1.263
24.280.000	4.332.552	-681.800	1.252
24.290.000	4.334.147	-682.021	1.243
24.300.000	4.335.886	-682.210	1.236
24.310.000	4.337.388	-682.440	1.232
24.320.000	4.339.158	-682.598	1.227
24.330.000	4.340.645	-682.870	1.222
24.340.000	4.342.478	-683.091	1.216
24.350.000	4.343.881	-683.270	1.213
24.360.000	4.345.737	-683.567	1.213
24.370.000	4.347.221	-683.706	1.214
24.380.000	4.348.938	-683.949	1.208
24.390.000	4.350.504	-684.154	1.201
24.400.000	4.352.210	-684.408	1.196
24.410.000	4.353.794	-684.554	1.191
24.420.000	4.355.391	-684.830	1.184
24.430.000	4.357.161	-685.010	1.177
24.440.000	4.358.593	-685.220	1.169
24.450.000	4.360.438	-685.474	1.161
24.460.000	4.361.915	-685.694	1.152
24.470.000	4.363.689	-685.881	1.142
24.480.000	4.365.152	-686.086	1.136
24.490.000	4.366.993	-686.357	1.137
24.500.000	4.368.472	-686.488	1.143
24.510.000	4.370.172	-686.770	1.146
24.520.000	4.371.788	-686.951	1.144
24.530.000	4.373.380	-687.180	1.143
24.540.000	4.375.069	-687.354	1.140

24.550.000	4.376.636	-687.675	1.136
24.560.000	4.378.414	-687.810	1.133
24.570.000	4.379.831	-688.059	1.129
24.580.000	4.381.709	-688.349	1.124
24.590.000	4.383.147	-688.497	1.119
24.600.000	4.384.945	-688.778	1.116
24.610.000	4.386.461	-688.996	1.112
24.620.000	4.388.211	-689.222	1.108
24.630.000	4.389.755	-689.430	1.105
24.640.000	4.391.426	-689.731	1.103
24.650.000	4.393.091	-689.874	1.099
24.660.000	4.394.620	-690.170	1.091
24.670.000	4.396.426	-690.382	1.085
24.680.000	4.397.917	-690.624	1.077
24.690.000	4.399.672	-690.882	1.066
24.700.000	4.401.185	-691.142	1.056
24.710.000	4.403.008	-691.390	1.051
24.720.000	4.404.429	-691.573	1.050
24.730.000	4.406.211	-691.882	1.047
24.740.000	4.407.756	-692.051	1.039
24.750.000	4.409.402	-692.311	1.027
24.760.000	4.411.056	-692.538	1.018
24.770.000	4.412.658	-692.805	1.016
24.780.000	4.414.351	-692.979	1.012
24.790.000	4.415.841	-693.301	1.004
24.800.000	4.417.675	-693.518	990
24.810.000	4.419.069	-693.755	982
24.820.000	4.420.925	-694.079	980
24.830.000	4.422.365	-694.302	980
24.840.000	4.424.107	-694.566	977

24.850.000	4.425.642	-694.803	974
24.860.000	4.427.402	-695.136	968
24.870.000	4.428.922	-695.284	963
24.880.000	4.430.567	-695.640	957
24.890.000	4.432.303	-695.858	951
24.900.000	4.433.801	-696.129	946
24.910.000	4.435.527	-696.379	941
24.920.000	4.437.072	-696.699	934
24.930.000	4.438.899	-696.952	930
24.940.000	4.440.306	-697.218	925
24.950.000	4.442.137	-697.549	918
24.960.000	4.443.554	-697.753	910
24.970.000	4.445.337	-698.087	899
24.980.000	4.446.884	-698.358	890
24.990.000	4.448.539	-698.632	882
25.000.000	4.450.178	-698.857	876
25.010.000	4.451.771	-699.208	870
25.020.000	4.453.481	-699.377	865
25.030.000	4.454.969	-699.705	861
25.040.000	4.456.821	-699.997	857
25.050.000	4.458.238	-700.207	855
25.060.000	4.460.032	-700.513	852
25.070.000	4.461.507	-700.776	849
25.080.000	4.463.299	-701.026	844
25.090.000	4.464.716	-701.255	841
25.100.000	4.466.536	-701.599	838
25.110.000	4.468.121	-701.785	834
25.120.000	4.469.716	-702.094	829
25.130.000	4.471.416	-702.331	825
25.140.000	4.472.997	-702.635	821

25.150.000	4.474.724	-702.850	817
25.160.000	4.476.188	-703.165	815
25.170.000	4.478.068	-703.451	812
25.180.000	4.479.423	-703.664	809
25.190.000	4.481.256	-703.996	805
25.200.000	4.482.748	-704.216	802
25.210.000	4.484.468	-704.492	800
25.220.000	4.486.011	-704.732	795
25.230.000	4.487.738	-705.056	790
25.240.000	4.489.352	-705.231	787
25.250.000	4.490.908	-705.563	782
25.260.000	4.492.704	-705.775	779
25.270.000	4.494.148	-706.044	777
25.280.000	4.495.975	-706.343	777
25.290.000	4.497.456	-706.625	778
25.300.000	4.499.232	-706.878	777
25.310.000	4.500.677	-707.144	777
25.320.000	4.502.536	-707.468	778
25.330.000	4.504.012	-707.670	778
25.340.000	4.505.715	-707.989	776
25.350.000	4.507.340	-708.221	775
25.360.000	4.508.940	-708.497	774
25.370.000	4.510.654	-708.709	775
25.380.000	4.512.193	-709.056	773
25.390.000	4.513.982	-709.234	769
25.400.000	4.515.393	-709.511	765
25.410.000	4.517.244	-709.827	760
25.420.000	4.518.628	-709.989	756
25.430.000	4.520.439	-710.284	753
25.440.000	4.521.966	-710.539	752

25.450.000	4.523.708	-710.768	749
25.460.000	4.525.208	-710.975	747
25.470.000	4.526.909	-711.286	745
25.480.000	4.528.562	-711.430	744
25.490.000	4.530.075	-711.731	744
25.500.000	4.531.831	-711.939	745
25.510.000	4.533.332	-712.188	747
25.520.000	4.535.094	-712.430	746
25.530.000	4.536.533	-712.666	744
25.540.000	4.538.396	-712.911	742
25.550.000	4.539.776	-713.103	739
25.560.000	4.541.614	-713.404	737
25.570.000	4.543.161	-713.575	734
25.580.000	4.544.756	-713.824	732
25.590.000	4.546.397	-714.019	732
25.600.000	4.548.058	-714.308	730
25.610.000	4.549.686	-714.429	725
25.620.000	4.551.222	-714.736	721
25.630.000	4.553.021	-714.936	718
25.640.000	4.554.416	-715.145	714
25.650.000	4.556.267	-715.418	709
25.660.000	4.557.738	-715.643	704
25.670.000	4.559.517	-715.863	697
25.680.000	4.561.020	-716.087	691
25.690.000	4.562.752	-716.373	684
25.700.000	4.564.267	-716.490	677
25.710.000	4.565.917	-716.801	674
25.720.000	4.567.650	-716.990	672
25.730.000	4.569.170	-717.226	671
25.740.000	4.570.908	-717.433	669

25.750.000	4.572.420	-717.714	665
25.760.000	4.574.195	-717.913	662
25.770.000	4.575.613	-718.145	659
25.780.000	4.577.473	-718.426	654
25.790.000	4.578.941	-718.590	650
25.800.000	4.580.644	-718.858	647
25.810.000	4.582.217	-719.072	646
25.820.000	4.583.921	-719.310	644
25.830.000	4.585.528	-719.480	639
25.840.000	4.587.163	-719.788	636
25.850.000	4.588.849	-719.913	631
25.860.000	4.590.328	-720.176	627
25.870.000	4.592.144	-720.437	624
25.880.000	4.593.606	-720.603	621
25.890.000	4.595.369	-720.843	619
25.900.000	4.596.856	-721.076	618
25.910.000	4.598.681	-721.301	616
25.920.000	4.600.141	-721.467	611
25.930.000	4.601.892	-721.780	605
25.940.000	4.603.488	-721.931	601
25.950.000	4.605.057	-722.171	595
25.960.000	4.606.713	-722.361	591
25.970.000	4.608.323	-722.630	589
25.980.000	4.610.020	-722.776	588
25.990.000	4.611.490	-723.028	588
26.000.000	4.613.359	-723.292	587
26.010.000	4.614.694	-723.439	586
26.020.000	4.616.525	-723.707	584
26.030.000	4.618.034	-723.926	583
26.040.000	4.619.718	-724.153	581

26.050.000	4.621.326	-724.354	579
26.060.000	4.623.018	-724.660	578
26.070.000	4.624.594	-724.756	579
26.080.000	4.626.203	-725.076	581
26.090.000	4.627.947	-725.266	580
26.100.000	4.629.440	-725.489	578
26.110.000	4.631.251	-725.738	579
26.120.000	4.632.688	-725.991	576
26.130.000	4.634.493	-726.198	573
26.140.000	4.635.899	-726.414	575
26.150.000	4.637.736	-726.704	573
26.160.000	4.639.279	-726.862	576
26.170.000	4.640.987	-727.151	574
26.180.000	4.642.581	-727.349	575
26.190.000	4.644.201	-727.576	575
26.200.000	4.645.821	-727.742	573
26.210.000	4.647.404	-728.062	573
26.220.000	4.649.208	-728.209	572
26.230.000	4.650.628	-728.457	573
26.240.000	4.652.477	-728.735	573
26.250.000	4.653.896	-728.902	575
26.260.000	4.655.667	-729.158	576
26.270.000	4.657.172	-729.387	574
26.280.000	4.658.951	-729.630	574
26.290.000	4.660.470	-729.795	577
26.300.000	4.662.166	-730.088	584
26.310.000	4.663.833	-730.238	593
26.320.000	4.665.362	-730.507	603
26.330.000	4.667.150	-730.717	614
26.340.000	4.668.649	-730.951	624

26.350.000	4.670.414	-731.179	632
26.360.000	4.671.832	-731.417	638
26.370.000	4.673.724	-731.680	644
26.380.000	4.675.132	-731.848	652
26.390.000	4.676.928	-732.144	665
26.400.000	4.678.491	-732.353	681
26.410.000	4.680.142	-732.592	699
26.420.000	4.681.738	-732.790	717
26.430.000	4.683.415	-733.107	733
26.440.000	4.685.084	-733.232	747
26.450.000	4.686.571	-733.525	756
26.460.000	4.688.400	-733.792	764
26.470.000	4.689.803	-733.977	773
26.480.000	4.691.634	-734.257	784
26.490.000	4.693.075	-734.491	797
26.500.000	4.694.879	-734.713	808
26.510.000	4.696.383	-734.926	821
26.520.000	4.698.153	-735.227	831
26.530.000	4.699.688	-735.341	838
26.540.000	4.701.334	-735.634	846
26.550.000	4.703.017	-735.823	853
26.560.000	4.704.563	-736.061	862
26.570.000	4.706.317	-736.268	873
26.580.000	4.707.801	-736.536	883
26.590.000	4.709.620	-736.742	892
26.600.000	4.710.988	-736.961	899
26.610.000	4.712.858	-737.253	904
26.620.000	4.714.322	-737.406	909
26.630.000	4.716.006	-737.679	912
26.640.000	4.717.612	-737.901	917

26.650.000	4.719.313	-738.167	923
26.660.000	4.720.878	-738.319	930
26.670.000	4.722.510	-738.631	936
26.680.000	4.724.253	-738.795	939
26.690.000	4.725.681	-739.048	940
26.700.000	4.727.543	-739.307	939
26.710.000	4.728.934	-739.524	938
26.720.000	4.730.753	-739.765	936
26.730.000	4.732.258	-740.003	936
26.740.000	4.734.062	-740.254	933
26.750.000	4.735.503	-740.403	930
26.760.000	4.737.258	-740.730	924
26.770.000	4.738.904	-740.900	916
26.780.000	4.740.483	-741.137	908
26.790.000	4.742.159	-741.339	900
26.800.000	4.743.722	-741.631	893
26.810.000	4.745.467	-741.783	887
26.820.000	4.746.889	-742.058	882
26.830.000	4.748.812	-742.339	879
26.840.000	4.750.180	-742.507	874
26.850.000	4.751.986	-742.793	869
26.860.000	4.753.513	-743.023	863
26.870.000	4.755.214	-743.247	859
26.880.000	4.756.775	-743.453	856
26.890.000	4.758.471	-743.764	853
26.900.000	4.760.049	-743.878	850
26.910.000	4.761.610	-744.185	847
26.920.000	4.763.400	-744.399	845
26.930.000	4.764.810	-744.600	845
26.940.000	4.766.629	-744.862	845

26.950.000	4.768.094	-745.112	844
26.960.000	4.769.931	-745.316	844
26.970.000	4.771.341	-745.518	845
26.980.000	4.773.133	-745.814	844
26.990.000	4.774.656	-745.946	842
27.000.000	4.776.292	-746.210	841
27.010.000	4.777.921	-746.417	840
27.020.000	4.779.551	-746.668	841
27.030.000	4.781.214	-746.830	842
27.040.000	4.782.723	-747.109	842
27.050.000	4.784.562	-747.327	842
27.060.000	4.785.964	-747.532	840
27.070.000	4.787.822	-747.803	838
27.080.000	4.789.290	-747.985	835
27.090.000	4.791.034	-748.231	834
27.100.000	4.792.585	-748.447	834
27.110.000	4.794.290	-748.725	834
27.120.000	4.795.829	-748.848	835
27.130.000	4.797.469	-749.142	834
27.140.000	4.799.153	-749.324	833
27.150.000	4.800.664	-749.567	832
27.160.000	4.802.426	-749.763	832
27.170.000	4.803.926	-750.044	832
27.180.000	4.805.742	-750.236	831
27.190.000	4.807.151	-750.480	831
27.200.000	4.808.982	-750.741	831
27.210.000	4.810.463	-750.898	831
27.220.000	4.812.200	-751.202	829
27.230.000	4.813.757	-751.398	828
27.240.000	4.815.399	-751.631	828

27.250.000	4.817.014	-751.817	827
27.260.000	4.818.647	-752.143	826
27.270.000	4.820.344	-752.257	825
27.280.000	4.821.812	-752.541	824
27.290.000	4.823.695	-752.801	823
27.300.000	4.825.071	-752.967	823
27.310.000	4.826.868	-753.231	822
27.320.000	4.828.361	-753.455	822
27.330.000	4.830.116	-753.675	822
27.340.000	4.831.586	-753.859	821
27.350.000	4.833.334	-754.148	820
27.360.000	4.834.940	-754.292	816
27.370.000	4.836.502	-754.554	814
27.380.000	4.838.203	-754.762	814
27.390.000	4.839.740	-754.995	814
27.400.000	4.841.492	-755.188	813
27.410.000	4.842.926	-755.454	812
27.420.000	4.844.804	-755.667	810
27.430.000	4.846.167	-755.857	808
27.440.000	4.847.998	-756.131	806
27.450.000	4.849.485	-756.320	803
27.460.000	4.851.175	-756.557	802
27.470.000	4.852.736	-756.753	802
27.480.000	4.854.430	-757.024	801
27.490.000	4.856.019	-757.149	800
27.500.000	4.857.627	-757.446	802
27.510.000	4.859.382	-757.637	801
27.520.000	4.860.798	-757.836	803
27.530.000	4.862.633	-758.086	804
27.540.000	4.864.092	-758.329	807

27.550.000	4.865.881	-758.515	808
27.560.000	4.867.343	-758.740	808
27.570.000	4.869.123	-759.025	808
27.580.000	4.870.618	-759.152	808
27.590.000	4.872.283	-759.428	807
27.600.000	4.873.881	-759.647	806
27.610.000	4.875.466	-759.865	807
27.620.000	4.877.179	-760.060	810
27.630.000	4.878.736	-760.357	810
27.640.000	4.880.511	-760.505	808
27.650.000	4.881.882	-760.778	806
27.660.000	4.883.777	-761.033	804
27.670.000	4.885.183	-761.187	803
27.680.000	4.886.929	-761.445	802
27.690.000	4.888.446	-761.658	800
27.700.000	4.890.171	-761.871	800
27.710.000	4.891.698	-762.068	803
27.720.000	4.893.393	-762.350	803
27.730.000	4.895.065	-762.503	800
27.740.000	4.896.533	-762.752	799
27.750.000	4.898.339	-762.976	800
27.760.000	4.899.816	-763.184	800
27.770.000	4.901.597	-763.426	800
27.780.000	4.903.063	-763.676	799
27.790.000	4.904.860	-763.899	799
27.800.000	4.906.288	-764.079	799
27.810.000	4.908.035	-764.382	797
27.820.000	4.909.594	-764.548	794
27.830.000	4.911.219	-764.793	793
27.840.000	4.912.833	-764.996	792

27.850.000	4.914.472	-765.272	792
27.860.000	4.916.135	-765.403	792
27.870.000	4.917.605	-765.686	792
27.880.000	4.919.492	-765.922	792
27.890.000	4.920.808	-766.083	791
27.900.000	4.922.642	-766.357	790
27.910.000	4.924.147	-766.565	789
27.920.000	4.925.831	-766.775	791
27.930.000	4.927.361	-766.972	791
27.940.000	4.929.098	-767.268	791
27.950.000	4.930.655	-767.370	792
27.960.000	4.932.280	-767.682	792
27.970.000	4.934.044	-767.874	792
27.980.000	4.935.555	-768.100	790
27.990.000	4.937.278	-768.330	788
28.000.000	4.938.764	-768.603	786
28.010.000	4.940.554	-768.792	783
28.020.000	4.941.962	-769.011	784
28.030.000	4.943.798	-769.305	784
28.040.000	4.945.262	-769.447	784
28.050.000	4.946.951	-769.722	784
28.060.000	4.948.540	-769.918	783
28.070.000	4.950.206	-770.159	783
28.080.000	4.951.825	-770.330	782
28.090.000	4.953.371	-770.614	782
28.100.000	4.955.187	-770.761	783
28.110.000	4.956.561	-771.002	784
28.120.000	4.958.433	-771.266	786
28.130.000	4.959.835	-771.435	786
28.140.000	4.961.565	-771.666	784

28.150.000	4.963.092	-771.874	782
28.160.000	4.964.839	-772.119	781
28.170.000	4.966.289	-772.243	780
28.180.000	4.967.976	-772.530	781
28.190.000	4.969.656	-772.691	783
28.200.000	4.971.194	-772.898	785
28.210.000	4.972.930	-773.069	785
28.220.000	4.974.437	-773.343	783
28.230.000	4.976.226	-773.506	781
28.240.000	4.977.614	-773.725	779
28.250.000	4.979.451	-773.974	776
28.260.000	4.980.890	-774.096	776
28.270.000	4.982.633	-774.375	779
28.280.000	4.984.190	-774.566	782
28.290.000	4.985.861	-774.765	785
28.300.000	4.987.434	-774.952	787
28.310.000	4.989.117	-775.216	788
28.320.000	4.990.761	-775.316	787
28.330.000	4.992.248	-775.574	784
28.340.000	4.994.082	-775.802	781
28.350.000	4.995.470	-775.954	779
28.360.000	4.997.266	-776.182	778
28.370.000	4.998.716	-776.385	776
28.380.000	5.000.465	-776.565	776
28.390.000	5.001.913	-776.725	777
28.400.000	5.003.671	-777.012	775
28.410.000	5.005.265	-777.126	773
28.420.000	5.006.873	-777.360	770
28.430.000	5.008.515	-777.529	769
28.440.000	5.010.074	-777.736	769

28.450.000	5.011.805	-777.884	770
28.460.000	5.013.266	-778.127	774
28.470.000	5.015.099	-778.293	777
28.480.000	5.016.465	-778.458	779
28.490.000	5.018.279	-778.695	780
28.500.000	5.019.768	-778.843	780
28.510.000	5.021.449	-779.052	778
28.520.000	5.022.983	-779.212	775
28.530.000	5.024.738	-779.458	773
28.540.000	5.026.258	-779.532	773
28.550.000	5.027.857	-779.792	773
28.560.000	5.029.631	-779.929	775
28.570.000	5.031.045	-780.096	776
28.580.000	5.032.829	-780.308	777
28.590.000	5.034.290	-780.516	779
28.600.000	5.036.059	-780.665	779
28.610.000	5.037.498	-780.838	776
28.620.000	5.039.295	-781.095	773
28.630.000	5.040.748	-781.172	769
28.640.000	5.042.512	-781.429	768
28.650.000	5.044.097	-781.595	769
28.660.000	5.045.702	-781.751	768
28.670.000	5.047.351	-781.915	770
28.680.000	5.048.901	-782.153	772
28.690.000	5.050.667	-782.259	775
28.700.000	5.052.080	-782.489	774
28.710.000	5.053.921	-782.714	772
28.720.000	5.055.351	-782.835	770
28.730.000	5.057.074	-783.062	769
28.740.000	5.058.642	-783.256	766

28.750.000	5.060.409	-783.469	766
28.760.000	5.061.922	-783.612	767
28.770.000	5.063.632	-783.872	769
28.780.000	5.065.234	-783.989	771
28.790.000	5.066.782	-784.217	771
28.800.000	5.068.554	-784.399	770
28.810.000	5.069.999	-784.606	770
28.820.000	5.071.825	-784.794	770
28.830.000	5.073.237	-785.011	770
28.840.000	5.075.071	-785.227	769
28.850.000	5.076.519	-785.381	770
28.860.000	5.078.362	-785.654	770
28.870.000	5.079.900	-785.807	768
28.880.000	5.081.552	-786.016	766
28.890.000	5.083.198	-786.211	764
28.900.000	5.084.832	-786.458	762
28.910.000	5.086.468	-786.562	761
28.920.000	5.087.990	-786.846	762
28.930.000	5.089.851	-787.067	764
28.940.000	5.091.205	-787.232	764
28.950.000	5.093.046	-787.498	764
28.960.000	5.094.559	-787.719	761
28.970.000	5.096.329	-787.948	759
28.980.000	5.097.882	-788.137	756
28.990.000	5.099.636	-788.423	755
29.000.000	5.101.159	-788.537	755
29.010.000	5.102.803	-788.814	756
29.020.000	5.104.482	-789.006	757
29.030.000	5.105.977	-789.196	757
29.040.000	5.107.714	-789.387	756

29.050.000	5.109.195	-789.654	753
29.060.000	5.110.981	-789.835	750
29.070.000	5.112.388	-790.035	748
29.080.000	5.114.212	-790.303	747
29.090.000	5.115.713	-790.444	745
29.100.000	5.117.432	-790.712	745
29.110.000	5.119.018	-790.893	748
29.120.000	5.120.694	-791.105	750
29.130.000	5.122.315	-791.260	751
29.140.000	5.123.864	-791.534	750
29.150.000	5.125.626	-791.662	750
29.160.000	5.127.018	-791.880	751
29.170.000	5.128.870	-792.129	749
29.180.000	5.130.293	-792.312	744
29.190.000	5.132.024	-792.508	742
29.200.000	5.133.559	-792.730	741
29.210.000	5.135.339	-792.968	739
29.220.000	5.136.819	-793.110	739
29.230.000	5.138.553	-793.400	742
29.240.000	5.140.124	-793.559	743
29.250.000	5.141.719	-793.784	745
29.260.000	5.143.427	-793.964	745
29.270.000	5.144.946	-794.234	746
29.280.000	5.146.713	-794.391	746
29.290.000	5.148.139	-794.646	745
29.300.000	5.150.016	-794.921	742
29.310.000	5.151.481	-795.070	740
29.320.000	5.153.218	-795.351	739
29.330.000	5.154.746	-795.550	738
29.340.000	5.156.439	-795.786	737

29.350.000	5.157.987	-795.970	737
29.360.000	5.159.633	-796.269	737
29.370.000	5.161.309	-796.399	737
29.380.000	5.162.832	-796.683	737
29.390.000	5.164.609	-796.900	736
29.400.000	5.166.057	-797.118	737
29.410.000	5.167.835	-797.345	737
29.420.000	5.169.341	-797.601	737
29.430.000	5.171.145	-797.811	736
29.440.000	5.172.580	-798.003	737
29.450.000	5.174.368	-798.313	734
29.460.000	5.175.900	-798.442	733
29.470.000	5.177.520	-798.727	732
29.480.000	5.179.189	-798.953	732
29.490.000	5.180.741	-799.201	734
29.500.000	5.182.458	-799.367	735
29.510.000	5.183.964	-799.692	734
29.520.000	5.185.788	-799.910	735
29.530.000	5.187.205	-800.132	735
29.540.000	5.189.041	-800.417	736
29.550.000	5.190.546	-800.632	736
29.560.000	5.192.262	-800.892	737
29.570.000	5.193.772	-801.103	740
29.580.000	5.195.504	-801.406	742
29.590.000	5.197.059	-801.560	741
29.600.000	5.198.643	-801.870	739
29.610.000	5.200.391	-802.047	739
29.620.000	5.201.838	-802.285	736
29.630.000	5.203.662	-802.528	735
29.640.000	5.205.124	-802.796	733

29.650.000	5.206.910	-803.003	734
29.660.000	5.208.408	-803.237	734
29.670.000	5.210.224	-803.550	733
29.680.000	5.211.672	-803.661	733
29.690.000	5.213.386	-803.972	733
29.700.000	5.214.982	-804.188	733
29.710.000	5.216.608	-804.435	733
29.720.000	5.218.259	-804.626	732
29.730.000	5.219.812	-804.939	734
29.740.000	5.221.609	-805.120	735
29.750.000	5.223.007	-805.394	734
29.760.000	5.224.867	-805.697	734
29.770.000	5.226.348	-805.888	734
29.780.000	5.228.070	-806.161	733
29.790.000	5.229.561	-806.406	733
29.800.000	5.231.304	-806.645	732
29.810.000	5.232.788	-806.834	732
29.820.000	5.234.500	-807.151	731
29.830.000	5.236.123	-807.302	730
29.840.000	5.237.660	-807.572	727
29.850.000	5.239.425	-807.776	724
29.860.000	5.240.905	-808.052	723
29.870.000	5.242.681	-808.251	722
29.880.000	5.244.154	-808.509	722
29.890.000	5.245.975	-808.775	724
29.900.000	5.247.409	-808.942	727
29.910.000	5.249.178	-809.232	731
29.920.000	5.250.689	-809.426	733
29.930.000	5.252.337	-809.667	733
29.940.000	5.253.927	-809.873	732

29.950.000	5.255.557	-810.161	730
29.960.000	5.257.223	-810.298	730
29.970.000	5.258.718	-810.597	730
29.980.000	5.260.590	-810.849	731
29.990.000	5.261.999	-811.041	734
30.000.000	5.263.803	-811.326	737
30.010.000	5.265.273	-811.568	738
30.020.000	5.267.004	-811.790	737
30.030.000	5.268.470	-811.996	735
30.040.000	5.270.247	-812.319	733
30.050.000	5.271.768	-812.449	731
30.060.000	5.273.350	-812.741	730
30.070.000	5.275.071	-812.961	730
30.080.000	5.276.595	-813.210	732
30.090.000	5.278.343	-813.413	733
30.100.000	5.279.850	-813.711	733
30.110.000	5.281.688	-813.935	731
30.120.000	5.283.094	-814.160	730
30.130.000	5.284.884	-814.465	729
30.140.000	5.286.365	-814.630	730
30.150.000	5.288.084	-814.923	731
30.160.000	5.289.631	-815.138	732
30.170.000	5.291.325	-815.409	733
30.180.000	5.292.917	-815.589	734
30.190.000	5.294.511	-815.910	732
30.200.000	5.296.294	-816.096	729
30.210.000	5.297.695	-816.333	727
30.220.000	5.299.559	-816.608	727
30.230.000	5.301.005	-816.850	728
30.240.000	5.302.744	-817.074	730

30.250.000	5.304.193	-817.317	732
30.260.000	5.305.963	-817.603	732
30.270.000	5.307.418	-817.753	731
30.280.000	5.309.163	-818.064	730
30.290.000	5.310.783	-818.288	730
30.300.000	5.312.365	-818.511	730
30.310.000	5.314.067	-818.716	730
30.320.000	5.315.593	-819.025	731
30.330.000	5.317.386	-819.180	732
30.340.000	5.318.814	-819.455	732
30.350.000	5.320.643	-819.754	732
30.360.000	5.322.079	-819.922	728
30.370.000	5.323.817	-820.209	727
30.380.000	5.325.311	-820.421	726
30.390.000	5.327.051	-820.663	725
30.400.000	5.328.556	-820.869	727
30.410.000	5.330.249	-821.167	729
30.420.000	5.331.921	-821.317	733
30.430.000	5.333.420	-821.610	735
30.440.000	5.335.244	-821.848	736
30.450.000	5.336.706	-822.063	735
30.460.000	5.338.453	-822.324	733
30.470.000	5.339.908	-822.552	731
30.480.000	5.341.723	-822.823	732
30.490.000	5.343.162	-822.998	733
30.500.000	5.344.919	-823.316	734
30.510.000	5.346.495	-823.503	737
30.520.000	5.348.106	-823.756	738
30.530.000	5.349.754	-823.968	737
30.540.000	5.351.333	-824.277	736

30.550.000	5.353.082	-824.434	734
30.560.000	5.354.563	-824.755	733
30.570.000	5.356.393	-825.016	733
30.580.000	5.357.813	-825.206	733
30.590.000	5.359.597	-825.508	737
30.600.000	5.361.110	-825.742	741
30.610.000	5.362.812	-825.968	744
30.620.000	5.364.314	-826.200	746
30.630.000	5.366.099	-826.528	746
30.640.000	5.367.667	-826.673	745
30.650.000	5.369.230	-826.983	742
30.660.000	5.370.997	-827.197	739
30.670.000	5.372.491	-827.455	739
30.680.000	5.374.258	-827.685	738
30.690.000	5.375.723	-827.969	740
30.700.000	5.377.534	-828.201	741
30.710.000	5.378.930	-828.428	741
30.720.000	5.380.735	-828.731	740
30.730.000	5.382.214	-828.899	739
30.740.000	5.383.919	-829.191	737
30.750.000	5.385.510	-829.431	734
30.760.000	5.387.130	-829.678	732
30.770.000	5.388.779	-829.860	732
30.780.000	5.390.372	-830.183	732
30.790.000	5.392.165	-830.361	733
30.800.000	5.393.512	-830.605	733
30.810.000	5.395.358	-830.894	732
30.820.000	5.396.813	-831.115	730
30.830.000	5.398.555	-831.356	728
30.840.000	5.400.032	-831.575	726

30.850.000	5.401.797	-831.865	726
30.860.000	5.403.292	-832.007	726
30.870.000	5.404.970	-832.309	727
30.880.000	5.406.626	-832.524	729
30.890.000	5.408.177	-832.773	730
30.900.000	5.409.937	-832.973	729
30.910.000	5.411.409	-833.274	728
30.920.000	5.413.175	-833.459	727
30.930.000	5.414.598	-833.708	726
30.940.000	5.416.400	-834.010	726
30.950.000	5.417.851	-834.148	726
30.960.000	5.419.592	-834.471	729
30.970.000	5.421.142	-834.698	732
30.980.000	5.422.821	-834.939	733
30.990.000	5.424.412	-835.138	735
31.000.000	5.426.012	-835.461	736
31.010.000	5.427.735	-835.592	734
31.020.000	5.429.177	-835.883	732
31.030.000	5.431.015	-836.145	729
31.040.000	5.432.438	-836.342	726
31.050.000	5.434.177	-836.589	723
31.060.000	5.435.642	-836.829	722
31.070.000	5.437.423	-837.068	721
31.080.000	5.438.887	-837.245	724
31.090.000	5.440.610	-837.577	726
31.100.000	5.442.198	-837.746	728
31.110.000	5.443.794	-838.008	730
31.120.000	5.445.473	-838.200	731
31.130.000	5.446.986	-838.458	730
31.140.000	5.448.705	-838.638	727

31.150.000	5.450.133	-838.911	724
31.160.000	5.452.004	-839.147	723
31.170.000	5.453.385	-839.335	723
31.180.000	5.455.175	-839.629	723
31.190.000	5.456.642	-839.829	723
31.200.000	5.458.336	-840.065	723
31.210.000	5.459.868	-840.289	721
31.220.000	5.461.587	-840.587	719
31.230.000	5.463.190	-840.729	716
31.240.000	5.464.698	-841.043	715
31.250.000	5.466.461	-841.250	716
31.260.000	5.467.898	-841.471	717
31.270.000	5.469.667	-841.733	720
31.280.000	5.471.102	-841.971	723
31.290.000	5.472.863	-842.195	724
31.300.000	5.474.337	-842.387	723
31.310.000	5.476.099	-842.707	721
31.320.000	5.477.587	-842.837	717
31.330.000	5.479.244	-843.128	714
31.340.000	5.480.893	-843.355	714
31.350.000	5.482.432	-843.582	714
31.360.000	5.484.111	-843.772	715
31.370.000	5.485.604	-844.085	715
31.380.000	5.487.385	-844.277	716
31.390.000	5.488.763	-844.495	714
31.400.000	5.490.561	-844.784	712
31.410.000	5.492.077	-844.980	711
31.420.000	5.493.787	-845.231	711
31.430.000	5.495.288	-845.449	712
31.440.000	5.497.019	-845.742	712

31.450.000	5.498.584	-845.902	713
31.460.000	5.500.199	-846.222	715
31.470.000	5.501.898	-846.385	715
31.480.000	5.503.339	-846.661	714
31.490.000	5.505.148	-846.900	713
31.500.000	5.506.588	-847.169	713
31.510.000	5.508.355	-847.394	712
31.520.000	5.509.812	-847.654	711
31.530.000	5.511.612	-847.942	709
31.540.000	5.513.037	-848.105	708
31.550.000	5.514.793	-848.431	708
31.560.000	5.516.391	-848.669	708
31.570.000	5.518.032	-848.908	708
31.580.000	5.519.635	-849.118	708
31.590.000	5.521.187	-849.445	708
31.600.000	5.522.935	-849.611	708
31.610.000	5.524.357	-849.914	707
31.620.000	5.526.195	-850.210	704
31.630.000	5.527.613	-850.415	704
31.640.000	5.529.359	-850.722	703
31.650.000	5.530.870	-850.960	705
31.660.000	5.532.587	-851.240	705
31.670.000	5.534.108	-851.456	705
31.680.000	5.535.818	-851.788	706
31.690.000	5.537.435	-851.972	705
31.700.000	5.538.933	-852.268	704
31.710.000	5.540.679	-852.488	704
31.720.000	5.542.169	-852.767	704
31.730.000	5.543.927	-852.997	704
31.740.000	5.545.368	-853.275	704

31.750.000	5.547.193	-853.530	703
31.760.000	5.548.590	-853.733	702
31.770.000	5.550.361	-854.060	702
31.780.000	5.551.915	-854.256	701
31.790.000	5.553.543	-854.522	702
31.800.000	5.555.149	-854.769	701
31.810.000	5.556.772	-855.075	700
31.820.000	5.558.429	-855.222	700
31.830.000	5.559.936	-855.556	700
31.840.000	5.561.723	-855.798	701
31.850.000	5.563.112	-856.023	703
31.860.000	5.564.922	-856.328	704
31.870.000	5.566.392	-856.585	706
31.880.000	5.568.141	-856.826	708
31.890.000	5.569.591	-857.056	708
31.900.000	5.571.354	-857.413	707
31.910.000	5.572.908	-857.575	706
31.920.000	5.574.504	-857.882	708
31.930.000	5.576.201	-858.125	708
31.940.000	5.577.721	-858.373	710
31.950.000	5.579.471	-858.599	711
31.960.000	5.580.893	-858.905	708
31.970.000	5.582.725	-859.144	709
31.980.000	5.584.094	-859.369	708
31.990.000	5.585.864	-859.697	707
32.000.000	5.587.345	-859.871	706
32.010.000	5.589.043	-860.176	705
32.020.000	5.590.621	-860.396	705
32.030.000	5.592.275	-860.663	705
32.040.000	5.593.863	-860.854	702

32.050.000	5.595.448	-861.197	700
32.060.000	5.597.202	-861.379	699
32.070.000	5.598.597	-861.648	699
32.080.000	5.600.408	-861.929	699
32.090.000	5.601.850	-862.178	700
32.100.000	5.603.561	-862.404	702
32.110.000	5.605.022	-862.646	703
32.120.000	5.606.804	-862.966	704
32.130.000	5.608.279	-863.118	704
32.140.000	5.609.982	-863.430	705
32.150.000	5.611.603	-863.662	705
32.160.000	5.613.113	-863.894	705
32.170.000	5.614.827	-864.107	703
32.180.000	5.616.326	-864.440	703
32.190.000	5.618.099	-864.628	702
32.200.000	5.619.496	-864.897	703
32.210.000	5.621.303	-865.203	702
32.220.000	5.622.745	-865.387	702
32.230.000	5.624.481	-865.682	702
32.240.000	5.626.018	-865.921	703
32.250.000	5.627.723	-866.179	704
32.260.000	5.629.249	-866.412	704
32.270.000	5.630.894	-866.712	705
32.280.000	5.632.571	-866.879	705
32.290.000	5.634.033	-867.196	706
32.300.000	5.635.854	-867.449	704
32.310.000	5.637.288	-867.702	704
32.320.000	5.639.063	-867.990	703
32.330.000	5.640.496	-868.249	703
32.340.000	5.642.295	-868.534	703

32.350.000	5.643.738	-868.743	703
32.360.000	5.645.501	-869.087	703
32.370.000	5.647.078	-869.293	702
32.380.000	5.648.662	-869.575	702
32.390.000	5.650.347	-869.821	703
32.400.000	5.651.906	-870.171	702
32.410.000	5.653.597	-870.350	703
32.420.000	5.655.073	-870.678	702
32.430.000	5.656.891	-870.983	701
32.440.000	5.658.256	-871.195	701
32.450.000	5.660.050	-871.531	701
32.460.000	5.661.592	-871.794	701
32.470.000	5.663.314	-872.048	702
32.480.000	5.664.786	-872.300	703
32.490.000	5.666.491	-872.631	705
32.500.000	5.668.070	-872.805	706
32.510.000	5.669.647	-873.131	706
32.520.000	5.671.407	-873.361	706
32.530.000	5.672.854	-873.615	706
32.540.000	5.674.613	-873.893	708
32.550.000	5.676.038	-874.181	710
32.560.000	5.677.843	-874.432	711
32.570.000	5.679.253	-874.659	711
32.580.000	5.681.089	-875.009	711
32.590.000	5.682.596	-875.186	710
32.600.000	5.684.265	-875.501	710
32.610.000	5.685.870	-875.743	710
32.620.000	5.687.481	-876.035	709
32.630.000	5.689.102	-876.236	710
32.640.000	5.690.655	-876.591	710

32.650.000	5.692.455	-876.837	710
32.660.000	5.693.852	-877.095	710
32.670.000	5.695.650	-877.424	710
32.680.000	5.697.121	-877.669	710
32.690.000	5.698.852	-877.951	710
32.700.000	5.700.358	-878.205	710
32.710.000	5.702.105	-878.543	711
32.720.000	5.703.634	-878.715	710
32.730.000	5.705.244	-879.058	710
32.740.000	5.706.981	-879.312	710
32.750.000	5.708.437	-879.577	709
32.760.000	5.710.244	-879.842	707
32.770.000	5.711.674	-880.163	706
32.780.000	5.713.456	-880.386	705
32.790.000	5.714.876	-880.683	706
32.800.000	5.716.692	-881.014	706
32.810.000	5.718.149	-881.192	707
32.820.000	5.719.870	-881.530	707
32.830.000	5.721.450	-881.793	708
32.840.000	5.723.101	-882.060	709
32.850.000	5.724.689	-882.289	710
32.860.000	5.726.270	-882.652	711
32.870.000	5.728.056	-882.853	712
32.880.000	5.729.435	-883.144	713
32.890.000	5.731.247	-883.456	715
32.900.000	5.732.680	-883.699	715
32.910.000	5.734.410	-883.984	713
32.920.000	5.735.897	-884.235	712
32.930.000	5.737.628	-884.536	713
32.940.000	5.739.088	-884.741	713

32.950.000	5.740.792	-885.060	715
32.960.000	5.742.419	-885.295	719
32.970.000	5.743.911	-885.558	721
32.980.000	5.745.675	-885.805	723
32.990.000	5.747.158	-886.131	724
33.000.000	5.748.909	-886.334	723
33.010.000	5.750.333	-886.639	721
33.020.000	5.752.177	-886.945	718
33.030.000	5.753.579	-887.145	717
33.040.000	5.755.348	-887.474	719
33.050.000	5.756.881	-887.724	719
33.060.000	5.758.549	-887.994	719
33.070.000	5.760.117	-888.243	721
33.080.000	5.761.749	-888.579	720
33.090.000	5.763.404	-888.751	719
33.100.000	5.764.853	-889.062	715
33.110.000	5.766.652	-889.349	712
33.120.000	5.768.058	-889.571	710
33.130.000	5.769.832	-889.879	709
33.140.000	5.771.315	-890.165	709
33.150.000	5.773.070	-890.427	710
33.160.000	5.774.497	-890.654	712
33.170.000	5.776.240	-890.999	714
33.180.000	5.777.845	-891.188	714
33.190.000	5.779.393	-891.475	713
33.200.000	5.781.074	-891.718	712
33.210.000	5.782.647	-892.019	711
33.220.000	5.784.363	-892.234	712
33.230.000	5.785.792	-892.548	714
33.240.000	5.787.618	-892.845	717

33.250.000	5.788.978	-893.060	721
33.260.000	5.790.820	-893.402	723
33.270.000	5.792.336	-893.628	725
33.280.000	5.794.005	-893.916	726
33.290.000	5.795.596	-894.172	725
33.300.000	5.797.246	-894.488	722
33.310.000	5.798.814	-894.649	720
33.320.000	5.800.401	-895.004	720
33.330.000	5.802.164	-895.252	720
33.340.000	5.803.589	-895.514	720
33.350.000	5.805.399	-895.808	720
33.360.000	5.806.871	-896.113	721
33.370.000	5.808.683	-896.376	721
33.380.000	5.810.122	-896.626	720
33.390.000	5.811.906	-896.980	718
33.400.000	5.813.435	-897.160	716
33.410.000	5.815.078	-897.482	718
33.420.000	5.816.678	-897.732	719
33.430.000	5.818.269	-897.998	720
33.440.000	5.819.955	-898.218	722
33.450.000	5.821.471	-898.563	724
33.460.000	5.823.280	-898.799	723
33.470.000	5.824.704	-899.067	721
33.480.000	5.826.550	-899.391	718
33.490.000	5.827.998	-899.626	715
33.500.000	5.829.722	-899.933	714
33.510.000	5.831.264	-900.197	714
33.520.000	5.832.966	-900.520	717
33.530.000	5.834.492	-900.702	720
33.540.000	5.836.105	-901.055	722

33.550.000	5.837.864	-901.288	725
33.560.000	5.839.266	-901.557	727
33.570.000	5.841.111	-901.847	726
33.580.000	5.842.548	-902.144	723
33.590.000	5.844.321	-902.398	719
33.600.000	5.845.799	-902.678	716
33.610.000	5.847.571	-903.023	713
33.620.000	5.849.026	-903.177	711
33.630.000	5.850.744	-903.533	711
33.640.000	5.852.329	-903.780	711
33.650.000	5.853.910	-904.035	712
33.660.000	5.855.556	-904.262	714
33.670.000	5.857.094	-904.617	713
33.680.000	5.858.893	-904.806	714
33.690.000	5.860.259	-905.097	715
33.700.000	5.862.117	-905.434	716
33.710.000	5.863.602	-905.644	716
33.720.000	5.865.326	-905.941	717
33.730.000	5.866.867	-906.221	720
33.740.000	5.868.574	-906.495	723
33.750.000	5.870.070	-906.713	724
33.760.000	5.871.759	-907.050	725
33.770.000	5.873.382	-907.247	726
33.780.000	5.874.884	-907.552	728
33.790.000	5.876.646	-907.799	726
33.800.000	5.878.105	-908.083	723
33.810.000	5.879.866	-908.341	723
33.820.000	5.881.356	-908.618	722
33.830.000	5.883.163	-908.918	720
33.840.000	5.884.566	-909.106	719

33.850.000	5.886.331	-909.458	720
33.860.000	5.887.897	-909.682	722
33.870.000	5.889.508	-909.949	722
33.880.000	5.891.130	-910.211	720
33.890.000	5.892.714	-910.534	719
33.900.000	5.894.397	-910.696	716
33.910.000	5.895.862	-911.021	715
33.920.000	5.897.712	-911.330	712
33.930.000	5.899.118	-911.532	714
33.940.000	5.900.900	-911.847	716
33.950.000	5.902.414	-912.129	718
33.960.000	5.904.166	-912.406	722
33.970.000	5.905.659	-912.623	724
33.980.000	5.907.385	-912.972	727
33.990.000	5.908.970	-913.150	727
34.000.000	5.910.548	-913.464	726
34.010.000	5.912.271	-913.708	726
34.020.000	5.913.766	-913.981	727
34.030.000	5.915.535	-914.257	728
34.040.000	5.916.995	-914.566	730
34.050.000	5.918.811	-914.834	730
34.060.000	5.920.214	-915.071	728
34.070.000	5.921.990	-915.401	727
34.080.000	5.923.527	-915.630	726
34.090.000	5.925.165	-915.907	725
34.100.000	5.926.738	-916.173	725
34.110.000	5.928.414	-916.503	726
34.120.000	5.930.021	-916.669	729
34.130.000	5.931.544	-917.017	731
34.140.000	5.933.348	-917.296	733

34.150.000	5.934.716	-917.544	733
34.160.000	5.936.574	-917.856	732
34.170.000	5.938.051	-918.135	730
34.180.000	5.939.758	-918.380	727
34.190.000	5.941.274	-918.624	725
34.200.000	5.943.009	-918.976	725
34.210.000	5.944.561	-919.129	725
34.220.000	5.946.146	-919.449	726
34.230.000	5.947.829	-919.710	726
34.240.000	5.949.363	-919.960	724
34.250.000	5.951.092	-920.206	724
34.260.000	5.952.546	-920.532	723
34.270.000	5.954.430	-920.788	724
34.280.000	5.955.828	-921.039	723
34.290.000	5.957.640	-921.373	723
34.300.000	5.959.132	-921.576	724
34.310.000	5.960.823	-921.895	723
34.320.000	5.962.370	-922.127	722
34.330.000	5.964.075	-922.435	722
34.340.000	5.965.614	-922.629	721
34.350.000	5.967.201	-922.981	722
34.360.000	5.968.988	-923.224	723
34.370.000	5.970.368	-923.491	725
34.380.000	5.972.246	-923.823	727
34.390.000	5.973.711	-924.098	729
34.400.000	5.975.456	-924.346	728
34.410.000	5.976.914	-924.606	727
34.420.000	5.978.685	-924.942	726
34.430.000	5.980.195	-925.110	725
34.440.000	5.981.870	-925.452	726

34.450.000	5.983.472	-925.684	727
34.460.000	5.985.040	-925.958	729
34.470.000	5.986.732	-926.184	732
34.480.000	5.988.238	-926.534	733
34.490.000	5.990.081	-926.778	734
34.500.000	5.991.482	-927.085	734
34.510.000	5.993.329	-927.394	734
34.520.000	5.994.771	-927.625	734
34.530.000	5.996.491	-927.956	734
34.540.000	5.998.063	-928.234	735
34.550.000	5.999.713	-928.533	736
34.560.000	6.001.274	-928.761	737
34.570.000	6.002.864	-929.114	737
34.580.000	6.004.577	-929.328	738
34.590.000	6.006.027	-929.633	738
34.600.000	6.007.867	-929.939	735
34.610.000	6.009.301	-930.207	734
34.620.000	6.011.082	-930.478	735
34.630.000	6.012.522	-930.753	733
34.640.000	6.014.299	-931.069	733
34.650.000	6.015.744	-931.262	733
34.660.000	6.017.432	-931.599	732
