# Supplementary material for: Data on statistical experimental design to formulate amphotericin B-loaded Eudragit RL100 nanoparticles coated with hyaluronic acid for the treatment of vulvovaginal candidiasis
Source: Data Brief. 2020 Mar 5;29:105311. doi: 10.1016/j.dib.2020.105311 (PMC7082528; doi:10.1016/j.dib.2020.105311)
Supplement: Multimedia component 7 [file mmc7.pdf]

File Name:	**AMP EUD nanoparticles HA**		
[Data]			
Time	Temp	**DTA**	**TGA**
sec	C	uV	mg
0	313.920	1.1159	1.4570
1.2000	313.899	1.1547	1.4569
2.2000	314.014	1.1744	1.4568
3.2000	314.194	1.2013	1.4567
4.2000	314.215	1.2266	1.4567
5.2000	314.382	1.2574	1.4567
6.2000	314.446	1.2700	1.4566
7.2000	314.725	1.3044	1.4564
8.2000	314.667	1.3178	1.4562
9.2000	315.185	1.3320	1.4560
10.2000	314.973	1.3587	1.4560
11.2000	315.494	1.3666	1.4558
12.2000	315.552	1.3793	1.4556
130.000	315.936	1.3917	1.4555
140.000	316.046	1.3989	1.4556
150.000	316.567	1.3932	1.4554
160.000	316.701	1.4050	1.4552
170.000	317.196	1.3897	1.4550
180.000	317.585	1.3877	1.4548
190.000	317.973	1.3723	1.4548
200.000	318.471	1.3601	1.4547
210.000	318.879	1.3310	1.4544
220.000	319.477	1.3154	1.4544
230.000	319.851	1.2710	1.4545
240.000	320.664	1.2447	1.4544

250.000	320.926	1.2036	1.4542
260.000	321.843	1.1522	1.4542
270.000	322.226	1.1134	1.4541
280.000	323.048	1.0593	1.4538
290.000	323.520	1.1934	1.4536
300.000	324.400	9.483	1.4536
310.000	324.884	8.910	1.4535
320.000	325.834	8.139	1.4534
330.000	326.417	7.619	1.4534
340.000	327.270	6.817	1.4533
350.000	328.070	6.166	1.4532
360.000	328.829	5.387	1.4528
370.000	329.708	4.671	1.4527
380.000	330.470	3.764	1.4526
390.000	331.476	3.079	1.4527
400.000	332.129	2.171	1.4527
410.000	333.354	1.248	1.4529
420.000	333.889	457	1.4532
430.000	335.101	-493	1.4534
440.000	335.816	-1.411	1.4534
450.000	336.994	-2.332	1.4533
460.000	337.807	-3.261	1.4534
470.000	339.001	-4.291	1.4534
480.000	339.787	-5.125	1.4533
490.000	340.926	-6.224	1.4532
500.000	341.944	-7.153	1.4533
510.000	342.981	-8.168	1.4534
520.000	344.115	-9.132	1.4538
530.000	345.124	-10.206	1.4539
540.000	346.303	-11.111	1.4539

550.000	347.225	-12.251	1.4538
560.000	348.656	-13.231	1.4536
570.000	349.448	-14.273	1.4535
580.000	350.898	-15.416	1.4531
590.000	351.788	-16.366	1.4528
600.000	353.127	-17.453	1.4525
610.000	354.130	-18.517	1.4521
620.000	355.554	-19.587	1.4520
630.000	356.555	-20.580	1.4521
640.000	357.921	-21.770	1.4520
650.000	359.089	-22.716	1.4519
660.000	360.325	-23.885	1.4519
670.000	361.618	-24.889	1.4518
680.000	362.850	-25.998	1.4515
690.000	364.182	-27.006	1.4512
700.000	365.373	-28.177	1.4508
710.000	366.802	-29.130	1.4505
720.000	367.815	-30.272	1.4501
730.000	369.479	-31.401	1.4503
740.000	370.531	-32.430	1.4503
750.000	372.095	-33.547	1.4502
760.000	373.266	-34.621	1.4502
770.000	374.761	-35.648	1.4500
780.000	375.982	-36.730	1.4498
790.000	377.542	-37.859	1.4495
800.000	378.684	-38.791	1.4490
810.000	380.186	-39.978	1.4486
820.000	381.611	-40.955	1.4485
830.000	382.926	-42.029	1.4482
840.000	384.409	-43.031	1.4479

850.000	385.755	-44.127	1.4476
860.000	387.340	-45.070	1.4474
870.000	388.590	-46.212	1.4473
880.000	390.272	-47.188	1.4472
890.000	391.428	-48.213	1.4470
900.000	393.211	-49.336	1.4470
910.000	394.371	-50.233	1.4470
920.000	396.021	-51.309	1.4471
930.000	397.313	-52.314	1.4474
940.000	398.959	-53.294	1.4474
950.000	400.258	-54.244	1.4475
960.000	401.915	-55.365	1.4478
970.000	403.387	-56.194	1.4478
980.000	404.817	-57.276	1.4472
990.000	406.390	-58.188	1.4468
1.000.000	407.810	-59.153	1.4465
1.010.000	409.372	-60.060	1.4462
1.020.000	410.782	-61.082	1.4455
1.030.000	412.408	-61.928	1.4450
1.040.000	413.668	-62.907	1.4451
1.050.000	415.488	-63.883	1.4450
1.060.000	416.622	-64.730	1.4448
1.070.000	418.445	-65.718	1.4449
1.080.000	419.824	-66.609	1.4453
1.090.000	421.498	-67.502	1.4454
1.100.000	422.894	-68.375	1.4451
1.110.000	424.617	-69.321	1.4450
1.120.000	425.955	-70.107	1.4450
1.130.000	427.619	-71.087	1.4447
1.140.000	429.168	-71.869	1.4442

1.150.000	430.629	-72.753	1.4440
1.160.000	432.300	-73.581	1.4440
1.170.000	433.810	-74.487	1.4436
1.180.000	435.459	-75.223	1.4433
1.190.000	436.863	-76.157	1.4429
1.200.000	438.706	-76.952	1.4427
1.210.000	439.974	-77.750	1.4422
1.220.000	441.881	-78.664	1.4417
1.230.000	443.176	-79.366	1.4414
1.240.000	445.007	-80.239	1.4412
1.250.000	446.451	-81.047	1.4410
1.260.000	448.181	-81.821	1.4408
1.270.000	449.631	-82.573	1.4408
1.280.000	451.391	-83.451	1.4408
1.290.000	452.891	-84.083	1.4410
1.300.000	454.539	-84.959	1.4408
1.310.000	456.227	-85.698	1.4406
1.320.000	457.772	-86.467	1.4404
1.330.000	459.503	-87.165	1.4402
1.340.000	461.011	-88.016	1.4397
1.350.000	462.848	-88.658	1.4391
1.360.000	464.218	-89.462	1.4391
1.370.000	466.191	-90.227	1.4393
1.380.000	467.466	-90.864	1.4395
1.390.000	469.347	-91.684	1.4397
1.400.000	470.893	-92.375	1.4400
1.410.000	472.643	-93.094	1.4403
1.420.000	474.168	-93.784	1.4402
1.430.000	476.011	-94.529	1.4400
1.440.000	477.503	-95.160	1.4396

1.450.000	479.303	-95.932	1.4392
1.460.000	480.900	-96.513	1.4390
1.470.000	482.515	-97.234	1.4390
1.480.000	484.301	-97.882	1.4390
1.490.000	485.842	-98.555	1.4389
1.500.000	487.657	-99.157	1.4388
1.510.000	489.193	-99.891	1.4391
1.520.000	491.083	-100.475	1.4390
1.530.000	492.458	-101.145	1.4386
1.540.000	494.479	-101.844	1.4385
1.550.000	495.855	-102.375	1.4386
1.560.000	497.769	-103.085	1.4385
1.570.000	499.346	-103.714	1.4382
1.580.000	501.161	-104.309	1.4380
1.590.000	502.682	-104.895	1.4384
1.600.000	504.517	-105.615	1.4387
1.610.000	506.059	-106.069	1.4384
1.620.000	507.780	-106.762	1.4384
1.630.000	509.476	-107.309	1.4386
1.640.000	511.114	-107.903	1.4386
1.650.000	512.885	-108.464	1.4382
1.660.000	514.465	-109.112	1.4377
1.670.000	516.289	-109.596	1.4377
1.680.000	517.730	-110.227	1.4379
1.690.000	519.712	-110.816	1.4379
1.700.000	521.071	-111.316	1.4376
1.710.000	523.018	-111.941	1.4377
1.720.000	524.494	-112.433	1.4379
1.730.000	526.316	-113.020	1.4377
1.740.000	527.852	-113.546	1.4371

1.750.000	529.761	-114.111	1.4368
1.760.000	531.246	-114.574	1.4367
1.770.000	533.105	-115.223	1.4366
1.780.000	534.757	-115.605	1.4363
1.790.000	536.370	-116.220	1.4362
1.800.000	538.173	-116.702	1.4362
1.810.000	539.764	-117.230	1.4359
1.820.000	541.550	-117.681	1.4356
1.830.000	543.139	-118.282	1.4356
1.840.000	545.028	-118.721	1.4355
1.850.000	546.477	-119.250	1.4354
1.860.000	548.503	-119.817	1.4353
1.870.000	549.901	-120.221	1.4351
1.880.000	551.863	-120.781	1.4350
1.890.000	553.426	-121.289	1.4346
1.900.000	555.256	-121.745	1.4342
1.910.000	556.814	-122.204	1.4338
1.920.000	558.668	-122.776	1.4333
1.930.000	560.243	-123.115	1.4328
1.940.000	562.009	-123.700	1.4327
1.950.000	563.733	-124.097	1.4326
1.960.000	565.361	-124.601	1.4324
1.970.000	567.235	-125.041	1.4323
1.980.000	568.837	-125.556	1.4325
1.990.000	570.687	-125.941	1.4328
2.000.000	572.229	-126.481	1.4327
2.010.000	574.203	-126.917	1.4324
2.020.000	575.594	-127.359	1.4324
2.030.000	577.583	-127.866	1.4324
2.040.000	579.105	-128.239	1.4319

2.050.000	580.957	-128.747	1.4315
2.060.000	582.518	-129.169	1.4313
2.070.000	584.433	-129.624	1.4313
2.080.000	585.974	-130.023	1.4312
2.090.000	587.860	-130.567	1.4309
2.100.000	589.559	-130.867	1.4310
2.110.000	591.253	-131.399	1.4312
2.120.000	593.115	-131.808	1.4310
2.130.000	594.658	-132.239	1.4307
2.140.000	596.549	-132.619	1.4305
2.150.000	598.168	-133.138	1.4305
2.160.000	600.083	-133.520	1.4300
2.170.000	601.525	-133.933	1.4298
2.180.000	603.593	-134.424	1.4299
2.190.000	604.982	-134.747	1.4301
2.200.000	606.951	-135.238	1.4301
2.210.000	608.481	-135.619	1.4303
2.220.000	610.311	-136.031	1.4307
2.230.000	611.939	-136.414	1.4309
2.240.000	613.788	-136.901	1.4309
2.250.000	615.342	-137.176	1.4309
2.260.000	617.154	-137.707	1.4308
2.270.000	618.862	-138.005	1.4306
2.280.000	620.487	-138.444	1.4304
2.290.000	622.348	-138.793	1.4302
2.300.000	623.867	-139.220	1.4298
2.310.000	625.794	-139.575	1.4295
2.320.000	627.311	-140.024	1.4293
2.330.000	629.303	-140.382	1.4291
2.340.000	630.671	-140.753	1.4290

2.350.000	632.732	-141.215	1.4289
2.360.000	634.221	-141.491	1.4288
2.370.000	636.060	-141.923	1.4286
2.380.000	637.646	-142.265	1.4283
2.390.000	639.474	-142.634	1.4279
2.400.000	641.047	-142.991	1.4275
2.410.000	642.922	-143.440	1.4270
2.420.000	644.550	-143.664	1.4265
2.430.000	646.264	-144.136	1.4262
2.440.000	648.113	-144.441	1.4259
2.450.000	649.691	-144.813	1.4259
2.460.000	651.566	-145.127	1.4260
2.470.000	653.120	-145.559	1.4258
2.480.000	655.010	-145.831	1.4256
2.490.000	656.494	-146.229	1.4257
2.500.000	658.534	-146.638	1.4258
2.510.000	659.922	-146.905	1.4256
2.520.000	661.939	-147.329	1.4253
2.530.000	663.496	-147.648	1.4253
2.540.000	665.338	-148.020	1.4253
2.550.000	667.015	-148.361	1.4250
2.560.000	668.840	-148.744	1.4247
2.570.000	670.435	-148.995	1.4245
2.580.000	672.248	-149.442	1.4245
2.590.000	673.969	-149.668	1.4245
2.600.000	675.599	-150.089	1.4243
2.610.000	677.491	-150.397	1.4242
2.620.000	679.072	-150.737	1.4242
2.630.000	680.955	-151.045	1.4241
2.640.000	682.512	-151.472	1.4240

2.650.000	684.502	-151.757	1.4240
2.660.000	685.910	-152.111	1.4237
2.670.000	687.950	-152.527	1.4238
2.680.000	689.442	-152.749	1.4237
2.690.000	691.352	-153.167	1.4237
2.700.000	692.944	-153.490	1.4237
2.710.000	694.822	-153.808	1.4235
2.720.000	696.395	-154.120	1.4234
2.730.000	698.260	-154.540	1.4234
2.740.000	699.895	-154.730	1.4235
2.750.000	701.606	-155.159	1.4236
2.760.000	703.472	-155.454	1.4232
2.770.000	705.085	-155.801	1.4232
2.780.000	706.928	-156.089	1.4231
2.790.000	708.544	-156.477	1.4230
2.800.000	710.362	-156.735	1.4229
2.810.000	711.808	-157.112	1.4228
2.820.000	713.888	-157.450	1.4230
2.830.000	715.190	-157.719	1.4232
2.840.000	717.215	-158.106	1.4234
2.850.000	718.735	-158.370	1.4235
2.860.000	720.563	-158.716	1.4236
2.870.000	722.179	-159.045	1.4237
2.880.000	724.070	-159.394	1.4238
2.890.000	725.625	-159.632	1.4239
2.900.000	727.433	-160.059	1.4242
2.910.000	729.095	-160.263	1.4245
2.920.000	730.758	-160.653	1.4251
2.930.000	732.595	-160.923	1.4254
2.940.000	734.165	-161.270	1.4256

2.950.000	736.028	-161.549	1.4258
2.960.000	737.556	-161.927	1.4258
2.970.000	739.459	-162.188	1.4256
2.980.000	740.913	-162.527	1.4254
2.990.000	742.935	-162.923	1.4254
3.000.000	744.411	-163.142	1.4253
3.010.000	746.348	-163.518	1.4251
3.020.000	747.915	-163.835	1.4250
3.030.000	749.764	-164.139	1.4249
3.040.000	751.331	-164.443	1.4249
3.050.000	753.195	-164.830	1.4248
3.060.000	754.769	-165.024	1.4248
3.070.000	756.515	-165.442	1.4249
3.080.000	758.297	-165.674	1.4247
3.090.000	759.897	-166.041	1.4247
3.100.000	761.750	-166.303	1.4247
3.110.000	763.421	-166.681	1.4246
3.120.000	765.272	-166.937	1.4245
3.130.000	766.736	-167.306	1.4246
3.140.000	768.737	-167.598	1.4248
3.150.000	770.146	-167.926	1.4248
3.160.000	772.166	-168.293	1.4248
3.170.000	773.663	-168.528	1.4251
3.180.000	775.506	-168.874	1.4251
3.190.000	777.144	-169.193	1.4248
3.200.000	779.008	-169.494	1.4246
3.210.000	780.551	-169.770	1.4244
3.220.000	782.445	-170.180	1.4241
3.230.000	784.097	-170.371	1.4239
3.240.000	785.768	-170.763	1.4238

3.250.000	787.604	-171.044	1.4238
3.260.000	789.185	-171.345	1.4237
3.270.000	791.085	-171.630	1.4239
3.280.000	792.644	-172.025	1.4242
3.290.000	794.543	-172.257	1.4241
3.300.000	796.022	-172.597	1.4240
3.310.000	798.041	-172.971	1.4242
3.320.000	799.449	-173.201	1.4244
3.330.000	801.476	-173.583	1.4242
3.340.000	803.019	-173.851	1.4243
3.350.000	804.839	-174.164	1.4246
3.360.000	806.421	-174.458	1.4246
3.370.000	808.276	-174.803	1.4243
3.380.000	809.818	-175.015	1.4243
3.390.000	811.605	-175.415	1.4245
3.400.000	813.311	-175.606	1.4244
3.410.000	814.903	-175.976	1.4241
3.420.000	816.735	-176.234	1.4240
3.430.000	818.339	-176.561	1.4242
3.440.000	820.204	-176.831	1.4239
3.450.000	821.748	-177.193	1.4236
3.460.000	823.666	-177.459	1.4238
3.470.000	825.113	-177.769	1.4239
3.480.000	827.123	-178.157	1.4236
3.490.000	828.556	-178.361	1.4233
3.500.000	830.416	-178.722	1.4232
3.510.000	831.999	-179.016	1.4231
3.520.000	833.823	-179.321	1.4227
3.530.000	835.372	-179.581	1.4223
3.540.000	837.222	-179.969	1.4222

3.550.000	838.844	-180.169	1.4222
3.560.000	840.632	-180.564	1.4219
3.570.000	842.371	-180.799	1.4217
3.580.000	843.982	-181.135	1.4218
3.590.000	845.833	-181.369	1.4215
3.600.000	847.383	-181.761	1.4212
3.610.000	849.253	-181.985	1.4209
3.620.000	850.711	-182.323	1.4208
3.630.000	852.738	-182.670	1.4205
3.640.000	854.100	-182.901	1.4202
3.650.000	856.090	-183.291	1.4201
3.660.000	857.620	-183.529	1.4204
3.670.000	859.526	-183.856	1.4205
3.680.000	861.079	-184.162	1.4206
3.690.000	862.912	-184.480	1.4210
3.700.000	864.504	-184.705	1.4214
3.710.000	866.281	-185.128	1.4214
3.720.000	868.032	-185.290	1.4215
3.730.000	869.625	-185.645	1.4218
3.740.000	871.453	-185.918	1.4223
3.750.000	873.023	-186.231	1.4225
3.760.000	874.895	-186.495	1.4225
3.770.000	876.455	-186.884	1.4228
3.780.000	878.422	-187.120	1.4231
3.790.000	879.855	-187.465	1.4230
3.800.000	881.829	-187.816	1.4231
3.810.000	883.323	-188.016	1.4234
3.820.000	885.236	-188.414	1.4238
3.830.000	886.800	-188.681	1.4237
3.840.000	888.622	-188.993	1.4238

3.850.000	890.223	-189.276	1.4240
3.860.000	892.109	-189.646	1.4240
3.870.000	893.673	-189.825	1.4234
3.880.000	895.454	-190.241	1.4232
3.890.000	897.271	-190.467	1.4231
3.900.000	898.829	-190.826	1.4231
3.910.000	900.739	-191.099	1.4228
3.920.000	902.320	-191.418	1.4223
3.930.000	904.113	-191.675	1.4226
3.940.000	905.656	-192.040	1.4227
3.950.000	907.625	-192.326	1.4224
3.960.000	909.010	-192.608	1.4222
3.970.000	910.980	-192.976	1.4223
3.980.000	912.405	-193.187	1.4226
3.990.000	914.283	-193.541	1.4222
4.000.000	915.888	-193.847	1.4217
4.010.000	917.700	-194.134	1.4215
4.020.000	919.300	-194.396	1.4213
4.030.000	921.090	-194.779	1.4207
4.040.000	922.695	-194.933	1.4202
4.050.000	924.391	-195.331	1.4201
4.060.000	926.148	-195.600	1.4200
4.070.000	927.696	-195.864	1.4197
4.080.000	929.575	-196.132	1.4194
4.090.000	931.087	-196.488	1.4193
4.100.000	933.001	-196.728	1.4194
4.110.000	934.487	-197.060	1.4191
4.120.000	936.443	-197.368	1.4188
4.130.000	937.842	-197.606	1.4187
4.140.000	939.794	-197.980	1.4185

4.150.000	941.292	-198.207	1.4182
4.160.000	943.132	-198.511	1.4178
4.170.000	944.672	-198.776	1.4177
4.180.000	946.505	-199.110	1.4175
4.190.000	948.086	-199.318	1.4173
4.200.000	949.832	-199.710	1.4172
4.210.000	951.567	-199.909	1.4174
4.220.000	953.230	-200.266	1.4174
4.230.000	955.079	-200.520	1.4172
4.240.000	956.663	-200.820	1.4170
4.250.000	958.440	-201.065	1.4169
4.260.000	959.988	-201.428	1.4165
4.270.000	961.892	-201.679	1.4163
4.280.000	963.302	-201.992	1.4164
4.290.000	965.313	-202.346	1.4163
4.300.000	966.737	-202.546	1.4163
4.310.000	968.636	-202.913	1.4165
4.320.000	970.230	-203.211	1.4167
4.330.000	972.066	-203.483	1.4167
4.340.000	973.684	-203.761	1.4167
4.350.000	975.535	-204.144	1.4171
4.360.000	977.130	-204.312	1.4173
4.370.000	978.872	-204.706	1.4172
4.380.000	980.605	-204.943	1.4174
4.390.000	982.157	-205.244	1.4173
4.400.000	984.037	-205.501	1.4172
4.410.000	985.589	-205.857	1.4169
4.420.000	987.439	-206.072	1.4168
4.430.000	988.938	-206.424	1.4170
4.440.000	990.914	-206.730	1.4167

4.450.000	992.372	-206.986	1.4167
4.460.000	994.361	-207.353	1.4169
4.470.000	995.840	-207.565	1.4169
4.480.000	997.678	-207.891	1.4169
4.490.000	999.289	-208.185	1.4168
4.500.000	1.001.112	-208.465	1.4168
4.510.000	1.002.659	-208.697	1.4165
4.520.000	1.004.442	-209.090	1.4167
4.530.000	1.006.120	-209.252	1.4170
4.540.000	1.007.742	-209.622	1.4173
4.550.000	1.009.481	-209.814	1.4171
4.560.000	1.011.066	-210.139	1.4173
4.570.000	1.012.921	-210.395	1.4179
4.580.000	1.014.419	-210.739	1.4178
4.590.000	1.016.277	-210.958	1.4175
4.600.000	1.017.677	-211.276	1.4174
4.610.000	1.019.694	-211.612	1.4172
4.620.000	1.021.087	-211.811	1.4170
4.630.000	1.022.957	-212.164	1.4165
4.640.000	1.024.484	-212.433	1.4164
4.650.000	1.026.263	-212.712	1.4159
4.660.000	1.027.892	-212.985	1.4155
4.670.000	1.029.726	-213.329	1.4152
4.680.000	1.031.226	-213.533	1.4149
4.690.000	1.033.017	-213.904	1.4147
4.700.000	1.034.754	-214.122	1.4146
4.710.000	1.036.302	-214.427	1.4147
4.720.000	1.038.061	-214.670	1.4148
4.730.000	1.039.626	-214.968	1.4151
4.740.000	1.041.427	-215.232	1.4154

4.750.000	1.043.019	-215.607	1.4153
4.760.000	1.044.930	-215.860	1.4156
4.770.000	1.046.318	-216.148	1.4159
4.780.000	1.048.317	-216.503	1.4156
4.790.000	1.049.749	-216.720	1.4153
4.800.000	1.051.683	-217.063	1.4153
4.810.000	1.053.174	-217.350	1.4154
4.820.000	1.054.957	-217.670	1.4154
4.830.000	1.056.594	-217.928	1.4150
4.840.000	1.058.391	-218.273	1.4148
4.850.000	1.059.936	-218.463	1.4146
4.860.000	1.061.692	-218.862	1.4140
4.870.000	1.063.501	-219.107	1.4137
4.880.000	1.065.073	-219.408	1.4131
4.890.000	1.066.770	-219.684	1.4127
4.900.000	1.068.515	-220.094	1.4123
4.910.000	1.070.300	-220.321	1.4122
4.920.000	1.071.760	-220.652	1.4123
4.930.000	1.073.768	-220.975	1.4118
4.940.000	1.075.151	-221.231	1.4116
4.950.000	1.077.103	-221.589	1.4113
4.960.000	1.078.692	-221.875	1.4111
4.970.000	1.080.462	-222.170	1.4112
4.980.000	1.082.031	-222.516	1.4112
4.990.000	1.083.919	-222.795	1.4114
5.000.000	1.085.428	-223.063	1.4114
5.010.000	1.087.310	-223.500	1.4115
5.020.000	1.088.972	-223.656	1.4120
5.030.000	1.090.648	-224.040	1.4122
5.040.000	1.092.381	-224.310	1.4125

5.050.000	1.093.996	-224.657	1.4127
5.060.000	1.095.817	-224.889	1.4130
5.070.000	1.097.417	-225.317	1.4133
5.080.000	1.099.301	-225.550	1.4134
5.090.000	1.100.727	-225.880	1.4135
5.100.000	1.102.759	-226.247	1.4135
5.110.000	1.104.147	-226.435	1.4134
5.120.000	1.106.141	-226.856	1.4136
5.130.000	1.107.645	-227.133	1.4137
5.140.000	1.109.440	-227.414	1.4135
5.150.000	1.110.986	-227.704	1.4136
5.160.000	1.112.810	-228.060	1.4135
5.170.000	1.114.368	-228.266	1.4131
5.180.000	1.116.149	-228.641	1.4128
5.190.000	1.117.818	-228.873	1.4123
5.200.000	1.119.445	-229.220	1.4118
5.210.000	1.121.253	-229.488	1.4112
5.220.000	1.122.819	-229.814	1.4107
5.230.000	1.124.629	-230.054	1.4103
5.240.000	1.126.162	-230.433	1.4099
5.250.000	1.128.107	-230.673	1.4095
5.260.000	1.129.470	-230.979	1.4091
5.270.000	1.131.437	-231.341	1.4088
5.280.000	1.132.909	-231.560	1.4087
5.290.000	1.134.750	-231.909	1.4087
5.300.000	1.136.299	-232.204	1.4088
5.310.000	1.138.141	-232.490	1.4089
5.320.000	1.139.650	-232.755	1.4092
5.330.000	1.141.519	-233.153	1.4093
5.340.000	1.143.108	-233.315	1.4095

5.350.000	1.144.822	-233.706	1.4098
5.360.000	1.146.586	-233.960	1.4100
5.370.000	1.148.202	-234.293	1.4100
5.380.000	1.149.981	-234.524	1.4101
5.390.000	1.151.561	-234.890	1.4103
5.400.000	1.153.393	-235.105	1.4102
5.410.000	1.154.898	-235.451	1.4097
5.420.000	1.156.862	-235.785	1.4094
5.430.000	1.158.255	-236.025	1.4092
5.440.000	1.160.207	-236.393	1.4089
5.450.000	1.161.729	-236.672	1.4085
5.460.000	1.163.571	-236.986	1.4083
5.470.000	1.165.155	-237.277	1.4082
5.480.000	1.167.004	-237.592	1.4082
5.490.000	1.168.532	-237.813	1.4084
5.500.000	1.170.374	-238.231	1.4086
5.510.000	1.171.990	-238.429	1.4087
5.520.000	1.173.651	-238.782	1.4090
5.530.000	1.175.443	-239.058	1.4095
5.540.000	1.177.073	-239.379	1.4098
5.550.000	1.178.866	-239.613	1.4097
5.560.000	1.180.422	-239.989	1.4097
5.570.000	1.182.341	-240.211	1.4096
5.580.000	1.183.783	-240.580	1.4096
5.590.000	1.185.780	-240.925	1.4095
5.600.000	1.187.227	-241.145	1.4093
5.610.000	1.189.149	-241.533	1.4093
5.620.000	1.190.666	-241.801	1.4093
5.630.000	1.192.507	-242.095	1.4093
5.640.000	1.194.066	-242.400	1.4093

5.650.000	1.195.952	-242.761	1.4091
5.660.000	1.197.520	-242.963	1.4090
5.670.000	1.199.278	-243.363	1.4089
5.680.000	1.201.042	-243.592	1.4087
5.690.000	1.202.643	-243.924	1.4085
5.700.000	1.204.423	-244.184	1.4084
5.710.000	1.206.066	-244.527	1.4081
5.720.000	1.207.789	-244.740	1.4079
5.730.000	1.209.365	-245.108	1.4078
5.740.000	1.211.248	-245.365	1.4074
5.750.000	1.212.638	-245.671	1.4070
5.760.000	1.214.648	-246.022	1.4071
5.770.000	1.215.998	-246.217	1.4070
5.780.000	1.217.912	-246.590	1.4067
5.790.000	1.219.462	-246.903	1.4066
5.800.000	1.221.301	-247.157	1.4070
5.810.000	1.222.832	-247.415	1.4071
5.820.000	1.224.722	-247.826	1.4070
5.830.000	1.226.273	-247.989	1.4070
5.840.000	1.228.005	-248.366	1.4072
5.850.000	1.229.702	-248.647	1.4070
5.860.000	1.231.332	-248.924	1.4065
5.870.000	1.233.103	-249.187	1.4061
5.880.000	1.234.708	-249.574	1.4059
5.890.000	1.236.541	-249.768	1.4056
5.900.000	1.238.027	-250.137	1.4053
5.910.000	1.239.998	-250.467	1.4051
5.920.000	1.241.422	-250.698	1.4049
5.930.000	1.243.364	-251.074	1.4047
5.940.000	1.244.858	-251.324	1.4046

5.950.000	1.246.685	-251.640	1.4046
5.960.000	1.248.257	-251.952	1.4044
5.970.000	1.250.115	-252.256	1.4043
5.980.000	1.251.626	-252.490	1.4044
5.990.000	1.253.444	-252.882	1.4046
6.000.000	1.255.092	-253.091	1.4044
6.010.000	1.256.769	-253.430	1.4042
6.020.000	1.258.521	-253.709	1.4041
6.030.000	1.260.184	-254.034	1.4044
6.040.000	1.261.973	-254.278	1.4041
6.050.000	1.263.561	-254.654	1.4043
6.060.000	1.265.421	-254.859	1.4047
6.070.000	1.266.864	-255.203	1.4050
6.080.000	1.268.851	-255.554	1.4052
6.090.000	1.270.285	-255.771	1.4052
6.100.000	1.272.172	-256.121	1.4056
6.110.000	1.273.730	-256.410	1.4056
6.120.000	1.275.555	-256.704	1.4053
6.130.000	1.277.063	-256.980	1.4050
6.140.000	1.279.033	-257.359	1.4050
6.150.000	1.280.522	-257.528	1.4049
6.160.000	1.282.340	-257.926	1.4046
6.170.000	1.284.048	-258.139	1.4045
6.180.000	1.285.631	-258.470	1.4044
6.190.000	1.287.441	-258.729	1.4043
6.200.000	1.289.109	-259.096	1.4040
6.210.000	1.290.827	-259.264	1.4039
6.220.000	1.292.411	-259.672	1.4040
6.230.000	1.294.332	-259.908	1.4039
6.240.000	1.295.782	-260.251	1.4037

6.250.000	1.297.793	-260.586	1.4035
6.260.000	1.299.245	-260.822	1.4035
6.270.000	1.301.154	-261.157	1.4035
6.280.000	1.302.681	-261.487	1.4033
6.290.000	1.304.548	-261.745	1.4030
6.300.000	1.306.014	-261.995	1.4031
6.310.000	1.307.846	-262.400	1.4032
6.320.000	1.309.454	-262.596	1.4030
6.330.000	1.311.206	-262.953	1.4028
6.340.000	1.312.875	-263.214	1.4029
6.350.000	1.314.522	-263.524	1.4031
6.360.000	1.316.284	-263.796	1.4031
6.370.000	1.317.914	-264.168	1.4030
6.380.000	1.319.669	-264.337	1.4031
6.390.000	1.321.200	-264.720	1.4029
6.400.000	1.323.147	-265.008	1.4027
6.410.000	1.324.492	-265.279	1.4025
6.420.000	1.326.484	-265.645	1.4026
6.430.000	1.327.989	-265.891	1.4026
6.440.000	1.329.812	-266.198	1.4025
6.450.000	1.331.294	-266.485	1.4026
6.460.000	1.333.260	-266.817	1.4028
6.470.000	1.334.706	-267.035	1.4027
6.480.000	1.336.578	-267.411	1.4025
6.490.000	1.338.154	-267.599	1.4024
6.500.000	1.339.838	-267.944	1.4022
6.510.000	1.341.560	-268.216	1.4020
6.520.000	1.343.233	-268.525	1.4018
6.530.000	1.344.955	-268.737	1.4019
6.540.000	1.346.585	-269.135	1.4021

6.550.000	1.348.428	-269.326	1.4021
6.560.000	1.349.882	-269.634	1.4020
6.570.000	1.351.781	-269.983	1.4023
6.580.000	1.353.245	-270.190	1.4026
6.590.000	1.355.143	-270.538	1.4025
6.600.000	1.356.688	-270.837	1.4023
6.610.000	1.358.521	-271.084	1.4022
6.620.000	1.360.040	-271.382	1.4022
6.630.000	1.361.937	-271.729	1.4019
6.640.000	1.363.478	-271.905	1.4014
6.650.000	1.365.263	-272.277	1.4012
6.660.000	1.366.922	-272.520	1.4012
6.670.000	1.368.591	-272.819	1.4010
6.680.000	1.370.312	-273.077	1.4007
6.690.000	1.371.994	-273.420	1.4005
6.700.000	1.373.761	-273.614	1.4007
6.710.000	1.375.334	-274.034	1.4008
6.720.000	1.377.258	-274.220	1.4005
6.730.000	1.378.641	-274.537	1.4002
6.740.000	1.380.630	-274.885	1.4003
6.750.000	1.382.115	-275.116	1.4005
6.760.000	1.383.959	-275.441	1.4003
6.770.000	1.385.489	-275.738	1.3999
6.780.000	1.387.391	-276.004	1.3998
6.790.000	1.388.858	-276.280	1.3999
6.800.000	1.390.776	-276.646	1.3995
6.810.000	1.392.327	-276.836	1.3993
6.820.000	1.394.105	-277.222	1.3992
6.830.000	1.395.798	-277.473	1.3993
6.840.000	1.397.455	-277.767	1.3990

6.850.000	1.399.203	-278.031	1.3987
6.860.000	1.400.864	-278.412	1.3986
6.870.000	1.402.633	-278.577	1.3986
6.880.000	1.404.204	-278.941	1.3984
6.890.000	1.406.099	-279.229	1.3980
6.900.000	1.407.508	-279.483	1.3981
6.910.000	1.409.461	-279.818	1.3982
6.920.000	1.410.956	-280.094	1.3982
6.930.000	1.412.808	-280.376	1.3979
6.940.000	1.414.353	-280.648	1.3979
6.950.000	1.416.197	-280.949	1.3975
6.960.000	1.417.679	-281.145	1.3974
6.970.000	1.419.554	-281.520	1.3972
6.980.000	1.421.123	-281.700	1.3973
6.990.000	1.422.824	-282.039	1.3973
7.000.000	1.424.531	-282.295	1.3975
7.010.000	1.426.178	-282.579	1.3979
7.020.000	1.427.933	-282.782	1.3981
7.030.000	1.429.518	-283.165	1.3979
7.040.000	1.431.348	-283.309	1.3979
7.050.000	1.432.876	-283.663	1.3981
7.060.000	1.434.820	-283.957	1.3982
7.070.000	1.436.246	-284.186	1.3979
7.080.000	1.438.117	-284.508	1.3980
7.090.000	1.439.630	-284.763	1.3983
7.100.000	1.441.510	-285.010	1.3982
7.110.000	1.442.992	-285.283	1.3982
7.120.000	1.444.897	-285.599	1.3984
7.130.000	1.446.426	-285.781	1.3986
7.140.000	1.448.180	-286.143	1.3986

7.150.000	1.449.864	-286.378	1.3987
7.160.000	1.451.565	-286.669	1.3990
7.170.000	1.453.263	-286.940	1.3989
7.180.000	1.454.973	-287.251	1.3983
7.190.000	1.456.664	-287.456	1.3982
7.200.000	1.458.265	-287.818	1.3983
7.210.000	1.460.142	-287.998	1.3980
7.220.000	1.461.569	-288.313	1.3978
7.230.000	1.463.557	-288.624	1.3979
7.240.000	1.464.980	-288.821	1.3981
7.250.000	1.466.840	-289.140	1.3981
7.260.000	1.468.392	-289.450	1.3979
7.270.000	1.470.294	-289.707	1.3978
7.280.000	1.471.758	-289.950	1.3976
7.290.000	1.473.692	-290.317	1.3975
7.300.000	1.475.203	-290.475	1.3974
7.310.000	1.476.996	-290.859	1.3973
7.320.000	1.478.691	-291.112	1.3972
7.330.000	1.480.370	-291.362	1.3973
7.340.000	1.482.065	-291.633	1.3973
7.350.000	1.483.805	-291.997	1.3970
7.360.000	1.485.491	-292.116	1.3970
7.370.000	1.487.070	-292.506	1.3969
7.380.000	1.489.023	-292.743	1.3967
7.390.000	1.490.466	-293.021	1.3965
7.400.000	1.492.412	-293.334	1.3966
7.410.000	1.493.873	-293.596	1.3964
7.420.000	1.495.761	-293.879	1.3958
7.430.000	1.497.267	-294.195	1.3956
7.440.000	1.499.223	-294.477	1.3954

7.450.000	1.500.670	-294.712	1.3950
7.460.000	1.502.550	-295.082	1.3948
7.470.000	1.504.136	-295.258	1.3945
7.480.000	1.505.865	-295.585	1.3943
7.490.000	1.507.542	-295.877	1.3941
7.500.000	1.509.257	-296.128	1.3936
7.510.000	1.510.914	-296.367	1.3932
7.520.000	1.512.598	-296.733	1.3925
7.530.000	1.514.393	-296.885	1.3920
7.540.000	1.515.873	-297.230	1.3920
7.550.000	1.517.800	-297.498	1.3917
7.560.000	1.519.252	-297.727	1.3915
7.570.000	1.521.123	-298.044	1.3914
7.580.000	1.522.637	-298.332	1.3916
7.590.000	1.524.515	-298.587	1.3917
7.600.000	1.525.985	-298.865	1.3915
7.610.000	1.527.941	-299.200	1.3914
7.620.000	1.529.363	-299.397	1.3914
7.630.000	1.531.223	-299.756	1.3911
7.640.000	1.532.848	-299.983	1.3909
7.650.000	1.534.563	-300.274	1.3908
7.660.000	1.536.217	-300.518	1.3910
7.670.000	1.537.940	-300.846	1.3910
7.680.000	1.539.570	-301.032	1.3909
7.690.000	1.541.261	-301.388	1.3909
7.700.000	1.543.057	-301.549	1.3908
7.710.000	1.544.553	-301.877	1.3905
7.720.000	1.546.486	-302.151	1.3898
7.730.000	1.547.935	-302.396	1.3894
7.740.000	1.549.807	-302.700	1.3891

7.750.000	1.551.325	-303.000	1.3890
7.760.000	1.553.210	-303.223	1.3889
7.770.000	1.554.693	-303.490	1.3889
7.780.000	1.556.610	-303.829	1.3891
7.790.000	1.558.073	-303.971	1.3893
7.800.000	1.559.866	-304.318	1.3894
7.810.000	1.561.470	-304.541	1.3897
7.820.000	1.563.207	-304.792	1.3899
7.830.000	1.564.883	-305.071	1.3899
7.840.000	1.566.601	-305.386	1.3901
7.850.000	1.568.310	-305.541	1.3902
7.860.000	1.569.912	-305.908	1.3905
7.870.000	1.571.770	-306.116	1.3906
7.880.000	1.573.253	-306.394	1.3905
7.890.000	1.575.150	-306.679	1.3906
7.900.000	1.576.655	-306.963	1.3905
7.910.000	1.578.503	-307.237	1.3905
7.920.000	1.579.989	-307.518	1.3904
7.930.000	1.581.922	-307.786	1.3900
7.940.000	1.583.343	-308.042	1.3899
7.950.000	1.585.283	-308.378	1.3902
7.960.000	1.586.833	-308.589	1.3904
7.970.000	1.588.625	-308.917	1.3902
7.980.000	1.590.277	-309.201	1.3902
7.990.000	1.591.991	-309.466	1.3902
8.000.000	1.593.652	-309.706	1.3901
8.010.000	1.595.381	-310.074	1.3895
8.020.000	1.597.092	-310.230	1.3888
8.030.000	1.598.684	-310.600	1.3883
8.040.000	1.600.574	-310.852	1.3875

8.050.000	1.602.063	-311.150	1.3872
8.060.000	1.603.996	-311.459	1.3870
8.070.000	1.605.493	-311.786	1.3865
8.080.000	1.607.343	-312.015	1.3860
8.090.000	1.608.818	-312.326	1.3855
8.100.000	1.610.720	-312.655	1.3850
8.110.000	1.612.185	-312.845	1.3848
8.120.000	1.614.043	-313.217	1.3844
8.130.000	1.615.597	-313.470	1.3841
8.140.000	1.617.356	-313.767	1.3842
8.150.000	1.618.947	-314.050	1.3843
8.160.000	1.620.724	-314.361	1.3848
8.170.000	1.622.386	-314.573	1.3848
8.180.000	1.624.079	-314.977	1.3847
8.190.000	1.625.868	-315.130	1.3849
8.200.000	1.627.385	-315.499	1.3849
8.210.000	1.629.240	-315.763	1.3849
8.220.000	1.630.759	-316.029	1.3847
8.230.000	1.632.605	-316.305	1.3845
8.240.000	1.634.128	-316.630	1.3845
8.250.000	1.636.002	-316.890	1.3842
8.260.000	1.637.424	-317.172	1.3839
8.270.000	1.639.395	-317.505	1.3837
8.280.000	1.640.876	-317.696	1.3837
8.290.000	1.642.710	-318.060	1.3834
8.300.000	1.644.319	-318.317	1.3834
8.310.000	1.646.070	-318.598	1.3834
8.320.000	1.647.678	-318.852	1.3831
8.330.000	1.649.474	-319.211	1.3830
8.340.000	1.651.084	-319.379	1.3827

8.350.000	1.652.763	-319.752	1.3825
8.360.000	1.654.560	-319.961	1.3823
8.370.000	1.656.059	-320.264	1.3821
8.380.000	1.657.950	-320.515	1.3824
8.390.000	1.659.468	-320.814	1.3826
8.400.000	1.661.316	-321.089	1.3825
8.410.000	1.662.846	-321.408	1.3826
8.420.000	1.664.748	-321.682	1.3828
8.430.000	1.666.211	-321.937	1.3828
8.440.000	1.668.141	-322.291	1.3825
8.450.000	1.669.626	-322.483	1.3825
8.460.000	1.671.461	-322.822	1.3825
8.470.000	1.673.042	-323.076	1.3824
8.480.000	1.674.819	-323.360	1.3821
8.490.000	1.676.432	-323.611	1.3824
8.500.000	1.678.184	-324.007	1.3826
8.510.000	1.679.870	-324.143	1.3825
8.520.000	1.681.530	-324.530	1.3824
8.530.000	1.683.318	-324.786	1.3825
8.540.000	1.684.943	-325.073	1.3828
8.550.000	1.686.751	-325.332	1.3826
8.560.000	1.688.275	-325.664	1.3821
8.570.000	1.690.171	-325.915	1.3819
8.580.000	1.691.622	-326.234	1.3815
8.590.000	1.693.573	-326.543	1.3809
8.600.000	1.695.060	-326.775	1.3802
8.610.000	1.696.893	-327.130	1.3799
8.620.000	1.698.511	-327.363	1.3797
8.630.000	1.700.292	-327.678	1.3793
8.640.000	1.701.884	-327.953	1.3789

8.650.000	1.703.701	-328.269	1.3788
8.660.000	1.705.275	-328.485	1.3788
8.670.000	1.707.022	-328.852	1.3783
8.680.000	1.708.729	-329.034	1.3779
8.690.000	1.710.295	-329.380	1.3778
8.700.000	1.712.121	-329.612	1.3778
8.710.000	1.713.649	-329.917	1.3777
8.720.000	1.715.488	-330.169	1.3775
8.730.000	1.717.042	-330.528	1.3779
8.740.000	1.718.916	-330.752	1.3782
8.750.000	1.720.357	-331.037	1.3782
8.760.000	1.722.310	-331.376	1.3781
8.770.000	1.723.759	-331.579	1.3781
8.780.000	1.725.631	-331.917	1.3783
8.790.000	1.727.192	-332.181	1.3782
8.800.000	1.728.967	-332.434	1.3778
8.810.000	1.730.532	-332.696	1.3777
8.820.000	1.732.328	-333.036	1.3780
8.830.000	1.733.898	-333.196	1.3781
8.840.000	1.735.635	-333.588	1.3779
8.850.000	1.737.383	-333.783	1.3780
8.860.000	1.738.994	-334.076	1.3781
8.870.000	1.740.802	-334.322	1.3781
8.880.000	1.742.366	-334.613	1.3778
8.890.000	1.744.182	-334.823	1.3774
8.900.000	1.745.678	-335.146	1.3774
8.910.000	1.747.580	-335.365	1.3773
8.920.000	1.749.004	-335.635	1.3770
8.930.000	1.751.000	-335.943	1.3768
8.940.000	1.752.331	-336.112	1.3767

8.950.000	1.754.287	-336.456	1.3767
8.960.000	1.755.839	-336.693	1.3763
8.970.000	1.757.676	-336.939	1.3761
8.980.000	1.759.203	-337.182	1.3760
8.990.000	1.760.980	-337.484	1.3755
9.000.000	1.762.624	-337.641	1.3751
9.010.000	1.764.325	-337.968	1.3748
9.020.000	1.766.082	-338.181	1.3746
9.030.000	1.767.628	-338.441	1.3741
9.040.000	1.769.431	-338.665	1.3737
9.050.000	1.771.024	-338.966	1.3735
9.060.000	1.772.859	-339.167	1.3735
9.070.000	1.774.330	-339.473	1.3732
9.080.000	1.776.322	-339.735	1.3730
9.090.000	1.777.735	-339.966	1.3731
9.100.000	1.779.665	-340.276	1.3729
9.110.000	1.781.154	-340.502	1.3725
9.120.000	1.783.000	-340.783	1.3723
9.130.000	1.784.575	-341.042	1.3723
9.140.000	1.786.424	-341.326	1.3720
9.150.000	1.787.938	-341.509	1.3715
9.160.000	1.789.719	-341.872	1.3712
9.170.000	1.791.408	-342.038	1.3711
9.180.000	1.793.077	-342.349	1.3707
9.190.000	1.794.870	-342.587	1.3701
9.200.000	1.796.469	-342.863	1.3696
9.210.000	1.798.264	-343.079	1.3695
9.220.000	1.799.866	-343.449	1.3692
9.230.000	1.801.678	-343.603	1.3687
9.240.000	1.803.187	-343.907	1.3684

9.250.000	1.805.087	-344.195	1.3682
9.260.000	1.806.536	-344.382	1.3678
9.270.000	1.808.440	-344.717	1.3674
9.280.000	1.809.946	-344.957	1.3671
9.290.000	1.811.764	-345.208	1.3668
9.300.000	1.813.298	-345.468	1.3665
9.310.000	1.815.184	-345.774	1.3664
9.320.000	1.816.696	-345.948	1.3665
9.330.000	1.818.438	-346.309	1.3662
9.340.000	1.820.167	-346.480	1.3658
9.350.000	1.821.773	-346.792	1.3655
9.360.000	1.823.512	-347.008	1.3655
9.370.000	1.825.112	-347.287	1.3652
9.380.000	1.826.920	-347.515	1.3646
9.390.000	1.828.427	-347.837	1.3644
9.400.000	1.830.358	-348.024	1.3643
9.410.000	1.831.776	-348.318	1.3642
9.420.000	1.833.717	-348.638	1.3636
9.430.000	1.835.141	-348.818	1.3636
9.440.000	1.837.015	-349.120	1.3637
9.450.000	1.838.549	-349.396	1.3638
9.460.000	1.840.364	-349.639	1.3637
9.470.000	1.841.887	-349.861	1.3639
9.480.000	1.843.738	-350.214	1.3640
9.490.000	1.845.292	-350.362	1.3639
9.500.000	1.847.006	-350.696	1.3636
9.510.000	1.848.721	-350.937	1.3631
9.520.000	1.850.390	-351.208	1.3627
9.530.000	1.852.142	-351.422	1.3622
9.540.000	1.853.777	-351.742	1.3618

9.550.000	1.855.491	-351.916	1.3615
9.560.000	1.857.049	-352.264	1.3611
9.570.000	1.858.960	-352.508	1.3607
9.580.000	1.860.374	-352.751	1.3605
9.590.000	1.862.341	-353.080	1.3600
9.600.000	1.863.797	-353.303	1.3595
9.610.000	1.865.649	-353.596	1.3591
9.620.000	1.867.161	-353.867	1.3589
9.630.000	1.869.087	-354.148	1.3587
9.640.000	1.870.612	-354.385	1.3586
9.650.000	1.872.453	-354.767	1.3586
9.660.000	1.874.055	-354.926	1.3586
9.670.000	1.875.755	-355.264	1.3586
9.680.000	1.877.444	-355.512	1.3587
9.690.000	1.879.155	-355.794	1.3588
9.700.000	1.880.864	-356.027	1.3587
9.710.000	1.882.484	-356.389	1.3587
9.720.000	1.884.292	-356.570	1.3590
9.730.000	1.885.808	-356.921	1.3589
9.740.000	1.887.743	-357.203	1.3586
9.750.000	1.889.181	-357.440	1.3585
9.760.000	1.891.103	-357.787	1.3584
9.770.000	1.892.651	-358.033	1.3582
9.780.000	1.894.453	-358.323	1.3579
9.790.000	1.895.989	-358.610	1.3579
9.800.000	1.897.900	-358.899	1.3578
9.810.000	1.899.361	-359.149	1.3575
9.820.000	1.901.202	-359.485	1.3571
9.830.000	1.902.804	-359.683	1.3568
9.840.000	1.904.468	-360.009	1.3562

9.850.000	1.906.212	-360.285	1.3556
9.860.000	1.907.919	-360.558	1.3553
9.870.000	1.909.602	-360.787	1.3548
9.880.000	1.911.273	-361.173	1.3543
9.890.000	1.913.039	-361.321	1.3540
9.900.000	1.914.522	-361.645	1.3539
9.910.000	1.916.479	-361.958	1.3535
9.920.000	1.917.794	-362.189	1.3533
9.930.000	1.919.778	-362.480	1.3531
9.940.000	1.921.266	-362.759	1.3528
9.950.000	1.923.062	-363.000	1.3525
9.960.000	1.924.626	-363.272	1.3519
9.970.000	1.926.493	-363.603	1.3513
9.980.000	1.928.000	-363.786	1.3508
9.990.000	1.929.819	-364.133	1.3503
10.000.000	1.931.408	-364.342	1.3499
10.010.000	1.933.097	-364.632	1.3495
10.020.000	1.934.789	-364.869	1.3493
10.030.000	1.936.462	-365.175	1.3491
10.040.000	1.938.158	-365.357	1.3487
10.050.000	1.939.754	-365.733	1.3481
10.060.000	1.941.599	-365.908	1.3478
10.070.000	1.943.057	-366.206	1.3474
10.080.000	1.945.004	-366.508	1.3471
10.090.000	1.946.450	-366.721	1.3468
10.100.000	1.948.339	-367.029	1.3467
10.110.000	1.949.829	-367.303	1.3465
10.120.000	1.951.691	-367.553	1.3461
10.130.000	1.953.165	-367.797	1.3459
10.140.000	1.955.056	-368.127	1.3458

10.150.000	1.956.545	-368.294	1.3454
10.160.000	1.958.315	-368.630	1.3452
10.170.000	1.960.001	-368.870	1.3450
10.180.000	1.961.653	-369.132	1.3447
10.190.000	1.963.359	-369.383	1.3444
10.200.000	1.965.124	-369.726	1.3440
10.210.000	1.966.790	-369.866	1.3436
10.220.000	1.968.400	-370.244	1.3430
10.230.000	1.970.282	-370.461	1.3425
10.240.000	1.971.693	-370.732	1.3421
10.250.000	1.973.634	-371.028	1.3417
10.260.000	1.975.097	-371.288	1.3414
10.270.000	1.976.939	-371.570	1.3413
10.280.000	1.978.472	-371.846	1.3410
10.290.000	1.980.361	-372.124	1.3405
10.300.000	1.981.819	-372.370	1.3403
10.310.000	1.983.770	-372.741	1.3401
10.320.000	1.985.297	-372.897	1.3399
10.330.000	1.987.067	-373.238	1.3396
10.340.000	1.988.709	-373.493	1.3393
10.350.000	1.990.410	-373.746	1.3391
10.360.000	1.992.075	-373.987	1.3389
10.370.000	1.993.777	-374.355	1.3388
10.380.000	1.995.478	-374.485	1.3386
10.390.000	1.997.096	-374.829	1.3387
10.400.000	1.998.915	-375.082	1.3389
10.410.000	2.000.414	-375.311	1.3391
10.420.000	2.002.323	-375.626	1.3391
10.430.000	2.003.855	-375.902	1.3390
10.440.000	2.005.686	-376.143	1.3389

10.450.000	2.007.169	-376.428	1.3388
10.460.000	2.009.063	-376.697	1.3385
10.470.000	2.010.509	-376.901	1.3381
10.480.000	2.012.361	-377.233	1.3378
10.490.000	2.013.942	-377.436	1.3376
10.500.000	2.015.678	-377.729	1.3372
10.510.000	2.017.314	-377.970	1.3366
10.520.000	2.019.088	-378.253	1.3360
10.530.000	2.020.698	-378.458	1.3354
10.540.000	2.022.389	-378.797	1.3351
10.550.000	2.024.173	-378.943	1.3346
10.560.000	2.025.704	-379.277	1.3343
10.570.000	2.027.587	-379.508	1.3343
10.580.000	2.029.037	-379.752	1.3342
10.590.000	2.030.881	-380.012	1.3338
10.600.000	2.032.403	-380.283	1.3335
10.610.000	2.034.234	-380.519	1.3331
10.620.000	2.035.691	-380.774	1.3326
10.630.000	2.037.659	-381.079	1.3317
10.640.000	2.039.123	-381.249	1.3313
10.650.000	2.041.019	-381.561	1.3309
10.660.000	2.042.554	-381.772	1.3306
10.670.000	2.044.320	-382.028	1.3305
10.680.000	2.045.951	-382.264	1.3303
10.690.000	2.047.695	-382.560	1.3301
10.700.000	2.049.290	-382.705	1.3298
10.710.000	2.050.974	-383.036	1.3295
10.720.000	2.052.757	-383.208	1.3293
10.730.000	2.054.279	-383.492	1.3289
10.740.000	2.056.163	-383.706	1.3286

10.750.000	2.057.626	-383.944	1.3283
10.760.000	2.059.505	-384.198	1.3282
10.770.000	2.061.005	-384.467	1.3280
10.780.000	2.062.893	-384.683	1.3279
10.790.000	2.064.344	-384.907	1.3277
10.800.000	2.066.218	-385.211	1.3273
10.810.000	2.067.744	-385.344	1.3270
10.820.000	2.069.542	-385.659	1.3269
10.830.000	2.071.138	-385.877	1.3266
10.840.000	2.072.885	-386.122	1.3261
10.850.000	2.074.513	-386.322	1.3257
10.860.000	2.076.288	-386.631	1.3254
10.870.000	2.077.927	-386.767	1.3249
10.880.000	2.079.610	-387.113	1.3245
10.890.000	2.081.434	-387.308	1.3241
10.900.000	2.082.933	-387.542	1.3238
10.910.000	2.084.796	-387.775	1.3233
10.920.000	2.086.369	-388.046	1.3231
10.930.000	2.088.134	-388.258	1.3231
10.940.000	2.089.639	-388.536	1.3229
10.950.000	2.091.596	-388.805	1.3225
10.960.000	2.093.042	-389.012	1.3223
10.970.000	2.094.940	-389.318	1.3221
10.980.000	2.096.556	-389.530	1.3217
10.990.000	2.098.315	-389.771	1.3208
11.000.000	2.099.927	-390.036	1.3203
11.010.000	2.101.709	-390.272	1.3199
11.020.000	2.103.314	-390.464	1.3192
11.030.000	2.105.045	-390.797	1.3185
11.040.000	2.106.734	-390.944	1.3180

11.050.000	2.108.282	-391.254	1.3174
11.060.000	2.110.091	-391.461	1.3165
11.070.000	2.111.636	-391.693	1.3162
11.080.000	2.113.481	-391.926	1.3162
11.090.000	2.114.979	-392.209	1.3165
11.100.000	2.116.904	-392.419	1.3165
11.110.000	2.118.309	-392.685	1.3162
11.120.000	2.120.255	-392.984	1.3167
11.130.000	2.121.685	-393.127	1.3169
11.140.000	2.123.568	-393.444	1.3164
11.150.000	2.125.106	-393.662	1.3161
11.160.000	2.126.897	-393.907	1.3157
11.170.000	2.128.435	-394.136	1.3160
11.180.000	2.130.269	-394.420	1.3158
11.190.000	2.131.845	-394.580	1.3152
11.200.000	2.133.609	-394.937	1.3148
11.210.000	2.135.313	-395.053	1.3145
11.220.000	2.136.869	-395.377	1.3141
11.230.000	2.138.773	-395.551	1.3133
11.240.000	2.140.331	-395.774	1.3125
11.250.000	2.142.073	-395.987	1.3122
11.260.000	2.143.624	-396.302	1.3120
11.270.000	2.145.504	-396.472	1.3114
11.280.000	2.146.912	-396.730	1.3108
11.290.000	2.148.877	-396.993	1.3107
11.300.000	2.150.286	-397.153	1.3106
11.310.000	2.152.106	-397.431	1.3101
11.320.000	2.153.726	-397.643	1.3097
11.330.000	2.155.474	-397.838	1.3095
11.340.000	2.157.034	-398.057	1.3092

11.350.000	2.158.892	-398.349	1.3086
11.360.000	2.160.462	-398.489	1.3082
11.370.000	2.162.114	-398.742	1.3082
11.380.000	2.163.932	-398.957	1.3079
11.390.000	2.165.501	-399.167	1.3075
11.400.000	2.167.297	-399.366	1.3071
11.410.000	2.168.861	-399.626	1.3069
11.420.000	2.170.638	-399.772	1.3069
11.430.000	2.172.221	-400.077	1.3061
11.440.000	2.174.101	-400.268	1.3056
11.450.000	2.175.534	-400.489	1.3055
11.460.000	2.177.461	-400.784	1.3056
11.470.000	2.178.929	-400.945	1.3050
11.480.000	2.180.752	-401.200	1.3042
11.490.000	2.182.290	-401.430	1.3034
11.500.000	2.184.141	-401.682	1.3031
11.510.000	2.185.703	-401.894	1.3024
11.520.000	2.187.478	-402.198	1.3011
11.530.000	2.189.112	-402.317	1.3008
11.540.000	2.190.828	-402.625	1.3003
11.550.000	2.192.571	-402.811	1.3005
11.560.000	2.194.186	-403.050	1.2999
11.570.000	2.195.985	-403.227	1.2994
11.580.000	2.197.547	-403.565	1.2989
11.590.000	2.199.355	-403.648	1.2981
11.600.000	2.200.873	-403.965	1.2976
11.610.000	2.202.815	-404.203	1.2970
11.620.000	2.204.206	-404.364	1.2963
11.630.000	2.206.012	-404.644	1.2960
11.640.000	2.207.676	-404.867	1.2956

11.650.000	2.209.434	-405.105	1.2955
11.660.000	2.211.010	-405.344	1.2951
11.670.000	2.212.867	-405.585	1.2945
11.680.000	2.214.383	-405.762	1.2941
11.690.000	2.216.152	-406.092	1.2937
11.700.000	2.217.815	-406.196	1.2932
11.710.000	2.219.422	-406.491	1.2927
11.720.000	2.221.218	-406.665	1.2925
11.730.000	2.222.737	-406.926	1.2920
11.740.000	2.224.513	-407.120	1.2914
11.750.000	2.226.175	-407.420	1.2911
11.760.000	2.227.959	-407.534	1.2908
11.770.000	2.229.454	-407.846	1.2904
11.780.000	2.231.382	-408.140	1.2899
11.790.000	2.232.750	-408.285	1.2897
11.800.000	2.234.671	-408.551	1.2894
11.810.000	2.236.212	-408.773	1.2889
11.820.000	2.237.992	-408.961	1.2882
11.830.000	2.239.573	-409.226	1.2878
11.840.000	2.241.425	-409.475	1.2870
11.850.000	2.242.931	-409.614	1.2862
11.860.000	2.244.698	-409.933	1.2857
11.870.000	2.246.335	-410.089	1.2854
11.880.000	2.247.991	-410.348	1.2849
11.890.000	2.249.726	-410.553	1.2844
11.900.000	2.251.342	-410.751	1.2843
11.910.000	2.253.076	-410.929	1.2839
11.920.000	2.254.763	-411.252	1.2836
11.930.000	2.256.500	-411.380	1.2833
11.940.000	2.257.973	-411.623	1.2830

11.950.000	2.259.870	-411.885	1.2829
11.960.000	2.261.356	-412.042	1.2826
11.970.000	2.263.241	-412.305	1.2826
11.980.000	2.264.750	-412.528	1.2824
11.990.000	2.266.608	-412.734	1.2819
12.000.000	2.268.142	-412.962	1.2814
12.010.000	2.270.011	-413.240	1.2812
12.020.000	2.271.532	-413.371	1.2806
12.030.000	2.273.291	-413.684	1.2797
12.040.000	2.274.943	-413.849	1.2792
12.050.000	2.276.621	-414.091	1.2789
12.060.000	2.278.311	-414.301	1.2785
12.070.000	2.279.980	-414.569	1.2778
12.080.000	2.281.720	-414.703	1.2774
12.090.000	2.283.279	-415.007	1.2772
12.100.000	2.285.190	-415.185	1.2767
12.110.000	2.286.610	-415.424	1.2761
12.120.000	2.288.561	-415.683	1.2758
12.130.000	2.290.033	-415.854	1.2754
12.140.000	2.291.863	-416.109	1.2748
12.150.000	2.293.434	-416.364	1.2743
12.160.000	2.295.229	-416.551	1.2739
12.170.000	2.296.731	-416.745	1.2736
12.180.000	2.298.706	-417.080	1.2732
12.190.000	2.300.172	-417.187	1.2728
12.200.000	2.301.909	-417.499	1.2724
12.210.000	2.303.619	-417.702	1.2720
12.220.000	2.305.261	-417.931	1.2715
12.230.000	2.306.987	-418.121	1.2710
12.240.000	2.308.621	-418.430	1.2702

12.250.000	2.310.383	-418.513	1.2695
12.260.000	2.311.905	-418.822	1.2690
12.270.000	2.313.760	-419.030	1.2684
12.280.000	2.315.181	-419.205	1.2677
12.290.000	2.317.163	-419.506	1.2671
12.300.000	2.318.596	-419.697	1.2664
12.310.000	2.320.417	-419.909	1.2659
12.320.000	2.321.942	-420.158	1.2653
12.330.000	2.323.803	-420.423	1.2649
12.340.000	2.325.342	-420.617	1.2645
12.350.000	2.327.193	-420.958	1.2643
12.360.000	2.328.760	-421.091	1.2642
12.370.000	2.330.467	-421.389	1.2640
12.380.000	2.332.114	-421.592	1.2637
12.390.000	2.333.810	-421.836	1.2633
12.400.000	2.335.467	-422.011	1.2630
12.410.000	2.337.117	-422.327	1.2624
12.420.000	2.338.916	-422.457	1.2619
12.430.000	2.340.399	-422.776	1.2614
12.440.000	2.342.288	-422.997	1.2607
12.450.000	2.343.774	-423.205	1.2601
12.460.000	2.345.654	-423.478	1.2594
12.470.000	2.347.180	-423.713	1.2587
12.480.000	2.348.967	-423.899	1.2581
12.490.000	2.350.438	-424.138	1.2576
12.500.000	2.352.369	-424.407	1.2571
12.510.000	2.353.834	-424.564	1.2567
12.520.000	2.355.676	-424.845	1.2563
12.530.000	2.357.233	-424.994	1.2560
12.540.000	2.358.924	-425.252	1.2554

12.550.000	2.360.599	-425.435	1.2550
12.560.000	2.362.383	-425.691	1.2546
12.570.000	2.363.990	-425.832	1.2538
12.580.000	2.365.702	-426.165	1.2531
12.590.000	2.367.452	-426.296	1.2525
12.600.000	2.368.948	-426.582	1.2518
12.610.000	2.370.896	-426.830	1.2513
12.620.000	2.372.383	-427.027	1.2507
12.630.000	2.374.239	-427.290	1.2501
12.640.000	2.375.749	-427.544	1.2496
12.650.000	2.377.564	-427.744	1.2492
12.660.000	2.379.085	-427.976	1.2486
12.670.000	2.380.947	-428.283	1.2482
12.680.000	2.382.439	-428.398	1.2478
12.690.000	2.384.285	-428.717	1.2476
12.700.000	2.385.891	-428.930	1.2472
12.710.000	2.387.631	-429.150	1.2470
12.720.000	2.389.279	-429.363	1.2469
12.730.000	2.391.032	-429.646	1.2469
12.740.000	2.392.647	-429.781	1.2467
12.750.000	2.394.314	-430.097	1.2465
12.760.000	2.396.089	-430.242	1.2464
12.770.000	2.397.637	-430.506	1.2462
12.780.000	2.399.501	-430.742	1.2459
12.790.000	2.401.041	-430.968	1.2457
12.800.000	2.402.882	-431.191	1.2454
12.810.000	2.404.349	-431.441	1.2449
12.820.000	2.406.235	-431.656	1.2443
12.830.000	2.407.706	-431.867	1.2439
12.840.000	2.409.630	-432.150	1.2434

12.850.000	2.411.092	-432.305	1.2428
12.860.000	2.412.896	-432.565	1.2422
12.870.000	2.414.428	-432.766	1.2419
12.880.000	2.416.222	-432.983	1.2416
12.890.000	2.417.771	-433.147	1.2411
12.900.000	2.419.562	-433.462	1.2405
12.910.000	2.421.238	-433.588	1.2400
12.920.000	2.422.867	-433.866	1.2395
12.930.000	2.424.613	-434.038	1.2388
12.940.000	2.426.136	-434.270	1.2381
12.950.000	2.427.996	-434.476	1.2377
12.960.000	2.429.530	-434.741	1.2372
12.970.000	2.431.365	-434.921	1.2368
12.980.000	2.432.842	-435.181	1.2365
12.990.000	2.434.771	-435.423	1.2362
13.000.000	2.436.145	-435.574	1.2358
13.010.000	2.438.064	-435.874	1.2355
13.020.000	2.439.581	-436.029	1.2353
13.030.000	2.441.361	-436.293	1.2350
13.040.000	2.442.968	-436.510	1.2345
13.050.000	2.444.724	-436.722	1.2341
13.060.000	2.446.318	-436.901	1.2335
13.070.000	2.448.065	-437.210	1.2326
13.080.000	2.449.737	-437.284	1.2318
13.090.000	2.451.340	-437.589	1.2312
13.100.000	2.453.120	-437.737	1.2307
13.110.000	2.454.666	-437.966	1.2303
13.120.000	2.456.453	-438.157	1.2301
13.130.000	2.458.012	-438.403	1.2299
13.140.000	2.459.867	-438.560	1.2297

13.150.000	2.461.342	-438.806	1.2295
13.160.000	2.463.245	-439.055	1.2292
13.170.000	2.464.706	-439.196	1.2289
13.180.000	2.466.564	-439.473	1.2287
13.190.000	2.468.107	-439.634	1.2285
13.200.000	2.469.854	-439.864	1.2281
13.210.000	2.471.418	-440.090	1.2278
13.220.000	2.473.207	-440.342	1.2275
13.230.000	2.474.823	-440.503	1.2270
13.240.000	2.476.576	-440.777	1.2263
13.250.000	2.478.228	-440.911	1.2258
13.260.000	2.479.828	-441.190	1.2254
13.270.000	2.481.613	-441.366	1.2250
13.280.000	2.483.200	-441.611	1.2246
13.290.000	2.484.974	-441.788	1.2247
13.300.000	2.486.549	-442.054	1.2247
13.310.000	2.488.408	-442.254	1.2245
13.320.000	2.489.837	-442.456	1.2247
13.330.000	2.491.801	-442.744	1.2246
13.340.000	2.493.225	-442.879	1.2243
13.350.000	2.495.069	-443.145	1.2239
13.360.000	2.496.651	-443.375	1.2237
13.370.000	2.498.458	-443.586	1.2235
13.380.000	2.500.001	-443.778	1.2228
13.390.000	2.501.822	-444.062	1.2221
13.400.000	2.503.405	-444.184	1.2218
13.410.000	2.505.149	-444.486	1.2216
13.420.000	2.506.795	-444.641	1.2210
13.430.000	2.508.395	-444.875	1.2203
13.440.000	2.510.155	-445.055	1.2197

13.450.000	2.511.743	-445.319	1.2192
13.460.000	2.513.530	-445.468	1.2183
13.470.000	2.515.080	-445.748	1.2173
13.480.000	2.516.948	-445.921	1.2167
13.490.000	2.518.367	-446.133	1.2163
13.500.000	2.520.250	-446.397	1.2158
13.510.000	2.521.739	-446.522	1.2154
13.520.000	2.523.580	-446.777	1.2152
13.530.000	2.525.102	-446.980	1.2151
13.540.000	2.526.952	-447.173	1.2147
13.550.000	2.528.478	-447.378	1.2141
13.560.000	2.530.243	-447.644	1.2139
13.570.000	2.531.835	-447.738	1.2136
13.580.000	2.533.522	-448.022	1.2131
13.590.000	2.535.258	-448.175	1.2130
13.600.000	2.536.881	-448.387	1.2129
13.610.000	2.538.624	-448.554	1.2125
13.620.000	2.540.262	-448.845	1.2120
13.630.000	2.542.025	-448.942	1.2117
13.640.000	2.543.502	-449.200	1.2115
13.650.000	2.545.407	-449.400	1.2109
13.660.000	2.546.795	-449.578	1.2105
13.670.000	2.548.717	-449.836	1.2105
13.680.000	2.550.237	-450.010	1.2103
13.690.000	2.552.044	-450.212	1.2098
13.700.000	2.553.597	-450.430	1.2096
13.710.000	2.555.450	-450.643	1.2095
13.720.000	2.556.929	-450.780	1.2092
13.730.000	2.558.779	-451.067	1.2086
13.740.000	2.560.339	-451.192	1.2083

13.750.000	2.562.043	-451.437	1.2082
13.760.000	2.563.744	-451.602	1.2079
13.770.000	2.565.357	-451.823	1.2073
13.780.000	2.567.079	-451.963	1.2068
13.790.000	2.568.718	-452.244	1.2065
13.800.000	2.570.503	-452.347	1.2061
13.810.000	2.572.000	-452.603	1.2057
13.820.000	2.573.884	-452.831	1.2055
13.830.000	2.575.344	-452.974	1.2051
13.840.000	2.577.230	-453.230	1.2045
13.850.000	2.578.721	-453.418	1.2040
13.860.000	2.580.550	-453.595	1.2039
13.870.000	2.582.054	-453.806	1.2034
13.880.000	2.583.902	-454.020	1.2027
13.890.000	2.585.393	-454.137	1.2022
13.900.000	2.587.182	-454.451	1.2019
13.910.000	2.588.878	-454.579	1.2013
13.920.000	2.590.529	-454.820	1.2006
13.930.000	2.592.232	-455.011	1.2001
13.940.000	2.593.924	-455.224	1.2000
13.950.000	2.595.622	-455.360	1.1997
13.960.000	2.597.254	-455.678	1.1993
13.970.000	2.599.036	-455.772	1.1994
13.980.000	2.600.527	-456.055	1.1994
13.990.000	2.602.444	-456.292	1.1991
14.000.000	2.603.872	-456.451	1.1985
14.010.000	2.605.736	-456.729	1.1982
14.020.000	2.607.272	-456.944	1.1980
14.030.000	2.609.083	-457.106	1.1975
14.040.000	2.610.547	-457.326	1.1967

14.050.000	2.612.441	-457.615	1.1962
14.060.000	2.613.918	-457.728	1.1960
14.070.000	2.615.705	-458.009	1.1959
14.080.000	2.617.329	-458.183	1.1955
14.090.000	2.618.985	-458.403	1.1950
14.100.000	2.620.678	-458.586	1.1949
14.110.000	2.622.334	-458.808	1.1951
14.120.000	2.624.009	-458.926	1.1947
14.130.000	2.625.620	-459.223	1.1940
14.140.000	2.627.452	-459.352	1.1938
14.150.000	2.628.924	-459.586	1.1937
14.160.000	2.630.829	-459.802	1.1934
14.170.000	2.632.276	-459.985	1.1928
14.180.000	2.634.133	-460.194	1.1924
14.190.000	2.635.628	-460.425	1.1925
14.200.000	2.637.480	-460.611	1.1922
14.210.000	2.638.954	-460.788	1.1916
14.220.000	2.640.815	-461.085	1.1912
14.230.000	2.642.313	-461.172	1.1911
14.240.000	2.644.072	-461.457	1.1912
14.250.000	2.645.752	-461.653	1.1908
14.260.000	2.647.430	-461.853	1.1903
14.270.000	2.649.092	-462.042	1.1902
14.280.000	2.650.795	-462.316	1.1898
14.290.000	2.652.467	-462.415	1.1894
14.300.000	2.654.048	-462.726	1.1888
14.310.000	2.655.894	-462.866	1.1882
14.320.000	2.657.357	-463.081	1.1876
14.330.000	2.659.210	-463.312	1.1870
14.340.000	2.660.714	-463.527	1.1864

14.350.000	2.662.574	-463.723	1.1859
14.360.000	2.664.021	-463.958	1.1853
14.370.000	2.665.966	-464.149	1.1850
14.380.000	2.667.411	-464.329	1.1849
14.390.000	2.669.275	-464.613	1.1846
14.400.000	2.670.817	-464.718	1.1845
14.410.000	2.672.573	-464.982	1.1844
14.420.000	2.674.156	-465.178	1.1841
14.430.000	2.675.897	-465.406	1.1837
14.440.000	2.677.489	-465.565	1.1832
14.450.000	2.679.282	-465.855	1.1829
14.460.000	2.680.947	-465.950	1.1825
14.470.000	2.682.521	-466.230	1.1821
14.480.000	2.684.331	-466.415	1.1819
14.490.000	2.685.852	-466.623	1.1815
14.500.000	2.687.741	-466.819	1.1812
14.510.000	2.689.248	-467.068	1.1807
14.520.000	2.691.021	-467.240	1.1802
14.530.000	2.692.563	-467.463	1.1797
14.540.000	2.694.446	-467.713	1.1790
14.550.000	2.695.899	-467.847	1.1783
14.560.000	2.697.750	-468.097	1.1776
14.570.000	2.699.312	-468.276	1.1770
14.580.000	2.701.057	-468.475	1.1767
14.590.000	2.702.661	-468.674	1.1762
14.600.000	2.704.417	-468.899	1.1756
14.610.000	2.706.027	-469.032	1.1751
14.620.000	2.707.729	-469.320	1.1749
14.630.000	2.709.420	-469.410	1.1743
14.640.000	2.710.974	-469.694	1.1740

14.650.000	2.712.686	-469.844	1.1737
14.660.000	2.714.289	-470.075	1.1734
14.670.000	2.716.109	-470.277	1.1733
14.680.000	2.717.590	-470.521	1.1734
14.690.000	2.719.469	-470.698	1.1736
14.700.000	2.720.854	-470.910	1.1736
14.710.000	2.722.838	-471.188	1.1735
14.720.000	2.724.292	-471.345	1.1735
14.730.000	2.726.157	-471.572	1.1734
14.740.000	2.727.701	-471.765	1.1731
14.750.000	2.729.414	-471.958	1.1727
14.760.000	2.731.039	-472.173	1.1722
14.770.000	2.732.777	-472.424	1.1718
14.780.000	2.734.282	-472.545	1.1714
14.790.000	2.736.053	-472.859	1.1710
14.800.000	2.737.753	-473.001	1.1707
14.810.000	2.739.339	-473.293	1.1705
14.820.000	2.741.230	-473.444	1.1704
14.830.000	2.742.662	-473.674	1.1704
14.840.000	2.744.434	-473.838	1.1704
14.850.000	2.746.005	-474.101	1.1702
14.860.000	2.747.887	-474.328	1.1698
14.870.000	2.749.311	-474.493	1.1693
14.880.000	2.751.246	-474.785	1.1687
14.890.000	2.752.624	-474.903	1.1680
14.900.000	2.754.482	-475.185	1.1672
14.910.000	2.756.010	-475.409	1.1667
14.920.000	2.757.822	-475.623	1.1663
14.930.000	2.759.347	-475.801	1.1661
14.940.000	2.761.185	-476.129	1.1659

14.950.000	2.762.718	-476.216	1.1656
14.960.000	2.764.459	-476.508	1.1653
14.970.000	2.766.139	-476.676	1.1651
14.980.000	2.767.743	-476.923	1.1647
14.990.000	2.769.540	-477.104	1.1643
15.000.000	2.771.086	-477.355	1.1637
15.010.000	2.772.890	-477.536	1.1630
15.020.000	2.774.295	-477.810	1.1625
15.030.000	2.776.252	-477.986	1.1615
15.040.000	2.777.734	-478.202	1.1608
15.050.000	2.779.606	-478.470	1.1602
15.060.000	2.781.130	-478.621	1.1599
15.070.000	2.782.900	-478.880	1.1595
15.080.000	2.784.425	-479.085	1.1593
15.090.000	2.786.239	-479.296	1.1591
15.100.000	2.787.733	-479.475	1.1592
15.110.000	2.789.537	-479.757	1.1592
15.120.000	2.791.140	-479.868	1.1592
15.130.000	2.792.796	-480.127	1.1593
15.140.000	2.794.506	-480.291	1.1594
15.150.000	2.796.118	-480.502	1.1594
15.160.000	2.797.862	-480.666	1.1593
15.170.000	2.799.518	-480.945	1.1591
15.180.000	2.801.259	-481.058	1.1591
15.190.000	2.802.758	-481.315	1.1587
15.200.000	2.804.637	-481.542	1.1581
15.210.000	2.806.048	-481.714	1.1576
15.220.000	2.807.952	-481.965	1.1573
15.230.000	2.809.442	-482.163	1.1565
15.240.000	2.811.208	-482.387	1.1558

15.250.000	2.812.782	-482.605	1.1550
15.260.000	2.814.603	-482.831	1.1545
15.270.000	2.816.094	-482.994	1.1540
15.280.000	2.817.906	-483.305	1.1536
15.290.000	2.819.519	-483.425	1.1536
15.300.000	2.821.170	-483.689	1.1535
15.310.000	2.822.887	-483.892	1.1533
15.320.000	2.824.497	-484.117	1.1530
15.330.000	2.826.213	-484.287	1.1528
15.340.000	2.827.780	-484.600	1.1526
15.350.000	2.829.587	-484.710	1.1522
15.360.000	2.831.061	-484.980	1.1517
15.370.000	2.832.961	-485.259	1.1514
15.380.000	2.834.443	-485.404	1.1513
15.390.000	2.836.324	-485.680	1.1514
15.400.000	2.837.863	-485.918	1.1510
15.410.000	2.839.606	-486.079	1.1505
15.420.000	2.841.120	-486.310	1.1502
15.430.000	2.842.997	-486.601	1.1497
15.440.000	2.844.481	-486.710	1.1491
15.450.000	2.846.246	-487.006	1.1486
15.460.000	2.847.853	-487.148	1.1483
15.470.000	2.849.500	-487.378	1.1482
15.480.000	2.851.251	-487.589	1.1481
15.490.000	2.852.911	-487.836	1.1482
15.500.000	2.854.596	-487.958	1.1481
15.510.000	2.856.171	-488.276	1.1480
15.520.000	2.857.999	-488.378	1.1480
15.530.000	2.859.402	-488.631	1.1479
15.540.000	2.861.327	-488.862	1.1478

15.550.000	2.862.763	-489.016	1.1475
15.560.000	2.864.598	-489.280	1.1476
15.570.000	2.866.149	-489.524	1.1475
15.580.000	2.867.966	-489.702	1.1472
15.590.000	2.869.454	-489.898	1.1472
15.600.000	2.871.343	-490.185	1.1470
15.610.000	2.872.851	-490.342	1.1469
15.620.000	2.874.666	-490.586	1.1467
15.630.000	2.876.231	-490.786	1.1466
15.640.000	2.877.874	-491.005	1.1463
15.650.000	2.879.544	-491.183	1.1456
15.660.000	2.881.249	-491.460	1.1451
15.670.000	2.882.898	-491.595	1.1445
15.680.000	2.884.482	-491.886	1.1435
15.690.000	2.886.300	-492.046	1.1425
15.700.000	2.887.788	-492.275	1.1420
15.710.000	2.889.714	-492.507	1.1414
15.720.000	2.891.198	-492.692	1.1407
15.730.000	2.892.998	-492.940	1.1403
15.740.000	2.894.528	-493.152	1.1400
15.750.000	2.896.378	-493.352	1.1397
15.760.000	2.897.833	-493.539	1.1395
15.770.000	2.899.668	-493.842	1.1394
15.780.000	2.901.187	-493.953	1.1393
15.790.000	2.902.983	-494.235	1.1390
15.800.000	2.904.561	-494.428	1.1387
15.810.000	2.906.255	-494.650	1.1388
15.820.000	2.907.918	-494.854	1.1386
15.830.000	2.909.609	-495.149	1.1376
15.840.000	2.911.281	-495.260	1.1372

15.850.000	2.912.897	-495.580	1.1369
15.860.000	2.914.714	-495.754	1.1364
15.870.000	2.916.158	-495.973	1.1359
15.880.000	2.917.984	-496.217	1.1357
15.890.000	2.919.525	-496.456	1.1357
15.900.000	2.921.300	-496.671	1.1355
15.910.000	2.922.789	-496.909	1.1352
15.920.000	2.924.652	-497.152	1.1349
15.930.000	2.926.093	-497.333	1.1348
15.940.000	2.927.972	-497.628	1.1346
15.950.000	2.929.522	-497.781	1.1344
15.960.000	2.931.245	-498.051	1.1344
15.970.000	2.932.828	-498.246	1.1341
15.980.000	2.934.542	-498.485	1.1340
15.990.000	2.936.143	-498.646	1.1341
16.000.000	2.937.834	-498.955	1.1335
16.010.000	2.939.495	-499.055	1.1332
16.020.000	2.941.039	-499.304	1.1328
16.030.000	2.942.862	-499.525	1.1325
16.040.000	2.944.399	-499.717	1.1320
16.050.000	2.946.234	-499.942	1.1317
16.060.000	2.947.770	-500.188	1.1317
16.070.000	2.949.611	-500.370	1.1311
16.080.000	2.951.055	-500.605	1.1310
16.090.000	2.952.966	-500.878	1.1309
16.100.000	2.954.372	-501.008	1.1304
16.110.000	2.956.234	-501.297	1.1297
16.120.000	2.957.750	-501.468	1.1290
16.130.000	2.959.505	-501.700	1.1287
16.140.000	2.961.117	-501.920	1.1279

16.150.000	2.962.879	-502.170	1.1270
16.160.000	2.964.450	-502.343	1.1267
16.170.000	2.966.185	-502.666	1.1267
16.180.000	2.967.861	-502.769	1.1262
16.190.000	2.969.418	-503.071	1.1256
16.200.000	2.971.212	-503.272	1.1255
16.210.000	2.972.768	-503.491	1.1253
16.220.000	2.974.538	-503.691	1.1245
16.230.000	2.976.031	-503.971	1.1241
16.240.000	2.977.920	-504.140	1.1239
16.250.000	2.979.359	-504.379	1.1237
16.260.000	2.981.260	-504.662	1.1231
16.270.000	2.982.694	-504.791	1.1226
16.280.000	2.984.560	-505.075	1.1222
16.290.000	2.986.111	-505.285	1.1216
16.300.000	2.987.899	-505.509	1.1208
16.310.000	2.989.419	-505.698	1.1203
16.320.000	2.991.193	-505.971	1.1200
16.330.000	2.992.756	-506.126	1.1196
16.340.000	2.994.411	-506.399	1.1192
16.350.000	2.996.137	-506.563	1.1190
16.360.000	2.997.703	-506.809	1.1188
16.370.000	2.999.505	-506.999	1.1184
16.380.000	3.001.070	-507.239	1.1180
16.390.000	3.002.840	-507.405	1.1179
16.400.000	3.004.357	-507.692	1.1177
16.410.000	3.006.210	-507.875	1.1173
16.420.000	3.007.617	-508.081	1.1171
16.430.000	3.009.525	-508.359	1.1172
16.440.000	3.010.946	-508.508	1.1169

16.450.000	3.012.780	-508.745	1.1163
16.460.000	3.014.317	-508.970	1.1159
16.470.000	3.016.072	-509.174	1.1155
16.480.000	3.017.602	-509.352	1.1150
16.490.000	3.019.394	-509.663	1.1144
16.500.000	3.020.989	-509.789	1.1139
16.510.000	3.022.692	-510.069	1.1136
16.520.000	3.024.333	-510.244	1.1130
16.530.000	3.025.929	-510.460	1.1124
16.540.000	3.027.642	-510.632	1.1119
16.550.000	3.029.239	-510.909	1.1117
16.560.000	3.030.999	-511.032	1.1112
16.570.000	3.032.498	-511.328	1.1106
16.580.000	3.034.347	-511.529	1.1104
16.590.000	3.035.758	-511.705	1.1105
16.600.000	3.037.638	-511.976	1.1102
16.610.000	3.039.150	-512.138	1.1096
16.620.000	3.040.943	-512.374	1.1091
16.630.000	3.042.480	-512.599	1.1085
16.640.000	3.044.292	-512.788	1.1078
16.650.000	3.045.747	-512.979	1.1069
16.660.000	3.047.548	-513.280	1.1065
16.670.000	3.049.143	-513.396	1.1063
16.680.000	3.050.788	-513.656	1.1061
16.690.000	3.052.495	-513.852	1.1055
16.700.000	3.054.083	-514.043	1.1053
16.710.000	3.055.789	-514.236	1.1048
16.720.000	3.057.446	-514.547	1.1043
16.730.000	3.059.217	-514.655	1.1039
16.740.000	3.060.731	-514.938	1.1035

16.750.000	3.062.608	-515.177	1.1031
16.760.000	3.063.995	-515.355	1.1030
16.770.000	3.065.909	-515.636	1.1033
16.780.000	3.067.402	-515.854	1.1031
16.790.000	3.069.167	-516.074	1.1027
16.800.000	3.070.682	-516.310	1.1023
16.810.000	3.072.525	-516.571	1.1021
16.820.000	3.074.000	-516.737	1.1013
16.830.000	3.075.822	-517.053	1.1006
16.840.000	3.077.429	-517.190	1.1001
16.850.000	3.079.123	-517.457	1.0994
16.860.000	3.080.816	-517.669	1.0989
16.870.000	3.082.452	-517.887	1.0985
16.880.000	3.084.125	-518.058	1.0983
16.890.000	3.085.771	-518.381	1.0981
16.900.000	3.087.509	-518.497	1.0978
16.910.000	3.089.013	-518.767	1.0975
16.920.000	3.090.884	-519.029	1.0973
16.930.000	3.092.345	-519.203	1.0970
16.940.000	3.094.231	-519.492	1.0968
16.950.000	3.095.718	-519.719	1.0965
16.960.000	3.097.589	-519.942	1.0960
16.970.000	3.099.022	-520.175	1.0956
16.980.000	3.100.888	-520.440	1.0952
16.990.000	3.102.365	-520.597	1.0945
17.000.000	3.104.140	-520.899	1.0941
17.010.000	3.105.745	-521.061	1.0936
17.020.000	3.107.409	-521.316	1.0932
17.030.000	3.109.035	-521.505	1.0926
17.040.000	3.110.760	-521.761	1.0923

17.050.000	3.112.394	-521.922	1.0918
17.060.000	3.114.021	-522.226	1.0912
17.070.000	3.115.798	-522.335	1.0907
17.080.000	3.117.270	-522.598	1.0904
17.090.000	3.119.125	-522.806	1.0898
17.100.000	3.120.574	-522.985	1.0892
17.110.000	3.122.407	-523.240	1.0887
17.120.000	3.123.885	-523.451	1.0881
17.130.000	3.125.731	-523.623	1.0874
17.140.000	3.127.177	-523.835	1.0866
17.150.000	3.129.057	-524.096	1.0859
17.160.000	3.130.544	-524.219	1.0854
17.170.000	3.132.353	-524.506	1.0849
17.180.000	3.133.969	-524.696	1.0846
17.190.000	3.135.657	-524.915	1.0842
17.200.000	3.137.268	-525.101	1.0839
17.210.000	3.138.964	-525.379	1.0838
17.220.000	3.140.576	-525.506	1.0838
17.230.000	3.142.230	-525.828	1.0833
17.240.000	3.143.974	-525.985	1.0830
17.250.000	3.145.498	-526.221	1.0827
17.260.000	3.147.305	-526.432	1.0823
17.270.000	3.148.816	-526.673	1.0816
17.280.000	3.150.655	-526.894	1.0810
17.290.000	3.152.155	-527.143	1.0804
17.300.000	3.154.002	-527.350	1.0797
17.310.000	3.155.448	-527.556	1.0787
17.320.000	3.157.327	-527.857	1.0782
17.330.000	3.158.809	-527.989	1.0775
17.340.000	3.160.597	-528.264	1.0769

17.350.000	3.162.163	-528.468	1.0764
17.360.000	3.163.916	-528.691	1.0760
17.370.000	3.165.485	-528.877	1.0757
17.380.000	3.167.211	-529.178	1.0755
17.390.000	3.168.856	-529.285	1.0752
17.400.000	3.170.488	-529.616	1.0746
17.410.000	3.172.274	-529.776	1.0743
17.420.000	3.173.809	-529.998	1.0740
17.430.000	3.175.571	-530.219	1.0737
17.440.000	3.177.163	-530.477	1.0730
17.450.000	3.178.951	-530.653	1.0725
17.460.000	3.180.423	-530.914	1.0721
17.470.000	3.182.355	-531.159	1.0715
17.480.000	3.183.717	-531.364	1.0709
17.490.000	3.185.603	-531.644	1.0704
17.500.000	3.187.143	-531.824	1.0700
17.510.000	3.188.854	-532.081	1.0693
17.520.000	3.190.516	-532.307	1.0688
17.530.000	3.192.234	-532.545	1.0682
17.540.000	3.193.791	-532.732	1.0677
17.550.000	3.195.542	-533.030	1.0671
17.560.000	3.197.187	-533.164	1.0667
17.570.000	3.198.788	-533.422	1.0661
17.580.000	3.200.518	-533.631	1.0654
17.590.000	3.202.093	-533.852	1.0648
17.600.000	3.203.889	-534.037	1.0641
17.610.000	3.205.408	-534.313	1.0633
17.620.000	3.207.186	-534.459	1.0625
17.630.000	3.208.686	-534.706	1.0618
17.640.000	3.210.571	-534.971	1.0613

17.650.000	3.211.974	-535.096	1.0608
17.660.000	3.213.813	-535.375	1.0601
17.670.000	3.215.323	-535.565	1.0595
17.680.000	3.217.090	-535.761	1.0589
17.690.000	3.218.625	-535.958	1.0583
17.700.000	3.220.409	-536.199	1.0576
17.710.000	3.221.958	-536.341	1.0569
17.720.000	3.223.681	-536.643	1.0562
17.730.000	3.225.332	-536.752	1.0559
17.740.000	3.226.952	-537.016	1.0554
17.750.000	3.228.695	-537.207	1.0549
17.760.000	3.230.284	-537.443	1.0546
17.770.000	3.232.014	-537.608	1.0542
17.780.000	3.233.564	-537.898	1.0538
17.790.000	3.235.403	-538.065	1.0533
17.800.000	3.236.792	-538.293	1.0527
17.810.000	3.238.700	-538.560	1.0522
17.820.000	3.240.138	-538.711	1.0517
17.830.000	3.241.948	-538.988	1.0513
17.840.000	3.243.476	-539.186	1.0508
17.850.000	3.245.252	-539.395	1.0502
17.860.000	3.246.779	-539.596	1.0498
17.870.000	3.248.611	-539.881	1.0491
17.880.000	3.250.085	-539.988	1.0484
17.890.000	3.251.803	-540.269	1.0478
17.900.000	3.253.488	-540.439	1.0474
17.910.000	3.255.058	-540.661	1.0469
17.920.000	3.256.778	-540.842	1.0466
17.930.000	3.258.421	-541.103	1.0464
17.940.000	3.260.148	-541.243	1.0461

17.950.000	3.261.700	-541.543	1.0457
17.960.000	3.263.518	-541.710	1.0455
17.970.000	3.264.948	-541.936	1.0452
17.980.000	3.266.866	-542.226	1.0448
17.990.000	3.268.341	-542.403	1.0442
18.000.000	3.270.152	-542.657	1.0436
18.010.000	3.271.642	-542.905	1.0430
18.020.000	3.273.450	-543.105	1.0422
18.030.000	3.274.933	-543.308	1.0417
18.040.000	3.276.761	-543.622	1.0409
18.050.000	3.278.291	-543.732	1.0403
18.060.000	3.280.033	-544.045	1.0399
18.070.000	3.281.662	-544.245	1.0394
18.080.000	3.283.312	-544.467	1.0390
18.090.000	3.285.034	-544.671	1.0386
18.100.000	3.286.658	-544.967	1.0381
18.110.000	3.288.359	-545.104	1.0376
18.120.000	3.289.911	-545.428	1.0369
18.130.000	3.291.788	-545.607	1.0365
18.140.000	3.293.180	-545.840	1.0362
18.150.000	3.295.063	-546.116	1.0356
18.160.000	3.296.553	-546.317	1.0351
18.170.000	3.298.371	-546.554	1.0348
18.180.000	3.299.825	-546.795	1.0343
18.190.000	3.301.706	-547.015	1.0337
18.200.000	3.303.167	-547.220	1.0332
18.210.000	3.304.977	-547.517	1.0328
18.220.000	3.306.542	-547.651	1.0323
18.230.000	3.308.225	-547.906	1.0319
18.240.000	3.309.856	-548.099	1.0318

18.250.000	3.311.530	-548.319	1.0317
18.260.000	3.313.174	-548.507	1.0312
18.270.000	3.314.828	-548.788	1.0309
18.280.000	3.316.529	-548.898	1.0306
18.290.000	3.318.069	-549.205	1.0302
18.300.000	3.319.923	-549.390	1.0296
18.310.000	3.321.355	-549.601	1.0292
18.320.000	3.323.189	-549.834	1.0288
18.330.000	3.324.686	-550.058	1.0282
18.340.000	3.326.473	-550.267	1.0274
18.350.000	3.327.970	-550.509	1.0268
18.360.000	3.329.824	-550.719	1.0264
18.370.000	3.331.250	-550.893	1.0256
18.380.000	3.333.101	-551.169	1.0249
18.390.000	3.334.652	-551.319	1.0246
18.400.000	3.336.339	-551.559	1.0244
18.410.000	3.337.971	-551.770	1.0238
18.420.000	3.339.668	-551.985	1.0229
18.430.000	3.341.285	-552.165	1.0226
18.440.000	3.342.980	-552.469	1.0222
18.450.000	3.344.629	-552.553	1.0215
18.460.000	3.346.207	-552.852	1.0206
18.470.000	3.347.996	-553.032	1.0201
18.480.000	3.349.493	-553.233	1.0197
18.490.000	3.351.340	-553.456	1.0190
18.500.000	3.352.814	-553.705	1.0181
18.510.000	3.354.627	-553.870	1.0171
18.520.000	3.356.093	-554.096	1.0164
18.530.000	3.357.996	-554.337	1.0155
18.540.000	3.359.447	-554.473	1.0145

18.550.000	3.361.272	-554.742	1.0137
18.560.000	3.362.810	-554.902	1.0130
18.570.000	3.364.532	-555.133	1.0123
18.580.000	3.366.083	-555.318	1.0114
18.590.000	3.367.856	-555.556	1.0109
18.600.000	3.369.438	-555.702	1.0104
18.610.000	3.371.110	-555.993	1.1997
18.620.000	3.372.833	-556.139	1.1990
18.630.000	3.374.375	-556.406	1.1984
18.640.000	3.376.195	-556.594	1.1978
18.650.000	3.377.706	-556.809	1.1971
18.660.000	3.379.498	-557.001	1.1965
18.670.000	3.381.003	-557.269	1.1960
18.680.000	3.382.857	-557.454	1.1953
18.690.000	3.384.262	-557.672	1.1947
18.700.000	3.386.183	-557.945	1.1944
18.710.000	3.387.643	-558.109	1.1943
18.720.000	3.389.479	-558.370	1.1940
18.730.000	3.390.979	-558.583	1.1937
18.740.000	3.392.739	-558.796	1.1936
18.750.000	3.394.298	-559.004	1.1937
18.760.000	3.396.113	-559.304	1.1936
18.770.000	3.397.632	-559.396	1.1932
18.780.000	3.399.322	-559.700	1.2029
18.790.000	3.401.031	-559.877	1.2028
18.800.000	3.402.585	-560.107	1.2023
18.810.000	3.404.370	-560.302	1.2016
18.820.000	3.405.936	-560.572	1.2007
18.830.000	3.407.697	-560.742	1.2000
18.840.000	3.409.194	-561.037	9.990

18.850.000	3.411.035	-561.233	9.977
18.860.000	3.412.460	-561.465	9.965
18.870.000	3.414.336	-561.759	9.953
18.880.000	3.415.818	-561.914	9.940
18.890.000	3.417.637	-562.193	9.928
18.900.000	3.419.120	-562.413	9.918
18.910.000	3.420.893	-562.623	9.909
18.920.000	3.422.423	-562.825	9.900
18.930.000	3.424.216	-563.128	9.890
18.940.000	3.425.739	-563.226	9.885
18.950.000	3.427.436	-563.547	9.881
18.960.000	3.429.138	-563.731	9.874
18.970.000	3.430.710	-563.969	9.865
18.980.000	3.432.432	-564.154	9.859
18.990.000	3.434.036	-564.456	9.853
19.000.000	3.435.764	-564.590	9.846
19.010.000	3.437.274	-564.896	9.837
19.020.000	3.439.166	-565.127	9.830
19.030.000	3.440.533	-565.340	9.823
19.040.000	3.442.416	-565.616	9.814
19.050.000	3.443.906	-565.812	9.804
19.060.000	3.445.683	-566.083	9.795
19.070.000	3.447.200	-566.309	9.788
19.080.000	3.448.994	-566.548	9.777
19.090.000	3.450.526	-566.750	9.770
19.100.000	3.452.330	-567.071	9.764
19.110.000	3.453.865	-567.223	9.756
19.120.000	3.455.544	-567.489	9.745
19.130.000	3.457.223	-567.691	9.737
19.140.000	3.458.825	-567.928	9.729

19.150.000	3.460.539	-568.120	9.720
19.160.000	3.462.152	-568.430	9.710
19.170.000	3.463.881	-568.557	9.706
19.180.000	3.465.351	-568.875	9.701
19.190.000	3.467.251	-569.142	9.693
19.200.000	3.468.647	-569.333	9.687
19.210.000	3.470.543	-569.618	9.681
19.220.000	3.472.055	-569.846	9.672
19.230.000	3.473.830	-570.067	9.662
19.240.000	3.475.305	-570.315	9.655
19.250.000	3.477.175	-570.569	9.651
19.260.000	3.478.618	-570.750	9.647
19.270.000	3.480.428	-571.081	9.639
19.280.000	3.481.987	-571.228	9.633
19.290.000	3.483.678	-571.521	9.628
19.300.000	3.485.329	-571.730	9.621
19.310.000	3.487.000	-571.990	9.610
19.320.000	3.488.633	-572.181	9.600
19.330.000	3.490.266	-572.518	9.591
19.340.000	3.492.032	-572.648	9.582
19.350.000	3.493.508	-572.978	9.573
19.360.000	3.495.402	-573.199	9.565
19.370.000	3.496.817	-573.408	9.557
19.380.000	3.498.679	-573.701	9.544
19.390.000	3.500.157	-573.966	9.535
19.400.000	3.501.959	-574.184	9.524
19.410.000	3.503.431	-574.429	9.514
19.420.000	3.505.269	-574.721	9.505
19.430.000	3.506.755	-574.904	9.498
19.440.000	3.508.524	-575.192	9.491

19.450.000	3.510.083	-575.382	9.482
19.460.000	3.511.751	-575.640	9.476
19.470.000	3.513.375	-575.864	9.471
19.480.000	3.515.063	-576.124	9.460
19.490.000	3.516.710	-576.296	9.451
19.500.000	3.518.330	-576.606	9.442
19.510.000	3.520.032	-576.774	9.433
19.520.000	3.521.555	-577.049	9.422
19.530.000	3.523.384	-577.291	9.413
19.540.000	3.524.868	-577.530	9.404
19.550.000	3.526.688	-577.781	9.390
19.560.000	3.528.187	-578.080	9.375
19.570.000	3.530.016	-578.304	9.363
19.580.000	3.531.428	-578.547	9.353
19.590.000	3.533.304	-578.863	9.340
19.600.000	3.534.791	-579.037	9.325
19.610.000	3.536.548	-579.375	9.312
19.620.000	3.538.122	-579.605	9.299
19.630.000	3.539.826	-579.864	9.286
19.640.000	3.541.401	-580.111	9.273
19.650.000	3.543.140	-580.451	9.259
19.660.000	3.544.793	-580.628	9.244
19.670.000	3.546.441	-580.994	9.231
19.680.000	3.548.162	-581.193	9.221
19.690.000	3.549.707	-581.503	9.211
19.700.000	3.551.454	-581.758	9.197
19.710.000	3.553.013	-582.053	9.186
19.720.000	3.554.794	-582.304	9.175
19.730.000	3.556.274	-582.631	9.163
19.740.000	3.558.164	-582.900	9.151

19.750.000	3.559.559	-583.170	9.139
19.760.000	3.561.447	-583.498	9.128
19.770.000	3.562.943	-583.721	9.118
19.780.000	3.564.718	-584.061	9.105
19.790.000	3.566.255	-584.312	9.094
19.800.000	3.568.035	-584.600	9.085
19.810.000	3.569.588	-584.855	9.075
19.820.000	3.571.354	-585.213	9.065
19.830.000	3.572.954	-585.405	9.056
19.840.000	3.574.613	-585.764	9.049
19.850.000	3.576.290	-585.999	9.043
19.860.000	3.577.846	-586.299	9.032
19.870.000	3.579.608	-586.541	9.023
19.880.000	3.581.190	-586.911	9.017
19.890.000	3.582.931	-587.116	9.009
19.900.000	3.584.423	-587.444	8.998
19.910.000	3.586.299	-587.743	8.989
19.920.000	3.587.689	-587.985	8.980
19.930.000	3.589.581	-588.326	8.972
19.940.000	3.591.050	-588.557	8.963
19.950.000	3.592.834	-588.852	8.952
19.960.000	3.594.350	-589.137	8.940
19.970.000	3.596.168	-589.439	8.928
19.980.000	3.597.679	-589.697	8.916
19.990.000	3.599.494	-590.104	8.902
20.000.000	3.601.088	-590.311	8.885
20.010.000	3.602.767	-590.663	8.871
20.020.000	3.604.423	-590.965	8.860
20.030.000	3.606.087	-591.290	8.847
20.040.000	3.607.790	-591.575	8.833

20.050.000	3.609.326	-591.947	8.823
20.060.000	3.611.099	-592.175	8.813
20.070.000	3.612.523	-592.560	8.801
20.080.000	3.614.413	-592.898	8.789
20.090.000	3.615.847	-593.157	8.778
20.100.000	3.617.669	-593.527	8.768
20.110.000	3.619.232	-593.819	8.754
20.120.000	3.621.000	-594.124	8.745
20.130.000	3.622.488	-594.450	8.737
20.140.000	3.624.360	-594.789	8.725
20.150.000	3.625.827	-595.063	8.713
20.160.000	3.627.569	-595.459	8.703
20.170.000	3.629.187	-595.714	8.692
20.180.000	3.630.785	-596.082	8.680
20.190.000	3.632.471	-596.392	8.669
20.200.000	3.634.075	-596.741	8.660
20.210.000	3.635.710	-597.010	8.650
20.220.000	3.637.375	-597.410	8.637
20.230.000	3.639.106	-597.637	8.627
20.240.000	3.640.602	-598.016	8.619
20.250.000	3.642.510	-598.371	8.608
20.260.000	3.643.942	-598.649	8.598
20.270.000	3.645.799	-599.022	8.588
20.280.000	3.647.302	-599.390	8.580
20.290.000	3.649.088	-599.712	8.570
20.300.000	3.650.591	-600.089	8.559
20.310.000	3.652.424	-600.494	8.546
20.320.000	3.653.901	-600.769	8.533
20.330.000	3.655.690	-601.239	8.520
20.340.000	3.657.302	-601.568	8.508

20.350.000	3.658.927	-601.959	8.494
20.360.000	3.660.634	-602.310	8.480
20.370.000	3.662.255	-602.724	8.466
20.380.000	3.663.933	-603.023	8.454
20.390.000	3.665.546	-603.490	8.437
20.400.000	3.667.333	-603.784	8.417
20.410.000	3.668.779	-604.203	8.396
20.420.000	3.670.659	-604.622	8.377
20.430.000	3.672.117	-604.979	8.359
20.440.000	3.673.961	-605.408	8.339
20.450.000	3.675.465	-605.838	8.318
20.460.000	3.677.316	-606.221	8.303
20.470.000	3.678.769	-606.633	8.288
20.480.000	3.680.615	-607.109	8.273
20.490.000	3.682.115	-607.451	8.256
20.500.000	3.683.838	-607.940	8.239
20.510.000	3.685.426	-608.343	8.226
20.520.000	3.687.126	-608.759	8.211
20.530.000	3.688.715	-609.164	8.196
20.540.000	3.690.429	-609.655	8.181
20.550.000	3.692.075	-609.983	8.166
20.560.000	3.693.682	-610.504	8.149
20.570.000	3.695.512	-610.869	8.134
20.580.000	3.696.978	-611.351	8.120
20.590.000	3.698.851	-611.784	8.105
20.600.000	3.700.332	-612.253	8.090
20.610.000	3.702.151	-612.724	8.076
20.620.000	3.703.659	-613.202	8.064
20.630.000	3.705.491	-613.668	8.048
20.640.000	3.706.876	-614.129	8.029

20.650.000	3.708.750	-614.665	8.010
20.660.000	3.710.235	-615.086	7.992
20.670.000	3.711.972	-615.601	7.970
20.680.000	3.713.576	-616.068	7.948
20.690.000	3.715.261	-616.533	7.928
20.700.000	3.716.882	-617.002	7.908
20.710.000	3.718.597	-617.550	7.887
20.720.000	3.720.216	-617.911	7.867
20.730.000	3.721.854	-618.505	7.845
20.740.000	3.723.602	-618.955	7.822
20.750.000	3.725.098	-619.442	7.798
20.760.000	3.726.921	-619.938	7.777
20.770.000	3.728.413	-620.481	7.757
20.780.000	3.730.270	-620.957	7.734
20.790.000	3.731.710	-621.477	7.714
20.800.000	3.733.588	-622.003	7.696
20.810.000	3.735.016	-622.474	7.679
20.820.000	3.736.867	-623.058	7.660
20.830.000	3.738.402	-623.509	7.640
20.840.000	3.740.151	-624.065	7.622
20.850.000	3.741.687	-624.579	7.601
20.860.000	3.743.468	-625.130	7.579
20.870.000	3.744.994	-625.598	7.556
20.880.000	3.746.709	-626.196	7.534
20.890.000	3.748.361	-626.649	7.512
20.900.000	3.749.960	-627.245	7.489
20.910.000	3.751.718	-627.742	7.468
20.920.000	3.753.277	-628.286	7.448
20.930.000	3.755.049	-628.797	7.426
20.940.000	3.756.598	-629.376	7.402

20.950.000	3.758.340	-629.879	7.381
20.960.000	3.759.800	-630.436	7.360
20.970.000	3.761.731	-631.031	7.339
20.980.000	3.763.157	-631.514	7.317
20.990.000	3.764.993	-632.100	7.295
21.000.000	3.766.504	-632.652	7.276
21.010.000	3.768.285	-633.214	7.255
21.020.000	3.769.824	-633.757	7.232
21.030.000	3.771.610	-634.357	7.209
21.040.000	3.773.112	-634.861	7.188
21.050.000	3.774.846	-635.494	7.167
21.060.000	3.776.487	-635.999	7.146
21.070.000	3.778.064	-636.566	7.125
21.080.000	3.779.783	-637.090	7.107
21.090.000	3.781.356	-637.680	7.085
21.100.000	3.783.083	-638.165	7.064
21.110.000	3.784.633	-638.797	7.044
21.120.000	3.786.507	-639.303	7.022
21.130.000	3.787.935	-639.895	7.000
21.140.000	3.789.835	-640.535	6.981
21.150.000	3.791.264	-641.028	6.961
21.160.000	3.793.081	-641.661	6.943
21.170.000	3.794.572	-642.266	6.923
21.180.000	3.796.370	-642.833	6.903
21.190.000	3.797.839	-643.407	6.882
21.200.000	3.799.629	-644.074	6.861
21.210.000	3.801.201	-644.546	6.838
21.220.000	3.802.877	-645.223	6.817
21.230.000	3.804.580	-645.772	6.795
21.240.000	3.806.223	-646.380	6.773

21.250.000	3.807.933	-646.953	6.753
21.260.000	3.809.580	-647.595	6.733
21.270.000	3.811.291	-648.118	6.713
21.280.000	3.812.822	-648.804	6.692
21.290.000	3.814.685	-649.395	6.672
21.300.000	3.816.112	-650.021	6.655
21.310.000	3.817.963	-650.696	6.636
21.320.000	3.819.402	-651.279	6.616
21.330.000	3.821.151	-651.924	6.596
21.340.000	3.822.713	-652.570	6.577
21.350.000	3.824.532	-653.185	6.558
21.360.000	3.826.005	-653.760	6.537
21.370.000	3.827.820	-654.435	6.516
21.380.000	3.829.328	-654.952	6.497
21.390.000	3.831.021	-655.620	6.478
21.400.000	3.832.673	-656.188	6.458
21.410.000	3.834.297	-656.797	6.439
21.420.000	3.835.968	-657.363	6.419
21.430.000	3.837.627	-658.048	6.400
21.440.000	3.839.282	-658.513	6.378
21.450.000	3.840.832	-659.215	6.357
21.460.000	3.842.715	-659.802	6.337
21.470.000	3.844.109	-660.405	6.318
21.480.000	3.845.990	-661.061	6.299
21.490.000	3.847.438	-661.661	6.279
21.500.000	3.849.191	-662.259	6.259
21.510.000	3.850.698	-662.896	6.242
21.520.000	3.852.533	-663.516	6.221
21.530.000	3.853.983	-664.078	6.200
21.540.000	3.855.787	-664.754	6.180

21.550.000	3.857.325	-665.284	6.161
21.560.000	3.859.053	-665.947	6.140
21.570.000	3.860.708	-666.539	6.119
21.580.000	3.862.433	-667.145	6.099
21.590.000	3.864.039	-667.738	6.080
21.600.000	3.865.732	-668.457	6.060
21.610.000	3.867.422	-668.953	6.040
21.620.000	3.868.971	-669.679	6.022
21.630.000	3.870.774	-670.255	6.004
21.640.000	3.872.215	-670.871	5.983
21.650.000	3.874.025	-671.515	5.962
21.660.000	3.875.508	-672.141	5.942
21.670.000	3.877.295	-672.721	5.921
21.680.000	3.878.782	-673.355	5.901
21.690.000	3.880.701	-673.987	5.881
21.700.000	3.882.150	-674.555	5.862
21.710.000	3.883.947	-675.206	5.845
21.720.000	3.885.496	-675.752	5.826
21.730.000	3.887.184	-676.385	5.807
21.740.000	3.888.807	-676.977	5.788
21.750.000	3.890.486	-677.590	5.769
21.760.000	3.892.104	-678.126	5.748
21.770.000	3.893.772	-678.814	5.728
21.780.000	3.895.488	-679.322	5.708
21.790.000	3.897.031	-679.967	5.688
21.800.000	3.898.890	-680.549	5.668
21.810.000	3.900.378	-681.145	5.648
21.820.000	3.902.210	-681.716	5.628
21.830.000	3.903.678	-682.339	5.607
21.840.000	3.905.512	-682.892	5.586

21.850.000	3.906.955	-683.473	5.566
21.860.000	3.908.849	-684.105	5.547
21.870.000	3.910.265	-684.582	5.527
21.880.000	3.912.084	-685.210	5.507
21.890.000	3.913.627	-685.765	5.488
21.900.000	3.915.376	-686.317	5.471
21.910.000	3.916.959	-686.848	5.453
21.920.000	3.918.714	-687.470	5.434
21.930.000	3.920.297	-687.927	5.417
21.940.000	3.921.955	-688.554	5.399
21.950.000	3.923.645	-688.995	5.380
21.960.000	3.925.206	-689.591	5.361
21.970.000	3.926.968	-690.074	5.342
21.980.000	3.928.498	-690.595	5.323
21.990.000	3.930.293	-691.069	5.302
22.000.000	3.931.746	-691.616	5.283
22.010.000	3.933.658	-692.080	5.263
22.020.000	3.935.075	-692.554	5.244
22.030.000	3.936.955	-693.106	5.225
22.040.000	3.938.442	-693.495	5.206
22.050.000	3.940.220	-694.026	5.187
22.060.000	3.941.756	-694.457	5.169
22.070.000	3.943.515	-694.917	5.150
22.080.000	3.945.070	-695.344	5.130
22.090.000	3.946.814	-695.870	5.110
22.100.000	3.948.362	-696.177	5.092
22.110.000	3.950.045	-696.686	5.074
22.120.000	3.951.765	-697.079	5.056
22.130.000	3.953.338	-697.515	5.037
22.140.000	3.955.128	-697.871	5.020

22.150.000	3.956.681	-698.325	5.003
22.160.000	3.958.438	-698.633	4.984
22.170.000	3.959.958	-699.080	4.967
22.180.000	3.961.835	-699.458	4.949
22.190.000	3.963.218	-699.804	4.930
22.200.000	3.965.117	-700.235	4.912
22.210.000	3.966.579	-700.570	4.893
22.220.000	3.968.374	-700.920	4.874
22.230.000	3.969.899	-701.272	4.852
22.240.000	3.971.732	-701.626	4.832
22.250.000	3.973.267	-701.909	4.813
22.260.000	3.975.028	-702.301	4.792
22.270.000	3.976.617	-702.529	4.772
22.280.000	3.978.307	-702.875	4.752
22.290.000	3.979.991	-703.150	4.732
22.300.000	3.981.573	-703.411	4.713
22.310.000	3.983.295	-703.624	4.692
22.320.000	3.984.904	-703.961	4.674
22.330.000	3.986.670	-704.082	4.656
22.340.000	3.988.158	-704.358	4.637
22.350.000	3.990.061	-704.597	4.620
22.360.000	3.991.509	-704.743	4.604
22.370.000	3.993.333	-705.008	4.588
22.380.000	3.994.837	-705.137	4.570
22.390.000	3.996.620	-705.285	4.554
22.400.000	3.998.066	-705.431	4.539
22.410.000	3.999.932	-705.592	4.522
22.420.000	4.001.389	-705.633	4.505
22.430.000	4.003.156	-705.838	4.487
22.440.000	4.004.795	-705.859	4.469

22.450.000	4.006.399	-706.014	4.451
22.460.000	4.008.105	-706.095	4.433
22.470.000	4.009.787	-706.201	4.414
22.480.000	4.011.381	-706.248	4.396
22.490.000	4.013.046	-706.469	4.380
22.500.000	4.014.769	-706.474	4.364
22.510.000	4.016.200	-706.647	4.347
22.520.000	4.018.111	-706.811	4.330
22.530.000	4.019.482	-706.923	4.313
22.540.000	4.021.329	-707.145	4.296
22.550.000	4.022.806	-707.384	4.275
22.560.000	4.024.590	-707.640	4.256
22.570.000	4.026.079	-707.970	4.236
22.580.000	4.027.926	-708.421	4.215
22.590.000	4.029.372	-708.766	4.193
22.600.000	4.031.164	-709.353	4.173
22.610.000	4.029.372	-708.766	4.193
22.620.000	4.032.731	-709.845	4.152
22.630.000	4.034.367	-710.440	4.131
22.640.000	4.035.984	-711.031	4.109
22.650.000	4.037.649	-711.722	4.088
22.660.000	4.039.272	-712.321	4.065
22.670.000	4.040.880	-713.120	4.042
22.680.000	4.042.628	-713.763	4.019
22.690.000	4.044.093	-714.562	3.995
22.700.000	4.045.926	-715.350	3.971
22.710.000	4.047.412	-716.112	3.947
22.720.000	4.049.250	-716.930	3.923
22.730.000	4.050.710	-717.735	3.897
22.740.000	4.052.554	-718.518	3.871

22.750.000	4.053.939	-719.274	3.846
22.760.000	4.055.792	-720.130	3.820
22.770.000	4.057.263	-720.821	3.796
22.780.000	4.058.997	-721.657	3.771
22.790.000	4.060.569	-722.386	3.747
22.800.000	4.062.220	-723.129	3.722
22.810.000	4.063.830	-723.863	3.698
22.820.000	4.065.584	-724.647	3.673
22.830.000	4.067.140	-725.278	3.647
22.840.000	4.068.765	-726.058	3.620
22.850.000	4.070.497	-726.675	3.594
22.860.000	4.071.947	-727.347	3.567
22.870.000	4.073.762	-727.961	3.539
22.880.000	4.075.255	-728.558	3.509
22.890.000	4.077.020	-729.117	3.481
22.900.000	4.078.519	-729.670	3.451
22.910.000	4.080.368	-730.185	3.422
22.920.000	4.081.781	-730.645	3.394
22.930.000	4.083.669	-731.183	3.365
22.940.000	4.085.147	-731.546	3.335
22.950.000	4.086.892	-732.007	3.306
22.960.000	4.088.470	-732.398	3.280
22.970.000	4.090.181	-732.792	3.251
22.980.000	4.091.742	-733.113	3.224
22.990.000	4.093.459	-733.540	3.197
23.000.000	4.095.057	-733.764	3.170
23.010.000	4.096.676	-734.163	3.142
23.020.000	4.098.431	-734.461	3.113
23.030.000	4.099.968	-734.765	3.087
23.040.000	4.101.766	-735.026	3.060

23.050.000	4.103.289	-735.359	3.030
23.060.000	4.105.085	-735.580	3.001
23.070.000	4.106.537	-735.876	2.975
23.080.000	4.108.394	-736.155	2.947
23.090.000	4.109.806	-736.358	2.918
23.100.000	4.111.640	-736.669	2.890
23.110.000	4.113.140	-736.862	2.863
23.120.000	4.114.868	-737.101	2.837
23.130.000	4.116.422	-737.310	2.811
23.140.000	4.118.183	-737.566	2.786
23.150.000	4.119.719	-737.714	2.761
23.160.000	4.121.461	-738.008	2.735
23.170.000	4.123.090	-738.113	2.707
23.180.000	4.124.676	-738.392	2.682
23.190.000	4.126.395	-738.552	2.657
23.200.000	4.127.965	-738.756	2.629
23.210.000	4.129.680	-738.899	2.602
23.220.000	4.131.223	-739.158	2.577
23.230.000	4.133.049	-739.266	2.552
23.240.000	4.134.430	-739.457	2.524
23.250.000	4.136.345	-739.660	2.498
23.260.000	4.137.727	-739.771	2.474
23.270.000	4.139.568	-739.973	2.448
23.280.000	4.141.091	-740.115	2.421
23.290.000	4.142.844	-740.258	2.394
23.300.000	4.144.371	-740.404	2.370
23.310.000	4.146.141	-740.611	2.346
23.320.000	4.147.640	-740.690	2.319
23.330.000	4.149.386	-740.933	2.292
23.340.000	4.151.001	-741.047	2.268

23.350.000	4.152.604	-741.233	2.242
23.360.000	4.154.355	-741.388	2.214
23.370.000	4.155.939	-741.621	2.188
23.380.000	4.157.660	-741.730	2.163
23.390.000	4.159.174	-741.989	2.140
23.400.000	4.160.989	-742.107	2.113
23.410.000	4.162.430	-742.305	2.088
23.420.000	4.164.300	-742.554	2.067
23.430.000	4.165.706	-742.650	2.043
23.440.000	4.167.517	-742.873	2.017
23.450.000	4.169.040	-743.072	1.990
23.460.000	4.170.802	-743.201	1.968
23.470.000	4.172.304	-743.379	1.947
23.480.000	4.174.157	-743.639	1.922
23.490.000	4.175.645	-743.716	1.896
23.500.000	4.177.394	-743.965	1.873
23.510.000	4.179.026	-744.118	1.851
23.520.000	4.180.646	-744.280	1.827
23.530.000	4.182.322	-744.460	1.800
23.540.000	4.183.950	-744.682	1.776
23.550.000	4.185.605	-744.771	1.755
23.560.000	4.187.154	-745.024	1.731
23.570.000	4.188.982	-745.159	1.705
23.580.000	4.190.423	-745.326	1.682
23.590.000	4.192.247	-745.517	1.664
23.600.000	4.193.719	-745.646	1.643
23.610.000	4.195.525	-745.834	1.618
23.620.000	4.197.018	-745.985	1.597
23.630.000	4.198.865	-746.129	1.579
23.640.000	4.200.271	-746.234	1.559

23.650.000	4.202.106	-746.454	1.535
23.660.000	4.203.631	-746.496	1.510
23.670.000	4.205.308	-746.696	1.488
23.680.000	4.206.948	-746.812	1.467
23.690.000	4.208.587	-746.963	1.440
23.700.000	4.210.180	-747.024	1.416
23.710.000	4.211.851	-747.241	1.396
23.720.000	4.213.538	-747.236	1.377
23.730.000	4.215.071	-747.435	1.354
23.740.000	4.216.838	-747.507	1.331
23.750.000	4.218.283	-747.613	1.315
23.760.000	4.220.130	-747.750	1.296
23.770.000	4.221.610	-747.876	1.272
23.780.000	4.223.362	-747.942	1.247
23.790.000	4.224.843	-748.053	1.225
23.800.000	4.226.700	-748.189	1.204
23.810.000	4.228.160	-748.242	1.180
23.820.000	4.229.946	-748.398	1.155
23.830.000	4.231.502	-748.457	1.133
23.840.000	4.233.231	-748.593	1.110
23.850.000	4.234.787	-748.691	1.087
23.860.000	4.236.486	-748.791	1.064
23.870.000	4.238.066	-748.834	1.042
23.880.000	4.239.704	-749.012	1.019
23.890.000	4.241.381	-748.990	996
23.900.000	4.242.898	-749.148	975
23.910.000	4.244.676	-749.197	953
23.920.000	4.246.162	-749.257	931
23.930.000	4.247.964	-749.365	910
23.940.000	4.249.412	-749.449	891

23.950.000	4.251.231	-749.484	871
23.960.000	4.252.657	-749.548	850
23.970.000	4.254.505	-749.639	829
23.980.000	4.255.890	-749.610	811
23.990.000	4.257.726	-749.734	791
24.000.000	4.259.256	-749.744	771
24.010.000	4.260.958	-749.801	753
24.020.000	4.262.524	-749.819	737
24.030.000	4.264.239	-749.928	719
24.040.000	4.265.756	-749.883	700
24.050.000	4.267.429	-750.007	685
24.060.000	4.269.137	-749.986	670
24.070.000	4.270.664	-750.054	652
24.080.000	4.272.441	-750.073	634
24.090.000	4.273.951	-750.104	620
24.100.000	4.275.676	-750.093	607
24.110.000	4.277.159	-750.164	590
24.120.000	4.279.010	-750.149	572
24.130.000	4.280.401	-750.130	559
24.140.000	4.282.266	-750.199	546
24.150.000	4.283.703	-750.119	531
24.160.000	4.285.484	-750.157	514
24.170.000	4.287.025	-750.163	501
24.180.000	4.288.780	-750.149	491
24.190.000	4.290.314	-750.105	475
24.200.000	4.292.025	-750.152	459
24.210.000	4.293.565	-750.024	447
24.220.000	4.295.208	-750.062	439
24.230.000	4.296.896	-749.990	426
24.240.000	4.298.462	-749.936	409

24.250.000	4.300.187	-749.860	397
24.260.000	4.301.728	-749.838	387
24.270.000	4.303.485	-749.694	372
24.280.000	4.304.937	-749.682	355
24.290.000	4.306.825	-749.585	342
24.300.000	4.308.190	-749.450	334
24.310.000	4.310.045	-749.406	320
24.320.000	4.311.481	-749.236	307
24.330.000	4.313.273	-749.153	299
24.340.000	4.314.766	-749.013	292
24.350.000	4.316.552	-748.871	279
24.360.000	4.318.029	-748.711	267
24.370.000	4.319.796	-748.632	258
24.380.000	4.321.360	-748.383	249
24.390.000	4.322.993	-748.291	236
24.400.000	4.324.703	-748.098	226
24.410.000	4.326.287	-747.943	218
24.420.000	4.327.951	-747.712	205
24.430.000	4.329.526	-747.606	192
24.440.000	4.331.247	-747.298	182
24.450.000	4.332.713	-747.152	172
24.460.000	4.334.586	-746.970	159
24.470.000	4.335.977	-746.687	146
24.480.000	4.337.858	-746.501	137
24.490.000	4.339.335	-746.269	125
24.500.000	4.341.064	-745.994	111
24.510.000	4.342.613	-745.739	101
24.520.000	4.344.397	-745.504	93
24.530.000	4.345.867	-745.145	81
24.540.000	4.347.608	-744.951	69

24.550.000	4.349.212	-744.573	60
24.560.000	4.350.837	-744.289	52
24.570.000	4.352.494	-743.960	41
24.580.000	4.354.085	-743.614	27
24.590.000	4.355.796	-743.216	16
24.600.000	4.357.341	-742.958	5
24.610.000	4.359.091	-742.484	-10
24.620.000	4.360.580	-742.161	-22
24.630.000	4.362.432	-741.802	-29
24.640.000	4.363.871	-741.356	-37
24.650.000	4.365.674	-740.992	-46
24.660.000	4.367.120	-740.558	-50
24.670.000	4.368.913	-740.097	-50
24.680.000	4.370.366	-739.651	-53
24.690.000	4.372.198	-739.234	-61
24.700.000	4.373.676	-738.660	-66
24.710.000	4.375.382	-738.264	-66
24.720.000	4.377.018	-737.710	-70
24.730.000	4.378.628	-737.198	-77
24.740.000	4.380.263	-736.648	-81
24.750.000	4.381.943	-736.187	-78
24.760.000	4.383.581	-735.544	-81
24.770.000	4.385.156	-735.078	-82
24.780.000	4.386.919	-734.445	-79
24.790.000	4.388.377	-733.897	-73
24.800.000	4.390.241	-733.341	-70
24.810.000	4.391.695	-732.707	-65
24.820.000	4.393.442	-732.107	-56
24.830.000	4.394.948	-731.526	-48
24.840.000	4.396.727	-730.886	-45

24.850.000	4.398.171	-730.220	-40
24.860.000	4.399.996	-729.649	-34
24.870.000	4.401.470	-728.913	-34
24.880.000	4.403.191	-728.327	-35
24.890.000	4.404.788	-727.635	-33
24.900.000	4.406.468	-726.966	-33
24.910.000	4.408.067	-726.271	-35
24.920.000	4.409.732	-725.676	-35
24.930.000	4.411.368	-724.880	-35
24.940.000	4.412.924	-724.300	-37
24.950.000	4.414.711	-723.580	-40
24.960.000	4.416.147	-722.895	-40
24.970.000	4.417.960	-722.219	-46
24.980.000	4.419.397	-721.556	-53
24.990.000	4.421.213	-720.853	-55
25.000.000	4.422.641	-720.182	-53
25.010.000	4.424.476	-719.488	-61
25.020.000	4.425.914	-718.760	-66
25.030.000	4.427.726	-718.134	-65
25.040.000	4.429.249	-717.367	-63
25.050.000	4.430.920	-716.707	-70
25.060.000	4.432.473	-715.998	-72
25.070.000	4.434.228	-715.322	-70
25.080.000	4.435.779	-714.603	-73
25.090.000	4.437.450	-713.981	-75
25.100.000	4.439.096	-713.185	-73
25.110.000	4.440.627	-712.578	-70
25.120.000	4.442.426	-711.856	-73
25.130.000	4.443.893	-711.164	-75
25.140.000	4.445.686	-710.469	-72

25.150.000	4.447.163	-709.834	-79
25.160.000	4.448.957	-709.143	-90
25.170.000	4.450.351	-708.461	-101
25.180.000	4.452.206	-707.848	-112
25.190.000	4.453.614	-707.112	-132
25.200.000	4.455.459	-706.501	-151
25.210.000	4.456.963	-705.838	-163
25.220.000	4.458.655	-705.182	-178
25.230.000	4.460.183	-704.525	-194
25.240.000	4.461.934	-703.965	-202
25.250.000	4.463.451	-703.257	-208
25.260.000	4.465.148	-702.710	-224
25.270.000	4.466.816	-702.035	-243
25.280.000	4.468.347	-701.465	-253
25.290.000	4.470.084	-700.862	-264
25.300.000	4.471.621	-700.308	-284
25.310.000	4.473.388	-699.707	-295
25.320.000	4.474.859	-699.202	-300
25.330.000	4.476.685	-698.598	-304
25.340.000	4.478.073	-698.062	-311
25.350.000	4.479.953	-697.597	-311
25.360.000	4.481.406	-696.986	-311
25.370.000	4.483.126	-696.516	-321
25.380.000	4.484.646	-696.012	-331
25.390.000	4.486.421	-695.514	-334
25.400.000	4.487.907	-695.018	-342
25.410.000	4.489.687	-694.610	-359
25.420.000	4.491.218	-694.059	-370
25.430.000	4.492.878	-693.699	-370
25.440.000	4.494.571	-693.217	-375

25.450.000	4.496.135	-692.825	-386
25.460.000	4.497.832	-692.377	-389
25.470.000	4.499.438	-692.037	-383
25.480.000	4.501.137	-691.578	-381
25.490.000	4.502.585	-691.243	-390
25.500.000	4.504.451	-690.900	-398
25.510.000	4.505.820	-690.519	-398
25.520.000	4.507.724	-690.222	-403
25.530.000	4.509.143	-689.833	-417
25.540.000	4.510.922	-689.537	-427
25.550.000	4.512.436	-689.228	-427
25.560.000	4.514.194	-688.902	-426
25.570.000	4.515.696	-688.568	-432
25.580.000	4.517.446	-688.368	-431
25.590.000	4.519.009	-687.983	-424
25.600.000	4.520.655	-687.767	-418
25.610.000	4.522.310	-687.508	-422
25.620.000	4.523.888	-687.269	-425
25.630.000	4.525.586	-686.999	-418
25.640.000	4.527.183	-686.868	-415
25.650.000	4.528.901	-686.519	-425
25.660.000	4.530.335	-686.369	-435
25.670.000	4.532.201	-686.196	-433
25.680.000	4.533.614	-685.944	-425
25.690.000	4.535.443	-685.801	-424
25.700.000	4.536.900	-685.610	-430
25.710.000	4.538.671	-685.414	-432
25.720.000	4.540.101	-685.262	-431
25.730.000	4.541.937	-685.135	-436
25.740.000	4.543.381	-684.921	-454

25.750.000	4.545.133	-684.853	-471
25.760.000	4.546.703	-684.645	-484
25.770.000	4.548.355	-684.561	-494
25.780.000	4.549.970	-684.417	-506
25.790.000	4.551.607	-684.344	-516
25.800.000	4.553.284	-684.186	-520
25.810.000	4.554.826	-684.183	-522
25.820.000	4.556.602	-684.006	-527
25.830.000	4.558.014	-683.986	-531
25.840.000	4.559.875	-683.947	-525
25.850.000	4.561.301	-683.842	-519
25.860.000	4.563.086	-683.823	-517
25.870.000	4.564.585	-683.783	-518
25.880.000	4.566.365	-683.716	-517
25.890.000	4.567.808	-683.663	-514
25.900.000	4.569.662	-683.687	-518
25.910.000	4.571.135	-683.556	-527
25.920.000	4.572.862	-683.586	-534
25.930.000	4.574.470	-683.544	-537
25.940.000	4.576.052	-683.521	-542
25.950.000	4.577.704	-683.500	-549
25.960.000	4.579.381	-683.563	-549
25.970.000	4.580.991	-683.465	-546
25.980.000	4.582.592	-683.557	-543
25.990.000	4.584.331	-683.501	-547
26.000.000	4.585.785	-683.526	-548
26.010.000	4.587.626	-683.563	-546
26.020.000	4.589.083	-683.558	-550
26.030.000	4.590.850	-683.605	-560
26.040.000	4.592.351	-683.653	-569

26.050.000	4.594.150	-683.655	-575
26.060.000	4.595.590	-683.688	-582
26.070.000	4.597.420	-683.813	-591
26.080.000	4.598.915	-683.765	-598
26.090.000	4.600.654	-683.884	-600
26.100.000	4.602.224	-683.934	-602
26.110.000	4.603.908	-683.991	-606
26.120.000	4.605.507	-684.026	-609
26.130.000	4.607.156	-684.172	-609
26.140.000	4.608.801	-684.142	-610
26.150.000	4.610.342	-684.308	-615
26.160.000	4.612.116	-684.343	-620
26.170.000	4.613.589	-684.430	-623
26.180.000	4.615.371	-684.512	-625
26.190.000	4.616.861	-684.624	-629
26.200.000	4.618.636	-684.679	-634
26.210.000	4.620.076	-684.780	-636
26.220.000	4.621.937	-684.898	-638
26.230.000	4.623.348	-684.948	-639
26.240.000	4.625.177	-685.088	-643
26.250.000	4.626.685	-685.137	-645
26.260.000	4.628.358	-685.253	-646
26.270.000	4.629.941	-685.347	-647
26.280.000	4.631.688	-685.469	-650
26.290.000	4.633.196	-685.497	-653
26.300.000	4.634.847	-685.688	-654
26.310.000	4.636.522	-685.698	-655
26.320.000	4.638.063	-685.839	-655
26.330.000	4.639.838	-685.931	-655
26.340.000	4.641.327	-686.034	-656

26.350.000	4.643.109	-686.127	-654
26.360.000	4.644.531	-686.262	-654
26.370.000	4.646.363	-686.342	-655
26.380.000	4.647.800	-686.460	-658
26.390.000	4.649.636	-686.621	-663
26.400.000	4.651.066	-686.644	-664
26.410.000	4.652.847	-686.823	-665
26.420.000	4.654.399	-686.937	-667
26.430.000	4.656.154	-687.044	-669
26.440.000	4.657.649	-687.156	-670
26.450.000	4.659.421	-687.350	-669
26.460.000	4.660.934	-687.371	-670
26.470.000	4.662.610	-687.603	-672
26.480.000	4.664.265	-687.664	-671
26.490.000	4.665.826	-687.827	-671
26.500.000	4.667.547	-687.935	-672
26.510.000	4.669.094	-688.087	-673
26.520.000	4.670.801	-688.170	-673
26.530.000	4.672.324	-688.383	-674
26.540.000	4.674.168	-688.487	-676
26.550.000	4.675.567	-688.624	-679
26.560.000	4.677.402	-688.807	-682
26.570.000	4.678.891	-688.880	-682
26.580.000	4.680.625	-689.049	-684
26.590.000	4.682.179	-689.186	-686
26.600.000	4.683.918	-689.329	-686
26.610.000	4.685.423	-689.449	-685
26.620.000	4.687.147	-689.667	-685
26.630.000	4.688.722	-689.723	-688
26.640.000	4.690.374	-689.935	-688

26.650.000	4.692.054	-690.053	-684
26.660.000	4.693.593	-690.196	-681
26.670.000	4.695.311	-690.307	-680
26.680.000	4.696.894	-690.514	-676
26.690.000	4.698.609	-690.573	-670
26.700.000	4.700.121	-690.764	-666
26.710.000	4.701.936	-690.910	-668
26.720.000	4.703.319	-691.002	-667
26.730.000	4.705.183	-691.189	-662
26.740.000	4.706.656	-691.294	-660
26.750.000	4.708.409	-691.413	-662
26.760.000	4.709.932	-691.561	-657
26.770.000	4.711.701	-691.697	-646
26.780.000	4.713.202	-691.786	-639
26.790.000	4.714.958	-692.005	-639
26.800.000	4.716.507	-692.041	-635
26.810.000	4.718.150	-692.225	-624
26.820.000	4.719.769	-692.339	-615
26.830.000	4.721.412	-692.460	-615
26.840.000	4.723.013	-692.540	-615
26.850.000	4.724.575	-692.767	-607
26.860.000	4.726.355	-692.820	-595
26.870.000	4.727.803	-693.001	-590
26.880.000	4.729.661	-693.171	-588
26.890.000	4.731.063	-693.255	-582
26.900.000	4.732.880	-693.450	-571
26.910.000	4.734.356	-693.580	-564
26.920.000	4.736.112	-693.711	-563
26.930.000	4.737.587	-693.856	-560
26.940.000	4.739.402	-694.051	-551

26.950.000	4.740.852	-694.107	-542
26.960.000	4.742.572	-694.310	-537
26.970.000	4.744.177	-694.412	-527
26.980.000	4.745.793	-694.553	-513
26.990.000	4.747.435	-694.661	-500
27.000.000	4.749.113	-694.836	-495
27.010.000	4.750.733	-694.883	-486
27.020.000	4.752.287	-695.096	-476
27.030.000	4.754.092	-695.149	-473
27.040.000	4.755.509	-695.314	-471
27.050.000	4.757.382	-695.471	-466
27.060.000	4.758.786	-695.564	-462
27.070.000	4.760.589	-695.710	-463
27.080.000	4.762.097	-695.862	-465
27.090.000	4.763.886	-695.982	-463
27.100.000	4.765.357	-696.096	-463
27.110.000	4.767.158	-696.274	-468
27.120.000	4.768.632	-696.312	-474
27.130.000	4.770.367	-696.516	-473
27.140.000	4.771.959	-696.629	-472
27.150.000	4.773.592	-696.730	-473
27.160.000	4.775.188	-696.827	-474
27.170.000	4.776.824	-697.017	-475
27.180.000	4.778.464	-697.036	-475
27.190.000	4.780.009	-697.228	-475
27.200.000	4.781.773	-697.321	-475
27.210.000	4.783.250	-697.431	-474
27.220.000	4.785.092	-697.583	-476
27.230.000	4.786.538	-697.697	-477
27.240.000	4.788.294	-697.810	-473

27.250.000	4.789.749	-697.938	-472
27.260.000	4.791.586	-698.065	-472
27.270.000	4.792.971	-698.153	-472
27.280.000	4.794.787	-698.345	-469
27.290.000	4.796.306	-698.395	-464
27.300.000	4.797.984	-698.569	-462
27.310.000	4.799.559	-698.651	-461
27.320.000	4.801.276	-698.774	-458
27.330.000	4.802.816	-698.852	-452
27.340.000	4.804.522	-699.037	-450
27.350.000	4.806.154	-699.052	-448
27.360.000	4.807.660	-699.224	-446
27.370.000	4.809.456	-699.308	-443
27.380.000	4.810.917	-699.430	-442
27.390.000	4.812.673	-699.495	-445
27.400.000	4.814.148	-699.633	-444
27.410.000	4.815.939	-699.693	-444
27.420.000	4.817.389	-699.792	-445
27.430.000	4.819.243	-699.932	-447
27.440.000	4.820.678	-699.961	-446
27.450.000	4.822.515	-700.121	-444
27.460.000	4.824.043	-700.218	-445
27.470.000	4.825.761	-700.324	-446
27.480.000	4.827.302	-700.434	-444
27.490.000	4.829.004	-700.595	-441
27.500.000	4.830.542	-700.645	-442
27.510.000	4.832.210	-700.854	-443
27.520.000	4.833.929	-700.915	-440
27.530.000	4.835.457	-701.086	-438
27.540.000	4.837.190	-701.186	-438

27.550.000	4.838.747	-701.328	-439
27.560.000	4.840.484	-701.425	-437
27.570.000	4.841.976	-701.609	-435
27.580.000	4.843.800	-701.695	-436
27.590.000	4.845.204	-701.830	-435
27.600.000	4.847.028	-702.000	-434
27.610.000	4.848.494	-702.077	-434
27.620.000	4.850.280	-702.262	-433
27.630.000	4.851.807	-702.369	-433
27.640.000	4.853.526	-702.478	-432
27.650.000	4.855.008	-702.578	-434
27.660.000	4.856.817	-702.753	-436
27.670.000	4.858.366	-702.759	-435
27.680.000	4.860.004	-702.928	-435
27.690.000	4.861.702	-702.999	-435
27.700.000	4.863.246	-703.105	-434
27.710.000	4.864.967	-703.164	-430
27.720.000	4.866.504	-703.325	-428
27.730.000	4.868.243	-703.351	-429
27.740.000	4.869.707	-703.492	-430
27.750.000	4.871.557	-703.599	-431
27.760.000	4.872.949	-703.658	-432
27.770.000	4.874.828	-703.810	-434
27.780.000	4.876.323	-703.875	-433
27.790.000	4.878.066	-703.966	-430
27.800.000	4.879.610	-704.070	-430
27.810.000	4.881.352	-704.170	-429
27.820.000	4.882.837	-704.217	-427
27.830.000	4.884.588	-704.397	-427
27.840.000	4.886.138	-704.401	-429

27.850.000	4.887.801	-704.548	-432
27.860.000	4.889.470	-704.624	-433
27.870.000	4.891.047	-704.717	-435
27.880.000	4.892.723	-704.775	-436
27.890.000	4.894.304	-704.961	-434
27.900.000	4.896.086	-704.970	-432
27.910.000	4.897.549	-705.124	-431
27.920.000	4.899.371	-705.258	-426
27.930.000	4.900.743	-705.303	-423
27.940.000	4.902.609	-705.478	-420
27.950.000	4.904.046	-705.575	-421
27.960.000	4.905.788	-705.654	-422
27.970.000	4.907.290	-705.766	-420
27.980.000	4.909.071	-705.902	-422
27.990.000	4.910.554	-705.933	-425
28.000.000	4.912.306	-706.110	-426
28.010.000	4.913.911	-706.170	-423
28.020.000	4.915.529	-706.303	-421
28.030.000	4.917.170	-706.385	-423
28.040.000	4.918.824	-706.533	-424
28.050.000	4.920.447	-706.572	-423
28.060.000	4.922.016	-706.770	-425
28.070.000	4.923.792	-706.833	-430
28.080.000	4.925.190	-706.970	-434
28.090.000	4.927.043	-707.124	-437
28.100.000	4.928.495	-707.215	-439
28.110.000	4.930.311	-707.362	-440
28.120.000	4.931.840	-707.488	-441
28.130.000	4.933.609	-707.580	-442
28.140.000	4.935.094	-707.668	-444

28.150.000	4.936.903	-707.832	-444
28.160.000	4.938.381	-707.830	-444
28.170.000	4.940.102	-708.002	-445
28.180.000	4.941.724	-708.091	-446
28.190.000	4.943.352	-708.164	-444
28.200.000	4.944.990	-708.248	-442
28.210.000	4.946.622	-708.412	-444
28.220.000	4.948.252	-708.406	-447
28.230.000	4.949.843	-708.609	-446
28.240.000	4.951.607	-708.652	-443
28.250.000	4.953.027	-708.745	-444
28.260.000	4.954.839	-708.885	-447
28.270.000	4.956.278	-708.968	-447
28.280.000	4.958.054	-709.058	-444
28.290.000	4.959.496	-709.173	-443
28.300.000	4.961.314	-709.256	-444
28.310.000	4.962.747	-709.319	-447
28.320.000	4.964.540	-709.477	-448
28.330.000	4.966.069	-709.491	-445
28.340.000	4.967.749	-709.640	-444
28.350.000	4.969.328	-709.727	-448
28.360.000	4.971.027	-709.820	-450
28.370.000	4.972.587	-709.901	-449
28.380.000	4.974.218	-710.070	-448
28.390.000	4.975.878	-710.061	-452
28.400.000	4.977.386	-710.241	-455
28.410.000	4.979.190	-710.326	-455
28.420.000	4.980.637	-710.417	-456
28.430.000	4.982.435	-710.537	-458
28.440.000	4.983.910	-710.694	-460

28.450.000	4.985.740	-710.802	-457
28.460.000	4.987.204	-710.938	-454
28.470.000	4.989.026	-711.120	-456
28.480.000	4.990.439	-711.183	-457
28.490.000	4.992.239	-711.372	-455
28.500.000	4.993.753	-711.488	-453
28.510.000	4.995.426	-711.629	-454
28.520.000	4.996.983	-711.756	-459
28.530.000	4.998.684	-711.923	-459
28.540.000	5.000.247	-711.966	-456
28.550.000	5.001.881	-712.177	-457
28.560.000	5.003.610	-712.220	-462
28.570.000	5.005.117	-712.373	-465
28.580.000	5.006.908	-712.473	-465
28.590.000	5.008.390	-712.591	-465
28.600.000	5.010.150	-712.690	-470
28.610.000	5.011.598	-712.872	-474
28.620.000	5.013.425	-712.957	-475
28.630.000	5.014.832	-713.056	-476
28.640.000	5.016.627	-713.242	-479
28.650.000	5.018.105	-713.298	-483
28.660.000	5.019.869	-713.469	-486
28.670.000	5.021.426	-713.589	-487
28.680.000	5.023.184	-713.693	-490
28.690.000	5.024.686	-713.783	-491
28.700.000	5.026.413	-713.968	-490
28.710.000	5.027.980	-713.969	-488
28.720.000	5.029.561	-714.153	-488
28.730.000	5.031.255	-714.223	-486
28.740.000	5.032.810	-714.330	-484

28.750.000	5.034.508	-714.417	-482
28.760.000	5.036.073	-714.588	-481
28.770.000	5.037.801	-714.632	-483
28.780.000	5.039.280	-714.793	-485
28.790.000	5.041.166	-714.902	-484
28.800.000	5.042.515	-714.975	-484
28.810.000	5.044.362	-715.122	-487
28.820.000	5.045.824	-715.204	-489
28.830.000	5.047.554	-715.334	-489
28.840.000	5.049.102	-715.450	-488
28.850.000	5.050.823	-715.582	-490
28.860.000	5.052.326	-715.648	-495
28.870.000	5.054.058	-715.849	-495
28.880.000	5.055.631	-715.869	-494
28.890.000	5.057.261	-716.049	-494
28.900.000	5.059.007	-716.180	-496
28.910.000	5.060.546	-716.300	-497
28.920.000	5.062.239	-716.396	-495
28.930.000	5.063.797	-716.630	-493
28.940.000	5.065.560	-716.662	-494
28.950.000	5.066.954	-716.824	-498
28.960.000	5.068.828	-717.013	-499
28.970.000	5.070.232	-717.065	-496
28.980.000	5.072.056	-717.246	-497
28.990.000	5.073.526	-717.374	-500
29.000.000	5.075.337	-717.470	-504
29.010.000	5.076.819	-717.600	-506
29.020.000	5.078.630	-717.764	-506
29.030.000	5.080.104	-717.817	-510
29.040.000	5.081.818	-718.020	-517

29.050.000	5.083.431	-718.091	-517
29.060.000	5.085.049	-718.260	-517
29.070.000	5.086.684	-718.380	-520
29.080.000	5.088.326	-718.562	-526
29.090.000	5.089.997	-718.655	-531
29.100.000	5.091.537	-718.853	-532
29.110.000	5.093.351	-718.952	-536
29.120.000	5.094.769	-719.134	-542
29.130.000	5.096.668	-719.304	-545
29.140.000	5.098.082	-719.395	-543
29.150.000	5.099.874	-719.553	-541
29.160.000	5.101.366	-719.706	-542
29.170.000	5.103.133	-719.816	-543
29.180.000	5.104.599	-719.917	-541
29.190.000	5.106.393	-720.128	-538
29.200.000	5.107.878	-720.146	-540
29.210.000	5.109.588	-720.329	-541
29.220.000	5.111.220	-720.445	-539
29.230.000	5.112.867	-720.551	-536
29.240.000	5.114.505	-720.657	-535
29.250.000	5.116.152	-720.866	-536
29.260.000	5.117.851	-720.886	-535
29.270.000	5.119.382	-721.097	-535
29.280.000	5.121.168	-721.203	-533
29.290.000	5.122.557	-721.324	-536
29.300.000	5.124.418	-721.493	-539
29.310.000	5.125.884	-721.612	-541
29.320.000	5.127.654	-721.738	-543
29.330.000	5.129.120	-721.877	-548
29.340.000	5.130.952	-722.022	-552

29.350.000	5.132.415	-722.089	-555
29.360.000	5.134.214	-722.315	-558
29.370.000	5.135.745	-722.348	-562
29.380.000	5.137.416	-722.496	-564
29.390.000	5.139.007	-722.612	-563
29.400.000	5.140.657	-722.725	-563
29.410.000	5.142.248	-722.782	-564
29.420.000	5.143.873	-722.980	-564
29.430.000	5.145.609	-722.984	-562
29.440.000	5.147.107	-723.172	-561
29.450.000	5.148.910	-723.247	-563
29.460.000	5.150.376	-723.341	-564
29.470.000	5.152.173	-723.487	-563
29.480.000	5.153.680	-723.621	-563
29.490.000	5.155.448	-723.705	-565
29.500.000	5.156.857	-723.836	-569
29.510.000	5.158.712	-723.995	-569
29.520.000	5.160.135	-724.062	-567
29.530.000	5.161.896	-724.238	-566
29.540.000	5.163.453	-724.340	-566
29.550.000	5.165.127	-724.463	-564
29.560.000	5.166.711	-724.573	-562
29.570.000	5.168.445	-724.732	-560
29.580.000	5.170.006	-724.784	-563
29.590.000	5.171.625	-725.007	-566
29.600.000	5.173.333	-725.088	-569
29.610.000	5.174.810	-725.243	-572
29.620.000	5.176.630	-725.374	-576
29.630.000	5.178.076	-725.538	-581
29.640.000	5.179.857	-725.679	-582

29.650.000	5.181.323	-725.860	-584
29.660.000	5.183.131	-725.995	-586
29.670.000	5.184.554	-726.122	-587
29.680.000	5.186.366	-726.334	-585
29.690.000	5.187.848	-726.400	-583
29.700.000	5.189.592	-726.585	-583
29.710.000	5.191.129	-726.701	-581
29.720.000	5.192.837	-726.830	-579
29.730.000	5.194.384	-726.934	-579
29.740.000	5.196.046	-727.142	-581
29.750.000	5.197.690	-727.158	-583
29.760.000	5.199.253	-727.383	-587
29.770.000	5.200.985	-727.471	-589
29.780.000	5.202.476	-727.609	-590
29.790.000	5.204.221	-727.731	-590
29.800.000	5.205.778	-727.926	-591
29.810.000	5.207.549	-728.016	-594
29.820.000	5.208.981	-728.195	-593
29.830.000	5.210.850	-728.370	-593
29.840.000	5.212.240	-728.488	-596
29.850.000	5.214.052	-728.679	-599
29.860.000	5.215.551	-728.814	-599
29.870.000	5.217.242	-728.989	-601
29.880.000	5.218.805	-729.135	-602
29.890.000	5.220.573	-729.333	-603
29.900.000	5.222.065	-729.448	-605
29.910.000	5.223.793	-729.695	-606
29.920.000	5.225.407	-729.793	-608
29.930.000	5.226.971	-730.004	-610
29.940.000	5.228.665	-730.159	-612

29.950.000	5.230.216	-730.345	-614
29.960.000	5.231.981	-730.463	-614
29.970.000	5.233.457	-730.701	-615
29.980.000	5.235.230	-730.806	-618
29.990.000	5.236.636	-730.984	-619
30.000.000	5.238.530	-731.201	-620
30.010.000	5.239.933	-731.294	-622
30.020.000	5.241.738	-731.498	-624
30.030.000	5.243.237	-731.651	-623
30.040.000	5.245.004	-731.799	-624
30.050.000	5.246.484	-731.954	-626
30.060.000	5.248.252	-732.191	-627
30.070.000	5.249.776	-732.261	-629
30.080.000	5.251.432	-732.514	-630
30.090.000	5.253.094	-732.636	-633
30.100.000	5.254.656	-732.794	-636
30.110.000	5.256.379	-732.938	-638
30.120.000	5.257.910	-733.142	-642
30.130.000	5.259.655	-733.234	-642
30.140.000	5.261.154	-733.456	-641
30.150.000	5.263.002	-733.613	-644
30.160.000	5.264.369	-733.771	-644
30.170.000	5.266.193	-733.973	-642
30.180.000	5.267.658	-734.100	-641
30.190.000	5.269.403	-734.290	-643
30.200.000	5.270.921	-734.443	-645
30.210.000	5.272.705	-734.621	-644
30.220.000	5.274.169	-734.744	-644
30.230.000	5.275.960	-735.000	-646
30.240.000	5.277.503	-735.073	-647

30.250.000	5.279.160	-735.285	-646
30.260.000	5.280.816	-735.425	-646
30.270.000	5.282.404	-735.603	-648
30.280.000	5.284.061	-735.712	-650
30.290.000	5.285.656	-735.946	-650
30.300.000	5.287.354	-736.005	-654
30.310.000	5.288.859	-736.231	-658
30.320.000	5.290.703	-736.405	-658
30.330.000	5.292.083	-736.500	-657
30.340.000	5.293.904	-736.712	-657
30.350.000	5.295.347	-736.873	-656
30.360.000	5.297.113	-736.989	-652
30.370.000	5.298.630	-737.165	-648
30.380.000	5.300.404	-737.365	-647
30.390.000	5.301.869	-737.426	-647
30.400.000	5.303.606	-737.646	-645
30.410.000	5.305.185	-737.763	-644
30.420.000	5.306.807	-737.939	-645
30.430.000	5.308.444	-738.089	-646
30.440.000	5.310.081	-738.273	-646
30.450.000	5.311.733	-738.365	-647
30.460.000	5.313.260	-738.607	-649
30.470.000	5.315.044	-738.699	-652
30.480.000	5.316.469	-738.896	-654
30.490.000	5.318.299	-739.081	-657
30.500.000	5.319.700	-739.212	-660
30.510.000	5.321.513	-739.419	-660
30.520.000	5.322.973	-739.576	-657
30.530.000	5.324.762	-739.719	-655
30.540.000	5.326.187	-739.863	-656

30.550.000	5.328.007	-740.092	-655
30.560.000	5.329.493	-740.157	-652
30.570.000	5.331.165	-740.392	-652
30.580.000	5.332.783	-740.536	-657
30.590.000	5.334.406	-740.703	-660
30.600.000	5.336.033	-740.821	-661
30.610.000	5.337.667	-741.073	-664
30.620.000	5.339.276	-741.120	-668
30.630.000	5.340.839	-741.360	-672
30.640.000	5.342.620	-741.480	-676
30.650.000	5.344.009	-741.622	-680
30.660.000	5.345.843	-741.805	-682
30.670.000	5.347.302	-742.006	-683
30.680.000	5.349.069	-742.132	-685
30.690.000	5.350.510	-742.314	-687
30.700.000	5.352.341	-742.508	-685
30.710.000	5.353.795	-742.622	-681
30.720.000	5.355.579	-742.862	-681
30.730.000	5.357.068	-742.966	-681
30.740.000	5.358.720	-743.172	-677
30.750.000	5.360.303	-743.316	-673
30.760.000	5.361.973	-743.494	-670
30.770.000	5.363.578	-743.603	-671
30.780.000	5.365.184	-743.842	-669
30.790.000	5.366.884	-743.900	-664
30.800.000	5.368.340	-744.096	-662
30.810.000	5.370.120	-744.244	-664
30.820.000	5.371.611	-744.393	-665
30.830.000	5.373.374	-744.547	-664
30.840.000	5.374.830	-744.740	-663

30.850.000	5.376.608	-744.868	-665
30.860.000	5.378.034	-745.025	-669
30.870.000	5.379.861	-745.229	-670
30.880.000	5.381.302	-745.325	-669
30.890.000	5.383.074	-745.541	-673
30.900.000	5.384.595	-745.682	-677
30.910.000	5.386.280	-745.840	-678
30.920.000	5.387.829	-745.998	-677
30.930.000	5.389.550	-746.222	-681
30.940.000	5.391.124	-746.291	-685
30.950.000	5.392.715	-746.525	-687
30.960.000	5.394.420	-746.641	-685
30.970.000	5.395.879	-746.834	-688
30.980.000	5.397.676	-746.994	-691
30.990.000	5.399.176	-747.181	-692
31.000.000	5.400.934	-747.335	-690
31.010.000	5.402.391	-747.541	-690
31.020.000	5.404.219	-747.729	-694
31.030.000	5.405.588	-747.863	-696
31.040.000	5.407.458	-748.113	-694
31.050.000	5.408.900	-748.230	-691
31.060.000	5.410.618	-748.446	-689
31.070.000	5.412.155	-748.625	-688
31.080.000	5.413.845	-748.795	-686
31.090.000	5.415.374	-748.934	-684
31.100.000	5.417.082	-749.199	-681
31.110.000	5.418.696	-749.264	-682
31.120.000	5.420.257	-749.518	-684
31.130.000	5.421.989	-749.672	-688
31.140.000	5.423.496	-749.851	-688

31.150.000	5.425.249	-750.016	-687
31.160.000	5.426.743	-750.268	-690
31.170.000	5.428.546	-750.389	-693
31.180.000	5.429.942	-750.613	-694
31.190.000	5.431.845	-750.822	-694
31.200.000	5.433.183	-750.925	-697
31.210.000	5.435.003	-751.165	-703
31.220.000	5.436.511	-751.337	-704
31.230.000	5.438.212	-751.501	-703
31.240.000	5.439.722	-751.656	-701
31.250.000	5.441.480	-751.901	-700
31.260.000	5.442.981	-752.008	-699
31.270.000	5.444.638	-752.237	-696
31.280.000	5.446.286	-752.365	-695
31.290.000	5.447.827	-752.569	-697
31.300.000	5.449.564	-752.699	-702
31.310.000	5.451.085	-752.920	-706
31.320.000	5.452.801	-753.038	-705
31.330.000	5.454.323	-753.270	-703
31.340.000	5.456.098	-753.420	-705
31.350.000	5.457.486	-753.580	-708
31.360.000	5.459.322	-753.823	-706
31.370.000	5.460.729	-753.942	-704
31.380.000	5.462.536	-754.159	-704
31.390.000	5.464.039	-754.365	-709
31.400.000	5.465.752	-754.516	-711
31.410.000	5.467.193	-754.661	-709
31.420.000	5.468.912	-754.919	-709
31.430.000	5.470.443	-754.977	-712
31.440.000	5.472.094	-755.212	-715

31.450.000	5.473.724	-755.365	-715
31.460.000	5.475.346	-755.546	-715
31.470.000	5.476.984	-755.675	-716
31.480.000	5.478.508	-755.915	-719
31.490.000	5.480.255	-755.998	-721
31.500.000	5.481.700	-756.243	-721
31.510.000	5.483.555	-756.442	-722
31.520.000	5.484.962	-756.577	-727
31.530.000	5.486.755	-756.813	-730
31.540.000	5.488.231	-756.976	-730
31.550.000	5.489.966	-757.160	-729
31.560.000	5.491.414	-757.329	-731
31.570.000	5.493.206	-757.559	-733
31.580.000	5.494.644	-757.683	-734
31.590.000	5.496.368	-757.937	-733
31.600.000	5.497.961	-758.059	-732
31.610.000	5.499.566	-758.287	-734
31.620.000	5.501.185	-758.440	-738
31.630.000	5.502.787	-758.660	-737
31.640.000	5.504.428	-758.777	-736
31.650.000	5.506.008	-759.050	-738
31.660.000	5.507.752	-759.166	-741
31.670.000	5.509.162	-759.385	-744
31.680.000	5.510.994	-759.612	-743
31.690.000	5.512.404	-759.754	-745
31.700.000	5.514.201	-759.984	-750
31.710.000	5.515.659	-760.196	-753
31.720.000	5.517.440	-760.363	-751
31.730.000	5.518.896	-760.555	-750
31.740.000	5.520.687	-760.802	-751

31.750.000	5.522.156	-760.899	-750
31.760.000	5.523.806	-761.168	-745
31.770.000	5.525.446	-761.343	-743
31.780.000	5.527.045	-761.521	-745
31.790.000	5.528.630	-761.686	-748
31.800.000	5.530.245	-761.941	-748
31.810.000	5.531.895	-762.026	-749
31.820.000	5.533.406	-762.302	-752
31.830.000	5.535.203	-762.441	-756
31.840.000	5.536.597	-762.609	-757
31.850.000	5.538.397	-762.832	-754
31.860.000	5.539.870	-762.998	-755
31.870.000	5.541.608	-763.182	-757
31.880.000	5.543.064	-763.364	-756
31.890.000	5.544.894	-763.563	-753
31.900.000	5.546.293	-763.712	-751
31.910.000	5.548.057	-763.961	-754
31.920.000	5.549.581	-764.094	-757
31.930.000	5.551.209	-764.333	-760
31.940.000	5.552.851	-764.500	-766
31.950.000	5.554.470	-764.714	-771
31.960.000	5.556.060	-764.861	-775
31.970.000	5.557.690	-765.145	-777
31.980.000	5.559.366	-765.223	-780
31.990.000	5.560.850	-765.492	-783
32.000.000	5.562.687	-765.670	-783
32.010.000	5.564.056	-765.827	-780
32.020.000	5.565.872	-766.054	-782
32.030.000	5.567.302	-766.263	-786
32.040.000	5.569.075	-766.410	-785

32.050.000	5.570.528	-766.621	-782
32.060.000	5.572.361	-766.880	-783
32.070.000	5.573.766	-766.949	-786
32.080.000	5.575.545	-767.224	-786
32.090.000	5.577.065	-767.386	-784
32.100.000	5.578.748	-767.572	-786
32.110.000	5.580.298	-767.729	-788
32.120.000	5.581.945	-767.971	-788
32.130.000	5.583.532	-768.058	-788
32.140.000	5.585.110	-768.323	-789
32.150.000	5.586.820	-768.438	-790
32.160.000	5.588.273	-768.643	-789
32.170.000	5.590.093	-768.834	-789
32.180.000	5.591.547	-769.013	-790
32.190.000	5.593.291	-769.202	-792
32.200.000	5.594.682	-769.404	-792
32.210.000	5.596.514	-769.588	-795
32.220.000	5.597.910	-769.767	-798
32.230.000	5.599.713	-770.009	-799
32.240.000	5.601.171	-770.123	-800
32.250.000	5.602.845	-770.362	-802
32.260.000	5.604.402	-770.537	-805
32.270.000	5.606.079	-770.716	-803
32.280.000	5.607.636	-770.870	-801
32.290.000	5.609.277	-771.129	-803
32.300.000	5.610.914	-771.228	-804
32.310.000	5.612.432	-771.467	-803
32.320.000	5.614.172	-771.629	-804
32.330.000	5.615.656	-771.839	-806
32.340.000	5.617.386	-771.997	-807

32.350.000	5.618.855	-772.245	-806
32.360.000	5.620.617	-772.409	-804
32.370.000	5.622.052	-772.608	-805
32.380.000	5.623.883	-772.865	-804
32.390.000	5.625.274	-773.008	-802
32.400.000	5.627.081	-773.256	-802
32.410.000	5.628.579	-773.462	-805
32.420.000	5.630.272	-773.655	-809
32.430.000	5.631.816	-773.856	-813
32.440.000	5.633.504	-774.127	-818
32.450.000	5.635.075	-774.248	-822
32.460.000	5.636.698	-774.530	-825
32.470.000	5.638.329	-774.677	-828
32.480.000	5.639.848	-774.902	-831
32.490.000	5.641.575	-775.103	-832
32.500.000	5.643.073	-775.320	-831
32.510.000	5.644.822	-775.494	-832
32.520.000	5.646.279	-775.740	-835
32.530.000	5.648.120	-775.923	-837
32.540.000	5.649.484	-776.107	-836
32.550.000	5.651.275	-776.363	-836
32.560.000	5.652.770	-776.501	-838
32.570.000	5.654.470	-776.739	-837
32.580.000	5.655.973	-776.934	-834
32.590.000	5.657.727	-777.141	-835
32.600.000	5.659.173	-777.274	-836
32.610.000	5.660.887	-777.561	-836
32.620.000	5.662.490	-777.654	-835
32.630.000	5.664.058	-777.899	-836
32.640.000	5.665.754	-778.048	-837

32.650.000	5.667.258	-778.247	-835
32.660.000	5.668.977	-778.397	-834
32.670.000	5.670.490	-778.647	-836
32.680.000	5.672.216	-778.759	-835
32.690.000	5.673.621	-778.963	-834
32.700.000	5.675.435	-779.186	-836
32.710.000	5.676.789	-779.292	-838
32.720.000	5.678.607	-779.542	-838
32.730.000	5.680.094	-779.701	-837
32.740.000	5.681.799	-779.860	-838
32.750.000	5.683.281	-780.037	-842
32.760.000	5.685.053	-780.251	-841
32.770.000	5.686.520	-780.347	-840
32.780.000	5.688.190	-780.616	-841
32.790.000	5.689.812	-780.721	-842
32.800.000	5.691.340	-780.933	-842
32.810.000	5.693.055	-781.105	-842
32.820.000	5.694.566	-781.318	-843
32.830.000	5.696.275	-781.455	-843
32.840.000	5.697.778	-781.729	-843
32.850.000	5.699.601	-781.885	-843
32.860.000	5.700.951	-782.093	-845
32.870.000	5.702.799	-782.350	-844
32.880.000	5.704.241	-782.484	-844
32.890.000	5.705.975	-782.725	-846
32.900.000	5.707.439	-782.941	-849
32.910.000	5.709.194	-783.124	-849
32.920.000	5.710.642	-783.303	-850
32.930.000	5.712.375	-783.582	-853
32.940.000	5.713.906	-783.698	-855

32.950.000	5.715.518	-783.961	-853
32.960.000	5.717.206	-784.143	-853
32.970.000	5.718.735	-784.337	-856
32.980.000	5.720.422	-784.504	-857
32.990.000	5.722.000	-784.778	-856
33.000.000	5.723.685	-784.884	-856
33.010.000	5.725.172	-785.152	-859
33.020.000	5.726.982	-785.373	-862
33.030.000	5.728.323	-785.525	-860
33.040.000	5.730.148	-785.786	-857
33.050.000	5.731.579	-785.980	-857
33.060.000	5.733.305	-786.172	-860
33.070.000	5.734.813	-786.362	-858
33.080.000	5.736.552	-786.585	-859
33.090.000	5.738.015	-786.710	-860
33.100.000	5.739.774	-786.962	-862
33.110.000	5.741.286	-787.094	-864
33.120.000	5.742.913	-787.307	-862
33.130.000	5.744.518	-787.470	-865
33.140.000	5.746.087	-787.673	-868
33.150.000	5.747.730	-787.795	-866
33.160.000	5.749.228	-788.073	-865
33.170.000	5.751.011	-788.150	-865
33.180.000	5.752.405	-788.383	-867
33.190.000	5.754.198	-788.616	-865
33.200.000	5.755.630	-788.742	-860
33.210.000	5.757.397	-788.963	-862
33.220.000	5.758.828	-789.139	-867
33.230.000	5.760.573	-789.274	-869
33.240.000	5.762.003	-789.465	-869

33.250.000	5.763.772	-789.702	-871
33.260.000	5.765.262	-789.786	-874
33.270.000	5.766.909	-790.032	-873
33.280.000	5.768.515	-790.186	-869
33.290.000	5.770.090	-790.358	-865
33.300.000	5.771.717	-790.524	-864
33.310.000	5.773.339	-790.804	-863
33.320.000	5.774.991	-790.894	-863
33.330.000	5.776.465	-791.134	-863
33.340.000	5.778.248	-791.327	-864
33.350.000	5.779.588	-791.497	-865
33.360.000	5.781.400	-791.736	-867
33.370.000	5.782.816	-791.928	-868
33.380.000	5.784.556	-792.107	-866
33.390.000	5.785.998	-792.317	-864
33.400.000	5.787.775	-792.540	-864
33.410.000	5.789.234	-792.680	-867
33.420.000	5.790.968	-792.940	-868
33.430.000	5.792.490	-793.083	-867
33.440.000	5.794.111	-793.295	-868
33.450.000	5.795.694	-793.473	-872
33.460.000	5.797.311	-793.690	-875
33.470.000	5.798.878	-793.830	-874
33.480.000	5.800.416	-794.122	-874
33.490.000	5.802.159	-794.237	-876
33.500.000	5.803.579	-794.492	-878
33.510.000	5.805.383	-794.715	-878
33.520.000	5.806.837	-794.888	-878
33.530.000	5.808.561	-795.117	-879
33.540.000	5.810.044	-795.339	-883

33.550.000	5.811.826	-795.536	-883
33.560.000	5.813.237	-795.727	-882
33.570.000	5.815.020	-795.993	-882
33.580.000	5.816.472	-796.138	-884
33.590.000	5.818.152	-796.407	-885
33.600.000	5.819.744	-796.606	-882
33.610.000	5.821.354	-796.835	-881
33.620.000	5.822.927	-797.016	-881
33.630.000	5.824.629	-797.314	-883
33.640.000	5.826.214	-797.427	-884
33.650.000	5.827.764	-797.721	-883
33.660.000	5.829.520	-797.894	-884
33.670.000	5.830.942	-798.110	-886
33.680.000	5.832.742	-798.332	-888
33.690.000	5.834.175	-798.584	-888
33.700.000	5.835.921	-798.758	-888
33.710.000	5.837.368	-798.999	-890
33.720.000	5.839.166	-799.243	-893
33.730.000	5.840.564	-799.389	-896
33.740.000	5.842.358	-799.678	-895
33.750.000	5.843.855	-799.821	-893
33.760.000	5.845.489	-800.056	-892
33.770.000	5.847.057	-800.246	-892
33.780.000	5.848.718	-800.458	-891
33.790.000	5.850.282	-800.592	-890
33.800.000	5.851.866	-800.877	-891
33.810.000	5.853.506	-800.958	-891
33.820.000	5.854.981	-801.208	-893
33.830.000	5.856.744	-801.382	-895
33.840.000	5.858.174	-801.535	-896

33.850.000	5.859.937	-801.757	-896
33.860.000	5.861.429	-801.983	-897
33.870.000	5.863.227	-802.151	-898
33.880.000	5.864.592	-802.335	-901
33.890.000	5.866.375	-802.571	-905
33.900.000	5.867.775	-802.683	-905
33.910.000	5.869.524	-802.947	-904
33.920.000	5.871.043	-803.114	-905
33.930.000	5.872.702	-803.299	-910
33.940.000	5.874.207	-803.466	-912
33.950.000	5.875.874	-803.735	-912
33.960.000	5.877.465	-803.831	-912
33.970.000	5.879.025	-804.108	-914
33.980.000	5.880.765	-804.243	-919
33.990.000	5.882.161	-804.435	-921
34.000.000	5.883.934	-804.620	-920
34.010.000	5.885.443	-804.838	-918
34.020.000	5.887.120	-804.991	-916
34.030.000	5.888.566	-805.218	-917
34.040.000	5.890.407	-805.428	-919
34.050.000	5.891.710	-805.558	-918
34.060.000	5.893.517	-805.825	-916
34.070.000	5.894.983	-805.995	-917
34.080.000	5.896.694	-806.210	-922
34.090.000	5.898.219	-806.402	-925
34.100.000	5.899.906	-806.629	-926
34.110.000	5.901.411	-806.773	-923
34.120.000	5.903.078	-807.063	-924
34.130.000	5.904.685	-807.166	-926
34.140.000	5.906.202	-807.434	-927

34.150.000	5.907.930	-807.624	-927
34.160.000	5.909.420	-807.830	-925
34.170.000	5.911.166	-808.025	-925
34.180.000	5.912.602	-808.276	-926
34.190.000	5.914.416	-808.468	-927
34.200.000	5.915.825	-808.692	-927
34.210.000	5.917.644	-808.962	-926
34.220.000	5.919.057	-809.113	-925
34.230.000	5.920.778	-809.403	-926
34.240.000	5.922.269	-809.595	-928
34.250.000	5.923.960	-809.814	-928
34.260.000	5.925.420	-810.011	-926
34.270.000	5.927.145	-810.298	-925
34.280.000	5.928.686	-810.408	-926
34.290.000	5.930.279	-810.669	-929
34.300.000	5.931.982	-810.859	-929
34.310.000	5.933.474	-811.079	-931
34.320.000	5.935.187	-811.247	-933
34.330.000	5.936.706	-811.508	-936
34.340.000	5.938.406	-811.665	-940
34.350.000	5.939.859	-811.918	-943
34.360.000	5.941.654	-812.163	-942
34.370.000	5.943.019	-812.315	-944
34.380.000	5.944.817	-812.600	-947
34.390.000	5.946.283	-812.792	-948
34.400.000	5.948.004	-813.011	-946
34.410.000	5.949.522	-813.232	-945
34.420.000	5.951.281	-813.477	-946
34.430.000	5.952.776	-813.637	-948
34.440.000	5.954.447	-813.938	-949

34.450.000	5.956.029	-814.078	-947
34.460.000	5.957.584	-814.350	-948
34.470.000	5.959.282	-814.535	-950
34.480.000	5.960.786	-814.780	-953
34.490.000	5.962.503	-814.973	-952
34.500.000	5.963.956	-815.259	-951
34.510.000	5.965.778	-815.452	-951
34.520.000	5.967.128	-815.675	-954
34.530.000	5.968.993	-815.976	-956
34.540.000	5.970.454	-816.139	-954
34.550.000	5.972.179	-816.394	-959
34.560.000	5.973.663	-816.632	-966
34.570.000	5.975.410	-816.858	-968
34.580.000	5.976.895	-817.066	-972
34.590.000	5.978.618	-817.369	-973
34.600.000	5.980.144	-817.513	-978
34.610.000	5.981.761	-817.805	-979
34.620.000	5.983.392	-818.023	-976
34.630.000	5.984.933	-818.251	-975
34.640.000	5.986.653	-818.457	-976
34.650.000	5.988.209	-818.763	-976
34.660.000	5.989.916	-818.893	-975
34.670.000	5.991.362	-819.170	-975
34.680.000	5.993.180	-819.397	-978
34.690.000	5.994.531	-819.551	-979
34.700.000	5.996.321	-819.810	-979
34.710.000	5.997.776	-820.010	-981
34.720.000	5.999.453	-820.191	-985
34.730.000	6.000.947	-820.387	-985
34.740.000	6.002.702	-820.642	-982

34.750.000	6.004.163	-820.760	-981
34.760.000	6.005.915	-821.052	-982
34.770.000	6.007.507	-821.186	-981
34.780.000	6.009.060	-821.421	-978
34.790.000	6.010.703	-821.607	-977
34.800.000	6.012.279	-821.848	-978
34.810.000	6.013.942	-821.993	-980
34.820.000	6.015.424	-822.295	-981
34.830.000	6.017.213	-822.449	-982
