# Supplementary material for: Data on statistical experimental design to formulate amphotericin B-loaded Eudragit RL100 nanoparticles coated with hyaluronic acid for the treatment of vulvovaginal candidiasis
Source: Data Brief. 2020 Mar 5;29:105311. doi: 10.1016/j.dib.2020.105311 (PMC7082528; doi:10.1016/j.dib.2020.105311)
Supplement: Multimedia component 8 [file mmc8.pdf]

File Name:	**pure EUD**	File Name:	**pure AMP**	File Name:	**pure HA**
[Data]		[Data]		[Data]	
Temp	**DSC**	Temp	**DSC**	Temp	**DSC**
C	mW	C	mW	C	mW
236.224	183	275.735	19	228.326	31
236.245	177	275.665	14	228.374	26
236.273	171	275.676	8	228.377	21
236.231	165	275.732	2	228.361	16
236.068	158	275.744	-4	228.384	10
236.047	152	275.791	-10	228.377	4
236.091	145	275.798	-17	228.358	-2
236.143	137	275.742	-24	228.337	-9
236.105	130	275.774	-31	228.361	-16
236.029	122	275.756	-38	228.405	-23
236.010	114	275.746	-46	228.398	-30
235.979	105	275.774	-54	228.389	-38
235.924	97	275.742	-62	228.354	-46
235.944	88	275.730	-71	228.351	-54
235.949	78	275.732	-80	228.384	-62
235.951	68	275.704	-89	228.388	-71
236.010	58	275.658	-98	228.379	-80
236.019	48	275.630	-108	228.354	-90
236.035	37	275.653	-118	228.319	-99
236.126	26	275.663	-128	228.300	-110
236.250	14	275.637	-139	228.276	-120
236.262	2	275.618	-150	228.270	-131
236.255	-10	275.627	-162	228.298	-142
236.232	-23	275.634	-174	228.340	-154
236.161	-36	275.660	-186	228.414	-166

236.190	-49	275.651	-198	228.447	-178
236.236	-63	275.632	-211	228.412	-191
236.252	-78	275.732	-225	228.374	-204
236.224	-93	275.761	-238	228.353	-217
236.127	-108	275.747	-253	228.344	-231
236.087	-124	275.723	-267	228.333	-246
236.019	-140	275.665	-282	228.314	-260
235.933	-156	275.669	-297	228.321	-276
235.879	-173	275.693	-313	228.333	-291
235.905	-191	275.728	-329	228.340	-307
236.014	-209	275.730	-346	228.272	-324
236.040	-227	275.697	-363	228.190	-341
236.061	-246	275.686	-380	228.186	-358
236.136	-266	275.721	-398	228.244	-376
236.210	-286	275.737	-417	228.293	-395
236.273	-306	275.737	-435	228.321	-414
236.297	-327	275.760	-455	228.379	-433
236.451	-349	275.730	-475	228.451	-453
236.530	-371	275.767	-495	228.561	-473
236.378	-394	275.821	-516	228.601	-494
236.255	-417	275.779	-537	228.622	-516
236.248	-441	275.753	-559	228.687	-537
236.327	-465	275.697	-581	228.715	-560
236.381	-490	275.632	-604	228.706	-583
236.597	-515	275.707	-627	228.715	-607
236.705	-541	275.777	-651	228.696	-631
236.507	-568	275.812	-675	228.675	-655
236.371	-595	275.854	-700	228.689	-680
236.567	-623	275.903	-725	228.671	-706
236.686	-651	275.938	-751	228.673	-732

236.630	-680	275.936	-777	228.699	-759
236.490	-710	275.968	-804	228.699	-787
236.460	-740	275.991	-831	228.750	-815
236.623	-771	276.038	-859	228.804	-843
236.787	-802	276.098	-888	228.844	-872
236.896	-834	276.094	-917	228.936	-902
236.943	-866	276.101	-946	228.953	-932
236.964	-899	276.141	-976	228.995	-963
237.024	-933	276.180	-1.007	229.114	-994
237.110	-967	276.171	-1.038	229.139	-1.026
237.103	-1.002	276.161	-1.070	229.102	-1.059
237.150	-1.038	276.182	-1.102	229.097	-1.092
237.183	-1.074	276.238	-1.134	229.135	-1.126
237.246	-1.110	276.271	-1.168	229.147	-1.160
237.292	-1.148	276.287	-1.201	229.177	-1.195
237.311	-1.185	276.318	-1.236	229.228	-1.230
237.330	-1.224	276.371	-1.271	229.291	-1.266
237.381	-1.263	276.409	-1.306	229.331	-1.302
237.435	-1.303	276.383	-1.342	229.328	-1.340
237.488	-1.343	276.379	-1.378	229.370	-1.377
237.568	-1.384	276.395	-1.415	229.457	-1.416
237.638	-1.425	276.432	-1.453	229.506	-1.454
237.752	-1.467	276.486	-1.491	229.545	-1.494
237.939	-1.510	276.542	-1.529	229.571	-1.534
238.111	-1.553	276.577	-1.568	229.596	-1.574
238.142	-1.597	276.645	-1.608	229.706	-1.615
238.109	-1.642	276.737	-1.648	229.757	-1.657
238.263	-1.687	276.838	-1.689	229.764	-1.699
238.233	-1.732	276.948	-1.730	229.879	-1.742
237.962	-1.779	277.045	-1.772	229.970	-1.785

237.931	-1.825	277.120	-1.814	230.007	-1.829
237.987	-1.873	277.122	-1.856	230.033	-1.873
238.046	-1.920	277.129	-1.900	230.044	-1.918
238.099	-1.969	277.165	-1.943	230.112	-1.964
238.169	-2.018	277.237	-1.987	230.186	-2.010
238.221	-2.068	277.274	-2.032	230.238	-2.056
238.328	-2.118	277.304	-2.077	230.282	-2.103
238.449	-2.168	277.395	-2.123	230.415	-2.151
238.503	-2.220	277.412	-2.169	230.508	-2.199
238.566	-2.271	277.374	-2.216	230.529	-2.247
238.657	-2.324	277.400	-2.263	230.511	-2.296
238.729	-2.376	277.435	-2.310	230.670	-2.346
238.809	-2.430	277.521	-2.358	231.049	-2.396
238.874	-2.484	277.582	-2.407	231.260	-2.447
239.056	-2.538	277.607	-2.456	231.304	-2.498
239.128	-2.593	277.736	-2.505	231.330	-2.549
239.049	-2.648	277.878	-2.555	231.266	-2.601
239.077	-2.704	277.950	-2.605	231.168	-2.654
239.072	-2.760	277.983	-2.656	231.131	-2.706
239.186	-2.817	278.043	-2.707	231.129	-2.760
239.354	-2.875	278.156	-2.758	231.147	-2.814
239.410	-2.932	278.244	-2.810	231.224	-2.868
239.436	-2.991	278.302	-2.862	231.301	-2.922
239.475	-3.049	278.363	-2.915	231.372	-2.977
239.576	-3.108	278.393	-2.968	231.486	-3.033
239.669	-3.168	278.393	-3.021	231.639	-3.089
239.793	-3.228	278.482	-3.075	231.773	-3.145
239.935	-3.288	278.654	-3.129	231.826	-3.202
239.977	-3.349	278.771	-3.184	231.913	-3.259
240.051	-3.410	278.829	-3.239	231.985	-3.316

240.205	-3.472	278.850	-3.294	232.076	-3.374
240.354	-3.534	278.913	-3.350	232.138	-3.432
240.459	-3.596	279.000	-3.406	232.166	-3.491
240.497	-3.659	279.086	-3.462	232.239	-3.549
240.504	-3.722	279.177	-3.519	232.295	-3.608
240.495	-3.785	279.216	-3.576	232.432	-3.668
240.567	-3.849	279.321	-3.633	232.533	-3.728
240.732	-3.913	279.445	-3.691	232.596	-3.788
240.819	-3.977	279.515	-3.749	232.679	-3.848
240.914	-4.042	279.630	-3.807	232.722	-3.909
240.951	-4.107	279.718	-3.865	232.766	-3.970
241.038	-4.172	279.739	-3.924	232.840	-4.031
241.203	-4.238	279.834	-3.983	233.011	-4.092
241.308	-4.304	279.960	-4.043	233.160	-4.154
241.383	-4.370	280.075	-4.102	233.253	-4.216
241.485	-4.437	280.222	-4.162	233.367	-4.278
241.551	-4.503	280.324	-4.222	233.496	-4.341
241.681	-4.570	280.408	-4.282	233.571	-4.403
241.859	-4.637	280.441	-4.343	233.608	-4.466
241.957	-4.705	280.487	-4.404	233.680	-4.529
242.025	-4.772	280.583	-4.465	233.781	-4.592
242.103	-4.840	280.662	-4.526	233.811	-4.656
242.150	-4.908	280.725	-4.587	233.802	-4.719
242.253	-4.976	280.791	-4.649	233.890	-4.783
242.360	-5.045	281.015	-4.710	234.049	-4.847
242.470	-5.113	281.421	-4.772	234.221	-4.911
242.626	-5.182	281.637	-4.834	234.349	-4.975
242.724	-5.251	281.667	-4.896	234.501	-5.039
242.861	-5.320	281.649	-4.959	234.585	-5.104
242.976	-5.389	281.649	-5.021	234.594	-5.168

243.092	-5.458	281.625	-5.084	234.711	-5.233
243.214	-5.527	281.689	-5.147	234.862	-5.297
243.249	-5.597	281.787	-5.210	234.991	-5.362
243.361	-5.666	281.805	-5.272	235.096	-5.427
243.456	-5.736	281.824	-5.336	235.184	-5.491
243.514	-5.806	281.861	-5.399	235.252	-5.556
243.655	-5.875	281.978	-5.462	235.303	-5.621
243.808	-5.945	282.120	-5.525	235.382	-5.686
243.993	-6.015	282.255	-5.589	235.501	-5.751
244.067	-6.085	282.360	-5.652	235.653	-5.816
244.128	-6.155	282.481	-5.715	235.853	-5.881
244.280	-6.225	282.570	-5.779	236.028	-5.946
244.342	-6.294	282.664	-5.842	236.140	-6.011
244.455	-6.364	282.808	-5.906	236.199	-6.076
244.638	-6.434	282.934	-5.970	236.227	-6.140
244.792	-6.504	282.994	-6.033	236.350	-6.205
244.888	-6.574	283.051	-6.097	236.530	-6.270
244.944	-6.644	283.183	-6.160	236.646	-6.335
245.042	-6.713	283.309	-6.224	236.740	-6.399
245.182	-6.783	283.396	-6.287	236.814	-6.464
245.289	-6.853	283.526	-6.351	236.873	-6.528
245.317	-6.922	283.619	-6.414	236.996	-6.593
245.392	-6.992	283.694	-6.478	237.146	-6.657
245.499	-7.061	283.797	-6.541	237.243	-6.721
245.592	-7.131	283.946	-6.605	237.367	-6.785
245.712	-7.200	284.049	-6.668	237.498	-6.849
245.907	-7.269	284.174	-6.731	237.600	-6.913
245.979	-7.338	284.349	-6.794	237.731	-6.977
246.038	-7.407	284.454	-6.857	237.880	-7.040
246.278	-7.476	284.564	-6.920	237.997	-7.104

246.474	-7.544	284.709	-6.983	238.072	-7.167
246.577	-7.613	284.862	-7.045	238.132	-7.230
246.677	-7.681	285.019	-7.108	238.190	-7.293
246.838	-7.749	285.126	-7.171	238.268	-7.356
247.062	-7.818	285.192	-7.233	238.358	-7.419
247.199	-7.885	285.261	-7.295	238.533	-7.481
247.216	-7.953	285.354	-7.357	238.685	-7.544
247.323	-8.021	285.508	-7.419	238.752	-7.606
247.428	-8.088	285.639	-7.481	238.808	-7.668
247.533	-8.155	285.726	-7.542	239.011	-7.729
247.677	-8.222	285.900	-7.604	239.312	-7.791
247.766	-8.289	286.089	-7.665	239.490	-7.852
247.845	-8.356	286.175	-7.726	239.634	-7.913
247.964	-8.422	286.222	-7.787	239.769	-7.974
248.146	-8.489	286.348	-7.848	239.855	-8.035
248.272	-8.555	286.528	-7.908	239.967	-8.096
248.368	-8.620	286.645	-7.968	240.114	-8.156
248.514	-8.686	286.715	-8.028	240.224	-8.216
248.718	-8.751	286.793	-8.088	240.326	-8.276
248.897	-8.816	286.933	-8.148	240.464	-8.335
248.960	-8.881	287.085	-8.208	240.558	-8.394
249.107	-8.946	287.178	-8.267	240.632	-8.453
249.251	-9.010	287.291	-8.326	240.754	-8.512
249.389	-9.074	287.491	-8.385	240.896	-8.571
249.480	-9.138	287.657	-8.443	240.998	-8.629
249.648	-9.202	287.740	-8.502	241.084	-8.687
249.727	-9.265	287.868	-8.560	241.227	-8.745
249.743	-9.329	288.039	-8.618	241.413	-8.802
250.021	-9.392	288.155	-8.675	241.588	-8.859
250.247	-9.454	288.254	-8.733	241.758	-8.916

250.404	-9.516	288.377	-8.790	241.931	-8.973
250.630	-9.579	288.540	-8.847	242.064	-9.029
250.854	-9.640	288.680	-8.903	242.204	-9.085
250.900	-9.702	288.762	-8.960	242.320	-9.141
250.914	-9.763	288.906	-9.016	242.423	-9.197
250.977	-9.824	289.049	-9.072	242.554	-9.252
251.117	-9.885	289.177	-9.127	242.680	-9.307
251.287	-9.945	289.354	-9.182	242.806	-9.362
251.441	-10.005	289.545	-9.237	242.915	-9.416
251.632	-10.065	289.615	-9.292	243.060	-9.470
251.691	-10.125	289.708	-9.347	243.286	-9.524
251.770	-10.184	289.886	-9.401	243.393	-9.577
251.976	-10.243	290.026	-9.455	243.444	-9.630
252.104	-10.301	290.142	-9.508	243.589	-9.683
252.209	-10.360	290.264	-9.561	243.794	-9.736
252.379	-10.418	290.410	-9.614	243.946	-9.788
252.586	-10.476	290.543	-9.667	244.027	-9.840
252.699	-10.533	290.632	-9.719	244.158	-9.892
252.771	-10.590	290.711	-9.772	244.342	-9.943
252.939	-10.647	290.877	-9.823	244.518	-9.994
253.155	-10.703	291.038	-9.875	244.632	-10.045
253.270	-10.760	291.173	-9.926	244.727	-10.096
253.342	-10.815	291.271	-9.977	244.883	-10.146
253.500	-10.871	291.371	-10.027	245.031	-10.196
253.661	-10.926	291.532	-10.077	245.112	-10.245
253.801	-10.981	291.696	-10.127	245.203	-10.294
253.984	-11.036	291.871	-10.177	245.366	-10.343
254.149	-11.090	292.078	-10.226	245.560	-10.392
254.198	-11.144	292.278	-10.275	245.728	-10.440
254.436	-11.198	292.470	-10.324	245.863	-10.488

254.764	-11.251	292.577	-10.372	246.008	-10.536
255.075	-11.304	292.645	-10.420	246.199	-10.583
255.364	-11.357	292.801	-10.468	246.329	-10.631
255.553	-11.409	292.929	-10.515	246.416	-10.677
255.557	-11.461	293.006	-10.562	246.595	-10.724
255.404	-11.513	293.146	-10.609	246.933	-10.770
255.306	-11.564	293.337	-10.655	247.311	-10.816
255.364	-11.615	293.442	-10.701	247.554	-10.861
255.539	-11.666	293.538	-10.747	247.643	-10.907
255.725	-11.717	293.657	-10.792	247.691	-10.952
255.875	-11.767	293.794	-10.837	247.638	-10.996
256.049	-11.817	293.983	-10.882	247.586	-11.041
256.152	-11.866	294.131	-10.926	247.701	-11.085
256.253	-11.915	294.207	-10.970	247.852	-11.128
256.441	-11.964	294.294	-11.014	247.950	-11.172
256.567	-12.013	294.466	-11.057	247.999	-11.215
256.679	-12.061	294.639	-11.100	248.142	-11.258
256.859	-12.109	294.844	-11.143	248.323	-11.300
257.039	-12.157	295.002	-11.185	248.458	-11.343
257.206	-12.204	295.128	-11.227	248.657	-11.385
257.290	-12.252	295.319	-11.269	248.813	-11.426
257.418	-12.298	295.516	-11.311	248.956	-11.468
257.600	-12.345	295.651	-11.352	249.165	-11.509
257.743	-12.391	295.739	-11.393	249.307	-11.550
257.927	-12.437	295.903	-11.433	249.436	-11.590
258.135	-12.482	296.077	-11.473	249.623	-11.631
258.267	-12.528	296.156	-11.513	249.783	-11.670
258.379	-12.573	296.279	-11.553	249.886	-11.710
258.451	-12.617	296.489	-11.592	250.012	-11.750
258.519	-12.662	296.654	-11.631	250.156	-11.789

258.692	-12.706	296.814	-11.669	250.296	-11.828
258.836	-12.750	296.956	-11.708	250.438	-11.866
259.032	-12.793	297.087	-11.746	250.560	-11.904
259.249	-12.836	297.215	-11.783	250.679	-11.942
259.433	-12.879	297.352	-11.821	250.819	-11.980
259.578	-12.922	297.525	-11.858	250.975	-12.018
259.760	-12.964	297.684	-11.894	251.105	-12.055
259.939	-13.006	297.872	-11.931	251.266	-12.092
260.038	-13.048	297.996	-11.967	251.446	-12.128
260.219	-13.089	298.141	-12.003	251.551	-12.165
260.373	-13.131	298.301	-12.039	251.749	-12.201
260.429	-13.171	298.398	-12.074	251.947	-12.237
260.473	-13.212	298.556	-12.109	252.022	-12.272
260.653	-13.252	298.649	-12.144	252.150	-12.307
260.858	-13.292	298.795	-12.178	252.314	-12.343
261.024	-13.332	299.027	-12.212	252.425	-12.377
261.133	-13.372	299.227	-12.246	252.537	-12.412
261.241	-13.411	299.382	-12.280	252.750	-12.446
261.448	-13.450	299.471	-12.313	252.966	-12.480
261.575	-13.489	299.507	-12.346	253.104	-12.514
261.681	-13.527	299.620	-12.379	253.212	-12.547
261.850	-13.565	299.880	-12.412	253.379	-12.581
262.020	-13.603	300.132	-12.444	253.580	-12.614
262.197	-13.641	300.418	-12.476	253.717	-12.647
262.306	-13.678	300.774	-12.508	253.834	-12.679
262.423	-13.715	301.014	-12.540	254.007	-12.711
262.638	-13.752	301.089	-12.571	254.149	-12.743
262.848	-13.788	301.147	-12.602	254.226	-12.775
262.983	-13.825	301.145	-12.633	254.354	-12.807
263.039	-13.861	301.144	-12.663	254.552	-12.838

263.160	-13.896	301.262	-12.694	254.774	-12.869
263.342	-13.932	301.392	-12.724	254.918	-12.900
263.515	-13.967	301.481	-12.753	255.019	-12.931
263.701	-14.002	301.597	-12.783	255.119	-12.961
263.885	-14.037	301.716	-12.812	255.245	-12.991
264.081	-14.071	301.862	-12.841	255.457	-13.021
264.289	-14.106	302.038	-12.870	255.613	-13.051
264.462	-14.140	302.208	-12.899	255.725	-13.081
264.609	-14.173	302.367	-12.927	255.868	-13.110
264.751	-14.207	302.535	-12.955	256.068	-13.139
264.844	-14.240	302.690	-12.983	256.217	-13.168
264.947	-14.273	302.857	-13.011	256.336	-13.196
265.166	-14.306	303.042	-13.038	256.518	-13.225
265.382	-14.338	303.204	-13.066	256.667	-13.253
265.527	-14.370	303.297	-13.093	256.822	-13.281
265.648	-14.402	303.398	-13.119	256.982	-13.309
265.781	-14.434	303.595	-13.146	257.164	-13.336
265.936	-14.465	303.754	-13.172	257.386	-13.364
266.117	-14.497	303.869	-13.198	257.524	-13.391
266.196	-14.528	304.043	-13.224	257.673	-13.418
266.232	-14.559	304.148	-13.250	257.817	-13.444
266.406	-14.589	304.269	-13.275	257.946	-13.471
266.618	-14.620	304.471	-13.301	258.118	-13.497
266.849	-14.650	304.617	-13.326	258.240	-13.523
266.997	-14.680	304.756	-13.351	258.347	-13.549
267.062	-14.709	304.970	-13.375	258.496	-13.575
267.169	-14.739	305.114	-13.400	258.638	-13.600
267.288	-14.768	305.263	-13.424	258.710	-13.626
267.395	-14.797	305.520	-13.448	258.790	-13.651
267.514	-14.826	305.738	-13.472	258.948	-13.676

267.685	-14.854	305.897	-13.496	259.116	-13.700
267.829	-14.882	306.037	-13.519	259.329	-13.725
268.022	-14.911	306.175	-13.542	259.560	-13.749
268.237	-14.938	306.252	-13.566	259.737	-13.773
268.345	-14.966	306.364	-13.588	259.944	-13.797
268.514	-14.994	306.506	-13.611	260.135	-13.821
268.750	-15.021	306.625	-13.634	260.301	-13.844
268.958	-15.048	306.784	-13.656	260.532	-13.868
269.193	-15.075	306.901	-13.678	260.739	-13.891
269.387	-15.101	307.049	-13.700	260.863	-13.914
269.531	-15.128	307.223	-13.722	261.022	-13.937
269.625	-15.154	307.377	-13.744	261.173	-13.959
269.779	-15.180	307.563	-13.765	261.227	-13.982
269.965	-15.206	307.708	-13.787	261.280	-14.004
270.093	-15.231	307.834	-13.808	261.392	-14.026
270.303	-15.256	308.022	-13.829	261.572	-14.048
270.457	-15.282	308.176	-13.850	261.793	-14.070
270.553	-15.307	308.280	-13.870	261.961	-14.091
270.662	-15.331	308.389	-13.891	262.078	-14.113
270.814	-15.356	308.499	-13.911	262.150	-14.134
271.019	-15.380	308.677	-13.931	262.318	-14.155
271.155	-15.404	308.905	-13.951	262.507	-14.176
271.297	-15.428	309.122	-13.971	262.701	-14.197
271.444	-15.452	309.290	-13.991	262.925	-14.217
271.590	-15.476	309.443	-14.010	263.069	-14.238
271.751	-15.499	309.592	-14.030	263.176	-14.258
271.908	-15.522	309.709	-14.049	263.305	-14.278
272.158	-15.545	309.894	-14.068	263.524	-14.298
272.292	-15.568	310.087	-14.087	263.731	-14.317
272.402	-15.591	310.165	-14.106	263.916	-14.337

272.642	-15.613	310.249	-14.125	263.995	-14.356
272.896	-15.635	310.413	-14.143	264.119	-14.375
273.093	-15.657	310.544	-14.162	264.349	-14.395
273.176	-15.679	310.710	-14.180	264.496	-14.413
273.319	-15.701	310.944	-14.198	264.613	-14.432
273.461	-15.722	311.150	-14.216	264.809	-14.451
273.533	-15.744	311.331	-14.234	265.056	-14.469
273.676	-15.765	311.476	-14.251	265.140	-14.488
273.904	-15.786	311.574	-14.269	265.257	-14.506
274.072	-15.806	311.709	-14.286	265.490	-14.524
274.188	-15.827	311.915	-14.303	265.662	-14.542
274.368	-15.847	312.102	-14.321	265.812	-14.559
274.524	-15.868	312.236	-14.338	266.000	-14.577
274.765	-15.888	312.347	-14.354	266.155	-14.594
274.956	-15.908	312.489	-14.371	266.285	-14.612
275.072	-15.927	312.690	-14.388	266.393	-14.629
275.226	-15.947	312.816	-14.404	266.535	-14.646
275.381	-15.966	312.894	-14.421	266.779	-14.663
275.490	-15.985	313.025	-14.437	266.968	-14.680
275.623	-16.004	313.137	-14.453	267.085	-14.696
275.791	-16.023	313.262	-14.469	267.213	-14.713
275.968	-16.042	313.451	-14.485	267.404	-14.729
276.190	-16.061	313.688	-14.500	267.614	-14.745
276.344	-16.079	313.893	-14.516	267.713	-14.761
276.504	-16.097	314.047	-14.531	267.838	-14.777
276.660	-16.115	314.202	-14.547	268.030	-14.793
276.758	-16.133	314.331	-14.562	268.202	-14.809
276.885	-16.151	314.480	-14.577	268.375	-14.825
277.039	-16.169	314.685	-14.592	268.498	-14.840
277.250	-16.186	314.871	-14.607	268.794	-14.855

277.477	-16.204	315.044	-14.621	269.249	-14.871
277.584	-16.221	315.212	-14.636	269.501	-14.886
277.710	-16.238	315.292	-14.650	269.622	-14.901
277.943	-16.255	315.416	-14.665	269.730	-14.916
278.053	-16.271	315.592	-14.679	269.711	-14.930
278.114	-16.288	315.762	-14.693	269.739	-14.945
278.340	-16.304	315.932	-14.707	269.865	-14.960
278.701	-16.321	316.056	-14.721	269.960	-14.974
279.109	-16.337	316.123	-14.735	270.030	-14.988
279.364	-16.353	316.262	-14.749	270.124	-15.002
279.457	-16.369	316.482	-14.762	270.301	-15.017
279.534	-16.385	316.635	-14.776	270.445	-15.031
279.587	-16.400	316.742	-14.789	270.627	-15.044
279.644	-16.416	316.896	-14.802	270.784	-15.058
279.729	-16.431	317.106	-14.815	270.944	-15.072
279.806	-16.447	317.283	-14.828	271.203	-15.085
279.909	-16.462	317.445	-14.841	271.350	-15.099
280.019	-16.477	317.684	-14.854	271.483	-15.112
280.105	-16.492	317.904	-14.867	271.681	-15.125
280.264	-16.506	318.001	-14.879	271.819	-15.138
280.490	-16.521	318.104	-14.892	271.915	-15.152
280.663	-16.535	318.273	-14.904	272.071	-15.164
280.793	-16.550	318.476	-14.916	272.253	-15.177
280.957	-16.564	318.659	-14.929	272.435	-15.190
281.164	-16.578	318.819	-14.941	272.549	-15.203
281.362	-16.592	318.971	-14.953	272.675	-15.215
281.518	-16.606	319.148	-14.965	272.885	-15.228
281.661	-16.620	319.308	-14.977	273.022	-15.240
281.852	-16.634	319.482	-14.988	273.125	-15.252
281.969	-16.647	319.648	-15.000	273.270	-15.265

282.137	-16.661	319.819	-15.011	273.478	-15.277
282.323	-16.674	319.979	-15.023	273.678	-15.289
282.449	-16.687	320.113	-15.034	273.795	-15.301
282.547	-16.701	320.315	-15.046	273.955	-15.313
282.670	-16.714	320.478	-15.057	274.144	-15.324
282.882	-16.727	320.646	-15.068	274.310	-15.336
283.020	-16.739	320.812	-15.079	274.539	-15.348
283.102	-16.752	320.943	-15.090	274.725	-15.359
283.251	-16.765	321.088	-15.101	274.802	-15.371
283.482	-16.777	321.263	-15.111	274.968	-15.382
283.680	-16.790	321.405	-15.122	275.170	-15.393
283.815	-16.802	321.520	-15.133	275.297	-15.405
283.970	-16.814	321.690	-15.143	275.429	-15.416
284.161	-16.827	321.797	-15.154	275.579	-15.427
284.249	-16.839	321.907	-15.164	275.744	-15.438
284.375	-16.851	322.117	-15.174	275.958	-15.449
284.620	-16.863	322.322	-15.184	276.140	-15.460
284.798	-16.874	322.476	-15.195	276.285	-15.471
284.974	-16.886	322.618	-15.205	276.449	-15.481
285.161	-16.898	322.789	-15.215	276.556	-15.492
285.310	-16.909	322.931	-15.224	276.665	-15.503
285.429	-16.921	323.063	-15.234	276.821	-15.513
285.560	-16.932	323.217	-15.244	277.024	-15.524
285.665	-16.943	323.509	-15.254	277.193	-15.534
285.830	-16.955	323.935	-15.263	277.267	-15.545
286.080	-16.966	324.177	-15.273	277.409	-15.555
286.208	-16.977	324.327	-15.282	277.670	-15.565
286.330	-16.988	324.426	-15.292	277.831	-15.575
286.532	-16.999	324.459	-15.301	277.894	-15.585
286.673	-17.010	324.518	-15.310	278.050	-15.596

286.770	-17.020	324.618	-15.319	278.258	-15.606
286.891	-17.031	324.711	-15.328	278.440	-15.616
286.973	-17.042	324.800	-15.337	278.589	-15.626
287.085	-17.052	324.906	-15.346	278.773	-15.635
287.341	-17.063	325.048	-15.355	278.981	-15.645
287.579	-17.073	325.205	-15.364	279.077	-15.655
287.701	-17.084	325.370	-15.373	279.151	-15.665
287.806	-17.094	325.596	-15.382	279.322	-15.674
287.915	-17.104	325.779	-15.390	279.566	-15.684
288.134	-17.114	325.933	-15.399	279.757	-15.694
288.349	-17.124	326.168	-15.407	279.872	-15.703
288.471	-17.134	326.376	-15.416	279.989	-15.713
288.657	-17.144	326.526	-15.424	280.145	-15.722
288.792	-17.154	326.643	-15.433	280.348	-15.731
288.955	-17.164	326.759	-15.441	280.520	-15.741
289.165	-17.174	326.870	-15.449	280.670	-15.750
289.329	-17.184	327.030	-15.457	280.865	-15.759
289.476	-17.193	327.203	-15.465	281.000	-15.768
289.629	-17.203	327.309	-15.473	281.017	-15.777
289.795	-17.212	327.486	-15.481	281.138	-15.787
289.963	-17.222	327.700	-15.489	281.364	-15.796
290.089	-17.231	327.861	-15.497	281.539	-15.805
290.210	-17.241	327.987	-15.505	281.658	-15.814
290.385	-17.250	328.103	-15.513	281.772	-15.822
290.567	-17.259	328.258	-15.521	281.934	-15.831
290.758	-17.269	328.406	-15.528	282.132	-15.840
290.914	-17.278	328.547	-15.536	282.241	-15.849
291.082	-17.287	328.782	-15.544	282.351	-15.858
291.234	-17.296	329.004	-15.551	282.650	-15.866
291.376	-17.305	329.190	-15.559	282.931	-15.875

291.567	-17.314	329.332	-15.566	283.118	-15.884
291.688	-17.323	329.485	-15.573	283.326	-15.892
291.742	-17.332	329.632	-15.581	283.564	-15.901
291.873	-17.341	329.745	-15.588	283.773	-15.909
292.113	-17.349	329.915	-15.595	283.853	-15.918
292.312	-17.358	330.048	-15.603	283.902	-15.926
292.437	-17.367	330.174	-15.610	283.981	-15.934
292.575	-17.375	330.336	-15.617	284.107	-15.943
292.664	-17.384	330.501	-15.624	284.252	-15.951
292.745	-17.392	330.687	-15.631	284.338	-15.959
292.958	-17.401	330.850	-15.638	284.457	-15.967
293.188	-17.409	330.996	-15.645	284.639	-15.976
293.368	-17.418	331.149	-15.652	284.805	-15.984
293.533	-17.426	331.313	-15.659	284.977	-15.992
293.743	-17.434	331.464	-15.666	285.173	-16.000
293.925	-17.442	331.620	-15.672	285.353	-16.008
294.107	-17.451	331.804	-15.679	285.420	-16.016
294.382	-17.459	331.997	-15.686	285.600	-16.024
294.548	-17.467	332.164	-15.693	285.856	-16.032
294.694	-17.475	332.277	-15.699	286.014	-16.040
294.928	-17.483	332.435	-15.706	286.180	-16.048
295.090	-17.491	332.574	-15.712	286.411	-16.056
295.130	-17.499	332.722	-15.719	286.551	-16.063
295.241	-17.507	332.899	-15.726	286.688	-16.071
295.461	-17.515	333.108	-15.732	286.852	-16.079
295.559	-17.522	333.309	-15.738	286.940	-16.087
295.670	-17.530	333.414	-15.745	287.092	-16.094
295.857	-17.538	333.628	-15.751	287.299	-16.102
295.950	-17.545	333.795	-15.758	287.486	-16.110
296.095	-17.553	333.908	-15.764	287.633	-16.117

296.290	-17.561	334.065	-15.770	287.771	-16.125
296.420	-17.568	334.233	-15.776	287.945	-16.133
296.575	-17.576	334.420	-15.783	288.134	-16.140
296.775	-17.583	334.544	-15.789	288.263	-16.148
296.920	-17.591	334.668	-15.795	288.422	-16.155
297.055	-17.598	334.870	-15.801	288.604	-16.163
297.194	-17.605	335.059	-15.807	288.785	-16.170
297.456	-17.613	335.194	-15.813	288.962	-16.177
297.699	-17.620	335.364	-15.819	289.088	-16.185
297.833	-17.627	335.548	-15.825	289.261	-16.192
297.951	-17.635	335.700	-15.831	289.426	-16.199
298.020	-17.642	335.871	-15.837	289.562	-16.207
298.174	-17.649	336.037	-15.843	289.749	-16.214
298.432	-17.656	336.168	-15.849	289.897	-16.221
298.580	-17.663	336.316	-15.855	290.010	-16.229
298.697	-17.670	336.475	-15.861	290.191	-16.236
298.907	-17.677	336.557	-15.867	290.404	-16.243
299.045	-17.684	336.680	-15.872	290.597	-16.250
299.219	-17.691	336.791	-15.878	290.697	-16.257
299.430	-17.698	336.892	-15.884	290.809	-16.264
299.536	-17.705	337.079	-15.890	291.014	-16.272
299.750	-17.712	337.247	-15.895	291.196	-16.279
299.957	-17.718	337.429	-15.901	291.315	-16.286
300.030	-17.725	337.634	-15.907	291.418	-16.293
300.180	-17.732	337.810	-15.912	291.747	-16.300
300.315	-17.739	337.947	-15.918	292.185	-16.307
300.520	-17.745	338.125	-15.923	292.456	-16.314
300.668	-17.752	338.356	-15.929	292.519	-16.321
300.837	-17.759	338.553	-15.934	292.551	-16.328
301.029	-17.765	338.793	-15.940	292.691	-16.335

301.169	-17.772	339.004	-15.945	292.752	-16.341
301.338	-17.778	339.116	-15.950	292.801	-16.348
301.495	-17.785	339.244	-15.956	292.910	-16.355
301.654	-17.791	339.389	-15.961	293.043	-16.362
301.821	-17.798	339.571	-15.966	293.151	-16.369
301.948	-17.804	339.746	-15.972	293.328	-16.376
302.137	-17.811	339.853	-15.977	293.541	-16.382
302.278	-17.817	339.917	-15.982	293.689	-16.389
302.404	-17.824	340.077	-15.987	293.841	-16.396
302.574	-17.830	340.257	-15.993	293.988	-16.403
302.726	-17.836	340.385	-15.998	294.145	-16.409
302.884	-17.843	340.545	-16.003	294.345	-16.416
303.066	-17.849	340.701	-16.008	294.543	-16.423
303.231	-17.855	340.891	-16.013	294.689	-16.430
303.387	-17.861	341.065	-16.018	294.852	-16.436
303.552	-17.868	341.209	-16.023	295.007	-16.443
303.902	-17.874	341.429	-16.028	295.155	-16.450
304.318	-17.880	341.620	-16.033	295.272	-16.456
304.522	-17.886	341.786	-16.038	295.404	-16.463
304.607	-17.892	342.025	-16.043	295.586	-16.469
304.695	-17.898	342.184	-16.048	295.734	-16.476
304.771	-17.904	342.285	-16.053	295.851	-16.482
304.740	-17.911	342.423	-16.058	295.955	-16.489
304.741	-17.917	342.566	-16.063	296.077	-16.496
304.840	-17.923	342.705	-16.068	296.282	-16.502
304.953	-17.929	342.850	-16.073	296.508	-16.509
305.077	-17.935	342.997	-16.077	296.705	-16.515
305.268	-17.941	343.184	-16.082	296.859	-16.522
305.508	-17.947	343.421	-16.087	297.022	-16.528
305.676	-17.953	343.621	-16.092	297.213	-16.535

305.808	-17.959	343.764	-16.096	297.415	-16.541
305.912	-17.965	343.871	-16.101	297.661	-16.548
306.065	-17.970	344.060	-16.106	297.843	-16.554
306.305	-17.976	344.216	-16.111	297.954	-16.561
306.505	-17.982	344.276	-16.115	297.994	-16.567
306.676	-17.988	344.482	-16.120	298.122	-16.573
306.863	-17.994	344.735	-16.125	298.281	-16.580
306.932	-18.000	344.887	-16.129	298.403	-16.586
306.977	-18.006	345.015	-16.134	298.585	-16.593
307.187	-18.012	345.170	-16.138	298.827	-16.599
307.453	-18.017	345.344	-16.143	299.038	-16.605
307.703	-18.023	345.460	-16.148	299.191	-16.612
307.849	-18.029	345.614	-16.152	299.331	-16.618
307.912	-18.035	345.827	-16.157	299.462	-16.625
307.989	-18.041	345.982	-16.161	299.634	-16.631
308.137	-18.047	346.141	-16.166	299.788	-16.637
308.309	-18.052	346.385	-16.170	299.877	-16.644
308.474	-18.058	346.597	-16.175	299.979	-16.650
308.642	-18.064	346.681	-16.179	300.177	-16.657
308.785	-18.070	346.761	-16.184	300.357	-16.663
308.973	-18.076	346.956	-16.188	300.501	-16.669
309.195	-18.081	347.164	-16.192	300.678	-16.676
309.414	-18.087	347.298	-16.197	300.838	-16.682
309.591	-18.093	347.379	-16.201	300.993	-16.688
309.722	-18.099	347.644	-16.206	301.181	-16.695
309.904	-18.105	348.066	-16.210	301.369	-16.701
310.068	-18.110	348.396	-16.214	301.491	-16.707
310.247	-18.116	348.553	-16.219	301.644	-16.714
310.408	-18.122	348.638	-16.223	301.814	-16.720
310.478	-18.128	348.708	-16.227	301.942	-16.726

310.529	-18.134	348.734	-16.232	302.133	-16.733
310.652	-18.139	348.729	-16.236	302.319	-16.739
310.797	-18.145	348.832	-16.240	302.425	-16.745
310.880	-18.151	348.952	-16.245	302.569	-16.751
310.903	-18.157	349.021	-16.249	302.719	-16.758
311.154	-18.162	349.198	-16.253	302.923	-16.764
311.458	-18.168	349.422	-16.258	303.127	-16.770
311.392	-18.174	349.609	-16.262	303.239	-16.777
311.532	-18.180	349.743	-16.266	303.409	-16.783
311.878	-18.186	349.938	-16.270	303.591	-16.789
312.097	-18.191	350.146	-16.275	303.655	-16.796
312.297	-18.197	350.331	-16.279	303.782	-16.802
312.465	-18.203	350.548	-16.283	303.995	-16.808
312.625	-18.209	350.728	-16.287	304.210	-16.814
312.805	-18.214	350.887	-16.292	304.362	-16.821
312.973	-18.220	351.024	-16.296	304.443	-16.827
313.118	-18.226	351.127	-16.300	304.584	-16.833
313.241	-18.232	351.255	-16.304	304.713	-16.839
313.413	-18.238	351.427	-16.308	304.822	-16.846
313.640	-18.243	351.575	-16.313	304.946	-16.852
313.779	-18.249	351.716	-16.317	305.133	-16.858
313.855	-18.255	351.855	-16.321	305.306	-16.865
313.995	-18.261	351.989	-16.325	305.432	-16.871
314.219	-18.267	352.132	-16.329	305.660	-16.877
314.461	-18.272	352.272	-16.334	305.884	-16.883
314.645	-18.278	352.462	-16.338	306.063	-16.890
314.633	-18.284	352.650	-16.342	306.234	-16.896
314.722	-18.290	352.817	-16.346	306.405	-16.902
314.953	-18.296	353.023	-16.350	306.602	-16.908
315.142	-18.301	353.224	-16.355	306.790	-16.914

315.356	-18.307	353.414	-16.359	307.002	-16.921
315.539	-18.313	353.572	-16.363	307.170	-16.927
315.624	-18.319	353.672	-16.367	307.236	-16.933
315.753	-18.325	353.817	-16.371	307.341	-16.939
315.985	-18.331	353.970	-16.376	307.493	-16.946
316.206	-18.336	354.089	-16.380	307.653	-16.952
316.433	-18.342	354.235	-16.384	307.745	-16.958
316.569	-18.348	354.366	-16.388	307.826	-16.964
316.683	-18.354	354.548	-16.392	308.003	-16.970
316.892	-18.360	354.761	-16.397	308.154	-16.977
317.037	-18.365	354.870	-16.401	308.333	-16.983
317.101	-18.371	355.046	-16.405	308.532	-16.989
317.284	-18.377	355.216	-16.409	308.661	-16.995
317.456	-18.383	355.337	-16.413	308.800	-17.001
317.530	-18.389	355.537	-16.418	308.967	-17.007
317.656	-18.394	355.724	-16.422	309.186	-17.014
317.805	-18.400	355.879	-16.426	309.370	-17.020
317.936	-18.406	356.057	-16.430	309.518	-17.026
318.081	-18.412	356.207	-16.435	309.731	-17.032
318.145	-18.417	356.287	-16.439	309.887	-17.038
318.285	-18.423	356.401	-16.443	310.021	-17.044
318.558	-18.429	356.585	-16.447	310.137	-17.051
318.725	-18.435	356.818	-16.452	310.236	-17.057
318.877	-18.441	356.987	-16.456	310.442	-17.063
319.048	-18.446	357.140	-16.460	310.679	-17.069
319.260	-18.452	357.337	-16.464	310.828	-17.075
319.489	-18.458	357.525	-16.469	310.968	-17.081
319.625	-18.463	357.719	-16.473	311.155	-17.088
319.787	-18.469	357.873	-16.477	311.258	-17.094
319.993	-18.475	358.003	-16.482	311.380	-17.100

320.128	-18.481	358.218	-16.486	311.608	-17.106
320.297	-18.486	358.415	-16.490	311.808	-17.112
320.523	-18.492	358.552	-16.494	311.975	-17.118
320.664	-18.498	358.660	-16.499	312.096	-17.124
320.760	-18.503	358.806	-16.503	312.232	-17.131
320.928	-18.509	358.988	-16.507	312.429	-17.137
321.146	-18.515	359.122	-16.512	312.568	-17.143
321.311	-18.520	359.273	-16.516	312.722	-17.149
321.411	-18.526	359.471	-16.520	312.950	-17.155
321.491	-18.532	359.590	-16.525	313.043	-17.161
321.616	-18.537	359.708	-16.529	313.165	-17.167
321.749	-18.543	359.911	-16.533	313.421	-17.173
321.932	-18.548	360.106	-16.537	313.625	-17.180
322.096	-18.554	360.245	-16.542	313.782	-17.186
322.263	-18.559	360.361	-16.546	313.930	-17.192
322.450	-18.565	360.545	-16.550	314.042	-17.198
322.577	-18.571	360.685	-16.555	314.186	-17.204
322.778	-18.576	360.786	-16.559	314.387	-17.210
323.011	-18.582	360.915	-16.563	314.545	-17.216
323.186	-18.587	361.082	-16.568	314.664	-17.222
323.312	-18.593	361.225	-16.572	314.792	-17.228
323.523	-18.598	361.344	-16.576	315.051	-17.235
323.691	-18.603	361.472	-16.580	315.511	-17.241
323.792	-18.609	361.617	-16.585	315.853	-17.247
323.990	-18.614	361.780	-16.589	315.937	-17.253
324.112	-18.620	361.933	-16.593	315.949	-17.259
324.276	-18.625	362.173	-16.598	316.005	-17.265
324.495	-18.631	362.396	-16.602	316.089	-17.271
324.664	-18.636	362.600	-16.606	316.173	-17.277
324.819	-18.641	362.844	-16.610	316.257	-17.284

324.986	-18.647	363.040	-16.615	316.331	-17.290
325.197	-18.652	363.176	-16.619	316.449	-17.296
325.323	-18.657	363.337	-16.623	316.634	-17.302
325.478	-18.662	363.527	-16.628	316.826	-17.308
325.673	-18.668	363.683	-16.632	316.988	-17.314
325.774	-18.673	363.873	-16.636	317.122	-17.320
325.907	-18.678	363.944	-16.640	317.265	-17.326
326.051	-18.683	364.012	-16.644	317.443	-17.332
326.207	-18.689	364.253	-16.649	317.680	-17.339
326.394	-18.694	364.399	-16.653	317.833	-17.345
326.577	-18.699	364.522	-16.657	317.945	-17.351
326.716	-18.704	364.707	-16.661	318.155	-17.357
326.841	-18.709	364.900	-16.666	318.380	-17.363
327.060	-18.714	365.025	-16.670	318.535	-17.369
327.200	-18.720	365.087	-16.674	318.647	-17.375
327.304	-18.725	365.250	-16.678	318.733	-17.381
327.439	-18.730	365.528	-16.682	318.841	-17.387
327.627	-18.735	365.751	-16.686	319.022	-17.393
327.869	-18.740	365.904	-16.691	319.219	-17.400
328.018	-18.745	366.107	-16.695	319.429	-17.406
328.184	-18.750	366.303	-16.699	319.583	-17.412
328.403	-18.755	366.453	-16.703	319.701	-17.418
328.607	-18.760	366.626	-16.707	319.861	-17.424
328.720	-18.765	366.765	-16.711	320.089	-17.430
328.795	-18.770	366.883	-16.715	320.305	-17.436
328.982	-18.775	367.079	-16.720	320.440	-17.442
329.334	-18.780	367.288	-16.724	320.596	-17.448
329.734	-18.785	367.449	-16.728	320.763	-17.455
330.032	-18.790	367.618	-16.732	320.973	-17.461
330.178	-18.794	367.745	-16.736	321.186	-17.467

330.190	-18.799	367.866	-16.740	321.266	-17.473
330.148	-18.804	368.063	-16.744	321.327	-17.479
330.282	-18.809	368.260	-16.748	321.470	-17.485
330.470	-18.814	368.372	-16.752	321.627	-17.491
330.493	-18.819	368.488	-16.756	321.728	-17.497
330.564	-18.823	368.656	-16.760	321.900	-17.504
330.678	-18.828	368.836	-16.764	322.129	-17.510
330.831	-18.833	368.967	-16.768	322.329	-17.516
331.065	-18.838	369.103	-16.772	322.492	-17.522
331.235	-18.842	369.295	-16.776	322.548	-17.528
331.366	-18.847	369.510	-16.780	322.691	-17.534
331.544	-18.852	369.678	-16.784	322.880	-17.540
331.754	-18.857	369.794	-16.788	322.995	-17.547
331.929	-18.861	369.919	-16.792	323.135	-17.553
332.036	-18.866	370.108	-16.796	323.268	-17.559
332.206	-18.871	370.294	-16.800	323.488	-17.565
332.367	-18.875	370.439	-16.804	323.664	-17.571
332.465	-18.880	370.666	-16.808	323.788	-17.577
332.705	-18.885	370.885	-16.812	323.955	-17.584
332.944	-18.889	370.970	-16.816	324.129	-17.590
333.081	-18.894	371.078	-16.820	324.309	-17.596
333.215	-18.898	371.286	-16.824	324.461	-17.602
333.294	-18.903	371.430	-16.828	324.615	-17.608
333.424	-18.908	371.504	-16.831	324.831	-17.614
333.651	-18.912	371.629	-16.835	325.017	-17.621
333.828	-18.917	371.957	-16.839	325.139	-17.627
333.910	-18.921	372.492	-16.843	325.335	-17.633
334.039	-18.926	372.838	-16.847	325.541	-17.639
334.255	-18.930	372.911	-16.851	325.654	-17.645
334.419	-18.935	372.975	-16.854	325.735	-17.652

334.582	-18.939	373.092	-16.858	325.880	-17.658
334.770	-18.944	373.146	-16.862	326.052	-17.664
334.908	-18.948	373.185	-16.866	326.262	-17.670
335.064	-18.953	373.289	-16.870	326.381	-17.676
335.260	-18.957	373.372	-16.873	326.457	-17.682
335.426	-18.962	373.457	-16.877	326.685	-17.689
335.614	-18.966	373.606	-16.881	326.927	-17.695
335.744	-18.971	373.806	-16.885	327.100	-17.701
335.888	-18.975	374.030	-16.888	327.257	-17.707
336.098	-18.980	374.255	-16.892	327.429	-17.713
336.194	-18.984	374.388	-16.896	327.548	-17.719
336.329	-18.989	374.495	-16.899	327.668	-17.726
336.439	-18.993	374.706	-16.903	327.811	-17.732
336.582	-18.997	374.852	-16.907	327.951	-17.738
336.763	-19.002	374.957	-16.910	328.093	-17.744
336.987	-19.006	375.178	-16.914	328.202	-17.750
337.284	-19.011	375.343	-16.918	328.399	-17.756
337.368	-19.015	375.482	-16.921	328.599	-17.763
337.376	-19.019	375.634	-16.925	328.751	-17.769
337.567	-19.024	375.763	-16.929	328.911	-17.775
337.799	-19.028	375.871	-16.932	329.066	-17.781
337.959	-19.033	376.012	-16.936	329.241	-17.787
338.094	-19.037	376.265	-16.940	329.397	-17.793
338.266	-19.041	376.437	-16.943	329.556	-17.799
338.454	-19.046	376.521	-16.947	329.784	-17.805
338.585	-19.050	376.631	-16.950	329.995	-17.811
338.745	-19.055	376.769	-16.954	330.169	-17.818
338.883	-19.059	376.982	-16.958	330.334	-17.824
339.038	-19.063	377.187	-16.961	330.513	-17.830
339.168	-19.068	377.370	-16.965	330.711	-17.836

339.186	-19.072	377.492	-16.969	330.849	-17.842
339.392	-19.076	377.628	-16.972	330.964	-17.848
339.739	-19.081	377.866	-16.976	331.076	-17.854
339.819	-19.085	378.073	-16.979	331.210	-17.860
339.944	-19.089	378.196	-16.983	331.342	-17.866
340.228	-19.094	378.349	-16.987	331.452	-17.872
340.445	-19.098	378.500	-16.990	331.593	-17.878
340.595	-19.102	378.621	-16.994	331.783	-17.884
340.687	-19.107	378.821	-16.997	331.944	-17.890
340.872	-19.111	379.002	-17.001	332.106	-17.896
341.047	-19.115	379.110	-17.004	332.306	-17.902
341.317	-19.120	379.188	-17.008	332.481	-17.908
341.591	-19.124	379.366	-17.012	332.676	-17.914
341.648	-19.128	379.603	-17.015	332.894	-17.920
341.665	-19.133	379.797	-17.019	333.038	-17.926
341.696	-19.137	379.978	-17.022	333.145	-17.932
341.958	-19.141	380.110	-17.026	333.345	-17.938
342.228	-19.146	380.232	-17.030	333.557	-17.944
342.318	-19.150	380.405	-17.033	333.709	-17.950
342.349	-19.154	380.611	-17.037	333.832	-17.956
342.466	-19.159	380.745	-17.040	333.911	-17.961
342.720	-19.163	380.864	-17.044	334.063	-17.967
342.957	-19.167	380.998	-17.048	334.271	-17.973
343.061	-19.172	381.099	-17.051	334.401	-17.979
343.170	-19.176	381.344	-17.055	334.515	-17.985
343.372	-19.180	381.622	-17.059	334.700	-17.991
343.497	-19.185	381.794	-17.062	334.958	-17.997
343.572	-19.189	381.917	-17.066	335.178	-18.002
343.787	-19.193	382.034	-17.069	335.274	-18.008
343.977	-19.197	382.172	-17.073	335.397	-18.014

343.996	-19.202	382.338	-17.077	335.600	-18.020
344.113	-19.206	382.543	-17.080	335.775	-18.026
344.375	-19.210	382.735	-17.084	335.894	-18.032
344.529	-19.215	382.865	-17.088	336.053	-18.037
344.603	-19.219	382.990	-17.091	336.267	-18.043
344.828	-19.223	383.127	-17.095	336.427	-18.049
345.136	-19.227	383.239	-17.098	336.600	-18.055
345.412	-19.232	383.375	-17.102	336.772	-18.060
345.559	-19.236	383.558	-17.106	336.865	-18.066
345.716	-19.240	383.735	-17.109	337.034	-18.072
345.940	-19.245	383.902	-17.113	337.256	-18.078
346.112	-19.249	384.081	-17.117	337.416	-18.083
346.225	-19.253	384.287	-17.120	337.583	-18.089
346.354	-19.257	384.482	-17.124	337.736	-18.095
346.462	-19.262	384.619	-17.127	337.865	-18.100
346.572	-19.266	384.762	-17.131	338.049	-18.106
346.724	-19.270	384.888	-17.135	338.211	-18.112
346.952	-19.274	384.994	-17.138	338.312	-18.118
347.107	-19.279	385.159	-17.142	338.467	-18.123
347.221	-19.283	385.313	-17.146	338.821	-18.129
347.412	-19.287	385.421	-17.149	339.247	-18.134
347.526	-19.291	385.557	-17.153	339.522	-18.140
347.675	-19.296	385.746	-17.156	339.625	-18.146
347.889	-19.300	385.865	-17.160	339.686	-18.151
348.067	-19.304	385.945	-17.164	339.779	-18.157
348.384	-19.308	386.133	-17.167	339.812	-18.163
348.508	-19.312	386.339	-17.171	339.800	-18.168
348.547	-19.317	386.544	-17.174	339.905	-18.174
348.859	-19.321	386.758	-17.178	340.055	-18.179
348.880	-19.325	386.880	-17.182	340.185	-18.185

348.997	-19.329	387.066	-17.185	340.361	-18.191
349.291	-19.333	387.343	-17.189	340.556	-18.196
349.412	-19.338	387.594	-17.192	340.705	-18.202
349.557	-19.342	387.758	-17.196	340.854	-18.207
349.713	-19.346	387.896	-17.200	341.008	-18.213
349.923	-19.350	388.025	-17.203	341.149	-18.218
350.124	-19.354	388.166	-17.207	341.310	-18.224
350.287	-19.359	388.333	-17.210	341.465	-18.230
350.315	-19.363	388.403	-17.214	341.605	-18.235
350.360	-19.367	388.458	-17.218	341.823	-18.241
350.525	-19.371	388.644	-17.221	342.092	-18.246
350.604	-19.375	388.887	-17.225	342.209	-18.252
350.795	-19.379	389.052	-17.228	342.337	-18.257
351.131	-19.384	389.179	-17.232	342.541	-18.263
351.347	-19.388	389.338	-17.235	342.656	-18.268
351.489	-19.392	389.518	-17.239	342.804	-18.274
351.590	-19.396	389.637	-17.242	342.969	-18.279
351.713	-19.400	389.777	-17.246	343.136	-18.285
351.990	-19.404	390.025	-17.250	343.352	-18.290
352.176	-19.408	390.283	-17.253	343.499	-18.296
352.249	-19.413	390.444	-17.257	343.626	-18.301
352.414	-19.417	390.559	-17.260	343.808	-18.307
352.615	-19.421	390.709	-17.264	344.021	-18.312
352.689	-19.425	390.871	-17.267	344.190	-18.318
352.829	-19.429	390.983	-17.271	344.300	-18.323
353.101	-19.433	391.147	-17.274	344.422	-18.329
353.268	-19.437	391.351	-17.278	344.574	-18.335
353.417	-19.441	391.522	-17.281	344.718	-18.340
353.625	-19.446	391.700	-17.285	344.898	-18.346
353.813	-19.450	391.879	-17.288	345.056	-18.351

353.895	-19.454	392.053	-17.292	345.174	-18.357
354.014	-19.458	392.222	-17.295	345.322	-18.362
354.252	-19.462	392.393	-17.299	345.515	-18.368
354.401	-19.466	392.532	-17.302	345.682	-18.373
354.536	-19.470	392.680	-17.306	345.823	-18.379
354.763	-19.474	392.798	-17.309	345.966	-18.384
354.959	-19.479	392.955	-17.313	346.146	-18.390
355.214	-19.483	393.142	-17.316	346.325	-18.395
355.611	-19.487	393.320	-17.320	346.516	-18.401
355.917	-19.491	393.537	-17.323	346.694	-18.407
356.080	-19.495	393.656	-17.327	346.829	-18.412
356.136	-19.499	393.738	-17.330	346.998	-18.418
356.141	-19.503	393.919	-17.334	347.145	-18.423
356.199	-19.507	394.124	-17.337	347.238	-18.429
356.256	-19.511	394.303	-17.341	347.350	-18.434
356.332	-19.516	394.453	-17.344	347.544	-18.440
356.453	-19.520	394.572	-17.347	347.753	-18.446
356.619	-19.524	394.629	-17.351	347.948	-18.451
356.836	-19.528	394.840	-17.354	348.133	-18.457
356.991	-19.532	395.087	-17.358	348.345	-18.462
357.122	-19.536	395.206	-17.361	348.494	-18.468
357.291	-19.540	395.390	-17.365	348.615	-18.474
357.445	-19.544	395.590	-17.368	348.759	-18.479
357.550	-19.548	395.710	-17.371	348.913	-18.485
357.647	-19.553	395.822	-17.375	349.126	-18.490
357.867	-19.557	395.978	-17.378	349.235	-18.496
358.091	-19.561	396.197	-17.382	349.325	-18.502
358.228	-19.565	396.535	-17.385	349.515	-18.507
358.414	-19.569	396.908	-17.389	349.732	-18.513
358.658	-19.573	397.167	-17.392	349.922	-18.519

358.770	-19.577	397.314	-17.395	350.025	-18.524
358.817	-19.581	397.384	-17.399	350.194	-18.530
359.058	-19.586	397.437	-17.402	350.377	-18.536
359.227	-19.590	397.464	-17.405	350.465	-18.541
359.262	-19.594	397.472	-17.409	350.634	-18.547
359.426	-19.598	397.566	-17.412	350.836	-18.553
359.651	-19.602	397.710	-17.416	350.994	-18.558
359.808	-19.606	397.902	-17.419	351.189	-18.564
359.917	-19.610	398.059	-17.422	351.406	-18.570
360.097	-19.614	398.183	-17.426	351.568	-18.575
360.315	-19.618	398.333	-17.429	351.653	-18.581
360.443	-19.623	398.478	-17.432	351.730	-18.587
360.645	-19.627	398.669	-17.436	351.887	-18.592
360.861	-19.631	398.909	-17.439	352.054	-18.598
360.987	-19.635	399.129	-17.443	352.188	-18.604
361.158	-19.639	399.290	-17.446	352.335	-18.609
361.363	-19.643	399.427	-17.449	352.484	-18.615
361.567	-19.647	399.577	-17.453	352.620	-18.621
361.689	-19.652	399.693	-17.456	352.806	-18.626
361.788	-19.656	399.828	-17.459	353.004	-18.632
362.013	-19.660	399.965	-17.463	353.189	-18.638
362.185	-19.664	400.150	-17.466	353.416	-18.643
362.275	-19.668	400.352	-17.469	353.582	-18.649
362.404	-19.672	400.494	-17.473	353.756	-18.655
362.530	-19.677	400.636	-17.476	353.913	-18.660
362.663	-19.681	400.775	-17.479	354.096	-18.666
362.840	-19.685	400.877	-17.483	354.296	-18.672
363.040	-19.689	401.012	-17.486	354.448	-18.677
363.221	-19.693	401.157	-17.489	354.591	-18.683
363.374	-19.697	401.357	-17.493	354.663	-18.689

363.550	-19.702	401.609	-17.496	354.817	-18.694
363.696	-19.706	401.792	-17.499	355.020	-18.700
363.821	-19.710	401.936	-17.503	355.159	-18.706
363.947	-19.714	402.112	-17.506	355.305	-18.712
364.086	-19.718	402.276	-17.509	355.441	-18.717
364.281	-19.723	402.437	-17.513	355.548	-18.723
364.473	-19.727	402.675	-17.516	355.711	-18.729
364.639	-19.731	402.828	-17.519	355.914	-18.734
364.754	-19.735	402.956	-17.523	356.110	-18.740
364.890	-19.739	403.122	-17.526	356.305	-18.746
365.077	-19.744	403.181	-17.530	356.510	-18.751
365.324	-19.748	403.309	-17.533	356.698	-18.757
365.487	-19.752	403.521	-17.536	356.865	-18.763
365.601	-19.756	403.683	-17.540	357.038	-18.768
365.824	-19.760	403.860	-17.543	357.190	-18.774
366.014	-19.765	404.044	-17.546	357.340	-18.780
366.173	-19.769	404.168	-17.550	357.482	-18.785
366.339	-19.773	404.327	-17.553	357.645	-18.791
366.488	-19.777	404.517	-17.557	357.822	-18.797
366.634	-19.781	404.667	-17.560	357.967	-18.802
366.811	-19.786	404.821	-17.563	358.135	-18.808
366.926	-19.790	404.955	-17.567	358.324	-18.813
367.188	-19.794	405.113	-17.570	358.483	-18.819
367.437	-19.798	405.328	-17.574	358.619	-18.825
367.411	-19.802	405.484	-17.577	358.771	-18.830
367.450	-19.807	405.571	-17.580	358.918	-18.836
367.718	-19.811	405.744	-17.584	359.075	-18.842
368.009	-19.815	405.941	-17.587	359.291	-18.847
368.141	-19.819	406.091	-17.591	359.467	-18.853
368.240	-19.824	406.246	-17.594	359.629	-18.858

368.362	-19.828	406.405	-17.597	359.816	-18.864
368.584	-19.832	406.580	-17.601	359.962	-18.870
368.836	-19.836	406.745	-17.604	360.147	-18.875
368.979	-19.840	406.898	-17.608	360.385	-18.881
369.144	-19.844	407.096	-17.611	360.501	-18.887
369.328	-19.849	407.240	-17.615	360.609	-18.892
369.442	-19.853	407.352	-17.618	360.776	-18.898
369.566	-19.857	407.546	-17.621	360.886	-18.903
369.647	-19.861	407.769	-17.625	361.066	-18.909
369.728	-19.865	407.949	-17.628	361.312	-18.914
369.878	-19.869	408.052	-17.632	361.478	-18.920
370.036	-19.874	408.183	-17.635	361.604	-18.926
370.184	-19.878	408.367	-17.639	361.736	-18.931
370.368	-19.882	408.571	-17.642	361.909	-18.937
370.615	-19.886	408.730	-17.646	362.088	-18.942
370.761	-19.890	408.829	-17.649	362.213	-18.948
370.872	-19.894	408.995	-17.652	362.345	-18.953
371.102	-19.898	409.146	-17.656	362.672	-18.959
371.427	-19.902	409.297	-17.659	363.095	-18.964
371.630	-19.906	409.488	-17.663	363.260	-18.970
371.711	-19.910	409.636	-17.666	363.314	-18.976
371.860	-19.915	409.766	-17.670	363.428	-18.981
372.038	-19.919	409.813	-17.673	363.515	-18.987
372.225	-19.923	409.936	-17.677	363.565	-18.992
372.336	-19.927	410.129	-17.680	363.621	-18.998
372.429	-19.931	410.283	-17.683	363.743	-19.003
372.596	-19.935	410.464	-17.687	363.896	-19.009
372.811	-19.939	410.626	-17.690	364.069	-19.014
372.971	-19.943	410.792	-17.694	364.246	-19.020
373.011	-19.947	410.992	-17.697	364.382	-19.025

373.110	-19.951	411.247	-17.700	364.506	-19.031
373.241	-19.955	411.425	-17.704	364.643	-19.036
373.400	-19.959	411.603	-17.707	364.860	-19.042
373.606	-19.963	411.824	-17.711	365.116	-19.047
373.778	-19.966	412.022	-17.714	365.278	-19.053
373.960	-19.970	412.172	-17.717	365.418	-19.058
374.159	-19.974	412.314	-17.721	365.587	-19.064
374.373	-19.978	412.516	-17.724	365.760	-19.069
374.583	-19.982	412.678	-17.728	365.907	-19.075
374.756	-19.986	412.780	-17.731	366.072	-19.080
374.912	-19.990	412.920	-17.734	366.261	-19.086
375.066	-19.994	413.083	-17.738	366.402	-19.091
375.192	-19.998	413.222	-17.741	366.531	-19.097
375.320	-20.001	413.324	-17.744	366.686	-19.102
375.524	-20.005	413.438	-17.748	366.832	-19.107
375.705	-20.009	413.593	-17.751	366.929	-19.113
375.833	-20.013	413.762	-17.754	367.070	-19.118
376.066	-20.017	413.929	-17.758	367.289	-19.124
376.295	-20.020	414.097	-17.761	367.513	-19.129
376.461	-20.024	414.284	-17.764	367.714	-19.135
376.600	-20.028	414.512	-17.768	367.885	-19.140
376.679	-20.032	414.775	-17.771	368.044	-19.146
376.821	-20.035	414.929	-17.774	368.219	-19.151
377.013	-20.039	415.014	-17.777	368.390	-19.157
377.161	-20.043	415.182	-17.781	368.550	-19.162
377.340	-20.047	415.382	-17.784	368.672	-19.168
377.534	-20.050	415.544	-17.787	368.768	-19.173
377.700	-20.054	415.708	-17.791	368.919	-19.179
377.809	-20.058	415.841	-17.794	369.072	-19.184
377.919	-20.061	415.964	-17.797	369.243	-19.190

378.082	-20.065	416.113	-17.800	369.414	-19.195
378.305	-20.069	416.305	-17.804	369.570	-19.200
378.515	-20.072	416.473	-17.807	369.686	-19.206
378.636	-20.076	416.603	-17.810	369.833	-19.211
378.812	-20.079	416.798	-17.813	370.067	-19.217
379.086	-20.083	416.961	-17.817	370.254	-19.222
379.257	-20.087	417.082	-17.820	370.351	-19.228
379.341	-20.090	417.200	-17.823	370.515	-19.233
379.498	-20.094	417.402	-17.826	370.727	-19.239
379.572	-20.097	417.587	-17.829	370.872	-19.244
379.702	-20.101	417.659	-17.833	370.991	-19.250
379.933	-20.104	417.837	-17.836	371.123	-19.255
380.103	-20.108	418.098	-17.839	371.270	-19.261
380.233	-20.111	418.334	-17.842	371.407	-19.266
380.448	-20.115	418.508	-17.845	371.522	-19.272
380.645	-20.118	418.610	-17.848	371.716	-19.277
380.783	-20.122	418.771	-17.852	371.948	-19.283
380.984	-20.125	418.952	-17.855	372.095	-19.288
381.309	-20.129	419.068	-17.858	372.237	-19.294
381.673	-20.132	419.229	-17.861	372.443	-19.299
381.969	-20.136	419.408	-17.864	372.666	-19.305
382.096	-20.139	419.603	-17.867	372.825	-19.310
382.086	-20.143	419.837	-17.871	372.976	-19.316
382.109	-20.146	419.970	-17.874	373.169	-19.321
382.195	-20.149	420.038	-17.877	373.363	-19.327
382.297	-20.153	420.171	-17.880	373.514	-19.332
382.383	-20.156	420.311	-17.883	373.672	-19.338
382.474	-20.160	420.419	-17.886	373.821	-19.343
382.622	-20.163	420.679	-17.889	373.945	-19.349
382.766	-20.166	421.124	-17.892	374.143	-19.354

382.885	-20.170	421.524	-17.896	374.331	-19.360
383.064	-20.173	421.715	-17.899	374.487	-19.365
383.211	-20.176	421.758	-17.902	374.660	-19.371
383.408	-20.180	421.777	-17.905	374.818	-19.376
383.588	-20.183	421.831	-17.908	374.953	-19.382
383.735	-20.186	421.886	-17.911	375.101	-19.387
383.945	-20.190	421.991	-17.914	375.262	-19.393
384.136	-20.193	422.127	-17.917	375.414	-19.398
384.292	-20.196	422.225	-17.920	375.540	-19.404
384.434	-20.200	422.362	-17.923	375.684	-19.409
384.566	-20.203	422.516	-17.926	375.824	-19.415
384.706	-20.206	422.672	-17.929	375.924	-19.420
384.904	-20.210	422.878	-17.932	376.027	-19.426
385.102	-20.213	423.115	-17.936	376.140	-19.431
385.237	-20.216	423.294	-17.939	376.303	-19.437
385.363	-20.220	423.439	-17.942	376.498	-19.442
385.578	-20.223	423.551	-17.945	376.707	-19.448
385.741	-20.226	423.674	-17.948	376.920	-19.453
385.812	-20.230	423.858	-17.951	377.084	-19.459
385.937	-20.233	424.055	-17.954	377.314	-19.464
386.143	-20.236	424.252	-17.957	377.606	-19.469
386.332	-20.239	424.409	-17.960	377.804	-19.475
386.523	-20.243	424.532	-17.963	377.884	-19.480
386.707	-20.246	424.609	-17.966	378.004	-19.486
386.859	-20.249	424.733	-17.969	378.195	-19.491
387.051	-20.253	424.971	-17.972	378.355	-19.497
387.239	-20.256	425.134	-17.975	378.515	-19.502
387.398	-20.259	425.170	-17.978	378.689	-19.507
387.536	-20.263	425.325	-17.981	378.802	-19.513
387.715	-20.266	425.559	-17.984	378.898	-19.518

387.847	-20.269	425.765	-17.987	378.983	-19.524
387.895	-20.273	425.964	-17.990	379.104	-19.529
388.003	-20.276	426.170	-17.993	379.287	-19.534
388.233	-20.279	426.351	-17.996	379.456	-19.540
388.420	-20.282	426.548	-17.999	379.611	-19.545
388.534	-20.286	426.755	-18.002	379.817	-19.550
388.713	-20.289	426.869	-18.005	380.041	-19.556
388.909	-20.292	427.017	-18.008	380.152	-19.561
389.046	-20.296	427.226	-18.011	380.337	-19.567
389.198	-20.299	427.367	-18.014	380.576	-19.572
389.368	-20.302	427.487	-18.017	380.757	-19.577
389.499	-20.306	427.620	-18.020	380.944	-19.583
389.684	-20.309	427.755	-18.024	381.075	-19.588
389.839	-20.313	427.911	-18.027	381.209	-19.593
389.903	-20.316	428.027	-18.030	381.388	-19.599
390.072	-20.319	428.153	-18.033	381.594	-19.604
390.315	-20.323	428.347	-18.036	381.748	-19.609
390.474	-20.326	428.576	-18.039	381.874	-19.614
390.580	-20.329	428.736	-18.042	382.068	-19.620
390.746	-20.333	428.859	-18.045	382.269	-19.625
390.985	-20.336	428.989	-18.048	382.440	-19.630
391.128	-20.340	429.102	-18.051	382.650	-19.635
391.223	-20.343	429.227	-18.054	382.824	-19.641
391.434	-20.346	429.420	-18.057	382.920	-19.646
391.671	-20.350	429.576	-18.060	383.028	-19.651
391.852	-20.353	429.723	-18.063	383.193	-19.656
391.971	-20.356	429.893	-18.066	383.397	-19.662
392.087	-20.360	430.049	-18.070	383.568	-19.667
392.217	-20.363	430.245	-18.073	383.680	-19.672
392.488	-20.367	430.408	-18.076	383.804	-19.677

392.769	-20.370	430.598	-18.079	384.000	-19.683
392.867	-20.374	430.821	-18.082	384.154	-19.688
392.994	-20.377	430.974	-18.085	384.273	-19.693
393.142	-20.380	431.108	-18.088	384.443	-19.698
393.321	-20.384	431.274	-18.091	384.593	-19.703
393.477	-20.387	431.440	-18.094	384.784	-19.709
393.620	-20.391	431.631	-18.098	385.029	-19.714
393.792	-20.394	431.804	-18.101	385.163	-19.719
393.904	-20.398	431.964	-18.104	385.283	-19.724
394.041	-20.401	432.128	-18.107	385.451	-19.729
394.266	-20.405	432.254	-18.110	385.637	-19.734
394.489	-20.408	432.404	-18.113	385.798	-19.739
394.630	-20.411	432.588	-18.116	385.877	-19.745
394.781	-20.415	432.742	-18.120	385.961	-19.750
394.876	-20.418	432.865	-18.123	386.174	-19.755
395.014	-20.422	432.954	-18.126	386.617	-19.760
395.139	-20.425	433.106	-18.129	387.036	-19.765
395.290	-20.429	433.340	-18.132	387.235	-19.770
395.452	-20.432	433.575	-18.135	387.324	-19.775
395.487	-20.436	433.753	-18.138	387.401	-19.780
395.588	-20.439	433.848	-18.142	387.438	-19.785
395.742	-20.443	433.963	-18.145	387.511	-19.791
395.913	-20.446	434.105	-18.148	387.634	-19.796
396.168	-20.449	434.247	-18.151	387.714	-19.801
396.348	-20.453	434.392	-18.154	387.749	-19.806
396.528	-20.456	434.560	-18.157	387.881	-19.811
396.756	-20.460	434.731	-18.161	388.109	-19.816
396.837	-20.463	434.866	-18.164	388.302	-19.821
397.068	-20.467	435.080	-18.167	388.492	-19.826
397.398	-20.470	435.263	-18.170	388.665	-19.831

397.618	-20.474	435.372	-18.173	388.833	-19.836
397.766	-20.477	435.517	-18.176	389.030	-19.841
397.892	-20.481	435.725	-18.180	389.210	-19.846
397.975	-20.484	435.983	-18.183	389.383	-19.851
398.086	-20.487	436.172	-18.186	389.574	-19.856
398.221	-20.491	436.348	-18.189	389.722	-19.861
398.323	-20.494	436.557	-18.192	389.819	-19.866
398.375	-20.498	436.699	-18.195	389.946	-19.871
398.567	-20.501	436.817	-18.199	390.130	-19.877
398.766	-20.505	436.942	-18.202	390.265	-19.882
398.902	-20.508	437.099	-18.205	390.426	-19.887
399.109	-20.511	437.239	-18.208	390.545	-19.892
399.216	-20.515	437.327	-18.211	390.681	-19.897
399.376	-20.518	437.524	-18.214	390.897	-19.902
399.614	-20.522	437.734	-18.218	391.049	-19.907
399.838	-20.525	437.863	-18.221	391.157	-19.912
400.001	-20.528	437.996	-18.224	391.319	-19.917
400.176	-20.532	438.096	-18.227	391.488	-19.922
400.378	-20.535	438.253	-18.230	391.686	-19.927
400.539	-20.539	438.463	-18.234	391.898	-19.932
400.722	-20.542	438.602	-18.237	392.041	-19.937
400.889	-20.545	438.763	-18.240	392.246	-19.942
401.066	-20.549	438.968	-18.243	392.428	-19.947
401.251	-20.552	439.180	-18.246	392.618	-19.952
401.360	-20.555	439.353	-18.249	392.809	-19.957
401.499	-20.559	439.502	-18.253	392.863	-19.962
401.659	-20.562	439.641	-18.256	392.984	-19.967
401.892	-20.565	439.805	-18.259	393.154	-19.972
402.078	-20.569	440.026	-18.262	393.315	-19.977
402.215	-20.572	440.181	-18.265	393.447	-19.982

402.400	-20.575	440.280	-18.268	393.495	-19.987
402.525	-20.579	440.405	-18.272	393.689	-19.992
402.663	-20.582	440.541	-18.275	393.983	-19.998
402.744	-20.585	440.725	-18.278	394.127	-20.003
402.916	-20.589	440.944	-18.281	394.220	-20.008
403.214	-20.592	441.132	-18.284	394.369	-20.013
403.412	-20.595	441.310	-18.287	394.540	-20.018
403.510	-20.598	441.480	-18.291	394.707	-20.023
403.623	-20.602	441.595	-18.294	394.884	-20.028
403.784	-20.605	441.736	-18.297	395.065	-20.033
403.921	-20.608	441.956	-18.300	395.235	-20.038
404.090	-20.611	442.189	-18.303	395.356	-20.043
404.327	-20.615	442.316	-18.306	395.450	-20.049
404.501	-20.618	442.396	-18.310	395.666	-20.054
404.643	-20.621	442.592	-18.313	395.861	-20.059
404.845	-20.624	442.819	-18.316	396.049	-20.064
405.045	-20.627	442.927	-18.319	396.267	-20.069
405.179	-20.631	443.029	-18.322	396.396	-20.074
405.325	-20.634	443.235	-18.326	396.542	-20.079
405.418	-20.637	443.433	-18.329	396.727	-20.084
405.587	-20.640	443.566	-18.332	396.885	-20.090
405.818	-20.643	443.757	-18.335	397.054	-20.095
405.928	-20.647	443.957	-18.338	397.234	-20.100
406.030	-20.650	444.153	-18.342	397.352	-20.105
406.128	-20.653	444.288	-18.345	397.495	-20.110
406.279	-20.656	444.369	-18.348	397.644	-20.115
406.478	-20.659	444.506	-18.351	397.803	-20.121
406.588	-20.662	444.651	-18.354	397.991	-20.126
406.804	-20.665	444.796	-18.358	398.191	-20.131
407.175	-20.669	444.954	-18.361	398.376	-20.136

407.548	-20.672	445.221	-18.364	398.508	-20.141
407.812	-20.675	445.517	-18.367	398.647	-20.147
407.959	-20.678	445.869	-18.371	398.799	-20.152
408.023	-20.681	446.113	-18.374	398.948	-20.157
408.170	-20.684	446.177	-18.377	399.109	-20.162
408.344	-20.687	446.276	-18.380	399.185	-20.167
408.342	-20.690	446.368	-18.384	399.279	-20.173
408.398	-20.693	446.450	-18.387	399.447	-20.178
408.443	-20.696	446.490	-18.390	399.611	-20.183
408.509	-20.700	446.551	-18.393	399.776	-20.188
408.690	-20.703	446.664	-18.397	399.868	-20.194
408.824	-20.706	446.784	-18.400	400.007	-20.199
409.031	-20.709	446.936	-18.403	400.243	-20.204
409.268	-20.712	447.127	-18.406	400.486	-20.209
409.417	-20.715	447.359	-18.410	400.650	-20.214
409.517	-20.718	447.576	-18.413	400.776	-20.220
409.702	-20.721	447.741	-18.416	400.978	-20.225
409.933	-20.724	447.862	-18.420	401.196	-20.230
410.135	-20.727	448.025	-18.423	401.432	-20.235
410.258	-20.730	448.206	-18.426	401.622	-20.241
410.376	-20.733	448.356	-18.430	401.721	-20.246
410.536	-20.736	448.593	-18.433	401.830	-20.251
410.723	-20.739	448.775	-18.436	402.008	-20.256
410.956	-20.742	448.851	-18.440	402.155	-20.261
411.097	-20.745	448.962	-18.443	402.293	-20.267
411.214	-20.749	449.121	-18.446	402.409	-20.272
411.409	-20.752	449.337	-18.450	402.497	-20.277
411.537	-20.755	449.508	-18.453	402.716	-20.282
411.668	-20.758	449.636	-18.456	402.960	-20.287
411.830	-20.761	449.791	-18.460	403.024	-20.293

411.903	-20.764	449.927	-18.463	403.072	-20.298
412.097	-20.767	450.051	-18.466	403.263	-20.303
412.262	-20.770	450.231	-18.470	403.457	-20.308
412.456	-20.773	450.438	-18.473	403.666	-20.313
412.702	-20.776	450.633	-18.476	403.885	-20.319
412.762	-20.779	450.855	-18.480	404.056	-20.324
412.881	-20.782	451.000	-18.483	404.227	-20.329
413.074	-20.785	451.142	-18.487	404.393	-20.334
413.231	-20.788	451.353	-18.490	404.558	-20.339
413.503	-20.791	451.536	-18.493	404.732	-20.344
413.678	-20.794	451.661	-18.497	404.956	-20.349
413.763	-20.797	451.774	-18.500	405.194	-20.355
413.984	-20.801	451.884	-18.504	405.305	-20.360
414.159	-20.804	452.045	-18.507	405.445	-20.365
414.211	-20.807	452.235	-18.511	405.622	-20.370
414.278	-20.810	452.396	-18.514	405.770	-20.375
414.468	-20.813	452.493	-18.518	405.920	-20.380
414.727	-20.816	452.620	-18.521	406.023	-20.385
414.850	-20.819	452.796	-18.525	406.224	-20.391
414.995	-20.822	452.970	-18.528	406.426	-20.396
415.198	-20.825	453.136	-18.532	406.545	-20.401
415.252	-20.828	453.350	-18.535	406.702	-20.406
415.360	-20.832	453.579	-18.539	406.887	-20.411
415.609	-20.835	453.721	-18.542	407.042	-20.416
415.840	-20.838	453.842	-18.546	407.193	-20.421
416.032	-20.841	453.954	-18.549	407.389	-20.426
416.222	-20.844	454.124	-18.553	407.525	-20.431
416.355	-20.847	454.315	-18.556	407.655	-20.436
416.553	-20.850	454.488	-18.560	407.831	-20.441
416.722	-20.854	454.685	-18.563	408.018	-20.447

416.816	-20.857	454.830	-18.567	408.204	-20.452
416.997	-20.860	454.978	-18.570	408.361	-20.457
417.183	-20.863	455.112	-18.574	408.520	-20.462
417.343	-20.866	455.276	-18.578	408.672	-20.467
417.479	-20.869	455.500	-18.581	408.873	-20.472
417.653	-20.873	455.682	-18.585	409.014	-20.477
417.816	-20.876	455.864	-18.588	409.110	-20.482
417.935	-20.879	456.016	-18.592	409.248	-20.487
418.121	-20.882	456.104	-18.596	409.402	-20.492
418.301	-20.885	456.244	-18.599	409.577	-20.497
418.430	-20.889	456.428	-18.603	409.712	-20.502
418.556	-20.892	456.594	-18.606	409.885	-20.507
418.657	-20.895	456.791	-18.610	410.237	-20.512
418.807	-20.898	456.960	-18.614	410.709	-20.517
419.015	-20.901	457.124	-18.617	411.013	-20.522
419.182	-20.905	457.313	-18.621	411.077	-20.527
419.457	-20.908	457.479	-18.624	411.129	-20.532
419.690	-20.911	457.629	-18.628	411.240	-20.537
419.760	-20.914	457.794	-18.632	411.288	-20.541
419.852	-20.918	457.954	-18.635	411.374	-20.546
419.975	-20.921	458.131	-18.639	411.477	-20.551
420.169	-20.924	458.254	-18.643	411.508	-20.556
420.441	-20.927	458.409	-18.646	411.619	-20.561
420.600	-20.931	458.565	-18.650	411.841	-20.566
420.712	-20.934	458.686	-18.654	412.016	-20.571
420.828	-20.937	458.820	-18.657	412.182	-20.576
420.977	-20.940	458.918	-18.661	412.382	-20.581
421.135	-20.943	459.057	-18.664	412.540	-20.586
421.302	-20.947	459.241	-18.668	412.716	-20.591
421.520	-20.950	459.402	-18.672	412.926	-20.595

421.652	-20.953	459.548	-18.675	413.102	-20.600
421.750	-20.956	459.701	-18.679	413.273	-20.605
421.778	-20.960	459.854	-18.683	413.435	-20.610
421.922	-20.963	460.019	-18.686	413.547	-20.615
422.156	-20.966	460.191	-18.690	413.695	-20.620
422.287	-20.969	460.409	-18.693	413.824	-20.625
422.397	-20.973	460.632	-18.697	413.985	-20.629
422.660	-20.976	460.841	-18.701	414.145	-20.634
422.955	-20.979	461.065	-18.704	414.304	-20.639
423.089	-20.982	461.276	-18.708	414.478	-20.644
423.301	-20.986	461.454	-18.712	414.610	-20.649
423.512	-20.989	461.552	-18.715	414.772	-20.654
423.708	-20.992	461.650	-18.719	414.915	-20.658
423.938	-20.995	461.785	-18.722	415.033	-20.663
424.023	-20.998	461.900	-18.726	415.143	-20.668
424.113	-21.002	462.048	-18.729	415.328	-20.673
424.275	-21.005	462.230	-18.733	415.584	-20.678
424.425	-21.008	462.356	-18.737	415.756	-20.682
424.610	-21.011	462.487	-18.740	415.892	-20.687
424.734	-21.014	462.659	-18.744	416.104	-20.692
424.852	-21.018	462.793	-18.747	416.294	-20.697
425.044	-21.021	462.967	-18.751	416.447	-20.702
425.290	-21.024	463.169	-18.754	416.596	-20.706
425.340	-21.027	463.308	-18.758	416.759	-20.711
425.292	-21.030	463.505	-18.761	416.905	-20.716
425.542	-21.033	463.709	-18.765	417.047	-20.721
425.760	-21.037	463.830	-18.768	417.198	-20.726
425.919	-21.040	464.014	-18.772	417.331	-20.730
426.085	-21.043	464.246	-18.775	417.460	-20.735
426.293	-21.046	464.424	-18.779	417.604	-20.740

426.536	-21.049	464.556	-18.782	417.795	-20.745
426.714	-21.052	464.706	-18.786	417.997	-20.750
426.916	-21.055	464.872	-18.789	418.176	-20.754
427.062	-21.058	465.077	-18.793	418.382	-20.759
427.242	-21.062	465.276	-18.796	418.460	-20.764
427.391	-21.065	465.427	-18.800	418.511	-20.769
427.546	-21.068	465.543	-18.803	418.722	-20.774
427.642	-21.071	465.641	-18.806	418.897	-20.779
427.808	-21.074	465.865	-18.810	419.012	-20.783
428.006	-21.077	466.136	-18.813	419.164	-20.788
428.085	-21.080	466.275	-18.817	419.377	-20.793
428.301	-21.083	466.371	-18.820	419.607	-20.798
428.548	-21.086	466.551	-18.823	419.793	-20.803
428.684	-21.089	466.732	-18.827	419.941	-20.808
428.772	-21.092	466.877	-18.830	420.100	-20.812
428.928	-21.095	467.072	-18.834	420.289	-20.817
429.199	-21.098	467.280	-18.837	420.499	-20.822
429.381	-21.101	467.399	-18.840	420.660	-20.827
429.451	-21.104	467.531	-18.844	420.782	-20.832
429.611	-21.107	467.680	-18.847	420.950	-20.837
429.768	-21.110	467.843	-18.850	421.095	-20.842
429.915	-21.113	467.995	-18.854	421.181	-20.847
430.153	-21.116	468.132	-18.857	421.351	-20.851
430.299	-21.119	468.295	-18.860	421.588	-20.856
430.383	-21.122	468.487	-18.864	421.798	-20.861
430.623	-21.125	468.712	-18.867	422.003	-20.866
430.856	-21.128	468.875	-18.870	422.113	-20.871
430.943	-21.131	468.991	-18.873	422.213	-20.876
431.122	-21.134	469.128	-18.877	422.349	-20.881
431.241	-21.137	469.288	-18.880	422.497	-20.886

431.332	-21.140	469.459	-18.883	422.628	-20.891
431.531	-21.143	469.555	-18.887	422.758	-20.896
431.708	-21.146	469.740	-18.890	422.955	-20.900
431.948	-21.148	470.099	-18.893	423.099	-20.905
432.041	-21.151	470.476	-18.896	423.222	-20.910
432.193	-21.154	470.781	-18.900	423.384	-20.915
432.539	-21.157	470.887	-18.903	423.499	-20.920
432.722	-21.160	470.927	-18.906	423.624	-20.925
432.792	-21.163	470.979	-18.909	423.831	-20.930
432.848	-21.166	471.026	-18.913	423.995	-20.935
432.983	-21.169	471.147	-18.916	424.083	-20.940
433.442	-21.172	471.272	-18.919	424.249	-20.945
433.872	-21.175	471.327	-18.922	424.543	-20.950
433.947	-21.177	471.393	-18.925	424.767	-20.955
434.041	-21.180	471.594	-18.929	424.936	-20.959
434.210	-21.183	471.810	-18.932	425.104	-20.964
434.254	-21.186	471.977	-18.935	425.280	-20.969
434.284	-21.189	472.175	-18.938	425.437	-20.974
434.444	-21.192	472.356	-18.941	425.546	-20.979
434.553	-21.195	472.536	-18.945	425.709	-20.984
434.555	-21.197	472.738	-18.948	425.926	-20.989
434.649	-21.200	472.902	-18.951	426.176	-20.994
434.868	-21.203	473.071	-18.954	426.321	-20.999
435.084	-21.206	473.273	-18.957	426.369	-21.004
435.165	-21.209	473.421	-18.960	426.474	-21.009
435.300	-21.212	473.552	-18.964	426.580	-21.014
435.560	-21.214	473.692	-18.967	426.754	-21.018
435.787	-21.217	473.831	-18.970	426.958	-21.023
435.939	-21.220	473.990	-18.973	427.121	-21.028
436.060	-21.223	474.123	-18.976	427.240	-21.033

436.268	-21.226	474.262	-18.979	427.401	-21.038
436.454	-21.229	474.430	-18.983	427.658	-21.043
436.548	-21.232	474.551	-18.986	427.810	-21.048
436.703	-21.234	474.702	-18.989	427.960	-21.053
436.866	-21.237	474.903	-18.992	428.166	-21.058
437.026	-21.240	475.067	-18.995	428.366	-21.063
437.176	-21.243	475.206	-18.998	428.560	-21.067
437.210	-21.246	475.403	-19.002	428.723	-21.072
437.335	-21.249	475.622	-19.005	428.897	-21.077
437.602	-21.252	475.809	-19.008	429.025	-21.082
437.818	-21.255	475.992	-19.011	429.130	-21.087
437.965	-21.257	476.152	-19.014	429.315	-21.092
438.096	-21.260	476.292	-19.017	429.502	-21.097
438.149	-21.263	476.424	-19.021	429.666	-21.102
438.311	-21.266	476.570	-19.024	429.842	-21.107
438.672	-21.269	476.714	-19.027	430.034	-21.112
438.835	-21.272	476.828	-19.030	430.171	-21.116
438.907	-21.275	476.949	-19.033	430.298	-21.121
439.053	-21.278	477.105	-19.036	430.449	-21.126
439.250	-21.281	477.238	-19.040	430.628	-21.131
439.547	-21.284	477.404	-19.043	430.840	-21.136
439.780	-21.287	477.557	-19.046	430.971	-21.141
439.849	-21.290	477.764	-19.049	431.136	-21.146
439.948	-21.293	478.027	-19.052	431.297	-21.151
440.162	-21.296	478.201	-19.055	431.450	-21.156
440.252	-21.299	478.346	-19.059	431.574	-21.161
440.326	-21.302	478.503	-19.062	431.680	-21.165
440.504	-21.305	478.656	-19.065	431.802	-21.170
440.700	-21.308	478.825	-19.068	432.000	-21.175
440.827	-21.311	478.953	-19.071	432.152	-21.180

440.953	-21.314	479.058	-19.075	432.310	-21.185
441.093	-21.317	479.105	-19.078	432.551	-21.190
441.278	-21.320	479.246	-19.081	432.746	-21.195
441.454	-21.323	479.553	-19.084	432.914	-21.200
441.695	-21.326	479.739	-19.087	433.070	-21.205
441.862	-21.329	479.893	-19.091	433.229	-21.209
441.943	-21.332	480.115	-19.094	433.399	-21.214
442.114	-21.335	480.334	-19.097	433.543	-21.219
442.272	-21.338	480.466	-19.100	433.705	-21.224
442.439	-21.342	480.576	-19.103	433.923	-21.229
442.625	-21.345	480.744	-19.107	434.268	-21.234
442.835	-21.348	480.908	-19.110	434.648	-21.239
442.979	-21.351	481.021	-19.113	434.852	-21.244
443.104	-21.354	481.204	-19.116	434.991	-21.249
443.270	-21.357	481.420	-19.120	435.045	-21.254
443.460	-21.361	481.553	-19.123	435.056	-21.258
443.681	-21.364	481.683	-19.126	435.076	-21.263
443.867	-21.367	481.851	-19.129	435.206	-21.268
444.048	-21.370	482.093	-19.133	435.390	-21.273
444.224	-21.374	482.252	-19.136	435.443	-21.278
444.379	-21.377	482.345	-19.139	435.553	-21.283
444.562	-21.380	482.445	-19.142	435.717	-21.288
444.797	-21.384	482.617	-19.146	435.855	-21.293
444.930	-21.387	482.810	-19.149	436.032	-21.298
444.908	-21.390	482.952	-19.152	436.268	-21.303
444.914	-21.394	483.109	-19.155	436.461	-21.308
445.094	-21.397	483.209	-19.159	436.625	-21.312
445.309	-21.400	483.350	-19.162	436.789	-21.317
445.538	-21.404	483.512	-19.165	437.036	-21.322
445.762	-21.407	483.605	-19.168	437.215	-21.327

445.899	-21.410	483.794	-19.172	437.259	-21.332
446.047	-21.414	484.026	-19.175	437.368	-21.337
446.248	-21.417	484.141	-19.178	437.522	-21.342
446.407	-21.421	484.260	-19.181	437.667	-21.347
446.538	-21.424	484.492	-19.184	437.828	-21.352
446.706	-21.427	484.664	-19.188	438.011	-21.357
446.880	-21.431	484.833	-19.191	438.152	-21.362
447.028	-21.434	485.078	-19.194	438.302	-21.367
447.225	-21.438	485.251	-19.197	438.501	-21.372
447.399	-21.441	485.436	-19.201	438.670	-21.377
447.495	-21.445	485.625	-19.204	438.781	-21.382
447.642	-21.448	485.777	-19.207	438.896	-21.387
447.759	-21.452	485.999	-19.210	439.053	-21.391
447.896	-21.455	486.149	-19.213	439.317	-21.396
448.050	-21.459	486.228	-19.217	439.524	-21.401
448.178	-21.462	486.378	-19.220	439.680	-21.406
448.328	-21.466	486.468	-19.223	439.845	-21.411
448.500	-21.469	486.612	-19.226	440.041	-21.416
448.649	-21.473	486.772	-19.229	440.203	-21.421
448.886	-21.476	486.896	-19.232	440.302	-21.427
449.129	-21.480	487.074	-19.235	440.428	-21.432
449.301	-21.483	487.232	-19.239	440.571	-21.437
449.397	-21.487	487.351	-19.242	440.711	-21.442
449.557	-21.490	487.534	-19.245	440.897	-21.447
449.791	-21.494	487.753	-19.248	441.033	-21.452
450.098	-21.497	487.945	-19.251	441.207	-21.457
450.586	-21.501	488.161	-19.254	441.443	-21.462
450.735	-21.504	488.327	-19.257	441.526	-21.467
450.418	-21.508	488.457	-19.260	441.646	-21.472
450.399	-21.512	488.647	-19.264	441.897	-21.477

450.645	-21.515	488.826	-19.267	442.096	-21.482
450.896	-21.519	488.977	-19.270	442.251	-21.488
451.059	-21.522	489.161	-19.273	442.364	-21.493
451.088	-21.526	489.318	-19.276	442.481	-21.498
451.221	-21.529	489.437	-19.279	442.642	-21.503
451.432	-21.533	489.607	-19.282	442.787	-21.508
451.768	-21.537	489.778	-19.285	442.939	-21.513
452.016	-21.540	489.951	-19.288	443.161	-21.519
451.959	-21.544	490.126	-19.291	443.368	-21.524
451.983	-21.547	490.322	-19.294	443.523	-21.529
452.157	-21.551	490.505	-19.297	443.688	-21.534
452.294	-21.555	490.616	-19.300	443.899	-21.540
452.426	-21.558	490.717	-19.303	444.091	-21.545
452.557	-21.562	490.888	-19.306	444.285	-21.550
452.900	-21.565	491.092	-19.309	444.436	-21.555
453.283	-21.569	491.249	-19.312	444.485	-21.561
453.466	-21.572	491.423	-19.315	444.595	-21.566
453.595	-21.576	491.598	-19.318	444.797	-21.571
453.709	-21.580	491.764	-19.321	445.013	-21.577
453.860	-21.583	491.927	-19.324	445.197	-21.582
454.152	-21.587	492.044	-19.327	445.362	-21.587
454.380	-21.590	492.212	-19.330	445.477	-21.593
454.462	-21.594	492.372	-19.333	445.634	-21.598
454.468	-21.598	492.554	-19.336	445.827	-21.603
454.536	-21.601	492.737	-19.339	445.987	-21.609
454.769	-21.605	492.918	-19.342	446.128	-21.614
454.932	-21.608	493.095	-19.344	446.277	-21.620
455.076	-21.612	493.316	-19.347	446.418	-21.625
455.268	-21.615	493.441	-19.350	446.547	-21.630
455.389	-21.619	493.525	-19.353	446.714	-21.636

455.523	-21.623	493.686	-19.356	446.921	-21.641
455.793	-21.626	493.783	-19.359	447.078	-21.647
455.991	-21.630	493.926	-19.362	447.156	-21.652
456.217	-21.633	494.129	-19.365	447.288	-21.657
456.389	-21.637	494.329	-19.368	447.512	-21.663
456.514	-21.640	494.609	-19.370	447.639	-21.668
456.648	-21.644	494.928	-19.373	447.723	-21.674
456.761	-21.648	495.229	-19.376	447.907	-21.679
456.973	-21.651	495.392	-19.379	448.092	-21.685
457.117	-21.655	495.488	-19.382	448.265	-21.690
457.235	-21.658	495.629	-19.385	448.448	-21.696
457.391	-21.662	495.688	-19.387	448.630	-21.701
457.600	-21.665	495.717	-19.390	448.807	-21.707
457.841	-21.669	495.813	-19.393	449.022	-21.712
457.922	-21.672	495.896	-19.396	449.228	-21.718
458.076	-21.676	496.014	-19.399	449.284	-21.723
458.324	-21.680	496.185	-19.402	449.389	-21.728
458.508	-21.683	496.361	-19.404	449.581	-21.734
458.698	-21.687	496.516	-19.407	449.782	-21.739
458.812	-21.690	496.710	-19.410	449.971	-21.745
458.871	-21.694	496.968	-19.413	450.171	-21.750
459.023	-21.697	497.132	-19.416	450.286	-21.756
459.485	-21.701	497.303	-19.418	450.405	-21.761
460.049	-21.704	497.469	-19.421	450.555	-21.767
460.359	-21.708	497.561	-19.424	450.703	-21.772
460.348	-21.711	497.727	-19.427	450.846	-21.778
460.300	-21.715	497.902	-19.429	450.974	-21.783
460.344	-21.718	498.027	-19.432	451.124	-21.789
460.355	-21.722	498.144	-19.435	451.351	-21.794
460.400	-21.726	498.315	-19.438	451.637	-21.800

460.492	-21.729	498.478	-19.441	451.785	-21.805
460.645	-21.733	498.586	-19.443	451.883	-21.811
460.698	-21.736	498.715	-19.446	452.065	-21.816
460.780	-21.740	498.948	-19.449	452.275	-21.821
461.069	-21.743	499.116	-19.452	452.450	-21.827
461.317	-21.747	499.292	-19.454	452.609	-21.832
461.494	-21.750	499.418	-19.457	452.779	-21.838
461.708	-21.754	499.561	-19.460	452.937	-21.843
461.844	-21.757	499.745	-19.463	453.039	-21.849
461.945	-21.761	499.969	-19.465	453.164	-21.854
462.135	-21.764	500.132	-19.468	453.326	-21.860
462.338	-21.768	500.290	-19.471	453.532	-21.865
462.498	-21.771	500.517	-19.474	453.762	-21.871
462.629	-21.775	500.673	-19.477	453.903	-21.876
462.801	-21.778	500.788	-19.479	454.008	-21.881
463.005	-21.782	500.925	-19.482	454.181	-21.887
463.057	-21.785	501.066	-19.485	454.356	-21.892
463.202	-21.789	501.229	-19.488	454.563	-21.898
463.432	-21.792	501.375	-19.490	454.835	-21.903
463.630	-21.796	501.517	-19.493	454.980	-21.909
463.853	-21.799	501.673	-19.496	455.068	-21.914
464.005	-21.803	501.812	-19.499	455.140	-21.919
464.080	-21.806	501.989	-19.502	455.247	-21.925
464.156	-21.810	502.193	-19.504	455.482	-21.930
464.220	-21.813	502.303	-19.507	455.696	-21.936
464.374	-21.817	502.482	-19.510	455.855	-21.941
464.599	-21.820	502.672	-19.513	455.994	-21.946
464.716	-21.824	502.846	-19.516	456.192	-21.952
465.025	-21.827	502.992	-19.519	456.414	-21.957
465.303	-21.831	503.105	-19.521	456.577	-21.962

465.452	-21.834	503.297	-19.524	456.724	-21.968
465.540	-21.838	503.495	-19.527	456.892	-21.973
465.712	-21.841	503.584	-19.530	457.016	-21.979
465.916	-21.845	503.740	-19.533	457.100	-21.984
466.104	-21.849	504.007	-19.536	457.259	-21.989
466.361	-21.852	504.141	-19.538	457.455	-21.995
466.387	-21.856	504.225	-19.541	457.610	-22.000
466.468	-21.859	504.433	-19.544	457.928	-22.005
466.683	-21.863	504.667	-19.547	458.394	-22.011
466.751	-21.866	504.814	-19.550	458.673	-22.016
466.954	-21.870	504.926	-19.553	458.789	-22.021
467.229	-21.874	505.088	-19.556	458.873	-22.027
467.389	-21.877	505.286	-19.558	458.980	-22.032
467.483	-21.881	505.459	-19.561	459.007	-22.037
467.650	-21.884	505.625	-19.564	459.008	-22.043
467.777	-21.888	505.733	-19.567	459.136	-22.048
467.927	-21.892	505.862	-19.570	459.289	-22.053
468.041	-21.895	506.012	-19.573	459.394	-22.058
468.170	-21.899	506.126	-19.576	459.485	-22.064
468.533	-21.903	506.341	-19.579	459.636	-22.069
468.658	-21.906	506.580	-19.582	459.798	-22.074
468.729	-21.910	506.755	-19.585	460.004	-22.079
468.886	-21.913	506.880	-19.587	460.245	-22.085
469.098	-21.917	507.023	-19.590	460.414	-22.090
469.323	-21.921	507.222	-19.593	460.628	-22.095
469.436	-21.925	507.430	-19.596	460.801	-22.100
469.498	-21.928	507.617	-19.599	460.905	-22.106
469.579	-21.932	507.721	-19.602	461.035	-22.111
469.853	-21.936	507.822	-19.605	461.204	-22.116
470.183	-21.939	507.942	-19.608	461.347	-22.121

470.345	-21.943	508.093	-19.611	461.477	-22.127
470.541	-21.947	508.205	-19.614	461.601	-22.132
470.706	-21.950	508.300	-19.617	461.784	-22.137
470.737	-21.954	508.467	-19.619	461.997	-22.142
470.915	-21.958	508.634	-19.622	462.153	-22.147
471.147	-21.962	508.769	-19.625	462.285	-22.153
471.329	-21.965	508.945	-19.628	462.469	-22.158
471.441	-21.969	509.094	-19.631	462.670	-22.163
471.488	-21.973	509.239	-19.634	462.773	-22.168
471.584	-21.977	509.485	-19.637	462.864	-22.173
471.779	-21.980	509.782	-19.640	463.054	-22.179
472.075	-21.984	509.929	-19.643	463.271	-22.184
472.346	-21.988	510.055	-19.646	463.456	-22.189
472.449	-21.992	510.318	-19.648	463.666	-22.194
472.493	-21.995	510.535	-19.651	463.834	-22.199
472.686	-21.999	510.659	-19.654	464.003	-22.205
472.774	-22.003	510.797	-19.657	464.123	-22.210
473.006	-22.007	510.990	-19.660	464.245	-22.215
473.268	-22.010	511.142	-19.663	464.460	-22.220
473.381	-22.014	511.265	-19.666	464.635	-22.225
473.566	-22.018	511.420	-19.669	464.669	-22.231
473.761	-22.022	511.532	-19.671	464.773	-22.236
473.857	-22.026	511.624	-19.674	464.969	-22.241
473.979	-22.029	511.808	-19.677	465.126	-22.246
474.065	-22.033	511.948	-19.680	465.329	-22.251
474.179	-22.037	512.085	-19.683	465.535	-22.257
474.345	-22.041	512.296	-19.686	465.702	-22.262
474.451	-22.044	512.463	-19.689	465.856	-22.267
474.550	-22.048	512.604	-19.691	466.029	-22.272
474.793	-22.052	512.842	-19.694	466.201	-22.278

475.024	-22.056	513.058	-19.697	466.347	-22.283
475.318	-22.060	513.227	-19.700	466.473	-22.288
475.528	-22.063	513.426	-19.703	466.601	-22.293
475.639	-22.067	513.578	-19.705	466.787	-22.298
475.772	-22.071	513.713	-19.708	466.986	-22.304
475.994	-22.075	513.885	-19.711	467.199	-22.309
476.174	-22.078	513.975	-19.714	467.390	-22.314
476.274	-22.082	514.119	-19.717	467.553	-22.319
476.444	-22.086	514.291	-19.719	467.721	-22.325
476.579	-22.090	514.463	-19.722	467.878	-22.330
476.702	-22.093	514.628	-19.725	468.021	-22.335
476.839	-22.097	514.778	-19.728	468.209	-22.340
476.972	-22.101	514.995	-19.730	468.357	-22.346
477.111	-22.105	515.166	-19.733	468.480	-22.351
477.294	-22.108	515.286	-19.736	468.637	-22.356
477.459	-22.112	515.470	-19.739	468.806	-22.361
477.593	-22.116	515.707	-19.741	469.006	-22.367
477.696	-22.120	515.865	-19.744	469.187	-22.372
477.943	-22.123	516.004	-19.747	469.336	-22.377
478.261	-22.127	516.178	-19.749	469.537	-22.382
478.404	-22.131	516.333	-19.752	469.692	-22.388
478.458	-22.135	516.472	-19.755	469.811	-22.393
478.540	-22.138	516.612	-19.757	469.981	-22.398
478.859	-22.142	516.798	-19.760	470.151	-22.403
479.109	-22.146	516.942	-19.763	470.301	-22.409
479.311	-22.150	517.071	-19.765	470.451	-22.414
479.390	-22.153	517.239	-19.768	470.585	-22.419
479.443	-22.157	517.397	-19.771	470.780	-22.424
479.616	-22.161	517.584	-19.773	470.965	-22.430
479.830	-22.164	517.694	-19.776	471.038	-22.435

479.919	-22.168	517.852	-19.779	471.101	-22.440
479.943	-22.172	518.134	-19.781	471.243	-22.445
480.232	-22.175	518.315	-19.784	471.340	-22.451
480.487	-22.179	518.396	-19.787	471.483	-22.456
480.650	-22.183	518.540	-19.789	471.719	-22.461
480.781	-22.186	518.681	-19.792	471.858	-22.466
480.890	-22.190	518.794	-19.794	472.016	-22.471
481.097	-22.194	518.963	-19.797	472.247	-22.477
481.407	-22.197	519.273	-19.800	472.442	-22.482
481.591	-22.201	519.693	-19.802	472.698	-22.487
481.571	-22.205	520.033	-19.805	472.924	-22.492
481.767	-22.208	520.157	-19.807	473.072	-22.498
482.010	-22.212	520.241	-19.810	473.310	-22.503
482.046	-22.216	520.338	-19.812	473.512	-22.508
482.203	-22.219	520.436	-19.815	473.651	-22.513
482.461	-22.223	520.458	-19.818	473.802	-22.518
482.610	-22.227	520.481	-19.820	473.962	-22.524
482.792	-22.230	520.584	-19.823	474.088	-22.529
483.019	-22.234	520.747	-19.825	474.163	-22.534
483.159	-22.237	520.899	-19.828	474.257	-22.539
483.184	-22.241	521.063	-19.830	474.410	-22.544
483.224	-22.245	521.314	-19.833	474.589	-22.549
483.458	-22.248	521.478	-19.835	474.769	-22.554
483.791	-22.252	521.593	-19.838	474.899	-22.560
483.898	-22.255	521.797	-19.840	475.037	-22.565
483.891	-22.259	521.971	-19.843	475.196	-22.570
484.021	-22.262	522.058	-19.845	475.346	-22.575
484.299	-22.266	522.228	-19.848	475.541	-22.580
484.520	-22.269	522.432	-19.850	475.826	-22.585
484.740	-22.273	522.607	-19.853	476.035	-22.590

484.894	-22.276	522.774	-19.855	476.206	-22.595
485.086	-22.280	522.909	-19.858	476.322	-22.601
485.210	-22.283	523.056	-19.860	476.418	-22.606
485.318	-22.287	523.216	-19.863	476.596	-22.611
485.658	-22.290	523.351	-19.865	476.790	-22.616
486.057	-22.294	523.525	-19.868	476.993	-22.621
486.258	-22.297	523.670	-19.870	477.139	-22.626
486.282	-22.301	523.784	-19.873	477.201	-22.631
486.420	-22.304	523.912	-19.875	477.369	-22.636
486.624	-22.308	524.028	-19.878	477.550	-22.641
486.832	-22.311	524.233	-19.880	477.747	-22.646
486.884	-22.315	524.500	-19.883	477.943	-22.651
486.900	-22.318	524.699	-19.885	478.081	-22.656
487.042	-22.322	524.836	-19.888	478.235	-22.661
487.178	-22.325	525.003	-19.890	478.396	-22.666
487.276	-22.329	525.201	-19.892	478.541	-22.671
487.381	-22.332	525.355	-19.895	478.662	-22.676
487.320	-22.335	525.423	-19.897	478.846	-22.681
487.518	-22.339	525.575	-19.900	479.038	-22.686
487.836	-22.342	525.840	-19.902	479.170	-22.691
488.124	-22.346	525.960	-19.905	479.347	-22.696
488.321	-22.349	526.135	-19.907	479.600	-22.701
488.358	-22.353	526.340	-19.910	479.753	-22.706
488.484	-22.356	526.475	-19.912	479.867	-22.711
488.607	-22.359	526.569	-19.915	480.058	-22.716
488.711	-22.363	526.772	-19.917	480.171	-22.721
488.918	-22.366	526.926	-19.920	480.342	-22.726
489.145	-22.369	527.085	-19.922	480.544	-22.731
489.442	-22.373	527.296	-19.925	480.679	-22.736
489.607	-22.376	527.443	-19.927	480.854	-22.741

489.746	-22.380	527.616	-19.930	481.021	-22.746
490.030	-22.383	527.749	-19.932	481.145	-22.751
490.180	-22.386	527.876	-19.935	481.271	-22.755
490.049	-22.390	527.989	-19.937	481.478	-22.760
489.980	-22.393	528.132	-19.940	481.702	-22.765
490.193	-22.397	528.326	-19.942	482.091	-22.770
490.452	-22.400	528.535	-19.945	482.472	-22.775
490.595	-22.403	528.732	-19.947	482.644	-22.780
490.894	-22.407	528.860	-19.950	482.735	-22.785
491.122	-22.410	528.994	-19.953	482.878	-22.790
491.114	-22.413	529.183	-19.955	482.991	-22.794
491.304	-22.417	529.379	-19.958	482.952	-22.799
491.494	-22.420	529.576	-19.960	483.006	-22.804
491.587	-22.424	529.732	-19.963	483.150	-22.809
491.787	-22.427	529.894	-19.965	483.245	-22.814
492.133	-22.430	530.029	-19.968	483.400	-22.819
492.289	-22.434	530.158	-19.970	483.637	-22.823
492.428	-22.437	530.306	-19.973	483.768	-22.828
492.468	-22.440	530.477	-19.976	483.883	-22.833
492.751	-22.444	530.650	-19.978	484.032	-22.838
493.052	-22.447	530.781	-19.981	484.148	-22.843
493.072	-22.451	530.865	-19.983	484.314	-22.848
493.207	-22.454	531.030	-19.986	484.556	-22.852
493.290	-22.457	531.239	-19.988	484.792	-22.857
493.403	-22.461	531.439	-19.991	484.940	-22.862
493.611	-22.464	531.625	-19.994	485.095	-22.867
493.873	-22.467	531.788	-19.996	485.235	-22.871
494.070	-22.471	531.936	-19.999	485.333	-22.876
493.968	-22.474	532.077	-20.001	485.494	-22.881
494.179	-22.478	532.223	-20.004	485.636	-22.886

494.464	-22.481	532.391	-20.007	485.806	-22.891
494.554	-22.484	532.488	-20.009	486.018	-22.895
494.679	-22.488	532.606	-20.012	486.160	-22.900
494.831	-22.491	532.765	-20.014	486.264	-22.905
494.961	-22.495	532.931	-20.017	486.408	-22.910
495.113	-22.498	533.116	-20.020	486.556	-22.915
495.408	-22.501	533.265	-20.022	486.735	-22.919
495.703	-22.505	533.395	-20.025	486.967	-22.924
495.728	-22.508	533.552	-20.027	487.137	-22.929
495.778	-22.512	533.714	-20.030	487.300	-22.934
496.129	-22.515	533.866	-20.033	487.492	-22.939
496.551	-22.518	534.036	-20.035	487.663	-22.943
496.647	-22.522	534.291	-20.038	487.820	-22.948
496.584	-22.525	534.533	-20.040	487.875	-22.953
496.689	-22.529	534.711	-20.043	488.026	-22.958
496.912	-22.532	534.885	-20.046	488.229	-22.963
497.108	-22.535	535.113	-20.048	488.310	-22.967
497.131	-22.539	535.313	-20.051	488.462	-22.972
497.290	-22.542	535.458	-20.053	488.707	-22.977
497.434	-22.546	535.592	-20.056	488.850	-22.982
497.590	-22.549	535.659	-20.058	489.024	-22.987
497.858	-22.553	535.746	-20.061	489.213	-22.991
498.050	-22.556	535.925	-20.064	489.357	-22.996
498.178	-22.559	536.152	-20.066	489.547	-23.001
498.483	-22.563	536.244	-20.069	489.718	-23.006
498.719	-22.566	536.311	-20.071	489.861	-23.011
498.741	-22.570	536.494	-20.074	490.058	-23.016
499.023	-22.573	536.734	-20.076	490.243	-23.020
499.100	-22.576	536.869	-20.079	490.359	-23.025
498.990	-22.580	536.925	-20.082	490.497	-23.030

499.439	-22.583	537.108	-20.084	490.638	-23.035
499.622	-22.587	537.350	-20.087	490.746	-23.040
499.612	-22.590	537.547	-20.089	490.916	-23.045
499.759	-22.593	537.728	-20.092	491.115	-23.050
499.841	-22.597	537.877	-20.094	491.262	-23.054
499.872	-22.600	538.041	-20.097	491.430	-23.059
500.000	-22.603	538.253	-20.099	491.624	-23.064
500.336	-22.607	538.420	-20.102	491.839	-23.069
500.690	-22.610	538.502	-20.104	492.013	-23.074
500.727	-22.614	538.659	-20.107	492.144	-23.079
500.834	-22.617	538.900	-20.109	492.308	-23.084
501.120	-22.620	539.043	-20.112	492.395	-23.089
501.336	-22.624	539.203	-20.114	492.504	-23.093
501.560	-22.627	539.402	-20.117	492.646	-23.098
501.888	-22.630	539.539	-20.119	492.874	-23.103
501.979	-22.634	539.674	-20.122	493.059	-23.108
501.745	-22.637	539.827	-20.124	493.240	-23.113
501.843	-22.640	539.997	-20.127	493.463	-23.118
502.129	-22.644	540.173	-20.129	493.636	-23.123
502.492	-22.647	540.354	-20.132	493.711	-23.128
502.776	-22.650	540.553	-20.134	493.843	-23.133
502.922	-22.654	540.731	-20.137	494.043	-23.137
502.882	-22.657	540.842	-20.139	494.211	-23.142
502.927	-22.660	540.952	-20.142	494.346	-23.147
503.151	-22.663	541.152	-20.144	494.475	-23.152
503.427	-22.667	541.394	-20.146	494.651	-23.157
503.441	-22.670	541.571	-20.149	494.833	-23.162
503.543	-22.673	541.704	-20.151	494.932	-23.167
503.776	-22.677	541.839	-20.154	495.025	-23.172
503.862	-22.680	541.976	-20.156	495.238	-23.177

504.235	-22.683	542.137	-20.158	495.341	-23.182
504.423	-22.686	542.329	-20.161	495.398	-23.187
504.614	-22.690	542.480	-20.163	495.553	-23.191
504.756	-22.693	542.616	-20.166	495.778	-23.196
504.887	-22.696	542.797	-20.168	496.023	-23.201
505.072	-22.699	542.970	-20.170	496.290	-23.206
505.172	-22.703	543.186	-20.173	496.479	-23.211
505.276	-22.706	543.393	-20.175	496.691	-23.216
505.344	-22.709	543.508	-20.177	496.866	-23.221
505.590	-22.712	543.605	-20.180	497.041	-23.226
505.813	-22.715	543.837	-20.182	497.257	-23.231
505.860	-22.719	544.251	-20.184	497.439	-23.236
506.219	-22.722	544.634	-20.187	497.600	-23.240
506.459	-22.725	544.818	-20.189	497.731	-23.245
506.674	-22.728	544.864	-20.191	497.838	-23.250
506.911	-22.731	544.943	-20.194	497.902	-23.255
506.700	-22.734	544.977	-20.196	497.995	-23.260
506.797	-22.738	545.003	-20.198	498.165	-23.265
507.046	-22.741	545.117	-20.201	498.299	-23.270
507.122	-22.744	545.231	-20.203	498.494	-23.274
507.574	-22.747	545.317	-20.205	498.717	-23.279
507.798	-22.750	545.494	-20.207	498.860	-23.284
507.676	-22.753	545.698	-20.210	499.029	-23.289
507.745	-22.756	545.815	-20.212	499.240	-23.294
507.993	-22.759	545.981	-20.214	499.409	-23.299
508.372	-22.762	546.215	-20.216	499.598	-23.303
508.678	-22.766	546.416	-20.219	499.777	-23.308
508.699	-22.769	546.563	-20.221	499.899	-23.313
508.805	-22.772	546.734	-20.223	500.063	-23.318
509.016	-22.775	546.897	-20.225	500.265	-23.323

508.973	-22.778	547.066	-20.228	500.422	-23.327
509.084	-22.781	547.264	-20.230	500.527	-23.332
509.397	-22.784	547.443	-20.232	500.714	-23.337
509.493	-22.787	547.575	-20.234	500.895	-23.342
509.558	-22.790	547.660	-20.236	501.040	-23.347
509.858	-22.793	547.783	-20.239	501.138	-23.351
510.167	-22.796	547.924	-20.241	501.254	-23.356
510.305	-22.799	548.135	-20.243	501.408	-23.361
510.546	-22.802	548.286	-20.245	501.562	-23.366
510.678	-22.805	548.326	-20.247	501.738	-23.370
510.614	-22.808	548.453	-20.249	501.937	-23.375
510.742	-22.811	548.685	-20.252	502.152	-23.380
511.029	-22.814	548.935	-20.254	502.312	-23.384
510.924	-22.817	549.081	-20.256	502.444	-23.389
511.008	-22.820	549.233	-20.258	502.608	-23.394
511.411	-22.822	549.449	-20.260	502.754	-23.398
512.107	-22.825	549.656	-20.262	502.940	-23.403
512.380	-22.828	549.802	-20.265	503.143	-23.408
512.228	-22.831	549.981	-20.267	503.233	-23.412
512.360	-22.834	550.153	-20.269	503.415	-23.417
512.773	-22.837	550.284	-20.271	503.654	-23.422
512.775	-22.840	550.437	-20.273	503.816	-23.426
512.616	-22.843	550.595	-20.275	503.985	-23.431
512.783	-22.845	550.776	-20.277	504.151	-23.436
512.996	-22.848	550.956	-20.280	504.324	-23.440
512.892	-22.851	551.077	-20.282	504.464	-23.445
512.696	-22.854	551.208	-20.284	504.651	-23.450
512.882	-22.857	551.380	-20.286	504.899	-23.454
513.423	-22.860	551.564	-20.288	505.062	-23.459
513.995	-22.862	551.742	-20.290	505.149	-23.463

513.983	-22.865	551.922	-20.292	505.222	-23.468
513.768	-22.868	552.046	-20.295	505.403	-23.473
514.078	-22.871	552.160	-20.297	505.640	-23.477
514.490	-22.873	552.328	-20.299	506.058	-23.482
514.730	-22.876	552.497	-20.301	506.486	-23.486
514.844	-22.879	552.702	-20.303	506.634	-23.491
514.653	-22.882	552.881	-20.305	506.698	-23.495
514.790	-22.884	552.991	-20.308	506.763	-23.500
515.398	-22.887	553.093	-20.310	506.840	-23.504
515.584	-22.890	553.294	-20.312	506.858	-23.509
515.440	-22.893	553.466	-20.314	506.828	-23.514
515.720	-22.895	553.613	-20.316	506.945	-23.518
515.968	-22.898	553.783	-20.318	507.145	-23.523
515.876	-22.901	553.959	-20.320	507.286	-23.527
516.006	-22.903	554.132	-20.323	507.432	-23.532
516.452	-22.906	554.260	-20.325	507.622	-23.536
516.587	-22.909	554.452	-20.327	507.800	-23.541
516.734	-22.911	554.669	-20.329	507.967	-23.545
516.891	-22.914	554.864	-20.331	508.107	-23.550
517.029	-22.917	554.990	-20.333	508.310	-23.554
517.119	-22.919	555.055	-20.336	508.530	-23.559
517.223	-22.922	555.170	-20.338	508.648	-23.563
517.686	-22.925	555.341	-20.340	508.837	-23.568
517.971	-22.927	555.530	-20.342	509.014	-23.572
517.863	-22.930	555.713	-20.344	509.154	-23.577
517.978	-22.932	555.855	-20.346	509.300	-23.581
518.325	-22.935	556.057	-20.349	509.399	-23.586
518.554	-22.938	556.335	-20.351	509.535	-23.590
518.482	-22.940	556.495	-20.353	509.748	-23.595
518.595	-22.943	556.618	-20.355	509.893	-23.599

518.897	-22.946	556.798	-20.357	509.972	-23.603
519.137	-22.948	556.950	-20.359	510.097	-23.608
519.295	-22.951	557.103	-20.361	510.263	-23.612
519.137	-22.953	557.223	-20.364	510.445	-23.617
519.273	-22.956	557.322	-20.366	510.643	-23.621
519.446	-22.958	557.474	-20.368	510.869	-23.626
519.494	-22.961	557.624	-20.370	511.071	-23.630
519.632	-22.964	557.739	-20.372	511.260	-23.635
519.801	-22.966	557.885	-20.374	511.414	-23.639
520.000	-22.969	558.031	-20.377	511.557	-23.644
520.162	-22.971	558.177	-20.379	511.722	-23.648
520.679	-22.974	558.357	-20.381	511.924	-23.653
520.863	-22.976	558.512	-20.383	512.131	-23.657
521.116	-22.979	558.672	-20.385	512.293	-23.662
521.268	-22.981	558.898	-20.387	512.400	-23.666
521.289	-22.984	559.128	-20.389	512.542	-23.671
521.378	-22.986	559.346	-20.392	512.643	-23.675
521.589	-22.989	559.544	-20.394	512.784	-23.680
521.726	-22.991	559.722	-20.396	513.001	-23.684
521.781	-22.994	559.864	-20.398	513.189	-23.689
522.074	-22.996	560.012	-20.400	513.363	-23.693
522.416	-22.999	560.184	-20.402	513.524	-23.698
522.570	-23.001	560.327	-20.404	513.657	-23.702
522.398	-23.004	560.454	-20.406	513.805	-23.707
522.490	-23.006	560.577	-20.409	513.966	-23.711
522.846	-23.009	560.717	-20.411	514.137	-23.716
523.096	-23.011	560.899	-20.413	514.344	-23.720
523.453	-23.013	561.014	-20.415	514.477	-23.725
523.484	-23.016	561.144	-20.417	514.578	-23.730
523.479	-23.018	561.307	-20.419	514.747	-23.734

523.630	-23.021	561.427	-20.421	514.947	-23.739
523.824	-23.023	561.571	-20.423	515.155	-23.743
524.013	-23.025	561.771	-20.425	515.312	-23.748
524.317	-23.028	562.017	-20.428	515.473	-23.752
524.238	-23.030	562.211	-20.430	515.627	-23.757
524.273	-23.033	562.369	-20.432	515.777	-23.762
524.809	-23.035	562.523	-20.434	515.907	-23.766
525.038	-23.037	562.654	-20.436	516.036	-23.771
525.045	-23.040	562.820	-20.438	516.149	-23.775
525.273	-23.042	563.021	-20.440	516.344	-23.780
525.414	-23.044	563.182	-20.442	516.548	-23.785
525.759	-23.047	563.400	-20.444	516.692	-23.789
525.766	-23.049	563.585	-20.446	516.864	-23.794
525.688	-23.051	563.689	-20.448	517.044	-23.799
525.922	-23.053	563.819	-20.450	517.191	-23.803
525.710	-23.056	563.954	-20.453	517.336	-23.808
525.877	-23.058	564.169	-20.455	517.500	-23.812
526.400	-23.060	564.428	-20.457	517.687	-23.817
526.466	-23.063	564.585	-20.459	517.827	-23.822
526.761	-23.065	564.739	-20.461	517.985	-23.826
527.066	-23.067	564.915	-20.463	518.131	-23.831
527.288	-23.069	565.018	-20.465	518.211	-23.836
527.209	-23.071	565.126	-20.467	518.389	-23.840
527.131	-23.074	565.334	-20.469	518.579	-23.845
527.355	-23.076	565.522	-20.471	518.636	-23.850
527.688	-23.078	565.681	-20.473	518.772	-23.854
527.745	-23.080	565.800	-20.475	518.955	-23.859
528.023	-23.082	565.953	-20.477	519.151	-23.864
528.507	-23.084	566.144	-20.479	519.316	-23.868
528.323	-23.086	566.326	-20.481	519.423	-23.873

528.401	-23.088	566.474	-20.483	519.558	-23.878
528.688	-23.091	566.644	-20.486	519.744	-23.883
528.610	-23.093	566.799	-20.488	519.978	-23.887
528.933	-23.095	566.995	-20.490	520.240	-23.892
529.347	-23.097	567.235	-20.492	520.474	-23.897
529.396	-23.099	567.381	-20.494	520.633	-23.901
529.212	-23.101	567.461	-20.496	520.744	-23.906
529.333	-23.103	567.606	-20.498	520.914	-23.911
529.622	-23.105	567.746	-20.500	521.157	-23.915
529.994	-23.107	567.905	-20.502	521.336	-23.920
530.082	-23.109	568.117	-20.504	521.445	-23.925
529.966	-23.111	568.200	-20.506	521.617	-23.929
530.033	-23.113	568.308	-20.508	521.738	-23.934
530.291	-23.115	568.563	-20.510	521.856	-23.939
530.652	-23.117	568.973	-20.512	522.032	-23.944
531.289	-23.118	569.354	-20.514	522.169	-23.948
531.330	-23.120	569.497	-20.516	522.265	-23.953
531.373	-23.122	569.529	-20.518	522.411	-23.958
531.808	-23.124	569.587	-20.520	522.580	-23.962
531.575	-23.126	569.692	-20.522	522.709	-23.967
531.561	-23.128	569.737	-20.524	522.881	-23.972
531.687	-23.130	569.784	-20.526	523.089	-23.976
531.649	-23.132	569.921	-20.528	523.306	-23.981
531.854	-23.133	570.104	-20.530	523.536	-23.986
532.281	-23.135	570.240	-20.532	523.726	-23.990
532.349	-23.137	570.370	-20.534	523.847	-23.995
532.609	-23.139	570.552	-20.536	523.945	-24.000
533.365	-23.141	570.757	-20.538	524.087	-24.004
533.233	-23.142	570.973	-20.540	524.291	-24.009
533.366	-23.144	571.150	-20.542	524.442	-24.014

533.587	-23.146	571.303	-20.543	524.579	-24.018
533.316	-23.148	571.527	-20.545	524.783	-24.023
533.407	-23.149	571.729	-20.547	524.994	-24.028
533.743	-23.151	571.859	-20.549	525.175	-24.032
533.928	-23.153	571.989	-20.551	525.323	-24.037
534.417	-23.154	572.108	-20.553	525.454	-24.042
534.504	-23.156	572.206	-20.555	525.621	-24.046
534.427	-23.158	572.427	-20.557	525.790	-24.051
534.397	-23.159	572.622	-20.559	525.976	-24.056
534.295	-23.161	572.755	-20.561	526.177	-24.060
534.865	-23.163	572.932	-20.563	526.323	-24.065
535.141	-23.164	573.064	-20.565	526.379	-24.069
534.883	-23.166	573.200	-20.567	526.504	-24.074
534.904	-23.167	573.352	-20.569	526.769	-24.079
535.198	-23.169	573.511	-20.571	526.978	-24.083
535.495	-23.171	573.653	-20.573	527.085	-24.088
535.250	-23.172	573.829	-20.575	527.260	-24.093
535.812	-23.174	574.060	-20.577	527.430	-24.097
536.301	-23.175	574.218	-20.579	527.555	-24.102
536.708	-23.177	574.426	-20.581	527.715	-24.106
536.859	-23.178	574.653	-20.583	527.875	-24.111
536.911	-23.180	574.816	-20.585	528.084	-24.116
537.064	-23.181	574.988	-20.587	528.287	-24.120
536.646	-23.183	575.156	-20.589	528.452	-24.125
536.701	-23.184	575.281	-20.591	528.638	-24.129
536.859	-23.186	575.356	-20.593	528.793	-24.134
537.103	-23.187	575.479	-20.595	528.890	-24.139
538.172	-23.189	575.660	-20.597	529.030	-24.143
538.631	-23.190	575.768	-20.599	529.193	-24.148
539.009	-23.192	575.957	-20.601	529.434	-24.152

539.225	-23.193	576.101	-20.603	529.823	-24.157
538.883	-23.194	576.211	-20.605	530.192	-24.161
538.702	-23.196	576.392	-20.607	530.353	-24.166
538.230	-23.197	576.614	-20.609	530.415	-24.170
538.649	-23.199	576.807	-20.611	530.509	-24.175
539.120	-23.200	576.937	-20.613	530.539	-24.180
539.168	-23.201	577.056	-20.615	530.559	-24.184
539.445	-23.203	577.280	-20.617	530.644	-24.189
539.617	-23.204	577.422	-20.619	530.707	-24.193
539.884	-23.206	577.535	-20.621	530.808	-24.198
539.804	-23.207	577.672	-20.623	531.001	-24.202
540.233	-23.208	577.839	-20.625	531.224	-24.207
540.416	-23.210	577.972	-20.627	531.393	-24.211
540.572	-23.211	578.142	-20.629	531.577	-24.216
540.691	-23.212	578.361	-20.631	531.769	-24.220
539.898	-23.214	578.559	-20.633	531.943	-24.225
540.356	-23.215	578.755	-20.635	532.066	-24.229
541.153	-23.216	578.955	-20.637	532.251	-24.234
541.436	-23.218	579.104	-20.639	532.486	-24.238
541.126	-23.219	579.216	-20.641	532.654	-24.243
541.432	-23.220	579.286	-20.643	532.785	-24.247
541.339	-23.222	579.487	-20.645	532.935	-24.252
541.497	-23.223	579.739	-20.647	533.062	-24.256
542.198	-23.224	579.893	-20.649	533.187	-24.261
541.846	-23.226	580.047	-20.651	533.360	-24.265
541.445	-23.227	580.154	-20.653	533.545	-24.270
542.275	-23.228	580.377	-20.655	533.693	-24.274
542.678	-23.229	580.641	-20.657	533.797	-24.279
542.782	-23.231	580.771	-20.659	533.890	-24.283
543.086	-23.232	580.912	-20.661	534.033	-24.288

543.121	-23.233	581.097	-20.663	534.263	-24.292
543.235	-23.235	581.212	-20.665	534.444	-24.297
543.332	-23.236	581.304	-20.667	534.620	-24.301
544.173	-23.237	581.470	-20.669	534.832	-24.306
543.713	-23.239	581.652	-20.671	535.052	-24.310
544.018	-23.240	581.827	-20.673	535.229	-24.314
544.577	-23.241	581.936	-20.675	535.413	-24.319
544.454	-23.242	582.052	-20.677	535.595	-24.323
544.397	-23.244	582.277	-20.679	535.746	-24.328
544.337	-23.245	582.434	-20.681	535.847	-24.332
544.711	-23.246	582.566	-20.683	535.973	-24.337
544.659	-23.248	582.720	-20.684	536.157	-24.341
544.640	-23.249	582.883	-20.686	536.321	-24.346
544.940	-23.250	583.010	-20.688	536.428	-24.350
545.294	-23.252	583.141	-20.690	536.565	-24.355
546.006	-23.253	583.317	-20.692	536.782	-24.359
546.154	-23.254	583.497	-20.694	536.927	-24.364
545.931	-23.256	583.742	-20.696	537.089	-24.368
545.832	-23.257	583.965	-20.698	537.286	-24.373
546.004	-23.258	584.127	-20.700	537.432	-24.377
546.358	-23.260	584.289	-20.701	537.533	-24.382
546.113	-23.261	584.488	-20.703	537.702	-24.386
546.556	-23.262	584.697	-20.705	537.909	-24.391
547.003	-23.264	584.803	-20.707	538.065	-24.395
547.603	-23.265	584.910	-20.709	538.148	-24.400
548.157	-23.266	585.108	-20.711	538.302	-24.404
547.791	-23.268	585.245	-20.712	538.505	-24.409
547.039	-23.269	585.352	-20.714	538.701	-24.414
547.616	-23.270	585.507	-20.716	538.877	-24.418
548.255	-23.272	585.645	-20.718	538.965	-24.423

547.836	-23.273	585.782	-20.719	539.108	-24.427
547.853	-23.275	585.944	-20.721	539.287	-24.432
547.992	-23.276	586.092	-20.723	539.495	-24.436
547.760	-23.277	586.256	-20.725	539.724	-24.441
548.125	-23.279	586.453	-20.726	539.839	-24.445
548.901	-23.280	586.619	-20.728	539.946	-24.450
548.827	-23.281	586.802	-20.730	540.050	-24.454
548.880	-23.283	586.966	-20.732	540.272	-24.459
549.551	-23.284	587.115	-20.733	540.542	-24.463
549.537	-23.285	587.314	-20.735	540.674	-24.468
549.530	-23.287	587.505	-20.737	540.816	-24.473
549.801	-23.288	587.671	-20.738	540.960	-24.477
549.607	-23.290	587.895	-20.740	541.088	-24.482
549.510	-23.291	588.081	-20.741	541.282	-24.486
550.113	-23.292	588.189	-20.743	541.494	-24.491
550.594	-23.294	588.344	-20.745	541.676	-24.495
550.217	-23.295	588.530	-20.746	541.775	-24.500
550.285	-23.297	588.711	-20.748	541.868	-24.504
550.962	-23.298	588.864	-20.749	542.026	-24.509
550.761	-23.299	588.995	-20.751	542.251	-24.514
550.967	-23.301	589.160	-20.752	542.405	-24.518
551.712	-23.302	589.384	-20.754	542.523	-24.523
551.436	-23.304	589.534	-20.755	542.664	-24.527
551.166	-23.305	589.636	-20.757	542.856	-24.532
551.840	-23.306	589.838	-20.758	543.038	-24.536
552.594	-23.308	590.026	-20.760	543.144	-24.541
552.800	-23.309	590.133	-20.761	543.290	-24.545
553.215	-23.311	590.321	-20.763	543.476	-24.550
552.347	-23.312	590.550	-20.764	543.622	-24.554
551.794	-23.313	590.661	-20.766	543.622	-24.559

552.343	-23.315	590.751	-20.767	543.990	-24.563
553.193	-23.316	590.921	-20.769	544.173	-24.568
553.200	-23.318	591.101	-20.770	544.394	-24.573
552.423	-23.319	591.265	-20.771	544.634	-24.577
552.768	-23.320	591.430	-20.773	544.854	-24.582
553.786	-23.322	591.573	-20.774	545.027	-24.586
553.675	-23.323	591.774	-20.775	545.144	-24.591
553.263	-23.324	592.003	-20.777	545.255	-24.595
553.960	-23.326	592.128	-20.778	545.412	-24.600
554.476	-23.327	592.262	-20.779	545.520	-24.604
554.241	-23.329	592.415	-20.781	545.615	-24.609
554.160	-23.330	592.574	-20.782	545.742	-24.613
554.732	-23.331	592.672	-20.783	545.862	-24.617
555.244	-23.333	592.810	-20.785	545.994	-24.622
555.329	-23.334	592.972	-20.786	546.152	-24.626
555.633	-23.335	593.294	-20.787	546.310	-24.631
555.900	-23.337	593.757	-20.788	546.445	-24.635
555.370	-23.338	594.101	-20.790	546.582	-24.640
555.799	-23.339	594.171	-20.791	546.860	-24.644
555.977	-23.341	594.218	-20.792	547.072	-24.649
555.768	-23.342	594.336	-20.793	547.248	-24.653
555.746	-23.343	594.414	-20.794	547.464	-24.657
556.328	-23.345	594.436	-20.796	547.671	-24.662
556.821	-23.346	594.484	-20.797	547.863	-24.666
556.606	-23.347	594.568	-20.798	547.982	-24.671
556.240	-23.349	594.689	-20.799	548.102	-24.675
556.772	-23.350	594.898	-20.800	548.285	-24.679
557.104	-23.351	595.068	-20.801	548.449	-24.684
557.563	-23.353	595.187	-20.803	548.590	-24.688
558.323	-23.354	595.363	-20.804	548.728	-24.693

557.861	-23.355	595.535	-20.805	548.922	-24.697
557.248	-23.357	595.739	-20.806	549.073	-24.701
557.917	-23.358	595.938	-20.807	549.203	-24.706
558.553	-23.359	596.108	-20.808	549.349	-24.710
558.237	-23.360	596.285	-20.809	549.459	-24.714
557.744	-23.362	596.503	-20.810	549.693	-24.719
558.278	-23.363	596.685	-20.811	549.976	-24.723
559.616	-23.364	596.848	-20.813	550.102	-24.727
559.360	-23.365	596.962	-20.814	550.272	-24.732
559.028	-23.367	597.040	-20.815	550.442	-24.736
559.724	-23.368	597.111	-20.816	550.546	-24.740
559.725	-23.369	597.250	-20.817	550.745	-24.745
560.012	-23.370	597.449	-20.818	550.946	-24.749
560.467	-23.372	597.649	-20.819	551.107	-24.753
560.448	-23.373	597.865	-20.820	551.235	-24.758
560.492	-23.374	598.061	-20.821	551.351	-24.762
560.053	-23.375	598.161	-20.822	551.545	-24.766
559.430	-23.376	598.282	-20.823	551.686	-24.770
560.527	-23.378	598.495	-20.824	551.909	-24.775
561.138	-23.379	598.677	-20.825	552.180	-24.779
560.754	-23.380	598.798	-20.826	552.289	-24.783
561.713	-23.381	598.939	-20.827	552.421	-24.787
561.936	-23.382	599.129	-20.828	552.607	-24.792
561.543	-23.383	599.294	-20.829	552.721	-24.796
561.342	-23.385	599.473	-20.830	552.844	-24.800
561.577	-23.386	599.625	-20.831	553.004	-24.804
562.187	-23.387	599.737	-20.832	553.168	-24.809
562.745	-23.388	599.933	-20.833	553.453	-24.813
562.703	-23.389	600.120	-20.834	553.879	-24.817
563.248	-23.390	600.213	-20.835	554.169	-24.821

563.011	-23.391	600.353	-20.836	554.239	-24.825
562.728	-23.392	600.548	-20.837	554.320	-24.830
562.819	-23.393	600.704	-20.839	554.413	-24.834
562.069	-23.395	600.862	-20.840	554.475	-24.838
562.895	-23.396	601.056	-20.841	554.580	-24.842
563.741	-23.397	601.265	-20.842	554.712	-24.846
564.042	-23.398	601.469	-20.843	554.812	-24.850
564.680	-23.399	601.627	-20.844	554.831	-24.855
564.045	-23.400	601.793	-20.845	554.963	-24.859
564.574	-23.401	601.955	-20.846	555.216	-24.863
564.955	-23.402	602.035	-20.847	555.403	-24.867
564.945	-23.403	602.146	-20.848	555.569	-24.871
564.993	-23.404	602.250	-20.849	555.712	-24.875
565.281	-23.405	602.382	-20.850	555.892	-24.879
565.158	-23.406	602.615	-20.851	556.126	-24.884
564.650	-23.407	602.838	-20.852	556.287	-24.888
564.901	-23.408	603.027	-20.853	556.471	-24.892
564.649	-23.409	603.227	-20.854	556.684	-24.896
564.508	-23.410	603.376	-20.855	556.747	-24.900
565.366	-23.411	603.514	-20.857	556.837	-24.904
565.506	-23.412	603.686	-20.858	557.008	-24.908
565.559	-23.413	603.843	-20.859	557.135	-24.912
565.880	-23.414	603.992	-20.860	557.315	-24.917
565.571	-23.415	604.140	-20.861	557.528	-24.921
565.627	-23.416	604.334	-20.862	557.657	-24.925
566.729	-23.417	604.536	-20.863	557.809	-24.929
567.799	-23.418	604.700	-20.864	557.950	-24.933
567.776	-23.419	604.830	-20.865	558.109	-24.937
567.891	-23.420	604.968	-20.866	558.329	-24.941
568.173	-23.421	605.139	-20.867	558.561	-24.945

567.598	-23.422	605.350	-20.868	558.725	-24.949
567.188	-23.423	605.561	-20.870	558.886	-24.953
567.992	-23.424	605.742	-20.871	559.100	-24.958
567.359	-23.425	605.899	-20.872	559.211	-24.962
567.403	-23.426	606.005	-20.873	559.346	-24.966
567.702	-23.427	606.082	-20.874	559.576	-24.970
568.020	-23.428	606.190	-20.875	559.724	-24.974
567.904	-23.429	606.390	-20.876	559.824	-24.978
568.229	-23.430	606.563	-20.877	559.993	-24.982
568.949	-23.431	606.716	-20.878	560.180	-24.986
568.777	-23.432	606.871	-20.879	560.294	-24.990
569.242	-23.433	606.935	-20.881	560.465	-24.994
569.762	-23.434	607.079	-20.882	560.616	-24.999
569.699	-23.435	607.251	-20.883	560.671	-25.003
569.744	-23.436	607.372	-20.884	560.852	-25.007
570.190	-23.437	607.545	-20.885	561.110	-25.011
570.294	-23.438	607.718	-20.886	561.256	-25.015
570.818	-23.439	607.899	-20.887	561.383	-25.019
571.316	-23.440	608.050	-20.888	561.510	-25.023
571.406	-23.441	608.213	-20.889	561.627	-25.027
571.558	-23.442	608.462	-20.890	561.831	-25.032
571.218	-23.443	608.740	-20.891	562.026	-25.036
571.102	-23.445	608.942	-20.892	562.181	-25.040
571.446	-23.446	609.079	-20.893	562.302	-25.044
571.726	-23.447	609.177	-20.894	562.399	-25.048
571.681	-23.448	609.306	-20.896	562.557	-25.052
572.479	-23.449	609.542	-20.897	562.814	-25.056
572.298	-23.450	609.748	-20.898	563.005	-25.061
572.047	-23.451	609.833	-20.899	563.224	-25.065
572.767	-23.452	609.928	-20.900	563.445	-25.069

572.353	-23.453	610.079	-20.901	563.529	-25.073
572.450	-23.454	610.261	-20.902	563.693	-25.077
572.821	-23.455	610.446	-20.903	563.925	-25.081
572.924	-23.456	610.547	-20.904	564.034	-25.086
573.257	-23.458	610.680	-20.905	564.123	-25.090
573.339	-23.459	610.866	-20.906	564.283	-25.094
572.969	-23.460	611.024	-20.907	564.467	-25.098
573.552	-23.461	611.248	-20.908	564.671	-25.102
573.366	-23.462	611.475	-20.909	564.886	-25.106
573.187	-23.463	611.617	-20.910	564.949	-25.111
574.361	-23.464	611.792	-20.911	565.054	-25.115
574.225	-23.465	611.993	-20.912	565.290	-25.119
573.767	-23.467	612.203	-20.913	565.467	-25.123
573.586	-23.468	612.339	-20.914	565.641	-25.127
573.878	-23.469	612.424	-20.915	565.837	-25.132
573.938	-23.470	612.567	-20.916	566.005	-25.136
574.191	-23.471	612.729	-20.917	566.158	-25.140
574.484	-23.472	612.901	-20.918	566.287	-25.144
575.312	-23.474	613.085	-20.918	566.471	-25.148
575.145	-23.475	613.277	-20.919	566.621	-25.153
575.198	-23.476	613.482	-20.920	566.712	-25.157
574.985	-23.477	613.652	-20.921	566.789	-25.161
574.966	-23.478	613.784	-20.922	566.871	-25.165
575.621	-23.480	613.915	-20.923	567.070	-25.169
576.066	-23.481	614.048	-20.924	567.334	-25.174
576.359	-23.482	614.259	-20.925	567.564	-25.178
576.046	-23.483	614.451	-20.926	567.712	-25.182
577.532	-23.485	614.608	-20.927	567.813	-25.186
576.935	-23.486	614.750	-20.928	567.958	-25.190
575.911	-23.487	614.903	-20.929	568.181	-25.195

577.327	-23.488	615.056	-20.929	568.506	-25.199
576.919	-23.490	615.201	-20.930	568.734	-25.203
576.838	-23.491	615.397	-20.931	568.864	-25.207
577.477	-23.492	615.565	-20.932	568.927	-25.211
577.216	-23.494	615.695	-20.933	569.083	-25.215
577.118	-23.495	615.849	-20.934	569.271	-25.220
577.774	-23.496	616.036	-20.935	569.361	-25.224
579.206	-23.497	616.229	-20.936	569.480	-25.228
578.706	-23.499	616.348	-20.936	569.614	-25.232
578.313	-23.500	616.518	-20.937	569.735	-25.236
579.098	-23.501	616.693	-20.938	569.911	-25.240
578.599	-23.503	616.856	-20.939	570.094	-25.244
578.591	-23.504	617.075	-20.940	570.302	-25.249
580.277	-23.505	617.263	-20.941	570.413	-25.253
579.865	-23.507	617.384	-20.941	570.513	-25.257
579.056	-23.508	617.470	-20.942	570.710	-25.261
579.515	-23.509	617.595	-20.943	570.920	-25.265
579.288	-23.510	617.927	-20.944	571.128	-25.269
580.293	-23.512	618.387	-20.945	571.294	-25.273
580.455	-23.513	618.688	-20.946	571.454	-25.277
579.524	-23.514	618.778	-20.946	571.640	-25.281
581.258	-23.516	618.817	-20.947	571.798	-25.286
582.036	-23.517	618.911	-20.948	571.962	-25.290
580.252	-23.518	618.943	-20.949	572.117	-25.294
580.532	-23.520	619.005	-20.949	572.259	-25.298
582.684	-23.521	619.125	-20.950	572.463	-25.302
581.845	-23.522	619.235	-20.951	572.662	-25.306
580.967	-23.524	619.375	-20.952	572.754	-25.310
582.328	-23.525	619.510	-20.953	572.884	-25.314
582.630	-23.526	619.695	-20.953	573.117	-25.318

580.994	-23.528	619.937	-20.954	573.317	-25.322
581.909	-23.529	620.117	-20.955	573.431	-25.326
583.635	-23.530	620.244	-20.956	573.569	-25.330
582.548	-23.531	620.386	-20.956	573.781	-25.334
581.930	-23.533	620.620	-20.957	573.956	-25.338
583.641	-23.534	620.891	-20.958	574.141	-25.342
583.471	-23.535	621.063	-20.959	574.334	-25.346
582.680	-23.537	621.163	-20.959	574.466	-25.350
583.473	-23.538	621.262	-20.960	574.605	-25.354
584.129	-23.539	621.371	-20.961	574.783	-25.358
582.619	-23.540	621.524	-20.962	574.990	-25.362
582.758	-23.542	621.704	-20.962	575.167	-25.366
584.466	-23.543	621.835	-20.963	575.282	-25.370
584.003	-23.544	621.992	-20.964	575.395	-25.374
583.059	-23.546	622.190	-20.965	575.565	-25.378
584.772	-23.547	622.290	-20.965	575.768	-25.382
585.734	-23.548	622.418	-20.966	575.913	-25.386
584.140	-23.549	622.591	-20.967	576.047	-25.390
583.593	-23.551	622.719	-20.968	576.263	-25.394
585.560	-23.552	622.914	-20.968	576.457	-25.398
586.262	-23.553	623.121	-20.969	576.580	-25.402
585.113	-23.554	623.312	-20.970	576.686	-25.406
584.045	-23.556	623.502	-20.971	576.828	-25.410
584.965	-23.557	623.669	-20.971	576.961	-25.413
586.779	-23.558	623.851	-20.972	577.247	-25.417
586.321	-23.559	623.943	-20.973	577.693	-25.421
585.145	-23.560	624.068	-20.974	577.961	-25.425
586.298	-23.562	624.286	-20.975	578.109	-25.429
587.493	-23.563	624.501	-20.975	578.210	-25.433
586.459	-23.564	624.646	-20.976	578.233	-25.437

585.132	-23.565	624.729	-20.977	578.305	-25.440
585.461	-23.566	624.904	-20.978	578.375	-25.444
586.377	-23.567	625.087	-20.978	578.442	-25.448
586.671	-23.569	625.248	-20.979	578.511	-25.452
585.839	-23.570	625.365	-20.980	578.659	-25.456
587.080	-23.571	625.492	-20.981	578.860	-25.460
588.942	-23.572	625.704	-20.982	579.026	-25.463
588.731	-23.573	625.858	-20.982	579.199	-25.467
587.099	-23.574	625.996	-20.983	579.358	-25.471
587.182	-23.575	626.196	-20.984	579.541	-25.475
589.571	-23.577	626.362	-20.985	579.769	-25.478
589.950	-23.578	626.494	-20.986	579.978	-25.482
588.885	-23.579	626.680	-20.986	580.152	-25.486
590.243	-23.580	626.870	-20.987	580.329	-25.490
589.955	-23.581	626.987	-20.988	580.450	-25.493
587.970	-23.582	627.118	-20.989	580.591	-25.497
588.872	-23.583	627.299	-20.990	580.802	-25.501
589.156	-23.584	627.485	-20.991	580.952	-25.505
589.396	-23.585	627.727	-20.991	581.086	-25.508
591.020	-23.586	627.954	-20.992	581.218	-25.512
591.075	-23.587	628.085	-20.993	581.361	-25.516
589.820	-23.588	628.216	-20.994	581.526	-25.519
591.436	-23.589	628.389	-20.995	581.662	-25.523
592.421	-23.591	628.541	-20.996	581.794	-25.527
590.629	-23.592	628.664	-20.997	581.939	-25.530
591.038	-23.593	628.810	-20.997	582.130	-25.534
592.711	-23.594	628.955	-20.998	582.375	-25.538
592.032	-23.595	629.138	-20.999	582.598	-25.541
590.405	-23.596	629.323	-21.000	582.788	-25.545
590.361	-23.597	629.481	-21.001	582.955	-25.549

592.446	-23.598	629.610	-21.002	583.112	-25.552
592.912	-23.599	629.743	-21.003	583.261	-25.556
591.196	-23.600	629.893	-21.003	583.383	-25.560
590.919	-23.601	630.072	-21.004	583.540	-25.563
592.519	-23.602	630.299	-21.005	583.713	-25.567
593.966	-23.603	630.454	-21.006	583.874	-25.571
594.311	-23.604	630.544	-21.007	583.991	-25.574
593.033	-23.605	630.700	-21.008	584.149	-25.578
592.862	-23.606	630.909	-21.009	584.328	-25.581
593.895	-23.607	631.032	-21.009	584.433	-25.585
592.697	-23.608	631.151	-21.010	584.573	-25.589
592.726	-23.609	631.384	-21.011	584.777	-25.592
594.968	-23.610	631.590	-21.012	584.934	-25.596
595.270	-23.611	631.648	-21.013	585.063	-25.599
593.523	-23.612	631.737	-21.014	585.277	-25.603
592.220	-23.613	631.904	-21.015	585.497	-25.606
591.860	-23.614	632.068	-21.015	585.636	-25.610
593.622	-23.615	632.241	-21.016	585.736	-25.614
595.644	-23.616	632.460	-21.017	585.897	-25.617
594.573	-23.617	632.659	-21.018	586.047	-25.621
593.598	-23.618	632.845	-21.019	586.207	-25.624
595.348	-23.619	633.041	-21.019	586.368	-25.628
596.257	-23.619	633.197	-21.020	586.519	-25.631
594.774	-23.620	633.384	-21.021	586.678	-25.635
593.744	-23.621	633.568	-21.022	586.887	-25.638
595.157	-23.622	633.733	-21.023	587.139	-25.642
597.152	-23.623	633.905	-21.023	587.315	-25.646
596.629	-23.624	634.071	-21.024	587.425	-25.649
595.477	-23.625	634.245	-21.025	587.560	-25.653
596.983	-23.626	634.412	-21.026	587.762	-25.656

597.142	-23.627	634.488	-21.026	587.945	-25.660
595.710	-23.628	634.608	-21.027	588.057	-25.663
596.919	-23.629	634.790	-21.028	588.148	-25.667
598.922	-23.630	634.958	-21.029	588.360	-25.670
599.477	-23.631	635.118	-21.029	588.549	-25.674
598.804	-23.632	635.250	-21.030	588.740	-25.677
597.032	-23.633	635.416	-21.031	588.976	-25.680
595.478	-23.634	635.586	-21.032	589.108	-25.684
595.468	-23.635	635.721	-21.032	589.211	-25.687
596.262	-23.636	635.826	-21.033	589.376	-25.691
597.981	-23.637	636.024	-21.034	589.539	-25.694
599.880	-23.638	636.322	-21.034	589.673	-25.698
599.358	-23.639	636.578	-21.035	589.880	-25.701
597.687	-23.640	636.747	-21.036	590.056	-25.705
598.826	-23.641	636.849	-21.036	590.184	-25.708
600.893	-23.642	636.942	-21.037	590.311	-25.711
601.191	-23.643	637.105	-21.038	590.398	-25.715
599.636	-23.644	637.292	-21.038	590.566	-25.718
598.235	-23.645	637.464	-21.039	590.756	-25.721
599.779	-23.646	637.655	-21.040	590.886	-25.725
601.167	-23.647	637.815	-21.040	591.056	-25.728
599.857	-23.648	637.938	-21.041	591.229	-25.732
598.332	-23.649	638.110	-21.041	591.404	-25.735
598.187	-23.650	638.293	-21.042	591.575	-25.738
599.888	-23.652	638.421	-21.043	591.746	-25.742
602.251	-23.653	638.560	-21.043	591.906	-25.745
603.220	-23.654	638.750	-21.044	592.057	-25.748
602.504	-23.655	638.890	-21.044	592.237	-25.751
600.767	-23.656	639.027	-21.045	592.415	-25.755
599.504	-23.657	639.263	-21.046	592.598	-25.758

600.799	-23.658	639.396	-21.046	592.828	-25.761
602.945	-23.659	639.476	-21.047	593.034	-25.764
602.615	-23.660	639.658	-21.047	593.182	-25.768
601.036	-23.661	639.862	-21.048	593.305	-25.771
600.254	-23.662	640.030	-21.048	593.453	-25.774
600.492	-23.663	640.217	-21.049	593.619	-25.777
602.057	-23.664	640.378	-21.049	593.732	-25.781
603.956	-23.665	640.554	-21.050	593.807	-25.784
604.010	-23.666	640.707	-21.050	593.951	-25.787
603.099	-23.667	640.804	-21.051	594.111	-25.790
602.519	-23.668	640.984	-21.051	594.259	-25.793
602.335	-23.669	641.236	-21.052	594.406	-25.796
603.048	-23.671	641.410	-21.052	594.543	-25.800
603.147	-23.672	641.524	-21.053	594.780	-25.803
602.343	-23.673	641.677	-21.053	595.029	-25.806
601.987	-23.674	641.836	-21.054	595.186	-25.809
603.327	-23.675	641.932	-21.054	595.331	-25.812
605.913	-23.676	642.114	-21.055	595.510	-25.815
607.183	-23.677	642.531	-21.055	595.708	-25.818
606.658	-23.678	642.938	-21.056	595.773	-25.821
605.557	-23.679	643.173	-21.056	595.957	-25.824
604.511	-23.680	643.254	-21.056	596.200	-25.827
603.533	-23.681	643.309	-21.057	596.356	-25.830
602.797	-23.682	643.368	-21.057	596.547	-25.833
602.768	-23.683	643.437	-21.058	596.671	-25.836
603.384	-23.684	643.527	-21.058	596.809	-25.839
605.338	-23.685	643.619	-21.059	597.058	-25.842
607.679	-23.686	643.686	-21.059	597.238	-25.845
608.151	-23.687	643.829	-21.059	597.334	-25.848
607.527	-23.688	644.062	-21.060	597.521	-25.851

606.414	-23.689	644.208	-21.060	597.711	-25.854
605.397	-23.690	644.333	-21.061	597.850	-25.857
605.111	-23.691	644.520	-21.061	597.974	-25.860
604.879	-23.692	644.641	-21.061	598.180	-25.863
604.729	-23.693	644.836	-21.062	598.397	-25.866
605.429	-23.694	645.090	-21.062	598.541	-25.869
606.403	-23.695	645.258	-21.063	598.709	-25.872
607.048	-23.696	645.389	-21.063	598.901	-25.874
608.229	-23.697	645.527	-21.063	599.067	-25.877
609.550	-23.698	645.667	-21.064	599.223	-25.880
610.096	-23.699	645.812	-21.064	599.358	-25.883
609.709	-23.700	645.974	-21.064	599.501	-25.886
608.805	-23.701	646.149	-21.065	599.644	-25.889
608.202	-23.702	646.339	-21.065	599.791	-25.891
608.307	-23.702	646.488	-21.066	599.942	-25.894
607.769	-23.703	646.626	-21.066	600.098	-25.897
606.947	-23.704	646.791	-21.066	600.321	-25.900
607.536	-23.705	646.943	-21.067	600.482	-25.903
608.691	-23.706	647.101	-21.067	600.612	-25.905
609.706	-23.707	647.286	-21.068	600.793	-25.908
610.075	-23.708	647.449	-21.068	600.923	-25.911
610.017	-23.708	647.591	-21.068	601.258	-25.914
610.774	-23.709	647.778	-21.069	601.741	-25.916
612.112	-23.710	647.973	-21.069	601.956	-25.919
613.012	-23.711	648.185	-21.070	601.998	-25.922
613.320	-23.712	648.336	-21.070	602.057	-25.924
613.041	-23.713	648.458	-21.070	602.135	-25.927
612.214	-23.713	648.648	-21.071	602.189	-25.930
612.549	-23.714	648.827	-21.071	602.220	-25.933
613.686	-23.715	648.988	-21.072	602.315	-25.935

613.899	-23.716	649.072	-21.072	602.483	-25.938
613.205	-23.716	649.207	-21.072	602.636	-25.941
611.962	-23.717	649.417	-21.073	602.795	-25.943
610.875	-23.718	649.567	-21.073	603.008	-25.946
610.266	-23.719	649.732	-21.074	603.182	-25.948
610.205	-23.719	649.892	-21.074	603.310	-25.951
610.197	-23.720	650.002	-21.075	603.422	-25.954
611.679	-23.721	650.169	-21.075	603.588	-25.956
613.832	-23.721	650.368	-21.076	603.848	-25.959
614.548	-23.722	650.572	-21.076	604.116	-25.962
613.649	-23.723	650.770	-21.076	604.284	-25.964
613.110	-23.723	650.891	-21.077	604.398	-25.967
614.018	-23.724	651.023	-21.077	604.536	-25.969
615.435	-23.725	651.162	-21.078	604.680	-25.972
615.356	-23.725	651.344	-21.078	604.800	-25.975
613.969	-23.726	651.553	-21.079	604.911	-25.977
612.586	-23.727	651.705	-21.079	605.088	-25.980
611.991	-23.727	651.798	-21.080	605.282	-25.982
612.209	-23.728	651.971	-21.080	605.435	-25.985
613.452	-23.728	652.170	-21.081	605.573	-25.988
614.748	-23.729	652.319	-21.082	605.728	-25.990
614.642	-23.730	652.501	-21.082	605.922	-25.993
614.504	-23.730	652.654	-21.083	606.141	-25.995
615.095	-23.731	652.825	-21.083	606.311	-25.998
616.512	-23.731	653.010	-21.084	606.463	-26.000
617.244	-23.732	653.174	-21.084	606.668	-26.003
615.695	-23.732	653.319	-21.085	606.850	-26.006
613.966	-23.733	653.471	-21.085	606.990	-26.008
613.523	-23.733	653.632	-21.086	607.128	-26.011
613.903	-23.734	653.742	-21.086	607.309	-26.013

614.405	-23.735	653.841	-21.087	607.482	-26.016
615.433	-23.735	653.989	-21.088	607.628	-26.018
617.651	-23.736	654.165	-21.088	607.780	-26.021
619.496	-23.736	654.389	-21.089	607.911	-26.024
619.905	-23.737	654.592	-21.089	608.038	-26.026
619.258	-23.737	654.730	-21.090	608.197	-26.029
618.905	-23.738	654.900	-21.090	608.393	-26.031
618.249	-23.738	655.126	-21.091	608.599	-26.034
617.671	-23.739	655.292	-21.092	608.762	-26.036
616.732	-23.739	655.411	-21.092	608.885	-26.039
616.228	-23.740	655.537	-21.093	609.045	-26.042
616.945	-23.740	655.681	-21.093	609.222	-26.044
618.689	-23.741	655.803	-21.094	609.373	-26.047
618.576	-23.741	655.910	-21.095	609.514	-26.049
616.920	-23.742	656.049	-21.095	609.725	-26.052
617.320	-23.742	656.199	-21.096	609.885	-26.054
618.537	-23.743	656.340	-21.096	609.977	-26.057
620.146	-23.743	656.545	-21.097	610.116	-26.060
621.403	-23.743	656.746	-21.097	610.255	-26.062
620.669	-23.744	656.907	-21.098	610.432	-26.065
619.107	-23.744	657.082	-21.099	610.664	-26.067
618.495	-23.745	657.290	-21.099	610.847	-26.070
619.487	-23.745	657.500	-21.100	610.976	-26.073
621.189	-23.746	657.673	-21.100	611.104	-26.075
622.405	-23.746	657.858	-21.101	611.243	-26.078
622.405	-23.747	658.022	-21.101	611.461	-26.080
621.157	-23.747	658.195	-21.102	611.685	-26.083
619.708	-23.748	658.386	-21.102	611.811	-26.086
619.681	-23.748	658.540	-21.103	611.980	-26.088
620.817	-23.749	658.664	-21.104	612.129	-26.091

622.073	-23.749	658.812	-21.104	612.197	-26.093
622.953	-23.750	658.948	-21.105	612.390	-26.096
622.621	-23.750	659.059	-21.105	612.648	-26.099
620.880	-23.751	659.201	-21.106	612.834	-26.101
619.593	-23.751	659.361	-21.106	612.973	-26.104
621.362	-23.752	659.487	-21.107	613.125	-26.107
623.426	-23.752	659.606	-21.107	613.321	-26.109
622.428	-23.753	659.796	-21.108	613.498	-26.112
620.594	-23.753	660.016	-21.108	613.621	-26.114
620.026	-23.754	660.192	-21.109	613.739	-26.117
620.370	-23.755	660.368	-21.109	613.898	-26.120
621.617	-23.755	660.580	-21.110	614.092	-26.122
623.859	-23.756	660.756	-21.110	614.236	-26.125
624.109	-23.756	660.912	-21.111	614.375	-26.127
622.039	-23.757	661.079	-21.111	614.516	-26.130
621.755	-23.758	661.185	-21.112	614.619	-26.133
623.383	-23.758	661.265	-21.112	614.748	-26.135
624.810	-23.759	661.477	-21.113	614.838	-26.138
624.086	-23.759	661.723	-21.113	614.975	-26.140
623.648	-23.760	661.833	-21.114	615.196	-26.143
625.413	-23.761	661.968	-21.114	615.360	-26.146
625.999	-23.761	662.204	-21.115	615.513	-26.148
624.455	-23.762	662.381	-21.115	615.781	-26.151
623.285	-23.763	662.548	-21.116	616.063	-26.153
624.399	-23.764	662.720	-21.116	616.241	-26.156
626.632	-23.764	662.885	-21.117	616.376	-26.159
627.470	-23.765	662.997	-21.117	616.562	-26.161
626.242	-23.766	663.120	-21.117	616.735	-26.164
624.645	-23.767	663.309	-21.118	616.861	-26.166
624.889	-23.767	663.467	-21.118	617.012	-26.169

626.971	-23.768	663.605	-21.119	617.192	-26.171
627.127	-23.769	663.778	-21.119	617.314	-26.174
625.552	-23.770	664.005	-21.120	617.445	-26.177
626.236	-23.770	664.194	-21.120	617.594	-26.179
627.753	-23.771	664.352	-21.120	617.715	-26.182
627.371	-23.772	664.510	-21.121	617.837	-26.184
625.897	-23.773	664.689	-21.121	618.002	-26.187
625.319	-23.774	664.834	-21.122	618.219	-26.189
627.166	-23.775	664.966	-21.122	618.465	-26.192
627.869	-23.776	665.138	-21.122	618.623	-26.194
626.671	-23.776	665.295	-21.123	618.754	-26.197
627.646	-23.777	665.436	-21.123	618.900	-26.199
628.742	-23.778	665.646	-21.124	619.049	-26.202
627.460	-23.779	665.847	-21.124	619.232	-26.204
627.046	-23.780	666.011	-21.124	619.376	-26.207
629.005	-23.781	666.217	-21.125	619.552	-26.209
629.568	-23.782	666.325	-21.125	619.732	-26.212
627.896	-23.783	666.393	-21.125	619.888	-26.214
628.001	-23.784	666.545	-21.126	620.040	-26.217
629.183	-23.785	666.856	-21.126	620.266	-26.219
628.651	-23.786	667.304	-21.126	620.452	-26.222
629.445	-23.787	667.606	-21.127	620.589	-26.224
630.956	-23.788	667.710	-21.127	620.778	-26.227
629.883	-23.789	667.792	-21.127	620.946	-26.229
629.701	-23.790	667.887	-21.128	621.114	-26.232
631.182	-23.791	667.933	-21.128	621.288	-26.234
630.225	-23.792	667.976	-21.128	621.418	-26.237
628.415	-23.793	668.067	-21.129	621.544	-26.239
628.886	-23.794	668.163	-21.129	621.727	-26.241
630.798	-23.795	668.342	-21.129	621.947	-26.244

630.641	-23.796	668.487	-21.130	622.091	-26.246
629.312	-23.797	668.618	-21.130	622.236	-26.249
630.770	-23.798	668.810	-21.130	622.386	-26.251
631.275	-23.799	668.980	-21.131	622.472	-26.254
630.022	-23.800	669.139	-21.131	622.625	-26.256
631.334	-23.801	669.291	-21.131	622.846	-26.258
632.162	-23.802	669.479	-21.131	622.973	-26.261
630.970	-23.803	669.655	-21.132	623.079	-26.263
631.937	-23.804	669.810	-21.132	623.270	-26.265
631.983	-23.806	670.029	-21.132	623.465	-26.268
631.534	-23.807	670.208	-21.133	623.689	-26.270
633.811	-23.808	670.299	-21.133	623.849	-26.273
633.601	-23.809	670.384	-21.133	623.948	-26.275
632.918	-23.810	670.478	-21.133	624.134	-26.277
633.483	-23.811	670.653	-21.134	624.295	-26.280
632.346	-23.812	670.854	-21.134	624.398	-26.282
633.193	-23.813	671.011	-21.134	624.619	-26.284
633.719	-23.814	671.129	-21.135	624.950	-26.287
633.829	-23.815	671.276	-21.135	625.333	-26.289
634.412	-23.817	671.512	-21.135	625.629	-26.291
634.396	-23.818	671.699	-21.135	625.762	-26.294
634.351	-23.819	671.910	-21.136	625.804	-26.296
633.718	-23.820	672.110	-21.136	625.784	-26.298
634.731	-23.821	672.225	-21.136	625.822	-26.301
634.043	-23.822	672.410	-21.137	625.951	-26.303
633.394	-23.823	672.613	-21.137	626.054	-26.305
635.299	-23.825	672.752	-21.137	626.163	-26.308
635.206	-23.826	672.943	-21.138	626.328	-26.310
635.320	-23.827	673.106	-21.138	626.432	-26.312
635.742	-23.828	673.204	-21.138	626.609	-26.315

636.328	-23.829	673.387	-21.139	626.923	-26.317
636.376	-23.830	673.557	-21.139	627.150	-26.319
635.272	-23.831	673.704	-21.139	627.221	-26.321
636.894	-23.832	673.853	-21.139	627.314	-26.324
638.252	-23.834	673.989	-21.140	627.513	-26.326
636.853	-23.835	674.137	-21.140	627.761	-26.328
636.474	-23.836	674.306	-21.140	627.997	-26.331
636.802	-23.837	674.516	-21.141	628.104	-26.333
636.506	-23.838	674.657	-21.141	628.176	-26.335
636.760	-23.839	674.783	-21.142	628.334	-26.337
636.880	-23.841	674.968	-21.142	628.535	-26.340
637.688	-23.842	675.117	-21.142	628.700	-26.342
637.677	-23.843	675.241	-21.143	628.794	-26.344
637.783	-23.844	675.425	-21.143	628.951	-26.346
637.567	-23.845	675.588	-21.143	629.144	-26.349
638.312	-23.846	675.716	-21.144	629.224	-26.351
639.124	-23.847	675.928	-21.144	629.319	-26.353
639.034	-23.849	676.155	-21.144	629.562	-26.356
638.593	-23.850	676.321	-21.145	629.848	-26.358
638.924	-23.851	676.459	-21.145	630.010	-26.360
639.314	-23.852	676.620	-21.145	630.134	-26.362
639.130	-23.853	676.754	-21.146	630.338	-26.365
639.366	-23.854	676.898	-21.146	630.530	-26.367
638.627	-23.855	677.076	-21.147	630.653	-26.369
638.774	-23.857	677.260	-21.147	630.821	-26.371
639.245	-23.858	677.449	-21.147	630.979	-26.374
639.981	-23.859	677.588	-21.148	631.076	-26.376
639.793	-23.860	677.713	-21.148	631.214	-26.378
639.506	-23.861	677.853	-21.149	631.392	-26.380
639.468	-23.862	677.990	-21.149	631.518	-26.383

640.668	-23.863	678.163	-21.149	631.667	-26.385
641.515	-23.865	678.354	-21.150	631.887	-26.387
639.870	-23.866	678.537	-21.150	632.020	-26.390
640.021	-23.867	678.690	-21.150	632.176	-26.392
640.613	-23.868	678.832	-21.151	632.382	-26.394
640.834	-23.869	679.018	-21.151	632.551	-26.396
641.155	-23.870	679.249	-21.152	632.716	-26.399
641.609	-23.871	679.457	-21.152	632.826	-26.401
642.701	-23.873	679.572	-21.152	632.946	-26.403
642.700	-23.874	679.680	-21.153	633.099	-26.406
643.109	-23.875	679.853	-21.153	633.261	-26.408
643.169	-23.876	680.059	-21.153	633.396	-26.410
643.333	-23.877	680.221	-21.154	633.548	-26.412
644.084	-23.878	680.351	-21.154	633.759	-26.415
643.783	-23.880	680.478	-21.155	633.956	-26.417
643.864	-23.881	680.602	-21.155	634.185	-26.419
644.022	-23.882	680.707	-21.155	634.369	-26.422
643.994	-23.883	680.833	-21.156	634.556	-26.424
643.371	-23.884	680.973	-21.156	634.711	-26.426
643.417	-23.885	681.133	-21.156	634.813	-26.429
643.389	-23.886	681.344	-21.157	634.972	-26.431
643.733	-23.888	681.542	-21.157	635.164	-26.433
645.179	-23.889	681.718	-21.157	635.301	-26.436
644.837	-23.890	681.917	-21.158	635.429	-26.438
644.396	-23.891	682.176	-21.158	635.616	-26.440
644.808	-23.892	682.385	-21.158	635.786	-26.443
644.743	-23.893	682.526	-21.158	635.959	-26.445
645.165	-23.895	682.715	-21.159	636.133	-26.447
645.047	-23.896	682.896	-21.159	636.287	-26.450
644.240	-23.897	683.008	-21.159	636.455	-26.452

645.210	-23.898	683.148	-21.160	636.630	-26.454
645.471	-23.899	683.336	-21.160	636.774	-26.456
645.677	-23.901	683.523	-21.160	636.919	-26.459
645.635	-23.902	683.632	-21.160	637.073	-26.461
644.926	-23.903	683.685	-21.160	637.272	-26.463
645.180	-23.904	683.799	-21.161	637.472	-26.466
645.655	-23.905	683.974	-21.161	637.621	-26.468
646.360	-23.907	684.137	-21.161	637.719	-26.470
646.600	-23.908	684.310	-21.161	637.833	-26.473
646.662	-23.909	684.504	-21.162	637.969	-26.475
646.629	-23.910	684.614	-21.162	638.057	-26.477
646.791	-23.912	684.807	-21.162	638.232	-26.480
646.993	-23.913	685.075	-21.162	638.396	-26.482
647.297	-23.914	685.231	-21.162	638.503	-26.484
648.176	-23.916	685.362	-21.162	638.695	-26.487
647.995	-23.917	685.573	-21.163	638.927	-26.489
647.812	-23.918	685.757	-21.163	639.153	-26.491
647.773	-23.920	685.880	-21.163	639.338	-26.493
647.603	-23.921	686.059	-21.163	639.480	-26.496
648.501	-23.922	686.216	-21.163	639.651	-26.498
648.840	-23.924	686.315	-21.163	639.846	-26.500
648.882	-23.925	686.488	-21.163	640.000	-26.502
648.913	-23.926	686.695	-21.163	640.180	-26.505
648.793	-23.928	686.886	-21.163	640.394	-26.507
650.061	-23.929	687.059	-21.163	640.539	-26.509
650.072	-23.930	687.217	-21.164	640.664	-26.511
649.743	-23.932	687.383	-21.164	640.798	-26.514
650.527	-23.933	687.543	-21.164	640.910	-26.516
650.229	-23.935	687.699	-21.164	641.035	-26.518
650.081	-23.936	687.883	-21.164	641.181	-26.520

649.779	-23.937	688.035	-21.164	641.309	-26.522
649.502	-23.939	688.147	-21.164	641.459	-26.525
650.161	-23.940	688.328	-21.164	641.618	-26.527
650.807	-23.942	688.511	-21.164	641.770	-26.529
651.138	-23.943	688.697	-21.164	642.009	-26.531
650.947	-23.945	688.869	-21.164	642.265	-26.533
650.784	-23.946	689.023	-21.164	642.466	-26.535
651.373	-23.948	689.134	-21.164	642.598	-26.538
651.998	-23.949	689.246	-21.164	642.722	-26.540
651.808	-23.951	689.441	-21.164	642.874	-26.542
651.547	-23.952	689.661	-21.164	643.080	-26.544
651.652	-23.954	689.884	-21.163	643.270	-26.546
651.609	-23.955	690.038	-21.163	643.410	-26.548
652.377	-23.957	690.168	-21.163	643.563	-26.550
652.781	-23.958	690.296	-21.163	643.698	-26.552
652.738	-23.960	690.432	-21.163	643.878	-26.554
652.887	-23.961	690.605	-21.163	644.102	-26.557
652.927	-23.963	690.774	-21.163	644.235	-26.559
653.368	-23.965	690.976	-21.163	644.380	-26.561
653.790	-23.966	691.149	-21.163	644.576	-26.563
654.044	-23.968	691.323	-21.163	644.753	-26.565
653.261	-23.969	691.700	-21.163	644.958	-26.567
653.046	-23.971	692.099	-21.162	645.114	-26.569
654.106	-23.973	692.304	-21.162	645.256	-26.571
654.911	-23.974	692.351	-21.162	645.407	-26.573
654.398	-23.976	692.381	-21.162	645.583	-26.575
653.417	-23.978	692.475	-21.162	645.742	-26.577
653.640	-23.979	692.515	-21.162	645.858	-26.579
654.294	-23.981	692.538	-21.162	646.011	-26.581
654.911	-23.982	692.640	-21.161	646.144	-26.583

655.172	-23.984	692.821	-21.161	646.325	-26.585
655.375	-23.986	693.023	-21.161	646.534	-26.587
655.208	-23.987	693.137	-21.161	646.662	-26.589
655.261	-23.989	693.256	-21.161	646.824	-26.591
655.608	-23.991	693.446	-21.161	647.010	-26.593
655.701	-23.992	693.630	-21.161	647.151	-26.595
655.765	-23.994	693.790	-21.160	647.288	-26.597
655.928	-23.996	693.937	-21.160	647.472	-26.599
656.203	-23.997	694.100	-21.160	647.681	-26.601
656.106	-23.999	694.277	-21.160	647.858	-26.602
656.119	-24.001	694.481	-21.160	647.959	-26.604
656.792	-24.002	694.674	-21.159	648.085	-26.606
657.360	-24.004	694.837	-21.159	648.305	-26.608
657.215	-24.006	694.967	-21.159	648.687	-26.610
657.376	-24.007	695.126	-21.159	649.080	-26.612
657.403	-24.009	695.300	-21.159	649.274	-26.614
656.876	-24.011	695.434	-21.158	649.381	-26.616
657.180	-24.012	695.610	-21.158	649.468	-26.618
658.090	-24.014	695.714	-21.158	649.553	-26.619
657.981	-24.016	695.854	-21.158	649.568	-26.621
657.661	-24.017	696.028	-21.158	649.605	-26.623
658.036	-24.019	696.189	-21.158	649.687	-26.625
658.930	-24.020	696.404	-21.157	649.881	-26.627
659.058	-24.022	696.620	-21.157	650.070	-26.629
658.511	-24.024	696.793	-21.157	650.208	-26.631
658.671	-24.025	696.944	-21.157	650.302	-26.632
658.832	-24.027	697.105	-21.157	650.461	-26.634
659.489	-24.029	697.309	-21.157	650.695	-26.636
660.442	-24.030	697.495	-21.156	650.919	-26.638
660.535	-24.032	697.616	-21.156	651.042	-26.640

660.094	-24.034	697.752	-21.156	651.206	-26.641
660.323	-24.035	697.840	-21.156	651.458	-26.643
661.068	-24.037	697.981	-21.156	651.608	-26.645
660.890	-24.038	698.157	-21.156	651.708	-26.647
660.663	-24.040	698.317	-21.156	651.882	-26.649
660.583	-24.041	698.512	-21.155	652.094	-26.650
660.601	-24.043	698.666	-21.155	652.233	-26.652
660.723	-24.045	698.827	-21.155	652.319	-26.654
660.886	-24.046	699.060	-21.155	652.481	-26.656
661.804	-24.048	699.212	-21.155	652.657	-26.658
661.863	-24.049	699.341	-21.155	652.803	-26.659
661.141	-24.051	699.501	-21.155	652.906	-26.661
661.693	-24.052	699.605	-21.155	653.064	-26.663
662.523	-24.054	699.797	-21.155	653.269	-26.665
662.243	-24.055	700.023	-21.155	653.412	-26.666
661.872	-24.057	700.121	-21.154	653.611	-26.668
662.450	-24.058	700.229	-21.154	653.798	-26.670
662.789	-24.060	700.414	-21.154	653.987	-26.672
662.913	-24.061	700.553	-21.154	654.190	-26.674
663.580	-24.063	700.743	-21.154	654.384	-26.675
663.811	-24.064	700.961	-21.154	654.591	-26.677
663.541	-24.066	701.126	-21.154	654.715	-26.679
663.518	-24.067	701.273	-21.154	654.777	-26.681
664.002	-24.069	701.420	-21.154	654.849	-26.683
664.065	-24.070	701.593	-21.154	654.975	-26.684
663.956	-24.072	701.712	-21.154	655.174	-26.686
664.315	-24.073	701.789	-21.154	655.368	-26.688
664.670	-24.074	702.026	-21.154	655.536	-26.690
664.811	-24.076	702.293	-21.154	655.704	-26.692
664.705	-24.077	702.427	-21.154	655.863	-26.694

665.030	-24.079	702.549	-21.154	656.004	-26.695
664.997	-24.080	702.705	-21.154	656.198	-26.697
664.587	-24.081	702.847	-21.154	656.382	-26.699
664.917	-24.083	702.992	-21.154	656.553	-26.701
665.962	-24.084	703.213	-21.154	656.728	-26.703
666.205	-24.085	703.462	-21.154	656.838	-26.705
665.617	-24.087	703.650	-21.154	656.964	-26.706
665.929	-24.088	703.770	-21.154	657.081	-26.708
666.087	-24.089	703.905	-21.154	657.287	-26.710
666.090	-24.091	704.056	-21.154	657.454	-26.712
666.772	-24.092	704.264	-21.155	657.577	-26.714
666.878	-24.093	704.436	-21.155	657.818	-26.716
666.273	-24.095	704.481	-21.155	658.022	-26.718
666.789	-24.096	704.603	-21.155	658.182	-26.719
667.442	-24.097	704.847	-21.155	658.359	-26.721
667.120	-24.098	704.961	-21.155	658.540	-26.723
667.053	-24.100	705.052	-21.155	658.720	-26.725
667.024	-24.101	705.210	-21.155	658.866	-26.727
667.208	-24.102	705.303	-21.155	658.979	-26.729
667.920	-24.103	705.487	-21.155	659.184	-26.731
668.109	-24.105	705.667	-21.155	659.314	-26.733
668.170	-24.106	705.808	-21.155	659.441	-26.735
668.687	-24.107	706.013	-21.155	659.628	-26.737
668.849	-24.108	706.189	-21.155	659.795	-26.739
669.133	-24.109	706.356	-21.155	659.939	-26.740
669.602	-24.111	706.563	-21.155	660.118	-26.742
669.187	-24.112	706.732	-21.155	660.315	-26.744
669.080	-24.113	706.936	-21.155	660.483	-26.746
670.125	-24.114	707.160	-21.155	660.634	-26.748
670.514	-24.115	707.291	-21.156	660.791	-26.750

669.687	-24.116	707.449	-21.156	660.896	-26.752
669.685	-24.117	707.629	-21.156	661.020	-26.754
669.662	-24.119	707.791	-21.156	661.198	-26.756
669.870	-24.120	707.940	-21.156	661.386	-26.758
670.679	-24.121	708.082	-21.156	661.498	-26.760
670.684	-24.122	708.203	-21.156	661.574	-26.762
670.742	-24.123	708.346	-21.156	661.703	-26.764
670.989	-24.124	708.508	-21.156	661.888	-26.765
671.069	-24.125	708.624	-21.156	662.021	-26.767
671.136	-24.126	708.690	-21.156	662.175	-26.769
671.665	-24.128	708.803	-21.156	662.336	-26.771
671.698	-24.129	709.018	-21.156	662.486	-26.773
671.527	-24.130	709.259	-21.156	662.726	-26.775
672.115	-24.131	709.396	-21.156	662.940	-26.777
672.638	-24.132	709.619	-21.156	663.160	-26.779
672.640	-24.133	709.892	-21.156	663.372	-26.781
672.338	-24.134	710.071	-21.156	663.534	-26.783
672.871	-24.135	710.282	-21.156	663.708	-26.785
673.378	-24.136	710.443	-21.156	663.895	-26.786
672.797	-24.137	710.562	-21.156	664.044	-26.788
672.796	-24.138	710.705	-21.156	664.166	-26.790
673.126	-24.140	710.879	-21.156	664.343	-26.792
673.024	-24.141	711.028	-21.156	664.447	-26.794
672.850	-24.142	711.160	-21.156	664.525	-26.796
672.785	-24.143	711.336	-21.156	664.616	-26.798
673.333	-24.144	711.509	-21.156	664.779	-26.800
674.100	-24.145	711.690	-21.156	665.000	-26.801
674.149	-24.146	711.887	-21.155	665.157	-26.803
674.323	-24.147	712.050	-21.155	665.369	-26.805
674.660	-24.148	712.236	-21.155	665.591	-26.807

674.612	-24.149	712.372	-21.155	665.762	-26.809
674.387	-24.150	712.489	-21.155	665.921	-26.811
674.550	-24.152	712.707	-21.155	666.105	-26.812
675.284	-24.153	712.927	-21.155	666.300	-26.814
675.243	-24.154	713.046	-21.155	666.461	-26.816
674.771	-24.155	713.138	-21.155	666.618	-26.818
675.115	-24.156	713.300	-21.155	666.785	-26.820
675.660	-24.157	713.492	-21.155	666.938	-26.822
675.958	-24.158	713.643	-21.155	667.056	-26.823
676.441	-24.159	713.813	-21.154	667.258	-26.825
676.378	-24.161	713.970	-21.154	667.430	-26.827
675.910	-24.162	714.106	-21.154	667.514	-26.829
676.069	-24.163	714.278	-21.154	667.682	-26.831
676.301	-24.164	714.444	-21.154	667.859	-26.832
676.611	-24.165	714.613	-21.154	668.009	-26.834
676.824	-24.166	714.804	-21.154	668.198	-26.836
676.947	-24.167	714.949	-21.154	668.336	-26.838
677.362	-24.168	715.075	-21.154	668.478	-26.839
677.283	-24.170	715.219	-21.153	668.639	-26.841
677.550	-24.171	715.377	-21.153	668.830	-26.843
677.682	-24.172	715.529	-21.153	669.053	-26.844
677.758	-24.173	715.681	-21.153	669.182	-26.846
677.787	-24.174	716.054	-21.153	669.293	-26.848
677.725	-24.175	716.415	-21.153	669.497	-26.849
678.160	-24.177	716.637	-21.152	669.726	-26.851
678.685	-24.178	716.805	-21.152	669.866	-26.853
679.111	-24.179	716.908	-21.152	670.029	-26.854
678.831	-24.180	717.002	-21.152	670.231	-26.856
678.742	-24.181	717.040	-21.152	670.387	-26.858
678.793	-24.183	717.111	-21.152	670.520	-26.859

679.078	-24.184	717.106	-21.151	670.655	-26.861
679.633	-24.185	717.197	-21.151	670.818	-26.863
679.771	-24.186	717.433	-21.151	670.999	-26.864
679.674	-24.187	717.612	-21.151	671.151	-26.866
680.113	-24.189	717.765	-21.151	671.353	-26.867
680.038	-24.190	717.912	-21.151	671.556	-26.869
679.994	-24.191	718.101	-21.150	671.685	-26.870
680.779	-24.192	718.322	-21.150	671.797	-26.872
680.877	-24.193	718.536	-21.150	671.919	-26.874
680.861	-24.195	718.720	-21.150	672.192	-26.875
680.970	-24.196	718.872	-21.150	672.680	-26.877
681.055	-24.197	719.007	-21.149	673.005	-26.878
681.386	-24.198	719.171	-21.149	673.071	-26.880
681.742	-24.200	719.388	-21.149	673.141	-26.881
681.785	-24.201	719.584	-21.149	673.211	-26.883
682.004	-24.202	719.703	-21.149	673.290	-26.884
682.059	-24.203	719.821	-21.148	673.375	-26.886
682.124	-24.205	719.900	-21.148	673.438	-26.887
681.763	-24.206	720.068	-21.148	673.520	-26.889
681.628	-24.207	720.254	-21.148	673.652	-26.890
682.365	-24.208	720.388	-21.148	673.819	-26.891
682.545	-24.210	720.573	-21.148	673.962	-26.893
683.088	-24.211	720.714	-21.147	674.114	-26.894
683.300	-24.212	720.858	-21.147	674.343	-26.896
683.310	-24.213	721.059	-21.147	674.613	-26.897
683.698	-24.215	721.233	-21.147	674.843	-26.898
683.850	-24.216	721.367	-21.147	675.010	-26.900
683.844	-24.217	721.542	-21.146	675.105	-26.901
684.092	-24.218	721.755	-21.146	675.213	-26.903
684.248	-24.220	721.935	-21.146	675.415	-26.904

684.496	-24.221	722.076	-21.146	675.636	-26.905
684.970	-24.222	722.241	-21.146	675.791	-26.907
685.146	-24.223	722.411	-21.145	675.911	-26.908
685.277	-24.225	722.539	-21.145	675.999	-26.909
685.113	-24.226	722.654	-21.145	676.114	-26.911
685.424	-24.227	722.776	-21.145	676.257	-26.912
685.685	-24.228	722.915	-21.145	676.419	-26.913
685.499	-24.229	723.049	-21.145	676.609	-26.915
685.479	-24.231	723.196	-21.144	676.756	-26.916
685.694	-24.232	723.439	-21.144	676.908	-26.917
685.910	-24.233	723.652	-21.144	677.091	-26.919
686.058	-24.234	723.792	-21.144	677.287	-26.920
686.328	-24.236	723.958	-21.144	677.481	-26.921
686.554	-24.237	724.135	-21.144	677.671	-26.922
686.578	-24.238	724.299	-21.144	677.886	-26.924
686.673	-24.239	724.435	-21.143	678.057	-26.925
686.581	-24.241	724.597	-21.143	678.176	-26.926
686.786	-24.242	724.680	-21.143	678.302	-26.928
687.104	-24.243	724.816	-21.143	678.437	-26.929
686.777	-24.244	725.069	-21.143	678.560	-26.930
687.140	-24.245	725.219	-21.143	678.678	-26.931
687.532	-24.247	725.348	-21.143	678.863	-26.933
687.750	-24.248	725.557	-21.142	679.077	-26.934
687.979	-24.249	725.747	-21.142	679.249	-26.935
687.877	-24.250	725.881	-21.142	679.470	-26.936
688.297	-24.251	726.097	-21.142	679.669	-26.938
688.520	-24.253	726.325	-21.142	679.781	-26.939
688.610	-24.254	726.483	-21.142	679.945	-26.940
688.843	-24.255	726.595	-21.142	680.123	-26.941
688.962	-24.256	726.754	-21.142	680.247	-26.943

689.005	-24.257	726.975	-21.141	680.408	-26.944
689.242	-24.258	727.095	-21.141	680.551	-26.945
689.445	-24.260	727.177	-21.141	680.634	-26.946
689.252	-24.261	727.370	-21.141	680.769	-26.948
689.376	-24.262	727.530	-21.141	680.943	-26.949
689.641	-24.263	727.698	-21.141	681.079	-26.950
690.110	-24.264	727.953	-21.141	681.242	-26.951
690.324	-24.265	728.144	-21.141	681.477	-26.953
690.396	-24.266	728.296	-21.141	681.705	-26.954
690.490	-24.267	728.437	-21.141	681.887	-26.955
690.954	-24.269	728.527	-21.140	682.026	-26.956
691.256	-24.270	728.651	-21.140	682.160	-26.958
691.186	-24.271	728.823	-21.140	682.355	-26.959
691.132	-24.272	729.021	-21.140	682.560	-26.960
691.188	-24.273	729.167	-21.140	682.716	-26.961
691.478	-24.274	729.228	-21.140	682.841	-26.962
692.111	-24.275	729.370	-21.140	682.971	-26.964
692.101	-24.276	729.535	-21.140	683.165	-26.965
691.952	-24.277	729.696	-21.139	683.337	-26.966
692.541	-24.278	729.889	-21.139	683.471	-26.967
692.691	-24.280	729.967	-21.139	683.601	-26.968
692.524	-24.281	730.070	-21.139	683.792	-26.970
692.719	-24.282	730.309	-21.139	684.035	-26.971
692.959	-24.283	730.483	-21.139	684.162	-26.972
693.027	-24.284	730.659	-21.139	684.275	-26.973
693.545	-24.285	730.872	-21.138	684.392	-26.974
693.801	-24.286	731.055	-21.138	684.538	-26.975
693.758	-24.287	731.292	-21.138	684.665	-26.977
693.918	-24.288	731.507	-21.138	684.843	-26.978
694.078	-24.289	731.662	-21.138	685.005	-26.979

694.201	-24.290	731.769	-21.138	685.144	-26.980
694.294	-24.291	731.935	-21.137	685.257	-26.981
694.722	-24.292	732.174	-21.137	685.378	-26.982
695.217	-24.293	732.290	-21.137	685.524	-26.983
695.355	-24.294	732.324	-21.137	685.658	-26.985
695.143	-24.295	732.448	-21.137	685.847	-26.986
695.097	-24.296	732.661	-21.136	686.039	-26.987
695.305	-24.298	732.857	-21.136	686.215	-26.988
695.392	-24.299	733.036	-21.136	686.419	-26.989
695.471	-24.300	733.178	-21.136	686.580	-26.990
695.789	-24.301	733.290	-21.135	686.736	-26.991
695.967	-24.302	733.458	-21.135	687.021	-26.992
696.032	-24.303	733.672	-21.135	687.245	-26.993
696.160	-24.304	733.850	-21.135	687.337	-26.995
696.207	-24.305	734.023	-21.134	687.467	-26.996
696.290	-24.306	734.191	-21.134	687.670	-26.997
696.516	-24.307	734.375	-21.134	687.828	-26.998
696.610	-24.308	734.586	-21.134	687.983	-26.999
697.059	-24.309	734.750	-21.133	688.126	-27.000
697.439	-24.310	734.883	-21.133	688.197	-27.001
697.302	-24.311	735.060	-21.133	688.399	-27.002
697.485	-24.312	735.230	-21.132	688.559	-27.003
697.839	-24.313	735.284	-21.132	688.692	-27.004
697.638	-24.314	735.415	-21.132	688.882	-27.005
697.735	-24.315	735.674	-21.131	689.071	-27.006
698.149	-24.316	735.857	-21.131	689.258	-27.007
698.253	-24.317	736.018	-21.131	689.423	-27.008
698.546	-24.318	736.121	-21.130	689.572	-27.009
698.745	-24.319	736.238	-21.130	689.743	-27.010
698.601	-24.320	736.395	-21.130	689.992	-27.011

698.743	-24.321	736.584	-21.129	690.160	-27.012
698.996	-24.322	736.808	-21.129	690.278	-27.013
699.196	-24.323	736.990	-21.129	690.389	-27.013
699.704	-24.324	737.103	-21.128	690.510	-27.014
699.702	-24.325	737.252	-21.128	690.684	-27.015
699.528	-24.326	737.488	-21.128	690.899	-27.016
699.788	-24.327	737.666	-21.127	691.082	-27.017
700.144	-24.329	737.785	-21.127	691.200	-27.018
700.425	-24.330	737.896	-21.126	691.347	-27.019
700.437	-24.331	738.067	-21.126	691.530	-27.020
700.781	-24.332	738.214	-21.126	691.677	-27.021
701.052	-24.333	738.363	-21.125	691.822	-27.021
700.646	-24.334	738.564	-21.125	691.972	-27.022
700.731	-24.335	738.763	-21.124	692.147	-27.023
701.339	-24.336	738.933	-21.124	692.360	-27.024
701.563	-24.337	739.070	-21.123	692.551	-27.025
701.684	-24.338	739.219	-21.123	692.727	-27.026
701.834	-24.339	739.401	-21.123	692.879	-27.026
701.768	-24.340	739.565	-21.122	693.025	-27.027
701.859	-24.342	739.706	-21.122	693.149	-27.028
702.255	-24.343	739.864	-21.121	693.321	-27.029
702.411	-24.344	739.950	-21.121	693.546	-27.029
702.587	-24.345	740.049	-21.121	693.705	-27.030
702.896	-24.346	740.301	-21.120	693.873	-27.031
702.731	-24.347	740.751	-21.120	694.014	-27.032
702.669	-24.348	741.186	-21.119	694.118	-27.032
703.042	-24.349	741.351	-21.119	694.259	-27.033
703.242	-24.350	741.402	-21.118	694.420	-27.034
703.434	-24.352	741.489	-21.118	694.610	-27.035
703.617	-24.353	741.582	-21.118	694.793	-27.035

703.738	-24.354	741.636	-21.117	694.966	-27.036
703.706	-24.355	741.745	-21.117	695.098	-27.037
703.827	-24.356	741.877	-21.116	695.255	-27.037
704.230	-24.357	741.945	-21.116	695.346	-27.038
704.433	-24.358	742.094	-21.116	695.492	-27.039
704.722	-24.360	742.326	-21.115	695.967	-27.039
704.873	-24.361	742.483	-21.115	696.435	-27.040
705.032	-24.362	742.617	-21.114	696.658	-27.041
705.266	-24.363	742.803	-21.114	696.712	-27.041
705.443	-24.364	742.976	-21.113	696.767	-27.042
705.660	-24.365	743.159	-21.113	696.841	-27.043
705.729	-24.367	743.344	-21.113	696.881	-27.043
705.970	-24.368	743.508	-21.112	696.950	-27.044
706.049	-24.369	743.677	-21.112	697.094	-27.044
706.049	-24.370	743.858	-21.111	697.268	-27.045
706.097	-24.371	743.951	-21.111	697.395	-27.046
706.282	-24.372	744.082	-21.110	697.562	-27.046
706.618	-24.374	744.291	-21.110	697.747	-27.047
706.980	-24.375	744.431	-21.110	697.886	-27.047
706.926	-24.376	744.548	-21.109	698.018	-27.048
707.187	-24.377	744.683	-21.109	698.195	-27.049
707.147	-24.378	744.773	-21.109	698.447	-27.049
707.178	-24.380	744.893	-21.108	698.650	-27.050
707.426	-24.381	745.088	-21.108	698.762	-27.050
707.515	-24.382	745.300	-21.107	698.927	-27.051
707.716	-24.383	745.485	-21.107	699.095	-27.051
707.905	-24.384	745.655	-21.107	699.232	-27.052
708.161	-24.385	745.834	-21.106	699.406	-27.052
708.195	-24.387	745.976	-21.106	699.576	-27.053
708.288	-24.388	746.129	-21.106	699.713	-27.054

708.743	-24.389	746.320	-21.105	699.809	-27.054
709.030	-24.390	746.535	-21.105	699.937	-27.055
709.251	-24.391	746.730	-21.105	700.104	-27.055
709.286	-24.392	746.877	-21.104	700.216	-27.056
709.335	-24.394	746.999	-21.104	700.387	-27.056
709.415	-24.395	747.102	-21.104	700.609	-27.057
709.426	-24.396	747.208	-21.104	700.818	-27.057
709.611	-24.397	747.361	-21.103	700.995	-27.058
709.864	-24.398	747.551	-21.103	701.154	-27.058
710.107	-24.399	747.710	-21.103	701.309	-27.059
710.152	-24.400	747.902	-21.103	701.476	-27.060
710.383	-24.401	748.098	-21.102	701.680	-27.060
710.724	-24.403	748.270	-21.102	701.786	-27.061
710.769	-24.404	748.455	-21.102	701.888	-27.061
710.836	-24.405	748.559	-21.102	702.054	-27.062
711.103	-24.406	748.714	-21.101	702.223	-27.062
711.299	-24.407	748.916	-21.101	702.346	-27.063
711.474	-24.408	749.060	-21.101	702.526	-27.063
711.662	-24.409	749.208	-21.101	702.700	-27.064
711.801	-24.410	749.373	-21.101	702.824	-27.065
711.985	-24.411	749.503	-21.101	702.955	-27.065
712.020	-24.412	749.639	-21.100	703.126	-27.066
712.095	-24.413	749.787	-21.100	703.334	-27.066
712.225	-24.414	749.986	-21.100	703.531	-27.067
712.336	-24.415	750.201	-21.100	703.717	-27.067
712.614	-24.416	750.367	-21.100	703.879	-27.068
712.783	-24.417	750.546	-21.100	704.017	-27.069
713.016	-24.418	750.758	-21.100	704.190	-27.069
713.160	-24.419	750.944	-21.100	704.360	-27.070
713.323	-24.420	751.075	-21.099	704.424	-27.070

713.504	-24.421	751.227	-21.099	704.546	-27.071
713.610	-24.422	751.386	-21.099	704.811	-27.072
713.977	-24.423	751.496	-21.099	705.049	-27.072
714.111	-24.424	751.603	-21.099	705.201	-27.073
714.022	-24.425	751.765	-21.099	705.297	-27.073
714.198	-24.426	751.939	-21.099	705.431	-27.074
714.516	-24.427	752.151	-21.099	705.656	-27.075
714.715	-24.428	752.312	-21.099	705.849	-27.075
714.834	-24.429	752.454	-21.099	706.022	-27.076
715.006	-24.430	752.645	-21.099	706.230	-27.076
715.231	-24.431	752.840	-21.099	706.366	-27.077
715.345	-24.432	753.008	-21.099	706.516	-27.078
715.591	-24.433	753.173	-21.099	706.645	-27.078
715.807	-24.434	753.355	-21.099	706.736	-27.079
715.730	-24.435	753.474	-21.099	706.885	-27.080
715.883	-24.435	753.568	-21.099	707.047	-27.080
716.186	-24.436	753.739	-21.099	707.159	-27.081
716.329	-24.437	753.913	-21.099	707.297	-27.082
716.511	-24.438	753.994	-21.099	707.522	-27.082
716.891	-24.439	754.072	-21.099	707.726	-27.083
717.176	-24.440	754.248	-21.099	707.855	-27.084
717.174	-24.441	754.437	-21.099	708.008	-27.084
717.286	-24.441	754.615	-21.099	708.207	-27.085
717.608	-24.442	754.812	-21.099	708.375	-27.086
717.698	-24.443	754.983	-21.099	708.496	-27.086
717.622	-24.444	755.107	-21.099	708.658	-27.087
717.719	-24.445	755.265	-21.099	708.795	-27.088
717.953	-24.446	755.506	-21.099	708.904	-27.088
718.128	-24.446	755.716	-21.099	709.028	-27.089
718.290	-24.447	755.902	-21.099	709.184	-27.090

718.462	-24.448	756.071	-21.099	709.367	-27.090
718.645	-24.449	756.224	-21.099	709.489	-27.091
718.765	-24.450	756.428	-21.099	709.632	-27.092
718.868	-24.450	756.633	-21.099	709.824	-27.092
719.119	-24.451	756.836	-21.099	710.020	-27.093
719.231	-24.452	757.020	-21.099	710.209	-27.094
719.351	-24.453	757.155	-21.099	710.406	-27.094
719.507	-24.454	757.272	-21.099	710.552	-27.095
719.555	-24.454	757.291	-21.099	710.812	-27.096
719.765	-24.455	757.410	-21.099	711.041	-27.096
720.081	-24.456	757.622	-21.099	711.199	-27.097
720.377	-24.457	757.757	-21.098	711.354	-27.098
720.758	-24.458	757.896	-21.098	711.484	-27.098
720.993	-24.458	758.061	-21.098	711.654	-27.099
721.049	-24.459	758.240	-21.098	711.802	-27.100
721.161	-24.460	758.444	-21.098	711.959	-27.100
721.181	-24.461	758.714	-21.098	712.047	-27.101
721.284	-24.461	758.884	-21.098	712.174	-27.102
721.392	-24.462	758.988	-21.098	712.348	-27.102
721.524	-24.463	759.138	-21.098	712.500	-27.103
721.892	-24.464	759.312	-21.098	712.657	-27.103
722.056	-24.464	759.493	-21.098	712.868	-27.104
722.001	-24.465	759.667	-21.098	713.047	-27.105
722.091	-24.466	759.797	-21.098	713.196	-27.105
722.100	-24.467	759.911	-21.097	713.393	-27.106
722.164	-24.468	760.123	-21.097	713.574	-27.106
722.348	-24.468	760.323	-21.097	713.762	-27.107
722.613	-24.469	760.433	-21.097	713.931	-27.108
723.008	-24.470	760.557	-21.097	714.068	-27.108
723.186	-24.471	760.757	-21.097	714.175	-27.109

723.324	-24.471	760.945	-21.097	714.338	-27.109
723.565	-24.472	761.075	-21.097	714.557	-27.110
723.713	-24.473	761.202	-21.096	714.686	-27.111
723.738	-24.474	761.359	-21.096	714.875	-27.111
723.854	-24.475	761.552	-21.096	715.096	-27.112
724.143	-24.476	761.737	-21.096	715.249	-27.112
724.310	-24.476	761.881	-21.096	715.402	-27.113
724.321	-24.477	762.095	-21.096	715.526	-27.113
724.538	-24.478	762.337	-21.095	715.646	-27.114
724.853	-24.479	762.442	-21.095	715.812	-27.114
725.132	-24.480	762.514	-21.095	716.028	-27.115
725.141	-24.481	762.670	-21.095	716.199	-27.116
725.115	-24.481	762.887	-21.095	716.365	-27.116
725.297	-24.482	763.064	-21.094	716.570	-27.117
725.443	-24.483	763.205	-21.094	716.752	-27.117
725.676	-24.484	763.331	-21.094	716.917	-27.118
725.931	-24.485	763.527	-21.094	717.039	-27.118
726.161	-24.486	763.747	-21.093	717.184	-27.119
726.530	-24.487	763.869	-21.093	717.346	-27.119
726.602	-24.488	764.044	-21.093	717.498	-27.120
726.558	-24.489	764.218	-21.093	717.654	-27.120
726.783	-24.490	764.362	-21.092	717.793	-27.121
726.852	-24.491	764.525	-21.092	717.982	-27.121
726.927	-24.492	764.591	-21.092	718.253	-27.121
727.127	-24.493	764.705	-21.092	718.370	-27.122
727.317	-24.494	765.021	-21.091	718.453	-27.122
727.496	-24.495	765.496	-21.091	718.623	-27.123
727.710	-24.496	765.849	-21.091	718.785	-27.123
727.831	-24.497	766.001	-21.090	718.932	-27.124
727.820	-24.498	766.053	-21.090	719.072	-27.124

727.815	-24.499	766.130	-21.090	719.323	-27.125
728.197	-24.500	766.235	-21.089	719.764	-27.125
728.483	-24.501	766.304	-21.089	720.135	-27.125
728.584	-24.502	766.416	-21.089	720.285	-27.126
728.851	-24.504	766.480	-21.088	720.383	-27.126
729.060	-24.505	766.526	-21.088	720.413	-27.127
729.040	-24.506	766.693	-21.088	720.439	-27.127
729.193	-24.507	766.862	-21.087	720.545	-27.127
729.377	-24.508	767.008	-21.087	720.676	-27.128
729.388	-24.509	767.178	-21.087	720.796	-27.128
729.504	-24.511	767.375	-21.086	720.929	-27.128
729.792	-24.512	767.584	-21.086	721.045	-27.129
729.976	-24.513	767.767	-21.085	721.204	-27.129
730.185	-24.514	767.962	-21.085	721.357	-27.129
730.457	-24.516	768.097	-21.085	721.509	-27.130
730.521	-24.517	768.251	-21.084	721.726	-27.130
730.667	-24.518	768.415	-21.084	721.914	-27.130
730.935	-24.519	768.531	-21.083	722.058	-27.131
731.143	-24.521	768.661	-21.083	722.213	-27.131
731.255	-24.522	768.799	-21.083	722.411	-27.131
731.187	-24.523	768.972	-21.082	722.603	-27.132
731.267	-24.525	769.127	-21.082	722.755	-27.132
731.581	-24.526	769.312	-21.082	722.887	-27.132
731.926	-24.527	769.482	-21.081	723.085	-27.133
732.169	-24.529	769.613	-21.081	723.258	-27.133
732.264	-24.530	769.759	-21.080	723.351	-27.133
732.292	-24.531	769.911	-21.080	723.449	-27.133
732.346	-24.533	770.060	-21.080	723.629	-27.134
732.592	-24.534	770.257	-21.079	723.773	-27.134
732.834	-24.536	770.530	-21.079	723.901	-27.134

732.944	-24.537	770.735	-21.078	724.107	-27.134
733.007	-24.538	770.896	-21.078	724.267	-27.135
733.196	-24.540	771.068	-21.078	724.448	-27.135
733.501	-24.541	771.239	-21.077	724.614	-27.135
733.652	-24.543	771.393	-21.077	724.784	-27.135
733.752	-24.544	771.526	-21.077	724.973	-27.136
733.897	-24.546	771.611	-21.076	725.109	-27.136
733.993	-24.547	771.749	-21.076	725.276	-27.136
734.245	-24.548	771.967	-21.075	725.435	-27.136
734.494	-24.550	772.142	-21.075	725.577	-27.137
734.540	-24.551	772.275	-21.075	725.759	-27.137
734.727	-24.553	772.436	-21.074	725.831	-27.137
734.860	-24.554	772.651	-21.074	725.927	-27.137
734.824	-24.556	772.795	-21.074	726.154	-27.138
735.032	-24.557	772.887	-21.073	726.348	-27.138
735.299	-24.559	773.065	-21.073	726.533	-27.138
735.496	-24.560	773.282	-21.073	726.675	-27.138
735.622	-24.562	773.465	-21.072	726.819	-27.139
735.724	-24.563	773.592	-21.072	727.026	-27.139
735.992	-24.565	773.730	-21.072	727.237	-27.139
736.213	-24.566	773.890	-21.072	727.444	-27.139
736.367	-24.568	774.073	-21.071	727.632	-27.140
736.575	-24.569	774.215	-21.071	727.783	-27.140
736.707	-24.571	774.295	-21.071	727.916	-27.140
736.954	-24.572	774.454	-21.070	728.032	-27.140
737.155	-24.573	774.622	-21.070	728.186	-27.141
737.178	-24.575	774.833	-21.070	728.364	-27.141
737.363	-24.576	775.033	-21.070	728.526	-27.141
737.647	-24.578	775.163	-21.069	728.697	-27.141
737.828	-24.579	775.338	-21.069	728.895	-27.142

737.820	-24.581	775.539	-21.069	729.052	-27.142
737.837	-24.582	775.707	-21.069	729.173	-27.142
737.965	-24.584	775.877	-21.068	729.343	-27.142
738.175	-24.585	776.040	-21.068	729.500	-27.143
738.444	-24.587	776.188	-21.068	729.646	-27.143
738.599	-24.588	776.318	-21.068	729.818	-27.143
738.725	-24.590	776.451	-21.067	729.992	-27.143
738.881	-24.591	776.616	-21.067	730.154	-27.144
739.184	-24.593	776.840	-21.067	730.323	-27.144
739.414	-24.594	776.954	-21.067	730.490	-27.144
739.566	-24.595	777.085	-21.067	730.632	-27.144
739.759	-24.597	777.233	-21.066	730.770	-27.144
739.837	-24.598	777.422	-21.066	730.945	-27.145
740.034	-24.600	777.636	-21.066	731.157	-27.145
740.238	-24.601	777.790	-21.066	731.304	-27.145
740.286	-24.603	777.899	-21.066	731.459	-27.145
740.481	-24.604	778.044	-21.065	731.633	-27.146
740.844	-24.605	778.122	-21.065	731.805	-27.146
740.958	-24.607	778.294	-21.065	731.936	-27.146
741.002	-24.608	778.592	-21.065	732.067	-27.146
741.172	-24.610	778.702	-21.065	732.223	-27.146
741.367	-24.611	778.806	-21.065	732.315	-27.147
741.523	-24.612	778.939	-21.065	732.396	-27.147
741.628	-24.614	779.091	-21.064	732.588	-27.147
741.810	-24.615	779.238	-21.064	732.740	-27.147
742.023	-24.616	779.435	-21.064	732.837	-27.147
742.212	-24.618	779.613	-21.064	733.010	-27.147
742.243	-24.619	779.760	-21.064	733.210	-27.148
742.283	-24.620	779.959	-21.064	733.456	-27.148
742.566	-24.622	780.203	-21.064	733.673	-27.148

742.764	-24.623	780.376	-21.064	733.814	-27.148
742.892	-24.624	780.557	-21.063	733.997	-27.148
743.204	-24.625	780.780	-21.063	734.200	-27.148
743.335	-24.627	780.919	-21.063	734.404	-27.148
743.422	-24.628	781.096	-21.063	734.574	-27.149
743.646	-24.629	781.239	-21.063	734.722	-27.149
743.745	-24.630	781.340	-21.063	734.895	-27.149
743.931	-24.632	781.513	-21.063	735.068	-27.149
744.202	-24.633	781.696	-21.063	735.253	-27.149
744.299	-24.634	781.832	-21.063	735.395	-27.149
744.426	-24.635	781.950	-21.063	735.495	-27.149
744.657	-24.636	782.088	-21.062	735.658	-27.149
744.796	-24.638	782.211	-21.062	735.847	-27.149
744.879	-24.639	782.346	-21.062	735.980	-27.149
744.998	-24.640	782.506	-21.062	736.019	-27.149
745.154	-24.641	782.645	-21.062	736.134	-27.149
745.288	-24.642	782.813	-21.062	736.367	-27.149
745.427	-24.644	783.043	-21.062	736.593	-27.149
745.646	-24.645	783.231	-21.062	736.786	-27.149
745.981	-24.646	783.391	-21.062	736.970	-27.149
746.491	-24.647	783.578	-21.062	737.153	-27.149
746.812	-24.648	783.772	-21.062	737.295	-27.149
746.944	-24.649	783.938	-21.062	737.483	-27.149
747.003	-24.650	784.088	-21.062	737.650	-27.149
747.048	-24.651	784.239	-21.062	737.791	-27.149
747.088	-24.653	784.434	-21.062	737.913	-27.149
747.113	-24.654	784.622	-21.062	738.027	-27.149
747.289	-24.655	784.713	-21.061	738.251	-27.149
747.297	-24.656	784.807	-21.061	738.487	-27.149
747.340	-24.657	784.993	-21.061	738.635	-27.149

747.645	-24.658	785.180	-21.061	738.774	-27.149
747.864	-24.659	785.323	-21.061	738.946	-27.149
748.015	-24.660	785.473	-21.061	739.106	-27.148
748.254	-24.661	785.640	-21.061	739.273	-27.148
748.381	-24.662	785.813	-21.061	739.439	-27.148
748.454	-24.663	785.969	-21.061	739.587	-27.148
748.610	-24.664	786.167	-21.061	739.797	-27.148
748.851	-24.665	786.388	-21.061	740.014	-27.148
749.027	-24.666	786.609	-21.061	740.168	-27.148
749.195	-24.667	786.721	-21.061	740.276	-27.147
749.267	-24.668	786.804	-21.061	740.369	-27.147
749.391	-24.669	786.948	-21.061	740.572	-27.147
749.637	-24.670	787.177	-21.061	740.753	-27.147
749.871	-24.671	787.358	-21.061	740.872	-27.147
749.979	-24.672	787.466	-21.061	741.034	-27.146
750.017	-24.673	787.590	-21.061	741.112	-27.146
750.172	-24.674	787.775	-21.061	741.265	-27.146
750.394	-24.675	787.952	-21.061	741.570	-27.146
750.505	-24.676	788.147	-21.061	741.805	-27.145
750.565	-24.677	788.303	-21.061	741.985	-27.145
750.742	-24.678	788.456	-21.061	742.155	-27.145
751.021	-24.679	788.597	-21.061	742.298	-27.145
751.236	-24.680	788.730	-21.061	742.435	-27.144
751.405	-24.681	788.900	-21.061	742.556	-27.144
751.544	-24.682	789.087	-21.061	742.643	-27.144
751.572	-24.683	789.221	-21.061	742.822	-27.144
751.771	-24.684	789.411	-21.061	743.230	-27.143
751.964	-24.685	789.724	-21.061	743.615	-27.143
752.096	-24.686	790.077	-21.061	743.903	-27.143
752.358	-24.687	790.322	-21.061	744.073	-27.142

752.500	-24.689	790.497	-21.061	744.069	-27.142
752.582	-24.690	790.575	-21.061	744.147	-27.141
752.712	-24.691	790.608	-21.061	744.280	-27.141
752.962	-24.692	790.673	-21.061	744.310	-27.141
753.165	-24.693	790.790	-21.061	744.351	-27.140
753.256	-24.694	790.891	-21.061	744.471	-27.140
753.350	-24.695	790.986	-21.060	744.625	-27.140
753.528	-24.696	791.131	-21.060	744.788	-27.139
753.743	-24.697	791.273	-21.060	744.930	-27.139
753.947	-24.698	791.412	-21.060	745.033	-27.138
754.179	-24.699	791.544	-21.060	745.173	-27.138
754.399	-24.700	791.714	-21.060	745.351	-27.138
754.519	-24.701	791.974	-21.060	745.564	-27.137
754.607	-24.702	792.224	-21.060	745.799	-27.137
754.787	-24.703	792.393	-21.060	746.014	-27.136
754.977	-24.705	792.540	-21.060	746.189	-27.136
755.173	-24.706	792.654	-21.060	746.321	-27.135
755.228	-24.707	792.785	-21.061	746.446	-27.135
755.247	-24.708	792.988	-21.061	746.579	-27.134
755.461	-24.709	793.142	-21.061	746.695	-27.134
755.706	-24.710	793.251	-21.061	746.784	-27.133
755.953	-24.711	793.373	-21.061	746.993	-27.133
756.177	-24.712	793.552	-21.061	747.261	-27.132
756.328	-24.714	793.743	-21.061	747.416	-27.132
756.426	-24.715	793.916	-21.061	747.533	-27.132
756.563	-24.716	794.103	-21.061	747.690	-27.131
756.784	-24.717	794.220	-21.061	747.777	-27.131
756.904	-24.718	794.306	-21.061	747.929	-27.130
756.985	-24.720	794.473	-21.061	748.176	-27.130
757.113	-24.721	794.675	-21.061	748.389	-27.129

757.281	-24.722	794.881	-21.061	748.536	-27.129
757.486	-24.723	795.072	-21.061	748.673	-27.128
757.618	-24.724	795.209	-21.061	748.769	-27.128
757.760	-24.726	795.394	-21.061	748.915	-27.127
757.943	-24.727	795.611	-21.061	749.207	-27.127
758.091	-24.728	795.801	-21.061	749.388	-27.126
758.288	-24.729	795.992	-21.061	749.494	-27.126
758.523	-24.731	796.158	-21.061	749.641	-27.125
758.658	-24.732	796.306	-21.061	749.805	-27.125
758.885	-24.733	796.441	-21.061	749.995	-27.124
759.091	-24.734	796.527	-21.061	750.165	-27.124
759.233	-24.736	796.649	-21.061	750.302	-27.123
759.326	-24.737	796.848	-21.061	750.473	-27.123
759.398	-24.738	797.029	-21.061	750.623	-27.122
759.678	-24.740	797.175	-21.061	750.742	-27.122
760.002	-24.741	797.373	-21.061	750.919	-27.121
760.042	-24.742	797.548	-21.061	751.136	-27.121
760.022	-24.744	797.719	-21.061	751.251	-27.120
760.084	-24.745	797.912	-21.061	751.388	-27.120
760.193	-24.746	798.063	-21.061	751.568	-27.119
760.435	-24.747	798.249	-21.061	751.674	-27.119
760.658	-24.749	798.397	-21.061	751.843	-27.118
760.794	-24.750	798.480	-21.061	752.078	-27.118
760.856	-24.751	798.615	-21.061	752.225	-27.118
760.975	-24.753	798.788	-21.061	752.336	-27.117
761.152	-24.754	798.916	-21.061	752.526	-27.117
761.286	-24.755	799.073	-21.061	752.732	-27.116
761.575	-24.757	799.282	-21.061	752.873	-27.116
761.916	-24.758	799.481	-21.061	753.017	-27.115
762.111	-24.759	799.678	-21.061	753.196	-27.115

762.245	-24.761	799.868	-21.061	753.363	-27.114
762.445	-24.762	799.961	-21.061	753.551	-27.114
762.724	-24.763	800.126	-21.061	753.715	-27.113
762.887	-24.765	800.328	-21.061	753.825	-27.113
762.980	-24.766	800.479	-21.061	753.981	-27.112
763.064	-24.768	800.620	-21.061	754.181	-27.112
763.197	-24.769	800.742	-21.061	754.344	-27.112
763.385	-24.770	800.944	-21.061	754.531	-27.111
763.410	-24.772	801.160	-21.061	754.734	-27.111
763.511	-24.773	801.309	-21.061	754.852	-27.110
763.818	-24.774	801.431	-21.061	754.994	-27.110
763.932	-24.776	801.583	-21.061	755.207	-27.109
764.002	-24.777	801.789	-21.061	755.378	-27.109
764.167	-24.778	801.981	-21.061	755.520	-27.108
764.350	-24.780	802.058	-21.061	755.654	-27.108
764.639	-24.781	802.144	-21.061	755.808	-27.108
764.846	-24.782	802.351	-21.061	755.991	-27.107
764.935	-24.784	802.523	-21.060	756.118	-27.107
765.103	-24.785	802.703	-21.060	756.208	-27.106
765.369	-24.786	802.899	-21.060	756.230	-27.106
765.588	-24.788	803.007	-21.060	756.381	-27.105
765.650	-24.789	803.187	-21.060	756.600	-27.105
765.790	-24.790	803.382	-21.060	756.752	-27.104
765.989	-24.792	803.465	-21.060	756.966	-27.104
766.148	-24.793	803.584	-21.059	757.141	-27.103
766.208	-24.794	803.757	-21.059	757.312	-27.103
766.329	-24.796	803.882	-21.059	757.529	-27.102
766.561	-24.797	804.035	-21.059	757.784	-27.102
766.822	-24.798	804.226	-21.059	758.030	-27.101
767.087	-24.800	804.417	-21.058	758.248	-27.101

767.175	-24.801	804.604	-21.058	758.400	-27.100
767.328	-24.802	804.836	-21.058	758.497	-27.100
767.505	-24.804	805.039	-21.058	758.691	-27.099
767.725	-24.805	805.213	-21.057	758.902	-27.099
767.934	-24.806	805.406	-21.057	759.035	-27.098
767.955	-24.808	805.582	-21.057	759.151	-27.098
768.072	-24.809	805.779	-21.056	759.268	-27.097
768.336	-24.810	805.891	-21.056	759.395	-27.097
768.422	-24.811	806.014	-21.056	759.548	-27.096
768.570	-24.813	806.200	-21.055	759.724	-27.096
768.839	-24.814	806.301	-21.055	759.863	-27.095
768.999	-24.815	806.365	-21.055	759.939	-27.094
769.096	-24.816	806.515	-21.054	760.081	-27.094
769.212	-24.818	806.715	-21.054	760.290	-27.093
769.391	-24.819	806.835	-21.053	760.490	-27.093
769.584	-24.820	806.969	-21.053	760.676	-27.092
769.735	-24.821	807.188	-21.052	760.799	-27.092
769.890	-24.823	807.368	-21.052	760.961	-27.091
770.042	-24.824	807.544	-21.052	761.097	-27.090
770.137	-24.825	807.707	-21.051	761.224	-27.090
770.302	-24.826	807.865	-21.051	761.469	-27.089
770.504	-24.827	808.066	-21.050	761.684	-27.089
770.747	-24.829	808.251	-21.050	761.820	-27.088
771.058	-24.830	808.427	-21.049	761.973	-27.087
771.188	-24.831	808.603	-21.048	762.131	-27.087
771.313	-24.832	808.661	-21.048	762.279	-27.086
771.423	-24.833	808.841	-21.047	762.428	-27.085
771.421	-24.835	809.114	-21.047	762.628	-27.085
771.606	-24.836	809.267	-21.046	762.809	-27.084
771.980	-24.837	809.455	-21.046	762.936	-27.084

772.446	-24.838	809.637	-21.045	763.065	-27.083
772.581	-24.839	809.801	-21.044	763.251	-27.082
772.650	-24.840	809.939	-21.044	763.489	-27.082
772.769	-24.842	810.096	-21.043	763.705	-27.081
772.909	-24.843	810.271	-21.042	763.884	-27.080
773.045	-24.844	810.443	-21.042	764.025	-27.079
773.090	-24.845	810.676	-21.041	764.200	-27.079
773.136	-24.846	810.877	-21.040	764.333	-27.078
773.242	-24.847	810.981	-21.040	764.432	-27.077
773.309	-24.848	811.124	-21.039	764.607	-27.077
773.469	-24.849	811.311	-21.038	764.759	-27.076
773.745	-24.851	811.432	-21.038	764.907	-27.075
773.946	-24.852	811.559	-21.037	765.126	-27.074
774.038	-24.853	811.754	-21.036	765.294	-27.074
774.118	-24.854	811.993	-21.035	765.444	-27.073
774.297	-24.855	812.157	-21.035	765.600	-27.072
774.563	-24.856	812.302	-21.034	765.763	-27.071
774.758	-24.857	812.454	-21.033	765.977	-27.071
774.905	-24.858	812.545	-21.032	766.163	-27.070
775.021	-24.859	812.698	-21.031	766.280	-27.069
775.157	-24.860	812.911	-21.031	766.345	-27.068
775.368	-24.861	813.046	-21.030	766.627	-27.068
775.448	-24.862	813.147	-21.029	767.116	-27.067
775.598	-24.863	813.313	-21.028	767.428	-27.066
775.860	-24.864	813.511	-21.027	767.503	-27.065
776.020	-24.866	813.664	-21.027	767.596	-27.064
776.219	-24.867	813.791	-21.026	767.682	-27.064
776.395	-24.868	813.966	-21.025	767.710	-27.063
776.396	-24.869	814.195	-21.024	767.748	-27.062
776.505	-24.870	814.599	-21.023	767.809	-27.061

776.777	-24.871	815.017	-21.022	767.918	-27.060
776.955	-24.872	815.162	-21.022	768.093	-27.059
777.202	-24.873	815.222	-21.021	768.278	-27.059
777.399	-24.874	815.273	-21.020	768.455	-27.058
777.623	-24.875	815.282	-21.019	768.601	-27.057
777.844	-24.876	815.316	-21.018	768.788	-27.056
778.005	-24.877	815.398	-21.017	768.949	-27.055
778.173	-24.878	815.507	-21.017	769.096	-27.054
778.252	-24.879	815.667	-21.016	769.254	-27.054
778.424	-24.880	815.843	-21.015	769.433	-27.053
778.615	-24.881	815.997	-21.014	769.644	-27.052
778.688	-24.882	816.142	-21.013	769.829	-27.051
778.758	-24.883	816.245	-21.012	769.935	-27.050
778.934	-24.884	816.420	-21.011	770.057	-27.049
779.061	-24.886	816.661	-21.011	770.224	-27.048
779.115	-24.887	816.897	-21.010	770.409	-27.048
779.361	-24.888	817.071	-21.009	770.569	-27.047
779.622	-24.889	817.221	-21.008	770.710	-27.046
779.746	-24.890	817.387	-21.007	770.912	-27.045
779.907	-24.891	817.515	-21.006	771.075	-27.044
780.036	-24.892	817.576	-21.006	771.204	-27.043
780.105	-24.893	817.783	-21.005	771.379	-27.042
780.340	-24.894	818.036	-21.004	771.558	-27.041
780.639	-24.895	818.196	-21.003	771.647	-27.041
780.748	-24.896	818.295	-21.002	771.851	-27.040
780.829	-24.897	818.431	-21.001	772.048	-27.039
780.938	-24.898	818.646	-21.001	772.178	-27.038
781.143	-24.899	818.828	-21.000	772.381	-27.037
781.311	-24.900	818.951	-20.999	772.609	-27.036
781.497	-24.901	819.061	-20.998	772.791	-27.036

781.724	-24.902	819.233	-20.998	772.907	-27.035
781.925	-24.904	819.478	-20.997	773.090	-27.034
782.069	-24.905	819.696	-20.996	773.279	-27.033
782.149	-24.906	819.847	-20.995	773.389	-27.032
782.381	-24.907	819.963	-20.995	773.496	-27.031
782.502	-24.908	820.090	-20.994	773.661	-27.031
782.629	-24.909	820.263	-20.993	773.818	-27.030
782.801	-24.910	820.452	-20.992	773.971	-27.029
783.158	-24.911	820.608	-20.992	774.134	-27.028
783.513	-24.912	820.697	-20.991	774.282	-27.028
783.717	-24.913	820.862	-20.990	774.445	-27.027
783.858	-24.914	821.032	-20.990	774.641	-27.026
783.831	-24.915	821.177	-20.989	774.874	-27.025
783.867	-24.917	821.388	-20.988	775.028	-27.024
783.896	-24.918	821.534	-20.988	775.117	-27.024
784.025	-24.919	821.665	-20.987	775.246	-27.023
784.286	-24.920	821.852	-20.986	775.399	-27.022
784.491	-24.921	821.997	-20.986	775.588	-27.021
784.773	-24.922	822.123	-20.985	775.739	-27.021
785.067	-24.923	822.320	-20.985	775.889	-27.020
785.154	-24.924	822.550	-20.984	776.111	-27.019
785.192	-24.925	822.734	-20.984	776.293	-27.019
785.338	-24.926	822.879	-20.983	776.446	-27.018
785.489	-24.927	823.025	-20.982	776.617	-27.017
785.572	-24.929	823.196	-20.982	776.755	-27.016
785.729	-24.930	823.297	-20.981	776.905	-27.016
785.859	-24.931	823.378	-20.981	777.068	-27.015
786.070	-24.932	823.592	-20.980	777.213	-27.014
786.227	-24.933	823.804	-20.980	777.439	-27.014
786.329	-24.934	823.910	-20.979	777.645	-27.013

786.491	-24.935	824.069	-20.979	777.721	-27.012
786.675	-24.936	824.285	-20.978	777.848	-27.012
786.805	-24.937	824.464	-20.978	778.041	-27.011
786.955	-24.938	824.667	-20.977	778.206	-27.010
787.217	-24.939	824.835	-20.977	778.379	-27.010
787.382	-24.940	824.940	-20.977	778.539	-27.009
787.504	-24.941	825.127	-20.976	778.661	-27.008
787.702	-24.942	825.331	-20.976	778.846	-27.008
787.916	-24.943	825.465	-20.975	779.060	-27.007
788.186	-24.944	825.646	-20.975	779.239	-27.006
788.413	-24.945	825.787	-20.975	779.361	-27.006
788.587	-24.946	825.920	-20.974	779.438	-27.005
788.653	-24.947	826.121	-20.974	779.633	-27.005
788.711	-24.948	826.328	-20.973	779.827	-27.004
788.845	-24.949	826.490	-20.973	779.913	-27.003
788.981	-24.950	826.637	-20.973	780.015	-27.003
789.177	-24.951	826.789	-20.972	780.184	-27.002
789.268	-24.952	826.979	-20.972	780.350	-27.001
789.395	-24.953	827.179	-20.972	780.433	-27.001
789.643	-24.954	827.363	-20.971	780.592	-27.000
789.873	-24.955	827.507	-20.971	780.814	-27.000
789.926	-24.956	827.600	-20.970	781.028	-26.999
790.156	-24.957	827.673	-20.970	781.276	-26.998
790.415	-24.958	827.776	-20.970	781.513	-26.998
790.418	-24.959	827.923	-20.969	781.706	-26.997
790.567	-24.960	828.094	-20.969	781.927	-26.996
790.754	-24.961	828.276	-20.969	782.043	-26.996
790.912	-24.962	828.389	-20.968	782.121	-26.995
791.170	-24.963	828.566	-20.968	782.197	-26.995
791.388	-24.964	828.805	-20.968	782.414	-26.994

791.581	-24.965	828.979	-20.967	782.635	-26.993
791.748	-24.966	829.116	-20.967	782.724	-26.993
791.889	-24.967	829.287	-20.967	782.813	-26.992
792.062	-24.968	829.494	-20.966	783.035	-26.991
792.212	-24.969	829.681	-20.966	783.173	-26.991
792.329	-24.970	829.811	-20.965	783.232	-26.990
792.550	-24.970	829.941	-20.965	783.350	-26.990
792.794	-24.971	830.128	-20.965	783.531	-26.989
792.935	-24.972	830.337	-20.964	783.702	-26.988
793.132	-24.973	830.520	-20.964	783.959	-26.988
793.317	-24.974	830.696	-20.964	784.131	-26.987
793.399	-24.975	830.828	-20.963	784.311	-26.986
793.481	-24.976	830.963	-20.963	784.536	-26.986
793.596	-24.977	831.059	-20.962	784.726	-26.985
793.854	-24.977	831.180	-20.962	784.859	-26.984
794.092	-24.978	831.397	-20.962	784.970	-26.984
794.210	-24.979	831.503	-20.961	785.089	-26.983
794.322	-24.980	831.615	-20.961	785.275	-26.983
794.426	-24.981	831.813	-20.960	785.381	-26.982
794.621	-24.981	832.036	-20.960	785.529	-26.981
794.791	-24.982	832.212	-20.959	785.744	-26.981
794.977	-24.983	832.376	-20.959	785.989	-26.980
795.159	-24.984	832.610	-20.959	786.172	-26.979
795.286	-24.985	832.784	-20.958	786.347	-26.979
795.392	-24.985	832.907	-20.958	786.479	-26.978
795.479	-24.986	833.102	-20.957	786.628	-26.977
795.722	-24.987	833.288	-20.957	786.758	-26.977
795.954	-24.988	833.440	-20.956	786.919	-26.976
796.144	-24.989	833.590	-20.956	787.091	-26.975
796.335	-24.989	833.756	-20.955	787.234	-26.975

796.438	-24.990	833.912	-20.955	787.502	-26.974
796.630	-24.991	834.099	-20.954	787.673	-26.973
796.831	-24.992	834.228	-20.954	787.771	-26.973
796.896	-24.992	834.363	-20.953	787.879	-26.972
796.972	-24.993	834.590	-20.953	788.030	-26.971
797.155	-24.994	834.775	-20.952	788.238	-26.970
797.440	-24.995	834.951	-20.952	788.418	-26.970
797.902	-24.995	835.072	-20.951	788.577	-26.969
798.299	-24.996	835.190	-20.951	788.696	-26.968
798.410	-24.997	835.393	-20.950	788.898	-26.967
798.471	-24.998	835.543	-20.949	789.137	-26.967
798.605	-24.998	835.611	-20.949	789.296	-26.966
798.636	-24.999	835.750	-20.948	789.424	-26.965
798.536	-25.000	835.936	-20.948	789.553	-26.964
798.577	-25.000	836.135	-20.947	789.728	-26.964
798.778	-25.001	836.339	-20.946	789.880	-26.963
798.998	-25.002	836.445	-20.946	790.032	-26.962
799.153	-25.003	836.590	-20.945	790.319	-26.961
799.258	-25.003	836.798	-20.944	790.710	-26.961
799.500	-25.004	836.953	-20.944	791.024	-26.960
799.686	-25.005	837.120	-20.943	791.164	-26.959
799.713	-25.005	837.330	-20.943	791.209	-26.958
799.847	-25.006	837.485	-20.942	791.251	-26.957
800.188	-25.007	837.634	-20.941	791.303	-26.956
800.514	-25.007	837.865	-20.940	791.364	-26.956
800.603	-25.008	838.051	-20.940	791.466	-26.955
800.661	-25.009	838.156	-20.939	791.639	-26.954
800.901	-25.009	838.279	-20.938	791.803	-26.953
801.069	-25.010	838.403	-20.938	791.900	-26.952
801.097	-25.011	838.618	-20.937	792.011	-26.951

801.165	-25.012	839.021	-20.936	792.219	-26.951
801.385	-25.012	839.360	-20.935	792.421	-26.950
801.584	-25.013	839.574	-20.935	792.637	-26.949
801.751	-25.014	839.639	-20.934	792.844	-26.948
801.961	-25.014	839.714	-20.933	792.974	-26.947
802.031	-25.015	839.870	-20.932	793.139	-26.946
802.109	-25.016	839.856	-20.932	793.301	-26.945
802.226	-25.017	839.871	-20.931	793.440	-26.944
802.402	-25.017	839.986	-20.930	793.574	-26.943
802.595	-25.018	840.108	-20.929	793.723	-26.942
802.859	-25.019	840.275	-20.928	793.888	-26.941
803.108	-25.019	840.442	-20.928	794.032	-26.941
803.145	-25.020	840.641	-20.927	794.209	-26.940
803.334	-25.021	840.874	-20.926	794.381	-26.939
803.617	-25.022	841.084	-20.925	794.498	-26.938
803.783	-25.022	841.272	-20.924	794.644	-26.937
803.916	-25.023	841.453	-20.924	794.865	-26.936
804.072	-25.024	841.618	-20.923	795.019	-26.935
804.233	-25.025	841.778	-20.922	795.183	-26.934
804.345	-25.025	841.939	-20.921	795.399	-26.933
804.513	-25.026	842.049	-20.920	795.605	-26.932
804.754	-25.027	842.195	-20.919	795.813	-26.931
804.877	-25.028	842.418	-20.919	795.954	-26.930
805.047	-25.029	842.609	-20.918	796.116	-26.929
805.235	-25.029	842.697	-20.917	796.309	-26.928
805.346	-25.030	842.711	-20.916	796.440	-26.927
805.527	-25.031	842.857	-20.915	796.565	-26.926
805.675	-25.032	843.076	-20.914	796.730	-26.925
805.808	-25.032	843.194	-20.914	796.883	-26.924
805.974	-25.033	843.329	-20.913	797.069	-26.923

806.124	-25.034	843.540	-20.912	797.225	-26.922
806.409	-25.035	843.716	-20.911	797.338	-26.921
806.590	-25.036	843.909	-20.910	797.512	-26.920
806.556	-25.036	844.153	-20.909	797.678	-26.919
806.631	-25.037	844.373	-20.909	797.859	-26.918
806.817	-25.038	844.513	-20.908	797.992	-26.916
806.982	-25.039	844.628	-20.907	798.100	-26.915
807.151	-25.040	844.796	-20.906	798.298	-26.914
807.338	-25.040	844.951	-20.905	798.490	-26.913
807.502	-25.041	845.110	-20.905	798.641	-26.912
807.718	-25.042	845.297	-20.904	798.818	-26.911
807.962	-25.043	845.476	-20.903	799.008	-26.910
808.159	-25.044	845.604	-20.902	799.097	-26.909
808.405	-25.045	845.712	-20.901	799.131	-26.908
808.671	-25.046	845.891	-20.901	799.326	-26.907
808.814	-25.046	846.049	-20.900	799.548	-26.906
808.901	-25.047	846.154	-20.899	799.719	-26.904
808.997	-25.048	846.330	-20.898	799.917	-26.903
809.099	-25.049	846.534	-20.898	800.155	-26.902
809.260	-25.050	846.738	-20.897	800.348	-26.901
809.410	-25.051	846.937	-20.896	800.400	-26.900
809.599	-25.052	847.095	-20.895	800.509	-26.899
809.790	-25.052	847.260	-20.895	800.713	-26.897
809.798	-25.053	847.346	-20.894	800.892	-26.896
809.945	-25.054	847.431	-20.893	801.076	-26.895
810.232	-25.055	847.573	-20.893	801.213	-26.894
810.385	-25.056	847.775	-20.892	801.334	-26.893
810.603	-25.057	847.923	-20.891	801.531	-26.891
810.838	-25.058	848.081	-20.891	801.730	-26.890
810.956	-25.059	848.288	-20.890	801.916	-26.889

811.030	-25.059	848.484	-20.889	802.045	-26.888
811.193	-25.060	848.633	-20.889	802.155	-26.886
811.396	-25.061	848.795	-20.888	802.354	-26.885
811.526	-25.062	849.008	-20.887	802.585	-26.884
811.691	-25.063	849.186	-20.887	802.785	-26.882
811.816	-25.064	849.331	-20.886	802.963	-26.881
811.892	-25.065	849.448	-20.885	803.111	-26.880
812.034	-25.066	849.539	-20.885	803.207	-26.879
812.214	-25.066	849.703	-20.884	803.285	-26.877
812.383	-25.067	849.940	-20.883	803.449	-26.876
812.476	-25.068	850.067	-20.883	803.643	-26.874
812.622	-25.069	850.178	-20.882	803.775	-26.873
812.908	-25.070	850.378	-20.882	803.900	-26.872
813.156	-25.071	850.491	-20.881	804.010	-26.870
813.277	-25.072	850.648	-20.881	804.163	-26.869
813.472	-25.072	850.837	-20.880	804.429	-26.868
813.454	-25.073	851.002	-20.879	804.700	-26.866
813.601	-25.074	851.204	-20.879	804.846	-26.865
814.205	-25.075	851.375	-20.878	804.969	-26.863
814.501	-25.076	851.540	-20.878	805.219	-26.862
814.492	-25.076	851.659	-20.877	805.473	-26.860
814.503	-25.077	851.788	-20.877	805.664	-26.859
814.652	-25.078	851.928	-20.876	805.798	-26.857
814.806	-25.079	852.079	-20.876	805.897	-26.856
814.876	-25.080	852.286	-20.875	806.027	-26.854
814.977	-25.080	852.428	-20.875	806.140	-26.853
815.241	-25.081	852.542	-20.874	806.264	-26.851
815.390	-25.082	852.702	-20.874	806.390	-26.850
815.443	-25.083	852.813	-20.873	806.538	-26.848
815.640	-25.084	852.915	-20.873	806.676	-26.846

815.952	-25.084	853.109	-20.872	806.848	-26.845
816.087	-25.085	853.298	-20.872	807.036	-26.843
816.067	-25.086	853.471	-20.871	807.196	-26.841
816.283	-25.087	853.743	-20.871	807.377	-26.840
816.531	-25.087	853.967	-20.870	807.567	-26.838
816.665	-25.088	854.103	-20.870	807.743	-26.837
816.950	-25.089	854.307	-20.869	807.898	-26.835
817.193	-25.090	854.437	-20.869	808.075	-26.833
817.250	-25.090	854.602	-20.869	808.263	-26.831
817.464	-25.091	854.821	-20.868	808.448	-26.830
817.682	-25.092	854.923	-20.868	808.635	-26.828
817.870	-25.092	855.023	-20.867	808.780	-26.826
818.008	-25.093	855.208	-20.867	808.932	-26.824
818.064	-25.094	855.396	-20.866	809.109	-26.823
818.150	-25.094	855.567	-20.866	809.268	-26.821
818.341	-25.095	855.692	-20.865	809.406	-26.819
818.517	-25.096	855.828	-20.865	809.550	-26.817
818.673	-25.096	856.017	-20.865	809.707	-26.815
818.833	-25.097	856.176	-20.864	809.864	-26.814
819.002	-25.098	856.291	-20.864	810.008	-26.812
819.230	-25.098	856.416	-20.863	810.238	-26.810
819.428	-25.099	856.616	-20.863	810.433	-26.808
819.633	-25.100	856.861	-20.863	810.514	-26.806
819.833	-25.100	857.048	-20.862	810.665	-26.804
820.014	-25.101	857.195	-20.862	810.872	-26.802
820.143	-25.101	857.382	-20.861	811.046	-26.800
820.172	-25.102	857.604	-20.861	811.219	-26.798
820.247	-25.103	857.776	-20.861	811.320	-26.797
820.360	-25.103	857.856	-20.860	811.469	-26.795
820.642	-25.104	857.952	-20.860	811.721	-26.793

820.871	-25.105	858.167	-20.859	811.926	-26.791
820.958	-25.105	858.412	-20.859	812.076	-26.789
820.933	-25.106	858.497	-20.859	812.199	-26.787
821.030	-25.106	858.611	-20.858	812.379	-26.785
821.227	-25.107	858.760	-20.858	812.629	-26.783
821.684	-25.108	858.948	-20.857	812.783	-26.781
821.899	-25.108	859.091	-20.857	812.880	-26.779
821.990	-25.109	859.230	-20.857	813.010	-26.777
822.106	-25.109	859.419	-20.856	813.160	-26.775
822.241	-25.110	859.616	-20.856	813.315	-26.773
822.346	-25.111	859.803	-20.855	813.506	-26.770
822.442	-25.111	859.915	-20.855	813.624	-26.768
822.553	-25.112	860.027	-20.855	813.780	-26.766
822.782	-25.112	860.236	-20.854	814.131	-26.764
823.095	-25.113	860.456	-20.854	814.566	-26.762
823.443	-25.114	860.560	-20.853	814.825	-26.760
823.719	-25.114	860.673	-20.853	814.855	-26.758
824.022	-25.115	860.901	-20.853	814.936	-26.756
824.250	-25.115	861.075	-20.852	815.047	-26.754
824.339	-25.116	861.219	-20.852	815.109	-26.752
824.454	-25.117	861.378	-20.851	815.164	-26.750
824.544	-25.117	861.553	-20.851	815.286	-26.748
824.587	-25.118	861.748	-20.851	815.436	-26.745
824.592	-25.118	861.898	-20.850	815.553	-26.743
824.648	-25.119	862.009	-20.850	815.667	-26.741
824.818	-25.120	862.185	-20.849	815.863	-26.739
824.960	-25.120	862.352	-20.849	816.087	-26.737
825.236	-25.121	862.497	-20.849	816.224	-26.735
825.546	-25.122	862.728	-20.848	816.364	-26.733
825.668	-25.122	862.906	-20.848	816.554	-26.731

825.604	-25.123	863.032	-20.848	816.771	-26.728
825.661	-25.124	863.363	-20.847	816.950	-26.726
825.902	-25.124	863.775	-20.847	817.041	-26.724
826.174	-25.125	864.035	-20.846	817.137	-26.722
826.329	-25.125	864.121	-20.846	817.353	-26.720
826.493	-25.126	864.195	-20.846	817.571	-26.718
826.563	-25.127	864.343	-20.845	817.708	-26.716
826.692	-25.127	864.432	-20.845	817.870	-26.713
826.883	-25.128	864.503	-20.844	818.017	-26.711
827.035	-25.129	864.549	-20.844	818.141	-26.709
827.081	-25.130	864.560	-20.844	818.303	-26.707
827.247	-25.130	864.714	-20.843	818.449	-26.705
827.491	-25.131	864.939	-20.843	818.597	-26.703
827.627	-25.132	865.120	-20.843	818.797	-26.701
827.753	-25.132	865.254	-20.842	818.988	-26.699
827.979	-25.133	865.386	-20.842	819.212	-26.696
828.267	-25.134	865.538	-20.842	819.406	-26.694
828.231	-25.135	865.691	-20.841	819.529	-26.692
828.130	-25.136	865.896	-20.841	819.708	-26.690
828.326	-25.136	866.098	-20.840	819.908	-26.688
828.650	-25.137	866.304	-20.840	820.095	-26.686
828.946	-25.138	866.456	-20.840	820.229	-26.684
829.155	-25.139	866.610	-20.839	820.349	-26.682
829.357	-25.140	866.812	-20.839	820.477	-26.680
829.507	-25.140	866.939	-20.839	820.602	-26.677
829.675	-25.141	867.044	-20.838	820.782	-26.675
829.908	-25.142	867.160	-20.838	820.971	-26.673
830.152	-25.143	867.306	-20.838	821.094	-26.671
830.294	-25.144	867.477	-20.837	821.236	-26.669
830.337	-25.145	867.603	-20.837	821.391	-26.667

830.456	-25.146	867.764	-20.837	821.516	-26.665
830.686	-25.147	867.925	-20.836	821.703	-26.663
830.845	-25.148	868.082	-20.836	821.898	-26.661
830.975	-25.149	868.298	-20.836	822.101	-26.659
831.218	-25.150	868.466	-20.836	822.239	-26.657
831.581	-25.151	868.626	-20.835	822.410	-26.655
831.750	-25.152	868.822	-20.835	822.617	-26.653
831.703	-25.153	869.010	-20.835	822.720	-26.651
831.624	-25.154	869.167	-20.835	822.865	-26.649
831.749	-25.155	869.334	-20.834	822.995	-26.647
832.104	-25.156	869.473	-20.834	823.127	-26.645
832.279	-25.157	869.654	-20.834	823.328	-26.643
832.532	-25.158	869.817	-20.834	823.528	-26.641
832.794	-25.159	869.971	-20.833	823.692	-26.639
832.855	-25.160	870.150	-20.833	823.883	-26.637
832.887	-25.161	870.282	-20.833	824.044	-26.635
833.058	-25.162	870.434	-20.833	824.180	-26.633
833.328	-25.163	870.620	-20.832	824.396	-26.631
833.410	-25.164	870.807	-20.832	824.540	-26.629
833.476	-25.165	870.955	-20.832	824.614	-26.627
833.633	-25.166	871.078	-20.832	824.825	-26.625
833.777	-25.168	871.239	-20.832	825.045	-26.623
833.993	-25.169	871.389	-20.832	825.103	-26.621
834.209	-25.170	871.513	-20.831	825.303	-26.619
834.386	-25.171	871.689	-20.831	825.577	-26.617
834.526	-25.172	871.939	-20.831	825.766	-26.615
834.718	-25.173	872.102	-20.831	825.903	-26.613
834.854	-25.175	872.157	-20.831	826.045	-26.611
834.837	-25.176	872.324	-20.831	826.164	-26.609
834.833	-25.177	872.535	-20.830	826.285	-26.608

835.078	-25.178	872.703	-20.830	826.393	-26.606
835.268	-25.179	872.847	-20.830	826.535	-26.604
835.497	-25.181	872.984	-20.830	826.730	-26.602
835.864	-25.182	873.161	-20.830	826.910	-26.600
835.910	-25.183	873.280	-20.830	827.044	-26.598
836.030	-25.184	873.443	-20.830	827.139	-26.596
836.322	-25.185	873.639	-20.830	827.333	-26.594
836.477	-25.187	873.829	-20.829	827.579	-26.592
836.525	-25.188	873.997	-20.829	827.691	-26.590
836.797	-25.189	874.156	-20.829	827.813	-26.588
837.073	-25.190	874.335	-20.829	828.008	-26.586
837.162	-25.191	874.450	-20.829	828.186	-26.585
837.293	-25.193	874.564	-20.829	828.412	-26.583
837.494	-25.194	874.740	-20.829	828.609	-26.581
837.591	-25.195	874.899	-20.829	828.716	-26.579
837.605	-25.196	875.079	-20.829	828.898	-26.577
837.805	-25.198	875.247	-20.829	829.175	-26.575
838.081	-25.199	875.430	-20.829	829.333	-26.573
838.232	-25.200	875.631	-20.829	829.401	-26.571
838.353	-25.201	875.759	-20.829	829.591	-26.569
838.501	-25.202	875.872	-20.829	829.778	-26.567
838.748	-25.204	876.021	-20.829	829.894	-26.565
838.999	-25.205	876.215	-20.829	830.024	-26.563
839.186	-25.206	876.413	-20.828	830.156	-26.561
839.398	-25.207	876.551	-20.828	830.331	-26.559
839.555	-25.208	876.666	-20.828	830.440	-26.557
839.666	-25.209	876.796	-20.828	830.539	-26.555
839.762	-25.211	876.902	-20.828	830.723	-26.553
839.886	-25.212	877.053	-20.828	830.880	-26.551
840.073	-25.213	877.225	-20.828	831.077	-26.549

840.210	-25.214	877.418	-20.828	831.301	-26.547
840.351	-25.215	877.586	-20.828	831.481	-26.545
840.667	-25.216	877.736	-20.828	831.726	-26.543
840.845	-25.217	877.899	-20.828	831.896	-26.541
840.900	-25.218	878.108	-20.828	832.014	-26.539
840.961	-25.219	878.366	-20.828	832.224	-26.537
841.103	-25.220	878.547	-20.828	832.345	-26.535
841.346	-25.222	878.708	-20.828	832.467	-26.533
841.513	-25.223	878.915	-20.828	832.678	-26.531
841.637	-25.224	879.068	-20.828	832.850	-26.529
841.776	-25.225	879.195	-20.828	833.036	-26.527
841.923	-25.226	879.409	-20.827	833.245	-26.525
842.130	-25.227	879.585	-20.827	833.382	-26.523
842.402	-25.228	879.677	-20.827	833.475	-26.521
842.567	-25.229	879.796	-20.827	833.594	-26.519
842.749	-25.230	879.924	-20.827	833.756	-26.517
842.934	-25.230	880.097	-20.827	833.935	-26.515
842.960	-25.231	880.264	-20.827	834.121	-26.513
843.105	-25.232	880.407	-20.827	834.352	-26.511
843.361	-25.233	880.536	-20.827	834.554	-26.508
843.475	-25.234	880.639	-20.827	834.683	-26.506
843.593	-25.235	880.807	-20.826	834.820	-26.504
843.690	-25.236	881.032	-20.826	835.027	-26.502
843.869	-25.237	881.239	-20.826	835.200	-26.500
844.088	-25.237	881.441	-20.826	835.285	-26.498
844.152	-25.238	881.633	-20.826	835.482	-26.496
844.391	-25.239	881.771	-20.826	835.675	-26.494
844.743	-25.240	881.901	-20.826	835.760	-26.491
844.957	-25.241	882.100	-20.826	835.969	-26.489
845.071	-25.241	882.291	-20.825	836.208	-26.487

845.123	-25.242	882.423	-20.825	836.364	-26.485
845.266	-25.243	882.578	-20.825	836.440	-26.483
845.450	-25.243	882.778	-20.825	836.586	-26.480
845.603	-25.244	882.916	-20.825	836.852	-26.478
845.814	-25.245	883.021	-20.824	836.994	-26.476
846.008	-25.245	883.188	-20.824	837.053	-26.474
846.161	-25.246	883.362	-20.824	837.148	-26.472
846.360	-25.247	883.493	-20.824	837.324	-26.469
846.581	-25.247	883.668	-20.824	837.741	-26.467
846.705	-25.248	883.849	-20.823	838.175	-26.465
846.849	-25.248	884.000	-20.823	838.361	-26.463
847.178	-25.249	884.169	-20.823	838.452	-26.460
847.377	-25.249	884.386	-20.823	838.539	-26.458
847.358	-25.250	884.563	-20.822	838.612	-26.456
847.421	-25.250	884.680	-20.822	838.633	-26.454
847.625	-25.251	884.854	-20.822	838.664	-26.451
847.804	-25.251	885.086	-20.822	838.799	-26.449
847.928	-25.252	885.271	-20.821	838.932	-26.447
848.116	-25.252	885.386	-20.821	839.092	-26.445
848.258	-25.253	885.547	-20.821	839.288	-26.442
848.407	-25.253	885.707	-20.820	839.484	-26.440
848.594	-25.253	885.847	-20.820	839.675	-26.438
848.689	-25.254	886.000	-20.820	839.815	-26.435
848.894	-25.254	886.187	-20.820	839.995	-26.433
849.438	-25.255	886.391	-20.819	840.085	-26.431
849.787	-25.255	886.581	-20.819	840.275	-26.429
849.786	-25.255	886.758	-20.819	840.501	-26.426
849.954	-25.255	886.914	-20.818	840.617	-26.424
850.228	-25.256	887.045	-20.818	840.852	-26.422
850.257	-25.256	887.225	-20.818	841.057	-26.419

850.272	-25.256	887.366	-20.817	841.179	-26.417
850.395	-25.257	887.503	-20.817	841.283	-26.415
850.443	-25.257	887.829	-20.817	841.367	-26.412
850.549	-25.257	888.221	-20.816	841.481	-26.410
850.652	-25.257	888.463	-20.816	841.618	-26.408
850.797	-25.257	888.598	-20.816	841.812	-26.405
851.049	-25.258	888.687	-20.815	842.017	-26.403
851.227	-25.258	888.766	-20.815	842.217	-26.401
851.347	-25.258	888.776	-20.814	842.427	-26.398
851.496	-25.258	888.800	-20.814	842.589	-26.396
851.672	-25.258	888.888	-20.814	842.738	-26.394
851.824	-25.259	889.035	-20.813	842.939	-26.391
851.984	-25.259	889.210	-20.813	843.102	-26.389
852.236	-25.259	889.313	-20.813	843.247	-26.387
852.372	-25.259	889.526	-20.812	843.438	-26.384
852.467	-25.259	889.765	-20.812	843.596	-26.382
852.689	-25.259	889.909	-20.811	843.742	-26.380
852.931	-25.259	890.053	-20.811	843.868	-26.377
853.025	-25.259	890.253	-20.811	844.004	-26.375
853.164	-25.259	890.409	-20.810	844.112	-26.373
853.342	-25.260	890.546	-20.810	844.237	-26.370
853.408	-25.260	890.720	-20.809	844.443	-26.368
853.540	-25.260	890.856	-20.809	844.600	-26.366
853.725	-25.260	890.936	-20.808	844.742	-26.364
853.856	-25.260	891.069	-20.808	844.893	-26.361
854.024	-25.260	891.284	-20.808	845.039	-26.359
854.271	-25.260	891.462	-20.807	845.240	-26.357
854.396	-25.260	891.619	-20.807	845.468	-26.354
854.585	-25.260	891.779	-20.806	845.674	-26.352
854.769	-25.260	891.934	-20.806	845.835	-26.350

855.015	-25.260	892.098	-20.806	845.974	-26.348
855.303	-25.261	892.217	-20.805	846.103	-26.345
855.315	-25.261	892.334	-20.805	846.283	-26.343
855.302	-25.261	892.523	-20.804	846.500	-26.341
855.414	-25.261	892.785	-20.804	846.666	-26.339
855.645	-25.261	893.009	-20.804	846.787	-26.336
855.881	-25.261	893.173	-20.803	846.919	-26.334
856.099	-25.261	893.315	-20.803	847.130	-26.332
856.249	-25.261	893.428	-20.802	847.352	-26.330
856.318	-25.261	893.613	-20.802	847.530	-26.327
856.353	-25.261	893.832	-20.802	847.667	-26.325
856.498	-25.261	893.985	-20.801	847.799	-26.323
856.796	-25.262	894.080	-20.801	847.924	-26.321
857.072	-25.262	894.246	-20.801	847.979	-26.319
857.210	-25.262	894.467	-20.800	848.151	-26.316
857.309	-25.262	894.625	-20.800	848.376	-26.314
857.511	-25.262	894.730	-20.800	848.504	-26.312
857.680	-25.262	894.887	-20.799	848.713	-26.310
857.818	-25.262	895.083	-20.799	848.869	-26.308
858.011	-25.262	895.185	-20.799	849.043	-26.305
858.216	-25.262	895.376	-20.798	849.234	-26.303
858.363	-25.263	895.614	-20.798	849.447	-26.301
858.483	-25.263	895.804	-20.798	849.629	-26.299
858.512	-25.263	895.913	-20.797	849.715	-26.297
858.670	-25.263	896.018	-20.797	849.830	-26.295
858.829	-25.263	896.197	-20.797	849.979	-26.292
859.044	-25.263	896.363	-20.796	850.132	-26.290
859.312	-25.264	896.460	-20.796	850.311	-26.288
859.409	-25.264	896.600	-20.796	850.499	-26.286
859.519	-25.264	896.736	-20.796	850.616	-26.284

859.648	-25.264	896.867	-20.795	850.736	-26.282
859.837	-25.265	897.086	-20.795	850.862	-26.279
860.020	-25.265	897.308	-20.795	850.962	-26.277
860.185	-25.265	897.467	-20.794	851.106	-26.275
860.375	-25.265	897.571	-20.794	851.285	-26.273
860.576	-25.266	897.745	-20.794	851.550	-26.271
860.751	-25.266	897.990	-20.794	851.740	-26.269
860.854	-25.266	898.150	-20.793	851.890	-26.266
861.005	-25.266	898.261	-20.793	852.112	-26.264
861.192	-25.267	898.410	-20.793	852.326	-26.262
861.366	-25.267	898.600	-20.793	852.520	-26.260
861.572	-25.267	898.773	-20.792	852.692	-26.258
861.734	-25.268	898.906	-20.792	852.840	-26.255
861.964	-25.268	899.043	-20.792	852.998	-26.253
862.132	-25.268	899.232	-20.792	853.146	-26.251
862.279	-25.268	899.381	-20.792	853.304	-26.249
862.465	-25.269	899.545	-20.791	853.449	-26.247
862.552	-25.269	899.771	-20.791	853.568	-26.244
862.687	-25.269	899.899	-20.791	853.705	-26.242
862.867	-25.270	900.010	-20.791	853.844	-26.240
862.965	-25.270	900.190	-20.790	853.996	-26.238
863.126	-25.271	900.405	-20.790	854.155	-26.236
863.350	-25.271	900.617	-20.790	854.295	-26.233
863.427	-25.271	900.779	-20.790	854.422	-26.231
863.542	-25.272	900.904	-20.790	854.596	-26.229
863.702	-25.272	901.009	-20.789	854.816	-26.227
863.800	-25.272	901.143	-20.789	855.045	-26.225
863.973	-25.273	901.288	-20.789	855.255	-26.222
864.147	-25.273	901.434	-20.789	855.415	-26.220
864.293	-25.274	901.578	-20.789	855.579	-26.218

864.519	-25.274	901.749	-20.788	855.639	-26.216
864.648	-25.274	901.928	-20.788	855.772	-26.213
864.836	-25.275	902.105	-20.788	856.041	-26.211
865.108	-25.275	902.287	-20.788	856.239	-26.209
865.294	-25.276	902.451	-20.787	856.393	-26.207
865.462	-25.276	902.648	-20.787	856.557	-26.204
865.624	-25.276	902.887	-20.787	856.733	-26.202
865.782	-25.277	903.116	-20.787	856.841	-26.200
865.939	-25.277	903.256	-20.787	856.993	-26.198
866.102	-25.278	903.391	-20.786	857.224	-26.195
866.272	-25.278	903.574	-20.786	857.383	-26.193
866.480	-25.279	903.739	-20.786	857.540	-26.191
866.566	-25.279	903.886	-20.785	857.699	-26.188
866.596	-25.279	904.008	-20.785	857.846	-26.186
866.675	-25.280	904.162	-20.785	858.026	-26.184
866.752	-25.280	904.304	-20.785	858.179	-26.181
866.973	-25.281	904.419	-20.784	858.344	-26.179
867.273	-25.281	904.558	-20.784	858.501	-26.177
867.466	-25.282	904.678	-20.784	858.655	-26.175
867.666	-25.282	904.781	-20.783	858.810	-26.172
867.813	-25.283	904.958	-20.783	858.977	-26.170
867.980	-25.283	905.147	-20.783	859.167	-26.168
868.249	-25.283	905.347	-20.783	859.366	-26.165
868.400	-25.284	905.553	-20.782	859.445	-26.163
868.514	-25.284	905.759	-20.782	859.595	-26.160
868.624	-25.285	905.938	-20.782	859.808	-26.158
868.840	-25.285	906.022	-20.781	859.966	-26.156
869.050	-25.286	906.197	-20.781	860.054	-26.153
869.221	-25.286	906.433	-20.780	860.209	-26.151
869.499	-25.287	906.620	-20.780	860.421	-26.149

869.663	-25.287	906.802	-20.780	860.608	-26.146
869.741	-25.287	906.965	-20.779	860.745	-26.144
869.842	-25.288	907.066	-20.779	860.909	-26.142
870.032	-25.288	907.169	-20.779	861.273	-26.139
870.215	-25.289	907.374	-20.778	861.643	-26.137
870.300	-25.289	907.496	-20.778	861.836	-26.134
870.417	-25.289	907.673	-20.777	861.915	-26.132
870.600	-25.290	907.926	-20.777	862.004	-26.130
870.800	-25.290	908.137	-20.776	862.124	-26.127
870.936	-25.291	908.281	-20.776	862.177	-26.125
871.062	-25.291	908.437	-20.775	862.212	-26.122
871.281	-25.291	908.572	-20.775	862.290	-26.120
871.443	-25.292	908.706	-20.775	862.439	-26.117
871.582	-25.292	908.911	-20.774	862.610	-26.115
871.777	-25.293	909.125	-20.774	862.735	-26.112
871.937	-25.293	909.308	-20.773	862.896	-26.110
872.083	-25.293	909.400	-20.773	863.157	-26.108
872.210	-25.294	909.538	-20.772	863.346	-26.105
872.393	-25.294	909.782	-20.771	863.480	-26.103
872.622	-25.295	909.998	-20.771	863.691	-26.100
872.755	-25.295	910.134	-20.770	863.881	-26.098
872.925	-25.295	910.263	-20.770	864.041	-26.095
873.057	-25.296	910.479	-20.769	864.167	-26.093
873.166	-25.296	910.657	-20.769	864.306	-26.090
873.366	-25.296	910.798	-20.768	864.502	-26.088
873.527	-25.297	910.966	-20.768	864.703	-26.085
873.699	-25.297	911.096	-20.767	864.883	-26.083
873.905	-25.298	911.190	-20.766	865.011	-26.081
873.950	-25.298	911.359	-20.766	865.135	-26.078
874.174	-25.298	911.552	-20.765	865.219	-26.076

874.510	-25.299	911.656	-20.765	865.349	-26.073
874.661	-25.299	911.739	-20.764	865.585	-26.071
874.954	-25.299	911.948	-20.763	865.772	-26.068
875.316	-25.300	912.354	-20.763	865.898	-26.066
875.637	-25.300	912.813	-20.762	866.080	-26.063
875.739	-25.300	913.040	-20.761	866.281	-26.061
875.639	-25.301	913.044	-20.761	866.471	-26.058
875.686	-25.301	913.117	-20.760	866.650	-26.056
875.781	-25.301	913.186	-20.759	866.853	-26.054
875.859	-25.302	913.194	-20.759	867.066	-26.051
875.943	-25.302	913.290	-20.758	867.193	-26.049
876.065	-25.302	913.410	-20.757	867.298	-26.046
876.233	-25.303	913.515	-20.757	867.403	-26.044
876.452	-25.303	913.661	-20.756	867.561	-26.041
876.715	-25.303	913.876	-20.755	867.715	-26.039
876.858	-25.304	914.074	-20.755	867.853	-26.036
876.915	-25.304	914.202	-20.754	868.024	-26.034
877.002	-25.304	914.346	-20.753	868.156	-26.032
877.235	-25.305	914.557	-20.752	868.291	-26.029
877.454	-25.305	914.782	-20.752	868.449	-26.027
877.673	-25.305	914.989	-20.751	868.616	-26.024
877.792	-25.306	915.127	-20.750	868.817	-26.022
877.911	-25.306	915.247	-20.749	869.007	-26.020
878.118	-25.306	915.449	-20.749	869.159	-26.017
878.318	-25.307	915.575	-20.748	869.314	-26.015
878.458	-25.307	915.695	-20.747	869.490	-26.012
878.550	-25.307	915.939	-20.746	869.593	-26.010
878.716	-25.307	916.152	-20.746	869.699	-26.008
878.878	-25.308	916.274	-20.745	869.871	-26.005
878.975	-25.308	916.375	-20.744	870.019	-26.003

879.204	-25.308	916.497	-20.743	870.229	-26.001
879.416	-25.309	916.656	-20.743	870.470	-25.998
879.506	-25.309	916.837	-20.742	870.702	-25.996
879.650	-25.309	916.970	-20.741	870.872	-25.994
879.783	-25.310	917.122	-20.740	870.863	-25.991
879.902	-25.310	917.331	-20.740	871.011	-25.989
880.104	-25.311	917.524	-20.739	871.388	-25.987
880.340	-25.311	917.702	-20.738	871.562	-25.984
880.512	-25.311	917.872	-20.737	871.593	-25.982
880.648	-25.312	918.042	-20.737	871.749	-25.980
880.743	-25.312	918.212	-20.736	871.939	-25.977
880.913	-25.312	918.370	-20.735	872.068	-25.975
881.180	-25.313	918.506	-20.734	872.250	-25.973
881.339	-25.313	918.649	-20.734	872.437	-25.970
881.485	-25.314	918.777	-20.733	872.620	-25.968
881.668	-25.314	918.860	-20.732	872.813	-25.966
881.866	-25.314	919.016	-20.732	872.990	-25.963
882.012	-25.315	919.197	-20.731	873.123	-25.961
882.008	-25.315	919.393	-20.730	873.271	-25.959
882.065	-25.316	919.632	-20.729	873.410	-25.957
882.141	-25.316	919.768	-20.729	873.537	-25.954
882.385	-25.316	919.902	-20.728	873.720	-25.952
882.550	-25.317	920.088	-20.727	873.873	-25.950
882.683	-25.317	920.234	-20.727	874.000	-25.947
882.959	-25.318	920.347	-20.726	874.120	-25.945
883.234	-25.318	920.499	-20.725	874.236	-25.943
883.374	-25.319	920.687	-20.725	874.370	-25.940
883.471	-25.319	920.833	-20.724	874.492	-25.938
883.598	-25.320	920.991	-20.723	874.563	-25.936
883.758	-25.320	921.174	-20.723	874.755	-25.934

883.960	-25.321	921.357	-20.722	875.076	-25.931
884.155	-25.321	921.552	-20.721	875.231	-25.929
884.392	-25.322	921.724	-20.721	875.402	-25.927
884.635	-25.322	921.824	-20.720	875.703	-25.924
884.771	-25.323	921.945	-20.719	875.915	-25.922
884.936	-25.323	922.166	-20.719	875.992	-25.920
885.165	-25.324	922.373	-20.718	876.080	-25.917
885.276	-25.324	922.524	-20.718	876.309	-25.915
885.338	-25.325	922.685	-20.717	876.451	-25.913
885.459	-25.326	922.838	-20.716	876.597	-25.911
885.631	-25.326	922.989	-20.716	876.778	-25.908
885.827	-25.327	923.193	-20.715	876.988	-25.906
886.037	-25.327	923.389	-20.715	877.131	-25.904
886.173	-25.328	923.534	-20.714	877.232	-25.901
886.313	-25.329	923.644	-20.714	877.401	-25.899
886.451	-25.329	923.804	-20.713	877.558	-25.897
886.561	-25.330	923.979	-20.712	877.663	-25.894
886.747	-25.331	924.144	-20.712	877.769	-25.892
886.878	-25.331	924.297	-20.711	877.962	-25.890
887.078	-25.332	924.486	-20.711	878.210	-25.887
887.361	-25.333	924.724	-20.710	878.408	-25.885
887.436	-25.333	924.852	-20.710	878.459	-25.882
887.553	-25.334	924.960	-20.709	878.679	-25.880
887.784	-25.335	925.152	-20.709	878.952	-25.878
887.906	-25.335	925.311	-20.708	879.102	-25.875
888.083	-25.336	925.414	-20.708	879.279	-25.873
888.266	-25.337	925.514	-20.707	879.398	-25.871
888.371	-25.338	925.685	-20.707	879.556	-25.868
888.506	-25.338	925.862	-20.706	879.774	-25.866
888.657	-25.339	925.958	-20.706	879.956	-25.864

888.879	-25.340	926.074	-20.705	880.088	-25.861
888.942	-25.341	926.221	-20.705	880.255	-25.859
889.036	-25.341	926.397	-20.704	880.437	-25.856
889.308	-25.342	926.615	-20.704	880.614	-25.854
889.467	-25.343	926.830	-20.703	880.828	-25.852
889.475	-25.344	927.008	-20.703	881.029	-25.849
889.594	-25.344	927.225	-20.702	881.174	-25.847
889.770	-25.345	927.412	-20.702	881.253	-25.844
890.001	-25.346	927.573	-20.701	881.376	-25.842
890.257	-25.347	927.726	-20.701	881.590	-25.839
890.487	-25.348	927.850	-20.700	881.803	-25.837
890.704	-25.348	928.023	-20.700	882.011	-25.835
890.882	-25.349	928.231	-20.700	882.190	-25.832
891.107	-25.350	928.411	-20.699	882.289	-25.830
891.288	-25.351	928.553	-20.699	882.356	-25.827
891.431	-25.352	928.671	-20.698	882.510	-25.825
891.578	-25.352	928.763	-20.698	882.663	-25.822
891.686	-25.353	928.904	-20.697	882.795	-25.820
891.853	-25.354	929.103	-20.697	882.962	-25.817
892.120	-25.355	929.222	-20.696	883.175	-25.815
892.325	-25.356	929.350	-20.696	883.391	-25.812
892.401	-25.356	929.537	-20.695	883.569	-25.810
892.504	-25.357	929.719	-20.695	883.708	-25.807
892.610	-25.358	929.911	-20.694	883.804	-25.805
892.734	-25.359	930.106	-20.694	883.982	-25.802
892.902	-25.360	930.305	-20.694	884.169	-25.800
893.066	-25.361	930.454	-20.693	884.277	-25.797
893.231	-25.361	930.591	-20.693	884.489	-25.795
893.399	-25.362	930.745	-20.692	884.952	-25.792
893.583	-25.363	930.986	-20.692	885.348	-25.790

893.695	-25.364	931.220	-20.691	885.486	-25.787
893.910	-25.365	931.364	-20.691	885.527	-25.785
894.091	-25.365	931.459	-20.690	885.570	-25.782
894.257	-25.366	931.616	-20.690	885.654	-25.780
894.486	-25.367	931.771	-20.690	885.701	-25.777
894.637	-25.368	931.880	-20.689	885.736	-25.775
894.790	-25.369	932.011	-20.689	885.878	-25.772
894.775	-25.369	932.203	-20.688	886.090	-25.770
894.902	-25.370	932.376	-20.688	886.286	-25.767
895.153	-25.371	932.542	-20.687	886.460	-25.764
895.322	-25.372	932.701	-20.687	886.625	-25.762
895.480	-25.373	932.840	-20.686	886.846	-25.759
895.618	-25.373	933.018	-20.686	886.999	-25.757
895.749	-25.374	933.193	-20.686	887.091	-25.754
896.040	-25.375	933.419	-20.685	887.207	-25.752
896.325	-25.376	933.624	-20.685	887.380	-25.749
896.403	-25.376	933.832	-20.684	887.577	-25.746
896.544	-25.377	933.930	-20.684	887.799	-25.744
896.755	-25.378	933.957	-20.683	888.005	-25.741
896.933	-25.379	934.109	-20.683	888.130	-25.739
897.051	-25.380	934.312	-20.682	888.239	-25.736
897.125	-25.380	934.493	-20.682	888.373	-25.733
897.299	-25.381	934.639	-20.681	888.571	-25.731
897.560	-25.382	934.846	-20.681	888.764	-25.728
897.766	-25.383	935.030	-20.681	888.850	-25.726
897.907	-25.383	935.207	-20.680	888.950	-25.723
898.040	-25.384	935.415	-20.680	889.174	-25.720
898.242	-25.385	935.583	-20.679	889.392	-25.718
898.422	-25.386	935.729	-20.679	889.554	-25.715
898.503	-25.386	935.862	-20.678	889.706	-25.713

898.702	-25.387	935.985	-20.678	889.854	-25.710
898.894	-25.388	936.089	-20.677	890.051	-25.707
899.024	-25.389	936.258	-20.677	890.208	-25.705
899.247	-25.389	936.590	-20.677	890.353	-25.702
899.372	-25.390	937.046	-20.676	890.450	-25.700
899.424	-25.391	937.324	-20.676	890.551	-25.697
899.685	-25.391	937.368	-20.675	890.720	-25.694
899.982	-25.392	937.430	-20.675	890.960	-25.692
900.117	-25.393	937.585	-20.674	891.163	-25.689
900.263	-25.394	937.663	-20.674	891.236	-25.687
900.441	-25.394	937.682	-20.673	891.411	-25.684
900.733	-25.395	937.739	-20.673	891.601	-25.682
901.040	-25.396	937.831	-20.673	891.736	-25.679
901.183	-25.396	938.002	-20.672	891.941	-25.676
901.419	-25.397	938.174	-20.672	892.107	-25.674
901.507	-25.398	938.331	-20.671	892.243	-25.671
901.568	-25.398	938.534	-20.671	892.446	-25.669
901.640	-25.399	938.768	-20.670	892.670	-25.666
901.669	-25.400	938.954	-20.670	892.803	-25.664
901.650	-25.400	939.028	-20.669	892.915	-25.661
901.855	-25.401	939.185	-20.669	893.064	-25.659
902.186	-25.401	939.380	-20.668	893.170	-25.656
902.325	-25.402	939.508	-20.668	893.317	-25.654
902.282	-25.403	939.660	-20.668	893.499	-25.651
902.418	-25.403	939.858	-20.667	893.621	-25.649
902.709	-25.404	940.086	-20.667	893.828	-25.646
902.868	-25.405	940.315	-20.666	894.089	-25.644
902.993	-25.405	940.459	-20.666	894.283	-25.641
903.189	-25.406	940.528	-20.665	894.453	-25.639
903.419	-25.406	940.605	-20.665	894.592	-25.636

903.616	-25.407	940.751	-20.664	894.732	-25.634
903.773	-25.407	940.920	-20.664	894.929	-25.631
903.930	-25.408	941.087	-20.664	895.070	-25.629
904.109	-25.409	941.245	-20.663	895.143	-25.626
904.221	-25.409	941.378	-20.663	895.293	-25.624
904.296	-25.410	941.544	-20.662	895.500	-25.621
904.266	-25.410	941.729	-20.662	895.672	-25.619
904.433	-25.411	941.937	-20.661	895.819	-25.616
904.799	-25.411	942.126	-20.661	896.015	-25.614
904.967	-25.412	942.301	-20.661	896.246	-25.611
904.998	-25.413	942.482	-20.660	896.410	-25.609
905.089	-25.413	942.630	-20.660	896.538	-25.606
905.358	-25.414	942.785	-20.660	896.693	-25.604
905.775	-25.414	942.907	-20.659	896.880	-25.601
905.945	-25.415	943.054	-20.659	897.063	-25.599
905.900	-25.415	943.196	-20.658	897.221	-25.596
906.067	-25.416	943.376	-20.658	897.309	-25.594
906.393	-25.416	943.575	-20.658	897.400	-25.591
906.600	-25.417	943.689	-20.657	897.567	-25.589
906.740	-25.418	943.809	-20.657	897.711	-25.586
906.939	-25.418	943.975	-20.657	897.835	-25.584
907.032	-25.419	944.192	-20.656	897.998	-25.581
907.174	-25.419	944.389	-20.656	898.116	-25.579
907.399	-25.420	944.501	-20.656	898.256	-25.576
907.539	-25.420	944.641	-20.655	898.478	-25.574
907.623	-25.421	944.807	-20.655	898.671	-25.571
907.757	-25.421	944.958	-20.655	898.841	-25.569
907.954	-25.422	945.165	-20.654	899.065	-25.566
908.159	-25.422	945.292	-20.654	899.270	-25.564
908.331	-25.423	945.385	-20.654	899.397	-25.561

908.472	-25.424	945.574	-20.654	899.588	-25.559
908.603	-25.424	945.758	-20.653	899.762	-25.556
908.764	-25.425	945.919	-20.653	899.966	-25.554
908.940	-25.425	946.062	-20.653	900.146	-25.551
909.068	-25.426	946.255	-20.653	900.276	-25.549
909.220	-25.426	946.473	-20.652	900.411	-25.546
909.391	-25.427	946.682	-20.652	900.495	-25.544
909.520	-25.427	946.869	-20.652	900.674	-25.541
909.678	-25.428	947.001	-20.652	900.845	-25.539
909.877	-25.428	947.124	-20.651	900.981	-25.536
910.086	-25.429	947.288	-20.651	901.136	-25.534
910.232	-25.430	947.464	-20.651	901.255	-25.531
910.331	-25.430	947.608	-20.651	901.386	-25.529
910.440	-25.431	947.785	-20.651	901.562	-25.526
910.646	-25.431	947.962	-20.650	901.799	-25.523
910.899	-25.432	948.117	-20.650	902.053	-25.521
911.075	-25.432	948.283	-20.650	902.209	-25.518
911.229	-25.433	948.408	-20.650	902.381	-25.516
911.346	-25.434	948.584	-20.650	902.596	-25.513
911.439	-25.434	948.812	-20.649	902.719	-25.511
911.570	-25.435	948.952	-20.649	902.841	-25.508
911.746	-25.435	949.044	-20.649	903.055	-25.505
911.941	-25.436	949.203	-20.649	903.263	-25.503
912.102	-25.436	949.378	-20.649	903.416	-25.500
912.312	-25.437	949.548	-20.648	903.565	-25.498
912.551	-25.438	949.629	-20.648	903.721	-25.495
912.641	-25.438	949.717	-20.648	903.867	-25.492
912.678	-25.439	949.948	-20.648	904.007	-25.490
912.946	-25.439	950.101	-20.648	904.189	-25.487
913.200	-25.440	950.212	-20.648	904.366	-25.484

913.323	-25.440	950.351	-20.647	904.556	-25.482
913.463	-25.441	950.538	-20.647	904.734	-25.479
913.673	-25.442	950.732	-20.647	904.820	-25.476
913.894	-25.442	950.853	-20.647	904.946	-25.474
914.023	-25.443	951.040	-20.647	905.190	-25.471
914.183	-25.443	951.236	-20.647	905.359	-25.468
914.380	-25.444	951.443	-20.646	905.462	-25.466
914.510	-25.444	951.656	-20.646	905.641	-25.463
914.544	-25.445	951.849	-20.646	905.871	-25.460
914.606	-25.446	952.010	-20.646	906.028	-25.458
914.769	-25.446	952.120	-20.646	906.171	-25.455
914.989	-25.447	952.317	-20.646	906.356	-25.452
915.131	-25.447	952.533	-20.645	906.504	-25.450
915.192	-25.448	952.710	-20.645	906.631	-25.447
915.347	-25.448	952.828	-20.645	906.786	-25.444
915.663	-25.449	952.876	-20.645	907.001	-25.442
915.909	-25.449	953.010	-20.645	907.149	-25.439
916.027	-25.450	953.215	-20.644	907.271	-25.436
916.217	-25.450	953.361	-20.644	907.423	-25.433
916.421	-25.451	953.485	-20.644	907.536	-25.431
916.603	-25.451	953.630	-20.644	907.651	-25.428
916.841	-25.452	953.811	-20.644	907.917	-25.425
916.993	-25.452	954.027	-20.644	908.408	-25.422
917.116	-25.453	954.218	-20.643	908.804	-25.420
917.279	-25.453	954.396	-20.643	908.925	-25.417
917.393	-25.454	954.615	-20.643	908.976	-25.414
917.648	-25.454	954.843	-20.643	909.064	-25.411
917.807	-25.455	954.929	-20.643	909.119	-25.409
917.831	-25.455	955.057	-20.642	909.128	-25.406
917.997	-25.456	955.341	-20.642	909.230	-25.403

918.131	-25.456	955.526	-20.642	909.340	-25.400
918.224	-25.457	955.581	-20.642	909.502	-25.398
918.424	-25.457	955.636	-20.642	909.725	-25.395
918.596	-25.458	955.847	-20.641	909.834	-25.392
918.708	-25.458	956.053	-20.641	909.995	-25.389
918.856	-25.459	956.208	-20.641	910.201	-25.386
919.041	-25.459	956.424	-20.641	910.381	-25.384
919.238	-25.460	956.593	-20.641	910.555	-25.381
919.429	-25.460	956.692	-20.640	910.711	-25.378
919.590	-25.460	956.829	-20.640	910.913	-25.375
919.731	-25.461	956.943	-20.640	911.092	-25.372
919.901	-25.461	957.130	-20.640	911.228	-25.370
920.119	-25.462	957.330	-20.640	911.411	-25.367
920.275	-25.462	957.512	-20.639	911.608	-25.364
920.450	-25.462	957.689	-20.639	911.753	-25.361
920.701	-25.463	957.820	-20.639	911.861	-25.358
920.817	-25.463	957.975	-20.639	911.963	-25.355
920.863	-25.464	958.202	-20.638	912.093	-25.353
921.090	-25.464	958.390	-20.638	912.319	-25.350
921.344	-25.464	958.506	-20.638	912.478	-25.347
921.404	-25.465	958.659	-20.638	912.589	-25.344
921.505	-25.465	958.792	-20.637	912.706	-25.341
921.722	-25.465	958.949	-20.637	912.862	-25.339
921.915	-25.466	959.153	-20.637	913.056	-25.336
922.160	-25.466	959.340	-20.637	913.238	-25.333
922.336	-25.466	959.489	-20.637	913.438	-25.330
922.441	-25.467	959.597	-20.636	913.652	-25.327
922.638	-25.467	959.759	-20.636	913.858	-25.324
922.859	-25.467	959.960	-20.636	914.012	-25.322
923.055	-25.467	960.117	-20.636	914.137	-25.319

923.214	-25.468	960.284	-20.635	914.292	-25.316
923.388	-25.468	960.452	-20.635	914.436	-25.313
923.493	-25.468	960.462	-20.635	914.566	-25.310
923.667	-25.469	960.686	-20.635	914.770	-25.308
923.865	-25.469	961.242	-20.634	914.943	-25.305
923.993	-25.469	961.575	-20.634	915.061	-25.302
924.065	-25.469	961.665	-20.634	915.222	-25.299
924.181	-25.470	961.725	-20.634	915.361	-25.296
924.371	-25.470	961.807	-20.633	915.474	-25.294
924.486	-25.470	961.898	-20.633	915.646	-25.291
924.678	-25.470	961.973	-20.633	915.812	-25.288
924.931	-25.470	961.994	-20.633	915.965	-25.285
925.109	-25.471	962.115	-20.632	916.152	-25.282
925.307	-25.471	962.286	-20.632	916.351	-25.280
925.489	-25.471	962.475	-20.632	916.535	-25.277
925.612	-25.471	962.680	-20.632	916.740	-25.274
925.751	-25.472	962.826	-20.631	916.871	-25.271
925.855	-25.472	962.995	-20.631	916.955	-25.268
925.971	-25.472	963.150	-20.631	917.116	-25.266
926.249	-25.472	963.278	-20.631	917.317	-25.263
926.706	-25.472	963.447	-20.631	917.539	-25.260
927.059	-25.472	963.669	-20.630	917.663	-25.257
927.174	-25.472	963.895	-20.630	917.787	-25.255
927.185	-25.473	964.074	-20.630	917.989	-25.252
927.272	-25.473	964.185	-20.630	918.194	-25.249
927.381	-25.473	964.265	-20.629	918.385	-25.246
927.460	-25.473	964.426	-20.629	918.548	-25.244
927.516	-25.473	964.607	-20.629	918.682	-25.241
927.553	-25.473	964.722	-20.629	918.813	-25.238
927.708	-25.473	964.835	-20.628	918.988	-25.235

927.885	-25.473	965.021	-20.628	919.187	-25.233
928.072	-25.474	965.193	-20.628	919.359	-25.230
928.284	-25.474	965.314	-20.628	919.517	-25.227
928.454	-25.474	965.413	-20.628	919.708	-25.225
928.674	-25.474	965.547	-20.627	919.837	-25.222
928.903	-25.474	965.750	-20.627	919.982	-25.219
929.050	-25.474	965.920	-20.627	920.184	-25.216
929.190	-25.474	966.097	-20.627	920.362	-25.214
929.309	-25.474	966.307	-20.627	920.474	-25.211
929.444	-25.474	966.516	-20.626	920.583	-25.208
929.653	-25.474	966.719	-20.626	920.757	-25.206
929.856	-25.474	966.915	-20.626	920.888	-25.203
930.005	-25.475	967.068	-20.626	921.031	-25.200
930.117	-25.475	967.170	-20.626	921.178	-25.197
930.248	-25.475	967.309	-20.626	921.301	-25.195
930.455	-25.475	967.447	-20.626	921.435	-25.192
930.673	-25.475	967.606	-20.626	921.591	-25.189
930.719	-25.475	967.756	-20.625	921.747	-25.186
930.805	-25.475	967.916	-20.625	921.864	-25.184
931.014	-25.475	968.092	-20.625	922.030	-25.181
931.174	-25.475	968.260	-20.625	922.270	-25.178
931.322	-25.475	968.411	-20.625	922.523	-25.176
931.480	-25.475	968.533	-20.625	922.747	-25.173
931.694	-25.475	968.690	-20.625	922.949	-25.170
931.949	-25.476	968.891	-20.625	923.145	-25.167
932.169	-25.476	969.113	-20.625	923.289	-25.165
932.263	-25.476	969.280	-20.625	923.439	-25.162
932.374	-25.476	969.407	-20.625	923.622	-25.159
932.559	-25.476	969.568	-20.625	923.770	-25.156
932.741	-25.476	969.766	-20.625	923.867	-25.154

932.872	-25.476	969.903	-20.624	924.033	-25.151
932.980	-25.476	970.010	-20.624	924.189	-25.148
933.129	-25.476	970.182	-20.624	924.299	-25.145
933.307	-25.476	970.353	-20.624	924.445	-25.142
933.431	-25.476	970.525	-20.624	924.569	-25.140
933.577	-25.477	970.755	-20.624	924.714	-25.137
933.776	-25.477	970.932	-20.624	924.919	-25.134
933.940	-25.477	971.044	-20.624	925.119	-25.131
934.037	-25.477	971.211	-20.624	925.323	-25.129
934.197	-25.477	971.388	-20.624	925.542	-25.126
934.493	-25.477	971.519	-20.624	925.704	-25.123
934.625	-25.477	971.638	-20.624	925.882	-25.120
934.723	-25.477	971.814	-20.624	926.042	-25.117
934.938	-25.478	971.991	-20.624	926.144	-25.115
935.119	-25.478	972.122	-20.624	926.320	-25.112
935.296	-25.478	972.269	-20.624	926.509	-25.109
935.462	-25.478	972.420	-20.625	926.666	-25.106
935.702	-25.478	972.567	-20.625	926.865	-25.103
935.901	-25.478	972.729	-20.625	927.050	-25.101
935.918	-25.478	972.911	-20.625	927.174	-25.098
935.928	-25.479	973.105	-20.625	927.367	-25.095
936.146	-25.479	973.260	-20.625	927.598	-25.092
936.376	-25.479	973.411	-20.625	927.707	-25.089
936.544	-25.479	973.562	-20.625	927.803	-25.087
936.720	-25.479	973.732	-20.625	927.980	-25.084
936.827	-25.480	973.878	-20.625	928.142	-25.081
937.038	-25.480	974.040	-20.625	928.304	-25.078
937.306	-25.480	974.187	-20.625	928.458	-25.075
937.433	-25.480	974.259	-20.625	928.615	-25.072
937.592	-25.480	974.389	-20.625	928.858	-25.070

937.715	-25.481	974.510	-20.625	929.016	-25.067
937.799	-25.481	974.696	-20.625	929.137	-25.064
937.943	-25.481	974.905	-20.625	929.317	-25.061
938.206	-25.481	975.051	-20.625	929.453	-25.058
938.360	-25.482	975.220	-20.625	929.574	-25.055
938.481	-25.482	975.368	-20.625	929.761	-25.052
938.727	-25.482	975.507	-20.625	929.931	-25.050
938.935	-25.482	975.711	-20.625	930.068	-25.047
939.008	-25.483	975.983	-20.625	930.275	-25.044
939.121	-25.483	976.211	-20.625	930.458	-25.041
939.280	-25.483	976.415	-20.625	930.579	-25.038
939.464	-25.483	976.600	-20.625	930.720	-25.035
939.665	-25.484	976.768	-20.625	930.852	-25.032
939.824	-25.484	976.929	-20.625	930.963	-25.029
939.956	-25.484	977.029	-20.625	931.122	-25.027
940.123	-25.484	977.157	-20.625	931.380	-25.024
940.320	-25.485	977.299	-20.624	931.824	-25.021
940.458	-25.485	977.413	-20.624	932.226	-25.018
940.524	-25.485	977.573	-20.624	932.311	-25.015
940.558	-25.485	977.702	-20.624	932.359	-25.012
940.669	-25.486	977.798	-20.624	932.527	-25.009
940.890	-25.486	977.980	-20.624	932.633	-25.006
941.133	-25.486	978.205	-20.624	932.654	-25.003
941.321	-25.487	978.388	-20.624	932.716	-25.001
941.434	-25.487	978.564	-20.624	932.805	-24.998
941.555	-25.487	978.791	-20.624	932.930	-24.995
941.793	-25.487	978.973	-20.624	933.136	-24.992
942.103	-25.488	979.131	-20.623	933.310	-24.989
942.263	-25.488	979.302	-20.623	933.493	-24.986
942.374	-25.488	979.437	-20.623	933.699	-24.983

942.552	-25.489	979.597	-20.623	933.873	-24.980
942.739	-25.489	979.749	-20.623	934.032	-24.977
942.951	-25.489	979.954	-20.623	934.190	-24.975
943.118	-25.489	980.153	-20.623	934.377	-24.972
943.196	-25.490	980.257	-20.622	934.557	-24.969
943.369	-25.490	980.419	-20.622	934.680	-24.966
943.534	-25.490	980.577	-20.622	934.808	-24.963
943.622	-25.491	980.736	-20.622	934.958	-24.960
943.780	-25.491	980.910	-20.622	935.100	-24.957
943.919	-25.491	981.068	-20.621	935.240	-24.954
944.100	-25.491	981.235	-20.621	935.369	-24.951
944.293	-25.492	981.319	-20.621	935.573	-24.949
944.417	-25.492	981.478	-20.621	935.736	-24.946
944.596	-25.492	981.709	-20.620	935.844	-24.943
944.784	-25.492	981.913	-20.620	935.960	-24.940
944.939	-25.493	982.085	-20.620	936.125	-24.937
945.207	-25.493	982.206	-20.620	936.338	-24.934
945.444	-25.493	982.349	-20.619	936.561	-24.931
945.554	-25.493	982.494	-20.619	936.872	-24.928
945.687	-25.494	982.672	-20.619	937.092	-24.926
945.816	-25.494	982.795	-20.618	937.171	-24.923
945.988	-25.494	982.936	-20.618	937.287	-24.920
946.252	-25.494	983.075	-20.618	937.394	-24.917
946.448	-25.495	983.217	-20.618	937.559	-24.914
946.527	-25.495	983.407	-20.617	937.718	-24.911
946.618	-25.495	983.699	-20.617	937.824	-24.909
946.728	-25.495	983.885	-20.617	937.993	-24.906
946.930	-25.495	984.035	-20.616	938.173	-24.903
947.172	-25.496	984.176	-20.616	938.388	-24.900
947.299	-25.496	984.363	-20.616	938.566	-24.897

947.410	-25.496	984.563	-20.615	938.747	-24.894
947.592	-25.496	984.688	-20.615	938.883	-24.892
947.850	-25.496	984.781	-20.614	938.979	-24.889
948.100	-25.497	984.892	-20.614	939.154	-24.886
948.206	-25.497	985.242	-20.614	939.370	-24.883
948.278	-25.497	985.706	-20.613	939.494	-24.881
948.502	-25.497	985.993	-20.613	939.616	-24.878
948.639	-25.497	986.121	-20.613	939.863	-24.875
948.727	-25.497	986.159	-20.612	940.025	-24.872
948.949	-25.498	986.195	-20.612	940.139	-24.869
949.144	-25.498	986.219	-20.611	940.303	-24.867
949.312	-25.498	986.289	-20.611	940.434	-24.864
949.423	-25.498	986.404	-20.611	940.580	-24.861
949.536	-25.498	986.516	-20.610	940.808	-24.858
949.705	-25.498	986.649	-20.610	941.011	-24.856
949.833	-25.499	986.759	-20.609	941.174	-24.853
949.977	-25.499	986.904	-20.609	941.324	-24.850
950.262	-25.499	987.097	-20.609	941.462	-24.847
950.548	-25.499	987.326	-20.608	941.636	-24.845
950.661	-25.499	987.518	-20.608	941.781	-24.842
950.652	-25.499	987.647	-20.607	941.945	-24.839
950.762	-25.499	987.853	-20.607	942.141	-24.837
951.012	-25.500	988.057	-20.607	942.282	-24.834
951.178	-25.500	988.248	-20.606	942.409	-24.831
951.291	-25.500	988.430	-20.606	942.598	-24.828
951.404	-25.500	988.555	-20.605	942.803	-24.826
951.669	-25.500	988.668	-20.605	942.958	-24.823
952.122	-25.500	988.827	-20.604	943.127	-24.820
952.505	-25.500	989.003	-20.604	943.285	-24.817
952.732	-25.500	989.148	-20.604	943.429	-24.815

952.898	-25.501	989.313	-20.603	943.560	-24.812
952.962	-25.501	989.501	-20.603	943.652	-24.809
952.996	-25.501	989.622	-20.603	943.773	-24.807
953.086	-25.501	989.756	-20.602	943.912	-24.804
953.167	-25.501	989.910	-20.602	944.128	-24.801
953.279	-25.501	990.029	-20.601	944.314	-24.798
953.352	-25.501	990.258	-20.601	944.390	-24.796
953.382	-25.501	990.490	-20.601	944.492	-24.793
953.509	-25.501	990.651	-20.600	944.637	-24.790
953.784	-25.501	990.776	-20.600	944.843	-24.788
954.005	-25.501	990.947	-20.600	944.984	-24.785
954.094	-25.501	991.125	-20.599	945.126	-24.782
954.306	-25.502	991.339	-20.599	945.365	-24.779
954.516	-25.502	991.527	-20.599	945.555	-24.777
954.621	-25.502	991.655	-20.598	945.789	-24.774
954.762	-25.502	991.748	-20.598	945.996	-24.771
954.914	-25.502	991.888	-20.598	946.150	-24.768
955.114	-25.502	992.078	-20.598	946.357	-24.766
955.257	-25.502	992.244	-20.597	946.524	-24.763
955.353	-25.502	992.362	-20.597	946.664	-24.760
955.564	-25.502	992.486	-20.597	946.812	-24.757
955.701	-25.502	992.692	-20.597	946.933	-24.755
955.800	-25.502	992.862	-20.596	947.103	-24.752
955.977	-25.502	993.019	-20.596	947.237	-24.749
956.189	-25.503	993.231	-20.596	947.377	-24.746
956.395	-25.503	993.352	-20.596	947.549	-24.743
956.519	-25.503	993.493	-20.595	947.661	-24.741
956.672	-25.503	993.642	-20.595	947.825	-24.738
956.809	-25.503	993.792	-20.595	948.014	-24.735
956.908	-25.503	994.029	-20.595	948.202	-24.732

957.029	-25.503	994.181	-20.595	948.357	-24.729
957.225	-25.503	994.253	-20.594	948.481	-24.727
957.483	-25.503	994.389	-20.594	948.696	-24.724
957.647	-25.504	994.576	-20.594	948.936	-24.721
957.767	-25.504	994.744	-20.594	949.066	-24.718
958.009	-25.504	994.943	-20.594	949.202	-24.715
958.219	-25.504	995.164	-20.594	949.383	-24.713
958.254	-25.504	995.377	-20.593	949.549	-24.710
958.399	-25.504	995.506	-20.593	949.688	-24.707
958.676	-25.504	995.592	-20.593	949.812	-24.704
958.982	-25.505	995.748	-20.593	950.010	-24.701
959.101	-25.505	995.920	-20.593	950.259	-24.698
959.196	-25.505	996.124	-20.593	950.415	-24.695
959.184	-25.505	996.296	-20.593	950.502	-24.693
959.298	-25.505	996.394	-20.593	950.683	-24.690
959.604	-25.506	996.563	-20.593	950.865	-24.687
959.744	-25.506	996.753	-20.593	951.011	-24.684
960.013	-25.506	996.913	-20.593	951.214	-24.681
960.232	-25.506	997.094	-20.592	951.402	-24.678
960.300	-25.506	997.278	-20.592	951.557	-24.675
960.473	-25.507	997.473	-20.592	951.739	-24.672
960.730	-25.507	997.608	-20.592	951.881	-24.669
960.791	-25.507	997.718	-20.592	952.016	-24.667
960.934	-25.507	997.879	-20.592	952.160	-24.664
961.121	-25.508	998.064	-20.592	952.314	-24.661
961.186	-25.508	998.226	-20.592	952.478	-24.658
961.329	-25.508	998.290	-20.592	952.641	-24.655
961.525	-25.508	998.400	-20.592	952.788	-24.652
961.751	-25.509	998.609	-20.592	952.956	-24.649
961.917	-25.509	998.755	-20.592	953.120	-24.646

961.998	-25.509	998.852	-20.592	953.263	-24.643
962.216	-25.510	999.037	-20.592	953.459	-24.640
962.396	-25.510	999.258	-20.592	953.637	-24.637
962.518	-25.510	999.417	-20.592	953.790	-24.634
962.721	-25.511	999.617	-20.592	953.957	-24.631
962.871	-25.511	999.798	-20.592	954.105	-24.628
962.891	-25.511	999.958	-20.592	954.233	-24.625
963.064	-25.512	1.000.126	-20.592	954.409	-24.623
963.428	-25.512	1.000.350	-20.592	954.559	-24.620
963.503	-25.512	1.000.589	-20.592	954.759	-24.617
963.615	-25.513	1.000.775	-20.591	955.173	-24.614
963.854	-25.513	1.000.919	-20.591	955.583	-24.611
963.873	-25.514	1.001.068	-20.591	955.740	-24.608
964.053	-25.514	1.001.245	-20.591	955.743	-24.605
964.343	-25.514	1.001.363	-20.591	955.813	-24.602
964.457	-25.515	1.001.505	-20.591	955.894	-24.599
964.601	-25.515	1.001.700	-20.591	955.943	-24.596
964.845	-25.516	1.001.829	-20.591	956.057	-24.593
965.063	-25.516	1.001.929	-20.591	956.181	-24.590
965.195	-25.516	1.001.998	-20.591	956.296	-24.587
965.426	-25.517	1.002.076	-20.591	956.469	-24.584
965.533	-25.517	1.002.294	-20.591	956.656	-24.581
965.637	-25.518	1.002.498	-20.591	956.817	-24.578
965.828	-25.518	1.002.648	-20.591	956.991	-24.575
965.873	-25.518	1.002.844	-20.591	957.217	-24.573
965.956	-25.519	1.003.057	-20.590	957.414	-24.570
965.965	-25.519	1.003.188	-20.590	957.517	-24.567
966.107	-25.520	1.003.395	-20.590	957.656	-24.564
966.367	-25.520	1.003.632	-20.590	957.842	-24.561
966.531	-25.521	1.003.801	-20.590	957.990	-24.558

966.625	-25.521	1.003.980	-20.590	958.128	-24.555
966.795	-25.522	1.004.128	-20.590	958.266	-24.552
967.082	-25.522	1.004.221	-20.590	958.445	-24.549
967.322	-25.522	1.004.364	-20.589	958.637	-24.547
967.507	-25.523	1.004.587	-20.589	958.794	-24.544
967.735	-25.523	1.004.753	-20.589	958.949	-24.541
967.977	-25.524	1.004.831	-20.589	959.082	-24.538
968.148	-25.524	1.004.960	-20.589	959.184	-24.535
968.342	-25.525	1.005.197	-20.589	959.268	-24.532
968.529	-25.525	1.005.363	-20.588	959.474	-24.530
968.669	-25.526	1.005.483	-20.588	959.669	-24.527
968.767	-25.526	1.005.708	-20.588	959.829	-24.524
968.905	-25.527	1.005.870	-20.588	960.051	-24.521
969.031	-25.527	1.006.055	-20.588	960.246	-24.518
969.100	-25.527	1.006.251	-20.587	960.434	-24.516
969.239	-25.528	1.006.364	-20.587	960.617	-24.513
969.358	-25.528	1.006.553	-20.587	960.815	-24.510
969.491	-25.529	1.006.719	-20.587	960.968	-24.507
969.647	-25.529	1.006.840	-20.586	961.084	-24.505
969.815	-25.530	1.006.982	-20.586	961.205	-24.502
969.997	-25.530	1.007.167	-20.586	961.369	-24.499
970.202	-25.531	1.007.368	-20.586	961.525	-24.497
970.367	-25.531	1.007.486	-20.585	961.652	-24.494
970.583	-25.532	1.007.592	-20.585	961.764	-24.491
970.828	-25.532	1.007.786	-20.585	961.924	-24.489
970.988	-25.532	1.007.980	-20.585	962.196	-24.486
971.128	-25.533	1.008.207	-20.584	962.359	-24.483
971.283	-25.533	1.008.402	-20.584	962.465	-24.481
971.396	-25.534	1.008.566	-20.584	962.610	-24.478
971.506	-25.534	1.008.698	-20.584	962.792	-24.476

971.635	-25.535	1.008.798	-20.583	962.979	-24.473
971.864	-25.535	1.008.987	-20.583	963.108	-24.470
972.085	-25.536	1.009.184	-20.583	963.316	-24.468
972.227	-25.536	1.009.311	-20.582	963.471	-24.465
972.413	-25.536	1.009.485	-20.582	963.577	-24.463
972.554	-25.537	1.009.837	-20.582	963.730	-24.460
972.729	-25.537	1.010.222	-20.581	963.900	-24.458
972.889	-25.538	1.010.462	-20.581	964.122	-24.455
973.009	-25.538	1.010.586	-20.581	964.321	-24.453
973.223	-25.538	1.010.642	-20.580	964.464	-24.450
973.402	-25.539	1.010.687	-20.580	964.643	-24.448
973.496	-25.539	1.010.755	-20.580	964.792	-24.446
973.656	-25.540	1.010.795	-20.580	964.889	-24.443
973.867	-25.540	1.010.845	-20.579	965.030	-24.441
974.074	-25.541	1.010.948	-20.579	965.235	-24.438
974.271	-25.541	1.011.141	-20.579	965.447	-24.436
974.451	-25.541	1.011.320	-20.578	965.603	-24.434
974.613	-25.542	1.011.422	-20.578	965.724	-24.431
974.769	-25.542	1.011.572	-20.577	965.835	-24.429
974.913	-25.542	1.011.778	-20.577	966.031	-24.427
974.983	-25.543	1.012.053	-20.577	966.247	-24.424
975.105	-25.543	1.012.281	-20.576	966.367	-24.422
975.338	-25.544	1.012.412	-20.576	966.535	-24.420
975.518	-25.544	1.012.520	-20.576	966.721	-24.417
975.720	-25.544	1.012.704	-20.575	966.852	-24.415
975.946	-25.545	1.012.886	-20.575	966.995	-24.413
976.127	-25.545	1.013.031	-20.575	967.142	-24.411
976.244	-25.546	1.013.161	-20.574	967.234	-24.408
976.280	-25.546	1.013.294	-20.574	967.364	-24.406
976.453	-25.546	1.013.435	-20.574	967.546	-24.404

976.753	-25.547	1.013.624	-20.573	967.647	-24.402
976.943	-25.547	1.013.837	-20.573	967.760	-24.400
977.001	-25.547	1.013.969	-20.573	967.991	-24.397
977.122	-25.548	1.014.044	-20.573	968.130	-24.395
977.467	-25.548	1.014.144	-20.572	968.260	-24.393
977.887	-25.548	1.014.324	-20.572	968.447	-24.391
978.123	-25.549	1.014.563	-20.572	968.549	-24.389
978.223	-25.549	1.014.782	-20.571	968.735	-24.386
978.233	-25.550	1.014.962	-20.571	968.979	-24.384
978.307	-25.550	1.015.154	-20.571	969.196	-24.382
978.427	-25.550	1.015.303	-20.571	969.398	-24.380
978.546	-25.551	1.015.456	-20.570	969.637	-24.378
978.661	-25.551	1.015.632	-20.570	969.808	-24.376
978.736	-25.551	1.015.825	-20.570	969.982	-24.373
978.807	-25.552	1.015.985	-20.569	970.209	-24.371
979.094	-25.552	1.016.116	-20.569	970.324	-24.369
979.122	-25.552	1.016.296	-20.569	970.394	-24.367
978.979	-25.553	1.016.461	-20.569	970.481	-24.365
979.283	-25.553	1.016.646	-20.568	970.631	-24.363
979.572	-25.553	1.016.827	-20.568	970.786	-24.361
979.756	-25.554	1.016.937	-20.568	970.941	-24.359
979.985	-25.554	1.017.022	-20.568	971.104	-24.357
980.188	-25.554	1.017.197	-20.568	971.262	-24.354
980.378	-25.555	1.017.353	-20.567	971.442	-24.352
980.713	-25.555	1.017.504	-20.567	971.626	-24.350
981.001	-25.556	1.017.680	-20.567	971.833	-24.348
981.074	-25.556	1.017.823	-20.567	971.966	-24.346
981.087	-25.556	1.017.993	-20.567	972.152	-24.344
981.252	-25.557	1.018.184	-20.566	972.368	-24.342
981.515	-25.557	1.018.369	-20.566	972.485	-24.340

981.618	-25.558	1.018.459	-20.566	972.656	-24.338
981.749	-25.558	1.018.587	-20.566	972.870	-24.335
981.703	-25.558	1.018.749	-20.566	973.047	-24.333
981.660	-25.559	1.018.902	-20.566	973.253	-24.331
981.880	-25.559	1.019.052	-20.566	973.381	-24.329
982.171	-25.560	1.019.292	-20.565	973.473	-24.327
982.451	-25.560	1.019.522	-20.565	973.625	-24.325
982.639	-25.560	1.019.634	-20.565	973.823	-24.323
982.831	-25.561	1.019.774	-20.565	974.013	-24.321
983.055	-25.561	1.019.927	-20.565	974.159	-24.319
983.180	-25.562	1.020.068	-20.565	974.342	-24.317
983.380	-25.562	1.020.201	-20.565	974.482	-24.314
983.616	-25.563	1.020.358	-20.565	974.598	-24.312
983.699	-25.563	1.020.548	-20.565	974.740	-24.310
983.766	-25.564	1.020.689	-20.565	974.942	-24.308
983.925	-25.564	1.020.850	-20.565	975.154	-24.306
984.152	-25.565	1.021.065	-20.565	975.288	-24.304
984.367	-25.565	1.021.221	-20.565	975.450	-24.302
984.462	-25.565	1.021.356	-20.565	975.614	-24.300
984.342	-25.566	1.021.584	-20.565	975.750	-24.297
984.541	-25.566	1.021.835	-20.565	975.919	-24.295
984.932	-25.567	1.021.983	-20.565	976.085	-24.293
985.055	-25.567	1.022.089	-20.564	976.234	-24.291
985.156	-25.568	1.022.189	-20.564	976.348	-24.289
985.340	-25.569	1.022.359	-20.564	976.500	-24.287
985.479	-25.569	1.022.530	-20.564	976.751	-24.285
985.652	-25.570	1.022.697	-20.564	976.930	-24.283
985.834	-25.570	1.022.824	-20.564	977.096	-24.280
985.978	-25.571	1.022.880	-20.564	977.216	-24.278
986.126	-25.571	1.023.040	-20.564	977.353	-24.276

986.183	-25.572	1.023.282	-20.564	977.495	-24.274
986.335	-25.572	1.023.521	-20.564	977.663	-24.272
986.529	-25.573	1.023.598	-20.564	977.868	-24.270
986.631	-25.573	1.023.651	-20.564	978.045	-24.268
986.861	-25.574	1.023.867	-20.564	978.419	-24.266
987.067	-25.575	1.024.112	-20.564	978.884	-24.263
987.165	-25.575	1.024.314	-20.564	979.051	-24.261
987.210	-25.576	1.024.485	-20.564	979.100	-24.259
987.525	-25.576	1.024.670	-20.564	979.182	-24.257
987.779	-25.577	1.024.843	-20.564	979.242	-24.255
987.669	-25.578	1.025.025	-20.564	979.270	-24.253
987.865	-25.578	1.025.148	-20.564	979.372	-24.250
988.128	-25.579	1.025.313	-20.564	979.489	-24.248
988.104	-25.580	1.025.483	-20.564	979.574	-24.246
988.385	-25.580	1.025.658	-20.563	979.753	-24.244
988.805	-25.581	1.025.839	-20.563	979.986	-24.242
989.016	-25.581	1.026.005	-20.563	980.161	-24.240
989.175	-25.582	1.026.100	-20.563	980.331	-24.238
989.289	-25.583	1.026.182	-20.563	980.535	-24.235
989.474	-25.583	1.026.379	-20.563	980.710	-24.233
989.690	-25.584	1.026.551	-20.563	980.875	-24.231
989.818	-25.585	1.026.640	-20.563	980.991	-24.229
990.021	-25.585	1.026.792	-20.563	981.096	-24.227
990.192	-25.586	1.026.918	-20.562	981.262	-24.224
990.281	-25.586	1.027.053	-20.562	981.417	-24.222
990.397	-25.587	1.027.278	-20.562	981.579	-24.220
990.571	-25.588	1.027.457	-20.562	981.764	-24.218
990.723	-25.588	1.027.596	-20.562	981.919	-24.216
990.866	-25.589	1.027.825	-20.562	982.081	-24.214
991.103	-25.590	1.028.012	-20.561	982.226	-24.211

991.216	-25.590	1.028.159	-20.561	982.358	-24.209
991.386	-25.591	1.028.285	-20.561	982.492	-24.207
991.609	-25.591	1.028.406	-20.561	982.611	-24.205
991.687	-25.592	1.028.615	-20.560	982.768	-24.203
991.805	-25.593	1.028.836	-20.560	982.970	-24.201
991.954	-25.593	1.029.024	-20.560	983.152	-24.198
992.055	-25.594	1.029.202	-20.560	983.317	-24.196
992.150	-25.594	1.029.374	-20.559	983.531	-24.194
992.315	-25.595	1.029.508	-20.559	983.747	-24.192
992.483	-25.596	1.029.642	-20.559	983.923	-24.190
992.695	-25.596	1.029.835	-20.559	984.101	-24.188
992.924	-25.597	1.030.044	-20.558	984.202	-24.186
993.173	-25.597	1.030.203	-20.558	984.280	-24.183
993.406	-25.598	1.030.335	-20.558	984.433	-24.181
993.584	-25.598	1.030.463	-20.557	984.613	-24.179
993.712	-25.599	1.030.634	-20.557	984.794	-24.177
993.865	-25.600	1.030.824	-20.557	984.941	-24.175
994.092	-25.600	1.030.993	-20.556	985.098	-24.173
994.308	-25.601	1.031.178	-20.556	985.284	-24.171
994.454	-25.601	1.031.327	-20.556	985.427	-24.168
994.526	-25.602	1.031.482	-20.555	985.581	-24.166
994.570	-25.602	1.031.675	-20.555	985.789	-24.164
994.743	-25.603	1.031.836	-20.554	985.956	-24.162
995.022	-25.603	1.031.980	-20.554	986.105	-24.160
995.194	-25.604	1.032.151	-20.554	986.272	-24.158
995.268	-25.604	1.032.303	-20.553	986.423	-24.156
995.358	-25.604	1.032.449	-20.553	986.604	-24.153
995.413	-25.605	1.032.608	-20.552	986.788	-24.151
995.536	-25.605	1.032.806	-20.552	986.936	-24.149
995.796	-25.606	1.033.006	-20.551	987.076	-24.147

996.009	-25.606	1.033.130	-20.551	987.255	-24.145
996.153	-25.606	1.033.244	-20.551	987.438	-24.143
996.350	-25.607	1.033.347	-20.550	987.597	-24.141
996.596	-25.607	1.033.474	-20.550	987.777	-24.139
996.767	-25.608	1.033.641	-20.549	987.955	-24.137
996.913	-25.608	1.033.989	-20.549	988.131	-24.135
997.114	-25.608	1.034.437	-20.548	988.283	-24.132
997.292	-25.608	1.034.710	-20.548	988.415	-24.130
997.387	-25.609	1.034.846	-20.547	988.565	-24.128
997.588	-25.609	1.034.937	-20.547	988.680	-24.126
997.790	-25.609	1.035.036	-20.546	988.867	-24.124
997.980	-25.610	1.035.117	-20.546	989.065	-24.122
998.158	-25.610	1.035.139	-20.545	989.211	-24.120
998.280	-25.610	1.035.175	-20.544	989.376	-24.118
998.410	-25.610	1.035.285	-20.544	989.584	-24.116
998.571	-25.611	1.035.431	-20.543	989.775	-24.114
998.801	-25.611	1.035.602	-20.543	989.877	-24.112
998.954	-25.611	1.035.803	-20.542	990.038	-24.110
999.127	-25.611	1.035.953	-20.542	990.229	-24.107
999.314	-25.611	1.036.115	-20.541	990.419	-24.105
999.386	-25.611	1.036.287	-20.540	990.571	-24.103
999.551	-25.612	1.036.435	-20.540	990.687	-24.101
999.949	-25.612	1.036.685	-20.539	990.829	-24.099
1.000.064	-25.612	1.036.919	-20.539	990.959	-24.097
1.000.237	-25.612	1.037.059	-20.538	991.109	-24.095
1.000.352	-25.612	1.037.160	-20.537	991.266	-24.093
1.000.322	-25.612	1.037.317	-20.537	991.384	-24.091
1.000.607	-25.612	1.037.511	-20.536	991.460	-24.089
1.000.810	-25.612	1.037.660	-20.536	991.610	-24.086
1.000.944	-25.612	1.037.745	-20.535	991.906	-24.084

1.001.081	-25.612	1.037.919	-20.534	992.091	-24.082
1.001.226	-25.612	1.038.146	-20.534	992.204	-24.080
1.001.366	-25.613	1.038.288	-20.533	992.378	-24.078
1.001.492	-25.613	1.038.430	-20.533	992.644	-24.076
1.001.681	-25.613	1.038.584	-20.532	992.902	-24.074
1.001.849	-25.613	1.038.719	-20.531	993.036	-24.071
1.001.971	-25.613	1.038.864	-20.531	993.189	-24.069
1.002.139	-25.613	1.039.042	-20.530	993.407	-24.067
1.002.345	-25.613	1.039.260	-20.530	993.568	-24.065
1.002.452	-25.613	1.039.414	-20.529	993.721	-24.063
1.002.548	-25.613	1.039.542	-20.529	993.872	-24.061
1.002.878	-25.613	1.039.725	-20.528	993.968	-24.058
1.003.288	-25.612	1.039.890	-20.527	994.063	-24.056
1.003.653	-25.612	1.040.082	-20.527	994.202	-24.054
1.003.821	-25.612	1.040.258	-20.526	994.411	-24.052
1.003.833	-25.612	1.040.378	-20.526	994.610	-24.049
1.003.964	-25.612	1.040.529	-20.525	994.724	-24.047
1.003.986	-25.612	1.040.711	-20.525	994.828	-24.045
1.004.025	-25.612	1.040.834	-20.524	994.997	-24.043
1.004.103	-25.612	1.040.910	-20.523	995.195	-24.041
1.004.316	-25.612	1.041.012	-20.523	995.394	-24.038
1.004.426	-25.612	1.041.187	-20.522	995.630	-24.036
1.004.490	-25.612	1.041.376	-20.522	995.838	-24.034
1.004.595	-25.611	1.041.537	-20.521	995.938	-24.031
1.004.849	-25.611	1.041.668	-20.521	996.069	-24.029
1.005.068	-25.611	1.041.917	-20.520	996.238	-24.027
1.005.206	-25.611	1.042.138	-20.520	996.407	-24.025
1.005.451	-25.611	1.042.193	-20.519	996.563	-24.022
1.005.583	-25.611	1.042.325	-20.519	996.759	-24.020
1.005.689	-25.611	1.042.542	-20.518	996.923	-24.018

1.005.890	-25.610	1.042.681	-20.518	997.028	-24.015
1.006.048	-25.610	1.042.833	-20.518	997.236	-24.013
1.006.170	-25.610	1.042.986	-20.517	997.449	-24.011
1.006.330	-25.610	1.043.132	-20.517	997.585	-24.008
1.006.563	-25.610	1.043.301	-20.516	997.725	-24.006
1.006.711	-25.610	1.043.436	-20.516	997.868	-24.004
1.006.825	-25.610	1.043.613	-20.515	998.040	-24.001
1.007.042	-25.609	1.043.879	-20.515	998.157	-23.999
1.007.198	-25.609	1.044.100	-20.515	998.325	-23.996
1.007.265	-25.609	1.044.305	-20.514	998.533	-23.994
1.007.357	-25.609	1.044.479	-20.514	998.720	-23.992
1.007.469	-25.609	1.044.600	-20.514	998.887	-23.989
1.007.611	-25.609	1.044.706	-20.513	998.964	-23.987
1.007.870	-25.609	1.044.806	-20.513	999.115	-23.984
1.008.074	-25.608	1.044.989	-20.513	999.352	-23.982
1.008.250	-25.608	1.045.204	-20.512	999.498	-23.979
1.008.469	-25.608	1.045.384	-20.512	999.606	-23.977
1.008.642	-25.608	1.045.511	-20.512	999.813	-23.975
1.008.805	-25.608	1.045.659	-20.511	1.000.023	-23.972
1.008.966	-25.608	1.045.811	-20.511	1.000.208	-23.970
1.009.137	-25.608	1.045.993	-20.511	1.000.433	-23.967
1.009.268	-25.607	1.046.236	-20.511	1.000.596	-23.965
1.009.353	-25.607	1.046.358	-20.510	1.000.695	-23.962
1.009.509	-25.607	1.046.460	-20.510	1.000.836	-23.960
1.009.669	-25.607	1.046.687	-20.510	1.001.024	-23.957
1.009.836	-25.607	1.046.901	-20.510	1.001.154	-23.955
1.010.001	-25.607	1.047.000	-20.509	1.001.275	-23.952
1.010.153	-25.607	1.047.100	-20.509	1.001.606	-23.950
1.010.285	-25.607	1.047.233	-20.509	1.002.057	-23.947
1.010.460	-25.607	1.047.402	-20.509	1.002.303	-23.945

1.010.678	-25.607	1.047.570	-20.508	1.002.413	-23.942
1.010.892	-25.607	1.047.645	-20.508	1.002.534	-23.940
1.011.024	-25.606	1.047.755	-20.508	1.002.586	-23.937
1.011.132	-25.606	1.047.875	-20.508	1.002.577	-23.935
1.011.227	-25.606	1.048.031	-20.508	1.002.626	-23.932
1.011.305	-25.606	1.048.238	-20.507	1.002.747	-23.930
1.011.523	-25.606	1.048.466	-20.507	1.002.893	-23.927
1.011.779	-25.606	1.048.675	-20.507	1.003.050	-23.925
1.011.991	-25.606	1.048.883	-20.507	1.003.197	-23.922
1.012.162	-25.606	1.049.079	-20.507	1.003.366	-23.920
1.012.339	-25.606	1.049.237	-20.507	1.003.557	-23.917
1.012.540	-25.606	1.049.367	-20.506	1.003.705	-23.915
1.012.686	-25.606	1.049.539	-20.506	1.003.847	-23.912
1.012.764	-25.606	1.049.750	-20.506	1.004.031	-23.910
1.012.879	-25.606	1.049.878	-20.506	1.004.210	-23.907
1.013.038	-25.606	1.050.023	-20.506	1.004.395	-23.905
1.013.203	-25.606	1.050.135	-20.506	1.004.530	-23.902
1.013.385	-25.606	1.050.281	-20.506	1.004.652	-23.899
1.013.563	-25.606	1.050.479	-20.505	1.004.797	-23.897
1.013.689	-25.606	1.050.605	-20.505	1.004.986	-23.894
1.013.893	-25.606	1.050.712	-20.505	1.005.176	-23.892
1.014.088	-25.606	1.050.761	-20.505	1.005.294	-23.889
1.014.240	-25.606	1.050.918	-20.505	1.005.446	-23.887
1.014.425	-25.606	1.051.156	-20.505	1.005.656	-23.884
1.014.535	-25.606	1.051.313	-20.505	1.005.781	-23.882
1.014.626	-25.606	1.051.504	-20.505	1.005.904	-23.879
1.014.790	-25.607	1.051.705	-20.504	1.006.091	-23.877
1.014.993	-25.607	1.051.891	-20.504	1.006.294	-23.874
1.015.163	-25.607	1.052.076	-20.504	1.006.519	-23.872
1.015.277	-25.607	1.052.299	-20.504	1.006.683	-23.869

1.015.458	-25.607	1.052.518	-20.504	1.006.799	-23.867
1.015.668	-25.607	1.052.652	-20.504	1.006.989	-23.864
1.015.832	-25.607	1.052.765	-20.504	1.007.187	-23.862
1.016.053	-25.607	1.052.905	-20.504	1.007.380	-23.859
1.016.205	-25.607	1.053.074	-20.504	1.007.474	-23.857
1.016.281	-25.607	1.053.260	-20.504	1.007.533	-23.854
1.016.368	-25.607	1.053.440	-20.503	1.007.758	-23.852
1.016.514	-25.607	1.053.577	-20.503	1.007.963	-23.849
1.016.684	-25.607	1.053.745	-20.503	1.008.137	-23.847
1.016.825	-25.607	1.053.907	-20.503	1.008.324	-23.844
1.016.938	-25.607	1.054.022	-20.503	1.008.456	-23.842
1.017.095	-25.607	1.054.239	-20.503	1.008.612	-23.839
1.017.279	-25.607	1.054.449	-20.503	1.008.754	-23.837
1.017.393	-25.607	1.054.604	-20.503	1.008.876	-23.834
1.017.560	-25.607	1.054.741	-20.503	1.009.093	-23.832
1.017.720	-25.607	1.054.914	-20.503	1.009.289	-23.830
1.017.884	-25.607	1.055.050	-20.502	1.009.423	-23.827
1.018.081	-25.607	1.055.183	-20.502	1.009.611	-23.825
1.018.303	-25.607	1.055.406	-20.502	1.009.766	-23.822
1.018.512	-25.607	1.055.600	-20.502	1.009.902	-23.820
1.018.687	-25.607	1.055.745	-20.502	1.010.015	-23.817
1.018.837	-25.607	1.055.867	-20.502	1.010.127	-23.815
1.019.099	-25.607	1.055.997	-20.502	1.010.303	-23.813
1.019.389	-25.607	1.056.145	-20.502	1.010.479	-23.810
1.019.502	-25.607	1.056.309	-20.502	1.010.695	-23.808
1.019.651	-25.607	1.056.457	-20.502	1.010.882	-23.806
1.019.817	-25.607	1.056.618	-20.502	1.011.009	-23.803
1.019.942	-25.607	1.056.824	-20.502	1.011.151	-23.801
1.020.083	-25.607	1.056.990	-20.501	1.011.336	-23.799
1.020.179	-25.607	1.057.150	-20.501	1.011.507	-23.796

1.020.245	-25.607	1.057.338	-20.501	1.011.650	-23.794
1.020.469	-25.607	1.057.518	-20.501	1.011.828	-23.792
1.020.695	-25.607	1.057.654	-20.501	1.011.982	-23.789
1.020.799	-25.607	1.057.795	-20.501	1.012.156	-23.787
1.020.932	-25.607	1.057.906	-20.501	1.012.359	-23.785
1.021.112	-25.607	1.058.046	-20.501	1.012.505	-23.782
1.021.236	-25.607	1.058.272	-20.501	1.012.641	-23.780
1.021.348	-25.607	1.058.654	-20.501	1.012.788	-23.778
1.021.576	-25.606	1.059.068	-20.501	1.012.998	-23.776
1.021.817	-25.606	1.059.176	-20.501	1.013.174	-23.773
1.021.991	-25.606	1.059.214	-20.501	1.013.319	-23.771
1.022.149	-25.606	1.059.322	-20.500	1.013.491	-23.769
1.022.334	-25.606	1.059.426	-20.500	1.013.653	-23.767
1.022.497	-25.606	1.059.455	-20.500	1.013.786	-23.764
1.022.607	-25.606	1.059.507	-20.500	1.013.887	-23.762
1.022.770	-25.605	1.059.636	-20.500	1.014.026	-23.760
1.022.973	-25.605	1.059.819	-20.500	1.014.205	-23.758
1.023.130	-25.605	1.059.992	-20.500	1.014.343	-23.756
1.023.314	-25.605	1.060.124	-20.500	1.014.434	-23.753
1.023.507	-25.605	1.060.294	-20.500	1.014.600	-23.751
1.023.632	-25.604	1.060.508	-20.500	1.014.791	-23.749
1.023.720	-25.604	1.060.678	-20.500	1.014.889	-23.747
1.023.872	-25.604	1.060.890	-20.500	1.015.062	-23.744
1.024.056	-25.604	1.061.090	-20.500	1.015.262	-23.742
1.024.228	-25.604	1.061.239	-20.499	1.015.414	-23.740
1.024.406	-25.603	1.061.395	-20.499	1.015.629	-23.738
1.024.533	-25.603	1.061.522	-20.499	1.015.906	-23.735
1.024.710	-25.603	1.061.658	-20.499	1.016.174	-23.733
1.024.985	-25.603	1.061.843	-20.499	1.016.366	-23.731
1.025.135	-25.602	1.061.984	-20.499	1.016.503	-23.729

1.025.229	-25.602	1.062.096	-20.499	1.016.641	-23.727
1.025.363	-25.602	1.062.224	-20.499	1.016.818	-23.724
1.025.511	-25.601	1.062.375	-20.499	1.016.991	-23.722
1.025.675	-25.601	1.062.537	-20.499	1.017.182	-23.720
1.025.861	-25.601	1.062.680	-20.499	1.017.281	-23.718
1.025.988	-25.601	1.062.831	-20.499	1.017.337	-23.715
1.026.145	-25.600	1.062.990	-20.499	1.017.478	-23.713
1.026.329	-25.600	1.063.161	-20.499	1.017.650	-23.711
1.026.553	-25.600	1.063.346	-20.499	1.017.797	-23.709
1.026.797	-25.599	1.063.570	-20.499	1.017.944	-23.706
1.026.878	-25.599	1.063.764	-20.499	1.018.101	-23.704
1.027.022	-25.599	1.063.895	-20.499	1.018.280	-23.702
1.027.243	-25.598	1.064.066	-20.499	1.018.463	-23.700
1.027.376	-25.598	1.064.244	-20.499	1.018.642	-23.697
1.027.497	-25.598	1.064.393	-20.499	1.018.818	-23.695
1.027.662	-25.597	1.064.572	-20.499	1.018.964	-23.693
1.027.847	-25.597	1.064.759	-20.499	1.019.121	-23.691
1.027.965	-25.597	1.064.885	-20.499	1.019.275	-23.688
1.028.068	-25.596	1.064.965	-20.499	1.019.441	-23.686
1.028.439	-25.596	1.065.105	-20.499	1.019.652	-23.684
1.028.942	-25.596	1.065.279	-20.499	1.019.801	-23.681
1.029.224	-25.595	1.065.403	-20.499	1.019.911	-23.679
1.029.294	-25.595	1.065.550	-20.499	1.020.051	-23.677
1.029.367	-25.595	1.065.760	-20.499	1.020.286	-23.675
1.029.490	-25.594	1.065.965	-20.499	1.020.486	-23.672
1.029.605	-25.594	1.066.075	-20.499	1.020.628	-23.670
1.029.584	-25.594	1.066.231	-20.499	1.020.767	-23.668
1.029.667	-25.593	1.066.442	-20.499	1.020.919	-23.665
1.029.827	-25.593	1.066.578	-20.499	1.021.117	-23.663
1.029.833	-25.593	1.066.743	-20.499	1.021.295	-23.661

1.029.863	-25.592	1.066.915	-20.500	1.021.420	-23.658
1.030.070	-25.592	1.067.047	-20.500	1.021.594	-23.656
1.030.329	-25.592	1.067.216	-20.500	1.021.818	-23.654
1.030.522	-25.591	1.067.377	-20.500	1.022.013	-23.651
1.030.649	-25.591	1.067.543	-20.500	1.022.166	-23.649
1.030.823	-25.591	1.067.730	-20.500	1.022.289	-23.646
1.031.027	-25.590	1.067.885	-20.500	1.022.421	-23.644
1.031.197	-25.590	1.068.033	-20.500	1.022.559	-23.642
1.031.410	-25.590	1.068.270	-20.500	1.022.672	-23.639
1.031.603	-25.589	1.068.531	-20.501	1.022.868	-23.637
1.031.720	-25.589	1.068.670	-20.501	1.023.114	-23.634
1.031.808	-25.589	1.068.761	-20.501	1.023.298	-23.632
1.031.974	-25.589	1.068.915	-20.501	1.023.511	-23.630
1.032.150	-25.588	1.069.132	-20.501	1.023.708	-23.627
1.032.262	-25.588	1.069.297	-20.501	1.023.822	-23.625
1.032.420	-25.588	1.069.398	-20.501	1.023.944	-23.622
1.032.583	-25.588	1.069.501	-20.502	1.024.158	-23.620
1.032.730	-25.587	1.069.631	-20.502	1.024.296	-23.617
1.032.896	-25.587	1.069.788	-20.502	1.024.410	-23.615
1.033.066	-25.587	1.069.967	-20.502	1.024.557	-23.613
1.033.280	-25.587	1.070.165	-20.502	1.024.841	-23.610
1.033.428	-25.586	1.070.317	-20.502	1.025.222	-23.608
1.033.553	-25.586	1.070.443	-20.503	1.025.503	-23.605
1.033.756	-25.586	1.070.637	-20.503	1.025.676	-23.603
1.033.885	-25.586	1.070.808	-20.503	1.025.747	-23.600
1.034.053	-25.586	1.070.973	-20.503	1.025.796	-23.598
1.034.264	-25.586	1.071.114	-20.503	1.025.855	-23.595
1.034.410	-25.585	1.071.206	-20.503	1.025.899	-23.593
1.034.592	-25.585	1.071.351	-20.503	1.025.965	-23.590
1.034.773	-25.585	1.071.476	-20.504	1.026.075	-23.588

1.034.938	-25.585	1.071.616	-20.504	1.026.174	-23.585
1.035.071	-25.585	1.071.777	-20.504	1.026.372	-23.582
1.035.176	-25.585	1.071.887	-20.504	1.026.631	-23.580
1.035.357	-25.585	1.072.046	-20.504	1.026.807	-23.577
1.035.483	-25.585	1.072.260	-20.504	1.026.970	-23.575
1.035.610	-25.585	1.072.431	-20.504	1.027.109	-23.572
1.035.867	-25.585	1.072.571	-20.505	1.027.261	-23.570
1.036.025	-25.585	1.072.733	-20.505	1.027.450	-23.567
1.036.136	-25.585	1.072.915	-20.505	1.027.622	-23.565
1.036.280	-25.585	1.073.104	-20.505	1.027.765	-23.562
1.036.451	-25.585	1.073.294	-20.505	1.027.941	-23.559
1.036.671	-25.585	1.073.467	-20.505	1.028.098	-23.557
1.036.795	-25.585	1.073.749	-20.505	1.028.205	-23.554
1.036.924	-25.585	1.073.988	-20.505	1.028.355	-23.552
1.037.110	-25.585	1.074.076	-20.506	1.028.530	-23.549
1.037.290	-25.585	1.074.212	-20.506	1.028.673	-23.546
1.037.422	-25.585	1.074.370	-20.506	1.028.831	-23.544
1.037.545	-25.585	1.074.545	-20.506	1.029.019	-23.541
1.037.702	-25.586	1.074.718	-20.506	1.029.154	-23.538
1.037.878	-25.586	1.074.865	-20.506	1.029.268	-23.536
1.038.070	-25.586	1.075.008	-20.506	1.029.402	-23.533
1.038.253	-25.586	1.075.095	-20.506	1.029.626	-23.531
1.038.397	-25.586	1.075.188	-20.506	1.029.863	-23.528
1.038.558	-25.587	1.075.328	-20.506	1.029.984	-23.525
1.038.705	-25.587	1.075.483	-20.506	1.030.092	-23.523
1.038.783	-25.587	1.075.702	-20.507	1.030.298	-23.520
1.038.919	-25.587	1.075.906	-20.507	1.030.535	-23.517
1.039.221	-25.588	1.076.044	-20.507	1.030.689	-23.515
1.039.492	-25.588	1.076.229	-20.507	1.030.861	-23.512
1.039.617	-25.588	1.076.503	-20.507	1.031.031	-23.510

1.039.701	-25.588	1.076.674	-20.507	1.031.168	-23.507
1.039.878	-25.589	1.076.762	-20.507	1.031.311	-23.504
1.040.085	-25.589	1.077.001	-20.507	1.031.432	-23.502
1.040.216	-25.589	1.077.185	-20.507	1.031.596	-23.499
1.040.366	-25.590	1.077.241	-20.507	1.031.788	-23.496
1.040.553	-25.590	1.077.412	-20.507	1.031.946	-23.494
1.040.753	-25.590	1.077.636	-20.507	1.032.100	-23.491
1.040.895	-25.591	1.077.756	-20.507	1.032.256	-23.489
1.041.033	-25.591	1.077.884	-20.507	1.032.492	-23.486
1.041.191	-25.592	1.078.066	-20.507	1.032.712	-23.483
1.041.348	-25.592	1.078.208	-20.507	1.032.786	-23.481
1.041.480	-25.592	1.078.375	-20.507	1.032.878	-23.478
1.041.637	-25.593	1.078.560	-20.507	1.033.018	-23.475
1.041.823	-25.593	1.078.731	-20.507	1.033.193	-23.473
1.041.957	-25.594	1.078.891	-20.507	1.033.351	-23.470
1.042.111	-25.594	1.079.052	-20.507	1.033.486	-23.468
1.042.310	-25.594	1.079.208	-20.506	1.033.683	-23.465
1.042.466	-25.595	1.079.339	-20.506	1.033.887	-23.462
1.042.562	-25.595	1.079.514	-20.506	1.034.025	-23.460
1.042.676	-25.596	1.079.733	-20.506	1.034.184	-23.457
1.042.818	-25.596	1.079.894	-20.506	1.034.379	-23.455
1.042.972	-25.597	1.080.026	-20.506	1.034.548	-23.452
1.043.180	-25.597	1.080.231	-20.506	1.034.746	-23.449
1.043.298	-25.598	1.080.398	-20.506	1.034.930	-23.447
1.043.445	-25.598	1.080.508	-20.506	1.035.042	-23.444
1.043.636	-25.598	1.080.657	-20.506	1.035.167	-23.442
1.043.800	-25.599	1.080.829	-20.506	1.035.340	-23.439
1.044.023	-25.599	1.080.942	-20.505	1.035.460	-23.437
1.044.293	-25.600	1.081.135	-20.505	1.035.605	-23.434
1.044.460	-25.600	1.081.339	-20.505	1.035.809	-23.431

1.044.641	-25.601	1.081.507	-20.505	1.035.977	-23.429
1.044.796	-25.601	1.081.660	-20.505	1.036.152	-23.426
1.044.946	-25.602	1.081.756	-20.505	1.036.348	-23.424
1.045.086	-25.602	1.081.936	-20.505	1.036.497	-23.421
1.045.202	-25.603	1.082.169	-20.505	1.036.640	-23.419
1.045.346	-25.603	1.082.329	-20.504	1.036.796	-23.416
1.045.459	-25.604	1.082.451	-20.504	1.036.938	-23.414
1.045.558	-25.604	1.082.735	-20.504	1.037.096	-23.411
1.045.710	-25.604	1.083.197	-20.504	1.037.278	-23.409
1.045.938	-25.605	1.083.484	-20.504	1.037.390	-23.406
1.046.119	-25.605	1.083.580	-20.504	1.037.514	-23.404
1.046.190	-25.606	1.083.689	-20.504	1.037.559	-23.401
1.046.342	-25.606	1.083.746	-20.503	1.037.701	-23.398
1.046.600	-25.607	1.083.730	-20.503	1.037.908	-23.396
1.046.749	-25.607	1.083.794	-20.503	1.038.042	-23.393
1.046.840	-25.607	1.083.968	-20.503	1.038.259	-23.391
1.047.040	-25.608	1.084.191	-20.503	1.038.435	-23.388
1.047.245	-25.608	1.084.276	-20.503	1.038.656	-23.386
1.047.400	-25.608	1.084.302	-20.502	1.038.830	-23.383
1.047.548	-25.609	1.084.433	-20.502	1.038.987	-23.381
1.047.741	-25.609	1.084.638	-20.502	1.039.242	-23.378
1.047.964	-25.610	1.084.855	-20.502	1.039.471	-23.376
1.048.148	-25.610	1.085.067	-20.502	1.039.662	-23.373
1.048.268	-25.610	1.085.213	-20.502	1.039.837	-23.371
1.048.390	-25.611	1.085.331	-20.502	1.039.985	-23.368
1.048.559	-25.611	1.085.502	-20.501	1.040.109	-23.366
1.048.803	-25.611	1.085.705	-20.501	1.040.264	-23.363
1.048.959	-25.612	1.085.903	-20.501	1.040.412	-23.361
1.049.044	-25.612	1.086.043	-20.501	1.040.515	-23.358
1.049.290	-25.612	1.086.170	-20.501	1.040.606	-23.356

1.049.501	-25.612	1.086.295	-20.501	1.040.725	-23.353
1.049.575	-25.613	1.086.398	-20.501	1.040.913	-23.351
1.049.766	-25.613	1.086.499	-20.501	1.041.104	-23.348
1.049.984	-25.613	1.086.675	-20.501	1.041.230	-23.346
1.050.108	-25.613	1.086.904	-20.501	1.041.356	-23.343
1.050.276	-25.614	1.087.088	-20.501	1.041.491	-23.341
1.050.434	-25.614	1.087.210	-20.500	1.041.671	-23.338
1.050.648	-25.614	1.087.339	-20.500	1.041.938	-23.336
1.050.790	-25.614	1.087.508	-20.500	1.042.147	-23.333
1.050.957	-25.614	1.087.718	-20.500	1.042.285	-23.331
1.051.108	-25.615	1.087.906	-20.500	1.042.457	-23.328
1.051.227	-25.615	1.088.083	-20.500	1.042.616	-23.326
1.051.321	-25.615	1.088.300	-20.500	1.042.768	-23.323
1.051.491	-25.615	1.088.478	-20.500	1.042.974	-23.321
1.051.648	-25.615	1.088.636	-20.500	1.043.156	-23.318
1.051.805	-25.615	1.088.780	-20.500	1.043.246	-23.316
1.052.010	-25.615	1.088.941	-20.501	1.043.348	-23.313
1.052.241	-25.616	1.089.094	-20.501	1.043.566	-23.311
1.052.434	-25.616	1.089.213	-20.501	1.043.830	-23.308
1.052.523	-25.616	1.089.378	-20.501	1.043.991	-23.306
1.052.593	-25.616	1.089.578	-20.501	1.044.103	-23.303
1.052.768	-25.616	1.089.672	-20.501	1.044.272	-23.301
1.052.938	-25.616	1.089.794	-20.501	1.044.397	-23.298
1.053.076	-25.616	1.090.043	-20.501	1.044.511	-23.296
1.053.227	-25.616	1.090.229	-20.501	1.044.665	-23.293
1.053.373	-25.616	1.090.413	-20.501	1.044.880	-23.291
1.053.621	-25.616	1.090.573	-20.502	1.045.052	-23.289
1.053.999	-25.616	1.090.676	-20.502	1.045.190	-23.286
1.054.374	-25.616	1.090.813	-20.502	1.045.353	-23.284
1.054.537	-25.616	1.090.920	-20.502	1.045.548	-23.281

1.054.675	-25.616	1.091.069	-20.502	1.045.764	-23.279
1.054.737	-25.616	1.091.225	-20.503	1.045.873	-23.276
1.054.770	-25.616	1.091.386	-20.503	1.046.033	-23.274
1.054.802	-25.616	1.091.598	-20.503	1.046.227	-23.271
1.054.837	-25.616	1.091.771	-20.503	1.046.377	-23.269
1.054.965	-25.616	1.091.908	-20.503	1.046.571	-23.267
1.055.117	-25.616	1.092.050	-20.504	1.046.699	-23.264
1.055.254	-25.616	1.092.226	-20.504	1.046.825	-23.262
1.055.432	-25.615	1.092.415	-20.504	1.047.001	-23.259
1.055.620	-25.615	1.092.617	-20.505	1.047.140	-23.257
1.055.755	-25.615	1.092.761	-20.505	1.047.318	-23.254
1.055.863	-25.615	1.092.887	-20.505	1.047.542	-23.252
1.056.021	-25.615	1.093.081	-20.506	1.047.664	-23.250
1.056.251	-25.615	1.093.254	-20.506	1.047.791	-23.247
1.056.388	-25.615	1.093.398	-20.506	1.048.166	-23.245
1.056.513	-25.615	1.093.552	-20.507	1.048.540	-23.242
1.056.736	-25.614	1.093.661	-20.507	1.048.716	-23.240
1.056.892	-25.614	1.093.800	-20.507	1.048.845	-23.238
1.057.068	-25.614	1.093.981	-20.508	1.048.953	-23.235
1.057.293	-25.614	1.094.149	-20.508	1.049.001	-23.233
1.057.419	-25.614	1.094.344	-20.509	1.049.024	-23.231
1.057.512	-25.614	1.094.510	-20.509	1.049.070	-23.228
1.057.669	-25.613	1.094.686	-20.510	1.049.124	-23.226
1.057.859	-25.613	1.094.910	-20.510	1.049.288	-23.224
1.057.963	-25.613	1.095.060	-20.510	1.049.492	-23.221
1.058.120	-25.613	1.095.180	-20.511	1.049.623	-23.219
1.058.332	-25.613	1.095.342	-20.511	1.049.801	-23.217
1.058.430	-25.612	1.095.462	-20.512	1.050.031	-23.214
1.058.599	-25.612	1.095.598	-20.512	1.050.219	-23.212
1.058.794	-25.612	1.095.801	-20.513	1.050.386	-23.210

1.058.911	-25.612	1.095.975	-20.513	1.050.549	-23.207
1.059.083	-25.612	1.096.098	-20.514	1.050.690	-23.205
1.059.316	-25.612	1.096.207	-20.514	1.050.838	-23.203
1.059.549	-25.611	1.096.397	-20.515	1.051.010	-23.200
1.059.734	-25.611	1.096.594	-20.515	1.051.256	-23.198
1.059.895	-25.611	1.096.674	-20.516	1.051.431	-23.196
1.060.037	-25.611	1.096.757	-20.516	1.051.544	-23.194
1.060.134	-25.611	1.096.899	-20.517	1.051.693	-23.191
1.060.297	-25.610	1.097.132	-20.517	1.051.769	-23.189
1.060.433	-25.610	1.097.403	-20.518	1.051.886	-23.187
1.060.571	-25.610	1.097.598	-20.518	1.052.031	-23.185
1.060.769	-25.610	1.097.757	-20.519	1.052.157	-23.183
1.060.921	-25.610	1.097.932	-20.519	1.052.298	-23.180
1.061.032	-25.610	1.098.137	-20.520	1.052.506	-23.178
1.061.193	-25.609	1.098.374	-20.520	1.052.738	-23.176
1.061.375	-25.609	1.098.603	-20.521	1.052.921	-23.174
1.061.507	-25.609	1.098.721	-20.521	1.053.108	-23.172
1.061.615	-25.609	1.098.748	-20.522	1.053.277	-23.170
1.061.739	-25.609	1.098.889	-20.522	1.053.506	-23.167
1.061.897	-25.609	1.099.107	-20.523	1.053.726	-23.165
1.062.085	-25.609	1.099.252	-20.523	1.053.849	-23.163
1.062.279	-25.608	1.099.380	-20.524	1.053.960	-23.161
1.062.443	-25.608	1.099.496	-20.524	1.054.056	-23.159
1.062.630	-25.608	1.099.612	-20.525	1.054.194	-23.157
1.062.775	-25.608	1.099.755	-20.525	1.054.379	-23.155
1.062.888	-25.608	1.099.905	-20.526	1.054.545	-23.153
1.063.037	-25.608	1.100.106	-20.526	1.054.700	-23.151
1.063.242	-25.608	1.100.275	-20.526	1.054.889	-23.149
1.063.355	-25.608	1.100.418	-20.527	1.055.065	-23.147
1.063.491	-25.608	1.100.617	-20.527	1.055.248	-23.145

1.063.643	-25.608	1.100.820	-20.528	1.055.407	-23.143
1.063.839	-25.608	1.100.982	-20.528	1.055.544	-23.141
1.064.007	-25.608	1.101.201	-20.529	1.055.707	-23.139
1.064.194	-25.608	1.101.451	-20.529	1.055.848	-23.137
1.064.408	-25.608	1.101.609	-20.529	1.056.027	-23.135
1.064.630	-25.608	1.101.678	-20.530	1.056.227	-23.133
1.064.779	-25.608	1.101.771	-20.530	1.056.332	-23.131
1.064.871	-25.607	1.101.971	-20.531	1.056.434	-23.129
1.065.011	-25.607	1.102.187	-20.531	1.056.603	-23.127
1.065.229	-25.607	1.102.324	-20.531	1.056.805	-23.125
1.065.419	-25.608	1.102.436	-20.532	1.056.988	-23.123
1.065.500	-25.608	1.102.654	-20.532	1.057.129	-23.122
1.065.623	-25.608	1.102.851	-20.532	1.057.268	-23.120
1.065.721	-25.608	1.103.012	-20.533	1.057.411	-23.118
1.065.902	-25.608	1.103.138	-20.533	1.057.592	-23.116
1.066.154	-25.608	1.103.229	-20.533	1.057.792	-23.114
1.066.339	-25.608	1.103.425	-20.534	1.057.943	-23.112
1.066.488	-25.608	1.103.565	-20.534	1.058.102	-23.111
1.066.648	-25.608	1.103.706	-20.534	1.058.291	-23.109
1.066.771	-25.608	1.103.948	-20.535	1.058.446	-23.107
1.066.947	-25.608	1.104.111	-20.535	1.058.593	-23.105
1.067.188	-25.608	1.104.290	-20.535	1.058.736	-23.103
1.067.274	-25.608	1.104.518	-20.535	1.058.895	-23.102
1.067.365	-25.608	1.104.657	-20.536	1.059.042	-23.100
1.067.505	-25.608	1.104.773	-20.536	1.059.204	-23.098
1.067.654	-25.608	1.104.924	-20.536	1.059.381	-23.096
1.067.832	-25.608	1.105.079	-20.536	1.059.566	-23.095
1.067.913	-25.608	1.105.231	-20.536	1.059.734	-23.093
1.068.046	-25.609	1.105.408	-20.537	1.059.880	-23.091
1.068.260	-25.609	1.105.639	-20.537	1.060.033	-23.089

1.068.381	-25.609	1.105.863	-20.537	1.060.138	-23.088
1.068.535	-25.609	1.106.009	-20.537	1.060.298	-23.086
1.068.764	-25.609	1.106.115	-20.537	1.060.487	-23.084
1.068.896	-25.609	1.106.249	-20.537	1.060.606	-23.083
1.069.016	-25.609	1.106.414	-20.537	1.060.708	-23.081
1.069.232	-25.609	1.106.625	-20.538	1.060.810	-23.079
1.069.473	-25.609	1.106.816	-20.538	1.060.931	-23.078
1.069.708	-25.609	1.106.960	-20.538	1.061.124	-23.076
1.069.904	-25.610	1.107.215	-20.538	1.061.339	-23.074
1.070.068	-25.610	1.107.583	-20.538	1.061.535	-23.073
1.070.225	-25.610	1.107.889	-20.538	1.061.750	-23.071
1.070.404	-25.610	1.108.041	-20.538	1.061.888	-23.069
1.070.544	-25.610	1.108.108	-20.538	1.062.072	-23.068
1.070.640	-25.610	1.108.158	-20.538	1.062.282	-23.066
1.070.777	-25.610	1.108.133	-20.538	1.062.473	-23.064
1.070.832	-25.610	1.108.148	-20.538	1.062.681	-23.063
1.070.983	-25.611	1.108.317	-20.538	1.062.819	-23.061
1.071.230	-25.611	1.108.490	-20.538	1.062.980	-23.059
1.071.462	-25.611	1.108.630	-20.538	1.063.164	-23.058
1.071.572	-25.611	1.108.747	-20.538	1.063.292	-23.056
1.071.655	-25.611	1.108.857	-20.538	1.063.450	-23.054
1.071.817	-25.611	1.108.998	-20.538	1.063.626	-23.053
1.071.964	-25.611	1.109.192	-20.538	1.063.777	-23.051
1.072.141	-25.611	1.109.498	-20.538	1.063.895	-23.049
1.072.346	-25.612	1.109.765	-20.538	1.063.998	-23.048
1.072.551	-25.612	1.109.911	-20.538	1.064.101	-23.046
1.072.705	-25.612	1.110.020	-20.538	1.064.214	-23.044
1.072.862	-25.612	1.110.135	-20.538	1.064.372	-23.043
1.073.083	-25.612	1.110.272	-20.538	1.064.575	-23.041
1.073.334	-25.612	1.110.404	-20.538	1.064.775	-23.039

1.073.509	-25.612	1.110.587	-20.538	1.065.029	-23.038
1.073.677	-25.612	1.110.779	-20.538	1.065.216	-23.036
1.073.871	-25.613	1.110.927	-20.538	1.065.323	-23.034
1.073.947	-25.613	1.110.965	-20.538	1.065.524	-23.032
1.074.075	-25.613	1.111.095	-20.538	1.065.680	-23.031
1.074.260	-25.613	1.111.368	-20.538	1.065.846	-23.029
1.074.401	-25.613	1.111.529	-20.538	1.066.063	-23.027
1.074.556	-25.613	1.111.663	-20.538	1.066.171	-23.026
1.074.743	-25.613	1.111.791	-20.537	1.066.290	-23.024
1.074.951	-25.613	1.111.973	-20.537	1.066.443	-23.022
1.075.079	-25.613	1.112.199	-20.537	1.066.604	-23.021
1.075.211	-25.614	1.112.418	-20.537	1.066.785	-23.019
1.075.362	-25.614	1.112.570	-20.537	1.066.993	-23.017
1.075.465	-25.614	1.112.697	-20.537	1.067.188	-23.016
1.075.600	-25.614	1.112.900	-20.537	1.067.330	-23.014
1.075.766	-25.614	1.113.158	-20.537	1.067.469	-23.012
1.075.935	-25.614	1.113.337	-20.537	1.067.671	-23.010
1.076.139	-25.614	1.113.440	-20.537	1.067.848	-23.009
1.076.301	-25.614	1.113.525	-20.537	1.067.998	-23.007
1.076.403	-25.614	1.113.674	-20.537	1.068.151	-23.005
1.076.572	-25.614	1.113.848	-20.537	1.068.277	-23.004
1.076.770	-25.615	1.113.997	-20.537	1.068.471	-23.002
1.076.957	-25.615	1.114.211	-20.536	1.068.674	-23.000
1.077.120	-25.615	1.114.390	-20.536	1.068.836	-22.998
1.077.271	-25.615	1.114.473	-20.536	1.068.967	-22.997
1.077.496	-25.615	1.114.536	-20.536	1.069.108	-22.995
1.077.587	-25.615	1.114.654	-20.536	1.069.296	-22.993
1.077.666	-25.615	1.114.866	-20.536	1.069.449	-22.991
1.077.859	-25.615	1.115.061	-20.536	1.069.600	-22.990
1.078.126	-25.615	1.115.286	-20.536	1.069.809	-22.988

1.078.298	-25.615	1.115.465	-20.536	1.069.995	-22.986
1.078.371	-25.616	1.115.520	-20.536	1.070.138	-22.984
1.078.503	-25.616	1.115.676	-20.536	1.070.270	-22.983
1.078.607	-25.616	1.115.855	-20.536	1.070.432	-22.981
1.078.857	-25.616	1.115.998	-20.536	1.070.587	-22.979
1.079.268	-25.616	1.116.184	-20.536	1.070.689	-22.977
1.079.650	-25.616	1.116.376	-20.536	1.070.854	-22.975
1.079.869	-25.616	1.116.590	-20.536	1.071.195	-22.974
1.079.973	-25.616	1.116.763	-20.535	1.071.546	-22.972
1.080.105	-25.616	1.116.889	-20.535	1.071.814	-22.970
1.080.179	-25.616	1.117.048	-20.535	1.072.000	-22.968
1.080.158	-25.617	1.117.233	-20.535	1.072.083	-22.966
1.080.201	-25.617	1.117.354	-20.535	1.072.125	-22.965
1.080.298	-25.617	1.117.486	-20.535	1.072.132	-22.963
1.080.446	-25.617	1.117.683	-20.535	1.072.131	-22.961
1.080.637	-25.617	1.117.854	-20.535	1.072.227	-22.959
1.080.798	-25.617	1.118.036	-20.535	1.072.405	-22.957
1.080.955	-25.617	1.118.197	-20.535	1.072.591	-22.956
1.081.133	-25.617	1.118.299	-20.535	1.072.744	-22.954
1.081.337	-25.618	1.118.365	-20.535	1.072.894	-22.952
1.081.518	-25.618	1.118.488	-20.535	1.073.123	-22.950
1.081.652	-25.618	1.118.763	-20.535	1.073.298	-22.948
1.081.848	-25.618	1.119.003	-20.535	1.073.406	-22.947
1.082.066	-25.618	1.119.129	-20.535	1.073.583	-22.945
1.082.197	-25.618	1.119.284	-20.535	1.073.823	-22.943
1.082.327	-25.618	1.119.466	-20.535	1.073.995	-22.941
1.082.470	-25.619	1.119.657	-20.535	1.074.157	-22.939
1.082.645	-25.619	1.119.827	-20.535	1.074.300	-22.937
1.082.833	-25.619	1.119.961	-20.535	1.074.421	-22.936
1.082.941	-25.619	1.120.105	-20.535	1.074.551	-22.934

1.083.074	-25.619	1.120.231	-20.535	1.074.689	-22.932
1.083.215	-25.619	1.120.394	-20.535	1.074.834	-22.930
1.083.367	-25.620	1.120.590	-20.535	1.074.989	-22.928
1.083.540	-25.620	1.120.720	-20.535	1.075.126	-22.926
1.083.663	-25.620	1.120.844	-20.535	1.075.258	-22.925
1.083.776	-25.620	1.121.020	-20.535	1.075.465	-22.923
1.083.942	-25.621	1.121.159	-20.535	1.075.611	-22.921
1.084.168	-25.621	1.121.246	-20.535	1.075.790	-22.919
1.084.393	-25.621	1.121.424	-20.534	1.076.053	-22.917
1.084.514	-25.621	1.121.638	-20.534	1.076.203	-22.916
1.084.643	-25.622	1.121.831	-20.534	1.076.324	-22.914
1.084.855	-25.622	1.122.067	-20.534	1.076.555	-22.912
1.085.025	-25.622	1.122.290	-20.534	1.076.792	-22.910
1.085.176	-25.623	1.122.466	-20.534	1.076.964	-22.908
1.085.361	-25.623	1.122.674	-20.534	1.077.104	-22.907
1.085.559	-25.623	1.122.868	-20.534	1.077.236	-22.905
1.085.686	-25.624	1.123.027	-20.534	1.077.358	-22.903
1.085.791	-25.624	1.123.167	-20.534	1.077.480	-22.901
1.085.932	-25.624	1.123.259	-20.533	1.077.666	-22.899
1.086.115	-25.625	1.123.354	-20.533	1.077.869	-22.898
1.086.274	-25.625	1.123.469	-20.533	1.078.019	-22.896
1.086.371	-25.625	1.123.602	-20.533	1.078.162	-22.894
1.086.566	-25.626	1.123.790	-20.533	1.078.300	-22.892
1.086.806	-25.626	1.123.925	-20.533	1.078.447	-22.891
1.086.983	-25.627	1.124.026	-20.532	1.078.637	-22.889
1.087.123	-25.627	1.124.175	-20.532	1.078.828	-22.887
1.087.264	-25.627	1.124.333	-20.532	1.078.995	-22.886
1.087.484	-25.628	1.124.521	-20.532	1.079.101	-22.884
1.087.680	-25.628	1.124.714	-20.531	1.079.256	-22.882
1.087.798	-25.629	1.124.937	-20.531	1.079.448	-22.880

1.087.940	-25.629	1.125.134	-20.531	1.079.628	-22.879
1.088.075	-25.630	1.125.278	-20.531	1.079.792	-22.877
1.088.238	-25.630	1.125.492	-20.530	1.079.862	-22.875
1.088.395	-25.631	1.125.723	-20.530	1.079.999	-22.874
1.088.526	-25.631	1.125.865	-20.530	1.080.212	-22.872
1.088.663	-25.632	1.125.985	-20.530	1.080.404	-22.870
1.088.831	-25.633	1.126.155	-20.529	1.080.574	-22.869
1.089.025	-25.633	1.126.335	-20.529	1.080.671	-22.867
1.089.221	-25.634	1.126.490	-20.529	1.080.838	-22.865
1.089.467	-25.634	1.126.627	-20.528	1.081.028	-22.864
1.089.706	-25.635	1.126.775	-20.528	1.081.214	-22.862
1.089.846	-25.636	1.126.957	-20.527	1.081.371	-22.860
1.089.952	-25.636	1.127.131	-20.527	1.081.471	-22.859
1.090.114	-25.637	1.127.262	-20.527	1.081.664	-22.857
1.090.250	-25.637	1.127.384	-20.526	1.081.877	-22.856
1.090.354	-25.638	1.127.520	-20.526	1.082.001	-22.854
1.090.503	-25.639	1.127.690	-20.525	1.082.151	-22.852
1.090.695	-25.639	1.127.954	-20.525	1.082.363	-22.851
1.090.819	-25.640	1.128.146	-20.525	1.082.551	-22.849
1.090.981	-25.641	1.128.279	-20.524	1.082.727	-22.848
1.091.167	-25.642	1.128.493	-20.524	1.082.822	-22.846
1.091.273	-25.642	1.128.627	-20.523	1.083.006	-22.844
1.091.343	-25.643	1.128.791	-20.523	1.083.235	-22.843
1.091.585	-25.644	1.128.973	-20.522	1.083.362	-22.841
1.091.910	-25.644	1.129.081	-20.522	1.083.470	-22.840
1.092.020	-25.645	1.129.232	-20.521	1.083.623	-22.838
1.092.074	-25.646	1.129.352	-20.521	1.083.820	-22.836
1.092.245	-25.647	1.129.527	-20.520	1.083.955	-22.835
1.092.474	-25.647	1.129.694	-20.520	1.084.085	-22.833
1.092.708	-25.648	1.129.846	-20.519	1.084.186	-22.832

1.092.838	-25.649	1.130.061	-20.518	1.084.281	-22.830
1.092.898	-25.650	1.130.186	-20.518	1.084.452	-22.829
1.093.024	-25.650	1.130.266	-20.517	1.084.624	-22.827
1.093.148	-25.651	1.130.437	-20.517	1.084.818	-22.825
1.093.285	-25.652	1.130.647	-20.516	1.085.043	-22.824
1.093.445	-25.653	1.130.867	-20.516	1.085.233	-22.822
1.093.632	-25.654	1.131.014	-20.515	1.085.452	-22.821
1.093.761	-25.654	1.131.069	-20.514	1.085.677	-22.819
1.093.851	-25.655	1.131.315	-20.514	1.085.851	-22.818
1.094.073	-25.656	1.131.813	-20.513	1.086.038	-22.816
1.094.311	-25.657	1.132.163	-20.512	1.086.215	-22.814
1.094.484	-25.658	1.132.255	-20.512	1.086.368	-22.813
1.094.703	-25.658	1.132.320	-20.511	1.086.525	-22.811
1.094.929	-25.659	1.132.425	-20.511	1.086.609	-22.810
1.095.085	-25.660	1.132.507	-20.510	1.086.668	-22.808
1.095.229	-25.661	1.132.545	-20.509	1.086.805	-22.806
1.095.435	-25.661	1.132.599	-20.509	1.086.956	-22.805
1.095.652	-25.662	1.132.671	-20.508	1.087.119	-22.803
1.095.782	-25.663	1.132.801	-20.507	1.087.270	-22.802
1.095.945	-25.664	1.132.999	-20.507	1.087.430	-22.800
1.096.111	-25.665	1.133.176	-20.506	1.087.596	-22.798
1.096.245	-25.665	1.133.310	-20.505	1.087.750	-22.797
1.096.320	-25.666	1.133.411	-20.505	1.087.934	-22.795
1.096.480	-25.667	1.133.635	-20.504	1.088.111	-22.794
1.096.635	-25.668	1.133.875	-20.503	1.088.277	-22.792
1.096.750	-25.669	1.134.053	-20.503	1.088.431	-22.790
1.096.928	-25.669	1.134.317	-20.502	1.088.617	-22.789
1.097.085	-25.670	1.134.481	-20.501	1.088.774	-22.787
1.097.224	-25.671	1.134.564	-20.500	1.088.943	-22.785
1.097.310	-25.672	1.134.728	-20.500	1.089.176	-22.784

1.097.418	-25.673	1.134.934	-20.499	1.089.389	-22.782
1.097.616	-25.673	1.135.060	-20.498	1.089.482	-22.780
1.097.895	-25.674	1.135.211	-20.498	1.089.563	-22.779
1.098.095	-25.675	1.135.384	-20.497	1.089.798	-22.777
1.098.243	-25.676	1.135.445	-20.496	1.090.023	-22.775
1.098.412	-25.676	1.135.539	-20.496	1.090.180	-22.774
1.098.613	-25.677	1.135.759	-20.495	1.090.333	-22.772
1.098.858	-25.678	1.135.962	-20.494	1.090.468	-22.770
1.099.025	-25.678	1.136.081	-20.494	1.090.615	-22.769
1.099.136	-25.679	1.136.191	-20.493	1.090.782	-22.767
1.099.274	-25.680	1.136.338	-20.493	1.090.914	-22.765
1.099.447	-25.681	1.136.632	-20.492	1.091.108	-22.764
1.099.641	-25.681	1.136.872	-20.491	1.091.333	-22.762
1.099.781	-25.682	1.136.992	-20.491	1.091.472	-22.760
1.099.940	-25.683	1.137.150	-20.490	1.091.608	-22.758
1.100.093	-25.683	1.137.294	-20.489	1.091.864	-22.757
1.100.227	-25.684	1.137.475	-20.489	1.092.038	-22.755
1.100.413	-25.685	1.137.607	-20.488	1.092.179	-22.753
1.100.534	-25.685	1.137.736	-20.488	1.092.381	-22.752
1.100.723	-25.686	1.137.909	-20.487	1.092.507	-22.750
1.100.941	-25.687	1.138.048	-20.487	1.092.597	-22.748
1.100.978	-25.687	1.138.183	-20.486	1.092.804	-22.746
1.101.167	-25.688	1.138.316	-20.485	1.092.969	-22.745
1.101.424	-25.689	1.138.466	-20.485	1.093.076	-22.743
1.101.562	-25.689	1.138.647	-20.484	1.093.232	-22.741
1.101.714	-25.690	1.138.847	-20.484	1.093.419	-22.739
1.101.877	-25.690	1.138.972	-20.483	1.093.637	-22.737
1.102.041	-25.691	1.139.074	-20.483	1.093.772	-22.736
1.102.179	-25.692	1.139.232	-20.482	1.093.885	-22.734
1.102.334	-25.692	1.139.457	-20.482	1.094.051	-22.732

1.102.518	-25.693	1.139.719	-20.481	1.094.404	-22.730
1.102.718	-25.694	1.139.841	-20.481	1.094.804	-22.729
1.102.897	-25.694	1.139.956	-20.480	1.095.052	-22.727
1.103.044	-25.695	1.140.124	-20.480	1.095.185	-22.725
1.103.225	-25.695	1.140.248	-20.480	1.095.238	-22.723
1.103.353	-25.696	1.140.353	-20.479	1.095.270	-22.721
1.103.475	-25.696	1.140.487	-20.479	1.095.329	-22.719
1.103.664	-25.697	1.140.647	-20.478	1.095.376	-22.718
1.103.917	-25.698	1.140.816	-20.478	1.095.494	-22.716
1.104.027	-25.698	1.141.038	-20.478	1.095.648	-22.714
1.104.054	-25.699	1.141.215	-20.477	1.095.723	-22.712
1.104.262	-25.699	1.141.382	-20.477	1.095.884	-22.710
1.104.688	-25.700	1.141.576	-20.477	1.096.104	-22.708
1.105.118	-25.700	1.141.728	-20.476	1.096.307	-22.707
1.105.335	-25.701	1.141.881	-20.476	1.096.507	-22.705
1.105.457	-25.701	1.142.031	-20.476	1.096.684	-22.703
1.105.491	-25.702	1.142.197	-20.475	1.096.887	-22.701
1.105.508	-25.702	1.142.374	-20.475	1.097.040	-22.699
1.105.515	-25.703	1.142.539	-20.475	1.097.143	-22.697
1.105.557	-25.703	1.142.735	-20.475	1.097.318	-22.696
1.105.753	-25.704	1.142.868	-20.474	1.097.465	-22.694
1.105.864	-25.704	1.143.025	-20.474	1.097.593	-22.692
1.105.975	-25.705	1.143.251	-20.474	1.097.768	-22.690
1.106.188	-25.705	1.143.413	-20.474	1.097.895	-22.688
1.106.378	-25.706	1.143.518	-20.473	1.098.061	-22.686
1.106.534	-25.706	1.143.590	-20.473	1.098.255	-22.684
1.106.679	-25.707	1.143.757	-20.473	1.098.356	-22.682
1.106.880	-25.707	1.143.997	-20.473	1.098.473	-22.681
1.107.048	-25.708	1.144.174	-20.473	1.098.663	-22.679
1.107.200	-25.708	1.144.333	-20.472	1.098.849	-22.677

1.107.367	-25.709	1.144.456	-20.472	1.099.023	-22.675
1.107.543	-25.709	1.144.544	-20.472	1.099.214	-22.673
1.107.746	-25.710	1.144.675	-20.472	1.099.402	-22.671
1.107.942	-25.710	1.144.826	-20.472	1.099.595	-22.669
1.108.071	-25.711	1.144.958	-20.472	1.099.801	-22.668
1.108.180	-25.711	1.145.111	-20.471	1.099.984	-22.666
1.108.335	-25.712	1.145.255	-20.471	1.100.111	-22.664
1.108.487	-25.712	1.145.471	-20.471	1.100.287	-22.662
1.108.653	-25.713	1.145.640	-20.471	1.100.469	-22.660
1.108.801	-25.713	1.145.745	-20.471	1.100.638	-22.658
1.108.792	-25.714	1.145.936	-20.471	1.100.785	-22.657
1.108.906	-25.714	1.146.102	-20.471	1.100.872	-22.655
1.109.162	-25.715	1.146.294	-20.471	1.101.023	-22.653
1.109.358	-25.715	1.146.508	-20.470	1.101.192	-22.651
1.109.538	-25.716	1.146.695	-20.470	1.101.314	-22.649
1.109.708	-25.716	1.146.917	-20.470	1.101.445	-22.647
1.109.856	-25.717	1.147.119	-20.470	1.101.674	-22.646
1.110.119	-25.717	1.147.271	-20.470	1.101.880	-22.644
1.110.325	-25.718	1.147.414	-20.470	1.102.010	-22.642
1.110.497	-25.718	1.147.509	-20.470	1.102.185	-22.640
1.110.714	-25.719	1.147.604	-20.470	1.102.309	-22.638
1.110.902	-25.719	1.147.774	-20.470	1.102.456	-22.637
1.111.046	-25.720	1.147.917	-20.469	1.102.718	-22.635
1.111.146	-25.720	1.148.071	-20.469	1.102.842	-22.633
1.111.263	-25.721	1.148.223	-20.469	1.102.922	-22.631
1.111.366	-25.721	1.148.307	-20.469	1.103.044	-22.629
1.111.506	-25.722	1.148.470	-20.469	1.103.202	-22.628
1.111.654	-25.722	1.148.700	-20.469	1.103.410	-22.626
1.111.778	-25.723	1.148.855	-20.469	1.103.589	-22.624
1.112.033	-25.723	1.149.063	-20.469	1.103.783	-22.622

1.112.292	-25.724	1.149.289	-20.469	1.103.980	-22.621
1.112.425	-25.724	1.149.439	-20.469	1.104.136	-22.619
1.112.546	-25.725	1.149.634	-20.469	1.104.277	-22.617
1.112.655	-25.725	1.149.859	-20.468	1.104.460	-22.615
1.112.791	-25.726	1.150.032	-20.468	1.104.583	-22.614
1.112.924	-25.727	1.150.152	-20.468	1.104.729	-22.612
1.113.102	-25.727	1.150.263	-20.468	1.104.900	-22.610
1.113.287	-25.728	1.150.413	-20.468	1.105.024	-22.608
1.113.420	-25.728	1.150.590	-20.468	1.105.165	-22.607
1.113.503	-25.729	1.150.761	-20.468	1.105.303	-22.605
1.113.671	-25.730	1.150.932	-20.468	1.105.515	-22.603
1.113.896	-25.730	1.151.077	-20.468	1.105.786	-22.602
1.114.018	-25.731	1.151.209	-20.467	1.105.919	-22.600
1.114.204	-25.731	1.151.429	-20.467	1.106.034	-22.598
1.114.398	-25.732	1.151.607	-20.467	1.106.236	-22.596
1.114.572	-25.733	1.151.707	-20.467	1.106.387	-22.595
1.114.756	-25.733	1.151.870	-20.467	1.106.538	-22.593
1.114.965	-25.734	1.152.084	-20.467	1.106.679	-22.591
1.115.189	-25.734	1.152.235	-20.467	1.106.842	-22.590
1.115.325	-25.735	1.152.338	-20.467	1.107.023	-22.588
1.115.424	-25.736	1.152.536	-20.466	1.107.116	-22.586
1.115.608	-25.736	1.152.704	-20.466	1.107.210	-22.585
1.115.869	-25.737	1.152.842	-20.466	1.107.355	-22.583
1.116.016	-25.738	1.152.996	-20.466	1.107.523	-22.581
1.116.086	-25.738	1.153.155	-20.466	1.107.719	-22.580
1.116.249	-25.739	1.153.345	-20.466	1.107.908	-22.578
1.116.423	-25.739	1.153.483	-20.466	1.108.085	-22.576
1.116.522	-25.740	1.153.640	-20.466	1.108.253	-22.574
1.116.710	-25.741	1.153.819	-20.465	1.108.468	-22.573
1.116.943	-25.741	1.153.998	-20.465	1.108.703	-22.571

1.117.119	-25.742	1.154.202	-20.465	1.108.899	-22.569
1.117.264	-25.743	1.154.323	-20.465	1.109.074	-22.568
1.117.392	-25.743	1.154.461	-20.465	1.109.202	-22.566
1.117.563	-25.744	1.154.626	-20.465	1.109.333	-22.564
1.117.674	-25.745	1.154.770	-20.465	1.109.501	-22.562
1.117.801	-25.745	1.154.937	-20.464	1.109.690	-22.561
1.117.991	-25.746	1.155.086	-20.464	1.109.825	-22.559
1.118.148	-25.746	1.155.197	-20.464	1.109.932	-22.557
1.118.333	-25.747	1.155.404	-20.464	1.110.060	-22.556
1.118.512	-25.748	1.155.820	-20.464	1.110.170	-22.554
1.118.642	-25.748	1.156.178	-20.464	1.110.293	-22.552
1.118.731	-25.749	1.156.306	-20.464	1.110.429	-22.550
1.118.899	-25.749	1.156.388	-20.463	1.110.630	-22.549
1.119.069	-25.750	1.156.520	-20.463	1.110.842	-22.547
1.119.203	-25.750	1.156.613	-20.463	1.110.969	-22.545
1.119.390	-25.751	1.156.660	-20.463	1.111.113	-22.543
1.119.563	-25.752	1.156.774	-20.463	1.111.330	-22.542
1.119.716	-25.752	1.156.884	-20.463	1.111.543	-22.540
1.119.855	-25.753	1.156.999	-20.462	1.111.730	-22.538
1.120.049	-25.753	1.157.184	-20.462	1.111.870	-22.536
1.120.285	-25.754	1.157.337	-20.462	1.112.029	-22.534
1.120.475	-25.754	1.157.490	-20.462	1.112.153	-22.533
1.120.664	-25.755	1.157.675	-20.462	1.112.264	-22.531
1.120.844	-25.755	1.157.831	-20.462	1.112.475	-22.529
1.120.997	-25.756	1.157.979	-20.462	1.112.719	-22.527
1.121.194	-25.756	1.158.125	-20.461	1.112.881	-22.525
1.121.367	-25.757	1.158.307	-20.461	1.113.027	-22.524
1.121.460	-25.757	1.158.507	-20.461	1.113.242	-22.522
1.121.538	-25.758	1.158.681	-20.461	1.113.391	-22.520
1.121.703	-25.758	1.158.827	-20.461	1.113.509	-22.518

1.121.893	-25.758	1.158.985	-20.461	1.113.671	-22.516
1.122.033	-25.759	1.159.137	-20.461	1.113.861	-22.515
1.122.172	-25.759	1.159.283	-20.461	1.114.048	-22.513
1.122.263	-25.760	1.159.420	-20.461	1.114.164	-22.511
1.122.391	-25.760	1.159.530	-20.460	1.114.359	-22.509
1.122.585	-25.760	1.159.706	-20.460	1.114.594	-22.507
1.122.751	-25.761	1.159.900	-20.460	1.114.751	-22.505
1.122.899	-25.761	1.160.050	-20.460	1.114.942	-22.503
1.123.127	-25.761	1.160.173	-20.460	1.115.098	-22.501
1.123.370	-25.762	1.160.274	-20.460	1.115.213	-22.500
1.123.491	-25.762	1.160.406	-20.460	1.115.370	-22.498
1.123.659	-25.762	1.160.591	-20.460	1.115.510	-22.496
1.123.832	-25.762	1.160.799	-20.460	1.115.618	-22.494
1.123.930	-25.763	1.160.986	-20.460	1.115.744	-22.492
1.124.103	-25.763	1.161.146	-20.460	1.115.947	-22.490
1.124.359	-25.763	1.161.401	-20.460	1.116.202	-22.488
1.124.576	-25.763	1.161.599	-20.460	1.116.383	-22.486
1.124.664	-25.763	1.161.718	-20.460	1.116.528	-22.484
1.124.846	-25.764	1.161.889	-20.460	1.116.612	-22.482
1.125.108	-25.764	1.162.001	-20.460	1.116.710	-22.480
1.125.200	-25.764	1.162.102	-20.460	1.116.917	-22.478
1.125.344	-25.764	1.162.216	-20.460	1.117.073	-22.476
1.125.510	-25.764	1.162.321	-20.460	1.117.309	-22.474
1.125.605	-25.764	1.162.526	-20.460	1.117.761	-22.473
1.125.766	-25.764	1.162.749	-20.460	1.118.162	-22.471
1.125.963	-25.764	1.162.934	-20.460	1.118.360	-22.469
1.126.142	-25.764	1.163.066	-20.460	1.118.398	-22.467
1.126.283	-25.764	1.163.150	-20.460	1.118.402	-22.465
1.126.435	-25.764	1.163.307	-20.460	1.118.428	-22.463
1.126.648	-25.764	1.163.542	-20.461	1.118.478	-22.461

1.126.865	-25.764	1.163.718	-20.461	1.118.554	-22.459
1.127.016	-25.764	1.163.869	-20.461	1.118.618	-22.457
1.127.143	-25.764	1.164.010	-20.461	1.118.800	-22.455
1.127.341	-25.764	1.164.116	-20.461	1.119.013	-22.453
1.127.494	-25.764	1.164.288	-20.461	1.119.199	-22.451
1.127.597	-25.764	1.164.538	-20.461	1.119.346	-22.449
1.127.711	-25.764	1.164.688	-20.462	1.119.489	-22.447
1.127.871	-25.764	1.164.790	-20.462	1.119.662	-22.445
1.128.036	-25.764	1.164.972	-20.462	1.119.806	-22.443
1.128.176	-25.763	1.165.115	-20.462	1.119.988	-22.440
1.128.382	-25.763	1.165.274	-20.462	1.120.203	-22.438
1.128.619	-25.763	1.165.490	-20.463	1.120.413	-22.436
1.128.829	-25.763	1.165.688	-20.463	1.120.499	-22.434
1.128.996	-25.763	1.165.844	-20.463	1.120.620	-22.432
1.129.102	-25.763	1.166.002	-20.463	1.120.890	-22.430
1.129.178	-25.762	1.166.180	-20.464	1.121.044	-22.428
1.129.362	-25.762	1.166.299	-20.464	1.121.132	-22.426
1.129.502	-25.762	1.166.467	-20.464	1.121.282	-22.424
1.129.717	-25.762	1.166.651	-20.464	1.121.457	-22.422
1.130.160	-25.761	1.166.730	-20.465	1.121.594	-22.420
1.130.584	-25.761	1.166.814	-20.465	1.121.752	-22.418
1.130.786	-25.761	1.167.029	-20.465	1.121.938	-22.416
1.130.807	-25.760	1.167.269	-20.466	1.122.085	-22.414
1.130.886	-25.760	1.167.394	-20.466	1.122.224	-22.412
1.130.975	-25.760	1.167.543	-20.466	1.122.409	-22.410
1.130.994	-25.759	1.167.752	-20.467	1.122.675	-22.408
1.131.047	-25.759	1.167.949	-20.467	1.122.843	-22.406
1.131.116	-25.759	1.168.120	-20.467	1.122.966	-22.404
1.131.244	-25.758	1.168.218	-20.468	1.123.139	-22.401
1.131.414	-25.758	1.168.335	-20.468	1.123.317	-22.399

1.131.496	-25.758	1.168.507	-20.468	1.123.493	-22.397
1.131.686	-25.757	1.168.693	-20.469	1.123.632	-22.395
1.132.004	-25.757	1.168.819	-20.469	1.123.756	-22.393
1.132.178	-25.756	1.168.924	-20.469	1.123.897	-22.391
1.132.263	-25.756	1.169.032	-20.470	1.124.025	-22.389
1.132.457	-25.755	1.169.131	-20.470	1.124.182	-22.387
1.132.676	-25.755	1.169.316	-20.471	1.124.393	-22.385
1.132.872	-25.755	1.169.520	-20.471	1.124.556	-22.383
1.133.018	-25.754	1.169.704	-20.471	1.124.656	-22.381
1.133.169	-25.754	1.169.877	-20.472	1.124.800	-22.379
1.133.360	-25.753	1.169.989	-20.472	1.124.983	-22.377
1.133.412	-25.753	1.170.183	-20.472	1.125.191	-22.375
1.133.501	-25.752	1.170.443	-20.473	1.125.339	-22.373
1.133.764	-25.752	1.170.633	-20.473	1.125.491	-22.371
1.133.960	-25.751	1.170.801	-20.474	1.125.680	-22.369
1.134.025	-25.751	1.171.039	-20.474	1.125.798	-22.367
1.134.132	-25.750	1.171.264	-20.474	1.125.939	-22.365
1.134.324	-25.750	1.171.417	-20.475	1.126.121	-22.363
1.134.499	-25.749	1.171.526	-20.475	1.126.290	-22.361
1.134.660	-25.749	1.171.631	-20.476	1.126.457	-22.359
1.134.825	-25.748	1.171.747	-20.476	1.126.561	-22.357
1.135.002	-25.748	1.171.884	-20.476	1.126.684	-22.355
1.135.163	-25.747	1.171.999	-20.477	1.126.879	-22.353
1.135.351	-25.747	1.172.155	-20.477	1.127.076	-22.351
1.135.518	-25.746	1.172.337	-20.477	1.127.288	-22.349
1.135.610	-25.746	1.172.477	-20.478	1.127.495	-22.347
1.135.778	-25.746	1.172.616	-20.478	1.127.614	-22.345
1.135.931	-25.745	1.172.761	-20.479	1.127.732	-22.343
1.136.089	-25.745	1.172.961	-20.479	1.127.887	-22.341
1.136.282	-25.744	1.173.148	-20.479	1.128.053	-22.339

1.136.460	-25.744	1.173.313	-20.480	1.128.225	-22.337
1.136.595	-25.743	1.173.456	-20.480	1.128.341	-22.335
1.136.729	-25.743	1.173.688	-20.480	1.128.520	-22.333
1.136.899	-25.742	1.173.926	-20.481	1.128.715	-22.331
1.137.030	-25.742	1.174.044	-20.481	1.128.895	-22.329
1.137.229	-25.741	1.174.135	-20.481	1.129.087	-22.327
1.137.427	-25.741	1.174.303	-20.482	1.129.246	-22.325
1.137.561	-25.741	1.174.482	-20.482	1.129.426	-22.323
1.137.713	-25.740	1.174.619	-20.482	1.129.603	-22.321
1.137.856	-25.740	1.174.762	-20.483	1.129.725	-22.319
1.138.005	-25.739	1.174.930	-20.483	1.129.841	-22.317
1.138.143	-25.739	1.175.115	-20.483	1.129.985	-22.315
1.138.305	-25.739	1.175.334	-20.484	1.130.147	-22.313
1.138.487	-25.738	1.175.488	-20.484	1.130.258	-22.311
1.138.643	-25.738	1.175.594	-20.484	1.130.396	-22.309
1.138.796	-25.737	1.175.733	-20.485	1.130.585	-22.307
1.138.954	-25.737	1.175.903	-20.485	1.130.673	-22.305
1.139.088	-25.737	1.176.086	-20.485	1.130.767	-22.303
1.139.227	-25.736	1.176.276	-20.486	1.130.983	-22.301
1.139.374	-25.736	1.176.454	-20.486	1.131.233	-22.299
1.139.535	-25.736	1.176.610	-20.486	1.131.366	-22.297
1.139.754	-25.735	1.176.805	-20.486	1.131.506	-22.295
1.139.993	-25.735	1.176.926	-20.487	1.131.729	-22.293
1.140.162	-25.735	1.177.035	-20.487	1.131.954	-22.291
1.140.343	-25.735	1.177.187	-20.487	1.132.170	-22.290
1.140.521	-25.734	1.177.335	-20.488	1.132.353	-22.288
1.140.652	-25.734	1.177.545	-20.488	1.132.579	-22.286
1.140.785	-25.734	1.177.757	-20.488	1.132.741	-22.284
1.140.957	-25.733	1.177.898	-20.489	1.132.837	-22.282
1.141.208	-25.733	1.178.003	-20.489	1.132.935	-22.279

1.141.347	-25.733	1.178.127	-20.489	1.133.056	-22.277
1.141.476	-25.733	1.178.417	-20.489	1.133.207	-22.275
1.141.665	-25.733	1.178.703	-20.490	1.133.330	-22.273
1.141.750	-25.732	1.178.812	-20.490	1.133.444	-22.271
1.141.803	-25.732	1.178.866	-20.490	1.133.575	-22.269
1.142.009	-25.732	1.179.009	-20.490	1.133.766	-22.267
1.142.247	-25.732	1.179.244	-20.491	1.133.913	-22.265
1.142.449	-25.732	1.179.389	-20.491	1.134.050	-22.263
1.142.579	-25.731	1.179.575	-20.491	1.134.297	-22.261
1.142.702	-25.731	1.179.893	-20.492	1.134.482	-22.259
1.142.879	-25.731	1.180.267	-20.492	1.134.617	-22.257
1.143.075	-25.731	1.180.566	-20.492	1.134.799	-22.255
1.143.204	-25.731	1.180.602	-20.492	1.134.969	-22.253
1.143.362	-25.731	1.180.622	-20.493	1.135.179	-22.251
1.143.483	-25.730	1.180.760	-20.493	1.135.361	-22.249
1.143.579	-25.730	1.180.858	-20.493	1.135.428	-22.247
1.143.740	-25.730	1.180.923	-20.493	1.135.573	-22.245
1.143.843	-25.730	1.180.990	-20.494	1.135.810	-22.243
1.143.972	-25.730	1.181.108	-20.494	1.136.019	-22.241
1.144.134	-25.730	1.181.276	-20.494	1.136.207	-22.239
1.144.284	-25.730	1.181.420	-20.494	1.136.354	-22.237
1.144.442	-25.730	1.181.483	-20.495	1.136.469	-22.235
1.144.561	-25.730	1.181.609	-20.495	1.136.637	-22.233
1.144.788	-25.729	1.181.809	-20.495	1.136.832	-22.230
1.144.979	-25.729	1.182.027	-20.495	1.136.975	-22.228
1.145.098	-25.729	1.182.253	-20.496	1.137.150	-22.226
1.145.359	-25.729	1.182.428	-20.496	1.137.313	-22.224
1.145.638	-25.729	1.182.605	-20.496	1.137.469	-22.222
1.145.807	-25.729	1.182.732	-20.497	1.137.702	-22.220
1.145.939	-25.729	1.182.830	-20.497	1.137.840	-22.218

1.146.115	-25.729	1.183.016	-20.497	1.137.940	-22.216
1.146.281	-25.729	1.183.217	-20.497	1.138.097	-22.214
1.146.306	-25.729	1.183.332	-20.498	1.138.260	-22.212
1.146.423	-25.729	1.183.426	-20.498	1.138.454	-22.210
1.146.695	-25.729	1.183.658	-20.498	1.138.604	-22.208
1.146.895	-25.728	1.183.861	-20.499	1.138.734	-22.206
1.146.999	-25.728	1.183.955	-20.499	1.138.895	-22.203
1.147.086	-25.728	1.184.081	-20.499	1.139.087	-22.201
1.147.226	-25.728	1.184.216	-20.500	1.139.214	-22.199
1.147.352	-25.728	1.184.399	-20.500	1.139.341	-22.197
1.147.488	-25.728	1.184.612	-20.500	1.139.533	-22.195
1.147.673	-25.728	1.184.775	-20.501	1.139.669	-22.193
1.147.858	-25.728	1.184.960	-20.501	1.139.812	-22.191
1.148.091	-25.728	1.185.155	-20.501	1.139.985	-22.189
1.148.278	-25.728	1.185.324	-20.502	1.140.118	-22.187
1.148.436	-25.728	1.185.539	-20.502	1.140.333	-22.185
1.148.590	-25.728	1.185.702	-20.502	1.140.739	-22.183
1.148.721	-25.728	1.185.852	-20.503	1.141.106	-22.181
1.148.950	-25.728	1.186.040	-20.503	1.141.311	-22.179
1.149.132	-25.727	1.186.194	-20.504	1.141.398	-22.176
1.149.306	-25.727	1.186.361	-20.504	1.141.433	-22.174
1.149.509	-25.727	1.186.500	-20.505	1.141.488	-22.172
1.149.718	-25.727	1.186.584	-20.505	1.141.552	-22.170
1.149.929	-25.727	1.186.716	-20.505	1.141.681	-22.168
1.150.042	-25.727	1.186.907	-20.506	1.141.766	-22.166
1.150.107	-25.727	1.187.125	-20.506	1.141.896	-22.164
1.150.228	-25.727	1.187.284	-20.507	1.142.125	-22.162
1.150.397	-25.727	1.187.409	-20.507	1.142.231	-22.160
1.150.565	-25.727	1.187.586	-20.508	1.142.301	-22.158
1.150.767	-25.727	1.187.742	-20.508	1.142.472	-22.156

1.150.919	-25.727	1.187.876	-20.509	1.142.684	-22.154
1.151.060	-25.727	1.188.037	-20.509	1.142.821	-22.152
1.151.209	-25.727	1.188.217	-20.510	1.143.011	-22.150
1.151.396	-25.726	1.188.311	-20.510	1.143.267	-22.148
1.151.531	-25.726	1.188.457	-20.511	1.143.469	-22.146
1.151.686	-25.726	1.188.670	-20.511	1.143.643	-22.144
1.151.892	-25.726	1.188.858	-20.512	1.143.773	-22.142
1.152.040	-25.726	1.189.044	-20.513	1.143.927	-22.140
1.152.184	-25.726	1.189.197	-20.513	1.144.080	-22.138
1.152.362	-25.726	1.189.327	-20.514	1.144.200	-22.136
1.152.562	-25.726	1.189.459	-20.514	1.144.334	-22.134
1.152.721	-25.726	1.189.650	-20.515	1.144.485	-22.132
1.152.823	-25.726	1.189.816	-20.516	1.144.636	-22.130
1.152.962	-25.726	1.189.986	-20.516	1.144.803	-22.128
1.153.170	-25.726	1.190.167	-20.517	1.144.993	-22.126
1.153.339	-25.726	1.190.300	-20.518	1.145.145	-22.124
1.153.488	-25.725	1.190.459	-20.518	1.145.260	-22.122
1.153.646	-25.725	1.190.573	-20.519	1.145.416	-22.120
1.153.782	-25.725	1.190.736	-20.520	1.145.566	-22.119
1.153.962	-25.725	1.190.959	-20.520	1.145.749	-22.117
1.154.150	-25.725	1.191.082	-20.521	1.145.991	-22.115
1.154.284	-25.725	1.191.252	-20.522	1.146.209	-22.113
1.154.411	-25.725	1.191.451	-20.522	1.146.398	-22.111
1.154.538	-25.725	1.191.560	-20.523	1.146.529	-22.109
1.154.736	-25.725	1.191.719	-20.524	1.146.656	-22.108
1.155.023	-25.725	1.191.898	-20.524	1.146.804	-22.106
1.155.419	-25.725	1.192.009	-20.525	1.146.954	-22.104
1.155.772	-25.724	1.192.163	-20.526	1.147.170	-22.102
1.155.959	-25.724	1.192.321	-20.526	1.147.318	-22.100
1.156.050	-25.724	1.192.458	-20.527	1.147.423	-22.099

1.156.149	-25.724	1.192.664	-20.528	1.147.599	-22.097
1.156.248	-25.724	1.192.866	-20.528	1.147.729	-22.095
1.156.314	-25.724	1.192.913	-20.529	1.147.862	-22.094
1.156.389	-25.724	1.192.990	-20.530	1.148.097	-22.092
1.156.431	-25.724	1.193.192	-20.531	1.148.214	-22.090
1.156.460	-25.724	1.193.332	-20.531	1.148.281	-22.088
1.156.546	-25.724	1.193.470	-20.532	1.148.441	-22.087
1.156.722	-25.724	1.193.641	-20.533	1.148.651	-22.085
1.156.982	-25.724	1.193.811	-20.533	1.148.854	-22.084
1.157.216	-25.724	1.194.014	-20.534	1.148.971	-22.082
1.157.408	-25.724	1.194.202	-20.535	1.149.055	-22.080
1.157.544	-25.723	1.194.387	-20.535	1.149.215	-22.079
1.157.668	-25.723	1.194.625	-20.536	1.149.466	-22.077
1.157.869	-25.723	1.194.874	-20.537	1.149.656	-22.076
1.158.019	-25.723	1.195.050	-20.538	1.149.781	-22.074
1.158.214	-25.723	1.195.223	-20.538	1.149.910	-22.073
1.158.335	-25.723	1.195.396	-20.539	1.150.074	-22.071
1.158.423	-25.723	1.195.546	-20.540	1.150.286	-22.070
1.158.559	-25.723	1.195.688	-20.540	1.150.413	-22.068
1.158.688	-25.723	1.195.825	-20.541	1.150.576	-22.067
1.158.882	-25.723	1.196.007	-20.542	1.150.823	-22.065
1.159.060	-25.723	1.196.178	-20.542	1.151.019	-22.064
1.159.202	-25.723	1.196.314	-20.543	1.151.198	-22.062
1.159.340	-25.723	1.196.404	-20.544	1.151.320	-22.061
1.159.524	-25.723	1.196.469	-20.544	1.151.416	-22.059
1.159.670	-25.723	1.196.558	-20.545	1.151.590	-22.058
1.159.781	-25.723	1.196.738	-20.545	1.151.788	-22.057
1.159.960	-25.724	1.196.956	-20.546	1.151.934	-22.055
1.160.164	-25.724	1.197.124	-20.547	1.152.093	-22.054
1.160.316	-25.724	1.197.285	-20.547	1.152.268	-22.052

1.160.513	-25.724	1.197.470	-20.548	1.152.408	-22.051
1.160.695	-25.724	1.197.688	-20.549	1.152.569	-22.050
1.160.841	-25.724	1.197.888	-20.549	1.152.720	-22.048
1.160.975	-25.724	1.198.069	-20.550	1.152.839	-22.047
1.161.158	-25.724	1.198.271	-20.550	1.153.008	-22.046
1.161.271	-25.724	1.198.412	-20.551	1.153.122	-22.044
1.161.472	-25.725	1.198.517	-20.551	1.153.214	-22.043
1.161.716	-25.725	1.198.665	-20.552	1.153.426	-22.042
1.161.852	-25.725	1.198.839	-20.553	1.153.606	-22.040
1.162.010	-25.725	1.199.060	-20.553	1.153.717	-22.039
1.162.143	-25.725	1.199.215	-20.554	1.153.890	-22.038
1.162.243	-25.726	1.199.342	-20.554	1.154.048	-22.037
1.162.411	-25.726	1.199.539	-20.555	1.154.201	-22.035
1.162.591	-25.726	1.199.676	-20.555	1.154.384	-22.034
1.162.738	-25.726	1.199.779	-20.556	1.154.570	-22.033
1.162.891	-25.727	1.199.964	-20.556	1.154.831	-22.031
1.163.052	-25.727	1.200.172	-20.557	1.155.071	-22.030
1.163.285	-25.727	1.200.359	-20.557	1.155.237	-22.029
1.163.449	-25.728	1.200.512	-20.558	1.155.367	-22.028
1.163.570	-25.728	1.200.655	-20.558	1.155.477	-22.026
1.163.731	-25.728	1.200.798	-20.559	1.155.611	-22.025
1.163.911	-25.729	1.200.929	-20.559	1.155.770	-22.024
1.164.048	-25.729	1.201.082	-20.560	1.155.886	-22.023
1.164.258	-25.729	1.201.293	-20.560	1.155.925	-22.021
1.164.410	-25.730	1.201.509	-20.561	1.156.041	-22.020
1.164.492	-25.730	1.201.613	-20.561	1.156.248	-22.019
1.164.645	-25.731	1.201.724	-20.561	1.156.401	-22.018
1.164.790	-25.731	1.201.970	-20.562	1.156.563	-22.016
1.164.923	-25.731	1.202.162	-20.562	1.156.749	-22.015
1.165.079	-25.732	1.202.275	-20.563	1.156.899	-22.014

1.165.302	-25.732	1.202.455	-20.563	1.157.074	-22.013
1.165.463	-25.733	1.202.620	-20.563	1.157.281	-22.011
1.165.593	-25.733	1.202.766	-20.564	1.157.486	-22.010
1.165.780	-25.734	1.202.969	-20.564	1.157.630	-22.009
1.166.034	-25.734	1.203.089	-20.565	1.157.766	-22.008
1.166.234	-25.735	1.203.150	-20.565	1.158.025	-22.006
1.166.314	-25.736	1.203.335	-20.565	1.158.257	-22.005
1.166.439	-25.736	1.203.500	-20.566	1.158.388	-22.004
1.166.659	-25.737	1.203.739	-20.566	1.158.483	-22.003
1.166.854	-25.737	1.204.142	-20.566	1.158.580	-22.001
1.166.954	-25.738	1.204.500	-20.567	1.158.768	-22.000
1.167.057	-25.738	1.204.705	-20.567	1.158.967	-21.999
1.167.276	-25.739	1.204.810	-20.568	1.159.111	-21.998
1.167.486	-25.740	1.204.871	-20.568	1.159.278	-21.996
1.167.628	-25.740	1.204.931	-20.568	1.159.492	-21.995
1.167.727	-25.741	1.204.983	-20.569	1.159.670	-21.994
1.167.798	-25.742	1.205.028	-20.569	1.159.802	-21.993
1.167.983	-25.742	1.205.135	-20.569	1.159.941	-21.991
1.168.167	-25.743	1.205.274	-20.569	1.160.105	-21.990
1.168.326	-25.744	1.205.405	-20.570	1.160.289	-21.989
1.168.526	-25.744	1.205.525	-20.570	1.160.466	-21.988
1.168.574	-25.745	1.205.676	-20.570	1.160.603	-21.986
1.168.623	-25.746	1.205.873	-20.571	1.160.743	-21.985
1.168.887	-25.746	1.206.059	-20.571	1.160.914	-21.984
1.169.085	-25.747	1.206.250	-20.571	1.161.102	-21.983
1.169.211	-25.748	1.206.443	-20.572	1.161.276	-21.981
1.169.414	-25.748	1.206.602	-20.572	1.161.471	-21.980
1.169.531	-25.749	1.206.770	-20.572	1.161.638	-21.979
1.169.620	-25.750	1.206.961	-20.573	1.161.742	-21.977
1.169.832	-25.750	1.207.084	-20.573	1.161.892	-21.976

1.170.087	-25.751	1.207.210	-20.573	1.162.089	-21.975
1.170.277	-25.752	1.207.403	-20.573	1.162.228	-21.974
1.170.502	-25.753	1.207.561	-20.574	1.162.349	-21.972
1.170.706	-25.753	1.207.676	-20.574	1.162.526	-21.971
1.170.844	-25.754	1.207.810	-20.574	1.162.705	-21.970
1.171.031	-25.755	1.207.937	-20.575	1.162.880	-21.968
1.171.236	-25.755	1.208.143	-20.575	1.163.027	-21.967
1.171.360	-25.756	1.208.325	-20.575	1.163.148	-21.966
1.171.460	-25.757	1.208.374	-20.576	1.163.395	-21.964
1.171.631	-25.757	1.208.549	-20.576	1.163.777	-21.963
1.171.852	-25.758	1.208.808	-20.576	1.164.103	-21.962
1.172.028	-25.759	1.209.034	-20.577	1.164.258	-21.960
1.172.113	-25.760	1.209.230	-20.577	1.164.281	-21.959
1.172.197	-25.760	1.209.444	-20.578	1.164.305	-21.958
1.172.299	-25.761	1.209.630	-20.578	1.164.392	-21.956
1.172.451	-25.762	1.209.765	-20.578	1.164.517	-21.955
1.172.629	-25.762	1.209.938	-20.579	1.164.556	-21.954
1.172.803	-25.763	1.210.084	-20.579	1.164.675	-21.952
1.172.950	-25.764	1.210.229	-20.579	1.164.837	-21.951
1.173.066	-25.764	1.210.351	-20.580	1.164.979	-21.950
1.173.213	-25.765	1.210.464	-20.580	1.165.148	-21.948
1.173.434	-25.766	1.210.628	-20.581	1.165.322	-21.947
1.173.655	-25.766	1.210.754	-20.581	1.165.574	-21.946
1.173.820	-25.767	1.210.900	-20.582	1.165.764	-21.944
1.173.997	-25.768	1.211.121	-20.582	1.165.939	-21.943
1.174.153	-25.768	1.211.298	-20.582	1.166.095	-21.942
1.174.309	-25.769	1.211.449	-20.583	1.166.233	-21.940
1.174.509	-25.770	1.211.607	-20.583	1.166.425	-21.939
1.174.667	-25.770	1.211.732	-20.584	1.166.581	-21.938
1.174.865	-25.771	1.211.869	-20.584	1.166.740	-21.936

1.175.080	-25.772	1.212.068	-20.585	1.166.939	-21.935
1.175.205	-25.772	1.212.248	-20.585	1.167.107	-21.934
1.175.283	-25.773	1.212.365	-20.586	1.167.198	-21.932
1.175.393	-25.773	1.212.561	-20.586	1.167.346	-21.931
1.175.552	-25.774	1.212.759	-20.587	1.167.513	-21.930
1.175.745	-25.775	1.212.890	-20.587	1.167.624	-21.928
1.175.874	-25.775	1.213.041	-20.588	1.167.726	-21.927
1.176.021	-25.776	1.213.186	-20.589	1.167.898	-21.926
1.176.216	-25.776	1.213.357	-20.589	1.168.058	-21.924
1.176.379	-25.777	1.213.532	-20.590	1.168.201	-21.923
1.176.566	-25.777	1.213.731	-20.590	1.168.465	-21.922
1.176.780	-25.778	1.213.960	-20.591	1.168.668	-21.920
1.176.994	-25.779	1.214.086	-20.592	1.168.754	-21.919
1.177.176	-25.779	1.214.185	-20.592	1.168.852	-21.918
1.177.281	-25.780	1.214.342	-20.593	1.169.060	-21.916
1.177.318	-25.780	1.214.518	-20.593	1.169.311	-21.915
1.177.515	-25.781	1.214.678	-20.594	1.169.438	-21.914
1.177.688	-25.781	1.214.825	-20.595	1.169.576	-21.912
1.177.840	-25.782	1.214.939	-20.595	1.169.768	-21.911
1.177.992	-25.782	1.215.107	-20.596	1.169.905	-21.910
1.178.254	-25.783	1.215.259	-20.597	1.170.026	-21.908
1.178.457	-25.783	1.215.289	-20.597	1.170.158	-21.907
1.178.551	-25.783	1.215.462	-20.598	1.170.300	-21.906
1.178.708	-25.784	1.215.764	-20.599	1.170.489	-21.905
1.178.904	-25.784	1.215.940	-20.599	1.170.651	-21.903
1.179.095	-25.785	1.216.097	-20.600	1.170.765	-21.902
1.179.258	-25.785	1.216.261	-20.601	1.170.931	-21.901
1.179.416	-25.786	1.216.404	-20.601	1.171.117	-21.900
1.179.548	-25.786	1.216.553	-20.602	1.171.289	-21.898
1.179.710	-25.787	1.216.770	-20.603	1.171.438	-21.897

1.179.865	-25.787	1.216.982	-20.604	1.171.583	-21.896
1.180.003	-25.787	1.217.077	-20.604	1.171.733	-21.895
1.180.344	-25.788	1.217.169	-20.605	1.171.921	-21.894
1.180.770	-25.788	1.217.326	-20.606	1.172.101	-21.892
1.181.044	-25.788	1.217.476	-20.606	1.172.225	-21.891
1.181.177	-25.789	1.217.655	-20.607	1.172.425	-21.890
1.181.208	-25.789	1.217.792	-20.608	1.172.605	-21.889
1.181.253	-25.789	1.217.849	-20.609	1.172.724	-21.888
1.181.315	-25.790	1.218.000	-20.609	1.172.887	-21.886
1.181.347	-25.790	1.218.150	-20.610	1.173.102	-21.885
1.181.411	-25.790	1.218.348	-20.611	1.173.259	-21.884
1.181.536	-25.791	1.218.661	-20.612	1.173.362	-21.883
1.181.674	-25.791	1.218.782	-20.612	1.173.520	-21.882
1.181.826	-25.791	1.218.849	-20.613	1.173.680	-21.881
1.181.966	-25.792	1.219.106	-20.614	1.173.854	-21.880
1.182.147	-25.792	1.219.356	-20.614	1.174.054	-21.878
1.182.390	-25.792	1.219.505	-20.615	1.174.272	-21.877
1.182.596	-25.793	1.219.648	-20.616	1.174.459	-21.876
1.182.772	-25.793	1.219.809	-20.617	1.174.630	-21.875
1.182.933	-25.793	1.219.938	-20.617	1.174.829	-21.874
1.183.080	-25.793	1.220.090	-20.618	1.174.979	-21.873
1.183.231	-25.794	1.220.238	-20.619	1.175.081	-21.872
1.183.419	-25.794	1.220.351	-20.619	1.175.214	-21.871
1.183.567	-25.794	1.220.482	-20.620	1.175.370	-21.870
1.183.684	-25.795	1.220.632	-20.621	1.175.516	-21.869
1.183.849	-25.795	1.220.704	-20.622	1.175.689	-21.867
1.184.025	-25.795	1.220.801	-20.622	1.175.805	-21.866
1.184.156	-25.795	1.221.048	-20.623	1.175.865	-21.865
1.184.279	-25.796	1.221.314	-20.624	1.176.040	-21.864
1.184.487	-25.796	1.221.489	-20.624	1.176.237	-21.863

1.184.669	-25.796	1.221.657	-20.625	1.176.337	-21.862
1.184.734	-25.797	1.221.892	-20.626	1.176.429	-21.861
1.184.871	-25.797	1.222.115	-20.627	1.176.610	-21.860
1.185.107	-25.797	1.222.223	-20.627	1.176.802	-21.859
1.185.217	-25.798	1.222.327	-20.628	1.177.014	-21.858
1.185.387	-25.798	1.222.505	-20.629	1.177.253	-21.857
1.185.690	-25.798	1.222.682	-20.629	1.177.470	-21.856
1.185.900	-25.798	1.222.806	-20.630	1.177.639	-21.855
1.186.052	-25.799	1.222.974	-20.631	1.177.785	-21.854
1.186.184	-25.799	1.223.158	-20.631	1.177.968	-21.853
1.186.347	-25.799	1.223.288	-20.632	1.178.131	-21.851
1.186.496	-25.800	1.223.431	-20.633	1.178.296	-21.850
1.186.684	-25.800	1.223.587	-20.633	1.178.493	-21.849
1.186.861	-25.800	1.223.793	-20.634	1.178.686	-21.848
1.187.004	-25.801	1.223.997	-20.635	1.178.806	-21.847
1.187.158	-25.801	1.224.156	-20.635	1.178.942	-21.846
1.187.239	-25.802	1.224.338	-20.636	1.179.075	-21.845
1.187.349	-25.802	1.224.542	-20.637	1.179.164	-21.844
1.187.497	-25.802	1.224.708	-20.637	1.179.322	-21.843
1.187.683	-25.803	1.224.828	-20.638	1.179.463	-21.842
1.187.819	-25.803	1.224.969	-20.639	1.179.566	-21.841
1.187.956	-25.803	1.225.163	-20.639	1.179.718	-21.840
1.188.101	-25.804	1.225.361	-20.640	1.179.826	-21.839
1.188.257	-25.804	1.225.527	-20.640	1.180.035	-21.838
1.188.459	-25.805	1.225.678	-20.641	1.180.371	-21.837
1.188.661	-25.805	1.225.809	-20.642	1.180.548	-21.836
1.188.847	-25.806	1.225.953	-20.642	1.180.695	-21.835
1.188.973	-25.806	1.226.124	-20.643	1.180.914	-21.833
1.189.109	-25.806	1.226.236	-20.644	1.181.094	-21.832
1.189.272	-25.807	1.226.355	-20.644	1.181.267	-21.831

1.189.452	-25.807	1.226.584	-20.645	1.181.459	-21.830
1.189.605	-25.808	1.226.756	-20.645	1.181.601	-21.829
1.189.815	-25.808	1.226.862	-20.646	1.181.771	-21.828
1.190.017	-25.809	1.227.042	-20.647	1.181.925	-21.827
1.190.081	-25.809	1.227.216	-20.647	1.182.015	-21.826
1.190.244	-25.810	1.227.329	-20.648	1.182.143	-21.825
1.190.477	-25.810	1.227.442	-20.648	1.182.365	-21.824
1.190.661	-25.811	1.227.609	-20.649	1.182.555	-21.823
1.190.839	-25.812	1.227.791	-20.650	1.182.730	-21.822
1.190.972	-25.812	1.228.093	-20.650	1.182.932	-21.821
1.191.085	-25.813	1.228.517	-20.651	1.183.006	-21.820
1.191.224	-25.813	1.228.834	-20.651	1.183.119	-21.818
1.191.443	-25.814	1.228.913	-20.652	1.183.379	-21.817
1.191.657	-25.814	1.228.936	-20.653	1.183.585	-21.816
1.191.814	-25.815	1.229.092	-20.653	1.183.717	-21.815
1.191.936	-25.816	1.229.176	-20.654	1.183.846	-21.814
1.192.106	-25.816	1.229.156	-20.654	1.183.957	-21.813
1.192.271	-25.817	1.229.240	-20.655	1.184.099	-21.812
1.192.456	-25.818	1.229.381	-20.656	1.184.261	-21.811
1.192.609	-25.818	1.229.532	-20.656	1.184.424	-21.810
1.192.752	-25.819	1.229.713	-20.657	1.184.583	-21.809
1.192.978	-25.819	1.229.841	-20.657	1.184.735	-21.808
1.193.176	-25.820	1.229.990	-20.658	1.184.934	-21.807
1.193.335	-25.821	1.230.196	-20.659	1.185.111	-21.805
1.193.465	-25.821	1.230.366	-20.659	1.185.218	-21.804
1.193.587	-25.822	1.230.507	-20.660	1.185.390	-21.803
1.193.700	-25.823	1.230.678	-20.660	1.185.571	-21.802
1.193.798	-25.823	1.230.827	-20.661	1.185.750	-21.801
1.193.935	-25.824	1.230.982	-20.662	1.185.937	-21.800
1.194.131	-25.825	1.231.146	-20.662	1.186.066	-21.799

1.194.298	-25.825	1.231.312	-20.663	1.186.326	-21.798
1.194.417	-25.826	1.231.558	-20.664	1.186.747	-21.797
1.194.537	-25.827	1.231.712	-20.664	1.187.071	-21.796
1.194.610	-25.827	1.231.840	-20.665	1.187.194	-21.795
1.194.750	-25.828	1.231.939	-20.666	1.187.234	-21.793
1.194.911	-25.829	1.232.069	-20.666	1.187.293	-21.792
1.195.028	-25.829	1.232.237	-20.667	1.187.366	-21.791
1.195.298	-25.830	1.232.449	-20.668	1.187.439	-21.790
1.195.575	-25.831	1.232.623	-20.668	1.187.523	-21.789
1.195.763	-25.831	1.232.732	-20.669	1.187.589	-21.788
1.195.988	-25.832	1.232.909	-20.670	1.187.751	-21.787
1.196.220	-25.833	1.233.094	-20.671	1.187.954	-21.786
1.196.399	-25.833	1.233.222	-20.671	1.188.074	-21.785
1.196.543	-25.834	1.233.430	-20.672	1.188.234	-21.784
1.196.699	-25.835	1.233.652	-20.673	1.188.438	-21.783
1.196.805	-25.835	1.233.833	-20.674	1.188.633	-21.782
1.196.930	-25.836	1.233.986	-20.674	1.188.792	-21.780
1.197.085	-25.837	1.234.117	-20.675	1.188.995	-21.779
1.197.206	-25.837	1.234.267	-20.676	1.189.212	-21.778
1.197.408	-25.838	1.234.426	-20.677	1.189.367	-21.777
1.197.570	-25.839	1.234.567	-20.678	1.189.502	-21.776
1.197.666	-25.839	1.234.658	-20.678	1.189.594	-21.775
1.197.820	-25.840	1.234.753	-20.679	1.189.695	-21.774
1.197.962	-25.841	1.234.906	-20.680	1.189.914	-21.773
1.198.057	-25.841	1.235.056	-20.681	1.190.095	-21.772
1.198.199	-25.842	1.235.221	-20.682	1.190.192	-21.771
1.198.413	-25.843	1.235.453	-20.683	1.190.378	-21.770
1.198.558	-25.843	1.235.643	-20.684	1.190.546	-21.769
1.198.770	-25.844	1.235.764	-20.685	1.190.677	-21.768
1.198.990	-25.845	1.235.880	-20.686	1.190.835	-21.767

1.199.094	-25.845	1.236.042	-20.687	1.191.030	-21.766
1.199.303	-25.846	1.236.276	-20.688	1.191.246	-21.765
1.199.504	-25.847	1.236.461	-20.689	1.191.395	-21.764
1.199.691	-25.847	1.236.591	-20.690	1.191.525	-21.763
1.199.840	-25.848	1.236.709	-20.691	1.191.703	-21.762
1.199.983	-25.848	1.236.812	-20.692	1.191.934	-21.761
1.200.126	-25.849	1.236.968	-20.693	1.192.121	-21.760
1.200.247	-25.850	1.237.143	-20.694	1.192.270	-21.759
1.200.424	-25.850	1.237.344	-20.695	1.192.415	-21.758
1.200.555	-25.851	1.237.552	-20.696	1.192.552	-21.757
1.200.708	-25.851	1.237.723	-20.697	1.192.709	-21.756
1.200.902	-25.852	1.237.873	-20.698	1.192.877	-21.755
1.201.131	-25.852	1.238.024	-20.699	1.193.026	-21.754
1.201.337	-25.853	1.238.192	-20.700	1.193.139	-21.753
1.201.430	-25.854	1.238.354	-20.702	1.193.212	-21.752
1.201.602	-25.854	1.238.580	-20.703	1.193.358	-21.751
1.201.846	-25.855	1.238.761	-20.704	1.193.587	-21.750
1.202.028	-25.855	1.238.837	-20.705	1.193.722	-21.749
1.202.183	-25.856	1.238.991	-20.706	1.193.859	-21.749
1.202.364	-25.856	1.239.118	-20.707	1.194.061	-21.748
1.202.478	-25.857	1.239.254	-20.709	1.194.253	-21.747
1.202.534	-25.857	1.239.454	-20.710	1.194.440	-21.746
1.202.721	-25.858	1.239.629	-20.711	1.194.601	-21.745
1.202.932	-25.858	1.239.817	-20.712	1.194.755	-21.744
1.203.046	-25.859	1.239.990	-20.714	1.194.934	-21.743
1.203.232	-25.859	1.240.157	-20.715	1.195.070	-21.743
1.203.479	-25.860	1.240.310	-20.716	1.195.163	-21.742
1.203.604	-25.860	1.240.448	-20.717	1.195.323	-21.741
1.203.678	-25.861	1.240.603	-20.719	1.195.516	-21.740
1.203.851	-25.861	1.240.768	-20.720	1.195.724	-21.739

1.204.051	-25.862	1.240.966	-20.721	1.195.926	-21.738
1.204.202	-25.862	1.241.123	-20.723	1.196.057	-21.738
1.204.343	-25.862	1.241.194	-20.724	1.196.208	-21.737
1.204.560	-25.863	1.241.265	-20.725	1.196.426	-21.736
1.204.762	-25.863	1.241.416	-20.726	1.196.613	-21.735
1.204.882	-25.864	1.241.542	-20.728	1.196.738	-21.735
1.205.021	-25.864	1.241.685	-20.729	1.196.843	-21.734
1.205.188	-25.865	1.241.908	-20.730	1.196.992	-21.733
1.205.412	-25.865	1.242.085	-20.732	1.197.178	-21.732
1.205.818	-25.865	1.242.222	-20.733	1.197.392	-21.732
1.206.182	-25.866	1.242.388	-20.734	1.197.630	-21.731
1.206.317	-25.866	1.242.595	-20.736	1.197.803	-21.730
1.206.403	-25.866	1.242.798	-20.737	1.197.906	-21.729
1.206.427	-25.867	1.242.978	-20.738	1.198.039	-21.729
1.206.556	-25.867	1.243.130	-20.740	1.198.215	-21.728
1.206.670	-25.868	1.243.320	-20.741	1.198.360	-21.727
1.206.656	-25.868	1.243.545	-20.742	1.198.542	-21.726
1.206.726	-25.868	1.243.758	-20.744	1.198.712	-21.726
1.206.908	-25.869	1.243.917	-20.745	1.198.782	-21.725
1.207.113	-25.869	1.244.024	-20.747	1.198.895	-21.724
1.207.222	-25.869	1.244.129	-20.748	1.199.026	-21.723
1.207.351	-25.870	1.244.249	-20.749	1.199.164	-21.723
1.207.523	-25.870	1.244.398	-20.751	1.199.351	-21.722
1.207.735	-25.870	1.244.549	-20.752	1.199.504	-21.721
1.208.001	-25.871	1.244.650	-20.753	1.199.638	-21.721
1.208.120	-25.871	1.244.771	-20.755	1.199.761	-21.720
1.208.237	-25.871	1.244.980	-20.756	1.199.912	-21.719
1.208.428	-25.872	1.245.190	-20.757	1.200.130	-21.718
1.208.644	-25.872	1.245.369	-20.759	1.200.399	-21.718
1.208.856	-25.872	1.245.517	-20.760	1.200.617	-21.717

1.208.950	-25.873	1.245.709	-20.761	1.200.781	-21.716
1.209.082	-25.873	1.245.897	-20.763	1.200.970	-21.715
1.209.244	-25.873	1.246.030	-20.764	1.201.127	-21.715
1.209.390	-25.874	1.246.192	-20.765	1.201.286	-21.714
1.209.515	-25.874	1.246.382	-20.767	1.201.429	-21.713
1.209.619	-25.874	1.246.573	-20.768	1.201.546	-21.712
1.209.791	-25.875	1.246.735	-20.769	1.201.718	-21.712
1.209.970	-25.875	1.246.882	-20.770	1.201.887	-21.711
1.210.145	-25.875	1.247.057	-20.772	1.202.014	-21.710
1.210.323	-25.876	1.247.200	-20.773	1.202.088	-21.709
1.210.464	-25.876	1.247.311	-20.774	1.202.236	-21.709
1.210.600	-25.877	1.247.495	-20.776	1.202.414	-21.708
1.210.820	-25.877	1.247.670	-20.777	1.202.560	-21.707
1.211.072	-25.877	1.247.852	-20.778	1.202.806	-21.706
1.211.263	-25.878	1.248.043	-20.779	1.203.053	-21.705
1.211.348	-25.878	1.248.233	-20.781	1.203.201	-21.705
1.211.446	-25.878	1.248.422	-20.782	1.203.336	-21.704
1.211.711	-25.879	1.248.543	-20.783	1.203.531	-21.703
1.211.873	-25.879	1.248.684	-20.784	1.203.763	-21.702
1.211.936	-25.880	1.248.803	-20.785	1.203.895	-21.701
1.212.127	-25.880	1.248.931	-20.787	1.204.007	-21.700
1.212.294	-25.880	1.249.119	-20.788	1.204.230	-21.700
1.212.458	-25.881	1.249.305	-20.789	1.204.393	-21.699
1.212.651	-25.881	1.249.431	-20.790	1.204.518	-21.698
1.212.760	-25.882	1.249.573	-20.791	1.204.671	-21.697
1.212.896	-25.882	1.249.788	-20.793	1.204.808	-21.696
1.213.009	-25.883	1.249.935	-20.794	1.204.970	-21.695
1.213.153	-25.883	1.250.091	-20.795	1.205.154	-21.694
1.213.372	-25.884	1.250.261	-20.796	1.205.330	-21.693
1.213.572	-25.884	1.250.417	-20.797	1.205.498	-21.692

1.213.789	-25.885	1.250.554	-20.798	1.205.657	-21.692
1.213.939	-25.885	1.250.688	-20.799	1.205.810	-21.691
1.214.048	-25.886	1.250.877	-20.801	1.205.960	-21.690
1.214.172	-25.886	1.251.072	-20.802	1.206.131	-21.689
1.214.285	-25.887	1.251.253	-20.803	1.206.327	-21.688
1.214.482	-25.887	1.251.404	-20.804	1.206.500	-21.687
1.214.686	-25.888	1.251.557	-20.805	1.206.613	-21.686
1.214.753	-25.888	1.251.666	-20.806	1.206.759	-21.685
1.214.939	-25.889	1.251.746	-20.807	1.206.966	-21.684
1.215.249	-25.889	1.251.924	-20.808	1.207.120	-21.683
1.215.445	-25.890	1.252.343	-20.809	1.207.239	-21.682
1.215.554	-25.890	1.252.824	-20.810	1.207.395	-21.681
1.215.727	-25.891	1.253.027	-20.811	1.207.540	-21.680
1.215.922	-25.892	1.253.015	-20.812	1.207.726	-21.679
1.216.029	-25.892	1.253.102	-20.813	1.207.925	-21.678
1.216.175	-25.893	1.253.239	-20.814	1.208.045	-21.677
1.216.358	-25.894	1.253.278	-20.815	1.208.249	-21.676
1.216.520	-25.894	1.253.316	-20.816	1.208.439	-21.675
1.216.638	-25.895	1.253.450	-20.817	1.208.543	-21.674
1.216.707	-25.895	1.253.659	-20.818	1.208.711	-21.673
1.216.852	-25.896	1.253.778	-20.819	1.208.865	-21.672
1.216.991	-25.897	1.253.815	-20.820	1.208.989	-21.671
1.217.183	-25.897	1.253.959	-20.821	1.209.235	-21.670
1.217.424	-25.898	1.254.210	-20.822	1.209.617	-21.669
1.217.546	-25.899	1.254.430	-20.823	1.210.003	-21.668
1.217.731	-25.899	1.254.635	-20.824	1.210.226	-21.666
1.217.953	-25.900	1.254.796	-20.825	1.210.266	-21.665
1.218.162	-25.901	1.254.930	-20.826	1.210.313	-21.664
1.218.349	-25.902	1.255.096	-20.827	1.210.356	-21.663
1.218.454	-25.902	1.255.258	-20.828	1.210.397	-21.662

1.218.534	-25.903	1.255.385	-20.829	1.210.482	-21.661
1.218.653	-25.904	1.255.522	-20.830	1.210.641	-21.660
1.218.836	-25.904	1.255.699	-20.831	1.210.795	-21.659
1.219.018	-25.905	1.255.876	-20.832	1.210.956	-21.658
1.219.122	-25.906	1.256.030	-20.832	1.211.103	-21.656
1.219.262	-25.906	1.256.147	-20.833	1.211.240	-21.655
1.219.481	-25.907	1.256.277	-20.834	1.211.464	-21.654
1.219.630	-25.908	1.256.431	-20.835	1.211.650	-21.653
1.219.757	-25.909	1.256.662	-20.836	1.211.839	-21.652
1.219.899	-25.909	1.256.823	-20.837	1.212.013	-21.651
1.220.004	-25.910	1.256.884	-20.838	1.212.129	-21.650
1.220.148	-25.911	1.257.003	-20.839	1.212.281	-21.648
1.220.343	-25.911	1.257.184	-20.840	1.212.487	-21.647
1.220.519	-25.912	1.257.386	-20.841	1.212.655	-21.646
1.220.661	-25.913	1.257.569	-20.842	1.212.803	-21.645
1.220.847	-25.914	1.257.758	-20.843	1.212.991	-21.644
1.221.017	-25.914	1.257.928	-20.844	1.213.160	-21.643
1.221.235	-25.915	1.258.116	-20.845	1.213.316	-21.641
1.221.516	-25.916	1.258.259	-20.846	1.213.459	-21.640
1.221.704	-25.917	1.258.385	-20.846	1.213.568	-21.639
1.221.843	-25.917	1.258.540	-20.847	1.213.661	-21.638
1.221.984	-25.918	1.258.703	-20.848	1.213.794	-21.637
1.222.143	-25.919	1.258.896	-20.849	1.213.945	-21.636
1.222.271	-25.919	1.259.034	-20.850	1.214.082	-21.634
1.222.446	-25.920	1.259.151	-20.851	1.214.305	-21.633
1.222.554	-25.921	1.259.348	-20.852	1.214.542	-21.632
1.222.666	-25.922	1.259.521	-20.853	1.214.710	-21.631
1.222.835	-25.922	1.259.706	-20.854	1.214.883	-21.630
1.222.979	-25.923	1.259.845	-20.855	1.215.053	-21.629
1.223.161	-25.924	1.259.933	-20.856	1.215.220	-21.628

1.223.334	-25.924	1.260.007	-20.857	1.215.405	-21.627
1.223.467	-25.925	1.260.235	-20.858	1.215.554	-21.625
1.223.599	-25.926	1.260.513	-20.859	1.215.678	-21.624
1.223.726	-25.926	1.260.587	-20.860	1.215.793	-21.623
1.223.817	-25.927	1.260.692	-20.861	1.215.921	-21.622
1.224.025	-25.928	1.260.892	-20.862	1.216.096	-21.621
1.224.296	-25.928	1.261.064	-20.863	1.216.217	-21.620
1.224.483	-25.929	1.261.245	-20.864	1.216.333	-21.619
1.224.689	-25.930	1.261.411	-20.865	1.216.500	-21.618
1.224.893	-25.930	1.261.566	-20.866	1.216.670	-21.616
1.225.041	-25.931	1.261.712	-20.867	1.216.891	-21.615
1.225.130	-25.932	1.261.841	-20.868	1.217.071	-21.614
1.225.266	-25.932	1.262.038	-20.869	1.217.210	-21.613
1.225.464	-25.933	1.262.281	-20.870	1.217.414	-21.612
1.225.641	-25.934	1.262.489	-20.871	1.217.515	-21.611
1.225.773	-25.934	1.262.614	-20.872	1.217.633	-21.610
1.225.877	-25.935	1.262.749	-20.873	1.217.832	-21.609
1.226.056	-25.935	1.262.896	-20.874	1.217.975	-21.608
1.226.253	-25.936	1.263.015	-20.875	1.218.127	-21.607
1.226.378	-25.937	1.263.165	-20.876	1.218.290	-21.606
1.226.490	-25.937	1.263.318	-20.877	1.218.496	-21.605
1.226.674	-25.938	1.263.497	-20.878	1.218.647	-21.604
1.226.892	-25.938	1.263.671	-20.879	1.218.786	-21.603
1.227.088	-25.939	1.263.799	-20.881	1.218.908	-21.602
1.227.237	-25.940	1.263.931	-20.882	1.219.106	-21.601
1.227.363	-25.940	1.264.102	-20.883	1.219.294	-21.600
1.227.534	-25.941	1.264.290	-20.884	1.219.464	-21.599
1.227.695	-25.941	1.264.509	-20.885	1.219.655	-21.598
1.227.860	-25.942	1.264.653	-20.886	1.219.808	-21.597
1.227.989	-25.942	1.264.763	-20.887	1.219.989	-21.596

1.228.186	-25.943	1.264.958	-20.888	1.220.137	-21.595
1.228.401	-25.944	1.265.105	-20.889	1.220.229	-21.594
1.228.487	-25.944	1.265.146	-20.890	1.220.323	-21.593
1.228.623	-25.945	1.265.298	-20.891	1.220.509	-21.592
1.228.873	-25.945	1.265.538	-20.892	1.220.760	-21.591
1.229.094	-25.946	1.265.730	-20.893	1.220.941	-21.590
1.229.231	-25.946	1.265.789	-20.895	1.221.105	-21.589
1.229.365	-25.947	1.265.891	-20.896	1.221.256	-21.589
1.229.501	-25.947	1.266.071	-20.897	1.221.415	-21.588
1.229.661	-25.948	1.266.172	-20.898	1.221.619	-21.587
1.229.826	-25.948	1.266.346	-20.899	1.221.803	-21.586
1.229.986	-25.949	1.266.586	-20.900	1.221.877	-21.585
1.230.154	-25.949	1.266.774	-20.901	1.221.895	-21.584
1.230.277	-25.950	1.266.967	-20.902	1.221.984	-21.583
1.230.379	-25.950	1.267.162	-20.903	1.222.194	-21.582
1.230.648	-25.951	1.267.361	-20.904	1.222.393	-21.581
1.231.115	-25.951	1.267.565	-20.905	1.222.511	-21.581
1.231.476	-25.952	1.267.738	-20.906	1.222.645	-21.580
1.231.627	-25.952	1.267.914	-20.907	1.222.890	-21.579
1.231.715	-25.953	1.268.074	-20.908	1.223.095	-21.578
1.231.768	-25.953	1.268.194	-20.910	1.223.204	-21.577
1.231.826	-25.954	1.268.329	-20.911	1.223.354	-21.576
1.231.883	-25.954	1.268.452	-20.912	1.223.600	-21.575
1.231.888	-25.955	1.268.596	-20.913	1.223.817	-21.574
1.231.954	-25.955	1.268.781	-20.914	1.223.962	-21.574
1.232.155	-25.956	1.268.916	-20.915	1.224.164	-21.573
1.232.346	-25.956	1.269.001	-20.916	1.224.370	-21.572
1.232.415	-25.957	1.269.135	-20.917	1.224.470	-21.571
1.232.572	-25.957	1.269.330	-20.918	1.224.538	-21.570
1.232.794	-25.958	1.269.541	-20.919	1.224.685	-21.569

1.232.964	-25.958	1.269.751	-20.920	1.224.840	-21.568
1.233.152	-25.959	1.269.924	-20.921	1.224.905	-21.567
1.233.324	-25.959	1.270.114	-20.922	1.225.024	-21.567
1.233.466	-25.960	1.270.271	-20.923	1.225.191	-21.566
1.233.589	-25.960	1.270.414	-20.924	1.225.368	-21.565
1.233.769	-25.961	1.270.625	-20.925	1.225.597	-21.564
1.233.957	-25.961	1.270.730	-20.926	1.225.791	-21.563
1.234.141	-25.961	1.270.854	-20.927	1.225.905	-21.562
1.234.277	-25.962	1.271.068	-20.928	1.226.050	-21.561
1.234.375	-25.962	1.271.248	-20.929	1.226.319	-21.561
1.234.513	-25.963	1.271.404	-20.930	1.226.579	-21.560
1.234.679	-25.964	1.271.555	-20.931	1.226.784	-21.559
1.234.846	-25.964	1.271.716	-20.932	1.226.886	-21.558
1.234.966	-25.965	1.271.911	-20.933	1.226.973	-21.557
1.235.166	-25.965	1.272.088	-20.934	1.227.166	-21.556
1.235.330	-25.966	1.272.225	-20.935	1.227.394	-21.555
1.235.440	-25.966	1.272.341	-20.936	1.227.523	-21.555
1.235.627	-25.967	1.272.480	-20.937	1.227.616	-21.554
1.235.821	-25.967	1.272.677	-20.938	1.227.822	-21.553
1.235.913	-25.968	1.272.820	-20.939	1.228.025	-21.552
1.236.088	-25.968	1.273.017	-20.940	1.228.177	-21.551
1.236.374	-25.969	1.273.186	-20.941	1.228.291	-21.550
1.236.582	-25.970	1.273.282	-20.942	1.228.386	-21.549
1.236.749	-25.970	1.273.436	-20.943	1.228.558	-21.549
1.236.912	-25.971	1.273.593	-20.944	1.228.737	-21.548
1.237.079	-25.971	1.273.792	-20.945	1.228.926	-21.547
1.237.219	-25.972	1.273.985	-20.946	1.229.145	-21.546
1.237.343	-25.973	1.274.135	-20.946	1.229.325	-21.545
1.237.506	-25.973	1.274.294	-20.947	1.229.470	-21.544
1.237.658	-25.974	1.274.444	-20.948	1.229.614	-21.543

1.237.798	-25.975	1.274.588	-20.949	1.229.772	-21.543
1.237.990	-25.975	1.274.740	-20.950	1.229.915	-21.542
1.238.194	-25.976	1.274.917	-20.951	1.230.049	-21.541
1.238.354	-25.977	1.275.033	-20.952	1.230.177	-21.540
1.238.464	-25.977	1.275.134	-20.953	1.230.335	-21.539
1.238.579	-25.978	1.275.255	-20.954	1.230.498	-21.538
1.238.712	-25.979	1.275.470	-20.955	1.230.664	-21.538
1.238.904	-25.979	1.275.739	-20.956	1.230.835	-21.537
1.239.103	-25.980	1.275.934	-20.957	1.230.987	-21.536
1.239.272	-25.981	1.276.071	-20.958	1.231.157	-21.535
1.239.370	-25.982	1.276.210	-20.959	1.231.341	-21.534
1.239.494	-25.982	1.276.563	-20.960	1.231.525	-21.533
1.239.644	-25.983	1.276.947	-20.961	1.231.698	-21.533
1.239.729	-25.984	1.277.220	-20.961	1.231.819	-21.532
1.239.893	-25.985	1.277.312	-20.962	1.231.910	-21.531
1.240.114	-25.986	1.277.327	-20.963	1.232.060	-21.530
1.240.258	-25.987	1.277.421	-20.964	1.232.447	-21.529
1.240.389	-25.987	1.277.511	-20.965	1.232.906	-21.528
1.240.641	-25.988	1.277.576	-20.966	1.233.204	-21.528
1.240.905	-25.989	1.277.596	-20.967	1.233.287	-21.527
1.241.049	-25.990	1.277.733	-20.968	1.233.250	-21.526
1.241.173	-25.991	1.277.919	-20.969	1.233.308	-21.525
1.241.290	-25.992	1.278.051	-20.970	1.233.388	-21.525
1.241.416	-25.993	1.278.194	-20.971	1.233.472	-21.524
1.241.608	-25.994	1.278.362	-20.972	1.233.563	-21.523
1.241.788	-25.995	1.278.533	-20.973	1.233.656	-21.522
1.241.908	-25.996	1.278.661	-20.974	1.233.855	-21.522
1.242.110	-25.997	1.278.799	-20.975	1.234.018	-21.521
1.242.367	-25.998	1.279.028	-20.976	1.234.118	-21.520
1.242.539	-25.999	1.279.243	-20.977	1.234.313	-21.519

1.242.632	-26.000	1.279.391	-20.978	1.234.568	-21.519
1.242.732	-26.001	1.279.489	-20.979	1.234.825	-21.518
1.242.920	-26.002	1.279.633	-20.980	1.234.977	-21.517
1.243.049	-26.003	1.279.843	-20.981	1.235.065	-21.516
1.243.205	-26.004	1.279.980	-20.982	1.235.176	-21.516
1.243.409	-26.005	1.280.121	-20.983	1.235.333	-21.515
1.243.538	-26.006	1.280.258	-20.984	1.235.547	-21.514
1.243.689	-26.007	1.280.381	-20.985	1.235.750	-21.514
1.243.876	-26.008	1.280.544	-20.987	1.235.888	-21.513
1.244.061	-26.009	1.280.760	-20.988	1.235.970	-21.512
1.244.195	-26.010	1.280.950	-20.989	1.236.087	-21.512
1.244.281	-26.011	1.281.118	-20.990	1.236.267	-21.511
1.244.415	-26.012	1.281.248	-20.991	1.236.415	-21.510
1.244.603	-26.013	1.281.398	-20.992	1.236.544	-21.510
1.244.747	-26.014	1.281.528	-20.993	1.236.685	-21.509
1.244.877	-26.016	1.281.691	-20.995	1.236.834	-21.509
1.244.999	-26.017	1.281.936	-20.996	1.237.039	-21.508
1.245.157	-26.018	1.282.135	-20.997	1.237.259	-21.507
1.245.325	-26.019	1.282.321	-20.998	1.237.448	-21.507
1.245.443	-26.020	1.282.428	-21.000	1.237.542	-21.506
1.245.676	-26.021	1.282.451	-21.001	1.237.655	-21.506
1.245.877	-26.022	1.282.632	-21.002	1.237.923	-21.505
1.245.986	-26.023	1.282.935	-21.004	1.238.128	-21.505
1.246.188	-26.024	1.283.107	-21.005	1.238.279	-21.504
1.246.404	-26.025	1.283.168	-21.006	1.238.373	-21.504
1.246.644	-26.026	1.283.275	-21.008	1.238.456	-21.504
1.246.863	-26.027	1.283.443	-21.009	1.238.562	-21.503
1.247.032	-26.029	1.283.589	-21.010	1.238.730	-21.503
1.247.123	-26.030	1.283.750	-21.012	1.238.997	-21.502
1.247.190	-26.031	1.283.943	-21.013	1.239.184	-21.502

1.247.411	-26.032	1.284.124	-21.015	1.239.268	-21.501
1.247.624	-26.033	1.284.252	-21.016	1.239.407	-21.501
1.247.735	-26.034	1.284.426	-21.018	1.239.584	-21.501
1.247.803	-26.035	1.284.639	-21.019	1.239.743	-21.500
1.247.931	-26.036	1.284.787	-21.020	1.239.784	-21.500
1.248.114	-26.037	1.284.897	-21.022	1.239.959	-21.500
1.248.307	-26.038	1.285.005	-21.024	1.240.294	-21.500
1.248.423	-26.039	1.285.179	-21.025	1.240.484	-21.499
1.248.521	-26.041	1.285.376	-21.027	1.240.606	-21.499
1.248.731	-26.042	1.285.526	-21.028	1.240.735	-21.499
1.248.979	-26.043	1.285.688	-21.030	1.240.883	-21.499
1.249.125	-26.044	1.285.912	-21.032	1.241.052	-21.498
1.249.239	-26.045	1.286.059	-21.033	1.241.187	-21.498
1.249.470	-26.046	1.286.178	-21.035	1.241.356	-21.498
1.249.651	-26.047	1.286.315	-21.037	1.241.534	-21.498
1.249.767	-26.048	1.286.456	-21.038	1.241.705	-21.498
1.249.894	-26.049	1.286.653	-21.040	1.241.881	-21.498
1.250.098	-26.050	1.286.833	-21.042	1.242.041	-21.498
1.250.336	-26.051	1.286.971	-21.043	1.242.222	-21.498
1.250.465	-26.052	1.287.108	-21.045	1.242.394	-21.497
1.250.611	-26.053	1.287.303	-21.047	1.242.550	-21.497
1.250.861	-26.054	1.287.440	-21.049	1.242.618	-21.497
1.250.978	-26.055	1.287.599	-21.051	1.242.745	-21.497
1.251.049	-26.056	1.287.785	-21.052	1.242.983	-21.497
1.251.250	-26.057	1.287.905	-21.054	1.243.188	-21.497
1.251.459	-26.058	1.288.044	-21.056	1.243.311	-21.497
1.251.609	-26.059	1.288.228	-21.058	1.243.421	-21.497
1.251.685	-26.060	1.288.445	-21.060	1.243.538	-21.497
1.251.840	-26.061	1.288.618	-21.062	1.243.681	-21.498
1.252.007	-26.062	1.288.801	-21.064	1.243.871	-21.498

1.252.178	-26.063	1.288.965	-21.065	1.244.072	-21.498
1.252.343	-26.064	1.289.068	-21.067	1.244.230	-21.498
1.252.489	-26.065	1.289.216	-21.069	1.244.374	-21.498
1.252.676	-26.066	1.289.348	-21.071	1.244.540	-21.498
1.252.881	-26.067	1.289.424	-21.073	1.244.680	-21.498
1.253.068	-26.068	1.289.509	-21.075	1.244.789	-21.498
1.253.204	-26.069	1.289.664	-21.077	1.244.921	-21.498
1.253.365	-26.070	1.289.837	-21.079	1.245.073	-21.498
1.253.506	-26.071	1.290.016	-21.081	1.245.257	-21.499
1.253.655	-26.072	1.290.154	-21.083	1.245.406	-21.499
1.253.855	-26.073	1.290.309	-21.085	1.245.476	-21.499
1.253.997	-26.073	1.290.540	-21.087	1.245.604	-21.499
1.254.167	-26.074	1.290.766	-21.089	1.245.842	-21.499
1.254.352	-26.075	1.290.945	-21.091	1.246.021	-21.499
1.254.493	-26.076	1.291.116	-21.093	1.246.203	-21.500
1.254.650	-26.077	1.291.299	-21.095	1.246.369	-21.500
1.254.789	-26.078	1.291.563	-21.097	1.246.646	-21.500
1.254.910	-26.079	1.291.805	-21.099	1.246.902	-21.500
1.255.081	-26.080	1.291.915	-21.101	1.247.007	-21.500
1.255.253	-26.081	1.292.032	-21.103	1.247.149	-21.501
1.255.417	-26.082	1.292.188	-21.105	1.247.332	-21.501
1.255.502	-26.082	1.292.397	-21.106	1.247.439	-21.501
1.255.616	-26.083	1.292.583	-21.108	1.247.521	-21.501
1.256.049	-26.084	1.292.635	-21.110	1.247.712	-21.502
1.256.494	-26.085	1.292.686	-21.112	1.247.908	-21.502
1.256.720	-26.086	1.292.905	-21.114	1.248.106	-21.502
1.256.852	-26.087	1.293.091	-21.116	1.248.286	-21.502
1.256.940	-26.088	1.293.167	-21.118	1.248.393	-21.503
1.256.973	-26.088	1.293.306	-21.120	1.248.521	-21.503
1.257.034	-26.089	1.293.448	-21.122	1.248.642	-21.503

1.257.095	-26.090	1.293.568	-21.124	1.248.814	-21.503
1.257.184	-26.091	1.293.696	-21.126	1.249.024	-21.503
1.257.289	-26.092	1.293.880	-21.128	1.249.210	-21.504
1.257.374	-26.093	1.294.104	-21.130	1.249.389	-21.504
1.257.572	-26.093	1.294.355	-21.132	1.249.579	-21.504
1.257.722	-26.094	1.294.599	-21.134	1.249.797	-21.504
1.257.847	-26.095	1.294.754	-21.136	1.249.951	-21.505
1.258.069	-26.096	1.294.843	-21.138	1.250.062	-21.505
1.258.251	-26.097	1.294.957	-21.140	1.250.190	-21.505
1.258.389	-26.097	1.295.112	-21.142	1.250.387	-21.505
1.258.586	-26.098	1.295.336	-21.144	1.250.601	-21.506
1.258.817	-26.099	1.295.517	-21.146	1.250.731	-21.506
1.258.965	-26.100	1.295.591	-21.148	1.250.864	-21.506
1.259.102	-26.101	1.295.733	-21.149	1.251.011	-21.506
1.259.200	-26.102	1.295.939	-21.151	1.251.143	-21.506
1.259.324	-26.103	1.296.158	-21.153	1.251.329	-21.507
1.259.507	-26.103	1.296.348	-21.155	1.251.537	-21.507
1.259.707	-26.104	1.296.425	-21.157	1.251.677	-21.507
1.259.868	-26.105	1.296.537	-21.159	1.251.771	-21.507
1.259.955	-26.106	1.296.767	-21.161	1.251.931	-21.507
1.260.114	-26.107	1.296.996	-21.163	1.252.180	-21.508
1.260.300	-26.108	1.297.166	-21.164	1.252.344	-21.508
1.260.443	-26.109	1.297.341	-21.166	1.252.427	-21.508
1.260.600	-26.110	1.297.522	-21.168	1.252.625	-21.508
1.260.786	-26.110	1.297.650	-21.170	1.252.802	-21.508
1.260.941	-26.111	1.297.753	-21.172	1.252.969	-21.508
1.261.080	-26.112	1.297.932	-21.174	1.253.071	-21.509
1.261.288	-26.113	1.298.134	-21.175	1.253.204	-21.509
1.261.440	-26.114	1.298.262	-21.177	1.253.419	-21.509
1.261.593	-26.115	1.298.466	-21.179	1.253.590	-21.509

1.261.799	-26.116	1.298.665	-21.181	1.253.728	-21.509
1.261.969	-26.117	1.298.770	-21.183	1.253.850	-21.509
1.262.115	-26.118	1.298.905	-21.184	1.253.986	-21.509
1.262.251	-26.119	1.299.063	-21.186	1.254.194	-21.509
1.262.381	-26.120	1.299.252	-21.188	1.254.398	-21.510
1.262.507	-26.121	1.299.438	-21.190	1.254.538	-21.510
1.262.717	-26.122	1.299.576	-21.192	1.254.693	-21.510
1.262.950	-26.123	1.299.767	-21.193	1.254.780	-21.510
1.263.067	-26.124	1.299.969	-21.195	1.254.924	-21.510
1.263.152	-26.125	1.300.096	-21.197	1.255.271	-21.510
1.263.307	-26.126	1.300.222	-21.199	1.255.723	-21.510
1.263.466	-26.127	1.300.296	-21.200	1.256.073	-21.510
1.263.611	-26.128	1.300.546	-21.202	1.256.198	-21.510
1.263.777	-26.129	1.301.049	-21.204	1.256.248	-21.510
1.263.969	-26.131	1.301.409	-21.206	1.256.324	-21.510
1.264.113	-26.132	1.301.519	-21.207	1.256.371	-21.510
1.264.308	-26.133	1.301.577	-21.209	1.256.387	-21.510
1.264.496	-26.134	1.301.660	-21.211	1.256.481	-21.510
1.264.613	-26.135	1.301.696	-21.213	1.256.647	-21.510
1.264.809	-26.136	1.301.745	-21.215	1.256.783	-21.510
1.264.948	-26.137	1.301.859	-21.216	1.256.904	-21.510
1.265.085	-26.139	1.301.958	-21.218	1.257.063	-21.510
1.265.258	-26.140	1.302.036	-21.220	1.257.211	-21.510
1.265.410	-26.141	1.302.180	-21.222	1.257.392	-21.510
1.265.616	-26.142	1.302.368	-21.223	1.257.617	-21.510
1.265.782	-26.143	1.302.466	-21.225	1.257.782	-21.510
1.265.894	-26.145	1.302.585	-21.227	1.257.952	-21.510
1.266.055	-26.146	1.302.852	-21.229	1.258.116	-21.510
1.266.273	-26.147	1.303.069	-21.230	1.258.255	-21.510
1.266.460	-26.149	1.303.181	-21.232	1.258.412	-21.510

1.266.586	-26.150	1.303.322	-21.234	1.258.555	-21.510
1.266.673	-26.151	1.303.530	-21.236	1.258.647	-21.510
1.266.862	-26.152	1.303.746	-21.237	1.258.793	-21.510
1.267.059	-26.154	1.303.895	-21.239	1.259.055	-21.510
1.267.231	-26.155	1.303.976	-21.241	1.259.200	-21.510
1.267.399	-26.156	1.304.155	-21.243	1.259.350	-21.510
1.267.518	-26.158	1.304.364	-21.245	1.259.498	-21.510
1.267.710	-26.159	1.304.482	-21.246	1.259.610	-21.510
1.267.896	-26.160	1.304.597	-21.248	1.259.745	-21.509
1.268.026	-26.162	1.304.747	-21.250	1.259.886	-21.509
1.268.179	-26.163	1.304.953	-21.252	1.260.083	-21.509
1.268.376	-26.164	1.304.987	-21.254	1.260.296	-21.509
1.268.524	-26.166	1.305.098	-21.256	1.260.437	-21.509
1.268.629	-26.167	1.305.300	-21.258	1.260.625	-21.509
1.268.839	-26.169	1.305.435	-21.259	1.260.889	-21.509
1.269.050	-26.170	1.305.688	-21.261	1.261.037	-21.509
1.269.191	-26.171	1.305.863	-21.263	1.261.145	-21.509
1.269.339	-26.173	1.306.019	-21.265	1.261.304	-21.509
1.269.480	-26.174	1.306.286	-21.267	1.261.463	-21.509
1.269.632	-26.175	1.306.451	-21.269	1.261.581	-21.509
1.269.796	-26.177	1.306.557	-21.271	1.261.707	-21.508
1.269.861	-26.178	1.306.723	-21.273	1.261.830	-21.508
1.269.935	-26.180	1.306.877	-21.275	1.261.983	-21.508
1.270.083	-26.181	1.307.023	-21.277	1.262.178	-21.508
1.270.231	-26.182	1.307.229	-21.279	1.262.330	-21.508
1.270.405	-26.184	1.307.383	-21.281	1.262.466	-21.508
1.270.574	-26.185	1.307.451	-21.283	1.262.639	-21.508
1.270.766	-26.186	1.307.549	-21.285	1.262.811	-21.508
1.270.974	-26.188	1.307.708	-21.287	1.262.948	-21.508
1.271.098	-26.189	1.307.910	-21.290	1.263.116	-21.508

1.271.217	-26.190	1.308.100	-21.292	1.263.340	-21.508
1.271.425	-26.192	1.308.289	-21.294	1.263.513	-21.508
1.271.682	-26.193	1.308.409	-21.296	1.263.640	-21.507
1.271.846	-26.194	1.308.524	-21.298	1.263.727	-21.507
1.271.994	-26.196	1.308.683	-21.300	1.263.880	-21.507
1.272.135	-26.197	1.308.848	-21.303	1.264.048	-21.507
1.272.312	-26.198	1.308.981	-21.305	1.264.229	-21.507
1.272.513	-26.200	1.309.104	-21.307	1.264.494	-21.507
1.272.596	-26.201	1.309.270	-21.309	1.264.720	-21.507
1.272.726	-26.202	1.309.444	-21.312	1.264.841	-21.507
1.272.930	-26.204	1.309.563	-21.314	1.264.953	-21.507
1.273.055	-26.205	1.309.713	-21.316	1.265.029	-21.507
1.273.194	-26.206	1.309.924	-21.319	1.265.159	-21.507
1.273.385	-26.207	1.310.146	-21.321	1.265.392	-21.507
1.273.533	-26.209	1.310.343	-21.324	1.265.592	-21.507
1.273.676	-26.210	1.310.473	-21.326	1.265.741	-21.507
1.273.813	-26.211	1.310.602	-21.328	1.265.835	-21.507
1.273.949	-26.212	1.310.730	-21.331	1.265.977	-21.507
1.274.117	-26.214	1.310.871	-21.333	1.266.151	-21.506
1.274.308	-26.215	1.311.078	-21.336	1.266.331	-21.506
1.274.462	-26.216	1.311.272	-21.338	1.266.521	-21.506
1.274.628	-26.217	1.311.422	-21.341	1.266.702	-21.506
1.274.805	-26.218	1.311.537	-21.343	1.266.868	-21.506
1.274.971	-26.219	1.311.631	-21.346	1.267.009	-21.506
1.275.190	-26.220	1.311.772	-21.348	1.267.169	-21.506
1.275.294	-26.222	1.311.991	-21.351	1.267.321	-21.506
1.275.471	-26.223	1.312.184	-21.354	1.267.453	-21.506
1.275.668	-26.224	1.312.359	-21.356	1.267.601	-21.506
1.275.869	-26.225	1.312.491	-21.359	1.267.813	-21.506
1.275.984	-26.226	1.312.560	-21.362	1.267.980	-21.506

1.276.104	-26.227	1.312.735	-21.364	1.268.011	-21.506
1.276.266	-26.228	1.312.937	-21.367	1.268.062	-21.506
1.276.387	-26.229	1.313.065	-21.369	1.268.217	-21.506
1.276.539	-26.230	1.313.241	-21.372	1.268.427	-21.506
1.276.747	-26.231	1.313.441	-21.375	1.268.575	-21.505
1.276.868	-26.232	1.313.627	-21.378	1.268.746	-21.505
1.276.949	-26.233	1.313.775	-21.380	1.268.954	-21.505
1.277.126	-26.233	1.313.878	-21.383	1.269.149	-21.505
1.277.325	-26.234	1.314.007	-21.386	1.269.377	-21.505
1.277.421	-26.235	1.314.126	-21.388	1.269.572	-21.505
1.277.583	-26.236	1.314.234	-21.391	1.269.736	-21.505
1.277.722	-26.237	1.314.427	-21.394	1.269.973	-21.505
1.277.905	-26.238	1.314.659	-21.397	1.270.170	-21.505
1.278.154	-26.239	1.314.830	-21.400	1.270.311	-21.505
1.278.315	-26.239	1.314.989	-21.402	1.270.513	-21.504
1.278.457	-26.240	1.315.177	-21.405	1.270.625	-21.504
1.278.580	-26.241	1.315.369	-21.408	1.270.708	-21.504
1.278.717	-26.242	1.315.513	-21.411	1.270.874	-21.504
1.278.918	-26.243	1.315.704	-21.413	1.271.035	-21.504
1.279.084	-26.243	1.315.952	-21.416	1.271.156	-21.504
1.279.220	-26.244	1.316.151	-21.419	1.271.255	-21.504
1.279.348	-26.245	1.316.306	-21.422	1.271.404	-21.504
1.279.547	-26.245	1.316.456	-21.425	1.271.618	-21.503
1.279.736	-26.246	1.316.582	-21.427	1.271.799	-21.503
1.279.843	-26.247	1.316.703	-21.430	1.271.974	-21.503
1.280.020	-26.247	1.316.877	-21.433	1.272.141	-21.503
1.280.206	-26.248	1.316.998	-21.436	1.272.301	-21.503
1.280.343	-26.249	1.317.108	-21.439	1.272.473	-21.503
1.280.529	-26.249	1.317.251	-21.441	1.272.645	-21.502
1.280.715	-26.250	1.317.422	-21.444	1.272.834	-21.502

1.280.730	-26.250	1.317.581	-21.447	1.272.990	-21.502
1.280.988	-26.251	1.317.690	-21.450	1.273.139	-21.502
1.281.519	-26.252	1.317.796	-21.452	1.273.313	-21.502
1.281.898	-26.252	1.317.944	-21.455	1.273.447	-21.501
1.282.119	-26.253	1.318.132	-21.458	1.273.595	-21.501
1.282.146	-26.253	1.318.297	-21.461	1.273.804	-21.501
1.282.195	-26.254	1.318.474	-21.463	1.273.967	-21.501
1.282.357	-26.254	1.318.687	-21.466	1.274.072	-21.501
1.282.451	-26.255	1.318.871	-21.469	1.274.218	-21.500
1.282.482	-26.255	1.319.059	-21.472	1.274.389	-21.500
1.282.513	-26.256	1.319.250	-21.474	1.274.579	-21.500
1.282.641	-26.256	1.319.391	-21.477	1.274.711	-21.500
1.282.796	-26.257	1.319.559	-21.480	1.274.827	-21.499
1.282.952	-26.257	1.319.727	-21.482	1.275.058	-21.499
1.283.082	-26.258	1.319.854	-21.485	1.275.266	-21.499
1.283.221	-26.258	1.319.991	-21.488	1.275.421	-21.499
1.283.392	-26.259	1.320.083	-21.490	1.275.558	-21.498
1.283.548	-26.259	1.320.202	-21.493	1.275.748	-21.498
1.283.721	-26.259	1.320.425	-21.495	1.275.963	-21.498
1.283.911	-26.260	1.320.650	-21.498	1.276.118	-21.498
1.284.196	-26.260	1.320.858	-21.501	1.276.214	-21.497
1.284.335	-26.261	1.320.990	-21.503	1.276.320	-21.497
1.284.344	-26.261	1.321.075	-21.506	1.276.472	-21.497
1.284.509	-26.262	1.321.261	-21.508	1.276.667	-21.496
1.284.740	-26.262	1.321.497	-21.511	1.276.859	-21.496
1.284.888	-26.263	1.321.671	-21.513	1.277.070	-21.496
1.284.989	-26.263	1.321.788	-21.516	1.277.240	-21.496
1.285.123	-26.264	1.321.967	-21.518	1.277.336	-21.495
1.285.305	-26.264	1.322.141	-21.521	1.277.495	-21.495
1.285.488	-26.264	1.322.289	-21.523	1.277.679	-21.495

1.285.609	-26.265	1.322.478	-21.526	1.277.827	-21.494
1.285.710	-26.265	1.322.646	-21.528	1.277.915	-21.494
1.285.858	-26.266	1.322.825	-21.531	1.278.056	-21.494
1.285.970	-26.266	1.322.975	-21.533	1.278.492	-21.494
1.286.145	-26.267	1.323.107	-21.536	1.278.983	-21.493
1.286.427	-26.267	1.323.223	-21.538	1.279.209	-21.493
1.286.618	-26.268	1.323.353	-21.540	1.279.286	-21.493
1.286.777	-26.269	1.323.530	-21.543	1.279.323	-21.493
1.286.976	-26.269	1.323.725	-21.545	1.279.341	-21.492
1.287.101	-26.270	1.323.878	-21.547	1.279.409	-21.492
1.287.197	-26.270	1.323.981	-21.550	1.279.543	-21.492
1.287.383	-26.271	1.324.128	-21.552	1.279.635	-21.492
1.287.572	-26.271	1.324.330	-21.554	1.279.707	-21.491
1.287.661	-26.272	1.324.474	-21.557	1.279.832	-21.491
1.287.822	-26.273	1.324.550	-21.559	1.279.942	-21.491
1.288.082	-26.273	1.324.736	-21.561	1.280.067	-21.491
1.288.223	-26.274	1.325.166	-21.563	1.280.253	-21.490
1.288.293	-26.275	1.325.589	-21.565	1.280.466	-21.490
1.288.400	-26.275	1.325.786	-21.568	1.280.661	-21.490
1.288.613	-26.276	1.325.876	-21.570	1.280.867	-21.490
1.288.833	-26.277	1.325.943	-21.572	1.281.104	-21.490
1.288.945	-26.278	1.326.010	-21.574	1.281.214	-21.489
1.289.075	-26.278	1.326.042	-21.576	1.281.348	-21.489
1.289.288	-26.279	1.326.111	-21.578	1.281.474	-21.489
1.289.474	-26.280	1.326.252	-21.581	1.281.602	-21.489
1.289.590	-26.281	1.326.436	-21.583	1.281.714	-21.489
1.289.754	-26.282	1.326.595	-21.585	1.281.875	-21.489
1.289.910	-26.282	1.326.682	-21.587	1.282.052	-21.489
1.290.081	-26.283	1.326.805	-21.589	1.282.206	-21.488
1.290.255	-26.284	1.327.023	-21.591	1.282.421	-21.488

1.290.379	-26.285	1.327.188	-21.593	1.282.542	-21.488
1.290.526	-26.286	1.327.269	-21.595	1.282.634	-21.488
1.290.726	-26.287	1.327.417	-21.597	1.282.793	-21.488
1.290.872	-26.288	1.327.643	-21.599	1.282.908	-21.488
1.290.956	-26.289	1.327.796	-21.601	1.283.109	-21.488
1.291.151	-26.290	1.327.939	-21.603	1.283.378	-21.488
1.291.398	-26.291	1.328.138	-21.605	1.283.539	-21.488
1.291.564	-26.292	1.328.293	-21.607	1.283.667	-21.488
1.291.676	-26.293	1.328.447	-21.609	1.283.839	-21.488
1.291.859	-26.294	1.328.651	-21.611	1.284.081	-21.488
1.291.978	-26.296	1.328.842	-21.613	1.284.308	-21.488
1.292.097	-26.297	1.328.970	-21.615	1.284.422	-21.488
1.292.281	-26.298	1.329.109	-21.617	1.284.529	-21.488
1.292.491	-26.299	1.329.281	-21.618	1.284.657	-21.488
1.292.681	-26.300	1.329.382	-21.620	1.284.702	-21.488
1.292.796	-26.302	1.329.496	-21.622	1.284.841	-21.488
1.292.932	-26.303	1.329.632	-21.624	1.285.087	-21.488
1.293.087	-26.304	1.329.803	-21.626	1.285.220	-21.489
1.293.295	-26.306	1.330.036	-21.628	1.285.345	-21.489
1.293.488	-26.307	1.330.233	-21.630	1.285.546	-21.489
1.293.658	-26.308	1.330.379	-21.632	1.285.757	-21.489
1.293.779	-26.310	1.330.546	-21.634	1.285.874	-21.489
1.293.920	-26.311	1.330.735	-21.635	1.286.046	-21.490
1.294.146	-26.313	1.330.959	-21.637	1.286.277	-21.490
1.294.323	-26.314	1.331.181	-21.639	1.286.438	-21.490
1.294.408	-26.315	1.331.333	-21.641	1.286.570	-21.490
1.294.507	-26.317	1.331.451	-21.643	1.286.720	-21.491
1.294.695	-26.318	1.331.534	-21.645	1.286.841	-21.491
1.294.848	-26.320	1.331.667	-21.647	1.286.994	-21.491
1.295.013	-26.322	1.331.817	-21.648	1.287.182	-21.492

1.295.168	-26.323	1.331.927	-21.650	1.287.316	-21.492
1.295.302	-26.325	1.332.099	-21.652	1.287.437	-21.492
1.295.439	-26.326	1.332.274	-21.654	1.287.619	-21.493
1.295.542	-26.328	1.332.446	-21.656	1.287.856	-21.493
1.295.674	-26.330	1.332.612	-21.658	1.288.076	-21.494
1.295.871	-26.331	1.332.699	-21.660	1.288.212	-21.494
1.296.035	-26.333	1.332.849	-21.661	1.288.315	-21.495
1.296.140	-26.335	1.333.020	-21.663	1.288.484	-21.495
1.296.313	-26.336	1.333.152	-21.665	1.288.640	-21.496
1.296.530	-26.338	1.333.309	-21.667	1.288.819	-21.496
1.296.810	-26.340	1.333.465	-21.669	1.289.005	-21.497
1.297.020	-26.341	1.333.622	-21.671	1.289.156	-21.497
1.297.141	-26.343	1.333.772	-21.673	1.289.283	-21.498
1.297.316	-26.345	1.333.934	-21.674	1.289.433	-21.498
1.297.545	-26.347	1.334.104	-21.676	1.289.650	-21.499
1.297.728	-26.349	1.334.272	-21.678	1.289.814	-21.499
1.297.849	-26.350	1.334.442	-21.680	1.289.957	-21.500
1.298.011	-26.352	1.334.601	-21.682	1.290.125	-21.501
1.298.170	-26.354	1.334.843	-21.684	1.290.285	-21.501
1.298.288	-26.356	1.335.036	-21.686	1.290.457	-21.502
1.298.457	-26.358	1.335.193	-21.688	1.290.596	-21.503
1.298.634	-26.360	1.335.383	-21.689	1.290.755	-21.503
1.298.723	-26.362	1.335.495	-21.691	1.290.918	-21.504
1.298.864	-26.364	1.335.641	-21.693	1.291.033	-21.505
1.299.023	-26.366	1.335.809	-21.695	1.291.138	-21.505
1.299.149	-26.368	1.335.943	-21.697	1.291.299	-21.506
1.299.315	-26.370	1.336.100	-21.699	1.291.402	-21.507
1.299.433	-26.372	1.336.227	-21.701	1.291.487	-21.507
1.299.577	-26.374	1.336.348	-21.703	1.291.658	-21.508
1.299.848	-26.376	1.336.537	-21.705	1.291.846	-21.509

1.300.043	-26.378	1.336.767	-21.707	1.292.027	-21.510
1.300.204	-26.380	1.336.945	-21.708	1.292.236	-21.510
1.300.349	-26.382	1.337.094	-21.710	1.292.458	-21.511
1.300.509	-26.384	1.337.271	-21.712	1.292.648	-21.512
1.300.703	-26.386	1.337.417	-21.714	1.292.863	-21.513
1.300.809	-26.388	1.337.589	-21.716	1.293.064	-21.513
1.300.912	-26.390	1.337.735	-21.718	1.293.248	-21.514
1.301.102	-26.392	1.337.852	-21.720	1.293.380	-21.515
1.301.329	-26.394	1.338.004	-21.722	1.293.503	-21.516
1.301.438	-26.396	1.338.121	-21.724	1.293.707	-21.516
1.301.568	-26.398	1.338.282	-21.726	1.293.889	-21.517
1.301.765	-26.400	1.338.466	-21.727	1.293.985	-21.518
1.301.882	-26.402	1.338.573	-21.729	1.294.100	-21.519
1.302.076	-26.405	1.338.681	-21.731	1.294.276	-21.519
1.302.285	-26.407	1.338.822	-21.733	1.294.415	-21.520
1.302.417	-26.409	1.338.972	-21.735	1.294.545	-21.521
1.302.560	-26.411	1.339.126	-21.737	1.294.725	-21.522
1.302.724	-26.413	1.339.267	-21.739	1.294.857	-21.522
1.302.874	-26.415	1.339.447	-21.741	1.294.982	-21.523
1.303.027	-26.418	1.339.668	-21.743	1.295.208	-21.524
1.303.223	-26.420	1.339.870	-21.744	1.295.437	-21.525
1.303.329	-26.422	1.340.045	-21.746	1.295.623	-21.525
1.303.436	-26.424	1.340.253	-21.748	1.295.786	-21.526
1.303.605	-26.426	1.340.450	-21.750	1.295.934	-21.527
1.303.820	-26.429	1.340.647	-21.752	1.296.082	-21.528
1.303.992	-26.431	1.340.833	-21.754	1.296.189	-21.528
1.304.151	-26.433	1.340.956	-21.756	1.296.322	-21.529
1.304.335	-26.435	1.341.075	-21.757	1.296.546	-21.530
1.304.473	-26.438	1.341.199	-21.759	1.296.736	-21.531
1.304.597	-26.440	1.341.324	-21.761	1.296.873	-21.531

1.304.742	-26.442	1.341.432	-21.763	1.297.068	-21.532
1.304.928	-26.445	1.341.595	-21.765	1.297.224	-21.533
1.305.179	-26.447	1.341.787	-21.767	1.297.316	-21.533
1.305.385	-26.449	1.341.933	-21.768	1.297.437	-21.534
1.305.497	-26.452	1.342.032	-21.770	1.297.612	-21.535
1.305.667	-26.454	1.342.126	-21.772	1.297.771	-21.536
1.305.871	-26.456	1.342.298	-21.774	1.297.937	-21.536
1.306.010	-26.459	1.342.513	-21.776	1.298.111	-21.537
1.306.134	-26.461	1.342.737	-21.777	1.298.282	-21.538
1.306.189	-26.463	1.342.865	-21.779	1.298.497	-21.538
1.306.375	-26.466	1.343.017	-21.781	1.298.665	-21.539
1.306.799	-26.468	1.343.275	-21.782	1.298.813	-21.540
1.307.222	-26.470	1.343.441	-21.784	1.298.972	-21.540
1.307.413	-26.473	1.343.636	-21.786	1.299.111	-21.541
1.307.446	-26.475	1.343.813	-21.788	1.299.254	-21.542
1.307.480	-26.478	1.343.958	-21.789	1.299.449	-21.542
1.307.518	-26.480	1.344.088	-21.791	1.299.639	-21.543
1.307.650	-26.483	1.344.258	-21.793	1.299.798	-21.544
1.307.753	-26.485	1.344.453	-21.794	1.299.951	-21.544
1.307.822	-26.487	1.344.585	-21.796	1.300.112	-21.545
1.307.935	-26.490	1.344.734	-21.798	1.300.287	-21.546
1.308.102	-26.492	1.344.919	-21.799	1.300.446	-21.546
1.308.270	-26.495	1.345.078	-21.801	1.300.627	-21.547
1.308.394	-26.497	1.345.231	-21.802	1.300.811	-21.548
1.308.586	-26.500	1.345.450	-21.804	1.300.954	-21.548
1.308.779	-26.502	1.345.621	-21.806	1.301.046	-21.549
1.308.871	-26.505	1.345.701	-21.807	1.301.194	-21.549
1.308.981	-26.507	1.345.849	-21.809	1.301.483	-21.550
1.309.151	-26.510	1.346.113	-21.810	1.301.866	-21.551
1.309.335	-26.512	1.346.304	-21.812	1.302.177	-21.551

1.309.501	-26.515	1.346.436	-21.813	1.302.319	-21.552
1.309.686	-26.518	1.346.572	-21.815	1.302.392	-21.553
1.309.874	-26.520	1.346.700	-21.816	1.302.525	-21.553
1.310.052	-26.523	1.346.833	-21.818	1.302.610	-21.554
1.310.206	-26.525	1.346.958	-21.819	1.302.661	-21.555
1.310.296	-26.528	1.347.159	-21.821	1.302.693	-21.555
1.310.466	-26.531	1.347.280	-21.822	1.302.749	-21.556
1.310.677	-26.533	1.347.381	-21.824	1.302.890	-21.556
1.310.755	-26.536	1.347.581	-21.825	1.302.993	-21.557
1.310.847	-26.539	1.347.751	-21.827	1.303.165	-21.558
1.311.030	-26.541	1.347.865	-21.828	1.303.367	-21.558
1.311.219	-26.544	1.348.033	-21.830	1.303.559	-21.559
1.311.425	-26.547	1.348.237	-21.831	1.303.745	-21.560
1.311.586	-26.550	1.348.412	-21.832	1.303.880	-21.560
1.311.723	-26.552	1.348.584	-21.834	1.304.070	-21.561
1.311.850	-26.555	1.348.799	-21.835	1.304.301	-21.562
1.311.980	-26.558	1.348.969	-21.837	1.304.420	-21.562
1.312.204	-26.561	1.349.050	-21.838	1.304.534	-21.563
1.312.473	-26.564	1.349.182	-21.839	1.304.727	-21.564
1.312.614	-26.566	1.349.456	-21.841	1.304.906	-21.564
1.312.740	-26.569	1.349.886	-21.842	1.305.033	-21.565
1.312.885	-26.572	1.350.170	-21.843	1.305.132	-21.566
1.313.020	-26.575	1.350.246	-21.845	1.305.262	-21.566
1.313.161	-26.578	1.350.378	-21.846	1.305.446	-21.567
1.313.284	-26.581	1.350.502	-21.847	1.305.665	-21.568
1.313.483	-26.584	1.350.574	-21.849	1.305.806	-21.568
1.313.649	-26.587	1.350.609	-21.850	1.305.941	-21.569
1.313.790	-26.589	1.350.629	-21.851	1.306.089	-21.570
1.313.960	-26.592	1.350.726	-21.852	1.306.196	-21.571
1.314.126	-26.595	1.350.921	-21.854	1.306.355	-21.571

1.314.301	-26.598	1.351.113	-21.855	1.306.503	-21.572
1.314.422	-26.601	1.351.279	-21.856	1.306.714	-21.573
1.314.532	-26.604	1.351.445	-21.857	1.306.969	-21.574
1.314.677	-26.607	1.351.629	-21.859	1.307.119	-21.575
1.314.816	-26.610	1.351.806	-21.860	1.307.238	-21.575
1.315.004	-26.613	1.351.962	-21.861	1.307.332	-21.576
1.315.206	-26.616	1.352.162	-21.862	1.307.525	-21.577
1.315.341	-26.620	1.352.350	-21.863	1.307.719	-21.578
1.315.500	-26.623	1.352.469	-21.865	1.307.852	-21.579
1.315.676	-26.626	1.352.623	-21.866	1.308.013	-21.580
1.315.804	-26.629	1.352.802	-21.867	1.308.147	-21.581
1.315.966	-26.632	1.352.977	-21.868	1.308.270	-21.581
1.316.143	-26.635	1.353.123	-21.869	1.308.443	-21.582
1.316.331	-26.638	1.353.277	-21.871	1.308.622	-21.583
1.316.514	-26.641	1.353.445	-21.872	1.308.739	-21.584
1.316.653	-26.645	1.353.551	-21.873	1.308.893	-21.585
1.316.823	-26.648	1.353.676	-21.874	1.309.115	-21.586
1.316.994	-26.651	1.353.846	-21.875	1.309.330	-21.587
1.317.173	-26.654	1.353.981	-21.876	1.309.485	-21.588
1.317.307	-26.657	1.354.075	-21.878	1.309.579	-21.589
1.317.419	-26.660	1.354.238	-21.879	1.309.729	-21.590
1.317.637	-26.664	1.354.440	-21.880	1.309.933	-21.591
1.317.813	-26.667	1.354.568	-21.881	1.310.023	-21.592
1.317.917	-26.670	1.354.720	-21.882	1.310.112	-21.594
1.318.132	-26.673	1.354.948	-21.884	1.310.309	-21.595
1.318.313	-26.677	1.355.157	-21.885	1.310.531	-21.596
1.318.342	-26.680	1.355.361	-21.886	1.310.690	-21.597
1.318.508	-26.683	1.355.549	-21.887	1.310.804	-21.598
1.318.741	-26.686	1.355.683	-21.888	1.311.026	-21.599
1.318.911	-26.690	1.355.757	-21.889	1.311.217	-21.601

1.319.129	-26.693	1.355.874	-21.891	1.311.342	-21.602
1.319.301	-26.696	1.356.044	-21.892	1.311.494	-21.603
1.319.413	-26.699	1.356.187	-21.893	1.311.684	-21.604
1.319.487	-26.703	1.356.357	-21.894	1.311.857	-21.606
1.319.632	-26.706	1.356.561	-21.895	1.311.940	-21.607
1.319.846	-26.709	1.356.680	-21.897	1.312.066	-21.608
1.320.009	-26.712	1.356.779	-21.898	1.312.253	-21.610
1.320.141	-26.716	1.356.942	-21.899	1.312.390	-21.611
1.320.298	-26.719	1.357.122	-21.900	1.312.560	-21.612
1.320.475	-26.722	1.357.312	-21.902	1.312.731	-21.614
1.320.609	-26.725	1.357.480	-21.903	1.312.872	-21.615
1.320.735	-26.729	1.357.632	-21.904	1.313.029	-21.616
1.320.853	-26.732	1.357.789	-21.905	1.313.217	-21.618
1.320.968	-26.735	1.357.961	-21.907	1.313.407	-21.619
1.321.158	-26.738	1.358.085	-21.908	1.313.568	-21.621
1.321.335	-26.742	1.358.223	-21.909	1.313.710	-21.622
1.321.472	-26.745	1.358.405	-21.910	1.313.869	-21.624
1.321.629	-26.748	1.358.555	-21.912	1.313.999	-21.625
1.321.803	-26.751	1.358.725	-21.913	1.314.104	-21.627
1.321.965	-26.754	1.358.922	-21.914	1.314.254	-21.628
1.322.137	-26.758	1.359.090	-21.916	1.314.404	-21.630
1.322.348	-26.761	1.359.212	-21.917	1.314.518	-21.631
1.322.563	-26.764	1.359.375	-21.918	1.314.648	-21.633
1.322.760	-26.767	1.359.572	-21.920	1.314.818	-21.634
1.322.943	-26.770	1.359.706	-21.921	1.314.966	-21.636
1.323.105	-26.773	1.359.893	-21.922	1.315.128	-21.637
1.323.286	-26.776	1.360.067	-21.924	1.315.318	-21.639
1.323.463	-26.780	1.360.190	-21.925	1.315.495	-21.640
1.323.602	-26.783	1.360.363	-21.926	1.315.676	-21.642
1.323.712	-26.786	1.360.558	-21.928	1.315.932	-21.643

1.323.786	-26.789	1.360.755	-21.929	1.316.169	-21.645
1.323.920	-26.792	1.360.844	-21.931	1.316.371	-21.647
1.324.066	-26.795	1.360.927	-21.932	1.316.546	-21.648
1.324.184	-26.798	1.361.095	-21.933	1.316.700	-21.650
1.324.339	-26.801	1.361.295	-21.935	1.316.861	-21.651
1.324.516	-26.804	1.361.541	-21.936	1.316.980	-21.653
1.324.682	-26.807	1.361.691	-21.938	1.317.077	-21.654
1.324.856	-26.810	1.361.788	-21.939	1.317.236	-21.656
1.325.022	-26.813	1.361.987	-21.941	1.317.383	-21.658
1.325.179	-26.816	1.362.168	-21.942	1.317.523	-21.659
1.325.381	-26.819	1.362.296	-21.943	1.317.701	-21.661
1.325.564	-26.822	1.362.435	-21.945	1.317.840	-21.662
1.325.768	-26.825	1.362.592	-21.946	1.318.002	-21.664
1.325.988	-26.827	1.362.720	-21.948	1.318.125	-21.666
1.326.104	-26.830	1.362.803	-21.949	1.318.226	-21.667
1.326.196	-26.833	1.362.894	-21.951	1.318.344	-21.669
1.326.369	-26.836	1.363.024	-21.952	1.318.562	-21.670
1.326.572	-26.839	1.363.208	-21.954	1.318.813	-21.672
1.326.750	-26.842	1.363.425	-21.955	1.318.976	-21.673
1.326.906	-26.844	1.363.557	-21.957	1.319.093	-21.675
1.327.014	-26.847	1.363.665	-21.958	1.319.279	-21.677
1.327.177	-26.850	1.363.844	-21.959	1.319.454	-21.678
1.327.370	-26.852	1.364.077	-21.961	1.319.642	-21.680
1.327.500	-26.855	1.364.283	-21.962	1.319.792	-21.681
1.327.661	-26.858	1.364.447	-21.964	1.319.913	-21.683
1.327.811	-26.860	1.364.639	-21.965	1.320.022	-21.684
1.327.984	-26.863	1.364.827	-21.967	1.320.181	-21.686
1.328.212	-26.866	1.365.038	-21.968	1.320.396	-21.687
1.328.383	-26.868	1.365.231	-21.970	1.320.591	-21.689
1.328.539	-26.871	1.365.376	-21.971	1.320.762	-21.690

1.328.685	-26.873	1.365.480	-21.973	1.320.938	-21.692
1.328.804	-26.876	1.365.593	-21.974	1.321.091	-21.693
1.329.014	-26.878	1.365.782	-21.976	1.321.198	-21.695
1.329.245	-26.881	1.365.905	-21.977	1.321.322	-21.696
1.329.375	-26.883	1.366.062	-21.979	1.321.609	-21.698
1.329.449	-26.886	1.366.268	-21.980	1.321.814	-21.699
1.329.594	-26.888	1.366.373	-21.982	1.321.850	-21.701
1.329.780	-26.891	1.366.451	-21.983	1.321.980	-21.702
1.329.911	-26.893	1.366.577	-21.985	1.322.141	-21.704
1.330.067	-26.895	1.366.749	-21.986	1.322.303	-21.705
1.330.266	-26.898	1.366.980	-21.988	1.322.435	-21.707
1.330.434	-26.900	1.367.200	-21.989	1.322.572	-21.708
1.330.531	-26.902	1.367.341	-21.990	1.322.784	-21.710
1.330.706	-26.905	1.367.480	-21.992	1.322.959	-21.711
1.330.907	-26.907	1.367.673	-21.993	1.323.109	-21.712
1.331.066	-26.909	1.367.903	-21.995	1.323.315	-21.714
1.331.232	-26.912	1.368.069	-21.996	1.323.490	-21.715
1.331.393	-26.914	1.368.210	-21.998	1.323.638	-21.717
1.331.550	-26.916	1.368.351	-21.999	1.323.795	-21.718
1.331.687	-26.918	1.368.481	-22.001	1.323.994	-21.719
1.331.814	-26.920	1.368.624	-22.002	1.324.156	-21.721
1.331.956	-26.923	1.368.788	-22.003	1.324.243	-21.722
1.332.350	-26.925	1.368.996	-22.005	1.324.352	-21.724
1.332.813	-26.927	1.369.151	-22.006	1.324.534	-21.725
1.333.076	-26.929	1.369.296	-22.008	1.324.881	-21.726
1.333.181	-26.931	1.369.523	-22.009	1.325.316	-21.728
1.333.165	-26.933	1.369.637	-22.010	1.325.672	-21.729
1.333.174	-26.935	1.369.722	-22.012	1.325.766	-21.730
1.333.235	-26.937	1.369.928	-22.013	1.325.773	-21.731
1.333.322	-26.939	1.370.116	-22.015	1.325.912	-21.733

1.333.463	-26.942	1.370.244	-22.016	1.325.981	-21.734
1.333.638	-26.944	1.370.405	-22.017	1.325.999	-21.735
1.333.736	-26.946	1.370.593	-22.019	1.326.071	-21.737
1.333.822	-26.948	1.370.737	-22.020	1.326.156	-21.738
1.333.974	-26.950	1.370.914	-22.021	1.326.261	-21.739
1.334.146	-26.952	1.371.089	-22.023	1.326.445	-21.740
1.334.348	-26.954	1.371.212	-22.024	1.326.599	-21.741
1.334.576	-26.956	1.371.387	-22.026	1.326.743	-21.743
1.334.745	-26.957	1.371.595	-22.027	1.326.953	-21.744
1.334.892	-26.959	1.371.756	-22.028	1.327.150	-21.745
1.335.067	-26.961	1.371.864	-22.030	1.327.318	-21.746
1.335.195	-26.963	1.372.009	-22.031	1.327.469	-21.747
1.335.302	-26.965	1.372.222	-22.032	1.327.677	-21.749
1.335.480	-26.967	1.372.415	-22.034	1.327.805	-21.750
1.335.699	-26.969	1.372.570	-22.035	1.327.912	-21.751
1.335.845	-26.971	1.372.686	-22.036	1.328.064	-21.752
1.336.024	-26.973	1.372.789	-22.038	1.328.208	-21.753
1.336.187	-26.975	1.372.943	-22.039	1.328.407	-21.754
1.336.279	-26.977	1.373.161	-22.040	1.328.573	-21.755
1.336.375	-26.979	1.373.347	-22.042	1.328.719	-21.757
1.336.483	-26.981	1.373.483	-22.043	1.328.862	-21.758
1.336.680	-26.982	1.373.640	-22.044	1.328.985	-21.759
1.336.882	-26.984	1.373.916	-22.045	1.329.126	-21.760
1.337.032	-26.986	1.374.333	-22.047	1.329.270	-21.761
1.337.135	-26.988	1.374.655	-22.048	1.329.447	-21.762
1.337.330	-26.990	1.374.767	-22.049	1.329.648	-21.763
1.337.574	-26.992	1.374.852	-22.051	1.329.821	-21.764
1.337.751	-26.994	1.374.953	-22.052	1.330.009	-21.765
1.337.939	-26.996	1.375.014	-22.053	1.330.114	-21.766
1.338.125	-26.998	1.375.045	-22.054	1.330.284	-21.768

1.338.279	-26.999	1.375.154	-22.056	1.330.544	-21.769
1.338.414	-27.001	1.375.264	-22.057	1.330.708	-21.770
1.338.575	-27.003	1.375.332	-22.058	1.330.815	-21.771
1.338.710	-27.005	1.375.504	-22.059	1.330.927	-21.772
1.338.869	-27.007	1.375.719	-22.061	1.331.077	-21.773
1.339.008	-27.009	1.375.891	-22.062	1.331.299	-21.774
1.339.160	-27.011	1.376.001	-22.063	1.331.517	-21.775
1.339.335	-27.013	1.376.187	-22.064	1.331.613	-21.776
1.339.449	-27.015	1.376.371	-22.066	1.331.740	-21.777
1.339.601	-27.016	1.376.514	-22.067	1.331.918	-21.778
1.339.789	-27.018	1.376.721	-22.068	1.332.068	-21.779
1.339.953	-27.020	1.376.891	-22.069	1.332.265	-21.780
1.340.094	-27.022	1.377.020	-22.070	1.332.442	-21.781
1.340.224	-27.024	1.377.146	-22.072	1.332.592	-21.782
1.340.327	-27.026	1.377.319	-22.073	1.332.735	-21.783
1.340.499	-27.028	1.377.504	-22.074	1.332.885	-21.784
1.340.746	-27.030	1.377.628	-22.075	1.333.056	-21.785
1.340.919	-27.032	1.377.733	-22.077	1.333.235	-21.786
1.341.089	-27.034	1.377.883	-22.078	1.333.304	-21.787
1.341.167	-27.036	1.378.042	-22.079	1.333.385	-21.788
1.341.293	-27.038	1.378.210	-22.080	1.333.559	-21.789
1.341.525	-27.040	1.378.376	-22.082	1.333.723	-21.790
1.341.595	-27.042	1.378.459	-22.083	1.333.884	-21.791
1.341.736	-27.044	1.378.591	-22.084	1.334.072	-21.792
1.342.012	-27.045	1.378.795	-22.085	1.334.263	-21.793
1.342.204	-27.047	1.378.985	-22.087	1.334.485	-21.795
1.342.363	-27.049	1.379.169	-22.088	1.334.659	-21.796
1.342.574	-27.051	1.379.402	-22.089	1.334.778	-21.797
1.342.715	-27.053	1.379.650	-22.090	1.334.984	-21.798
1.342.849	-27.055	1.379.762	-22.092	1.335.161	-21.799

1.343.051	-27.057	1.379.852	-22.093	1.335.316	-21.800
1.343.203	-27.059	1.379.996	-22.094	1.335.448	-21.801
1.343.331	-27.062	1.380.141	-22.096	1.335.585	-21.802
1.343.512	-27.064	1.380.294	-22.097	1.335.784	-21.803
1.343.656	-27.066	1.380.488	-22.098	1.335.878	-21.804
1.343.828	-27.068	1.380.623	-22.100	1.336.033	-21.805
1.343.958	-27.070	1.380.730	-22.101	1.336.232	-21.806
1.344.052	-27.072	1.380.867	-22.102	1.336.355	-21.807
1.344.220	-27.074	1.380.961	-22.103	1.336.507	-21.808
1.344.404	-27.076	1.381.227	-22.105	1.336.696	-21.809
1.344.615	-27.078	1.381.474	-22.106	1.336.852	-21.810
1.344.807	-27.080	1.381.613	-22.107	1.336.969	-21.811
1.344.930	-27.082	1.381.803	-22.109	1.337.153	-21.812
1.345.060	-27.084	1.381.935	-22.110	1.337.341	-21.813
1.345.188	-27.086	1.382.106	-22.112	1.337.424	-21.814
1.345.381	-27.088	1.382.303	-22.113	1.337.529	-21.814
1.345.583	-27.090	1.382.428	-22.114	1.337.693	-21.815
1.345.690	-27.092	1.382.522	-22.116	1.337.874	-21.816
1.345.795	-27.094	1.382.661	-22.117	1.338.069	-21.817
1.345.947	-27.096	1.382.825	-22.118	1.338.199	-21.818
1.346.131	-27.098	1.382.979	-22.120	1.338.338	-21.819
1.346.268	-27.100	1.383.125	-22.121	1.338.508	-21.820
1.346.355	-27.103	1.383.302	-22.123	1.338.674	-21.821
1.346.487	-27.105	1.383.530	-22.124	1.338.844	-21.822
1.346.651	-27.107	1.383.736	-22.126	1.339.039	-21.823
1.346.808	-27.109	1.383.880	-22.127	1.339.249	-21.823
1.346.989	-27.111	1.384.017	-22.129	1.339.424	-21.824
1.347.169	-27.113	1.384.169	-22.130	1.339.608	-21.825
1.347.312	-27.115	1.384.371	-22.132	1.339.753	-21.826
1.347.451	-27.117	1.384.541	-22.133	1.339.933	-21.827

1.347.666	-27.119	1.384.682	-22.135	1.340.161	-21.828
1.347.915	-27.121	1.384.828	-22.136	1.340.327	-21.828
1.348.118	-27.123	1.384.977	-22.138	1.340.452	-21.829
1.348.282	-27.125	1.385.159	-22.139	1.340.542	-21.830
1.348.423	-27.127	1.385.307	-22.141	1.340.654	-21.831
1.348.575	-27.129	1.385.443	-22.142	1.340.847	-21.831
1.348.797	-27.131	1.385.611	-22.144	1.341.059	-21.832
1.348.916	-27.133	1.385.780	-22.145	1.341.161	-21.833
1.349.035	-27.135	1.385.956	-22.147	1.341.261	-21.834
1.349.198	-27.137	1.386.120	-22.148	1.341.407	-21.834
1.349.348	-27.139	1.386.329	-22.150	1.341.512	-21.835
1.349.577	-27.141	1.386.555	-22.152	1.341.675	-21.836
1.349.767	-27.143	1.386.635	-22.153	1.341.830	-21.836
1.349.845	-27.145	1.386.707	-22.155	1.341.956	-21.837
1.349.879	-27.147	1.386.828	-22.156	1.342.191	-21.838
1.350.022	-27.149	1.386.962	-22.158	1.342.431	-21.838
1.350.249	-27.151	1.387.124	-22.159	1.342.626	-21.839
1.350.394	-27.153	1.387.301	-22.161	1.342.861	-21.839
1.350.499	-27.155	1.387.437	-22.163	1.343.040	-21.840
1.350.733	-27.157	1.387.563	-22.164	1.343.201	-21.841
1.351.006	-27.159	1.387.688	-22.166	1.343.345	-21.841
1.351.165	-27.160	1.387.840	-22.167	1.343.430	-21.842
1.351.333	-27.162	1.388.011	-22.169	1.343.542	-21.842
1.351.510	-27.164	1.388.147	-22.171	1.343.712	-21.843
1.351.684	-27.166	1.388.353	-22.172	1.343.923	-21.843
1.351.861	-27.168	1.388.513	-22.174	1.344.079	-21.844
1.351.980	-27.170	1.388.624	-22.175	1.344.245	-21.844
1.352.142	-27.172	1.388.885	-22.177	1.344.415	-21.845
1.352.339	-27.173	1.389.164	-22.179	1.344.529	-21.845
1.352.451	-27.175	1.389.380	-22.180	1.344.688	-21.845

1.352.500	-27.177	1.389.599	-22.182	1.344.924	-21.846
1.352.646	-27.179	1.389.758	-22.183	1.345.134	-21.846
1.352.934	-27.181	1.389.917	-22.185	1.345.262	-21.847
1.353.139	-27.182	1.390.065	-22.187	1.345.289	-21.847
1.353.302	-27.184	1.390.150	-22.188	1.345.443	-21.847
1.353.407	-27.186	1.390.251	-22.190	1.345.750	-21.848
1.353.447	-27.187	1.390.388	-22.192	1.345.943	-21.848
1.353.685	-27.189	1.390.533	-22.193	1.346.050	-21.849
1.353.916	-27.191	1.390.679	-22.195	1.346.203	-21.849
1.354.005	-27.193	1.390.802	-22.196	1.346.375	-21.849
1.354.155	-27.194	1.390.965	-22.198	1.346.485	-21.849
1.354.321	-27.196	1.391.162	-22.200	1.346.635	-21.850
1.354.445	-27.198	1.391.286	-22.201	1.346.841	-21.850
1.354.626	-27.199	1.391.431	-22.203	1.347.059	-21.850
1.354.834	-27.201	1.391.671	-22.204	1.347.206	-21.851
1.355.016	-27.202	1.391.870	-22.206	1.347.336	-21.851
1.355.172	-27.204	1.392.030	-22.207	1.347.533	-21.851
1.355.316	-27.206	1.392.150	-22.209	1.347.733	-21.851
1.355.468	-27.207	1.392.294	-22.211	1.347.845	-21.852
1.355.630	-27.209	1.392.446	-22.212	1.347.930	-21.852
1.355.804	-27.210	1.392.603	-22.214	1.348.082	-21.852
1.355.954	-27.212	1.392.796	-22.215	1.348.382	-21.852
1.356.082	-27.213	1.393.017	-22.217	1.348.797	-21.852
1.356.257	-27.215	1.393.264	-22.218	1.349.108	-21.853
1.356.451	-27.216	1.393.396	-22.220	1.349.279	-21.853
1.356.590	-27.218	1.393.499	-22.222	1.349.342	-21.853
1.356.765	-27.219	1.393.676	-22.223	1.349.364	-21.853
1.356.931	-27.221	1.393.828	-22.225	1.349.391	-21.853
1.357.068	-27.222	1.394.012	-22.226	1.349.431	-21.853
1.357.271	-27.224	1.394.106	-22.228	1.349.505	-21.853

1.357.374	-27.225	1.394.174	-22.229	1.349.594	-21.853
1.357.507	-27.227	1.394.310	-22.231	1.349.715	-21.854
1.357.890	-27.228	1.394.510	-22.232	1.349.901	-21.854
1.358.279	-27.230	1.394.749	-22.234	1.350.103	-21.854
1.358.533	-27.231	1.394.928	-22.236	1.350.300	-21.854
1.358.681	-27.232	1.395.072	-22.237	1.350.405	-21.854
1.358.766	-27.234	1.395.211	-22.239	1.350.533	-21.854
1.358.880	-27.235	1.395.331	-22.240	1.350.708	-21.854
1.358.941	-27.236	1.395.533	-22.242	1.350.909	-21.854
1.359.001	-27.238	1.395.719	-22.243	1.351.129	-21.854
1.359.108	-27.239	1.395.845	-22.245	1.351.337	-21.854
1.359.151	-27.240	1.396.057	-22.246	1.351.496	-21.854
1.359.240	-27.242	1.396.263	-22.248	1.351.644	-21.854
1.359.435	-27.243	1.396.308	-22.249	1.351.774	-21.854
1.359.603	-27.244	1.396.434	-22.251	1.351.895	-21.854
1.359.785	-27.246	1.396.714	-22.252	1.352.045	-21.855
1.360.018	-27.247	1.396.877	-22.254	1.352.182	-21.855
1.360.206	-27.248	1.397.003	-22.255	1.352.359	-21.855
1.360.285	-27.250	1.397.213	-22.257	1.352.538	-21.855
1.360.363	-27.251	1.397.401	-22.258	1.352.681	-21.855
1.360.547	-27.252	1.397.579	-22.260	1.352.807	-21.855
1.360.741	-27.253	1.397.722	-22.262	1.352.901	-21.855
1.360.921	-27.255	1.397.785	-22.263	1.353.094	-21.855
1.361.098	-27.256	1.397.905	-22.265	1.353.358	-21.855
1.361.189	-27.257	1.398.076	-22.266	1.353.548	-21.855
1.361.286	-27.258	1.398.436	-22.268	1.353.683	-21.855
1.361.483	-27.260	1.398.925	-22.269	1.353.860	-21.855
1.361.709	-27.261	1.399.245	-22.271	1.354.026	-21.855
1.361.884	-27.262	1.399.339	-22.272	1.354.214	-21.855
1.362.034	-27.263	1.399.357	-22.274	1.354.384	-21.855

1.362.180	-27.265	1.399.431	-22.275	1.354.496	-21.855
1.362.299	-27.266	1.399.507	-22.277	1.354.592	-21.856
1.362.411	-27.267	1.399.518	-22.278	1.354.718	-21.856
1.362.556	-27.268	1.399.579	-22.280	1.354.874	-21.856
1.362.755	-27.270	1.399.711	-22.281	1.355.070	-21.856
1.362.923	-27.271	1.399.854	-22.283	1.355.260	-21.856
1.363.067	-27.272	1.399.939	-22.284	1.355.419	-21.856
1.363.244	-27.273	1.400.072	-22.286	1.355.596	-21.856
1.363.447	-27.275	1.400.298	-22.287	1.355.728	-21.856
1.363.640	-27.276	1.400.471	-22.289	1.355.889	-21.856
1.363.766	-27.277	1.400.600	-22.290	1.356.037	-21.857
1.363.900	-27.278	1.400.757	-22.292	1.356.201	-21.857
1.364.021	-27.280	1.400.903	-22.293	1.356.405	-21.857
1.364.169	-27.281	1.401.109	-22.295	1.356.568	-21.857
1.364.395	-27.282	1.401.362	-22.296	1.356.671	-21.857
1.364.532	-27.283	1.401.572	-22.298	1.356.790	-21.857
1.364.733	-27.285	1.401.745	-22.299	1.356.989	-21.858
1.364.910	-27.286	1.401.879	-22.301	1.357.130	-21.858
1.364.998	-27.287	1.401.989	-22.302	1.357.204	-21.858
1.365.168	-27.288	1.402.083	-22.304	1.357.365	-21.858
1.365.349	-27.290	1.402.200	-22.305	1.357.583	-21.859
1.365.495	-27.291	1.402.383	-22.307	1.357.760	-21.859
1.365.605	-27.292	1.402.634	-22.308	1.357.970	-21.859
1.365.751	-27.294	1.402.753	-22.310	1.358.179	-21.859
1.365.959	-27.295	1.402.791	-22.311	1.358.273	-21.860
1.366.189	-27.296	1.402.896	-22.313	1.358.389	-21.860
1.366.351	-27.297	1.403.096	-22.314	1.358.578	-21.860
1.366.467	-27.299	1.403.315	-22.316	1.358.707	-21.861
1.366.622	-27.300	1.403.503	-22.317	1.358.851	-21.861
1.366.768	-27.301	1.403.685	-22.319	1.359.044	-21.861

1.366.911	-27.303	1.403.866	-22.320	1.359.207	-21.862
1.367.032	-27.304	1.404.008	-22.322	1.359.333	-21.862
1.367.144	-27.306	1.404.117	-22.324	1.359.462	-21.862
1.367.314	-27.307	1.404.323	-22.325	1.359.644	-21.863
1.367.460	-27.308	1.404.529	-22.327	1.359.877	-21.863
1.367.666	-27.310	1.404.659	-22.328	1.360.067	-21.864
1.367.863	-27.311	1.404.832	-22.330	1.360.182	-21.864
1.368.056	-27.313	1.405.051	-22.331	1.360.316	-21.864
1.368.237	-27.314	1.405.222	-22.333	1.360.473	-21.865
1.368.351	-27.315	1.405.327	-22.335	1.360.593	-21.865
1.368.499	-27.317	1.405.432	-22.336	1.360.766	-21.866
1.368.743	-27.318	1.405.645	-22.338	1.360.956	-21.866
1.368.938	-27.320	1.405.833	-22.339	1.361.078	-21.866
1.369.016	-27.321	1.405.977	-22.341	1.361.254	-21.867
1.369.155	-27.323	1.406.167	-22.343	1.361.409	-21.867
1.369.270	-27.324	1.406.324	-22.344	1.361.548	-21.868
1.369.406	-27.326	1.406.474	-22.346	1.361.702	-21.868
1.369.644	-27.327	1.406.579	-22.347	1.361.808	-21.869
1.369.812	-27.329	1.406.637	-22.349	1.361.908	-21.869
1.369.886	-27.330	1.406.756	-22.351	1.362.036	-21.869
1.370.036	-27.332	1.407.011	-22.352	1.362.227	-21.870
1.370.264	-27.333	1.407.271	-22.354	1.362.469	-21.870
1.370.437	-27.335	1.407.397	-22.356	1.362.682	-21.871
1.370.614	-27.336	1.407.500	-22.357	1.362.930	-21.871
1.370.804	-27.338	1.407.661	-22.359	1.363.190	-21.872
1.370.952	-27.340	1.407.784	-22.361	1.363.342	-21.872
1.371.098	-27.341	1.407.935	-22.362	1.363.474	-21.873
1.371.248	-27.343	1.408.167	-22.364	1.363.647	-21.873
1.371.382	-27.344	1.408.391	-22.366	1.363.811	-21.874
1.371.534	-27.346	1.408.562	-22.367	1.363.952	-21.874

1.371.626	-27.347	1.408.689	-22.369	1.364.072	-21.874
1.371.812	-27.349	1.408.811	-22.371	1.364.146	-21.875
1.372.021	-27.351	1.408.922	-22.373	1.364.254	-21.875
1.372.146	-27.352	1.409.052	-22.374	1.364.371	-21.876
1.372.318	-27.354	1.409.232	-22.376	1.364.496	-21.876
1.372.424	-27.355	1.409.339	-22.378	1.364.706	-21.877
1.372.524	-27.357	1.409.474	-22.379	1.364.906	-21.877
1.372.634	-27.359	1.409.686	-22.381	1.365.043	-21.878
1.372.818	-27.360	1.409.912	-22.383	1.365.257	-21.878
1.373.067	-27.362	1.410.116	-22.384	1.365.468	-21.879
1.373.230	-27.363	1.410.224	-22.386	1.365.574	-21.879
1.373.398	-27.365	1.410.350	-22.388	1.365.723	-21.879
1.373.629	-27.367	1.410.524	-22.389	1.365.867	-21.880
1.373.826	-27.368	1.410.695	-22.391	1.366.033	-21.880
1.374.005	-27.370	1.410.896	-22.393	1.366.270	-21.881
1.374.155	-27.372	1.411.069	-22.394	1.366.507	-21.881
1.374.303	-27.373	1.411.189	-22.396	1.366.637	-21.882
1.374.451	-27.375	1.411.279	-22.398	1.366.705	-21.882
1.374.606	-27.376	1.411.400	-22.399	1.366.837	-21.882
1.374.828	-27.378	1.411.573	-22.401	1.367.000	-21.883
1.374.996	-27.380	1.411.774	-22.402	1.367.206	-21.883
1.375.074	-27.381	1.411.870	-22.404	1.367.401	-21.884
1.375.150	-27.383	1.411.951	-22.406	1.367.529	-21.884
1.375.257	-27.384	1.412.155	-22.407	1.367.603	-21.884
1.375.376	-27.386	1.412.399	-22.409	1.367.755	-21.885
1.375.511	-27.388	1.412.590	-22.410	1.368.040	-21.885
1.375.726	-27.389	1.412.693	-22.412	1.368.129	-21.886
1.375.968	-27.391	1.412.820	-22.413	1.368.239	-21.886
1.376.156	-27.392	1.413.015	-22.415	1.368.537	-21.886
1.376.283	-27.394	1.413.250	-22.417	1.368.692	-21.887

1.376.416	-27.396	1.413.459	-22.418	1.368.857	-21.887
1.376.604	-27.397	1.413.620	-22.420	1.368.992	-21.887
1.376.819	-27.399	1.413.837	-22.421	1.369.135	-21.888
1.377.029	-27.400	1.414.063	-22.423	1.369.352	-21.888
1.377.260	-27.402	1.414.250	-22.424	1.369.511	-21.889
1.377.406	-27.403	1.414.377	-22.425	1.369.635	-21.889
1.377.500	-27.405	1.414.503	-22.427	1.369.792	-21.889
1.377.657	-27.406	1.414.648	-22.428	1.370.040	-21.890
1.377.785	-27.408	1.414.801	-22.430	1.370.233	-21.890
1.377.957	-27.409	1.415.007	-22.431	1.370.363	-21.890
1.378.120	-27.411	1.415.119	-22.432	1.370.513	-21.891
1.378.286	-27.413	1.415.173	-22.434	1.370.701	-21.891
1.378.512	-27.414	1.415.309	-22.435	1.370.849	-21.891
1.378.660	-27.416	1.415.500	-22.437	1.370.903	-21.891
1.378.792	-27.417	1.415.647	-22.438	1.371.031	-21.892
1.378.951	-27.418	1.415.780	-22.439	1.371.252	-21.892
1.379.072	-27.420	1.415.957	-22.440	1.371.440	-21.892
1.379.185	-27.421	1.416.140	-22.442	1.371.586	-21.893
1.379.312	-27.423	1.416.317	-22.443	1.371.931	-21.893
1.379.489	-27.424	1.416.523	-22.444	1.372.359	-21.893
1.379.706	-27.426	1.416.669	-22.445	1.372.554	-21.894
1.379.919	-27.427	1.416.808	-22.447	1.372.661	-21.894
1.380.110	-27.429	1.416.985	-22.448	1.372.764	-21.894
1.380.275	-27.430	1.417.150	-22.449	1.372.822	-21.894
1.380.499	-27.432	1.417.296	-22.450	1.372.859	-21.895
1.380.730	-27.433	1.417.449	-22.451	1.372.881	-21.895
1.380.858	-27.434	1.417.635	-22.452	1.372.980	-21.895
1.380.963	-27.436	1.417.791	-22.453	1.373.087	-21.895
1.381.075	-27.437	1.417.919	-22.455	1.373.147	-21.896
1.381.234	-27.438	1.418.096	-22.456	1.373.289	-21.896

1.381.432	-27.440	1.418.329	-22.457	1.373.495	-21.896
1.381.572	-27.441	1.418.465	-22.458	1.373.723	-21.896
1.381.736	-27.443	1.418.562	-22.459	1.373.896	-21.897
1.381.911	-27.444	1.418.770	-22.460	1.374.050	-21.897
1.382.010	-27.445	1.418.931	-22.461	1.374.247	-21.897
1.382.198	-27.447	1.419.120	-22.462	1.374.444	-21.897
1.382.399	-27.448	1.419.252	-22.463	1.374.626	-21.897
1.382.531	-27.449	1.419.368	-22.464	1.374.789	-21.898
1.382.710	-27.451	1.419.474	-22.465	1.374.933	-21.898
1.382.829	-27.452	1.419.744	-22.466	1.375.002	-21.898
1.382.968	-27.453	1.419.948	-22.467	1.375.166	-21.898
1.383.139	-27.454	1.420.099	-22.467	1.375.428	-21.899
1.383.373	-27.456	1.420.231	-22.468	1.375.558	-21.899
1.383.766	-27.457	1.420.374	-22.469	1.375.629	-21.899
1.384.093	-27.458	1.420.573	-22.470	1.375.784	-21.899
1.384.288	-27.459	1.420.748	-22.471	1.375.990	-21.899
1.384.402	-27.461	1.420.905	-22.472	1.376.185	-21.900
1.384.536	-27.462	1.421.068	-22.473	1.376.328	-21.900
1.384.619	-27.463	1.421.199	-22.473	1.376.436	-21.900
1.384.586	-27.464	1.421.367	-22.474	1.376.606	-21.900
1.384.606	-27.465	1.421.563	-22.475	1.376.799	-21.901
1.384.702	-27.467	1.421.752	-22.476	1.376.969	-21.901
1.384.843	-27.468	1.421.918	-22.476	1.377.148	-21.901
1.384.948	-27.469	1.422.001	-22.477	1.377.381	-21.901
1.385.119	-27.470	1.422.041	-22.478	1.377.617	-21.902
1.385.334	-27.471	1.422.178	-22.479	1.377.738	-21.902
1.385.493	-27.473	1.422.451	-22.479	1.377.834	-21.902
1.385.623	-27.474	1.422.692	-22.480	1.377.961	-21.902
1.385.802	-27.475	1.422.986	-22.481	1.378.058	-21.903
1.385.984	-27.476	1.423.329	-22.482	1.378.212	-21.903

1.386.142	-27.477	1.423.564	-22.482	1.378.400	-21.903
1.386.344	-27.478	1.423.660	-22.483	1.378.564	-21.904
1.386.534	-27.479	1.423.752	-22.484	1.378.723	-21.904
1.386.711	-27.481	1.423.911	-22.484	1.378.799	-21.904
1.386.891	-27.482	1.423.963	-22.485	1.378.914	-21.904
1.386.976	-27.483	1.423.949	-22.485	1.379.147	-21.905
1.387.077	-27.484	1.424.044	-22.486	1.379.368	-21.905
1.387.251	-27.485	1.424.162	-22.487	1.379.521	-21.905
1.387.421	-27.486	1.424.285	-22.487	1.379.650	-21.906
1.387.581	-27.487	1.424.426	-22.488	1.379.803	-21.906
1.387.782	-27.489	1.424.601	-22.489	1.379.977	-21.907
1.387.959	-27.490	1.424.814	-22.489	1.380.107	-21.907
1.388.071	-27.491	1.424.984	-22.490	1.380.275	-21.907
1.388.208	-27.492	1.425.110	-22.490	1.380.479	-21.908
1.388.392	-27.493	1.425.296	-22.491	1.380.629	-21.908
1.388.584	-27.494	1.425.520	-22.492	1.380.784	-21.908
1.388.703	-27.495	1.425.708	-22.492	1.380.927	-21.909
1.388.849	-27.496	1.425.869	-22.493	1.381.091	-21.909
1.389.070	-27.498	1.426.030	-22.493	1.381.277	-21.910
1.389.254	-27.499	1.426.185	-22.494	1.381.467	-21.910
1.389.386	-27.500	1.426.324	-22.495	1.381.676	-21.911
1.389.566	-27.501	1.426.478	-22.495	1.381.877	-21.911
1.389.722	-27.502	1.426.559	-22.496	1.381.991	-21.912
1.389.901	-27.503	1.426.640	-22.497	1.382.065	-21.912
1.390.146	-27.505	1.426.779	-22.497	1.382.231	-21.913
1.390.244	-27.506	1.426.951	-22.498	1.382.424	-21.913
1.390.327	-27.507	1.427.126	-22.498	1.382.536	-21.914
1.390.455	-27.508	1.427.292	-22.499	1.382.686	-21.914
1.390.578	-27.509	1.427.437	-22.500	1.382.847	-21.915
1.390.739	-27.511	1.427.594	-22.500	1.382.980	-21.915

1.390.918	-27.512	1.427.820	-22.501	1.383.170	-21.916
1.391.100	-27.513	1.427.964	-22.502	1.383.311	-21.916
1.391.281	-27.514	1.428.112	-22.502	1.383.465	-21.917
1.391.418	-27.515	1.428.351	-22.503	1.383.683	-21.917
1.391.544	-27.517	1.428.544	-22.504	1.383.857	-21.918
1.391.729	-27.518	1.428.708	-22.505	1.384.046	-21.918
1.391.938	-27.519	1.428.857	-22.505	1.384.203	-21.919
1.392.090	-27.521	1.429.041	-22.506	1.384.332	-21.920
1.392.253	-27.522	1.429.187	-22.507	1.384.476	-21.920
1.392.410	-27.523	1.429.317	-22.508	1.384.597	-21.921
1.392.520	-27.525	1.429.501	-22.508	1.384.754	-21.921
1.392.672	-27.526	1.429.657	-22.509	1.384.893	-21.922
1.392.784	-27.527	1.429.769	-22.510	1.385.036	-21.922
1.392.894	-27.529	1.429.895	-22.511	1.385.233	-21.923
1.393.069	-27.530	1.430.098	-22.512	1.385.394	-21.924
1.393.280	-27.531	1.430.298	-22.512	1.385.513	-21.924
1.393.495	-27.533	1.430.412	-22.513	1.385.672	-21.925
1.393.629	-27.534	1.430.515	-22.514	1.385.867	-21.925
1.393.772	-27.536	1.430.654	-22.515	1.386.055	-21.926
1.393.938	-27.537	1.430.795	-22.516	1.386.237	-21.927
1.394.129	-27.538	1.430.983	-22.517	1.386.445	-21.927
1.394.321	-27.540	1.431.163	-22.518	1.386.660	-21.928
1.394.449	-27.541	1.431.311	-22.519	1.386.850	-21.928
1.394.572	-27.543	1.431.409	-22.520	1.386.989	-21.929
1.394.765	-27.544	1.431.539	-22.521	1.387.121	-21.929
1.394.977	-27.546	1.431.731	-22.522	1.387.310	-21.930
1.395.159	-27.547	1.431.920	-22.523	1.387.448	-21.931
1.395.341	-27.549	1.432.056	-22.524	1.387.592	-21.931
1.395.453	-27.551	1.432.236	-22.525	1.387.706	-21.932
1.395.580	-27.552	1.432.466	-22.526	1.387.809	-21.932

1.395.723	-27.554	1.432.636	-22.527	1.387.962	-21.933
1.395.941	-27.555	1.432.766	-22.528	1.388.127	-21.934
1.396.205	-27.557	1.432.903	-22.529	1.388.295	-21.934
1.396.364	-27.558	1.433.083	-22.531	1.388.436	-21.935
1.396.474	-27.560	1.433.251	-22.532	1.388.553	-21.935
1.396.575	-27.562	1.433.400	-22.533	1.388.692	-21.936
1.396.741	-27.563	1.433.541	-22.534	1.388.907	-21.936
1.396.973	-27.565	1.433.685	-22.535	1.389.106	-21.937
1.397.057	-27.567	1.433.824	-22.537	1.389.258	-21.938
1.397.128	-27.568	1.434.043	-22.538	1.389.440	-21.938
1.397.269	-27.570	1.434.202	-22.539	1.389.623	-21.939
1.397.449	-27.572	1.434.261	-22.540	1.389.807	-21.939
1.397.583	-27.573	1.434.391	-22.542	1.390.011	-21.940
1.397.731	-27.575	1.434.613	-22.543	1.390.143	-21.940
1.397.876	-27.577	1.434.834	-22.544	1.390.244	-21.941
1.398.038	-27.578	1.434.984	-22.545	1.390.397	-21.941
1.398.147	-27.580	1.435.148	-22.547	1.390.600	-21.942
1.398.250	-27.582	1.435.300	-22.548	1.390.818	-21.942
1.398.376	-27.583	1.435.439	-22.550	1.390.977	-21.943
1.398.503	-27.585	1.435.598	-22.551	1.391.031	-21.943
1.398.699	-27.587	1.435.750	-22.552	1.391.185	-21.944
1.398.909	-27.589	1.435.966	-22.554	1.391.425	-21.944
1.399.138	-27.590	1.436.134	-22.555	1.391.570	-21.945
1.399.384	-27.592	1.436.214	-22.556	1.391.785	-21.945
1.399.583	-27.594	1.436.326	-22.558	1.391.960	-21.946
1.399.781	-27.596	1.436.476	-22.559	1.392.085	-21.946
1.399.957	-27.597	1.436.593	-22.561	1.392.231	-21.947
1.400.078	-27.599	1.436.745	-22.562	1.392.392	-21.947
1.400.213	-27.601	1.436.927	-22.564	1.392.569	-21.948
1.400.401	-27.603	1.437.011	-22.565	1.392.701	-21.948

1.400.522	-27.605	1.437.200	-22.566	1.392.883	-21.949
1.400.639	-27.606	1.437.458	-22.568	1.393.044	-21.949
1.400.840	-27.608	1.437.634	-22.569	1.393.206	-21.950
1.400.948	-27.610	1.437.827	-22.571	1.393.398	-21.950
1.401.057	-27.612	1.438.031	-22.572	1.393.577	-21.951
1.401.234	-27.613	1.438.259	-22.574	1.393.743	-21.951
1.401.376	-27.615	1.438.410	-22.575	1.393.934	-21.951
1.401.476	-27.617	1.438.492	-22.577	1.394.068	-21.952
1.401.638	-27.619	1.438.685	-22.578	1.394.194	-21.952
1.401.846	-27.621	1.438.853	-22.580	1.394.366	-21.953
1.401.998	-27.622	1.438.940	-22.581	1.394.496	-21.953
1.402.110	-27.624	1.439.099	-22.583	1.394.671	-21.953
1.402.375	-27.626	1.439.218	-22.584	1.394.836	-21.954
1.402.641	-27.628	1.439.344	-22.586	1.394.933	-21.954
1.402.822	-27.629	1.439.510	-22.587	1.395.110	-21.955
1.403.002	-27.631	1.439.585	-22.589	1.395.520	-21.955
1.403.152	-27.633	1.439.691	-22.591	1.395.954	-21.955
1.403.304	-27.635	1.439.895	-22.592	1.396.176	-21.956
1.403.484	-27.636	1.440.060	-22.594	1.396.246	-21.956
1.403.643	-27.638	1.440.240	-22.595	1.396.295	-21.957
1.403.759	-27.640	1.440.484	-22.597	1.396.382	-21.957
1.403.885	-27.642	1.440.723	-22.598	1.396.436	-21.957
1.404.052	-27.643	1.440.896	-22.600	1.396.452	-21.958
1.404.249	-27.645	1.441.030	-22.601	1.396.465	-21.958
1.404.435	-27.647	1.441.212	-22.603	1.396.568	-21.958
1.404.617	-27.648	1.441.342	-22.604	1.396.752	-21.959
1.404.794	-27.650	1.441.483	-22.606	1.396.960	-21.959
1.404.962	-27.652	1.441.689	-22.607	1.397.186	-21.959
1.405.103	-27.654	1.441.875	-22.609	1.397.337	-21.960
1.405.257	-27.655	1.441.969	-22.610	1.397.511	-21.960

1.405.477	-27.657	1.442.103	-22.612	1.397.628	-21.960
1.405.609	-27.659	1.442.319	-22.613	1.397.717	-21.961
1.405.739	-27.660	1.442.471	-22.615	1.397.896	-21.961
1.405.941	-27.662	1.442.610	-22.616	1.398.076	-21.961
1.406.071	-27.664	1.442.789	-22.618	1.398.306	-21.961
1.406.178	-27.665	1.442.953	-22.620	1.398.510	-21.962
1.406.317	-27.667	1.443.118	-22.621	1.398.676	-21.962
1.406.492	-27.668	1.443.253	-22.623	1.398.725	-21.962
1.406.651	-27.670	1.443.387	-22.624	1.398.831	-21.963
1.406.790	-27.672	1.443.606	-22.626	1.399.104	-21.963
1.407.018	-27.673	1.443.842	-22.627	1.399.279	-21.963
1.407.233	-27.675	1.444.053	-22.629	1.399.379	-21.964
1.407.332	-27.676	1.444.209	-22.630	1.399.489	-21.964
1.407.500	-27.678	1.444.352	-22.632	1.399.691	-21.964
1.407.668	-27.679	1.444.534	-22.633	1.399.901	-21.964
1.407.802	-27.681	1.444.668	-22.634	1.400.049	-21.965
1.407.906	-27.682	1.444.762	-22.636	1.400.202	-21.965
1.408.035	-27.684	1.444.870	-22.637	1.400.419	-21.965
1.408.259	-27.685	1.445.023	-22.639	1.400.611	-21.966
1.408.512	-27.687	1.445.304	-22.640	1.400.784	-21.966
1.408.643	-27.688	1.445.497	-22.642	1.400.968	-21.966
1.408.665	-27.690	1.445.621	-22.643	1.401.165	-21.967
1.408.842	-27.691	1.445.793	-22.645	1.401.400	-21.967
1.409.263	-27.693	1.445.961	-22.646	1.401.474	-21.967
1.409.700	-27.694	1.446.102	-22.648	1.401.492	-21.968
1.409.865	-27.696	1.446.203	-22.649	1.401.644	-21.968
1.409.872	-27.697	1.446.344	-22.651	1.401.852	-21.968
1.409.977	-27.698	1.446.548	-22.652	1.402.029	-21.969
1.410.096	-27.700	1.446.703	-22.653	1.402.112	-21.969
1.410.191	-27.701	1.446.805	-22.655	1.402.247	-21.969

1.410.307	-27.702	1.446.996	-22.656	1.402.466	-21.970
1.410.419	-27.704	1.447.395	-22.658	1.402.616	-21.970
1.410.522	-27.705	1.447.729	-22.659	1.402.733	-21.970
1.410.618	-27.706	1.447.919	-22.661	1.402.894	-21.971
1.410.704	-27.708	1.448.074	-22.662	1.403.080	-21.971
1.410.863	-27.709	1.448.163	-22.663	1.403.233	-21.972
1.411.113	-27.710	1.448.165	-22.665	1.403.409	-21.972
1.411.279	-27.712	1.448.165	-22.666	1.403.568	-21.972
1.411.483	-27.713	1.448.257	-22.668	1.403.770	-21.973
1.411.673	-27.714	1.448.418	-22.669	1.403.938	-21.973
1.411.850	-27.715	1.448.542	-22.670	1.404.025	-21.974
1.411.980	-27.717	1.448.652	-22.672	1.404.149	-21.974
1.412.097	-27.718	1.448.750	-22.673	1.404.274	-21.974
1.412.298	-27.719	1.448.925	-22.675	1.404.449	-21.975
1.412.473	-27.720	1.449.108	-22.676	1.404.657	-21.975
1.412.643	-27.722	1.449.301	-22.677	1.404.834	-21.976
1.412.769	-27.723	1.449.487	-22.679	1.405.020	-21.976
1.412.883	-27.724	1.449.606	-22.680	1.405.199	-21.977
1.413.051	-27.725	1.449.825	-22.682	1.405.294	-21.977
1.413.197	-27.726	1.450.101	-22.683	1.405.464	-21.977
1.413.297	-27.727	1.450.264	-22.684	1.405.656	-21.978
1.413.450	-27.729	1.450.439	-22.686	1.405.815	-21.978
1.413.660	-27.730	1.450.605	-22.687	1.405.997	-21.979
1.413.835	-27.731	1.450.695	-22.688	1.406.145	-21.979
1.413.943	-27.732	1.450.768	-22.690	1.406.252	-21.980
1.414.068	-27.733	1.450.916	-22.691	1.406.420	-21.980
1.414.245	-27.734	1.451.089	-22.693	1.406.664	-21.981
1.414.473	-27.736	1.451.203	-22.694	1.406.855	-21.981
1.414.650	-27.737	1.451.330	-22.695	1.407.000	-21.982
1.414.818	-27.738	1.451.474	-22.697	1.407.119	-21.982

1.414.989	-27.739	1.451.624	-22.698	1.407.238	-21.983
1.415.096	-27.740	1.451.805	-22.700	1.407.401	-21.983
1.415.269	-27.741	1.452.048	-22.701	1.407.560	-21.984
1.415.497	-27.742	1.452.222	-22.703	1.407.699	-21.984
1.415.594	-27.744	1.452.348	-22.704	1.407.874	-21.985
1.415.728	-27.745	1.452.554	-22.706	1.408.051	-21.985
1.415.945	-27.746	1.452.751	-22.707	1.408.143	-21.986
1.416.044	-27.747	1.452.910	-22.708	1.408.291	-21.986
1.416.133	-27.748	1.453.084	-22.710	1.408.441	-21.987
1.416.333	-27.749	1.453.221	-22.711	1.408.580	-21.987
1.416.521	-27.750	1.453.335	-22.713	1.408.741	-21.988
1.416.709	-27.751	1.453.490	-22.714	1.408.855	-21.988
1.416.911	-27.753	1.453.674	-22.716	1.409.001	-21.989
1.416.991	-27.754	1.453.810	-22.717	1.409.238	-21.989
1.417.061	-27.755	1.453.911	-22.719	1.409.447	-21.990
1.417.235	-27.756	1.454.068	-22.720	1.409.693	-21.990
1.417.421	-27.757	1.454.267	-22.722	1.409.910	-21.991
1.417.645	-27.758	1.454.391	-22.723	1.410.004	-21.991
1.417.861	-27.759	1.454.550	-22.725	1.410.116	-21.992
1.417.959	-27.761	1.454.729	-22.727	1.410.334	-21.992
1.418.127	-27.762	1.454.879	-22.728	1.410.569	-21.992
1.418.275	-27.763	1.455.024	-22.730	1.410.703	-21.993
1.418.391	-27.764	1.455.190	-22.731	1.410.840	-21.993
1.418.562	-27.765	1.455.327	-22.733	1.410.950	-21.994
1.418.754	-27.767	1.455.459	-22.734	1.411.100	-21.994
1.418.969	-27.768	1.455.645	-22.736	1.411.277	-21.995
1.419.160	-27.769	1.455.802	-22.738	1.411.398	-21.995
1.419.272	-27.770	1.455.872	-22.739	1.411.519	-21.995
1.419.413	-27.771	1.456.037	-22.741	1.411.673	-21.996
1.419.585	-27.773	1.456.243	-22.743	1.411.749	-21.996

1.419.776	-27.774	1.456.375	-22.744	1.411.855	-21.997
1.419.935	-27.775	1.456.523	-22.746	1.412.090	-21.997
1.420.070	-27.776	1.456.723	-22.748	1.412.258	-21.997
1.420.238	-27.778	1.456.942	-22.749	1.412.386	-21.998
1.420.381	-27.779	1.457.106	-22.751	1.412.580	-21.998
1.420.546	-27.780	1.457.256	-22.753	1.412.847	-21.999
1.420.744	-27.781	1.457.442	-22.754	1.413.006	-21.999
1.420.874	-27.783	1.457.641	-22.756	1.413.159	-21.999
1.421.035	-27.784	1.457.771	-22.758	1.413.369	-22.000
1.421.194	-27.785	1.457.901	-22.760	1.413.539	-22.000
1.421.328	-27.787	1.458.058	-22.761	1.413.696	-22.000
1.421.521	-27.788	1.458.165	-22.763	1.413.875	-22.000
1.421.709	-27.789	1.458.271	-22.765	1.413.985	-22.001
1.421.850	-27.790	1.458.459	-22.767	1.414.111	-22.001
1.421.967	-27.792	1.458.671	-22.768	1.414.315	-22.001
1.422.137	-27.793	1.458.808	-22.770	1.414.498	-22.002
1.422.381	-27.794	1.459.032	-22.772	1.414.700	-22.002
1.422.493	-27.796	1.459.290	-22.774	1.414.915	-22.002
1.422.549	-27.797	1.459.395	-22.776	1.415.013	-22.002
1.422.724	-27.798	1.459.503	-22.777	1.415.132	-22.003
1.422.937	-27.800	1.459.691	-22.779	1.415.284	-22.003
1.423.080	-27.801	1.459.847	-22.781	1.415.502	-22.003
1.423.181	-27.802	1.459.955	-22.783	1.415.688	-22.003
1.423.264	-27.804	1.460.112	-22.785	1.415.782	-22.004
1.423.362	-27.805	1.460.271	-22.787	1.415.921	-22.004
1.423.528	-27.807	1.460.405	-22.788	1.416.124	-22.004
1.423.696	-27.808	1.460.528	-22.790	1.416.308	-22.004
1.423.824	-27.809	1.460.647	-22.792	1.416.490	-22.004
1.424.007	-27.811	1.460.807	-22.794	1.416.629	-22.005
1.424.191	-27.812	1.460.925	-22.796	1.416.702	-22.005

1.424.332	-27.814	1.461.044	-22.798	1.416.877	-22.005
1.424.554	-27.815	1.461.199	-22.799	1.417.088	-22.005
1.424.789	-27.816	1.461.400	-22.801	1.417.287	-22.005
1.424.951	-27.818	1.461.620	-22.803	1.417.440	-22.005
1.425.114	-27.819	1.461.819	-22.805	1.417.576	-22.006
1.425.320	-27.821	1.462.010	-22.807	1.417.753	-22.006
1.425.495	-27.822	1.462.206	-22.809	1.417.849	-22.006
1.425.661	-27.824	1.462.399	-22.811	1.417.973	-22.006
1.425.876	-27.825	1.462.567	-22.812	1.418.143	-22.006
1.426.013	-27.826	1.462.775	-22.814	1.418.295	-22.006
1.426.124	-27.828	1.462.914	-22.816	1.418.423	-22.006
1.426.322	-27.829	1.462.993	-22.818	1.418.423	-22.006
1.426.488	-27.831	1.463.195	-22.820	1.419.110	-22.006
1.426.563	-27.832	1.463.360	-22.822	1.419.453	-22.006
1.426.653	-27.834	1.463.477	-22.824	1.419.626	-22.007
1.426.841	-27.835	1.463.546	-22.825	1.419.655	-22.007
1.427.034	-27.837	1.463.694	-22.827	1.419.729	-22.007
1.427.133	-27.838	1.463.931	-22.829	1.419.818	-22.007
1.427.312	-27.840	1.464.075	-22.831	1.419.852	-22.007
1.427.516	-27.841	1.464.099	-22.833	1.419.933	-22.007
1.427.581	-27.842	1.464.230	-22.835	1.420.070	-22.007
1.427.706	-27.844	1.464.462	-22.837	1.420.192	-22.007
1.427.988	-27.845	1.464.565	-22.838	1.420.352	-22.007
1.428.241	-27.847	1.464.769	-22.840	1.420.555	-22.007
1.428.383	-27.848	1.465.083	-22.842	1.420.730	-22.007
1.428.515	-27.850	1.465.197	-22.844	1.420.882	-22.007
1.428.725	-27.851	1.465.331	-22.846	1.421.008	-22.007
1.428.918	-27.853	1.465.443	-22.848	1.421.127	-22.007
1.429.048	-27.854	1.465.576	-22.849	1.421.299	-22.007
1.429.126	-27.856	1.465.815	-22.851	1.421.548	-22.007

1.429.234	-27.857	1.466.035	-22.853	1.421.705	-22.007
1.429.393	-27.859	1.466.189	-22.855	1.421.870	-22.007
1.429.595	-27.860	1.466.362	-22.857	1.422.097	-22.007
1.429.760	-27.861	1.466.528	-22.858	1.422.175	-22.007
1.429.924	-27.863	1.466.680	-22.860	1.422.262	-22.007
1.430.130	-27.864	1.466.812	-22.862	1.422.431	-22.007
1.430.298	-27.866	1.466.967	-22.864	1.422.612	-22.007
1.430.446	-27.867	1.467.151	-22.866	1.422.787	-22.007
1.430.603	-27.869	1.467.272	-22.867	1.422.977	-22.007
1.430.739	-27.870	1.467.419	-22.869	1.423.129	-22.007
1.430.865	-27.872	1.467.592	-22.871	1.423.201	-22.007
1.431.075	-27.873	1.467.755	-22.873	1.423.318	-22.007
1.431.284	-27.875	1.467.897	-22.874	1.423.512	-22.007
1.431.407	-27.876	1.468.125	-22.876	1.423.719	-22.007
1.431.552	-27.877	1.468.358	-22.878	1.423.969	-22.007
1.431.738	-27.879	1.468.457	-22.880	1.424.151	-22.007
1.431.850	-27.880	1.468.602	-22.881	1.424.236	-22.007
1.431.989	-27.882	1.468.730	-22.883	1.424.409	-22.007
1.432.215	-27.883	1.468.853	-22.885	1.424.603	-22.007
1.432.366	-27.885	1.469.055	-22.887	1.424.758	-22.007
1.432.511	-27.886	1.469.270	-22.888	1.424.942	-22.007
1.432.598	-27.888	1.469.438	-22.890	1.425.094	-22.007
1.432.760	-27.889	1.469.592	-22.892	1.425.164	-22.007
1.433.022	-27.890	1.469.751	-22.893	1.425.313	-22.007
1.433.141	-27.892	1.469.872	-22.895	1.425.551	-22.007
1.433.295	-27.893	1.470.031	-22.897	1.425.683	-22.007
1.433.528	-27.895	1.470.208	-22.899	1.425.795	-22.007
1.433.642	-27.896	1.470.311	-22.900	1.425.977	-22.007
1.433.783	-27.898	1.470.444	-22.902	1.426.151	-22.007
1.434.023	-27.899	1.470.663	-22.904	1.426.270	-22.007

1.434.187	-27.900	1.470.840	-22.905	1.426.411	-22.007
1.434.243	-27.902	1.470.939	-22.907	1.426.622	-22.007
1.434.422	-27.903	1.471.069	-22.909	1.426.824	-22.007
1.434.832	-27.905	1.471.344	-22.910	1.426.951	-22.007
1.435.264	-27.906	1.471.758	-22.912	1.427.085	-22.008
1.435.484	-27.907	1.472.061	-22.914	1.427.267	-22.008
1.435.594	-27.909	1.472.236	-22.915	1.427.395	-22.008
1.435.710	-27.910	1.472.332	-22.917	1.427.538	-22.008
1.435.757	-27.911	1.472.375	-22.919	1.427.728	-22.008
1.435.773	-27.913	1.472.421	-22.920	1.427.856	-22.008
1.435.822	-27.914	1.472.457	-22.922	1.427.999	-22.008
1.435.896	-27.916	1.472.612	-22.924	1.428.145	-22.008
1.436.042	-27.917	1.472.731	-22.925	1.428.257	-22.009
1.436.111	-27.918	1.472.816	-22.927	1.428.479	-22.009
1.436.183	-27.920	1.472.953	-22.929	1.428.701	-22.009
1.436.319	-27.921	1.473.116	-22.930	1.428.853	-22.009
1.436.572	-27.922	1.473.298	-22.932	1.429.053	-22.009
1.436.770	-27.924	1.473.495	-22.934	1.429.207	-22.010
1.436.976	-27.925	1.473.651	-22.935	1.429.312	-22.010
1.437.159	-27.926	1.473.828	-22.937	1.429.489	-22.010
1.437.299	-27.928	1.474.026	-22.939	1.429.718	-22.010
1.437.507	-27.929	1.474.189	-22.940	1.429.852	-22.010
1.437.708	-27.931	1.474.377	-22.942	1.429.968	-22.011
1.437.794	-27.932	1.474.608	-22.943	1.430.179	-22.011
1.437.917	-27.933	1.474.747	-22.945	1.430.320	-22.011
1.438.136	-27.935	1.474.856	-22.947	1.430.444	-22.011
1.438.242	-27.936	1.474.948	-22.948	1.430.605	-22.012
1.438.338	-27.937	1.475.005	-22.950	1.430.759	-22.012
1.438.515	-27.939	1.475.188	-22.952	1.430.956	-22.012
1.438.658	-27.940	1.475.464	-22.953	1.431.125	-22.012

1.438.757	-27.941	1.475.618	-22.955	1.431.288	-22.013
1.438.945	-27.943	1.475.728	-22.957	1.431.423	-22.013
1.439.135	-27.944	1.475.829	-22.958	1.431.526	-22.013
1.439.258	-27.946	1.475.981	-22.960	1.431.693	-22.013
1.439.431	-27.947	1.476.176	-22.962	1.431.810	-22.014
1.439.585	-27.948	1.476.288	-22.964	1.431.900	-22.014
1.439.713	-27.950	1.476.488	-22.965	1.432.094	-22.014
1.439.919	-27.951	1.476.738	-22.967	1.432.278	-22.015
1.440.154	-27.953	1.476.884	-22.969	1.432.366	-22.015
1.440.257	-27.954	1.477.014	-22.970	1.432.477	-22.015
1.440.415	-27.955	1.477.193	-22.972	1.432.628	-22.015
1.440.710	-27.957	1.477.386	-22.974	1.432.820	-22.016
1.440.880	-27.958	1.477.551	-22.976	1.433.015	-22.016
1.441.026	-27.960	1.477.701	-22.977	1.433.226	-22.016
1.441.149	-27.961	1.477.827	-22.979	1.433.506	-22.016
1.441.301	-27.963	1.477.917	-22.981	1.433.725	-22.017
1.441.451	-27.964	1.478.027	-22.983	1.433.895	-22.017
1.441.577	-27.965	1.478.190	-22.984	1.434.057	-22.017
1.441.741	-27.967	1.478.391	-22.986	1.434.209	-22.018
1.441.882	-27.968	1.478.591	-22.988	1.434.317	-22.018
1.441.978	-27.970	1.478.748	-22.990	1.434.408	-22.018
1.442.121	-27.971	1.478.913	-22.991	1.434.548	-22.018
1.442.325	-27.973	1.479.028	-22.993	1.434.704	-22.019
1.442.540	-27.974	1.479.182	-22.995	1.434.816	-22.019
1.442.710	-27.976	1.479.371	-22.997	1.434.921	-22.019
1.442.818	-27.977	1.479.476	-22.999	1.435.085	-22.019
1.442.972	-27.979	1.479.679	-23.001	1.435.276	-22.020
1.443.197	-27.980	1.479.888	-23.002	1.435.439	-22.020
1.443.360	-27.982	1.479.968	-23.004	1.435.567	-22.020
1.443.432	-27.983	1.480.089	-23.006	1.435.726	-22.020

1.443.562	-27.985	1.480.262	-23.008	1.435.938	-22.021
1.443.750	-27.986	1.480.385	-23.010	1.436.156	-22.021
1.443.866	-27.988	1.480.569	-23.012	1.436.308	-22.021
1.444.010	-27.990	1.480.858	-23.014	1.436.469	-22.021
1.444.180	-27.991	1.481.057	-23.016	1.436.653	-22.022
1.444.321	-27.993	1.481.169	-23.018	1.436.761	-22.022
1.444.501	-27.994	1.481.339	-23.019	1.436.906	-22.022
1.444.682	-27.996	1.481.478	-23.021	1.437.094	-22.022
1.444.841	-27.998	1.481.626	-23.023	1.437.299	-22.023
1.445.114	-27.999	1.481.752	-23.025	1.437.507	-22.023
1.445.267	-28.001	1.481.898	-23.027	1.437.715	-22.023
1.445.340	-28.003	1.482.083	-23.029	1.437.916	-22.023
1.445.499	-28.004	1.482.276	-23.031	1.438.022	-22.023
1.445.674	-28.006	1.482.424	-23.033	1.438.129	-22.024
1.445.811	-28.007	1.482.507	-23.035	1.438.322	-22.024
1.445.981	-28.009	1.482.683	-23.037	1.438.488	-22.024
1.446.169	-28.011	1.482.879	-23.039	1.438.615	-22.024
1.446.319	-28.012	1.483.017	-23.041	1.438.806	-22.024
1.446.476	-28.014	1.483.203	-23.043	1.438.927	-22.024
1.446.599	-28.016	1.483.445	-23.045	1.439.104	-22.025
1.446.732	-28.018	1.483.624	-23.047	1.439.308	-22.025
1.446.889	-28.019	1.483.763	-23.049	1.439.429	-22.025
1.447.041	-28.021	1.483.938	-23.051	1.439.563	-22.025
1.447.229	-28.023	1.484.104	-23.053	1.439.733	-22.025
1.447.466	-28.024	1.484.198	-23.055	1.439.933	-22.025
1.447.635	-28.026	1.484.301	-23.057	1.440.096	-22.026
1.447.702	-28.028	1.484.420	-23.059	1.440.244	-22.026
1.447.809	-28.030	1.484.552	-23.061	1.440.352	-22.026
1.448.033	-28.031	1.484.693	-23.063	1.440.504	-22.026
1.448.255	-28.033	1.484.832	-23.065	1.440.658	-22.026

1.448.439	-28.035	1.484.937	-23.067	1.440.806	-22.026
1.448.553	-28.037	1.485.085	-23.069	1.441.004	-22.027
1.448.631	-28.038	1.485.311	-23.071	1.441.201	-22.027
1.448.765	-28.040	1.485.455	-23.073	1.441.335	-22.027
1.448.893	-28.042	1.485.549	-23.075	1.441.481	-22.027
1.449.019	-28.044	1.485.692	-23.077	1.441.573	-22.027
1.449.180	-28.045	1.485.905	-23.079	1.441.712	-22.027
1.449.382	-28.047	1.486.178	-23.081	1.441.920	-22.027
1.449.563	-28.049	1.486.400	-23.083	1.442.267	-22.027
1.449.662	-28.051	1.486.553	-23.085	1.442.702	-22.028
1.449.805	-28.052	1.486.767	-23.087	1.442.997	-22.028
1.450.065	-28.054	1.486.973	-23.088	1.443.109	-22.028
1.450.309	-28.056	1.487.132	-23.090	1.443.096	-22.028
1.450.522	-28.058	1.487.283	-23.092	1.443.125	-22.028
1.450.697	-28.059	1.487.408	-23.094	1.443.201	-22.028
1.450.894	-28.061	1.487.536	-23.096	1.443.257	-22.028
1.451.077	-28.063	1.487.666	-23.098	1.443.365	-22.028
1.451.163	-28.065	1.487.796	-23.100	1.443.475	-22.028
1.451.315	-28.067	1.487.957	-23.102	1.443.549	-22.029
1.451.555	-28.068	1.488.120	-23.104	1.443.716	-22.029
1.451.705	-28.070	1.488.273	-23.106	1.443.931	-22.029
1.451.750	-28.072	1.488.423	-23.108	1.444.109	-22.029
1.451.837	-28.074	1.488.566	-23.109	1.444.361	-22.029
1.451.991	-28.075	1.488.717	-23.111	1.444.469	-22.029
1.452.130	-28.077	1.488.889	-23.113	1.444.559	-22.029
1.452.233	-28.079	1.489.097	-23.115	1.444.789	-22.029
1.452.390	-28.081	1.489.281	-23.117	1.445.002	-22.029
1.452.583	-28.083	1.489.465	-23.119	1.445.159	-22.029
1.452.726	-28.084	1.489.590	-23.120	1.445.287	-22.030
1.452.868	-28.086	1.489.765	-23.122	1.445.453	-22.030

1.453.111	-28.088	1.489.995	-23.124	1.445.593	-22.030
1.453.297	-28.090	1.490.157	-23.126	1.445.712	-22.030
1.453.400	-28.091	1.490.298	-23.127	1.445.840	-22.030
1.453.631	-28.093	1.490.408	-23.129	1.445.993	-22.030
1.453.806	-28.095	1.490.584	-23.131	1.446.227	-22.030
1.453.965	-28.096	1.490.840	-23.133	1.446.420	-22.030
1.454.137	-28.098	1.490.995	-23.134	1.446.532	-22.030
1.454.308	-28.100	1.491.053	-23.136	1.446.680	-22.031
1.454.476	-28.102	1.491.125	-23.138	1.446.828	-22.031
1.454.669	-28.103	1.491.299	-23.139	1.446.962	-22.031
1.454.827	-28.105	1.491.510	-23.141	1.447.175	-22.031
1.454.917	-28.107	1.491.720	-23.143	1.447.321	-22.031
1.455.141	-28.108	1.491.875	-23.144	1.447.511	-22.031
1.455.356	-28.110	1.492.063	-23.146	1.447.771	-22.032
1.455.343	-28.112	1.492.253	-23.148	1.447.961	-22.032
1.455.500	-28.114	1.492.375	-23.149	1.448.073	-22.032
1.455.759	-28.115	1.492.536	-23.151	1.448.179	-22.032
1.455.878	-28.117	1.492.731	-23.152	1.448.360	-22.032
1.456.124	-28.119	1.492.923	-23.154	1.448.495	-22.032
1.456.315	-28.120	1.493.015	-23.155	1.448.634	-22.033
1.456.362	-28.122	1.493.111	-23.157	1.448.775	-22.033
1.456.510	-28.124	1.493.306	-23.158	1.448.907	-22.033
1.456.732	-28.125	1.493.470	-23.160	1.449.100	-22.033
1.456.924	-28.127	1.493.625	-23.161	1.449.270	-22.034
1.457.088	-28.129	1.493.790	-23.163	1.449.404	-22.034
1.457.258	-28.130	1.493.983	-23.164	1.449.511	-22.034
1.457.419	-28.132	1.494.194	-23.166	1.449.680	-22.034
1.457.567	-28.133	1.494.366	-23.167	1.449.886	-22.035
1.457.766	-28.135	1.494.505	-23.169	1.450.024	-22.035
1.457.952	-28.137	1.494.641	-23.170	1.450.177	-22.035

1.458.064	-28.138	1.494.776	-23.172	1.450.361	-22.035
1.458.096	-28.140	1.494.935	-23.173	1.450.488	-22.036
1.458.217	-28.142	1.495.117	-23.174	1.450.652	-22.036
1.458.474	-28.143	1.495.242	-23.176	1.450.780	-22.036
1.458.716	-28.145	1.495.360	-23.177	1.450.943	-22.037
1.458.891	-28.146	1.495.625	-23.179	1.451.064	-22.037
1.459.028	-28.148	1.496.131	-23.180	1.451.192	-22.038
1.459.138	-28.150	1.496.507	-23.181	1.451.422	-22.038
1.459.299	-28.151	1.496.651	-23.183	1.451.647	-22.038
1.459.471	-28.153	1.496.709	-23.184	1.451.846	-22.039
1.459.583	-28.154	1.496.718	-23.185	1.451.976	-22.039
1.459.688	-28.156	1.496.727	-23.186	1.452.092	-22.040
1.459.846	-28.158	1.496.720	-23.188	1.452.272	-22.040
1.460.217	-28.159	1.496.758	-23.189	1.452.413	-22.041
1.460.674	-28.161	1.496.938	-23.190	1.452.554	-22.041
1.460.903	-28.162	1.497.114	-23.192	1.452.789	-22.041
1.460.977	-28.164	1.497.234	-23.193	1.452.984	-22.042
1.461.075	-28.165	1.497.390	-23.194	1.453.127	-22.042
1.461.129	-28.167	1.497.551	-23.195	1.453.235	-22.043
1.461.154	-28.169	1.497.719	-23.196	1.453.354	-22.043
1.461.194	-28.170	1.497.946	-23.198	1.453.512	-22.044
1.461.310	-28.172	1.498.105	-23.199	1.453.703	-22.045
1.461.526	-28.173	1.498.224	-23.200	1.453.880	-22.045
1.461.689	-28.175	1.498.447	-23.201	1.454.061	-22.046
1.461.790	-28.176	1.498.692	-23.202	1.454.214	-22.046
1.461.917	-28.178	1.498.855	-23.204	1.454.377	-22.047
1.462.076	-28.180	1.498.958	-23.205	1.454.574	-22.047
1.462.247	-28.181	1.499.093	-23.206	1.454.706	-22.048
1.462.310	-28.183	1.499.261	-23.207	1.454.814	-22.049
1.462.457	-28.184	1.499.370	-23.208	1.454.951	-22.049

1.462.715	-28.186	1.499.454	-23.209	1.455.058	-22.050
1.462.896	-28.187	1.499.601	-23.211	1.455.172	-22.050
1.463.091	-28.189	1.499.834	-23.212	1.455.303	-22.051
1.463.288	-28.191	1.499.971	-23.213	1.455.408	-22.052
1.463.403	-28.192	1.500.134	-23.214	1.455.513	-22.052
1.463.589	-28.194	1.500.320	-23.215	1.455.647	-22.053
1.463.808	-28.195	1.500.423	-23.216	1.455.824	-22.053
1.463.880	-28.197	1.500.573	-23.217	1.456.097	-22.054
1.463.981	-28.199	1.500.710	-23.219	1.456.306	-22.055
1.464.146	-28.200	1.500.867	-23.220	1.456.485	-22.055
1.464.245	-28.202	1.501.033	-23.221	1.456.736	-22.056
1.464.402	-28.204	1.501.241	-23.222	1.456.926	-22.057
1.464.632	-28.205	1.501.454	-23.223	1.457.048	-22.057
1.464.818	-28.207	1.501.644	-23.224	1.457.209	-22.058
1.465.002	-28.209	1.501.776	-23.225	1.457.413	-22.059
1.465.154	-28.210	1.501.973	-23.226	1.457.542	-22.060
1.465.242	-28.212	1.502.122	-23.228	1.457.621	-22.060
1.465.414	-28.214	1.502.206	-23.229	1.457.769	-22.061
1.465.607	-28.215	1.502.368	-23.230	1.457.966	-22.062
1.465.759	-28.217	1.502.502	-23.231	1.458.129	-22.062
1.465.927	-28.219	1.502.619	-23.232	1.458.284	-22.063
1.466.176	-28.220	1.502.760	-23.233	1.458.387	-22.064
1.466.420	-28.222	1.502.917	-23.234	1.458.452	-22.065
1.466.555	-28.224	1.503.134	-23.236	1.458.636	-22.065
1.466.655	-28.226	1.503.391	-23.237	1.458.804	-22.066
1.466.796	-28.227	1.503.537	-23.238	1.458.923	-22.067
1.466.931	-28.229	1.503.669	-23.239	1.459.084	-22.067
1.467.090	-28.231	1.503.810	-23.240	1.459.308	-22.068
1.467.262	-28.233	1.503.981	-23.241	1.459.509	-22.069
1.467.357	-28.235	1.504.142	-23.242	1.459.722	-22.070

1.467.395	-28.236	1.504.274	-23.244	1.459.931	-22.070
1.467.538	-28.238	1.504.469	-23.245	1.460.114	-22.071
1.467.737	-28.240	1.504.677	-23.246	1.460.302	-22.072
1.467.906	-28.242	1.504.825	-23.247	1.460.441	-22.073
1.468.089	-28.244	1.504.899	-23.248	1.460.587	-22.073
1.468.293	-28.246	1.505.063	-23.250	1.460.710	-22.074
1.468.427	-28.248	1.505.237	-23.251	1.460.845	-22.075
1.468.544	-28.249	1.505.390	-23.252	1.461.033	-22.076
1.468.685	-28.251	1.505.634	-23.253	1.461.156	-22.076
1.468.882	-28.253	1.505.835	-23.254	1.461.324	-22.077
1.469.093	-28.255	1.506.021	-23.256	1.461.546	-22.078
1.469.256	-28.257	1.506.254	-23.257	1.461.723	-22.079
1.469.400	-28.259	1.506.373	-23.258	1.461.895	-22.080
1.469.494	-28.261	1.506.494	-23.259	1.462.048	-22.080
1.469.572	-28.263	1.506.660	-23.260	1.462.191	-22.081
1.469.697	-28.265	1.506.767	-23.262	1.462.384	-22.082
1.469.944	-28.267	1.506.870	-23.263	1.462.569	-22.083
1.470.148	-28.269	1.507.000	-23.264	1.462.719	-22.083
1.470.294	-28.271	1.507.141	-23.265	1.462.856	-22.084
1.470.428	-28.273	1.507.417	-23.266	1.463.009	-22.085
1.470.638	-28.275	1.507.681	-23.268	1.463.176	-22.086
1.470.934	-28.277	1.507.762	-23.269	1.463.295	-22.087
1.471.098	-28.279	1.507.845	-23.270	1.463.457	-22.087
1.471.187	-28.281	1.508.031	-23.271	1.463.607	-22.088
1.471.281	-28.283	1.508.165	-23.272	1.463.746	-22.089
1.471.436	-28.286	1.508.306	-23.274	1.463.873	-22.090
1.471.680	-28.288	1.508.521	-23.275	1.463.997	-22.090
1.471.796	-28.290	1.508.665	-23.276	1.464.173	-22.091
1.471.837	-28.292	1.508.764	-23.277	1.464.366	-22.092
1.472.036	-28.294	1.508.933	-23.278	1.464.563	-22.093

1.472.251	-28.296	1.509.086	-23.280	1.464.716	-22.094
1.472.403	-28.298	1.509.153	-23.281	1.464.836	-22.094
1.472.626	-28.301	1.509.279	-23.282	1.464.984	-22.095
1.472.832	-28.303	1.509.442	-23.283	1.465.105	-22.096
1.472.950	-28.305	1.509.547	-23.284	1.465.219	-22.097
1.473.042	-28.307	1.509.686	-23.285	1.465.551	-22.098
1.473.185	-28.309	1.509.912	-23.287	1.466.022	-22.098
1.473.340	-28.311	1.510.134	-23.288	1.466.369	-22.099
1.473.564	-28.314	1.510.376	-23.289	1.466.458	-22.100
1.473.736	-28.316	1.510.605	-23.290	1.466.478	-22.101
1.473.813	-28.318	1.510.804	-23.291	1.466.496	-22.102
1.473.913	-28.320	1.511.008	-23.292	1.466.559	-22.103
1.474.039	-28.322	1.511.169	-23.293	1.466.682	-22.103
1.474.225	-28.325	1.511.331	-23.295	1.466.776	-22.104
1.474.413	-28.327	1.511.469	-23.296	1.466.888	-22.105
1.474.527	-28.329	1.511.573	-23.297	1.467.018	-22.106
1.474.666	-28.331	1.511.740	-23.298	1.467.104	-22.107
1.474.841	-28.333	1.511.917	-23.299	1.467.233	-22.107
1.474.930	-28.336	1.512.041	-23.300	1.467.460	-22.108
1.475.094	-28.338	1.512.175	-23.301	1.467.704	-22.109
1.475.316	-28.340	1.512.316	-23.302	1.467.912	-22.110
1.475.484	-28.342	1.512.477	-23.303	1.468.044	-22.111
1.475.670	-28.345	1.512.639	-23.304	1.468.221	-22.111
1.475.862	-28.347	1.512.796	-23.305	1.468.423	-22.112
1.476.037	-28.349	1.512.919	-23.306	1.468.553	-22.113
1.476.158	-28.351	1.513.004	-23.307	1.468.712	-22.114
1.476.337	-28.353	1.513.147	-23.308	1.468.911	-22.115
1.476.579	-28.356	1.513.409	-23.309	1.469.048	-22.116
1.476.786	-28.358	1.513.645	-23.310	1.469.149	-22.116
1.476.949	-28.360	1.513.802	-23.311	1.469.362	-22.117

1.477.048	-28.362	1.513.969	-23.312	1.469.568	-22.118
1.477.159	-28.364	1.514.088	-23.313	1.469.612	-22.119
1.477.303	-28.367	1.514.232	-23.314	1.469.722	-22.120
1.477.435	-28.369	1.514.476	-23.315	1.469.886	-22.121
1.477.574	-28.371	1.514.653	-23.316	1.470.000	-22.121
1.477.758	-28.373	1.514.762	-23.317	1.470.201	-22.122
1.477.946	-28.375	1.514.897	-23.318	1.470.480	-22.123
1.478.044	-28.377	1.515.098	-23.319	1.470.623	-22.124
1.478.183	-28.380	1.515.282	-23.320	1.470.724	-22.125
1.478.432	-28.382	1.515.383	-23.321	1.470.936	-22.126
1.478.669	-28.384	1.515.558	-23.321	1.471.192	-22.127
1.478.801	-28.386	1.515.773	-23.322	1.471.339	-22.127
1.478.942	-28.388	1.515.945	-23.323	1.471.451	-22.128
1.479.115	-28.390	1.516.106	-23.324	1.471.566	-22.129
1.479.229	-28.392	1.516.236	-23.325	1.471.651	-22.130
1.479.386	-28.395	1.516.355	-23.326	1.471.803	-22.131
1.479.556	-28.397	1.516.507	-23.327	1.471.902	-22.132
1.479.753	-28.399	1.516.687	-23.327	1.472.016	-22.133
1.479.984	-28.401	1.516.904	-23.328	1.472.224	-22.134
1.480.166	-28.403	1.517.045	-23.329	1.472.430	-22.135
1.480.311	-28.405	1.517.186	-23.330	1.472.563	-22.135
1.480.446	-28.407	1.517.375	-23.331	1.472.690	-22.136
1.480.564	-28.409	1.517.542	-23.331	1.472.903	-22.137
1.480.748	-28.411	1.517.726	-23.332	1.473.138	-22.138
1.480.930	-28.413	1.517.899	-23.333	1.473.280	-22.139
1.481.059	-28.415	1.517.964	-23.334	1.473.394	-22.140
1.481.180	-28.417	1.518.060	-23.334	1.473.501	-22.141
1.481.375	-28.419	1.518.223	-23.335	1.473.698	-22.142
1.481.575	-28.421	1.518.479	-23.336	1.473.887	-22.143
1.481.736	-28.423	1.518.620	-23.337	1.474.005	-22.144

1.481.835	-28.425	1.518.802	-23.338	1.474.126	-22.145
1.481.965	-28.427	1.518.988	-23.338	1.474.218	-22.146
1.482.092	-28.429	1.519.117	-23.339	1.474.404	-22.146
1.482.254	-28.431	1.519.306	-23.340	1.474.615	-22.147
1.482.428	-28.433	1.519.494	-23.341	1.474.758	-22.148
1.482.605	-28.435	1.519.621	-23.341	1.474.993	-22.149
1.482.878	-28.437	1.519.910	-23.342	1.475.179	-22.150
1.483.121	-28.439	1.520.302	-23.343	1.475.298	-22.151
1.483.239	-28.441	1.520.585	-23.343	1.475.446	-22.152
1.483.302	-28.443	1.520.760	-23.344	1.475.643	-22.153
1.483.416	-28.445	1.520.820	-23.345	1.475.824	-22.154
1.483.553	-28.447	1.520.865	-23.346	1.475.934	-22.155
1.483.773	-28.448	1.520.916	-23.346	1.476.138	-22.156
1.483.884	-28.450	1.521.003	-23.347	1.476.264	-22.157
1.484.039	-28.452	1.521.082	-23.348	1.476.319	-22.158
1.484.238	-28.454	1.521.207	-23.349	1.476.523	-22.159
1.484.424	-28.456	1.521.351	-23.349	1.476.767	-22.160
1.484.621	-28.458	1.521.481	-23.350	1.476.973	-22.161
1.484.744	-28.460	1.521.662	-23.351	1.477.188	-22.162
1.484.796	-28.462	1.521.796	-23.352	1.477.345	-22.163
1.484.955	-28.463	1.521.985	-23.352	1.477.442	-22.164
1.485.188	-28.465	1.522.188	-23.353	1.477.599	-22.165
1.485.287	-28.467	1.522.298	-23.354	1.477.822	-22.166
1.485.556	-28.469	1.522.406	-23.355	1.477.962	-22.167
1.486.035	-28.471	1.522.554	-23.355	1.478.062	-22.168
1.486.391	-28.472	1.522.757	-23.356	1.478.185	-22.169
1.486.535	-28.474	1.523.015	-23.357	1.478.326	-22.170
1.486.588	-28.476	1.523.246	-23.358	1.478.452	-22.171
1.486.700	-28.478	1.523.329	-23.359	1.478.528	-22.171
1.486.815	-28.480	1.523.371	-23.359	1.478.658	-22.172

1.486.832	-28.481	1.523.494	-23.360	1.478.860	-22.173
1.486.888	-28.483	1.523.689	-23.361	1.479.055	-22.174
1.486.994	-28.485	1.523.925	-23.362	1.479.180	-22.175
1.487.106	-28.487	1.524.050	-23.363	1.479.292	-22.176
1.487.218	-28.488	1.524.144	-23.364	1.479.449	-22.177
1.487.408	-28.490	1.524.330	-23.365	1.479.763	-22.178
1.487.576	-28.492	1.524.489	-23.365	1.479.980	-22.179
1.487.738	-28.494	1.524.619	-23.366	1.480.128	-22.180
1.487.964	-28.495	1.524.798	-23.367	1.480.365	-22.181
1.488.111	-28.497	1.525.011	-23.368	1.480.549	-22.182
1.488.284	-28.499	1.525.166	-23.369	1.480.674	-22.182
1.488.488	-28.501	1.525.285	-23.370	1.480.851	-22.183
1.488.631	-28.503	1.525.459	-23.371	1.481.019	-22.184
1.488.781	-28.504	1.525.621	-23.372	1.481.136	-22.185
1.488.947	-28.506	1.525.858	-23.373	1.481.207	-22.186
1.489.147	-28.508	1.526.104	-23.374	1.481.321	-22.187
1.489.258	-28.510	1.526.254	-23.375	1.481.523	-22.188
1.489.366	-28.511	1.526.328	-23.376	1.481.676	-22.188
1.489.567	-28.513	1.526.411	-23.378	1.481.727	-22.189
1.489.693	-28.515	1.526.581	-23.379	1.481.899	-22.190
1.489.819	-28.517	1.526.718	-23.380	1.482.130	-22.191
1.490.052	-28.518	1.526.868	-23.381	1.482.169	-22.192
1.490.157	-28.520	1.527.000	-23.382	1.482.283	-22.192
1.490.285	-28.522	1.527.146	-23.383	1.482.641	-22.193
1.490.471	-28.524	1.527.411	-23.384	1.482.906	-22.194
1.490.665	-28.526	1.527.650	-23.386	1.483.064	-22.195
1.490.842	-28.527	1.527.729	-23.387	1.483.215	-22.195
1.490.966	-28.529	1.527.874	-23.388	1.483.421	-22.196
1.491.104	-28.531	1.528.055	-23.390	1.483.591	-22.197
1.491.263	-28.533	1.528.156	-23.391	1.483.732	-22.198

1.491.451	-28.535	1.528.308	-23.392	1.483.871	-22.198
1.491.666	-28.537	1.528.493	-23.394	1.484.001	-22.199
1.491.850	-28.539	1.528.652	-23.395	1.484.115	-22.200
1.492.014	-28.540	1.528.801	-23.396	1.484.290	-22.201
1.492.144	-28.542	1.528.945	-23.398	1.484.518	-22.201
1.492.289	-28.544	1.529.109	-23.399	1.484.679	-22.202
1.492.408	-28.546	1.529.223	-23.401	1.484.843	-22.203
1.492.538	-28.548	1.529.370	-23.402	1.485.007	-22.203
1.492.675	-28.550	1.529.543	-23.404	1.485.157	-22.204
1.492.813	-28.552	1.529.747	-23.405	1.485.347	-22.205
1.492.995	-28.554	1.529.919	-23.407	1.485.527	-22.205
1.493.172	-28.556	1.530.078	-23.409	1.485.636	-22.206
1.493.353	-28.557	1.530.219	-23.410	1.485.766	-22.207
1.493.492	-28.559	1.530.379	-23.412	1.485.943	-22.207
1.493.671	-28.561	1.530.546	-23.414	1.486.046	-22.208
1.493.851	-28.563	1.530.694	-23.415	1.486.192	-22.209
1.493.938	-28.565	1.530.824	-23.417	1.486.357	-22.209
1.494.061	-28.567	1.530.984	-23.419	1.486.525	-22.210
1.494.261	-28.569	1.531.140	-23.420	1.486.698	-22.210
1.494.476	-28.571	1.531.272	-23.422	1.486.850	-22.211
1.494.612	-28.573	1.531.483	-23.424	1.487.020	-22.212
1.494.715	-28.575	1.531.665	-23.426	1.487.216	-22.212
1.494.850	-28.577	1.531.824	-23.428	1.487.404	-22.213
1.495.013	-28.579	1.531.994	-23.429	1.487.511	-22.214
1.495.166	-28.582	1.532.119	-23.431	1.487.635	-22.214
1.495.372	-28.584	1.532.211	-23.433	1.487.827	-22.215
1.495.580	-28.586	1.532.415	-23.435	1.488.028	-22.215
1.495.798	-28.588	1.532.648	-23.437	1.488.201	-22.216
1.496.003	-28.590	1.532.807	-23.439	1.488.364	-22.216
1.496.143	-28.592	1.532.939	-23.441	1.488.526	-22.217

1.496.255	-28.594	1.533.000	-23.443	1.488.638	-22.218
1.496.362	-28.596	1.533.107	-23.445	1.488.784	-22.218
1.496.514	-28.598	1.533.313	-23.447	1.489.160	-22.219
1.496.702	-28.600	1.533.419	-23.449	1.489.576	-22.219
1.496.931	-28.603	1.533.524	-23.451	1.489.805	-22.220
1.497.081	-28.605	1.533.703	-23.453	1.489.921	-22.220
1.497.202	-28.607	1.533.940	-23.455	1.489.960	-22.221
1.497.325	-28.609	1.534.126	-23.457	1.489.977	-22.222
1.497.435	-28.611	1.534.268	-23.459	1.489.984	-22.222
1.497.583	-28.613	1.534.462	-23.461	1.489.989	-22.223
1.497.776	-28.616	1.534.679	-23.463	1.490.119	-22.223
1.497.941	-28.618	1.534.910	-23.465	1.490.403	-22.224
1.498.114	-28.620	1.535.132	-23.468	1.490.576	-22.224
1.498.329	-28.622	1.535.275	-23.470	1.490.663	-22.225
1.498.515	-28.624	1.535.405	-23.472	1.490.818	-22.226
1.498.654	-28.626	1.535.600	-23.474	1.491.015	-22.226
1.498.784	-28.629	1.535.777	-23.476	1.491.237	-22.227
1.498.963	-28.631	1.535.938	-23.478	1.491.447	-22.227
1.499.126	-28.633	1.536.046	-23.481	1.491.521	-22.228
1.499.274	-28.635	1.536.180	-23.483	1.491.646	-22.228
1.499.454	-28.637	1.536.342	-23.485	1.491.857	-22.229
1.499.539	-28.639	1.536.449	-23.487	1.492.039	-22.230
1.499.657	-28.642	1.536.559	-23.489	1.492.213	-22.230
1.499.816	-28.644	1.536.711	-23.492	1.492.321	-22.231
1.499.966	-28.646	1.536.859	-23.494	1.492.444	-22.231
1.500.081	-28.648	1.537.036	-23.496	1.492.603	-22.232
1.500.228	-28.650	1.537.229	-23.498	1.492.758	-22.232
1.500.412	-28.653	1.537.386	-23.501	1.492.950	-22.233
1.500.551	-28.655	1.537.565	-23.503	1.493.091	-22.234
1.500.692	-28.657	1.537.764	-23.505	1.493.233	-22.234

1.500.871	-28.659	1.537.907	-23.507	1.493.403	-22.235
1.501.084	-28.661	1.538.029	-23.510	1.493.503	-22.235
1.501.373	-28.663	1.538.205	-23.512	1.493.638	-22.236
1.501.628	-28.665	1.538.376	-23.514	1.493.862	-22.237
1.501.736	-28.668	1.538.537	-23.517	1.494.048	-22.237
1.501.824	-28.670	1.538.714	-23.519	1.494.243	-22.238
1.501.985	-28.672	1.538.882	-23.521	1.494.417	-22.239
1.502.224	-28.674	1.539.050	-23.523	1.494.581	-22.239
1.502.431	-28.676	1.539.238	-23.526	1.494.796	-22.240
1.502.560	-28.678	1.539.393	-23.528	1.494.971	-22.241
1.502.715	-28.680	1.539.538	-23.530	1.495.112	-22.241
1.502.872	-28.682	1.539.722	-23.533	1.495.231	-22.242
1.502.977	-28.684	1.539.883	-23.535	1.495.345	-22.243
1.503.123	-28.686	1.540.058	-23.537	1.495.457	-22.243
1.503.255	-28.688	1.540.242	-23.540	1.495.571	-22.244
1.503.340	-28.690	1.540.349	-23.542	1.495.744	-22.245
1.503.493	-28.692	1.540.506	-23.544	1.495.936	-22.245
1.503.690	-28.694	1.540.621	-23.547	1.496.106	-22.246
1.503.873	-28.696	1.540.764	-23.549	1.496.281	-22.247
1.504.041	-28.698	1.541.015	-23.551	1.496.467	-22.248
1.504.216	-28.700	1.541.180	-23.554	1.496.582	-22.248
1.504.404	-28.702	1.541.337	-23.556	1.496.678	-22.249
1.504.590	-28.704	1.541.505	-23.558	1.496.902	-22.250
1.504.724	-28.706	1.541.671	-23.561	1.497.092	-22.251
1.504.886	-28.708	1.541.821	-23.563	1.497.218	-22.251
1.505.130	-28.710	1.541.965	-23.565	1.497.370	-22.252
1.505.264	-28.712	1.542.159	-23.568	1.497.486	-22.253
1.505.396	-28.713	1.542.303	-23.570	1.497.637	-22.254
1.505.598	-28.715	1.542.498	-23.572	1.497.854	-22.255
1.505.699	-28.717	1.542.697	-23.575	1.498.031	-22.255

1.505.791	-28.719	1.542.818	-23.577	1.498.134	-22.256
1.505.977	-28.721	1.542.924	-23.579	1.498.244	-22.257
1.506.189	-28.722	1.543.116	-23.582	1.498.450	-22.258
1.506.362	-28.724	1.543.286	-23.584	1.498.719	-22.259
1.506.479	-28.726	1.543.398	-23.586	1.498.941	-22.259
1.506.626	-28.727	1.543.472	-23.589	1.499.063	-22.260
1.506.821	-28.729	1.543.658	-23.591	1.499.229	-22.261
1.507.038	-28.731	1.544.016	-23.593	1.499.415	-22.262
1.507.215	-28.732	1.544.462	-23.596	1.499.529	-22.263
1.507.377	-28.734	1.544.767	-23.598	1.499.659	-22.263
1.507.540	-28.736	1.544.834	-23.600	1.499.816	-22.264
1.507.708	-28.737	1.544.875	-23.603	1.499.962	-22.265
1.507.865	-28.739	1.544.915	-23.605	1.500.195	-22.266
1.507.982	-28.740	1.545.015	-23.607	1.500.352	-22.267
1.508.148	-28.742	1.545.105	-23.610	1.500.408	-22.268
1.508.244	-28.743	1.545.157	-23.612	1.500.609	-22.268
1.508.418	-28.745	1.545.204	-23.615	1.500.892	-22.269
1.508.618	-28.746	1.545.331	-23.617	1.501.086	-22.270
1.508.761	-28.748	1.545.513	-23.619	1.501.189	-22.271
1.508.976	-28.749	1.545.647	-23.622	1.501.302	-22.272
1.509.158	-28.751	1.545.762	-23.624	1.501.431	-22.272
1.509.270	-28.752	1.545.919	-23.626	1.501.548	-22.273
1.509.404	-28.753	1.546.180	-23.629	1.501.669	-22.274
1.509.610	-28.755	1.546.380	-23.631	1.501.801	-22.275
1.509.872	-28.756	1.546.510	-23.633	1.501.946	-22.276
1.510.036	-28.757	1.546.707	-23.636	1.502.063	-22.276
1.510.135	-28.759	1.546.873	-23.638	1.502.211	-22.277
1.510.320	-28.760	1.547.014	-23.640	1.502.406	-22.278
1.510.468	-28.761	1.547.195	-23.643	1.502.581	-22.279
1.510.567	-28.762	1.547.316	-23.645	1.502.724	-22.279

1.510.717	-28.764	1.547.384	-23.647	1.502.852	-22.280
1.510.905	-28.765	1.547.560	-23.650	1.503.042	-22.281
1.511.118	-28.766	1.547.804	-23.652	1.503.232	-22.282
1.511.418	-28.767	1.547.950	-23.655	1.503.398	-22.282
1.511.834	-28.769	1.548.125	-23.657	1.503.618	-22.283
1.512.043	-28.770	1.548.194	-23.659	1.503.862	-22.284
1.512.079	-28.771	1.548.351	-23.662	1.504.068	-22.284
1.512.188	-28.772	1.548.615	-23.664	1.504.225	-22.285
1.512.265	-28.773	1.548.745	-23.667	1.504.371	-22.286
1.512.319	-28.774	1.548.907	-23.669	1.504.518	-22.286
1.512.399	-28.775	1.549.070	-23.671	1.504.628	-22.287
1.512.457	-28.776	1.549.268	-23.674	1.504.735	-22.287
1.512.585	-28.777	1.549.458	-23.676	1.504.886	-22.288
1.512.755	-28.778	1.549.567	-23.679	1.505.060	-22.289
1.512.901	-28.780	1.549.736	-23.681	1.505.105	-22.289
1.513.031	-28.781	1.549.944	-23.684	1.505.294	-22.290
1.513.192	-28.782	1.550.117	-23.686	1.505.620	-22.290
1.513.403	-28.783	1.550.278	-23.689	1.505.753	-22.291
1.513.553	-28.784	1.550.405	-23.691	1.505.858	-22.291
1.513.723	-28.785	1.550.537	-23.694	1.505.947	-22.291
1.513.938	-28.786	1.550.726	-23.696	1.506.122	-22.292
1.514.119	-28.787	1.550.907	-23.698	1.506.377	-22.292
1.514.256	-28.788	1.550.999	-23.701	1.506.552	-22.293
1.514.397	-28.789	1.551.102	-23.703	1.506.785	-22.293
1.514.599	-28.790	1.551.306	-23.706	1.506.980	-22.293
1.514.767	-28.791	1.551.460	-23.708	1.507.095	-22.294
1.514.870	-28.792	1.551.620	-23.711	1.507.265	-22.294
1.514.991	-28.792	1.551.810	-23.714	1.507.468	-22.294
1.515.152	-28.793	1.551.973	-23.716	1.507.643	-22.295
1.515.257	-28.794	1.552.092	-23.719	1.507.776	-22.295

1.515.401	-28.795	1.552.215	-23.721	1.507.914	-22.295
1.515.605	-28.796	1.552.392	-23.724	1.508.033	-22.296
1.515.770	-28.797	1.552.538	-23.726	1.508.196	-22.296
1.515.927	-28.798	1.552.675	-23.729	1.508.362	-22.296
1.516.062	-28.799	1.552.840	-23.731	1.508.521	-22.296
1.516.163	-28.800	1.552.999	-23.734	1.508.692	-22.296
1.516.348	-28.801	1.553.192	-23.737	1.508.851	-22.296
1.516.568	-28.802	1.553.340	-23.739	1.509.068	-22.297
1.516.711	-28.803	1.553.474	-23.742	1.509.238	-22.297
1.516.890	-28.804	1.553.671	-23.744	1.509.382	-22.297
1.517.130	-28.805	1.553.904	-23.747	1.509.554	-22.297
1.517.305	-28.806	1.554.104	-23.750	1.509.695	-22.297
1.517.457	-28.807	1.554.207	-23.752	1.509.865	-22.297
1.517.672	-28.808	1.554.375	-23.755	1.509.991	-22.297
1.517.827	-28.809	1.554.536	-23.758	1.510.123	-22.297
1.517.914	-28.810	1.554.713	-23.760	1.510.284	-22.297
1.518.022	-28.811	1.554.899	-23.763	1.510.475	-22.297
1.518.168	-28.812	1.555.043	-23.766	1.510.679	-22.297
1.518.300	-28.813	1.555.233	-23.768	1.510.804	-22.297
1.518.456	-28.814	1.555.392	-23.771	1.510.948	-22.297
1.518.674	-28.816	1.555.553	-23.773	1.511.120	-22.296
1.518.864	-28.817	1.555.728	-23.776	1.511.274	-22.296
1.518.965	-28.818	1.555.775	-23.779	1.511.447	-22.296
1.519.093	-28.819	1.555.872	-23.781	1.511.624	-22.296
1.519.333	-28.820	1.556.122	-23.784	1.511.783	-22.296
1.519.511	-28.821	1.556.330	-23.787	1.511.935	-22.296
1.519.552	-28.822	1.556.492	-23.789	1.512.038	-22.295
1.519.702	-28.823	1.556.667	-23.792	1.512.193	-22.295
1.519.902	-28.825	1.556.808	-23.795	1.512.524	-22.295
1.520.067	-28.826	1.556.906	-23.797	1.512.928	-22.295

1.520.197	-28.827	1.557.020	-23.800	1.513.242	-22.294
1.520.349	-28.828	1.557.220	-23.803	1.513.373	-22.294
1.520.522	-28.830	1.557.415	-23.805	1.513.438	-22.294
1.520.663	-28.831	1.557.504	-23.808	1.513.508	-22.294
1.520.806	-28.832	1.557.646	-23.811	1.513.519	-22.293
1.521.015	-28.833	1.557.762	-23.813	1.513.633	-22.293
1.521.192	-28.835	1.557.937	-23.816	1.513.781	-22.293
1.521.317	-28.836	1.558.125	-23.819	1.513.829	-22.292
1.521.432	-28.837	1.558.302	-23.821	1.513.938	-22.292
1.521.622	-28.839	1.558.499	-23.824	1.514.128	-22.291
1.521.794	-28.840	1.558.636	-23.826	1.514.296	-22.291
1.521.969	-28.842	1.558.880	-23.829	1.514.433	-22.291
1.522.170	-28.843	1.559.138	-23.832	1.514.603	-22.290
1.522.370	-28.845	1.559.312	-23.834	1.514.816	-22.290
1.522.498	-28.846	1.559.476	-23.837	1.515.011	-22.289
1.522.581	-28.847	1.559.635	-23.839	1.515.188	-22.289
1.522.733	-28.849	1.559.736	-23.842	1.515.417	-22.289
1.522.905	-28.850	1.559.859	-23.844	1.515.587	-22.288
1.523.049	-28.852	1.560.040	-23.847	1.515.726	-22.288
1.523.242	-28.854	1.560.204	-23.850	1.515.820	-22.287
1.523.485	-28.855	1.560.313	-23.852	1.515.896	-22.287
1.523.647	-28.857	1.560.406	-23.855	1.516.037	-22.286
1.523.766	-28.858	1.560.517	-23.857	1.516.190	-22.286
1.523.871	-28.860	1.560.692	-23.860	1.516.384	-22.285
1.524.052	-28.862	1.560.920	-23.862	1.516.510	-22.285
1.524.301	-28.863	1.561.075	-23.865	1.516.600	-22.284
1.524.482	-28.865	1.561.176	-23.867	1.516.740	-22.284
1.524.617	-28.866	1.561.299	-23.870	1.516.962	-22.283
1.524.689	-28.868	1.561.488	-23.872	1.517.189	-22.283
1.524.855	-28.870	1.561.718	-23.875	1.517.368	-22.282

1.525.007	-28.872	1.561.938	-23.877	1.517.531	-22.282
1.525.179	-28.873	1.562.099	-23.879	1.517.675	-22.282
1.525.367	-28.875	1.562.240	-23.882	1.517.841	-22.281
1.525.462	-28.877	1.562.428	-23.884	1.518.017	-22.281
1.525.605	-28.879	1.562.601	-23.887	1.518.190	-22.280
1.525.728	-28.880	1.562.699	-23.889	1.518.394	-22.280
1.525.862	-28.882	1.562.869	-23.892	1.518.578	-22.279
1.526.030	-28.884	1.563.096	-23.894	1.518.696	-22.279
1.526.111	-28.886	1.563.273	-23.896	1.518.801	-22.278
1.526.257	-28.887	1.563.436	-23.899	1.518.916	-22.278
1.526.510	-28.889	1.563.575	-23.901	1.519.077	-22.277
1.526.740	-28.891	1.563.736	-23.903	1.519.268	-22.277
1.526.965	-28.893	1.563.907	-23.906	1.519.411	-22.276
1.527.162	-28.895	1.564.050	-23.908	1.519.523	-22.276
1.527.341	-28.897	1.564.252	-23.910	1.519.650	-22.276
1.527.547	-28.899	1.564.422	-23.913	1.519.801	-22.275
1.527.688	-28.901	1.564.565	-23.915	1.519.973	-22.275
1.527.753	-28.902	1.564.776	-23.917	1.520.197	-22.274
1.527.840	-28.904	1.564.906	-23.919	1.520.343	-22.274
1.528.024	-28.906	1.565.027	-23.922	1.520.432	-22.274
1.528.277	-28.908	1.565.201	-23.924	1.520.658	-22.273
1.528.441	-28.910	1.565.368	-23.926	1.520.876	-22.273
1.528.539	-28.912	1.565.592	-23.928	1.521.017	-22.273
1.528.647	-28.914	1.565.755	-23.931	1.521.154	-22.272
1.528.775	-28.916	1.565.833	-23.933	1.521.293	-22.272
1.528.902	-28.918	1.565.999	-23.935	1.521.490	-22.272
1.529.059	-28.920	1.566.216	-23.937	1.521.629	-22.271
1.529.212	-28.922	1.566.391	-23.939	1.521.720	-22.271
1.529.305	-28.924	1.566.545	-23.941	1.521.931	-22.271
1.529.435	-28.926	1.566.711	-23.944	1.522.150	-22.271

1.529.655	-28.927	1.566.835	-23.946	1.522.332	-22.270
1.529.870	-28.929	1.566.904	-23.948	1.522.522	-22.270
1.530.067	-28.931	1.567.021	-23.950	1.522.682	-22.270
1.530.309	-28.933	1.567.260	-23.952	1.522.820	-22.270
1.530.515	-28.935	1.567.455	-23.954	1.522.991	-22.270
1.530.696	-28.937	1.567.601	-23.956	1.523.167	-22.269
1.530.798	-28.939	1.567.701	-23.959	1.523.304	-22.269
1.530.862	-28.941	1.567.881	-23.961	1.523.443	-22.269
1.531.053	-28.943	1.568.300	-23.963	1.523.606	-22.269
1.531.243	-28.945	1.568.755	-23.965	1.523.817	-22.269
1.531.447	-28.947	1.568.954	-23.967	1.523.976	-22.269
1.531.610	-28.949	1.568.988	-23.969	1.524.059	-22.269
1.531.637	-28.951	1.569.048	-23.971	1.524.187	-22.269
1.531.801	-28.953	1.569.073	-23.973	1.524.368	-22.268
1.532.063	-28.955	1.569.124	-23.975	1.524.590	-22.268
1.532.242	-28.957	1.569.193	-23.977	1.524.760	-22.268
1.532.399	-28.958	1.569.287	-23.979	1.524.868	-22.268
1.532.608	-28.960	1.569.386	-23.981	1.525.016	-22.268
1.532.802	-28.962	1.569.509	-23.983	1.525.173	-22.268
1.532.897	-28.964	1.569.720	-23.985	1.525.311	-22.268
1.533.033	-28.966	1.569.953	-23.987	1.525.426	-22.268
1.533.208	-28.968	1.570.137	-23.989	1.525.580	-22.268
1.533.365	-28.970	1.570.264	-23.991	1.525.737	-22.268
1.533.524	-28.972	1.570.406	-23.993	1.525.878	-22.268
1.533.764	-28.974	1.570.598	-23.995	1.526.019	-22.268
1.533.945	-28.975	1.570.833	-23.997	1.526.201	-22.269
1.534.063	-28.977	1.571.028	-23.999	1.526.429	-22.269
1.534.171	-28.979	1.571.109	-24.001	1.526.548	-22.269
1.534.342	-28.981	1.571.221	-24.003	1.526.619	-22.269
1.534.612	-28.983	1.571.429	-24.005	1.526.819	-22.269

1.534.798	-28.985	1.571.582	-24.007	1.527.058	-22.269
1.534.939	-28.986	1.571.709	-24.009	1.527.298	-22.269
1.535.031	-28.988	1.571.855	-24.011	1.527.531	-22.269
1.535.143	-28.990	1.572.016	-24.013	1.527.661	-22.269
1.535.322	-28.992	1.572.155	-24.015	1.527.769	-22.270
1.535.499	-28.994	1.572.330	-24.017	1.527.935	-22.270
1.535.639	-28.995	1.572.480	-24.019	1.528.112	-22.270
1.535.807	-28.997	1.572.634	-24.021	1.528.217	-22.270
1.535.975	-28.999	1.572.831	-24.023	1.528.324	-22.270
1.536.104	-29.001	1.573.064	-24.025	1.528.515	-22.271
1.536.264	-29.003	1.573.255	-24.027	1.528.678	-22.271
1.536.398	-29.004	1.573.378	-24.029	1.528.813	-22.271
1.536.653	-29.006	1.573.559	-24.031	1.528.936	-22.271
1.537.103	-29.008	1.573.690	-24.033	1.529.025	-22.271
1.537.421	-29.010	1.573.848	-24.035	1.529.212	-22.272
1.537.536	-29.011	1.574.097	-24.037	1.529.417	-22.272
1.537.612	-29.013	1.574.305	-24.039	1.529.675	-22.272
1.537.677	-29.015	1.574.447	-24.041	1.529.846	-22.272
1.537.751	-29.016	1.574.554	-24.043	1.529.978	-22.273
1.537.840	-29.018	1.574.626	-24.045	1.530.166	-22.273
1.537.921	-29.020	1.574.747	-24.047	1.530.368	-22.273
1.537.993	-29.021	1.574.902	-24.049	1.530.551	-22.274
1.538.105	-29.023	1.575.134	-24.051	1.530.717	-22.274
1.538.226	-29.025	1.575.343	-24.053	1.530.869	-22.274
1.538.331	-29.026	1.575.457	-24.055	1.530.979	-22.275
1.538.503	-29.028	1.575.605	-24.058	1.531.107	-22.275
1.538.768	-29.030	1.575.858	-24.060	1.531.328	-22.275
1.538.996	-29.031	1.576.015	-24.062	1.531.507	-22.276
1.539.093	-29.033	1.576.050	-24.064	1.531.584	-22.276
1.539.231	-29.035	1.576.178	-24.066	1.531.662	-22.276

1.539.482	-29.036	1.576.418	-24.068	1.531.861	-22.277
1.539.648	-29.038	1.576.613	-24.070	1.532.090	-22.277
1.539.868	-29.040	1.576.693	-24.073	1.532.244	-22.277
1.540.033	-29.041	1.576.776	-24.075	1.532.406	-22.278
1.540.175	-29.043	1.576.937	-24.077	1.532.657	-22.278
1.540.300	-29.045	1.577.144	-24.079	1.532.869	-22.279
1.540.446	-29.046	1.577.325	-24.081	1.532.980	-22.279
1.540.672	-29.048	1.577.478	-24.084	1.533.051	-22.279
1.540.779	-29.049	1.577.682	-24.086	1.533.203	-22.280
1.540.889	-29.051	1.577.905	-24.088	1.533.376	-22.280
1.541.035	-29.053	1.578.102	-24.091	1.533.510	-22.281
1.541.241	-29.054	1.578.246	-24.093	1.533.678	-22.281
1.541.386	-29.056	1.578.398	-24.095	1.533.849	-22.282
1.541.552	-29.058	1.578.564	-24.097	1.534.063	-22.282
1.541.711	-29.059	1.578.633	-24.100	1.534.261	-22.283
1.541.799	-29.061	1.578.804	-24.102	1.534.398	-22.283
1.541.998	-29.063	1.579.045	-24.104	1.534.462	-22.284
1.542.271	-29.064	1.579.173	-24.107	1.534.608	-22.284
1.542.399	-29.066	1.579.276	-24.109	1.534.868	-22.285
1.542.558	-29.068	1.579.444	-24.111	1.535.009	-22.285
1.542.699	-29.069	1.579.630	-24.114	1.535.134	-22.286
1.542.831	-29.071	1.579.789	-24.116	1.535.294	-22.286
1.543.064	-29.073	1.579.987	-24.118	1.535.423	-22.287
1.543.271	-29.074	1.580.208	-24.121	1.535.567	-22.287
1.543.409	-29.076	1.580.374	-24.123	1.535.941	-22.288
1.543.539	-29.078	1.580.504	-24.126	1.536.427	-22.288
1.543.651	-29.080	1.580.623	-24.128	1.536.664	-22.289
1.543.734	-29.081	1.580.811	-24.130	1.536.774	-22.290
1.543.819	-29.083	1.580.988	-24.133	1.536.877	-22.290
1.543.922	-29.085	1.581.082	-24.135	1.536.870	-22.291

1.544.193	-29.087	1.581.232	-24.138	1.536.861	-22.291
1.544.476	-29.088	1.581.391	-24.140	1.536.926	-22.292
1.544.610	-29.090	1.581.544	-24.142	1.537.000	-22.293
1.544.700	-29.092	1.581.664	-24.145	1.537.146	-22.293
1.544.818	-29.094	1.581.781	-24.147	1.537.312	-22.294
1.545.009	-29.096	1.581.913	-24.150	1.537.486	-22.294
1.545.248	-29.097	1.582.061	-24.152	1.537.744	-22.295
1.545.390	-29.099	1.582.244	-24.154	1.537.926	-22.296
1.545.459	-29.101	1.582.401	-24.157	1.538.078	-22.296
1.545.546	-29.103	1.582.545	-24.159	1.538.291	-22.297
1.545.800	-29.105	1.582.746	-24.162	1.538.443	-22.298
1.546.006	-29.107	1.582.943	-24.164	1.538.535	-22.298
1.546.127	-29.109	1.583.118	-24.167	1.538.714	-22.299
1.546.315	-29.111	1.583.372	-24.169	1.538.909	-22.300
1.546.479	-29.113	1.583.584	-24.171	1.539.017	-22.300
1.546.510	-29.115	1.583.707	-24.174	1.539.147	-22.301
1.546.640	-29.117	1.583.849	-24.176	1.539.326	-22.302
1.546.911	-29.119	1.584.043	-24.179	1.539.487	-22.302
1.547.108	-29.121	1.584.171	-24.181	1.539.628	-22.303
1.547.244	-29.123	1.584.259	-24.183	1.539.742	-22.304
1.547.469	-29.125	1.584.435	-24.186	1.539.868	-22.305
1.547.607	-29.127	1.584.612	-24.188	1.540.029	-22.305
1.547.746	-29.129	1.584.729	-24.191	1.540.220	-22.306
1.547.890	-29.131	1.584.841	-24.193	1.540.397	-22.307
1.548.067	-29.133	1.585.011	-24.195	1.540.553	-22.308
1.548.293	-29.135	1.585.186	-24.198	1.540.704	-22.308
1.548.463	-29.137	1.585.341	-24.200	1.540.809	-22.309
1.548.636	-29.139	1.585.488	-24.203	1.540.934	-22.310
1.548.723	-29.142	1.585.699	-24.205	1.541.192	-22.311
1.548.795	-29.144	1.585.909	-24.207	1.541.358	-22.312

1.548.976	-29.146	1.586.082	-24.210	1.541.463	-22.313
1.549.155	-29.148	1.586.209	-24.212	1.541.702	-22.313
1.549.352	-29.150	1.586.382	-24.215	1.541.949	-22.314
1.549.536	-29.152	1.586.628	-24.217	1.542.047	-22.315
1.549.657	-29.155	1.586.821	-24.219	1.542.184	-22.316
1.549.850	-29.157	1.586.953	-24.222	1.542.325	-22.317
1.550.009	-29.159	1.587.079	-24.224	1.542.428	-22.318
1.550.114	-29.161	1.587.303	-24.226	1.542.625	-22.319
1.550.303	-29.164	1.587.471	-24.229	1.542.713	-22.319
1.550.473	-29.166	1.587.587	-24.231	1.542.858	-22.320
1.550.584	-29.168	1.587.735	-24.233	1.543.031	-22.321
1.550.721	-29.171	1.587.905	-24.236	1.543.248	-22.322
1.550.892	-29.173	1.588.125	-24.238	1.543.427	-22.323
1.551.026	-29.175	1.588.309	-24.240	1.543.593	-22.324
1.551.124	-29.177	1.588.428	-24.243	1.543.745	-22.325
1.551.286	-29.180	1.588.615	-24.245	1.543.887	-22.326
1.551.467	-29.182	1.588.822	-24.247	1.544.079	-22.327
1.551.620	-29.184	1.588.963	-24.249	1.544.209	-22.328
1.551.722	-29.187	1.589.093	-24.252	1.544.341	-22.329
1.551.917	-29.189	1.589.259	-24.254	1.544.525	-22.330
1.552.142	-29.192	1.589.454	-24.256	1.544.673	-22.331
1.552.341	-29.194	1.589.626	-24.259	1.544.818	-22.332
1.552.558	-29.196	1.589.783	-24.261	1.544.984	-22.333
1.552.800	-29.199	1.589.922	-24.263	1.545.191	-22.334
1.552.944	-29.201	1.590.065	-24.265	1.545.410	-22.335
1.553.004	-29.203	1.590.222	-24.267	1.545.553	-22.336
1.553.194	-29.206	1.590.336	-24.270	1.545.674	-22.337
1.553.409	-29.208	1.590.471	-24.272	1.545.806	-22.338
1.553.553	-29.211	1.590.661	-24.274	1.545.959	-22.339
1.553.716	-29.213	1.590.887	-24.276	1.546.160	-22.340

1.553.851	-29.215	1.591.055	-24.278	1.546.369	-22.341
1.553.949	-29.218	1.591.199	-24.281	1.546.510	-22.342
1.554.072	-29.220	1.591.357	-24.283	1.546.640	-22.343
1.554.209	-29.223	1.591.476	-24.285	1.546.794	-22.344
1.554.371	-29.225	1.591.602	-24.287	1.546.889	-22.345
1.554.539	-29.227	1.591.716	-24.289	1.547.059	-22.346
1.554.742	-29.230	1.591.884	-24.291	1.547.301	-22.347
1.554.944	-29.232	1.592.153	-24.294	1.547.444	-22.348
1.555.130	-29.235	1.592.589	-24.296	1.547.614	-22.350
1.555.215	-29.237	1.592.984	-24.298	1.547.773	-22.351
1.555.376	-29.239	1.593.197	-24.300	1.547.899	-22.352
1.555.625	-29.242	1.593.275	-24.302	1.547.970	-22.353
1.555.773	-29.244	1.593.309	-24.304	1.548.112	-22.354
1.555.970	-29.247	1.593.356	-24.306	1.548.315	-22.355
1.556.174	-29.249	1.593.414	-24.308	1.548.470	-22.356
1.556.290	-29.251	1.593.456	-24.311	1.548.640	-22.357
1.556.407	-29.254	1.593.508	-24.313	1.548.748	-22.358
1.556.548	-29.256	1.593.678	-24.315	1.548.936	-22.359
1.556.741	-29.258	1.593.815	-24.317	1.549.138	-22.360
1.556.924	-29.261	1.593.913	-24.319	1.549.216	-22.361
1.557.092	-29.263	1.594.124	-24.321	1.549.375	-22.362
1.557.271	-29.266	1.594.342	-24.323	1.549.583	-22.363
1.557.408	-29.268	1.594.541	-24.325	1.549.724	-22.364
1.557.571	-29.270	1.594.778	-24.327	1.549.955	-22.365
1.557.766	-29.272	1.594.962	-24.329	1.550.210	-22.366
1.557.935	-29.275	1.595.161	-24.331	1.550.401	-22.367
1.558.073	-29.277	1.595.383	-24.333	1.550.558	-22.368
1.558.219	-29.279	1.595.470	-24.335	1.550.692	-22.369
1.558.356	-29.282	1.595.542	-24.337	1.550.793	-22.370
1.558.548	-29.284	1.595.714	-24.340	1.550.952	-22.371

1.558.719	-29.286	1.595.889	-24.342	1.551.196	-22.372
1.558.882	-29.289	1.596.048	-24.344	1.551.331	-22.373
1.559.077	-29.291	1.596.185	-24.346	1.551.465	-22.374
1.559.223	-29.293	1.596.353	-24.348	1.551.647	-22.375
1.559.426	-29.295	1.596.521	-24.350	1.551.799	-22.376
1.559.603	-29.298	1.596.642	-24.352	1.551.929	-22.377
1.559.731	-29.300	1.596.743	-24.354	1.552.061	-22.378
1.559.801	-29.302	1.596.902	-24.356	1.552.218	-22.379
1.559.904	-29.304	1.597.137	-24.358	1.552.354	-22.380
1.560.152	-29.306	1.597.289	-24.360	1.552.502	-22.381
1.560.331	-29.309	1.597.475	-24.363	1.552.690	-22.382
1.560.426	-29.311	1.597.666	-24.365	1.552.903	-22.383
1.560.546	-29.313	1.597.831	-24.367	1.553.102	-22.384
1.560.824	-29.315	1.598.002	-24.369	1.553.275	-22.385
1.561.093	-29.317	1.598.183	-24.371	1.553.436	-22.386
1.561.198	-29.319	1.598.347	-24.373	1.553.602	-22.386
1.561.308	-29.321	1.598.452	-24.375	1.553.772	-22.387
1.561.461	-29.324	1.598.602	-24.378	1.553.907	-22.388
1.561.637	-29.326	1.598.815	-24.380	1.554.037	-22.389
1.561.817	-29.328	1.598.956	-24.382	1.554.209	-22.390
1.561.891	-29.330	1.599.057	-24.384	1.554.388	-22.391
1.562.133	-29.332	1.599.209	-24.386	1.554.585	-22.392
1.562.601	-29.334	1.599.328	-24.389	1.554.745	-22.392
1.562.934	-29.336	1.599.453	-24.391	1.554.872	-22.393
1.563.033	-29.338	1.599.659	-24.393	1.555.067	-22.394
1.563.062	-29.340	1.599.868	-24.395	1.555.204	-22.395
1.563.208	-29.342	1.600.051	-24.398	1.555.278	-22.396
1.563.349	-29.344	1.600.237	-24.400	1.555.414	-22.396
1.563.363	-29.346	1.600.417	-24.402	1.555.582	-22.397
1.563.382	-29.348	1.600.527	-24.404	1.555.768	-22.398

1.563.510	-29.350	1.600.621	-24.407	1.555.954	-22.399
1.563.680	-29.352	1.600.759	-24.409	1.556.154	-22.400
1.563.842	-29.354	1.600.898	-24.411	1.556.326	-22.400
1.564.019	-29.356	1.601.055	-24.414	1.556.413	-22.401
1.564.178	-29.358	1.601.279	-24.416	1.556.572	-22.402
1.564.328	-29.360	1.601.472	-24.418	1.556.796	-22.403
1.564.487	-29.362	1.601.579	-24.421	1.556.922	-22.403
1.564.626	-29.364	1.601.763	-24.423	1.557.101	-22.404
1.564.834	-29.365	1.601.953	-24.426	1.557.303	-22.405
1.565.049	-29.367	1.602.103	-24.428	1.557.422	-22.405
1.565.244	-29.369	1.602.299	-24.431	1.557.545	-22.406
1.565.444	-29.371	1.602.489	-24.433	1.557.704	-22.407
1.565.596	-29.373	1.602.654	-24.435	1.557.892	-22.408
1.565.732	-29.375	1.602.804	-24.438	1.558.093	-22.408
1.565.815	-29.377	1.602.953	-24.440	1.558.302	-22.409
1.565.910	-29.379	1.603.111	-24.443	1.558.376	-22.410
1.566.116	-29.381	1.603.282	-24.445	1.558.544	-22.410
1.566.337	-29.382	1.603.450	-24.448	1.558.788	-22.411
1.566.523	-29.384	1.603.636	-24.450	1.558.914	-22.412
1.566.644	-29.386	1.603.777	-24.453	1.559.156	-22.412
1.566.721	-29.388	1.603.898	-24.455	1.559.541	-22.413
1.566.850	-29.390	1.604.068	-24.458	1.559.892	-22.414
1.567.018	-29.392	1.604.250	-24.461	1.560.060	-22.415
1.567.173	-29.394	1.604.447	-24.463	1.560.134	-22.415
1.567.312	-29.396	1.604.678	-24.466	1.560.228	-22.416
1.567.487	-29.397	1.604.890	-24.468	1.560.303	-22.417
1.567.717	-29.399	1.604.969	-24.471	1.560.253	-22.417
1.567.906	-29.401	1.605.051	-24.474	1.560.246	-22.418
1.568.044	-29.403	1.605.188	-24.476	1.560.421	-22.419
1.568.228	-29.405	1.605.313	-24.479	1.560.665	-22.419

1.568.428	-29.407	1.605.472	-24.481	1.560.831	-22.420
1.568.600	-29.409	1.605.649	-24.484	1.560.972	-22.421
1.568.737	-29.411	1.605.802	-24.487	1.561.131	-22.421
1.568.885	-29.412	1.605.938	-24.489	1.561.237	-22.422
1.569.043	-29.414	1.606.089	-24.492	1.561.377	-22.423
1.569.180	-29.416	1.606.237	-24.495	1.561.530	-22.423
1.569.312	-29.418	1.606.339	-24.497	1.561.718	-22.424
1.569.384	-29.420	1.606.433	-24.500	1.561.924	-22.425
1.569.520	-29.422	1.606.590	-24.503	1.562.119	-22.425
1.569.747	-29.424	1.606.881	-24.505	1.562.274	-22.426
1.569.912	-29.426	1.607.108	-24.508	1.562.424	-22.427
1.570.034	-29.428	1.607.314	-24.511	1.562.542	-22.427
1.570.217	-29.430	1.607.565	-24.514	1.562.728	-22.428
1.570.363	-29.432	1.607.753	-24.516	1.562.923	-22.429
1.570.508	-29.433	1.607.937	-24.519	1.563.037	-22.430
1.570.723	-29.435	1.608.065	-24.522	1.563.111	-22.430
1.570.927	-29.437	1.608.176	-24.524	1.563.262	-22.431
1.571.115	-29.439	1.608.336	-24.527	1.563.461	-22.432
1.571.228	-29.441	1.608.461	-24.530	1.563.613	-22.432
1.571.326	-29.443	1.608.625	-24.533	1.563.728	-22.433
1.571.458	-29.445	1.608.773	-24.535	1.563.846	-22.434
1.571.620	-29.447	1.608.902	-24.538	1.564.068	-22.435
1.571.821	-29.449	1.609.055	-24.541	1.564.281	-22.435
1.571.980	-29.451	1.609.144	-24.544	1.564.496	-22.436
1.572.110	-29.453	1.609.272	-24.546	1.564.722	-22.437
1.572.307	-29.456	1.609.449	-24.549	1.564.881	-22.438
1.572.560	-29.458	1.609.662	-24.552	1.564.991	-22.439
1.572.800	-29.460	1.609.816	-24.555	1.565.134	-22.439
1.572.915	-29.462	1.609.986	-24.557	1.565.293	-22.440
1.572.999	-29.464	1.610.220	-24.560	1.565.397	-22.441

1.573.161	-29.466	1.610.423	-24.563	1.565.506	-22.442
1.573.326	-29.468	1.610.600	-24.565	1.565.661	-22.443
1.573.468	-29.470	1.610.797	-24.568	1.565.871	-22.444
1.573.591	-29.472	1.611.008	-24.571	1.566.019	-22.445
1.573.728	-29.475	1.611.102	-24.574	1.566.165	-22.445
1.573.893	-29.477	1.611.216	-24.576	1.566.389	-22.446
1.574.050	-29.479	1.611.391	-24.579	1.566.602	-22.447
1.574.225	-29.481	1.611.575	-24.582	1.566.752	-22.448
1.574.418	-29.483	1.611.792	-24.585	1.566.850	-22.449
1.574.583	-29.485	1.611.991	-24.587	1.567.050	-22.450
1.574.738	-29.488	1.612.123	-24.590	1.567.289	-22.451
1.574.933	-29.490	1.612.256	-24.593	1.567.448	-22.452
1.575.089	-29.492	1.612.406	-24.595	1.567.659	-22.453
1.575.264	-29.494	1.612.570	-24.598	1.567.803	-22.454
1.575.437	-29.496	1.612.766	-24.601	1.567.858	-22.455
1.575.620	-29.499	1.612.926	-24.603	1.567.961	-22.456
1.575.777	-29.501	1.613.024	-24.606	1.568.096	-22.457
1.575.851	-29.503	1.613.205	-24.609	1.568.284	-22.458
1.575.981	-29.505	1.613.360	-24.612	1.568.493	-22.459
1.576.102	-29.508	1.613.488	-24.614	1.568.669	-22.460
1.576.230	-29.510	1.613.676	-24.617	1.568.808	-22.461
1.576.342	-29.512	1.613.840	-24.620	1.568.996	-22.462
1.576.483	-29.514	1.614.001	-24.622	1.569.164	-22.463
1.576.694	-29.517	1.614.216	-24.625	1.569.292	-22.464
1.576.821	-29.519	1.614.400	-24.627	1.569.427	-22.465
1.576.949	-29.521	1.614.500	-24.630	1.569.557	-22.467
1.577.204	-29.524	1.614.668	-24.633	1.569.729	-22.468
1.577.392	-29.526	1.614.818	-24.635	1.569.883	-22.469
1.577.569	-29.528	1.614.906	-24.638	1.570.013	-22.470
1.577.798	-29.531	1.615.072	-24.641	1.570.213	-22.471

1.577.966	-29.533	1.615.340	-24.643	1.570.356	-22.472
1.578.105	-29.535	1.615.486	-24.646	1.570.529	-22.473
1.578.329	-29.538	1.615.593	-24.648	1.570.775	-22.475
1.578.537	-29.540	1.615.717	-24.651	1.570.972	-22.476
1.578.653	-29.542	1.615.896	-24.654	1.571.127	-22.477
1.578.810	-29.545	1.616.107	-24.656	1.571.290	-22.478
1.578.967	-29.547	1.616.237	-24.659	1.571.467	-22.479
1.579.100	-29.549	1.616.402	-24.661	1.571.586	-22.481
1.579.229	-29.552	1.616.810	-24.664	1.571.694	-22.482
1.579.406	-29.554	1.617.242	-24.666	1.571.803	-22.483
1.579.545	-29.556	1.617.365	-24.669	1.571.936	-22.484
1.579.639	-29.559	1.617.404	-24.672	1.572.086	-22.486
1.579.798	-29.561	1.617.580	-24.674	1.572.153	-22.487
1.580.000	-29.563	1.617.708	-24.677	1.572.244	-22.488
1.580.145	-29.566	1.617.757	-24.679	1.572.402	-22.489
1.580.302	-29.568	1.617.805	-24.682	1.572.583	-22.490
1.580.450	-29.570	1.617.888	-24.684	1.572.775	-22.492
1.580.616	-29.573	1.618.056	-24.687	1.572.993	-22.493
1.580.822	-29.575	1.618.228	-24.689	1.573.185	-22.494
1.581.026	-29.577	1.618.331	-24.692	1.573.358	-22.495
1.581.208	-29.580	1.618.441	-24.695	1.573.604	-22.497
1.581.369	-29.582	1.618.571	-24.697	1.573.862	-22.498
1.581.528	-29.584	1.618.775	-24.700	1.574.072	-22.499
1.581.675	-29.587	1.619.052	-24.702	1.574.191	-22.500
1.581.848	-29.589	1.619.231	-24.705	1.574.350	-22.502
1.582.066	-29.591	1.619.408	-24.707	1.574.520	-22.503
1.582.238	-29.594	1.619.612	-24.710	1.574.637	-22.504
1.582.381	-29.596	1.619.742	-24.712	1.574.776	-22.505
1.582.522	-29.598	1.619.877	-24.715	1.574.962	-22.507
1.582.697	-29.601	1.619.996	-24.717	1.575.081	-22.508

1.582.879	-29.603	1.620.132	-24.720	1.575.157	-22.509
1.583.006	-29.605	1.620.309	-24.722	1.575.294	-22.510
1.583.154	-29.608	1.620.473	-24.725	1.575.462	-22.512
1.583.289	-29.610	1.620.582	-24.728	1.575.587	-22.513
1.583.443	-29.612	1.620.717	-24.730	1.575.759	-22.514
1.583.676	-29.615	1.620.860	-24.733	1.575.907	-22.515
1.583.875	-29.617	1.621.059	-24.735	1.576.104	-22.516
1.584.048	-29.619	1.621.241	-24.738	1.576.357	-22.518
1.584.175	-29.622	1.621.384	-24.740	1.576.519	-22.519
1.584.303	-29.624	1.621.579	-24.743	1.576.727	-22.520
1.584.478	-29.626	1.621.783	-24.746	1.576.926	-22.521
1.584.731	-29.629	1.621.958	-24.748	1.577.094	-22.522
1.584.883	-29.631	1.622.119	-24.751	1.577.224	-22.524
1.585.024	-29.633	1.622.312	-24.753	1.577.365	-22.525
1.585.238	-29.635	1.622.471	-24.756	1.577.493	-22.526
1.585.356	-29.638	1.622.641	-24.759	1.577.618	-22.527
1.585.486	-29.640	1.622.796	-24.761	1.577.829	-22.528
1.585.614	-29.642	1.622.975	-24.764	1.577.961	-22.529
1.585.755	-29.645	1.623.158	-24.766	1.578.123	-22.531
1.585.907	-29.647	1.623.286	-24.769	1.578.318	-22.532
1.586.120	-29.649	1.623.389	-24.772	1.578.459	-22.533
1.586.344	-29.651	1.623.557	-24.774	1.578.667	-22.534
1.586.499	-29.654	1.623.736	-24.777	1.578.848	-22.535
1.586.703	-29.656	1.623.842	-24.780	1.578.988	-22.536
1.586.859	-29.658	1.624.001	-24.782	1.579.146	-22.537
1.586.944	-29.661	1.624.194	-24.785	1.579.312	-22.539
1.587.054	-29.663	1.624.343	-24.788	1.579.482	-22.540
1.587.188	-29.665	1.624.409	-24.790	1.579.612	-22.541
1.587.336	-29.667	1.624.550	-24.793	1.579.740	-22.542
1.587.576	-29.670	1.624.823	-24.796	1.579.902	-22.543

1.587.971	-29.672	1.625.034	-24.798	1.580.083	-22.544
1.588.389	-29.674	1.625.152	-24.801	1.580.269	-22.545
1.588.618	-29.676	1.625.275	-24.804	1.580.479	-22.546
1.588.636	-29.679	1.625.407	-24.807	1.580.681	-22.547
1.588.685	-29.681	1.625.518	-24.809	1.580.802	-22.548
1.588.781	-29.683	1.625.645	-24.812	1.580.914	-22.549
1.588.808	-29.685	1.625.869	-24.815	1.581.122	-22.551
1.588.895	-29.688	1.626.125	-24.818	1.581.256	-22.552
1.588.992	-29.690	1.626.286	-24.821	1.581.368	-22.553
1.589.075	-29.692	1.626.458	-24.823	1.581.602	-22.554
1.589.254	-29.694	1.626.709	-24.826	1.581.790	-22.555
1.589.406	-29.697	1.626.890	-24.829	1.581.888	-22.556
1.589.534	-29.699	1.627.061	-24.832	1.582.047	-22.557
1.589.691	-29.701	1.627.233	-24.835	1.582.216	-22.558
1.589.870	-29.703	1.627.343	-24.837	1.582.408	-22.559
1.590.076	-29.705	1.627.489	-24.840	1.582.850	-22.560
1.590.273	-29.708	1.627.648	-24.843	1.583.235	-22.561
1.590.457	-29.710	1.627.843	-24.846	1.583.365	-22.562
1.590.630	-29.712	1.627.979	-24.849	1.583.461	-22.563
1.590.782	-29.714	1.628.089	-24.852	1.583.557	-22.564
1.590.943	-29.717	1.628.243	-24.855	1.583.593	-22.565
1.591.122	-29.719	1.628.409	-24.858	1.583.652	-22.566
1.591.297	-29.721	1.628.580	-24.860	1.583.763	-22.567
1.591.357	-29.723	1.628.750	-24.863	1.583.835	-22.568
1.591.467	-29.726	1.628.933	-24.866	1.583.949	-22.569
1.591.716	-29.728	1.629.095	-24.869	1.584.120	-22.569
1.591.924	-29.730	1.629.256	-24.872	1.584.285	-22.570
1.592.066	-29.732	1.629.424	-24.875	1.584.433	-22.571
1.592.213	-29.735	1.629.567	-24.878	1.584.603	-22.572
1.592.251	-29.737	1.629.693	-24.881	1.584.771	-22.573

1.592.343	-29.739	1.629.843	-24.884	1.584.971	-22.574
1.592.599	-29.741	1.629.931	-24.887	1.585.172	-22.575
1.592.813	-29.744	1.630.049	-24.890	1.585.350	-22.576
1.592.986	-29.746	1.630.188	-24.893	1.585.502	-22.577
1.593.172	-29.748	1.630.331	-24.896	1.585.658	-22.578
1.593.297	-29.751	1.630.475	-24.899	1.585.813	-22.579
1.593.434	-29.753	1.630.640	-24.901	1.585.995	-22.580
1.593.638	-29.755	1.630.822	-24.904	1.586.183	-22.581
1.593.810	-29.757	1.630.986	-24.907	1.586.322	-22.581
1.594.014	-29.760	1.631.196	-24.910	1.586.481	-22.582
1.594.252	-29.762	1.631.454	-24.913	1.586.602	-22.583
1.594.447	-29.764	1.631.662	-24.916	1.586.716	-22.584
1.594.527	-29.767	1.631.855	-24.919	1.586.868	-22.585
1.594.632	-29.769	1.632.041	-24.922	1.587.016	-22.586
1.594.805	-29.771	1.632.150	-24.925	1.587.148	-22.587
1.594.919	-29.774	1.632.254	-24.928	1.587.301	-22.588
1.595.052	-29.776	1.632.458	-24.931	1.587.468	-22.589
1.595.231	-29.779	1.632.641	-24.934	1.587.654	-22.590
1.595.432	-29.781	1.632.809	-24.937	1.587.854	-22.591
1.595.611	-29.783	1.632.970	-24.940	1.588.071	-22.592
1.595.753	-29.786	1.633.078	-24.943	1.588.268	-22.592
1.595.905	-29.788	1.633.224	-24.946	1.588.391	-22.593
1.596.091	-29.791	1.633.391	-24.949	1.588.550	-22.594
1.596.250	-29.793	1.633.522	-24.952	1.588.718	-22.595
1.596.371	-29.796	1.633.654	-24.955	1.588.849	-22.596
1.596.507	-29.798	1.633.804	-24.958	1.588.987	-22.597
1.596.693	-29.801	1.633.969	-24.961	1.589.135	-22.598
1.596.908	-29.803	1.634.167	-24.964	1.589.299	-22.599
1.597.032	-29.805	1.634.334	-24.967	1.589.516	-22.600
1.597.130	-29.808	1.634.563	-24.969	1.589.753	-22.601

1.597.305	-29.810	1.634.765	-24.972	1.589.910	-22.602
1.597.498	-29.813	1.634.903	-24.975	1.590.067	-22.603
1.597.720	-29.815	1.635.108	-24.978	1.590.217	-22.604
1.597.961	-29.818	1.635.325	-24.981	1.590.343	-22.605
1.598.123	-29.821	1.635.491	-24.984	1.590.459	-22.606
1.598.194	-29.823	1.635.592	-24.987	1.590.620	-22.607
1.598.365	-29.826	1.635.741	-24.990	1.590.813	-22.608
1.598.553	-29.828	1.635.876	-24.993	1.590.985	-22.609
1.598.627	-29.831	1.635.999	-24.995	1.591.147	-22.610
1.598.819	-29.833	1.636.214	-24.998	1.591.239	-22.611
1.599.025	-29.836	1.636.353	-25.001	1.591.400	-22.612
1.599.120	-29.838	1.636.503	-25.004	1.591.604	-22.613
1.599.229	-29.841	1.636.700	-25.007	1.591.758	-22.614
1.599.431	-29.844	1.636.888	-25.010	1.591.962	-22.615
1.599.622	-29.846	1.637.043	-25.013	1.592.170	-22.616
1.599.765	-29.849	1.637.159	-25.015	1.592.292	-22.617
1.599.845	-29.851	1.637.361	-25.018	1.592.404	-22.619
1.600.011	-29.854	1.637.578	-25.021	1.592.565	-22.620
1.600.289	-29.857	1.637.757	-25.024	1.592.706	-22.621
1.600.508	-29.859	1.637.915	-25.027	1.592.838	-22.622
1.600.663	-29.862	1.638.055	-25.029	1.593.044	-22.623
1.600.858	-29.864	1.638.201	-25.032	1.593.248	-22.624
1.601.044	-29.867	1.638.351	-25.035	1.593.391	-22.625
1.601.189	-29.869	1.638.490	-25.038	1.593.571	-22.626
1.601.337	-29.872	1.638.609	-25.041	1.593.748	-22.627
1.601.483	-29.875	1.638.777	-25.043	1.593.860	-22.629
1.601.613	-29.877	1.639.008	-25.046	1.594.021	-22.630
1.601.687	-29.880	1.639.185	-25.049	1.594.227	-22.631
1.601.772	-29.882	1.639.269	-25.052	1.594.330	-22.632
1.601.918	-29.885	1.639.444	-25.054	1.594.444	-22.633

1.602.061	-29.887	1.639.686	-25.057	1.594.657	-22.634
1.602.278	-29.890	1.639.894	-25.060	1.594.825	-22.635
1.602.433	-29.893	1.639.987	-25.063	1.594.987	-22.637
1.602.536	-29.895	1.640.103	-25.065	1.595.121	-22.638
1.602.704	-29.898	1.640.300	-25.068	1.595.219	-22.639
1.602.919	-29.900	1.640.401	-25.071	1.595.378	-22.640
1.603.179	-29.903	1.640.533	-25.073	1.595.531	-22.641
1.603.392	-29.905	1.640.862	-25.076	1.595.679	-22.642
1.603.562	-29.908	1.641.364	-25.079	1.595.840	-22.644
1.603.774	-29.910	1.641.702	-25.082	1.595.990	-22.645
1.603.983	-29.913	1.641.796	-25.084	1.596.129	-22.646
1.604.140	-29.915	1.641.839	-25.087	1.596.243	-22.647
1.604.265	-29.918	1.641.890	-25.090	1.596.382	-22.648
1.604.397	-29.920	1.641.942	-25.092	1.596.606	-22.649
1.604.594	-29.923	1.642.012	-25.095	1.596.830	-22.651
1.604.807	-29.925	1.642.108	-25.098	1.597.074	-22.652
1.604.926	-29.928	1.642.234	-25.100	1.597.318	-22.653
1.605.009	-29.930	1.642.377	-25.103	1.597.471	-22.654
1.605.101	-29.933	1.642.457	-25.106	1.597.598	-22.655
1.605.251	-29.935	1.642.608	-25.108	1.597.827	-22.656
1.605.416	-29.938	1.642.859	-25.111	1.598.044	-22.658
1.605.578	-29.940	1.643.062	-25.114	1.598.176	-22.659
1.605.766	-29.943	1.643.242	-25.117	1.598.380	-22.660
1.605.932	-29.945	1.643.387	-25.119	1.598.562	-22.661
1.606.131	-29.947	1.643.506	-25.122	1.598.593	-22.662
1.606.252	-29.950	1.643.703	-25.125	1.598.687	-22.663
1.606.346	-29.952	1.643.871	-25.127	1.598.867	-22.664
1.606.519	-29.954	1.643.990	-25.130	1.598.998	-22.666
1.606.749	-29.957	1.644.169	-25.133	1.599.146	-22.667
1.607.012	-29.959	1.644.353	-25.135	1.599.243	-22.668

1.607.222	-29.961	1.644.500	-25.138	1.599.375	-22.669
1.607.392	-29.964	1.644.650	-25.141	1.599.668	-22.670
1.607.529	-29.966	1.644.774	-25.143	1.599.906	-22.671
1.607.626	-29.968	1.644.881	-25.146	1.600.094	-22.672
1.607.713	-29.971	1.645.052	-25.149	1.600.253	-22.673
1.607.924	-29.973	1.645.262	-25.152	1.600.383	-22.674
1.608.150	-29.975	1.645.423	-25.154	1.600.520	-22.676
1.608.331	-29.977	1.645.529	-25.157	1.600.706	-22.677
1.608.465	-29.979	1.645.670	-25.160	1.600.898	-22.678
1.608.607	-29.982	1.645.891	-25.163	1.601.049	-22.679
1.608.799	-29.984	1.646.125	-25.165	1.601.171	-22.680
1.609.005	-29.986	1.646.243	-25.168	1.601.286	-22.681
1.609.160	-29.988	1.646.416	-25.171	1.601.413	-22.682
1.609.296	-29.990	1.646.653	-25.174	1.601.514	-22.683
1.609.482	-29.992	1.646.810	-25.177	1.601.689	-22.684
1.609.614	-29.994	1.647.014	-25.179	1.602.029	-22.685
1.609.801	-29.997	1.647.159	-25.182	1.602.222	-22.686
1.609.966	-29.999	1.647.256	-25.185	1.602.321	-22.687
1.610.154	-30.001	1.647.437	-25.188	1.602.486	-22.689
1.610.372	-30.003	1.647.547	-25.191	1.602.626	-22.690
1.610.560	-30.005	1.647.683	-25.194	1.602.845	-22.691
1.610.699	-30.007	1.647.906	-25.196	1.603.047	-22.692
1.610.797	-30.009	1.648.064	-25.199	1.603.138	-22.693
1.610.939	-30.011	1.648.221	-25.202	1.603.190	-22.694
1.611.073	-30.013	1.648.363	-25.205	1.603.329	-22.695
1.611.232	-30.015	1.648.544	-25.208	1.603.577	-22.696
1.611.458	-30.017	1.648.777	-25.211	1.603.773	-22.697
1.611.644	-30.018	1.648.902	-25.214	1.603.931	-22.698
1.611.770	-30.020	1.649.014	-25.217	1.604.102	-22.699
1.611.915	-30.022	1.649.144	-25.220	1.604.308	-22.700

1.612.038	-30.024	1.649.299	-25.223	1.604.493	-22.701
1.612.153	-30.026	1.649.527	-25.226	1.604.554	-22.702
1.612.350	-30.028	1.649.709	-25.229	1.604.758	-22.703
1.612.585	-30.030	1.649.857	-25.232	1.604.967	-22.704
1.612.755	-30.031	1.649.991	-25.235	1.605.094	-22.705
1.612.890	-30.033	1.650.087	-25.238	1.605.264	-22.706
1.613.013	-30.035	1.650.235	-25.241	1.605.397	-22.707
1.613.210	-30.037	1.650.439	-25.244	1.605.546	-22.708
1.613.580	-30.039	1.650.672	-25.247	1.605.733	-22.709
1.613.974	-30.040	1.650.898	-25.250	1.605.891	-22.710
1.614.167	-30.042	1.651.071	-25.253	1.606.299	-22.711
1.614.213	-30.044	1.651.172	-25.257	1.606.790	-22.712
1.614.265	-30.045	1.651.270	-25.260	1.606.980	-22.713
1.614.321	-30.047	1.651.433	-25.263	1.607.030	-22.714
1.614.406	-30.049	1.651.610	-25.266	1.607.016	-22.714
1.614.511	-30.050	1.651.803	-25.269	1.607.023	-22.715
1.614.579	-30.052	1.652.016	-25.272	1.607.117	-22.716
1.614.700	-30.054	1.652.179	-25.276	1.607.260	-22.717
1.614.818	-30.055	1.652.325	-25.279	1.607.386	-22.718
1.614.942	-30.057	1.652.421	-25.282	1.607.462	-22.719
1.615.132	-30.059	1.652.538	-25.285	1.607.558	-22.720
1.615.296	-30.060	1.652.760	-25.289	1.607.751	-22.721
1.615.499	-30.062	1.652.943	-25.292	1.607.941	-22.722
1.615.732	-30.063	1.653.071	-25.295	1.608.129	-22.723
1.615.876	-30.065	1.653.264	-25.298	1.608.289	-22.724
1.616.075	-30.067	1.653.450	-25.302	1.608.414	-22.725
1.616.277	-30.068	1.653.577	-25.305	1.608.602	-22.725
1.616.396	-30.070	1.653.705	-25.308	1.608.802	-22.726
1.616.536	-30.071	1.653.839	-25.311	1.609.003	-22.727
1.616.709	-30.073	1.654.039	-25.315	1.609.227	-22.728

1.616.895	-30.075	1.654.167	-25.318	1.609.408	-22.729
1.617.094	-30.076	1.654.230	-25.321	1.609.552	-22.730
1.617.224	-30.078	1.654.395	-25.325	1.609.671	-22.731
1.617.370	-30.079	1.654.554	-25.328	1.609.760	-22.732
1.617.522	-30.081	1.654.661	-25.331	1.609.922	-22.733
1.617.635	-30.082	1.654.841	-25.335	1.610.058	-22.734
1.617.757	-30.084	1.654.982	-25.338	1.610.193	-22.735
1.617.919	-30.086	1.655.154	-25.341	1.610.374	-22.735
1.618.055	-30.087	1.655.423	-25.345	1.610.542	-22.736
1.618.190	-30.089	1.655.591	-25.348	1.610.701	-22.737
1.618.439	-30.090	1.655.782	-25.351	1.610.835	-22.738
1.618.665	-30.092	1.656.012	-25.355	1.610.959	-22.739
1.618.848	-30.094	1.656.223	-25.358	1.611.120	-22.740
1.619.012	-30.095	1.656.389	-25.361	1.611.324	-22.741
1.619.175	-30.097	1.656.572	-25.365	1.611.577	-22.742
1.619.361	-30.098	1.656.732	-25.368	1.611.712	-22.743
1.619.554	-30.100	1.656.846	-25.371	1.611.812	-22.744
1.619.700	-30.102	1.657.021	-25.375	1.612.002	-22.745
1.619.767	-30.103	1.657.209	-25.378	1.612.251	-22.746
1.619.888	-30.105	1.657.303	-25.381	1.612.480	-22.747
1.620.036	-30.106	1.657.417	-25.385	1.612.639	-22.748
1.620.181	-30.108	1.657.529	-25.388	1.612.711	-22.749
1.620.323	-30.110	1.657.657	-25.391	1.612.798	-22.750
1.620.435	-30.111	1.657.849	-25.395	1.612.957	-22.751
1.620.638	-30.113	1.657.982	-25.398	1.613.138	-22.752
1.620.887	-30.115	1.658.131	-25.402	1.613.329	-22.753
1.621.075	-30.116	1.658.340	-25.405	1.613.497	-22.754
1.621.208	-30.118	1.658.503	-25.408	1.613.647	-22.755
1.621.333	-30.120	1.658.669	-25.412	1.613.804	-22.756
1.621.530	-30.121	1.658.891	-25.415	1.613.983	-22.757

1.621.752	-30.123	1.659.061	-25.418	1.614.144	-22.758
1.621.904	-30.125	1.659.267	-25.421	1.614.278	-22.759
1.621.992	-30.127	1.659.476	-25.425	1.614.444	-22.760
1.622.092	-30.128	1.659.599	-25.428	1.614.621	-22.761
1.622.222	-30.130	1.659.738	-25.431	1.614.790	-22.762
1.622.408	-30.132	1.659.908	-25.435	1.614.942	-22.763
1.622.574	-30.134	1.660.096	-25.438	1.615.118	-22.764
1.622.690	-30.136	1.660.210	-25.441	1.615.307	-22.765
1.622.769	-30.137	1.660.311	-25.445	1.615.421	-22.767
1.623.004	-30.139	1.660.515	-25.448	1.615.591	-22.768
1.623.364	-30.141	1.660.704	-25.451	1.615.766	-22.769
1.623.515	-30.143	1.660.802	-25.454	1.615.947	-22.770
1.623.627	-30.145	1.660.952	-25.458	1.616.093	-22.771
1.623.819	-30.147	1.661.200	-25.461	1.616.194	-22.772
1.623.967	-30.149	1.661.423	-25.464	1.616.409	-22.773
1.624.101	-30.151	1.661.579	-25.468	1.616.649	-22.774
1.624.272	-30.153	1.661.696	-25.471	1.616.796	-22.776
1.624.408	-30.154	1.661.841	-25.474	1.616.879	-22.777
1.624.543	-30.156	1.662.005	-25.477	1.617.054	-22.778
1.624.731	-30.158	1.662.182	-25.481	1.617.256	-22.779
1.624.995	-30.160	1.662.390	-25.484	1.617.394	-22.780
1.625.139	-30.162	1.662.545	-25.487	1.617.610	-22.781
1.625.210	-30.164	1.662.715	-25.490	1.617.805	-22.783
1.625.397	-30.166	1.662.856	-25.493	1.617.935	-22.784
1.625.551	-30.168	1.663.037	-25.497	1.618.031	-22.785
1.625.676	-30.171	1.663.223	-25.500	1.618.139	-22.786
1.625.858	-30.173	1.663.378	-25.503	1.618.329	-22.787
1.626.044	-30.175	1.663.535	-25.506	1.618.540	-22.788
1.626.248	-30.177	1.663.642	-25.509	1.618.725	-22.790
1.626.418	-30.179	1.663.826	-25.513	1.618.848	-22.791

1.626.510	-30.181	1.664.039	-25.516	1.618.947	-22.792
1.626.637	-30.183	1.664.202	-25.519	1.619.075	-22.793
1.626.796	-30.185	1.664.359	-25.522	1.619.175	-22.794
1.626.967	-30.187	1.664.529	-25.525	1.619.196	-22.795
1.627.160	-30.190	1.664.691	-25.528	1.619.388	-22.796
1.627.260	-30.192	1.664.810	-25.532	1.619.698	-22.798
1.627.307	-30.194	1.664.930	-25.535	1.619.865	-22.799
1.627.498	-30.196	1.665.341	-25.538	1.620.029	-22.800
1.627.693	-30.198	1.665.849	-25.541	1.620.182	-22.801
1.627.845	-30.201	1.666.066	-25.544	1.620.352	-22.802
1.628.042	-30.203	1.666.145	-25.547	1.620.632	-22.803
1.628.248	-30.205	1.666.228	-25.550	1.620.849	-22.804
1.628.438	-30.207	1.666.281	-25.554	1.621.017	-22.805
1.628.580	-30.210	1.666.324	-25.557	1.621.225	-22.806
1.628.763	-30.212	1.666.420	-25.560	1.621.424	-22.808
1.628.920	-30.214	1.666.496	-25.563	1.621.577	-22.809
1.629.133	-30.217	1.666.579	-25.566	1.621.745	-22.810
1.629.386	-30.219	1.666.684	-25.569	1.621.917	-22.811
1.629.574	-30.221	1.666.846	-25.572	1.622.025	-22.812
1.629.718	-30.223	1.667.059	-25.575	1.622.112	-22.813
1.629.855	-30.226	1.667.247	-25.579	1.622.226	-22.814
1.630.014	-30.228	1.667.412	-25.582	1.622.350	-22.815
1.630.173	-30.230	1.667.556	-25.585	1.622.538	-22.816
1.630.307	-30.233	1.667.657	-25.588	1.622.708	-22.817
1.630.450	-30.235	1.667.803	-25.591	1.622.874	-22.818
1.630.546	-30.238	1.668.027	-25.594	1.623.056	-22.819
1.630.650	-30.240	1.668.205	-25.597	1.623.163	-22.820
1.630.820	-30.242	1.668.355	-25.600	1.623.331	-22.821
1.630.923	-30.245	1.668.521	-25.603	1.623.540	-22.822
1.631.151	-30.247	1.668.680	-25.606	1.623.709	-22.823

1.631.395	-30.250	1.668.829	-25.610	1.623.851	-22.824
1.631.472	-30.252	1.669.016	-25.613	1.624.081	-22.825
1.631.570	-30.254	1.669.169	-25.616	1.624.306	-22.825
1.631.774	-30.257	1.669.250	-25.619	1.624.491	-22.826
1.631.942	-30.259	1.669.395	-25.622	1.624.653	-22.827
1.632.099	-30.262	1.669.594	-25.625	1.624.734	-22.828
1.632.343	-30.264	1.669.747	-25.628	1.624.955	-22.829
1.632.551	-30.266	1.669.890	-25.632	1.625.121	-22.830
1.632.771	-30.269	1.670.061	-25.635	1.625.184	-22.831
1.632.896	-30.271	1.670.275	-25.638	1.625.367	-22.832
1.633.053	-30.274	1.670.414	-25.641	1.625.569	-22.832
1.633.266	-30.276	1.670.558	-25.644	1.625.739	-22.833
1.633.363	-30.279	1.670.815	-25.647	1.625.903	-22.834
1.633.456	-30.281	1.671.082	-25.651	1.625.975	-22.835
1.633.692	-30.284	1.671.190	-25.654	1.626.120	-22.836
1.633.947	-30.286	1.671.288	-25.657	1.626.398	-22.836
1.634.095	-30.288	1.671.456	-25.660	1.626.570	-22.837
1.634.236	-30.291	1.671.637	-25.664	1.626.696	-22.838
1.634.399	-30.293	1.671.806	-25.667	1.626.857	-22.839
1.634.541	-30.296	1.671.980	-25.670	1.627.072	-22.839
1.634.680	-30.298	1.672.081	-25.673	1.627.211	-22.840
1.634.841	-30.301	1.672.238	-25.677	1.627.363	-22.841
1.634.975	-30.303	1.672.390	-25.680	1.627.562	-22.841
1.635.190	-30.306	1.672.551	-25.683	1.627.690	-22.842
1.635.394	-30.308	1.672.811	-25.687	1.627.836	-22.843
1.635.502	-30.311	1.672.952	-25.690	1.628.040	-22.843
1.635.677	-30.313	1.673.022	-25.693	1.628.194	-22.844
1.635.903	-30.316	1.673.176	-25.697	1.628.322	-22.845
1.636.053	-30.318	1.673.322	-25.700	1.628.493	-22.845
1.636.153	-30.320	1.673.490	-25.703	1.628.661	-22.846

1.636.288	-30.323	1.673.721	-25.707	1.628.918	-22.846
1.636.438	-30.325	1.673.884	-25.710	1.629.097	-22.847
1.636.566	-30.328	1.674.010	-25.714	1.629.148	-22.848
1.636.703	-30.330	1.674.153	-25.717	1.629.299	-22.848
1.636.888	-30.333	1.674.267	-25.721	1.629.612	-22.849
1.637.090	-30.335	1.674.431	-25.724	1.630.040	-22.849
1.637.290	-30.338	1.674.641	-25.727	1.630.378	-22.850
1.637.484	-30.340	1.674.803	-25.731	1.630.565	-22.850
1.637.655	-30.343	1.674.973	-25.734	1.630.611	-22.851
1.637.825	-30.345	1.675.170	-25.738	1.630.558	-22.851
1.637.997	-30.348	1.675.351	-25.742	1.630.623	-22.852
1.638.100	-30.350	1.675.500	-25.745	1.630.733	-22.852
1.638.194	-30.353	1.675.661	-25.749	1.630.849	-22.853
1.638.282	-30.355	1.675.826	-25.752	1.630.894	-22.853
1.638.447	-30.358	1.675.979	-25.756	1.631.001	-22.854
1.638.810	-30.360	1.676.113	-25.759	1.631.272	-22.854
1.639.274	-30.363	1.676.243	-25.763	1.631.398	-22.855
1.639.623	-30.365	1.676.436	-25.766	1.631.483	-22.855
1.639.740	-30.368	1.676.631	-25.770	1.631.656	-22.855
1.639.776	-30.370	1.676.835	-25.774	1.631.859	-22.856
1.639.852	-30.373	1.677.014	-25.777	1.632.049	-22.856
1.639.973	-30.375	1.677.112	-25.781	1.632.258	-22.857
1.640.045	-30.378	1.677.287	-25.785	1.632.477	-22.857
1.640.067	-30.380	1.677.471	-25.788	1.632.623	-22.857
1.640.219	-30.383	1.677.634	-25.792	1.632.769	-22.858
1.640.359	-30.385	1.677.766	-25.795	1.632.934	-22.858
1.640.392	-30.388	1.677.903	-25.799	1.633.062	-22.859
1.640.576	-30.390	1.678.098	-25.803	1.633.206	-22.859
1.640.793	-30.393	1.678.255	-25.806	1.633.362	-22.859
1.640.975	-30.395	1.678.351	-25.810	1.633.515	-22.860

1.641.105	-30.398	1.678.481	-25.814	1.633.622	-22.860
1.641.255	-30.400	1.678.669	-25.817	1.633.757	-22.860
1.641.490	-30.403	1.678.824	-25.821	1.633.956	-22.861
1.641.707	-30.405	1.678.965	-25.825	1.634.124	-22.861
1.641.853	-30.408	1.679.115	-25.828	1.634.245	-22.861
1.641.985	-30.410	1.679.303	-25.832	1.634.462	-22.862
1.642.159	-30.413	1.679.493	-25.836	1.634.684	-22.862
1.642.352	-30.415	1.679.632	-25.839	1.634.771	-22.862
1.642.518	-30.418	1.679.836	-25.843	1.634.937	-22.863
1.642.646	-30.420	1.680.080	-25.846	1.635.186	-22.863
1.642.747	-30.423	1.680.255	-25.850	1.635.323	-22.863
1.642.869	-30.426	1.680.459	-25.854	1.635.468	-22.864
1.643.073	-30.428	1.680.683	-25.857	1.635.663	-22.864
1.643.270	-30.431	1.680.864	-25.861	1.635.826	-22.864
1.643.474	-30.433	1.681.008	-25.865	1.635.988	-22.865
1.643.568	-30.436	1.681.152	-25.868	1.636.151	-22.865
1.643.651	-30.438	1.681.297	-25.872	1.636.328	-22.866
1.643.819	-30.441	1.681.449	-25.876	1.636.488	-22.866
1.643.996	-30.444	1.681.590	-25.879	1.636.633	-22.866
1.644.182	-30.446	1.681.734	-25.883	1.636.819	-22.867
1.644.335	-30.449	1.681.819	-25.886	1.636.978	-22.867
1.644.527	-30.451	1.681.888	-25.890	1.637.148	-22.867
1.644.731	-30.454	1.682.043	-25.894	1.637.290	-22.868
1.644.881	-30.457	1.682.222	-25.897	1.637.402	-22.868
1.645.036	-30.459	1.682.415	-25.901	1.637.554	-22.869
1.645.173	-30.462	1.682.571	-25.904	1.637.773	-22.869
1.645.347	-30.465	1.682.715	-25.908	1.637.966	-22.869
1.645.564	-30.467	1.682.924	-25.911	1.638.055	-22.870
1.645.728	-30.470	1.683.161	-25.915	1.638.201	-22.870
1.645.847	-30.473	1.683.320	-25.919	1.638.270	-22.871

1.645.965	-30.475	1.683.457	-25.922	1.638.423	-22.871
1.646.198	-30.478	1.683.649	-25.926	1.638.642	-22.872
1.646.422	-30.481	1.683.819	-25.929	1.638.896	-22.872
1.646.480	-30.483	1.683.983	-25.933	1.639.030	-22.872
1.646.584	-30.486	1.684.155	-25.936	1.639.109	-22.873
1.646.805	-30.489	1.684.337	-25.939	1.639.314	-22.873
1.646.991	-30.491	1.684.483	-25.943	1.639.527	-22.874
1.647.130	-30.494	1.684.657	-25.946	1.639.709	-22.874
1.647.274	-30.497	1.684.841	-25.950	1.639.843	-22.875
1.647.464	-30.500	1.685.027	-25.953	1.639.946	-22.875
1.647.636	-30.503	1.685.235	-25.957	1.640.108	-22.876
1.647.755	-30.505	1.685.390	-25.960	1.640.285	-22.876
1.647.968	-30.508	1.685.535	-25.963	1.640.416	-22.877
1.648.116	-30.511	1.685.658	-25.967	1.640.583	-22.877
1.648.210	-30.514	1.685.789	-25.970	1.640.793	-22.878
1.648.392	-30.517	1.685.965	-25.974	1.640.950	-22.878
1.648.571	-30.519	1.686.221	-25.977	1.641.109	-22.879
1.648.662	-30.522	1.686.409	-25.980	1.641.319	-22.880
1.648.801	-30.525	1.686.535	-25.984	1.641.492	-22.880
1.649.066	-30.528	1.686.723	-25.987	1.641.566	-22.881
1.649.343	-30.531	1.686.873	-25.990	1.641.713	-22.881
1.649.505	-30.534	1.687.005	-25.993	1.641.866	-22.882
1.649.599	-30.537	1.687.153	-25.997	1.642.014	-22.882
1.649.736	-30.539	1.687.332	-26.000	1.642.164	-22.883
1.649.939	-30.542	1.687.430	-26.003	1.642.307	-22.883
1.650.107	-30.545	1.687.574	-26.006	1.642.440	-22.884
1.650.188	-30.548	1.687.827	-26.010	1.642.598	-22.884
1.650.300	-30.551	1.688.049	-26.013	1.642.773	-22.885
1.650.506	-30.554	1.688.246	-26.016	1.642.896	-22.885
1.650.768	-30.557	1.688.411	-26.019	1.643.037	-22.886

1.650.938	-30.560	1.688.586	-26.022	1.643.288	-22.886
1.651.046	-30.563	1.688.725	-26.026	1.643.454	-22.887
1.651.127	-30.566	1.688.835	-26.029	1.643.580	-22.888
1.651.308	-30.569	1.688.980	-26.032	1.643.759	-22.888
1.651.510	-30.572	1.689.117	-26.035	1.643.956	-22.889
1.651.666	-30.575	1.689.294	-26.038	1.644.157	-22.889
1.651.877	-30.578	1.689.688	-26.041	1.644.330	-22.890
1.652.043	-30.581	1.690.130	-26.044	1.644.514	-22.890
1.652.180	-30.583	1.690.374	-26.047	1.644.713	-22.891
1.652.361	-30.586	1.690.452	-26.050	1.644.861	-22.891
1.652.505	-30.589	1.690.488	-26.053	1.645.036	-22.892
1.652.619	-30.592	1.690.549	-26.056	1.645.246	-22.892
1.652.728	-30.595	1.690.591	-26.059	1.645.372	-22.893
1.652.878	-30.598	1.690.670	-26.062	1.645.499	-22.893
1.653.100	-30.601	1.690.770	-26.065	1.645.643	-22.894
1.653.181	-30.604	1.690.853	-26.068	1.645.761	-22.894
1.653.252	-30.607	1.691.003	-26.071	1.645.878	-22.895
1.653.356	-30.610	1.691.165	-26.074	1.646.031	-22.895
1.653.517	-30.613	1.691.396	-26.077	1.646.140	-22.896
1.653.741	-30.616	1.691.595	-26.080	1.646.277	-22.896
1.653.956	-30.619	1.691.705	-26.083	1.646.512	-22.897
1.654.184	-30.622	1.691.922	-26.086	1.646.696	-22.897
1.654.442	-30.625	1.692.101	-26.089	1.646.853	-22.898
1.654.662	-30.628	1.692.285	-26.092	1.647.018	-22.898
1.654.818	-30.631	1.692.475	-26.094	1.647.206	-22.899
1.655.004	-30.633	1.692.581	-26.097	1.647.482	-22.899
1.655.193	-30.636	1.692.753	-26.100	1.647.659	-22.900
1.655.352	-30.639	1.692.919	-26.103	1.647.764	-22.900
1.655.542	-30.642	1.693.010	-26.106	1.647.941	-22.901
1.655.721	-30.645	1.693.181	-26.109	1.648.112	-22.901

1.655.838	-30.648	1.693.416	-26.112	1.648.268	-22.902
1.655.974	-30.651	1.693.589	-26.114	1.648.407	-22.902
1.656.127	-30.653	1.693.678	-26.117	1.648.542	-22.903
1.656.252	-30.656	1.693.839	-26.120	1.648.658	-22.903
1.656.358	-30.659	1.694.008	-26.123	1.648.848	-22.903
1.656.499	-30.662	1.694.110	-26.126	1.649.063	-22.904
1.656.673	-30.665	1.694.274	-26.129	1.649.211	-22.904
1.656.855	-30.667	1.694.435	-26.132	1.649.303	-22.905
1.657.020	-30.670	1.694.642	-26.134	1.649.437	-22.905
1.657.162	-30.673	1.694.834	-26.137	1.649.621	-22.906
1.657.294	-30.676	1.694.997	-26.140	1.649.805	-22.906
1.657.404	-30.678	1.695.231	-26.143	1.649.984	-22.907
1.657.529	-30.681	1.695.457	-26.146	1.650.208	-22.907
1.657.747	-30.684	1.695.618	-26.149	1.650.414	-22.908
1.657.986	-30.687	1.695.719	-26.151	1.650.542	-22.908
1.658.163	-30.689	1.695.864	-26.154	1.650.719	-22.909
1.658.360	-30.692	1.696.028	-26.157	1.650.887	-22.909
1.658.575	-30.695	1.696.230	-26.160	1.651.069	-22.909
1.658.703	-30.697	1.696.398	-26.163	1.651.279	-22.910
1.658.790	-30.700	1.696.489	-26.166	1.651.404	-22.910
1.658.954	-30.702	1.696.658	-26.169	1.651.494	-22.911
1.659.131	-30.705	1.696.837	-26.172	1.651.675	-22.911
1.659.323	-30.708	1.696.982	-26.174	1.651.889	-22.912
1.659.491	-30.710	1.697.101	-26.177	1.652.036	-22.912
1.659.632	-30.713	1.697.220	-26.180	1.652.159	-22.913
1.659.834	-30.715	1.697.385	-26.183	1.652.346	-22.913
1.659.993	-30.718	1.697.598	-26.186	1.652.504	-22.914
1.660.125	-30.720	1.697.834	-26.189	1.652.632	-22.914
1.660.266	-30.723	1.698.015	-26.192	1.652.773	-22.915
1.660.379	-30.725	1.698.147	-26.195	1.652.946	-22.915

1.660.457	-30.728	1.698.313	-26.198	1.653.353	-22.916
1.660.690	-30.730	1.698.448	-26.201	1.653.781	-22.916
1.660.883	-30.732	1.698.564	-26.204	1.653.965	-22.917
1.661.033	-30.735	1.698.671	-26.207	1.654.026	-22.917
1.661.236	-30.737	1.698.781	-26.209	1.654.133	-22.918
1.661.407	-30.739	1.699.007	-26.212	1.654.213	-22.918
1.661.600	-30.742	1.699.236	-26.215	1.654.258	-22.919
1.661.776	-30.744	1.699.381	-26.218	1.654.314	-22.919
1.661.877	-30.746	1.699.516	-26.221	1.654.370	-22.920
1.662.030	-30.749	1.699.731	-26.224	1.654.498	-22.921
1.662.189	-30.751	1.699.960	-26.227	1.654.635	-22.921
1.662.415	-30.753	1.700.116	-26.230	1.654.798	-22.922
1.662.674	-30.755	1.700.229	-26.233	1.654.967	-22.922
1.662.836	-30.758	1.700.334	-26.236	1.655.078	-22.923
1.662.995	-30.760	1.700.439	-26.239	1.655.246	-22.923
1.663.143	-30.762	1.700.620	-26.242	1.655.502	-22.924
1.663.288	-30.764	1.700.744	-26.245	1.655.752	-22.925
1.663.497	-30.766	1.700.966	-26.248	1.655.981	-22.925
1.663.656	-30.769	1.701.183	-26.251	1.656.095	-22.926
1.663.763	-30.771	1.701.333	-26.254	1.656.156	-22.926
1.663.819	-30.773	1.701.510	-26.258	1.656.324	-22.927
1.663.932	-30.775	1.701.644	-26.261	1.656.499	-22.928
1.664.102	-30.777	1.701.812	-26.264	1.656.640	-22.928
1.664.563	-30.779	1.701.991	-26.267	1.656.760	-22.929
1.665.071	-30.781	1.702.166	-26.270	1.656.917	-22.930
1.665.282	-30.783	1.702.318	-26.273	1.657.150	-22.931
1.665.331	-30.785	1.702.480	-26.276	1.657.294	-22.931
1.665.374	-30.787	1.702.632	-26.279	1.657.428	-22.932
1.665.424	-30.789	1.702.720	-26.282	1.657.608	-22.933
1.665.488	-30.791	1.702.841	-26.285	1.657.717	-22.934

1.665.555	-30.793	1.702.944	-26.288	1.657.906	-22.934
1.665.602	-30.795	1.703.109	-26.291	1.658.141	-22.935
1.665.719	-30.797	1.703.363	-26.294	1.658.331	-22.936
1.665.858	-30.799	1.703.553	-26.297	1.658.530	-22.937
1.666.001	-30.801	1.703.633	-26.300	1.658.635	-22.938
1.666.198	-30.803	1.703.745	-26.303	1.658.766	-22.938
1.666.357	-30.805	1.703.951	-26.306	1.658.987	-22.939
1.666.476	-30.807	1.704.211	-26.309	1.659.213	-22.940
1.666.633	-30.809	1.704.446	-26.312	1.659.325	-22.941
1.666.830	-30.810	1.704.632	-26.315	1.659.438	-22.942
1.667.030	-30.812	1.704.859	-26.318	1.659.614	-22.943
1.667.209	-30.814	1.705.063	-26.321	1.659.711	-22.944
1.667.448	-30.816	1.705.246	-26.324	1.659.839	-22.945
1.667.657	-30.818	1.705.394	-26.327	1.660.029	-22.946
1.667.726	-30.820	1.705.522	-26.330	1.660.186	-22.947
1.667.818	-30.822	1.705.622	-26.333	1.660.365	-22.948
1.667.975	-30.823	1.705.748	-26.336	1.660.553	-22.949
1.668.141	-30.825	1.705.889	-26.339	1.660.703	-22.950
1.668.302	-30.827	1.706.017	-26.342	1.660.896	-22.951
1.668.421	-30.829	1.706.196	-26.345	1.661.080	-22.952
1.668.530	-30.831	1.706.357	-26.348	1.661.208	-22.954
1.668.692	-30.833	1.706.463	-26.351	1.661.362	-22.955
1.668.808	-30.834	1.706.582	-26.354	1.661.463	-22.956
1.669.030	-30.836	1.706.758	-26.357	1.661.579	-22.957
1.669.285	-30.838	1.706.920	-26.360	1.661.803	-22.958
1.669.406	-30.840	1.707.095	-26.363	1.661.982	-22.960
1.669.606	-30.842	1.707.290	-26.365	1.662.099	-22.961
1.669.845	-30.843	1.707.455	-26.368	1.662.258	-22.962
1.670.058	-30.845	1.707.652	-26.371	1.662.468	-22.963
1.670.197	-30.847	1.707.881	-26.374	1.662.635	-22.965

1.670.300	-30.849	1.708.069	-26.377	1.662.834	-22.966
1.670.461	-30.851	1.708.250	-26.380	1.663.040	-22.967
1.670.683	-30.853	1.708.432	-26.382	1.663.187	-22.969
1.670.878	-30.855	1.708.560	-26.385	1.663.309	-22.970
1.670.990	-30.856	1.708.689	-26.388	1.663.394	-22.971
1.671.129	-30.858	1.708.880	-26.391	1.663.533	-22.973
1.671.326	-30.860	1.709.109	-26.394	1.663.779	-22.974
1.671.447	-30.862	1.709.263	-26.396	1.664.007	-22.976
1.671.608	-30.864	1.709.377	-26.399	1.664.142	-22.977
1.671.801	-30.866	1.709.545	-26.402	1.664.290	-22.978
1.671.893	-30.868	1.709.724	-26.404	1.664.424	-22.980
1.672.025	-30.870	1.709.895	-26.407	1.664.520	-22.981
1.672.195	-30.872	1.710.121	-26.410	1.664.677	-22.983
1.672.296	-30.874	1.710.325	-26.413	1.664.877	-22.984
1.672.430	-30.876	1.710.484	-26.415	1.665.058	-22.986
1.672.618	-30.878	1.710.620	-26.418	1.665.210	-22.987
1.672.813	-30.880	1.710.721	-26.420	1.665.284	-22.989
1.673.076	-30.882	1.710.880	-26.423	1.665.526	-22.990
1.673.298	-30.884	1.711.057	-26.426	1.665.768	-22.992
1.673.382	-30.886	1.711.214	-26.428	1.665.862	-22.993
1.673.425	-30.888	1.711.346	-26.431	1.665.997	-22.995
1.673.550	-30.890	1.711.465	-26.433	1.666.106	-22.996
1.673.728	-30.892	1.711.615	-26.436	1.666.216	-22.998
1.673.927	-30.894	1.711.866	-26.438	1.666.328	-23.000
1.674.129	-30.896	1.712.119	-26.441	1.666.476	-23.001
1.674.303	-30.898	1.712.272	-26.443	1.666.732	-23.003
1.674.458	-30.900	1.712.412	-26.446	1.666.917	-23.004
1.674.617	-30.903	1.712.570	-26.448	1.667.048	-23.006
1.674.774	-30.905	1.712.767	-26.450	1.667.262	-23.007
1.674.926	-30.907	1.712.939	-26.453	1.667.462	-23.009

1.675.154	-30.909	1.713.037	-26.455	1.667.717	-23.011
1.675.336	-30.912	1.713.206	-26.457	1.667.937	-23.012
1.675.493	-30.914	1.713.331	-26.460	1.668.049	-23.014
1.675.667	-30.916	1.713.418	-26.462	1.668.223	-23.015
1.675.777	-30.918	1.713.687	-26.464	1.668.407	-23.017
1.675.887	-30.921	1.714.131	-26.467	1.668.564	-23.019
1.676.064	-30.923	1.714.534	-26.469	1.668.727	-23.020
1.676.265	-30.925	1.714.697	-26.471	1.668.884	-23.022
1.676.413	-30.928	1.714.794	-26.473	1.668.990	-23.023
1.676.517	-30.930	1.714.875	-26.475	1.669.077	-23.025
1.676.707	-30.933	1.714.919	-26.478	1.669.261	-23.027
1.676.898	-30.935	1.714.966	-26.480	1.669.451	-23.028
1.677.066	-30.937	1.715.072	-26.482	1.669.557	-23.030
1.677.253	-30.940	1.715.211	-26.484	1.669.706	-23.031
1.677.415	-30.942	1.715.278	-26.486	1.669.868	-23.033
1.677.605	-30.945	1.715.387	-26.488	1.670.011	-23.034
1.677.800	-30.947	1.715.544	-26.490	1.670.181	-23.036
1.677.952	-30.950	1.715.712	-26.492	1.670.374	-23.038
1.678.006	-30.952	1.715.853	-26.494	1.670.578	-23.039
1.678.118	-30.955	1.716.030	-26.497	1.670.741	-23.041
1.678.306	-30.957	1.716.212	-26.499	1.670.907	-23.042
1.678.467	-30.960	1.716.398	-26.501	1.671.084	-23.044
1.678.618	-30.963	1.716.617	-26.502	1.671.261	-23.045
1.678.759	-30.965	1.716.747	-26.504	1.671.460	-23.047
1.678.902	-30.968	1.716.911	-26.506	1.671.615	-23.048
1.679.054	-30.970	1.717.083	-26.508	1.671.763	-23.050
1.679.196	-30.973	1.717.242	-26.510	1.671.967	-23.051
1.679.303	-30.976	1.717.462	-26.512	1.672.130	-23.053
1.679.534	-30.978	1.717.612	-26.514	1.672.251	-23.054
1.679.787	-30.981	1.717.755	-26.516	1.672.403	-23.056

1.679.980	-30.984	1.717.901	-26.518	1.672.556	-23.057
1.680.206	-30.986	1.718.065	-26.520	1.672.740	-23.059
1.680.365	-30.989	1.718.259	-26.522	1.672.905	-23.060
1.680.553	-30.992	1.718.349	-26.523	1.673.064	-23.062
1.680.755	-30.994	1.718.459	-26.525	1.673.286	-23.063
1.680.909	-30.997	1.718.671	-26.527	1.673.474	-23.065
1.681.048	-31.000	1.718.929	-26.529	1.673.611	-23.066
1.681.214	-31.003	1.719.122	-26.531	1.673.803	-23.068
1.681.386	-31.005	1.719.286	-26.532	1.673.974	-23.069
1.681.541	-31.008	1.719.487	-26.534	1.674.144	-23.070
1.681.669	-31.011	1.719.633	-26.536	1.674.332	-23.072
1.681.756	-31.014	1.719.751	-26.538	1.674.465	-23.073
1.681.911	-31.017	1.719.939	-26.539	1.674.610	-23.075
1.682.108	-31.019	1.720.123	-26.541	1.674.717	-23.076
1.682.276	-31.022	1.720.277	-26.543	1.674.809	-23.077
1.682.334	-31.025	1.720.376	-26.544	1.675.069	-23.079
1.682.406	-31.028	1.720.457	-26.546	1.675.325	-23.080
1.682.603	-31.031	1.720.640	-26.548	1.675.466	-23.081
1.682.825	-31.033	1.720.874	-26.550	1.675.631	-23.083
1.683.008	-31.036	1.721.019	-26.551	1.675.739	-23.084
1.683.185	-31.039	1.721.102	-26.553	1.675.876	-23.086
1.683.420	-31.042	1.721.277	-26.555	1.676.091	-23.087
1.683.635	-31.045	1.721.458	-26.556	1.676.277	-23.088
1.683.775	-31.048	1.721.624	-26.558	1.676.409	-23.089
1.683.914	-31.050	1.721.783	-26.559	1.676.700	-23.091
1.684.052	-31.053	1.721.933	-26.561	1.677.146	-23.092
1.684.205	-31.056	1.722.126	-26.563	1.677.424	-23.093
1.684.428	-31.059	1.722.305	-26.564	1.677.516	-23.095
1.684.648	-31.062	1.722.462	-26.566	1.677.525	-23.096
1.684.830	-31.065	1.722.592	-26.567	1.677.536	-23.097

1.684.991	-31.068	1.722.778	-26.569	1.677.630	-23.098
1.685.123	-31.071	1.722.939	-26.571	1.677.751	-23.100
1.685.244	-31.073	1.723.040	-26.572	1.677.809	-23.101
1.685.410	-31.076	1.723.201	-26.574	1.677.905	-23.102
1.685.566	-31.079	1.723.398	-26.575	1.678.089	-23.103
1.685.694	-31.082	1.723.624	-26.577	1.678.277	-23.105
1.685.948	-31.085	1.723.819	-26.579	1.678.427	-23.106
1.686.178	-31.088	1.723.958	-26.580	1.678.584	-23.107
1.686.330	-31.091	1.724.124	-26.582	1.678.786	-23.108
1.686.460	-31.094	1.724.245	-26.583	1.678.976	-23.110
1.686.604	-31.096	1.724.409	-26.585	1.679.157	-23.111
1.686.785	-31.099	1.724.655	-26.586	1.679.344	-23.112
1.686.902	-31.102	1.724.789	-26.588	1.679.492	-23.113
1.687.105	-31.105	1.724.980	-26.589	1.679.635	-23.114
1.687.341	-31.108	1.725.172	-26.591	1.679.751	-23.116
1.687.556	-31.111	1.725.294	-26.592	1.679.890	-23.117
1.687.679	-31.114	1.725.403	-26.594	1.680.025	-23.118
1.687.695	-31.117	1.725.555	-26.595	1.680.181	-23.119
1.687.823	-31.119	1.725.759	-26.597	1.680.455	-23.120
1.688.026	-31.122	1.725.934	-26.598	1.680.640	-23.122
1.688.192	-31.125	1.726.127	-26.599	1.680.737	-23.123
1.688.322	-31.128	1.726.288	-26.601	1.680.907	-23.124
1.688.484	-31.131	1.726.431	-26.602	1.681.109	-23.125
1.688.691	-31.134	1.726.669	-26.604	1.681.236	-23.126
1.688.898	-31.137	1.726.833	-26.605	1.681.355	-23.128
1.689.100	-31.139	1.726.928	-26.606	1.681.514	-23.129
1.689.202	-31.142	1.727.094	-26.608	1.681.721	-23.130
1.689.301	-31.145	1.727.186	-26.609	1.681.949	-23.131
1.689.476	-31.148	1.727.247	-26.611	1.682.178	-23.132
1.689.655	-31.151	1.727.386	-26.612	1.682.334	-23.134

1.689.997	-31.154	1.727.598	-26.613	1.682.469	-23.135
1.690.414	-31.156	1.727.744	-26.615	1.682.666	-23.136
1.690.676	-31.159	1.727.894	-26.616	1.682.798	-23.137
1.690.782	-31.162	1.728.107	-26.617	1.682.948	-23.139
1.690.882	-31.165	1.728.286	-26.618	1.683.176	-23.140
1.690.981	-31.168	1.728.452	-26.620	1.683.322	-23.141
1.691.023	-31.171	1.728.661	-26.621	1.683.394	-23.142
1.691.131	-31.173	1.728.943	-26.622	1.683.503	-23.143
1.691.201	-31.176	1.729.149	-26.624	1.683.654	-23.145
1.691.236	-31.179	1.729.348	-26.625	1.683.889	-23.146
1.691.328	-31.182	1.729.541	-26.626	1.684.066	-23.147
1.691.474	-31.185	1.729.655	-26.627	1.684.223	-23.148
1.691.682	-31.187	1.729.762	-26.628	1.684.399	-23.150
1.691.837	-31.190	1.729.899	-26.630	1.684.505	-23.151
1.691.976	-31.193	1.730.085	-26.631	1.684.659	-23.152
1.692.200	-31.196	1.730.226	-26.632	1.684.848	-23.153
1.692.412	-31.199	1.730.291	-26.633	1.685.049	-23.155
1.692.596	-31.201	1.730.389	-26.634	1.685.233	-23.156
1.692.829	-31.204	1.730.589	-26.635	1.685.323	-23.157
1.693.022	-31.207	1.730.804	-26.636	1.685.401	-23.159
1.693.145	-31.210	1.730.963	-26.638	1.685.571	-23.160
1.693.275	-31.212	1.731.115	-26.639	1.685.788	-23.161
1.693.429	-31.215	1.731.252	-26.640	1.685.970	-23.163
1.693.597	-31.218	1.731.384	-26.641	1.686.158	-23.164
1.693.770	-31.221	1.731.600	-26.642	1.686.364	-23.165
1.693.931	-31.224	1.731.799	-26.643	1.686.481	-23.167
1.694.057	-31.226	1.731.947	-26.644	1.686.620	-23.168
1.694.178	-31.229	1.732.077	-26.645	1.686.830	-23.169
1.694.346	-31.232	1.732.280	-26.646	1.687.057	-23.171
1.694.510	-31.235	1.732.542	-26.647	1.687.247	-23.172

1.694.659	-31.238	1.732.744	-26.648	1.687.318	-23.173
1.694.816	-31.240	1.732.885	-26.649	1.687.468	-23.175
1.695.018	-31.243	1.732.997	-26.650	1.687.654	-23.176
1.695.237	-31.246	1.733.183	-26.651	1.687.789	-23.177
1.695.387	-31.249	1.733.336	-26.652	1.687.887	-23.179
1.695.562	-31.252	1.733.474	-26.652	1.688.087	-23.180
1.695.771	-31.254	1.733.685	-26.653	1.688.313	-23.181
1.695.918	-31.257	1.733.858	-26.654	1.688.360	-23.183
1.696.086	-31.260	1.734.012	-26.655	1.688.519	-23.184
1.696.248	-31.263	1.734.218	-26.656	1.688.730	-23.186
1.696.422	-31.266	1.734.366	-26.657	1.688.909	-23.187
1.696.610	-31.269	1.734.474	-26.658	1.689.106	-23.188
1.696.700	-31.271	1.734.644	-26.659	1.689.202	-23.190
1.696.796	-31.274	1.734.792	-26.659	1.689.330	-23.191
1.696.945	-31.277	1.734.930	-26.660	1.689.518	-23.192
1.697.072	-31.280	1.735.094	-26.661	1.689.666	-23.194
1.697.245	-31.283	1.735.273	-26.662	1.689.814	-23.195
1.697.426	-31.286	1.735.479	-26.663	1.689.939	-23.197
1.697.565	-31.289	1.735.647	-26.663	1.690.056	-23.198
1.697.724	-31.292	1.735.734	-26.664	1.690.224	-23.199
1.697.925	-31.294	1.735.887	-26.665	1.690.481	-23.201
1.698.121	-31.297	1.736.040	-26.666	1.690.685	-23.202
1.698.268	-31.300	1.736.209	-26.666	1.690.743	-23.203
1.698.409	-31.303	1.736.436	-26.667	1.690.967	-23.205
1.698.517	-31.306	1.736.613	-26.668	1.691.270	-23.206
1.698.615	-31.309	1.736.796	-26.669	1.691.469	-23.207
1.698.875	-31.312	1.736.955	-26.669	1.691.588	-23.209
1.699.115	-31.315	1.737.121	-26.670	1.691.769	-23.210
1.699.180	-31.318	1.737.262	-26.671	1.691.964	-23.211
1.699.254	-31.321	1.737.383	-26.671	1.692.076	-23.213

1.699.353	-31.324	1.737.527	-26.672	1.692.265	-23.214
1.699.637	-31.327	1.737.722	-26.673	1.692.383	-23.215
1.699.984	-31.330	1.737.838	-26.673	1.692.505	-23.217
1.700.121	-31.333	1.738.026	-26.674	1.692.645	-23.218
1.700.190	-31.336	1.738.398	-26.675	1.692.757	-23.219
1.700.368	-31.339	1.738.864	-26.675	1.692.899	-23.220
1.700.531	-31.342	1.739.019	-26.676	1.693.100	-23.222
1.700.721	-31.345	1.739.052	-26.677	1.693.284	-23.223
1.700.963	-31.348	1.739.129	-26.677	1.693.387	-23.224
1.701.116	-31.351	1.739.216	-26.678	1.693.523	-23.226
1.701.288	-31.354	1.739.272	-26.679	1.693.768	-23.227
1.701.458	-31.357	1.739.359	-26.679	1.693.990	-23.228
1.701.624	-31.360	1.739.473	-26.680	1.694.135	-23.229
1.701.734	-31.363	1.739.545	-26.680	1.694.310	-23.231
1.701.870	-31.366	1.739.605	-26.681	1.694.512	-23.232
1.702.018	-31.369	1.739.816	-26.682	1.694.677	-23.233
1.702.157	-31.372	1.740.118	-26.682	1.694.845	-23.234
1.702.292	-31.375	1.740.284	-26.683	1.695.009	-23.235
1.702.462	-31.378	1.740.401	-26.683	1.695.159	-23.237
1.702.684	-31.381	1.740.578	-26.684	1.695.358	-23.238
1.702.888	-31.384	1.740.788	-26.685	1.695.504	-23.239
1.703.078	-31.387	1.740.972	-26.685	1.695.639	-23.240
1.703.217	-31.390	1.741.122	-26.686	1.695.757	-23.241
1.703.396	-31.393	1.741.270	-26.686	1.695.898	-23.242
1.703.571	-31.396	1.741.440	-26.687	1.696.019	-23.244
1.703.700	-31.399	1.741.617	-26.688	1.696.198	-23.245
1.703.853	-31.402	1.741.740	-26.688	1.696.536	-23.246
1.703.983	-31.405	1.741.875	-26.689	1.696.754	-23.247
1.704.092	-31.408	1.742.099	-26.689	1.696.877	-23.248
1.704.223	-31.411	1.742.283	-26.690	1.696.987	-23.249

1.704.370	-31.415	1.742.393	-26.691	1.697.177	-23.250
1.704.518	-31.418	1.742.518	-26.691	1.697.348	-23.252
1.704.624	-31.421	1.742.581	-26.692	1.697.480	-23.253
1.704.825	-31.424	1.742.780	-26.692	1.697.645	-23.254
1.705.049	-31.427	1.743.051	-26.693	1.697.791	-23.255
1.705.224	-31.430	1.743.214	-26.694	1.698.008	-23.256
1.705.361	-31.433	1.743.398	-26.694	1.698.212	-23.257
1.705.551	-31.436	1.743.571	-26.695	1.698.363	-23.258
1.705.797	-31.439	1.743.709	-26.696	1.698.530	-23.259
1.705.990	-31.442	1.743.889	-26.696	1.698.672	-23.260
1.706.178	-31.445	1.744.068	-26.697	1.698.795	-23.261
1.706.351	-31.448	1.744.164	-26.697	1.698.954	-23.262
1.706.488	-31.451	1.744.292	-26.698	1.699.146	-23.264
1.706.664	-31.454	1.744.498	-26.699	1.699.326	-23.265
1.706.821	-31.457	1.744.659	-26.699	1.699.438	-23.266
1.706.969	-31.460	1.744.792	-26.700	1.699.561	-23.267
1.707.170	-31.464	1.744.888	-26.701	1.699.738	-23.268
1.707.191	-31.467	1.745.002	-26.701	1.699.906	-23.269
1.707.269	-31.470	1.745.220	-26.702	1.700.208	-23.270
1.707.498	-31.473	1.745.459	-26.703	1.700.667	-23.271
1.707.616	-31.476	1.745.627	-26.703	1.700.948	-23.272
1.707.831	-31.479	1.745.726	-26.704	1.701.030	-23.273
1.708.092	-31.482	1.745.887	-26.704	1.701.079	-23.274
1.708.223	-31.485	1.746.078	-26.705	1.701.147	-23.275
1.708.317	-31.488	1.746.259	-26.706	1.701.216	-23.276
1.708.414	-31.491	1.746.460	-26.706	1.701.292	-23.277
1.708.588	-31.494	1.746.543	-26.707	1.701.438	-23.278
1.708.828	-31.497	1.746.665	-26.708	1.701.514	-23.279
1.709.053	-31.500	1.746.837	-26.708	1.701.617	-23.280
1.709.232	-31.503	1.746.953	-26.709	1.701.810	-23.281

1.709.384	-31.506	1.747.119	-26.710	1.702.000	-23.282
1.709.626	-31.509	1.747.345	-26.710	1.702.249	-23.283
1.709.834	-31.512	1.747.527	-26.711	1.702.439	-23.284
1.709.951	-31.515	1.747.726	-26.712	1.702.536	-23.285
1.710.040	-31.518	1.747.905	-26.712	1.702.668	-23.286
1.710.177	-31.521	1.748.114	-26.713	1.702.876	-23.287
1.710.396	-31.524	1.748.266	-26.714	1.703.060	-23.288
1.710.629	-31.527	1.748.418	-26.714	1.703.219	-23.289
1.710.851	-31.530	1.748.540	-26.715	1.703.380	-23.289
1.710.970	-31.533	1.748.736	-26.715	1.703.550	-23.290
1.711.059	-31.535	1.748.923	-26.716	1.703.736	-23.291
1.711.214	-31.538	1.749.037	-26.717	1.703.882	-23.292
1.711.413	-31.541	1.749.178	-26.717	1.704.014	-23.293
1.711.559	-31.544	1.749.382	-26.718	1.704.160	-23.294
1.711.707	-31.547	1.749.529	-26.718	1.704.323	-23.295
1.711.864	-31.550	1.749.671	-26.719	1.704.437	-23.296
1.712.034	-31.553	1.749.816	-26.720	1.704.592	-23.297
1.712.287	-31.556	1.750.033	-26.720	1.704.745	-23.298
1.712.399	-31.559	1.750.233	-26.721	1.704.928	-23.299
1.712.518	-31.562	1.750.387	-26.721	1.705.148	-23.300
1.712.701	-31.565	1.750.587	-26.722	1.705.309	-23.301
1.712.890	-31.568	1.750.755	-26.722	1.705.502	-23.302
1.713.006	-31.571	1.750.878	-26.723	1.705.683	-23.303
1.713.138	-31.574	1.751.017	-26.723	1.705.887	-23.304
1.713.322	-31.577	1.751.140	-26.723	1.706.040	-23.305
1.713.475	-31.580	1.751.281	-26.724	1.706.210	-23.307
1.713.605	-31.583	1.751.438	-26.724	1.706.420	-23.308
1.713.806	-31.586	1.751.553	-26.725	1.706.555	-23.309
1.713.956	-31.589	1.751.642	-26.725	1.706.694	-23.310
1.714.084	-31.592	1.751.805	-26.725	1.706.799	-23.311

1.714.227	-31.595	1.752.030	-26.725	1.706.931	-23.312
1.714.438	-31.598	1.752.193	-26.726	1.707.159	-23.313
1.714.657	-31.601	1.752.348	-26.726	1.707.332	-23.314
1.714.772	-31.604	1.752.509	-26.726	1.707.431	-23.315
1.714.776	-31.607	1.752.690	-26.726	1.707.561	-23.316
1.714.872	-31.610	1.752.874	-26.727	1.707.740	-23.317
1.715.127	-31.613	1.753.046	-26.727	1.707.915	-23.318
1.715.484	-31.616	1.753.268	-26.727	1.708.060	-23.320
1.715.961	-31.619	1.753.508	-26.727	1.708.228	-23.321
1.716.234	-31.622	1.753.721	-26.727	1.708.416	-23.322
1.716.415	-31.625	1.753.895	-26.727	1.708.611	-23.323
1.716.534	-31.628	1.754.077	-26.727	1.708.819	-23.324
1.716.608	-31.631	1.754.288	-26.727	1.708.969	-23.325
1.716.646	-31.634	1.754.397	-26.727	1.709.081	-23.327
1.716.684	-31.637	1.754.464	-26.726	1.709.249	-23.328
1.716.777	-31.640	1.754.579	-26.726	1.709.373	-23.329
1.716.871	-31.643	1.754.776	-26.726	1.709.527	-23.330
1.717.014	-31.646	1.754.953	-26.726	1.709.771	-23.331
1.717.180	-31.649	1.755.114	-26.726	1.709.933	-23.333
1.717.253	-31.652	1.755.228	-26.725	1.710.105	-23.334
1.717.359	-31.655	1.755.374	-26.725	1.710.247	-23.335
1.717.572	-31.659	1.755.602	-26.724	1.710.399	-23.336
1.717.755	-31.662	1.755.733	-26.724	1.710.587	-23.338
1.717.975	-31.665	1.755.858	-26.724	1.710.770	-23.339
1.718.179	-31.668	1.756.046	-26.723	1.710.909	-23.340
1.718.293	-31.671	1.756.275	-26.722	1.711.015	-23.342
1.718.457	-31.674	1.756.517	-26.722	1.711.156	-23.343
1.718.658	-31.678	1.756.713	-26.721	1.711.346	-23.344
1.718.777	-31.681	1.756.855	-26.720	1.711.452	-23.345
1.718.924	-31.684	1.757.030	-26.720	1.711.644	-23.347

1.719.041	-31.687	1.757.209	-26.719	1.711.944	-23.348
1.719.218	-31.690	1.757.354	-26.718	1.712.112	-23.349
1.719.380	-31.694	1.757.538	-26.717	1.712.240	-23.351
1.719.561	-31.697	1.757.706	-26.716	1.712.412	-23.352
1.719.729	-31.700	1.757.840	-26.715	1.712.551	-23.353
1.719.924	-31.704	1.757.973	-26.714	1.712.706	-23.355
1.720.114	-31.707	1.758.145	-26.713	1.712.876	-23.356
1.720.222	-31.710	1.758.360	-26.712	1.712.975	-23.357
1.720.313	-31.714	1.758.546	-26.711	1.713.118	-23.358
1.720.466	-31.717	1.758.741	-26.710	1.713.264	-23.360
1.720.593	-31.720	1.758.868	-26.709	1.713.400	-23.361
1.720.739	-31.724	1.758.978	-26.707	1.713.535	-23.362
1.720.914	-31.727	1.759.153	-26.706	1.713.647	-23.364
1.721.236	-31.731	1.759.305	-26.705	1.713.765	-23.365
1.721.456	-31.734	1.759.469	-26.703	1.713.940	-23.366
1.721.563	-31.738	1.759.635	-26.702	1.714.176	-23.368
1.721.664	-31.741	1.759.809	-26.700	1.714.431	-23.369
1.721.808	-31.745	1.760.011	-26.699	1.714.679	-23.370
1.722.003	-31.748	1.760.217	-26.697	1.714.879	-23.371
1.722.175	-31.752	1.760.383	-26.696	1.715.031	-23.373
1.722.289	-31.755	1.760.475	-26.694	1.715.177	-23.374
1.722.361	-31.759	1.760.573	-26.692	1.715.394	-23.375
1.722.450	-31.763	1.760.813	-26.691	1.715.555	-23.376
1.722.668	-31.766	1.761.010	-26.689	1.715.672	-23.378
1.722.870	-31.770	1.761.199	-26.687	1.715.869	-23.379
1.723.031	-31.774	1.761.329	-26.685	1.715.950	-23.380
1.723.257	-31.777	1.761.429	-26.683	1.716.057	-23.381
1.723.443	-31.781	1.761.618	-26.681	1.716.239	-23.383
1.723.618	-31.785	1.761.786	-26.679	1.716.366	-23.384
1.723.777	-31.788	1.761.967	-26.677	1.716.523	-23.385

1.723.952	-31.792	1.762.128	-26.675	1.716.703	-23.386
1.724.191	-31.796	1.762.247	-26.673	1.716.877	-23.388
1.724.350	-31.800	1.762.426	-26.670	1.717.036	-23.389
1.724.476	-31.804	1.762.843	-26.668	1.717.193	-23.390
1.724.646	-31.808	1.763.244	-26.666	1.717.359	-23.391
1.724.790	-31.811	1.763.465	-26.663	1.717.579	-23.392
1.724.955	-31.815	1.763.539	-26.661	1.717.773	-23.394
1.725.103	-31.819	1.763.624	-26.658	1.717.923	-23.395
1.725.251	-31.823	1.763.732	-26.656	1.718.143	-23.396
1.725.425	-31.827	1.763.768	-26.653	1.718.313	-23.397
1.725.591	-31.831	1.763.891	-26.651	1.718.439	-23.398
1.725.757	-31.835	1.763.918	-26.648	1.718.607	-23.399
1.725.956	-31.839	1.764.012	-26.646	1.718.723	-23.400
1.726.216	-31.843	1.764.213	-26.643	1.718.828	-23.401
1.726.402	-31.847	1.764.324	-26.640	1.719.005	-23.403
1.726.499	-31.851	1.764.464	-26.637	1.719.174	-23.404
1.726.655	-31.855	1.764.684	-26.635	1.719.422	-23.405
1.726.833	-31.859	1.764.908	-26.632	1.719.632	-23.406
1.726.940	-31.863	1.765.109	-26.629	1.719.794	-23.407
1.727.112	-31.867	1.765.295	-26.626	1.719.964	-23.408
1.727.271	-31.871	1.765.392	-26.623	1.720.083	-23.409
1.727.374	-31.875	1.765.522	-26.620	1.720.229	-23.410
1.727.556	-31.879	1.765.708	-26.617	1.720.435	-23.411
1.727.737	-31.883	1.765.882	-26.614	1.720.672	-23.412
1.727.905	-31.888	1.766.059	-26.611	1.720.858	-23.413
1.728.091	-31.892	1.766.252	-26.607	1.720.990	-23.414
1.728.241	-31.896	1.766.445	-26.604	1.721.122	-23.415
1.728.354	-31.900	1.766.608	-26.601	1.721.286	-23.416
1.728.557	-31.904	1.766.761	-26.598	1.721.467	-23.417
1.728.763	-31.908	1.766.906	-26.595	1.721.662	-23.418

1.728.918	-31.912	1.766.998	-26.591	1.721.790	-23.419
1.729.083	-31.916	1.767.079	-26.588	1.721.927	-23.420
1.729.174	-31.920	1.767.285	-26.585	1.722.096	-23.421
1.729.278	-31.925	1.767.482	-26.581	1.722.309	-23.421
1.729.436	-31.929	1.767.639	-26.578	1.722.502	-23.422
1.729.581	-31.933	1.767.803	-26.574	1.722.652	-23.423
1.729.738	-31.937	1.768.006	-26.571	1.722.816	-23.424
1.729.921	-31.941	1.768.237	-26.568	1.722.979	-23.425
1.730.089	-31.945	1.768.430	-26.564	1.723.154	-23.426
1.730.251	-31.949	1.768.618	-26.561	1.723.241	-23.427
1.730.385	-31.953	1.768.784	-26.557	1.723.360	-23.428
1.730.510	-31.957	1.768.914	-26.553	1.723.587	-23.428
1.730.627	-31.962	1.769.019	-26.550	1.723.989	-23.429
1.730.795	-31.966	1.769.184	-26.546	1.724.384	-23.430
1.731.059	-31.970	1.769.361	-26.543	1.724.588	-23.431
1.731.398	-31.974	1.769.516	-26.539	1.724.678	-23.432
1.731.664	-31.978	1.769.655	-26.535	1.724.751	-23.432
1.731.776	-31.982	1.769.791	-26.532	1.724.814	-23.433
1.731.965	-31.986	1.769.978	-26.528	1.724.859	-23.434
1.732.132	-31.990	1.770.154	-26.524	1.724.935	-23.435
1.732.262	-31.994	1.770.302	-26.520	1.725.038	-23.436
1.732.435	-31.998	1.770.464	-26.517	1.725.141	-23.436
1.732.560	-32.002	1.770.639	-26.513	1.725.284	-23.437
1.732.693	-32.006	1.770.721	-26.509	1.725.464	-23.438
1.732.832	-32.010	1.770.898	-26.505	1.725.607	-23.439
1.732.919	-32.014	1.771.120	-26.502	1.725.770	-23.439
1.733.049	-32.018	1.771.265	-26.498	1.725.965	-23.440
1.733.243	-32.021	1.771.433	-26.494	1.726.144	-23.441
1.733.432	-32.025	1.771.573	-26.490	1.726.335	-23.441
1.733.593	-32.029	1.771.758	-26.486	1.726.552	-23.442

1.733.689	-32.033	1.772.007	-26.482	1.726.727	-23.443
1.733.840	-32.037	1.772.202	-26.479	1.726.810	-23.444
1.734.090	-32.041	1.772.350	-26.475	1.726.906	-23.444
1.734.241	-32.044	1.772.458	-26.471	1.727.090	-23.445
1.734.395	-32.048	1.772.612	-26.467	1.727.330	-23.446
1.734.590	-32.052	1.772.850	-26.463	1.727.531	-23.446
1.734.754	-32.056	1.773.073	-26.459	1.727.650	-23.447
1.734.921	-32.059	1.773.195	-26.455	1.727.771	-23.448
1.735.159	-32.063	1.773.364	-26.451	1.727.887	-23.449
1.735.351	-32.067	1.773.537	-26.447	1.727.997	-23.449
1.735.428	-32.070	1.773.640	-26.443	1.728.167	-23.450
1.735.540	-32.074	1.773.725	-26.439	1.728.374	-23.451
1.735.746	-32.077	1.773.884	-26.435	1.728.582	-23.451
1.735.934	-32.081	1.774.157	-26.431	1.728.801	-23.452
1.736.044	-32.085	1.774.312	-26.427	1.729.014	-23.453
1.736.227	-32.088	1.774.438	-26.423	1.729.198	-23.454
1.736.436	-32.092	1.774.652	-26.419	1.729.290	-23.454
1.736.566	-32.095	1.774.816	-26.415	1.729.431	-23.455
1.736.760	-32.099	1.774.917	-26.411	1.729.666	-23.456
1.736.976	-32.102	1.775.045	-26.407	1.729.848	-23.457
1.737.130	-32.105	1.775.282	-26.403	1.729.984	-23.457
1.737.303	-32.109	1.775.484	-26.399	1.730.134	-23.458
1.737.419	-32.112	1.775.607	-26.395	1.730.320	-23.459
1.737.574	-32.116	1.775.804	-26.390	1.730.484	-23.460
1.737.737	-32.119	1.775.927	-26.386	1.730.627	-23.461
1.737.874	-32.122	1.776.039	-26.382	1.730.724	-23.461
1.738.071	-32.125	1.776.135	-26.378	1.730.864	-23.462
1.738.275	-32.129	1.776.208	-26.374	1.731.089	-23.463
1.738.515	-32.132	1.776.373	-26.369	1.731.263	-23.464
1.738.698	-32.135	1.776.588	-26.365	1.731.373	-23.465

1.738.783	-32.138	1.776.768	-26.361	1.731.526	-23.465
1.738.936	-32.141	1.776.908	-26.357	1.731.759	-23.466
1.739.139	-32.145	1.777.048	-26.352	1.731.904	-23.467
1.739.306	-32.148	1.777.216	-26.348	1.731.998	-23.468
1.739.469	-32.151	1.777.403	-26.344	1.732.152	-23.469
1.739.608	-32.154	1.777.645	-26.339	1.732.301	-23.470
1.739.790	-32.157	1.777.887	-26.335	1.732.397	-23.471
1.739.968	-32.160	1.778.058	-26.331	1.732.516	-23.472
1.740.119	-32.163	1.778.210	-26.326	1.732.704	-23.473
1.740.269	-32.166	1.778.405	-26.322	1.732.932	-23.474
1.740.403	-32.169	1.778.530	-26.317	1.733.163	-23.474
1.740.575	-32.172	1.778.663	-26.313	1.733.376	-23.475
1.740.784	-32.175	1.778.904	-26.308	1.733.508	-23.476
1.740.907	-32.177	1.779.090	-26.304	1.733.634	-23.477
1.740.972	-32.180	1.779.185	-26.300	1.733.828	-23.478
1.741.321	-32.183	1.779.267	-26.295	1.734.007	-23.479
1.741.801	-32.186	1.779.413	-26.290	1.734.209	-23.480
1.742.128	-32.189	1.779.612	-26.286	1.734.388	-23.482
1.742.220	-32.191	1.779.828	-26.281	1.734.487	-23.483
1.742.213	-32.194	1.779.971	-26.277	1.734.599	-23.484
1.742.318	-32.197	1.780.107	-26.272	1.734.805	-23.485
1.742.450	-32.199	1.780.300	-26.268	1.734.964	-23.486
1.742.540	-32.202	1.780.475	-26.263	1.735.121	-23.487
1.742.590	-32.205	1.780.654	-26.258	1.735.280	-23.488
1.742.704	-32.207	1.780.829	-26.254	1.735.470	-23.489
1.742.809	-32.210	1.781.026	-26.249	1.735.636	-23.490
1.742.899	-32.213	1.781.214	-26.244	1.735.836	-23.491
1.743.004	-32.215	1.781.400	-26.239	1.736.003	-23.493
1.743.167	-32.218	1.781.626	-26.235	1.736.118	-23.494
1.743.441	-32.220	1.781.781	-26.230	1.736.230	-23.495

1.743.645	-32.223	1.781.940	-26.225	1.736.387	-23.496
1.743.828	-32.225	1.782.076	-26.220	1.736.561	-23.497
1.744.046	-32.228	1.782.184	-26.215	1.736.702	-23.498
1.744.175	-32.230	1.782.345	-26.211	1.736.882	-23.500
1.744.292	-32.233	1.782.523	-26.206	1.737.005	-23.501
1.744.492	-32.235	1.782.708	-26.201	1.737.079	-23.502
1.744.673	-32.237	1.782.888	-26.196	1.737.236	-23.503
1.744.807	-32.240	1.783.078	-26.191	1.737.415	-23.505
1.744.922	-32.242	1.783.264	-26.186	1.737.645	-23.506
1.745.110	-32.245	1.783.420	-26.181	1.737.867	-23.507
1.745.271	-32.247	1.783.524	-26.176	1.738.022	-23.508
1.745.352	-32.249	1.783.667	-26.171	1.738.235	-23.510
1.745.515	-32.252	1.783.868	-26.166	1.738.483	-23.511
1.745.634	-32.254	1.784.091	-26.161	1.738.674	-23.512
1.745.851	-32.256	1.784.249	-26.156	1.738.831	-23.513
1.746.095	-32.259	1.784.400	-26.151	1.739.030	-23.515
1.746.237	-32.261	1.784.565	-26.146	1.739.193	-23.516
1.746.402	-32.264	1.784.726	-26.141	1.739.332	-23.517
1.746.595	-32.266	1.784.852	-26.136	1.739.440	-23.519
1.746.743	-32.268	1.785.016	-26.130	1.739.489	-23.520
1.746.884	-32.271	1.785.186	-26.125	1.739.664	-23.521
1.747.056	-32.273	1.785.342	-26.120	1.739.859	-23.523
1.747.234	-32.275	1.785.493	-26.115	1.740.000	-23.524
1.747.491	-32.278	1.785.676	-26.110	1.740.152	-23.525
1.747.701	-32.280	1.785.880	-26.104	1.740.282	-23.527
1.747.885	-32.282	1.786.049	-26.099	1.740.410	-23.528
1.748.053	-32.284	1.786.281	-26.094	1.740.600	-23.529
1.748.174	-32.287	1.786.358	-26.089	1.740.807	-23.531
1.748.271	-32.289	1.786.461	-26.083	1.741.021	-23.532
1.748.396	-32.291	1.786.669	-26.078	1.741.207	-23.533

1.748.513	-32.294	1.786.828	-26.073	1.741.329	-23.535
1.748.665	-32.296	1.787.198	-26.067	1.741.451	-23.536
1.748.907	-32.298	1.787.672	-26.062	1.741.575	-23.537
1.749.086	-32.301	1.787.972	-26.057	1.741.738	-23.539
1.749.205	-32.303	1.788.091	-26.051	1.741.976	-23.540
1.749.308	-32.306	1.788.107	-26.046	1.742.206	-23.541
1.749.482	-32.308	1.788.143	-26.040	1.742.278	-23.543
1.749.657	-32.310	1.788.201	-26.035	1.742.448	-23.544
1.749.780	-32.313	1.788.243	-26.030	1.742.659	-23.545
1.749.978	-32.315	1.788.324	-26.024	1.742.823	-23.547
1.750.192	-32.318	1.788.447	-26.019	1.743.053	-23.548
1.750.323	-32.320	1.788.667	-26.013	1.743.210	-23.549
1.750.471	-32.322	1.788.858	-26.008	1.743.354	-23.551
1.750.629	-32.325	1.788.974	-26.002	1.743.517	-23.552
1.750.770	-32.327	1.789.128	-25.997	1.743.642	-23.553
1.750.932	-32.330	1.789.346	-25.991	1.743.820	-23.555
1.751.048	-32.332	1.789.576	-25.986	1.744.068	-23.556
1.751.246	-32.335	1.789.782	-25.980	1.744.195	-23.557
1.751.537	-32.337	1.789.941	-25.975	1.744.370	-23.559
1.751.727	-32.340	1.790.051	-25.969	1.744.637	-23.560
1.751.862	-32.342	1.790.217	-25.964	1.744.809	-23.562
1.752.056	-32.345	1.790.396	-25.958	1.744.906	-23.563
1.752.233	-32.347	1.790.544	-25.953	1.745.022	-23.564
1.752.314	-32.350	1.790.656	-25.947	1.745.123	-23.566
1.752.393	-32.352	1.790.809	-25.942	1.745.277	-23.567
1.752.565	-32.355	1.790.938	-25.936	1.745.453	-23.568
1.752.751	-32.358	1.791.048	-25.931	1.745.621	-23.570
1.752.952	-32.360	1.791.227	-25.925	1.745.813	-23.571
1.753.060	-32.363	1.791.378	-25.920	1.746.017	-23.572
1.753.275	-32.365	1.791.499	-25.914	1.746.180	-23.574

1.753.383	-32.368	1.791.648	-25.909	1.746.326	-23.575
1.753.519	-32.371	1.791.882	-25.903	1.746.485	-23.577
1.753.741	-32.373	1.792.049	-25.898	1.746.651	-23.578
1.753.976	-32.376	1.792.200	-25.893	1.746.826	-23.579
1.754.093	-32.378	1.792.450	-25.887	1.746.922	-23.581
1.754.119	-32.381	1.792.686	-25.882	1.747.122	-23.582
1.754.268	-32.384	1.792.827	-25.876	1.747.612	-23.583
1.754.527	-32.386	1.792.959	-25.871	1.748.026	-23.585
1.754.733	-32.389	1.793.176	-25.866	1.748.129	-23.586
1.754.854	-32.392	1.793.342	-25.860	1.748.181	-23.588
1.754.926	-32.394	1.793.521	-25.855	1.748.232	-23.589
1.755.078	-32.397	1.793.653	-25.849	1.748.282	-23.590
1.755.271	-32.400	1.793.766	-25.844	1.748.398	-23.592
1.755.444	-32.403	1.793.931	-25.839	1.748.549	-23.593
1.755.627	-32.405	1.793.996	-25.834	1.748.638	-23.594
1.755.766	-32.408	1.794.142	-25.828	1.748.770	-23.596
1.755.811	-32.411	1.794.426	-25.823	1.748.860	-23.597
1.755.939	-32.413	1.794.632	-25.818	1.748.951	-23.599
1.756.100	-32.416	1.794.745	-25.812	1.749.104	-23.600
1.756.315	-32.419	1.794.910	-25.807	1.749.339	-23.601
1.756.600	-32.422	1.795.074	-25.802	1.749.543	-23.603
1.756.796	-32.424	1.795.197	-25.797	1.749.641	-23.604
1.756.890	-32.427	1.795.385	-25.792	1.749.852	-23.606
1.757.065	-32.430	1.795.611	-25.787	1.749.995	-23.607
1.757.356	-32.433	1.795.797	-25.781	1.750.134	-23.608
1.757.580	-32.436	1.795.925	-25.776	1.750.392	-23.610
1.757.726	-32.438	1.796.086	-25.771	1.750.578	-23.611
1.757.905	-32.441	1.796.270	-25.766	1.750.723	-23.613
1.758.000	-32.444	1.796.434	-25.761	1.750.844	-23.614
1.758.136	-32.447	1.796.557	-25.756	1.750.985	-23.616

1.758.391	-32.450	1.796.720	-25.751	1.751.153	-23.617
1.758.577	-32.453	1.796.899	-25.746	1.751.324	-23.619
1.758.669	-32.455	1.797.050	-25.741	1.751.490	-23.620
1.758.768	-32.458	1.797.209	-25.737	1.751.669	-23.622
1.758.900	-32.461	1.797.392	-25.732	1.751.815	-23.623
1.759.041	-32.464	1.797.567	-25.727	1.751.962	-23.625
1.759.079	-32.467	1.797.730	-25.722	1.752.168	-23.626
1.759.285	-32.469	1.797.889	-25.717	1.752.375	-23.628
1.759.487	-32.472	1.798.042	-25.712	1.752.520	-23.629
1.759.644	-32.475	1.798.264	-25.708	1.752.691	-23.631
1.759.902	-32.478	1.798.407	-25.703	1.752.950	-23.632
1.760.096	-32.481	1.798.485	-25.698	1.753.177	-23.634
1.760.266	-32.484	1.798.669	-25.694	1.753.313	-23.635
1.760.457	-32.487	1.798.871	-25.689	1.753.479	-23.637
1.760.659	-32.489	1.799.016	-25.684	1.753.613	-23.638
1.760.802	-32.492	1.799.160	-25.680	1.753.690	-23.640
1.760.948	-32.495	1.799.404	-25.675	1.753.790	-23.642
1.761.084	-32.498	1.799.572	-25.670	1.753.884	-23.643
1.761.270	-32.501	1.799.664	-25.666	1.754.053	-23.645
1.761.483	-32.504	1.799.834	-25.661	1.754.212	-23.647
1.761.624	-32.506	1.800.049	-25.657	1.754.370	-23.648
1.761.797	-32.509	1.800.213	-25.652	1.754.485	-23.650
1.762.032	-32.512	1.800.363	-25.648	1.754.673	-23.651
1.762.229	-32.515	1.800.495	-25.643	1.754.930	-23.653
1.762.374	-32.518	1.800.584	-25.639	1.755.101	-23.655
1.762.486	-32.521	1.800.685	-25.634	1.755.192	-23.656
1.762.578	-32.523	1.800.847	-25.630	1.755.345	-23.658
1.762.728	-32.526	1.800.961	-25.626	1.755.547	-23.660
1.762.912	-32.529	1.801.093	-25.621	1.755.773	-23.662
1.763.087	-32.532	1.801.303	-25.617	1.755.979	-23.663

1.763.232	-32.535	1.801.499	-25.612	1.756.079	-23.665
1.763.472	-32.538	1.801.618	-25.608	1.756.147	-23.667
1.763.727	-32.540	1.801.839	-25.604	1.756.304	-23.669
1.763.896	-32.543	1.802.150	-25.599	1.756.503	-23.670
1.764.075	-32.546	1.802.370	-25.595	1.756.689	-23.672
1.764.176	-32.549	1.802.560	-25.591	1.756.823	-23.674
1.764.292	-32.552	1.802.728	-25.587	1.756.976	-23.676
1.764.454	-32.554	1.802.881	-25.582	1.757.204	-23.678
1.764.617	-32.557	1.803.049	-25.578	1.757.428	-23.680
1.764.758	-32.560	1.803.181	-25.574	1.757.536	-23.681
1.764.897	-32.563	1.803.342	-25.570	1.757.554	-23.683
1.765.083	-32.565	1.803.484	-25.565	1.757.683	-23.685
1.765.282	-32.568	1.803.615	-25.561	1.757.888	-23.687
1.765.461	-32.571	1.803.774	-25.557	1.758.154	-23.689
1.765.634	-32.574	1.803.909	-25.553	1.758.365	-23.691
1.765.789	-32.576	1.804.066	-25.549	1.758.481	-23.693
1.765.907	-32.579	1.804.230	-25.545	1.758.674	-23.695
1.766.073	-32.582	1.804.404	-25.541	1.758.915	-23.697
1.766.210	-32.584	1.804.536	-25.536	1.759.070	-23.698
1.766.297	-32.587	1.804.702	-25.532	1.759.225	-23.700
1.766.440	-32.590	1.804.928	-25.528	1.759.361	-23.702
1.766.631	-32.593	1.805.096	-25.524	1.759.493	-23.704
1.766.933	-32.595	1.805.284	-25.520	1.759.646	-23.706
1.767.370	-32.598	1.805.481	-25.516	1.759.832	-23.708
1.767.719	-32.601	1.805.665	-25.512	1.760.062	-23.710
1.767.863	-32.603	1.805.871	-25.508	1.760.164	-23.712
1.767.941	-32.606	1.806.039	-25.504	1.760.193	-23.714
1.767.977	-32.609	1.806.198	-25.500	1.760.336	-23.716
1.767.997	-32.611	1.806.311	-25.496	1.760.542	-23.718
1.768.049	-32.614	1.806.451	-25.491	1.760.665	-23.720

1.768.201	-32.616	1.806.626	-25.487	1.760.797	-23.722
1.768.369	-32.619	1.806.842	-25.483	1.760.947	-23.724
1.768.445	-32.622	1.807.025	-25.479	1.761.091	-23.726
1.768.521	-32.624	1.807.150	-25.475	1.761.317	-23.728
1.768.680	-32.627	1.807.318	-25.471	1.761.534	-23.730
1.768.848	-32.630	1.807.406	-25.467	1.761.704	-23.732
1.769.059	-32.632	1.807.547	-25.463	1.761.904	-23.734
1.769.332	-32.635	1.807.769	-25.459	1.762.132	-23.736
1.769.552	-32.637	1.807.980	-25.455	1.762.314	-23.738
1.769.693	-32.640	1.808.178	-25.451	1.762.500	-23.740
1.769.800	-32.642	1.808.351	-25.447	1.762.695	-23.742
1.769.937	-32.645	1.808.519	-25.443	1.762.840	-23.744
1.770.184	-32.648	1.808.676	-25.439	1.762.954	-23.746
1.770.394	-32.650	1.808.868	-25.435	1.763.078	-23.748
1.770.542	-32.653	1.809.079	-25.432	1.763.224	-23.749
1.770.690	-32.655	1.809.191	-25.428	1.763.344	-23.751
1.770.782	-32.658	1.809.294	-25.424	1.763.513	-23.753
1.770.882	-32.661	1.809.491	-25.420	1.763.710	-23.755
1.770.999	-32.663	1.809.613	-25.416	1.763.885	-23.757
1.771.218	-32.666	1.809.792	-25.412	1.764.026	-23.759
1.771.424	-32.668	1.810.051	-25.408	1.764.133	-23.761
1.771.561	-32.671	1.810.201	-25.404	1.764.277	-23.763
1.771.696	-32.674	1.810.356	-25.400	1.764.480	-23.765
1.771.881	-32.676	1.810.499	-25.396	1.764.716	-23.767
1.772.076	-32.679	1.810.641	-25.392	1.764.937	-23.768
1.772.225	-32.681	1.810.838	-25.388	1.765.107	-23.770
1.772.368	-32.684	1.810.992	-25.384	1.765.237	-23.772
1.772.574	-32.687	1.811.091	-25.381	1.765.332	-23.774
1.772.827	-32.689	1.811.321	-25.377	1.765.502	-23.776
1.773.018	-32.692	1.811.781	-25.373	1.765.815	-23.777

1.773.172	-32.694	1.812.254	-25.369	1.765.999	-23.779
1.773.284	-32.697	1.812.460	-25.365	1.766.106	-23.781
1.773.405	-32.700	1.812.488	-25.361	1.766.292	-23.783
1.773.611	-32.702	1.812.504	-25.357	1.766.476	-23.784
1.773.817	-32.705	1.812.542	-25.354	1.766.667	-23.786
1.773.911	-32.708	1.812.607	-25.350	1.766.832	-23.788
1.774.017	-32.710	1.812.710	-25.346	1.766.953	-23.790
1.774.146	-32.713	1.812.834	-25.342	1.767.123	-23.791
1.774.294	-32.715	1.812.975	-25.338	1.767.280	-23.793
1.774.552	-32.718	1.813.134	-25.335	1.767.431	-23.795
1.774.765	-32.721	1.813.257	-25.331	1.767.598	-23.796
1.774.876	-32.723	1.813.349	-25.327	1.767.802	-23.798
1.774.926	-32.726	1.813.506	-25.323	1.768.031	-23.799
1.775.034	-32.729	1.813.709	-25.320	1.768.174	-23.801
1.775.226	-32.732	1.813.970	-25.316	1.768.266	-23.803
1.775.423	-32.734	1.814.156	-25.312	1.768.452	-23.804
1.775.582	-32.737	1.814.285	-25.308	1.768.682	-23.806
1.775.788	-32.740	1.814.473	-25.305	1.768.833	-23.807
1.776.051	-32.742	1.814.623	-25.301	1.768.909	-23.809
1.776.210	-32.745	1.814.773	-25.297	1.769.052	-23.810
1.776.257	-32.748	1.814.935	-25.294	1.769.312	-23.811
1.776.407	-32.751	1.815.047	-25.290	1.769.500	-23.813
1.776.490	-32.753	1.815.208	-25.286	1.769.644	-23.814
1.776.637	-32.756	1.815.408	-25.283	1.769.764	-23.816
1.776.886	-32.759	1.815.542	-25.279	1.769.885	-23.817
1.777.110	-32.762	1.815.681	-25.276	1.770.076	-23.818
1.777.357	-32.765	1.815.831	-25.272	1.770.295	-23.820
1.777.525	-32.767	1.815.983	-25.269	1.770.425	-23.821
1.777.654	-32.770	1.816.109	-25.265	1.770.510	-23.822
1.777.860	-32.773	1.816.272	-25.262	1.770.665	-23.824

1.778.062	-32.776	1.816.496	-25.258	1.771.017	-23.825
1.778.156	-32.779	1.816.707	-25.255	1.771.442	-23.826
1.778.290	-32.782	1.816.954	-25.251	1.771.729	-23.828
1.778.472	-32.785	1.817.135	-25.248	1.771.880	-23.829
1.778.665	-32.787	1.817.276	-25.245	1.771.904	-23.830
1.778.867	-32.790	1.817.413	-25.241	1.771.956	-23.831
1.779.023	-32.793	1.817.558	-25.238	1.772.043	-23.832
1.779.122	-32.796	1.817.758	-25.235	1.772.058	-23.834
1.779.258	-32.799	1.817.908	-25.231	1.772.099	-23.835
1.779.435	-32.802	1.818.064	-25.228	1.772.267	-23.836
1.779.673	-32.805	1.818.194	-25.225	1.772.514	-23.837
1.779.879	-32.808	1.818.362	-25.222	1.772.692	-23.838
1.780.025	-32.810	1.818.579	-25.219	1.772.836	-23.839
1.780.181	-32.813	1.818.754	-25.216	1.772.997	-23.840
1.780.271	-32.816	1.818.915	-25.213	1.773.143	-23.841
1.780.401	-32.819	1.819.054	-25.209	1.773.347	-23.842
1.780.557	-32.822	1.819.209	-25.206	1.773.568	-23.843
1.780.715	-32.825	1.819.350	-25.203	1.773.727	-23.844
1.780.896	-32.828	1.819.536	-25.201	1.773.907	-23.845
1.781.066	-32.831	1.819.722	-25.198	1.774.162	-23.846
1.781.198	-32.834	1.819.829	-25.195	1.774.361	-23.847
1.781.339	-32.837	1.819.969	-25.192	1.774.493	-23.848
1.781.478	-32.840	1.820.139	-25.189	1.774.585	-23.849
1.781.557	-32.843	1.820.284	-25.186	1.774.675	-23.850
1.781.729	-32.846	1.820.457	-25.183	1.774.846	-23.851
1.781.953	-32.849	1.820.647	-25.181	1.775.029	-23.852
1.782.132	-32.852	1.820.811	-25.178	1.775.163	-23.853
1.782.274	-32.855	1.821.010	-25.175	1.775.318	-23.854
1.782.473	-32.857	1.821.192	-25.173	1.775.459	-23.854
1.782.740	-32.860	1.821.369	-25.170	1.775.614	-23.855

1.782.959	-32.863	1.821.552	-25.167	1.775.820	-23.856
1.783.132	-32.866	1.821.732	-25.165	1.776.003	-23.857
1.783.335	-32.869	1.821.868	-25.162	1.776.189	-23.858
1.783.546	-32.872	1.821.949	-25.160	1.776.415	-23.859
1.783.671	-32.875	1.822.122	-25.158	1.776.608	-23.860
1.783.802	-32.878	1.822.339	-25.155	1.776.745	-23.860
1.783.965	-32.881	1.822.498	-25.153	1.776.895	-23.861
1.784.088	-32.884	1.822.609	-25.150	1.777.045	-23.862
1.784.184	-32.887	1.822.796	-25.148	1.777.197	-23.863
1.784.321	-32.890	1.822.984	-25.146	1.777.406	-23.864
1.784.586	-32.893	1.823.129	-25.144	1.777.567	-23.864
1.784.785	-32.896	1.823.248	-25.141	1.777.650	-23.865
1.784.861	-32.899	1.823.418	-25.139	1.777.773	-23.866
1.784.977	-32.902	1.823.651	-25.137	1.777.934	-23.867
1.785.192	-32.905	1.823.846	-25.135	1.778.121	-23.868
1.785.367	-32.908	1.824.016	-25.133	1.778.326	-23.868
1.785.470	-32.910	1.824.131	-25.131	1.778.479	-23.869
1.785.618	-32.913	1.824.251	-25.129	1.778.604	-23.870
1.785.836	-32.916	1.824.413	-25.127	1.778.748	-23.871
1.786.008	-32.919	1.824.574	-25.125	1.778.915	-23.871
1.786.203	-32.922	1.824.787	-25.123	1.779.068	-23.872
1.786.501	-32.925	1.824.919	-25.121	1.779.220	-23.873
1.786.682	-32.928	1.824.937	-25.119	1.779.426	-23.874
1.786.736	-32.931	1.825.119	-25.117	1.779.572	-23.874
1.786.895	-32.934	1.825.318	-25.115	1.779.679	-23.875
1.787.086	-32.936	1.825.425	-25.113	1.779.881	-23.876
1.787.198	-32.939	1.825.629	-25.111	1.780.087	-23.877
1.787.321	-32.942	1.825.795	-25.109	1.780.289	-23.878
1.787.462	-32.945	1.825.943	-25.108	1.780.423	-23.878
1.787.648	-32.948	1.826.171	-25.106	1.780.544	-23.879

1.787.860	-32.951	1.826.416	-25.104	1.780.737	-23.880
1.787.986	-32.954	1.826.646	-25.102	1.780.851	-23.881
1.788.056	-32.956	1.826.884	-25.101	1.781.023	-23.881
1.788.201	-32.959	1.827.083	-25.099	1.781.250	-23.882
1.788.441	-32.962	1.827.202	-25.097	1.781.409	-23.883
1.788.631	-32.965	1.827.365	-25.096	1.781.602	-23.884
1.788.820	-32.968	1.827.596	-25.094	1.781.743	-23.885
1.788.978	-32.970	1.827.731	-25.092	1.781.864	-23.885
1.789.184	-32.973	1.827.789	-25.091	1.782.020	-23.886
1.789.380	-32.976	1.827.915	-25.089	1.782.188	-23.887
1.789.540	-32.979	1.828.100	-25.087	1.782.393	-23.888
1.789.720	-32.982	1.828.243	-25.086	1.782.598	-23.889
1.789.865	-32.984	1.828.349	-25.084	1.782.776	-23.889
1.790.022	-32.987	1.828.503	-25.083	1.782.964	-23.890
1.790.177	-32.990	1.828.634	-25.081	1.783.178	-23.891
1.790.284	-32.993	1.828.799	-25.079	1.783.333	-23.892
1.790.417	-32.995	1.828.965	-25.078	1.783.441	-23.892
1.790.607	-32.998	1.829.135	-25.076	1.783.568	-23.893
1.790.799	-33.001	1.829.370	-25.075	1.783.663	-23.894
1.790.975	-33.004	1.829.543	-25.073	1.783.815	-23.895
1.791.152	-33.006	1.829.707	-25.072	1.784.035	-23.895
1.791.348	-33.009	1.829.892	-25.070	1.784.187	-23.896
1.791.487	-33.012	1.830.011	-25.069	1.784.285	-23.897
1.791.618	-33.015	1.830.184	-25.067	1.784.381	-23.898
1.791.783	-33.017	1.830.426	-25.066	1.784.469	-23.899
1.791.951	-33.020	1.830.639	-25.064	1.784.651	-23.899
1.792.032	-33.023	1.830.788	-25.063	1.784.874	-23.900
1.792.132	-33.025	1.830.907	-25.061	1.785.074	-23.901
1.792.374	-33.028	1.831.077	-25.060	1.785.293	-23.902
1.792.730	-33.031	1.831.283	-25.059	1.785.488	-23.902

1.793.131	-33.034	1.831.454	-25.057	1.785.701	-23.903
1.793.430	-33.036	1.831.579	-25.056	1.785.869	-23.904
1.793.609	-33.039	1.831.752	-25.054	1.786.003	-23.904
1.793.694	-33.042	1.831.935	-25.053	1.786.136	-23.905
1.793.748	-33.044	1.832.070	-25.051	1.786.351	-23.906
1.793.786	-33.047	1.832.224	-25.050	1.786.492	-23.907
1.793.842	-33.050	1.832.410	-25.049	1.786.642	-23.907
1.793.947	-33.052	1.832.587	-25.047	1.786.803	-23.908
1.794.057	-33.055	1.832.686	-25.046	1.786.938	-23.909
1.794.171	-33.058	1.832.865	-25.045	1.787.072	-23.909
1.794.368	-33.060	1.833.113	-25.043	1.787.184	-23.910
1.794.588	-33.063	1.833.275	-25.042	1.787.359	-23.911
1.794.729	-33.065	1.833.421	-25.040	1.787.558	-23.912
1.794.894	-33.068	1.833.559	-25.039	1.787.706	-23.912
1.795.058	-33.071	1.833.687	-25.038	1.787.827	-23.913
1.795.246	-33.073	1.833.830	-25.036	1.787.939	-23.914
1.795.443	-33.076	1.834.019	-25.035	1.788.100	-23.914
1.795.582	-33.079	1.834.243	-25.034	1.788.358	-23.915
1.795.799	-33.081	1.834.464	-25.033	1.788.564	-23.916
1.795.954	-33.084	1.834.639	-25.031	1.788.737	-23.916
1.796.048	-33.087	1.834.758	-25.030	1.788.967	-23.917
1.796.149	-33.089	1.834.876	-25.029	1.789.182	-23.917
1.796.324	-33.092	1.835.100	-25.027	1.789.366	-23.918
1.796.548	-33.094	1.835.309	-25.026	1.789.507	-23.919
1.796.685	-33.097	1.835.434	-25.025	1.789.633	-23.919
1.796.826	-33.100	1.835.556	-25.023	1.789.820	-23.920
1.796.962	-33.102	1.835.849	-25.022	1.790.034	-23.921
1.797.146	-33.105	1.836.310	-25.021	1.790.166	-23.921
1.797.312	-33.108	1.836.610	-25.020	1.790.286	-23.922
1.797.426	-33.110	1.836.727	-25.018	1.790.477	-23.922

1.797.565	-33.113	1.836.799	-25.017	1.790.625	-23.923
1.797.762	-33.116	1.836.844	-25.016	1.790.789	-23.923
1.797.980	-33.118	1.836.877	-25.015	1.790.988	-23.924
1.798.195	-33.121	1.836.919	-25.014	1.791.207	-23.925
1.798.383	-33.124	1.836.960	-25.012	1.791.422	-23.925
1.798.488	-33.126	1.837.061	-25.011	1.791.552	-23.926
1.798.573	-33.129	1.837.208	-25.010	1.791.682	-23.926
1.798.773	-33.132	1.837.395	-25.009	1.791.855	-23.927
1.799.016	-33.135	1.837.632	-25.008	1.792.036	-23.927
1.799.229	-33.137	1.837.863	-25.006	1.792.148	-23.928
1.799.400	-33.140	1.838.035	-25.005	1.792.267	-23.928
1.799.440	-33.143	1.838.161	-25.004	1.792.450	-23.929
1.799.545	-33.145	1.838.344	-25.003	1.792.643	-23.929
1.799.713	-33.148	1.838.553	-25.002	1.792.813	-23.930
1.799.870	-33.151	1.838.698	-25.001	1.792.923	-23.930
1.800.076	-33.154	1.838.895	-25.000	1.793.091	-23.931
1.800.318	-33.156	1.839.084	-24.999	1.793.293	-23.931
1.800.524	-33.159	1.839.198	-24.997	1.793.515	-23.932
1.800.648	-33.162	1.839.323	-24.996	1.793.692	-23.932
1.800.782	-33.165	1.839.493	-24.995	1.793.808	-23.933
1.800.920	-33.168	1.839.641	-24.994	1.793.947	-23.933
1.801.098	-33.170	1.839.782	-24.993	1.794.077	-23.934
1.801.303	-33.173	1.839.955	-24.992	1.794.230	-23.934
1.801.469	-33.176	1.840.108	-24.991	1.794.480	-23.935
1.801.680	-33.179	1.840.260	-24.990	1.794.883	-23.935
1.801.816	-33.182	1.840.354	-24.989	1.795.291	-23.936
1.801.908	-33.185	1.840.553	-24.988	1.795.502	-23.936
1.802.067	-33.188	1.840.809	-24.987	1.795.500	-23.936
1.802.227	-33.190	1.840.974	-24.986	1.795.531	-23.937
1.802.402	-33.193	1.841.111	-24.985	1.795.616	-23.937

1.802.534	-33.196	1.841.310	-24.985	1.795.659	-23.938
1.802.693	-33.199	1.841.528	-24.984	1.795.764	-23.938
1.802.874	-33.202	1.841.698	-24.983	1.795.909	-23.939
1.803.105	-33.205	1.841.861	-24.982	1.796.086	-23.939
1.803.295	-33.208	1.841.996	-24.981	1.796.232	-23.939
1.803.439	-33.211	1.842.162	-24.980	1.796.266	-23.940
1.803.633	-33.214	1.842.281	-24.979	1.796.395	-23.940
1.803.779	-33.217	1.842.426	-24.979	1.796.684	-23.940
1.803.938	-33.220	1.842.643	-24.978	1.796.890	-23.941
1.804.077	-33.223	1.842.834	-24.977	1.797.057	-23.941
1.804.229	-33.226	1.842.971	-24.976	1.797.289	-23.942
1.804.359	-33.229	1.843.114	-24.976	1.797.455	-23.942
1.804.520	-33.232	1.843.280	-24.975	1.797.579	-23.942
1.804.740	-33.235	1.843.434	-24.974	1.797.769	-23.943
1.804.942	-33.238	1.843.618	-24.974	1.797.968	-23.943
1.805.135	-33.241	1.843.754	-24.973	1.798.109	-23.943
1.805.313	-33.244	1.843.876	-24.973	1.798.214	-23.944
1.805.432	-33.246	1.844.070	-24.972	1.798.329	-23.944
1.805.565	-33.249	1.844.303	-24.971	1.798.508	-23.945
1.805.784	-33.252	1.844.467	-24.971	1.798.699	-23.945
1.805.898	-33.255	1.844.547	-24.970	1.798.828	-23.945
1.806.014	-33.258	1.844.671	-24.970	1.798.969	-23.946
1.806.190	-33.261	1.844.792	-24.969	1.799.160	-23.946
1.806.368	-33.264	1.844.919	-24.969	1.799.368	-23.947
1.806.507	-33.267	1.845.145	-24.969	1.799.576	-23.947
1.806.639	-33.270	1.845.390	-24.968	1.799.753	-23.947
1.806.808	-33.273	1.845.580	-24.968	1.799.908	-23.948
1.806.922	-33.276	1.845.741	-24.967	1.800.023	-23.948
1.807.054	-33.279	1.845.921	-24.967	1.800.137	-23.949
1.807.247	-33.282	1.846.122	-24.967	1.800.372	-23.949

1.807.395	-33.285	1.846.292	-24.966	1.800.596	-23.950
1.807.505	-33.288	1.846.429	-24.966	1.800.694	-23.950
1.807.643	-33.291	1.846.610	-24.966	1.800.811	-23.950
1.807.874	-33.294	1.846.787	-24.966	1.800.956	-23.951
1.808.183	-33.297	1.846.915	-24.965	1.801.136	-23.951
1.808.373	-33.299	1.847.052	-24.965	1.801.333	-23.952
1.808.501	-33.302	1.847.238	-24.965	1.801.505	-23.952
1.808.643	-33.305	1.847.444	-24.965	1.801.609	-23.953
1.808.846	-33.308	1.847.583	-24.965	1.801.772	-23.953
1.809.155	-33.311	1.847.746	-24.964	1.801.984	-23.954
1.809.424	-33.314	1.847.943	-24.964	1.802.146	-23.954
1.809.534	-33.317	1.848.132	-24.964	1.802.294	-23.955
1.809.619	-33.320	1.848.192	-24.964	1.802.457	-23.955
1.809.805	-33.322	1.848.322	-24.964	1.802.616	-23.956
1.809.911	-33.325	1.848.584	-24.964	1.802.778	-23.956
1.809.935	-33.328	1.848.752	-24.964	1.802.953	-23.957
1.810.067	-33.331	1.848.876	-24.963	1.803.076	-23.958
1.810.327	-33.334	1.849.043	-24.963	1.803.246	-23.958
1.810.565	-33.336	1.849.203	-24.963	1.803.448	-23.959
1.810.701	-33.339	1.849.363	-24.963	1.803.609	-23.959
1.810.781	-33.342	1.849.547	-24.963	1.803.781	-23.960
1.810.889	-33.345	1.849.632	-24.963	1.803.987	-23.960
1.811.030	-33.347	1.849.749	-24.963	1.804.120	-23.961
1.811.200	-33.350	1.849.935	-24.963	1.804.290	-23.962
1.811.422	-33.353	1.850.105	-24.963	1.804.521	-23.962
1.811.600	-33.355	1.850.307	-24.963	1.804.677	-23.963
1.811.790	-33.358	1.850.520	-24.963	1.804.834	-23.964
1.812.009	-33.361	1.850.748	-24.963	1.804.964	-23.964
1.812.152	-33.363	1.850.936	-24.962	1.805.083	-23.965
1.812.318	-33.366	1.851.089	-24.962	1.805.235	-23.966

1.812.500	-33.369	1.851.290	-24.962	1.805.432	-23.966
1.812.599	-33.371	1.851.503	-24.962	1.805.576	-23.967
1.812.730	-33.374	1.851.678	-24.962	1.805.694	-23.968
1.812.907	-33.376	1.851.832	-24.962	1.805.903	-23.968
1.813.114	-33.379	1.851.964	-24.962	1.806.151	-23.969
1.813.320	-33.381	1.852.090	-24.962	1.806.358	-23.970
1.813.513	-33.384	1.852.152	-24.962	1.806.552	-23.970
1.813.692	-33.386	1.852.298	-24.962	1.806.709	-23.971
1.813.857	-33.389	1.852.480	-24.961	1.806.785	-23.972
1.814.057	-33.391	1.852.585	-24.961	1.806.913	-23.972
1.814.225	-33.394	1.852.748	-24.961	1.807.065	-23.973
1.814.355	-33.396	1.852.903	-24.961	1.807.224	-23.974
1.814.523	-33.399	1.853.055	-24.961	1.807.386	-23.974
1.814.764	-33.401	1.853.243	-24.961	1.807.505	-23.975
1.814.868	-33.404	1.853.398	-24.960	1.807.666	-23.976
1.814.946	-33.406	1.853.559	-24.960	1.807.820	-23.977
1.815.182	-33.408	1.853.752	-24.960	1.807.930	-23.977
1.815.408	-33.411	1.853.925	-24.960	1.808.073	-23.978
1.815.553	-33.413	1.854.126	-24.959	1.808.241	-23.979
1.815.647	-33.415	1.854.332	-24.959	1.808.439	-23.979
1.815.781	-33.418	1.854.541	-24.959	1.808.596	-23.980
1.815.997	-33.420	1.854.740	-24.959	1.808.727	-23.981
1.816.216	-33.422	1.854.901	-24.958	1.808.941	-23.981
1.816.346	-33.425	1.855.038	-24.958	1.809.160	-23.982
1.816.445	-33.427	1.855.154	-24.958	1.809.406	-23.983
1.816.660	-33.429	1.855.320	-24.957	1.809.655	-23.983
1.816.877	-33.431	1.855.495	-24.957	1.809.810	-23.984
1.817.002	-33.434	1.855.705	-24.957	1.809.971	-23.985
1.817.164	-33.436	1.855.885	-24.956	1.810.092	-23.986
1.817.350	-33.438	1.856.001	-24.956	1.810.193	-23.986

1.817.558	-33.440	1.856.236	-24.955	1.810.345	-23.987
1.817.746	-33.442	1.856.405	-24.955	1.810.511	-23.988
1.817.908	-33.445	1.856.543	-24.954	1.810.658	-23.988
1.818.029	-33.447	1.856.734	-24.954	1.810.748	-23.989
1.818.154	-33.449	1.856.832	-24.953	1.810.905	-23.989
1.818.584	-33.451	1.856.971	-24.953	1.811.105	-23.990
1.819.063	-33.453	1.857.218	-24.952	1.811.246	-23.991
1.819.245	-33.455	1.857.419	-24.952	1.811.393	-23.991
1.819.276	-33.457	1.857.542	-24.951	1.811.586	-23.992
1.819.355	-33.460	1.857.731	-24.951	1.811.731	-23.993
1.819.445	-33.462	1.857.870	-24.950	1.811.870	-23.993
1.819.492	-33.464	1.857.982	-24.950	1.812.141	-23.994
1.819.547	-33.466	1.858.178	-24.949	1.812.410	-23.994
1.819.570	-33.468	1.858.396	-24.948	1.812.570	-23.995
1.819.682	-33.470	1.858.526	-24.948	1.812.719	-23.995
1.819.838	-33.472	1.858.662	-24.947	1.812.919	-23.996
1.820.018	-33.474	1.858.889	-24.946	1.813.087	-23.997
1.820.230	-33.476	1.859.097	-24.946	1.813.214	-23.997
1.820.397	-33.478	1.859.285	-24.945	1.813.376	-23.998
1.820.497	-33.480	1.859.438	-24.944	1.813.564	-23.998
1.820.710	-33.482	1.859.516	-24.944	1.813.714	-23.999
1.820.965	-33.484	1.859.563	-24.943	1.813.880	-23.999
1.821.095	-33.486	1.859.713	-24.942	1.814.075	-24.000
1.821.230	-33.488	1.860.062	-24.941	1.814.240	-24.000
1.821.431	-33.490	1.860.497	-24.941	1.814.368	-24.001
1.821.628	-33.492	1.860.869	-24.940	1.814.518	-24.001
1.821.788	-33.494	1.861.040	-24.939	1.814.691	-24.002
1.821.846	-33.496	1.861.095	-24.938	1.814.821	-24.002
1.821.953	-33.498	1.861.172	-24.937	1.815.018	-24.003
1.822.193	-33.500	1.861.176	-24.937	1.815.249	-24.003

1.822.379	-33.502	1.861.189	-24.936	1.815.421	-24.003
1.822.511	-33.504	1.861.317	-24.935	1.815.553	-24.004
1.822.677	-33.506	1.861.416	-24.934	1.815.699	-24.004
1.822.800	-33.508	1.861.478	-24.933	1.815.869	-24.005
1.822.869	-33.510	1.861.630	-24.932	1.816.040	-24.005
1.822.993	-33.512	1.861.810	-24.931	1.816.171	-24.005
1.823.204	-33.515	1.862.043	-24.930	1.816.331	-24.006
1.823.430	-33.517	1.862.294	-24.929	1.816.510	-24.006
1.823.611	-33.519	1.862.451	-24.928	1.816.680	-24.006
1.823.820	-33.521	1.862.614	-24.928	1.816.870	-24.007
1.824.019	-33.523	1.862.820	-24.927	1.817.020	-24.007
1.824.195	-33.525	1.863.020	-24.926	1.817.179	-24.008
1.824.395	-33.527	1.863.214	-24.925	1.817.388	-24.008
1.824.565	-33.529	1.863.372	-24.924	1.817.545	-24.008
1.824.727	-33.532	1.863.470	-24.923	1.817.621	-24.008
1.824.883	-33.534	1.863.542	-24.922	1.817.717	-24.009
1.825.056	-33.536	1.863.667	-24.921	1.817.910	-24.009
1.825.260	-33.538	1.863.882	-24.920	1.818.382	-24.009
1.825.356	-33.540	1.864.115	-24.919	1.818.858	-24.010
1.825.477	-33.543	1.864.301	-24.918	1.819.021	-24.010
1.825.708	-33.545	1.864.409	-24.917	1.819.061	-24.010
1.825.878	-33.547	1.864.507	-24.916	1.819.082	-24.010
1.825.990	-33.550	1.864.642	-24.915	1.819.142	-24.011
1.826.122	-33.552	1.864.825	-24.914	1.819.200	-24.011
1.826.263	-33.554	1.865.006	-24.913	1.819.287	-24.011
1.826.451	-33.557	1.865.217	-24.912	1.819.393	-24.011
1.826.584	-33.559	1.865.454	-24.911	1.819.498	-24.012
1.826.745	-33.561	1.865.663	-24.910	1.819.668	-24.012
1.826.947	-33.564	1.865.831	-24.909	1.819.821	-24.012
1.827.090	-33.566	1.865.968	-24.908	1.819.989	-24.012

1.827.269	-33.569	1.866.120	-24.907	1.820.161	-24.013
1.827.424	-33.571	1.866.290	-24.906	1.820.340	-24.013
1.827.592	-33.574	1.866.431	-24.905	1.820.564	-24.013
1.827.755	-33.576	1.866.544	-24.904	1.820.732	-24.013
1.827.885	-33.579	1.866.711	-24.903	1.820.918	-24.013
1.828.022	-33.581	1.866.913	-24.902	1.821.111	-24.014
1.828.214	-33.584	1.867.074	-24.902	1.821.277	-24.014
1.828.383	-33.587	1.867.182	-24.901	1.821.409	-24.014
1.828.557	-33.589	1.867.339	-24.900	1.821.564	-24.014
1.828.788	-33.592	1.867.547	-24.899	1.821.754	-24.014
1.828.980	-33.595	1.867.699	-24.898	1.821.937	-24.015
1.829.160	-33.597	1.867.878	-24.897	1.822.074	-24.015
1.829.339	-33.600	1.867.988	-24.896	1.822.188	-24.015
1.829.433	-33.603	1.868.102	-24.895	1.822.379	-24.015
1.829.509	-33.606	1.868.336	-24.895	1.822.547	-24.016
1.829.715	-33.609	1.868.517	-24.894	1.822.708	-24.016
1.829.986	-33.611	1.868.685	-24.893	1.822.883	-24.016
1.830.161	-33.614	1.868.839	-24.892	1.823.022	-24.016
1.830.244	-33.617	1.868.960	-24.892	1.823.212	-24.016
1.830.378	-33.620	1.869.075	-24.891	1.823.411	-24.017
1.830.499	-33.623	1.869.198	-24.890	1.823.555	-24.017
1.830.665	-33.626	1.869.435	-24.889	1.823.761	-24.017
1.830.889	-33.629	1.869.646	-24.889	1.824.005	-24.018
1.831.053	-33.632	1.869.789	-24.888	1.824.182	-24.018
1.831.238	-33.635	1.869.949	-24.887	1.824.279	-24.018
1.831.398	-33.638	1.870.074	-24.887	1.824.391	-24.018
1.831.521	-33.641	1.870.282	-24.886	1.824.545	-24.019
1.831.696	-33.644	1.870.527	-24.885	1.824.669	-24.019
1.831.915	-33.647	1.870.721	-24.885	1.824.843	-24.019
1.832.063	-33.650	1.870.883	-24.884	1.825.011	-24.020

1.832.195	-33.653	1.871.021	-24.884	1.825.089	-24.020
1.832.386	-33.656	1.871.169	-24.883	1.825.199	-24.020
1.832.475	-33.660	1.871.225	-24.883	1.825.398	-24.021
1.832.531	-33.663	1.871.317	-24.882	1.825.654	-24.021
1.832.742	-33.666	1.871.521	-24.882	1.825.858	-24.022
1.832.944	-33.669	1.871.770	-24.881	1.826.003	-24.022
1.833.053	-33.672	1.871.973	-24.881	1.826.144	-24.022
1.833.152	-33.676	1.872.186	-24.880	1.826.288	-24.023
1.833.371	-33.679	1.872.368	-24.880	1.826.447	-24.023
1.833.642	-33.682	1.872.516	-24.879	1.826.564	-24.024
1.833.817	-33.685	1.872.661	-24.879	1.826.716	-24.024
1.833.974	-33.689	1.872.838	-24.878	1.826.929	-24.025
1.834.122	-33.692	1.872.993	-24.878	1.827.050	-24.025
1.834.296	-33.695	1.873.181	-24.878	1.827.146	-24.026
1.834.531	-33.699	1.873.360	-24.877	1.827.350	-24.026
1.834.787	-33.702	1.873.474	-24.877	1.827.592	-24.027
1.834.953	-33.705	1.873.597	-24.876	1.827.793	-24.027
1.835.065	-33.709	1.873.712	-24.876	1.827.952	-24.028
1.835.266	-33.712	1.873.880	-24.876	1.828.100	-24.028
1.835.415	-33.715	1.874.048	-24.875	1.828.322	-24.029
1.835.500	-33.719	1.874.175	-24.875	1.828.524	-24.029
1.835.645	-33.722	1.874.355	-24.875	1.828.640	-24.030
1.835.797	-33.726	1.874.574	-24.875	1.828.831	-24.031
1.835.976	-33.729	1.874.778	-24.874	1.828.996	-24.031
1.836.131	-33.732	1.874.939	-24.874	1.829.117	-24.032
1.836.212	-33.736	1.875.105	-24.874	1.829.251	-24.033
1.836.331	-33.739	1.875.329	-24.874	1.829.451	-24.033
1.836.519	-33.743	1.875.551	-24.873	1.829.671	-24.034
1.836.711	-33.746	1.875.723	-24.873	1.829.827	-24.035
1.836.924	-33.750	1.875.844	-24.873	1.829.951	-24.035

1.837.159	-33.753	1.875.938	-24.873	1.830.094	-24.036
1.837.350	-33.757	1.876.100	-24.872	1.830.282	-24.037
1.837.455	-33.760	1.876.270	-24.872	1.830.450	-24.038
1.837.549	-33.764	1.876.429	-24.872	1.830.556	-24.038
1.837.755	-33.767	1.876.566	-24.872	1.830.696	-24.039
1.837.945	-33.771	1.876.696	-24.872	1.830.840	-24.040
1.838.176	-33.774	1.876.828	-24.872	1.830.965	-24.041
1.838.474	-33.778	1.877.001	-24.871	1.831.165	-24.041
1.838.544	-33.781	1.877.193	-24.871	1.831.324	-24.042
1.838.584	-33.785	1.877.316	-24.871	1.831.441	-24.043
1.838.718	-33.788	1.877.477	-24.871	1.831.579	-24.044
1.838.900	-33.792	1.877.650	-24.871	1.831.682	-24.045
1.839.115	-33.795	1.877.838	-24.871	1.831.810	-24.045
1.839.312	-33.799	1.878.069	-24.871	1.832.067	-24.046
1.839.514	-33.802	1.878.266	-24.870	1.832.316	-24.047
1.839.622	-33.806	1.878.391	-24.870	1.832.502	-24.048
1.839.769	-33.809	1.878.578	-24.870	1.832.728	-24.049
1.839.946	-33.813	1.878.817	-24.870	1.832.914	-24.050
1.840.110	-33.816	1.878.989	-24.870	1.833.069	-24.050
1.840.318	-33.820	1.879.108	-24.870	1.833.232	-24.051
1.840.475	-33.823	1.879.218	-24.870	1.833.447	-24.052
1.840.663	-33.827	1.879.436	-24.870	1.833.591	-24.053
1.840.847	-33.830	1.879.698	-24.870	1.833.690	-24.054
1.840.997	-33.834	1.879.839	-24.869	1.833.851	-24.055
1.841.182	-33.837	1.879.975	-24.869	1.833.976	-24.056
1.841.319	-33.841	1.880.154	-24.869	1.834.043	-24.057
1.841.449	-33.844	1.880.276	-24.869	1.834.148	-24.057
1.841.639	-33.848	1.880.414	-24.869	1.834.361	-24.058
1.841.812	-33.851	1.880.580	-24.869	1.834.547	-24.059
1.841.982	-33.855	1.880.753	-24.869	1.834.686	-24.060

1.842.101	-33.858	1.880.932	-24.869	1.834.843	-24.061
1.842.258	-33.861	1.881.115	-24.869	1.835.033	-24.062
1.842.471	-33.865	1.881.288	-24.869	1.835.282	-24.063
1.842.641	-33.868	1.881.488	-24.869	1.835.527	-24.064
1.842.824	-33.872	1.881.700	-24.869	1.835.771	-24.065
1.842.966	-33.875	1.881.834	-24.869	1.835.952	-24.066
1.843.169	-33.879	1.881.960	-24.869	1.836.060	-24.067
1.843.362	-33.882	1.882.126	-24.869	1.836.232	-24.067
1.843.477	-33.886	1.882.299	-24.869	1.836.434	-24.068
1.843.620	-33.889	1.882.462	-24.869	1.836.588	-24.069
1.843.777	-33.893	1.882.603	-24.869	1.836.765	-24.070
1.844.026	-33.896	1.882.822	-24.869	1.836.946	-24.071
1.844.418	-33.900	1.883.040	-24.868	1.837.063	-24.072
1.844.753	-33.903	1.883.165	-24.868	1.837.225	-24.073
1.844.816	-33.906	1.883.284	-24.868	1.837.453	-24.074
1.844.957	-33.910	1.883.468	-24.868	1.837.590	-24.075
1.845.154	-33.913	1.883.635	-24.868	1.837.654	-24.076
1.845.177	-33.917	1.883.745	-24.868	1.837.778	-24.077
1.845.096	-33.920	1.883.864	-24.868	1.837.966	-24.078
1.845.150	-33.923	1.884.005	-24.868	1.838.152	-24.079
1.845.313	-33.927	1.884.353	-24.868	1.838.338	-24.080
1.845.466	-33.930	1.884.828	-24.868	1.838.521	-24.080
1.845.676	-33.934	1.885.089	-24.868	1.838.661	-24.081
1.845.851	-33.937	1.885.183	-24.868	1.838.859	-24.082
1.845.939	-33.940	1.885.313	-24.868	1.839.057	-24.083
1.846.091	-33.944	1.885.416	-24.868	1.839.202	-24.084
1.846.348	-33.947	1.885.470	-24.868	1.839.384	-24.085
1.846.584	-33.950	1.885.535	-24.868	1.839.534	-24.086
1.846.760	-33.954	1.885.565	-24.868	1.839.632	-24.087
1.846.908	-33.957	1.885.636	-24.868	1.839.798	-24.088

1.847.099	-33.961	1.885.804	-24.868	1.839.946	-24.089
1.847.296	-33.964	1.886.001	-24.868	1.840.067	-24.090
1.847.401	-33.967	1.886.156	-24.868	1.840.262	-24.091
1.847.542	-33.971	1.886.259	-24.868	1.840.455	-24.092
1.847.735	-33.974	1.886.443	-24.868	1.840.607	-24.092
1.847.859	-33.977	1.886.651	-24.868	1.840.755	-24.093
1.847.961	-33.981	1.886.854	-24.869	1.840.914	-24.094
1.848.132	-33.984	1.886.971	-24.869	1.841.042	-24.095
1.848.313	-33.988	1.887.152	-24.869	1.841.189	-24.096
1.848.490	-33.991	1.887.388	-24.869	1.841.414	-24.097
1.848.669	-33.994	1.887.592	-24.869	1.841.844	-24.098
1.848.770	-33.998	1.887.773	-24.869	1.842.296	-24.099
1.848.954	-34.001	1.887.919	-24.869	1.842.468	-24.100
1.849.118	-34.005	1.888.015	-24.869	1.842.511	-24.101
1.849.319	-34.008	1.888.143	-24.869	1.842.556	-24.102
1.849.563	-34.011	1.888.313	-24.869	1.842.617	-24.103
1.849.758	-34.015	1.888.488	-24.869	1.842.690	-24.104
1.849.919	-34.018	1.888.611	-24.870	1.842.751	-24.105
1.850.089	-34.022	1.888.725	-24.870	1.842.879	-24.105
1.850.282	-34.025	1.888.918	-24.870	1.843.044	-24.106
1.850.452	-34.028	1.889.157	-24.870	1.843.190	-24.107
1.850.605	-34.032	1.889.357	-24.870	1.843.356	-24.108
1.850.735	-34.035	1.889.442	-24.870	1.843.577	-24.109
1.850.885	-34.039	1.889.583	-24.871	1.843.763	-24.110
1.851.048	-34.042	1.889.865	-24.871	1.843.940	-24.111
1.851.167	-34.046	1.890.114	-24.871	1.844.113	-24.112
1.851.288	-34.049	1.890.287	-24.871	1.844.274	-24.113
1.851.438	-34.053	1.890.399	-24.871	1.844.455	-24.114
1.851.626	-34.056	1.890.562	-24.872	1.844.637	-24.115
1.851.777	-34.060	1.890.719	-24.872	1.844.810	-24.116

1.851.906	-34.063	1.890.827	-24.872	1.844.919	-24.117
1.852.067	-34.067	1.891.017	-24.873	1.845.011	-24.118
1.852.311	-34.070	1.891.194	-24.873	1.845.213	-24.119
1.852.484	-34.074	1.891.270	-24.873	1.845.426	-24.120
1.852.659	-34.077	1.891.359	-24.873	1.845.553	-24.121
1.852.901	-34.081	1.891.543	-24.874	1.845.737	-24.122
1.853.019	-34.084	1.891.680	-24.874	1.845.905	-24.123
1.853.116	-34.088	1.891.868	-24.875	1.845.997	-24.124
1.853.217	-34.091	1.892.052	-24.875	1.846.144	-24.125
1.853.293	-34.095	1.892.166	-24.875	1.846.328	-24.126
1.853.474	-34.099	1.892.356	-24.876	1.846.449	-24.127
1.853.591	-34.102	1.892.533	-24.876	1.846.573	-24.128
1.853.792	-34.106	1.892.717	-24.877	1.846.823	-24.129
1.854.063	-34.109	1.892.948	-24.877	1.847.007	-24.130
1.854.312	-34.113	1.893.143	-24.878	1.847.135	-24.131
1.854.446	-34.117	1.893.251	-24.878	1.847.314	-24.132
1.854.597	-34.120	1.893.324	-24.879	1.847.480	-24.133
1.854.789	-34.124	1.893.493	-24.879	1.847.650	-24.134
1.854.977	-34.128	1.893.671	-24.880	1.847.854	-24.135
1.855.161	-34.131	1.893.873	-24.880	1.847.995	-24.136
1.855.367	-34.135	1.894.101	-24.881	1.848.109	-24.138
1.855.556	-34.139	1.894.272	-24.881	1.848.268	-24.139
1.855.708	-34.142	1.894.359	-24.882	1.848.389	-24.140
1.855.882	-34.146	1.894.532	-24.883	1.848.532	-24.141
1.856.080	-34.150	1.894.742	-24.883	1.848.750	-24.142
1.856.216	-34.153	1.894.821	-24.884	1.848.918	-24.143
1.856.317	-34.157	1.895.020	-24.884	1.849.061	-24.145
1.856.481	-34.161	1.895.266	-24.885	1.849.231	-24.146
1.856.678	-34.164	1.895.412	-24.886	1.849.337	-24.147
1.856.792	-34.168	1.895.551	-24.887	1.849.465	-24.148

1.856.949	-34.172	1.895.714	-24.887	1.849.697	-24.149
1.857.146	-34.175	1.895.873	-24.888	1.849.926	-24.151
1.857.269	-34.179	1.896.030	-24.889	1.850.070	-24.152
1.857.408	-34.183	1.896.255	-24.889	1.850.197	-24.153
1.857.558	-34.186	1.896.492	-24.890	1.850.383	-24.154
1.857.692	-34.190	1.896.669	-24.891	1.850.546	-24.156
1.857.845	-34.194	1.896.801	-24.892	1.850.735	-24.157
1.858.042	-34.197	1.896.980	-24.892	1.850.905	-24.158
1.858.178	-34.201	1.897.142	-24.893	1.851.032	-24.160
1.858.270	-34.205	1.897.238	-24.894	1.851.230	-24.161
1.858.382	-34.208	1.897.390	-24.895	1.851.438	-24.162
1.858.553	-34.212	1.897.560	-24.896	1.851.665	-24.164
1.858.761	-34.216	1.897.726	-24.896	1.851.839	-24.165
1.858.927	-34.219	1.897.831	-24.897	1.852.014	-24.166
1.859.091	-34.223	1.897.963	-24.898	1.852.132	-24.168
1.859.272	-34.227	1.898.111	-24.899	1.852.229	-24.169
1.859.485	-34.230	1.898.183	-24.900	1.852.411	-24.171
1.859.686	-34.234	1.898.351	-24.900	1.852.592	-24.172
1.859.859	-34.238	1.898.513	-24.901	1.852.773	-24.174
1.860.058	-34.241	1.898.681	-24.902	1.852.907	-24.175
1.860.220	-34.245	1.898.887	-24.903	1.853.071	-24.176
1.860.383	-34.248	1.899.052	-24.904	1.853.275	-24.178
1.860.594	-34.252	1.899.287	-24.905	1.853.474	-24.179
1.860.688	-34.255	1.899.543	-24.906	1.853.663	-24.181
1.860.869	-34.259	1.899.760	-24.906	1.853.817	-24.182
1.861.105	-34.263	1.899.946	-24.907	1.853.929	-24.184
1.861.238	-34.266	1.900.107	-24.908	1.854.043	-24.185
1.861.406	-34.270	1.900.215	-24.909	1.854.185	-24.187
1.861.470	-34.273	1.900.363	-24.910	1.854.285	-24.188
1.861.527	-34.277	1.900.569	-24.911	1.854.451	-24.189

1.861.691	-34.280	1.900.719	-24.912	1.854.621	-24.191
1.861.922	-34.283	1.900.822	-24.913	1.854.734	-24.192
1.862.186	-34.287	1.900.925	-24.913	1.854.834	-24.194
1.862.316	-34.290	1.901.093	-24.914	1.854.980	-24.195
1.862.471	-34.294	1.901.207	-24.915	1.855.211	-24.197
1.862.688	-34.297	1.901.364	-24.916	1.855.432	-24.198
1.862.850	-34.300	1.901.563	-24.917	1.855.647	-24.200
1.863.024	-34.304	1.901.720	-24.918	1.855.786	-24.201
1.863.172	-34.307	1.901.881	-24.919	1.855.979	-24.203
1.863.345	-34.310	1.902.032	-24.919	1.856.198	-24.204
1.863.555	-34.314	1.902.251	-24.920	1.856.402	-24.206
1.863.734	-34.317	1.902.515	-24.921	1.856.611	-24.207
1.863.889	-34.320	1.902.695	-24.922	1.856.799	-24.209
1.864.086	-34.323	1.902.863	-24.923	1.856.971	-24.210
1.864.263	-34.327	1.903.038	-24.924	1.857.099	-24.212
1.864.408	-34.330	1.903.165	-24.924	1.857.217	-24.213
1.864.581	-34.333	1.903.318	-24.925	1.857.337	-24.215
1.864.789	-34.336	1.903.461	-24.926	1.857.475	-24.216
1.864.957	-34.339	1.903.651	-24.927	1.857.583	-24.217
1.865.090	-34.342	1.903.891	-24.928	1.857.755	-24.219
1.865.237	-34.345	1.904.090	-24.928	1.857.885	-24.220
1.865.370	-34.348	1.904.307	-24.929	1.858.055	-24.222
1.865.510	-34.351	1.904.458	-24.930	1.858.257	-24.223
1.865.726	-34.354	1.904.604	-24.931	1.858.344	-24.225
1.865.921	-34.357	1.904.785	-24.931	1.858.595	-24.226
1.866.091	-34.360	1.904.911	-24.932	1.858.878	-24.227
1.866.337	-34.363	1.905.011	-24.933	1.859.045	-24.229
1.866.461	-34.366	1.905.155	-24.934	1.859.225	-24.230
1.866.521	-34.369	1.905.356	-24.934	1.859.379	-24.232
1.866.682	-34.372	1.905.576	-24.935	1.859.492	-24.233

1.866.877	-34.375	1.905.753	-24.936	1.859.713	-24.234
1.867.070	-34.377	1.905.896	-24.936	1.859.946	-24.236
1.867.227	-34.380	1.906.064	-24.937	1.860.143	-24.237
1.867.334	-34.383	1.906.191	-24.938	1.860.255	-24.238
1.867.495	-34.386	1.906.294	-24.938	1.860.363	-24.240
1.867.641	-34.388	1.906.487	-24.939	1.860.546	-24.241
1.867.791	-34.391	1.906.723	-24.939	1.860.674	-24.243
1.868.035	-34.394	1.906.891	-24.940	1.860.844	-24.244
1.868.270	-34.396	1.907.018	-24.941	1.861.080	-24.245
1.868.479	-34.399	1.907.206	-24.941	1.861.270	-24.246
1.868.636	-34.401	1.907.401	-24.942	1.861.380	-24.248
1.868.763	-34.404	1.907.560	-24.942	1.861.561	-24.249
1.868.869	-34.406	1.907.695	-24.943	1.861.712	-24.250
1.869.039	-34.409	1.907.825	-24.943	1.861.868	-24.252
1.869.178	-34.411	1.907.977	-24.944	1.862.072	-24.253
1.869.193	-34.414	1.908.143	-24.944	1.862.225	-24.254
1.869.417	-34.416	1.908.275	-24.945	1.862.441	-24.255
1.869.984	-34.419	1.908.609	-24.945	1.862.612	-24.257
1.870.410	-34.421	1.909.090	-24.946	1.862.726	-24.258
1.870.576	-34.423	1.909.335	-24.946	1.862.896	-24.259
1.870.665	-34.426	1.909.389	-24.947	1.863.082	-24.260
1.870.764	-34.428	1.909.467	-24.947	1.863.181	-24.261
1.870.845	-34.430	1.909.574	-24.947	1.863.320	-24.263
1.870.854	-34.432	1.909.594	-24.948	1.863.521	-24.264
1.870.900	-34.435	1.909.644	-24.948	1.863.633	-24.265
1.871.017	-34.437	1.909.762	-24.949	1.863.850	-24.266
1.871.163	-34.439	1.909.846	-24.949	1.864.088	-24.267
1.871.348	-34.441	1.910.036	-24.949	1.864.184	-24.268
1.871.552	-34.443	1.910.273	-24.949	1.864.315	-24.270
1.871.678	-34.445	1.910.403	-24.950	1.864.491	-24.271

1.871.778	-34.447	1.910.574	-24.950	1.864.630	-24.272
1.871.956	-34.449	1.910.797	-24.950	1.864.765	-24.273
1.872.188	-34.451	1.910.997	-24.951	1.865.098	-24.274
1.872.395	-34.453	1.911.160	-24.951	1.865.488	-24.275
1.872.612	-34.455	1.911.292	-24.951	1.865.759	-24.276
1.872.760	-34.457	1.911.440	-24.951	1.865.927	-24.277
1.872.885	-34.459	1.911.644	-24.951	1.866.022	-24.278
1.873.051	-34.461	1.911.819	-24.952	1.866.070	-24.279
1.873.214	-34.463	1.911.962	-24.952	1.866.124	-24.280
1.873.398	-34.465	1.912.119	-24.952	1.866.256	-24.282
1.873.468	-34.467	1.912.249	-24.952	1.866.333	-24.283
1.873.578	-34.469	1.912.385	-24.952	1.866.413	-24.284
1.873.766	-34.470	1.912.498	-24.952	1.866.588	-24.285
1.873.918	-34.472	1.912.686	-24.953	1.866.776	-24.286
1.874.131	-34.474	1.912.906	-24.953	1.866.962	-24.287
1.874.307	-34.476	1.913.080	-24.953	1.867.150	-24.288
1.874.390	-34.478	1.913.148	-24.953	1.867.328	-24.289
1.874.538	-34.479	1.913.295	-24.953	1.867.471	-24.290
1.874.726	-34.481	1.913.465	-24.953	1.867.639	-24.291
1.874.841	-34.483	1.913.658	-24.953	1.867.861	-24.292
1.875.022	-34.485	1.913.864	-24.953	1.868.013	-24.292
1.875.309	-34.486	1.914.018	-24.953	1.868.167	-24.293
1.875.471	-34.488	1.914.189	-24.954	1.868.351	-24.294
1.875.604	-34.490	1.914.377	-24.954	1.868.513	-24.295
1.875.773	-34.491	1.914.556	-24.954	1.868.613	-24.296
1.875.990	-34.493	1.914.722	-24.954	1.868.786	-24.297
1.876.214	-34.495	1.914.838	-24.954	1.868.997	-24.298
1.876.265	-34.496	1.914.969	-24.954	1.869.142	-24.299
1.876.344	-34.498	1.915.221	-24.954	1.869.298	-24.300
1.876.530	-34.500	1.915.423	-24.954	1.869.444	-24.301

1.876.742	-34.501	1.915.522	-24.954	1.869.565	-24.302
1.876.931	-34.503	1.915.630	-24.954	1.869.697	-24.303
1.877.103	-34.505	1.915.813	-24.954	1.869.870	-24.304
1.877.251	-34.506	1.915.979	-24.954	1.870.065	-24.305
1.877.393	-34.508	1.916.079	-24.954	1.870.197	-24.306
1.877.560	-34.509	1.916.250	-24.954	1.870.390	-24.307
1.877.733	-34.511	1.916.424	-24.954	1.870.605	-24.308
1.877.876	-34.513	1.916.611	-24.954	1.870.773	-24.309
1.877.961	-34.514	1.916.806	-24.954	1.870.938	-24.310
1.878.167	-34.516	1.916.926	-24.954	1.871.124	-24.311
1.878.400	-34.517	1.917.036	-24.954	1.871.313	-24.312
1.878.561	-34.519	1.917.235	-24.954	1.871.405	-24.313
1.878.746	-34.521	1.917.404	-24.954	1.871.503	-24.314
1.878.869	-34.522	1.917.533	-24.954	1.871.711	-24.315
1.878.992	-34.524	1.917.677	-24.954	1.871.944	-24.316
1.879.180	-34.525	1.917.859	-24.954	1.872.088	-24.317
1.879.395	-34.527	1.918.096	-24.954	1.872.229	-24.318
1.879.596	-34.529	1.918.297	-24.954	1.872.406	-24.319
1.879.749	-34.530	1.918.492	-24.954	1.872.569	-24.320
1.879.901	-34.532	1.918.660	-24.954	1.872.721	-24.321
1.880.119	-34.533	1.918.877	-24.954	1.872.872	-24.322
1.880.331	-34.535	1.919.046	-24.954	1.873.013	-24.323
1.880.428	-34.537	1.919.176	-24.954	1.873.167	-24.324
1.880.553	-34.538	1.919.310	-24.954	1.873.281	-24.325
1.880.724	-34.540	1.919.379	-24.954	1.873.443	-24.326
1.880.863	-34.541	1.919.574	-24.954	1.873.613	-24.327
1.880.988	-34.543	1.919.785	-24.954	1.873.741	-24.328
1.881.133	-34.545	1.919.944	-24.954	1.873.911	-24.329
1.881.313	-34.546	1.920.107	-24.954	1.874.095	-24.331
1.881.469	-34.548	1.920.251	-24.954	1.874.324	-24.332

1.881.611	-34.549	1.920.445	-24.954	1.874.534	-24.333
1.881.833	-34.551	1.920.618	-24.954	1.874.724	-24.334
1.882.038	-34.553	1.920.831	-24.954	1.874.912	-24.335
1.882.179	-34.554	1.921.003	-24.954	1.875.098	-24.336
1.882.341	-34.556	1.921.095	-24.954	1.875.295	-24.337
1.882.527	-34.557	1.921.241	-24.954	1.875.414	-24.339
1.882.695	-34.559	1.921.378	-24.954	1.875.517	-24.340
1.882.849	-34.561	1.921.519	-24.954	1.875.748	-24.341
1.883.015	-34.562	1.921.736	-24.953	1.875.932	-24.342
1.883.167	-34.564	1.921.913	-24.953	1.876.061	-24.343
1.883.299	-34.565	1.922.014	-24.953	1.876.216	-24.344
1.883.419	-34.567	1.922.119	-24.953	1.876.368	-24.346
1.883.566	-34.569	1.922.242	-24.953	1.876.555	-24.347
1.883.691	-34.570	1.922.350	-24.953	1.876.730	-24.348
1.883.806	-34.572	1.922.511	-24.953	1.876.897	-24.349
1.883.976	-34.574	1.922.724	-24.953	1.877.101	-24.350
1.884.194	-34.575	1.922.917	-24.953	1.877.231	-24.352
1.884.359	-34.577	1.923.109	-24.953	1.877.327	-24.353
1.884.505	-34.578	1.923.337	-24.953	1.877.520	-24.354
1.884.621	-34.580	1.923.575	-24.953	1.877.672	-24.355
1.884.865	-34.582	1.923.736	-24.953	1.877.789	-24.356
1.885.109	-34.583	1.923.947	-24.953	1.877.915	-24.358
1.885.215	-34.585	1.924.191	-24.953	1.878.082	-24.359
1.885.415	-34.586	1.924.319	-24.953	1.878.197	-24.360
1.885.665	-34.588	1.924.386	-24.953	1.878.284	-24.361
1.885.880	-34.590	1.924.510	-24.953	1.878.461	-24.362
1.886.057	-34.591	1.924.697	-24.953	1.878.673	-24.363
1.886.203	-34.593	1.924.859	-24.953	1.878.842	-24.365
1.886.335	-34.594	1.925.016	-24.952	1.878.954	-24.366
1.886.512	-34.596	1.925.141	-24.952	1.879.173	-24.367

1.886.691	-34.597	1.925.253	-24.952	1.879.413	-24.368
1.886.880	-34.599	1.925.435	-24.952	1.879.637	-24.369
1.887.076	-34.601	1.925.620	-24.952	1.879.846	-24.371
1.887.159	-34.602	1.925.811	-24.952	1.880.038	-24.372
1.887.229	-34.604	1.925.981	-24.952	1.880.246	-24.373
1.887.350	-34.605	1.926.127	-24.952	1.880.401	-24.374
1.887.486	-34.607	1.926.310	-24.952	1.880.558	-24.375
1.887.619	-34.608	1.926.539	-24.952	1.880.699	-24.376
1.887.827	-34.610	1.926.718	-24.951	1.880.842	-24.377
1.888.053	-34.611	1.926.833	-24.951	1.880.992	-24.379
1.888.158	-34.613	1.927.018	-24.951	1.881.104	-24.380
1.888.324	-34.615	1.927.197	-24.951	1.881.252	-24.381
1.888.539	-34.616	1.927.330	-24.951	1.881.443	-24.382
1.888.696	-34.618	1.927.560	-24.951	1.881.581	-24.383
1.888.848	-34.619	1.927.708	-24.951	1.881.687	-24.384
1.889.054	-34.621	1.927.814	-24.951	1.881.786	-24.385
1.889.216	-34.622	1.927.961	-24.950	1.882.005	-24.386
1.889.375	-34.624	1.928.145	-24.950	1.882.291	-24.387
1.889.507	-34.625	1.928.362	-24.950	1.882.506	-24.388
1.889.619	-34.627	1.928.542	-24.950	1.882.701	-24.390
1.889.841	-34.628	1.928.763	-24.950	1.882.838	-24.391
1.890.040	-34.630	1.928.887	-24.950	1.882.997	-24.392
1.890.233	-34.631	1.928.997	-24.949	1.883.212	-24.393
1.890.421	-34.633	1.929.184	-24.949	1.883.382	-24.394
1.890.547	-34.634	1.929.362	-24.949	1.883.598	-24.395
1.890.670	-34.636	1.929.527	-24.949	1.883.745	-24.396
1.890.883	-34.637	1.929.688	-24.949	1.883.850	-24.397
1.891.113	-34.638	1.929.827	-24.949	1.883.985	-24.398
1.891.237	-34.640	1.929.984	-24.948	1.884.104	-24.399
1.891.355	-34.641	1.930.186	-24.948	1.884.297	-24.400

1.891.494	-34.643	1.930.401	-24.948	1.884.505	-24.401
1.891.722	-34.644	1.930.556	-24.948	1.884.678	-24.402
1.891.945	-34.646	1.930.715	-24.948	1.884.825	-24.402
1.892.124	-34.647	1.930.910	-24.947	1.884.993	-24.403
1.892.296	-34.649	1.931.062	-24.947	1.885.163	-24.404
1.892.422	-34.650	1.931.178	-24.947	1.885.322	-24.405
1.892.486	-34.651	1.931.312	-24.947	1.885.519	-24.406
1.892.634	-34.653	1.931.519	-24.947	1.885.683	-24.407
1.892.885	-34.654	1.931.680	-24.946	1.885.822	-24.408
1.893.064	-34.656	1.931.810	-24.946	1.885.925	-24.409
1.893.203	-34.657	1.932.003	-24.946	1.886.096	-24.410
1.893.363	-34.658	1.932.197	-24.946	1.886.265	-24.411
1.893.533	-34.660	1.932.314	-24.946	1.886.400	-24.412
1.893.678	-34.661	1.932.401	-24.945	1.886.633	-24.412
1.893.873	-34.663	1.932.733	-24.945	1.886.794	-24.413
1.894.059	-34.664	1.933.259	-24.945	1.886.881	-24.414
1.894.225	-34.665	1.933.550	-24.945	1.887.047	-24.415
1.894.409	-34.667	1.933.645	-24.944	1.887.251	-24.416
1.894.628	-34.668	1.933.747	-24.944	1.887.473	-24.417
1.894.841	-34.670	1.933.837	-24.944	1.887.691	-24.417
1.894.924	-34.671	1.933.909	-24.944	1.887.827	-24.418
1.895.089	-34.672	1.933.956	-24.944	1.887.878	-24.419
1.895.484	-34.674	1.933.997	-24.943	1.888.046	-24.420
1.895.912	-34.675	1.934.111	-24.943	1.888.262	-24.421
1.896.167	-34.676	1.934.272	-24.943	1.888.373	-24.421
1.896.252	-34.678	1.934.433	-24.943	1.888.682	-24.422
1.896.266	-34.679	1.934.628	-24.942	1.889.091	-24.423
1.896.297	-34.680	1.934.818	-24.942	1.889.330	-24.424
1.896.315	-34.682	1.934.957	-24.942	1.889.500	-24.424
1.896.387	-34.683	1.935.099	-24.942	1.889.594	-24.425

1.896.548	-34.684	1.935.248	-24.941	1.889.623	-24.426
1.896.649	-34.685	1.935.423	-24.941	1.889.677	-24.427
1.896.815	-34.687	1.935.627	-24.941	1.889.781	-24.427
1.896.942	-34.688	1.935.764	-24.941	1.889.915	-24.428
1.897.126	-34.689	1.935.930	-24.941	1.890.061	-24.429
1.897.361	-34.691	1.936.107	-24.940	1.890.204	-24.430
1.897.446	-34.692	1.936.265	-24.940	1.890.332	-24.430
1.897.542	-34.693	1.936.445	-24.940	1.890.450	-24.431
1.897.789	-34.694	1.936.568	-24.940	1.890.592	-24.432
1.898.062	-34.696	1.936.718	-24.940	1.890.815	-24.432
1.898.150	-34.697	1.936.893	-24.939	1.891.021	-24.433
1.898.270	-34.698	1.937.020	-24.939	1.891.227	-24.434
1.898.499	-34.699	1.937.146	-24.939	1.891.431	-24.435
1.898.651	-34.701	1.937.352	-24.939	1.891.583	-24.435
1.898.794	-34.702	1.937.533	-24.939	1.891.707	-24.436
1.898.976	-34.703	1.937.706	-24.939	1.891.911	-24.437
1.899.109	-34.704	1.937.919	-24.939	1.892.083	-24.437
1.899.220	-34.706	1.938.082	-24.938	1.892.182	-24.438
1.899.402	-34.707	1.938.250	-24.938	1.892.334	-24.439
1.899.619	-34.708	1.938.384	-24.938	1.892.500	-24.439
1.899.798	-34.709	1.938.497	-24.938	1.892.652	-24.440
1.899.957	-34.710	1.938.719	-24.938	1.892.829	-24.441
1.900.087	-34.712	1.938.931	-24.938	1.892.981	-24.442
1.900.210	-34.713	1.939.102	-24.938	1.893.083	-24.442
1.900.378	-34.714	1.939.218	-24.938	1.893.217	-24.443
1.900.665	-34.715	1.939.319	-24.938	1.893.411	-24.444
1.900.876	-34.717	1.939.467	-24.938	1.893.696	-24.445
1.900.983	-34.718	1.939.606	-24.937	1.893.875	-24.445
1.901.190	-34.719	1.939.745	-24.937	1.893.981	-24.446
1.901.382	-34.720	1.939.935	-24.937	1.894.198	-24.447

1.901.507	-34.721	1.940.118	-24.937	1.894.422	-24.448
1.901.691	-34.723	1.940.277	-24.937	1.894.565	-24.448
1.901.839	-34.724	1.940.475	-24.937	1.894.621	-24.449
1.901.935	-34.725	1.940.584	-24.937	1.894.688	-24.450
1.902.144	-34.726	1.940.760	-24.937	1.894.848	-24.451
1.902.350	-34.727	1.940.974	-24.937	1.895.009	-24.452
1.902.437	-34.728	1.941.131	-24.937	1.895.165	-24.453
1.902.529	-34.730	1.941.333	-24.937	1.895.336	-24.453
1.902.702	-34.731	1.941.490	-24.937	1.895.517	-24.454
1.902.912	-34.732	1.941.595	-24.938	1.895.715	-24.455
1.903.067	-34.733	1.941.704	-24.938	1.895.853	-24.456
1.903.232	-34.734	1.941.886	-24.938	1.896.044	-24.457
1.903.483	-34.735	1.942.101	-24.938	1.896.238	-24.458
1.903.710	-34.737	1.942.319	-24.938	1.896.324	-24.459
1.903.866	-34.738	1.942.442	-24.938	1.896.521	-24.460
1.903.985	-34.739	1.942.625	-24.938	1.896.761	-24.460
1.904.101	-34.740	1.942.892	-24.938	1.896.955	-24.461
1.904.232	-34.741	1.943.036	-24.938	1.897.162	-24.462
1.904.398	-34.742	1.943.143	-24.938	1.897.267	-24.463
1.904.498	-34.743	1.943.315	-24.939	1.897.367	-24.464
1.904.610	-34.745	1.943.474	-24.939	1.897.496	-24.465
1.904.791	-34.746	1.943.656	-24.939	1.897.665	-24.466
1.904.926	-34.747	1.943.846	-24.939	1.897.941	-24.467
1.905.121	-34.748	1.943.988	-24.939	1.898.165	-24.468
1.905.291	-34.749	1.944.109	-24.939	1.898.309	-24.469
1.905.482	-34.750	1.944.286	-24.940	1.898.481	-24.470
1.905.748	-34.751	1.944.465	-24.940	1.898.631	-24.471
1.905.976	-34.753	1.944.643	-24.940	1.898.784	-24.472
1.906.086	-34.754	1.944.798	-24.940	1.898.960	-24.473
1.906.212	-34.755	1.944.953	-24.940	1.899.108	-24.475

1.906.406	-34.756	1.945.161	-24.940	1.899.218	-24.476
1.906.588	-34.757	1.945.311	-24.941	1.899.363	-24.477
1.906.781	-34.758	1.945.425	-24.941	1.899.552	-24.478
1.906.879	-34.759	1.945.574	-24.941	1.899.760	-24.479
1.906.933	-34.760	1.945.735	-24.941	1.899.969	-24.480
1.907.047	-34.761	1.945.889	-24.941	1.900.078	-24.481
1.907.264	-34.762	1.946.064	-24.941	1.900.177	-24.482
1.907.444	-34.763	1.946.214	-24.942	1.900.365	-24.483
1.907.603	-34.764	1.946.349	-24.942	1.900.533	-24.485
1.907.901	-34.765	1.946.447	-24.942	1.900.706	-24.486
1.908.179	-34.766	1.946.600	-24.942	1.900.903	-24.487
1.908.277	-34.767	1.946.824	-24.942	1.901.053	-24.488
1.908.327	-34.768	1.946.987	-24.943	1.901.214	-24.489
1.908.458	-34.769	1.947.108	-24.943	1.901.350	-24.490
1.908.640	-34.770	1.947.271	-24.943	1.901.460	-24.491
1.908.792	-34.770	1.947.556	-24.943	1.901.600	-24.493
1.908.969	-34.771	1.947.809	-24.943	1.901.765	-24.494
1.909.122	-34.772	1.947.992	-24.943	1.901.889	-24.495
1.909.249	-34.773	1.948.114	-24.943	1.902.000	-24.496
1.909.341	-34.774	1.948.237	-24.944	1.902.108	-24.497
1.909.444	-34.775	1.948.405	-24.944	1.902.276	-24.498
1.909.697	-34.775	1.948.616	-24.944	1.902.507	-24.499
1.909.944	-34.776	1.948.822	-24.944	1.902.735	-24.501
1.910.044	-34.777	1.948.969	-24.944	1.902.921	-24.502
1.910.173	-34.778	1.949.110	-24.944	1.903.112	-24.503
1.910.358	-34.778	1.949.254	-24.944	1.903.333	-24.504
1.910.573	-34.779	1.949.406	-24.944	1.903.571	-24.505
1.910.809	-34.780	1.949.556	-24.944	1.903.797	-24.506
1.911.017	-34.780	1.949.740	-24.944	1.903.956	-24.507
1.911.225	-34.781	1.949.881	-24.944	1.904.101	-24.508

1.911.321	-34.781	1.949.966	-24.944	1.904.198	-24.510
1.911.463	-34.782	1.950.080	-24.944	1.904.337	-24.511
1.911.666	-34.782	1.950.331	-24.944	1.904.554	-24.512
1.911.803	-34.783	1.950.567	-24.944	1.904.693	-24.513
1.911.964	-34.783	1.950.735	-24.944	1.904.787	-24.514
1.912.146	-34.784	1.950.949	-24.944	1.904.951	-24.515
1.912.245	-34.784	1.951.158	-24.944	1.905.150	-24.516
1.912.390	-34.785	1.951.288	-24.944	1.905.349	-24.517
1.912.538	-34.785	1.951.474	-24.944	1.905.555	-24.518
1.912.668	-34.786	1.951.674	-24.944	1.905.712	-24.519
1.912.861	-34.786	1.951.805	-24.944	1.905.838	-24.520
1.912.981	-34.786	1.951.935	-24.944	1.905.988	-24.521
1.913.139	-34.787	1.952.068	-24.944	1.906.138	-24.522
1.913.353	-34.787	1.952.182	-24.943	1.906.378	-24.523
1.913.493	-34.787	1.952.320	-24.943	1.906.640	-24.524
1.913.712	-34.787	1.952.554	-24.943	1.906.842	-24.525
1.913.949	-34.788	1.952.720	-24.943	1.906.967	-24.526
1.914.088	-34.788	1.952.807	-24.943	1.907.048	-24.527
1.914.198	-34.788	1.953.022	-24.942	1.907.175	-24.528
1.914.328	-34.788	1.953.307	-24.942	1.907.348	-24.529
1.914.574	-34.788	1.953.456	-24.942	1.907.554	-24.530
1.914.791	-34.788	1.953.578	-24.942	1.907.706	-24.531
1.914.901	-34.789	1.953.772	-24.941	1.907.870	-24.532
1.915.087	-34.789	1.953.931	-24.941	1.907.984	-24.533
1.915.251	-34.789	1.954.063	-24.941	1.908.098	-24.534
1.915.441	-34.789	1.954.189	-24.941	1.908.304	-24.534
1.915.571	-34.789	1.954.363	-24.940	1.908.436	-24.535
1.915.719	-34.789	1.954.547	-24.940	1.908.587	-24.536
1.915.979	-34.789	1.954.734	-24.940	1.908.815	-24.537
1.916.120	-34.789	1.954.843	-24.939	1.909.026	-24.538

1.916.256	-34.789	1.954.980	-24.939	1.909.175	-24.539
1.916.411	-34.789	1.955.192	-24.939	1.909.321	-24.539
1.916.586	-34.789	1.955.406	-24.938	1.909.501	-24.540
1.916.763	-34.789	1.955.571	-24.938	1.909.691	-24.541
1.916.884	-34.788	1.955.704	-24.937	1.909.789	-24.542
1.917.003	-34.788	1.955.880	-24.937	1.909.967	-24.542
1.917.197	-34.788	1.956.078	-24.937	1.910.181	-24.543
1.917.471	-34.788	1.956.265	-24.936	1.910.304	-24.544
1.917.710	-34.788	1.956.434	-24.936	1.910.504	-24.545
1.917.818	-34.788	1.956.552	-24.935	1.910.672	-24.545
1.918.013	-34.788	1.956.720	-24.935	1.910.824	-24.546
1.918.152	-34.787	1.957.110	-24.934	1.911.046	-24.547
1.918.234	-34.787	1.957.547	-24.934	1.911.207	-24.547
1.918.349	-34.787	1.957.764	-24.933	1.911.310	-24.548
1.918.501	-34.787	1.957.834	-24.933	1.911.480	-24.548
1.918.752	-34.787	1.957.878	-24.932	1.911.647	-24.549
1.918.956	-34.787	1.957.984	-24.932	1.911.747	-24.550
1.919.090	-34.786	1.958.092	-24.931	1.911.846	-24.550
1.919.265	-34.786	1.958.140	-24.931	1.912.249	-24.551
1.919.451	-34.786	1.958.186	-24.930	1.912.820	-24.551
1.919.587	-34.786	1.958.300	-24.930	1.913.040	-24.552
1.919.743	-34.786	1.958.445	-24.929	1.913.075	-24.553
1.919.884	-34.785	1.958.629	-24.929	1.913.158	-24.553
1.920.067	-34.785	1.958.857	-24.928	1.913.259	-24.554
1.920.217	-34.785	1.959.097	-24.928	1.913.315	-24.554
1.920.316	-34.785	1.959.267	-24.927	1.913.421	-24.555
1.920.544	-34.785	1.959.442	-24.927	1.913.539	-24.555
1.920.943	-34.784	1.959.594	-24.926	1.913.596	-24.556
1.921.335	-34.784	1.959.713	-24.926	1.913.721	-24.556
1.921.554	-34.784	1.959.903	-24.925	1.913.887	-24.557

1.921.635	-34.784	1.960.067	-24.925	1.914.072	-24.557
1.921.685	-34.784	1.960.246	-24.924	1.914.328	-24.557
1.921.767	-34.784	1.960.455	-24.924	1.914.557	-24.558
1.921.805	-34.783	1.960.612	-24.923	1.914.711	-24.558
1.921.906	-34.783	1.960.721	-24.922	1.914.827	-24.559
1.921.975	-34.783	1.960.831	-24.922	1.915.002	-24.559
1.922.067	-34.783	1.960.947	-24.921	1.915.228	-24.560
1.922.263	-34.783	1.961.060	-24.921	1.915.439	-24.560
1.922.390	-34.783	1.961.173	-24.921	1.915.556	-24.560
1.922.531	-34.783	1.961.304	-24.920	1.915.636	-24.561
1.922.683	-34.783	1.961.530	-24.920	1.915.768	-24.561
1.922.863	-34.782	1.961.686	-24.919	1.915.912	-24.562
1.923.123	-34.782	1.961.819	-24.919	1.916.118	-24.562
1.923.358	-34.782	1.962.018	-24.918	1.916.308	-24.562
1.923.549	-34.782	1.962.227	-24.918	1.916.431	-24.563
1.923.752	-34.782	1.962.455	-24.918	1.916.543	-24.563
1.923.904	-34.782	1.962.668	-24.917	1.916.644	-24.564
1.924.016	-34.782	1.962.809	-24.917	1.916.756	-24.564
1.924.155	-34.782	1.962.968	-24.916	1.916.922	-24.564
1.924.332	-34.783	1.963.145	-24.916	1.917.142	-24.565
1.924.511	-34.783	1.963.288	-24.916	1.917.365	-24.565
1.924.592	-34.783	1.963.461	-24.915	1.917.542	-24.566
1.924.718	-34.783	1.963.640	-24.915	1.917.753	-24.566
1.924.888	-34.783	1.963.671	-24.915	1.917.990	-24.566
1.925.031	-34.783	1.963.841	-24.914	1.918.152	-24.567
1.925.154	-34.784	1.964.153	-24.914	1.918.335	-24.567
1.925.298	-34.784	1.964.305	-24.914	1.918.445	-24.568
1.925.555	-34.784	1.964.377	-24.914	1.918.533	-24.568
1.925.802	-34.785	1.964.492	-24.913	1.918.662	-24.569
1.925.919	-34.785	1.964.722	-24.913	1.918.785	-24.569

1.926.039	-34.785	1.964.966	-24.913	1.919.012	-24.569
1.926.252	-34.786	1.965.080	-24.913	1.919.207	-24.570
1.926.496	-34.786	1.965.258	-24.913	1.919.353	-24.570
1.926.727	-34.787	1.965.450	-24.913	1.919.440	-24.571
1.926.933	-34.787	1.965.604	-24.913	1.919.549	-24.571
1.926.994	-34.788	1.965.742	-24.912	1.919.845	-24.572
1.927.104	-34.789	1.965.891	-24.912	1.920.157	-24.572
1.927.316	-34.789	1.966.062	-24.912	1.920.365	-24.573
1.927.421	-34.790	1.966.187	-24.912	1.920.457	-24.573
1.927.565	-34.791	1.966.310	-24.912	1.920.542	-24.574
1.927.777	-34.791	1.966.534	-24.912	1.920.726	-24.574
1.927.924	-34.792	1.966.783	-24.912	1.920.916	-24.575
1.928.091	-34.793	1.967.003	-24.912	1.921.022	-24.575
1.928.214	-34.794	1.967.162	-24.912	1.921.162	-24.576
1.928.335	-34.794	1.967.305	-24.912	1.921.317	-24.576
1.928.559	-34.795	1.967.439	-24.912	1.921.514	-24.577
1.928.718	-34.796	1.967.531	-24.913	1.921.794	-24.577
1.928.889	-34.797	1.967.701	-24.913	1.922.000	-24.578
1.929.044	-34.798	1.967.898	-24.913	1.922.184	-24.579
1.929.184	-34.799	1.968.028	-24.913	1.922.316	-24.579
1.929.353	-34.801	1.968.197	-24.913	1.922.384	-24.580
1.929.541	-34.802	1.968.380	-24.913	1.922.544	-24.580
1.929.698	-34.803	1.968.526	-24.913	1.922.735	-24.581
1.929.825	-34.804	1.968.748	-24.914	1.922.919	-24.581
1.929.984	-34.805	1.968.983	-24.914	1.923.104	-24.582
1.930.109	-34.807	1.969.108	-24.914	1.923.252	-24.583
1.930.255	-34.808	1.969.231	-24.914	1.923.402	-24.583
1.930.468	-34.809	1.969.357	-24.914	1.923.512	-24.584
1.930.672	-34.811	1.969.487	-24.915	1.923.649	-24.585
1.930.791	-34.812	1.969.657	-24.915	1.923.873	-24.585

1.930.997	-34.814	1.969.807	-24.915	1.924.053	-24.586
1.931.217	-34.815	1.969.935	-24.915	1.924.171	-24.586
1.931.375	-34.817	1.970.065	-24.916	1.924.299	-24.587
1.931.546	-34.818	1.970.229	-24.916	1.924.484	-24.588
1.931.714	-34.820	1.970.374	-24.916	1.924.626	-24.588
1.931.897	-34.822	1.970.517	-24.917	1.924.756	-24.589
1.932.056	-34.823	1.970.685	-24.917	1.924.946	-24.590
1.932.215	-34.825	1.970.840	-24.917	1.925.116	-24.590
1.932.363	-34.827	1.970.983	-24.918	1.925.233	-24.591
1.932.495	-34.829	1.971.189	-24.918	1.925.347	-24.592
1.932.715	-34.830	1.971.445	-24.918	1.925.524	-24.592
1.932.935	-34.832	1.971.648	-24.918	1.925.692	-24.593
1.933.024	-34.834	1.971.848	-24.919	1.925.849	-24.594
1.933.168	-34.836	1.972.063	-24.919	1.925.993	-24.594
1.933.432	-34.838	1.972.287	-24.919	1.926.149	-24.595
1.933.609	-34.840	1.972.495	-24.920	1.926.333	-24.596
1.933.649	-34.842	1.972.650	-24.920	1.926.557	-24.596
1.933.772	-34.844	1.972.800	-24.920	1.926.821	-24.597
1.933.934	-34.846	1.972.975	-24.921	1.927.038	-24.598
1.934.162	-34.848	1.973.141	-24.921	1.927.188	-24.598
1.934.315	-34.850	1.973.232	-24.921	1.927.316	-24.599
1.934.397	-34.852	1.973.272	-24.922	1.927.500	-24.600
1.934.552	-34.854	1.973.400	-24.922	1.927.686	-24.600
1.934.675	-34.856	1.973.609	-24.922	1.927.834	-24.601
1.934.846	-34.858	1.973.759	-24.923	1.927.988	-24.602
1.935.023	-34.861	1.973.905	-24.923	1.928.131	-24.602
1.935.130	-34.863	1.974.075	-24.923	1.928.242	-24.603
1.935.211	-34.865	1.974.184	-24.923	1.928.342	-24.604
1.935.436	-34.867	1.974.337	-24.924	1.928.479	-24.604
1.935.714	-34.869	1.974.568	-24.924	1.928.652	-24.605

1.935.893	-34.872	1.974.838	-24.924	1.928.862	-24.606
1.936.116	-34.874	1.975.029	-24.924	1.929.072	-24.606
1.936.328	-34.876	1.975.206	-24.924	1.929.234	-24.607
1.936.479	-34.878	1.975.369	-24.925	1.929.428	-24.607
1.936.602	-34.881	1.975.472	-24.925	1.929.684	-24.608
1.936.803	-34.883	1.975.645	-24.925	1.929.868	-24.609
1.936.994	-34.885	1.975.822	-24.925	1.930.011	-24.609
1.937.168	-34.888	1.976.012	-24.925	1.930.152	-24.610
1.937.325	-34.890	1.976.207	-24.925	1.930.282	-24.610
1.937.464	-34.892	1.976.414	-24.925	1.930.441	-24.611
1.937.621	-34.894	1.976.572	-24.926	1.930.602	-24.612
1.937.796	-34.897	1.976.721	-24.926	1.930.797	-24.612
1.937.936	-34.899	1.976.899	-24.926	1.930.968	-24.613
1.938.053	-34.901	1.976.991	-24.926	1.931.104	-24.613
1.938.201	-34.904	1.977.132	-24.926	1.931.227	-24.614
1.938.367	-34.906	1.977.338	-24.926	1.931.418	-24.614
1.938.508	-34.908	1.977.507	-24.926	1.931.604	-24.615
1.938.700	-34.911	1.977.699	-24.925	1.931.781	-24.616
1.938.949	-34.913	1.977.896	-24.925	1.931.975	-24.616
1.939.082	-34.915	1.978.026	-24.925	1.932.117	-24.617
1.939.186	-34.918	1.978.147	-24.925	1.932.280	-24.617
1.939.379	-34.920	1.978.302	-24.925	1.932.453	-24.618
1.939.612	-34.922	1.978.485	-24.925	1.932.648	-24.618
1.939.755	-34.924	1.978.678	-24.924	1.932.832	-24.619
1.939.883	-34.927	1.978.806	-24.924	1.932.991	-24.619
1.940.139	-34.929	1.978.969	-24.924	1.933.120	-24.620
1.940.403	-34.931	1.979.070	-24.923	1.933.252	-24.620
1.940.535	-34.934	1.979.200	-24.923	1.933.447	-24.621
1.940.721	-34.936	1.979.449	-24.923	1.933.674	-24.621
1.940.891	-34.938	1.979.628	-24.922	1.933.826	-24.621

1.941.032	-34.940	1.979.818	-24.922	1.933.922	-24.622
1.941.185	-34.943	1.980.020	-24.921	1.934.113	-24.622
1.941.279	-34.945	1.980.172	-24.921	1.934.344	-24.623
1.941.389	-34.947	1.980.361	-24.920	1.934.514	-24.623
1.941.561	-34.949	1.980.591	-24.920	1.934.664	-24.624
1.941.810	-34.951	1.980.706	-24.919	1.934.810	-24.624
1.942.056	-34.954	1.980.793	-24.919	1.934.946	-24.624
1.942.168	-34.956	1.981.031	-24.918	1.935.053	-24.625
1.942.231	-34.958	1.981.494	-24.917	1.935.204	-24.625
1.942.352	-34.960	1.981.919	-24.917	1.935.434	-24.626
1.942.556	-34.962	1.982.072	-24.916	1.935.773	-24.626
1.942.793	-34.964	1.982.108	-24.915	1.936.194	-24.626
1.942.979	-34.966	1.982.153	-24.914	1.936.469	-24.627
1.943.109	-34.968	1.982.209	-24.913	1.936.615	-24.627
1.943.242	-34.970	1.982.281	-24.913	1.936.689	-24.627
1.943.387	-34.972	1.982.401	-24.912	1.936.765	-24.628
1.943.561	-34.974	1.982.505	-24.911	1.936.833	-24.628
1.943.710	-34.976	1.982.625	-24.910	1.936.888	-24.628
1.943.858	-34.978	1.982.791	-24.909	1.936.991	-24.629
1.944.065	-34.980	1.982.979	-24.908	1.937.130	-24.629
1.944.229	-34.982	1.983.107	-24.907	1.937.292	-24.629
1.944.417	-34.984	1.983.251	-24.906	1.937.477	-24.630
1.944.599	-34.986	1.983.458	-24.905	1.937.632	-24.630
1.944.738	-34.988	1.983.596	-24.903	1.937.755	-24.630
1.944.959	-34.990	1.983.759	-24.902	1.937.937	-24.631
1.945.121	-34.992	1.983.934	-24.901	1.938.067	-24.631
1.945.298	-34.994	1.984.081	-24.900	1.938.224	-24.631
1.945.495	-34.995	1.984.258	-24.899	1.938.354	-24.632
1.945.553	-34.997	1.984.473	-24.897	1.938.573	-24.632
1.945.636	-34.999	1.984.621	-24.896	1.938.734	-24.632

1.945.853	-35.001	1.984.749	-24.895	1.938.923	-24.632
1.946.221	-35.003	1.984.924	-24.894	1.939.109	-24.633
1.946.680	-35.004	1.985.116	-24.892	1.939.227	-24.633
1.946.895	-35.006	1.985.273	-24.891	1.939.319	-24.633
1.946.982	-35.008	1.985.396	-24.889	1.939.465	-24.634
1.947.117	-35.009	1.985.531	-24.888	1.939.650	-24.634
1.947.240	-35.011	1.985.670	-24.887	1.939.828	-24.634
1.947.320	-35.013	1.985.853	-24.885	1.939.953	-24.634
1.947.318	-35.014	1.986.032	-24.884	1.940.078	-24.635
1.947.399	-35.016	1.986.212	-24.882	1.940.211	-24.635
1.947.523	-35.018	1.986.461	-24.881	1.940.381	-24.635
1.947.668	-35.019	1.986.684	-24.879	1.940.480	-24.636
1.947.876	-35.021	1.986.857	-24.878	1.940.652	-24.636
1.948.087	-35.022	1.986.938	-24.876	1.940.898	-24.636
1.948.272	-35.024	1.987.034	-24.875	1.941.098	-24.637
1.948.358	-35.025	1.987.269	-24.873	1.941.319	-24.637
1.948.490	-35.027	1.987.449	-24.872	1.941.527	-24.637
1.948.709	-35.028	1.987.576	-24.870	1.941.687	-24.638
1.948.875	-35.030	1.987.776	-24.868	1.941.812	-24.638
1.949.045	-35.031	1.988.006	-24.867	1.941.962	-24.639
1.949.207	-35.033	1.988.163	-24.865	1.942.096	-24.639
1.949.350	-35.034	1.988.257	-24.863	1.942.235	-24.639
1.949.561	-35.036	1.988.441	-24.862	1.942.424	-24.640
1.949.754	-35.037	1.988.642	-24.860	1.942.617	-24.640
1.949.861	-35.038	1.988.756	-24.858	1.942.784	-24.640
1.950.018	-35.040	1.988.896	-24.857	1.942.930	-24.641
1.950.181	-35.041	1.989.082	-24.855	1.943.078	-24.641
1.950.331	-35.043	1.989.321	-24.853	1.943.244	-24.642
1.950.479	-35.044	1.989.510	-24.852	1.943.421	-24.642
1.950.652	-35.045	1.989.637	-24.850	1.943.595	-24.643

1.950.757	-35.047	1.989.744	-24.848	1.943.732	-24.643
1.950.892	-35.048	1.989.892	-24.846	1.943.871	-24.643
1.951.089	-35.049	1.990.078	-24.845	1.944.072	-24.644
1.951.308	-35.051	1.990.231	-24.843	1.944.211	-24.644
1.951.487	-35.052	1.990.368	-24.841	1.944.348	-24.645
1.951.648	-35.054	1.990.527	-24.839	1.944.558	-24.645
1.951.877	-35.055	1.990.742	-24.838	1.944.722	-24.646
1.952.052	-35.056	1.990.860	-24.836	1.944.883	-24.646
1.952.193	-35.057	1.991.022	-24.834	1.945.121	-24.647
1.952.350	-35.059	1.991.236	-24.832	1.945.356	-24.648
1.952.549	-35.060	1.991.440	-24.830	1.945.488	-24.648
1.952.703	-35.061	1.991.570	-24.829	1.945.611	-24.649
1.952.847	-35.063	1.991.714	-24.827	1.945.804	-24.649
1.952.979	-35.064	1.991.834	-24.825	1.945.990	-24.650
1.953.033	-35.065	1.991.974	-24.823	1.946.147	-24.650
1.953.147	-35.067	1.992.195	-24.822	1.946.308	-24.651
1.953.331	-35.068	1.992.381	-24.820	1.946.443	-24.652
1.953.465	-35.069	1.992.507	-24.818	1.946.544	-24.652
1.953.620	-35.070	1.992.655	-24.816	1.946.704	-24.653
1.953.797	-35.072	1.992.871	-24.814	1.946.897	-24.653
1.953.958	-35.073	1.993.127	-24.813	1.947.101	-24.654
1.954.178	-35.074	1.993.270	-24.811	1.947.296	-24.655
1.954.366	-35.076	1.993.338	-24.809	1.947.397	-24.655
1.954.561	-35.077	1.993.506	-24.807	1.947.525	-24.656
1.954.776	-35.078	1.993.689	-24.805	1.947.661	-24.657
1.954.921	-35.080	1.993.878	-24.804	1.947.878	-24.657
1.955.078	-35.081	1.994.061	-24.802	1.948.017	-24.658
1.955.244	-35.082	1.994.142	-24.800	1.948.216	-24.659
1.955.370	-35.084	1.994.287	-24.798	1.948.423	-24.659
1.955.531	-35.085	1.994.462	-24.796	1.948.503	-24.660

1.955.650	-35.086	1.994.585	-24.795	1.948.580	-24.661
1.955.795	-35.088	1.994.767	-24.793	1.948.716	-24.661
1.956.048	-35.089	1.994.897	-24.791	1.948.848	-24.662
1.956.259	-35.091	1.994.996	-24.789	1.949.016	-24.663
1.956.422	-35.092	1.995.145	-24.788	1.949.209	-24.664
1.956.591	-35.093	1.995.343	-24.786	1.949.382	-24.664
1.956.778	-35.095	1.995.582	-24.784	1.949.634	-24.665
1.956.980	-35.096	1.995.828	-24.782	1.949.801	-24.666
1.957.193	-35.098	1.996.060	-24.781	1.949.917	-24.666
1.957.320	-35.099	1.996.227	-24.779	1.950.208	-24.667
1.957.422	-35.100	1.996.409	-24.777	1.950.432	-24.668
1.957.547	-35.102	1.996.620	-24.775	1.950.591	-24.669
1.957.605	-35.103	1.996.817	-24.774	1.950.762	-24.669
1.957.711	-35.105	1.996.980	-24.772	1.950.882	-24.670
1.957.975	-35.106	1.997.179	-24.770	1.951.022	-24.671
1.958.237	-35.107	1.997.323	-24.769	1.951.208	-24.672
1.958.295	-35.109	1.997.384	-24.767	1.951.368	-24.672
1.958.438	-35.110	1.997.506	-24.765	1.951.499	-24.673
1.958.705	-35.112	1.997.670	-24.764	1.951.653	-24.674
1.958.871	-35.113	1.997.838	-24.762	1.951.770	-24.675
1.959.050	-35.115	1.997.957	-24.760	1.951.895	-24.675
1.959.162	-35.116	1.998.037	-24.759	1.952.047	-24.676
1.959.267	-35.118	1.998.156	-24.757	1.952.213	-24.677
1.959.458	-35.119	1.998.322	-24.755	1.952.426	-24.678
1.959.622	-35.120	1.998.564	-24.754	1.952.666	-24.678
1.959.742	-35.122	1.998.768	-24.752	1.952.854	-24.679
1.959.917	-35.123	1.998.911	-24.751	1.953.006	-24.680
1.960.085	-35.125	1.999.095	-24.749	1.953.183	-24.680
1.960.210	-35.126	1.999.286	-24.748	1.953.335	-24.681
1.960.378	-35.128	1.999.456	-24.746	1.953.519	-24.682

1.960.459	-35.129	1.999.646	-24.744	1.953.745	-24.683
1.960.542	-35.131	1.999.814	-24.743	1.953.931	-24.683
1.960.690	-35.132	1.999.946	-24.741	1.954.050	-24.684
1.960.878	-35.134	2.000.114	-24.740	1.954.088	-24.685
1.961.138	-35.135	2.000.311	-24.738	1.954.245	-24.686
1.961.357	-35.137	2.000.468	-24.737	1.954.498	-24.686
1.961.474	-35.138	2.000.634	-24.735	1.954.648	-24.687
1.961.658	-35.140	2.000.849	-24.734	1.954.740	-24.688
1.961.889	-35.141	2.001.030	-24.732	1.954.911	-24.688
1.962.134	-35.143	2.001.165	-24.731	1.955.155	-24.689
1.962.385	-35.145	2.001.337	-24.729	1.955.350	-24.690
1.962.507	-35.146	2.001.519	-24.728	1.955.490	-24.691
1.962.641	-35.148	2.001.669	-24.726	1.955.582	-24.691
1.962.818	-35.149	2.001.817	-24.725	1.955.757	-24.692
1.963.013	-35.151	2.001.989	-24.723	1.955.961	-24.693
1.963.165	-35.152	2.002.188	-24.722	1.956.080	-24.693
1.963.302	-35.154	2.002.278	-24.721	1.956.237	-24.694
1.963.459	-35.155	2.002.455	-24.719	1.956.462	-24.695
1.963.566	-35.157	2.002.711	-24.718	1.956.671	-24.696
1.963.676	-35.158	2.002.834	-24.716	1.956.861	-24.696
1.963.929	-35.160	2.002.973	-24.715	1.957.003	-24.697
1.964.120	-35.161	2.003.113	-24.713	1.957.137	-24.698
1.964.198	-35.163	2.003.281	-24.712	1.957.298	-24.698
1.964.353	-35.164	2.003.461	-24.711	1.957.486	-24.699
1.964.525	-35.166	2.003.633	-24.709	1.957.655	-24.700
1.964.729	-35.167	2.003.811	-24.708	1.957.791	-24.700
1.964.944	-35.169	2.003.985	-24.707	1.957.977	-24.701
1.965.123	-35.170	2.004.173	-24.705	1.958.127	-24.702
1.965.251	-35.172	2.004.389	-24.704	1.958.297	-24.702
1.965.423	-35.173	2.004.605	-24.702	1.958.508	-24.703

1.965.652	-35.175	2.004.749	-24.701	1.958.638	-24.704
1.965.818	-35.176	2.004.906	-24.700	1.958.770	-24.704
1.965.941	-35.178	2.005.000	-24.698	1.958.965	-24.705
1.966.113	-35.179	2.005.168	-24.697	1.959.390	-24.706
1.966.234	-35.181	2.005.629	-24.696	1.959.825	-24.707
1.966.359	-35.182	2.006.068	-24.694	1.960.007	-24.707
1.966.544	-35.184	2.006.190	-24.693	1.960.089	-24.708
1.966.745	-35.185	2.006.207	-24.692	1.960.139	-24.709
1.966.911	-35.187	2.006.264	-24.691	1.960.192	-24.709
1.967.101	-35.188	2.006.308	-24.689	1.960.215	-24.710
1.967.299	-35.190	2.006.342	-24.688	1.960.320	-24.711
1.967.424	-35.191	2.006.445	-24.687	1.960.479	-24.711
1.967.589	-35.193	2.006.546	-24.685	1.960.564	-24.712
1.967.800	-35.194	2.006.669	-24.684	1.960.687	-24.713
1.967.952	-35.195	2.006.850	-24.683	1.960.876	-24.713
1.968.125	-35.197	2.007.076	-24.681	1.961.091	-24.714
1.968.235	-35.198	2.007.258	-24.680	1.961.301	-24.715
1.968.385	-35.200	2.007.403	-24.679	1.961.447	-24.715
1.968.618	-35.201	2.007.621	-24.678	1.961.595	-24.716
1.968.736	-35.203	2.007.809	-24.676	1.961.765	-24.717
1.968.900	-35.204	2.008.011	-24.675	1.961.886	-24.717
1.969.106	-35.205	2.008.131	-24.674	1.962.021	-24.718
1.969.254	-35.207	2.008.266	-24.673	1.962.195	-24.719
1.969.350	-35.208	2.008.484	-24.671	1.962.402	-24.719
1.969.545	-35.210	2.008.633	-24.670	1.962.556	-24.720
1.969.819	-35.211	2.008.747	-24.669	1.962.753	-24.721
1.969.977	-35.212	2.008.885	-24.668	1.962.908	-24.722
1.970.150	-35.214	2.009.057	-24.666	1.962.997	-24.722
1.970.340	-35.215	2.009.238	-24.665	1.963.125	-24.723
1.970.502	-35.216	2.009.406	-24.664	1.963.300	-24.724

1.970.681	-35.218	2.009.586	-24.663	1.963.512	-24.724
1.970.822	-35.219	2.009.742	-24.662	1.963.689	-24.725
1.970.959	-35.221	2.009.801	-24.660	1.963.822	-24.726
1.971.079	-35.222	2.010.011	-24.659	1.964.003	-24.727
1.971.194	-35.223	2.010.302	-24.658	1.964.223	-24.727
1.971.487	-35.224	2.010.470	-24.657	1.964.395	-24.728
1.971.910	-35.226	2.010.676	-24.656	1.964.588	-24.729
1.972.258	-35.227	2.010.854	-24.655	1.964.763	-24.730
1.972.419	-35.228	2.010.995	-24.653	1.964.843	-24.731
1.972.513	-35.230	2.011.178	-24.652	1.965.034	-24.731
1.972.607	-35.231	2.011.377	-24.651	1.965.244	-24.732
1.972.710	-35.232	2.011.548	-24.650	1.965.341	-24.733
1.972.811	-35.233	2.011.660	-24.649	1.965.513	-24.734
1.972.894	-35.235	2.011.828	-24.648	1.965.703	-24.735
1.972.959	-35.236	2.011.982	-24.647	1.965.770	-24.735
1.973.022	-35.237	2.012.067	-24.646	1.965.898	-24.736
1.973.105	-35.238	2.012.238	-24.644	1.966.129	-24.737
1.973.257	-35.239	2.012.444	-24.643	1.966.308	-24.738
1.973.400	-35.240	2.012.587	-24.642	1.966.497	-24.739
1.973.571	-35.242	2.012.748	-24.641	1.966.712	-24.740
1.973.873	-35.243	2.012.889	-24.640	1.966.877	-24.741
1.974.097	-35.244	2.013.015	-24.639	1.967.014	-24.741
1.974.213	-35.245	2.013.244	-24.638	1.967.166	-24.742
1.974.348	-35.246	2.013.447	-24.637	1.967.332	-24.743
1.974.566	-35.247	2.013.593	-24.636	1.967.493	-24.744
1.974.754	-35.248	2.013.752	-24.635	1.967.639	-24.745
1.974.901	-35.250	2.013.880	-24.634	1.967.798	-24.746
1.975.054	-35.251	2.013.997	-24.633	1.967.968	-24.747
1.975.206	-35.252	2.014.193	-24.632	1.968.192	-24.748
1.975.323	-35.253	2.014.393	-24.631	1.968.389	-24.749

1.975.517	-35.254	2.014.529	-24.630	1.968.488	-24.750
1.975.757	-35.255	2.014.664	-24.629	1.968.658	-24.751
1.975.853	-35.256	2.014.866	-24.628	1.968.907	-24.752
1.975.932	-35.257	2.015.043	-24.627	1.969.086	-24.753
1.976.064	-35.258	2.015.220	-24.626	1.969.247	-24.754
1.976.171	-35.259	2.015.435	-24.625	1.969.391	-24.755
1.976.342	-35.260	2.015.598	-24.624	1.969.471	-24.756
1.976.561	-35.261	2.015.739	-24.623	1.969.587	-24.757
1.976.774	-35.262	2.015.854	-24.622	1.969.760	-24.758
1.976.975	-35.263	2.016.031	-24.622	1.969.978	-24.759
1.977.126	-35.264	2.016.252	-24.621	1.970.152	-24.760
1.977.318	-35.265	2.016.393	-24.620	1.970.277	-24.761
1.977.520	-35.266	2.016.516	-24.619	1.970.450	-24.762
1.977.751	-35.267	2.016.637	-24.618	1.970.656	-24.763
1.977.934	-35.268	2.016.817	-24.617	1.970.833	-24.764
1.978.073	-35.269	2.017.045	-24.616	1.970.977	-24.765
1.978.272	-35.270	2.017.198	-24.615	1.971.173	-24.766
1.978.405	-35.271	2.017.359	-24.614	1.971.339	-24.767
1.978.496	-35.272	2.017.556	-24.613	1.971.478	-24.768
1.978.629	-35.273	2.017.690	-24.613	1.971.675	-24.769
1.978.763	-35.274	2.017.901	-24.612	1.971.803	-24.770
1.978.953	-35.275	2.018.156	-24.611	1.971.898	-24.771
1.979.126	-35.275	2.018.304	-24.610	1.972.039	-24.772
1.979.305	-35.276	2.018.358	-24.609	1.972.182	-24.773
1.979.462	-35.277	2.018.447	-24.608	1.972.305	-24.774
1.979.599	-35.278	2.018.605	-24.607	1.972.437	-24.775
1.979.794	-35.279	2.018.734	-24.606	1.972.603	-24.776
1.979.953	-35.279	2.018.817	-24.606	1.972.775	-24.777
1.980.132	-35.280	2.019.001	-24.605	1.972.910	-24.778
1.980.336	-35.281	2.019.225	-24.604	1.973.060	-24.779

1.980.488	-35.282	2.019.355	-24.603	1.973.304	-24.780
1.980.602	-35.283	2.019.520	-24.602	1.973.559	-24.781
1.980.751	-35.283	2.019.769	-24.601	1.973.833	-24.782
1.980.907	-35.284	2.019.998	-24.600	1.974.065	-24.783
1.980.994	-35.285	2.020.181	-24.600	1.974.212	-24.784
1.981.234	-35.285	2.020.403	-24.599	1.974.377	-24.785
1.981.499	-35.286	2.020.648	-24.598	1.974.483	-24.786
1.981.626	-35.287	2.020.793	-24.597	1.974.646	-24.787
1.981.779	-35.287	2.020.918	-24.596	1.974.821	-24.787
1.981.958	-35.288	2.021.169	-24.595	1.974.939	-24.788
1.982.112	-35.289	2.021.337	-24.595	1.975.100	-24.789
1.982.234	-35.289	2.021.378	-24.594	1.975.284	-24.790
1.982.435	-35.290	2.021.470	-24.593	1.975.405	-24.791
1.982.639	-35.290	2.021.637	-24.592	1.975.468	-24.792
1.982.766	-35.291	2.021.799	-24.591	1.975.623	-24.792
1.982.888	-35.291	2.021.917	-24.590	1.975.780	-24.793
1.983.069	-35.292	2.022.067	-24.590	1.976.010	-24.794
1.983.250	-35.292	2.022.224	-24.589	1.976.241	-24.795
1.983.391	-35.293	2.022.419	-24.588	1.976.416	-24.796
1.983.591	-35.293	2.022.598	-24.587	1.976.552	-24.796
1.983.810	-35.293	2.022.776	-24.586	1.976.696	-24.797
1.984.008	-35.294	2.022.982	-24.585	1.976.861	-24.798
1.984.122	-35.294	2.023.161	-24.585	1.977.045	-24.798
1.984.265	-35.294	2.023.331	-24.584	1.977.234	-24.799
1.984.420	-35.294	2.023.521	-24.583	1.977.395	-24.800
1.984.554	-35.295	2.023.736	-24.582	1.977.600	-24.800
1.984.707	-35.295	2.023.971	-24.581	1.977.802	-24.801
1.984.852	-35.295	2.024.169	-24.581	1.977.939	-24.802
1.985.031	-35.295	2.024.263	-24.580	1.978.118	-24.802
1.985.157	-35.295	2.024.337	-24.579	1.978.289	-24.803

1.985.365	-35.295	2.024.531	-24.578	1.978.427	-24.803
1.985.598	-35.295	2.024.758	-24.577	1.978.577	-24.804
1.985.668	-35.295	2.024.937	-24.577	1.978.730	-24.804
1.985.730	-35.295	2.025.089	-24.576	1.978.923	-24.805
1.985.911	-35.295	2.025.226	-24.575	1.979.124	-24.805
1.986.116	-35.295	2.025.370	-24.574	1.979.287	-24.806
1.986.273	-35.295	2.025.508	-24.574	1.979.406	-24.806
1.986.402	-35.295	2.025.687	-24.573	1.979.548	-24.807
1.986.541	-35.295	2.025.865	-24.572	1.979.760	-24.807
1.986.769	-35.294	2.026.070	-24.571	1.979.948	-24.807
1.987.010	-35.294	2.026.292	-24.571	1.980.108	-24.808
1.987.206	-35.294	2.026.418	-24.570	1.980.226	-24.808
1.987.348	-35.293	2.026.546	-24.569	1.980.343	-24.809
1.987.576	-35.293	2.026.729	-24.568	1.980.540	-24.809
1.987.838	-35.293	2.026.917	-24.568	1.980.780	-24.809
1.987.964	-35.292	2.027.108	-24.567	1.980.983	-24.810
1.988.134	-35.292	2.027.289	-24.566	1.981.100	-24.810
1.988.340	-35.291	2.027.430	-24.565	1.981.285	-24.810
1.988.573	-35.291	2.027.621	-24.565	1.981.555	-24.810
1.988.757	-35.290	2.027.807	-24.564	1.981.749	-24.811
1.988.806	-35.289	2.027.919	-24.563	1.981.826	-24.811
1.988.855	-35.289	2.028.085	-24.563	1.981.962	-24.811
1.988.918	-35.288	2.028.252	-24.562	1.982.189	-24.811
1.989.064	-35.287	2.028.425	-24.561	1.982.281	-24.811
1.989.247	-35.286	2.028.618	-24.561	1.982.477	-24.811
1.989.435	-35.285	2.028.725	-24.560	1.982.936	-24.812
1.989.621	-35.284	2.028.859	-24.560	1.983.315	-24.812
1.989.762	-35.284	2.029.010	-24.559	1.983.519	-24.812
1.990.007	-35.283	2.029.155	-24.558	1.983.635	-24.812
1.990.211	-35.282	2.029.422	-24.558	1.983.647	-24.812

1.990.338	-35.280	2.029.881	-24.557	1.983.651	-24.812
1.990.528	-35.279	2.030.257	-24.557	1.983.745	-24.812
1.990.703	-35.278	2.030.359	-24.556	1.983.882	-24.812
1.990.862	-35.277	2.030.424	-24.556	1.983.945	-24.812
1.991.037	-35.276	2.030.513	-24.555	1.984.007	-24.812
1.991.192	-35.275	2.030.564	-24.555	1.984.173	-24.812
1.991.396	-35.273	2.030.638	-24.554	1.984.362	-24.812
1.991.582	-35.272	2.030.762	-24.554	1.984.543	-24.812
1.991.729	-35.271	2.030.851	-24.553	1.984.702	-24.812
1.991.870	-35.269	2.030.952	-24.553	1.984.890	-24.812
1.991.967	-35.268	2.031.115	-24.552	1.985.081	-24.812
1.992.139	-35.266	2.031.288	-24.552	1.985.230	-24.812
1.992.352	-35.265	2.031.413	-24.552	1.985.379	-24.812
1.992.469	-35.263	2.031.563	-24.551	1.985.536	-24.812
1.992.626	-35.262	2.031.797	-24.551	1.985.695	-24.812
1.992.872	-35.260	2.032.018	-24.550	1.985.858	-24.811
1.993.033	-35.259	2.032.231	-24.550	1.986.052	-24.811
1.993.136	-35.257	2.032.408	-24.550	1.986.250	-24.811
1.993.271	-35.255	2.032.543	-24.550	1.986.447	-24.811
1.993.505	-35.253	2.032.724	-24.549	1.986.621	-24.811
1.993.808	-35.252	2.032.910	-24.549	1.986.747	-24.811
1.994.027	-35.250	2.033.091	-24.549	1.986.835	-24.811
1.994.137	-35.248	2.033.207	-24.549	1.986.927	-24.811
1.994.272	-35.246	2.033.353	-24.549	1.987.083	-24.810
1.994.411	-35.244	2.033.524	-24.548	1.987.244	-24.810
1.994.566	-35.242	2.033.617	-24.548	1.987.410	-24.810
1.994.677	-35.240	2.033.792	-24.548	1.987.567	-24.810
1.994.846	-35.238	2.033.934	-24.548	1.987.764	-24.810
1.995.060	-35.236	2.034.059	-24.548	1.987.980	-24.810
1.995.186	-35.234	2.034.294	-24.548	1.988.181	-24.810

1.995.367	-35.232	2.034.509	-24.548	1.988.372	-24.810
1.995.612	-35.230	2.034.713	-24.548	1.988.528	-24.810
1.995.817	-35.228	2.034.910	-24.548	1.988.696	-24.809
1.995.967	-35.225	2.035.110	-24.549	1.988.893	-24.809
1.996.086	-35.223	2.035.300	-24.549	1.989.025	-24.809
1.996.255	-35.221	2.035.471	-24.549	1.989.156	-24.809
1.996.423	-35.219	2.035.649	-24.549	1.989.333	-24.809
1.996.530	-35.216	2.035.764	-24.549	1.989.484	-24.809
1.996.689	-35.214	2.035.873	-24.550	1.989.657	-24.809
1.996.911	-35.212	2.036.013	-24.550	1.989.861	-24.809
1.997.290	-35.209	2.036.201	-24.550	1.990.058	-24.809
1.997.677	-35.207	2.036.384	-24.551	1.990.217	-24.809
1.997.865	-35.205	2.036.479	-24.551	1.990.368	-24.809
1.997.932	-35.202	2.036.579	-24.552	1.990.471	-24.809
1.997.988	-35.200	2.036.751	-24.552	1.990.613	-24.809
1.998.047	-35.197	2.036.987	-24.553	1.990.811	-24.809
1.998.123	-35.195	2.037.142	-24.553	1.990.961	-24.809
1.998.266	-35.192	2.037.222	-24.554	1.991.113	-24.809
1.998.434	-35.190	2.037.372	-24.554	1.991.276	-24.809
1.998.537	-35.187	2.037.605	-24.555	1.991.443	-24.809
1.998.591	-35.185	2.037.767	-24.556	1.991.662	-24.809
1.998.743	-35.182	2.037.863	-24.556	1.991.870	-24.809
1.998.985	-35.179	2.038.033	-24.557	1.992.036	-24.809
1.999.146	-35.177	2.038.221	-24.558	1.992.204	-24.809
1.999.301	-35.174	2.038.385	-24.559	1.992.356	-24.809
1.999.485	-35.172	2.038.517	-24.560	1.992.488	-24.809
1.999.695	-35.169	2.038.692	-24.561	1.992.677	-24.809
1.999.899	-35.166	2.038.914	-24.562	1.992.820	-24.810
2.000.051	-35.164	2.039.092	-24.563	1.992.966	-24.810
2.000.139	-35.161	2.039.292	-24.564	1.993.129	-24.810

2.000.238	-35.159	2.039.453	-24.565	1.993.250	-24.810
2.000.437	-35.156	2.039.583	-24.566	1.993.412	-24.810
2.000.647	-35.153	2.039.774	-24.567	1.993.622	-24.811
2.000.820	-35.151	2.039.979	-24.568	1.993.820	-24.811
2.000.963	-35.148	2.040.126	-24.569	1.993.952	-24.811
2.001.145	-35.146	2.040.246	-24.571	1.994.108	-24.811
2.001.357	-35.143	2.040.383	-24.572	1.994.285	-24.812
2.001.541	-35.140	2.040.553	-24.573	1.994.415	-24.812
2.001.673	-35.138	2.040.755	-24.575	1.994.601	-24.812
2.001.749	-35.135	2.040.931	-24.576	1.994.807	-24.812
2.001.835	-35.133	2.041.082	-24.577	1.994.932	-24.813
2.002.034	-35.130	2.041.256	-24.579	1.995.083	-24.813
2.002.294	-35.127	2.041.409	-24.580	1.995.235	-24.813
2.002.514	-35.125	2.041.563	-24.582	1.995.349	-24.814
2.002.721	-35.122	2.041.718	-24.583	1.995.520	-24.814
2.002.897	-35.120	2.041.843	-24.585	1.995.723	-24.814
2.003.044	-35.117	2.042.031	-24.586	1.995.833	-24.815
2.003.215	-35.115	2.042.222	-24.588	1.995.961	-24.815
2.003.394	-35.112	2.042.358	-24.590	1.996.120	-24.815
2.003.584	-35.110	2.042.486	-24.591	1.996.268	-24.816
2.003.727	-35.107	2.042.681	-24.593	1.996.449	-24.816
2.003.826	-35.105	2.042.887	-24.595	1.996.678	-24.817
2.003.953	-35.102	2.043.022	-24.597	1.996.918	-24.817
2.004.057	-35.100	2.043.131	-24.598	1.997.134	-24.817
2.004.245	-35.097	2.043.239	-24.600	1.997.296	-24.818
2.004.509	-35.095	2.043.400	-24.602	1.997.423	-24.818
2.004.630	-35.092	2.043.551	-24.604	1.997.635	-24.819
2.004.745	-35.090	2.043.768	-24.606	1.997.840	-24.819
2.004.964	-35.087	2.044.016	-24.608	1.997.944	-24.820
2.005.087	-35.085	2.044.182	-24.610	1.998.098	-24.820

2.005.184	-35.083	2.044.382	-24.612	1.998.286	-24.820
2.005.396	-35.080	2.044.576	-24.614	1.998.400	-24.821
2.005.540	-35.078	2.044.774	-24.616	1.998.513	-24.821
2.005.668	-35.076	2.044.948	-24.618	1.998.672	-24.822
2.005.889	-35.073	2.045.121	-24.620	1.998.866	-24.822
2.006.049	-35.071	2.045.282	-24.622	1.999.026	-24.823
2.006.164	-35.069	2.045.424	-24.624	1.999.186	-24.823
2.006.311	-35.067	2.045.591	-24.626	1.999.348	-24.823
2.006.530	-35.064	2.045.753	-24.628	1.999.494	-24.824
2.006.749	-35.062	2.045.876	-24.630	1.999.684	-24.824
2.006.949	-35.060	2.046.012	-24.632	1.999.867	-24.825
2.007.143	-35.058	2.046.115	-24.634	2.000.022	-24.825
2.007.260	-35.056	2.046.248	-24.637	2.000.215	-24.826
2.007.402	-35.054	2.046.447	-24.639	2.000.415	-24.826
2.007.621	-35.052	2.046.604	-24.641	2.000.609	-24.827
2.007.829	-35.050	2.046.767	-24.643	2.000.775	-24.827
2.007.905	-35.047	2.046.966	-24.645	2.000.923	-24.828
2.008.024	-35.045	2.047.132	-24.648	2.001.084	-24.828
2.008.277	-35.043	2.047.294	-24.650	2.001.254	-24.829
2.008.447	-35.041	2.047.491	-24.652	2.001.402	-24.829
2.008.613	-35.039	2.047.706	-24.655	2.001.557	-24.830
2.008.799	-35.037	2.047.919	-24.657	2.001.718	-24.830
2.008.978	-35.035	2.048.011	-24.659	2.001.848	-24.831
2.009.144	-35.033	2.048.161	-24.661	2.002.045	-24.831
2.009.270	-35.031	2.048.400	-24.664	2.002.204	-24.832
2.009.422	-35.030	2.048.486	-24.666	2.002.392	-24.832
2.009.628	-35.028	2.048.645	-24.669	2.002.605	-24.832
2.009.845	-35.026	2.048.882	-24.671	2.002.782	-24.833
2.010.022	-35.024	2.049.063	-24.673	2.002.952	-24.833
2.010.208	-35.022	2.049.241	-24.676	2.003.065	-24.834

2.010.309	-35.020	2.049.413	-24.678	2.003.219	-24.834
2.010.423	-35.018	2.049.590	-24.680	2.003.391	-24.835
2.010.600	-35.016	2.049.715	-24.683	2.003.557	-24.835
2.010.780	-35.015	2.049.874	-24.685	2.003.710	-24.836
2.010.958	-35.013	2.050.116	-24.688	2.003.867	-24.836
2.011.102	-35.011	2.050.271	-24.690	2.004.075	-24.837
2.011.219	-35.009	2.050.434	-24.693	2.004.267	-24.837
2.011.290	-35.007	2.050.636	-24.695	2.004.401	-24.838
2.011.371	-35.005	2.050.804	-24.697	2.004.601	-24.838
2.011.526	-35.004	2.050.968	-24.700	2.004.792	-24.839
2.011.756	-35.002	2.051.113	-24.702	2.004.894	-24.839
2.011.969	-35.000	2.051.261	-24.705	2.005.052	-24.840
2.012.142	-34.998	2.051.375	-24.707	2.005.239	-24.840
2.012.363	-34.997	2.051.496	-24.710	2.005.462	-24.841
2.012.596	-34.995	2.051.673	-24.712	2.005.576	-24.841
2.012.728	-34.993	2.051.845	-24.715	2.005.681	-24.842
2.012.863	-34.992	2.051.978	-24.717	2.006.039	-24.842
2.013.100	-34.990	2.052.148	-24.720	2.006.443	-24.843
2.013.315	-34.988	2.052.294	-24.722	2.006.696	-24.843
2.013.443	-34.986	2.052.457	-24.725	2.006.821	-24.843
2.013.629	-34.985	2.052.691	-24.727	2.006.924	-24.844
2.013.846	-34.983	2.052.824	-24.730	2.007.016	-24.844
2.014.018	-34.981	2.052.954	-24.732	2.007.077	-24.845
2.014.173	-34.979	2.053.091	-24.735	2.007.101	-24.845
2.014.279	-34.978	2.053.375	-24.737	2.007.148	-24.846
2.014.395	-34.976	2.053.826	-24.740	2.007.343	-24.846
2.014.489	-34.974	2.054.185	-24.742	2.007.514	-24.847
2.014.581	-34.973	2.054.373	-24.745	2.007.623	-24.847
2.014.713	-34.971	2.054.406	-24.747	2.007.829	-24.848
2.014.850	-34.969	2.054.440	-24.750	2.008.051	-24.848

2.015.040	-34.967	2.054.536	-24.753	2.008.185	-24.848
2.015.284	-34.966	2.054.592	-24.755	2.008.351	-24.849
2.015.513	-34.964	2.054.661	-24.758	2.008.553	-24.849
2.015.719	-34.962	2.054.773	-24.760	2.008.756	-24.850
2.015.896	-34.960	2.054.892	-24.763	2.008.983	-24.850
2.016.037	-34.959	2.055.058	-24.765	2.009.138	-24.851
2.016.189	-34.957	2.055.266	-24.768	2.009.227	-24.851
2.016.368	-34.955	2.055.430	-24.770	2.009.363	-24.852
2.016.582	-34.953	2.055.582	-24.773	2.009.548	-24.852
2.016.819	-34.952	2.055.761	-24.776	2.009.634	-24.852
2.017.009	-34.950	2.055.987	-24.778	2.009.764	-24.853
2.017.179	-34.948	2.056.086	-24.781	2.009.987	-24.853
2.017.327	-34.946	2.056.232	-24.783	2.010.161	-24.854
2.017.446	-34.944	2.056.541	-24.786	2.010.271	-24.854
2.017.659	-34.943	2.056.721	-24.789	2.010.408	-24.855
2.017.865	-34.941	2.056.803	-24.791	2.010.627	-24.855
2.018.026	-34.939	2.056.906	-24.794	2.010.817	-24.856
2.018.136	-34.937	2.057.094	-24.796	2.010.954	-24.856
2.018.215	-34.935	2.057.293	-24.799	2.011.124	-24.857
2.018.439	-34.934	2.057.523	-24.802	2.011.288	-24.857
2.018.712	-34.932	2.057.715	-24.804	2.011.463	-24.857
2.018.896	-34.930	2.057.795	-24.807	2.011.687	-24.858
2.019.025	-34.928	2.057.849	-24.810	2.011.893	-24.858
2.019.164	-34.926	2.057.943	-24.812	2.012.061	-24.859
2.019.279	-34.924	2.058.147	-24.815	2.012.234	-24.859
2.019.404	-34.922	2.058.380	-24.818	2.012.374	-24.860
2.019.605	-34.920	2.058.602	-24.821	2.012.498	-24.860
2.019.769	-34.919	2.058.826	-24.823	2.012.690	-24.861
2.019.967	-34.917	2.058.987	-24.826	2.012.874	-24.862
2.020.204	-34.915	2.059.162	-24.829	2.012.959	-24.862

2.020.313	-34.913	2.059.384	-24.832	2.013.092	-24.863
2.020.392	-34.911	2.059.583	-24.834	2.013.262	-24.863
2.020.553	-34.909	2.059.747	-24.837	2.013.372	-24.864
2.020.786	-34.907	2.059.879	-24.840	2.013.566	-24.864
2.021.017	-34.905	2.060.018	-24.843	2.013.773	-24.865
2.021.158	-34.903	2.060.125	-24.846	2.014.007	-24.865
2.021.301	-34.901	2.060.255	-24.848	2.014.160	-24.866
2.021.472	-34.899	2.060.423	-24.851	2.014.254	-24.867
2.021.581	-34.897	2.060.555	-24.854	2.014.437	-24.867
2.021.767	-34.895	2.060.683	-24.857	2.014.612	-24.868
2.021.960	-34.893	2.060.853	-24.860	2.014.776	-24.869
2.022.007	-34.891	2.061.046	-24.863	2.014.917	-24.869
2.022.197	-34.889	2.061.243	-24.866	2.015.081	-24.870
2.022.634	-34.887	2.061.384	-24.869	2.015.286	-24.871
2.023.040	-34.885	2.061.530	-24.872	2.015.461	-24.871
2.023.246	-34.883	2.061.691	-24.875	2.015.656	-24.872
2.023.320	-34.881	2.061.812	-24.878	2.015.827	-24.873
2.023.384	-34.879	2.061.962	-24.881	2.015.983	-24.873
2.023.477	-34.877	2.062.117	-24.884	2.016.129	-24.874
2.023.541	-34.875	2.062.258	-24.887	2.016.248	-24.875
2.023.629	-34.873	2.062.469	-24.890	2.016.423	-24.875
2.023.794	-34.871	2.062.635	-24.893	2.016.664	-24.876
2.023.896	-34.869	2.062.854	-24.896	2.016.861	-24.877
2.024.016	-34.867	2.063.104	-24.899	2.016.949	-24.878
2.024.169	-34.865	2.063.232	-24.902	2.017.067	-24.878
2.024.330	-34.863	2.063.345	-24.906	2.017.254	-24.879
2.024.476	-34.861	2.063.512	-24.909	2.017.417	-24.880
2.024.662	-34.859	2.063.703	-24.912	2.017.569	-24.881
2.024.908	-34.857	2.063.810	-24.915	2.017.703	-24.881
2.025.110	-34.855	2.063.967	-24.918	2.017.959	-24.882

2.025.253	-34.853	2.064.187	-24.922	2.018.183	-24.883
2.025.405	-34.851	2.064.384	-24.925	2.018.288	-24.884
2.025.569	-34.849	2.064.601	-24.928	2.018.438	-24.884
2.025.714	-34.847	2.064.706	-24.931	2.018.526	-24.885
2.025.802	-34.845	2.064.834	-24.935	2.018.644	-24.886
2.025.952	-34.843	2.065.076	-24.938	2.018.799	-24.887
2.026.199	-34.842	2.065.280	-24.941	2.018.920	-24.887
2.026.380	-34.840	2.065.423	-24.945	2.019.115	-24.888
2.026.519	-34.838	2.065.571	-24.948	2.019.303	-24.889
2.026.642	-34.836	2.065.707	-24.951	2.019.417	-24.890
2.026.814	-34.834	2.065.855	-24.955	2.019.534	-24.890
2.027.005	-34.832	2.066.059	-24.958	2.019.794	-24.891
2.027.079	-34.830	2.066.265	-24.961	2.020.094	-24.892
2.027.238	-34.828	2.066.414	-24.965	2.020.257	-24.893
2.027.439	-34.827	2.066.523	-24.968	2.020.457	-24.893
2.027.565	-34.825	2.066.635	-24.972	2.020.686	-24.894
2.027.802	-34.823	2.066.798	-24.975	2.020.842	-24.895
2.028.019	-34.821	2.066.942	-24.978	2.021.010	-24.896
2.028.156	-34.819	2.067.061	-24.982	2.021.153	-24.896
2.028.313	-34.818	2.067.211	-24.985	2.021.295	-24.897
2.028.506	-34.816	2.067.372	-24.989	2.021.483	-24.898
2.028.705	-34.814	2.067.544	-24.992	2.021.618	-24.898
2.028.891	-34.812	2.067.739	-24.996	2.021.751	-24.899
2.029.061	-34.811	2.067.912	-24.999	2.021.888	-24.900
2.029.164	-34.809	2.068.103	-25.002	2.022.011	-24.900
2.029.283	-34.807	2.068.313	-25.006	2.022.144	-24.901
2.029.494	-34.806	2.068.533	-25.009	2.022.292	-24.902
2.029.664	-34.804	2.068.741	-25.013	2.022.455	-24.902
2.029.798	-34.802	2.068.931	-25.016	2.022.643	-24.903
2.029.847	-34.801	2.069.097	-25.020	2.022.876	-24.904

2.030.000	-34.799	2.069.202	-25.023	2.023.037	-24.904
2.030.210	-34.798	2.069.357	-25.027	2.023.195	-24.905
2.030.358	-34.796	2.069.505	-25.030	2.023.428	-24.905
2.030.515	-34.795	2.069.646	-25.033	2.023.602	-24.906
2.030.744	-34.793	2.069.811	-25.037	2.023.759	-24.907
2.030.948	-34.792	2.069.958	-25.040	2.023.947	-24.907
2.031.108	-34.790	2.070.127	-25.044	2.024.075	-24.908
2.031.324	-34.789	2.070.266	-25.047	2.024.230	-24.908
2.031.440	-34.788	2.070.430	-25.051	2.024.462	-24.909
2.031.595	-34.786	2.070.580	-25.054	2.024.648	-24.909
2.031.752	-34.785	2.070.685	-25.057	2.024.772	-24.910
2.031.886	-34.784	2.070.851	-25.061	2.024.917	-24.910
2.032.020	-34.782	2.071.111	-25.064	2.025.074	-24.911
2.032.146	-34.781	2.071.283	-25.068	2.025.239	-24.911
2.032.355	-34.780	2.071.415	-25.071	2.025.412	-24.912
2.032.581	-34.779	2.071.660	-25.074	2.025.538	-24.912
2.032.803	-34.778	2.071.877	-25.078	2.025.704	-24.913
2.033.024	-34.776	2.071.994	-25.081	2.025.941	-24.913
2.033.163	-34.775	2.072.161	-25.084	2.026.131	-24.914
2.033.356	-34.774	2.072.296	-25.088	2.026.293	-24.914
2.033.548	-34.773	2.072.448	-25.091	2.026.447	-24.915
2.033.716	-34.772	2.072.641	-25.094	2.026.572	-24.915
2.033.855	-34.771	2.072.802	-25.098	2.026.727	-24.916
2.034.041	-34.770	2.072.977	-25.101	2.026.924	-24.916
2.034.241	-34.769	2.073.156	-25.104	2.027.074	-24.917
2.034.377	-34.768	2.073.338	-25.107	2.027.199	-24.917
2.034.563	-34.767	2.073.504	-25.111	2.027.341	-24.917
2.034.619	-34.766	2.073.685	-25.114	2.027.529	-24.918
2.034.740	-34.765	2.073.900	-25.117	2.027.724	-24.918
2.034.991	-34.765	2.074.113	-25.120	2.027.906	-24.919

2.035.157	-34.764	2.074.220	-25.124	2.028.127	-24.919
2.035.224	-34.763	2.074.276	-25.127	2.028.295	-24.919
2.035.367	-34.762	2.074.498	-25.130	2.028.461	-24.920
2.035.598	-34.761	2.074.718	-25.133	2.028.681	-24.920
2.035.751	-34.760	2.074.917	-25.136	2.028.840	-24.920
2.035.912	-34.760	2.075.087	-25.139	2.028.913	-24.921
2.036.080	-34.759	2.075.194	-25.142	2.028.985	-24.921
2.036.173	-34.758	2.075.376	-25.145	2.029.315	-24.922
2.036.344	-34.757	2.075.557	-25.149	2.029.825	-24.922
2.036.492	-34.757	2.075.708	-25.152	2.030.163	-24.922
2.036.570	-34.756	2.075.838	-25.155	2.030.280	-24.923
2.036.678	-34.755	2.076.004	-25.158	2.030.343	-24.923
2.036.897	-34.755	2.076.208	-25.161	2.030.412	-24.923
2.037.146	-34.754	2.076.384	-25.164	2.030.425	-24.924
2.037.321	-34.753	2.076.556	-25.167	2.030.471	-24.924
2.037.479	-34.753	2.076.653	-25.169	2.030.591	-24.924
2.037.655	-34.752	2.076.767	-25.172	2.030.708	-24.925
2.037.836	-34.751	2.076.991	-25.175	2.030.851	-24.925
2.038.039	-34.751	2.077.137	-25.178	2.031.026	-24.925
2.038.246	-34.750	2.077.229	-25.181	2.031.198	-24.926
2.038.427	-34.750	2.077.578	-25.184	2.031.373	-24.926
2.038.618	-34.749	2.078.058	-25.187	2.031.572	-24.926
2.038.841	-34.748	2.078.307	-25.190	2.031.779	-24.926
2.039.050	-34.748	2.078.369	-25.192	2.031.931	-24.927
2.039.242	-34.747	2.078.456	-25.195	2.032.016	-24.927
2.039.404	-34.747	2.078.575	-25.198	2.032.153	-24.927
2.039.501	-34.746	2.078.691	-25.201	2.032.403	-24.928
2.039.608	-34.745	2.078.752	-25.203	2.032.592	-24.928
2.039.742	-34.745	2.078.770	-25.206	2.032.717	-24.928
2.039.904	-34.744	2.078.880	-25.209	2.032.804	-24.929

2.040.067	-34.744	2.079.073	-25.211	2.032.905	-24.929
2.040.219	-34.743	2.079.261	-25.214	2.033.109	-24.929
2.040.374	-34.742	2.079.440	-25.216	2.033.331	-24.929
2.040.567	-34.742	2.079.642	-25.219	2.033.499	-24.930
2.040.728	-34.741	2.079.776	-25.222	2.033.629	-24.930
2.040.845	-34.741	2.079.953	-25.224	2.033.763	-24.930
2.041.010	-34.740	2.080.182	-25.227	2.033.923	-24.931
2.041.229	-34.740	2.080.334	-25.229	2.034.081	-24.931
2.041.453	-34.739	2.080.502	-25.232	2.034.258	-24.931
2.041.669	-34.738	2.080.701	-25.234	2.034.464	-24.932
2.041.870	-34.738	2.080.876	-25.236	2.034.644	-24.932
2.042.025	-34.737	2.081.017	-25.239	2.034.865	-24.932
2.042.150	-34.737	2.081.138	-25.241	2.035.076	-24.933
2.042.246	-34.736	2.081.313	-25.244	2.035.193	-24.933
2.042.330	-34.735	2.081.505	-25.246	2.035.325	-24.934
2.042.505	-34.735	2.081.684	-25.248	2.035.576	-24.934
2.042.749	-34.734	2.081.854	-25.251	2.035.777	-24.934
2.042.997	-34.733	2.081.908	-25.253	2.035.887	-24.935
2.043.207	-34.733	2.081.989	-25.255	2.036.010	-24.935
2.043.380	-34.732	2.082.161	-25.258	2.036.158	-24.936
2.043.517	-34.731	2.082.341	-25.260	2.036.368	-24.936
2.043.597	-34.731	2.082.547	-25.262	2.036.507	-24.937
2.043.741	-34.730	2.082.762	-25.264	2.036.581	-24.937
2.043.941	-34.729	2.082.916	-25.267	2.036.792	-24.937
2.044.171	-34.729	2.083.047	-25.269	2.036.989	-24.938
2.044.343	-34.728	2.083.210	-25.271	2.037.166	-24.938
2.044.465	-34.727	2.083.398	-25.273	2.037.399	-24.939
2.044.635	-34.726	2.083.620	-25.275	2.037.561	-24.939
2.044.807	-34.726	2.083.817	-25.278	2.037.668	-24.940
2.044.982	-34.725	2.083.949	-25.280	2.037.795	-24.941

2.045.147	-34.724	2.084.052	-25.282	2.037.943	-24.941
2.045.258	-34.723	2.084.234	-25.284	2.038.109	-24.942
2.045.387	-34.723	2.084.379	-25.286	2.038.358	-24.942
2.045.594	-34.722	2.084.518	-25.288	2.038.532	-24.943
2.045.744	-34.721	2.084.709	-25.290	2.038.627	-24.943
2.045.882	-34.720	2.084.875	-25.292	2.038.755	-24.944
2.046.052	-34.719	2.085.069	-25.294	2.038.927	-24.945
2.046.279	-34.718	2.085.264	-25.297	2.039.151	-24.945
2.046.454	-34.718	2.085.418	-25.299	2.039.352	-24.946
2.046.653	-34.717	2.085.538	-25.301	2.039.520	-24.946
2.046.935	-34.716	2.085.696	-25.303	2.039.664	-24.947
2.047.069	-34.715	2.085.891	-25.305	2.039.821	-24.948
2.047.153	-34.714	2.086.057	-25.307	2.039.962	-24.948
2.047.253	-34.713	2.086.214	-25.309	2.040.089	-24.949
2.047.448	-34.712	2.086.353	-25.311	2.040.296	-24.950
2.047.641	-34.711	2.086.503	-25.313	2.040.508	-24.950
2.047.787	-34.710	2.086.693	-25.315	2.040.658	-24.951
2.048.138	-34.709	2.086.774	-25.317	2.040.780	-24.952
2.048.589	-34.708	2.086.924	-25.319	2.040.938	-24.952
2.048.822	-34.707	2.087.164	-25.321	2.041.115	-24.953
2.048.958	-34.706	2.087.311	-25.323	2.041.245	-24.954
2.049.118	-34.705	2.087.473	-25.325	2.041.416	-24.954
2.049.180	-34.704	2.087.634	-25.327	2.041.642	-24.955
2.049.126	-34.703	2.087.869	-25.329	2.041.819	-24.956
2.049.137	-34.702	2.088.127	-25.331	2.041.938	-24.957
2.049.278	-34.701	2.088.311	-25.333	2.042.069	-24.957
2.049.366	-34.699	2.088.418	-25.335	2.042.181	-24.958
2.049.501	-34.698	2.088.564	-25.337	2.042.309	-24.959
2.049.673	-34.697	2.088.738	-25.339	2.042.518	-24.959
2.049.866	-34.696	2.088.850	-25.341	2.042.610	-24.960

2.050.099	-34.695	2.089.025	-25.343	2.042.757	-24.961
2.050.242	-34.694	2.089.236	-25.345	2.042.957	-24.961
2.050.381	-34.692	2.089.354	-25.347	2.043.139	-24.962
2.050.569	-34.691	2.089.492	-25.349	2.043.391	-24.963
2.050.770	-34.690	2.089.688	-25.351	2.043.598	-24.963
2.050.966	-34.689	2.089.876	-25.352	2.043.831	-24.964
2.051.124	-34.687	2.090.089	-25.354	2.044.030	-24.965
2.051.264	-34.686	2.090.220	-25.356	2.044.189	-24.965
2.051.438	-34.685	2.090.347	-25.358	2.044.339	-24.966
2.051.592	-34.684	2.090.509	-25.360	2.044.498	-24.967
2.051.786	-34.682	2.090.571	-25.362	2.044.639	-24.967
2.051.957	-34.681	2.090.665	-25.364	2.044.767	-24.968
2.052.090	-34.680	2.090.855	-25.366	2.044.939	-24.969
2.052.227	-34.679	2.091.064	-25.367	2.045.138	-24.969
2.052.330	-34.677	2.091.239	-25.369	2.045.329	-24.970
2.052.491	-34.676	2.091.313	-25.371	2.045.470	-24.970
2.052.650	-34.675	2.091.429	-25.373	2.045.567	-24.971
2.052.840	-34.673	2.091.665	-25.375	2.045.716	-24.971
2.053.037	-34.672	2.091.862	-25.376	2.045.928	-24.972
2.053.163	-34.671	2.092.061	-25.378	2.046.111	-24.973
2.053.353	-34.669	2.092.289	-25.380	2.046.221	-24.973
2.053.546	-34.668	2.092.518	-25.382	2.046.376	-24.974
2.053.746	-34.667	2.092.764	-25.383	2.046.597	-24.974
2.053.974	-34.665	2.092.966	-25.385	2.046.801	-24.975
2.054.151	-34.664	2.093.136	-25.387	2.046.973	-24.975
2.054.292	-34.663	2.093.291	-25.389	2.047.106	-24.975
2.054.460	-34.661	2.093.398	-25.390	2.047.264	-24.976
2.054.594	-34.660	2.093.474	-25.392	2.047.453	-24.976
2.054.722	-34.659	2.093.604	-25.394	2.047.648	-24.977
2.054.903	-34.657	2.093.747	-25.395	2.047.816	-24.977

2.055.015	-34.656	2.093.924	-25.397	2.047.926	-24.978
2.055.103	-34.655	2.094.095	-25.399	2.048.073	-24.978
2.055.293	-34.653	2.094.247	-25.400	2.048.250	-24.978
2.055.432	-34.652	2.094.384	-25.402	2.048.459	-24.979
2.055.629	-34.651	2.094.532	-25.404	2.048.660	-24.979
2.055.784	-34.649	2.094.771	-25.405	2.048.826	-24.979
2.055.996	-34.648	2.094.977	-25.407	2.048.983	-24.980
2.056.223	-34.647	2.095.094	-25.408	2.049.109	-24.980
2.056.335	-34.645	2.095.271	-25.410	2.049.310	-24.980
2.056.523	-34.644	2.095.524	-25.412	2.049.501	-24.981
2.056.745	-34.643	2.095.750	-25.413	2.049.655	-24.981
2.056.848	-34.641	2.095.896	-25.415	2.049.807	-24.981
2.056.975	-34.640	2.096.057	-25.416	2.049.962	-24.981
2.057.119	-34.639	2.096.241	-25.418	2.050.110	-24.982
2.057.211	-34.637	2.096.341	-25.419	2.050.244	-24.982
2.057.401	-34.636	2.096.483	-25.421	2.050.380	-24.982
2.057.626	-34.635	2.096.651	-25.422	2.050.557	-24.982
2.057.822	-34.633	2.096.832	-25.424	2.050.813	-24.982
2.057.991	-34.632	2.096.991	-25.425	2.051.019	-24.982
2.058.167	-34.631	2.097.104	-25.427	2.051.180	-24.983
2.058.401	-34.630	2.097.287	-25.428	2.051.344	-24.983
2.058.604	-34.628	2.097.502	-25.430	2.051.469	-24.983
2.058.741	-34.627	2.097.738	-25.431	2.051.630	-24.983
2.058.866	-34.626	2.097.952	-25.433	2.051.841	-24.983
2.058.989	-34.625	2.098.064	-25.434	2.052.066	-24.983
2.059.086	-34.623	2.098.167	-25.436	2.052.258	-24.983
2.059.227	-34.622	2.098.373	-25.437	2.052.357	-24.983
2.059.397	-34.621	2.098.553	-25.438	2.052.504	-24.983
2.059.579	-34.620	2.098.676	-25.440	2.052.811	-24.983
2.059.767	-34.618	2.098.860	-25.441	2.053.259	-24.983

2.059.996	-34.617	2.099.079	-25.443	2.053.564	-24.983
2.060.096	-34.616	2.099.234	-25.444	2.053.642	-24.983
2.060.190	-34.614	2.099.334	-25.445	2.053.681	-24.983
2.060.374	-34.613	2.099.491	-25.447	2.053.745	-24.983
2.060.602	-34.612	2.099.690	-25.448	2.053.884	-24.983
2.060.755	-34.611	2.099.845	-25.449	2.053.952	-24.983
2.060.927	-34.609	2.100.038	-25.451	2.053.952	-24.983
2.061.102	-34.608	2.100.224	-25.452	2.053.985	-24.983
2.061.245	-34.607	2.100.354	-25.453	2.054.133	-24.983
2.061.378	-34.606	2.100.526	-25.454	2.054.362	-24.983
2.061.534	-34.604	2.100.656	-25.456	2.054.558	-24.983
2.061.655	-34.603	2.100.815	-25.457	2.054.726	-24.983
2.061.765	-34.602	2.101.013	-25.458	2.054.877	-24.983
2.061.996	-34.601	2.101.176	-25.460	2.055.065	-24.982
2.062.168	-34.599	2.101.350	-25.461	2.055.294	-24.982
2.062.314	-34.598	2.101.673	-25.462	2.055.457	-24.982
2.062.433	-34.597	2.102.106	-25.463	2.055.609	-24.982
2.062.607	-34.595	2.102.401	-25.465	2.055.761	-24.982
2.062.852	-34.594	2.102.536	-25.466	2.055.918	-24.982
2.063.011	-34.593	2.102.581	-25.467	2.056.070	-24.982
2.063.152	-34.592	2.102.578	-25.468	2.056.245	-24.982
2.063.331	-34.590	2.102.619	-25.469	2.056.429	-24.982
2.063.557	-34.589	2.102.728	-25.471	2.056.583	-24.981
2.063.783	-34.588	2.102.806	-25.472	2.056.754	-24.981
2.063.967	-34.586	2.102.921	-25.473	2.056.888	-24.981
2.064.223	-34.585	2.103.127	-25.474	2.056.989	-24.981
2.064.473	-34.584	2.103.297	-25.475	2.057.076	-24.981
2.064.585	-34.582	2.103.463	-25.476	2.057.233	-24.981
2.064.682	-34.581	2.103.685	-25.477	2.057.466	-24.981
2.064.820	-34.580	2.103.830	-25.479	2.057.728	-24.981

2.065.002	-34.578	2.104.019	-25.480	2.057.966	-24.981
2.065.242	-34.577	2.104.212	-25.481	2.058.190	-24.981
2.065.374	-34.575	2.104.332	-25.482	2.058.372	-24.981
2.065.435	-34.574	2.104.489	-25.483	2.058.515	-24.980
2.065.522	-34.572	2.104.722	-25.484	2.058.690	-24.980
2.065.670	-34.571	2.104.881	-25.485	2.058.853	-24.980
2.065.806	-34.569	2.104.966	-25.486	2.058.974	-24.980
2.065.934	-34.568	2.105.177	-25.488	2.059.117	-24.980
2.066.098	-34.566	2.105.362	-25.489	2.059.306	-24.980
2.066.299	-34.565	2.105.504	-25.490	2.059.465	-24.980
2.066.503	-34.563	2.105.694	-25.491	2.059.572	-24.980
2.066.677	-34.562	2.105.844	-25.492	2.059.740	-24.980
2.066.864	-34.560	2.105.966	-25.493	2.059.962	-24.980
2.067.070	-34.559	2.106.077	-25.494	2.060.082	-24.980
2.067.314	-34.557	2.106.203	-25.495	2.060.201	-24.980
2.067.458	-34.556	2.106.402	-25.496	2.060.345	-24.981
2.067.630	-34.554	2.106.635	-25.498	2.060.531	-24.981
2.067.840	-34.553	2.106.859	-25.499	2.060.771	-24.981
2.067.988	-34.551	2.107.002	-25.500	2.060.954	-24.981
2.068.159	-34.549	2.107.173	-25.501	2.061.097	-24.981
2.068.331	-34.548	2.107.395	-25.502	2.061.203	-24.981
2.068.470	-34.546	2.107.569	-25.503	2.061.337	-24.981
2.068.622	-34.544	2.107.682	-25.504	2.061.465	-24.981
2.068.792	-34.543	2.107.802	-25.506	2.061.647	-24.982
2.068.918	-34.541	2.107.948	-25.507	2.061.868	-24.982
2.069.048	-34.539	2.108.098	-25.508	2.062.005	-24.982
2.069.220	-34.537	2.108.250	-25.509	2.062.204	-24.982
2.069.362	-34.536	2.108.438	-25.510	2.062.419	-24.982
2.069.487	-34.534	2.108.624	-25.511	2.062.614	-24.983
2.069.755	-34.532	2.108.736	-25.513	2.062.793	-24.983

2.070.027	-34.530	2.108.891	-25.514	2.062.916	-24.983
2.070.154	-34.529	2.109.099	-25.515	2.063.040	-24.983
2.070.280	-34.527	2.109.238	-25.516	2.063.181	-24.984
2.070.450	-34.525	2.109.352	-25.518	2.063.315	-24.984
2.070.649	-34.523	2.109.532	-25.519	2.063.501	-24.984
2.070.855	-34.521	2.109.729	-25.520	2.063.690	-24.985
2.071.030	-34.519	2.109.865	-25.521	2.063.815	-24.985
2.071.120	-34.517	2.110.007	-25.523	2.063.953	-24.985
2.071.257	-34.515	2.110.181	-25.524	2.064.126	-24.986
2.071.503	-34.514	2.110.343	-25.525	2.064.339	-24.986
2.071.673	-34.512	2.110.524	-25.526	2.064.525	-24.987
2.071.797	-34.510	2.110.748	-25.528	2.064.717	-24.987
2.072.047	-34.508	2.110.988	-25.529	2.064.908	-24.987
2.072.253	-34.506	2.111.147	-25.530	2.065.022	-24.988
2.072.372	-34.504	2.111.265	-25.532	2.065.114	-24.988
2.072.502	-34.502	2.111.480	-25.533	2.065.280	-24.989
2.072.677	-34.500	2.111.682	-25.535	2.065.443	-24.989
2.072.865	-34.498	2.111.817	-25.536	2.065.578	-24.990
2.072.999	-34.496	2.111.996	-25.537	2.065.649	-24.990
2.073.121	-34.494	2.112.135	-25.539	2.065.831	-24.991
2.073.299	-34.492	2.112.305	-25.540	2.066.062	-24.991
2.073.674	-34.490	2.112.533	-25.542	2.066.238	-24.992
2.074.115	-34.488	2.112.701	-25.543	2.066.402	-24.992
2.074.323	-34.485	2.112.750	-25.544	2.066.516	-24.993
2.074.418	-34.483	2.112.854	-25.546	2.066.714	-24.993
2.074.498	-34.481	2.113.111	-25.547	2.066.909	-24.994
2.074.561	-34.479	2.113.354	-25.549	2.067.164	-24.994
2.074.639	-34.477	2.113.522	-25.550	2.067.444	-24.995
2.074.724	-34.475	2.113.685	-25.552	2.067.661	-24.995
2.074.807	-34.473	2.113.830	-25.553	2.067.843	-24.996

2.074.919	-34.471	2.113.967	-25.555	2.067.966	-24.996
2.075.067	-34.468	2.114.081	-25.556	2.068.071	-24.997
2.075.262	-34.466	2.114.191	-25.558	2.068.205	-24.997
2.075.421	-34.464	2.114.397	-25.559	2.068.400	-24.998
2.075.522	-34.462	2.114.594	-25.561	2.068.579	-24.998
2.075.750	-34.460	2.114.760	-25.562	2.068.691	-24.999
2.076.010	-34.457	2.114.875	-25.564	2.068.846	-24.999
2.076.203	-34.455	2.114.951	-25.565	2.068.996	-25.000
2.076.402	-34.453	2.115.110	-25.567	2.069.124	-25.001
2.076.541	-34.451	2.115.188	-25.568	2.069.281	-25.001
2.076.633	-34.449	2.115.304	-25.570	2.069.435	-25.002
2.076.842	-34.446	2.115.529	-25.571	2.069.565	-25.002
2.077.077	-34.444	2.115.725	-25.573	2.069.718	-25.003
2.077.242	-34.442	2.115.975	-25.574	2.069.962	-25.003
2.077.274	-34.440	2.116.241	-25.576	2.070.217	-25.004
2.077.366	-34.438	2.116.432	-25.578	2.070.379	-25.004
2.077.562	-34.435	2.116.572	-25.579	2.070.517	-25.005
2.077.692	-34.433	2.116.743	-25.581	2.070.665	-25.005
2.077.840	-34.431	2.116.931	-25.582	2.070.826	-25.006
2.078.022	-34.429	2.117.142	-25.584	2.071.012	-25.006
2.078.197	-34.427	2.117.346	-25.585	2.071.151	-25.007
2.078.419	-34.424	2.117.455	-25.587	2.071.277	-25.007
2.078.615	-34.422	2.117.551	-25.588	2.071.420	-25.008
2.078.707	-34.420	2.117.690	-25.590	2.071.621	-25.008
2.078.900	-34.418	2.117.899	-25.591	2.071.859	-25.009
2.079.144	-34.416	2.118.082	-25.593	2.072.041	-25.009
2.079.307	-34.413	2.118.148	-25.595	2.072.215	-25.010
2.079.509	-34.411	2.118.318	-25.596	2.072.349	-25.010
2.079.702	-34.409	2.118.552	-25.598	2.072.462	-25.011
2.079.838	-34.407	2.118.654	-25.599	2.072.600	-25.011

2.080.058	-34.405	2.118.767	-25.601	2.072.793	-25.011
2.080.226	-34.403	2.119.005	-25.602	2.073.001	-25.012
2.080.246	-34.401	2.119.198	-25.604	2.073.186	-25.012
2.080.340	-34.399	2.119.413	-25.605	2.073.351	-25.013
2.080.587	-34.396	2.119.641	-25.607	2.073.465	-25.013
2.080.789	-34.394	2.119.805	-25.608	2.073.573	-25.013
2.080.918	-34.392	2.119.957	-25.610	2.073.768	-25.014
2.080.997	-34.390	2.120.130	-25.611	2.073.987	-25.014
2.081.160	-34.388	2.120.260	-25.613	2.074.218	-25.014
2.081.409	-34.386	2.120.423	-25.614	2.074.401	-25.015
2.081.581	-34.384	2.120.576	-25.616	2.074.514	-25.015
2.081.724	-34.382	2.120.715	-25.617	2.074.632	-25.015
2.081.861	-34.380	2.120.916	-25.619	2.074.849	-25.016
2.082.013	-34.378	2.121.111	-25.620	2.075.067	-25.016
2.082.193	-34.376	2.121.322	-25.622	2.075.212	-25.016
2.082.320	-34.374	2.121.485	-25.623	2.075.446	-25.017
2.082.450	-34.372	2.121.638	-25.625	2.075.629	-25.017
2.082.650	-34.370	2.121.799	-25.626	2.075.710	-25.017
2.082.862	-34.369	2.121.989	-25.628	2.075.858	-25.017
2.083.029	-34.367	2.122.204	-25.629	2.076.120	-25.018
2.083.190	-34.365	2.122.283	-25.631	2.076.501	-25.018
2.083.363	-34.363	2.122.385	-25.632	2.076.859	-25.018
2.083.519	-34.361	2.122.583	-25.634	2.077.020	-25.018
2.083.728	-34.360	2.122.782	-25.635	2.077.114	-25.018
2.083.882	-34.358	2.122.968	-25.637	2.077.269	-25.018
2.083.992	-34.356	2.123.104	-25.638	2.077.285	-25.019
2.084.171	-34.354	2.123.266	-25.639	2.077.242	-25.019
2.084.328	-34.353	2.123.436	-25.641	2.077.337	-25.019
2.084.482	-34.351	2.123.568	-25.642	2.077.444	-25.019
2.084.668	-34.349	2.123.743	-25.644	2.077.598	-25.019

2.084.776	-34.348	2.123.974	-25.645	2.077.791	-25.019
2.084.966	-34.346	2.124.140	-25.646	2.077.885	-25.019
2.085.235	-34.344	2.124.265	-25.648	2.078.066	-25.020
2.085.418	-34.343	2.124.395	-25.649	2.078.333	-25.020
2.085.539	-34.341	2.124.559	-25.651	2.078.487	-25.020
2.085.638	-34.340	2.124.776	-25.652	2.078.595	-25.020
2.085.855	-34.338	2.124.967	-25.653	2.078.741	-25.020
2.086.075	-34.337	2.125.128	-25.655	2.078.945	-25.020
2.086.225	-34.335	2.125.273	-25.656	2.079.124	-25.020
2.086.406	-34.334	2.125.495	-25.657	2.079.245	-25.020
2.086.541	-34.332	2.125.943	-25.659	2.079.354	-25.020
2.086.676	-34.331	2.126.265	-25.660	2.079.514	-25.020
2.086.871	-34.329	2.126.355	-25.661	2.079.801	-25.020
2.087.063	-34.328	2.126.445	-25.662	2.080.029	-25.020
2.087.222	-34.327	2.126.557	-25.664	2.080.141	-25.020
2.087.285	-34.325	2.126.642	-25.665	2.080.228	-25.020
2.087.421	-34.324	2.126.673	-25.666	2.080.351	-25.020
2.087.556	-34.322	2.126.794	-25.668	2.080.501	-25.020
2.087.652	-34.321	2.126.971	-25.669	2.080.647	-25.020
2.087.832	-34.320	2.127.081	-25.670	2.080.846	-25.020
2.088.060	-34.319	2.127.184	-25.671	2.081.095	-25.020
2.088.235	-34.317	2.127.392	-25.672	2.081.341	-25.020
2.088.358	-34.316	2.127.598	-25.674	2.081.474	-25.020
2.088.555	-34.315	2.127.811	-25.675	2.081.628	-25.020
2.088.766	-34.313	2.128.001	-25.676	2.081.796	-25.020
2.088.922	-34.312	2.128.080	-25.677	2.081.994	-25.020
2.089.133	-34.311	2.128.205	-25.678	2.082.130	-25.020
2.089.368	-34.310	2.128.438	-25.679	2.082.253	-25.020
2.089.543	-34.309	2.128.661	-25.681	2.082.430	-25.020
2.089.720	-34.307	2.128.806	-25.682	2.082.625	-25.020

2.089.941	-34.306	2.128.933	-25.683	2.082.822	-25.020
2.090.150	-34.305	2.129.068	-25.684	2.082.926	-25.021
2.090.268	-34.304	2.129.238	-25.685	2.083.049	-25.021
2.090.403	-34.303	2.129.431	-25.686	2.083.250	-25.021
2.090.555	-34.302	2.129.601	-25.687	2.083.396	-25.021
2.090.645	-34.300	2.129.735	-25.688	2.083.550	-25.021
2.090.761	-34.299	2.129.852	-25.689	2.083.747	-25.021
2.090.835	-34.298	2.130.031	-25.690	2.083.891	-25.021
2.090.988	-34.297	2.130.203	-25.691	2.084.025	-25.021
2.091.237	-34.296	2.130.414	-25.692	2.084.258	-25.021
2.091.369	-34.295	2.130.614	-25.693	2.084.460	-25.021
2.091.436	-34.294	2.130.813	-25.695	2.084.522	-25.021
2.091.601	-34.293	2.130.986	-25.696	2.084.653	-25.021
2.091.871	-34.292	2.131.100	-25.697	2.084.841	-25.021
2.092.070	-34.290	2.131.290	-25.698	2.084.980	-25.021
2.092.238	-34.289	2.131.449	-25.699	2.085.145	-25.021
2.092.412	-34.288	2.131.619	-25.700	2.085.338	-25.021
2.092.621	-34.287	2.131.824	-25.701	2.085.490	-25.022
2.092.932	-34.286	2.131.962	-25.702	2.085.704	-25.022
2.093.118	-34.285	2.132.103	-25.703	2.085.931	-25.022
2.093.233	-34.284	2.132.287	-25.704	2.086.105	-25.022
2.093.391	-34.283	2.132.435	-25.705	2.086.276	-25.022
2.093.546	-34.282	2.132.558	-25.706	2.086.404	-25.022
2.093.732	-34.281	2.132.677	-25.707	2.086.517	-25.022
2.093.895	-34.280	2.132.871	-25.708	2.086.655	-25.022
2.094.025	-34.279	2.132.984	-25.709	2.086.801	-25.023
2.094.207	-34.278	2.133.159	-25.710	2.086.929	-25.023
2.094.422	-34.277	2.133.393	-25.711	2.087.085	-25.023
2.094.556	-34.276	2.133.513	-25.712	2.087.370	-25.023
2.094.648	-34.275	2.133.729	-25.713	2.087.569	-25.023

2.094.832	-34.274	2.133.911	-25.714	2.087.740	-25.023
2.095.069	-34.273	2.134.053	-25.715	2.087.945	-25.024
2.095.202	-34.272	2.134.211	-25.716	2.088.074	-25.024
2.095.293	-34.271	2.134.312	-25.717	2.088.210	-25.024
2.095.423	-34.270	2.134.460	-25.718	2.088.380	-25.024
2.095.629	-34.269	2.134.675	-25.719	2.088.490	-25.024
2.095.853	-34.268	2.134.875	-25.720	2.088.606	-25.024
2.096.039	-34.267	2.135.056	-25.720	2.088.748	-25.025
2.096.178	-34.266	2.135.221	-25.721	2.088.898	-25.025
2.096.297	-34.265	2.135.394	-25.722	2.089.065	-25.025
2.096.453	-34.264	2.135.573	-25.723	2.089.242	-25.025
2.096.665	-34.263	2.135.706	-25.724	2.089.377	-25.025
2.096.852	-34.262	2.135.824	-25.725	2.089.462	-25.026
2.096.982	-34.261	2.136.021	-25.726	2.089.623	-25.026
2.097.112	-34.260	2.136.232	-25.727	2.089.874	-25.026
2.097.258	-34.259	2.136.422	-25.728	2.090.121	-25.026
2.097.469	-34.258	2.136.581	-25.729	2.090.367	-25.026
2.097.654	-34.257	2.136.677	-25.730	2.090.571	-25.027
2.097.794	-34.256	2.136.846	-25.731	2.090.737	-25.027
2.098.001	-34.255	2.137.054	-25.732	2.090.949	-25.027
2.098.203	-34.254	2.137.216	-25.733	2.091.176	-25.027
2.098.349	-34.253	2.137.388	-25.734	2.091.359	-25.027
2.098.439	-34.252	2.137.558	-25.735	2.091.503	-25.027
2.098.634	-34.251	2.137.794	-25.736	2.091.628	-25.028
2.099.043	-34.250	2.138.015	-25.737	2.091.740	-25.028
2.099.475	-34.249	2.138.141	-25.737	2.091.825	-25.028
2.099.695	-34.248	2.138.251	-25.738	2.091.949	-25.028
2.099.767	-34.247	2.138.353	-25.739	2.092.130	-25.028
2.099.834	-34.246	2.138.494	-25.740	2.092.307	-25.028
2.099.911	-34.245	2.138.593	-25.741	2.092.495	-25.029

2.099.980	-34.244	2.138.710	-25.742	2.092.679	-25.029
2.099.995	-34.243	2.138.915	-25.743	2.092.815	-25.029
2.100.053	-34.242	2.139.066	-25.743	2.092.930	-25.029
2.100.121	-34.241	2.139.224	-25.744	2.093.091	-25.029
2.100.259	-34.240	2.139.382	-25.745	2.093.288	-25.029
2.100.495	-34.239	2.139.511	-25.746	2.093.521	-25.029
2.100.706	-34.238	2.139.693	-25.747	2.093.714	-25.029
2.100.931	-34.237	2.139.923	-25.747	2.093.875	-25.029
2.101.125	-34.236	2.140.114	-25.748	2.094.053	-25.029
2.101.252	-34.235	2.140.327	-25.749	2.094.176	-25.029
2.101.362	-34.234	2.140.569	-25.750	2.094.317	-25.030
2.101.570	-34.233	2.140.687	-25.750	2.094.522	-25.030
2.101.837	-34.232	2.140.856	-25.751	2.094.678	-25.030
2.102.063	-34.231	2.141.129	-25.752	2.094.785	-25.030
2.102.215	-34.230	2.141.281	-25.753	2.094.942	-25.030
2.102.325	-34.229	2.141.409	-25.753	2.095.123	-25.030
2.102.453	-34.228	2.141.599	-25.754	2.095.304	-25.030
2.102.612	-34.227	2.141.747	-25.755	2.095.492	-25.030
2.102.751	-34.226	2.141.846	-25.755	2.095.659	-25.030
2.102.865	-34.225	2.142.002	-25.756	2.095.824	-25.030
2.103.033	-34.224	2.142.144	-25.757	2.095.981	-25.030
2.103.185	-34.223	2.142.242	-25.757	2.096.140	-25.030
2.103.401	-34.222	2.142.393	-25.758	2.096.357	-25.030
2.103.662	-34.221	2.142.580	-25.759	2.096.604	-25.030
2.103.767	-34.220	2.142.845	-25.759	2.096.774	-25.029
2.103.866	-34.219	2.143.082	-25.760	2.096.913	-25.029
2.104.055	-34.218	2.143.230	-25.761	2.097.065	-25.029
2.104.265	-34.217	2.143.315	-25.761	2.097.206	-25.029
2.104.426	-34.216	2.143.463	-25.762	2.097.404	-25.029
2.104.569	-34.215	2.143.690	-25.762	2.097.608	-25.029

2.104.747	-34.214	2.143.864	-25.763	2.097.737	-25.029
2.104.964	-34.213	2.144.068	-25.763	2.097.859	-25.029
2.105.157	-34.212	2.144.231	-25.764	2.098.035	-25.029
2.105.271	-34.211	2.144.372	-25.765	2.098.257	-25.029
2.105.372	-34.210	2.144.563	-25.765	2.098.457	-25.028
2.105.526	-34.209	2.144.715	-25.766	2.098.631	-25.028
2.105.734	-34.208	2.144.914	-25.766	2.098.783	-25.028
2.105.916	-34.207	2.145.078	-25.767	2.098.914	-25.028
2.106.064	-34.207	2.145.201	-25.767	2.099.050	-25.028
2.106.164	-34.206	2.145.359	-25.768	2.099.164	-25.027
2.106.293	-34.205	2.145.529	-25.768	2.099.509	-25.027
2.106.527	-34.204	2.145.698	-25.769	2.099.977	-25.027
2.106.738	-34.203	2.145.806	-25.769	2.100.267	-25.027
2.106.884	-34.202	2.146.012	-25.770	2.100.394	-25.027
2.107.067	-34.201	2.146.236	-25.770	2.100.378	-25.026
2.107.227	-34.200	2.146.376	-25.771	2.100.398	-25.026
2.107.332	-34.200	2.146.561	-25.771	2.100.492	-25.026
2.107.453	-34.199	2.146.767	-25.771	2.100.586	-25.026
2.107.632	-34.198	2.146.935	-25.772	2.100.712	-25.025
2.107.838	-34.197	2.147.078	-25.772	2.100.858	-25.025
2.107.995	-34.196	2.147.206	-25.773	2.100.938	-25.025
2.108.152	-34.196	2.147.356	-25.773	2.101.051	-25.024
2.108.367	-34.195	2.147.570	-25.774	2.101.241	-25.024
2.108.575	-34.194	2.147.789	-25.774	2.101.458	-25.024
2.108.708	-34.193	2.147.903	-25.775	2.101.656	-25.024
2.108.866	-34.193	2.148.022	-25.775	2.101.845	-25.023
2.109.055	-34.192	2.148.241	-25.775	2.102.052	-25.023
2.109.211	-34.191	2.148.400	-25.776	2.102.240	-25.023
2.109.406	-34.190	2.148.479	-25.776	2.102.370	-25.022
2.109.632	-34.190	2.148.673	-25.777	2.102.565	-25.022

2.109.787	-34.189	2.148.896	-25.777	2.102.766	-25.021
2.109.899	-34.188	2.149.088	-25.777	2.102.852	-25.021
2.110.051	-34.188	2.149.224	-25.778	2.102.988	-25.021
2.110.255	-34.187	2.149.415	-25.778	2.103.174	-25.020
2.110.416	-34.186	2.149.810	-25.778	2.103.335	-25.020
2.110.486	-34.186	2.150.247	-25.779	2.103.474	-25.020
2.110.632	-34.185	2.150.479	-25.779	2.103.617	-25.019
2.110.826	-34.184	2.150.509	-25.780	2.103.783	-25.019
2.111.042	-34.184	2.150.576	-25.780	2.103.909	-25.019
2.111.180	-34.183	2.150.627	-25.780	2.104.061	-25.018
2.111.317	-34.182	2.150.715	-25.781	2.104.236	-25.018
2.111.548	-34.182	2.150.835	-25.781	2.104.480	-25.017
2.111.644	-34.181	2.150.900	-25.781	2.104.734	-25.017
2.111.727	-34.180	2.150.987	-25.782	2.104.908	-25.017
2.111.928	-34.180	2.151.145	-25.782	2.105.107	-25.016
2.112.160	-34.179	2.151.328	-25.783	2.105.289	-25.016
2.112.381	-34.178	2.151.483	-25.783	2.105.410	-25.016
2.112.488	-34.178	2.151.642	-25.783	2.105.538	-25.015
2.112.573	-34.177	2.151.870	-25.784	2.105.685	-25.015
2.112.757	-34.176	2.152.031	-25.784	2.105.860	-25.015
2.112.923	-34.176	2.152.150	-25.784	2.106.073	-25.014
2.113.080	-34.175	2.152.392	-25.785	2.106.232	-25.014
2.113.210	-34.174	2.152.599	-25.785	2.106.397	-25.014
2.113.308	-34.174	2.152.759	-25.785	2.106.566	-25.013
2.113.441	-34.173	2.152.899	-25.786	2.106.665	-25.013
2.113.562	-34.172	2.153.006	-25.786	2.106.805	-25.013
2.113.766	-34.172	2.153.185	-25.787	2.106.964	-25.012
2.114.057	-34.171	2.153.396	-25.787	2.107.117	-25.012
2.114.269	-34.170	2.153.523	-25.787	2.107.330	-25.012
2.114.408	-34.170	2.153.647	-25.788	2.107.489	-25.012

2.114.606	-34.169	2.153.767	-25.788	2.107.594	-25.011
2.114.841	-34.168	2.153.922	-25.789	2.107.719	-25.011
2.115.056	-34.168	2.154.081	-25.789	2.107.910	-25.011
2.115.260	-34.167	2.154.227	-25.789	2.108.082	-25.010
2.115.406	-34.166	2.154.492	-25.790	2.108.194	-25.010
2.115.578	-34.165	2.154.678	-25.790	2.108.414	-25.010
2.115.694	-34.165	2.154.818	-25.791	2.108.644	-25.010
2.115.817	-34.164	2.155.038	-25.791	2.108.803	-25.010
2.115.983	-34.163	2.155.219	-25.792	2.108.971	-25.009
2.116.087	-34.162	2.155.406	-25.792	2.109.133	-25.009
2.116.236	-34.161	2.155.578	-25.793	2.109.299	-25.009
2.116.366	-34.160	2.155.746	-25.793	2.109.436	-25.009
2.116.518	-34.160	2.155.896	-25.794	2.109.599	-25.009
2.116.770	-34.159	2.155.983	-25.794	2.109.866	-25.008
2.116.945	-34.158	2.156.163	-25.795	2.110.047	-25.008
2.117.108	-34.157	2.156.339	-25.795	2.110.143	-25.008
2.117.242	-34.156	2.156.480	-25.796	2.110.309	-25.008
2.117.424	-34.155	2.156.655	-25.796	2.110.506	-25.008
2.117.713	-34.154	2.156.814	-25.797	2.110.705	-25.008
2.117.827	-34.153	2.156.969	-25.798	2.110.898	-25.008
2.117.921	-34.152	2.157.132	-25.798	2.111.057	-25.007
2.118.156	-34.151	2.157.307	-25.799	2.111.196	-25.007
2.118.387	-34.150	2.157.466	-25.799	2.111.339	-25.007
2.118.568	-34.149	2.157.641	-25.800	2.111.505	-25.007
2.118.719	-34.148	2.157.831	-25.801	2.111.734	-25.007
2.118.907	-34.147	2.157.979	-25.801	2.111.927	-25.007
2.119.115	-34.146	2.158.167	-25.802	2.111.886	-25.007
2.119.231	-34.145	2.158.315	-25.803	2.112.000	-25.007
2.119.335	-34.144	2.158.438	-25.803	2.112.242	-25.007
2.119.505	-34.142	2.158.634	-25.804	2.112.363	-25.007

2.119.700	-34.141	2.158.822	-25.805	2.112.495	-25.006
2.119.867	-34.140	2.158.935	-25.805	2.112.632	-25.006
2.120.036	-34.139	2.159.074	-25.806	2.112.753	-25.006
2.120.210	-34.138	2.159.352	-25.807	2.112.919	-25.006
2.120.396	-34.136	2.159.566	-25.808	2.113.150	-25.006
2.120.576	-34.135	2.159.684	-25.808	2.113.362	-25.006
2.120.723	-34.134	2.159.843	-25.809	2.113.548	-25.006
2.120.884	-34.133	2.159.986	-25.810	2.113.716	-25.006
2.121.030	-34.131	2.160.155	-25.811	2.113.936	-25.006
2.121.176	-34.130	2.160.349	-25.812	2.114.211	-25.006
2.121.371	-34.129	2.160.511	-25.812	2.114.454	-25.006
2.121.503	-34.127	2.160.629	-25.813	2.114.612	-25.006
2.121.664	-34.126	2.160.763	-25.814	2.114.722	-25.005
2.121.892	-34.125	2.160.976	-25.815	2.114.838	-25.005
2.122.056	-34.123	2.161.158	-25.816	2.115.006	-25.005
2.122.179	-34.122	2.161.290	-25.816	2.115.168	-25.005
2.122.370	-34.120	2.161.407	-25.817	2.115.295	-25.005
2.122.554	-34.119	2.161.606	-25.818	2.115.430	-25.005
2.122.721	-34.117	2.161.798	-25.819	2.115.551	-25.005
2.122.914	-34.116	2.161.955	-25.820	2.115.705	-25.005
2.123.030	-34.114	2.162.139	-25.821	2.115.856	-25.005
2.123.205	-34.113	2.162.300	-25.821	2.116.040	-25.005
2.123.309	-34.111	2.162.468	-25.822	2.116.285	-25.004
2.123.454	-34.110	2.162.598	-25.823	2.116.451	-25.004
2.123.690	-34.108	2.162.701	-25.824	2.116.595	-25.004
2.123.853	-34.107	2.162.859	-25.825	2.116.805	-25.004
2.124.090	-34.105	2.163.035	-25.826	2.116.991	-25.004
2.124.424	-34.103	2.163.139	-25.827	2.117.130	-25.004
2.124.810	-34.102	2.163.263	-25.827	2.117.316	-25.004
2.125.047	-34.100	2.163.470	-25.828	2.117.479	-25.003

2.125.089	-34.098	2.163.616	-25.829	2.117.634	-25.003
2.125.085	-34.097	2.163.785	-25.830	2.117.879	-25.003
2.125.172	-34.095	2.164.063	-25.831	2.118.044	-25.003
2.125.370	-34.093	2.164.321	-25.832	2.118.158	-25.003
2.125.466	-34.092	2.164.540	-25.832	2.118.337	-25.003
2.125.574	-34.090	2.164.734	-25.833	2.118.499	-25.002
2.125.715	-34.088	2.164.888	-25.834	2.118.671	-25.002
2.125.840	-34.086	2.165.051	-25.835	2.118.817	-25.002
2.126.001	-34.084	2.165.231	-25.836	2.118.949	-25.002
2.126.138	-34.083	2.165.372	-25.836	2.119.165	-25.002
2.126.292	-34.081	2.165.517	-25.837	2.119.384	-25.001
2.126.517	-34.079	2.165.627	-25.838	2.119.570	-25.001
2.126.751	-34.077	2.165.751	-25.839	2.119.680	-25.001
2.126.864	-34.075	2.165.974	-25.840	2.119.771	-25.001
2.127.003	-34.073	2.166.153	-25.840	2.120.000	-25.000
2.127.161	-34.072	2.166.245	-25.841	2.120.226	-25.000
2.127.285	-34.070	2.166.402	-25.842	2.120.383	-25.000
2.127.504	-34.068	2.166.586	-25.843	2.120.452	-25.000
2.127.708	-34.066	2.166.732	-25.843	2.120.585	-24.999
2.127.905	-34.064	2.166.906	-25.844	2.120.815	-24.999
2.128.111	-34.062	2.167.077	-25.845	2.120.997	-24.999
2.128.210	-34.060	2.167.260	-25.846	2.121.153	-24.999
2.128.282	-34.058	2.167.484	-25.846	2.121.315	-24.998
2.128.371	-34.057	2.167.688	-25.847	2.121.519	-24.998
2.128.537	-34.055	2.167.802	-25.848	2.121.662	-24.998
2.128.712	-34.053	2.167.905	-25.848	2.121.799	-24.997
2.128.920	-34.051	2.168.075	-25.849	2.122.009	-24.997
2.129.104	-34.049	2.168.243	-25.850	2.122.215	-24.997
2.129.211	-34.047	2.168.448	-25.850	2.122.374	-24.996
2.129.436	-34.045	2.168.663	-25.851	2.122.509	-24.996

2.129.646	-34.043	2.168.772	-25.851	2.122.863	-24.996
2.129.843	-34.042	2.168.902	-25.852	2.123.331	-24.995
2.130.040	-34.040	2.169.130	-25.853	2.123.559	-24.995
2.130.199	-34.038	2.169.342	-25.853	2.123.582	-24.995
2.130.392	-34.036	2.169.487	-25.854	2.123.625	-24.994
2.130.551	-34.034	2.169.632	-25.854	2.123.690	-24.994
2.130.703	-34.032	2.169.789	-25.855	2.123.770	-24.993
2.130.892	-34.030	2.169.966	-25.856	2.123.871	-24.993
2.130.927	-34.028	2.170.136	-25.856	2.123.960	-24.993
2.130.999	-34.026	2.170.282	-25.857	2.124.077	-24.992
2.131.218	-34.025	2.170.445	-25.857	2.124.272	-24.992
2.131.373	-34.023	2.170.683	-25.858	2.124.471	-24.991
2.131.568	-34.021	2.170.804	-25.858	2.124.614	-24.991
2.131.783	-34.019	2.170.925	-25.859	2.124.792	-24.990
2.131.978	-34.017	2.171.151	-25.859	2.124.975	-24.990
2.132.130	-34.015	2.171.315	-25.860	2.125.192	-24.989
2.132.325	-34.014	2.171.391	-25.860	2.125.367	-24.989
2.132.526	-34.012	2.171.523	-25.860	2.125.491	-24.988
2.132.682	-34.010	2.171.774	-25.861	2.125.651	-24.988
2.132.838	-34.008	2.172.011	-25.861	2.125.806	-24.987
2.133.009	-34.007	2.172.211	-25.862	2.125.968	-24.987
2.133.181	-34.005	2.172.352	-25.862	2.126.185	-24.986
2.133.322	-34.003	2.172.526	-25.862	2.126.335	-24.986
2.133.410	-34.002	2.172.731	-25.863	2.126.438	-24.985
2.133.598	-34.000	2.172.912	-25.863	2.126.599	-24.985
2.133.801	-33.998	2.173.047	-25.863	2.126.778	-24.984
2.133.913	-33.997	2.173.149	-25.864	2.126.951	-24.984
2.134.097	-33.995	2.173.353	-25.864	2.127.117	-24.983
2.134.297	-33.993	2.173.738	-25.864	2.127.186	-24.983
2.134.491	-33.992	2.174.204	-25.865	2.127.334	-24.982

2.134.653	-33.990	2.174.456	-25.865	2.127.626	-24.982
2.134.763	-33.989	2.174.525	-25.865	2.127.827	-24.981
2.134.937	-33.987	2.174.550	-25.866	2.128.026	-24.981
2.135.179	-33.985	2.174.540	-25.866	2.128.197	-24.980
2.135.356	-33.984	2.174.568	-25.866	2.128.362	-24.980
2.135.526	-33.982	2.174.680	-25.866	2.128.535	-24.979
2.135.692	-33.981	2.174.829	-25.867	2.128.678	-24.979
2.135.777	-33.979	2.174.959	-25.867	2.128.837	-24.978
2.135.919	-33.978	2.175.089	-25.867	2.128.974	-24.978
2.136.113	-33.976	2.175.226	-25.867	2.129.070	-24.977
2.136.303	-33.975	2.175.414	-25.868	2.129.168	-24.977
2.136.508	-33.973	2.175.564	-25.868	2.129.381	-24.976
2.136.687	-33.972	2.175.761	-25.868	2.129.660	-24.976
2.136.801	-33.970	2.175.952	-25.868	2.129.837	-24.975
2.136.942	-33.969	2.176.066	-25.868	2.129.962	-24.975
2.137.117	-33.967	2.176.297	-25.869	2.130.112	-24.974
2.137.213	-33.966	2.176.498	-25.869	2.130.251	-24.974
2.137.397	-33.964	2.176.639	-25.869	2.130.452	-24.973
2.137.605	-33.963	2.176.756	-25.869	2.130.676	-24.973
2.137.719	-33.961	2.176.893	-25.869	2.130.851	-24.972
2.137.849	-33.960	2.177.074	-25.869	2.130.970	-24.972
2.138.020	-33.958	2.177.244	-25.870	2.131.080	-24.971
2.138.216	-33.957	2.177.408	-25.870	2.131.200	-24.971
2.138.378	-33.956	2.177.605	-25.870	2.131.396	-24.970
2.138.429	-33.954	2.177.766	-25.870	2.131.617	-24.970
2.138.566	-33.953	2.177.901	-25.870	2.131.759	-24.970
2.138.826	-33.951	2.178.060	-25.870	2.131.936	-24.969
2.138.990	-33.950	2.178.207	-25.871	2.132.139	-24.969
2.139.135	-33.948	2.178.344	-25.871	2.132.289	-24.968
2.139.348	-33.947	2.178.551	-25.871	2.132.458	-24.968

2.139.610	-33.945	2.178.806	-25.871	2.132.674	-24.968
2.139.836	-33.944	2.178.997	-25.871	2.132.870	-24.967
2.140.013	-33.942	2.179.135	-25.871	2.132.997	-24.967
2.140.242	-33.941	2.179.274	-25.872	2.133.058	-24.966
2.140.441	-33.940	2.179.404	-25.872	2.133.203	-24.966
2.140.539	-33.938	2.179.552	-25.872	2.133.411	-24.966
2.140.640	-33.937	2.179.738	-25.872	2.133.606	-24.965
2.140.893	-33.935	2.179.875	-25.872	2.133.808	-24.965
2.141.140	-33.934	2.180.074	-25.873	2.134.023	-24.964
2.141.247	-33.932	2.180.269	-25.873	2.134.173	-24.964
2.141.302	-33.930	2.180.397	-25.873	2.134.319	-24.964
2.141.366	-33.929	2.180.564	-25.873	2.134.457	-24.963
2.141.521	-33.927	2.180.768	-25.873	2.134.568	-24.963
2.141.729	-33.926	2.180.975	-25.874	2.134.756	-24.962
2.141.910	-33.924	2.181.122	-25.874	2.134.917	-24.962
2.142.029	-33.923	2.181.225	-25.874	2.135.082	-24.962
2.142.141	-33.921	2.181.393	-25.874	2.135.285	-24.961
2.142.305	-33.919	2.181.613	-25.875	2.135.418	-24.961
2.142.563	-33.918	2.181.736	-25.875	2.135.542	-24.961
2.142.825	-33.916	2.181.823	-25.875	2.135.677	-24.960
2.142.988	-33.914	2.182.007	-25.875	2.135.784	-24.960
2.143.100	-33.913	2.182.224	-25.876	2.135.938	-24.959
2.143.297	-33.911	2.182.404	-25.876	2.136.125	-24.959
2.143.521	-33.909	2.182.505	-25.876	2.136.272	-24.959
2.143.680	-33.907	2.182.686	-25.877	2.136.463	-24.958
2.143.792	-33.906	2.182.883	-25.877	2.136.736	-24.958
2.143.956	-33.904	2.183.015	-25.877	2.136.971	-24.957
2.144.162	-33.902	2.183.246	-25.878	2.137.178	-24.957
2.144.344	-33.900	2.183.441	-25.878	2.137.359	-24.957
2.144.547	-33.899	2.183.582	-25.878	2.137.511	-24.956

2.144.709	-33.897	2.183.732	-25.879	2.137.654	-24.956
2.144.863	-33.895	2.183.876	-25.879	2.137.845	-24.955
2.145.058	-33.893	2.184.017	-25.880	2.138.060	-24.955
2.145.203	-33.891	2.184.153	-25.880	2.138.192	-24.955
2.145.370	-33.889	2.184.328	-25.880	2.138.320	-24.954
2.145.535	-33.888	2.184.460	-25.881	2.138.427	-24.954
2.145.692	-33.886	2.184.657	-25.881	2.138.553	-24.953
2.145.831	-33.884	2.184.888	-25.882	2.138.660	-24.953
2.146.003	-33.882	2.185.076	-25.882	2.138.828	-24.952
2.146.273	-33.880	2.185.275	-25.882	2.139.010	-24.952
2.146.418	-33.878	2.185.425	-25.883	2.139.129	-24.951
2.146.431	-33.876	2.185.569	-25.883	2.139.325	-24.951
2.146.568	-33.874	2.185.728	-25.884	2.139.540	-24.950
2.146.837	-33.872	2.185.905	-25.884	2.139.738	-24.950
2.147.101	-33.870	2.186.021	-25.885	2.139.904	-24.949
2.147.255	-33.868	2.186.142	-25.885	2.140.121	-24.949
2.147.361	-33.866	2.186.344	-25.886	2.140.336	-24.948
2.147.549	-33.863	2.186.510	-25.886	2.140.488	-24.948
2.147.699	-33.861	2.186.608	-25.887	2.140.622	-24.947
2.147.879	-33.859	2.186.714	-25.887	2.140.763	-24.947
2.148.078	-33.857	2.186.879	-25.888	2.140.972	-24.946
2.148.246	-33.855	2.187.058	-25.888	2.141.131	-24.946
2.148.450	-33.853	2.187.226	-25.889	2.141.241	-24.945
2.148.624	-33.850	2.187.370	-25.889	2.141.382	-24.945
2.148.786	-33.848	2.187.531	-25.890	2.141.564	-24.944
2.148.947	-33.846	2.187.703	-25.890	2.141.774	-24.943
2.149.059	-33.844	2.187.945	-25.891	2.142.009	-24.943
2.149.146	-33.841	2.188.242	-25.892	2.142.197	-24.942
2.149.467	-33.839	2.188.474	-25.892	2.142.209	-24.942
2.149.959	-33.837	2.188.634	-25.893	2.142.392	-24.941

2.150.264	-33.835	2.188.784	-25.893	2.142.623	-24.941
2.150.380	-33.832	2.188.965	-25.894	2.142.701	-24.940
2.150.509	-33.830	2.189.180	-25.894	2.142.930	-24.939
2.150.575	-33.828	2.189.348	-25.895	2.143.163	-24.939
2.150.558	-33.825	2.189.469	-25.896	2.143.358	-24.938
2.150.583	-33.823	2.189.596	-25.896	2.143.546	-24.938
2.150.730	-33.820	2.189.689	-25.897	2.143.698	-24.937
2.150.909	-33.818	2.189.807	-25.897	2.143.862	-24.936
2.150.943	-33.815	2.189.973	-25.898	2.143.999	-24.936
2.151.055	-33.813	2.190.129	-25.898	2.144.160	-24.935
2.151.310	-33.810	2.190.271	-25.899	2.144.328	-24.934
2.151.451	-33.808	2.190.436	-25.900	2.144.511	-24.934
2.151.550	-33.805	2.190.604	-25.900	2.144.720	-24.933
2.151.758	-33.803	2.190.777	-25.901	2.144.861	-24.932
2.151.980	-33.800	2.190.927	-25.901	2.145.022	-24.932
2.152.130	-33.798	2.191.115	-25.902	2.145.215	-24.931
2.152.293	-33.795	2.191.364	-25.903	2.145.341	-24.930
2.152.441	-33.793	2.191.552	-25.903	2.145.436	-24.930
2.152.600	-33.790	2.191.684	-25.904	2.145.557	-24.929
2.152.791	-33.787	2.191.798	-25.905	2.145.894	-24.928
2.152.918	-33.785	2.192.003	-25.905	2.146.364	-24.928
2.153.071	-33.782	2.192.280	-25.906	2.146.736	-24.927
2.153.280	-33.780	2.192.426	-25.907	2.146.908	-24.926
2.153.472	-33.777	2.192.522	-25.907	2.146.944	-24.925
2.153.606	-33.774	2.192.655	-25.908	2.146.996	-24.925
2.153.712	-33.772	2.192.840	-25.908	2.147.029	-24.924
2.153.873	-33.769	2.193.017	-25.909	2.147.038	-24.923
2.153.949	-33.767	2.193.156	-25.910	2.147.081	-24.923
2.154.121	-33.764	2.193.353	-25.911	2.147.272	-24.922
2.154.402	-33.761	2.193.543	-25.911	2.147.475	-24.921

2.154.601	-33.759	2.193.694	-25.912	2.147.607	-24.920
2.154.814	-33.756	2.193.880	-25.913	2.147.780	-24.920
2.155.014	-33.754	2.194.053	-25.913	2.147.915	-24.919
2.155.183	-33.751	2.194.247	-25.914	2.148.055	-24.918
2.155.318	-33.749	2.194.411	-25.915	2.148.233	-24.917
2.155.524	-33.746	2.194.437	-25.915	2.148.420	-24.916
2.155.741	-33.743	2.194.581	-25.916	2.148.631	-24.916
2.155.961	-33.741	2.194.823	-25.917	2.148.826	-24.915
2.156.154	-33.738	2.195.025	-25.918	2.149.030	-24.914
2.156.259	-33.736	2.195.226	-25.918	2.149.185	-24.913
2.156.438	-33.733	2.195.398	-25.919	2.149.297	-24.913
2.156.530	-33.730	2.195.537	-25.920	2.149.427	-24.912
2.156.595	-33.728	2.195.681	-25.920	2.149.576	-24.911
2.156.792	-33.725	2.195.845	-25.921	2.149.735	-24.910
2.156.875	-33.723	2.195.983	-25.922	2.149.877	-24.909
2.156.993	-33.720	2.196.191	-25.923	2.150.014	-24.909
2.157.282	-33.718	2.196.328	-25.923	2.150.177	-24.908
2.157.526	-33.715	2.196.496	-25.924	2.150.324	-24.907
2.157.711	-33.713	2.196.642	-25.925	2.150.502	-24.906
2.157.854	-33.710	2.196.780	-25.926	2.150.703	-24.906
2.157.941	-33.708	2.196.953	-25.926	2.150.925	-24.905
2.158.113	-33.706	2.197.086	-25.927	2.151.135	-24.904
2.158.315	-33.703	2.197.426	-25.928	2.151.321	-24.903
2.158.418	-33.701	2.197.865	-25.929	2.151.514	-24.902
2.158.543	-33.698	2.198.165	-25.930	2.151.698	-24.902
2.158.716	-33.696	2.198.354	-25.930	2.151.830	-24.901
2.158.896	-33.694	2.198.389	-25.931	2.151.918	-24.900
2.159.090	-33.691	2.198.383	-25.932	2.152.087	-24.899
2.159.308	-33.689	2.198.434	-25.933	2.152.281	-24.899
2.159.534	-33.687	2.198.495	-25.934	2.152.431	-24.898

2.159.698	-33.685	2.198.591	-25.935	2.152.578	-24.897
2.159.818	-33.682	2.198.674	-25.935	2.152.735	-24.897
2.159.979	-33.680	2.198.802	-25.936	2.152.952	-24.896
2.160.135	-33.678	2.199.032	-25.937	2.153.176	-24.895
2.160.347	-33.676	2.199.247	-25.938	2.153.335	-24.894
2.160.544	-33.673	2.199.335	-25.939	2.153.481	-24.894
2.160.634	-33.671	2.199.543	-25.940	2.153.640	-24.893
2.160.797	-33.669	2.199.760	-25.941	2.153.781	-24.892
2.161.015	-33.667	2.199.921	-25.942	2.153.895	-24.892
2.161.171	-33.665	2.200.045	-25.943	2.154.041	-24.891
2.161.301	-33.663	2.200.213	-25.944	2.154.223	-24.890
2.161.470	-33.661	2.200.387	-25.945	2.154.411	-24.890
2.161.642	-33.658	2.200.506	-25.945	2.154.550	-24.889
2.161.698	-33.656	2.200.688	-25.946	2.154.677	-24.888
2.161.826	-33.654	2.200.916	-25.947	2.154.859	-24.888
2.162.103	-33.652	2.201.082	-25.948	2.155.038	-24.887
2.162.307	-33.650	2.201.198	-25.949	2.155.244	-24.887
2.162.471	-33.648	2.201.402	-25.950	2.155.453	-24.886
2.162.684	-33.646	2.201.581	-25.951	2.155.600	-24.885
2.162.856	-33.644	2.201.647	-25.952	2.155.762	-24.885
2.162.954	-33.642	2.201.783	-25.953	2.155.905	-24.884
2.163.051	-33.640	2.201.938	-25.955	2.156.075	-24.884
2.163.190	-33.638	2.202.114	-25.956	2.156.243	-24.883
2.163.414	-33.636	2.202.316	-25.957	2.156.393	-24.882
2.163.526	-33.634	2.202.542	-25.958	2.156.532	-24.882
2.163.662	-33.632	2.202.741	-25.959	2.156.736	-24.881
2.163.895	-33.630	2.202.897	-25.960	2.156.996	-24.881
2.164.037	-33.629	2.203.098	-25.961	2.157.128	-24.880
2.164.139	-33.627	2.203.297	-25.962	2.157.272	-24.880
2.164.350	-33.625	2.203.465	-25.963	2.157.464	-24.879

2.164.628	-33.623	2.203.627	-25.965	2.157.625	-24.879
2.164.828	-33.621	2.203.741	-25.966	2.157.802	-24.878
2.164.982	-33.619	2.203.826	-25.967	2.157.959	-24.878
2.165.179	-33.617	2.203.981	-25.968	2.158.020	-24.877
2.165.381	-33.615	2.204.202	-25.969	2.158.160	-24.877
2.165.547	-33.614	2.204.406	-25.971	2.158.360	-24.876
2.165.739	-33.612	2.204.605	-25.972	2.158.535	-24.876
2.165.885	-33.610	2.204.776	-25.973	2.158.685	-24.875
2.166.014	-33.608	2.204.924	-25.974	2.158.848	-24.874
2.166.180	-33.606	2.205.092	-25.976	2.158.994	-24.874
2.166.360	-33.604	2.205.220	-25.977	2.159.086	-24.873
2.166.441	-33.603	2.205.340	-25.978	2.159.267	-24.873
2.166.579	-33.601	2.205.502	-25.980	2.159.455	-24.872
2.166.720	-33.599	2.205.714	-25.981	2.159.622	-24.872
2.166.855	-33.597	2.205.858	-25.982	2.159.814	-24.871
2.167.029	-33.595	2.205.943	-25.984	2.160.061	-24.871
2.167.180	-33.593	2.206.129	-25.985	2.160.329	-24.870
2.167.363	-33.592	2.206.301	-25.987	2.160.548	-24.870
2.167.563	-33.590	2.206.458	-25.988	2.160.706	-24.869
2.167.710	-33.588	2.206.666	-25.989	2.160.837	-24.869
2.167.887	-33.586	2.206.821	-25.991	2.161.001	-24.868
2.168.114	-33.585	2.206.987	-25.992	2.161.229	-24.867
2.168.297	-33.583	2.207.184	-25.994	2.161.438	-24.867
2.168.425	-33.581	2.207.374	-25.995	2.161.539	-24.866
2.168.568	-33.579	2.207.531	-25.996	2.161.618	-24.866
2.168.777	-33.578	2.207.683	-25.998	2.161.720	-24.865
2.168.981	-33.576	2.207.811	-25.999	2.161.864	-24.865
2.169.160	-33.574	2.207.948	-26.001	2.162.034	-24.864
2.169.298	-33.572	2.208.116	-26.002	2.162.169	-24.863
2.169.398	-33.571	2.208.349	-26.004	2.162.253	-24.863

2.169.587	-33.569	2.208.497	-26.005	2.162.415	-24.862
2.169.852	-33.567	2.208.642	-26.007	2.162.663	-24.862
2.170.023	-33.565	2.208.841	-26.008	2.162.876	-24.861
2.170.148	-33.564	2.208.969	-26.010	2.163.076	-24.860
2.170.327	-33.562	2.209.126	-26.011	2.163.266	-24.860
2.170.508	-33.560	2.209.312	-26.013	2.163.474	-24.859
2.170.707	-33.559	2.209.484	-26.014	2.163.655	-24.859
2.170.898	-33.557	2.209.680	-26.016	2.163.840	-24.858
2.171.040	-33.555	2.209.774	-26.017	2.163.998	-24.857
2.171.181	-33.553	2.209.910	-26.019	2.164.162	-24.857
2.171.321	-33.552	2.210.092	-26.020	2.164.330	-24.856
2.171.435	-33.550	2.210.248	-26.022	2.164.427	-24.855
2.171.610	-33.548	2.210.405	-26.023	2.164.612	-24.855
2.171.880	-33.547	2.210.526	-26.024	2.164.825	-24.854
2.172.081	-33.545	2.210.668	-26.026	2.164.984	-24.853
2.172.195	-33.543	2.210.864	-26.027	2.165.134	-24.853
2.172.305	-33.542	2.211.055	-26.029	2.165.318	-24.852
2.172.457	-33.540	2.211.216	-26.030	2.165.457	-24.851
2.172.688	-33.538	2.211.375	-26.032	2.165.574	-24.850
2.172.906	-33.537	2.211.505	-26.033	2.165.728	-24.850
2.173.044	-33.535	2.211.696	-26.035	2.165.876	-24.849
2.173.185	-33.533	2.211.964	-26.036	2.166.105	-24.848
2.173.362	-33.532	2.212.193	-26.037	2.166.288	-24.847
2.173.517	-33.530	2.212.349	-26.039	2.166.454	-24.847
2.173.698	-33.528	2.212.563	-26.040	2.166.628	-24.846
2.173.898	-33.527	2.212.722	-26.042	2.166.797	-24.845
2.174.065	-33.525	2.212.872	-26.043	2.167.002	-24.844
2.174.160	-33.523	2.213.053	-26.044	2.167.155	-24.844
2.174.308	-33.522	2.213.214	-26.046	2.167.287	-24.843
2.174.603	-33.520	2.213.409	-26.047	2.167.460	-24.842

2.175.031	-33.518	2.213.550	-26.048	2.167.623	-24.841
2.175.361	-33.517	2.213.642	-26.049	2.167.789	-24.840
2.175.468	-33.515	2.213.772	-26.051	2.168.058	-24.840
2.175.533	-33.514	2.213.967	-26.052	2.168.232	-24.839
2.175.600	-33.512	2.214.103	-26.053	2.168.335	-24.838
2.175.676	-33.510	2.214.182	-26.054	2.168.481	-24.837
2.175.750	-33.509	2.214.334	-26.056	2.168.638	-24.836
2.175.822	-33.507	2.214.558	-26.057	2.168.804	-24.835
2.175.934	-33.505	2.214.754	-26.058	2.168.997	-24.834
2.176.075	-33.504	2.214.899	-26.059	2.169.326	-24.834
2.176.236	-33.502	2.215.100	-26.060	2.169.796	-24.833
2.176.387	-33.501	2.215.282	-26.061	2.170.067	-24.832
2.176.546	-33.499	2.215.448	-26.062	2.170.085	-24.831
2.176.714	-33.497	2.215.623	-26.064	2.170.154	-24.830
2.176.924	-33.496	2.215.784	-26.065	2.170.235	-24.829
2.177.132	-33.494	2.215.992	-26.066	2.170.255	-24.828
2.177.242	-33.493	2.216.223	-26.067	2.170.255	-24.827
2.177.449	-33.491	2.216.349	-26.068	2.170.371	-24.826
2.177.681	-33.490	2.216.503	-26.069	2.170.571	-24.825
2.177.847	-33.488	2.216.705	-26.070	2.170.750	-24.824
2.178.024	-33.487	2.216.787	-26.071	2.170.892	-24.823
2.178.216	-33.485	2.216.942	-26.072	2.171.019	-24.822
2.178.358	-33.484	2.217.171	-26.072	2.171.187	-24.821
2.178.416	-33.482	2.217.343	-26.073	2.171.384	-24.820
2.178.578	-33.481	2.217.477	-26.074	2.171.595	-24.819
2.178.832	-33.479	2.217.670	-26.075	2.171.789	-24.818
2.178.960	-33.478	2.217.858	-26.076	2.171.919	-24.817
2.179.095	-33.476	2.217.979	-26.077	2.172.065	-24.816
2.179.229	-33.475	2.218.118	-26.077	2.172.298	-24.815
2.179.368	-33.473	2.218.295	-26.078	2.172.486	-24.814

2.179.630	-33.472	2.218.466	-26.079	2.172.629	-24.813
2.179.827	-33.471	2.218.669	-26.080	2.172.764	-24.812
2.179.968	-33.469	2.218.882	-26.080	2.172.897	-24.811
2.180.108	-33.468	2.219.052	-26.081	2.173.051	-24.810
2.180.285	-33.467	2.219.240	-26.082	2.173.207	-24.808
2.180.589	-33.465	2.219.424	-26.082	2.173.393	-24.807
2.180.847	-33.464	2.219.572	-26.083	2.173.539	-24.806
2.180.928	-33.463	2.219.695	-26.083	2.173.707	-24.805
2.180.956	-33.462	2.219.855	-26.084	2.173.862	-24.804
2.181.052	-33.460	2.220.022	-26.084	2.173.965	-24.803
2.181.191	-33.459	2.220.197	-26.085	2.174.142	-24.802
2.181.378	-33.458	2.220.340	-26.085	2.174.397	-24.801
2.181.617	-33.457	2.220.459	-26.086	2.174.625	-24.800
2.181.780	-33.456	2.220.660	-26.086	2.174.867	-24.798
2.181.946	-33.454	2.220.827	-26.087	2.175.063	-24.797
2.182.122	-33.453	2.220.958	-26.087	2.175.172	-24.796
2.182.298	-33.452	2.221.116	-26.087	2.175.287	-24.795
2.182.467	-33.451	2.221.479	-26.088	2.175.462	-24.794
2.182.630	-33.450	2.221.913	-26.088	2.175.616	-24.793
2.182.746	-33.449	2.222.116	-26.088	2.175.782	-24.792
2.182.853	-33.448	2.222.235	-26.089	2.175.948	-24.790
2.183.069	-33.447	2.222.294	-26.089	2.176.071	-24.789
2.183.246	-33.446	2.222.355	-26.089	2.176.270	-24.788
2.183.356	-33.445	2.222.437	-26.089	2.176.431	-24.787
2.183.505	-33.444	2.222.493	-26.090	2.176.521	-24.786
2.183.689	-33.443	2.222.630	-26.090	2.176.723	-24.785
2.183.812	-33.442	2.222.737	-26.090	2.176.958	-24.783
2.183.969	-33.442	2.222.898	-26.090	2.177.148	-24.782
2.184.193	-33.441	2.223.085	-26.090	2.177.327	-24.781
2.184.353	-33.440	2.223.248	-26.091	2.177.453	-24.780

2.184.482	-33.439	2.223.419	-26.091	2.177.569	-24.779
2.184.718	-33.438	2.223.615	-26.091	2.177.681	-24.778
2.184.861	-33.438	2.223.759	-26.091	2.177.823	-24.776
2.184.984	-33.437	2.223.877	-26.091	2.178.046	-24.775
2.185.275	-33.436	2.224.090	-26.091	2.178.288	-24.774
2.185.472	-33.436	2.224.361	-26.091	2.178.483	-24.773
2.185.649	-33.435	2.224.583	-26.091	2.178.627	-24.772
2.185.813	-33.434	2.224.693	-26.091	2.178.759	-24.770
2.185.938	-33.434	2.224.825	-26.091	2.178.974	-24.769
2.186.134	-33.433	2.224.986	-26.091	2.179.153	-24.768
2.186.315	-33.433	2.225.107	-26.091	2.179.265	-24.767
2.186.461	-33.432	2.225.217	-26.091	2.179.398	-24.766
2.186.612	-33.432	2.225.378	-26.091	2.179.493	-24.764
2.186.799	-33.431	2.225.577	-26.091	2.179.686	-24.763
2.187.016	-33.431	2.225.737	-26.091	2.179.971	-24.762
2.187.168	-33.430	2.225.883	-26.091	2.180.129	-24.761
2.187.325	-33.430	2.226.019	-26.091	2.180.197	-24.760
2.187.526	-33.429	2.226.194	-26.091	2.180.342	-24.758
2.187.641	-33.429	2.226.458	-26.091	2.180.533	-24.757
2.187.742	-33.428	2.226.674	-26.091	2.180.755	-24.756
2.187.901	-33.428	2.226.870	-26.091	2.180.934	-24.754
2.188.107	-33.428	2.227.025	-26.091	2.181.064	-24.753
2.188.304	-33.427	2.227.160	-26.091	2.181.120	-24.752
2.188.407	-33.427	2.227.311	-26.091	2.181.315	-24.751
2.188.465	-33.427	2.227.504	-26.091	2.181.617	-24.749
2.188.615	-33.426	2.227.728	-26.091	2.181.756	-24.748
2.188.801	-33.426	2.227.865	-26.091	2.181.864	-24.747
2.188.934	-33.426	2.227.970	-26.091	2.181.974	-24.745
2.189.131	-33.426	2.228.062	-26.090	2.182.119	-24.744
2.189.336	-33.425	2.228.219	-26.090	2.182.260	-24.743

2.189.437	-33.425	2.228.448	-26.090	2.182.345	-24.741
2.189.556	-33.425	2.228.640	-26.090	2.182.540	-24.740
2.189.846	-33.425	2.228.779	-26.090	2.182.789	-24.739
2.190.150	-33.425	2.228.893	-26.090	2.182.955	-24.737
2.190.311	-33.425	2.229.041	-26.090	2.183.113	-24.736
2.190.430	-33.424	2.229.214	-26.090	2.183.398	-24.734
2.190.546	-33.424	2.229.464	-26.090	2.183.694	-24.733
2.190.723	-33.424	2.229.632	-26.090	2.183.813	-24.731
2.190.918	-33.424	2.229.699	-26.090	2.183.969	-24.730
2.191.091	-33.424	2.229.843	-26.090	2.184.137	-24.729
2.191.281	-33.424	2.230.016	-26.090	2.184.278	-24.727
2.191.353	-33.424	2.230.150	-26.090	2.184.466	-24.726
2.191.485	-33.424	2.230.289	-26.090	2.184.621	-24.724
2.191.715	-33.424	2.230.468	-26.091	2.184.771	-24.723
2.191.843	-33.423	2.230.667	-26.091	2.184.896	-24.721
2.191.895	-33.423	2.230.914	-26.091	2.185.038	-24.720
2.192.116	-33.423	2.231.129	-26.091	2.185.188	-24.718
2.192.385	-33.423	2.231.270	-26.091	2.185.340	-24.716
2.192.417	-33.423	2.231.423	-26.091	2.185.499	-24.715
2.192.560	-33.423	2.231.559	-26.091	2.185.603	-24.713
2.192.796	-33.423	2.231.702	-26.091	2.185.748	-24.712
2.192.950	-33.423	2.231.904	-26.091	2.185.922	-24.710
2.193.172	-33.423	2.232.041	-26.092	2.186.144	-24.708
2.193.418	-33.423	2.232.155	-26.092	2.186.382	-24.707
2.193.629	-33.423	2.232.294	-26.092	2.186.552	-24.705
2.193.737	-33.423	2.232.477	-26.092	2.186.767	-24.703
2.193.844	-33.423	2.232.724	-26.092	2.186.935	-24.702
2.194.008	-33.423	2.232.867	-26.092	2.187.013	-24.700
2.194.215	-33.423	2.232.923	-26.093	2.187.164	-24.698
2.194.411	-33.423	2.233.174	-26.093	2.187.361	-24.697

2.194.540	-33.424	2.233.443	-26.093	2.187.558	-24.695
2.194.650	-33.424	2.233.577	-26.093	2.187.751	-24.693
2.194.859	-33.424	2.233.746	-26.094	2.187.880	-24.692
2.195.100	-33.424	2.233.891	-26.094	2.188.053	-24.690
2.195.228	-33.424	2.234.068	-26.094	2.188.241	-24.688
2.195.365	-33.424	2.234.260	-26.094	2.188.396	-24.686
2.195.592	-33.424	2.234.388	-26.095	2.188.604	-24.685
2.195.690	-33.424	2.234.567	-26.095	2.188.763	-24.683
2.195.818	-33.424	2.234.764	-26.095	2.188.862	-24.681
2.196.090	-33.424	2.234.854	-26.095	2.189.007	-24.679
2.196.272	-33.424	2.234.953	-26.096	2.189.185	-24.678
2.196.409	-33.424	2.235.098	-26.096	2.189.368	-24.676
2.196.579	-33.425	2.235.226	-26.096	2.189.586	-24.674
2.196.720	-33.425	2.235.385	-26.097	2.189.781	-24.672
2.196.857	-33.425	2.235.596	-26.097	2.189.951	-24.670
2.196.978	-33.425	2.235.762	-26.097	2.190.108	-24.668
2.197.095	-33.425	2.235.963	-26.098	2.190.244	-24.667
2.197.327	-33.425	2.236.252	-26.098	2.190.430	-24.665
2.197.567	-33.425	2.236.443	-26.098	2.190.607	-24.663
2.197.730	-33.425	2.236.579	-26.099	2.190.761	-24.661
2.197.862	-33.425	2.236.778	-26.099	2.190.986	-24.659
2.198.011	-33.426	2.236.984	-26.099	2.191.221	-24.657
2.198.183	-33.426	2.237.128	-26.100	2.191.344	-24.656
2.198.360	-33.426	2.237.249	-26.100	2.191.418	-24.654
2.198.533	-33.426	2.237.402	-26.101	2.191.572	-24.652
2.198.689	-33.426	2.237.529	-26.101	2.191.718	-24.650
2.198.891	-33.426	2.237.674	-26.101	2.191.841	-24.648
2.199.041	-33.427	2.237.820	-26.102	2.192.018	-24.646
2.199.124	-33.427	2.237.955	-26.102	2.192.274	-24.644
2.199.278	-33.427	2.238.082	-26.103	2.192.713	-24.643

2.199.592	-33.427	2.238.192	-26.103	2.193.078	-24.641
2.200.011	-33.427	2.238.371	-26.103	2.193.221	-24.639
2.200.365	-33.427	2.238.615	-26.104	2.193.299	-24.637
2.200.533	-33.428	2.238.876	-26.104	2.193.328	-24.635
2.200.591	-33.428	2.239.023	-26.105	2.193.358	-24.633
2.200.681	-33.428	2.239.144	-26.105	2.193.429	-24.631
2.200.712	-33.428	2.239.307	-26.106	2.193.531	-24.629
2.200.777	-33.428	2.239.484	-26.106	2.193.667	-24.628
2.200.905	-33.429	2.239.698	-26.106	2.193.824	-24.626
2.200.996	-33.429	2.239.865	-26.107	2.193.974	-24.624
2.201.120	-33.429	2.240.031	-26.107	2.194.164	-24.622
2.201.225	-33.429	2.240.228	-26.108	2.194.357	-24.620
2.201.369	-33.430	2.240.414	-26.108	2.194.550	-24.618
2.201.588	-33.430	2.240.586	-26.108	2.194.749	-24.617
2.201.738	-33.430	2.240.698	-26.109	2.194.921	-24.615
2.201.931	-33.430	2.240.831	-26.109	2.195.061	-24.613
2.202.164	-33.431	2.241.057	-26.110	2.195.208	-24.611
2.202.354	-33.431	2.241.248	-26.110	2.195.399	-24.609
2.202.540	-33.431	2.241.349	-26.111	2.195.602	-24.607
2.202.728	-33.431	2.241.543	-26.111	2.195.717	-24.606
2.202.883	-33.432	2.241.799	-26.111	2.195.824	-24.604
2.202.997	-33.432	2.241.964	-26.112	2.196.005	-24.602
2.203.116	-33.432	2.242.134	-26.112	2.196.180	-24.600
2.203.293	-33.433	2.242.327	-26.113	2.196.344	-24.599
2.203.512	-33.433	2.242.493	-26.113	2.196.425	-24.597
2.203.661	-33.433	2.242.628	-26.114	2.196.544	-24.595
2.203.799	-33.434	2.242.803	-26.114	2.196.781	-24.593
2.203.947	-33.434	2.243.008	-26.114	2.197.009	-24.592
2.204.086	-33.434	2.243.163	-26.115	2.197.213	-24.590
2.204.214	-33.435	2.243.275	-26.115	2.197.352	-24.588

2.204.379	-33.435	2.243.403	-26.116	2.197.496	-24.587
2.204.558	-33.436	2.243.591	-26.116	2.197.737	-24.585
2.204.736	-33.436	2.243.770	-26.116	2.197.932	-24.583
2.204.919	-33.436	2.243.947	-26.117	2.198.042	-24.582
2.205.134	-33.437	2.244.126	-26.117	2.198.242	-24.580
2.205.298	-33.437	2.244.307	-26.117	2.198.418	-24.579
2.205.461	-33.438	2.244.457	-26.118	2.198.539	-24.577
2.205.668	-33.438	2.244.583	-26.118	2.198.699	-24.576
2.205.853	-33.439	2.244.745	-26.118	2.198.889	-24.574
2.206.008	-33.439	2.244.890	-26.119	2.199.059	-24.573
2.206.095	-33.440	2.245.016	-26.119	2.199.180	-24.571
2.206.225	-33.441	2.245.233	-26.119	2.199.323	-24.570
2.206.423	-33.441	2.245.660	-26.120	2.199.473	-24.568
2.206.575	-33.442	2.246.037	-26.120	2.199.605	-24.567
2.206.702	-33.442	2.246.194	-26.120	2.199.758	-24.565
2.206.901	-33.443	2.246.299	-26.121	2.199.939	-24.564
2.207.067	-33.444	2.246.387	-26.121	2.200.146	-24.562
2.207.251	-33.444	2.246.397	-26.121	2.200.289	-24.561
2.207.367	-33.445	2.246.447	-26.122	2.200.441	-24.560
2.207.466	-33.445	2.246.579	-26.122	2.200.622	-24.558
2.207.692	-33.446	2.246.718	-26.122	2.200.766	-24.557
2.207.850	-33.447	2.246.875	-26.123	2.200.941	-24.556
2.207.959	-33.448	2.246.995	-26.123	2.201.070	-24.555
2.208.142	-33.448	2.247.108	-26.123	2.201.203	-24.553
2.208.344	-33.449	2.247.249	-26.123	2.201.397	-24.552
2.208.535	-33.450	2.247.413	-26.124	2.201.595	-24.551
2.208.723	-33.451	2.247.641	-26.124	2.201.770	-24.550
2.208.893	-33.452	2.247.894	-26.124	2.201.951	-24.549
2.209.030	-33.452	2.248.121	-26.124	2.202.114	-24.547
2.209.146	-33.453	2.248.309	-26.125	2.202.256	-24.546

2.209.281	-33.454	2.248.467	-26.125	2.202.432	-24.545
2.209.516	-33.455	2.248.584	-26.125	2.202.600	-24.544
2.209.799	-33.456	2.248.707	-26.125	2.202.746	-24.543
2.209.950	-33.457	2.248.895	-26.126	2.202.865	-24.542
2.210.081	-33.458	2.249.045	-26.126	2.203.047	-24.541
2.210.249	-33.459	2.249.173	-26.126	2.203.255	-24.540
2.210.398	-33.460	2.249.341	-26.126	2.203.372	-24.539
2.210.553	-33.461	2.249.543	-26.126	2.203.551	-24.538
2.210.743	-33.462	2.249.725	-26.127	2.203.773	-24.537
2.210.965	-33.463	2.249.856	-26.127	2.203.971	-24.536
2.211.142	-33.464	2.250.020	-26.127	2.204.160	-24.535
2.211.270	-33.465	2.250.237	-26.127	2.204.240	-24.534
2.211.393	-33.466	2.250.378	-26.128	2.204.393	-24.533
2.211.570	-33.468	2.250.542	-26.128	2.204.588	-24.532
2.211.749	-33.469	2.250.733	-26.128	2.204.717	-24.531
2.211.883	-33.470	2.250.910	-26.128	2.204.874	-24.530
2.212.085	-33.471	2.251.117	-26.129	2.205.042	-24.529
2.212.271	-33.472	2.251.319	-26.129	2.205.184	-24.528
2.212.377	-33.473	2.251.498	-26.129	2.205.293	-24.527
2.212.504	-33.475	2.251.595	-26.129	2.205.448	-24.526
2.212.672	-33.476	2.251.727	-26.130	2.205.609	-24.525
2.212.838	-33.477	2.251.841	-26.130	2.205.759	-24.524
2.212.980	-33.478	2.252.000	-26.130	2.205.865	-24.523
2.213.148	-33.479	2.252.175	-26.130	2.206.044	-24.522
2.213.353	-33.481	2.252.357	-26.131	2.206.355	-24.521
2.213.503	-33.482	2.252.489	-26.131	2.206.583	-24.520
2.213.559	-33.483	2.252.621	-26.131	2.206.736	-24.520
2.213.723	-33.485	2.252.836	-26.131	2.206.940	-24.519
2.213.920	-33.486	2.253.033	-26.132	2.207.189	-24.518
2.214.077	-33.487	2.253.186	-26.132	2.207.352	-24.517

2.214.209	-33.488	2.253.315	-26.132	2.207.513	-24.516
2.214.336	-33.490	2.253.485	-26.132	2.207.692	-24.515
2.214.534	-33.491	2.253.669	-26.133	2.207.767	-24.514
2.214.762	-33.493	2.253.783	-26.133	2.207.867	-24.513
2.214.997	-33.494	2.253.931	-26.133	2.208.021	-24.512
2.215.154	-33.495	2.254.097	-26.134	2.208.201	-24.511
2.215.320	-33.497	2.254.213	-26.134	2.208.383	-24.511
2.215.524	-33.498	2.254.371	-26.134	2.208.486	-24.510
2.215.678	-33.499	2.254.612	-26.134	2.208.575	-24.509
2.215.925	-33.501	2.254.793	-26.135	2.208.781	-24.508
2.216.201	-33.502	2.254.939	-26.135	2.208.974	-24.507
2.216.329	-33.504	2.255.145	-26.135	2.209.155	-24.506
2.216.429	-33.505	2.255.309	-26.136	2.209.362	-24.505
2.216.553	-33.506	2.255.518	-26.136	2.209.525	-24.504
2.216.659	-33.508	2.255.677	-26.136	2.209.708	-24.503
2.216.771	-33.509	2.255.813	-26.137	2.209.908	-24.502
2.216.929	-33.510	2.255.988	-26.137	2.210.069	-24.501
2.217.092	-33.512	2.256.086	-26.138	2.210.233	-24.500
2.217.271	-33.513	2.256.293	-26.138	2.210.423	-24.499
2.217.431	-33.515	2.256.532	-26.138	2.210.618	-24.498
2.217.612	-33.516	2.256.666	-26.139	2.210.746	-24.497
2.217.805	-33.517	2.256.803	-26.139	2.210.835	-24.496
2.217.915	-33.519	2.256.910	-26.139	2.211.021	-24.495
2.218.098	-33.520	2.257.107	-26.140	2.211.201	-24.494
2.218.345	-33.522	2.257.334	-26.140	2.211.353	-24.493
2.218.532	-33.523	2.257.498	-26.140	2.211.573	-24.492
2.218.736	-33.524	2.257.666	-26.141	2.211.774	-24.491
2.218.949	-33.526	2.257.856	-26.141	2.211.951	-24.490
2.219.102	-33.527	2.258.024	-26.141	2.212.139	-24.489
2.219.218	-33.529	2.258.151	-26.142	2.212.327	-24.488

2.219.366	-33.530	2.258.297	-26.142	2.212.482	-24.487
2.219.514	-33.531	2.258.434	-26.143	2.212.682	-24.486
2.219.664	-33.533	2.258.609	-26.143	2.212.854	-24.484
2.219.901	-33.534	2.258.777	-26.143	2.212.965	-24.483
2.220.089	-33.536	2.258.916	-26.144	2.213.116	-24.482
2.220.164	-33.537	2.259.057	-26.144	2.213.266	-24.481
2.220.340	-33.538	2.259.198	-26.144	2.213.474	-24.480
2.220.560	-33.540	2.259.368	-26.145	2.213.681	-24.478
2.220.739	-33.541	2.259.545	-26.145	2.213.819	-24.477
2.220.858	-33.543	2.259.782	-26.145	2.213.947	-24.476
2.220.994	-33.544	2.259.939	-26.146	2.214.053	-24.475
2.221.171	-33.545	2.260.099	-26.146	2.214.207	-24.473
2.221.313	-33.547	2.260.322	-26.146	2.214.503	-24.472
2.221.435	-33.548	2.260.501	-26.147	2.214.693	-24.471
2.221.559	-33.550	2.260.674	-26.147	2.214.734	-24.470
2.221.777	-33.551	2.260.858	-26.147	2.214.895	-24.468
2.221.980	-33.552	2.261.066	-26.148	2.215.079	-24.467
2.222.175	-33.554	2.261.237	-26.148	2.215.240	-24.465
2.222.377	-33.555	2.261.377	-26.148	2.215.474	-24.464
2.222.523	-33.556	2.261.512	-26.148	2.215.810	-24.463
2.222.675	-33.558	2.261.631	-26.149	2.216.147	-24.461
2.222.820	-33.559	2.261.763	-26.149	2.216.328	-24.460
2.222.977	-33.560	2.261.899	-26.149	2.216.461	-24.458
2.223.174	-33.562	2.262.056	-26.150	2.216.550	-24.457
2.223.293	-33.563	2.262.215	-26.150	2.216.629	-24.456
2.223.439	-33.564	2.262.374	-26.150	2.216.698	-24.454
2.223.669	-33.566	2.262.526	-26.150	2.216.812	-24.453
2.223.864	-33.567	2.262.679	-26.150	2.216.913	-24.451
2.224.032	-33.568	2.262.909	-26.151	2.216.971	-24.449
2.224.187	-33.570	2.263.104	-26.151	2.217.146	-24.448

2.224.330	-33.571	2.263.277	-26.151	2.217.348	-24.446
2.224.487	-33.573	2.263.448	-26.151	2.217.531	-24.445
2.224.881	-33.574	2.263.578	-26.152	2.217.715	-24.443
2.225.266	-33.575	2.263.725	-26.152	2.217.854	-24.442
2.225.524	-33.577	2.263.905	-26.152	2.218.008	-24.440
2.225.636	-33.578	2.264.142	-26.152	2.218.221	-24.438
2.225.743	-33.579	2.264.350	-26.152	2.218.448	-24.437
2.225.838	-33.581	2.264.485	-26.152	2.218.635	-24.435
2.225.891	-33.582	2.264.632	-26.153	2.218.772	-24.433
2.225.952	-33.583	2.264.790	-26.153	2.218.907	-24.432
2.226.039	-33.585	2.264.966	-26.153	2.219.043	-24.430
2.226.190	-33.586	2.265.235	-26.153	2.219.133	-24.428
2.226.349	-33.587	2.265.418	-26.153	2.219.279	-24.427
2.226.476	-33.589	2.265.482	-26.153	2.219.496	-24.425
2.226.583	-33.590	2.265.686	-26.153	2.219.619	-24.423
2.226.749	-33.591	2.265.884	-26.153	2.219.767	-24.422
2.226.991	-33.592	2.266.026	-26.154	2.220.015	-24.420
2.227.171	-33.594	2.266.243	-26.154	2.220.161	-24.418
2.227.263	-33.595	2.266.424	-26.154	2.220.275	-24.416
2.227.396	-33.596	2.266.565	-26.154	2.220.497	-24.415
2.227.605	-33.598	2.266.682	-26.154	2.220.748	-24.413
2.227.862	-33.599	2.266.814	-26.154	2.220.920	-24.411
2.228.040	-33.600	2.266.996	-26.154	2.221.086	-24.409
2.228.154	-33.602	2.267.178	-26.154	2.221.310	-24.407
2.228.326	-33.603	2.267.379	-26.154	2.221.481	-24.406
2.228.546	-33.604	2.267.491	-26.154	2.221.622	-24.404
2.228.699	-33.606	2.267.605	-26.154	2.221.756	-24.402
2.228.795	-33.607	2.267.803	-26.154	2.221.872	-24.400
2.228.887	-33.608	2.267.997	-26.154	2.222.020	-24.398
2.229.059	-33.610	2.268.178	-26.154	2.222.168	-24.397

2.229.231	-33.611	2.268.375	-26.154	2.222.314	-24.395
2.229.341	-33.613	2.268.570	-26.154	2.222.491	-24.393
2.229.534	-33.614	2.268.741	-26.154	2.222.661	-24.391
2.229.735	-33.615	2.268.879	-26.154	2.222.867	-24.389
2.229.932	-33.617	2.269.023	-26.154	2.223.083	-24.387
2.230.129	-33.618	2.269.149	-26.154	2.223.241	-24.386
2.230.298	-33.620	2.269.410	-26.154	2.223.369	-24.384
2.230.439	-33.621	2.269.841	-26.154	2.223.492	-24.382
2.230.641	-33.623	2.270.154	-26.154	2.223.629	-24.380
2.230.878	-33.624	2.270.280	-26.154	2.223.859	-24.378
2.231.019	-33.626	2.270.371	-26.154	2.224.056	-24.376
2.231.111	-33.627	2.270.472	-26.154	2.224.169	-24.375
2.231.295	-33.629	2.270.479	-26.154	2.224.326	-24.373
2.231.512	-33.630	2.270.546	-26.154	2.224.473	-24.371
2.231.631	-33.632	2.270.687	-26.154	2.224.650	-24.369
2.231.738	-33.633	2.270.795	-26.154	2.224.836	-24.367
2.231.899	-33.635	2.270.892	-26.154	2.225.013	-24.365
2.232.085	-33.636	2.271.030	-26.154	2.225.168	-24.363
2.232.264	-33.638	2.271.207	-26.154	2.225.329	-24.361
2.232.426	-33.639	2.271.404	-26.154	2.225.526	-24.359
2.232.571	-33.641	2.271.584	-26.153	2.225.694	-24.358
2.232.796	-33.642	2.271.734	-26.153	2.225.862	-24.356
2.233.004	-33.644	2.271.939	-26.153	2.225.981	-24.354
2.233.130	-33.646	2.272.128	-26.153	2.226.126	-24.352
2.233.306	-33.647	2.272.296	-26.153	2.226.335	-24.350
2.233.508	-33.649	2.272.540	-26.153	2.226.500	-24.348
2.233.653	-33.651	2.272.744	-26.153	2.226.648	-24.346
2.233.759	-33.652	2.272.853	-26.153	2.226.799	-24.344
2.233.887	-33.654	2.273.013	-26.153	2.227.012	-24.342
2.234.124	-33.656	2.273.203	-26.153	2.227.186	-24.340

2.234.324	-33.658	2.273.298	-26.153	2.227.245	-24.338
2.234.435	-33.659	2.273.396	-26.153	2.227.414	-24.336
2.234.543	-33.661	2.273.589	-26.153	2.227.659	-24.334
2.234.735	-33.663	2.273.788	-26.153	2.227.838	-24.332
2.234.940	-33.665	2.273.953	-26.153	2.227.980	-24.330
2.235.105	-33.666	2.274.122	-26.153	2.228.141	-24.328
2.235.293	-33.668	2.274.249	-26.153	2.228.313	-24.326
2.235.441	-33.670	2.274.386	-26.153	2.228.443	-24.324
2.235.596	-33.672	2.274.616	-26.153	2.228.555	-24.322
2.235.807	-33.674	2.274.792	-26.153	2.228.734	-24.320
2.236.001	-33.676	2.274.919	-26.153	2.228.920	-24.318
2.236.154	-33.678	2.275.159	-26.153	2.229.061	-24.316
2.236.264	-33.679	2.275.361	-26.153	2.229.216	-24.313
2.236.357	-33.681	2.275.482	-26.153	2.229.400	-24.311
2.236.526	-33.683	2.275.678	-26.153	2.229.585	-24.309
2.236.740	-33.685	2.275.818	-26.153	2.229.764	-24.307
2.236.899	-33.687	2.275.930	-26.153	2.230.002	-24.305
2.237.083	-33.689	2.276.129	-26.153	2.230.179	-24.303
2.237.265	-33.691	2.276.273	-26.154	2.230.380	-24.300
2.237.437	-33.693	2.276.402	-26.154	2.230.633	-24.298
2.237.677	-33.695	2.276.547	-26.154	2.230.833	-24.296
2.237.818	-33.697	2.276.691	-26.154	2.230.936	-24.294
2.237.887	-33.699	2.276.870	-26.154	2.231.003	-24.291
2.238.020	-33.701	2.277.038	-26.154	2.231.142	-24.289
2.238.154	-33.703	2.277.228	-26.154	2.231.272	-24.287
2.238.326	-33.705	2.277.393	-26.155	2.231.449	-24.284
2.238.530	-33.707	2.277.549	-26.155	2.231.628	-24.282
2.238.647	-33.709	2.277.735	-26.155	2.231.720	-24.280
2.238.732	-33.711	2.277.885	-26.155	2.231.852	-24.277
2.238.876	-33.713	2.278.013	-26.155	2.232.043	-24.275

2.239.039	-33.715	2.278.139	-26.156	2.232.244	-24.273
2.239.209	-33.717	2.278.304	-26.156	2.232.394	-24.270
2.239.442	-33.719	2.278.524	-26.156	2.232.534	-24.268
2.239.675	-33.721	2.278.728	-26.156	2.232.771	-24.265
2.239.834	-33.723	2.278.844	-26.156	2.232.912	-24.263
2.239.980	-33.725	2.278.956	-26.157	2.233.065	-24.260
2.240.229	-33.727	2.279.128	-26.157	2.233.309	-24.258
2.240.486	-33.729	2.279.299	-26.157	2.233.508	-24.255
2.240.681	-33.731	2.279.439	-26.157	2.233.690	-24.253
2.240.842	-33.733	2.279.648	-26.158	2.233.929	-24.250
2.240.974	-33.735	2.279.881	-26.158	2.234.119	-24.247
2.241.133	-33.737	2.280.058	-26.158	2.234.185	-24.245
2.241.283	-33.739	2.280.237	-26.158	2.234.310	-24.242
2.241.400	-33.741	2.280.398	-26.158	2.234.464	-24.239
2.241.579	-33.743	2.280.536	-26.159	2.234.644	-24.237
2.241.743	-33.745	2.280.688	-26.159	2.234.823	-24.234
2.241.924	-33.747	2.280.887	-26.159	2.235.070	-24.231
2.242.041	-33.749	2.281.099	-26.159	2.235.226	-24.229
2.242.081	-33.751	2.281.232	-26.160	2.235.318	-24.226
2.242.283	-33.752	2.281.418	-26.160	2.235.490	-24.223
2.242.473	-33.754	2.281.644	-26.160	2.235.670	-24.220
2.242.524	-33.756	2.281.779	-26.160	2.235.865	-24.217
2.242.648	-33.758	2.281.901	-26.160	2.236.031	-24.215
2.242.894	-33.760	2.282.094	-26.160	2.236.173	-24.212
2.243.151	-33.762	2.282.208	-26.161	2.236.333	-24.209
2.243.382	-33.764	2.282.321	-26.161	2.236.516	-24.206
2.243.615	-33.766	2.282.513	-26.161	2.236.741	-24.203
2.243.867	-33.768	2.282.674	-26.161	2.236.935	-24.200
2.244.009	-33.770	2.282.773	-26.161	2.237.051	-24.197
2.244.090	-33.772	2.282.918	-26.161	2.237.155	-24.194

2.244.301	-33.773	2.283.131	-26.161	2.237.350	-24.191
2.244.478	-33.775	2.283.223	-26.161	2.237.554	-24.188
2.244.583	-33.777	2.283.391	-26.161	2.237.717	-24.185
2.244.769	-33.779	2.283.596	-26.161	2.237.863	-24.182
2.244.944	-33.781	2.283.739	-26.161	2.238.029	-24.179
2.245.058	-33.783	2.283.893	-26.161	2.238.243	-24.176
2.245.197	-33.785	2.284.063	-26.161	2.238.427	-24.173
2.245.403	-33.786	2.284.303	-26.161	2.238.559	-24.169
2.245.621	-33.788	2.284.501	-26.161	2.238.689	-24.166
2.245.797	-33.790	2.284.706	-26.161	2.239.041	-24.163
2.245.940	-33.792	2.284.946	-26.161	2.239.460	-24.160
2.246.086	-33.793	2.285.081	-26.161	2.239.698	-24.157
2.246.241	-33.795	2.285.145	-26.161	2.239.809	-24.153
2.246.438	-33.797	2.285.302	-26.160	2.239.852	-24.150
2.246.595	-33.799	2.285.544	-26.160	2.239.919	-24.147
2.246.715	-33.800	2.285.690	-26.160	2.239.986	-24.143
2.246.960	-33.802	2.285.791	-26.160	2.240.017	-24.140
2.247.177	-33.804	2.285.947	-26.160	2.240.087	-24.137
2.247.292	-33.805	2.286.102	-26.159	2.240.201	-24.133
2.247.479	-33.807	2.286.232	-26.159	2.240.383	-24.130
2.247.697	-33.809	2.286.395	-26.159	2.240.612	-24.127
2.247.829	-33.810	2.286.521	-26.159	2.240.760	-24.123
2.247.961	-33.812	2.286.684	-26.158	2.240.918	-24.120
2.248.136	-33.813	2.286.841	-26.158	2.241.153	-24.116
2.248.354	-33.815	2.287.072	-26.158	2.241.360	-24.113
2.248.542	-33.816	2.287.309	-26.157	2.241.548	-24.109
2.248.707	-33.818	2.287.527	-26.157	2.241.785	-24.106
2.248.878	-33.820	2.287.699	-26.157	2.241.953	-24.102
2.249.048	-33.821	2.287.851	-26.156	2.242.076	-24.099
2.249.229	-33.823	2.288.017	-26.156	2.242.233	-24.095

2.249.352	-33.824	2.288.107	-26.155	2.242.428	-24.092
2.249.435	-33.826	2.288.225	-26.155	2.242.574	-24.088
2.249.565	-33.827	2.288.456	-26.154	2.242.688	-24.085
2.249.867	-33.829	2.288.672	-26.154	2.242.809	-24.081
2.250.350	-33.830	2.288.801	-26.153	2.242.952	-24.078
2.250.694	-33.832	2.288.913	-26.153	2.243.185	-24.074
2.250.802	-33.833	2.289.092	-26.152	2.243.320	-24.071
2.250.876	-33.834	2.289.315	-26.152	2.243.385	-24.067
2.251.019	-33.836	2.289.548	-26.151	2.243.566	-24.063
2.251.093	-33.837	2.289.724	-26.151	2.243.802	-24.060
2.251.102	-33.839	2.289.872	-26.150	2.244.003	-24.056
2.251.173	-33.840	2.290.051	-26.150	2.244.213	-24.053
2.251.290	-33.841	2.290.183	-26.149	2.244.442	-24.049
2.251.409	-33.843	2.290.318	-26.149	2.244.595	-24.045
2.251.500	-33.844	2.290.540	-26.148	2.244.736	-24.042
2.251.665	-33.845	2.290.759	-26.147	2.244.863	-24.038
2.251.834	-33.847	2.290.936	-26.147	2.244.989	-24.035
2.252.043	-33.848	2.291.034	-26.146	2.245.159	-24.031
2.252.226	-33.849	2.291.164	-26.145	2.245.320	-24.027
2.252.402	-33.851	2.291.324	-26.145	2.245.495	-24.024
2.252.686	-33.852	2.291.416	-26.144	2.245.674	-24.020
2.252.888	-33.853	2.291.572	-26.143	2.245.865	-24.017
2.253.006	-33.854	2.291.788	-26.143	2.246.026	-24.013
2.253.129	-33.856	2.291.971	-26.142	2.246.185	-24.010
2.253.254	-33.857	2.292.135	-26.141	2.246.396	-24.006
2.253.374	-33.858	2.292.265	-26.140	2.246.535	-24.002
2.253.526	-33.859	2.292.422	-26.140	2.246.630	-23.999
2.253.714	-33.861	2.292.589	-26.139	2.246.797	-23.995
2.253.929	-33.862	2.292.803	-26.138	2.246.951	-23.992
2.254.066	-33.863	2.293.006	-26.137	2.247.083	-23.988

2.254.215	-33.864	2.293.091	-26.137	2.247.284	-23.984
2.254.408	-33.866	2.293.195	-26.136	2.247.457	-23.981
2.254.521	-33.867	2.293.506	-26.135	2.247.609	-23.977
2.254.606	-33.868	2.293.974	-26.134	2.247.836	-23.974
2.254.704	-33.870	2.294.330	-26.134	2.248.022	-23.970
2.254.930	-33.871	2.294.431	-26.133	2.248.122	-23.967
2.255.175	-33.872	2.294.476	-26.132	2.248.266	-23.963
2.255.345	-33.873	2.294.599	-26.131	2.248.452	-23.960
2.255.521	-33.875	2.294.633	-26.131	2.248.633	-23.956
2.255.779	-33.876	2.294.678	-26.130	2.248.783	-23.953
2.256.044	-33.877	2.294.799	-26.129	2.248.922	-23.949
2.256.201	-33.878	2.294.928	-26.129	2.249.129	-23.946
2.256.322	-33.880	2.295.067	-26.128	2.249.328	-23.942
2.256.445	-33.881	2.295.224	-26.127	2.249.489	-23.939
2.256.600	-33.882	2.295.353	-26.127	2.249.601	-23.935
2.256.854	-33.884	2.295.536	-26.126	2.249.734	-23.932
2.256.985	-33.885	2.295.716	-26.126	2.249.921	-23.928
2.257.043	-33.886	2.295.891	-26.125	2.250.132	-23.925
2.257.202	-33.887	2.296.089	-26.125	2.250.313	-23.921
2.257.419	-33.889	2.296.324	-26.124	2.250.464	-23.918
2.257.641	-33.890	2.296.523	-26.124	2.250.705	-23.914
2.257.778	-33.891	2.296.633	-26.123	2.250.849	-23.911
2.257.861	-33.893	2.296.772	-26.123	2.250.965	-23.907
2.258.024	-33.894	2.296.948	-26.122	2.251.140	-23.904
2.258.226	-33.895	2.297.172	-26.122	2.251.283	-23.900
2.258.360	-33.897	2.297.339	-26.122	2.251.393	-23.897
2.258.495	-33.898	2.297.379	-26.122	2.251.525	-23.893
2.258.660	-33.899	2.297.522	-26.121	2.251.720	-23.890
2.258.866	-33.901	2.297.750	-26.121	2.251.843	-23.886
2.259.059	-33.902	2.297.888	-26.121	2.251.927	-23.883

2.259.193	-33.904	2.298.042	-26.121	2.252.094	-23.879
2.259.402	-33.905	2.298.210	-26.121	2.252.278	-23.876
2.259.512	-33.907	2.298.378	-26.121	2.252.437	-23.872
2.259.633	-33.908	2.298.608	-26.121	2.252.612	-23.869
2.259.843	-33.909	2.298.801	-26.121	2.252.775	-23.865
2.259.987	-33.911	2.298.976	-26.121	2.252.984	-23.862
2.260.228	-33.912	2.299.124	-26.121	2.253.266	-23.858
2.260.427	-33.914	2.299.229	-26.122	2.253.449	-23.855
2.260.580	-33.915	2.299.348	-26.122	2.253.629	-23.851
2.260.728	-33.917	2.299.545	-26.122	2.253.864	-23.848
2.260.842	-33.918	2.299.809	-26.123	2.254.026	-23.844
2.260.984	-33.920	2.299.957	-26.123	2.254.135	-23.841
2.261.199	-33.921	2.300.067	-26.124	2.254.312	-23.837
2.261.376	-33.923	2.300.224	-26.124	2.254.523	-23.833
2.261.503	-33.924	2.300.365	-26.125	2.254.662	-23.830
2.261.700	-33.926	2.300.466	-26.126	2.254.747	-23.826
2.261.836	-33.927	2.300.600	-26.127	2.254.890	-23.823
2.261.875	-33.929	2.300.820	-26.127	2.255.049	-23.819
2.262.113	-33.931	2.301.030	-26.128	2.255.163	-23.815
2.262.446	-33.932	2.301.208	-26.129	2.255.331	-23.812
2.262.628	-33.934	2.301.378	-26.130	2.255.502	-23.808
2.262.732	-33.935	2.301.523	-26.131	2.255.609	-23.805
2.262.829	-33.937	2.301.613	-26.132	2.255.818	-23.801
2.262.977	-33.938	2.301.769	-26.134	2.256.084	-23.797
2.263.149	-33.940	2.301.884	-26.135	2.256.295	-23.794
2.263.317	-33.942	2.302.122	-26.136	2.256.449	-23.790
2.263.422	-33.943	2.302.219	-26.137	2.256.637	-23.786
2.263.532	-33.945	2.302.345	-26.139	2.256.853	-23.783
2.263.708	-33.946	2.302.552	-26.140	2.256.983	-23.779
2.263.875	-33.948	2.302.791	-26.142	2.257.097	-23.775

2.264.070	-33.950	2.302.950	-26.143	2.257.296	-23.771
2.264.303	-33.951	2.303.066	-26.145	2.257.461	-23.768
2.264.436	-33.953	2.303.284	-26.146	2.257.600	-23.764
2.264.565	-33.954	2.303.463	-26.148	2.257.804	-23.760
2.264.810	-33.956	2.303.622	-26.150	2.257.954	-23.756
2.264.946	-33.958	2.303.803	-26.152	2.258.091	-23.752
2.265.060	-33.959	2.303.949	-26.154	2.258.174	-23.749
2.265.276	-33.961	2.304.148	-26.155	2.258.347	-23.745
2.265.466	-33.963	2.304.343	-26.157	2.258.671	-23.741
2.265.670	-33.964	2.304.469	-26.159	2.258.902	-23.737
2.265.880	-33.966	2.304.608	-26.161	2.259.068	-23.733
2.266.084	-33.967	2.304.785	-26.163	2.259.229	-23.729
2.266.270	-33.969	2.304.997	-26.166	2.259.361	-23.725
2.266.382	-33.971	2.305.155	-26.168	2.259.480	-23.721
2.266.485	-33.972	2.305.278	-26.170	2.259.634	-23.718
2.266.709	-33.974	2.305.423	-26.172	2.259.807	-23.714
2.266.933	-33.975	2.305.629	-26.174	2.259.991	-23.710
2.266.969	-33.977	2.305.789	-26.177	2.260.172	-23.706
2.267.029	-33.978	2.305.912	-26.179	2.260.361	-23.702
2.267.215	-33.980	2.306.104	-26.181	2.260.574	-23.698
2.267.384	-33.981	2.306.273	-26.184	2.260.706	-23.693
2.267.520	-33.983	2.306.395	-26.186	2.260.860	-23.689
2.267.724	-33.984	2.306.497	-26.188	2.261.022	-23.685
2.267.907	-33.986	2.306.617	-26.191	2.261.169	-23.681
2.268.058	-33.987	2.306.770	-26.193	2.261.376	-23.677
2.268.266	-33.989	2.306.922	-26.196	2.261.582	-23.673
2.268.519	-33.990	2.307.096	-26.198	2.261.738	-23.669
2.268.743	-33.992	2.307.242	-26.201	2.261.835	-23.665
2.268.882	-33.993	2.307.358	-26.204	2.261.960	-23.660
2.269.090	-33.995	2.307.538	-26.206	2.262.157	-23.656

2.269.256	-33.996	2.307.748	-26.209	2.262.505	-23.652
2.269.350	-33.998	2.307.896	-26.211	2.262.959	-23.648
2.269.543	-33.999	2.308.038	-26.214	2.263.167	-23.643
2.269.749	-34.000	2.308.253	-26.217	2.263.221	-23.639
2.269.855	-34.002	2.308.409	-26.220	2.263.270	-23.635
2.269.980	-34.003	2.308.605	-26.222	2.263.280	-23.630
2.270.179	-34.004	2.308.846	-26.225	2.263.344	-23.626
2.270.331	-34.006	2.309.048	-26.228	2.263.438	-23.621
2.270.513	-34.007	2.309.265	-26.231	2.263.593	-23.617
2.270.763	-34.008	2.309.429	-26.233	2.263.799	-23.613
2.270.963	-34.010	2.309.541	-26.236	2.263.962	-23.608
2.271.131	-34.011	2.309.661	-26.239	2.264.075	-23.604
2.271.285	-34.012	2.309.829	-26.242	2.264.207	-23.599
2.271.434	-34.013	2.309.957	-26.245	2.264.386	-23.595
2.271.646	-34.015	2.310.042	-26.248	2.264.601	-23.590
2.271.825	-34.016	2.310.141	-26.251	2.264.807	-23.586
2.271.953	-34.017	2.310.320	-26.253	2.265.004	-23.581
2.272.081	-34.018	2.310.528	-26.256	2.265.179	-23.576
2.272.225	-34.019	2.310.712	-26.259	2.265.363	-23.572
2.272.339	-34.020	2.310.931	-26.262	2.265.470	-23.567
2.272.498	-34.021	2.311.096	-26.265	2.265.567	-23.563
2.272.730	-34.022	2.311.281	-26.268	2.265.725	-23.558
2.272.930	-34.024	2.311.479	-26.271	2.265.891	-23.553
2.273.029	-34.025	2.311.635	-26.273	2.266.070	-23.548
2.273.147	-34.026	2.311.783	-26.276	2.266.281	-23.544
2.273.400	-34.027	2.311.984	-26.279	2.266.476	-23.539
2.273.602	-34.028	2.312.180	-26.282	2.266.591	-23.534
2.273.756	-34.029	2.312.340	-26.285	2.266.729	-23.529
2.273.902	-34.030	2.312.430	-26.288	2.266.944	-23.525
2.274.008	-34.030	2.312.639	-26.290	2.267.133	-23.520

2.274.204	-34.031	2.312.865	-26.293	2.267.302	-23.515
2.274.422	-34.032	2.313.024	-26.296	2.267.468	-23.510
2.274.566	-34.033	2.313.154	-26.299	2.267.638	-23.505
2.274.684	-34.034	2.313.311	-26.301	2.267.802	-23.500
2.274.913	-34.035	2.313.510	-26.304	2.267.993	-23.496
2.275.304	-34.036	2.313.702	-26.307	2.268.228	-23.491
2.275.665	-34.037	2.313.839	-26.310	2.268.449	-23.486
2.275.800	-34.037	2.313.976	-26.312	2.268.589	-23.481
2.275.876	-34.038	2.314.115	-26.315	2.268.634	-23.476
2.276.019	-34.039	2.314.278	-26.318	2.268.739	-23.471
2.276.107	-34.040	2.314.442	-26.320	2.268.942	-23.466
2.276.189	-34.041	2.314.601	-26.323	2.269.167	-23.461
2.276.273	-34.041	2.314.805	-26.325	2.269.372	-23.456
2.276.396	-34.042	2.315.029	-26.328	2.269.568	-23.451
2.276.525	-34.043	2.315.182	-26.330	2.269.709	-23.446
2.276.633	-34.043	2.315.306	-26.333	2.269.819	-23.441
2.276.832	-34.044	2.315.459	-26.335	2.269.901	-23.436
2.276.995	-34.045	2.315.607	-26.338	2.269.980	-23.431
2.277.104	-34.045	2.315.739	-26.340	2.270.190	-23.426
2.277.321	-34.046	2.315.887	-26.343	2.270.423	-23.421
2.277.430	-34.047	2.316.059	-26.345	2.270.580	-23.416
2.277.603	-34.047	2.316.252	-26.347	2.270.730	-23.411
2.277.885	-34.048	2.316.391	-26.350	2.270.934	-23.406
2.278.058	-34.049	2.316.523	-26.352	2.271.087	-23.401
2.278.237	-34.049	2.316.776	-26.354	2.271.198	-23.396
2.278.392	-34.050	2.317.009	-26.356	2.271.400	-23.391
2.278.555	-34.050	2.317.087	-26.359	2.271.588	-23.385
2.278.775	-34.051	2.317.255	-26.361	2.271.814	-23.380
2.278.932	-34.052	2.317.432	-26.363	2.271.998	-23.375
2.278.972	-34.052	2.317.511	-26.365	2.272.103	-23.370

2.279.079	-34.053	2.317.800	-26.367	2.272.301	-23.365
2.279.324	-34.053	2.318.300	-26.369	2.272.467	-23.360
2.279.513	-34.054	2.318.611	-26.371	2.272.648	-23.355
2.279.590	-34.055	2.318.658	-26.373	2.272.795	-23.350
2.279.776	-34.055	2.318.721	-26.375	2.272.948	-23.344
2.279.937	-34.056	2.318.898	-26.377	2.273.158	-23.339
2.280.049	-34.056	2.318.979	-26.379	2.273.268	-23.334
2.280.271	-34.057	2.319.003	-26.381	2.273.392	-23.329
2.280.506	-34.058	2.319.034	-26.383	2.273.577	-23.324
2.280.717	-34.058	2.319.169	-26.384	2.273.743	-23.318
2.280.846	-34.059	2.319.339	-26.386	2.273.909	-23.313
2.280.994	-34.059	2.319.502	-26.388	2.274.151	-23.308
2.281.185	-34.060	2.319.702	-26.390	2.274.321	-23.303
2.281.393	-34.061	2.319.913	-26.391	2.274.489	-23.297
2.281.570	-34.061	2.320.109	-26.393	2.274.704	-23.292
2.281.682	-34.062	2.320.238	-26.395	2.274.861	-23.287
2.281.763	-34.062	2.320.388	-26.396	2.275.009	-23.282
2.281.888	-34.063	2.320.607	-26.398	2.275.141	-23.276
2.282.119	-34.064	2.320.779	-26.400	2.275.219	-23.271
2.282.328	-34.064	2.320.918	-26.401	2.275.282	-23.266
2.282.466	-34.065	2.321.084	-26.403	2.275.445	-23.260
2.282.638	-34.066	2.321.243	-26.404	2.275.650	-23.255
2.282.822	-34.066	2.321.376	-26.406	2.275.849	-23.250
2.282.950	-34.067	2.321.541	-26.407	2.276.012	-23.244
2.283.078	-34.068	2.321.693	-26.409	2.276.127	-23.239
2.283.194	-34.069	2.321.861	-26.410	2.276.274	-23.233
2.283.329	-34.069	2.322.038	-26.412	2.276.471	-23.228
2.283.533	-34.070	2.322.155	-26.413	2.276.758	-23.223
2.283.654	-34.071	2.322.240	-26.414	2.277.007	-23.217
2.283.802	-34.072	2.322.336	-26.416	2.277.137	-23.212

2.283.974	-34.072	2.322.525	-26.417	2.277.372	-23.206
2.284.090	-34.073	2.322.719	-26.419	2.277.623	-23.201
2.284.195	-34.074	2.322.870	-26.420	2.277.735	-23.195
2.284.406	-34.075	2.323.122	-26.421	2.277.842	-23.190
2.284.597	-34.076	2.323.354	-26.423	2.277.995	-23.184
2.284.825	-34.077	2.323.488	-26.424	2.278.156	-23.179
2.285.089	-34.078	2.323.674	-26.425	2.278.210	-23.173
2.285.190	-34.079	2.323.916	-26.427	2.278.270	-23.168
2.285.347	-34.080	2.324.032	-26.428	2.278.393	-23.162
2.285.551	-34.081	2.324.150	-26.429	2.278.548	-23.156
2.285.656	-34.082	2.324.361	-26.431	2.278.840	-23.151
2.285.819	-34.083	2.324.520	-26.432	2.279.099	-23.145
2.286.048	-34.084	2.324.650	-26.433	2.279.193	-23.140
2.286.265	-34.085	2.324.814	-26.435	2.279.348	-23.134
2.286.440	-34.086	2.324.950	-26.436	2.279.543	-23.128
2.286.600	-34.088	2.325.042	-26.437	2.279.713	-23.122
2.286.759	-34.089	2.325.235	-26.438	2.279.899	-23.117
2.286.911	-34.090	2.325.441	-26.440	2.280.123	-23.111
2.287.050	-34.091	2.325.586	-26.441	2.280.302	-23.105
2.287.180	-34.092	2.325.733	-26.442	2.280.415	-23.100
2.287.397	-34.094	2.325.934	-26.444	2.280.518	-23.094
2.287.646	-34.095	2.326.044	-26.445	2.280.668	-23.088
2.287.800	-34.096	2.326.190	-26.446	2.280.863	-23.082
2.287.878	-34.098	2.326.413	-26.447	2.281.032	-23.076
2.287.965	-34.099	2.326.523	-26.449	2.281.219	-23.071
2.288.085	-34.100	2.326.633	-26.450	2.281.414	-23.065
2.288.304	-34.102	2.326.863	-26.451	2.281.519	-23.059
2.288.539	-34.103	2.327.092	-26.452	2.281.675	-23.053
2.288.651	-34.105	2.327.275	-26.454	2.281.886	-23.047
2.288.783	-34.106	2.327.453	-26.455	2.282.081	-23.041

2.288.926	-34.108	2.327.630	-26.456	2.282.316	-23.035
2.288.994	-34.109	2.327.818	-26.457	2.282.500	-23.030
2.289.144	-34.111	2.328.008	-26.459	2.282.670	-23.024
2.289.335	-34.112	2.328.197	-26.460	2.282.840	-23.018
2.289.510	-34.114	2.328.385	-26.461	2.282.941	-23.012
2.289.671	-34.115	2.328.566	-26.463	2.283.015	-23.006
2.289.854	-34.117	2.328.665	-26.464	2.283.177	-23.000
2.290.069	-34.119	2.328.792	-26.465	2.283.380	-22.994
2.290.217	-34.120	2.328.929	-26.466	2.283.604	-22.988
2.290.430	-34.122	2.329.077	-26.468	2.283.822	-22.982
2.290.683	-34.124	2.329.265	-26.469	2.284.003	-22.976
2.290.872	-34.126	2.329.458	-26.470	2.284.200	-22.970
2.291.028	-34.127	2.329.651	-26.471	2.284.418	-22.964
2.291.208	-34.129	2.329.778	-26.473	2.284.530	-22.958
2.291.414	-34.131	2.329.890	-26.474	2.284.624	-22.952
2.291.575	-34.133	2.330.070	-26.475	2.284.798	-22.946
2.291.720	-34.134	2.330.284	-26.476	2.284.935	-22.939
2.291.868	-34.136	2.330.423	-26.478	2.285.082	-22.933
2.291.980	-34.138	2.330.555	-26.479	2.285.228	-22.927
2.292.099	-34.140	2.330.703	-26.480	2.285.405	-22.921
2.292.262	-34.142	2.330.905	-26.481	2.285.730	-22.915
2.292.444	-34.144	2.331.075	-26.482	2.286.111	-22.909
2.292.587	-34.146	2.331.173	-26.484	2.286.350	-22.903
2.292.665	-34.148	2.331.355	-26.485	2.286.447	-22.897
2.292.753	-34.149	2.331.496	-26.486	2.286.508	-22.890
2.292.971	-34.151	2.331.601	-26.487	2.286.577	-22.884
2.293.203	-34.153	2.331.796	-26.488	2.286.703	-22.878
2.293.400	-34.155	2.331.922	-26.490	2.286.785	-22.872
2.293.669	-34.157	2.332.043	-26.491	2.286.861	-22.866
2.293.893	-34.159	2.332.258	-26.492	2.286.944	-22.859

2.294.009	-34.161	2.332.493	-26.493	2.287.072	-22.853
2.294.130	-34.163	2.332.708	-26.494	2.287.251	-22.847
2.294.334	-34.165	2.332.910	-26.495	2.287.421	-22.840
2.294.567	-34.168	2.333.113	-26.496	2.287.679	-22.834
2.294.771	-34.170	2.333.259	-26.498	2.287.943	-22.828
2.294.899	-34.172	2.333.432	-26.499	2.288.111	-22.822
2.295.035	-34.174	2.333.667	-26.500	2.288.196	-22.815
2.295.215	-34.176	2.333.885	-26.501	2.288.328	-22.809
2.295.367	-34.178	2.333.983	-26.502	2.288.568	-22.803
2.295.468	-34.180	2.334.059	-26.503	2.288.765	-22.796
2.295.604	-34.182	2.334.229	-26.504	2.288.873	-22.790
2.295.835	-34.185	2.334.401	-26.505	2.288.987	-22.784
2.296.090	-34.187	2.334.559	-26.506	2.289.142	-22.777
2.296.272	-34.189	2.334.743	-26.507	2.289.209	-22.771
2.296.384	-34.191	2.334.875	-26.508	2.289.437	-22.764
2.296.527	-34.193	2.335.017	-26.509	2.289.720	-22.758
2.296.693	-34.196	2.335.253	-26.510	2.289.892	-22.752
2.296.879	-34.198	2.335.353	-26.511	2.290.074	-22.745
2.297.001	-34.200	2.335.428	-26.512	2.290.206	-22.739
2.297.090	-34.202	2.335.591	-26.513	2.290.392	-22.733
2.297.291	-34.204	2.335.900	-26.514	2.290.592	-22.726
2.297.511	-34.207	2.336.120	-26.515	2.290.719	-22.720
2.297.646	-34.209	2.336.279	-26.516	2.290.849	-22.713
2.297.773	-34.211	2.336.505	-26.517	2.291.102	-22.707
2.297.914	-34.214	2.336.714	-26.518	2.291.310	-22.700
2.298.089	-34.216	2.336.827	-26.519	2.291.465	-22.694
2.298.318	-34.218	2.336.973	-26.519	2.291.649	-22.688
2.298.514	-34.221	2.337.146	-26.520	2.291.765	-22.681
2.298.716	-34.223	2.337.348	-26.521	2.291.859	-22.675
2.298.929	-34.225	2.337.468	-26.522	2.291.998	-22.668

2.299.075	-34.228	2.337.594	-26.523	2.292.166	-22.662
2.299.236	-34.230	2.337.793	-26.524	2.292.388	-22.655
2.299.397	-34.233	2.337.964	-26.524	2.292.603	-22.649
2.299.523	-34.235	2.338.107	-26.525	2.292.755	-22.643
2.299.595	-34.237	2.338.219	-26.526	2.292.906	-22.636
2.299.706	-34.240	2.338.389	-26.527	2.293.013	-22.630
2.299.886	-34.242	2.338.566	-26.527	2.293.118	-22.623
2.300.215	-34.245	2.338.671	-26.528	2.293.290	-22.617
2.300.676	-34.247	2.338.922	-26.529	2.293.484	-22.610
2.300.936	-34.250	2.339.178	-26.530	2.293.654	-22.604
2.301.015	-34.252	2.339.330	-26.530	2.293.821	-22.597
2.301.105	-34.255	2.339.453	-26.531	2.293.994	-22.591
2.301.273	-34.257	2.339.581	-26.532	2.294.156	-22.584
2.301.294	-34.260	2.339.782	-26.532	2.294.285	-22.578
2.301.266	-34.262	2.339.975	-26.533	2.294.413	-22.571
2.301.319	-34.265	2.340.121	-26.533	2.294.577	-22.565
2.301.449	-34.267	2.340.284	-26.534	2.294.814	-22.559
2.301.691	-34.270	2.340.405	-26.535	2.295.029	-22.552
2.301.906	-34.273	2.340.548	-26.535	2.295.241	-22.546
2.302.076	-34.275	2.340.734	-26.536	2.295.430	-22.539
2.302.305	-34.278	2.340.894	-26.536	2.295.556	-22.533
2.302.460	-34.280	2.341.077	-26.537	2.295.694	-22.526
2.302.625	-34.283	2.341.299	-26.537	2.295.820	-22.520
2.302.766	-34.286	2.341.472	-26.538	2.295.954	-22.513
2.302.928	-34.288	2.341.650	-26.538	2.296.169	-22.507
2.303.080	-34.291	2.341.837	-26.539	2.296.349	-22.500
2.303.232	-34.294	2.341.879	-26.539	2.296.485	-22.494
2.303.503	-34.297	2.341.947	-26.540	2.296.622	-22.487
2.303.658	-34.299	2.342.177	-26.540	2.296.771	-22.481
2.303.716	-34.302	2.342.643	-26.541	2.296.964	-22.474

2.303.826	-34.305	2.342.970	-26.541	2.297.128	-22.468
2.304.012	-34.308	2.343.167	-26.541	2.297.301	-22.461
2.304.225	-34.311	2.343.347	-26.542	2.297.453	-22.455
2.304.382	-34.314	2.343.465	-26.542	2.297.583	-22.448
2.304.503	-34.316	2.343.531	-26.542	2.297.822	-22.442
2.304.643	-34.319	2.343.600	-26.543	2.298.048	-22.435
2.304.782	-34.322	2.343.689	-26.543	2.298.163	-22.429
2.304.899	-34.325	2.343.756	-26.544	2.298.291	-22.422
2.305.052	-34.328	2.343.855	-26.544	2.298.487	-22.416
2.305.259	-34.331	2.344.050	-26.544	2.298.584	-22.409
2.305.499	-34.334	2.344.241	-26.544	2.298.644	-22.402
2.305.750	-34.338	2.344.310	-26.545	2.298.817	-22.396
2.305.916	-34.341	2.344.445	-26.545	2.299.023	-22.389
2.306.068	-34.344	2.344.711	-26.545	2.299.220	-22.383
2.306.248	-34.347	2.344.930	-26.546	2.299.406	-22.376
2.306.324	-34.350	2.345.143	-26.546	2.299.558	-22.369
2.306.434	-34.353	2.345.302	-26.546	2.299.740	-22.363
2.306.620	-34.357	2.345.421	-26.546	2.299.977	-22.356
2.306.816	-34.360	2.345.600	-26.546	2.300.190	-22.349
2.306.994	-34.363	2.345.771	-26.547	2.300.392	-22.343
2.307.108	-34.367	2.345.907	-26.547	2.300.600	-22.336
2.307.274	-34.370	2.346.053	-26.547	2.300.757	-22.329
2.307.417	-34.374	2.346.210	-26.547	2.300.902	-22.322
2.307.529	-34.377	2.346.329	-26.548	2.301.082	-22.316
2.307.764	-34.381	2.346.507	-26.548	2.301.234	-22.309
2.307.977	-34.384	2.346.702	-26.548	2.301.272	-22.302
2.308.111	-34.388	2.346.857	-26.548	2.301.328	-22.295
2.308.289	-34.391	2.346.978	-26.548	2.301.503	-22.289
2.308.439	-34.395	2.347.107	-26.549	2.301.702	-22.282
2.308.534	-34.399	2.347.294	-26.549	2.301.886	-22.275

2.308.620	-34.402	2.347.493	-26.549	2.302.049	-22.268
2.308.889	-34.406	2.347.659	-26.549	2.302.159	-22.261
2.309.088	-34.410	2.347.853	-26.550	2.302.325	-22.254
2.309.241	-34.414	2.348.062	-26.550	2.302.484	-22.247
2.309.411	-34.418	2.348.257	-26.550	2.302.600	-22.240
2.309.585	-34.421	2.348.468	-26.550	2.302.785	-22.233
2.309.762	-34.425	2.348.640	-26.550	2.303.000	-22.226
2.309.892	-34.429	2.348.741	-26.551	2.303.271	-22.219
2.310.058	-34.433	2.348.909	-26.551	2.303.470	-22.212
2.310.267	-34.438	2.349.097	-26.551	2.303.561	-22.205
2.310.446	-34.442	2.349.218	-26.551	2.303.748	-22.198
2.310.584	-34.446	2.349.375	-26.552	2.303.907	-22.191
2.310.746	-34.450	2.349.471	-26.552	2.304.104	-22.184
2.310.925	-34.454	2.349.585	-26.552	2.304.354	-22.177
2.311.084	-34.458	2.349.726	-26.552	2.304.464	-22.170
2.311.265	-34.463	2.349.958	-26.553	2.304.592	-22.163
2.311.426	-34.467	2.350.191	-26.553	2.304.785	-22.156
2.311.553	-34.471	2.350.325	-26.553	2.304.840	-22.149
2.311.674	-34.476	2.350.535	-26.554	2.305.013	-22.141
2.311.812	-34.480	2.350.739	-26.554	2.305.336	-22.134
2.312.034	-34.485	2.350.920	-26.554	2.305.502	-22.127
2.312.206	-34.489	2.351.046	-26.554	2.305.630	-22.120
2.312.361	-34.494	2.351.196	-26.555	2.305.732	-22.113
2.312.509	-34.498	2.351.362	-26.555	2.305.909	-22.105
2.312.657	-34.503	2.351.532	-26.555	2.306.187	-22.098
2.312.844	-34.507	2.351.687	-26.556	2.306.384	-22.091
2.312.971	-34.512	2.351.808	-26.556	2.306.505	-22.083
2.313.169	-34.517	2.351.989	-26.557	2.306.665	-22.076
2.313.382	-34.521	2.352.215	-26.557	2.306.806	-22.069
2.313.447	-34.526	2.352.381	-26.557	2.306.958	-22.061

2.313.543	-34.531	2.352.560	-26.558	2.307.103	-22.054
2.313.763	-34.536	2.352.711	-26.558	2.307.289	-22.047
2.313.909	-34.540	2.352.856	-26.558	2.307.467	-22.039
2.314.065	-34.545	2.353.019	-26.559	2.307.605	-22.032
2.314.209	-34.550	2.353.212	-26.559	2.307.775	-22.024
2.314.344	-34.555	2.353.434	-26.560	2.307.970	-22.017
2.314.521	-34.560	2.353.526	-26.560	2.308.145	-22.009
2.314.704	-34.565	2.353.631	-26.561	2.308.333	-22.002
2.314.865	-34.570	2.353.831	-26.561	2.308.517	-21.994
2.315.017	-34.575	2.354.005	-26.562	2.308.588	-21.987
2.315.215	-34.580	2.354.169	-26.562	2.308.662	-21.979
2.315.426	-34.585	2.354.379	-26.563	2.308.835	-21.972
2.315.571	-34.590	2.354.572	-26.563	2.309.202	-21.964
2.315.775	-34.595	2.354.729	-26.563	2.309.673	-21.957
2.315.983	-34.600	2.354.881	-26.564	2.309.948	-21.949
2.316.160	-34.605	2.355.022	-26.564	2.310.058	-21.941
2.316.404	-34.610	2.355.145	-26.565	2.310.134	-21.934
2.316.603	-34.615	2.355.278	-26.565	2.310.156	-21.926
2.316.751	-34.620	2.355.465	-26.566	2.310.172	-21.918
2.316.898	-34.625	2.355.672	-26.566	2.310.246	-21.911
2.317.034	-34.630	2.355.788	-26.567	2.310.385	-21.903
2.317.184	-34.635	2.355.860	-26.567	2.310.480	-21.895
2.317.323	-34.641	2.356.014	-26.568	2.310.629	-21.887
2.317.446	-34.646	2.356.171	-26.568	2.310.779	-21.880
2.317.594	-34.651	2.356.312	-26.569	2.310.885	-21.872
2.317.728	-34.656	2.356.503	-26.569	2.311.135	-21.864
2.317.910	-34.661	2.356.624	-26.570	2.311.384	-21.856
2.318.067	-34.666	2.356.765	-26.570	2.311.552	-21.848
2.318.185	-34.672	2.357.038	-26.570	2.311.702	-21.841
2.318.333	-34.677	2.357.312	-26.571	2.311.870	-21.833

2.318.499	-34.682	2.357.487	-26.571	2.312.052	-21.825
2.318.745	-34.687	2.357.623	-26.572	2.312.217	-21.817
2.318.895	-34.692	2.357.779	-26.572	2.312.358	-21.809
2.319.052	-34.698	2.358.031	-26.573	2.312.495	-21.801
2.319.289	-34.703	2.358.248	-26.573	2.312.650	-21.793
2.319.482	-34.708	2.358.360	-26.574	2.312.809	-21.785
2.319.641	-34.713	2.358.466	-26.574	2.312.977	-21.777
2.319.849	-34.719	2.358.702	-26.574	2.313.167	-21.769
2.320.020	-34.724	2.358.913	-26.575	2.313.354	-21.761
2.320.154	-34.729	2.359.027	-26.575	2.313.522	-21.753
2.320.314	-34.734	2.359.173	-26.576	2.313.665	-21.745
2.320.484	-34.739	2.359.357	-26.576	2.313.820	-21.737
2.320.605	-34.745	2.359.529	-26.576	2.313.998	-21.729
2.320.723	-34.750	2.359.659	-26.577	2.314.142	-21.721
2.320.863	-34.755	2.359.818	-26.577	2.314.314	-21.713
2.321.043	-34.760	2.359.950	-26.578	2.314.522	-21.705
2.321.335	-34.765	2.360.033	-26.578	2.314.684	-21.697
2.321.552	-34.770	2.360.255	-26.578	2.314.843	-21.688
2.321.729	-34.776	2.360.518	-26.579	2.315.031	-21.680
2.321.850	-34.781	2.360.665	-26.579	2.315.202	-21.672
2.321.974	-34.786	2.360.875	-26.579	2.315.369	-21.664
2.322.092	-34.791	2.361.104	-26.579	2.315.513	-21.656
2.322.249	-34.796	2.361.257	-26.580	2.315.656	-21.648
2.322.459	-34.801	2.361.393	-26.580	2.315.849	-21.639
2.322.675	-34.806	2.361.500	-26.580	2.316.014	-21.631
2.322.844	-34.811	2.361.680	-26.580	2.316.174	-21.623
2.323.028	-34.816	2.361.908	-26.581	2.316.370	-21.615
2.323.212	-34.821	2.362.148	-26.581	2.316.467	-21.606
2.323.322	-34.826	2.362.347	-26.581	2.316.570	-21.598
2.323.391	-34.831	2.362.469	-26.581	2.316.780	-21.590

2.323.552	-34.836	2.362.617	-26.581	2.317.004	-21.581
2.323.767	-34.841	2.362.818	-26.581	2.317.146	-21.573
2.323.947	-34.846	2.362.935	-26.581	2.317.263	-21.565
2.324.158	-34.851	2.363.060	-26.582	2.317.412	-21.556
2.324.236	-34.856	2.363.257	-26.582	2.317.612	-21.548
2.324.413	-34.861	2.363.405	-26.582	2.317.769	-21.539
2.324.718	-34.866	2.363.557	-26.582	2.317.896	-21.531
2.324.926	-34.871	2.363.725	-26.582	2.318.082	-21.523
2.325.014	-34.875	2.363.849	-26.582	2.318.252	-21.514
2.325.096	-34.880	2.364.014	-26.582	2.318.461	-21.506
2.325.257	-34.885	2.364.223	-26.582	2.318.642	-21.497
2.325.477	-34.890	2.364.372	-26.582	2.318.795	-21.489
2.325.948	-34.895	2.364.516	-26.582	2.318.969	-21.480
2.326.369	-34.899	2.364.686	-26.582	2.319.124	-21.472
2.326.563	-34.904	2.364.867	-26.582	2.319.316	-21.463
2.326.622	-34.909	2.365.045	-26.581	2.319.545	-21.454
2.326.671	-34.913	2.365.179	-26.581	2.319.725	-21.446
2.326.769	-34.918	2.365.353	-26.581	2.319.823	-21.437
2.326.845	-34.923	2.365.573	-26.581	2.319.966	-21.428
2.326.902	-34.927	2.365.683	-26.581	2.320.063	-21.420
2.326.974	-34.932	2.365.768	-26.581	2.320.197	-21.411
2.327.094	-34.936	2.366.008	-26.580	2.320.459	-21.402
2.327.301	-34.941	2.366.283	-26.580	2.320.627	-21.394
2.327.431	-34.945	2.366.491	-26.580	2.320.810	-21.385
2.327.573	-34.950	2.366.629	-26.580	2.320.999	-21.376
2.327.757	-34.954	2.366.716	-26.579	2.321.196	-21.367
2.327.924	-34.959	2.366.841	-26.579	2.321.422	-21.358
2.328.089	-34.963	2.367.179	-26.579	2.321.530	-21.349
2.328.246	-34.967	2.367.598	-26.579	2.321.637	-21.340
2.328.438	-34.972	2.367.880	-26.578	2.321.827	-21.331

2.328.615	-34.976	2.368.074	-26.578	2.322.009	-21.322
2.328.752	-34.980	2.368.214	-26.577	2.322.116	-21.313
2.328.915	-34.985	2.368.286	-26.577	2.322.217	-21.304
2.329.118	-34.989	2.368.280	-26.577	2.322.357	-21.295
2.329.274	-34.993	2.368.324	-26.576	2.322.535	-21.286
2.329.417	-34.997	2.368.362	-26.576	2.322.695	-21.276
2.329.596	-35.002	2.368.490	-26.575	2.322.836	-21.267
2.329.760	-35.006	2.368.642	-26.575	2.323.033	-21.258
2.329.902	-35.010	2.368.811	-26.574	2.323.242	-21.248
2.329.966	-35.014	2.368.893	-26.574	2.323.363	-21.239
2.330.163	-35.018	2.369.036	-26.573	2.323.504	-21.230
2.330.392	-35.022	2.369.267	-26.573	2.323.781	-21.220
2.330.600	-35.027	2.369.469	-26.572	2.324.025	-21.211
2.330.775	-35.031	2.369.704	-26.571	2.324.250	-21.201
2.330.835	-35.035	2.369.910	-26.571	2.324.389	-21.191
2.330.983	-35.039	2.370.020	-26.570	2.324.547	-21.182
2.331.194	-35.043	2.370.163	-26.570	2.324.801	-21.172
2.331.405	-35.047	2.370.358	-26.569	2.324.948	-21.162
2.331.638	-35.051	2.370.504	-26.568	2.325.033	-21.153
2.331.808	-35.055	2.370.656	-26.568	2.325.170	-21.143
2.331.967	-35.059	2.370.833	-26.567	2.325.300	-21.133
2.332.148	-35.063	2.370.995	-26.566	2.325.370	-21.123
2.332.204	-35.067	2.371.149	-26.565	2.325.491	-21.113
2.332.352	-35.071	2.371.359	-26.565	2.325.719	-21.103
2.332.620	-35.075	2.371.541	-26.564	2.325.866	-21.093
2.332.747	-35.079	2.371.630	-26.563	2.326.080	-21.083
2.332.861	-35.082	2.371.751	-26.563	2.326.279	-21.072
2.333.026	-35.086	2.371.956	-26.562	2.326.375	-21.062
2.333.207	-35.090	2.372.188	-26.561	2.326.516	-21.052
2.333.398	-35.094	2.372.376	-26.560	2.326.777	-21.041

2.333.595	-35.098	2.372.531	-26.559	2.327.058	-21.031
2.333.790	-35.102	2.372.648	-26.559	2.327.243	-21.020
2.333.904	-35.106	2.372.800	-26.558	2.327.352	-21.010
2.334.003	-35.109	2.373.042	-26.557	2.327.505	-20.999
2.334.121	-35.113	2.373.270	-26.556	2.327.641	-20.989
2.334.321	-35.117	2.373.445	-26.555	2.327.791	-20.978
2.334.606	-35.121	2.373.622	-26.555	2.328.006	-20.967
2.334.789	-35.124	2.373.716	-26.554	2.328.196	-20.957
2.334.926	-35.128	2.373.738	-26.553	2.328.340	-20.946
2.335.067	-35.132	2.373.820	-26.552	2.328.490	-20.935
2.335.127	-35.136	2.374.084	-26.551	2.328.635	-20.924
2.335.266	-35.139	2.374.256	-26.550	2.328.794	-20.913
2.335.501	-35.143	2.374.449	-26.550	2.329.007	-20.902
2.335.719	-35.147	2.374.622	-26.549	2.329.202	-20.891
2.335.864	-35.150	2.374.772	-26.548	2.329.413	-20.879
2.336.003	-35.154	2.374.973	-26.547	2.329.560	-20.868
2.336.167	-35.158	2.375.123	-26.546	2.329.651	-20.857
2.336.393	-35.161	2.375.304	-26.545	2.329.872	-20.845
2.336.586	-35.165	2.375.495	-26.544	2.330.063	-20.834
2.336.707	-35.168	2.375.652	-26.543	2.330.217	-20.823
2.336.877	-35.172	2.375.835	-26.542	2.330.341	-20.811
2.337.027	-35.175	2.376.008	-26.542	2.330.520	-20.799
2.337.164	-35.179	2.376.140	-26.541	2.330.735	-20.788
2.337.347	-35.182	2.376.243	-26.540	2.330.909	-20.776
2.337.491	-35.186	2.376.391	-26.539	2.331.082	-20.764
2.337.616	-35.189	2.376.573	-26.538	2.331.216	-20.753
2.337.833	-35.193	2.376.723	-26.537	2.331.393	-20.741
2.338.015	-35.196	2.376.915	-26.536	2.331.597	-20.729
2.338.174	-35.200	2.377.130	-26.535	2.331.778	-20.717
2.338.342	-35.203	2.377.338	-26.534	2.331.965	-20.705

2.338.517	-35.206	2.377.549	-26.533	2.332.141	-20.693
2.338.674	-35.210	2.377.704	-26.532	2.332.253	-20.680
2.338.808	-35.213	2.377.853	-26.532	2.332.381	-20.668
2.338.914	-35.216	2.377.979	-26.531	2.332.574	-20.656
2.339.128	-35.219	2.378.112	-26.530	2.332.693	-20.644
2.339.390	-35.223	2.378.300	-26.529	2.332.889	-20.631
2.339.560	-35.226	2.378.468	-26.528	2.333.248	-20.619
2.339.682	-35.229	2.378.611	-26.527	2.333.656	-20.606
2.339.796	-35.232	2.378.749	-26.526	2.333.944	-20.594
2.339.843	-35.235	2.378.917	-26.525	2.334.001	-20.581
2.339.978	-35.239	2.379.072	-26.524	2.334.017	-20.569
2.340.224	-35.242	2.379.281	-26.523	2.334.099	-20.556
2.340.455	-35.245	2.379.474	-26.522	2.334.171	-20.543
2.340.618	-35.248	2.379.612	-26.521	2.334.207	-20.530
2.340.800	-35.251	2.379.778	-26.520	2.334.306	-20.517
2.340.970	-35.254	2.379.948	-26.519	2.334.491	-20.505
2.341.075	-35.257	2.380.186	-26.518	2.334.630	-20.492
2.341.236	-35.260	2.380.358	-26.517	2.334.810	-20.479
2.341.456	-35.263	2.380.439	-26.516	2.335.014	-20.465
2.341.684	-35.265	2.380.551	-26.515	2.335.165	-20.452
2.341.969	-35.268	2.380.699	-26.514	2.335.276	-20.439
2.342.143	-35.271	2.380.876	-26.513	2.335.461	-20.426
2.342.246	-35.274	2.381.049	-26.512	2.335.712	-20.413
2.342.435	-35.277	2.381.156	-26.511	2.335.894	-20.399
2.342.579	-35.279	2.381.326	-26.510	2.336.064	-20.386
2.342.663	-35.282	2.381.447	-26.509	2.336.234	-20.372
2.342.795	-35.285	2.381.602	-26.508	2.336.413	-20.359
2.342.991	-35.287	2.381.842	-26.507	2.336.586	-20.345
2.343.177	-35.290	2.382.010	-26.506	2.336.776	-20.332
2.343.376	-35.293	2.382.226	-26.505	2.336.964	-20.318

2.343.541	-35.295	2.382.419	-26.504	2.337.068	-20.305
2.343.671	-35.298	2.382.578	-26.503	2.337.162	-20.291
2.343.819	-35.300	2.382.693	-26.502	2.337.334	-20.277
2.343.902	-35.303	2.382.956	-26.501	2.337.442	-20.263
2.344.016	-35.305	2.383.192	-26.499	2.337.596	-20.250
2.344.252	-35.307	2.383.407	-26.498	2.337.791	-20.236
2.344.542	-35.310	2.383.533	-26.497	2.337.943	-20.222
2.344.758	-35.312	2.383.618	-26.496	2.338.129	-20.208
2.344.946	-35.314	2.383.781	-26.495	2.338.362	-20.194
2.345.098	-35.316	2.383.965	-26.494	2.338.571	-20.180
2.345.284	-35.319	2.384.126	-26.492	2.338.746	-20.166
2.345.461	-35.321	2.384.274	-26.491	2.338.902	-20.152
2.345.592	-35.323	2.384.374	-26.490	2.339.070	-20.137
2.345.732	-35.325	2.384.512	-26.489	2.339.272	-20.123
2.345.875	-35.327	2.384.735	-26.488	2.339.428	-20.109
2.345.983	-35.329	2.384.881	-26.486	2.339.579	-20.095
2.346.149	-35.331	2.384.982	-26.485	2.339.722	-20.081
2.346.362	-35.333	2.385.150	-26.484	2.339.870	-20.066
2.346.518	-35.335	2.385.331	-26.482	2.340.056	-20.052
2.346.740	-35.337	2.385.573	-26.481	2.340.224	-20.037
2.346.955	-35.339	2.385.792	-26.480	2.340.409	-20.023
2.347.072	-35.341	2.385.927	-26.479	2.340.638	-20.009
2.347.235	-35.342	2.386.120	-26.477	2.340.795	-19.994
2.347.379	-35.344	2.386.355	-26.476	2.340.871	-19.980
2.347.496	-35.346	2.386.507	-26.474	2.340.999	-19.965
2.347.704	-35.348	2.386.671	-26.473	2.341.187	-19.950
2.347.950	-35.349	2.386.827	-26.472	2.341.373	-19.936
2.348.098	-35.351	2.386.945	-26.470	2.341.557	-19.921
2.348.203	-35.352	2.387.077	-26.469	2.341.734	-19.906
2.348.307	-35.354	2.387.284	-26.468	2.341.819	-19.892

2.348.579	-35.355	2.387.509	-26.466	2.341.897	-19.877
2.348.741	-35.357	2.387.652	-26.465	2.342.097	-19.862
2.348.873	-35.358	2.387.746	-26.463	2.342.345	-19.847
2.349.072	-35.360	2.387.936	-26.462	2.342.555	-19.833
2.349.294	-35.361	2.388.174	-26.460	2.342.712	-19.818
2.349.422	-35.362	2.388.326	-26.459	2.342.813	-19.803
2.349.534	-35.364	2.388.463	-26.457	2.342.982	-19.788
2.349.713	-35.365	2.388.631	-26.456	2.343.181	-19.773
2.349.987	-35.366	2.388.799	-26.454	2.343.356	-19.758
2.350.264	-35.368	2.389.029	-26.453	2.343.543	-19.743
2.350.400	-35.369	2.389.220	-26.451	2.343.709	-19.728
2.350.472	-35.370	2.389.372	-26.450	2.343.840	-19.713
2.350.594	-35.371	2.389.520	-26.448	2.343.978	-19.698
2.350.768	-35.372	2.389.550	-26.447	2.344.121	-19.683
2.350.884	-35.373	2.389.668	-26.445	2.344.361	-19.668
2.351.001	-35.374	2.389.957	-26.444	2.344.540	-19.653
2.351.362	-35.375	2.390.176	-26.442	2.344.651	-19.637
2.351.886	-35.376	2.390.356	-26.440	2.344.794	-19.622
2.352.186	-35.377	2.390.576	-26.439	2.345.016	-19.607
2.352.302	-35.378	2.390.701	-26.437	2.345.246	-19.592
2.352.346	-35.379	2.390.799	-26.436	2.345.450	-19.576
2.352.408	-35.380	2.391.028	-26.434	2.345.592	-19.561
2.352.455	-35.381	2.391.218	-26.432	2.345.737	-19.546
2.352.549	-35.382	2.391.283	-26.431	2.345.947	-19.530
2.352.617	-35.382	2.391.369	-26.429	2.346.091	-19.515
2.352.690	-35.383	2.391.534	-26.428	2.346.187	-19.499
2.352.912	-35.384	2.391.933	-26.426	2.346.335	-19.484
2.353.120	-35.385	2.392.415	-26.424	2.346.474	-19.468
2.353.235	-35.385	2.392.710	-26.423	2.346.613	-19.453
2.353.313	-35.386	2.392.868	-26.421	2.346.703	-19.437

2.353.454	-35.387	2.392.903	-26.419	2.346.868	-19.421
2.353.638	-35.387	2.392.943	-26.418	2.347.115	-19.406
2.353.826	-35.388	2.393.033	-26.416	2.347.354	-19.390
2.353.996	-35.389	2.393.111	-26.414	2.347.504	-19.374
2.354.147	-35.389	2.393.216	-26.413	2.347.703	-19.359
2.354.305	-35.390	2.393.295	-26.411	2.347.923	-19.343
2.354.525	-35.390	2.393.382	-26.409	2.348.065	-19.327
2.354.726	-35.391	2.393.526	-26.408	2.348.232	-19.311
2.354.852	-35.392	2.393.705	-26.406	2.348.436	-19.295
2.355.014	-35.392	2.393.866	-26.404	2.348.602	-19.279
2.355.255	-35.393	2.394.061	-26.403	2.348.768	-19.263
2.355.417	-35.393	2.394.256	-26.401	2.348.907	-19.247
2.355.464	-35.394	2.394.384	-26.399	2.349.117	-19.231
2.355.580	-35.394	2.394.588	-26.398	2.349.306	-19.215
2.355.737	-35.395	2.394.771	-26.396	2.349.406	-19.199
2.355.905	-35.395	2.394.905	-26.395	2.349.547	-19.183
2.356.060	-35.396	2.395.103	-26.393	2.349.724	-19.166
2.356.270	-35.396	2.395.325	-26.391	2.349.863	-19.150
2.356.462	-35.397	2.395.436	-26.390	2.350.026	-19.134
2.356.588	-35.397	2.395.560	-26.388	2.350.228	-19.118
2.356.803	-35.398	2.395.788	-26.386	2.350.347	-19.101
2.357.054	-35.398	2.395.956	-26.385	2.350.405	-19.085
2.357.290	-35.399	2.396.084	-26.383	2.350.591	-19.068
2.357.478	-35.399	2.396.210	-26.382	2.350.847	-19.052
2.357.639	-35.400	2.396.315	-26.380	2.351.099	-19.036
2.357.759	-35.400	2.396.433	-26.379	2.351.305	-19.019
2.357.907	-35.401	2.396.680	-26.377	2.351.465	-19.002
2.358.129	-35.402	2.396.888	-26.375	2.351.644	-18.986
2.358.261	-35.402	2.397.034	-26.374	2.351.794	-18.969
2.358.405	-35.403	2.397.307	-26.372	2.351.954	-18.952

2.358.573	-35.403	2.397.500	-26.371	2.352.078	-18.936
2.358.685	-35.404	2.397.592	-26.369	2.352.195	-18.919
2.358.805	-35.404	2.397.776	-26.368	2.352.402	-18.902
2.359.030	-35.405	2.397.970	-26.367	2.352.585	-18.885
2.359.259	-35.405	2.398.129	-26.365	2.352.679	-18.869
2.359.359	-35.406	2.398.295	-26.364	2.352.883	-18.852
2.359.489	-35.406	2.398.452	-26.362	2.353.167	-18.835
2.359.698	-35.407	2.398.602	-26.361	2.353.371	-18.818
2.359.861	-35.408	2.398.764	-26.360	2.353.496	-18.801
2.359.989	-35.408	2.398.926	-26.358	2.353.581	-18.784
2.360.157	-35.409	2.399.070	-26.357	2.353.788	-18.767
2.360.343	-35.409	2.399.180	-26.356	2.354.023	-18.750
2.360.510	-35.410	2.399.297	-26.354	2.354.274	-18.733
2.360.634	-35.411	2.399.503	-26.353	2.354.442	-18.715
2.360.754	-35.412	2.399.722	-26.352	2.354.585	-18.698
2.360.959	-35.412	2.399.870	-26.350	2.354.733	-18.681
2.361.144	-35.413	2.400.031	-26.349	2.354.810	-18.664
2.361.272	-35.414	2.400.199	-26.348	2.355.016	-18.647
2.361.456	-35.414	2.400.349	-26.347	2.355.275	-18.629
2.361.624	-35.415	2.400.527	-26.346	2.355.448	-18.612
2.361.790	-35.416	2.400.707	-26.345	2.355.620	-18.595
2.362.011	-35.417	2.400.838	-26.343	2.355.755	-18.577
2.362.197	-35.418	2.400.981	-26.342	2.355.925	-18.560
2.362.309	-35.418	2.401.169	-26.341	2.356.109	-18.542
2.362.446	-35.419	2.401.340	-26.340	2.356.270	-18.525
2.362.650	-35.420	2.401.514	-26.339	2.356.389	-18.507
2.362.827	-35.421	2.401.693	-26.338	2.356.536	-18.490
2.362.999	-35.422	2.401.901	-26.337	2.356.745	-18.472
2.363.206	-35.423	2.402.040	-26.336	2.356.805	-18.455
2.363.425	-35.424	2.402.168	-26.335	2.356.879	-18.437

2.363.544	-35.424	2.402.428	-26.334	2.357.289	-18.420
2.363.643	-35.425	2.402.605	-26.333	2.357.796	-18.402
2.363.767	-35.426	2.402.729	-26.332	2.358.006	-18.385
2.363.909	-35.427	2.402.896	-26.332	2.358.141	-18.367
2.364.126	-35.428	2.403.017	-26.331	2.358.232	-18.349
2.364.352	-35.429	2.403.189	-26.330	2.358.232	-18.332
2.364.534	-35.430	2.403.349	-26.329	2.358.313	-18.314
2.364.745	-35.431	2.403.457	-26.328	2.358.438	-18.296
2.364.879	-35.432	2.403.698	-26.327	2.358.521	-18.278
2.364.979	-35.433	2.403.885	-26.327	2.358.624	-18.261
2.365.166	-35.434	2.403.998	-26.326	2.358.797	-18.243
2.365.347	-35.435	2.404.200	-26.325	2.359.044	-18.225
2.365.405	-35.436	2.404.357	-26.324	2.359.218	-18.207
2.365.531	-35.437	2.404.504	-26.324	2.359.298	-18.190
2.365.719	-35.438	2.404.689	-26.323	2.359.437	-18.172
2.365.918	-35.439	2.404.899	-26.322	2.359.588	-18.154
2.366.055	-35.440	2.405.083	-26.322	2.359.773	-18.136
2.366.176	-35.442	2.405.224	-26.321	2.360.011	-18.119
2.366.380	-35.443	2.405.338	-26.320	2.360.217	-18.101
2.366.621	-35.444	2.405.423	-26.320	2.360.358	-18.083
2.366.781	-35.445	2.405.564	-26.319	2.360.538	-18.065
2.366.909	-35.446	2.405.730	-26.318	2.360.728	-18.047
2.367.098	-35.447	2.405.894	-26.318	2.360.894	-18.030
2.367.345	-35.448	2.406.041	-26.317	2.361.079	-18.012
2.367.542	-35.449	2.406.212	-26.316	2.361.276	-17.994
2.367.742	-35.451	2.406.425	-26.316	2.361.411	-17.976
2.367.955	-35.452	2.406.628	-26.315	2.361.547	-17.959
2.368.124	-35.453	2.406.809	-26.315	2.361.709	-17.941
2.368.309	-35.454	2.407.016	-26.314	2.361.895	-17.923
2.368.466	-35.455	2.407.240	-26.313	2.362.039	-17.905

2.368.586	-35.456	2.407.411	-26.313	2.362.139	-17.888
2.368.757	-35.458	2.407.580	-26.312	2.362.269	-17.870
2.368.913	-35.459	2.407.829	-26.312	2.362.462	-17.852
2.369.045	-35.460	2.408.039	-26.311	2.362.711	-17.835
2.369.133	-35.461	2.408.163	-26.310	2.362.934	-17.817
2.369.280	-35.462	2.408.250	-26.310	2.363.118	-17.800
2.369.469	-35.464	2.408.328	-26.309	2.363.291	-17.782
2.369.619	-35.465	2.408.506	-26.309	2.363.465	-17.765
2.369.709	-35.466	2.408.729	-26.308	2.363.618	-17.747
2.369.796	-35.467	2.408.846	-26.307	2.363.739	-17.730
2.369.893	-35.468	2.408.949	-26.307	2.363.913	-17.712
2.370.085	-35.470	2.409.128	-26.306	2.364.118	-17.695
2.370.374	-35.471	2.409.280	-26.306	2.364.271	-17.677
2.370.618	-35.472	2.409.456	-26.305	2.364.390	-17.660
2.370.784	-35.473	2.409.622	-26.304	2.364.518	-17.643
2.370.988	-35.474	2.409.780	-26.304	2.364.704	-17.626
2.371.176	-35.476	2.409.961	-26.303	2.364.843	-17.609
2.371.317	-35.477	2.410.152	-26.302	2.364.984	-17.591
2.371.550	-35.478	2.410.370	-26.302	2.365.181	-17.574
2.371.767	-35.479	2.410.549	-26.301	2.365.331	-17.557
2.371.910	-35.480	2.410.717	-26.301	2.365.499	-17.540
2.371.924	-35.482	2.410.936	-26.300	2.365.724	-17.523
2.372.018	-35.483	2.411.090	-26.299	2.365.940	-17.506
2.372.303	-35.484	2.411.234	-26.299	2.366.044	-17.490
2.372.506	-35.485	2.411.380	-26.298	2.366.155	-17.473
2.372.664	-35.486	2.411.528	-26.297	2.366.313	-17.456
2.372.802	-35.487	2.411.736	-26.296	2.366.489	-17.440
2.373.010	-35.489	2.411.939	-26.296	2.366.680	-17.423
2.373.210	-35.490	2.412.074	-26.295	2.366.872	-17.406
2.373.438	-35.491	2.412.200	-26.294	2.367.057	-17.390

2.373.598	-35.492	2.412.426	-26.294	2.367.226	-17.374
2.373.723	-35.493	2.412.645	-26.293	2.367.332	-17.357
2.373.873	-35.494	2.412.847	-26.292	2.367.522	-17.341
2.374.046	-35.496	2.413.003	-26.291	2.367.710	-17.325
2.374.195	-35.497	2.413.163	-26.291	2.367.842	-17.309
2.374.391	-35.498	2.413.366	-26.290	2.368.004	-17.293
2.374.662	-35.499	2.413.513	-26.289	2.368.163	-17.277
2.374.811	-35.500	2.413.647	-26.288	2.368.298	-17.261
2.374.876	-35.501	2.413.776	-26.287	2.368.472	-17.245
2.375.006	-35.502	2.413.933	-26.287	2.368.604	-17.229
2.375.136	-35.503	2.414.144	-26.286	2.368.764	-17.214
2.375.370	-35.504	2.414.339	-26.285	2.369.038	-17.198
2.375.605	-35.505	2.414.516	-26.284	2.369.277	-17.183
2.375.771	-35.506	2.414.650	-26.283	2.369.413	-17.167
2.375.909	-35.508	2.414.751	-26.282	2.369.534	-17.152
2.376.021	-35.509	2.414.937	-26.281	2.369.710	-17.137
2.376.201	-35.510	2.415.145	-26.280	2.369.884	-17.122
2.376.422	-35.511	2.415.345	-26.279	2.370.052	-17.107
2.376.575	-35.512	2.415.557	-26.279	2.370.159	-17.092
2.376.664	-35.513	2.415.725	-26.278	2.370.324	-17.077
2.376.725	-35.514	2.415.842	-26.277	2.370.515	-17.062
2.377.048	-35.515	2.415.992	-26.276	2.370.688	-17.048
2.377.594	-35.516	2.416.091	-26.275	2.370.867	-17.033
2.377.892	-35.517	2.416.227	-26.274	2.371.003	-17.018
2.378.046	-35.518	2.416.586	-26.273	2.371.142	-17.004
2.378.105	-35.519	2.417.081	-26.272	2.371.290	-16.990
2.378.158	-35.520	2.417.347	-26.271	2.371.411	-16.976
2.378.239	-35.521	2.417.403	-26.270	2.371.539	-16.961
2.378.317	-35.522	2.417.467	-26.269	2.371.783	-16.948
2.378.393	-35.523	2.417.556	-26.268	2.372.094	-16.934

2.378.530	-35.523	2.417.619	-26.266	2.372.341	-16.920
2.378.643	-35.524	2.417.594	-26.265	2.372.486	-16.906
2.378.712	-35.525	2.417.654	-26.264	2.372.715	-16.893
2.378.949	-35.526	2.417.825	-26.263	2.372.905	-16.879
2.379.191	-35.527	2.417.995	-26.262	2.373.038	-16.866
2.379.308	-35.528	2.418.204	-26.261	2.373.140	-16.853
2.379.453	-35.529	2.418.411	-26.260	2.373.215	-16.839
2.379.688	-35.530	2.418.557	-26.259	2.373.398	-16.826
2.379.883	-35.531	2.418.748	-26.257	2.373.604	-16.814
2.379.933	-35.531	2.418.954	-26.256	2.373.714	-16.801
2.380.082	-35.532	2.419.182	-26.255	2.373.853	-16.788
2.380.394	-35.533	2.419.344	-26.254	2.374.072	-16.776
2.380.636	-35.534	2.419.473	-26.253	2.374.204	-16.763
2.380.777	-35.535	2.419.659	-26.252	2.374.296	-16.751
2.380.833	-35.536	2.419.801	-26.250	2.374.471	-16.739
2.380.972	-35.537	2.419.950	-26.249	2.374.630	-16.726
2.381.155	-35.537	2.420.128	-26.248	2.374.794	-16.714
2.381.285	-35.538	2.420.293	-26.247	2.375.047	-16.703
2.381.429	-35.539	2.420.396	-26.245	2.375.309	-16.691
2.381.626	-35.540	2.420.524	-26.244	2.375.430	-16.679
2.381.789	-35.541	2.420.665	-26.243	2.375.557	-16.668
2.381.890	-35.542	2.420.817	-26.242	2.375.712	-16.656
2.382.076	-35.542	2.420.997	-26.241	2.375.907	-16.645
2.382.293	-35.543	2.421.187	-26.239	2.376.108	-16.634
2.382.460	-35.544	2.421.413	-26.238	2.376.239	-16.623
2.382.654	-35.545	2.421.622	-26.237	2.376.367	-16.612
2.382.840	-35.546	2.421.833	-26.236	2.376.538	-16.602
2.382.999	-35.547	2.422.010	-26.235	2.376.624	-16.591
2.383.221	-35.547	2.422.184	-26.233	2.376.833	-16.581
2.383.438	-35.548	2.422.349	-26.232	2.377.159	-16.570

2.383.593	-35.549	2.422.508	-26.231	2.377.357	-16.560
2.383.746	-35.550	2.422.654	-26.230	2.377.535	-16.550
2.383.935	-35.551	2.422.802	-26.228	2.377.697	-16.540
2.384.100	-35.551	2.422.966	-26.227	2.377.852	-16.531
2.384.212	-35.552	2.423.118	-26.226	2.378.021	-16.521
2.384.336	-35.553	2.423.219	-26.225	2.378.228	-16.512
2.384.482	-35.554	2.423.362	-26.224	2.378.402	-16.502
2.384.603	-35.555	2.423.600	-26.223	2.378.568	-16.493
2.384.719	-35.555	2.423.776	-26.221	2.378.745	-16.484
2.384.887	-35.556	2.423.913	-26.220	2.378.801	-16.475
2.385.033	-35.557	2.424.068	-26.219	2.378.911	-16.467
2.385.096	-35.558	2.424.224	-26.218	2.379.138	-16.458
2.385.264	-35.558	2.424.390	-26.217	2.379.444	-16.450
2.385.531	-35.559	2.424.576	-26.216	2.379.592	-16.441
2.385.773	-35.560	2.424.798	-26.214	2.379.662	-16.433
2.385.967	-35.561	2.424.995	-26.213	2.379.816	-16.425
2.386.077	-35.561	2.425.117	-26.212	2.380.008	-16.417
2.386.234	-35.562	2.425.237	-26.211	2.380.211	-16.410
2.386.456	-35.563	2.425.358	-26.210	2.380.369	-16.402
2.386.564	-35.564	2.425.455	-26.209	2.380.486	-16.395
2.386.637	-35.565	2.425.649	-26.208	2.380.634	-16.387
2.386.843	-35.565	2.425.900	-26.207	2.380.808	-16.380
2.387.079	-35.566	2.426.062	-26.206	2.380.909	-16.373
2.387.245	-35.567	2.426.212	-26.205	2.381.079	-16.367
2.387.411	-35.568	2.426.431	-26.204	2.381.432	-16.360
2.387.630	-35.568	2.426.568	-26.203	2.381.790	-16.354
2.387.775	-35.569	2.426.673	-26.202	2.382.001	-16.347
2.387.840	-35.570	2.426.853	-26.201	2.382.177	-16.341
2.388.068	-35.571	2.427.036	-26.200	2.382.298	-16.335
2.388.357	-35.571	2.427.283	-26.199	2.382.415	-16.329

2.388.530	-35.572	2.427.459	-26.198	2.382.516	-16.324
2.388.717	-35.573	2.427.598	-26.198	2.382.545	-16.318
2.388.819	-35.574	2.427.775	-26.197	2.382.573	-16.313
2.388.889	-35.574	2.427.941	-26.196	2.382.679	-16.308
2.389.065	-35.575	2.428.080	-26.195	2.382.891	-16.303
2.389.308	-35.576	2.428.179	-26.194	2.383.102	-16.298
2.389.445	-35.576	2.428.310	-26.193	2.383.235	-16.293
2.389.610	-35.577	2.428.528	-26.192	2.383.363	-16.288
2.389.801	-35.578	2.428.727	-26.191	2.383.593	-16.284
2.390.024	-35.578	2.428.938	-26.191	2.383.829	-16.280
2.390.288	-35.579	2.429.073	-26.190	2.383.965	-16.276
2.390.387	-35.579	2.429.232	-26.189	2.384.088	-16.272
2.390.479	-35.580	2.429.483	-26.188	2.384.250	-16.268
2.390.674	-35.581	2.429.617	-26.187	2.384.404	-16.265
2.390.842	-35.581	2.429.738	-26.187	2.384.585	-16.261
2.390.941	-35.582	2.429.881	-26.186	2.384.731	-16.258
2.391.003	-35.582	2.430.042	-26.185	2.384.888	-16.255
2.391.120	-35.583	2.430.123	-26.184	2.385.096	-16.252
2.391.292	-35.583	2.430.248	-26.183	2.385.248	-16.249
2.391.411	-35.584	2.430.499	-26.183	2.385.412	-16.247
2.391.559	-35.584	2.430.665	-26.182	2.385.569	-16.244
2.391.736	-35.584	2.430.764	-26.181	2.385.674	-16.242
2.391.879	-35.585	2.430.887	-26.180	2.385.759	-16.240
2.392.069	-35.585	2.431.019	-26.180	2.385.903	-16.238
2.392.282	-35.586	2.431.183	-26.179	2.386.117	-16.236
2.392.488	-35.586	2.431.420	-26.178	2.386.342	-16.234
2.392.721	-35.586	2.431.711	-26.178	2.386.626	-16.233
2.392.926	-35.586	2.431.964	-26.177	2.386.810	-16.232
2.393.150	-35.587	2.432.245	-26.176	2.387.013	-16.231
2.393.369	-35.587	2.432.449	-26.175	2.387.249	-16.230

2.393.593	-35.587	2.432.563	-26.175	2.387.355	-16.229
2.393.754	-35.587	2.432.697	-26.174	2.387.464	-16.228
2.393.880	-35.588	2.432.824	-26.173	2.387.628	-16.228
2.394.023	-35.588	2.432.956	-26.173	2.387.813	-16.228
2.394.135	-35.588	2.433.132	-26.172	2.387.980	-16.228
2.394.294	-35.588	2.433.311	-26.171	2.388.149	-16.228
2.394.473	-35.588	2.433.436	-26.170	2.388.243	-16.228
2.394.595	-35.588	2.433.543	-26.170	2.388.398	-16.228
2.394.717	-35.588	2.433.640	-26.169	2.388.586	-16.229
2.394.866	-35.588	2.433.773	-26.168	2.388.748	-16.230
2.394.988	-35.588	2.433.938	-26.167	2.388.905	-16.230
2.395.083	-35.588	2.434.097	-26.167	2.389.085	-16.231
2.395.217	-35.588	2.434.274	-26.166	2.389.222	-16.233
2.395.468	-35.587	2.434.489	-26.165	2.389.357	-16.234
2.395.677	-35.587	2.434.789	-26.165	2.389.487	-16.236
2.395.846	-35.587	2.435.046	-26.164	2.389.666	-16.237
2.396.025	-35.587	2.435.123	-26.163	2.389.884	-16.239
2.396.196	-35.586	2.435.175	-26.162	2.390.080	-16.241
2.396.422	-35.586	2.435.365	-26.162	2.390.179	-16.243
2.396.579	-35.586	2.435.558	-26.161	2.390.333	-16.245
2.396.706	-35.585	2.435.679	-26.160	2.390.501	-16.248
2.396.962	-35.585	2.435.949	-26.159	2.390.695	-16.251
2.397.216	-35.584	2.436.172	-26.159	2.390.898	-16.253
2.397.385	-35.584	2.436.290	-26.158	2.391.062	-16.256
2.397.479	-35.583	2.436.496	-26.157	2.391.243	-16.259
2.397.643	-35.583	2.436.648	-26.156	2.391.400	-16.263
2.397.862	-35.582	2.436.797	-26.155	2.391.532	-16.266
2.397.997	-35.582	2.436.935	-26.155	2.391.693	-16.269
2.398.129	-35.581	2.437.041	-26.154	2.391.902	-16.273
2.398.295	-35.580	2.437.307	-26.153	2.392.134	-16.277

2.398.505	-35.580	2.437.511	-26.152	2.392.311	-16.281
2.398.678	-35.579	2.437.679	-26.152	2.392.419	-16.285
2.398.792	-35.578	2.437.813	-26.151	2.392.540	-16.289
2.398.940	-35.577	2.437.849	-26.150	2.392.699	-16.294
2.399.147	-35.576	2.438.089	-26.149	2.392.838	-16.298
2.399.380	-35.575	2.438.374	-26.148	2.393.010	-16.303
2.399.530	-35.574	2.438.559	-26.147	2.393.221	-16.308
2.399.695	-35.573	2.438.700	-26.147	2.393.389	-16.313
2.399.930	-35.572	2.438.788	-26.146	2.393.548	-16.318
2.400.118	-35.571	2.438.958	-26.145	2.393.743	-16.323
2.400.221	-35.570	2.439.155	-26.144	2.393.940	-16.328
2.400.297	-35.569	2.439.256	-26.143	2.394.110	-16.334
2.400.471	-35.568	2.439.379	-26.142	2.394.279	-16.339
2.400.656	-35.567	2.439.598	-26.141	2.394.426	-16.345
2.400.761	-35.565	2.439.823	-26.141	2.394.561	-16.351
2.400.948	-35.564	2.439.986	-26.140	2.394.675	-16.357
2.401.183	-35.563	2.440.201	-26.139	2.394.816	-16.363
2.401.387	-35.561	2.440.441	-26.138	2.394.975	-16.369
2.401.498	-35.560	2.440.636	-26.137	2.395.089	-16.375
2.401.617	-35.559	2.440.760	-26.136	2.395.241	-16.382
2.401.806	-35.557	2.440.753	-26.135	2.395.459	-16.388
2.401.974	-35.556	2.440.952	-26.134	2.395.707	-16.395
2.402.180	-35.554	2.441.422	-26.133	2.395.892	-16.402
2.402.325	-35.552	2.441.776	-26.132	2.396.049	-16.408
2.402.437	-35.551	2.441.974	-26.131	2.396.245	-16.415
2.402.755	-35.549	2.442.074	-26.130	2.396.435	-16.423
2.403.198	-35.548	2.442.115	-26.129	2.396.566	-16.430
2.403.542	-35.546	2.442.143	-26.129	2.396.733	-16.437
2.403.754	-35.544	2.442.159	-26.128	2.397.005	-16.444
2.403.841	-35.542	2.442.273	-26.127	2.397.213	-16.452

2.403.820	-35.541	2.442.399	-26.126	2.397.305	-16.459
2.403.880	-35.539	2.442.531	-26.125	2.397.439	-16.467
2.403.958	-35.537	2.442.708	-26.124	2.397.573	-16.475
2.404.068	-35.535	2.442.887	-26.123	2.397.650	-16.483
2.404.222	-35.533	2.443.073	-26.122	2.397.791	-16.491
2.404.332	-35.531	2.443.214	-26.120	2.397.961	-16.499
2.404.487	-35.529	2.443.378	-26.119	2.398.134	-16.507
2.404.639	-35.527	2.443.604	-26.118	2.398.282	-16.515
2.404.774	-35.525	2.443.817	-26.117	2.398.400	-16.524
2.404.961	-35.523	2.443.983	-26.116	2.398.559	-16.532
2.405.156	-35.521	2.444.164	-26.115	2.398.776	-16.541
2.405.278	-35.519	2.444.375	-26.114	2.399.052	-16.549
2.405.468	-35.517	2.444.529	-26.113	2.399.274	-16.558
2.405.719	-35.514	2.444.604	-26.112	2.399.448	-16.567
2.405.921	-35.512	2.444.725	-26.111	2.399.617	-16.575
2.406.113	-35.510	2.444.816	-26.110	2.399.760	-16.584
2.406.312	-35.508	2.444.989	-26.109	2.399.977	-16.593
2.406.443	-35.506	2.445.249	-26.108	2.400.146	-16.602
2.406.509	-35.503	2.445.374	-26.107	2.400.262	-16.611
2.406.601	-35.501	2.445.499	-26.106	2.400.432	-16.621
2.406.781	-35.499	2.445.640	-26.105	2.400.586	-16.630
2.407.012	-35.496	2.445.759	-26.103	2.400.723	-16.639
2.407.193	-35.494	2.445.967	-26.102	2.400.892	-16.649
2.407.357	-35.492	2.446.274	-26.101	2.401.120	-16.658
2.407.468	-35.489	2.446.454	-26.100	2.401.283	-16.668
2.407.625	-35.487	2.446.570	-26.099	2.401.416	-16.677
2.407.865	-35.485	2.446.761	-26.098	2.401.550	-16.687
2.408.024	-35.482	2.446.973	-26.097	2.401.767	-16.697
2.408.129	-35.480	2.447.188	-26.096	2.401.974	-16.706
2.408.282	-35.478	2.447.374	-26.095	2.402.143	-16.716

2.408.483	-35.475	2.447.484	-26.094	2.402.280	-16.726
2.408.725	-35.473	2.447.591	-26.093	2.402.406	-16.736
2.408.897	-35.470	2.447.701	-26.092	2.402.641	-16.746
2.409.106	-35.468	2.447.861	-26.091	2.402.805	-16.756
2.409.323	-35.466	2.448.115	-26.090	2.402.948	-16.766
2.409.501	-35.463	2.448.268	-26.089	2.403.130	-16.777
2.409.581	-35.461	2.448.394	-26.088	2.403.281	-16.787
2.409.707	-35.458	2.448.571	-26.087	2.403.427	-16.797
2.409.874	-35.456	2.448.723	-26.086	2.403.575	-16.807
2.409.943	-35.453	2.448.895	-26.085	2.403.757	-16.818
2.410.078	-35.451	2.449.091	-26.084	2.404.012	-16.828
2.410.309	-35.448	2.449.263	-26.083	2.404.193	-16.839
2.410.529	-35.446	2.449.368	-26.082	2.404.332	-16.849
2.410.690	-35.443	2.449.533	-26.081	2.404.489	-16.860
2.410.766	-35.441	2.449.790	-26.080	2.404.675	-16.870
2.410.898	-35.439	2.449.955	-26.079	2.404.785	-16.881
2.411.147	-35.436	2.450.036	-26.078	2.404.843	-16.892
2.411.351	-35.434	2.450.158	-26.077	2.405.134	-16.902
2.411.485	-35.431	2.450.379	-26.076	2.405.661	-16.913
2.411.629	-35.429	2.450.595	-26.076	2.405.992	-16.924
2.411.747	-35.426	2.450.757	-26.075	2.406.028	-16.935
2.411.810	-35.424	2.450.894	-26.074	2.406.137	-16.945
2.412.031	-35.422	2.451.095	-26.073	2.406.256	-16.956
2.412.260	-35.419	2.451.315	-26.072	2.406.297	-16.967
2.412.311	-35.417	2.451.478	-26.071	2.406.353	-16.978
2.412.466	-35.414	2.451.622	-26.070	2.406.441	-16.989
2.412.775	-35.412	2.451.767	-26.070	2.406.525	-17.000
2.412.999	-35.410	2.451.928	-26.069	2.406.653	-17.011
2.413.214	-35.407	2.452.094	-26.068	2.406.830	-17.022
2.413.393	-35.405	2.452.200	-26.067	2.407.041	-17.033

2.413.530	-35.403	2.452.338	-26.066	2.407.240	-17.044
2.413.763	-35.400	2.452.527	-26.066	2.407.439	-17.055
2.413.994	-35.398	2.452.641	-26.065	2.407.629	-17.067
2.414.130	-35.396	2.452.814	-26.064	2.407.782	-17.078
2.414.200	-35.393	2.453.051	-26.063	2.407.995	-17.089
2.414.326	-35.391	2.453.289	-26.063	2.408.221	-17.100
2.414.542	-35.389	2.453.514	-26.062	2.408.416	-17.111
2.414.686	-35.386	2.453.651	-26.061	2.408.490	-17.123
2.414.766	-35.384	2.453.799	-26.060	2.408.604	-17.134
2.414.914	-35.382	2.454.012	-26.059	2.408.784	-17.145
2.415.134	-35.380	2.454.240	-26.059	2.408.920	-17.157
2.415.327	-35.377	2.454.344	-26.058	2.409.095	-17.168
2.415.578	-35.375	2.454.438	-26.057	2.409.227	-17.179
2.415.761	-35.373	2.454.619	-26.056	2.409.375	-17.191
2.415.806	-35.370	2.454.733	-26.056	2.409.583	-17.202
2.416.016	-35.368	2.454.809	-26.055	2.409.745	-17.213
2.416.219	-35.366	2.454.989	-26.054	2.409.852	-17.225
2.416.387	-35.364	2.455.168	-26.053	2.410.026	-17.236
2.416.550	-35.361	2.455.247	-26.053	2.410.271	-17.248
2.416.649	-35.359	2.455.452	-26.052	2.410.500	-17.259
2.416.814	-35.357	2.455.683	-26.051	2.410.624	-17.271
2.416.962	-35.355	2.455.793	-26.050	2.410.707	-17.282
2.417.092	-35.352	2.456.010	-26.050	2.410.977	-17.294
2.417.228	-35.350	2.456.228	-26.049	2.411.173	-17.305
2.417.352	-35.348	2.456.404	-26.048	2.411.313	-17.317
2.417.504	-35.346	2.456.617	-26.048	2.411.485	-17.328
2.417.614	-35.344	2.456.854	-26.047	2.411.610	-17.340
2.417.717	-35.341	2.457.009	-26.046	2.411.754	-17.351
2.417.932	-35.339	2.457.123	-26.045	2.411.931	-17.363
2.418.237	-35.337	2.457.264	-26.044	2.412.086	-17.375

2.418.512	-35.335	2.457.402	-26.044	2.412.222	-17.386
2.418.694	-35.333	2.457.607	-26.043	2.412.363	-17.398
2.418.808	-35.330	2.457.771	-26.042	2.412.533	-17.409
2.418.994	-35.328	2.457.891	-26.041	2.412.724	-17.421
2.419.234	-35.326	2.458.048	-26.040	2.412.880	-17.433
2.419.415	-35.324	2.458.185	-26.040	2.413.071	-17.444
2.419.559	-35.321	2.458.301	-26.039	2.413.254	-17.456
2.419.613	-35.319	2.458.411	-26.038	2.413.380	-17.468
2.419.794	-35.317	2.458.595	-26.037	2.413.548	-17.479
2.420.076	-35.315	2.458.823	-26.036	2.413.714	-17.491
2.420.192	-35.313	2.459.052	-26.035	2.413.819	-17.503
2.420.296	-35.310	2.459.294	-26.035	2.414.012	-17.514
2.420.475	-35.308	2.459.471	-26.034	2.414.225	-17.526
2.420.600	-35.306	2.459.628	-26.033	2.414.408	-17.538
2.420.766	-35.304	2.459.809	-26.032	2.414.597	-17.549
2.420.983	-35.301	2.459.993	-26.031	2.414.744	-17.561
2.421.122	-35.299	2.460.137	-26.030	2.414.872	-17.573
2.421.335	-35.297	2.460.222	-26.029	2.415.016	-17.585
2.421.550	-35.295	2.460.378	-26.028	2.415.128	-17.596
2.421.669	-35.293	2.460.602	-26.027	2.415.307	-17.608
2.421.833	-35.290	2.460.712	-26.026	2.415.591	-17.620
2.422.115	-35.288	2.460.862	-26.025	2.415.768	-17.631
2.422.322	-35.286	2.461.111	-26.024	2.415.909	-17.643
2.422.423	-35.284	2.461.293	-26.023	2.416.059	-17.655
2.422.526	-35.281	2.461.432	-26.022	2.416.198	-17.666
2.422.666	-35.279	2.461.617	-26.021	2.416.339	-17.678
2.422.879	-35.277	2.461.794	-26.020	2.416.492	-17.690
2.423.133	-35.274	2.461.971	-26.019	2.416.696	-17.701
2.423.318	-35.272	2.462.144	-26.018	2.416.938	-17.713
2.423.396	-35.270	2.462.312	-26.017	2.417.146	-17.724

2.423.503	-35.268	2.462.467	-26.016	2.417.251	-17.736
2.423.671	-35.265	2.462.573	-26.015	2.417.408	-17.748
2.423.839	-35.263	2.462.775	-26.014	2.417.551	-17.759
2.424.053	-35.261	2.462.957	-26.013	2.417.632	-17.771
2.424.271	-35.258	2.463.102	-26.012	2.417.845	-17.782
2.424.489	-35.256	2.463.288	-26.011	2.418.074	-17.794
2.424.650	-35.254	2.463.484	-26.010	2.418.219	-17.805
2.424.807	-35.251	2.463.665	-26.009	2.418.320	-17.817
2.425.094	-35.249	2.463.822	-26.008	2.418.475	-17.828
2.425.259	-35.246	2.463.998	-26.006	2.418.687	-17.840
2.425.331	-35.244	2.464.164	-26.005	2.418.792	-17.851
2.425.472	-35.242	2.464.323	-26.004	2.418.882	-17.862
2.425.618	-35.239	2.464.489	-26.003	2.419.045	-17.874
2.425.751	-35.237	2.464.641	-26.002	2.419.214	-17.885
2.425.916	-35.234	2.464.805	-26.001	2.419.406	-17.897
2.426.057	-35.232	2.465.013	-25.999	2.419.657	-17.908
2.426.216	-35.230	2.465.170	-25.998	2.419.872	-17.919
2.426.400	-35.227	2.465.276	-25.997	2.420.053	-17.930
2.426.581	-35.225	2.465.488	-25.996	2.420.239	-17.942
2.426.810	-35.222	2.465.864	-25.994	2.420.426	-17.953
2.426.992	-35.220	2.466.265	-25.993	2.420.638	-17.964
2.427.116	-35.217	2.466.482	-25.992	2.420.801	-17.975
2.427.267	-35.215	2.466.593	-25.991	2.420.975	-17.987
2.427.422	-35.212	2.466.723	-25.989	2.421.169	-17.998
2.427.533	-35.210	2.466.765	-25.988	2.421.284	-18.009
2.427.688	-35.207	2.466.747	-25.987	2.421.317	-18.020
2.427.825	-35.205	2.466.857	-25.985	2.421.441	-18.031
2.427.990	-35.202	2.466.991	-25.984	2.421.660	-18.042
2.428.398	-35.199	2.467.081	-25.983	2.421.803	-18.053
2.428.858	-35.197	2.467.236	-25.981	2.421.935	-18.064

2.429.068	-35.194	2.467.401	-25.980	2.422.126	-18.075
2.429.225	-35.192	2.467.603	-25.979	2.422.289	-18.086
2.429.388	-35.189	2.467.813	-25.977	2.422.466	-18.097
2.429.500	-35.187	2.467.997	-25.976	2.422.703	-18.108
2.429.527	-35.184	2.468.165	-25.975	2.422.901	-18.118
2.429.545	-35.181	2.468.353	-25.973	2.423.011	-18.129
2.429.594	-35.179	2.468.559	-25.972	2.423.187	-18.140
2.429.700	-35.176	2.468.647	-25.970	2.423.407	-18.151
2.429.890	-35.173	2.468.763	-25.969	2.423.599	-18.161
2.430.025	-35.171	2.468.996	-25.968	2.423.793	-18.172
2.430.170	-35.168	2.469.200	-25.966	2.423.911	-18.183
2.430.351	-35.165	2.469.330	-25.965	2.424.039	-18.193
2.430.499	-35.163	2.469.493	-25.963	2.424.168	-18.204
2.430.630	-35.160	2.469.689	-25.962	2.424.373	-18.214
2.430.856	-35.158	2.469.828	-25.960	2.424.590	-18.225
2.431.122	-35.155	2.469.928	-25.959	2.424.713	-18.235
2.431.326	-35.152	2.470.078	-25.958	2.424.887	-18.246
2.431.467	-35.150	2.470.282	-25.956	2.425.107	-18.256
2.431.538	-35.147	2.470.435	-25.955	2.425.284	-18.267
2.431.696	-35.144	2.470.551	-25.953	2.425.416	-18.277
2.431.915	-35.142	2.470.748	-25.952	2.425.547	-18.287
2.432.092	-35.139	2.470.979	-25.950	2.425.717	-18.298
2.432.314	-35.136	2.471.236	-25.949	2.425.889	-18.308
2.432.455	-35.134	2.471.444	-25.948	2.426.039	-18.318
2.432.531	-35.131	2.471.552	-25.946	2.426.240	-18.328
2.432.686	-35.129	2.471.682	-25.945	2.426.438	-18.339
2.432.869	-35.126	2.471.828	-25.943	2.426.593	-18.349
2.432.982	-35.123	2.472.018	-25.942	2.426.796	-18.359
2.433.121	-35.121	2.472.188	-25.941	2.426.987	-18.369
2.433.248	-35.118	2.472.298	-25.939	2.427.154	-18.379

2.433.391	-35.116	2.472.453	-25.938	2.427.343	-18.389
2.433.693	-35.113	2.472.594	-25.936	2.427.500	-18.399
2.433.893	-35.111	2.472.744	-25.935	2.427.650	-18.409
2.433.998	-35.108	2.472.903	-25.934	2.427.793	-18.419
2.434.168	-35.106	2.473.078	-25.932	2.427.950	-18.429
2.434.410	-35.103	2.473.301	-25.931	2.428.127	-18.439
2.434.601	-35.101	2.473.472	-25.929	2.428.244	-18.448
2.434.742	-35.098	2.473.649	-25.928	2.428.373	-18.458
2.434.971	-35.096	2.473.774	-25.927	2.428.561	-18.468
2.435.107	-35.093	2.473.900	-25.925	2.428.694	-18.478
2.435.186	-35.091	2.474.068	-25.924	2.428.996	-18.487
2.435.300	-35.088	2.474.220	-25.923	2.429.509	-18.497
2.435.391	-35.086	2.474.413	-25.921	2.429.801	-18.506
2.435.537	-35.084	2.474.585	-25.920	2.429.840	-18.516
2.435.815	-35.081	2.474.708	-25.919	2.429.885	-18.526
2.435.969	-35.079	2.474.883	-25.918	2.429.939	-18.535
2.436.097	-35.076	2.475.089	-25.916	2.429.997	-18.545
2.436.314	-35.074	2.475.246	-25.915	2.430.067	-18.554
2.436.583	-35.072	2.475.369	-25.914	2.430.215	-18.564
2.436.780	-35.070	2.475.524	-25.913	2.430.390	-18.573
2.436.904	-35.067	2.475.714	-25.912	2.430.486	-18.582
2.437.036	-35.065	2.475.929	-25.910	2.430.602	-18.592
2.437.157	-35.063	2.476.164	-25.909	2.430.746	-18.601
2.437.262	-35.061	2.476.288	-25.908	2.430.976	-18.610
2.437.412	-35.059	2.476.355	-25.907	2.431.193	-18.619
2.437.594	-35.057	2.476.544	-25.906	2.431.366	-18.629
2.437.820	-35.055	2.476.792	-25.905	2.431.507	-18.638
2.438.022	-35.052	2.476.942	-25.904	2.431.659	-18.647
2.438.100	-35.050	2.477.085	-25.902	2.431.808	-18.656
2.438.262	-35.048	2.477.280	-25.901	2.432.000	-18.665

2.438.474	-35.046	2.477.443	-25.900	2.432.190	-18.674
2.438.663	-35.044	2.477.522	-25.899	2.432.359	-18.683
2.438.897	-35.043	2.477.664	-25.898	2.432.533	-18.692
2.439.117	-35.041	2.477.890	-25.897	2.432.691	-18.701
2.439.279	-35.039	2.478.033	-25.896	2.432.784	-18.710
2.439.402	-35.037	2.478.243	-25.895	2.432.905	-18.719
2.439.505	-35.035	2.478.460	-25.894	2.433.049	-18.728
2.439.697	-35.033	2.478.595	-25.893	2.433.270	-18.737
2.439.843	-35.031	2.478.812	-25.892	2.433.420	-18.746
2.439.958	-35.030	2.479.009	-25.891	2.433.504	-18.754
2.440.152	-35.028	2.479.092	-25.890	2.433.758	-18.763
2.440.311	-35.026	2.479.177	-25.889	2.434.021	-18.772
2.440.464	-35.024	2.479.274	-25.888	2.434.194	-18.781
2.440.645	-35.023	2.479.462	-25.887	2.434.357	-18.789
2.440.806	-35.021	2.479.643	-25.886	2.434.538	-18.798
2.440.966	-35.019	2.479.733	-25.885	2.434.677	-18.807
2.441.142	-35.018	2.479.930	-25.884	2.434.850	-18.815
2.441.311	-35.016	2.480.126	-25.883	2.435.031	-18.824
2.441.523	-35.014	2.480.284	-25.882	2.435.206	-18.833
2.441.696	-35.013	2.480.486	-25.881	2.435.376	-18.841
2.441.823	-35.011	2.480.591	-25.880	2.435.513	-18.850
2.441.986	-35.010	2.480.822	-25.879	2.435.688	-18.858
2.442.116	-35.008	2.481.064	-25.878	2.435.836	-18.867
2.442.271	-35.007	2.481.217	-25.877	2.435.954	-18.875
2.442.464	-35.005	2.481.357	-25.876	2.436.169	-18.884
2.442.652	-35.004	2.481.552	-25.875	2.436.314	-18.892
2.442.762	-35.002	2.481.756	-25.874	2.436.357	-18.901
2.442.836	-35.001	2.481.960	-25.873	2.436.516	-18.909
2.443.048	-35.000	2.482.130	-25.872	2.436.729	-18.918
2.443.227	-34.998	2.482.254	-25.871	2.436.949	-18.926

2.443.373	-34.997	2.482.367	-25.870	2.437.119	-18.934
2.443.515	-34.996	2.482.498	-25.869	2.437.231	-18.943
2.443.728	-34.994	2.482.646	-25.868	2.437.399	-18.951
2.443.952	-34.993	2.482.780	-25.867	2.437.565	-18.959
2.444.126	-34.992	2.482.953	-25.866	2.437.647	-18.967
2.444.332	-34.990	2.483.118	-25.865	2.437.843	-18.976
2.444.551	-34.989	2.483.207	-25.865	2.438.089	-18.984
2.444.816	-34.988	2.483.346	-25.863	2.438.205	-18.992
2.445.065	-34.987	2.483.581	-25.862	2.438.378	-19.000
2.445.181	-34.985	2.483.849	-25.861	2.438.608	-19.008
2.445.282	-34.984	2.484.014	-25.860	2.438.819	-19.017
2.445.427	-34.983	2.484.162	-25.859	2.439.008	-19.025
2.445.600	-34.982	2.484.330	-25.858	2.439.189	-19.033
2.445.741	-34.981	2.484.529	-25.857	2.439.348	-19.041
2.445.835	-34.979	2.484.758	-25.856	2.439.525	-19.049
2.445.988	-34.978	2.484.933	-25.855	2.439.700	-19.057
2.446.127	-34.977	2.485.079	-25.854	2.439.839	-19.065
2.446.217	-34.976	2.485.257	-25.853	2.439.922	-19.073
2.446.371	-34.975	2.485.414	-25.852	2.440.135	-19.081
2.446.600	-34.974	2.485.548	-25.851	2.440.376	-19.089
2.446.792	-34.973	2.485.716	-25.850	2.440.455	-19.097
2.446.964	-34.971	2.485.900	-25.849	2.440.585	-19.104
2.447.166	-34.970	2.486.113	-25.848	2.440.742	-19.112
2.447.358	-34.969	2.486.281	-25.847	2.440.936	-19.120
2.447.522	-34.968	2.486.413	-25.846	2.441.149	-19.128
2.447.730	-34.967	2.486.582	-25.845	2.441.326	-19.136
2.447.941	-34.966	2.486.821	-25.844	2.441.467	-19.143
2.448.026	-34.965	2.487.012	-25.843	2.441.581	-19.151
2.448.176	-34.964	2.487.161	-25.842	2.441.733	-19.159
2.448.393	-34.963	2.487.262	-25.841	2.441.900	-19.166

2.448.606	-34.962	2.487.383	-25.840	2.442.058	-19.174
2.448.770	-34.961	2.487.652	-25.838	2.442.184	-19.181
2.448.898	-34.960	2.487.854	-25.837	2.442.328	-19.189
2.449.034	-34.959	2.487.981	-25.836	2.442.475	-19.196
2.449.174	-34.958	2.488.165	-25.835	2.442.607	-19.204
2.449.366	-34.957	2.488.322	-25.834	2.442.776	-19.211
2.449.549	-34.956	2.488.425	-25.833	2.443.013	-19.218
2.449.762	-34.955	2.488.519	-25.832	2.443.190	-19.226
2.449.946	-34.954	2.488.669	-25.831	2.443.344	-19.233
2.450.056	-34.953	2.488.886	-25.829	2.443.537	-19.240
2.450.253	-34.952	2.489.121	-25.828	2.443.752	-19.248
2.450.419	-34.951	2.489.310	-25.827	2.443.940	-19.255
2.450.616	-34.950	2.489.498	-25.826	2.444.081	-19.262
2.450.848	-34.949	2.489.615	-25.825	2.444.287	-19.269
2.451.051	-34.948	2.489.661	-25.824	2.444.507	-19.276
2.451.134	-34.947	2.489.775	-25.823	2.444.659	-19.283
2.451.241	-34.946	2.490.020	-25.821	2.444.820	-19.290
2.451.380	-34.945	2.490.425	-25.820	2.444.977	-19.297
2.451.521	-34.944	2.490.809	-25.819	2.445.089	-19.304
2.451.707	-34.943	2.491.070	-25.818	2.445.217	-19.311
2.451.913	-34.942	2.491.173	-25.817	2.445.356	-19.318
2.452.135	-34.941	2.491.196	-25.816	2.445.548	-19.325
2.452.242	-34.940	2.491.263	-25.814	2.445.741	-19.331
2.452.361	-34.939	2.491.355	-25.813	2.445.813	-19.338
2.452.534	-34.938	2.491.440	-25.812	2.445.948	-19.345
2.452.699	-34.937	2.491.516	-25.811	2.446.176	-19.351
2.452.903	-34.936	2.491.631	-25.810	2.446.387	-19.358
2.453.118	-34.935	2.491.835	-25.809	2.446.554	-19.365
2.453.297	-34.934	2.492.018	-25.807	2.446.706	-19.371
2.453.422	-34.933	2.492.179	-25.806	2.446.926	-19.378

2.453.578	-34.932	2.492.395	-25.805	2.447.121	-19.384
2.453.929	-34.931	2.492.560	-25.804	2.447.216	-19.390
2.454.330	-34.930	2.492.728	-25.803	2.447.365	-19.397
2.454.628	-34.929	2.492.923	-25.802	2.447.525	-19.403
2.454.782	-34.929	2.493.138	-25.800	2.447.679	-19.409
2.454.854	-34.928	2.493.301	-25.799	2.447.867	-19.415
2.454.980	-34.927	2.493.418	-25.798	2.448.056	-19.422
2.455.011	-34.926	2.493.595	-25.797	2.448.230	-19.428
2.454.971	-34.925	2.493.795	-25.796	2.448.438	-19.434
2.455.072	-34.924	2.493.900	-25.795	2.448.622	-19.440
2.455.197	-34.923	2.494.028	-25.793	2.448.767	-19.446
2.455.327	-34.922	2.494.261	-25.792	2.448.996	-19.452
2.455.522	-34.921	2.494.430	-25.791	2.449.182	-19.458
2.455.712	-34.920	2.494.563	-25.790	2.449.346	-19.464
2.455.907	-34.919	2.494.677	-25.789	2.449.504	-19.469
2.456.061	-34.918	2.494.743	-25.788	2.449.635	-19.475
2.456.198	-34.917	2.494.799	-25.787	2.449.854	-19.481
2.456.386	-34.916	2.494.970	-25.786	2.450.008	-19.486
2.456.490	-34.915	2.495.201	-25.784	2.450.145	-19.492
2.456.620	-34.914	2.495.406	-25.783	2.450.385	-19.498
2.456.877	-34.913	2.495.612	-25.782	2.450.571	-19.503
2.457.113	-34.912	2.495.869	-25.781	2.450.688	-19.509
2.457.327	-34.911	2.496.075	-25.780	2.450.811	-19.514
2.457.511	-34.910	2.496.243	-25.779	2.451.001	-19.520
2.457.601	-34.909	2.496.418	-25.778	2.451.205	-19.525
2.457.685	-34.908	2.496.512	-25.777	2.451.332	-19.530
2.457.945	-34.907	2.496.630	-25.776	2.451.478	-19.536
2.458.210	-34.906	2.496.789	-25.775	2.451.648	-19.541
2.458.309	-34.906	2.496.935	-25.774	2.451.814	-19.546
2.458.376	-34.905	2.497.027	-25.773	2.451.982	-19.551

2.458.487	-34.904	2.497.228	-25.772	2.452.119	-19.556
2.458.622	-34.903	2.497.475	-25.772	2.452.211	-19.561
2.458.792	-34.902	2.497.701	-25.771	2.452.355	-19.566
2.459.025	-34.901	2.497.903	-25.770	2.452.618	-19.571
2.459.115	-34.900	2.497.981	-25.769	2.453.017	-19.576
2.459.309	-34.899	2.498.095	-25.768	2.453.369	-19.581
2.459.587	-34.898	2.498.354	-25.767	2.453.523	-19.586
2.459.805	-34.897	2.498.578	-25.766	2.453.658	-19.591
2.459.996	-34.897	2.498.661	-25.766	2.453.743	-19.596
2.460.123	-34.896	2.498.775	-25.765	2.453.745	-19.600
2.460.269	-34.895	2.499.003	-25.764	2.453.811	-19.605
2.460.406	-34.894	2.499.117	-25.763	2.453.904	-19.610
2.460.589	-34.893	2.499.156	-25.763	2.454.025	-19.614
2.460.748	-34.892	2.499.350	-25.762	2.454.200	-19.619
2.460.905	-34.891	2.499.589	-25.761	2.454.342	-19.623
2.461.039	-34.891	2.499.805	-25.761	2.454.500	-19.628
2.461.167	-34.890	2.499.995	-25.760	2.454.646	-19.632
2.461.386	-34.889	2.500.164	-25.760	2.454.861	-19.636
2.461.590	-34.888	2.500.385	-25.759	2.455.067	-19.641
2.461.747	-34.887	2.500.527	-25.759	2.455.258	-19.645
2.461.917	-34.887	2.500.642	-25.758	2.455.513	-19.649
2.462.087	-34.886	2.500.880	-25.758	2.455.681	-19.654
2.462.207	-34.885	2.501.133	-25.757	2.455.744	-19.658
2.462.376	-34.884	2.501.272	-25.757	2.455.947	-19.662
2.462.587	-34.884	2.501.351	-25.756	2.456.158	-19.666
2.462.750	-34.883	2.501.462	-25.756	2.456.227	-19.670
2.462.876	-34.882	2.501.622	-25.756	2.456.395	-19.674
2.463.008	-34.882	2.501.805	-25.755	2.456.563	-19.678
2.463.145	-34.881	2.502.002	-25.755	2.456.749	-19.682
2.463.257	-34.880	2.502.197	-25.754	2.456.962	-19.686

2.463.432	-34.880	2.502.375	-25.754	2.457.056	-19.690
2.463.665	-34.879	2.502.500	-25.754	2.457.169	-19.694
2.463.802	-34.878	2.502.599	-25.754	2.457.423	-19.698
2.463.998	-34.878	2.502.717	-25.753	2.457.645	-19.701
2.464.202	-34.877	2.502.935	-25.753	2.457.827	-19.705
2.464.276	-34.877	2.503.165	-25.753	2.458.037	-19.709
2.464.413	-34.876	2.503.373	-25.752	2.458.204	-19.713
2.464.621	-34.876	2.503.542	-25.752	2.458.384	-19.716
2.464.859	-34.875	2.503.608	-25.752	2.458.530	-19.720
2.465.006	-34.874	2.503.763	-25.752	2.458.687	-19.724
2.465.107	-34.874	2.503.857	-25.752	2.458.822	-19.727
2.465.282	-34.873	2.503.938	-25.752	2.458.925	-19.731
2.465.490	-34.873	2.504.072	-25.751	2.459.139	-19.734
2.465.623	-34.872	2.504.243	-25.751	2.459.336	-19.738
2.465.777	-34.872	2.504.455	-25.751	2.459.444	-19.741
2.465.925	-34.871	2.504.603	-25.751	2.459.583	-19.745
2.466.040	-34.871	2.504.867	-25.751	2.459.745	-19.748
2.466.238	-34.870	2.505.111	-25.751	2.459.908	-19.751
2.466.442	-34.870	2.505.282	-25.751	2.460.079	-19.755
2.466.635	-34.870	2.505.497	-25.751	2.460.220	-19.758
2.466.861	-34.869	2.505.695	-25.751	2.460.388	-19.761
2.467.085	-34.869	2.505.878	-25.751	2.460.565	-19.765
2.467.112	-34.868	2.506.037	-25.751	2.460.697	-19.768
2.467.233	-34.868	2.506.218	-25.751	2.460.862	-19.771
2.467.469	-34.867	2.506.400	-25.751	2.461.008	-19.775
2.467.567	-34.867	2.506.528	-25.751	2.461.160	-19.778
2.467.820	-34.867	2.506.673	-25.751	2.461.357	-19.781
2.468.022	-34.866	2.506.798	-25.751	2.461.494	-19.784
2.468.080	-34.866	2.506.947	-25.751	2.461.656	-19.787
2.468.197	-34.865	2.507.088	-25.751	2.461.848	-19.790

2.468.346	-34.865	2.507.229	-25.751	2.462.063	-19.793
2.468.519	-34.865	2.507.329	-25.751	2.462.281	-19.796
2.468.655	-34.864	2.507.486	-25.751	2.462.439	-19.800
2.468.861	-34.864	2.507.697	-25.751	2.462.587	-19.803
2.469.032	-34.863	2.507.847	-25.751	2.462.708	-19.806
2.469.200	-34.863	2.508.075	-25.751	2.462.831	-19.809
2.469.453	-34.863	2.508.328	-25.751	2.463.055	-19.811
2.469.720	-34.862	2.508.485	-25.751	2.463.280	-19.814
2.469.986	-34.862	2.508.629	-25.751	2.463.414	-19.817
2.470.217	-34.861	2.508.819	-25.751	2.463.499	-19.820
2.470.329	-34.861	2.509.012	-25.751	2.463.698	-19.823
2.470.387	-34.861	2.509.156	-25.751	2.463.922	-19.826
2.470.571	-34.860	2.509.296	-25.751	2.464.029	-19.829
2.470.742	-34.860	2.509.498	-25.751	2.464.209	-19.831
2.470.889	-34.859	2.509.731	-25.752	2.464.404	-19.834
2.471.022	-34.859	2.509.955	-25.752	2.464.608	-19.837
2.471.107	-34.859	2.510.098	-25.752	2.464.825	-19.839
2.471.198	-34.858	2.510.229	-25.752	2.464.982	-19.842
2.471.313	-34.858	2.510.385	-25.752	2.465.132	-19.845
2.471.491	-34.857	2.510.490	-25.752	2.465.304	-19.847
2.471.635	-34.857	2.510.636	-25.752	2.465.480	-19.850
2.471.833	-34.856	2.510.853	-25.752	2.465.591	-19.852
2.472.020	-34.856	2.511.046	-25.752	2.465.706	-19.855
2.472.186	-34.856	2.511.241	-25.752	2.465.851	-19.857
2.472.458	-34.855	2.511.408	-25.752	2.465.992	-19.860
2.472.621	-34.855	2.511.559	-25.752	2.466.131	-19.862
2.472.824	-34.854	2.511.700	-25.752	2.466.270	-19.865
2.473.060	-34.854	2.511.861	-25.752	2.466.469	-19.867
2.473.214	-34.853	2.512.022	-25.752	2.466.705	-19.869
2.473.355	-34.853	2.512.175	-25.752	2.466.853	-19.871

2.473.523	-34.852	2.512.321	-25.752	2.467.016	-19.874
2.473.720	-34.852	2.512.502	-25.752	2.467.244	-19.876
2.473.868	-34.851	2.512.684	-25.752	2.467.466	-19.878
2.474.044	-34.851	2.512.813	-25.752	2.467.655	-19.880
2.474.243	-34.850	2.512.939	-25.752	2.467.847	-19.882
2.474.388	-34.850	2.513.167	-25.752	2.468.029	-19.884
2.474.554	-34.849	2.513.347	-25.752	2.468.177	-19.886
2.474.695	-34.849	2.513.463	-25.752	2.468.335	-19.888
2.474.787	-34.848	2.513.596	-25.752	2.468.465	-19.890
2.474.948	-34.848	2.513.766	-25.752	2.468.546	-19.892
2.475.182	-34.847	2.513.926	-25.752	2.468.729	-19.894
2.475.320	-34.847	2.514.099	-25.752	2.468.942	-19.896
2.475.518	-34.846	2.514.315	-25.752	2.469.109	-19.898
2.475.631	-34.846	2.514.445	-25.752	2.469.215	-19.900
2.475.809	-34.845	2.514.697	-25.751	2.469.332	-19.902
2.476.033	-34.845	2.515.110	-25.751	2.469.511	-19.903
2.476.194	-34.844	2.515.495	-25.751	2.469.660	-19.905
2.476.335	-34.844	2.515.701	-25.751	2.469.859	-19.907
2.476.512	-34.843	2.515.699	-25.751	2.470.085	-19.908
2.476.718	-34.842	2.515.797	-25.751	2.470.226	-19.910
2.476.888	-34.842	2.515.932	-25.751	2.470.371	-19.911
2.477.066	-34.841	2.515.997	-25.750	2.470.618	-19.913
2.477.218	-34.841	2.516.093	-25.750	2.470.771	-19.914
2.477.388	-34.840	2.516.151	-25.750	2.470.916	-19.916
2.477.581	-34.840	2.516.245	-25.750	2.471.102	-19.917
2.477.724	-34.839	2.516.402	-25.750	2.471.237	-19.918
2.477.859	-34.838	2.516.591	-25.749	2.471.449	-19.920
2.478.035	-34.838	2.516.821	-25.749	2.471.656	-19.921
2.478.254	-34.837	2.517.023	-25.749	2.471.777	-19.922
2.478.384	-34.837	2.517.283	-25.749	2.471.938	-19.923

2.478.577	-34.836	2.517.502	-25.748	2.472.101	-19.924
2.478.781	-34.836	2.517.650	-25.748	2.472.271	-19.926
2.478.895	-34.835	2.517.742	-25.748	2.472.493	-19.927
2.478.967	-34.834	2.517.838	-25.747	2.472.655	-19.928
2.479.209	-34.834	2.518.042	-25.747	2.472.777	-19.929
2.479.681	-34.833	2.518.241	-25.747	2.472.980	-19.930
2.479.978	-34.833	2.518.416	-25.746	2.473.143	-19.931
2.480.096	-34.832	2.518.564	-25.746	2.473.306	-19.932
2.480.262	-34.831	2.518.708	-25.746	2.473.472	-19.932
2.480.359	-34.831	2.518.873	-25.745	2.473.604	-19.933
2.480.412	-34.830	2.519.035	-25.745	2.473.806	-19.934
2.480.518	-34.829	2.519.232	-25.745	2.474.010	-19.935
2.480.562	-34.829	2.519.352	-25.744	2.474.146	-19.935
2.480.567	-34.828	2.519.427	-25.744	2.474.262	-19.936
2.480.670	-34.828	2.519.570	-25.744	2.474.449	-19.937
2.480.958	-34.827	2.519.720	-25.743	2.474.653	-19.937
2.481.142	-34.826	2.519.952	-25.743	2.474.866	-19.938
2.481.228	-34.826	2.520.217	-25.742	2.475.036	-19.939
2.481.429	-34.825	2.520.378	-25.742	2.475.154	-19.939
2.481.603	-34.824	2.520.510	-25.742	2.475.326	-19.940
2.481.776	-34.824	2.520.710	-25.741	2.475.461	-19.940
2.481.957	-34.823	2.520.943	-25.741	2.475.625	-19.940
2.482.151	-34.823	2.521.142	-25.740	2.475.779	-19.941
2.482.352	-34.822	2.521.258	-25.740	2.475.900	-19.941
2.482.479	-34.821	2.521.344	-25.740	2.476.091	-19.941
2.482.609	-34.821	2.521.512	-25.739	2.476.395	-19.942
2.482.815	-34.820	2.521.697	-25.739	2.476.910	-19.942
2.482.907	-34.820	2.521.812	-25.738	2.477.238	-19.942
2.483.046	-34.819	2.521.962	-25.738	2.477.249	-19.942
2.483.329	-34.818	2.522.141	-25.737	2.477.291	-19.942

2.483.513	-34.818	2.522.290	-25.737	2.477.455	-19.943
2.483.672	-34.817	2.522.444	-25.736	2.477.544	-19.943
2.483.805	-34.817	2.522.634	-25.736	2.477.536	-19.943
2.483.857	-34.816	2.522.814	-25.736	2.477.598	-19.943
2.484.038	-34.816	2.522.927	-25.735	2.477.694	-19.943
2.484.290	-34.815	2.523.049	-25.735	2.477.870	-19.943
2.484.477	-34.815	2.523.226	-25.734	2.478.053	-19.942
2.484.596	-34.814	2.523.366	-25.734	2.478.176	-19.942
2.484.713	-34.814	2.523.522	-25.733	2.478.391	-19.942
2.484.890	-34.813	2.523.723	-25.733	2.478.616	-19.942
2.485.293	-34.813	2.523.922	-25.732	2.478.773	-19.942
2.485.508	-34.812	2.524.168	-25.732	2.478.953	-19.942
2.485.632	-34.812	2.524.316	-25.732	2.479.186	-19.941
2.485.783	-34.812	2.524.404	-25.731	2.479.352	-19.941
2.485.847	-34.811	2.524.608	-25.731	2.479.424	-19.941
2.485.970	-34.811	2.524.875	-25.730	2.479.604	-19.940
2.486.147	-34.810	2.525.070	-25.730	2.479.760	-19.940
2.486.310	-34.810	2.525.226	-25.729	2.479.910	-19.940
2.486.375	-34.810	2.525.401	-25.729	2.480.158	-19.939
2.486.429	-34.809	2.525.526	-25.729	2.480.300	-19.939
2.486.655	-34.809	2.525.641	-25.728	2.480.427	-19.938
2.486.892	-34.809	2.525.854	-25.728	2.480.620	-19.938
2.487.012	-34.808	2.526.030	-25.727	2.480.804	-19.937
2.487.144	-34.808	2.526.149	-25.727	2.480.976	-19.937
2.487.293	-34.808	2.526.332	-25.727	2.481.153	-19.936
2.487.458	-34.808	2.526.523	-25.726	2.481.326	-19.936
2.487.686	-34.808	2.526.613	-25.726	2.481.487	-19.935
2.487.883	-34.807	2.526.778	-25.725	2.481.649	-19.935
2.487.986	-34.807	2.527.087	-25.725	2.481.843	-19.934
2.488.076	-34.807	2.527.283	-25.724	2.482.029	-19.933

2.488.234	-34.807	2.527.402	-25.724	2.482.224	-19.933
2.488.416	-34.807	2.527.497	-25.723	2.482.431	-19.932
2.488.606	-34.807	2.527.658	-25.723	2.482.569	-19.931
2.488.804	-34.807	2.527.822	-25.722	2.482.681	-19.931
2.489.007	-34.807	2.527.975	-25.722	2.482.845	-19.930
2.489.211	-34.807	2.528.120	-25.722	2.482.980	-19.929
2.489.393	-34.807	2.528.232	-25.721	2.483.114	-19.929
2.489.585	-34.807	2.528.423	-25.721	2.483.250	-19.928
2.489.805	-34.807	2.528.517	-25.720	2.483.362	-19.927
2.489.951	-34.807	2.528.667	-25.720	2.483.543	-19.926
2.490.042	-34.808	2.528.902	-25.719	2.483.705	-19.926
2.490.201	-34.808	2.529.065	-25.719	2.483.923	-19.925
2.490.450	-34.808	2.529.196	-25.718	2.484.150	-19.924
2.490.587	-34.808	2.529.314	-25.718	2.484.258	-19.923
2.490.715	-34.809	2.529.507	-25.717	2.484.386	-19.922
2.490.872	-34.809	2.529.757	-25.717	2.484.610	-19.922
2.491.016	-34.809	2.529.969	-25.716	2.484.818	-19.921
2.491.246	-34.809	2.530.129	-25.716	2.484.905	-19.920
2.491.535	-34.810	2.530.365	-25.715	2.485.020	-19.919
2.491.709	-34.810	2.530.609	-25.714	2.485.186	-19.918
2.491.805	-34.810	2.530.748	-25.714	2.485.349	-19.917
2.491.995	-34.811	2.530.871	-25.713	2.485.535	-19.917
2.492.164	-34.811	2.531.049	-25.713	2.485.712	-19.916
2.492.233	-34.812	2.531.190	-25.712	2.485.920	-19.915
2.492.348	-34.812	2.531.270	-25.712	2.486.128	-19.914
2.492.523	-34.812	2.531.460	-25.711	2.486.279	-19.913
2.492.719	-34.813	2.531.617	-25.711	2.486.435	-19.912
2.492.908	-34.813	2.531.734	-25.710	2.486.593	-19.911
2.493.069	-34.814	2.531.897	-25.709	2.486.787	-19.911
2.493.221	-34.814	2.531.998	-25.709	2.486.953	-19.910

2.493.331	-34.815	2.532.181	-25.708	2.487.094	-19.909
2.493.428	-34.815	2.532.399	-25.707	2.487.211	-19.908
2.493.535	-34.816	2.532.556	-25.707	2.487.379	-19.907
2.493.692	-34.817	2.532.793	-25.706	2.487.605	-19.906
2.493.962	-34.817	2.533.040	-25.705	2.487.735	-19.905
2.494.157	-34.818	2.533.237	-25.705	2.487.918	-19.904
2.494.269	-34.818	2.533.385	-25.704	2.488.129	-19.903
2.494.408	-34.819	2.533.557	-25.703	2.488.304	-19.903
2.494.590	-34.820	2.533.723	-25.703	2.488.425	-19.902
2.494.857	-34.820	2.533.911	-25.702	2.488.542	-19.901
2.495.051	-34.821	2.534.156	-25.701	2.488.727	-19.900
2.495.224	-34.822	2.534.288	-25.701	2.488.922	-19.899
2.495.434	-34.822	2.534.375	-25.700	2.489.115	-19.898
2.495.574	-34.823	2.534.491	-25.699	2.489.214	-19.897
2.495.724	-34.824	2.534.658	-25.698	2.489.317	-19.896
2.495.941	-34.824	2.534.840	-25.698	2.489.525	-19.895
2.496.061	-34.825	2.535.020	-25.697	2.489.731	-19.894
2.496.223	-34.826	2.535.209	-25.696	2.489.895	-19.893
2.496.339	-34.826	2.535.371	-25.695	2.490.002	-19.892
2.496.411	-34.827	2.535.537	-25.694	2.490.070	-19.891
2.496.509	-34.828	2.535.685	-25.694	2.490.247	-19.890
2.496.759	-34.828	2.535.815	-25.693	2.490.482	-19.889
2.496.998	-34.829	2.536.011	-25.692	2.490.728	-19.888
2.497.126	-34.830	2.536.221	-25.691	2.490.968	-19.887
2.497.204	-34.831	2.536.421	-25.690	2.491.146	-19.886
2.497.397	-34.831	2.536.604	-25.690	2.491.297	-19.885
2.497.634	-34.832	2.536.739	-25.689	2.491.476	-19.883
2.497.820	-34.833	2.536.899	-25.688	2.491.684	-19.882
2.498.029	-34.833	2.537.050	-25.687	2.491.826	-19.881
2.498.148	-34.834	2.537.196	-25.686	2.491.942	-19.880

2.498.297	-34.835	2.537.415	-25.685	2.492.095	-19.879
2.498.559	-34.835	2.537.563	-25.684	2.492.242	-19.878
2.498.680	-34.836	2.537.643	-25.683	2.492.414	-19.877
2.498.826	-34.837	2.537.859	-25.682	2.492.578	-19.876
2.499.074	-34.838	2.538.102	-25.681	2.492.706	-19.874
2.499.200	-34.838	2.538.239	-25.680	2.492.852	-19.873
2.499.368	-34.839	2.538.381	-25.679	2.492.950	-19.872
2.499.581	-34.840	2.538.585	-25.678	2.493.080	-19.871
2.499.737	-34.840	2.538.759	-25.677	2.493.277	-19.870
2.499.872	-34.841	2.538.904	-25.676	2.493.513	-19.868
2.500.049	-34.842	2.539.042	-25.675	2.493.712	-19.867
2.500.250	-34.842	2.539.207	-25.674	2.493.902	-19.866
2.500.412	-34.843	2.539.551	-25.673	2.494.068	-19.865
2.500.622	-34.843	2.539.880	-25.672	2.494.220	-19.863
2.500.761	-34.844	2.540.158	-25.671	2.494.395	-19.862
2.500.866	-34.845	2.540.321	-25.670	2.494.615	-19.861
2.501.075	-34.845	2.540.352	-25.669	2.494.814	-19.860
2.501.283	-34.846	2.540.464	-25.668	2.494.917	-19.858
2.501.416	-34.846	2.540.563	-25.667	2.495.049	-19.857
2.501.521	-34.847	2.540.660	-25.666	2.495.233	-19.856
2.501.687	-34.848	2.540.746	-25.665	2.495.444	-19.854
2.501.857	-34.848	2.540.861	-25.664	2.495.616	-19.853
2.502.033	-34.849	2.541.000	-25.662	2.495.815	-19.852
2.502.190	-34.849	2.541.216	-25.661	2.496.023	-19.850
2.502.365	-34.850	2.541.436	-25.660	2.496.149	-19.849
2.502.572	-34.850	2.541.570	-25.659	2.496.252	-19.848
2.502.691	-34.851	2.541.750	-25.658	2.496.456	-19.846
2.502.847	-34.851	2.541.949	-25.657	2.496.673	-19.845
2.503.104	-34.852	2.542.130	-25.655	2.496.835	-19.843
2.503.307	-34.852	2.542.346	-25.654	2.497.001	-19.842

2.503.396	-34.852	2.542.542	-25.653	2.497.164	-19.841
2.503.544	-34.853	2.542.683	-25.652	2.497.316	-19.839
2.503.746	-34.853	2.542.819	-25.651	2.497.471	-19.838
2.503.864	-34.854	2.542.955	-25.649	2.497.618	-19.836
2.503.967	-34.854	2.543.091	-25.648	2.497.735	-19.835
2.504.184	-34.854	2.543.239	-25.647	2.497.968	-19.833
2.504.420	-34.855	2.543.394	-25.646	2.498.174	-19.832
2.504.707	-34.855	2.543.525	-25.645	2.498.275	-19.831
2.505.098	-34.855	2.543.654	-25.643	2.498.459	-19.829
2.505.428	-34.856	2.543.811	-25.642	2.498.661	-19.828
2.505.602	-34.856	2.543.976	-25.641	2.498.833	-19.826
2.505.624	-34.856	2.544.066	-25.640	2.499.005	-19.825
2.505.659	-34.857	2.544.219	-25.639	2.499.180	-19.823
2.505.748	-34.857	2.544.482	-25.637	2.499.327	-19.822
2.505.820	-34.857	2.544.792	-25.636	2.499.435	-19.820
2.505.891	-34.857	2.545.003	-25.635	2.499.601	-19.819
2.506.086	-34.857	2.545.126	-25.634	2.499.801	-19.817
2.506.285	-34.858	2.545.262	-25.633	2.500.099	-19.816
2.506.380	-34.858	2.545.415	-25.631	2.500.528	-19.814
2.506.546	-34.858	2.545.594	-25.630	2.500.742	-19.813
2.506.697	-34.858	2.545.720	-25.629	2.500.858	-19.811
2.506.821	-34.858	2.545.880	-25.628	2.501.039	-19.810
2.506.989	-34.858	2.546.090	-25.627	2.501.131	-19.808
2.507.184	-34.858	2.546.266	-25.626	2.501.125	-19.807
2.507.422	-34.858	2.546.386	-25.624	2.501.142	-19.805
2.507.639	-34.858	2.546.444	-25.623	2.501.184	-19.804
2.507.785	-34.858	2.546.618	-25.622	2.501.306	-19.802
2.507.926	-34.858	2.546.780	-25.621	2.501.494	-19.801
2.508.069	-34.858	2.546.946	-25.620	2.501.689	-19.799
2.508.244	-34.858	2.547.195	-25.619	2.501.855	-19.798

2.508.407	-34.858	2.547.364	-25.618	2.502.029	-19.796
2.508.569	-34.858	2.547.441	-25.617	2.502.217	-19.795
2.508.743	-34.858	2.547.592	-25.616	2.502.428	-19.793
2.508.851	-34.858	2.547.798	-25.615	2.502.675	-19.792
2.508.958	-34.858	2.547.979	-25.613	2.502.874	-19.790
2.509.196	-34.858	2.548.202	-25.612	2.503.042	-19.789
2.509.421	-34.858	2.548.334	-25.611	2.503.127	-19.787
2.509.474	-34.858	2.548.457	-25.610	2.503.272	-19.786
2.509.607	-34.858	2.548.647	-25.609	2.503.476	-19.784
2.509.895	-34.858	2.548.793	-25.608	2.503.611	-19.783
2.510.167	-34.858	2.548.983	-25.607	2.503.783	-19.782
2.510.318	-34.858	2.549.172	-25.607	2.503.929	-19.780
2.510.417	-34.858	2.549.340	-25.606	2.504.100	-19.779
2.510.593	-34.858	2.549.588	-25.605	2.504.240	-19.777
2.510.694	-34.858	2.549.756	-25.604	2.504.375	-19.776
2.510.770	-34.858	2.549.832	-25.603	2.504.585	-19.774
2.510.976	-34.858	2.550.016	-25.602	2.504.769	-19.773
2.511.153	-34.858	2.550.240	-25.601	2.504.967	-19.772
2.511.310	-34.858	2.550.410	-25.600	2.505.167	-19.770
2.511.494	-34.858	2.550.580	-25.600	2.505.382	-19.769
2.511.666	-34.858	2.550.708	-25.599	2.505.602	-19.767
2.511.780	-34.857	2.550.798	-25.598	2.505.703	-19.766
2.511.877	-34.857	2.550.985	-25.597	2.505.763	-19.765
2.512.083	-34.857	2.551.223	-25.596	2.505.892	-19.763
2.512.274	-34.857	2.551.367	-25.596	2.506.111	-19.762
2.512.499	-34.857	2.551.497	-25.595	2.506.311	-19.761
2.512.679	-34.857	2.551.703	-25.594	2.506.464	-19.759
2.512.786	-34.857	2.551.853	-25.593	2.506.582	-19.758
2.512.917	-34.857	2.551.996	-25.592	2.506.680	-19.757
2.513.121	-34.857	2.552.202	-25.592	2.506.808	-19.755

2.513.266	-34.857	2.552.364	-25.591	2.507.016	-19.754
2.513.410	-34.857	2.552.503	-25.590	2.507.269	-19.753
2.513.691	-34.857	2.552.661	-25.590	2.507.453	-19.752
2.513.788	-34.857	2.552.809	-25.589	2.507.590	-19.750
2.513.849	-34.857	2.552.942	-25.588	2.507.701	-19.749
2.514.109	-34.858	2.553.052	-25.588	2.507.859	-19.748
2.514.271	-34.858	2.553.163	-25.587	2.508.065	-19.747
2.514.352	-34.858	2.553.261	-25.586	2.508.277	-19.746
2.514.659	-34.858	2.553.405	-25.586	2.508.422	-19.745
2.514.890	-34.858	2.553.609	-25.585	2.508.544	-19.743
2.515.029	-34.858	2.553.803	-25.584	2.508.720	-19.742
2.515.172	-34.858	2.553.939	-25.584	2.508.859	-19.741
2.515.329	-34.859	2.554.150	-25.583	2.508.988	-19.740
2.515.546	-34.859	2.554.416	-25.583	2.509.141	-19.739
2.515.692	-34.859	2.554.644	-25.582	2.509.341	-19.738
2.515.770	-34.859	2.554.889	-25.582	2.509.494	-19.737
2.515.967	-34.860	2.555.128	-25.581	2.509.681	-19.736
2.516.223	-34.860	2.555.321	-25.580	2.509.888	-19.735
2.516.357	-34.860	2.555.421	-25.580	2.510.081	-19.734
2.516.470	-34.861	2.555.515	-25.579	2.510.288	-19.733
2.516.665	-34.861	2.555.692	-25.579	2.510.423	-19.732
2.516.945	-34.861	2.555.876	-25.578	2.510.499	-19.731
2.517.197	-34.862	2.556.076	-25.578	2.510.634	-19.730
2.517.350	-34.862	2.556.201	-25.577	2.510.827	-19.729
2.517.388	-34.862	2.556.313	-25.577	2.511.032	-19.728
2.517.490	-34.863	2.556.458	-25.576	2.511.216	-19.727
2.517.728	-34.863	2.556.574	-25.576	2.511.385	-19.726
2.517.905	-34.863	2.556.752	-25.575	2.511.572	-19.725
2.518.053	-34.864	2.556.954	-25.575	2.511.797	-19.725
2.518.189	-34.864	2.557.134	-25.574	2.511.987	-19.724

2.518.326	-34.865	2.557.290	-25.574	2.512.116	-19.723
2.518.443	-34.865	2.557.437	-25.574	2.512.275	-19.722
2.518.551	-34.866	2.557.617	-25.573	2.512.460	-19.721
2.518.707	-34.866	2.557.841	-25.573	2.512.632	-19.720
2.518.871	-34.867	2.558.004	-25.572	2.512.764	-19.719
2.519.059	-34.867	2.558.105	-25.572	2.512.925	-19.718
2.519.254	-34.868	2.558.322	-25.571	2.513.091	-19.717
2.519.406	-34.869	2.558.565	-25.571	2.513.183	-19.717
2.519.527	-34.869	2.558.746	-25.570	2.513.250	-19.716
2.519.708	-34.870	2.558.874	-25.570	2.513.421	-19.715
2.519.942	-34.870	2.559.020	-25.570	2.513.600	-19.714
2.520.132	-34.871	2.559.199	-25.569	2.513.799	-19.713
2.520.329	-34.872	2.559.313	-25.569	2.514.014	-19.712
2.520.531	-34.872	2.559.445	-25.568	2.514.153	-19.711
2.520.741	-34.873	2.559.640	-25.568	2.514.317	-19.710
2.520.958	-34.874	2.559.858	-25.568	2.514.487	-19.710
2.521.135	-34.874	2.560.067	-25.567	2.514.646	-19.709
2.521.183	-34.875	2.560.233	-25.567	2.514.852	-19.708
2.521.293	-34.876	2.560.363	-25.566	2.515.058	-19.707
2.521.487	-34.876	2.560.498	-25.566	2.515.282	-19.706
2.521.657	-34.877	2.560.685	-25.566	2.515.441	-19.705
2.521.844	-34.878	2.560.876	-25.565	2.515.589	-19.704
2.521.944	-34.879	2.561.058	-25.565	2.515.697	-19.703
2.521.983	-34.879	2.561.248	-25.564	2.515.818	-19.702
2.522.087	-34.880	2.561.412	-25.564	2.515.934	-19.701
2.522.361	-34.881	2.561.549	-25.564	2.516.063	-19.700
2.522.576	-34.882	2.561.690	-25.563	2.516.227	-19.700
2.522.682	-34.882	2.561.876	-25.563	2.516.432	-19.699
2.522.824	-34.883	2.562.022	-25.562	2.516.615	-19.698
2.523.035	-34.884	2.562.135	-25.562	2.516.752	-19.697

2.523.281	-34.885	2.562.311	-25.562	2.516.977	-19.696
2.523.461	-34.885	2.562.484	-25.561	2.517.215	-19.695
2.523.620	-34.886	2.562.637	-25.561	2.517.314	-19.694
2.523.830	-34.887	2.562.879	-25.560	2.517.458	-19.693
2.523.980	-34.888	2.563.141	-25.560	2.517.708	-19.692
2.524.146	-34.889	2.563.264	-25.560	2.517.892	-19.691
2.524.395	-34.890	2.563.413	-25.559	2.518.057	-19.690
2.524.504	-34.890	2.563.506	-25.559	2.518.187	-19.689
2.524.642	-34.891	2.563.643	-25.558	2.518.342	-19.688
2.524.847	-34.892	2.563.875	-25.558	2.518.584	-19.687
2.525.013	-34.893	2.564.301	-25.557	2.518.732	-19.686
2.525.223	-34.894	2.564.663	-25.557	2.518.783	-19.685
2.525.349	-34.895	2.564.789	-25.556	2.518.976	-19.683
2.525.414	-34.896	2.564.826	-25.556	2.519.211	-19.682
2.525.625	-34.897	2.565.009	-25.556	2.519.391	-19.681
2.525.869	-34.897	2.565.189	-25.555	2.519.613	-19.680
2.526.039	-34.898	2.565.184	-25.555	2.519.818	-19.679
2.526.211	-34.899	2.565.182	-25.554	2.519.975	-19.678
2.526.355	-34.900	2.565.309	-25.554	2.520.126	-19.677
2.526.526	-34.901	2.565.537	-25.553	2.520.286	-19.676
2.526.650	-34.902	2.565.703	-25.553	2.520.453	-19.675
2.526.763	-34.903	2.565.832	-25.552	2.520.636	-19.674
2.527.083	-34.904	2.565.996	-25.551	2.520.779	-19.672
2.527.318	-34.905	2.566.147	-25.551	2.520.907	-19.671
2.527.393	-34.905	2.566.349	-25.550	2.521.066	-19.670
2.527.545	-34.906	2.566.587	-25.550	2.521.234	-19.669
2.527.747	-34.907	2.566.725	-25.549	2.521.382	-19.668
2.527.936	-34.908	2.566.863	-25.549	2.521.529	-19.667
2.528.094	-34.909	2.567.033	-25.548	2.521.749	-19.665
2.528.205	-34.910	2.567.195	-25.547	2.521.962	-19.664

2.528.371	-34.911	2.567.355	-25.547	2.522.119	-19.663
2.528.539	-34.912	2.567.498	-25.546	2.522.303	-19.662
2.528.723	-34.913	2.567.646	-25.546	2.522.466	-19.661
2.528.896	-34.914	2.567.806	-25.545	2.522.580	-19.660
2.529.081	-34.914	2.567.904	-25.544	2.522.731	-19.658
2.529.276	-34.915	2.568.083	-25.544	2.522.888	-19.657
2.529.357	-34.916	2.568.294	-25.543	2.523.089	-19.656
2.529.455	-34.917	2.568.415	-25.542	2.523.235	-19.655
2.529.668	-34.918	2.568.531	-25.542	2.523.496	-19.654
2.530.070	-34.919	2.568.712	-25.541	2.523.953	-19.652
2.530.526	-34.920	2.568.952	-25.541	2.524.283	-19.651
2.530.643	-34.921	2.569.106	-25.540	2.524.426	-19.650
2.530.762	-34.921	2.569.236	-25.539	2.524.482	-19.649
2.530.990	-34.922	2.569.471	-25.539	2.524.556	-19.648
2.531.044	-34.923	2.569.731	-25.538	2.524.615	-19.646
2.531.031	-34.924	2.569.897	-25.537	2.524.651	-19.645
2.531.057	-34.925	2.570.067	-25.537	2.524.693	-19.644
2.531.202	-34.926	2.570.245	-25.536	2.524.816	-19.643
2.531.344	-34.927	2.570.450	-25.535	2.525.058	-19.642
2.531.497	-34.927	2.570.605	-25.535	2.525.226	-19.640
2.531.665	-34.928	2.570.703	-25.534	2.525.365	-19.639
2.531.817	-34.929	2.570.796	-25.533	2.525.544	-19.638
2.532.010	-34.930	2.570.959	-25.532	2.525.715	-19.637
2.532.175	-34.931	2.571.140	-25.532	2.525.891	-19.636
2.532.329	-34.931	2.571.255	-25.531	2.526.052	-19.635
2.532.533	-34.932	2.571.465	-25.530	2.526.205	-19.633
2.532.668	-34.933	2.571.656	-25.530	2.526.402	-19.632
2.532.804	-34.934	2.571.797	-25.529	2.526.615	-19.631
2.533.013	-34.934	2.572.023	-25.528	2.526.801	-19.630
2.533.178	-34.935	2.572.209	-25.527	2.526.980	-19.629

2.533.283	-34.936	2.572.354	-25.527	2.527.113	-19.628
2.533.515	-34.937	2.572.534	-25.526	2.527.215	-19.627
2.533.772	-34.938	2.572.701	-25.525	2.527.367	-19.626
2.533.855	-34.938	2.572.859	-25.525	2.527.538	-19.625
2.533.949	-34.939	2.573.022	-25.524	2.527.699	-19.624
2.534.054	-34.940	2.573.195	-25.523	2.527.825	-19.623
2.534.204	-34.941	2.573.336	-25.522	2.527.986	-19.622
2.534.444	-34.942	2.573.488	-25.522	2.528.156	-19.621
2.534.617	-34.942	2.573.709	-25.521	2.528.326	-19.620
2.534.745	-34.943	2.573.963	-25.520	2.528.571	-19.619
2.534.942	-34.944	2.574.171	-25.520	2.528.750	-19.618
2.535.191	-34.945	2.574.283	-25.519	2.528.925	-19.617
2.535.350	-34.946	2.574.442	-25.518	2.529.132	-19.617
2.535.491	-34.947	2.574.622	-25.517	2.529.339	-19.616
2.535.681	-34.947	2.574.734	-25.517	2.529.503	-19.615
2.535.876	-34.948	2.574.815	-25.516	2.529.661	-19.614
2.536.107	-34.949	2.574.919	-25.515	2.529.772	-19.614
2.536.273	-34.950	2.575.169	-25.514	2.529.925	-19.613
2.536.353	-34.951	2.575.435	-25.514	2.530.040	-19.612
2.536.468	-34.952	2.575.508	-25.513	2.530.188	-19.612
2.536.599	-34.952	2.575.536	-25.512	2.530.408	-19.611
2.536.729	-34.953	2.575.713	-25.511	2.530.522	-19.611
2.536.911	-34.954	2.575.944	-25.511	2.530.665	-19.610
2.537.113	-34.955	2.576.219	-25.510	2.530.824	-19.610
2.537.231	-34.956	2.576.408	-25.509	2.530.981	-19.609
2.537.341	-34.957	2.576.488	-25.508	2.531.185	-19.609
2.537.556	-34.958	2.576.614	-25.508	2.531.395	-19.608
2.537.790	-34.959	2.576.775	-25.507	2.531.574	-19.608
2.537.946	-34.959	2.576.976	-25.506	2.531.756	-19.608
2.538.047	-34.960	2.577.155	-25.505	2.531.845	-19.607

2.538.228	-34.961	2.577.292	-25.504	2.531.949	-19.607
2.538.468	-34.962	2.577.458	-25.504	2.532.181	-19.607
2.538.651	-34.963	2.577.626	-25.503	2.532.356	-19.607
2.538.835	-34.964	2.577.755	-25.502	2.532.443	-19.606
2.539.052	-34.965	2.577.874	-25.501	2.532.634	-19.606
2.539.236	-34.966	2.578.022	-25.500	2.532.924	-19.606
2.539.298	-34.967	2.578.186	-25.500	2.533.093	-19.606
2.539.355	-34.968	2.578.391	-25.499	2.533.197	-19.606
2.539.605	-34.969	2.578.564	-25.498	2.533.319	-19.606
2.539.814	-34.970	2.578.647	-25.497	2.533.458	-19.606
2.539.902	-34.971	2.578.853	-25.496	2.533.638	-19.606
2.540.130	-34.972	2.579.150	-25.496	2.533.830	-19.607
2.540.319	-34.974	2.579.382	-25.495	2.533.999	-19.607
2.540.493	-34.975	2.579.559	-25.494	2.534.126	-19.607
2.540.666	-34.976	2.579.779	-25.493	2.534.259	-19.607
2.540.757	-34.977	2.580.012	-25.492	2.534.412	-19.607
2.540.912	-34.978	2.580.148	-25.492	2.534.641	-19.608
2.541.170	-34.979	2.580.254	-25.491	2.534.859	-19.608
2.541.361	-34.980	2.580.368	-25.490	2.534.999	-19.609
2.541.528	-34.981	2.580.544	-25.489	2.535.262	-19.609
2.541.657	-34.983	2.580.718	-25.488	2.535.424	-19.609
2.541.747	-34.984	2.580.848	-25.487	2.535.522	-19.610
2.541.911	-34.985	2.580.966	-25.487	2.535.670	-19.610
2.542.126	-34.986	2.581.111	-25.486	2.535.885	-19.611
2.542.344	-34.987	2.581.299	-25.485	2.536.054	-19.611
2.542.518	-34.989	2.581.510	-25.484	2.536.151	-19.612
2.542.643	-34.990	2.581.703	-25.483	2.536.237	-19.613
2.542.777	-34.991	2.581.895	-25.482	2.536.338	-19.613
2.542.957	-34.992	2.582.065	-25.482	2.536.544	-19.614
2.543.146	-34.993	2.582.207	-25.481	2.536.722	-19.615

2.543.358	-34.995	2.582.442	-25.480	2.536.876	-19.615
2.543.532	-34.996	2.582.578	-25.479	2.536.978	-19.616
2.543.647	-34.997	2.582.673	-25.478	2.537.127	-19.617
2.543.738	-34.998	2.582.856	-25.478	2.537.398	-19.618
2.543.846	-34.999	2.583.063	-25.477	2.537.596	-19.619
2.543.919	-35.001	2.583.236	-25.476	2.537.766	-19.619
2.544.051	-35.002	2.583.414	-25.475	2.537.968	-19.620
2.544.308	-35.003	2.583.598	-25.474	2.538.156	-19.621
2.544.532	-35.004	2.583.730	-25.474	2.538.377	-19.622
2.544.688	-35.006	2.583.860	-25.473	2.538.571	-19.623
2.544.850	-35.007	2.584.064	-25.472	2.538.728	-19.624
2.545.031	-35.008	2.584.225	-25.471	2.538.933	-19.625
2.545.186	-35.010	2.584.380	-25.471	2.539.143	-19.626
2.545.372	-35.011	2.584.558	-25.470	2.539.290	-19.627
2.545.598	-35.012	2.584.658	-25.469	2.539.366	-19.628
2.545.830	-35.013	2.584.786	-25.468	2.539.417	-19.629
2.546.010	-35.015	2.584.992	-25.468	2.539.521	-19.630
2.546.165	-35.016	2.585.193	-25.467	2.539.691	-19.631
2.546.422	-35.017	2.585.345	-25.466	2.539.868	-19.633
2.546.609	-35.018	2.585.515	-25.466	2.540.060	-19.634
2.546.710	-35.020	2.585.673	-25.465	2.540.233	-19.635
2.546.895	-35.021	2.585.775	-25.464	2.540.352	-19.636
2.547.036	-35.022	2.585.891	-25.464	2.540.563	-19.637
2.547.180	-35.023	2.586.078	-25.463	2.540.852	-19.638
2.547.354	-35.025	2.586.281	-25.462	2.541.074	-19.639
2.547.440	-35.026	2.586.516	-25.462	2.541.221	-19.641
2.547.527	-35.027	2.586.734	-25.461	2.541.346	-19.642
2.547.654	-35.028	2.586.895	-25.461	2.541.519	-19.643
2.547.874	-35.029	2.587.070	-25.460	2.541.696	-19.644
2.548.091	-35.031	2.587.245	-25.460	2.541.844	-19.645

2.548.251	-35.032	2.587.397	-25.459	2.542.011	-19.647
2.548.460	-35.033	2.587.592	-25.458	2.542.173	-19.648
2.548.661	-35.034	2.587.791	-25.458	2.542.275	-19.649
2.548.867	-35.035	2.587.931	-25.457	2.542.473	-19.650
2.549.082	-35.036	2.588.009	-25.457	2.542.726	-19.652
2.549.165	-35.038	2.588.141	-25.457	2.542.846	-19.653
2.549.369	-35.039	2.588.464	-25.456	2.543.000	-19.654
2.549.544	-35.040	2.588.887	-25.456	2.543.192	-19.655
2.549.617	-35.041	2.589.229	-25.455	2.543.390	-19.657
2.549.810	-35.042	2.589.384	-25.455	2.543.544	-19.658
2.549.955	-35.043	2.589.446	-25.455	2.543.721	-19.659
2.550.104	-35.044	2.589.514	-25.454	2.543.952	-19.661
2.550.330	-35.046	2.589.573	-25.454	2.544.095	-19.662
2.550.490	-35.047	2.589.577	-25.454	2.544.221	-19.663
2.550.652	-35.048	2.589.631	-25.454	2.544.322	-19.664
2.550.820	-35.049	2.589.739	-25.453	2.544.536	-19.666
2.550.967	-35.050	2.589.879	-25.453	2.544.739	-19.667
2.551.212	-35.051	2.590.057	-25.453	2.544.893	-19.668
2.551.404	-35.052	2.590.256	-25.453	2.545.038	-19.669
2.551.510	-35.053	2.590.410	-25.453	2.545.190	-19.671
2.551.681	-35.054	2.590.536	-25.452	2.545.354	-19.672
2.551.918	-35.055	2.590.718	-25.452	2.545.594	-19.673
2.552.072	-35.056	2.590.971	-25.452	2.545.805	-19.674
2.552.162	-35.057	2.591.165	-25.452	2.545.903	-19.676
2.552.318	-35.058	2.591.358	-25.452	2.546.006	-19.677
2.552.507	-35.059	2.591.473	-25.452	2.546.123	-19.678
2.552.701	-35.060	2.591.647	-25.452	2.546.260	-19.679
2.552.914	-35.061	2.591.777	-25.452	2.546.444	-19.680
2.553.038	-35.062	2.591.896	-25.452	2.546.640	-19.682
2.553.126	-35.063	2.592.047	-25.453	2.546.846	-19.683

2.553.271	-35.063	2.592.232	-25.453	2.547.220	-19.684
2.553.511	-35.064	2.592.476	-25.453	2.547.654	-19.685
2.553.752	-35.065	2.592.630	-25.453	2.547.870	-19.686
2.553.835	-35.066	2.592.758	-25.453	2.547.939	-19.687
2.553.914	-35.067	2.592.875	-25.454	2.548.036	-19.689
2.554.142	-35.068	2.592.961	-25.454	2.548.099	-19.690
2.554.414	-35.068	2.593.131	-25.454	2.548.113	-19.691
2.554.576	-35.069	2.593.352	-25.455	2.548.226	-19.692
2.554.723	-35.070	2.593.567	-25.455	2.548.358	-19.693
2.554.826	-35.071	2.593.782	-25.455	2.548.500	-19.694
2.554.998	-35.072	2.593.922	-25.456	2.548.683	-19.695
2.555.385	-35.072	2.594.084	-25.456	2.548.864	-19.696
2.555.833	-35.073	2.594.283	-25.457	2.549.013	-19.697
2.556.083	-35.074	2.594.486	-25.457	2.549.222	-19.699
2.556.096	-35.075	2.594.604	-25.458	2.549.424	-19.700
2.556.170	-35.075	2.594.724	-25.459	2.549.504	-19.701
2.556.288	-35.076	2.594.898	-25.459	2.549.641	-19.702
2.556.403	-35.077	2.595.103	-25.460	2.549.804	-19.703
2.556.582	-35.077	2.595.263	-25.461	2.549.974	-19.704
2.556.736	-35.078	2.595.443	-25.461	2.550.137	-19.705
2.556.748	-35.079	2.595.534	-25.462	2.550.305	-19.706
2.556.768	-35.079	2.595.668	-25.463	2.550.533	-19.707
2.556.911	-35.080	2.595.851	-25.464	2.550.758	-19.708
2.557.153	-35.080	2.596.079	-25.465	2.550.895	-19.709
2.557.387	-35.081	2.596.241	-25.465	2.551.033	-19.710
2.557.528	-35.082	2.596.340	-25.466	2.551.170	-19.711
2.557.624	-35.082	2.596.476	-25.467	2.551.277	-19.712
2.557.813	-35.083	2.596.715	-25.468	2.551.430	-19.713
2.558.074	-35.083	2.596.938	-25.469	2.551.631	-19.714
2.558.251	-35.084	2.597.108	-25.470	2.551.838	-19.715

2.558.383	-35.084	2.597.217	-25.471	2.552.065	-19.716
2.558.515	-35.085	2.597.351	-25.472	2.552.261	-19.716
2.558.611	-35.085	2.597.478	-25.473	2.552.411	-19.717
2.558.770	-35.086	2.597.669	-25.475	2.552.626	-19.718
2.559.064	-35.086	2.597.824	-25.476	2.552.754	-19.719
2.559.261	-35.087	2.597.965	-25.477	2.552.960	-19.720
2.559.320	-35.087	2.598.170	-25.478	2.553.034	-19.721
2.559.445	-35.088	2.598.394	-25.479	2.553.167	-19.722
2.559.634	-35.088	2.598.596	-25.481	2.553.376	-19.723
2.559.817	-35.089	2.598.795	-25.482	2.553.517	-19.724
2.559.929	-35.089	2.598.896	-25.483	2.553.632	-19.725
2.560.042	-35.089	2.599.017	-25.484	2.553.752	-19.726
2.560.251	-35.090	2.599.167	-25.486	2.553.907	-19.727
2.560.508	-35.090	2.599.326	-25.487	2.554.140	-19.728
2.560.721	-35.091	2.599.500	-25.488	2.554.362	-19.729
2.560.891	-35.091	2.599.652	-25.490	2.554.488	-19.730
2.561.064	-35.092	2.599.818	-25.491	2.554.615	-19.731
2.561.234	-35.092	2.600.016	-25.493	2.554.812	-19.732
2.561.441	-35.093	2.600.137	-25.494	2.555.009	-19.732
2.561.616	-35.093	2.600.323	-25.495	2.555.164	-19.733
2.561.776	-35.094	2.600.533	-25.497	2.555.302	-19.734
2.561.925	-35.094	2.600.681	-25.498	2.555.443	-19.735
2.562.061	-35.094	2.600.793	-25.500	2.555.577	-19.736
2.562.196	-35.095	2.600.971	-25.501	2.555.690	-19.737
2.562.278	-35.095	2.601.132	-25.503	2.555.836	-19.738
2.562.466	-35.096	2.601.289	-25.504	2.556.065	-19.739
2.562.680	-35.096	2.601.457	-25.506	2.556.284	-19.740
2.562.885	-35.097	2.601.606	-25.507	2.556.468	-19.741
2.563.052	-35.097	2.601.726	-25.509	2.556.628	-19.742
2.563.175	-35.097	2.601.841	-25.510	2.556.812	-19.743

2.563.316	-35.098	2.602.035	-25.512	2.557.047	-19.744
2.563.535	-35.098	2.602.247	-25.513	2.557.235	-19.745
2.563.746	-35.099	2.602.411	-25.515	2.557.426	-19.746
2.563.943	-35.099	2.602.552	-25.516	2.557.545	-19.747
2.564.106	-35.100	2.602.675	-25.517	2.557.579	-19.748
2.564.174	-35.100	2.602.827	-25.519	2.557.701	-19.749
2.564.294	-35.101	2.603.078	-25.520	2.557.911	-19.750
2.564.456	-35.101	2.603.319	-25.522	2.558.117	-19.751
2.564.619	-35.102	2.603.528	-25.523	2.558.343	-19.752
2.564.763	-35.102	2.603.706	-25.525	2.558.560	-19.753
2.564.978	-35.103	2.603.905	-25.526	2.558.665	-19.754
2.565.226	-35.103	2.604.136	-25.527	2.558.796	-19.755
2.565.417	-35.104	2.604.334	-25.529	2.559.063	-19.756
2.565.522	-35.104	2.604.528	-25.530	2.559.254	-19.757
2.565.667	-35.105	2.604.698	-25.531	2.559.311	-19.758
2.565.883	-35.105	2.604.828	-25.533	2.559.486	-19.759
2.566.057	-35.106	2.604.977	-25.534	2.559.707	-19.760
2.566.170	-35.106	2.605.099	-25.535	2.559.904	-19.761
2.566.289	-35.107	2.605.154	-25.537	2.560.075	-19.762
2.566.473	-35.107	2.605.291	-25.538	2.560.147	-19.763
2.566.640	-35.108	2.605.458	-25.539	2.560.236	-19.764
2.566.787	-35.108	2.605.627	-25.540	2.560.364	-19.765
2.566.932	-35.109	2.605.804	-25.541	2.560.511	-19.767
2.567.086	-35.109	2.605.966	-25.542	2.560.773	-19.768
2.567.277	-35.110	2.606.154	-25.543	2.560.941	-19.769
2.567.514	-35.110	2.606.371	-25.545	2.560.977	-19.770
2.567.697	-35.111	2.606.545	-25.546	2.561.053	-19.771
2.567.838	-35.112	2.606.697	-25.547	2.561.299	-19.772
2.568.058	-35.112	2.606.940	-25.547	2.561.577	-19.773
2.568.260	-35.113	2.607.148	-25.548	2.561.783	-19.774

2.568.390	-35.113	2.607.294	-25.549	2.562.021	-19.775
2.568.471	-35.114	2.607.478	-25.550	2.562.263	-19.776
2.568.633	-35.114	2.607.657	-25.551	2.562.456	-19.777
2.568.857	-35.115	2.607.798	-25.552	2.562.532	-19.778
2.568.972	-35.115	2.607.960	-25.553	2.562.614	-19.779
2.569.031	-35.116	2.608.145	-25.553	2.562.773	-19.780
2.569.240	-35.117	2.608.260	-25.554	2.562.926	-19.781
2.569.409	-35.117	2.608.381	-25.555	2.563.080	-19.782
2.569.535	-35.118	2.608.594	-25.555	2.563.249	-19.783
2.569.707	-35.118	2.608.824	-25.556	2.563.379	-19.784
2.569.879	-35.119	2.608.973	-25.556	2.563.525	-19.785
2.570.027	-35.119	2.609.089	-25.557	2.563.676	-19.787
2.570.233	-35.120	2.609.305	-25.557	2.563.857	-19.788
2.570.447	-35.121	2.609.512	-25.558	2.564.052	-19.789
2.570.583	-35.121	2.609.633	-25.558	2.564.203	-19.790
2.570.728	-35.122	2.609.778	-25.559	2.564.373	-19.791
2.570.984	-35.122	2.609.969	-25.559	2.564.566	-19.792
2.571.245	-35.123	2.610.121	-25.559	2.564.796	-19.793
2.571.451	-35.123	2.610.327	-25.559	2.565.020	-19.794
2.571.628	-35.124	2.610.497	-25.560	2.565.182	-19.795
2.571.758	-35.125	2.610.610	-25.560	2.565.345	-19.796
2.571.913	-35.125	2.610.773	-25.560	2.565.472	-19.797
2.572.112	-35.126	2.610.930	-25.560	2.565.645	-19.798
2.572.265	-35.126	2.611.136	-25.560	2.565.801	-19.799
2.572.366	-35.127	2.611.317	-25.560	2.565.934	-19.800
2.572.462	-35.127	2.611.483	-25.560	2.566.158	-19.801
2.572.592	-35.128	2.611.703	-25.560	2.566.320	-19.802
2.572.814	-35.128	2.611.862	-25.560	2.566.441	-19.803
2.573.051	-35.129	2.612.010	-25.559	2.566.610	-19.804
2.573.159	-35.129	2.612.207	-25.559	2.566.841	-19.805

2.573.235	-35.130	2.612.379	-25.559	2.567.102	-19.806
2.573.430	-35.130	2.612.520	-25.559	2.567.282	-19.807
2.573.608	-35.131	2.612.704	-25.558	2.567.394	-19.808
2.573.752	-35.131	2.612.987	-25.558	2.567.561	-19.809
2.573.947	-35.132	2.613.365	-25.557	2.567.754	-19.810
2.574.167	-35.132	2.613.730	-25.557	2.567.924	-19.811
2.574.385	-35.133	2.613.896	-25.556	2.568.106	-19.813
2.574.593	-35.133	2.613.945	-25.556	2.568.273	-19.814
2.574.778	-35.134	2.614.034	-25.555	2.568.428	-19.815
2.574.913	-35.134	2.614.088	-25.555	2.568.567	-19.816
2.575.054	-35.135	2.614.081	-25.554	2.568.727	-19.817
2.575.241	-35.135	2.614.142	-25.553	2.568.894	-19.818
2.575.366	-35.136	2.614.250	-25.552	2.569.041	-19.819
2.575.471	-35.136	2.614.346	-25.552	2.569.225	-19.820
2.575.638	-35.137	2.614.507	-25.551	2.569.424	-19.821
2.575.779	-35.137	2.614.705	-25.550	2.569.606	-19.822
2.575.992	-35.137	2.614.927	-25.549	2.569.743	-19.823
2.576.242	-35.138	2.615.128	-25.548	2.569.904	-19.824
2.576.440	-35.138	2.615.323	-25.547	2.570.025	-19.825
2.576.559	-35.139	2.615.493	-25.546	2.570.136	-19.826
2.576.699	-35.139	2.615.664	-25.545	2.570.287	-19.827
2.576.863	-35.140	2.615.865	-25.544	2.570.631	-19.828
2.577.069	-35.140	2.616.038	-25.543	2.571.071	-19.829
2.577.278	-35.141	2.616.249	-25.542	2.571.320	-19.830
2.577.401	-35.141	2.616.349	-25.541	2.571.450	-19.831
2.577.556	-35.141	2.616.461	-25.539	2.571.552	-19.832
2.577.680	-35.142	2.616.617	-25.538	2.571.632	-19.833
2.577.816	-35.142	2.616.822	-25.537	2.571.678	-19.834
2.578.033	-35.143	2.616.929	-25.536	2.571.746	-19.835
2.578.174	-35.143	2.617.066	-25.534	2.571.831	-19.836

2.578.343	-35.144	2.617.260	-25.533	2.571.909	-19.837
2.578.594	-35.144	2.617.447	-25.532	2.572.021	-19.838
2.578.760	-35.144	2.617.624	-25.530	2.572.234	-19.839
2.578.818	-35.145	2.617.729	-25.529	2.572.430	-19.841
2.578.954	-35.145	2.617.870	-25.528	2.572.632	-19.842
2.579.146	-35.146	2.618.098	-25.526	2.572.798	-19.843
2.579.342	-35.146	2.618.282	-25.525	2.572.982	-19.844
2.579.561	-35.147	2.618.435	-25.523	2.573.184	-19.845
2.579.731	-35.147	2.618.618	-25.522	2.573.391	-19.846
2.579.908	-35.147	2.618.828	-25.520	2.573.582	-19.847
2.580.083	-35.148	2.619.023	-25.519	2.573.691	-19.848
2.580.200	-35.148	2.619.276	-25.517	2.573.875	-19.849
2.580.375	-35.149	2.619.398	-25.516	2.574.008	-19.850
2.580.685	-35.149	2.619.474	-25.514	2.574.167	-19.852
2.581.065	-35.150	2.619.591	-25.512	2.574.342	-19.853
2.581.405	-35.150	2.619.734	-25.511	2.574.431	-19.854
2.581.537	-35.151	2.619.936	-25.509	2.574.595	-19.855
2.581.598	-35.151	2.620.113	-25.508	2.574.818	-19.856
2.581.638	-35.152	2.620.269	-25.506	2.574.971	-19.858
2.581.602	-35.152	2.620.497	-25.504	2.575.107	-19.859
2.581.656	-35.153	2.620.724	-25.503	2.575.284	-19.860
2.581.823	-35.153	2.620.892	-25.501	2.575.511	-19.861
2.581.947	-35.154	2.620.984	-25.499	2.575.769	-19.863
2.582.062	-35.154	2.621.075	-25.498	2.575.970	-19.864
2.582.274	-35.155	2.621.199	-25.496	2.576.122	-19.865
2.582.465	-35.155	2.621.376	-25.494	2.576.266	-19.866
2.582.567	-35.156	2.621.613	-25.493	2.576.357	-19.868
2.582.776	-35.156	2.621.779	-25.491	2.576.527	-19.869
2.582.986	-35.157	2.621.925	-25.489	2.576.740	-19.871
2.583.139	-35.157	2.622.105	-25.488	2.576.837	-19.872

2.583.318	-35.158	2.622.293	-25.486	2.577.021	-19.873
2.583.434	-35.158	2.622.460	-25.485	2.577.213	-19.875
2.583.629	-35.159	2.622.603	-25.483	2.577.364	-19.876
2.583.867	-35.159	2.622.787	-25.481	2.577.530	-19.878
2.583.973	-35.160	2.622.935	-25.480	2.577.680	-19.879
2.584.031	-35.161	2.623.150	-25.478	2.577.809	-19.881
2.584.264	-35.161	2.623.380	-25.477	2.577.906	-19.882
2.584.568	-35.162	2.623.543	-25.475	2.578.083	-19.884
2.584.700	-35.163	2.623.679	-25.473	2.578.341	-19.886
2.584.770	-35.163	2.623.839	-25.472	2.578.517	-19.887
2.584.859	-35.164	2.624.001	-25.470	2.578.665	-19.889
2.584.996	-35.165	2.624.167	-25.469	2.578.831	-19.891
2.585.168	-35.166	2.624.322	-25.467	2.578.951	-19.892
2.585.325	-35.166	2.624.447	-25.465	2.579.118	-19.894
2.585.479	-35.167	2.624.609	-25.464	2.579.298	-19.896
2.585.681	-35.168	2.624.746	-25.462	2.579.395	-19.898
2.585.931	-35.169	2.624.940	-25.461	2.579.510	-19.899
2.586.180	-35.170	2.625.193	-25.459	2.579.763	-19.901
2.586.382	-35.171	2.625.417	-25.458	2.579.958	-19.903
2.586.535	-35.172	2.625.529	-25.456	2.580.124	-19.905
2.586.703	-35.172	2.625.612	-25.455	2.580.302	-19.907
2.586.867	-35.173	2.625.764	-25.453	2.580.406	-19.909
2.587.035	-35.174	2.625.928	-25.452	2.580.551	-19.911
2.587.195	-35.175	2.626.080	-25.450	2.580.757	-19.913
2.587.307	-35.176	2.626.198	-25.449	2.580.917	-19.915
2.587.467	-35.177	2.626.369	-25.447	2.581.107	-19.917
2.587.690	-35.178	2.626.552	-25.446	2.581.245	-19.919
2.587.854	-35.179	2.626.692	-25.444	2.581.462	-19.922
2.588.014	-35.181	2.626.851	-25.443	2.581.642	-19.924
2.588.199	-35.182	2.627.072	-25.441	2.581.697	-19.926

2.588.326	-35.183	2.627.267	-25.440	2.581.884	-19.928
2.588.457	-35.184	2.627.373	-25.439	2.582.123	-19.930
2.588.633	-35.185	2.627.477	-25.437	2.582.234	-19.933
2.588.811	-35.186	2.627.661	-25.436	2.582.393	-19.935
2.588.986	-35.188	2.627.944	-25.434	2.582.618	-19.937
2.589.080	-35.189	2.628.211	-25.433	2.582.789	-19.940
2.589.234	-35.190	2.628.410	-25.432	2.582.962	-19.942
2.589.431	-35.192	2.628.636	-25.430	2.583.049	-19.945
2.589.551	-35.193	2.628.818	-25.429	2.583.157	-19.947
2.589.653	-35.194	2.628.947	-25.428	2.583.366	-19.950
2.589.789	-35.196	2.629.084	-25.426	2.583.513	-19.952
2.590.035	-35.197	2.629.175	-25.425	2.583.641	-19.955
2.590.273	-35.199	2.629.312	-25.424	2.583.792	-19.957
2.590.439	-35.200	2.629.499	-25.422	2.583.976	-19.960
2.590.554	-35.202	2.629.681	-25.421	2.584.142	-19.962
2.590.742	-35.204	2.629.810	-25.420	2.584.230	-19.965
2.590.956	-35.205	2.629.877	-25.418	2.584.360	-19.968
2.591.123	-35.207	2.630.032	-25.417	2.584.583	-19.970
2.591.324	-35.208	2.630.234	-25.416	2.584.887	-19.973
2.591.486	-35.210	2.630.424	-25.415	2.585.146	-19.976
2.591.638	-35.212	2.630.526	-25.413	2.585.303	-19.979
2.591.811	-35.213	2.630.650	-25.412	2.585.453	-19.981
2.591.954	-35.215	2.630.905	-25.411	2.585.642	-19.984
2.592.095	-35.217	2.631.151	-25.409	2.585.815	-19.987
2.592.265	-35.219	2.631.346	-25.408	2.585.928	-19.990
2.592.375	-35.220	2.631.487	-25.407	2.586.107	-19.993
2.592.514	-35.222	2.631.631	-25.406	2.586.252	-19.996
2.592.784	-35.224	2.631.819	-25.405	2.586.332	-19.999
2.593.000	-35.226	2.632.028	-25.403	2.586.458	-20.002
2.593.061	-35.228	2.632.176	-25.402	2.586.602	-20.005

2.593.193	-35.229	2.632.297	-25.401	2.586.797	-20.008
2.593.495	-35.231	2.632.509	-25.400	2.587.011	-20.011
2.593.662	-35.233	2.632.740	-25.399	2.587.137	-20.014
2.593.778	-35.235	2.632.875	-25.397	2.587.227	-20.017
2.593.965	-35.237	2.633.000	-25.396	2.587.408	-20.020
2.594.109	-35.239	2.633.186	-25.395	2.587.646	-20.023
2.594.243	-35.241	2.633.402	-25.394	2.587.842	-20.026
2.594.341	-35.243	2.633.553	-25.393	2.588.002	-20.030
2.594.508	-35.245	2.633.686	-25.392	2.588.217	-20.033
2.594.739	-35.247	2.633.896	-25.391	2.588.380	-20.036
2.594.861	-35.249	2.634.055	-25.389	2.588.554	-20.039
2.594.977	-35.251	2.634.156	-25.388	2.588.741	-20.042
2.595.140	-35.253	2.634.378	-25.387	2.588.886	-20.046
2.595.386	-35.255	2.634.630	-25.386	2.589.019	-20.049
2.595.602	-35.257	2.634.767	-25.385	2.589.147	-20.052
2.595.642	-35.259	2.634.922	-25.384	2.589.305	-20.056
2.595.797	-35.261	2.635.135	-25.383	2.589.537	-20.059
2.596.104	-35.263	2.635.247	-25.382	2.589.702	-20.062
2.596.407	-35.265	2.635.378	-25.381	2.589.839	-20.066
2.596.624	-35.267	2.635.587	-25.380	2.590.060	-20.069
2.596.740	-35.269	2.635.764	-25.379	2.590.269	-20.073
2.596.874	-35.271	2.635.897	-25.378	2.590.374	-20.076
2.597.021	-35.273	2.636.057	-25.377	2.590.555	-20.079
2.597.135	-35.275	2.636.278	-25.375	2.590.782	-20.083
2.597.332	-35.277	2.636.408	-25.374	2.590.927	-20.086
2.597.467	-35.280	2.636.515	-25.373	2.591.086	-20.090
2.597.520	-35.282	2.636.728	-25.372	2.591.212	-20.093
2.597.672	-35.284	2.636.896	-25.371	2.591.367	-20.097
2.597.879	-35.286	2.636.974	-25.370	2.591.538	-20.100
2.597.980	-35.288	2.637.205	-25.369	2.591.750	-20.104

2.598.087	-35.290	2.637.677	-25.368	2.591.899	-20.108
2.598.318	-35.292	2.638.072	-25.367	2.591.978	-20.111
2.598.546	-35.294	2.638.225	-25.366	2.592.160	-20.115
2.598.755	-35.296	2.638.287	-25.365	2.592.384	-20.118
2.598.972	-35.297	2.638.348	-25.364	2.592.563	-20.122
2.599.100	-35.299	2.638.401	-25.363	2.592.700	-20.126
2.599.161	-35.301	2.638.431	-25.362	2.592.903	-20.129
2.599.374	-35.303	2.638.509	-25.361	2.593.137	-20.133
2.599.706	-35.305	2.638.605	-25.361	2.593.294	-20.136
2.599.942	-35.307	2.638.731	-25.360	2.593.412	-20.140
2.600.027	-35.309	2.638.992	-25.359	2.593.522	-20.144
2.600.092	-35.311	2.639.225	-25.358	2.593.626	-20.147
2.600.212	-35.313	2.639.381	-25.357	2.593.923	-20.151
2.600.421	-35.315	2.639.554	-25.356	2.594.378	-20.155
2.600.648	-35.317	2.639.691	-25.355	2.594.710	-20.158
2.600.847	-35.318	2.639.859	-25.354	2.594.824	-20.162
2.601.013	-35.320	2.640.013	-25.353	2.594.850	-20.166
2.601.024	-35.322	2.640.182	-25.352	2.594.937	-20.169
2.601.183	-35.324	2.640.396	-25.351	2.595.028	-20.173
2.601.473	-35.325	2.640.569	-25.350	2.595.005	-20.177
2.601.664	-35.327	2.640.757	-25.349	2.595.150	-20.181
2.601.812	-35.329	2.640.934	-25.349	2.595.327	-20.184
2.601.985	-35.330	2.641.065	-25.348	2.595.410	-20.188
2.602.188	-35.332	2.641.180	-25.347	2.595.567	-20.192
2.602.333	-35.334	2.641.293	-25.346	2.595.754	-20.195
2.602.491	-35.335	2.641.415	-25.345	2.595.910	-20.199
2.602.682	-35.337	2.641.606	-25.344	2.596.096	-20.203
2.602.795	-35.338	2.641.826	-25.343	2.596.317	-20.207
2.602.913	-35.340	2.641.905	-25.343	2.596.488	-20.210
2.603.156	-35.341	2.642.101	-25.342	2.596.604	-20.214

2.603.434	-35.343	2.642.361	-25.341	2.596.812	-20.218
2.603.586	-35.344	2.642.507	-25.340	2.597.054	-20.222
2.603.742	-35.346	2.642.722	-25.340	2.597.166	-20.225
2.603.954	-35.347	2.642.958	-25.339	2.597.303	-20.229
2.604.039	-35.348	2.643.113	-25.338	2.597.451	-20.233
2.604.128	-35.350	2.643.315	-25.337	2.597.654	-20.236
2.604.367	-35.351	2.643.496	-25.337	2.597.835	-20.240
2.604.622	-35.352	2.643.600	-25.336	2.597.993	-20.244
2.604.764	-35.354	2.643.773	-25.335	2.598.217	-20.248
2.604.832	-35.355	2.643.930	-25.335	2.598.384	-20.251
2.604.958	-35.356	2.644.084	-25.334	2.598.484	-20.255
2.605.189	-35.357	2.644.241	-25.333	2.598.591	-20.259
2.605.382	-35.358	2.644.355	-25.333	2.598.756	-20.262
2.605.509	-35.359	2.644.496	-25.332	2.598.961	-20.266
2.605.721	-35.360	2.644.670	-25.332	2.599.149	-20.270
2.606.109	-35.361	2.644.864	-25.331	2.599.363	-20.274
2.606.516	-35.362	2.645.065	-25.330	2.599.533	-20.277
2.606.665	-35.363	2.645.244	-25.330	2.599.706	-20.281
2.606.703	-35.364	2.645.359	-25.329	2.599.918	-20.285
2.606.734	-35.365	2.645.525	-25.329	2.600.110	-20.288
2.606.777	-35.366	2.645.675	-25.328	2.600.296	-20.292
2.606.878	-35.367	2.645.782	-25.328	2.600.429	-20.296
2.606.963	-35.368	2.645.952	-25.327	2.600.544	-20.299
2.607.068	-35.368	2.646.176	-25.327	2.600.696	-20.303
2.607.225	-35.369	2.646.346	-25.327	2.600.894	-20.307
2.607.371	-35.370	2.646.451	-25.326	2.601.079	-20.310
2.607.458	-35.371	2.646.616	-25.326	2.601.227	-20.314
2.607.625	-35.371	2.646.852	-25.325	2.601.310	-20.318
2.607.906	-35.372	2.647.065	-25.325	2.601.479	-20.321
2.608.146	-35.372	2.647.166	-25.325	2.601.678	-20.325

2.608.319	-35.373	2.647.339	-25.324	2.601.859	-20.329
2.608.453	-35.373	2.647.567	-25.324	2.602.044	-20.332
2.608.597	-35.374	2.647.697	-25.324	2.602.194	-20.336
2.608.908	-35.374	2.647.859	-25.324	2.602.411	-20.339
2.609.070	-35.375	2.648.051	-25.323	2.602.564	-20.343
2.609.183	-35.375	2.648.199	-25.323	2.602.655	-20.347
2.609.442	-35.376	2.648.376	-25.323	2.602.823	-20.350
2.609.519	-35.376	2.648.571	-25.323	2.603.025	-20.354
2.609.627	-35.376	2.648.739	-25.322	2.603.167	-20.357
2.609.868	-35.377	2.648.883	-25.322	2.603.356	-20.361
2.610.045	-35.377	2.649.026	-25.322	2.603.525	-20.364
2.610.157	-35.377	2.649.218	-25.322	2.603.683	-20.368
2.610.294	-35.378	2.649.367	-25.322	2.603.854	-20.371
2.610.500	-35.378	2.649.523	-25.322	2.604.001	-20.375
2.610.708	-35.378	2.649.663	-25.321	2.604.172	-20.378
2.610.818	-35.378	2.649.764	-25.321	2.604.366	-20.382
2.610.960	-35.379	2.649.995	-25.321	2.604.557	-20.385
2.611.209	-35.379	2.650.193	-25.321	2.604.688	-20.389
2.611.404	-35.379	2.650.367	-25.321	2.604.837	-20.392
2.611.541	-35.379	2.650.493	-25.321	2.605.046	-20.395
2.611.707	-35.379	2.650.583	-25.321	2.605.237	-20.399
2.611.900	-35.379	2.650.776	-25.321	2.605.384	-20.402
2.612.095	-35.379	2.650.935	-25.320	2.605.551	-20.405
2.612.203	-35.379	2.651.062	-25.320	2.605.779	-20.409
2.612.324	-35.380	2.651.172	-25.320	2.605.953	-20.412
2.612.548	-35.380	2.651.343	-25.320	2.606.064	-20.415
2.612.759	-35.380	2.651.581	-25.320	2.606.245	-20.419
2.612.859	-35.380	2.651.758	-25.320	2.606.429	-20.422
2.612.939	-35.380	2.651.928	-25.320	2.606.549	-20.425
2.613.141	-35.380	2.652.178	-25.320	2.606.703	-20.428

2.613.323	-35.380	2.652.424	-25.320	2.606.848	-20.431
2.613.439	-35.380	2.652.552	-25.320	2.606.999	-20.435
2.613.589	-35.380	2.652.725	-25.320	2.607.141	-20.438
2.613.710	-35.380	2.653.008	-25.320	2.607.236	-20.441
2.613.882	-35.380	2.653.188	-25.320	2.607.369	-20.444
2.614.153	-35.380	2.653.319	-25.320	2.607.593	-20.447
2.614.388	-35.380	2.653.470	-25.320	2.607.870	-20.450
2.614.507	-35.380	2.653.591	-25.320	2.608.065	-20.453
2.614.631	-35.379	2.653.713	-25.320	2.608.232	-20.456
2.614.804	-35.379	2.653.861	-25.320	2.608.417	-20.459
2.614.930	-35.379	2.654.023	-25.320	2.608.626	-20.462
2.615.119	-35.379	2.654.178	-25.320	2.608.831	-20.465
2.615.298	-35.379	2.654.349	-25.320	2.608.994	-20.468
2.615.437	-35.379	2.654.503	-25.320	2.609.171	-20.471
2.615.608	-35.379	2.654.637	-25.320	2.609.323	-20.473
2.615.761	-35.379	2.654.760	-25.320	2.609.460	-20.476
2.615.906	-35.379	2.654.901	-25.320	2.609.630	-20.479
2.616.104	-35.379	2.655.103	-25.320	2.609.779	-20.482
2.616.310	-35.379	2.655.346	-25.320	2.609.901	-20.484
2.616.422	-35.379	2.655.549	-25.320	2.610.041	-20.487
2.616.663	-35.379	2.655.735	-25.320	2.610.125	-20.490
2.616.967	-35.379	2.655.903	-25.320	2.610.237	-20.492
2.617.040	-35.379	2.656.072	-25.320	2.610.439	-20.495
2.617.093	-35.379	2.656.274	-25.320	2.610.594	-20.498
2.617.299	-35.379	2.656.425	-25.320	2.610.771	-20.500
2.617.422	-35.379	2.656.528	-25.320	2.610.985	-20.503
2.617.531	-35.379	2.656.656	-25.320	2.611.108	-20.505
2.617.774	-35.379	2.656.826	-25.320	2.611.307	-20.508
2.618.015	-35.379	2.657.023	-25.320	2.611.555	-20.510
2.618.170	-35.379	2.657.213	-25.320	2.611.751	-20.513

2.618.275	-35.379	2.657.434	-25.320	2.611.945	-20.515
2.618.491	-35.379	2.657.647	-25.320	2.612.090	-20.518
2.618.669	-35.379	2.657.817	-25.320	2.612.252	-20.520
2.618.807	-35.378	2.658.002	-25.320	2.612.430	-20.522
2.619.027	-35.378	2.658.196	-25.319	2.612.617	-20.525
2.619.237	-35.378	2.658.278	-25.319	2.612.744	-20.527
2.619.378	-35.378	2.658.397	-25.319	2.612.932	-20.529
2.619.470	-35.378	2.658.564	-25.319	2.613.195	-20.532
2.619.588	-35.378	2.658.734	-25.319	2.613.326	-20.534
2.619.714	-35.378	2.658.915	-25.319	2.613.354	-20.536
2.619.930	-35.378	2.659.077	-25.319	2.613.515	-20.538
2.620.067	-35.378	2.659.272	-25.319	2.613.825	-20.540
2.620.110	-35.378	2.659.427	-25.319	2.613.997	-20.543
2.620.274	-35.378	2.659.529	-25.319	2.614.045	-20.545
2.620.472	-35.378	2.659.688	-25.319	2.614.219	-20.547
2.620.679	-35.378	2.659.922	-25.318	2.614.421	-20.549
2.620.840	-35.378	2.660.168	-25.318	2.614.609	-20.551
2.621.025	-35.378	2.660.384	-25.318	2.614.821	-20.553
2.621.244	-35.378	2.660.509	-25.318	2.615.016	-20.555
2.621.389	-35.378	2.660.642	-25.318	2.615.180	-20.557
2.621.665	-35.378	2.660.779	-25.318	2.615.319	-20.559
2.621.917	-35.378	2.660.977	-25.317	2.615.508	-20.561
2.621.992	-35.378	2.661.194	-25.317	2.615.681	-20.563
2.622.142	-35.378	2.661.320	-25.317	2.615.811	-20.565
2.622.383	-35.378	2.661.437	-25.317	2.615.942	-20.567
2.622.488	-35.378	2.661.682	-25.317	2.616.138	-20.569
2.622.668	-35.379	2.662.094	-25.316	2.616.335	-20.571
2.622.925	-35.379	2.662.459	-25.316	2.616.459	-20.572
2.622.993	-35.379	2.662.565	-25.316	2.616.599	-20.574
2.623.135	-35.379	2.662.593	-25.316	2.616.798	-20.576

2.623.282	-35.379	2.662.729	-25.316	2.617.017	-20.578
2.623.419	-35.379	2.662.805	-25.315	2.617.129	-20.580
2.623.556	-35.379	2.662.888	-25.315	2.617.249	-20.581
2.623.698	-35.379	2.662.979	-25.315	2.617.675	-20.583
2.623.811	-35.379	2.663.095	-25.314	2.618.156	-20.585
2.623.882	-35.379	2.663.240	-25.314	2.618.361	-20.587
2.624.181	-35.379	2.663.362	-25.314	2.618.441	-20.588
2.624.478	-35.379	2.663.564	-25.314	2.618.456	-20.590
2.624.541	-35.379	2.663.792	-25.313	2.618.467	-20.592
2.624.662	-35.379	2.664.012	-25.313	2.618.560	-20.594
2.624.955	-35.379	2.664.193	-25.313	2.618.654	-20.595
2.625.182	-35.379	2.664.299	-25.312	2.618.741	-20.597
2.625.378	-35.379	2.664.457	-25.312	2.618.878	-20.599
2.625.569	-35.379	2.664.641	-25.312	2.619.008	-20.600
2.625.692	-35.379	2.664.818	-25.311	2.619.183	-20.602
2.625.805	-35.379	2.665.031	-25.311	2.619.395	-20.604
2.625.959	-35.379	2.665.146	-25.311	2.619.612	-20.605
2.626.132	-35.379	2.665.251	-25.310	2.619.841	-20.607
2.626.338	-35.379	2.665.449	-25.310	2.619.971	-20.608
2.626.539	-35.379	2.665.525	-25.310	2.620.135	-20.610
2.626.660	-35.379	2.665.685	-25.309	2.620.370	-20.612
2.626.826	-35.379	2.665.974	-25.309	2.620.509	-20.613
2.627.005	-35.380	2.666.132	-25.309	2.620.652	-20.615
2.627.156	-35.380	2.666.252	-25.308	2.620.833	-20.616
2.627.321	-35.380	2.666.418	-25.308	2.620.949	-20.618
2.627.451	-35.380	2.666.581	-25.308	2.621.137	-20.620
2.627.589	-35.380	2.666.734	-25.307	2.621.349	-20.621
2.627.856	-35.380	2.666.893	-25.307	2.621.510	-20.623
2.628.138	-35.380	2.667.091	-25.307	2.621.620	-20.624
2.628.251	-35.380	2.667.359	-25.306	2.621.719	-20.626

2.628.362	-35.380	2.667.570	-25.306	2.621.846	-20.628
2.628.511	-35.380	2.667.689	-25.306	2.621.978	-20.629
2.628.677	-35.380	2.667.875	-25.305	2.622.167	-20.631
2.628.781	-35.380	2.668.083	-25.305	2.622.390	-20.632
2.628.940	-35.380	2.668.228	-25.305	2.622.614	-20.634
2.629.099	-35.380	2.668.409	-25.304	2.622.796	-20.636
2.629.284	-35.380	2.668.531	-25.304	2.622.962	-20.637
2.629.514	-35.380	2.668.625	-25.304	2.623.193	-20.639
2.629.720	-35.380	2.668.777	-25.303	2.623.372	-20.641
2.629.875	-35.380	2.668.925	-25.303	2.623.532	-20.642
2.630.034	-35.380	2.669.153	-25.303	2.623.698	-20.644
2.630.191	-35.381	2.669.348	-25.302	2.623.856	-20.645
2.630.382	-35.381	2.669.485	-25.302	2.624.022	-20.647
2.630.511	-35.381	2.669.633	-25.302	2.624.124	-20.649
2.630.598	-35.381	2.669.786	-25.302	2.624.230	-20.650
2.630.808	-35.381	2.669.879	-25.301	2.624.438	-20.652
2.631.217	-35.381	2.670.031	-25.301	2.624.660	-20.654
2.631.739	-35.381	2.670.234	-25.301	2.624.848	-20.656
2.632.007	-35.381	2.670.335	-25.300	2.624.988	-20.657
2.632.036	-35.381	2.670.497	-25.300	2.625.082	-20.659
2.632.039	-35.382	2.670.710	-25.300	2.625.236	-20.661
2.632.149	-35.382	2.670.883	-25.300	2.625.396	-20.663
2.632.214	-35.382	2.671.060	-25.299	2.625.556	-20.664
2.632.216	-35.382	2.671.256	-25.299	2.625.750	-20.666
2.632.429	-35.382	2.671.436	-25.299	2.625.941	-20.668
2.632.561	-35.382	2.671.657	-25.299	2.626.122	-20.670
2.632.517	-35.383	2.671.820	-25.299	2.626.231	-20.672
2.632.633	-35.383	2.671.986	-25.298	2.626.374	-20.673
2.632.823	-35.383	2.672.166	-25.298	2.626.596	-20.675
2.632.953	-35.383	2.672.318	-25.298	2.626.783	-20.677

2.633.280	-35.383	2.672.483	-25.298	2.626.978	-20.679
2.633.590	-35.384	2.672.625	-25.298	2.627.178	-20.681
2.633.672	-35.384	2.672.789	-25.297	2.627.328	-20.683
2.633.836	-35.384	2.672.926	-25.297	2.627.492	-20.685
2.634.010	-35.385	2.673.045	-25.297	2.627.676	-20.687
2.634.301	-35.385	2.673.242	-25.297	2.627.831	-20.689
2.634.518	-35.385	2.673.401	-25.297	2.627.928	-20.691
2.634.546	-35.386	2.673.533	-25.296	2.628.030	-20.693
2.634.691	-35.386	2.673.752	-25.296	2.628.197	-20.695
2.634.803	-35.387	2.674.001	-25.296	2.628.374	-20.697
2.634.911	-35.387	2.674.149	-25.296	2.628.575	-20.698
2.635.036	-35.388	2.674.346	-25.296	2.628.810	-20.701
2.635.168	-35.388	2.674.501	-25.296	2.628.980	-20.703
2.635.334	-35.389	2.674.583	-25.295	2.629.062	-20.705
2.635.552	-35.389	2.674.745	-25.295	2.629.236	-20.707
2.635.720	-35.390	2.674.900	-25.295	2.629.470	-20.709
2.635.804	-35.391	2.675.038	-25.295	2.629.633	-20.711
2.635.948	-35.391	2.675.202	-25.295	2.629.789	-20.713
2.636.172	-35.392	2.675.338	-25.294	2.629.933	-20.715
2.636.364	-35.393	2.675.491	-25.294	2.630.038	-20.717
2.636.549	-35.394	2.675.656	-25.294	2.630.201	-20.719
2.636.671	-35.394	2.675.804	-25.294	2.630.435	-20.721
2.636.873	-35.395	2.675.996	-25.294	2.630.580	-20.723
2.637.111	-35.396	2.676.190	-25.294	2.630.684	-20.725
2.637.426	-35.397	2.676.338	-25.293	2.630.825	-20.727
2.637.611	-35.398	2.676.555	-25.293	2.631.006	-20.730
2.637.635	-35.399	2.676.776	-25.293	2.631.208	-20.732
2.637.687	-35.400	2.676.939	-25.293	2.631.408	-20.734
2.637.859	-35.401	2.677.105	-25.293	2.631.611	-20.736
2.638.102	-35.402	2.677.322	-25.293	2.631.815	-20.738

2.638.293	-35.403	2.677.545	-25.292	2.632.036	-20.740
2.638.327	-35.405	2.677.715	-25.292	2.632.214	-20.742
2.638.387	-35.406	2.677.859	-25.292	2.632.408	-20.745
2.638.553	-35.407	2.678.029	-25.292	2.632.625	-20.747
2.638.839	-35.409	2.678.134	-25.292	2.632.799	-20.749
2.638.988	-35.410	2.678.237	-25.291	2.632.888	-20.751
2.639.161	-35.411	2.678.359	-25.291	2.632.989	-20.753
2.639.344	-35.413	2.678.492	-25.291	2.633.159	-20.756
2.639.598	-35.414	2.678.676	-25.291	2.633.329	-20.758
2.639.763	-35.416	2.678.821	-25.291	2.633.470	-20.760
2.639.846	-35.417	2.678.984	-25.290	2.633.574	-20.762
2.640.064	-35.419	2.679.194	-25.290	2.633.684	-20.764
2.640.251	-35.421	2.679.337	-25.290	2.633.817	-20.767
2.640.262	-35.423	2.679.503	-25.290	2.633.983	-20.769
2.640.348	-35.424	2.679.692	-25.289	2.634.147	-20.771
2.640.572	-35.426	2.679.824	-25.289	2.634.298	-20.773
2.640.779	-35.428	2.680.003	-25.289	2.634.532	-20.775
2.640.966	-35.430	2.680.244	-25.289	2.634.774	-20.778
2.641.158	-35.432	2.680.353	-25.288	2.634.942	-20.780
2.641.461	-35.434	2.680.548	-25.288	2.635.110	-20.782
2.641.653	-35.436	2.680.735	-25.288	2.635.320	-20.784
2.641.859	-35.438	2.680.869	-25.288	2.635.493	-20.787
2.642.112	-35.441	2.681.076	-25.287	2.635.653	-20.789
2.642.243	-35.443	2.681.268	-25.287	2.635.815	-20.791
2.642.377	-35.445	2.681.412	-25.287	2.635.919	-20.793
2.642.470	-35.447	2.681.567	-25.287	2.636.071	-20.796
2.642.502	-35.450	2.681.740	-25.286	2.636.190	-20.798
2.642.679	-35.452	2.681.931	-25.286	2.636.357	-20.800
2.642.867	-35.455	2.682.129	-25.286	2.636.548	-20.802
2.643.034	-35.457	2.682.289	-25.286	2.636.703	-20.805

2.643.112	-35.460	2.682.470	-25.285	2.636.917	-20.807
2.643.322	-35.462	2.682.662	-25.285	2.637.098	-20.809
2.643.591	-35.465	2.682.806	-25.285	2.637.271	-20.812
2.643.817	-35.468	2.683.001	-25.284	2.637.491	-20.814
2.643.893	-35.470	2.683.157	-25.284	2.637.646	-20.816
2.644.084	-35.473	2.683.287	-25.284	2.637.787	-20.818
2.644.186	-35.476	2.683.484	-25.283	2.637.921	-20.821
2.644.236	-35.479	2.683.633	-25.283	2.638.131	-20.823
2.644.405	-35.482	2.683.793	-25.283	2.638.325	-20.825
2.644.586	-35.485	2.683.961	-25.282	2.638.471	-20.828
2.644.767	-35.488	2.684.100	-25.282	2.638.615	-20.830
2.644.951	-35.491	2.684.287	-25.282	2.638.836	-20.832
2.645.078	-35.494	2.684.428	-25.281	2.639.023	-20.835
2.645.193	-35.497	2.684.587	-25.281	2.639.194	-20.837
2.645.266	-35.500	2.684.778	-25.281	2.639.388	-20.839
2.645.421	-35.503	2.684.970	-25.280	2.639.518	-20.841
2.645.536	-35.506	2.685.144	-25.280	2.639.713	-20.844
2.645.833	-35.510	2.685.240	-25.280	2.639.900	-20.846
2.646.154	-35.513	2.685.338	-25.279	2.640.056	-20.848
2.646.307	-35.516	2.685.491	-25.279	2.640.262	-20.851
2.646.356	-35.519	2.685.673	-25.278	2.640.399	-20.853
2.646.549	-35.523	2.685.869	-25.278	2.640.457	-20.855
2.646.819	-35.526	2.686.176	-25.278	2.640.596	-20.858
2.647.043	-35.530	2.686.563	-25.277	2.640.870	-20.860
2.647.279	-35.533	2.686.902	-25.277	2.641.286	-20.862
2.647.455	-35.536	2.687.025	-25.277	2.641.592	-20.865
2.647.552	-35.540	2.687.050	-25.276	2.641.718	-20.867
2.647.722	-35.543	2.687.083	-25.276	2.641.853	-20.870
2.647.935	-35.547	2.687.159	-25.275	2.641.909	-20.872
2.648.065	-35.550	2.687.227	-25.275	2.641.945	-20.874

2.648.149	-35.554	2.687.315	-25.275	2.641.985	-20.877
2.648.166	-35.557	2.687.531	-25.274	2.642.105	-20.879
2.648.365	-35.561	2.687.737	-25.274	2.642.243	-20.881
2.648.619	-35.565	2.687.841	-25.273	2.642.314	-20.884
2.648.719	-35.568	2.687.971	-25.273	2.642.463	-20.886
2.648.886	-35.572	2.688.143	-25.272	2.642.636	-20.888
2.649.042	-35.575	2.688.351	-25.272	2.642.830	-20.891
2.649.179	-35.579	2.688.596	-25.272	2.643.102	-20.893
2.649.355	-35.583	2.688.753	-25.271	2.643.369	-20.896
2.649.485	-35.586	2.688.886	-25.271	2.643.558	-20.898
2.649.659	-35.590	2.689.049	-25.270	2.643.694	-20.900
2.649.944	-35.594	2.689.211	-25.270	2.643.827	-20.903
2.650.110	-35.597	2.689.362	-25.270	2.644.012	-20.905
2.650.278	-35.601	2.689.532	-25.269	2.644.181	-20.908
2.650.421	-35.604	2.689.716	-25.269	2.644.322	-20.910
2.650.587	-35.608	2.689.887	-25.268	2.644.431	-20.913
2.650.858	-35.612	2.690.006	-25.268	2.644.588	-20.915
2.651.082	-35.615	2.690.125	-25.268	2.644.774	-20.917
2.651.206	-35.619	2.690.288	-25.267	2.644.898	-20.920
2.651.322	-35.622	2.690.437	-25.267	2.645.039	-20.922
2.651.440	-35.626	2.690.578	-25.266	2.645.200	-20.925
2.651.664	-35.630	2.690.783	-25.266	2.645.417	-20.927
2.651.907	-35.633	2.691.035	-25.266	2.645.645	-20.930
2.652.059	-35.637	2.691.233	-25.265	2.645.829	-20.932
2.652.095	-35.640	2.691.400	-25.265	2.645.975	-20.935
2.652.289	-35.644	2.691.577	-25.265	2.646.083	-20.937
2.652.484	-35.647	2.691.754	-25.264	2.646.248	-20.940
2.652.639	-35.651	2.691.971	-25.264	2.646.501	-20.942
2.652.805	-35.654	2.692.152	-25.264	2.646.704	-20.945
2.652.946	-35.658	2.692.350	-25.263	2.646.862	-20.947

2.653.094	-35.661	2.692.531	-25.263	2.647.079	-20.950
2.653.351	-35.665	2.692.651	-25.263	2.647.224	-20.952
2.653.518	-35.668	2.692.763	-25.262	2.647.361	-20.955
2.653.687	-35.671	2.692.903	-25.262	2.647.521	-20.958
2.653.849	-35.675	2.692.983	-25.262	2.647.644	-20.960
2.654.049	-35.678	2.693.098	-25.262	2.647.776	-20.963
2.654.203	-35.681	2.693.365	-25.261	2.647.955	-20.965
2.654.277	-35.685	2.693.557	-25.261	2.648.173	-20.968
2.654.380	-35.688	2.693.650	-25.261	2.648.289	-20.971
2.654.557	-35.691	2.693.818	-25.261	2.648.383	-20.973
2.654.796	-35.694	2.694.015	-25.260	2.648.554	-20.976
2.655.041	-35.697	2.694.194	-25.260	2.648.716	-20.979
2.655.169	-35.700	2.694.366	-25.260	2.648.869	-20.981
2.655.339	-35.704	2.694.510	-25.260	2.649.100	-20.984
2.655.536	-35.707	2.694.664	-25.260	2.649.305	-20.987
2.655.713	-35.710	2.694.792	-25.259	2.649.425	-20.989
2.655.863	-35.713	2.694.882	-25.259	2.649.554	-20.992
2.656.024	-35.716	2.695.042	-25.259	2.649.725	-20.995
2.656.158	-35.719	2.695.274	-25.259	2.649.940	-20.997
2.656.387	-35.721	2.695.518	-25.259	2.650.130	-21.000
2.656.852	-35.724	2.695.735	-25.259	2.650.298	-21.003
2.657.210	-35.727	2.695.869	-25.259	2.650.461	-21.006
2.657.437	-35.730	2.695.993	-25.259	2.650.652	-21.008
2.657.596	-35.733	2.696.186	-25.258	2.650.876	-21.011
2.657.593	-35.736	2.696.432	-25.258	2.651.062	-21.014
2.657.682	-35.738	2.696.642	-25.258	2.651.179	-21.017
2.657.850	-35.741	2.696.754	-25.258	2.651.260	-21.019
2.657.877	-35.743	2.696.866	-25.258	2.651.411	-21.022
2.657.900	-35.746	2.696.949	-25.258	2.651.581	-21.025
2.658.022	-35.749	2.697.111	-25.258	2.651.757	-21.028

2.658.137	-35.751	2.697.335	-25.258	2.651.920	-21.031
2.658.275	-35.754	2.697.494	-25.258	2.652.091	-21.033
2.658.397	-35.756	2.697.673	-25.258	2.652.341	-21.036
2.658.593	-35.758	2.697.935	-25.258	2.652.538	-21.039
2.658.803	-35.761	2.698.148	-25.258	2.652.647	-21.042
2.659.044	-35.763	2.698.311	-25.258	2.652.747	-21.045
2.659.263	-35.765	2.698.497	-25.258	2.652.897	-21.047
2.659.460	-35.767	2.698.607	-25.257	2.653.096	-21.050
2.659.706	-35.770	2.698.698	-25.257	2.653.275	-21.053
2.659.826	-35.772	2.698.864	-25.257	2.653.470	-21.056
2.659.919	-35.774	2.699.099	-25.257	2.653.622	-21.058
2.660.052	-35.776	2.699.248	-25.257	2.653.699	-21.061
2.660.220	-35.778	2.699.352	-25.257	2.653.813	-21.064
2.660.401	-35.780	2.699.438	-25.257	2.653.950	-21.067
2.660.493	-35.782	2.699.568	-25.257	2.654.124	-21.070
2.660.634	-35.784	2.699.799	-25.257	2.654.284	-21.072
2.660.743	-35.786	2.699.984	-25.257	2.654.421	-21.075
2.660.935	-35.788	2.700.183	-25.257	2.654.594	-21.078
2.661.165	-35.790	2.700.330	-25.256	2.654.806	-21.080
2.661.292	-35.792	2.700.424	-25.256	2.655.028	-21.083
2.661.403	-35.794	2.700.617	-25.256	2.655.262	-21.086
2.661.679	-35.795	2.700.848	-25.256	2.655.526	-21.089
2.661.975	-35.797	2.701.042	-25.256	2.655.746	-21.091
2.662.086	-35.799	2.701.194	-25.256	2.655.920	-21.094
2.662.162	-35.801	2.701.408	-25.256	2.656.115	-21.097
2.662.307	-35.802	2.701.621	-25.256	2.656.228	-21.099
2.662.554	-35.804	2.701.797	-25.255	2.656.282	-21.102
2.662.776	-35.806	2.701.997	-25.255	2.656.400	-21.105
2.662.968	-35.807	2.702.151	-25.255	2.656.592	-21.107
2.663.102	-35.809	2.702.245	-25.255	2.656.744	-21.110

2.663.217	-35.810	2.702.355	-25.255	2.656.863	-21.113
2.663.340	-35.812	2.702.473	-25.255	2.657.079	-21.115
2.663.497	-35.813	2.702.556	-25.254	2.657.202	-21.118
2.663.654	-35.815	2.702.713	-25.254	2.657.379	-21.120
2.663.770	-35.816	2.702.935	-25.254	2.657.595	-21.123
2.663.932	-35.818	2.703.175	-25.254	2.657.682	-21.126
2.664.052	-35.819	2.703.349	-25.254	2.657.832	-21.128
2.664.142	-35.821	2.703.427	-25.253	2.658.061	-21.131
2.664.425	-35.822	2.703.557	-25.253	2.658.278	-21.133
2.664.617	-35.823	2.703.730	-25.253	2.658.448	-21.136
2.664.803	-35.825	2.703.955	-25.253	2.658.623	-21.138
2.664.981	-35.826	2.704.190	-25.252	2.658.820	-21.141
2.665.194	-35.828	2.704.366	-25.252	2.659.001	-21.143
2.665.408	-35.829	2.704.544	-25.252	2.659.172	-21.146
2.665.549	-35.830	2.704.723	-25.252	2.659.319	-21.148
2.665.747	-35.831	2.704.806	-25.251	2.659.418	-21.150
2.665.895	-35.833	2.704.980	-25.251	2.659.609	-21.153
2.665.939	-35.834	2.705.229	-25.251	2.659.807	-21.155
2.666.074	-35.835	2.705.406	-25.250	2.659.937	-21.158
2.666.273	-35.837	2.705.555	-25.250	2.660.164	-21.160
2.666.462	-35.838	2.705.742	-25.250	2.660.392	-21.162
2.666.643	-35.839	2.705.930	-25.249	2.660.573	-21.165
2.666.841	-35.840	2.706.065	-25.249	2.660.703	-21.167
2.667.119	-35.842	2.706.277	-25.249	2.660.818	-21.169
2.667.296	-35.843	2.706.454	-25.248	2.660.970	-21.172
2.667.411	-35.844	2.706.624	-25.248	2.661.151	-21.174
2.667.646	-35.845	2.706.761	-25.248	2.661.389	-21.176
2.667.820	-35.847	2.706.938	-25.247	2.661.542	-21.179
2.667.868	-35.848	2.707.177	-25.247	2.661.715	-21.181
2.667.935	-35.849	2.707.369	-25.247	2.661.996	-21.183

2.668.152	-35.850	2.707.520	-25.246	2.662.185	-21.185
2.668.341	-35.851	2.707.643	-25.246	2.662.267	-21.187
2.668.445	-35.853	2.707.798	-25.245	2.662.362	-21.190
2.668.582	-35.854	2.707.950	-25.245	2.662.542	-21.192
2.668.781	-35.855	2.708.087	-25.245	2.662.762	-21.194
2.668.977	-35.856	2.708.228	-25.244	2.662.944	-21.196
2.669.185	-35.857	2.708.391	-25.244	2.663.090	-21.198
2.669.409	-35.858	2.708.576	-25.243	2.663.224	-21.200
2.669.644	-35.860	2.708.746	-25.243	2.663.391	-21.203
2.669.810	-35.861	2.708.889	-25.243	2.663.543	-21.205
2.669.929	-35.862	2.709.062	-25.242	2.663.690	-21.207
2.670.155	-35.863	2.709.275	-25.242	2.663.831	-21.209
2.670.277	-35.864	2.709.427	-25.241	2.663.958	-21.211
2.670.325	-35.865	2.709.522	-25.241	2.664.299	-21.213
2.670.493	-35.866	2.709.720	-25.240	2.664.792	-21.215
2.670.627	-35.867	2.709.859	-25.240	2.665.016	-21.217
2.670.735	-35.868	2.710.078	-25.239	2.665.041	-21.219
2.670.851	-35.869	2.710.522	-25.239	2.665.171	-21.221
2.671.010	-35.871	2.710.887	-25.238	2.665.331	-21.223
2.671.231	-35.872	2.711.086	-25.238	2.665.401	-21.225
2.671.378	-35.873	2.711.144	-25.237	2.665.414	-21.227
2.671.578	-35.874	2.711.209	-25.237	2.665.519	-21.229
2.671.869	-35.875	2.711.300	-25.236	2.665.670	-21.231
2.671.993	-35.876	2.711.393	-25.236	2.665.787	-21.233
2.672.188	-35.877	2.711.510	-25.235	2.665.920	-21.235
2.672.534	-35.878	2.711.584	-25.235	2.666.109	-21.237
2.672.729	-35.879	2.711.628	-25.234	2.666.339	-21.238
2.672.839	-35.880	2.711.799	-25.234	2.666.501	-21.240
2.672.997	-35.881	2.711.960	-25.233	2.666.668	-21.242
2.673.163	-35.882	2.712.055	-25.233	2.666.870	-21.244

2.673.305	-35.883	2.712.283	-25.232	2.667.041	-21.246
2.673.493	-35.884	2.712.512	-25.232	2.667.206	-21.248
2.673.647	-35.884	2.712.661	-25.231	2.667.350	-21.250
2.673.727	-35.885	2.712.875	-25.231	2.667.563	-21.252
2.673.860	-35.886	2.713.123	-25.230	2.667.747	-21.253
2.674.019	-35.887	2.713.280	-25.230	2.667.885	-21.255
2.674.160	-35.888	2.713.416	-25.229	2.668.062	-21.257
2.674.366	-35.889	2.713.580	-25.229	2.668.195	-21.259
2.674.609	-35.890	2.713.750	-25.228	2.668.322	-21.261
2.674.712	-35.890	2.713.973	-25.228	2.668.416	-21.263
2.674.837	-35.891	2.714.131	-25.227	2.668.597	-21.265
2.675.103	-35.892	2.714.232	-25.227	2.668.809	-21.267
2.675.289	-35.893	2.714.381	-25.226	2.668.929	-21.268
2.675.480	-35.893	2.714.550	-25.226	2.669.142	-21.270
2.675.664	-35.894	2.714.752	-25.225	2.669.378	-21.272
2.675.750	-35.895	2.714.901	-25.225	2.669.546	-21.274
2.675.872	-35.895	2.715.034	-25.224	2.669.727	-21.276
2.676.072	-35.896	2.715.245	-25.224	2.669.904	-21.278
2.676.281	-35.897	2.715.396	-25.224	2.670.052	-21.280
2.676.400	-35.897	2.715.496	-25.223	2.670.219	-21.282
2.676.610	-35.898	2.715.720	-25.223	2.670.411	-21.284
2.676.781	-35.899	2.715.986	-25.222	2.670.556	-21.286
2.676.938	-35.899	2.716.170	-25.222	2.670.717	-21.288
2.677.198	-35.900	2.716.317	-25.222	2.670.906	-21.289
2.677.412	-35.900	2.716.419	-25.221	2.671.056	-21.291
2.677.521	-35.901	2.716.573	-25.221	2.671.166	-21.293
2.677.595	-35.901	2.716.723	-25.221	2.671.259	-21.295
2.677.785	-35.902	2.716.855	-25.220	2.671.454	-21.297
2.678.013	-35.902	2.717.035	-25.220	2.671.645	-21.299
2.678.188	-35.903	2.717.158	-25.220	2.671.759	-21.301

2.678.365	-35.903	2.717.294	-25.220	2.671.918	-21.303
2.678.571	-35.903	2.717.473	-25.219	2.672.117	-21.305
2.678.739	-35.904	2.717.612	-25.219	2.672.236	-21.308
2.678.872	-35.904	2.717.805	-25.219	2.672.383	-21.310
2.679.026	-35.904	2.718.040	-25.219	2.672.565	-21.312
2.679.156	-35.904	2.718.178	-25.218	2.672.770	-21.314
2.679.323	-35.905	2.718.301	-25.218	2.672.968	-21.316
2.679.489	-35.905	2.718.486	-25.218	2.673.085	-21.318
2.679.666	-35.905	2.718.658	-25.218	2.673.246	-21.320
2.679.797	-35.905	2.718.795	-25.218	2.673.532	-21.322
2.679.933	-35.905	2.718.961	-25.218	2.673.755	-21.325
2.680.115	-35.906	2.719.107	-25.218	2.673.840	-21.327
2.680.346	-35.906	2.719.326	-25.217	2.673.973	-21.329
2.680.630	-35.906	2.719.507	-25.217	2.674.156	-21.331
2.680.809	-35.906	2.719.655	-25.217	2.674.293	-21.333
2.680.966	-35.906	2.719.846	-25.217	2.674.449	-21.336
2.681.075	-35.906	2.719.985	-25.217	2.674.626	-21.338
2.681.183	-35.906	2.720.157	-25.217	2.674.847	-21.340
2.681.335	-35.906	2.720.363	-25.217	2.675.005	-21.343
2.681.492	-35.906	2.720.577	-25.217	2.675.140	-21.345
2.681.693	-35.906	2.720.750	-25.217	2.675.338	-21.347
2.681.986	-35.906	2.720.883	-25.217	2.675.544	-21.350
2.682.415	-35.905	2.721.010	-25.217	2.675.711	-21.352
2.682.798	-35.905	2.721.221	-25.217	2.675.811	-21.354
2.682.908	-35.905	2.721.430	-25.217	2.675.945	-21.357
2.682.918	-35.905	2.721.544	-25.217	2.676.150	-21.359
2.682.979	-35.905	2.721.714	-25.217	2.676.227	-21.361
2.683.058	-35.904	2.721.882	-25.217	2.676.367	-21.364
2.683.090	-35.904	2.722.061	-25.217	2.676.635	-21.366
2.683.224	-35.904	2.722.272	-25.217	2.676.765	-21.369

2.683.397	-35.903	2.722.465	-25.217	2.676.916	-21.371
2.683.448	-35.903	2.722.644	-25.218	2.677.079	-21.374
2.683.618	-35.903	2.722.781	-25.218	2.677.205	-21.376
2.683.800	-35.902	2.722.906	-25.218	2.677.384	-21.378
2.683.980	-35.902	2.723.066	-25.218	2.677.531	-21.381
2.684.146	-35.901	2.723.250	-25.218	2.677.581	-21.383
2.684.283	-35.901	2.723.424	-25.218	2.677.776	-21.386
2.684.442	-35.900	2.723.553	-25.218	2.678.080	-21.388
2.684.595	-35.900	2.723.674	-25.218	2.678.265	-21.391
2.684.864	-35.899	2.723.864	-25.218	2.678.431	-21.394
2.685.194	-35.899	2.724.006	-25.218	2.678.670	-21.396
2.685.287	-35.898	2.724.107	-25.218	2.678.835	-21.399
2.685.334	-35.897	2.724.263	-25.218	2.679.028	-21.401
2.685.487	-35.897	2.724.432	-25.218	2.679.276	-21.404
2.685.675	-35.896	2.724.562	-25.218	2.679.445	-21.406
2.685.849	-35.896	2.724.703	-25.218	2.679.599	-21.409
2.685.924	-35.895	2.724.944	-25.218	2.679.736	-21.411
2.686.056	-35.894	2.725.173	-25.218	2.679.876	-21.414
2.686.224	-35.894	2.725.384	-25.218	2.680.031	-21.417
2.686.369	-35.893	2.725.624	-25.219	2.680.162	-21.419
2.686.549	-35.892	2.725.814	-25.219	2.680.258	-21.422
2.686.718	-35.891	2.725.977	-25.219	2.680.388	-21.424
2.686.929	-35.891	2.726.115	-25.219	2.680.526	-21.427
2.687.094	-35.890	2.726.208	-25.219	2.680.682	-21.430
2.687.239	-35.889	2.726.374	-25.219	2.680.865	-21.432
2.687.514	-35.888	2.726.584	-25.218	2.681.053	-21.435
2.687.827	-35.888	2.726.719	-25.218	2.681.230	-21.437
2.687.928	-35.887	2.726.822	-25.218	2.681.465	-21.440
2.688.005	-35.886	2.726.936	-25.218	2.681.671	-21.443
2.688.242	-35.885	2.727.099	-25.218	2.681.804	-21.445

2.688.445	-35.885	2.727.260	-25.218	2.682.021	-21.448
2.688.578	-35.884	2.727.424	-25.218	2.682.207	-21.450
2.688.760	-35.883	2.727.633	-25.218	2.682.348	-21.453
2.688.864	-35.882	2.727.823	-25.218	2.682.463	-21.456
2.688.990	-35.881	2.727.968	-25.218	2.682.603	-21.458
2.689.176	-35.881	2.728.112	-25.218	2.682.794	-21.461
2.689.265	-35.880	2.728.282	-25.217	2.682.953	-21.463
2.689.449	-35.879	2.728.524	-25.217	2.683.117	-21.466
2.689.638	-35.878	2.728.752	-25.217	2.683.268	-21.469
2.689.778	-35.877	2.728.894	-25.217	2.683.424	-21.471
2.689.962	-35.876	2.729.085	-25.217	2.683.615	-21.474
2.690.190	-35.876	2.729.265	-25.216	2.683.786	-21.476
2.690.392	-35.875	2.729.330	-25.216	2.683.997	-21.479
2.690.515	-35.874	2.729.452	-25.216	2.684.190	-21.482
2.690.641	-35.873	2.729.658	-25.216	2.684.391	-21.484
2.690.746	-35.872	2.729.901	-25.215	2.684.580	-21.487
2.690.917	-35.872	2.730.130	-25.215	2.684.717	-21.489
2.691.105	-35.871	2.730.277	-25.215	2.684.908	-21.492
2.691.317	-35.870	2.730.429	-25.215	2.685.042	-21.495
2.691.559	-35.869	2.730.619	-25.214	2.685.193	-21.497
2.691.624	-35.869	2.730.780	-25.214	2.685.352	-21.500
2.691.743	-35.868	2.730.995	-25.214	2.685.469	-21.502
2.691.910	-35.867	2.731.205	-25.213	2.685.617	-21.505
2.692.047	-35.867	2.731.266	-25.213	2.685.775	-21.507
2.692.426	-35.866	2.731.405	-25.213	2.685.950	-21.510
2.692.722	-35.865	2.731.624	-25.212	2.686.198	-21.512
2.692.788	-35.864	2.731.772	-25.212	2.686.477	-21.515
2.692.924	-35.864	2.731.953	-25.211	2.686.598	-21.517
2.693.063	-35.863	2.732.147	-25.211	2.686.711	-21.520
2.693.195	-35.862	2.732.307	-25.210	2.686.891	-21.522

2.693.326	-35.862	2.732.441	-25.210	2.686.971	-21.525
2.693.485	-35.861	2.732.596	-25.209	2.687.010	-21.527
2.693.697	-35.860	2.732.783	-25.209	2.687.322	-21.530
2.693.878	-35.860	2.732.968	-25.208	2.687.841	-21.532
2.693.994	-35.859	2.733.163	-25.208	2.688.192	-21.535
2.694.106	-35.858	2.733.347	-25.207	2.688.340	-21.537
2.694.335	-35.858	2.733.506	-25.207	2.688.417	-21.540
2.694.453	-35.857	2.733.659	-25.206	2.688.466	-21.542
2.694.562	-35.856	2.733.780	-25.205	2.688.477	-21.545
2.694.749	-35.856	2.733.927	-25.205	2.688.549	-21.547
2.694.882	-35.855	2.734.088	-25.204	2.688.647	-21.549
2.695.028	-35.854	2.734.346	-25.203	2.688.695	-21.552
2.695.183	-35.854	2.734.800	-25.203	2.688.835	-21.554
2.695.421	-35.853	2.735.132	-25.202	2.689.026	-21.556
2.695.624	-35.853	2.735.240	-25.201	2.689.263	-21.559
2.695.753	-35.852	2.735.283	-25.201	2.689.501	-21.561
2.695.873	-35.851	2.735.406	-25.200	2.689.658	-21.564
2.696.028	-35.851	2.735.486	-25.199	2.689.781	-21.566
2.696.174	-35.850	2.735.457	-25.198	2.689.967	-21.568
2.696.288	-35.850	2.735.559	-25.197	2.690.208	-21.571
2.696.495	-35.849	2.735.715	-25.197	2.690.390	-21.573
2.696.783	-35.848	2.735.852	-25.196	2.690.533	-21.575
2.696.947	-35.848	2.736.013	-25.195	2.690.710	-21.577
2.697.058	-35.847	2.736.208	-25.194	2.690.852	-21.580
2.697.272	-35.847	2.736.454	-25.193	2.690.979	-21.582
2.697.528	-35.846	2.736.640	-25.192	2.691.155	-21.584
2.697.734	-35.845	2.736.786	-25.191	2.691.299	-21.586
2.697.918	-35.845	2.736.928	-25.190	2.691.419	-21.589
2.698.134	-35.844	2.737.137	-25.189	2.691.533	-21.591
2.698.283	-35.844	2.737.383	-25.188	2.691.676	-21.593

2.698.387	-35.843	2.737.531	-25.187	2.691.860	-21.595
2.698.549	-35.842	2.737.589	-25.186	2.692.057	-21.598
2.698.735	-35.842	2.737.733	-25.185	2.692.173	-21.600
2.698.853	-35.841	2.737.987	-25.184	2.692.340	-21.602
2.698.950	-35.840	2.738.143	-25.183	2.692.630	-21.604
2.699.117	-35.840	2.738.286	-25.182	2.692.814	-21.607
2.699.304	-35.839	2.738.474	-25.181	2.692.984	-21.609
2.699.483	-35.838	2.738.609	-25.180	2.693.186	-21.611
2.699.615	-35.838	2.738.748	-25.179	2.693.341	-21.613
2.699.749	-35.837	2.738.864	-25.178	2.693.463	-21.615
2.699.877	-35.836	2.738.926	-25.177	2.693.615	-21.618
2.700.100	-35.836	2.739.088	-25.176	2.693.788	-21.620
2.700.168	-35.835	2.739.385	-25.175	2.693.958	-21.622
2.700.327	-35.834	2.739.605	-25.174	2.694.093	-21.624
2.700.616	-35.834	2.739.742	-25.173	2.694.252	-21.626
2.700.859	-35.833	2.739.895	-25.172	2.694.447	-21.629
2.701.071	-35.832	2.740.031	-25.170	2.694.630	-21.631
2.701.234	-35.831	2.740.241	-25.169	2.694.810	-21.633
2.701.331	-35.831	2.740.502	-25.168	2.694.966	-21.635
2.701.457	-35.830	2.740.656	-25.167	2.695.135	-21.637
2.701.600	-35.829	2.740.786	-25.166	2.695.320	-21.639
2.701.710	-35.828	2.740.948	-25.165	2.695.457	-21.642
2.702.001	-35.827	2.741.073	-25.164	2.695.598	-21.644
2.702.365	-35.827	2.741.223	-25.162	2.695.786	-21.646
2.702.470	-35.826	2.741.383	-25.161	2.695.930	-21.648
2.702.527	-35.825	2.741.516	-25.160	2.696.091	-21.650
2.702.680	-35.824	2.741.649	-25.159	2.696.295	-21.653
2.702.796	-35.823	2.741.795	-25.158	2.696.483	-21.655
2.702.987	-35.822	2.741.972	-25.157	2.696.617	-21.657
2.703.237	-35.821	2.742.199	-25.155	2.696.765	-21.659

2.703.387	-35.821	2.742.427	-25.154	2.697.007	-21.661
2.703.504	-35.820	2.742.610	-25.153	2.697.213	-21.664
2.703.605	-35.819	2.742.766	-25.152	2.697.361	-21.666
2.703.806	-35.818	2.742.899	-25.151	2.697.455	-21.668
2.704.041	-35.817	2.743.030	-25.150	2.697.597	-21.670
2.704.156	-35.816	2.743.168	-25.149	2.697.816	-21.672
2.704.348	-35.815	2.743.304	-25.148	2.697.942	-21.674
2.704.554	-35.814	2.743.466	-25.146	2.698.098	-21.677
2.704.778	-35.813	2.743.654	-25.145	2.698.347	-21.679
2.704.962	-35.812	2.743.846	-25.144	2.698.495	-21.681
2.705.064	-35.810	2.743.979	-25.143	2.698.622	-21.683
2.705.085	-35.809	2.744.179	-25.142	2.698.791	-21.685
2.705.168	-35.808	2.744.406	-25.141	2.699.008	-21.688
2.705.331	-35.807	2.744.503	-25.140	2.699.211	-21.690
2.705.566	-35.806	2.744.685	-25.139	2.699.333	-21.692
2.705.836	-35.805	2.744.858	-25.138	2.699.464	-21.694
2.706.114	-35.804	2.745.042	-25.137	2.699.591	-21.696
2.706.237	-35.802	2.745.237	-25.136	2.699.667	-21.698
2.706.335	-35.801	2.745.413	-25.134	2.699.801	-21.701
2.706.491	-35.800	2.745.511	-25.133	2.700.009	-21.703
2.706.678	-35.799	2.745.643	-25.132	2.700.215	-21.705
2.706.964	-35.797	2.745.876	-25.131	2.700.417	-21.707
2.707.278	-35.796	2.746.080	-25.130	2.700.551	-21.709
2.707.541	-35.795	2.746.245	-25.129	2.700.614	-21.711
2.707.845	-35.794	2.746.414	-25.128	2.700.749	-21.714
2.707.990	-35.792	2.746.555	-25.127	2.700.949	-21.716
2.708.005	-35.791	2.746.723	-25.126	2.701.161	-21.718
2.708.105	-35.789	2.746.888	-25.125	2.701.349	-21.720
2.708.135	-35.788	2.747.057	-25.124	2.701.527	-21.722
2.708.192	-35.787	2.747.202	-25.123	2.701.773	-21.724

2.708.365	-35.785	2.747.354	-25.121	2.702.010	-21.726
2.708.582	-35.784	2.747.488	-25.120	2.702.229	-21.728
2.708.670	-35.782	2.747.560	-25.119	2.702.398	-21.731
2.708.820	-35.781	2.747.701	-25.118	2.702.521	-21.733
2.708.937	-35.780	2.747.845	-25.117	2.702.673	-21.735
2.709.066	-35.778	2.748.000	-25.116	2.702.856	-21.737
2.709.283	-35.777	2.748.160	-25.115	2.703.027	-21.739
2.709.446	-35.775	2.748.297	-25.114	2.703.155	-21.741
2.709.525	-35.774	2.748.473	-25.113	2.703.279	-21.743
2.709.555	-35.772	2.748.705	-25.112	2.703.434	-21.745
2.709.973	-35.771	2.748.916	-25.111	2.703.575	-21.747
2.710.245	-35.769	2.749.110	-25.110	2.703.687	-21.749
2.710.258	-35.768	2.749.360	-25.109	2.703.821	-21.751
2.710.302	-35.766	2.749.518	-25.108	2.704.015	-21.753
2.710.446	-35.765	2.749.673	-25.107	2.704.258	-21.755
2.710.812	-35.763	2.749.857	-25.105	2.704.434	-21.757
2.710.829	-35.762	2.750.045	-25.104	2.704.588	-21.759
2.711.075	-35.760	2.750.237	-25.103	2.704.767	-21.761
2.711.430	-35.759	2.750.403	-25.102	2.704.959	-21.763
2.711.581	-35.757	2.750.580	-25.101	2.705.140	-21.765
2.711.698	-35.756	2.750.732	-25.100	2.705.276	-21.767
2.711.651	-35.754	2.750.859	-25.099	2.705.444	-21.769
2.711.758	-35.753	2.750.957	-25.098	2.705.609	-21.771
2.712.007	-35.751	2.751.089	-25.097	2.705.764	-21.773
2.712.354	-35.750	2.751.259	-25.096	2.705.952	-21.775
2.712.532	-35.748	2.751.369	-25.095	2.706.129	-21.777
2.712.711	-35.747	2.751.495	-25.094	2.706.296	-21.779
2.712.931	-35.745	2.751.671	-25.093	2.706.466	-21.781
2.713.022	-35.744	2.751.842	-25.092	2.706.638	-21.783
2.713.102	-35.742	2.752.086	-25.090	2.706.823	-21.785

2.713.224	-35.741	2.752.366	-25.089	2.706.967	-21.787
2.713.493	-35.740	2.752.571	-25.088	2.707.126	-21.789
2.713.802	-35.738	2.752.693	-25.087	2.707.315	-21.791
2.713.720	-35.737	2.752.832	-25.086	2.707.513	-21.793
2.713.637	-35.736	2.753.029	-25.085	2.707.684	-21.795
2.713.908	-35.734	2.753.217	-25.084	2.707.813	-21.797
2.714.240	-35.733	2.753.354	-25.083	2.707.998	-21.798
2.714.355	-35.732	2.753.435	-25.082	2.708.170	-21.800
2.714.471	-35.730	2.753.643	-25.081	2.708.329	-21.802
2.714.818	-35.729	2.753.918	-25.080	2.708.515	-21.804
2.715.099	-35.728	2.754.078	-25.079	2.708.724	-21.806
2.715.162	-35.727	2.754.232	-25.078	2.708.918	-21.808
2.715.309	-35.725	2.754.442	-25.077	2.709.030	-21.810
2.715.367	-35.724	2.754.575	-25.076	2.709.165	-21.811
2.715.451	-35.723	2.754.706	-25.075	2.709.344	-21.813
2.715.742	-35.722	2.754.865	-25.074	2.709.517	-21.815
2.715.909	-35.721	2.754.981	-25.073	2.709.653	-21.817
2.716.105	-35.720	2.755.191	-25.072	2.709.799	-21.819
2.716.191	-35.719	2.755.421	-25.071	2.709.971	-21.821
2.716.282	-35.718	2.755.576	-25.070	2.710.133	-21.822
2.716.483	-35.717	2.755.767	-25.069	2.710.241	-21.824
2.716.700	-35.716	2.755.923	-25.068	2.710.426	-21.826
2.716.918	-35.715	2.756.039	-25.067	2.710.910	-21.828
2.717.050	-35.714	2.756.192	-25.066	2.711.385	-21.829
2.717.144	-35.714	2.756.361	-25.065	2.711.600	-21.831
2.717.364	-35.713	2.756.488	-25.064	2.711.642	-21.833
2.717.628	-35.712	2.756.678	-25.064	2.711.638	-21.835
2.717.791	-35.711	2.756.906	-25.063	2.711.645	-21.836
2.717.969	-35.711	2.757.051	-25.062	2.711.668	-21.838
2.718.094	-35.710	2.757.227	-25.061	2.711.830	-21.840

2.718.168	-35.709	2.757.351	-25.060	2.711.999	-21.841
2.718.293	-35.709	2.757.460	-25.059	2.712.101	-21.843
2.718.514	-35.708	2.757.608	-25.058	2.712.184	-21.845
2.718.666	-35.708	2.757.792	-25.057	2.712.369	-21.847
2.718.822	-35.707	2.758.012	-25.056	2.712.625	-21.848
2.718.974	-35.707	2.758.146	-25.056	2.712.806	-21.850
2.719.143	-35.707	2.758.379	-25.055	2.712.980	-21.852
2.719.519	-35.706	2.758.824	-25.054	2.713.141	-21.853
2.719.688	-35.706	2.759.146	-25.053	2.713.311	-21.855
2.719.664	-35.706	2.759.266	-25.052	2.713.475	-21.857
2.719.861	-35.705	2.759.364	-25.051	2.713.634	-21.858
2.720.035	-35.705	2.759.417	-25.051	2.713.784	-21.860
2.720.168	-35.705	2.759.507	-25.050	2.713.976	-21.862
2.720.411	-35.705	2.759.602	-25.049	2.714.160	-21.863
2.720.555	-35.705	2.759.680	-25.048	2.714.312	-21.865
2.720.671	-35.705	2.759.775	-25.047	2.714.492	-21.867
2.720.833	-35.705	2.759.912	-25.047	2.714.682	-21.868
2.721.029	-35.705	2.760.114	-25.046	2.714.760	-21.870
2.721.163	-35.705	2.760.299	-25.045	2.714.846	-21.871
2.721.241	-35.705	2.760.426	-25.044	2.715.052	-21.873
2.721.329	-35.705	2.760.543	-25.043	2.715.251	-21.875
2.721.450	-35.705	2.760.740	-25.043	2.715.397	-21.876
2.721.667	-35.705	2.760.975	-25.042	2.715.601	-21.878
2.721.880	-35.706	2.761.216	-25.041	2.715.890	-21.880
2.722.019	-35.706	2.761.414	-25.041	2.716.028	-21.881
2.722.348	-35.706	2.761.538	-25.040	2.716.137	-21.883
2.722.687	-35.707	2.761.660	-25.039	2.716.347	-21.885
2.722.864	-35.707	2.761.805	-25.039	2.716.512	-21.886
2.723.108	-35.707	2.761.983	-25.038	2.716.640	-21.888
2.723.239	-35.708	2.762.180	-25.037	2.716.812	-21.890

2.723.329	-35.708	2.762.383	-25.037	2.716.978	-21.891
2.723.499	-35.709	2.762.491	-25.036	2.717.159	-21.893
2.723.749	-35.709	2.762.574	-25.035	2.717.299	-21.895
2.723.759	-35.710	2.762.773	-25.035	2.717.393	-21.896
2.723.734	-35.711	2.762.962	-25.034	2.717.593	-21.898
2.723.973	-35.711	2.763.092	-25.034	2.717.814	-21.900
2.724.142	-35.712	2.763.242	-25.033	2.717.900	-21.901
2.724.317	-35.713	2.763.441	-25.033	2.718.033	-21.903
2.724.454	-35.713	2.763.677	-25.032	2.718.275	-21.905
2.724.478	-35.714	2.763.897	-25.032	2.718.448	-21.906
2.724.656	-35.715	2.764.044	-25.031	2.718.596	-21.908
2.724.915	-35.716	2.764.203	-25.031	2.718.821	-21.910
2.725.039	-35.717	2.764.413	-25.031	2.719.006	-21.911
2.725.230	-35.717	2.764.598	-25.030	2.719.179	-21.913
2.725.537	-35.718	2.764.695	-25.030	2.719.323	-21.915
2.725.790	-35.719	2.764.803	-25.029	2.719.427	-21.916
2.725.917	-35.720	2.765.002	-25.029	2.719.529	-21.918
2.726.015	-35.721	2.765.164	-25.029	2.719.656	-21.920
2.726.205	-35.722	2.765.307	-25.028	2.719.883	-21.922
2.726.382	-35.723	2.765.471	-25.028	2.720.111	-21.923
2.726.606	-35.724	2.765.609	-25.028	2.720.294	-21.925
2.726.808	-35.725	2.765.776	-25.028	2.720.450	-21.927
2.726.927	-35.726	2.765.938	-25.027	2.720.648	-21.928
2.726.953	-35.727	2.766.121	-25.027	2.720.825	-21.930
2.727.073	-35.728	2.766.317	-25.027	2.720.916	-21.932
2.727.353	-35.729	2.766.448	-25.027	2.721.035	-21.934
2.727.543	-35.731	2.766.570	-25.027	2.721.255	-21.935
2.727.737	-35.732	2.766.748	-25.027	2.721.462	-21.937
2.727.875	-35.733	2.766.972	-25.026	2.721.591	-21.939
2.727.975	-35.734	2.767.162	-25.026	2.721.754	-21.941

2.728.206	-35.735	2.767.214	-25.026	2.721.938	-21.942
2.728.488	-35.736	2.767.285	-25.026	2.722.094	-21.944
2.728.655	-35.738	2.767.554	-25.026	2.722.259	-21.946
2.728.763	-35.739	2.767.856	-25.026	2.722.451	-21.947
2.728.880	-35.740	2.768.052	-25.026	2.722.626	-21.949
2.729.122	-35.741	2.768.210	-25.026	2.722.798	-21.951
2.729.290	-35.743	2.768.361	-25.026	2.722.918	-21.953
2.729.362	-35.744	2.768.542	-25.026	2.723.070	-21.954
2.729.505	-35.745	2.768.766	-25.026	2.723.196	-21.956
2.729.743	-35.746	2.768.923	-25.027	2.723.344	-21.958
2.729.973	-35.748	2.769.056	-25.027	2.723.596	-21.959
2.730.117	-35.749	2.769.248	-25.027	2.723.730	-21.961
2.730.325	-35.750	2.769.387	-25.027	2.723.842	-21.963
2.730.512	-35.752	2.769.467	-25.027	2.724.016	-21.964
2.730.653	-35.753	2.769.599	-25.027	2.724.174	-21.966
2.730.804	-35.754	2.769.771	-25.027	2.724.399	-21.968
2.730.988	-35.756	2.769.857	-25.028	2.724.641	-21.969
2.731.138	-35.757	2.770.010	-25.028	2.724.789	-21.971
2.731.220	-35.758	2.770.284	-25.028	2.724.923	-21.973
2.731.415	-35.760	2.770.559	-25.028	2.725.114	-21.974
2.731.607	-35.761	2.770.674	-25.028	2.725.359	-21.976
2.731.758	-35.762	2.770.798	-25.029	2.725.581	-21.977
2.731.989	-35.764	2.770.960	-25.029	2.725.785	-21.979
2.732.406	-35.765	2.771.075	-25.029	2.725.967	-21.981
2.732.770	-35.767	2.771.250	-25.029	2.726.078	-21.982
2.732.885	-35.768	2.771.432	-25.030	2.726.173	-21.984
2.732.968	-35.769	2.771.571	-25.030	2.726.317	-21.985
2.733.033	-35.771	2.771.687	-25.030	2.726.468	-21.987
2.733.128	-35.772	2.771.812	-25.031	2.726.660	-21.988
2.733.196	-35.773	2.771.936	-25.031	2.726.823	-21.990

2.733.333	-35.775	2.772.083	-25.031	2.726.911	-21.991
2.733.448	-35.776	2.772.245	-25.031	2.727.035	-21.993
2.733.405	-35.777	2.772.469	-25.032	2.727.213	-21.994
2.733.504	-35.779	2.772.687	-25.032	2.727.420	-21.995
2.733.681	-35.780	2.772.819	-25.032	2.727.626	-21.997
2.733.990	-35.782	2.772.986	-25.033	2.727.802	-21.998
2.734.222	-35.783	2.773.206	-25.033	2.727.955	-22.000
2.734.338	-35.784	2.773.426	-25.033	2.728.117	-22.001
2.734.530	-35.786	2.773.647	-25.034	2.728.286	-22.002
2.734.620	-35.787	2.773.867	-25.034	2.728.484	-22.004
2.734.969	-35.788	2.774.008	-25.034	2.728.681	-22.005
2.735.348	-35.790	2.774.193	-25.035	2.728.876	-22.006
2.735.475	-35.791	2.774.366	-25.035	2.729.080	-22.008
2.735.533	-35.792	2.774.512	-25.035	2.729.239	-22.009
2.735.562	-35.794	2.774.649	-25.036	2.729.353	-22.010
2.735.671	-35.795	2.774.753	-25.036	2.729.494	-22.011
2.735.854	-35.797	2.774.951	-25.036	2.729.684	-22.013
2.736.060	-35.798	2.775.067	-25.037	2.729.871	-22.014
2.736.285	-35.799	2.775.136	-25.037	2.730.038	-22.015
2.736.427	-35.801	2.775.327	-25.037	2.730.209	-22.016
2.736.589	-35.802	2.775.513	-25.038	2.730.368	-22.017
2.736.862	-35.804	2.775.645	-25.038	2.730.522	-22.018
2.736.996	-35.805	2.775.859	-25.038	2.730.692	-22.019
2.737.117	-35.807	2.776.058	-25.039	2.730.836	-22.021
2.737.263	-35.808	2.776.255	-25.039	2.730.999	-22.022
2.737.325	-35.809	2.776.501	-25.039	2.731.190	-22.023
2.737.527	-35.811	2.776.667	-25.039	2.731.398	-22.024
2.737.756	-35.812	2.776.791	-25.040	2.731.646	-22.025
2.737.972	-35.814	2.776.953	-25.040	2.731.830	-22.026
2.738.250	-35.815	2.777.163	-25.040	2.731.992	-22.027

2.738.452	-35.817	2.777.377	-25.040	2.732.119	-22.028
2.738.634	-35.818	2.777.521	-25.041	2.732.236	-22.029
2.738.724	-35.820	2.777.672	-25.041	2.732.437	-22.030
2.738.750	-35.821	2.777.867	-25.041	2.732.639	-22.030
2.738.867	-35.822	2.778.065	-25.041	2.732.838	-22.031
2.739.197	-35.824	2.778.244	-25.042	2.733.004	-22.032
2.739.427	-35.825	2.778.402	-25.042	2.733.131	-22.033
2.739.445	-35.827	2.778.521	-25.042	2.733.271	-22.034
2.739.579	-35.828	2.778.652	-25.042	2.733.424	-22.035
2.739.815	-35.830	2.778.824	-25.042	2.733.568	-22.035
2.739.977	-35.831	2.778.954	-25.042	2.733.778	-22.036
2.740.027	-35.833	2.779.131	-25.042	2.734.218	-22.037
2.740.147	-35.835	2.779.323	-25.043	2.734.561	-22.038
2.740.341	-35.836	2.779.529	-25.043	2.734.662	-22.038
2.740.520	-35.838	2.779.741	-25.043	2.734.771	-22.039
2.740.667	-35.839	2.779.862	-25.043	2.734.922	-22.040
2.740.815	-35.841	2.779.974	-25.043	2.735.002	-22.041
2.740.997	-35.843	2.780.153	-25.043	2.735.023	-22.041
2.741.183	-35.844	2.780.303	-25.043	2.735.081	-22.042
2.741.378	-35.846	2.780.453	-25.043	2.735.173	-22.043
2.741.539	-35.848	2.780.675	-25.043	2.735.280	-22.043
2.741.643	-35.849	2.780.848	-25.043	2.735.421	-22.044
2.741.719	-35.851	2.781.010	-25.043	2.735.603	-22.044
2.741.902	-35.853	2.781.180	-25.043	2.735.815	-22.045
2.742.200	-35.854	2.781.342	-25.042	2.736.042	-22.046
2.742.332	-35.856	2.781.481	-25.042	2.736.205	-22.046
2.742.466	-35.858	2.781.631	-25.042	2.736.400	-22.047
2.742.661	-35.860	2.781.820	-25.042	2.736.628	-22.047
2.742.896	-35.861	2.781.956	-25.042	2.736.751	-22.048
2.743.130	-35.863	2.782.095	-25.042	2.736.898	-22.048

2.743.260	-35.865	2.782.328	-25.042	2.737.106	-22.049
2.743.380	-35.867	2.782.791	-25.041	2.737.232	-22.049
2.743.533	-35.869	2.783.192	-25.041	2.737.365	-22.050
2.743.672	-35.871	2.783.343	-25.041	2.737.516	-22.050
2.743.784	-35.873	2.783.329	-25.041	2.737.668	-22.050
2.744.031	-35.874	2.783.401	-25.040	2.737.838	-22.051
2.744.223	-35.876	2.783.560	-25.040	2.737.993	-22.051
2.744.442	-35.878	2.783.594	-25.040	2.738.148	-22.052
2.744.674	-35.880	2.783.680	-25.039	2.738.264	-22.052
2.744.782	-35.882	2.783.770	-25.039	2.738.460	-22.053
2.744.951	-35.884	2.783.909	-25.038	2.738.672	-22.053
2.745.157	-35.886	2.784.122	-25.038	2.738.860	-22.053
2.745.231	-35.888	2.784.308	-25.038	2.739.023	-22.054
2.745.280	-35.890	2.784.464	-25.037	2.739.233	-22.054
2.745.482	-35.892	2.784.645	-25.037	2.739.496	-22.055
2.745.642	-35.894	2.784.839	-25.036	2.739.641	-22.055
2.745.855	-35.896	2.784.998	-25.036	2.739.782	-22.056
2.746.098	-35.898	2.785.211	-25.035	2.739.922	-22.056
2.746.223	-35.900	2.785.406	-25.035	2.740.068	-22.056
2.746.295	-35.902	2.785.512	-25.034	2.740.226	-22.057
2.746.392	-35.904	2.785.656	-25.034	2.740.403	-22.057
2.746.555	-35.906	2.785.815	-25.033	2.740.552	-22.058
2.746.719	-35.908	2.785.941	-25.033	2.740.679	-22.058
2.746.940	-35.910	2.786.130	-25.032	2.740.854	-22.058
2.747.140	-35.912	2.786.331	-25.031	2.740.997	-22.059
2.747.267	-35.914	2.786.510	-25.031	2.741.154	-22.059
2.747.442	-35.916	2.786.653	-25.030	2.741.349	-22.060
2.747.676	-35.918	2.786.786	-25.030	2.741.479	-22.060
2.747.866	-35.920	2.786.920	-25.029	2.741.614	-22.061
2.748.058	-35.922	2.787.065	-25.028	2.741.799	-22.061

2.748.320	-35.924	2.787.299	-25.028	2.742.021	-22.062
2.748.503	-35.926	2.787.485	-25.027	2.742.216	-22.062
2.748.666	-35.929	2.787.632	-25.026	2.742.336	-22.063
2.748.809	-35.931	2.787.845	-25.026	2.742.455	-22.063
2.748.918	-35.933	2.788.004	-25.025	2.742.592	-22.064
2.749.102	-35.935	2.788.167	-25.024	2.742.767	-22.064
2.749.218	-35.937	2.788.412	-25.024	2.742.997	-22.065
2.749.310	-35.939	2.788.544	-25.023	2.743.199	-22.065
2.749.490	-35.941	2.788.634	-25.022	2.743.398	-22.066
2.749.659	-35.943	2.788.804	-25.022	2.743.556	-22.066
2.749.797	-35.945	2.788.970	-25.021	2.743.673	-22.067
2.749.958	-35.947	2.789.055	-25.020	2.743.878	-22.067
2.750.158	-35.949	2.789.200	-25.019	2.744.080	-22.068
2.750.361	-35.951	2.789.449	-25.019	2.744.243	-22.069
2.750.390	-35.953	2.789.651	-25.018	2.744.346	-22.069
2.750.522	-35.955	2.789.765	-25.017	2.744.440	-22.070
2.750.832	-35.957	2.789.911	-25.017	2.744.612	-22.070
2.751.085	-35.959	2.790.096	-25.016	2.744.779	-22.071
2.751.270	-35.961	2.790.266	-25.015	2.744.944	-22.072
2.751.443	-35.963	2.790.393	-25.014	2.745.160	-22.072
2.751.628	-35.965	2.790.543	-25.014	2.745.388	-22.073
2.751.791	-35.967	2.790.689	-25.013	2.745.548	-22.074
2.751.871	-35.969	2.790.896	-25.012	2.745.675	-22.074
2.751.974	-35.971	2.791.072	-25.012	2.745.826	-22.075
2.752.209	-35.973	2.791.170	-25.011	2.746.003	-22.076
2.752.401	-35.975	2.791.364	-25.010	2.746.187	-22.077
2.752.560	-35.977	2.791.534	-25.010	2.746.317	-22.077
2.752.767	-35.978	2.791.740	-25.009	2.746.425	-22.078
2.752.960	-35.980	2.791.957	-25.008	2.746.552	-22.079
2.753.138	-35.982	2.792.083	-25.008	2.746.711	-22.080

2.753.327	-35.984	2.792.265	-25.007	2.746.862	-22.080
2.753.507	-35.986	2.792.496	-25.006	2.747.029	-22.081
2.753.688	-35.987	2.792.684	-25.006	2.747.227	-22.082
2.753.849	-35.989	2.792.859	-25.005	2.747.335	-22.083
2.754.052	-35.991	2.793.051	-25.004	2.747.500	-22.084
2.754.252	-35.993	2.793.157	-25.004	2.747.763	-22.084
2.754.281	-35.994	2.793.320	-25.003	2.747.961	-22.085
2.754.330	-35.996	2.793.506	-25.003	2.748.167	-22.086
2.754.609	-35.998	2.793.625	-25.002	2.748.421	-22.087
2.754.886	-35.999	2.793.773	-25.001	2.748.615	-22.088
2.755.002	-36.001	2.793.972	-25.001	2.748.771	-22.088
2.755.065	-36.003	2.794.150	-25.000	2.748.925	-22.089
2.755.157	-36.004	2.794.295	-25.000	2.749.091	-22.090
2.755.400	-36.006	2.794.460	-24.999	2.749.234	-22.091
2.755.648	-36.007	2.794.631	-24.998	2.749.352	-22.092
2.755.790	-36.009	2.794.821	-24.998	2.749.494	-22.093
2.755.981	-36.010	2.794.940	-24.997	2.749.694	-22.094
2.756.122	-36.012	2.795.077	-24.997	2.749.850	-22.095
2.756.310	-36.013	2.795.303	-24.996	2.749.955	-22.095
2.756.537	-36.015	2.795.468	-24.996	2.750.122	-22.096
2.756.538	-36.016	2.795.574	-24.995	2.750.299	-22.097
2.756.563	-36.017	2.795.720	-24.995	2.750.446	-22.098
2.756.768	-36.019	2.795.786	-24.994	2.750.578	-22.099
2.757.195	-36.020	2.795.884	-24.994	2.750.789	-22.100
2.757.741	-36.021	2.796.083	-24.993	2.751.021	-22.101
2.758.116	-36.023	2.796.255	-24.993	2.751.183	-22.102
2.758.137	-36.024	2.796.379	-24.992	2.751.375	-22.103
2.758.051	-36.025	2.796.571	-24.992	2.751.573	-22.103
2.758.170	-36.026	2.796.812	-24.991	2.751.686	-22.104
2.758.260	-36.027	2.796.945	-24.991	2.751.804	-22.105

2.758.338	-36.029	2.797.131	-24.991	2.752.012	-22.106
2.758.365	-36.030	2.797.401	-24.990	2.752.177	-22.107
2.758.515	-36.031	2.797.650	-24.990	2.752.358	-22.108
2.758.738	-36.032	2.797.870	-24.989	2.752.516	-22.109
2.758.864	-36.033	2.798.002	-24.989	2.752.642	-22.110
2.759.092	-36.034	2.798.135	-24.989	2.752.816	-22.111
2.759.276	-36.035	2.798.319	-24.988	2.752.980	-22.112
2.759.431	-36.036	2.798.449	-24.988	2.753.123	-22.113
2.759.612	-36.037	2.798.477	-24.988	2.753.344	-22.114
2.759.807	-36.038	2.798.594	-24.987	2.753.557	-22.114
2.759.985	-36.038	2.798.846	-24.987	2.753.708	-22.115
2.760.166	-36.039	2.799.019	-24.987	2.753.896	-22.116
2.760.410	-36.040	2.799.165	-24.987	2.754.103	-22.117
2.760.580	-36.041	2.799.351	-24.986	2.754.241	-22.118
2.760.718	-36.042	2.799.489	-24.986	2.754.373	-22.119
2.760.847	-36.043	2.799.624	-24.986	2.754.565	-22.120
2.760.945	-36.043	2.799.783	-24.985	2.754.700	-22.121
2.761.042	-36.044	2.799.989	-24.985	2.754.886	-22.122
2.761.231	-36.045	2.800.249	-24.985	2.755.086	-22.123
2.761.470	-36.046	2.800.432	-24.985	2.755.237	-22.124
2.761.578	-36.046	2.800.605	-24.984	2.755.411	-22.125
2.761.743	-36.047	2.800.818	-24.984	2.755.570	-22.126
2.761.927	-36.048	2.800.968	-24.984	2.755.758	-22.127
2.762.014	-36.048	2.801.089	-24.984	2.755.944	-22.127
2.762.216	-36.049	2.801.212	-24.984	2.756.089	-22.128
2.762.478	-36.050	2.801.336	-24.984	2.756.213	-22.129
2.762.597	-36.050	2.801.508	-24.983	2.756.400	-22.130
2.762.730	-36.051	2.801.746	-24.983	2.756.577	-22.131
2.762.935	-36.051	2.802.001	-24.983	2.756.693	-22.132
2.763.177	-36.052	2.802.163	-24.983	2.757.003	-22.133

2.763.401	-36.053	2.802.285	-24.983	2.757.487	-22.134
2.763.515	-36.053	2.802.397	-24.983	2.757.748	-22.135
2.763.627	-36.054	2.802.517	-24.983	2.757.827	-22.135
2.763.835	-36.054	2.802.733	-24.983	2.757.955	-22.136
2.764.052	-36.055	2.802.996	-24.982	2.758.058	-22.137
2.764.265	-36.055	2.803.141	-24.982	2.758.062	-22.138
2.764.442	-36.056	2.803.239	-24.982	2.758.092	-22.139
2.764.445	-36.056	2.803.387	-24.982	2.758.195	-22.140
2.764.492	-36.057	2.803.567	-24.982	2.758.347	-22.141
2.764.656	-36.057	2.803.793	-24.982	2.758.477	-22.141
2.764.862	-36.058	2.804.019	-24.982	2.758.598	-22.142
2.765.100	-36.058	2.804.145	-24.982	2.758.768	-22.143
2.765.313	-36.059	2.804.246	-24.982	2.758.961	-22.144
2.765.453	-36.059	2.804.442	-24.982	2.759.175	-22.145
2.765.583	-36.060	2.804.623	-24.982	2.759.338	-22.145
2.765.677	-36.060	2.804.752	-24.982	2.759.507	-22.146
2.765.780	-36.061	2.804.966	-24.982	2.759.685	-22.147
2.765.995	-36.061	2.805.182	-24.982	2.759.843	-22.148
2.766.090	-36.062	2.805.291	-24.982	2.760.052	-22.149
2.766.263	-36.062	2.805.451	-24.982	2.760.182	-22.149
2.766.515	-36.063	2.805.594	-24.982	2.760.349	-22.150
2.766.685	-36.063	2.805.728	-24.982	2.760.516	-22.151
2.766.887	-36.064	2.805.863	-24.982	2.760.659	-22.152
2.767.130	-36.064	2.805.981	-24.982	2.760.815	-22.152
2.767.379	-36.065	2.806.271	-24.982	2.761.020	-22.153
2.767.491	-36.065	2.806.658	-24.982	2.761.125	-22.154
2.767.588	-36.066	2.807.079	-24.982	2.761.233	-22.155
2.767.773	-36.066	2.807.375	-24.982	2.761.385	-22.155
2.767.914	-36.067	2.807.430	-24.982	2.761.581	-22.156
2.768.117	-36.067	2.807.483	-24.982	2.761.808	-22.157

2.768.275	-36.068	2.807.595	-24.982	2.762.022	-22.158
2.768.341	-36.068	2.807.599	-24.982	2.762.263	-22.158
2.768.533	-36.069	2.807.658	-24.982	2.762.451	-22.159
2.768.785	-36.069	2.807.834	-24.982	2.762.489	-22.160
2.768.983	-36.070	2.807.904	-24.983	2.762.658	-22.161
2.769.048	-36.070	2.808.011	-24.983	2.762.910	-22.161
2.769.201	-36.071	2.808.195	-24.983	2.763.073	-22.162
2.769.406	-36.071	2.808.394	-24.983	2.763.239	-22.163
2.769.599	-36.072	2.808.608	-24.983	2.763.380	-22.164
2.769.778	-36.072	2.808.782	-24.983	2.763.528	-22.164
2.769.915	-36.073	2.808.947	-24.983	2.763.706	-22.165
2.770.088	-36.073	2.809.118	-24.983	2.763.872	-22.166
2.770.298	-36.074	2.809.265	-24.983	2.764.016	-22.166
2.770.424	-36.074	2.809.467	-24.983	2.764.174	-22.167
2.770.574	-36.075	2.809.710	-24.983	2.764.326	-22.168
2.770.854	-36.076	2.809.829	-24.983	2.764.475	-22.169
2.770.988	-36.076	2.809.953	-24.984	2.764.659	-22.169
2.771.105	-36.077	2.810.157	-24.984	2.764.887	-22.170
2.771.220	-36.077	2.810.270	-24.984	2.765.107	-22.171
2.771.259	-36.078	2.810.399	-24.984	2.765.238	-22.171
2.771.380	-36.078	2.810.558	-24.984	2.765.305	-22.172
2.771.577	-36.079	2.810.695	-24.984	2.765.428	-22.173
2.771.773	-36.079	2.810.862	-24.984	2.765.667	-22.174
2.771.953	-36.080	2.811.003	-24.984	2.765.891	-22.174
2.772.137	-36.080	2.811.165	-24.985	2.766.064	-22.175
2.772.332	-36.081	2.811.351	-24.985	2.766.198	-22.176
2.772.574	-36.081	2.811.563	-24.985	2.766.313	-22.177
2.772.784	-36.082	2.811.801	-24.985	2.766.526	-22.177
2.773.060	-36.082	2.812.025	-24.985	2.766.721	-22.178
2.773.286	-36.083	2.812.152	-24.985	2.766.895	-22.179

2.773.351	-36.083	2.812.292	-24.986	2.767.101	-22.179
2.773.460	-36.084	2.812.495	-24.986	2.767.253	-22.180
2.773.593	-36.084	2.812.632	-24.986	2.767.440	-22.181
2.773.789	-36.085	2.812.812	-24.986	2.767.597	-22.181
2.773.977	-36.085	2.812.975	-24.986	2.767.736	-22.182
2.774.015	-36.086	2.813.092	-24.987	2.767.870	-22.183
2.774.203	-36.086	2.813.251	-24.987	2.767.968	-22.184
2.774.479	-36.087	2.813.385	-24.987	2.768.116	-22.184
2.774.523	-36.087	2.813.528	-24.987	2.768.297	-22.185
2.774.627	-36.087	2.813.677	-24.988	2.768.497	-22.186
2.774.859	-36.088	2.813.889	-24.988	2.768.680	-22.186
2.775.059	-36.088	2.814.142	-24.988	2.768.854	-22.187
2.775.242	-36.089	2.814.359	-24.988	2.769.026	-22.188
2.775.489	-36.089	2.814.534	-24.989	2.769.096	-22.188
2.775.643	-36.090	2.814.615	-24.989	2.769.283	-22.189
2.775.684	-36.090	2.814.761	-24.989	2.769.530	-22.190
2.775.879	-36.090	2.814.937	-24.989	2.769.629	-22.190
2.776.147	-36.091	2.815.038	-24.990	2.769.720	-22.191
2.776.295	-36.091	2.815.153	-24.990	2.769.873	-22.191
2.776.425	-36.092	2.815.349	-24.990	2.770.081	-22.192
2.776.585	-36.092	2.815.562	-24.990	2.770.247	-22.193
2.776.791	-36.092	2.815.733	-24.991	2.770.392	-22.193
2.776.917	-36.093	2.815.944	-24.991	2.770.608	-22.194
2.776.970	-36.093	2.816.184	-24.991	2.770.757	-22.194
2.777.192	-36.094	2.816.364	-24.992	2.770.869	-22.195
2.777.498	-36.094	2.816.528	-24.992	2.771.086	-22.195
2.777.679	-36.094	2.816.689	-24.992	2.771.364	-22.196
2.777.810	-36.095	2.816.815	-24.993	2.771.602	-22.196
2.777.942	-36.095	2.816.971	-24.993	2.771.808	-22.197
2.778.092	-36.095	2.817.122	-24.994	2.771.981	-22.197

2.778.298	-36.096	2.817.249	-24.994	2.772.104	-22.198
2.778.423	-36.096	2.817.433	-24.994	2.772.256	-22.198
2.778.506	-36.096	2.817.617	-24.995	2.772.426	-22.199
2.778.734	-36.097	2.817.744	-24.995	2.772.581	-22.199
2.778.997	-36.097	2.817.867	-24.995	2.772.737	-22.200
2.779.163	-36.097	2.818.051	-24.996	2.772.867	-22.200
2.779.268	-36.097	2.818.269	-24.996	2.772.957	-22.201
2.779.414	-36.098	2.818.455	-24.997	2.773.114	-22.201
2.779.630	-36.098	2.818.598	-24.997	2.773.304	-22.201
2.779.738	-36.098	2.818.763	-24.997	2.773.409	-22.202
2.779.821	-36.098	2.819.020	-24.998	2.773.506	-22.202
2.780.046	-36.099	2.819.189	-24.998	2.773.706	-22.202
2.780.244	-36.099	2.819.268	-24.998	2.773.955	-22.203
2.780.419	-36.099	2.819.449	-24.999	2.774.157	-22.203
2.780.627	-36.099	2.819.620	-24.999	2.774.312	-22.203
2.780.820	-36.099	2.819.709	-25.000	2.774.475	-22.204
2.780.970	-36.099	2.819.814	-25.000	2.774.732	-22.204
2.781.151	-36.100	2.819.984	-25.000	2.774.949	-22.204
2.781.358	-36.100	2.820.143	-25.001	2.775.074	-22.204
2.781.517	-36.100	2.820.341	-25.001	2.775.166	-22.205
2.781.719	-36.100	2.820.515	-25.001	2.775.320	-22.205
2.781.905	-36.100	2.820.630	-25.002	2.775.478	-22.205
2.781.893	-36.100	2.820.829	-25.002	2.775.646	-22.205
2.782.149	-36.100	2.821.049	-25.002	2.775.876	-22.205
2.782.626	-36.101	2.821.241	-25.003	2.776.053	-22.206
2.782.961	-36.101	2.821.441	-25.003	2.776.168	-22.206
2.783.181	-36.101	2.821.646	-25.004	2.776.335	-22.206
2.783.255	-36.101	2.821.824	-25.004	2.776.548	-22.206
2.783.279	-36.101	2.821.974	-25.004	2.776.722	-22.206
2.783.327	-36.101	2.822.090	-25.005	2.776.911	-22.206

2.783.361	-36.101	2.822.286	-25.005	2.777.079	-22.206
2.783.430	-36.101	2.822.488	-25.005	2.777.214	-22.206
2.783.604	-36.101	2.822.596	-25.005	2.777.346	-22.206
2.783.688	-36.101	2.822.726	-25.006	2.777.500	-22.206
2.783.762	-36.101	2.822.885	-25.006	2.777.666	-22.206
2.784.008	-36.101	2.823.033	-25.006	2.777.809	-22.206
2.784.223	-36.101	2.823.178	-25.007	2.777.994	-22.206
2.784.337	-36.101	2.823.406	-25.007	2.778.112	-22.206
2.784.478	-36.101	2.823.612	-25.007	2.778.271	-22.206
2.784.659	-36.101	2.823.778	-25.007	2.778.533	-22.206
2.784.872	-36.101	2.823.954	-25.008	2.778.755	-22.206
2.785.204	-36.101	2.824.131	-25.008	2.778.952	-22.206
2.785.426	-36.101	2.824.330	-25.008	2.779.085	-22.206
2.785.486	-36.101	2.824.536	-25.008	2.779.244	-22.206
2.785.643	-36.101	2.824.702	-25.008	2.779.420	-22.206
2.785.787	-36.101	2.824.859	-25.009	2.779.532	-22.206
2.785.920	-36.101	2.824.998	-25.009	2.779.660	-22.205
2.786.031	-36.101	2.825.137	-25.009	2.779.850	-22.205
2.786.174	-36.101	2.825.334	-25.009	2.780.171	-22.205
2.786.408	-36.101	2.825.518	-25.009	2.780.605	-22.205
2.786.520	-36.101	2.825.693	-25.009	2.780.852	-22.205
2.786.599	-36.101	2.825.849	-25.009	2.780.941	-22.204
2.786.748	-36.101	2.825.964	-25.010	2.781.064	-22.204
2.786.880	-36.101	2.826.108	-25.010	2.781.209	-22.204
2.787.083	-36.101	2.826.302	-25.010	2.781.278	-22.204
2.787.390	-36.101	2.826.528	-25.010	2.781.295	-22.203
2.787.680	-36.101	2.826.703	-25.010	2.781.383	-22.203
2.787.724	-36.101	2.826.830	-25.010	2.781.454	-22.203
2.787.756	-36.101	2.827.051	-25.010	2.781.614	-22.202
2.788.052	-36.101	2.827.263	-25.010	2.781.798	-22.202

2.788.340	-36.101	2.827.391	-25.010	2.782.001	-22.202
2.788.531	-36.101	2.827.535	-25.010	2.782.171	-22.201
2.788.636	-36.101	2.827.794	-25.010	2.782.322	-22.201
2.788.764	-36.101	2.828.011	-25.010	2.782.531	-22.200
2.788.951	-36.101	2.828.090	-25.010	2.782.707	-22.200
2.789.055	-36.101	2.828.210	-25.010	2.782.856	-22.200
2.789.160	-36.101	2.828.398	-25.010	2.783.062	-22.199
2.789.272	-36.101	2.828.607	-25.010	2.783.275	-22.199
2.789.413	-36.102	2.828.752	-25.010	2.783.395	-22.198
2.789.652	-36.102	2.828.880	-25.010	2.783.496	-22.198
2.789.883	-36.102	2.829.017	-25.010	2.783.684	-22.197
2.790.070	-36.102	2.829.178	-25.010	2.783.875	-22.197
2.790.255	-36.102	2.829.382	-25.010	2.784.060	-22.197
2.790.432	-36.102	2.829.570	-25.010	2.784.197	-22.196
2.790.569	-36.103	2.829.753	-25.010	2.784.273	-22.196
2.790.726	-36.103	2.829.888	-25.009	2.784.420	-22.195
2.790.910	-36.103	2.830.014	-25.009	2.784.630	-22.195
2.791.056	-36.103	2.830.222	-25.009	2.784.817	-22.194
2.791.159	-36.104	2.830.608	-25.009	2.784.966	-22.194
2.791.277	-36.104	2.831.064	-25.009	2.785.161	-22.193
2.791.494	-36.104	2.831.280	-25.009	2.785.370	-22.193
2.791.667	-36.105	2.831.320	-25.008	2.785.526	-22.192
2.791.852	-36.105	2.831.397	-25.008	2.785.692	-22.192
2.792.044	-36.106	2.831.452	-25.008	2.785.905	-22.191
2.792.221	-36.106	2.831.479	-25.008	2.785.959	-22.191
2.792.416	-36.107	2.831.595	-25.008	2.786.108	-22.191
2.792.581	-36.107	2.831.732	-25.007	2.786.300	-22.190
2.792.794	-36.108	2.831.840	-25.007	2.786.459	-22.190
2.792.943	-36.108	2.832.010	-25.007	2.786.657	-22.189
2.793.123	-36.109	2.832.198	-25.007	2.786.797	-22.189

2.793.273	-36.109	2.832.351	-25.006	2.787.000	-22.188
2.793.374	-36.110	2.832.470	-25.006	2.787.195	-22.188
2.793.531	-36.110	2.832.647	-25.006	2.787.364	-22.187
2.793.661	-36.111	2.832.868	-25.006	2.787.518	-22.187
2.793.770	-36.112	2.833.094	-25.005	2.787.661	-22.187
2.793.909	-36.112	2.833.312	-25.005	2.787.794	-22.186
2.794.124	-36.113	2.833.474	-25.005	2.787.939	-22.186
2.794.391	-36.114	2.833.604	-25.004	2.788.125	-22.186
2.794.587	-36.114	2.833.719	-25.004	2.788.345	-22.185
2.794.743	-36.115	2.833.860	-25.004	2.788.538	-22.185
2.794.915	-36.116	2.833.983	-25.003	2.788.607	-22.184
2.795.036	-36.116	2.834.138	-25.003	2.788.699	-22.184
2.795.168	-36.117	2.834.276	-25.003	2.788.918	-22.184
2.795.280	-36.118	2.834.406	-25.002	2.789.164	-22.183
2.795.450	-36.119	2.834.644	-25.002	2.789.356	-22.183
2.795.678	-36.120	2.834.818	-25.002	2.789.552	-22.183
2.795.749	-36.120	2.834.911	-25.001	2.789.753	-22.183
2.795.854	-36.121	2.835.104	-25.001	2.789.872	-22.182
2.796.053	-36.122	2.835.338	-25.001	2.790.005	-22.182
2.796.219	-36.123	2.835.566	-25.000	2.790.164	-22.182
2.796.351	-36.124	2.835.808	-25.000	2.790.298	-22.182
2.796.486	-36.125	2.835.952	-25.000	2.790.422	-22.182
2.796.701	-36.126	2.836.053	-24.999	2.790.583	-22.181
2.796.880	-36.127	2.836.221	-24.999	2.790.776	-22.181
2.797.041	-36.128	2.836.432	-24.999	2.790.956	-22.181
2.797.218	-36.129	2.836.582	-24.998	2.791.140	-22.181
2.797.364	-36.130	2.836.700	-24.998	2.791.300	-22.181
2.797.644	-36.131	2.836.848	-24.998	2.791.444	-22.181
2.797.947	-36.132	2.837.015	-24.997	2.791.586	-22.181
2.798.128	-36.133	2.837.160	-24.997	2.791.813	-22.180

2.798.282	-36.134	2.837.337	-24.997	2.792.043	-22.180
2.798.515	-36.135	2.837.476	-24.997	2.792.218	-22.180
2.798.690	-36.136	2.837.571	-24.996	2.792.337	-22.180
2.798.803	-36.137	2.837.799	-24.996	2.792.427	-22.180
2.798.995	-36.138	2.838.077	-24.996	2.792.589	-22.180
2.799.059	-36.139	2.838.232	-24.996	2.792.762	-22.180
2.799.113	-36.140	2.838.372	-24.995	2.792.830	-22.180
2.799.281	-36.141	2.838.558	-24.995	2.792.942	-22.180
2.799.532	-36.142	2.838.681	-24.995	2.793.112	-22.180
2.799.664	-36.143	2.838.825	-24.995	2.793.271	-22.180
2.799.775	-36.144	2.839.005	-24.994	2.793.499	-22.180
2.799.924	-36.145	2.839.139	-24.994	2.793.698	-22.180
2.800.049	-36.146	2.839.288	-24.994	2.793.867	-22.180
2.800.153	-36.147	2.839.471	-24.994	2.794.077	-22.180
2.800.341	-36.149	2.839.709	-24.994	2.794.294	-22.180
2.800.596	-36.150	2.839.915	-24.994	2.794.478	-22.180
2.800.778	-36.151	2.840.031	-24.993	2.794.656	-22.180
2.800.992	-36.152	2.840.190	-24.993	2.794.865	-22.180
2.801.112	-36.153	2.840.366	-24.993	2.795.042	-22.181
2.801.227	-36.154	2.840.555	-24.993	2.795.212	-22.181
2.801.523	-36.155	2.840.743	-24.993	2.795.396	-22.181
2.801.725	-36.157	2.840.908	-24.993	2.795.540	-22.181
2.801.909	-36.158	2.841.105	-24.993	2.795.672	-22.181
2.802.098	-36.159	2.841.300	-24.993	2.795.807	-22.181
2.802.120	-36.160	2.841.451	-24.993	2.795.946	-22.181
2.802.231	-36.161	2.841.580	-24.993	2.796.097	-22.182
2.802.463	-36.162	2.841.690	-24.993	2.796.268	-22.182
2.802.679	-36.164	2.841.853	-24.993	2.796.375	-22.182
2.802.860	-36.165	2.842.018	-24.993	2.796.465	-22.182
2.803.016	-36.166	2.842.198	-24.993	2.796.638	-22.182

2.803.214	-36.167	2.842.383	-24.993	2.796.906	-22.182
2.803.363	-36.168	2.842.525	-24.993	2.797.137	-22.183
2.803.467	-36.169	2.842.751	-24.993	2.797.401	-22.183
2.803.726	-36.171	2.842.903	-24.993	2.797.538	-22.183
2.803.961	-36.172	2.843.004	-24.993	2.797.618	-22.183
2.804.096	-36.173	2.843.185	-24.993	2.797.787	-22.184
2.804.211	-36.174	2.843.332	-24.993	2.798.000	-22.184
2.804.392	-36.175	2.843.428	-24.993	2.798.175	-22.184
2.804.619	-36.176	2.843.542	-24.993	2.798.311	-22.184
2.804.718	-36.177	2.843.715	-24.993	2.798.437	-22.185
2.804.828	-36.179	2.843.947	-24.993	2.798.681	-22.185
2.804.973	-36.180	2.844.066	-24.993	2.798.893	-22.185
2.805.187	-36.181	2.844.164	-24.993	2.798.955	-22.186
2.805.341	-36.182	2.844.366	-24.993	2.799.080	-22.186
2.805.560	-36.183	2.844.543	-24.993	2.799.248	-22.186
2.805.851	-36.184	2.844.716	-24.993	2.799.388	-22.187
2.805.941	-36.185	2.844.952	-24.993	2.799.583	-22.187
2.806.093	-36.187	2.845.169	-24.993	2.799.793	-22.187
2.806.292	-36.188	2.845.348	-24.993	2.800.002	-22.188
2.806.439	-36.189	2.845.471	-24.993	2.800.204	-22.188
2.806.526	-36.190	2.845.693	-24.993	2.800.298	-22.188
2.806.610	-36.191	2.845.935	-24.993	2.800.410	-22.189
2.806.826	-36.192	2.846.080	-24.993	2.800.576	-22.189
2.807.299	-36.193	2.846.230	-24.994	2.800.742	-22.190
2.807.762	-36.194	2.846.422	-24.994	2.800.981	-22.190
2.807.987	-36.196	2.846.584	-24.994	2.801.147	-22.190
2.808.131	-36.197	2.846.672	-24.994	2.801.306	-22.191
2.808.149	-36.198	2.846.813	-24.994	2.801.508	-22.191
2.808.163	-36.199	2.846.974	-24.994	2.801.693	-22.192
2.808.255	-36.200	2.847.105	-24.994	2.801.925	-22.192

2.808.338	-36.201	2.847.303	-24.994	2.802.093	-22.192
2.808.412	-36.202	2.847.527	-24.994	2.802.191	-22.193
2.808.468	-36.203	2.847.664	-24.994	2.802.325	-22.193
2.808.643	-36.204	2.847.798	-24.994	2.802.465	-22.194
2.808.883	-36.205	2.848.008	-24.994	2.802.647	-22.194
2.809.004	-36.206	2.848.197	-24.994	2.802.821	-22.194
2.809.122	-36.207	2.848.355	-24.994	2.803.025	-22.195
2.809.330	-36.208	2.848.554	-24.994	2.803.437	-22.195
2.809.576	-36.210	2.848.730	-24.994	2.803.853	-22.196
2.809.800	-36.211	2.848.914	-24.994	2.804.056	-22.196
2.810.049	-36.212	2.849.085	-24.994	2.804.140	-22.197
2.810.171	-36.213	2.849.233	-24.994	2.804.226	-22.197
2.810.309	-36.214	2.849.377	-24.994	2.804.279	-22.197
2.810.486	-36.215	2.849.537	-24.994	2.804.308	-22.198
2.810.567	-36.216	2.849.720	-24.994	2.804.400	-22.198
2.810.722	-36.217	2.849.904	-24.994	2.804.504	-22.199
2.810.840	-36.218	2.850.057	-24.994	2.804.641	-22.199
2.810.981	-36.219	2.850.204	-24.994	2.804.846	-22.200
2.811.188	-36.220	2.850.421	-24.994	2.805.028	-22.200
2.811.307	-36.221	2.850.538	-24.994	2.805.198	-22.200
2.811.425	-36.222	2.850.670	-24.994	2.805.325	-22.201
2.811.653	-36.223	2.850.870	-24.994	2.805.446	-22.201
2.811.813	-36.224	2.851.033	-24.994	2.805.578	-22.202
2.811.924	-36.225	2.851.208	-24.994	2.805.760	-22.202
2.812.112	-36.226	2.851.414	-24.994	2.806.062	-22.202
2.812.323	-36.227	2.851.621	-24.993	2.806.281	-22.203
2.812.581	-36.228	2.851.793	-24.993	2.806.411	-22.203
2.812.758	-36.230	2.851.989	-24.993	2.806.570	-22.204
2.812.924	-36.231	2.852.159	-24.993	2.806.710	-22.204
2.813.235	-36.232	2.852.271	-24.993	2.806.828	-22.204

2.813.426	-36.233	2.852.382	-24.993	2.806.994	-22.205
2.813.506	-36.234	2.852.567	-24.993	2.807.188	-22.205
2.813.676	-36.235	2.852.778	-24.992	2.807.346	-22.206
2.813.829	-36.236	2.852.957	-24.992	2.807.484	-22.206
2.813.969	-36.237	2.853.138	-24.992	2.807.635	-22.206
2.814.089	-36.238	2.853.279	-24.992	2.807.784	-22.207
2.814.211	-36.240	2.853.408	-24.991	2.807.943	-22.207
2.814.422	-36.241	2.853.611	-24.991	2.808.185	-22.207
2.814.546	-36.242	2.853.810	-24.991	2.808.432	-22.208
2.814.651	-36.243	2.853.908	-24.990	2.808.623	-22.208
2.814.875	-36.244	2.854.038	-24.990	2.808.750	-22.209
2.814.987	-36.245	2.854.435	-24.990	2.808.905	-22.209
2.815.147	-36.247	2.854.873	-24.989	2.809.116	-22.209
2.815.428	-36.248	2.855.128	-24.989	2.809.255	-22.210
2.815.601	-36.249	2.855.238	-24.989	2.809.338	-22.210
2.815.728	-36.250	2.855.292	-24.988	2.809.501	-22.211
2.815.873	-36.251	2.855.396	-24.988	2.809.700	-22.211
2.816.076	-36.253	2.855.482	-24.987	2.809.789	-22.211
2.816.259	-36.254	2.855.548	-24.987	2.809.948	-22.212
2.816.350	-36.255	2.855.625	-24.986	2.810.155	-22.212
2.816.517	-36.257	2.855.787	-24.986	2.810.305	-22.213
2.816.638	-36.258	2.855.931	-24.985	2.810.519	-22.213
2.816.747	-36.259	2.856.078	-24.985	2.810.692	-22.213
2.816.967	-36.261	2.856.248	-24.984	2.810.840	-22.214
2.817.253	-36.262	2.856.443	-24.983	2.810.984	-22.214
2.817.465	-36.263	2.856.642	-24.983	2.811.133	-22.214
2.817.603	-36.265	2.856.808	-24.982	2.811.315	-22.215
2.817.749	-36.266	2.856.992	-24.982	2.811.468	-22.215
2.817.899	-36.268	2.857.156	-24.981	2.811.658	-22.216
2.818.065	-36.269	2.857.310	-24.980	2.811.858	-22.216

2.818.217	-36.271	2.857.459	-24.980	2.812.029	-22.216
2.818.333	-36.272	2.857.564	-24.979	2.812.214	-22.217
2.818.513	-36.273	2.857.718	-24.978	2.812.390	-22.217
2.818.701	-36.275	2.857.921	-24.977	2.812.534	-22.218
2.818.799	-36.276	2.858.101	-24.977	2.812.741	-22.218
2.818.972	-36.278	2.858.249	-24.976	2.812.954	-22.218
2.819.236	-36.279	2.858.392	-24.975	2.813.060	-22.219
2.819.420	-36.281	2.858.540	-24.974	2.813.143	-22.219
2.819.583	-36.282	2.858.665	-24.974	2.813.291	-22.220
2.819.772	-36.284	2.858.810	-24.973	2.813.492	-22.220
2.819.846	-36.285	2.858.994	-24.972	2.813.654	-22.220
2.819.922	-36.287	2.859.194	-24.971	2.813.828	-22.221
2.820.092	-36.289	2.859.353	-24.970	2.814.004	-22.221
2.820.320	-36.290	2.859.562	-24.970	2.814.133	-22.222
2.820.551	-36.292	2.859.803	-24.969	2.814.317	-22.222
2.820.718	-36.293	2.860.038	-24.968	2.814.499	-22.222
2.820.690	-36.295	2.860.212	-24.967	2.814.677	-22.223
2.820.862	-36.297	2.860.319	-24.966	2.814.857	-22.223
2.821.169	-36.298	2.860.431	-24.965	2.815.071	-22.223
2.821.223	-36.300	2.860.574	-24.965	2.815.237	-22.224
2.821.375	-36.301	2.860.706	-24.964	2.815.299	-22.224
2.821.570	-36.303	2.860.834	-24.963	2.815.421	-22.224
2.821.714	-36.305	2.860.959	-24.962	2.815.613	-22.225
2.821.917	-36.306	2.861.163	-24.961	2.815.775	-22.225
2.822.127	-36.308	2.861.412	-24.960	2.815.887	-22.225
2.822.390	-36.310	2.861.577	-24.959	2.816.013	-22.226
2.822.671	-36.311	2.861.757	-24.959	2.816.158	-22.226
2.822.809	-36.313	2.861.922	-24.958	2.816.274	-22.226
2.822.910	-36.314	2.862.066	-24.957	2.816.436	-22.226
2.823.134	-36.316	2.862.261	-24.956	2.816.661	-22.227

2.823.401	-36.318	2.862.424	-24.955	2.816.827	-22.227
2.823.544	-36.319	2.862.542	-24.954	2.816.985	-22.227
2.823.627	-36.321	2.862.711	-24.953	2.817.267	-22.227
2.823.741	-36.323	2.862.870	-24.953	2.817.563	-22.228
2.823.880	-36.324	2.863.029	-24.952	2.817.755	-22.228
2.824.089	-36.326	2.863.235	-24.951	2.817.899	-22.228
2.824.252	-36.328	2.863.434	-24.950	2.818.045	-22.228
2.824.337	-36.329	2.863.596	-24.949	2.818.217	-22.228
2.824.483	-36.331	2.863.742	-24.948	2.818.362	-22.229
2.824.695	-36.332	2.863.903	-24.948	2.818.517	-22.229
2.824.864	-36.334	2.864.131	-24.947	2.818.676	-22.229
2.824.991	-36.335	2.864.370	-24.946	2.818.817	-22.229
2.825.078	-36.337	2.864.521	-24.945	2.819.019	-22.229
2.825.234	-36.339	2.864.623	-24.944	2.819.211	-22.229
2.825.473	-36.340	2.864.709	-24.944	2.819.322	-22.229
2.825.739	-36.342	2.864.835	-24.943	2.819.406	-22.229
2.825.935	-36.343	2.865.057	-24.942	2.819.590	-22.229
2.826.056	-36.345	2.865.259	-24.941	2.819.810	-22.230
2.826.172	-36.346	2.865.415	-24.940	2.819.938	-22.230
2.826.307	-36.348	2.865.554	-24.940	2.820.108	-22.230
2.826.473	-36.349	2.865.659	-24.939	2.820.346	-22.230
2.826.786	-36.351	2.865.833	-24.938	2.820.540	-22.230
2.826.985	-36.352	2.866.031	-24.937	2.820.718	-22.230
2.827.086	-36.354	2.866.199	-24.937	2.820.876	-22.230
2.827.285	-36.355	2.866.407	-24.936	2.821.035	-22.230
2.827.442	-36.357	2.866.544	-24.935	2.821.230	-22.230
2.827.600	-36.358	2.866.710	-24.934	2.821.426	-22.229
2.827.747	-36.360	2.866.947	-24.934	2.821.553	-22.229
2.827.856	-36.361	2.867.070	-24.933	2.821.645	-22.229
2.828.060	-36.362	2.867.149	-24.932	2.821.852	-22.229

2.828.280	-36.364	2.867.307	-24.932	2.822.084	-22.229
2.828.427	-36.365	2.867.488	-24.931	2.822.238	-22.229
2.828.600	-36.367	2.867.601	-24.930	2.822.387	-22.229
2.828.658	-36.368	2.867.715	-24.929	2.822.577	-22.229
2.828.828	-36.369	2.867.871	-24.929	2.822.805	-22.229
2.829.077	-36.370	2.868.020	-24.928	2.822.931	-22.228
2.829.258	-36.372	2.868.193	-24.927	2.823.040	-22.228
2.829.511	-36.373	2.868.379	-24.927	2.823.229	-22.228
2.829.685	-36.374	2.868.582	-24.926	2.823.401	-22.228
2.829.814	-36.375	2.868.719	-24.926	2.823.594	-22.228
2.829.948	-36.377	2.868.945	-24.925	2.823.745	-22.228
2.830.100	-36.378	2.869.245	-24.924	2.823.846	-22.227
2.830.248	-36.379	2.869.471	-24.924	2.824.037	-22.227
2.830.406	-36.380	2.869.645	-24.923	2.824.222	-22.227
2.830.612	-36.381	2.869.768	-24.922	2.824.380	-22.227
2.830.771	-36.382	2.869.930	-24.922	2.824.557	-22.226
2.831.056	-36.384	2.870.110	-24.921	2.824.743	-22.226
2.831.342	-36.385	2.870.256	-24.921	2.824.929	-22.226
2.831.439	-36.386	2.870.352	-24.920	2.825.005	-22.225
2.831.555	-36.387	2.870.500	-24.920	2.825.175	-22.225
2.831.726	-36.388	2.870.677	-24.919	2.825.498	-22.225
2.831.844	-36.389	2.870.880	-24.918	2.825.710	-22.224
2.831.996	-36.390	2.871.094	-24.918	2.825.812	-22.224
2.832.451	-36.391	2.871.270	-24.917	2.825.901	-22.224
2.832.890	-36.392	2.871.407	-24.917	2.826.078	-22.223
2.832.928	-36.393	2.871.486	-24.916	2.826.479	-22.223
2.832.935	-36.394	2.871.651	-24.916	2.826.882	-22.222
2.833.070	-36.394	2.871.891	-24.915	2.827.101	-22.222
2.833.124	-36.395	2.872.076	-24.915	2.827.198	-22.221
2.833.210	-36.396	2.872.209	-24.914	2.827.228	-22.221

2.833.356	-36.397	2.872.397	-24.914	2.827.296	-22.220
2.833.445	-36.398	2.872.589	-24.913	2.827.339	-22.220
2.833.600	-36.399	2.872.724	-24.913	2.827.470	-22.219
2.833.817	-36.399	2.872.872	-24.912	2.827.640	-22.219
2.833.918	-36.400	2.873.027	-24.912	2.827.713	-22.218
2.833.995	-36.401	2.873.229	-24.911	2.827.849	-22.218
2.834.221	-36.402	2.873.431	-24.911	2.828.005	-22.217
2.834.457	-36.402	2.873.590	-24.911	2.828.174	-22.217
2.834.651	-36.403	2.873.734	-24.910	2.828.427	-22.216
2.834.890	-36.403	2.873.882	-24.910	2.828.615	-22.216
2.835.092	-36.404	2.874.077	-24.909	2.828.713	-22.215
2.835.237	-36.405	2.874.283	-24.909	2.828.864	-22.215
2.835.306	-36.405	2.874.422	-24.908	2.829.085	-22.214
2.835.382	-36.406	2.874.529	-24.908	2.829.275	-22.213
2.835.576	-36.406	2.874.676	-24.908	2.829.457	-22.213
2.835.771	-36.407	2.874.872	-24.907	2.829.623	-22.212
2.836.042	-36.407	2.875.075	-24.907	2.829.765	-22.211
2.836.220	-36.408	2.875.287	-24.907	2.829.955	-22.211
2.836.252	-36.408	2.875.475	-24.906	2.830.093	-22.210
2.836.440	-36.409	2.875.650	-24.906	2.830.222	-22.210
2.836.541	-36.409	2.875.793	-24.905	2.830.382	-22.209
2.836.638	-36.410	2.875.849	-24.905	2.830.495	-22.208
2.836.833	-36.410	2.876.011	-24.905	2.830.612	-22.208
2.837.025	-36.411	2.876.263	-24.904	2.830.761	-22.207
2.837.184	-36.411	2.876.479	-24.904	2.830.946	-22.206
2.837.340	-36.411	2.876.595	-24.904	2.831.184	-22.206
2.837.596	-36.412	2.876.768	-24.903	2.831.434	-22.205
2.837.769	-36.412	2.877.000	-24.903	2.831.591	-22.204
2.837.883	-36.413	2.877.159	-24.903	2.831.678	-22.204
2.838.156	-36.413	2.877.322	-24.902	2.831.853	-22.203

2.838.304	-36.413	2.877.467	-24.902	2.832.076	-22.202
2.838.445	-36.414	2.877.622	-24.902	2.832.213	-22.202
2.838.703	-36.414	2.877.755	-24.901	2.832.347	-22.201
2.838.919	-36.414	2.877.903	-24.901	2.832.509	-22.200
2.839.075	-36.415	2.878.279	-24.900	2.832.672	-22.200
2.839.092	-36.415	2.878.723	-24.900	2.832.801	-22.199
2.839.189	-36.415	2.878.974	-24.900	2.832.964	-22.198
2.839.406	-36.415	2.879.045	-24.899	2.833.231	-22.198
2.839.504	-36.416	2.879.091	-24.899	2.833.423	-22.197
2.839.767	-36.416	2.879.158	-24.899	2.833.569	-22.196
2.840.071	-36.416	2.879.259	-24.898	2.833.737	-22.196
2.840.101	-36.417	2.879.344	-24.898	2.833.905	-22.195
2.840.182	-36.417	2.879.427	-24.898	2.834.081	-22.194
2.840.323	-36.417	2.879.521	-24.897	2.834.243	-22.194
2.840.493	-36.417	2.879.696	-24.897	2.834.366	-22.193
2.840.679	-36.418	2.879.933	-24.896	2.834.539	-22.193
2.840.873	-36.418	2.880.089	-24.896	2.834.680	-22.192
2.841.126	-36.418	2.880.280	-24.896	2.834.788	-22.191
2.841.350	-36.419	2.880.483	-24.895	2.835.028	-22.191
2.841.470	-36.419	2.880.624	-24.895	2.835.316	-22.190
2.841.658	-36.419	2.880.800	-24.894	2.835.490	-22.189
2.841.852	-36.420	2.881.013	-24.894	2.835.577	-22.189
2.841.953	-36.420	2.881.155	-24.894	2.835.750	-22.188
2.842.142	-36.420	2.881.288	-24.893	2.835.984	-22.188
2.842.348	-36.421	2.881.427	-24.893	2.836.140	-22.187
2.842.429	-36.421	2.881.553	-24.892	2.836.277	-22.187
2.842.561	-36.421	2.881.729	-24.892	2.836.441	-22.186
2.842.737	-36.422	2.881.906	-24.891	2.836.613	-22.185
2.842.935	-36.422	2.882.104	-24.891	2.836.744	-22.185
2.843.087	-36.423	2.882.283	-24.891	2.836.856	-22.184

2.843.170	-36.423	2.882.390	-24.890	2.837.017	-22.184
2.843.307	-36.423	2.882.531	-24.890	2.837.213	-22.183
2.843.535	-36.424	2.882.671	-24.889	2.837.406	-22.182
2.843.759	-36.424	2.882.798	-24.889	2.837.578	-22.182
2.843.911	-36.425	2.883.013	-24.888	2.837.759	-22.181
2.844.169	-36.425	2.883.239	-24.888	2.837.924	-22.181
2.844.304	-36.425	2.883.428	-24.888	2.838.094	-22.180
2.844.284	-36.426	2.883.607	-24.887	2.838.253	-22.180
2.844.572	-36.426	2.883.789	-24.887	2.838.383	-22.179
2.844.877	-36.427	2.883.969	-24.886	2.838.557	-22.178
2.845.005	-36.427	2.884.070	-24.886	2.838.723	-22.178
2.845.046	-36.428	2.884.214	-24.885	2.838.782	-22.177
2.845.200	-36.428	2.884.413	-24.885	2.838.885	-22.177
2.845.302	-36.428	2.884.472	-24.884	2.839.102	-22.176
2.845.341	-36.429	2.884.631	-24.884	2.839.269	-22.175
2.845.500	-36.429	2.884.894	-24.884	2.839.405	-22.175
2.845.819	-36.430	2.885.081	-24.883	2.839.555	-22.174
2.846.039	-36.430	2.885.263	-24.883	2.839.727	-22.174
2.846.162	-36.431	2.885.410	-24.882	2.839.908	-22.173
2.846.320	-36.431	2.885.540	-24.882	2.840.106	-22.172
2.846.477	-36.432	2.885.708	-24.881	2.840.348	-22.172
2.846.620	-36.432	2.885.895	-24.881	2.840.476	-22.171
2.846.732	-36.433	2.886.096	-24.880	2.840.649	-22.170
2.846.875	-36.433	2.886.255	-24.880	2.840.992	-22.170
2.847.029	-36.434	2.886.454	-24.880	2.841.201	-22.169
2.847.390	-36.435	2.886.640	-24.879	2.841.315	-22.168
2.847.672	-36.435	2.886.750	-24.879	2.841.447	-22.168
2.847.777	-36.436	2.886.888	-24.878	2.841.555	-22.167
2.847.968	-36.436	2.887.057	-24.878	2.841.700	-22.166
2.848.206	-36.437	2.887.158	-24.877	2.841.856	-22.166

2.848.448	-36.437	2.887.279	-24.877	2.841.987	-22.165
2.848.438	-36.438	2.887.513	-24.877	2.842.126	-22.164
2.848.457	-36.439	2.887.722	-24.876	2.842.286	-22.163
2.848.773	-36.439	2.887.924	-24.876	2.842.437	-22.163
2.848.914	-36.440	2.888.099	-24.875	2.842.588	-22.162
2.848.811	-36.440	2.888.214	-24.875	2.842.843	-22.161
2.848.997	-36.441	2.888.362	-24.875	2.843.051	-22.160
2.849.305	-36.442	2.888.557	-24.874	2.843.185	-22.160
2.849.388	-36.442	2.888.738	-24.874	2.843.401	-22.159
2.849.572	-36.443	2.888.944	-24.874	2.843.633	-22.158
2.849.702	-36.443	2.889.124	-24.873	2.843.810	-22.157
2.849.958	-36.444	2.889.214	-24.873	2.844.013	-22.156
2.850.270	-36.444	2.889.326	-24.872	2.844.143	-22.155
2.850.407	-36.445	2.889.501	-24.872	2.844.210	-22.155
2.850.595	-36.446	2.889.685	-24.872	2.844.402	-22.154
2.850.852	-36.446	2.889.898	-24.871	2.844.626	-22.153
2.851.067	-36.447	2.890.078	-24.871	2.844.776	-22.152
2.851.155	-36.448	2.890.213	-24.871	2.844.904	-22.151
2.851.195	-36.448	2.890.332	-24.870	2.845.042	-22.150
2.851.392	-36.449	2.890.466	-24.870	2.845.179	-22.149
2.851.701	-36.450	2.890.679	-24.869	2.845.392	-22.148
2.851.777	-36.450	2.890.841	-24.869	2.845.596	-22.148
2.851.835	-36.451	2.890.977	-24.869	2.845.753	-22.147
2.852.012	-36.451	2.891.082	-24.868	2.845.885	-22.146
2.852.079	-36.452	2.891.249	-24.868	2.846.044	-22.145
2.852.224	-36.453	2.891.371	-24.867	2.846.259	-22.144
2.852.491	-36.453	2.891.526	-24.867	2.846.400	-22.143
2.852.701	-36.454	2.891.711	-24.867	2.846.521	-22.142
2.852.792	-36.455	2.891.830	-24.866	2.846.700	-22.141
2.852.979	-36.455	2.891.928	-24.866	2.846.899	-22.140

2.853.203	-36.456	2.892.133	-24.865	2.847.010	-22.139
2.853.401	-36.457	2.892.444	-24.865	2.847.080	-22.138
2.853.636	-36.457	2.892.686	-24.865	2.847.299	-22.137
2.853.897	-36.458	2.892.885	-24.864	2.847.590	-22.136
2.853.987	-36.459	2.893.084	-24.864	2.847.813	-22.134
2.854.073	-36.460	2.893.300	-24.863	2.847.968	-22.133
2.854.163	-36.460	2.893.478	-24.863	2.848.116	-22.132
2.854.313	-36.461	2.893.627	-24.863	2.848.301	-22.131
2.854.518	-36.462	2.893.782	-24.862	2.848.426	-22.130
2.854.772	-36.462	2.893.950	-24.862	2.848.598	-22.129
2.855.046	-36.463	2.894.127	-24.861	2.848.828	-22.128
2.855.212	-36.464	2.894.229	-24.861	2.849.039	-22.126
2.855.424	-36.464	2.894.319	-24.860	2.849.218	-22.125
2.855.545	-36.465	2.894.489	-24.860	2.849.518	-22.124
2.855.786	-36.466	2.894.695	-24.859	2.849.902	-22.123
2.855.981	-36.466	2.894.865	-24.859	2.850.057	-22.122
2.855.993	-36.467	2.895.009	-24.859	2.850.180	-22.120
2.856.161	-36.468	2.895.150	-24.858	2.850.277	-22.119
2.856.335	-36.468	2.895.313	-24.858	2.850.320	-22.118
2.856.575	-36.469	2.895.518	-24.857	2.850.450	-22.117
2.856.705	-36.470	2.895.718	-24.857	2.850.538	-22.115
2.856.729	-36.470	2.895.883	-24.856	2.850.609	-22.114
2.857.134	-36.471	2.896.028	-24.856	2.850.753	-22.113
2.857.643	-36.472	2.896.205	-24.855	2.850.913	-22.111
2.857.878	-36.472	2.896.401	-24.855	2.851.057	-22.110
2.857.972	-36.473	2.896.638	-24.854	2.851.231	-22.109
2.857.980	-36.474	2.896.849	-24.854	2.851.396	-22.107
2.858.081	-36.474	2.896.968	-24.853	2.851.593	-22.106
2.858.123	-36.475	2.897.122	-24.853	2.851.765	-22.105
2.858.112	-36.476	2.897.290	-24.852	2.851.922	-22.103

2.858.250	-36.476	2.897.415	-24.851	2.852.054	-22.102
2.858.279	-36.477	2.897.586	-24.851	2.852.200	-22.100
2.858.463	-36.478	2.897.762	-24.850	2.852.437	-22.099
2.858.773	-36.478	2.897.921	-24.850	2.852.604	-22.098
2.858.918	-36.479	2.898.113	-24.849	2.852.708	-22.096
2.858.995	-36.479	2.898.275	-24.849	2.852.849	-22.095
2.859.163	-36.480	2.898.402	-24.848	2.853.043	-22.093
2.859.420	-36.481	2.898.578	-24.848	2.853.246	-22.092
2.859.681	-36.481	2.898.795	-24.847	2.853.403	-22.091
2.859.804	-36.482	2.898.987	-24.846	2.853.536	-22.089
2.859.817	-36.483	2.899.153	-24.846	2.853.690	-22.088
2.860.009	-36.483	2.899.279	-24.845	2.853.831	-22.086
2.860.179	-36.484	2.899.420	-24.845	2.853.970	-22.085
2.860.399	-36.484	2.899.599	-24.844	2.854.157	-22.083
2.860.558	-36.485	2.899.771	-24.844	2.854.410	-22.082
2.860.787	-36.486	2.899.955	-24.843	2.854.626	-22.081
2.860.981	-36.486	2.900.092	-24.842	2.854.772	-22.079
2.861.044	-36.487	2.900.237	-24.842	2.854.919	-22.078
2.861.122	-36.487	2.900.493	-24.841	2.855.106	-22.076
2.861.336	-36.488	2.900.695	-24.840	2.855.287	-22.075
2.861.497	-36.489	2.900.829	-24.840	2.855.484	-22.074
2.861.667	-36.489	2.900.974	-24.839	2.855.657	-22.072
2.861.862	-36.490	2.901.150	-24.839	2.855.743	-22.071
2.862.012	-36.491	2.901.304	-24.838	2.855.872	-22.069
2.862.288	-36.491	2.901.458	-24.837	2.856.021	-22.068
2.862.542	-36.492	2.901.582	-24.837	2.856.148	-22.067
2.862.816	-36.493	2.901.817	-24.836	2.856.349	-22.065
2.863.000	-36.493	2.902.281	-24.835	2.856.566	-22.064
2.863.027	-36.494	2.902.672	-24.835	2.856.758	-22.063
2.863.179	-36.495	2.902.845	-24.834	2.856.949	-22.061

2.863.402	-36.495	2.902.878	-24.833	2.857.104	-22.060
2.863.705	-36.496	2.902.950	-24.833	2.857.228	-22.059
2.863.822	-36.497	2.902.973	-24.832	2.857.365	-22.058
2.863.777	-36.497	2.902.995	-24.831	2.857.487	-22.056
2.863.919	-36.498	2.903.130	-24.831	2.857.590	-22.055
2.864.178	-36.499	2.903.251	-24.830	2.857.726	-22.054
2.864.306	-36.499	2.903.370	-24.829	2.857.918	-22.053
2.864.395	-36.500	2.903.538	-24.828	2.858.204	-22.052
2.864.518	-36.501	2.903.745	-24.828	2.858.435	-22.051
2.864.666	-36.502	2.903.921	-24.827	2.858.585	-22.049
2.864.857	-36.502	2.904.051	-24.826	2.858.752	-22.048
2.865.124	-36.503	2.904.251	-24.826	2.858.878	-22.047
2.865.330	-36.504	2.904.474	-24.825	2.858.995	-22.046
2.865.565	-36.505	2.904.612	-24.824	2.859.175	-22.045
2.865.690	-36.505	2.904.792	-24.823	2.859.374	-22.044
2.865.746	-36.506	2.904.935	-24.823	2.859.548	-22.043
2.865.970	-36.507	2.905.043	-24.822	2.859.698	-22.042
2.866.125	-36.508	2.905.244	-24.821	2.859.893	-22.041
2.866.212	-36.509	2.905.446	-24.820	2.860.065	-22.040
2.866.340	-36.510	2.905.609	-24.820	2.860.222	-22.039
2.866.509	-36.511	2.905.786	-24.819	2.860.396	-22.039
2.866.698	-36.512	2.905.901	-24.818	2.860.547	-22.038
2.866.913	-36.513	2.906.033	-24.817	2.860.714	-22.037
2.867.213	-36.514	2.906.201	-24.816	2.860.935	-22.036
2.867.267	-36.515	2.906.380	-24.816	2.861.129	-22.035
2.867.380	-36.516	2.906.538	-24.815	2.861.252	-22.035
2.867.635	-36.517	2.906.676	-24.814	2.861.433	-22.034
2.867.874	-36.518	2.906.848	-24.813	2.861.519	-22.033
2.867.899	-36.519	2.907.070	-24.813	2.861.600	-22.032
2.867.939	-36.520	2.907.321	-24.812	2.861.777	-22.032

2.868.283	-36.521	2.907.505	-24.811	2.861.920	-22.031
2.868.520	-36.522	2.907.657	-24.810	2.862.069	-22.030
2.868.748	-36.523	2.907.863	-24.810	2.862.254	-22.030
2.868.839	-36.524	2.908.016	-24.809	2.862.361	-22.029
2.868.945	-36.525	2.908.174	-24.808	2.862.458	-22.029
2.869.128	-36.526	2.908.326	-24.807	2.862.672	-22.028
2.869.304	-36.527	2.908.424	-24.807	2.862.892	-22.028
2.869.533	-36.529	2.908.568	-24.806	2.863.047	-22.027
2.869.707	-36.530	2.908.691	-24.805	2.863.228	-22.027
2.869.778	-36.531	2.908.875	-24.804	2.863.432	-22.026
2.869.915	-36.532	2.909.106	-24.804	2.863.658	-22.026
2.870.193	-36.533	2.909.286	-24.803	2.863.886	-22.025
2.870.294	-36.534	2.909.482	-24.802	2.864.062	-22.025
2.870.435	-36.536	2.909.611	-24.801	2.864.304	-22.025
2.870.630	-36.537	2.909.768	-24.801	2.864.481	-22.025
2.870.637	-36.538	2.909.948	-24.800	2.864.612	-22.024
2.870.587	-36.539	2.910.096	-24.799	2.864.767	-22.024
2.870.892	-36.541	2.910.263	-24.799	2.864.937	-22.024
2.871.278	-36.542	2.910.364	-24.798	2.865.047	-22.024
2.871.336	-36.543	2.910.515	-24.797	2.865.130	-22.023
2.871.439	-36.544	2.910.710	-24.796	2.865.321	-22.023
2.871.545	-36.546	2.910.928	-24.796	2.865.507	-22.023
2.871.721	-36.547	2.911.161	-24.795	2.865.685	-22.023
2.871.969	-36.548	2.911.318	-24.794	2.865.820	-22.023
2.872.070	-36.550	2.911.483	-24.794	2.865.992	-22.023
2.872.347	-36.551	2.911.603	-24.793	2.866.264	-22.023
2.872.722	-36.552	2.911.729	-24.792	2.866.447	-22.023
2.872.834	-36.553	2.911.920	-24.792	2.866.707	-22.023
2.873.027	-36.555	2.912.061	-24.791	2.866.932	-22.023
2.873.221	-36.556	2.912.206	-24.790	2.866.840	-22.023

2.873.316	-36.557	2.912.369	-24.790	2.866.867	-22.023
2.873.452	-36.559	2.912.568	-24.789	2.867.174	-22.023
2.873.557	-36.560	2.912.760	-24.788	2.867.364	-22.023
2.873.814	-36.561	2.912.957	-24.788	2.867.536	-22.024
2.874.070	-36.563	2.913.145	-24.787	2.867.729	-22.024
2.874.222	-36.564	2.913.264	-24.787	2.867.827	-22.024
2.874.257	-36.566	2.913.419	-24.786	2.867.885	-22.024
2.874.291	-36.567	2.913.615	-24.785	2.868.152	-22.024
2.874.446	-36.568	2.913.785	-24.785	2.868.431	-22.025
2.874.682	-36.570	2.913.932	-24.784	2.868.531	-22.025
2.874.850	-36.571	2.914.110	-24.783	2.868.708	-22.025
2.874.926	-36.572	2.914.276	-24.783	2.868.909	-22.026
2.875.056	-36.574	2.914.403	-24.782	2.869.073	-22.026
2.875.294	-36.575	2.914.561	-24.781	2.869.214	-22.026
2.875.475	-36.576	2.914.698	-24.781	2.869.370	-22.027
2.875.692	-36.578	2.914.825	-24.780	2.869.529	-22.027
2.876.006	-36.579	2.914.985	-24.780	2.869.724	-22.028
2.876.108	-36.580	2.915.147	-24.779	2.869.974	-22.028
2.876.232	-36.582	2.915.334	-24.778	2.870.125	-22.029
2.876.534	-36.583	2.915.390	-24.778	2.870.244	-22.029
2.876.761	-36.584	2.915.479	-24.777	2.870.381	-22.030
2.876.899	-36.586	2.915.743	-24.776	2.870.446	-22.030
2.877.094	-36.587	2.915.982	-24.776	2.870.610	-22.031
2.877.217	-36.588	2.916.187	-24.775	2.870.804	-22.031
2.877.297	-36.590	2.916.425	-24.774	2.870.928	-22.032
2.877.478	-36.591	2.916.657	-24.774	2.871.130	-22.032
2.877.672	-36.593	2.916.852	-24.773	2.871.282	-22.033
2.877.741	-36.594	2.917.061	-24.772	2.871.469	-22.034
2.877.859	-36.595	2.917.235	-24.771	2.871.646	-22.034
2.878.094	-36.597	2.917.359	-24.771	2.871.676	-22.035

2.878.273	-36.598	2.917.502	-24.770	2.871.882	-22.036
2.878.319	-36.599	2.917.680	-24.769	2.872.368	-22.036
2.878.579	-36.600	2.917.853	-24.769	2.872.823	-22.037
2.878.900	-36.602	2.917.939	-24.768	2.872.980	-22.037
2.879.151	-36.603	2.918.012	-24.767	2.873.070	-22.038
2.879.189	-36.604	2.918.177	-24.766	2.873.195	-22.039
2.879.174	-36.606	2.918.294	-24.765	2.873.282	-22.039
2.879.377	-36.607	2.918.399	-24.765	2.873.332	-22.040
2.879.655	-36.608	2.918.630	-24.764	2.873.474	-22.041
2.879.788	-36.609	2.918.882	-24.763	2.873.608	-22.042
2.880.045	-36.611	2.919.099	-24.762	2.873.651	-22.042
2.880.507	-36.612	2.919.268	-24.761	2.873.757	-22.043
2.880.523	-36.613	2.919.399	-24.761	2.873.941	-22.044
2.880.504	-36.614	2.919.586	-24.760	2.874.161	-22.044
2.880.655	-36.616	2.919.779	-24.759	2.874.359	-22.045
2.880.822	-36.617	2.919.995	-24.758	2.874.550	-22.046
2.881.058	-36.618	2.920.165	-24.757	2.874.757	-22.047
2.881.245	-36.619	2.920.313	-24.756	2.874.944	-22.047
2.881.389	-36.620	2.920.493	-24.755	2.875.114	-22.048
2.881.535	-36.622	2.920.638	-24.754	2.875.216	-22.049
2.881.672	-36.623	2.920.825	-24.753	2.875.381	-22.049
2.881.853	-36.624	2.921.036	-24.752	2.875.495	-22.050
2.882.236	-36.625	2.921.180	-24.751	2.875.562	-22.051
2.882.603	-36.626	2.921.322	-24.751	2.875.807	-22.052
2.882.679	-36.627	2.921.461	-24.750	2.876.036	-22.052
2.882.752	-36.628	2.921.578	-24.749	2.876.216	-22.053
2.882.868	-36.629	2.921.770	-24.748	2.876.389	-22.054
2.882.990	-36.630	2.921.939	-24.747	2.876.541	-22.054
2.883.083	-36.632	2.922.108	-24.745	2.876.624	-22.055
2.883.239	-36.633	2.922.350	-24.744	2.876.777	-22.056

2.883.416	-36.634	2.922.512	-24.743	2.877.001	-22.056
2.883.351	-36.635	2.922.633	-24.742	2.877.254	-22.057
2.883.423	-36.636	2.922.838	-24.741	2.877.556	-22.058
2.883.697	-36.637	2.923.009	-24.740	2.877.740	-22.058
2.883.880	-36.638	2.923.192	-24.739	2.877.842	-22.059
2.884.073	-36.639	2.923.307	-24.738	2.877.946	-22.060
2.884.349	-36.640	2.923.414	-24.737	2.878.040	-22.060
2.884.588	-36.641	2.923.637	-24.736	2.878.185	-22.061
2.884.821	-36.642	2.923.825	-24.734	2.878.333	-22.062
2.884.924	-36.642	2.923.959	-24.733	2.878.484	-22.062
2.885.046	-36.643	2.924.088	-24.732	2.878.701	-22.063
2.885.162	-36.644	2.924.310	-24.731	2.878.893	-22.064
2.885.231	-36.645	2.924.500	-24.730	2.879.048	-22.064
2.885.417	-36.646	2.924.617	-24.728	2.879.237	-22.065
2.885.558	-36.647	2.924.774	-24.727	2.879.388	-22.065
2.885.796	-36.648	2.924.980	-24.726	2.879.440	-22.066
2.886.015	-36.649	2.925.118	-24.725	2.879.537	-22.066
2.886.103	-36.650	2.925.224	-24.723	2.879.796	-22.067
2.886.306	-36.651	2.925.584	-24.722	2.880.061	-22.067
2.886.559	-36.652	2.926.073	-24.721	2.880.204	-22.068
2.886.790	-36.653	2.926.389	-24.719	2.880.336	-22.068
2.886.938	-36.653	2.926.515	-24.718	2.880.548	-22.069
2.886.947	-36.654	2.926.530	-24.717	2.880.732	-22.069
2.887.079	-36.655	2.926.559	-24.715	2.880.896	-22.070
2.887.314	-36.656	2.926.678	-24.714	2.881.181	-22.070
2.887.534	-36.657	2.926.754	-24.713	2.881.440	-22.071
2.887.698	-36.658	2.926.815	-24.711	2.881.575	-22.071
2.887.843	-36.659	2.926.969	-24.710	2.881.698	-22.071
2.888.058	-36.660	2.927.177	-24.708	2.881.877	-22.072
2.888.217	-36.661	2.927.293	-24.707	2.882.083	-22.072

2.888.423	-36.662	2.927.412	-24.705	2.882.188	-22.072
2.888.538	-36.662	2.927.570	-24.704	2.882.206	-22.072
2.888.676	-36.663	2.927.758	-24.703	2.882.278	-22.073
2.888.856	-36.664	2.928.000	-24.701	2.882.528	-22.073
2.888.932	-36.665	2.928.202	-24.700	2.882.805	-22.073
2.889.149	-36.666	2.928.377	-24.698	2.882.892	-22.073
2.889.375	-36.667	2.928.503	-24.696	2.882.961	-22.074
2.889.436	-36.668	2.928.688	-24.695	2.883.150	-22.074
2.889.478	-36.669	2.928.868	-24.693	2.883.374	-22.074
2.889.727	-36.670	2.929.019	-24.692	2.883.499	-22.074
2.889.933	-36.671	2.929.171	-24.690	2.883.654	-22.074
2.889.996	-36.672	2.929.305	-24.689	2.883.856	-22.074
2.890.142	-36.673	2.929.406	-24.687	2.884.037	-22.074
2.890.280	-36.674	2.929.569	-24.686	2.884.182	-22.074
2.890.552	-36.675	2.929.761	-24.684	2.884.334	-22.074
2.890.844	-36.676	2.929.922	-24.682	2.884.503	-22.074
2.890.887	-36.677	2.930.112	-24.681	2.884.684	-22.074
2.890.970	-36.678	2.930.269	-24.679	2.884.796	-22.074
2.891.079	-36.679	2.930.471	-24.678	2.884.891	-22.074
2.891.183	-36.680	2.930.707	-24.676	2.885.061	-22.073
2.891.560	-36.681	2.930.903	-24.674	2.885.248	-22.073
2.891.793	-36.682	2.931.036	-24.673	2.885.400	-22.073
2.891.874	-36.683	2.931.076	-24.671	2.885.442	-22.073
2.892.062	-36.684	2.931.280	-24.669	2.885.617	-22.073
2.892.350	-36.685	2.931.582	-24.668	2.885.859	-22.072
2.892.577	-36.686	2.931.721	-24.666	2.886.014	-22.072
2.892.774	-36.687	2.931.824	-24.664	2.886.252	-22.072
2.892.903	-36.688	2.931.987	-24.663	2.886.512	-22.071
2.893.055	-36.689	2.932.153	-24.661	2.886.736	-22.071
2.893.246	-36.690	2.932.294	-24.660	2.886.965	-22.070

2.893.392	-36.691	2.932.430	-24.658	2.887.144	-22.070
2.893.460	-36.692	2.932.589	-24.656	2.887.258	-22.069
2.893.579	-36.693	2.932.802	-24.655	2.887.397	-22.069
2.893.720	-36.694	2.932.997	-24.653	2.887.561	-22.068
2.893.864	-36.696	2.933.149	-24.651	2.887.754	-22.068
2.894.107	-36.697	2.933.309	-24.650	2.887.892	-22.067
2.894.309	-36.698	2.933.513	-24.648	2.888.043	-22.067
2.894.539	-36.699	2.933.697	-24.646	2.888.233	-22.066
2.894.814	-36.700	2.933.835	-24.645	2.888.471	-22.065
2.894.883	-36.701	2.933.940	-24.643	2.888.694	-22.065
2.894.988	-36.702	2.934.075	-24.641	2.888.814	-22.064
2.895.183	-36.703	2.934.280	-24.640	2.888.864	-22.063
2.895.334	-36.704	2.934.449	-24.638	2.889.086	-22.062
2.895.563	-36.705	2.934.590	-24.637	2.889.279	-22.062
2.895.693	-36.707	2.934.732	-24.635	2.889.375	-22.061
2.895.695	-36.708	2.934.906	-24.633	2.889.637	-22.060
2.895.772	-36.709	2.935.112	-24.632	2.889.783	-22.059
2.896.093	-36.710	2.935.245	-24.630	2.889.902	-22.058
2.896.310	-36.711	2.935.426	-24.629	2.890.031	-22.057
2.896.264	-36.712	2.935.677	-24.627	2.890.165	-22.056
2.896.436	-36.713	2.935.862	-24.625	2.890.357	-22.055
2.896.674	-36.714	2.935.989	-24.624	2.890.533	-22.054
2.896.834	-36.715	2.936.127	-24.622	2.890.715	-22.053
2.897.090	-36.716	2.936.278	-24.621	2.890.894	-22.052
2.897.328	-36.717	2.936.452	-24.619	2.891.006	-22.051
2.897.517	-36.718	2.936.646	-24.618	2.891.228	-22.050
2.897.792	-36.719	2.936.820	-24.616	2.891.350	-22.049
2.897.892	-36.721	2.936.913	-24.615	2.891.425	-22.048
2.897.937	-36.722	2.937.046	-24.613	2.891.693	-22.047
2.898.188	-36.723	2.937.307	-24.611	2.891.931	-22.046

2.898.322	-36.724	2.937.538	-24.610	2.892.073	-22.045
2.898.477	-36.725	2.937.690	-24.608	2.892.260	-22.044
2.898.734	-36.726	2.937.767	-24.607	2.892.422	-22.043
2.898.835	-36.727	2.937.903	-24.605	2.892.575	-22.042
2.898.974	-36.728	2.938.170	-24.604	2.892.774	-22.040
2.899.081	-36.728	2.938.315	-24.602	2.892.892	-22.039
2.899.216	-36.729	2.938.336	-24.601	2.893.084	-22.038
2.899.457	-36.730	2.938.477	-24.599	2.893.333	-22.037
2.899.637	-36.731	2.938.687	-24.598	2.893.464	-22.036
2.899.781	-36.732	2.938.785	-24.596	2.893.594	-22.034
2.899.944	-36.733	2.938.915	-24.595	2.893.785	-22.033
2.900.166	-36.734	2.939.146	-24.593	2.893.911	-22.032
2.900.360	-36.735	2.939.317	-24.592	2.894.037	-22.031
2.900.511	-36.736	2.939.532	-24.590	2.894.236	-22.029
2.900.670	-36.737	2.939.745	-24.589	2.894.413	-22.028
2.900.901	-36.737	2.939.900	-24.587	2.894.583	-22.027
2.901.049	-36.738	2.940.139	-24.586	2.894.727	-22.026
2.901.150	-36.739	2.940.338	-24.584	2.894.879	-22.024
2.901.317	-36.740	2.940.495	-24.583	2.895.060	-22.023
2.901.570	-36.741	2.940.670	-24.581	2.895.443	-22.022
2.901.755	-36.741	2.940.874	-24.580	2.895.909	-22.021
2.901.920	-36.742	2.941.068	-24.578	2.896.042	-22.019
2.902.062	-36.743	2.941.215	-24.577	2.896.096	-22.018
2.902.225	-36.744	2.941.396	-24.575	2.896.237	-22.017
2.902.462	-36.744	2.941.544	-24.574	2.896.317	-22.015
2.902.643	-36.745	2.941.674	-24.573	2.896.296	-22.014
2.902.820	-36.746	2.941.816	-24.571	2.896.346	-22.013
2.902.969	-36.746	2.941.947	-24.570	2.896.491	-22.012
2.903.105	-36.747	2.942.057	-24.568	2.896.667	-22.010
2.903.239	-36.747	2.942.229	-24.567	2.896.857	-22.009

2.903.387	-36.748	2.942.415	-24.565	2.897.032	-22.008
2.903.593	-36.749	2.942.545	-24.564	2.897.272	-22.006
2.903.784	-36.749	2.942.686	-24.562	2.897.505	-22.005
2.903.969	-36.750	2.942.870	-24.561	2.897.575	-22.004
2.904.127	-36.750	2.943.124	-24.560	2.897.659	-22.003
2.904.196	-36.750	2.943.372	-24.558	2.897.843	-22.002
2.904.297	-36.751	2.943.549	-24.557	2.898.022	-22.000
2.904.505	-36.751	2.943.712	-24.555	2.898.177	-21.999
2.904.644	-36.752	2.943.860	-24.554	2.898.340	-21.998
2.904.774	-36.752	2.943.983	-24.552	2.898.492	-21.997
2.904.988	-36.752	2.944.140	-24.551	2.898.636	-21.996
2.905.195	-36.753	2.944.301	-24.550	2.898.814	-21.994
2.905.471	-36.753	2.944.453	-24.548	2.899.019	-21.993
2.905.685	-36.753	2.944.613	-24.547	2.899.185	-21.992
2.905.743	-36.754	2.944.799	-24.545	2.899.272	-21.991
2.905.898	-36.754	2.944.976	-24.544	2.899.421	-21.990
2.906.154	-36.754	2.945.110	-24.542	2.899.641	-21.989
2.906.307	-36.754	2.945.271	-24.541	2.899.823	-21.988
2.906.367	-36.755	2.945.480	-24.540	2.899.996	-21.987
2.906.564	-36.755	2.945.725	-24.538	2.900.146	-21.986
2.907.046	-36.755	2.945.874	-24.537	2.900.381	-21.985
2.907.337	-36.755	2.945.986	-24.536	2.900.599	-21.984
2.907.419	-36.755	2.946.192	-24.534	2.900.726	-21.983
2.907.604	-36.755	2.946.369	-24.533	2.900.861	-21.982
2.907.776	-36.755	2.946.541	-24.532	2.901.021	-21.981
2.907.805	-36.755	2.946.693	-24.530	2.901.174	-21.980
2.907.791	-36.755	2.946.823	-24.529	2.901.340	-21.979
2.907.925	-36.756	2.946.954	-24.527	2.901.555	-21.979
2.908.058	-36.755	2.947.094	-24.526	2.901.693	-21.978
2.908.163	-36.755	2.947.305	-24.525	2.901.806	-21.977

2.908.312	-36.755	2.947.563	-24.524	2.901.974	-21.976
2.908.532	-36.755	2.947.697	-24.522	2.902.142	-21.975
2.908.752	-36.755	2.947.809	-24.521	2.902.328	-21.975
2.908.929	-36.755	2.948.012	-24.520	2.902.470	-21.974
2.909.099	-36.755	2.948.157	-24.518	2.902.608	-21.973
2.909.252	-36.755	2.948.304	-24.517	2.902.776	-21.973
2.909.409	-36.755	2.948.507	-24.516	2.902.960	-21.972
2.909.559	-36.755	2.948.637	-24.514	2.903.174	-21.972
2.909.757	-36.754	2.948.753	-24.513	2.903.358	-21.971
2.909.915	-36.754	2.948.962	-24.512	2.903.538	-21.971
2.910.028	-36.754	2.949.345	-24.511	2.903.668	-21.970
2.910.281	-36.754	2.949.823	-24.509	2.903.820	-21.970
2.910.504	-36.754	2.950.096	-24.508	2.904.013	-21.969
2.910.630	-36.753	2.950.164	-24.507	2.904.190	-21.969
2.910.758	-36.753	2.950.211	-24.506	2.904.382	-21.968
2.910.869	-36.753	2.950.276	-24.504	2.904.562	-21.968
2.910.941	-36.753	2.950.341	-24.503	2.904.738	-21.968
2.911.090	-36.752	2.950.411	-24.502	2.904.901	-21.967
2.911.357	-36.752	2.950.516	-24.501	2.905.047	-21.967
2.911.516	-36.752	2.950.638	-24.500	2.905.215	-21.967
2.911.676	-36.751	2.950.754	-24.498	2.905.399	-21.967
2.911.882	-36.751	2.950.909	-24.497	2.905.504	-21.966
2.912.008	-36.751	2.951.101	-24.496	2.905.679	-21.966
2.912.214	-36.751	2.951.266	-24.495	2.905.858	-21.966
2.912.418	-36.750	2.951.369	-24.494	2.906.067	-21.966
2.912.516	-36.750	2.951.549	-24.492	2.906.256	-21.966
2.912.633	-36.750	2.951.773	-24.491	2.906.411	-21.966
2.912.824	-36.749	2.951.940	-24.490	2.906.578	-21.966
2.913.123	-36.749	2.952.146	-24.489	2.906.639	-21.966
2.913.246	-36.749	2.952.350	-24.488	2.906.757	-21.966

2.913.322	-36.749	2.952.513	-24.487	2.907.003	-21.966
2.913.535	-36.748	2.952.668	-24.485	2.907.234	-21.966
2.913.753	-36.748	2.952.785	-24.484	2.907.325	-21.966
2.913.908	-36.748	2.952.957	-24.483	2.907.435	-21.966
2.913.972	-36.748	2.953.137	-24.482	2.907.630	-21.966
2.914.164	-36.747	2.953.273	-24.481	2.907.770	-21.966
2.914.362	-36.747	2.953.466	-24.480	2.907.883	-21.966
2.914.496	-36.747	2.953.608	-24.479	2.908.002	-21.966
2.914.648	-36.747	2.953.748	-24.478	2.908.168	-21.966
2.914.869	-36.746	2.953.945	-24.477	2.908.349	-21.966
2.915.101	-36.746	2.954.089	-24.476	2.908.499	-21.966
2.915.201	-36.746	2.954.250	-24.475	2.908.694	-21.967
2.915.305	-36.746	2.954.395	-24.474	2.908.954	-21.967
2.915.462	-36.746	2.954.616	-24.472	2.909.182	-21.967
2.915.657	-36.745	2.954.846	-24.471	2.909.344	-21.967
2.915.844	-36.745	2.955.016	-24.470	2.909.496	-21.967
2.915.934	-36.745	2.955.216	-24.469	2.909.698	-21.968
2.916.129	-36.745	2.955.367	-24.468	2.909.924	-21.968
2.916.347	-36.745	2.955.497	-24.467	2.910.096	-21.968
2.916.552	-36.745	2.955.614	-24.466	2.910.236	-21.968
2.916.776	-36.745	2.955.775	-24.466	2.910.435	-21.969
2.916.940	-36.745	2.956.006	-24.465	2.910.620	-21.969
2.917.126	-36.745	2.956.190	-24.464	2.910.684	-21.969
2.917.343	-36.745	2.956.300	-24.463	2.910.811	-21.969
2.917.596	-36.745	2.956.454	-24.462	2.910.995	-21.970
2.917.690	-36.745	2.956.643	-24.461	2.911.108	-21.970
2.917.776	-36.745	2.956.794	-24.460	2.911.256	-21.970
2.917.914	-36.745	2.956.913	-24.459	2.911.474	-21.971
2.917.975	-36.745	2.957.090	-24.458	2.911.685	-21.971
2.918.098	-36.745	2.957.279	-24.457	2.911.828	-21.971

2.918.323	-36.745	2.957.495	-24.456	2.912.018	-21.972
2.918.575	-36.745	2.957.726	-24.455	2.912.247	-21.972
2.918.791	-36.745	2.957.881	-24.454	2.912.455	-21.972
2.918.987	-36.745	2.958.029	-24.453	2.912.610	-21.973
2.919.139	-36.745	2.958.130	-24.453	2.912.751	-21.973
2.919.272	-36.745	2.958.264	-24.452	2.912.950	-21.973
2.919.424	-36.745	2.958.450	-24.451	2.913.123	-21.974
2.919.612	-36.746	2.958.607	-24.450	2.913.315	-21.974
2.919.735	-36.746	2.958.777	-24.449	2.913.474	-21.974
2.919.869	-36.746	2.958.969	-24.448	2.913.609	-21.975
2.920.052	-36.746	2.959.193	-24.447	2.913.767	-21.975
2.920.213	-36.746	2.959.366	-24.447	2.913.948	-21.975
2.920.327	-36.747	2.959.469	-24.446	2.914.149	-21.976
2.920.444	-36.747	2.959.633	-24.445	2.914.276	-21.976
2.920.591	-36.747	2.959.810	-24.444	2.914.399	-21.976
2.920.775	-36.747	2.959.984	-24.443	2.914.559	-21.977
2.920.959	-36.748	2.960.154	-24.442	2.914.752	-21.977
2.921.060	-36.748	2.960.309	-24.441	2.914.893	-21.977
2.921.136	-36.748	2.960.448	-24.440	2.915.007	-21.977
2.921.367	-36.749	2.960.630	-24.440	2.915.229	-21.978
2.921.690	-36.749	2.960.848	-24.439	2.915.457	-21.978
2.921.842	-36.749	2.961.002	-24.438	2.915.614	-21.978
2.921.960	-36.750	2.961.162	-24.437	2.915.707	-21.979
2.922.252	-36.750	2.961.310	-24.436	2.915.887	-21.979
2.922.471	-36.750	2.961.456	-24.435	2.916.137	-21.979
2.922.557	-36.751	2.961.618	-24.434	2.916.318	-21.979
2.922.708	-36.751	2.961.802	-24.433	2.916.472	-21.980
2.922.892	-36.752	2.962.008	-24.432	2.916.646	-21.980
2.923.052	-36.752	2.962.198	-24.431	2.916.862	-21.980
2.923.228	-36.752	2.962.325	-24.430	2.916.992	-21.980

2.923.387	-36.753	2.962.394	-24.429	2.917.109	-21.981
2.923.489	-36.753	2.962.523	-24.428	2.917.328	-21.981
2.923.645	-36.754	2.962.657	-24.427	2.917.513	-21.981
2.923.796	-36.754	2.962.799	-24.426	2.917.640	-21.981
2.923.926	-36.755	2.962.971	-24.425	2.917.730	-21.981
2.924.128	-36.755	2.963.202	-24.424	2.917.882	-21.981
2.924.380	-36.755	2.963.406	-24.423	2.918.269	-21.982
2.924.559	-36.756	2.963.553	-24.422	2.918.723	-21.982
2.924.624	-36.756	2.963.791	-24.420	2.918.904	-21.982
2.924.771	-36.757	2.964.017	-24.419	2.918.988	-21.982
2.924.973	-36.757	2.964.221	-24.418	2.919.167	-21.982
2.925.139	-36.758	2.964.431	-24.417	2.919.265	-21.982
2.925.298	-36.759	2.964.599	-24.416	2.919.196	-21.982
2.925.525	-36.759	2.964.729	-24.414	2.919.174	-21.982
2.925.760	-36.760	2.964.854	-24.413	2.919.355	-21.982
2.925.945	-36.760	2.965.050	-24.412	2.919.575	-21.982
2.926.046	-36.761	2.965.161	-24.410	2.919.775	-21.982
2.926.180	-36.761	2.965.255	-24.409	2.919.974	-21.982
2.926.313	-36.762	2.965.466	-24.408	2.920.094	-21.982
2.926.441	-36.763	2.965.656	-24.406	2.920.252	-21.982
2.926.661	-36.763	2.965.765	-24.405	2.920.426	-21.982
2.926.851	-36.764	2.965.910	-24.403	2.920.587	-21.982
2.926.978	-36.765	2.966.076	-24.402	2.920.804	-21.982
2.927.133	-36.765	2.966.194	-24.400	2.920.952	-21.982
2.927.350	-36.766	2.966.358	-24.399	2.921.158	-21.982
2.927.570	-36.767	2.966.568	-24.397	2.921.354	-21.982
2.927.716	-36.767	2.966.815	-24.395	2.921.451	-21.982
2.927.863	-36.768	2.967.050	-24.394	2.921.653	-21.982
2.928.138	-36.769	2.967.232	-24.392	2.921.866	-21.981
2.928.377	-36.769	2.967.430	-24.390	2.921.974	-21.981

2.928.488	-36.770	2.967.622	-24.389	2.922.124	-21.981
2.928.623	-36.771	2.967.769	-24.387	2.922.303	-21.981
2.928.813	-36.772	2.967.928	-24.385	2.922.393	-21.981
2.928.876	-36.772	2.968.051	-24.383	2.922.578	-21.981
2.928.979	-36.773	2.968.182	-24.381	2.922.747	-21.980
2.929.261	-36.774	2.968.351	-24.380	2.922.877	-21.980
2.929.438	-36.775	2.968.546	-24.378	2.923.055	-21.980
2.929.615	-36.775	2.968.694	-24.376	2.923.164	-21.980
2.929.804	-36.776	2.968.828	-24.374	2.923.481	-21.979
2.929.877	-36.777	2.969.016	-24.372	2.923.828	-21.979
2.930.060	-36.778	2.969.174	-24.370	2.923.926	-21.979
2.930.211	-36.779	2.969.352	-24.368	2.923.983	-21.978
2.930.345	-36.779	2.969.514	-24.366	2.924.127	-21.978
2.930.641	-36.780	2.969.656	-24.364	2.924.298	-21.978
2.930.823	-36.781	2.969.851	-24.361	2.924.416	-21.977
2.930.896	-36.782	2.970.054	-24.359	2.924.510	-21.977
2.930.964	-36.783	2.970.226	-24.357	2.924.671	-21.977
2.931.190	-36.784	2.970.435	-24.355	2.924.865	-21.976
2.931.708	-36.785	2.970.659	-24.353	2.924.991	-21.976
2.932.173	-36.786	2.970.749	-24.350	2.925.182	-21.976
2.932.220	-36.787	2.970.851	-24.348	2.925.482	-21.975
2.932.198	-36.788	2.971.036	-24.346	2.925.638	-21.975
2.932.344	-36.788	2.971.249	-24.343	2.925.746	-21.974
2.932.517	-36.789	2.971.434	-24.341	2.925.941	-21.974
2.932.549	-36.790	2.971.549	-24.338	2.926.100	-21.974
2.932.599	-36.791	2.971.711	-24.336	2.926.215	-21.973
2.932.760	-36.792	2.971.906	-24.333	2.926.357	-21.973
2.932.885	-36.793	2.972.093	-24.331	2.926.596	-21.972
2.933.019	-36.794	2.972.281	-24.328	2.926.723	-21.972
2.933.149	-36.795	2.972.453	-24.326	2.926.743	-21.971

2.933.293	-36.796	2.972.570	-24.323	2.926.972	-21.971
2.933.502	-36.797	2.972.727	-24.321	2.927.312	-21.970
2.933.665	-36.798	2.973.083	-24.318	2.927.433	-21.970
2.933.773	-36.799	2.973.543	-24.315	2.927.520	-21.969
2.933.972	-36.800	2.973.815	-24.313	2.927.788	-21.969
2.934.180	-36.801	2.973.853	-24.310	2.928.091	-21.968
2.934.294	-36.803	2.973.911	-24.307	2.928.244	-21.968
2.934.402	-36.804	2.973.976	-24.305	2.928.351	-21.967
2.934.633	-36.805	2.974.030	-24.302	2.928.557	-21.967
2.934.821	-36.806	2.974.138	-24.299	2.928.702	-21.966
2.934.940	-36.807	2.974.240	-24.296	2.928.705	-21.966
2.935.153	-36.808	2.974.334	-24.293	2.928.802	-21.965
2.935.327	-36.809	2.974.435	-24.290	2.929.015	-21.964
2.935.415	-36.810	2.974.572	-24.288	2.929.254	-21.964
2.935.596	-36.812	2.974.804	-24.285	2.929.512	-21.963
2.935.755	-36.813	2.975.063	-24.282	2.929.716	-21.963
2.935.827	-36.814	2.975.233	-24.279	2.929.908	-21.962
2.936.013	-36.815	2.975.413	-24.276	2.930.049	-21.961
2.936.259	-36.817	2.975.580	-24.273	2.930.129	-21.961
2.936.461	-36.818	2.975.761	-24.270	2.930.247	-21.960
2.936.621	-36.819	2.975.923	-24.267	2.930.415	-21.959
2.936.762	-36.821	2.976.046	-24.264	2.930.602	-21.959
2.936.996	-36.822	2.976.231	-24.261	2.930.704	-21.958
2.937.264	-36.823	2.976.378	-24.258	2.930.797	-21.957
2.937.397	-36.825	2.976.526	-24.255	2.930.930	-21.957
2.937.473	-36.826	2.976.710	-24.252	2.931.087	-21.956
2.937.643	-36.827	2.976.882	-24.249	2.931.259	-21.955
2.937.834	-36.829	2.977.039	-24.246	2.931.461	-21.954
2.937.910	-36.830	2.977.178	-24.243	2.931.631	-21.954
2.938.081	-36.832	2.977.272	-24.240	2.931.770	-21.953

2.938.305	-36.833	2.977.427	-24.237	2.932.026	-21.952
2.938.428	-36.835	2.977.633	-24.234	2.932.256	-21.951
2.938.542	-36.836	2.977.762	-24.231	2.932.435	-21.950
2.938.821	-36.838	2.978.000	-24.228	2.932.654	-21.950
2.939.068	-36.839	2.978.224	-24.225	2.932.848	-21.949
2.939.157	-36.841	2.978.401	-24.222	2.932.995	-21.948
2.939.337	-36.843	2.978.604	-24.219	2.933.188	-21.947
2.939.605	-36.844	2.978.777	-24.216	2.933.345	-21.946
2.939.729	-36.846	2.978.914	-24.213	2.933.408	-21.945
2.939.750	-36.848	2.979.059	-24.210	2.933.542	-21.944
2.939.971	-36.849	2.979.279	-24.207	2.933.738	-21.944
2.940.202	-36.851	2.979.447	-24.204	2.933.813	-21.943
2.940.350	-36.853	2.979.595	-24.201	2.933.886	-21.942
2.940.508	-36.855	2.979.772	-24.198	2.934.149	-21.941
2.940.714	-36.856	2.979.938	-24.196	2.934.326	-21.940
2.940.945	-36.858	2.980.092	-24.193	2.934.422	-21.939
2.941.122	-36.860	2.980.240	-24.190	2.934.667	-21.938
2.941.230	-36.862	2.980.339	-24.187	2.934.935	-21.937
2.941.346	-36.864	2.980.486	-24.184	2.935.089	-21.936
2.941.606	-36.866	2.980.721	-24.181	2.935.231	-21.935
2.941.783	-36.868	2.980.901	-24.178	2.935.460	-21.934
2.941.869	-36.870	2.981.043	-24.176	2.935.670	-21.933
2.942.040	-36.872	2.981.192	-24.173	2.935.811	-21.932
2.942.203	-36.874	2.981.343	-24.170	2.935.992	-21.931
2.942.326	-36.876	2.981.542	-24.167	2.936.161	-21.930
2.942.516	-36.878	2.981.711	-24.165	2.936.339	-21.929
2.942.766	-36.880	2.981.801	-24.162	2.936.506	-21.928
2.942.914	-36.882	2.981.965	-24.159	2.936.631	-21.927
2.943.022	-36.884	2.982.195	-24.157	2.936.827	-21.926
2.943.105	-36.886	2.982.397	-24.154	2.936.935	-21.925

2.943.237	-36.888	2.982.564	-24.152	2.937.066	-21.924
2.943.408	-36.890	2.982.767	-24.149	2.937.319	-21.923
2.943.634	-36.892	2.982.937	-24.147	2.937.480	-21.922
2.943.868	-36.894	2.983.091	-24.144	2.937.624	-21.921
2.944.031	-36.897	2.983.261	-24.142	2.937.823	-21.920
2.944.164	-36.899	2.983.373	-24.139	2.938.029	-21.918
2.944.270	-36.901	2.983.514	-24.137	2.938.185	-21.917
2.944.463	-36.903	2.983.637	-24.134	2.938.314	-21.916
2.944.713	-36.905	2.983.766	-24.132	2.938.463	-21.915
2.944.825	-36.907	2.983.955	-24.129	2.938.654	-21.914
2.944.913	-36.909	2.984.200	-24.127	2.938.802	-21.913
2.945.099	-36.912	2.984.413	-24.125	2.938.950	-21.912
2.945.321	-36.914	2.984.559	-24.122	2.939.093	-21.911
2.945.403	-36.916	2.984.698	-24.120	2.939.244	-21.910
2.945.422	-36.918	2.984.861	-24.118	2.939.469	-21.909
2.945.616	-36.920	2.985.025	-24.116	2.939.655	-21.908
2.945.916	-36.922	2.985.175	-24.114	2.939.843	-21.906
2.946.191	-36.925	2.985.334	-24.111	2.939.995	-21.905
2.946.349	-36.927	2.985.502	-24.109	2.940.157	-21.904
2.946.552	-36.929	2.985.628	-24.107	2.940.316	-21.903
2.946.770	-36.931	2.985.768	-24.105	2.940.424	-21.902
2.946.902	-36.933	2.985.960	-24.103	2.940.567	-21.901
2.947.115	-36.936	2.986.094	-24.101	2.940.797	-21.900
2.947.358	-36.938	2.986.260	-24.099	2.941.199	-21.899
2.947.506	-36.940	2.986.411	-24.097	2.941.649	-21.898
2.947.610	-36.942	2.986.530	-24.095	2.941.870	-21.897
2.947.771	-36.944	2.986.697	-24.093	2.941.891	-21.896
2.947.929	-36.946	2.986.902	-24.091	2.941.994	-21.894
2.948.058	-36.948	2.987.130	-24.089	2.941.992	-21.893
2.948.271	-36.950	2.987.339	-24.087	2.941.982	-21.892

2.948.345	-36.952	2.987.547	-24.085	2.942.180	-21.891
2.948.347	-36.954	2.987.734	-24.083	2.942.312	-21.890
2.948.459	-36.956	2.987.925	-24.081	2.942.453	-21.889
2.948.683	-36.959	2.988.132	-24.079	2.942.608	-21.888
2.949.021	-36.961	2.988.311	-24.078	2.942.716	-21.887
2.949.164	-36.963	2.988.474	-24.076	2.942.842	-21.886
2.949.237	-36.965	2.988.600	-24.074	2.943.051	-21.885
2.949.384	-36.967	2.988.730	-24.072	2.943.322	-21.884
2.949.565	-36.969	2.988.897	-24.071	2.943.482	-21.883
2.949.803	-36.970	2.989.001	-24.069	2.943.643	-21.882
2.950.092	-36.972	2.989.056	-24.067	2.943.860	-21.881
2.950.309	-36.974	2.989.198	-24.066	2.944.070	-21.880
2.950.464	-36.976	2.989.404	-24.064	2.944.254	-21.879
2.950.638	-36.978	2.989.588	-24.062	2.944.380	-21.878
2.950.773	-36.980	2.989.803	-24.061	2.944.482	-21.877
2.950.884	-36.982	2.990.002	-24.059	2.944.619	-21.876
2.951.127	-36.984	2.990.150	-24.058	2.944.832	-21.875
2.951.306	-36.986	2.990.364	-24.056	2.944.994	-21.875
2.951.434	-36.988	2.990.565	-24.054	2.945.071	-21.874
2.951.585	-36.989	2.990.737	-24.053	2.945.184	-21.873
2.951.672	-36.991	2.990.905	-24.051	2.945.374	-21.872
2.951.801	-36.993	2.991.049	-24.050	2.945.516	-21.871
2.952.028	-36.995	2.991.216	-24.048	2.945.696	-21.870
2.952.328	-36.996	2.991.378	-24.047	2.945.921	-21.870
2.952.456	-36.998	2.991.537	-24.046	2.946.098	-21.869
2.952.654	-37.000	2.991.689	-24.044	2.946.293	-21.868
2.952.925	-37.001	2.991.880	-24.043	2.946.470	-21.868
2.953.044	-37.003	2.992.040	-24.041	2.946.606	-21.867
2.953.102	-37.005	2.992.133	-24.040	2.946.744	-21.866
2.953.135	-37.006	2.992.357	-24.039	2.946.898	-21.866

2.953.308	-37.008	2.992.570	-24.037	2.947.062	-21.865
2.953.597	-37.010	2.992.716	-24.036	2.947.290	-21.864
2.953.795	-37.011	2.992.935	-24.034	2.947.469	-21.864
2.953.893	-37.013	2.993.112	-24.033	2.947.571	-21.863
2.954.131	-37.014	2.993.275	-24.032	2.947.712	-21.863
2.954.411	-37.016	2.993.417	-24.031	2.947.867	-21.862
2.954.425	-37.017	2.993.640	-24.029	2.948.037	-21.862
2.954.503	-37.019	2.993.839	-24.028	2.948.199	-21.861
2.954.811	-37.020	2.993.926	-24.027	2.948.365	-21.861
2.955.082	-37.021	2.994.093	-24.025	2.948.568	-21.860
2.955.204	-37.023	2.994.310	-24.024	2.948.702	-21.860
2.955.241	-37.024	2.994.496	-24.023	2.948.875	-21.860
2.955.352	-37.025	2.994.652	-24.022	2.949.067	-21.859
2.955.587	-37.027	2.994.864	-24.020	2.949.207	-21.859
2.955.804	-37.028	2.995.047	-24.019	2.949.352	-21.859
2.956.150	-37.029	2.995.186	-24.018	2.949.504	-21.858
2.956.600	-37.031	2.995.359	-24.017	2.949.676	-21.858
2.956.799	-37.032	2.995.515	-24.015	2.949.857	-21.858
2.956.900	-37.033	2.995.672	-24.014	2.950.070	-21.858
2.956.956	-37.034	2.995.865	-24.013	2.950.269	-21.858
2.957.001	-37.035	2.996.013	-24.012	2.950.440	-21.857
2.957.196	-37.037	2.996.096	-24.010	2.950.587	-21.857
2.957.372	-37.038	2.996.203	-24.009	2.950.764	-21.857
2.957.448	-37.039	2.996.602	-24.008	2.950.941	-21.857
2.957.514	-37.040	2.997.098	-24.007	2.951.126	-21.857
2.957.488	-37.041	2.997.346	-24.005	2.951.342	-21.857
2.957.589	-37.042	2.997.397	-24.004	2.951.432	-21.857
2.957.736	-37.043	2.997.466	-24.003	2.951.549	-21.857
2.957.874	-37.044	2.997.531	-24.002	2.951.798	-21.857
2.958.106	-37.045	2.997.557	-24.001	2.951.974	-21.857

2.958.361	-37.046	2.997.639	-23.999	2.952.101	-21.858
2.958.640	-37.047	2.997.738	-23.998	2.952.307	-21.858
2.958.853	-37.047	2.997.893	-23.997	2.952.459	-21.858
2.959.092	-37.048	2.998.040	-23.996	2.952.567	-21.858
2.959.265	-37.049	2.998.188	-23.995	2.952.788	-21.858
2.959.328	-37.050	2.998.340	-23.993	2.952.972	-21.858
2.959.413	-37.051	2.998.484	-23.992	2.953.116	-21.859
2.959.586	-37.052	2.998.694	-23.991	2.953.304	-21.859
2.959.779	-37.052	2.998.926	-23.990	2.953.402	-21.859
2.959.935	-37.053	2.999.106	-23.988	2.953.474	-21.859
2.960.139	-37.054	2.999.297	-23.987	2.953.586	-21.860
2.960.284	-37.055	2.999.458	-23.986	2.953.738	-21.860
2.960.352	-37.056	2.999.576	-23.985	2.953.894	-21.860
2.960.374	-37.056	2.999.698	-23.984	2.954.046	-21.861
2.960.693	-37.057	2.999.836	-23.982	2.954.240	-21.861
2.960.780	-37.058	3.000.023	-23.981	2.954.471	-21.861
2.960.885	-37.058	3.000.223	-23.980	2.954.705	-21.862
2.961.315	-37.059	3.000.401	-23.979	2.954.854	-21.862
2.961.412	-37.060	3.000.555	-23.977	2.954.981	-21.863
2.961.694	-37.060	3.000.689	-23.976	2.955.187	-21.863
2.962.086	-37.061	3.000.796	-23.975	2.955.428	-21.863
2.962.249	-37.062	3.000.917	-23.974	2.955.612	-21.864
2.962.290	-37.062	3.001.093	-23.972	2.955.772	-21.864
2.962.447	-37.063	3.001.335	-23.971	2.955.973	-21.865
2.962.419	-37.064	3.001.568	-23.970	2.956.190	-21.865
2.962.249	-37.064	3.001.754	-23.969	2.956.328	-21.866
2.962.633	-37.065	3.001.975	-23.968	2.956.371	-21.866
2.962.745	-37.066	3.002.180	-23.966	2.956.477	-21.867
2.962.827	-37.066	3.002.328	-23.965	2.956.646	-21.867
2.963.221	-37.067	3.002.476	-23.964	2.956.797	-21.868

2.963.401	-37.068	3.002.629	-23.963	2.957.001	-21.868
2.963.508	-37.068	3.002.770	-23.962	2.957.155	-21.869
2.963.651	-37.069	3.002.908	-23.960	2.957.242	-21.870
2.963.990	-37.069	3.003.044	-23.959	2.957.419	-21.870
2.964.092	-37.070	3.003.222	-23.958	2.957.693	-21.871
2.964.015	-37.071	3.003.420	-23.957	2.957.950	-21.871
2.963.994	-37.071	3.003.568	-23.956	2.958.078	-21.872
2.964.359	-37.072	3.003.719	-23.954	2.958.197	-21.872
2.964.764	-37.073	3.003.854	-23.953	2.958.380	-21.873
2.964.857	-37.073	3.004.030	-23.952	2.958.558	-21.874
2.965.031	-37.074	3.004.232	-23.951	2.958.726	-21.874
2.965.219	-37.075	3.004.468	-23.950	2.958.929	-21.875
2.965.269	-37.075	3.004.655	-23.949	2.959.096	-21.876
2.965.408	-37.076	3.004.782	-23.947	2.959.247	-21.876
2.965.703	-37.077	3.004.955	-23.946	2.959.396	-21.877
2.965.941	-37.077	3.005.092	-23.945	2.959.565	-21.877
2.966.205	-37.078	3.005.258	-23.944	2.959.797	-21.878
2.966.313	-37.079	3.005.406	-23.943	2.959.908	-21.879
2.966.585	-37.080	3.005.520	-23.942	2.960.060	-21.879
2.966.743	-37.080	3.005.710	-23.940	2.960.265	-21.880
2.966.614	-37.081	3.005.899	-23.939	2.960.475	-21.881
2.966.873	-37.082	3.006.079	-23.938	2.960.746	-21.881
2.967.169	-37.083	3.006.270	-23.937	2.960.836	-21.882
2.967.278	-37.083	3.006.447	-23.936	2.960.914	-21.883
2.967.379	-37.084	3.006.640	-23.935	2.961.089	-21.883
2.967.610	-37.085	3.006.797	-23.933	2.961.259	-21.884
2.967.776	-37.086	3.006.907	-23.932	2.961.418	-21.885
2.967.976	-37.086	3.007.065	-23.931	2.961.546	-21.885
2.968.156	-37.087	3.007.238	-23.930	2.961.700	-21.886
2.968.204	-37.088	3.007.429	-23.929	2.961.918	-21.887

2.968.322	-37.089	3.007.632	-23.928	2.962.105	-21.887
2.968.723	-37.089	3.007.776	-23.926	2.962.292	-21.888
2.968.846	-37.090	3.007.953	-23.925	2.962.419	-21.888
2.968.829	-37.091	3.008.130	-23.924	2.962.553	-21.889
2.969.044	-37.092	3.008.237	-23.923	2.962.781	-21.890
2.969.190	-37.092	3.008.398	-23.922	2.962.991	-21.890
2.969.327	-37.093	3.008.597	-23.921	2.963.096	-21.891
2.969.565	-37.094	3.008.734	-23.919	2.963.193	-21.892
2.969.826	-37.095	3.008.886	-23.918	2.963.354	-21.892
2.969.873	-37.095	3.009.080	-23.917	2.963.549	-21.893
2.969.850	-37.096	3.009.272	-23.916	2.963.952	-21.893
2.969.989	-37.097	3.009.438	-23.914	2.964.366	-21.894
2.970.245	-37.098	3.009.536	-23.913	2.964.512	-21.894
2.970.621	-37.099	3.009.680	-23.912	2.964.609	-21.895
2.970.768	-37.099	3.009.870	-23.911	2.964.695	-21.896
2.970.887	-37.100	3.010.005	-23.909	2.964.796	-21.896
2.970.801	-37.101	3.010.180	-23.908	2.964.922	-21.897
2.970.992	-37.102	3.010.388	-23.907	2.964.963	-21.897
2.971.486	-37.102	3.010.547	-23.905	2.965.002	-21.898
2.971.698	-37.103	3.010.688	-23.904	2.965.172	-21.898
2.971.934	-37.104	3.010.919	-23.903	2.965.411	-21.899
2.972.025	-37.105	3.011.190	-23.901	2.965.598	-21.899
2.972.064	-37.105	3.011.400	-23.900	2.965.768	-21.900
2.972.272	-37.106	3.011.528	-23.899	2.965.885	-21.900
2.972.469	-37.107	3.011.646	-23.897	2.966.071	-21.901
2.972.720	-37.108	3.011.815	-23.896	2.966.264	-21.901
2.972.867	-37.108	3.011.968	-23.895	2.966.436	-21.902
2.972.935	-37.109	3.012.156	-23.893	2.966.707	-21.902
2.973.001	-37.110	3.012.272	-23.892	2.966.873	-21.903
2.973.202	-37.110	3.012.408	-23.890	2.966.964	-21.903

2.973.470	-37.111	3.012.599	-23.889	2.967.148	-21.904
2.973.549	-37.112	3.012.698	-23.887	2.967.289	-21.904
2.973.792	-37.113	3.012.856	-23.886	2.967.404	-21.904
2.973.969	-37.113	3.013.045	-23.884	2.967.574	-21.905
2.973.969	-37.114	3.013.206	-23.883	2.967.784	-21.905
2.974.218	-37.115	3.013.388	-23.881	2.967.933	-21.906
2.974.655	-37.115	3.013.578	-23.880	2.968.098	-21.906
2.974.788	-37.116	3.013.781	-23.878	2.968.369	-21.906
2.974.832	-37.117	3.013.907	-23.877	2.968.531	-21.907
2.975.273	-37.117	3.014.106	-23.875	2.968.586	-21.907
2.975.486	-37.118	3.014.304	-23.874	2.968.709	-21.908
2.975.686	-37.119	3.014.481	-23.872	2.968.932	-21.908
2.975.829	-37.119	3.014.727	-23.870	2.969.142	-21.908
2.975.598	-37.120	3.014.828	-23.869	2.969.301	-21.909
2.975.628	-37.121	3.014.947	-23.867	2.969.443	-21.909
2.975.920	-37.121	3.015.179	-23.866	2.969.605	-21.909
2.976.177	-37.122	3.015.388	-23.864	2.969.783	-21.910
2.976.340	-37.122	3.015.504	-23.862	2.969.922	-21.910
2.976.353	-37.123	3.015.628	-23.861	2.970.052	-21.910
2.976.599	-37.124	3.015.833	-23.859	2.970.197	-21.911
2.976.898	-37.124	3.016.003	-23.857	2.970.342	-21.911
2.976.938	-37.125	3.016.148	-23.855	2.970.523	-21.911
2.977.117	-37.125	3.016.346	-23.854	2.970.681	-21.912
2.977.369	-37.126	3.016.523	-23.852	2.970.836	-21.912
2.977.498	-37.127	3.016.611	-23.850	2.971.017	-21.912
2.977.776	-37.127	3.016.783	-23.849	2.971.223	-21.913
2.978.011	-37.128	3.017.029	-23.847	2.971.439	-21.913
2.978.040	-37.128	3.017.264	-23.845	2.971.632	-21.914
2.978.273	-37.129	3.017.474	-23.843	2.971.794	-21.914
2.978.394	-37.129	3.017.565	-23.841	2.971.952	-21.914

2.978.356	-37.130	3.017.708	-23.840	2.972.079	-21.915
2.978.557	-37.130	3.017.929	-23.838	2.972.191	-21.915
2.978.838	-37.131	3.018.070	-23.836	2.972.315	-21.915
2.979.035	-37.131	3.018.219	-23.834	2.972.463	-21.916
2.979.270	-37.132	3.018.401	-23.832	2.972.639	-21.916
2.979.409	-37.133	3.018.590	-23.830	2.972.841	-21.916
2.979.622	-37.133	3.018.773	-23.829	2.973.078	-21.917
2.979.906	-37.134	3.018.936	-23.827	2.973.250	-21.917
2.979.995	-37.134	3.019.106	-23.825	2.973.401	-21.917
2.980.047	-37.135	3.019.240	-23.823	2.973.558	-21.918
2.980.211	-37.135	3.019.362	-23.821	2.973.802	-21.918
2.980.330	-37.136	3.019.510	-23.819	2.973.981	-21.918
2.980.513	-37.136	3.019.673	-23.817	2.974.096	-21.919
2.980.930	-37.137	3.019.912	-23.815	2.974.284	-21.919
2.981.144	-37.137	3.020.245	-23.813	2.974.493	-21.919
2.981.350	-37.137	3.020.594	-23.812	2.974.676	-21.919
2.981.541	-37.138	3.020.833	-23.810	2.974.785	-21.920
2.981.685	-37.138	3.020.919	-23.808	2.974.938	-21.920
2.981.938	-37.139	3.021.046	-23.806	2.975.157	-21.920
2.981.960	-37.139	3.021.125	-23.804	2.975.325	-21.921
2.981.848	-37.140	3.021.127	-23.802	2.975.439	-21.921
2.981.841	-37.140	3.021.208	-23.800	2.975.587	-21.921
2.982.059	-37.141	3.021.324	-23.798	2.975.808	-21.922
2.982.281	-37.141	3.021.445	-23.796	2.976.071	-21.922
2.982.329	-37.141	3.021.570	-23.794	2.976.239	-21.922
2.982.527	-37.142	3.021.730	-23.792	2.976.288	-21.922
2.982.882	-37.142	3.021.947	-23.790	2.976.357	-21.923
2.983.060	-37.143	3.022.166	-23.788	2.976.491	-21.923
2.983.166	-37.143	3.022.341	-23.786	2.976.650	-21.923
2.983.326	-37.144	3.022.510	-23.784	2.976.855	-21.923

2.983.529	-37.144	3.022.693	-23.782	2.977.004	-21.923
2.983.710	-37.144	3.022.901	-23.780	2.977.112	-21.924
2.983.867	-37.145	3.023.060	-23.778	2.977.236	-21.924
2.984.019	-37.145	3.023.200	-23.776	2.977.422	-21.924
2.984.117	-37.146	3.023.345	-23.774	2.977.704	-21.924
2.984.178	-37.146	3.023.495	-23.772	2.977.921	-21.924
2.984.369	-37.147	3.023.690	-23.770	2.978.065	-21.924
2.984.693	-37.147	3.023.864	-23.768	2.978.297	-21.925
2.984.906	-37.147	3.023.999	-23.766	2.978.484	-21.925
2.985.013	-37.148	3.024.136	-23.764	2.978.614	-21.925
2.985.155	-37.148	3.024.292	-23.762	2.978.802	-21.925
2.985.234	-37.149	3.024.416	-23.760	2.979.008	-21.925
2.985.359	-37.149	3.024.611	-23.758	2.979.167	-21.925
2.985.612	-37.150	3.024.812	-23.756	2.979.236	-21.925
2.985.744	-37.151	3.025.000	-23.755	2.979.356	-21.925
2.985.822	-37.151	3.025.244	-23.753	2.979.537	-21.925
2.986.061	-37.152	3.025.403	-23.751	2.979.658	-21.925
2.986.353	-37.152	3.025.580	-23.749	2.979.727	-21.925
2.986.523	-37.153	3.025.761	-23.747	2.979.922	-21.925
2.986.844	-37.153	3.025.905	-23.745	2.980.186	-21.925
2.987.025	-37.154	3.026.071	-23.743	2.980.327	-21.925
2.987.123	-37.155	3.026.230	-23.742	2.980.422	-21.925
2.987.470	-37.155	3.026.393	-23.740	2.980.657	-21.925
2.987.484	-37.156	3.026.527	-23.738	2.980.973	-21.925
2.987.455	-37.157	3.026.689	-23.736	2.981.176	-21.925
2.987.499	-37.157	3.026.895	-23.735	2.981.289	-21.925
2.987.646	-37.158	3.027.030	-23.733	2.981.368	-21.925
2.987.859	-37.159	3.027.192	-23.731	2.981.526	-21.925
2.988.044	-37.159	3.027.379	-23.729	2.981.729	-21.925
2.988.293	-37.160	3.027.516	-23.728	2.981.924	-21.925

2.988.448	-37.161	3.027.639	-23.726	2.982.101	-21.925
2.988.553	-37.162	3.027.801	-23.724	2.982.234	-21.925
2.988.670	-37.162	3.027.971	-23.723	2.982.437	-21.925
2.988.824	-37.163	3.028.106	-23.721	2.982.630	-21.924
2.988.992	-37.164	3.028.289	-23.720	2.982.754	-21.924
2.989.128	-37.165	3.028.489	-23.718	2.982.939	-21.924
2.989.375	-37.166	3.028.640	-23.717	2.983.137	-21.924
2.989.577	-37.167	3.028.809	-23.715	2.983.319	-21.924
2.989.711	-37.168	3.028.970	-23.714	2.983.495	-21.924
2.989.843	-37.169	3.029.103	-23.712	2.983.651	-21.923
2.989.948	-37.170	3.029.268	-23.711	2.983.842	-21.923
2.990.136	-37.171	3.029.438	-23.709	2.984.030	-21.923
2.990.367	-37.172	3.029.604	-23.708	2.984.201	-21.923
2.990.605	-37.173	3.029.818	-23.707	2.984.301	-21.923
2.990.816	-37.174	3.030.036	-23.705	2.984.409	-21.922
2.990.901	-37.175	3.030.204	-23.704	2.984.626	-21.922
2.991.068	-37.176	3.030.334	-23.703	2.984.844	-21.922
2.991.314	-37.178	3.030.457	-23.701	2.984.971	-21.922
2.991.401	-37.179	3.030.674	-23.700	2.985.140	-21.921
2.991.573	-37.180	3.030.869	-23.699	2.985.356	-21.921
2.991.761	-37.181	3.030.999	-23.698	2.985.508	-21.921
2.991.870	-37.182	3.031.167	-23.697	2.985.648	-21.921
2.992.025	-37.184	3.031.335	-23.695	2.985.764	-21.920
2.992.279	-37.185	3.031.505	-23.694	2.985.974	-21.920
2.992.462	-37.186	3.031.667	-23.693	2.986.158	-21.920
2.992.571	-37.188	3.031.860	-23.692	2.986.253	-21.919
2.992.805	-37.189	3.032.050	-23.691	2.986.479	-21.919
2.993.042	-37.190	3.032.148	-23.690	2.986.891	-21.919
2.993.141	-37.192	3.032.281	-23.689	2.987.245	-21.918
2.993.225	-37.193	3.032.507	-23.688	2.987.361	-21.918

2.993.406	-37.194	3.032.744	-23.687	2.987.451	-21.918
2.993.638	-37.196	3.032.864	-23.686	2.987.553	-21.917
2.993.824	-37.197	3.032.935	-23.685	2.987.572	-21.917
2.993.915	-37.199	3.033.102	-23.684	2.987.643	-21.916
2.994.113	-37.200	3.033.251	-23.683	2.987.760	-21.916
2.994.190	-37.202	3.033.395	-23.682	2.987.888	-21.916
2.994.208	-37.203	3.033.571	-23.681	2.988.049	-21.915
2.994.360	-37.205	3.033.737	-23.680	2.988.206	-21.915
2.994.637	-37.206	3.033.903	-23.679	2.988.351	-21.914
2.994.859	-37.208	3.034.044	-23.678	2.988.504	-21.914
2.995.049	-37.209	3.034.241	-23.678	2.988.723	-21.914
2.995.242	-37.211	3.034.503	-23.677	2.988.952	-21.913
2.995.425	-37.212	3.034.705	-23.676	2.989.136	-21.913
2.995.556	-37.214	3.034.879	-23.675	2.989.352	-21.912
2.995.692	-37.215	3.035.081	-23.674	2.989.597	-21.912
2.995.920	-37.217	3.035.309	-23.673	2.989.721	-21.911
2.996.197	-37.219	3.035.545	-23.673	2.989.821	-21.911
2.996.516	-37.220	3.035.710	-23.672	2.989.970	-21.910
2.996.653	-37.222	3.035.804	-23.671	2.990.130	-21.910
2.996.690	-37.224	3.035.924	-23.670	2.990.273	-21.909
2.996.841	-37.225	3.036.065	-23.669	2.990.414	-21.909
2.997.034	-37.227	3.036.172	-23.669	2.990.602	-21.908
2.997.151	-37.228	3.036.288	-23.668	2.990.800	-21.908
2.997.249	-37.230	3.036.443	-23.667	2.990.939	-21.907
2.997.359	-37.232	3.036.592	-23.667	2.991.021	-21.907
2.997.489	-37.233	3.036.700	-23.666	2.991.180	-21.907
2.997.643	-37.235	3.036.864	-23.665	2.991.400	-21.906
2.997.816	-37.237	3.037.102	-23.664	2.991.603	-21.906
2.998.012	-37.238	3.037.283	-23.664	2.991.765	-21.905
2.998.073	-37.240	3.037.481	-23.663	2.991.931	-21.905

2.998.244	-37.242	3.037.704	-23.662	2.992.090	-21.904
2.998.515	-37.243	3.037.848	-23.662	2.992.249	-21.904
2.998.658	-37.245	3.038.026	-23.661	2.992.447	-21.903
2.998.832	-37.247	3.038.207	-23.660	2.992.615	-21.903
2.999.175	-37.248	3.038.423	-23.659	2.992.748	-21.902
2.999.328	-37.250	3.038.615	-23.659	2.992.877	-21.902
2.999.348	-37.252	3.038.749	-23.658	2.993.054	-21.901
2.999.566	-37.253	3.038.954	-23.657	2.993.260	-21.901
2.999.750	-37.255	3.039.142	-23.657	2.993.405	-21.901
2.999.886	-37.257	3.039.241	-23.656	2.993.589	-21.900
3.000.009	-37.258	3.039.381	-23.656	2.993.791	-21.900
3.000.065	-37.260	3.039.561	-23.655	2.993.900	-21.899
3.000.353	-37.262	3.039.727	-23.654	2.994.026	-21.899
3.000.648	-37.263	3.039.891	-23.654	2.994.204	-21.899
3.000.649	-37.265	3.040.020	-23.653	2.994.355	-21.898
3.000.776	-37.267	3.040.201	-23.652	2.994.546	-21.898
3.001.143	-37.268	3.040.417	-23.652	2.994.731	-21.897
3.001.426	-37.270	3.040.560	-23.651	2.994.862	-21.897
3.001.519	-37.272	3.040.724	-23.650	2.995.020	-21.897
3.001.639	-37.273	3.040.924	-23.650	2.995.176	-21.896
3.001.841	-37.275	3.041.130	-23.649	2.995.359	-21.896
3.002.083	-37.277	3.041.328	-23.649	2.995.533	-21.896
3.002.220	-37.278	3.041.512	-23.648	2.995.666	-21.896
3.002.242	-37.280	3.041.654	-23.647	2.995.898	-21.895
3.002.481	-37.282	3.041.776	-23.647	2.996.143	-21.895
3.002.852	-37.283	3.041.910	-23.646	2.996.349	-21.895
3.002.950	-37.285	3.042.141	-23.645	2.996.516	-21.895
3.002.975	-37.286	3.042.366	-23.645	2.996.613	-21.894
3.003.196	-37.288	3.042.451	-23.644	2.996.743	-21.894
3.003.392	-37.290	3.042.564	-23.643	2.996.893	-21.894

3.003.468	-37.291	3.042.765	-23.643	2.997.088	-21.894
3.003.661	-37.293	3.042.975	-23.642	2.997.265	-21.893
3.003.896	-37.294	3.043.124	-23.641	2.997.401	-21.893
3.003.984	-37.296	3.043.231	-23.641	2.997.560	-21.893
3.004.276	-37.297	3.043.388	-23.640	2.997.748	-21.893
3.004.547	-37.299	3.043.784	-23.640	2.997.971	-21.893
3.004.601	-37.300	3.044.196	-23.639	2.998.137	-21.892
3.004.788	-37.302	3.044.356	-23.638	2.998.315	-21.892
3.004.893	-37.303	3.044.446	-23.638	2.998.468	-21.892
3.005.281	-37.305	3.044.543	-23.637	2.998.590	-21.892
3.005.657	-37.306	3.044.624	-23.636	2.998.717	-21.892
3.005.811	-37.308	3.044.735	-23.636	2.998.860	-21.892
3.006.060	-37.309	3.044.835	-23.635	2.999.023	-21.891
3.006.068	-37.311	3.044.888	-23.634	2.999.161	-21.891
3.006.118	-37.312	3.045.006	-23.634	2.999.305	-21.891
3.006.282	-37.314	3.045.154	-23.633	2.999.469	-21.891
3.006.327	-37.315	3.045.301	-23.632	2.999.601	-21.891
3.006.361	-37.317	3.045.490	-23.631	2.999.746	-21.891
3.006.537	-37.318	3.045.739	-23.631	2.999.911	-21.891
3.006.690	-37.319	3.045.977	-23.630	3.000.111	-21.890
3.006.824	-37.321	3.046.165	-23.629	3.000.316	-21.890
3.006.909	-37.322	3.046.335	-23.629	3.000.520	-21.890
3.006.997	-37.324	3.046.479	-23.628	3.000.739	-21.890
3.007.224	-37.325	3.046.606	-23.627	3.000.982	-21.890
3.007.496	-37.326	3.046.761	-23.627	3.001.161	-21.890
3.007.669	-37.328	3.046.906	-23.626	3.001.317	-21.890
3.007.839	-37.329	3.047.104	-23.625	3.001.483	-21.890
3.008.098	-37.330	3.047.305	-23.624	3.001.620	-21.889
3.008.330	-37.331	3.047.429	-23.624	3.001.777	-21.889
3.008.468	-37.333	3.047.556	-23.623	3.001.887	-21.889

3.008.650	-37.334	3.047.666	-23.622	3.001.964	-21.889
3.008.809	-37.335	3.047.816	-23.621	3.002.151	-21.889
3.008.842	-37.337	3.048.020	-23.621	3.002.404	-21.889
3.008.944	-37.338	3.048.159	-23.620	3.002.546	-21.889
3.009.142	-37.339	3.048.348	-23.619	3.002.665	-21.889
3.009.384	-37.340	3.048.627	-23.619	3.002.801	-21.888
3.009.492	-37.342	3.048.791	-23.618	3.002.935	-21.888
3.009.575	-37.343	3.048.947	-23.617	3.003.174	-21.888
3.009.781	-37.344	3.049.146	-23.616	3.003.410	-21.888
3.010.002	-37.345	3.049.334	-23.616	3.003.582	-21.888
3.010.150	-37.347	3.049.503	-23.615	3.003.770	-21.888
3.010.321	-37.348	3.049.613	-23.614	3.003.955	-21.888
3.010.511	-37.349	3.049.747	-23.614	3.004.122	-21.887
3.010.598	-37.350	3.049.969	-23.613	3.004.323	-21.887
3.010.811	-37.352	3.050.183	-23.612	3.004.467	-21.887
3.011.176	-37.353	3.050.312	-23.611	3.004.634	-21.887
3.011.401	-37.354	3.050.410	-23.611	3.004.778	-21.887
3.011.452	-37.355	3.050.558	-23.610	3.004.876	-21.887
3.011.620	-37.357	3.050.739	-23.609	3.005.114	-21.887
3.011.824	-37.358	3.050.935	-23.609	3.005.334	-21.887
3.011.805	-37.359	3.051.082	-23.608	3.005.479	-21.886
3.011.931	-37.360	3.051.224	-23.607	3.005.670	-21.886
3.012.180	-37.361	3.051.374	-23.607	3.005.892	-21.886
3.012.281	-37.363	3.051.476	-23.606	3.006.036	-21.886
3.012.478	-37.364	3.051.654	-23.605	3.006.161	-21.886
3.012.806	-37.365	3.051.887	-23.605	3.006.400	-21.886
3.013.049	-37.366	3.052.086	-23.604	3.006.575	-21.885
3.013.081	-37.367	3.052.231	-23.603	3.006.668	-21.885
3.013.029	-37.369	3.052.386	-23.603	3.006.794	-21.885
3.013.211	-37.370	3.052.550	-23.602	3.007.005	-21.885

3.013.504	-37.371	3.052.762	-23.601	3.007.239	-21.885
3.013.716	-37.372	3.052.982	-23.601	3.007.301	-21.885
3.013.835	-37.374	3.053.170	-23.600	3.007.404	-21.884
3.013.954	-37.375	3.053.351	-23.600	3.007.617	-21.884
3.014.081	-37.376	3.053.459	-23.599	3.007.827	-21.884
3.014.232	-37.377	3.053.549	-23.598	3.008.019	-21.884
3.014.500	-37.379	3.053.697	-23.598	3.008.145	-21.884
3.014.705	-37.380	3.053.907	-23.597	3.008.283	-21.883
3.014.832	-37.381	3.054.120	-23.597	3.008.463	-21.883
3.014.998	-37.382	3.054.306	-23.596	3.008.656	-21.883
3.015.157	-37.384	3.054.435	-23.595	3.008.818	-21.883
3.015.386	-37.385	3.054.547	-23.595	3.008.940	-21.882
3.015.653	-37.386	3.054.702	-23.594	3.009.158	-21.882
3.015.789	-37.387	3.054.886	-23.594	3.009.575	-21.882
3.015.804	-37.389	3.055.079	-23.593	3.009.963	-21.882
3.016.003	-37.390	3.055.262	-23.592	3.010.114	-21.881
3.016.328	-37.391	3.055.363	-23.592	3.010.154	-21.881
3.016.408	-37.393	3.055.565	-23.591	3.010.238	-21.881
3.016.533	-37.394	3.055.786	-23.591	3.010.327	-21.881
3.016.758	-37.395	3.055.955	-23.590	3.010.403	-21.880
3.016.949	-37.397	3.056.138	-23.590	3.010.490	-21.880
3.016.999	-37.398	3.056.248	-23.589	3.010.594	-21.880
3.017.109	-37.399	3.056.376	-23.588	3.010.765	-21.879
3.017.368	-37.400	3.056.538	-23.588	3.010.921	-21.879
3.017.516	-37.402	3.056.708	-23.587	3.011.031	-21.879
3.017.711	-37.403	3.056.870	-23.587	3.011.213	-21.878
3.017.897	-37.404	3.057.029	-23.586	3.011.494	-21.878
3.018.084	-37.405	3.057.159	-23.585	3.011.689	-21.878
3.018.261	-37.407	3.057.278	-23.585	3.011.852	-21.877
3.018.428	-37.408	3.057.408	-23.584	3.012.062	-21.877

3.018.554	-37.409	3.057.612	-23.584	3.012.202	-21.877
3.018.580	-37.410	3.057.874	-23.583	3.012.368	-21.876
3.018.792	-37.412	3.058.105	-23.582	3.012.542	-21.876
3.019.059	-37.413	3.058.268	-23.582	3.012.640	-21.876
3.019.121	-37.414	3.058.484	-23.581	3.012.813	-21.875
3.019.185	-37.415	3.058.724	-23.580	3.012.983	-21.875
3.019.369	-37.417	3.058.886	-23.580	3.013.124	-21.874
3.019.534	-37.418	3.059.034	-23.579	3.013.264	-21.874
3.019.746	-37.419	3.059.111	-23.578	3.013.410	-21.874
3.020.016	-37.420	3.059.229	-23.578	3.013.596	-21.873
3.020.173	-37.422	3.059.400	-23.577	3.013.727	-21.873
3.020.291	-37.423	3.059.581	-23.576	3.013.959	-21.873
3.020.525	-37.424	3.059.753	-23.576	3.014.189	-21.872
3.020.747	-37.425	3.059.865	-23.575	3.014.371	-21.872
3.020.883	-37.426	3.060.005	-23.574	3.014.562	-21.871
3.021.039	-37.428	3.060.154	-23.573	3.014.721	-21.871
3.021.201	-37.429	3.060.302	-23.573	3.014.924	-21.871
3.021.477	-37.430	3.060.495	-23.572	3.015.112	-21.870
3.021.693	-37.431	3.060.710	-23.571	3.015.202	-21.870
3.021.758	-37.432	3.060.923	-23.570	3.015.298	-21.869
3.021.873	-37.433	3.061.093	-23.569	3.015.505	-21.869
3.022.019	-37.434	3.061.224	-23.568	3.015.684	-21.869
3.022.177	-37.435	3.061.378	-23.568	3.015.768	-21.868
3.022.265	-37.437	3.061.578	-23.567	3.015.927	-21.868
3.022.466	-37.438	3.061.743	-23.566	3.016.134	-21.867
3.022.657	-37.439	3.061.859	-23.565	3.016.343	-21.867
3.022.759	-37.440	3.062.050	-23.564	3.016.490	-21.867
3.022.939	-37.441	3.062.246	-23.563	3.016.566	-21.866
3.023.134	-37.442	3.062.416	-23.562	3.016.783	-21.866
3.023.301	-37.443	3.062.586	-23.561	3.016.974	-21.865

3.023.427	-37.444	3.062.766	-23.560	3.017.099	-21.865
3.023.665	-37.445	3.062.953	-23.559	3.017.301	-21.865
3.023.977	-37.446	3.063.123	-23.558	3.017.514	-21.864
3.024.151	-37.447	3.063.221	-23.558	3.017.676	-21.864
3.024.261	-37.448	3.063.391	-23.557	3.017.809	-21.864
3.024.342	-37.449	3.063.616	-23.556	3.018.009	-21.863
3.024.474	-37.449	3.063.788	-23.554	3.018.131	-21.863
3.024.669	-37.450	3.064.003	-23.553	3.018.236	-21.863
3.024.775	-37.451	3.064.187	-23.552	3.018.431	-21.862
3.024.952	-37.452	3.064.328	-23.551	3.018.575	-21.862
3.025.233	-37.453	3.064.566	-23.550	3.018.699	-21.862
3.025.446	-37.454	3.064.752	-23.549	3.018.882	-21.861
3.025.585	-37.455	3.064.836	-23.548	3.019.128	-21.861
3.025.769	-37.455	3.064.981	-23.547	3.019.290	-21.861
3.025.937	-37.456	3.065.187	-23.546	3.019.458	-21.860
3.026.082	-37.457	3.065.378	-23.545	3.019.649	-21.860
3.026.241	-37.458	3.065.533	-23.544	3.019.793	-21.860
3.026.440	-37.459	3.065.724	-23.542	3.019.980	-21.859
3.026.620	-37.459	3.065.931	-23.541	3.020.146	-21.859
3.026.808	-37.460	3.066.086	-23.540	3.020.345	-21.859
3.027.061	-37.461	3.066.227	-23.539	3.020.576	-21.858
3.027.227	-37.461	3.066.414	-23.538	3.020.710	-21.858
3.027.314	-37.462	3.066.585	-23.536	3.020.862	-21.858
3.027.444	-37.463	3.066.683	-23.535	3.021.040	-21.857
3.027.516	-37.464	3.066.736	-23.534	3.021.190	-21.857
3.027.646	-37.464	3.067.025	-23.533	3.021.383	-21.857
3.027.871	-37.465	3.067.534	-23.531	3.021.528	-21.856
3.028.073	-37.465	3.067.871	-23.530	3.021.628	-21.856
3.028.239	-37.466	3.067.957	-23.529	3.021.768	-21.856
3.028.380	-37.467	3.068.022	-23.527	3.021.906	-21.855

3.028.564	-37.467	3.068.123	-23.526	3.022.050	-21.855
3.028.896	-37.468	3.068.132	-23.525	3.022.249	-21.855
3.029.132	-37.468	3.068.153	-23.523	3.022.401	-21.854
3.029.232	-37.469	3.068.268	-23.522	3.022.513	-21.854
3.029.398	-37.469	3.068.406	-23.520	3.022.653	-21.854
3.029.435	-37.470	3.068.575	-23.519	3.022.824	-21.853
3.029.465	-37.471	3.068.767	-23.518	3.023.000	-21.853
3.029.840	-37.471	3.068.936	-23.516	3.023.214	-21.852
3.030.357	-37.472	3.069.111	-23.515	3.023.466	-21.852
3.030.616	-37.472	3.069.266	-23.513	3.023.719	-21.852
3.030.664	-37.473	3.069.453	-23.512	3.023.939	-21.851
3.030.724	-37.473	3.069.666	-23.510	3.024.064	-21.851
3.030.872	-37.473	3.069.847	-23.509	3.024.132	-21.851
3.031.017	-37.474	3.069.977	-23.507	3.024.294	-21.850
3.031.054	-37.474	3.070.099	-23.506	3.024.453	-21.850
3.031.089	-37.475	3.070.274	-23.504	3.024.534	-21.849
3.031.234	-37.475	3.070.442	-23.503	3.024.682	-21.849
3.031.411	-37.476	3.070.659	-23.501	3.024.857	-21.849
3.031.549	-37.476	3.070.870	-23.500	3.025.057	-21.848
3.031.653	-37.476	3.070.975	-23.498	3.025.266	-21.848
3.031.819	-37.477	3.071.089	-23.497	3.025.406	-21.847
3.032.050	-37.477	3.071.241	-23.495	3.025.505	-21.847
3.032.285	-37.477	3.071.434	-23.494	3.025.688	-21.847
3.032.516	-37.478	3.071.609	-23.492	3.025.932	-21.846
3.032.641	-37.478	3.071.730	-23.491	3.026.107	-21.846
3.032.713	-37.478	3.071.924	-23.489	3.026.275	-21.845
3.032.906	-37.479	3.072.202	-23.488	3.026.469	-21.845
3.033.058	-37.479	3.072.415	-23.486	3.026.654	-21.844
3.033.206	-37.479	3.072.574	-23.485	3.026.848	-21.844
3.033.394	-37.480	3.072.697	-23.483	3.027.007	-21.844

3.033.517	-37.480	3.072.834	-23.482	3.027.145	-21.843
3.033.770	-37.480	3.072.993	-23.480	3.027.332	-21.843
3.034.075	-37.481	3.073.134	-23.479	3.027.510	-21.842
3.034.230	-37.481	3.073.341	-23.477	3.027.733	-21.842
3.034.288	-37.481	3.073.522	-23.476	3.027.943	-21.841
3.034.391	-37.482	3.073.615	-23.474	3.028.080	-21.841
3.034.601	-37.482	3.073.760	-23.472	3.028.268	-21.841
3.034.776	-37.482	3.073.976	-23.471	3.028.408	-21.840
3.034.884	-37.483	3.074.174	-23.469	3.028.564	-21.840
3.035.027	-37.483	3.074.304	-23.468	3.028.755	-21.839
3.035.230	-37.483	3.074.449	-23.466	3.028.921	-21.839
3.035.472	-37.484	3.074.605	-23.465	3.029.109	-21.838
3.035.689	-37.484	3.074.778	-23.464	3.029.290	-21.838
3.035.896	-37.485	3.074.970	-23.462	3.029.454	-21.837
3.036.137	-37.485	3.075.096	-23.461	3.029.586	-21.837
3.036.237	-37.485	3.075.218	-23.459	3.029.740	-21.837
3.036.264	-37.486	3.075.436	-23.458	3.029.884	-21.836
3.036.503	-37.486	3.075.670	-23.456	3.030.024	-21.836
3.036.705	-37.487	3.075.807	-23.455	3.030.255	-21.835
3.036.649	-37.487	3.075.941	-23.454	3.030.451	-21.835
3.036.770	-37.488	3.076.071	-23.452	3.030.558	-21.834
3.037.109	-37.488	3.076.216	-23.451	3.030.695	-21.834
3.037.353	-37.488	3.076.463	-23.449	3.030.844	-21.833
3.037.527	-37.489	3.076.646	-23.448	3.030.999	-21.833
3.037.574	-37.489	3.076.736	-23.447	3.031.162	-21.833
3.037.662	-37.490	3.076.906	-23.445	3.031.318	-21.832
3.037.820	-37.490	3.077.167	-23.444	3.031.505	-21.832
3.038.120	-37.491	3.077.416	-23.443	3.031.657	-21.831
3.038.396	-37.492	3.077.578	-23.442	3.031.885	-21.831
3.038.412	-37.492	3.077.664	-23.440	3.032.321	-21.830

3.038.492	-37.493	3.077.838	-23.439	3.032.702	-21.830
3.038.685	-37.493	3.078.029	-23.438	3.032.849	-21.830
3.038.839	-37.494	3.078.132	-23.437	3.032.908	-21.829
3.039.039	-37.495	3.078.286	-23.435	3.032.961	-21.829
3.039.275	-37.495	3.078.481	-23.434	3.032.990	-21.828
3.039.499	-37.496	3.078.680	-23.433	3.033.087	-21.828
3.039.594	-37.497	3.078.832	-23.432	3.033.188	-21.828
3.039.640	-37.498	3.078.969	-23.431	3.033.296	-21.827
3.039.778	-37.498	3.079.088	-23.430	3.033.434	-21.827
3.039.988	-37.499	3.079.265	-23.428	3.033.627	-21.826
3.040.271	-37.500	3.079.485	-23.427	3.033.803	-21.826
3.040.462	-37.501	3.079.691	-23.426	3.033.981	-21.826
3.040.552	-37.502	3.079.839	-23.425	3.034.178	-21.825
3.040.751	-37.503	3.079.908	-23.424	3.034.304	-21.825
3.041.061	-37.503	3.079.991	-23.423	3.034.440	-21.825
3.041.194	-37.504	3.080.132	-23.422	3.034.713	-21.824
3.041.315	-37.505	3.080.341	-23.421	3.034.846	-21.824
3.041.501	-37.506	3.080.476	-23.419	3.034.917	-21.823
3.041.596	-37.507	3.080.595	-23.418	3.035.177	-21.823
3.041.812	-37.508	3.080.798	-23.417	3.035.382	-21.823
3.041.982	-37.509	3.081.017	-23.416	3.035.504	-21.823
3.042.080	-37.510	3.081.208	-23.415	3.035.623	-21.822
3.042.343	-37.511	3.081.401	-23.414	3.035.807	-21.822
3.042.570	-37.512	3.081.613	-23.413	3.035.981	-21.822
3.042.738	-37.513	3.081.892	-23.412	3.036.098	-21.821
3.042.939	-37.514	3.082.094	-23.411	3.036.259	-21.821
3.043.095	-37.515	3.082.205	-23.410	3.036.454	-21.821
3.043.091	-37.516	3.082.351	-23.409	3.036.606	-21.821
3.043.207	-37.517	3.082.514	-23.408	3.036.790	-21.820
3.043.470	-37.518	3.082.690	-23.407	3.037.014	-21.820

3.043.651	-37.519	3.082.842	-23.406	3.037.213	-21.820
3.043.666	-37.521	3.082.971	-23.405	3.037.350	-21.820
3.043.694	-37.522	3.083.145	-23.404	3.037.502	-21.820
3.043.878	-37.523	3.083.331	-23.403	3.037.704	-21.820
3.044.150	-37.524	3.083.423	-23.402	3.037.903	-21.820
3.044.291	-37.525	3.083.524	-23.401	3.038.081	-21.819
3.044.363	-37.526	3.083.719	-23.401	3.038.232	-21.819
3.044.678	-37.527	3.083.903	-23.400	3.038.343	-21.819
3.044.987	-37.529	3.084.052	-23.399	3.038.515	-21.819
3.045.220	-37.530	3.084.179	-23.398	3.038.720	-21.819
3.045.465	-37.531	3.084.421	-23.397	3.038.871	-21.819
3.045.692	-37.532	3.084.648	-23.396	3.039.002	-21.819
3.045.829	-37.534	3.084.799	-23.395	3.039.147	-21.819
3.045.822	-37.535	3.085.005	-23.394	3.039.331	-21.819
3.045.938	-37.536	3.085.179	-23.393	3.039.500	-21.819
3.046.100	-37.537	3.085.345	-23.392	3.039.667	-21.819
3.046.212	-37.539	3.085.483	-23.391	3.039.843	-21.819
3.046.382	-37.540	3.085.627	-23.390	3.040.016	-21.820
3.046.596	-37.541	3.085.797	-23.390	3.040.157	-21.820
3.046.595	-37.542	3.085.952	-23.389	3.040.309	-21.820
3.046.645	-37.544	3.086.083	-23.388	3.040.465	-21.820
3.046.870	-37.545	3.086.292	-23.387	3.040.605	-21.820
3.047.081	-37.546	3.086.513	-23.386	3.040.786	-21.821
3.047.372	-37.548	3.086.671	-23.385	3.040.963	-21.821
3.047.469	-37.549	3.086.816	-23.384	3.041.147	-21.821
3.047.590	-37.550	3.086.967	-23.383	3.041.303	-21.821
3.047.859	-37.552	3.087.156	-23.383	3.041.444	-21.822
3.048.023	-37.553	3.087.347	-23.382	3.041.617	-21.822
3.048.202	-37.554	3.087.513	-23.381	3.041.806	-21.822
3.048.445	-37.556	3.087.697	-23.380	3.041.979	-21.823

3.048.608	-37.557	3.087.910	-23.379	3.042.141	-21.823
3.048.723	-37.558	3.088.139	-23.378	3.042.296	-21.823
3.048.911	-37.560	3.088.273	-23.378	3.042.469	-21.824
3.049.132	-37.561	3.088.336	-23.377	3.042.625	-21.824
3.049.410	-37.563	3.088.515	-23.376	3.042.778	-21.825
3.049.649	-37.564	3.088.766	-23.375	3.042.986	-21.825
3.049.757	-37.565	3.088.954	-23.374	3.043.185	-21.826
3.049.861	-37.567	3.089.089	-23.373	3.043.331	-21.826
3.050.005	-37.568	3.089.221	-23.373	3.043.484	-21.827
3.050.255	-37.569	3.089.375	-23.372	3.043.705	-21.827
3.050.413	-37.571	3.089.543	-23.371	3.043.925	-21.828
3.050.447	-37.572	3.089.742	-23.370	3.044.071	-21.828
3.050.617	-37.574	3.089.929	-23.369	3.044.167	-21.829
3.050.872	-37.575	3.090.075	-23.368	3.044.287	-21.829
3.051.129	-37.576	3.090.175	-23.368	3.044.476	-21.830
3.051.288	-37.578	3.090.316	-23.367	3.044.631	-21.830
3.051.339	-37.579	3.090.682	-23.366	3.044.691	-21.831
3.051.443	-37.581	3.091.086	-23.365	3.044.818	-21.832
3.051.584	-37.582	3.091.321	-23.364	3.044.992	-21.832
3.051.772	-37.583	3.091.476	-23.363	3.045.118	-21.833
3.051.954	-37.585	3.091.570	-23.363	3.045.287	-21.833
3.052.144	-37.586	3.091.610	-23.362	3.045.518	-21.834
3.052.232	-37.588	3.091.620	-23.361	3.045.674	-21.835
3.052.369	-37.589	3.091.694	-23.360	3.045.912	-21.835
3.052.601	-37.590	3.091.808	-23.359	3.046.165	-21.836
3.052.763	-37.592	3.091.953	-23.358	3.046.368	-21.837
3.053.018	-37.593	3.092.094	-23.357	3.046.568	-21.837
3.053.286	-37.594	3.092.227	-23.357	3.046.751	-21.838
3.053.484	-37.596	3.092.444	-23.356	3.046.884	-21.839
3.053.618	-37.597	3.092.653	-23.355	3.047.000	-21.840

3.053.687	-37.599	3.092.820	-23.354	3.047.202	-21.840
3.053.806	-37.600	3.092.968	-23.353	3.047.358	-21.841
3.054.078	-37.601	3.093.142	-23.352	3.047.482	-21.842
3.054.316	-37.603	3.093.359	-23.352	3.047.597	-21.842
3.054.543	-37.604	3.093.580	-23.351	3.047.704	-21.843
3.054.973	-37.605	3.093.757	-23.350	3.047.856	-21.844
3.054.958	-37.607	3.093.889	-23.349	3.048.027	-21.845
3.055.025	-37.608	3.094.073	-23.348	3.048.189	-21.845
3.055.222	-37.609	3.094.171	-23.347	3.048.365	-21.846
3.055.231	-37.611	3.094.240	-23.347	3.048.565	-21.847
3.055.489	-37.612	3.094.457	-23.346	3.048.832	-21.847
3.055.661	-37.613	3.094.638	-23.345	3.049.048	-21.848
3.055.757	-37.615	3.094.782	-23.344	3.049.190	-21.849
3.055.873	-37.616	3.094.969	-23.343	3.049.328	-21.850
3.056.013	-37.617	3.095.135	-23.343	3.049.449	-21.850
3.056.202	-37.619	3.095.267	-23.342	3.049.631	-21.851
3.056.404	-37.620	3.095.482	-23.341	3.049.807	-21.852
3.056.407	-37.621	3.095.746	-23.340	3.049.966	-21.853
3.056.653	-37.622	3.095.949	-23.340	3.050.139	-21.853
3.056.924	-37.624	3.096.114	-23.339	3.050.303	-21.854
3.056.952	-37.625	3.096.288	-23.338	3.050.473	-21.855
3.057.242	-37.626	3.096.421	-23.337	3.050.674	-21.856
3.057.417	-37.628	3.096.555	-23.337	3.050.845	-21.856
3.057.380	-37.629	3.096.704	-23.336	3.051.013	-21.857
3.057.448	-37.630	3.096.824	-23.335	3.051.248	-21.858
3.057.525	-37.631	3.097.018	-23.334	3.051.422	-21.859
3.057.922	-37.633	3.097.188	-23.334	3.051.546	-21.859
3.058.237	-37.634	3.097.350	-23.333	3.051.676	-21.860
3.058.312	-37.635	3.097.518	-23.332	3.051.819	-21.861
3.058.341	-37.636	3.097.729	-23.332	3.052.036	-21.861

3.058.502	-37.638	3.097.932	-23.331	3.052.276	-21.862
3.058.861	-37.639	3.098.042	-23.330	3.052.473	-21.863
3.059.015	-37.640	3.098.175	-23.330	3.052.597	-21.864
3.059.023	-37.642	3.098.331	-23.329	3.052.747	-21.864
3.059.020	-37.643	3.098.492	-23.328	3.052.899	-21.865
3.059.374	-37.644	3.098.654	-23.328	3.053.026	-21.866
3.059.735	-37.646	3.098.802	-23.327	3.053.243	-21.866
3.059.753	-37.647	3.098.977	-23.327	3.053.434	-21.867
3.059.901	-37.648	3.099.128	-23.326	3.053.622	-21.868
3.060.094	-37.649	3.099.277	-23.325	3.053.797	-21.868
3.060.284	-37.651	3.099.486	-23.325	3.053.891	-21.869
3.060.518	-37.652	3.099.662	-23.324	3.054.109	-21.870
3.060.753	-37.653	3.099.833	-23.324	3.054.283	-21.870
3.060.885	-37.655	3.100.027	-23.323	3.054.402	-21.871
3.061.008	-37.656	3.100.209	-23.323	3.054.692	-21.871
3.061.325	-37.657	3.100.367	-23.322	3.055.023	-21.872
3.061.443	-37.659	3.100.545	-23.322	3.055.334	-21.873
3.061.415	-37.660	3.100.714	-23.321	3.055.594	-21.873
3.061.526	-37.662	3.100.854	-23.321	3.055.657	-21.874
3.061.928	-37.663	3.100.997	-23.320	3.055.642	-21.874
3.062.408	-37.664	3.101.180	-23.319	3.055.699	-21.875
3.062.412	-37.666	3.101.357	-23.319	3.055.807	-21.876
3.062.635	-37.667	3.101.502	-23.318	3.055.903	-21.876
3.063.083	-37.668	3.101.685	-23.318	3.055.974	-21.877
3.063.081	-37.670	3.101.909	-23.318	3.056.096	-21.877
3.062.973	-37.671	3.102.080	-23.317	3.056.288	-21.878
3.062.881	-37.673	3.102.245	-23.317	3.056.451	-21.878
3.063.174	-37.674	3.102.422	-23.316	3.056.632	-21.879
3.063.481	-37.676	3.102.541	-23.316	3.056.844	-21.879
3.063.420	-37.677	3.102.715	-23.315	3.057.072	-21.880

3.063.473	-37.679	3.102.896	-23.315	3.057.242	-21.880
3.063.643	-37.680	3.103.023	-23.314	3.057.377	-21.881
3.063.963	-37.682	3.103.163	-23.314	3.057.563	-21.881
3.064.104	-37.683	3.103.334	-23.313	3.057.713	-21.881
3.064.268	-37.685	3.103.478	-23.313	3.057.892	-21.882
3.064.468	-37.686	3.103.579	-23.312	3.058.051	-21.882
3.064.638	-37.688	3.103.683	-23.312	3.058.188	-21.883
3.064.953	-37.689	3.103.814	-23.311	3.058.383	-21.883
3.065.047	-37.691	3.103.987	-23.311	3.058.539	-21.883
3.064.941	-37.692	3.104.205	-23.310	3.058.706	-21.884
3.065.106	-37.694	3.104.402	-23.310	3.058.887	-21.884
3.065.367	-37.695	3.104.539	-23.309	3.059.027	-21.885
3.065.690	-37.697	3.104.734	-23.309	3.059.156	-21.885
3.065.982	-37.698	3.105.045	-23.308	3.059.333	-21.885
3.066.064	-37.700	3.105.345	-23.308	3.059.548	-21.886
3.066.409	-37.702	3.105.511	-23.307	3.059.747	-21.886
3.066.537	-37.703	3.105.596	-23.307	3.059.919	-21.886
3.066.508	-37.705	3.105.721	-23.306	3.060.101	-21.887
3.066.716	-37.706	3.105.949	-23.305	3.060.354	-21.887
3.066.833	-37.708	3.106.133	-23.305	3.060.504	-21.887
3.066.985	-37.709	3.106.208	-23.304	3.060.580	-21.888
3.067.213	-37.711	3.106.335	-23.304	3.060.740	-21.888
3.067.422	-37.712	3.106.472	-23.303	3.060.984	-21.888
3.067.525	-37.714	3.106.603	-23.303	3.061.154	-21.889
3.067.514	-37.715	3.106.813	-23.302	3.061.194	-21.889
3.067.661	-37.717	3.107.011	-23.301	3.061.327	-21.889
3.067.942	-37.718	3.107.140	-23.301	3.061.575	-21.889
3.067.906	-37.720	3.107.223	-23.300	3.061.750	-21.890
3.067.986	-37.722	3.107.386	-23.299	3.061.914	-21.890
3.068.217	-37.723	3.107.625	-23.299	3.062.054	-21.890

3.068.467	-37.725	3.107.827	-23.298	3.062.198	-21.890
3.068.699	-37.726	3.108.065	-23.297	3.062.379	-21.891
3.068.970	-37.728	3.108.304	-23.297	3.062.520	-21.891
3.069.071	-37.729	3.108.455	-23.296	3.062.722	-21.891
3.069.091	-37.731	3.108.619	-23.295	3.062.928	-21.892
3.069.391	-37.732	3.108.762	-23.295	3.063.011	-21.892
3.069.605	-37.734	3.108.872	-23.294	3.063.123	-21.892
3.069.713	-37.735	3.109.081	-23.293	3.063.298	-21.892
3.069.890	-37.737	3.109.241	-23.292	3.063.448	-21.892
3.069.967	-37.738	3.109.364	-23.292	3.063.647	-21.893
3.070.063	-37.740	3.109.487	-23.291	3.063.872	-21.893
3.070.544	-37.741	3.109.645	-23.290	3.064.060	-21.893
3.070.901	-37.743	3.109.843	-23.289	3.064.275	-21.893
3.070.966	-37.744	3.110.034	-23.289	3.064.474	-21.894
3.071.028	-37.746	3.110.186	-23.288	3.064.597	-21.894
3.071.213	-37.747	3.110.302	-23.287	3.064.729	-21.894
3.071.454	-37.748	3.110.508	-23.286	3.064.877	-21.894
3.071.418	-37.750	3.110.782	-23.285	3.065.010	-21.895
3.071.526	-37.751	3.110.968	-23.284	3.065.211	-21.895
3.071.559	-37.753	3.111.150	-23.283	3.065.378	-21.895
3.071.602	-37.754	3.111.346	-23.283	3.065.536	-21.895
3.072.113	-37.755	3.111.517	-23.282	3.065.779	-21.895
3.072.509	-37.757	3.111.640	-23.281	3.065.942	-21.896
3.072.523	-37.758	3.111.777	-23.280	3.066.133	-21.896
3.072.892	-37.759	3.111.976	-23.279	3.066.331	-21.896
3.073.109	-37.761	3.112.202	-23.278	3.066.432	-21.896
3.073.195	-37.762	3.112.370	-23.277	3.066.560	-21.896
3.073.434	-37.763	3.112.531	-23.276	3.066.685	-21.897
3.073.683	-37.765	3.112.683	-23.275	3.066.849	-21.897
3.073.735	-37.766	3.112.798	-23.274	3.067.015	-21.897

3.073.644	-37.767	3.113.016	-23.273	3.067.167	-21.897
3.073.721	-37.768	3.113.218	-23.272	3.067.325	-21.897
3.073.792	-37.770	3.113.289	-23.271	3.067.430	-21.897
3.074.333	-37.771	3.113.441	-23.270	3.067.563	-21.898
3.074.818	-37.772	3.113.616	-23.269	3.067.755	-21.898
3.074.924	-37.773	3.113.757	-23.268	3.067.944	-21.898
3.074.825	-37.774	3.114.092	-23.267	3.068.091	-21.898
3.074.796	-37.776	3.114.482	-23.266	3.068.251	-21.898
3.075.059	-37.777	3.114.727	-23.264	3.068.471	-21.898
3.075.281	-37.778	3.114.832	-23.263	3.068.723	-21.898
3.075.179	-37.779	3.114.944	-23.262	3.068.955	-21.898
3.075.172	-37.780	3.115.063	-23.261	3.069.136	-21.898
3.075.536	-37.781	3.115.099	-23.260	3.069.292	-21.898
3.075.859	-37.782	3.115.168	-23.259	3.069.464	-21.898
3.076.162	-37.783	3.115.281	-23.258	3.069.606	-21.898
3.076.249	-37.784	3.115.410	-23.257	3.069.779	-21.898
3.076.495	-37.785	3.115.563	-23.255	3.069.931	-21.898
3.076.651	-37.786	3.115.714	-23.254	3.070.061	-21.898
3.076.725	-37.787	3.115.906	-23.253	3.070.222	-21.898
3.076.447	-37.788	3.116.073	-23.252	3.070.349	-21.898
3.076.982	-37.789	3.116.220	-23.251	3.070.457	-21.898
3.077.641	-37.790	3.116.408	-23.250	3.070.602	-21.898
3.077.325	-37.791	3.116.591	-23.249	3.070.773	-21.898
3.077.177	-37.792	3.116.765	-23.247	3.070.967	-21.898
3.077.596	-37.793	3.116.943	-23.246	3.071.179	-21.898
3.077.806	-37.794	3.117.170	-23.245	3.071.335	-21.898
3.077.849	-37.795	3.117.368	-23.244	3.071.510	-21.898
3.078.055	-37.796	3.117.502	-23.243	3.071.715	-21.898
3.078.464	-37.797	3.117.611	-23.242	3.071.892	-21.898
3.078.455	-37.797	3.117.744	-23.240	3.072.130	-21.898

3.078.421	-37.798	3.117.953	-23.239	3.072.307	-21.898
3.079.063	-37.799	3.118.123	-23.238	3.072.393	-21.898
3.079.781	-37.800	3.118.307	-23.237	3.072.593	-21.898
3.080.010	-37.801	3.118.463	-23.236	3.072.785	-21.898
3.079.995	-37.801	3.118.587	-23.235	3.072.907	-21.898
3.080.255	-37.802	3.118.744	-23.234	3.073.076	-21.898
3.079.980	-37.803	3.118.900	-23.233	3.073.275	-21.897
3.079.792	-37.804	3.119.114	-23.232	3.073.475	-21.897
3.080.064	-37.804	3.119.334	-23.231	3.073.650	-21.897
3.080.382	-37.805	3.119.539	-23.229	3.073.763	-21.897
3.080.583	-37.806	3.119.745	-23.228	3.073.903	-21.897
3.080.706	-37.806	3.119.883	-23.227	3.074.169	-21.897
3.080.786	-37.807	3.119.945	-23.226	3.074.392	-21.897
3.080.872	-37.807	3.120.072	-23.225	3.074.514	-21.897
3.081.096	-37.808	3.120.263	-23.224	3.074.646	-21.896
3.081.183	-37.809	3.120.476	-23.224	3.074.836	-21.896
3.081.282	-37.809	3.120.667	-23.223	3.075.021	-21.896
3.081.700	-37.810	3.120.793	-23.222	3.075.179	-21.896
3.081.907	-37.810	3.120.966	-23.221	3.075.320	-21.896
3.081.646	-37.811	3.121.105	-23.220	3.075.425	-21.896
3.081.812	-37.811	3.121.224	-23.219	3.075.573	-21.895
3.081.898	-37.812	3.121.372	-23.218	3.075.769	-21.895
3.082.184	-37.812	3.121.519	-23.217	3.075.995	-21.895
3.082.705	-37.813	3.121.714	-23.217	3.076.219	-21.895
3.082.552	-37.813	3.121.909	-23.216	3.076.375	-21.895
3.082.614	-37.814	3.122.043	-23.215	3.076.476	-21.895
3.083.362	-37.814	3.122.202	-23.215	3.076.632	-21.894
3.083.275	-37.815	3.122.380	-23.214	3.076.815	-21.894
3.082.781	-37.815	3.122.507	-23.213	3.076.961	-21.894
3.083.598	-37.816	3.122.619	-23.213	3.077.086	-21.894

3.084.067	-37.816	3.122.856	-23.212	3.077.234	-21.894
3.083.771	-37.817	3.123.072	-23.212	3.077.614	-21.894
3.084.041	-37.817	3.123.195	-23.211	3.078.047	-21.893
3.084.522	-37.818	3.123.380	-23.211	3.078.251	-21.893
3.084.525	-37.818	3.123.573	-23.210	3.078.351	-21.893
3.084.344	-37.819	3.123.781	-23.210	3.078.450	-21.893
3.084.768	-37.819	3.123.961	-23.209	3.078.504	-21.893
3.085.013	-37.820	3.124.066	-23.209	3.078.508	-21.892
3.085.260	-37.820	3.124.176	-23.209	3.078.601	-21.892
3.085.576	-37.821	3.124.330	-23.208	3.078.742	-21.892
3.085.461	-37.821	3.124.563	-23.208	3.078.894	-21.892
3.085.248	-37.822	3.124.783	-23.208	3.079.125	-21.892
3.085.295	-37.822	3.124.944	-23.208	3.079.306	-21.891
3.085.677	-37.823	3.125.072	-23.207	3.079.406	-21.891
3.085.891	-37.823	3.125.233	-23.207	3.079.547	-21.891
3.086.556	-37.824	3.125.482	-23.207	3.079.770	-21.891
3.086.324	-37.825	3.125.614	-23.207	3.080.020	-21.891
3.085.892	-37.825	3.125.740	-23.207	3.080.184	-21.890
3.086.745	-37.826	3.125.939	-23.207	3.080.302	-21.890
3.087.010	-37.826	3.126.112	-23.207	3.080.460	-21.890
3.087.036	-37.827	3.126.234	-23.207	3.080.602	-21.890
3.087.216	-37.828	3.126.342	-23.207	3.080.778	-21.890
3.087.456	-37.828	3.126.519	-23.207	3.080.957	-21.889
3.087.904	-37.829	3.126.690	-23.207	3.081.098	-21.889
3.087.885	-37.830	3.126.830	-23.207	3.081.259	-21.889
3.087.724	-37.830	3.126.982	-23.207	3.081.386	-21.889
3.088.159	-37.831	3.127.133	-23.207	3.081.562	-21.889
3.088.289	-37.832	3.127.274	-23.208	3.081.744	-21.889
3.088.231	-37.833	3.127.452	-23.208	3.081.929	-21.888
3.088.510	-37.834	3.127.639	-23.208	3.082.142	-21.888

3.088.885	-37.834	3.127.817	-23.208	3.082.318	-21.888
3.088.979	-37.835	3.127.977	-23.209	3.082.524	-21.888
3.089.002	-37.836	3.128.167	-23.209	3.082.676	-21.888
3.089.359	-37.837	3.128.450	-23.209	3.082.852	-21.888
3.089.467	-37.838	3.128.692	-23.210	3.083.051	-21.888
3.089.846	-37.839	3.128.876	-23.210	3.083.214	-21.888
3.089.978	-37.840	3.128.997	-23.210	3.083.381	-21.888
3.089.393	-37.841	3.129.138	-23.211	3.083.515	-21.888
3.089.922	-37.842	3.129.348	-23.211	3.083.677	-21.887
3.090.659	-37.843	3.129.499	-23.212	3.083.826	-21.887
3.090.345	-37.844	3.129.595	-23.212	3.083.932	-21.887
3.090.430	-37.845	3.129.709	-23.213	3.084.031	-21.887
3.090.475	-37.846	3.129.894	-23.213	3.084.236	-21.887
3.090.589	-37.847	3.130.081	-23.214	3.084.482	-21.887
3.091.145	-37.848	3.130.187	-23.214	3.084.624	-21.887
3.091.466	-37.849	3.130.317	-23.215	3.084.808	-21.887
3.091.509	-37.850	3.130.494	-23.215	3.085.034	-21.887
3.091.537	-37.851	3.130.708	-23.216	3.085.136	-21.887
3.092.061	-37.852	3.130.865	-23.216	3.085.218	-21.887
3.092.256	-37.854	3.130.975	-23.217	3.085.410	-21.887
3.091.799	-37.855	3.131.216	-23.218	3.085.566	-21.888
3.092.142	-37.856	3.131.419	-23.218	3.085.697	-21.888
3.092.687	-37.857	3.131.557	-23.219	3.085.843	-21.888
3.092.679	-37.859	3.131.732	-23.220	3.085.973	-21.888
3.092.990	-37.860	3.131.873	-23.220	3.086.162	-21.888
3.092.947	-37.861	3.132.046	-23.221	3.086.411	-21.888
3.092.874	-37.862	3.132.261	-23.222	3.086.629	-21.888
3.093.026	-37.864	3.132.419	-23.222	3.086.795	-21.888
3.093.127	-37.865	3.132.547	-23.223	3.086.922	-21.889
3.093.282	-37.867	3.132.722	-23.224	3.087.093	-21.889

3.093.409	-37.868	3.132.849	-23.225	3.087.336	-21.889
3.093.701	-37.869	3.133.067	-23.225	3.087.474	-21.889
3.093.697	-37.871	3.133.340	-23.226	3.087.554	-21.889
3.093.875	-37.872	3.133.482	-23.227	3.087.734	-21.890
3.094.171	-37.874	3.133.670	-23.228	3.087.964	-21.890
3.094.265	-37.875	3.133.838	-23.228	3.088.114	-21.890
3.094.516	-37.877	3.133.941	-23.229	3.088.250	-21.890
3.095.014	-37.878	3.134.109	-23.230	3.088.452	-21.891
3.095.115	-37.880	3.134.323	-23.231	3.088.665	-21.891
3.095.065	-37.881	3.134.516	-23.232	3.088.814	-21.891
3.095.220	-37.883	3.134.662	-23.232	3.088.976	-21.891
3.095.464	-37.884	3.134.869	-23.233	3.089.146	-21.892
3.095.916	-37.886	3.134.980	-23.234	3.089.283	-21.892
3.095.961	-37.888	3.135.110	-23.235	3.089.476	-21.892
3.095.668	-37.889	3.135.309	-23.236	3.089.635	-21.892
3.095.802	-37.891	3.135.484	-23.236	3.089.774	-21.893
3.096.401	-37.892	3.135.650	-23.237	3.089.931	-21.893
3.096.517	-37.894	3.135.840	-23.238	3.090.029	-21.893
3.096.544	-37.896	3.136.049	-23.239	3.090.168	-21.894
3.096.834	-37.897	3.136.279	-23.239	3.090.341	-21.894
3.096.949	-37.899	3.136.398	-23.240	3.090.475	-21.894
3.097.177	-37.901	3.136.490	-23.241	3.090.643	-21.894
3.097.495	-37.903	3.136.643	-23.242	3.090.809	-21.895
3.097.630	-37.904	3.136.848	-23.243	3.091.006	-21.895
3.097.849	-37.906	3.137.011	-23.243	3.091.220	-21.895
3.098.109	-37.908	3.137.091	-23.244	3.091.433	-21.896
3.098.054	-37.910	3.137.498	-23.245	3.091.664	-21.896
3.098.365	-37.911	3.137.965	-23.246	3.091.859	-21.896
3.098.695	-37.913	3.138.103	-23.246	3.092.033	-21.896
3.098.503	-37.915	3.138.199	-23.247	3.092.216	-21.897

3.098.770	-37.917	3.138.319	-23.248	3.092.380	-21.897
3.098.947	-37.919	3.138.347	-23.249	3.092.535	-21.897
3.099.084	-37.920	3.138.369	-23.249	3.092.665	-21.897
3.098.918	-37.922	3.138.456	-23.250	3.092.802	-21.898
3.099.183	-37.924	3.138.568	-23.251	3.092.973	-21.898
3.099.547	-37.926	3.138.685	-23.251	3.093.091	-21.898
3.099.617	-37.928	3.138.857	-23.252	3.093.177	-21.898
3.099.962	-37.930	3.139.091	-23.253	3.093.348	-21.899
3.100.222	-37.932	3.139.320	-23.253	3.093.477	-21.899
3.100.140	-37.933	3.139.454	-23.254	3.093.634	-21.899
3.100.209	-37.935	3.139.604	-23.255	3.093.923	-21.899
3.100.436	-37.937	3.139.837	-23.255	3.094.129	-21.899
3.100.684	-37.939	3.140.031	-23.256	3.094.272	-21.899
3.101.036	-37.941	3.140.159	-23.256	3.094.424	-21.900
3.101.096	-37.943	3.140.296	-23.257	3.094.591	-21.900
3.101.136	-37.945	3.140.466	-23.258	3.094.796	-21.900
3.101.433	-37.947	3.140.591	-23.258	3.095.010	-21.900
3.101.755	-37.949	3.140.762	-23.259	3.095.148	-21.900
3.101.875	-37.951	3.140.971	-23.259	3.095.291	-21.900
3.101.904	-37.953	3.141.143	-23.260	3.095.472	-21.900
3.102.288	-37.954	3.141.317	-23.260	3.095.668	-21.901
3.102.442	-37.956	3.141.479	-23.261	3.095.811	-21.901
3.102.455	-37.958	3.141.627	-23.261	3.095.966	-21.901
3.102.599	-37.960	3.141.788	-23.262	3.096.154	-21.901
3.102.785	-37.962	3.141.971	-23.262	3.096.281	-21.901
3.102.930	-37.964	3.142.097	-23.262	3.096.398	-21.901
3.103.074	-37.966	3.142.256	-23.263	3.096.580	-21.901
3.103.307	-37.968	3.142.451	-23.263	3.096.752	-21.901
3.103.779	-37.970	3.142.637	-23.264	3.096.914	-21.901
3.104.117	-37.972	3.142.846	-23.264	3.097.152	-21.901

3.104.316	-37.974	3.143.055	-23.264	3.097.372	-21.901
3.104.404	-37.976	3.143.228	-23.265	3.097.552	-21.901
3.104.428	-37.977	3.143.394	-23.265	3.097.658	-21.901
3.104.499	-37.979	3.143.586	-23.265	3.097.802	-21.901
3.104.608	-37.981	3.143.694	-23.266	3.097.990	-21.901
3.104.716	-37.983	3.143.836	-23.266	3.098.186	-21.901
3.104.776	-37.985	3.144.041	-23.266	3.098.351	-21.901
3.104.987	-37.987	3.144.160	-23.267	3.098.482	-21.901
3.105.262	-37.989	3.144.237	-23.267	3.098.663	-21.901
3.105.309	-37.991	3.144.366	-23.267	3.098.871	-21.901
3.105.212	-37.992	3.144.559	-23.267	3.099.120	-21.901
3.105.385	-37.994	3.144.721	-23.268	3.099.315	-21.900
3.105.780	-37.996	3.144.862	-23.268	3.099.445	-21.900
3.105.703	-37.998	3.145.096	-23.268	3.099.576	-21.900
3.105.753	-38.000	3.145.284	-23.268	3.099.684	-21.900
3.106.371	-38.001	3.145.476	-23.268	3.099.812	-21.900
3.106.574	-38.003	3.145.677	-23.268	3.100.223	-21.900
3.106.729	-38.005	3.145.800	-23.269	3.100.689	-21.899
3.106.758	-38.007	3.145.917	-23.269	3.100.851	-21.899
3.106.492	-38.008	3.146.075	-23.269	3.100.895	-21.899
3.107.040	-38.010	3.146.249	-23.269	3.100.992	-21.899
3.107.484	-38.012	3.146.411	-23.269	3.101.096	-21.899
3.107.404	-38.014	3.146.607	-23.269	3.101.134	-21.898
3.107.527	-38.015	3.146.783	-23.269	3.101.216	-21.898
3.107.856	-38.017	3.146.967	-23.269	3.101.318	-21.898
3.107.936	-38.019	3.147.163	-23.269	3.101.436	-21.898
3.108.015	-38.021	3.147.317	-23.269	3.101.595	-21.897
3.108.062	-38.022	3.147.491	-23.270	3.101.791	-21.897
3.108.020	-38.024	3.147.646	-23.270	3.101.987	-21.897
3.108.381	-38.026	3.147.821	-23.270	3.102.148	-21.896

3.108.792	-38.027	3.147.998	-23.270	3.102.339	-21.896
3.108.997	-38.029	3.148.154	-23.270	3.102.507	-21.896
3.108.875	-38.031	3.148.320	-23.270	3.102.693	-21.895
3.109.218	-38.032	3.148.466	-23.270	3.102.918	-21.895
3.109.604	-38.034	3.148.683	-23.269	3.103.121	-21.895
3.109.742	-38.036	3.148.885	-23.269	3.103.302	-21.894
3.110.177	-38.037	3.149.042	-23.269	3.103.378	-21.894
3.110.404	-38.039	3.149.189	-23.269	3.103.417	-21.894
3.110.231	-38.041	3.149.306	-23.269	3.103.561	-21.893
3.110.193	-38.042	3.149.454	-23.269	3.103.731	-21.893
3.110.316	-38.044	3.149.656	-23.269	3.103.940	-21.893
3.110.367	-38.046	3.149.840	-23.269	3.104.114	-21.892
3.110.620	-38.047	3.150.000	-23.269	3.104.287	-21.892
3.110.908	-38.049	3.150.150	-23.268	3.104.526	-21.891
3.111.049	-38.050	3.150.265	-23.268	3.104.696	-21.891
3.111.147	-38.052	3.150.410	-23.268	3.104.828	-21.891
3.111.380	-38.054	3.150.598	-23.268	3.105.009	-21.890
3.111.528	-38.055	3.150.719	-23.268	3.105.234	-21.890
3.111.538	-38.057	3.150.831	-23.267	3.105.439	-21.890
3.111.678	-38.058	3.151.006	-23.267	3.105.601	-21.889
3.111.819	-38.060	3.151.238	-23.267	3.105.768	-21.889
3.112.046	-38.062	3.151.451	-23.266	3.105.931	-21.888
3.112.639	-38.063	3.151.578	-23.266	3.106.091	-21.888
3.112.976	-38.065	3.151.765	-23.266	3.106.194	-21.888
3.112.639	-38.067	3.152.016	-23.265	3.106.288	-21.887
3.112.806	-38.068	3.152.224	-23.265	3.106.519	-21.887
3.113.300	-38.070	3.152.386	-23.265	3.106.714	-21.887
3.113.641	-38.071	3.152.606	-23.264	3.106.841	-21.886
3.113.802	-38.073	3.152.776	-23.264	3.107.057	-21.886
3.113.557	-38.075	3.152.871	-23.263	3.107.236	-21.886

3.113.806	-38.076	3.153.019	-23.263	3.107.339	-21.885
3.114.385	-38.078	3.153.181	-23.263	3.107.480	-21.885
3.114.357	-38.079	3.153.291	-23.262	3.107.682	-21.885
3.114.559	-38.081	3.153.401	-23.262	3.107.870	-21.884
3.114.489	-38.083	3.153.582	-23.261	3.108.008	-21.884
3.114.232	-38.084	3.153.752	-23.261	3.108.135	-21.884
3.115.016	-38.086	3.153.869	-23.260	3.108.255	-21.883
3.115.504	-38.087	3.154.035	-23.260	3.108.409	-21.883
3.115.454	-38.089	3.154.251	-23.259	3.108.611	-21.883
3.115.251	-38.090	3.154.486	-23.258	3.108.810	-21.883
3.115.276	-38.092	3.154.673	-23.258	3.108.997	-21.882
3.115.367	-38.094	3.154.840	-23.257	3.109.196	-21.882
3.115.569	-38.095	3.155.045	-23.257	3.109.330	-21.882
3.115.849	-38.097	3.155.186	-23.256	3.109.519	-21.882
3.116.198	-38.098	3.155.342	-23.256	3.109.760	-21.882
3.116.530	-38.100	3.155.519	-23.255	3.109.879	-21.881
3.116.696	-38.101	3.155.681	-23.254	3.109.964	-21.881
3.116.824	-38.103	3.155.883	-23.254	3.110.121	-21.881
3.116.853	-38.104	3.156.073	-23.253	3.110.331	-21.881
3.117.228	-38.106	3.156.162	-23.252	3.110.493	-21.881
3.117.279	-38.107	3.156.284	-23.252	3.110.667	-21.881
3.117.271	-38.109	3.156.490	-23.251	3.110.826	-21.881
3.117.330	-38.110	3.156.685	-23.250	3.111.003	-21.880
3.117.575	-38.112	3.156.882	-23.250	3.111.180	-21.880
3.117.607	-38.113	3.157.021	-23.249	3.111.315	-21.880
3.117.723	-38.114	3.157.196	-23.248	3.111.520	-21.880
3.118.033	-38.116	3.157.411	-23.248	3.111.683	-21.880
3.118.403	-38.117	3.157.606	-23.247	3.111.768	-21.880
3.118.795	-38.119	3.157.777	-23.246	3.112.000	-21.880
3.118.879	-38.120	3.157.907	-23.246	3.112.210	-21.880

3.119.045	-38.121	3.158.067	-23.245	3.112.260	-21.880
3.119.232	-38.123	3.158.260	-23.244	3.112.430	-21.880
3.119.687	-38.124	3.158.497	-23.243	3.112.621	-21.880
3.119.606	-38.126	3.158.634	-23.243	3.112.755	-21.880
3.119.290	-38.127	3.158.773	-23.242	3.112.881	-21.880
3.119.846	-38.128	3.158.973	-23.241	3.113.027	-21.880
3.120.144	-38.130	3.159.109	-23.241	3.113.250	-21.880
3.120.338	-38.131	3.159.301	-23.240	3.113.470	-21.880
3.120.082	-38.132	3.159.485	-23.239	3.113.615	-21.880
3.120.005	-38.134	3.159.630	-23.238	3.113.824	-21.880
3.120.198	-38.135	3.159.754	-23.238	3.114.095	-21.880
3.120.346	-38.136	3.159.904	-23.237	3.114.319	-21.880
3.120.684	-38.137	3.160.111	-23.236	3.114.521	-21.880
3.121.150	-38.139	3.160.278	-23.236	3.114.640	-21.880
3.121.503	-38.140	3.160.389	-23.235	3.114.804	-21.880
3.121.545	-38.141	3.160.569	-23.234	3.114.919	-21.880
3.121.649	-38.142	3.160.986	-23.233	3.114.995	-21.880
3.121.759	-38.144	3.161.378	-23.233	3.115.165	-21.880
3.121.635	-38.145	3.161.513	-23.232	3.115.258	-21.880
3.121.679	-38.146	3.161.555	-23.231	3.115.436	-21.880
3.122.552	-38.147	3.161.620	-23.231	3.115.616	-21.880
3.123.052	-38.149	3.161.675	-23.230	3.115.754	-21.881
3.122.995	-38.150	3.161.768	-23.229	3.115.927	-21.881
3.122.579	-38.151	3.161.916	-23.229	3.116.071	-21.881
3.122.671	-38.152	3.162.030	-23.228	3.116.213	-21.881
3.122.973	-38.153	3.162.162	-23.227	3.116.447	-21.881
3.122.950	-38.154	3.162.355	-23.226	3.116.647	-21.881
3.123.268	-38.155	3.162.541	-23.226	3.116.866	-21.881
3.123.506	-38.157	3.162.686	-23.225	3.117.068	-21.881
3.123.799	-38.158	3.162.856	-23.224	3.117.221	-21.881

3.123.916	-38.159	3.163.022	-23.224	3.117.422	-21.881
3.124.019	-38.160	3.163.243	-23.223	3.117.592	-21.881
3.124.121	-38.161	3.163.445	-23.222	3.117.711	-21.881
3.124.305	-38.162	3.163.614	-23.222	3.117.800	-21.881
3.124.344	-38.163	3.163.770	-23.221	3.117.975	-21.881
3.123.919	-38.164	3.163.864	-23.221	3.118.179	-21.881
3.124.410	-38.166	3.164.005	-23.220	3.118.362	-21.882
3.124.952	-38.167	3.164.182	-23.219	3.118.532	-21.882
3.124.945	-38.168	3.164.315	-23.219	3.118.680	-21.882
3.125.439	-38.169	3.164.521	-23.218	3.118.856	-21.882
3.125.646	-38.170	3.164.745	-23.218	3.119.052	-21.882
3.125.534	-38.171	3.164.933	-23.217	3.119.214	-21.882
3.126.015	-38.172	3.165.088	-23.217	3.119.374	-21.882
3.126.364	-38.173	3.165.198	-23.216	3.119.552	-21.882
3.126.440	-38.174	3.165.377	-23.215	3.119.727	-21.882
3.126.649	-38.175	3.165.599	-23.215	3.119.893	-21.882
3.126.649	-38.176	3.165.783	-23.215	3.119.948	-21.882
3.126.964	-38.177	3.165.928	-23.214	3.120.099	-21.882
3.127.102	-38.178	3.166.141	-23.214	3.120.349	-21.882
3.126.960	-38.179	3.166.365	-23.213	3.120.544	-21.882
3.127.182	-38.180	3.166.517	-23.213	3.120.742	-21.882
3.127.408	-38.181	3.166.686	-23.212	3.120.945	-21.882
3.127.524	-38.182	3.166.815	-23.212	3.121.098	-21.882
3.127.701	-38.183	3.166.936	-23.212	3.121.266	-21.882
3.128.123	-38.184	3.167.122	-23.211	3.121.517	-21.882
3.128.571	-38.185	3.167.290	-23.211	3.121.633	-21.882
3.128.615	-38.186	3.167.438	-23.210	3.121.690	-21.882
3.128.648	-38.187	3.167.531	-23.210	3.121.844	-21.882
3.129.023	-38.188	3.167.657	-23.210	3.122.083	-21.882
3.129.121	-38.189	3.167.838	-23.210	3.122.290	-21.882

3.128.979	-38.190	3.167.986	-23.209	3.122.478	-21.882
3.129.002	-38.191	3.168.137	-23.209	3.122.831	-21.882
3.129.120	-38.192	3.168.369	-23.209	3.123.199	-21.882
3.129.270	-38.193	3.168.571	-23.209	3.123.366	-21.882
3.129.619	-38.194	3.168.698	-23.208	3.123.441	-21.882
3.129.725	-38.195	3.168.864	-23.208	3.123.529	-21.882
3.129.678	-38.196	3.169.019	-23.208	3.123.629	-21.882
3.130.046	-38.197	3.169.165	-23.208	3.123.709	-21.882
3.130.157	-38.198	3.169.339	-23.208	3.123.810	-21.882
3.130.137	-38.199	3.169.518	-23.208	3.123.911	-21.882
3.130.443	-38.200	3.169.659	-23.207	3.124.041	-21.882
3.130.908	-38.201	3.169.854	-23.207	3.124.221	-21.882
3.131.035	-38.202	3.170.083	-23.207	3.124.338	-21.882
3.130.948	-38.203	3.170.204	-23.207	3.124.446	-21.882
3.131.216	-38.204	3.170.320	-23.207	3.124.635	-21.881
3.131.358	-38.205	3.170.558	-23.207	3.124.862	-21.881
3.131.443	-38.206	3.170.793	-23.207	3.125.096	-21.881
3.131.999	-38.206	3.170.903	-23.207	3.125.262	-21.881
3.132.189	-38.207	3.171.058	-23.207	3.125.415	-21.881
3.132.151	-38.208	3.171.259	-23.207	3.125.592	-21.881
3.132.170	-38.209	3.171.361	-23.207	3.125.771	-21.881
3.132.759	-38.210	3.171.450	-23.207	3.125.916	-21.881
3.132.373	-38.211	3.171.664	-23.208	3.125.995	-21.880
3.132.113	-38.212	3.171.920	-23.208	3.126.112	-21.880
3.132.805	-38.213	3.172.063	-23.208	3.126.299	-21.880
3.133.221	-38.214	3.172.211	-23.208	3.126.474	-21.880
3.133.080	-38.215	3.172.437	-23.208	3.126.584	-21.880
3.133.268	-38.216	3.172.592	-23.208	3.126.733	-21.880
3.133.821	-38.217	3.172.766	-23.208	3.126.935	-21.880
3.133.918	-38.219	3.172.946	-23.208	3.127.175	-21.879

3.133.997	-38.220	3.173.091	-23.208	3.127.372	-21.879
3.134.006	-38.221	3.173.272	-23.209	3.127.495	-21.879
3.133.900	-38.222	3.173.394	-23.209	3.127.628	-21.879
3.134.568	-38.223	3.173.510	-23.209	3.127.836	-21.879
3.134.861	-38.224	3.173.695	-23.209	3.128.132	-21.879
3.134.844	-38.225	3.173.839	-23.209	3.128.309	-21.878
3.135.244	-38.226	3.173.968	-23.209	3.128.427	-21.878
3.135.175	-38.227	3.174.132	-23.209	3.128.585	-21.878
3.135.193	-38.228	3.174.268	-23.210	3.128.745	-21.878
3.135.552	-38.229	3.174.445	-23.210	3.128.923	-21.878
3.135.296	-38.230	3.174.674	-23.210	3.129.037	-21.878
3.135.583	-38.232	3.174.944	-23.210	3.129.171	-21.878
3.136.057	-38.233	3.175.135	-23.210	3.129.427	-21.877
3.135.941	-38.234	3.175.263	-23.210	3.129.660	-21.877
3.136.219	-38.235	3.175.455	-23.210	3.129.776	-21.877
3.136.895	-38.236	3.175.664	-23.211	3.129.879	-21.877
3.136.862	-38.237	3.175.891	-23.211	3.130.081	-21.877
3.136.705	-38.239	3.176.035	-23.211	3.130.314	-21.877
3.137.063	-38.240	3.176.114	-23.211	3.130.469	-21.877
3.137.263	-38.241	3.176.273	-23.211	3.130.602	-21.877
3.136.914	-38.242	3.176.450	-23.211	3.130.735	-21.877
3.137.184	-38.244	3.176.566	-23.211	3.130.887	-21.876
3.137.686	-38.245	3.176.678	-23.211	3.131.078	-21.876
3.137.921	-38.246	3.176.824	-23.212	3.131.267	-21.876
3.138.041	-38.247	3.176.971	-23.212	3.131.418	-21.876
3.137.821	-38.249	3.177.116	-23.212	3.131.546	-21.876
3.137.957	-38.250	3.177.239	-23.212	3.131.725	-21.876
3.138.300	-38.251	3.177.386	-23.212	3.131.914	-21.876
3.138.589	-38.253	3.177.688	-23.212	3.132.034	-21.876
3.138.770	-38.254	3.177.944	-23.212	3.132.177	-21.876

3.138.965	-38.255	3.178.077	-23.212	3.132.361	-21.876
3.138.927	-38.257	3.178.243	-23.212	3.132.546	-21.876
3.139.084	-38.258	3.178.385	-23.212	3.132.729	-21.876
3.139.547	-38.260	3.178.561	-23.212	3.132.859	-21.876
3.139.641	-38.261	3.178.784	-23.212	3.133.020	-21.876
3.139.557	-38.262	3.178.919	-23.212	3.133.163	-21.876
3.139.687	-38.264	3.179.071	-23.212	3.133.305	-21.876
3.140.099	-38.265	3.179.240	-23.212	3.133.511	-21.876
3.140.444	-38.266	3.179.398	-23.213	3.133.730	-21.876
3.140.556	-38.268	3.179.609	-23.213	3.133.916	-21.876
3.140.837	-38.269	3.179.821	-23.213	3.134.055	-21.876
3.140.974	-38.271	3.179.971	-23.213	3.134.226	-21.877
3.140.746	-38.272	3.180.093	-23.213	3.134.409	-21.877
3.140.677	-38.273	3.180.332	-23.212	3.134.534	-21.877
3.140.990	-38.275	3.180.493	-23.212	3.134.688	-21.877
3.141.057	-38.276	3.180.616	-23.212	3.134.825	-21.877
3.141.255	-38.278	3.180.822	-23.212	3.134.931	-21.877
3.141.486	-38.279	3.180.963	-23.212	3.135.078	-21.877
3.141.533	-38.280	3.181.136	-23.212	3.135.234	-21.877
3.141.826	-38.282	3.181.335	-23.212	3.135.388	-21.877
3.142.119	-38.283	3.181.494	-23.212	3.135.581	-21.877
3.142.281	-38.284	3.181.667	-23.212	3.135.765	-21.878
3.142.127	-38.286	3.181.826	-23.212	3.135.901	-21.878
3.142.422	-38.287	3.181.942	-23.212	3.136.065	-21.878
3.143.015	-38.288	3.182.072	-23.212	3.136.291	-21.878
3.143.040	-38.290	3.182.211	-23.212	3.136.614	-21.878
3.143.077	-38.291	3.182.406	-23.212	3.136.895	-21.878
3.143.496	-38.292	3.182.628	-23.211	3.137.030	-21.878
3.143.755	-38.294	3.182.787	-23.211	3.137.137	-21.878
3.144.192	-38.295	3.182.964	-23.211	3.137.335	-21.879

3.144.194	-38.296	3.183.145	-23.211	3.137.502	-21.879
3.144.151	-38.298	3.183.322	-23.211	3.137.597	-21.879
3.144.464	-38.299	3.183.517	-23.211	3.137.688	-21.879
3.144.164	-38.300	3.183.662	-23.211	3.137.841	-21.879
3.144.330	-38.301	3.183.778	-23.210	3.137.993	-21.879
3.144.633	-38.303	3.184.167	-23.210	3.138.096	-21.879
3.144.787	-38.304	3.184.637	-23.210	3.138.224	-21.879
3.145.316	-38.305	3.184.801	-23.210	3.138.457	-21.879
3.145.020	-38.306	3.184.861	-23.210	3.138.676	-21.880
3.144.872	-38.307	3.184.980	-23.210	3.138.833	-21.880
3.145.508	-38.308	3.185.023	-23.209	3.139.006	-21.880
3.145.679	-38.310	3.184.974	-23.209	3.139.153	-21.880
3.145.861	-38.311	3.185.059	-23.209	3.139.323	-21.880
3.146.101	-38.312	3.185.194	-23.209	3.139.547	-21.880
3.146.358	-38.313	3.185.323	-23.209	3.139.721	-21.880
3.146.578	-38.314	3.185.480	-23.208	3.139.847	-21.880
3.146.680	-38.315	3.185.724	-23.208	3.140.053	-21.880
3.146.812	-38.316	3.185.963	-23.208	3.140.265	-21.880
3.146.852	-38.317	3.186.086	-23.208	3.140.424	-21.880
3.146.938	-38.318	3.186.263	-23.208	3.140.544	-21.880
3.147.149	-38.319	3.186.492	-23.207	3.140.690	-21.880
3.147.358	-38.320	3.186.708	-23.207	3.140.876	-21.881
3.147.477	-38.321	3.186.859	-23.207	3.141.075	-21.881
3.147.641	-38.322	3.187.007	-23.207	3.141.282	-21.881
3.147.950	-38.323	3.187.152	-23.207	3.141.403	-21.881
3.147.968	-38.324	3.187.239	-23.206	3.141.567	-21.881
3.147.987	-38.325	3.187.430	-23.206	3.141.772	-21.881
3.148.568	-38.326	3.187.650	-23.206	3.141.902	-21.881
3.148.672	-38.327	3.187.816	-23.206	3.142.059	-21.881
3.148.685	-38.328	3.188.005	-23.206	3.142.234	-21.881

3.149.023	-38.329	3.188.139	-23.205	3.142.419	-21.881
3.149.269	-38.330	3.188.242	-23.205	3.142.615	-21.881
3.149.492	-38.331	3.188.372	-23.205	3.142.791	-21.881
3.149.510	-38.331	3.188.578	-23.205	3.142.921	-21.881
3.149.653	-38.332	3.188.799	-23.205	3.143.062	-21.881
3.149.929	-38.333	3.188.976	-23.205	3.143.253	-21.881
3.149.951	-38.334	3.189.153	-23.205	3.143.437	-21.881
3.150.031	-38.335	3.189.286	-23.205	3.143.615	-21.881
3.150.303	-38.335	3.189.445	-23.204	3.143.795	-21.881
3.150.433	-38.336	3.189.664	-23.204	3.144.005	-21.881
3.150.648	-38.337	3.189.875	-23.204	3.144.189	-21.881
3.150.878	-38.338	3.190.006	-23.204	3.144.301	-21.881
3.151.082	-38.339	3.190.144	-23.204	3.144.446	-21.881
3.151.248	-38.339	3.190.296	-23.204	3.144.655	-21.881
3.151.320	-38.340	3.190.446	-23.204	3.144.810	-21.881
3.151.422	-38.341	3.190.639	-23.204	3.144.976	-21.881
3.151.606	-38.342	3.190.744	-23.204	3.145.330	-21.881
3.151.747	-38.342	3.190.827	-23.204	3.145.722	-21.881
3.151.831	-38.343	3.191.042	-23.204	3.145.977	-21.881
3.152.177	-38.344	3.191.241	-23.205	3.146.111	-21.880
3.152.567	-38.344	3.191.393	-23.205	3.146.152	-21.880
3.152.809	-38.345	3.191.577	-23.205	3.146.145	-21.880
3.153.150	-38.346	3.191.726	-23.205	3.146.176	-21.880
3.153.319	-38.347	3.191.902	-23.205	3.146.284	-21.880
3.153.127	-38.347	3.192.133	-23.205	3.146.436	-21.880
3.153.076	-38.348	3.192.285	-23.206	3.146.575	-21.880
3.153.463	-38.349	3.192.382	-23.206	3.146.700	-21.880
3.153.755	-38.349	3.192.523	-23.206	3.146.892	-21.880
3.153.739	-38.350	3.192.677	-23.206	3.147.080	-21.879
3.153.529	-38.351	3.192.823	-23.207	3.147.224	-21.879

3.153.571	-38.351	3.193.044	-23.207	3.147.412	-21.879
3.153.744	-38.352	3.193.297	-23.207	3.147.597	-21.879
3.154.031	-38.353	3.193.457	-23.208	3.147.798	-21.879
3.154.425	-38.353	3.193.618	-23.208	3.147.976	-21.879
3.154.745	-38.354	3.193.803	-23.209	3.148.128	-21.878
3.154.952	-38.355	3.193.943	-23.209	3.148.300	-21.878
3.155.009	-38.355	3.194.102	-23.209	3.148.423	-21.878
3.154.991	-38.356	3.194.247	-23.210	3.148.616	-21.878
3.155.191	-38.357	3.194.337	-23.210	3.148.817	-21.878
3.155.554	-38.357	3.194.528	-23.211	3.148.940	-21.878
3.155.881	-38.358	3.194.691	-23.212	3.149.095	-21.877
3.155.606	-38.359	3.194.857	-23.212	3.149.275	-21.877
3.155.558	-38.359	3.195.050	-23.213	3.149.434	-21.877
3.156.112	-38.360	3.195.195	-23.213	3.149.597	-21.877
3.156.440	-38.361	3.195.381	-23.214	3.149.742	-21.877
3.156.718	-38.361	3.195.576	-23.215	3.149.906	-21.876
3.156.817	-38.362	3.195.728	-23.215	3.150.090	-21.876
3.156.740	-38.363	3.195.909	-23.216	3.150.283	-21.876
3.156.862	-38.364	3.196.118	-23.217	3.150.497	-21.876
3.157.033	-38.364	3.196.268	-23.218	3.150.667	-21.876
3.157.195	-38.365	3.196.440	-23.218	3.150.825	-21.875
3.157.383	-38.366	3.196.599	-23.219	3.150.974	-21.875
3.157.553	-38.367	3.196.719	-23.220	3.151.137	-21.875
3.157.766	-38.367	3.196.822	-23.221	3.151.282	-21.875
3.157.971	-38.368	3.196.947	-23.222	3.151.418	-21.875
3.158.152	-38.369	3.197.065	-23.223	3.151.592	-21.874
3.158.302	-38.370	3.197.220	-23.223	3.151.775	-21.874
3.158.354	-38.371	3.197.433	-23.224	3.151.961	-21.874
3.158.679	-38.371	3.197.646	-23.225	3.152.117	-21.874
3.158.947	-38.372	3.197.835	-23.226	3.152.245	-21.874

3.158.938	-38.373	3.197.995	-23.227	3.152.384	-21.874
3.158.965	-38.374	3.198.210	-23.228	3.152.520	-21.873
3.159.162	-38.375	3.198.391	-23.229	3.152.719	-21.873
3.159.420	-38.376	3.198.579	-23.230	3.152.917	-21.873
3.159.568	-38.377	3.198.793	-23.231	3.153.080	-21.873
3.159.767	-38.378	3.198.979	-23.232	3.153.304	-21.873
3.159.866	-38.378	3.199.091	-23.233	3.153.486	-21.873
3.159.985	-38.379	3.199.185	-23.235	3.153.633	-21.873
3.160.255	-38.380	3.199.427	-23.236	3.153.755	-21.873
3.160.406	-38.381	3.199.655	-23.237	3.153.896	-21.873
3.160.410	-38.382	3.199.767	-23.238	3.154.073	-21.873
3.160.613	-38.384	3.199.846	-23.239	3.154.244	-21.873
3.160.924	-38.385	3.199.973	-23.240	3.154.482	-21.873
3.161.025	-38.386	3.200.139	-23.241	3.154.670	-21.873
3.161.149	-38.387	3.200.259	-23.243	3.154.785	-21.873
3.161.286	-38.388	3.200.383	-23.244	3.154.919	-21.873
3.161.409	-38.389	3.200.544	-23.245	3.155.092	-21.873
3.161.585	-38.390	3.200.728	-23.246	3.155.294	-21.873
3.161.721	-38.392	3.200.956	-23.248	3.155.447	-21.873
3.162.021	-38.393	3.201.176	-23.249	3.155.565	-21.873
3.162.285	-38.394	3.201.364	-23.250	3.155.754	-21.873
3.162.340	-38.395	3.201.510	-23.251	3.155.973	-21.873
3.162.495	-38.397	3.201.668	-23.253	3.156.127	-21.873
3.162.741	-38.398	3.201.857	-23.254	3.156.252	-21.873
3.162.921	-38.399	3.202.036	-23.255	3.156.425	-21.873
3.162.994	-38.401	3.202.202	-23.257	3.156.606	-21.873
3.163.233	-38.402	3.202.307	-23.258	3.156.776	-21.873
3.163.585	-38.403	3.202.430	-23.260	3.156.945	-21.873
3.163.755	-38.405	3.202.665	-23.261	3.157.057	-21.874
3.163.952	-38.406	3.202.870	-23.262	3.157.187	-21.874

3.163.997	-38.408	3.203.044	-23.264	3.157.328	-21.874
3.163.984	-38.409	3.203.254	-23.265	3.157.445	-21.874
3.164.268	-38.410	3.203.408	-23.267	3.157.594	-21.874
3.164.508	-38.412	3.203.582	-23.268	3.157.747	-21.874
3.164.604	-38.413	3.203.768	-23.270	3.157.903	-21.874
3.164.738	-38.415	3.203.929	-23.271	3.158.096	-21.875
3.164.973	-38.416	3.204.100	-23.273	3.158.272	-21.875
3.165.182	-38.418	3.204.261	-23.274	3.158.401	-21.875
3.165.276	-38.419	3.204.454	-23.276	3.158.573	-21.875
3.165.363	-38.421	3.204.633	-23.277	3.158.857	-21.875
3.165.522	-38.423	3.204.796	-23.279	3.159.093	-21.875
3.165.641	-38.424	3.204.924	-23.280	3.159.218	-21.876
3.165.710	-38.426	3.205.101	-23.282	3.159.391	-21.876
3.165.966	-38.427	3.205.263	-23.284	3.159.594	-21.876
3.166.201	-38.429	3.205.364	-23.285	3.159.774	-21.876
3.166.264	-38.431	3.205.555	-23.287	3.159.941	-21.876
3.166.375	-38.432	3.205.758	-23.288	3.160.092	-21.877
3.166.555	-38.434	3.205.973	-23.290	3.160.244	-21.877
3.166.815	-38.436	3.206.122	-23.292	3.160.383	-21.877
3.167.032	-38.437	3.206.246	-23.293	3.160.576	-21.877
3.167.213	-38.439	3.206.386	-23.295	3.160.735	-21.877
3.167.477	-38.441	3.206.534	-23.297	3.160.891	-21.878
3.167.690	-38.442	3.206.676	-23.298	3.161.078	-21.878
3.167.831	-38.444	3.206.776	-23.300	3.161.253	-21.878
3.168.045	-38.446	3.207.041	-23.302	3.161.450	-21.878
3.168.189	-38.447	3.207.498	-23.303	3.161.556	-21.879
3.168.330	-38.449	3.207.849	-23.305	3.161.733	-21.879
3.168.528	-38.451	3.207.997	-23.307	3.161.986	-21.879
3.168.625	-38.452	3.208.037	-23.308	3.162.170	-21.879
3.168.737	-38.454	3.208.154	-23.310	3.162.322	-21.879

3.168.915	-38.456	3.208.240	-23.312	3.162.473	-21.880
3.169.095	-38.457	3.208.290	-23.313	3.162.599	-21.880
3.169.218	-38.459	3.208.425	-23.315	3.162.752	-21.880
3.169.370	-38.460	3.208.518	-23.317	3.162.932	-21.880
3.169.541	-38.462	3.208.609	-23.319	3.163.091	-21.881
3.169.709	-38.464	3.208.742	-23.320	3.163.290	-21.881
3.169.873	-38.465	3.208.940	-23.322	3.163.493	-21.881
3.169.964	-38.467	3.209.160	-23.324	3.163.641	-21.881
3.170.129	-38.469	3.209.346	-23.325	3.163.773	-21.881
3.170.428	-38.470	3.209.529	-23.327	3.163.903	-21.882
3.170.710	-38.472	3.209.691	-23.329	3.164.099	-21.882
3.170.880	-38.474	3.209.901	-23.331	3.164.272	-21.882
3.171.046	-38.475	3.210.100	-23.332	3.164.396	-21.882
3.171.208	-38.477	3.210.191	-23.334	3.164.597	-21.883
3.171.349	-38.479	3.210.371	-23.336	3.164.843	-21.883
3.171.476	-38.480	3.210.648	-23.338	3.165.014	-21.883
3.171.617	-38.482	3.210.773	-23.339	3.165.182	-21.883
3.171.799	-38.484	3.210.845	-23.341	3.165.336	-21.883
3.171.906	-38.485	3.211.080	-23.343	3.165.421	-21.884
3.172.086	-38.487	3.211.273	-23.345	3.165.594	-21.884
3.172.200	-38.488	3.211.421	-23.346	3.165.800	-21.884
3.172.411	-38.490	3.211.620	-23.348	3.165.999	-21.884
3.172.712	-38.492	3.211.788	-23.350	3.166.165	-21.884
3.172.900	-38.493	3.211.880	-23.352	3.166.321	-21.885
3.173.062	-38.495	3.211.979	-23.354	3.166.429	-21.885
3.173.172	-38.496	3.212.173	-23.355	3.166.555	-21.885
3.173.313	-38.498	3.212.382	-23.357	3.166.831	-21.885
3.173.417	-38.500	3.212.640	-23.359	3.167.007	-21.885
3.173.549	-38.501	3.212.839	-23.361	3.167.106	-21.886
3.173.705	-38.503	3.212.986	-23.362	3.167.270	-21.886

3.173.907	-38.505	3.213.119	-23.364	3.167.477	-21.886
3.174.124	-38.506	3.213.240	-23.366	3.167.878	-21.886
3.174.309	-38.508	3.213.392	-23.368	3.168.297	-21.886
3.174.557	-38.509	3.213.597	-23.370	3.168.481	-21.886
3.174.702	-38.511	3.213.835	-23.371	3.168.557	-21.887
3.174.724	-38.513	3.213.900	-23.373	3.168.626	-21.887
3.174.879	-38.514	3.213.980	-23.375	3.168.702	-21.887
3.175.143	-38.516	3.214.164	-23.377	3.168.796	-21.887
3.175.270	-38.517	3.214.319	-23.379	3.168.885	-21.887
3.175.415	-38.519	3.214.476	-23.380	3.168.965	-21.887
3.175.659	-38.521	3.214.684	-23.382	3.169.136	-21.887
3.175.866	-38.522	3.214.915	-23.384	3.169.304	-21.888
3.176.049	-38.524	3.215.108	-23.386	3.169.442	-21.888
3.176.174	-38.525	3.215.247	-23.387	3.169.669	-21.888
3.176.248	-38.527	3.215.414	-23.389	3.169.894	-21.888
3.176.549	-38.529	3.215.606	-23.391	3.170.052	-21.888
3.177.003	-38.530	3.215.744	-23.393	3.170.173	-21.888
3.177.198	-38.532	3.215.870	-23.395	3.170.360	-21.888
3.177.242	-38.534	3.216.017	-23.396	3.170.567	-21.888
3.177.366	-38.535	3.216.165	-23.398	3.170.726	-21.888
3.177.528	-38.537	3.216.367	-23.400	3.170.910	-21.888
3.177.632	-38.539	3.216.600	-23.402	3.171.042	-21.889
3.177.618	-38.540	3.216.769	-23.404	3.171.133	-21.889
3.177.679	-38.542	3.216.920	-23.405	3.171.303	-21.889
3.177.828	-38.544	3.217.077	-23.407	3.171.438	-21.889
3.177.975	-38.545	3.217.224	-23.409	3.171.589	-21.889
3.178.166	-38.547	3.217.339	-23.411	3.171.772	-21.889
3.178.403	-38.549	3.217.552	-23.412	3.171.974	-21.889
3.178.513	-38.550	3.217.817	-23.414	3.172.149	-21.889
3.178.676	-38.552	3.217.947	-23.416	3.172.242	-21.889

3.179.001	-38.554	3.218.011	-23.418	3.172.429	-21.889
3.179.161	-38.555	3.218.190	-23.419	3.172.639	-21.889
3.179.247	-38.557	3.218.387	-23.421	3.172.794	-21.890
3.179.497	-38.559	3.218.520	-23.423	3.172.990	-21.890
3.179.754	-38.560	3.218.734	-23.425	3.173.175	-21.890
3.179.869	-38.562	3.218.921	-23.426	3.173.340	-21.890
3.180.067	-38.564	3.219.073	-23.428	3.173.518	-21.890
3.180.211	-38.566	3.219.243	-23.430	3.173.705	-21.890
3.180.313	-38.567	3.219.429	-23.431	3.173.921	-21.890
3.180.483	-38.569	3.219.616	-23.433	3.174.027	-21.890
3.180.574	-38.571	3.219.796	-23.434	3.174.057	-21.891
3.180.710	-38.573	3.219.944	-23.436	3.174.236	-21.891
3.180.830	-38.575	3.220.038	-23.438	3.174.471	-21.891
3.180.966	-38.577	3.220.215	-23.439	3.174.680	-21.891
3.181.122	-38.578	3.220.350	-23.441	3.174.864	-21.891
3.181.286	-38.580	3.220.469	-23.442	3.174.995	-21.891
3.181.517	-38.582	3.220.663	-23.444	3.175.136	-21.892
3.181.631	-38.584	3.220.798	-23.445	3.175.305	-21.892
3.181.867	-38.586	3.220.967	-23.447	3.175.489	-21.892
3.182.243	-38.588	3.221.208	-23.448	3.175.719	-21.892
3.182.415	-38.590	3.221.385	-23.450	3.175.891	-21.892
3.182.524	-38.592	3.221.555	-23.451	3.175.963	-21.893
3.182.637	-38.594	3.221.777	-23.452	3.176.098	-21.893
3.182.798	-38.596	3.221.998	-23.454	3.176.326	-21.893
3.182.991	-38.598	3.222.156	-23.455	3.176.472	-21.893
3.183.110	-38.600	3.222.292	-23.456	3.176.606	-21.894
3.183.254	-38.602	3.222.478	-23.458	3.176.839	-21.894
3.183.326	-38.604	3.222.654	-23.459	3.177.032	-21.894
3.183.558	-38.606	3.222.799	-23.460	3.177.156	-21.894
3.183.785	-38.609	3.222.950	-23.461	3.177.281	-21.895

3.183.822	-38.611	3.223.036	-23.463	3.177.451	-21.895
3.184.044	-38.613	3.223.178	-23.464	3.177.606	-21.895
3.184.305	-38.615	3.223.322	-23.465	3.177.763	-21.896
3.184.440	-38.617	3.223.424	-23.466	3.177.960	-21.896
3.184.497	-38.620	3.223.618	-23.467	3.178.156	-21.896
3.184.576	-38.622	3.223.817	-23.468	3.178.334	-21.897
3.184.814	-38.624	3.223.990	-23.469	3.178.507	-21.897
3.185.045	-38.627	3.224.219	-23.470	3.178.658	-21.897
3.185.248	-38.629	3.224.362	-23.471	3.178.858	-21.898
3.185.421	-38.632	3.224.533	-23.472	3.179.019	-21.898
3.185.466	-38.634	3.224.736	-23.473	3.179.156	-21.899
3.185.670	-38.637	3.224.890	-23.474	3.179.309	-21.899
3.185.941	-38.639	3.225.115	-23.475	3.179.458	-21.899
3.186.161	-38.642	3.225.313	-23.476	3.179.631	-21.900
3.186.389	-38.644	3.225.435	-23.477	3.179.763	-21.900
3.186.437	-38.647	3.225.583	-23.478	3.179.904	-21.901
3.186.494	-38.649	3.225.701	-23.478	3.180.041	-21.901
3.186.721	-38.652	3.225.830	-23.479	3.180.211	-21.901
3.186.918	-38.655	3.226.015	-23.480	3.180.383	-21.902
3.187.142	-38.658	3.226.263	-23.481	3.180.548	-21.902
3.187.419	-38.660	3.226.473	-23.481	3.180.736	-21.903
3.187.576	-38.663	3.226.629	-23.482	3.180.863	-21.903
3.187.622	-38.666	3.226.761	-23.483	3.181.017	-21.903
3.187.677	-38.669	3.226.945	-23.483	3.181.245	-21.904
3.187.831	-38.672	3.227.180	-23.484	3.181.479	-21.904
3.188.069	-38.674	3.227.354	-23.485	3.181.683	-21.905
3.188.286	-38.677	3.227.426	-23.485	3.181.806	-21.905
3.188.508	-38.680	3.227.610	-23.486	3.181.990	-21.905
3.188.708	-38.683	3.227.838	-23.486	3.182.210	-21.906
3.188.889	-38.686	3.228.029	-23.487	3.182.379	-21.906

3.189.063	-38.689	3.228.225	-23.488	3.182.503	-21.907
3.189.176	-38.692	3.228.351	-23.488	3.182.646	-21.907
3.189.317	-38.695	3.228.421	-23.488	3.182.816	-21.907
3.189.449	-38.698	3.228.561	-23.489	3.182.993	-21.908
3.189.537	-38.701	3.228.761	-23.489	3.183.157	-21.908
3.189.674	-38.704	3.228.965	-23.490	3.183.282	-21.908
3.189.774	-38.707	3.229.171	-23.490	3.183.459	-21.909
3.189.833	-38.710	3.229.364	-23.491	3.183.644	-21.909
3.190.032	-38.713	3.229.539	-23.491	3.183.778	-21.910
3.190.233	-38.717	3.229.673	-23.491	3.183.936	-21.910
3.190.446	-38.720	3.229.808	-23.492	3.184.158	-21.910
3.190.645	-38.723	3.229.926	-23.492	3.184.330	-21.911
3.190.794	-38.726	3.230.028	-23.492	3.184.489	-21.911
3.190.984	-38.729	3.230.176	-23.492	3.184.666	-21.911
3.191.237	-38.733	3.230.498	-23.493	3.184.819	-21.912
3.191.472	-38.736	3.230.941	-23.493	3.185.018	-21.912
3.191.571	-38.739	3.231.288	-23.493	3.185.215	-21.913
3.191.685	-38.742	3.231.436	-23.493	3.185.397	-21.913
3.191.841	-38.746	3.231.455	-23.494	3.185.562	-21.913
3.192.076	-38.749	3.231.534	-23.494	3.185.696	-21.914
3.192.246	-38.752	3.231.639	-23.494	3.185.836	-21.914
3.192.384	-38.755	3.231.675	-23.494	3.186.003	-21.914
3.192.636	-38.759	3.231.714	-23.494	3.186.183	-21.915
3.192.765	-38.762	3.231.920	-23.494	3.186.309	-21.915
3.192.810	-38.765	3.232.126	-23.494	3.186.447	-21.915
3.192.944	-38.769	3.232.185	-23.495	3.186.689	-21.916
3.193.101	-38.772	3.232.310	-23.495	3.186.895	-21.916
3.193.239	-38.775	3.232.556	-23.495	3.187.018	-21.916
3.193.399	-38.779	3.232.720	-23.495	3.187.171	-21.917
3.193.633	-38.782	3.232.874	-23.495	3.187.372	-21.917

3.193.811	-38.785	3.233.083	-23.495	3.187.528	-21.917
3.193.921	-38.789	3.233.297	-23.495	3.187.657	-21.917
3.194.136	-38.792	3.233.536	-23.495	3.187.809	-21.918
3.194.449	-38.795	3.233.645	-23.495	3.187.950	-21.918
3.194.659	-38.799	3.233.799	-23.495	3.188.101	-21.918
3.194.804	-38.802	3.234.037	-23.495	3.188.304	-21.919
3.194.974	-38.805	3.234.162	-23.495	3.188.532	-21.919
3.195.096	-38.809	3.234.286	-23.495	3.188.705	-21.919
3.195.288	-38.812	3.234.438	-23.495	3.188.849	-21.920
3.195.529	-38.815	3.234.584	-23.495	3.189.039	-21.920
3.195.695	-38.819	3.234.756	-23.495	3.189.240	-21.920
3.195.772	-38.822	3.234.886	-23.495	3.189.395	-21.921
3.195.887	-38.826	3.234.999	-23.495	3.189.536	-21.921
3.196.089	-38.829	3.235.186	-23.495	3.189.622	-21.921
3.196.293	-38.832	3.235.415	-23.495	3.189.810	-21.921
3.196.407	-38.836	3.235.649	-23.495	3.190.208	-21.922
3.196.548	-38.839	3.235.859	-23.495	3.190.623	-21.922
3.196.736	-38.843	3.236.057	-23.495	3.190.935	-21.922
3.196.953	-38.846	3.236.232	-23.495	3.191.085	-21.923
3.197.093	-38.849	3.236.351	-23.495	3.191.140	-21.923
3.197.267	-38.853	3.236.454	-23.495	3.191.132	-21.923
3.197.485	-38.856	3.236.613	-23.495	3.191.129	-21.923
3.197.588	-38.859	3.236.801	-23.495	3.191.270	-21.924
3.197.711	-38.863	3.237.003	-23.495	3.191.429	-21.924
3.197.890	-38.866	3.237.159	-23.495	3.191.531	-21.924
3.198.116	-38.870	3.237.256	-23.495	3.191.658	-21.925
3.198.297	-38.873	3.237.405	-23.495	3.191.809	-21.925
3.198.408	-38.876	3.237.593	-23.495	3.192.007	-21.925
3.198.573	-38.880	3.237.769	-23.495	3.192.195	-21.926
3.198.755	-38.883	3.237.921	-23.496	3.192.301	-21.926

3.198.959	-38.886	3.238.078	-23.496	3.192.487	-21.926
3.199.093	-38.890	3.238.257	-23.496	3.192.683	-21.927
3.199.252	-38.893	3.238.457	-23.496	3.192.874	-21.927
3.199.471	-38.896	3.238.659	-23.496	3.193.077	-21.927
3.199.577	-38.900	3.238.792	-23.496	3.193.213	-21.928
3.199.706	-38.903	3.238.922	-23.496	3.193.334	-21.928
3.199.879	-38.907	3.239.005	-23.496	3.193.484	-21.928
3.200.045	-38.910	3.239.143	-23.497	3.193.679	-21.929
3.200.277	-38.913	3.239.324	-23.497	3.193.878	-21.929
3.200.415	-38.917	3.239.512	-23.497	3.194.026	-21.929
3.200.504	-38.920	3.239.729	-23.497	3.194.114	-21.930
3.200.862	-38.923	3.239.937	-23.497	3.194.233	-21.930
3.201.335	-38.927	3.240.103	-23.498	3.194.406	-21.931
3.201.685	-38.930	3.240.238	-23.498	3.194.606	-21.931
3.201.751	-38.933	3.240.442	-23.498	3.194.793	-21.931
3.201.725	-38.936	3.240.653	-23.498	3.194.991	-21.932
3.201.819	-38.940	3.240.791	-23.499	3.195.226	-21.932
3.201.819	-38.943	3.240.949	-23.499	3.195.385	-21.933
3.201.889	-38.946	3.241.079	-23.499	3.195.495	-21.933
3.202.025	-38.949	3.241.158	-23.499	3.195.684	-21.934
3.202.191	-38.953	3.241.324	-23.500	3.195.862	-21.934
3.202.333	-38.956	3.241.591	-23.500	3.196.049	-21.935
3.202.412	-38.959	3.241.815	-23.500	3.196.202	-21.935
3.202.641	-38.962	3.241.958	-23.501	3.196.257	-21.936
3.202.839	-38.966	3.242.116	-23.501	3.196.389	-21.937
3.203.015	-38.969	3.242.310	-23.501	3.196.574	-21.937
3.203.213	-38.972	3.242.442	-23.502	3.196.730	-21.938
3.203.374	-38.975	3.242.588	-23.502	3.196.925	-21.938
3.203.546	-38.978	3.242.805	-23.502	3.197.187	-21.939
3.203.800	-38.981	3.242.980	-23.503	3.197.321	-21.940

3.204.030	-38.985	3.243.106	-23.503	3.197.404	-21.941
3.204.132	-38.988	3.243.235	-23.503	3.197.596	-21.941
3.204.233	-38.991	3.243.391	-23.504	3.197.736	-21.942
3.204.396	-38.994	3.243.557	-23.504	3.197.896	-21.943
3.204.548	-38.997	3.243.681	-23.504	3.198.065	-21.944
3.204.761	-39.000	3.243.818	-23.505	3.198.179	-21.944
3.205.005	-39.003	3.243.945	-23.505	3.198.365	-21.945
3.205.125	-39.007	3.244.081	-23.505	3.198.576	-21.946
3.205.222	-39.010	3.244.320	-23.506	3.198.680	-21.947
3.205.378	-39.013	3.244.501	-23.506	3.198.853	-21.948
3.205.491	-39.016	3.244.662	-23.506	3.199.122	-21.949
3.205.537	-39.019	3.244.873	-23.507	3.199.328	-21.950
3.205.740	-39.022	3.245.083	-23.507	3.199.516	-21.951
3.206.080	-39.025	3.245.300	-23.508	3.199.656	-21.952
3.206.304	-39.028	3.245.476	-23.508	3.199.821	-21.953
3.206.486	-39.032	3.245.660	-23.508	3.199.984	-21.954
3.206.748	-39.035	3.245.840	-23.509	3.200.177	-21.955
3.206.945	-39.038	3.245.981	-23.509	3.200.357	-21.956
3.207.004	-39.041	3.246.143	-23.509	3.200.453	-21.957
3.207.141	-39.044	3.246.315	-23.510	3.200.596	-21.958
3.207.257	-39.047	3.246.454	-23.510	3.200.747	-21.959
3.207.359	-39.050	3.246.599	-23.510	3.200.899	-21.960
3.207.600	-39.053	3.246.700	-23.511	3.201.061	-21.961
3.207.796	-39.056	3.246.773	-23.511	3.201.217	-21.962
3.207.868	-39.059	3.246.950	-23.511	3.201.444	-21.963
3.207.987	-39.062	3.247.149	-23.511	3.201.599	-21.965
3.208.123	-39.065	3.247.307	-23.512	3.201.693	-21.966
3.208.293	-39.069	3.247.524	-23.512	3.201.848	-21.967
3.208.489	-39.072	3.247.715	-23.512	3.202.025	-21.968
3.208.681	-39.075	3.247.878	-23.513	3.202.185	-21.969

3.208.955	-39.078	3.248.060	-23.513	3.202.310	-21.971
3.209.128	-39.081	3.248.233	-23.513	3.202.444	-21.972
3.209.254	-39.084	3.248.410	-23.513	3.202.644	-21.973
3.209.357	-39.087	3.248.611	-23.513	3.202.751	-21.974
3.209.458	-39.090	3.248.793	-23.514	3.202.859	-21.976
3.209.706	-39.093	3.248.974	-23.514	3.203.054	-21.977
3.209.873	-39.096	3.249.092	-23.514	3.203.224	-21.978
3.209.980	-39.099	3.249.298	-23.514	3.203.459	-21.979
3.210.141	-39.102	3.249.555	-23.514	3.203.645	-21.981
3.210.294	-39.105	3.249.678	-23.514	3.203.836	-21.982
3.210.500	-39.109	3.249.850	-23.515	3.204.078	-21.983
3.210.750	-39.112	3.250.029	-23.515	3.204.268	-21.985
3.210.984	-39.115	3.250.188	-23.515	3.204.460	-21.986
3.211.219	-39.118	3.250.327	-23.515	3.204.681	-21.987
3.211.400	-39.121	3.250.495	-23.515	3.204.891	-21.989
3.211.517	-39.124	3.250.679	-23.515	3.205.003	-21.990
3.211.690	-39.127	3.250.826	-23.515	3.205.024	-21.991
3.211.851	-39.130	3.250.984	-23.515	3.205.136	-21.993
3.211.982	-39.133	3.251.144	-23.515	3.205.363	-21.994
3.212.148	-39.136	3.251.360	-23.515	3.205.555	-21.995
3.212.274	-39.139	3.251.559	-23.515	3.205.685	-21.996
3.212.354	-39.142	3.251.661	-23.515	3.205.826	-21.998
3.212.499	-39.145	3.251.768	-23.515	3.205.995	-21.999
3.212.726	-39.148	3.251.938	-23.515	3.206.163	-22.000
3.213.001	-39.151	3.252.191	-23.515	3.206.326	-22.002
3.213.188	-39.154	3.252.435	-23.515	3.206.506	-22.003
3.213.315	-39.157	3.252.636	-23.515	3.206.718	-22.004
3.213.484	-39.160	3.252.795	-23.515	3.206.956	-22.006
3.213.586	-39.163	3.252.921	-23.515	3.207.169	-22.007
3.213.770	-39.166	3.253.074	-23.514	3.207.357	-22.008

3.214.012	-39.169	3.253.206	-23.514	3.207.534	-22.009
3.214.088	-39.172	3.253.383	-23.514	3.207.676	-22.011
3.214.154	-39.175	3.253.536	-23.514	3.207.836	-22.012
3.214.331	-39.178	3.253.643	-23.514	3.208.018	-22.013
3.214.496	-39.181	3.253.972	-23.513	3.208.199	-22.014
3.214.622	-39.184	3.254.453	-23.513	3.208.419	-22.016
3.214.685	-39.186	3.254.695	-23.513	3.208.575	-22.017
3.214.868	-39.189	3.254.760	-23.512	3.208.651	-22.018
3.215.135	-39.192	3.254.870	-23.512	3.208.774	-22.019
3.215.327	-39.195	3.254.947	-23.512	3.208.962	-22.021
3.215.540	-39.198	3.255.007	-23.511	3.209.198	-22.022
3.215.754	-39.201	3.255.063	-23.511	3.209.369	-22.023
3.215.913	-39.203	3.255.125	-23.510	3.209.464	-22.024
3.216.057	-39.206	3.255.235	-23.510	3.209.664	-22.025
3.216.232	-39.209	3.255.378	-23.509	3.209.875	-22.026
3.216.459	-39.211	3.255.581	-23.509	3.210.013	-22.028
3.216.629	-39.214	3.255.791	-23.508	3.210.188	-22.029
3.216.862	-39.217	3.255.955	-23.508	3.210.334	-22.030
3.217.110	-39.219	3.256.154	-23.507	3.210.462	-22.031
3.217.200	-39.222	3.256.357	-23.507	3.210.677	-22.032
3.217.305	-39.225	3.256.508	-23.506	3.210.902	-22.033
3.217.491	-39.227	3.256.667	-23.505	3.211.054	-22.034
3.217.665	-39.230	3.256.805	-23.505	3.211.165	-22.035
3.217.719	-39.232	3.256.971	-23.504	3.211.273	-22.036
3.217.838	-39.235	3.257.151	-23.503	3.211.461	-22.037
3.218.070	-39.237	3.257.312	-23.503	3.211.721	-22.038
3.218.224	-39.240	3.257.513	-23.502	3.211.880	-22.039
3.218.388	-39.242	3.257.619	-23.501	3.211.994	-22.040
3.218.633	-39.245	3.257.749	-23.500	3.212.146	-22.041
3.218.824	-39.247	3.257.954	-23.499	3.212.271	-22.042

3.218.994	-39.250	3.258.127	-23.499	3.212.607	-22.043
3.219.174	-39.252	3.258.284	-23.498	3.213.022	-22.044
3.219.297	-39.254	3.258.466	-23.497	3.213.258	-22.045
3.219.449	-39.257	3.258.645	-23.496	3.213.439	-22.046
3.219.626	-39.259	3.258.835	-23.495	3.213.553	-22.046
3.219.821	-39.261	3.259.008	-23.494	3.213.568	-22.047
3.220.078	-39.264	3.259.207	-23.493	3.213.620	-22.048
3.220.237	-39.266	3.259.396	-23.492	3.213.730	-22.049
3.220.381	-39.268	3.259.554	-23.491	3.213.842	-22.050
3.220.534	-39.270	3.259.756	-23.490	3.213.983	-22.051
3.220.645	-39.273	3.259.945	-23.489	3.214.091	-22.051
3.220.811	-39.275	3.260.075	-23.488	3.214.193	-22.052
3.220.938	-39.277	3.260.219	-23.487	3.214.373	-22.053
3.221.039	-39.279	3.260.381	-23.486	3.214.623	-22.054
3.221.210	-39.281	3.260.479	-23.485	3.214.800	-22.054
3.221.425	-39.284	3.260.603	-23.484	3.214.995	-22.055
3.221.595	-39.286	3.260.883	-23.483	3.215.208	-22.056
3.221.809	-39.288	3.261.084	-23.482	3.215.359	-22.056
3.222.046	-39.290	3.261.143	-23.481	3.215.497	-22.057
3.222.196	-39.292	3.261.313	-23.480	3.215.619	-22.058
3.222.341	-39.294	3.261.469	-23.478	3.215.789	-22.058
3.222.502	-39.296	3.261.628	-23.477	3.216.003	-22.059
3.222.610	-39.298	3.261.849	-23.476	3.216.154	-22.059
3.222.709	-39.300	3.262.032	-23.475	3.216.279	-22.060
3.222.900	-39.302	3.262.133	-23.474	3.216.436	-22.061
3.223.113	-39.304	3.262.270	-23.472	3.216.573	-22.061
3.223.236	-39.306	3.262.463	-23.471	3.216.718	-22.062
3.223.391	-39.308	3.262.589	-23.470	3.216.904	-22.062
3.223.656	-39.309	3.262.727	-23.468	3.217.086	-22.063
3.223.914	-39.311	3.262.906	-23.467	3.217.232	-22.064

3.224.039	-39.313	3.263.146	-23.466	3.217.411	-22.064
3.224.150	-39.315	3.263.362	-23.464	3.217.657	-22.065
3.224.327	-39.317	3.263.553	-23.463	3.217.872	-22.065
3.224.443	-39.319	3.263.724	-23.462	3.218.049	-22.066
3.224.583	-39.320	3.263.807	-23.460	3.218.164	-22.066
3.224.799	-39.322	3.263.992	-23.459	3.218.287	-22.067
3.225.056	-39.324	3.264.223	-23.457	3.218.417	-22.068
3.225.371	-39.326	3.264.356	-23.456	3.218.567	-22.068
3.225.764	-39.328	3.264.518	-23.454	3.218.760	-22.069
3.226.010	-39.329	3.264.706	-23.453	3.218.908	-22.069
3.226.068	-39.331	3.264.864	-23.451	3.219.073	-22.070
3.226.125	-39.333	3.265.027	-23.450	3.219.225	-22.070
3.226.159	-39.334	3.265.234	-23.448	3.219.398	-22.071
3.226.191	-39.336	3.265.389	-23.447	3.219.587	-22.072
3.226.291	-39.338	3.265.500	-23.445	3.219.734	-22.072
3.226.448	-39.340	3.265.692	-23.443	3.219.912	-22.073
3.226.629	-39.341	3.265.840	-23.442	3.220.024	-22.073
3.226.759	-39.343	3.265.952	-23.440	3.220.220	-22.074
3.226.848	-39.344	3.266.118	-23.438	3.220.411	-22.075
3.227.025	-39.346	3.266.336	-23.437	3.220.551	-22.075
3.227.319	-39.348	3.266.520	-23.435	3.220.757	-22.076
3.227.557	-39.349	3.266.632	-23.433	3.220.902	-22.076
3.227.673	-39.351	3.266.797	-23.431	3.221.075	-22.077
3.227.784	-39.353	3.266.986	-23.429	3.221.227	-22.078
3.227.966	-39.354	3.267.079	-23.427	3.221.365	-22.078
3.228.206	-39.356	3.267.171	-23.425	3.221.557	-22.079
3.228.401	-39.357	3.267.372	-23.424	3.221.736	-22.080
3.228.496	-39.359	3.267.614	-23.422	3.221.931	-22.080
3.228.661	-39.360	3.267.817	-23.420	3.222.162	-22.081
3.228.904	-39.362	3.267.997	-23.418	3.222.325	-22.082

3.229.028	-39.364	3.268.199	-23.415	3.222.412	-22.082
3.229.128	-39.365	3.268.394	-23.413	3.222.608	-22.083
3.229.268	-39.367	3.268.575	-23.411	3.222.795	-22.084
3.229.398	-39.369	3.268.732	-23.409	3.223.001	-22.084
3.229.554	-39.370	3.268.915	-23.407	3.223.167	-22.085
3.229.703	-39.372	3.269.095	-23.405	3.223.253	-22.086
3.229.888	-39.373	3.269.244	-23.402	3.223.437	-22.087
3.230.146	-39.375	3.269.403	-23.400	3.223.658	-22.087
3.230.357	-39.377	3.269.496	-23.398	3.223.784	-22.088
3.230.433	-39.378	3.269.573	-23.396	3.223.922	-22.089
3.230.530	-39.380	3.269.768	-23.393	3.224.117	-22.090
3.230.760	-39.382	3.269.971	-23.391	3.224.283	-22.090
3.231.093	-39.383	3.270.057	-23.388	3.224.464	-22.091
3.231.321	-39.385	3.270.184	-23.386	3.224.630	-22.092
3.231.407	-39.387	3.270.406	-23.383	3.224.774	-22.093
3.231.537	-39.389	3.270.587	-23.381	3.224.893	-22.093
3.231.664	-39.390	3.270.779	-23.378	3.224.999	-22.094
3.231.750	-39.392	3.271.013	-23.376	3.225.132	-22.095
3.231.921	-39.394	3.271.203	-23.373	3.225.306	-22.096
3.232.148	-39.396	3.271.404	-23.370	3.225.464	-22.097
3.232.328	-39.398	3.271.584	-23.368	3.225.619	-22.097
3.232.542	-39.399	3.271.709	-23.365	3.225.768	-22.098
3.232.680	-39.401	3.271.869	-23.362	3.225.925	-22.099
3.232.718	-39.403	3.272.047	-23.359	3.226.152	-22.100
3.232.755	-39.405	3.272.263	-23.357	3.226.407	-22.100
3.232.904	-39.407	3.272.347	-23.354	3.226.687	-22.101
3.233.123	-39.409	3.272.527	-23.351	3.226.857	-22.102
3.233.337	-39.411	3.272.852	-23.348	3.227.012	-22.103
3.233.572	-39.413	3.273.000	-23.345	3.227.191	-22.103
3.233.797	-39.415	3.273.170	-23.342	3.227.321	-22.104

3.234.023	-39.417	3.273.336	-23.339	3.227.456	-22.105
3.234.227	-39.419	3.273.488	-23.336	3.227.601	-22.106
3.234.284	-39.421	3.273.658	-23.333	3.227.729	-22.106
3.234.384	-39.423	3.273.792	-23.330	3.227.881	-22.107
3.234.648	-39.425	3.273.987	-23.327	3.228.069	-22.108
3.234.840	-39.427	3.274.156	-23.324	3.228.208	-22.109
3.234.992	-39.429	3.274.312	-23.320	3.228.354	-22.109
3.235.212	-39.431	3.274.489	-23.317	3.228.502	-22.110
3.235.388	-39.434	3.274.666	-23.314	3.228.637	-22.111
3.235.482	-39.436	3.274.846	-23.311	3.228.828	-22.112
3.235.630	-39.438	3.274.998	-23.307	3.229.030	-22.112
3.235.826	-39.440	3.275.115	-23.304	3.229.236	-22.113
3.236.010	-39.443	3.275.267	-23.301	3.229.467	-22.114
3.236.108	-39.445	3.275.473	-23.297	3.229.644	-22.114
3.236.226	-39.447	3.275.648	-23.294	3.229.800	-22.115
3.236.456	-39.449	3.275.772	-23.290	3.229.930	-22.116
3.236.679	-39.452	3.275.927	-23.287	3.230.052	-22.116
3.236.860	-39.454	3.276.143	-23.283	3.230.271	-22.117
3.236.983	-39.457	3.276.326	-23.280	3.230.482	-22.117
3.237.123	-39.459	3.276.425	-23.276	3.230.635	-22.118
3.237.376	-39.461	3.276.534	-23.273	3.230.807	-22.119
3.237.557	-39.464	3.276.705	-23.269	3.230.960	-22.119
3.237.684	-39.466	3.276.877	-23.265	3.231.104	-22.120
3.237.835	-39.469	3.277.202	-23.262	3.231.293	-22.120
3.238.030	-39.471	3.277.669	-23.258	3.231.472	-22.121
3.238.156	-39.473	3.277.957	-23.254	3.231.628	-22.122
3.238.185	-39.476	3.278.047	-23.251	3.231.823	-22.122
3.238.304	-39.478	3.278.134	-23.247	3.232.005	-22.123
3.238.493	-39.481	3.278.213	-23.243	3.232.158	-22.123
3.238.661	-39.483	3.278.246	-23.239	3.232.307	-22.124

3.238.820	-39.486	3.278.286	-23.235	3.232.466	-22.124
3.238.961	-39.489	3.278.349	-23.231	3.232.673	-22.125
3.239.150	-39.491	3.278.482	-23.227	3.232.871	-22.125
3.239.261	-39.494	3.278.688	-23.224	3.233.026	-22.126
3.239.385	-39.496	3.278.806	-23.220	3.233.153	-22.126
3.239.580	-39.499	3.278.896	-23.216	3.233.286	-22.127
3.239.854	-39.501	3.279.091	-23.212	3.233.450	-22.127
3.240.141	-39.504	3.279.333	-23.208	3.233.656	-22.127
3.240.323	-39.507	3.279.528	-23.204	3.233.907	-22.128
3.240.490	-39.509	3.279.699	-23.200	3.234.055	-22.128
3.240.569	-39.512	3.279.894	-23.196	3.234.145	-22.128
3.240.751	-39.515	3.280.104	-23.192	3.234.346	-22.129
3.241.035	-39.517	3.280.247	-23.187	3.234.590	-22.129
3.241.197	-39.520	3.280.408	-23.183	3.234.721	-22.129
3.241.376	-39.522	3.280.580	-23.179	3.234.725	-22.130
3.241.541	-39.525	3.280.688	-23.175	3.234.969	-22.130
3.241.669	-39.528	3.280.859	-23.171	3.235.466	-22.130
3.241.822	-39.530	3.281.040	-23.167	3.235.844	-22.130
3.242.044	-39.533	3.281.201	-23.163	3.236.014	-22.131
3.242.158	-39.536	3.281.309	-23.159	3.236.049	-22.131
3.242.332	-39.538	3.281.451	-23.155	3.236.068	-22.131
3.242.507	-39.541	3.281.642	-23.150	3.236.118	-22.131
3.242.567	-39.544	3.281.833	-23.146	3.236.201	-22.131
3.242.747	-39.547	3.282.039	-23.142	3.236.365	-22.131
3.242.971	-39.549	3.282.202	-23.138	3.236.501	-22.132
3.243.177	-39.552	3.282.380	-23.134	3.236.628	-22.132
3.243.318	-39.555	3.282.550	-23.130	3.236.783	-22.132
3.243.470	-39.557	3.282.742	-23.125	3.236.931	-22.132
3.243.681	-39.560	3.282.991	-23.121	3.237.122	-22.132
3.243.885	-39.563	3.283.177	-23.117	3.237.305	-22.132

3.244.099	-39.565	3.283.294	-23.113	3.237.502	-22.132
3.244.286	-39.568	3.283.421	-23.109	3.237.695	-22.132
3.244.341	-39.571	3.283.629	-23.105	3.237.896	-22.132
3.244.403	-39.574	3.283.775	-23.101	3.238.091	-22.132
3.244.615	-39.576	3.283.861	-23.096	3.238.266	-22.131
3.244.850	-39.579	3.283.987	-23.092	3.238.412	-22.131
3.245.050	-39.582	3.284.150	-23.088	3.238.520	-22.131
3.245.159	-39.584	3.284.330	-23.084	3.238.687	-22.131
3.245.248	-39.587	3.284.514	-23.080	3.238.846	-22.131
3.245.432	-39.590	3.284.655	-23.076	3.239.002	-22.131
3.245.583	-39.592	3.284.775	-23.072	3.239.175	-22.131
3.245.789	-39.595	3.284.984	-23.068	3.239.291	-22.130
3.246.008	-39.598	3.285.154	-23.064	3.239.474	-22.130
3.246.177	-39.601	3.285.294	-23.060	3.239.641	-22.130
3.246.347	-39.603	3.285.435	-23.056	3.239.752	-22.130
3.246.487	-39.606	3.285.617	-23.052	3.239.922	-22.129
3.246.660	-39.609	3.285.782	-23.048	3.240.165	-22.129
3.246.875	-39.611	3.285.920	-23.044	3.240.414	-22.129
3.247.066	-39.614	3.286.140	-23.040	3.240.573	-22.129
3.247.286	-39.617	3.286.313	-23.036	3.240.757	-22.128
3.247.379	-39.619	3.286.498	-23.032	3.240.935	-22.128
3.247.534	-39.622	3.286.701	-23.028	3.241.071	-22.128
3.247.723	-39.625	3.286.871	-23.024	3.241.241	-22.127
3.247.896	-39.627	3.286.996	-23.020	3.241.378	-22.127
3.247.973	-39.630	3.287.158	-23.016	3.241.521	-22.127
3.248.199	-39.633	3.287.373	-23.012	3.241.667	-22.126
3.248.380	-39.635	3.287.491	-23.008	3.241.805	-22.126
3.248.438	-39.638	3.287.628	-23.005	3.241.938	-22.126
3.248.591	-39.640	3.287.812	-23.001	3.242.099	-22.125
3.248.799	-39.643	3.287.947	-22.997	3.242.347	-22.125

3.249.064	-39.646	3.288.160	-22.993	3.242.543	-22.125
3.249.266	-39.648	3.288.377	-22.989	3.242.661	-22.124
3.249.391	-39.651	3.288.524	-22.986	3.242.780	-22.124
3.249.689	-39.653	3.288.677	-22.982	3.242.885	-22.124
3.250.164	-39.656	3.288.857	-22.978	3.243.083	-22.123
3.250.450	-39.659	3.289.049	-22.974	3.243.283	-22.123
3.250.516	-39.661	3.289.205	-22.971	3.243.428	-22.123
3.250.520	-39.664	3.289.299	-22.967	3.243.632	-22.122
3.250.518	-39.666	3.289.427	-22.963	3.243.727	-22.122
3.250.620	-39.669	3.289.635	-22.960	3.243.907	-22.122
3.250.782	-39.671	3.289.794	-22.956	3.244.110	-22.121
3.250.937	-39.674	3.289.933	-22.952	3.244.272	-22.121
3.251.020	-39.676	3.290.063	-22.949	3.244.529	-22.121
3.251.076	-39.679	3.290.182	-22.945	3.244.700	-22.120
3.251.248	-39.681	3.290.334	-22.942	3.244.836	-22.120
3.251.573	-39.684	3.290.490	-22.938	3.244.995	-22.120
3.251.739	-39.686	3.290.641	-22.935	3.245.126	-22.119
3.251.674	-39.689	3.290.764	-22.931	3.245.269	-22.119
3.251.956	-39.691	3.291.007	-22.928	3.245.475	-22.119
3.252.209	-39.694	3.291.314	-22.924	3.245.659	-22.118
3.252.296	-39.696	3.291.485	-22.921	3.245.784	-22.118
3.252.448	-39.699	3.291.631	-22.917	3.245.952	-22.118
3.252.476	-39.701	3.291.851	-22.914	3.246.187	-22.117
3.252.686	-39.703	3.292.052	-22.910	3.246.387	-22.117
3.252.921	-39.706	3.292.242	-22.907	3.246.495	-22.117
3.253.107	-39.708	3.292.451	-22.904	3.246.635	-22.117
3.253.325	-39.711	3.292.599	-22.900	3.246.816	-22.116
3.253.515	-39.713	3.292.762	-22.897	3.246.972	-22.116
3.253.608	-39.716	3.292.867	-22.894	3.247.153	-22.116
3.253.784	-39.718	3.292.972	-22.890	3.247.305	-22.115

3.253.951	-39.720	3.293.109	-22.887	3.247.429	-22.115
3.254.063	-39.723	3.293.218	-22.884	3.247.505	-22.115
3.254.290	-39.725	3.293.398	-22.880	3.247.646	-22.115
3.254.539	-39.728	3.293.589	-22.877	3.247.814	-22.114
3.254.709	-39.730	3.293.713	-22.874	3.247.950	-22.114
3.254.846	-39.733	3.293.820	-22.871	3.248.167	-22.114
3.255.021	-39.735	3.294.044	-22.868	3.248.327	-22.114
3.255.383	-39.738	3.294.317	-22.865	3.248.474	-22.113
3.255.544	-39.740	3.294.530	-22.861	3.248.705	-22.113
3.255.721	-39.742	3.294.658	-22.858	3.248.956	-22.113
3.255.891	-39.745	3.294.785	-22.855	3.249.162	-22.113
3.255.649	-39.747	3.295.014	-22.852	3.249.323	-22.113
3.255.816	-39.750	3.295.169	-22.849	3.249.525	-22.112
3.256.169	-39.752	3.295.271	-22.846	3.249.717	-22.112
3.256.329	-39.755	3.295.464	-22.843	3.249.876	-22.112
3.256.463	-39.757	3.295.620	-22.840	3.250.027	-22.112
3.256.642	-39.759	3.295.815	-22.837	3.250.137	-22.112
3.256.754	-39.762	3.296.058	-22.834	3.250.269	-22.112
3.256.823	-39.764	3.296.255	-22.831	3.250.401	-22.111
3.257.007	-39.767	3.296.423	-22.828	3.250.520	-22.111
3.257.307	-39.769	3.296.557	-22.826	3.250.667	-22.111
3.257.390	-39.772	3.296.679	-22.823	3.250.815	-22.111
3.257.390	-39.774	3.296.849	-22.820	3.251.000	-22.111
3.257.556	-39.777	3.297.047	-22.817	3.251.219	-22.111
3.257.845	-39.779	3.297.218	-22.814	3.251.349	-22.111
3.258.208	-39.782	3.297.368	-22.812	3.251.483	-22.111
3.258.537	-39.784	3.297.529	-22.809	3.251.736	-22.110
3.258.644	-39.787	3.297.711	-22.806	3.251.989	-22.110
3.258.724	-39.789	3.297.892	-22.803	3.252.202	-22.110
3.258.936	-39.792	3.298.018	-22.801	3.252.394	-22.110

3.258.970	-39.794	3.298.156	-22.798	3.252.546	-22.110
3.259.156	-39.797	3.298.325	-22.796	3.252.708	-22.110
3.259.458	-39.799	3.298.459	-22.793	3.252.860	-22.110
3.259.717	-39.802	3.298.625	-22.790	3.253.016	-22.110
3.259.879	-39.804	3.298.883	-22.788	3.253.175	-22.110
3.259.964	-39.807	3.299.117	-22.786	3.253.338	-22.110
3.260.023	-39.809	3.299.324	-22.783	3.253.528	-22.110
3.260.300	-39.812	3.299.472	-22.781	3.253.708	-22.110
3.260.487	-39.814	3.299.626	-22.778	3.253.925	-22.110
3.260.476	-39.816	3.299.786	-22.776	3.254.120	-22.110
3.260.619	-39.819	3.299.875	-22.774	3.254.284	-22.110
3.260.912	-39.821	3.299.944	-22.771	3.254.422	-22.110
3.261.190	-39.824	3.300.099	-22.769	3.254.522	-22.110
3.261.212	-39.826	3.300.484	-22.767	3.254.746	-22.110
3.261.281	-39.829	3.300.925	-22.765	3.254.977	-22.110
3.261.378	-39.831	3.301.192	-22.762	3.255.081	-22.110
3.261.779	-39.833	3.301.271	-22.760	3.255.208	-22.110
3.262.077	-39.836	3.301.325	-22.758	3.255.381	-22.110
3.262.047	-39.838	3.301.411	-22.756	3.255.591	-22.110
3.262.207	-39.841	3.301.494	-22.754	3.255.704	-22.110
3.262.267	-39.843	3.301.559	-22.752	3.255.833	-22.110
3.262.299	-39.845	3.301.570	-22.750	3.256.100	-22.110
3.262.561	-39.847	3.301.700	-22.748	3.256.257	-22.110
3.262.795	-39.850	3.301.878	-22.746	3.256.375	-22.110
3.262.935	-39.852	3.302.007	-22.745	3.256.577	-22.110
3.263.067	-39.854	3.302.245	-22.743	3.256.779	-22.110
3.263.343	-39.856	3.302.484	-22.741	3.256.939	-22.110
3.263.571	-39.859	3.302.632	-22.739	3.257.083	-22.110
3.263.632	-39.861	3.302.780	-22.738	3.257.222	-22.110
3.263.712	-39.863	3.302.943	-22.736	3.257.348	-22.111

3.263.872	-39.865	3.303.134	-22.734	3.257.648	-22.111
3.264.189	-39.867	3.303.293	-22.733	3.258.088	-22.111
3.264.341	-39.869	3.303.481	-22.731	3.258.341	-22.111
3.264.382	-39.871	3.303.702	-22.730	3.258.463	-22.111
3.264.803	-39.873	3.303.826	-22.728	3.258.578	-22.111
3.265.045	-39.875	3.303.925	-22.727	3.258.645	-22.111
3.264.865	-39.877	3.304.059	-22.725	3.258.684	-22.111
3.264.877	-39.879	3.304.219	-22.724	3.258.694	-22.111
3.265.335	-39.881	3.304.398	-22.723	3.258.763	-22.111
3.265.971	-39.883	3.304.561	-22.722	3.258.972	-22.111
3.266.093	-39.885	3.304.720	-22.721	3.259.160	-22.111
3.266.035	-39.887	3.304.922	-22.720	3.259.245	-22.112
3.266.177	-39.889	3.305.132	-22.718	3.259.450	-22.112
3.266.104	-39.890	3.305.283	-22.717	3.259.727	-22.112
3.266.151	-39.892	3.305.422	-22.717	3.259.894	-22.112
3.266.437	-39.894	3.305.574	-22.716	3.260.070	-22.112
3.266.575	-39.896	3.305.766	-22.715	3.260.260	-22.112
3.266.674	-39.897	3.305.977	-22.714	3.260.461	-22.112
3.266.939	-39.899	3.306.134	-22.713	3.260.638	-22.112
3.267.391	-39.901	3.306.268	-22.713	3.260.797	-22.112
3.267.621	-39.902	3.306.437	-22.712	3.260.942	-22.112
3.267.470	-39.904	3.306.570	-22.711	3.261.102	-22.112
3.267.553	-39.905	3.306.700	-22.711	3.261.281	-22.112
3.267.697	-39.907	3.306.942	-22.710	3.261.419	-22.113
3.267.641	-39.908	3.307.130	-22.710	3.261.562	-22.113
3.267.883	-39.910	3.307.249	-22.710	3.261.747	-22.113
3.268.456	-39.911	3.307.368	-22.709	3.261.954	-22.113
3.268.875	-39.913	3.307.518	-22.709	3.262.104	-22.113
3.268.802	-39.914	3.307.691	-22.709	3.262.231	-22.113
3.268.860	-39.915	3.307.830	-22.709	3.262.357	-22.113

3.269.085	-39.916	3.307.984	-22.709	3.262.528	-22.113
3.269.009	-39.918	3.308.192	-22.708	3.262.760	-22.113
3.269.162	-39.919	3.308.362	-22.708	3.262.982	-22.113
3.269.789	-39.920	3.308.511	-22.709	3.263.224	-22.113
3.269.817	-39.921	3.308.687	-22.709	3.263.347	-22.114
3.269.429	-39.923	3.308.789	-22.709	3.263.413	-22.114
3.269.656	-39.924	3.308.923	-22.709	3.263.540	-22.114
3.270.193	-39.925	3.309.117	-22.709	3.263.667	-22.114
3.270.483	-39.926	3.309.308	-22.710	3.263.811	-22.114
3.270.585	-39.927	3.309.490	-22.710	3.263.999	-22.114
3.270.818	-39.928	3.309.664	-22.711	3.264.208	-22.114
3.270.921	-39.929	3.309.870	-22.711	3.264.362	-22.114
3.271.155	-39.930	3.310.039	-22.712	3.264.588	-22.114
3.271.461	-39.931	3.310.179	-22.712	3.264.789	-22.114
3.271.520	-39.932	3.310.331	-22.713	3.264.872	-22.114
3.271.801	-39.933	3.310.497	-22.714	3.265.000	-22.114
3.271.943	-39.934	3.310.686	-22.715	3.265.206	-22.114
3.272.205	-39.934	3.310.807	-22.716	3.265.381	-22.115
3.272.554	-39.935	3.310.894	-22.716	3.265.551	-22.115
3.272.492	-39.936	3.311.058	-22.717	3.265.789	-22.115
3.272.559	-39.937	3.311.304	-22.718	3.265.919	-22.115
3.272.655	-39.938	3.311.519	-22.719	3.266.040	-22.115
3.272.561	-39.938	3.311.678	-22.721	3.266.248	-22.115
3.272.726	-39.939	3.311.859	-22.722	3.266.408	-22.115
3.273.074	-39.940	3.312.011	-22.723	3.266.530	-22.115
3.273.257	-39.940	3.312.153	-22.724	3.266.712	-22.115
3.273.414	-39.941	3.312.321	-22.725	3.266.909	-22.115
3.273.479	-39.942	3.312.494	-22.727	3.267.063	-22.115
3.273.591	-39.942	3.312.675	-22.728	3.267.225	-22.115
3.273.988	-39.943	3.312.864	-22.729	3.267.402	-22.115

3.274.543	-39.944	3.312.993	-22.731	3.267.604	-22.115
3.274.875	-39.944	3.313.139	-22.732	3.267.816	-22.115
3.274.977	-39.945	3.313.322	-22.734	3.267.924	-22.115
3.275.092	-39.945	3.313.441	-22.736	3.267.986	-22.116
3.275.052	-39.946	3.313.575	-22.737	3.268.200	-22.116
3.275.017	-39.946	3.313.734	-22.739	3.268.435	-22.116
3.275.166	-39.947	3.313.847	-22.740	3.268.580	-22.116
3.275.276	-39.947	3.314.002	-22.742	3.268.763	-22.116
3.275.317	-39.948	3.314.204	-22.744	3.268.927	-22.116
3.275.432	-39.948	3.314.391	-22.746	3.269.071	-22.116
3.275.674	-39.949	3.314.628	-22.748	3.269.247	-22.116
3.275.816	-39.949	3.314.810	-22.749	3.269.398	-22.116
3.275.818	-39.950	3.314.973	-22.751	3.269.555	-22.116
3.276.151	-39.950	3.315.188	-22.753	3.269.724	-22.116
3.276.519	-39.951	3.315.372	-22.755	3.269.850	-22.116
3.276.591	-39.951	3.315.545	-22.757	3.270.031	-22.116
3.276.528	-39.952	3.315.711	-22.759	3.270.200	-22.116
3.276.610	-39.952	3.315.847	-22.761	3.270.339	-22.115
3.277.073	-39.953	3.316.013	-22.763	3.270.446	-22.115
3.277.334	-39.953	3.316.161	-22.765	3.270.558	-22.115
3.277.383	-39.954	3.316.266	-22.767	3.270.729	-22.115
3.277.394	-39.954	3.316.415	-22.769	3.270.934	-22.115
3.277.610	-39.955	3.316.622	-22.771	3.271.154	-22.115
3.277.852	-39.955	3.316.772	-22.774	3.271.385	-22.115
3.277.943	-39.956	3.316.891	-22.776	3.271.643	-22.115
3.277.922	-39.957	3.317.068	-22.778	3.271.794	-22.115
3.277.975	-39.957	3.317.239	-22.780	3.271.945	-22.115
3.278.203	-39.958	3.317.379	-22.782	3.272.099	-22.115
3.278.446	-39.959	3.317.553	-22.785	3.272.297	-22.115
3.278.768	-39.959	3.317.744	-22.787	3.272.456	-22.115

3.279.010	-39.960	3.317.930	-22.789	3.272.621	-22.114
3.279.214	-39.961	3.318.139	-22.791	3.272.817	-22.114
3.279.543	-39.962	3.318.294	-22.794	3.272.900	-22.114
3.279.599	-39.963	3.318.472	-22.796	3.272.979	-22.114
3.279.605	-39.963	3.318.670	-22.798	3.273.131	-22.114
3.279.886	-39.964	3.318.843	-22.800	3.273.304	-22.114
3.280.256	-39.965	3.318.997	-22.803	3.273.459	-22.114
3.280.511	-39.966	3.319.124	-22.805	3.273.665	-22.113
3.280.435	-39.967	3.319.250	-22.807	3.273.857	-22.113
3.280.551	-39.968	3.319.429	-22.810	3.274.026	-22.113
3.280.710	-39.969	3.319.633	-22.812	3.274.283	-22.113
3.280.883	-39.970	3.319.787	-22.815	3.274.438	-22.113
3.281.216	-39.971	3.319.949	-22.817	3.274.583	-22.113
3.281.232	-39.973	3.320.139	-22.819	3.274.832	-22.112
3.281.187	-39.974	3.320.332	-22.822	3.274.897	-22.112
3.281.591	-39.975	3.320.472	-22.824	3.275.011	-22.112
3.281.609	-39.976	3.320.630	-22.826	3.275.265	-22.112
3.281.665	-39.978	3.320.780	-22.829	3.275.443	-22.112
3.282.041	-39.979	3.320.887	-22.831	3.275.613	-22.112
3.282.141	-39.981	3.321.060	-22.834	3.275.798	-22.112
3.282.336	-39.982	3.321.297	-22.836	3.276.035	-22.111
3.282.547	-39.984	3.321.479	-22.838	3.276.252	-22.111
3.282.686	-39.985	3.321.656	-22.841	3.276.333	-22.111
3.282.809	-39.987	3.321.819	-22.843	3.276.411	-22.111
3.282.908	-39.989	3.321.945	-22.845	3.276.603	-22.111
3.282.986	-39.991	3.322.124	-22.848	3.276.798	-22.111
3.283.080	-39.992	3.322.328	-22.850	3.276.920	-22.110
3.283.296	-39.994	3.322.509	-22.852	3.277.128	-22.110
3.283.616	-39.996	3.322.704	-22.855	3.277.373	-22.110
3.283.887	-39.998	3.322.856	-22.857	3.277.487	-22.110

3.283.976	-40.000	3.322.979	-22.860	3.277.592	-22.110
3.284.024	-40.002	3.323.128	-22.862	3.277.783	-22.110
3.284.193	-40.004	3.323.322	-22.864	3.277.990	-22.110
3.284.514	-40.007	3.323.464	-22.867	3.278.196	-22.110
3.284.677	-40.009	3.323.580	-22.869	3.278.383	-22.109
3.284.833	-40.011	3.323.824	-22.871	3.278.466	-22.109
3.284.810	-40.013	3.324.243	-22.874	3.278.561	-22.109
3.284.897	-40.016	3.324.576	-22.876	3.278.742	-22.109
3.285.172	-40.018	3.324.662	-22.878	3.278.972	-22.109
3.285.370	-40.021	3.324.731	-22.881	3.279.164	-22.109
3.285.614	-40.023	3.324.855	-22.883	3.279.299	-22.109
3.285.721	-40.026	3.324.924	-22.885	3.279.418	-22.109
3.285.782	-40.028	3.324.971	-22.887	3.279.570	-22.109
3.285.949	-40.031	3.325.065	-22.890	3.279.722	-22.109
3.286.174	-40.034	3.325.177	-22.892	3.279.879	-22.108
3.286.544	-40.036	3.325.316	-22.894	3.280.233	-22.108
3.286.804	-40.039	3.325.508	-22.897	3.280.648	-22.108
3.286.992	-40.042	3.325.693	-22.899	3.280.912	-22.108
3.287.041	-40.045	3.325.855	-22.901	3.281.013	-22.108
3.287.061	-40.048	3.325.996	-22.903	3.281.060	-22.108
3.286.863	-40.051	3.326.198	-22.906	3.281.147	-22.108
3.286.935	-40.054	3.326.490	-22.908	3.281.278	-22.108
3.287.588	-40.057	3.326.714	-22.910	3.281.373	-22.108
3.287.871	-40.060	3.326.846	-22.912	3.281.403	-22.108
3.287.809	-40.063	3.326.986	-22.915	3.281.483	-22.108
3.287.895	-40.066	3.327.115	-22.917	3.281.649	-22.108
3.288.517	-40.069	3.327.267	-22.919	3.281.846	-22.108
3.288.311	-40.072	3.327.422	-22.921	3.282.021	-22.108
3.287.979	-40.076	3.327.521	-22.923	3.282.180	-22.108
3.288.793	-40.079	3.327.679	-22.926	3.282.370	-22.109

3.289.118	-40.082	3.327.872	-22.928	3.282.579	-22.109
3.288.868	-40.086	3.328.049	-22.930	3.282.762	-22.109
3.289.272	-40.089	3.328.195	-22.932	3.282.953	-22.109
3.289.619	-40.092	3.328.329	-22.934	3.283.145	-22.109
3.289.678	-40.096	3.328.515	-22.937	3.283.268	-22.109
3.289.756	-40.099	3.328.744	-22.939	3.283.416	-22.109
3.290.122	-40.103	3.328.936	-22.941	3.283.572	-22.109
3.290.541	-40.106	3.329.081	-22.943	3.283.698	-22.110
3.290.316	-40.110	3.329.286	-22.945	3.283.865	-22.110
3.290.365	-40.113	3.329.474	-22.948	3.284.005	-22.110
3.290.555	-40.117	3.329.671	-22.950	3.284.175	-22.110
3.290.612	-40.121	3.329.857	-22.952	3.284.375	-22.110
3.290.811	-40.124	3.329.998	-22.954	3.284.557	-22.111
3.290.899	-40.128	3.330.170	-22.956	3.284.724	-22.111
3.291.049	-40.132	3.330.289	-22.959	3.284.870	-22.111
3.291.284	-40.135	3.330.392	-22.961	3.285.099	-22.111
3.291.448	-40.139	3.330.574	-22.963	3.285.316	-22.112
3.291.689	-40.143	3.330.779	-22.965	3.285.473	-22.112
3.291.936	-40.146	3.330.872	-22.968	3.285.612	-22.112
3.292.209	-40.150	3.331.017	-22.970	3.285.762	-22.113
3.292.242	-40.154	3.331.181	-22.972	3.285.941	-22.113
3.292.518	-40.158	3.331.299	-22.974	3.286.109	-22.114
3.292.856	-40.161	3.331.510	-22.977	3.286.259	-22.114
3.292.865	-40.165	3.331.714	-22.979	3.286.398	-22.114
3.293.047	-40.169	3.331.869	-22.981	3.286.550	-22.115
3.293.221	-40.173	3.332.065	-22.983	3.286.718	-22.115
3.293.315	-40.177	3.332.221	-22.986	3.286.857	-22.116
3.293.481	-40.181	3.332.354	-22.988	3.286.990	-22.116
3.293.952	-40.184	3.332.536	-22.990	3.287.130	-22.117
3.294.039	-40.188	3.332.682	-22.992	3.287.299	-22.117

3.293.871	-40.192	3.332.818	-22.995	3.287.529	-22.118
3.294.063	-40.196	3.332.971	-22.997	3.287.737	-22.118
3.294.274	-40.200	3.333.123	-22.999	3.287.870	-22.119
3.294.489	-40.204	3.333.344	-23.002	3.288.034	-22.120
3.294.602	-40.208	3.333.567	-23.004	3.288.170	-22.120
3.294.789	-40.211	3.333.726	-23.006	3.288.289	-22.121
3.294.909	-40.215	3.333.867	-23.009	3.288.481	-22.121
3.295.024	-40.219	3.334.005	-23.011	3.288.627	-22.122
3.295.412	-40.223	3.334.189	-23.013	3.288.813	-22.123
3.295.648	-40.227	3.334.389	-23.016	3.289.030	-22.123
3.295.710	-40.231	3.334.529	-23.018	3.289.180	-22.124
3.295.718	-40.235	3.334.698	-23.021	3.289.370	-22.125
3.295.928	-40.239	3.334.894	-23.023	3.289.586	-22.125
3.296.234	-40.243	3.335.046	-23.025	3.289.711	-22.126
3.296.433	-40.246	3.335.158	-23.028	3.289.847	-22.127
3.296.638	-40.250	3.335.365	-23.030	3.290.034	-22.128
3.296.754	-40.254	3.335.581	-23.033	3.290.157	-22.128
3.296.920	-40.258	3.335.737	-23.035	3.290.346	-22.129
3.297.198	-40.262	3.335.938	-23.037	3.290.529	-22.130
3.297.409	-40.266	3.336.082	-23.040	3.290.663	-22.131
3.297.543	-40.270	3.336.208	-23.042	3.290.829	-22.131
3.297.676	-40.273	3.336.386	-23.045	3.291.010	-22.132
3.297.680	-40.277	3.336.563	-23.047	3.291.263	-22.133
3.297.730	-40.281	3.336.721	-23.050	3.291.443	-22.134
3.297.954	-40.285	3.336.880	-23.052	3.291.593	-22.135
3.298.148	-40.289	3.337.018	-23.054	3.291.787	-22.135
3.298.356	-40.293	3.337.177	-23.057	3.291.906	-22.136
3.298.676	-40.296	3.337.311	-23.059	3.292.029	-22.137
3.299.048	-40.300	3.337.455	-23.062	3.292.216	-22.138
3.299.232	-40.304	3.337.630	-23.064	3.292.379	-22.139

3.299.214	-40.308	3.337.780	-23.067	3.292.518	-22.139
3.299.371	-40.312	3.337.954	-23.069	3.292.635	-22.140
3.299.536	-40.315	3.338.181	-23.072	3.292.784	-22.141
3.299.601	-40.319	3.338.417	-23.074	3.292.965	-22.142
3.299.714	-40.323	3.338.608	-23.076	3.293.096	-22.143
3.299.814	-40.327	3.338.791	-23.079	3.293.239	-22.143
3.300.031	-40.330	3.338.938	-23.081	3.293.405	-22.144
3.300.016	-40.334	3.339.081	-23.084	3.293.625	-22.145
3.300.018	-40.338	3.339.276	-23.086	3.293.857	-22.146
3.300.216	-40.342	3.339.410	-23.089	3.294.016	-22.146
3.300.341	-40.345	3.339.540	-23.091	3.294.225	-22.147
3.300.663	-40.349	3.339.724	-23.094	3.294.449	-22.148
3.301.024	-40.353	3.339.870	-23.096	3.294.608	-22.149
3.300.768	-40.356	3.339.964	-23.098	3.294.778	-22.149
3.300.818	-40.360	3.340.081	-23.101	3.294.917	-22.150
3.301.360	-40.364	3.340.294	-23.103	3.295.032	-22.151
3.301.703	-40.368	3.340.461	-23.106	3.295.188	-22.152
3.301.835	-40.371	3.340.587	-23.108	3.295.361	-22.152
3.301.993	-40.375	3.340.739	-23.110	3.295.480	-22.153
3.302.162	-40.379	3.340.956	-23.113	3.295.580	-22.154
3.302.442	-40.383	3.341.190	-23.115	3.295.729	-22.155
3.302.535	-40.386	3.341.379	-23.118	3.295.934	-22.155
3.302.361	-40.390	3.341.555	-23.120	3.296.151	-22.156
3.302.534	-40.394	3.341.700	-23.122	3.296.318	-22.157
3.302.776	-40.398	3.341.851	-23.125	3.296.487	-22.157
3.302.729	-40.401	3.342.007	-23.127	3.296.651	-22.158
3.302.809	-40.405	3.342.162	-23.129	3.296.810	-22.159
3.303.289	-40.409	3.342.347	-23.132	3.296.960	-22.159
3.303.716	-40.413	3.342.578	-23.134	3.297.148	-22.160
3.303.882	-40.416	3.342.749	-23.136	3.297.370	-22.160

3.303.965	-40.420	3.342.878	-23.139	3.297.541	-22.161
3.304.344	-40.424	3.342.968	-23.141	3.297.711	-22.162
3.304.609	-40.428	3.343.109	-23.143	3.297.853	-22.162
3.304.395	-40.432	3.343.338	-23.146	3.298.040	-22.163
3.304.483	-40.436	3.343.520	-23.148	3.298.161	-22.163
3.304.557	-40.440	3.343.720	-23.150	3.298.322	-22.164
3.304.691	-40.443	3.343.915	-23.153	3.298.553	-22.165
3.305.266	-40.447	3.344.115	-23.155	3.298.708	-22.165
3.305.302	-40.451	3.344.315	-23.157	3.298.857	-22.166
3.305.363	-40.455	3.344.482	-23.160	3.299.059	-22.166
3.305.350	-40.459	3.344.617	-23.162	3.299.230	-22.167
3.305.401	-40.463	3.344.752	-23.164	3.299.381	-22.167
3.305.822	-40.467	3.344.927	-23.166	3.299.557	-22.168
3.306.169	-40.471	3.345.110	-23.168	3.299.720	-22.168
3.306.067	-40.475	3.345.298	-23.171	3.299.899	-22.169
3.305.841	-40.479	3.345.483	-23.173	3.300.101	-22.169
3.306.479	-40.483	3.345.646	-23.175	3.300.237	-22.170
3.306.780	-40.487	3.345.813	-23.177	3.300.381	-22.171
3.307.094	-40.491	3.345.967	-23.179	3.300.599	-22.171
3.307.623	-40.496	3.346.167	-23.182	3.300.838	-22.172
3.307.444	-40.500	3.346.328	-23.184	3.300.941	-22.172
3.306.928	-40.504	3.346.483	-23.186	3.301.042	-22.173
3.306.920	-40.508	3.346.661	-23.188	3.301.275	-22.173
3.307.477	-40.512	3.346.788	-23.190	3.301.468	-22.173
3.307.318	-40.517	3.346.953	-23.192	3.301.596	-22.174
3.307.518	-40.521	3.347.080	-23.194	3.301.797	-22.174
3.307.835	-40.525	3.347.198	-23.196	3.301.972	-22.175
3.307.603	-40.530	3.347.549	-23.198	3.302.090	-22.175
3.308.260	-40.534	3.347.990	-23.200	3.302.167	-22.176
3.308.817	-40.539	3.348.278	-23.202	3.302.240	-22.176

3.308.701	-40.543	3.348.471	-23.205	3.302.528	-22.177
3.308.938	-40.548	3.348.497	-23.207	3.302.975	-22.177
3.309.295	-40.552	3.348.497	-23.209	3.303.341	-22.178
3.309.154	-40.557	3.348.586	-23.210	3.303.441	-22.178
3.309.009	-40.561	3.348.681	-23.212	3.303.535	-22.179
3.309.483	-40.566	3.348.773	-23.214	3.303.573	-22.179
3.309.587	-40.571	3.348.873	-23.216	3.303.652	-22.180
3.309.518	-40.575	3.348.997	-23.218	3.303.724	-22.180
3.309.899	-40.580	3.349.174	-23.220	3.303.785	-22.181
3.310.208	-40.585	3.349.384	-23.222	3.303.882	-22.181
3.310.623	-40.589	3.349.579	-23.224	3.304.017	-22.182
3.310.883	-40.594	3.349.804	-23.226	3.304.180	-22.182
3.311.239	-40.599	3.349.953	-23.228	3.304.348	-22.183
3.311.006	-40.604	3.350.045	-23.230	3.304.486	-22.183
3.311.013	-40.608	3.350.255	-23.231	3.304.601	-22.184
3.311.776	-40.613	3.350.484	-23.233	3.304.812	-22.184
3.311.654	-40.618	3.350.656	-23.235	3.305.103	-22.185
3.311.682	-40.623	3.350.786	-23.237	3.305.260	-22.185
3.312.126	-40.628	3.350.855	-23.239	3.305.392	-22.186
3.311.942	-40.633	3.350.974	-23.241	3.305.549	-22.187
3.311.719	-40.638	3.351.203	-23.242	3.305.721	-22.187
3.312.122	-40.643	3.351.411	-23.244	3.305.881	-22.188
3.312.531	-40.648	3.351.546	-23.246	3.305.970	-22.188
3.312.741	-40.653	3.351.667	-23.248	3.306.132	-22.189
3.312.552	-40.658	3.351.786	-23.249	3.306.400	-22.190
3.312.534	-40.663	3.352.012	-23.251	3.306.566	-22.190
3.312.626	-40.668	3.352.253	-23.253	3.306.711	-22.191
3.312.281	-40.673	3.352.430	-23.255	3.306.866	-22.192
3.312.903	-40.678	3.352.621	-23.257	3.306.992	-22.192
3.314.039	-40.684	3.352.803	-23.258	3.307.138	-22.193

3.313.983	-40.689	3.352.957	-23.260	3.307.314	-22.194
3.313.448	-40.694	3.353.128	-23.262	3.307.535	-22.195
3.314.274	-40.699	3.353.365	-23.264	3.307.706	-22.195
3.315.092	-40.704	3.353.553	-23.265	3.307.817	-22.196
3.315.157	-40.710	3.353.680	-23.267	3.307.995	-22.197
3.315.334	-40.715	3.353.785	-23.269	3.308.250	-22.198
3.315.094	-40.720	3.353.901	-23.271	3.308.438	-22.199
3.314.851	-40.725	3.354.084	-23.272	3.308.607	-22.200
3.315.417	-40.731	3.354.254	-23.274	3.308.759	-22.201
3.315.591	-40.736	3.354.399	-23.276	3.308.868	-22.202
3.315.404	-40.741	3.354.561	-23.278	3.309.009	-22.203
3.315.822	-40.747	3.354.749	-23.279	3.309.132	-22.204
3.315.891	-40.752	3.354.902	-23.281	3.309.301	-22.205
3.315.630	-40.757	3.355.035	-23.283	3.309.492	-22.206
3.315.908	-40.763	3.355.136	-23.285	3.309.608	-22.207
3.316.285	-40.768	3.355.341	-23.287	3.309.787	-22.208
3.316.792	-40.773	3.355.583	-23.288	3.309.942	-22.209
3.316.711	-40.779	3.355.704	-23.290	3.310.078	-22.210
3.316.071	-40.784	3.355.841	-23.292	3.310.307	-22.211
3.316.869	-40.789	3.356.013	-23.294	3.310.545	-22.213
3.317.698	-40.795	3.356.205	-23.295	3.310.732	-22.214
3.317.406	-40.800	3.356.365	-23.297	3.310.822	-22.215
3.317.160	-40.806	3.356.487	-23.299	3.310.919	-22.216
3.317.543	-40.811	3.356.660	-23.301	3.311.114	-22.218
3.318.130	-40.816	3.356.874	-23.303	3.311.349	-22.219
3.318.185	-40.822	3.357.057	-23.305	3.311.498	-22.221
3.318.178	-40.827	3.357.220	-23.306	3.311.599	-22.222
3.318.326	-40.833	3.357.386	-23.308	3.311.768	-22.223
3.318.282	-40.838	3.357.599	-23.310	3.311.974	-22.225
3.318.647	-40.843	3.357.796	-23.312	3.312.166	-22.226

3.319.182	-40.849	3.357.944	-23.314	3.312.314	-22.228
3.318.767	-40.854	3.358.083	-23.316	3.312.433	-22.229
3.318.311	-40.860	3.358.232	-23.318	3.312.581	-22.231
3.319.240	-40.865	3.358.445	-23.319	3.312.769	-22.233
3.319.695	-40.870	3.358.445	-23.321	3.312.947	-22.234
3.319.756	-40.876	3.358.750	-23.323	3.313.096	-22.236
3.320.075	-40.881	3.358.757	-23.325	3.313.264	-22.237
3.319.901	-40.887	3.358.943	-23.327	3.313.430	-22.239
3.320.164	-40.892	3.359.247	-23.329	3.313.611	-22.241
3.320.247	-40.897	3.359.438	-23.331	3.313.802	-22.242
3.320.370	-40.903	3.359.570	-23.332	3.313.955	-22.244
3.320.826	-40.908	3.359.720	-23.334	3.314.135	-22.246
3.321.026	-40.913	3.359.875	-23.336	3.314.328	-22.248
3.321.425	-40.919	3.360.071	-23.338	3.314.498	-22.250
3.321.378	-40.924	3.360.248	-23.340	3.314.602	-22.251
3.320.818	-40.929	3.360.338	-23.342	3.314.750	-22.253
3.321.097	-40.935	3.360.491	-23.343	3.314.924	-22.255
3.321.304	-40.940	3.360.688	-23.345	3.315.074	-22.257
3.321.347	-40.945	3.360.815	-23.347	3.315.204	-22.259
3.321.172	-40.950	3.360.880	-23.349	3.315.371	-22.261
3.321.687	-40.956	3.361.006	-23.351	3.315.541	-22.263
3.322.159	-40.961	3.361.198	-23.353	3.315.623	-22.264
3.321.837	-40.966	3.361.398	-23.354	3.315.743	-22.266
3.323.416	-40.971	3.361.618	-23.356	3.315.938	-22.268
3.323.829	-40.977	3.361.821	-23.358	3.316.187	-22.270
3.322.841	-40.982	3.362.021	-23.360	3.316.461	-22.272
3.323.768	-40.987	3.362.242	-23.362	3.316.671	-22.274
3.324.541	-40.992	3.362.419	-23.364	3.316.823	-22.276
3.324.435	-40.997	3.362.543	-23.365	3.317.000	-22.278
3.324.041	-41.003	3.362.784	-23.367	3.317.274	-22.280

3.323.467	-41.008	3.362.983	-23.369	3.317.481	-22.282
3.323.927	-41.013	3.363.076	-23.371	3.317.585	-22.284
3.324.042	-41.018	3.363.174	-23.373	3.317.670	-22.286
3.324.391	-41.023	3.363.327	-23.374	3.317.809	-22.288
3.324.384	-41.028	3.363.490	-23.376	3.317.982	-22.290
3.324.208	-41.033	3.363.654	-23.378	3.318.092	-22.292
3.325.067	-41.038	3.363.818	-23.380	3.318.183	-22.294
3.324.964	-41.043	3.364.027	-23.381	3.318.354	-22.296
3.324.850	-41.048	3.364.169	-23.383	3.318.499	-22.298
3.326.021	-41.053	3.364.254	-23.385	3.318.597	-22.300
3.326.127	-41.058	3.364.393	-23.387	3.318.768	-22.302
3.326.268	-41.063	3.364.615	-23.389	3.319.008	-22.304
3.326.194	-41.068	3.364.792	-23.390	3.319.236	-22.306
3.325.789	-41.072	3.364.996	-23.392	3.319.382	-22.308
3.326.505	-41.077	3.365.206	-23.394	3.319.497	-22.310
3.326.639	-41.082	3.365.381	-23.396	3.319.671	-22.312
3.326.754	-41.087	3.365.567	-23.397	3.319.868	-22.314
3.326.389	-41.092	3.365.697	-23.399	3.320.083	-22.316
3.326.310	-41.096	3.365.898	-23.401	3.320.271	-22.318
3.326.996	-41.101	3.366.072	-23.403	3.320.399	-22.320
3.327.191	-41.106	3.366.226	-23.404	3.320.555	-22.321
3.327.460	-41.111	3.366.385	-23.406	3.320.744	-22.323
3.328.164	-41.115	3.366.542	-23.408	3.320.916	-22.325
3.328.358	-41.120	3.366.761	-23.410	3.321.108	-22.327
3.327.758	-41.125	3.366.909	-23.411	3.321.285	-22.329
3.327.430	-41.129	3.367.116	-23.413	3.321.444	-22.331
3.328.031	-41.134	3.367.337	-23.415	3.321.570	-22.333
3.327.983	-41.139	3.367.442	-23.416	3.321.714	-22.335
3.327.950	-41.143	3.367.590	-23.418	3.321.902	-22.336
3.328.222	-41.148	3.367.784	-23.420	3.322.077	-22.338

3.328.495	-41.153	3.367.979	-23.421	3.322.278	-22.340
3.329.016	-41.157	3.368.134	-23.423	3.322.442	-22.342
3.329.432	-41.162	3.368.318	-23.425	3.322.599	-22.344
3.329.443	-41.166	3.368.471	-23.427	3.322.777	-22.345
3.329.431	-41.171	3.368.636	-23.428	3.322.874	-22.347
3.329.622	-41.175	3.368.867	-23.430	3.323.066	-22.349
3.329.774	-41.180	3.369.030	-23.432	3.323.378	-22.351
3.330.100	-41.184	3.369.147	-23.433	3.323.502	-22.352
3.330.049	-41.189	3.369.297	-23.435	3.323.601	-22.354
3.330.094	-41.193	3.369.505	-23.436	3.323.734	-22.356
3.330.504	-41.198	3.369.666	-23.438	3.323.897	-22.357
3.330.619	-41.202	3.369.778	-23.440	3.324.171	-22.359
3.330.748	-41.207	3.369.966	-23.441	3.324.367	-22.361
3.330.923	-41.211	3.370.190	-23.443	3.324.508	-22.362
3.331.011	-41.216	3.370.347	-23.445	3.324.663	-22.364
3.331.331	-41.220	3.370.482	-23.446	3.324.826	-22.365
3.331.643	-41.224	3.370.605	-23.448	3.325.024	-22.367
3.331.620	-41.229	3.370.754	-23.449	3.325.161	-22.368
3.331.552	-41.233	3.370.872	-23.451	3.325.323	-22.370
3.331.838	-41.238	3.371.100	-23.453	3.325.699	-22.371
3.331.982	-41.242	3.371.555	-23.454	3.326.046	-22.373
3.332.109	-41.246	3.371.877	-23.456	3.326.203	-22.374
3.332.175	-41.251	3.372.014	-23.457	3.326.296	-22.375
3.332.347	-41.255	3.372.149	-23.459	3.326.393	-22.377
3.332.615	-41.259	3.372.268	-23.460	3.326.452	-22.378
3.332.827	-41.264	3.372.303	-23.462	3.326.459	-22.379
3.333.116	-41.268	3.372.333	-23.464	3.326.537	-22.381
3.333.237	-41.273	3.372.412	-23.465	3.326.758	-22.382
3.333.376	-41.277	3.372.517	-23.467	3.326.949	-22.383
3.333.459	-41.281	3.372.664	-23.468	3.326.996	-22.384

3.333.717	-41.286	3.372.854	-23.470	3.327.115	-22.386
3.333.911	-41.290	3.373.063	-23.471	3.327.332	-22.387
3.333.962	-41.294	3.373.232	-23.473	3.327.581	-22.388
3.334.136	-41.299	3.373.410	-23.474	3.327.765	-22.389
3.334.431	-41.303	3.373.586	-23.476	3.327.857	-22.390
3.334.623	-41.307	3.373.709	-23.477	3.328.022	-22.391
3.334.792	-41.312	3.373.912	-23.479	3.328.210	-22.392
3.334.891	-41.316	3.374.127	-23.480	3.328.394	-22.393
3.335.038	-41.320	3.374.269	-23.482	3.328.627	-22.394
3.335.144	-41.325	3.374.442	-23.483	3.328.781	-22.395
3.335.334	-41.329	3.374.597	-23.485	3.328.887	-22.396
3.335.726	-41.333	3.374.673	-23.486	3.329.042	-22.397
3.335.860	-41.337	3.374.808	-23.488	3.329.179	-22.398
3.335.953	-41.342	3.375.085	-23.489	3.329.346	-22.399
3.336.180	-41.346	3.375.313	-23.491	3.329.548	-22.400
3.336.282	-41.350	3.375.454	-23.492	3.329.677	-22.401
3.336.360	-41.354	3.375.612	-23.494	3.329.837	-22.402
3.336.527	-41.359	3.375.715	-23.495	3.330.079	-22.403
3.336.798	-41.363	3.375.848	-23.497	3.330.255	-22.404
3.336.965	-41.367	3.376.035	-23.498	3.330.401	-22.405
3.336.951	-41.371	3.376.216	-23.500	3.330.598	-22.406
3.337.083	-41.376	3.376.430	-23.502	3.330.783	-22.406
3.337.130	-41.380	3.376.620	-23.503	3.330.949	-22.407
3.337.142	-41.384	3.376.787	-23.505	3.331.163	-22.408
3.337.614	-41.388	3.376.961	-23.506	3.331.344	-22.409
3.337.831	-41.392	3.377.169	-23.508	3.331.473	-22.410
3.338.109	-41.397	3.377.365	-23.509	3.331.628	-22.410
3.338.367	-41.401	3.377.532	-23.511	3.331.750	-22.411
3.338.319	-41.405	3.377.659	-23.513	3.331.873	-22.412
3.338.549	-41.409	3.377.755	-23.514	3.332.004	-22.413

3.338.611	-41.413	3.377.888	-23.516	3.332.153	-22.413
3.338.788	-41.417	3.378.098	-23.518	3.332.312	-22.414
3.339.492	-41.422	3.378.293	-23.519	3.332.487	-22.415
3.339.727	-41.426	3.378.424	-23.521	3.332.690	-22.416
3.339.417	-41.430	3.378.560	-23.523	3.332.879	-22.416
3.339.327	-41.434	3.378.708	-23.525	3.333.036	-22.417
3.339.794	-41.438	3.378.903	-23.526	3.333.185	-22.418
3.340.147	-41.442	3.379.131	-23.528	3.333.326	-22.419
3.340.045	-41.446	3.379.341	-23.530	3.333.500	-22.419
3.340.347	-41.451	3.379.516	-23.531	3.333.658	-22.420
3.340.216	-41.455	3.379.677	-23.533	3.333.792	-22.421
3.340.294	-41.459	3.379.765	-23.535	3.333.991	-22.421
3.340.540	-41.463	3.379.844	-23.537	3.334.179	-22.422
3.340.536	-41.467	3.380.002	-23.539	3.334.346	-22.423
3.340.737	-41.471	3.380.150	-23.541	3.334.523	-22.423
3.340.901	-41.475	3.380.302	-23.542	3.334.698	-22.424
3.341.306	-41.479	3.380.505	-23.544	3.334.848	-22.425
3.341.653	-41.483	3.380.729	-23.546	3.335.031	-22.426
3.341.578	-41.487	3.380.895	-23.548	3.335.249	-22.426
3.341.758	-41.491	3.381.040	-23.550	3.335.424	-22.427
3.342.081	-41.495	3.381.216	-23.552	3.335.558	-22.428
3.342.267	-41.499	3.381.438	-23.554	3.335.692	-22.428
3.342.325	-41.503	3.381.640	-23.556	3.335.901	-22.429
3.342.357	-41.507	3.381.779	-23.558	3.336.098	-22.430
3.342.471	-41.512	3.381.896	-23.560	3.336.230	-22.430
3.342.892	-41.516	3.382.026	-23.561	3.336.328	-22.431
3.343.120	-41.520	3.382.246	-23.563	3.336.481	-22.432
3.343.092	-41.524	3.382.419	-23.565	3.336.708	-22.433
3.343.226	-41.528	3.382.570	-23.567	3.336.895	-22.433
3.343.446	-41.532	3.382.705	-23.569	3.337.093	-22.434

3.343.623	-41.536	3.382.886	-23.571	3.337.252	-22.435
3.343.818	-41.540	3.383.134	-23.573	3.337.397	-22.435
3.344.093	-41.544	3.383.279	-23.575	3.337.576	-22.436
3.344.356	-41.548	3.383.456	-23.577	3.337.697	-22.437
3.344.493	-41.552	3.383.637	-23.579	3.337.791	-22.437
3.344.548	-41.556	3.383.741	-23.581	3.337.964	-22.438
3.344.677	-41.559	3.383.883	-23.583	3.338.134	-22.439
3.344.870	-41.563	3.384.073	-23.585	3.338.279	-22.439
3.345.143	-41.567	3.384.232	-23.587	3.338.423	-22.440
3.345.266	-41.571	3.384.316	-23.589	3.338.546	-22.441
3.345.374	-41.575	3.384.416	-23.591	3.338.726	-22.441
3.345.598	-41.579	3.384.615	-23.593	3.338.907	-22.442
3.345.681	-41.583	3.384.823	-23.595	3.339.073	-22.443
3.345.831	-41.587	3.384.973	-23.597	3.339.237	-22.443
3.345.981	-41.591	3.385.146	-23.599	3.339.519	-22.444
3.346.165	-41.595	3.385.345	-23.601	3.339.839	-22.445
3.346.438	-41.599	3.385.534	-23.603	3.339.998	-22.446
3.346.624	-41.603	3.385.750	-23.605	3.340.107	-22.446
3.346.776	-41.607	3.385.953	-23.607	3.340.280	-22.447
3.346.880	-41.611	3.386.136	-23.609	3.340.459	-22.448
3.346.918	-41.614	3.386.339	-23.611	3.340.624	-22.448
3.347.110	-41.618	3.386.521	-23.613	3.340.815	-22.449
3.347.305	-41.622	3.386.672	-23.615	3.340.945	-22.450
3.347.542	-41.626	3.386.830	-23.617	3.341.002	-22.450
3.347.737	-41.630	3.386.972	-23.619	3.341.058	-22.451
3.347.769	-41.634	3.387.105	-23.621	3.341.214	-22.452
3.348.135	-41.638	3.387.222	-23.623	3.341.382	-22.452
3.348.461	-41.642	3.387.368	-23.625	3.341.499	-22.453
3.348.716	-41.645	3.387.535	-23.626	3.341.701	-22.454
3.348.860	-41.649	3.387.659	-23.628	3.341.932	-22.454

3.348.970	-41.653	3.387.758	-23.630	3.342.083	-22.455
3.349.060	-41.657	3.387.910	-23.632	3.342.263	-22.456
3.349.150	-41.661	3.388.112	-23.634	3.342.429	-22.457
3.349.261	-41.665	3.388.329	-23.636	3.342.600	-22.457
3.349.428	-41.668	3.388.553	-23.637	3.342.785	-22.458
3.349.532	-41.672	3.388.748	-23.639	3.342.961	-22.459
3.349.612	-41.676	3.388.893	-23.641	3.343.132	-22.459
3.349.765	-41.680	3.389.080	-23.643	3.343.311	-22.460
3.349.937	-41.683	3.389.272	-23.644	3.343.481	-22.461
3.350.074	-41.687	3.389.421	-23.646	3.343.573	-22.461
3.350.233	-41.691	3.389.579	-23.648	3.343.734	-22.462
3.350.364	-41.695	3.389.743	-23.650	3.343.955	-22.463
3.350.670	-41.698	3.389.908	-23.651	3.344.189	-22.464
3.350.960	-41.702	3.390.068	-23.653	3.344.424	-22.464
3.351.158	-41.706	3.390.258	-23.655	3.344.572	-22.465
3.351.284	-41.710	3.390.472	-23.656	3.344.742	-22.466
3.351.227	-41.713	3.390.659	-23.658	3.344.886	-22.467
3.351.526	-41.717	3.390.775	-23.660	3.345.053	-22.467
3.351.882	-41.721	3.390.836	-23.661	3.345.255	-22.468
3.351.752	-41.724	3.391.004	-23.663	3.345.352	-22.469
3.351.846	-41.728	3.391.277	-23.664	3.345.417	-22.470
3.352.180	-41.732	3.391.526	-23.666	3.345.634	-22.470
3.352.372	-41.736	3.391.636	-23.667	3.345.941	-22.471
3.352.582	-41.739	3.391.791	-23.669	3.346.143	-22.472
3.352.646	-41.743	3.391.994	-23.670	3.346.315	-22.473
3.352.849	-41.747	3.392.191	-23.672	3.346.468	-22.473
3.353.044	-41.750	3.392.321	-23.673	3.346.575	-22.474
3.353.145	-41.754	3.392.482	-23.674	3.346.714	-22.475
3.353.387	-41.758	3.392.693	-23.676	3.346.875	-22.476
3.353.560	-41.761	3.392.842	-23.677	3.347.072	-22.476

3.353.575	-41.765	3.392.983	-23.679	3.347.285	-22.477
3.353.784	-41.769	3.393.161	-23.680	3.347.433	-22.478
3.354.028	-41.772	3.393.297	-23.681	3.347.585	-22.479
3.354.026	-41.776	3.393.442	-23.682	3.347.749	-22.479
3.354.128	-41.780	3.393.648	-23.684	3.347.911	-22.480
3.354.529	-41.783	3.393.803	-23.685	3.348.056	-22.481
3.354.823	-41.787	3.393.980	-23.686	3.348.402	-22.482
3.354.992	-41.790	3.394.156	-23.687	3.348.850	-22.482
3.355.086	-41.794	3.394.212	-23.688	3.349.068	-22.483
3.355.074	-41.798	3.394.274	-23.689	3.349.143	-22.484
3.355.157	-41.801	3.394.384	-23.690	3.349.223	-22.484
3.355.306	-41.805	3.394.640	-23.692	3.349.286	-22.485
3.355.588	-41.808	3.395.118	-23.693	3.349.315	-22.486
3.355.802	-41.812	3.395.533	-23.694	3.349.389	-22.487
3.355.851	-41.816	3.395.666	-23.695	3.349.504	-22.487
3.356.022	-41.819	3.395.725	-23.696	3.349.601	-22.488
3.356.086	-41.823	3.395.813	-23.697	3.349.758	-22.489
3.356.241	-41.826	3.395.924	-23.697	3.349.976	-22.489
3.356.844	-41.830	3.395.986	-23.698	3.350.135	-22.490
3.357.148	-41.834	3.396.035	-23.699	3.350.313	-22.491
3.357.128	-41.837	3.396.184	-23.700	3.350.498	-22.492
3.357.169	-41.841	3.396.271	-23.701	3.350.670	-22.492
3.357.304	-41.844	3.396.385	-23.702	3.350.837	-22.493
3.357.603	-41.848	3.396.620	-23.702	3.351.075	-22.494
3.357.798	-41.851	3.396.828	-23.703	3.351.235	-22.494
3.357.917	-41.855	3.396.981	-23.704	3.351.353	-22.495
3.357.870	-41.859	3.397.146	-23.705	3.351.555	-22.496
3.358.076	-41.862	3.397.361	-23.705	3.351.653	-22.496
3.358.423	-41.866	3.397.527	-23.706	3.351.765	-22.497
3.358.526	-41.869	3.397.650	-23.707	3.351.975	-22.498

3.358.706	-41.873	3.397.870	-23.707	3.352.148	-22.498
3.358.788	-41.876	3.398.065	-23.708	3.352.310	-22.499
3.359.028	-41.880	3.398.231	-23.709	3.352.451	-22.500
3.359.259	-41.883	3.398.370	-23.709	3.352.534	-22.500
3.359.305	-41.887	3.398.466	-23.710	3.352.664	-22.501
3.358.973	-41.890	3.398.547	-23.710	3.352.914	-22.502
3.359.286	-41.894	3.398.730	-23.711	3.353.190	-22.502
3.359.817	-41.897	3.398.979	-23.711	3.353.367	-22.503
3.359.745	-41.901	3.399.104	-23.712	3.353.464	-22.504
3.360.088	-41.904	3.399.164	-23.713	3.353.710	-22.504
3.360.555	-41.907	3.399.290	-23.713	3.353.961	-22.505
3.360.858	-41.911	3.399.497	-23.714	3.354.096	-22.506
3.361.035	-41.914	3.399.746	-23.714	3.354.236	-22.506
3.361.172	-41.917	3.399.998	-23.715	3.354.352	-22.507
3.361.133	-41.921	3.400.219	-23.715	3.354.515	-22.508
3.361.416	-41.924	3.400.336	-23.716	3.354.680	-22.508
3.361.380	-41.927	3.400.493	-23.716	3.354.810	-22.509
3.361.281	-41.931	3.400.692	-23.717	3.355.006	-22.510
3.361.855	-41.934	3.400.833	-23.717	3.355.190	-22.510
3.362.351	-41.937	3.400.972	-23.718	3.355.338	-22.511
3.362.235	-41.940	3.401.154	-23.718	3.355.522	-22.511
3.362.054	-41.944	3.401.313	-23.719	3.355.696	-22.512
3.362.232	-41.947	3.401.422	-23.719	3.355.840	-22.513
3.362.364	-41.950	3.401.562	-23.720	3.355.995	-22.513
3.362.365	-41.953	3.401.776	-23.720	3.356.194	-22.514
3.362.498	-41.956	3.402.003	-23.721	3.356.375	-22.515
3.362.930	-41.959	3.402.137	-23.721	3.356.503	-22.515
3.363.146	-41.962	3.402.256	-23.722	3.356.662	-22.516
3.363.535	-41.965	3.402.380	-23.722	3.356.896	-22.517
3.363.828	-41.968	3.402.570	-23.723	3.357.072	-22.517

3.363.506	-41.972	3.402.787	-23.723	3.357.213	-22.518
3.363.273	-41.975	3.402.971	-23.724	3.357.379	-22.519
3.363.687	-41.978	3.403.123	-23.725	3.357.546	-22.519
3.364.193	-41.980	3.403.214	-23.725	3.357.718	-22.520
3.364.425	-41.983	3.403.347	-23.726	3.357.882	-22.521
3.364.544	-41.986	3.403.521	-23.726	3.358.041	-22.521
3.364.471	-41.989	3.403.669	-23.727	3.358.185	-22.522
3.365.191	-41.992	3.403.850	-23.728	3.358.334	-22.522
3.365.379	-41.995	3.404.063	-23.729	3.358.492	-22.523
3.364.865	-41.998	3.404.252	-23.729	3.358.669	-22.524
3.365.182	-42.001	3.404.388	-23.730	3.358.839	-22.524
3.365.663	-42.003	3.404.566	-23.731	3.358.983	-22.525
3.365.885	-42.006	3.404.836	-23.731	3.359.149	-22.526
3.366.098	-42.009	3.405.005	-23.732	3.359.348	-22.526
3.366.125	-42.011	3.405.104	-23.733	3.359.532	-22.527
3.366.210	-42.014	3.405.280	-23.734	3.359.732	-22.527
3.366.458	-42.017	3.405.432	-23.735	3.359.948	-22.528
3.366.456	-42.019	3.405.533	-23.736	3.360.097	-22.528
3.366.503	-42.022	3.405.703	-23.737	3.360.212	-22.529
3.366.881	-42.025	3.405.876	-23.737	3.360.385	-22.529
3.367.166	-42.027	3.406.025	-23.738	3.360.531	-22.530
3.367.141	-42.030	3.406.234	-23.739	3.360.663	-22.530
3.367.346	-42.032	3.406.422	-23.740	3.360.797	-22.531
3.367.503	-42.035	3.406.581	-23.741	3.360.930	-22.531
3.367.583	-42.037	3.406.774	-23.742	3.361.093	-22.532
3.367.892	-42.040	3.406.943	-23.743	3.361.223	-22.532
3.368.412	-42.042	3.407.088	-23.744	3.361.277	-22.533
3.368.432	-42.045	3.407.222	-23.745	3.361.447	-22.533
3.368.497	-42.047	3.407.362	-23.746	3.361.701	-22.534
3.368.698	-42.049	3.407.552	-23.748	3.361.870	-22.534

3.368.865	-42.052	3.407.677	-23.749	3.362.083	-22.534
3.369.020	-42.054	3.407.788	-23.750	3.362.314	-22.535
3.369.165	-42.056	3.407.973	-23.751	3.362.520	-22.535
3.369.113	-42.059	3.408.134	-23.752	3.362.747	-22.536
3.369.281	-42.061	3.408.271	-23.753	3.362.912	-22.536
3.369.828	-42.063	3.408.470	-23.755	3.363.051	-22.536
3.370.206	-42.065	3.408.672	-23.756	3.363.286	-22.536
3.370.424	-42.067	3.408.853	-23.757	3.363.486	-22.537
3.370.083	-42.070	3.409.028	-23.758	3.363.640	-22.537
3.370.027	-42.072	3.409.175	-23.760	3.363.789	-22.537
3.370.475	-42.074	3.409.356	-23.761	3.363.905	-22.537
3.370.736	-42.076	3.409.584	-23.763	3.363.984	-22.538
3.370.919	-42.078	3.409.799	-23.764	3.364.060	-22.538
3.370.848	-42.080	3.409.946	-23.765	3.364.211	-22.538
3.370.620	-42.082	3.410.081	-23.767	3.364.433	-22.538
3.371.107	-42.084	3.410.230	-23.768	3.364.601	-22.538
3.371.317	-42.086	3.410.415	-23.770	3.364.760	-22.539
3.371.433	-42.088	3.410.594	-23.771	3.364.933	-22.539
3.372.072	-42.090	3.410.703	-23.773	3.365.128	-22.539
3.372.285	-42.092	3.410.809	-23.775	3.365.292	-22.539
3.372.346	-42.094	3.410.906	-23.776	3.365.450	-22.539
3.372.026	-42.096	3.411.065	-23.778	3.365.701	-22.539
3.372.094	-42.098	3.411.252	-23.780	3.365.927	-22.539
3.372.417	-42.100	3.411.414	-23.781	3.366.080	-22.539
3.372.451	-42.102	3.411.593	-23.783	3.366.245	-22.539
3.372.835	-42.104	3.411.795	-23.785	3.366.432	-22.539
3.373.367	-42.106	3.411.975	-23.786	3.366.566	-22.539
3.373.923	-42.108	3.412.144	-23.788	3.366.747	-22.539
3.374.110	-42.110	3.412.350	-23.790	3.366.946	-22.539
3.374.219	-42.111	3.412.542	-23.792	3.367.058	-22.539

3.374.240	-42.113	3.412.704	-23.794	3.367.159	-22.539
3.374.395	-42.115	3.412.888	-23.796	3.367.334	-22.539
3.374.439	-42.117	3.413.034	-23.798	3.367.549	-22.539
3.374.352	-42.119	3.413.186	-23.800	3.367.680	-22.539
3.374.398	-42.121	3.413.370	-23.802	3.367.820	-22.539
3.374.804	-42.122	3.413.565	-23.804	3.367.993	-22.538
3.374.826	-42.124	3.413.719	-23.806	3.368.189	-22.538
3.374.389	-42.126	3.413.855	-23.808	3.368.354	-22.538
3.375.010	-42.128	3.414.032	-23.810	3.368.522	-22.538
3.375.660	-42.129	3.414.178	-23.812	3.368.727	-22.538
3.375.516	-42.131	3.414.315	-23.814	3.368.882	-22.538
3.375.215	-42.133	3.414.562	-23.817	3.369.102	-22.537
3.375.135	-42.134	3.414.814	-23.819	3.369.297	-22.537
3.375.855	-42.136	3.414.958	-23.821	3.369.388	-22.537
3.376.823	-42.138	3.415.128	-23.823	3.369.572	-22.537
3.376.430	-42.140	3.415.251	-23.826	3.369.764	-22.536
3.375.907	-42.141	3.415.367	-23.828	3.369.923	-22.536
3.376.672	-42.143	3.415.555	-23.830	3.370.117	-22.536
3.377.268	-42.145	3.415.739	-23.833	3.370.328	-22.536
3.377.076	-42.146	3.415.919	-23.835	3.370.533	-22.535
3.376.914	-42.148	3.416.093	-23.838	3.370.624	-22.535
3.377.611	-42.150	3.416.252	-23.840	3.370.710	-22.535
3.378.080	-42.152	3.416.382	-23.843	3.370.858	-22.534
3.377.677	-42.153	3.416.524	-23.845	3.371.061	-22.534
3.377.343	-42.155	3.416.714	-23.848	3.371.477	-22.534
3.377.878	-42.157	3.416.889	-23.850	3.371.989	-22.533
3.378.445	-42.158	3.417.084	-23.853	3.372.177	-22.533
3.378.322	-42.160	3.417.269	-23.856	3.372.164	-22.532
3.378.683	-42.162	3.417.422	-23.858	3.372.245	-22.532
3.378.773	-42.164	3.417.636	-23.861	3.372.286	-22.532

3.378.633	-42.166	3.417.791	-23.864	3.372.322	-22.531
3.378.525	-42.167	3.417.856	-23.866	3.372.440	-22.531
3.378.795	-42.169	3.418.058	-23.869	3.372.614	-22.530
3.379.221	-42.171	3.418.529	-23.872	3.372.727	-22.530
3.379.467	-42.173	3.418.914	-23.875	3.372.825	-22.529
3.379.978	-42.175	3.419.052	-23.877	3.373.047	-22.529
3.379.298	-42.176	3.419.103	-23.880	3.373.250	-22.528
3.379.378	-42.178	3.419.146	-23.883	3.373.366	-22.528
3.379.745	-42.180	3.419.245	-23.886	3.373.479	-22.527
3.379.662	-42.182	3.419.302	-23.889	3.373.680	-22.527
3.380.042	-42.184	3.419.366	-23.892	3.373.889	-22.526
3.381.145	-42.186	3.419.425	-23.895	3.374.070	-22.526
3.381.201	-42.188	3.419.566	-23.898	3.374.254	-22.525
3.381.593	-42.189	3.419.778	-23.901	3.374.409	-22.525
3.381.703	-42.191	3.420.005	-23.904	3.374.551	-22.524
3.380.475	-42.193	3.420.187	-23.907	3.374.753	-22.524
3.381.904	-42.195	3.420.368	-23.910	3.374.926	-22.523
3.382.672	-42.197	3.420.566	-23.913	3.375.058	-22.523
3.382.518	-42.199	3.420.739	-23.916	3.375.201	-22.522
3.382.440	-42.201	3.420.972	-23.919	3.375.309	-22.522
3.382.496	-42.203	3.421.122	-23.922	3.375.454	-22.521
3.383.213	-42.205	3.421.274	-23.925	3.375.620	-22.520
3.382.791	-42.207	3.421.416	-23.928	3.375.834	-22.520
3.381.485	-42.209	3.421.538	-23.931	3.376.013	-22.519
3.382.881	-42.212	3.421.709	-23.935	3.376.140	-22.519
3.383.243	-42.214	3.421.863	-23.938	3.376.342	-22.518
3.381.683	-42.216	3.421.997	-23.941	3.376.541	-22.518
3.382.474	-42.218	3.422.090	-23.944	3.376.672	-22.517
3.383.390	-42.220	3.422.249	-23.948	3.376.866	-22.517
3.382.856	-42.222	3.422.451	-23.951	3.377.057	-22.516

3.384.117	-42.224	3.422.619	-23.954	3.377.245	-22.516
3.384.168	-42.227	3.422.836	-23.958	3.377.420	-22.515
3.382.422	-42.229	3.423.055	-23.961	3.377.572	-22.515
3.383.466	-42.231	3.423.221	-23.965	3.377.748	-22.514
3.385.193	-42.233	3.423.338	-23.968	3.377.878	-22.513
3.384.727	-42.236	3.423.542	-23.972	3.378.006	-22.513
3.385.006	-42.238	3.423.774	-23.975	3.378.211	-22.512
3.385.338	-42.240	3.423.954	-23.979	3.378.419	-22.512
3.383.832	-42.242	3.424.095	-23.982	3.378.524	-22.512
3.384.803	-42.245	3.424.244	-23.986	3.378.673	-22.511
3.386.024	-42.247	3.424.468	-23.989	3.378.860	-22.511
3.385.347	-42.249	3.424.663	-23.993	3.379.016	-22.510
3.386.241	-42.252	3.424.792	-23.996	3.379.190	-22.510
3.386.331	-42.254	3.424.926	-24.000	3.379.370	-22.509
3.385.107	-42.256	3.425.079	-24.004	3.379.539	-22.509
3.386.111	-42.259	3.425.150	-24.007	3.379.696	-22.508
3.387.682	-42.261	3.425.227	-24.011	3.379.865	-22.508
3.386.411	-42.264	3.425.439	-24.015	3.379.946	-22.507
3.384.912	-42.266	3.425.695	-24.019	3.380.110	-22.507
3.386.003	-42.268	3.425.860	-24.022	3.380.242	-22.507
3.388.078	-42.271	3.425.981	-24.026	3.380.429	-22.506
3.389.388	-42.273	3.426.137	-24.030	3.380.697	-22.506
3.389.247	-42.276	3.426.310	-24.034	3.380.880	-22.505
3.388.575	-42.278	3.426.515	-24.038	3.380.981	-22.505
3.387.592	-42.280	3.426.690	-24.041	3.381.141	-22.505
3.386.534	-42.283	3.426.844	-24.045	3.381.329	-22.504
3.387.205	-42.285	3.426.972	-24.049	3.381.454	-22.504
3.389.146	-42.288	3.427.120	-24.053	3.381.638	-22.503
3.389.057	-42.290	3.427.281	-24.057	3.381.819	-22.503
3.387.300	-42.293	3.427.467	-24.061	3.381.938	-22.503

3.388.291	-42.295	3.427.655	-24.065	3.382.080	-22.502
3.390.620	-42.298	3.427.796	-24.069	3.382.278	-22.502
3.390.429	-42.300	3.428.018	-24.073	3.382.513	-22.502
3.389.099	-42.302	3.428.218	-24.077	3.382.711	-22.501
3.389.295	-42.305	3.428.372	-24.081	3.382.805	-22.501
3.389.330	-42.307	3.428.623	-24.085	3.382.919	-22.501
3.389.930	-42.310	3.428.799	-24.089	3.383.146	-22.500
3.390.179	-42.312	3.428.929	-24.093	3.383.283	-22.500
3.389.364	-42.315	3.429.088	-24.097	3.383.403	-22.500
3.390.343	-42.317	3.429.186	-24.101	3.383.567	-22.499
3.390.454	-42.320	3.429.323	-24.105	3.383.719	-22.499
3.389.734	-42.322	3.429.472	-24.109	3.383.875	-22.499
3.390.139	-42.325	3.429.642	-24.113	3.384.023	-22.499
3.390.454	-42.327	3.429.858	-24.117	3.384.153	-22.498
3.391.227	-42.329	3.430.070	-24.121	3.384.294	-22.498
3.391.490	-42.332	3.430.208	-24.125	3.384.475	-22.498
3.391.555	-42.334	3.430.354	-24.129	3.384.675	-22.497
3.391.273	-42.337	3.430.536	-24.133	3.384.839	-22.497
3.390.703	-42.339	3.430.675	-24.137	3.385.023	-22.497
3.392.570	-42.342	3.430.812	-24.141	3.385.222	-22.496
3.393.009	-42.344	3.430.978	-24.145	3.385.454	-22.496
3.392.011	-42.347	3.431.115	-24.149	3.385.668	-22.495
3.392.785	-42.349	3.431.237	-24.153	3.385.854	-22.495
3.392.481	-42.352	3.431.385	-24.157	3.386.035	-22.495
3.392.498	-42.354	3.431.491	-24.161	3.386.166	-22.494
3.393.102	-42.357	3.431.644	-24.165	3.386.295	-22.494
3.392.778	-42.359	3.431.833	-24.169	3.386.443	-22.494
3.392.993	-42.362	3.432.003	-24.173	3.386.636	-22.493
3.393.103	-42.364	3.432.206	-24.177	3.386.768	-22.493
3.393.116	-42.367	3.432.467	-24.181	3.386.899	-22.492

3.393.221	-42.369	3.432.722	-24.185	3.387.058	-22.492
3.393.580	-42.372	3.432.877	-24.189	3.387.236	-22.492
3.393.546	-42.374	3.433.018	-24.192	3.387.408	-22.491
3.394.078	-42.377	3.433.221	-24.196	3.387.547	-22.491
3.394.837	-42.379	3.433.424	-24.200	3.387.751	-22.490
3.394.764	-42.382	3.433.598	-24.204	3.387.955	-22.490
3.394.753	-42.384	3.433.717	-24.208	3.388.123	-22.490
3.394.417	-42.387	3.433.817	-24.212	3.388.276	-22.489
3.394.297	-42.389	3.433.932	-24.216	3.388.481	-22.489
3.395.155	-42.392	3.434.081	-24.220	3.388.649	-22.488
3.395.843	-42.394	3.434.232	-24.223	3.388.782	-22.488
3.395.724	-42.397	3.434.406	-24.227	3.388.999	-22.487
3.396.064	-42.399	3.434.590	-24.231	3.389.122	-22.487
3.395.717	-42.402	3.434.717	-24.235	3.389.265	-22.486
3.395.556	-42.404	3.434.904	-24.238	3.389.503	-22.486
3.395.909	-42.407	3.435.099	-24.242	3.389.649	-22.486
3.395.135	-42.409	3.435.258	-24.246	3.389.772	-22.485
3.396.239	-42.412	3.435.446	-24.249	3.389.951	-22.485
3.397.059	-42.414	3.435.630	-24.253	3.390.141	-22.484
3.396.297	-42.417	3.435.842	-24.257	3.390.325	-22.484
3.397.234	-42.419	3.436.015	-24.260	3.390.468	-22.483
3.397.924	-42.422	3.436.154	-24.264	3.390.602	-22.483
3.396.581	-42.424	3.436.333	-24.268	3.390.782	-22.482
3.396.297	-42.426	3.436.469	-24.271	3.390.960	-22.482
3.397.601	-42.429	3.436.628	-24.275	3.391.143	-22.481
3.399.590	-42.431	3.436.833	-24.278	3.391.314	-22.480
3.400.685	-42.434	3.437.030	-24.282	3.391.456	-22.480
3.399.337	-42.436	3.437.200	-24.285	3.391.615	-22.479
3.397.619	-42.439	3.437.282	-24.289	3.391.727	-22.479
3.397.061	-42.441	3.437.433	-24.292	3.391.862	-22.478

3.398.096	-42.444	3.437.657	-24.296	3.392.122	-22.478
3.400.085	-42.446	3.437.817	-24.299	3.392.279	-22.477
3.399.709	-42.448	3.437.953	-24.303	3.392.388	-22.476
3.397.906	-42.451	3.438.167	-24.306	3.392.597	-22.476
3.398.795	-42.453	3.438.362	-24.309	3.392.774	-22.475
3.399.192	-42.456	3.438.510	-24.313	3.392.947	-22.475
3.399.019	-42.458	3.438.741	-24.316	3.393.077	-22.474
3.400.070	-42.460	3.438.864	-24.319	3.393.163	-22.473
3.400.401	-42.463	3.438.911	-24.323	3.393.380	-22.473
3.399.367	-42.465	3.439.092	-24.326	3.393.605	-22.472
3.400.895	-42.467	3.439.360	-24.329	3.393.723	-22.471
3.402.648	-42.470	3.439.580	-24.332	3.393.865	-22.471
3.401.566	-42.472	3.439.724	-24.335	3.394.255	-22.470
3.400.009	-42.474	3.439.872	-24.339	3.394.671	-22.469
3.399.846	-42.477	3.440.049	-24.342	3.394.881	-22.469
3.401.429	-42.479	3.440.193	-24.345	3.394.993	-22.468
3.402.977	-42.481	3.440.372	-24.348	3.395.085	-22.467
3.402.471	-42.484	3.440.583	-24.351	3.395.115	-22.466
3.400.471	-42.486	3.440.715	-24.354	3.395.161	-22.466
3.400.266	-42.488	3.440.833	-24.357	3.395.334	-22.465
3.401.763	-42.491	3.440.948	-24.360	3.395.493	-22.464
3.402.676	-42.493	3.441.104	-24.363	3.395.601	-22.463
3.403.040	-42.495	3.441.300	-24.366	3.395.742	-22.463
3.403.969	-42.498	3.441.487	-24.369	3.395.910	-22.462
3.404.247	-42.500	3.441.815	-24.372	3.396.082	-22.461
3.402.850	-42.502	3.442.153	-24.375	3.396.259	-22.460
3.402.687	-42.504	3.442.426	-24.378	3.396.441	-22.459
3.404.214	-42.507	3.442.581	-24.381	3.396.647	-22.459
3.403.793	-42.509	3.442.628	-24.384	3.396.831	-22.458
3.403.297	-42.511	3.442.695	-24.387	3.396.957	-22.457

3.403.123	-42.514	3.442.765	-24.390	3.397.066	-22.456
3.403.615	-42.516	3.442.863	-24.393	3.397.231	-22.455
3.403.813	-42.518	3.442.993	-24.396	3.397.411	-22.454
3.403.041	-42.521	3.443.136	-24.399	3.397.565	-22.453
3.404.583	-42.523	3.443.298	-24.401	3.397.730	-22.452
3.405.646	-42.525	3.443.442	-24.404	3.397.965	-22.452
3.404.081	-42.528	3.443.604	-24.407	3.398.177	-22.451
3.403.295	-42.530	3.443.820	-24.410	3.398.250	-22.450
3.404.703	-42.532	3.443.980	-24.413	3.398.348	-22.449
3.404.804	-42.535	3.444.124	-24.416	3.398.499	-22.448
3.404.872	-42.537	3.444.294	-24.418	3.398.691	-22.447
3.404.776	-42.539	3.444.507	-24.421	3.398.941	-22.446
3.404.156	-42.542	3.444.684	-24.424	3.399.109	-22.445
3.405.925	-42.544	3.444.808	-24.427	3.399.218	-22.444
3.406.602	-42.546	3.444.904	-24.429	3.399.409	-22.443
3.404.898	-42.549	3.445.063	-24.432	3.399.586	-22.442
3.404.109	-42.551	3.445.255	-24.435	3.399.742	-22.441
3.404.474	-42.553	3.445.407	-24.438	3.399.969	-22.440
3.405.280	-42.556	3.445.580	-24.441	3.400.161	-22.439
3.407.081	-42.558	3.445.724	-24.443	3.400.291	-22.438
3.409.035	-42.561	3.445.870	-24.446	3.400.429	-22.438
3.409.453	-42.563	3.446.024	-24.449	3.400.584	-22.437
3.409.489	-42.565	3.446.205	-24.452	3.400.713	-22.436
3.409.899	-42.568	3.446.415	-24.454	3.400.874	-22.435
3.410.208	-42.570	3.446.602	-24.457	3.401.033	-22.434
3.410.529	-42.573	3.446.763	-24.460	3.401.221	-22.433
3.409.901	-42.575	3.446.954	-24.463	3.401.436	-22.432
3.408.326	-42.578	3.447.130	-24.466	3.401.591	-22.431
3.408.533	-42.580	3.447.333	-24.468	3.401.736	-22.430
3.408.831	-42.583	3.447.509	-24.471	3.401.917	-22.429

3.407.384	-42.585	3.447.686	-24.474	3.402.145	-22.428
3.407.119	-42.587	3.447.791	-24.477	3.402.315	-22.427
3.408.557	-42.590	3.447.921	-24.480	3.402.365	-22.426
3.410.280	-42.592	3.448.067	-24.483	3.402.482	-22.425
3.411.179	-42.595	3.448.214	-24.486	3.402.708	-22.424
3.411.628	-42.597	3.448.381	-24.488	3.402.890	-22.423
3.410.634	-42.600	3.448.553	-24.491	3.403.150	-22.422
3.409.169	-42.602	3.448.739	-24.494	3.403.365	-22.421
3.409.368	-42.605	3.448.880	-24.497	3.403.489	-22.420
3.411.217	-42.607	3.449.008	-24.500	3.403.633	-22.419
3.411.194	-42.610	3.449.236	-24.503	3.403.778	-22.418
3.409.620	-42.612	3.449.425	-24.506	3.403.907	-22.417
3.409.171	-42.614	3.449.562	-24.509	3.404.109	-22.416
3.410.202	-42.617	3.449.738	-24.512	3.404.327	-22.415
3.412.091	-42.619	3.449.905	-24.515	3.404.456	-22.414
3.412.128	-42.622	3.450.028	-24.518	3.404.601	-22.413
3.410.410	-42.624	3.450.154	-24.521	3.404.792	-22.412
3.411.047	-42.627	3.450.282	-24.524	3.404.952	-22.411
3.411.371	-42.629	3.450.453	-24.527	3.405.093	-22.410
3.411.029	-42.631	3.450.630	-24.530	3.405.266	-22.409
3.411.169	-42.634	3.450.793	-24.533	3.405.415	-22.408
3.411.622	-42.636	3.451.010	-24.536	3.405.565	-22.407
3.412.104	-42.639	3.451.184	-24.539	3.405.736	-22.406
3.412.156	-42.641	3.451.357	-24.543	3.405.938	-22.405
3.412.296	-42.644	3.451.567	-24.546	3.406.122	-22.404
3.412.045	-42.646	3.451.755	-24.549	3.406.293	-22.403
3.412.877	-42.649	3.451.913	-24.552	3.406.422	-22.402
3.413.206	-42.651	3.452.065	-24.555	3.406.567	-22.401
3.413.427	-42.654	3.452.220	-24.558	3.406.723	-22.400
3.414.060	-42.656	3.452.332	-24.562	3.406.823	-22.399

3.413.727	-42.658	3.452.458	-24.565	3.406.933	-22.398
3.413.869	-42.661	3.452.639	-24.568	3.407.094	-22.397
3.413.904	-42.663	3.452.841	-24.571	3.407.268	-22.395
3.413.927	-42.666	3.453.044	-24.574	3.407.440	-22.394
3.414.113	-42.668	3.453.199	-24.578	3.407.664	-22.393
3.414.572	-42.671	3.453.356	-24.581	3.407.864	-22.392
3.414.700	-42.673	3.453.535	-24.584	3.408.004	-22.391
3.414.698	-42.676	3.453.706	-24.587	3.408.185	-22.390
3.414.962	-42.678	3.453.858	-24.591	3.408.344	-22.389
3.414.839	-42.680	3.453.991	-24.594	3.408.546	-22.388
3.414.937	-42.683	3.454.142	-24.597	3.408.781	-22.386
3.415.406	-42.685	3.454.312	-24.601	3.408.904	-22.385
3.415.486	-42.688	3.454.485	-24.604	3.409.024	-22.384
3.415.554	-42.690	3.454.663	-24.607	3.409.272	-22.383
3.415.874	-42.693	3.454.828	-24.611	3.409.480	-22.382
3.416.006	-42.695	3.454.930	-24.614	3.409.497	-22.380
3.416.332	-42.698	3.455.079	-24.617	3.409.547	-22.379
3.416.690	-42.700	3.455.280	-24.621	3.409.702	-22.378
3.416.917	-42.703	3.455.419	-24.624	3.409.924	-22.377
3.417.120	-42.705	3.455.574	-24.627	3.410.092	-22.376
3.417.310	-42.708	3.455.765	-24.631	3.410.284	-22.374
3.417.343	-42.710	3.455.981	-24.634	3.410.471	-22.373
3.417.151	-42.713	3.456.230	-24.637	3.410.605	-22.372
3.417.332	-42.715	3.456.425	-24.641	3.410.818	-22.371
3.417.608	-42.718	3.456.508	-24.644	3.411.017	-22.369
3.417.590	-42.720	3.456.660	-24.647	3.411.219	-22.368
3.417.789	-42.723	3.456.826	-24.651	3.411.458	-22.367
3.418.199	-42.725	3.457.016	-24.654	3.411.631	-22.365
3.418.279	-42.728	3.457.204	-24.658	3.411.780	-22.364
3.418.394	-42.731	3.457.393	-24.661	3.411.888	-22.363

3.418.684	-42.733	3.457.477	-24.664	3.412.079	-22.361
3.418.767	-42.736	3.457.561	-24.668	3.412.245	-22.360
3.418.887	-42.739	3.457.740	-24.671	3.412.402	-22.359
3.419.034	-42.741	3.457.859	-24.675	3.412.570	-22.357
3.419.041	-42.744	3.458.011	-24.678	3.412.733	-22.356
3.419.193	-42.747	3.458.195	-24.681	3.412.926	-22.355
3.419.612	-42.750	3.458.333	-24.685	3.413.048	-22.353
3.419.879	-42.752	3.458.546	-24.688	3.413.215	-22.352
3.420.082	-42.755	3.458.752	-24.691	3.413.414	-22.350
3.420.182	-42.758	3.458.936	-24.695	3.413.571	-22.349
3.420.133	-42.761	3.459.133	-24.698	3.413.684	-22.348
3.420.284	-42.764	3.459.326	-24.702	3.413.818	-22.346
3.420.440	-42.766	3.459.487	-24.705	3.414.039	-22.345
3.420.494	-42.769	3.459.608	-24.708	3.414.229	-22.343
3.420.786	-42.772	3.459.789	-24.712	3.414.371	-22.342
3.421.014	-42.775	3.460.011	-24.715	3.414.606	-22.341
3.420.981	-42.778	3.460.195	-24.719	3.414.805	-22.339
3.421.252	-42.781	3.460.378	-24.722	3.414.897	-22.338
3.421.627	-42.784	3.460.540	-24.725	3.415.052	-22.336
3.421.711	-42.787	3.460.638	-24.729	3.415.206	-22.335
3.421.733	-42.790	3.460.793	-24.732	3.415.334	-22.333
3.421.824	-42.793	3.460.952	-24.735	3.415.567	-22.332
3.422.326	-42.796	3.461.104	-24.739	3.415.764	-22.331
3.422.737	-42.799	3.461.271	-24.742	3.415.873	-22.329
3.422.962	-42.803	3.461.481	-24.746	3.415.984	-22.328
3.423.276	-42.806	3.461.629	-24.749	3.416.172	-22.326
3.423.322	-42.809	3.461.761	-24.752	3.416.387	-22.325
3.423.336	-42.812	3.461.994	-24.756	3.416.472	-22.323
3.423.547	-42.815	3.462.216	-24.759	3.416.665	-22.322
3.423.492	-42.819	3.462.365	-24.762	3.417.039	-22.320

3.423.503	-42.822	3.462.495	-24.766	3.417.449	-22.319
3.423.753	-42.825	3.462.713	-24.769	3.417.709	-22.317
3.423.958	-42.829	3.462.886	-24.773	3.417.773	-22.316
3.423.625	-42.832	3.463.044	-24.776	3.417.836	-22.314
3.423.560	-42.835	3.463.200	-24.779	3.417.859	-22.313
3.424.698	-42.839	3.463.336	-24.783	3.417.924	-22.311
3.424.731	-42.842	3.463.521	-24.786	3.418.048	-22.310
3.424.345	-42.846	3.463.728	-24.789	3.418.130	-22.308
3.424.709	-42.849	3.463.925	-24.793	3.418.266	-22.307
3.425.067	-42.853	3.464.059	-24.796	3.418.492	-22.305
3.425.490	-42.856	3.464.113	-24.799	3.418.716	-22.303
3.425.728	-42.860	3.464.247	-24.803	3.418.940	-22.302
3.425.772	-42.863	3.464.418	-24.806	3.419.120	-22.300
3.425.732	-42.867	3.464.579	-24.809	3.419.272	-22.299
3.425.632	-42.871	3.464.782	-24.813	3.419.460	-22.297
3.425.742	-42.874	3.465.166	-24.816	3.419.587	-22.296
3.425.784	-42.878	3.465.649	-24.820	3.419.729	-22.294
3.425.620	-42.882	3.465.924	-24.823	3.419.933	-22.293
3.426.237	-42.885	3.466.008	-24.826	3.420.086	-22.291
3.426.985	-42.889	3.466.066	-24.830	3.420.251	-22.290
3.426.515	-42.893	3.466.104	-24.833	3.420.421	-22.288
3.426.397	-42.897	3.466.112	-24.836	3.420.520	-22.286
3.427.614	-42.901	3.466.183	-24.840	3.420.634	-22.285
3.427.910	-42.905	3.466.349	-24.843	3.420.837	-22.283
3.427.350	-42.909	3.466.502	-24.847	3.421.028	-22.282
3.427.641	-42.913	3.466.625	-24.850	3.421.133	-22.280
3.428.316	-42.917	3.466.745	-24.853	3.421.248	-22.279
3.428.127	-42.921	3.466.882	-24.857	3.421.441	-22.277
3.427.723	-42.925	3.467.061	-24.860	3.421.600	-22.276
3.428.356	-42.929	3.467.258	-24.864	3.421.762	-22.274

3.428.893	-42.933	3.467.477	-24.867	3.421.987	-22.273
3.429.117	-42.938	3.467.641	-24.870	3.422.205	-22.271
3.428.851	-42.942	3.467.788	-24.874	3.422.404	-22.270
3.428.962	-42.946	3.467.951	-24.877	3.422.553	-22.268
3.429.399	-42.950	3.468.124	-24.881	3.422.701	-22.267
3.429.245	-42.955	3.468.276	-24.884	3.422.823	-22.265
3.429.201	-42.959	3.468.452	-24.888	3.422.971	-22.264
3.429.261	-42.964	3.468.643	-24.891	3.423.118	-22.263
3.429.721	-42.968	3.468.853	-24.895	3.423.289	-22.261
3.429.875	-42.972	3.468.990	-24.898	3.423.502	-22.260
3.429.998	-42.977	3.469.052	-24.902	3.423.723	-22.258
3.430.083	-42.981	3.469.225	-24.905	3.423.854	-22.257
3.430.241	-42.986	3.469.368	-24.909	3.423.927	-22.255
3.430.652	-42.991	3.469.456	-24.913	3.424.056	-22.254
3.430.999	-42.995	3.469.633	-24.916	3.424.239	-22.253
3.431.101	-43.000	3.469.846	-24.920	3.424.422	-22.251
3.431.039	-43.004	3.470.032	-24.923	3.424.640	-22.250
3.431.340	-43.009	3.470.269	-24.927	3.424.834	-22.249
3.431.389	-43.014	3.470.501	-24.931	3.424.976	-22.247
3.431.676	-43.019	3.470.683	-24.935	3.425.081	-22.246
3.431.909	-43.023	3.470.859	-24.938	3.425.212	-22.245
3.431.651	-43.028	3.470.967	-24.942	3.425.410	-22.244
3.431.821	-43.033	3.471.087	-24.946	3.425.609	-22.242
3.432.068	-43.038	3.471.250	-24.950	3.425.854	-22.241
3.432.236	-43.043	3.471.429	-24.953	3.426.021	-22.240
3.432.823	-43.048	3.471.593	-24.957	3.426.125	-22.239
3.432.951	-43.053	3.471.780	-24.961	3.426.273	-22.237
3.432.708	-43.058	3.471.974	-24.965	3.426.443	-22.236
3.432.575	-43.063	3.472.120	-24.969	3.426.693	-22.235
3.432.701	-43.068	3.472.239	-24.973	3.426.892	-22.234

3.433.601	-43.073	3.472.343	-24.977	3.427.050	-22.233
3.434.237	-43.078	3.472.498	-24.981	3.427.199	-22.231
3.434.283	-43.083	3.472.690	-24.985	3.427.372	-22.230
3.434.628	-43.088	3.472.874	-24.989	3.427.531	-22.229
3.435.457	-43.093	3.473.055	-24.993	3.427.639	-22.228
3.435.188	-43.098	3.473.232	-24.997	3.427.799	-22.227
3.434.568	-43.103	3.473.414	-25.001	3.428.029	-22.226
3.435.247	-43.108	3.473.589	-25.005	3.428.222	-22.225
3.435.067	-43.113	3.473.727	-25.009	3.428.381	-22.223
3.435.415	-43.118	3.473.896	-25.013	3.428.531	-22.222
3.435.869	-43.123	3.474.045	-25.017	3.428.679	-22.221
3.435.404	-43.128	3.474.230	-25.021	3.428.828	-22.220
3.435.435	-43.133	3.474.413	-25.026	3.428.994	-22.219
3.435.616	-43.138	3.474.568	-25.030	3.429.118	-22.218
3.435.797	-43.143	3.474.781	-25.034	3.429.241	-22.217
3.436.862	-43.148	3.474.926	-25.038	3.429.395	-22.216
3.436.631	-43.153	3.475.035	-25.043	3.429.541	-22.214
3.435.356	-43.159	3.475.188	-25.047	3.429.641	-22.213
3.436.618	-43.164	3.475.375	-25.051	3.429.702	-22.212
3.436.563	-43.169	3.475.558	-25.056	3.429.908	-22.211
3.436.342	-43.174	3.475.719	-25.060	3.430.126	-22.210
3.437.478	-43.179	3.475.845	-25.064	3.430.335	-22.209
3.437.493	-43.183	3.476.017	-25.069	3.430.558	-22.208
3.437.834	-43.188	3.476.220	-25.073	3.430.735	-22.207
3.436.792	-43.193	3.476.400	-25.078	3.430.945	-22.206
3.435.844	-43.198	3.476.581	-25.082	3.431.082	-22.204
3.437.581	-43.203	3.476.754	-25.086	3.431.187	-22.203
3.439.125	-43.208	3.476.909	-25.091	3.431.361	-22.202
3.439.518	-43.213	3.477.079	-25.095	3.431.638	-22.201
3.440.201	-43.218	3.477.256	-25.100	3.431.851	-22.200

3.440.455	-43.223	3.477.369	-25.104	3.431.963	-22.199
3.438.676	-43.227	3.477.520	-25.109	3.432.079	-22.198
3.437.202	-43.232	3.477.769	-25.113	3.432.202	-22.196
3.437.180	-43.237	3.477.939	-25.118	3.432.343	-22.195
3.438.608	-43.242	3.478.008	-25.122	3.432.481	-22.194
3.440.068	-43.246	3.478.107	-25.127	3.432.603	-22.193
3.438.800	-43.251	3.478.266	-25.131	3.432.841	-22.192
3.437.323	-43.256	3.478.441	-25.136	3.433.084	-22.191
3.437.495	-43.260	3.478.615	-25.140	3.433.278	-22.189
3.438.271	-43.265	3.478.813	-25.145	3.433.390	-22.188
3.438.943	-43.270	3.478.998	-25.150	3.433.488	-22.187
3.439.539	-43.274	3.479.157	-25.154	3.433.708	-22.186
3.439.996	-43.279	3.479.337	-25.159	3.433.920	-22.185
3.440.281	-43.283	3.479.617	-25.163	3.434.099	-22.183
3.439.711	-43.288	3.479.876	-25.168	3.434.290	-22.182
3.438.887	-43.292	3.480.023	-25.172	3.434.500	-22.181
3.439.738	-43.297	3.480.146	-25.177	3.434.684	-22.180
3.440.966	-43.301	3.480.267	-25.181	3.434.840	-22.179
3.440.516	-43.305	3.480.461	-25.186	3.435.009	-22.177
3.439.897	-43.310	3.480.653	-25.191	3.435.179	-22.176
3.440.984	-43.314	3.480.794	-25.195	3.435.363	-22.175
3.442.983	-43.318	3.480.894	-25.200	3.435.493	-22.174
3.444.103	-43.323	3.481.011	-25.204	3.435.659	-22.172
3.442.943	-43.327	3.481.187	-25.209	3.435.837	-22.171
3.442.043	-43.331	3.481.335	-25.213	3.435.972	-22.170
3.443.112	-43.335	3.481.461	-25.218	3.436.104	-22.169
3.442.565	-43.340	3.481.628	-25.222	3.436.306	-22.167
3.442.449	-43.344	3.481.784	-25.227	3.436.523	-22.166
3.443.294	-43.348	3.481.974	-25.231	3.436.638	-22.165
3.442.184	-43.352	3.482.206	-25.236	3.436.790	-22.164

3.441.385	-43.356	3.482.358	-25.240	3.437.000	-22.162
3.441.967	-43.360	3.482.507	-25.245	3.437.166	-22.161
3.444.223	-43.364	3.482.701	-25.249	3.437.305	-22.160
3.445.833	-43.368	3.482.937	-25.254	3.437.476	-22.159
3.445.617	-43.372	3.483.136	-25.258	3.437.669	-22.157
3.445.439	-43.376	3.483.260	-25.263	3.437.817	-22.156
3.446.032	-43.380	3.483.394	-25.267	3.437.939	-22.155
3.446.516	-43.384	3.483.555	-25.272	3.438.015	-22.153
3.445.057	-43.388	3.483.752	-25.276	3.438.178	-22.152
3.444.021	-43.391	3.483.947	-25.280	3.438.430	-22.151
3.445.457	-43.395	3.484.095	-25.285	3.438.629	-22.149
3.446.527	-43.399	3.484.229	-25.289	3.438.775	-22.148
3.445.291	-43.403	3.484.402	-25.294	3.439.008	-22.147
3.445.602	-43.406	3.484.606	-25.298	3.439.175	-22.146
3.447.637	-43.410	3.484.767	-25.302	3.439.263	-22.144
3.447.328	-43.413	3.484.870	-25.307	3.439.429	-22.143
3.445.847	-43.417	3.485.035	-25.311	3.439.792	-22.142
3.444.933	-43.421	3.485.194	-25.315	3.440.187	-22.140
3.444.590	-43.424	3.485.400	-25.320	3.440.444	-22.139
3.445.516	-43.428	3.485.643	-25.324	3.440.538	-22.138
3.447.370	-43.431	3.485.823	-25.328	3.440.573	-22.136
3.447.542	-43.435	3.486.021	-25.333	3.440.576	-22.135
3.446.725	-43.438	3.486.143	-25.337	3.440.627	-22.134
3.447.451	-43.442	3.486.273	-25.341	3.440.765	-22.132
3.448.341	-43.445	3.486.451	-25.346	3.440.866	-22.131
3.448.799	-43.448	3.486.632	-25.350	3.441.031	-22.130
3.449.642	-43.452	3.486.797	-25.354	3.441.242	-22.128
3.449.677	-43.455	3.486.917	-25.358	3.441.393	-22.127
3.448.355	-43.458	3.487.112	-25.362	3.441.571	-22.126
3.447.025	-43.462	3.487.300	-25.367	3.441.737	-22.124

3.446.797	-43.465	3.487.446	-25.371	3.441.953	-22.123
3.447.401	-43.468	3.487.653	-25.375	3.442.144	-22.122
3.448.741	-43.471	3.487.870	-25.379	3.442.267	-22.120
3.450.482	-43.475	3.488.008	-25.383	3.442.419	-22.119
3.450.682	-43.478	3.488.112	-25.388	3.442.597	-22.118
3.449.352	-43.481	3.488.289	-25.392	3.442.771	-22.116
3.448.174	-43.484	3.488.691	-25.396	3.442.921	-22.115
3.447.646	-43.487	3.489.111	-25.400	3.443.094	-22.114
3.447.639	-43.490	3.489.283	-25.404	3.443.257	-22.113
3.448.962	-43.493	3.489.348	-25.408	3.443.401	-22.111
3.451.143	-43.496	3.489.403	-25.413	3.443.589	-22.110
3.450.856	-43.500	3.489.501	-25.417	3.443.750	-22.109
3.450.177	-43.503	3.489.575	-25.421	3.443.889	-22.108
3.451.794	-43.506	3.489.583	-25.425	3.444.091	-22.106
3.452.279	-43.509	3.489.651	-25.429	3.444.276	-22.105
3.450.547	-43.512	3.489.799	-25.433	3.444.416	-22.104
3.449.522	-43.515	3.490.005	-25.437	3.444.608	-22.103
3.449.977	-43.518	3.490.252	-25.441	3.444.789	-22.101
3.450.588	-43.521	3.490.424	-25.445	3.444.949	-22.100
3.452.408	-43.524	3.490.544	-25.450	3.445.150	-22.099
3.453.739	-43.527	3.490.713	-25.454	3.445.327	-22.098
3.453.308	-43.530	3.490.909	-25.458	3.445.498	-22.097
3.453.670	-43.533	3.491.093	-25.462	3.445.663	-22.096
3.454.633	-43.536	3.491.231	-25.466	3.445.764	-22.095
3.454.837	-43.539	3.491.414	-25.470	3.445.920	-22.094
3.453.923	-43.542	3.491.602	-25.474	3.446.123	-22.093
3.452.102	-43.545	3.491.676	-25.478	3.446.277	-22.092
3.451.595	-43.548	3.491.844	-25.483	3.446.411	-22.091
3.453.344	-43.551	3.492.101	-25.487	3.446.523	-22.090
3.453.708	-43.554	3.492.263	-25.491	3.446.642	-22.089

3.452.686	-43.557	3.492.446	-25.495	3.446.837	-22.088
3.453.699	-43.561	3.492.593	-25.499	3.447.090	-22.087
3.453.789	-43.564	3.492.715	-25.503	3.447.250	-22.086
3.452.507	-43.567	3.492.874	-25.507	3.447.323	-22.085
3.452.846	-43.570	3.493.012	-25.512	3.447.463	-22.085
3.454.877	-43.573	3.493.188	-25.516	3.447.672	-22.084
3.456.115	-43.576	3.493.409	-25.520	3.447.860	-22.083
3.454.893	-43.579	3.493.665	-25.524	3.448.031	-22.082
3.454.724	-43.582	3.493.813	-25.529	3.448.192	-22.082
3.455.455	-43.586	3.493.912	-25.533	3.448.247	-22.081
3.454.328	-43.589	3.494.109	-25.537	3.448.423	-22.080
3.454.722	-43.592	3.494.378	-25.541	3.448.735	-22.080
3.455.682	-43.595	3.494.536	-25.546	3.448.948	-22.079
3.454.586	-43.599	3.494.634	-25.550	3.449.120	-22.079
3.455.031	-43.602	3.494.753	-25.554	3.449.254	-22.078
3.455.891	-43.605	3.494.926	-25.559	3.449.399	-22.078
3.454.839	-43.608	3.495.067	-25.563	3.449.597	-22.077
3.454.157	-43.612	3.495.182	-25.567	3.449.742	-22.077
3.454.577	-43.615	3.495.309	-25.572	3.449.868	-22.076
3.455.334	-43.619	3.495.466	-25.576	3.450.074	-22.076
3.456.750	-43.622	3.495.671	-25.580	3.450.253	-22.076
3.458.246	-43.625	3.495.870	-25.585	3.450.435	-22.075
3.457.919	-43.629	3.496.086	-25.589	3.450.594	-22.075
3.456.044	-43.632	3.496.230	-25.594	3.450.743	-22.075
3.455.735	-43.636	3.496.373	-25.598	3.450.924	-22.075
3.458.015	-43.639	3.496.549	-25.603	3.451.049	-22.074
3.458.691	-43.643	3.496.679	-25.607	3.451.205	-22.074
3.456.725	-43.646	3.496.812	-25.612	3.451.373	-22.074
3.456.508	-43.650	3.496.950	-25.617	3.451.570	-22.074
3.458.573	-43.653	3.497.094	-25.621	3.451.790	-22.074

3.459.395	-43.657	3.497.250	-25.626	3.451.907	-22.074
3.457.654	-43.660	3.497.477	-25.631	3.452.011	-22.074
3.456.495	-43.664	3.497.637	-25.635	3.452.195	-22.074
3.457.101	-43.668	3.497.794	-25.640	3.452.351	-22.073
3.459.114	-43.671	3.498.004	-25.645	3.452.481	-22.073
3.460.135	-43.675	3.498.157	-25.649	3.452.621	-22.074
3.459.276	-43.679	3.498.282	-25.654	3.452.729	-22.074
3.460.295	-43.682	3.498.423	-25.659	3.452.890	-22.074
3.461.141	-43.686	3.498.565	-25.664	3.453.105	-22.074
3.459.485	-43.690	3.498.770	-25.669	3.453.294	-22.074
3.458.271	-43.693	3.498.900	-25.673	3.453.475	-22.074
3.459.279	-43.697	3.499.131	-25.678	3.453.738	-22.074
3.461.490	-43.701	3.499.344	-25.683	3.453.952	-22.074
3.462.090	-43.705	3.499.500	-25.688	3.454.120	-22.074
3.460.650	-43.708	3.499.651	-25.693	3.454.287	-22.075
3.459.602	-43.712	3.499.821	-25.698	3.454.467	-22.075
3.459.713	-43.716	3.499.981	-25.703	3.454.586	-22.075
3.460.294	-43.720	3.500.093	-25.708	3.454.754	-22.075
3.461.801	-43.724	3.500.294	-25.713	3.454.995	-22.075
3.461.925	-43.727	3.500.495	-25.718	3.455.112	-22.076
3.461.611	-43.731	3.500.616	-25.723	3.455.179	-22.076
3.461.971	-43.735	3.500.764	-25.728	3.455.336	-22.076
3.462.216	-43.739	3.500.899	-25.733	3.455.469	-22.077
3.461.768	-43.743	3.501.013	-25.738	3.455.609	-22.077
3.462.123	-43.747	3.501.202	-25.743	3.455.841	-22.077
3.463.880	-43.751	3.501.397	-25.748	3.456.051	-22.077
3.463.213	-43.754	3.501.481	-25.753	3.456.163	-22.078
3.463.522	-43.758	3.501.613	-25.758	3.456.255	-22.078
3.463.786	-43.762	3.501.805	-25.763	3.456.474	-22.079
3.462.820	-43.766	3.502.029	-25.769	3.456.714	-22.079

3.464.031	-43.770	3.502.202	-25.774	3.456.898	-22.079
3.463.933	-43.774	3.502.318	-25.779	3.457.137	-22.080
3.463.271	-43.778	3.502.495	-25.784	3.457.304	-22.080
3.464.839	-43.782	3.502.738	-25.789	3.457.390	-22.080
3.464.709	-43.786	3.503.009	-25.794	3.457.565	-22.081
3.464.400	-43.790	3.503.232	-25.799	3.457.794	-22.081
3.465.067	-43.794	3.503.394	-25.805	3.457.961	-22.082
3.463.800	-43.798	3.503.596	-25.810	3.458.092	-22.082
3.462.787	-43.802	3.503.807	-25.815	3.458.258	-22.083
3.462.979	-43.806	3.503.930	-25.820	3.458.459	-22.083
3.462.932	-43.810	3.504.071	-25.825	3.458.638	-22.084
3.462.997	-43.814	3.504.186	-25.830	3.458.806	-22.084
3.464.317	-43.818	3.504.315	-25.835	3.458.966	-22.084
3.466.307	-43.822	3.504.489	-25.840	3.459.097	-22.085
3.465.888	-43.826	3.504.604	-25.846	3.459.236	-22.085
3.465.168	-43.830	3.504.742	-25.851	3.459.461	-22.086
3.466.902	-43.834	3.504.836	-25.856	3.459.635	-22.086
3.467.279	-43.838	3.504.964	-25.861	3.459.753	-22.087
3.465.906	-43.842	3.505.213	-25.866	3.459.916	-22.087
3.465.016	-43.846	3.505.435	-25.871	3.460.097	-22.088
3.465.347	-43.850	3.505.589	-25.876	3.460.282	-22.088
3.467.480	-43.854	3.505.768	-25.881	3.460.403	-22.089
3.469.416	-43.858	3.505.954	-25.886	3.460.565	-22.090
3.469.738	-43.862	3.506.125	-25.891	3.460.804	-22.090
3.468.539	-43.866	3.506.296	-25.896	3.461.007	-22.091
3.466.498	-43.870	3.506.479	-25.901	3.461.172	-22.091
3.465.536	-43.874	3.506.609	-25.906	3.461.371	-22.092
3.466.014	-43.878	3.506.750	-25.911	3.461.527	-22.092
3.467.723	-43.883	3.506.976	-25.916	3.461.649	-22.093
3.467.874	-43.887	3.507.144	-25.921	3.461.826	-22.093

3.467.742	-43.891	3.507.321	-25.926	3.462.003	-22.094
3.469.720	-43.895	3.507.505	-25.931	3.462.101	-22.094
3.469.703	-43.899	3.507.672	-25.936	3.462.263	-22.095
3.469.587	-43.903	3.507.842	-25.941	3.462.673	-22.096
3.469.449	-43.907	3.507.980	-25.946	3.463.055	-22.096
3.468.600	-43.911	3.508.102	-25.950	3.463.253	-22.097
3.470.009	-43.915	3.508.286	-25.955	3.463.354	-22.097
3.471.680	-43.919	3.508.542	-25.960	3.463.423	-22.098
3.472.149	-43.924	3.508.748	-25.965	3.463.497	-22.098
3.471.172	-43.928	3.508.967	-25.970	3.463.524	-22.099
3.469.716	-43.932	3.509.081	-25.975	3.463.554	-22.099
3.468.922	-43.936	3.509.120	-25.979	3.463.698	-22.100
3.468.413	-43.940	3.509.317	-25.984	3.463.860	-22.100
3.468.890	-43.944	3.509.568	-25.989	3.464.023	-22.101
3.470.363	-43.948	3.509.720	-25.994	3.464.189	-22.102
3.471.725	-43.952	3.509.848	-25.998	3.464.339	-22.102
3.471.719	-43.957	3.510.056	-26.003	3.464.532	-22.103
3.470.511	-43.961	3.510.237	-26.008	3.464.739	-22.103
3.469.810	-43.965	3.510.378	-26.012	3.464.897	-22.104
3.471.194	-43.969	3.510.544	-26.017	3.465.103	-22.104
3.473.399	-43.973	3.510.773	-26.022	3.465.332	-22.105
3.474.214	-43.977	3.510.988	-26.026	3.465.502	-22.105
3.473.846	-43.982	3.511.143	-26.031	3.465.649	-22.106
3.472.673	-43.986	3.511.274	-26.035	3.465.807	-22.106
3.471.660	-43.990	3.511.427	-26.040	3.465.953	-22.107
3.471.379	-43.994	3.511.548	-26.045	3.466.053	-22.107
3.471.342	-43.998	3.511.703	-26.049	3.466.266	-22.108
3.471.349	-44.002	3.512.112	-26.054	3.466.505	-22.109
3.471.206	-44.006	3.512.542	-26.058	3.466.617	-22.109
3.471.127	-44.011	3.512.770	-26.063	3.466.732	-22.110

3.471.539	-44.015	3.512.867	-26.067	3.466.904	-22.110
3.471.838	-44.019	3.512.889	-26.072	3.467.083	-22.111
3.471.774	-44.023	3.512.946	-26.076	3.467.305	-22.111
3.471.902	-44.027	3.513.037	-26.081	3.467.509	-22.112
3.473.539	-44.032	3.513.179	-26.085	3.467.670	-22.112
3.475.630	-44.036	3.513.333	-26.089	3.467.894	-22.113
3.476.477	-44.040	3.513.388	-26.094	3.468.056	-22.113
3.476.412	-44.044	3.513.459	-26.098	3.468.149	-22.114
3.476.499	-44.048	3.513.638	-26.103	3.468.230	-22.114
3.476.812	-44.053	3.513.864	-26.107	3.468.387	-22.115
3.477.011	-44.057	3.514.046	-26.111	3.468.636	-22.115
3.477.285	-44.061	3.514.182	-26.116	3.468.810	-22.116
3.477.505	-44.065	3.514.378	-26.120	3.468.929	-22.116
3.476.415	-44.070	3.514.609	-26.124	3.469.092	-22.117
3.474.393	-44.074	3.514.779	-26.129	3.469.316	-22.117
3.473.543	-44.078	3.514.902	-26.133	3.469.454	-22.118
3.474.006	-44.082	3.515.061	-26.137	3.469.570	-22.118
3.475.453	-44.087	3.515.193	-26.142	3.469.789	-22.118
3.477.435	-44.091	3.515.347	-26.146	3.469.964	-22.119
3.478.217	-44.095	3.515.569	-26.150	3.470.117	-22.119
3.476.931	-44.100	3.515.713	-26.154	3.470.294	-22.120
3.476.794	-44.104	3.515.818	-26.159	3.470.428	-22.120
3.477.845	-44.108	3.515.909	-26.163	3.470.556	-22.121
3.476.609	-44.113	3.516.120	-26.167	3.470.748	-22.121
3.475.495	-44.117	3.516.308	-26.171	3.470.937	-22.122
3.476.544	-44.122	3.516.469	-26.176	3.471.079	-22.122
3.477.366	-44.126	3.516.579	-26.180	3.471.155	-22.123
3.476.987	-44.130	3.516.697	-26.184	3.471.306	-22.123
3.477.070	-44.135	3.516.918	-26.188	3.471.579	-22.124
3.477.599	-44.139	3.517.162	-26.193	3.471.781	-22.124

3.477.282	-44.144	3.517.330	-26.197	3.471.939	-22.125
3.476.597	-44.148	3.517.589	-26.201	3.472.098	-22.125
3.476.378	-44.153	3.517.832	-26.205	3.472.278	-22.125
3.476.411	-44.158	3.517.930	-26.210	3.472.464	-22.126
3.476.671	-44.162	3.518.063	-26.214	3.472.630	-22.126
3.477.097	-44.167	3.518.235	-26.218	3.472.816	-22.127
3.477.896	-44.171	3.518.391	-26.222	3.472.984	-22.127
3.478.690	-44.176	3.518.578	-26.227	3.473.109	-22.128
3.478.604	-44.181	3.518.750	-26.231	3.473.286	-22.128
3.478.396	-44.186	3.518.885	-26.235	3.473.430	-22.128
3.480.331	-44.190	3.519.031	-26.239	3.473.601	-22.129
3.482.397	-44.195	3.519.207	-26.243	3.473.850	-22.129
3.481.823	-44.200	3.519.348	-26.248	3.474.023	-22.130
3.480.099	-44.205	3.519.469	-26.252	3.474.171	-22.130
3.479.109	-44.209	3.519.641	-26.256	3.474.341	-22.130
3.480.674	-44.214	3.519.797	-26.260	3.474.566	-22.131
3.482.340	-44.219	3.520.034	-26.265	3.474.758	-22.131
3.481.556	-44.224	3.520.242	-26.269	3.474.858	-22.131
3.480.683	-44.229	3.520.328	-26.273	3.474.969	-22.131
3.480.450	-44.234	3.520.483	-26.277	3.475.052	-22.132
3.480.443	-44.239	3.520.689	-26.282	3.475.175	-22.132
3.480.783	-44.244	3.520.825	-26.286	3.475.417	-22.132
3.482.354	-44.249	3.520.957	-26.290	3.475.576	-22.132
3.483.442	-44.254	3.521.131	-26.294	3.475.701	-22.133
3.481.978	-44.259	3.521.320	-26.299	3.475.870	-22.133
3.480.683	-44.264	3.521.530	-26.303	3.476.004	-22.133
3.481.184	-44.269	3.521.736	-26.307	3.476.195	-22.133
3.481.911	-44.274	3.521.939	-26.311	3.476.412	-22.133
3.482.019	-44.279	3.522.134	-26.316	3.476.582	-22.133
3.482.384	-44.284	3.522.283	-26.320	3.476.809	-22.133

3.483.569	-44.290	3.522.408	-26.324	3.477.047	-22.134
3.485.046	-44.295	3.522.589	-26.328	3.477.224	-22.134
3.486.083	-44.300	3.522.771	-26.333	3.477.406	-22.134
3.485.807	-44.305	3.522.907	-26.337	3.477.575	-22.134
3.483.997	-44.310	3.523.000	-26.341	3.477.688	-22.134
3.483.272	-44.315	3.523.163	-26.346	3.477.799	-22.134
3.484.814	-44.321	3.523.378	-26.350	3.477.986	-22.133
3.484.843	-44.326	3.523.497	-26.354	3.478.157	-22.133
3.484.302	-44.331	3.523.654	-26.358	3.478.239	-22.133
3.485.417	-44.336	3.523.842	-26.362	3.478.366	-22.133
3.486.458	-44.342	3.524.035	-26.367	3.478.478	-22.133
3.486.736	-44.347	3.524.239	-26.371	3.478.641	-22.133
3.485.910	-44.352	3.524.409	-26.375	3.478.846	-22.133
3.484.539	-44.357	3.524.563	-26.379	3.479.015	-22.132
3.484.363	-44.363	3.524.706	-26.383	3.479.225	-22.132
3.485.032	-44.368	3.524.897	-26.387	3.479.467	-22.132
3.485.670	-44.373	3.525.060	-26.392	3.479.646	-22.131
3.486.636	-44.379	3.525.128	-26.396	3.479.821	-22.131
3.486.506	-44.384	3.525.173	-26.400	3.479.995	-22.131
3.485.444	-44.389	3.525.320	-26.404	3.480.078	-22.130
3.485.862	-44.395	3.525.519	-26.408	3.480.295	-22.130
3.487.376	-44.400	3.525.743	-26.412	3.480.574	-22.130
3.486.906	-44.405	3.525.934	-26.416	3.480.717	-22.129
3.485.555	-44.411	3.526.115	-26.420	3.480.837	-22.129
3.485.364	-44.416	3.526.351	-26.424	3.481.024	-22.128
3.485.663	-44.421	3.526.579	-26.428	3.481.198	-22.128
3.486.867	-44.427	3.526.827	-26.432	3.481.373	-22.127
3.488.926	-44.432	3.527.022	-26.435	3.481.602	-22.127
3.488.854	-44.437	3.527.137	-26.439	3.481.779	-22.126
3.487.690	-44.443	3.527.263	-26.443	3.481.924	-22.126

3.488.990	-44.448	3.527.452	-26.447	3.482.050	-22.125
3.489.577	-44.453	3.527.592	-26.451	3.482.185	-22.125
3.488.297	-44.459	3.527.688	-26.454	3.482.343	-22.124
3.488.557	-44.464	3.527.879	-26.458	3.482.558	-22.124
3.489.395	-44.469	3.528.038	-26.462	3.482.752	-22.123
3.488.846	-44.474	3.528.098	-26.465	3.482.926	-22.122
3.488.403	-44.480	3.528.233	-26.469	3.483.089	-22.122
3.488.871	-44.485	3.528.456	-26.473	3.483.250	-22.121
3.489.312	-44.490	3.528.607	-26.476	3.483.365	-22.120
3.489.818	-44.496	3.528.763	-26.480	3.483.448	-22.120
3.490.502	-44.501	3.528.932	-26.483	3.483.644	-22.119
3.490.222	-44.506	3.529.149	-26.487	3.483.858	-22.118
3.490.370	-44.511	3.529.388	-26.490	3.484.055	-22.117
3.490.426	-44.517	3.529.577	-26.493	3.484.218	-22.117
3.490.518	-44.522	3.529.768	-26.497	3.484.417	-22.116
3.491.205	-44.527	3.529.944	-26.500	3.484.623	-22.115
3.491.501	-44.532	3.530.082	-26.503	3.484.729	-22.114
3.491.100	-44.537	3.530.176	-26.507	3.484.804	-22.114
3.491.279	-44.543	3.530.349	-26.510	3.484.955	-22.113
3.493.286	-44.548	3.530.576	-26.513	3.485.244	-22.112
3.494.160	-44.553	3.530.725	-26.516	3.485.667	-22.111
3.492.646	-44.558	3.530.841	-26.519	3.486.022	-22.110
3.491.841	-44.563	3.530.974	-26.522	3.486.161	-22.110
3.492.881	-44.569	3.531.167	-26.526	3.486.220	-22.109
3.492.131	-44.574	3.531.385	-26.529	3.486.299	-22.108
3.491.115	-44.579	3.531.521	-26.532	3.486.317	-22.107
3.491.683	-44.584	3.531.665	-26.535	3.486.383	-22.106
3.493.188	-44.589	3.531.849	-26.538	3.486.530	-22.105
3.494.370	-44.594	3.532.075	-26.541	3.486.689	-22.105
3.493.474	-44.599	3.532.303	-26.543	3.486.792	-22.104

3.493.387	-44.604	3.532.452	-26.546	3.486.895	-22.103
3.493.909	-44.609	3.532.629	-26.549	3.487.079	-22.102
3.492.975	-44.614	3.532.762	-26.552	3.487.238	-22.101
3.493.905	-44.619	3.532.910	-26.555	3.487.420	-22.100
3.494.622	-44.624	3.533.128	-26.558	3.487.628	-22.100
3.494.687	-44.629	3.533.260	-26.560	3.487.800	-22.099
3.494.651	-44.634	3.533.427	-26.563	3.487.990	-22.098
3.495.083	-44.639	3.533.683	-26.566	3.488.163	-22.097
3.495.100	-44.644	3.533.876	-26.569	3.488.268	-22.096
3.494.226	-44.649	3.533.976	-26.571	3.488.484	-22.096
3.495.985	-44.654	3.534.124	-26.574	3.488.661	-22.095
3.497.430	-44.659	3.534.294	-26.577	3.488.811	-22.094
3.496.510	-44.664	3.534.433	-26.579	3.488.908	-22.093
3.494.924	-44.669	3.534.609	-26.582	3.489.102	-22.093
3.494.579	-44.674	3.534.797	-26.584	3.489.315	-22.092
3.495.764	-44.679	3.534.995	-26.587	3.489.438	-22.091
3.497.413	-44.683	3.535.191	-26.590	3.489.583	-22.091
3.498.753	-44.688	3.535.389	-26.592	3.489.778	-22.090
3.499.262	-44.693	3.535.760	-26.595	3.489.938	-22.089
3.498.896	-44.698	3.536.122	-26.597	3.490.164	-22.089
3.498.195	-44.703	3.536.310	-26.600	3.490.396	-22.088
3.498.135	-44.707	3.536.354	-26.602	3.490.559	-22.087
3.498.732	-44.712	3.536.394	-26.605	3.490.656	-22.087
3.498.496	-44.717	3.536.494	-26.607	3.490.747	-22.086
3.498.721	-44.722	3.536.553	-26.610	3.490.941	-22.086
3.499.630	-44.726	3.536.635	-26.612	3.491.155	-22.085
3.498.843	-44.731	3.536.722	-26.614	3.491.346	-22.085
3.496.914	-44.736	3.536.852	-26.617	3.491.510	-22.084
3.497.670	-44.740	3.537.008	-26.619	3.491.656	-22.084
3.499.360	-44.745	3.537.211	-26.622	3.491.846	-22.083

3.498.354	-44.750	3.537.430	-26.624	3.491.975	-22.083
3.498.331	-44.754	3.537.607	-26.627	3.492.062	-22.083
3.498.745	-44.759	3.537.769	-26.629	3.492.278	-22.082
3.498.568	-44.764	3.537.921	-26.631	3.492.424	-22.082
3.498.594	-44.768	3.538.148	-26.634	3.492.547	-22.082
3.499.045	-44.773	3.538.349	-26.636	3.492.781	-22.081
3.499.472	-44.778	3.538.506	-26.639	3.492.957	-22.081
3.499.175	-44.782	3.538.629	-26.641	3.493.083	-22.081
3.500.352	-44.787	3.538.764	-26.644	3.493.275	-22.081
3.500.038	-44.792	3.538.932	-26.646	3.493.452	-22.081
3.498.748	-44.796	3.539.113	-26.649	3.493.558	-22.081
3.500.428	-44.801	3.539.305	-26.651	3.493.723	-22.081
3.501.116	-44.806	3.539.479	-26.654	3.493.844	-22.081
3.499.805	-44.810	3.539.651	-26.656	3.494.017	-22.081
3.500.620	-44.815	3.539.764	-26.659	3.494.333	-22.081
3.501.212	-44.820	3.539.911	-26.661	3.494.587	-22.081
3.500.544	-44.825	3.540.074	-26.664	3.494.714	-22.081
3.500.133	-44.829	3.540.216	-26.667	3.494.834	-22.081
3.501.414	-44.834	3.540.406	-26.669	3.494.963	-22.081
3.502.498	-44.839	3.540.576	-26.672	3.495.125	-22.081
3.503.347	-44.844	3.540.746	-26.675	3.495.273	-22.082
3.502.701	-44.849	3.541.021	-26.677	3.495.428	-22.082
3.501.560	-44.853	3.541.242	-26.680	3.495.649	-22.082
3.502.664	-44.858	3.541.375	-26.683	3.495.834	-22.082
3.503.934	-44.863	3.541.503	-26.686	3.495.984	-22.083
3.503.474	-44.868	3.541.597	-26.688	3.496.129	-22.083
3.501.856	-44.873	3.541.776	-26.691	3.496.331	-22.083
3.502.520	-44.878	3.541.907	-26.694	3.496.487	-22.084
3.504.760	-44.883	3.542.072	-26.697	3.496.643	-22.084
3.505.551	-44.888	3.542.279	-26.700	3.496.819	-22.085

3.504.696	-44.893	3.542.458	-26.703	3.496.967	-22.085
3.503.698	-44.898	3.542.570	-26.706	3.497.159	-22.086
3.503.120	-44.903	3.542.705	-26.709	3.497.341	-22.086
3.503.775	-44.908	3.542.926	-26.712	3.497.517	-22.087
3.505.489	-44.913	3.543.091	-26.715	3.497.659	-22.087
3.505.266	-44.918	3.543.171	-26.719	3.497.782	-22.088
3.503.409	-44.923	3.543.329	-26.722	3.497.912	-22.088
3.503.994	-44.928	3.543.558	-26.725	3.498.098	-22.089
3.505.291	-44.934	3.543.678	-26.728	3.498.244	-22.089
3.505.329	-44.939	3.543.842	-26.732	3.498.333	-22.090
3.504.840	-44.944	3.544.034	-26.735	3.498.481	-22.091
3.504.323	-44.949	3.544.208	-26.738	3.498.680	-22.091
3.504.428	-44.955	3.544.338	-26.742	3.498.844	-22.092
3.505.681	-44.960	3.544.467	-26.745	3.499.093	-22.093
3.506.143	-44.966	3.544.680	-26.749	3.499.377	-22.093
3.505.043	-44.971	3.544.898	-26.752	3.499.529	-22.094
3.504.456	-44.977	3.545.063	-26.756	3.499.659	-22.095
3.506.255	-44.982	3.545.253	-26.760	3.499.828	-22.095
3.507.126	-44.988	3.545.383	-26.763	3.500.043	-22.096
3.506.563	-44.994	3.545.537	-26.767	3.500.213	-22.097
3.505.894	-44.999	3.545.695	-26.771	3.500.329	-22.097
3.507.651	-45.005	3.545.789	-26.775	3.500.441	-22.098
3.507.789	-45.011	3.545.992	-26.779	3.500.614	-22.099
3.506.638	-45.017	3.546.154	-26.782	3.500.786	-22.099
3.507.275	-45.022	3.546.263	-26.786	3.500.924	-22.100
3.507.639	-45.028	3.546.432	-26.790	3.501.036	-22.101
3.506.674	-45.034	3.546.653	-26.794	3.501.274	-22.101
3.507.709	-45.040	3.546.831	-26.798	3.501.411	-22.102
3.508.651	-45.046	3.546.971	-26.802	3.501.501	-22.103
3.507.794	-45.052	3.547.133	-26.806	3.501.664	-22.103

3.507.867	-45.058	3.547.308	-26.810	3.501.904	-22.104
3.508.329	-45.065	3.547.484	-26.815	3.502.110	-22.104
3.508.228	-45.071	3.547.632	-26.819	3.502.286	-22.105
3.509.086	-45.077	3.547.827	-26.823	3.502.422	-22.106
3.508.983	-45.083	3.548.022	-26.827	3.502.621	-22.106
3.509.445	-45.090	3.548.165	-26.831	3.502.864	-22.107
3.510.294	-45.096	3.548.301	-26.836	3.503.048	-22.108
3.509.375	-45.102	3.548.323	-26.840	3.503.203	-22.108
3.509.677	-45.109	3.548.470	-26.844	3.503.378	-22.109
3.509.946	-45.115	3.548.766	-26.848	3.503.547	-22.109
3.509.667	-45.122	3.548.871	-26.853	3.503.698	-22.110
3.509.864	-45.128	3.548.995	-26.857	3.503.832	-22.110
3.509.735	-45.135	3.549.276	-26.861	3.503.934	-22.111
3.510.497	-45.141	3.549.507	-26.866	3.504.189	-22.111
3.511.362	-45.148	3.549.637	-26.870	3.504.403	-22.112
3.510.574	-45.155	3.549.804	-26.874	3.504.534	-22.112
3.510.757	-45.162	3.550.096	-26.879	3.504.724	-22.113
3.511.650	-45.168	3.550.353	-26.883	3.504.884	-22.113
3.510.930	-45.175	3.550.515	-26.887	3.505.078	-22.114
3.511.299	-45.182	3.550.642	-26.892	3.505.255	-22.114
3.512.556	-45.189	3.550.801	-26.896	3.505.394	-22.115
3.512.509	-45.196	3.550.970	-26.900	3.505.576	-22.115
3.512.546	-45.203	3.551.112	-26.905	3.505.725	-22.115
3.513.123	-45.210	3.551.214	-26.909	3.505.862	-22.116
3.512.625	-45.217	3.551.284	-26.914	3.506.071	-22.116
3.512.347	-45.224	3.551.458	-26.918	3.506.261	-22.116
3.511.927	-45.231	3.551.603	-26.922	3.506.386	-22.117
3.512.206	-45.239	3.551.754	-26.927	3.506.534	-22.117
3.512.589	-45.246	3.551.953	-26.931	3.506.727	-22.117
3.512.326	-45.253	3.552.116	-26.935	3.506.924	-22.117

3.513.042	-45.260	3.552.297	-26.940	3.507.079	-22.118
3.514.092	-45.267	3.552.470	-26.944	3.507.281	-22.118
3.514.046	-45.275	3.552.665	-26.948	3.507.488	-22.118
3.513.749	-45.282	3.552.893	-26.953	3.507.599	-22.118
3.513.629	-45.290	3.553.101	-26.957	3.507.665	-22.118
3.513.615	-45.297	3.553.232	-26.961	3.507.870	-22.118
3.513.973	-45.304	3.553.424	-26.965	3.508.295	-22.119
3.514.136	-45.312	3.553.640	-26.970	3.508.676	-22.119
3.514.096	-45.319	3.553.699	-26.974	3.508.862	-22.119
3.514.774	-45.327	3.553.808	-26.978	3.508.922	-22.119
3.514.598	-45.334	3.554.091	-26.982	3.508.958	-22.119
3.513.990	-45.342	3.554.288	-26.986	3.509.056	-22.119
3.515.346	-45.349	3.554.366	-26.991	3.509.157	-22.119
3.515.555	-45.357	3.554.515	-26.995	3.509.232	-22.118
3.514.785	-45.365	3.554.687	-26.999	3.509.398	-22.118
3.515.804	-45.372	3.554.917	-27.003	3.509.507	-22.118
3.516.028	-45.380	3.555.146	-27.007	3.509.572	-22.118
3.514.913	-45.387	3.555.277	-27.011	3.509.754	-22.118
3.515.182	-45.395	3.555.446	-27.015	3.509.962	-22.118
3.516.942	-45.403	3.555.649	-27.019	3.510.186	-22.117
3.518.410	-45.410	3.555.844	-27.023	3.510.392	-22.117
3.517.047	-45.418	3.555.977	-27.027	3.510.565	-22.117
3.515.826	-45.426	3.556.089	-27.031	3.510.743	-22.117
3.516.595	-45.434	3.556.270	-27.035	3.510.894	-22.116
3.516.763	-45.441	3.556.354	-27.038	3.511.082	-22.116
3.516.165	-45.449	3.556.516	-27.042	3.511.275	-22.116
3.516.942	-45.457	3.556.740	-27.046	3.511.403	-22.115
3.517.997	-45.465	3.556.931	-27.050	3.511.498	-22.115
3.518.643	-45.472	3.557.151	-27.054	3.511.581	-22.114
3.519.151	-45.480	3.557.304	-27.057	3.511.772	-22.114

3.519.931	-45.488	3.557.491	-27.061	3.512.023	-22.113
3.520.476	-45.496	3.557.684	-27.065	3.512.198	-22.113
3.519.338	-45.503	3.557.823	-27.068	3.512.269	-22.112
3.517.868	-45.511	3.558.002	-27.072	3.512.460	-22.112
3.517.077	-45.519	3.558.193	-27.075	3.512.672	-22.111
3.517.079	-45.527	3.558.347	-27.079	3.512.861	-22.111
3.517.386	-45.535	3.558.507	-27.082	3.513.067	-22.110
3.518.958	-45.542	3.558.651	-27.086	3.513.286	-22.109
3.520.813	-45.550	3.558.938	-27.089	3.513.486	-22.109
3.521.224	-45.558	3.559.400	-27.093	3.513.658	-22.108
3.519.450	-45.566	3.559.713	-27.096	3.513.777	-22.107
3.518.092	-45.573	3.559.819	-27.100	3.513.886	-22.107
3.518.373	-45.581	3.559.888	-27.103	3.514.059	-22.106
3.518.710	-45.589	3.559.940	-27.106	3.514.258	-22.105
3.518.802	-45.597	3.559.984	-27.110	3.514.409	-22.104
3.519.078	-45.604	3.559.988	-27.113	3.514.518	-22.104
3.520.414	-45.612	3.560.118	-27.116	3.514.673	-22.103
3.521.014	-45.620	3.560.289	-27.119	3.514.857	-22.102
3.521.022	-45.628	3.560.424	-27.123	3.515.067	-22.101
3.522.185	-45.635	3.560.583	-27.126	3.515.234	-22.101
3.524.167	-45.643	3.560.736	-27.129	3.515.383	-22.100
3.524.292	-45.651	3.560.914	-27.132	3.515.596	-22.099
3.524.333	-45.658	3.561.065	-27.135	3.515.769	-22.098
3.523.560	-45.666	3.561.255	-27.138	3.515.943	-22.097
3.522.016	-45.674	3.561.443	-27.141	3.516.083	-22.096
3.521.039	-45.681	3.561.592	-27.144	3.516.212	-22.096
3.521.039	-45.689	3.561.786	-27.147	3.516.383	-22.095
3.522.025	-45.696	3.561.985	-27.150	3.516.550	-22.094
3.523.127	-45.704	3.562.169	-27.153	3.516.709	-22.093
3.524.154	-45.711	3.562.293	-27.156	3.516.900	-22.092

3.525.125	-45.719	3.562.434	-27.159	3.517.094	-22.091
3.524.757	-45.726	3.562.625	-27.162	3.517.229	-22.090
3.523.240	-45.734	3.562.781	-27.165	3.517.401	-22.090
3.522.271	-45.741	3.562.975	-27.168	3.517.625	-22.089
3.523.051	-45.749	3.563.177	-27.171	3.517.802	-22.088
3.523.445	-45.756	3.563.262	-27.173	3.517.929	-22.087
3.522.679	-45.764	3.563.377	-27.176	3.518.060	-22.086
3.524.261	-45.771	3.563.568	-27.179	3.518.190	-22.085
3.526.134	-45.779	3.563.763	-27.182	3.518.341	-22.084
3.526.267	-45.786	3.563.961	-27.185	3.518.495	-22.083
3.524.743	-45.793	3.564.151	-27.188	3.518.670	-22.082
3.523.528	-45.801	3.564.301	-27.190	3.518.883	-22.081
3.523.943	-45.808	3.564.498	-27.193	3.519.096	-22.080
3.524.031	-45.816	3.564.713	-27.196	3.519.306	-22.079
3.523.623	-45.823	3.564.880	-27.199	3.519.483	-22.079
3.523.999	-45.830	3.565.046	-27.202	3.519.624	-22.078
3.524.962	-45.837	3.565.157	-27.205	3.519.702	-22.077
3.526.351	-45.845	3.565.298	-27.207	3.519.846	-22.076
3.526.173	-45.852	3.565.428	-27.210	3.520.070	-22.075
3.525.170	-45.859	3.565.620	-27.213	3.520.244	-22.074
3.524.442	-45.867	3.565.808	-27.216	3.520.349	-22.073
3.524.515	-45.874	3.565.956	-27.219	3.520.483	-22.072
3.524.895	-45.881	3.566.137	-27.222	3.520.674	-22.071
3.526.019	-45.888	3.566.255	-27.224	3.520.812	-22.070
3.527.126	-45.895	3.566.328	-27.227	3.520.946	-22.069
3.528.168	-45.902	3.566.537	-27.230	3.521.091	-22.068
3.528.569	-45.910	3.566.761	-27.233	3.521.259	-22.067
3.527.178	-45.917	3.566.982	-27.236	3.521.433	-22.066
3.526.425	-45.924	3.567.159	-27.239	3.521.667	-22.065
3.528.008	-45.931	3.567.238	-27.242	3.521.864	-22.064

3.529.297	-45.938	3.567.361	-27.245	3.522.011	-22.063
3.528.698	-45.945	3.567.575	-27.248	3.522.247	-22.062
3.527.744	-45.952	3.567.763	-27.251	3.522.520	-22.061
3.529.316	-45.959	3.567.914	-27.254	3.522.666	-22.060
3.529.735	-45.966	3.568.012	-27.257	3.522.778	-22.059
3.528.395	-45.973	3.568.177	-27.260	3.523.037	-22.058
3.528.531	-45.980	3.568.427	-27.263	3.523.269	-22.056
3.528.748	-45.987	3.568.607	-27.266	3.523.396	-22.055
3.527.932	-45.994	3.568.708	-27.269	3.523.456	-22.054
3.528.419	-46.001	3.568.857	-27.272	3.523.517	-22.053
3.529.754	-46.008	3.569.088	-27.276	3.523.640	-22.052
3.531.437	-46.015	3.569.222	-27.279	3.523.840	-22.051
3.531.733	-46.022	3.569.355	-27.282	3.524.001	-22.050
3.530.078	-46.029	3.569.532	-27.285	3.524.189	-22.049
3.529.857	-46.035	3.569.727	-27.288	3.524.353	-22.048
3.531.407	-46.042	3.569.865	-27.292	3.524.490	-22.046
3.532.820	-46.049	3.569.982	-27.295	3.524.696	-22.045
3.533.018	-46.056	3.570.147	-27.298	3.524.865	-22.044
3.532.402	-46.063	3.570.345	-27.301	3.525.052	-22.043
3.531.902	-46.070	3.570.533	-27.305	3.525.262	-22.042
3.531.195	-46.076	3.570.757	-27.308	3.525.430	-22.041
3.530.433	-46.083	3.570.925	-27.312	3.525.616	-22.039
3.530.912	-46.090	3.571.035	-27.315	3.525.758	-22.038
3.532.798	-46.097	3.571.208	-27.318	3.525.858	-22.037
3.533.753	-46.103	3.571.376	-27.322	3.526.060	-22.036
3.533.979	-46.110	3.571.526	-27.325	3.526.274	-22.035
3.533.887	-46.117	3.571.691	-27.329	3.526.452	-22.033
3.532.332	-46.123	3.571.852	-27.332	3.526.577	-22.032
3.531.530	-46.130	3.571.945	-27.335	3.526.718	-22.031
3.533.373	-46.137	3.572.007	-27.339	3.526.900	-22.030

3.534.344	-46.143	3.572.144	-27.342	3.527.087	-22.029
3.533.463	-46.150	3.572.352	-27.346	3.527.279	-22.027
3.532.982	-46.156	3.572.583	-27.350	3.527.456	-22.026
3.534.734	-46.163	3.572.758	-27.353	3.527.583	-22.025
3.534.984	-46.169	3.572.961	-27.357	3.527.705	-22.024
3.534.132	-46.176	3.573.179	-27.360	3.527.908	-22.023
3.533.170	-46.182	3.573.396	-27.364	3.528.095	-22.021
3.534.771	-46.189	3.573.573	-27.367	3.528.277	-22.020
3.536.149	-46.195	3.573.750	-27.371	3.528.525	-22.019
3.534.967	-46.202	3.573.962	-27.374	3.528.727	-22.018
3.533.572	-46.208	3.574.133	-27.378	3.528.900	-22.016
3.533.049	-46.214	3.574.299	-27.382	3.529.110	-22.015
3.534.627	-46.221	3.574.432	-27.385	3.529.305	-22.014
3.536.206	-46.227	3.574.594	-27.389	3.529.409	-22.013
3.535.544	-46.233	3.574.747	-27.393	3.529.487	-22.012
3.535.830	-46.239	3.574.836	-27.396	3.529.666	-22.010
3.535.558	-46.246	3.574.962	-27.400	3.529.880	-22.009
3.534.877	-46.252	3.575.164	-27.403	3.530.035	-22.008
3.535.880	-46.258	3.575.331	-27.407	3.530.144	-22.007
3.535.923	-46.264	3.575.446	-27.411	3.530.306	-22.006
3.536.008	-46.270	3.575.623	-27.414	3.530.435	-22.004
3.536.564	-46.276	3.575.819	-27.418	3.530.692	-22.003
3.536.562	-46.282	3.576.010	-27.421	3.531.118	-22.002
3.537.109	-46.289	3.576.203	-27.425	3.531.487	-22.001
3.537.592	-46.295	3.576.383	-27.429	3.531.714	-22.000
3.536.950	-46.301	3.576.568	-27.432	3.531.772	-21.999
3.536.823	-46.307	3.576.751	-27.436	3.531.791	-21.997
3.538.081	-46.312	3.576.873	-27.440	3.531.801	-21.996
3.537.727	-46.318	3.577.040	-27.443	3.531.860	-21.995
3.536.750	-46.324	3.577.231	-27.447	3.531.956	-21.994

3.538.217	-46.330	3.577.408	-27.450	3.532.094	-21.993
3.539.093	-46.336	3.577.592	-27.454	3.532.225	-21.992
3.538.282	-46.342	3.577.755	-27.458	3.532.350	-21.991
3.538.828	-46.348	3.577.917	-27.461	3.532.586	-21.990
3.539.427	-46.353	3.578.065	-27.465	3.532.839	-21.989
3.538.817	-46.359	3.578.235	-27.468	3.533.019	-21.988
3.538.533	-46.365	3.578.383	-27.472	3.533.156	-21.987
3.539.106	-46.370	3.578.554	-27.476	3.533.304	-21.986
3.538.645	-46.376	3.578.752	-27.479	3.533.472	-21.985
3.539.251	-46.382	3.578.920	-27.483	3.533.647	-21.984
3.539.674	-46.387	3.579.099	-27.486	3.533.825	-21.983
3.539.150	-46.393	3.579.308	-27.490	3.533.988	-21.982
3.539.836	-46.398	3.579.475	-27.493	3.534.143	-21.981
3.540.267	-46.404	3.579.626	-27.497	3.534.294	-21.980
3.540.363	-46.409	3.579.794	-27.501	3.534.359	-21.979
3.540.678	-46.415	3.579.984	-27.504	3.534.505	-21.978
3.540.634	-46.420	3.580.139	-27.508	3.534.729	-21.977
3.540.472	-46.426	3.580.248	-27.511	3.534.891	-21.976
3.541.091	-46.431	3.580.418	-27.514	3.535.031	-21.975
3.541.156	-46.436	3.580.645	-27.518	3.535.206	-21.975
3.541.125	-46.442	3.580.837	-27.521	3.535.407	-21.974
3.540.747	-46.447	3.580.971	-27.525	3.535.581	-21.973
3.541.397	-46.452	3.581.127	-27.528	3.535.829	-21.972
3.542.105	-46.458	3.581.300	-27.532	3.536.042	-21.971
3.541.444	-46.463	3.581.450	-27.535	3.536.169	-21.971
3.542.014	-46.468	3.581.617	-27.538	3.536.310	-21.970
3.543.730	-46.473	3.581.723	-27.541	3.536.538	-21.969
3.543.821	-46.478	3.581.852	-27.545	3.536.776	-21.969
3.542.264	-46.483	3.582.072	-27.548	3.536.885	-21.968
3.543.130	-46.489	3.582.426	-27.551	3.536.993	-21.967

3.543.690	-46.494	3.582.852	-27.554	3.537.153	-21.967
3.542.829	-46.499	3.583.170	-27.557	3.537.350	-21.966
3.544.208	-46.504	3.583.277	-27.561	3.537.484	-21.966
3.544.832	-46.509	3.583.311	-27.564	3.537.610	-21.965
3.543.161	-46.514	3.583.398	-27.567	3.537.788	-21.965
3.542.863	-46.519	3.583.456	-27.570	3.537.975	-21.964
3.544.955	-46.524	3.583.579	-27.573	3.538.110	-21.964
3.545.775	-46.529	3.583.723	-27.576	3.538.235	-21.963
3.544.128	-46.534	3.583.855	-27.578	3.538.490	-21.963
3.543.388	-46.539	3.583.943	-27.581	3.538.698	-21.963
3.544.747	-46.544	3.584.086	-27.584	3.538.797	-21.962
3.544.938	-46.549	3.584.290	-27.587	3.538.965	-21.962
3.544.124	-46.554	3.584.510	-27.590	3.539.124	-21.962
3.544.977	-46.558	3.584.669	-27.592	3.539.203	-21.961
3.546.137	-46.563	3.584.834	-27.595	3.539.297	-21.961
3.547.299	-46.568	3.584.988	-27.598	3.539.456	-21.961
3.546.552	-46.573	3.585.139	-27.600	3.539.689	-21.961
3.546.111	-46.578	3.585.335	-27.603	3.539.858	-21.961
3.547.729	-46.583	3.585.486	-27.606	3.540.052	-21.960
3.546.996	-46.587	3.585.591	-27.608	3.540.177	-21.960
3.545.526	-46.592	3.585.755	-27.611	3.540.367	-21.960
3.546.852	-46.597	3.585.956	-27.613	3.540.602	-21.960
3.548.275	-46.602	3.586.075	-27.616	3.540.815	-21.960
3.547.596	-46.607	3.586.230	-27.618	3.540.967	-21.960
3.547.341	-46.611	3.586.375	-27.620	3.541.047	-21.960
3.547.484	-46.616	3.586.477	-27.623	3.541.144	-21.960
3.547.487	-46.621	3.586.629	-27.625	3.541.346	-21.960
3.547.982	-46.625	3.586.855	-27.627	3.541.548	-21.960
3.547.362	-46.630	3.587.068	-27.630	3.541.722	-21.961
3.547.578	-46.635	3.587.254	-27.632	3.541.902	-21.961

3.548.102	-46.640	3.587.467	-27.634	3.542.041	-21.961
3.548.431	-46.644	3.587.676	-27.636	3.542.224	-21.961
3.548.658	-46.649	3.587.827	-27.638	3.542.451	-21.961
3.548.255	-46.654	3.587.946	-27.641	3.542.585	-21.961
3.548.763	-46.659	3.588.098	-27.643	3.542.657	-21.962
3.549.622	-46.663	3.588.301	-27.645	3.542.818	-21.962
3.549.469	-46.668	3.588.492	-27.647	3.543.030	-21.962
3.548.820	-46.673	3.588.631	-27.649	3.543.197	-21.963
3.549.209	-46.678	3.588.810	-27.651	3.543.311	-21.963
3.549.433	-46.683	3.588.979	-27.653	3.543.420	-21.963
3.550.175	-46.687	3.589.171	-27.655	3.543.564	-21.964
3.551.440	-46.692	3.589.330	-27.657	3.543.721	-21.964
3.550.602	-46.697	3.589.417	-27.659	3.543.904	-21.964
3.550.663	-46.702	3.589.601	-27.661	3.544.096	-21.965
3.551.161	-46.707	3.589.691	-27.663	3.544.341	-21.965
3.551.217	-46.712	3.589.804	-27.665	3.544.583	-21.966
3.551.358	-46.716	3.590.009	-27.667	3.544.799	-21.966
3.551.568	-46.721	3.590.208	-27.669	3.544.995	-21.967
3.552.011	-46.726	3.590.392	-27.671	3.545.204	-21.967
3.552.881	-46.731	3.590.558	-27.673	3.545.426	-21.967
3.551.756	-46.736	3.590.699	-27.675	3.545.641	-21.968
3.551.331	-46.741	3.590.888	-27.678	3.545.811	-21.968
3.551.703	-46.746	3.591.060	-27.680	3.545.924	-21.969
3.551.495	-46.751	3.591.181	-27.682	3.546.051	-21.969
3.552.290	-46.756	3.591.385	-27.684	3.546.205	-21.970
3.552.422	-46.761	3.591.563	-27.686	3.546.315	-21.970
3.552.643	-46.767	3.591.693	-27.688	3.546.400	-21.971
3.552.832	-46.772	3.591.860	-27.690	3.546.570	-21.972
3.553.586	-46.777	3.592.059	-27.692	3.546.714	-21.972
3.553.741	-46.782	3.592.286	-27.694	3.546.855	-21.973

3.553.658	-46.787	3.592.498	-27.696	3.547.050	-21.973
3.553.271	-46.793	3.592.651	-27.698	3.547.202	-21.974
3.553.781	-46.798	3.592.729	-27.700	3.547.376	-21.974
3.553.727	-46.803	3.592.823	-27.702	3.547.621	-21.975
3.553.076	-46.809	3.593.034	-27.704	3.547.849	-21.975
3.553.994	-46.814	3.593.232	-27.706	3.548.052	-21.976
3.554.568	-46.819	3.593.401	-27.708	3.548.232	-21.976
3.554.725	-46.825	3.593.618	-27.711	3.548.348	-21.977
3.555.023	-46.830	3.593.775	-27.713	3.548.478	-21.978
3.554.822	-46.836	3.593.934	-27.715	3.548.667	-21.978
3.555.392	-46.842	3.594.131	-27.717	3.548.868	-21.979
3.555.504	-46.847	3.594.272	-27.719	3.549.001	-21.979
3.555.282	-46.853	3.594.439	-27.721	3.549.174	-21.980
3.556.079	-46.859	3.594.598	-27.723	3.549.338	-21.980
3.556.024	-46.864	3.594.760	-27.725	3.549.451	-21.981
3.556.389	-46.870	3.594.973	-27.728	3.549.610	-21.981
3.556.339	-46.876	3.595.118	-27.730	3.549.794	-21.982
3.555.869	-46.882	3.595.193	-27.732	3.549.946	-21.982
3.556.441	-46.888	3.595.325	-27.734	3.550.130	-21.983
3.556.714	-46.894	3.595.426	-27.736	3.550.341	-21.983
3.556.874	-46.900	3.595.537	-27.738	3.550.527	-21.984
3.556.523	-46.906	3.595.689	-27.740	3.550.735	-21.984
3.557.014	-46.912	3.595.862	-27.742	3.550.916	-21.985
3.557.275	-46.918	3.596.090	-27.745	3.551.043	-21.985
3.557.690	-46.924	3.596.326	-27.747	3.551.145	-21.986
3.557.462	-46.930	3.596.534	-27.749	3.551.241	-21.986
3.557.879	-46.937	3.596.704	-27.751	3.551.418	-21.987
3.558.402	-46.943	3.596.945	-27.753	3.551.700	-21.987
3.557.990	-46.949	3.597.126	-27.755	3.551.907	-21.987
3.557.726	-46.955	3.597.278	-27.758	3.552.032	-21.988

3.558.040	-46.962	3.597.437	-27.760	3.552.209	-21.988
3.558.507	-46.968	3.597.612	-27.762	3.552.394	-21.989
3.558.759	-46.975	3.597.763	-27.764	3.552.547	-21.989
3.558.839	-46.981	3.597.894	-27.767	3.552.752	-21.989
3.559.189	-46.988	3.598.125	-27.769	3.552.947	-21.990
3.559.310	-46.994	3.598.286	-27.771	3.553.088	-21.990
3.559.480	-47.001	3.598.377	-27.773	3.553.189	-21.991
3.559.790	-47.008	3.598.529	-27.776	3.553.363	-21.991
3.559.313	-47.014	3.598.656	-27.778	3.553.792	-21.991
3.558.918	-47.021	3.598.788	-27.780	3.554.248	-21.992
3.559.243	-47.028	3.599.006	-27.783	3.554.429	-21.992
3.559.793	-47.035	3.599.254	-27.785	3.554.436	-21.992
3.559.771	-47.041	3.599.415	-27.787	3.554.460	-21.993
3.560.215	-47.048	3.599.597	-27.790	3.554.464	-21.993
3.560.642	-47.055	3.599.782	-27.792	3.554.514	-21.993
3.560.777	-47.062	3.599.967	-27.795	3.554.680	-21.994
3.560.765	-47.069	3.600.125	-27.797	3.554.816	-21.994
3.561.241	-47.076	3.600.276	-27.800	3.554.959	-21.994
3.561.019	-47.083	3.600.426	-27.802	3.555.139	-21.995
3.561.263	-47.090	3.600.592	-27.805	3.555.309	-21.995
3.561.308	-47.097	3.600.773	-27.807	3.555.506	-21.995
3.561.700	-47.104	3.600.941	-27.810	3.555.692	-21.996
3.562.202	-47.112	3.601.126	-27.812	3.555.880	-21.996
3.562.344	-47.119	3.601.285	-27.815	3.556.076	-21.996
3.562.726	-47.126	3.601.420	-27.817	3.556.238	-21.996
3.562.679	-47.133	3.601.581	-27.820	3.556.372	-21.997
3.562.720	-47.140	3.601.779	-27.822	3.556.553	-21.997
3.563.096	-47.148	3.601.972	-27.825	3.556.733	-21.997
3.563.130	-47.155	3.602.119	-27.828	3.556.884	-21.997
3.563.130	-47.162	3.602.274	-27.830	3.557.059	-21.998

3.563.240	-47.170	3.602.475	-27.833	3.557.178	-21.998
3.563.340	-47.177	3.602.679	-27.835	3.557.299	-21.998
3.563.392	-47.184	3.602.828	-27.838	3.557.433	-21.998
3.563.489	-47.192	3.602.979	-27.841	3.557.582	-21.999
3.564.131	-47.199	3.603.141	-27.843	3.557.738	-21.999
3.563.720	-47.206	3.603.277	-27.846	3.557.878	-21.999
3.564.024	-47.214	3.603.388	-27.849	3.558.047	-21.999
3.564.276	-47.221	3.603.553	-27.851	3.558.282	-21.999
3.564.727	-47.229	3.603.695	-27.854	3.558.511	-22.000
3.564.201	-47.236	3.603.826	-27.857	3.558.724	-22.000
3.564.045	-47.244	3.604.067	-27.859	3.558.976	-22.000
3.565.475	-47.251	3.604.324	-27.862	3.559.121	-22.000
3.565.603	-47.259	3.604.559	-27.865	3.559.274	-22.001
3.566.217	-47.267	3.604.745	-27.868	3.559.429	-22.001
3.566.199	-47.274	3.604.868	-27.870	3.559.554	-22.001
3.566.537	-47.282	3.604.963	-27.873	3.559.763	-22.001
3.566.477	-47.289	3.605.107	-27.875	3.559.930	-22.002
3.565.761	-47.297	3.605.247	-27.878	3.560.081	-22.002
3.566.437	-47.305	3.605.462	-27.881	3.560.258	-22.002
3.566.597	-47.312	3.605.889	-27.883	3.560.374	-22.002
3.566.011	-47.320	3.606.295	-27.886	3.560.530	-22.003
3.567.330	-47.327	3.606.513	-27.888	3.560.703	-22.003
3.567.883	-47.335	3.606.555	-27.891	3.560.836	-22.003
3.566.745	-47.343	3.606.644	-27.894	3.560.981	-22.004
3.567.151	-47.350	3.606.734	-27.896	3.561.140	-22.004
3.567.542	-47.358	3.606.788	-27.899	3.561.321	-22.004
3.567.625	-47.366	3.606.947	-27.901	3.561.512	-22.004
3.568.398	-47.373	3.607.025	-27.903	3.561.656	-22.005
3.568.034	-47.381	3.607.069	-27.906	3.561.846	-22.005
3.568.181	-47.389	3.607.220	-27.908	3.562.034	-22.005

3.568.528	-47.396	3.607.430	-27.911	3.562.119	-22.006
3.567.711	-47.404	3.607.570	-27.913	3.562.322	-22.006
3.568.221	-47.411	3.607.796	-27.915	3.562.487	-22.006
3.569.050	-47.419	3.608.019	-27.917	3.562.665	-22.007
3.569.482	-47.427	3.608.139	-27.920	3.562.810	-22.007
3.568.511	-47.434	3.608.282	-27.922	3.563.011	-22.007
3.568.539	-47.442	3.608.416	-27.924	3.563.215	-22.008
3.568.680	-47.450	3.608.622	-27.926	3.563.312	-22.008
3.569.373	-47.457	3.608.896	-27.928	3.563.421	-22.008
3.569.854	-47.465	3.609.017	-27.930	3.563.546	-22.009
3.570.161	-47.473	3.609.110	-27.932	3.563.712	-22.009
3.570.490	-47.480	3.609.276	-27.934	3.563.970	-22.010
3.570.815	-47.488	3.609.445	-27.936	3.564.197	-22.010
3.571.014	-47.495	3.609.579	-27.938	3.564.370	-22.010
3.571.073	-47.503	3.609.695	-27.940	3.564.537	-22.011
3.570.671	-47.510	3.609.876	-27.942	3.564.688	-22.011
3.570.822	-47.518	3.610.064	-27.944	3.564.912	-22.012
3.571.176	-47.526	3.610.233	-27.946	3.565.088	-22.012
3.571.292	-47.533	3.610.365	-27.948	3.565.175	-22.012
3.571.314	-47.541	3.610.529	-27.949	3.565.328	-22.013
3.571.425	-47.548	3.610.758	-27.951	3.565.515	-22.013
3.571.635	-47.556	3.610.970	-27.953	3.565.690	-22.014
3.571.678	-47.563	3.611.107	-27.955	3.565.836	-22.014
3.571.826	-47.570	3.611.295	-27.956	3.565.961	-22.015
3.571.903	-47.578	3.611.484	-27.958	3.566.093	-22.015
3.572.220	-47.585	3.611.555	-27.960	3.566.242	-22.016
3.572.329	-47.593	3.611.718	-27.961	3.566.414	-22.016
3.572.362	-47.600	3.611.889	-27.963	3.566.574	-22.016
3.572.899	-47.607	3.612.057	-27.965	3.566.729	-22.017
3.573.276	-47.615	3.612.227	-27.966	3.566.874	-22.017

3.573.518	-47.622	3.612.354	-27.968	3.566.996	-22.018
3.573.421	-47.629	3.612.549	-27.969	3.567.174	-22.018
3.573.542	-47.636	3.612.668	-27.971	3.567.456	-22.019
3.573.806	-47.644	3.612.834	-27.972	3.567.716	-22.019
3.574.120	-47.651	3.613.055	-27.974	3.567.903	-22.020
3.574.031	-47.658	3.613.247	-27.975	3.568.101	-22.020
3.574.099	-47.665	3.613.466	-27.977	3.568.279	-22.020
3.574.335	-47.672	3.613.669	-27.978	3.568.387	-22.021
3.574.191	-47.679	3.613.860	-27.980	3.568.553	-22.021
3.573.979	-47.687	3.613.970	-27.981	3.568.774	-22.022
3.574.639	-47.694	3.614.118	-27.982	3.568.860	-22.022
3.574.872	-47.701	3.614.299	-27.984	3.568.901	-22.023
3.575.204	-47.708	3.614.458	-27.985	3.569.102	-22.023
3.575.266	-47.715	3.614.608	-27.986	3.569.286	-22.023
3.574.890	-47.722	3.614.797	-27.988	3.569.418	-22.024
3.575.078	-47.729	3.615.085	-27.989	3.569.586	-22.024
3.575.312	-47.736	3.615.215	-27.990	3.569.709	-22.024
3.575.849	-47.742	3.615.258	-27.991	3.569.883	-22.025
3.576.387	-47.749	3.615.401	-27.993	3.570.168	-22.025
3.576.718	-47.756	3.615.609	-27.994	3.570.415	-22.026
3.576.490	-47.763	3.615.800	-27.995	3.570.565	-22.026
3.577.153	-47.770	3.615.988	-27.996	3.570.740	-22.026
3.577.480	-47.777	3.616.145	-27.997	3.570.907	-22.027
3.576.920	-47.783	3.616.281	-27.998	3.571.053	-22.027
3.577.155	-47.790	3.616.416	-27.999	3.571.227	-22.027
3.577.440	-47.797	3.616.635	-28.000	3.571.407	-22.028
3.577.440	-47.803	3.616.833	-28.001	3.571.563	-22.028
3.577.582	-47.810	3.616.986	-28.002	3.571.691	-22.028
3.577.871	-47.817	3.617.162	-28.003	3.571.874	-22.028
3.578.407	-47.823	3.617.346	-28.004	3.572.051	-22.029

3.578.537	-47.830	3.617.480	-28.005	3.572.211	-22.029
3.578.542	-47.836	3.617.572	-28.006	3.572.387	-22.029
3.578.472	-47.843	3.617.729	-28.007	3.572.546	-22.030
3.578.217	-47.849	3.617.930	-28.008	3.572.709	-22.030
3.578.254	-47.856	3.618.087	-28.009	3.572.827	-22.030
3.579.303	-47.862	3.618.221	-28.010	3.572.990	-22.030
3.579.825	-47.869	3.618.369	-28.011	3.573.150	-22.031
3.580.132	-47.875	3.618.528	-28.011	3.573.315	-22.031
3.579.942	-47.881	3.618.709	-28.012	3.573.477	-22.031
3.579.843	-47.888	3.618.810	-28.013	3.573.688	-22.031
3.579.685	-47.894	3.618.883	-28.014	3.573.876	-22.031
3.579.801	-47.900	3.619.085	-28.015	3.574.023	-22.032
3.579.731	-47.907	3.619.261	-28.016	3.574.222	-22.032
3.579.993	-47.913	3.619.425	-28.016	3.574.407	-22.032
3.580.488	-47.919	3.619.675	-28.017	3.574.505	-22.032
3.580.346	-47.925	3.619.919	-28.018	3.574.648	-22.032
3.580.977	-47.932	3.620.101	-28.019	3.574.826	-22.033
3.580.753	-47.938	3.620.255	-28.019	3.575.042	-22.033
3.580.959	-47.944	3.620.410	-28.020	3.575.177	-22.033
3.581.853	-47.950	3.620.620	-28.021	3.575.323	-22.033
3.582.231	-47.956	3.620.813	-28.022	3.575.583	-22.033
3.582.227	-47.963	3.620.968	-28.023	3.575.782	-22.033
3.582.151	-47.969	3.621.147	-28.023	3.575.874	-22.033
3.582.054	-47.975	3.621.293	-28.024	3.576.079	-22.034
3.582.456	-47.981	3.621.423	-28.025	3.576.505	-22.034
3.582.657	-47.987	3.621.563	-28.026	3.576.907	-22.034
3.582.340	-47.993	3.621.716	-28.027	3.577.130	-22.034
3.582.677	-47.999	3.621.852	-28.027	3.577.188	-22.034
3.583.063	-48.005	3.622.004	-28.028	3.577.171	-22.034
3.583.174	-48.011	3.622.180	-28.029	3.577.221	-22.034

3.583.341	-48.017	3.622.325	-28.030	3.577.332	-22.034
3.583.385	-48.023	3.622.495	-28.031	3.577.449	-22.034
3.583.208	-48.028	3.622.719	-28.032	3.577.538	-22.034
3.583.553	-48.034	3.622.964	-28.033	3.577.648	-22.035
3.584.073	-48.040	3.623.168	-28.034	3.577.859	-22.035
3.584.236	-48.046	3.623.298	-28.035	3.578.056	-22.035
3.584.439	-48.052	3.623.492	-28.036	3.578.242	-22.035
3.584.027	-48.058	3.623.654	-28.037	3.578.432	-22.035
3.584.073	-48.064	3.623.745	-28.038	3.578.584	-22.035
3.584.478	-48.070	3.623.911	-28.039	3.578.730	-22.035
3.584.789	-48.075	3.624.026	-28.040	3.578.889	-22.035
3.585.397	-48.081	3.624.207	-28.041	3.579.044	-22.035
3.585.399	-48.087	3.624.449	-28.042	3.579.182	-22.035
3.585.530	-48.093	3.624.550	-28.044	3.579.355	-22.035
3.585.681	-48.099	3.624.716	-28.045	3.579.533	-22.035
3.585.819	-48.104	3.624.915	-28.046	3.579.652	-22.035
3.586.279	-48.110	3.625.052	-28.047	3.579.781	-22.036
3.586.531	-48.116	3.625.230	-28.049	3.579.969	-22.036
3.586.624	-48.122	3.625.394	-28.050	3.580.187	-22.036
3.586.362	-48.127	3.625.603	-28.051	3.580.371	-22.036
3.586.613	-48.133	3.625.818	-28.053	3.580.458	-22.036
3.587.406	-48.139	3.626.011	-28.054	3.580.610	-22.036
3.587.607	-48.145	3.626.187	-28.055	3.580.869	-22.036
3.587.282	-48.150	3.626.302	-28.057	3.581.054	-22.036
3.586.603	-48.156	3.626.375	-28.058	3.581.209	-22.036
3.586.703	-48.162	3.626.502	-28.060	3.581.373	-22.036
3.587.713	-48.167	3.626.722	-28.061	3.581.548	-22.037
3.587.672	-48.173	3.626.949	-28.063	3.581.690	-22.037
3.587.466	-48.179	3.627.115	-28.064	3.581.833	-22.037
3.587.894	-48.184	3.627.267	-28.066	3.581.982	-22.037

3.587.932	-48.190	3.627.505	-28.067	3.582.148	-22.037
3.588.109	-48.196	3.627.697	-28.069	3.582.346	-22.037
3.588.748	-48.201	3.627.828	-28.071	3.582.495	-22.038
3.589.641	-48.207	3.627.958	-28.072	3.582.643	-22.038
3.589.409	-48.213	3.628.074	-28.074	3.582.857	-22.038
3.588.748	-48.219	3.628.271	-28.075	3.582.983	-22.038
3.589.250	-48.224	3.628.471	-28.077	3.583.167	-22.038
3.590.118	-48.230	3.628.623	-28.079	3.583.452	-22.039
3.590.089	-48.236	3.628.882	-28.080	3.583.546	-22.039
3.590.011	-48.242	3.629.299	-28.082	3.583.648	-22.039
3.590.520	-48.247	3.629.637	-28.084	3.583.869	-22.039
3.589.695	-48.253	3.629.710	-28.085	3.584.014	-22.040
3.589.980	-48.259	3.629.721	-28.087	3.584.092	-22.040
3.590.747	-48.265	3.629.904	-28.089	3.584.200	-22.040
3.589.988	-48.270	3.630.031	-28.091	3.584.378	-22.041
3.591.105	-48.276	3.630.039	-28.092	3.584.561	-22.041
3.591.577	-48.282	3.630.110	-28.094	3.584.716	-22.041
3.591.915	-48.288	3.630.226	-28.096	3.584.944	-22.042
3.592.076	-48.294	3.630.341	-28.098	3.585.215	-22.042
3.590.298	-48.299	3.630.486	-28.100	3.585.350	-22.042
3.589.886	-48.305	3.630.732	-28.101	3.585.527	-22.043
3.591.122	-48.311	3.630.931	-28.103	3.585.706	-22.043
3.592.882	-48.317	3.631.069	-28.105	3.585.872	-22.044
3.594.221	-48.323	3.631.239	-28.107	3.586.024	-22.044
3.593.513	-48.329	3.631.436	-28.109	3.586.180	-22.045
3.591.859	-48.335	3.631.581	-28.111	3.586.376	-22.045
3.591.982	-48.341	3.631.763	-28.113	3.586.582	-22.046
3.592.791	-48.347	3.631.974	-28.114	3.586.721	-22.046
3.592.307	-48.353	3.632.115	-28.116	3.586.856	-22.047
3.592.827	-48.359	3.632.292	-28.118	3.587.048	-22.047

3.592.973	-48.365	3.632.458	-28.120	3.587.256	-22.048
3.593.479	-48.371	3.632.547	-28.122	3.587.449	-22.048
3.593.471	-48.377	3.632.697	-28.124	3.587.553	-22.049
3.593.456	-48.383	3.632.888	-28.126	3.587.676	-22.049
3.593.549	-48.389	3.633.013	-28.128	3.587.809	-22.050
3.592.401	-48.395	3.633.177	-28.130	3.588.024	-22.051
3.593.705	-48.401	3.633.295	-28.133	3.588.264	-22.051
3.595.825	-48.408	3.633.456	-28.135	3.588.369	-22.052
3.595.513	-48.414	3.633.691	-28.137	3.588.461	-22.052
3.595.058	-48.420	3.633.882	-28.139	3.588.636	-22.053
3.595.934	-48.426	3.634.013	-28.141	3.588.775	-22.054
3.595.583	-48.432	3.634.216	-28.143	3.588.886	-22.054
3.595.045	-48.439	3.634.451	-28.146	3.588.997	-22.055
3.596.361	-48.445	3.634.660	-28.148	3.589.135	-22.055
3.596.192	-48.451	3.634.832	-28.150	3.589.368	-22.056
3.595.746	-48.458	3.634.951	-28.153	3.589.551	-22.057
3.596.169	-48.464	3.635.093	-28.155	3.589.740	-22.057
3.596.230	-48.470	3.635.128	-28.157	3.589.893	-22.058
3.597.332	-48.477	3.635.170	-28.160	3.590.061	-22.059
3.596.956	-48.483	3.635.382	-28.162	3.590.289	-22.059
3.596.761	-48.490	3.635.646	-28.164	3.590.450	-22.060
3.597.500	-48.496	3.635.876	-28.167	3.590.635	-22.061
3.597.328	-48.503	3.636.053	-28.169	3.590.880	-22.061
3.596.827	-48.509	3.636.231	-28.172	3.591.013	-22.062
3.597.326	-48.516	3.636.411	-28.174	3.591.180	-22.063
3.597.549	-48.522	3.636.544	-28.177	3.591.367	-22.063
3.598.120	-48.529	3.636.715	-28.179	3.591.469	-22.064
3.598.443	-48.535	3.636.904	-28.182	3.591.629	-22.065
3.597.697	-48.542	3.637.087	-28.185	3.591.815	-22.065
3.598.387	-48.549	3.637.258	-28.187	3.591.967	-22.066

3.598.331	-48.555	3.637.334	-28.190	3.592.128	-22.067
3.598.112	-48.562	3.637.480	-28.192	3.592.265	-22.067
3.598.565	-48.569	3.637.682	-28.195	3.592.408	-22.068
3.598.730	-48.575	3.637.827	-28.198	3.592.585	-22.069
3.598.795	-48.582	3.637.990	-28.200	3.592.794	-22.069
3.599.399	-48.589	3.638.214	-28.203	3.593.026	-22.070
3.599.810	-48.596	3.638.431	-28.206	3.593.253	-22.071
3.599.615	-48.602	3.638.663	-28.208	3.593.414	-22.071
3.600.002	-48.609	3.638.839	-28.211	3.593.536	-22.072
3.600.070	-48.616	3.638.952	-28.214	3.593.698	-22.072
3.600.975	-48.623	3.639.128	-28.216	3.593.839	-22.073
3.601.042	-48.630	3.639.287	-28.219	3.593.977	-22.074
3.599.837	-48.637	3.639.425	-28.221	3.594.189	-22.074
3.601.053	-48.644	3.639.561	-28.224	3.594.439	-22.075
3.602.560	-48.650	3.639.702	-28.227	3.594.649	-22.076
3.601.971	-48.657	3.639.823	-28.229	3.594.779	-22.076
3.600.424	-48.664	3.639.977	-28.232	3.594.890	-22.077
3.599.938	-48.671	3.640.227	-28.234	3.595.049	-22.077
3.601.292	-48.678	3.640.382	-28.237	3.595.212	-22.078
3.603.248	-48.685	3.640.518	-28.240	3.595.357	-22.079
3.603.838	-48.692	3.640.671	-28.242	3.595.493	-22.079
3.602.529	-48.699	3.640.840	-28.245	3.595.707	-22.080
3.600.757	-48.706	3.641.022	-28.247	3.595.842	-22.080
3.600.981	-48.713	3.641.216	-28.250	3.595.995	-22.081
3.603.099	-48.720	3.641.396	-28.252	3.596.148	-22.082
3.604.005	-48.727	3.641.516	-28.254	3.596.329	-22.082
3.602.267	-48.734	3.641.679	-28.257	3.596.570	-22.083
3.600.757	-48.741	3.641.837	-28.259	3.596.718	-22.083
3.601.040	-48.748	3.641.965	-28.262	3.596.841	-22.084
3.601.541	-48.755	3.642.112	-28.264	3.597.025	-22.084

3.603.116	-48.763	3.642.299	-28.266	3.597.169	-22.085
3.605.016	-48.770	3.642.435	-28.269	3.597.310	-22.086
3.605.600	-48.777	3.642.607	-28.271	3.597.543	-22.086
3.604.402	-48.784	3.642.885	-28.273	3.597.762	-22.087
3.603.481	-48.791	3.643.062	-28.276	3.597.876	-22.087
3.603.620	-48.798	3.643.246	-28.278	3.598.005	-22.088
3.603.380	-48.805	3.643.454	-28.280	3.598.197	-22.088
3.604.255	-48.813	3.643.579	-28.282	3.598.363	-22.089
3.606.393	-48.820	3.643.724	-28.284	3.598.535	-22.089
3.607.253	-48.827	3.643.938	-28.287	3.598.885	-22.090
3.606.813	-48.834	3.644.131	-28.289	3.599.243	-22.090
3.606.823	-48.841	3.644.341	-28.291	3.599.496	-22.091
3.606.758	-48.848	3.644.565	-28.293	3.599.652	-22.091
3.605.742	-48.856	3.644.682	-28.295	3.599.666	-22.092
3.604.547	-48.863	3.644.729	-28.297	3.599.704	-22.092
3.604.360	-48.870	3.644.839	-28.299	3.599.803	-22.093
3.604.844	-48.877	3.644.955	-28.301	3.599.922	-22.093
3.604.800	-48.884	3.645.024	-28.303	3.600.078	-22.094
3.605.323	-48.892	3.645.202	-28.305	3.600.194	-22.094
3.607.332	-48.899	3.645.476	-28.307	3.600.278	-22.094
3.608.395	-48.906	3.645.692	-28.309	3.600.450	-22.095
3.606.656	-48.913	3.645.840	-28.311	3.600.642	-22.095
3.605.034	-48.920	3.645.993	-28.313	3.600.845	-22.096
3.605.139	-48.927	3.646.213	-28.315	3.601.060	-22.096
3.605.859	-48.934	3.646.432	-28.317	3.601.271	-22.097
3.606.842	-48.941	3.646.628	-28.319	3.601.436	-22.097
3.608.547	-48.949	3.646.792	-28.321	3.601.609	-22.097
3.609.703	-48.956	3.646.925	-28.323	3.601.779	-22.098
3.608.103	-48.963	3.647.051	-28.325	3.601.895	-22.098
3.606.758	-48.970	3.647.204	-28.327	3.602.040	-22.098

3.608.186	-48.977	3.647.408	-28.329	3.602.271	-22.099
3.609.422	-48.984	3.647.581	-28.331	3.602.431	-22.099
3.609.396	-48.991	3.647.752	-28.333	3.602.527	-22.100
3.608.633	-48.998	3.647.957	-28.334	3.602.604	-22.100
3.608.781	-49.005	3.648.116	-28.336	3.602.719	-22.100
3.609.110	-49.012	3.648.266	-28.338	3.602.906	-22.101
3.608.839	-49.019	3.648.419	-28.340	3.603.051	-22.101
3.609.613	-49.026	3.648.542	-28.342	3.603.275	-22.102
3.609.290	-49.033	3.648.661	-28.344	3.603.532	-22.102
3.609.631	-49.040	3.648.850	-28.346	3.603.680	-22.102
3.610.338	-49.047	3.649.064	-28.347	3.603.829	-22.103
3.609.872	-49.054	3.649.265	-28.349	3.604.023	-22.103
3.610.693	-49.061	3.649.482	-28.351	3.604.234	-22.103
3.610.807	-49.068	3.649.602	-28.353	3.604.392	-22.104
3.609.933	-49.074	3.649.720	-28.355	3.604.586	-22.104
3.610.685	-49.081	3.649.880	-28.356	3.604.787	-22.105
3.611.548	-49.088	3.650.032	-28.358	3.604.915	-22.105
3.611.495	-49.095	3.650.182	-28.360	3.605.065	-22.105
3.611.664	-49.102	3.650.356	-28.361	3.605.197	-22.106
3.611.260	-49.108	3.650.605	-28.363	3.605.307	-22.106
3.611.606	-49.115	3.650.797	-28.365	3.605.471	-22.107
3.612.004	-49.122	3.650.898	-28.366	3.605.674	-22.107
3.611.718	-49.129	3.651.028	-28.368	3.605.852	-22.107
3.611.770	-49.135	3.651.234	-28.369	3.606.013	-22.108
3.612.325	-49.142	3.651.476	-28.371	3.606.156	-22.108
3.612.896	-49.149	3.651.704	-28.372	3.606.385	-22.109
3.611.414	-49.155	3.651.790	-28.374	3.606.591	-22.109
3.611.629	-49.162	3.651.884	-28.375	3.606.662	-22.109
3.613.703	-49.169	3.652.267	-28.377	3.606.844	-22.110
3.615.146	-49.175	3.652.708	-28.378	3.607.070	-22.110

3.614.868	-49.182	3.652.939	-28.379	3.607.203	-22.111
3.612.953	-49.188	3.653.033	-28.381	3.607.344	-22.111
3.611.862	-49.195	3.653.120	-28.382	3.607.534	-22.112
3.612.581	-49.201	3.653.199	-28.383	3.607.729	-22.112
3.613.446	-49.208	3.653.257	-28.384	3.607.846	-22.113
3.613.537	-49.215	3.653.319	-28.386	3.607.982	-22.113
3.613.467	-49.221	3.653.384	-28.387	3.608.159	-22.114
3.613.524	-49.228	3.653.499	-28.388	3.608.315	-22.114
3.613.170	-49.234	3.653.604	-28.389	3.608.517	-22.115
3.612.751	-49.241	3.653.773	-28.390	3.608.720	-22.115
3.613.160	-49.247	3.654.001	-28.391	3.608.875	-22.116
3.614.941	-49.254	3.654.211	-28.392	3.609.055	-22.116
3.615.524	-49.260	3.654.421	-28.393	3.609.219	-22.117
3.614.458	-49.267	3.654.572	-28.394	3.609.384	-22.117
3.615.361	-49.273	3.654.731	-28.395	3.609.602	-22.118
3.616.943	-49.280	3.654.931	-28.396	3.609.812	-22.118
3.616.996	-49.286	3.655.068	-28.396	3.610.009	-22.119
3.616.111	-49.293	3.655.164	-28.397	3.610.166	-22.119
3.615.018	-49.299	3.655.299	-28.398	3.610.253	-22.120
3.614.395	-49.305	3.655.506	-28.399	3.610.356	-22.120
3.614.283	-49.312	3.655.712	-28.399	3.610.508	-22.121
3.614.669	-49.318	3.655.884	-28.400	3.610.707	-22.121
3.616.506	-49.325	3.655.988	-28.401	3.610.865	-22.122
3.618.401	-49.331	3.656.137	-28.401	3.611.031	-22.122
3.617.749	-49.338	3.656.332	-28.402	3.611.194	-22.123
3.616.033	-49.344	3.656.544	-28.403	3.611.290	-22.123
3.615.432	-49.351	3.656.693	-28.403	3.611.425	-22.124
3.615.533	-49.357	3.656.792	-28.404	3.611.581	-22.124
3.616.346	-49.364	3.656.964	-28.405	3.611.707	-22.125
3.618.083	-49.370	3.657.159	-28.405	3.611.826	-22.125

3.618.094	-49.377	3.657.412	-28.406	3.611.963	-22.126
3.616.707	-49.384	3.657.570	-28.406	3.612.231	-22.126
3.617.010	-49.390	3.657.708	-28.407	3.612.550	-22.127
3.619.338	-49.397	3.657.893	-28.407	3.612.747	-22.127
3.620.746	-49.403	3.658.044	-28.408	3.612.950	-22.128
3.619.778	-49.410	3.658.210	-28.408	3.613.141	-22.128
3.620.374	-49.417	3.658.399	-28.409	3.613.282	-22.128
3.620.736	-49.423	3.658.536	-28.410	3.613.401	-22.129
3.619.016	-49.430	3.658.626	-28.410	3.613.536	-22.129
3.619.749	-49.437	3.658.738	-28.411	3.613.688	-22.130
3.620.187	-49.443	3.658.923	-28.411	3.613.792	-22.130
3.619.754	-49.450	3.659.178	-28.412	3.613.929	-22.131
3.620.768	-49.457	3.659.366	-28.412	3.614.067	-22.131
3.622.054	-49.464	3.659.489	-28.413	3.614.254	-22.131
3.622.727	-49.470	3.659.622	-28.413	3.614.435	-22.132
3.621.929	-49.477	3.659.818	-28.414	3.614.550	-22.132
3.621.104	-49.484	3.660.029	-28.415	3.614.692	-22.133
3.620.500	-49.491	3.660.152	-28.415	3.614.884	-22.133
3.620.359	-49.498	3.660.313	-28.416	3.615.118	-22.133
3.621.962	-49.505	3.660.511	-28.416	3.615.370	-22.134
3.623.784	-49.512	3.660.652	-28.417	3.615.606	-22.134
3.623.448	-49.519	3.660.805	-28.418	3.615.764	-22.134
3.621.790	-49.526	3.660.971	-28.418	3.615.887	-22.135
3.621.826	-49.533	3.661.155	-28.419	3.616.024	-22.135
3.623.915	-49.540	3.661.364	-28.420	3.616.198	-22.135
3.623.927	-49.547	3.661.553	-28.421	3.616.385	-22.136
3.623.506	-49.554	3.661.726	-28.421	3.616.618	-22.136
3.623.275	-49.561	3.661.867	-28.422	3.616.795	-22.136
3.621.940	-49.569	3.662.025	-28.423	3.616.870	-22.137
3.621.674	-49.576	3.662.211	-28.423	3.617.023	-22.137

3.623.365	-49.583	3.662.350	-28.424	3.617.193	-22.137
3.624.019	-49.591	3.662.491	-28.425	3.617.361	-22.138
3.622.903	-49.598	3.662.679	-28.426	3.617.523	-22.138
3.624.316	-49.606	3.662.805	-28.427	3.617.720	-22.138
3.626.057	-49.613	3.662.957	-28.427	3.617.939	-22.139
3.625.086	-49.621	3.663.161	-28.428	3.618.102	-22.139
3.623.492	-49.628	3.663.294	-28.429	3.618.264	-22.139
3.624.102	-49.636	3.663.376	-28.430	3.618.380	-22.139
3.624.935	-49.643	3.663.549	-28.430	3.618.583	-22.140
3.623.967	-49.651	3.663.788	-28.431	3.618.781	-22.140
3.624.724	-49.659	3.663.911	-28.432	3.618.867	-22.140
3.625.288	-49.667	3.664.023	-28.433	3.618.981	-22.141
3.625.551	-49.675	3.664.252	-28.433	3.619.149	-22.141
3.626.888	-49.682	3.664.458	-28.434	3.619.380	-22.141
3.625.613	-49.690	3.664.608	-28.435	3.619.572	-22.141
3.624.649	-49.698	3.664.754	-28.436	3.619.745	-22.142
3.625.150	-49.706	3.664.923	-28.436	3.619.926	-22.142
3.626.080	-49.715	3.665.092	-28.437	3.620.070	-22.142
3.626.660	-49.723	3.665.182	-28.438	3.620.231	-22.142
3.625.901	-49.731	3.665.269	-28.438	3.620.453	-22.143
3.626.997	-49.739	3.665.399	-28.439	3.620.598	-22.143
3.627.329	-49.747	3.665.600	-28.440	3.620.686	-22.143
3.626.443	-49.756	3.665.829	-28.440	3.620.793	-22.143
3.626.308	-49.764	3.666.017	-28.441	3.620.995	-22.144
3.627.094	-49.773	3.666.223	-28.441	3.621.394	-22.144
3.627.550	-49.781	3.666.423	-28.442	3.621.798	-22.144
3.627.061	-49.790	3.666.624	-28.443	3.622.007	-22.144
3.628.395	-49.798	3.666.893	-28.443	3.622.109	-22.145
3.628.089	-49.807	3.667.134	-28.444	3.622.204	-22.145
3.627.202	-49.816	3.667.242	-28.444	3.622.202	-22.145

3.627.829	-49.825	3.667.334	-28.445	3.622.214	-22.145
3.627.679	-49.833	3.667.451	-28.445	3.622.310	-22.146
3.628.130	-49.842	3.667.599	-28.446	3.622.495	-22.146
3.628.517	-49.851	3.667.769	-28.446	3.622.693	-22.146
3.629.445	-49.860	3.667.946	-28.447	3.622.829	-22.146
3.629.677	-49.869	3.668.062	-28.448	3.623.004	-22.146
3.627.957	-49.878	3.668.195	-28.448	3.623.235	-22.147
3.627.565	-49.888	3.668.394	-28.449	3.623.394	-22.147
3.628.262	-49.897	3.668.561	-28.449	3.623.434	-22.147
3.629.428	-49.906	3.668.723	-28.450	3.623.611	-22.147
3.630.950	-49.915	3.668.893	-28.450	3.623.875	-22.148
3.630.335	-49.925	3.669.074	-28.451	3.624.044	-22.148
3.629.277	-49.934	3.669.243	-28.451	3.624.237	-22.148
3.630.117	-49.943	3.669.407	-28.452	3.624.428	-22.148
3.631.054	-49.953	3.669.602	-28.452	3.624.562	-22.149
3.629.673	-49.963	3.669.789	-28.453	3.624.749	-22.149
3.629.030	-49.972	3.669.975	-28.453	3.624.940	-22.149
3.629.059	-49.982	3.670.141	-28.454	3.625.028	-22.150
3.629.147	-49.991	3.670.332	-28.454	3.625.135	-22.150
3.629.566	-50.001	3.670.513	-28.455	3.625.241	-22.150
3.631.232	-50.011	3.670.627	-28.456	3.625.403	-22.150
3.633.366	-50.021	3.670.793	-28.456	3.625.667	-22.151
3.633.836	-50.031	3.670.979	-28.457	3.625.830	-22.151
3.632.966	-50.041	3.671.100	-28.457	3.625.957	-22.151
3.633.059	-50.050	3.671.230	-28.458	3.626.156	-22.152
3.633.846	-50.060	3.671.343	-28.459	3.626.378	-22.152
3.633.566	-50.071	3.671.497	-28.459	3.626.568	-22.152
3.632.626	-50.081	3.671.736	-28.460	3.626.672	-22.153
3.631.546	-50.091	3.671.931	-28.461	3.626.774	-22.153
3.630.883	-50.101	3.672.043	-28.461	3.626.932	-22.153

3.631.761	-50.111	3.672.214	-28.462	3.627.169	-22.154
3.633.670	-50.121	3.672.398	-28.463	3.627.376	-22.154
3.633.745	-50.132	3.672.592	-28.464	3.627.496	-22.155
3.632.780	-50.142	3.672.796	-28.464	3.627.635	-22.155
3.634.176	-50.152	3.672.950	-28.465	3.627.809	-22.156
3.634.608	-50.163	3.673.085	-28.466	3.627.993	-22.156
3.633.264	-50.173	3.673.235	-28.467	3.628.142	-22.156
3.634.760	-50.184	3.673.424	-28.468	3.628.333	-22.157
3.636.018	-50.194	3.673.602	-28.469	3.628.528	-22.157
3.634.236	-50.205	3.673.745	-28.469	3.628.618	-22.158
3.632.915	-50.216	3.673.878	-28.470	3.628.748	-22.158
3.633.952	-50.226	3.674.038	-28.471	3.628.932	-22.159
3.635.100	-50.237	3.674.225	-28.472	3.629.109	-22.159
3.634.548	-50.248	3.674.399	-28.473	3.629.315	-22.160
3.635.143	-50.258	3.674.554	-28.474	3.629.476	-22.161
3.635.352	-50.269	3.674.692	-28.475	3.629.641	-22.161
3.635.281	-50.280	3.674.876	-28.476	3.629.812	-22.162
3.636.414	-50.291	3.675.148	-28.477	3.629.945	-22.162
3.635.471	-50.302	3.675.556	-28.478	3.630.027	-22.163
3.635.541	-50.313	3.675.892	-28.479	3.630.220	-22.164
3.636.487	-50.324	3.676.035	-28.480	3.630.500	-22.164
3.636.154	-50.335	3.676.100	-28.481	3.630.664	-22.165
3.637.193	-50.346	3.676.210	-28.482	3.630.813	-22.165
3.638.193	-50.357	3.676.279	-28.483	3.630.966	-22.166
3.637.202	-50.368	3.676.271	-28.483	3.631.119	-22.167
3.636.900	-50.379	3.676.390	-28.484	3.631.284	-22.168
3.638.341	-50.390	3.676.559	-28.485	3.631.501	-22.168
3.637.861	-50.401	3.676.687	-28.486	3.631.680	-22.169
3.637.401	-50.413	3.676.855	-28.487	3.631.810	-22.170
3.637.664	-50.424	3.677.029	-28.488	3.631.975	-22.170

3.637.946	-50.435	3.677.242	-28.489	3.632.220	-22.171
3.638.441	-50.447	3.677.471	-28.490	3.632.458	-22.172
3.638.452	-50.458	3.677.639	-28.491	3.632.578	-22.173
3.638.820	-50.470	3.677.818	-28.492	3.632.712	-22.174
3.639.146	-50.481	3.677.975	-28.493	3.632.861	-22.174
3.639.320	-50.493	3.678.114	-28.494	3.633.000	-22.175
3.639.276	-50.504	3.678.308	-28.495	3.633.141	-22.176
3.639.774	-50.516	3.678.470	-28.495	3.633.275	-22.177
3.640.206	-50.528	3.678.557	-28.496	3.633.463	-22.178
3.639.716	-50.540	3.678.738	-28.497	3.633.605	-22.178
3.639.284	-50.551	3.678.914	-28.498	3.633.719	-22.179
3.639.492	-50.563	3.679.067	-28.499	3.633.873	-22.180
3.639.926	-50.575	3.679.240	-28.500	3.634.019	-22.181
3.640.329	-50.587	3.679.348	-28.500	3.634.194	-22.182
3.640.497	-50.599	3.679.449	-28.501	3.634.373	-22.182
3.640.903	-50.611	3.679.577	-28.502	3.634.518	-22.183
3.641.227	-50.623	3.679.803	-28.503	3.634.710	-22.184
3.641.224	-50.635	3.680.040	-28.504	3.634.948	-22.185
3.641.296	-50.648	3.680.258	-28.505	3.635.146	-22.186
3.641.582	-50.660	3.680.483	-28.505	3.635.411	-22.187
3.641.606	-50.672	3.680.653	-28.506	3.635.613	-22.188
3.641.693	-50.685	3.680.800	-28.507	3.635.732	-22.188
3.641.812	-50.697	3.680.954	-28.508	3.635.854	-22.189
3.642.108	-50.710	3.681.105	-28.509	3.635.966	-22.190
3.642.686	-50.722	3.681.245	-28.510	3.636.120	-22.191
3.642.847	-50.735	3.681.426	-28.511	3.636.270	-22.192
3.642.299	-50.748	3.681.578	-28.511	3.636.444	-22.193
3.642.430	-50.761	3.681.691	-28.512	3.636.552	-22.194
3.643.503	-50.774	3.681.880	-28.513	3.636.638	-22.194
3.643.217	-50.786	3.682.072	-28.514	3.636.772	-22.195

3.642.964	-50.799	3.682.180	-28.515	3.637.015	-22.196
3.643.643	-50.813	3.682.372	-28.516	3.637.238	-22.197
3.643.943	-50.826	3.682.610	-28.517	3.637.394	-22.198
3.644.279	-50.839	3.682.799	-28.518	3.637.565	-22.199
3.644.855	-50.852	3.682.935	-28.519	3.637.780	-22.200
3.644.873	-50.866	3.683.040	-28.520	3.637.929	-22.201
3.644.498	-50.879	3.683.232	-28.521	3.638.099	-22.201
3.644.886	-50.893	3.683.387	-28.522	3.638.344	-22.202
3.644.749	-50.906	3.683.561	-28.523	3.638.492	-22.203
3.643.999	-50.920	3.683.719	-28.524	3.638.669	-22.204
3.644.247	-50.934	3.683.833	-28.525	3.638.911	-22.205
3.644.337	-50.948	3.683.991	-28.526	3.639.077	-22.206
3.645.299	-50.961	3.684.179	-28.527	3.639.169	-22.207
3.645.453	-50.975	3.684.382	-28.528	3.639.292	-22.208
3.645.251	-50.990	3.684.592	-28.529	3.639.474	-22.209
3.645.509	-51.004	3.684.775	-28.531	3.639.675	-22.209
3.645.542	-51.018	3.684.873	-28.532	3.639.846	-22.210
3.645.986	-51.032	3.685.000	-28.533	3.640.023	-22.211
3.646.674	-51.047	3.685.247	-28.534	3.640.183	-22.212
3.646.571	-51.062	3.685.446	-28.535	3.640.370	-22.213
3.646.382	-51.076	3.685.602	-28.536	3.640.596	-22.214
3.646.842	-51.091	3.685.751	-28.538	3.640.695	-22.215
3.647.054	-51.106	3.685.841	-28.539	3.640.760	-22.216
3.647.240	-51.121	3.686.028	-28.540	3.640.988	-22.217
3.647.769	-51.136	3.686.271	-28.541	3.641.216	-22.218
3.647.437	-51.151	3.686.409	-28.542	3.641.335	-22.219
3.647.234	-51.166	3.686.526	-28.544	3.641.466	-22.220
3.647.251	-51.182	3.686.685	-28.545	3.641.646	-22.221
3.647.216	-51.197	3.686.870	-28.546	3.641.826	-22.222
3.647.845	-51.213	3.687.101	-28.547	3.641.996	-22.223

3.648.261	-51.228	3.687.283	-28.548	3.642.146	-22.224
3.648.018	-51.244	3.687.390	-28.549	3.642.310	-22.225
3.648.141	-51.260	3.687.583	-28.550	3.642.495	-22.226
3.648.661	-51.276	3.687.717	-28.552	3.642.654	-22.227
3.649.099	-51.292	3.687.831	-28.553	3.642.818	-22.228
3.649.247	-51.308	3.688.004	-28.554	3.643.037	-22.229
3.648.991	-51.324	3.688.049	-28.555	3.643.136	-22.230
3.649.207	-51.341	3.688.106	-28.556	3.643.224	-22.231
3.649.967	-51.357	3.688.268	-28.557	3.643.608	-22.232
3.650.439	-51.374	3.688.517	-28.558	3.644.059	-22.233
3.650.428	-51.390	3.688.770	-28.559	3.644.359	-22.235
3.650.500	-51.407	3.688.934	-28.560	3.644.494	-22.236
3.650.486	-51.424	3.689.064	-28.561	3.644.539	-22.237
3.650.945	-51.441	3.689.215	-28.562	3.644.622	-22.238
3.651.297	-51.458	3.689.498	-28.563	3.644.619	-22.239
3.650.988	-51.475	3.689.734	-28.563	3.644.677	-22.240
3.651.373	-51.493	3.689.909	-28.564	3.644.781	-22.241
3.651.577	-51.510	3.690.094	-28.565	3.644.848	-22.243
3.651.277	-51.528	3.690.251	-28.566	3.645.020	-22.244
3.651.647	-51.545	3.690.448	-28.567	3.645.220	-22.245
3.652.281	-51.563	3.690.588	-28.567	3.645.415	-22.246
3.652.633	-51.581	3.690.722	-28.568	3.645.616	-22.248
3.652.204	-51.599	3.690.905	-28.568	3.645.802	-22.249
3.651.682	-51.617	3.691.079	-28.569	3.645.949	-22.250
3.651.974	-51.635	3.691.206	-28.570	3.646.158	-22.251
3.651.773	-51.653	3.691.296	-28.570	3.646.389	-22.253
3.652.234	-51.672	3.691.371	-28.571	3.646.544	-22.254
3.652.988	-51.690	3.691.505	-28.571	3.646.716	-22.255
3.653.351	-51.709	3.691.721	-28.571	3.646.850	-22.257
3.653.466	-51.727	3.691.964	-28.572	3.646.968	-22.258

3.653.186	-51.746	3.692.157	-28.572	3.647.149	-22.260
3.653.420	-51.765	3.692.368	-28.572	3.647.305	-22.261
3.653.933	-51.784	3.692.632	-28.573	3.647.466	-22.262
3.654.233	-51.803	3.692.802	-28.573	3.647.622	-22.264
3.654.208	-51.822	3.692.953	-28.573	3.647.763	-22.265
3.653.987	-51.841	3.693.085	-28.573	3.647.899	-22.267
3.653.843	-51.860	3.693.248	-28.573	3.648.154	-22.268
3.654.458	-51.880	3.693.464	-28.573	3.648.369	-22.270
3.655.067	-51.899	3.693.643	-28.573	3.648.419	-22.271
3.654.778	-51.919	3.693.779	-28.573	3.648.575	-22.273
3.654.602	-51.938	3.693.894	-28.573	3.648.817	-22.275
3.654.926	-51.958	3.694.038	-28.573	3.649.013	-22.276
3.655.224	-51.978	3.694.157	-28.573	3.649.205	-22.278
3.655.129	-51.998	3.694.344	-28.573	3.649.344	-22.280
3.655.345	-52.017	3.694.516	-28.573	3.649.493	-22.281
3.655.772	-52.037	3.694.678	-28.573	3.649.627	-22.283
3.655.903	-52.057	3.694.913	-28.573	3.649.750	-22.285
3.655.899	-52.077	3.695.132	-28.573	3.649.901	-22.287
3.656.101	-52.097	3.695.291	-28.573	3.650.074	-22.288
3.656.360	-52.118	3.695.365	-28.572	3.650.316	-22.290
3.656.355	-52.138	3.695.502	-28.572	3.650.475	-22.292
3.656.534	-52.158	3.695.656	-28.572	3.650.587	-22.294
3.656.691	-52.178	3.695.830	-28.572	3.650.747	-22.296
3.657.094	-52.199	3.695.995	-28.571	3.650.931	-22.298
3.657.206	-52.219	3.696.165	-28.571	3.651.065	-22.300
3.657.227	-52.240	3.696.339	-28.571	3.651.198	-22.302
3.657.516	-52.260	3.696.487	-28.570	3.651.365	-22.304
3.657.214	-52.280	3.696.653	-28.570	3.651.469	-22.306
3.657.329	-52.301	3.696.844	-28.570	3.651.624	-22.308
3.657.811	-52.322	3.697.055	-28.569	3.651.846	-22.310

3.658.149	-52.342	3.697.229	-28.569	3.651.979	-22.312
3.658.547	-52.363	3.697.328	-28.568	3.652.163	-22.314
3.658.905	-52.383	3.697.433	-28.568	3.652.365	-22.317
3.658.672	-52.404	3.697.607	-28.567	3.652.509	-22.319
3.658.654	-52.425	3.697.771	-28.567	3.652.670	-22.321
3.658.937	-52.445	3.697.842	-28.566	3.652.914	-22.323
3.659.201	-52.466	3.698.112	-28.566	3.653.120	-22.326
3.659.461	-52.487	3.698.566	-28.565	3.653.275	-22.328
3.659.487	-52.507	3.698.944	-28.565	3.653.497	-22.330
3.659.839	-52.528	3.699.151	-28.564	3.653.640	-22.333
3.660.132	-52.549	3.699.266	-28.564	3.653.730	-22.335
3.660.081	-52.570	3.699.335	-28.563	3.653.900	-22.337
3.660.158	-52.590	3.699.345	-28.562	3.654.146	-22.340
3.660.751	-52.611	3.699.414	-28.562	3.654.352	-22.342
3.660.930	-52.632	3.699.539	-28.561	3.654.482	-22.345
3.661.061	-52.652	3.699.612	-28.560	3.654.682	-22.347
3.660.999	-52.673	3.699.691	-28.560	3.654.861	-22.350
3.660.887	-52.694	3.699.854	-28.559	3.655.005	-22.352
3.661.212	-52.714	3.700.056	-28.558	3.655.155	-22.355
3.661.361	-52.735	3.700.270	-28.557	3.655.273	-22.357
3.661.270	-52.755	3.700.403	-28.557	3.655.424	-22.360
3.661.566	-52.776	3.700.552	-28.556	3.655.614	-22.363
3.661.918	-52.797	3.700.764	-28.555	3.655.732	-22.365
3.662.112	-52.817	3.700.913	-28.554	3.655.855	-22.368
3.662.333	-52.838	3.701.094	-28.553	3.656.035	-22.370
3.662.442	-52.858	3.701.329	-28.553	3.656.190	-22.373
3.662.469	-52.879	3.701.527	-28.552	3.656.364	-22.376
3.662.741	-52.899	3.701.664	-28.551	3.656.516	-22.378
3.662.776	-52.919	3.701.773	-28.550	3.656.599	-22.381
3.662.817	-52.940	3.701.931	-28.549	3.656.810	-22.384

3.663.163	-52.960	3.702.112	-28.549	3.657.098	-22.387
3.663.391	-52.980	3.702.218	-28.548	3.657.263	-22.389
3.663.532	-53.001	3.702.334	-28.547	3.657.452	-22.392
3.663.694	-53.021	3.702.538	-28.546	3.657.690	-22.395
3.663.753	-53.041	3.702.731	-28.545	3.657.875	-22.397
3.663.983	-53.061	3.702.857	-28.545	3.658.016	-22.400
3.664.146	-53.081	3.703.063	-28.544	3.658.120	-22.403
3.664.167	-53.101	3.703.264	-28.543	3.658.277	-22.406
3.664.070	-53.121	3.703.508	-28.542	3.658.450	-22.408
3.664.236	-53.141	3.703.721	-28.542	3.658.598	-22.411
3.664.857	-53.161	3.703.820	-28.541	3.658.781	-22.414
3.665.093	-53.181	3.703.969	-28.540	3.658.869	-22.417
3.664.987	-53.201	3.704.183	-28.540	3.658.979	-22.419
3.665.172	-53.220	3.704.377	-28.539	3.659.157	-22.422
3.665.562	-53.240	3.704.449	-28.538	3.659.277	-22.425
3.665.695	-53.260	3.704.583	-28.538	3.659.384	-22.427
3.665.757	-53.279	3.704.753	-28.537	3.659.591	-22.430
3.665.899	-53.299	3.704.963	-28.537	3.659.870	-22.433
3.666.201	-53.318	3.705.143	-28.536	3.660.081	-22.436
3.666.253	-53.338	3.705.215	-28.536	3.660.208	-22.438
3.666.227	-53.357	3.705.345	-28.535	3.660.397	-22.441
3.666.651	-53.376	3.705.533	-28.535	3.660.618	-22.444
3.666.848	-53.396	3.705.681	-28.534	3.660.744	-22.447
3.667.019	-53.415	3.705.854	-28.534	3.660.844	-22.449
3.667.351	-53.434	3.706.098	-28.534	3.661.017	-22.452
3.667.760	-53.453	3.706.304	-28.534	3.661.234	-22.455
3.668.116	-53.472	3.706.434	-28.533	3.661.407	-22.457
3.668.326	-53.491	3.706.625	-28.533	3.661.530	-22.460
3.668.598	-53.510	3.706.772	-28.533	3.661.740	-22.463
3.668.459	-53.529	3.706.859	-28.533	3.661.982	-22.466

3.668.083	-53.548	3.707.000	-28.533	3.662.095	-22.468
3.668.102	-53.566	3.707.189	-28.533	3.662.195	-22.471
3.668.358	-53.585	3.707.398	-28.533	3.662.395	-22.474
3.668.672	-53.604	3.707.581	-28.533	3.662.626	-22.476
3.668.984	-53.622	3.707.760	-28.534	3.662.883	-22.479
3.669.032	-53.641	3.707.950	-28.534	3.663.074	-22.482
3.668.994	-53.659	3.708.183	-28.534	3.663.197	-22.484
3.668.904	-53.677	3.708.369	-28.534	3.663.323	-22.487
3.669.089	-53.696	3.708.423	-28.535	3.663.456	-22.490
3.669.727	-53.714	3.708.501	-28.535	3.663.593	-22.492
3.669.923	-53.732	3.708.695	-28.536	3.663.737	-22.495
3.670.129	-53.750	3.708.911	-28.536	3.663.907	-22.497
3.670.328	-53.768	3.709.100	-28.537	3.664.118	-22.500
3.670.126	-53.786	3.709.283	-28.538	3.664.262	-22.503
3.670.435	-53.804	3.709.435	-28.538	3.664.370	-22.505
3.670.802	-53.822	3.709.574	-28.539	3.664.574	-22.508
3.670.964	-53.840	3.709.714	-28.540	3.664.821	-22.510
3.670.971	-53.858	3.709.915	-28.541	3.665.014	-22.513
3.671.031	-53.876	3.710.078	-28.541	3.665.154	-22.515
3.671.429	-53.894	3.710.205	-28.542	3.665.289	-22.518
3.671.770	-53.911	3.710.408	-28.543	3.665.389	-22.520
3.671.871	-53.929	3.710.559	-28.544	3.665.490	-22.523
3.671.754	-53.946	3.710.663	-28.545	3.665.714	-22.525
3.671.714	-53.964	3.710.800	-28.546	3.666.149	-22.528
3.671.893	-53.982	3.710.988	-28.547	3.666.606	-22.530
3.672.260	-53.999	3.711.172	-28.549	3.666.790	-22.533
3.672.467	-54.016	3.711.331	-28.550	3.666.816	-22.535
3.672.630	-54.034	3.711.451	-28.551	3.666.903	-22.537
3.672.896	-54.051	3.711.560	-28.552	3.666.914	-22.540
3.673.192	-54.068	3.711.690	-28.553	3.666.909	-22.542

3.673.450	-54.086	3.711.852	-28.555	3.667.080	-22.545
3.673.427	-54.103	3.712.059	-28.556	3.667.235	-22.547
3.673.544	-54.120	3.712.236	-28.557	3.667.339	-22.549
3.673.672	-54.137	3.712.466	-28.559	3.667.499	-22.551
3.673.828	-54.154	3.712.658	-28.560	3.667.699	-22.554
3.674.207	-54.171	3.712.824	-28.562	3.667.935	-22.556
3.674.291	-54.189	3.713.044	-28.563	3.668.102	-22.558
3.674.279	-54.206	3.713.224	-28.565	3.668.246	-22.560
3.674.619	-54.223	3.713.374	-28.566	3.668.456	-22.563
3.674.783	-54.239	3.713.445	-28.568	3.668.684	-22.565
3.674.787	-54.256	3.713.598	-28.569	3.668.880	-22.567
3.674.941	-54.273	3.713.846	-28.571	3.669.045	-22.569
3.675.105	-54.290	3.714.059	-28.572	3.669.126	-22.571
3.675.056	-54.307	3.714.219	-28.574	3.669.245	-22.574
3.675.235	-54.324	3.714.277	-28.576	3.669.341	-22.576
3.675.466	-54.341	3.714.388	-28.577	3.669.489	-22.578
3.675.641	-54.358	3.714.554	-28.579	3.669.681	-22.580
3.675.932	-54.374	3.714.699	-28.581	3.669.774	-22.582
3.676.064	-54.391	3.714.901	-28.582	3.669.940	-22.584
3.676.136	-54.408	3.715.103	-28.584	3.670.093	-22.586
3.676.416	-54.425	3.715.298	-28.586	3.670.266	-22.588
3.676.602	-54.441	3.715.486	-28.588	3.670.526	-22.590
3.676.730	-54.458	3.715.714	-28.589	3.670.650	-22.592
3.677.005	-54.475	3.715.939	-28.591	3.670.837	-22.594
3.677.047	-54.491	3.716.096	-28.593	3.671.105	-22.596
3.677.352	-54.508	3.716.263	-28.595	3.671.314	-22.598
3.677.415	-54.525	3.716.407	-28.597	3.671.505	-22.600
3.677.427	-54.541	3.716.514	-28.598	3.671.705	-22.602
3.677.802	-54.558	3.716.643	-28.600	3.671.844	-22.604
3.678.008	-54.574	3.716.917	-28.602	3.671.933	-22.605

3.678.148	-54.591	3.717.043	-28.604	3.672.088	-22.607
3.678.336	-54.607	3.717.081	-28.606	3.672.278	-22.609
3.678.531	-54.624	3.717.344	-28.608	3.672.434	-22.611
3.678.680	-54.641	3.717.594	-28.610	3.672.542	-22.613
3.678.712	-54.657	3.717.679	-28.612	3.672.720	-22.615
3.678.708	-54.673	3.717.831	-28.614	3.672.912	-22.616
3.678.940	-54.690	3.718.096	-28.615	3.673.091	-22.618
3.679.009	-54.706	3.718.208	-28.617	3.673.295	-22.620
3.679.252	-54.723	3.718.315	-28.619	3.673.405	-22.622
3.679.622	-54.739	3.718.536	-28.621	3.673.529	-22.624
3.679.778	-54.756	3.718.737	-28.623	3.673.662	-22.625
3.680.085	-54.772	3.718.889	-28.625	3.673.832	-22.627
3.680.183	-54.788	3.719.005	-28.627	3.674.059	-22.629
3.680.302	-54.805	3.719.189	-28.629	3.674.197	-22.630
3.680.338	-54.821	3.719.424	-28.630	3.674.334	-22.632
3.680.530	-54.837	3.719.547	-28.632	3.674.550	-22.634
3.680.876	-54.854	3.719.674	-28.634	3.674.725	-22.636
3.680.872	-54.870	3.719.893	-28.636	3.674.919	-22.637
3.681.054	-54.886	3.720.064	-28.638	3.675.182	-22.639
3.681.093	-54.902	3.720.247	-28.640	3.675.374	-22.640
3.680.877	-54.919	3.720.443	-28.641	3.675.458	-22.642
3.681.451	-54.935	3.720.612	-28.643	3.675.536	-22.644
3.682.134	-54.951	3.720.795	-28.645	3.675.750	-22.645
3.682.193	-54.967	3.720.936	-28.647	3.675.974	-22.647
3.682.010	-54.983	3.721.003	-28.649	3.676.140	-22.648
3.682.222	-54.999	3.721.300	-28.650	3.676.277	-22.650
3.682.592	-55.015	3.721.715	-28.652	3.676.408	-22.651
3.682.847	-55.032	3.722.086	-28.654	3.676.541	-22.653
3.682.972	-55.048	3.722.200	-28.656	3.676.691	-22.654
3.683.019	-55.064	3.722.117	-28.657	3.676.828	-22.656

3.683.423	-55.080	3.722.387	-28.659	3.677.033	-22.657
3.683.648	-55.096	3.722.552	-28.661	3.677.169	-22.659
3.683.622	-55.112	3.722.496	-28.662	3.677.263	-22.660
3.683.616	-55.127	3.722.596	-28.664	3.677.511	-22.662
3.683.658	-55.143	3.722.747	-28.666	3.677.709	-22.663
3.683.698	-55.159	3.722.847	-28.667	3.677.924	-22.664
3.683.898	-55.175	3.723.035	-28.669	3.678.112	-22.666
3.684.223	-55.191	3.723.228	-28.670	3.678.214	-22.667
3.684.403	-55.207	3.723.374	-28.672	3.678.347	-22.668
3.684.283	-55.222	3.723.604	-28.673	3.678.495	-22.670
3.684.458	-55.238	3.723.799	-28.675	3.678.612	-22.671
3.684.834	-55.254	3.723.947	-28.676	3.678.756	-22.672
3.685.424	-55.269	3.724.073	-28.678	3.678.941	-22.673
3.685.136	-55.285	3.724.174	-28.679	3.679.109	-22.675
3.685.376	-55.301	3.724.348	-28.681	3.679.297	-22.676
3.685.636	-55.316	3.724.597	-28.682	3.679.525	-22.677
3.685.692	-55.332	3.724.781	-28.683	3.679.745	-22.678
3.685.977	-55.347	3.724.873	-28.685	3.679.969	-22.679
3.686.488	-55.363	3.725.018	-28.686	3.680.147	-22.680
3.686.747	-55.378	3.725.211	-28.688	3.680.318	-22.682
3.686.812	-55.394	3.725.397	-28.689	3.680.536	-22.683
3.687.073	-55.409	3.725.490	-28.690	3.680.755	-22.684
3.686.980	-55.424	3.725.569	-28.692	3.680.909	-22.685
3.687.084	-55.440	3.725.769	-28.693	3.680.988	-22.686
3.687.463	-55.455	3.725.964	-28.694	3.681.104	-22.687
3.687.428	-55.470	3.726.158	-28.695	3.681.260	-22.688
3.687.272	-55.486	3.726.368	-28.697	3.681.438	-22.688
3.687.798	-55.501	3.726.568	-28.698	3.681.603	-22.689
3.688.240	-55.516	3.726.723	-28.699	3.681.703	-22.690
3.688.200	-55.531	3.726.902	-28.701	3.681.828	-22.691

3.688.171	-55.546	3.727.083	-28.702	3.682.008	-22.692
3.688.636	-55.561	3.727.175	-28.703	3.682.149	-22.693
3.688.826	-55.576	3.727.336	-28.704	3.682.336	-22.694
3.688.383	-55.591	3.727.539	-28.706	3.682.574	-22.694
3.688.730	-55.606	3.727.723	-28.707	3.682.735	-22.695
3.689.373	-55.621	3.727.856	-28.708	3.682.921	-22.696
3.689.568	-55.636	3.727.975	-28.709	3.683.135	-22.697
3.690.096	-55.651	3.728.127	-28.711	3.683.257	-22.697
3.689.812	-55.666	3.728.312	-28.712	3.683.398	-22.698
3.689.407	-55.681	3.728.434	-28.713	3.683.640	-22.699
3.689.389	-55.696	3.728.566	-28.714	3.683.792	-22.699
3.689.916	-55.710	3.728.749	-28.716	3.683.862	-22.700
3.690.399	-55.725	3.728.922	-28.717	3.683.987	-22.701
3.690.038	-55.740	3.729.068	-28.718	3.684.207	-22.701
3.689.812	-55.755	3.729.276	-28.719	3.684.400	-22.702
3.690.023	-55.769	3.729.496	-28.721	3.684.554	-22.703
3.690.150	-55.784	3.729.648	-28.722	3.684.781	-22.703
3.690.717	-55.799	3.729.789	-28.723	3.684.991	-22.704
3.691.534	-55.813	3.729.919	-28.725	3.685.108	-22.704
3.691.653	-55.828	3.730.104	-28.726	3.685.241	-22.705
3.691.754	-55.843	3.730.309	-28.727	3.685.466	-22.705
3.692.192	-55.857	3.730.486	-28.728	3.685.696	-22.706
3.692.445	-55.872	3.730.630	-28.730	3.685.834	-22.706
3.692.408	-55.886	3.730.823	-28.731	3.685.979	-22.707
3.692.195	-55.901	3.730.982	-28.732	3.686.188	-22.707
3.692.206	-55.916	3.731.143	-28.734	3.686.324	-22.708
3.692.791	-55.930	3.731.320	-28.735	3.686.467	-22.708
3.692.889	-55.945	3.731.523	-28.736	3.686.665	-22.709
3.692.596	-55.959	3.731.740	-28.738	3.686.873	-22.709
3.692.842	-55.974	3.731.902	-28.739	3.686.993	-22.709

3.693.069	-55.988	3.731.987	-28.740	3.687.127	-22.710
3.693.262	-56.003	3.732.128	-28.742	3.687.365	-22.710
3.693.217	-56.017	3.732.365	-28.743	3.687.570	-22.711
3.693.384	-56.032	3.732.585	-28.744	3.687.752	-22.711
3.693.835	-56.046	3.732.769	-28.746	3.687.903	-22.711
3.693.853	-56.061	3.732.878	-28.747	3.687.988	-22.712
3.694.005	-56.075	3.733.045	-28.748	3.688.124	-22.712
3.693.925	-56.090	3.733.255	-28.749	3.688.535	-22.712
3.693.963	-56.104	3.733.399	-28.751	3.688.934	-22.713
3.694.464	-56.119	3.733.543	-28.752	3.689.142	-22.713
3.695.023	-56.133	3.733.705	-28.753	3.689.229	-22.713
3.695.204	-56.148	3.733.831	-28.755	3.689.245	-22.714
3.694.897	-56.162	3.733.947	-28.756	3.689.334	-22.714
3.694.978	-56.177	3.734.038	-28.757	3.689.371	-22.714
3.695.211	-56.191	3.734.138	-28.758	3.689.398	-22.715
3.695.570	-56.206	3.734.308	-28.760	3.689.551	-22.715
3.695.953	-56.221	3.734.505	-28.761	3.689.745	-22.715
3.696.167	-56.235	3.734.634	-28.762	3.689.901	-22.715
3.696.073	-56.250	3.734.804	-28.764	3.690.087	-22.716
3.696.061	-56.265	3.734.953	-28.765	3.690.258	-22.716
3.696.048	-56.280	3.735.177	-28.766	3.690.418	-22.716
3.696.310	-56.294	3.735.399	-28.767	3.690.645	-22.716
3.696.555	-56.309	3.735.642	-28.768	3.690.880	-22.717
3.696.581	-56.324	3.735.894	-28.770	3.691.004	-22.717
3.696.917	-56.339	3.736.032	-28.771	3.691.084	-22.717
3.697.271	-56.354	3.736.163	-28.772	3.691.320	-22.717
3.697.500	-56.369	3.736.326	-28.773	3.691.544	-22.718
3.697.899	-56.384	3.736.519	-28.774	3.691.646	-22.718
3.698.013	-56.399	3.736.660	-28.775	3.691.734	-22.718
3.697.784	-56.414	3.736.779	-28.776	3.691.833	-22.718

3.697.971	-56.429	3.736.924	-28.778	3.691.967	-22.719
3.698.145	-56.444	3.737.055	-28.779	3.692.155	-22.719
3.698.472	-56.459	3.737.224	-28.780	3.692.330	-22.719
3.698.752	-56.474	3.737.361	-28.781	3.692.516	-22.720
3.698.496	-56.490	3.737.455	-28.782	3.692.690	-22.720
3.698.584	-56.505	3.737.644	-28.783	3.692.897	-22.720
3.699.088	-56.520	3.737.876	-28.784	3.693.077	-22.720
3.699.415	-56.536	3.738.084	-28.785	3.693.251	-22.721
3.699.645	-56.551	3.738.308	-28.786	3.693.492	-22.721
3.699.781	-56.567	3.738.486	-28.786	3.693.672	-22.721
3.699.905	-56.582	3.738.604	-28.787	3.693.778	-22.722
3.700.144	-56.598	3.738.719	-28.788	3.693.974	-22.722
3.700.271	-56.614	3.738.867	-28.789	3.694.201	-22.722
3.700.462	-56.629	3.739.092	-28.790	3.694.320	-22.722
3.700.451	-56.645	3.739.338	-28.791	3.694.392	-22.723
3.700.692	-56.661	3.739.475	-28.791	3.694.543	-22.723
3.700.771	-56.677	3.739.615	-28.792	3.694.720	-22.723
3.700.657	-56.693	3.739.798	-28.793	3.694.852	-22.724
3.700.927	-56.709	3.739.951	-28.794	3.695.016	-22.724
3.701.241	-56.725	3.740.147	-28.794	3.695.238	-22.725
3.701.389	-56.741	3.740.345	-28.795	3.695.441	-22.725
3.701.526	-56.757	3.740.453	-28.796	3.695.583	-22.725
3.701.705	-56.774	3.740.623	-28.796	3.695.760	-22.726
3.701.927	-56.790	3.740.812	-28.797	3.695.943	-22.726
3.702.139	-56.807	3.740.996	-28.798	3.696.141	-22.727
3.702.151	-56.823	3.741.166	-28.798	3.696.364	-22.727
3.701.968	-56.840	3.741.310	-28.799	3.696.461	-22.727
3.702.388	-56.856	3.741.461	-28.799	3.696.506	-22.728
3.703.178	-56.873	3.741.636	-28.800	3.696.671	-22.728
3.702.914	-56.890	3.741.837	-28.800	3.696.862	-22.729

3.702.845	-56.906	3.741.997	-28.801	3.697.008	-22.729
3.703.232	-56.923	3.742.142	-28.801	3.697.189	-22.730
3.703.412	-56.940	3.742.278	-28.802	3.697.387	-22.730
3.703.688	-56.957	3.742.446	-28.802	3.697.628	-22.731
3.703.705	-56.974	3.742.607	-28.802	3.697.878	-22.731
3.704.095	-56.991	3.742.704	-28.803	3.698.065	-22.732
3.704.370	-57.008	3.742.868	-28.803	3.698.165	-22.732
3.704.142	-57.026	3.743.145	-28.803	3.698.266	-22.733
3.703.943	-57.043	3.743.264	-28.803	3.698.428	-22.733
3.704.061	-57.060	3.743.398	-28.804	3.698.568	-22.734
3.704.565	-57.078	3.743.708	-28.804	3.698.741	-22.735
3.704.807	-57.095	3.743.850	-28.804	3.698.944	-22.735
3.705.100	-57.113	3.743.886	-28.804	3.699.055	-22.736
3.705.292	-57.130	3.744.059	-28.804	3.699.250	-22.736
3.705.143	-57.148	3.744.397	-28.804	3.699.442	-22.737
3.705.223	-57.166	3.744.844	-28.805	3.699.529	-22.737
3.705.441	-57.184	3.745.139	-28.805	3.699.673	-22.738
3.705.588	-57.201	3.745.186	-28.805	3.699.922	-22.739
3.705.996	-57.219	3.745.280	-28.805	3.700.144	-22.739
3.705.984	-57.237	3.745.435	-28.805	3.700.299	-22.740
3.706.071	-57.255	3.745.530	-28.805	3.700.465	-22.740
3.706.528	-57.273	3.745.584	-28.804	3.700.594	-22.741
3.706.443	-57.291	3.745.693	-28.804	3.700.630	-22.742
3.706.584	-57.309	3.745.819	-28.804	3.700.714	-22.742
3.707.159	-57.327	3.745.892	-28.804	3.700.907	-22.743
3.707.084	-57.346	3.746.035	-28.804	3.701.051	-22.743
3.707.184	-57.364	3.746.243	-28.804	3.701.245	-22.744
3.707.683	-57.382	3.746.444	-28.803	3.701.491	-22.745
3.707.735	-57.400	3.746.624	-28.803	3.701.705	-22.745
3.707.823	-57.419	3.746.855	-28.803	3.701.892	-22.746

3.707.787	-57.437	3.747.048	-28.802	3.702.055	-22.747
3.707.944	-57.456	3.747.192	-28.802	3.702.260	-22.747
3.708.347	-57.474	3.747.325	-28.802	3.702.524	-22.748
3.708.860	-57.492	3.747.513	-28.801	3.702.716	-22.748
3.708.947	-57.511	3.747.687	-28.801	3.702.829	-22.749
3.708.558	-57.529	3.747.835	-28.800	3.702.980	-22.750
3.708.782	-57.548	3.748.055	-28.800	3.703.192	-22.750
3.709.178	-57.566	3.748.244	-28.799	3.703.372	-22.751
3.708.952	-57.585	3.748.389	-28.799	3.703.478	-22.751
3.709.129	-57.604	3.748.486	-28.798	3.703.531	-22.752
3.709.489	-57.622	3.748.597	-28.798	3.703.670	-22.753
3.709.451	-57.641	3.748.757	-28.797	3.703.883	-22.753
3.710.112	-57.659	3.748.938	-28.797	3.704.030	-22.754
3.710.609	-57.678	3.749.128	-28.796	3.704.117	-22.754
3.710.670	-57.697	3.749.281	-28.796	3.704.312	-22.755
3.710.711	-57.715	3.749.449	-28.795	3.704.530	-22.755
3.710.956	-57.734	3.749.682	-28.794	3.704.677	-22.756
3.711.296	-57.752	3.749.935	-28.794	3.704.935	-22.757
3.711.266	-57.771	3.750.137	-28.793	3.705.168	-22.757
3.711.282	-57.790	3.750.314	-28.792	3.705.320	-22.758
3.711.538	-57.808	3.750.459	-28.792	3.705.512	-22.758
3.712.036	-57.827	3.750.562	-28.791	3.705.667	-22.759
3.712.061	-57.845	3.750.670	-28.791	3.705.779	-22.759
3.712.120	-57.864	3.750.800	-28.790	3.705.919	-22.760
3.712.175	-57.882	3.750.972	-28.789	3.706.040	-22.760
3.711.986	-57.901	3.751.125	-28.789	3.706.183	-22.761
3.712.390	-57.919	3.751.255	-28.788	3.706.432	-22.762
3.712.481	-57.937	3.751.469	-28.787	3.706.611	-22.762
3.712.209	-57.956	3.751.707	-28.787	3.706.733	-22.763
3.712.802	-57.974	3.751.848	-28.786	3.706.895	-22.763

3.713.623	-57.992	3.751.969	-28.785	3.707.070	-22.764
3.713.345	-58.011	3.752.151	-28.785	3.707.242	-22.764
3.712.784	-58.029	3.752.329	-28.784	3.707.416	-22.765
3.713.467	-58.047	3.752.464	-28.784	3.707.621	-22.765
3.713.926	-58.065	3.752.607	-28.783	3.707.794	-22.766
3.713.900	-58.083	3.752.762	-28.783	3.707.922	-22.766
3.714.107	-58.102	3.752.881	-28.782	3.708.071	-22.767
3.714.209	-58.120	3.753.044	-28.781	3.708.235	-22.767
3.714.540	-58.138	3.753.228	-28.781	3.708.385	-22.768
3.715.017	-58.156	3.753.412	-28.780	3.708.503	-22.768
3.714.987	-58.173	3.753.618	-28.780	3.708.721	-22.769
3.714.899	-58.191	3.753.813	-28.780	3.708.988	-22.770
3.715.299	-58.209	3.754.010	-28.779	3.709.182	-22.770
3.715.602	-58.227	3.754.142	-28.779	3.709.319	-22.771
3.715.577	-58.245	3.754.310	-28.778	3.709.471	-22.771
3.716.019	-58.262	3.754.547	-28.778	3.709.674	-22.772
3.716.138	-58.280	3.754.702	-28.778	3.709.868	-22.772
3.716.177	-58.297	3.754.850	-28.777	3.710.009	-22.773
3.716.449	-58.315	3.754.981	-28.777	3.710.161	-22.773
3.716.567	-58.332	3.755.117	-28.777	3.710.281	-22.774
3.716.502	-58.350	3.755.259	-28.776	3.710.443	-22.774
3.716.400	-58.367	3.755.433	-28.776	3.710.865	-22.775
3.716.436	-58.384	3.755.652	-28.776	3.711.264	-22.775
3.716.599	-58.401	3.755.851	-28.776	3.711.459	-22.776
3.716.913	-58.418	3.756.044	-28.775	3.711.600	-22.776
3.717.257	-58.436	3.756.176	-28.775	3.711.639	-22.777
3.717.520	-58.452	3.756.332	-28.775	3.711.654	-22.777
3.717.737	-58.469	3.756.527	-28.775	3.711.675	-22.778
3.717.657	-58.486	3.756.650	-28.775	3.711.754	-22.778
3.717.643	-58.503	3.756.758	-28.775	3.711.949	-22.779

3.718.015	-58.520	3.756.939	-28.775	3.712.135	-22.779
3.718.430	-58.536	3.757.109	-28.774	3.712.257	-22.780
3.718.589	-58.553	3.757.249	-28.774	3.712.376	-22.780
3.718.553	-58.569	3.757.413	-28.774	3.712.539	-22.781
3.718.855	-58.586	3.757.549	-28.774	3.712.693	-22.781
3.718.836	-58.602	3.757.657	-28.774	3.712.888	-22.782
3.718.885	-58.618	3.757.841	-28.774	3.713.146	-22.782
3.718.951	-58.635	3.758.047	-28.774	3.713.360	-22.783
3.719.111	-58.651	3.758.182	-28.774	3.713.501	-22.783
3.719.429	-58.667	3.758.260	-28.774	3.713.665	-22.784
3.719.569	-58.683	3.758.434	-28.774	3.713.831	-22.784
3.719.810	-58.699	3.758.695	-28.774	3.713.980	-22.785
3.719.971	-58.715	3.758.991	-28.773	3.714.115	-22.785
3.720.050	-58.730	3.759.182	-28.773	3.714.218	-22.786
3.720.168	-58.746	3.759.317	-28.773	3.714.400	-22.786
3.720.688	-58.762	3.759.536	-28.773	3.714.580	-22.787
3.720.583	-58.777	3.759.716	-28.773	3.714.713	-22.787
3.720.513	-58.793	3.759.908	-28.773	3.714.854	-22.788
3.721.071	-58.808	3.760.081	-28.772	3.715.063	-22.788
3.721.335	-58.823	3.760.194	-28.772	3.715.270	-22.789
3.721.393	-58.838	3.760.321	-28.772	3.715.417	-22.790
3.721.512	-58.854	3.760.410	-28.772	3.715.627	-22.790
3.721.398	-58.869	3.760.555	-28.771	3.715.816	-22.791
3.721.802	-58.884	3.760.765	-28.771	3.715.992	-22.791
3.722.120	-58.898	3.760.968	-28.771	3.716.116	-22.792
3.721.799	-58.913	3.761.111	-28.770	3.716.315	-22.792
3.722.204	-58.928	3.761.234	-28.770	3.716.443	-22.793
3.722.727	-58.943	3.761.425	-28.770	3.716.581	-22.794
3.722.861	-58.957	3.761.628	-28.769	3.716.769	-22.794
3.722.847	-58.972	3.761.817	-28.769	3.716.886	-22.795

3.722.654	-58.986	3.762.008	-28.768	3.717.057	-22.795
3.722.979	-59.000	3.762.206	-28.768	3.717.251	-22.796
3.723.540	-59.014	3.762.375	-28.767	3.717.375	-22.797
3.723.600	-59.028	3.762.560	-28.766	3.717.536	-22.797
3.723.849	-59.042	3.762.712	-28.766	3.717.697	-22.798
3.724.061	-59.056	3.762.863	-28.765	3.717.827	-22.798
3.724.084	-59.070	3.762.983	-28.764	3.718.062	-22.799
3.724.203	-59.084	3.763.130	-28.764	3.718.297	-22.800
3.724.421	-59.098	3.763.311	-28.763	3.718.466	-22.800
3.724.662	-59.111	3.763.486	-28.762	3.718.613	-22.801
3.724.590	-59.125	3.763.723	-28.761	3.718.735	-22.801
3.724.696	-59.138	3.763.876	-28.760	3.718.878	-22.802
3.724.995	-59.151	3.763.985	-28.759	3.719.032	-22.803
3.725.479	-59.165	3.764.164	-28.758	3.719.185	-22.803
3.725.693	-59.178	3.764.373	-28.757	3.719.364	-22.804
3.725.959	-59.191	3.764.565	-28.756	3.719.574	-22.805
3.725.984	-59.204	3.764.680	-28.755	3.719.772	-22.805
3.725.765	-59.217	3.764.865	-28.754	3.719.991	-22.806
3.725.881	-59.229	3.765.125	-28.753	3.720.190	-22.807
3.726.021	-59.242	3.765.251	-28.752	3.720.356	-22.807
3.726.469	-59.255	3.765.324	-28.750	3.720.455	-22.808
3.726.828	-59.267	3.765.490	-28.749	3.720.559	-22.808
3.726.725	-59.279	3.765.688	-28.748	3.720.692	-22.809
3.726.726	-59.292	3.765.845	-28.746	3.720.771	-22.810
3.726.774	-59.304	3.766.001	-28.745	3.720.961	-22.810
3.726.975	-59.316	3.766.143	-28.744	3.721.176	-22.811
3.727.487	-59.328	3.766.317	-28.742	3.721.369	-22.811
3.727.453	-59.340	3.766.524	-28.740	3.721.556	-22.812
3.727.578	-59.352	3.766.714	-28.739	3.721.705	-22.813
3.727.697	-59.363	3.766.906	-28.737	3.721.936	-22.813

3.727.816	-59.375	3.767.051	-28.736	3.722.141	-22.814
3.728.233	-59.386	3.767.163	-28.734	3.722.209	-22.814
3.728.442	-59.398	3.767.235	-28.732	3.722.281	-22.815
3.728.354	-59.409	3.767.534	-28.730	3.722.467	-22.816
3.728.434	-59.420	3.768.015	-28.728	3.722.658	-22.816
3.728.792	-59.431	3.768.271	-28.727	3.722.777	-22.817
3.728.904	-59.442	3.768.391	-28.725	3.722.904	-22.817
3.728.988	-59.453	3.768.484	-28.723	3.723.067	-22.818
3.729.273	-59.464	3.768.598	-28.721	3.723.210	-22.818
3.729.453	-59.475	3.768.698	-28.719	3.723.377	-22.819
3.729.706	-59.485	3.768.772	-28.716	3.723.593	-22.819
3.729.544	-59.496	3.768.846	-28.714	3.723.757	-22.820
3.729.492	-59.506	3.768.972	-28.712	3.723.962	-22.820
3.730.129	-59.516	3.769.128	-28.710	3.724.102	-22.821
3.730.313	-59.527	3.769.268	-28.707	3.724.256	-22.821
3.730.233	-59.537	3.769.406	-28.705	3.724.536	-22.821
3.730.480	-59.547	3.769.580	-28.703	3.724.750	-22.822
3.730.686	-59.557	3.769.782	-28.700	3.724.883	-22.822
3.730.887	-59.566	3.770.010	-28.698	3.725.042	-22.823
3.730.977	-59.576	3.770.235	-28.695	3.725.285	-22.823
3.731.028	-59.586	3.770.367	-28.693	3.725.464	-22.823
3.731.354	-59.595	3.770.500	-28.690	3.725.613	-22.824
3.731.683	-59.604	3.770.667	-28.687	3.725.764	-22.824
3.731.860	-59.614	3.770.833	-28.685	3.725.869	-22.824
3.731.943	-59.623	3.770.984	-28.682	3.725.959	-22.825
3.732.207	-59.632	3.771.107	-28.679	3.726.068	-22.825
3.732.599	-59.641	3.771.284	-28.676	3.726.248	-22.825
3.732.621	-59.650	3.771.488	-28.673	3.726.438	-22.825
3.732.535	-59.658	3.771.675	-28.670	3.726.644	-22.826
3.732.655	-59.667	3.771.844	-28.667	3.726.844	-22.826

3.733.019	-59.676	3.771.935	-28.664	3.727.050	-22.826
3.733.228	-59.684	3.772.110	-28.661	3.727.246	-22.826
3.733.186	-59.692	3.772.310	-28.658	3.727.426	-22.826
3.733.233	-59.700	3.772.428	-28.655	3.727.611	-22.827
3.733.734	-59.708	3.772.607	-28.652	3.727.780	-22.827
3.734.173	-59.716	3.772.795	-28.649	3.727.911	-22.827
3.734.232	-59.724	3.773.004	-28.646	3.728.070	-22.827
3.734.136	-59.732	3.773.214	-28.643	3.728.260	-22.827
3.734.230	-59.740	3.773.391	-28.639	3.728.416	-22.827
3.734.294	-59.747	3.773.521	-28.636	3.728.576	-22.827
3.734.330	-59.755	3.773.710	-28.633	3.728.727	-22.827
3.734.595	-59.762	3.773.901	-28.629	3.728.867	-22.827
3.734.832	-59.769	3.774.034	-28.626	3.729.056	-22.827
3.734.955	-59.777	3.774.178	-28.623	3.729.261	-22.828
3.735.338	-59.784	3.774.319	-28.619	3.729.447	-22.828
3.735.551	-59.791	3.774.523	-28.616	3.729.627	-22.828
3.735.479	-59.797	3.774.663	-28.613	3.729.779	-22.828
3.735.678	-59.804	3.774.768	-28.609	3.729.935	-22.828
3.736.015	-59.811	3.774.928	-28.606	3.730.079	-22.828
3.736.409	-59.817	3.775.150	-28.603	3.730.235	-22.828
3.736.646	-59.824	3.775.327	-28.599	3.730.440	-22.828
3.736.557	-59.830	3.775.475	-28.596	3.730.672	-22.828
3.736.617	-59.836	3.775.670	-28.593	3.730.851	-22.828
3.736.895	-59.843	3.775.822	-28.589	3.730.974	-22.828
3.737.015	-59.849	3.775.974	-28.586	3.731.093	-22.828
3.737.122	-59.855	3.776.064	-28.583	3.731.239	-22.827
3.737.256	-59.861	3.776.208	-28.580	3.731.394	-22.827
3.737.441	-59.866	3.776.422	-28.576	3.731.592	-22.827
3.737.777	-59.872	3.776.609	-28.573	3.731.812	-22.827
3.737.915	-59.878	3.776.787	-28.570	3.731.978	-22.827

3.738.083	-59.883	3.777.036	-28.567	3.732.108	-22.827
3.738.327	-59.889	3.777.260	-28.564	3.732.264	-22.827
3.738.372	-59.894	3.777.404	-28.561	3.732.415	-22.827
3.738.319	-59.899	3.777.612	-28.558	3.732.538	-22.827
3.738.467	-59.905	3.777.777	-28.555	3.732.771	-22.827
3.738.954	-59.910	3.777.879	-28.553	3.733.221	-22.827
3.739.179	-59.915	3.778.062	-28.550	3.733.607	-22.827
3.739.204	-59.920	3.778.253	-28.547	3.733.698	-22.826
3.739.579	-59.925	3.778.409	-28.544	3.733.802	-22.826
3.739.942	-59.930	3.778.564	-28.542	3.733.886	-22.826
3.740.141	-59.935	3.778.661	-28.539	3.733.896	-22.826
3.740.159	-59.940	3.778.808	-28.537	3.733.956	-22.826
3.740.253	-59.944	3.779.026	-28.535	3.734.091	-22.826
3.740.363	-59.949	3.779.215	-28.532	3.734.243	-22.826
3.740.342	-59.954	3.779.380	-28.530	3.734.355	-22.826
3.740.529	-59.958	3.779.548	-28.528	3.734.504	-22.826
3.740.685	-59.963	3.779.641	-28.526	3.734.660	-22.825
3.740.787	-59.968	3.779.792	-28.524	3.734.865	-22.825
3.740.891	-59.972	3.779.960	-28.522	3.735.057	-22.825
3.741.137	-59.977	3.780.081	-28.520	3.735.191	-22.825
3.741.646	-59.981	3.780.255	-28.518	3.735.410	-22.825
3.741.712	-59.985	3.780.374	-28.517	3.735.616	-22.825
3.741.495	-59.990	3.780.483	-28.515	3.735.819	-22.825
3.741.441	-59.994	3.780.660	-28.514	3.736.004	-22.825
3.741.701	-59.998	3.780.872	-28.512	3.736.136	-22.825
3.742.099	-60.003	3.781.061	-28.511	3.736.282	-22.825
3.742.218	-60.007	3.781.181	-28.510	3.736.389	-22.825
3.742.612	-60.011	3.781.339	-28.509	3.736.502	-22.824
3.742.983	-60.016	3.781.520	-28.508	3.736.635	-22.824
3.742.893	-60.020	3.781.730	-28.507	3.736.783	-22.824

3.743.083	-60.024	3.781.936	-28.506	3.736.972	-22.824
3.743.407	-60.028	3.782.088	-28.505	3.737.119	-22.824
3.743.596	-60.033	3.782.235	-28.504	3.737.267	-22.824
3.743.604	-60.037	3.782.491	-28.504	3.737.416	-22.824
3.743.423	-60.041	3.782.751	-28.503	3.737.634	-22.824
3.743.598	-60.046	3.782.937	-28.503	3.737.841	-22.824
3.743.731	-60.050	3.783.037	-28.503	3.737.975	-22.825
3.743.748	-60.054	3.783.102	-28.503	3.738.174	-22.825
3.744.096	-60.059	3.783.269	-28.503	3.738.394	-22.825
3.744.270	-60.063	3.783.417	-28.503	3.738.528	-22.825
3.744.570	-60.068	3.783.572	-28.503	3.738.694	-22.825
3.744.742	-60.072	3.783.723	-28.503	3.738.876	-22.825
3.744.868	-60.077	3.783.878	-28.504	3.739.030	-22.825
3.745.094	-60.081	3.784.050	-28.504	3.739.193	-22.825
3.745.403	-60.086	3.784.189	-28.505	3.739.301	-22.826
3.745.759	-60.090	3.784.404	-28.505	3.739.375	-22.826
3.746.024	-60.095	3.784.612	-28.506	3.739.624	-22.826
3.746.284	-60.099	3.784.751	-28.507	3.739.843	-22.826
3.746.141	-60.104	3.784.865	-28.508	3.739.970	-22.827
3.746.136	-60.109	3.785.031	-28.509	3.740.188	-22.827
3.746.532	-60.114	3.785.277	-28.510	3.740.407	-22.827
3.746.801	-60.118	3.785.473	-28.511	3.740.589	-22.827
3.746.794	-60.123	3.785.630	-28.513	3.740.700	-22.828
3.747.167	-60.128	3.785.826	-28.514	3.740.819	-22.828
3.747.408	-60.133	3.786.024	-28.516	3.740.968	-22.828
3.747.399	-60.138	3.786.190	-28.517	3.741.152	-22.829
3.747.505	-60.143	3.786.342	-28.519	3.741.322	-22.829
3.747.669	-60.148	3.786.469	-28.521	3.741.485	-22.829
3.747.697	-60.154	3.786.624	-28.523	3.741.690	-22.830
3.747.727	-60.159	3.786.830	-28.525	3.741.902	-22.830

3.747.879	-60.164	3.786.990	-28.527	3.742.090	-22.831
3.747.776	-60.170	3.787.106	-28.529	3.742.286	-22.831
3.747.951	-60.175	3.787.214	-28.532	3.742.471	-22.832
3.748.428	-60.181	3.787.363	-28.534	3.742.594	-22.832
3.748.389	-60.186	3.787.592	-28.537	3.742.734	-22.833
3.748.481	-60.192	3.787.824	-28.539	3.742.939	-22.833
3.748.818	-60.198	3.788.029	-28.542	3.743.116	-22.834
3.749.073	-60.204	3.788.163	-28.545	3.743.248	-22.834
3.749.724	-60.210	3.788.340	-28.548	3.743.401	-22.835
3.749.908	-60.216	3.788.483	-28.551	3.743.569	-22.835
3.749.861	-60.222	3.788.575	-28.554	3.743.737	-22.836
3.750.148	-60.228	3.788.738	-28.557	3.743.933	-22.836
3.750.117	-60.234	3.788.938	-28.561	3.744.106	-22.837
3.750.459	-60.241	3.789.153	-28.564	3.744.218	-22.837
3.750.584	-60.247	3.789.333	-28.568	3.744.377	-22.838
3.750.652	-60.254	3.789.503	-28.571	3.744.562	-22.838
3.750.903	-60.261	3.789.637	-28.575	3.744.724	-22.839
3.750.827	-60.267	3.789.818	-28.579	3.744.884	-22.839
3.750.956	-60.274	3.789.980	-28.582	3.745.054	-22.840
3.751.138	-60.281	3.790.114	-28.586	3.745.175	-22.840
3.751.223	-60.288	3.790.288	-28.590	3.745.215	-22.841
3.751.270	-60.296	3.790.469	-28.594	3.745.359	-22.841
3.751.414	-60.303	3.790.820	-28.599	3.745.603	-22.842
3.751.595	-60.310	3.791.187	-28.603	3.745.764	-22.842
3.752.240	-60.318	3.791.409	-28.607	3.745.867	-22.843
3.752.323	-60.326	3.791.548	-28.612	3.746.040	-22.843
3.752.105	-60.333	3.791.613	-28.616	3.746.225	-22.844
3.752.560	-60.341	3.791.653	-28.621	3.746.432	-22.844
3.753.193	-60.349	3.791.693	-28.625	3.746.667	-22.845
3.753.036	-60.357	3.791.732	-28.630	3.746.902	-22.845

3.752.405	-60.366	3.791.895	-28.635	3.747.079	-22.846
3.753.181	-60.374	3.792.097	-28.639	3.747.246	-22.846
3.754.149	-60.382	3.792.242	-28.644	3.747.478	-22.847
3.753.116	-60.391	3.792.423	-28.649	3.747.646	-22.847
3.753.371	-60.400	3.792.600	-28.654	3.747.697	-22.847
3.754.454	-60.409	3.792.780	-28.659	3.747.818	-22.848
3.753.416	-60.418	3.792.933	-28.664	3.748.062	-22.848
3.754.254	-60.427	3.793.161	-28.669	3.748.182	-22.849
3.755.361	-60.436	3.793.396	-28.675	3.748.253	-22.849
3.754.359	-60.446	3.793.515	-28.680	3.748.441	-22.849
3.754.294	-60.455	3.793.665	-28.685	3.748.596	-22.850
3.754.642	-60.465	3.793.818	-28.690	3.748.731	-22.850
3.755.031	-60.475	3.793.952	-28.696	3.748.891	-22.850
3.754.572	-60.485	3.794.113	-28.701	3.749.067	-22.851
3.754.828	-60.496	3.794.315	-28.706	3.749.333	-22.851
3.755.948	-60.506	3.794.494	-28.712	3.749.554	-22.851
3.755.751	-60.516	3.794.572	-28.717	3.749.677	-22.852
3.755.495	-60.527	3.794.709	-28.723	3.749.837	-22.852
3.755.730	-60.538	3.794.890	-28.728	3.750.110	-22.852
3.755.807	-60.549	3.795.072	-28.734	3.750.309	-22.853
3.756.342	-60.560	3.795.224	-28.739	3.750.436	-22.853
3.756.617	-60.572	3.795.385	-28.745	3.750.641	-22.853
3.757.043	-60.583	3.795.609	-28.750	3.750.820	-22.853
3.756.931	-60.595	3.795.746	-28.756	3.750.959	-22.854
3.756.881	-60.607	3.795.896	-28.761	3.751.126	-22.854
3.757.133	-60.619	3.796.080	-28.767	3.751.273	-22.854
3.757.322	-60.631	3.796.255	-28.772	3.751.389	-22.854
3.758.131	-60.644	3.796.452	-28.778	3.751.544	-22.854
3.757.086	-60.657	3.796.661	-28.784	3.751.718	-22.855
3.757.668	-60.670	3.796.787	-28.789	3.751.896	-22.855

3.758.506	-60.683	3.796.866	-28.795	3.752.088	-22.855
3.757.210	-60.696	3.797.041	-28.800	3.752.221	-22.855
3.757.004	-60.710	3.797.290	-28.806	3.752.387	-22.855
3.757.834	-60.723	3.797.426	-28.811	3.752.633	-22.855
3.758.442	-60.737	3.797.463	-28.817	3.752.827	-22.856
3.759.464	-60.752	3.797.592	-28.822	3.752.929	-22.856
3.760.569	-60.766	3.797.817	-28.827	3.753.063	-22.856
3.759.926	-60.781	3.798.018	-28.833	3.753.210	-22.856
3.758.452	-60.796	3.798.204	-28.838	3.753.412	-22.856
3.758.822	-60.811	3.798.371	-28.844	3.753.587	-22.856
3.760.534	-60.826	3.798.536	-28.849	3.753.699	-22.856
3.759.953	-60.842	3.798.677	-28.854	3.753.884	-22.856
3.759.283	-60.858	3.798.800	-28.859	3.754.067	-22.856
3.761.259	-60.874	3.798.972	-28.864	3.754.204	-22.856
3.761.181	-60.890	3.799.161	-28.870	3.754.368	-22.856
3.760.107	-60.907	3.799.323	-28.875	3.754.539	-22.856
3.760.276	-60.924	3.799.485	-28.880	3.754.757	-22.856
3.760.757	-60.941	3.799.689	-28.885	3.754.960	-22.856
3.761.750	-60.959	3.799.922	-28.890	3.755.231	-22.856
3.761.396	-60.976	3.800.139	-28.895	3.755.650	-22.856
3.761.561	-60.995	3.800.269	-28.899	3.755.896	-22.856
3.761.719	-61.013	3.800.436	-28.904	3.755.913	-22.856
3.761.808	-61.032	3.800.609	-28.909	3.756.021	-22.856
3.762.558	-61.051	3.800.750	-28.914	3.756.169	-22.856
3.762.286	-61.070	3.800.902	-28.918	3.756.208	-22.856
3.762.134	-61.089	3.801.073	-28.923	3.756.279	-22.856
3.762.644	-61.109	3.801.219	-28.927	3.756.405	-22.856
3.764.279	-61.130	3.801.402	-28.932	3.756.561	-22.856
3.763.611	-61.150	3.801.545	-28.936	3.756.707	-22.856
3.761.860	-61.171	3.801.687	-28.940	3.756.855	-22.856

3.761.853	-61.192	3.801.945	-28.944	3.757.005	-22.856
3.763.647	-61.214	3.802.222	-28.949	3.757.206	-22.855
3.763.412	-61.236	3.802.409	-28.953	3.757.420	-22.855
3.762.477	-61.258	3.802.492	-28.957	3.757.664	-22.855
3.764.518	-61.281	3.802.621	-28.961	3.757.863	-22.855
3.766.107	-61.304	3.802.820	-28.964	3.757.930	-22.855
3.764.497	-61.327	3.803.000	-28.968	3.758.052	-22.855
3.763.277	-61.351	3.803.113	-28.972	3.758.250	-22.855
3.764.736	-61.375	3.803.203	-28.975	3.758.412	-22.854
3.765.681	-61.399	3.803.279	-28.979	3.758.560	-22.854
3.765.400	-61.424	3.803.456	-28.982	3.758.710	-22.854
3.765.306	-61.449	3.803.652	-28.985	3.758.882	-22.854
3.765.522	-61.475	3.803.793	-28.989	3.759.046	-22.854
3.764.958	-61.501	3.803.974	-28.992	3.759.198	-22.853
3.765.070	-61.528	3.804.124	-28.995	3.759.319	-22.853
3.765.453	-61.554	3.804.259	-28.998	3.759.461	-22.853
3.765.255	-61.582	3.804.460	-29.001	3.759.660	-22.853
3.765.211	-61.609	3.804.706	-29.004	3.759.826	-22.853
3.765.415	-61.637	3.804.935	-29.006	3.760.063	-22.853
3.767.193	-61.666	3.805.140	-29.009	3.760.229	-22.852
3.766.917	-61.695	3.805.332	-29.011	3.760.354	-22.852
3.766.411	-61.724	3.805.458	-29.014	3.760.573	-22.852
3.766.671	-61.754	3.805.612	-29.016	3.760.791	-22.852
3.766.358	-61.784	3.805.798	-29.019	3.760.938	-22.852
3.766.181	-61.815	3.805.952	-29.021	3.761.024	-22.852
3.766.412	-61.846	3.806.083	-29.023	3.761.190	-22.852
3.767.000	-61.878	3.806.223	-29.025	3.761.339	-22.851
3.767.025	-61.910	3.806.382	-29.027	3.761.440	-22.851
3.767.336	-61.943	3.806.537	-29.029	3.761.588	-22.851
3.767.274	-61.976	3.806.671	-29.030	3.761.866	-22.851

3.767.134	-62.010	3.806.795	-29.032	3.762.010	-22.851
3.767.825	-62.044	3.806.954	-29.034	3.762.130	-22.851
3.767.961	-62.079	3.807.104	-29.035	3.762.384	-22.851
3.767.935	-62.114	3.807.287	-29.037	3.762.554	-22.851
3.769.020	-62.149	3.807.531	-29.038	3.762.690	-22.851
3.768.806	-62.186	3.807.766	-29.039	3.762.820	-22.851
3.768.250	-62.222	3.807.955	-29.040	3.762.954	-22.851
3.768.553	-62.260	3.808.160	-29.041	3.763.121	-22.851
3.769.230	-62.297	3.808.351	-29.042	3.763.235	-22.851
3.769.344	-62.336	3.808.474	-29.043	3.763.383	-22.851
3.770.070	-62.375	3.808.600	-29.044	3.763.625	-22.851
3.769.789	-62.414	3.808.738	-29.045	3.763.824	-22.851
3.769.783	-62.454	3.808.901	-29.046	3.763.983	-22.851
3.769.555	-62.495	3.809.085	-29.046	3.764.169	-22.851
3.770.589	-62.536	3.809.254	-29.047	3.764.334	-22.851
3.771.007	-62.578	3.809.439	-29.048	3.764.540	-22.851
3.770.320	-62.620	3.809.639	-29.048	3.764.767	-22.851
3.770.549	-62.663	3.809.823	-29.048	3.764.958	-22.851
3.771.100	-62.707	3.810.018	-29.049	3.765.121	-22.851
3.770.865	-62.751	3.810.195	-29.049	3.765.277	-22.851
3.770.320	-62.796	3.810.325	-29.049	3.765.410	-22.851
3.771.703	-62.841	3.810.516	-29.049	3.765.574	-22.851
3.771.785	-62.887	3.810.682	-29.049	3.765.830	-22.851
3.770.576	-62.934	3.810.822	-29.049	3.765.977	-22.851
3.770.771	-62.981	3.810.963	-29.049	3.766.064	-22.851
3.772.599	-63.029	3.811.155	-29.049	3.766.227	-22.851
3.772.820	-63.078	3.811.304	-29.049	3.766.407	-22.852
3.771.325	-63.127	3.811.448	-29.049	3.766.577	-22.852
3.770.576	-63.177	3.811.631	-29.048	3.766.755	-22.852
3.771.147	-63.227	3.811.763	-29.048	3.766.956	-22.852

3.772.529	-63.279	3.811.935	-29.048	3.767.068	-22.852
3.774.034	-63.331	3.812.120	-29.047	3.767.141	-22.852
3.774.608	-63.383	3.812.329	-29.047	3.767.303	-22.852
3.773.949	-63.436	3.812.511	-29.046	3.767.527	-22.852
3.773.042	-63.490	3.812.695	-29.046	3.767.701	-22.852
3.772.422	-63.545	3.812.878	-29.045	3.767.814	-22.852
3.773.546	-63.600	3.812.987	-29.045	3.767.946	-22.852
3.775.164	-63.656	3.813.141	-29.044	3.768.089	-22.852
3.774.227	-63.713	3.813.260	-29.043	3.768.290	-22.852
3.773.130	-63.770	3.813.496	-29.042	3.768.484	-22.852
3.774.120	-63.828	3.813.972	-29.042	3.768.651	-22.851
3.774.487	-63.887	3.814.297	-29.041	3.768.849	-22.851
3.775.553	-63.946	3.814.420	-29.040	3.769.063	-22.851
3.775.746	-64.007	3.814.483	-29.039	3.769.297	-22.851
3.773.849	-64.067	3.814.527	-29.038	3.769.507	-22.851
3.774.532	-64.129	3.814.579	-29.038	3.769.638	-22.851
3.774.940	-64.191	3.814.680	-29.037	3.769.804	-22.851
3.774.984	-64.254	3.814.763	-29.036	3.769.998	-22.850
3.775.642	-64.318	3.814.841	-29.035	3.770.111	-22.850
3.776.158	-64.383	3.814.962	-29.034	3.770.219	-22.850
3.776.288	-64.448	3.815.132	-29.033	3.770.357	-22.850
3.775.971	-64.514	3.815.345	-29.032	3.770.518	-22.849
3.776.026	-64.580	3.815.500	-29.031	3.770.634	-22.849
3.775.920	-64.648	3.815.701	-29.030	3.770.764	-22.849
3.776.292	-64.716	3.815.919	-29.029	3.770.930	-22.848
3.777.044	-64.785	3.816.098	-29.028	3.771.031	-22.848
3.777.563	-64.855	3.816.343	-29.027	3.771.250	-22.847
3.777.032	-64.925	3.816.564	-29.026	3.771.599	-22.847
3.776.987	-64.996	3.816.711	-29.025	3.771.833	-22.847
3.777.397	-65.068	3.816.874	-29.025	3.771.994	-22.846

3.777.269	-65.141	3.817.003	-29.024	3.772.159	-22.846
3.777.397	-65.214	3.817.135	-29.023	3.772.318	-22.845
3.777.906	-65.289	3.817.296	-29.022	3.772.482	-22.845
3.778.708	-65.364	3.817.405	-29.021	3.772.618	-22.844
3.778.674	-65.440	3.817.502	-29.020	3.772.758	-22.844
3.779.042	-65.516	3.817.686	-29.019	3.772.881	-22.843
3.779.471	-65.593	3.817.864	-29.019	3.773.051	-22.842
3.779.147	-65.672	3.818.000	-29.018	3.773.282	-22.842
3.779.268	-65.750	3.818.156	-29.017	3.773.472	-22.841
3.779.055	-65.830	3.818.392	-29.016	3.773.629	-22.840
3.779.078	-65.911	3.818.611	-29.016	3.773.779	-22.840
3.779.431	-65.992	3.818.768	-29.015	3.773.955	-22.839
3.779.562	-66.074	3.818.987	-29.014	3.774.149	-22.838
3.779.500	-66.157	3.819.139	-29.014	3.774.319	-22.838
3.779.834	-66.241	3.819.279	-29.013	3.774.442	-22.837
3.779.975	-66.325	3.819.457	-29.013	3.774.622	-22.836
3.780.176	-66.411	3.819.591	-29.012	3.774.821	-22.835
3.780.856	-66.497	3.819.710	-29.012	3.774.944	-22.834
3.781.017	-66.584	3.819.884	-29.011	3.775.050	-22.833
3.780.862	-66.672	3.820.060	-29.011	3.775.202	-22.832
3.781.303	-66.760	3.820.180	-29.010	3.775.406	-22.832
3.781.344	-66.850	3.820.364	-29.010	3.775.577	-22.831
3.781.315	-66.940	3.820.573	-29.010	3.775.725	-22.830
3.781.425	-67.031	3.820.711	-29.010	3.775.909	-22.829
3.781.868	-67.123	3.820.836	-29.009	3.776.078	-22.828
3.782.184	-67.216	3.821.015	-29.009	3.776.288	-22.827
3.782.014	-67.310	3.821.212	-29.009	3.776.472	-22.826
3.782.043	-67.404	3.821.389	-29.009	3.776.608	-22.825
3.782.144	-67.499	3.821.591	-29.009	3.776.750	-22.823
3.782.350	-67.596	3.821.759	-29.009	3.776.927	-22.822

3.782.372	-67.693	3.821.897	-29.009	3.777.086	-22.821
3.782.796	-67.791	3.822.022	-29.009	3.777.243	-22.820
3.782.980	-67.889	3.822.138	-29.009	3.777.644	-22.819
3.783.087	-67.989	3.822.326	-29.009	3.778.102	-22.818
3.783.136	-68.089	3.822.531	-29.009	3.778.297	-22.816
3.783.127	-68.190	3.822.684	-29.009	3.778.301	-22.815
3.783.835	-68.292	3.822.836	-29.009	3.778.324	-22.814
3.783.869	-68.395	3.823.110	-29.009	3.778.354	-22.813
3.783.417	-68.499	3.823.387	-29.010	3.778.430	-22.811
3.783.799	-68.604	3.823.526	-29.010	3.778.564	-22.810
3.784.286	-68.709	3.823.665	-29.010	3.778.727	-22.809
3.784.395	-68.815	3.823.835	-29.010	3.778.901	-22.807
3.784.927	-68.922	3.823.934	-29.010	3.779.023	-22.806
3.784.854	-69.030	3.824.088	-29.011	3.779.185	-22.804
3.784.579	-69.139	3.824.261	-29.011	3.779.418	-22.803
3.784.872	-69.248	3.824.397	-29.011	3.779.638	-22.801
3.784.933	-69.358	3.824.576	-29.012	3.779.828	-22.800
3.785.273	-69.469	3.824.814	-29.012	3.779.992	-22.798
3.785.471	-69.581	3.825.039	-29.012	3.780.126	-22.797
3.785.353	-69.694	3.825.209	-29.012	3.780.302	-22.795
3.785.556	-69.807	3.825.371	-29.013	3.780.511	-22.793
3.786.014	-69.921	3.825.508	-29.013	3.780.682	-22.792
3.785.963	-70.036	3.825.616	-29.013	3.780.829	-22.790
3.786.058	-70.152	3.825.760	-29.013	3.780.909	-22.788
3.786.833	-70.268	3.825.946	-29.014	3.781.053	-22.787
3.787.361	-70.385	3.826.079	-29.014	3.781.275	-22.785
3.787.210	-70.503	3.826.243	-29.014	3.781.415	-22.783
3.787.368	-70.621	3.826.405	-29.014	3.781.553	-22.782
3.787.552	-70.740	3.826.519	-29.014	3.781.733	-22.780
3.787.249	-70.860	3.826.628	-29.014	3.781.896	-22.778

3.787.159	-70.981	3.826.737	-29.014	3.782.069	-22.776
3.787.665	-71.102	3.826.891	-29.014	3.782.289	-22.775
3.787.680	-71.224	3.827.079	-29.014	3.782.460	-22.773
3.787.492	-71.347	3.827.249	-29.014	3.782.596	-22.771
3.787.903	-71.470	3.827.452	-29.014	3.782.773	-22.769
3.788.221	-71.594	3.827.729	-29.014	3.782.964	-22.767
3.788.201	-71.718	3.827.935	-29.014	3.783.109	-22.765
3.788.301	-71.844	3.828.123	-29.013	3.783.280	-22.764
3.788.315	-71.969	3.828.327	-29.013	3.783.423	-22.762
3.788.521	-72.096	3.828.471	-29.013	3.783.549	-22.760
3.788.887	-72.222	3.828.625	-29.012	3.783.742	-22.758
3.788.925	-72.350	3.828.779	-29.012	3.783.865	-22.756
3.789.001	-72.478	3.828.943	-29.011	3.784.005	-22.754
3.788.907	-72.607	3.829.102	-29.011	3.784.219	-22.752
3.789.183	-72.736	3.829.211	-29.010	3.784.388	-22.750
3.789.822	-72.865	3.829.334	-29.009	3.784.548	-22.748
3.790.212	-72.996	3.829.492	-29.008	3.784.746	-22.746
3.790.027	-73.126	3.829.634	-29.007	3.784.941	-22.744
3.790.056	-73.257	3.829.774	-29.006	3.785.110	-22.742
3.790.194	-73.389	3.829.970	-29.005	3.785.251	-22.741
3.790.170	-73.521	3.830.227	-29.004	3.785.388	-22.739
3.790.415	-73.654	3.830.446	-29.002	3.785.509	-22.737
3.790.421	-73.787	3.830.635	-29.001	3.785.681	-22.735
3.790.670	-73.920	3.830.837	-28.999	3.785.885	-22.733
3.791.436	-74.054	3.830.993	-28.998	3.786.065	-22.731
3.791.494	-74.189	3.831.136	-28.996	3.786.239	-22.729
3.791.039	-74.323	3.831.292	-28.994	3.786.459	-22.727
3.791.556	-74.458	3.831.434	-28.992	3.786.628	-22.725
3.792.097	-74.594	3.831.610	-28.990	3.786.756	-22.723
3.792.151	-74.730	3.831.802	-28.988	3.786.886	-22.721

3.792.326	-74.866	3.831.985	-28.985	3.787.068	-22.719
3.792.004	-75.002	3.832.159	-28.983	3.787.256	-22.717
3.792.293	-75.139	3.832.351	-28.980	3.787.386	-22.715
3.792.701	-75.277	3.832.520	-28.978	3.787.572	-22.713
3.792.619	-75.414	3.832.629	-28.975	3.787.784	-22.711
3.792.506	-75.552	3.832.799	-28.972	3.787.951	-22.709
3.792.802	-75.690	3.832.980	-28.969	3.788.073	-22.707
3.793.282	-75.828	3.833.189	-28.966	3.788.250	-22.705
3.793.394	-75.967	3.833.338	-28.963	3.788.430	-22.703
3.793.720	-76.106	3.833.438	-28.959	3.788.629	-22.702
3.794.023	-76.245	3.833.582	-28.956	3.788.842	-22.700
3.793.979	-76.384	3.833.755	-28.952	3.788.967	-22.698
3.793.795	-76.524	3.833.949	-28.948	3.789.092	-22.696
3.794.012	-76.664	3.834.169	-28.944	3.789.280	-22.694
3.794.555	-76.804	3.834.352	-28.940	3.789.435	-22.692
3.794.898	-76.944	3.834.467	-28.936	3.789.559	-22.690
3.794.778	-77.085	3.834.642	-28.931	3.789.702	-22.688
3.795.013	-77.225	3.834.810	-28.927	3.789.847	-22.686
3.795.182	-77.366	3.834.971	-28.922	3.790.006	-22.684
3.794.594	-77.507	3.835.170	-28.918	3.790.121	-22.682
3.794.958	-77.648	3.835.338	-28.913	3.790.262	-22.680
3.795.699	-77.789	3.835.515	-28.908	3.790.490	-22.678
3.796.051	-77.930	3.835.656	-28.903	3.790.627	-22.676
3.796.014	-78.071	3.835.789	-28.898	3.790.798	-22.674
3.796.061	-78.213	3.835.959	-28.892	3.790.981	-22.672
3.796.234	-78.354	3.836.060	-28.887	3.791.170	-22.670
3.795.948	-78.496	3.836.196	-28.881	3.791.398	-22.668
3.796.161	-78.637	3.836.527	-28.875	3.791.651	-22.666
3.796.855	-78.779	3.836.935	-28.869	3.791.808	-22.664
3.797.315	-78.921	3.837.236	-28.863	3.791.921	-22.663

3.796.978	-79.062	3.837.401	-28.857	3.792.025	-22.661
3.797.026	-79.204	3.837.457	-28.851	3.792.218	-22.659
3.797.175	-79.346	3.837.498	-28.845	3.792.440	-22.657
3.797.007	-79.487	3.837.556	-28.838	3.792.505	-22.655
3.797.596	-79.629	3.837.581	-28.832	3.792.676	-22.653
3.797.998	-79.771	3.837.628	-28.825	3.792.889	-22.651
3.798.221	-79.912	3.837.771	-28.818	3.792.961	-22.649
3.798.174	-80.054	3.837.971	-28.811	3.793.049	-22.647
3.797.890	-80.195	3.838.175	-28.804	3.793.195	-22.645
3.798.165	-80.337	3.838.380	-28.797	3.793.352	-22.643
3.798.503	-80.478	3.838.626	-28.790	3.793.496	-22.641
3.798.688	-80.619	3.838.824	-28.782	3.793.691	-22.640
3.798.752	-80.760	3.838.976	-28.775	3.793.987	-22.638
3.798.842	-80.901	3.839.193	-28.767	3.794.160	-22.636
3.798.849	-81.042	3.839.364	-28.760	3.794.341	-22.634
3.799.073	-81.183	3.839.503	-28.752	3.794.554	-22.632
3.799.417	-81.324	3.839.664	-28.744	3.794.746	-22.630
3.799.460	-81.464	3.839.845	-28.736	3.794.904	-22.629
3.799.218	-81.605	3.840.020	-28.728	3.795.054	-22.627
3.799.427	-81.745	3.840.179	-28.720	3.795.223	-22.625
3.799.693	-81.885	3.840.318	-28.712	3.795.426	-22.623
3.799.883	-82.025	3.840.443	-28.704	3.795.610	-22.621
3.800.053	-82.165	3.840.663	-28.696	3.795.771	-22.620
3.800.264	-82.305	3.840.833	-28.688	3.795.956	-22.618
3.800.813	-82.444	3.840.930	-28.679	3.796.035	-22.616
3.800.869	-82.584	3.841.053	-28.671	3.796.136	-22.615
3.800.899	-82.723	3.841.188	-28.662	3.796.425	-22.613
3.801.187	-82.862	3.841.385	-28.654	3.796.693	-22.611
3.801.443	-83.001	3.841.625	-28.646	3.796.841	-22.610
3.801.458	-83.139	3.841.877	-28.637	3.796.989	-22.608

3.801.770	-83.278	3.842.077	-28.629	3.797.095	-22.606
3.802.048	-83.416	3.842.239	-28.620	3.797.148	-22.605
3.801.920	-83.554	3.842.361	-28.612	3.797.325	-22.603
3.801.827	-83.692	3.842.487	-28.603	3.797.520	-22.602
3.802.127	-83.830	3.842.639	-28.594	3.797.697	-22.600
3.802.482	-83.967	3.842.803	-28.586	3.797.932	-22.598
3.802.227	-84.104	3.842.961	-28.577	3.798.120	-22.597
3.802.390	-84.241	3.843.069	-28.569	3.798.291	-22.595
3.802.831	-84.378	3.843.221	-28.560	3.798.452	-22.594
3.802.794	-84.514	3.843.419	-28.552	3.798.629	-22.592
3.802.933	-84.651	3.843.584	-28.543	3.798.784	-22.591
3.803.221	-84.787	3.843.724	-28.535	3.798.912	-22.589
3.803.497	-84.923	3.843.873	-28.526	3.799.084	-22.588
3.804.049	-85.058	3.844.063	-28.518	3.799.221	-22.587
3.804.171	-85.194	3.844.268	-28.509	3.799.389	-22.585
3.804.171	-85.329	3.844.424	-28.501	3.799.793	-22.584
3.804.536	-85.464	3.844.572	-28.493	3.800.197	-22.582
3.804.570	-85.599	3.844.716	-28.484	3.800.388	-22.581
3.804.583	-85.734	3.844.855	-28.476	3.800.465	-22.580
3.804.775	-85.868	3.845.003	-28.468	3.800.534	-22.578
3.804.767	-86.002	3.845.175	-28.460	3.800.562	-22.577
3.804.861	-86.136	3.845.439	-28.452	3.800.632	-22.576
3.805.253	-86.270	3.845.654	-28.444	3.800.706	-22.575
3.805.620	-86.403	3.845.811	-28.436	3.800.784	-22.573
3.805.793	-86.536	3.845.952	-28.428	3.800.970	-22.572
3.805.800	-86.670	3.846.155	-28.420	3.801.147	-22.571
3.805.838	-86.802	3.846.299	-28.412	3.801.263	-22.570
3.806.108	-86.935	3.846.463	-28.405	3.801.449	-22.569
3.806.398	-87.067	3.846.665	-28.397	3.801.656	-22.567
3.806.549	-87.200	3.846.803	-28.390	3.801.790	-22.566

3.806.456	-87.332	3.846.944	-28.382	3.801.978	-22.565
3.806.438	-87.464	3.847.144	-28.375	3.802.181	-22.564
3.806.646	-87.595	3.847.376	-28.368	3.802.355	-22.563
3.806.935	-87.727	3.847.477	-28.361	3.802.528	-22.562
3.807.115	-87.858	3.847.633	-28.354	3.802.701	-22.561
3.807.209	-87.989	3.847.870	-28.347	3.802.843	-22.560
3.807.511	-88.120	3.848.047	-28.340	3.803.004	-22.559
3.807.535	-88.250	3.848.156	-28.333	3.803.235	-22.558
3.807.672	-88.381	3.848.246	-28.327	3.803.394	-22.557
3.808.161	-88.511	3.848.438	-28.320	3.803.519	-22.556
3.808.438	-88.641	3.848.660	-28.314	3.803.685	-22.555
3.808.885	-88.771	3.848.804	-28.308	3.803.846	-22.554
3.808.618	-88.901	3.848.965	-28.302	3.804.023	-22.553
3.808.484	-89.030	3.849.091	-28.296	3.804.196	-22.552
3.809.006	-89.160	3.849.175	-28.290	3.804.381	-22.551
3.808.987	-89.289	3.849.285	-28.284	3.804.598	-22.551
3.809.113	-89.418	3.849.424	-28.278	3.804.754	-22.550
3.809.573	-89.547	3.849.621	-28.273	3.804.909	-22.549
3.810.024	-89.675	3.849.781	-28.267	3.805.117	-22.548
3.810.206	-89.804	3.849.906	-28.262	3.805.342	-22.547
3.810.281	-89.932	3.850.057	-28.257	3.805.491	-22.547
3.810.263	-90.060	3.850.284	-28.251	3.805.631	-22.546
3.810.237	-90.188	3.850.552	-28.246	3.805.797	-22.545
3.810.365	-90.315	3.850.815	-28.241	3.805.914	-22.544
3.810.522	-90.443	3.851.017	-28.237	3.806.026	-22.544
3.810.490	-90.570	3.851.202	-28.232	3.806.172	-22.543
3.810.620	-90.697	3.851.389	-28.227	3.806.335	-22.542
3.810.815	-90.824	3.851.519	-28.223	3.806.501	-22.542
3.810.909	-90.951	3.851.624	-28.219	3.806.714	-22.541
3.811.125	-91.078	3.851.768	-28.214	3.806.897	-22.541

3.811.402	-91.204	3.851.880	-28.210	3.807.014	-22.540
3.811.588	-91.330	3.852.007	-28.206	3.807.184	-22.539
3.811.526	-91.457	3.852.180	-28.202	3.807.368	-22.539
3.811.588	-91.583	3.852.314	-28.198	3.807.520	-22.538
3.811.740	-91.708	3.852.455	-28.195	3.807.662	-22.538
3.812.131	-91.834	3.852.625	-28.191	3.807.803	-22.537
3.812.242	-91.959	3.852.802	-28.188	3.807.968	-22.536
3.812.267	-92.085	3.852.976	-28.184	3.808.145	-22.536
3.812.419	-92.210	3.853.134	-28.181	3.808.333	-22.535
3.812.416	-92.335	3.853.352	-28.178	3.808.500	-22.535
3.812.672	-92.460	3.853.605	-28.175	3.808.692	-22.534
3.813.033	-92.585	3.853.774	-28.172	3.808.882	-22.534
3.813.269	-92.709	3.853.914	-28.170	3.809.079	-22.533
3.813.371	-92.834	3.853.976	-28.167	3.809.274	-22.533
3.813.499	-92.958	3.854.147	-28.164	3.809.415	-22.532
3.813.674	-93.082	3.854.457	-28.162	3.809.590	-22.532
3.813.695	-93.206	3.854.673	-28.160	3.809.785	-22.531
3.813.974	-93.330	3.854.841	-28.158	3.809.913	-22.531
3.814.321	-93.454	3.855.028	-28.156	3.810.002	-22.530
3.814.149	-93.578	3.855.172	-28.154	3.810.161	-22.530
3.814.125	-93.701	3.855.296	-28.152	3.810.350	-22.529
3.814.525	-93.825	3.855.484	-28.150	3.810.558	-22.529
3.814.887	-93.948	3.855.605	-28.148	3.810.732	-22.528
3.814.915	-94.071	3.855.710	-28.147	3.810.852	-22.528
3.814.951	-94.194	3.855.876	-28.146	3.811.010	-22.527
3.815.295	-94.317	3.856.047	-28.144	3.811.209	-22.527
3.815.595	-94.440	3.856.184	-28.143	3.811.436	-22.526
3.815.682	-94.563	3.856.371	-28.142	3.811.599	-22.526
3.815.559	-94.685	3.856.599	-28.141	3.811.747	-22.525
3.815.780	-94.808	3.856.761	-28.140	3.811.888	-22.524

3.815.949	-94.931	3.856.910	-28.139	3.811.979	-22.524
3.815.820	-95.053	3.857.092	-28.138	3.812.120	-22.523
3.816.172	-95.175	3.857.238	-28.138	3.812.278	-22.522
3.816.181	-95.297	3.857.383	-28.137	3.812.431	-22.522
3.816.444	-95.420	3.857.552	-28.137	3.812.625	-22.521
3.817.074	-95.542	3.857.748	-28.136	3.812.836	-22.520
3.817.094	-95.664	3.857.906	-28.136	3.813.051	-22.520
3.817.033	-95.786	3.858.048	-28.136	3.813.262	-22.519
3.817.502	-95.907	3.858.250	-28.136	3.813.450	-22.518
3.817.925	-96.029	3.858.414	-28.136	3.813.578	-22.517
3.817.889	-96.151	3.858.571	-28.136	3.813.753	-22.517
3.817.885	-96.273	3.858.742	-28.136	3.813.904	-22.516
3.817.451	-96.395	3.858.952	-28.136	3.813.994	-22.515
3.817.856	-96.516	3.859.078	-28.136	3.814.225	-22.514
3.818.143	-96.638	3.859.321	-28.136	3.814.424	-22.513
3.818.171	-96.760	3.859.760	-28.137	3.814.557	-22.512
3.818.479	-96.881	3.860.063	-28.137	3.814.742	-22.512
3.818.481	-97.003	3.860.226	-28.138	3.814.893	-22.511
3.818.896	-97.125	3.860.309	-28.138	3.815.007	-22.510
3.819.128	-97.247	3.860.349	-28.139	3.815.121	-22.509
3.819.200	-97.368	3.860.446	-28.140	3.815.287	-22.508
3.819.475	-97.490	3.860.544	-28.140	3.815.462	-22.507
3.819.641	-97.612	3.860.544	-28.141	3.815.613	-22.506
3.819.767	-97.734	3.860.653	-28.142	3.815.800	-22.505
3.819.815	-97.856	3.860.869	-28.143	3.816.004	-22.504
3.820.065	-97.978	3.861.057	-28.144	3.816.245	-22.503
3.819.963	-98.100	3.861.199	-28.145	3.816.436	-22.502
3.820.063	-98.222	3.861.306	-28.146	3.816.555	-22.501
3.820.717	-98.344	3.861.523	-28.147	3.816.696	-22.500
3.821.115	-98.466	3.861.711	-28.148	3.816.911	-22.499

3.821.357	-98.588	3.861.917	-28.149	3.817.076	-22.497
3.821.365	-98.711	3.862.117	-28.150	3.817.142	-22.496
3.821.243	-98.833	3.862.265	-28.152	3.817.314	-22.495
3.821.349	-98.956	3.862.469	-28.153	3.817.493	-22.494
3.821.136	-99.079	3.862.611	-28.155	3.817.741	-22.493
3.821.441	-99.201	3.862.709	-28.156	3.817.982	-22.492
3.821.638	-99.324	3.862.926	-28.158	3.818.053	-22.490
3.821.354	-99.448	3.863.148	-28.159	3.818.171	-22.489
3.821.499	-99.571	3.863.221	-28.161	3.818.392	-22.488
3.821.790	-99.694	3.863.391	-28.162	3.818.539	-22.487
3.822.303	-99.818	3.863.595	-28.164	3.818.741	-22.486
3.822.791	-99.942	3.863.694	-28.166	3.818.952	-22.484
3.823.042	-100.066	3.863.808	-28.168	3.819.099	-22.483
3.823.147	-100.190	3.864.016	-28.170	3.819.272	-22.482
3.823.269	-100.314	3.864.301	-28.172	3.819.431	-22.481
3.823.441	-100.438	3.864.485	-28.174	3.819.536	-22.479
3.823.449	-100.563	3.864.613	-28.176	3.819.714	-22.478
3.823.792	-100.687	3.864.819	-28.178	3.819.933	-22.477
3.824.067	-100.812	3.865.057	-28.180	3.820.104	-22.475
3.824.164	-100.937	3.865.233	-28.182	3.820.271	-22.474
3.824.283	-101.063	3.865.356	-28.184	3.820.392	-22.473
3.824.537	-101.188	3.865.469	-28.187	3.820.580	-22.471
3.824.669	-101.314	3.865.616	-28.189	3.820.765	-22.470
3.824.857	-101.440	3.865.742	-28.192	3.820.920	-22.469
3.824.872	-101.566	3.865.858	-28.194	3.821.094	-22.467
3.824.829	-101.692	3.866.028	-28.197	3.821.249	-22.466
3.825.125	-101.819	3.866.191	-28.200	3.821.368	-22.465
3.825.072	-101.946	3.866.400	-28.203	3.821.593	-22.463
3.824.779	-102.072	3.866.549	-28.206	3.822.036	-22.462
3.825.285	-102.200	3.866.621	-28.209	3.822.410	-22.461

3.825.736	-102.327	3.866.826	-28.212	3.822.556	-22.459
3.825.710	-102.455	3.867.016	-28.215	3.822.563	-22.458
3.826.144	-102.583	3.867.144	-28.218	3.822.611	-22.457
3.826.133	-102.711	3.867.373	-28.221	3.822.709	-22.455
3.826.036	-102.839	3.867.589	-28.225	3.822.787	-22.454
3.826.703	-102.968	3.867.675	-28.229	3.822.907	-22.452
3.826.813	-103.096	3.867.742	-28.232	3.823.092	-22.451
3.826.768	-103.225	3.867.906	-28.236	3.823.206	-22.450
3.827.177	-103.355	3.868.135	-28.240	3.823.255	-22.448
3.827.752	-103.484	3.868.356	-28.244	3.823.420	-22.447
3.827.759	-103.614	3.868.481	-28.248	3.823.637	-22.445
3.827.189	-103.744	3.868.663	-28.252	3.823.793	-22.444
3.827.191	-103.874	3.868.883	-28.256	3.823.991	-22.442
3.827.834	-104.005	3.869.061	-28.260	3.824.233	-22.441
3.828.033	-104.135	3.869.219	-28.265	3.824.467	-22.440
3.828.106	-104.266	3.869.371	-28.269	3.824.616	-22.438
3.828.457	-104.398	3.869.554	-28.274	3.824.727	-22.437
3.828.401	-104.529	3.869.727	-28.278	3.824.873	-22.435
3.828.514	-104.661	3.869.898	-28.283	3.824.995	-22.434
3.828.862	-104.792	3.870.032	-28.288	3.825.103	-22.433
3.829.238	-104.924	3.870.175	-28.293	3.825.263	-22.431
3.829.483	-105.056	3.870.343	-28.298	3.825.465	-22.430
3.829.168	-105.189	3.870.488	-28.303	3.825.641	-22.429
3.829.048	-105.321	3.870.648	-28.308	3.825.732	-22.427
3.829.301	-105.454	3.870.873	-28.313	3.825.884	-22.426
3.829.060	-105.587	3.871.115	-28.318	3.826.090	-22.424
3.829.901	-105.720	3.871.286	-28.324	3.826.266	-22.423
3.830.063	-105.853	3.871.393	-28.329	3.826.488	-22.422
3.829.924	-105.986	3.871.508	-28.335	3.826.705	-22.421
3.829.998	-106.120	3.871.678	-28.340	3.826.906	-22.419

3.829.363	-106.254	3.871.859	-28.346	3.827.112	-22.418
3.830.700	-106.387	3.872.018	-28.351	3.827.304	-22.417
3.832.000	-106.521	3.872.119	-28.357	3.827.487	-22.415
3.830.845	-106.655	3.872.173	-28.363	3.827.672	-22.414
3.830.461	-106.789	3.872.346	-28.368	3.827.802	-22.413
3.831.332	-106.924	3.872.599	-28.374	3.827.903	-22.412
3.831.347	-107.058	3.872.778	-28.380	3.828.060	-22.411
3.830.894	-107.192	3.872.865	-28.386	3.828.250	-22.409
3.830.089	-107.327	3.873.036	-28.392	3.828.448	-22.408
3.829.826	-107.461	3.873.336	-28.397	3.828.591	-22.407
3.830.219	-107.595	3.873.531	-28.403	3.828.743	-22.406
3.830.596	-107.730	3.873.673	-28.409	3.828.882	-22.405
3.831.957	-107.864	3.873.878	-28.415	3.829.026	-22.404
3.834.205	-107.999	3.874.084	-28.421	3.829.204	-22.403
3.835.173	-108.134	3.874.272	-28.427	3.829.344	-22.402
3.834.829	-108.268	3.874.454	-28.433	3.829.460	-22.401
3.834.710	-108.402	3.874.565	-28.440	3.829.581	-22.400
3.833.970	-108.537	3.874.673	-28.446	3.829.774	-22.399
3.832.182	-108.671	3.874.844	-28.452	3.829.930	-22.398
3.831.602	-108.805	3.874.937	-28.458	3.830.063	-22.397
3.833.441	-108.940	3.875.052	-28.464	3.830.281	-22.396
3.835.773	-109.074	3.875.252	-28.470	3.830.531	-22.395
3.836.544	-109.208	3.875.437	-28.476	3.830.775	-22.394
3.836.454	-109.342	3.875.603	-28.482	3.830.909	-22.393
3.835.234	-109.475	3.875.753	-28.489	3.831.008	-22.392
3.833.445	-109.609	3.875.912	-28.495	3.831.180	-22.391
3.834.557	-109.742	3.876.125	-28.501	3.831.382	-22.390
3.836.238	-109.875	3.876.371	-28.507	3.831.560	-22.389
3.834.811	-110.008	3.876.567	-28.513	3.831.671	-22.389
3.834.985	-110.141	3.876.683	-28.519	3.831.815	-22.388

3.836.364	-110.274	3.876.787	-28.526	3.831.980	-22.387
3.834.956	-110.406	3.876.965	-28.532	3.832.142	-22.386
3.833.987	-110.538	3.877.216	-28.538	3.832.310	-22.385
3.835.107	-110.670	3.877.343	-28.544	3.832.516	-22.385
3.835.695	-110.801	3.877.491	-28.550	3.832.726	-22.384
3.835.822	-110.933	3.877.784	-28.556	3.832.858	-22.383
3.837.000	-111.064	3.877.919	-28.563	3.833.033	-22.382
3.837.924	-111.194	3.878.037	-28.569	3.833.160	-22.382
3.837.003	-111.325	3.878.221	-28.575	3.833.315	-22.381
3.835.504	-111.454	3.878.398	-28.581	3.833.503	-22.380
3.835.296	-111.584	3.878.550	-28.587	3.833.535	-22.380
3.836.909	-111.713	3.878.684	-28.593	3.833.672	-22.379
3.839.068	-111.842	3.878.882	-28.599	3.833.911	-22.378
3.839.928	-111.970	3.879.078	-28.606	3.834.131	-22.377
3.840.110	-112.098	3.879.284	-28.612	3.834.294	-22.377
3.839.756	-112.226	3.879.453	-28.618	3.834.429	-22.376
3.837.742	-112.353	3.879.556	-28.624	3.834.590	-22.375
3.836.013	-112.479	3.879.709	-28.630	3.834.709	-22.375
3.835.952	-112.606	3.879.850	-28.636	3.834.886	-22.374
3.836.577	-112.731	3.880.031	-28.642	3.835.140	-22.373
3.837.574	-112.856	3.880.258	-28.648	3.835.360	-22.372
3.838.188	-112.981	3.880.399	-28.654	3.835.504	-22.372
3.838.531	-113.105	3.880.551	-28.659	3.835.743	-22.371
3.838.203	-113.229	3.880.737	-28.665	3.835.981	-22.370
3.837.932	-113.352	3.880.930	-28.671	3.836.169	-22.370
3.839.245	-113.474	3.881.118	-28.677	3.836.394	-22.369
3.840.544	-113.596	3.881.234	-28.682	3.836.467	-22.368
3.841.355	-113.718	3.881.416	-28.688	3.836.494	-22.367
3.841.867	-113.839	3.881.606	-28.694	3.836.615	-22.367
3.841.626	-113.959	3.881.750	-28.699	3.836.756	-22.366

3.840.175	-114.078	3.881.864	-28.705	3.836.927	-22.365
3.838.355	-114.198	3.882.062	-28.710	3.837.104	-22.364
3.837.975	-114.316	3.882.491	-28.715	3.837.272	-22.364
3.838.377	-114.434	3.882.856	-28.721	3.837.433	-22.363
3.840.072	-114.551	3.883.009	-28.726	3.837.596	-22.362
3.842.405	-114.667	3.883.098	-28.731	3.837.798	-22.361
3.842.430	-114.783	3.883.145	-28.736	3.837.971	-22.361
3.841.709	-114.899	3.883.185	-28.741	3.838.164	-22.360
3.842.593	-115.013	3.883.291	-28.746	3.838.338	-22.359
3.842.451	-115.127	3.883.340	-28.750	3.838.445	-22.358
3.840.614	-115.240	3.883.410	-28.755	3.838.564	-22.358
3.840.728	-115.353	3.883.535	-28.759	3.838.708	-22.357
3.842.625	-115.465	3.883.708	-28.764	3.838.914	-22.356
3.843.315	-115.576	3.883.955	-28.768	3.839.191	-22.355
3.842.054	-115.686	3.884.121	-28.772	3.839.421	-22.355
3.840.638	-115.796	3.884.331	-28.776	3.839.514	-22.354
3.840.856	-115.905	3.884.568	-28.780	3.839.641	-22.353
3.842.853	-116.013	3.884.742	-28.784	3.839.810	-22.352
3.844.500	-116.121	3.884.905	-28.787	3.840.002	-22.352
3.843.962	-116.227	3.885.056	-28.791	3.840.168	-22.351
3.842.357	-116.333	3.885.122	-28.794	3.840.341	-22.350
3.842.117	-116.439	3.885.280	-28.797	3.840.527	-22.350
3.844.201	-116.543	3.885.552	-28.800	3.840.740	-22.349
3.845.851	-116.647	3.885.710	-28.803	3.840.932	-22.348
3.845.241	-116.750	3.885.862	-28.806	3.841.049	-22.348
3.843.446	-116.852	3.886.031	-28.809	3.841.223	-22.347
3.842.498	-116.953	3.886.151	-28.811	3.841.328	-22.346
3.844.187	-117.054	3.886.270	-28.814	3.841.419	-22.346
3.844.861	-117.153	3.886.436	-28.816	3.841.647	-22.345
3.844.160	-117.253	3.886.624	-28.818	3.841.910	-22.344

3.845.284	-117.351	3.886.805	-28.820	3.842.087	-22.344
3.844.576	-117.448	3.886.956	-28.822	3.842.200	-22.343
3.842.939	-117.545	3.887.180	-28.823	3.842.419	-22.342
3.843.015	-117.641	3.887.449	-28.825	3.842.575	-22.342
3.845.064	-117.736	3.887.535	-28.826	3.842.709	-22.341
3.847.294	-117.830	3.887.658	-28.827	3.842.970	-22.340
3.847.719	-117.924	3.887.903	-28.828	3.843.145	-22.340
3.848.253	-118.017	3.888.073	-28.829	3.843.265	-22.339
3.848.685	-118.109	3.888.185	-28.830	3.843.336	-22.339
3.848.815	-118.200	3.888.329	-28.831	3.843.504	-22.338
3.849.153	-118.290	3.888.489	-28.831	3.843.907	-22.338
3.849.133	-118.380	3.888.638	-28.832	3.844.323	-22.337
3.847.958	-118.469	3.888.735	-28.832	3.844.541	-22.336
3.846.620	-118.557	3.888.882	-28.832	3.844.568	-22.336
3.846.181	-118.644	3.889.131	-28.832	3.844.503	-22.335
3.846.140	-118.731	3.889.283	-28.831	3.844.498	-22.335
3.845.999	-118.817	3.889.378	-28.831	3.844.630	-22.334
3.845.667	-118.902	3.889.572	-28.831	3.844.810	-22.334
3.846.720	-118.987	3.889.821	-28.830	3.844.935	-22.333
3.848.583	-119.070	3.889.955	-28.829	3.845.075	-22.333
3.849.789	-119.153	3.890.148	-28.828	3.845.276	-22.332
3.848.890	-119.236	3.890.318	-28.827	3.845.519	-22.332
3.847.162	-119.317	3.890.393	-28.826	3.845.682	-22.331
3.848.316	-119.398	3.890.565	-28.825	3.845.797	-22.331
3.850.356	-119.478	3.890.675	-28.823	3.845.963	-22.330
3.851.332	-119.558	3.890.800	-28.821	3.846.173	-22.330
3.850.754	-119.637	3.891.028	-28.820	3.846.369	-22.330
3.848.996	-119.715	3.891.223	-28.818	3.846.548	-22.329
3.848.324	-119.792	3.891.378	-28.816	3.846.715	-22.329
3.848.504	-119.869	3.891.646	-28.814	3.846.899	-22.328

3.848.293	-119.946	3.891.904	-28.811	3.847.069	-22.328
3.848.094	-120.021	3.892.010	-28.809	3.847.175	-22.328
3.849.743	-120.096	3.892.087	-28.806	3.847.379	-22.327
3.850.537	-120.170	3.892.240	-28.804	3.847.496	-22.327
3.849.104	-120.244	3.892.456	-28.801	3.847.575	-22.327
3.848.412	-120.317	3.892.646	-28.798	3.847.733	-22.327
3.848.537	-120.390	3.892.730	-28.795	3.847.890	-22.326
3.850.432	-120.462	3.892.799	-28.791	3.848.078	-22.326
3.852.648	-120.533	3.893.049	-28.788	3.848.300	-22.326
3.852.582	-120.604	3.893.302	-28.784	3.848.503	-22.326
3.852.045	-120.674	3.893.418	-28.781	3.848.698	-22.325
3.851.845	-120.744	3.893.593	-28.777	3.848.879	-22.325
3.851.674	-120.813	3.893.835	-28.773	3.849.061	-22.325
3.851.013	-120.881	3.894.017	-28.769	3.849.167	-22.325
3.850.338	-120.949	3.894.200	-28.764	3.849.346	-22.325
3.850.540	-121.017	3.894.392	-28.760	3.849.594	-22.325
3.850.896	-121.083	3.894.474	-28.755	3.849.733	-22.324
3.851.387	-121.150	3.894.545	-28.751	3.849.811	-22.324
3.851.273	-121.216	3.894.698	-28.746	3.849.879	-22.324
3.850.798	-121.281	3.894.818	-28.741	3.850.107	-22.324
3.850.826	-121.346	3.894.969	-28.736	3.850.338	-22.324
3.851.935	-121.410	3.895.165	-28.730	3.850.473	-22.324
3.854.204	-121.473	3.895.309	-28.725	3.850.609	-22.324
3.855.751	-121.537	3.895.443	-28.719	3.850.789	-22.324
3.855.393	-121.599	3.895.613	-28.713	3.850.984	-22.324
3.853.464	-121.661	3.895.798	-28.707	3.851.166	-22.324
3.852.556	-121.723	3.896.036	-28.701	3.851.364	-22.324
3.854.522	-121.784	3.896.299	-28.695	3.851.531	-22.325
3.855.883	-121.845	3.896.498	-28.688	3.851.738	-22.325
3.855.034	-121.905	3.896.689	-28.682	3.851.913	-22.325

3.853.861	-121.965	3.896.805	-28.675	3.851.978	-22.325
3.853.224	-122.024	3.896.956	-28.668	3.852.076	-22.325
3.853.312	-122.082	3.897.202	-28.661	3.852.236	-22.326
3.854.815	-122.141	3.897.401	-28.654	3.852.511	-22.326
3.857.372	-122.198	3.897.524	-28.647	3.852.712	-22.326
3.857.629	-122.255	3.897.621	-28.639	3.852.762	-22.326
3.855.504	-122.312	3.897.785	-28.632	3.852.917	-22.327
3.854.630	-122.368	3.897.958	-28.624	3.853.077	-22.327
3.855.959	-122.424	3.898.065	-28.616	3.853.236	-22.327
3.855.688	-122.479	3.898.143	-28.608	3.853.459	-22.328
3.855.804	-122.534	3.898.333	-28.600	3.853.600	-22.328
3.856.766	-122.588	3.898.582	-28.592	3.853.743	-22.328
3.855.609	-122.641	3.898.698	-28.584	3.853.936	-22.329
3.856.064	-122.695	3.898.806	-28.575	3.854.136	-22.329
3.857.617	-122.747	3.899.024	-28.567	3.854.319	-22.330
3.856.904	-122.799	3.899.272	-28.558	3.854.494	-22.330
3.856.899	-122.851	3.899.496	-28.549	3.854.659	-22.331
3.858.872	-122.902	3.899.644	-28.540	3.854.832	-22.331
3.858.763	-122.953	3.899.789	-28.532	3.854.998	-22.332
3.856.924	-123.003	3.899.975	-28.523	3.855.101	-22.332
3.856.425	-123.053	3.900.190	-28.514	3.855.249	-22.333
3.858.371	-123.102	3.900.358	-28.504	3.855.465	-22.333
3.860.071	-123.150	3.900.498	-28.495	3.855.605	-22.334
3.859.244	-123.199	3.900.552	-28.486	3.855.695	-22.334
3.858.185	-123.246	3.900.627	-28.477	3.855.862	-22.335
3.857.814	-123.293	3.900.896	-28.467	3.856.046	-22.335
3.859.107	-123.340	3.901.192	-28.458	3.856.143	-22.336
3.859.964	-123.386	3.901.354	-28.449	3.856.264	-22.337
3.858.604	-123.432	3.901.461	-28.439	3.856.461	-22.337
3.859.248	-123.477	3.901.674	-28.430	3.856.643	-22.338

3.860.652	-123.522	3.901.848	-28.420	3.856.758	-22.338
3.859.960	-123.566	3.902.000	-28.411	3.857.000	-22.339
3.859.474	-123.610	3.902.188	-28.401	3.857.355	-22.339
3.860.558	-123.653	3.902.357	-28.391	3.857.574	-22.340
3.860.205	-123.696	3.902.556	-28.382	3.857.737	-22.341
3.858.976	-123.738	3.902.741	-28.372	3.857.940	-22.341
3.859.447	-123.780	3.902.870	-28.363	3.858.127	-22.342
3.860.403	-123.822	3.903.049	-28.353	3.858.257	-22.342
3.861.707	-123.863	3.903.203	-28.343	3.858.365	-22.343
3.862.888	-123.903	3.903.370	-28.334	3.858.526	-22.344
3.861.790	-123.943	3.903.549	-28.324	3.858.669	-22.344
3.859.904	-123.983	3.903.663	-28.315	3.858.770	-22.345
3.859.315	-124.022	3.903.795	-28.305	3.858.911	-22.345
3.859.453	-124.061	3.903.954	-28.296	3.859.103	-22.346
3.859.841	-124.099	3.904.167	-28.286	3.859.258	-22.346
3.860.022	-124.137	3.904.392	-28.277	3.859.359	-22.347
3.860.173	-124.174	3.904.565	-28.267	3.859.478	-22.348
3.861.273	-124.211	3.904.637	-28.258	3.859.688	-22.348
3.863.439	-124.248	3.904.843	-28.249	3.859.912	-22.349
3.864.648	-124.284	3.905.287	-28.239	3.860.126	-22.349
3.864.398	-124.319	3.905.632	-28.230	3.860.376	-22.350
3.864.161	-124.354	3.905.707	-28.221	3.860.508	-22.350
3.863.810	-124.389	3.905.782	-28.212	3.860.565	-22.351
3.863.268	-124.423	3.905.928	-28.203	3.860.721	-22.351
3.862.470	-124.457	3.906.025	-28.194	3.860.898	-22.352
3.861.687	-124.491	3.906.055	-28.185	3.861.069	-22.353
3.861.610	-124.523	3.906.083	-28.177	3.861.282	-22.353
3.862.026	-124.556	3.906.252	-28.168	3.861.459	-22.354
3.863.446	-124.588	3.906.467	-28.159	3.861.625	-22.354
3.865.493	-124.619	3.906.602	-28.151	3.861.815	-22.355

3.865.667	-124.651	3.906.718	-28.142	3.862.010	-22.355
3.864.045	-124.681	3.906.873	-28.134	3.862.159	-22.356
3.863.694	-124.711	3.907.126	-28.126	3.862.321	-22.356
3.865.826	-124.741	3.907.381	-28.117	3.862.516	-22.357
3.867.287	-124.770	3.907.511	-28.109	3.862.719	-22.358
3.867.462	-124.799	3.907.657	-28.101	3.862.929	-22.358
3.868.044	-124.827	3.907.817	-28.093	3.863.077	-22.359
3.868.272	-124.855	3.907.982	-28.086	3.863.136	-22.359
3.867.889	-124.882	3.908.148	-28.078	3.863.232	-22.360
3.866.975	-124.909	3.908.277	-28.070	3.863.419	-22.360
3.866.001	-124.935	3.908.438	-28.063	3.863.618	-22.361
3.865.484	-124.960	3.908.667	-28.056	3.863.847	-22.361
3.867.000	-124.986	3.908.868	-28.048	3.864.077	-22.362
3.867.893	-125.010	3.908.967	-28.041	3.864.245	-22.362
3.866.902	-125.034	3.909.084	-28.034	3.864.348	-22.363
3.866.892	-125.058	3.909.238	-28.027	3.864.464	-22.363
3.867.117	-125.081	3.909.438	-28.021	3.864.606	-22.364
3.866.834	-125.103	3.909.642	-28.014	3.864.733	-22.365
3.866.313	-125.125	3.909.836	-28.008	3.865.005	-22.365
3.867.018	-125.147	3.910.011	-28.001	3.865.271	-22.366
3.868.683	-125.167	3.910.151	-27.995	3.865.418	-22.366
3.868.239	-125.188	3.910.323	-27.989	3.865.617	-22.367
3.867.556	-125.207	3.910.555	-27.983	3.865.949	-22.367
3.868.898	-125.227	3.910.729	-27.978	3.866.257	-22.368
3.868.666	-125.245	3.910.916	-27.972	3.866.477	-22.368
3.867.365	-125.263	3.911.095	-27.967	3.866.584	-22.369
3.867.393	-125.281	3.911.166	-27.961	3.866.646	-22.369
3.867.435	-125.298	3.911.307	-27.956	3.866.718	-22.370
3.867.263	-125.314	3.911.448	-27.951	3.866.725	-22.371
3.868.636	-125.330	3.911.559	-27.947	3.866.787	-22.371

3.870.654	-125.345	3.911.765	-27.942	3.866.960	-22.372
3.869.839	-125.360	3.911.971	-27.938	3.867.148	-22.372
3.868.564	-125.374	3.912.115	-27.933	3.867.289	-22.373
3.870.170	-125.388	3.912.294	-27.929	3.867.452	-22.374
3.870.818	-125.401	3.912.426	-27.925	3.867.611	-22.374
3.869.783	-125.413	3.912.589	-27.922	3.867.803	-22.375
3.870.860	-125.425	3.912.807	-27.918	3.868.035	-22.375
3.871.603	-125.436	3.912.932	-27.915	3.868.243	-22.376
3.871.405	-125.446	3.913.038	-27.912	3.868.369	-22.377
3.871.205	-125.456	3.913.203	-27.909	3.868.445	-22.377
3.871.781	-125.466	3.913.365	-27.906	3.868.594	-22.378
3.871.626	-125.475	3.913.492	-27.903	3.868.784	-22.379
3.870.847	-125.483	3.913.615	-27.901	3.868.999	-22.379
3.872.124	-125.491	3.913.768	-27.898	3.869.176	-22.380
3.871.711	-125.498	3.914.039	-27.896	3.869.294	-22.381
3.870.172	-125.505	3.914.292	-27.895	3.869.507	-22.381
3.870.025	-125.511	3.914.469	-27.893	3.869.693	-22.382
3.870.558	-125.516	3.914.645	-27.891	3.869.841	-22.383
3.870.898	-125.521	3.914.805	-27.890	3.869.964	-22.384
3.870.918	-125.525	3.914.962	-27.889	3.870.130	-22.384
3.872.051	-125.529	3.915.152	-27.888	3.870.323	-22.385
3.874.276	-125.532	3.915.302	-27.887	3.870.461	-22.386
3.875.424	-125.535	3.915.388	-27.887	3.870.678	-22.387
3.875.173	-125.537	3.915.589	-27.887	3.870.894	-22.388
3.873.774	-125.539	3.915.757	-27.886	3.871.087	-22.388
3.872.823	-125.540	3.915.858	-27.886	3.871.223	-22.389
3.874.009	-125.541	3.915.988	-27.887	3.871.339	-22.390
3.873.972	-125.541	3.916.250	-27.887	3.871.532	-22.391
3.873.262	-125.540	3.916.472	-27.887	3.871.678	-22.392
3.875.274	-125.539	3.916.541	-27.888	3.871.816	-22.393

3.876.306	-125.538	3.916.755	-27.889	3.871.978	-22.394
3.875.529	-125.536	3.916.989	-27.890	3.872.122	-22.395
3.875.045	-125.533	3.917.079	-27.891	3.872.287	-22.396
3.874.309	-125.530	3.917.166	-27.892	3.872.456	-22.397
3.873.504	-125.527	3.917.336	-27.894	3.872.610	-22.398
3.873.952	-125.523	3.917.502	-27.895	3.872.858	-22.399
3.876.314	-125.519	3.917.583	-27.897	3.873.011	-22.400
3.877.740	-125.514	3.917.766	-27.899	3.873.142	-22.401
3.876.223	-125.509	3.917.975	-27.901	3.873.378	-22.402
3.874.858	-125.503	3.918.143	-27.903	3.873.522	-22.403
3.875.949	-125.497	3.918.359	-27.906	3.873.630	-22.404
3.876.664	-125.490	3.918.510	-27.908	3.873.759	-22.405
3.876.331	-125.483	3.918.687	-27.911	3.873.914	-22.406
3.877.516	-125.475	3.918.911	-27.913	3.874.052	-22.408
3.877.279	-125.467	3.919.126	-27.916	3.874.211	-22.409
3.876.701	-125.458	3.919.370	-27.919	3.874.413	-22.410
3.877.746	-125.449	3.919.565	-27.922	3.874.620	-22.411
3.877.148	-125.440	3.919.713	-27.925	3.874.829	-22.413
3.877.401	-125.430	3.919.840	-27.929	3.875.009	-22.414
3.878.412	-125.420	3.919.944	-27.932	3.875.159	-22.415
3.877.633	-125.409	3.920.078	-27.935	3.875.277	-22.416
3.877.984	-125.398	3.920.253	-27.939	3.875.428	-22.418
3.878.605	-125.386	3.920.462	-27.942	3.875.667	-22.419
3.878.716	-125.374	3.920.610	-27.946	3.875.818	-22.420
3.879.102	-125.362	3.920.703	-27.950	3.875.934	-22.422
3.879.357	-125.349	3.920.800	-27.954	3.876.127	-22.423
3.879.509	-125.336	3.920.910	-27.958	3.876.310	-22.425
3.879.911	-125.322	3.921.097	-27.962	3.876.517	-22.426
3.880.446	-125.308	3.921.277	-27.966	3.876.714	-22.427
3.878.568	-125.294	3.921.440	-27.970	3.876.832	-22.429

3.878.947	-125.279	3.921.660	-27.974	3.876.924	-22.430
3.880.034	-125.264	3.921.838	-27.978	3.877.061	-22.432
3.879.045	-125.249	3.921.982	-27.983	3.877.285	-22.433
3.879.393	-125.233	3.922.133	-27.987	3.877.502	-22.435
3.879.749	-125.217	3.922.341	-27.991	3.877.596	-22.436
3.880.177	-125.201	3.922.568	-27.996	3.877.686	-22.438
3.881.868	-125.184	3.922.780	-28.000	3.877.784	-22.439
3.881.223	-125.167	3.922.965	-28.005	3.877.921	-22.441
3.879.944	-125.149	3.923.112	-28.010	3.878.102	-22.442
3.880.479	-125.132	3.923.262	-28.014	3.878.287	-22.444
3.882.332	-125.114	3.923.423	-28.019	3.878.461	-22.446
3.883.658	-125.096	3.923.595	-28.024	3.878.641	-22.447
3.883.401	-125.077	3.923.727	-28.029	3.878.855	-22.449
3.883.199	-125.058	3.923.840	-28.034	3.879.074	-22.450
3.883.489	-125.039	3.924.031	-28.038	3.879.262	-22.452
3.882.337	-125.020	3.924.218	-28.043	3.879.418	-22.453
3.881.219	-125.000	3.924.371	-28.048	3.879.680	-22.455
3.882.706	-124.980	3.924.579	-28.053	3.879.908	-22.457
3.884.489	-124.960	3.924.826	-28.058	3.880.009	-22.458
3.883.890	-124.940	3.925.021	-28.063	3.880.132	-22.460
3.882.285	-124.920	3.925.164	-28.068	3.880.244	-22.461
3.882.518	-124.899	3.925.253	-28.074	3.880.352	-22.463
3.883.633	-124.878	3.925.342	-28.079	3.880.553	-22.465
3.884.684	-124.857	3.925.531	-28.084	3.880.719	-22.466
3.884.668	-124.836	3.925.728	-28.089	3.880.872	-22.468
3.883.623	-124.815	3.925.891	-28.094	3.881.019	-22.470
3.883.103	-124.793	3.926.094	-28.099	3.881.190	-22.471
3.883.539	-124.771	3.926.289	-28.105	3.881.365	-22.473
3.885.390	-124.749	3.926.436	-28.110	3.881.559	-22.474
3.886.531	-124.727	3.926.615	-28.115	3.881.765	-22.476

3.884.946	-124.705	3.926.777	-28.120	3.881.898	-22.478
3.883.871	-124.683	3.926.947	-28.126	3.882.063	-22.479
3.885.653	-124.661	3.927.102	-28.131	3.882.285	-22.481
3.885.963	-124.638	3.927.210	-28.137	3.882.511	-22.483
3.885.264	-124.616	3.927.420	-28.142	3.882.658	-22.484
3.886.743	-124.593	3.927.741	-28.147	3.882.784	-22.486
3.886.933	-124.571	3.928.106	-28.153	3.882.987	-22.488
3.885.288	-124.548	3.928.362	-28.158	3.883.175	-22.489
3.884.338	-124.525	3.928.459	-28.164	3.883.306	-22.491
3.885.166	-124.503	3.928.500	-28.169	3.883.497	-22.493
3.887.092	-124.480	3.928.529	-28.174	3.883.684	-22.494
3.886.870	-124.457	3.928.586	-28.180	3.883.858	-22.496
3.886.888	-124.434	3.928.709	-28.185	3.884.037	-22.498
3.887.011	-124.411	3.928.860	-28.191	3.884.124	-22.499
3.885.592	-124.388	3.928.976	-28.196	3.884.178	-22.501
3.885.180	-124.365	3.929.104	-28.202	3.884.362	-22.503
3.885.177	-124.343	3.929.276	-28.207	3.884.604	-22.504
3.885.252	-124.320	3.929.474	-28.213	3.884.790	-22.506
3.885.754	-124.297	3.929.627	-28.218	3.885.000	-22.507
3.887.257	-124.275	3.929.787	-28.224	3.885.126	-22.509
3.888.334	-124.252	3.930.005	-28.229	3.885.244	-22.511
3.887.863	-124.230	3.930.137	-28.235	3.885.443	-22.512
3.887.842	-124.207	3.930.336	-28.240	3.885.616	-22.514
3.888.951	-124.185	3.930.606	-28.245	3.885.771	-22.516
3.890.060	-124.163	3.930.721	-28.251	3.885.977	-22.517
3.890.421	-124.141	3.930.801	-28.256	3.886.151	-22.519
3.890.269	-124.119	3.930.984	-28.262	3.886.263	-22.520
3.890.495	-124.097	3.931.180	-28.267	3.886.443	-22.522
3.890.422	-124.075	3.931.350	-28.273	3.886.654	-22.524
3.890.477	-124.054	3.931.514	-28.278	3.886.830	-22.525

3.891.279	-124.032	3.931.675	-28.283	3.886.993	-22.527
3.892.138	-124.011	3.931.828	-28.289	3.887.123	-22.528
3.892.231	-123.990	3.931.917	-28.294	3.887.231	-22.530
3.891.564	-123.969	3.932.090	-28.299	3.887.460	-22.532
3.890.845	-123.948	3.932.312	-28.304	3.887.838	-22.533
3.889.734	-123.928	3.932.531	-28.310	3.888.210	-22.535
3.889.975	-123.908	3.932.693	-28.315	3.888.389	-22.536
3.891.577	-123.888	3.932.857	-28.320	3.888.398	-22.538
3.892.883	-123.868	3.933.065	-28.325	3.888.406	-22.539
3.893.398	-123.848	3.933.207	-28.330	3.888.504	-22.541
3.892.599	-123.829	3.933.394	-28.336	3.888.640	-22.542
3.890.907	-123.809	3.933.598	-28.341	3.888.719	-22.544
3.889.639	-123.790	3.933.749	-28.346	3.888.818	-22.545
3.890.036	-123.771	3.933.875	-28.351	3.888.987	-22.547
3.890.822	-123.753	3.934.019	-28.356	3.889.232	-22.548
3.890.642	-123.735	3.934.185	-28.361	3.889.469	-22.550
3.890.762	-123.717	3.934.292	-28.366	3.889.573	-22.551
3.892.108	-123.699	3.934.482	-28.371	3.889.675	-22.553
3.892.863	-123.681	3.934.696	-28.376	3.889.923	-22.554
3.891.927	-123.664	3.934.847	-28.381	3.890.164	-22.556
3.891.777	-123.647	3.935.053	-28.386	3.890.285	-22.557
3.892.437	-123.630	3.935.220	-28.391	3.890.462	-22.559
3.891.871	-123.613	3.935.328	-28.396	3.890.688	-22.560
3.891.452	-123.597	3.935.486	-28.402	3.890.794	-22.562
3.891.942	-123.581	3.935.707	-28.407	3.890.876	-22.563
3.892.362	-123.565	3.935.851	-28.412	3.891.062	-22.564
3.892.234	-123.549	3.935.945	-28.417	3.891.248	-22.566
3.892.113	-123.534	3.936.136	-28.422	3.891.396	-22.567
3.892.213	-123.518	3.936.315	-28.427	3.891.528	-22.569
3.892.308	-123.504	3.936.479	-28.432	3.891.606	-22.570

3.893.298	-123.489	3.936.721	-28.437	3.891.808	-22.571
3.895.086	-123.474	3.936.936	-28.442	3.892.072	-22.573
3.895.972	-123.460	3.937.086	-28.447	3.892.238	-22.574
3.896.707	-123.446	3.937.206	-28.452	3.892.452	-22.576
3.897.381	-123.432	3.937.355	-28.457	3.892.673	-22.577
3.896.931	-123.418	3.937.560	-28.462	3.892.838	-22.578
3.896.013	-123.405	3.937.688	-28.467	3.893.015	-22.580
3.894.617	-123.392	3.937.805	-28.472	3.893.194	-22.581
3.894.793	-123.378	3.938.018	-28.477	3.893.300	-22.582
3.897.274	-123.365	3.938.164	-28.482	3.893.396	-22.584
3.898.891	-123.352	3.938.300	-28.487	3.893.535	-22.585
3.898.990	-123.340	3.938.485	-28.493	3.893.673	-22.586
3.898.515	-123.327	3.938.696	-28.498	3.893.835	-22.588
3.897.239	-123.315	3.938.843	-28.503	3.894.014	-22.589
3.895.609	-123.302	3.938.951	-28.508	3.894.207	-22.590
3.896.255	-123.290	3.939.124	-28.513	3.894.391	-22.592
3.898.699	-123.278	3.939.285	-28.518	3.894.565	-22.593
3.900.045	-123.266	3.939.485	-28.523	3.894.745	-22.594
3.900.381	-123.253	3.939.619	-28.528	3.894.866	-22.596
3.899.826	-123.241	3.939.760	-28.533	3.895.017	-22.597
3.898.749	-123.229	3.939.955	-28.538	3.895.222	-22.598
3.898.087	-123.217	3.940.076	-28.543	3.895.363	-22.600
3.898.077	-123.206	3.940.201	-28.548	3.895.476	-22.601
3.898.369	-123.194	3.940.358	-28.554	3.895.625	-22.602
3.898.168	-123.182	3.940.515	-28.559	3.895.819	-22.603
3.897.776	-123.170	3.940.706	-28.564	3.896.019	-22.605
3.898.641	-123.158	3.940.873	-28.569	3.896.224	-22.606
3.899.220	-123.146	3.941.046	-28.574	3.896.376	-22.607
3.899.174	-123.134	3.941.266	-28.579	3.896.530	-22.609
3.900.179	-123.122	3.941.393	-28.584	3.896.765	-22.610

3.899.684	-123.110	3.941.578	-28.589	3.896.961	-22.611
3.898.970	-123.098	3.941.850	-28.594	3.897.112	-22.612
3.900.605	-123.086	3.942.036	-28.599	3.897.296	-22.614
3.900.603	-123.074	3.942.171	-28.603	3.897.487	-22.615
3.899.281	-123.062	3.942.362	-28.608	3.897.668	-22.616
3.900.542	-123.049	3.942.553	-28.613	3.897.799	-22.617
3.901.180	-123.037	3.942.676	-28.618	3.897.932	-22.618
3.899.858	-123.024	3.942.791	-28.623	3.898.094	-22.620
3.900.961	-123.012	3.942.897	-28.628	3.898.268	-22.621
3.902.928	-122.999	3.943.066	-28.632	3.898.445	-22.622
3.902.763	-122.986	3.943.226	-28.637	3.898.579	-22.623
3.901.335	-122.973	3.943.359	-28.642	3.898.734	-22.624
3.901.335	-122.959	3.943.488	-28.646	3.898.956	-22.625
3.903.630	-122.946	3.943.637	-28.651	3.899.133	-22.627
3.904.380	-122.932	3.943.792	-28.656	3.899.245	-22.628
3.903.026	-122.918	3.943.979	-28.660	3.899.352	-22.629
3.903.539	-122.904	3.944.223	-28.664	3.899.465	-22.630
3.904.258	-122.890	3.944.445	-28.669	3.899.616	-22.631
3.902.986	-122.876	3.944.599	-28.673	3.899.810	-22.632
3.901.802	-122.861	3.944.796	-28.678	3.899.975	-22.633
3.901.248	-122.846	3.945.029	-28.682	3.900.111	-22.634
3.902.406	-122.831	3.945.076	-28.686	3.900.247	-22.635
3.904.630	-122.816	3.945.188	-28.690	3.900.410	-22.636
3.905.092	-122.800	3.945.408	-28.694	3.900.621	-22.637
3.903.774	-122.784	3.945.599	-28.698	3.900.815	-22.638
3.902.863	-122.768	3.945.801	-28.702	3.900.995	-22.639
3.903.904	-122.752	3.945.995	-28.706	3.901.212	-22.640
3.904.150	-122.735	3.946.152	-28.710	3.901.481	-22.641
3.904.431	-122.718	3.946.259	-28.714	3.901.679	-22.641
3.905.054	-122.701	3.946.349	-28.718	3.901.750	-22.642

3.904.565	-122.683	3.946.494	-28.722	3.901.845	-22.643
3.905.447	-122.666	3.946.722	-28.725	3.902.052	-22.644
3.904.769	-122.647	3.946.866	-28.729	3.902.261	-22.645
3.904.445	-122.629	3.946.929	-28.732	3.902.377	-22.646
3.906.466	-122.610	3.947.168	-28.736	3.902.474	-22.646
3.906.293	-122.590	3.947.487	-28.739	3.902.661	-22.647
3.906.180	-122.571	3.947.684	-28.743	3.902.807	-22.648
3.906.492	-122.550	3.947.853	-28.746	3.902.900	-22.649
3.905.426	-122.530	3.948.031	-28.749	3.903.099	-22.649
3.906.546	-122.509	3.948.127	-28.752	3.903.265	-22.650
3.907.570	-122.488	3.948.163	-28.755	3.903.380	-22.651
3.906.024	-122.466	3.948.348	-28.758	3.903.569	-22.651
3.905.627	-122.443	3.948.578	-28.761	3.903.860	-22.652
3.907.336	-122.421	3.948.724	-28.764	3.904.102	-22.653
3.907.957	-122.397	3.948.912	-28.767	3.904.287	-22.654
3.906.949	-122.373	3.949.126	-28.770	3.904.526	-22.654
3.907.672	-122.349	3.949.341	-28.773	3.904.656	-22.655
3.908.485	-122.324	3.949.545	-28.775	3.904.765	-22.656
3.907.482	-122.299	3.949.622	-28.778	3.904.930	-22.656
3.907.896	-122.273	3.949.667	-28.780	3.905.031	-22.657
3.908.396	-122.247	3.949.825	-28.783	3.905.204	-22.657
3.908.481	-122.220	3.950.147	-28.785	3.905.393	-22.658
3.908.626	-122.192	3.950.578	-28.788	3.905.534	-22.659
3.909.403	-122.164	3.950.912	-28.790	3.905.694	-22.659
3.909.315	-122.135	3.951.078	-28.792	3.905.917	-22.660
3.908.360	-122.106	3.951.097	-28.794	3.906.129	-22.660
3.908.705	-122.076	3.951.098	-28.796	3.906.250	-22.661
3.907.993	-122.046	3.951.143	-28.798	3.906.387	-22.662
3.908.627	-122.014	3.951.252	-28.800	3.906.591	-22.662
3.909.785	-121.983	3.951.342	-28.802	3.906.880	-22.663

3.909.598	-121.950	3.951.454	-28.804	3.907.152	-22.663
3.910.307	-121.917	3.951.644	-28.805	3.907.276	-22.664
3.910.215	-121.884	3.951.841	-28.807	3.907.263	-22.665
3.910.831	-121.849	3.952.047	-28.809	3.907.375	-22.665
3.910.931	-121.814	3.952.193	-28.810	3.907.545	-22.666
3.909.641	-121.778	3.952.358	-28.811	3.907.701	-22.666
3.910.724	-121.742	3.952.540	-28.813	3.907.914	-22.667
3.910.665	-121.705	3.952.766	-28.814	3.908.134	-22.667
3.910.249	-121.667	3.953.015	-28.815	3.908.302	-22.668
3.911.307	-121.629	3.953.178	-28.816	3.908.425	-22.668
3.910.802	-121.589	3.953.324	-28.817	3.908.607	-22.669
3.911.064	-121.550	3.953.431	-28.818	3.908.734	-22.670
3.911.385	-121.509	3.953.548	-28.819	3.908.849	-22.670
3.911.368	-121.468	3.953.752	-28.819	3.908.947	-22.671
3.911.892	-121.426	3.953.961	-28.820	3.909.151	-22.671
3.912.039	-121.383	3.954.075	-28.821	3.909.606	-22.672
3.911.035	-121.340	3.954.144	-28.821	3.910.031	-22.672
3.912.283	-121.295	3.954.287	-28.822	3.910.208	-22.673
3.913.905	-121.251	3.954.480	-28.822	3.910.303	-22.674
3.912.744	-121.205	3.954.659	-28.822	3.910.432	-22.674
3.912.762	-121.159	3.954.880	-28.823	3.910.446	-22.675
3.914.854	-121.112	3.955.081	-28.823	3.910.453	-22.675
3.914.641	-121.064	3.955.233	-28.823	3.910.563	-22.676
3.913.333	-121.016	3.955.388	-28.823	3.910.665	-22.677
3.914.113	-120.967	3.955.524	-28.823	3.910.780	-22.677
3.914.290	-120.917	3.955.740	-28.823	3.910.942	-22.678
3.914.555	-120.866	3.955.975	-28.823	3.911.105	-22.678
3.914.811	-120.815	3.956.198	-28.823	3.911.302	-22.679
3.915.027	-120.763	3.956.404	-28.823	3.911.514	-22.680
3.915.151	-120.711	3.956.494	-28.823	3.911.665	-22.680

3.915.161	-120.657	3.956.534	-28.823	3.911.839	-22.681
3.915.193	-120.603	3.956.642	-28.823	3.912.081	-22.682
3.915.002	-120.549	3.956.824	-28.822	3.912.321	-22.682
3.915.063	-120.494	3.957.054	-28.822	3.912.487	-22.683
3.915.399	-120.438	3.957.260	-28.822	3.912.581	-22.684
3.915.462	-120.381	3.957.363	-28.821	3.912.677	-22.684
3.914.651	-120.324	3.957.520	-28.821	3.912.834	-22.685
3.915.999	-120.266	3.957.745	-28.820	3.913.065	-22.686
3.916.903	-120.208	3.957.882	-28.820	3.913.264	-22.686
3.916.080	-120.149	3.958.055	-28.819	3.913.305	-22.687
3.916.864	-120.089	3.958.257	-28.819	3.913.398	-22.688
3.916.870	-120.029	3.958.396	-28.818	3.913.626	-22.689
3.916.608	-119.968	3.958.564	-28.817	3.913.835	-22.689
3.917.630	-119.907	3.958.723	-28.817	3.914.023	-22.690
3.917.704	-119.845	3.958.896	-28.816	3.914.218	-22.691
3.917.174	-119.782	3.959.079	-28.815	3.914.418	-22.692
3.917.391	-119.719	3.959.218	-28.814	3.914.612	-22.693
3.917.711	-119.655	3.959.406	-28.813	3.914.814	-22.694
3.917.702	-119.591	3.959.579	-28.813	3.914.951	-22.694
3.917.795	-119.526	3.959.763	-28.812	3.915.045	-22.695
3.918.872	-119.460	3.959.948	-28.811	3.915.190	-22.696
3.919.073	-119.394	3.960.136	-28.810	3.915.345	-22.697
3.918.528	-119.328	3.960.287	-28.809	3.915.464	-22.698
3.919.638	-119.261	3.960.392	-28.808	3.915.562	-22.699
3.918.773	-119.193	3.960.538	-28.807	3.915.726	-22.700
3.919.041	-119.125	3.960.659	-28.806	3.915.956	-22.701
3.920.278	-119.057	3.960.827	-28.805	3.916.086	-22.702
3.919.026	-118.988	3.961.033	-28.804	3.916.237	-22.703
3.918.510	-118.918	3.961.198	-28.802	3.916.440	-22.704
3.919.421	-118.848	3.961.425	-28.801	3.916.615	-22.705

3.920.186	-118.778	3.961.653	-28.800	3.916.779	-22.706
3.919.908	-118.707	3.961.784	-28.799	3.916.910	-22.708
3.919.014	-118.635	3.961.906	-28.798	3.917.095	-22.709
3.919.207	-118.563	3.962.068	-28.796	3.917.256	-22.710
3.920.891	-118.491	3.962.163	-28.795	3.917.386	-22.711
3.922.376	-118.418	3.962.271	-28.794	3.917.554	-22.712
3.920.945	-118.345	3.962.368	-28.792	3.917.758	-22.714
3.920.605	-118.272	3.962.488	-28.791	3.917.951	-22.715
3.922.545	-118.198	3.962.777	-28.789	3.918.084	-22.716
3.922.039	-118.123	3.962.957	-28.788	3.918.260	-22.717
3.920.414	-118.049	3.963.033	-28.786	3.918.450	-22.719
3.921.609	-117.974	3.963.214	-28.785	3.918.612	-22.720
3.923.600	-117.898	3.963.412	-28.783	3.918.792	-22.721
3.923.289	-117.822	3.963.586	-28.781	3.918.886	-22.723
3.921.792	-117.746	3.963.828	-28.780	3.919.012	-22.724
3.921.066	-117.670	3.964.021	-28.778	3.919.245	-22.725
3.920.786	-117.593	3.964.151	-28.776	3.919.420	-22.727
3.920.899	-117.516	3.964.389	-28.775	3.919.604	-22.728
3.921.792	-117.438	3.964.680	-28.773	3.919.828	-22.730
3.923.745	-117.361	3.964.841	-28.771	3.919.987	-22.731
3.925.541	-117.283	3.964.949	-28.769	3.920.154	-22.732
3.925.816	-117.205	3.965.049	-28.767	3.920.363	-22.734
3.924.702	-117.126	3.965.168	-28.765	3.920.522	-22.735
3.922.835	-117.048	3.965.364	-28.763	3.920.634	-22.737
3.922.063	-116.969	3.965.540	-28.761	3.920.771	-22.738
3.922.883	-116.890	3.965.688	-28.759	3.920.967	-22.740
3.924.557	-116.811	3.965.851	-28.756	3.921.192	-22.741
3.924.417	-116.731	3.965.999	-28.754	3.921.340	-22.743
3.923.528	-116.652	3.966.076	-28.752	3.921.456	-22.744
3.924.742	-116.572	3.966.115	-28.750	3.921.596	-22.746

3.925.631	-116.493	3.966.271	-28.747	3.921.766	-22.747
3.925.459	-116.413	3.966.552	-28.745	3.921.882	-22.749
3.926.268	-116.333	3.966.824	-28.743	3.921.969	-22.750
3.927.586	-116.253	3.967.012	-28.740	3.922.159	-22.752
3.928.262	-116.173	3.967.151	-28.738	3.922.375	-22.753
3.928.550	-116.093	3.967.318	-28.735	3.922.520	-22.755
3.927.838	-116.013	3.967.416	-28.733	3.922.719	-22.756
3.926.398	-115.933	3.967.550	-28.731	3.923.006	-22.758
3.925.634	-115.853	3.967.755	-28.728	3.923.208	-22.759
3.925.276	-115.772	3.967.914	-28.725	3.923.337	-22.761
3.924.797	-115.692	3.968.059	-28.723	3.923.484	-22.762
3.924.765	-115.612	3.968.204	-28.720	3.923.637	-22.764
3.925.114	-115.532	3.968.460	-28.718	3.923.802	-22.765
3.925.963	-115.452	3.968.642	-28.715	3.923.991	-22.766
3.927.509	-115.372	3.968.800	-28.713	3.924.164	-22.768
3.929.328	-115.293	3.968.922	-28.710	3.924.261	-22.769
3.929.933	-115.213	3.969.074	-28.707	3.924.353	-22.771
3.928.618	-115.134	3.969.286	-28.705	3.924.493	-22.772
3.928.145	-115.054	3.969.402	-28.702	3.924.711	-22.774
3.928.564	-114.975	3.969.637	-28.699	3.924.892	-22.775
3.927.762	-114.896	3.969.872	-28.697	3.924.987	-22.777
3.926.716	-114.817	3.970.046	-28.694	3.925.143	-22.778
3.927.267	-114.739	3.970.130	-28.691	3.925.383	-22.780
3.929.666	-114.661	3.970.219	-28.689	3.925.613	-22.781
3.931.408	-114.582	3.970.562	-28.686	3.925.783	-22.783
3.931.592	-114.505	3.970.764	-28.683	3.925.938	-22.784
3.931.228	-114.427	3.970.836	-28.680	3.926.082	-22.785
3.929.810	-114.350	3.971.046	-28.678	3.926.263	-22.787
3.929.868	-114.273	3.971.252	-28.675	3.926.484	-22.788
3.931.911	-114.196	3.971.401	-28.672	3.926.671	-22.790

3.932.792	-114.120	3.971.555	-28.669	3.926.788	-22.791
3.931.766	-114.044	3.971.740	-28.666	3.926.921	-22.793
3.929.872	-113.968	3.971.911	-28.663	3.927.174	-22.794
3.928.821	-113.893	3.972.058	-28.660	3.927.387	-22.795
3.928.934	-113.818	3.972.174	-28.658	3.927.473	-22.797
3.929.402	-113.743	3.972.325	-28.655	3.927.581	-22.798
3.930.110	-113.669	3.972.546	-28.652	3.927.762	-22.800
3.932.043	-113.595	3.972.716	-28.649	3.927.964	-22.801
3.933.434	-113.521	3.973.081	-28.646	3.928.138	-22.802
3.932.832	-113.448	3.973.460	-28.643	3.928.318	-22.804
3.931.219	-113.376	3.973.607	-28.640	3.928.526	-22.805
3.931.893	-113.303	3.973.745	-28.637	3.928.690	-22.807
3.933.661	-113.232	3.973.853	-28.634	3.928.786	-22.808
3.934.649	-113.160	3.973.900	-28.631	3.928.990	-22.810
3.934.691	-113.089	3.973.987	-28.628	3.929.245	-22.811
3.933.749	-113.019	3.974.055	-28.625	3.929.415	-22.812
3.932.110	-112.948	3.974.157	-28.622	3.929.558	-22.814
3.931.284	-112.879	3.974.288	-28.619	3.929.727	-22.815
3.931.307	-112.810	3.974.360	-28.615	3.929.908	-22.817
3.931.711	-112.741	3.974.507	-28.612	3.930.014	-22.818
3.932.567	-112.672	3.974.753	-28.609	3.930.193	-22.819
3.932.747	-112.605	3.974.985	-28.606	3.930.365	-22.821
3.932.831	-112.537	3.975.212	-28.603	3.930.469	-22.822
3.933.742	-112.470	3.975.316	-28.600	3.930.652	-22.824
3.935.005	-112.404	3.975.436	-28.597	3.930.836	-22.825
3.936.068	-112.338	3.975.623	-28.594	3.930.953	-22.827
3.936.831	-112.272	3.975.725	-28.591	3.931.198	-22.828
3.937.173	-112.207	3.975.903	-28.588	3.931.651	-22.829
3.937.162	-112.143	3.976.158	-28.585	3.931.957	-22.831
3.937.328	-112.079	3.976.378	-28.582	3.932.086	-22.832

3.937.130	-112.015	3.976.560	-28.579	3.932.164	-22.834
3.935.642	-111.952	3.976.711	-28.577	3.932.198	-22.835
3.934.767	-111.890	3.976.785	-28.574	3.932.217	-22.837
3.936.108	-111.827	3.976.899	-28.571	3.932.300	-22.838
3.935.863	-111.766	3.977.050	-28.568	3.932.383	-22.839
3.934.714	-111.705	3.977.227	-28.566	3.932.510	-22.841
3.935.847	-111.644	3.977.435	-28.563	3.932.740	-22.842
3.937.762	-111.584	3.977.654	-28.560	3.932.904	-22.844
3.938.015	-111.524	3.977.863	-28.558	3.933.040	-22.845
3.936.418	-111.465	3.977.994	-28.555	3.933.235	-22.847
3.936.017	-111.406	3.978.193	-28.553	3.933.431	-22.848
3.937.856	-111.348	3.978.405	-28.550	3.933.601	-22.850
3.937.755	-111.290	3.978.477	-28.548	3.933.833	-22.851
3.936.185	-111.233	3.978.640	-28.546	3.934.048	-22.853
3.937.593	-111.176	3.978.835	-28.543	3.934.171	-22.854
3.938.620	-111.119	3.978.896	-28.541	3.934.315	-22.856
3.937.139	-111.064	3.979.060	-28.539	3.934.496	-22.857
3.937.199	-111.008	3.979.254	-28.537	3.934.608	-22.859
3.939.122	-110.953	3.979.373	-28.535	3.934.725	-22.860
3.938.638	-110.899	3.979.554	-28.534	3.934.886	-22.862
3.937.227	-110.844	3.979.751	-28.532	3.935.093	-22.863
3.938.378	-110.791	3.979.879	-28.530	3.935.288	-22.865
3.940.186	-110.738	3.980.074	-28.528	3.935.414	-22.866
3.939.620	-110.685	3.980.316	-28.527	3.935.580	-22.868
3.938.463	-110.633	3.980.414	-28.526	3.935.793	-22.869
3.939.940	-110.581	3.980.565	-28.524	3.936.021	-22.871
3.941.225	-110.529	3.980.754	-28.523	3.936.155	-22.873
3.941.118	-110.478	3.980.870	-28.522	3.936.261	-22.874
3.941.115	-110.428	3.981.048	-28.521	3.936.470	-22.876
3.940.396	-110.377	3.981.272	-28.520	3.936.646	-22.877

3.939.590	-110.327	3.981.483	-28.519	3.936.839	-22.879
3.938.943	-110.278	3.981.617	-28.518	3.937.008	-22.881
3.938.489	-110.229	3.981.744	-28.518	3.937.112	-22.882
3.939.099	-110.180	3.981.929	-28.517	3.937.242	-22.884
3.940.656	-110.132	3.982.170	-28.517	3.937.416	-22.886
3.940.786	-110.084	3.982.325	-28.517	3.937.641	-22.887
3.941.010	-110.036	3.982.440	-28.516	3.937.820	-22.889
3.942.021	-109.989	3.982.612	-28.516	3.937.979	-22.891
3.941.293	-109.942	3.982.762	-28.516	3.938.190	-22.893
3.941.539	-109.896	3.982.954	-28.516	3.938.324	-22.894
3.942.451	-109.849	3.983.106	-28.516	3.938.484	-22.896
3.942.487	-109.803	3.983.212	-28.517	3.938.651	-22.898
3.942.448	-109.758	3.983.418	-28.517	3.938.774	-22.899
3.942.278	-109.712	3.983.643	-28.517	3.938.885	-22.901
3.942.457	-109.667	3.983.800	-28.518	3.938.976	-22.903
3.942.257	-109.623	3.983.948	-28.519	3.939.160	-22.905
3.941.526	-109.578	3.984.118	-28.519	3.939.331	-22.907
3.942.932	-109.534	3.984.297	-28.520	3.939.544	-22.908
3.943.896	-109.490	3.984.443	-28.521	3.939.807	-22.910
3.942.299	-109.446	3.984.583	-28.522	3.939.944	-22.912
3.942.970	-109.403	3.984.702	-28.523	3.940.090	-22.914
3.943.837	-109.360	3.984.821	-28.524	3.940.281	-22.916
3.944.138	-109.317	3.985.009	-28.525	3.940.404	-22.918
3.944.872	-109.274	3.985.190	-28.527	3.940.551	-22.919
3.944.521	-109.231	3.985.300	-28.528	3.940.717	-22.921
3.944.529	-109.189	3.985.370	-28.530	3.940.884	-22.923
3.943.730	-109.147	3.985.501	-28.531	3.941.055	-22.925
3.943.611	-109.105	3.985.699	-28.533	3.941.190	-22.927
3.944.431	-109.063	3.985.884	-28.534	3.941.333	-22.929
3.944.895	-109.022	3.986.047	-28.536	3.941.521	-22.930

3.944.834	-108.980	3.986.275	-28.538	3.941.765	-22.932
3.946.029	-108.939	3.986.544	-28.540	3.941.962	-22.934
3.946.372	-108.898	3.986.745	-28.542	3.942.072	-22.936
3.945.090	-108.857	3.986.960	-28.544	3.942.167	-22.938
3.946.350	-108.816	3.987.177	-28.546	3.942.354	-22.940
3.946.675	-108.775	3.987.326	-28.548	3.942.549	-22.942
3.946.461	-108.735	3.987.491	-28.551	3.942.701	-22.943
3.946.669	-108.694	3.987.650	-28.553	3.942.899	-22.945
3.946.750	-108.654	3.987.751	-28.555	3.943.016	-22.947
3.947.416	-108.614	3.987.899	-28.558	3.943.120	-22.949
3.946.472	-108.573	3.988.019	-28.561	3.943.340	-22.951
3.946.196	-108.533	3.988.170	-28.563	3.943.467	-22.953
3.947.625	-108.493	3.988.316	-28.566	3.943.515	-22.954
3.948.163	-108.453	3.988.452	-28.569	3.943.666	-22.956
3.947.549	-108.414	3.988.651	-28.571	3.943.901	-22.958
3.948.113	-108.374	3.988.777	-28.574	3.944.131	-22.960
3.948.911	-108.334	3.988.930	-28.577	3.944.378	-22.961
3.947.575	-108.294	3.989.124	-28.580	3.944.612	-22.963
3.947.575	-108.255	3.989.310	-28.583	3.944.774	-22.965
3.949.053	-108.215	3.989.496	-28.587	3.944.952	-22.967
3.948.192	-108.176	3.989.686	-28.590	3.945.172	-22.968
3.947.571	-108.136	3.989.933	-28.593	3.945.327	-22.970
3.949.497	-108.097	3.990.141	-28.596	3.945.440	-22.972
3.949.684	-108.057	3.990.298	-28.600	3.945.677	-22.973
3.948.979	-108.018	3.990.486	-28.603	3.945.899	-22.975
3.950.797	-107.979	3.990.635	-28.606	3.945.920	-22.977
3.952.039	-107.939	3.990.712	-28.610	3.945.974	-22.978
3.950.537	-107.900	3.990.849	-28.613	3.946.154	-22.980
3.949.091	-107.861	3.991.082	-28.617	3.946.268	-22.982
3.949.627	-107.822	3.991.311	-28.621	3.946.342	-22.983

3.951.740	-107.782	3.991.449	-28.624	3.946.570	-22.985
3.952.971	-107.743	3.991.494	-28.628	3.946.833	-22.986
3.952.883	-107.704	3.991.693	-28.632	3.946.956	-22.988
3.952.112	-107.665	3.991.978	-28.636	3.947.167	-22.989
3.951.448	-107.626	3.992.109	-28.639	3.947.449	-22.991
3.952.474	-107.586	3.992.239	-28.643	3.947.619	-22.992
3.953.612	-107.547	3.992.404	-28.647	3.947.769	-22.994
3.954.027	-107.508	3.992.552	-28.651	3.947.953	-22.995
3.953.652	-107.469	3.992.730	-28.655	3.948.112	-22.997
3.951.913	-107.430	3.992.911	-28.659	3.948.210	-22.998
3.950.729	-107.391	3.993.065	-28.663	3.948.320	-23.000
3.952.036	-107.352	3.993.214	-28.667	3.948.508	-23.001
3.953.876	-107.312	3.993.337	-28.671	3.948.698	-23.003
3.953.197	-107.273	3.993.546	-28.675	3.948.866	-23.004
3.951.852	-107.234	3.993.766	-28.679	3.949.048	-23.005
3.953.275	-107.195	3.993.900	-28.683	3.949.238	-23.007
3.954.102	-107.156	3.994.066	-28.687	3.949.393	-23.008
3.952.316	-107.117	3.994.214	-28.691	3.949.556	-23.009
3.952.982	-107.078	3.994.381	-28.695	3.949.789	-23.011
3.954.348	-107.039	3.994.588	-28.699	3.949.998	-23.012
3.954.153	-107.000	3.994.709	-28.703	3.950.115	-23.013
3.954.393	-106.961	3.994.803	-28.707	3.950.296	-23.015
3.954.951	-106.922	3.994.958	-28.711	3.950.491	-23.016
3.955.024	-106.883	3.995.170	-28.715	3.950.569	-23.017
3.954.108	-106.844	3.995.614	-28.719	3.950.670	-23.019
3.953.860	-106.805	3.996.079	-28.723	3.950.856	-23.020
3.954.374	-106.766	3.996.281	-28.728	3.951.089	-23.021
3.954.527	-106.727	3.996.384	-28.732	3.951.306	-23.022
3.954.695	-106.688	3.996.404	-28.736	3.951.454	-23.024
3.955.211	-106.649	3.996.350	-28.740	3.951.548	-23.025

3.955.896	-106.610	3.996.407	-28.744	3.951.743	-23.026
3.955.252	-106.571	3.996.476	-28.748	3.951.946	-23.027
3.955.580	-106.533	3.996.586	-28.752	3.952.133	-23.029
3.957.814	-106.494	3.996.729	-28.756	3.952.325	-23.030
3.958.413	-106.455	3.996.862	-28.760	3.952.466	-23.031
3.957.141	-106.416	3.997.069	-28.764	3.952.621	-23.032
3.955.285	-106.377	3.997.329	-28.767	3.952.722	-23.034
3.954.927	-106.338	3.997.464	-28.771	3.952.961	-23.035
3.955.233	-106.299	3.997.567	-28.775	3.953.435	-23.036
3.956.163	-106.261	3.997.813	-28.779	3.953.770	-23.037
3.957.921	-106.222	3.998.070	-28.783	3.953.894	-23.039
3.958.112	-106.183	3.998.273	-28.787	3.953.958	-23.040
3.956.831	-106.145	3.998.409	-28.791	3.953.969	-23.041
3.957.673	-106.106	3.998.510	-28.795	3.953.990	-23.042
3.958.243	-106.067	3.998.667	-28.798	3.954.039	-23.043
3.956.526	-106.029	3.998.824	-28.802	3.954.109	-23.045
3.956.784	-105.990	3.998.915	-28.806	3.954.240	-23.046
3.958.492	-105.951	3.999.109	-28.810	3.954.458	-23.047
3.959.789	-105.913	3.999.327	-28.813	3.954.655	-23.048
3.958.745	-105.875	3.999.471	-28.817	3.954.828	-23.050
3.957.553	-105.836	3.999.619	-28.821	3.955.027	-23.051
3.959.411	-105.798	3.999.742	-28.825	3.955.216	-23.052
3.959.966	-105.759	3.999.901	-28.828	3.955.368	-23.054
3.958.427	-105.721	4.000.122	-28.832	3.955.519	-23.055
3.957.522	-105.683	4.000.303	-28.835	3.955.714	-23.056
3.958.013	-105.645	4.000.488	-28.839	3.955.892	-23.058
3.960.125	-105.606	4.000.636	-28.843	3.956.061	-23.059
3.961.299	-105.568	4.000.789	-28.846	3.956.259	-23.060
3.959.768	-105.530	4.000.923	-28.850	3.956.465	-23.062
3.959.139	-105.492	4.001.068	-28.853	3.956.570	-23.063

3.961.337	-105.454	4.001.304	-28.857	3.956.647	-23.064
3.961.608	-105.416	4.001.461	-28.860	3.956.783	-23.066
3.959.486	-105.378	4.001.590	-28.864	3.956.929	-23.067
3.960.383	-105.340	4.001.785	-28.867	3.957.134	-23.069
3.962.332	-105.303	4.001.920	-28.870	3.957.307	-23.070
3.961.227	-105.265	4.002.052	-28.874	3.957.448	-23.072
3.961.022	-105.227	4.002.224	-28.877	3.957.646	-23.073
3.962.935	-105.189	4.002.430	-28.880	3.957.789	-23.075
3.963.571	-105.152	4.002.640	-28.884	3.958.002	-23.076
3.962.729	-105.114	4.002.805	-28.887	3.958.334	-23.078
3.961.158	-105.077	4.002.972	-28.890	3.958.488	-23.079
3.960.580	-105.039	4.003.109	-28.893	3.958.564	-23.081
3.962.664	-105.002	4.003.283	-28.896	3.958.681	-23.083
3.963.656	-104.964	4.003.441	-28.899	3.958.875	-23.084
3.961.870	-104.927	4.003.521	-28.902	3.959.014	-23.086
3.961.685	-104.890	4.003.607	-28.905	3.959.096	-23.088
3.963.663	-104.852	4.003.774	-28.908	3.959.338	-23.089
3.963.854	-104.815	4.004.064	-28.911	3.959.544	-23.091
3.962.610	-104.778	4.004.355	-28.914	3.959.617	-23.093
3.963.320	-104.741	4.004.574	-28.917	3.959.749	-23.095
3.964.624	-104.704	4.004.648	-28.919	3.959.953	-23.096
3.963.644	-104.667	4.004.678	-28.922	3.960.168	-23.098
3.963.853	-104.630	4.004.836	-28.925	3.960.352	-23.100
3.964.763	-104.593	4.005.107	-28.927	3.960.531	-23.102
3.964.052	-104.557	4.005.332	-28.930	3.960.681	-23.104
3.965.327	-104.520	4.005.435	-28.932	3.960.747	-23.106
3.966.940	-104.483	4.005.634	-28.935	3.960.954	-23.108
3.965.536	-104.447	4.005.847	-28.937	3.961.185	-23.110
3.964.124	-104.410	4.005.931	-28.940	3.961.320	-23.112
3.964.919	-104.374	4.006.014	-28.942	3.961.465	-23.114

3.966.057	-104.337	4.006.210	-28.944	3.961.595	-23.116
3.967.101	-104.301	4.006.436	-28.947	3.961.750	-23.118
3.966.696	-104.264	4.006.564	-28.949	3.961.942	-23.120
3.964.604	-104.228	4.006.687	-28.951	3.962.198	-23.122
3.964.023	-104.192	4.006.906	-28.953	3.962.451	-23.124
3.965.457	-104.156	4.007.106	-28.955	3.962.564	-23.126
3.967.083	-104.120	4.007.249	-28.957	3.962.661	-23.128
3.966.568	-104.083	4.007.419	-28.959	3.962.760	-23.130
3.965.647	-104.047	4.007.549	-28.961	3.962.870	-23.132
3.967.167	-104.012	4.007.619	-28.963	3.963.034	-23.135
3.968.904	-103.976	4.007.766	-28.965	3.963.192	-23.137
3.968.785	-103.940	4.007.961	-28.967	3.963.392	-23.139
3.967.334	-103.904	4.008.074	-28.969	3.963.546	-23.141
3.966.071	-103.868	4.008.185	-28.970	3.963.790	-23.143
3.966.195	-103.833	4.008.399	-28.972	3.963.909	-23.145
3.967.975	-103.797	4.008.597	-28.974	3.964.102	-23.148
3.969.323	-103.761	4.008.775	-28.975	3.964.350	-23.150
3.968.728	-103.726	4.009.023	-28.977	3.964.460	-23.152
3.967.333	-103.691	4.009.218	-28.978	3.964.622	-23.154
3.967.590	-103.655	4.009.377	-28.980	3.964.786	-23.157
3.969.153	-103.620	4.009.604	-28.981	3.964.911	-23.159
3.970.228	-103.585	4.009.812	-28.982	3.965.060	-23.161
3.971.033	-103.549	4.009.969	-28.984	3.965.213	-23.163
3.971.315	-103.514	4.010.117	-28.985	3.965.343	-23.165
3.970.972	-103.479	4.010.280	-28.986	3.965.443	-23.168
3.971.102	-103.444	4.010.411	-28.987	3.965.636	-23.170
3.971.762	-103.409	4.010.533	-28.988	3.965.830	-23.172
3.972.218	-103.374	4.010.719	-28.989	3.966.030	-23.174
3.972.522	-103.339	4.010.851	-28.990	3.966.292	-23.177
3.972.682	-103.304	4.010.993	-28.991	3.966.526	-23.179

3.971.302	-103.269	4.011.154	-28.992	3.966.729	-23.181
3.969.323	-103.234	4.011.317	-28.993	3.966.898	-23.183
3.969.775	-103.199	4.011.508	-28.994	3.967.088	-23.185
3.971.430	-103.164	4.011.638	-28.995	3.967.281	-23.187
3.972.632	-103.130	4.011.801	-28.996	3.967.458	-23.190
3.973.593	-103.095	4.012.047	-28.997	3.967.589	-23.192
3.974.043	-103.060	4.012.235	-28.997	3.967.708	-23.194
3.973.831	-103.026	4.012.401	-28.998	3.967.875	-23.196
3.972.185	-102.991	4.012.604	-28.999	3.968.000	-23.198
3.971.125	-102.957	4.012.738	-28.999	3.968.080	-23.200
3.972.545	-102.922	4.012.852	-29.000	3.968.232	-23.202
3.972.690	-102.888	4.013.011	-29.001	3.968.376	-23.204
3.971.501	-102.853	4.013.134	-29.001	3.968.553	-23.206
3.971.465	-102.819	4.013.279	-29.002	3.968.773	-23.208
3.971.510	-102.785	4.013.504	-29.002	3.968.947	-23.210
3.971.696	-102.751	4.013.702	-29.003	3.969.095	-23.212
3.972.200	-102.716	4.013.866	-29.003	3.969.254	-23.214
3.972.081	-102.682	4.013.995	-29.003	3.969.479	-23.216
3.971.944	-102.648	4.014.156	-29.004	3.969.659	-23.218
3.972.164	-102.614	4.014.321	-29.004	3.969.843	-23.220
3.973.160	-102.580	4.014.439	-29.004	3.970.004	-23.222
3.975.097	-102.546	4.014.653	-29.005	3.970.105	-23.224
3.975.430	-102.512	4.014.843	-29.005	3.970.276	-23.226
3.973.750	-102.478	4.014.957	-29.005	3.970.502	-23.228
3.973.652	-102.445	4.015.110	-29.005	3.970.706	-23.229
3.975.535	-102.411	4.015.270	-29.005	3.970.800	-23.231
3.976.638	-102.377	4.015.451	-29.005	3.970.892	-23.233
3.977.054	-102.344	4.015.681	-29.005	3.971.037	-23.235
3.977.482	-102.310	4.015.891	-29.005	3.971.223	-23.237
3.977.267	-102.276	4.016.026	-29.005	3.971.429	-23.238

3.976.270	-102.243	4.016.207	-29.005	3.971.655	-23.240
3.975.569	-102.209	4.016.355	-29.005	3.971.828	-23.242
3.975.919	-102.176	4.016.455	-29.005	3.971.954	-23.243
3.975.295	-102.142	4.016.643	-29.005	3.972.159	-23.245
3.974.258	-102.109	4.016.852	-29.005	3.972.344	-23.247
3.974.652	-102.076	4.017.088	-29.005	3.972.466	-23.248
3.975.924	-102.043	4.017.245	-29.005	3.972.654	-23.250
3.977.782	-102.009	4.017.366	-29.004	3.972.849	-23.251
3.979.138	-101.976	4.017.532	-29.004	3.973.044	-23.253
3.979.344	-101.943	4.017.878	-29.004	3.973.228	-23.254
3.978.636	-101.910	4.018.312	-29.004	3.973.293	-23.256
3.976.928	-101.877	4.018.542	-29.003	3.973.405	-23.257
3.975.455	-101.844	4.018.633	-29.003	3.973.683	-23.259
3.975.414	-101.812	4.018.683	-29.003	3.973.940	-23.260
3.976.902	-101.779	4.018.759	-29.002	3.974.034	-23.262
3.978.135	-101.746	4.018.803	-29.002	3.974.164	-23.263
3.977.401	-101.713	4.018.912	-29.001	3.974.268	-23.264
3.976.451	-101.681	4.019.034	-29.001	3.974.363	-23.266
3.976.149	-101.648	4.019.169	-29.001	3.974.745	-23.267
3.976.490	-101.616	4.019.324	-29.000	3.975.204	-23.268
3.977.668	-101.583	4.019.503	-29.000	3.975.488	-23.270
3.979.728	-101.551	4.019.698	-28.999	3.975.583	-23.271
3.981.438	-101.519	4.019.786	-28.999	3.975.609	-23.272
3.980.988	-101.487			3.975.674	-23.273
3.979.237	-101.454			3.975.743	-23.275
3.979.772	-101.422			3.975.822	-23.276
3.981.906	-101.390			3.975.935	-23.277
3.982.475	-101.358			3.976.030	-23.278
3.981.632	-101.326			3.976.194	-23.280
3.979.931	-101.294			3.976.389	-23.281

3.979.829	-101.262			3.976.531	-23.282
3.981.680	-101.231			3.976.733	-23.283
3.982.007	-101.199			3.976.985	-23.284
3.980.219	-101.167			3.977.184	-23.285
3.978.974	-101.135			3.977.265	-23.287
3.979.584	-101.104			3.977.405	-23.288
3.981.387	-101.072			3.977.605	-23.289
3.983.085	-101.041			3.977.758	-23.290
3.983.420	-101.009			3.977.868	-23.291
3.983.394	-100.978			3.977.936	-23.292
3.983.992	-100.946			3.978.143	-23.294
3.982.932	-100.915			3.978.439	-23.295
3.981.028	-100.884			3.978.659	-23.296
3.981.485	-100.852			3.978.788	-23.297
3.981.833	-100.821			3.978.831	-23.298
3.981.066	-100.790			3.978.966	-23.300
3.981.855	-100.759			3.979.161	-23.301
3.984.205	-100.727			3.979.388	-23.302
3.984.151	-100.696			3.979.630	-23.303
3.982.249	-100.665			3.979.776	-23.305
3.982.048	-100.634			3.979.933	-23.306
3.983.041	-100.603			3.980.089	-23.307
3.984.942	-100.572			3.980.219	-23.308
3.984.920	-100.541			3.980.381	-23.310
3.983.153	-100.510			3.980.504	-23.311
3.983.658	-100.479			3.980.663	-23.312
3.985.844	-100.448			3.980.847	-23.314
3.986.130	-100.417			3.980.942	-23.315
3.984.583	-100.386			3.981.120	-23.317
3.984.469	-100.355			3.981.362	-23.318

3.986.095	-100.324			3.981.557	-23.319
3.987.043	-100.293			3.981.721	-23.321
3.986.827	-100.262			3.981.895	-23.322
3.986.517	-100.231			3.982.084	-23.324
3.987.047	-100.200			3.982.247	-23.326
3.988.253	-100.169			3.982.411	-23.327
3.988.885	-100.137			3.982.542	-23.329
3.987.680	-100.106			3.982.683	-23.330
3.985.542	-100.075			3.982.853	-23.332
3.985.407	-100.044			3.983.027	-23.334
3.987.505	-100.013			3.983.242	-23.335
3.989.160	-99.982			3.983.401	-23.337
3.987.885	-99.951			3.983.528	-23.339
3.986.100	-99.920			3.983.696	-23.340
3.986.073	-99.889			3.983.947	-23.342
3.986.620	-99.858			3.984.164	-23.344
3.987.079	-99.826			3.984.301	-23.346
3.987.026	-99.795			3.984.449	-23.348
3.986.873	-99.764			3.984.550	-23.350
3.987.094	-99.733			3.984.674	-23.352
3.987.134	-99.702			3.984.815	-23.353
3.986.956	-99.670			3.984.966	-23.355
3.988.150	-99.639			3.985.111	-23.357
3.990.423	-99.608			3.985.299	-23.359
3.991.245	-99.576			3.985.606	-23.361
3.990.988	-99.545			3.985.789	-23.363
3.991.524	-99.513			3.985.833	-23.365
3.992.433	-99.482			3.986.019	-23.368
3.992.762	-99.451			3.986.196	-23.370
3.992.634	-99.419			3.986.243	-23.372

3.992.086	-99.387			3.986.337	-23.374
3.990.804	-99.356			3.986.466	-23.376
3.989.577	-99.324			3.986.600	-23.378
3.990.664	-99.292			3.986.751	-23.380
3.992.858	-99.261			3.986.982	-23.382
3.992.686	-99.229			3.987.246	-23.385
3.990.746	-99.197			3.987.419	-23.387
3.989.908	-99.165			3.987.565	-23.389
3.991.744	-99.133			3.987.720	-23.391
3.993.972	-99.101			3.987.948	-23.394
3.993.584	-99.069			3.988.174	-23.396
3.991.575	-99.037			3.988.323	-23.398
3.991.304	-99.005			3.988.518	-23.401
3.993.618	-98.973			3.988.690	-23.403
3.995.128	-98.941			3.988.829	-23.405
3.994.924	-98.909			3.988.990	-23.408
3.994.391	-98.877			3.989.113	-23.410
3.994.135	-98.845			3.989.270	-23.412
3.993.918	-98.812			3.989.451	-23.415
3.993.648	-98.780			3.989.594	-23.417
3.994.277	-98.748			3.989.728	-23.420
3.995.677	-98.715			3.989.857	-23.422
3.996.534	-98.683			3.990.027	-23.425
3.996.451	-98.650			3.990.271	-23.427
3.995.721	-98.618			3.990.462	-23.429
3.994.749	-98.585			3.990.555	-23.432
3.994.471	-98.552			3.990.728	-23.434
3.994.350	-98.520			3.990.970	-23.437
3.994.255	-98.487			3.991.172	-23.439
3.993.994	-98.454			3.991.326	-23.442

3.993.622	-98.421			3.991.474	-23.444
3.993.710	-98.389			3.991.585	-23.447
3.993.713	-98.356			3.991.772	-23.450
3.994.991	-98.323			3.992.012	-23.452
3.997.554	-98.290			3.992.169	-23.455
3.998.961	-98.257			3.992.293	-23.457
3.998.098	-98.224			3.992.469	-23.460
3.996.058	-98.191			3.992.652	-23.462
3.996.064	-98.158			3.992.827	-23.465
3.998.221	-98.125			3.992.961	-23.468
3.998.141	-98.091			3.993.128	-23.470
3.996.158	-98.058			3.993.344	-23.473
3.995.332	-98.025			3.993.481	-23.475
3.995.331	-97.992			3.993.600	-23.478
3.995.488	-97.959			3.993.759	-23.480
3.995.805	-97.925			3.993.940	-23.483
3.996.003	-97.892			3.994.096	-23.486
3.996.438	-97.859			3.994.290	-23.488
3.998.544	-97.825			3.994.487	-23.491
4.000.630	-97.792			3.994.580	-23.493
4.000.744	-97.758			3.994.742	-23.496
3.999.414	-97.725			3.994.981	-23.498
3.998.055	-97.692			3.995.118	-23.501
3.998.672	-97.658			3.995.285	-23.504
4.000.939	-97.625			3.995.479	-23.506
4.002.263	-97.591			3.995.656	-23.509
4.002.094	-97.557			3.995.775	-23.511
4.001.559	-97.524			3.995.877	-23.514
4.000.764	-97.490			3.996.144	-23.516
3.999.841	-97.457			3.996.593	-23.519

3.999.301	-97.423			3.996.855	-23.521
3.999.171	-97.389			3.997.003	-23.524
3.998.796	-97.356			3.997.120	-23.527
3.998.705	-97.322			3.997.169	-23.529
4.000.144	-97.289			3.997.240	-23.532
4.002.285	-97.255			3.997.303	-23.534
4.003.289	-97.221			3.997.381	-23.537
4.003.271	-97.188			3.997.542	-23.539
4.002.446	-97.154			3.997.740	-23.542
4.001.187	-97.120			3.997.921	-23.544
4.001.873	-97.087			3.998.146	-23.547
4.003.515	-97.053			3.998.341	-23.549
4.002.639	-97.019			3.998.456	-23.551
4.001.295	-96.986			3.998.619	-23.554
4.001.447	-96.952			3.998.849	-23.556
4.002.701	-96.918			3.998.961	-23.559
4.004.299	-96.885			3.999.053	-23.561
4.004.980	-96.851			3.999.218	-23.564
4.005.184	-96.817			3.999.364	-23.566
4.003.582	-96.784			3.999.608	-23.569
4.002.986	-96.750			3.999.868	-23.571
4.004.547	-96.717			3.999.969	-23.573
4.005.588	-96.683			4.000.068	-23.576
4.006.194	-96.650			4.000.183	-23.578
4.005.941	-96.617			4.000.352	-23.581
4.006.126	-96.583			4.000.515	-23.583
4.006.595	-96.550			4.000.627	-23.586
4.007.032	-96.517			4.000.830	-23.588
4.007.081	-96.484			4.001.053	-23.591
4.005.381	-96.451			4.001.243	-23.593

4.004.443	-96.418			4.001.368	-23.595
4.005.115	-96.385			4.001.501	-23.598
4.006.297	-96.352			4.001.723	-23.600
4.007.791	-96.319			4.001.949	-23.603
4.008.002	-96.286			4.002.092	-23.605
4.006.563	-96.254			4.002.210	-23.608
4.005.078	-96.221			4.002.329	-23.610
4.005.248	-96.189			4.002.455	-23.613
4.006.238	-96.156			4.002.630	-23.615
4.006.101	-96.124			4.002.847	-23.618
4.005.563	-96.092			4.003.038	-23.621
4.005.883	-96.060			4.003.127	-23.623
4.007.137	-96.028			4.003.282	-23.626
4.008.608	-95.996			4.003.506	-23.628
4.009.360	-95.964			4.003.654	-23.631
4.010.074	-95.933			4.003.842	-23.633
4.010.591	-95.901			4.004.012	-23.636
4.009.919	-95.870			4.004.125	-23.639
4.008.171	-95.839			4.004.349	-23.641
4.006.816	-95.807			4.004.573	-23.644
4.006.719	-95.776			4.004.710	-23.646
4.006.940	-95.746			4.004.886	-23.649
4.006.991	-95.715			4.005.036	-23.652
4.007.110	-95.684			4.005.158	-23.654
4.007.717	-95.654			4.005.365	-23.657
4.008.754	-95.624			4.005.562	-23.660
4.009.558	-95.593			4.005.689	-23.662
4.010.827	-95.563			4.005.869	-23.665
4.012.297	-95.534			4.006.047	-23.668
4.011.602	-95.504			4.006.188	-23.670

4.010.408	-95.474			4.006.358	-23.673
4.009.646	-95.445			4.006.538	-23.676
4.010.158	-95.416			4.006.638	-23.678
4.012.010	-95.387			4.006.765	-23.681
4.012.881	-95.358			4.006.998	-23.684
4.012.788	-95.330			4.007.195	-23.686
4.013.071	-95.302			4.007.379	-23.689
4.014.128	-95.275			4.007.516	-23.692
4.014.283	-95.248			4.007.636	-23.694
4.014.247	-95.221			4.007.803	-23.697
4.014.751	-95.194			4.007.966	-23.700
4.014.733	-95.168			4.008.074	-23.702
4.014.265	-95.142			4.008.091	-23.705
4.014.074	-95.117			4.008.228	-23.708
4.012.619	-95.092			4.008.483	-23.710
4.011.819	-95.067			4.008.692	-23.713
4.013.814	-95.042			4.008.865	-23.716
4.015.529	-95.018			4.009.037	-23.718
4.015.936	-94.994			4.009.202	-23.721
4.015.771	-94.971			4.009.391	-23.724
4.015.735	-94.947			4.009.662	-23.726
4.015.137	-94.924			4.009.851	-23.729
4.014.523	-94.902			4.009.946	-23.731
4.015.605	-94.880			4.010.096	-23.734
4.016.676	-94.858			4.010.309	-23.737
4.015.978	-94.836			4.010.464	-23.739
4.014.566	-94.815			4.010.616	-23.742
4.013.839	-94.794			4.010.768	-23.744
4.013.407	-94.774			4.010.876	-23.747
4.013.262	-94.753			4.011.022	-23.749

4.013.878	-94.733			4.011.194	-23.752
4.014.692	-94.714			4.011.347	-23.754
4.016.115	-94.695			4.011.544	-23.757
4.017.531	-94.676			4.011.653	-23.759
4.016.436	-94.657			4.011.828	-23.762
4.015.928	-94.639			4.012.076	-23.764
4.017.940	-94.621			4.012.267	-23.766
4.018.376	-94.603			4.012.467	-23.769
4.016.434	-94.586			4.012.623	-23.771
4.015.475	-94.569			4.012.789	-23.773
4.016.834	-94.552			4.012.972	-23.776
4.017.946	-94.536			4.013.145	-23.778
4.017.961	-94.520			4.013.326	-23.780
4.018.511	-94.505			4.013.488	-23.782
4.017.870	-94.489			4.013.651	-23.785
4.017.538	-94.474			4.013.815	-23.787
4.018.022	-94.460			4.013.947	-23.789
4.017.300	-94.445			4.014.070	-23.791
4.017.871	-94.431			4.014.240	-23.793
4.019.393	-94.417			4.014.451	-23.795
4.018.831	-94.404			4.014.700	-23.797
4.018.434	-94.391			4.014.883	-23.799
4.019.563	-94.378			4.014.984	-23.801
				4.015.211	-23.803
				4.015.403	-23.805
				4.015.547	-23.807
				4.015.715	-23.809
				4.015.844	-23.811
				4.016.042	-23.813
				4.016.209	-23.814

				4.016.261	-23.816
				4.016.393	-23.818
				4.016.613	-23.819
				4.016.795	-23.821
				4.016.938	-23.823
				4.017.061	-23.824
				4.017.256	-23.826
				4.017.401	-23.827
				4.017.675	-23.829
				4.018.147	-23.830
				4.018.442	-23.832
				4.018.477	-23.833
				4.018.503	-23.835
				4.018.660	-23.836
				4.018.756	-23.837
				4.018.846	-23.839
				4.018.954	-23.840
				4.019.030	-23.841
				4.019.211	-23.842
				4.019.362	-23.844
				4.019.469	-23.845
				4.019.652	-23.846
				4.019.883	-23.847
