# Supplementary material for: Data on statistical experimental design to formulate amphotericin B-loaded Eudragit RL100 nanoparticles coated with hyaluronic acid for the treatment of vulvovaginal candidiasis
Source: Data Brief. 2020 Mar 5;29:105311. doi: 10.1016/j.dib.2020.105311 (PMC7082528; doi:10.1016/j.dib.2020.105311)
Supplement: Multimedia component 9 [file mmc9.pdf]

File Name:	**EUD nanoparticles**	File Name:	**AMP EUD nano**	File Name:	**EUD nano HA**
[Data]		[Data]		[Data]	
Temp	**DSC**	Temp	**DSC**	Temp	**DSC**
C	mW	C	mW	C	mW
237.999	-549	261.927	-95	214.314	-49
238.006	-562	261.973	-101	214.258	-56
238.001	-575	261.880	-108	214.200	-62
237.983	-589	261.784	-115	214.212	-70
237.980	-603	261.646	-122	214.251	-77
237.936	-618	261.479	-130	214.277	-84
237.896	-632	261.341	-138	214.230	-92
237.910	-647	261.274	-146	214.182	-100
237.959	-662	261.327	-155	214.235	-109
238.027	-677	261.360	-163	214.291	-118
238.071	-693	261.371	-173	214.258	-127
238.067	-709	261.364	-182	214.251	-136
238.060	-725	261.323	-192	214.247	-146
238.074	-742	261.264	-202	214.223	-156
238.088	-758	261.313	-213	214.105	-167
238.086	-775	261.402	-224	213.999	-178
238.043	-793	261.465	-235	214.119	-189
238.102	-810	261.458	-247	214.184	-201
238.165	-828	261.395	-259	214.121	-213
238.165	-846	261.383	-272	214.069	-225
238.153	-864	261.399	-285	214.041	-238
238.121	-883	261.430	-298	214.104	-251
238.149	-902	261.386	-312	214.172	-265
238.181	-921	261.346	-327	214.207	-279
238.179	-941	261.327	-341	214.226	-293

238.156	-961	261.309	-356	214.216	-308
238.116	-981	261.276	-372	214.280	-323
238.083	-1.001	261.238	-388	214.366	-339
238.088	-1.022	261.323	-405	214.429	-355
238.102	-1.043	261.434	-422	214.454	-372
238.120	-1.064	261.455	-439	214.456	-389
238.137	-1.086	261.439	-457	214.422	-406
238.163	-1.108	261.486	-476	214.389	-424
238.151	-1.130	261.511	-495	214.401	-443
238.106	-1.152	261.484	-515	214.391	-462
238.169	-1.175	261.516	-535	214.328	-482
238.240	-1.198	261.539	-555	214.265	-502
238.242	-1.221	261.479	-577	214.251	-522
238.211	-1.245	261.376	-598	214.287	-543
238.265	-1.269	261.381	-621	214.308	-565
238.314	-1.293	261.472	-643	214.328	-587
238.260	-1.318	261.526	-667	214.315	-610
238.216	-1.343	261.602	-691	214.300	-633
238.200	-1.368	261.632	-715	214.399	-657
238.340	-1.393	261.581	-741	214.403	-681
238.634	-1.419	261.572	-766	214.384	-706
238.865	-1.445	261.614	-793	214.473	-731
238.892	-1.471	261.639	-820	214.436	-758
238.832	-1.498	261.640	-848	214.454	-784
238.727	-1.525	261.658	-876	214.599	-811
238.589	-1.552	261.709	-905	214.666	-839
238.496	-1.580	261.707	-934	214.615	-868
238.463	-1.608	261.693	-965	214.601	-897
238.468	-1.636	261.735	-995	214.685	-926
238.475	-1.664	261.779	-1.027	214.715	-956

238.517	-1.693	261.808	-1.059	214.739	-987
238.542	-1.722	261.882	-1.092	214.757	-1.018
238.533	-1.752	261.896	-1.125	214.776	-1.050
238.608	-1.781	261.913	-1.159	214.848	-1.083
238.704	-1.811	261.996	-1.194	214.877	-1.116
238.762	-1.841	261.989	-1.229	214.916	-1.150
238.780	-1.872	261.987	-1.265	214.907	-1.184
238.769	-1.903	262.043	-1.301	214.925	-1.219
238.848	-1.934	262.085	-1.339	214.979	-1.254
238.946	-1.966	262.137	-1.377	214.998	-1.290
238.960	-1.997	262.183	-1.415	215.012	-1.327
238.967	-2.029	262.148	-1.454	215.044	-1.364
238.937	-2.062	262.157	-1.494	215.159	-1.402
238.957	-2.094	262.251	-1.535	215.280	-1.440
239.023	-2.127	262.323	-1.576	215.292	-1.479
239.088	-2.161	262.344	-1.618	215.261	-1.519
239.212	-2.194	262.351	-1.660	215.334	-1.559
239.261	-2.228	262.374	-1.703	215.420	-1.600
239.198	-2.262	262.386	-1.747	215.429	-1.641
239.226	-2.297	262.379	-1.792	215.457	-1.683
239.326	-2.331	262.446	-1.837	215.541	-1.725
239.357	-2.366	262.458	-1.882	215.590	-1.768
239.427	-2.402	262.442	-1.929	215.616	-1.812
239.531	-2.437	262.461	-1.976	215.632	-1.856
239.599	-2.473	262.509	-2.024	215.693	-1.901
239.648	-2.509	262.603	-2.072	215.786	-1.946
239.646	-2.546	262.650	-2.121	215.844	-1.992
239.664	-2.582	262.708	-2.171	215.914	-2.038
239.725	-2.619	262.833	-2.221	215.933	-2.085
239.741	-2.656	262.957	-2.272	215.956	-2.133

239.835	-2.694	263.016	-2.323	216.005	-2.181
239.905	-2.732	263.090	-2.375	216.026	-2.230
239.914	-2.770	263.244	-2.428	216.180	-2.279
240.016	-2.808	263.314	-2.481	216.485	-2.328
240.114	-2.847	263.310	-2.535	216.745	-2.378
240.164	-2.886	263.349	-2.590	216.840	-2.429
240.221	-2.925	263.365	-2.645	216.800	-2.480
240.275	-2.965	263.396	-2.701	216.737	-2.532
240.301	-3.005	263.433	-2.757	216.698	-2.584
240.341	-3.045	263.475	-2.814	216.649	-2.637
240.368	-3.085	263.493	-2.872	216.618	-2.690
240.403	-3.125	263.508	-2.930	216.616	-2.744
240.483	-3.166	263.615	-2.988	216.635	-2.798
240.501	-3.207	263.757	-3.048	216.712	-2.852
240.614	-3.249	263.829	-3.107	216.730	-2.907
240.767	-3.290	263.843	-3.168	216.758	-2.963
240.868	-3.332	263.925	-3.228	216.884	-3.019
240.958	-3.374	264.074	-3.290	216.964	-3.075
241.007	-3.417	264.198	-3.351	217.057	-3.132
241.035	-3.459	264.266	-3.414	217.146	-3.189
241.059	-3.502	264.326	-3.477	217.230	-3.247
241.150	-3.545	264.403	-3.540	217.384	-3.305
241.243	-3.589	264.468	-3.604	217.481	-3.363
241.343	-3.632	264.538	-3.668	217.481	-3.422
241.464	-3.676	264.622	-3.733	217.514	-3.481
241.525	-3.720	264.618	-3.798	217.595	-3.541
241.591	-3.764	264.648	-3.864	217.668	-3.601
241.688	-3.808	264.781	-3.930	217.773	-3.661
241.765	-3.853	264.897	-3.997	217.843	-3.722
241.824	-3.897	264.956	-4.064	217.845	-3.783

241.840	-3.942	265.019	-4.131	217.908	-3.844
241.945	-3.987	265.149	-4.199	218.008	-3.906
242.101	-4.033	265.252	-4.267	218.127	-3.968
242.157	-4.078	265.275	-4.336	218.206	-4.030
242.201	-4.124	265.331	-4.405	218.225	-4.093
242.272	-4.170	265.415	-4.475	218.330	-4.156
242.362	-4.216	265.515	-4.544	218.472	-4.219
242.446	-4.262	265.644	-4.614	218.554	-4.282
242.516	-4.308	265.728	-4.685	218.657	-4.346
242.556	-4.355	265.770	-4.756	218.759	-4.410
242.612	-4.402	265.833	-4.827	218.811	-4.474
242.656	-4.448	265.956	-4.898	218.922	-4.539
242.731	-4.495	266.073	-4.970	219.076	-4.604
242.847	-4.543	266.187	-5.042	219.210	-4.668
243.027	-4.590	266.278	-5.115	219.314	-4.734
243.183	-4.637	266.413	-5.187	219.356	-4.799
243.344	-4.685	266.448	-5.260	219.443	-4.864
243.477	-4.733	266.428	-5.334	219.513	-4.930
243.561	-4.781	266.667	-5.407	219.491	-4.996
243.675	-4.829	267.074	-5.481	219.557	-5.062
243.739	-4.877	267.344	-5.555	219.681	-5.128
243.815	-4.925	267.397	-5.629	219.792	-5.194
243.925	-4.974	267.418	-5.703	219.960	-5.261
244.020	-5.022	267.386	-5.778	220.072	-5.327
244.121	-5.071	267.360	-5.853	220.128	-5.394
244.158	-5.120	267.355	-5.928	220.236	-5.461
244.160	-5.169	267.367	-6.003	220.361	-5.527
244.244	-5.218	267.458	-6.078	220.431	-5.594
244.394	-5.267	267.551	-6.153	220.508	-5.661
244.517	-5.316	267.624	-6.229	220.606	-5.728

244.606	-5.365	267.740	-6.305	220.730	-5.795
244.751	-5.414	267.882	-6.380	220.875	-5.862
244.928	-5.464	268.032	-6.456	221.000	-5.930
245.089	-5.513	268.120	-6.532	221.063	-5.997
245.196	-5.563	268.167	-6.608	221.106	-6.064
245.261	-5.613	268.328	-6.685	221.187	-6.131
245.366	-5.662	268.521	-6.761	221.281	-6.198
245.509	-5.712	268.626	-6.837	221.467	-6.266
245.627	-5.762	268.701	-6.913	221.623	-6.333
245.651	-5.812	268.834	-6.990	221.681	-6.400
245.774	-5.862	268.958	-7.066	221.784	-6.467
245.958	-5.912	269.039	-7.142	221.978	-6.534
246.066	-5.962	269.093	-7.219	222.073	-6.601
246.194	-6.012	269.121	-7.295	222.153	-6.668
246.327	-6.062	269.221	-7.372	222.255	-6.735
246.425	-6.112	269.373	-7.448	222.311	-6.802
246.462	-6.163	269.487	-7.524	222.430	-6.868
246.539	-6.213	269.587	-7.600	222.577	-6.935
246.654	-6.263	269.730	-7.677	222.729	-7.002
246.831	-6.313	269.844	-7.753	222.805	-7.068
247.031	-6.364	269.942	-7.829	222.873	-7.134
247.157	-6.414	270.105	-7.905	222.990	-7.201
247.265	-6.464	270.231	-7.981	223.116	-7.267
247.362	-6.514	270.338	-8.057	223.288	-7.333
247.470	-6.565	270.452	-8.133	223.445	-7.398
247.568	-6.615	270.558	-8.208	223.563	-7.464
247.705	-6.665	270.658	-8.284	223.715	-7.530
247.857	-6.716	270.712	-8.359	223.834	-7.595
247.967	-6.766	270.805	-8.434	223.913	-7.660
248.071	-6.816	270.982	-8.510	223.972	-7.725

248.146	-6.866	271.105	-8.585	224.062	-7.790
248.221	-6.916	271.222	-8.659	224.174	-7.855
248.366	-6.966	271.413	-8.734	224.268	-7.920
248.496	-7.016	271.488	-8.809	224.352	-7.984
248.596	-7.066	271.572	-8.883	224.447	-8.048
248.715	-7.116	271.758	-8.957	224.590	-8.112
248.827	-7.166	271.873	-9.031	224.722	-8.176
249.044	-7.216	271.936	-9.105	224.795	-8.239
249.378	-7.266	272.080	-9.179	224.816	-8.302
249.664	-7.316	272.264	-9.252	224.923	-8.366
249.818	-7.365	272.328	-9.325	225.093	-8.428
249.874	-7.415	272.423	-9.398	225.252	-8.491
249.897	-7.464	272.549	-9.471	225.422	-8.553
249.916	-7.514	272.715	-9.544	225.620	-8.615
249.932	-7.563	272.899	-9.616	225.779	-8.677
249.970	-7.613	273.039	-9.688	225.921	-8.739
250.079	-7.662	273.156	-9.760	226.119	-8.800
250.205	-7.711	273.284	-9.831	226.257	-8.861
250.312	-7.760	273.410	-9.903	226.348	-8.922
250.448	-7.809	273.494	-9.974	226.490	-8.983
250.590	-7.858	273.603	-10.045	226.625	-9.043
250.733	-7.906	273.738	-10.115	226.775	-9.103
250.872	-7.955	273.860	-10.185	226.863	-9.163
251.050	-8.003	273.991	-10.255	226.875	-9.222
251.150	-8.052	274.191	-10.325	227.034	-9.282
251.241	-8.100	274.387	-10.395	227.227	-9.340
251.420	-8.148	274.485	-10.464	227.278	-9.399
251.586	-8.196	274.531	-10.533	227.383	-9.457
251.752	-8.244	274.629	-10.601	227.535	-9.515
251.868	-8.292	274.785	-10.670	227.663	-9.573

251.924	-8.339	274.886	-10.738	227.857	-9.630
252.048	-8.387	274.970	-10.806	227.997	-9.688
252.241	-8.434	275.080	-10.873	228.097	-9.744
252.376	-8.481	275.196	-10.940	228.230	-9.801
252.460	-8.529	275.292	-11.007	228.386	-9.857
252.540	-8.575	275.397	-11.074	228.575	-9.913
252.668	-8.622	275.541	-11.140	228.746	-9.968
252.840	-8.669	275.667	-11.206	228.832	-10.024
253.015	-8.715	275.786	-11.271	228.946	-10.078
253.186	-8.761	275.984	-11.336	229.182	-10.133
253.302	-8.808	276.199	-11.401	229.342	-10.187
253.424	-8.854	276.351	-11.466	229.413	-10.241
253.557	-8.899	276.504	-11.530	229.564	-10.295
253.717	-8.945	276.665	-11.594	229.715	-10.348
253.834	-8.990	276.814	-11.658	229.844	-10.401
253.916	-9.036	276.978	-11.721	229.961	-10.453
254.079	-9.081	277.083	-11.784	230.077	-10.506
254.181	-9.126	277.151	-11.846	230.208	-10.558
254.326	-9.170	277.281	-11.908	230.347	-10.609
254.501	-9.215	277.442	-11.970	230.441	-10.660
254.674	-9.259	277.500	-12.032	230.520	-10.711
254.755	-9.304	277.568	-12.093	230.688	-10.762
254.860	-9.348	277.710	-12.153	230.814	-10.812
255.038	-9.391	277.857	-12.214	230.926	-10.862
255.157	-9.435	278.037	-12.274	231.141	-10.912
255.245	-9.478	278.181	-12.334	231.301	-10.961
255.345	-9.522	278.237	-12.393	231.439	-11.010
255.546	-9.565	278.442	-12.452	231.581	-11.058
255.683	-9.608	278.708	-12.510	231.728	-11.106
255.774	-9.650	278.844	-12.569	231.875	-11.154

255.926	-9.693	278.993	-12.627	232.020	-11.202
256.056	-9.735	279.152	-12.684	232.155	-11.249
256.229	-9.777	279.249	-12.741	232.276	-11.296
256.374	-9.819	279.436	-12.798	232.432	-11.342
256.504	-9.860	279.639	-12.854	232.528	-11.388
256.689	-9.902	279.746	-12.910	232.638	-11.434
256.884	-9.943	279.800	-12.966	232.782	-11.480
257.069	-9.984	279.863	-13.021	232.962	-11.525
257.239	-10.025	280.047	-13.076	233.188	-11.570
257.388	-10.065	280.229	-13.131	233.335	-11.614
257.528	-10.105	280.389	-13.185	233.454	-11.659
257.638	-10.146	280.557	-13.239	233.584	-11.702
257.713	-10.185	280.697	-13.292	233.736	-11.746
257.817	-10.225	280.824	-13.345	233.944	-11.789
257.899	-10.265	280.933	-13.398	234.083	-11.832
258.093	-10.304	281.068	-13.450	234.165	-11.874
258.354	-10.343	281.227	-13.502	234.452	-11.917
258.545	-10.382	281.355	-13.554	234.862	-11.958
258.676	-10.420	281.486	-13.605	235.114	-12.000
258.787	-10.459	281.661	-13.656	235.240	-12.041
258.899	-10.497	281.840	-13.707	235.354	-12.082
259.035	-10.535	281.990	-13.757	235.408	-12.123
259.202	-10.572	282.127	-13.807	235.466	-12.163
259.312	-10.610	282.227	-13.856	235.541	-12.203
259.434	-10.647	282.372	-13.905	235.548	-12.242
259.538	-10.684	282.547	-13.954	235.599	-12.282
259.653	-10.721	282.673	-14.002	235.749	-12.321
259.750	-10.757	282.845	-14.050	235.914	-12.359
259.865	-10.794	282.994	-14.098	236.043	-12.398
260.038	-10.830	283.071	-14.145	236.176	-12.436

260.182	-10.865	283.190	-14.192	236.367	-12.473
260.373	-10.901	283.349	-14.239	236.516	-12.511
260.541	-10.937	283.664	-14.285	236.646	-12.548
260.728	-10.972	284.086	-14.331	236.798	-12.585
260.935	-11.007	284.345	-14.377	236.924	-12.621
261.113	-11.041	284.420	-14.422	237.106	-12.657
261.281	-11.076	284.475	-14.467	237.241	-12.693
261.418	-11.110	284.487	-14.511	237.358	-12.729
261.586	-11.144	284.531	-14.556	237.512	-12.764
261.730	-11.178	284.693	-14.599	237.691	-12.799
261.857	-11.212	284.800	-14.643	237.864	-12.834
261.985	-11.245	284.881	-14.686	237.988	-12.868
262.123	-11.278	285.002	-14.729	238.109	-12.902
262.276	-11.311	285.157	-14.772	238.204	-12.936
262.369	-11.344	285.353	-14.814	238.396	-12.969
262.446	-11.376	285.564	-14.856	238.613	-13.003
262.565	-11.408	285.723	-14.897	238.778	-13.036
262.712	-11.440	285.842	-14.938	238.892	-13.068
262.850	-11.472	286.000	-14.979	238.935	-13.101
263.041	-11.504	286.150	-15.020	239.118	-13.133
263.170	-11.535	286.260	-15.060	239.333	-13.165
263.333	-11.566	286.434	-15.100	239.485	-13.196
263.584	-11.597	286.635	-15.139	239.624	-13.227
263.785	-11.628	286.768	-15.179	239.781	-13.258
263.976	-11.658	286.861	-15.217	239.986	-13.289
264.142	-11.689	287.015	-15.256	240.135	-13.320
264.287	-11.719	287.158	-15.294	240.296	-13.350
264.405	-11.748	287.276	-15.332	240.429	-13.380
264.536	-11.778	287.400	-15.370	240.513	-13.410
264.688	-11.807	287.503	-15.407	240.681	-13.439

264.846	-11.836	287.668	-15.444	240.900	-13.468
264.984	-11.865	287.869	-15.481	241.043	-13.497
265.114	-11.894	288.046	-15.518	241.134	-13.526
265.266	-11.923	288.221	-15.554	241.269	-13.555
265.445	-11.951	288.359	-15.590	241.434	-13.583
265.618	-11.979	288.519	-15.625	241.607	-13.611
265.747	-12.007	288.697	-15.660	241.756	-13.639
265.917	-12.034	288.848	-15.695	241.889	-13.666
266.124	-12.062	288.970	-15.730	242.041	-13.693
266.294	-12.089	289.035	-15.764	242.194	-13.721
266.439	-12.116	289.189	-15.798	242.334	-13.747
266.570	-12.143	289.410	-15.832	242.477	-13.774
266.719	-12.170	289.499	-15.866	242.678	-13.800
266.852	-12.196	289.627	-15.899	242.873	-13.826
266.997	-12.222	289.846	-15.932	242.985	-13.852
267.123	-12.248	289.981	-15.964	243.095	-13.878
267.276	-12.274	290.101	-15.997	243.214	-13.904
267.460	-12.299	290.268	-16.029	243.337	-13.929
267.640	-12.325	290.432	-16.061	243.515	-13.954
267.818	-12.350	290.560	-16.092	243.729	-13.979
267.922	-12.375	290.735	-16.123	243.867	-14.004
268.055	-12.400	290.898	-16.154	243.986	-14.028
268.246	-12.424	290.956	-16.185	244.200	-14.052
268.421	-12.449	291.164	-16.216	244.426	-14.076
268.561	-12.473	291.395	-16.246	244.560	-14.100
268.696	-12.497	291.526	-16.276	244.725	-14.124
268.876	-12.521	291.680	-16.306	244.883	-14.147
269.230	-12.544	291.812	-16.335	244.981	-14.170
269.613	-12.568	291.966	-16.364	245.138	-14.194
269.813	-12.591	292.150	-16.393	245.271	-14.216

269.947	-12.614	292.340	-16.422	245.387	-14.239
269.967	-12.637	292.486	-16.450	245.555	-14.262
269.960	-12.660	292.614	-16.479	245.686	-14.284
270.052	-12.682	292.771	-16.506	245.819	-14.306
270.145	-12.704	292.930	-16.534	246.003	-14.328
270.173	-12.727	293.078	-16.562	246.162	-14.350
270.243	-12.748	293.246	-16.589	246.329	-14.371
270.422	-12.770	293.435	-16.616	246.495	-14.393
270.639	-12.792	293.576	-16.643	246.672	-14.414
270.830	-12.813	293.701	-16.669	246.819	-14.435
270.965	-12.834	293.867	-16.696	246.929	-14.456
271.147	-12.855	294.023	-16.722	247.108	-14.476
271.329	-12.876	294.209	-16.748	247.241	-14.497
271.441	-12.897	294.335	-16.773	247.377	-14.517
271.572	-12.917	294.459	-16.799	247.573	-14.537
271.754	-12.938	294.634	-16.824	247.747	-14.557
271.919	-12.958	294.792	-16.849	247.890	-14.577
272.104	-12.978	294.960	-16.873	247.999	-14.597
272.313	-12.998	295.021	-16.898	248.113	-14.616
272.463	-13.018	295.133	-16.922	248.242	-14.636
272.586	-13.037	295.273	-16.946	248.310	-14.655
272.759	-13.056	295.337	-16.970	248.454	-14.674
272.861	-13.075	295.458	-16.994	248.650	-14.693
272.918	-13.094	295.636	-17.018	248.839	-14.711
273.107	-13.113	295.899	-17.041	249.013	-14.730
273.342	-13.132	296.121	-17.064	249.137	-14.748
273.510	-13.151	296.319	-17.087	249.336	-14.767
273.708	-13.169	296.547	-17.109	249.503	-14.785
273.923	-13.187	296.715	-17.132	249.688	-14.803
274.069	-13.205	296.818	-17.154	249.886	-14.821

274.170	-13.223	296.947	-17.176	250.049	-14.838
274.319	-13.241	297.101	-17.198	250.250	-14.856
274.478	-13.258	297.261	-17.220	250.445	-14.873
274.597	-13.276	297.415	-17.241	250.630	-14.890
274.732	-13.293	297.535	-17.263	250.763	-14.907
274.879	-13.310	297.666	-17.284	250.933	-14.924
275.070	-13.327	297.781	-17.305	251.078	-14.941
275.182	-13.344	297.890	-17.325	251.182	-14.958
275.250	-13.361	298.029	-17.346	251.266	-14.974
275.436	-13.377	298.189	-17.366	251.371	-14.991
275.644	-13.394	298.350	-17.386	251.546	-15.007
275.803	-13.410	298.468	-17.406	251.712	-15.023
275.994	-13.426	298.679	-17.426	251.854	-15.039
276.119	-13.442	298.925	-17.446	252.006	-15.055
276.222	-13.458	299.073	-17.465	252.176	-15.071
276.439	-13.474	299.286	-17.485	252.321	-15.086
276.639	-13.489	299.475	-17.504	252.472	-15.102
276.784	-13.505	299.573	-17.523	252.647	-15.117
276.894	-13.520	299.741	-17.542	252.875	-15.133
277.024	-13.535	299.919	-17.560	253.079	-15.148
277.234	-13.550	300.054	-17.579	253.183	-15.163
277.391	-13.565	300.231	-17.597	253.293	-15.178
277.474	-13.580	300.432	-17.615	253.493	-15.192
277.621	-13.594	300.598	-17.633	253.647	-15.207
277.811	-13.609	300.772	-17.651	253.830	-15.221
277.997	-13.623	300.921	-17.669	254.063	-15.236
278.170	-13.638	301.041	-17.686	254.198	-15.250
278.356	-13.652	301.187	-17.704	254.300	-15.264
278.478	-13.666	301.314	-17.721	254.389	-15.278
278.580	-13.680	301.444	-17.738	254.553	-15.292

278.792	-13.693	301.638	-17.755	254.795	-15.306
279.014	-13.707	301.794	-17.772	254.991	-15.320
279.144	-13.720	301.944	-17.788	255.068	-15.334
279.219	-13.734	302.144	-17.805	255.217	-15.347
279.380	-13.747	302.277	-17.821	255.488	-15.360
279.637	-13.760	302.364	-17.837	255.620	-15.374
279.792	-13.773	302.540	-17.853	255.721	-15.387
279.916	-13.786	302.754	-17.869	255.870	-15.400
280.105	-13.799	302.902	-17.885	256.063	-15.413
280.238	-13.812	303.010	-17.901	256.248	-15.426
280.357	-13.824	303.169	-17.916	256.442	-15.439
280.522	-13.837	303.328	-17.932	256.635	-15.451
280.700	-13.849	303.453	-17.947	256.761	-15.464
280.817	-13.862	303.612	-17.962	256.910	-15.476
280.910	-13.874	303.776	-17.977	257.083	-15.489
281.008	-13.886	303.925	-17.992	257.288	-15.501
281.140	-13.898	304.048	-18.007	257.468	-15.513
281.281	-13.910	304.335	-18.022	257.523	-15.525
281.411	-13.921	304.813	-18.036	257.591	-15.537
281.560	-13.933	305.123	-18.051	257.768	-15.549
281.763	-13.945	305.249	-18.065	258.001	-15.561
281.989	-13.956	305.295	-18.079	258.258	-15.573
282.160	-13.967	305.295	-18.093	258.424	-15.584
282.342	-13.979	305.288	-18.107	258.515	-15.596
282.556	-13.990	305.337	-18.121	258.594	-15.607
282.689	-14.001	305.455	-18.135	258.734	-15.619
282.867	-14.012	305.516	-18.148	258.951	-15.630
283.086	-14.023	305.683	-18.162	259.151	-15.641
283.240	-14.033	305.898	-18.175	259.300	-15.652
283.363	-14.044	306.056	-18.188	259.420	-15.663

283.478	-14.055	306.220	-18.201	259.608	-15.674
283.678	-14.065	306.433	-18.215	259.977	-15.685
283.848	-14.075	306.635	-18.228	260.403	-15.696
283.962	-14.086	306.831	-18.240	260.611	-15.706
284.079	-14.096	307.007	-18.253	260.679	-15.717
284.200	-14.106	307.152	-18.266	260.758	-15.727
284.340	-14.116	307.297	-18.278	260.849	-15.738
284.469	-14.126	307.419	-18.291	260.919	-15.748
284.639	-14.136	307.564	-18.303	261.017	-15.758
284.879	-14.146	307.735	-18.315	261.141	-15.769
285.108	-14.156	307.933	-18.328	261.227	-15.779
285.278	-14.165	308.020	-18.340	261.315	-15.789
285.424	-14.175	308.152	-18.352	261.439	-15.799
285.597	-14.184	308.362	-18.364	261.574	-15.808
285.793	-14.194	308.490	-18.375	261.712	-15.818
285.979	-14.203	308.659	-18.387	261.915	-15.828
286.094	-14.212	308.809	-18.399	262.099	-15.838
286.166	-14.221	308.941	-18.410	262.271	-15.847
286.320	-14.230	309.116	-18.422	262.425	-15.857
286.535	-14.239	309.283	-18.433	262.565	-15.866
286.728	-14.248	309.465	-18.444	262.796	-15.876
286.831	-14.257	309.611	-18.456	262.945	-15.885
286.985	-14.266	309.736	-18.467	263.076	-15.894
287.183	-14.275	309.896	-18.478	263.268	-15.903
287.370	-14.283	310.054	-18.489	263.398	-15.913
287.549	-14.292	310.240	-18.500	263.508	-15.922
287.677	-14.300	310.396	-18.511	263.692	-15.931
287.804	-14.309	310.508	-18.521	263.858	-15.940
287.974	-14.317	310.624	-18.532	264.004	-15.948
288.144	-14.326	310.814	-18.543	264.139	-15.957

288.296	-14.334	311.027	-18.553	264.272	-15.966
288.468	-14.342	311.145	-18.564	264.457	-15.975
288.601	-14.350	311.280	-18.574	264.606	-15.983
288.732	-14.358	311.429	-18.584	264.757	-15.992
288.962	-14.366	311.580	-18.595	264.958	-16.001
289.166	-14.374	311.750	-18.605	265.140	-16.009
289.249	-14.382	311.948	-18.615	265.301	-16.017
289.352	-14.390	312.129	-18.625	265.473	-16.026
289.515	-14.397	312.232	-18.635	265.672	-16.034
289.704	-14.405	312.349	-18.645	265.842	-16.042
289.895	-14.413	312.539	-18.655	265.993	-16.051
290.091	-14.420	312.669	-18.665	266.182	-16.059
290.266	-14.428	312.796	-18.675	266.350	-16.067
290.345	-14.435	312.979	-18.684	266.456	-16.075
290.437	-14.442	313.129	-18.694	266.577	-16.083
290.560	-14.450	313.320	-18.704	266.716	-16.091
290.858	-14.457	313.533	-18.713	266.892	-16.099
291.344	-14.464	313.681	-18.723	267.059	-16.107
291.663	-14.471	313.832	-18.732	267.158	-16.115
291.780	-14.478	313.990	-18.742	267.314	-16.122
291.882	-14.486	314.149	-18.751	267.484	-16.130
291.931	-14.493	314.298	-18.760	267.600	-16.138
291.903	-14.499	314.410	-18.769	267.748	-16.145
291.954	-14.506	314.563	-18.779	267.924	-16.153
292.104	-14.513	314.738	-18.788	268.079	-16.161
292.248	-14.520	314.973	-18.797	268.239	-16.168
292.400	-14.527	315.176	-18.806	268.396	-16.176
292.558	-14.533	315.274	-18.815	268.594	-16.183
292.698	-14.540	315.426	-18.824	268.780	-16.190
292.899	-14.547	315.618	-18.832	268.941	-16.198

293.069	-14.553	315.763	-18.841	269.103	-16.205
293.172	-14.560	315.892	-18.850	269.252	-16.212
293.289	-14.566	315.997	-18.859	269.331	-16.220
293.419	-14.573	316.090	-18.867	269.459	-16.227
293.613	-14.579	316.215	-18.876	269.671	-16.234
293.836	-14.585	316.333	-18.884	269.793	-16.241
293.974	-14.591	316.489	-18.893	269.928	-16.248
294.130	-14.598	316.615	-18.901	270.159	-16.255
294.338	-14.604	316.806	-18.910	270.392	-16.262
294.541	-14.610	317.023	-18.918	270.588	-16.269
294.681	-14.616	317.162	-18.927	270.774	-16.276
294.824	-14.622	317.311	-18.935	270.961	-16.283
294.981	-14.628	317.559	-18.943	271.117	-16.290
295.090	-14.634	317.821	-18.951	271.229	-16.297
295.251	-14.640	317.980	-18.959	271.367	-16.303
295.417	-14.646	318.146	-18.967	271.576	-16.310
295.580	-14.652	318.314	-18.975	271.735	-16.317
295.781	-14.658	318.455	-18.983	271.828	-16.324
295.982	-14.663	318.594	-18.991	271.987	-16.330
296.147	-14.669	318.717	-18.999	272.183	-16.337
296.290	-14.675	318.841	-19.007	272.400	-16.343
296.441	-14.681	318.986	-19.015	272.537	-16.350
296.604	-14.686	319.143	-19.023	272.614	-16.357
296.751	-14.692	319.270	-19.030	272.792	-16.363
296.849	-14.697	319.449	-19.038	273.018	-16.369
297.026	-14.703	319.635	-19.046	273.193	-16.376
297.219	-14.708	319.761	-19.053	273.314	-16.382
297.373	-14.714	319.873	-19.061	273.417	-16.389
297.525	-14.719	320.029	-19.069	273.577	-16.395
297.680	-14.725	320.239	-19.076	273.734	-16.401

297.835	-14.730	320.421	-19.083	273.846	-16.407
297.949	-14.735	320.581	-19.091	274.007	-16.414
298.109	-14.740	320.743	-19.098	274.184	-16.420
298.263	-14.746	320.882	-19.105	274.321	-16.426
298.409	-14.751	321.021	-19.113	274.422	-16.432
298.561	-14.756	321.192	-19.120	274.538	-16.438
298.723	-14.761	321.401	-19.127	274.697	-16.444
298.892	-14.766	321.594	-19.134	274.862	-16.450
299.068	-14.772	321.727	-19.141	275.000	-16.456
299.209	-14.777	321.884	-19.149	275.161	-16.462
299.391	-14.782	322.050	-19.156	275.380	-16.468
299.564	-14.787	322.222	-19.163	275.574	-16.474
299.675	-14.792	322.365	-19.170	275.746	-16.480
299.839	-14.797	322.540	-19.176	275.959	-16.486
300.049	-14.802	322.782	-19.183	276.175	-16.492
300.197	-14.806	322.940	-19.190	276.357	-16.498
300.353	-14.811	323.063	-19.197	276.504	-16.503
300.591	-14.816	323.214	-19.204	276.668	-16.509
300.738	-14.821	323.369	-19.211	276.856	-16.515
300.836	-14.826	323.559	-19.217	277.015	-16.521
300.983	-14.831	323.752	-19.224	277.153	-16.526
301.177	-14.835	323.872	-19.231	277.337	-16.532
301.333	-14.840	323.992	-19.237	277.453	-16.537
301.471	-14.845	324.198	-19.244	277.561	-16.543
301.655	-14.849	324.377	-19.250	277.722	-16.549
301.827	-14.854	324.524	-19.257	277.845	-16.554
302.003	-14.859	324.673	-19.263	278.027	-16.560
302.171	-14.863	324.830	-19.270	278.183	-16.565
302.288	-14.868	325.009	-19.276	278.324	-16.571
302.418	-14.872	325.116	-19.282	278.512	-16.576

302.577	-14.877	325.225	-19.289	278.622	-16.581
302.725	-14.881	325.371	-19.295	278.745	-16.587
302.842	-14.886	325.518	-19.301	278.974	-16.592
303.029	-14.890	325.726	-19.308	279.173	-16.597
303.240	-14.895	326.121	-19.314	279.373	-16.603
303.390	-14.899	326.486	-19.320	279.590	-16.608
303.462	-14.904	326.652	-19.326	279.774	-16.613
303.537	-14.908	326.759	-19.332	279.933	-16.618
303.669	-14.912	326.862	-19.338	280.037	-16.624
303.833	-14.917	326.945	-19.344	280.159	-16.629
303.990	-14.921	326.987	-19.350	280.380	-16.634
304.130	-14.925	327.086	-19.356	280.632	-16.639
304.342	-14.930	327.225	-19.362	280.791	-16.644
304.611	-14.934	327.258	-19.368	280.865	-16.649
304.813	-14.938	327.338	-19.374	280.915	-16.654
304.988	-14.942	327.558	-19.380	281.094	-16.659
305.153	-14.947	327.757	-19.386	281.285	-16.664
305.304	-14.951	327.943	-19.391	281.367	-16.669
305.496	-14.955	328.157	-19.397	281.526	-16.674
305.671	-14.959	328.351	-19.403	281.751	-16.679
305.814	-14.963	328.525	-19.408	281.929	-16.684
305.945	-14.968	328.691	-19.414	282.055	-16.689
306.024	-14.972	328.789	-19.420	282.209	-16.694
306.136	-14.976	328.933	-19.425	282.407	-16.699
306.301	-14.980	329.122	-19.431	282.591	-16.704
306.434	-14.984	329.258	-19.436	282.754	-16.709
306.559	-14.988	329.409	-19.442	282.875	-16.713
306.662	-14.992	329.605	-19.447	283.006	-16.718
306.809	-14.996	329.799	-19.453	283.239	-16.723
307.037	-15.000	329.900	-19.458	283.436	-16.728

307.203	-15.004	329.975	-19.464	283.596	-16.732
307.371	-15.008	330.135	-19.469	283.706	-16.737
307.549	-15.012	330.300	-19.474	283.799	-16.742
307.686	-15.016	330.492	-19.480	283.937	-16.747
307.931	-15.020	330.725	-19.485	284.128	-16.751
308.184	-15.024	330.913	-19.490	284.321	-16.756
308.330	-15.028	331.091	-19.495	284.508	-16.761
308.443	-15.032	331.299	-19.501	284.643	-16.765
308.549	-15.036	331.467	-19.506	284.788	-16.770
308.712	-15.040	331.599	-19.511	284.984	-16.774
308.917	-15.043	331.739	-19.516	285.152	-16.779
309.117	-15.047	331.846	-19.521	285.322	-16.784
309.280	-15.051	331.995	-19.526	285.406	-16.788
309.420	-15.055	332.170	-19.531	285.557	-16.793
309.551	-15.059	332.303	-19.536	285.707	-16.797
309.722	-15.063	332.448	-19.541	285.816	-16.802
309.918	-15.066	332.507	-19.546	286.017	-16.806
310.077	-15.070	332.664	-19.551	286.390	-16.811
310.212	-15.074	332.876	-19.556	286.840	-16.815
310.385	-15.078	333.005	-19.561	287.083	-16.820
310.561	-15.082	333.200	-19.566	287.244	-16.824
310.679	-15.085	333.317	-19.571	287.349	-16.829
310.813	-15.089	333.476	-19.576	287.367	-16.833
310.955	-15.093	333.734	-19.581	287.421	-16.837
311.094	-15.097	333.945	-19.585	287.503	-16.842
311.275	-15.100	334.061	-19.590	287.535	-16.846
311.396	-15.104	334.170	-19.595	287.575	-16.851
311.517	-15.108	334.376	-19.600	287.759	-16.855
311.716	-15.111	334.546	-19.604	287.955	-16.859
311.901	-15.115	334.650	-19.609	288.076	-16.864

312.073	-15.119	334.860	-19.614	288.235	-16.868
312.241	-15.122	335.061	-19.618	288.386	-16.873
312.434	-15.126	335.218	-19.623	288.524	-16.877
312.595	-15.130	335.367	-19.628	288.648	-16.881
312.709	-15.133	335.531	-19.632	288.834	-16.886
312.801	-15.137	335.709	-19.637	289.100	-16.890
312.949	-15.141	335.813	-19.642	289.270	-16.894
313.278	-15.144	335.954	-19.646	289.394	-16.899
313.687	-15.148	336.160	-19.651	289.562	-16.903
314.004	-15.151	336.325	-19.655	289.732	-16.907
314.144	-15.155	336.447	-19.660	289.890	-16.912
314.205	-15.159	336.595	-19.664	290.084	-16.916
314.261	-15.162	336.783	-19.669	290.217	-16.920
314.330	-15.166	336.974	-19.673	290.366	-16.925
314.410	-15.169	337.151	-19.677	290.525	-16.929
314.452	-15.173	337.188	-19.682	290.628	-16.933
314.519	-15.176	337.366	-19.686	290.795	-16.938
314.642	-15.180	337.654	-19.691	290.938	-16.942
314.805	-15.184	337.746	-19.695	290.986	-16.946
315.069	-15.187	337.860	-19.699	291.159	-16.950
315.256	-15.191	338.056	-19.704	291.416	-16.955
315.394	-15.194	338.225	-19.708	291.609	-16.959
315.591	-15.198	338.313	-19.712	291.803	-16.963
315.769	-15.201	338.465	-19.716	291.969	-16.968
315.972	-15.205	338.662	-19.721	292.118	-16.972
316.153	-15.208	338.786	-19.725	292.321	-16.976
316.308	-15.212	338.923	-19.729	292.474	-16.980
316.462	-15.215	339.169	-19.733	292.642	-16.985
316.621	-15.218	339.362	-19.737	292.874	-16.989
316.760	-15.222	339.548	-19.742	293.001	-16.993

316.876	-15.225	339.805	-19.746	293.109	-16.998
316.965	-15.229	339.964	-19.750	293.230	-17.002
317.069	-15.232	340.135	-19.754	293.382	-17.006
317.241	-15.236	340.300	-19.758	293.512	-17.011
317.439	-15.239	340.402	-19.762	293.638	-17.015
317.590	-15.242	340.570	-19.766	293.837	-17.019
317.755	-15.246	340.744	-19.770	293.993	-17.023
317.973	-15.249	340.850	-19.774	294.091	-17.028
318.106	-15.253	340.908	-19.778	294.279	-17.032
318.268	-15.256	341.046	-19.782	294.492	-17.036
318.491	-15.259	341.158	-19.786	294.693	-17.041
318.644	-15.263	341.262	-19.790	294.886	-17.045
318.790	-15.266	341.439	-19.794	295.022	-17.049
318.977	-15.270	341.662	-19.798	295.102	-17.054
319.136	-15.273	341.882	-19.802	295.251	-17.058
319.257	-15.276	342.065	-19.805	295.469	-17.062
319.387	-15.280	342.221	-19.809	295.637	-17.066
319.523	-15.283	342.383	-19.813	295.767	-17.071
319.654	-15.286	342.597	-19.817	295.870	-17.075
319.797	-15.290	342.772	-19.821	296.027	-17.079
320.002	-15.293	342.932	-19.824	296.244	-17.084
320.222	-15.296	343.084	-19.828	296.408	-17.088
320.359	-15.299	343.253	-19.832	296.551	-17.092
320.458	-15.303	343.384	-19.836	296.739	-17.097
320.611	-15.306	343.529	-19.839	296.962	-17.101
320.795	-15.309	343.693	-19.843	297.138	-17.105
320.942	-15.313	343.887	-19.847	297.270	-17.110
321.081	-15.316	343.995	-19.850	297.418	-17.114
321.225	-15.319	344.111	-19.854	297.573	-17.118
321.335	-15.322	344.323	-19.858	297.728	-17.123

321.520	-15.326	344.485	-19.861	297.908	-17.127
321.713	-15.329	344.708	-19.865	298.053	-17.131
321.835	-15.332	344.830	-19.869	298.156	-17.136
321.978	-15.335	344.964	-19.872	298.336	-17.140
322.208	-15.338	345.249	-19.876	298.514	-17.144
322.396	-15.342	345.403	-19.879	298.654	-17.149
322.535	-15.345	345.492	-19.883	298.791	-17.153
322.730	-15.348	345.659	-19.886	298.925	-17.157
322.885	-15.351	345.829	-19.890	299.093	-17.162
323.059	-15.354	345.971	-19.894	299.283	-17.166
323.239	-15.358	346.130	-19.897	299.468	-17.170
323.403	-15.361	346.293	-19.901	299.628	-17.174
323.586	-15.364	346.486	-19.904	299.742	-17.179
323.733	-15.367	346.709	-19.908	299.847	-17.183
323.887	-15.370	346.848	-19.911	300.036	-17.187
324.060	-15.373	346.983	-19.914	300.231	-17.192
324.200	-15.377	347.180	-19.918	300.436	-17.196
324.365	-15.380	347.363	-19.921	300.572	-17.200
324.523	-15.383	347.518	-19.925	300.637	-17.205
324.675	-15.386	347.631	-19.928	300.785	-17.209
324.825	-15.389	347.778	-19.932	300.936	-17.213
324.988	-15.392	348.110	-19.935	301.081	-17.218
325.121	-15.395	348.496	-19.938	301.234	-17.222
325.218	-15.398	348.698	-19.942	301.374	-17.226
325.394	-15.402	348.772	-19.945	301.543	-17.230
325.574	-15.405	348.834	-19.949	301.729	-17.235
325.734	-15.408	348.886	-19.952	301.896	-17.239
325.871	-15.411	348.937	-19.955	302.065	-17.243
325.993	-15.414	349.039	-19.959	302.227	-17.248
326.132	-15.417	349.207	-19.962	302.390	-17.252

326.277	-15.420	349.333	-19.965	302.551	-17.256
326.447	-15.423	349.456	-19.969	302.740	-17.260
326.624	-15.426	349.616	-19.972	302.973	-17.265
326.776	-15.429	349.759	-19.976	303.241	-17.269
326.963	-15.432	349.939	-19.979	303.394	-17.273
327.172	-15.435	350.128	-19.982	303.514	-17.277
327.383	-15.438	350.323	-19.985	303.659	-17.281
327.553	-15.441	350.522	-19.989	303.776	-17.286
327.746	-15.444	350.621	-19.992	303.962	-17.290
327.968	-15.447	350.747	-19.995	304.139	-17.294
328.022	-15.450	350.936	-19.999	304.270	-17.298
328.097	-15.453	351.151	-20.002	304.312	-17.303
328.364	-15.456	351.314	-20.005	304.386	-17.307
328.547	-15.459	351.461	-20.009	304.601	-17.311
328.566	-15.462	351.653	-20.012	304.813	-17.315
328.646	-15.465	351.727	-20.015	304.999	-17.319
328.843	-15.468	351.824	-20.018	305.166	-17.323
329.025	-15.471	351.991	-20.022	305.260	-17.328
329.199	-15.474	352.153	-20.025	305.385	-17.332
329.306	-15.477	352.383	-20.028	305.548	-17.336
329.442	-15.480	352.605	-20.032	305.744	-17.340
329.623	-15.483	352.762	-20.035	305.960	-17.344
329.820	-15.486	352.970	-20.038	306.130	-17.348
330.025	-15.489	353.134	-20.041	306.266	-17.352
330.208	-15.492	353.222	-20.045	306.374	-17.357
330.413	-15.495	353.376	-20.048	306.550	-17.361
330.597	-15.498	353.572	-20.051	306.772	-17.365
330.780	-15.501	353.755	-20.054	306.904	-17.369
330.915	-15.504	353.890	-20.058	307.059	-17.373
331.091	-15.507	354.002	-20.061	307.265	-17.377

331.295	-15.509	354.177	-20.064	307.395	-17.381
331.487	-15.512	354.333	-20.068	307.546	-17.385
331.642	-15.515	354.454	-20.071	307.736	-17.389
331.737	-15.518	354.662	-20.074	307.928	-17.394
331.889	-15.521	354.856	-20.077	308.138	-17.398
332.074	-15.524	355.061	-20.081	308.254	-17.402
332.229	-15.527	355.215	-20.084	308.341	-17.406
332.429	-15.530	355.285	-20.087	308.519	-17.410
332.602	-15.533	355.411	-20.091	308.711	-17.414
332.721	-15.535	355.599	-20.094	308.850	-17.418
332.908	-15.538	355.800	-20.097	309.005	-17.422
333.073	-15.541	355.918	-20.101	309.218	-17.426
333.235	-15.544	356.007	-20.104	309.405	-17.430
333.391	-15.547	356.122	-20.107	309.528	-17.434
333.570	-15.550	356.305	-20.110	309.686	-17.438
333.782	-15.553	356.574	-20.114	309.887	-17.442
333.898	-15.555	356.741	-20.117	310.023	-17.446
333.995	-15.558	356.848	-20.120	310.171	-17.450
334.129	-15.561	357.079	-20.124	310.376	-17.455
334.292	-15.564	357.261	-20.127	310.515	-17.459
334.451	-15.567	357.389	-20.130	310.618	-17.463
334.577	-15.570	357.580	-20.134	310.785	-17.467
334.754	-15.572	357.738	-20.137	310.969	-17.471
334.958	-15.575	357.849	-20.140	311.090	-17.475
335.115	-15.578	357.970	-20.144	311.245	-17.479
335.249	-15.581	358.153	-20.147	311.459	-17.483
335.373	-15.584	358.358	-20.151	311.630	-17.487
335.477	-15.586	358.497	-20.154	311.733	-17.491
335.696	-15.589	358.628	-20.157	311.851	-17.495
336.135	-15.592	358.830	-20.161	312.013	-17.499

336.545	-15.595	359.028	-20.164	312.205	-17.503
336.759	-15.597	359.196	-20.167	312.387	-17.507
336.801	-15.600	359.315	-20.171	312.508	-17.511
336.833	-15.603	359.435	-20.174	312.661	-17.515
336.913	-15.606	359.656	-20.178	312.963	-17.519
336.951	-15.608	359.825	-20.181	313.371	-17.523
337.057	-15.611	359.881	-20.184	313.673	-17.527
337.135	-15.614	360.025	-20.188	313.813	-17.531
337.214	-15.617	360.222	-20.191	313.883	-17.535
337.393	-15.619	360.403	-20.194	313.940	-17.539
337.573	-15.622	360.518	-20.198	314.022	-17.543
337.777	-15.625	360.587	-20.201	314.085	-17.547
337.941	-15.628	360.754	-20.205	314.171	-17.551
338.064	-15.630	360.981	-20.208	314.294	-17.555
338.264	-15.633	361.185	-20.211	314.376	-17.559
338.528	-15.636	361.341	-20.215	314.471	-17.564
338.706	-15.638	361.552	-20.218	314.653	-17.568
338.852	-15.641	361.788	-20.222	314.830	-17.572
339.065	-15.644	362.022	-20.225	314.925	-17.576
339.230	-15.646	362.154	-20.228	315.100	-17.580
339.349	-15.649	362.277	-20.232	315.332	-17.584
339.490	-15.652	362.505	-20.235	315.522	-17.588
339.677	-15.654	362.643	-20.239	315.743	-17.592
339.827	-15.657	362.733	-20.242	315.925	-17.596
339.949	-15.660	362.857	-20.245	316.021	-17.600
340.088	-15.662	363.032	-20.249	316.142	-17.604
340.213	-15.665	363.198	-20.252	316.333	-17.608
340.391	-15.668	363.314	-20.256	316.466	-17.612
340.583	-15.670	363.452	-20.259	316.597	-17.617
340.736	-15.673	363.609	-20.262	316.764	-17.621

340.898	-15.676	363.731	-20.266	316.925	-17.625
341.075	-15.678	363.871	-20.269	317.120	-17.629
341.250	-15.681	364.074	-20.272	317.305	-17.633
341.428	-15.684	364.339	-20.276	317.429	-17.637
341.627	-15.686	364.505	-20.279	317.541	-17.641
341.737	-15.689	364.644	-20.282	317.691	-17.645
341.829	-15.691	364.826	-20.286	317.838	-17.650
341.994	-15.694	364.957	-20.289	318.029	-17.654
342.106	-15.697	365.105	-20.292	318.216	-17.658
342.252	-15.699	365.286	-20.296	318.325	-17.662
342.435	-15.702	365.438	-20.299	318.459	-17.666
342.574	-15.704	365.606	-20.302	318.678	-17.670
342.737	-15.707	365.837	-20.306	318.803	-17.675
342.921	-15.710	366.003	-20.309	318.922	-17.679
343.074	-15.712	366.169	-20.312	319.158	-17.683
343.263	-15.715	366.321	-20.316	319.396	-17.687
343.469	-15.717	366.432	-20.319	319.533	-17.691
343.631	-15.720	366.594	-20.322	319.584	-17.696
343.758	-15.722	366.812	-20.326	319.741	-17.700
343.914	-15.725	367.001	-20.329	319.951	-17.704
344.052	-15.727	367.054	-20.332	320.090	-17.708
344.154	-15.730	367.211	-20.335	320.219	-17.713
344.302	-15.733	367.477	-20.339	320.415	-17.717
344.455	-15.735	367.643	-20.342	320.601	-17.721
344.653	-15.738	367.797	-20.345	320.743	-17.725
344.867	-15.740	367.935	-20.348	320.883	-17.730
345.102	-15.743	368.039	-20.352	321.046	-17.734
345.321	-15.745	368.199	-20.355	321.245	-17.738
345.450	-15.748	368.437	-20.358	321.412	-17.743
345.581	-15.750	368.653	-20.361	321.582	-17.747

345.700	-15.753	368.814	-20.365	321.729	-17.751
345.772	-15.755	368.940	-20.368	321.864	-17.756
345.974	-15.758	369.074	-20.371	322.031	-17.760
346.201	-15.760	369.261	-20.374	322.192	-17.764
346.327	-15.763	369.429	-20.377	322.319	-17.768
346.453	-15.765	369.630	-20.381	322.489	-17.773
346.645	-15.768	369.780	-20.384	322.602	-17.777
346.849	-15.770	369.982	-20.387	322.726	-17.782
346.993	-15.773	370.436	-20.390	322.983	-17.786
347.152	-15.775	370.764	-20.393	323.194	-17.790
347.377	-15.778	370.871	-20.397	323.357	-17.795
347.586	-15.780	370.953	-20.400	323.563	-17.799
347.710	-15.783	371.012	-20.403	323.760	-17.803
347.820	-15.785	371.051	-20.406	323.884	-17.808
347.992	-15.788	371.064	-20.409	324.011	-17.812
348.140	-15.790	371.160	-20.412	324.186	-17.816
348.221	-15.792	371.308	-20.415	324.339	-17.821
348.350	-15.795	371.450	-20.419	324.490	-17.825
348.495	-15.797	371.612	-20.422	324.699	-17.830
348.616	-15.800	371.770	-20.425	324.840	-17.834
348.852	-15.802	371.974	-20.428	324.929	-17.838
349.039	-15.805	372.192	-20.431	325.087	-17.843
349.154	-15.807	372.350	-20.434	325.263	-17.847
349.234	-15.810	372.488	-20.437	325.422	-17.851
349.326	-15.812	372.640	-20.440	325.605	-17.856
349.579	-15.814	372.771	-20.443	325.840	-17.860
349.821	-15.817	372.939	-20.446	326.012	-17.865
350.030	-15.819	373.134	-20.449	326.119	-17.869
350.246	-15.822	373.282	-20.453	326.234	-17.873
350.442	-15.824	373.431	-20.456	326.349	-17.878

350.565	-15.827	373.573	-20.459	326.512	-17.882
350.684	-15.829	373.684	-20.462	326.707	-17.886
350.861	-15.831	373.893	-20.465	326.886	-17.891
351.065	-15.834	374.086	-20.468	327.069	-17.895
351.221	-15.836	374.186	-20.471	327.243	-17.899
351.312	-15.839	374.279	-20.474	327.363	-17.904
351.431	-15.841	374.436	-20.477	327.517	-17.908
351.589	-15.844	374.598	-20.480	327.637	-17.912
351.705	-15.846	374.753	-20.483	327.736	-17.917
351.830	-15.848	375.012	-20.486	327.867	-17.921
352.043	-15.851	375.263	-20.489	327.965	-17.925
352.221	-15.853	375.404	-20.492	328.169	-17.929
352.340	-15.856	375.561	-20.495	328.403	-17.934
352.500	-15.858	375.789	-20.498	328.531	-17.938
352.700	-15.860	375.917	-20.501	328.672	-17.942
352.894	-15.863	376.057	-20.503	328.824	-17.946
353.060	-15.865	376.191	-20.506	329.009	-17.951
353.247	-15.868	376.302	-20.509	329.199	-17.955
353.438	-15.870	376.467	-20.512	329.401	-17.959
353.629	-15.872	376.641	-20.515	329.599	-17.963
353.785	-15.875	376.819	-20.518	329.749	-17.967
353.906	-15.877	376.963	-20.521	329.946	-17.972
354.106	-15.880	377.147	-20.524	330.121	-17.976
354.284	-15.882	377.349	-20.527	330.252	-17.980
354.367	-15.885	377.489	-20.530	330.438	-17.984
354.508	-15.887	377.609	-20.533	330.693	-17.988
354.740	-15.889	377.749	-20.536	330.871	-17.992
354.920	-15.892	377.883	-20.539	330.991	-17.996
355.083	-15.894	378.033	-20.542	331.147	-18.001
355.244	-15.897	378.189	-20.545	331.266	-18.005

355.366	-15.899	378.312	-20.548	331.357	-18.009
355.532	-15.901	378.479	-20.550	331.502	-18.013
355.811	-15.904	378.692	-20.553	331.659	-18.017
356.038	-15.906	378.866	-20.556	331.775	-18.021
356.140	-15.909	379.025	-20.559	331.913	-18.025
356.262	-15.911	379.200	-20.562	332.081	-18.029
356.443	-15.913	379.384	-20.565	332.286	-18.033
356.589	-15.916	379.585	-20.568	332.474	-18.037
356.745	-15.918	379.746	-20.571	332.628	-18.040
356.888	-15.921	379.880	-20.574	332.824	-18.044
357.021	-15.923	379.988	-20.577	332.995	-18.048
357.208	-15.925	380.167	-20.579	333.134	-18.052
357.380	-15.928	380.354	-20.582	333.280	-18.056
357.524	-15.930	380.455	-20.585	333.467	-18.060
357.687	-15.933	380.603	-20.588	333.710	-18.064
357.844	-15.935	380.797	-20.591	333.869	-18.068
357.987	-15.938	380.984	-20.594	333.994	-18.071
358.151	-15.940	381.130	-20.597	334.167	-18.075
358.307	-15.942	381.282	-20.600	334.308	-18.079
358.484	-15.945	381.469	-20.603	334.460	-18.083
358.765	-15.947	381.622	-20.605	334.638	-18.086
359.174	-15.950	381.741	-20.608	334.788	-18.090
359.498	-15.952	381.890	-20.611	334.901	-18.094
359.594	-15.954	382.048	-20.614	335.073	-18.098
359.649	-15.957	382.202	-20.617	335.255	-18.101
359.741	-15.959	382.319	-20.620	335.416	-18.105
359.756	-15.962	382.431	-20.622	335.544	-18.109
359.802	-15.964	382.621	-20.625	335.686	-18.112
359.925	-15.966	382.813	-20.628	335.848	-18.116
360.082	-15.969	382.976	-20.631	336.037	-18.120

360.218	-15.971	383.173	-20.634	336.246	-18.123
360.318	-15.974	383.371	-20.637	336.410	-18.127
360.486	-15.976	383.552	-20.639	336.557	-18.130
360.643	-15.979	383.728	-20.642	336.745	-18.134
360.804	-15.981	383.929	-20.645	336.922	-18.137
361.037	-15.983	384.127	-20.648	337.047	-18.141
361.163	-15.986	384.269	-20.650	337.187	-18.144
361.318	-15.988	384.416	-20.653	337.340	-18.148
361.573	-15.991	384.576	-20.656	337.510	-18.151
361.744	-15.993	384.699	-20.659	337.700	-18.155
361.910	-15.995	384.834	-20.662	337.811	-18.158
362.095	-15.998	385.013	-20.664	337.861	-18.162
362.222	-16.000	385.131	-20.667	338.025	-18.165
362.357	-16.003	385.169	-20.670	338.254	-18.169
362.507	-16.005	385.323	-20.672	338.463	-18.172
362.661	-16.007	385.500	-20.675	338.678	-18.175
362.841	-16.010	385.644	-20.678	338.910	-18.179
363.030	-16.012	385.855	-20.681	339.080	-18.182
363.157	-16.015	386.082	-20.683	339.166	-18.186
363.280	-16.017	386.280	-20.686	339.277	-18.189
363.461	-16.019	386.439	-20.689	339.441	-18.192
363.641	-16.022	386.634	-20.691	339.628	-18.196
363.833	-16.024	386.785	-20.694	339.824	-18.199
364.019	-16.027	386.944	-20.697	340.201	-18.202
364.171	-16.029	387.123	-20.699	340.613	-18.206
364.349	-16.031	387.283	-20.702	340.822	-18.209
364.536	-16.034	387.474	-20.705	340.918	-18.212
364.716	-16.036	387.631	-20.707	341.023	-18.216
364.845	-16.039	387.743	-20.710	341.137	-18.219
364.930	-16.041	387.886	-20.712	341.175	-18.222

365.106	-16.043	388.063	-20.715	341.212	-18.225
365.264	-16.046	388.192	-20.718	341.310	-18.229
365.401	-16.048	388.363	-20.720	341.392	-18.232
365.591	-16.051	388.598	-20.723	341.469	-18.235
365.702	-16.053	388.781	-20.725	341.631	-18.238
365.810	-16.055	388.893	-20.728	341.774	-18.242
365.983	-16.058	389.053	-20.731	341.925	-18.245
366.150	-16.060	389.290	-20.733	342.116	-18.248
366.360	-16.063	389.484	-20.736	342.311	-18.251
366.538	-16.065	389.608	-20.738	342.484	-18.255
366.682	-16.067	389.742	-20.741	342.641	-18.258
366.867	-16.070	389.881	-20.743	342.799	-18.261
367.000	-16.072	390.057	-20.746	342.940	-18.264
367.103	-16.074	390.204	-20.749	343.101	-18.268
367.207	-16.077	390.279	-20.751	343.265	-18.271
367.375	-16.079	390.419	-20.754	343.474	-18.274
367.598	-16.081	390.685	-20.756	343.640	-18.277
367.836	-16.084	390.968	-20.759	343.749	-18.281
368.062	-16.086	391.109	-20.761	343.889	-18.284
368.212	-16.089	391.201	-20.764	343.982	-18.287
368.359	-16.091	391.323	-20.766	344.124	-18.290
368.501	-16.093	391.506	-20.769	344.318	-18.293
368.688	-16.096	391.722	-20.771	344.509	-18.297
368.889	-16.098	391.837	-20.774	344.688	-18.300
368.942	-16.100	392.112	-20.776	344.833	-18.303
369.081	-16.103	392.598	-20.779	344.977	-18.307
369.311	-16.105	392.865	-20.781	345.112	-18.310
369.442	-16.107	392.921	-20.784	345.306	-18.313
369.618	-16.110	393.019	-20.786	345.513	-18.316
369.834	-16.112	393.103	-20.789	345.713	-18.320

370.015	-16.114	393.142	-20.791	345.851	-18.323
370.186	-16.117	393.196	-20.793	345.950	-18.326
370.378	-16.119	393.257	-20.796	346.188	-18.330
370.512	-16.121	393.428	-20.798	346.411	-18.333
370.643	-16.124	393.609	-20.801	346.551	-18.336
370.812	-16.126	393.701	-20.803	346.698	-18.339
370.946	-16.128	393.841	-20.806	346.857	-18.343
371.093	-16.131	394.027	-20.808	347.007	-18.346
371.208	-16.133	394.222	-20.811	347.114	-18.350
371.333	-16.135	394.393	-20.813	347.232	-18.353
371.448	-16.138	394.587	-20.815	347.436	-18.356
371.529	-16.140	394.772	-20.818	347.644	-18.360
371.744	-16.142	394.883	-20.820	347.782	-18.363
371.898	-16.145	394.982	-20.823	347.909	-18.367
371.984	-16.147	395.139	-20.825	348.074	-18.370
372.228	-16.149	395.398	-20.827	348.262	-18.373
372.455	-16.151	395.634	-20.830	348.382	-18.377
372.579	-16.154	395.724	-20.832	348.504	-18.380
372.742	-16.156	395.821	-20.835	348.708	-18.384
372.981	-16.158	395.965	-20.837	348.920	-18.387
373.199	-16.161	396.100	-20.839	349.079	-18.391
373.387	-16.163	396.270	-20.842	349.235	-18.394
373.582	-16.165	396.412	-20.844	349.398	-18.398
373.725	-16.168	396.584	-20.847	349.524	-18.401
373.819	-16.170	396.791	-20.849	349.666	-18.405
373.959	-16.172	396.988	-20.851	349.845	-18.408
374.159	-16.174	397.165	-20.854	350.010	-18.412
374.307	-16.177	397.316	-20.856	350.206	-18.415
374.395	-16.179	397.447	-20.859	350.408	-18.419
374.543	-16.181	397.615	-20.861	350.517	-18.423

374.693	-16.183	397.792	-20.863	350.657	-18.426
374.837	-16.186	397.932	-20.866	350.888	-18.430
374.998	-16.188	398.055	-20.868	351.036	-18.433
375.138	-16.190	398.214	-20.871	351.130	-18.437
375.310	-16.193	398.450	-20.873	351.317	-18.441
375.530	-16.195	398.669	-20.875	351.535	-18.444
375.728	-16.197	398.805	-20.878	351.684	-18.448
375.897	-16.199	398.900	-20.880	351.854	-18.451
376.064	-16.202	399.046	-20.883	352.065	-18.455
376.272	-16.204	399.202	-20.885	352.213	-18.459
376.466	-16.206	399.336	-20.887	352.306	-18.462
376.573	-16.208	399.459	-20.890	352.434	-18.466
376.754	-16.211	399.667	-20.892	352.604	-18.470
376.976	-16.213	399.911	-20.895	352.755	-18.473
377.122	-16.215	400.059	-20.897	352.910	-18.477
377.307	-16.217	400.203	-20.899	353.072	-18.481
377.469	-16.220	400.313	-20.902	353.229	-18.485
377.607	-16.222	400.409	-20.904	353.405	-18.488
377.722	-16.224	400.622	-20.907	353.601	-18.492
377.864	-16.226	400.838	-20.909	353.741	-18.496
378.054	-16.228	401.001	-20.912	353.916	-18.499
378.206	-16.231	401.155	-20.914	354.132	-18.503
378.371	-16.233	401.314	-20.917	354.295	-18.507
378.546	-16.235	401.508	-20.919	354.423	-18.511
378.711	-16.237	401.650	-20.921	354.528	-18.514
378.907	-16.240	401.714	-20.924	354.645	-18.518
379.117	-16.242	401.876	-20.926	354.825	-18.522
379.303	-16.244	402.088	-20.929	355.002	-18.526
379.454	-16.246	402.228	-20.931	355.110	-18.529
379.578	-16.249	402.384	-20.934	355.232	-18.533

379.726	-16.251	402.540	-20.936	355.366	-18.537
379.867	-16.253	402.759	-20.939	355.524	-18.541
380.025	-16.255	402.956	-20.941	355.693	-18.544
380.198	-16.257	403.073	-20.944	355.876	-18.548
380.344	-16.260	403.217	-20.946	356.071	-18.552
380.512	-16.262	403.362	-20.949	356.265	-18.556
380.709	-16.264	403.498	-20.951	356.466	-18.559
380.863	-16.266	403.656	-20.953	356.678	-18.563
380.964	-16.268	403.778	-20.956	356.848	-18.567
381.113	-16.271	403.935	-20.958	357.028	-18.571
381.227	-16.273	404.168	-20.961	357.195	-18.574
381.389	-16.275	404.263	-20.963	357.347	-18.578
381.787	-16.277	404.349	-20.966	357.533	-18.582
382.267	-16.279	404.517	-20.968	357.652	-18.586
382.483	-16.281	404.681	-20.971	357.792	-18.589
382.533	-16.284	404.846	-20.973	357.993	-18.593
382.618	-16.286	405.025	-20.976	358.170	-18.597
382.654	-16.288	405.202	-20.978	358.244	-18.600
382.685	-16.290	405.358	-20.980	358.357	-18.604
382.776	-16.292	405.596	-20.983	358.553	-18.608
382.896	-16.294	405.803	-20.985	358.648	-18.612
383.037	-16.297	405.991	-20.988	358.783	-18.615
383.211	-16.299	406.220	-20.990	358.998	-18.619
383.375	-16.301	406.368	-20.993	359.115	-18.623
383.528	-16.303	406.488	-20.995	359.261	-18.626
383.714	-16.305	406.679	-20.997	359.454	-18.630
383.909	-16.307	406.844	-21.000	359.625	-18.634
384.055	-16.310	406.987	-21.002	359.813	-18.637
384.173	-16.312	407.090	-21.005	360.052	-18.641
384.289	-16.314	407.159	-21.007	360.255	-18.645

384.460	-16.316	407.288	-21.009	360.402	-18.648
384.688	-16.318	407.491	-21.012	360.577	-18.652
384.896	-16.320	407.727	-21.014	360.770	-18.656
385.034	-16.322	407.916	-21.016	360.964	-18.659
385.189	-16.324	408.061	-21.019	361.048	-18.663
385.370	-16.327	408.182	-21.021	361.164	-18.666
385.496	-16.329	408.298	-21.024	361.395	-18.670
385.612	-16.331	408.494	-21.026	361.527	-18.674
385.762	-16.333	408.705	-21.028	361.701	-18.677
385.931	-16.335	408.888	-21.031	361.930	-18.681
386.085	-16.337	409.075	-21.033	362.039	-18.684
386.240	-16.339	409.196	-21.035	362.224	-18.688
386.416	-16.341	409.336	-21.038	362.364	-18.691
386.577	-16.343	409.569	-21.040	362.477	-18.695
386.748	-16.346	409.765	-21.042	362.666	-18.698
386.946	-16.348	409.888	-21.045	362.872	-18.702
387.118	-16.350	410.056	-21.047	363.120	-18.705
387.313	-16.352	410.187	-21.049	363.271	-18.709
387.488	-16.354	410.322	-21.051	363.434	-18.712
387.695	-16.356	410.534	-21.054	363.602	-18.716
387.860	-16.358	410.704	-21.056	363.717	-18.719
387.891	-16.360	410.866	-21.058	363.870	-18.723
388.007	-16.362	411.062	-21.061	364.008	-18.726
388.153	-16.364	411.205	-21.063	364.144	-18.729
388.282	-16.366	411.346	-21.065	364.338	-18.733
388.501	-16.368	411.557	-21.067	364.529	-18.736
388.680	-16.370	411.729	-21.070	364.673	-18.740
388.819	-16.372	411.824	-21.072	364.801	-18.743
388.994	-16.374	411.937	-21.074	364.940	-18.746
389.141	-16.376	412.112	-21.076	365.101	-18.750

389.293	-16.378	412.287	-21.078	365.272	-18.753
389.444	-16.380	412.498	-21.081	365.438	-18.756
389.580	-16.382	412.664	-21.083	365.593	-18.760
389.743	-16.384	412.839	-21.085	365.785	-18.763
389.934	-16.386	413.027	-21.087	365.996	-18.766
390.100	-16.388	413.150	-21.089	366.124	-18.769
390.276	-16.390	413.294	-21.092	366.257	-18.773
390.493	-16.392	413.442	-21.094	366.443	-18.776
390.632	-16.394	413.601	-21.096	366.587	-18.779
390.767	-16.396	413.723	-21.098	366.685	-18.782
390.942	-16.398	413.941	-21.100	366.856	-18.786
391.080	-16.400	414.391	-21.102	367.130	-18.789
391.241	-16.402	414.790	-21.105	367.530	-18.792
391.391	-16.404	414.947	-21.107	367.891	-18.795
391.582	-16.406	414.991	-21.109	368.021	-18.798
391.781	-16.408	415.057	-21.111	368.064	-18.802
391.908	-16.410	415.099	-21.113	368.174	-18.805
392.014	-16.412	415.083	-21.115	368.318	-18.808
392.197	-16.414	415.121	-21.117	368.379	-18.811
392.365	-16.416	415.259	-21.119	368.444	-18.814
392.529	-16.418	415.461	-21.121	368.526	-18.817
392.722	-16.420	415.693	-21.124	368.631	-18.821
392.839	-16.422	415.924	-21.126	368.798	-18.824
393.045	-16.424	416.068	-21.128	368.910	-18.827
393.242	-16.426	416.153	-21.130	369.075	-18.830
393.334	-16.428	416.349	-21.132	369.261	-18.833
393.513	-16.430	416.592	-21.134	369.455	-18.836
393.684	-16.432	416.723	-21.136	369.706	-18.839
393.854	-16.434	416.814	-21.138	369.879	-18.842
394.006	-16.436	416.938	-21.140	369.987	-18.845

394.105	-16.437	417.072	-21.142	370.147	-18.849
394.243	-16.439	417.255	-21.144	370.329	-18.852
394.422	-16.441	417.455	-21.146	370.470	-18.855
394.556	-16.443	417.588	-21.148	370.626	-18.858
394.669	-16.445	417.765	-21.150	370.793	-18.861
394.862	-16.447	417.949	-21.152	370.975	-18.864
395.050	-16.449	418.063	-21.154	371.172	-18.867
395.229	-16.451	418.190	-21.156	371.315	-18.870
395.436	-16.453	418.385	-21.158	371.438	-18.873
395.624	-16.454	418.592	-21.160	371.563	-18.876
395.789	-16.456	418.736	-21.162	371.693	-18.879
395.968	-16.458	418.887	-21.164	371.846	-18.882
396.169	-16.460	419.076	-21.166	372.014	-18.885
396.364	-16.462	419.227	-21.168	372.146	-18.888
396.524	-16.464	419.406	-21.170	372.263	-18.891
396.631	-16.466	419.578	-21.172	372.421	-18.894
396.762	-16.468	419.717	-21.174	372.607	-18.897
396.977	-16.469	419.845	-21.176	372.851	-18.900
397.135	-16.471	420.001	-21.178	373.049	-18.903
397.219	-16.473	420.164	-21.180	373.223	-18.906
397.320	-16.475	420.311	-21.182	373.450	-18.909
397.452	-16.477	420.526	-21.184	373.677	-18.912
397.630	-16.479	420.696	-21.186	373.839	-18.915
397.764	-16.481	420.807	-21.188	374.002	-18.918
397.906	-16.482	420.988	-21.191	374.103	-18.922
398.152	-16.484	421.172	-21.193	374.201	-18.925
398.339	-16.486	421.331	-21.195	374.393	-18.928
398.466	-16.488	421.533	-21.197	374.472	-18.931
398.672	-16.490	421.704	-21.199	374.577	-18.934
398.876	-16.492	421.833	-21.201	374.755	-18.937

399.026	-16.493	421.982	-21.203	374.936	-18.940
399.185	-16.495	422.149	-21.205	375.102	-18.943
399.347	-16.497	422.259	-21.207	375.210	-18.946
399.534	-16.499	422.381	-21.209	375.398	-18.949
399.662	-16.501	422.563	-21.211	375.586	-18.952
399.816	-16.502	422.764	-21.213	375.761	-18.955
400.021	-16.504	422.980	-21.215	375.960	-18.958
400.168	-16.506	423.161	-21.217	376.065	-18.961
400.326	-16.508	423.335	-21.219	376.194	-18.964
400.502	-16.510	423.525	-21.222	376.327	-18.967
400.673	-16.512	423.656	-21.224	376.492	-18.970
400.823	-16.513	423.772	-21.226	376.730	-18.973
400.953	-16.515	423.914	-21.228	376.879	-18.976
401.084	-16.517	424.078	-21.230	377.042	-18.979
401.243	-16.519	424.213	-21.232	377.228	-18.982
401.441	-16.521	424.383	-21.234	377.360	-18.985
401.633	-16.522	424.635	-21.236	377.532	-18.989
401.809	-16.524	424.775	-21.239	377.656	-18.992
401.971	-16.526	424.861	-21.241	377.810	-18.995
402.139	-16.528	425.032	-21.243	378.059	-18.998
402.290	-16.529	425.144	-21.245	378.308	-19.001
402.408	-16.531	425.262	-21.247	378.469	-19.004
402.601	-16.533	425.465	-21.249	378.628	-19.007
402.789	-16.535	425.655	-21.251	378.826	-19.010
402.932	-16.537	425.841	-21.254	378.897	-19.013
403.108	-16.538	426.008	-21.256	378.989	-19.016
403.288	-16.540	426.137	-21.258	379.177	-19.019
403.459	-16.542	426.276	-21.260	379.340	-19.022
403.587	-16.544	426.406	-21.262	379.533	-19.025
403.763	-16.546	426.541	-21.264	379.753	-19.029

403.896	-16.547	426.681	-21.266	379.872	-19.032
403.960	-16.549	426.839	-21.269	379.980	-19.035
404.125	-16.551	427.030	-21.271	380.098	-19.038
404.407	-16.553	427.196	-21.273	380.228	-19.041
404.896	-16.554	427.381	-21.275	380.438	-19.044
405.265	-16.556	427.583	-21.277	380.620	-19.047
405.342	-16.558	427.775	-21.279	380.789	-19.050
405.412	-16.560	427.942	-21.282	380.923	-19.053
405.514	-16.561	428.163	-21.284	381.035	-19.056
405.585	-16.563	428.369	-21.286	381.218	-19.059
405.617	-16.565	428.487	-21.288	381.432	-19.062
405.686	-16.567	428.554	-21.290	381.618	-19.066
405.772	-16.568	428.678	-21.292	381.714	-19.069
405.905	-16.570	428.868	-21.295	381.837	-19.072
406.077	-16.572	429.019	-21.297	382.009	-19.075
406.276	-16.574	429.189	-21.299	382.097	-19.078
406.509	-16.575	429.344	-21.301	382.220	-19.081
406.626	-16.577	429.457	-21.303	382.415	-19.084
406.749	-16.579	429.599	-21.305	382.554	-19.087
406.963	-16.581	429.742	-21.308	382.750	-19.090
407.150	-16.582	429.940	-21.310	382.939	-19.093
407.357	-16.584	430.166	-21.312	383.108	-19.096
407.485	-16.586	430.352	-21.314	383.276	-19.099
407.584	-16.588	430.542	-21.316	383.412	-19.102
407.798	-16.589	430.687	-21.318	383.563	-19.105
407.960	-16.591	430.866	-21.321	383.741	-19.109
408.099	-16.593	431.048	-21.323	383.981	-19.112
408.289	-16.595	431.197	-21.325	384.184	-19.115
408.439	-16.596	431.377	-21.327	384.416	-19.118
408.592	-16.598	431.502	-21.329	384.647	-19.121

408.751	-16.600	431.587	-21.331	384.774	-19.124
408.902	-16.602	431.758	-21.334	384.885	-19.127
409.088	-16.603	431.940	-21.336	385.044	-19.130
409.244	-16.605	432.150	-21.338	385.189	-19.133
409.398	-16.607	432.342	-21.340	385.325	-19.136
409.575	-16.608	432.418	-21.342	385.500	-19.139
409.789	-16.610	432.509	-21.344	385.700	-19.142
409.956	-16.612	432.747	-21.347	385.831	-19.145
410.102	-16.614	432.961	-21.349	385.883	-19.148
410.294	-16.615	433.150	-21.351	386.041	-19.151
410.436	-16.617	433.357	-21.353	386.244	-19.154
410.590	-16.619	433.453	-21.355	386.388	-19.157
410.720	-16.620	433.568	-21.357	386.505	-19.160
410.841	-16.622	433.728	-21.360	386.647	-19.163
411.059	-16.624	433.908	-21.362	386.864	-19.166
411.263	-16.626	434.115	-21.364	387.062	-19.169
411.343	-16.627	434.294	-21.366	387.220	-19.171
411.417	-16.629	434.427	-21.368	387.399	-19.174
411.553	-16.631	434.585	-21.370	387.583	-19.177
411.717	-16.632	434.794	-21.373	387.766	-19.180
411.892	-16.634	434.944	-21.375	387.917	-19.183
412.091	-16.636	435.072	-21.377	388.012	-19.186
412.297	-16.638	435.205	-21.379	388.200	-19.189
412.464	-16.639	435.373	-21.381	388.427	-19.192
412.629	-16.641	435.568	-21.383	388.583	-19.195
412.782	-16.643	435.738	-21.385	388.737	-19.198
412.909	-16.644	435.892	-21.388	388.895	-19.200
413.021	-16.646	436.188	-21.390	389.039	-19.203
413.182	-16.648	436.557	-21.392	389.146	-19.206
413.395	-16.649	436.788	-21.394	389.277	-19.209

413.616	-16.651	436.941	-21.396	389.497	-19.212
413.821	-16.653	437.058	-21.398	389.694	-19.215
413.948	-16.654	437.132	-21.401	389.830	-19.218
414.090	-16.656	437.195	-21.403	390.001	-19.220
414.264	-16.658	437.227	-21.405	390.213	-19.223
414.408	-16.660	437.319	-21.407	390.370	-19.226
414.550	-16.661	437.481	-21.409	390.521	-19.229
414.710	-16.663	437.633	-21.411	390.674	-19.232
414.883	-16.665	437.794	-21.413	390.783	-19.234
415.004	-16.666	437.975	-21.416	390.960	-19.237
415.138	-16.668	438.171	-21.418	391.138	-19.240
415.362	-16.670	438.381	-21.420	391.283	-19.243
415.562	-16.671	438.546	-21.422	391.453	-19.246
415.731	-16.673	438.659	-21.424	391.604	-19.248
415.939	-16.675	438.800	-21.426	391.727	-19.251
416.074	-16.676	438.996	-21.429	391.960	-19.254
416.206	-16.678	439.144	-21.431	392.146	-19.257
416.376	-16.680	439.292	-21.433	392.236	-19.259
416.540	-16.681	439.490	-21.435	392.442	-19.262
416.708	-16.683	439.602	-21.437	392.654	-19.265
416.837	-16.685	439.756	-21.439	392.838	-19.268
416.940	-16.686	439.961	-21.442	392.999	-19.270
417.072	-16.688	440.098	-21.444	393.172	-19.273
417.257	-16.690	440.146	-21.446	393.342	-19.276
417.440	-16.691	440.286	-21.448	393.463	-19.279
417.514	-16.693	440.525	-21.450	393.606	-19.281
417.561	-16.695	440.621	-21.452	393.795	-19.284
417.738	-16.696	440.726	-21.455	393.947	-19.287
418.012	-16.698	441.000	-21.457	394.026	-19.290
418.227	-16.700	441.260	-21.459	394.226	-19.292

418.324	-16.701	441.416	-21.461	394.645	-19.295
418.539	-16.703	441.562	-21.463	395.025	-19.298
418.822	-16.705	441.735	-21.466	395.231	-19.300
418.964	-16.706	441.832	-21.468	395.308	-19.303
419.104	-16.708	441.912	-21.470	395.378	-19.306
419.307	-16.710	442.092	-21.472	395.493	-19.308
419.489	-16.711	442.277	-21.475	395.518	-19.311
419.598	-16.713	442.487	-21.477	395.533	-19.314
419.706	-16.715	442.655	-21.479	395.633	-19.317
419.842	-16.716	442.776	-21.481	395.794	-19.319
419.995	-16.718	442.912	-21.484	395.916	-19.322
420.139	-16.720	443.097	-21.486	396.024	-19.325
420.231	-16.721	443.329	-21.488	396.211	-19.327
420.380	-16.723	443.457	-21.491	396.422	-19.330
420.617	-16.725	443.596	-21.493	396.623	-19.333
420.782	-16.726	443.783	-21.495	396.801	-19.335
420.883	-16.728	443.942	-21.498	396.940	-19.338
421.048	-16.730	444.073	-21.500	397.047	-19.341
421.267	-16.731	444.224	-21.502	397.240	-19.344
421.440	-16.733	444.354	-21.505	397.441	-19.346
421.570	-16.735	444.476	-21.507	397.595	-19.349
421.744	-16.737	444.659	-21.509	397.767	-19.352
421.940	-16.738	444.839	-21.512	397.946	-19.354
422.103	-16.740	445.001	-21.514	398.126	-19.357
422.270	-16.742	445.203	-21.517	398.272	-19.360
422.466	-16.743	445.429	-21.519	398.402	-19.362
422.628	-16.745	445.591	-21.521	398.478	-19.365
422.783	-16.747	445.716	-21.524	398.606	-19.368
422.964	-16.748	445.889	-21.526	398.781	-19.371
423.143	-16.750	446.086	-21.529	398.925	-19.373

423.323	-16.752	446.265	-21.531	399.111	-19.376
423.445	-16.753	446.413	-21.534	399.279	-19.379
423.583	-16.755	446.543	-21.536	399.381	-19.382
423.781	-16.757	446.684	-21.539	399.588	-19.384
423.958	-16.759	446.840	-21.541	399.866	-19.387
424.123	-16.760	447.084	-21.544	400.032	-19.390
424.305	-16.762	447.328	-21.546	400.153	-19.392
424.495	-16.764	447.422	-21.549	400.309	-19.395
424.654	-16.765	447.513	-21.552	400.460	-19.398
424.806	-16.767	447.665	-21.554	400.639	-19.401
424.967	-16.769	447.822	-21.557	400.863	-19.404
425.066	-16.771	447.992	-21.559	401.074	-19.406
425.215	-16.772	448.127	-21.562	401.258	-19.409
425.431	-16.774	448.245	-21.564	401.423	-19.412
425.619	-16.776	448.380	-21.567	401.512	-19.415
425.783	-16.777	448.538	-21.570	401.616	-19.417
425.944	-16.779	448.689	-21.572	401.829	-19.420
426.112	-16.781	448.799	-21.575	402.002	-19.423
426.255	-16.783	448.916	-21.578	402.115	-19.426
426.409	-16.784	449.086	-21.580	402.265	-19.429
426.583	-16.786	449.344	-21.583	402.421	-19.432
426.714	-16.788	449.536	-21.585	402.613	-19.434
426.785	-16.790	449.719	-21.588	402.797	-19.437
426.916	-16.791	449.919	-21.591	402.905	-19.440
427.188	-16.793	450.147	-21.593	403.045	-19.443
427.642	-16.795	450.342	-21.596	403.226	-19.446
428.029	-16.797	450.465	-21.599	403.373	-19.449
428.151	-16.798	450.566	-21.602	403.543	-19.451
428.196	-16.800	450.693	-21.604	403.697	-19.454
428.261	-16.802	450.839	-21.607	403.808	-19.457

428.317	-16.804	450.996	-21.610	403.980	-19.460
428.381	-16.805	451.142	-21.612	404.183	-19.463
428.456	-16.807	451.273	-21.615	404.347	-19.466
428.522	-16.809	451.459	-21.618	404.483	-19.469
428.635	-16.811	451.546	-21.620	404.612	-19.472
428.856	-16.812	451.698	-21.623	404.797	-19.474
429.098	-16.814	452.008	-21.626	404.988	-19.477
429.251	-16.816	452.222	-21.629	405.105	-19.480
429.411	-16.818	452.374	-21.631	405.263	-19.483
429.634	-16.819	452.501	-21.634	405.460	-19.486
429.824	-16.821	452.642	-21.637	405.643	-19.489
430.008	-16.823	452.847	-21.640	405.844	-19.492
430.161	-16.825	453.048	-21.642	405.956	-19.495
430.259	-16.827	453.182	-21.645	406.090	-19.498
430.413	-16.828	453.280	-21.648	406.282	-19.501
430.578	-16.830	453.444	-21.650	406.451	-19.504
430.719	-16.832	453.637	-21.653	406.644	-19.507
430.849	-16.834	453.838	-21.656	406.798	-19.509
430.981	-16.836	454.051	-21.659	406.926	-19.512
431.161	-16.837	454.184	-21.661	407.008	-19.515
431.358	-16.839	454.316	-21.664	407.191	-19.518
431.531	-16.841	454.478	-21.667	407.425	-19.521
431.681	-16.843	454.639	-21.670	407.587	-19.524
431.846	-16.845	454.804	-21.672	407.789	-19.527
432.009	-16.846	454.982	-21.675	407.960	-19.530
432.128	-16.848	455.189	-21.678	408.079	-19.533
432.332	-16.850	455.390	-21.681	408.233	-19.536
432.538	-16.852	455.546	-21.683	408.381	-19.539
432.732	-16.854	455.650	-21.686	408.554	-19.542
432.922	-16.855	455.764	-21.689	408.697	-19.545

433.050	-16.857	455.923	-21.692	408.817	-19.548
433.193	-16.859	456.120	-21.694	409.010	-19.550
433.336	-16.861	456.304	-21.697	409.215	-19.553
433.455	-16.863	456.448	-21.700	409.369	-19.556
433.605	-16.865	456.630	-21.703	409.500	-19.559
433.796	-16.866	456.810	-21.705	409.621	-19.562
433.961	-16.868	456.918	-21.708	409.711	-19.565
434.100	-16.870	457.040	-21.711	409.839	-19.568
434.257	-16.872	457.211	-21.714	410.035	-19.571
434.372	-16.874	457.436	-21.716	410.210	-19.574
434.534	-16.876	457.593	-21.719	410.317	-19.577
434.773	-16.877	457.707	-21.722	410.463	-19.580
434.939	-16.879	457.861	-21.724	410.664	-19.582
435.095	-16.881	458.041	-21.727	410.881	-19.585
435.294	-16.883	458.394	-21.730	411.121	-19.588
435.445	-16.885	458.815	-21.733	411.328	-19.591
435.529	-16.887	459.100	-21.735	411.482	-19.594
435.610	-16.889	459.165	-21.738	411.701	-19.597
435.815	-16.890	459.196	-21.741	411.871	-19.600
435.981	-16.892	459.322	-21.743	411.987	-19.602
436.121	-16.894	459.351	-21.746	412.172	-19.605
436.347	-16.896	459.350	-21.749	412.294	-19.608
436.593	-16.898	459.463	-21.751	412.397	-19.611
436.762	-16.900	459.607	-21.754	412.575	-19.614
436.911	-16.902	459.733	-21.757	412.772	-19.616
437.080	-16.903	459.948	-21.759	412.914	-19.619
437.171	-16.905	460.177	-21.762	413.021	-19.622
437.328	-16.907	460.317	-21.765	413.180	-19.625
437.502	-16.909	460.468	-21.767	413.354	-19.628
437.657	-16.911	460.696	-21.770	413.494	-19.630

437.823	-16.913	460.905	-21.773	413.622	-19.633
437.988	-16.915	461.038	-21.775	413.744	-19.636
438.093	-16.917	461.155	-21.778	413.916	-19.639
438.244	-16.919	461.290	-21.781	414.085	-19.641
438.518	-16.920	461.467	-21.783	414.239	-19.644
438.716	-16.922	461.677	-21.786	414.461	-19.647
438.844	-16.924	461.850	-21.789	414.674	-19.649
438.963	-16.926	461.963	-21.791	414.853	-19.652
439.107	-16.928	462.108	-21.794	415.074	-19.655
439.269	-16.930	462.254	-21.796	415.249	-19.657
439.420	-16.932	462.398	-21.799	415.361	-19.660
439.600	-16.934	462.566	-21.802	415.530	-19.663
439.720	-16.936	462.697	-21.804	415.716	-19.665
439.794	-16.938	462.862	-21.807	415.865	-19.668
439.959	-16.940	463.068	-21.809	416.005	-19.671
440.133	-16.941	463.239	-21.812	416.169	-19.673
440.274	-16.943	463.438	-21.814	416.347	-19.676
440.411	-16.945	463.631	-21.817	416.511	-19.678
440.574	-16.947	463.807	-21.820	416.721	-19.681
440.737	-16.949	463.973	-21.822	416.886	-19.684
440.927	-16.951	464.093	-21.825	417.013	-19.686
441.134	-16.953	464.205	-21.827	417.144	-19.689
441.342	-16.955	464.328	-21.830	417.260	-19.691
441.550	-16.957	464.520	-21.832	417.439	-19.694
441.749	-16.959	464.727	-21.835	417.631	-19.696
441.927	-16.961	464.900	-21.838	417.757	-19.699
442.046	-16.963	465.068	-21.840	417.934	-19.701
442.218	-16.965	465.224	-21.843	418.162	-19.704
442.374	-16.967	465.319	-21.845	418.340	-19.706
442.507	-16.969	465.396	-21.848	418.473	-19.709

442.607	-16.971	465.561	-21.850	418.581	-19.711
442.745	-16.972	465.773	-21.853	418.710	-19.714
442.924	-16.974	465.951	-21.855	418.887	-19.716
443.114	-16.976	466.099	-21.858	419.061	-19.719
443.262	-16.978	466.240	-21.860	419.211	-19.721
443.375	-16.980	466.366	-21.863	419.334	-19.723
443.529	-16.982	466.512	-21.865	419.482	-19.726
443.688	-16.984	466.694	-21.868	419.655	-19.728
443.848	-16.986	466.948	-21.870	419.826	-19.731
444.068	-16.988	467.149	-21.873	420.015	-19.733
444.299	-16.990	467.322	-21.875	420.207	-19.735
444.491	-16.992	467.535	-21.878	420.411	-19.738
444.658	-16.994	467.695	-21.880	420.617	-19.740
444.811	-16.996	467.866	-21.883	420.709	-19.743
444.930	-16.998	468.000	-21.885	420.813	-19.745
445.083	-17.000	468.095	-21.888	421.028	-19.747
445.261	-17.002	468.300	-21.890	421.179	-19.750
445.440	-17.004	468.490	-21.893	421.276	-19.752
445.634	-17.006	468.609	-21.895	421.493	-19.754
445.774	-17.008	468.797	-21.898	421.914	-19.757
445.878	-17.010	468.978	-21.900	422.343	-19.759
446.054	-17.012	469.162	-21.903	422.527	-19.761
446.293	-17.014	469.351	-21.905	422.536	-19.764
446.419	-17.016	469.486	-21.908	422.603	-19.766
446.535	-17.018	469.654	-21.910	422.681	-19.768
446.710	-17.020	469.836	-21.913	422.726	-19.771
446.885	-17.022	469.959	-21.915	422.817	-19.773
447.031	-17.024	470.069	-21.918	422.946	-19.775
447.229	-17.026	470.241	-21.920	423.133	-19.778
447.416	-17.028	470.399	-21.923	423.272	-19.780

447.590	-17.031	470.542	-21.925	423.349	-19.782
447.749	-17.033	470.684	-21.927	423.473	-19.785
447.920	-17.035	470.787	-21.930	423.640	-19.787
448.109	-17.037	470.873	-21.932	423.804	-19.789
448.247	-17.039	470.991	-21.935	423.994	-19.792
448.418	-17.041	471.162	-21.937	424.204	-19.794
448.613	-17.043	471.357	-21.939	424.374	-19.796
448.747	-17.045	471.569	-21.942	424.554	-19.799
448.900	-17.047	471.756	-21.944	424.750	-19.801
449.123	-17.049	471.981	-21.947	424.898	-19.803
449.312	-17.051	472.148	-21.949	425.058	-19.806
449.450	-17.053	472.289	-21.951	425.263	-19.808
449.562	-17.055	472.546	-21.954	425.423	-19.810
449.713	-17.057	472.757	-21.956	425.553	-19.813
449.855	-17.059	472.896	-21.958	425.725	-19.815
450.112	-17.061	473.040	-21.961	425.910	-19.817
450.537	-17.064	473.195	-21.963	426.063	-19.820
450.827	-17.066	473.319	-21.965	426.207	-19.822
450.947	-17.068	473.398	-21.968	426.317	-19.824
451.079	-17.070	473.532	-21.970	426.438	-19.827
451.194	-17.072	473.673	-21.972	426.570	-19.829
451.222	-17.074	473.818	-21.975	426.752	-19.831
451.213	-17.076	474.035	-21.977	426.935	-19.834
451.242	-17.078	474.238	-21.979	427.077	-19.836
451.347	-17.080	474.344	-21.981	427.265	-19.838
451.559	-17.082	474.517	-21.984	427.440	-19.841
451.752	-17.084	474.787	-21.986	427.598	-19.843
451.900	-17.087	474.933	-21.988	427.792	-19.846
452.043	-17.089	475.092	-21.990	427.965	-19.848
452.240	-17.091	475.312	-21.993	428.098	-19.850

452.493	-17.093	475.502	-21.995	428.276	-19.853
452.665	-17.095	475.685	-21.997	428.456	-19.855
452.778	-17.097	475.880	-21.999	428.631	-19.858
452.951	-17.099	475.982	-22.002	428.796	-19.860
453.136	-17.101	476.162	-22.004	428.956	-19.862
453.280	-17.103	476.308	-22.006	429.126	-19.865
453.449	-17.106	476.458	-22.008	429.238	-19.867
453.588	-17.108	476.625	-22.010	429.376	-19.870
453.777	-17.110	476.754	-22.012	429.523	-19.872
453.946	-17.112	476.876	-22.015	429.677	-19.875
454.079	-17.114	477.076	-22.017	429.861	-19.877
454.257	-17.116	477.302	-22.019	430.038	-19.880
454.366	-17.118	477.484	-22.021	430.139	-19.882
454.445	-17.120	477.601	-22.023	430.314	-19.885
454.590	-17.123	477.700	-22.025	430.540	-19.887
454.902	-17.125	477.884	-22.027	430.706	-19.890
455.126	-17.127	478.099	-22.030	430.889	-19.893
455.254	-17.129	478.280	-22.032	431.018	-19.895
455.415	-17.131	478.423	-22.034	431.132	-19.898
455.602	-17.133	478.550	-22.036	431.277	-19.900
455.761	-17.135	478.712	-22.038	431.478	-19.903
455.956	-17.137	478.870	-22.040	431.616	-19.905
456.100	-17.140	479.011	-22.042	431.762	-19.908
456.213	-17.142	479.210	-22.044	431.993	-19.911
456.333	-17.144	479.367	-22.046	432.135	-19.913
456.447	-17.146	479.499	-22.048	432.284	-19.916
456.608	-17.148	479.694	-22.050	432.460	-19.919
456.823	-17.150	479.863	-22.052	432.633	-19.921
457.011	-17.152	479.933	-22.054	432.793	-19.924
457.136	-17.154	480.054	-22.056	432.971	-19.927

457.283	-17.157	480.276	-22.059	433.174	-19.929
457.429	-17.159	480.590	-22.061	433.300	-19.932
457.568	-17.161	481.010	-22.063	433.439	-19.935
457.736	-17.163	481.308	-22.065	433.601	-19.938
457.925	-17.165	481.418	-22.067	433.758	-19.940
458.096	-17.167	481.474	-22.069	433.889	-19.943
458.268	-17.169	481.523	-22.071	434.065	-19.946
458.435	-17.171	481.585	-22.073	434.253	-19.948
458.543	-17.173	481.655	-22.074	434.327	-19.951
458.669	-17.176	481.689	-22.076	434.455	-19.954
458.820	-17.178	481.756	-22.078	434.699	-19.957
459.010	-17.180	481.910	-22.080	434.911	-19.960
459.187	-17.182	482.109	-22.082	435.087	-19.962
459.366	-17.184	482.300	-22.084	435.299	-19.965
459.557	-17.186	482.525	-22.086	435.414	-19.968
459.719	-17.188	482.702	-22.088	435.508	-19.971
459.900	-17.190	482.876	-22.090	435.662	-19.974
460.094	-17.192	483.091	-22.092	435.818	-19.976
460.285	-17.195	483.283	-22.094	435.968	-19.979
460.431	-17.197	483.469	-22.096	436.100	-19.982
460.492	-17.199	483.622	-22.098	436.292	-19.985
460.639	-17.201	483.744	-22.100	436.513	-19.988
460.866	-17.203	483.890	-22.102	436.619	-19.991
461.028	-17.205	484.054	-22.104	436.736	-19.993
461.159	-17.207	484.190	-22.106	436.933	-19.996
461.279	-17.209	484.307	-22.107	437.047	-19.999
461.473	-17.211	484.505	-22.109	437.172	-20.002
461.689	-17.214	484.656	-22.111	437.333	-20.005
461.863	-17.216	484.733	-22.113	437.494	-20.008
462.068	-17.218	484.895	-22.115	437.607	-20.011

462.235	-17.220	485.105	-22.117	437.767	-20.013
462.360	-17.222	485.273	-22.119	437.991	-20.016
462.477	-17.224	485.474	-22.121	438.263	-20.019
462.571	-17.226	485.724	-22.123	438.531	-20.022
462.715	-17.228	485.902	-22.125	438.703	-20.025
462.953	-17.230	486.058	-22.127	438.841	-20.028
463.097	-17.232	486.211	-22.128	438.964	-20.031
463.200	-17.234	486.363	-22.130	439.129	-20.033
463.353	-17.236	486.526	-22.132	439.356	-20.036
463.531	-17.239	486.637	-22.134	439.523	-20.039
463.675	-17.241	486.803	-22.136	439.650	-20.042
463.792	-17.243	486.919	-22.138	439.810	-20.045
463.987	-17.245	487.010	-22.140	439.969	-20.048
464.277	-17.247	487.190	-22.142	440.067	-20.051
464.479	-17.249	487.394	-22.144	440.232	-20.053
464.591	-17.251	487.561	-22.146	440.427	-20.056
464.748	-17.253	487.729	-22.148	440.531	-20.059
464.886	-17.255	487.862	-22.150	440.626	-20.062
465.056	-17.257	487.965	-22.151	440.783	-20.065
465.276	-17.259	488.143	-22.153	441.005	-20.068
465.406	-17.261	488.316	-22.155	441.152	-20.070
465.534	-17.263	488.495	-22.157	441.280	-20.073
465.631	-17.265	488.685	-22.159	441.467	-20.076
465.787	-17.268	488.809	-22.161	441.605	-20.079
465.986	-17.270	488.952	-22.163	441.782	-20.082
466.113	-17.272	489.154	-22.165	441.972	-20.085
466.277	-17.274	489.390	-22.167	442.123	-20.088
466.426	-17.276	489.541	-22.169	442.312	-20.090
466.585	-17.278	489.688	-22.171	442.511	-20.093
466.759	-17.280	489.805	-22.173	442.685	-20.096

466.934	-17.282	489.901	-22.175	442.827	-20.099
467.135	-17.284	490.137	-22.177	442.969	-20.102
467.350	-17.286	490.271	-22.179	443.106	-20.104
467.521	-17.288	490.375	-22.181	443.253	-20.107
467.664	-17.290	490.645	-22.183	443.433	-20.110
467.794	-17.292	490.826	-22.185	443.660	-20.113
467.935	-17.294	490.967	-22.187	443.829	-20.116
468.122	-17.296	491.093	-22.189	443.979	-20.118
468.310	-17.298	491.271	-22.191	444.140	-20.121
468.492	-17.300	491.485	-22.193	444.273	-20.124
468.634	-17.302	491.609	-22.195	444.473	-20.127
468.817	-17.304	491.767	-22.197	444.630	-20.130
469.012	-17.306	491.885	-22.199	444.781	-20.132
469.142	-17.308	492.030	-22.201	445.003	-20.135
469.338	-17.310	492.289	-22.203	445.184	-20.138
469.526	-17.312	492.475	-22.205	445.284	-20.141
469.689	-17.314	492.580	-22.207	445.389	-20.143
469.871	-17.316	492.665	-22.209	445.537	-20.146
470.010	-17.318	492.786	-22.211	445.718	-20.149
470.135	-17.320	492.960	-22.213	445.886	-20.152
470.332	-17.322	493.109	-22.215	446.033	-20.154
470.495	-17.324	493.242	-22.217	446.250	-20.157
470.646	-17.326	493.441	-22.219	446.448	-20.160
470.851	-17.328	493.620	-22.221	446.587	-20.163
471.055	-17.330	493.723	-22.223	446.735	-20.165
471.211	-17.332	493.907	-22.225	446.912	-20.168
471.359	-17.334	494.175	-22.227	447.095	-20.171
471.514	-17.336	494.376	-22.229	447.270	-20.173
471.717	-17.338	494.541	-22.231	447.423	-20.176
471.879	-17.340	494.694	-22.233	447.593	-20.179

471.933	-17.342	494.840	-22.235	447.788	-20.182
472.065	-17.344	495.034	-22.237	447.923	-20.184
472.272	-17.346	495.201	-22.239	448.020	-20.187
472.435	-17.348	495.363	-22.241	448.198	-20.190
472.593	-17.350	495.518	-22.243	448.401	-20.192
472.754	-17.352	495.624	-22.245	448.550	-20.195
473.021	-17.354	495.755	-22.247	448.681	-20.198
473.461	-17.356	495.923	-22.249	448.947	-20.200
473.805	-17.358	496.073	-22.251	449.400	-20.203
473.945	-17.360	496.226	-22.253	449.762	-20.206
474.002	-17.362	496.359	-22.255	449.863	-20.208
474.022	-17.364	496.510	-22.257	449.939	-20.211
474.059	-17.366	496.700	-22.259	450.074	-20.214
474.129	-17.368	496.874	-22.261	450.175	-20.216
474.244	-17.370	497.050	-22.263	450.189	-20.219
474.372	-17.372	497.264	-22.265	450.192	-20.222
474.496	-17.374	497.453	-22.267	450.310	-20.224
474.664	-17.376	497.617	-22.269	450.443	-20.227
474.849	-17.378	497.727	-22.271	450.576	-20.230
475.007	-17.380	497.887	-22.273	450.770	-20.232
475.152	-17.382	497.994	-22.275	450.912	-20.235
475.315	-17.384	498.182	-22.277	451.043	-20.238
475.539	-17.386	498.408	-22.279	451.242	-20.240
475.700	-17.388	498.597	-22.281	451.445	-20.243
475.842	-17.390	498.791	-22.283	451.622	-20.246
476.063	-17.392	498.925	-22.285	451.776	-20.248
476.207	-17.394	499.105	-22.287	451.925	-20.251
476.317	-17.396	499.253	-22.289	452.072	-20.254
476.441	-17.397	499.372	-22.291	452.230	-20.256
476.583	-17.399	499.594	-22.293	452.404	-20.259

476.697	-17.401	499.800	-22.295	452.564	-20.262
476.838	-17.403	499.953	-22.297	452.766	-20.264
477.100	-17.405	500.128	-22.299	452.977	-20.267
477.298	-17.407	500.301	-22.301	453.096	-20.270
477.459	-17.409	500.429	-22.303	453.170	-20.273
477.583	-17.411	500.616	-22.305	453.306	-20.275
477.720	-17.413	500.772	-22.307	453.499	-20.278
477.933	-17.415	500.853	-22.308	453.665	-20.281
478.142	-17.417	500.982	-22.310	453.844	-20.283
478.366	-17.418	501.124	-22.312	454.017	-20.286
478.534	-17.420	501.373	-22.314	454.162	-20.289
478.619	-17.422	501.554	-22.316	454.321	-20.291
478.740	-17.424	501.693	-22.318	454.472	-20.294
478.869	-17.426	501.851	-22.320	454.667	-20.297
479.060	-17.428	501.999	-22.322	454.872	-20.300
479.256	-17.430	502.096	-22.324	455.028	-20.302
479.396	-17.432	502.242	-22.326	455.174	-20.305
479.550	-17.433	502.430	-22.327	455.336	-20.308
479.764	-17.435	502.755	-22.329	455.578	-20.311
479.925	-17.437	503.182	-22.331	455.788	-20.314
480.090	-17.439	503.431	-22.333	455.914	-20.316
480.256	-17.441	503.551	-22.335	456.031	-20.319
480.363	-17.443	503.649	-22.337	456.157	-20.322
480.459	-17.445	503.711	-22.339	456.284	-20.325
480.590	-17.446	503.754	-22.340	456.460	-20.328
480.797	-17.448	503.854	-22.342	456.611	-20.330
481.009	-17.450	503.959	-22.344	456.719	-20.333
481.200	-17.452	504.003	-22.346	456.842	-20.336
481.300	-17.454	504.144	-22.348	457.047	-20.339
481.413	-17.456	504.345	-22.350	457.271	-20.342

481.595	-17.458	504.503	-22.351	457.378	-20.345
481.767	-17.459	504.716	-22.353	457.513	-20.348
481.946	-17.461	504.871	-22.355	457.714	-20.351
482.129	-17.463	505.042	-22.357	457.904	-20.353
482.296	-17.465	505.260	-22.359	458.104	-20.356
482.468	-17.467	505.447	-22.360	458.236	-20.359
482.638	-17.468	505.601	-22.362	458.349	-20.362
482.777	-17.470	505.718	-22.364	458.532	-20.365
482.946	-17.472	505.836	-22.366	458.709	-20.368
483.120	-17.474	506.039	-22.367	458.854	-20.371
483.276	-17.476	506.223	-22.369	458.982	-20.374
483.471	-17.477	506.392	-22.371	459.103	-20.377
483.632	-17.479	506.557	-22.373	459.255	-20.380
483.758	-17.481	506.644	-22.374	459.449	-20.383
483.924	-17.483	506.770	-22.376	459.656	-20.386
484.055	-17.485	506.957	-22.378	459.838	-20.389
484.236	-17.486	507.122	-22.380	459.991	-20.392
484.468	-17.488	507.322	-22.381	460.170	-20.395
484.639	-17.490	507.572	-22.383	460.374	-20.398
484.777	-17.492	507.744	-22.385	460.531	-20.401
484.931	-17.493	507.892	-22.386	460.670	-20.404
485.060	-17.495	508.065	-22.388	460.812	-20.407
485.156	-17.497	508.193	-22.390	460.904	-20.410
485.282	-17.499	508.375	-22.392	461.083	-20.413
485.460	-17.500	508.551	-22.393	461.328	-20.416
485.667	-17.502	508.687	-22.395	461.550	-20.419
485.743	-17.504	508.801	-22.397	461.743	-20.422
485.819	-17.506	508.942	-22.398	461.871	-20.425
486.013	-17.507	509.133	-22.400	461.991	-20.428
486.193	-17.509	509.324	-22.402	462.126	-20.431

486.346	-17.511	509.466	-22.403	462.325	-20.434
486.526	-17.513	509.563	-22.405	462.541	-20.437
486.764	-17.514	509.737	-22.407	462.687	-20.440
486.964	-17.516	509.930	-22.409	462.833	-20.444
487.186	-17.518	510.094	-22.410	462.975	-20.447
487.367	-17.520	510.251	-22.412	463.156	-20.450
487.575	-17.521	510.403	-22.414	463.353	-20.453
487.784	-17.523	510.611	-22.415	463.484	-20.456
487.907	-17.525	510.755	-22.417	463.634	-20.459
488.070	-17.527	510.850	-22.419	463.821	-20.462
488.180	-17.528	510.994	-22.420	463.950	-20.465
488.302	-17.530	511.138	-22.422	464.077	-20.468
488.472	-17.532	511.321	-22.424	464.203	-20.471
488.588	-17.533	511.532	-22.425	464.317	-20.474
488.728	-17.535	511.692	-22.427	464.450	-20.477
488.888	-17.537	511.820	-22.429	464.557	-20.480
489.033	-17.539	512.024	-22.430	464.672	-20.483
489.173	-17.540	512.242	-22.432	464.828	-20.487
489.309	-17.542	512.367	-22.434	465.023	-20.490
489.448	-17.544	512.430	-22.435	465.259	-20.493
489.647	-17.545	512.595	-22.437	465.441	-20.496
489.872	-17.547	512.828	-22.439	465.628	-20.499
490.027	-17.549	512.997	-22.440	465.868	-20.502
490.205	-17.550	513.111	-22.442	466.079	-20.505
490.386	-17.552	513.270	-22.443	466.267	-20.508
490.533	-17.554	513.499	-22.445	466.460	-20.511
490.728	-17.556	513.684	-22.447	466.636	-20.514
490.860	-17.557	513.800	-22.448	466.761	-20.517
490.983	-17.559	513.945	-22.450	466.875	-20.520
491.161	-17.561	514.138	-22.452	467.073	-20.523

491.333	-17.562	514.329	-22.453	467.321	-20.526
491.538	-17.564	514.497	-22.455	467.430	-20.529
491.680	-17.566	514.664	-22.457	467.507	-20.532
491.831	-17.567	514.771	-22.458	467.633	-20.535
492.035	-17.569	514.836	-22.460	467.758	-20.538
492.144	-17.571	514.982	-22.461	467.874	-20.540
492.294	-17.572	515.203	-22.463	468.013	-20.543
492.537	-17.574	515.343	-22.465	468.207	-20.546
492.724	-17.576	515.452	-22.466	468.329	-20.549
492.903	-17.578	515.621	-22.468	468.439	-20.552
493.080	-17.579	515.813	-22.469	468.666	-20.555
493.257	-17.581	515.978	-22.471	468.940	-20.558
493.428	-17.583	516.103	-22.473	469.195	-20.561
493.538	-17.584	516.345	-22.474	469.363	-20.563
493.701	-17.586	516.568	-22.476	469.503	-20.566
493.881	-17.588	516.771	-22.477	469.638	-20.569
494.026	-17.589	517.006	-22.479	469.794	-20.572
494.163	-17.591	517.125	-22.480	469.966	-20.575
494.267	-17.593	517.303	-22.482	470.135	-20.578
494.424	-17.594	517.483	-22.484	470.386	-20.580
494.669	-17.596	517.551	-22.485	470.522	-20.583
494.870	-17.598	517.679	-22.487	470.593	-20.586
495.036	-17.599	517.801	-22.488	470.748	-20.589
495.188	-17.601	517.892	-22.490	470.974	-20.591
495.323	-17.603	518.026	-22.491	471.183	-20.594
495.459	-17.604	518.216	-22.493	471.309	-20.597
495.599	-17.606	518.425	-22.494	471.442	-20.599
495.767	-17.608	518.584	-22.496	471.616	-20.602
496.124	-17.609	518.735	-22.497	471.799	-20.605
496.563	-17.611	518.946	-22.499	471.950	-20.607

496.787	-17.613	519.143	-22.500	472.104	-20.610
496.832	-17.614	519.311	-22.502	472.316	-20.613
496.918	-17.616	519.472	-22.503	472.535	-20.615
496.999	-17.618	519.636	-22.505	472.674	-20.618
497.045	-17.619	519.817	-22.506	472.807	-20.620
497.141	-17.621	519.967	-22.508	472.977	-20.623
497.230	-17.623	520.108	-22.509	473.137	-20.625
497.296	-17.624	520.261	-22.511	473.237	-20.628
497.453	-17.626	520.459	-22.512	473.394	-20.630
497.622	-17.628	520.656	-22.514	473.608	-20.633
497.789	-17.629	520.774	-22.515	473.770	-20.635
497.989	-17.631	520.967	-22.516	473.933	-20.638
498.136	-17.632	521.154	-22.518	474.091	-20.640
498.246	-17.634	521.303	-22.519	474.203	-20.643
498.473	-17.636	521.478	-22.521	474.351	-20.645
498.758	-17.637	521.669	-22.522	474.498	-20.648
498.915	-17.639	521.871	-22.524	474.664	-20.650
499.041	-17.641	521.971	-22.525	474.879	-20.653
499.198	-17.642	522.129	-22.526	475.027	-20.655
499.300	-17.644	522.409	-22.528	475.215	-20.657
499.436	-17.646	522.581	-22.529	475.416	-20.660
499.624	-17.647	522.675	-22.531	475.639	-20.662
499.791	-17.649	522.809	-22.532	475.836	-20.664
499.876	-17.650	522.942	-22.533	475.899	-20.667
499.997	-17.652	523.070	-22.535	475.965	-20.669
500.199	-17.654	523.233	-22.536	476.130	-20.671
500.359	-17.655	523.426	-22.538	476.377	-20.674
500.553	-17.657	523.609	-22.539	476.748	-20.676
500.745	-17.659	523.797	-22.540	477.158	-20.678
500.885	-17.660	523.972	-22.542	477.400	-20.680

501.067	-17.662	524.130	-22.543	477.494	-20.683
501.288	-17.663	524.264	-22.544	477.569	-20.685
501.456	-17.665	524.413	-22.546	477.628	-20.687
501.593	-17.667	524.533	-22.547	477.629	-20.689
501.752	-17.668	524.758	-22.548	477.701	-20.692
501.922	-17.670	525.185	-22.550	477.849	-20.694
502.080	-17.672	525.533	-22.551	477.979	-20.696
502.213	-17.673	525.704	-22.552	478.098	-20.698
502.377	-17.675	525.810	-22.554	478.194	-20.700
502.491	-17.676	525.889	-22.555	478.347	-20.703
502.632	-17.678	525.920	-22.556	478.523	-20.705
502.803	-17.680	525.942	-22.558	478.748	-20.707
502.992	-17.681	526.022	-22.559	478.964	-20.709
503.150	-17.683	526.072	-22.561	479.101	-20.711
503.313	-17.684	526.190	-22.562	479.243	-20.714
503.489	-17.686	526.414	-22.563	479.454	-20.716
503.672	-17.688	526.606	-22.565	479.720	-20.718
503.817	-17.689	526.740	-22.566	479.928	-20.720
503.924	-17.691	526.876	-22.567	480.066	-20.722
504.125	-17.692	527.049	-22.568	480.094	-20.724
504.289	-17.694	527.281	-22.570	480.172	-20.726
504.412	-17.695	527.452	-22.571	480.282	-20.728
504.607	-17.697	527.571	-22.572	480.453	-20.731
504.754	-17.699	527.745	-22.574	480.714	-20.733
504.935	-17.700	527.925	-22.575	480.891	-20.735
505.150	-17.702	528.093	-22.576	480.989	-20.737
505.308	-17.703	528.257	-22.578	481.144	-20.739
505.472	-17.705	528.385	-22.579	481.306	-20.741
505.629	-17.706	528.535	-22.580	481.448	-20.743
505.785	-17.708	528.749	-22.582	481.587	-20.745

505.957	-17.710	528.880	-22.583	481.697	-20.748
506.104	-17.711	529.035	-22.584	481.884	-20.750
506.250	-17.713	529.209	-22.586	482.086	-20.752
506.417	-17.714	529.312	-22.587	482.302	-20.754
506.623	-17.716	529.508	-22.588	482.506	-20.756
506.799	-17.717	529.728	-22.590	482.651	-20.758
506.898	-17.719	529.892	-22.591	482.833	-20.760
507.033	-17.721	530.000	-22.593	482.987	-20.762
507.218	-17.722	530.177	-22.594	483.142	-20.765
507.375	-17.724	530.363	-22.595	483.354	-20.767
507.527	-17.725	530.502	-22.597	483.499	-20.769
507.691	-17.727	530.689	-22.598	483.613	-20.771
507.842	-17.728	530.862	-22.599	483.782	-20.773
508.016	-17.730	531.032	-22.601	483.976	-20.775
508.209	-17.731	531.215	-22.602	484.023	-20.777
508.359	-17.733	531.379	-22.604	484.130	-20.780
508.497	-17.734	531.536	-22.605	484.363	-20.782
508.626	-17.736	531.602	-22.606	484.504	-20.784
508.707	-17.738	531.694	-22.608	484.664	-20.786
508.838	-17.739	531.900	-22.609	484.853	-20.788
509.040	-17.741	532.114	-22.611	485.018	-20.790
509.177	-17.742	532.319	-22.612	485.201	-20.793
509.365	-17.744	532.437	-22.614	485.412	-20.795
509.635	-17.745	532.556	-22.615	485.589	-20.797
509.901	-17.747	532.733	-22.616	485.766	-20.799
510.102	-17.748	532.845	-22.618	485.904	-20.802
510.227	-17.750	532.991	-22.619	485.997	-20.804
510.371	-17.751	533.163	-22.621	486.200	-20.806
510.507	-17.753	533.330	-22.622	486.376	-20.808
510.678	-17.754	533.544	-22.624	486.473	-20.810

510.896	-17.756	533.694	-22.625	486.618	-20.813
511.059	-17.757	533.829	-22.627	486.794	-20.815
511.179	-17.759	534.068	-22.629	486.995	-20.817
511.285	-17.761	534.253	-22.630	487.181	-20.819
511.416	-17.762	534.426	-22.632	487.356	-20.822
511.570	-17.764	534.577	-22.633	487.544	-20.824
511.672	-17.765	534.660	-22.635	487.783	-20.826
511.798	-17.767	534.792	-22.636	487.927	-20.828
511.969	-17.768	534.954	-22.638	488.004	-20.831
512.175	-17.770	535.138	-22.639	488.134	-20.833
512.313	-17.771	535.287	-22.641	488.279	-20.835
512.461	-17.773	535.409	-22.643	488.466	-20.838
512.612	-17.774	535.512	-22.644	488.644	-20.840
512.820	-17.776	535.670	-22.646	488.782	-20.842
513.095	-17.777	535.956	-22.648	488.973	-20.844
513.292	-17.779	536.211	-22.649	489.169	-20.847
513.418	-17.780	536.377	-22.651	489.314	-20.849
513.549	-17.782	536.463	-22.652	489.415	-20.851
513.710	-17.783	536.573	-22.654	489.561	-20.854
513.873	-17.785	536.760	-22.656	489.799	-20.856
514.005	-17.786	536.932	-22.658	489.967	-20.858
514.176	-17.788	537.055	-22.659	490.121	-20.861
514.384	-17.789	537.132	-22.661	490.347	-20.863
514.560	-17.791	537.220	-22.663	490.519	-20.865
514.719	-17.792	537.375	-22.664	490.654	-20.868
514.903	-17.794	537.549	-22.666	490.782	-20.870
514.983	-17.795	537.710	-22.668	490.894	-20.872
515.139	-17.797	537.895	-22.669	491.081	-20.875
515.290	-17.798	538.087	-22.671	491.222	-20.877
515.421	-17.800	538.323	-22.673	491.262	-20.879

515.638	-17.801	538.532	-22.675	491.327	-20.881
515.869	-17.803	538.740	-22.676	491.440	-20.884
516.039	-17.805	538.934	-22.678	491.615	-20.886
516.166	-17.806	539.139	-22.680	491.784	-20.888
516.307	-17.808	539.332	-22.682	492.011	-20.891
516.469	-17.809	539.454	-22.683	492.124	-20.893
516.619	-17.811	539.569	-22.685	492.281	-20.895
516.807	-17.812	539.740	-22.687	492.502	-20.898
516.958	-17.814	539.867	-22.689	492.688	-20.900
517.105	-17.815	539.961	-22.690	492.891	-20.902
517.284	-17.817	540.062	-22.692	493.067	-20.905
517.481	-17.818	540.147	-22.694	493.234	-20.907
517.694	-17.820	540.343	-22.696	493.428	-20.909
517.853	-17.821	540.580	-22.698	493.633	-20.911
517.990	-17.823	540.695	-22.699	493.876	-20.914
518.149	-17.824	540.849	-22.701	494.073	-20.916
518.265	-17.826	541.121	-22.703	494.193	-20.918
518.385	-17.827	541.251	-22.705	494.379	-20.921
518.570	-17.829	541.362	-22.707	494.579	-20.923
518.972	-17.830	541.561	-22.708	494.677	-20.925
519.424	-17.832	541.785	-22.710	494.773	-20.927
519.588	-17.833	541.942	-22.712	494.960	-20.930
519.623	-17.835	542.062	-22.714	495.137	-20.932
519.751	-17.836	542.242	-22.716	495.253	-20.934
519.862	-17.838	542.453	-22.718	495.438	-20.936
519.873	-17.839	542.615	-22.719	495.610	-20.939
519.918	-17.841	542.783	-22.721	495.686	-20.941
520.054	-17.842	542.990	-22.723	495.848	-20.943
520.161	-17.844	543.130	-22.725	496.004	-20.945
520.307	-17.845	543.294	-22.727	496.117	-20.947

520.546	-17.847	543.469	-22.729	496.222	-20.950
520.711	-17.849	543.606	-22.730	496.435	-20.952
520.847	-17.850	543.754	-22.732	496.694	-20.954
520.971	-17.852	543.902	-22.734	496.880	-20.956
521.130	-17.853	544.117	-22.736	497.032	-20.958
521.371	-17.855	544.253	-22.738	497.155	-20.961
521.613	-17.856	544.433	-22.740	497.246	-20.963
521.798	-17.858	544.653	-22.742	497.461	-20.965
521.910	-17.859	544.755	-22.743	497.683	-20.967
522.040	-17.861	544.893	-22.745	497.787	-20.969
522.206	-17.862	545.048	-22.747	497.971	-20.971
522.333	-17.864	545.233	-22.749	498.228	-20.973
522.437	-17.865	545.389	-22.751	498.346	-20.976
522.580	-17.867	545.567	-22.753	498.450	-20.978
522.763	-17.868	545.727	-22.755	498.635	-20.980
522.957	-17.870	545.863	-22.757	498.820	-20.982
523.029	-17.871	546.022	-22.758	498.990	-20.984
523.161	-17.873	546.205	-22.760	499.157	-20.986
523.351	-17.874	546.409	-22.762	499.345	-20.988
523.532	-17.876	546.593	-22.764	499.447	-20.990
523.732	-17.877	546.706	-22.766	499.590	-20.992
523.901	-17.879	546.829	-22.768	499.859	-20.994
524.095	-17.880	547.178	-22.770	500.044	-20.996
524.319	-17.882	547.532	-22.772	500.136	-20.999
524.527	-17.883	547.754	-22.774	500.306	-21.001
524.722	-17.885	547.899	-22.775	500.524	-21.003
524.855	-17.886	548.006	-22.777	500.685	-21.005
524.943	-17.888	548.060	-22.779	500.828	-21.007
525.059	-17.889	548.084	-22.781	500.986	-21.009
525.242	-17.891	548.159	-22.783	501.177	-21.011

525.401	-17.892	548.251	-22.785	501.312	-21.013
525.567	-17.894	548.438	-22.787	501.409	-21.015
525.723	-17.895	548.591	-22.789	501.589	-21.017
525.861	-17.897	548.698	-22.791	501.785	-21.019
526.013	-17.898	548.856	-22.793	501.924	-21.021
526.184	-17.900	549.082	-22.794	502.086	-21.023
526.374	-17.901	549.308	-22.796	502.320	-21.025
526.550	-17.903	549.457	-22.798	502.544	-21.027
526.687	-17.904	549.636	-22.800	502.675	-21.029
526.852	-17.906	549.845	-22.802	502.828	-21.031
527.031	-17.907	550.043	-22.804	502.980	-21.033
527.167	-17.909	550.141	-22.806	503.089	-21.035
527.273	-17.910	550.232	-22.808	503.204	-21.037
527.400	-17.912	550.388	-22.810	503.365	-21.039
527.552	-17.913	550.568	-22.811	503.505	-21.041
527.758	-17.915	550.721	-22.813	503.674	-21.043
527.963	-17.916	550.861	-22.815	504.000	-21.044
528.141	-17.918	551.026	-22.817	504.479	-21.046
528.320	-17.919	551.092	-22.819	504.715	-21.048
528.509	-17.921	551.211	-22.821	504.849	-21.050
528.695	-17.922	551.414	-22.823	504.932	-21.052
528.817	-17.924	551.649	-22.825	505.021	-21.054
528.918	-17.925	551.891	-22.827	505.101	-21.056
529.077	-17.927	552.040	-22.829	505.161	-21.058
529.265	-17.928	552.238	-22.831	505.220	-21.060
529.439	-17.930	552.409	-22.832	505.270	-21.062
529.601	-17.931	552.551	-22.834	505.388	-21.064
529.728	-17.932	552.751	-22.836	505.593	-21.066
529.882	-17.934	552.878	-22.838	505.766	-21.068
530.059	-17.935	552.989	-22.840	505.907	-21.070

530.215	-17.937	553.100	-22.842	506.044	-21.072
530.378	-17.938	553.269	-22.844	506.188	-21.074
530.519	-17.940	553.452	-22.846	506.353	-21.076
530.664	-17.941	553.617	-22.848	506.520	-21.078
530.825	-17.943	553.774	-22.850	506.740	-21.080
531.049	-17.944	553.974	-22.852	506.972	-21.082
531.176	-17.946	554.150	-22.854	507.153	-21.084
531.339	-17.947	554.319	-22.856	507.296	-21.086
531.516	-17.949	554.513	-22.858	507.434	-21.088
531.595	-17.950	554.664	-22.860	507.612	-21.090
531.645	-17.952	554.786	-22.862	507.787	-21.092
531.740	-17.953	554.872	-22.864	507.874	-21.094
531.938	-17.955	554.935	-22.866	507.994	-21.096
532.179	-17.956	555.102	-22.868	508.219	-21.098
532.375	-17.958	555.370	-22.870	508.365	-21.100
532.559	-17.959	555.531	-22.872	508.505	-21.102
532.734	-17.961	555.687	-22.874	508.658	-21.104
532.964	-17.962	555.877	-22.876	508.790	-21.106
533.193	-17.964	556.044	-22.878	508.971	-21.108
533.370	-17.965	556.245	-22.880	509.142	-21.110
533.594	-17.967	556.436	-22.882	509.325	-21.112
533.777	-17.968	556.561	-22.884	509.525	-21.114
533.910	-17.970	556.674	-22.886	509.694	-21.116
534.049	-17.971	556.847	-22.888	509.863	-21.118
534.151	-17.973	557.018	-22.890	510.030	-21.120
534.256	-17.974	557.145	-22.892	510.151	-21.122
534.409	-17.975	557.306	-22.894	510.287	-21.125
534.539	-17.977	557.520	-22.896	510.503	-21.127
534.723	-17.978	557.666	-22.898	510.705	-21.129
534.909	-17.980	557.833	-22.900	510.846	-21.131

535.021	-17.981	558.000	-22.902	510.960	-21.133
535.135	-17.983	558.132	-22.904	511.118	-21.135
535.248	-17.984	558.315	-22.906	511.304	-21.138
535.477	-17.986	558.486	-22.908	511.449	-21.140
535.724	-17.987	558.623	-22.910	511.623	-21.142
535.902	-17.989	558.780	-22.912	511.792	-21.144
536.094	-17.990	558.943	-22.914	511.933	-21.146
536.244	-17.992	559.043	-22.916	512.045	-21.149
536.331	-17.993	559.160	-22.918	512.157	-21.151
536.485	-17.995	559.310	-22.920	512.335	-21.153
536.716	-17.996	559.423	-22.922	512.484	-21.155
536.902	-17.998	559.524	-22.924	512.646	-21.158
537.084	-17.999	559.714	-22.926	512.879	-21.160
537.211	-18.001	559.981	-22.928	513.072	-21.162
537.321	-18.002	560.170	-22.930	513.229	-21.165
537.532	-18.004	560.338	-22.932	513.356	-21.167
537.731	-18.005	560.500	-22.934	513.467	-21.169
537.872	-18.007	560.716	-22.937	513.633	-21.172
538.007	-18.008	560.920	-22.939	513.771	-21.174
538.166	-18.010	561.086	-22.941	513.897	-21.176
538.345	-18.011	561.263	-22.943	514.031	-21.179
538.411	-18.013	561.459	-22.945	514.200	-21.181
538.549	-18.014	561.573	-22.947	514.391	-21.183
538.839	-18.016	561.694	-22.949	514.587	-21.186
539.071	-18.017	561.932	-22.951	514.778	-21.188
539.269	-18.019	562.098	-22.953	514.944	-21.191
539.418	-18.020	562.150	-22.955	515.184	-21.193
539.601	-18.022	562.238	-22.957	515.377	-21.196
539.739	-18.024	562.384	-22.959	515.517	-21.198
539.892	-18.025	562.555	-22.961	515.682	-21.201

540.017	-18.027	562.729	-22.963	515.860	-21.203
540.154	-18.028	562.928	-22.965	516.008	-21.205
540.351	-18.030	563.117	-22.967	516.138	-21.208
540.513	-18.031	563.263	-22.969	516.332	-21.210
540.698	-18.033	563.418	-22.971	516.486	-21.213
540.904	-18.034	563.620	-22.973	516.633	-21.215
541.047	-18.036	563.822	-22.975	516.763	-21.218
541.142	-18.037	563.958	-22.977	516.902	-21.220
541.266	-18.039	564.078	-22.979	517.115	-21.223
541.415	-18.040	564.244	-22.981	517.325	-21.225
541.665	-18.042	564.418	-22.983	517.484	-21.228
542.112	-18.043	564.645	-22.985	517.643	-21.231
542.453	-18.045	564.847	-22.986	517.774	-21.233
542.549	-18.046	564.993	-22.988	517.954	-21.236
542.605	-18.048	565.128	-22.990	518.191	-21.238
542.635	-18.050	565.307	-22.992	518.359	-21.241
542.670	-18.051	565.475	-22.994	518.459	-21.243
542.802	-18.053	565.610	-22.996	518.511	-21.246
542.951	-18.054	565.744	-22.998	518.639	-21.248
543.057	-18.056	565.906	-23.000	518.848	-21.251
543.179	-18.057	566.068	-23.002	518.971	-21.253
543.328	-18.059	566.252	-23.004	519.109	-21.256
543.508	-18.060	566.447	-23.006	519.257	-21.259
543.701	-18.062	566.603	-23.007	519.339	-21.261
543.891	-18.063	566.747	-23.009	519.514	-21.264
544.084	-18.065	566.919	-23.011	519.736	-21.266
544.247	-18.066	567.125	-23.013	519.889	-21.269
544.384	-18.068	567.233	-23.015	520.009	-21.271
544.534	-18.070	567.349	-23.017	520.144	-21.274
544.691	-18.071	567.587	-23.019	520.370	-21.276

544.853	-18.073	567.775	-23.020	520.533	-21.279
545.079	-18.074	567.920	-23.022	520.722	-21.282
545.263	-18.076	568.122	-23.024	520.960	-21.284
545.365	-18.077	568.294	-23.026	521.198	-21.287
545.453	-18.079	568.369	-23.028	521.451	-21.289
545.582	-18.080	568.541	-23.030	521.630	-21.292
545.762	-18.082	568.813	-23.031	521.747	-21.294
545.943	-18.083	568.927	-23.033	521.879	-21.297
546.126	-18.085	568.984	-23.035	522.024	-21.299
546.304	-18.086	569.277	-23.037	522.146	-21.302
546.444	-18.088	569.657	-23.038	522.295	-21.304
546.619	-18.090	569.943	-23.040	522.458	-21.307
546.813	-18.091	570.095	-23.042	522.517	-21.309
547.006	-18.093	570.187	-23.044	522.649	-21.312
547.182	-18.094	570.234	-23.045	522.869	-21.314
547.331	-18.096	570.304	-23.047	523.044	-21.317
547.525	-18.097	570.413	-23.049	523.200	-21.319
547.714	-18.099	570.470	-23.051	523.345	-21.322
547.845	-18.100	570.587	-23.052	523.506	-21.324
547.948	-18.102	570.754	-23.054	523.641	-21.327
548.059	-18.103	570.902	-23.056	523.738	-21.329
548.221	-18.105	571.093	-23.057	523.909	-21.331
548.396	-18.106	571.296	-23.059	524.187	-21.334
548.564	-18.108	571.522	-23.061	524.411	-21.336
548.756	-18.110	571.691	-23.062	524.576	-21.339
548.937	-18.111	571.790	-23.064	524.706	-21.341
549.072	-18.113	571.958	-23.066	524.914	-21.343
549.193	-18.114	572.182	-23.067	525.089	-21.346
549.360	-18.116	572.351	-23.069	525.254	-21.348
549.584	-18.117	572.461	-23.071	525.452	-21.351

549.754	-18.119	572.600	-23.072	525.584	-21.353
549.908	-18.120	572.790	-23.074	525.771	-21.355
550.067	-18.122	572.924	-23.076	525.936	-21.358
550.183	-18.123	573.092	-23.077	526.082	-21.360
550.286	-18.125	573.198	-23.079	526.249	-21.362
550.483	-18.126	573.283	-23.080	526.404	-21.365
550.703	-18.128	573.457	-23.082	526.547	-21.367
550.870	-18.129	573.680	-23.084	526.699	-21.369
551.025	-18.131	573.858	-23.085	526.864	-21.371
551.140	-18.133	574.049	-23.087	527.039	-21.374
551.296	-18.134	574.208	-23.088	527.177	-21.376
551.490	-18.136	574.319	-23.090	527.329	-21.378
551.685	-18.137	574.493	-23.091	527.559	-21.380
551.909	-18.139	574.679	-23.093	527.688	-21.383
552.074	-18.140	574.908	-23.095	527.828	-21.385
552.123	-18.142	575.087	-23.096	527.993	-21.387
552.274	-18.143	575.162	-23.098	528.165	-21.389
552.490	-18.145	575.313	-23.099	528.378	-21.391
552.617	-18.146	575.498	-23.101	528.533	-21.394
552.811	-18.148	575.637	-23.102	528.669	-21.396
553.009	-18.149	575.819	-23.104	528.750	-21.398
553.103	-18.151	575.982	-23.105	528.920	-21.400
553.250	-18.152	576.099	-23.107	529.128	-21.402
553.481	-18.154	576.247	-23.109	529.288	-21.404
553.676	-18.155	576.435	-23.110	529.431	-21.406
553.830	-18.157	576.624	-23.112	529.613	-21.408
553.932	-18.158	576.819	-23.113	529.803	-21.411
554.035	-18.160	576.996	-23.115	529.903	-21.413
554.221	-18.161	577.170	-23.116	530.061	-21.415
554.401	-18.163	577.327	-23.118	530.205	-21.417

554.511	-18.164	577.445	-23.119	530.368	-21.419
554.604	-18.166	577.584	-23.121	530.597	-21.421
554.735	-18.167	577.734	-23.122	530.777	-21.423
554.918	-18.169	577.877	-23.124	530.905	-21.425
555.051	-18.170	578.099	-23.125	531.057	-21.427
555.197	-18.172	578.287	-23.127	531.357	-21.429
555.413	-18.173	578.470	-23.128	531.788	-21.431
555.650	-18.175	578.611	-23.130	532.139	-21.433
555.891	-18.177	578.758	-23.131	532.292	-21.435
556.068	-18.178	578.920	-23.133	532.367	-21.437
556.268	-18.180	579.031	-23.134	532.444	-21.439
556.460	-18.181	579.197	-23.136	532.492	-21.441
556.643	-18.183	579.422	-23.137	532.563	-21.443
556.807	-18.184	579.579	-23.139	532.679	-21.445
556.906	-18.186	579.659	-23.140	532.807	-21.447
557.046	-18.187	579.806	-23.142	532.842	-21.449
557.231	-18.189	580.037	-23.143	532.940	-21.451
557.356	-18.190	580.176	-23.145	533.129	-21.453
557.457	-18.192	580.295	-23.146	533.305	-21.455
557.605	-18.193	580.493	-23.148	533.480	-21.457
557.747	-18.195	580.714	-23.149	533.684	-21.459
557.853	-18.196	580.874	-23.151	533.838	-21.461
558.045	-18.198	580.967	-23.152	533.917	-21.463
558.239	-18.199	581.068	-23.154	534.113	-21.465
558.404	-18.201	581.232	-23.155	534.292	-21.467
558.626	-18.202	581.415	-23.156	534.396	-21.469
558.798	-18.204	581.594	-23.158	534.607	-21.471
558.980	-18.205	581.715	-23.159	534.842	-21.473
559.184	-18.207	581.800	-23.161	535.017	-21.474
559.351	-18.208	581.904	-23.162	535.165	-21.476

559.490	-18.210	582.063	-23.164	535.335	-21.478
559.600	-18.211	582.259	-23.165	535.483	-21.480
559.742	-18.213	582.409	-23.167	535.627	-21.482
559.879	-18.214	582.619	-23.168	535.794	-21.484
560.028	-18.216	582.868	-23.169	535.944	-21.486
560.203	-18.217	583.088	-23.171	536.039	-21.488
560.395	-18.219	583.318	-23.172	536.148	-21.490
560.572	-18.220	583.522	-23.174	536.367	-21.492
560.726	-18.222	583.688	-23.175	536.568	-21.494
560.889	-18.223	583.849	-23.177	536.687	-21.496
561.086	-18.225	583.984	-23.178	536.821	-21.498
561.268	-18.226	584.103	-23.179	536.995	-21.500
561.420	-18.228	584.291	-23.181	537.176	-21.502
561.581	-18.229	584.463	-23.182	537.341	-21.504
561.753	-18.231	584.561	-23.183	537.547	-21.506
561.949	-18.232	584.606	-23.185	537.729	-21.508
562.141	-18.234	584.773	-23.186	537.905	-21.510
562.284	-18.236	585.028	-23.188	537.988	-21.512
562.381	-18.237	585.195	-23.189	538.135	-21.514
562.538	-18.239	585.330	-23.190	538.364	-21.516
562.720	-18.240	585.529	-23.192	538.516	-21.518
562.823	-18.242	585.726	-23.193	538.675	-21.520
562.986	-18.243	585.865	-23.194	538.831	-21.523
563.227	-18.245	586.077	-23.195	538.990	-21.525
563.434	-18.246	586.278	-23.197	539.114	-21.527
563.590	-18.248	586.400	-23.198	539.208	-21.529
563.744	-18.249	586.522	-23.199	539.393	-21.531
563.929	-18.251	586.601	-23.201	539.671	-21.533
564.136	-18.252	586.802	-23.202	539.835	-21.535
564.277	-18.254	587.028	-23.203	539.910	-21.537

564.360	-18.255	587.188	-23.204	540.101	-21.539
564.581	-18.257	587.361	-23.206	540.366	-21.542
564.959	-18.258	587.454	-23.207	540.543	-21.544
565.345	-18.260	587.622	-23.208	540.695	-21.546
565.617	-18.261	587.854	-23.209	540.796	-21.548
565.700	-18.263	588.018	-23.210	540.963	-21.550
565.674	-18.264	588.219	-23.212	541.205	-21.552
565.646	-18.266	588.381	-23.213	541.344	-21.555
565.727	-18.268	588.509	-23.214	541.389	-21.557
565.908	-18.269	588.664	-23.215	541.454	-21.559
565.987	-18.271	588.813	-23.216	541.652	-21.561
566.089	-18.272	588.918	-23.218	541.840	-21.563
566.254	-18.274	589.074	-23.219	542.014	-21.566
566.396	-18.275	589.323	-23.220	542.282	-21.568
566.590	-18.277	589.468	-23.221	542.460	-21.570
566.810	-18.278	589.566	-23.222	542.553	-21.572
566.975	-18.280	589.765	-23.223	542.697	-21.575
567.188	-18.281	589.927	-23.224	542.874	-21.577
567.381	-18.283	590.107	-23.225	543.029	-21.579
567.497	-18.284	590.342	-23.226	543.155	-21.581
567.626	-18.286	590.542	-23.228	543.298	-21.584
567.753	-18.287	590.588	-23.229	543.474	-21.586
567.908	-18.289	590.721	-23.230	543.670	-21.588
568.095	-18.290	591.001	-23.231	543.814	-21.591
568.214	-18.292	591.154	-23.232	543.938	-21.593
568.316	-18.293	591.264	-23.233	544.104	-21.595
568.487	-18.295	591.573	-23.234	544.247	-21.598
568.644	-18.296	592.004	-23.235	544.398	-21.600
568.826	-18.298	592.240	-23.236	544.614	-21.602
569.039	-18.299	592.309	-23.237	544.845	-21.605

569.200	-18.301	592.343	-23.238	545.048	-21.607
569.388	-18.302	592.393	-23.239	545.252	-21.609
569.557	-18.304	592.474	-23.240	545.355	-21.612
569.699	-18.305	592.561	-23.241	545.424	-21.614
569.886	-18.307	592.728	-23.242	545.579	-21.617
570.040	-18.308	592.914	-23.243	545.667	-21.619
570.190	-18.310	593.026	-23.244	545.863	-21.621
570.359	-18.311	593.161	-23.245	546.193	-21.624
570.553	-18.313	593.351	-23.246	546.302	-21.626
570.766	-18.314	593.524	-23.246	546.428	-21.629
570.900	-18.316	593.713	-23.247	546.552	-21.631
570.991	-18.317	593.914	-23.248	546.617	-21.633
571.090	-18.319	594.107	-23.249	546.742	-21.636
571.244	-18.320	594.280	-23.250	546.907	-21.638
571.449	-18.322	594.384	-23.251	547.068	-21.641
571.616	-18.323	594.488	-23.252	547.315	-21.643
571.800	-18.325	594.661	-23.253	547.529	-21.645
571.982	-18.326	594.835	-23.253	547.664	-21.648
572.177	-18.328	595.023	-23.254	547.866	-21.650
572.366	-18.329	595.158	-23.255	548.103	-21.653
572.473	-18.331	595.274	-23.256	548.308	-21.655
572.610	-18.332	595.412	-23.257	548.498	-21.657
572.780	-18.334	595.588	-23.258	548.661	-21.660
572.966	-18.335	595.741	-23.258	548.817	-21.662
573.062	-18.336	595.896	-23.259	549.012	-21.665
573.138	-18.338	596.109	-23.260	549.149	-21.667
573.302	-18.339	596.292	-23.261	549.301	-21.669
573.534	-18.341	596.388	-23.262	549.480	-21.672
573.794	-18.342	596.581	-23.262	549.582	-21.674
573.980	-18.344	596.776	-23.263	549.732	-21.676

574.082	-18.345	596.909	-23.264	549.919	-21.679
574.248	-18.347	597.082	-23.265	550.080	-21.681
574.447	-18.348	597.264	-23.265	550.189	-21.684
574.591	-18.349	597.433	-23.266	550.271	-21.686
574.719	-18.351	597.620	-23.267	550.427	-21.688
574.836	-18.352	597.777	-23.268	550.611	-21.691
574.977	-18.354	597.920	-23.268	550.732	-21.693
575.157	-18.355	598.033	-23.269	550.859	-21.695
575.301	-18.357	598.168	-23.270	551.018	-21.698
575.417	-18.358	598.282	-23.270	551.208	-21.700
575.660	-18.359	598.466	-23.271	551.401	-21.702
575.957	-18.361	598.655	-23.272	551.582	-21.705
576.113	-18.362	598.831	-23.273	551.793	-21.707
576.213	-18.364	599.037	-23.273	552.034	-21.709
576.312	-18.365	599.203	-23.274	552.234	-21.711
576.413	-18.366	599.353	-23.275	552.352	-21.714
576.626	-18.368	599.435	-23.275	552.457	-21.716
576.854	-18.369	599.520	-23.276	552.691	-21.718
576.972	-18.371	599.700	-23.277	552.875	-21.721
577.069	-18.372	599.922	-23.277	552.957	-21.723
577.185	-18.373	600.124	-23.278	553.047	-21.725
577.329	-18.375	600.317	-23.279	553.218	-21.727
577.524	-18.376	600.486	-23.279	553.326	-21.730
577.682	-18.377	600.661	-23.280	553.477	-21.732
577.796	-18.379	600.863	-23.281	553.756	-21.734
577.911	-18.380	600.966	-23.281	553.995	-21.736
578.115	-18.381	601.107	-23.282	554.157	-21.738
578.305	-18.383	601.333	-23.283	554.262	-21.741
578.509	-18.384	601.533	-23.283	554.422	-21.743
578.788	-18.386	601.684	-23.284	554.561	-21.745

578.992	-18.387	601.818	-23.285	554.730	-21.747
579.176	-18.388	601.913	-23.285	554.961	-21.749
579.337	-18.390	602.019	-23.286	555.201	-21.752
579.491	-18.391	602.212	-23.286	555.355	-21.754
579.638	-18.392	602.415	-23.287	555.473	-21.756
579.777	-18.394	602.589	-23.288	555.666	-21.758
579.934	-18.395	602.753	-23.288	555.824	-21.760
580.080	-18.396	602.933	-23.289	555.965	-21.762
580.246	-18.398	603.046	-23.289	556.124	-21.764
580.415	-18.399	603.192	-23.290	556.256	-21.766
580.527	-18.400	603.401	-23.291	556.350	-21.769
580.633	-18.402	603.533	-23.291	556.480	-21.771
580.806	-18.403	603.681	-23.292	556.724	-21.773
580.912	-18.404	603.828	-23.292	556.965	-21.775
581.045	-18.405	603.923	-23.293	557.098	-21.777
581.233	-18.407	604.032	-23.293	557.270	-21.779
581.441	-18.408	604.217	-23.294	557.441	-21.781
581.662	-18.409	604.396	-23.295	557.575	-21.783
581.825	-18.411	604.547	-23.295	557.690	-21.785
582.014	-18.412	604.755	-23.296	557.831	-21.787
582.224	-18.413	604.956	-23.296	558.026	-21.789
582.403	-18.414	605.159	-23.297	558.182	-21.791
582.557	-18.416	605.387	-23.297	558.310	-21.793
582.687	-18.417	605.586	-23.298	558.462	-21.795
582.775	-18.418	605.759	-23.298	558.731	-21.797
582.939	-18.420	605.924	-23.299	559.127	-21.799
583.153	-18.421	606.009	-23.299	559.489	-21.801
583.322	-18.422	606.168	-23.299	559.676	-21.803
583.511	-18.423	606.381	-23.300	559.766	-21.805
583.626	-18.425	606.481	-23.300	559.831	-21.807

583.782	-18.426	606.593	-23.301	559.846	-21.809
583.995	-18.427	606.758	-23.301	559.922	-21.811
584.194	-18.428	606.963	-23.302	560.107	-21.813
584.357	-18.430	607.102	-23.302	560.209	-21.815
584.491	-18.431	607.218	-23.302	560.308	-21.817
584.639	-18.432	607.395	-23.303	560.444	-21.819
584.825	-18.433	607.578	-23.303	560.558	-21.821
584.929	-18.435	607.792	-23.304	560.704	-21.823
585.053	-18.436	607.998	-23.304	560.902	-21.825
585.177	-18.437	608.195	-23.304	561.045	-21.827
585.409	-18.438	608.379	-23.305	561.140	-21.829
585.622	-18.440	608.490	-23.305	561.305	-21.831
585.785	-18.441	608.661	-23.305	561.539	-21.833
585.952	-18.442	608.865	-23.306	561.752	-21.835
586.144	-18.443	609.021	-23.306	561.940	-21.836
586.355	-18.444	609.150	-23.306	562.128	-21.838
586.561	-18.446	609.256	-23.306	562.308	-21.840
586.690	-18.447	609.416	-23.307	562.443	-21.842
586.774	-18.448	609.603	-23.307	562.584	-21.844
586.840	-18.449	609.771	-23.307	562.743	-21.846
587.063	-18.451	609.896	-23.307	562.865	-21.848
587.298	-18.452	610.075	-23.308	562.998	-21.850
587.503	-18.453	610.251	-23.308	563.223	-21.852
587.822	-18.454	610.382	-23.308	563.441	-21.854
588.219	-18.455	610.561	-23.308	563.510	-21.856
588.457	-18.456	610.721	-23.308	563.597	-21.858
588.537	-18.458	610.894	-23.308	563.780	-21.860
588.594	-18.459	611.085	-23.309	563.935	-21.862
588.622	-18.460	611.244	-23.309	564.115	-21.863
588.650	-18.461	611.447	-23.309	564.304	-21.865

588.744	-18.462	611.630	-23.309	564.505	-21.867
588.845	-18.464	611.754	-23.309	564.693	-21.869
588.980	-18.465	611.839	-23.309	564.857	-21.871
589.106	-18.466	611.980	-23.309	565.014	-21.873
589.234	-18.467	612.212	-23.309	565.177	-21.875
589.462	-18.468	612.410	-23.310	565.331	-21.877
589.666	-18.469	612.559	-23.310	565.503	-21.879
589.824	-18.470	612.746	-23.310	565.701	-21.881
590.033	-18.472	612.946	-23.310	565.866	-21.883
590.186	-18.473	613.121	-23.310	566.019	-21.885
590.357	-18.474	613.230	-23.310	566.108	-21.887
590.562	-18.475	613.286	-23.310	566.217	-21.889
590.717	-18.476	613.572	-23.310	566.446	-21.891
590.800	-18.477	614.021	-23.310	566.630	-21.893
590.919	-18.478	614.293	-23.310	566.735	-21.895
591.115	-18.479	614.427	-23.310	566.866	-21.897
591.271	-18.480	614.469	-23.310	567.072	-21.899
591.444	-18.482	614.500	-23.310	567.291	-21.901
591.656	-18.483	614.543	-23.310	567.451	-21.903
591.821	-18.484	614.658	-23.310	567.609	-21.905
591.904	-18.485	614.761	-23.310	567.749	-21.907
591.960	-18.486	614.856	-23.310	567.865	-21.909
592.118	-18.487	615.047	-23.309	567.994	-21.911
592.343	-18.488	615.220	-23.309	568.202	-21.913
592.530	-18.489	615.336	-23.309	568.397	-21.915
592.710	-18.490	615.537	-23.309	568.532	-21.917
592.958	-18.491	615.794	-23.309	568.676	-21.919
593.149	-18.492	615.998	-23.309	568.793	-21.921
593.246	-18.493	616.158	-23.309	568.930	-21.923
593.419	-18.494	616.275	-23.309	569.133	-21.925

593.652	-18.495	616.459	-23.309	569.338	-21.927
593.805	-18.497	616.686	-23.309	569.547	-21.929
593.936	-18.498	616.831	-23.308	569.735	-21.931
594.060	-18.499	616.978	-23.308	569.893	-21.933
594.132	-18.500	617.120	-23.308	570.080	-21.935
594.301	-18.501	617.249	-23.308	570.243	-21.937
594.511	-18.502	617.386	-23.308	570.360	-21.939
594.687	-18.503	617.568	-23.308	570.506	-21.941
594.895	-18.504	617.705	-23.308	570.758	-21.943
595.088	-18.505	617.775	-23.307	570.912	-21.945
595.243	-18.506	617.926	-23.307	571.012	-21.947
595.369	-18.507	618.125	-23.307	571.194	-21.949
595.512	-18.508	618.258	-23.307	571.397	-21.951
595.730	-18.509	618.445	-23.307	571.560	-21.953
595.940	-18.510	618.675	-23.307	571.629	-21.956
596.058	-18.511	618.890	-23.306	571.721	-21.958
596.102	-18.511	619.065	-23.306	571.884	-21.960
596.206	-18.512	619.180	-23.306	572.102	-21.962
596.417	-18.513	619.354	-23.306	572.314	-21.964
596.584	-18.514	619.494	-23.306	572.487	-21.966
596.749	-18.515	619.619	-23.306	572.663	-21.968
596.972	-18.516	619.785	-23.306	572.851	-21.970
597.192	-18.517	619.947	-23.305	572.989	-21.972
597.365	-18.518	620.084	-23.305	573.190	-21.974
597.486	-18.519	620.246	-23.305	573.340	-21.976
597.566	-18.520	620.431	-23.305	573.438	-21.978
597.695	-18.521	620.552	-23.305	573.606	-21.980
597.892	-18.522	620.666	-23.305	573.798	-21.982
598.107	-18.523	620.841	-23.305	573.949	-21.984
598.229	-18.524	621.045	-23.304	574.081	-21.986

598.324	-18.524	621.263	-23.304	574.201	-21.988
598.502	-18.525	621.377	-23.304	574.322	-21.990
598.720	-18.526	621.495	-23.304	574.491	-21.992
598.913	-18.527	621.661	-23.304	574.660	-21.994
599.107	-18.528	621.838	-23.304	574.807	-21.996
599.257	-18.529	622.055	-23.304	574.907	-21.998
599.393	-18.530	622.221	-23.304	575.021	-22.000
599.533	-18.531	622.395	-23.304	575.287	-22.002
599.695	-18.531	622.628	-23.304	575.511	-22.004
599.895	-18.532	622.789	-23.303	575.686	-22.006
599.989	-18.533	622.855	-23.303	575.890	-22.008
600.091	-18.534	623.028	-23.303	576.064	-22.010
600.207	-18.535	623.237	-23.303	576.225	-22.012
600.291	-18.536	623.398	-23.303	576.419	-22.014
600.432	-18.537	623.614	-23.303	576.671	-22.016
600.680	-18.537	623.748	-23.303	576.867	-22.018
600.835	-18.538	623.846	-23.303	576.964	-22.020
601.012	-18.539	623.978	-23.303	577.117	-22.022
601.181	-18.540	624.135	-23.303	577.229	-22.024
601.420	-18.541	624.282	-23.303	577.342	-22.026
601.680	-18.541	624.435	-23.303	577.489	-22.028
601.858	-18.542	624.687	-23.303	577.647	-22.030
601.991	-18.543	624.922	-23.303	577.772	-22.032
602.176	-18.544	625.037	-23.303	577.910	-22.034
602.345	-18.545	625.134	-23.303	578.056	-22.036
602.556	-18.545	625.272	-23.303	578.219	-22.037
602.714	-18.546	625.436	-23.303	578.347	-22.039
602.899	-18.547	625.600	-23.303	578.521	-22.041
603.018	-18.548	625.743	-23.303	578.759	-22.043
603.113	-18.549	625.860	-23.303	578.956	-22.045

603.284	-18.549	625.967	-23.303	579.079	-22.047
603.450	-18.550	626.141	-23.304	579.252	-22.049
603.586	-18.551	626.275	-23.304	579.422	-22.050
603.699	-18.552	626.428	-23.304	579.616	-22.052
603.807	-18.552	626.627	-23.304	579.849	-22.054
604.001	-18.553	626.780	-23.304	580.021	-22.056
604.173	-18.554	626.973	-23.304	580.182	-22.058
604.299	-18.555	627.222	-23.304	580.336	-22.060
604.497	-18.555	627.401	-23.304	580.460	-22.061
604.730	-18.556	627.560	-23.304	580.609	-22.063
604.882	-18.557	627.755	-23.305	580.784	-22.065
605.042	-18.558	627.892	-23.305	580.967	-22.067
605.232	-18.558	628.060	-23.305	581.117	-22.068
605.385	-18.559	628.225	-23.305	581.290	-22.070
605.543	-18.560	628.346	-23.305	581.489	-22.072
605.684	-18.561	628.498	-23.305	581.590	-22.074
605.793	-18.561	628.612	-23.305	581.685	-22.075
605.947	-18.562	628.728	-23.306	581.839	-22.077
606.153	-18.563	628.812	-23.306	582.009	-22.079
606.270	-18.564	628.958	-23.306	582.183	-22.081
606.409	-18.564	629.250	-23.306	582.360	-22.082
606.667	-18.565	629.462	-23.306	582.579	-22.084
606.851	-18.566	629.674	-23.307	582.802	-22.086
606.984	-18.566	629.874	-23.307	582.944	-22.087
607.159	-18.567	630.017	-23.307	583.059	-22.089
607.343	-18.568	630.177	-23.307	583.233	-22.091
607.493	-18.569	630.376	-23.308	583.361	-22.092
607.687	-18.569	630.520	-23.308	583.506	-22.094
607.882	-18.570	630.676	-23.308	583.649	-22.096
608.025	-18.571	630.880	-23.308	583.800	-22.097

608.113	-18.571	631.028	-23.309	583.980	-22.099
608.271	-18.572	631.163	-23.309	584.231	-22.101
608.488	-18.573	631.315	-23.309	584.412	-22.102
608.635	-18.574	631.562	-23.309	584.536	-22.104
608.837	-18.574	631.761	-23.310	584.736	-22.106
609.054	-18.575	631.826	-23.310	584.916	-22.107
609.237	-18.576	631.918	-23.310	585.010	-22.109
609.404	-18.576	632.088	-23.311	585.164	-22.111
609.513	-18.577	632.343	-23.311	585.322	-22.112
609.617	-18.578	632.565	-23.311	585.471	-22.114
609.829	-18.578	632.708	-23.311	585.592	-22.116
610.028	-18.579	632.902	-23.312	585.774	-22.117
610.129	-18.580	633.045	-23.312	585.917	-22.119
610.311	-18.580	633.154	-23.312	586.057	-22.120
610.740	-18.581	633.298	-23.313	586.370	-22.122
611.149	-18.582	633.474	-23.313	586.822	-22.124
611.331	-18.583	633.648	-23.313	587.233	-22.125
611.373	-18.583	633.778	-23.314	587.384	-22.127
611.432	-18.584	633.900	-23.314	587.412	-22.128
611.528	-18.585	634.061	-23.314	587.529	-22.130
611.521	-18.585	634.307	-23.315	587.656	-22.132
611.576	-18.586	634.536	-23.315	587.706	-22.133
611.786	-18.587	634.658	-23.316	587.739	-22.135
611.937	-18.587	634.732	-23.316	587.826	-22.137
612.003	-18.588	634.839	-23.316	587.957	-22.138
612.145	-18.589	635.016	-23.317	588.047	-22.140
612.353	-18.589	635.161	-23.317	588.139	-22.141
612.554	-18.590	635.380	-23.317	588.327	-22.143
612.741	-18.591	635.800	-23.318	588.524	-22.145
612.878	-18.591	636.188	-23.318	588.669	-22.146

613.020	-18.592	636.381	-23.319	588.853	-22.148
613.232	-18.593	636.462	-23.319	589.018	-22.149
613.439	-18.593	636.493	-23.319	589.220	-22.151
613.586	-18.594	636.507	-23.320	589.405	-22.153
613.712	-18.595	636.608	-23.320	589.531	-22.154
613.868	-18.595	636.717	-23.321	589.718	-22.156
614.058	-18.596	636.828	-23.321	589.901	-22.158
614.184	-18.597	636.972	-23.321	590.083	-22.159
614.298	-18.597	637.143	-23.322	590.271	-22.161
614.494	-18.598	637.358	-23.322	590.373	-22.163
614.664	-18.599	637.528	-23.323	590.425	-22.164
614.785	-18.599	637.666	-23.323	590.585	-22.166
614.926	-18.600	637.855	-23.324	590.866	-22.168
615.076	-18.601	638.039	-23.324	591.001	-22.169
615.273	-18.601	638.219	-23.324	591.081	-22.171
615.483	-18.602	638.382	-23.325	591.226	-22.173
615.684	-18.603	638.489	-23.325	591.374	-22.174
615.802	-18.603	638.630	-23.326	591.495	-22.176
615.987	-18.604	638.803	-23.326	591.657	-22.178
616.225	-18.605	638.932	-23.327	591.926	-22.180
616.369	-18.605	639.089	-23.327	592.059	-22.181
616.502	-18.606	639.228	-23.328	592.201	-22.183
616.618	-18.607	639.395	-23.328	592.469	-22.185
616.765	-18.607	639.581	-23.329	592.703	-22.187
616.948	-18.608	639.716	-23.329	592.867	-22.188
617.188	-18.609	639.909	-23.330	592.970	-22.190
617.349	-18.609	640.106	-23.330	593.123	-22.192
617.448	-18.610	640.256	-23.331	593.333	-22.194
617.604	-18.610	640.385	-23.331	593.478	-22.196
617.781	-18.611	640.525	-23.332	593.690	-22.197

618.002	-18.612	640.733	-23.332	593.888	-22.199
618.165	-18.612	640.958	-23.333	593.985	-22.201
618.290	-18.613	641.110	-23.333	594.095	-22.203
618.449	-18.614	641.256	-23.334	594.296	-22.205
618.570	-18.614	641.385	-23.334	594.486	-22.207
618.741	-18.615	641.516	-23.335	594.597	-22.209
618.928	-18.616	641.672	-23.335	594.719	-22.211
619.020	-18.616	641.800	-23.336	594.893	-22.212
619.136	-18.617	641.979	-23.336	595.084	-22.214
619.352	-18.618	642.164	-23.337	595.248	-22.216
619.526	-18.618	642.300	-23.338	595.371	-22.218
619.684	-18.619	642.435	-23.338	595.494	-22.220
619.843	-18.619	642.587	-23.339	595.646	-22.222
619.973	-18.620	642.728	-23.339	595.858	-22.224
620.155	-18.621	642.885	-23.340	596.025	-22.226
620.313	-18.621	643.063	-23.341	596.151	-22.228
620.425	-18.622	643.262	-23.341	596.280	-22.230
620.614	-18.623	643.416	-23.342	596.395	-22.232
620.816	-18.623	643.554	-23.343	596.588	-22.234
620.948	-18.624	643.697	-23.343	596.821	-22.236
621.091	-18.625	643.885	-23.344	596.994	-22.238
621.287	-18.625	644.099	-23.345	597.112	-22.240
621.483	-18.626	644.236	-23.345	597.240	-22.242
621.589	-18.627	644.398	-23.346	597.462	-22.244
621.742	-18.627	644.600	-23.347	597.705	-22.247
621.993	-18.628	644.805	-23.347	597.835	-22.249
622.153	-18.628	644.966	-23.348	597.914	-22.251
622.248	-18.629	645.132	-23.349	598.044	-22.253
622.397	-18.630	645.271	-23.350	598.252	-22.255
622.579	-18.630	645.387	-23.350	598.463	-22.257

622.759	-18.631	645.588	-23.351	598.653	-22.259
622.938	-18.632	645.760	-23.352	598.780	-22.261
623.046	-18.632	645.878	-23.352	598.904	-22.264
623.136	-18.633	646.037	-23.353	599.055	-22.266
623.193	-18.634	646.255	-23.354	599.226	-22.268
623.344	-18.634	646.455	-23.355	599.344	-22.270
623.580	-18.635	646.623	-23.356	599.498	-22.272
623.753	-18.636	646.749	-23.356	599.727	-22.274
623.980	-18.636	646.842	-23.357	599.915	-22.277
624.205	-18.637	646.998	-23.358	600.121	-22.279
624.423	-18.637	647.174	-23.359	600.296	-22.281
624.643	-18.638	647.351	-23.359	600.444	-22.283
624.810	-18.639	647.516	-23.360	600.589	-22.286
625.004	-18.639	647.661	-23.361	600.747	-22.288
625.168	-18.640	647.746	-23.362	600.888	-22.290
625.273	-18.641	647.836	-23.363	601.004	-22.292
625.411	-18.641	648.028	-23.363	601.200	-22.295
625.569	-18.642	648.225	-23.364	601.364	-22.297
625.724	-18.643	648.354	-23.365	601.477	-22.299
625.861	-18.643	648.508	-23.366	601.677	-22.301
625.984	-18.644	648.719	-23.367	601.845	-22.304
626.143	-18.645	648.940	-23.368	601.911	-22.306
626.304	-18.645	649.154	-23.368	602.025	-22.308
626.487	-18.646	649.338	-23.369	602.175	-22.310
626.607	-18.647	649.496	-23.370	602.319	-22.313
626.708	-18.647	649.649	-23.371	602.476	-22.315
626.887	-18.648	649.815	-23.372	602.632	-22.317
627.057	-18.649	649.958	-23.373	602.832	-22.319
627.278	-18.649	650.108	-23.373	603.045	-22.322
627.528	-18.650	650.286	-23.374	603.279	-22.324

627.697	-18.651	650.423	-23.375	603.478	-22.326
627.840	-18.651	650.515	-23.376	603.630	-22.329
628.013	-18.652	650.648	-23.377	603.826	-22.331
628.146	-18.653	650.827	-23.378	604.063	-22.333
628.284	-18.653	650.970	-23.378	604.246	-22.335
628.475	-18.654	651.095	-23.379	604.330	-22.338
628.691	-18.655	651.274	-23.380	604.449	-22.340
628.851	-18.656	651.459	-23.381	604.682	-22.342
629.000	-18.656	651.643	-23.382	604.838	-22.344
629.104	-18.657	651.852	-23.383	604.924	-22.347
629.276	-18.658	652.019	-23.383	605.081	-22.349
629.490	-18.658	652.224	-23.384	605.209	-22.351
629.592	-18.659	652.410	-23.385	605.335	-22.353
629.786	-18.660	652.521	-23.386	605.527	-22.356
629.991	-18.660	652.731	-23.387	605.631	-22.358
630.065	-18.661	652.950	-23.388	605.764	-22.360
630.229	-18.662	653.099	-23.388	605.998	-22.363
630.494	-18.663	653.241	-23.389	606.183	-22.365
630.683	-18.663	653.363	-23.390	606.296	-22.367
630.806	-18.664	653.501	-23.391	606.421	-22.369
630.936	-18.665	653.654	-23.392	606.686	-22.372
631.047	-18.666	653.840	-23.393	606.916	-22.374
631.183	-18.666	654.043	-23.394	607.065	-22.376
631.385	-18.667	654.194	-23.394	607.221	-22.378
631.589	-18.668	654.344	-23.395	607.391	-22.380
631.791	-18.668	654.549	-23.396	607.447	-22.383
631.945	-18.669	654.741	-23.397	607.606	-22.385
632.077	-18.670	654.912	-23.398	607.869	-22.387
632.227	-18.671	655.039	-23.399	608.054	-22.389
632.412	-18.671	655.173	-23.399	608.230	-22.391

632.587	-18.672	655.366	-23.400	608.418	-22.394
632.719	-18.673	655.572	-23.401	608.544	-22.396
632.846	-18.674	655.725	-23.402	608.688	-22.398
633.114	-18.674	655.806	-23.403	608.887	-22.400
633.537	-18.675	655.943	-23.404	609.065	-22.402
633.899	-18.676	656.134	-23.404	609.174	-22.404
634.061	-18.677	656.312	-23.405	609.366	-22.407
634.070	-18.677	656.481	-23.406	609.541	-22.409
634.095	-18.678	656.626	-23.407	609.756	-22.411
634.158	-18.679	656.764	-23.408	609.940	-22.413
634.207	-18.680	656.926	-23.409	610.070	-22.415
634.319	-18.681	657.064	-23.410	610.211	-22.417
634.479	-18.681	657.281	-23.410	610.370	-22.419
634.610	-18.682	657.691	-23.411	610.566	-22.421
634.744	-18.683	658.028	-23.412	610.774	-22.424
634.860	-18.684	658.175	-23.413	610.984	-22.426
635.033	-18.684	658.315	-23.414	611.117	-22.428
635.266	-18.685	658.388	-23.415	611.241	-22.430
635.448	-18.686	658.431	-23.415	611.298	-22.432
635.631	-18.687	658.489	-23.416	611.366	-22.434
635.800	-18.688	658.505	-23.417	611.625	-22.436
635.914	-18.688	658.596	-23.418	611.778	-22.438
636.092	-18.689	658.800	-23.419	611.984	-22.440
636.290	-18.690	658.965	-23.420	612.134	-22.442
636.421	-18.691	659.133	-23.420	612.243	-22.444
636.590	-18.692	659.367	-23.421	612.404	-22.446
636.747	-18.692	659.565	-23.422	612.595	-22.448
636.885	-18.693	659.650	-23.423	612.845	-22.450
636.997	-18.694	659.797	-23.424	613.029	-22.452
637.192	-18.695	660.040	-23.425	613.104	-22.454

637.438	-18.696	660.211	-23.425	613.267	-22.456
637.556	-18.696	660.359	-23.426	613.407	-22.458
637.678	-18.697	660.485	-23.427	613.550	-22.460
637.855	-18.698	660.644	-23.428	613.719	-22.462
638.025	-18.699	660.834	-23.429	614.047	-22.464
638.183	-18.700	660.994	-23.430	614.437	-22.466
638.366	-18.701	661.147	-23.430	614.680	-22.468
638.574	-18.701	661.311	-23.431	614.866	-22.470
638.737	-18.702	661.430	-23.432	615.009	-22.472
638.906	-18.703	661.573	-23.433	615.093	-22.474
639.061	-18.704	661.698	-23.434	615.112	-22.476
639.198	-18.705	661.812	-23.435	615.140	-22.478
639.357	-18.706	662.073	-23.435	615.255	-22.480
639.496	-18.706	662.310	-23.436	615.392	-22.482
639.625	-18.707	662.432	-23.437	615.499	-22.484
639.800	-18.708	662.619	-23.438	615.626	-22.485
639.964	-18.709	662.830	-23.439	615.802	-22.487
640.136	-18.710	662.945	-23.440	615.966	-22.489
640.349	-18.711	663.116	-23.441	616.141	-22.491
640.466	-18.711	663.271	-23.441	616.319	-22.493
640.592	-18.712	663.422	-23.442	616.455	-22.495
640.752	-18.713	663.580	-23.443	616.615	-22.497
640.894	-18.714	663.720	-23.444	616.836	-22.498
641.055	-18.715	663.897	-23.445	617.052	-22.500
641.222	-18.716	664.018	-23.446	617.243	-22.502
641.422	-18.717	664.155	-23.447	617.407	-22.504
641.628	-18.717	664.334	-23.448	617.562	-22.506
641.805	-18.718	664.468	-23.449	617.705	-22.508
641.951	-18.719	664.599	-23.449	617.856	-22.509
642.044	-18.720	664.849	-23.450	618.023	-22.511

642.181	-18.721	665.099	-23.451	618.111	-22.513
642.369	-18.722	665.175	-23.452	618.225	-22.515
642.534	-18.723	665.229	-23.453	618.384	-22.517
642.744	-18.723	665.397	-23.454	618.576	-22.519
642.946	-18.724	665.574	-23.455	618.770	-22.520
643.118	-18.725	665.723	-23.456	618.887	-22.522
643.242	-18.726	665.882	-23.457	618.937	-22.524
643.405	-18.727	666.106	-23.458	619.089	-22.526
643.583	-18.728	666.310	-23.459	619.343	-22.528
643.709	-18.729	666.488	-23.460	619.519	-22.529
643.839	-18.730	666.693	-23.461	619.655	-22.531
643.993	-18.730	666.833	-23.462	619.772	-22.533
644.250	-18.731	666.965	-23.463	620.005	-22.535
644.492	-18.732	667.133	-23.464	620.212	-22.536
644.548	-18.733	667.221	-23.465	620.412	-22.538
644.615	-18.734	667.352	-23.466	620.621	-22.540
644.853	-18.735	667.528	-23.467	620.748	-22.542
645.068	-18.736	667.717	-23.468	620.884	-22.544
645.191	-18.737	667.934	-23.469	621.055	-22.545
645.300	-18.738	668.099	-23.470	621.204	-22.547
645.405	-18.739	668.312	-23.471	621.318	-22.549
645.546	-18.739	668.530	-23.472	621.477	-22.551
645.700	-18.740	668.614	-23.474	621.688	-22.552
645.810	-18.741	668.694	-23.475	621.807	-22.554
645.957	-18.742	668.919	-23.476	621.909	-22.556
646.117	-18.743	669.084	-23.477	622.028	-22.558
646.224	-18.744	669.237	-23.478	622.218	-22.559
646.416	-18.745	669.403	-23.479	622.397	-22.561
646.680	-18.746	669.502	-23.480	622.569	-22.563
646.904	-18.747	669.604	-23.481	622.755	-22.565

647.149	-18.748	669.722	-23.482	622.895	-22.566
647.408	-18.749	669.837	-23.483	623.097	-22.568
647.601	-18.750	669.975	-23.485	623.235	-22.570
647.736	-18.751	670.181	-23.486	623.338	-22.572
647.868	-18.751	670.415	-23.487	623.498	-22.573
648.018	-18.752	670.622	-23.488	623.732	-22.575
648.148	-18.753	670.821	-23.489	623.854	-22.577
648.298	-18.754	671.004	-23.490	623.947	-22.579
648.492	-18.755	671.162	-23.492	624.133	-22.580
648.625	-18.756	671.350	-23.493	624.326	-22.582
648.692	-18.757	671.530	-23.494	624.478	-22.584
648.823	-18.758	671.673	-23.495	624.664	-22.586
648.996	-18.759	671.832	-23.496	624.881	-22.587
649.123	-18.760	672.002	-23.497	625.101	-22.589
649.298	-18.761	672.121	-23.498	625.293	-22.591
649.503	-18.762	672.211	-23.500	625.444	-22.593
649.692	-18.763	672.393	-23.501	625.565	-22.594
649.855	-18.764	672.597	-23.502	625.723	-22.596
650.051	-18.765	672.717	-23.503	625.921	-22.598
650.205	-18.766	672.757	-23.504	626.168	-22.600
650.269	-18.767	672.914	-23.506	626.323	-22.601
650.403	-18.768	673.160	-23.507	626.415	-22.603
650.594	-18.769	673.314	-23.508	626.549	-22.605
650.835	-18.770	673.539	-23.509	626.688	-22.606
651.057	-18.771	673.706	-23.510	626.824	-22.608
651.226	-18.772	673.822	-23.511	626.990	-22.610
651.383	-18.773	674.014	-23.513	627.164	-22.612
651.544	-18.774	674.202	-23.514	627.329	-22.613
651.673	-18.775	674.340	-23.515	627.547	-22.615
651.800	-18.776	674.475	-23.516	627.724	-22.617

651.985	-18.777	674.690	-23.517	627.868	-22.618
652.197	-18.778	674.879	-23.518	627.994	-22.620
652.336	-18.779	675.039	-23.520	628.160	-22.622
652.475	-18.780	675.233	-23.521	628.400	-22.623
652.621	-18.781	675.431	-23.522	628.560	-22.625
652.792	-18.782	675.597	-23.523	628.662	-22.626
652.954	-18.783	675.751	-23.524	628.742	-22.628
653.122	-18.784	675.905	-23.526	628.843	-22.630
653.298	-18.785	676.014	-23.527	629.016	-22.631
653.457	-18.786	676.187	-23.528	629.204	-22.633
653.613	-18.787	676.384	-23.529	629.345	-22.634
653.767	-18.788	676.527	-23.530	629.480	-22.636
653.965	-18.789	676.677	-23.532	629.657	-22.638
654.023	-18.790	676.863	-23.533	629.834	-22.639
654.161	-18.791	677.049	-23.534	630.019	-22.641
654.409	-18.792	677.232	-23.535	630.199	-22.642
654.606	-18.793	677.372	-23.536	630.361	-22.644
654.829	-18.794	677.467	-23.537	630.457	-22.645
655.011	-18.795	677.585	-23.539	630.626	-22.647
655.152	-18.796	677.774	-23.540	630.906	-22.648
655.330	-18.797	677.981	-23.541	631.141	-22.650
655.443	-18.798	678.215	-23.542	631.360	-22.651
655.574	-18.799	678.430	-23.543	631.523	-22.653
655.925	-18.800	678.528	-23.545	631.670	-22.654
656.345	-18.802	678.646	-23.546	631.881	-22.656
656.608	-18.803	678.791	-23.547	632.097	-22.657
656.672	-18.804	678.925	-23.548	632.234	-22.658
656.730	-18.805	679.196	-23.549	632.342	-22.660
656.814	-18.806	679.615	-23.551	632.498	-22.661
656.889	-18.807	679.975	-23.552	632.629	-22.663

656.959	-18.808	680.112	-23.553	632.772	-22.664
657.058	-18.809	680.163	-23.554	632.934	-22.665
657.189	-18.810	680.280	-23.555	633.103	-22.667
657.339	-18.811	680.305	-23.556	633.233	-22.668
657.453	-18.812	680.304	-23.558	633.303	-22.669
657.601	-18.813	680.376	-23.559	633.492	-22.671
657.821	-18.814	680.513	-23.560	633.683	-22.672
658.000	-18.815	680.706	-23.561	633.835	-22.673
658.183	-18.816	680.789	-23.562	634.023	-22.675
658.340	-18.817	680.920	-23.563	634.204	-22.676
658.478	-18.819	681.171	-23.565	634.384	-22.677
658.641	-18.820	681.414	-23.566	634.555	-22.678
658.835	-18.821	681.580	-23.567	634.657	-22.680
659.030	-18.822	681.724	-23.568	634.843	-22.681
659.133	-18.823	681.916	-23.569	635.031	-22.682
659.236	-18.824	682.102	-23.570	635.164	-22.683
659.444	-18.825	682.218	-23.572	635.355	-22.684
659.632	-18.826	682.342	-23.573	635.558	-22.686
659.744	-18.827	682.495	-23.574	635.650	-22.687
659.900	-18.828	682.679	-23.575	635.750	-22.688
660.022	-18.829	682.897	-23.576	635.937	-22.689
660.154	-18.830	683.041	-23.577	636.210	-22.690
660.342	-18.831	683.167	-23.578	636.395	-22.691
660.561	-18.832	683.335	-23.580	636.533	-22.692
660.776	-18.833	683.422	-23.581	636.633	-22.694
660.925	-18.835	683.536	-23.582	636.808	-22.695
661.093	-18.836	683.726	-23.583	637.031	-22.696
661.245	-18.837	683.846	-23.584	637.178	-22.697
661.457	-18.838	684.056	-23.585	637.318	-22.698
661.709	-18.839	684.261	-23.586	637.451	-22.699

661.775	-18.840	684.504	-23.588	637.570	-22.700
661.804	-18.841	684.711	-23.589	637.785	-22.701
661.997	-18.842	684.832	-23.590	637.998	-22.702
662.197	-18.843	684.939	-23.591	638.134	-22.703
662.384	-18.844	685.069	-23.592	638.218	-22.704
662.532	-18.845	685.224	-23.593	638.406	-22.705
662.681	-18.846	685.374	-23.594	638.629	-22.706
662.856	-18.847	685.509	-23.595	638.772	-22.707
663.041	-18.848	685.623	-23.597	638.930	-22.708
663.237	-18.849	685.761	-23.598	639.087	-22.709
663.416	-18.851	685.972	-23.599	639.222	-22.710
663.568	-18.852	686.115	-23.600	639.435	-22.711
663.685	-18.853	686.245	-23.601	639.620	-22.712
663.841	-18.854	686.433	-23.602	639.824	-22.713
663.976	-18.855	686.624	-23.603	640.039	-22.714
664.081	-18.856	686.843	-23.605	640.157	-22.714
664.245	-18.857	687.026	-23.606	640.246	-22.715
664.464	-18.858	687.136	-23.607	640.402	-22.716
664.623	-18.859	687.276	-23.608	640.622	-22.717
664.771	-18.860	687.402	-23.609	640.826	-22.718
664.928	-18.861	687.540	-23.610	640.959	-22.719
665.080	-18.862	687.743	-23.611	641.042	-22.720
665.285	-18.863	687.941	-23.612	641.160	-22.721
665.481	-18.864	688.126	-23.614	641.498	-22.722
665.627	-18.865	688.305	-23.615	641.947	-22.722
665.787	-18.866	688.496	-23.616	642.216	-22.723
665.949	-18.867	688.650	-23.617	642.363	-22.724
666.096	-18.868	688.807	-23.618	642.482	-22.725
666.228	-18.869	688.996	-23.619	642.598	-22.726
666.356	-18.870	689.145	-23.620	642.685	-22.727

666.528	-18.871	689.286	-23.622	642.730	-22.728
666.733	-18.873	689.387	-23.623	642.810	-22.728
666.935	-18.874	689.488	-23.624	642.926	-22.729
667.118	-18.875	689.688	-23.625	642.969	-22.730
667.271	-18.876	689.867	-23.626	643.083	-22.731
667.361	-18.877	690.011	-23.627	643.276	-22.732
667.530	-18.878	690.110	-23.628	643.374	-22.732
667.703	-18.879	690.244	-23.629	643.503	-22.733
667.845	-18.880	690.515	-23.631	643.775	-22.734
668.008	-18.881	690.719	-23.632	644.015	-22.735
668.148	-18.882	690.836	-23.633	644.174	-22.736
668.291	-18.883	690.998	-23.634	644.334	-22.736
668.393	-18.884	691.171	-23.635	644.471	-22.737
668.577	-18.885	691.304	-23.636	644.618	-22.738
668.727	-18.886	691.441	-23.637	644.817	-22.739
668.783	-18.887	691.586	-23.638	645.005	-22.740
668.949	-18.888	691.693	-23.639	645.151	-22.741
669.156	-18.889	691.802	-23.640	645.296	-22.741
669.398	-18.890	692.002	-23.642	645.434	-22.742
669.597	-18.891	692.208	-23.643	645.593	-22.743
669.754	-18.892	692.383	-23.644	645.719	-22.744
669.967	-18.893	692.597	-23.645	645.856	-22.745
670.184	-18.894	692.822	-23.646	645.956	-22.746
670.390	-18.895	693.017	-23.647	646.125	-22.746
670.527	-18.896	693.210	-23.648	646.412	-22.747
670.675	-18.897	693.356	-23.649	646.581	-22.748
670.900	-18.898	693.531	-23.650	646.693	-22.749
671.042	-18.899	693.744	-23.651	646.860	-22.750
671.135	-18.900	693.851	-23.652	646.985	-22.751
671.264	-18.901	693.951	-23.653	647.148	-22.752

671.373	-18.902	694.084	-23.654	647.380	-22.753
671.530	-18.903	694.223	-23.655	647.593	-22.753
671.717	-18.904	694.333	-23.656	647.786	-22.754
671.859	-18.905	694.531	-23.657	647.951	-22.755
672.032	-18.906	694.736	-23.658	648.111	-22.756
672.204	-18.907	694.851	-23.659	648.271	-22.757
672.354	-18.908	694.948	-23.660	648.474	-22.758
672.543	-18.909	695.075	-23.661	648.648	-22.759
672.712	-18.910	695.284	-23.662	648.771	-22.760
672.881	-18.911	695.511	-23.663	648.913	-22.761
673.116	-18.912	695.730	-23.664	649.088	-22.762
673.291	-18.913	695.940	-23.665	649.288	-22.763
673.394	-18.914	696.115	-23.666	649.426	-22.764
673.541	-18.915	696.219	-23.667	649.483	-22.765
673.759	-18.916	696.363	-23.668	649.656	-22.766
673.913	-18.917	696.535	-23.669	649.869	-22.767
674.058	-18.918	696.673	-23.670	650.014	-22.768
674.246	-18.919	696.820	-23.671	650.163	-22.769
674.408	-18.920	697.006	-23.672	650.339	-22.770
674.564	-18.921	697.172	-23.673	650.507	-22.771
674.734	-18.922	697.316	-23.674	650.602	-22.772
674.899	-18.923	697.479	-23.674	650.796	-22.773
675.056	-18.924	697.615	-23.675	651.021	-22.774
675.229	-18.925	697.719	-23.676	651.141	-22.775
675.411	-18.926	697.962	-23.677	651.293	-22.776
675.576	-18.927	698.263	-23.678	651.437	-22.777
675.729	-18.928	698.424	-23.679	651.552	-22.778
675.842	-18.929	698.538	-23.680	651.660	-22.780
676.051	-18.930	698.671	-23.681	651.853	-22.781
676.267	-18.931	698.863	-23.682	652.069	-22.782

676.395	-18.932	699.047	-23.682	652.237	-22.783
676.532	-18.933	699.200	-23.683	652.397	-22.784
676.662	-18.934	699.368	-23.684	652.557	-22.785
676.859	-18.935	699.503	-23.685	652.757	-22.786
677.010	-18.936	699.622	-23.686	652.961	-22.787
677.166	-18.937	699.817	-23.687	653.120	-22.789
677.371	-18.938	700.020	-23.688	653.242	-22.790
677.523	-18.939	700.212	-23.689	653.443	-22.791
677.692	-18.940	700.355	-23.689	653.668	-22.792
677.855	-18.941	700.464	-23.690	653.778	-22.793
677.968	-18.942	700.625	-23.691	653.887	-22.795
678.007	-18.943	700.759	-23.692	654.089	-22.796
678.277	-18.944	700.891	-23.693	654.267	-22.797
678.753	-18.945	701.210	-23.694	654.451	-22.798
679.114	-18.946	701.701	-23.694	654.620	-22.799
679.265	-18.947	701.993	-23.695	654.695	-22.801
679.338	-18.948	702.035	-23.696	654.827	-22.802
679.387	-18.949	702.044	-23.697	655.044	-22.803
679.481	-18.950	702.113	-23.698	655.201	-22.804
679.567	-18.951	702.123	-23.699	655.365	-22.805
679.605	-18.952	702.174	-23.699	655.600	-22.807
679.641	-18.953	702.258	-23.700	655.798	-22.808
679.753	-18.954	702.439	-23.701	655.954	-22.809
679.968	-18.955	702.654	-23.702	656.123	-22.810
680.115	-18.956	702.829	-23.703	656.244	-22.812
680.303	-18.957	702.993	-23.704	656.318	-22.813
680.487	-18.958	703.166	-23.704	656.449	-22.814
680.650	-18.959	703.374	-23.705	656.653	-22.815
680.785	-18.960	703.578	-23.706	656.813	-22.817
680.966	-18.961	703.726	-23.707	656.905	-22.818

681.161	-18.962	703.864	-23.708	657.064	-22.819
681.331	-18.963	704.031	-23.708	657.197	-22.820
681.536	-18.964	704.212	-23.709	657.289	-22.822
681.676	-18.965	704.380	-23.710	657.402	-22.823
681.798	-18.966	704.515	-23.711	657.595	-22.824
681.917	-18.966	704.690	-23.712	657.804	-22.825
682.057	-18.967	704.840	-23.713	657.952	-22.827
682.230	-18.968	704.946	-23.713	658.154	-22.828
682.382	-18.969	705.038	-23.714	658.445	-22.829
682.502	-18.970	705.173	-23.715	658.718	-22.830
682.661	-18.971	705.381	-23.716	658.941	-22.832
682.864	-18.972	705.573	-23.717	659.058	-22.833
683.067	-18.973	705.779	-23.718	659.174	-22.834
683.266	-18.974	706.019	-23.718	659.349	-22.835
683.468	-18.975	706.176	-23.719	659.485	-22.837
683.617	-18.976	706.328	-23.720	659.638	-22.838
683.790	-18.977	706.479	-23.721	659.781	-22.839
683.933	-18.978	706.671	-23.722	659.930	-22.840
684.070	-18.979	706.897	-23.723	660.054	-22.842
684.273	-18.980	706.997	-23.724	660.201	-22.843
684.448	-18.980	707.081	-23.725	660.376	-22.844
684.586	-18.981	707.230	-23.726	660.533	-22.845
684.674	-18.982	707.420	-23.726	660.666	-22.847
684.845	-18.983	707.550	-23.727	660.800	-22.848
685.041	-18.984	707.694	-23.728	660.924	-22.849
685.163	-18.985	707.855	-23.729	661.067	-22.850
685.360	-18.986	707.998	-23.730	661.189	-22.851
685.555	-18.987	708.163	-23.731	661.429	-22.853
685.707	-18.988	708.298	-23.732	661.656	-22.854
685.865	-18.989	708.444	-23.733	661.851	-22.855

685.976	-18.990	708.639	-23.734	662.020	-22.856
686.099	-18.990	708.856	-23.735	662.172	-22.858
686.291	-18.991	708.952	-23.736	662.292	-22.859
686.497	-18.992	709.119	-23.737	662.423	-22.860
686.621	-18.993	709.242	-23.738	662.663	-22.861
686.752	-18.994	709.334	-23.739	662.869	-22.862
686.978	-18.995	709.583	-23.740	663.006	-22.863
687.200	-18.996	709.830	-23.741	663.159	-22.865
687.324	-18.997	709.959	-23.742	663.278	-22.866
687.440	-18.998	710.166	-23.743	663.461	-22.867
687.627	-18.998	710.351	-23.744	663.692	-22.868
687.838	-18.999	710.481	-23.745	663.863	-22.869
688.043	-19.000	710.679	-23.746	664.061	-22.871
688.166	-19.001	710.789	-23.747	664.212	-22.872
688.254	-19.002	710.900	-23.748	664.323	-22.873
688.389	-19.003	711.108	-23.749	664.492	-22.874
688.547	-19.004	711.266	-23.751	664.651	-22.875
688.688	-19.005	711.410	-23.752	664.826	-22.876
688.834	-19.005	711.570	-23.753	664.956	-22.877
689.006	-19.006	711.746	-23.754	665.069	-22.879
689.175	-19.007	711.885	-23.755	665.255	-22.880
689.369	-19.008	712.077	-23.756	665.458	-22.881
689.552	-19.009	712.281	-23.757	665.666	-22.882
689.711	-19.010	712.409	-23.758	665.827	-22.883
689.940	-19.011	712.538	-23.759	665.950	-22.884
690.131	-19.011	712.701	-23.761	666.115	-22.885
690.215	-19.012	712.860	-23.762	666.317	-22.886
690.340	-19.013	712.924	-23.763	666.483	-22.887
690.406	-19.014	713.038	-23.764	666.637	-22.889
690.530	-19.015	713.267	-23.765	666.786	-22.890

690.774	-19.016	713.410	-23.766	666.884	-22.891
690.930	-19.017	713.494	-23.768	667.040	-22.892
691.021	-19.017	713.708	-23.769	667.267	-22.893
691.144	-19.018	713.931	-23.770	667.433	-22.894
691.308	-19.019	714.131	-23.771	667.625	-22.895
691.463	-19.020	714.296	-23.772	667.813	-22.896
691.655	-19.021	714.452	-23.773	667.914	-22.897
691.833	-19.022	714.662	-23.775	668.082	-22.898
692.046	-19.022	714.944	-23.776	668.284	-22.900
692.278	-19.023	715.174	-23.777	668.393	-22.901
692.484	-19.024	715.312	-23.778	668.465	-22.902
692.697	-19.025	715.501	-23.779	668.621	-22.903
692.878	-19.026	715.626	-23.780	668.968	-22.904
693.064	-19.027	715.707	-23.782	669.292	-22.905
693.234	-19.028	715.826	-23.783	669.603	-22.906
693.355	-19.028	715.921	-23.784	669.838	-22.907
693.509	-19.029	716.085	-23.785	669.937	-22.908
693.623	-19.030	716.275	-23.786	670.038	-22.909
693.736	-19.031	716.419	-23.787	670.151	-22.910
693.892	-19.032	716.577	-23.788	670.240	-22.911
693.999	-19.033	716.714	-23.790	670.284	-22.912
694.161	-19.033	716.826	-23.791	670.415	-22.913
694.386	-19.034	717.020	-23.792	670.564	-22.914
694.513	-19.035	717.253	-23.793	670.638	-22.916
694.664	-19.036	717.419	-23.794	670.748	-22.917
694.883	-19.037	717.582	-23.795	670.968	-22.918
695.077	-19.038	717.768	-23.797	671.179	-22.919
695.259	-19.038	717.906	-23.798	671.395	-22.920
695.436	-19.039	718.125	-23.799	671.533	-22.921
695.592	-19.040	718.337	-23.800	671.646	-22.922

695.742	-19.041	718.458	-23.801	671.833	-22.923
695.875	-19.042	718.627	-23.802	671.991	-22.924
696.004	-19.043	718.809	-23.803	672.167	-22.925
696.198	-19.044	718.933	-23.804	672.383	-22.926
696.412	-19.044	719.062	-23.806	672.548	-22.927
696.562	-19.045	719.199	-23.807	672.696	-22.928
696.712	-19.046	719.399	-23.808	672.880	-22.929
696.870	-19.047	719.573	-23.809	673.014	-22.930
696.968	-19.048	719.678	-23.810	673.078	-22.931
697.141	-19.049	719.868	-23.811	673.186	-22.932
697.354	-19.050	720.091	-23.812	673.384	-22.933
697.505	-19.050	720.243	-23.813	673.588	-22.935
697.681	-19.051	720.358	-23.815	673.661	-22.936
697.898	-19.052	720.572	-23.816	673.753	-22.937
698.029	-19.053	720.724	-23.817	673.904	-22.938
698.144	-19.054	720.872	-23.818	674.064	-22.939
698.321	-19.055	721.057	-23.819	674.230	-22.940
698.477	-19.056	721.185	-23.820	674.398	-22.941
698.651	-19.056	721.341	-23.821	674.583	-22.942
698.867	-19.057	721.547	-23.822	674.771	-22.943
699.026	-19.058	721.696	-23.823	674.969	-22.944
699.121	-19.059	721.840	-23.824	675.185	-22.945
699.288	-19.060	722.051	-23.825	675.421	-22.947
699.489	-19.061	722.248	-23.827	675.621	-22.948
699.689	-19.062	722.410	-23.828	675.756	-22.949
699.893	-19.062	722.528	-23.829	675.918	-22.950
699.994	-19.063	722.671	-23.830	676.047	-22.951
700.095	-19.064	722.825	-23.831	676.143	-22.952
700.337	-19.065	723.239	-23.832	676.292	-22.953
700.562	-19.066	723.706	-23.833	676.469	-22.954

700.655	-19.067	723.838	-23.834	676.638	-22.956
700.810	-19.068	723.904	-23.835	676.718	-22.957
701.202	-19.069	723.975	-23.836	676.854	-22.958
701.598	-19.069	724.044	-23.837	677.101	-22.959
701.810	-19.070	724.107	-23.838	677.296	-22.960
701.906	-19.071	724.148	-23.839	677.433	-22.961
701.942	-19.072	724.247	-23.840	677.589	-22.963
701.981	-19.073	724.371	-23.841	677.669	-22.964
702.027	-19.074	724.519	-23.842	677.905	-22.965
702.093	-19.075	724.740	-23.843	678.081	-22.966
702.234	-19.076	724.958	-23.844	678.219	-22.967
702.391	-19.076	725.135	-23.845	678.333	-22.968
702.530	-19.077	725.320	-23.846	678.409	-22.970
702.645	-19.078	725.463	-23.847	678.613	-22.971
702.809	-19.079	725.592	-23.848	678.859	-22.972
703.120	-19.080	725.776	-23.849	678.988	-22.973
703.334	-19.081	725.919	-23.850	679.149	-22.974
703.399	-19.082	726.079	-23.851	679.343	-22.975
703.511	-19.083	726.244	-23.852	679.490	-22.977
703.670	-19.083	726.367	-23.853	679.632	-22.978
703.839	-19.084	726.564	-23.854	679.823	-22.979
703.994	-19.085	726.712	-23.855	679.988	-22.980
704.183	-19.086	726.815	-23.856	680.145	-22.981
704.340	-19.087	726.976	-23.857	680.329	-22.983
704.500	-19.088	727.123	-23.858	680.463	-22.984
704.681	-19.089	727.282	-23.859	680.676	-22.985
704.809	-19.090	727.457	-23.860	680.838	-22.986
704.911	-19.091	727.600	-23.860	680.976	-22.987
705.042	-19.091	727.805	-23.861	681.093	-22.989
705.212	-19.092	727.961	-23.862	681.245	-22.990

705.402	-19.093	728.089	-23.863	681.419	-22.991
705.557	-19.094	728.298	-23.864	681.563	-22.992
705.730	-19.095	728.463	-23.865	681.740	-22.994
705.957	-19.096	728.644	-23.866	681.961	-22.995
706.170	-19.097	728.813	-23.867	682.087	-22.996
706.364	-19.098	728.938	-23.868	682.215	-22.997
706.526	-19.099	729.114	-23.869	682.349	-22.998
706.670	-19.099	729.285	-23.870	682.488	-23.000
706.821	-19.100	729.429	-23.871	682.722	-23.001
706.965	-19.101	729.583	-23.872	682.930	-23.002
707.106	-19.102	729.786	-23.873	683.062	-23.003
707.197	-19.103	729.949	-23.874	683.183	-23.004
707.337	-19.104	730.036	-23.875	683.347	-23.005
707.430	-19.105	730.241	-23.876	683.478	-23.007
707.591	-19.106	730.474	-23.877	683.696	-23.008
707.833	-19.107	730.619	-23.878	683.871	-23.009
707.971	-19.107	730.798	-23.879	683.976	-23.010
708.201	-19.108	730.961	-23.880	684.068	-23.011
708.484	-19.109	731.085	-23.881	684.200	-23.012
708.691	-19.110	731.255	-23.882	684.348	-23.014
708.814	-19.111	731.404	-23.883	684.510	-23.015
708.881	-19.112	731.528	-23.884	684.750	-23.016
709.030	-19.113	731.677	-23.885	684.935	-23.017
709.192	-19.114	731.835	-23.886	685.037	-23.018
709.286	-19.114	732.071	-23.887	685.188	-23.019
709.454	-19.115	732.239	-23.888	685.385	-23.020
709.633	-19.116	732.351	-23.889	685.618	-23.021
709.756	-19.117	732.550	-23.890	685.803	-23.022
709.933	-19.118	732.707	-23.891	685.957	-23.024
710.132	-19.119	732.795	-23.892	686.178	-23.025

710.330	-19.120	732.942	-23.893	686.414	-23.026
710.507	-19.121	733.161	-23.895	686.618	-23.027
710.695	-19.121	733.384	-23.896	686.740	-23.028
710.899	-19.122	733.517	-23.897	686.908	-23.029
711.040	-19.123	733.660	-23.898	687.094	-23.030
711.133	-19.124	733.849	-23.899	687.165	-23.031
711.233	-19.125	734.035	-23.900	687.258	-23.032
711.388	-19.126	734.168	-23.901	687.445	-23.033
711.583	-19.127	734.305	-23.902	687.596	-23.034
711.740	-19.128	734.510	-23.903	687.744	-23.035
711.889	-19.128	734.627	-23.904	687.879	-23.036
712.049	-19.129	734.725	-23.905	687.981	-23.037
712.215	-19.130	734.929	-23.906	688.101	-23.038
712.392	-19.131	735.126	-23.908	688.202	-23.039
712.561	-19.132	735.221	-23.909	688.354	-23.040
712.765	-19.133	735.264	-23.910	688.603	-23.041
712.899	-19.134	735.406	-23.911	688.789	-23.042
713.015	-19.135	735.631	-23.912	688.916	-23.043
713.172	-19.135	735.769	-23.913	689.126	-23.044
713.328	-19.136	735.903	-23.914	689.323	-23.045
713.471	-19.137	736.153	-23.915	689.479	-23.045
713.602	-19.138	736.409	-23.916	689.668	-23.046
713.700	-19.139	736.575	-23.917	689.846	-23.047
713.836	-19.140	736.813	-23.919	690.033	-23.048
714.009	-19.141	737.040	-23.920	690.237	-23.049
714.159	-19.141	737.183	-23.921	690.401	-23.050
714.372	-19.142	737.310	-23.922	690.571	-23.051
714.545	-19.143	737.445	-23.923	690.754	-23.051
714.734	-19.144	737.590	-23.924	690.859	-23.052
715.023	-19.145	737.715	-23.925	691.003	-23.053

715.275	-19.146	737.841	-23.926	691.201	-23.054
715.474	-19.147	737.974	-23.927	691.321	-23.055
715.622	-19.148	738.167	-23.928	691.423	-23.055
715.749	-19.148	738.364	-23.930	691.618	-23.056
715.910	-19.149	738.484	-23.931	691.887	-23.057
716.094	-19.150	738.594	-23.932	692.073	-23.057
716.199	-19.151	738.745	-23.933	692.127	-23.058
716.270	-19.152	738.914	-23.934	692.150	-23.059
716.413	-19.153	739.124	-23.935	692.375	-23.060
716.561	-19.154	739.349	-23.936	692.657	-23.060
716.735	-19.154	739.529	-23.937	692.803	-23.061
716.929	-19.155	739.664	-23.938	692.972	-23.062
717.072	-19.156	739.835	-23.939	693.190	-23.062
717.204	-19.157	740.021	-23.940	693.304	-23.063
717.401	-19.158	740.138	-23.942	693.416	-23.063
717.596	-19.159	740.249	-23.943	693.635	-23.064
717.803	-19.160	740.409	-23.944	693.811	-23.065
718.018	-19.161	740.664	-23.945	693.939	-23.065
718.193	-19.161	740.901	-23.946	694.093	-23.066
718.355	-19.162	740.994	-23.947	694.289	-23.066
718.504	-19.163	741.125	-23.948	694.417	-23.067
718.689	-19.164	741.310	-23.949	694.591	-23.068
718.825	-19.165	741.444	-23.950	694.807	-23.068
718.958	-19.166	741.645	-23.951	694.949	-23.069
719.069	-19.167	741.805	-23.953	695.106	-23.069
719.200	-19.168	741.964	-23.954	695.240	-23.070
719.415	-19.169	742.178	-23.955	695.378	-23.070
719.602	-19.169	742.272	-23.956	695.611	-23.071
719.744	-19.170	742.433	-23.957	695.780	-23.071
719.903	-19.171	742.642	-23.958	695.869	-23.072

720.101	-19.172	742.776	-23.959	696.013	-23.073
720.282	-19.173	742.906	-23.960	696.263	-23.073
720.415	-19.174	743.070	-23.961	696.437	-23.074
720.508	-19.175	743.193	-23.962	696.816	-23.074
720.707	-19.176	743.375	-23.963	697.111	-23.075
720.942	-19.177	743.584	-23.965	697.290	-23.075
721.107	-19.177	743.704	-23.966	697.383	-23.076
721.306	-19.178	743.872	-23.967	697.493	-23.076
721.449	-19.179	744.072	-23.968	697.562	-23.076
721.554	-19.180	744.182	-23.969	697.579	-23.077
721.703	-19.181	744.371	-23.970	697.696	-23.077
721.812	-19.182	744.504	-23.971	697.783	-23.078
721.964	-19.183	744.659	-23.972	697.859	-23.078
722.194	-19.184	745.061	-23.973	698.008	-23.079
722.401	-19.185	745.497	-23.974	698.206	-23.079
722.552	-19.185	745.695	-23.976	698.373	-23.080
722.673	-19.186	745.745	-23.977	698.474	-23.080
722.819	-19.187	745.814	-23.978	698.594	-23.081
723.004	-19.188	745.859	-23.979	698.820	-23.081
723.185	-19.189	745.922	-23.980	699.034	-23.082
723.289	-19.190	746.055	-23.981	699.205	-23.082
723.580	-19.191	746.161	-23.982	699.345	-23.082
724.015	-19.192	746.253	-23.983	699.526	-23.083
724.285	-19.193	746.383	-23.984	699.722	-23.083
724.443	-19.194	746.569	-23.986	699.866	-23.084
724.508	-19.194	746.827	-23.987	700.025	-23.084
724.508	-19.195	747.016	-23.988	700.226	-23.085
724.549	-19.196	747.132	-23.989	700.388	-23.085
724.655	-19.197	747.279	-23.990	700.548	-23.086
724.812	-19.198	747.475	-23.991	700.637	-23.086

724.950	-19.199	747.628	-23.992	700.743	-23.087
725.098	-19.200	747.741	-23.993	700.906	-23.087
725.280	-19.201	747.911	-23.995	701.061	-23.088
725.424	-19.202	748.063	-23.996	701.263	-23.088
725.569	-19.203	748.228	-23.997	701.448	-23.089
725.784	-19.203	748.407	-23.998	701.571	-23.089
726.049	-19.204	748.498	-23.999	701.684	-23.090
726.241	-19.205	748.649	-24.000	701.885	-23.090
726.383	-19.206	748.822	-24.001	702.148	-23.091
726.488	-19.207	748.914	-24.003	702.327	-23.091
726.593	-19.208	749.042	-24.004	702.467	-23.092
726.693	-19.209	749.266	-24.005	702.642	-23.092
726.892	-19.210	749.464	-24.006	702.794	-23.093
727.100	-19.211	749.660	-24.007	702.965	-23.094
727.220	-19.212	749.852	-24.009	703.167	-23.094
727.375	-19.212	750.094	-24.010	703.341	-23.095
727.562	-19.213	750.312	-24.011	703.437	-23.095
727.713	-19.214	750.434	-24.012	703.557	-23.096
727.802	-19.215	750.530	-24.013	703.733	-23.097
727.897	-19.216	750.630	-24.015	703.906	-23.097
728.055	-19.217	750.763	-24.016	704.017	-23.098
728.251	-19.218	750.919	-24.017	704.110	-23.099
728.425	-19.219	751.058	-24.018	704.301	-23.099
728.660	-19.220	751.180	-24.019	704.492	-23.100
728.838	-19.221	751.357	-24.021	704.648	-23.101
728.957	-19.221	751.563	-24.022	704.790	-23.101
729.129	-19.222	751.730	-24.023	704.970	-23.102
729.348	-19.223	751.888	-24.025	705.168	-23.103
729.563	-19.224	752.076	-24.026	705.321	-23.103
729.690	-19.225	752.226	-24.027	705.516	-23.104

729.773	-19.226	752.337	-24.028	705.686	-23.105
729.944	-19.227	752.523	-24.030	705.797	-23.106
730.141	-19.228	752.685	-24.031	705.984	-23.107
730.292	-19.228	752.838	-24.032	706.191	-23.107
730.431	-19.229	753.063	-24.034	706.348	-23.108
730.547	-19.230	753.259	-24.035	706.428	-23.109
730.723	-19.231	753.459	-24.036	706.544	-23.110
730.859	-19.232	753.668	-24.038	706.740	-23.111
730.991	-19.233	753.831	-24.039	706.928	-23.112
731.222	-19.234	753.962	-24.040	707.157	-23.113
731.421	-19.235	754.080	-24.042	707.344	-23.113
731.580	-19.235	754.245	-24.043	707.364	-23.114
731.704	-19.236	754.415	-24.045	707.539	-23.115
731.829	-19.237	754.576	-24.046	707.847	-23.116
732.030	-19.238	754.784	-24.047	708.060	-23.117
732.196	-19.239	754.931	-24.049	708.173	-23.118
732.413	-19.240	755.010	-24.050	708.211	-23.119
732.645	-19.241	755.162	-24.052	708.382	-23.120
732.794	-19.241	755.338	-24.053	708.613	-23.121
732.934	-19.242	755.526	-24.054	708.835	-23.122
733.056	-19.243	755.697	-24.056	709.039	-23.123
733.195	-19.244	755.870	-24.057	709.165	-23.124
733.368	-19.245	756.076	-24.059	709.269	-23.125
733.587	-19.246	756.217	-24.060	709.398	-23.126
733.764	-19.247	756.307	-24.062	709.581	-23.127
733.903	-19.247	756.454	-24.063	709.802	-23.129
734.008	-19.248	756.639	-24.065	709.965	-23.130
734.144	-19.249	756.750	-24.066	710.128	-23.131
734.310	-19.250	756.877	-24.067	710.274	-23.132
734.480	-19.251	757.034	-24.069	710.362	-23.133

734.677	-19.252	757.179	-24.070	710.526	-23.134
734.827	-19.252	757.304	-24.072	710.756	-23.135
734.982	-19.253	757.454	-24.073	710.965	-23.136
735.114	-19.254	757.620	-24.075	711.108	-23.138
735.265	-19.255	757.808	-24.076	711.213	-23.139
735.476	-19.256	758.018	-24.078	711.342	-23.140
735.629	-19.257	758.188	-24.079	711.465	-23.141
735.726	-19.257	758.400	-24.081	711.596	-23.142
735.866	-19.258	758.638	-24.082	711.758	-23.143
736.017	-19.259	758.802	-24.084	711.859	-23.145
736.174	-19.260	758.935	-24.085	711.997	-23.146
736.323	-19.261	759.109	-24.087	712.128	-23.147
736.449	-19.262	759.298	-24.088	712.261	-23.148
736.581	-19.262	759.422	-24.090	712.489	-23.149
736.701	-19.263	759.549	-24.091	712.680	-23.151
736.899	-19.264	759.657	-24.093	712.880	-23.152
737.188	-19.265	759.776	-24.094	713.110	-23.153
737.388	-19.266	759.991	-24.095	713.272	-23.154
737.516	-19.267	760.189	-24.097	713.476	-23.156
737.718	-19.267	760.334	-24.098	713.709	-23.157
737.958	-19.268	760.508	-24.100	713.906	-23.158
738.122	-19.269	760.681	-24.101	714.102	-23.159
738.222	-19.270	760.842	-24.103	714.240	-23.161
738.373	-19.271	761.023	-24.104	714.336	-23.162
738.471	-19.272	761.201	-24.106	714.455	-23.163
738.640	-19.272	761.372	-24.107	714.584	-23.164
738.766	-19.273	761.570	-24.108	714.748	-23.166
738.929	-19.274	761.777	-24.110	714.888	-23.167
739.139	-19.275	761.967	-24.111	714.922	-23.168
739.228	-19.276	762.155	-24.113	715.104	-23.169

739.358	-19.276	762.320	-24.114	715.291	-23.171
739.537	-19.277	762.442	-24.116	715.441	-23.172
739.688	-19.278	762.521	-24.117	715.642	-23.173
739.876	-19.279	762.601	-24.118	715.850	-23.174
740.085	-19.280	762.790	-24.120	716.015	-23.176
740.299	-19.281	762.992	-24.121	716.159	-23.177
740.468	-19.281	763.156	-24.122	716.354	-23.178
740.621	-19.282	763.326	-24.124	716.512	-23.180
740.771	-19.283	763.447	-24.125	716.699	-23.181
740.925	-19.284	763.605	-24.127	716.926	-23.182
741.132	-19.285	763.798	-24.128	717.095	-23.183
741.348	-19.286	763.960	-24.129	717.246	-23.185
741.488	-19.286	764.165	-24.131	717.475	-23.186
741.656	-19.287	764.394	-24.132	717.682	-23.187
741.852	-19.288	764.513	-24.133	717.769	-23.188
741.989	-19.289	764.619	-24.135	717.884	-23.190
742.174	-19.290	764.764	-24.136	718.055	-23.191
742.382	-19.291	764.936	-24.137	718.191	-23.192
742.526	-19.291	765.118	-24.139	718.321	-23.193
742.579	-19.292	765.239	-24.140	718.502	-23.195
742.758	-19.293	765.408	-24.141	718.719	-23.196
743.021	-19.294	765.562	-24.142	718.914	-23.197
743.207	-19.295	765.723	-24.144	719.078	-23.198
743.347	-19.296	765.910	-24.145	719.221	-23.200
743.493	-19.296	766.099	-24.146	719.390	-23.201
743.672	-19.297	766.264	-24.147	719.531	-23.202
743.801	-19.298	766.426	-24.149	719.656	-23.203
743.929	-19.299	766.538	-24.150	719.835	-23.205
744.095	-19.300	766.862	-24.151	720.053	-23.206
744.260	-19.301	767.363	-24.152	720.230	-23.207

744.447	-19.301	767.534	-24.154	720.373	-23.208
744.594	-19.302	767.577	-24.155	720.536	-23.210
744.694	-19.303	767.675	-24.156	720.677	-23.211
744.862	-19.304	767.719	-24.157	720.767	-23.212
745.071	-19.305	767.748	-24.158	720.929	-23.213
745.283	-19.306	767.842	-24.160	721.180	-23.214
745.431	-19.307	767.928	-24.161	721.381	-23.216
745.476	-19.307	768.083	-24.162	721.509	-23.217
745.613	-19.308	768.290	-24.163	721.646	-23.218
745.852	-19.309	768.489	-24.164	721.808	-23.219
746.004	-19.310	768.697	-24.165	721.971	-23.220
746.395	-19.311	768.907	-24.167	722.149	-23.222
746.853	-19.312	769.070	-24.168	722.292	-23.223
746.954	-19.313	769.164	-24.169	722.401	-23.224
746.995	-19.313	769.303	-24.170	722.591	-23.225
747.051	-19.314	769.529	-24.171	722.749	-23.226
747.049	-19.315	769.731	-24.172	722.914	-23.228
747.133	-19.316	769.914	-24.173	723.090	-23.229
747.325	-19.317	770.080	-24.174	723.213	-23.230
747.502	-19.318	770.173	-24.175	723.470	-23.231
747.619	-19.319	770.294	-24.176	723.798	-23.232
747.729	-19.319	770.432	-24.178	724.164	-23.234
747.878	-19.320	770.590	-24.179	724.487	-23.235
748.058	-19.321	770.742	-24.180	724.609	-23.236
748.288	-19.322	770.883	-24.181	724.683	-23.237
748.441	-19.323	771.068	-24.182	724.741	-23.238
748.583	-19.324	771.275	-24.183	724.786	-23.239
748.776	-19.325	771.402	-24.184	724.866	-23.240
748.960	-19.325	771.552	-24.185	724.954	-23.242
749.123	-19.326	771.796	-24.186	725.126	-23.243

749.240	-19.327	771.947	-24.187	725.263	-23.244
749.413	-19.328	772.055	-24.188	725.336	-23.245
749.546	-19.329	772.255	-24.189	725.415	-23.246
749.593	-19.330	772.486	-24.190	725.565	-23.247
749.778	-19.331	772.616	-24.191	725.862	-23.248
750.017	-19.332	772.725	-24.192	726.095	-23.250
750.185	-19.332	772.865	-24.193	726.191	-23.251
750.368	-19.333	773.021	-24.194	726.349	-23.252
750.535	-19.334	773.204	-24.195	726.512	-23.253
750.644	-19.335	773.349	-24.196	726.664	-23.254
750.779	-19.336	773.528	-24.197	726.864	-23.255
750.977	-19.337	773.711	-24.198	727.028	-23.256
751.197	-19.338	773.816	-24.199	727.159	-23.258
751.378	-19.338	773.959	-24.200	727.318	-23.259
751.508	-19.339	774.094	-24.202	727.480	-23.260
751.662	-19.340	774.277	-24.203	727.635	-23.261
751.833	-19.341	774.566	-24.204	727.774	-23.262
752.017	-19.342	774.774	-24.205	727.967	-23.264
752.139	-19.343	774.863	-24.206	728.146	-23.265
752.169	-19.344	774.918	-24.207	728.294	-23.266
752.314	-19.345	775.085	-24.208	728.452	-23.267
752.517	-19.345	775.293	-24.209	728.564	-23.268
752.692	-19.346	775.443	-24.210	728.724	-23.270
752.887	-19.347	775.653	-24.211	728.905	-23.271
753.055	-19.348	775.869	-24.212	729.088	-23.272
753.219	-19.349	776.039	-24.213	729.249	-23.273
753.354	-19.350	776.200	-24.214	729.422	-23.274
753.509	-19.351	776.367	-24.215	729.549	-23.276
753.728	-19.352	776.553	-24.216	729.665	-23.277
753.860	-19.352	776.677	-24.217	729.937	-23.278

754.043	-19.353	776.786	-24.218	730.169	-23.279
754.217	-19.354	776.911	-24.219	730.320	-23.281
754.342	-19.355	777.058	-24.220	730.431	-23.282
754.474	-19.356	777.234	-24.221	730.558	-23.283
754.661	-19.357	777.402	-24.222	730.704	-23.285
754.836	-19.358	777.569	-24.223	730.851	-23.286
755.011	-19.358	777.691	-24.224	731.018	-23.287
755.237	-19.359	777.851	-24.225	731.170	-23.289
755.406	-19.360	778.056	-24.226	731.356	-23.290
755.552	-19.361	778.216	-24.227	731.514	-23.291
755.752	-19.362	778.402	-24.228	731.644	-23.292
755.880	-19.363	778.568	-24.229	731.806	-23.294
755.965	-19.364	778.669	-24.230	731.998	-23.295
756.144	-19.365	778.773	-24.231	732.164	-23.297
756.338	-19.365	778.922	-24.232	732.325	-23.298
756.496	-19.366	779.097	-24.233	732.557	-23.299
756.685	-19.367	779.270	-24.234	732.786	-23.301
756.824	-19.368	779.446	-24.235	732.904	-23.302
756.937	-19.369	779.587	-24.236	732.974	-23.303
757.140	-19.370	779.719	-24.236	733.111	-23.305
757.335	-19.371	779.877	-24.237	733.296	-23.306
757.494	-19.371	780.094	-24.238	733.490	-23.308
757.635	-19.372	780.282	-24.239	733.618	-23.309
757.760	-19.373	780.469	-24.240	733.727	-23.311
757.916	-19.374	780.759	-24.241	733.895	-23.312
758.113	-19.375	780.968	-24.242	734.069	-23.313
758.282	-19.376	781.084	-24.243	734.284	-23.315
758.429	-19.377	781.235	-24.244	734.497	-23.316
758.538	-19.378	781.323	-24.245	734.676	-23.318
758.649	-19.378	781.424	-24.246	734.840	-23.319

758.809	-19.379	781.622	-24.247	734.937	-23.321
758.961	-19.380	781.756	-24.248	735.089	-23.322
759.135	-19.381	781.913	-24.249	735.303	-23.324
759.296	-19.382	782.097	-24.250	735.458	-23.325
759.407	-19.383	782.240	-24.251	735.648	-23.327
759.556	-19.384	782.383	-24.252	735.782	-23.328
759.781	-19.385	782.581	-24.253	735.910	-23.330
759.986	-19.385	782.815	-24.254	736.117	-23.331
760.210	-19.386	782.949	-24.255	736.266	-23.333
760.442	-19.387	783.064	-24.256	736.359	-23.334
760.577	-19.388	783.250	-24.257	736.486	-23.336
760.679	-19.389	783.440	-24.258	736.708	-23.337
760.837	-19.390	783.670	-24.258	736.918	-23.339
760.985	-19.391	783.882	-24.259	737.064	-23.341
761.151	-19.392	783.944	-24.260	737.232	-23.342
761.347	-19.392	784.082	-24.261	737.427	-23.344
761.506	-19.393	784.324	-24.262	737.541	-23.345
761.647	-19.394	784.480	-24.263	737.664	-23.347
761.757	-19.395	784.622	-24.264	737.889	-23.348
761.897	-19.396	784.737	-24.265	738.026	-23.350
762.045	-19.397	784.856	-24.266	738.195	-23.351
762.217	-19.398	785.091	-24.267	738.393	-23.353
762.366	-19.399	785.311	-24.268	738.481	-23.354
762.572	-19.400	785.437	-24.269	738.643	-23.356
762.841	-19.400	785.609	-24.270	738.808	-23.357
763.043	-19.401	785.812	-24.270	738.948	-23.359
763.212	-19.402	785.967	-24.271	739.101	-23.361
763.342	-19.403	786.152	-24.272	739.227	-23.362
763.487	-19.404	786.297	-24.273	739.389	-23.364
763.652	-19.405	786.398	-24.274	739.551	-23.365

763.836	-19.406	786.590	-24.275	739.745	-23.367
763.985	-19.407	786.768	-24.276	739.865	-23.368
764.148	-19.408	786.890	-24.277	740.030	-23.370
764.344	-19.409	787.029	-24.278	740.292	-23.371
764.493	-19.409	787.161	-24.278	740.469	-23.373
764.610	-19.410	787.340	-24.279	740.646	-23.374
764.697	-19.411	787.542	-24.280	740.844	-23.376
764.865	-19.412	787.664	-24.281	741.061	-23.377
765.076	-19.413	787.837	-24.282	741.214	-23.379
765.236	-19.414	787.990	-24.283	741.345	-23.380
765.405	-19.415	788.140	-24.284	741.569	-23.382
765.598	-19.416	788.278	-24.285	741.768	-23.383
765.742	-19.417	788.605	-24.285	741.867	-23.385
765.936	-19.418	789.035	-24.286	741.964	-23.386
766.161	-19.419	789.353	-24.287	742.057	-23.388
766.288	-19.420	789.520	-24.288	742.124	-23.389
766.413	-19.420	789.509	-24.289	742.249	-23.390
766.561	-19.421	789.538	-24.290	742.455	-23.392
766.739	-19.422	789.587	-24.291	742.651	-23.393
766.943	-19.423	789.694	-24.291	742.822	-23.395
767.064	-19.424	789.840	-24.292	742.970	-23.396
767.221	-19.425	789.977	-24.293	743.139	-23.398
767.475	-19.426	790.134	-24.294	743.328	-23.399
767.678	-19.427	790.227	-24.295	743.518	-23.400
767.804	-19.428	790.336	-24.296	743.748	-23.402
767.911	-19.429	790.546	-24.296	743.890	-23.403
768.010	-19.430	790.759	-24.297	744.019	-23.404
768.142	-19.431	790.940	-24.298	744.183	-23.406
768.299	-19.432	791.169	-24.299	744.370	-23.407
768.566	-19.433	791.368	-24.300	744.548	-23.408

768.883	-19.434	791.484	-24.301	744.748	-23.410
769.258	-19.435	791.603	-24.302	744.931	-23.411
769.528	-19.435	791.778	-24.302	745.068	-23.412
769.538	-19.436	791.950	-24.303	745.210	-23.414
769.590	-19.437	792.073	-24.304	745.337	-23.415
769.628	-19.438	792.189	-24.305	745.536	-23.416
769.699	-19.439	792.339	-24.306	745.715	-23.417
769.849	-19.440	792.545	-24.307	745.936	-23.419
769.984	-19.441	792.673	-24.308	746.120	-23.420
770.145	-19.442	792.814	-24.308	746.199	-23.421
770.293	-19.443	793.045	-24.309	746.377	-23.422
770.417	-19.444	793.158	-24.310	746.577	-23.424
770.609	-19.445	793.288	-24.311	746.739	-23.425
770.857	-19.446	793.512	-24.312	746.909	-23.426
771.015	-19.447	793.704	-24.313	747.095	-23.427
771.189	-19.448	793.901	-24.314	747.254	-23.428
771.358	-19.449	794.059	-24.314	747.411	-23.429
771.522	-19.450	794.159	-24.315	747.597	-23.431
771.692	-19.451	794.278	-24.316	747.746	-23.432
771.806	-19.452	794.454	-24.317	747.974	-23.433
771.982	-19.453	794.659	-24.318	748.176	-23.434
772.124	-19.453	794.805	-24.319	748.231	-23.435
772.251	-19.454	794.964	-24.320	748.327	-23.436
772.390	-19.455	795.090	-24.321	748.557	-23.437
772.529	-19.456	795.224	-24.322	748.742	-23.438
772.707	-19.457	795.399	-24.323	748.834	-23.439
772.872	-19.458	795.589	-24.324	749.052	-23.440
773.032	-19.459	795.770	-24.324	749.241	-23.442
773.169	-19.460	795.944	-24.325	749.342	-23.443
773.332	-19.461	796.111	-24.326	749.475	-23.444

773.545	-19.462	796.248	-24.327	749.646	-23.445
773.717	-19.463	796.409	-24.328	749.800	-23.446
773.921	-19.464	796.561	-24.329	749.992	-23.447
774.102	-19.465	796.695	-24.330	750.202	-23.448
774.249	-19.466	796.824	-24.331	750.301	-23.449
774.397	-19.467	796.903	-24.332	750.388	-23.450
774.535	-19.468	797.105	-24.333	750.592	-23.451
774.684	-19.469	797.432	-24.334	750.834	-23.452
774.799	-19.470	797.584	-24.335	751.234	-23.453
774.973	-19.471	797.688	-24.336	751.677	-23.454
775.212	-19.471	797.868	-24.337	751.775	-23.455
775.378	-19.472	798.085	-24.338	751.756	-23.456
775.454	-19.473	798.267	-24.339	751.857	-23.457
775.594	-19.474	798.430	-24.340	752.008	-23.457
775.765	-19.475	798.578	-24.341	752.101	-23.458
775.971	-19.476	798.701	-24.342	752.196	-23.459
776.166	-19.477	798.848	-24.343	752.270	-23.460
776.258	-19.478	799.038	-24.344	752.304	-23.461
776.425	-19.479	799.273	-24.345	752.430	-23.462
776.693	-19.480	799.394	-24.347	752.632	-23.463
776.829	-19.481	799.479	-24.348	752.813	-23.464
776.898	-19.482	799.609	-24.349	752.990	-23.465
777.060	-19.483	799.792	-24.350	753.175	-23.466
777.284	-19.484	799.986	-24.351	753.241	-23.467
777.465	-19.484	800.182	-24.352	753.411	-23.468
777.568	-19.485	800.306	-24.353	753.676	-23.469
777.785	-19.486	800.404	-24.354	753.874	-23.469
778.010	-19.487	800.508	-24.355	754.069	-23.470
778.111	-19.488	800.659	-24.356	754.265	-23.471
778.291	-19.489	800.835	-24.357	754.460	-23.472

778.510	-19.490	801.003	-24.358	754.594	-23.473
778.608	-19.491	801.092	-24.359	754.691	-23.474
778.735	-19.492	801.245	-24.360	754.848	-23.475
778.912	-19.493	801.491	-24.362	755.039	-23.476
779.094	-19.494	801.708	-24.363	755.156	-23.477
779.254	-19.495	801.844	-24.364	755.322	-23.478
779.426	-19.495	802.046	-24.365	755.538	-23.479
779.612	-19.496	802.262	-24.366	755.694	-23.480
779.787	-19.497	802.403	-24.367	755.796	-23.481
779.928	-19.498	802.575	-24.368	755.879	-23.482
780.038	-19.499	802.753	-24.369	756.068	-23.483
780.175	-19.500	802.853	-24.370	756.297	-23.484
780.360	-19.501	803.057	-24.371	756.452	-23.484
780.563	-19.502	803.266	-24.372	756.611	-23.485
780.713	-19.503	803.437	-24.374	756.778	-23.486
780.803	-19.504	803.533	-24.375	756.938	-23.487
780.907	-19.505	803.645	-24.376	757.104	-23.488
781.075	-19.505	803.780	-24.377	757.328	-23.489
781.169	-19.506	803.820	-24.378	757.541	-23.490
781.316	-19.507	803.957	-24.379	757.674	-23.492
781.519	-19.508	804.202	-24.380	757.794	-23.493
781.622	-19.509	804.417	-24.381	757.963	-23.494
781.761	-19.510	804.605	-24.382	758.147	-23.495
781.937	-19.511	804.739	-24.383	758.319	-23.496
782.196	-19.512	804.911	-24.385	758.473	-23.497
782.438	-19.513	805.125	-24.386	758.590	-23.498
782.609	-19.513	805.313	-24.387	758.780	-23.499
782.811	-19.514	805.520	-24.388	758.985	-23.500
783.029	-19.515	805.656	-24.389	759.061	-23.501
783.182	-19.516	805.824	-24.390	759.088	-23.502

783.317	-19.517	806.006	-24.391	759.275	-23.503
783.494	-19.518	806.150	-24.392	759.493	-23.504
783.635	-19.519	806.309	-24.393	759.631	-23.506
783.750	-19.520	806.489	-24.394	759.772	-23.507
783.817	-19.521	806.665	-24.395	759.956	-23.508
783.968	-19.521	806.855	-24.397	760.150	-23.509
784.189	-19.522	807.047	-24.398	760.285	-23.510
784.381	-19.523	807.140	-24.399	760.418	-23.511
784.499	-19.524	807.279	-24.400	760.611	-23.513
784.592	-19.525	807.443	-24.401	760.849	-23.514
784.768	-19.526	807.617	-24.402	760.965	-23.515
784.964	-19.527	807.808	-24.403	761.077	-23.516
785.173	-19.528	807.974	-24.404	761.256	-23.518
785.406	-19.529	808.159	-24.405	761.425	-23.519
785.627	-19.529	808.393	-24.406	761.610	-23.520
785.738	-19.530	808.524	-24.407	761.796	-23.521
785.870	-19.531	808.585	-24.408	761.980	-23.523
786.042	-19.532	808.739	-24.410	762.197	-23.524
786.232	-19.533	808.924	-24.411	762.442	-23.525
786.421	-19.534	809.139	-24.412	762.589	-23.527
786.600	-19.535	809.335	-24.413	762.701	-23.528
786.777	-19.536	809.508	-24.414	762.870	-23.529
786.938	-19.536	809.652	-24.415	763.051	-23.531
787.124	-19.537	809.754	-24.416	763.185	-23.532
787.263	-19.538	809.896	-24.417	763.298	-23.533
787.361	-19.539	810.069	-24.418	763.457	-23.535
787.476	-19.540	810.179	-24.419	763.642	-23.536
787.650	-19.541	810.423	-24.420	763.773	-23.537
787.837	-19.542	810.930	-24.421	763.975	-23.539
788.054	-19.543	811.283	-24.422	764.174	-23.540

788.209	-19.544	811.375	-24.423	764.334	-23.542
788.301	-19.544	811.413	-24.425	764.479	-23.543
788.512	-19.545	811.385	-24.426	764.622	-23.544
788.736	-19.546	811.359	-24.427	764.772	-23.546
788.857	-19.547	811.435	-24.428	764.926	-23.547
788.979	-19.548	811.620	-24.429	765.119	-23.549
789.146	-19.549	811.797	-24.430	765.294	-23.550
789.290	-19.550	811.918	-24.431	765.368	-23.552
789.444	-19.551	812.122	-24.432	765.529	-23.553
789.633	-19.552	812.256	-24.433	765.692	-23.554
789.809	-19.552	812.429	-24.434	765.775	-23.556
789.997	-19.553	812.679	-24.435	765.946	-23.557
790.209	-19.554	812.869	-24.436	766.129	-23.559
790.357	-19.555	813.070	-24.437	766.244	-23.560
790.464	-19.556	813.247	-24.438	766.342	-23.562
790.618	-19.557	813.418	-24.439	766.466	-23.563
790.767	-19.558	813.564	-24.440	766.583	-23.564
790.861	-19.559	813.661	-24.441	766.742	-23.566
791.108	-19.559	813.771	-24.442	766.987	-23.567
791.525	-19.560	813.951	-24.443	767.231	-23.569
791.915	-19.561	814.130	-24.444	767.411	-23.570
792.045	-19.562	814.310	-24.445	767.576	-23.572
792.088	-19.563	814.463	-24.446	767.720	-23.573
792.247	-19.564	814.564	-24.447	767.903	-23.574
792.306	-19.565	814.718	-24.448	768.128	-23.576
792.325	-19.566	814.867	-24.449	768.312	-23.577
792.410	-19.567	815.016	-24.450	768.513	-23.579
792.530	-19.567	815.232	-24.451	768.664	-23.580
792.710	-19.568	815.435	-24.453	768.812	-23.582
792.853	-19.569	815.586	-24.454	769.012	-23.583

792.989	-19.570	815.763	-24.455	769.178	-23.584
793.174	-19.571	815.945	-24.456	769.312	-23.586
793.346	-19.572	816.099	-24.457	769.448	-23.587
793.579	-19.573	816.269	-24.458	769.657	-23.589
793.808	-19.574	816.419	-24.459	769.763	-23.590
794.023	-19.575	816.522	-24.460	769.808	-23.592
794.199	-19.575	816.644	-24.461	769.974	-23.593
794.271	-19.576	816.836	-24.462	770.150	-23.594
794.387	-19.577	817.044	-24.463	770.314	-23.596
794.544	-19.578	817.165	-24.464	770.463	-23.597
794.685	-19.579	817.236	-24.465	770.654	-23.599
794.793	-19.580	817.430	-24.466	770.905	-23.600
794.942	-19.581	817.650	-24.467	771.033	-23.601
795.128	-19.582	817.773	-24.468	771.135	-23.603
795.238	-19.582	817.934	-24.469	771.309	-23.604
795.374	-19.583	818.176	-24.471	771.549	-23.605
795.562	-19.584	818.369	-24.472	771.765	-23.607
795.706	-19.585	818.496	-24.473	771.944	-23.608
795.910	-19.586	818.615	-24.474	772.075	-23.609
796.116	-19.587	818.692	-24.475	772.163	-23.611
796.321	-19.588	818.851	-24.476	772.365	-23.612
796.523	-19.589	819.038	-24.477	772.596	-23.613
796.686	-19.589	819.268	-24.478	772.752	-23.615
796.843	-19.590	819.423	-24.479	772.856	-23.616
796.991	-19.591	819.562	-24.481	772.962	-23.617
797.155	-19.592	819.783	-24.482	773.158	-23.618
797.260	-19.593	819.940	-24.483	773.366	-23.620
797.376	-19.594	820.049	-24.484	773.520	-23.621
797.507	-19.595	820.247	-24.485	773.688	-23.622
797.661	-19.595	820.430	-24.486	773.830	-23.623

797.842	-19.596	820.583	-24.487	773.966	-23.625
797.999	-19.597	820.698	-24.489	774.192	-23.626
798.149	-19.598	820.846	-24.490	774.396	-23.627
798.295	-19.599	821.012	-24.491	774.543	-23.628
798.456	-19.600	821.255	-24.492	774.700	-23.630
798.615	-19.601	821.432	-24.493	774.899	-23.631
798.795	-19.601	821.563	-24.494	775.045	-23.632
798.969	-19.602	821.765	-24.496	775.140	-23.633
799.103	-19.603	821.898	-24.497	775.306	-23.634
799.361	-19.604	822.050	-24.498	775.508	-23.635
799.581	-19.605	822.209	-24.499	775.670	-23.637
799.652	-19.606	822.348	-24.500	775.786	-23.638
799.800	-19.607	822.478	-24.502	775.977	-23.639
799.969	-19.607	822.604	-24.503	776.148	-23.640
800.146	-19.608	822.743	-24.504	776.201	-23.641
800.306	-19.609	822.903	-24.505	776.375	-23.642
800.443	-19.610	823.075	-24.506	776.693	-23.644
800.619	-19.611	823.206	-24.507	776.894	-23.645
800.809	-19.612	823.370	-24.509	777.002	-23.646
801.001	-19.612	823.577	-24.510	777.160	-23.647
801.177	-19.613	823.738	-24.511	777.290	-23.648
801.323	-19.614	823.948	-24.512	777.438	-23.649
801.472	-19.615	824.202	-24.513	777.597	-23.650
801.610	-19.616	824.371	-24.514	777.787	-23.651
801.731	-19.617	824.534	-24.516	777.932	-23.652
801.898	-19.617	824.723	-24.517	778.170	-23.653
802.116	-19.618	824.831	-24.518	778.512	-23.654
802.337	-19.619	824.915	-24.519	778.849	-23.656
802.530	-19.620	825.022	-24.520	779.126	-23.657
802.709	-19.621	825.159	-24.521	779.230	-23.658

802.860	-19.622	825.353	-24.523	779.342	-23.659
802.986	-19.622	825.526	-24.524	779.415	-23.660
803.137	-19.623	825.675	-24.525	779.414	-23.661
803.281	-19.624	825.779	-24.526	779.510	-23.662
803.405	-19.625	825.958	-24.527	779.676	-23.663
803.462	-19.626	826.161	-24.528	779.770	-23.664
803.622	-19.627	826.316	-24.529	779.870	-23.665
803.870	-19.627	826.489	-24.530	780.028	-23.666
804.006	-19.628	826.674	-24.531	780.182	-23.667
804.109	-19.629	826.830	-24.533	780.295	-23.668
804.246	-19.630	827.012	-24.534	780.496	-23.669
804.410	-19.631	827.190	-24.535	780.702	-23.670
804.595	-19.632	827.326	-24.536	780.871	-23.671
804.779	-19.632	827.486	-24.537	781.068	-23.672
804.977	-19.633	827.635	-24.538	781.256	-23.673
805.169	-19.634	827.763	-24.539	781.379	-23.674
805.344	-19.635	827.927	-24.540	781.516	-23.675
805.583	-19.636	828.160	-24.541	781.742	-23.676
805.807	-19.636	828.389	-24.542	781.917	-23.677
805.913	-19.637	828.567	-24.543	782.041	-23.678
806.000	-19.638	828.713	-24.544	782.186	-23.679
806.181	-19.639	828.912	-24.545	782.355	-23.680
806.383	-19.640	829.118	-24.546	782.503	-23.681
806.519	-19.641	829.249	-24.547	782.627	-23.682
806.602	-19.641	829.411	-24.548	782.815	-23.683
806.684	-19.642	829.595	-24.549	783.005	-23.684
806.880	-19.643	829.777	-24.550	783.149	-23.685
807.066	-19.644	829.937	-24.551	783.297	-23.686
807.219	-19.645	830.090	-24.552	783.424	-23.687
807.403	-19.646	830.249	-24.553	783.564	-23.688

807.599	-19.646	830.378	-24.554	783.680	-23.689
807.828	-19.647	830.521	-24.555	783.804	-23.690
808.009	-19.648	830.707	-24.556	784.043	-23.691
808.158	-19.649	830.903	-24.557	784.246	-23.692
808.318	-19.650	831.075	-24.558	784.415	-23.693
808.476	-19.651	831.224	-24.559	784.681	-23.694
808.631	-19.651	831.367	-24.560	784.847	-23.695
808.822	-19.652	831.507	-24.560	784.910	-23.697
809.026	-19.653	831.659	-24.561	785.054	-23.698
809.219	-19.654	831.817	-24.562	785.285	-23.699
809.368	-19.655	831.956	-24.563	785.432	-23.700
809.494	-19.656	832.140	-24.564	785.575	-23.701
809.606	-19.656	832.449	-24.565	785.723	-23.702
809.772	-19.657	832.886	-24.566	785.878	-23.703
810.014	-19.658	833.197	-24.566	786.060	-23.704
810.188	-19.659	833.213	-24.567	786.190	-23.705
810.325	-19.660	833.239	-24.568	786.379	-23.706
810.445	-19.661	833.355	-24.569	786.600	-23.707
810.583	-19.662	833.424	-24.570	786.722	-23.708
810.778	-19.662	833.434	-24.570	786.799	-23.710
810.969	-19.663	833.505	-24.571	786.988	-23.711
811.115	-19.664	833.697	-24.572	787.190	-23.712
811.247	-19.665	833.894	-24.573	787.368	-23.713
811.418	-19.666	834.053	-24.573	787.548	-23.714
811.571	-19.667	834.219	-24.574	787.694	-23.715
811.706	-19.668	834.363	-24.575	787.874	-23.716
811.862	-19.668	834.529	-24.576	788.012	-23.717
812.001	-19.669	834.767	-24.576	788.099	-23.719
812.246	-19.670	834.965	-24.577	788.173	-23.720
812.458	-19.671	835.171	-24.578	788.354	-23.721

812.625	-19.672	835.267	-24.578	788.602	-23.722
812.809	-19.673	835.381	-24.579	788.707	-23.723
812.976	-19.674	835.574	-24.580	788.860	-23.724
813.116	-19.674	835.742	-24.580	789.009	-23.726
813.205	-19.675	835.891	-24.581	789.186	-23.727
813.351	-19.676	836.010	-24.582	789.434	-23.728
813.472	-19.677	836.198	-24.582	789.671	-23.729
813.660	-19.678	836.358	-24.583	789.739	-23.730
814.078	-19.679	836.451	-24.584	789.834	-23.732
814.499	-19.680	836.575	-24.584	790.087	-23.733
814.678	-19.681	836.756	-24.585	790.275	-23.734
814.722	-19.681	836.928	-24.586	790.427	-23.735
814.801	-19.682	837.133	-24.586	790.602	-23.736
814.883	-19.683	837.364	-24.587	790.769	-23.738
814.949	-19.684	837.598	-24.587	790.884	-23.739
815.061	-19.685	837.766	-24.588	790.993	-23.740
815.165	-19.686	837.906	-24.588	791.114	-23.741
815.212	-19.687	838.148	-24.589	791.278	-23.743
815.359	-19.688	838.279	-24.590	791.423	-23.744
815.556	-19.688	838.364	-24.590	791.633	-23.745
815.799	-19.689	838.533	-24.591	791.899	-23.746
815.959	-19.690	838.698	-24.591	792.020	-23.747
816.087	-19.691	838.805	-24.592	792.105	-23.749
816.311	-19.692	838.874	-24.592	792.281	-23.750
816.487	-19.693	839.035	-24.593	792.498	-23.751
816.670	-19.694	839.260	-24.593	792.701	-23.752
816.796	-19.695	839.427	-24.594	792.897	-23.753
816.889	-19.695	839.568	-24.594	793.021	-23.755
817.054	-19.696	839.717	-24.595	793.142	-23.756
817.203	-19.697	839.893	-24.596	793.342	-23.757

817.346	-19.698	840.008	-24.596	793.532	-23.758
817.515	-19.699	840.070	-24.597	793.632	-23.759
817.753	-19.700	840.268	-24.597	793.678	-23.761
817.959	-19.701	840.495	-24.598	793.838	-23.762
818.029	-19.702	840.658	-24.598	794.090	-23.763
818.094	-19.702	840.834	-24.599	794.261	-23.764
818.265	-19.703	841.014	-24.599	794.324	-23.765
818.496	-19.704	841.245	-24.600	794.484	-23.767
818.689	-19.705	841.441	-24.600	794.733	-23.768
818.857	-19.706	841.551	-24.601	794.907	-23.769
819.064	-19.707	841.693	-24.601	795.086	-23.770
819.240	-19.708	841.867	-24.602	795.306	-23.771
819.405	-19.709	842.010	-24.602	795.517	-23.772
819.512	-19.709	842.130	-24.603	795.666	-23.773
819.650	-19.710	842.290	-24.603	795.756	-23.775
819.809	-19.711	842.516	-24.604	795.919	-23.776
819.958	-19.712	842.727	-24.604	796.126	-23.777
820.156	-19.713	842.792	-24.605	796.284	-23.778
820.294	-19.714	842.948	-24.605	796.466	-23.779
820.426	-19.715	843.244	-24.606	796.661	-23.780
820.588	-19.716	843.410	-24.606	796.730	-23.781
820.796	-19.716	843.456	-24.607	796.818	-23.782
820.971	-19.717	843.576	-24.607	797.091	-23.783
821.104	-19.718	843.798	-24.608	797.301	-23.785
821.242	-19.719	844.023	-24.608	797.366	-23.786
821.339	-19.720	844.192	-24.609	797.457	-23.787
821.532	-19.721	844.294	-24.609	797.642	-23.788
821.761	-19.722	844.389	-24.610	797.808	-23.789
821.909	-19.722	844.473	-24.610	797.952	-23.790
822.020	-19.723	844.613	-24.611	798.086	-23.791

822.153	-19.724	844.823	-24.611	798.219	-23.792
822.375	-19.725	844.995	-24.612	798.428	-23.793
822.575	-19.726	845.148	-24.612	798.641	-23.794
822.701	-19.727	845.366	-24.613	798.850	-23.795
822.877	-19.728	845.536	-24.613	799.105	-23.796
823.111	-19.728	845.687	-24.614	799.246	-23.797
823.356	-19.729	845.883	-24.614	799.382	-23.798
823.529	-19.730	846.133	-24.614	799.650	-23.799
823.601	-19.731	846.372	-24.615	799.838	-23.800
823.681	-19.732	846.537	-24.615	799.977	-23.801
823.809	-19.733	846.661	-24.616	800.155	-23.802
823.966	-19.733	846.798	-24.616	800.299	-23.803
824.161	-19.734	846.961	-24.617	800.381	-23.804
824.307	-19.735	847.066	-24.617	800.558	-23.805
824.453	-19.736	847.194	-24.618	800.782	-23.806
824.634	-19.737	847.364	-24.618	800.919	-23.806
824.801	-19.738	847.473	-24.618	801.049	-23.807
825.017	-19.738	847.563	-24.619	801.162	-23.808
825.220	-19.739	847.701	-24.619	801.310	-23.809
825.356	-19.740	847.883	-24.620	801.488	-23.810
825.514	-19.741	848.016	-24.620	801.677	-23.811
825.684	-19.742	848.180	-24.621	801.802	-23.812
825.799	-19.742	848.430	-24.621	801.953	-23.813
825.957	-19.743	848.652	-24.621	802.124	-23.814
826.124	-19.744	848.797	-24.622	802.334	-23.815
826.239	-19.745	849.000	-24.622	802.561	-23.815
826.340	-19.746	849.234	-24.623	802.676	-23.816
826.440	-19.747	849.396	-24.623	802.858	-23.817
826.589	-19.747	849.573	-24.623	803.040	-23.818
826.801	-19.748	849.724	-24.624	803.093	-23.819

826.961	-19.749	849.835	-24.624	803.259	-23.820
827.121	-19.750	849.989	-24.624	803.403	-23.821
827.306	-19.751	850.126	-24.625	803.536	-23.822
827.505	-19.751	850.296	-24.625	803.667	-23.822
827.699	-19.752	850.486	-24.626	803.902	-23.823
827.885	-19.753	850.590	-24.626	804.180	-23.824
828.118	-19.754	850.796	-24.626	804.305	-23.825
828.300	-19.755	851.019	-24.627	804.341	-23.826
828.466	-19.755	851.141	-24.627	804.528	-23.827
828.654	-19.756	851.295	-24.627	804.779	-23.828
828.785	-19.757	851.477	-24.628	804.907	-23.829
828.923	-19.758	851.650	-24.628	805.034	-23.829
829.080	-19.759	851.881	-24.628	805.174	-23.830
829.184	-19.759	852.020	-24.629	805.349	-23.831
829.308	-19.760	852.180	-24.629	805.730	-23.832
829.465	-19.761	852.385	-24.630	806.155	-23.833
829.567	-19.762	852.524	-24.630	806.467	-23.834
829.725	-19.762	852.693	-24.630	806.609	-23.835
829.960	-19.763	852.832	-24.631	806.631	-23.836
830.090	-19.764	852.979	-24.631	806.689	-23.836
830.218	-19.765	853.146	-24.631	806.736	-23.837
830.436	-19.766	853.294	-24.632	806.824	-23.838
830.688	-19.766	853.456	-24.632	806.881	-23.839
830.881	-19.767	853.632	-24.632	806.963	-23.840
831.040	-19.768	853.761	-24.633	807.104	-23.841
831.249	-19.769	853.854	-24.633	807.275	-23.842
831.442	-19.769	854.035	-24.633	807.488	-23.843
831.591	-19.770	854.388	-24.633	807.664	-23.844
831.719	-19.771	854.771	-24.634	807.842	-23.845
831.905	-19.772	855.003	-24.634	808.004	-23.846

832.065	-19.772	855.098	-24.634	808.103	-23.847
832.195	-19.773	855.200	-24.635	808.286	-23.848
832.352	-19.774	855.291	-24.635	808.487	-23.849
832.510	-19.775	855.325	-24.635	808.652	-23.850
832.645	-19.776	855.358	-24.636	808.860	-23.851
832.832	-19.776	855.443	-24.636	809.047	-23.852
833.048	-19.777	855.595	-24.636	809.213	-23.853
833.186	-19.778	855.723	-24.636	809.417	-23.854
833.325	-19.779	855.883	-24.637	809.592	-23.855
833.465	-19.779	856.085	-24.637	809.635	-23.856
833.669	-19.780	856.276	-24.637	809.707	-23.857
833.895	-19.781	856.467	-24.638	809.812	-23.858
833.997	-19.782	856.662	-24.638	809.983	-23.859
834.125	-19.782	856.826	-24.638	810.219	-23.860
834.276	-19.783	856.993	-24.639	810.470	-23.862
834.414	-19.784	857.218	-24.639	810.630	-23.863
834.593	-19.785	857.370	-24.639	810.655	-23.864
834.777	-19.785	857.477	-24.639	810.753	-23.865
834.950	-19.786	857.622	-24.640	810.947	-23.866
835.155	-19.787	857.775	-24.640	811.124	-23.868
835.340	-19.787	857.962	-24.640	811.307	-23.869
835.459	-19.788	858.111	-24.641	811.527	-23.870
835.642	-19.789	858.262	-24.641	811.681	-23.871
835.854	-19.790	858.419	-24.641	811.805	-23.873
835.983	-19.790	858.553	-24.642	812.000	-23.874
836.013	-19.791	858.678	-24.642	812.172	-23.875
836.145	-19.792	858.853	-24.642	812.330	-23.876
836.578	-19.793	859.054	-24.643	812.477	-23.878
837.037	-19.793	859.249	-24.643	812.700	-23.879
837.222	-19.794	859.498	-24.643	812.890	-23.881

837.259	-19.795	859.679	-24.644	813.028	-23.882
837.354	-19.796	859.837	-24.644	813.120	-23.883
837.411	-19.796	859.991	-24.644	813.237	-23.885
837.418	-19.797	860.123	-24.645	813.438	-23.886
837.566	-19.798	860.265	-24.645	813.626	-23.888
837.706	-19.798	860.400	-24.645	813.736	-23.889
837.790	-19.799	860.487	-24.646	813.846	-23.891
837.969	-19.800	860.618	-24.646	814.003	-23.892
838.158	-19.801	860.790	-24.647	814.240	-23.894
838.323	-19.801	860.974	-24.647	814.374	-23.895
838.474	-19.802	861.177	-24.647	814.475	-23.897
838.660	-19.803	861.348	-24.648	814.546	-23.898
838.911	-19.803	861.495	-24.648	814.797	-23.900
839.107	-19.804	861.655	-24.649	815.049	-23.902
839.220	-19.805	861.810	-24.649	815.225	-23.903
839.355	-19.805	861.919	-24.649	815.393	-23.905
839.567	-19.806	862.032	-24.650	815.536	-23.907
839.735	-19.807	862.204	-24.650	815.660	-23.908
839.868	-19.808	862.420	-24.651	815.758	-23.910
840.007	-19.808	862.577	-24.651	815.913	-23.912
840.115	-19.809	862.747	-24.652	816.143	-23.913
840.277	-19.810	862.937	-24.652	816.275	-23.915
840.460	-19.810	863.107	-24.653	816.457	-23.917
840.580	-19.811	863.232	-24.653	816.672	-23.919
840.695	-19.812	863.352	-24.654	816.851	-23.920
840.833	-19.812	863.545	-24.654	817.006	-23.922
841.003	-19.813	863.743	-24.655	817.175	-23.924
841.225	-19.814	863.915	-24.655	817.345	-23.926
841.405	-19.814	864.053	-24.656	817.520	-23.928
841.578	-19.815	864.194	-24.657	817.643	-23.930

841.740	-19.816	864.374	-24.657	817.818	-23.932
841.946	-19.816	864.567	-24.658	818.005	-23.933
842.148	-19.817	864.738	-24.658	818.113	-23.935
842.323	-19.818	864.889	-24.659	818.251	-23.937
842.513	-19.818	865.039	-24.659	818.496	-23.939
842.608	-19.819	865.191	-24.660	818.699	-23.941
842.683	-19.820	865.422	-24.661	818.789	-23.943
842.811	-19.820	865.583	-24.661	818.844	-23.945
842.982	-19.821	865.641	-24.662	818.986	-23.947
843.173	-19.822	865.792	-24.663	819.232	-23.949
843.371	-19.822	865.965	-24.663	819.409	-23.951
843.521	-19.823	866.105	-24.664	819.566	-23.953
843.685	-19.823	866.289	-24.665	819.744	-23.955
843.884	-19.824	866.438	-24.665	819.885	-23.957
844.042	-19.825	866.563	-24.666	820.036	-23.959
844.168	-19.825	866.695	-24.667	820.162	-23.961
844.314	-19.826	866.807	-24.667	820.266	-23.962
844.447	-19.827	866.983	-24.668	820.498	-23.964
844.578	-19.827	867.185	-24.669	820.682	-23.966
844.781	-19.828	867.362	-24.669	820.805	-23.968
844.963	-19.828	867.575	-24.670	820.995	-23.970
845.111	-19.829	867.775	-24.671	821.137	-23.972
845.283	-19.830	867.970	-24.671	821.234	-23.974
845.439	-19.830	868.164	-24.672	821.345	-23.976
845.622	-19.831	868.362	-24.673	821.523	-23.978
845.790	-19.832	868.545	-24.674	821.732	-23.980
845.968	-19.832	868.690	-24.674	821.901	-23.982
846.169	-19.833	868.876	-24.675	821.993	-23.984
846.335	-19.833	869.008	-24.676	822.204	-23.986
846.494	-19.834	869.090	-24.677	822.519	-23.988

846.656	-19.835	869.194	-24.677	822.719	-23.990
846.814	-19.835	869.268	-24.678	822.861	-23.992
846.945	-19.836	869.435	-24.679	823.026	-23.994
847.067	-19.836	869.557	-24.680	823.182	-23.996
847.271	-19.837	869.649	-24.680	823.361	-23.998
847.465	-19.838	869.827	-24.681	823.594	-24.000
847.609	-19.838	870.100	-24.682	823.779	-24.001
847.786	-19.839	870.347	-24.683	823.870	-24.003
847.963	-19.839	870.516	-24.683	823.940	-24.005
848.102	-19.840	870.686	-24.684	824.072	-24.007
848.205	-19.840	870.844	-24.685	824.267	-24.009
848.338	-19.841	871.018	-24.686	824.475	-24.011
848.510	-19.842	871.215	-24.686	824.622	-24.013
848.636	-19.842	871.345	-24.687	824.718	-24.014
848.805	-19.843	871.487	-24.688	824.830	-24.016
848.962	-19.843	871.662	-24.689	825.058	-24.018
849.065	-19.844	871.855	-24.690	825.294	-24.020
849.162	-19.845	872.013	-24.690	825.472	-24.021
849.333	-19.845	872.167	-24.691	825.581	-24.023
849.572	-19.846	872.337	-24.692	825.749	-24.025
849.705	-19.846	872.514	-24.693	825.918	-24.026
849.837	-19.847	872.669	-24.694	826.040	-24.028
850.068	-19.847	872.779	-24.694	826.236	-24.030
850.301	-19.848	872.905	-24.695	826.391	-24.031
850.461	-19.849	873.037	-24.696	826.534	-24.033
850.622	-19.849	873.257	-24.697	826.723	-24.034
850.808	-19.850	873.485	-24.698	826.929	-24.036
851.018	-19.850	873.634	-24.699	827.094	-24.038
851.201	-19.851	873.844	-24.699	827.218	-24.039
851.357	-19.851	874.036	-24.700	827.410	-24.041

851.522	-19.852	874.137	-24.701	827.568	-24.042
851.672	-19.853	874.257	-24.702	827.716	-24.043
851.740	-19.853	874.457	-24.703	827.876	-24.045
851.842	-19.854	874.680	-24.703	828.047	-24.046
852.039	-19.854	874.884	-24.704	828.250	-24.048
852.197	-19.855	875.027	-24.705	828.394	-24.049
852.321	-19.855	875.164	-24.706	828.551	-24.050
852.519	-19.856	875.338	-24.707	828.714	-24.052
852.656	-19.857	875.507	-24.708	828.895	-24.053
852.787	-19.857	875.676	-24.708	829.072	-24.054
853.057	-19.858	875.772	-24.709	829.192	-24.056
853.213	-19.858	875.845	-24.710	829.368	-24.057
853.335	-19.859	876.130	-24.711	829.606	-24.058
853.520	-19.860	876.641	-24.712	829.712	-24.059
853.716	-19.860	876.964	-24.713	829.830	-24.060
853.893	-19.861	876.992	-24.714	830.090	-24.061
854.026	-19.861	876.994	-24.714	830.224	-24.063
854.214	-19.862	877.065	-24.715	830.406	-24.064
854.417	-19.862	877.186	-24.716	830.661	-24.065
854.547	-19.863	877.279	-24.717	830.771	-24.066
854.670	-19.864	877.349	-24.718	830.900	-24.067
854.803	-19.864	877.478	-24.719	831.065	-24.068
855.001	-19.865	877.605	-24.720	831.229	-24.069
855.185	-19.865	877.721	-24.720	831.413	-24.070
855.361	-19.866	877.958	-24.721	831.535	-24.071
855.495	-19.867	878.196	-24.722	831.683	-24.071
855.672	-19.867	878.410	-24.723	831.910	-24.072
855.891	-19.868	878.654	-24.724	832.090	-24.073
856.053	-19.868	878.795	-24.725	832.234	-24.074
856.221	-19.869	878.876	-24.726	832.372	-24.075

856.368	-19.870	878.967	-24.727	832.507	-24.075
856.494	-19.870	879.096	-24.727	832.648	-24.076
856.642	-19.871	879.317	-24.728	832.907	-24.077
856.805	-19.871	879.470	-24.729	833.343	-24.078
856.957	-19.872	879.576	-24.730	833.639	-24.078
857.111	-19.873	879.699	-24.731	833.856	-24.079
857.284	-19.873	879.880	-24.732	833.989	-24.079
857.430	-19.874	880.010	-24.733	834.042	-24.080
857.594	-19.874	880.174	-24.734	834.106	-24.081
857.776	-19.875	880.357	-24.735	834.190	-24.081
857.942	-19.876	880.508	-24.736	834.231	-24.082
858.096	-19.876	880.667	-24.737	834.288	-24.082
858.214	-19.877	880.843	-24.737	834.471	-24.083
858.402	-19.877	881.079	-24.738	834.604	-24.083
858.571	-19.878	881.280	-24.739	834.707	-24.084
858.788	-19.879	881.437	-24.740	834.858	-24.084
859.212	-19.879	881.580	-24.741	834.993	-24.084
859.603	-19.880	881.717	-24.742	835.136	-24.085
859.778	-19.881	881.878	-24.743	835.357	-24.085
859.828	-19.881	882.020	-24.744	835.537	-24.085
859.873	-19.882	882.156	-24.745	835.649	-24.086
859.961	-19.883	882.324	-24.746	835.819	-24.086
860.031	-19.883	882.555	-24.747	836.000	-24.086
860.072	-19.884	882.700	-24.748	836.151	-24.086
860.150	-19.884	882.829	-24.749	836.311	-24.087
860.299	-19.885	882.969	-24.750	836.478	-24.087
860.445	-19.886	883.121	-24.751	836.635	-24.087
860.612	-19.886	883.344	-24.752	836.820	-24.087
860.801	-19.887	883.542	-24.753	837.017	-24.087
861.010	-19.888	883.683	-24.754	837.157	-24.088

861.235	-19.888	883.809	-24.755	837.271	-24.088
861.414	-19.889	883.977	-24.756	837.328	-24.088
861.605	-19.890	884.149	-24.757	837.525	-24.088
861.741	-19.890	884.283	-24.758	837.791	-24.088
861.852	-19.891	884.411	-24.760	837.943	-24.088
862.029	-19.892	884.597	-24.761	838.114	-24.088
862.198	-19.892	884.818	-24.762	838.278	-24.088
862.362	-19.893	884.921	-24.763	838.436	-24.088
862.542	-19.894	885.058	-24.764	838.621	-24.088
862.701	-19.894	885.208	-24.765	838.828	-24.088
862.833	-19.895	885.416	-24.766	838.969	-24.088
862.955	-19.896	885.591	-24.767	839.158	-24.088
863.081	-19.896	885.733	-24.769	839.308	-24.088
863.217	-19.897	885.900	-24.770	839.438	-24.088
863.379	-19.898	886.061	-24.771	839.637	-24.088
863.546	-19.898	886.215	-24.772	839.843	-24.088
863.736	-19.899	886.381	-24.773	839.993	-24.088
863.932	-19.900	886.533	-24.774	840.129	-24.088
864.120	-19.900	886.691	-24.776	840.277	-24.088
864.279	-19.901	886.855	-24.777	840.380	-24.088
864.480	-19.902	887.022	-24.778	840.549	-24.088
864.713	-19.902	887.228	-24.779	840.715	-24.088
864.792	-19.903	887.405	-24.780	840.874	-24.088
864.849	-19.904	887.542	-24.782	841.038	-24.088
865.027	-19.904	887.741	-24.783	841.129	-24.088
865.197	-19.905	887.900	-24.784	841.286	-24.088
865.328	-19.906	887.990	-24.785	841.514	-24.088
865.443	-19.906	888.131	-24.787	841.684	-24.088
865.621	-19.907	888.183	-24.788	841.839	-24.087
865.827	-19.908	888.323	-24.789	842.019	-24.087

866.009	-19.908	888.592	-24.790	842.199	-24.087
866.249	-19.909	888.702	-24.792	842.421	-24.087
866.375	-19.910	888.817	-24.793	842.538	-24.087
866.438	-19.910	888.981	-24.794	842.636	-24.087
866.575	-19.911	889.193	-24.796	842.812	-24.087
866.749	-19.912	889.416	-24.797	842.978	-24.087
866.924	-19.913	889.632	-24.798	843.150	-24.087
867.060	-19.913	889.835	-24.799	843.278	-24.087
867.214	-19.914	890.034	-24.801	843.398	-24.087
867.449	-19.915	890.219	-24.802	843.596	-24.087
867.632	-19.915	890.350	-24.803	843.781	-24.087
867.821	-19.916	890.533	-24.805	843.933	-24.087
868.008	-19.917	890.722	-24.806	844.119	-24.086
868.121	-19.917	890.855	-24.807	844.268	-24.086
868.313	-19.918	890.969	-24.808	844.455	-24.086
868.492	-19.919	891.024	-24.810	844.634	-24.086
868.677	-19.919	891.079	-24.811	844.818	-24.086
868.814	-19.920	891.229	-24.812	844.973	-24.086
868.950	-19.921	891.434	-24.814	845.081	-24.086
869.158	-19.922	891.634	-24.815	845.229	-24.086
869.279	-19.922	891.853	-24.816	845.393	-24.086
869.442	-19.923	892.025	-24.817	845.579	-24.086
869.635	-19.924	892.193	-24.819	845.761	-24.086
869.754	-19.924	892.409	-24.820	845.936	-24.086
869.936	-19.925	892.598	-24.821	846.056	-24.086
870.126	-19.926	892.801	-24.823	846.209	-24.086
870.247	-19.926	892.972	-24.824	846.402	-24.086
870.338	-19.927	893.040	-24.825	846.615	-24.086
870.505	-19.928	893.163	-24.826	846.814	-24.086
870.750	-19.929	893.352	-24.828	846.992	-24.086

870.969	-19.929	893.477	-24.829	847.138	-24.086
871.094	-19.930	893.624	-24.830	847.243	-24.086
871.176	-19.931	893.846	-24.832	847.428	-24.086
871.298	-19.931	893.990	-24.833	847.587	-24.086
871.430	-19.932	894.105	-24.834	847.731	-24.086
871.505	-19.933	894.267	-24.835	847.868	-24.086
871.605	-19.934	894.464	-24.837	848.023	-24.086
871.790	-19.934	894.644	-24.838	848.112	-24.086
871.979	-19.935	894.806	-24.839	848.196	-24.086
872.111	-19.936	894.989	-24.840	848.382	-24.086
872.311	-19.936	895.213	-24.842	848.494	-24.086
872.560	-19.937	895.458	-24.843	848.618	-24.086
872.781	-19.938	895.559	-24.844	848.818	-24.086
872.995	-19.939	895.672	-24.845	849.012	-24.086
873.176	-19.939	895.856	-24.847	849.162	-24.086
873.334	-19.940	895.957	-24.848	849.299	-24.086
873.512	-19.941	896.096	-24.849	849.467	-24.086
873.653	-19.941	896.272	-24.850	849.657	-24.086
873.801	-19.942	896.435	-24.851	849.921	-24.086
873.930	-19.943	896.637	-24.853	850.191	-24.086
874.046	-19.944	896.798	-24.854	850.368	-24.086
874.178	-19.944	896.945	-24.855	850.449	-24.086
874.293	-19.945	897.110	-24.856	850.567	-24.086
874.428	-19.946	897.381	-24.857	850.771	-24.086
874.612	-19.947	897.526	-24.859	850.936	-24.086
874.755	-19.947	897.618	-24.860	851.057	-24.086
874.880	-19.948	897.767	-24.861	851.205	-24.087
875.049	-19.949	898.003	-24.862	851.407	-24.087
875.240	-19.950	898.364	-24.863	851.554	-24.087
875.465	-19.950	898.756	-24.864	851.684	-24.087

875.714	-19.951	898.952	-24.866	851.790	-24.087
875.955	-19.952	898.980	-24.867	851.870	-24.087
876.078	-19.953	899.019	-24.868	852.031	-24.087
876.170	-19.953	899.054	-24.869	852.272	-24.087
876.339	-19.954	899.194	-24.870	852.407	-24.087
876.553	-19.955	899.289	-24.871	852.521	-24.087
876.741	-19.956	899.359	-24.872	852.738	-24.087
876.914	-19.956	899.508	-24.873	852.923	-24.087
877.134	-19.957	899.652	-24.874	853.090	-24.087
877.252	-19.958	899.834	-24.875	853.307	-24.087
877.374	-19.959	900.061	-24.876	853.576	-24.088
877.589	-19.959	900.271	-24.877	853.727	-24.088
877.700	-19.960	900.416	-24.879	853.816	-24.088
877.791	-19.961	900.582	-24.880	853.979	-24.088
877.980	-19.962	900.736	-24.881	854.142	-24.088
878.212	-19.962	900.891	-24.882	854.329	-24.088
878.412	-19.963	901.043	-24.883	854.539	-24.088
878.615	-19.964	901.193	-24.884	854.658	-24.088
878.749	-19.965	901.299	-24.885	854.837	-24.088
878.883	-19.966	901.424	-24.886	855.096	-24.088
879.028	-19.966	901.599	-24.887	855.243	-24.088
879.191	-19.967	901.715	-24.888	855.359	-24.088
879.385	-19.968	901.879	-24.889	855.495	-24.088
879.516	-19.969	902.078	-24.890	855.646	-24.088
879.638	-19.969	902.217	-24.891	855.822	-24.088
879.777	-19.970	902.408	-24.891	855.962	-24.089
879.958	-19.971	902.645	-24.892	856.096	-24.089
880.110	-19.972	902.816	-24.893	856.298	-24.089
880.327	-19.973	902.947	-24.894	856.450	-24.089
880.562	-19.973	903.103	-24.895	856.599	-24.089

880.691	-19.974	903.239	-24.896	856.797	-24.089
880.805	-19.975	903.447	-24.897	856.908	-24.089
880.934	-19.976	903.604	-24.898	857.068	-24.089
881.077	-19.976	903.754	-24.899	857.259	-24.089
881.407	-19.977	903.979	-24.900	857.429	-24.089
881.824	-19.978	904.106	-24.901	857.629	-24.089
882.122	-19.979	904.222	-24.902	857.763	-24.089
882.238	-19.980	904.325	-24.902	857.900	-24.089
882.251	-19.980	904.511	-24.903	858.046	-24.089
882.289	-19.981	904.728	-24.904	858.200	-24.089
882.337	-19.982	904.911	-24.905	858.372	-24.090
882.431	-19.983	905.042	-24.906	858.553	-24.090
882.583	-19.984	905.212	-24.907	858.730	-24.090
882.688	-19.984	905.428	-24.908	858.863	-24.090
882.737	-19.985	905.587	-24.908	859.016	-24.090
882.891	-19.986	905.777	-24.909	859.181	-24.090
883.146	-19.987	905.982	-24.910	859.364	-24.090
883.343	-19.988	906.069	-24.911	859.482	-24.090
883.544	-19.988	906.168	-24.912	859.635	-24.090
883.773	-19.989	906.328	-24.912	859.736	-24.090
883.948	-19.990	906.450	-24.913	859.947	-24.090
884.101	-19.991	906.583	-24.914	860.367	-24.090
884.329	-19.992	906.785	-24.915	860.736	-24.091
884.473	-19.992	907.025	-24.916	860.957	-24.091
884.581	-19.993	907.213	-24.916	861.075	-24.091
884.715	-19.994	907.318	-24.917	861.144	-24.091
884.893	-19.995	907.485	-24.918	861.227	-24.091
885.049	-19.996	907.758	-24.919	861.298	-24.091
885.154	-19.996	907.895	-24.919	861.361	-24.091
885.286	-19.997	907.971	-24.920	861.501	-24.091

885.417	-19.998	908.131	-24.921	861.645	-24.091
885.551	-19.999	908.298	-24.922	861.752	-24.091
885.742	-20.000	908.416	-24.922	861.830	-24.092
885.980	-20.000	908.551	-24.923	862.003	-24.092
886.150	-20.001	908.795	-24.924	862.196	-24.092
886.301	-20.002	909.025	-24.925	862.330	-24.092
886.458	-20.003	909.206	-24.925	862.439	-24.092
886.644	-20.004	909.365	-24.926	862.589	-24.092
886.841	-20.004	909.527	-24.927	862.825	-24.092
886.950	-20.005	909.634	-24.927	863.028	-24.093
887.073	-20.006	909.750	-24.928	863.204	-24.093
887.253	-20.007	909.943	-24.929	863.406	-24.093
887.449	-20.008	910.089	-24.929	863.618	-24.093
887.551	-20.008	910.160	-24.930	863.729	-24.093
887.633	-20.009	910.287	-24.931	863.800	-24.093
887.766	-20.010	910.454	-24.931	863.956	-24.094
887.984	-20.011	910.577	-24.932	864.121	-24.094
888.159	-20.011	910.732	-24.933	864.265	-24.094
888.307	-20.012	910.936	-24.933	864.454	-24.094
888.465	-20.013	911.092	-24.934	864.634	-24.094
888.586	-20.014	911.290	-24.935	864.745	-24.095
888.821	-20.015	911.574	-24.935	864.911	-24.095
889.074	-20.015	911.767	-24.936	865.038	-24.095
889.232	-20.016	911.950	-24.936	865.151	-24.095
889.342	-20.017	912.125	-24.937	865.337	-24.096
889.457	-20.018	912.253	-24.937	865.514	-24.096
889.602	-20.018	912.447	-24.938	865.696	-24.096
889.770	-20.019	912.606	-24.939	865.885	-24.097
889.970	-20.020	912.720	-24.939	866.013	-24.097
890.122	-20.021	912.883	-24.940	866.180	-24.097

890.286	-20.022	913.047	-24.940	866.423	-24.098
890.398	-20.022	913.185	-24.941	866.621	-24.098
890.562	-20.023	913.276	-24.941	866.754	-24.098
890.817	-20.024	913.369	-24.942	866.898	-24.099
890.975	-20.025	913.510	-24.942	867.038	-24.099
891.137	-20.025	913.709	-24.943	867.208	-24.099
891.310	-20.026	913.958	-24.943	867.356	-24.100
891.528	-20.027	914.176	-24.944	867.487	-24.100
891.677	-20.028	914.360	-24.944	867.646	-24.101
891.789	-20.028	914.539	-24.945	867.768	-24.101
891.954	-20.029	914.730	-24.945	867.940	-24.101
892.129	-20.030	914.863	-24.946	868.104	-24.102
892.321	-20.031	914.962	-24.946	868.221	-24.102
892.478	-20.031	915.119	-24.947	868.391	-24.103
892.638	-20.032	915.273	-24.947	868.601	-24.103
892.806	-20.033	915.435	-24.948	868.774	-24.104
892.980	-20.034	915.605	-24.948	868.944	-24.104
893.148	-20.034	915.791	-24.948	869.097	-24.105
893.232	-20.035	915.984	-24.949	869.230	-24.106
893.326	-20.036	916.111	-24.949	869.406	-24.106
893.537	-20.037	916.301	-24.950	869.586	-24.107
893.729	-20.037	916.499	-24.950	869.727	-24.107
893.812	-20.038	916.646	-24.950	869.883	-24.108
893.924	-20.039	916.796	-24.951	870.048	-24.109
894.124	-20.040	916.917	-24.951	870.213	-24.109
894.257	-20.040	917.116	-24.952	870.341	-24.110
894.326	-20.041	917.300	-24.952	870.462	-24.110
894.455	-20.042	917.411	-24.952	870.701	-24.111
894.679	-20.042	917.568	-24.953	870.975	-24.112
894.937	-20.043	917.778	-24.953	871.174	-24.113

895.173	-20.044	917.962	-24.953	871.305	-24.113
895.330	-20.045	918.107	-24.954	871.402	-24.114
895.519	-20.045	918.288	-24.954	871.521	-24.115
895.743	-20.046	918.431	-24.954	871.739	-24.116
895.865	-20.047	918.612	-24.955	871.945	-24.116
896.050	-20.048	918.773	-24.955	872.086	-24.117
896.235	-20.048	918.875	-24.955	872.233	-24.118
896.337	-20.049	919.092	-24.956	872.355	-24.119
896.460	-20.050	919.324	-24.956	872.475	-24.120
896.618	-20.050	919.486	-24.956	872.645	-24.120
896.724	-20.051	919.594	-24.957	872.825	-24.121
896.873	-20.052	919.660	-24.957	872.971	-24.122
897.068	-20.053	919.904	-24.957	873.066	-24.123
897.245	-20.053	920.411	-24.957	873.170	-24.124
897.408	-20.054	920.742	-24.958	873.450	-24.125
897.503	-20.055	920.857	-24.958	873.714	-24.126
897.645	-20.055	920.945	-24.958	873.885	-24.127
897.831	-20.056	920.975	-24.958	874.075	-24.128
897.999	-20.057	921.027	-24.959	874.130	-24.129
898.267	-20.058	921.095	-24.959	874.191	-24.130
898.492	-20.058	921.162	-24.959	874.332	-24.131
898.641	-20.059	921.312	-24.960	874.602	-24.131
898.809	-20.060	921.503	-24.960	874.824	-24.132
898.967	-20.060	921.658	-24.960	874.851	-24.133
899.094	-20.061	921.837	-24.960	874.954	-24.134
899.258	-20.062	922.044	-24.961	875.137	-24.135
899.389	-20.062	922.260	-24.961	875.296	-24.137
899.548	-20.063	922.395	-24.961	875.411	-24.138
899.709	-20.064	922.479	-24.961	875.585	-24.139
899.858	-20.065	922.618	-24.961	875.835	-24.140

900.063	-20.065	922.785	-24.962	876.020	-24.141
900.268	-20.066	922.950	-24.962	876.143	-24.142
900.422	-20.067	923.111	-24.962	876.252	-24.143
900.548	-20.067	923.310	-24.962	876.423	-24.144
900.674	-20.068	923.508	-24.963	876.729	-24.145
900.868	-20.069	923.663	-24.963	876.897	-24.146
901.128	-20.069	923.820	-24.963	877.016	-24.147
901.320	-20.070	923.952	-24.963	877.231	-24.148
901.413	-20.071	924.077	-24.964	877.436	-24.149
901.534	-20.072	924.210	-24.964	877.513	-24.150
901.687	-20.072	924.350	-24.964	877.692	-24.151
901.829	-20.073	924.548	-24.964	877.937	-24.153
902.013	-20.074	924.734	-24.965	878.065	-24.154
902.212	-20.074	924.890	-24.965	878.179	-24.155
902.395	-20.075	925.081	-24.965	878.335	-24.156
902.579	-20.076	925.271	-24.965	878.477	-24.157
902.755	-20.076	925.426	-24.966	878.664	-24.158
902.911	-20.077	925.585	-24.966	878.794	-24.159
903.046	-20.078	925.806	-24.966	878.848	-24.160
903.172	-20.078	925.931	-24.967	878.966	-24.162
903.307	-20.079	926.020	-24.967	879.106	-24.163
903.459	-20.080	926.180	-24.967	879.307	-24.164
903.665	-20.080	926.284	-24.967	879.480	-24.165
904.072	-20.081	926.489	-24.968	879.635	-24.166
904.510	-20.082	926.693	-24.968	879.846	-24.167
904.666	-20.083	926.825	-24.968	880.077	-24.169
904.662	-20.083	927.004	-24.969	880.301	-24.170
904.708	-20.084	927.185	-24.969	880.403	-24.171
904.811	-20.085	927.364	-24.969	880.551	-24.172
904.865	-20.085	927.496	-24.969	880.739	-24.173

904.966	-20.086	927.663	-24.970	880.910	-24.174
905.146	-20.087	927.874	-24.970	881.102	-24.176
905.256	-20.087	927.987	-24.970	881.269	-24.177
905.372	-20.088	928.093	-24.971	881.470	-24.178
905.542	-20.089	928.231	-24.971	881.643	-24.179
905.679	-20.089	928.370	-24.971	881.714	-24.180
905.891	-20.090	928.580	-24.972	881.832	-24.182
906.124	-20.091	928.790	-24.972	881.988	-24.183
906.272	-20.091	928.957	-24.973	882.193	-24.184
906.452	-20.092	929.130	-24.973	882.419	-24.185
906.600	-20.093	929.305	-24.973	882.541	-24.186
906.752	-20.093	929.493	-24.974	882.680	-24.188
906.948	-20.094	929.672	-24.974	882.818	-24.189
907.138	-20.095	929.809	-24.974	882.954	-24.190
907.298	-20.095	929.932	-24.975	883.182	-24.191
907.439	-20.096	930.078	-24.975	883.360	-24.192
907.655	-20.097	930.222	-24.976	883.513	-24.194
907.805	-20.097	930.391	-24.976	883.670	-24.195
907.887	-20.098	930.576	-24.976	883.795	-24.196
908.016	-20.099	930.698	-24.977	883.980	-24.197
908.188	-20.099	930.851	-24.977	884.167	-24.199
908.342	-20.100	931.078	-24.978	884.330	-24.200
908.549	-20.100	931.291	-24.978	884.508	-24.201
908.839	-20.101	931.421	-24.978	884.721	-24.202
909.040	-20.102	931.527	-24.979	884.951	-24.203
909.165	-20.102	931.694	-24.979	885.095	-24.205
909.303	-20.103	931.850	-24.980	885.215	-24.206
909.473	-20.104	931.962	-24.980	885.433	-24.207
909.598	-20.104	932.044	-24.980	885.620	-24.208
909.714	-20.105	932.177	-24.981	885.749	-24.210

909.865	-20.106	932.386	-24.981	885.881	-24.211
909.969	-20.106	932.560	-24.982	886.064	-24.212
910.099	-20.107	932.730	-24.982	886.232	-24.213
910.276	-20.107	932.893	-24.982	886.395	-24.215
910.496	-20.108	933.074	-24.983	886.513	-24.216
910.694	-20.109	933.286	-24.983	886.606	-24.217
910.822	-20.109	933.492	-24.984	886.777	-24.219
910.948	-20.110	933.674	-24.984	886.986	-24.220
911.112	-20.110	933.788	-24.985	887.290	-24.221
911.289	-20.111	933.921	-24.985	887.655	-24.222
911.433	-20.112	934.167	-24.985	887.926	-24.224
911.593	-20.112	934.377	-24.986	888.071	-24.225
911.721	-20.113	934.484	-24.986	888.147	-24.226
911.862	-20.113	934.624	-24.987	888.251	-24.227
912.053	-20.114	934.777	-24.987	888.360	-24.229
912.261	-20.115	934.881	-24.988	888.393	-24.230
912.447	-20.115	935.013	-24.988	888.456	-24.231
912.617	-20.116	935.210	-24.989	888.550	-24.232
912.822	-20.116	935.376	-24.989	888.658	-24.234
912.987	-20.117	935.478	-24.989	888.753	-24.235
913.120	-20.118	935.648	-24.990	888.913	-24.236
913.228	-20.118	935.820	-24.990	889.097	-24.238
913.385	-20.119	935.982	-24.991	889.280	-24.239
913.599	-20.119	936.227	-24.991	889.417	-24.240
913.747	-20.120	936.487	-24.992	889.622	-24.241
913.894	-20.120	936.620	-24.992	889.849	-24.243
914.072	-20.121	936.740	-24.993	890.020	-24.244
914.231	-20.122	936.892	-24.993	890.178	-24.245
914.369	-20.122	937.057	-24.993	890.306	-24.247
914.545	-20.123	937.253	-24.994	890.424	-24.248

914.734	-20.123	937.431	-24.994	890.608	-24.249
914.884	-20.124	937.577	-24.995	890.789	-24.251
915.029	-20.124	937.711	-24.995	890.993	-24.252
915.243	-20.125	937.891	-24.996	891.186	-24.253
915.434	-20.125	938.044	-24.996	891.297	-24.255
915.617	-20.126	938.189	-24.997	891.431	-24.256
915.786	-20.127	938.343	-24.997	891.584	-24.257
915.868	-20.127	938.519	-24.997	891.719	-24.259
915.953	-20.128	938.721	-24.998	891.917	-24.260
916.167	-20.128	938.898	-24.998	892.101	-24.261
916.274	-20.129	939.081	-24.999	892.258	-24.263
916.397	-20.129	939.177	-24.999	892.446	-24.264
916.547	-20.130	939.321	-25.000	892.601	-24.265
916.649	-20.130	939.529	-25.000	892.732	-24.267
916.748	-20.131	939.667	-25.000	892.907	-24.268
916.908	-20.131	939.837	-25.001	893.085	-24.270
917.143	-20.132	940.068	-25.001	893.213	-24.271
917.348	-20.132	940.275	-25.002	893.381	-24.273
917.541	-20.133	940.424	-25.002	893.624	-24.274
917.776	-20.133	940.508	-25.003	893.865	-24.275
918.025	-20.134	940.660	-25.003	894.028	-24.277
918.189	-20.134	940.888	-25.004	894.153	-24.278
918.326	-20.135	941.049	-25.004	894.296	-24.280
918.443	-20.135	941.188	-25.004	894.453	-24.281
918.578	-20.136	941.358	-25.005	894.613	-24.283
918.762	-20.136	941.484	-25.005	894.705	-24.284
918.923	-20.137	941.559	-25.006	894.774	-24.286
919.085	-20.137	941.795	-25.006	894.991	-24.287
919.187	-20.138	942.176	-25.007	895.163	-24.289
919.342	-20.138	942.553	-25.007	895.257	-24.290

919.576	-20.139	942.756	-25.007	895.462	-24.292
919.656	-20.139	942.775	-25.008	895.689	-24.293
919.684	-20.140	942.829	-25.008	895.879	-24.295
919.866	-20.140	942.868	-25.009	895.983	-24.296
920.107	-20.141	942.956	-25.009	896.088	-24.298
920.312	-20.141	943.085	-25.010	896.274	-24.299
920.526	-20.142	943.183	-25.010	896.480	-24.301
920.702	-20.142	943.336	-25.010	896.690	-24.302
920.786	-20.143	943.510	-25.011	896.785	-24.304
920.980	-20.143	943.711	-25.011	896.797	-24.305
921.215	-20.144	943.929	-25.012	896.955	-24.307
921.370	-20.144	944.110	-25.012	897.205	-24.308
921.560	-20.145	944.239	-25.013	897.440	-24.310
921.740	-20.145	944.350	-25.013	897.578	-24.312
921.853	-20.146	944.501	-25.013	897.722	-24.313
922.021	-20.146	944.728	-25.014	897.933	-24.315
922.243	-20.147	944.899	-25.014	898.062	-24.316
922.458	-20.147	945.003	-25.015	898.262	-24.318
922.608	-20.148	945.129	-25.015	898.521	-24.320
922.709	-20.148	945.268	-25.016	898.678	-24.321
922.857	-20.149	945.446	-25.016	898.794	-24.323
923.020	-20.149	945.576	-25.017	898.953	-24.324
923.202	-20.150	945.759	-25.017	899.175	-24.326
923.380	-20.150	945.941	-25.017	899.318	-24.328
923.553	-20.150	946.018	-25.018	899.445	-24.329
923.758	-20.151	946.165	-25.018	899.635	-24.331
923.944	-20.151	946.382	-25.019	899.753	-24.332
924.118	-20.152	946.496	-25.019	899.877	-24.334
924.247	-20.152	946.652	-25.020	900.051	-24.336
924.301	-20.153	946.917	-25.020	900.254	-24.337

924.437	-20.153	947.109	-25.021	900.458	-24.339
924.644	-20.154	947.261	-25.021	900.610	-24.340
924.835	-20.154	947.443	-25.022	900.775	-24.342
924.982	-20.155	947.639	-25.022	900.918	-24.343
925.080	-20.155	947.768	-25.023	901.078	-24.345
925.251	-20.156	947.833	-25.023	901.317	-24.347
925.443	-20.156	947.950	-25.024	901.458	-24.348
925.595	-20.157	948.183	-25.024	901.533	-24.350
925.722	-20.157	948.417	-25.025	901.637	-24.351
925.850	-20.157	948.528	-25.025	901.812	-24.353
926.226	-20.158	948.663	-25.026	901.975	-24.354
926.714	-20.158	948.820	-25.026	902.125	-24.356
927.003	-20.159	948.968	-25.027	902.293	-24.357
927.075	-20.159	949.128	-25.028	902.415	-24.359
927.094	-20.160	949.373	-25.028	902.555	-24.360
927.097	-20.160	949.567	-25.029	902.753	-24.362
927.118	-20.161	949.663	-25.029	902.891	-24.363
927.219	-20.161	949.824	-25.030	903.016	-24.365
927.393	-20.161	949.969	-25.030	903.207	-24.366
927.544	-20.162	950.087	-25.031	903.410	-24.368
927.694	-20.162	950.254	-25.032	903.600	-24.369
927.898	-20.163	950.428	-25.032	903.780	-24.371
928.055	-20.163	950.612	-25.033	903.941	-24.372
928.204	-20.164	950.852	-25.034	904.098	-24.374
928.392	-20.164	951.032	-25.034	904.286	-24.375
928.540	-20.165	951.228	-25.035	904.488	-24.376
928.734	-20.165	951.419	-25.035	904.660	-24.378
928.932	-20.165	951.532	-25.036	904.826	-24.379
929.054	-20.166	951.684	-25.037	905.020	-24.381
929.236	-20.166	951.861	-25.037	905.180	-24.382

929.425	-20.167	952.035	-25.038	905.294	-24.383
929.569	-20.167	952.190	-25.039	905.396	-24.385
929.725	-20.168	952.334	-25.039	905.490	-24.386
929.842	-20.168	952.457	-25.040	905.667	-24.387
929.988	-20.168	952.617	-25.041	905.896	-24.389
930.190	-20.169	952.785	-25.042	906.038	-24.390
930.329	-20.169	952.956	-25.042	906.162	-24.391
930.436	-20.170	953.131	-25.043	906.281	-24.393
930.614	-20.170	953.295	-25.044	906.412	-24.394
930.826	-20.171	953.438	-25.044	906.611	-24.395
930.954	-20.171	953.570	-25.045	906.816	-24.396
931.068	-20.171	953.738	-25.046	907.001	-24.398
931.279	-20.172	953.859	-25.047	907.215	-24.399
931.509	-20.172	953.988	-25.047	907.439	-24.400
931.652	-20.173	954.185	-25.048	907.573	-24.401
931.820	-20.173	954.317	-25.049	907.766	-24.402
931.989	-20.173	954.365	-25.049	907.999	-24.403
932.113	-20.174	954.489	-25.050	908.178	-24.404
932.274	-20.174	954.669	-25.051	908.326	-24.406
932.449	-20.175	954.880	-25.052	908.455	-24.407
932.582	-20.175	955.151	-25.052	908.577	-24.408
932.725	-20.175	955.379	-25.053	908.760	-24.409
932.897	-20.176	955.611	-25.054	908.996	-24.410
933.019	-20.176	955.779	-25.055	909.128	-24.411
933.157	-20.176	955.904	-25.055	909.249	-24.412
933.341	-20.177	956.078	-25.056	909.388	-24.413
933.492	-20.177	956.207	-25.057	909.527	-24.414
933.623	-20.178	956.294	-25.058	909.704	-24.415
933.796	-20.178	956.441	-25.058	909.889	-24.416
934.012	-20.178	956.541	-25.059	910.082	-24.417

934.164	-20.179	956.697	-25.060	910.230	-24.417
934.301	-20.179	956.934	-25.061	910.394	-24.418
934.444	-20.179	957.051	-25.061	910.637	-24.419
934.570	-20.180	957.138	-25.062	910.813	-24.420
934.784	-20.180	957.291	-25.063	910.918	-24.421
934.952	-20.180	957.549	-25.064	911.024	-24.422
935.061	-20.181	957.763	-25.064	911.205	-24.423
935.237	-20.181	957.946	-25.065	911.376	-24.423
935.474	-20.181	958.146	-25.066	911.495	-24.424
935.695	-20.182	958.313	-25.066	911.692	-24.425
935.891	-20.182	958.438	-25.067	911.880	-24.426
936.080	-20.182	958.578	-25.068	912.013	-24.427
936.209	-20.183	958.776	-25.069	912.190	-24.427
936.358	-20.183	958.960	-25.069	912.336	-24.428
936.561	-20.183	959.096	-25.070	912.438	-24.429
936.711	-20.184	959.189	-25.071	912.604	-24.430
936.832	-20.184	959.341	-25.072	912.824	-24.430
937.053	-20.184	959.538	-25.072	913.044	-24.431
937.226	-20.185	959.688	-25.073	913.186	-24.432
937.354	-20.185	959.852	-25.074	913.257	-24.432
937.512	-20.185	960.042	-25.074	913.439	-24.433
937.670	-20.186	960.239	-25.075	913.719	-24.434
937.818	-20.186	960.407	-25.076	913.883	-24.434
937.952	-20.186	960.547	-25.077	913.965	-24.435
938.107	-20.187	960.745	-25.077	914.090	-24.435
938.245	-20.187	960.965	-25.078	914.426	-24.436
938.376	-20.187	961.143	-25.079	914.861	-24.437
938.491	-20.187	961.267	-25.079	915.175	-24.437
938.644	-20.188	961.339	-25.080	915.315	-24.438
938.842	-20.188	961.429	-25.081	915.387	-24.438

938.970	-20.188	961.605	-25.082	915.490	-24.439
939.025	-20.189	961.854	-25.082	915.543	-24.439
939.183	-20.189	961.991	-25.083	915.559	-24.440
939.406	-20.189	962.105	-25.084	915.639	-24.440
939.577	-20.189	962.336	-25.084	915.789	-24.441
939.752	-20.190	962.493	-25.085	915.916	-24.441
939.982	-20.190	962.641	-25.086	916.012	-24.442
940.247	-20.190	962.834	-25.087	916.125	-24.442
940.408	-20.191	962.977	-25.087	916.277	-24.443
940.528	-20.191	963.161	-25.088	916.518	-24.443
940.714	-20.191	963.335	-25.089	916.727	-24.444
940.913	-20.191	963.438	-25.089	916.873	-24.444
941.078	-20.192	963.736	-25.090	917.040	-24.445
941.191	-20.192	964.167	-25.091	917.256	-24.445
941.288	-20.192	964.508	-25.091	917.464	-24.446
941.459	-20.192	964.576	-25.092	917.616	-24.446
941.644	-20.193	964.513	-25.093	917.795	-24.447
941.755	-20.193	964.605	-25.093	917.947	-24.447
941.908	-20.193	964.669	-25.094	918.059	-24.448
942.072	-20.194	964.750	-25.095	918.229	-24.448
942.221	-20.194	964.921	-25.095	918.420	-24.449
942.413	-20.194	965.074	-25.096	918.558	-24.449
942.620	-20.194	965.194	-25.097	918.661	-24.450
942.782	-20.195	965.359	-25.098	918.792	-24.450
942.962	-20.195	965.549	-25.098	918.952	-24.451
943.176	-20.195	965.673	-25.099	919.093	-24.451
943.334	-20.195	965.817	-25.100	919.264	-24.452
943.513	-20.196	966.028	-25.100	919.456	-24.452
943.643	-20.196	966.245	-25.101	919.634	-24.453
943.777	-20.196	966.489	-25.102	919.781	-24.453

943.991	-20.196	966.664	-25.102	919.913	-24.454
944.167	-20.197	966.789	-25.103	920.107	-24.454
944.341	-20.197	966.946	-25.104	920.287	-24.455
944.480	-20.197	967.007	-25.104	920.477	-24.456
944.650	-20.197	967.125	-25.105	920.638	-24.456
944.827	-20.198	967.346	-25.106	920.790	-24.457
944.975	-20.198	967.524	-25.106	920.952	-24.457
945.116	-20.198	967.667	-25.107	921.165	-24.458
945.239	-20.198	967.815	-25.108	921.383	-24.458
945.368	-20.199	967.919	-25.108	921.505	-24.459
945.564	-20.199	968.062	-25.109	921.677	-24.460
945.803	-20.199	968.262	-25.110	921.870	-24.460
945.988	-20.199	968.401	-25.110	921.936	-24.461
946.115	-20.200	968.616	-25.111	922.034	-24.462
946.251	-20.200	968.859	-25.112	922.191	-24.462
946.436	-20.200	969.037	-25.112	922.355	-24.463
946.612	-20.200	969.172	-25.113	922.558	-24.464
946.774	-20.201	969.320	-25.114	922.692	-24.464
946.926	-20.201	969.483	-25.114	922.796	-24.465
947.122	-20.201	969.615	-25.115	923.074	-24.466
947.326	-20.202	969.755	-25.116	923.256	-24.466
947.458	-20.202	969.889	-25.117	923.372	-24.467
947.588	-20.202	970.051	-25.117	923.543	-24.468
947.738	-20.202	970.167	-25.118	923.701	-24.468
947.939	-20.203	970.285	-25.119	923.864	-24.469
948.079	-20.203	970.463	-25.119	924.036	-24.470
948.150	-20.203	970.612	-25.120	924.158	-24.471
948.367	-20.203	970.787	-25.121	924.246	-24.472
948.762	-20.204	971.031	-25.122	924.408	-24.472
949.046	-20.204	971.210	-25.122	924.631	-24.473

949.221	-20.204	971.335	-25.123	924.820	-24.474
949.326	-20.204	971.478	-25.124	924.939	-24.475
949.387	-20.205	971.626	-25.125	925.138	-24.476
949.499	-20.205	971.779	-25.125	925.320	-24.476
949.546	-20.205	971.923	-25.126	925.493	-24.477
949.592	-20.205	972.085	-25.127	925.679	-24.478
949.713	-20.206	972.226	-25.128	925.831	-24.479
949.903	-20.206	972.411	-25.129	926.001	-24.480
950.061	-20.206	972.626	-25.129	926.179	-24.481
950.181	-20.207	972.773	-25.130	926.328	-24.482
950.357	-20.207	972.956	-25.131	926.423	-24.483
950.586	-20.207	973.127	-25.132	926.578	-24.484
950.753	-20.207	973.285	-25.133	926.795	-24.484
950.883	-20.208	973.438	-25.134	926.954	-24.485
951.041	-20.208	973.599	-25.134	927.053	-24.486
951.197	-20.208	973.802	-25.135	927.190	-24.487
951.423	-20.209	973.941	-25.136	927.419	-24.488
951.581	-20.209	974.084	-25.137	927.635	-24.489
951.649	-20.209	974.275	-25.138	927.809	-24.490
951.832	-20.209	974.482	-25.139	927.950	-24.491
952.020	-20.210	974.672	-25.140	928.055	-24.492
952.188	-20.210	974.846	-25.140	928.207	-24.493
952.358	-20.210	974.984	-25.141	928.350	-24.494
952.503	-20.211	975.081	-25.142	928.524	-24.495
952.616	-20.211	975.216	-25.143	928.746	-24.496
952.777	-20.211	975.360	-25.144	928.883	-24.497
952.996	-20.211	975.465	-25.145	928.970	-24.498
953.155	-20.212	975.627	-25.146	929.168	-24.499
953.352	-20.212	975.814	-25.147	929.364	-24.500
953.539	-20.212	975.908	-25.148	929.457	-24.501

953.671	-20.213	976.034	-25.149	929.547	-24.502
953.868	-20.213	976.178	-25.150	929.680	-24.503
954.044	-20.213	976.330	-25.150	929.862	-24.504
954.158	-20.214	976.522	-25.151	930.055	-24.504
954.341	-20.214	976.714	-25.152	930.211	-24.505
954.540	-20.214	976.913	-25.153	930.439	-24.506
954.660	-20.214	977.131	-25.154	930.643	-24.507
954.756	-20.215	977.333	-25.155	930.837	-24.508
954.884	-20.215	977.510	-25.156	931.077	-24.509
955.052	-20.215	977.722	-25.157	931.227	-24.510
955.229	-20.216	977.923	-25.158	931.356	-24.511
955.359	-20.216	978.059	-25.159	931.550	-24.512
955.480	-20.216	978.178	-25.160	931.789	-24.513
955.659	-20.217	978.307	-25.161	931.983	-24.514
955.845	-20.217	978.439	-25.162	932.094	-24.515
956.018	-20.217	978.572	-25.163	932.149	-24.516
956.208	-20.218	978.718	-25.164	932.289	-24.517
956.391	-20.218	978.864	-25.165	932.494	-24.518
956.534	-20.218	979.036	-25.166	932.654	-24.519
956.647	-20.219	979.168	-25.167	932.747	-24.520
956.777	-20.219	979.322	-25.168	932.860	-24.521
956.939	-20.219	979.574	-25.169	933.020	-24.522
957.120	-20.220	979.800	-25.170	933.167	-24.523
957.319	-20.220	979.969	-25.171	933.351	-24.523
957.485	-20.220	980.106	-25.172	933.527	-24.524
957.718	-20.221	980.292	-25.173	933.672	-24.525
957.946	-20.221	980.453	-25.174	933.838	-24.526
958.074	-20.221	980.614	-25.175	934.017	-24.527
958.235	-20.222	980.819	-25.176	934.184	-24.528
958.374	-20.222	981.004	-25.177	934.354	-24.529

958.505	-20.222	981.152	-25.178	934.565	-24.529
958.653	-20.223	981.235	-25.179	934.727	-24.530
958.780	-20.223	981.385	-25.180	934.867	-24.531
958.989	-20.223	981.572	-25.181	935.057	-24.532
959.182	-20.224	981.712	-25.182	935.230	-24.533
959.337	-20.224	981.886	-25.183	935.385	-24.533
959.486	-20.225	982.074	-25.184	935.539	-24.534
959.619	-20.225	982.261	-25.185	935.757	-24.535
959.771	-20.225	982.450	-25.186	935.987	-24.536
959.941	-20.226	982.641	-25.187	936.046	-24.536
960.118	-20.226	982.821	-25.188	936.176	-24.537
960.294	-20.226	982.942	-25.189	936.404	-24.538
960.441	-20.227	983.070	-25.189	936.575	-24.539
960.545	-20.227	983.214	-25.190	936.771	-24.539
960.649	-20.228	983.360	-25.191	936.969	-24.540
960.807	-20.228	983.579	-25.192	937.169	-24.541
961.032	-20.228	983.761	-25.193	937.309	-24.541
961.105	-20.229	983.903	-25.194	937.390	-24.542
961.196	-20.229	984.090	-25.195	937.496	-24.543
961.357	-20.230	984.224	-25.196	937.706	-24.543
961.544	-20.230	984.355	-25.197	937.933	-24.544
961.758	-20.231	984.522	-25.198	938.038	-24.545
961.976	-20.231	984.722	-25.199	938.208	-24.545
962.187	-20.231	984.918	-25.200	938.425	-24.546
962.391	-20.232	985.001	-25.201	938.577	-24.546
962.546	-20.232	985.097	-25.202	938.756	-24.547
962.701	-20.233	985.239	-25.203	938.944	-24.548
962.925	-20.233	985.523	-25.204	939.111	-24.548
963.083	-20.234	985.955	-25.205	939.249	-24.549
963.167	-20.234	986.257	-25.206	939.362	-24.549

963.279	-20.234	986.428	-25.207	939.495	-24.550
963.428	-20.235	986.554	-25.208	939.642	-24.550
963.549	-20.235	986.615	-25.209	939.802	-24.551
963.745	-20.236	986.612	-25.210	939.993	-24.552
963.941	-20.236	986.682	-25.211	940.189	-24.552
964.072	-20.237	986.764	-25.212	940.339	-24.553
964.249	-20.237	986.857	-25.213	940.538	-24.553
964.392	-20.238	987.036	-25.214	940.721	-24.554
964.557	-20.238	987.199	-25.216	940.843	-24.554
964.741	-20.239	987.389	-25.217	940.929	-24.555
964.931	-20.239	987.580	-25.218	941.012	-24.555
965.101	-20.240	987.697	-25.219	941.212	-24.556
965.250	-20.240	987.871	-25.220	941.681	-24.556
965.464	-20.241	988.079	-25.221	942.129	-24.557
965.663	-20.241	988.230	-25.222	942.305	-24.557
965.790	-20.242	988.400	-25.223	942.391	-24.557
965.940	-20.242	988.572	-25.224	942.504	-24.558
966.123	-20.243	988.692	-25.225	942.636	-24.558
966.291	-20.243	988.839	-25.227	942.651	-24.559
966.441	-20.244	989.035	-25.228	942.623	-24.559
966.578	-20.245	989.237	-25.229	942.693	-24.560
966.709	-20.245	989.374	-25.230	942.809	-24.560
966.860	-20.246	989.479	-25.231	942.930	-24.561
967.029	-20.246	989.610	-25.233	943.101	-24.561
967.225	-20.247	989.805	-25.234	943.257	-24.562
967.402	-20.247	990.048	-25.235	943.425	-24.562
967.533	-20.248	990.232	-25.236	943.633	-24.563
967.739	-20.249	990.333	-25.238	943.859	-24.563
967.932	-20.249	990.539	-25.239	944.047	-24.564
968.070	-20.250	990.745	-25.240	944.204	-24.564

968.252	-20.250	990.863	-25.242	944.392	-24.564
968.419	-20.251	991.013	-25.243	944.517	-24.565
968.551	-20.252	991.158	-25.245	944.634	-24.565
968.738	-20.252	991.320	-25.246	944.855	-24.566
968.913	-20.253	991.460	-25.248	945.025	-24.566
969.082	-20.254	991.626	-25.249	945.117	-24.567
969.220	-20.254	991.781	-25.251	945.265	-24.567
969.380	-20.255	991.908	-25.252	945.462	-24.568
969.555	-20.255	992.058	-25.254	945.658	-24.569
969.727	-20.256	992.217	-25.255	945.825	-24.569
969.899	-20.257	992.405	-25.257	945.960	-24.570
970.039	-20.257	992.574	-25.258	946.133	-24.570
970.203	-20.258	992.766	-25.260	946.298	-24.571
970.294	-20.259	992.921	-25.262	946.449	-24.571
970.371	-20.259	993.040	-25.264	946.626	-24.572
970.678	-20.260	993.210	-25.265	946.787	-24.572
971.134	-20.261	993.372	-25.267	946.919	-24.573
971.490	-20.262	993.531	-25.269	947.012	-24.574
971.666	-20.262	993.699	-25.271	947.163	-24.574
971.694	-20.263	993.840	-25.273	947.382	-24.575
971.751	-20.264	993.968	-25.274	947.622	-24.576
971.778	-20.264	994.158	-25.276	947.795	-24.576
971.805	-20.265	994.362	-25.278	947.955	-24.577
971.890	-20.266	994.573	-25.280	948.168	-24.578
972.031	-20.267	994.746	-25.282	948.249	-24.578
972.215	-20.267	994.880	-25.284	948.392	-24.579
972.354	-20.268	995.043	-25.286	948.612	-24.580
972.479	-20.269	995.212	-25.288	948.692	-24.580
972.692	-20.269	995.371	-25.290	948.844	-24.581
972.896	-20.270	995.547	-25.292	948.987	-24.582

973.015	-20.271	995.699	-25.294	949.110	-24.583
973.155	-20.272	995.889	-25.296	949.283	-24.583
973.315	-20.273	996.069	-25.299	949.455	-24.584
973.436	-20.273	996.180	-25.301	949.638	-24.585
973.605	-20.274	996.348	-25.303	949.799	-24.586
973.737	-20.275	996.556	-25.305	949.946	-24.587
973.904	-20.276	996.769	-25.307	950.089	-24.588
974.153	-20.276	996.937	-25.309	950.314	-24.588
974.309	-20.277	997.053	-25.312	950.484	-24.589
974.466	-20.278	997.182	-25.314	950.613	-24.590
974.632	-20.279	997.353	-25.316	950.808	-24.591
974.753	-20.280	997.564	-25.318	950.967	-24.592
974.876	-20.280	997.635	-25.321	951.042	-24.593
975.016	-20.281	997.703	-25.323	951.163	-24.594
975.189	-20.282	997.810	-25.325	951.342	-24.595
975.452	-20.283	997.964	-25.328	951.572	-24.596
975.696	-20.284	998.065	-25.330	951.802	-24.597
975.862	-20.284	998.241	-25.332	951.962	-24.598
976.028	-20.285	998.460	-25.335	952.146	-24.599
976.133	-20.286	998.607	-25.337	952.266	-24.600
976.270	-20.287	998.754	-25.339	952.370	-24.601
976.470	-20.288	998.971	-25.342	952.566	-24.602
976.615	-20.289	999.193	-25.344	952.775	-24.603
976.803	-20.289	999.360	-25.347	952.946	-24.604
976.930	-20.290	999.597	-25.349	953.111	-24.605
977.051	-20.291	999.816	-25.352	953.299	-24.606
977.235	-20.292	999.931	-25.354	953.484	-24.607
977.338	-20.293	1.000.049	-25.356	953.633	-24.609
977.467	-20.294	1.000.164	-25.359	953.747	-24.610
977.663	-20.295	1.000.291	-25.361	953.840	-24.611

977.823	-20.295	1.000.438	-25.364	953.919	-24.612
978.010	-20.296	1.000.539	-25.366	954.154	-24.613
978.186	-20.297	1.000.639	-25.369	954.396	-24.614
978.324	-20.298	1.000.805	-25.371	954.492	-24.615
978.457	-20.299	1.001.048	-25.374	954.676	-24.617
978.586	-20.300	1.001.263	-25.376	954.889	-24.618
978.752	-20.301	1.001.415	-25.379	955.063	-24.619
978.917	-20.302	1.001.570	-25.381	955.214	-24.620
979.063	-20.302	1.001.734	-25.384	955.334	-24.621
979.223	-20.303	1.001.852	-25.386	955.425	-24.623
979.444	-20.304	1.002.078	-25.389	955.584	-24.624
979.651	-20.305	1.002.324	-25.391	955.770	-24.625
979.810	-20.306	1.002.478	-25.394	955.947	-24.626
979.987	-20.307	1.002.632	-25.396	956.123	-24.627
980.171	-20.308	1.002.770	-25.399	956.318	-24.629
980.305	-20.309	1.002.927	-25.401	956.464	-24.630
980.450	-20.310	1.003.082	-25.404	956.522	-24.631
980.631	-20.311	1.003.212	-25.406	956.636	-24.632
980.806	-20.311	1.003.367	-25.408	956.783	-24.634
980.958	-20.312	1.003.581	-25.411	956.966	-24.635
981.079	-20.313	1.003.749	-25.413	957.149	-24.636
981.234	-20.314	1.003.951	-25.416	957.290	-24.637
981.369	-20.315	1.004.159	-25.418	957.480	-24.639
981.500	-20.316	1.004.297	-25.420	957.654	-24.640
981.746	-20.317	1.004.439	-25.423	957.766	-24.641
981.993	-20.318	1.004.616	-25.425	957.995	-24.642
982.150	-20.319	1.004.762	-25.427	958.264	-24.644
982.270	-20.320	1.004.933	-25.429	958.456	-24.645
982.428	-20.321	1.005.113	-25.432	958.632	-24.646
982.609	-20.322	1.005.230	-25.434	958.773	-24.647

982.766	-20.323	1.005.404	-25.436	958.959	-24.649
982.906	-20.323	1.005.592	-25.438	959.171	-24.650
983.027	-20.324	1.005.772	-25.440	959.301	-24.651
983.110	-20.325	1.005.951	-25.442	959.388	-24.652
983.297	-20.326	1.006.114	-25.444	959.519	-24.654
983.495	-20.327	1.006.292	-25.446	959.677	-24.655
983.635	-20.328	1.006.405	-25.448	959.806	-24.656
983.768	-20.329	1.006.497	-25.450	959.949	-24.657
983.930	-20.330	1.006.687	-25.452	960.106	-24.659
984.137	-20.331	1.006.861	-25.454	960.264	-24.660
984.339	-20.332	1.006.971	-25.456	960.415	-24.661
984.551	-20.333	1.007.201	-25.458	960.595	-24.662
984.749	-20.334	1.007.643	-25.460	960.770	-24.663
984.902	-20.335	1.008.068	-25.461	960.917	-24.665
985.074	-20.336	1.008.176	-25.463	961.115	-24.666
985.290	-20.337	1.008.168	-25.465	961.339	-24.667
985.462	-20.338	1.008.255	-25.466	961.479	-24.668
985.590	-20.339	1.008.323	-25.468	961.631	-24.669
985.727	-20.340	1.008.390	-25.469	961.835	-24.670
985.838	-20.341	1.008.491	-25.471	962.010	-24.672
985.944	-20.342	1.008.589	-25.472	962.198	-24.673
986.080	-20.343	1.008.727	-25.473	962.348	-24.674
986.236	-20.344	1.008.936	-25.475	962.455	-24.675
986.363	-20.345	1.009.111	-25.476	962.633	-24.676
986.516	-20.346	1.009.234	-25.477	962.841	-24.677
986.748	-20.346	1.009.388	-25.478	963.014	-24.678
986.889	-20.347	1.009.512	-25.479	963.129	-24.679
986.997	-20.348	1.009.717	-25.481	963.269	-24.680
987.189	-20.349	1.009.966	-25.482	963.504	-24.681
987.411	-20.350	1.010.163	-25.483	963.693	-24.682

987.634	-20.351	1.010.315	-25.483	963.793	-24.684
987.838	-20.352	1.010.419	-25.484	963.917	-24.685
987.998	-20.353	1.010.566	-25.485	964.057	-24.686
988.150	-20.354	1.010.712	-25.486	964.251	-24.687
988.308	-20.355	1.010.908	-25.487	964.461	-24.688
988.436	-20.356	1.011.124	-25.488	964.592	-24.689
988.587	-20.357	1.011.244	-25.488	964.730	-24.690
988.695	-20.358	1.011.373	-25.489	964.898	-24.691
988.803	-20.359	1.011.544	-25.490	965.098	-24.692
989.003	-20.360	1.011.732	-25.490	965.247	-24.693
989.126	-20.361	1.011.905	-25.491	965.389	-24.694
989.324	-20.362	1.012.070	-25.491	965.567	-24.695
989.541	-20.364	1.012.299	-25.492	965.693	-24.696
989.744	-20.365	1.012.483	-25.492	965.900	-24.697
989.902	-20.366	1.012.634	-25.492	966.152	-24.697
990.050	-20.367	1.012.816	-25.493	966.275	-24.698
990.273	-20.368	1.012.971	-25.493	966.369	-24.699
990.454	-20.369	1.013.124	-25.494	966.522	-24.700
990.536	-20.370	1.013.253	-25.494	966.713	-24.701
990.659	-20.371	1.013.350	-25.494	966.899	-24.702
990.891	-20.372	1.013.475	-25.494	967.095	-24.703
991.105	-20.373	1.013.625	-25.495	967.272	-24.704
991.219	-20.374	1.013.764	-25.495	967.408	-24.705
991.330	-20.375	1.013.913	-25.495	967.551	-24.706
991.480	-20.376	1.014.088	-25.495	967.675	-24.707
991.684	-20.377	1.014.274	-25.495	967.846	-24.707
991.901	-20.378	1.014.441	-25.495	967.984	-24.708
992.078	-20.379	1.014.624	-25.495	968.057	-24.709
992.242	-20.380	1.014.837	-25.495	968.283	-24.710
992.365	-20.381	1.015.002	-25.495	968.704	-24.711

992.490	-20.382	1.015.101	-25.495	969.032	-24.712
992.607	-20.383	1.015.230	-25.495	969.259	-24.713
992.806	-20.384	1.015.428	-25.495	969.449	-24.713
993.203	-20.385	1.015.573	-25.495	969.492	-24.714
993.605	-20.386	1.015.685	-25.495	969.587	-24.715
993.789	-20.387	1.015.804	-25.495	969.676	-24.716
993.869	-20.388	1.016.020	-25.495	969.708	-24.717
993.938	-20.389	1.016.258	-25.495	969.796	-24.718
993.995	-20.390	1.016.406	-25.494	969.883	-24.718
994.016	-20.391	1.016.507	-25.494	970.008	-24.719
994.074	-20.392	1.016.668	-25.494	970.143	-24.720
994.249	-20.394	1.016.861	-25.494	970.305	-24.721
994.371	-20.395	1.017.000	-25.494	970.468	-24.722
994.492	-20.396	1.017.156	-25.493	970.591	-24.722
994.658	-20.397	1.017.329	-25.493	970.753	-24.723
994.794	-20.398	1.017.520	-25.493	970.968	-24.724
994.968	-20.399	1.017.711	-25.493	971.133	-24.725
995.199	-20.400	1.017.871	-25.492	971.245	-24.726
995.381	-20.401	1.018.002	-25.492	971.456	-24.726
995.555	-20.402	1.018.148	-25.492	971.665	-24.727
995.754	-20.403	1.018.353	-25.491	971.846	-24.728
995.904	-20.404	1.018.551	-25.491	972.027	-24.729
996.040	-20.405	1.018.669	-25.491	972.132	-24.730
996.159	-20.406	1.018.811	-25.490	972.309	-24.731
996.309	-20.407	1.019.063	-25.490	972.523	-24.731
996.508	-20.408	1.019.224	-25.489	972.629	-24.732
996.648	-20.409	1.019.302	-25.489	972.734	-24.733
996.813	-20.410	1.019.370	-25.489	972.851	-24.734
997.007	-20.411	1.019.441	-25.488	972.995	-24.735
997.122	-20.412	1.019.593	-25.488	973.187	-24.736

997.217	-20.414	1.019.849	-25.487	973.359	-24.736
997.386	-20.415	1.020.091	-25.487	973.507	-24.737
997.563	-20.416	1.020.254	-25.487	973.589	-24.738
997.729	-20.417	1.020.435	-25.486	973.743	-24.739
997.899	-20.418	1.020.664	-25.486	973.976	-24.740
998.092	-20.419	1.020.827	-25.485	974.172	-24.741
998.338	-20.420	1.020.971	-25.485	974.359	-24.742
998.520	-20.421	1.021.124	-25.484	974.535	-24.742
998.677	-20.422	1.021.284	-25.484	974.662	-24.743
998.867	-20.423	1.021.432	-25.484	974.808	-24.744
999.037	-20.424	1.021.557	-25.483	975.060	-24.745
999.177	-20.425	1.021.752	-25.483	975.266	-24.746
999.323	-20.426	1.021.883	-25.482	975.387	-24.747
999.405	-20.427	1.022.013	-25.482	975.564	-24.748
999.538	-20.428	1.022.132	-25.481	975.735	-24.749
999.697	-20.429	1.022.244	-25.481	975.863	-24.750
999.889	-20.430	1.022.408	-25.481	976.016	-24.751
1.000.086	-20.431	1.022.567	-25.480	976.143	-24.751
1.000.231	-20.432	1.022.746	-25.480	976.292	-24.752
1.000.419	-20.433	1.022.963	-25.480	976.482	-24.753
1.000.602	-20.434	1.023.183	-25.479	976.678	-24.754
1.000.701	-20.435	1.023.382	-25.479	976.886	-24.755
1.000.825	-20.436	1.023.581	-25.479	976.982	-24.756
1.001.023	-20.437	1.023.744	-25.478	977.084	-24.757
1.001.215	-20.439	1.023.877	-25.478	977.327	-24.758
1.001.385	-20.440	1.024.019	-25.478	977.507	-24.759
1.001.514	-20.441	1.024.165	-25.477	977.630	-24.760
1.001.646	-20.442	1.024.323	-25.477	977.801	-24.761
1.001.794	-20.443	1.024.432	-25.477	977.951	-24.762
1.001.986	-20.444	1.024.566	-25.476	978.067	-24.763

1.002.201	-20.445	1.024.801	-25.476	978.189	-24.764
1.002.374	-20.446	1.025.005	-25.476	978.369	-24.765
1.002.502	-20.447	1.025.191	-25.476	978.570	-24.766
1.002.676	-20.448	1.025.365	-25.476	978.745	-24.768
1.002.868	-20.449	1.025.552	-25.475	978.867	-24.769
1.003.036	-20.450	1.025.680	-25.475	979.003	-24.770
1.003.193	-20.451	1.025.826	-25.475	979.199	-24.771
1.003.303	-20.452	1.026.041	-25.475	979.407	-24.772
1.003.439	-20.453	1.026.221	-25.475	979.574	-24.773
1.003.615	-20.454	1.026.382	-25.475	979.716	-24.774
1.003.816	-20.455	1.026.579	-25.475	979.940	-24.775
1.004.057	-20.456	1.026.723	-25.475	980.138	-24.776
1.004.196	-20.457	1.026.794	-25.474	980.223	-24.777
1.004.342	-20.458	1.026.966	-25.474	980.349	-24.778
1.004.500	-20.459	1.027.185	-25.474	980.544	-24.780
1.004.607	-20.460	1.027.321	-25.474	980.717	-24.781
1.004.783	-20.461	1.027.430	-25.474	980.882	-24.782
1.004.956	-20.462	1.027.622	-25.474	981.059	-24.783
1.005.096	-20.463	1.027.856	-25.474	981.235	-24.784
1.005.201	-20.464	1.028.021	-25.474	981.359	-24.785
1.005.313	-20.465	1.028.120	-25.474	981.468	-24.786
1.005.491	-20.466	1.028.288	-25.474	981.620	-24.787
1.005.677	-20.467	1.028.455	-25.474	981.814	-24.789
1.005.803	-20.468	1.028.587	-25.475	981.944	-24.790
1.005.953	-20.469	1.028.697	-25.475	982.135	-24.791
1.006.118	-20.470	1.028.949	-25.475	982.381	-24.792
1.006.270	-20.471	1.029.358	-25.475	982.542	-24.793
1.006.496	-20.472	1.029.722	-25.475	982.700	-24.794
1.006.715	-20.473	1.029.885	-25.475	982.824	-24.795
1.006.911	-20.474	1.029.952	-25.475	982.938	-24.797

1.007.148	-20.475	1.030.005	-25.475	983.133	-24.798
1.007.326	-20.476	1.030.001	-25.475	983.254	-24.799
1.007.505	-20.477	1.030.058	-25.476	983.353	-24.800
1.007.667	-20.478	1.030.182	-25.476	983.493	-24.801
1.007.796	-20.479	1.030.225	-25.476	983.632	-24.802
1.007.925	-20.480	1.030.341	-25.476	983.764	-24.803
1.008.064	-20.481	1.030.564	-25.476	983.897	-24.804
1.008.174	-20.482	1.030.798	-25.476	984.096	-24.805
1.008.259	-20.483	1.030.993	-25.477	984.350	-24.806
1.008.400	-20.483	1.031.094	-25.477	984.484	-24.808
1.008.577	-20.484	1.031.205	-25.477	984.614	-24.809
1.008.721	-20.485	1.031.429	-25.477	984.827	-24.810
1.008.857	-20.486	1.031.652	-25.477	985.051	-24.811
1.009.007	-20.487	1.031.818	-25.478	985.266	-24.812
1.009.137	-20.488	1.031.982	-25.478	985.417	-24.813
1.009.357	-20.489	1.032.020	-25.478	985.559	-24.814
1.009.574	-20.490	1.032.197	-25.479	985.753	-24.815
1.009.803	-20.491	1.032.473	-25.479	985.931	-24.816
1.009.982	-20.492	1.032.655	-25.479	986.115	-24.817
1.010.104	-20.493	1.032.832	-25.479	986.274	-24.818
1.010.266	-20.494	1.032.898	-25.480	986.401	-24.819
1.010.459	-20.495	1.033.001	-25.480	986.544	-24.820
1.010.627	-20.496	1.033.178	-25.480	986.723	-24.821
1.010.787	-20.497	1.033.363	-25.481	986.873	-24.822
1.010.995	-20.498	1.033.568	-25.481	986.988	-24.823
1.011.158	-20.499	1.033.725	-25.481	987.067	-24.824
1.011.270	-20.500	1.033.895	-25.482	987.207	-24.825
1.011.429	-20.501	1.034.102	-25.482	987.462	-24.826
1.011.617	-20.502	1.034.281	-25.482	987.624	-24.827
1.011.736	-20.503	1.034.475	-25.483	987.760	-24.828

1.011.879	-20.504	1.034.660	-25.483	987.959	-24.828
1.012.132	-20.505	1.034.775	-25.483	988.167	-24.829
1.012.352	-20.506	1.034.897	-25.484	988.345	-24.830
1.012.480	-20.507	1.035.027	-25.484	988.520	-24.831
1.012.665	-20.508	1.035.230	-25.485	988.682	-24.832
1.012.824	-20.508	1.035.422	-25.485	988.861	-24.833
1.012.938	-20.509	1.035.518	-25.486	989.037	-24.834
1.013.087	-20.510	1.035.661	-25.486	989.190	-24.835
1.013.279	-20.511	1.035.829	-25.486	989.370	-24.835
1.013.463	-20.512	1.035.983	-25.487	989.604	-24.836
1.013.626	-20.513	1.036.142	-25.487	989.787	-24.837
1.013.809	-20.514	1.036.291	-25.488	989.866	-24.838
1.013.977	-20.515	1.036.438	-25.488	989.990	-24.839
1.014.156	-20.516	1.036.603	-25.489	990.137	-24.839
1.014.316	-20.517	1.036.779	-25.489	990.316	-24.840
1.014.445	-20.518	1.036.904	-25.490	990.525	-24.841
1.014.566	-20.519	1.037.028	-25.490	990.670	-24.842
1.014.721	-20.520	1.037.215	-25.491	990.819	-24.842
1.014.933	-20.521	1.037.394	-25.492	991.056	-24.843
1.015.072	-20.522	1.037.570	-25.492	991.295	-24.844
1.015.267	-20.523	1.037.779	-25.493	991.412	-24.844
1.015.658	-20.523	1.037.963	-25.493	991.482	-24.845
1.016.038	-20.524	1.038.102	-25.494	991.625	-24.846
1.016.227	-20.525	1.038.235	-25.494	991.781	-24.847
1.016.240	-20.526	1.038.388	-25.495	991.986	-24.847
1.016.291	-20.527	1.038.572	-25.496	992.197	-24.848
1.016.342	-20.528	1.038.730	-25.496	992.340	-24.849
1.016.378	-20.529	1.038.875	-25.497	992.473	-24.849
1.016.506	-20.530	1.039.028	-25.497	992.642	-24.850
1.016.638	-20.531	1.039.215	-25.498	992.785	-24.850

1.016.724	-20.532	1.039.381	-25.499	992.882	-24.851
1.016.898	-20.533	1.039.520	-25.499	993.059	-24.852
1.017.088	-20.534	1.039.667	-25.500	993.276	-24.852
1.017.281	-20.534	1.039.890	-25.501	993.466	-24.853
1.017.502	-20.535	1.040.130	-25.501	993.600	-24.854
1.017.667	-20.536	1.040.244	-25.502	993.772	-24.854
1.017.842	-20.537	1.040.376	-25.503	993.959	-24.855
1.018.033	-20.538	1.040.505	-25.503	994.082	-24.855
1.018.211	-20.539	1.040.626	-25.504	994.257	-24.856
1.018.332	-20.540	1.040.801	-25.505	994.425	-24.857
1.018.465	-20.541	1.040.950	-25.506	994.554	-24.857
1.018.637	-20.542	1.041.067	-25.506	994.705	-24.858
1.018.772	-20.542	1.041.217	-25.507	994.893	-24.858
1.018.911	-20.543	1.041.368	-25.508	995.051	-24.859
1.019.087	-20.544	1.041.478	-25.508	995.142	-24.859
1.019.229	-20.545	1.041.587	-25.509	995.340	-24.860
1.019.354	-20.546	1.041.814	-25.510	995.747	-24.860
1.019.531	-20.547	1.041.960	-25.511	996.195	-24.861
1.019.751	-20.548	1.042.144	-25.511	996.420	-24.862
1.019.865	-20.549	1.042.410	-25.512	996.494	-24.862
1.019.975	-20.549	1.042.679	-25.513	996.524	-24.863
1.020.105	-20.550	1.042.843	-25.514	996.622	-24.863
1.020.294	-20.551	1.042.935	-25.514	996.701	-24.864
1.020.518	-20.552	1.043.058	-25.515	996.724	-24.864
1.020.694	-20.553	1.043.232	-25.516	996.780	-24.865
1.020.932	-20.554	1.043.376	-25.517	996.961	-24.866
1.021.153	-20.554	1.043.513	-25.517	997.116	-24.866
1.021.273	-20.555	1.043.733	-25.518	997.234	-24.867
1.021.413	-20.556	1.043.886	-25.519	997.382	-24.867
1.021.544	-20.557	1.043.976	-25.520	997.518	-24.868

1.021.648	-20.558	1.044.062	-25.520	997.728	-24.868
1.021.835	-20.559	1.044.192	-25.521	997.947	-24.869
1.021.998	-20.559	1.044.410	-25.522	998.112	-24.870
1.022.135	-20.560	1.044.600	-25.523	998.262	-24.870
1.022.362	-20.561	1.044.790	-25.524	998.450	-24.871
1.022.586	-20.562	1.045.040	-25.524	998.671	-24.871
1.022.672	-20.563	1.045.232	-25.525	998.829	-24.872
1.022.783	-20.563	1.045.293	-25.526	998.990	-24.873
1.022.959	-20.564	1.045.443	-25.527	999.132	-24.873
1.023.115	-20.565	1.045.662	-25.528	999.262	-24.874
1.023.234	-20.566	1.045.780	-25.529	999.398	-24.875
1.023.357	-20.567	1.045.948	-25.529	999.544	-24.875
1.023.551	-20.567	1.046.147	-25.530	999.723	-24.876
1.023.715	-20.568	1.046.292	-25.531	999.865	-24.877
1.023.865	-20.569	1.046.426	-25.532	999.957	-24.877
1.024.081	-20.570	1.046.582	-25.533	1.000.115	-24.878
1.024.275	-20.571	1.046.795	-25.534	1.000.279	-24.879
1.024.395	-20.571	1.046.994	-25.534	1.000.368	-24.880
1.024.548	-20.572	1.047.077	-25.535	1.000.536	-24.880
1.024.765	-20.573	1.047.215	-25.536	1.000.706	-24.881
1.024.933	-20.574	1.047.461	-25.537	1.000.834	-24.882
1.025.053	-20.574	1.047.617	-25.538	1.001.021	-24.883
1.025.237	-20.575	1.047.805	-25.539	1.001.275	-24.884
1.025.398	-20.576	1.047.990	-25.540	1.001.450	-24.884
1.025.502	-20.577	1.048.115	-25.541	1.001.620	-24.885
1.025.659	-20.577	1.048.270	-25.541	1.001.821	-24.886
1.025.834	-20.578	1.048.475	-25.542	1.002.019	-24.887
1.025.958	-20.579	1.048.671	-25.543	1.002.196	-24.888
1.026.147	-20.580	1.048.827	-25.544	1.002.312	-24.889
1.026.432	-20.580	1.048.970	-25.545	1.002.479	-24.890

1.026.656	-20.581	1.049.113	-25.546	1.002.673	-24.891
1.026.787	-20.582	1.049.276	-25.547	1.002.862	-24.892
1.026.932	-20.583	1.049.433	-25.548	1.003.005	-24.893
1.027.040	-20.583	1.049.594	-25.549	1.003.062	-24.894
1.027.126	-20.584	1.049.749	-25.550	1.003.173	-24.895
1.027.274	-20.585	1.049.892	-25.551	1.003.365	-24.896
1.027.460	-20.585	1.050.032	-25.551	1.003.528	-24.897
1.027.641	-20.586	1.050.171	-25.552	1.003.712	-24.898
1.027.760	-20.587	1.050.357	-25.553	1.003.891	-24.899
1.027.893	-20.588	1.050.594	-25.554	1.003.997	-24.900
1.028.033	-20.588	1.050.988	-25.555	1.004.168	-24.901
1.028.217	-20.589	1.051.314	-25.556	1.004.327	-24.903
1.028.469	-20.590	1.051.407	-25.557	1.004.492	-24.904
1.028.561	-20.590	1.051.471	-25.558	1.004.682	-24.905
1.028.667	-20.591	1.051.535	-25.559	1.004.827	-24.906
1.028.981	-20.592	1.051.609	-25.560	1.005.020	-24.907
1.029.249	-20.593	1.051.667	-25.561	1.005.183	-24.909
1.029.410	-20.593	1.051.769	-25.562	1.005.256	-24.910
1.029.564	-20.594	1.051.902	-25.563	1.005.349	-24.911
1.029.695	-20.595	1.052.039	-25.564	1.005.549	-24.912
1.029.852	-20.595	1.052.185	-25.565	1.005.787	-24.914
1.030.024	-20.596	1.052.377	-25.566	1.005.967	-24.915
1.030.174	-20.597	1.052.571	-25.567	1.006.171	-24.916
1.030.277	-20.597	1.052.704	-25.568	1.006.352	-24.918
1.030.393	-20.598	1.052.842	-25.569	1.006.524	-24.919
1.030.538	-20.599	1.052.981	-25.570	1.006.658	-24.921
1.030.647	-20.599	1.053.221	-25.571	1.006.796	-24.922
1.030.816	-20.600	1.053.473	-25.572	1.007.009	-24.923
1.030.987	-20.601	1.053.604	-25.573	1.007.218	-24.925
1.031.124	-20.601	1.053.736	-25.574	1.007.337	-24.926

1.031.245	-20.602	1.053.902	-25.575	1.007.433	-24.928
1.031.469	-20.603	1.054.004	-25.576	1.007.629	-24.929
1.031.665	-20.603	1.054.134	-25.577	1.007.832	-24.931
1.031.816	-20.604	1.054.328	-25.578	1.007.987	-24.932
1.032.045	-20.605	1.054.439	-25.579	1.008.109	-24.934
1.032.265	-20.605	1.054.540	-25.580	1.008.234	-24.935
1.032.430	-20.606	1.054.751	-25.581	1.008.409	-24.937
1.032.522	-20.607	1.054.969	-25.582	1.008.603	-24.939
1.032.646	-20.607	1.055.107	-25.583	1.008.774	-24.940
1.032.848	-20.608	1.055.291	-25.584	1.008.964	-24.942
1.033.025	-20.609	1.055.531	-25.585	1.009.146	-24.943
1.033.176	-20.609	1.055.713	-25.586	1.009.268	-24.945
1.033.370	-20.610	1.055.897	-25.588	1.009.393	-24.946
1.033.532	-20.611	1.056.065	-25.589	1.009.518	-24.948
1.033.688	-20.611	1.056.219	-25.590	1.009.688	-24.950
1.033.820	-20.612	1.056.336	-25.591	1.009.894	-24.951
1.033.880	-20.613	1.056.451	-25.592	1.010.033	-24.953
1.034.110	-20.613	1.056.617	-25.593	1.010.204	-24.954
1.034.324	-20.614	1.056.779	-25.594	1.010.359	-24.956
1.034.423	-20.615	1.056.948	-25.595	1.010.462	-24.958
1.034.622	-20.615	1.057.059	-25.597	1.010.592	-24.959
1.034.892	-20.616	1.057.208	-25.598	1.010.753	-24.961
1.035.087	-20.617	1.057.374	-25.599	1.010.916	-24.963
1.035.207	-20.617	1.057.565	-25.600	1.011.106	-24.964
1.035.307	-20.618	1.057.769	-25.601	1.011.276	-24.966
1.035.453	-20.619	1.057.974	-25.602	1.011.330	-24.967
1.035.639	-20.619	1.058.120	-25.604	1.011.528	-24.969
1.035.815	-20.620	1.058.204	-25.605	1.011.794	-24.971
1.036.032	-20.621	1.058.328	-25.606	1.011.927	-24.972
1.036.227	-20.621	1.058.481	-25.607	1.012.094	-24.974

1.036.355	-20.622	1.058.619	-25.609	1.012.287	-24.975
1.036.517	-20.623	1.058.823	-25.610	1.012.441	-24.977
1.036.710	-20.623	1.059.025	-25.611	1.012.679	-24.979
1.036.825	-20.624	1.059.149	-25.612	1.012.896	-24.980
1.036.945	-20.625	1.059.320	-25.614	1.013.016	-24.982
1.037.124	-20.625	1.059.473	-25.615	1.013.196	-24.983
1.037.272	-20.626	1.059.621	-25.616	1.013.326	-24.985
1.037.479	-20.627	1.059.762	-25.617	1.013.501	-24.987
1.037.906	-20.627	1.059.910	-25.619	1.013.755	-24.988
1.038.269	-20.628	1.060.051	-25.620	1.013.839	-24.990
1.038.404	-20.629	1.060.255	-25.621	1.013.913	-24.991
1.038.466	-20.629	1.060.474	-25.623	1.014.057	-24.993
1.038.496	-20.630	1.060.578	-25.624	1.014.222	-24.994
1.038.555	-20.631	1.060.727	-25.625	1.014.428	-24.996
1.038.655	-20.631	1.060.926	-25.627	1.014.604	-24.997
1.038.746	-20.632	1.061.102	-25.628	1.014.741	-24.999
1.038.852	-20.633	1.061.305	-25.629	1.014.838	-25.000
1.039.015	-20.633	1.061.483	-25.631	1.015.014	-25.002
1.039.139	-20.634	1.061.610	-25.632	1.015.165	-25.003
1.039.270	-20.635	1.061.751	-25.634	1.015.331	-25.005
1.039.476	-20.635	1.061.921	-25.635	1.015.494	-25.006
1.039.669	-20.636	1.062.020	-25.636	1.015.742	-25.008
1.039.827	-20.637	1.062.142	-25.638	1.015.929	-25.009
1.040.009	-20.637	1.062.296	-25.639	1.016.012	-25.010
1.040.223	-20.638	1.062.448	-25.640	1.016.198	-25.012
1.040.389	-20.639	1.062.615	-25.642	1.016.393	-25.013
1.040.528	-20.639	1.062.751	-25.643	1.016.563	-25.015
1.040.649	-20.640	1.062.925	-25.645	1.016.756	-25.016
1.040.758	-20.641	1.063.050	-25.646	1.016.927	-25.017
1.040.864	-20.641	1.063.187	-25.647	1.017.048	-25.019

1.041.033	-20.642	1.063.391	-25.649	1.017.188	-25.020
1.041.234	-20.643	1.063.604	-25.650	1.017.355	-25.021
1.041.385	-20.643	1.063.782	-25.652	1.017.532	-25.022
1.041.592	-20.644	1.063.994	-25.653	1.017.678	-25.024
1.041.712	-20.645	1.064.253	-25.654	1.017.810	-25.025
1.041.845	-20.645	1.064.464	-25.656	1.018.024	-25.026
1.042.013	-20.646	1.064.613	-25.657	1.018.204	-25.027
1.042.235	-20.647	1.064.729	-25.659	1.018.334	-25.028
1.042.469	-20.647	1.064.836	-25.660	1.018.499	-25.030
1.042.609	-20.648	1.064.962	-25.661	1.018.699	-25.031
1.042.819	-20.649	1.065.154	-25.663	1.018.923	-25.032
1.043.018	-20.649	1.065.330	-25.664	1.019.056	-25.033
1.043.156	-20.650	1.065.494	-25.666	1.019.143	-25.034
1.043.296	-20.651	1.065.594	-25.667	1.019.388	-25.035
1.043.428	-20.651	1.065.636	-25.668	1.019.572	-25.036
1.043.587	-20.652	1.065.782	-25.670	1.019.705	-25.037
1.043.754	-20.653	1.065.950	-25.671	1.019.884	-25.038
1.043.864	-20.653	1.066.139	-25.673	1.020.040	-25.039
1.044.003	-20.654	1.066.310	-25.674	1.020.217	-25.040
1.044.231	-20.655	1.066.478	-25.675	1.020.340	-25.041
1.044.362	-20.655	1.066.720	-25.677	1.020.473	-25.042
1.044.454	-20.656	1.066.852	-25.678	1.020.658	-25.043
1.044.630	-20.657	1.067.001	-25.679	1.020.864	-25.044
1.044.836	-20.657	1.067.241	-25.681	1.021.091	-25.045
1.044.997	-20.658	1.067.405	-25.682	1.021.248	-25.046
1.045.107	-20.659	1.067.525	-25.683	1.021.391	-25.047
1.045.267	-20.659	1.067.667	-25.685	1.021.565	-25.048
1.045.395	-20.660	1.067.787	-25.686	1.021.751	-25.049
1.045.511	-20.661	1.067.947	-25.688	1.021.846	-25.050
1.045.680	-20.662	1.068.113	-25.689	1.021.956	-25.051

1.045.860	-20.662	1.068.372	-25.690	1.022.159	-25.051
1.046.027	-20.663	1.068.631	-25.692	1.022.319	-25.052
1.046.195	-20.664	1.068.721	-25.693	1.022.541	-25.053
1.046.363	-20.664	1.068.842	-25.694	1.022.935	-25.054
1.046.548	-20.665	1.068.999	-25.696	1.023.298	-25.055
1.046.762	-20.666	1.069.163	-25.697	1.023.445	-25.055
1.047.011	-20.666	1.069.324	-25.698	1.023.523	-25.056
1.047.196	-20.667	1.069.477	-25.700	1.023.627	-25.057
1.047.271	-20.668	1.069.613	-25.701	1.023.673	-25.058
1.047.362	-20.668	1.069.849	-25.702	1.023.720	-25.058
1.047.512	-20.669	1.070.119	-25.704	1.023.774	-25.059
1.047.708	-20.670	1.070.227	-25.705	1.023.866	-25.060
1.047.862	-20.670	1.070.313	-25.706	1.024.023	-25.060
1.047.989	-20.671	1.070.466	-25.708	1.024.146	-25.061
1.048.176	-20.672	1.070.627	-25.709	1.024.214	-25.062
1.048.353	-20.672	1.070.799	-25.710	1.024.350	-25.063
1.048.501	-20.673	1.070.985	-25.711	1.024.574	-25.063
1.048.715	-20.674	1.071.154	-25.713	1.024.744	-25.064
1.048.933	-20.674	1.071.307	-25.714	1.024.924	-25.065
1.049.014	-20.675	1.071.479	-25.715	1.025.145	-25.065
1.049.121	-20.676	1.071.611	-25.717	1.025.341	-25.066
1.049.286	-20.676	1.071.786	-25.718	1.025.497	-25.066
1.049.409	-20.677	1.071.958	-25.719	1.025.674	-25.067
1.049.591	-20.678	1.072.097	-25.721	1.025.841	-25.068
1.049.770	-20.678	1.072.465	-25.722	1.025.956	-25.068
1.049.951	-20.679	1.072.885	-25.723	1.026.091	-25.069
1.050.095	-20.680	1.073.131	-25.724	1.026.299	-25.070
1.050.205	-20.681	1.073.234	-25.726	1.026.457	-25.070
1.050.322	-20.681	1.073.279	-25.727	1.026.563	-25.071
1.050.468	-20.682	1.073.318	-25.728	1.026.739	-25.072

1.050.676	-20.683	1.073.353	-25.729	1.026.965	-25.072
1.050.898	-20.683	1.073.397	-25.731	1.027.153	-25.073
1.051.090	-20.684	1.073.477	-25.732	1.027.347	-25.073
1.051.273	-20.685	1.073.652	-25.733	1.027.459	-25.074
1.051.464	-20.685	1.073.802	-25.735	1.027.503	-25.075
1.051.665	-20.686	1.073.944	-25.736	1.027.628	-25.075
1.051.842	-20.687	1.074.127	-25.737	1.027.803	-25.076
1.052.004	-20.688	1.074.266	-25.738	1.027.988	-25.077
1.052.152	-20.688	1.074.445	-25.740	1.028.236	-25.077
1.052.306	-20.689	1.074.630	-25.741	1.028.472	-25.078
1.052.434	-20.690	1.074.773	-25.742	1.028.646	-25.079
1.052.558	-20.690	1.074.945	-25.743	1.028.756	-25.079
1.052.679	-20.691	1.075.135	-25.745	1.028.913	-25.080
1.052.740	-20.692	1.075.321	-25.746	1.029.144	-25.081
1.052.859	-20.693	1.075.443	-25.747	1.029.312	-25.081
1.053.036	-20.693	1.075.589	-25.748	1.029.432	-25.082
1.053.260	-20.694	1.075.772	-25.750	1.029.593	-25.083
1.053.447	-20.695	1.075.940	-25.751	1.029.771	-25.083
1.053.591	-20.695	1.076.112	-25.752	1.029.905	-25.084
1.053.761	-20.696	1.076.266	-25.753	1.030.024	-25.085
1.053.979	-20.697	1.076.370	-25.755	1.030.190	-25.086
1.054.205	-20.698	1.076.539	-25.756	1.030.369	-25.086
1.054.379	-20.698	1.076.701	-25.757	1.030.510	-25.087
1.054.554	-20.699	1.076.852	-25.758	1.030.683	-25.088
1.054.681	-20.700	1.077.037	-25.760	1.030.874	-25.088
1.054.821	-20.701	1.077.214	-25.761	1.030.993	-25.089
1.054.996	-20.701	1.077.439	-25.762	1.031.113	-25.090
1.055.136	-20.702	1.077.665	-25.763	1.031.245	-25.091
1.055.319	-20.703	1.077.877	-25.765	1.031.398	-25.092
1.055.494	-20.704	1.077.978	-25.766	1.031.578	-25.092

1.055.642	-20.704	1.078.042	-25.767	1.031.739	-25.093
1.055.826	-20.705	1.078.183	-25.768	1.031.891	-25.094
1.055.964	-20.706	1.078.325	-25.770	1.032.075	-25.095
1.056.100	-20.707	1.078.506	-25.771	1.032.206	-25.096
1.056.253	-20.707	1.078.701	-25.772	1.032.305	-25.097
1.056.410	-20.708	1.078.822	-25.773	1.032.514	-25.097
1.056.637	-20.709	1.078.931	-25.775	1.032.731	-25.098
1.056.843	-20.710	1.079.094	-25.776	1.032.921	-25.099
1.056.908	-20.710	1.079.342	-25.777	1.033.083	-25.100
1.057.042	-20.711	1.079.544	-25.779	1.033.270	-25.101
1.057.265	-20.712	1.079.668	-25.780	1.033.466	-25.102
1.057.468	-20.713	1.079.886	-25.781	1.033.596	-25.103
1.057.656	-20.713	1.080.081	-25.783	1.033.748	-25.104
1.057.846	-20.714	1.080.166	-25.784	1.033.939	-25.105
1.058.020	-20.715	1.080.315	-25.785	1.034.063	-25.106
1.058.123	-20.716	1.080.481	-25.787	1.034.138	-25.107
1.058.277	-20.717	1.080.610	-25.788	1.034.358	-25.108
1.058.465	-20.717	1.080.753	-25.789	1.034.596	-25.109
1.058.645	-20.718	1.080.925	-25.791	1.034.764	-25.110
1.058.805	-20.719	1.081.076	-25.792	1.034.964	-25.111
1.058.967	-20.720	1.081.249	-25.793	1.035.087	-25.112
1.059.099	-20.721	1.081.485	-25.795	1.035.216	-25.113
1.059.193	-20.721	1.081.656	-25.796	1.035.413	-25.114
1.059.316	-20.722	1.081.805	-25.797	1.035.574	-25.115
1.059.520	-20.723	1.081.938	-25.799	1.035.707	-25.116
1.059.893	-20.724	1.082.013	-25.800	1.035.827	-25.117
1.060.326	-20.725	1.082.166	-25.802	1.035.978	-25.118
1.060.549	-20.725	1.082.375	-25.803	1.036.193	-25.119
1.060.602	-20.726	1.082.504	-25.804	1.036.380	-25.120
1.060.665	-20.727	1.082.504	-25.806	1.036.510	-25.121

1.060.693	-20.728	1.082.897	-25.807	1.036.686	-25.122
1.060.723	-20.729	1.083.054	-25.809	1.036.860	-25.123
1.060.867	-20.729	1.083.280	-25.810	1.036.985	-25.124
1.061.013	-20.730	1.083.484	-25.811	1.037.096	-25.125
1.061.115	-20.731	1.083.586	-25.813	1.037.262	-25.126
1.061.243	-20.732	1.083.717	-25.814	1.037.460	-25.128
1.061.450	-20.733	1.083.839	-25.816	1.037.522	-25.129
1.061.640	-20.733	1.083.957	-25.817	1.037.598	-25.130
1.061.774	-20.734	1.084.099	-25.819	1.037.738	-25.131
1.061.944	-20.735	1.084.210	-25.820	1.037.907	-25.132
1.062.084	-20.736	1.084.370	-25.822	1.038.049	-25.133
1.062.241	-20.737	1.084.493	-25.823	1.038.211	-25.134
1.062.427	-20.738	1.084.632	-25.825	1.038.435	-25.135
1.062.620	-20.738	1.084.850	-25.826	1.038.621	-25.136
1.062.824	-20.739	1.085.060	-25.827	1.038.795	-25.137
1.062.986	-20.740	1.085.261	-25.829	1.039.000	-25.138
1.063.139	-20.741	1.085.482	-25.830	1.039.256	-25.139
1.063.286	-20.742	1.085.670	-25.832	1.039.440	-25.141
1.063.350	-20.743	1.085.899	-25.833	1.039.632	-25.142
1.063.490	-20.743	1.086.133	-25.835	1.039.829	-25.143
1.063.715	-20.744	1.086.285	-25.836	1.039.919	-25.144
1.063.820	-20.745	1.086.389	-25.838	1.040.043	-25.145
1.063.926	-20.746	1.086.462	-25.839	1.040.211	-25.146
1.064.145	-20.747	1.086.635	-25.841	1.040.423	-25.147
1.064.349	-20.748	1.086.873	-25.842	1.040.615	-25.148
1.064.504	-20.748	1.087.057	-25.844	1.040.702	-25.149
1.064.658	-20.749	1.087.159	-25.845	1.040.794	-25.150
1.064.848	-20.750	1.087.243	-25.847	1.040.959	-25.151
1.065.042	-20.751	1.087.321	-25.848	1.041.161	-25.152
1.065.236	-20.752	1.087.452	-25.850	1.041.296	-25.153

1.065.418	-20.753	1.087.701	-25.851	1.041.392	-25.154
1.065.573	-20.753	1.087.909	-25.853	1.041.470	-25.156
1.065.695	-20.754	1.088.064	-25.854	1.041.629	-25.157
1.065.811	-20.755	1.088.202	-25.856	1.041.862	-25.158
1.065.974	-20.756	1.088.369	-25.857	1.042.057	-25.159
1.066.130	-20.757	1.088.603	-25.859	1.042.244	-25.160
1.066.282	-20.758	1.088.820	-25.860	1.042.455	-25.161
1.066.451	-20.758	1.088.973	-25.862	1.042.654	-25.162
1.066.639	-20.759	1.089.100	-25.863	1.042.760	-25.163
1.066.833	-20.760	1.089.265	-25.865	1.042.912	-25.164
1.066.989	-20.761	1.089.437	-25.866	1.043.170	-25.165
1.067.104	-20.762	1.089.583	-25.868	1.043.397	-25.166
1.067.194	-20.763	1.089.743	-25.869	1.043.542	-25.167
1.067.354	-20.763	1.089.908	-25.871	1.043.644	-25.168
1.067.578	-20.764	1.090.065	-25.872	1.043.785	-25.169
1.067.757	-20.765	1.090.219	-25.874	1.043.952	-25.170
1.067.909	-20.766	1.090.358	-25.875	1.044.138	-25.171
1.068.040	-20.767	1.090.536	-25.877	1.044.321	-25.172
1.068.176	-20.768	1.090.686	-25.878	1.044.498	-25.173
1.068.383	-20.768	1.090.832	-25.880	1.044.657	-25.174
1.068.569	-20.769	1.091.038	-25.881	1.044.811	-25.175
1.068.738	-20.770	1.091.249	-25.883	1.044.956	-25.175
1.068.877	-20.771	1.091.413	-25.884	1.045.111	-25.176
1.069.010	-20.772	1.091.550	-25.886	1.045.277	-25.177
1.069.216	-20.773	1.091.693	-25.887	1.045.378	-25.178
1.069.415	-20.774	1.091.807	-25.889	1.045.542	-25.179
1.069.557	-20.774	1.091.994	-25.890	1.045.723	-25.180
1.069.679	-20.775	1.092.184	-25.892	1.045.865	-25.181
1.069.857	-20.776	1.092.273	-25.893	1.046.038	-25.182
1.070.060	-20.777	1.092.447	-25.895	1.046.208	-25.183

1.070.191	-20.778	1.092.716	-25.896	1.046.410	-25.184
1.070.297	-20.779	1.092.887	-25.898	1.046.612	-25.185
1.070.471	-20.779	1.093.022	-25.899	1.046.709	-25.186
1.070.683	-20.780	1.093.194	-25.901	1.046.814	-25.187
1.070.837	-20.781	1.093.356	-25.902	1.047.001	-25.187
1.070.984	-20.782	1.093.521	-25.904	1.047.165	-25.188
1.071.160	-20.783	1.093.696	-25.905	1.047.305	-25.189
1.071.305	-20.784	1.093.826	-25.907	1.047.467	-25.190
1.071.425	-20.785	1.094.093	-25.908	1.047.654	-25.191
1.071.570	-20.785	1.094.529	-25.910	1.047.856	-25.192
1.071.749	-20.786	1.094.790	-25.911	1.048.016	-25.193
1.071.842	-20.787	1.094.873	-25.913	1.048.127	-25.194
1.071.976	-20.788	1.094.927	-25.914	1.048.252	-25.194
1.072.198	-20.789	1.094.980	-25.916	1.048.483	-25.195
1.072.355	-20.790	1.095.065	-25.917	1.048.703	-25.196
1.072.465	-20.791	1.095.189	-25.919	1.048.850	-25.197
1.072.570	-20.791	1.095.292	-25.921	1.048.947	-25.198
1.072.750	-20.792	1.095.396	-25.922	1.049.035	-25.199
1.072.970	-20.793	1.095.521	-25.924	1.049.243	-25.200
1.073.204	-20.794	1.095.641	-25.925	1.049.650	-25.200
1.073.364	-20.795	1.095.867	-25.927	1.050.039	-25.201
1.073.530	-20.796	1.096.088	-25.928	1.050.253	-25.202
1.073.755	-20.797	1.096.173	-25.930	1.050.372	-25.203
1.073.916	-20.797	1.096.328	-25.931	1.050.436	-25.204
1.074.132	-20.798	1.096.554	-25.933	1.050.536	-25.205
1.074.363	-20.799	1.096.762	-25.934	1.050.615	-25.205
1.074.484	-20.800	1.096.982	-25.936	1.050.704	-25.206
1.074.576	-20.801	1.097.132	-25.938	1.050.826	-25.207
1.074.698	-20.802	1.097.207	-25.939	1.050.936	-25.208
1.074.839	-20.803	1.097.335	-25.941	1.051.065	-25.209

1.075.006	-20.804	1.097.495	-25.942	1.051.209	-25.210
1.075.136	-20.805	1.097.636	-25.944	1.051.320	-25.210
1.075.272	-20.805	1.097.793	-25.946	1.051.438	-25.211
1.075.396	-20.806	1.097.907	-25.947	1.051.626	-25.212
1.075.537	-20.807	1.098.018	-25.949	1.051.820	-25.213
1.075.733	-20.808	1.098.186	-25.950	1.051.996	-25.214
1.075.956	-20.809	1.098.402	-25.952	1.052.196	-25.215
1.076.130	-20.810	1.098.618	-25.954	1.052.394	-25.216
1.076.335	-20.811	1.098.763	-25.955	1.052.570	-25.217
1.076.598	-20.812	1.098.904	-25.957	1.052.699	-25.217
1.076.733	-20.813	1.099.094	-25.959	1.052.840	-25.218
1.076.841	-20.814	1.099.324	-25.960	1.053.003	-25.219
1.076.981	-20.814	1.099.492	-25.962	1.053.199	-25.220
1.077.138	-20.815	1.099.595	-25.964	1.053.412	-25.221
1.077.236	-20.816	1.099.712	-25.965	1.053.568	-25.222
1.077.413	-20.817	1.099.908	-25.967	1.053.697	-25.223
1.077.621	-20.818	1.100.089	-25.969	1.053.789	-25.224
1.077.765	-20.819	1.100.240	-25.970	1.053.895	-25.225
1.077.870	-20.820	1.100.414	-25.972	1.054.066	-25.226
1.078.026	-20.821	1.100.586	-25.974	1.054.236	-25.227
1.078.232	-20.822	1.100.789	-25.975	1.054.361	-25.228
1.078.413	-20.823	1.100.957	-25.977	1.054.446	-25.229
1.078.596	-20.824	1.101.090	-25.979	1.054.608	-25.230
1.078.777	-20.825	1.101.217	-25.981	1.054.818	-25.231
1.078.973	-20.826	1.101.401	-25.983	1.055.017	-25.232
1.079.149	-20.827	1.101.583	-25.984	1.055.217	-25.233
1.079.296	-20.827	1.101.701	-25.986	1.055.382	-25.234
1.079.463	-20.828	1.101.871	-25.988	1.055.564	-25.235
1.079.608	-20.829	1.102.079	-25.990	1.055.723	-25.236
1.079.793	-20.830	1.102.192	-25.992	1.055.922	-25.237

1.079.932	-20.831	1.102.287	-25.993	1.056.108	-25.238
1.080.067	-20.832	1.102.459	-25.995	1.056.255	-25.239
1.080.287	-20.833	1.102.688	-25.997	1.056.435	-25.240
1.080.404	-20.834	1.102.869	-25.999	1.056.594	-25.241
1.080.575	-20.835	1.103.015	-26.001	1.056.716	-25.242
1.080.856	-20.836	1.103.223	-26.003	1.056.858	-25.243
1.081.003	-20.837	1.103.404	-26.005	1.057.024	-25.244
1.081.043	-20.838	1.103.542	-26.007	1.057.206	-25.246
1.081.170	-20.839	1.103.680	-26.009	1.057.363	-25.247
1.081.376	-20.840	1.103.838	-26.011	1.057.495	-25.248
1.081.574	-20.841	1.103.966	-26.013	1.057.669	-25.249
1.081.703	-20.842	1.104.107	-26.015	1.057.801	-25.250
1.081.967	-20.843	1.104.344	-26.017	1.057.973	-25.251
1.082.368	-20.844	1.104.507	-26.019	1.058.151	-25.252
1.082.624	-20.845	1.104.619	-26.021	1.058.310	-25.254
1.082.741	-20.846	1.104.801	-26.023	1.058.486	-25.255
1.082.802	-20.847	1.104.996	-26.025	1.058.664	-25.256
1.082.902	-20.848	1.105.194	-26.027	1.058.861	-25.257
1.082.953	-20.849	1.105.341	-26.029	1.059.020	-25.258
1.083.009	-20.850	1.105.461	-26.031	1.059.137	-25.260
1.083.123	-20.851	1.105.617	-26.033	1.059.229	-25.261
1.083.214	-20.852	1.105.768	-26.035	1.059.317	-25.262
1.083.386	-20.853	1.105.846	-26.037	1.059.428	-25.263
1.083.572	-20.854	1.105.974	-26.039	1.059.645	-25.265
1.083.726	-20.855	1.106.154	-26.041	1.059.891	-25.266
1.083.896	-20.856	1.106.264	-26.044	1.060.095	-25.267
1.084.110	-20.857	1.106.331	-26.046	1.060.261	-25.268
1.084.322	-20.858	1.106.482	-26.048	1.060.365	-25.270
1.084.464	-20.859	1.106.756	-26.050	1.060.519	-25.271
1.084.675	-20.860	1.106.988	-26.052	1.060.727	-25.272

1.084.824	-20.861	1.107.158	-26.055	1.060.941	-25.273
1.084.919	-20.862	1.107.331	-26.057	1.061.103	-25.275
1.085.110	-20.863	1.107.589	-26.059	1.061.205	-25.276
1.085.264	-20.864	1.107.826	-26.061	1.061.401	-25.277
1.085.433	-20.865	1.107.943	-26.064	1.061.576	-25.278
1.085.587	-20.866	1.108.118	-26.066	1.061.678	-25.280
1.085.689	-20.867	1.108.268	-26.068	1.061.848	-25.281
1.085.852	-20.868	1.108.400	-26.071	1.062.022	-25.282
1.085.995	-20.870	1.108.624	-26.073	1.062.156	-25.283
1.086.135	-20.871	1.108.737	-26.075	1.062.310	-25.285
1.086.305	-20.872	1.108.809	-26.078	1.062.488	-25.286
1.086.478	-20.873	1.108.942	-26.080	1.062.703	-25.287
1.086.685	-20.874	1.109.085	-26.082	1.062.866	-25.289
1.086.824	-20.875	1.109.229	-26.085	1.062.969	-25.290
1.086.976	-20.876	1.109.380	-26.087	1.063.136	-25.291
1.087.223	-20.877	1.109.549	-26.089	1.063.316	-25.292
1.087.441	-20.878	1.109.771	-26.092	1.063.459	-25.293
1.087.580	-20.879	1.109.997	-26.094	1.063.633	-25.295
1.087.727	-20.880	1.110.161	-26.097	1.063.845	-25.296
1.087.877	-20.881	1.110.350	-26.099	1.063.990	-25.297
1.088.044	-20.882	1.110.549	-26.102	1.064.046	-25.298
1.088.177	-20.883	1.110.690	-26.104	1.064.109	-25.299
1.088.298	-20.884	1.110.857	-26.107	1.064.294	-25.301
1.088.507	-20.885	1.111.030	-26.109	1.064.481	-25.302
1.088.631	-20.886	1.111.144	-26.112	1.064.639	-25.303
1.088.763	-20.888	1.111.283	-26.114	1.064.837	-25.304
1.088.954	-20.889	1.111.460	-26.117	1.064.984	-25.305
1.089.113	-20.890	1.111.591	-26.119	1.065.095	-25.306
1.089.277	-20.891	1.111.744	-26.122	1.065.260	-25.308
1.089.415	-20.892	1.111.947	-26.125	1.065.452	-25.309

1.089.535	-20.893	1.112.085	-26.127	1.065.591	-25.310
1.089.753	-20.894	1.112.254	-26.130	1.065.704	-25.311
1.089.947	-20.895	1.112.424	-26.132	1.065.917	-25.312
1.090.068	-20.896	1.112.561	-26.135	1.066.147	-25.313
1.090.205	-20.897	1.112.697	-26.138	1.066.348	-25.314
1.090.368	-20.898	1.112.847	-26.140	1.066.579	-25.315
1.090.529	-20.900	1.113.027	-26.143	1.066.710	-25.316
1.090.673	-20.901	1.113.214	-26.146	1.066.856	-25.317
1.090.903	-20.902	1.113.376	-26.149	1.067.063	-25.318
1.091.135	-20.903	1.113.528	-26.151	1.067.260	-25.319
1.091.294	-20.904	1.113.705	-26.154	1.067.453	-25.320
1.091.440	-20.905	1.113.908	-26.157	1.067.561	-25.321
1.091.597	-20.906	1.114.073	-26.160	1.067.658	-25.322
1.091.786	-20.907	1.114.188	-26.163	1.067.770	-25.323
1.091.921	-20.908	1.114.378	-26.165	1.067.887	-25.324
1.092.070	-20.909	1.114.577	-26.168	1.068.084	-25.325
1.092.225	-20.911	1.114.681	-26.171	1.068.233	-25.326
1.092.364	-20.912	1.114.791	-26.174	1.068.375	-25.326
1.092.505	-20.913	1.114.970	-26.177	1.068.596	-25.327
1.092.646	-20.914	1.115.167	-26.180	1.068.737	-25.328
1.092.868	-20.915	1.115.338	-26.183	1.068.877	-25.329
1.093.083	-20.916	1.115.530	-26.186	1.069.042	-25.330
1.093.182	-20.917	1.115.883	-26.189	1.069.206	-25.330
1.093.285	-20.918	1.116.283	-26.192	1.069.408	-25.331
1.093.500	-20.920	1.116.464	-26.195	1.069.561	-25.332
1.093.661	-20.921	1.116.488	-26.198	1.069.706	-25.333
1.093.813	-20.922	1.116.582	-26.201	1.069.858	-25.333
1.093.999	-20.923	1.116.601	-26.204	1.070.091	-25.334
1.094.088	-20.924	1.116.656	-26.208	1.070.342	-25.335
1.094.155	-20.925	1.116.816	-26.211	1.070.469	-25.335

1.094.268	-20.926	1.116.916	-26.214	1.070.620	-25.336
1.094.440	-20.928	1.117.024	-26.217	1.070.782	-25.337
1.094.643	-20.929	1.117.163	-26.221	1.070.874	-25.337
1.094.817	-20.930	1.117.339	-26.224	1.070.977	-25.338
1.094.967	-20.931	1.117.537	-26.227	1.071.202	-25.338
1.095.135	-20.932	1.117.772	-26.231	1.071.405	-25.339
1.095.347	-20.933	1.117.924	-26.234	1.071.476	-25.339
1.095.594	-20.934	1.118.062	-26.238	1.071.478	-25.340
1.095.788	-20.936	1.118.260	-26.241	1.071.693	-25.340
1.095.972	-20.937	1.118.428	-26.245	1.071.973	-25.341
1.096.181	-20.938	1.118.567	-26.248	1.072.156	-25.341
1.096.350	-20.939	1.118.756	-26.252	1.072.344	-25.342
1.096.471	-20.940	1.118.948	-26.255	1.072.459	-25.342
1.096.629	-20.941	1.119.080	-26.259	1.072.617	-25.343
1.096.805	-20.943	1.119.216	-26.263	1.072.820	-25.343
1.096.970	-20.944	1.119.380	-26.266	1.073.001	-25.343
1.097.115	-20.945	1.119.527	-26.270	1.073.196	-25.344
1.097.199	-20.946	1.119.654	-26.274	1.073.371	-25.344
1.097.290	-20.947	1.119.836	-26.278	1.073.512	-25.345
1.097.448	-20.948	1.119.978	-26.282	1.073.714	-25.345
1.097.642	-20.950	1.120.118	-26.286	1.073.912	-25.345
1.097.792	-20.951	1.120.300	-26.290	1.074.053	-25.346
1.097.937	-20.952	1.120.462	-26.294	1.074.185	-25.346
1.098.072	-20.953	1.120.609	-26.298	1.074.305	-25.346
1.098.236	-20.954	1.120.834	-26.302	1.074.464	-25.347
1.098.466	-20.956	1.121.038	-26.306	1.074.640	-25.347
1.098.671	-20.957	1.121.171	-26.310	1.074.829	-25.347
1.098.829	-20.958	1.121.314	-26.314	1.075.005	-25.348
1.099.005	-20.959	1.121.445	-26.319	1.075.138	-25.348
1.099.218	-20.960	1.121.617	-26.323	1.075.275	-25.348

1.099.397	-20.962	1.121.784	-26.328	1.075.415	-25.349
1.099.548	-20.963	1.121.918	-26.332	1.075.561	-25.349
1.099.671	-20.964	1.122.003	-26.337	1.075.710	-25.349
1.099.803	-20.965	1.122.038	-26.341	1.075.890	-25.349
1.099.920	-20.967	1.122.234	-26.346	1.076.070	-25.350
1.100.078	-20.968	1.122.572	-26.350	1.076.332	-25.350
1.100.264	-20.969	1.122.789	-26.355	1.076.765	-25.350
1.100.442	-20.970	1.122.915	-26.360	1.077.077	-25.350
1.100.619	-20.971	1.123.055	-26.365	1.077.218	-25.351
1.100.783	-20.973	1.123.223	-26.370	1.077.318	-25.351
1.100.941	-20.974	1.123.392	-26.375	1.077.411	-25.351
1.101.091	-20.975	1.123.493	-26.380	1.077.496	-25.351
1.101.302	-20.976	1.123.593	-26.385	1.077.595	-25.352
1.101.492	-20.978	1.123.759	-26.390	1.077.646	-25.352
1.101.671	-20.979	1.123.986	-26.395	1.077.684	-25.352
1.101.814	-20.980	1.124.177	-26.401	1.077.774	-25.353
1.101.887	-20.981	1.124.316	-26.406	1.077.869	-25.353
1.102.022	-20.983	1.124.476	-26.411	1.077.978	-25.353
1.102.231	-20.984	1.124.622	-26.417	1.078.146	-25.353
1.102.477	-20.985	1.124.810	-26.422	1.078.292	-25.354
1.102.635	-20.986	1.125.018	-26.428	1.078.491	-25.354
1.102.694	-20.987	1.125.167	-26.434	1.078.690	-25.354
1.102.914	-20.989	1.125.307	-26.440	1.078.888	-25.355
1.103.165	-20.990	1.125.483	-26.446	1.079.073	-25.355
1.103.300	-20.991	1.125.636	-26.451	1.079.203	-25.355
1.103.403	-20.992	1.125.760	-26.458	1.079.447	-25.356
1.103.559	-20.994	1.125.910	-26.464	1.079.696	-25.356
1.103.699	-20.995	1.126.098	-26.470	1.079.817	-25.356
1.103.897	-20.996	1.126.312	-26.476	1.079.921	-25.357
1.104.272	-20.997	1.126.506	-26.482	1.080.109	-25.357

1.104.684	-20.999	1.126.654	-26.489	1.080.311	-25.358
1.104.945	-21.000	1.126.753	-26.495	1.080.458	-25.358
1.105.012	-21.001	1.126.889	-26.502	1.080.602	-25.358
1.105.051	-21.002	1.127.047	-26.509	1.080.741	-25.359
1.105.118	-21.004	1.127.162	-26.516	1.080.857	-25.359
1.105.182	-21.005	1.127.279	-26.522	1.081.001	-25.360
1.105.251	-21.006	1.127.468	-26.529	1.081.191	-25.360
1.105.350	-21.007	1.127.611	-26.536	1.081.363	-25.361
1.105.451	-21.009	1.127.720	-26.544	1.081.495	-25.361
1.105.587	-21.010	1.127.876	-26.551	1.081.636	-25.362
1.105.732	-21.011	1.128.021	-26.558	1.081.797	-25.362
1.105.882	-21.012	1.128.149	-26.566	1.081.981	-25.363
1.106.052	-21.014	1.128.323	-26.573	1.082.176	-25.364
1.106.271	-21.015	1.128.525	-26.581	1.082.328	-25.364
1.106.451	-21.016	1.128.700	-26.589	1.082.526	-25.365
1.106.566	-21.017	1.128.884	-26.597	1.082.731	-25.365
1.106.733	-21.019	1.129.136	-26.605	1.082.940	-25.366
1.106.935	-21.020	1.129.313	-26.613	1.083.124	-25.367
1.107.111	-21.021	1.129.446	-26.621	1.083.208	-25.367
1.107.250	-21.022	1.129.613	-26.629	1.083.336	-25.368
1.107.358	-21.024	1.129.772	-26.637	1.083.466	-25.369
1.107.524	-21.025	1.129.980	-26.646	1.083.645	-25.370
1.107.657	-21.026	1.130.151	-26.655	1.083.840	-25.370
1.107.731	-21.027	1.130.285	-26.663	1.083.952	-25.371
1.107.994	-21.028	1.130.414	-26.672	1.084.110	-25.372
1.108.227	-21.030	1.130.501	-26.681	1.084.275	-25.373
1.108.306	-21.031	1.130.629	-26.690	1.084.427	-25.374
1.108.476	-21.032	1.130.795	-26.700	1.084.611	-25.374
1.108.680	-21.033	1.130.986	-26.709	1.084.775	-25.375
1.108.845	-21.035	1.131.143	-26.718	1.084.922	-25.376

1.109.022	-21.036	1.131.305	-26.728	1.085.128	-25.377
1.109.222	-21.037	1.131.537	-26.738	1.085.340	-25.378
1.109.448	-21.038	1.131.750	-26.748	1.085.508	-25.379
1.109.575	-21.039	1.131.930	-26.758	1.085.642	-25.380
1.109.684	-21.041	1.132.067	-26.768	1.085.704	-25.381
1.109.891	-21.042	1.132.206	-26.778	1.085.835	-25.382
1.110.070	-21.043	1.132.394	-26.788	1.085.977	-25.383
1.110.236	-21.044	1.132.579	-26.799	1.086.098	-25.384
1.110.386	-21.045	1.132.736	-26.809	1.086.246	-25.385
1.110.533	-21.047	1.132.886	-26.820	1.086.432	-25.386
1.110.704	-21.048	1.133.074	-26.831	1.086.595	-25.387
1.110.867	-21.049	1.133.224	-26.842	1.086.784	-25.388
1.110.984	-21.050	1.133.327	-26.854	1.087.009	-25.389
1.111.155	-21.051	1.133.457	-26.865	1.087.154	-25.391
1.111.404	-21.052	1.133.635	-26.877	1.087.389	-25.392
1.111.544	-21.054	1.133.847	-26.888	1.087.606	-25.393
1.111.620	-21.055	1.134.007	-26.900	1.087.748	-25.394
1.111.786	-21.056	1.134.185	-26.912	1.087.923	-25.395
1.111.973	-21.057	1.134.363	-26.924	1.088.093	-25.397
1.112.122	-21.058	1.134.491	-26.937	1.088.240	-25.398
1.112.255	-21.059	1.134.640	-26.949	1.088.356	-25.399
1.112.410	-21.060	1.134.827	-26.962	1.088.495	-25.400
1.112.587	-21.062	1.135.005	-26.975	1.088.612	-25.402
1.112.745	-21.063	1.135.188	-26.988	1.088.823	-25.403
1.112.886	-21.064	1.135.342	-27.001	1.089.042	-25.404
1.113.061	-21.065	1.135.464	-27.014	1.089.157	-25.406
1.113.238	-21.066	1.135.603	-27.028	1.089.290	-25.407
1.113.411	-21.067	1.135.725	-27.041	1.089.542	-25.408
1.113.609	-21.068	1.135.869	-27.055	1.089.745	-25.410
1.113.775	-21.070	1.136.039	-27.069	1.089.900	-25.411

1.113.896	-21.071	1.136.169	-27.083	1.090.020	-25.412
1.114.044	-21.072	1.136.363	-27.098	1.090.145	-25.414
1.114.171	-21.073	1.136.610	-27.112	1.090.257	-25.415
1.114.358	-21.074	1.136.731	-27.127	1.090.390	-25.416
1.114.594	-21.075	1.136.803	-27.142	1.090.564	-25.418
1.114.698	-21.076	1.136.966	-27.157	1.090.694	-25.419
1.114.839	-21.077	1.137.312	-27.172	1.090.892	-25.421
1.115.032	-21.078	1.137.775	-27.188	1.091.059	-25.422
1.115.225	-21.079	1.138.005	-27.204	1.091.183	-25.424
1.115.406	-21.081	1.138.063	-27.219	1.091.320	-25.425
1.115.535	-21.082	1.138.187	-27.235	1.091.397	-25.426
1.115.693	-21.083	1.138.190	-27.252	1.091.525	-25.428
1.115.863	-21.084	1.138.142	-27.268	1.091.703	-25.429
1.115.998	-21.085	1.138.259	-27.285	1.091.884	-25.431
1.116.095	-21.086	1.138.413	-27.302	1.092.060	-25.432
1.116.189	-21.087	1.138.550	-27.319	1.092.186	-25.434
1.116.374	-21.088	1.138.714	-27.336	1.092.347	-25.435
1.116.578	-21.089	1.138.895	-27.353	1.092.581	-25.437
1.116.681	-21.090	1.139.107	-27.371	1.092.761	-25.438
1.116.753	-21.091	1.139.306	-27.388	1.092.936	-25.440
1.116.895	-21.092	1.139.521	-27.406	1.093.140	-25.441
1.117.093	-21.093	1.139.695	-27.424	1.093.348	-25.443
1.117.349	-21.094	1.139.807	-27.443	1.093.532	-25.444
1.117.527	-21.095	1.139.966	-27.461	1.093.646	-25.446
1.117.703	-21.096	1.140.120	-27.480	1.093.764	-25.447
1.118.001	-21.097	1.140.248	-27.499	1.093.969	-25.449
1.118.223	-21.098	1.140.382	-27.518	1.094.113	-25.450
1.118.361	-21.100	1.140.478	-27.537	1.094.243	-25.452
1.118.468	-21.101	1.140.594	-27.557	1.094.417	-25.454
1.118.644	-21.102	1.140.758	-27.576	1.094.635	-25.455

1.118.866	-21.103	1.140.941	-27.596	1.094.813	-25.457
1.119.006	-21.104	1.141.109	-27.616	1.094.931	-25.458
1.119.090	-21.105	1.141.255	-27.636	1.095.049	-25.460
1.119.190	-21.106	1.141.379	-27.656	1.095.154	-25.461
1.119.360	-21.107	1.141.573	-27.677	1.095.317	-25.463
1.119.510	-21.108	1.141.796	-27.697	1.095.483	-25.464
1.119.614	-21.109	1.141.953	-27.718	1.095.621	-25.466
1.119.731	-21.110	1.142.140	-27.739	1.095.853	-25.467
1.119.958	-21.111	1.142.360	-27.760	1.096.065	-25.469
1.120.172	-21.112	1.142.493	-27.782	1.096.224	-25.470
1.120.284	-21.113	1.142.561	-27.803	1.096.389	-25.472
1.120.433	-21.114	1.142.725	-27.825	1.096.584	-25.473
1.120.712	-21.115	1.142.878	-27.846	1.096.797	-25.475
1.120.955	-21.116	1.143.038	-27.868	1.096.936	-25.476
1.121.035	-21.117	1.143.244	-27.890	1.097.111	-25.478
1.121.182	-21.118	1.143.389	-27.913	1.097.290	-25.479
1.121.324	-21.119	1.143.510	-27.935	1.097.425	-25.481
1.121.475	-21.120	1.143.638	-27.957	1.097.567	-25.482
1.121.692	-21.121	1.143.771	-27.980	1.097.698	-25.484
1.121.874	-21.122	1.143.923	-28.003	1.097.812	-25.485
1.122.019	-21.123	1.144.095	-28.026	1.098.001	-25.486
1.122.139	-21.124	1.144.285	-28.049	1.098.220	-25.488
1.122.283	-21.125	1.144.479	-28.072	1.098.357	-25.489
1.122.471	-21.126	1.144.660	-28.095	1.098.535	-25.491
1.122.636	-21.127	1.144.809	-28.118	1.098.723	-25.492
1.122.784	-21.128	1.144.918	-28.142	1.098.882	-25.494
1.122.921	-21.129	1.145.003	-28.166	1.099.037	-25.495
1.123.120	-21.130	1.145.184	-28.189	1.099.140	-25.496
1.123.326	-21.131	1.145.431	-28.213	1.099.267	-25.498
1.123.474	-21.132	1.145.613	-28.237	1.099.438	-25.499

1.123.641	-21.133	1.145.735	-28.261	1.099.617	-25.501
1.123.831	-21.134	1.145.886	-28.285	1.099.832	-25.502
1.123.932	-21.135	1.146.059	-28.310	1.100.030	-25.504
1.124.029	-21.136	1.146.238	-28.334	1.100.146	-25.505
1.124.240	-21.137	1.146.408	-28.358	1.100.276	-25.506
1.124.510	-21.138	1.146.566	-28.383	1.100.480	-25.508
1.124.698	-21.139	1.146.731	-28.408	1.100.696	-25.509
1.124.799	-21.140	1.146.889	-28.432	1.100.856	-25.511
1.124.969	-21.141	1.147.014	-28.457	1.100.992	-25.512
1.125.160	-21.142	1.147.197	-28.482	1.101.123	-25.513
1.125.323	-21.143	1.147.421	-28.507	1.101.268	-25.515
1.125.501	-21.144	1.147.552	-28.532	1.101.494	-25.516
1.125.665	-21.145	1.147.649	-28.557	1.101.697	-25.518
1.125.797	-21.146	1.147.801	-28.582	1.101.750	-25.519
1.125.906	-21.147	1.148.013	-28.608	1.101.923	-25.520
1.126.040	-21.148	1.148.131	-28.633	1.102.083	-25.522
1.126.299	-21.149	1.148.231	-28.658	1.102.195	-25.523
1.126.693	-21.150	1.148.343	-28.684	1.102.401	-25.524
1.126.995	-21.151	1.148.530	-28.709	1.102.561	-25.526
1.127.169	-21.152	1.148.689	-28.735	1.102.741	-25.527
1.127.321	-21.153	1.148.830	-28.761	1.102.881	-25.529
1.127.390	-21.154	1.148.995	-28.786	1.103.040	-25.530
1.127.343	-21.155	1.149.080	-28.812	1.103.380	-25.531
1.127.359	-21.156	1.149.160	-28.838	1.103.785	-25.533
1.127.515	-21.157	1.149.279	-28.863	1.104.074	-25.534
1.127.661	-21.158	1.149.466	-28.889	1.104.230	-25.536
1.127.757	-21.159	1.149.656	-28.915	1.104.306	-25.537
1.127.904	-21.160	1.149.818	-28.941	1.104.359	-25.538
1.128.049	-21.161	1.149.959	-28.967	1.104.448	-25.540
1.128.227	-21.162	1.150.167	-28.993	1.104.573	-25.541

1.128.469	-21.163	1.150.459	-29.018	1.104.640	-25.542
1.128.631	-21.164	1.150.686	-29.044	1.104.687	-25.544
1.128.774	-21.165	1.150.876	-29.070	1.104.791	-25.545
1.128.988	-21.166	1.151.034	-29.096	1.104.951	-25.547
1.129.177	-21.167	1.151.133	-29.122	1.105.129	-25.548
1.129.266	-21.168	1.151.270	-29.148	1.105.236	-25.549
1.129.412	-21.169	1.151.416	-29.174	1.105.359	-25.551
1.129.657	-21.170	1.151.526	-29.200	1.105.575	-25.552
1.129.844	-21.171	1.151.628	-29.225	1.105.751	-25.554
1.129.939	-21.172	1.151.770	-29.251	1.105.931	-25.555
1.130.005	-21.173	1.151.942	-29.277	1.106.105	-25.556
1.130.107	-21.174	1.152.045	-29.303	1.106.226	-25.558
1.130.289	-21.175	1.152.191	-29.328	1.106.375	-25.559
1.130.510	-21.176	1.152.414	-29.354	1.106.561	-25.561
1.130.646	-21.178	1.152.639	-29.379	1.106.757	-25.562
1.130.789	-21.179	1.152.820	-29.405	1.106.964	-25.564
1.130.992	-21.180	1.152.999	-29.430	1.107.102	-25.565
1.131.161	-21.181	1.153.214	-29.456	1.107.219	-25.567
1.131.402	-21.182	1.153.384	-29.481	1.107.438	-25.568
1.131.608	-21.183	1.153.487	-29.506	1.107.606	-25.570
1.131.678	-21.184	1.153.627	-29.531	1.107.725	-25.571
1.131.784	-21.185	1.153.839	-29.556	1.107.898	-25.572
1.131.958	-21.186	1.154.029	-29.581	1.108.036	-25.574
1.132.148	-21.187	1.154.165	-29.606	1.108.148	-25.575
1.132.368	-21.188	1.154.274	-29.631	1.108.268	-25.577
1.132.533	-21.190	1.154.374	-29.656	1.108.417	-25.578
1.132.630	-21.191	1.154.569	-29.680	1.108.599	-25.580
1.132.761	-21.192	1.154.816	-29.705	1.108.743	-25.581
1.132.935	-21.193	1.154.997	-29.729	1.108.861	-25.583
1.133.067	-21.194	1.155.125	-29.753	1.109.017	-25.584

1.133.204	-21.195	1.155.236	-29.778	1.109.293	-25.586
1.133.394	-21.196	1.155.399	-29.802	1.109.576	-25.587
1.133.584	-21.197	1.155.605	-29.825	1.109.743	-25.589
1.133.758	-21.198	1.155.768	-29.849	1.109.846	-25.590
1.133.995	-21.200	1.155.900	-29.873	1.110.011	-25.592
1.134.107	-21.201	1.156.057	-29.896	1.110.212	-25.594
1.134.246	-21.202	1.156.208	-29.920	1.110.434	-25.595
1.134.443	-21.203	1.156.371	-29.943	1.110.555	-25.597
1.134.524	-21.204	1.156.535	-29.966	1.110.627	-25.598
1.134.650	-21.205	1.156.718	-29.989	1.110.774	-25.600
1.134.865	-21.206	1.156.880	-30.012	1.110.906	-25.601
1.135.083	-21.208	1.157.031	-30.034	1.111.091	-25.603
1.135.247	-21.209	1.157.228	-30.057	1.111.298	-25.605
1.135.352	-21.210	1.157.390	-30.079	1.111.472	-25.606
1.135.483	-21.211	1.157.523	-30.101	1.111.655	-25.608
1.135.631	-21.212	1.157.663	-30.123	1.111.788	-25.609
1.135.776	-21.213	1.157.843	-30.145	1.111.906	-25.611
1.135.967	-21.215	1.158.033	-30.167	1.112.048	-25.612
1.136.153	-21.216	1.158.147	-30.188	1.112.206	-25.614
1.136.286	-21.217	1.158.318	-30.210	1.112.377	-25.616
1.136.417	-21.218	1.158.615	-30.231	1.112.551	-25.617
1.136.522	-21.219	1.158.982	-30.252	1.112.686	-25.619
1.136.693	-21.221	1.159.266	-30.273	1.112.813	-25.621
1.136.914	-21.222	1.159.363	-30.293	1.112.934	-25.622
1.137.115	-21.223	1.159.408	-30.314	1.113.076	-25.624
1.137.269	-21.224	1.159.333	-30.334	1.113.262	-25.626
1.137.421	-21.225	1.159.384	-30.354	1.113.427	-25.627
1.137.582	-21.227	1.159.597	-30.374	1.113.612	-25.629
1.137.766	-21.228	1.159.680	-30.394	1.113.879	-25.631
1.137.940	-21.229	1.159.752	-30.414	1.114.126	-25.632

1.138.073	-21.230	1.159.900	-30.433	1.114.225	-25.634
1.138.223	-21.231	1.160.074	-30.452	1.114.286	-25.636
1.138.372	-21.233	1.160.245	-30.471	1.114.470	-25.637
1.138.485	-21.234	1.160.530	-30.490	1.114.668	-25.639
1.138.585	-21.235	1.160.748	-30.509	1.114.797	-25.641
1.138.736	-21.236	1.160.892	-30.527	1.114.932	-25.642
1.138.916	-21.237	1.161.082	-30.546	1.115.079	-25.644
1.139.045	-21.239	1.161.249	-30.564	1.115.222	-25.646
1.139.165	-21.240	1.161.434	-30.582	1.115.387	-25.648
1.139.300	-21.241	1.161.617	-30.599	1.115.536	-25.649
1.139.528	-21.242	1.161.730	-30.617	1.115.692	-25.651
1.139.824	-21.244	1.161.799	-30.634	1.115.908	-25.653
1.139.992	-21.245	1.161.903	-30.651	1.116.020	-25.654
1.140.155	-21.246	1.162.041	-30.667	1.116.170	-25.656
1.140.340	-21.247	1.162.229	-30.684	1.116.401	-25.658
1.140.465	-21.249	1.162.356	-30.700	1.116.572	-25.659
1.140.589	-21.250	1.162.498	-30.716	1.116.730	-25.661
1.140.807	-21.251	1.162.681	-30.732	1.116.937	-25.663
1.141.015	-21.252	1.162.796	-30.748	1.117.191	-25.665
1.141.143	-21.253	1.162.959	-30.763	1.117.298	-25.666
1.141.227	-21.255	1.163.183	-30.778	1.117.406	-25.668
1.141.357	-21.256	1.163.391	-30.793	1.117.585	-25.670
1.141.570	-21.257	1.163.539	-30.808	1.117.686	-25.671
1.141.733	-21.258	1.163.669	-30.822	1.117.803	-25.673
1.141.877	-21.260	1.163.885	-30.836	1.117.997	-25.675
1.142.028	-21.261	1.164.061	-30.850	1.118.183	-25.676
1.142.189	-21.262	1.164.175	-30.863	1.118.283	-25.678
1.142.378	-21.263	1.164.287	-30.877	1.118.413	-25.680
1.142.588	-21.264	1.164.455	-30.890	1.118.594	-25.681
1.142.805	-21.266	1.164.662	-30.902	1.118.711	-25.683

1.142.996	-21.267	1.164.791	-30.915	1.118.867	-25.685
1.143.083	-21.268	1.164.902	-30.927	1.119.064	-25.686
1.143.155	-21.269	1.165.074	-30.939	1.119.206	-25.688
1.143.365	-21.271	1.165.220	-30.950	1.119.409	-25.689
1.143.615	-21.272	1.165.418	-30.962	1.119.623	-25.691
1.143.767	-21.273	1.165.620	-30.973	1.119.872	-25.693
1.143.885	-21.274	1.165.733	-30.983	1.120.068	-25.694
1.144.011	-21.275	1.165.848	-30.994	1.120.198	-25.696
1.144.174	-21.277	1.165.990	-31.004	1.120.375	-25.697
1.144.368	-21.278	1.166.178	-31.014	1.120.508	-25.699
1.144.481	-21.279	1.166.355	-31.023	1.120.658	-25.700
1.144.656	-21.280	1.166.436	-31.032	1.120.819	-25.702
1.144.895	-21.281	1.166.566	-31.041	1.120.938	-25.703
1.145.051	-21.282	1.166.791	-31.049	1.121.087	-25.705
1.145.180	-21.284	1.167.001	-31.058	1.121.308	-25.706
1.145.339	-21.285	1.167.253	-31.065	1.121.479	-25.708
1.145.534	-21.286	1.167.420	-31.073	1.121.532	-25.709
1.145.693	-21.287	1.167.525	-31.080	1.121.656	-25.711
1.145.835	-21.288	1.167.662	-31.087	1.121.845	-25.712
1.145.991	-21.289	1.167.796	-31.094	1.122.008	-25.714
1.146.182	-21.290	1.167.926	-31.100	1.122.119	-25.715
1.146.338	-21.292	1.168.085	-31.106	1.122.271	-25.716
1.146.461	-21.293	1.168.279	-31.111	1.122.410	-25.718
1.146.646	-21.294	1.168.468	-31.117	1.122.592	-25.719
1.146.814	-21.295	1.168.652	-31.122	1.122.848	-25.720
1.146.908	-21.296	1.168.802	-31.126	1.123.034	-25.722
1.147.085	-21.297	1.168.978	-31.130	1.123.216	-25.723
1.147.294	-21.298	1.169.169	-31.134	1.123.374	-25.724
1.147.430	-21.299	1.169.342	-31.138	1.123.567	-25.726
1.147.642	-21.300	1.169.461	-31.141	1.123.745	-25.727

1.147.833	-21.301	1.169.558	-31.144	1.123.843	-25.728
1.147.943	-21.302	1.169.722	-31.147	1.123.968	-25.729
1.148.050	-21.303	1.169.875	-31.149	1.124.109	-25.731
1.148.332	-21.304	1.170.090	-31.151	1.124.330	-25.732
1.148.662	-21.305	1.170.250	-31.153	1.124.529	-25.733
1.148.971	-21.306	1.170.249	-31.154	1.124.592	-25.734
1.149.188	-21.307	1.170.391	-31.155	1.124.761	-25.735
1.149.286	-21.308	1.170.580	-31.156	1.125.004	-25.736
1.149.368	-21.309	1.170.713	-31.156	1.125.170	-25.738
1.149.398	-21.310	1.170.889	-31.156	1.125.345	-25.739
1.149.399	-21.311	1.171.025	-31.156	1.125.536	-25.740
1.149.499	-21.312	1.171.225	-31.155	1.125.682	-25.741
1.149.596	-21.313	1.171.441	-31.154	1.125.824	-25.742
1.149.760	-21.314	1.171.674	-31.153	1.125.976	-25.743
1.149.951	-21.315	1.171.898	-31.151	1.126.154	-25.744
1.150.121	-21.316	1.172.066	-31.149	1.126.339	-25.745
1.150.352	-21.317	1.172.243	-31.147	1.126.571	-25.746
1.150.516	-21.318	1.172.408	-31.145	1.126.739	-25.747
1.150.667	-21.318	1.172.592	-31.142	1.126.831	-25.748
1.150.811	-21.319	1.172.746	-31.139	1.126.999	-25.749
1.150.934	-21.320	1.172.852	-31.135	1.127.172	-25.750
1.151.114	-21.321	1.172.927	-31.132	1.127.406	-25.751
1.151.313	-21.322	1.173.028	-31.128	1.127.630	-25.752
1.151.469	-21.323	1.173.159	-31.123	1.127.751	-25.753
1.151.603	-21.323	1.173.316	-31.119	1.127.837	-25.754
1.151.785	-21.324	1.173.484	-31.114	1.127.978	-25.755
1.151.918	-21.325	1.173.627	-31.109	1.128.110	-25.756
1.152.030	-21.326	1.173.804	-31.103	1.128.230	-25.757
1.152.150	-21.326	1.174.035	-31.098	1.128.434	-25.758
1.152.281	-21.327	1.174.268	-31.092	1.128.609	-25.759

1.152.462	-21.328	1.174.476	-31.085	1.128.796	-25.760
1.152.637	-21.328	1.174.660	-31.079	1.128.973	-25.760
1.152.828	-21.329	1.174.780	-31.072	1.129.075	-25.761
1.153.000	-21.330	1.174.930	-31.065	1.129.220	-25.762
1.153.211	-21.330	1.175.125	-31.057	1.129.426	-25.763
1.153.407	-21.331	1.175.283	-31.050	1.129.605	-25.764
1.153.521	-21.332	1.175.449	-31.042	1.129.690	-25.765
1.153.659	-21.332	1.175.685	-31.034	1.129.781	-25.766
1.153.857	-21.333	1.175.855	-31.025	1.130.067	-25.766
1.154.074	-21.334	1.175.981	-31.017	1.130.552	-25.767
1.154.188	-21.334	1.176.134	-31.008	1.130.979	-25.768
1.154.288	-21.335	1.176.317	-30.999	1.131.134	-25.769
1.154.473	-21.335	1.176.501	-30.989	1.131.122	-25.770
1.154.692	-21.336	1.176.625	-30.980	1.131.159	-25.771
1.154.848	-21.336	1.176.732	-30.970	1.131.242	-25.771
1.154.909	-21.337	1.176.887	-30.960	1.131.337	-25.772
1.155.023	-21.337	1.177.098	-30.949	1.131.423	-25.773
1.155.229	-21.338	1.177.279	-30.939	1.131.531	-25.774
1.155.473	-21.338	1.177.414	-30.928	1.131.625	-25.775
1.155.625	-21.339	1.177.527	-30.917	1.131.737	-25.775
1.155.765	-21.339	1.177.701	-30.906	1.131.910	-25.776
1.155.967	-21.340	1.177.879	-30.895	1.132.103	-25.777
1.156.161	-21.340	1.178.029	-30.883	1.132.246	-25.778
1.156.327	-21.340	1.178.223	-30.871	1.132.421	-25.779
1.156.397	-21.341	1.178.372	-30.859	1.132.665	-25.779
1.156.499	-21.341	1.178.550	-30.847	1.132.834	-25.780
1.156.742	-21.341	1.178.727	-30.835	1.133.021	-25.781
1.156.928	-21.342	1.178.884	-30.822	1.133.193	-25.782
1.157.064	-21.342	1.179.066	-30.810	1.133.335	-25.783
1.157.220	-21.342	1.179.219	-30.797	1.133.444	-25.784

1.157.353	-21.343	1.179.359	-30.784	1.133.559	-25.785
1.157.517	-21.343	1.179.507	-30.771	1.133.747	-25.785
1.157.682	-21.343	1.179.649	-30.757	1.133.933	-25.786
1.157.840	-21.343	1.179.854	-30.744	1.134.098	-25.787
1.157.952	-21.344	1.180.287	-30.730	1.134.250	-25.788
1.158.105	-21.344	1.180.665	-30.717	1.134.331	-25.789
1.158.344	-21.344	1.180.817	-30.703	1.134.489	-25.790
1.158.481	-21.344	1.180.901	-30.689	1.134.703	-25.791
1.158.562	-21.344	1.180.905	-30.675	1.134.810	-25.792
1.158.731	-21.344	1.180.947	-30.661	1.134.968	-25.793
1.158.969	-21.345	1.181.031	-30.646	1.135.115	-25.794
1.159.212	-21.345	1.181.132	-30.632	1.135.226	-25.795
1.159.370	-21.345	1.181.285	-30.617	1.135.359	-25.796
1.159.494	-21.345	1.181.425	-30.603	1.135.536	-25.797
1.159.657	-21.345	1.181.523	-30.588	1.135.729	-25.798
1.159.803	-21.345	1.181.738	-30.573	1.135.930	-25.799
1.159.938	-21.345	1.181.971	-30.558	1.136.136	-25.800
1.160.079	-21.345	1.182.127	-30.544	1.136.286	-25.801
1.160.205	-21.345	1.182.322	-30.529	1.136.414	-25.802
1.160.320	-21.345	1.182.520	-30.513	1.136.595	-25.803
1.160.426	-21.345	1.182.652	-30.498	1.136.779	-25.804
1.160.587	-21.345	1.182.876	-30.483	1.136.917	-25.805
1.160.729	-21.345	1.183.111	-30.468	1.137.087	-25.806
1.160.828	-21.345	1.183.224	-30.453	1.137.256	-25.808
1.161.044	-21.345	1.183.329	-30.437	1.137.383	-25.809
1.161.234	-21.345	1.183.441	-30.422	1.137.562	-25.810
1.161.405	-21.345	1.183.638	-30.407	1.137.747	-25.811
1.161.579	-21.345	1.183.848	-30.391	1.137.854	-25.812
1.161.723	-21.344	1.183.977	-30.376	1.138.039	-25.814
1.161.968	-21.344	1.184.091	-30.361	1.138.236	-25.815

1.162.261	-21.344	1.184.232	-30.345	1.138.361	-25.816
1.162.517	-21.344	1.184.370	-30.330	1.138.457	-25.818
1.162.716	-21.344	1.184.552	-30.315	1.138.635	-25.819
1.162.816	-21.344	1.184.783	-30.299	1.138.852	-25.820
1.162.896	-21.343	1.184.981	-30.284	1.138.977	-25.822
1.163.012	-21.343	1.185.112	-30.268	1.139.128	-25.823
1.163.106	-21.343	1.185.242	-30.253	1.139.277	-25.825
1.163.292	-21.343	1.185.426	-30.238	1.139.440	-25.826
1.163.469	-21.342	1.185.592	-30.223	1.139.641	-25.828
1.163.514	-21.342	1.185.709	-30.207	1.139.790	-25.829
1.163.617	-21.342	1.185.883	-30.192	1.139.914	-25.831
1.163.828	-21.342	1.186.080	-30.177	1.140.052	-25.832
1.163.996	-21.341	1.186.231	-30.162	1.140.221	-25.834
1.164.154	-21.341	1.186.320	-30.147	1.140.404	-25.836
1.164.365	-21.341	1.186.438	-30.132	1.140.566	-25.837
1.164.549	-21.340	1.186.664	-30.117	1.140.742	-25.839
1.164.709	-21.340	1.186.851	-30.102	1.140.908	-25.841
1.164.955	-21.340	1.186.973	-30.087	1.141.142	-25.842
1.165.136	-21.339	1.187.119	-30.072	1.141.363	-25.844
1.165.227	-21.339	1.187.310	-30.057	1.141.451	-25.846
1.165.454	-21.338	1.187.464	-30.042	1.141.566	-25.848
1.165.640	-21.338	1.187.628	-30.028	1.141.727	-25.849
1.165.697	-21.338	1.187.817	-30.013	1.141.896	-25.851
1.165.818	-21.337	1.187.935	-29.999	1.142.067	-25.853
1.165.978	-21.337	1.188.053	-29.984	1.142.259	-25.855
1.166.132	-21.336	1.188.170	-29.970	1.142.431	-25.857
1.166.367	-21.336	1.188.361	-29.956	1.142.520	-25.859
1.166.553	-21.335	1.188.577	-29.942	1.142.661	-25.860
1.166.688	-21.335	1.188.795	-29.928	1.142.875	-25.862
1.166.877	-21.334	1.188.982	-29.914	1.142.989	-25.864

1.167.024	-21.334	1.189.073	-29.900	1.143.151	-25.866
1.167.161	-21.333	1.189.284	-29.886	1.143.357	-25.868
1.167.340	-21.333	1.189.514	-29.872	1.143.482	-25.870
1.167.531	-21.332	1.189.620	-29.859	1.143.654	-25.872
1.167.703	-21.332	1.189.753	-29.845	1.143.839	-25.874
1.167.895	-21.331	1.189.977	-29.832	1.143.990	-25.876
1.167.996	-21.331	1.190.179	-29.818	1.144.148	-25.878
1.168.075	-21.330	1.190.308	-29.805	1.144.289	-25.880
1.168.295	-21.330	1.190.499	-29.792	1.144.454	-25.882
1.168.501	-21.329	1.190.705	-29.779	1.144.620	-25.884
1.168.669	-21.328	1.190.851	-29.766	1.144.724	-25.887
1.168.820	-21.328	1.191.004	-29.753	1.144.841	-25.889
1.168.960	-21.327	1.191.200	-29.740	1.145.014	-25.891
1.169.139	-21.327	1.191.347	-29.728	1.145.138	-25.893
1.169.277	-21.326	1.191.472	-29.715	1.145.251	-25.895
1.169.443	-21.325	1.191.553	-29.703	1.145.385	-25.897
1.169.610	-21.325	1.191.675	-29.690	1.145.556	-25.899
1.169.718	-21.324	1.191.840	-29.678	1.145.745	-25.901
1.169.862	-21.324	1.191.990	-29.666	1.145.872	-25.904
1.170.035	-21.323	1.192.188	-29.654	1.146.076	-25.906
1.170.380	-21.322	1.192.316	-29.642	1.146.315	-25.908
1.170.829	-21.322	1.192.346	-29.630	1.146.511	-25.910
1.171.134	-21.321	1.192.436	-29.619	1.146.737	-25.912
1.171.221	-21.321	1.192.642	-29.607	1.146.918	-25.914
1.171.274	-21.320	1.192.895	-29.596	1.147.060	-25.917
1.171.332	-21.319	1.193.102	-29.584	1.147.226	-25.919
1.171.367	-21.319	1.193.292	-29.573	1.147.359	-25.921
1.171.473	-21.318	1.193.557	-29.562	1.147.539	-25.923
1.171.609	-21.317	1.193.772	-29.551	1.147.704	-25.925
1.171.755	-21.317	1.193.895	-29.540	1.147.833	-25.928

1.171.871	-21.316	1.194.062	-29.529	1.148.033	-25.930
1.171.945	-21.315	1.194.262	-29.519	1.148.201	-25.932
1.172.084	-21.315	1.194.356	-29.508	1.148.260	-25.934
1.172.317	-21.314	1.194.448	-29.498	1.148.412	-25.936
1.172.521	-21.314	1.194.620	-29.487	1.148.643	-25.938
1.172.654	-21.313	1.194.774	-29.477	1.148.774	-25.941
1.172.828	-21.312	1.194.881	-29.467	1.148.957	-25.943
1.173.015	-21.312	1.195.006	-29.457	1.149.107	-25.945
1.173.161	-21.311	1.195.128	-29.448	1.149.242	-25.947
1.173.337	-21.310	1.195.237	-29.438	1.149.417	-25.949
1.173.523	-21.310	1.195.410	-29.428	1.149.604	-25.951
1.173.698	-21.309	1.195.612	-29.419	1.149.753	-25.954
1.173.821	-21.309	1.195.807	-29.409	1.149.931	-25.956
1.173.978	-21.308	1.196.031	-29.400	1.150.184	-25.958
1.174.147	-21.307	1.196.283	-29.391	1.150.338	-25.960
1.174.224	-21.307	1.196.476	-29.382	1.150.462	-25.962
1.174.353	-21.306	1.196.630	-29.373	1.150.642	-25.964
1.174.503	-21.306	1.196.757	-29.364	1.150.823	-25.966
1.174.684	-21.305	1.196.898	-29.356	1.150.929	-25.968
1.174.909	-21.305	1.197.073	-29.347	1.151.063	-25.970
1.175.116	-21.304	1.197.226	-29.339	1.151.208	-25.972
1.175.313	-21.303	1.197.397	-29.331	1.151.320	-25.974
1.175.470	-21.303	1.197.563	-29.322	1.151.497	-25.977
1.175.612	-21.302	1.197.726	-29.314	1.151.749	-25.979
1.175.778	-21.302	1.197.922	-29.306	1.151.924	-25.981
1.175.919	-21.301	1.198.068	-29.299	1.152.043	-25.983
1.176.012	-21.301	1.198.231	-29.291	1.152.199	-25.984
1.176.131	-21.300	1.198.398	-29.283	1.152.352	-25.986
1.176.292	-21.300	1.198.561	-29.276	1.152.562	-25.988
1.176.439	-21.299	1.198.758	-29.268	1.152.747	-25.990

1.176.599	-21.299	1.198.905	-29.261	1.152.830	-25.992
1.176.765	-21.299	1.199.078	-29.254	1.152.965	-25.994
1.176.989	-21.298	1.199.281	-29.247	1.153.106	-25.996
1.177.167	-21.298	1.199.433	-29.240	1.153.336	-25.998
1.177.304	-21.297	1.199.604	-29.233	1.153.587	-26.000
1.177.411	-21.297	1.199.780	-29.227	1.153.705	-26.002
1.177.604	-21.297	1.199.890	-29.220	1.153.842	-26.004
1.177.852	-21.296	1.200.013	-29.214	1.153.962	-26.005
1.177.960	-21.296	1.200.160	-29.207	1.154.081	-26.007
1.178.102	-21.296	1.200.291	-29.201	1.154.286	-26.009
1.178.272	-21.295	1.200.469	-29.195	1.154.526	-26.011
1.178.436	-21.295	1.200.667	-29.189	1.154.686	-26.012
1.178.593	-21.295	1.200.800	-29.183	1.154.776	-26.014
1.178.768	-21.295	1.201.010	-29.177	1.154.915	-26.016
1.178.979	-21.294	1.201.252	-29.171	1.155.145	-26.018
1.179.169	-21.294	1.201.356	-29.166	1.155.359	-26.019
1.179.352	-21.294	1.201.471	-29.160	1.155.545	-26.021
1.179.529	-21.294	1.201.833	-29.155	1.155.688	-26.023
1.179.634	-21.294	1.202.216	-29.149	1.155.735	-26.024
1.179.815	-21.294	1.202.399	-29.144	1.155.874	-26.026
1.180.002	-21.293	1.202.453	-29.139	1.156.063	-26.028
1.180.148	-21.293	1.202.516	-29.134	1.156.278	-26.029
1.180.315	-21.293	1.202.608	-29.129	1.156.455	-26.031
1.180.433	-21.293	1.202.667	-29.124	1.156.544	-26.032
1.180.546	-21.293	1.202.724	-29.119	1.156.786	-26.034
1.180.686	-21.293	1.202.826	-29.114	1.157.186	-26.036
1.180.835	-21.293	1.203.011	-29.110	1.157.550	-26.037
1.181.028	-21.293	1.203.186	-29.105	1.157.781	-26.039
1.181.208	-21.293	1.203.391	-29.101	1.157.837	-26.040
1.181.399	-21.293	1.203.594	-29.097	1.157.898	-26.042

1.181.594	-21.293	1.203.734	-29.092	1.158.039	-26.043
1.181.787	-21.293	1.203.906	-29.088	1.158.100	-26.045
1.181.927	-21.294	1.204.094	-29.084	1.158.094	-26.046
1.182.014	-21.294	1.204.202	-29.080	1.158.167	-26.047
1.182.195	-21.294	1.204.370	-29.076	1.158.314	-26.049
1.182.354	-21.294	1.204.582	-29.072	1.158.423	-26.050
1.182.438	-21.294	1.204.762	-29.068	1.158.506	-26.052
1.182.551	-21.295	1.204.892	-29.065	1.158.615	-26.053
1.182.670	-21.295	1.205.000	-29.061	1.158.780	-26.055
1.182.802	-21.295	1.205.174	-29.057	1.158.998	-26.056
1.183.002	-21.295	1.205.356	-29.054	1.159.188	-26.057
1.183.181	-21.296	1.205.536	-29.050	1.159.364	-26.059
1.183.338	-21.296	1.205.666	-29.047	1.159.547	-26.060
1.183.548	-21.296	1.205.760	-29.044	1.159.735	-26.062
1.183.766	-21.297	1.205.949	-29.041	1.159.967	-26.063
1.183.935	-21.297	1.206.151	-29.037	1.160.177	-26.064
1.184.146	-21.298	1.206.339	-29.034	1.160.326	-26.066
1.184.303	-21.298	1.206.544	-29.031	1.160.432	-26.067
1.184.428	-21.299	1.206.707	-29.028	1.160.505	-26.068
1.184.664	-21.299	1.206.859	-29.025	1.160.664	-26.070
1.184.894	-21.300	1.207.005	-29.023	1.160.835	-26.071
1.185.051	-21.300	1.207.169	-29.020	1.160.977	-26.073
1.185.162	-21.301	1.207.306	-29.017	1.161.140	-26.074
1.185.274	-21.301	1.207.480	-29.014	1.161.314	-26.075
1.185.370	-21.302	1.207.680	-29.012	1.161.485	-26.077
1.185.484	-21.303	1.207.805	-29.009	1.161.607	-26.078
1.185.632	-21.303	1.207.955	-29.007	1.161.749	-26.079
1.185.830	-21.304	1.208.100	-29.004	1.161.896	-26.081
1.186.020	-21.305	1.208.226	-29.002	1.162.087	-26.082
1.186.162	-21.305	1.208.388	-28.999	1.162.293	-26.084

1.186.346	-21.306	1.208.553	-28.997	1.162.457	-26.085
1.186.566	-21.307	1.208.708	-28.995	1.162.642	-26.086
1.186.740	-21.308	1.208.908	-28.993	1.162.850	-26.088
1.186.870	-21.308	1.209.058	-28.991	1.163.035	-26.089
1.187.089	-21.309	1.209.212	-28.988	1.163.178	-26.090
1.187.343	-21.310	1.209.412	-28.986	1.163.395	-26.092
1.187.472	-21.311	1.209.510	-28.984	1.163.551	-26.093
1.187.568	-21.312	1.209.602	-28.982	1.163.638	-26.095
1.187.699	-21.313	1.209.787	-28.980	1.163.820	-26.096
1.187.843	-21.313	1.210.005	-28.978	1.163.967	-26.098
1.187.980	-21.314	1.210.191	-28.976	1.164.048	-26.099
1.188.151	-21.315	1.210.321	-28.975	1.164.191	-26.100
1.188.287	-21.316	1.210.516	-28.973	1.164.381	-26.102
1.188.430	-21.317	1.210.714	-28.971	1.164.557	-26.103
1.188.661	-21.318	1.210.863	-28.969	1.164.695	-26.105
1.188.883	-21.319	1.210.979	-28.967	1.164.838	-26.106
1.189.076	-21.320	1.211.128	-28.966	1.165.060	-26.108
1.189.210	-21.321	1.211.283	-28.964	1.165.229	-26.109
1.189.323	-21.322	1.211.460	-28.962	1.165.340	-26.111
1.189.510	-21.323	1.211.557	-28.961	1.165.470	-26.112
1.189.707	-21.324	1.211.725	-28.959	1.165.648	-26.114
1.189.827	-21.325	1.211.973	-28.957	1.165.821	-26.115
1.190.000	-21.326	1.212.070	-28.956	1.165.934	-26.117
1.190.221	-21.328	1.212.282	-28.954	1.166.068	-26.119
1.190.320	-21.329	1.212.505	-28.953	1.166.252	-26.120
1.190.456	-21.330	1.212.612	-28.951	1.166.443	-26.122
1.190.620	-21.331	1.212.747	-28.949	1.166.637	-26.123
1.190.773	-21.332	1.212.955	-28.948	1.166.808	-26.125
1.190.911	-21.333	1.213.114	-28.946	1.166.903	-26.127
1.191.040	-21.334	1.213.222	-28.945	1.167.018	-26.128

1.191.219	-21.336	1.213.347	-28.943	1.167.141	-26.130
1.191.426	-21.337	1.213.481	-28.942	1.167.293	-26.132
1.191.625	-21.338	1.213.645	-28.940	1.167.500	-26.133
1.191.739	-21.339	1.213.795	-28.939	1.167.732	-26.135
1.191.884	-21.340	1.213.860	-28.937	1.167.934	-26.137
1.192.089	-21.342	1.213.996	-28.936	1.168.046	-26.139
1.192.331	-21.343	1.214.170	-28.934	1.168.181	-26.140
1.192.711	-21.344	1.214.399	-28.933	1.168.357	-26.142
1.193.053	-21.345	1.214.625	-28.931	1.168.554	-26.144
1.193.213	-21.347	1.214.804	-28.930	1.168.701	-26.146
1.193.288	-21.348	1.214.971	-28.928	1.168.826	-26.147
1.193.360	-21.349	1.215.188	-28.927	1.169.046	-26.149
1.193.412	-21.350	1.215.387	-28.925	1.169.215	-26.151
1.193.457	-21.352	1.215.561	-28.924	1.169.333	-26.153
1.193.526	-21.353	1.215.721	-28.922	1.169.508	-26.155
1.193.661	-21.354	1.215.828	-28.920	1.169.648	-26.157
1.193.804	-21.355	1.215.956	-28.919	1.169.818	-26.159
1.193.989	-21.357	1.216.093	-28.917	1.170.023	-26.160
1.194.130	-21.358	1.216.247	-28.916	1.170.227	-26.162
1.194.269	-21.359	1.216.434	-28.914	1.170.403	-26.164
1.194.448	-21.361	1.216.586	-28.912	1.170.543	-26.166
1.194.598	-21.362	1.216.739	-28.911	1.170.681	-26.168
1.194.806	-21.363	1.216.853	-28.909	1.170.800	-26.170
1.195.031	-21.364	1.216.987	-28.908	1.170.954	-26.172
1.195.188	-21.366	1.217.154	-28.906	1.171.137	-26.174
1.195.322	-21.367	1.217.289	-28.904	1.171.242	-26.176
1.195.450	-21.368	1.217.481	-28.903	1.171.399	-26.178
1.195.527	-21.370	1.217.680	-28.901	1.171.524	-26.180
1.195.668	-21.371	1.217.861	-28.899	1.171.588	-26.182
1.195.899	-21.372	1.218.043	-28.897	1.171.816	-26.184

1.196.042	-21.374	1.218.207	-28.896	1.172.009	-26.186
1.196.180	-21.375	1.218.356	-28.894	1.172.101	-26.188
1.196.348	-21.376	1.218.469	-28.892	1.172.208	-26.190
1.196.529	-21.378	1.218.578	-28.890	1.172.364	-26.192
1.196.774	-21.379	1.218.764	-28.889	1.172.599	-26.194
1.196.852	-21.380	1.218.972	-28.887	1.172.759	-26.196
1.196.908	-21.381	1.219.151	-28.885	1.172.936	-26.198
1.197.172	-21.383	1.219.365	-28.883	1.173.190	-26.200
1.197.344	-21.384	1.219.548	-28.881	1.173.391	-26.202
1.197.439	-21.385	1.219.715	-28.880	1.173.538	-26.204
1.197.656	-21.387	1.219.867	-28.878	1.173.652	-26.207
1.197.897	-21.388	1.220.024	-28.876	1.173.832	-26.209
1.198.073	-21.389	1.220.211	-28.874	1.174.037	-26.211
1.198.250	-21.390	1.220.342	-28.872	1.174.204	-26.213
1.198.399	-21.392	1.220.487	-28.870	1.174.363	-26.215
1.198.542	-21.393	1.220.642	-28.868	1.174.535	-26.217
1.198.630	-21.394	1.220.811	-28.866	1.174.709	-26.219
1.198.680	-21.396	1.221.031	-28.864	1.174.862	-26.221
1.198.876	-21.397	1.221.246	-28.862	1.174.946	-26.223
1.199.090	-21.398	1.221.382	-28.860	1.175.028	-26.225
1.199.205	-21.399	1.221.525	-28.858	1.175.170	-26.227
1.199.378	-21.401	1.221.697	-28.856	1.175.294	-26.229
1.199.554	-21.402	1.221.842	-28.854	1.175.441	-26.232
1.199.710	-21.403	1.221.972	-28.852	1.175.600	-26.234
1.199.883	-21.404	1.222.143	-28.850	1.175.745	-26.236
1.200.005	-21.406	1.222.327	-28.848	1.175.947	-26.238
1.200.155	-21.407	1.222.469	-28.846	1.176.058	-26.240
1.200.360	-21.408	1.222.637	-28.844	1.176.295	-26.242
1.200.538	-21.409	1.222.752	-28.842	1.176.609	-26.244
1.200.662	-21.411	1.222.798	-28.840	1.176.809	-26.246

1.200.891	-21.412	1.222.985	-28.838	1.177.007	-26.248
1.201.112	-21.413	1.223.419	-28.836	1.177.143	-26.250
1.201.243	-21.414	1.223.840	-28.834	1.177.310	-26.252
1.201.392	-21.415	1.224.047	-28.832	1.177.453	-26.254
1.201.589	-21.417	1.224.118	-28.830	1.177.617	-26.256
1.201.740	-21.418	1.224.206	-28.828	1.177.766	-26.258
1.201.851	-21.419	1.224.267	-28.825	1.177.896	-26.260
1.202.035	-21.420	1.224.284	-28.823	1.178.124	-26.262
1.202.228	-21.421	1.224.377	-28.821	1.178.313	-26.264
1.202.356	-21.422	1.224.510	-28.819	1.178.466	-26.266
1.202.498	-21.424	1.224.690	-28.817	1.178.642	-26.268
1.202.664	-21.425	1.224.785	-28.815	1.178.811	-26.270
1.202.820	-21.426	1.224.847	-28.813	1.178.906	-26.272
1.202.964	-21.427	1.225.070	-28.811	1.179.014	-26.274
1.203.139	-21.428	1.225.293	-28.809	1.179.203	-26.276
1.203.338	-21.429	1.225.440	-28.806	1.179.398	-26.278
1.203.519	-21.430	1.225.631	-28.804	1.179.595	-26.280
1.203.682	-21.432	1.225.843	-28.802	1.179.716	-26.282
1.203.857	-21.433	1.225.982	-28.800	1.179.804	-26.284
1.203.980	-21.434	1.226.150	-28.798	1.179.978	-26.286
1.204.097	-21.435	1.226.328	-28.796	1.180.191	-26.288
1.204.303	-21.436	1.226.472	-28.794	1.180.357	-26.290
1.204.445	-21.437	1.226.595	-28.792	1.180.549	-26.292
1.204.519	-21.438	1.226.714	-28.790	1.180.733	-26.294
1.204.598	-21.439	1.226.890	-28.788	1.180.867	-26.296
1.204.737	-21.440	1.227.060	-28.786	1.181.051	-26.298
1.204.897	-21.441	1.227.223	-28.783	1.181.181	-26.300
1.205.058	-21.443	1.227.358	-28.781	1.181.293	-26.302
1.205.280	-21.444	1.227.456	-28.779	1.181.495	-26.304
1.205.508	-21.445	1.227.657	-28.777	1.181.634	-26.306

1.205.676	-21.446	1.227.852	-28.775	1.181.785	-26.308
1.205.872	-21.447	1.227.989	-28.773	1.181.981	-26.310
1.206.103	-21.448	1.228.212	-28.771	1.182.166	-26.312
1.206.248	-21.449	1.228.430	-28.769	1.182.377	-26.314
1.206.410	-21.450	1.228.560	-28.767	1.182.542	-26.316
1.206.604	-21.451	1.228.697	-28.766	1.182.664	-26.318
1.206.755	-21.452	1.228.904	-28.764	1.182.814	-26.320
1.206.955	-21.453	1.229.042	-28.762	1.182.951	-26.322
1.207.152	-21.454	1.229.155	-28.760	1.183.027	-26.324
1.207.257	-21.455	1.229.333	-28.758	1.183.176	-26.326
1.207.390	-21.456	1.229.493	-28.756	1.183.569	-26.327
1.207.584	-21.457	1.229.649	-28.754	1.184.008	-26.329
1.207.713	-21.458	1.229.834	-28.753	1.184.174	-26.331
1.207.791	-21.459	1.229.956	-28.751	1.184.296	-26.333
1.207.924	-21.460	1.230.035	-28.749	1.184.482	-26.335
1.208.072	-21.461	1.230.214	-28.747	1.184.594	-26.337
1.208.290	-21.462	1.230.394	-28.745	1.184.648	-26.339
1.208.505	-21.463	1.230.548	-28.744	1.184.700	-26.341
1.208.647	-21.464	1.230.741	-28.742	1.184.754	-26.343
1.208.827	-21.465	1.230.914	-28.740	1.184.831	-26.345
1.209.042	-21.466	1.231.013	-28.739	1.185.005	-26.347
1.209.219	-21.466	1.231.137	-28.737	1.185.210	-26.349
1.209.377	-21.467	1.231.317	-28.736	1.185.381	-26.351
1.209.564	-21.468	1.231.462	-28.734	1.185.506	-26.353
1.209.679	-21.469	1.231.607	-28.733	1.185.655	-26.355
1.209.865	-21.470	1.231.782	-28.731	1.185.817	-26.357
1.210.100	-21.471	1.231.982	-28.730	1.185.955	-26.359
1.210.190	-21.472	1.232.155	-28.728	1.186.156	-26.361
1.210.320	-21.473	1.232.303	-28.727	1.186.371	-26.363
1.210.470	-21.474	1.232.458	-28.725	1.186.487	-26.365

1.210.660	-21.474	1.232.635	-28.724	1.186.638	-26.367
1.210.875	-21.475	1.232.813	-28.722	1.186.892	-26.369
1.211.006	-21.476	1.232.972	-28.721	1.187.078	-26.371
1.211.128	-21.477	1.233.168	-28.720	1.187.136	-26.373
1.211.306	-21.478	1.233.288	-28.718	1.187.253	-26.375
1.211.479	-21.479	1.233.394	-28.717	1.187.382	-26.377
1.211.625	-21.480	1.233.532	-28.716	1.187.521	-26.379
1.211.767	-21.480	1.233.717	-28.715	1.187.691	-26.381
1.211.902	-21.481	1.233.908	-28.713	1.187.875	-26.383
1.212.097	-21.482	1.234.028	-28.712	1.188.064	-26.385
1.212.243	-21.483	1.234.261	-28.711	1.188.223	-26.388
1.212.363	-21.484	1.234.464	-28.710	1.188.360	-26.390
1.212.577	-21.484	1.234.639	-28.709	1.188.531	-26.392
1.212.750	-21.485	1.234.773	-28.708	1.188.677	-26.394
1.212.887	-21.486	1.234.874	-28.707	1.188.786	-26.396
1.213.066	-21.487	1.235.015	-28.706	1.188.980	-26.398
1.213.197	-21.487	1.235.145	-28.705	1.189.219	-26.400
1.213.314	-21.488	1.235.322	-28.704	1.189.361	-26.403
1.213.484	-21.489	1.235.508	-28.703	1.189.467	-26.405
1.213.679	-21.490	1.235.611	-28.702	1.189.648	-26.407
1.213.843	-21.490	1.235.714	-28.701	1.189.848	-26.409
1.213.988	-21.491	1.235.921	-28.700	1.190.042	-26.411
1.214.240	-21.492	1.236.091	-28.699	1.190.209	-26.414
1.214.660	-21.492	1.236.269	-28.698	1.190.371	-26.416
1.215.092	-21.493	1.236.476	-28.697	1.190.543	-26.418
1.215.267	-21.494	1.236.656	-28.696	1.190.668	-26.421
1.215.273	-21.494	1.236.851	-28.695	1.190.813	-26.423
1.215.260	-21.495	1.237.051	-28.695	1.190.951	-26.425
1.215.222	-21.496	1.237.290	-28.694	1.191.067	-26.428
1.215.385	-21.496	1.237.452	-28.693	1.191.182	-26.430

1.215.546	-21.497	1.237.519	-28.692	1.191.357	-26.432
1.215.627	-21.498	1.237.628	-28.692	1.191.599	-26.435
1.215.764	-21.498	1.237.765	-28.691	1.191.731	-26.437
1.215.920	-21.499	1.237.908	-28.690	1.191.903	-26.439
1.216.115	-21.499	1.238.053	-28.690	1.192.135	-26.442
1.216.259	-21.500	1.238.177	-28.689	1.192.251	-26.444
1.216.448	-21.501	1.238.316	-28.688	1.192.406	-26.447
1.216.609	-21.501	1.238.476	-28.688	1.192.608	-26.449
1.216.743	-21.502	1.238.689	-28.687	1.192.803	-26.452
1.216.903	-21.502	1.238.908	-28.687	1.192.959	-26.454
1.217.135	-21.503	1.239.042	-28.686	1.193.056	-26.457
1.217.377	-21.503	1.239.232	-28.685	1.193.199	-26.459
1.217.472	-21.504	1.239.403	-28.685	1.193.328	-26.462
1.217.558	-21.504	1.239.514	-28.684	1.193.482	-26.464
1.217.714	-21.505	1.239.746	-28.684	1.193.689	-26.467
1.217.845	-21.506	1.239.935	-28.683	1.193.838	-26.469
1.217.990	-21.506	1.240.070	-28.683	1.194.000	-26.472
1.218.197	-21.507	1.240.265	-28.682	1.194.269	-26.475
1.218.313	-21.507	1.240.472	-28.682	1.194.517	-26.477
1.218.414	-21.508	1.240.594	-28.682	1.194.638	-26.480
1.218.529	-21.508	1.240.693	-28.681	1.194.731	-26.483
1.218.671	-21.509	1.240.851	-28.681	1.194.864	-26.485
1.218.914	-21.509	1.241.014	-28.680	1.194.989	-26.488
1.219.142	-21.509	1.241.216	-28.680	1.195.111	-26.491
1.219.358	-21.510	1.241.385	-28.680	1.195.341	-26.493
1.219.548	-21.510	1.241.516	-28.679	1.195.527	-26.496
1.219.705	-21.511	1.241.682	-28.679	1.195.604	-26.499
1.219.832	-21.511	1.241.892	-28.679	1.195.731	-26.502
1.219.948	-21.512	1.242.038	-28.678	1.195.923	-26.504
1.220.112	-21.512	1.242.089	-28.678	1.196.138	-26.507

1.220.280	-21.513	1.242.243	-28.678	1.196.323	-26.510
1.220.419	-21.513	1.242.468	-28.677	1.196.448	-26.513
1.220.583	-21.514	1.242.624	-28.677	1.196.572	-26.515
1.220.770	-21.514	1.242.761	-28.677	1.196.701	-26.518
1.220.898	-21.514	1.242.988	-28.677	1.196.823	-26.521
1.221.056	-21.515	1.243.150	-28.676	1.196.986	-26.524
1.221.236	-21.515	1.243.276	-28.676	1.197.183	-26.527
1.221.426	-21.516	1.243.465	-28.676	1.197.383	-26.529
1.221.570	-21.516	1.243.622	-28.676	1.197.570	-26.532
1.221.685	-21.516	1.243.838	-28.675	1.197.784	-26.535
1.221.860	-21.517	1.244.051	-28.675	1.197.914	-26.538
1.222.048	-21.517	1.244.177	-28.675	1.197.981	-26.541
1.222.256	-21.518	1.244.323	-28.675	1.198.062	-26.543
1.222.368	-21.518	1.244.481	-28.674	1.198.167	-26.546
1.222.443	-21.518	1.244.594	-28.674	1.198.327	-26.549
1.222.633	-21.519	1.244.863	-28.674	1.198.496	-26.552
1.222.833	-21.519	1.245.285	-28.674	1.198.673	-26.555
1.222.976	-21.520	1.245.565	-28.674	1.198.870	-26.558
1.223.192	-21.520	1.245.714	-28.673	1.199.070	-26.560
1.223.353	-21.520	1.245.761	-28.673	1.199.249	-26.563
1.223.458	-21.521	1.245.763	-28.673	1.199.423	-26.566
1.223.625	-21.521	1.245.807	-28.673	1.199.613	-26.569
1.223.837	-21.521	1.245.872	-28.672	1.199.875	-26.572
1.224.028	-21.522	1.245.984	-28.672	1.200.087	-26.574
1.224.160	-21.522	1.246.094	-28.672	1.200.227	-26.577
1.224.318	-21.523	1.246.273	-28.672	1.200.413	-26.580
1.224.494	-21.523	1.246.452	-28.672	1.200.552	-26.583
1.224.630	-21.523	1.246.583	-28.671	1.200.709	-26.586
1.224.745	-21.524	1.246.787	-28.671	1.200.958	-26.589
1.224.929	-21.524	1.247.010	-28.671	1.201.099	-26.591

1.225.147	-21.525	1.247.166	-28.671	1.201.189	-26.594
1.225.361	-21.525	1.247.282	-28.670	1.201.358	-26.597
1.225.500	-21.525	1.247.452	-28.670	1.201.555	-26.600
1.225.612	-21.526	1.247.639	-28.670	1.201.666	-26.603
1.225.768	-21.526	1.247.789	-28.670	1.201.740	-26.606
1.225.856	-21.527	1.247.947	-28.669	1.201.926	-26.608
1.225.992	-21.527	1.248.073	-28.669	1.202.089	-26.611
1.226.198	-21.527	1.248.167	-28.669	1.202.250	-26.614
1.226.351	-21.528	1.248.334	-28.668	1.202.390	-26.617
1.226.459	-21.528	1.248.512	-28.668	1.202.478	-26.620
1.226.593	-21.529	1.248.682	-28.668	1.202.636	-26.623
1.226.716	-21.529	1.248.857	-28.668	1.202.831	-26.626
1.226.856	-21.530	1.248.859	-28.667	1.203.056	-26.629
1.227.027	-21.530	1.249.029	-28.667	1.203.277	-26.632
1.227.120	-21.531	1.249.317	-28.667	1.203.481	-26.635
1.227.280	-21.531	1.249.530	-28.666	1.203.624	-26.638
1.227.480	-21.531	1.249.711	-28.666	1.203.743	-26.641
1.227.701	-21.532	1.249.858	-28.666	1.203.914	-26.644
1.227.972	-21.532	1.249.958	-28.665	1.204.073	-26.647
1.228.165	-21.533	1.250.110	-28.665	1.204.175	-26.650
1.228.300	-21.533	1.250.345	-28.665	1.204.325	-26.653
1.228.502	-21.534	1.250.529	-28.664	1.204.519	-26.656
1.228.699	-21.534	1.250.643	-28.664	1.204.707	-26.659
1.228.822	-21.535	1.250.780	-28.663	1.204.929	-26.663
1.228.957	-21.535	1.250.947	-28.663	1.205.088	-26.666
1.229.045	-21.536	1.251.136	-28.663	1.205.142	-26.669
1.229.185	-21.536	1.251.261	-28.662	1.205.295	-26.673
1.229.397	-21.537	1.251.357	-28.662	1.205.509	-26.676
1.229.565	-21.538	1.251.560	-28.661	1.205.701	-26.679
1.229.738	-21.538	1.251.758	-28.661	1.205.854	-26.683

1.229.924	-21.539	1.251.920	-28.660	1.205.928	-26.687
1.230.084	-21.539	1.252.114	-28.660	1.206.115	-26.690
1.230.211	-21.540	1.252.261	-28.659	1.206.349	-26.694
1.230.283	-21.540	1.252.356	-28.659	1.206.507	-26.697
1.230.379	-21.541	1.252.486	-28.658	1.206.667	-26.701
1.230.564	-21.542	1.252.638	-28.658	1.206.831	-26.705
1.230.826	-21.542	1.252.775	-28.657	1.206.972	-26.709
1.231.077	-21.543	1.252.927	-28.657	1.207.132	-26.713
1.231.252	-21.543	1.253.058	-28.656	1.207.377	-26.717
1.231.392	-21.544	1.253.236	-28.656	1.207.568	-26.721
1.231.536	-21.545	1.253.487	-28.655	1.207.693	-26.725
1.231.707	-21.545	1.253.762	-28.654	1.207.833	-26.729
1.231.815	-21.546	1.253.969	-28.654	1.207.996	-26.733
1.231.990	-21.547	1.254.075	-28.653	1.208.186	-26.738
1.232.204	-21.547	1.254.191	-28.652	1.208.316	-26.742
1.232.351	-21.548	1.254.268	-28.652	1.208.429	-26.746
1.232.500	-21.549	1.254.368	-28.651	1.208.640	-26.751
1.232.665	-21.550	1.254.532	-28.650	1.208.857	-26.756
1.232.843	-21.550	1.254.749	-28.650	1.208.988	-26.760
1.232.980	-21.551	1.254.940	-28.649	1.209.147	-26.765
1.233.087	-21.552	1.255.051	-28.648	1.209.345	-26.770
1.233.270	-21.552	1.255.166	-28.647	1.209.487	-26.774
1.233.476	-21.553	1.255.378	-28.646	1.209.602	-26.779
1.233.644	-21.554	1.255.648	-28.646	1.209.686	-26.784
1.233.787	-21.555	1.255.798	-28.645	1.209.841	-26.789
1.233.995	-21.556	1.255.932	-28.644	1.210.275	-26.794
1.234.184	-21.556	1.256.073	-28.643	1.210.689	-26.800
1.234.322	-21.557	1.256.201	-28.642	1.210.873	-26.805
1.234.484	-21.558	1.256.371	-28.641	1.210.973	-26.810
1.234.570	-21.559	1.256.429	-28.640	1.211.080	-26.816

1.234.695	-21.559	1.256.600	-28.639	1.211.208	-26.821
1.234.877	-21.560	1.256.835	-28.638	1.211.250	-26.826
1.235.080	-21.561	1.256.857	-28.637	1.211.259	-26.832
1.235.263	-21.562	1.256.956	-28.636	1.211.364	-26.838
1.235.389	-21.563	1.257.211	-28.635	1.211.490	-26.843
1.235.521	-21.564	1.257.355	-28.634	1.211.621	-26.849
1.235.696	-21.564	1.257.518	-28.633	1.211.767	-26.855
1.235.863	-21.565	1.257.670	-28.632	1.211.895	-26.861
1.235.984	-21.566	1.257.867	-28.631	1.212.048	-26.867
1.236.149	-21.567	1.258.083	-28.630	1.212.222	-26.873
1.236.524	-21.568	1.258.282	-28.629	1.212.399	-26.879
1.236.935	-21.569	1.258.508	-28.627	1.212.565	-26.885
1.237.119	-21.569	1.258.728	-28.626	1.212.745	-26.891
1.237.207	-21.570	1.258.880	-28.625	1.212.916	-26.898
1.237.319	-21.571	1.259.016	-28.624	1.213.101	-26.904
1.237.355	-21.572	1.259.173	-28.623	1.213.342	-26.910
1.237.383	-21.573	1.259.342	-28.621	1.213.571	-26.917
1.237.498	-21.574	1.259.471	-28.620	1.213.706	-26.923
1.237.581	-21.575	1.259.579	-28.619	1.213.849	-26.930
1.237.677	-21.576	1.259.682	-28.617	1.214.009	-26.936
1.237.840	-21.576	1.259.821	-28.616	1.214.113	-26.943
1.238.037	-21.577	1.259.982	-28.615	1.214.222	-26.950
1.238.220	-21.578	1.260.099	-28.613	1.214.351	-26.957
1.238.365	-21.579	1.260.202	-28.612	1.214.528	-26.963
1.238.586	-21.580	1.260.385	-28.610	1.214.652	-26.970
1.238.820	-21.581	1.260.627	-28.609	1.214.752	-26.977
1.238.987	-21.582	1.260.842	-28.608	1.214.862	-26.984
1.239.135	-21.583	1.261.015	-28.606	1.215.048	-26.991
1.239.322	-21.584	1.261.185	-28.605	1.215.283	-26.999
1.239.459	-21.585	1.261.373	-28.603	1.215.424	-27.006

1.239.577	-21.585	1.261.541	-28.602	1.215.549	-27.013
1.239.735	-21.586	1.261.669	-28.600	1.215.787	-27.020
1.239.844	-21.587	1.261.801	-28.599	1.216.007	-27.028
1.239.974	-21.588	1.261.960	-28.597	1.216.196	-27.035
1.240.114	-21.589	1.262.112	-28.596	1.216.336	-27.042
1.240.301	-21.590	1.262.309	-28.594	1.216.469	-27.050
1.240.540	-21.591	1.262.514	-28.592	1.216.628	-27.057
1.240.612	-21.592	1.262.648	-28.591	1.216.765	-27.065
1.240.639	-21.593	1.262.789	-28.589	1.216.906	-27.073
1.240.842	-21.593	1.262.962	-28.588	1.217.057	-27.080
1.241.098	-21.594	1.263.168	-28.586	1.217.166	-27.088
1.241.333	-21.595	1.263.329	-28.584	1.217.307	-27.096
1.241.516	-21.596	1.263.452	-28.583	1.217.474	-27.104
1.241.663	-21.597	1.263.618	-28.581	1.217.663	-27.111
1.241.843	-21.598	1.263.839	-28.580	1.217.859	-27.119
1.241.967	-21.599	1.263.936	-28.578	1.217.979	-27.127
1.242.076	-21.600	1.264.064	-28.576	1.218.114	-27.135
1.242.253	-21.601	1.264.326	-28.575	1.218.241	-27.143
1.242.418	-21.601	1.264.471	-28.573	1.218.336	-27.151
1.242.592	-21.602	1.264.622	-28.571	1.218.484	-27.159
1.242.784	-21.603	1.264.818	-28.570	1.218.753	-27.167
1.242.882	-21.604	1.265.004	-28.568	1.218.982	-27.175
1.242.942	-21.605	1.265.157	-28.566	1.219.148	-27.183
1.243.058	-21.606	1.265.316	-28.565	1.219.296	-27.191
1.243.270	-21.607	1.265.490	-28.563	1.219.481	-27.199
1.243.538	-21.607	1.265.598	-28.561	1.219.594	-27.207
1.243.706	-21.608	1.265.723	-28.559	1.219.660	-27.215
1.243.858	-21.609	1.265.845	-28.558	1.219.823	-27.223
1.244.025	-21.610	1.266.003	-28.556	1.219.997	-27.231
1.244.119	-21.611	1.266.230	-28.554	1.220.123	-27.239

1.244.241	-21.612	1.266.573	-28.553	1.220.242	-27.247
1.244.336	-21.612	1.266.987	-28.551	1.220.445	-27.255
1.244.462	-21.613	1.267.224	-28.549	1.220.642	-27.263
1.244.708	-21.614	1.267.283	-28.548	1.220.777	-27.271
1.244.963	-21.615	1.267.336	-28.546	1.220.899	-27.279
1.245.126	-21.616	1.267.397	-28.544	1.221.095	-27.287
1.245.231	-21.617	1.267.466	-28.543	1.221.311	-27.295
1.245.410	-21.617	1.267.547	-28.541	1.221.498	-27.303
1.245.644	-21.618	1.267.594	-28.539	1.221.686	-27.311
1.245.826	-21.619	1.267.740	-28.538	1.221.843	-27.318
1.245.953	-21.620	1.267.921	-28.536	1.222.003	-27.326
1.246.136	-21.621	1.268.085	-28.534	1.222.094	-27.334
1.246.284	-21.621	1.268.286	-28.533	1.222.247	-27.342
1.246.346	-21.622	1.268.430	-28.531	1.222.440	-27.349
1.246.494	-21.623	1.268.620	-28.529	1.222.567	-27.357
1.246.622	-21.624	1.268.848	-28.528	1.222.735	-27.364
1.246.805	-21.624	1.268.990	-28.526	1.222.935	-27.372
1.247.098	-21.625	1.269.093	-28.524	1.223.180	-27.379
1.247.297	-21.626	1.269.252	-28.523	1.223.355	-27.386
1.247.336	-21.627	1.269.397	-28.521	1.223.493	-27.394
1.247.465	-21.627	1.269.507	-28.520	1.223.587	-27.401
1.247.663	-21.628	1.269.666	-28.518	1.223.736	-27.408
1.247.798	-21.629	1.269.857	-28.516	1.223.923	-27.415
1.247.948	-21.630	1.270.033	-28.515	1.224.090	-27.422
1.248.158	-21.630	1.270.184	-28.513	1.224.262	-27.429
1.248.293	-21.631	1.270.363	-28.512	1.224.374	-27.436
1.248.382	-21.632	1.270.508	-28.510	1.224.536	-27.443
1.248.503	-21.632	1.270.647	-28.509	1.224.681	-27.449
1.248.637	-21.633	1.270.791	-28.507	1.224.728	-27.456
1.248.738	-21.634	1.270.909	-28.506	1.224.837	-27.462

1.248.859	-21.634	1.271.118	-28.504	1.225.058	-27.469
1.249.072	-21.635	1.271.337	-28.503	1.225.248	-27.475
1.249.307	-21.636	1.271.463	-28.501	1.225.416	-27.482
1.249.547	-21.636	1.271.693	-28.500	1.225.592	-27.488
1.249.747	-21.637	1.271.947	-28.499	1.225.727	-27.494
1.249.927	-21.638	1.272.108	-28.497	1.225.883	-27.500
1.250.126	-21.638	1.272.234	-28.496	1.226.078	-27.506
1.250.238	-21.639	1.272.345	-28.494	1.226.202	-27.512
1.250.400	-21.640	1.272.502	-28.493	1.226.397	-27.518
1.250.640	-21.640	1.272.621	-28.492	1.226.697	-27.524
1.250.779	-21.641	1.272.751	-28.490	1.226.875	-27.529
1.250.909	-21.642	1.272.977	-28.489	1.227.010	-27.535
1.251.056	-21.642	1.273.170	-28.488	1.227.247	-27.540
1.251.226	-21.643	1.273.329	-28.487	1.227.458	-27.546
1.251.377	-21.644	1.273.459	-28.485	1.227.584	-27.551
1.251.488	-21.644	1.273.591	-28.484	1.227.699	-27.557
1.251.622	-21.645	1.273.768	-28.483	1.227.861	-27.562
1.251.765	-21.645	1.273.996	-28.482	1.227.999	-27.567
1.251.912	-21.646	1.274.169	-28.480	1.228.013	-27.572
1.252.056	-21.647	1.274.258	-28.479	1.228.105	-27.577
1.252.228	-21.647	1.274.352	-28.478	1.228.223	-27.582
1.252.427	-21.648	1.274.440	-28.477	1.228.361	-27.587
1.252.625	-21.649	1.274.617	-28.476	1.228.606	-27.592
1.252.785	-21.649	1.274.814	-28.475	1.228.814	-27.596
1.252.984	-21.650	1.274.953	-28.473	1.228.998	-27.601
1.253.212	-21.650	1.275.141	-28.472	1.229.155	-27.606
1.253.358	-21.651	1.275.303	-28.471	1.229.306	-27.610
1.253.477	-21.652	1.275.470	-28.470	1.229.452	-27.615
1.253.622	-21.652	1.275.699	-28.469	1.229.597	-27.619
1.253.778	-21.653	1.275.871	-28.468	1.229.871	-27.624

1.253.926	-21.653	1.276.040	-28.467	1.230.092	-27.628
1.254.100	-21.654	1.276.201	-28.466	1.230.239	-27.632
1.254.206	-21.654	1.276.315	-28.465	1.230.396	-27.637
1.254.361	-21.655	1.276.472	-28.464	1.230.536	-27.641
1.254.595	-21.656	1.276.633	-28.463	1.230.758	-27.645
1.254.805	-21.656	1.276.792	-28.462	1.230.947	-27.649
1.254.953	-21.657	1.277.010	-28.461	1.231.068	-27.653
1.255.040	-21.657	1.277.162	-28.460	1.231.190	-27.657
1.255.145	-21.658	1.277.283	-28.459	1.231.375	-27.661
1.255.343	-21.658	1.277.437	-28.458	1.231.558	-27.665
1.255.526	-21.659	1.277.583	-28.458	1.231.685	-27.669
1.255.697	-21.660	1.277.769	-28.457	1.231.821	-27.672
1.255.948	-21.660	1.277.894	-28.456	1.231.954	-27.676
1.256.084	-21.661	1.278.006	-28.455	1.232.113	-27.680
1.256.174	-21.661	1.278.167	-28.454	1.232.346	-27.684
1.256.375	-21.662	1.278.297	-28.453	1.232.480	-27.687
1.256.532	-21.662	1.278.434	-28.452	1.232.561	-27.691
1.256.638	-21.663	1.278.535	-28.451	1.232.765	-27.694
1.256.855	-21.663	1.278.613	-28.450	1.232.888	-27.698
1.257.090	-21.664	1.278.734	-28.450	1.233.017	-27.701
1.257.216	-21.664	1.278.943	-28.449	1.233.258	-27.705
1.257.314	-21.665	1.279.106	-28.448	1.233.400	-27.708
1.257.466	-21.665	1.279.314	-28.447	1.233.525	-27.712
1.257.657	-21.666	1.279.581	-28.446	1.233.738	-27.715
1.257.803	-21.666	1.279.816	-28.445	1.233.923	-27.718
1.257.863	-21.667	1.279.978	-28.444	1.234.023	-27.722
1.258.020	-21.668	1.280.128	-28.444	1.234.109	-27.725
1.258.456	-21.668	1.280.313	-28.443	1.234.238	-27.728
1.258.927	-21.668	1.280.504	-28.442	1.234.388	-27.731
1.259.113	-21.669	1.280.650	-28.441	1.234.621	-27.734

1.259.194	-21.669	1.280.755	-28.440	1.234.782	-27.737
1.259.240	-21.670	1.280.892	-28.439	1.234.967	-27.741
1.259.229	-21.670	1.281.075	-28.438	1.235.155	-27.744
1.259.297	-21.671	1.281.190	-28.438	1.235.328	-27.747
1.259.382	-21.671	1.281.277	-28.437	1.235.524	-27.750
1.259.487	-21.672	1.281.393	-28.436	1.235.686	-27.753
1.259.675	-21.672	1.281.550	-28.435	1.235.770	-27.756
1.259.919	-21.673	1.281.705	-28.434	1.235.888	-27.758
1.260.061	-21.673	1.281.886	-28.433	1.236.088	-27.761
1.260.199	-21.674	1.282.092	-28.433	1.236.212	-27.764
1.260.383	-21.674	1.282.323	-28.432	1.236.442	-27.767
1.260.538	-21.674	1.282.511	-28.431	1.236.884	-27.770
1.260.697	-21.675	1.282.639	-28.430	1.237.254	-27.773
1.260.853	-21.675	1.282.816	-28.429	1.237.503	-27.775
1.260.999	-21.676	1.283.011	-28.428	1.237.645	-27.778
1.261.131	-21.676	1.283.150	-28.428	1.237.660	-27.781
1.261.317	-21.677	1.283.291	-28.427	1.237.739	-27.783
1.261.494	-21.677	1.283.495	-28.426	1.237.857	-27.786
1.261.689	-21.677	1.283.694	-28.425	1.237.898	-27.789
1.261.888	-21.678	1.283.851	-28.424	1.238.010	-27.791
1.262.050	-21.678	1.284.025	-28.423	1.238.088	-27.794
1.262.204	-21.679	1.284.202	-28.423	1.238.167	-27.796
1.262.265	-21.679	1.284.337	-28.422	1.238.300	-27.799
1.262.381	-21.679	1.284.464	-28.421	1.238.500	-27.801
1.262.563	-21.680	1.284.606	-28.420	1.238.706	-27.804
1.262.733	-21.680	1.284.711	-28.419	1.238.855	-27.806
1.262.903	-21.680	1.284.879	-28.419	1.238.925	-27.809
1.263.087	-21.681	1.285.085	-28.418	1.239.048	-27.811
1.263.300	-21.681	1.285.257	-28.417	1.239.280	-27.813
1.263.421	-21.681	1.285.441	-28.416	1.239.461	-27.816

1.263.548	-21.682	1.285.620	-28.416	1.239.622	-27.818
1.263.716	-21.682	1.285.775	-28.415	1.239.799	-27.820
1.263.900	-21.682	1.285.921	-28.414	1.239.940	-27.823
1.264.109	-21.683	1.286.124	-28.413	1.240.079	-27.825
1.264.285	-21.683	1.286.263	-28.413	1.240.210	-27.827
1.264.413	-21.683	1.286.339	-28.412	1.240.342	-27.829
1.264.534	-21.684	1.286.452	-28.411	1.240.515	-27.832
1.264.601	-21.684	1.286.676	-28.411	1.240.704	-27.834
1.264.733	-21.684	1.286.918	-28.410	1.240.851	-27.836
1.264.944	-21.685	1.287.076	-28.409	1.240.984	-27.838
1.265.116	-21.685	1.287.229	-28.409	1.241.180	-27.840
1.265.229	-21.685	1.287.410	-28.408	1.241.342	-27.842
1.265.361	-21.686	1.287.612	-28.407	1.241.459	-27.844
1.265.587	-21.686	1.287.708	-28.407	1.241.582	-27.846
1.265.802	-21.686	1.287.791	-28.406	1.241.707	-27.848
1.265.943	-21.686	1.288.150	-28.406	1.241.870	-27.850
1.266.062	-21.687	1.288.656	-28.405	1.242.041	-27.852
1.266.205	-21.687	1.288.943	-28.404	1.242.253	-27.854
1.266.431	-21.687	1.288.947	-28.404	1.242.465	-27.856
1.266.561	-21.688	1.288.927	-28.403	1.242.727	-27.858
1.266.631	-21.688	1.289.039	-28.403	1.242.906	-27.860
1.266.839	-21.688	1.289.128	-28.403	1.243.027	-27.862
1.267.065	-21.688	1.289.180	-28.402	1.243.297	-27.864
1.267.204	-21.689	1.289.274	-28.402	1.243.465	-27.866
1.267.334	-21.689	1.289.420	-28.401	1.243.615	-27.868
1.267.525	-21.689	1.289.594	-28.401	1.243.755	-27.870
1.267.719	-21.690	1.289.715	-28.401	1.243.882	-27.871
1.267.921	-21.690	1.289.814	-28.400	1.243.959	-27.873
1.268.091	-21.690	1.290.004	-28.400	1.244.106	-27.875
1.268.217	-21.690	1.290.219	-28.400	1.244.337	-27.877

1.268.345	-21.691	1.290.434	-28.400	1.244.446	-27.878
1.268.512	-21.691	1.290.598	-28.399	1.244.539	-27.880
1.268.721	-21.691	1.290.737	-28.399	1.244.642	-27.882
1.268.864	-21.691	1.290.961	-28.399	1.244.858	-27.883
1.268.954	-21.692	1.291.111	-28.399	1.245.098	-27.885
1.269.088	-21.692	1.291.180	-28.399	1.245.143	-27.887
1.269.294	-21.692	1.291.317	-28.399	1.245.280	-27.888
1.269.570	-21.693	1.291.505	-28.399	1.245.554	-27.890
1.269.713	-21.693	1.291.707	-28.399	1.245.708	-27.891
1.269.812	-21.693	1.291.884	-28.399	1.245.833	-27.893
1.269.982	-21.693	1.291.989	-28.399	1.245.956	-27.894
1.270.101	-21.694	1.292.115	-28.399	1.246.085	-27.896
1.270.222	-21.694	1.292.258	-28.399	1.246.255	-27.897
1.270.345	-21.694	1.292.431	-28.400	1.246.434	-27.898
1.270.437	-21.695	1.292.672	-28.400	1.246.572	-27.900
1.270.585	-21.695	1.292.867	-28.400	1.246.713	-27.901
1.270.748	-21.695	1.293.015	-28.401	1.246.887	-27.902
1.270.891	-21.695	1.293.143	-28.401	1.247.122	-27.903
1.271.086	-21.696	1.293.304	-28.401	1.247.308	-27.904
1.271.279	-21.696	1.293.504	-28.402	1.247.458	-27.906
1.271.470	-21.696	1.293.660	-28.402	1.247.660	-27.907
1.271.685	-21.697	1.293.781	-28.403	1.247.803	-27.908
1.271.879	-21.697	1.293.951	-28.404	1.247.932	-27.909
1.272.066	-21.697	1.294.084	-28.404	1.248.111	-27.910
1.272.263	-21.698	1.294.221	-28.405	1.248.323	-27.911
1.272.473	-21.698	1.294.379	-28.406	1.248.451	-27.912
1.272.596	-21.698	1.294.501	-28.407	1.248.540	-27.913
1.272.684	-21.699	1.294.657	-28.408	1.248.682	-27.913
1.272.802	-21.699	1.294.821	-28.409	1.248.858	-27.914
1.272.968	-21.699	1.294.998	-28.410	1.249.046	-27.915

1.273.136	-21.700	1.295.157	-28.411	1.249.150	-27.916
1.273.264	-21.700	1.295.381	-28.412	1.249.313	-27.916
1.273.443	-21.701	1.295.555	-28.413	1.249.492	-27.917
1.273.582	-21.701	1.295.679	-28.414	1.249.617	-27.917
1.273.642	-21.701	1.295.842	-28.416	1.249.811	-27.918
1.273.788	-21.702	1.295.970	-28.417	1.249.993	-27.918
1.274.019	-21.702	1.296.131	-28.419	1.250.185	-27.919
1.274.225	-21.703	1.296.317	-28.420	1.250.367	-27.919
1.274.415	-21.703	1.296.499	-28.422	1.250.481	-27.920
1.274.579	-21.704	1.296.682	-28.423	1.250.611	-27.920
1.274.736	-21.704	1.296.824	-28.425	1.250.825	-27.920
1.274.958	-21.705	1.296.996	-28.427	1.251.042	-27.921
1.275.164	-21.705	1.297.188	-28.429	1.251.171	-27.921
1.275.336	-21.706	1.297.298	-28.431	1.251.286	-27.921
1.275.518	-21.706	1.297.448	-28.433	1.251.423	-27.921
1.275.667	-21.707	1.297.630	-28.435	1.251.557	-27.921
1.275.835	-21.707	1.297.785	-28.437	1.251.701	-27.921
1.276.015	-21.708	1.297.926	-28.439	1.251.824	-27.921
1.276.158	-21.708	1.298.064	-28.441	1.251.953	-27.921
1.276.270	-21.709	1.298.235	-28.444	1.252.136	-27.921
1.276.387	-21.709	1.298.412	-28.446	1.252.318	-27.921
1.276.561	-21.710	1.298.615	-28.448	1.252.503	-27.921
1.276.772	-21.711	1.298.792	-28.451	1.252.652	-27.921
1.276.978	-21.711	1.298.914	-28.454	1.252.850	-27.921
1.277.159	-21.712	1.299.048	-28.456	1.253.088	-27.921
1.277.330	-21.712	1.299.174	-28.459	1.253.318	-27.920
1.277.462	-21.713	1.299.362	-28.462	1.253.477	-27.920
1.277.585	-21.714	1.299.527	-28.465	1.253.633	-27.920
1.277.722	-21.714	1.299.682	-28.468	1.253.801	-27.919
1.277.807	-21.715	1.299.921	-28.471	1.253.930	-27.919

1.278.013	-21.716	1.300.081	-28.474	1.254.071	-27.919
1.278.217	-21.717	1.300.117	-28.477	1.254.200	-27.918
1.278.340	-21.717	1.300.193	-28.480	1.254.330	-27.918
1.278.544	-21.718	1.300.320	-28.483	1.254.453	-27.917
1.278.710	-21.719	1.300.432	-28.487	1.254.628	-27.917
1.278.811	-21.720	1.300.603	-28.490	1.254.756	-27.916
1.278.947	-21.720	1.300.802	-28.493	1.254.850	-27.916
1.279.151	-21.721	1.300.970	-28.497	1.254.984	-27.915
1.279.285	-21.722	1.301.165	-28.500	1.255.145	-27.915
1.279.427	-21.723	1.301.360	-28.504	1.255.336	-27.914
1.279.668	-21.724	1.301.595	-28.508	1.255.540	-27.913
1.279.830	-21.724	1.301.817	-28.511	1.255.701	-27.913
1.279.928	-21.725	1.301.960	-28.515	1.255.838	-27.912
1.280.058	-21.726	1.302.202	-28.519	1.255.988	-27.911
1.280.435	-21.727	1.302.404	-28.523	1.256.151	-27.911
1.280.900	-21.728	1.302.462	-28.527	1.256.384	-27.910
1.281.086	-21.729	1.302.527	-28.531	1.256.584	-27.909
1.281.145	-21.730	1.302.648	-28.535	1.256.741	-27.909
1.281.196	-21.731	1.302.780	-28.539	1.256.918	-27.908
1.281.254	-21.732	1.302.930	-28.543	1.257.159	-27.907
1.281.317	-21.733	1.303.089	-28.547	1.257.379	-27.906
1.281.331	-21.734	1.303.214	-28.551	1.257.440	-27.906
1.281.416	-21.735	1.303.358	-28.555	1.257.556	-27.905
1.281.622	-21.736	1.303.526	-28.560	1.257.728	-27.904
1.281.812	-21.737	1.303.743	-28.564	1.257.861	-27.903
1.281.964	-21.738	1.303.951	-28.568	1.258.056	-27.902
1.282.119	-21.739	1.304.086	-28.573	1.258.186	-27.902
1.282.290	-21.740	1.304.256	-28.577	1.258.275	-27.901
1.282.417	-21.741	1.304.467	-28.582	1.258.521	-27.900
1.282.563	-21.742	1.304.671	-28.586	1.258.725	-27.899

1.282.811	-21.744	1.304.776	-28.591	1.258.875	-27.898
1.283.047	-21.745	1.304.906	-28.595	1.259.066	-27.897
1.283.197	-21.746	1.305.087	-28.600	1.259.211	-27.897
1.283.329	-21.747	1.305.240	-28.604	1.259.357	-27.896
1.283.512	-21.748	1.305.365	-28.609	1.259.507	-27.895
1.283.685	-21.749	1.305.495	-28.614	1.259.686	-27.894
1.283.885	-21.751	1.305.668	-28.619	1.259.866	-27.893
1.284.012	-21.752	1.305.835	-28.623	1.260.029	-27.892
1.284.077	-21.753	1.306.006	-28.628	1.260.208	-27.891
1.284.221	-21.754	1.306.154	-28.633	1.260.378	-27.890
1.284.366	-21.756	1.306.306	-28.638	1.260.578	-27.889
1.284.496	-21.757	1.306.503	-28.643	1.260.793	-27.889
1.284.677	-21.758	1.306.725	-28.648	1.260.914	-27.888
1.284.828	-21.760	1.306.884	-28.653	1.261.051	-27.887
1.284.958	-21.761	1.307.007	-28.658	1.261.210	-27.886
1.285.145	-21.762	1.307.171	-28.663	1.261.304	-27.885
1.285.376	-21.764	1.307.348	-28.668	1.261.474	-27.884
1.285.549	-21.765	1.307.533	-28.673	1.261.653	-27.883
1.285.694	-21.767	1.307.655	-28.678	1.261.781	-27.882
1.285.793	-21.768	1.307.785	-28.683	1.261.942	-27.881
1.285.947	-21.769	1.307.973	-28.688	1.262.076	-27.880
1.286.192	-21.771	1.308.139	-28.693	1.262.262	-27.879
1.286.335	-21.772	1.308.297	-28.698	1.262.467	-27.878
1.286.395	-21.774	1.308.445	-28.703	1.262.605	-27.877
1.286.499	-21.775	1.308.620	-28.709	1.262.757	-27.877
1.286.651	-21.777	1.308.775	-28.714	1.262.876	-27.876
1.286.819	-21.778	1.308.938	-28.719	1.263.065	-27.875
1.287.005	-21.780	1.309.099	-28.724	1.263.387	-27.874
1.287.215	-21.781	1.309.249	-28.729	1.263.792	-27.873
1.287.413	-21.783	1.309.426	-28.735	1.264.081	-27.872

1.287.589	-21.785	1.309.552	-28.740	1.264.196	-27.871
1.287.715	-21.786	1.309.872	-28.745	1.264.276	-27.870
1.287.787	-21.788	1.310.280	-28.750	1.264.308	-27.869
1.287.952	-21.789	1.310.484	-28.756	1.264.402	-27.868
1.288.161	-21.791	1.310.542	-28.761	1.264.529	-27.867
1.288.315	-21.793	1.310.650	-28.766	1.264.559	-27.866
1.288.466	-21.794	1.310.777	-28.771	1.264.615	-27.866
1.288.611	-21.796	1.310.813	-28.777	1.264.776	-27.865
1.288.723	-21.798	1.310.880	-28.782	1.264.911	-27.864
1.288.923	-21.800	1.311.008	-28.787	1.265.042	-27.863
1.289.144	-21.801	1.311.129	-28.793	1.265.148	-27.862
1.289.256	-21.803	1.311.266	-28.798	1.265.343	-27.861
1.289.422	-21.805	1.311.413	-28.803	1.265.569	-27.860
1.289.597	-21.807	1.311.548	-28.809	1.265.643	-27.859
1.289.749	-21.808	1.311.788	-28.814	1.265.842	-27.859
1.289.955	-21.810	1.311.960	-28.819	1.266.127	-27.858
1.290.119	-21.812	1.312.106	-28.824	1.266.283	-27.857
1.290.280	-21.814	1.312.298	-28.830	1.266.409	-27.856
1.290.457	-21.816	1.312.406	-28.835	1.266.590	-27.855
1.290.639	-21.818	1.312.549	-28.840	1.266.783	-27.855
1.290.789	-21.820	1.312.697	-28.846	1.266.918	-27.854
1.290.939	-21.821	1.312.836	-28.851	1.267.047	-27.853
1.291.158	-21.823	1.313.044	-28.856	1.267.168	-27.852
1.291.317	-21.825	1.313.233	-28.861	1.267.321	-27.851
1.291.431	-21.827	1.313.380	-28.867	1.267.522	-27.851
1.291.579	-21.829	1.313.503	-28.872	1.267.697	-27.850
1.291.741	-21.831	1.313.593	-28.877	1.267.881	-27.849
1.291.891	-21.833	1.313.741	-28.882	1.268.015	-27.849
1.292.066	-21.835	1.313.904	-28.888	1.268.116	-27.848
1.292.213	-21.837	1.314.044	-28.893	1.268.282	-27.847

1.292.339	-21.839	1.314.285	-28.898	1.268.391	-27.847
1.292.493	-21.841	1.314.480	-28.903	1.268.517	-27.846
1.292.576	-21.843	1.314.574	-28.908	1.268.768	-27.846
1.292.664	-21.845	1.314.744	-28.913	1.269.001	-27.845
1.292.829	-21.847	1.314.973	-28.919	1.269.187	-27.844
1.292.977	-21.850	1.315.188	-28.924	1.269.364	-27.844
1.293.154	-21.852	1.315.336	-28.929	1.269.491	-27.843
1.293.353	-21.854	1.315.428	-28.934	1.269.662	-27.843
1.293.573	-21.856	1.315.569	-28.939	1.269.825	-27.842
1.293.831	-21.858	1.315.746	-28.944	1.269.937	-27.842
1.294.072	-21.860	1.315.872	-28.949	1.270.139	-27.841
1.294.218	-21.862	1.315.993	-28.954	1.270.320	-27.841
1.294.319	-21.865	1.316.156	-28.959	1.270.434	-27.840
1.294.482	-21.867	1.316.331	-28.964	1.270.602	-27.840
1.294.691	-21.869	1.316.512	-28.969	1.270.759	-27.840
1.294.906	-21.871	1.316.684	-28.974	1.270.851	-27.839
1.295.038	-21.873	1.316.886	-28.979	1.271.030	-27.839
1.295.101	-21.876	1.317.079	-28.984	1.271.270	-27.838
1.295.193	-21.878	1.317.211	-28.988	1.271.440	-27.838
1.295.345	-21.880	1.317.372	-28.993	1.271.577	-27.838
1.295.531	-21.882	1.317.574	-28.998	1.271.738	-27.837
1.295.658	-21.885	1.317.663	-29.003	1.271.900	-27.837
1.295.737	-21.887	1.317.744	-29.008	1.271.998	-27.837
1.295.912	-21.889	1.317.928	-29.012	1.272.110	-27.836
1.296.140	-21.892	1.318.056	-29.017	1.272.337	-27.836
1.296.192	-21.894	1.318.161	-29.022	1.272.513	-27.836
1.296.360	-21.896	1.318.327	-29.026	1.272.607	-27.836
1.296.687	-21.899	1.318.546	-29.031	1.272.773	-27.836
1.296.918	-21.901	1.318.764	-29.036	1.272.917	-27.835
1.297.020	-21.903	1.318.920	-29.040	1.272.988	-27.835

1.297.146	-21.906	1.319.095	-29.045	1.273.152	-27.835
1.297.316	-21.908	1.319.240	-29.049	1.273.340	-27.835
1.297.451	-21.910	1.319.362	-29.054	1.273.508	-27.835
1.297.556	-21.913	1.319.559	-29.058	1.273.741	-27.835
1.297.760	-21.915	1.319.706	-29.063	1.273.960	-27.835
1.298.026	-21.918	1.319.825	-29.067	1.274.086	-27.834
1.298.156	-21.920	1.319.969	-29.071	1.274.241	-27.834
1.298.286	-21.922	1.320.135	-29.076	1.274.388	-27.834
1.298.409	-21.925	1.320.341	-29.080	1.274.557	-27.834
1.298.589	-21.927	1.320.500	-29.084	1.274.734	-27.834
1.298.790	-21.930	1.320.625	-29.089	1.274.904	-27.834
1.298.958	-21.932	1.320.802	-29.093	1.275.074	-27.834
1.299.124	-21.935	1.321.037	-29.097	1.275.195	-27.834
1.299.220	-21.937	1.321.194	-29.101	1.275.323	-27.834
1.299.371	-21.939	1.321.266	-29.105	1.275.518	-27.834
1.299.644	-21.942	1.321.396	-29.109	1.275.679	-27.834
1.299.839	-21.944	1.321.568	-29.113	1.275.820	-27.834
1.299.942	-21.947	1.321.707	-29.117	1.276.015	-27.834
1.300.022	-21.949	1.321.812	-29.121	1.276.167	-27.834
1.300.112	-21.952	1.321.929	-29.125	1.276.313	-27.835
1.300.282	-21.954	1.322.074	-29.129	1.276.505	-27.835
1.300.468	-21.957	1.322.225	-29.133	1.276.678	-27.835
1.300.656	-21.959	1.322.332	-29.136	1.276.808	-27.835
1.300.820	-21.962	1.322.486	-29.140	1.276.971	-27.835
1.300.997	-21.964	1.322.713	-29.144	1.277.142	-27.835
1.301.190	-21.967	1.322.912	-29.147	1.277.337	-27.835
1.301.371	-21.969	1.323.150	-29.151	1.277.502	-27.835
1.301.521	-21.972	1.323.387	-29.155	1.277.594	-27.835
1.301.638	-21.974	1.323.542	-29.158	1.277.679	-27.836
1.301.770	-21.977	1.323.699	-29.162	1.277.840	-27.836

1.301.926	-21.979	1.323.902	-29.165	1.278.020	-27.836
1.302.117	-21.982	1.324.104	-29.168	1.278.127	-27.836
1.302.451	-21.984	1.324.240	-29.172	1.278.313	-27.836
1.302.852	-21.987	1.324.341	-29.175	1.278.475	-27.836
1.303.127	-21.989	1.324.478	-29.178	1.278.580	-27.837
1.303.311	-21.992	1.324.579	-29.181	1.278.689	-27.837
1.303.322	-21.994	1.324.650	-29.184	1.278.869	-27.837
1.303.320	-21.997	1.324.805	-29.187	1.279.088	-27.837
1.303.391	-21.999	1.324.946	-29.190	1.279.263	-27.837
1.303.385	-22.001	1.325.076	-29.193	1.279.456	-27.837
1.303.412	-22.004	1.325.260	-29.196	1.279.706	-27.838
1.303.575	-22.006	1.325.448	-29.199	1.279.935	-27.838
1.303.759	-22.009	1.325.634	-29.202	1.280.049	-27.838
1.303.934	-22.011	1.325.901	-29.205	1.280.235	-27.838
1.304.109	-22.014	1.326.133	-29.207	1.280.448	-27.838
1.304.285	-22.016	1.326.301	-29.210	1.280.605	-27.839
1.304.480	-22.019	1.326.472	-29.213	1.280.775	-27.839
1.304.677	-22.021	1.326.593	-29.215	1.280.860	-27.839
1.304.901	-22.024	1.326.711	-29.218	1.280.950	-27.839
1.305.049	-22.026	1.326.911	-29.220	1.281.151	-27.839
1.305.166	-22.028	1.327.074	-29.222	1.281.320	-27.840
1.305.323	-22.031	1.327.166	-29.225	1.281.451	-27.840
1.305.453	-22.033	1.327.366	-29.227	1.281.581	-27.840
1.305.578	-22.036	1.327.567	-29.229	1.281.673	-27.840
1.305.744	-22.038	1.327.717	-29.231	1.281.873	-27.840
1.305.952	-22.040	1.327.849	-29.234	1.282.113	-27.841
1.306.149	-22.043	1.328.033	-29.236	1.282.220	-27.841
1.306.317	-22.045	1.328.264	-29.238	1.282.305	-27.841
1.306.429	-22.048	1.328.394	-29.239	1.282.444	-27.841
1.306.526	-22.050	1.328.517	-29.241	1.282.578	-27.842

1.306.684	-22.052	1.328.681	-29.243	1.282.773	-27.842
1.306.850	-22.055	1.328.819	-29.245	1.283.026	-27.842
1.307.059	-22.057	1.329.028	-29.247	1.283.248	-27.842
1.307.262	-22.059	1.329.223	-29.248	1.283.447	-27.842
1.307.421	-22.062	1.329.317	-29.250	1.283.598	-27.842
1.307.594	-22.064	1.329.447	-29.251	1.283.795	-27.843
1.307.733	-22.066	1.329.585	-29.253	1.283.974	-27.843
1.307.865	-22.068	1.329.753	-29.254	1.284.102	-27.843
1.308.015	-22.071	1.329.944	-29.256	1.284.205	-27.843
1.308.206	-22.073	1.330.148	-29.257	1.284.323	-27.843
1.308.378	-22.075	1.330.260	-29.258	1.284.527	-27.844
1.308.490	-22.077	1.330.376	-29.259	1.284.731	-27.844
1.308.602	-22.080	1.330.612	-29.261	1.284.881	-27.844
1.308.757	-22.082	1.330.791	-29.262	1.285.069	-27.844
1.308.976	-22.084	1.330.934	-29.263	1.285.264	-27.844
1.309.198	-22.086	1.331.093	-29.264	1.285.336	-27.844
1.309.359	-22.088	1.331.192	-29.265	1.285.455	-27.845
1.309.503	-22.091	1.331.339	-29.266	1.285.665	-27.845
1.309.612	-22.093	1.331.676	-29.266	1.285.858	-27.845
1.309.711	-22.095	1.332.137	-29.267	1.286.006	-27.845
1.309.877	-22.097	1.332.404	-29.268	1.286.176	-27.845
1.310.085	-22.099	1.332.473	-29.269	1.286.366	-27.846
1.310.296	-22.101	1.332.507	-29.269	1.286.541	-27.846
1.310.455	-22.103	1.332.560	-29.270	1.286.669	-27.846
1.310.538	-22.106	1.332.666	-29.270	1.286.765	-27.846
1.310.636	-22.108	1.332.713	-29.271	1.286.904	-27.846
1.310.759	-22.110	1.332.749	-29.271	1.287.056	-27.847
1.310.923	-22.112	1.332.843	-29.272	1.287.215	-27.847
1.311.118	-22.114	1.332.997	-29.272	1.287.399	-27.847
1.311.301	-22.116	1.333.206	-29.273	1.287.567	-27.847

1.311.436	-22.118	1.333.427	-29.273	1.287.704	-27.847
1.311.646	-22.120	1.333.600	-29.273	1.287.863	-27.848
1.311.877	-22.122	1.333.775	-29.273	1.288.051	-27.848
1.312.023	-22.124	1.334.019	-29.273	1.288.154	-27.848
1.312.184	-22.126	1.334.223	-29.274	1.288.340	-27.848
1.312.332	-22.128	1.334.315	-29.274	1.288.615	-27.849
1.312.433	-22.130	1.334.433	-29.274	1.288.835	-27.849
1.312.569	-22.132	1.334.635	-29.274	1.289.017	-27.849
1.312.708	-22.134	1.334.796	-29.274	1.289.160	-27.849
1.312.854	-22.136	1.334.913	-29.274	1.289.272	-27.850
1.313.065	-22.137	1.335.085	-29.274	1.289.364	-27.850
1.313.284	-22.139	1.335.235	-29.274	1.289.529	-27.850
1.313.474	-22.141	1.335.341	-29.273	1.289.682	-27.850
1.313.613	-22.143	1.335.457	-29.273	1.289.855	-27.851
1.313.736	-22.145	1.335.623	-29.273	1.290.282	-27.851
1.313.884	-22.147	1.335.770	-29.273	1.290.714	-27.851
1.314.070	-22.149	1.335.914	-29.273	1.290.909	-27.852
1.314.234	-22.150	1.336.084	-29.272	1.290.992	-27.852
1.314.348	-22.152	1.336.232	-29.272	1.291.035	-27.852
1.314.496	-22.154	1.336.427	-29.272	1.291.100	-27.853
1.314.675	-22.156	1.336.626	-29.272	1.291.105	-27.853
1.314.796	-22.158	1.336.787	-29.271	1.291.163	-27.853
1.314.922	-22.159	1.336.976	-29.271	1.291.349	-27.854
1.315.058	-22.161	1.337.153	-29.270	1.291.499	-27.854
1.315.206	-22.163	1.337.283	-29.270	1.291.590	-27.854
1.315.347	-22.164	1.337.406	-29.270	1.291.667	-27.855
1.315.479	-22.166	1.337.531	-29.269	1.291.796	-27.855
1.315.618	-22.168	1.337.643	-29.269	1.291.951	-27.856
1.315.862	-22.170	1.337.838	-29.268	1.292.126	-27.856
1.316.113	-22.171	1.338.029	-29.268	1.292.330	-27.857

1.316.279	-22.173	1.338.163	-29.267	1.292.540	-27.857
1.316.483	-22.175	1.338.306	-29.267	1.292.728	-27.858
1.316.635	-22.176	1.338.454	-29.267	1.292.883	-27.858
1.316.743	-22.178	1.338.611	-29.266	1.293.076	-27.859
1.316.900	-22.179	1.338.754	-29.266	1.293.268	-27.859
1.317.059	-22.181	1.338.954	-29.265	1.293.436	-27.860
1.317.173	-22.183	1.339.155	-29.265	1.293.602	-27.861
1.317.276	-22.184	1.339.319	-29.264	1.293.678	-27.861
1.317.471	-22.186	1.339.480	-29.264	1.293.727	-27.862
1.317.677	-22.187	1.339.637	-29.263	1.293.922	-27.863
1.317.784	-22.189	1.339.774	-29.263	1.294.111	-27.863
1.317.883	-22.190	1.339.906	-29.262	1.294.249	-27.864
1.318.046	-22.192	1.340.098	-29.262	1.294.415	-27.865
1.318.190	-22.193	1.340.289	-29.261	1.294.585	-27.866
1.318.342	-22.195	1.340.459	-29.261	1.294.747	-27.866
1.318.533	-22.196	1.340.612	-29.260	1.294.906	-27.867
1.318.766	-22.198	1.340.806	-29.260	1.295.051	-27.868
1.318.940	-22.199	1.340.977	-29.259	1.295.132	-27.869
1.319.030	-22.201	1.341.113	-29.259	1.295.294	-27.870
1.319.238	-22.202	1.341.283	-29.258	1.295.506	-27.871
1.319.462	-22.203	1.341.474	-29.258	1.295.616	-27.872
1.319.642	-22.205	1.341.633	-29.257	1.295.818	-27.873
1.319.816	-22.206	1.341.743	-29.257	1.296.053	-27.874
1.319.964	-22.208	1.341.913	-29.256	1.296.207	-27.875
1.320.145	-22.209	1.342.076	-29.256	1.296.432	-27.876
1.320.356	-22.210	1.342.258	-29.255	1.296.671	-27.877
1.320.490	-22.212	1.342.516	-29.255	1.296.777	-27.878
1.320.583	-22.213	1.342.659	-29.254	1.296.902	-27.880
1.320.773	-22.214	1.342.735	-29.254	1.297.020	-27.881
1.320.979	-22.216	1.342.887	-29.254	1.297.150	-27.882

1.321.138	-22.217	1.343.027	-29.253	1.297.336	-27.883
1.321.299	-22.218	1.343.136	-29.253	1.297.502	-27.885
1.321.494	-22.220	1.343.253	-29.252	1.297.672	-27.886
1.321.664	-22.221	1.343.400	-29.252	1.297.758	-27.887
1.321.768	-22.222	1.343.573	-29.252	1.297.881	-27.889
1.321.895	-22.223	1.343.694	-29.251	1.298.067	-27.890
1.322.068	-22.225	1.343.824	-29.251	1.298.230	-27.892
1.322.238	-22.226	1.344.014	-29.251	1.298.434	-27.893
1.322.410	-22.227	1.344.135	-29.250	1.298.678	-27.895
1.322.563	-22.228	1.344.247	-29.250	1.298.828	-27.896
1.322.677	-22.230	1.344.391	-29.250	1.298.945	-27.898
1.322.803	-22.231	1.344.599	-29.249	1.299.048	-27.900
1.323.013	-22.232	1.344.821	-29.249	1.299.158	-27.901
1.323.215	-22.233	1.345.011	-29.249	1.299.285	-27.903
1.323.365	-22.234	1.345.193	-29.249	1.299.433	-27.905
1.323.501	-22.235	1.345.428	-29.248	1.299.651	-27.907
1.323.634	-22.237	1.345.652	-29.248	1.299.837	-27.908
1.323.862	-22.238	1.345.840	-29.248	1.299.977	-27.910
1.324.057	-22.239	1.346.001	-29.248	1.300.177	-27.912
1.324.155	-22.240	1.346.068	-29.248	1.300.349	-27.914
1.324.243	-22.241	1.346.192	-29.247	1.300.448	-27.916
1.324.373	-22.242	1.346.351	-29.247	1.300.598	-27.918
1.324.756	-22.243	1.346.483	-29.247	1.300.815	-27.920
1.325.177	-22.244	1.346.628	-29.247	1.300.997	-27.922
1.325.363	-22.245	1.346.738	-29.247	1.301.129	-27.924
1.325.490	-22.246	1.346.837	-29.247	1.301.308	-27.926
1.325.594	-22.247	1.347.030	-29.247	1.301.516	-27.929
1.325.627	-22.248	1.347.242	-29.247	1.301.684	-27.931
1.325.665	-22.250	1.347.415	-29.247	1.301.898	-27.933
1.325.800	-22.251	1.347.567	-29.246	1.302.034	-27.935

1.325.901	-22.252	1.347.773	-29.246	1.302.101	-27.938
1.325.932	-22.253	1.347.968	-29.246	1.302.225	-27.940
1.326.071	-22.254	1.348.120	-29.246	1.302.368	-27.942
1.326.317	-22.255	1.348.273	-29.246	1.302.583	-27.945
1.326.534	-22.256	1.348.436	-29.246	1.302.710	-27.947
1.326.655	-22.256	1.348.595	-29.246	1.302.838	-27.949
1.326.785	-22.257	1.348.759	-29.246	1.303.055	-27.952
1.326.985	-22.258	1.348.951	-29.247	1.303.143	-27.954
1.327.213	-22.259	1.349.171	-29.247	1.303.298	-27.957
1.327.401	-22.260	1.349.346	-29.247	1.303.540	-27.959
1.327.547	-22.261	1.349.469	-29.247	1.303.705	-27.962
1.327.708	-22.262	1.349.604	-29.247	1.303.871	-27.965
1.327.841	-22.263	1.349.738	-29.247	1.303.981	-27.967
1.327.964	-22.264	1.349.926	-29.247	1.304.081	-27.970
1.328.100	-22.265	1.350.061	-29.247	1.304.249	-27.972
1.328.223	-22.266	1.350.222	-29.247	1.304.480	-27.975
1.328.367	-22.267	1.350.441	-29.248	1.304.662	-27.978
1.328.569	-22.267	1.350.529	-29.248	1.304.740	-27.980
1.328.788	-22.268	1.350.667	-29.248	1.304.814	-27.983
1.328.940	-22.269	1.350.887	-29.248	1.304.942	-27.986
1.329.070	-22.270	1.350.995	-29.248	1.305.114	-27.989
1.329.229	-22.271	1.351.138	-29.248	1.305.246	-27.991
1.329.377	-22.272	1.351.389	-29.249	1.305.428	-27.994
1.329.529	-22.272	1.351.507	-29.249	1.305.629	-27.997
1.329.789	-22.273	1.351.642	-29.249	1.305.757	-28.000
1.330.013	-22.274	1.351.877	-29.249	1.305.945	-28.002
1.330.134	-22.275	1.352.023	-29.250	1.306.248	-28.005
1.330.282	-22.276	1.352.150	-29.250	1.306.501	-28.008
1.330.430	-22.276	1.352.254	-29.250	1.306.649	-28.011
1.330.551	-22.277	1.352.395	-29.250	1.306.756	-28.014

1.330.629	-22.278	1.352.558	-29.251	1.306.871	-28.017
1.330.793	-22.279	1.352.706	-29.251	1.307.065	-28.020
1.331.012	-22.279	1.352.867	-29.251	1.307.243	-28.022
1.331.174	-22.280	1.353.024	-29.251	1.307.417	-28.025
1.331.348	-22.281	1.353.172	-29.252	1.307.543	-28.028
1.331.503	-22.282	1.353.470	-29.252	1.307.645	-28.031
1.331.622	-22.282	1.353.941	-29.252	1.307.849	-28.034
1.331.763	-22.283	1.354.234	-29.253	1.308.033	-28.037
1.331.908	-22.284	1.354.330	-29.253	1.308.123	-28.040
1.332.088	-22.285	1.354.413	-29.253	1.308.219	-28.043
1.332.220	-22.285	1.354.476	-29.254	1.308.374	-28.046
1.332.309	-22.286	1.354.547	-29.254	1.308.524	-28.049
1.332.514	-22.287	1.354.603	-29.254	1.308.654	-28.052
1.332.711	-22.287	1.354.505	-29.255	1.308.792	-28.055
1.332.840	-22.288	1.354.646	-29.255	1.308.918	-28.058
1.333.018	-22.289	1.354.948	-29.255	1.309.147	-28.060
1.333.170	-22.289	1.355.076	-29.256	1.309.359	-28.063
1.333.273	-22.290	1.355.249	-29.256	1.309.469	-28.066
1.333.436	-22.291	1.355.419	-29.256	1.309.641	-28.069
1.333.620	-22.291	1.355.578	-29.257	1.309.895	-28.072
1.333.808	-22.292	1.355.809	-29.257	1.310.020	-28.075
1.334.046	-22.293	1.356.026	-29.257	1.310.181	-28.078
1.334.216	-22.293	1.356.208	-29.258	1.310.374	-28.081
1.334.279	-22.294	1.356.375	-29.258	1.310.486	-28.084
1.334.440	-22.295	1.356.532	-29.258	1.310.676	-28.087
1.334.633	-22.295	1.356.655	-29.259	1.310.856	-28.090
1.334.798	-22.296	1.356.756	-29.259	1.311.013	-28.092
1.334.986	-22.297	1.356.920	-29.259	1.311.176	-28.095
1.335.128	-22.297	1.357.103	-29.260	1.311.310	-28.098
1.335.235	-22.298	1.357.229	-29.260	1.311.451	-28.101

1.335.383	-22.299	1.357.397	-29.260	1.311.619	-28.104
1.335.587	-22.299	1.357.551	-29.261	1.311.832	-28.107
1.335.811	-22.300	1.357.704	-29.261	1.311.951	-28.109
1.336.033	-22.300	1.357.910	-29.262	1.312.065	-28.112
1.336.129	-22.301	1.358.058	-29.262	1.312.327	-28.115
1.336.216	-22.302	1.358.230	-29.262	1.312.502	-28.118
1.336.423	-22.302	1.358.436	-29.263	1.312.587	-28.121
1.336.660	-22.303	1.358.586	-29.263	1.312.775	-28.123
1.336.759	-22.304	1.358.696	-29.263	1.312.999	-28.126
1.336.808	-22.304	1.358.806	-29.264	1.313.165	-28.129
1.336.974	-22.305	1.358.974	-29.264	1.313.284	-28.132
1.337.072	-22.305	1.359.171	-29.265	1.313.436	-28.134
1.337.188	-22.306	1.359.308	-29.265	1.313.622	-28.137
1.337.392	-22.307	1.359.465	-29.265	1.313.736	-28.140
1.337.594	-22.307	1.359.678	-29.266	1.313.896	-28.142
1.337.787	-22.308	1.359.776	-29.266	1.314.075	-28.145
1.337.912	-22.308	1.359.845	-29.267	1.314.185	-28.148
1.338.037	-22.309	1.360.034	-29.267	1.314.375	-28.150
1.338.271	-22.310	1.360.202	-29.268	1.314.615	-28.153
1.338.497	-22.310	1.360.313	-29.268	1.314.805	-28.155
1.338.692	-22.311	1.360.464	-29.268	1.314.884	-28.158
1.338.891	-22.312	1.360.649	-29.269	1.314.971	-28.161
1.339.052	-22.312	1.360.807	-29.269	1.315.193	-28.163
1.339.202	-22.313	1.360.961	-29.270	1.315.403	-28.166
1.339.373	-22.313	1.361.171	-29.270	1.315.565	-28.168
1.339.581	-22.314	1.361.373	-29.271	1.315.683	-28.171
1.339.709	-22.315	1.361.465	-29.271	1.315.800	-28.173
1.339.725	-22.315	1.361.548	-29.272	1.315.990	-28.176
1.339.877	-22.316	1.361.736	-29.272	1.316.156	-28.178
1.340.092	-22.316	1.361.945	-29.273	1.316.279	-28.180

1.340.130	-22.317	1.362.155	-29.273	1.316.378	-28.183
1.340.231	-22.318	1.362.318	-29.274	1.316.548	-28.185
1.340.443	-22.318	1.362.466	-29.274	1.316.935	-28.188
1.340.647	-22.319	1.362.677	-29.275	1.317.323	-28.190
1.340.849	-22.320	1.362.818	-29.275	1.317.605	-28.192
1.341.015	-22.320	1.362.961	-29.276	1.317.782	-28.195
1.341.198	-22.321	1.363.148	-29.277	1.317.859	-28.197
1.341.385	-22.321	1.363.304	-29.277	1.317.923	-28.199
1.341.559	-22.322	1.363.447	-29.278	1.317.977	-28.201
1.341.667	-22.323	1.363.654	-29.278	1.318.017	-28.204
1.341.761	-22.323	1.363.802	-29.279	1.318.074	-28.206
1.341.918	-22.324	1.363.882	-29.280	1.318.219	-28.208
1.342.086	-22.325	1.364.048	-29.280	1.318.318	-28.210
1.342.251	-22.325	1.364.265	-29.281	1.318.391	-28.213
1.342.477	-22.326	1.364.482	-29.281	1.318.600	-28.215
1.342.681	-22.327	1.364.639	-29.282	1.318.792	-28.217
1.342.813	-22.327	1.364.778	-29.283	1.318.947	-28.219
1.342.973	-22.328	1.364.964	-29.283	1.319.102	-28.221
1.343.123	-22.329	1.365.145	-29.284	1.319.230	-28.224
1.343.246	-22.329	1.365.284	-29.285	1.319.424	-28.226
1.343.416	-22.330	1.365.381	-29.285	1.319.630	-28.228
1.343.645	-22.331	1.365.482	-29.286	1.319.852	-28.230
1.343.869	-22.331	1.365.614	-29.287	1.320.031	-28.232
1.344.032	-22.332	1.365.768	-29.288	1.320.135	-28.234
1.344.167	-22.333	1.365.889	-29.288	1.320.289	-28.236
1.344.321	-22.333	1.365.921	-29.289	1.320.452	-28.238
1.344.417	-22.334	1.366.037	-29.290	1.320.551	-28.241
1.344.538	-22.335	1.366.232	-29.290	1.320.699	-28.243
1.344.716	-22.335	1.366.474	-29.291	1.320.945	-28.245
1.344.904	-22.336	1.366.684	-29.292	1.321.077	-28.247

1.345.085	-22.337	1.366.899	-29.293	1.321.174	-28.249
1.345.251	-22.337	1.367.150	-29.293	1.321.293	-28.251
1.345.421	-22.338	1.367.339	-29.294	1.321.476	-28.253
1.345.556	-22.339	1.367.504	-29.295	1.321.691	-28.255
1.345.719	-22.340	1.367.704	-29.296	1.321.794	-28.257
1.345.867	-22.340	1.367.843	-29.296	1.321.906	-28.259
1.346.053	-22.341	1.367.905	-29.297	1.322.054	-28.261
1.346.245	-22.342	1.368.056	-29.298	1.322.198	-28.263
1.346.344	-22.342	1.368.244	-29.299	1.322.352	-28.265
1.346.487	-22.343	1.368.360	-29.300	1.322.552	-28.267
1.346.646	-22.344	1.368.479	-29.301	1.322.796	-28.269
1.346.823	-22.345	1.368.611	-29.302	1.322.988	-28.271
1.347.231	-22.345	1.368.734	-29.303	1.323.136	-28.273
1.347.641	-22.346	1.368.837	-29.304	1.323.306	-28.275
1.347.791	-22.347	1.368.999	-29.305	1.323.481	-28.277
1.347.861	-22.348	1.369.238	-29.306	1.323.647	-28.279
1.347.941	-22.348	1.369.415	-29.307	1.323.761	-28.281
1.347.982	-22.349	1.369.547	-29.308	1.323.893	-28.283
1.348.004	-22.350	1.369.716	-29.310	1.324.055	-28.285
1.348.094	-22.351	1.369.897	-29.311	1.324.169	-28.287
1.348.208	-22.351	1.370.065	-29.313	1.324.281	-28.289
1.348.380	-22.352	1.370.235	-29.314	1.324.464	-28.291
1.348.559	-22.353	1.370.428	-29.316	1.324.709	-28.293
1.348.699	-22.354	1.370.636	-29.317	1.324.861	-28.295
1.348.882	-22.354	1.370.829	-29.319	1.325.052	-28.297
1.348.988	-22.355	1.370.979	-29.321	1.325.235	-28.299
1.349.122	-22.356	1.371.162	-29.323	1.325.276	-28.301
1.349.357	-22.357	1.371.320	-29.325	1.325.434	-28.303
1.349.521	-22.358	1.371.460	-29.327	1.325.648	-28.305
1.349.675	-22.358	1.371.617	-29.329	1.325.815	-28.307

1.349.879	-22.359	1.371.749	-29.331	1.325.916	-28.309
1.350.047	-22.360	1.371.944	-29.334	1.326.095	-28.311
1.350.206	-22.361	1.372.220	-29.336	1.326.306	-28.313
1.350.354	-22.362	1.372.397	-29.339	1.326.431	-28.315
1.350.490	-22.362	1.372.435	-29.342	1.326.553	-28.317
1.350.618	-22.363	1.372.576	-29.345	1.326.702	-28.319
1.350.780	-22.364	1.372.755	-29.348	1.326.888	-28.321
1.350.988	-22.365	1.372.943	-29.351	1.327.065	-28.323
1.351.118	-22.366	1.373.114	-29.354	1.327.260	-28.325
1.351.227	-22.367	1.373.230	-29.358	1.327.419	-28.326
1.351.413	-22.367	1.373.409	-29.361	1.327.567	-28.328
1.351.615	-22.368	1.373.571	-29.365	1.327.766	-28.330
1.351.815	-22.369	1.373.671	-29.369	1.327.935	-28.332
1.351.960	-22.370	1.373.795	-29.373	1.328.073	-28.334
1.352.092	-22.371	1.373.958	-29.377	1.328.284	-28.336
1.352.276	-22.372	1.374.124	-29.381	1.328.447	-28.338
1.352.466	-22.373	1.374.296	-29.386	1.328.571	-28.340
1.352.663	-22.373	1.374.478	-29.390	1.328.721	-28.342
1.352.758	-22.374	1.374.691	-29.395	1.328.947	-28.344
1.352.868	-22.375	1.374.821	-29.400	1.329.131	-28.346
1.353.044	-22.376	1.374.935	-29.405	1.329.220	-28.347
1.353.194	-22.377	1.375.117	-29.411	1.329.344	-28.349
1.353.333	-22.378	1.375.322	-29.416	1.329.505	-28.351
1.353.494	-22.379	1.375.674	-29.422	1.329.660	-28.353
1.353.647	-22.380	1.376.071	-29.428	1.329.818	-28.355
1.353.763	-22.380	1.376.254	-29.434	1.329.958	-28.357
1.353.951	-22.381	1.376.290	-29.440	1.330.126	-28.358
1.354.164	-22.382	1.376.357	-29.446	1.330.291	-28.360
1.354.296	-22.383	1.376.382	-29.453	1.330.434	-28.362
1.354.449	-22.384	1.376.494	-29.460	1.330.596	-28.364

1.354.659	-22.385	1.376.673	-29.467	1.330.818	-28.366
1.354.809	-22.386	1.376.738	-29.474	1.330.979	-28.367
1.354.951	-22.387	1.376.808	-29.481	1.331.046	-28.369
1.355.145	-22.388	1.376.949	-29.488	1.331.232	-28.371
1.355.228	-22.389	1.377.130	-29.496	1.331.443	-28.373
1.355.334	-22.390	1.377.318	-29.504	1.331.557	-28.374
1.355.490	-22.390	1.377.437	-29.512	1.331.640	-28.376
1.355.656	-22.391	1.377.585	-29.520	1.331.738	-28.378
1.355.874	-22.392	1.377.825	-29.528	1.331.893	-28.379
1.356.057	-22.393	1.378.004	-29.536	1.332.074	-28.381
1.356.196	-22.394	1.378.176	-29.545	1.332.216	-28.383
1.356.351	-22.395	1.378.371	-29.554	1.332.319	-28.384
1.356.534	-22.396	1.378.528	-29.563	1.332.496	-28.386
1.356.716	-22.397	1.378.703	-29.572	1.332.713	-28.388
1.356.886	-22.398	1.378.886	-29.581	1.332.923	-28.389
1.357.020	-22.399	1.379.028	-29.590	1.333.069	-28.391
1.357.148	-22.400	1.379.146	-29.600	1.333.219	-28.392
1.357.290	-22.401	1.379.254	-29.610	1.333.438	-28.394
1.357.478	-22.402	1.379.393	-29.619	1.333.589	-28.395
1.357.697	-22.403	1.379.525	-29.629	1.333.826	-28.397
1.357.887	-22.404	1.379.666	-29.640	1.334.144	-28.398
1.358.026	-22.405	1.379.843	-29.650	1.334.343	-28.400
1.358.145	-22.406	1.380.056	-29.660	1.334.444	-28.401
1.358.309	-22.407	1.380.235	-29.671	1.334.574	-28.403
1.358.474	-22.408	1.380.443	-29.681	1.334.778	-28.404
1.358.613	-22.409	1.380.656	-29.692	1.334.906	-28.406
1.358.750	-22.410	1.380.818	-29.703	1.334.960	-28.407
1.358.943	-22.411	1.380.981	-29.714	1.335.069	-28.408
1.359.113	-22.412	1.381.091	-29.726	1.335.237	-28.410
1.359.261	-22.413	1.381.207	-29.737	1.335.390	-28.411

1.359.391	-22.414	1.381.407	-29.748	1.335.555	-28.412
1.359.496	-22.415	1.381.563	-29.760	1.335.715	-28.413
1.359.637	-22.416	1.381.698	-29.772	1.335.842	-28.415
1.359.767	-22.417	1.381.866	-29.784	1.335.950	-28.416
1.359.921	-22.418	1.382.014	-29.796	1.336.075	-28.417
1.360.112	-22.419	1.382.133	-29.808	1.336.252	-28.418
1.360.322	-22.420	1.382.258	-29.820	1.336.438	-28.419
1.360.497	-22.421	1.382.399	-29.832	1.336.638	-28.421
1.360.645	-22.422	1.382.558	-29.845	1.336.853	-28.422
1.360.824	-22.423	1.382.731	-29.858	1.337.056	-28.423
1.361.057	-22.424	1.382.892	-29.870	1.337.191	-28.424
1.361.273	-22.425	1.383.071	-29.883	1.337.330	-28.425
1.361.440	-22.426	1.383.212	-29.896	1.337.507	-28.426
1.361.604	-22.427	1.383.356	-29.909	1.337.684	-28.427
1.361.718	-22.428	1.383.508	-29.922	1.337.905	-28.428
1.361.823	-22.429	1.383.615	-29.935	1.338.040	-28.429
1.361.953	-22.430	1.383.699	-29.949	1.338.103	-28.430
1.362.124	-22.431	1.383.918	-29.962	1.338.246	-28.431
1.362.296	-22.433	1.384.137	-29.976	1.338.445	-28.431
1.362.406	-22.434	1.384.299	-29.989	1.338.680	-28.432
1.362.538	-22.435	1.384.436	-30.003	1.338.851	-28.433
1.362.657	-22.436	1.384.574	-30.016	1.339.010	-28.434
1.362.751	-22.437	1.384.756	-30.030	1.339.185	-28.435
1.362.896	-22.438	1.384.908	-30.044	1.339.259	-28.435
1.363.123	-22.439	1.384.969	-30.058	1.339.323	-28.436
1.363.358	-22.440	1.385.098	-30.072	1.339.561	-28.437
1.363.530	-22.441	1.385.340	-30.085	1.339.807	-28.438
1.363.718	-22.442	1.385.520	-30.099	1.339.989	-28.438
1.363.925	-22.444	1.385.667	-30.113	1.340.105	-28.439
1.364.073	-22.445	1.385.856	-30.127	1.340.201	-28.440

1.364.216	-22.446	1.385.981	-30.141	1.340.399	-28.440
1.364.339	-22.447	1.386.167	-30.154	1.340.621	-28.441
1.364.496	-22.448	1.386.355	-30.168	1.340.777	-28.442
1.364.610	-22.449	1.386.460	-30.182	1.340.882	-28.442
1.364.709	-22.450	1.386.646	-30.195	1.341.049	-28.443
1.364.960	-22.451	1.386.828	-30.209	1.341.176	-28.443
1.365.177	-22.452	1.387.010	-30.223	1.341.344	-28.444
1.365.327	-22.454	1.387.220	-30.236	1.341.546	-28.444
1.365.504	-22.455	1.387.345	-30.249	1.341.698	-28.445
1.365.681	-22.456	1.387.422	-30.263	1.341.879	-28.446
1.365.800	-22.457	1.387.511	-30.276	1.342.115	-28.446
1.365.999	-22.458	1.387.635	-30.289	1.342.307	-28.447
1.366.237	-22.459	1.387.793	-30.302	1.342.392	-28.447
1.366.387	-22.460	1.387.999	-30.314	1.342.507	-28.447
1.366.497	-22.462	1.388.179	-30.327	1.342.641	-28.448
1.366.628	-22.463	1.388.333	-30.339	1.342.793	-28.448
1.366.812	-22.464	1.388.472	-30.352	1.342.984	-28.449
1.366.982	-22.465	1.388.589	-30.364	1.343.138	-28.449
1.367.141	-22.466	1.388.783	-30.376	1.343.302	-28.450
1.367.292	-22.467	1.389.066	-30.387	1.343.461	-28.450
1.367.410	-22.469	1.389.303	-30.399	1.343.598	-28.450
1.367.567	-22.470	1.389.509	-30.410	1.343.916	-28.451
1.367.764	-22.471	1.389.673	-30.422	1.344.361	-28.451
1.367.943	-22.472	1.389.816	-30.433	1.344.668	-28.451
1.368.120	-22.473	1.389.910	-30.443	1.344.798	-28.452
1.368.253	-22.474	1.389.995	-30.454	1.344.855	-28.452
1.368.371	-22.476	1.390.148	-30.464	1.344.911	-28.453
1.368.499	-22.477	1.390.313	-30.474	1.344.980	-28.453
1.368.649	-22.478	1.390.417	-30.484	1.345.060	-28.453
1.368.893	-22.479	1.390.535	-30.494	1.345.098	-28.454

1.369.057	-22.480	1.390.663	-30.503	1.345.226	-28.454
1.369.263	-22.482	1.390.797	-30.512	1.345.336	-28.454
1.369.641	-22.483	1.391.021	-30.521	1.345.390	-28.455
1.369.942	-22.484	1.391.281	-30.529	1.345.538	-28.455
1.370.128	-22.485	1.391.458	-30.537	1.345.755	-28.455
1.370.206	-22.486	1.391.557	-30.545	1.345.993	-28.456
1.370.264	-22.487	1.391.660	-30.553	1.346.176	-28.456
1.370.340	-22.489	1.391.866	-30.560	1.346.322	-28.457
1.370.338	-22.490	1.392.104	-30.567	1.346.487	-28.457
1.370.343	-22.491	1.392.312	-30.574	1.346.635	-28.457
1.370.540	-22.492	1.392.505	-30.580	1.346.794	-28.458
1.370.775	-22.493	1.392.587	-30.586	1.346.982	-28.458
1.370.907	-22.495	1.392.701	-30.592	1.347.122	-28.458
1.371.078	-22.496	1.392.950	-30.598	1.347.269	-28.459
1.371.257	-22.497	1.393.127	-30.603	1.347.458	-28.459
1.371.409	-22.498	1.393.297	-30.608	1.347.645	-28.460
1.371.637	-22.499	1.393.479	-30.613	1.347.760	-28.460
1.371.819	-22.500	1.393.631	-30.617	1.347.843	-28.461
1.371.931	-22.502	1.393.777	-30.621	1.348.006	-28.461
1.372.074	-22.503	1.393.925	-30.625	1.348.190	-28.461
1.372.218	-22.504	1.394.095	-30.628	1.348.322	-28.462
1.372.348	-22.505	1.394.249	-30.632	1.348.472	-28.462
1.372.498	-22.506	1.394.442	-30.635	1.348.587	-28.463
1.372.682	-22.507	1.394.619	-30.638	1.348.667	-28.463
1.372.840	-22.509	1.394.751	-30.640	1.348.882	-28.464
1.372.944	-22.510	1.394.951	-30.642	1.349.050	-28.464
1.373.065	-22.511	1.395.155	-30.644	1.349.221	-28.465
1.373.226	-22.512	1.395.269	-30.646	1.349.509	-28.466
1.373.418	-22.513	1.395.403	-30.647	1.349.697	-28.466
1.373.645	-22.514	1.395.611	-30.648	1.349.861	-28.467

1.373.808	-22.516	1.395.777	-30.649	1.350.067	-28.467
1.373.958	-22.517	1.395.909	-30.650	1.350.228	-28.468
1.374.117	-22.518	1.396.013	-30.650	1.350.421	-28.469
1.374.303	-22.519	1.396.189	-30.651	1.350.611	-28.469
1.374.518	-22.520	1.396.362	-30.651	1.350.723	-28.470
1.374.678	-22.521	1.396.429	-30.650	1.350.829	-28.471
1.374.852	-22.523	1.396.581	-30.650	1.350.943	-28.472
1.375.031	-22.524	1.396.805	-30.649	1.351.080	-28.472
1.375.143	-22.525	1.396.973	-30.648	1.351.275	-28.473
1.375.260	-22.526	1.397.117	-30.647	1.351.431	-28.474
1.375.479	-22.527	1.397.437	-30.645	1.351.537	-28.475
1.375.654	-22.528	1.397.858	-30.643	1.351.705	-28.476
1.375.766	-22.529	1.398.176	-30.642	1.351.859	-28.477
1.375.858	-22.530	1.398.291	-30.639	1.352.027	-28.477
1.376.003	-22.532	1.398.335	-30.637	1.352.180	-28.478
1.376.158	-22.533	1.398.363	-30.635	1.352.327	-28.479
1.376.301	-22.534	1.398.371	-30.632	1.352.509	-28.480
1.376.512	-22.535	1.398.429	-30.629	1.352.592	-28.481
1.376.711	-22.536	1.398.530	-30.626	1.352.749	-28.482
1.376.839	-22.537	1.398.674	-30.622	1.352.970	-28.483
1.376.931	-22.538	1.398.788	-30.619	1.353.148	-28.484
1.377.106	-22.539	1.398.992	-30.615	1.353.277	-28.486
1.377.307	-22.540	1.399.234	-30.611	1.353.427	-28.487
1.377.417	-22.542	1.399.377	-30.607	1.353.622	-28.488
1.377.522	-22.543	1.399.563	-30.602	1.353.743	-28.489
1.377.684	-22.544	1.399.776	-30.598	1.353.855	-28.490
1.377.847	-22.545	1.399.892	-30.593	1.354.037	-28.491
1.378.035	-22.546	1.400.005	-30.588	1.354.259	-28.492
1.378.217	-22.547	1.400.157	-30.583	1.354.451	-28.494
1.378.410	-22.548	1.400.302	-30.578	1.354.570	-28.495

1.378.611	-22.549	1.400.490	-30.572	1.354.702	-28.496
1.378.813	-22.550	1.400.704	-30.567	1.354.924	-28.498
1.378.999	-22.551	1.400.878	-30.561	1.355.130	-28.499
1.379.122	-22.552	1.401.026	-30.555	1.355.229	-28.500
1.379.265	-22.553	1.401.136	-30.549	1.355.385	-28.502
1.379.337	-22.554	1.401.270	-30.543	1.355.562	-28.503
1.379.429	-22.556	1.401.458	-30.537	1.355.674	-28.504
1.379.630	-22.557	1.401.552	-30.530	1.355.827	-28.506
1.379.807	-22.558	1.401.644	-30.524	1.356.026	-28.507
1.379.966	-22.559	1.401.864	-30.517	1.356.257	-28.509
1.380.215	-22.560	1.402.094	-30.510	1.356.324	-28.510
1.380.412	-22.561	1.402.258	-30.504	1.356.375	-28.512
1.380.602	-22.562	1.402.384	-30.497	1.356.611	-28.513
1.380.739	-22.563	1.402.551	-30.490	1.356.803	-28.515
1.380.871	-22.564	1.402.764	-30.482	1.356.947	-28.516
1.381.053	-22.565	1.403.002	-30.475	1.357.092	-28.518
1.381.145	-22.566	1.403.172	-30.468	1.357.253	-28.519
1.381.205	-22.567	1.403.302	-30.461	1.357.475	-28.521
1.381.416	-22.568	1.403.495	-30.453	1.357.628	-28.523
1.381.669	-22.569	1.403.604	-30.446	1.357.697	-28.524
1.381.787	-22.570	1.403.703	-30.438	1.357.852	-28.526
1.381.848	-22.571	1.403.920	-30.430	1.358.089	-28.527
1.381.938	-22.572	1.404.104	-30.423	1.358.270	-28.529
1.382.081	-22.573	1.404.185	-30.415	1.358.405	-28.531
1.382.271	-22.574	1.404.303	-30.407	1.358.573	-28.532
1.382.551	-22.575	1.404.538	-30.400	1.358.687	-28.534
1.382.751	-22.576	1.404.729	-30.392	1.358.828	-28.536
1.382.867	-22.577	1.404.827	-30.384	1.358.990	-28.538
1.383.093	-22.578	1.404.924	-30.376	1.359.099	-28.539
1.383.307	-22.579	1.405.116	-30.369	1.359.261	-28.541

1.383.450	-22.580	1.405.303	-30.361	1.359.426	-28.543
1.383.618	-22.581	1.405.466	-30.353	1.359.588	-28.544
1.383.775	-22.582	1.405.647	-30.345	1.359.758	-28.546
1.383.920	-22.583	1.405.804	-30.338	1.359.919	-28.548
1.384.104	-22.584	1.405.928	-30.330	1.360.114	-28.550
1.384.258	-22.585	1.406.035	-30.323	1.360.273	-28.552
1.384.357	-22.586	1.406.196	-30.315	1.360.452	-28.553
1.384.485	-22.587	1.406.393	-30.308	1.360.688	-28.555
1.384.628	-22.588	1.406.546	-30.300	1.360.867	-28.557
1.384.740	-22.589	1.406.765	-30.293	1.361.068	-28.559
1.384.899	-22.590	1.406.915	-30.286	1.361.237	-28.560
1.385.069	-22.591	1.407.027	-30.278	1.361.328	-28.562
1.385.217	-22.592	1.407.182	-30.271	1.361.501	-28.564
1.385.408	-22.593	1.407.363	-30.264	1.361.617	-28.566
1.385.618	-22.594	1.407.540	-30.257	1.361.752	-28.568
1.385.809	-22.595	1.407.717	-30.251	1.361.945	-28.569
1.385.977	-22.596	1.407.845	-30.244	1.362.052	-28.571
1.386.131	-22.597	1.407.943	-30.237	1.362.142	-28.573
1.386.259	-22.598	1.408.165	-30.231	1.362.283	-28.575
1.386.409	-22.599	1.408.376	-30.225	1.362.516	-28.577
1.386.597	-22.600	1.408.562	-30.218	1.362.679	-28.578
1.386.765	-22.601	1.408.775	-30.212	1.362.742	-28.580
1.386.922	-22.602	1.408.911	-30.206	1.362.849	-28.582
1.387.052	-22.603	1.409.037	-30.200	1.363.022	-28.584
1.387.253	-22.604	1.409.236	-30.194	1.363.230	-28.585
1.387.430	-22.605	1.409.357	-30.189	1.363.414	-28.587
1.387.509	-22.606	1.409.400	-30.183	1.363.613	-28.589
1.387.701	-22.607	1.409.503	-30.178	1.363.761	-28.591
1.387.959	-22.608	1.409.669	-30.173	1.363.864	-28.593
1.388.156	-22.609	1.409.828	-30.168	1.364.106	-28.594

1.388.284	-22.610	1.409.946	-30.163	1.364.377	-28.596
1.388.376	-22.611	1.410.105	-30.158	1.364.590	-28.598
1.388.559	-22.612	1.410.246	-30.153	1.364.767	-28.600
1.388.779	-22.613	1.410.392	-30.148	1.364.879	-28.601
1.388.974	-22.614	1.410.605	-30.144	1.365.018	-28.603
1.389.126	-22.615	1.410.869	-30.140	1.365.208	-28.605
1.389.250	-22.616	1.411.111	-30.136	1.365.325	-28.607
1.389.357	-22.617	1.411.283	-30.131	1.365.450	-28.608
1.389.507	-22.618	1.411.427	-30.128	1.365.683	-28.610
1.389.722	-22.619	1.411.617	-30.124	1.365.820	-28.612
1.389.879	-22.620	1.411.790	-30.120	1.365.972	-28.614
1.390.018	-22.621	1.411.938	-30.117	1.366.160	-28.615
1.390.210	-22.622	1.412.083	-30.113	1.366.304	-28.617
1.390.385	-22.623	1.412.251	-30.110	1.366.476	-28.619
1.390.558	-22.624	1.412.359	-30.107	1.366.642	-28.620
1.390.735	-22.625	1.412.455	-30.104	1.366.803	-28.622
1.390.903	-22.626	1.412.625	-30.101	1.366.960	-28.624
1.391.060	-22.627	1.412.816	-30.099	1.367.132	-28.625
1.391.134	-22.628	1.412.910	-30.096	1.367.323	-28.627
1.391.205	-22.629	1.413.037	-30.094	1.367.460	-28.629
1.391.355	-22.630	1.413.255	-30.091	1.367.585	-28.630
1.391.667	-22.631	1.413.425	-30.089	1.367.760	-28.632
1.392.160	-22.632	1.413.553	-30.087	1.367.856	-28.634
1.392.451	-22.633	1.413.681	-30.085	1.367.979	-28.635
1.392.498	-22.634	1.413.932	-30.084	1.368.132	-28.637
1.392.589	-22.635	1.414.175	-30.082	1.368.226	-28.639
1.392.654	-22.636	1.414.370	-30.080	1.368.403	-28.640
1.392.724	-22.637	1.414.482	-30.079	1.368.613	-28.642
1.392.804	-22.638	1.414.639	-30.078	1.368.754	-28.644
1.392.867	-22.639	1.414.852	-30.077	1.368.947	-28.645

1.393.009	-22.641	1.414.977	-30.076	1.369.137	-28.647
1.393.147	-22.642	1.415.108	-30.075	1.369.352	-28.648
1.393.248	-22.643	1.415.307	-30.074	1.369.541	-28.650
1.393.436	-22.644	1.415.484	-30.073	1.369.632	-28.652
1.393.656	-22.645	1.415.542	-30.073	1.369.767	-28.653
1.393.846	-22.646	1.415.699	-30.073	1.369.910	-28.655
1.394.034	-22.647	1.415.988	-30.072	1.370.051	-28.656
1.394.214	-22.648	1.416.120	-30.072	1.370.182	-28.658
1.394.337	-22.649	1.416.245	-30.072	1.370.311	-28.660
1.394.483	-22.650	1.416.411	-30.073	1.370.495	-28.661
1.394.659	-22.651	1.416.620	-30.073	1.370.907	-28.663
1.394.805	-22.652	1.416.786	-30.074	1.371.391	-28.664
1.394.957	-22.653	1.416.949	-30.074	1.371.642	-28.666
1.395.130	-22.654	1.417.213	-30.075	1.371.734	-28.668
1.395.217	-22.656	1.417.379	-30.076	1.371.761	-28.669
1.395.381	-22.657	1.417.428	-30.077	1.371.866	-28.671
1.395.585	-22.658	1.417.574	-30.078	1.372.012	-28.672
1.395.710	-22.659	1.417.744	-30.080	1.372.079	-28.674
1.395.894	-22.660	1.417.921	-30.081	1.372.128	-28.675
1.396.026	-22.661	1.418.062	-30.083	1.372.213	-28.677
1.396.140	-22.662	1.418.138	-30.085	1.372.285	-28.679
1.396.353	-22.663	1.418.275	-30.087	1.372.361	-28.680
1.396.649	-22.664	1.418.447	-30.089	1.372.538	-28.682
1.396.852	-22.665	1.418.636	-30.091	1.372.755	-28.683
1.396.940	-22.666	1.418.837	-30.094	1.372.986	-28.685
1.397.103	-22.668	1.419.005	-30.096	1.373.165	-28.687
1.397.303	-22.669	1.419.137	-30.099	1.373.251	-28.688
1.397.453	-22.670	1.419.317	-30.102	1.373.403	-28.690
1.397.565	-22.671	1.419.734	-30.105	1.373.559	-28.692
1.397.717	-22.672	1.420.103	-30.109	1.373.748	-28.693

1.397.874	-22.673	1.420.269	-30.112	1.373.992	-28.695
1.398.015	-22.674	1.420.247	-30.116	1.374.187	-28.696
1.398.165	-22.675	1.420.266	-30.119	1.374.332	-28.698
1.398.313	-22.676	1.420.379	-30.123	1.374.417	-28.700
1.398.445	-22.677	1.420.452	-30.128	1.374.543	-28.701
1.398.607	-22.679	1.420.538	-30.132	1.374.724	-28.703
1.398.790	-22.680	1.420.632	-30.136	1.374.919	-28.705
1.398.918	-22.681	1.420.773	-30.141	1.375.092	-28.706
1.399.052	-22.682	1.420.932	-30.146	1.375.181	-28.708
1.399.211	-22.683	1.421.080	-30.151	1.375.318	-28.710
1.399.399	-22.684	1.421.259	-30.156	1.375.470	-28.711
1.399.597	-22.685	1.421.429	-30.161	1.375.564	-28.713
1.399.698	-22.686	1.421.570	-30.167	1.375.726	-28.715
1.399.767	-22.687	1.421.729	-30.172	1.375.912	-28.717
1.399.948	-22.689	1.421.942	-30.178	1.376.095	-28.718
1.400.112	-22.690	1.422.130	-30.184	1.376.230	-28.720
1.400.246	-22.691	1.422.263	-30.190	1.376.328	-28.722
1.400.491	-22.692	1.422.426	-30.196	1.376.514	-28.724
1.400.730	-22.693	1.422.563	-30.203	1.376.740	-28.725
1.400.849	-22.694	1.422.722	-30.209	1.376.886	-28.727
1.400.977	-22.695	1.422.923	-30.216	1.377.059	-28.729
1.401.171	-22.696	1.423.058	-30.222	1.377.243	-28.731
1.401.362	-22.697	1.423.197	-30.229	1.377.386	-28.733
1.401.516	-22.699	1.423.403	-30.236	1.377.565	-28.734
1.401.673	-22.700	1.423.580	-30.243	1.377.813	-28.736
1.401.783	-22.701	1.423.631	-30.251	1.377.995	-28.738
1.401.938	-22.702	1.423.745	-30.258	1.378.044	-28.740
1.402.101	-22.703	1.423.981	-30.265	1.378.143	-28.742
1.402.242	-22.704	1.424.180	-30.273	1.378.318	-28.744
1.402.489	-22.705	1.424.350	-30.281	1.378.481	-28.745

1.402.650	-22.706	1.424.543	-30.289	1.378.647	-28.747
1.402.737	-22.707	1.424.711	-30.296	1.378.817	-28.749
1.402.789	-22.709	1.424.847	-30.304	1.379.032	-28.751
1.402.964	-22.710	1.425.049	-30.313	1.379.189	-28.753
1.403.208	-22.711	1.425.266	-30.321	1.379.279	-28.755
1.403.376	-22.712	1.425.367	-30.329	1.379.426	-28.757
1.403.539	-22.713	1.425.462	-30.337	1.379.617	-28.759
1.403.712	-22.714	1.425.647	-30.346	1.379.807	-28.761
1.403.922	-22.715	1.425.811	-30.354	1.379.933	-28.763
1.404.093	-22.716	1.425.849	-30.363	1.380.040	-28.765
1.404.142	-22.717	1.425.952	-30.372	1.380.170	-28.767
1.404.270	-22.719	1.426.183	-30.380	1.380.397	-28.768
1.404.435	-22.720	1.426.382	-30.389	1.380.648	-28.770
1.404.520	-22.721	1.426.599	-30.398	1.380.761	-28.772
1.404.688	-22.722	1.426.740	-30.407	1.380.894	-28.774
1.404.897	-22.723	1.426.839	-30.416	1.381.125	-28.776
1.405.033	-22.724	1.427.016	-30.425	1.381.292	-28.778
1.405.238	-22.725	1.427.200	-30.434	1.381.416	-28.780
1.405.522	-22.726	1.427.350	-30.443	1.381.546	-28.782
1.405.701	-22.727	1.427.480	-30.452	1.381.698	-28.784
1.405.845	-22.729	1.427.639	-30.461	1.381.897	-28.786
1.406.086	-22.730	1.427.793	-30.470	1.382.088	-28.788
1.406.270	-22.731	1.427.941	-30.480	1.382.220	-28.790
1.406.404	-22.732	1.428.062	-30.489	1.382.426	-28.792
1.406.548	-22.733	1.428.235	-30.498	1.382.636	-28.794
1.406.604	-22.734	1.428.463	-30.508	1.382.789	-28.796
1.406.673	-22.735	1.428.636	-30.517	1.382.905	-28.798
1.406.841	-22.736	1.428.773	-30.526	1.383.033	-28.800
1.406.953	-22.738	1.429.023	-30.536	1.383.210	-28.802
1.407.068	-22.739	1.429.187	-30.545	1.383.315	-28.804

1.407.285	-22.740	1.429.272	-30.555	1.383.385	-28.806
1.407.437	-22.741	1.429.408	-30.564	1.383.622	-28.808
1.407.578	-22.742	1.429.529	-30.574	1.383.913	-28.810
1.407.784	-22.743	1.429.695	-30.583	1.384.048	-28.811
1.407.952	-22.744	1.429.877	-30.593	1.384.122	-28.813
1.408.091	-22.745	1.430.067	-30.602	1.384.249	-28.815
1.408.329	-22.747	1.430.287	-30.612	1.384.395	-28.817
1.408.535	-22.748	1.430.388	-30.621	1.384.583	-28.819
1.408.687	-22.749	1.430.499	-30.631	1.384.734	-28.821
1.408.882	-22.750	1.430.636	-30.641	1.384.888	-28.823
1.409.034	-22.751	1.430.797	-30.650	1.385.098	-28.824
1.409.193	-22.752	1.430.974	-30.660	1.385.269	-28.826
1.409.359	-22.753	1.431.107	-30.670	1.385.408	-28.828
1.409.478	-22.755	1.431.216	-30.679	1.385.578	-28.830
1.409.604	-22.756	1.431.386	-30.689	1.385.728	-28.831
1.409.830	-22.757	1.431.557	-30.699	1.385.816	-28.833
1.409.980	-22.758	1.431.665	-30.708	1.385.912	-28.835
1.410.076	-22.759	1.431.754	-30.718	1.386.078	-28.836
1.410.305	-22.760	1.431.888	-30.728	1.386.230	-28.838
1.410.486	-22.761	1.432.090	-30.737	1.386.384	-28.840
1.410.616	-22.762	1.432.247	-30.747	1.386.528	-28.841
1.410.802	-22.764	1.432.403	-30.756	1.386.617	-28.843
1.410.990	-22.765	1.432.621	-30.766	1.386.796	-28.844
1.411.207	-22.766	1.432.834	-30.775	1.387.038	-28.846
1.411.380	-22.767	1.433.067	-30.785	1.387.220	-28.848
1.411.530	-22.768	1.433.291	-30.794	1.387.468	-28.849
1.411.586	-22.769	1.433.443	-30.803	1.387.735	-28.850
1.411.705	-22.771	1.433.540	-30.813	1.387.910	-28.852
1.411.965	-22.772	1.433.665	-30.822	1.388.013	-28.853
1.412.151	-22.773	1.433.878	-30.831	1.388.161	-28.855

1.412.300	-22.774	1.434.028	-30.840	1.388.434	-28.856
1.412.460	-22.775	1.434.151	-30.849	1.388.606	-28.857
1.412.634	-22.776	1.434.308	-30.858	1.388.710	-28.859
1.412.805	-22.777	1.434.426	-30.867	1.388.860	-28.860
1.412.919	-22.779	1.434.534	-30.875	1.389.003	-28.861
1.412.993	-22.780	1.434.666	-30.884	1.389.122	-28.862
1.413.145	-22.781	1.434.769	-30.892	1.389.240	-28.863
1.413.347	-22.782	1.434.881	-30.901	1.389.402	-28.864
1.413.517	-22.783	1.435.096	-30.909	1.389.595	-28.865
1.413.674	-22.784	1.435.341	-30.917	1.389.700	-28.867
1.413.826	-22.785	1.435.546	-30.925	1.389.796	-28.868
1.414.171	-22.787	1.435.737	-30.933	1.389.962	-28.868
1.414.650	-22.788	1.435.844	-30.941	1.390.150	-28.869
1.414.865	-22.789	1.435.979	-30.949	1.390.278	-28.870
1.414.924	-22.790	1.436.201	-30.956	1.390.432	-28.871
1.415.033	-22.791	1.436.429	-30.964	1.390.607	-28.872
1.415.087	-22.792	1.436.577	-30.971	1.390.800	-28.873
1.415.080	-22.794	1.436.664	-30.978	1.390.992	-28.874
1.415.150	-22.795	1.436.824	-30.985	1.391.185	-28.874
1.415.253	-22.796	1.437.054	-30.992	1.391.364	-28.875
1.415.325	-22.797	1.437.231	-30.998	1.391.521	-28.876
1.415.509	-22.798	1.437.352	-31.005	1.391.783	-28.876
1.415.690	-22.799	1.437.484	-31.011	1.391.944	-28.877
1.415.851	-22.800	1.437.668	-31.018	1.392.020	-28.877
1.416.046	-22.802	1.437.773	-31.024	1.392.180	-28.878
1.416.234	-22.803	1.437.858	-31.029	1.392.352	-28.878
1.416.348	-22.804	1.438.089	-31.035	1.392.484	-28.879
1.416.512	-22.805	1.438.235	-31.041	1.392.628	-28.879
1.416.700	-22.806	1.438.398	-31.046	1.392.782	-28.879
1.416.873	-22.807	1.438.627	-31.052	1.392.964	-28.880

1.417.072	-22.808	1.438.806	-31.057	1.393.174	-28.880
1.417.260	-22.810	1.439.005	-31.062	1.393.288	-28.880
1.417.390	-22.811	1.439.216	-31.066	1.393.436	-28.880
1.417.495	-22.812	1.439.324	-31.071	1.393.629	-28.881
1.417.569	-22.813	1.439.413	-31.075	1.393.766	-28.881
1.417.720	-22.814	1.439.622	-31.080	1.393.916	-28.881
1.417.937	-22.815	1.439.781	-31.084	1.394.108	-28.881
1.418.049	-22.816	1.439.948	-31.088	1.394.296	-28.881
1.418.141	-22.817	1.440.116	-31.091	1.394.460	-28.881
1.418.365	-22.819	1.440.251	-31.095	1.394.633	-28.881
1.418.568	-22.820	1.440.401	-31.098	1.394.812	-28.881
1.418.779	-22.821	1.440.607	-31.102	1.394.986	-28.881
1.418.981	-22.822	1.440.755	-31.105	1.395.105	-28.881
1.419.118	-22.823	1.440.903	-31.108	1.395.238	-28.881
1.419.323	-22.824	1.441.037	-31.111	1.395.403	-28.880
1.419.525	-22.825	1.441.292	-31.113	1.395.596	-28.880
1.419.688	-22.826	1.441.721	-31.116	1.395.795	-28.880
1.419.789	-22.827	1.442.086	-31.118	1.395.903	-28.880
1.419.921	-22.828	1.442.193	-31.121	1.396.022	-28.879
1.420.083	-22.829	1.442.162	-31.123	1.396.190	-28.879
1.420.233	-22.831	1.442.206	-31.125	1.396.422	-28.879
1.420.414	-22.832	1.442.281	-31.126	1.396.646	-28.878
1.420.623	-22.833	1.442.343	-31.128	1.396.796	-28.878
1.420.730	-22.834	1.442.419	-31.130	1.396.902	-28.878
1.420.847	-22.835	1.442.592	-31.131	1.397.052	-28.877
1.420.992	-22.836	1.442.764	-31.132	1.397.206	-28.877
1.421.160	-22.837	1.442.854	-31.133	1.397.332	-28.876
1.421.357	-22.838	1.443.017	-31.134	1.397.475	-28.876
1.421.483	-22.839	1.443.212	-31.135	1.397.610	-28.876
1.421.599	-22.840	1.443.347	-31.136	1.397.865	-28.875

1.421.772	-22.841	1.443.530	-31.137	1.398.244	-28.875
1.421.944	-22.842	1.443.690	-31.137	1.398.553	-28.874
1.422.101	-22.843	1.443.846	-31.137	1.398.721	-28.874
1.422.224	-22.844	1.444.032	-31.138	1.398.844	-28.873
1.422.366	-22.845	1.444.167	-31.138	1.398.947	-28.872
1.422.556	-22.846	1.444.343	-31.138	1.398.976	-28.872
1.422.729	-22.847	1.444.565	-31.138	1.398.987	-28.871
1.422.906	-22.848	1.444.769	-31.138	1.399.072	-28.871
1.423.053	-22.849	1.444.874	-31.137	1.399.229	-28.870
1.423.143	-22.850	1.444.939	-31.137	1.399.368	-28.870
1.423.326	-22.851	1.445.136	-31.136	1.399.498	-28.869
1.423.542	-22.852	1.445.284	-31.136	1.399.601	-28.868
1.423.678	-22.853	1.445.349	-31.135	1.399.790	-28.868
1.423.857	-22.854	1.445.499	-31.134	1.400.013	-28.867
1.424.026	-22.855	1.445.744	-31.133	1.400.155	-28.867
1.424.155	-22.856	1.445.981	-31.132	1.400.340	-28.866
1.424.317	-22.857	1.446.131	-31.131	1.400.553	-28.866
1.424.478	-22.858	1.446.292	-31.129	1.400.724	-28.865
1.424.657	-22.859	1.446.449	-31.128	1.400.903	-28.864
1.424.825	-22.860	1.446.595	-31.126	1.401.068	-28.864
1.424.948	-22.861	1.446.810	-31.125	1.401.180	-28.863
1.425.103	-22.861	1.446.996	-31.123	1.401.339	-28.863
1.425.318	-22.862	1.447.144	-31.121	1.401.465	-28.862
1.425.479	-22.863	1.447.300	-31.119	1.401.613	-28.862
1.425.600	-22.864	1.447.435	-31.117	1.401.817	-28.861
1.425.775	-22.865	1.447.531	-31.115	1.401.947	-28.861
1.425.948	-22.866	1.447.731	-31.112	1.402.079	-28.860
1.426.028	-22.867	1.447.950	-31.110	1.402.209	-28.860
1.426.163	-22.868	1.448.055	-31.107	1.402.390	-28.859
1.426.371	-22.869	1.448.176	-31.105	1.402.607	-28.859

1.426.505	-22.870	1.448.349	-31.102	1.402.778	-28.859
1.426.595	-22.870	1.448.481	-31.099	1.402.905	-28.858
1.426.678	-22.871	1.448.633	-31.096	1.403.055	-28.858
1.426.832	-22.872	1.448.819	-31.093	1.403.201	-28.858
1.427.056	-22.873	1.449.021	-31.090	1.403.356	-28.857
1.427.227	-22.874	1.449.180	-31.087	1.403.557	-28.857
1.427.386	-22.875	1.449.314	-31.084	1.403.728	-28.857
1.427.623	-22.876	1.449.465	-31.080	1.403.914	-28.857
1.427.787	-22.876	1.449.574	-31.077	1.404.102	-28.857
1.428.046	-22.877	1.449.686	-31.074	1.404.247	-28.857
1.428.266	-22.878	1.449.892	-31.070	1.404.386	-28.857
1.428.365	-22.879	1.450.060	-31.066	1.404.621	-28.857
1.428.559	-22.880	1.450.204	-31.063	1.404.763	-28.857
1.428.777	-22.881	1.450.423	-31.059	1.404.866	-28.857
1.428.898	-22.881	1.450.578	-31.055	1.405.056	-28.857
1.429.014	-22.882	1.450.638	-31.051	1.405.190	-28.857
1.429.185	-22.883	1.450.824	-31.047	1.405.325	-28.857
1.429.328	-22.884	1.451.021	-31.043	1.405.480	-28.858
1.429.391	-22.885	1.451.158	-31.039	1.405.625	-28.858
1.429.462	-22.885	1.451.344	-31.035	1.405.804	-28.859
1.429.619	-22.886	1.451.476	-31.031	1.405.961	-28.859
1.429.798	-22.887	1.451.649	-31.027	1.406.120	-28.860
1.429.944	-22.888	1.451.814	-31.023	1.406.257	-28.861
1.430.157	-22.889	1.451.989	-31.019	1.406.391	-28.861
1.430.345	-22.889	1.452.155	-31.014	1.406.597	-28.862
1.430.542	-22.890	1.452.316	-31.010	1.406.788	-28.863
1.430.724	-22.891	1.452.444	-31.006	1.406.976	-28.864
1.430.862	-22.892	1.452.558	-31.001	1.407.157	-28.865
1.431.084	-22.893	1.452.746	-30.997	1.407.260	-28.866
1.431.261	-22.893	1.452.924	-30.992	1.407.386	-28.868

1.431.402	-22.894	1.453.037	-30.988	1.407.551	-28.869
1.431.581	-22.895	1.453.176	-30.983	1.407.717	-28.870
1.431.716	-22.896	1.453.313	-30.979	1.407.890	-28.872
1.431.873	-22.897	1.453.407	-30.974	1.407.988	-28.874
1.432.009	-22.897	1.453.582	-30.970	1.408.111	-28.875
1.432.141	-22.898	1.453.804	-30.965	1.408.273	-28.877
1.432.327	-22.899	1.453.913	-30.961	1.408.438	-28.879
1.432.487	-22.900	1.454.039	-30.956	1.408.680	-28.881
1.432.614	-22.901	1.454.252	-30.951	1.408.864	-28.883
1.432.762	-22.901	1.454.480	-30.947	1.408.983	-28.885
1.432.986	-22.902	1.454.675	-30.942	1.409.167	-28.888
1.433.226	-22.903	1.454.870	-30.937	1.409.375	-28.890
1.433.378	-22.904	1.455.007	-30.932	1.409.527	-28.893
1.433.477	-22.904	1.455.148	-30.928	1.409.599	-28.895
1.433.604	-22.905	1.455.340	-30.923	1.409.731	-28.898
1.433.835	-22.906	1.455.546	-30.918	1.409.928	-28.901
1.434.019	-22.907	1.455.739	-30.913	1.410.072	-28.904
1.434.193	-22.908	1.455.912	-30.909	1.410.266	-28.907
1.434.319	-22.908	1.456.049	-30.904	1.410.414	-28.910
1.434.429	-22.909	1.456.154	-30.899	1.410.598	-28.913
1.434.606	-22.910	1.456.295	-30.894	1.410.833	-28.917
1.434.753	-22.911	1.456.414	-30.890	1.410.972	-28.920
1.434.881	-22.912	1.456.521	-30.885	1.411.098	-28.924
1.435.047	-22.912	1.456.660	-30.880	1.411.227	-28.928
1.435.262	-22.913	1.456.819	-30.875	1.411.360	-28.932
1.435.450	-22.914	1.457.034	-30.870	1.411.584	-28.936
1.435.589	-22.915	1.457.222	-30.865	1.411.768	-28.940
1.435.706	-22.916	1.457.350	-30.861	1.411.926	-28.944
1.435.791	-22.916	1.457.520	-30.856	1.412.110	-28.948
1.435.914	-22.917	1.457.697	-30.851	1.412.287	-28.953

1.436.086	-22.918	1.457.876	-30.846	1.412.422	-28.957
1.436.402	-22.919	1.458.028	-30.841	1.412.522	-28.962
1.436.873	-22.920	1.458.172	-30.836	1.412.632	-28.967
1.437.177	-22.920	1.458.326	-30.831	1.412.800	-28.971
1.437.336	-22.921	1.458.528	-30.826	1.412.890	-28.976
1.437.343	-22.922	1.458.732	-30.821	1.413.040	-28.981
1.437.352	-22.923	1.458.900	-30.816	1.413.214	-28.987
1.437.437	-22.924	1.459.017	-30.811	1.413.286	-28.992
1.437.522	-22.925	1.459.187	-30.806	1.413.416	-28.997
1.437.540	-22.925	1.459.366	-30.801	1.413.598	-29.003
1.437.643	-22.926	1.459.520	-30.796	1.413.736	-29.008
1.437.858	-22.927	1.459.693	-30.791	1.413.904	-29.014
1.438.033	-22.928	1.459.809	-30.786	1.414.151	-29.020
1.438.212	-22.929	1.459.939	-30.781	1.414.317	-29.026
1.438.382	-22.929	1.460.135	-30.776	1.414.476	-29.032
1.438.542	-22.930	1.460.345	-30.771	1.414.745	-29.038
1.438.685	-22.931	1.460.517	-30.766	1.414.967	-29.044
1.438.878	-22.932	1.460.618	-30.760	1.415.135	-29.050
1.439.075	-22.933	1.460.674	-30.755	1.415.313	-29.056
1.439.160	-22.934	1.460.885	-30.750	1.415.466	-29.063
1.439.294	-22.935	1.461.151	-30.745	1.415.562	-29.069
1.439.491	-22.935	1.461.344	-30.740	1.415.703	-29.076
1.439.680	-22.936	1.461.474	-30.735	1.415.873	-29.082
1.439.861	-22.937	1.461.613	-30.729	1.416.031	-29.089
1.440.002	-22.938	1.461.796	-30.724	1.416.133	-29.096
1.440.134	-22.939	1.461.962	-30.719	1.416.219	-29.103
1.440.266	-22.940	1.462.160	-30.714	1.416.409	-29.110
1.440.399	-22.940	1.462.334	-30.708	1.416.588	-29.117
1.440.517	-22.941	1.462.403	-30.703	1.416.698	-29.124
1.440.706	-22.942	1.462.480	-30.698	1.416.868	-29.131

1.440.878	-22.943	1.462.645	-30.693	1.417.000	-29.138
1.441.017	-22.944	1.462.885	-30.687	1.417.106	-29.145
1.441.214	-22.945	1.463.116	-30.682	1.417.319	-29.153
1.441.355	-22.946	1.463.501	-30.677	1.417.489	-29.160
1.441.575	-22.947	1.463.866	-30.672	1.417.594	-29.168
1.441.792	-22.947	1.464.032	-30.666	1.417.811	-29.175
1.441.940	-22.948	1.464.025	-30.661	1.418.040	-29.183
1.442.068	-22.949	1.464.077	-30.656	1.418.244	-29.190
1.442.206	-22.950	1.464.169	-30.651	1.418.419	-29.198
1.442.381	-22.951	1.464.218	-30.645	1.418.559	-29.206
1.442.569	-22.952	1.464.335	-30.640	1.418.708	-29.214
1.442.706	-22.953	1.464.424	-30.635	1.418.940	-29.222
1.442.831	-22.954	1.464.539	-30.629	1.419.142	-29.230
1.442.959	-22.954	1.464.738	-30.624	1.419.249	-29.238
1.443.116	-22.955	1.464.919	-30.619	1.419.391	-29.246
1.443.327	-22.956	1.465.119	-30.614	1.419.541	-29.254
1.443.490	-22.957	1.465.291	-30.608	1.419.664	-29.262
1.443.591	-22.958	1.465.410	-30.603	1.419.794	-29.270
1.443.741	-22.959	1.465.537	-30.598	1.419.939	-29.278
1.443.932	-22.960	1.465.737	-30.593	1.420.117	-29.286
1.444.086	-22.961	1.465.885	-30.587	1.420.296	-29.294
1.444.274	-22.962	1.465.995	-30.582	1.420.477	-29.302
1.444.471	-22.963	1.466.149	-30.577	1.420.634	-29.311
1.444.623	-22.963	1.466.355	-30.571	1.420.753	-29.319
1.444.727	-22.964	1.466.497	-30.566	1.420.901	-29.327
1.444.798	-22.965	1.466.664	-30.561	1.421.073	-29.335
1.444.946	-22.966	1.466.828	-30.556	1.421.185	-29.343
1.445.175	-22.967	1.467.009	-30.550	1.421.335	-29.351
1.445.340	-22.968	1.467.224	-30.545	1.421.526	-29.360
1.445.486	-22.969	1.467.363	-30.540	1.421.709	-29.368

1.445.638	-22.970	1.467.455	-30.534	1.421.888	-29.376
1.445.811	-22.971	1.467.545	-30.529	1.422.090	-29.384
1.445.984	-22.972	1.467.607	-30.524	1.422.280	-29.392
1.446.156	-22.973	1.467.820	-30.519	1.422.386	-29.400
1.446.351	-22.973	1.468.098	-30.514	1.422.540	-29.408
1.446.496	-22.974	1.468.282	-30.508	1.422.715	-29.416
1.446.637	-22.975	1.468.436	-30.503	1.422.887	-29.424
1.446.801	-22.976	1.468.589	-30.498	1.423.073	-29.432
1.446.971	-22.977	1.468.685	-30.493	1.423.221	-29.440
1.447.108	-22.978	1.468.898	-30.487	1.423.365	-29.448
1.447.300	-22.979	1.469.124	-30.482	1.423.517	-29.456
1.447.500	-22.980	1.469.243	-30.477	1.423.669	-29.463
1.447.643	-22.981	1.469.386	-30.472	1.423.810	-29.471
1.447.746	-22.982	1.469.538	-30.467	1.423.945	-29.478
1.447.818	-22.983	1.469.695	-30.462	1.424.113	-29.486
1.447.928	-22.984	1.469.901	-30.456	1.424.263	-29.493
1.448.134	-22.985	1.470.063	-30.451	1.424.377	-29.501
1.448.369	-22.986	1.470.210	-30.446	1.424.592	-29.508
1.448.490	-22.987	1.470.360	-30.441	1.424.895	-29.515
1.448.573	-22.988	1.470.522	-30.436	1.425.300	-29.522
1.448.674	-22.989	1.470.723	-30.431	1.425.648	-29.529
1.448.857	-22.990	1.470.892	-30.426	1.425.724	-29.536
1.449.030	-22.991	1.471.050	-30.421	1.425.741	-29.543
1.449.187	-22.991	1.471.185	-30.416	1.425.844	-29.550
1.449.384	-22.992	1.471.254	-30.411	1.425.923	-29.557
1.449.511	-22.993	1.471.360	-30.406	1.425.981	-29.563
1.449.682	-22.994	1.471.490	-30.401	1.426.113	-29.570
1.449.951	-22.995	1.471.685	-30.396	1.426.243	-29.576
1.450.114	-22.996	1.471.978	-30.391	1.426.353	-29.582
1.450.280	-22.997	1.472.188	-30.386	1.426.483	-29.588

1.450.528	-22.998	1.472.296	-30.382	1.426.620	-29.594
1.450.732	-22.999	1.472.421	-30.377	1.426.855	-29.600
1.450.872	-23.000	1.472.589	-30.372	1.427.047	-29.606
1.450.997	-23.001	1.472.717	-30.367	1.427.088	-29.612
1.451.124	-23.002	1.472.811	-30.363	1.427.298	-29.617
1.451.288	-23.003	1.472.977	-30.358	1.427.569	-29.623
1.451.465	-23.004	1.473.215	-30.353	1.427.673	-29.628
1.451.608	-23.005	1.473.363	-30.349	1.427.887	-29.633
1.451.756	-23.006	1.473.483	-30.344	1.428.024	-29.638
1.451.850	-23.007	1.473.676	-30.340	1.428.150	-29.643
1.451.960	-23.008	1.473.898	-30.335	1.428.279	-29.648
1.452.110	-23.009	1.474.097	-30.331	1.428.414	-29.653
1.452.236	-23.010	1.474.169	-30.326	1.428.577	-29.657
1.452.417	-23.012	1.474.278	-30.322	1.428.727	-29.662
1.452.654	-23.013	1.474.431	-30.318	1.428.880	-29.666
1.452.838	-23.014	1.474.637	-30.314	1.429.061	-29.671
1.452.977	-23.015	1.474.857	-30.309	1.429.207	-29.675
1.453.134	-23.016	1.474.986	-30.305	1.429.335	-29.679
1.453.297	-23.017	1.475.117	-30.301	1.429.480	-29.683
1.453.438	-23.018	1.475.210	-30.297	1.429.581	-29.687
1.453.627	-23.019	1.475.354	-30.293	1.429.713	-29.691
1.453.811	-23.020	1.475.497	-30.289	1.429.874	-29.694
1.453.996	-23.021	1.475.609	-30.285	1.430.067	-29.698
1.454.191	-23.022	1.475.782	-30.281	1.430.249	-29.701
1.454.272	-23.023	1.475.970	-30.278	1.430.425	-29.705
1.454.408	-23.024	1.476.138	-30.274	1.430.659	-29.708
1.454.623	-23.026	1.476.283	-30.270	1.430.809	-29.711
1.454.769	-23.027	1.476.483	-30.266	1.430.943	-29.714
1.454.931	-23.028	1.476.698	-30.263	1.431.118	-29.717
1.455.094	-23.029	1.476.861	-30.259	1.431.290	-29.720

1.455.298	-23.030	1.477.050	-30.256	1.431.476	-29.723
1.455.464	-23.031	1.477.294	-30.252	1.431.613	-29.726
1.455.600	-23.032	1.477.525	-30.249	1.431.783	-29.729
1.455.714	-23.033	1.477.648	-30.246	1.431.938	-29.731
1.455.907	-23.035	1.477.719	-30.243	1.432.108	-29.734
1.456.051	-23.036	1.477.856	-30.239	1.432.274	-29.736
1.456.127	-23.037	1.478.018	-30.236	1.432.285	-29.738
1.456.329	-23.038	1.478.156	-30.233	1.432.435	-29.741
1.456.517	-23.039	1.478.297	-30.230	1.432.706	-29.743
1.456.655	-23.040	1.478.432	-30.227	1.432.849	-29.745
1.456.832	-23.042	1.478.582	-30.224	1.432.968	-29.747
1.456.942	-23.043	1.478.765	-30.221	1.433.085	-29.749
1.457.106	-23.044	1.478.909	-30.219	1.433.286	-29.751
1.457.321	-23.045	1.479.073	-30.216	1.433.533	-29.752
1.457.444	-23.046	1.479.285	-30.213	1.433.685	-29.754
1.457.589	-23.048	1.479.480	-30.211	1.433.779	-29.756
1.457.841	-23.049	1.479.612	-30.208	1.433.934	-29.757
1.458.017	-23.050	1.479.713	-30.206	1.434.097	-29.759
1.458.159	-23.051	1.479.863	-30.203	1.434.256	-29.761
1.458.340	-23.052	1.480.087	-30.201	1.434.478	-29.762
1.458.450	-23.054	1.480.309	-30.199	1.434.693	-29.763
1.458.739	-23.055	1.480.488	-30.196	1.434.823	-29.765
1.459.212	-23.056	1.480.612	-30.194	1.434.960	-29.766
1.459.442	-23.057	1.480.739	-30.192	1.435.076	-29.767
1.459.556	-23.059	1.480.932	-30.190	1.435.217	-29.768
1.459.718	-23.060	1.481.066	-30.188	1.435.479	-29.769
1.459.803	-23.061	1.481.183	-30.186	1.435.685	-29.770
1.459.765	-23.063	1.481.353	-30.184	1.435.773	-29.772
1.459.821	-23.064	1.481.474	-30.182	1.435.869	-29.772
1.459.924	-23.065	1.481.631	-30.181	1.436.015	-29.773

1.460.038	-23.066	1.481.861	-30.179	1.436.205	-29.774
1.460.168	-23.068	1.482.032	-30.177	1.436.382	-29.775
1.460.303	-23.069	1.482.189	-30.176	1.436.528	-29.776
1.460.497	-23.070	1.482.356	-30.174	1.436.689	-29.777
1.460.650	-23.072	1.482.464	-30.173	1.436.815	-29.778
1.460.773	-23.073	1.482.603	-30.172	1.436.996	-29.778
1.460.952	-23.074	1.482.794	-30.170	1.437.177	-29.779
1.461.158	-23.075	1.482.934	-30.169	1.437.290	-29.780
1.461.304	-23.077	1.483.105	-30.168	1.437.442	-29.780
1.461.436	-23.078	1.483.280	-30.167	1.437.549	-29.781
1.461.617	-23.079	1.483.421	-30.166	1.437.728	-29.782
1.461.830	-23.081	1.483.607	-30.165	1.437.935	-29.782
1.461.987	-23.082	1.483.837	-30.164	1.438.062	-29.783
1.462.074	-23.083	1.483.969	-30.163	1.438.194	-29.783
1.462.234	-23.085	1.484.075	-30.162	1.438.349	-29.784
1.462.431	-23.086	1.484.285	-30.161	1.438.508	-29.784
1.462.523	-23.087	1.484.473	-30.161	1.438.654	-29.785
1.462.661	-23.089	1.484.581	-30.160	1.438.898	-29.785
1.462.778	-23.090	1.484.724	-30.159	1.439.108	-29.786
1.462.937	-23.091	1.485.011	-30.159	1.439.227	-29.786
1.463.125	-23.093	1.485.428	-30.159	1.439.321	-29.786
1.463.306	-23.094	1.485.789	-30.158	1.439.433	-29.787
1.463.557	-23.095	1.485.947	-30.158	1.439.570	-29.787
1.463.685	-23.097	1.485.977	-30.158	1.439.707	-29.788
1.463.821	-23.098	1.485.983	-30.157	1.439.827	-29.788
1.464.064	-23.099	1.485.994	-30.157	1.439.977	-29.789
1.464.211	-23.101	1.486.102	-30.157	1.440.184	-29.789
1.464.335	-23.102	1.486.277	-30.157	1.440.354	-29.789
1.464.518	-23.103	1.486.382	-30.157	1.440.511	-29.790
1.464.695	-23.105	1.486.467	-30.157	1.440.690	-29.790

1.464.866	-23.106	1.486.615	-30.157	1.440.938	-29.791
1.465.007	-23.107	1.486.801	-30.158	1.441.140	-29.791
1.465.128	-23.109	1.486.958	-30.158	1.441.275	-29.792
1.465.244	-23.110	1.487.139	-30.158	1.441.442	-29.792
1.465.387	-23.111	1.487.336	-30.158	1.441.665	-29.792
1.465.524	-23.113	1.487.574	-30.159	1.441.857	-29.793
1.465.723	-23.114	1.487.722	-30.159	1.441.982	-29.793
1.465.968	-23.115	1.487.818	-30.160	1.442.139	-29.794
1.466.095	-23.117	1.488.026	-30.160	1.442.361	-29.794
1.466.268	-23.118	1.488.167	-30.161	1.442.522	-29.795
1.466.429	-23.119	1.488.250	-30.162	1.442.605	-29.795
1.466.514	-23.121	1.488.391	-30.162	1.442.679	-29.796
1.466.680	-23.122	1.488.568	-30.163	1.442.836	-29.796
1.466.853	-23.123	1.488.788	-30.164	1.443.031	-29.797
1.466.998	-23.125	1.488.904	-30.164	1.443.212	-29.797
1.467.086	-23.126	1.489.021	-30.165	1.443.342	-29.798
1.467.204	-23.127	1.489.196	-30.166	1.443.452	-29.799
1.467.451	-23.129	1.489.272	-30.167	1.443.573	-29.799
1.467.655	-23.130	1.489.373	-30.168	1.443.768	-29.800
1.467.814	-23.131	1.489.599	-30.169	1.443.911	-29.800
1.468.024	-23.133	1.489.796	-30.170	1.444.044	-29.801
1.468.130	-23.134	1.489.964	-30.171	1.444.205	-29.802
1.468.246	-23.135	1.490.177	-30.172	1.444.368	-29.802
1.468.438	-23.136	1.490.336	-30.173	1.444.570	-29.803
1.468.569	-23.138	1.490.500	-30.174	1.444.794	-29.804
1.468.689	-23.139	1.490.710	-30.175	1.444.971	-29.804
1.468.907	-23.140	1.490.827	-30.176	1.445.060	-29.805
1.469.106	-23.142	1.490.985	-30.178	1.445.195	-29.806
1.469.176	-23.143	1.491.161	-30.179	1.445.365	-29.806
1.469.312	-23.144	1.491.333	-30.180	1.445.540	-29.807

1.469.529	-23.146	1.491.492	-30.181	1.445.779	-29.808
1.469.702	-23.147	1.491.521	-30.182	1.445.994	-29.808
1.469.886	-23.148	1.491.626	-30.184	1.446.084	-29.809
1.469.998	-23.149	1.491.778	-30.185	1.446.131	-29.810
1.470.101	-23.151	1.492.023	-30.186	1.446.288	-29.810
1.470.242	-23.152	1.492.280	-30.188	1.446.537	-29.811
1.470.425	-23.153	1.492.457	-30.189	1.446.774	-29.812
1.470.623	-23.154	1.492.576	-30.190	1.446.931	-29.813
1.470.784	-23.156	1.492.715	-30.192	1.446.987	-29.813
1.470.921	-23.157	1.492.883	-30.193	1.447.126	-29.814
1.471.102	-23.158	1.493.028	-30.195	1.447.350	-29.815
1.471.160	-23.160	1.493.197	-30.196	1.447.469	-29.815
1.471.230	-23.161	1.493.409	-30.197	1.447.639	-29.816
1.471.470	-23.162	1.493.591	-30.199	1.447.859	-29.817
1.471.660	-23.163	1.493.725	-30.200	1.447.961	-29.817
1.471.821	-23.164	1.493.880	-30.202	1.448.152	-29.818
1.472.005	-23.166	1.494.041	-30.203	1.448.407	-29.819
1.472.213	-23.167	1.494.160	-30.205	1.448.559	-29.819
1.472.433	-23.168	1.494.274	-30.206	1.448.678	-29.820
1.472.641	-23.169	1.494.390	-30.207	1.448.853	-29.821
1.472.820	-23.171	1.494.613	-30.209	1.449.032	-29.821
1.472.959	-23.172	1.494.846	-30.210	1.449.135	-29.822
1.473.127	-23.173	1.494.980	-30.212	1.449.247	-29.823
1.473.326	-23.174	1.495.098	-30.213	1.449.402	-29.823
1.473.494	-23.175	1.495.280	-30.215	1.449.574	-29.824
1.473.613	-23.177	1.495.493	-30.216	1.449.707	-29.824
1.473.754	-23.178	1.495.636	-30.218	1.449.868	-29.825
1.473.828	-23.179	1.495.766	-30.219	1.450.078	-29.825
1.473.965	-23.180	1.495.985	-30.220	1.450.193	-29.826
1.474.100	-23.181	1.496.122	-30.222	1.450.358	-29.826

1.474.220	-23.183	1.496.299	-30.223	1.450.632	-29.827
1.474.404	-23.184	1.496.476	-30.225	1.450.862	-29.827
1.474.633	-23.185	1.496.572	-30.226	1.450.993	-29.828
1.474.812	-23.186	1.496.702	-30.228	1.451.078	-29.828
1.474.863	-23.187	1.496.848	-30.229	1.451.205	-29.829
1.474.989	-23.189	1.497.023	-30.230	1.451.315	-29.829
1.475.246	-23.190	1.497.193	-30.232	1.451.463	-29.829
1.475.437	-23.191	1.497.294	-30.233	1.451.740	-29.830
1.475.591	-23.192	1.497.379	-30.235	1.452.155	-29.830
1.475.741	-23.193	1.497.480	-30.236	1.452.455	-29.830
1.475.901	-23.194	1.497.666	-30.237	1.452.594	-29.831
1.476.167	-23.196	1.497.883	-30.239	1.452.697	-29.831
1.476.315	-23.197	1.498.040	-30.240	1.452.769	-29.831
1.476.447	-23.198	1.498.190	-30.241	1.452.847	-29.832
1.476.619	-23.199	1.498.468	-30.243	1.452.939	-29.832
1.476.756	-23.200	1.498.694	-30.244	1.453.073	-29.832
1.476.891	-23.201	1.498.830	-30.245	1.453.154	-29.832
1.477.065	-23.202	1.499.007	-30.247	1.453.210	-29.832
1.477.216	-23.204	1.499.218	-30.248	1.453.279	-29.832
1.477.375	-23.205	1.499.310	-30.249	1.453.427	-29.833
1.477.540	-23.206	1.499.483	-30.250	1.453.624	-29.833
1.477.704	-23.207	1.499.621	-30.252	1.453.792	-29.833
1.477.899	-23.208	1.499.731	-30.253	1.454.016	-29.833
1.478.091	-23.209	1.499.908	-30.254	1.454.158	-29.833
1.478.270	-23.210	1.499.991	-30.255	1.454.281	-29.833
1.478.391	-23.212	1.500.107	-30.257	1.454.474	-29.833
1.478.530	-23.213	1.500.336	-30.258	1.454.669	-29.833
1.478.727	-23.214	1.500.471	-30.259	1.454.859	-29.833
1.478.846	-23.215	1.500.607	-30.260	1.455.031	-29.833
1.478.951	-23.216	1.500.838	-30.261	1.455.197	-29.833

1.479.144	-23.217	1.501.073	-30.262	1.455.318	-29.833
1.479.335	-23.218	1.501.217	-30.263	1.455.444	-29.833
1.479.494	-23.219	1.501.369	-30.264	1.455.578	-29.833
1.479.675	-23.221	1.501.530	-30.265	1.455.742	-29.833
1.479.827	-23.222	1.501.725	-30.266	1.455.927	-29.833
1.479.975	-23.223	1.501.958	-30.267	1.456.026	-29.832
1.480.177	-23.224	1.502.132	-30.268	1.456.194	-29.832
1.480.358	-23.225	1.502.249	-30.269	1.456.393	-29.832
1.480.444	-23.226	1.502.399	-30.270	1.456.541	-29.832
1.480.533	-23.227	1.502.580	-30.271	1.456.678	-29.832
1.480.732	-23.228	1.502.686	-30.272	1.456.821	-29.832
1.481.129	-23.229	1.502.780	-30.273	1.456.996	-29.831
1.481.590	-23.230	1.502.939	-30.274	1.457.216	-29.831
1.481.850	-23.231	1.503.149	-30.275	1.457.355	-29.831
1.481.895	-23.233	1.503.266	-30.276	1.457.513	-29.831
1.481.902	-23.234	1.503.423	-30.276	1.457.688	-29.831
1.481.933	-23.235	1.503.660	-30.277	1.457.885	-29.830
1.482.034	-23.236	1.503.893	-30.278	1.458.082	-29.830
1.482.197	-23.237	1.504.034	-30.279	1.458.246	-29.830
1.482.276	-23.238	1.504.149	-30.280	1.458.434	-29.830
1.482.321	-23.239	1.504.274	-30.280	1.458.604	-29.829
1.482.473	-23.240	1.504.400	-30.281	1.458.662	-29.829
1.482.650	-23.241	1.504.566	-30.282	1.458.721	-29.829
1.482.787	-23.242	1.504.734	-30.282	1.458.954	-29.829
1.482.970	-23.243	1.504.872	-30.283	1.459.081	-29.828
1.483.181	-23.244	1.505.034	-30.284	1.459.218	-29.828
1.483.389	-23.245	1.505.233	-30.284	1.459.404	-29.828
1.483.605	-23.246	1.505.415	-30.285	1.459.525	-29.828
1.483.759	-23.247	1.505.564	-30.286	1.459.744	-29.827
1.483.866	-23.248	1.505.683	-30.286	1.459.899	-29.827

1.483.997	-23.249	1.505.809	-30.287	1.460.054	-29.827
1.484.133	-23.250	1.506.006	-30.287	1.460.233	-29.827
1.484.225	-23.251	1.506.228	-30.288	1.460.300	-29.826
1.484.359	-23.252	1.506.418	-30.288	1.460.426	-29.826
1.484.550	-23.253	1.506.516	-30.289	1.460.616	-29.826
1.484.749	-23.254	1.506.640	-30.289	1.460.775	-29.825
1.484.908	-23.255	1.506.982	-30.290	1.460.963	-29.825
1.485.004	-23.256	1.507.417	-30.290	1.461.165	-29.825
1.485.201	-23.257	1.507.672	-30.291	1.461.265	-29.825
1.485.424	-23.258	1.507.749	-30.291	1.461.393	-29.824
1.485.536	-23.259	1.507.827	-30.291	1.461.543	-29.824
1.485.704	-23.260	1.507.932	-30.292	1.461.678	-29.824
1.485.938	-23.261	1.507.959	-30.292	1.461.875	-29.824
1.486.109	-23.262	1.508.017	-30.292	1.462.027	-29.824
1.486.257	-23.263	1.508.116	-30.293	1.462.265	-29.823
1.486.418	-23.264	1.508.248	-30.293	1.462.509	-29.823
1.486.572	-23.265	1.508.438	-30.293	1.462.525	-29.823
1.486.702	-23.266	1.508.627	-30.293	1.462.666	-29.823
1.486.850	-23.267	1.508.790	-30.294	1.462.914	-29.822
1.487.005	-23.268	1.508.954	-30.294	1.463.087	-29.822
1.487.159	-23.269	1.509.194	-30.294	1.463.333	-29.822
1.487.318	-23.270	1.509.386	-30.294	1.463.466	-29.822
1.487.437	-23.271	1.509.529	-30.294	1.463.562	-29.822
1.487.540	-23.272	1.509.688	-30.294	1.463.770	-29.822
1.487.739	-23.272	1.509.825	-30.295	1.463.891	-29.821
1.487.966	-23.273	1.509.939	-30.295	1.463.963	-29.821
1.488.139	-23.274	1.510.094	-30.295	1.464.137	-29.821
1.488.297	-23.275	1.510.278	-30.295	1.464.382	-29.821
1.488.494	-23.276	1.510.430	-30.295	1.464.559	-29.821
1.488.698	-23.277	1.510.592	-30.295	1.464.646	-29.821

1.488.864	-23.278	1.510.768	-30.295	1.464.812	-29.821
1.489.005	-23.279	1.510.858	-30.294	1.465.054	-29.821
1.489.041	-23.279	1.510.983	-30.294	1.465.210	-29.821
1.489.102	-23.280	1.511.169	-30.294	1.465.304	-29.821
1.489.317	-23.281	1.511.317	-30.294	1.465.437	-29.821
1.489.558	-23.282	1.511.489	-30.294	1.465.638	-29.821
1.489.702	-23.283	1.511.676	-30.294	1.465.815	-29.820
1.489.837	-23.284	1.511.875	-30.294	1.465.999	-29.820
1.490.070	-23.284	1.512.032	-30.293	1.466.153	-29.820
1.490.269	-23.285	1.512.166	-30.293	1.466.232	-29.821
1.490.408	-23.286	1.512.332	-30.293	1.466.299	-29.821
1.490.542	-23.287	1.512.460	-30.292	1.466.467	-29.821
1.490.674	-23.288	1.512.621	-30.292	1.466.646	-29.821
1.490.929	-23.288	1.512.811	-30.292	1.466.756	-29.821
1.491.091	-23.289	1.512.961	-30.291	1.466.904	-29.821
1.491.167	-23.290	1.513.082	-30.291	1.467.061	-29.821
1.491.315	-23.291	1.513.226	-30.290	1.467.198	-29.821
1.491.474	-23.291	1.513.349	-30.290	1.467.305	-29.821
1.491.638	-23.292	1.513.501	-30.289	1.467.406	-29.821
1.491.801	-23.293	1.513.714	-30.289	1.467.610	-29.821
1.491.976	-23.294	1.513.871	-30.288	1.467.892	-29.822
1.492.164	-23.294	1.514.016	-30.287	1.468.148	-29.822
1.492.337	-23.295	1.514.198	-30.287	1.468.340	-29.822
1.492.527	-23.296	1.514.375	-30.286	1.468.512	-29.822
1.492.733	-23.296	1.514.529	-30.285	1.468.678	-29.822
1.492.863	-23.297	1.514.727	-30.284	1.468.871	-29.823
1.492.944	-23.298	1.514.877	-30.284	1.468.981	-29.823
1.493.027	-23.298	1.514.971	-30.283	1.469.138	-29.823
1.493.158	-23.299	1.515.143	-30.282	1.469.323	-29.824
1.493.336	-23.300	1.515.347	-30.281	1.469.453	-29.824

1.493.465	-23.300	1.515.493	-30.280	1.469.619	-29.824
1.493.591	-23.301	1.515.611	-30.279	1.469.792	-29.824
1.493.710	-23.302	1.515.815	-30.278	1.469.868	-29.825
1.493.801	-23.302	1.516.062	-30.277	1.469.987	-29.825
1.493.967	-23.303	1.516.225	-30.276	1.470.114	-29.826
1.494.212	-23.304	1.516.360	-30.275	1.470.260	-29.826
1.494.447	-23.304	1.516.572	-30.274	1.470.488	-29.826
1.494.733	-23.305	1.516.772	-30.273	1.470.630	-29.827
1.494.944	-23.306	1.516.949	-30.272	1.470.762	-29.827
1.495.078	-23.306	1.517.097	-30.271	1.470.943	-29.828
1.495.251	-23.307	1.517.177	-30.269	1.471.131	-29.828
1.495.475	-23.307	1.517.267	-30.268	1.471.313	-29.829
1.495.667	-23.308	1.517.426	-30.267	1.471.474	-29.829
1.495.779	-23.309	1.517.675	-30.266	1.471.613	-29.830
1.495.856	-23.309	1.517.899	-30.264	1.471.783	-29.830
1.495.927	-23.310	1.518.040	-30.263	1.471.982	-29.831
1.496.100	-23.310	1.518.168	-30.261	1.472.182	-29.831
1.496.301	-23.311	1.518.286	-30.260	1.472.390	-29.832
1.496.460	-23.311	1.518.403	-30.259	1.472.536	-29.832
1.496.599	-23.312	1.518.557	-30.257	1.472.648	-29.833
1.496.669	-23.312	1.518.707	-30.256	1.472.751	-29.834
1.496.765	-23.313	1.518.848	-30.254	1.472.941	-29.834
1.496.969	-23.313	1.518.974	-30.252	1.473.181	-29.835
1.497.209	-23.314	1.519.046	-30.251	1.473.362	-29.836
1.497.403	-23.315	1.519.180	-30.249	1.473.537	-29.836
1.497.592	-23.315	1.519.386	-30.248	1.473.654	-29.837
1.497.784	-23.316	1.519.576	-30.246	1.473.721	-29.838
1.497.917	-23.316	1.519.762	-30.244	1.473.871	-29.838
1.498.078	-23.317	1.519.942	-30.242	1.474.077	-29.839
1.498.266	-23.317	1.520.152	-30.241	1.474.270	-29.840

1.498.441	-23.318	1.520.356	-30.239	1.474.357	-29.841
1.498.589	-23.318	1.520.526	-30.237	1.474.482	-29.842
1.498.737	-23.319	1.520.701	-30.235	1.474.695	-29.842
1.498.909	-23.319	1.520.827	-30.233	1.474.845	-29.843
1.499.063	-23.319	1.520.970	-30.231	1.475.000	-29.844
1.499.169	-23.320	1.521.181	-30.229	1.475.217	-29.845
1.499.229	-23.320	1.521.353	-30.228	1.475.381	-29.846
1.499.408	-23.321	1.521.497	-30.226	1.475.490	-29.847
1.499.621	-23.321	1.521.629	-30.224	1.475.580	-29.848
1.499.790	-23.322	1.521.740	-30.222	1.475.742	-29.849
1.499.955	-23.322	1.521.832	-30.219	1.475.952	-29.850
1.500.098	-23.323	1.521.915	-30.217	1.476.122	-29.851
1.500.257	-23.323	1.522.097	-30.215	1.476.302	-29.852
1.500.450	-23.324	1.522.309	-30.213	1.476.526	-29.853
1.500.618	-23.324	1.522.475	-30.211	1.476.700	-29.854
1.500.708	-23.324	1.522.668	-30.209	1.476.846	-29.855
1.500.842	-23.325	1.522.859	-30.207	1.477.018	-29.856
1.501.111	-23.325	1.523.024	-30.205	1.477.191	-29.857
1.501.333	-23.326	1.523.226	-30.202	1.477.321	-29.858
1.501.456	-23.326	1.523.423	-30.200	1.477.458	-29.859
1.501.581	-23.326	1.523.656	-30.198	1.477.616	-29.860
1.501.658	-23.327	1.523.840	-30.196	1.477.675	-29.861
1.501.882	-23.327	1.523.985	-30.193	1.477.847	-29.862
1.502.164	-23.328	1.524.135	-30.191	1.478.064	-29.863
1.502.339	-23.328	1.524.236	-30.189	1.478.154	-29.865
1.502.509	-23.328	1.524.420	-30.186	1.478.338	-29.866
1.502.623	-23.329	1.524.601	-30.184	1.478.662	-29.867
1.502.719	-23.329	1.524.742	-30.182	1.479.066	-29.868
1.502.861	-23.329	1.524.870	-30.179	1.479.467	-29.869
1.503.004	-23.330	1.525.029	-30.177	1.479.633	-29.870

1.503.313	-23.330	1.525.219	-30.174	1.479.666	-29.872
1.503.757	-23.331	1.525.351	-30.172	1.479.767	-29.873
1.504.086	-23.331	1.525.477	-30.170	1.479.839	-29.874
1.504.281	-23.331	1.525.571	-30.167	1.479.846	-29.875
1.504.366	-23.332	1.525.813	-30.165	1.479.828	-29.877
1.504.375	-23.332	1.526.022	-30.162	1.479.987	-29.878
1.504.370	-23.332	1.526.153	-30.160	1.480.199	-29.879
1.504.427	-23.333	1.526.302	-30.157	1.480.300	-29.880
1.504.487	-23.333	1.526.496	-30.155	1.480.385	-29.882
1.504.621	-23.333	1.526.657	-30.152	1.480.491	-29.883
1.504.823	-23.334	1.526.790	-30.150	1.480.670	-29.884
1.504.959	-23.334	1.526.909	-30.147	1.480.943	-29.885
1.505.099	-23.334	1.527.066	-30.145	1.481.156	-29.887
1.505.246	-23.334	1.527.253	-30.142	1.481.284	-29.888
1.505.434	-23.335	1.527.374	-30.140	1.481.395	-29.889
1.505.607	-23.335	1.527.520	-30.137	1.481.608	-29.890
1.505.762	-23.335	1.527.724	-30.135	1.481.814	-29.892
1.505.934	-23.336	1.527.856	-30.132	1.481.971	-29.893
1.506.120	-23.336	1.527.986	-30.130	1.482.148	-29.894
1.506.295	-23.336	1.528.205	-30.127	1.482.290	-29.896
1.506.434	-23.336	1.528.349	-30.125	1.482.426	-29.897
1.506.637	-23.337	1.528.555	-30.122	1.482.560	-29.898
1.506.848	-23.337	1.529.025	-30.120	1.482.733	-29.899
1.506.958	-23.337	1.529.427	-30.117	1.482.865	-29.901
1.507.034	-23.337	1.529.548	-30.115	1.482.988	-29.902
1.507.193	-23.338	1.529.523	-30.112	1.483.129	-29.903
1.507.374	-23.338	1.529.529	-30.110	1.483.297	-29.905
1.507.486	-23.338	1.529.556	-30.107	1.483.427	-29.906
1.507.627	-23.338	1.529.597	-30.105	1.483.499	-29.907
1.507.850	-23.338	1.529.774	-30.103	1.483.694	-29.908

1.508.067	-23.339	1.529.977	-30.100	1.483.967	-29.910
1.508.237	-23.339	1.530.107	-30.098	1.484.133	-29.911
1.508.425	-23.339	1.530.235	-30.095	1.484.270	-29.912
1.508.560	-23.339	1.530.376	-30.093	1.484.464	-29.913
1.508.689	-23.339	1.530.571	-30.090	1.484.641	-29.915
1.508.927	-23.340	1.530.732	-30.088	1.484.809	-29.916
1.509.108	-23.340	1.530.892	-30.086	1.484.973	-29.917
1.509.200	-23.340	1.531.133	-30.083	1.485.143	-29.918
1.509.211	-23.340	1.531.344	-30.081	1.485.282	-29.920
1.509.326	-23.340	1.531.521	-30.078	1.485.441	-29.921
1.509.572	-23.340	1.531.644	-30.076	1.485.650	-29.922
1.509.758	-23.340	1.531.794	-30.074	1.485.800	-29.923
1.509.955	-23.341	1.531.964	-30.071	1.485.916	-29.924
1.510.101	-23.341	1.532.123	-30.069	1.486.089	-29.926
1.510.183	-23.341	1.532.263	-30.067	1.486.275	-29.927
1.510.387	-23.341	1.532.363	-30.064	1.486.395	-29.928
1.510.632	-23.341	1.532.468	-30.062	1.486.505	-29.929
1.510.751	-23.341	1.532.634	-30.060	1.486.653	-29.930
1.510.871	-23.341	1.532.851	-30.058	1.486.792	-29.932
1.511.059	-23.341	1.532.975	-30.055	1.486.973	-29.933
1.511.208	-23.341	1.533.109	-30.053	1.487.186	-29.934
1.511.348	-23.342	1.533.313	-30.051	1.487.352	-29.935
1.511.517	-23.342	1.533.481	-30.049	1.487.545	-29.936
1.511.640	-23.342	1.533.609	-30.047	1.487.682	-29.937
1.511.783	-23.342	1.533.764	-30.044	1.487.800	-29.938
1.511.969	-23.342	1.533.960	-30.042	1.487.950	-29.940
1.512.159	-23.342	1.534.162	-30.040	1.488.018	-29.941
1.512.377	-23.342	1.534.332	-30.038	1.488.205	-29.942
1.512.549	-23.342	1.534.431	-30.036	1.488.425	-29.943
1.512.729	-23.342	1.534.552	-30.034	1.488.530	-29.944

1.512.928	-23.342	1.534.740	-30.032	1.488.652	-29.945
1.513.020	-23.342	1.534.915	-30.030	1.488.821	-29.946
1.513.163	-23.342	1.535.022	-30.028	1.489.001	-29.948
1.513.331	-23.342	1.535.181	-30.026	1.489.174	-29.949
1.513.436	-23.342	1.535.379	-30.024	1.489.368	-29.950
1.513.596	-23.342	1.535.499	-30.022	1.489.623	-29.951
1.513.754	-23.342	1.535.667	-30.020	1.489.816	-29.952
1.513.886	-23.342	1.535.795	-30.018	1.489.897	-29.953
1.514.068	-23.342	1.535.961	-30.016	1.490.040	-29.954
1.514.231	-23.342	1.536.203	-30.014	1.490.213	-29.955
1.514.382	-23.342	1.536.286	-30.012	1.490.345	-29.956
1.514.648	-23.342	1.536.405	-30.010	1.490.444	-29.957
1.514.809	-23.342	1.536.552	-30.008	1.490.620	-29.958
1.514.879	-23.342	1.536.622	-30.006	1.490.833	-29.959
1.515.056	-23.342	1.536.797	-30.004	1.490.979	-29.960
1.515.244	-23.342	1.536.983	-30.003	1.491.129	-29.962
1.515.396	-23.342	1.537.146	-30.001	1.491.295	-29.963
1.515.526	-23.342	1.537.361	-29.999	1.491.478	-29.964
1.515.583	-23.342	1.537.592	-29.997	1.491.637	-29.965
1.515.649	-23.342	1.537.769	-29.995	1.491.750	-29.966
1.515.806	-23.342	1.537.906	-29.994	1.491.902	-29.967
1.515.983	-23.341	1.538.044	-29.992	1.492.092	-29.968
1.516.189	-23.341	1.538.183	-29.990	1.492.267	-29.969
1.516.367	-23.341	1.538.389	-29.989	1.492.426	-29.970
1.516.581	-23.341	1.538.638	-29.987	1.492.533	-29.971
1.516.787	-23.341	1.538.763	-29.985	1.492.733	-29.971
1.516.951	-23.341	1.538.849	-29.984	1.492.910	-29.972
1.517.137	-23.341	1.539.017	-29.982	1.493.031	-29.973
1.517.339	-23.341	1.539.207	-29.981	1.493.139	-29.974
1.517.556	-23.341	1.539.397	-29.979	1.493.300	-29.975

1.517.692	-23.341	1.539.572	-29.977	1.493.450	-29.976
1.517.874	-23.341	1.539.753	-29.976	1.493.656	-29.977
1.518.080	-23.341	1.539.897	-29.974	1.493.824	-29.978
1.518.186	-23.340	1.539.989	-29.973	1.493.933	-29.979
1.518.326	-23.340	1.540.117	-29.972	1.494.088	-29.980
1.518.497	-23.340	1.540.311	-29.970	1.494.263	-29.981
1.518.591	-23.340	1.540.462	-29.969	1.494.418	-29.982
1.518.683	-23.340	1.540.584	-29.967	1.494.545	-29.982
1.518.853	-23.340	1.540.688	-29.966	1.494.727	-29.983
1.519.070	-23.340	1.540.786	-29.965	1.494.948	-29.984
1.519.232	-23.340	1.540.965	-29.963	1.495.166	-29.985
1.519.339	-23.340	1.541.120	-29.962	1.495.307	-29.986
1.519.469	-23.340	1.541.228	-29.961	1.495.434	-29.987
1.519.659	-23.339	1.541.418	-29.959	1.495.667	-29.987
1.519.834	-23.339	1.541.597	-29.958	1.495.865	-29.988
1.520.038	-23.339	1.541.794	-29.957	1.496.026	-29.989
1.520.242	-23.339	1.542.045	-29.956	1.496.236	-29.990
1.520.419	-23.339	1.542.249	-29.954	1.496.395	-29.991
1.520.553	-23.339	1.542.484	-29.953	1.496.463	-29.991
1.520.679	-23.339	1.542.612	-29.952	1.496.590	-29.992
1.520.845	-23.339	1.542.701	-29.951	1.496.781	-29.993
1.520.947	-23.339	1.542.876	-29.950	1.496.911	-29.994
1.521.124	-23.339	1.543.078	-29.949	1.497.036	-29.995
1.521.362	-23.338	1.543.255	-29.948	1.497.211	-29.995
1.521.514	-23.338	1.543.371	-29.947	1.497.310	-29.996
1.521.667	-23.338	1.543.461	-29.946	1.497.408	-29.997
1.521.805	-23.338	1.543.569	-29.945	1.497.522	-29.998
1.521.953	-23.338	1.543.714	-29.944	1.497.664	-29.998
1.522.103	-23.338	1.543.862	-29.943	1.497.836	-29.999
1.522.309	-23.338	1.543.996	-29.942	1.498.069	-30.000

1.522.504	-23.338	1.544.140	-29.941	1.498.279	-30.001
1.522.589	-23.338	1.544.348	-29.941	1.498.468	-30.001
1.522.701	-23.338	1.544.604	-29.940	1.498.618	-30.002
1.522.930	-23.338	1.544.821	-29.939	1.498.806	-30.003
1.523.109	-23.338	1.544.989	-29.938	1.499.016	-30.004
1.523.224	-23.338	1.545.139	-29.938	1.499.211	-30.004
1.523.382	-23.338	1.545.271	-29.937	1.499.393	-30.005
1.523.611	-23.338	1.545.441	-29.936	1.499.556	-30.006
1.523.745	-23.338	1.545.598	-29.936	1.499.673	-30.006
1.523.839	-23.338	1.545.733	-29.935	1.499.810	-30.007
1.524.034	-23.337	1.545.860	-29.935	1.499.982	-30.008
1.524.230	-23.337	1.546.015	-29.934	1.500.121	-30.009
1.524.344	-23.337	1.546.208	-29.934	1.500.257	-30.009
1.524.453	-23.337	1.546.411	-29.933	1.500.417	-30.010
1.524.644	-23.337	1.546.552	-29.933	1.500.547	-30.011
1.524.890	-23.337	1.546.646	-29.932	1.500.699	-30.011
1.525.054	-23.337	1.546.819	-29.932	1.500.874	-30.012
1.525.159	-23.337	1.547.066	-29.932	1.501.059	-30.013
1.525.298	-23.337	1.547.216	-29.931	1.501.268	-30.014
1.525.540	-23.337	1.547.332	-29.931	1.501.478	-30.014
1.525.883	-23.337	1.547.462	-29.931	1.501.604	-30.015
1.526.243	-23.338	1.547.650	-29.931	1.501.727	-30.016
1.526.458	-23.338	1.547.858	-29.930	1.501.902	-30.016
1.526.507	-23.338	1.548.060	-29.930	1.502.002	-30.017
1.526.588	-23.338	1.548.246	-29.930	1.502.179	-30.018
1.526.647	-23.338	1.548.345	-29.930	1.502.415	-30.019
1.526.722	-23.338	1.548.503	-29.930	1.502.589	-30.019
1.526.895	-23.338	1.548.651	-29.930	1.502.764	-30.020
1.526.992	-23.338	1.548.773	-29.930	1.502.924	-30.021
1.527.032	-23.338	1.548.958	-29.930	1.503.093	-30.021

1.527.235	-23.338	1.549.097	-29.930	1.503.261	-30.022
1.527.457	-23.338	1.549.281	-29.931	1.503.365	-30.023
1.527.589	-23.338	1.549.480	-29.931	1.503.468	-30.024
1.527.760	-23.338	1.549.624	-29.931	1.503.553	-30.024
1.527.939	-23.339	1.549.783	-29.931	1.503.821	-30.025
1.528.147	-23.339	1.549.899	-29.932	1.504.052	-30.026
1.528.271	-23.339	1.550.033	-29.932	1.504.133	-30.026
1.528.389	-23.339	1.550.275	-29.932	1.504.272	-30.027
1.528.598	-23.339	1.550.618	-29.933	1.504.451	-30.028
1.528.786	-23.339	1.550.995	-29.933	1.504.650	-30.029
1.528.875	-23.339	1.551.138	-29.934	1.504.794	-30.029
1.528.974	-23.340	1.551.230	-29.934	1.504.939	-30.030
1.529.133	-23.340	1.551.286	-29.935	1.505.125	-30.031
1.529.267	-23.340	1.551.257	-29.936	1.505.291	-30.031
1.529.442	-23.340	1.551.299	-29.936	1.505.540	-30.032
1.529.677	-23.340	1.551.441	-29.937	1.505.889	-30.033
1.529.841	-23.340	1.551.539	-29.938	1.506.237	-30.034
1.529.930	-23.341	1.551.730	-29.939	1.506.449	-30.034
1.530.070	-23.341	1.551.911	-29.940	1.506.553	-30.035
1.530.257	-23.341	1.552.036	-29.940	1.506.685	-30.036
1.530.453	-23.341	1.552.117	-29.941	1.506.828	-30.036
1.530.656	-23.342	1.552.309	-29.942	1.506.897	-30.037
1.530.849	-23.342	1.552.605	-29.943	1.506.954	-30.038
1.530.997	-23.342	1.552.825	-29.945	1.507.009	-30.038
1.531.115	-23.342	1.553.011	-29.946	1.507.066	-30.039
1.531.292	-23.343	1.553.152	-29.947	1.507.119	-30.040
1.531.488	-23.343	1.553.297	-29.948	1.507.256	-30.040
1.531.606	-23.343	1.553.423	-29.949	1.507.426	-30.041
1.531.682	-23.344	1.553.559	-29.951	1.507.592	-30.042
1.531.819	-23.344	1.553.721	-29.952	1.507.740	-30.042

1.531.987	-23.344	1.553.853	-29.953	1.507.831	-30.043
1.532.155	-23.345	1.553.988	-29.955	1.507.975	-30.043
1.532.305	-23.345	1.554.153	-29.956	1.508.156	-30.044
1.532.468	-23.345	1.554.326	-29.958	1.508.423	-30.045
1.532.663	-23.346	1.554.489	-29.959	1.508.689	-30.045
1.532.825	-23.346	1.554.610	-29.961	1.508.837	-30.046
1.533.011	-23.346	1.554.776	-29.963	1.509.025	-30.047
1.533.167	-23.347	1.554.968	-29.964	1.509.256	-30.047
1.533.300	-23.347	1.555.103	-29.966	1.509.379	-30.048
1.533.450	-23.347	1.555.300	-29.968	1.509.458	-30.048
1.533.616	-23.348	1.555.573	-29.970	1.509.577	-30.049
1.533.774	-23.348	1.555.755	-29.972	1.509.758	-30.050
1.533.891	-23.349	1.555.820	-29.974	1.509.971	-30.050
1.534.075	-23.349	1.555.947	-29.976	1.510.072	-30.051
1.534.314	-23.349	1.556.093	-29.978	1.510.282	-30.051
1.534.500	-23.350	1.556.190	-29.980	1.510.493	-30.052
1.534.592	-23.350	1.556.304	-29.982	1.510.558	-30.053
1.534.756	-23.351	1.556.523	-29.984	1.510.630	-30.053
1.535.013	-23.351	1.556.705	-29.986	1.510.759	-30.054
1.535.201	-23.352	1.556.866	-29.988	1.510.932	-30.054
1.535.376	-23.352	1.557.029	-29.991	1.511.134	-30.055
1.535.486	-23.353	1.557.211	-29.993	1.511.382	-30.055
1.535.513	-23.353	1.557.334	-29.995	1.511.597	-30.056
1.535.654	-23.354	1.557.437	-29.998	1.511.738	-30.057
1.535.869	-23.354	1.557.652	-30.000	1.511.922	-30.057
1.536.021	-23.355	1.557.795	-30.003	1.512.023	-30.058
1.536.181	-23.355	1.557.943	-30.005	1.512.193	-30.058
1.536.328	-23.356	1.558.149	-30.008	1.512.372	-30.059
1.536.490	-23.356	1.558.340	-30.010	1.512.482	-30.059
1.536.693	-23.357	1.558.450	-30.013	1.512.612	-30.060

1.536.855	-23.358	1.558.535	-30.016	1.512.843	-30.061
1.537.007	-23.358	1.558.745	-30.018	1.512.993	-30.061
1.537.121	-23.359	1.558.927	-30.021	1.513.123	-30.062
1.537.309	-23.359	1.559.072	-30.024	1.513.237	-30.062
1.537.513	-23.360	1.559.268	-30.026	1.513.331	-30.063
1.537.612	-23.361	1.559.415	-30.029	1.513.533	-30.063
1.537.724	-23.361	1.559.565	-30.032	1.513.663	-30.064
1.537.852	-23.362	1.559.854	-30.035	1.513.837	-30.064
1.538.004	-23.363	1.559.939	-30.038	1.513.999	-30.065
1.538.147	-23.363	1.560.000	-30.041	1.514.088	-30.066
1.538.284	-23.364	1.560.202	-30.044	1.514.285	-30.066
1.538.423	-23.365	1.560.356	-30.047	1.514.455	-30.067
1.538.562	-23.365	1.560.473	-30.050	1.514.644	-30.067
1.538.739	-23.366	1.560.659	-30.052	1.514.828	-30.068
1.538.970	-23.367	1.560.853	-30.056	1.514.966	-30.068
1.539.167	-23.367	1.561.003	-30.059	1.515.168	-30.069
1.539.348	-23.368	1.561.129	-30.062	1.515.341	-30.069
1.539.568	-23.369	1.561.326	-30.065	1.515.390	-30.070
1.539.796	-23.370	1.561.510	-30.068	1.515.495	-30.070
1.540.004	-23.370	1.561.657	-30.071	1.515.627	-30.071
1.540.197	-23.371	1.561.797	-30.074	1.515.853	-30.071
1.540.320	-23.372	1.561.958	-30.077	1.516.185	-30.072
1.540.443	-23.373	1.562.108	-30.080	1.516.321	-30.072
1.540.600	-23.373	1.562.265	-30.083	1.516.409	-30.073
1.540.697	-23.374	1.562.390	-30.087	1.516.582	-30.073
1.540.777	-23.375	1.562.471	-30.090	1.516.709	-30.074
1.540.941	-23.376	1.562.607	-30.093	1.516.850	-30.074
1.541.125	-23.377	1.562.780	-30.096	1.517.070	-30.075
1.541.243	-23.377	1.562.919	-30.099	1.517.243	-30.075
1.541.384	-23.378	1.563.096	-30.102	1.517.372	-30.076

1.541.512	-23.379	1.563.208	-30.106	1.517.509	-30.076
1.541.629	-23.380	1.563.349	-30.109	1.517.742	-30.077
1.541.853	-23.381	1.563.633	-30.112	1.517.943	-30.077
1.542.047	-23.382	1.563.918	-30.115	1.518.047	-30.078
1.542.249	-23.383	1.564.100	-30.118	1.518.156	-30.078
1.542.498	-23.384	1.564.263	-30.122	1.518.306	-30.078
1.542.679	-23.384	1.564.415	-30.125	1.518.497	-30.079
1.542.825	-23.385	1.564.525	-30.128	1.518.683	-30.079
1.542.981	-23.386	1.564.666	-30.131	1.518.851	-30.080
1.543.129	-23.387	1.564.819	-30.134	1.519.057	-30.080
1.543.208	-23.388	1.564.977	-30.137	1.519.173	-30.080
1.543.277	-23.389	1.565.103	-30.140	1.519.245	-30.081
1.543.474	-23.390	1.565.282	-30.144	1.519.408	-30.081
1.543.687	-23.391	1.565.443	-30.147	1.519.585	-30.081
1.543.855	-23.392	1.565.533	-30.150	1.519.818	-30.082
1.544.061	-23.393	1.565.679	-30.153	1.520.018	-30.082
1.544.250	-23.394	1.565.847	-30.156	1.520.103	-30.082
1.544.426	-23.395	1.566.010	-30.159	1.520.186	-30.083
1.544.559	-23.396	1.566.217	-30.162	1.520.365	-30.083
1.544.682	-23.397	1.566.445	-30.165	1.520.529	-30.083
1.544.765	-23.398	1.566.633	-30.168	1.520.643	-30.084
1.544.926	-23.399	1.566.833	-30.171	1.520.750	-30.084
1.545.157	-23.400	1.567.002	-30.174	1.520.869	-30.084
1.545.360	-23.401	1.567.106	-30.177	1.521.059	-30.084
1.545.477	-23.402	1.567.242	-30.180	1.521.272	-30.085
1.545.625	-23.403	1.567.408	-30.183	1.521.416	-30.085
1.545.811	-23.404	1.567.654	-30.186	1.521.599	-30.085
1.545.959	-23.406	1.567.881	-30.189	1.521.803	-30.085
1.546.084	-23.407	1.567.961	-30.192	1.522.016	-30.086
1.546.252	-23.408	1.568.116	-30.195	1.522.166	-30.086

1.546.351	-23.409	1.568.324	-30.197	1.522.327	-30.086
1.546.465	-23.410	1.568.436	-30.200	1.522.524	-30.086
1.546.716	-23.411	1.568.566	-30.203	1.522.764	-30.086
1.546.926	-23.412	1.568.806	-30.206	1.522.972	-30.087
1.547.048	-23.413	1.569.052	-30.209	1.523.084	-30.087
1.547.211	-23.415	1.569.193	-30.211	1.523.244	-30.087
1.547.359	-23.416	1.569.296	-30.214	1.523.316	-30.087
1.547.486	-23.417	1.569.420	-30.217	1.523.436	-30.087
1.547.645	-23.418	1.569.532	-30.219	1.523.653	-30.087
1.548.044	-23.419	1.569.706	-30.222	1.523.831	-30.087
1.548.463	-23.420	1.569.912	-30.225	1.523.927	-30.087
1.548.707	-23.422	1.570.092	-30.227	1.524.007	-30.088
1.548.853	-23.423	1.570.233	-30.230	1.524.169	-30.088
1.548.911	-23.424	1.570.354	-30.232	1.524.368	-30.088
1.548.961	-23.425	1.570.533	-30.235	1.524.523	-30.088
1.548.996	-23.426	1.570.750	-30.237	1.524.650	-30.088
1.549.044	-23.428	1.570.887	-30.240	1.524.857	-30.088
1.549.164	-23.429	1.571.001	-30.242	1.525.040	-30.088
1.549.305	-23.430	1.571.144	-30.245	1.525.184	-30.088
1.549.433	-23.431	1.571.293	-30.247	1.525.461	-30.088
1.549.559	-23.433	1.571.487	-30.249	1.525.670	-30.088
1.549.700	-23.434	1.571.631	-30.252	1.525.757	-30.088
1.549.901	-23.435	1.571.714	-30.254	1.525.878	-30.088
1.550.128	-23.436	1.571.893	-30.256	1.526.069	-30.088
1.550.345	-23.438	1.572.300	-30.258	1.526.232	-30.088
1.550.515	-23.439	1.572.753	-30.261	1.526.389	-30.088
1.550.611	-23.440	1.572.986	-30.263	1.526.618	-30.088
1.550.728	-23.442	1.573.064	-30.265	1.526.761	-30.088
1.550.952	-23.443	1.573.145	-30.267	1.526.846	-30.088
1.551.176	-23.444	1.573.167	-30.269	1.526.954	-30.088

1.551.299	-23.445	1.573.217	-30.271	1.527.063	-30.088
1.551.411	-23.447	1.573.326	-30.273	1.527.243	-30.088
1.551.548	-23.448	1.573.403	-30.275	1.527.482	-30.088
1.551.694	-23.449	1.573.522	-30.277	1.527.670	-30.088
1.551.835	-23.451	1.573.685	-30.279	1.527.881	-30.088
1.551.994	-23.452	1.573.853	-30.281	1.528.044	-30.088
1.552.177	-23.453	1.574.082	-30.283	1.528.167	-30.088
1.552.318	-23.455	1.574.258	-30.285	1.528.317	-30.088
1.552.471	-23.456	1.574.357	-30.287	1.528.407	-30.088
1.552.675	-23.457	1.574.476	-30.289	1.528.539	-30.088
1.552.883	-23.459	1.574.677	-30.291	1.528.739	-30.088
1.553.026	-23.460	1.574.883	-30.293	1.528.916	-30.088
1.553.167	-23.461	1.575.110	-30.295	1.529.077	-30.088
1.553.311	-23.463	1.575.251	-30.296	1.529.223	-30.088
1.553.472	-23.464	1.575.365	-30.298	1.529.328	-30.088
1.553.643	-23.465	1.575.544	-30.300	1.529.480	-30.088
1.553.828	-23.467	1.575.737	-30.302	1.529.720	-30.088
1.554.008	-23.468	1.575.943	-30.303	1.529.877	-30.089
1.554.137	-23.469	1.576.075	-30.305	1.529.915	-30.089
1.554.283	-23.471	1.576.185	-30.307	1.530.074	-30.089
1.554.436	-23.472	1.576.337	-30.308	1.530.307	-30.089
1.554.588	-23.473	1.576.458	-30.310	1.530.448	-30.089
1.554.707	-23.475	1.576.577	-30.312	1.530.536	-30.089
1.554.852	-23.476	1.576.799	-30.313	1.530.786	-30.089
1.555.076	-23.477	1.577.085	-30.315	1.530.891	-30.089
1.555.210	-23.479	1.577.267	-30.316	1.531.116	-30.089
1.555.374	-23.480	1.577.356	-30.318	1.531.436	-30.090
1.555.605	-23.482	1.577.549	-30.319	1.531.481	-30.090
1.555.786	-23.483	1.577.747	-30.321	1.531.602	-30.090
1.555.918	-23.484	1.577.863	-30.323	1.531.837	-30.090

1.556.026	-23.486	1.578.004	-30.324	1.531.962	-30.090
1.556.178	-23.487	1.578.203	-30.326	1.532.012	-30.091
1.556.308	-23.488	1.578.372	-30.327	1.532.238	-30.091
1.556.463	-23.490	1.578.443	-30.329	1.532.603	-30.091
1.556.691	-23.491	1.578.557	-30.330	1.532.999	-30.091
1.556.837	-23.492	1.578.725	-30.332	1.533.279	-30.092
1.556.985	-23.494	1.578.925	-30.333	1.533.387	-30.092
1.557.229	-23.495	1.579.109	-30.334	1.533.490	-30.092
1.557.426	-23.496	1.579.202	-30.336	1.533.613	-30.093
1.557.599	-23.498	1.579.379	-30.337	1.533.652	-30.093
1.557.701	-23.499	1.579.547	-30.339	1.533.647	-30.094
1.557.813	-23.501	1.579.678	-30.340	1.533.714	-30.094
1.558.031	-23.502	1.579.901	-30.342	1.533.819	-30.094
1.558.179	-23.503	1.580.049	-30.343	1.534.019	-30.095
1.558.306	-23.505	1.580.130	-30.345	1.534.259	-30.095
1.558.396	-23.506	1.580.258	-30.346	1.534.310	-30.096
1.558.503	-23.507	1.580.412	-30.348	1.534.390	-30.096
1.558.703	-23.509	1.580.582	-30.349	1.534.599	-30.097
1.558.925	-23.510	1.580.770	-30.351	1.534.747	-30.098
1.559.113	-23.511	1.580.970	-30.352	1.534.921	-30.098
1.559.278	-23.513	1.581.154	-30.354	1.535.087	-30.099
1.559.355	-23.514	1.581.321	-30.355	1.535.264	-30.100
1.559.438	-23.515	1.581.537	-30.357	1.535.434	-30.100
1.559.648	-23.517	1.581.682	-30.358	1.535.533	-30.101
1.559.832	-23.518	1.581.815	-30.360	1.535.676	-30.102
1.559.940	-23.519	1.581.982	-30.361	1.535.887	-30.103
1.560.074	-23.521	1.582.108	-30.363	1.536.044	-30.103
1.560.253	-23.522	1.582.229	-30.364	1.536.210	-30.104
1.560.338	-23.523	1.582.372	-30.366	1.536.290	-30.105
1.560.457	-23.525	1.582.605	-30.367	1.536.425	-30.106

1.560.600	-23.526	1.582.746	-30.369	1.536.631	-30.107
1.560.766	-23.527	1.582.928	-30.371	1.536.803	-30.108
1.560.968	-23.529	1.583.167	-30.372	1.536.931	-30.109
1.561.095	-23.530	1.583.302	-30.374	1.537.077	-30.110
1.561.264	-23.531	1.583.456	-30.376	1.537.267	-30.111
1.561.451	-23.533	1.583.595	-30.377	1.537.406	-30.112
1.561.689	-23.534	1.583.752	-30.379	1.537.621	-30.114
1.561.965	-23.535	1.583.916	-30.381	1.537.858	-30.115
1.562.189	-23.537	1.584.037	-30.383	1.538.004	-30.116
1.562.377	-23.538	1.584.211	-30.385	1.538.147	-30.117
1.562.570	-23.539	1.584.333	-30.386	1.538.298	-30.118
1.562.677	-23.541	1.584.431	-30.388	1.538.447	-30.120
1.562.789	-23.542	1.584.608	-30.390	1.538.595	-30.121
1.562.944	-23.543	1.584.787	-30.392	1.538.815	-30.122
1.563.083	-23.544	1.584.937	-30.394	1.539.012	-30.124
1.563.194	-23.546	1.585.092	-30.396	1.539.106	-30.125
1.563.308	-23.547	1.585.224	-30.398	1.539.220	-30.127
1.563.459	-23.548	1.585.396	-30.400	1.539.370	-30.128
1.563.618	-23.550	1.585.685	-30.402	1.539.538	-30.130
1.563.754	-23.551	1.585.914	-30.404	1.539.763	-30.132
1.563.918	-23.552	1.586.080	-30.406	1.539.866	-30.133
1.564.115	-23.554	1.586.266	-30.408	1.540.023	-30.135
1.564.303	-23.555	1.586.451	-30.410	1.540.253	-30.136
1.564.467	-23.556	1.586.640	-30.412	1.540.358	-30.138
1.564.628	-23.557	1.586.801	-30.414	1.540.531	-30.140
1.564.881	-23.559	1.586.881	-30.416	1.540.694	-30.142
1.565.065	-23.560	1.586.940	-30.419	1.540.779	-30.144
1.565.184	-23.561	1.587.070	-30.421	1.540.941	-30.145
1.565.367	-23.563	1.587.188	-30.423	1.541.091	-30.147
1.565.542	-23.564	1.587.316	-30.425	1.541.232	-30.149

1.565.712	-23.565	1.587.527	-30.428	1.541.456	-30.151
1.565.862	-23.566	1.587.701	-30.430	1.541.637	-30.153
1.565.977	-23.568	1.587.841	-30.432	1.541.799	-30.155
1.566.093	-23.569	1.588.047	-30.435	1.541.940	-30.157
1.566.212	-23.570	1.588.260	-30.437	1.542.027	-30.159
1.566.360	-23.571	1.588.461	-30.440	1.542.188	-30.161
1.566.582	-23.573	1.588.640	-30.442	1.542.394	-30.163
1.566.823	-23.574	1.588.810	-30.445	1.542.514	-30.166
1.566.971	-23.575	1.589.019	-30.447	1.542.626	-30.168
1.567.092	-23.577	1.589.138	-30.450	1.542.825	-30.170
1.567.280	-23.578	1.589.252	-30.452	1.542.986	-30.172
1.567.428	-23.579	1.589.453	-30.455	1.543.134	-30.175
1.567.576	-23.580	1.589.577	-30.458	1.543.340	-30.177
1.567.742	-23.582	1.589.715	-30.460	1.543.550	-30.179
1.567.924	-23.583	1.589.926	-30.463	1.543.719	-30.181
1.568.154	-23.584	1.590.078	-30.466	1.543.895	-30.184
1.568.311	-23.585	1.590.282	-30.469	1.544.048	-30.186
1.568.376	-23.587	1.590.486	-30.472	1.544.088	-30.189
1.568.508	-23.588	1.590.630	-30.475	1.544.146	-30.191
1.568.719	-23.589	1.590.804	-30.477	1.544.393	-30.193
1.568.909	-23.590	1.590.939	-30.480	1.544.628	-30.196
1.569.061	-23.592	1.591.131	-30.483	1.544.760	-30.198
1.569.263	-23.593	1.591.274	-30.486	1.544.967	-30.201
1.569.436	-23.594	1.591.445	-30.489	1.545.107	-30.203
1.569.541	-23.595	1.591.635	-30.492	1.545.217	-30.206
1.569.669	-23.596	1.591.765	-30.495	1.545.387	-30.208
1.569.845	-23.598	1.591.922	-30.499	1.545.520	-30.211
1.569.940	-23.599	1.592.110	-30.502	1.545.602	-30.213
1.570.269	-23.600	1.592.243	-30.505	1.545.788	-30.216
1.570.775	-23.601	1.592.439	-30.508	1.545.974	-30.219

1.571.039	-23.603	1.592.639	-30.511	1.546.178	-30.221
1.571.153	-23.604	1.592.782	-30.515	1.546.427	-30.224
1.571.171	-23.605	1.592.916	-30.518	1.546.508	-30.226
1.571.254	-23.606	1.593.031	-30.521	1.546.613	-30.229
1.571.351	-23.608	1.593.174	-30.525	1.546.814	-30.231
1.571.398	-23.609	1.593.365	-30.528	1.547.020	-30.234
1.571.494	-23.610	1.593.584	-30.532	1.547.166	-30.237
1.571.604	-23.611	1.593.745	-30.535	1.547.249	-30.239
1.571.714	-23.612	1.593.884	-30.539	1.547.379	-30.242
1.571.871	-23.614	1.594.119	-30.542	1.547.513	-30.244
1.572.090	-23.615	1.594.500	-30.546	1.547.601	-30.247
1.572.309	-23.616	1.594.872	-30.549	1.547.778	-30.250
1.572.502	-23.617	1.594.989	-30.553	1.547.999	-30.252
1.572.663	-23.618	1.595.018	-30.557	1.548.174	-30.255
1.572.811	-23.620	1.595.060	-30.560	1.548.405	-30.257
1.572.984	-23.621	1.595.090	-30.564	1.548.602	-30.260
1.573.201	-23.622	1.595.141	-30.568	1.548.779	-30.262
1.573.371	-23.623	1.595.284	-30.571	1.549.007	-30.265
1.573.477	-23.624	1.595.439	-30.575	1.549.173	-30.267
1.573.605	-23.626	1.595.547	-30.579	1.549.357	-30.270
1.573.712	-23.627	1.595.672	-30.583	1.549.572	-30.273
1.573.786	-23.628	1.595.824	-30.587	1.549.697	-30.275
1.573.918	-23.629	1.596.013	-30.591	1.549.818	-30.278
1.574.146	-23.630	1.596.239	-30.595	1.550.007	-30.280
1.574.332	-23.631	1.596.478	-30.599	1.550.161	-30.282
1.574.420	-23.633	1.596.684	-30.603	1.550.247	-30.285
1.574.561	-23.634	1.596.841	-30.607	1.550.441	-30.287
1.574.774	-23.635	1.596.938	-30.611	1.550.616	-30.290
1.575.029	-23.636	1.597.047	-30.615	1.550.724	-30.292
1.575.217	-23.637	1.597.197	-30.619	1.550.860	-30.295

1.575.318	-23.638	1.597.341	-30.623	1.550.950	-30.297
1.575.488	-23.640	1.597.478	-30.627	1.551.026	-30.299
1.575.690	-23.641	1.597.641	-30.631	1.551.189	-30.302
1.575.824	-23.642	1.597.834	-30.635	1.551.393	-30.304
1.576.026	-23.643	1.597.999	-30.640	1.551.579	-30.306
1.576.230	-23.644	1.598.075	-30.644	1.551.738	-30.309
1.576.315	-23.645	1.598.239	-30.648	1.551.940	-30.311
1.576.409	-23.646	1.598.456	-30.652	1.552.142	-30.313
1.576.568	-23.648	1.598.647	-30.656	1.552.285	-30.315
1.576.743	-23.649	1.598.849	-30.661	1.552.433	-30.318
1.576.915	-23.650	1.599.023	-30.665	1.552.664	-30.320
1.577.070	-23.651	1.599.209	-30.669	1.552.894	-30.322
1.577.200	-23.652	1.599.368	-30.673	1.552.997	-30.324
1.577.350	-23.653	1.599.527	-30.677	1.553.136	-30.326
1.577.477	-23.654	1.599.727	-30.682	1.553.295	-30.328
1.577.659	-23.655	1.599.865	-30.686	1.553.486	-30.330
1.577.867	-23.656	1.599.969	-30.690	1.553.613	-30.333
1.578.040	-23.658	1.600.130	-30.694	1.553.692	-30.335
1.578.212	-23.659	1.600.295	-30.699	1.553.978	-30.337
1.578.306	-23.660	1.600.484	-30.703	1.554.216	-30.339
1.578.450	-23.661	1.600.611	-30.707	1.554.323	-30.341
1.578.652	-23.662	1.600.737	-30.711	1.554.453	-30.343
1.578.822	-23.663	1.600.938	-30.715	1.554.608	-30.345
1.579.023	-23.664	1.601.059	-30.719	1.554.780	-30.347
1.579.193	-23.665	1.601.223	-30.724	1.554.906	-30.348
1.579.350	-23.666	1.601.440	-30.728	1.555.070	-30.350
1.579.516	-23.667	1.601.606	-30.732	1.555.271	-30.352
1.579.742	-23.668	1.601.794	-30.736	1.555.446	-30.354
1.579.975	-23.670	1.601.931	-30.740	1.555.623	-30.356
1.580.117	-23.671	1.602.030	-30.744	1.555.849	-30.358

1.580.215	-23.672	1.602.150	-30.748	1.556.010	-30.360
1.580.387	-23.673	1.602.267	-30.752	1.556.109	-30.361
1.580.558	-23.674	1.602.475	-30.756	1.556.259	-30.363
1.580.728	-23.675	1.602.708	-30.760	1.556.375	-30.365
1.580.798	-23.676	1.602.910	-30.764	1.556.496	-30.367
1.580.932	-23.677	1.603.093	-30.768	1.556.713	-30.368
1.581.118	-23.678	1.603.230	-30.772	1.556.911	-30.370
1.581.248	-23.679	1.603.385	-30.776	1.557.077	-30.372
1.581.420	-23.680	1.603.537	-30.780	1.557.195	-30.373
1.581.651	-23.681	1.603.705	-30.784	1.557.312	-30.375
1.581.803	-23.682	1.603.882	-30.788	1.557.502	-30.377
1.581.976	-23.683	1.604.019	-30.791	1.557.701	-30.378
1.582.153	-23.684	1.604.189	-30.795	1.557.867	-30.380
1.582.278	-23.685	1.604.370	-30.799	1.557.995	-30.382
1.582.413	-23.686	1.604.514	-30.802	1.558.138	-30.383
1.582.545	-23.687	1.604.675	-30.806	1.558.304	-30.385
1.582.648	-23.688	1.604.845	-30.810	1.558.432	-30.386
1.582.728	-23.689	1.604.991	-30.813	1.558.539	-30.388
1.582.892	-23.690	1.605.172	-30.817	1.558.680	-30.389
1.583.085	-23.691	1.605.367	-30.820	1.558.880	-30.391
1.583.194	-23.692	1.605.502	-30.823	1.559.128	-30.392
1.583.317	-23.693	1.605.578	-30.827	1.559.464	-30.394
1.583.540	-23.694	1.605.674	-30.830	1.559.890	-30.395
1.583.745	-23.695	1.605.925	-30.833	1.560.103	-30.397
1.583.936	-23.696	1.606.154	-30.836	1.560.181	-30.399
1.584.191	-23.697	1.606.180	-30.840	1.560.170	-30.400
1.584.379	-23.698	1.606.221	-30.843	1.560.273	-30.402
1.584.543	-23.699	1.606.380	-30.846	1.560.392	-30.403
1.584.749	-23.700	1.606.570	-30.849	1.560.435	-30.405
1.584.917	-23.701	1.606.736	-30.852	1.560.558	-30.406

1.585.051	-23.702	1.606.935	-30.854	1.560.668	-30.408
1.585.179	-23.703	1.607.130	-30.857	1.560.748	-30.409
1.585.278	-23.704	1.607.332	-30.860	1.560.872	-30.411
1.585.378	-23.705	1.607.628	-30.863	1.561.060	-30.413
1.585.497	-23.706	1.607.870	-30.865	1.561.292	-30.414
1.585.674	-23.707	1.608.044	-30.868	1.561.438	-30.416
1.585.869	-23.708	1.608.214	-30.871	1.561.599	-30.418
1.586.012	-23.709	1.608.351	-30.873	1.561.814	-30.420
1.586.165	-23.710	1.608.445	-30.875	1.562.016	-30.421
1.586.351	-23.711	1.608.546	-30.878	1.562.175	-30.423
1.586.548	-23.712	1.608.709	-30.880	1.562.334	-30.425
1.586.758	-23.713	1.608.913	-30.882	1.562.489	-30.427
1.586.958	-23.714	1.609.054	-30.885	1.562.598	-30.429
1.587.148	-23.715	1.609.162	-30.887	1.562.769	-30.431
1.587.323	-23.716	1.609.263	-30.889	1.562.914	-30.433
1.587.491	-23.717	1.609.408	-30.891	1.563.067	-30.435
1.587.722	-23.718	1.609.590	-30.893	1.563.282	-30.438
1.587.849	-23.719	1.609.758	-30.895	1.563.494	-30.440
1.587.937	-23.720	1.609.921	-30.896	1.563.604	-30.442
1.588.152	-23.721	1.610.125	-30.898	1.563.716	-30.445
1.588.317	-23.722	1.610.350	-30.900	1.563.819	-30.447
1.588.448	-23.723	1.610.542	-30.901	1.563.882	-30.450
1.588.647	-23.724	1.610.663	-30.903	1.564.059	-30.452
1.588.802	-23.725	1.610.813	-30.905	1.564.301	-30.455
1.588.942	-23.725	1.610.995	-30.906	1.564.471	-30.458
1.589.135	-23.726	1.611.156	-30.907	1.564.518	-30.461
1.589.352	-23.727	1.611.286	-30.909	1.564.650	-30.464
1.589.500	-23.728	1.611.447	-30.910	1.564.877	-30.467
1.589.606	-23.729	1.611.637	-30.911	1.565.110	-30.470
1.589.734	-23.730	1.611.833	-30.912	1.565.347	-30.473

1.589.926	-23.731	1.612.001	-30.913	1.565.519	-30.476
1.590.118	-23.732	1.612.133	-30.914	1.565.648	-30.480
1.590.289	-23.733	1.612.309	-30.915	1.565.822	-30.484
1.590.423	-23.734	1.612.502	-30.916	1.565.990	-30.487
1.590.556	-23.735	1.612.733	-30.917	1.566.127	-30.491
1.590.726	-23.736	1.612.892	-30.918	1.566.283	-30.495
1.590.858	-23.737	1.613.013	-30.919	1.566.440	-30.499
1.591.026	-23.738	1.613.179	-30.919	1.566.595	-30.503
1.591.223	-23.739	1.613.317	-30.920	1.566.722	-30.507
1.591.384	-23.740	1.613.436	-30.920	1.566.817	-30.512
1.591.584	-23.741	1.613.629	-30.921	1.566.994	-30.516
1.591.738	-23.742	1.613.869	-30.921	1.567.191	-30.521
1.591.848	-23.743	1.614.010	-30.922	1.567.328	-30.526
1.592.007	-23.743	1.614.151	-30.922	1.567.524	-30.531
1.592.204	-23.744	1.614.342	-30.922	1.567.706	-30.536
1.592.394	-23.745	1.614.494	-30.923	1.567.890	-30.541
1.592.540	-23.746	1.614.668	-30.923	1.568.049	-30.546
1.592.829	-23.747	1.614.855	-30.923	1.568.228	-30.552
1.593.273	-23.748	1.614.971	-30.923	1.568.391	-30.557
1.593.559	-23.749	1.615.076	-30.923	1.568.481	-30.563
1.593.602	-23.750	1.615.231	-30.923	1.568.629	-30.569
1.593.680	-23.751	1.615.444	-30.923	1.568.703	-30.575
1.593.676	-23.752	1.615.621	-30.923	1.568.835	-30.581
1.593.662	-23.753	1.615.764	-30.923	1.569.097	-30.587
1.593.781	-23.754	1.616.140	-30.923	1.569.301	-30.594
1.593.913	-23.755	1.616.610	-30.922	1.569.382	-30.600
1.594.055	-23.756	1.616.781	-30.922	1.569.476	-30.607
1.594.203	-23.757	1.616.786	-30.922	1.569.686	-30.614
1.594.328	-23.758	1.616.880	-30.922	1.569.899	-30.621
1.594.538	-23.758	1.616.976	-30.921	1.570.199	-30.628

1.594.785	-23.759	1.617.034	-30.921	1.570.432	-30.635
1.594.939	-23.760	1.617.139	-30.921	1.570.535	-30.642
1.595.051	-23.761	1.617.249	-30.920	1.570.679	-30.650
1.595.204	-23.762	1.617.343	-30.920	1.570.833	-30.658
1.595.417	-23.763	1.617.455	-30.919	1.571.003	-30.665
1.595.632	-23.764	1.617.601	-30.919	1.571.152	-30.673
1.595.807	-23.765	1.617.814	-30.918	1.571.277	-30.681
1.595.918	-23.766	1.618.092	-30.918	1.571.425	-30.689
1.596.001	-23.767	1.618.237	-30.917	1.571.561	-30.698
1.596.095	-23.768	1.618.347	-30.917	1.571.702	-30.706
1.596.243	-23.769	1.618.508	-30.916	1.571.904	-30.715
1.596.436	-23.769	1.618.649	-30.915	1.572.099	-30.723
1.596.626	-23.770	1.618.804	-30.915	1.572.256	-30.732
1.596.792	-23.771	1.618.969	-30.914	1.572.428	-30.741
1.596.962	-23.772	1.619.147	-30.913	1.572.598	-30.750
1.597.112	-23.773	1.619.319	-30.913	1.572.726	-30.759
1.597.233	-23.774	1.619.451	-30.912	1.572.883	-30.769
1.597.415	-23.775	1.619.558	-30.912	1.572.973	-30.778
1.597.601	-23.776	1.619.765	-30.911	1.573.107	-30.788
1.597.805	-23.777	1.619.980	-30.910	1.573.311	-30.797
1.598.022	-23.778	1.620.089	-30.910	1.573.407	-30.807
1.598.165	-23.778	1.620.204	-30.909	1.573.571	-30.817
1.598.308	-23.779	1.620.415	-30.908	1.573.763	-30.827
1.598.497	-23.780	1.620.576	-30.908	1.573.918	-30.837
1.598.672	-23.781	1.620.712	-30.907	1.574.109	-30.847
1.598.829	-23.782	1.620.912	-30.906	1.574.258	-30.858
1.598.940	-23.783	1.621.037	-30.906	1.574.301	-30.868
1.599.037	-23.784	1.621.201	-30.905	1.574.370	-30.879
1.599.137	-23.785	1.621.436	-30.904	1.574.523	-30.889
1.599.299	-23.786	1.621.615	-30.904	1.574.686	-30.900

1.599.529	-23.787	1.621.819	-30.903	1.574.866	-30.911
1.599.744	-23.787	1.622.018	-30.903	1.575.061	-30.922
1.599.901	-23.788	1.622.155	-30.902	1.575.220	-30.933
1.600.027	-23.789	1.622.226	-30.902	1.575.365	-30.944
1.600.201	-23.790	1.622.343	-30.901	1.575.593	-30.955
1.600.415	-23.791	1.622.527	-30.900	1.575.845	-30.967
1.600.491	-23.792	1.622.733	-30.900	1.575.984	-30.978
1.600.591	-23.793	1.622.926	-30.900	1.576.181	-30.989
1.600.817	-23.794	1.623.031	-30.899	1.576.460	-31.001
1.600.994	-23.794	1.623.168	-30.899	1.576.586	-31.012
1.601.129	-23.795	1.623.369	-30.898	1.576.653	-31.024
1.601.236	-23.796	1.623.564	-30.898	1.576.819	-31.035
1.601.463	-23.797	1.623.705	-30.897	1.576.971	-31.047
1.601.703	-23.798	1.623.839	-30.897	1.577.159	-31.059
1.601.889	-23.799	1.623.958	-30.897	1.577.278	-31.071
1.602.072	-23.800	1.624.104	-30.896	1.577.383	-31.082
1.602.222	-23.800	1.624.294	-30.896	1.577.529	-31.094
1.602.363	-23.801	1.624.487	-30.896	1.577.744	-31.106
1.602.516	-23.802	1.624.691	-30.896	1.577.897	-31.118
1.602.659	-23.803	1.624.856	-30.895	1.578.056	-31.130
1.602.816	-23.804	1.624.993	-30.895	1.578.167	-31.141
1.602.993	-23.805	1.625.177	-30.895	1.578.302	-31.153
1.603.140	-23.806	1.625.378	-30.895	1.578.499	-31.165
1.603.304	-23.807	1.625.535	-30.895	1.578.654	-31.177
1.603.405	-23.807	1.625.656	-30.895	1.578.795	-31.189
1.603.531	-23.808	1.625.780	-30.895	1.578.987	-31.200
1.603.788	-23.809	1.625.934	-30.895	1.579.258	-31.212
1.603.981	-23.810	1.626.109	-30.895	1.579.471	-31.224
1.604.117	-23.811	1.626.331	-30.895	1.579.630	-31.235
1.604.254	-23.812	1.626.492	-30.895	1.579.749	-31.247

1.604.408	-23.812	1.626.608	-30.895	1.579.855	-31.259
1.604.543	-23.813	1.626.819	-30.895	1.579.973	-31.270
1.604.702	-23.814	1.627.021	-30.895	1.580.083	-31.281
1.604.861	-23.815	1.627.128	-30.896	1.580.278	-31.293
1.604.977	-23.816	1.627.258	-30.896	1.580.488	-31.304
1.605.101	-23.817	1.627.428	-30.896	1.580.663	-31.315
1.605.215	-23.818	1.627.531	-30.896	1.580.860	-31.326
1.605.426	-23.818	1.627.710	-30.897	1.581.044	-31.337
1.605.625	-23.819	1.627.897	-30.897	1.581.196	-31.348
1.605.760	-23.820	1.628.040	-30.897	1.581.337	-31.359
1.605.907	-23.821	1.628.179	-30.898	1.581.400	-31.370
1.606.019	-23.822	1.628.279	-30.898	1.581.521	-31.380
1.606.209	-23.823	1.628.448	-30.899	1.581.711	-31.391
1.606.465	-23.824	1.628.566	-30.899	1.581.933	-31.401
1.606.698	-23.824	1.628.696	-30.899	1.582.144	-31.411
1.606.902	-23.825	1.628.905	-30.900	1.582.276	-31.421
1.607.097	-23.826	1.629.099	-30.901	1.582.372	-31.431
1.607.233	-23.827	1.629.312	-30.901	1.582.625	-31.441
1.607.393	-23.828	1.629.547	-30.902	1.582.827	-31.451
1.607.592	-23.829	1.629.756	-30.902	1.582.955	-31.460
1.607.744	-23.830	1.629.893	-30.903	1.583.100	-31.470
1.607.870	-23.830	1.630.047	-30.904	1.583.221	-31.479
1.607.957	-23.831	1.630.262	-30.904	1.583.367	-31.488
1.607.997	-23.832	1.630.464	-30.905	1.583.544	-31.497
1.608.132	-23.833	1.630.620	-30.906	1.583.710	-31.505
1.608.340	-23.834	1.630.750	-30.906	1.583.929	-31.514
1.608.566	-23.835	1.630.903	-30.907	1.584.023	-31.522
1.608.777	-23.836	1.631.030	-30.908	1.584.200	-31.530
1.608.929	-23.836	1.631.160	-30.909	1.584.467	-31.538
1.609.081	-23.837	1.631.277	-30.910	1.584.561	-31.546

1.609.249	-23.838	1.631.333	-30.911	1.584.706	-31.554
1.609.433	-23.839	1.631.476	-30.911	1.584.973	-31.561
1.609.682	-23.840	1.631.664	-30.912	1.585.179	-31.568
1.609.879	-23.841	1.631.886	-30.913	1.585.271	-31.575
1.609.982	-23.842	1.632.083	-30.914	1.585.376	-31.582
1.610.157	-23.843	1.632.267	-30.915	1.585.551	-31.589
1.610.370	-23.843	1.632.509	-30.916	1.585.614	-31.595
1.610.558	-23.844	1.632.630	-30.917	1.585.674	-31.601
1.610.667	-23.845	1.632.755	-30.918	1.585.952	-31.607
1.610.775	-23.846	1.632.954	-30.919	1.586.474	-31.613
1.610.911	-23.847	1.633.152	-30.920	1.586.967	-31.619
1.611.075	-23.848	1.633.322	-30.921	1.587.220	-31.624
1.611.234	-23.849	1.633.463	-30.922	1.587.229	-31.630
1.611.422	-23.850	1.633.625	-30.923	1.587.186	-31.635
1.611.588	-23.851	1.633.783	-30.924	1.587.269	-31.639
1.611.749	-23.852	1.633.931	-30.925	1.587.345	-31.644
1.611.924	-23.852	1.634.095	-30.927	1.587.428	-31.649
1.612.144	-23.853	1.634.214	-30.928	1.587.498	-31.653
1.612.332	-23.854	1.634.319	-30.929	1.587.570	-31.657
1.612.453	-23.855	1.634.509	-30.930	1.587.720	-31.661
1.612.587	-23.856	1.634.771	-30.931	1.587.856	-31.665
1.612.699	-23.857	1.634.912	-30.932	1.587.981	-31.668
1.612.856	-23.858	1.635.034	-30.933	1.588.109	-31.672
1.613.028	-23.859	1.635.217	-30.935	1.588.261	-31.675
1.613.201	-23.860	1.635.379	-30.936	1.588.389	-31.678
1.613.331	-23.861	1.635.517	-30.937	1.588.591	-31.681
1.613.467	-23.862	1.635.690	-30.938	1.588.871	-31.683
1.613.687	-23.862	1.635.930	-30.940	1.589.144	-31.686
1.613.911	-23.863	1.636.158	-30.941	1.589.288	-31.688
1.614.059	-23.864	1.636.250	-30.942	1.589.390	-31.690

1.614.205	-23.865	1.636.304	-30.943	1.589.543	-31.692
1.614.408	-23.866	1.636.463	-30.944	1.589.662	-31.694
1.614.549	-23.867	1.636.680	-30.946	1.589.834	-31.695
1.614.713	-23.868	1.636.868	-30.947	1.589.998	-31.697
1.614.810	-23.869	1.637.045	-30.948	1.590.117	-31.698
1.614.960	-23.870	1.637.249	-30.950	1.590.300	-31.699
1.615.365	-23.871	1.637.403	-30.951	1.590.448	-31.700
1.615.739	-23.872	1.637.506	-30.952	1.590.578	-31.701
1.615.920	-23.873	1.637.690	-30.953	1.590.750	-31.701
1.616.037	-23.874	1.638.082	-30.955	1.590.968	-31.702
1.616.104	-23.875	1.638.564	-30.956	1.591.149	-31.702
1.616.115	-23.876	1.638.773	-30.957	1.591.225	-31.702
1.616.250	-23.876	1.638.784	-30.959	1.591.344	-31.702
1.616.373	-23.877	1.638.902	-30.960	1.591.548	-31.702
1.616.474	-23.878	1.638.925	-30.961	1.591.660	-31.701
1.616.640	-23.879	1.638.893	-30.962	1.591.881	-31.701
1.616.767	-23.880	1.638.974	-30.964	1.592.135	-31.700
1.616.886	-23.881	1.639.090	-30.965	1.592.258	-31.700
1.617.056	-23.882	1.639.229	-30.966	1.592.406	-31.699
1.617.242	-23.883	1.639.384	-30.968	1.592.605	-31.698
1.617.408	-23.884	1.639.644	-30.969	1.592.867	-31.697
1.617.590	-23.885	1.639.803	-30.970	1.592.995	-31.695
1.617.780	-23.886	1.639.854	-30.972	1.593.098	-31.694
1.617.975	-23.887	1.640.018	-30.973	1.593.250	-31.692
1.618.167	-23.888	1.640.237	-30.974	1.593.414	-31.691
1.618.259	-23.889	1.640.452	-30.976	1.593.535	-31.689
1.618.356	-23.890	1.640.616	-30.977	1.593.690	-31.687
1.618.530	-23.891	1.640.777	-30.978	1.593.907	-31.685
1.618.716	-23.892	1.640.988	-30.980	1.594.041	-31.683
1.618.916	-23.893	1.641.129	-30.981	1.594.135	-31.681

1.619.077	-23.893	1.641.230	-30.982	1.594.286	-31.679
1.619.234	-23.894	1.641.424	-30.984	1.594.498	-31.676
1.619.341	-23.895	1.641.563	-30.985	1.594.670	-31.674
1.619.440	-23.896	1.641.662	-30.986	1.594.836	-31.672
1.619.614	-23.897	1.641.834	-30.988	1.595.011	-31.669
1.619.780	-23.898	1.642.034	-30.989	1.595.197	-31.666
1.619.989	-23.899	1.642.173	-30.991	1.595.347	-31.664
1.620.204	-23.900	1.642.296	-30.992	1.595.480	-31.661
1.620.363	-23.901	1.642.507	-30.993	1.595.679	-31.658
1.620.519	-23.902	1.642.701	-30.995	1.595.815	-31.655
1.620.710	-23.903	1.642.800	-30.996	1.595.932	-31.652
1.620.858	-23.904	1.642.981	-30.997	1.596.087	-31.649
1.620.995	-23.905	1.643.244	-30.999	1.596.234	-31.646
1.621.192	-23.906	1.643.400	-31.000	1.596.440	-31.643
1.621.333	-23.907	1.643.539	-31.002	1.596.671	-31.639
1.621.492	-23.908	1.643.701	-31.003	1.596.805	-31.636
1.621.666	-23.909	1.643.831	-31.004	1.596.895	-31.633
1.621.763	-23.909	1.643.924	-31.006	1.597.047	-31.630
1.621.888	-23.910	1.643.990	-31.007	1.597.278	-31.626
1.622.049	-23.911	1.644.171	-31.009	1.597.480	-31.623
1.622.198	-23.912	1.644.395	-31.010	1.597.645	-31.619
1.622.397	-23.913	1.644.605	-31.012	1.597.769	-31.616
1.622.585	-23.914	1.644.828	-31.013	1.597.932	-31.612
1.622.710	-23.915	1.644.957	-31.014	1.598.152	-31.609
1.622.901	-23.916	1.645.076	-31.016	1.598.235	-31.605
1.623.069	-23.917	1.645.244	-31.017	1.598.389	-31.602
1.623.212	-23.918	1.645.423	-31.019	1.598.571	-31.598
1.623.316	-23.919	1.645.614	-31.020	1.598.732	-31.595
1.623.358	-23.920	1.645.755	-31.022	1.598.902	-31.591
1.623.571	-23.921	1.645.925	-31.023	1.599.053	-31.587

1.623.868	-23.922	1.646.111	-31.025	1.599.221	-31.584
1.624.068	-23.922	1.646.286	-31.026	1.599.339	-31.580
1.624.198	-23.923	1.646.458	-31.028	1.599.482	-31.576
1.624.348	-23.924	1.646.586	-31.029	1.599.700	-31.573
1.624.603	-23.925	1.646.767	-31.031	1.599.924	-31.569
1.624.782	-23.926	1.646.971	-31.032	1.600.063	-31.566
1.624.910	-23.927	1.647.137	-31.034	1.600.186	-31.562
1.625.101	-23.928	1.647.327	-31.035	1.600.426	-31.558
1.625.248	-23.929	1.647.495	-31.037	1.600.600	-31.555
1.625.419	-23.930	1.647.562	-31.038	1.600.685	-31.551
1.625.536	-23.931	1.647.681	-31.040	1.600.804	-31.548
1.625.674	-23.932	1.647.822	-31.042	1.600.959	-31.544
1.625.911	-23.932	1.647.963	-31.043	1.601.082	-31.541
1.626.149	-23.933	1.648.165	-31.045	1.601.255	-31.537
1.626.376	-23.934	1.648.344	-31.046	1.601.387	-31.534
1.626.505	-23.935	1.648.560	-31.048	1.601.505	-31.530
1.626.575	-23.936	1.648.714	-31.049	1.601.651	-31.527
1.626.689	-23.937	1.648.842	-31.051	1.601.801	-31.523
1.626.866	-23.938	1.649.032	-31.053	1.601.996	-31.520
1.627.041	-23.939	1.649.193	-31.054	1.602.186	-31.516
1.627.160	-23.940	1.649.324	-31.056	1.602.377	-31.513
1.627.323	-23.940	1.649.494	-31.057	1.602.601	-31.510
1.627.422	-23.941	1.649.650	-31.059	1.602.791	-31.506
1.627.484	-23.942	1.649.776	-31.060	1.602.939	-31.503
1.627.652	-23.943	1.649.888	-31.062	1.603.138	-31.500
1.627.827	-23.944	1.650.013	-31.064	1.603.336	-31.496
1.627.995	-23.945	1.650.139	-31.065	1.603.553	-31.493
1.628.208	-23.946	1.650.313	-31.067	1.603.741	-31.490
1.628.434	-23.947	1.650.502	-31.068	1.603.826	-31.486
1.628.569	-23.948	1.650.661	-31.070	1.603.996	-31.483

1.628.665	-23.948	1.650.815	-31.071	1.604.227	-31.480
1.628.869	-23.949	1.650.981	-31.073	1.604.370	-31.477
1.629.106	-23.950	1.651.246	-31.075	1.604.523	-31.474
1.629.290	-23.951	1.651.499	-31.076	1.604.650	-31.470
1.629.471	-23.952	1.651.673	-31.078	1.604.754	-31.467
1.629.673	-23.953	1.651.826	-31.079	1.604.906	-31.464
1.629.850	-23.954	1.652.005	-31.081	1.605.005	-31.461
1.630.011	-23.955	1.652.182	-31.082	1.605.108	-31.458
1.630.163	-23.955	1.652.309	-31.084	1.605.300	-31.455
1.630.289	-23.956	1.652.431	-31.085	1.605.506	-31.452
1.630.406	-23.957	1.652.578	-31.087	1.605.683	-31.449
1.630.524	-23.958	1.652.697	-31.088	1.605.815	-31.446
1.630.618	-23.959	1.652.820	-31.090	1.605.876	-31.443
1.630.759	-23.960	1.652.973	-31.091	1.606.077	-31.440
1.630.929	-23.961	1.653.205	-31.093	1.606.385	-31.437
1.630.994	-23.962	1.653.389	-31.094	1.606.557	-31.434
1.631.205	-23.962	1.653.490	-31.096	1.606.698	-31.431
1.631.487	-23.963	1.653.662	-31.097	1.606.955	-31.428
1.631.698	-23.964	1.653.846	-31.099	1.607.148	-31.425
1.631.915	-23.965	1.654.036	-31.100	1.607.269	-31.422
1.632.065	-23.966	1.654.240	-31.102	1.607.446	-31.419
1.632.207	-23.967	1.654.426	-31.103	1.607.639	-31.416
1.632.388	-23.968	1.654.606	-31.105	1.607.786	-31.413
1.632.502	-23.969	1.654.767	-31.106	1.607.888	-31.411
1.632.612	-23.970	1.654.910	-31.107	1.608.035	-31.408
1.632.840	-23.970	1.655.101	-31.109	1.608.253	-31.405
1.633.076	-23.971	1.655.251	-31.110	1.608.405	-31.402
1.633.260	-23.972	1.655.397	-31.112	1.608.488	-31.399
1.633.423	-23.973	1.655.562	-31.113	1.608.714	-31.396
1.633.550	-23.974	1.655.705	-31.114	1.608.866	-31.394

1.633.678	-23.975	1.655.894	-31.116	1.608.947	-31.391
1.633.839	-23.976	1.656.053	-31.117	1.609.176	-31.388
1.634.030	-23.977	1.656.187	-31.118	1.609.402	-31.385
1.634.231	-23.978	1.656.355	-31.120	1.609.610	-31.383
1.634.384	-23.978	1.656.541	-31.121	1.609.763	-31.380
1.634.556	-23.979	1.656.714	-31.122	1.609.906	-31.377
1.634.720	-23.980	1.656.882	-31.123	1.610.065	-31.375
1.634.890	-23.981	1.657.045	-31.125	1.610.222	-31.372
1.635.069	-23.982	1.657.189	-31.126	1.610.416	-31.369
1.635.222	-23.983	1.657.347	-31.127	1.610.544	-31.367
1.635.374	-23.984	1.657.491	-31.129	1.610.632	-31.364
1.635.519	-23.985	1.657.625	-31.130	1.610.824	-31.361
1.635.656	-23.986	1.657.818	-31.131	1.610.976	-31.359
1.635.793	-23.987	1.658.018	-31.132	1.611.151	-31.356
1.635.981	-23.987	1.658.186	-31.133	1.611.297	-31.353
1.636.181	-23.988	1.658.324	-31.135	1.611.494	-31.351
1.636.368	-23.989	1.658.494	-31.136	1.611.776	-31.348
1.636.514	-23.990	1.658.716	-31.137	1.611.871	-31.346
1.636.687	-23.991	1.658.866	-31.138	1.611.993	-31.343
1.636.839	-23.992	1.658.967	-31.139	1.612.218	-31.341
1.636.944	-23.993	1.659.118	-31.140	1.612.428	-31.338
1.637.072	-23.994	1.659.301	-31.142	1.612.529	-31.336
1.637.195	-23.995	1.659.427	-31.143	1.612.674	-31.333
1.637.366	-23.996	1.659.734	-31.144	1.612.860	-31.331
1.637.699	-23.997	1.660.210	-31.145	1.612.986	-31.328
1.638.161	-23.997	1.660.495	-31.146	1.613.073	-31.326
1.638.445	-23.998	1.660.596	-31.147	1.613.353	-31.324
1.638.528	-23.999	1.660.667	-31.148	1.613.790	-31.321
1.638.596	-24.000	1.660.719	-31.149	1.614.122	-31.319
1.638.649	-24.001	1.660.714	-31.150	1.614.281	-31.317

1.638.736	-24.002	1.660.751	-31.151	1.614.368	-31.314
1.638.819	-24.003	1.660.838	-31.152	1.614.523	-31.312
1.638.864	-24.004	1.660.990	-31.153	1.614.610	-31.310
1.638.958	-24.005	1.661.216	-31.154	1.614.615	-31.308
1.639.122	-24.006	1.661.411	-31.155	1.614.614	-31.305
1.639.346	-24.007	1.661.597	-31.156	1.614.671	-31.303
1.639.523	-24.008	1.661.767	-31.157	1.614.798	-31.301
1.639.644	-24.009	1.661.899	-31.158	1.614.926	-31.299
1.639.852	-24.009	1.662.076	-31.159	1.615.105	-31.297
1.640.040	-24.010	1.662.245	-31.160	1.615.331	-31.295
1.640.215	-24.011	1.662.444	-31.161	1.615.495	-31.293
1.640.403	-24.012	1.662.650	-31.162	1.615.656	-31.290
1.640.592	-24.013	1.662.805	-31.163	1.615.851	-31.288
1.640.719	-24.014	1.662.939	-31.164	1.616.073	-31.286
1.640.759	-24.015	1.663.020	-31.165	1.616.227	-31.285
1.640.952	-24.016	1.663.102	-31.166	1.616.429	-31.283
1.641.176	-24.017	1.663.275	-31.167	1.616.680	-31.281
1.641.308	-24.018	1.663.483	-31.168	1.616.841	-31.279
1.641.467	-24.019	1.663.676	-31.169	1.616.933	-31.277
1.641.662	-24.020	1.663.804	-31.170	1.617.083	-31.275
1.641.758	-24.021	1.663.900	-31.170	1.617.249	-31.273
1.641.884	-24.022	1.664.066	-31.171	1.617.442	-31.272
1.642.095	-24.023	1.664.249	-31.172	1.617.558	-31.270
1.642.240	-24.023	1.664.485	-31.173	1.617.657	-31.268
1.642.392	-24.024	1.664.695	-31.174	1.617.798	-31.267
1.642.578	-24.025	1.664.861	-31.175	1.617.980	-31.265
1.642.760	-24.026	1.665.033	-31.176	1.618.138	-31.263
1.642.953	-24.027	1.665.152	-31.177	1.618.226	-31.262
1.643.154	-24.028	1.665.284	-31.178	1.618.291	-31.260
1.643.299	-24.029	1.665.448	-31.179	1.618.506	-31.259

1.643.436	-24.030	1.665.571	-31.180	1.618.754	-31.257
1.643.569	-24.031	1.665.752	-31.180	1.618.896	-31.256
1.643.725	-24.032	1.665.986	-31.181	1.619.073	-31.254
1.643.916	-24.033	1.666.187	-31.182	1.619.310	-31.253
1.644.037	-24.034	1.666.290	-31.183	1.619.498	-31.252
1.644.175	-24.035	1.666.375	-31.184	1.619.655	-31.250
1.644.364	-24.036	1.666.575	-31.185	1.619.874	-31.249
1.644.489	-24.037	1.666.770	-31.186	1.620.042	-31.248
1.644.603	-24.038	1.666.924	-31.187	1.620.170	-31.247
1.644.776	-24.038	1.667.077	-31.188	1.620.365	-31.245
1.644.958	-24.039	1.667.186	-31.189	1.620.508	-31.244
1.645.136	-24.040	1.667.336	-31.190	1.620.611	-31.243
1.645.295	-24.041	1.667.522	-31.191	1.620.733	-31.242
1.645.453	-24.042	1.667.711	-31.192	1.620.896	-31.241
1.645.598	-24.043	1.667.912	-31.193	1.621.086	-31.240
1.645.753	-24.044	1.668.046	-31.194	1.621.205	-31.239
1.645.885	-24.045	1.668.201	-31.195	1.621.351	-31.238
1.646.033	-24.046	1.668.353	-31.196	1.621.516	-31.237
1.646.239	-24.047	1.668.499	-31.197	1.621.687	-31.236
1.646.395	-24.048	1.668.649	-31.198	1.621.835	-31.235
1.646.561	-24.049	1.668.828	-31.199	1.621.949	-31.235
1.646.754	-24.050	1.669.050	-31.200	1.622.094	-31.234
1.647.001	-24.051	1.669.222	-31.201	1.622.272	-31.233
1.647.137	-24.052	1.669.404	-31.203	1.622.406	-31.232
1.647.242	-24.053	1.669.572	-31.204	1.622.614	-31.232
1.647.419	-24.054	1.669.684	-31.205	1.622.883	-31.231
1.647.605	-24.055	1.669.782	-31.206	1.623.044	-31.230
1.647.762	-24.056	1.669.935	-31.207	1.623.203	-31.230
1.647.874	-24.057	1.670.172	-31.208	1.623.315	-31.229
1.648.046	-24.058	1.670.419	-31.210	1.623.436	-31.229

1.648.255	-24.058	1.670.585	-31.211	1.623.588	-31.228
1.648.411	-24.059	1.670.683	-31.212	1.623.770	-31.228
1.648.604	-24.060	1.670.791	-31.213	1.623.913	-31.227
1.648.786	-24.061	1.670.968	-31.215	1.624.117	-31.227
1.648.960	-24.062	1.671.149	-31.216	1.624.381	-31.226
1.649.081	-24.063	1.671.308	-31.217	1.624.507	-31.226
1.649.218	-24.064	1.671.472	-31.218	1.624.659	-31.225
1.649.373	-24.065	1.671.579	-31.220	1.624.803	-31.225
1.649.527	-24.066	1.671.725	-31.221	1.624.933	-31.225
1.649.704	-24.067	1.671.933	-31.222	1.625.134	-31.225
1.649.827	-24.068	1.672.054	-31.224	1.625.316	-31.224
1.649.915	-24.069	1.672.150	-31.225	1.625.536	-31.224
1.650.076	-24.070	1.672.254	-31.226	1.625.775	-31.224
1.650.260	-24.071	1.672.384	-31.228	1.625.905	-31.224
1.650.307	-24.072	1.672.634	-31.229	1.625.992	-31.223
1.650.461	-24.073	1.672.860	-31.231	1.626.118	-31.223
1.650.710	-24.074	1.673.073	-31.232	1.626.301	-31.223
1.650.842	-24.075	1.673.324	-31.234	1.626.443	-31.223
1.651.030	-24.076	1.673.541	-31.235	1.626.566	-31.223
1.651.252	-24.077	1.673.689	-31.236	1.626.716	-31.223
1.651.449	-24.078	1.673.829	-31.238	1.626.940	-31.223
1.651.727	-24.079	1.673.996	-31.239	1.627.126	-31.223
1.651.908	-24.080	1.674.110	-31.241	1.627.334	-31.223
1.652.018	-24.081	1.674.259	-31.242	1.627.522	-31.223
1.652.180	-24.082	1.674.469	-31.244	1.627.652	-31.222
1.652.352	-24.083	1.674.655	-31.245	1.627.722	-31.222
1.652.477	-24.084	1.674.733	-31.247	1.627.847	-31.222
1.652.605	-24.085	1.674.836	-31.248	1.628.031	-31.222
1.652.818	-24.086	1.674.977	-31.250	1.628.181	-31.222
1.652.959	-24.087	1.675.148	-31.251	1.628.316	-31.222

1.653.058	-24.088	1.675.303	-31.253	1.628.443	-31.222
1.653.158	-24.089	1.675.437	-31.254	1.628.584	-31.222
1.653.286	-24.090	1.675.681	-31.256	1.628.689	-31.222
1.653.443	-24.091	1.675.947	-31.257	1.628.808	-31.223
1.653.640	-24.092	1.676.098	-31.259	1.628.933	-31.223
1.653.848	-24.093	1.676.200	-31.260	1.629.097	-31.223
1.653.951	-24.094	1.676.429	-31.262	1.629.294	-31.223
1.654.088	-24.095	1.676.647	-31.264	1.629.518	-31.223
1.654.361	-24.097	1.676.806	-31.265	1.629.729	-31.223
1.654.630	-24.098	1.676.920	-31.267	1.629.874	-31.223
1.654.785	-24.099	1.677.032	-31.268	1.629.982	-31.223
1.654.888	-24.100	1.677.160	-31.270	1.630.195	-31.223
1.655.081	-24.101	1.677.365	-31.271	1.630.490	-31.223
1.655.307	-24.102	1.677.558	-31.273	1.630.721	-31.223
1.655.452	-24.103	1.677.731	-31.274	1.630.901	-31.223
1.655.567	-24.104	1.677.937	-31.276	1.631.050	-31.223
1.655.704	-24.105	1.678.075	-31.278	1.631.180	-31.223
1.655.847	-24.106	1.678.210	-31.279	1.631.376	-31.223
1.656.006	-24.107	1.678.382	-31.281	1.631.501	-31.223
1.656.208	-24.108	1.678.495	-31.282	1.631.606	-31.223
1.656.420	-24.109	1.678.649	-31.284	1.631.799	-31.223
1.656.604	-24.111	1.678.884	-31.285	1.631.918	-31.223
1.656.788	-24.112	1.679.039	-31.287	1.631.954	-31.223
1.656.964	-24.113	1.679.147	-31.288	1.632.056	-31.223
1.657.061	-24.114	1.679.312	-31.290	1.632.217	-31.223
1.657.236	-24.115	1.679.505	-31.291	1.632.428	-31.223
1.657.478	-24.116	1.679.642	-31.293	1.632.614	-31.223
1.657.605	-24.117	1.679.796	-31.295	1.632.776	-31.223
1.657.728	-24.118	1.680.009	-31.296	1.632.923	-31.223
1.657.887	-24.120	1.680.159	-31.298	1.633.118	-31.223

1.658.040	-24.121	1.680.291	-31.299	1.633.329	-31.223
1.658.186	-24.122	1.680.486	-31.301	1.633.472	-31.223
1.658.306	-24.123	1.680.676	-31.302	1.633.618	-31.223
1.658.479	-24.124	1.680.827	-31.304	1.633.846	-31.223
1.658.683	-24.125	1.680.963	-31.305	1.634.008	-31.223
1.658.860	-24.126	1.681.109	-31.307	1.634.117	-31.223
1.659.041	-24.128	1.681.236	-31.308	1.634.254	-31.222
1.659.243	-24.129	1.681.414	-31.310	1.634.426	-31.222
1.659.397	-24.130	1.681.783	-31.311	1.634.686	-31.222
1.659.536	-24.131	1.682.213	-31.312	1.634.884	-31.222
1.659.718	-24.132	1.682.451	-31.314	1.634.980	-31.222
1.659.809	-24.133	1.682.533	-31.315	1.635.117	-31.222
1.660.029	-24.135	1.682.569	-31.317	1.635.316	-31.222
1.660.479	-24.136	1.682.632	-31.318	1.635.491	-31.222
1.660.811	-24.137	1.682.715	-31.319	1.635.663	-31.221
1.660.950	-24.138	1.682.782	-31.321	1.635.802	-31.221
1.661.049	-24.139	1.682.930	-31.322	1.635.925	-31.221
1.661.105	-24.141	1.683.085	-31.324	1.636.118	-31.221
1.661.104	-24.142	1.683.223	-31.325	1.636.326	-31.221
1.661.129	-24.143	1.683.396	-31.326	1.636.483	-31.221
1.661.228	-24.144	1.683.591	-31.328	1.636.642	-31.221
1.661.398	-24.145	1.683.790	-31.329	1.636.790	-31.220
1.661.517	-24.147	1.683.936	-31.330	1.636.920	-31.220
1.661.673	-24.148	1.684.054	-31.331	1.637.085	-31.220
1.661.828	-24.149	1.684.252	-31.333	1.637.265	-31.220
1.661.996	-24.150	1.684.480	-31.334	1.637.469	-31.220
1.662.242	-24.152	1.684.621	-31.335	1.637.657	-31.220
1.662.441	-24.153	1.684.774	-31.336	1.637.809	-31.219
1.662.650	-24.154	1.684.948	-31.338	1.637.912	-31.219
1.662.805	-24.155	1.685.094	-31.339	1.638.087	-31.219

1.662.921	-24.156	1.685.228	-31.340	1.638.313	-31.219
1.663.123	-24.158	1.685.388	-31.341	1.638.479	-31.219
1.663.279	-24.159	1.685.549	-31.342	1.638.618	-31.219
1.663.372	-24.160	1.685.748	-31.343	1.638.784	-31.218
1.663.540	-24.161	1.685.914	-31.345	1.639.007	-31.218
1.663.727	-24.163	1.685.997	-31.346	1.639.153	-31.218
1.663.898	-24.164	1.686.122	-31.347	1.639.312	-31.218
1.664.048	-24.165	1.686.313	-31.348	1.639.512	-31.218
1.664.222	-24.166	1.686.514	-31.349	1.639.606	-31.218
1.664.377	-24.168	1.686.725	-31.350	1.639.727	-31.218
1.664.510	-24.169	1.686.956	-31.351	1.639.931	-31.217
1.664.650	-24.170	1.687.160	-31.352	1.640.079	-31.217
1.664.848	-24.171	1.687.280	-31.353	1.640.139	-31.217
1.665.054	-24.173	1.687.350	-31.354	1.640.287	-31.217
1.665.226	-24.174	1.687.455	-31.355	1.640.672	-31.217
1.665.448	-24.175	1.687.560	-31.356	1.641.097	-31.217
1.665.641	-24.176	1.687.726	-31.357	1.641.362	-31.217
1.665.739	-24.178	1.687.878	-31.358	1.641.552	-31.216
1.665.874	-24.179	1.688.080	-31.359	1.641.635	-31.216
1.666.057	-24.180	1.688.338	-31.359	1.641.658	-31.216
1.666.160	-24.181	1.688.472	-31.360	1.641.687	-31.216
1.666.263	-24.183	1.688.627	-31.361	1.641.772	-31.216
1.666.470	-24.184	1.688.855	-31.362	1.641.855	-31.216
1.666.662	-24.185	1.689.010	-31.363	1.641.913	-31.216
1.666.799	-24.186	1.689.106	-31.364	1.642.034	-31.216
1.666.960	-24.188	1.689.297	-31.364	1.642.217	-31.216
1.667.103	-24.189	1.689.478	-31.365	1.642.442	-31.216
1.667.217	-24.190	1.689.604	-31.366	1.642.623	-31.216
1.667.361	-24.191	1.689.778	-31.367	1.642.764	-31.216
1.667.621	-24.193	1.689.962	-31.368	1.642.928	-31.216

1.667.798	-24.194	1.690.004	-31.368	1.643.078	-31.216
1.667.905	-24.195	1.690.121	-31.369	1.643.195	-31.216
1.668.073	-24.196	1.690.432	-31.370	1.643.333	-31.216
1.668.232	-24.198	1.690.674	-31.370	1.643.523	-31.216
1.668.382	-24.199	1.690.804	-31.371	1.643.752	-31.216
1.668.501	-24.200	1.690.894	-31.372	1.643.974	-31.216
1.668.642	-24.201	1.691.062	-31.373	1.644.158	-31.216
1.668.833	-24.203	1.691.281	-31.373	1.644.294	-31.217
1.669.008	-24.204	1.691.456	-31.374	1.644.402	-31.217
1.669.184	-24.205	1.691.577	-31.374	1.644.491	-31.217
1.669.357	-24.206	1.691.749	-31.375	1.644.648	-31.217
1.669.458	-24.207	1.691.931	-31.376	1.644.854	-31.217
1.669.675	-24.209	1.692.099	-31.376	1.645.036	-31.218
1.669.893	-24.210	1.692.296	-31.377	1.645.174	-31.218
1.670.052	-24.211	1.692.478	-31.377	1.645.282	-31.218
1.670.208	-24.212	1.692.641	-31.378	1.645.396	-31.218
1.670.338	-24.214	1.692.780	-31.379	1.645.600	-31.219
1.670.513	-24.215	1.692.868	-31.379	1.645.854	-31.219
1.670.636	-24.216	1.693.004	-31.380	1.646.046	-31.219
1.670.845	-24.217	1.693.288	-31.380	1.646.205	-31.220
1.671.040	-24.219	1.693.474	-31.381	1.646.357	-31.220
1.671.237	-24.220	1.693.528	-31.381	1.646.480	-31.221
1.671.407	-24.221	1.693.669	-31.382	1.646.624	-31.221
1.671.472	-24.222	1.693.810	-31.382	1.646.893	-31.222
1.671.653	-24.224	1.693.945	-31.383	1.647.083	-31.222
1.671.855	-24.225	1.694.072	-31.383	1.647.204	-31.223
1.672.056	-24.226	1.694.204	-31.384	1.647.359	-31.223
1.672.197	-24.227	1.694.346	-31.384	1.647.471	-31.224
1.672.280	-24.228	1.694.500	-31.384	1.647.652	-31.224
1.672.455	-24.230	1.694.709	-31.385	1.647.874	-31.225

1.672.623	-24.231	1.694.921	-31.385	1.648.056	-31.226
1.672.706	-24.232	1.695.177	-31.386	1.648.179	-31.226
1.672.865	-24.233	1.695.396	-31.386	1.648.347	-31.227
1.673.051	-24.234	1.695.571	-31.386	1.648.485	-31.228
1.673.170	-24.236	1.695.779	-31.387	1.648.519	-31.228
1.673.304	-24.237	1.695.909	-31.387	1.648.671	-31.229
1.673.470	-24.238	1.696.023	-31.387	1.648.891	-31.230
1.673.730	-24.239	1.696.199	-31.388	1.649.044	-31.231
1.673.929	-24.241	1.696.322	-31.388	1.649.256	-31.231
1.674.043	-24.242	1.696.438	-31.388	1.649.474	-31.232
1.674.283	-24.243	1.696.638	-31.389	1.649.630	-31.233
1.674.554	-24.244	1.696.777	-31.389	1.649.742	-31.234
1.674.733	-24.245	1.696.855	-31.389	1.649.837	-31.235
1.674.850	-24.247	1.696.987	-31.389	1.649.946	-31.236
1.674.971	-24.248	1.697.182	-31.390	1.650.081	-31.237
1.675.108	-24.249	1.697.370	-31.390	1.650.264	-31.238
1.675.204	-24.250	1.697.531	-31.390	1.650.407	-31.239
1.675.367	-24.251	1.697.670	-31.390	1.650.607	-31.240
1.675.519	-24.253	1.697.912	-31.391	1.650.791	-31.241
1.675.665	-24.254	1.698.226	-31.391	1.650.934	-31.242
1.675.816	-24.255	1.698.396	-31.391	1.651.153	-31.243
1.675.965	-24.256	1.698.517	-31.391	1.651.337	-31.244
1.676.107	-24.257	1.698.694	-31.391	1.651.514	-31.245
1.676.243	-24.258	1.698.810	-31.392	1.651.674	-31.247
1.676.454	-24.260	1.698.947	-31.392	1.651.797	-31.248
1.676.680	-24.261	1.699.156	-31.392	1.651.991	-31.249
1.676.871	-24.262	1.699.355	-31.392	1.652.177	-31.250
1.677.105	-24.263	1.699.527	-31.392	1.652.327	-31.251
1.677.280	-24.264	1.699.653	-31.393	1.652.489	-31.253
1.677.426	-24.265	1.699.836	-31.393	1.652.627	-31.254

1.677.601	-24.267	1.700.022	-31.393	1.652.793	-31.255
1.677.755	-24.268	1.700.150	-31.393	1.652.955	-31.256
1.677.961	-24.269	1.700.304	-31.393	1.652.990	-31.258
1.678.118	-24.270	1.700.495	-31.393	1.653.116	-31.259
1.678.277	-24.271	1.700.663	-31.394	1.653.349	-31.260
1.678.454	-24.272	1.700.851	-31.394	1.653.571	-31.262
1.678.618	-24.274	1.701.044	-31.394	1.653.757	-31.263
1.678.725	-24.275	1.701.153	-31.394	1.653.940	-31.264
1.678.871	-24.276	1.701.248	-31.394	1.654.137	-31.266
1.679.025	-24.277	1.701.393	-31.394	1.654.303	-31.267
1.679.189	-24.278	1.701.543	-31.394	1.654.413	-31.268
1.679.438	-24.279	1.701.747	-31.394	1.654.566	-31.270
1.679.668	-24.281	1.701.971	-31.395	1.654.756	-31.271
1.679.781	-24.282	1.702.128	-31.395	1.654.908	-31.272
1.679.875	-24.283	1.702.220	-31.395	1.655.074	-31.274
1.680.009	-24.284	1.702.392	-31.395	1.655.251	-31.275
1.680.152	-24.285	1.702.639	-31.395	1.655.403	-31.277
1.680.359	-24.286	1.702.793	-31.395	1.655.471	-31.278
1.680.578	-24.287	1.702.959	-31.395	1.655.602	-31.279
1.680.793	-24.289	1.703.156	-31.395	1.655.760	-31.281
1.680.905	-24.290	1.703.282	-31.395	1.655.851	-31.282
1.680.988	-24.291	1.703.365	-31.395	1.656.012	-31.284
1.681.129	-24.292	1.703.671	-31.395	1.656.234	-31.285
1.681.382	-24.293	1.704.117	-31.395	1.656.377	-31.287
1.681.629	-24.294	1.704.420	-31.396	1.656.485	-31.288
1.681.723	-24.295	1.704.556	-31.396	1.656.657	-31.289
1.681.832	-24.296	1.704.574	-31.396	1.656.873	-31.291
1.682.009	-24.297	1.704.621	-31.396	1.657.032	-31.292
1.682.144	-24.299	1.704.720	-31.396	1.657.207	-31.294
1.682.265	-24.300	1.704.760	-31.396	1.657.383	-31.295

1.682.515	-24.301	1.704.830	-31.396	1.657.585	-31.296
1.682.937	-24.302	1.704.966	-31.396	1.657.805	-31.298
1.683.306	-24.303	1.705.141	-31.396	1.657.988	-31.299
1.683.416	-24.304	1.705.356	-31.396	1.658.085	-31.301
1.683.456	-24.305	1.705.540	-31.396	1.658.297	-31.302
1.683.537	-24.306	1.705.719	-31.396	1.658.420	-31.304
1.683.618	-24.307	1.705.858	-31.396	1.658.537	-31.305
1.683.671	-24.308	1.706.068	-31.396	1.658.718	-31.306
1.683.770	-24.310	1.706.295	-31.396	1.658.860	-31.308
1.683.951	-24.311	1.706.458	-31.396	1.659.010	-31.309
1.684.157	-24.312	1.706.604	-31.396	1.659.144	-31.311
1.684.352	-24.313	1.706.767	-31.396	1.659.243	-31.312
1.684.471	-24.314	1.706.904	-31.396	1.659.370	-31.313
1.684.534	-24.315	1.707.023	-31.396	1.659.610	-31.315
1.684.668	-24.316	1.707.198	-31.396	1.659.789	-31.316
1.684.845	-24.317	1.707.381	-31.396	1.659.903	-31.317
1.685.107	-24.318	1.707.563	-31.396	1.660.067	-31.319
1.685.332	-24.319	1.707.719	-31.396	1.660.219	-31.320
1.685.441	-24.320	1.707.818	-31.396	1.660.401	-31.321
1.685.596	-24.321	1.707.946	-31.396	1.660.580	-31.323
1.685.755	-24.322	1.708.140	-31.396	1.660.708	-31.324
1.685.905	-24.323	1.708.286	-31.396	1.660.885	-31.325
1.686.021	-24.324	1.708.466	-31.396	1.661.120	-31.327
1.686.161	-24.325	1.708.672	-31.396	1.661.310	-31.328
1.686.320	-24.326	1.708.833	-31.396	1.661.514	-31.329
1.686.559	-24.327	1.709.079	-31.396	1.661.657	-31.330
1.686.745	-24.328	1.709.240	-31.396	1.661.812	-31.332
1.686.837	-24.329	1.709.375	-31.397	1.661.980	-31.333
1.686.955	-24.330	1.709.552	-31.397	1.662.112	-31.334
1.687.130	-24.331	1.709.666	-31.397	1.662.280	-31.335

1.687.381	-24.332	1.709.778	-31.397	1.662.500	-31.337
1.687.612	-24.333	1.709.953	-31.397	1.662.626	-31.338
1.687.731	-24.334	1.710.130	-31.397	1.662.791	-31.339
1.687.887	-24.335	1.710.262	-31.397	1.662.948	-31.340
1.688.120	-24.336	1.710.426	-31.397	1.663.125	-31.342
1.688.288	-24.337	1.710.616	-31.397	1.663.335	-31.343
1.688.411	-24.338	1.710.768	-31.397	1.663.497	-31.344
1.688.539	-24.339	1.710.889	-31.397	1.663.683	-31.345
1.688.743	-24.340	1.711.089	-31.398	1.663.851	-31.346
1.688.871	-24.341	1.711.279	-31.398	1.663.985	-31.347
1.688.929	-24.342	1.711.386	-31.398	1.664.180	-31.349
1.689.050	-24.343	1.711.521	-31.398	1.664.324	-31.350
1.689.277	-24.344	1.711.698	-31.398	1.664.395	-31.351
1.689.482	-24.345	1.711.857	-31.398	1.664.529	-31.352
1.689.612	-24.346	1.712.007	-31.399	1.664.805	-31.353
1.689.801	-24.347	1.712.173	-31.399	1.664.991	-31.354
1.690.009	-24.348	1.712.372	-31.399	1.664.997	-31.355
1.690.161	-24.349	1.712.576	-31.399	1.665.132	-31.356
1.690.280	-24.350	1.712.715	-31.400	1.665.397	-31.358
1.690.434	-24.351	1.712.883	-31.400	1.665.654	-31.359
1.690.609	-24.352	1.713.118	-31.400	1.665.755	-31.360
1.690.721	-24.353	1.713.259	-31.400	1.665.802	-31.361
1.690.844	-24.353	1.713.382	-31.401	1.666.006	-31.362
1.691.060	-24.354	1.713.571	-31.401	1.666.236	-31.363
1.691.221	-24.355	1.713.719	-31.401	1.666.431	-31.364
1.691.407	-24.356	1.713.855	-31.402	1.666.548	-31.365
1.691.618	-24.357	1.714.050	-31.402	1.666.648	-31.366
1.691.823	-24.358	1.714.243	-31.402	1.666.848	-31.367
1.691.989	-24.359	1.714.413	-31.403	1.667.016	-31.368
1.692.121	-24.360	1.714.603	-31.403	1.667.132	-31.369

1.692.282	-24.361	1.714.758	-31.403	1.667.294	-31.370
1.692.431	-24.362	1.714.881	-31.404	1.667.453	-31.371
1.692.538	-24.363	1.715.056	-31.404	1.667.782	-31.372
1.692.663	-24.363	1.715.230	-31.405	1.668.235	-31.373
1.692.887	-24.364	1.715.385	-31.405	1.668.506	-31.374
1.693.064	-24.365	1.715.529	-31.406	1.668.635	-31.375
1.693.210	-24.366	1.715.643	-31.406	1.668.752	-31.376
1.693.418	-24.367	1.715.750	-31.407	1.668.745	-31.377
1.693.564	-24.368	1.715.874	-31.407	1.668.757	-31.378
1.693.689	-24.369	1.715.992	-31.408	1.668.884	-31.379
1.693.804	-24.370	1.716.106	-31.408	1.669.043	-31.380
1.694.052	-24.371	1.716.283	-31.409	1.669.196	-31.381
1.694.250	-24.371	1.716.525	-31.409	1.669.301	-31.382
1.694.375	-24.372	1.716.758	-31.410	1.669.464	-31.384
1.694.514	-24.373	1.716.880	-31.411	1.669.557	-31.385
1.694.671	-24.374	1.716.987	-31.411	1.669.628	-31.386
1.694.821	-24.375	1.717.218	-31.412	1.669.776	-31.387
1.694.960	-24.376	1.717.468	-31.412	1.670.029	-31.388
1.695.134	-24.377	1.717.665	-31.413	1.670.280	-31.389
1.695.275	-24.378	1.717.869	-31.414	1.670.428	-31.390
1.695.349	-24.378	1.718.062	-31.414	1.670.593	-31.391
1.695.475	-24.379	1.718.192	-31.415	1.670.742	-31.392
1.695.694	-24.380	1.718.326	-31.416	1.670.851	-31.393
1.695.896	-24.381	1.718.438	-31.417	1.671.048	-31.394
1.696.050	-24.382	1.718.528	-31.417	1.671.281	-31.395
1.696.205	-24.383	1.718.694	-31.418	1.671.497	-31.396
1.696.458	-24.384	1.718.806	-31.419	1.671.707	-31.397
1.696.776	-24.385	1.718.882	-31.420	1.671.870	-31.398
1.696.987	-24.385	1.719.055	-31.421	1.671.958	-31.399
1.697.070	-24.386	1.719.277	-31.422	1.672.056	-31.400

1.697.132	-24.387	1.719.444	-31.422	1.672.148	-31.402
1.697.359	-24.388	1.719.650	-31.423	1.672.287	-31.403
1.697.531	-24.389	1.719.921	-31.424	1.672.514	-31.404
1.697.655	-24.390	1.720.125	-31.425	1.672.670	-31.405
1.697.794	-24.391	1.720.316	-31.426	1.672.805	-31.406
1.697.906	-24.392	1.720.493	-31.427	1.672.932	-31.408
1.698.069	-24.393	1.720.667	-31.428	1.673.049	-31.409
1.698.148	-24.393	1.720.838	-31.429	1.673.203	-31.410
1.698.235	-24.394	1.720.872	-31.430	1.673.405	-31.411
1.698.454	-24.395	1.720.984	-31.431	1.673.555	-31.412
1.698.651	-24.396	1.721.268	-31.432	1.673.701	-31.414
1.698.795	-24.397	1.721.483	-31.433	1.673.933	-31.415
1.699.028	-24.398	1.721.662	-31.434	1.674.138	-31.416
1.699.263	-24.399	1.721.801	-31.436	1.674.350	-31.418
1.699.429	-24.400	1.721.906	-31.437	1.674.525	-31.419
1.699.585	-24.401	1.722.040	-31.438	1.674.709	-31.420
1.699.781	-24.402	1.722.189	-31.439	1.674.874	-31.422
1.699.966	-24.403	1.722.392	-31.440	1.674.966	-31.423
1.700.081	-24.403	1.722.616	-31.442	1.675.130	-31.424
1.700.258	-24.404	1.722.829	-31.443	1.675.298	-31.426
1.700.425	-24.405	1.722.953	-31.444	1.675.468	-31.427
1.700.486	-24.406	1.723.121	-31.445	1.675.620	-31.429
1.700.643	-24.407	1.723.324	-31.447	1.675.681	-31.430
1.700.862	-24.408	1.723.458	-31.448	1.675.844	-31.432
1.701.060	-24.409	1.723.551	-31.450	1.676.010	-31.433
1.701.248	-24.410	1.723.692	-31.451	1.676.156	-31.435
1.701.451	-24.411	1.723.940	-31.452	1.676.402	-31.436
1.701.610	-24.412	1.724.090	-31.454	1.676.584	-31.438
1.701.785	-24.413	1.724.245	-31.455	1.676.684	-31.439
1.701.973	-24.414	1.724.438	-31.457	1.676.879	-31.441

1.702.119	-24.415	1.724.594	-31.458	1.677.110	-31.443
1.702.251	-24.416	1.724.749	-31.460	1.677.243	-31.444
1.702.402	-24.417	1.724.883	-31.461	1.677.419	-31.446
1.702.583	-24.418	1.724.993	-31.463	1.677.601	-31.448
1.702.778	-24.419	1.725.130	-31.464	1.677.780	-31.449
1.702.937	-24.420	1.725.275	-31.466	1.677.948	-31.451
1.703.036	-24.421	1.725.419	-31.468	1.678.026	-31.453
1.703.172	-24.422	1.725.786	-31.469	1.678.188	-31.454
1.703.367	-24.423	1.726.284	-31.471	1.678.481	-31.456
1.703.553	-24.424	1.726.508	-31.473	1.678.691	-31.458
1.703.721	-24.425	1.726.579	-31.474	1.678.795	-31.459
1.703.918	-24.426	1.726.658	-31.476	1.678.886	-31.461
1.704.091	-24.427	1.726.682	-31.478	1.679.019	-31.463
1.704.229	-24.428	1.726.741	-31.479	1.679.243	-31.465
1.704.368	-24.429	1.726.785	-31.481	1.679.458	-31.467
1.704.523	-24.430	1.726.805	-31.483	1.679.603	-31.468
1.704.635	-24.431	1.726.996	-31.485	1.679.713	-31.470
1.704.767	-24.432	1.727.238	-31.486	1.679.838	-31.472
1.705.049	-24.433	1.727.440	-31.488	1.679.984	-31.474
1.705.517	-24.434	1.727.547	-31.490	1.680.159	-31.476
1.705.869	-24.435	1.727.569	-31.492	1.680.325	-31.477
1.705.979	-24.436	1.727.751	-31.494	1.680.479	-31.479
1.706.041	-24.437	1.728.044	-31.496	1.680.632	-31.481
1.706.082	-24.439	1.728.288	-31.497	1.680.768	-31.483
1.706.122	-24.440	1.728.488	-31.499	1.680.918	-31.485
1.706.187	-24.441	1.728.609	-31.501	1.681.078	-31.487
1.706.301	-24.442	1.728.734	-31.503	1.681.255	-31.489
1.706.438	-24.443	1.728.924	-31.505	1.681.378	-31.490
1.706.584	-24.444	1.729.088	-31.507	1.681.570	-31.492
1.706.727	-24.445	1.729.198	-31.509	1.681.844	-31.494

1.706.888	-24.446	1.729.333	-31.511	1.681.973	-31.496
1.707.047	-24.447	1.729.487	-31.513	1.682.108	-31.498
1.707.222	-24.449	1.729.623	-31.515	1.682.258	-31.500
1.707.430	-24.450	1.729.791	-31.517	1.682.366	-31.502
1.707.646	-24.451	1.729.863	-31.519	1.682.547	-31.503
1.707.860	-24.452	1.730.038	-31.521	1.682.742	-31.505
1.708.042	-24.453	1.730.294	-31.523	1.682.885	-31.507
1.708.206	-24.454	1.730.510	-31.525	1.682.959	-31.509
1.708.360	-24.455	1.730.678	-31.527	1.683.046	-31.511
1.708.486	-24.457	1.730.820	-31.529	1.683.168	-31.513
1.708.602	-24.458	1.730.968	-31.531	1.683.374	-31.515
1.708.783	-24.459	1.731.167	-31.533	1.683.540	-31.516
1.708.923	-24.460	1.731.339	-31.535	1.683.768	-31.518
1.709.077	-24.461	1.731.489	-31.537	1.683.963	-31.520
1.709.276	-24.462	1.731.604	-31.539	1.684.079	-31.522
1.709.399	-24.464	1.731.788	-31.541	1.684.334	-31.524
1.709.558	-24.465	1.732.016	-31.543	1.684.597	-31.526
1.709.735	-24.466	1.732.137	-31.545	1.684.773	-31.527
1.709.906	-24.467	1.732.269	-31.547	1.684.926	-31.529
1.710.126	-24.468	1.732.419	-31.549	1.685.130	-31.531
1.710.365	-24.470	1.732.545	-31.551	1.685.303	-31.533
1.710.531	-24.471	1.732.730	-31.553	1.685.416	-31.535
1.710.667	-24.472	1.732.892	-31.555	1.685.571	-31.536
1.710.833	-24.473	1.733.008	-31.557	1.685.795	-31.538
1.710.952	-24.474	1.733.129	-31.559	1.685.974	-31.540
1.711.053	-24.476	1.733.331	-31.561	1.686.096	-31.542
1.711.218	-24.477	1.733.551	-31.563	1.686.209	-31.543
1.711.396	-24.478	1.733.725	-31.565	1.686.371	-31.545
1.711.523	-24.479	1.733.866	-31.567	1.686.481	-31.547
1.711.629	-24.481	1.733.967	-31.569	1.686.563	-31.548

1.711.772	-24.482	1.734.086	-31.571	1.686.790	-31.550
1.711.940	-24.483	1.734.344	-31.573	1.687.016	-31.552
1.712.155	-24.484	1.734.556	-31.575	1.687.188	-31.553
1.712.386	-24.486	1.734.702	-31.577	1.687.298	-31.555
1.712.529	-24.487	1.734.792	-31.579	1.687.402	-31.557
1.712.661	-24.488	1.734.977	-31.581	1.687.654	-31.558
1.712.809	-24.489	1.735.159	-31.583	1.687.845	-31.560
1.712.952	-24.491	1.735.331	-31.585	1.687.934	-31.561
1.713.131	-24.492	1.735.493	-31.587	1.688.122	-31.563
1.713.293	-24.493	1.735.598	-31.589	1.688.344	-31.564
1.713.456	-24.494	1.735.750	-31.591	1.688.557	-31.566
1.713.642	-24.496	1.735.970	-31.593	1.688.658	-31.567
1.713.756	-24.497	1.736.158	-31.595	1.688.719	-31.569
1.713.869	-24.498	1.736.302	-31.597	1.688.907	-31.570
1.714.118	-24.499	1.736.418	-31.599	1.689.212	-31.572
1.714.391	-24.501	1.736.613	-31.600	1.689.404	-31.573
1.714.583	-24.502	1.736.794	-31.602	1.689.500	-31.574
1.714.778	-24.503	1.736.944	-31.604	1.689.635	-31.576
1.714.892	-24.505	1.737.135	-31.606	1.689.809	-31.577
1.714.964	-24.506	1.737.273	-31.608	1.689.966	-31.578
1.715.101	-24.507	1.737.449	-31.609	1.690.103	-31.580
1.715.221	-24.509	1.737.574	-31.611	1.690.311	-31.581
1.715.396	-24.510	1.737.674	-31.613	1.690.484	-31.582
1.715.526	-24.511	1.737.794	-31.614	1.690.638	-31.583
1.715.692	-24.512	1.737.876	-31.616	1.690.782	-31.585
1.715.967	-24.514	1.738.049	-31.618	1.690.934	-31.586
1.716.165	-24.515	1.738.277	-31.619	1.691.131	-31.587
1.716.339	-24.516	1.738.490	-31.621	1.691.284	-31.588
1.716.510	-24.518	1.738.678	-31.623	1.691.306	-31.589
1.716.649	-24.519	1.738.786	-31.624	1.691.469	-31.591

1.716.812	-24.520	1.738.936	-31.626	1.691.698	-31.592
1.716.938	-24.522	1.739.175	-31.627	1.691.842	-31.593
1.717.108	-24.523	1.739.467	-31.629	1.692.023	-31.594
1.717.296	-24.524	1.739.715	-31.630	1.692.207	-31.595
1.717.417	-24.526	1.739.863	-31.631	1.692.390	-31.596
1.717.513	-24.527	1.740.004	-31.633	1.692.630	-31.597
1.717.663	-24.528	1.740.134	-31.634	1.692.849	-31.598
1.717.791	-24.530	1.740.303	-31.635	1.692.946	-31.599
1.717.923	-24.531	1.740.473	-31.637	1.693.107	-31.600
1.718.026	-24.533	1.740.670	-31.638	1.693.275	-31.601
1.718.170	-24.534	1.740.777	-31.639	1.693.423	-31.602
1.718.376	-24.535	1.740.880	-31.640	1.693.624	-31.603
1.718.593	-24.537	1.741.028	-31.641	1.693.828	-31.604
1.718.811	-24.538	1.741.149	-31.642	1.693.992	-31.605
1.718.971	-24.539	1.741.261	-31.643	1.694.108	-31.606
1.719.173	-24.541	1.741.371	-31.644	1.694.245	-31.607
1.719.442	-24.542	1.741.479	-31.645	1.694.438	-31.608
1.719.613	-24.544	1.741.680	-31.646	1.694.583	-31.609
1.719.782	-24.545	1.741.949	-31.647	1.694.657	-31.610
1.719.926	-24.546	1.742.119	-31.648	1.694.944	-31.611
1.720.040	-24.548	1.742.316	-31.649	1.695.423	-31.611
1.720.168	-24.549	1.742.553	-31.650	1.695.730	-31.612
1.720.295	-24.550	1.742.708	-31.651	1.695.806	-31.613
1.720.435	-24.552	1.742.834	-31.651	1.695.882	-31.614
1.720.576	-24.553	1.742.986	-31.652	1.695.986	-31.615
1.720.714	-24.555	1.743.163	-31.653	1.696.093	-31.616
1.720.849	-24.556	1.743.360	-31.654	1.696.208	-31.617
1.720.984	-24.558	1.743.530	-31.654	1.696.310	-31.617
1.721.102	-24.559	1.743.676	-31.655	1.696.358	-31.618
1.721.283	-24.560	1.743.853	-31.655	1.696.405	-31.619

1.721.496	-24.562	1.744.027	-31.656	1.696.507	-31.620
1.721.736	-24.563	1.744.165	-31.656	1.696.633	-31.621
1.721.906	-24.565	1.744.326	-31.657	1.696.830	-31.622
1.722.043	-24.566	1.744.491	-31.657	1.697.074	-31.622
1.722.267	-24.568	1.744.666	-31.657	1.697.211	-31.623
1.722.464	-24.569	1.744.877	-31.658	1.697.421	-31.624
1.722.596	-24.570	1.745.036	-31.658	1.697.621	-31.625
1.722.728	-24.572	1.745.168	-31.658	1.697.803	-31.626
1.722.961	-24.573	1.745.336	-31.658	1.698.024	-31.627
1.723.134	-24.575	1.745.546	-31.658	1.698.131	-31.627
1.723.235	-24.576	1.745.746	-31.659	1.698.264	-31.628
1.723.391	-24.578	1.745.900	-31.659	1.698.378	-31.629
1.723.618	-24.579	1.746.105	-31.659	1.698.541	-31.630
1.723.842	-24.581	1.746.283	-31.659	1.698.732	-31.631
1.723.987	-24.582	1.746.380	-31.659	1.698.898	-31.632
1.724.182	-24.584	1.746.545	-31.659	1.699.010	-31.633
1.724.424	-24.585	1.746.772	-31.658	1.699.204	-31.633
1.724.527	-24.586	1.746.928	-31.658	1.699.377	-31.634
1.724.664	-24.588	1.747.052	-31.658	1.699.424	-31.635
1.724.870	-24.589	1.747.245	-31.658	1.699.601	-31.636
1.725.023	-24.591	1.747.455	-31.658	1.699.816	-31.637
1.725.179	-24.592	1.747.565	-31.657	1.699.969	-31.638
1.725.280	-24.594	1.747.670	-31.657	1.700.141	-31.639
1.725.403	-24.595	1.747.997	-31.656	1.700.305	-31.640
1.725.580	-24.597	1.748.457	-31.656	1.700.495	-31.641
1.725.795	-24.598	1.748.689	-31.656	1.700.665	-31.642
1.725.941	-24.600	1.748.756	-31.655	1.700.835	-31.643
1.726.084	-24.601	1.748.866	-31.655	1.701.044	-31.644
1.726.273	-24.603	1.748.940	-31.654	1.701.284	-31.645
1.726.479	-24.604	1.748.970	-31.653	1.701.485	-31.646

1.726.705	-24.606	1.749.001	-31.653	1.701.595	-31.647
1.726.850	-24.607	1.749.100	-31.652	1.701.727	-31.648
1.726.983	-24.609	1.749.254	-31.651	1.701.826	-31.649
1.727.065	-24.610	1.749.435	-31.651	1.701.956	-31.650
1.727.216	-24.612	1.749.599	-31.650	1.702.128	-31.651
1.727.372	-24.613	1.749.724	-31.649	1.702.310	-31.652
1.727.690	-24.615	1.749.931	-31.648	1.702.495	-31.653
1.728.158	-24.616	1.750.121	-31.647	1.702.607	-31.655
1.728.452	-24.618	1.750.354	-31.646	1.702.766	-31.656
1.728.620	-24.619	1.750.583	-31.645	1.702.941	-31.657
1.728.698	-24.621	1.750.686	-31.644	1.703.107	-31.658
1.728.689	-24.622	1.750.849	-31.643	1.703.288	-31.659
1.728.674	-24.624	1.751.051	-31.642	1.703.454	-31.661
1.728.761	-24.626	1.751.205	-31.641	1.703.627	-31.662
1.728.876	-24.627	1.751.328	-31.640	1.703.775	-31.663
1.729.001	-24.629	1.751.458	-31.639	1.703.878	-31.664
1.729.164	-24.630	1.751.609	-31.638	1.704.070	-31.666
1.729.346	-24.632	1.751.803	-31.637	1.704.342	-31.667
1.729.518	-24.633	1.751.908	-31.635	1.704.454	-31.668
1.729.666	-24.635	1.752.068	-31.634	1.704.530	-31.670
1.729.861	-24.636	1.752.350	-31.633	1.704.711	-31.671
1.730.036	-24.638	1.752.533	-31.632	1.704.874	-31.672
1.730.154	-24.639	1.752.668	-31.630	1.705.047	-31.674
1.730.251	-24.641	1.752.809	-31.629	1.705.219	-31.675
1.730.398	-24.642	1.753.022	-31.628	1.705.363	-31.677
1.730.650	-24.644	1.753.212	-31.626	1.705.555	-31.678
1.730.835	-24.645	1.753.364	-31.625	1.705.728	-31.680
1.730.950	-24.647	1.753.533	-31.623	1.705.885	-31.681
1.731.064	-24.648	1.753.705	-31.622	1.706.059	-31.683
1.731.254	-24.650	1.753.891	-31.620	1.706.237	-31.684

1.731.488	-24.651	1.754.030	-31.619	1.706.422	-31.686
1.731.676	-24.653	1.754.124	-31.617	1.706.609	-31.687
1.731.812	-24.654	1.754.230	-31.616	1.706.734	-31.689
1.731.960	-24.656	1.754.373	-31.614	1.706.843	-31.690
1.732.128	-24.657	1.754.529	-31.613	1.707.010	-31.692
1.732.217	-24.659	1.754.760	-31.611	1.707.233	-31.693
1.732.361	-24.660	1.754.899	-31.609	1.707.455	-31.695
1.732.618	-24.662	1.755.108	-31.608	1.707.626	-31.697
1.732.794	-24.663	1.755.269	-31.606	1.707.769	-31.698
1.732.970	-24.665	1.755.367	-31.604	1.707.865	-31.700
1.733.174	-24.666	1.755.560	-31.603	1.707.980	-31.701
1.733.360	-24.668	1.755.790	-31.601	1.708.158	-31.703
1.733.508	-24.669	1.755.981	-31.599	1.708.372	-31.705
1.733.665	-24.671	1.756.162	-31.597	1.708.606	-31.706
1.733.868	-24.672	1.756.252	-31.596	1.708.829	-31.708
1.734.052	-24.674	1.756.286	-31.594	1.708.960	-31.710
1.734.189	-24.675	1.756.474	-31.592	1.709.146	-31.711
1.734.272	-24.676	1.756.709	-31.590	1.709.364	-31.713
1.734.420	-24.678	1.756.796	-31.589	1.709.478	-31.715
1.734.628	-24.679	1.756.949	-31.587	1.709.563	-31.716
1.734.781	-24.681	1.757.184	-31.585	1.709.713	-31.718
1.734.888	-24.682	1.757.449	-31.583	1.709.841	-31.720
1.735.108	-24.684	1.757.632	-31.581	1.709.955	-31.721
1.735.296	-24.685	1.757.782	-31.580	1.710.119	-31.723
1.735.388	-24.687	1.757.901	-31.578	1.710.260	-31.725
1.735.549	-24.688	1.758.004	-31.576	1.710.316	-31.726
1.735.728	-24.690	1.758.087	-31.574	1.710.461	-31.728
1.735.858	-24.691	1.758.223	-31.572	1.710.576	-31.730
1.735.988	-24.692	1.758.493	-31.570	1.710.750	-31.731
1.736.142	-24.694	1.758.690	-31.568	1.710.988	-31.733

1.736.299	-24.695	1.758.817	-31.566	1.711.189	-31.734
1.736.497	-24.697	1.758.920	-31.564	1.711.364	-31.736
1.736.705	-24.698	1.759.115	-31.563	1.711.507	-31.738
1.736.898	-24.700	1.759.306	-31.561	1.711.741	-31.739
1.737.072	-24.701	1.759.446	-31.559	1.711.984	-31.741
1.737.238	-24.703	1.759.608	-31.557	1.712.144	-31.743
1.737.421	-24.704	1.759.691	-31.555	1.712.343	-31.744
1.737.553	-24.705	1.759.818	-31.553	1.712.518	-31.746
1.737.666	-24.707	1.760.069	-31.551	1.712.632	-31.747
1.737.847	-24.708	1.760.219	-31.549	1.712.744	-31.749
1.738.051	-24.710	1.760.329	-31.547	1.712.872	-31.750
1.738.203	-24.711	1.760.461	-31.545	1.713.046	-31.752
1.738.356	-24.712	1.760.627	-31.543	1.713.271	-31.754
1.738.578	-24.714	1.760.802	-31.541	1.713.448	-31.755
1.738.690	-24.715	1.761.028	-31.539	1.713.537	-31.757
1.738.797	-24.717	1.761.218	-31.537	1.713.633	-31.758
1.739.068	-24.718	1.761.409	-31.535	1.713.819	-31.760
1.739.254	-24.720	1.761.642	-31.533	1.713.954	-31.761
1.739.364	-24.721	1.761.832	-31.531	1.714.115	-31.763
1.739.539	-24.722	1.762.047	-31.529	1.714.285	-31.764
1.739.717	-24.724	1.762.280	-31.527	1.714.411	-31.766
1.739.859	-24.725	1.762.457	-31.525	1.714.668	-31.767
1.739.971	-24.727	1.762.583	-31.523	1.714.855	-31.769
1.740.114	-24.728	1.762.654	-31.521	1.715.029	-31.770
1.740.222	-24.729	1.762.807	-31.519	1.715.249	-31.771
1.740.356	-24.731	1.762.971	-31.517	1.715.405	-31.773
1.740.513	-24.732	1.763.078	-31.515	1.715.560	-31.774
1.740.605	-24.734	1.763.232	-31.513	1.715.759	-31.776
1.740.741	-24.735	1.763.380	-31.511	1.715.988	-31.777
1.741.019	-24.736	1.763.481	-31.509	1.716.187	-31.778

1.741.245	-24.738	1.763.647	-31.507	1.716.353	-31.780
1.741.471	-24.739	1.763.839	-31.505	1.716.480	-31.781
1.741.673	-24.740	1.764.014	-31.502	1.716.613	-31.782
1.741.870	-24.742	1.764.263	-31.500	1.716.754	-31.784
1.742.056	-24.743	1.764.478	-31.498	1.716.875	-31.785
1.742.260	-24.745	1.764.659	-31.496	1.717.088	-31.786
1.742.459	-24.746	1.764.845	-31.494	1.717.258	-31.788
1.742.527	-24.747	1.764.984	-31.492	1.717.388	-31.789
1.742.578	-24.749	1.765.123	-31.490	1.717.673	-31.790
1.742.786	-24.750	1.765.269	-31.488	1.717.883	-31.791
1.742.995	-24.752	1.765.374	-31.486	1.717.952	-31.793
1.743.121	-24.753	1.765.524	-31.484	1.718.053	-31.794
1.743.219	-24.754	1.765.757	-31.482	1.718.161	-31.795
1.743.338	-24.756	1.765.961	-31.480	1.718.340	-31.796
1.743.513	-24.757	1.766.120	-31.478	1.718.604	-31.797
1.743.703	-24.759	1.766.281	-31.476	1.718.826	-31.799
1.743.833	-24.760	1.766.454	-31.474	1.718.949	-31.800
1.743.913	-24.761	1.766.604	-31.472	1.719.050	-31.801
1.744.115	-24.763	1.766.729	-31.470	1.719.216	-31.802
1.744.377	-24.764	1.766.908	-31.468	1.719.386	-31.803
1.744.502	-24.766	1.767.130	-31.466	1.719.518	-31.804
1.744.639	-24.767	1.767.320	-31.464	1.719.633	-31.805
1.744.771	-24.768	1.767.421	-31.462	1.719.857	-31.807
1.744.924	-24.770	1.767.558	-31.460	1.720.096	-31.808
1.745.174	-24.771	1.767.739	-31.458	1.720.191	-31.809
1.745.392	-24.773	1.767.912	-31.456	1.720.341	-31.810
1.745.524	-24.774	1.768.107	-31.454	1.720.499	-31.811
1.745.625	-24.776	1.768.291	-31.452	1.720.663	-31.812
1.745.742	-24.777	1.768.416	-31.450	1.720.822	-31.813
1.745.947	-24.779	1.768.544	-31.448	1.720.999	-31.814

1.746.149	-24.780	1.768.732	-31.446	1.721.225	-31.815
1.746.337	-24.781	1.768.947	-31.444	1.721.380	-31.816
1.746.391	-24.783	1.769.151	-31.442	1.721.512	-31.817
1.746.570	-24.784	1.769.333	-31.440	1.721.660	-31.818
1.746.812	-24.786	1.769.514	-31.439	1.721.810	-31.819
1.746.991	-24.787	1.769.675	-31.437	1.722.043	-31.820
1.747.144	-24.789	1.769.827	-31.435	1.722.359	-31.821
1.747.309	-24.790	1.769.988	-31.433	1.722.793	-31.822
1.747.489	-24.792	1.770.405	-31.431	1.723.087	-31.823
1.747.695	-24.793	1.770.824	-31.429	1.723.177	-31.824
1.747.865	-24.795	1.770.954	-31.427	1.723.309	-31.825
1.748.018	-24.796	1.771.042	-31.425	1.723.373	-31.826
1.748.190	-24.798	1.771.106	-31.423	1.723.387	-31.827
1.748.447	-24.799	1.771.077	-31.421	1.723.461	-31.828
1.748.652	-24.801	1.771.068	-31.420	1.723.593	-31.829
1.748.752	-24.802	1.771.223	-31.418	1.723.676	-31.830
1.748.871	-24.804	1.771.380	-31.416	1.723.774	-31.831
1.749.027	-24.805	1.771.544	-31.414	1.723.885	-31.832
1.749.180	-24.807	1.771.747	-31.412	1.724.030	-31.833
1.749.325	-24.808	1.771.915	-31.410	1.724.252	-31.834
1.749.500	-24.810	1.772.050	-31.409	1.724.429	-31.835
1.749.668	-24.811	1.772.213	-31.407	1.724.608	-31.836
1.749.790	-24.813	1.772.401	-31.405	1.724.803	-31.837
1.749.948	-24.815	1.772.612	-31.403	1.724.930	-31.838
1.750.322	-24.816	1.772.803	-31.401	1.725.033	-31.839
1.750.757	-24.818	1.772.961	-31.400	1.725.195	-31.840
1.750.974	-24.819	1.773.140	-31.398	1.725.421	-31.841
1.751.059	-24.821	1.773.297	-31.396	1.725.679	-31.842
1.751.140	-24.822	1.773.423	-31.394	1.725.882	-31.843
1.751.189	-24.824	1.773.559	-31.393	1.726.008	-31.844

1.751.214	-24.826	1.773.750	-31.391	1.726.106	-31.845
1.751.272	-24.827	1.773.885	-31.389	1.726.241	-31.847
1.751.376	-24.829	1.773.958	-31.388	1.726.413	-31.848
1.751.505	-24.830	1.774.115	-31.386	1.726.516	-31.849
1.751.680	-24.832	1.774.328	-31.384	1.726.626	-31.850
1.751.855	-24.834	1.774.501	-31.383	1.726.774	-31.851
1.752.036	-24.835	1.774.661	-31.381	1.726.960	-31.852
1.752.242	-24.837	1.774.807	-31.379	1.727.168	-31.853
1.752.350	-24.839	1.775.020	-31.378	1.727.337	-31.854
1.752.529	-24.840	1.775.219	-31.376	1.727.424	-31.855
1.752.787	-24.842	1.775.313	-31.375	1.727.543	-31.857
1.752.977	-24.843	1.775.555	-31.373	1.727.720	-31.858
1.753.102	-24.845	1.775.768	-31.372	1.727.905	-31.859
1.753.177	-24.847	1.775.874	-31.370	1.728.129	-31.860
1.753.331	-24.848	1.776.084	-31.368	1.728.335	-31.861
1.753.532	-24.850	1.776.254	-31.367	1.728.512	-31.863
1.753.727	-24.852	1.776.353	-31.365	1.728.721	-31.864
1.753.844	-24.853	1.776.451	-31.364	1.728.904	-31.865
1.753.990	-24.855	1.776.611	-31.363	1.729.032	-31.866
1.754.100	-24.857	1.776.790	-31.361	1.729.066	-31.868
1.754.247	-24.858	1.776.982	-31.360	1.729.162	-31.869
1.754.538	-24.860	1.777.168	-31.358	1.729.301	-31.870
1.754.675	-24.862	1.777.375	-31.357	1.729.454	-31.872
1.754.832	-24.864	1.777.553	-31.355	1.729.684	-31.873
1.755.033	-24.865	1.777.686	-31.354	1.729.839	-31.874
1.755.226	-24.867	1.777.851	-31.353	1.729.982	-31.876
1.755.441	-24.869	1.778.001	-31.351	1.730.197	-31.877
1.755.575	-24.870	1.778.120	-31.350	1.730.417	-31.878
1.755.730	-24.872	1.778.277	-31.349	1.730.630	-31.880
1.755.902	-24.874	1.778.481	-31.348	1.730.797	-31.881

1.756.073	-24.875	1.778.685	-31.346	1.730.957	-31.883
1.756.169	-24.877	1.778.817	-31.345	1.731.151	-31.884
1.756.272	-24.879	1.778.949	-31.344	1.731.293	-31.886
1.756.441	-24.881	1.779.099	-31.343	1.731.391	-31.887
1.756.628	-24.882	1.779.274	-31.341	1.731.563	-31.889
1.756.770	-24.884	1.779.559	-31.340	1.731.651	-31.890
1.756.949	-24.886	1.779.751	-31.339	1.731.783	-31.892
1.757.079	-24.888	1.779.825	-31.338	1.732.034	-31.893
1.757.235	-24.889	1.779.928	-31.337	1.732.251	-31.895
1.757.462	-24.891	1.780.114	-31.336	1.732.323	-31.896
1.757.661	-24.893	1.780.313	-31.335	1.732.424	-31.898
1.757.816	-24.894	1.780.457	-31.334	1.732.630	-31.900
1.757.943	-24.896	1.780.566	-31.333	1.732.901	-31.901
1.758.064	-24.898	1.780.779	-31.332	1.733.149	-31.903
1.758.266	-24.900	1.781.040	-31.331	1.733.250	-31.904
1.758.458	-24.901	1.781.178	-31.330	1.733.351	-31.906
1.758.595	-24.903	1.781.313	-31.329	1.733.472	-31.908
1.758.661	-24.905	1.781.514	-31.328	1.733.640	-31.909
1.758.781	-24.907	1.781.691	-31.327	1.733.851	-31.911
1.758.983	-24.908	1.781.805	-31.326	1.734.012	-31.913
1.759.195	-24.910	1.781.915	-31.325	1.734.146	-31.915
1.759.411	-24.912	1.782.039	-31.324	1.734.339	-31.916
1.759.534	-24.914	1.782.202	-31.323	1.734.507	-31.918
1.759.697	-24.915	1.782.327	-31.322	1.734.626	-31.920
1.759.877	-24.917	1.782.415	-31.321	1.734.778	-31.921
1.760.031	-24.919	1.782.534	-31.320	1.734.957	-31.923
1.760.230	-24.921	1.782.672	-31.319	1.735.172	-31.925
1.760.365	-24.922	1.782.898	-31.319	1.735.331	-31.927
1.760.466	-24.924	1.783.136	-31.318	1.735.428	-31.928
1.760.582	-24.926	1.783.311	-31.317	1.735.556	-31.930

1.760.824	-24.927	1.783.539	-31.316	1.735.777	-31.932
1.761.006	-24.929	1.783.801	-31.315	1.736.014	-31.934
1.761.120	-24.931	1.784.014	-31.315	1.736.178	-31.935
1.761.279	-24.933	1.784.185	-31.314	1.736.266	-31.937
1.761.526	-24.934	1.784.417	-31.313	1.736.378	-31.939
1.761.695	-24.936	1.784.612	-31.312	1.736.584	-31.941
1.761.861	-24.938	1.784.689	-31.312	1.736.752	-31.942
1.761.933	-24.940	1.784.776	-31.311	1.736.917	-31.944
1.762.079	-24.941	1.784.874	-31.310	1.737.108	-31.946
1.762.345	-24.943	1.784.991	-31.310	1.737.280	-31.948
1.762.527	-24.945	1.785.087	-31.309	1.737.381	-31.949
1.762.596	-24.946	1.785.251	-31.308	1.737.513	-31.951
1.762.695	-24.948	1.785.453	-31.308	1.737.673	-31.953
1.762.811	-24.950	1.785.634	-31.307	1.737.823	-31.955
1.762.963	-24.951	1.785.842	-31.306	1.738.024	-31.956
1.763.121	-24.953	1.786.037	-31.306	1.738.161	-31.958
1.763.297	-24.955	1.786.191	-31.305	1.738.279	-31.960
1.763.490	-24.957	1.786.367	-31.304	1.738.463	-31.962
1.763.672	-24.958	1.786.527	-31.304	1.738.647	-31.963
1.763.826	-24.960	1.786.703	-31.303	1.738.857	-31.965
1.764.095	-24.962	1.786.909	-31.303	1.739.050	-31.967
1.764.350	-24.963	1.787.095	-31.302	1.739.198	-31.968
1.764.445	-24.965	1.787.262	-31.302	1.739.453	-31.970
1.764.583	-24.967	1.787.385	-31.301	1.739.749	-31.972
1.764.847	-24.968	1.787.520	-31.301	1.739.904	-31.973
1.765.070	-24.970	1.787.708	-31.300	1.739.982	-31.975
1.765.262	-24.972	1.787.919	-31.300	1.740.126	-31.977
1.765.423	-24.973	1.788.148	-31.299	1.740.303	-31.978
1.765.538	-24.975	1.788.306	-31.299	1.740.445	-31.980
1.765.672	-24.977	1.788.456	-31.298	1.740.591	-31.982

1.765.773	-24.978	1.788.575	-31.298	1.740.768	-31.983
1.765.824	-24.980	1.788.674	-31.297	1.740.905	-31.985
1.765.894	-24.981	1.788.851	-31.297	1.741.039	-31.986
1.766.048	-24.983	1.789.025	-31.297	1.741.142	-31.988
1.766.263	-24.985	1.789.223	-31.296	1.741.217	-31.989
1.766.458	-24.986	1.789.420	-31.296	1.741.386	-31.991
1.766.637	-24.988	1.789.612	-31.296	1.741.535	-31.992
1.766.767	-24.990	1.789.747	-31.295	1.741.665	-31.994
1.766.987	-24.991	1.789.845	-31.295	1.741.821	-31.995
1.767.289	-24.993	1.790.042	-31.295	1.742.036	-31.997
1.767.533	-24.994	1.790.253	-31.294	1.742.289	-31.998
1.767.630	-24.996	1.790.428	-31.294	1.742.561	-32.000
1.767.728	-24.998	1.790.611	-31.294	1.742.755	-32.001
1.767.892	-24.999	1.790.730	-31.294	1.742.897	-32.003
1.768.013	-25.001	1.790.873	-31.293	1.743.089	-32.004
1.768.201	-25.002	1.791.086	-31.293	1.743.226	-32.006
1.768.396	-25.004	1.791.257	-31.293	1.743.362	-32.007
1.768.540	-25.005	1.791.427	-31.293	1.743.510	-32.008
1.768.699	-25.007	1.791.563	-31.293	1.743.696	-32.010
1.768.909	-25.008	1.791.718	-31.292	1.743.885	-32.011
1.769.072	-25.010	1.791.868	-31.292	1.744.052	-32.012
1.769.247	-25.011	1.792.092	-31.292	1.744.230	-32.014
1.769.460	-25.013	1.792.471	-31.292	1.744.323	-32.015
1.769.608	-25.015	1.792.834	-31.292	1.744.469	-32.016
1.769.771	-25.016	1.793.047	-31.292	1.744.601	-32.017
1.769.915	-25.018	1.793.121	-31.292	1.744.805	-32.019
1.770.047	-25.019	1.793.156	-31.292	1.745.020	-32.020
1.770.224	-25.021	1.793.206	-31.292	1.745.127	-32.021
1.770.475	-25.022	1.793.351	-31.292	1.745.217	-32.022
1.770.652	-25.023	1.793.452	-31.292	1.745.439	-32.024

1.770.743	-25.025	1.793.495	-31.292	1.745.695	-32.025
1.770.910	-25.026	1.793.605	-31.292	1.745.788	-32.026
1.771.102	-25.028	1.793.799	-31.292	1.745.894	-32.027
1.771.252	-25.029	1.794.017	-31.292	1.746.122	-32.028
1.771.424	-25.031	1.794.178	-31.292	1.746.366	-32.030
1.771.658	-25.032	1.794.332	-31.292	1.746.539	-32.031
1.771.868	-25.034	1.794.505	-31.292	1.746.635	-32.032
1.772.021	-25.035	1.794.693	-31.292	1.746.760	-32.033
1.772.197	-25.037	1.794.855	-31.292	1.746.973	-32.034
1.772.350	-25.038	1.795.002	-31.293	1.747.135	-32.035
1.772.464	-25.039	1.795.154	-31.293	1.747.271	-32.036
1.772.542	-25.041	1.795.351	-31.293	1.747.464	-32.037
1.772.699	-25.042	1.795.493	-31.293	1.747.652	-32.038
1.773.176	-25.044	1.795.614	-31.293	1.747.800	-32.040
1.773.615	-25.045	1.795.755	-31.294	1.747.932	-32.041
1.773.759	-25.046	1.795.909	-31.294	1.748.089	-32.042
1.773.815	-25.048	1.796.060	-31.294	1.748.308	-32.043
1.773.821	-25.049	1.796.243	-31.294	1.748.503	-32.044
1.773.873	-25.050	1.796.391	-31.295	1.748.620	-32.045
1.773.992	-25.052	1.796.523	-31.295	1.748.752	-32.046
1.774.059	-25.053	1.796.671	-31.295	1.748.909	-32.047
1.774.135	-25.054	1.796.889	-31.296	1.749.010	-32.048
1.774.310	-25.056	1.797.045	-31.296	1.749.165	-32.049
1.774.431	-25.057	1.797.238	-31.297	1.749.554	-32.050
1.774.588	-25.058	1.797.482	-31.297	1.749.959	-32.051
1.774.816	-25.060	1.797.710	-31.297	1.750.277	-32.052
1.775.004	-25.061	1.797.838	-31.298	1.750.421	-32.053
1.775.199	-25.062	1.797.897	-31.298	1.750.434	-32.054
1.775.379	-25.063	1.798.078	-31.299	1.750.537	-32.055
1.775.549	-25.065	1.798.264	-31.299	1.750.636	-32.056

1.775.678	-25.066	1.798.372	-31.300	1.750.730	-32.057
1.775.844	-25.067	1.798.495	-31.300	1.750.768	-32.058
1.776.066	-25.068	1.798.662	-31.301	1.750.811	-32.059
1.776.223	-25.070	1.798.893	-31.302	1.750.909	-32.060
1.776.369	-25.071	1.799.091	-31.302	1.751.046	-32.061
1.776.505	-25.072	1.799.225	-31.303	1.751.227	-32.062
1.776.592	-25.073	1.799.379	-31.303	1.751.432	-32.063
1.776.761	-25.074	1.799.545	-31.304	1.751.626	-32.064
1.776.984	-25.076	1.799.702	-31.305	1.751.777	-32.065
1.777.193	-25.077	1.799.919	-31.306	1.751.924	-32.066
1.777.379	-25.078	1.800.087	-31.306	1.752.132	-32.067
1.777.444	-25.079	1.800.148	-31.307	1.752.321	-32.068
1.777.592	-25.080	1.800.284	-31.308	1.752.439	-32.069
1.777.876	-25.081	1.800.461	-31.308	1.752.587	-32.070
1.778.076	-25.083	1.800.679	-31.309	1.752.711	-32.071
1.778.199	-25.084	1.800.851	-31.310	1.752.906	-32.072
1.778.358	-25.085	1.800.977	-31.311	1.753.150	-32.073
1.778.550	-25.086	1.801.183	-31.312	1.753.351	-32.074
1.778.718	-25.087	1.801.368	-31.313	1.753.519	-32.075
1.778.844	-25.088	1.801.510	-31.313	1.753.683	-32.076
1.779.010	-25.089	1.801.676	-31.314	1.753.833	-32.077
1.779.281	-25.090	1.801.870	-31.315	1.753.970	-32.078
1.779.500	-25.091	1.802.007	-31.316	1.754.115	-32.079
1.779.583	-25.092	1.802.085	-31.317	1.754.216	-32.080
1.779.688	-25.093	1.802.300	-31.318	1.754.384	-32.081
1.779.892	-25.094	1.802.518	-31.319	1.754.525	-32.082
1.780.069	-25.096	1.802.650	-31.320	1.754.655	-32.084
1.780.224	-25.097	1.802.793	-31.321	1.754.870	-32.085
1.780.331	-25.098	1.803.046	-31.322	1.755.060	-32.086
1.780.477	-25.099	1.803.277	-31.323	1.755.210	-32.087

1.780.667	-25.100	1.803.414	-31.324	1.755.356	-32.088
1.780.807	-25.100	1.803.508	-31.325	1.755.510	-32.090
1.780.977	-25.101	1.803.651	-31.326	1.755.710	-32.091
1.781.180	-25.102	1.803.855	-31.327	1.755.950	-32.092
1.781.317	-25.103	1.803.981	-31.328	1.756.169	-32.093
1.781.387	-25.104	1.804.070	-31.329	1.756.326	-32.095
1.781.516	-25.105	1.804.218	-31.330	1.756.490	-32.096
1.781.722	-25.106	1.804.377	-31.331	1.756.644	-32.097
1.782.027	-25.107	1.804.518	-31.332	1.756.761	-32.099
1.782.260	-25.108	1.804.623	-31.333	1.756.886	-32.100
1.782.352	-25.109	1.804.823	-31.334	1.757.036	-32.101
1.782.473	-25.110	1.805.022	-31.335	1.757.177	-32.103
1.782.688	-25.111	1.805.183	-31.337	1.757.233	-32.104
1.782.836	-25.111	1.805.394	-31.338	1.757.413	-32.105
1.782.959	-25.112	1.805.600	-31.339	1.757.652	-32.107
1.783.105	-25.113	1.805.804	-31.340	1.757.764	-32.108
1.783.264	-25.114	1.806.062	-31.341	1.757.915	-32.110
1.783.425	-25.115	1.806.203	-31.342	1.758.064	-32.111
1.783.577	-25.116	1.806.304	-31.343	1.758.234	-32.113
1.783.772	-25.116	1.806.588	-31.344	1.758.479	-32.114
1.783.960	-25.117	1.806.790	-31.345	1.758.651	-32.116
1.784.148	-25.118	1.806.915	-31.346	1.758.783	-32.117
1.784.333	-25.119	1.807.052	-31.347	1.758.913	-32.119
1.784.456	-25.120	1.807.146	-31.348	1.759.106	-32.120
1.784.604	-25.120	1.807.272	-31.349	1.759.276	-32.122
1.784.801	-25.121	1.807.462	-31.350	1.759.420	-32.124
1.784.937	-25.122	1.807.614	-31.351	1.759.525	-32.125
1.785.123	-25.123	1.807.758	-31.352	1.759.688	-32.127
1.785.238	-25.123	1.807.948	-31.353	1.759.899	-32.129
1.785.271	-25.124	1.808.037	-31.354	1.760.074	-32.130

1.785.468	-25.125	1.808.219	-31.355	1.760.285	-32.132
1.785.643	-25.126	1.808.432	-31.356	1.760.450	-32.134
1.785.739	-25.126	1.808.566	-31.357	1.760.592	-32.135
1.785.887	-25.127	1.808.750	-31.358	1.760.793	-32.137
1.786.032	-25.128	1.808.994	-31.359	1.760.981	-32.139
1.786.227	-25.128	1.809.205	-31.360	1.761.149	-32.141
1.786.496	-25.129	1.809.330	-31.361	1.761.335	-32.142
1.786.703	-25.130	1.809.502	-31.362	1.761.508	-32.144
1.786.933	-25.130	1.809.637	-31.363	1.761.648	-32.146
1.787.189	-25.131	1.809.785	-31.364	1.761.835	-32.148
1.787.370	-25.132	1.809.975	-31.365	1.761.985	-32.149
1.787.554	-25.132	1.810.130	-31.366	1.762.113	-32.151
1.787.719	-25.133	1.810.282	-31.367	1.762.278	-32.153
1.787.809	-25.134	1.810.419	-31.368	1.762.482	-32.155
1.787.932	-25.134	1.810.582	-31.368	1.762.730	-32.157
1.788.053	-25.135	1.810.746	-31.369	1.762.856	-32.159
1.788.172	-25.135	1.810.950	-31.370	1.762.939	-32.160
1.788.362	-25.136	1.811.153	-31.371	1.763.093	-32.162
1.788.532	-25.137	1.811.279	-31.372	1.763.273	-32.164
1.788.691	-25.137	1.811.474	-31.373	1.763.445	-32.166
1.788.797	-25.138	1.811.700	-31.373	1.763.595	-32.168
1.788.898	-25.138	1.811.828	-31.374	1.763.743	-32.170
1.789.072	-25.139	1.811.935	-31.375	1.763.876	-32.172
1.789.207	-25.140	1.812.050	-31.376	1.764.017	-32.174
1.789.363	-25.140	1.812.256	-31.376	1.764.135	-32.175
1.789.612	-25.141	1.812.477	-31.377	1.764.301	-32.177
1.789.854	-25.141	1.812.614	-31.378	1.764.493	-32.179
1.790.031	-25.142	1.812.800	-31.379	1.764.621	-32.181
1.790.186	-25.142	1.812.984	-31.379	1.764.798	-32.183
1.790.293	-25.143	1.813.141	-31.380	1.764.968	-32.185

1.790.468	-25.144	1.813.298	-31.381	1.765.076	-32.187
1.790.687	-25.144	1.813.423	-31.381	1.765.186	-32.188
1.790.836	-25.145	1.813.593	-31.382	1.765.329	-32.190
1.791.001	-25.145	1.813.781	-31.383	1.765.510	-32.192
1.791.171	-25.146	1.813.806	-31.383	1.765.744	-32.194
1.791.339	-25.146	1.814.088	-31.384	1.765.997	-32.196
1.791.526	-25.147	1.814.675	-31.384	1.766.169	-32.198
1.791.712	-25.147	1.815.009	-31.385	1.766.288	-32.200
1.791.924	-25.148	1.815.114	-31.386	1.766.487	-32.201
1.792.068	-25.148	1.815.188	-31.386	1.766.721	-32.203
1.792.206	-25.149	1.815.273	-31.387	1.766.933	-32.205
1.792.384	-25.150	1.815.336	-31.387	1.767.085	-32.207
1.792.567	-25.150	1.815.389	-31.388	1.767.235	-32.209
1.792.728	-25.151	1.815.448	-31.388	1.767.406	-32.210
1.792.820	-25.151	1.815.531	-31.389	1.767.531	-32.212
1.792.881	-25.152	1.815.663	-31.389	1.767.641	-32.214
1.793.083	-25.152	1.815.860	-31.390	1.767.775	-32.216
1.793.364	-25.153	1.816.059	-31.390	1.767.934	-32.217
1.793.541	-25.153	1.816.236	-31.391	1.768.037	-32.219
1.793.714	-25.154	1.816.431	-31.391	1.768.141	-32.221
1.793.965	-25.154	1.816.629	-31.392	1.768.400	-32.223
1.794.149	-25.155	1.816.796	-31.392	1.768.633	-32.224
1.794.249	-25.155	1.816.975	-31.392	1.768.743	-32.226
1.794.408	-25.156	1.817.132	-31.393	1.768.846	-32.228
1.794.603	-25.156	1.817.287	-31.393	1.769.039	-32.229
1.794.736	-25.157	1.817.460	-31.394	1.769.218	-32.231
1.794.841	-25.157	1.817.621	-31.394	1.769.417	-32.233
1.794.973	-25.158	1.817.762	-31.394	1.769.662	-32.234
1.795.107	-25.158	1.817.917	-31.395	1.769.809	-32.236
1.795.343	-25.159	1.818.118	-31.395	1.769.973	-32.238

1.795.826	-25.159	1.818.199	-31.396	1.770.099	-32.239
1.796.246	-25.160	1.818.317	-31.396	1.770.278	-32.241
1.796.376	-25.160	1.818.514	-31.396	1.770.556	-32.242
1.796.433	-25.161	1.818.727	-31.397	1.770.766	-32.244
1.796.546	-25.161	1.818.956	-31.397	1.770.898	-32.245
1.796.597	-25.162	1.819.106	-31.398	1.770.958	-32.247
1.796.590	-25.163	1.819.258	-31.398	1.771.102	-32.248
1.796.631	-25.163	1.819.422	-31.398	1.771.310	-32.250
1.796.720	-25.164	1.819.567	-31.399	1.771.478	-32.251
1.796.917	-25.164	1.819.754	-31.399	1.771.673	-32.253
1.797.083	-25.165	1.819.928	-31.399	1.771.844	-32.254
1.797.290	-25.165	1.820.067	-31.400	1.771.936	-32.256
1.797.525	-25.166	1.820.237	-31.400	1.772.043	-32.257
1.797.661	-25.166	1.820.372	-31.400	1.772.253	-32.259
1.797.827	-25.167	1.820.513	-31.401	1.772.460	-32.260
1.797.957	-25.167	1.820.704	-31.401	1.772.645	-32.262
1.798.112	-25.168	1.820.885	-31.401	1.772.800	-32.263
1.798.297	-25.168	1.821.030	-31.402	1.772.968	-32.264
1.798.443	-25.169	1.821.187	-31.402	1.773.186	-32.266
1.798.622	-25.169	1.821.359	-31.403	1.773.342	-32.267
1.798.821	-25.170	1.821.478	-31.403	1.773.486	-32.268
1.798.934	-25.170	1.821.599	-31.403	1.773.644	-32.270
1.799.095	-25.171	1.821.808	-31.404	1.773.754	-32.271
1.799.258	-25.171	1.822.023	-31.404	1.773.889	-32.272
1.799.348	-25.172	1.822.128	-31.404	1.774.084	-32.274
1.799.529	-25.172	1.822.249	-31.405	1.774.276	-32.275
1.799.740	-25.173	1.822.428	-31.405	1.774.447	-32.276
1.799.928	-25.174	1.822.646	-31.406	1.774.621	-32.278
1.800.103	-25.174	1.822.851	-31.406	1.774.769	-32.279
1.800.246	-25.175	1.823.051	-31.406	1.774.997	-32.280

1.800.367	-25.175	1.823.203	-31.407	1.775.186	-32.281
1.800.611	-25.176	1.823.308	-31.407	1.775.253	-32.283
1.800.806	-25.176	1.823.494	-31.408	1.775.332	-32.284
1.800.957	-25.177	1.823.738	-31.408	1.775.535	-32.285
1.801.165	-25.177	1.823.938	-31.409	1.775.777	-32.286
1.801.295	-25.178	1.824.068	-31.409	1.775.869	-32.288
1.801.431	-25.178	1.824.202	-31.409	1.776.030	-32.289
1.801.599	-25.179	1.824.344	-31.410	1.776.234	-32.290
1.801.758	-25.180	1.824.462	-31.410	1.776.447	-32.291
1.801.900	-25.180	1.824.635	-31.411	1.776.810	-32.292
1.802.085	-25.181	1.824.879	-31.411	1.777.231	-32.294
1.802.264	-25.181	1.825.042	-31.412	1.777.529	-32.295
1.802.449	-25.182	1.825.195	-31.412	1.777.630	-32.296
1.802.634	-25.182	1.825.329	-31.413	1.777.699	-32.297
1.802.794	-25.183	1.825.423	-31.413	1.777.721	-32.298
1.802.944	-25.183	1.825.598	-31.414	1.777.753	-32.300
1.803.092	-25.184	1.825.773	-31.414	1.777.805	-32.301
1.803.248	-25.185	1.825.929	-31.415	1.777.881	-32.302
1.803.428	-25.185	1.826.049	-31.416	1.778.069	-32.303
1.803.602	-25.186	1.826.161	-31.416	1.778.210	-32.304
1.803.691	-25.186	1.826.377	-31.417	1.778.369	-32.305
1.803.850	-25.187	1.826.582	-31.417	1.778.542	-32.307
1.804.072	-25.187	1.826.691	-31.418	1.778.636	-32.308
1.804.220	-25.188	1.826.808	-31.418	1.778.824	-32.309
1.804.370	-25.189	1.826.958	-31.419	1.779.063	-32.310
1.804.574	-25.189	1.827.142	-31.420	1.779.220	-32.311
1.804.774	-25.190	1.827.352	-31.420	1.779.332	-32.312
1.804.950	-25.190	1.827.553	-31.421	1.779.496	-32.314
1.805.105	-25.191	1.827.760	-31.421	1.779.664	-32.315
1.805.233	-25.191	1.827.921	-31.422	1.779.809	-32.316

1.805.410	-25.192	1.828.098	-31.423	1.780.006	-32.317
1.805.602	-25.193	1.828.322	-31.423	1.780.165	-32.318
1.805.751	-25.193	1.828.508	-31.424	1.780.303	-32.319
1.805.851	-25.194	1.828.689	-31.425	1.780.459	-32.321
1.805.947	-25.194	1.828.864	-31.425	1.780.631	-32.322
1.806.140	-25.195	1.828.996	-31.426	1.780.842	-32.323
1.806.328	-25.195	1.829.160	-31.427	1.780.984	-32.324
1.806.487	-25.196	1.829.334	-31.427	1.781.070	-32.326
1.806.702	-25.197	1.829.393	-31.428	1.781.236	-32.327
1.806.871	-25.197	1.829.440	-31.429	1.781.445	-32.328
1.807.029	-25.198	1.829.622	-31.429	1.781.640	-32.330
1.807.166	-25.198	1.829.809	-31.430	1.781.745	-32.331
1.807.339	-25.199	1.829.980	-31.431	1.781.880	-32.332
1.807.529	-25.200	1.830.184	-31.432	1.782.146	-32.333
1.807.691	-25.200	1.830.428	-31.432	1.782.361	-32.335
1.807.820	-25.201	1.830.600	-31.433	1.782.538	-32.336
1.807.919	-25.201	1.830.755	-31.434	1.782.693	-32.337
1.808.037	-25.202	1.830.952	-31.435	1.782.843	-32.339
1.808.203	-25.202	1.831.087	-31.435	1.782.986	-32.340
1.808.293	-25.203	1.831.245	-31.436	1.783.156	-32.342
1.808.432	-25.204	1.831.452	-31.437	1.783.295	-32.343
1.808.638	-25.204	1.831.606	-31.438	1.783.484	-32.345
1.808.813	-25.205	1.831.720	-31.438	1.783.605	-32.346
1.809.063	-25.205	1.831.888	-31.439	1.783.718	-32.347
1.809.337	-25.206	1.832.088	-31.440	1.783.929	-32.349
1.809.554	-25.207	1.832.249	-31.441	1.784.059	-32.350
1.809.704	-25.207	1.832.403	-31.441	1.784.200	-32.352
1.809.915	-25.208	1.832.621	-31.442	1.784.406	-32.353
1.810.155	-25.208	1.832.784	-31.443	1.784.554	-32.355
1.810.293	-25.209	1.832.901	-31.444	1.784.635	-32.357

1.810.390	-25.210	1.833.047	-31.445	1.784.791	-32.358
1.810.547	-25.210	1.833.226	-31.446	1.785.020	-32.360
1.810.717	-25.211	1.833.461	-31.446	1.785.161	-32.361
1.810.853	-25.211	1.833.604	-31.447	1.785.379	-32.363
1.810.941	-25.212	1.833.754	-31.448	1.785.544	-32.365
1.811.046	-25.213	1.833.900	-31.449	1.785.719	-32.366
1.811.171	-25.213	1.834.066	-31.450	1.785.902	-32.368
1.811.357	-25.214	1.834.294	-31.451	1.786.046	-32.370
1.811.573	-25.215	1.834.433	-31.452	1.786.138	-32.371
1.811.769	-25.215	1.834.547	-31.452	1.786.250	-32.373
1.811.964	-25.216	1.834.706	-31.453	1.786.366	-32.375
1.812.099	-25.216	1.834.930	-31.454	1.786.530	-32.377
1.812.287	-25.217	1.835.141	-31.455	1.786.747	-32.378
1.812.495	-25.218	1.835.295	-31.456	1.786.954	-32.380
1.812.663	-25.218	1.835.423	-31.457	1.787.085	-32.382
1.812.881	-25.219	1.835.557	-31.458	1.787.263	-32.384
1.812.972	-25.219	1.835.714	-31.459	1.787.410	-32.386
1.813.082	-25.220	1.835.867	-31.460	1.787.625	-32.387
1.813.327	-25.221	1.836.057	-31.461	1.787.854	-32.389
1.813.506	-25.221	1.836.471	-31.462	1.787.995	-32.391
1.813.625	-25.222	1.836.933	-31.463	1.788.149	-32.393
1.813.772	-25.223	1.837.153	-31.464	1.788.365	-32.395
1.813.938	-25.223	1.837.200	-31.465	1.788.517	-32.397
1.814.073	-25.224	1.837.243	-31.466	1.788.622	-32.399
1.814.276	-25.225	1.837.276	-31.467	1.788.774	-32.400
1.814.491	-25.225	1.837.253	-31.468	1.788.927	-32.402
1.814.669	-25.226	1.837.372	-31.469	1.789.106	-32.404
1.814.827	-25.226	1.837.574	-31.470	1.789.292	-32.406
1.814.953	-25.227	1.837.681	-31.471	1.789.496	-32.408
1.815.123	-25.228	1.837.791	-31.472	1.789.603	-32.410

1.815.325	-25.228	1.837.966	-31.473	1.789.758	-32.412
1.815.497	-25.229	1.838.147	-31.474	1.789.971	-32.414
1.815.616	-25.230	1.838.358	-31.475	1.790.121	-32.416
1.815.779	-25.230	1.838.560	-31.476	1.790.293	-32.418
1.815.965	-25.231	1.838.709	-31.477	1.790.484	-32.420
1.816.113	-25.232	1.838.895	-31.478	1.790.663	-32.422
1.816.234	-25.232	1.839.101	-31.479	1.790.797	-32.424
1.816.375	-25.233	1.839.281	-31.481	1.790.916	-32.426
1.816.590	-25.234	1.839.473	-31.482	1.791.115	-32.428
1.816.861	-25.234	1.839.570	-31.483	1.791.234	-32.429
1.817.057	-25.235	1.839.668	-31.484	1.791.378	-32.431
1.817.139	-25.236	1.839.837	-31.485	1.791.588	-32.433
1.817.338	-25.236	1.840.009	-31.486	1.791.673	-32.435
1.817.565	-25.237	1.840.181	-31.488	1.791.850	-32.437
1.817.652	-25.238	1.840.331	-31.489	1.791.989	-32.439
1.817.776	-25.238	1.840.454	-31.490	1.792.117	-32.441
1.818.042	-25.239	1.840.631	-31.491	1.792.292	-32.443
1.818.457	-25.240	1.840.746	-31.493	1.792.410	-32.445
1.818.792	-25.240	1.840.916	-31.494	1.792.561	-32.447
1.818.947	-25.241	1.841.181	-31.495	1.792.818	-32.449
1.819.077	-25.242	1.841.353	-31.497	1.793.062	-32.450
1.819.104	-25.242	1.841.485	-31.498	1.793.246	-32.452
1.819.039	-25.243	1.841.702	-31.499	1.793.434	-32.454
1.819.102	-25.244	1.841.868	-31.501	1.793.555	-32.456
1.819.241	-25.244	1.841.969	-31.502	1.793.703	-32.458
1.819.431	-25.245	1.842.161	-31.503	1.793.954	-32.460
1.819.659	-25.246	1.842.318	-31.505	1.794.204	-32.461
1.819.783	-25.247	1.842.402	-31.506	1.794.395	-32.463
1.819.921	-25.247	1.842.520	-31.508	1.794.514	-32.465
1.820.083	-25.248	1.842.746	-31.509	1.794.639	-32.467

1.820.228	-25.249	1.842.937	-31.511	1.794.780	-32.469
1.820.414	-25.249	1.843.071	-31.512	1.794.899	-32.470
1.820.618	-25.250	1.843.201	-31.513	1.795.014	-32.472
1.820.734	-25.251	1.843.392	-31.515	1.795.210	-32.474
1.820.925	-25.251	1.843.588	-31.517	1.795.397	-32.476
1.821.122	-25.252	1.843.730	-31.518	1.795.501	-32.477
1.821.288	-25.253	1.843.875	-31.520	1.795.650	-32.479
1.821.478	-25.253	1.844.010	-31.521	1.795.813	-32.481
1.821.570	-25.254	1.844.207	-31.523	1.795.983	-32.482
1.821.687	-25.255	1.844.363	-31.524	1.796.140	-32.484
1.821.895	-25.255	1.844.538	-31.526	1.796.277	-32.485
1.822.130	-25.256	1.844.632	-31.528	1.796.431	-32.487
1.822.238	-25.257	1.844.789	-31.529	1.796.599	-32.489
1.822.316	-25.258	1.845.126	-31.531	1.796.768	-32.490
1.822.464	-25.258	1.845.334	-31.533	1.797.014	-32.492
1.822.646	-25.259	1.845.484	-31.534	1.797.224	-32.493
1.822.861	-25.260	1.845.607	-31.536	1.797.376	-32.495
1.823.074	-25.260	1.845.762	-31.538	1.797.524	-32.496
1.823.241	-25.261	1.845.972	-31.540	1.797.626	-32.498
1.823.392	-25.262	1.846.163	-31.541	1.797.757	-32.499
1.823.568	-25.262	1.846.292	-31.543	1.797.896	-32.500
1.823.741	-25.263	1.846.467	-31.545	1.798.074	-32.502
1.823.896	-25.264	1.846.637	-31.547	1.798.297	-32.503
1.824.075	-25.265	1.846.747	-31.548	1.798.508	-32.504
1.824.272	-25.265	1.846.937	-31.550	1.798.708	-32.506
1.824.406	-25.266	1.847.101	-31.552	1.798.920	-32.507
1.824.576	-25.267	1.847.271	-31.554	1.799.072	-32.508
1.824.740	-25.267	1.847.482	-31.556	1.799.222	-32.510
1.824.895	-25.268	1.847.621	-31.558	1.799.388	-32.511
1.825.058	-25.269	1.847.721	-31.559	1.799.563	-32.512

1.825.235	-25.269	1.847.876	-31.561	1.799.698	-32.513
1.825.341	-25.270	1.848.114	-31.563	1.799.834	-32.515
1.825.421	-25.271	1.848.257	-31.565	1.800.020	-32.516
1.825.650	-25.271	1.848.304	-31.567	1.800.177	-32.517
1.825.903	-25.272	1.848.391	-31.569	1.800.385	-32.518
1.826.057	-25.273	1.848.522	-31.571	1.800.580	-32.519
1.826.185	-25.274	1.848.689	-31.573	1.800.730	-32.520
1.826.344	-25.274	1.848.864	-31.575	1.800.928	-32.521
1.826.445	-25.275	1.848.954	-31.576	1.801.082	-32.522
1.826.586	-25.276	1.849.124	-31.578	1.801.161	-32.524
1.826.781	-25.276	1.849.368	-31.580	1.801.335	-32.525
1.827.000	-25.277	1.849.592	-31.582	1.801.568	-32.526
1.827.278	-25.278	1.849.807	-31.584	1.801.738	-32.527
1.827.451	-25.278	1.850.027	-31.586	1.801.857	-32.528
1.827.502	-25.279	1.850.271	-31.588	1.802.039	-32.529
1.827.592	-25.280	1.850.470	-31.590	1.802.262	-32.529
1.827.760	-25.281	1.850.565	-31.592	1.802.471	-32.530
1.827.941	-25.281	1.850.710	-31.594	1.802.641	-32.531
1.828.154	-25.282	1.850.902	-31.596	1.802.751	-32.532
1.828.387	-25.283	1.851.012	-31.598	1.802.865	-32.533
1.828.530	-25.283	1.851.118	-31.600	1.803.019	-32.534
1.828.658	-25.284	1.851.212	-31.601	1.803.165	-32.535
1.828.819	-25.285	1.851.367	-31.603	1.803.315	-32.536
1.828.954	-25.285	1.851.543	-31.605	1.803.436	-32.536
1.829.140	-25.286	1.851.700	-31.607	1.803.685	-32.537
1.829.366	-25.287	1.851.866	-31.609	1.804.164	-32.538
1.829.548	-25.288	1.852.077	-31.611	1.804.547	-32.539
1.829.729	-25.288	1.852.305	-31.613	1.804.720	-32.540
1.829.881	-25.289	1.852.466	-31.615	1.804.738	-32.540
1.829.973	-25.290	1.852.664	-31.617	1.804.799	-32.541

1.830.105	-25.290	1.852.854	-31.619	1.804.931	-32.542
1.830.257	-25.291	1.853.011	-31.621	1.805.027	-32.543
1.830.399	-25.292	1.853.178	-31.622	1.805.051	-32.543
1.830.484	-25.292	1.853.394	-31.624	1.805.125	-32.544
1.830.580	-25.293	1.853.546	-31.626	1.805.246	-32.545
1.830.755	-25.294	1.853.698	-31.628	1.805.425	-32.546
1.830.958	-25.295	1.853.876	-31.630	1.805.596	-32.546
1.831.162	-25.295	1.854.043	-31.632	1.805.670	-32.547
1.831.337	-25.296	1.854.213	-31.634	1.805.797	-32.548
1.831.451	-25.297	1.854.388	-31.635	1.805.997	-32.548
1.831.611	-25.297	1.854.635	-31.637	1.806.243	-32.549
1.831.889	-25.298	1.854.820	-31.639	1.806.416	-32.550
1.832.143	-25.299	1.854.975	-31.641	1.806.503	-32.551
1.832.334	-25.300	1.855.132	-31.643	1.806.610	-32.551
1.832.484	-25.300	1.855.229	-31.645	1.806.893	-32.552
1.832.677	-25.301	1.855.350	-31.646	1.807.177	-32.553
1.832.872	-25.302	1.855.517	-31.648	1.807.296	-32.553
1.832.977	-25.303	1.855.708	-31.650	1.807.421	-32.554
1.833.116	-25.303	1.855.914	-31.652	1.807.650	-32.555
1.833.317	-25.304	1.856.091	-31.654	1.807.885	-32.555
1.833.436	-25.305	1.856.227	-31.655	1.807.995	-32.556
1.833.526	-25.305	1.856.387	-31.657	1.808.105	-32.557
1.833.696	-25.306	1.856.541	-31.659	1.808.203	-32.558
1.833.855	-25.307	1.856.660	-31.661	1.808.346	-32.558
1.834.005	-25.308	1.856.841	-31.662	1.808.541	-32.559
1.834.207	-25.308	1.857.070	-31.664	1.808.721	-32.560
1.834.384	-25.309	1.857.287	-31.666	1.808.893	-32.561
1.834.583	-25.310	1.857.482	-31.667	1.808.983	-32.561
1.834.825	-25.311	1.857.612	-31.669	1.809.113	-32.562
1.834.957	-25.311	1.857.740	-31.671	1.809.319	-32.563

1.835.047	-25.312	1.857.867	-31.672	1.809.538	-32.564
1.835.233	-25.313	1.858.006	-31.674	1.809.722	-32.565
1.835.446	-25.314	1.858.212	-31.676	1.809.859	-32.565
1.835.616	-25.315	1.858.414	-31.677	1.810.042	-32.566
1.835.771	-25.315	1.858.756	-31.679	1.810.314	-32.567
1.835.925	-25.316	1.859.093	-31.681	1.810.475	-32.568
1.836.134	-25.317	1.859.276	-31.682	1.810.549	-32.569
1.836.330	-25.318	1.859.337	-31.684	1.810.670	-32.570
1.836.458	-25.318	1.859.328	-31.685	1.810.855	-32.571
1.836.619	-25.319	1.859.343	-31.687	1.811.008	-32.572
1.836.777	-25.320	1.859.473	-31.688	1.811.172	-32.573
1.836.942	-25.321	1.859.613	-31.690	1.811.375	-32.574
1.837.155	-25.322	1.859.743	-31.692	1.811.516	-32.575
1.837.343	-25.322	1.859.904	-31.693	1.811.635	-32.576
1.837.455	-25.323	1.860.031	-31.695	1.811.803	-32.577
1.837.603	-25.324	1.860.143	-31.696	1.811.931	-32.578
1.837.818	-25.325	1.860.369	-31.698	1.812.081	-32.579
1.837.946	-25.326	1.860.652	-31.699	1.812.309	-32.580
1.838.053	-25.327	1.860.855	-31.701	1.812.495	-32.581
1.838.246	-25.327	1.861.021	-31.702	1.812.682	-32.582
1.838.461	-25.328	1.861.194	-31.704	1.812.865	-32.583
1.838.649	-25.329	1.861.367	-31.705	1.813.031	-32.585
1.838.808	-25.330	1.861.523	-31.707	1.813.219	-32.586
1.838.940	-25.331	1.861.656	-31.708	1.813.383	-32.587
1.839.126	-25.332	1.861.816	-31.709	1.813.436	-32.588
1.839.332	-25.332	1.861.926	-31.711	1.813.535	-32.589
1.839.458	-25.333	1.862.038	-31.712	1.813.754	-32.591
1.839.581	-25.334	1.862.226	-31.714	1.813.936	-32.592
1.839.789	-25.335	1.862.379	-31.715	1.814.095	-32.593
1.839.975	-25.336	1.862.515	-31.717	1.814.299	-32.595

1.840.094	-25.337	1.862.674	-31.718	1.814.496	-32.596
1.840.155	-25.338	1.862.816	-31.719	1.814.619	-32.597
1.840.347	-25.339	1.862.944	-31.721	1.814.816	-32.599
1.840.826	-25.339	1.863.176	-31.722	1.814.995	-32.600
1.841.292	-25.340	1.863.443	-31.724	1.815.137	-32.602
1.841.483	-25.341	1.863.667	-31.725	1.815.338	-32.603
1.841.530	-25.342	1.863.867	-31.726	1.815.510	-32.605
1.841.622	-25.343	1.864.030	-31.728	1.815.694	-32.606
1.841.680	-25.344	1.864.160	-31.729	1.815.867	-32.608
1.841.721	-25.345	1.864.260	-31.731	1.816.008	-32.609
1.841.852	-25.346	1.864.357	-31.732	1.816.104	-32.611
1.841.890	-25.347	1.864.527	-31.734	1.816.232	-32.612
1.841.933	-25.348	1.864.699	-31.735	1.816.436	-32.614
1.842.169	-25.349	1.864.859	-31.736	1.816.622	-32.615
1.842.399	-25.349	1.865.014	-31.738	1.816.768	-32.617
1.842.565	-25.350	1.865.152	-31.739	1.816.922	-32.619
1.842.753	-25.351	1.865.338	-31.741	1.817.101	-32.620
1.842.872	-25.352	1.865.470	-31.742	1.817.361	-32.622
1.843.040	-25.353	1.865.656	-31.743	1.817.585	-32.624
1.843.201	-25.354	1.865.887	-31.745	1.817.673	-32.625
1.843.356	-25.355	1.866.048	-31.746	1.817.800	-32.627
1.843.542	-25.356	1.866.216	-31.748	1.817.921	-32.629
1.843.689	-25.357	1.866.391	-31.749	1.818.075	-32.630
1.843.904	-25.358	1.866.561	-31.750	1.818.277	-32.632
1.844.113	-25.359	1.866.725	-31.752	1.818.411	-32.634
1.844.259	-25.360	1.866.832	-31.753	1.818.541	-32.635
1.844.362	-25.361	1.866.969	-31.755	1.818.658	-32.637
1.844.518	-25.362	1.867.177	-31.756	1.818.799	-32.639
1.844.711	-25.363	1.867.320	-31.758	1.818.945	-32.641
1.844.881	-25.364	1.867.439	-31.759	1.819.034	-32.642

1.845.004	-25.365	1.867.648	-31.760	1.819.133	-32.644
1.845.143	-25.366	1.867.879	-31.762	1.819.276	-32.646
1.845.318	-25.367	1.868.017	-31.763	1.819.465	-32.648
1.845.497	-25.368	1.868.199	-31.765	1.819.691	-32.649
1.845.701	-25.369	1.868.419	-31.766	1.819.895	-32.651
1.845.889	-25.370	1.868.522	-31.768	1.820.096	-32.653
1.846.048	-25.371	1.868.651	-31.769	1.820.291	-32.655
1.846.237	-25.372	1.868.830	-31.771	1.820.501	-32.657
1.846.454	-25.373	1.869.016	-31.772	1.820.674	-32.659
1.846.583	-25.374	1.869.209	-31.773	1.820.853	-32.660
1.846.671	-25.375	1.869.406	-31.775	1.821.028	-32.662
1.846.816	-25.376	1.869.581	-31.776	1.821.214	-32.664
1.847.003	-25.377	1.869.731	-31.778	1.821.389	-32.666
1.847.206	-25.378	1.869.883	-31.779	1.821.510	-32.668
1.847.401	-25.379	1.870.027	-31.781	1.821.676	-32.669
1.847.554	-25.380	1.870.213	-31.782	1.821.880	-32.671
1.847.623	-25.381	1.870.345	-31.784	1.821.933	-32.673
1.847.719	-25.382	1.870.491	-31.785	1.822.025	-32.675
1.847.939	-25.383	1.870.658	-31.787	1.822.276	-32.677
1.848.152	-25.384	1.870.721	-31.788	1.822.453	-32.679
1.848.333	-25.385	1.870.849	-31.789	1.822.578	-32.680
1.848.510	-25.386	1.870.994	-31.791	1.822.701	-32.682
1.848.647	-25.387	1.871.203	-31.792	1.822.836	-32.684
1.848.759	-25.388	1.871.432	-31.794	1.823.017	-32.686
1.848.896	-25.389	1.871.572	-31.795	1.823.219	-32.688
1.849.055	-25.390	1.871.700	-31.797	1.823.416	-32.689
1.849.303	-25.391	1.871.886	-31.798	1.823.616	-32.691
1.849.543	-25.392	1.872.157	-31.799	1.823.795	-32.693
1.849.635	-25.393	1.872.422	-31.801	1.823.989	-32.695
1.849.778	-25.394	1.872.650	-31.802	1.824.176	-32.697

1.849.949	-25.395	1.872.769	-31.804	1.824.357	-32.698
1.850.163	-25.396	1.872.836	-31.805	1.824.523	-32.700
1.850.347	-25.397	1.872.984	-31.807	1.824.678	-32.702
1.850.511	-25.398	1.873.210	-31.808	1.824.828	-32.704
1.850.652	-25.399	1.873.351	-31.809	1.824.996	-32.706
1.850.791	-25.400	1.873.472	-31.811	1.825.132	-32.707
1.850.927	-25.401	1.873.593	-31.812	1.825.300	-32.709
1.851.035	-25.402	1.873.669	-31.813	1.825.529	-32.711
1.851.174	-25.403	1.873.817	-31.815	1.825.696	-32.713
1.851.360	-25.405	1.873.998	-31.816	1.825.813	-32.715
1.851.588	-25.406	1.874.187	-31.818	1.825.986	-32.716
1.851.825	-25.407	1.874.319	-31.819	1.826.172	-32.718
1.852.005	-25.408	1.874.473	-31.820	1.826.353	-32.720
1.852.122	-25.409	1.874.767	-31.822	1.826.532	-32.722
1.852.240	-25.410	1.875.027	-31.823	1.826.693	-32.723
1.852.359	-25.411	1.875.159	-31.824	1.826.824	-32.725
1.852.525	-25.412	1.875.271	-31.826	1.826.922	-32.727
1.852.686	-25.413	1.875.434	-31.827	1.827.110	-32.729
1.852.861	-25.414	1.875.605	-31.828	1.827.352	-32.730
1.853.102	-25.415	1.875.766	-31.830	1.827.565	-32.732
1.853.230	-25.416	1.876.015	-31.831	1.827.722	-32.734
1.853.264	-25.417	1.876.261	-31.832	1.827.870	-32.735
1.853.306	-25.418	1.876.393	-31.833	1.828.004	-32.737
1.853.510	-25.419	1.876.478	-31.835	1.828.145	-32.739
1.853.700	-25.420	1.876.626	-31.836	1.828.318	-32.740
1.853.839	-25.421	1.876.819	-31.837	1.828.508	-32.742
1.854.124	-25.423	1.876.982	-31.839	1.828.689	-32.744
1.854.364	-25.424	1.877.177	-31.840	1.828.829	-32.746
1.854.525	-25.425	1.877.374	-31.841	1.828.954	-32.747
1.854.718	-25.426	1.877.536	-31.842	1.829.050	-32.749

1.854.877	-25.427	1.877.692	-31.844	1.829.187	-32.751
1.855.047	-25.428	1.877.869	-31.845	1.829.319	-32.752
1.855.291	-25.429	1.878.066	-31.846	1.829.558	-32.754
1.855.436	-25.430	1.878.219	-31.847	1.829.812	-32.756
1.855.593	-25.431	1.878.345	-31.848	1.830.023	-32.757
1.855.744	-25.432	1.878.492	-31.850	1.830.219	-32.759
1.855.840	-25.433	1.878.671	-31.851	1.830.313	-32.760
1.855.956	-25.434	1.878.871	-31.852	1.830.398	-32.762
1.856.115	-25.435	1.879.059	-31.853	1.830.546	-32.764
1.856.277	-25.436	1.879.180	-31.854	1.830.800	-32.765
1.856.405	-25.438	1.879.371	-31.856	1.831.223	-32.767
1.856.579	-25.439	1.879.561	-31.857	1.831.635	-32.769
1.856.720	-25.440	1.879.632	-31.858	1.831.839	-32.770
1.856.951	-25.441	1.879.745	-31.859	1.831.908	-32.772
1.857.283	-25.442	1.879.913	-31.860	1.831.962	-32.773
1.857.441	-25.443	1.880.043	-31.861	1.832.027	-32.775
1.857.547	-25.444	1.880.224	-31.863	1.832.173	-32.777
1.857.706	-25.445	1.880.652	-31.864	1.832.294	-32.778
1.857.876	-25.446	1.881.158	-31.865	1.832.334	-32.780
1.858.019	-25.447	1.881.393	-31.866	1.832.459	-32.781
1.858.187	-25.448	1.881.402	-31.867	1.832.553	-32.783
1.858.465	-25.450	1.881.423	-31.868	1.832.612	-32.785
1.858.647	-25.451	1.881.487	-31.869	1.832.798	-32.786
1.858.759	-25.452	1.881.478	-31.870	1.832.993	-32.788
1.858.864	-25.453	1.881.561	-31.872	1.833.221	-32.789
1.858.976	-25.454	1.881.718	-31.873	1.833.428	-32.791
1.859.130	-25.455	1.881.857	-31.874	1.833.521	-32.792
1.859.305	-25.456	1.881.989	-31.875	1.833.660	-32.794
1.859.460	-25.457	1.882.160	-31.876	1.833.859	-32.796
1.859.651	-25.458	1.882.366	-31.877	1.834.032	-32.797

1.859.893	-25.459	1.882.540	-31.878	1.834.148	-32.799
1.860.054	-25.460	1.882.726	-31.879	1.834.344	-32.801
1.860.195	-25.462	1.882.856	-31.880	1.834.561	-32.802
1.860.365	-25.463	1.883.017	-31.881	1.834.686	-32.804
1.860.571	-25.464	1.883.210	-31.883	1.834.794	-32.805
1.860.704	-25.465	1.883.401	-31.884	1.834.949	-32.807
1.860.829	-25.466	1.883.649	-31.885	1.835.107	-32.809
1.861.033	-25.467	1.883.806	-31.886	1.835.309	-32.810
1.861.214	-25.468	1.883.927	-31.887	1.835.513	-32.812
1.861.382	-25.469	1.884.081	-31.888	1.835.643	-32.814
1.861.519	-25.470	1.884.229	-31.889	1.835.788	-32.815
1.861.647	-25.471	1.884.422	-31.890	1.835.927	-32.817
1.861.848	-25.473	1.884.559	-31.891	1.836.079	-32.819
1.862.030	-25.474	1.884.642	-31.892	1.836.286	-32.820
1.862.206	-25.475	1.884.816	-31.893	1.836.415	-32.822
1.862.410	-25.476	1.885.031	-31.895	1.836.626	-32.824
1.862.475	-25.477	1.885.197	-31.896	1.836.904	-32.826
1.862.549	-25.478	1.885.394	-31.897	1.837.043	-32.827
1.862.847	-25.479	1.885.600	-31.898	1.837.146	-32.829
1.863.197	-25.480	1.885.795	-31.899	1.837.339	-32.831
1.863.479	-25.481	1.885.999	-31.900	1.837.496	-32.833
1.863.802	-25.483	1.886.131	-31.901	1.837.677	-32.834
1.863.965	-25.484	1.886.205	-31.902	1.837.894	-32.836
1.864.017	-25.485	1.886.348	-31.903	1.838.056	-32.838
1.864.086	-25.486	1.886.602	-31.905	1.838.172	-32.840
1.864.115	-25.487	1.886.756	-31.906	1.838.299	-32.841
1.864.256	-25.488	1.886.833	-31.907	1.838.434	-32.843
1.864.330	-25.489	1.886.924	-31.908	1.838.600	-32.845
1.864.397	-25.490	1.887.085	-31.909	1.838.806	-32.847
1.864.594	-25.491	1.887.336	-31.910	1.839.005	-32.849

1.864.820	-25.492	1.887.522	-31.911	1.839.144	-32.851
1.865.009	-25.494	1.887.661	-31.913	1.839.238	-32.853
1.865.164	-25.495	1.887.782	-31.914	1.839.404	-32.854
1.865.350	-25.496	1.887.948	-31.915	1.839.642	-32.856
1.865.553	-25.497	1.888.186	-31.916	1.839.794	-32.858
1.865.741	-25.498	1.888.329	-31.917	1.839.966	-32.860
1.865.947	-25.499	1.888.465	-31.919	1.840.047	-32.862
1.866.127	-25.500	1.888.595	-31.920	1.840.192	-32.864
1.866.225	-25.501	1.888.741	-31.921	1.840.394	-32.866
1.866.331	-25.502	1.888.932	-31.922	1.840.546	-32.868
1.866.492	-25.503	1.889.097	-31.923	1.840.696	-32.870
1.866.662	-25.504	1.889.243	-31.925	1.840.811	-32.872
1.866.770	-25.506	1.889.390	-31.926	1.841.003	-32.874
1.866.897	-25.507	1.889.579	-31.927	1.841.217	-32.876
1.866.985	-25.508	1.889.720	-31.928	1.841.404	-32.878
1.867.115	-25.509	1.889.879	-31.930	1.841.575	-32.880
1.867.423	-25.510	1.890.099	-31.931	1.841.687	-32.882
1.867.702	-25.511	1.890.278	-31.932	1.841.846	-32.884
1.867.861	-25.512	1.890.437	-31.933	1.842.041	-32.886
1.868.002	-25.513	1.890.614	-31.935	1.842.198	-32.888
1.868.098	-25.514	1.890.795	-31.936	1.842.421	-32.890
1.868.145	-25.515	1.890.997	-31.937	1.842.628	-32.892
1.868.383	-25.516	1.891.104	-31.938	1.842.814	-32.894
1.868.624	-25.517	1.891.198	-31.940	1.843.006	-32.896
1.868.750	-25.518	1.891.415	-31.941	1.843.089	-32.898
1.868.970	-25.519	1.891.642	-31.942	1.843.148	-32.900
1.869.171	-25.520	1.891.839	-31.944	1.843.299	-32.902
1.869.270	-25.521	1.891.976	-31.945	1.843.497	-32.904
1.869.420	-25.522	1.892.122	-31.946	1.843.709	-32.906
1.869.588	-25.523	1.892.296	-31.947	1.843.906	-32.908

1.869.749	-25.524	1.892.419	-31.949	1.844.032	-32.910
1.869.946	-25.526	1.892.536	-31.950	1.844.229	-32.912
1.870.072	-25.527	1.892.674	-31.951	1.844.489	-32.914
1.870.172	-25.528	1.892.818	-31.953	1.844.677	-32.916
1.870.408	-25.529	1.892.984	-31.954	1.844.760	-32.918
1.870.620	-25.530	1.893.145	-31.955	1.844.921	-32.920
1.870.728	-25.531	1.893.275	-31.956	1.845.173	-32.922
1.870.943	-25.532	1.893.452	-31.958	1.845.235	-32.924
1.871.138	-25.533	1.893.683	-31.959	1.845.325	-32.926
1.871.268	-25.534	1.893.878	-31.960	1.845.497	-32.928
1.871.378	-25.535	1.894.068	-31.962	1.845.649	-32.930
1.871.496	-25.536	1.894.283	-31.963	1.845.836	-32.932
1.871.713	-25.537	1.894.529	-31.964	1.846.026	-32.934
1.871.951	-25.538	1.894.704	-31.965	1.846.207	-32.936
1.872.070	-25.538	1.894.827	-31.967	1.846.346	-32.938
1.872.204	-25.539	1.894.957	-31.968	1.846.467	-32.939
1.872.449	-25.540	1.895.103	-31.969	1.846.593	-32.941
1.872.659	-25.541	1.895.260	-31.970	1.846.723	-32.943
1.872.762	-25.542	1.895.367	-31.971	1.846.875	-32.945
1.872.861	-25.543	1.895.459	-31.973	1.847.065	-32.947
1.873.048	-25.544	1.895.583	-31.974	1.847.280	-32.949
1.873.177	-25.545	1.895.775	-31.975	1.847.491	-32.951
1.873.329	-25.546	1.895.970	-31.976	1.847.730	-32.953
1.873.586	-25.547	1.896.129	-31.977	1.847.975	-32.955
1.873.826	-25.548	1.896.306	-31.979	1.848.091	-32.956
1.873.965	-25.549	1.896.492	-31.980	1.848.241	-32.958
1.874.101	-25.550	1.896.680	-31.981	1.848.448	-32.960
1.874.272	-25.551	1.896.853	-31.982	1.848.611	-32.962
1.874.422	-25.552	1.897.032	-31.983	1.848.721	-32.964
1.874.545	-25.553	1.897.234	-31.984	1.848.855	-32.965

1.874.776	-25.554	1.897.411	-31.985	1.849.014	-32.967
1.875.011	-25.554	1.897.558	-31.986	1.849.117	-32.969
1.875.159	-25.555	1.897.710	-31.987	1.849.236	-32.971
1.875.329	-25.556	1.897.870	-31.989	1.849.417	-32.973
1.875.368	-25.557	1.898.031	-31.990	1.849.594	-32.974
1.875.490	-25.558	1.898.228	-31.991	1.849.772	-32.976
1.875.620	-25.559	1.898.396	-31.992	1.849.879	-32.978
1.875.777	-25.560	1.898.540	-31.993	1.850.013	-32.979
1.875.981	-25.561	1.898.727	-31.994	1.850.212	-32.981
1.876.075	-25.562	1.898.801	-31.995	1.850.430	-32.983
1.876.189	-25.562	1.898.960	-31.996	1.850.616	-32.984
1.876.353	-25.563	1.899.277	-31.997	1.850.733	-32.986
1.876.590	-25.564	1.899.498	-31.998	1.850.889	-32.988
1.876.720	-25.565	1.899.693	-31.999	1.851.071	-32.989
1.876.769	-25.566	1.899.825	-31.999	1.851.297	-32.991
1.877.038	-25.567	1.899.942	-32.000	1.851.507	-32.992
1.877.462	-25.568	1.900.085	-32.001	1.851.646	-32.994
1.877.634	-25.568	1.900.247	-32.002	1.851.806	-32.995
1.877.733	-25.569	1.900.424	-32.003	1.851.983	-32.997
1.877.899	-25.570	1.900.556	-32.004	1.852.146	-32.999
1.877.952	-25.571	1.900.714	-32.005	1.852.303	-33.000
1.878.098	-25.572	1.900.952	-32.006	1.852.464	-33.002
1.878.255	-25.573	1.901.129	-32.006	1.852.643	-33.003
1.878.378	-25.574	1.901.246	-32.007	1.852.811	-33.004
1.878.564	-25.574	1.901.402	-32.008	1.852.961	-33.006
1.878.613	-25.575	1.901.548	-32.009	1.853.116	-33.007
1.878.705	-25.576	1.901.759	-32.010	1.853.252	-33.009
1.879.021	-25.577	1.901.964	-32.010	1.853.465	-33.010
1.879.286	-25.578	1.902.139	-32.011	1.853.699	-33.012
1.879.465	-25.578	1.902.280	-32.012	1.853.857	-33.013

1.879.725	-25.579	1.902.549	-32.013	1.854.003	-33.014
1.880.009	-25.580	1.902.953	-32.013	1.854.124	-33.016
1.880.173	-25.581	1.903.246	-32.014	1.854.268	-33.017
1.880.238	-25.582	1.903.382	-32.015	1.854.493	-33.018
1.880.410	-25.582	1.903.416	-32.015	1.854.628	-33.020
1.880.627	-25.583	1.903.474	-32.016	1.854.697	-33.021
1.880.777	-25.584	1.903.515	-32.017	1.854.836	-33.022
1.880.855	-25.585	1.903.587	-32.017	1.855.051	-33.024
1.880.835	-25.586	1.903.691	-32.018	1.855.230	-33.025
1.881.057	-25.586	1.903.781	-32.019	1.855.396	-33.026
1.881.288	-25.587	1.903.952	-32.019	1.855.602	-33.028
1.881.414	-25.588	1.904.104	-32.020	1.855.798	-33.029
1.881.723	-25.589	1.904.245	-32.020	1.855.966	-33.030
1.881.945	-25.590	1.904.409	-32.021	1.856.102	-33.031
1.882.054	-25.590	1.904.635	-32.022	1.856.317	-33.033
1.882.130	-25.591	1.904.845	-32.022	1.856.532	-33.034
1.882.251	-25.592	1.905.018	-32.023	1.856.678	-33.035
1.882.352	-25.593	1.905.201	-32.023	1.856.808	-33.037
1.882.641	-25.593	1.905.408	-32.024	1.856.958	-33.038
1.882.954	-25.594	1.905.578	-32.024	1.857.112	-33.039
1.883.048	-25.595	1.905.649	-32.025	1.857.296	-33.040
1.883.163	-25.596	1.905.795	-32.025	1.857.444	-33.041
1.883.279	-25.597	1.905.963	-32.026	1.857.567	-33.043
1.883.450	-25.597	1.906.145	-32.026	1.857.728	-33.044
1.883.584	-25.598	1.906.328	-32.027	1.857.903	-33.045
1.883.741	-25.599	1.906.447	-32.027	1.858.244	-33.046
1.883.974	-25.600	1.906.557	-32.028	1.858.649	-33.048
1.884.025	-25.600	1.906.729	-32.029	1.858.969	-33.049
1.884.137	-25.601	1.906.980	-32.029	1.859.109	-33.050
1.884.489	-25.602	1.907.155	-32.030	1.859.106	-33.051

1.884.729	-25.603	1.907.307	-32.030	1.859.160	-33.052
1.884.877	-25.603	1.907.515	-32.031	1.859.283	-33.054
1.885.051	-25.604	1.907.681	-32.031	1.859.334	-33.055
1.885.177	-25.605	1.907.858	-32.032	1.859.372	-33.056
1.885.300	-25.606	1.908.102	-32.032	1.859.491	-33.057
1.885.564	-25.606	1.908.286	-32.033	1.859.642	-33.059
1.886.008	-25.607	1.908.372	-32.033	1.859.856	-33.060
1.886.335	-25.608	1.908.472	-32.034	1.860.031	-33.061
1.886.359	-25.609	1.908.640	-32.035	1.860.172	-33.062
1.886.431	-25.609	1.908.860	-32.035	1.860.324	-33.064
1.886.532	-25.610	1.909.066	-32.036	1.860.520	-33.065
1.886.586	-25.611	1.909.196	-32.036	1.860.704	-33.066
1.886.734	-25.612	1.909.292	-32.037	1.860.829	-33.067
1.886.837	-25.612	1.909.487	-32.038	1.860.999	-33.069
1.886.872	-25.613	1.909.686	-32.038	1.861.207	-33.070
1.886.908	-25.614	1.909.838	-32.039	1.861.423	-33.071
1.887.137	-25.614	1.909.944	-32.039	1.861.610	-33.072
1.887.430	-25.615	1.910.067	-32.040	1.861.716	-33.074
1.887.645	-25.616	1.910.289	-32.041	1.861.843	-33.075
1.887.887	-25.617	1.910.441	-32.041	1.862.023	-33.076
1.888.091	-25.617	1.910.495	-32.042	1.862.179	-33.078
1.888.223	-25.618	1.910.640	-32.043	1.862.381	-33.079
1.888.344	-25.619	1.910.869	-32.044	1.862.589	-33.080
1.888.528	-25.620	1.911.088	-32.044	1.862.737	-33.082
1.888.714	-25.620	1.911.281	-32.045	1.862.880	-33.083
1.888.810	-25.621	1.911.400	-32.046	1.863.060	-33.085
1.888.915	-25.622	1.911.532	-32.047	1.863.178	-33.086
1.889.104	-25.622	1.911.763	-32.047	1.863.248	-33.087
1.889.288	-25.623	1.911.944	-32.048	1.863.439	-33.089
1.889.424	-25.624	1.912.103	-32.049	1.863.633	-33.090

1.889.595	-25.624	1.912.283	-32.050	1.863.810	-33.092
1.889.785	-25.625	1.912.419	-32.051	1.863.971	-33.093
1.889.899	-25.626	1.912.574	-32.051	1.864.097	-33.095
1.890.031	-25.627	1.912.710	-32.052	1.864.247	-33.096
1.890.224	-25.627	1.912.833	-32.053	1.864.409	-33.098
1.890.426	-25.628	1.912.995	-32.054	1.864.623	-33.099
1.890.506	-25.629	1.913.174	-32.055	1.864.805	-33.101
1.890.650	-25.629	1.913.315	-32.056	1.865.023	-33.102
1.890.860	-25.630	1.913.514	-32.057	1.865.141	-33.104
1.891.133	-25.631	1.913.714	-32.058	1.865.217	-33.105
1.891.353	-25.631	1.913.855	-32.059	1.865.398	-33.107
1.891.472	-25.632	1.914.048	-32.060	1.865.600	-33.108
1.891.747	-25.633	1.914.131	-32.061	1.865.771	-33.110
1.891.835	-25.633	1.914.240	-32.062	1.865.900	-33.111
1.891.828	-25.634	1.914.458	-32.063	1.865.992	-33.113
1.891.984	-25.635	1.914.612	-32.064	1.866.165	-33.115
1.892.220	-25.635	1.914.771	-32.065	1.866.357	-33.116
1.892.370	-25.636	1.914.946	-32.066	1.866.471	-33.118
1.892.354	-25.637	1.915.078	-32.067	1.866.647	-33.120
1.892.531	-25.637	1.915.210	-32.068	1.866.886	-33.121
1.892.840	-25.638	1.915.430	-32.070	1.867.016	-33.123
1.893.042	-25.639	1.915.638	-32.071	1.867.135	-33.125
1.893.277	-25.639	1.915.894	-32.072	1.867.430	-33.126
1.893.362	-25.640	1.916.138	-32.073	1.867.668	-33.128
1.893.438	-25.641	1.916.286	-32.074	1.867.766	-33.130
1.893.609	-25.641	1.916.471	-32.075	1.867.878	-33.131
1.893.748	-25.642	1.916.657	-32.076	1.868.015	-33.133
1.893.951	-25.643	1.916.797	-32.078	1.868.123	-33.135
1.894.140	-25.643	1.916.919	-32.079	1.868.232	-33.137
1.894.323	-25.644	1.917.086	-32.080	1.868.450	-33.138

1.894.502	-25.645	1.917.244	-32.081	1.868.723	-33.140
1.894.650	-25.645	1.917.365	-32.083	1.868.962	-33.142
1.894.870	-25.646	1.917.435	-32.084	1.869.148	-33.144
1.895.069	-25.647	1.917.579	-32.085	1.869.296	-33.145
1.895.215	-25.647	1.917.753	-32.086	1.869.442	-33.147
1.895.358	-25.648	1.917.943	-32.088	1.869.679	-33.149
1.895.531	-25.649	1.918.042	-32.089	1.869.906	-33.151
1.895.728	-25.649	1.918.201	-32.090	1.870.036	-33.152
1.895.804	-25.650	1.918.423	-32.092	1.870.154	-33.154
1.895.943	-25.650	1.918.573	-32.093	1.870.289	-33.156
1.896.171	-25.651	1.918.772	-32.094	1.870.445	-33.158
1.896.339	-25.652	1.918.985	-32.096	1.870.603	-33.159
1.896.548	-25.652	1.919.180	-32.097	1.870.750	-33.161
1.896.768	-25.653	1.919.353	-32.098	1.870.838	-33.163
1.896.821	-25.654	1.919.491	-32.100	1.871.001	-33.165
1.896.900	-25.654	1.919.615	-32.101	1.871.194	-33.166
1.897.117	-25.655	1.919.807	-32.102	1.871.393	-33.168
1.897.300	-25.655	1.920.042	-32.104	1.871.597	-33.170
1.897.464	-25.656	1.920.202	-32.105	1.871.756	-33.171
1.897.641	-25.657	1.920.336	-32.107	1.871.969	-33.173
1.897.789	-25.657	1.920.457	-32.108	1.872.105	-33.175
1.897.898	-25.658	1.920.583	-32.109	1.872.213	-33.177
1.898.004	-25.659	1.920.779	-32.111	1.872.440	-33.178
1.898.160	-25.659	1.920.990	-32.112	1.872.632	-33.180
1.898.331	-25.660	1.921.187	-32.114	1.872.731	-33.181
1.898.465	-25.660	1.921.339	-32.115	1.872.816	-33.183
1.898.626	-25.661	1.921.441	-32.117	1.872.912	-33.185
1.898.864	-25.662	1.921.577	-32.118	1.873.145	-33.186
1.899.100	-25.662	1.921.740	-32.120	1.873.358	-33.188
1.899.299	-25.663	1.921.955	-32.121	1.873.458	-33.189

1.899.462	-25.664	1.922.193	-32.123	1.873.600	-33.191
1.899.689	-25.664	1.922.383	-32.124	1.873.734	-33.192
1.899.935	-25.665	1.922.489	-32.126	1.873.877	-33.194
1.900.052	-25.665	1.922.594	-32.127	1.874.010	-33.195
1.900.161	-25.666	1.922.766	-32.129	1.874.157	-33.197
1.900.276	-25.667	1.922.959	-32.130	1.874.366	-33.198
1.900.475	-25.667	1.923.139	-32.132	1.874.565	-33.200
1.900.630	-25.668	1.923.307	-32.133	1.874.769	-33.201
1.900.690	-25.668	1.923.461	-32.135	1.874.975	-33.203
1.900.898	-25.669	1.923.645	-32.137	1.875.202	-33.204
1.901.042	-25.670	1.923.848	-32.138	1.875.439	-33.205
1.901.160	-25.670	1.924.034	-32.140	1.875.600	-33.207
1.901.416	-25.671	1.924.180	-32.141	1.875.712	-33.208
1.901.593	-25.672	1.924.415	-32.143	1.875.826	-33.209
1.901.689	-25.672	1.924.823	-32.144	1.876.050	-33.210
1.901.868	-25.673	1.925.206	-32.146	1.876.257	-33.212
1.902.077	-25.673	1.925.342	-32.148	1.876.395	-33.213
1.902.301	-25.674	1.925.365	-32.149	1.876.519	-33.214
1.902.533	-25.675	1.925.426	-32.151	1.876.689	-33.215
1.902.643	-25.675	1.925.454	-32.152	1.876.846	-33.216
1.902.778	-25.676	1.925.515	-32.154	1.876.937	-33.217
1.902.968	-25.677	1.925.622	-32.156	1.877.110	-33.218
1.903.165	-25.677	1.925.692	-32.157	1.877.278	-33.219
1.903.315	-25.678	1.925.822	-32.159	1.877.370	-33.220
1.903.438	-25.679	1.926.084	-32.160	1.877.504	-33.221
1.903.602	-25.679	1.926.357	-32.162	1.877.706	-33.222
1.903.736	-25.680	1.926.474	-32.164	1.877.962	-33.223
1.903.884	-25.680	1.926.561	-32.165	1.878.188	-33.224
1.904.097	-25.681	1.926.729	-32.167	1.878.358	-33.225
1.904.283	-25.682	1.926.940	-32.168	1.878.465	-33.226

1.904.442	-25.682	1.927.139	-32.170	1.878.622	-33.227
1.904.565	-25.683	1.927.289	-32.172	1.878.824	-33.228
1.904.720	-25.684	1.927.471	-32.173	1.878.969	-33.228
1.904.942	-25.684	1.927.618	-32.175	1.879.117	-33.229
1.905.116	-25.685	1.927.775	-32.176	1.879.335	-33.230
1.905.286	-25.686	1.927.997	-32.178	1.879.541	-33.231
1.905.499	-25.686	1.928.196	-32.180	1.879.709	-33.231
1.905.638	-25.687	1.928.358	-32.181	1.879.861	-33.232
1.905.748	-25.688	1.928.436	-32.183	1.880.011	-33.233
1.905.907	-25.688	1.928.517	-32.185	1.880.155	-33.233
1.906.012	-25.689	1.928.617	-32.186	1.880.240	-33.234
1.906.180	-25.690	1.928.777	-32.188	1.880.401	-33.234
1.906.391	-25.690	1.928.999	-32.190	1.880.638	-33.235
1.906.503	-25.691	1.929.204	-32.191	1.880.838	-33.235
1.906.747	-25.692	1.929.413	-32.193	1.880.929	-33.236
1.907.016	-25.692	1.929.632	-32.194	1.881.088	-33.236
1.907.162	-25.693	1.929.749	-32.196	1.881.297	-33.237
1.907.379	-25.694	1.929.867	-32.198	1.881.400	-33.237
1.907.535	-25.694	1.930.044	-32.199	1.881.548	-33.238
1.907.659	-25.695	1.930.215	-32.201	1.881.682	-33.238
1.907.773	-25.696	1.930.383	-32.203	1.881.853	-33.239
1.907.984	-25.696	1.930.567	-32.205	1.882.058	-33.239
1.908.398	-25.697	1.930.764	-32.206	1.882.240	-33.239
1.908.647	-25.698	1.930.925	-32.208	1.882.410	-33.240
1.908.877	-25.698	1.931.060	-32.210	1.882.655	-33.240
1.909.048	-25.699	1.931.223	-32.211	1.882.885	-33.240
1.909.091	-25.700	1.931.376	-32.213	1.882.962	-33.241
1.908.974	-25.701	1.931.521	-32.215	1.883.065	-33.241
1.908.876	-25.701	1.931.695	-32.216	1.883.289	-33.241
1.909.084	-25.702	1.931.888	-32.218	1.883.512	-33.242

1.909.366	-25.703	1.932.076	-32.220	1.883.669	-33.242
1.909.579	-25.703	1.932.213	-32.222	1.883.745	-33.242
1.909.697	-25.704	1.932.309	-32.223	1.883.885	-33.242
1.909.778	-25.705	1.932.435	-32.225	1.884.086	-33.243
1.909.937	-25.705	1.932.607	-32.227	1.884.268	-33.243
1.910.181	-25.706	1.932.784	-32.229	1.884.492	-33.243
1.910.401	-25.707	1.933.010	-32.230	1.884.612	-33.243
1.910.618	-25.708	1.933.190	-32.232	1.884.715	-33.243
1.910.835	-25.708	1.933.364	-32.234	1.884.876	-33.243
1.911.021	-25.709	1.933.521	-32.236	1.885.094	-33.244
1.911.158	-25.710	1.933.676	-32.238	1.885.471	-33.244
1.911.245	-25.710	1.933.802	-32.239	1.885.876	-33.244
1.911.371	-25.711	1.933.927	-32.241	1.886.147	-33.244
1.911.498	-25.712	1.934.093	-32.243	1.886.299	-33.244
1.911.613	-25.713	1.934.314	-32.245	1.886.409	-33.244
1.911.872	-25.713	1.934.543	-32.247	1.886.492	-33.245
1.912.132	-25.714	1.934.682	-32.249	1.886.471	-33.245
1.912.175	-25.715	1.934.758	-32.250	1.886.487	-33.245
1.912.179	-25.715	1.934.902	-32.252	1.886.586	-33.245
1.912.386	-25.716	1.935.168	-32.254	1.886.684	-33.245
1.912.610	-25.717	1.935.358	-32.256	1.886.846	-33.245
1.912.793	-25.717	1.935.513	-32.258	1.886.998	-33.246
1.913.031	-25.718	1.935.658	-32.260	1.887.092	-33.246
1.913.210	-25.719	1.935.753	-32.261	1.887.294	-33.246
1.913.387	-25.720	1.935.905	-32.263	1.887.500	-33.246
1.913.555	-25.720	1.936.055	-32.265	1.887.618	-33.246
1.913.638	-25.721	1.936.176	-32.267	1.887.841	-33.246
1.913.775	-25.722	1.936.340	-32.269	1.888.040	-33.247
1.913.996	-25.722	1.936.528	-32.271	1.888.098	-33.247
1.914.182	-25.723	1.936.622	-32.273	1.888.286	-33.247

1.914.384	-25.724	1.936.772	-32.275	1.888.548	-33.247
1.914.507	-25.725	1.936.973	-32.276	1.888.716	-33.247
1.914.566	-25.725	1.937.132	-32.278	1.888.889	-33.248
1.914.796	-25.726	1.937.296	-32.280	1.889.072	-33.248
1.914.986	-25.727	1.937.479	-32.282	1.889.189	-33.248
1.915.121	-25.727	1.937.659	-32.284	1.889.298	-33.249
1.915.293	-25.728	1.937.910	-32.286	1.889.456	-33.249
1.915.407	-25.729	1.938.178	-32.288	1.889.608	-33.249
1.915.576	-25.730	1.938.320	-32.289	1.889.791	-33.250
1.915.771	-25.730	1.938.476	-32.291	1.890.011	-33.250
1.915.957	-25.731	1.938.689	-32.293	1.890.126	-33.250
1.916.104	-25.732	1.938.858	-32.295	1.890.199	-33.251
1.916.265	-25.732	1.938.998	-32.297	1.890.378	-33.251
1.916.378	-25.733	1.939.166	-32.299	1.890.576	-33.252
1.916.510	-25.734	1.939.290	-32.300	1.890.701	-33.252
1.916.747	-25.735	1.939.406	-32.302	1.890.862	-33.253
1.916.933	-25.735	1.939.587	-32.304	1.891.013	-33.253
1.917.096	-25.736	1.939.751	-32.306	1.891.165	-33.254
1.917.280	-25.737	1.939.901	-32.308	1.891.396	-33.254
1.917.523	-25.737	1.940.020	-32.309	1.891.644	-33.255
1.917.682	-25.738	1.940.199	-32.311	1.891.828	-33.255
1.917.784	-25.739	1.940.470	-32.313	1.891.996	-33.256
1.917.970	-25.739	1.940.625	-32.315	1.892.161	-33.257
1.918.139	-25.740	1.940.750	-32.316	1.892.291	-33.257
1.918.264	-25.741	1.940.860	-32.318	1.892.430	-33.258
1.918.333	-25.742	1.941.064	-32.320	1.892.625	-33.259
1.918.458	-25.742	1.941.330	-32.322	1.892.747	-33.260
1.918.710	-25.743	1.941.523	-32.323	1.892.854	-33.260
1.918.976	-25.744	1.941.642	-32.325	1.893.093	-33.261
1.919.153	-25.744	1.941.803	-32.327	1.893.275	-33.262

1.919.261	-25.745	1.941.906	-32.328	1.893.407	-33.263
1.919.373	-25.746	1.942.025	-32.330	1.893.562	-33.264
1.919.540	-25.746	1.942.255	-32.332	1.893.667	-33.265
1.919.744	-25.747	1.942.435	-32.333	1.893.822	-33.266
1.919.846	-25.748	1.942.562	-32.335	1.894.016	-33.266
1.919.982	-25.749	1.942.749	-32.337	1.894.164	-33.267
1.920.175	-25.749	1.942.959	-32.338	1.894.372	-33.268
1.920.313	-25.750	1.943.078	-32.340	1.894.601	-33.269
1.920.370	-25.751	1.943.208	-32.342	1.894.754	-33.271
1.920.522	-25.751	1.943.374	-32.343	1.894.899	-33.272
1.920.703	-25.752	1.943.593	-32.345	1.895.031	-33.273
1.920.869	-25.753	1.943.783	-32.347	1.895.170	-33.274
1.921.053	-25.754	1.943.949	-32.348	1.895.318	-33.275
1.921.283	-25.754	1.944.138	-32.350	1.895.455	-33.276
1.921.544	-25.755	1.944.274	-32.351	1.895.602	-33.277
1.921.745	-25.756	1.944.442	-32.353	1.895.833	-33.279
1.922.018	-25.756	1.944.612	-32.355	1.896.037	-33.280
1.922.218	-25.757	1.944.734	-32.356	1.896.214	-33.281
1.922.314	-25.758	1.944.888	-32.358	1.896.367	-33.282
1.922.462	-25.758	1.945.044	-32.359	1.896.499	-33.284
1.922.668	-25.759	1.945.228	-32.361	1.896.644	-33.285
1.922.876	-25.760	1.945.425	-32.362	1.896.776	-33.286
1.923.071	-25.761	1.945.603	-32.364	1.896.991	-33.288
1.923.145	-25.761	1.945.788	-32.365	1.897.188	-33.289
1.923.172	-25.762	1.945.932	-32.367	1.897.328	-33.291
1.923.260	-25.763	1.946.059	-32.368	1.897.469	-33.292
1.923.387	-25.764	1.946.294	-32.370	1.897.648	-33.293
1.923.541	-25.764	1.946.763	-32.371	1.897.820	-33.295
1.923.678	-25.765	1.947.137	-32.373	1.897.910	-33.296
1.923.840	-25.766	1.947.233	-32.374	1.898.091	-33.298

1.924.068	-25.766	1.947.258	-32.376	1.898.313	-33.299
1.924.236	-25.767	1.947.314	-32.377	1.898.508	-33.301
1.924.410	-25.768	1.947.365	-32.379	1.898.680	-33.302
1.924.641	-25.769	1.947.473	-32.380	1.898.839	-33.304
1.924.830	-25.769	1.947.587	-32.381	1.898.951	-33.305
1.925.040	-25.770	1.947.617	-32.383	1.899.138	-33.307
1.925.206	-25.771	1.947.728	-32.384	1.899.431	-33.308
1.925.327	-25.772	1.947.934	-32.386	1.899.559	-33.310
1.925.488	-25.772	1.948.186	-32.387	1.899.594	-33.311
1.925.667	-25.773	1.948.410	-32.388	1.899.684	-33.313
1.925.846	-25.774	1.948.607	-32.390	1.899.928	-33.315
1.926.022	-25.775	1.948.801	-32.391	1.900.101	-33.316
1.926.225	-25.775	1.948.922	-32.392	1.900.206	-33.318
1.926.404	-25.776	1.949.048	-32.394	1.900.408	-33.319
1.926.555	-25.777	1.949.209	-32.395	1.900.594	-33.321
1.926.666	-25.778	1.949.399	-32.396	1.900.748	-33.322
1.926.801	-25.778	1.949.536	-32.398	1.900.876	-33.324
1.927.025	-25.779	1.949.651	-32.399	1.901.032	-33.326
1.927.227	-25.780	1.949.829	-32.400	1.901.198	-33.327
1.927.449	-25.781	1.950.058	-32.402	1.901.351	-33.329
1.927.637	-25.781	1.950.206	-32.403	1.901.488	-33.330
1.927.786	-25.782	1.950.334	-32.404	1.901.711	-33.332
1.927.959	-25.783	1.950.457	-32.405	1.901.924	-33.334
1.928.091	-25.784	1.950.618	-32.407	1.902.123	-33.335
1.928.228	-25.785	1.950.842	-32.408	1.902.356	-33.337
1.928.400	-25.785	1.950.981	-32.409	1.902.536	-33.338
1.928.513	-25.786	1.951.102	-32.411	1.902.737	-33.340
1.928.625	-25.787	1.951.277	-32.412	1.902.948	-33.341
1.928.768	-25.788	1.951.532	-32.413	1.903.064	-33.343
1.928.970	-25.789	1.951.774	-32.414	1.903.172	-33.344

1.929.187	-25.789	1.951.931	-32.415	1.903.311	-33.346
1.929.442	-25.790	1.952.043	-32.417	1.903.423	-33.347
1.929.614	-25.791	1.952.139	-32.418	1.903.508	-33.349
1.929.722	-25.792	1.952.229	-32.419	1.903.636	-33.350
1.929.868	-25.793	1.952.372	-32.420	1.903.831	-33.352
1.929.893	-25.793	1.952.529	-32.422	1.903.990	-33.353
1.930.020	-25.794	1.952.715	-32.423	1.904.131	-33.355
1.930.468	-25.795	1.953.017	-32.424	1.904.310	-33.356
1.930.948	-25.796	1.953.230	-32.425	1.904.435	-33.358
1.931.187	-25.797	1.953.344	-32.427	1.904.522	-33.359
1.931.275	-25.797	1.953.477	-32.428	1.904.743	-33.361
1.931.315	-25.798	1.953.591	-32.429	1.905.027	-33.362
1.931.351	-25.799	1.953.765	-32.430	1.905.255	-33.364
1.931.400	-25.800	1.953.940	-32.431	1.905.441	-33.365
1.931.528	-25.801	1.954.104	-32.433	1.905.592	-33.367
1.931.664	-25.802	1.954.294	-32.434	1.905.701	-33.368
1.931.758	-25.802	1.954.386	-32.435	1.905.885	-33.369
1.931.898	-25.803	1.954.498	-32.436	1.906.088	-33.371
1.932.074	-25.804	1.954.718	-32.437	1.906.187	-33.372
1.932.251	-25.805	1.954.935	-32.439	1.906.348	-33.374
1.932.448	-25.806	1.955.099	-32.440	1.906.543	-33.375
1.932.647	-25.807	1.955.266	-32.441	1.906.620	-33.376
1.932.831	-25.807	1.955.488	-32.442	1.906.834	-33.378
1.933.006	-25.808	1.955.654	-32.444	1.907.083	-33.379
1.933.206	-25.809	1.955.799	-32.445	1.907.189	-33.380
1.933.414	-25.810	1.955.934	-32.446	1.907.345	-33.382
1.933.555	-25.811	1.956.111	-32.447	1.907.579	-33.383
1.933.681	-25.812	1.956.295	-32.448	1.907.755	-33.384
1.933.844	-25.812	1.956.447	-32.450	1.907.905	-33.386
1.934.036	-25.813	1.956.568	-32.451	1.908.091	-33.387

1.934.193	-25.814	1.956.768	-32.452	1.908.257	-33.388
1.934.337	-25.815	1.956.954	-32.453	1.908.416	-33.390
1.934.514	-25.816	1.957.101	-32.455	1.908.568	-33.391
1.934.585	-25.817	1.957.202	-32.456	1.908.747	-33.392
1.934.699	-25.817	1.957.343	-32.457	1.908.974	-33.393
1.934.928	-25.818	1.957.594	-32.458	1.909.139	-33.395
1.935.101	-25.819	1.957.764	-32.460	1.909.285	-33.396
1.935.269	-25.820	1.957.921	-32.461	1.909.422	-33.397
1.935.455	-25.821	1.958.139	-32.462	1.909.585	-33.398
1.935.656	-25.822	1.958.281	-32.463	1.909.755	-33.399
1.935.849	-25.823	1.958.356	-32.464	1.909.884	-33.401
1.936.001	-25.823	1.958.485	-32.466	1.910.098	-33.402
1.936.174	-25.824	1.958.640	-32.467	1.910.280	-33.403
1.936.335	-25.825	1.958.763	-32.468	1.910.399	-33.404
1.936.469	-25.826	1.958.929	-32.469	1.910.582	-33.405
1.936.629	-25.827	1.959.099	-32.470	1.910.759	-33.407
1.936.833	-25.828	1.959.216	-32.472	1.910.916	-33.408
1.936.944	-25.828	1.959.384	-32.473	1.911.088	-33.409
1.937.011	-25.829	1.959.657	-32.474	1.911.245	-33.410
1.937.166	-25.830	1.959.867	-32.475	1.911.373	-33.411
1.937.307	-25.831	1.960.025	-32.476	1.911.559	-33.412
1.937.491	-25.832	1.960.235	-32.477	1.911.713	-33.414
1.937.681	-25.833	1.960.450	-32.478	1.911.792	-33.415
1.937.833	-25.834	1.960.565	-32.480	1.912.081	-33.416
1.938.024	-25.834	1.960.755	-32.481	1.912.515	-33.417
1.938.178	-25.835	1.960.954	-32.482	1.912.861	-33.418
1.938.349	-25.836	1.961.084	-32.483	1.913.037	-33.419
1.938.454	-25.837	1.961.236	-32.484	1.913.042	-33.420
1.938.678	-25.838	1.961.395	-32.485	1.913.148	-33.422
1.938.905	-25.839	1.961.550	-32.486	1.913.322	-33.423

1.939.133	-25.839	1.961.675	-32.487	1.913.358	-33.424
1.939.337	-25.840	1.961.803	-32.488	1.913.394	-33.425
1.939.482	-25.841	1.961.938	-32.489	1.913.472	-33.426
1.939.594	-25.842	1.962.122	-32.490	1.913.577	-33.427
1.939.740	-25.843	1.962.348	-32.491	1.913.700	-33.428
1.939.910	-25.844	1.962.538	-32.492	1.913.831	-33.429
1.940.027	-25.845	1.962.742	-32.493	1.914.044	-33.430
1.940.130	-25.845	1.962.977	-32.494	1.914.202	-33.432
1.940.336	-25.846	1.963.120	-32.495	1.914.306	-33.433
1.940.511	-25.847	1.963.262	-32.496	1.914.576	-33.434
1.940.667	-25.848	1.963.353	-32.497	1.914.781	-33.435
1.940.762	-25.849	1.963.528	-32.498	1.914.942	-33.436
1.940.902	-25.850	1.963.763	-32.499	1.915.165	-33.437
1.941.161	-25.850	1.963.880	-32.500	1.915.383	-33.438
1.941.348	-25.851	1.964.035	-32.501	1.915.585	-33.439
1.941.518	-25.852	1.964.241	-32.502	1.915.725	-33.441
1.941.716	-25.853	1.964.359	-32.503	1.915.865	-33.442
1.941.895	-25.854	1.964.520	-32.504	1.916.022	-33.443
1.942.034	-25.855	1.964.673	-32.505	1.916.138	-33.444
1.942.141	-25.855	1.964.850	-32.506	1.916.270	-33.445
1.942.282	-25.856	1.965.092	-32.506	1.916.453	-33.446
1.942.428	-25.857	1.965.259	-32.507	1.916.570	-33.448
1.942.634	-25.858	1.965.399	-32.508	1.916.711	-33.449
1.942.771	-25.859	1.965.578	-32.509	1.916.906	-33.450
1.942.887	-25.860	1.965.790	-32.510	1.917.040	-33.451
1.942.997	-25.860	1.965.952	-32.510	1.917.195	-33.452
1.943.143	-25.861	1.966.084	-32.511	1.917.395	-33.454
1.943.311	-25.862	1.966.259	-32.512	1.917.596	-33.455
1.943.499	-25.863	1.966.402	-32.513	1.917.749	-33.456
1.943.613	-25.864	1.966.527	-32.513	1.917.823	-33.457

1.943.732	-25.865	1.966.677	-32.514	1.917.973	-33.459
1.943.904	-25.865	1.966.870	-32.515	1.918.194	-33.460
1.944.137	-25.866	1.967.088	-32.516	1.918.385	-33.461
1.944.353	-25.867	1.967.280	-32.516	1.918.550	-33.462
1.944.541	-25.868	1.967.408	-32.517	1.918.727	-33.464
1.944.755	-25.869	1.967.536	-32.518	1.918.942	-33.465
1.944.915	-25.870	1.967.661	-32.518	1.919.097	-33.466
1.945.094	-25.870	1.967.842	-32.519	1.919.211	-33.468
1.945.238	-25.871	1.968.017	-32.520	1.919.363	-33.469
1.945.351	-25.872	1.968.223	-32.520	1.919.520	-33.470
1.945.491	-25.873	1.968.660	-32.521	1.919.673	-33.472
1.945.672	-25.874	1.969.110	-32.522	1.919.820	-33.473
1.945.845	-25.875	1.969.283	-32.522	1.919.971	-33.475
1.945.958	-25.875	1.969.296	-32.523	1.920.150	-33.476
1.946.098	-25.876	1.969.330	-32.524	1.920.327	-33.477
1.946.257	-25.877	1.969.388	-32.524	1.920.522	-33.479
1.946.420	-25.878	1.969.406	-32.525	1.920.647	-33.480
1.946.592	-25.879	1.969.471	-32.525	1.920.741	-33.482
1.946.790	-25.880	1.969.587	-32.526	1.921.001	-33.483
1.946.984	-25.881	1.969.753	-32.526	1.921.203	-33.484
1.947.150	-25.881	1.969.967	-32.527	1.921.299	-33.486
1.947.350	-25.882	1.970.181	-32.528	1.921.458	-33.487
1.947.558	-25.883	1.970.417	-32.528	1.921.642	-33.489
1.947.706	-25.884	1.970.585	-32.529	1.921.790	-33.490
1.947.928	-25.885	1.970.710	-32.529	1.921.918	-33.492
1.948.122	-25.886	1.970.887	-32.530	1.922.122	-33.493
1.948.195	-25.886	1.971.033	-32.530	1.922.305	-33.495
1.948.327	-25.887	1.971.241	-32.531	1.922.430	-33.496
1.948.496	-25.888	1.971.460	-32.531	1.922.571	-33.498
1.948.696	-25.889	1.971.568	-32.532	1.922.825	-33.499

1.948.906	-25.890	1.971.732	-32.532	1.923.035	-33.501
1.949.095	-25.891	1.971.884	-32.533	1.923.136	-33.502
1.949.245	-25.892	1.972.010	-32.533	1.923.336	-33.504
1.949.359	-25.892	1.972.180	-32.534	1.923.566	-33.506
1.949.540	-25.893	1.972.319	-32.534	1.923.734	-33.507
1.949.742	-25.894	1.972.402	-32.535	1.923.873	-33.509
1.949.865	-25.895	1.972.556	-32.535	1.924.030	-33.510
1.950.002	-25.896	1.972.784	-32.536	1.924.195	-33.512
1.950.237	-25.897	1.972.988	-32.536	1.924.346	-33.513
1.950.448	-25.898	1.973.138	-32.536	1.924.514	-33.515
1.950.629	-25.898	1.973.360	-32.537	1.924.637	-33.516
1.950.813	-25.899	1.973.600	-32.537	1.924.800	-33.518
1.950.936	-25.900	1.973.734	-32.538	1.924.923	-33.519
1.951.102	-25.901	1.973.860	-32.538	1.925.067	-33.521
1.951.261	-25.902	1.974.032	-32.539	1.925.278	-33.522
1.951.362	-25.903	1.974.178	-32.539	1.925.466	-33.524
1.951.411	-25.904	1.974.286	-32.540	1.925.607	-33.525
1.951.512	-25.905	1.974.413	-32.540	1.925.779	-33.527
1.951.803	-25.905	1.974.630	-32.541	1.926.028	-33.528
1.952.052	-25.906	1.974.845	-32.541	1.926.225	-33.530
1.952.197	-25.907	1.974.960	-32.542	1.926.351	-33.531
1.952.381	-25.908	1.975.112	-32.542	1.926.429	-33.532
1.952.435	-25.909	1.975.255	-32.543	1.926.537	-33.534
1.952.594	-25.910	1.975.416	-32.543	1.926.664	-33.535
1.953.100	-25.911	1.975.598	-32.544	1.926.848	-33.537
1.953.492	-25.912	1.975.732	-32.544	1.927.018	-33.538
1.953.622	-25.912	1.975.889	-32.545	1.927.117	-33.540
1.953.694	-25.913	1.976.073	-32.545	1.927.262	-33.541
1.953.743	-25.914	1.976.217	-32.546	1.927.446	-33.542
1.953.802	-25.915	1.976.344	-32.547	1.927.558	-33.544

1.953.824	-25.916	1.976.517	-32.547	1.927.690	-33.545
1.953.857	-25.917	1.976.700	-32.548	1.927.917	-33.546
1.953.969	-25.918	1.976.898	-32.548	1.928.080	-33.548
1.954.220	-25.918	1.977.094	-32.549	1.928.271	-33.549
1.954.444	-25.919	1.977.234	-32.549	1.928.501	-33.550
1.954.565	-25.920	1.977.399	-32.550	1.928.708	-33.552
1.954.763	-25.921	1.977.571	-32.551	1.928.896	-33.553
1.954.917	-25.922	1.977.786	-32.551	1.929.045	-33.554
1.955.103	-25.923	1.977.988	-32.552	1.929.234	-33.556
1.955.262	-25.924	1.978.159	-32.553	1.929.467	-33.557
1.955.454	-25.925	1.978.299	-32.553	1.929.742	-33.558
1.955.712	-25.925	1.978.393	-32.554	1.929.819	-33.559
1.955.874	-25.926	1.978.575	-32.555	1.929.884	-33.560
1.956.019	-25.927	1.978.793	-32.555	1.930.092	-33.562
1.956.091	-25.928	1.978.990	-32.556	1.930.231	-33.563
1.956.189	-25.929	1.979.157	-32.557	1.930.349	-33.564
1.956.407	-25.930	1.979.274	-32.557	1.930.491	-33.565
1.956.570	-25.930	1.979.400	-32.558	1.930.645	-33.566
1.956.723	-25.931	1.979.576	-32.559	1.930.760	-33.567
1.956.918	-25.932	1.979.702	-32.560	1.930.947	-33.569
1.957.132	-25.933	1.979.827	-32.560	1.931.105	-33.570
1.957.290	-25.934	1.980.031	-32.561	1.931.270	-33.571
1.957.451	-25.935	1.980.235	-32.562	1.931.474	-33.572
1.957.668	-25.935	1.980.378	-32.563	1.931.604	-33.573
1.957.854	-25.936	1.980.491	-32.564	1.931.875	-33.574
1.958.031	-25.937	1.980.621	-32.564	1.932.189	-33.575
1.958.219	-25.938	1.980.755	-32.565	1.932.314	-33.576
1.958.407	-25.939	1.980.884	-32.566	1.932.423	-33.577
1.958.519	-25.940	1.981.055	-32.567	1.932.576	-33.578
1.958.600	-25.940	1.981.288	-32.568	1.932.690	-33.579

1.958.737	-25.941	1.981.462	-32.568	1.932.874	-33.580
1.958.909	-25.942	1.981.615	-32.569	1.933.055	-33.581
1.959.091	-25.943	1.981.816	-32.570	1.933.221	-33.582
1.959.213	-25.944	1.982.034	-32.571	1.933.378	-33.583
1.959.357	-25.944	1.982.244	-32.572	1.933.470	-33.584
1.959.534	-25.945	1.982.444	-32.573	1.933.640	-33.585
1.959.688	-25.946	1.982.618	-32.574	1.933.848	-33.586
1.959.897	-25.947	1.982.773	-32.574	1.934.007	-33.587
1.960.049	-25.948	1.982.892	-32.575	1.934.202	-33.588
1.960.182	-25.948	1.983.015	-32.576	1.934.332	-33.589
1.960.333	-25.949	1.983.165	-32.577	1.934.446	-33.589
1.960.535	-25.950	1.983.313	-32.578	1.934.702	-33.590
1.960.710	-25.951	1.983.423	-32.579	1.934.953	-33.591
1.960.822	-25.951	1.983.582	-32.580	1.935.056	-33.592
1.960.976	-25.952	1.983.774	-32.581	1.935.116	-33.593
1.961.145	-25.953	1.983.936	-32.582	1.935.338	-33.594
1.961.311	-25.954	1.984.155	-32.582	1.935.571	-33.595
1.961.463	-25.955	1.984.345	-32.583	1.935.706	-33.595
1.961.628	-25.955	1.984.505	-32.584	1.935.954	-33.596
1.961.839	-25.956	1.984.666	-32.585	1.936.173	-33.597
1.962.040	-25.957	1.984.872	-32.586	1.936.238	-33.598
1.962.198	-25.957	1.985.078	-32.587	1.936.292	-33.599
1.962.368	-25.958	1.985.282	-32.588	1.936.400	-33.599
1.962.509	-25.959	1.985.457	-32.589	1.936.552	-33.600
1.962.659	-25.960	1.985.594	-32.590	1.936.785	-33.601
1.962.858	-25.960	1.985.804	-32.590	1.936.996	-33.602
1.962.959	-25.961	1.985.996	-32.591	1.937.171	-33.603
1.963.158	-25.962	1.986.138	-32.592	1.937.350	-33.604
1.963.443	-25.963	1.986.311	-32.593	1.937.486	-33.604
1.963.537	-25.963	1.986.462	-32.594	1.937.664	-33.605

1.963.615	-25.964	1.986.606	-32.595	1.937.874	-33.606
1.963.812	-25.965	1.986.763	-32.596	1.938.027	-33.607
1.964.025	-25.965	1.986.933	-32.597	1.938.167	-33.608
1.964.229	-25.966	1.987.123	-32.598	1.938.318	-33.608
1.964.409	-25.967	1.987.323	-32.599	1.938.456	-33.609
1.964.543	-25.967	1.987.451	-32.599	1.938.582	-33.610
1.964.682	-25.968	1.987.549	-32.600	1.938.747	-33.611
1.964.850	-25.969	1.987.733	-32.601	1.939.046	-33.612
1.964.967	-25.969	1.987.932	-32.602	1.939.485	-33.612
1.965.154	-25.970	1.988.066	-32.603	1.939.816	-33.613
1.965.334	-25.971	1.988.255	-32.604	1.939.948	-33.614
1.965.414	-25.971	1.988.477	-32.605	1.940.025	-33.615
1.965.501	-25.972	1.988.604	-32.606	1.940.101	-33.616
1.965.647	-25.973	1.988.732	-32.607	1.940.182	-33.617
1.965.842	-25.973	1.988.947	-32.607	1.940.267	-33.618
1.966.005	-25.974	1.989.157	-32.608	1.940.322	-33.618
1.966.217	-25.975	1.989.292	-32.609	1.940.430	-33.619
1.966.413	-25.975	1.989.433	-32.610	1.940.578	-33.620
1.966.557	-25.976	1.989.588	-32.611	1.940.748	-33.621
1.966.750	-25.977	1.989.762	-32.612	1.940.905	-33.622
1.966.951	-25.977	1.989.911	-32.612	1.941.037	-33.623
1.967.186	-25.978	1.990.024	-32.613	1.941.149	-33.624
1.967.377	-25.978	1.990.264	-32.614	1.941.335	-33.625
1.967.565	-25.979	1.990.724	-32.615	1.941.584	-33.626
1.967.715	-25.980	1.991.100	-32.616	1.941.747	-33.627
1.967.832	-25.980	1.991.216	-32.617	1.941.936	-33.628
1.967.912	-25.981	1.991.245	-32.617	1.942.146	-33.629
1.968.040	-25.981	1.991.303	-32.618	1.942.323	-33.630
1.968.150	-25.982	1.991.333	-32.619	1.942.468	-33.631
1.968.326	-25.983	1.991.306	-32.620	1.942.576	-33.632

1.968.539	-25.983	1.991.460	-32.621	1.942.636	-33.633
1.968.658	-25.984	1.991.704	-32.621	1.942.820	-33.634
1.968.828	-25.984	1.991.857	-32.622	1.943.067	-33.635
1.969.059	-25.985	1.992.041	-32.623	1.943.190	-33.636
1.969.259	-25.986	1.992.231	-32.624	1.943.337	-33.637
1.969.259	-25.986	1.992.377	-32.624	1.943.504	-33.638
1.969.634	-25.987	1.992.560	-32.625	1.943.662	-33.640
1.969.769	-25.987	1.992.735	-32.626	1.943.808	-33.641
1.969.955	-25.988	1.992.888	-32.627	1.943.925	-33.642
1.970.137	-25.988	1.993.098	-32.627	1.944.120	-33.643
1.970.258	-25.989	1.993.243	-32.628	1.944.354	-33.644
1.970.403	-25.990	1.993.380	-32.629	1.944.501	-33.646
1.970.614	-25.990	1.993.568	-32.630	1.944.576	-33.647
1.970.817	-25.991	1.993.718	-32.630	1.944.743	-33.648
1.970.997	-25.991	1.993.837	-32.631	1.945.002	-33.650
1.971.115	-25.992	1.993.983	-32.632	1.945.266	-33.651
1.971.256	-25.992	1.994.160	-32.633	1.945.426	-33.652
1.971.451	-25.993	1.994.315	-32.633	1.945.557	-33.654
1.971.628	-25.993	1.994.471	-32.634	1.945.764	-33.655
1.971.805	-25.994	1.994.643	-32.635	1.945.910	-33.657
1.971.989	-25.995	1.994.828	-32.635	1.946.091	-33.658
1.972.224	-25.995	1.994.980	-32.636	1.946.227	-33.659
1.972.430	-25.996	1.995.179	-32.637	1.946.328	-33.661
1.972.520	-25.996	1.995.401	-32.638	1.946.406	-33.662
1.972.691	-25.997	1.995.524	-32.638	1.946.523	-33.664
1.972.899	-25.997	1.995.652	-32.639	1.946.768	-33.665
1.973.008	-25.998	1.995.844	-32.640	1.946.949	-33.667
1.973.129	-25.998	1.996.087	-32.640	1.947.110	-33.669
1.973.268	-25.999	1.996.252	-32.641	1.947.276	-33.670
1.973.448	-25.999	1.996.330	-32.642	1.947.419	-33.672

1.973.643	-26.000	1.996.478	-32.642	1.947.605	-33.673
1.973.811	-26.000	1.996.588	-32.643	1.947.807	-33.675
1.974.017	-26.001	1.996.729	-32.644	1.947.930	-33.677
1.974.182	-26.001	1.996.946	-32.644	1.948.083	-33.679
1.974.285	-26.002	1.997.144	-32.645	1.948.344	-33.680
1.974.433	-26.002	1.997.296	-32.646	1.948.474	-33.682
1.974.637	-26.003	1.997.419	-32.646	1.948.571	-33.684
1.974.800	-26.003	1.997.583	-32.647	1.948.801	-33.685
1.974.883	-26.004	1.997.769	-32.648	1.948.934	-33.687
1.975.114	-26.004	1.998.004	-32.648	1.949.027	-33.689
1.975.587	-26.005	1.998.168	-32.649	1.949.200	-33.691
1.975.952	-26.005	1.998.230	-32.650	1.949.415	-33.693
1.976.129	-26.006	1.998.369	-32.650	1.949.578	-33.695
1.976.156	-26.006	1.998.553	-32.651	1.949.740	-33.696
1.976.165	-26.007	1.998.741	-32.652	1.949.987	-33.698
1.976.261	-26.007	1.998.846	-32.652	1.950.163	-33.700
1.976.301	-26.008	1.999.074	-32.653	1.950.271	-33.702
1.976.409	-26.008	1.999.337	-32.654	1.950.468	-33.704
1.976.514	-26.009	1.999.480	-32.654	1.950.629	-33.706
1.976.621	-26.009	1.999.678	-32.655	1.950.704	-33.708
1.976.828	-26.010	1.999.861	-32.656	1.950.869	-33.710
1.977.020	-26.010	2.000.044	-32.656	1.951.115	-33.712
1.977.206	-26.011	2.000.206	-32.657	1.951.292	-33.714
1.977.421	-26.011	2.000.343	-32.658	1.951.342	-33.716
1.977.621	-26.012	2.000.455	-32.658	1.951.467	-33.718
1.977.811	-26.012	2.000.604	-32.659	1.951.718	-33.720
1.977.999	-26.012	2.000.797	-32.660	1.951.937	-33.722
1.978.125	-26.013	2.001.013	-32.660	1.952.078	-33.724
1.978.275	-26.013	2.001.185	-32.661	1.952.188	-33.726
1.978.490	-26.014	2.001.317	-32.662	1.952.381	-33.728

1.978.691	-26.014	2.001.494	-32.662	1.952.682	-33.729
1.978.839	-26.015	2.001.626	-32.663	1.952.901	-33.731
1.978.945	-26.015	2.001.760	-32.664	1.952.986	-33.733
1.979.090	-26.016	2.001.933	-32.664	1.953.028	-33.735
1.979.231	-26.016	2.002.094	-32.665	1.953.152	-33.737
1.979.321	-26.016	2.002.242	-32.666	1.953.371	-33.739
1.979.507	-26.017	2.002.406	-32.666	1.953.537	-33.741
1.979.699	-26.017	2.002.464	-32.667	1.953.689	-33.743
1.979.852	-26.018	2.002.547	-32.668	1.953.806	-33.745
1.980.038	-26.018	2.002.782	-32.668	1.953.911	-33.747
1.980.228	-26.018	2.002.962	-32.669	1.954.104	-33.749
1.980.443	-26.019	2.003.089	-32.669	1.954.288	-33.751
1.980.661	-26.019	2.003.306	-32.670	1.954.420	-33.753
1.980.853	-26.020	2.003.519	-32.671	1.954.601	-33.755
1.980.975	-26.020	2.003.698	-32.671	1.954.818	-33.757
1.981.109	-26.020	2.003.902	-32.672	1.954.968	-33.759
1.981.279	-26.021	2.004.090	-32.673	1.955.065	-33.761
1.981.407	-26.021	2.004.332	-32.673	1.955.244	-33.763
1.981.548	-26.022	2.004.529	-32.674	1.955.517	-33.765
1.981.731	-26.022	2.004.682	-32.674	1.955.705	-33.767
1.981.868	-26.022	2.004.820	-32.675	1.955.840	-33.768
1.982.005	-26.023	2.004.977	-32.675	1.956.053	-33.770
1.982.141	-26.023	2.005.159	-32.676	1.956.283	-33.772
1.982.251	-26.024	2.005.262	-32.676	1.956.445	-33.774
1.982.430	-26.024	2.005.389	-32.677	1.956.600	-33.776
1.982.664	-26.024	2.005.529	-32.678	1.956.770	-33.778
1.982.854	-26.025	2.005.627	-32.678	1.956.864	-33.780
1.983.004	-26.025	2.005.734	-32.679	1.956.983	-33.781
1.983.181	-26.025	2.005.903	-32.679	1.957.179	-33.783
1.983.393	-26.026	2.006.111	-32.680	1.957.337	-33.785

1.983.503	-26.026	2.006.337	-32.680	1.957.458	-33.787
1.983.607	-26.026	2.006.561	-32.681	1.957.590	-33.788
1.983.817	-26.027	2.006.742	-32.681	1.957.773	-33.790
1.983.980	-26.027	2.006.913	-32.682	1.957.919	-33.792
1.984.117	-26.027	2.007.101	-32.682	1.958.048	-33.793
1.984.312	-26.028	2.007.292	-32.683	1.958.199	-33.795
1.984.500	-26.028	2.007.433	-32.683	1.958.308	-33.797
1.984.697	-26.028	2.007.614	-32.683	1.958.493	-33.798
1.984.863	-26.029	2.007.824	-32.684	1.958.730	-33.800
1.984.960	-26.029	2.007.943	-32.684	1.958.909	-33.802
1.985.109	-26.029	2.008.082	-32.685	1.959.135	-33.803
1.985.295	-26.030	2.008.297	-32.685	1.959.337	-33.805
1.985.486	-26.030	2.008.479	-32.686	1.959.493	-33.806
1.985.620	-26.030	2.008.615	-32.686	1.959.655	-33.808
1.985.815	-26.031	2.008.763	-32.686	1.959.819	-33.810
1.986.037	-26.031	2.008.915	-32.687	1.960.047	-33.811
1.986.221	-26.031	2.009.074	-32.687	1.960.226	-33.813
1.986.389	-26.031	2.009.258	-32.688	1.960.304	-33.814
1.986.492	-26.032	2.009.440	-32.688	1.960.430	-33.815
1.986.655	-26.032	2.009.628	-32.688	1.960.652	-33.817
1.986.862	-26.032	2.009.870	-32.689	1.960.862	-33.818
1.987.081	-26.033	2.010.043	-32.689	1.961.004	-33.820
1.987.238	-26.033	2.010.121	-32.689	1.961.156	-33.821
1.987.383	-26.033	2.010.246	-32.690	1.961.324	-33.823
1.987.518	-26.034	2.010.425	-32.690	1.961.476	-33.824
1.987.612	-26.034	2.010.618	-32.690	1.961.619	-33.825
1.987.697	-26.034	2.010.768	-32.691	1.961.814	-33.827
1.987.784	-26.034	2.010.947	-32.691	1.962.059	-33.828
1.987.977	-26.035	2.011.154	-32.691	1.962.209	-33.829
1.988.201	-26.035	2.011.320	-32.692	1.962.280	-33.830

1.988.389	-26.035	2.011.476	-32.692	1.962.471	-33.832
1.988.522	-26.036	2.011.647	-32.692	1.962.738	-33.833
1.988.649	-26.036	2.011.817	-32.692	1.962.899	-33.834
1.988.864	-26.036	2.011.922	-32.693	1.962.997	-33.836
1.989.118	-26.036	2.012.048	-32.693	1.963.138	-33.837
1.989.265	-26.037	2.012.415	-32.693	1.963.304	-33.838
1.989.469	-26.037	2.012.863	-32.693	1.963.505	-33.839
1.989.715	-26.037	2.013.095	-32.694	1.963.660	-33.840
1.989.855	-26.037	2.013.172	-32.694	1.963.867	-33.842
1.990.014	-26.038	2.013.244	-32.694	1.964.099	-33.843
1.990.199	-26.038	2.013.331	-32.694	1.964.174	-33.844
1.990.307	-26.038	2.013.331	-32.695	1.964.263	-33.845
1.990.410	-26.039	2.013.382	-32.695	1.964.464	-33.846
1.990.580	-26.039	2.013.540	-32.695	1.964.648	-33.847
1.990.710	-26.039	2.013.653	-32.695	1.964.827	-33.848
1.990.835	-26.039	2.013.761	-32.696	1.964.987	-33.849
1.990.940	-26.040	2.013.936	-32.696	1.965.094	-33.851
1.991.174	-26.040	2.014.220	-32.696	1.965.341	-33.852
1.991.501	-26.040	2.014.366	-32.696	1.965.501	-33.853
1.991.702	-26.040	2.014.498	-32.696	1.965.517	-33.854
1.991.819	-26.041	2.014.796	-32.697	1.965.732	-33.855
1.991.951	-26.041	2.014.948	-32.697	1.966.140	-33.856
1.992.110	-26.041	2.015.070	-32.697	1.966.488	-33.857
1.992.276	-26.042	2.015.244	-32.697	1.966.736	-33.858
1.992.437	-26.042	2.015.385	-32.697	1.966.915	-33.859
1.992.618	-26.042	2.015.529	-32.698	1.967.027	-33.860
1.992.798	-26.042	2.015.703	-32.698	1.967.113	-33.861
1.992.932	-26.043	2.015.872	-32.698	1.967.133	-33.862
1.993.017	-26.043	2.016.068	-32.698	1.967.124	-33.863
1.993.223	-26.043	2.016.214	-32.699	1.967.220	-33.864

1.993.423	-26.043	2.016.368	-32.699	1.967.365	-33.865
1.993.531	-26.044	2.016.527	-32.699	1.967.484	-33.866
1.993.719	-26.044	2.016.608	-32.699	1.967.610	-33.867
1.993.940	-26.044	2.016.803	-32.699	1.967.753	-33.868
1.994.186	-26.045	2.017.067	-32.700	1.967.878	-33.869
1.994.377	-26.045	2.017.300	-32.700	1.968.118	-33.870
1.994.525	-26.045	2.017.477	-32.700	1.968.344	-33.870
1.994.688	-26.045	2.017.614	-32.700	1.968.486	-33.871
1.994.827	-26.046	2.017.827	-32.701	1.968.716	-33.872
1.994.960	-26.046	2.018.015	-32.701	1.968.794	-33.873
1.995.179	-26.046	2.018.058	-32.701	1.968.902	-33.874
1.995.329	-26.047	2.018.178	-32.701	1.969.147	-33.875
1.995.457	-26.047	2.018.392	-32.702	1.969.348	-33.876
1.995.623	-26.047	2.018.551	-32.702	1.969.485	-33.877
1.995.842	-26.047	2.018.669	-32.702	1.969.668	-33.878
1.996.008	-26.048	2.018.808	-32.703	1.969.845	-33.879
1.996.068	-26.048	2.019.003	-32.703	1.970.004	-33.880
1.996.237	-26.048	2.019.162	-32.703	1.970.154	-33.881
1.996.433	-26.049	2.019.299	-32.704	1.970.331	-33.882
1.996.696	-26.049	2.019.440	-32.704	1.970.533	-33.883
1.996.893	-26.049	2.019.590	-32.704	1.970.708	-33.884
1.996.985	-26.049	2.019.829	-32.705	1.970.813	-33.885
1.997.117	-26.050	2.020.080	-32.705	1.970.907	-33.886
1.997.242	-26.050	2.020.193	-32.705	1.971.066	-33.887
1.997.435	-26.050	2.020.242	-32.706	1.971.308	-33.888
1.997.802	-26.051	2.020.427	-32.706	1.971.485	-33.889
1.998.203	-26.051	2.020.616	-32.707	1.971.583	-33.890
1.998.465	-26.051	2.020.775	-32.707	1.971.774	-33.891
1.998.539	-26.052	2.020.947	-32.708	1.971.982	-33.892
1.998.541	-26.052	2.021.113	-32.708	1.972.108	-33.893

1.998.566	-26.052	2.021.362	-32.709	1.972.282	-33.894
1.998.604	-26.052	2.021.528	-32.709	1.972.511	-33.895
1.998.660	-26.053	2.021.667	-32.710	1.972.706	-33.896
1.998.783	-26.053	2.021.839	-32.710	1.972.809	-33.897
1.999.003	-26.053	2.021.982	-32.711	1.972.885	-33.898
1.999.216	-26.054	2.022.142	-32.711	1.973.044	-33.900
1.999.319	-26.054	2.022.280	-32.712	1.973.277	-33.901
1.999.453	-26.054	2.022.455	-32.712	1.973.407	-33.902
1.999.671	-26.055	2.022.681	-32.713	1.973.521	-33.903
1.999.836	-26.055	2.022.914	-32.713	1.973.717	-33.904
1.999.980	-26.055	2.023.046	-32.714	1.973.904	-33.905
2.000.186	-26.056	2.023.186	-32.715	1.974.055	-33.906
2.000.414	-26.056	2.023.367	-32.715	1.974.177	-33.907
2.000.564	-26.056	2.023.505	-32.716	1.974.294	-33.909
2.000.647	-26.057	2.023.689	-32.716	1.974.474	-33.910
2.000.829	-26.057	2.023.888	-32.717	1.974.734	-33.911
2.001.075	-26.057	2.024.039	-32.718	1.974.971	-33.912
2.001.212	-26.058	2.024.180	-32.718	1.975.078	-33.913
2.001.355	-26.058	2.024.344	-32.719	1.975.210	-33.914
2.001.510	-26.058	2.024.469	-32.720	1.975.452	-33.916
2.001.613	-26.058	2.024.536	-32.720	1.975.607	-33.917
2.001.785	-26.059	2.024.637	-32.721	1.975.639	-33.918
2.001.971	-26.059	2.024.754	-32.722	1.975.795	-33.919
2.002.141	-26.059	2.024.935	-32.722	1.976.041	-33.920
2.002.289	-26.060	2.025.190	-32.723	1.976.265	-33.922
2.002.446	-26.060	2.025.394	-32.724	1.976.474	-33.923
2.002.706	-26.060	2.025.508	-32.724	1.976.584	-33.924
2.002.983	-26.061	2.025.656	-32.725	1.976.732	-33.925
2.003.145	-26.061	2.025.916	-32.726	1.976.906	-33.927
2.003.242	-26.061	2.026.178	-32.726	1.977.103	-33.928

2.003.441	-26.062	2.026.353	-32.727	1.977.280	-33.929
2.003.624	-26.062	2.026.465	-32.728	1.977.399	-33.930
2.003.689	-26.062	2.026.621	-32.729	1.977.632	-33.932
2.003.797	-26.063	2.026.789	-32.729	1.977.823	-33.933
2.003.952	-26.063	2.026.926	-32.730	1.977.917	-33.934
2.004.106	-26.063	2.027.047	-32.731	1.978.039	-33.935
2.004.301	-26.064	2.027.224	-32.731	1.978.203	-33.937
2.004.545	-26.064	2.027.453	-32.732	1.978.372	-33.938
2.004.733	-26.064	2.027.612	-32.733	1.978.493	-33.939
2.004.848	-26.065	2.027.713	-32.734	1.978.682	-33.940
2.004.982	-26.065	2.027.831	-32.734	1.978.902	-33.942
2.005.107	-26.065	2.027.961	-32.735	1.979.111	-33.943
2.005.259	-26.066	2.028.167	-32.736	1.979.265	-33.944
2.005.432	-26.066	2.028.378	-32.737	1.979.386	-33.945
2.005.620	-26.066	2.028.575	-32.737	1.979.540	-33.947
2.005.781	-26.067	2.028.806	-32.738	1.979.628	-33.948
2.005.947	-26.067	2.028.989	-32.739	1.979.805	-33.949
2.006.138	-26.067	2.029.077	-32.740	1.980.045	-33.950
2.006.286	-26.068	2.029.182	-32.740	1.980.224	-33.951
2.006.436	-26.068	2.029.388	-32.741	1.980.389	-33.953
2.006.615	-26.068	2.029.585	-32.742	1.980.533	-33.954
2.006.801	-26.069	2.029.697	-32.742	1.980.625	-33.955
2.006.937	-26.069	2.029.870	-32.743	1.980.721	-33.956
2.007.123	-26.069	2.030.098	-32.744	1.980.914	-33.957
2.007.341	-26.070	2.030.311	-32.745	1.981.062	-33.958
2.007.526	-26.070	2.030.452	-32.745	1.981.100	-33.960
2.007.706	-26.070	2.030.593	-32.746	1.981.261	-33.961
2.007.796	-26.071	2.030.772	-32.747	1.981.485	-33.962
2.007.883	-26.071	2.030.954	-32.748	1.981.693	-33.963
2.008.048	-26.071	2.031.136	-32.748	1.981.859	-33.964

2.008.194	-26.072	2.031.281	-32.749	1.981.992	-33.965
2.008.409	-26.072	2.031.494	-32.750	1.982.213	-33.966
2.008.660	-26.073	2.031.718	-32.750	1.982.459	-33.967
2.008.799	-26.073	2.031.872	-32.751	1.982.621	-33.968
2.008.974	-26.073	2.031.945	-32.752	1.982.795	-33.969
2.009.151	-26.074	2.032.114	-32.753	1.982.959	-33.970
2.009.283	-26.074	2.032.314	-32.753	1.983.093	-33.971
2.009.502	-26.074	2.032.421	-32.754	1.983.315	-33.972
2.009.643	-26.075	2.032.574	-32.755	1.983.604	-33.973
2.009.731	-26.075	2.032.793	-32.755	1.983.777	-33.974
2.009.877	-26.075	2.033.031	-32.756	1.983.812	-33.975
2.010.043	-26.076	2.033.221	-32.757	1.983.876	-33.976
2.010.193	-26.076	2.033.351	-32.757	1.984.030	-33.977
2.010.302	-26.076	2.033.465	-32.758	1.984.272	-33.978
2.010.477	-26.077	2.033.609	-32.759	1.984.469	-33.979
2.010.661	-26.077	2.033.732	-32.759	1.984.576	-33.980
2.010.845	-26.078	2.033.888	-32.760	1.984.704	-33.980
2.011.080	-26.078	2.034.286	-32.761	1.984.874	-33.981
2.011.288	-26.078	2.034.742	-32.761	1.985.007	-33.982
2.011.436	-26.079	2.034.967	-32.762	1.985.199	-33.983
2.011.628	-26.079	2.035.052	-32.763	1.985.419	-33.984
2.011.877	-26.079	2.035.108	-32.763	1.985.589	-33.984
2.012.117	-26.080	2.035.172	-32.764	1.985.764	-33.985
2.012.269	-26.080	2.035.174	-32.765	1.985.988	-33.986
2.012.332	-26.081	2.035.259	-32.765	1.986.218	-33.987
2.012.455	-26.081	2.035.419	-32.766	1.986.387	-33.987
2.012.581	-26.081	2.035.524	-32.766	1.986.534	-33.988
2.012.706	-26.082	2.035.701	-32.767	1.986.685	-33.989
2.012.932	-26.082	2.035.918	-32.768	1.986.864	-33.989
2.013.174	-26.082	2.036.115	-32.768	1.986.973	-33.990

2.013.322	-26.083	2.036.332	-32.769	1.987.097	-33.991
2.013.363	-26.083	2.036.465	-32.769	1.987.296	-33.991
2.013.470	-26.084	2.036.563	-32.770	1.987.431	-33.992
2.013.669	-26.084	2.036.772	-32.771	1.987.626	-33.992
2.013.860	-26.084	2.036.956	-32.771	1.987.869	-33.993
2.014.021	-26.085	2.037.153	-32.772	1.988.020	-33.994
2.014.193	-26.085	2.037.289	-32.772	1.988.147	-33.994
2.014.350	-26.086	2.037.303	-32.773	1.988.293	-33.995
2.014.514	-26.086	2.037.500	-32.773	1.988.407	-33.995
2.014.758	-26.086	2.037.749	-32.774	1.988.540	-33.996
2.014.989	-26.087	2.037.917	-32.775	1.988.705	-33.996
2.015.139	-26.087	2.038.051	-32.775	1.988.877	-33.997
2.015.258	-26.088	2.038.181	-32.776	1.989.032	-33.997
2.015.406	-26.088	2.038.389	-32.776	1.989.189	-33.998
2.015.593	-26.089	2.038.533	-32.777	1.989.415	-33.998
2.015.788	-26.089	2.038.689	-32.777	1.989.622	-33.999
2.015.952	-26.089	2.038.918	-32.778	1.989.809	-33.999
2.016.095	-26.090	2.039.099	-32.778	1.989.955	-34.000
2.016.274	-26.090	2.039.292	-32.779	1.990.063	-34.000
2.016.344	-26.091	2.039.501	-32.779	1.990.183	-34.001
2.016.490	-26.091	2.039.653	-32.780	1.990.322	-34.001
2.016.848	-26.092	2.039.805	-32.780	1.990.551	-34.001
2.017.020	-26.092	2.039.939	-32.781	1.990.773	-34.002
2.017.124	-26.092	2.040.087	-32.782	1.990.943	-34.002
2.017.272	-26.093	2.040.231	-32.782	1.990.975	-34.003
2.017.394	-26.093	2.040.367	-32.783	1.991.133	-34.003
2.017.561	-26.094	2.040.540	-32.783	1.991.431	-34.004
2.017.762	-26.094	2.040.692	-32.784	1.991.604	-34.004
2.017.977	-26.095	2.040.806	-32.784	1.991.786	-34.004
2.018.138	-26.095	2.040.932	-32.785	1.991.982	-34.005

2.018.251	-26.096	2.041.138	-32.785	1.992.159	-34.005
2.018.463	-26.096	2.041.375	-32.786	1.992.265	-34.006
2.018.678	-26.096	2.041.554	-32.786	1.992.388	-34.006
2.018.839	-26.097	2.041.686	-32.787	1.992.502	-34.007
2.019.028	-26.097	2.041.866	-32.787	1.992.627	-34.007
2.019.173	-26.098	2.042.085	-32.788	1.993.046	-34.007
2.019.267	-26.098	2.042.299	-32.788	1.993.586	-34.008
2.019.393	-26.099	2.042.388	-32.789	1.993.876	-34.008
2.019.587	-26.099	2.042.444	-32.790	1.993.951	-34.009
2.019.791	-26.100	2.042.623	-32.790	1.994.014	-34.009
2.020.145	-26.100	2.042.822	-32.791	1.994.081	-34.009
2.020.569	-26.100	2.042.970	-32.791	1.994.124	-34.010
2.020.835	-26.101	2.043.147	-32.792	1.994.186	-34.010
2.020.923	-26.101	2.043.349	-32.792	1.994.250	-34.011
2.020.912	-26.102	2.043.492	-32.793	1.994.366	-34.011
2.020.984	-26.102	2.043.673	-32.793	1.994.493	-34.012
2.021.069	-26.103	2.043.875	-32.794	1.994.628	-34.012
2.021.091	-26.103	2.044.005	-32.794	1.994.782	-34.013
2.021.147	-26.104	2.044.121	-32.795	1.994.959	-34.013
2.021.313	-26.104	2.044.287	-32.796	1.995.154	-34.014
2.021.545	-26.105	2.044.464	-32.796	1.995.329	-34.014
2.021.703	-26.105	2.044.605	-32.797	1.995.508	-34.015
2.021.866	-26.106	2.044.807	-32.797	1.995.694	-34.015
2.022.038	-26.106	2.045.085	-32.798	1.995.851	-34.016
2.022.186	-26.106	2.045.138	-32.798	1.995.972	-34.017
2.022.401	-26.107	2.045.230	-32.799	1.996.124	-34.017
2.022.574	-26.107	2.045.480	-32.799	1.996.346	-34.018
2.022.691	-26.108	2.045.649	-32.800	1.996.534	-34.018
2.022.833	-26.108	2.045.808	-32.801	1.996.673	-34.019
2.023.046	-26.109	2.045.947	-32.801	1.996.803	-34.020

2.023.241	-26.109	2.046.097	-32.802	1.996.983	-34.020
2.023.405	-26.110	2.046.248	-32.802	1.997.168	-34.021
2.023.548	-26.110	2.046.386	-32.803	1.997.338	-34.022
2.023.680	-26.111	2.046.496	-32.803	1.997.480	-34.022
2.023.855	-26.111	2.046.633	-32.804	1.997.518	-34.023
2.024.026	-26.112	2.046.776	-32.804	1.997.673	-34.024
2.024.171	-26.112	2.046.935	-32.805	1.997.897	-34.025
2.024.384	-26.112	2.047.179	-32.805	1.998.048	-34.026
2.024.563	-26.113	2.047.440	-32.806	1.998.176	-34.026
2.024.651	-26.113	2.047.605	-32.806	1.998.383	-34.027
2.024.836	-26.114	2.047.757	-32.807	1.998.634	-34.028
2.025.079	-26.114	2.047.968	-32.807	1.998.745	-34.029
2.025.302	-26.115	2.048.178	-32.808	1.998.920	-34.030
2.025.488	-26.115	2.048.358	-32.808	1.999.128	-34.031
2.025.634	-26.116	2.048.513	-32.809	1.999.294	-34.032
2.025.773	-26.116	2.048.694	-32.809	1.999.478	-34.033
2.025.882	-26.116	2.048.864	-32.810	1.999.693	-34.034
2.026.019	-26.117	2.048.961	-32.810	1.999.890	-34.035
2.026.234	-26.117	2.049.034	-32.811	2.000.024	-34.036
2.026.427	-26.118	2.049.175	-32.811	2.000.172	-34.037
2.026.573	-26.118	2.049.319	-32.811	2.000.298	-34.038
2.026.709	-26.119	2.049.473	-32.812	2.000.453	-34.039
2.026.866	-26.119	2.049.642	-32.812	2.000.618	-34.041
2.027.036	-26.120	2.049.818	-32.813	2.000.784	-34.042
2.027.211	-26.120	2.050.006	-32.813	2.000.970	-34.043
2.027.404	-26.120	2.050.150	-32.813	2.001.149	-34.044
2.027.565	-26.121	2.050.327	-32.814	2.001.279	-34.045
2.027.722	-26.121	2.050.573	-32.814	2.001.411	-34.047
2.027.898	-26.122	2.050.777	-32.814	2.001.559	-34.048
2.028.039	-26.122	2.050.947	-32.815	2.001.693	-34.049

2.028.139	-26.123	2.051.138	-32.815	2.001.893	-34.051
2.028.259	-26.123	2.051.310	-32.815	2.002.148	-34.052
2.028.405	-26.124	2.051.422	-32.816	2.002.354	-34.053
2.028.604	-26.124	2.051.577	-32.816	2.002.401	-34.055
2.028.815	-26.124	2.051.740	-32.816	2.002.547	-34.056
2.028.994	-26.125	2.051.900	-32.817	2.002.728	-34.058
2.029.209	-26.125	2.052.076	-32.817	2.002.869	-34.059
2.029.408	-26.126	2.052.217	-32.817	2.003.019	-34.060
2.029.545	-26.126	2.052.411	-32.817	2.003.185	-34.062
2.029.677	-26.127	2.052.599	-32.818	2.003.382	-34.063
2.029.820	-26.127	2.052.759	-32.818	2.003.573	-34.065
2.029.978	-26.127	2.052.903	-32.818	2.003.741	-34.067
2.030.105	-26.128	2.053.064	-32.818	2.003.897	-34.068
2.030.224	-26.128	2.053.293	-32.819	2.004.099	-34.070
2.030.401	-26.129	2.053.510	-32.819	2.004.268	-34.071
2.030.558	-26.129	2.053.689	-32.819	2.004.391	-34.073
2.030.802	-26.130	2.053.774	-32.819	2.004.574	-34.075
2.031.075	-26.130	2.053.932	-32.819	2.004.659	-34.076
2.031.236	-26.131	2.054.169	-32.820	2.004.802	-34.078
2.031.373	-26.131	2.054.377	-32.820	2.005.047	-34.079
2.031.503	-26.131	2.054.505	-32.820	2.005.181	-34.081
2.031.653	-26.132	2.054.637	-32.820	2.005.314	-34.083
2.031.758	-26.132	2.054.816	-32.820	2.005.486	-34.085
2.031.895	-26.133	2.055.029	-32.820	2.005.647	-34.086
2.032.110	-26.133	2.055.257	-32.820	2.005.873	-34.088
2.032.280	-26.134	2.055.372	-32.821	2.006.167	-34.090
2.032.357	-26.134	2.055.459	-32.821	2.006.315	-34.091
2.032.462	-26.134	2.055.611	-32.821	2.006.366	-34.093
2.032.712	-26.135	2.055.768	-32.821	2.006.499	-34.095
2.032.897	-26.135	2.055.993	-32.821	2.006.673	-34.096

2.033.058	-26.136	2.056.389	-32.821	2.006.853	-34.098
2.033.266	-26.136	2.056.770	-32.821	2.007.000	-34.100
2.033.465	-26.137	2.056.904	-32.821	2.007.117	-34.102
2.033.669	-26.137	2.056.895	-32.821	2.007.269	-34.103
2.033.848	-26.138	2.056.978	-32.821	2.007.435	-34.105
2.034.041	-26.138	2.057.101	-32.822	2.007.626	-34.107
2.034.245	-26.138	2.057.175	-32.822	2.007.786	-34.108
2.034.408	-26.139	2.057.253	-32.822	2.007.917	-34.110
2.034.536	-26.139	2.057.343	-32.822	2.008.078	-34.112
2.034.677	-26.140	2.057.500	-32.822	2.008.219	-34.113
2.034.845	-26.140	2.057.672	-32.822	2.008.353	-34.115
2.034.967	-26.141	2.057.831	-32.822	2.008.544	-34.117
2.035.107	-26.141	2.058.024	-32.822	2.008.743	-34.118
2.035.276	-26.142	2.058.251	-32.822	2.008.900	-34.120
2.035.358	-26.142	2.058.436	-32.822	2.009.095	-34.122
2.035.481	-26.142	2.058.600	-32.822	2.009.290	-34.123
2.035.676	-26.143	2.058.811	-32.822	2.009.466	-34.125
2.035.827	-26.143	2.058.988	-32.822	2.009.661	-34.127
2.036.008	-26.144	2.059.158	-32.822	2.009.890	-34.128
2.036.220	-26.144	2.059.294	-32.823	2.010.089	-34.130
2.036.415	-26.145	2.059.415	-32.823	2.010.298	-34.131
2.036.577	-26.145	2.059.492	-32.823	2.010.472	-34.133
2.036.749	-26.146	2.059.625	-32.823	2.010.600	-34.134
2.037.009	-26.146	2.059.774	-32.823	2.010.755	-34.136
2.037.190	-26.147	2.059.964	-32.823	2.010.869	-34.138
2.037.289	-26.147	2.060.098	-32.823	2.010.965	-34.139
2.037.493	-26.148	2.060.181	-32.823	2.011.084	-34.140
2.037.681	-26.148	2.060.289	-32.824	2.011.176	-34.142
2.037.791	-26.149	2.060.513	-32.824	2.011.337	-34.143
2.037.903	-26.149	2.060.813	-32.824	2.011.561	-34.145

2.038.074	-26.150	2.061.060	-32.824	2.011.747	-34.146
2.038.243	-26.150	2.061.275	-32.824	2.011.888	-34.148
2.038.430	-26.151	2.061.462	-32.824	2.011.991	-34.149
2.038.624	-26.151	2.061.624	-32.825	2.012.166	-34.150
2.038.785	-26.152	2.061.745	-32.825	2.012.388	-34.152
2.038.989	-26.152	2.061.866	-32.825	2.012.561	-34.153
2.039.144	-26.153	2.062.014	-32.825	2.012.739	-34.154
2.039.308	-26.153	2.062.209	-32.826	2.012.939	-34.155
2.039.492	-26.154	2.062.350	-32.826	2.013.120	-34.157
2.039.659	-26.154	2.062.477	-32.826	2.013.322	-34.158
2.039.847	-26.155	2.062.699	-32.826	2.013.595	-34.159
2.040.034	-26.155	2.062.923	-32.827	2.013.788	-34.160
2.040.165	-26.156	2.063.074	-32.827	2.013.878	-34.161
2.040.316	-26.156	2.063.210	-32.827	2.013.976	-34.162
2.040.506	-26.157	2.063.279	-32.828	2.014.088	-34.164
2.040.658	-26.157	2.063.447	-32.828	2.014.321	-34.165
2.040.822	-26.158	2.063.685	-32.829	2.014.561	-34.166
2.040.983	-26.158	2.063.862	-32.829	2.014.686	-34.167
2.041.127	-26.159	2.063.996	-32.829	2.014.798	-34.168
2.041.319	-26.160	2.064.117	-32.830	2.014.966	-34.169
2.041.496	-26.160	2.064.214	-32.830	2.015.179	-34.170
2.041.673	-26.161	2.064.389	-32.831	2.015.365	-34.171
2.041.834	-26.161	2.064.608	-32.831	2.015.527	-34.172
2.041.993	-26.162	2.064.749	-32.832	2.015.665	-34.173
2.042.148	-26.162	2.064.971	-32.832	2.015.820	-34.173
2.042.325	-26.163	2.065.172	-32.833	2.016.098	-34.174
2.042.762	-26.163	2.065.282	-32.834	2.016.302	-34.175
2.043.187	-26.164	2.065.450	-32.834	2.016.429	-34.176
2.043.335	-26.165	2.065.620	-32.835	2.016.599	-34.177
2.043.327	-26.165	2.065.790	-32.835	2.016.747	-34.177

2.043.327	-26.166	2.065.932	-32.836	2.016.906	-34.178
2.043.443	-26.166	2.066.008	-32.837	2.017.074	-34.179
2.043.526	-26.167	2.066.156	-32.837	2.017.179	-34.180
2.043.596	-26.167	2.066.359	-32.838	2.017.357	-34.180
2.043.727	-26.168	2.066.564	-32.839	2.017.576	-34.181
2.043.905	-26.168	2.066.768	-32.840	2.017.654	-34.182
2.044.030	-26.169	2.066.936	-32.840	2.017.787	-34.182
2.044.191	-26.170	2.067.121	-32.841	2.017.993	-34.183
2.044.436	-26.170	2.067.269	-32.842	2.018.165	-34.184
2.044.567	-26.171	2.067.384	-32.843	2.018.360	-34.184
2.044.713	-26.171	2.067.509	-32.843	2.018.551	-34.185
2.044.960	-26.172	2.067.681	-32.844	2.018.738	-34.185
2.045.175	-26.172	2.067.851	-32.845	2.018.889	-34.186
2.045.313	-26.173	2.067.977	-32.846	2.018.989	-34.186
2.045.434	-26.174	2.068.143	-32.847	2.019.072	-34.187
2.045.611	-26.174	2.068.315	-32.848	2.019.221	-34.187
2.045.777	-26.175	2.068.461	-32.849	2.019.406	-34.188
2.045.916	-26.175	2.068.640	-32.850	2.019.590	-34.188
2.046.032	-26.176	2.068.828	-32.851	2.019.935	-34.189
2.046.178	-26.176	2.069.050	-32.851	2.020.336	-34.189
2.046.332	-26.177	2.069.231	-32.852	2.020.651	-34.189
2.046.494	-26.178	2.069.379	-32.853	2.020.800	-34.190
2.046.667	-26.178	2.069.576	-32.854	2.020.842	-34.190
2.046.846	-26.179	2.069.734	-32.855	2.020.934	-34.191
2.047.056	-26.179	2.069.964	-32.856	2.020.994	-34.191
2.047.204	-26.180	2.070.139	-32.857	2.021.057	-34.191
2.047.334	-26.180	2.070.222	-32.858	2.021.172	-34.192
2.047.556	-26.181	2.070.394	-32.859	2.021.255	-34.192
2.047.757	-26.182	2.070.625	-32.861	2.021.339	-34.192
2.047.937	-26.182	2.070.775	-32.862	2.021.487	-34.192

2.048.092	-26.183	2.070.919	-32.863	2.021.573	-34.193
2.048.250	-26.183	2.071.104	-32.864	2.021.756	-34.193
2.048.407	-26.184	2.071.216	-32.865	2.022.058	-34.193
2.048.548	-26.184	2.071.413	-32.866	2.022.251	-34.194
2.048.714	-26.185	2.071.563	-32.867	2.022.422	-34.194
2.048.889	-26.185	2.071.673	-32.868	2.022.578	-34.194
2.049.039	-26.186	2.071.891	-32.869	2.022.768	-34.195
2.049.189	-26.187	2.072.065	-32.870	2.022.953	-34.195
2.049.397	-26.187	2.072.189	-32.871	2.023.076	-34.195
2.049.554	-26.188	2.072.323	-32.872	2.023.239	-34.195
2.049.695	-26.188	2.072.542	-32.873	2.023.443	-34.196
2.049.832	-26.189	2.072.760	-32.875	2.023.654	-34.196
2.049.991	-26.189	2.072.948	-32.876	2.023.831	-34.196
2.050.222	-26.190	2.073.145	-32.877	2.023.953	-34.197
2.050.365	-26.190	2.073.246	-32.878	2.024.106	-34.197
2.050.475	-26.191	2.073.329	-32.879	2.024.286	-34.197
2.050.645	-26.191	2.073.543	-32.880	2.024.507	-34.197
2.050.798	-26.192	2.073.777	-32.881	2.024.643	-34.198
2.050.910	-26.193	2.073.875	-32.882	2.024.704	-34.198
2.051.073	-26.193	2.074.016	-32.883	2.024.843	-34.198
2.051.263	-26.194	2.074.216	-32.885	2.025.004	-34.199
2.051.433	-26.194	2.074.342	-32.886	2.025.166	-34.199
2.051.655	-26.195	2.074.545	-32.887	2.025.374	-34.200
2.051.805	-26.195	2.074.780	-32.888	2.025.573	-34.200
2.051.960	-26.196	2.074.914	-32.889	2.025.728	-34.200
2.052.148	-26.196	2.075.081	-32.890	2.025.889	-34.201
2.052.323	-26.197	2.075.235	-32.891	2.026.053	-34.201
2.052.470	-26.197	2.075.369	-32.892	2.026.255	-34.202
2.052.600	-26.198	2.075.546	-32.893	2.026.472	-34.202
2.052.782	-26.198	2.075.717	-32.894	2.026.674	-34.203

2.052.986	-26.199	2.075.880	-32.895	2.026.814	-34.203
2.053.141	-26.199	2.076.044	-32.896	2.026.940	-34.204
2.053.327	-26.200	2.076.270	-32.897	2.027.094	-34.204
2.053.519	-26.200	2.076.453	-32.898	2.027.240	-34.205
2.053.692	-26.201	2.076.597	-32.899	2.027.352	-34.205
2.053.866	-26.201	2.076.740	-32.901	2.027.453	-34.206
2.053.989	-26.202	2.076.906	-32.902	2.027.625	-34.207
2.054.135	-26.202	2.077.103	-32.902	2.027.789	-34.207
2.054.328	-26.203	2.077.289	-32.903	2.027.961	-34.208
2.054.482	-26.203	2.077.457	-32.904	2.028.089	-34.209
2.054.604	-26.204	2.077.565	-32.905	2.028.271	-34.209
2.054.704	-26.204	2.077.726	-32.906	2.028.510	-34.210
2.054.856	-26.205	2.078.096	-32.907	2.028.690	-34.211
2.055.004	-26.205	2.078.541	-32.908	2.028.869	-34.212
2.055.119	-26.206	2.078.738	-32.909	2.029.016	-34.213
2.055.260	-26.206	2.078.802	-32.910	2.029.191	-34.213
2.055.517	-26.207	2.078.885	-32.911	2.029.326	-34.214
2.055.712	-26.207	2.078.893	-32.912	2.029.462	-34.215
2.055.869	-26.208	2.078.929	-32.913	2.029.641	-34.216
2.056.077	-26.208	2.079.092	-32.913	2.029.796	-34.217
2.056.301	-26.209	2.079.238	-32.914	2.029.955	-34.218
2.056.546	-26.209	2.079.345	-32.915	2.030.082	-34.219
2.056.734	-26.210	2.079.447	-32.916	2.030.302	-34.220
2.056.843	-26.210	2.079.646	-32.917	2.030.511	-34.221
2.057.048	-26.211	2.079.892	-32.918	2.030.672	-34.222
2.057.238	-26.211	2.080.060	-32.918	2.030.824	-34.223
2.057.363	-26.212	2.080.184	-32.919	2.030.972	-34.224
2.057.529	-26.212	2.080.342	-32.920	2.031.203	-34.226
2.057.639	-26.213	2.080.558	-32.921	2.031.380	-34.227
2.057.744	-26.213	2.080.714	-32.921	2.031.487	-34.228

2.057.934	-26.214	2.080.853	-32.922	2.031.662	-34.229
2.058.111	-26.214	2.081.035	-32.923	2.031.877	-34.231
2.058.257	-26.215	2.081.205	-32.923	2.032.034	-34.232
2.058.319	-26.215	2.081.378	-32.924	2.032.195	-34.233
2.058.428	-26.216	2.081.583	-32.925	2.032.370	-34.234
2.058.653	-26.216	2.081.714	-32.925	2.032.520	-34.236
2.058.841	-26.217	2.081.808	-32.926	2.032.692	-34.237
2.059.012	-26.217	2.081.949	-32.927	2.032.787	-34.239
2.059.225	-26.217	2.082.143	-32.927	2.032.937	-34.240
2.059.427	-26.218	2.082.336	-32.928	2.033.174	-34.241
2.059.599	-26.218	2.082.513	-32.929	2.033.382	-34.243
2.059.776	-26.219	2.082.749	-32.929	2.033.575	-34.244
2.059.955	-26.219	2.082.959	-32.930	2.033.719	-34.246
2.060.130	-26.220	2.083.107	-32.930	2.033.824	-34.247
2.060.282	-26.220	2.083.328	-32.931	2.033.974	-34.249
2.060.443	-26.221	2.083.474	-32.932	2.034.079	-34.250
2.060.658	-26.221	2.083.602	-32.932	2.034.207	-34.252
2.060.788	-26.222	2.083.761	-32.933	2.034.371	-34.254
2.060.891	-26.222	2.083.924	-32.933	2.034.496	-34.255
2.061.131	-26.223	2.084.090	-32.934	2.034.605	-34.257
2.061.292	-26.223	2.084.186	-32.934	2.034.718	-34.258
2.061.433	-26.224	2.084.332	-32.935	2.034.910	-34.260
2.061.647	-26.224	2.084.525	-32.935	2.035.107	-34.262
2.061.855	-26.225	2.084.738	-32.936	2.035.266	-34.263
2.061.978	-26.225	2.084.910	-32.936	2.035.470	-34.265
2.062.130	-26.225	2.085.031	-32.937	2.035.639	-34.267
2.062.352	-26.226	2.085.137	-32.937	2.035.777	-34.268
2.062.482	-26.226	2.085.280	-32.938	2.035.952	-34.270
2.062.540	-26.227	2.085.490	-32.938	2.036.176	-34.272
2.062.721	-26.227	2.085.714	-32.939	2.036.436	-34.274

2.063.020	-26.228	2.085.909	-32.939	2.036.660	-34.275
2.063.154	-26.228	2.086.060	-32.940	2.036.906	-34.277
2.063.243	-26.229	2.086.196	-32.940	2.037.049	-34.279
2.063.436	-26.229	2.086.283	-32.941	2.037.090	-34.281
2.063.689	-26.230	2.086.474	-32.941	2.037.336	-34.283
2.063.848	-26.230	2.086.700	-32.942	2.037.542	-34.284
2.063.987	-26.230	2.086.879	-32.942	2.037.643	-34.286
2.064.153	-26.231	2.087.050	-32.943	2.037.822	-34.288
2.064.310	-26.231	2.087.181	-32.943	2.037.946	-34.290
2.064.419	-26.232	2.087.349	-32.944	2.038.100	-34.292
2.064.576	-26.232	2.087.525	-32.944	2.038.253	-34.294
2.064.982	-26.233	2.087.683	-32.944	2.038.335	-34.295
2.065.455	-26.233	2.087.829	-32.945	2.038.483	-34.297
2.065.669	-26.234	2.087.992	-32.945	2.038.669	-34.299
2.065.697	-26.234	2.088.174	-32.946	2.038.849	-34.301
2.065.750	-26.235	2.088.282	-32.946	2.039.019	-34.303
2.065.798	-26.235	2.088.378	-32.947	2.039.146	-34.305
2.065.854	-26.235	2.088.575	-32.947	2.039.287	-34.307
2.065.952	-26.236	2.088.820	-32.948	2.039.522	-34.309
2.066.046	-26.236	2.088.965	-32.948	2.039.745	-34.311
2.066.171	-26.237	2.089.079	-32.948	2.039.922	-34.313
2.066.346	-26.237	2.089.267	-32.949	2.040.107	-34.314
2.066.516	-26.238	2.089.446	-32.949	2.040.260	-34.316
2.066.682	-26.238	2.089.599	-32.950	2.040.430	-34.318
2.066.888	-26.238	2.089.836	-32.950	2.040.575	-34.320
2.067.076	-26.239	2.089.984	-32.951	2.040.763	-34.322
2.067.224	-26.239	2.090.060	-32.951	2.040.979	-34.324
2.067.441	-26.240	2.090.244	-32.951	2.041.203	-34.326
2.067.616	-26.240	2.090.376	-32.952	2.041.371	-34.328
2.067.789	-26.240	2.090.470	-32.952	2.041.461	-34.330

2.067.995	-26.241	2.090.620	-32.953	2.041.604	-34.332
2.068.174	-26.241	2.090.824	-32.953	2.041.727	-34.334
2.068.328	-26.242	2.091.051	-32.953	2.041.854	-34.336
2.068.409	-26.242	2.091.214	-32.954	2.042.094	-34.338
2.068.501	-26.242	2.091.425	-32.954	2.042.280	-34.340
2.068.683	-26.243	2.091.704	-32.955	2.042.419	-34.342
2.068.909	-26.243	2.091.861	-32.955	2.042.614	-34.344
2.069.072	-26.244	2.091.965	-32.955	2.042.827	-34.346
2.069.162	-26.244	2.092.171	-32.956	2.042.968	-34.348
2.069.335	-26.244	2.092.383	-32.956	2.043.058	-34.350
2.069.549	-26.245	2.092.552	-32.956	2.043.185	-34.351
2.069.751	-26.245	2.092.675	-32.957	2.043.367	-34.353
2.069.946	-26.246	2.092.777	-32.957	2.043.596	-34.355
2.070.157	-26.246	2.092.914	-32.958	2.043.779	-34.357
2.070.341	-26.246	2.093.098	-32.958	2.043.956	-34.359
2.070.486	-26.247	2.093.244	-32.958	2.044.075	-34.361
2.070.629	-26.247	2.093.382	-32.959	2.044.144	-34.363
2.070.730	-26.247	2.093.571	-32.959	2.044.292	-34.365
2.070.916	-26.248	2.093.745	-32.959	2.044.525	-34.367
2.071.111	-26.248	2.093.951	-32.960	2.044.682	-34.369
2.071.268	-26.248	2.094.113	-32.960	2.044.820	-34.371
2.071.422	-26.249	2.094.323	-32.960	2.045.043	-34.373
2.071.550	-26.249	2.094.556	-32.961	2.045.300	-34.375
2.071.707	-26.249	2.094.669	-32.961	2.045.486	-34.377
2.071.908	-26.250	2.094.764	-32.961	2.045.598	-34.379
2.072.066	-26.250	2.094.953	-32.962	2.045.793	-34.381
2.072.224	-26.250	2.095.141	-32.962	2.045.976	-34.383
2.072.422	-26.251	2.095.300	-32.962	2.046.087	-34.384
2.072.592	-26.251	2.095.486	-32.963	2.046.203	-34.386
2.072.757	-26.251	2.095.618	-32.963	2.046.333	-34.388

2.072.885	-26.252	2.095.772	-32.963	2.046.462	-34.390
2.073.018	-26.252	2.095.927	-32.964	2.046.792	-34.392
2.073.192	-26.252	2.096.115	-32.964	2.047.258	-34.394
2.073.380	-26.253	2.096.339	-32.964	2.047.598	-34.396
2.073.550	-26.253	2.096.481	-32.965	2.047.757	-34.398
2.073.748	-26.253	2.096.633	-32.965	2.047.793	-34.400
2.073.956	-26.253	2.096.772	-32.965	2.047.861	-34.402
2.074.050	-26.254	2.096.933	-32.966	2.047.986	-34.403
2.074.189	-26.254	2.097.069	-32.966	2.048.116	-34.405
2.074.431	-26.254	2.097.225	-32.966	2.048.177	-34.407
2.074.581	-26.254	2.097.509	-32.967	2.048.165	-34.409
2.074.720	-26.255	2.097.666	-32.967	2.048.271	-34.411
2.074.892	-26.255	2.097.784	-32.967	2.048.452	-34.413
2.075.096	-26.255	2.097.952	-32.967	2.048.642	-34.415
2.075.275	-26.256	2.098.080	-32.968	2.048.820	-34.416
2.075.397	-26.256	2.098.250	-32.968	2.048.967	-34.418
2.075.546	-26.256	2.098.454	-32.968	2.049.139	-34.420
2.075.668	-26.256	2.098.672	-32.969	2.049.314	-34.422
2.075.849	-26.256	2.098.808	-32.969	2.049.491	-34.424
2.076.041	-26.257	2.098.844	-32.969	2.049.689	-34.426
2.076.243	-26.257	2.099.079	-32.969	2.049.858	-34.427
2.076.452	-26.257	2.099.381	-32.970	2.049.991	-34.429
2.076.556	-26.257	2.099.518	-32.970	2.050.186	-34.431
2.076.702	-26.258	2.099.686	-32.970	2.050.332	-34.433
2.076.875	-26.258	2.100.105	-32.970	2.050.472	-34.435
2.077.000	-26.258	2.100.484	-32.971	2.050.634	-34.437
2.077.137	-26.258	2.100.584	-32.971	2.050.763	-34.438
2.077.283	-26.259	2.100.658	-32.971	2.050.864	-34.440
2.077.414	-26.259	2.100.773	-32.971	2.050.999	-34.442
2.077.525	-26.259	2.100.813	-32.972	2.051.207	-34.444

2.077.693	-26.259	2.100.831	-32.972	2.051.422	-34.446
2.077.816	-26.259	2.100.948	-32.972	2.051.592	-34.447
2.077.984	-26.260	2.101.095	-32.972	2.051.749	-34.449
2.078.239	-26.260	2.101.238	-32.973	2.051.879	-34.451
2.078.440	-26.260	2.101.400	-32.973	2.051.989	-34.453
2.078.647	-26.260	2.101.544	-32.973	2.052.186	-34.455
2.078.867	-26.260	2.101.724	-32.973	2.052.424	-34.457
2.079.137	-26.261	2.101.954	-32.974	2.052.598	-34.458
2.079.321	-26.261	2.102.113	-32.974	2.052.747	-34.460
2.079.454	-26.261	2.102.332	-32.974	2.052.903	-34.462
2.079.613	-26.261	2.102.529	-32.974	2.053.069	-34.464
2.079.798	-26.261	2.102.596	-32.974	2.053.270	-34.466
2.079.987	-26.262	2.102.721	-32.975	2.053.476	-34.468
2.080.096	-26.262	2.102.945	-32.975	2.053.663	-34.470
2.080.212	-26.262	2.103.113	-32.975	2.053.797	-34.471
2.080.329	-26.262	2.103.252	-32.975	2.053.988	-34.473
2.080.486	-26.262	2.103.402	-32.975	2.054.184	-34.475
2.080.640	-26.263	2.103.570	-32.976	2.054.314	-34.477
2.080.757	-26.263	2.103.775	-32.976	2.054.460	-34.479
2.080.939	-26.263	2.103.898	-32.976	2.054.585	-34.481
2.081.134	-26.263	2.104.012	-32.976	2.054.722	-34.483
2.081.312	-26.263	2.104.212	-32.976	2.054.875	-34.485
2.081.472	-26.264	2.104.370	-32.977	2.055.045	-34.486
2.081.666	-26.264	2.104.588	-32.977	2.055.161	-34.488
2.081.908	-26.264	2.104.829	-32.977	2.055.322	-34.490
2.082.103	-26.264	2.104.991	-32.977	2.055.569	-34.492
2.082.262	-26.264	2.105.148	-32.978	2.055.732	-34.494
2.082.319	-26.265	2.105.253	-32.978	2.055.918	-34.496
2.082.471	-26.265	2.105.453	-32.978	2.056.108	-34.498
2.082.701	-26.265	2.105.592	-32.978	2.056.237	-34.500

2.082.869	-26.265	2.105.704	-32.978	2.056.341	-34.502
2.083.055	-26.265	2.105.905	-32.979	2.056.505	-34.504
2.083.208	-26.266	2.106.078	-32.979	2.056.703	-34.505
2.083.378	-26.266	2.106.275	-32.979	2.056.825	-34.507
2.083.526	-26.266	2.106.438	-32.979	2.057.000	-34.509
2.083.665	-26.266	2.106.465	-32.980	2.057.143	-34.511
2.083.848	-26.267	2.106.631	-32.980	2.057.334	-34.513
2.084.021	-26.267	2.106.875	-32.980	2.057.576	-34.515
2.084.185	-26.267	2.107.025	-32.980	2.057.701	-34.517
2.084.352	-26.267	2.107.213	-32.981	2.057.798	-34.519
2.084.530	-26.267	2.107.381	-32.981	2.058.024	-34.521
2.084.755	-26.268	2.107.565	-32.981	2.058.190	-34.522
2.084.955	-26.268	2.107.747	-32.981	2.058.378	-34.524
2.085.074	-26.268	2.107.910	-32.982	2.058.604	-34.526
2.085.226	-26.268	2.108.078	-32.982	2.058.723	-34.528
2.085.383	-26.268	2.108.298	-32.982	2.058.839	-34.530
2.085.515	-26.269	2.108.447	-32.983	2.059.039	-34.532
2.085.670	-26.269	2.108.561	-32.983	2.059.240	-34.534
2.085.815	-26.269	2.108.754	-32.983	2.059.406	-34.535
2.085.968	-26.269	2.108.983	-32.983	2.059.559	-34.537
2.086.178	-26.269	2.109.162	-32.984	2.059.729	-34.539
2.086.409	-26.270	2.109.276	-32.984	2.059.939	-34.541
2.086.536	-26.270	2.109.393	-32.984	2.060.016	-34.543
2.086.629	-26.270	2.109.548	-32.985	2.060.092	-34.544
2.086.819	-26.270	2.109.780	-32.985	2.060.300	-34.546
2.087.101	-26.271	2.110.013	-32.985	2.060.515	-34.548
2.087.478	-26.271	2.110.141	-32.986	2.060.728	-34.550
2.087.856	-26.271	2.110.242	-32.986	2.060.874	-34.551
2.088.062	-26.271	2.110.392	-32.986	2.061.015	-34.553
2.088.102	-26.271	2.110.584	-32.987	2.061.225	-34.555

2.088.152	-26.272	2.110.804	-32.987	2.061.445	-34.556
2.088.230	-26.272	2.110.938	-32.987	2.061.539	-34.558
2.088.263	-26.272	2.111.046	-32.988	2.061.592	-34.560
2.088.349	-26.272	2.111.189	-32.988	2.061.729	-34.561
2.088.484	-26.273	2.111.406	-32.988	2.061.928	-34.563
2.088.616	-26.273	2.111.590	-32.989	2.062.108	-34.564
2.088.781	-26.273	2.111.668	-32.989	2.062.216	-34.566
2.088.947	-26.273	2.111.736	-32.989	2.062.359	-34.567
2.089.128	-26.274	2.111.879	-32.990	2.062.522	-34.569
2.089.379	-26.274	2.112.065	-32.990	2.062.766	-34.570
2.089.567	-26.274	2.112.207	-32.991	2.062.966	-34.572
2.089.722	-26.274	2.112.325	-32.991	2.063.107	-34.573
2.089.872	-26.274	2.112.535	-32.991	2.063.271	-34.575
2.090.027	-26.275	2.112.742	-32.992	2.063.477	-34.576
2.090.224	-26.275	2.112.941	-32.992	2.063.644	-34.578
2.090.412	-26.275	2.113.176	-32.992	2.063.835	-34.579
2.090.524	-26.275	2.113.405	-32.993	2.064.059	-34.580
2.090.658	-26.276	2.113.608	-32.993	2.064.254	-34.582
2.090.826	-26.276	2.113.776	-32.993	2.064.465	-34.583
2.090.927	-26.276	2.113.954	-32.994	2.064.641	-34.584
2.091.077	-26.276	2.114.133	-32.994	2.064.735	-34.585
2.091.268	-26.276	2.114.247	-32.994	2.064.825	-34.587
2.091.385	-26.277	2.114.413	-32.995	2.064.926	-34.588
2.091.525	-26.277	2.114.621	-32.995	2.065.042	-34.589
2.091.740	-26.277	2.114.762	-32.996	2.065.203	-34.590
2.091.951	-26.277	2.114.825	-32.996	2.065.354	-34.591
2.092.146	-26.278	2.114.897	-32.996	2.065.473	-34.593
2.092.336	-26.278	2.115.083	-32.997	2.065.614	-34.594
2.092.498	-26.278	2.115.268	-32.997	2.065.789	-34.595
2.092.621	-26.278	2.115.408	-32.997	2.066.021	-34.596

2.092.782	-26.278	2.115.603	-32.998	2.066.216	-34.597
2.092.972	-26.279	2.115.875	-32.998	2.066.416	-34.598
2.093.163	-26.279	2.116.091	-32.998	2.066.637	-34.599
2.093.290	-26.279	2.116.292	-32.999	2.066.850	-34.600
2.093.383	-26.279	2.116.552	-32.999	2.066.998	-34.601
2.093.577	-26.279	2.116.685	-32.999	2.067.059	-34.602
2.093.781	-26.280	2.116.756	-33.000	2.067.207	-34.603
2.093.916	-26.280	2.116.870	-33.000	2.067.417	-34.603
2.094.073	-26.280	2.117.025	-33.000	2.067.679	-34.604
2.094.256	-26.280	2.117.224	-33.001	2.067.892	-34.605
2.094.438	-26.280	2.117.403	-33.001	2.067.980	-34.606
2.094.610	-26.281	2.117.596	-33.001	2.068.091	-34.607
2.094.736	-26.281	2.117.744	-33.002	2.068.286	-34.607
2.094.854	-26.281	2.117.908	-33.002	2.068.481	-34.608
2.095.090	-26.281	2.118.078	-33.002	2.068.667	-34.609
2.095.354	-26.281	2.118.203	-33.003	2.068.795	-34.610
2.095.470	-26.282	2.118.412	-33.003	2.068.951	-34.610
2.095.560	-26.282	2.118.570	-33.004	2.069.180	-34.611
2.095.725	-26.282	2.118.696	-33.004	2.069.345	-34.612
2.095.918	-26.282	2.118.969	-33.004	2.069.466	-34.612
2.096.097	-26.282	2.119.160	-33.005	2.069.572	-34.613
2.096.304	-26.283	2.119.198	-33.005	2.069.765	-34.613
2.096.490	-26.283	2.119.353	-33.005	2.070.060	-34.614
2.096.664	-26.283	2.119.569	-33.006	2.070.311	-34.614
2.096.835	-26.283	2.119.708	-33.006	2.070.466	-34.615
2.096.974	-26.283	2.119.856	-33.006	2.070.557	-34.615
2.097.191	-26.283	2.120.062	-33.007	2.070.616	-34.616
2.097.336	-26.284	2.120.242	-33.007	2.070.790	-34.616
2.097.415	-26.284	2.120.401	-33.007	2.071.015	-34.617
2.097.590	-26.284	2.120.495	-33.008	2.071.169	-34.617

2.097.813	-26.284	2.120.636	-33.008	2.071.359	-34.618
2.098.008	-26.284	2.120.875	-33.009	2.071.514	-34.618
2.098.132	-26.284	2.121.073	-33.009	2.071.640	-34.619
2.098.293	-26.285	2.121.182	-33.009	2.071.830	-34.619
2.098.439	-26.285	2.121.315	-33.010	2.072.005	-34.619
2.098.631	-26.285	2.121.651	-33.010	2.072.130	-34.620
2.098.835	-26.285	2.122.103	-33.010	2.072.285	-34.620
2.098.936	-26.285	2.122.478	-33.011	2.072.522	-34.620
2.099.077	-26.285	2.122.529	-33.011	2.072.697	-34.621
2.099.186	-26.286	2.122.520	-33.012	2.072.825	-34.621
2.099.310	-26.286	2.122.610	-33.012	2.072.966	-34.621
2.099.548	-26.286	2.122.659	-33.012	2.073.122	-34.622
2.099.693	-26.286	2.122.768	-33.013	2.073.288	-34.622
2.099.758	-26.286	2.122.905	-33.013	2.073.434	-34.622
2.099.838	-26.286	2.123.002	-33.014	2.073.705	-34.623
2.100.047	-26.286	2.123.125	-33.014	2.074.164	-34.623
2.100.291	-26.287	2.123.319	-33.014	2.074.532	-34.623
2.100.470	-26.287	2.123.557	-33.015	2.074.634	-34.623
2.100.668	-26.287	2.123.774	-33.015	2.074.670	-34.624
2.100.927	-26.287	2.123.974	-33.016	2.074.799	-34.624
2.101.113	-26.287	2.124.137	-33.016	2.074.955	-34.624
2.101.274	-26.287	2.124.328	-33.017	2.074.980	-34.624
2.101.509	-26.287	2.124.502	-33.017	2.074.988	-34.625
2.101.702	-26.287	2.124.628	-33.018	2.075.098	-34.625
2.101.866	-26.288	2.124.780	-33.018	2.075.247	-34.625
2.102.048	-26.288	2.124.926	-33.018	2.075.320	-34.625
2.102.160	-26.288	2.125.069	-33.019	2.075.479	-34.625
2.102.222	-26.288	2.125.231	-33.019	2.075.667	-34.626
2.102.417	-26.288	2.125.398	-33.020	2.075.817	-34.626
2.102.551	-26.288	2.125.547	-33.020	2.076.043	-34.626

2.102.636	-26.288	2.125.629	-33.021	2.076.230	-34.626
2.102.858	-26.289	2.125.717	-33.021	2.076.449	-34.627
2.103.122	-26.289	2.125.905	-33.022	2.076.673	-34.627
2.103.295	-26.289	2.126.115	-33.023	2.076.845	-34.627
2.103.380	-26.289	2.126.317	-33.023	2.076.938	-34.627
2.103.490	-26.289	2.126.503	-33.024	2.077.049	-34.628
2.103.699	-26.289	2.126.671	-33.024	2.077.233	-34.628
2.103.953	-26.289	2.126.839	-33.025	2.077.432	-34.628
2.104.173	-26.289	2.127.022	-33.025	2.077.623	-34.629
2.104.350	-26.290	2.127.191	-33.026	2.077.746	-34.629
2.104.496	-26.290	2.127.312	-33.027	2.077.896	-34.629
2.104.625	-26.290	2.127.448	-33.027	2.078.091	-34.629
2.104.765	-26.290	2.127.647	-33.028	2.078.272	-34.630
2.104.915	-26.290	2.127.845	-33.029	2.078.452	-34.630
2.105.074	-26.290	2.128.009	-33.029	2.078.593	-34.630
2.105.282	-26.290	2.128.122	-33.030	2.078.691	-34.631
2.105.446	-26.290	2.128.224	-33.031	2.078.820	-34.631
2.105.607	-26.291	2.128.398	-33.031	2.079.007	-34.632
2.105.802	-26.291	2.128.537	-33.032	2.079.194	-34.632
2.105.923	-26.291	2.128.664	-33.033	2.079.353	-34.632
2.106.070	-26.291	2.128.932	-33.034	2.079.527	-34.633
2.106.239	-26.291	2.129.211	-33.034	2.079.713	-34.633
2.106.418	-26.291	2.129.361	-33.035	2.079.899	-34.634
2.106.586	-26.291	2.129.448	-33.036	2.080.074	-34.634
2.106.689	-26.291	2.129.577	-33.037	2.080.282	-34.635
2.106.879	-26.292	2.129.755	-33.038	2.080.524	-34.635
2.107.105	-26.292	2.129.928	-33.038	2.080.681	-34.636
2.107.291	-26.292	2.130.123	-33.039	2.080.737	-34.636
2.107.486	-26.292	2.130.336	-33.040	2.080.858	-34.637
2.107.673	-26.292	2.130.493	-33.041	2.081.077	-34.637

2.107.796	-26.292	2.130.679	-33.042	2.081.227	-34.638
2.107.943	-26.292	2.130.884	-33.043	2.081.326	-34.639
2.108.089	-26.292	2.131.008	-33.044	2.081.512	-34.639
2.108.223	-26.293	2.131.134	-33.045	2.081.693	-34.640
2.108.387	-26.293	2.131.283	-33.045	2.081.785	-34.641
2.108.593	-26.293	2.131.447	-33.046	2.081.890	-34.641
2.108.709	-26.293	2.131.622	-33.047	2.082.099	-34.642
2.108.895	-26.293	2.131.837	-33.048	2.082.319	-34.643
2.109.112	-26.293	2.132.034	-33.049	2.082.473	-34.643
2.109.280	-26.293	2.132.171	-33.050	2.082.571	-34.644
2.109.558	-26.293	2.132.330	-33.051	2.082.789	-34.645
2.109.967	-26.294	2.132.480	-33.052	2.082.932	-34.646
2.110.293	-26.294	2.132.693	-33.053	2.083.029	-34.647
2.110.459	-26.294	2.132.905	-33.054	2.083.241	-34.647
2.110.538	-26.294	2.133.044	-33.055	2.083.458	-34.648
2.110.592	-26.294	2.133.183	-33.056	2.083.599	-34.649
2.110.623	-26.294	2.133.290	-33.057	2.083.747	-34.650
2.110.618	-26.294	2.133.391	-33.058	2.083.934	-34.651
2.110.751	-26.294	2.133.552	-33.059	2.084.108	-34.652
2.110.885	-26.294	2.133.689	-33.060	2.084.290	-34.653
2.111.028	-26.295	2.133.832	-33.062	2.084.478	-34.654
2.111.225	-26.295	2.134.054	-33.063	2.084.648	-34.655
2.111.406	-26.295	2.134.216	-33.064	2.084.832	-34.656
2.111.600	-26.295	2.134.355	-33.065	2.085.011	-34.657
2.111.821	-26.295	2.134.549	-33.066	2.085.197	-34.658
2.112.029	-26.295	2.134.742	-33.067	2.085.329	-34.659
2.112.173	-26.295	2.134.957	-33.068	2.085.468	-34.660
2.112.354	-26.295	2.135.175	-33.069	2.085.636	-34.661
2.112.542	-26.296	2.135.325	-33.070	2.085.798	-34.662
2.112.659	-26.296	2.135.510	-33.071	2.085.963	-34.663

2.112.728	-26.296	2.135.734	-33.072	2.086.098	-34.664
2.112.867	-26.296	2.135.954	-33.073	2.086.303	-34.665
2.113.022	-26.296	2.136.104	-33.074	2.086.516	-34.666
2.113.194	-26.296	2.136.198	-33.075	2.086.644	-34.667
2.113.391	-26.296	2.136.333	-33.076	2.086.761	-34.668
2.113.559	-26.296	2.136.458	-33.077	2.086.900	-34.669
2.113.651	-26.296	2.136.579	-33.079	2.087.077	-34.671
2.113.761	-26.296	2.136.700	-33.080	2.087.345	-34.672
2.113.942	-26.297	2.136.868	-33.081	2.087.513	-34.673
2.114.142	-26.297	2.137.063	-33.082	2.087.619	-34.674
2.114.384	-26.297	2.137.211	-33.083	2.087.791	-34.675
2.114.655	-26.297	2.137.370	-33.084	2.087.946	-34.676
2.114.850	-26.297	2.137.547	-33.085	2.088.091	-34.677
2.114.995	-26.297	2.137.796	-33.086	2.088.214	-34.678
2.115.143	-26.297	2.138.015	-33.087	2.088.378	-34.680
2.115.273	-26.297	2.138.165	-33.088	2.088.515	-34.681
2.115.417	-26.297	2.138.358	-33.089	2.088.631	-34.682
2.115.569	-26.297	2.138.560	-33.090	2.088.797	-34.683
2.115.674	-26.297	2.138.717	-33.091	2.088.954	-34.684
2.115.842	-26.297	2.138.873	-33.092	2.089.117	-34.685
2.116.062	-26.298	2.139.057	-33.093	2.089.286	-34.686
2.116.236	-26.298	2.139.205	-33.094	2.089.475	-34.688
2.116.373	-26.298	2.139.370	-33.095	2.089.628	-34.689
2.116.530	-26.298	2.139.525	-33.096	2.089.762	-34.690
2.116.711	-26.298	2.139.668	-33.096	2.090.005	-34.691
2.116.882	-26.298	2.139.836	-33.097	2.090.224	-34.692
2.117.038	-26.298	2.140.036	-33.098	2.090.414	-34.693
2.117.186	-26.298	2.140.202	-33.099	2.090.692	-34.694
2.117.384	-26.298	2.140.365	-33.100	2.090.869	-34.695
2.117.581	-26.298	2.140.591	-33.101	2.091.046	-34.696

2.117.733	-26.298	2.140.750	-33.102	2.091.208	-34.697
2.117.854	-26.298	2.140.885	-33.103	2.091.342	-34.699
2.118.015	-26.298	2.141.040	-33.104	2.091.521	-34.700
2.118.216	-26.298	2.141.152	-33.105	2.091.617	-34.701
2.118.398	-26.298	2.141.290	-33.105	2.091.796	-34.702
2.118.605	-26.299	2.141.497	-33.106	2.091.956	-34.703
2.118.840	-26.299	2.141.657	-33.107	2.092.117	-34.704
2.118.998	-26.299	2.141.789	-33.108	2.092.278	-34.705
2.119.088	-26.299	2.141.966	-33.109	2.092.399	-34.706
2.119.220	-26.299	2.142.175	-33.109	2.092.574	-34.707
2.119.458	-26.299	2.142.343	-33.110	2.092.769	-34.708
2.119.657	-26.299	2.142.495	-33.111	2.092.905	-34.709
2.119.789	-26.299	2.142.722	-33.112	2.093.024	-34.710
2.119.958	-26.299	2.142.934	-33.112	2.093.194	-34.711
2.120.067	-26.299	2.143.038	-33.113	2.093.347	-34.712
2.120.138	-26.299	2.143.136	-33.114	2.093.521	-34.713
2.120.244	-26.299	2.143.356	-33.115	2.093.732	-34.714
2.120.443	-26.299	2.143.810	-33.115	2.093.990	-34.714
2.120.681	-26.299	2.144.198	-33.116	2.094.130	-34.715
2.120.869	-26.299	2.144.308	-33.117	2.094.180	-34.716
2.121.048	-26.299	2.144.335	-33.117	2.094.330	-34.717
2.121.190	-26.299	2.144.368	-33.118	2.094.599	-34.718
2.121.321	-26.299	2.144.424	-33.119	2.094.823	-34.719
2.121.507	-26.299	2.144.467	-33.119	2.094.977	-34.720
2.121.725	-26.299	2.144.529	-33.120	2.095.123	-34.721
2.121.826	-26.299	2.144.682	-33.121	2.095.278	-34.721
2.121.900	-26.300	2.144.883	-33.121	2.095.387	-34.722
2.122.067	-26.300	2.145.112	-33.122	2.095.544	-34.723
2.122.234	-26.300	2.145.327	-33.122	2.095.768	-34.724
2.122.363	-26.300	2.145.454	-33.123	2.095.901	-34.725

2.122.513	-26.300	2.145.591	-33.123	2.096.003	-34.726
2.122.697	-26.300	2.145.797	-33.124	2.096.203	-34.726
2.122.887	-26.300	2.146.030	-33.124	2.096.462	-34.727
2.123.138	-26.300	2.146.279	-33.125	2.096.620	-34.728
2.123.363	-26.300	2.146.411	-33.126	2.096.747	-34.729
2.123.530	-26.300	2.146.499	-33.126	2.096.933	-34.729
2.123.730	-26.300	2.146.702	-33.127	2.097.090	-34.730
2.123.873	-26.300	2.146.821	-33.127	2.097.255	-34.731
2.123.969	-26.300	2.146.924	-33.128	2.097.426	-34.732
2.124.117	-26.300	2.147.128	-33.128	2.097.576	-34.732
2.124.370	-26.300	2.147.349	-33.128	2.097.724	-34.733
2.124.608	-26.300	2.147.509	-33.129	2.097.924	-34.734
2.124.690	-26.300	2.147.612	-33.129	2.098.082	-34.735
2.124.811	-26.300	2.147.759	-33.130	2.098.277	-34.735
2.124.958	-26.301	2.147.990	-33.130	2.098.573	-34.736
2.125.098	-26.301	2.148.216	-33.131	2.098.707	-34.737
2.125.284	-26.301	2.148.373	-33.131	2.098.754	-34.738
2.125.443	-26.301	2.148.484	-33.132	2.098.909	-34.738
2.125.582	-26.301	2.148.640	-33.132	2.099.124	-34.739
2.125.741	-26.301	2.148.837	-33.133	2.099.281	-34.740
2.126.008	-26.301	2.148.996	-33.133	2.099.419	-34.740
2.126.259	-26.301	2.149.173	-33.133	2.099.608	-34.741
2.126.465	-26.301	2.149.333	-33.134	2.099.716	-34.742
2.126.685	-26.301	2.149.487	-33.134	2.099.843	-34.742
2.126.806	-26.301	2.149.662	-33.135	2.100.052	-34.743
2.126.906	-26.302	2.149.772	-33.135	2.100.199	-34.744
2.127.041	-26.302	2.149.861	-33.136	2.100.350	-34.744
2.127.238	-26.302	2.150.018	-33.136	2.100.683	-34.745
2.127.444	-26.302	2.150.249	-33.136	2.101.127	-34.746
2.127.626	-26.302	2.150.432	-33.137	2.101.463	-34.746

2.127.758	-26.302	2.150.566	-33.137	2.101.586	-34.747
2.127.901	-26.302	2.150.746	-33.138	2.101.673	-34.748
2.128.047	-26.302	2.150.943	-33.138	2.101.810	-34.749
2.128.181	-26.302	2.151.126	-33.139	2.101.913	-34.749
2.128.362	-26.303	2.151.245	-33.139	2.101.953	-34.750
2.128.510	-26.303	2.151.376	-33.140	2.101.985	-34.751
2.128.676	-26.303	2.151.618	-33.140	2.102.065	-34.751
2.128.893	-26.303	2.151.751	-33.140	2.102.152	-34.752
2.129.014	-26.303	2.151.913	-33.141	2.102.320	-34.753
2.129.166	-26.303	2.152.173	-33.141	2.102.516	-34.753
2.129.406	-26.303	2.152.372	-33.142	2.102.686	-34.754
2.129.585	-26.304	2.152.495	-33.142	2.102.861	-34.755
2.129.720	-26.304	2.152.560	-33.143	2.103.040	-34.756
2.129.885	-26.304	2.152.762	-33.143	2.103.174	-34.756
2.130.047	-26.304	2.153.011	-33.144	2.103.297	-34.757
2.130.211	-26.304	2.153.170	-33.144	2.103.512	-34.758
2.130.392	-26.304	2.153.304	-33.145	2.103.737	-34.759
2.130.544	-26.305	2.153.431	-33.145	2.103.915	-34.759
2.130.723	-26.305	2.153.638	-33.146	2.104.065	-34.760
2.130.918	-26.305	2.153.844	-33.146	2.104.185	-34.761
2.131.077	-26.305	2.153.988	-33.147	2.104.314	-34.762
2.131.250	-26.305	2.154.118	-33.148	2.104.460	-34.763
2.131.400	-26.306	2.154.294	-33.148	2.104.644	-34.763
2.131.471	-26.306	2.154.484	-33.149	2.104.832	-34.764
2.131.664	-26.306	2.154.690	-33.149	2.104.995	-34.765
2.132.032	-26.306	2.154.883	-33.150	2.105.148	-34.766
2.132.460	-26.306	2.155.051	-33.150	2.105.325	-34.767
2.132.728	-26.307	2.155.182	-33.151	2.105.473	-34.768
2.132.766	-26.307	2.155.303	-33.151	2.105.598	-34.769
2.132.803	-26.307	2.155.436	-33.152	2.105.779	-34.770

2.132.870	-26.307	2.155.549	-33.152	2.105.952	-34.771
2.132.943	-26.307	2.155.690	-33.153	2.106.142	-34.772
2.133.029	-26.308	2.155.849	-33.154	2.106.310	-34.773
2.133.161	-26.308	2.156.003	-33.154	2.106.516	-34.774
2.133.342	-26.308	2.156.162	-33.155	2.106.702	-34.775
2.133.537	-26.308	2.156.317	-33.155	2.106.853	-34.776
2.133.745	-26.309	2.156.474	-33.156	2.107.020	-34.777
2.133.927	-26.309	2.156.655	-33.156	2.107.198	-34.778
2.134.147	-26.309	2.156.908	-33.157	2.107.435	-34.779
2.134.339	-26.309	2.157.141	-33.158	2.107.594	-34.780
2.134.462	-26.310	2.157.278	-33.158	2.107.701	-34.781
2.134.605	-26.310	2.157.448	-33.159	2.107.838	-34.782
2.134.787	-26.310	2.157.661	-33.159	2.108.011	-34.783
2.134.975	-26.310	2.157.834	-33.160	2.108.183	-34.785
2.135.130	-26.311	2.157.991	-33.160	2.108.369	-34.786
2.135.233	-26.311	2.158.116	-33.161	2.108.504	-34.787
2.135.340	-26.311	2.158.246	-33.162	2.108.651	-34.788
2.135.480	-26.311	2.158.396	-33.162	2.108.817	-34.789
2.135.557	-26.312	2.158.461	-33.163	2.108.996	-34.791
2.135.692	-26.312	2.158.540	-33.163	2.109.191	-34.792
2.135.934	-26.312	2.158.732	-33.164	2.109.372	-34.793
2.136.151	-26.312	2.158.997	-33.165	2.109.520	-34.795
2.136.286	-26.313	2.159.236	-33.165	2.109.621	-34.796
2.136.449	-26.313	2.159.342	-33.166	2.109.796	-34.797
2.136.707	-26.313	2.159.523	-33.166	2.110.013	-34.799
2.136.917	-26.313	2.159.756	-33.167	2.110.184	-34.800
2.137.039	-26.314	2.159.910	-33.167	2.110.376	-34.801
2.137.168	-26.314	2.160.070	-33.168	2.110.510	-34.803
2.137.334	-26.314	2.160.257	-33.169	2.110.580	-34.804
2.137.567	-26.314	2.160.466	-33.169	2.110.706	-34.806

2.137.762	-26.315	2.160.654	-33.170	2.110.921	-34.807
2.137.868	-26.315	2.160.795	-33.170	2.111.181	-34.809
2.138.000	-26.315	2.160.970	-33.171	2.111.395	-34.810
2.138.170	-26.316	2.161.169	-33.171	2.111.514	-34.812
2.138.313	-26.316	2.161.319	-33.172	2.111.637	-34.813
2.138.472	-26.316	2.161.485	-33.173	2.111.808	-34.815
2.138.676	-26.316	2.161.653	-33.173	2.111.975	-34.816
2.138.839	-26.317	2.161.823	-33.174	2.112.159	-34.818
2.138.969	-26.317	2.162.040	-33.174	2.112.323	-34.820
2.139.149	-26.317	2.162.197	-33.175	2.112.473	-34.821
2.139.361	-26.317	2.162.352	-33.175	2.112.661	-34.823
2.139.433	-26.318	2.162.536	-33.176	2.112.793	-34.825
2.139.572	-26.318	2.162.684	-33.176	2.112.899	-34.826
2.139.805	-26.318	2.162.822	-33.177	2.113.065	-34.828
2.139.964	-26.318	2.162.943	-33.177	2.113.268	-34.830
2.140.123	-26.319	2.163.130	-33.178	2.113.450	-34.831
2.140.267	-26.319	2.163.344	-33.179	2.113.528	-34.833
2.140.405	-26.319	2.163.512	-33.179	2.113.707	-34.835
2.140.600	-26.319	2.163.707	-33.180	2.113.994	-34.836
2.140.828	-26.320	2.163.869	-33.180	2.114.180	-34.838
2.140.992	-26.320	2.164.032	-33.181	2.114.375	-34.840
2.141.187	-26.320	2.164.185	-33.181	2.114.516	-34.841
2.141.395	-26.321	2.164.303	-33.182	2.114.669	-34.843
2.141.588	-26.321	2.164.496	-33.182	2.114.818	-34.845
2.141.758	-26.321	2.164.691	-33.183	2.114.942	-34.847
2.141.926	-26.321	2.164.794	-33.183	2.115.172	-34.848
2.142.122	-26.322	2.164.866	-33.184	2.115.368	-34.850
2.142.231	-26.322	2.165.112	-33.184	2.115.506	-34.852
2.142.267	-26.322	2.165.602	-33.185	2.115.629	-34.854
2.142.437	-26.322	2.166.015	-33.185	2.115.786	-34.855

2.142.699	-26.323	2.166.171	-33.186	2.115.947	-34.857
2.142.851	-26.323	2.166.174	-33.186	2.116.030	-34.859
2.142.941	-26.323	2.166.207	-33.186	2.116.142	-34.861
2.143.109	-26.323	2.166.273	-33.187	2.116.330	-34.862
2.143.309	-26.324	2.166.348	-33.187	2.116.559	-34.864
2.143.467	-26.324	2.166.440	-33.188	2.116.740	-34.866
2.143.611	-26.324	2.166.547	-33.188	2.116.877	-34.868
2.143.786	-26.324	2.166.756	-33.189	2.117.052	-34.869
2.143.941	-26.325	2.166.893	-33.189	2.117.243	-34.871
2.144.028	-26.325	2.166.992	-33.189	2.117.453	-34.873
2.144.171	-26.325	2.167.229	-33.190	2.117.645	-34.875
2.144.350	-26.325	2.167.448	-33.190	2.117.813	-34.876
2.144.516	-26.326	2.167.596	-33.190	2.117.977	-34.878
2.144.655	-26.326	2.167.766	-33.191	2.118.116	-34.880
2.144.771	-26.326	2.167.939	-33.191	2.118.315	-34.881
2.144.932	-26.326	2.168.122	-33.192	2.118.484	-34.883
2.145.173	-26.327	2.168.295	-33.192	2.118.633	-34.885
2.145.399	-26.327	2.168.456	-33.192	2.118.817	-34.886
2.145.573	-26.327	2.168.685	-33.193	2.118.947	-34.888
2.145.775	-26.327	2.168.900	-33.193	2.119.032	-34.890
2.146.050	-26.328	2.169.016	-33.193	2.119.151	-34.891
2.146.211	-26.328	2.169.124	-33.194	2.119.286	-34.893
2.146.348	-26.328	2.169.290	-33.194	2.119.411	-34.895
2.146.557	-26.328	2.169.415	-33.194	2.119.603	-34.896
2.146.698	-26.329	2.169.567	-33.195	2.119.746	-34.898
2.146.817	-26.329	2.169.778	-33.195	2.119.893	-34.900
2.146.965	-26.329	2.169.928	-33.195	2.120.125	-34.901
2.147.054	-26.329	2.170.074	-33.195	2.120.338	-34.903
2.147.199	-26.330	2.170.323	-33.196	2.120.457	-34.904
2.147.467	-26.330	2.170.605	-33.196	2.120.642	-34.906

2.147.635	-26.330	2.170.771	-33.196	2.120.815	-34.907
2.147.766	-26.330	2.170.891	-33.197	2.121.064	-34.909
2.147.914	-26.331	2.171.043	-33.197	2.121.252	-34.910
2.148.082	-26.331	2.171.176	-33.197	2.121.349	-34.912
2.148.261	-26.331	2.171.358	-33.198	2.121.509	-34.913
2.148.436	-26.331	2.171.496	-33.198	2.121.704	-34.915
2.148.652	-26.332	2.171.561	-33.198	2.121.884	-34.916
2.148.889	-26.332	2.171.720	-33.198	2.122.130	-34.918
2.149.061	-26.332	2.171.929	-33.199	2.122.262	-34.919
2.149.198	-26.333	2.172.137	-33.199	2.122.399	-34.921
2.149.370	-26.333	2.172.287	-33.199	2.122.558	-34.922
2.149.547	-26.333	2.172.442	-33.199	2.122.701	-34.924
2.149.677	-26.333	2.172.574	-33.200	2.122.887	-34.925
2.149.812	-26.334	2.172.704	-33.200	2.123.075	-34.927
2.149.991	-26.334	2.172.950	-33.200	2.123.275	-34.928
2.150.163	-26.334	2.173.143	-33.201	2.123.457	-34.929
2.150.347	-26.334	2.173.210	-33.201	2.123.609	-34.931
2.150.567	-26.335	2.173.286	-33.201	2.123.770	-34.932
2.150.757	-26.335	2.173.492	-33.201	2.123.918	-34.933
2.150.898	-26.335	2.173.694	-33.202	2.124.091	-34.935
2.151.051	-26.335	2.173.877	-33.202	2.124.312	-34.936
2.151.180	-26.336	2.174.028	-33.202	2.124.516	-34.937
2.151.315	-26.336	2.174.182	-33.203	2.124.715	-34.939
2.151.508	-26.336	2.174.440	-33.203	2.124.836	-34.940
2.151.740	-26.337	2.174.664	-33.203	2.124.939	-34.941
2.151.931	-26.337	2.174.764	-33.204	2.125.128	-34.943
2.152.059	-26.337	2.174.874	-33.204	2.125.305	-34.944
2.152.211	-26.337	2.175.096	-33.204	2.125.486	-34.945
2.152.379	-26.338	2.175.280	-33.204	2.125.640	-34.947
2.152.533	-26.338	2.175.398	-33.205	2.125.804	-34.948

2.152.679	-26.338	2.175.574	-33.205	2.125.961	-34.949
2.152.865	-26.338	2.175.733	-33.205	2.126.111	-34.951
2.153.030	-26.339	2.175.876	-33.206	2.126.277	-34.952
2.153.230	-26.339	2.176.079	-33.206	2.126.393	-34.953
2.153.450	-26.339	2.176.288	-33.206	2.126.572	-34.954
2.153.595	-26.340	2.176.474	-33.207	2.126.767	-34.956
2.153.721	-26.340	2.176.612	-33.207	2.126.893	-34.957
2.153.815	-26.340	2.176.752	-33.207	2.127.036	-34.958
2.153.941	-26.340	2.176.926	-33.208	2.127.202	-34.959
2.154.310	-26.341	2.177.070	-33.208	2.127.408	-34.961
2.154.743	-26.341	2.177.224	-33.208	2.127.773	-34.962
2.154.991	-26.341	2.177.363	-33.208	2.128.221	-34.963
2.155.069	-26.342	2.177.446	-33.209	2.128.501	-34.964
2.155.107	-26.342	2.177.571	-33.209	2.128.559	-34.966
2.155.188	-26.342	2.177.737	-33.209	2.128.606	-34.967
2.155.324	-26.342	2.177.909	-33.210	2.128.785	-34.968
2.155.455	-26.343	2.178.053	-33.210	2.128.853	-34.969
2.155.557	-26.343	2.178.259	-33.210	2.128.819	-34.970
2.155.651	-26.343	2.178.490	-33.211	2.128.913	-34.972
2.155.780	-26.344	2.178.703	-33.211	2.129.083	-34.973
2.155.945	-26.344	2.178.956	-33.211	2.129.243	-34.974
2.156.140	-26.344	2.179.144	-33.211	2.129.361	-34.975
2.156.333	-26.344	2.179.268	-33.212	2.129.536	-34.976
2.156.539	-26.345	2.179.417	-33.212	2.129.707	-34.978
2.156.774	-26.345	2.179.619	-33.212	2.129.837	-34.979
2.156.948	-26.345	2.179.884	-33.212	2.130.020	-34.980
2.157.137	-26.346	2.180.079	-33.213	2.130.235	-34.981
2.157.309	-26.346	2.180.116	-33.213	2.130.412	-34.982
2.157.426	-26.346	2.180.174	-33.213	2.130.544	-34.984
2.157.553	-26.346	2.180.354	-33.213	2.130.661	-34.985

2.157.708	-26.347	2.180.526	-33.214	2.130.887	-34.986
2.157.862	-26.347	2.180.658	-33.214	2.131.078	-34.987
2.158.027	-26.347	2.180.784	-33.214	2.131.221	-34.989
2.158.172	-26.347	2.180.894	-33.214	2.131.344	-34.990
2.158.279	-26.348	2.181.068	-33.215	2.131.505	-34.991
2.158.447	-26.348	2.181.286	-33.215	2.131.758	-34.992
2.158.611	-26.348	2.181.532	-33.215	2.131.948	-34.994
2.158.757	-26.348	2.181.689	-33.215	2.132.106	-34.995
2.158.976	-26.349	2.181.880	-33.215	2.132.225	-34.996
2.159.168	-26.349	2.182.101	-33.216	2.132.359	-34.997
2.159.323	-26.349	2.182.249	-33.216	2.132.526	-34.999
2.159.520	-26.349	2.182.417	-33.216	2.132.713	-35.000
2.159.728	-26.350	2.182.591	-33.216	2.132.859	-35.001
2.159.913	-26.350	2.182.740	-33.216	2.132.984	-35.002
2.160.049	-26.350	2.182.923	-33.216	2.133.136	-35.004
2.160.168	-26.350	2.183.147	-33.217	2.133.360	-35.005
2.160.307	-26.351	2.183.279	-33.217	2.133.571	-35.006
2.160.444	-26.351	2.183.335	-33.217	2.133.714	-35.008
2.160.600	-26.351	2.183.514	-33.217	2.133.915	-35.009
2.160.827	-26.351	2.183.741	-33.217	2.134.135	-35.010
2.161.001	-26.352	2.183.904	-33.217	2.134.328	-35.012
2.161.097	-26.352	2.184.103	-33.218	2.134.447	-35.013
2.161.261	-26.352	2.184.242	-33.218	2.134.516	-35.014
2.161.449	-26.352	2.184.370	-33.218	2.134.681	-35.016
2.161.546	-26.353	2.184.557	-33.218	2.134.802	-35.017
2.161.689	-26.353	2.184.745	-33.218	2.134.899	-35.019
2.161.929	-26.353	2.184.905	-33.218	2.135.096	-35.020
2.162.112	-26.353	2.185.069	-33.218	2.135.333	-35.021
2.162.262	-26.353	2.185.230	-33.218	2.135.499	-35.023
2.162.453	-26.354	2.185.423	-33.218	2.135.620	-35.024

2.162.608	-26.354	2.185.659	-33.219	2.135.779	-35.026
2.162.735	-26.354	2.185.802	-33.219	2.135.963	-35.027
2.162.953	-26.354	2.185.934	-33.219	2.136.133	-35.029
2.163.201	-26.354	2.186.144	-33.219	2.136.250	-35.030
2.163.385	-26.355	2.186.329	-33.219	2.136.407	-35.032
2.163.549	-26.355	2.186.487	-33.219	2.136.604	-35.033
2.163.676	-26.355	2.186.644	-33.219	2.136.752	-35.035
2.163.804	-26.355	2.186.743	-33.219	2.136.919	-35.036
2.163.976	-26.355	2.186.861	-33.219	2.137.119	-35.038
2.164.162	-26.356	2.187.188	-33.219	2.137.307	-35.039
2.164.328	-26.356	2.187.670	-33.219	2.137.495	-35.041
2.164.484	-26.356	2.187.937	-33.219	2.137.652	-35.042
2.164.650	-26.356	2.187.968	-33.220	2.137.782	-35.044
2.164.754	-26.356	2.188.039	-33.220	2.137.977	-35.045
2.164.922	-26.356	2.188.098	-33.220	2.138.181	-35.047
2.165.134	-26.357	2.188.145	-33.220	2.138.346	-35.048
2.165.297	-26.357	2.188.252	-33.220	2.138.521	-35.050
2.165.435	-26.357	2.188.309	-33.220	2.138.676	-35.051
2.165.595	-26.357	2.188.398	-33.220	2.138.841	-35.053
2.165.849	-26.357	2.188.608	-33.220	2.139.086	-35.055
2.166.003	-26.357	2.188.850	-33.220	2.139.272	-35.056
2.166.109	-26.358	2.189.021	-33.220	2.139.350	-35.058
2.166.248	-26.358	2.189.180	-33.220	2.139.473	-35.059
2.166.382	-26.358	2.189.379	-33.220	2.139.621	-35.061
2.166.561	-26.358	2.189.563	-33.220	2.139.780	-35.062
2.166.673	-26.358	2.189.749	-33.220	2.140.013	-35.064
2.166.780	-26.358	2.189.874	-33.220	2.140.197	-35.065
2.166.948	-26.358	2.190.004	-33.220	2.140.311	-35.067
2.167.144	-26.359	2.190.143	-33.221	2.140.455	-35.069
2.167.310	-26.359	2.190.293	-33.221	2.140.616	-35.070

2.167.466	-26.359	2.190.486	-33.221	2.140.842	-35.072
2.167.654	-26.359	2.190.636	-33.221	2.141.061	-35.073
2.167.863	-26.359	2.190.790	-33.221	2.141.216	-35.075
2.168.071	-26.359	2.190.925	-33.221	2.141.335	-35.076
2.168.275	-26.359	2.191.116	-33.221	2.141.431	-35.078
2.168.533	-26.360	2.191.331	-33.221	2.141.548	-35.079
2.168.723	-26.360	2.191.494	-33.221	2.141.777	-35.081
2.168.858	-26.360	2.191.702	-33.222	2.141.998	-35.082
2.168.960	-26.360	2.191.895	-33.222	2.142.168	-35.084
2.169.102	-26.360	2.192.095	-33.222	2.142.287	-35.085
2.169.308	-26.360	2.192.272	-33.222	2.142.379	-35.086
2.169.444	-26.360	2.192.426	-33.222	2.142.558	-35.088
2.169.579	-26.360	2.192.621	-33.222	2.142.749	-35.089
2.169.742	-26.361	2.192.780	-33.222	2.142.832	-35.091
2.169.843	-26.361	2.192.885	-33.223	2.143.010	-35.092
2.169.973	-26.361	2.193.019	-33.223	2.143.230	-35.093
2.170.220	-26.361	2.193.194	-33.223	2.143.362	-35.095
2.170.430	-26.361	2.193.358	-33.223	2.143.488	-35.096
2.170.640	-26.361	2.193.519	-33.223	2.143.647	-35.097
2.170.856	-26.361	2.193.667	-33.224	2.143.884	-35.099
2.171.064	-26.362	2.193.784	-33.224	2.144.075	-35.100
2.171.216	-26.362	2.194.001	-33.224	2.144.272	-35.101
2.171.375	-26.362	2.194.202	-33.225	2.144.449	-35.102
2.171.537	-26.362	2.194.335	-33.225	2.144.695	-35.104
2.171.659	-26.362	2.194.462	-33.225	2.144.955	-35.105
2.171.843	-26.362	2.194.601	-33.225	2.145.082	-35.106
2.172.047	-26.362	2.194.758	-33.226	2.145.229	-35.107
2.172.235	-26.362	2.194.944	-33.226	2.145.425	-35.108
2.172.383	-26.363	2.195.098	-33.226	2.145.622	-35.109
2.172.533	-26.363	2.195.203	-33.227	2.145.780	-35.110

2.172.672	-26.363	2.195.365	-33.227	2.145.909	-35.111
2.172.825	-26.363	2.195.524	-33.228	2.145.979	-35.113
2.172.957	-26.363	2.195.708	-33.228	2.146.113	-35.114
2.173.065	-26.363	2.195.901	-33.228	2.146.301	-35.115
2.173.291	-26.363	2.196.075	-33.229	2.146.427	-35.116
2.173.548	-26.363	2.196.279	-33.229	2.146.586	-35.116
2.173.730	-26.364	2.196.456	-33.230	2.146.776	-35.117
2.173.842	-26.364	2.196.615	-33.230	2.146.951	-35.118
2.173.949	-26.364	2.196.810	-33.231	2.147.112	-35.119
2.174.095	-26.364	2.196.975	-33.232	2.147.234	-35.120
2.174.308	-26.364	2.197.148	-33.232	2.147.439	-35.121
2.174.493	-26.364	2.197.291	-33.233	2.147.685	-35.122
2.174.612	-26.364	2.197.390	-33.233	2.147.865	-35.123
2.174.758	-26.365	2.197.542	-33.234	2.148.009	-35.123
2.174.967	-26.365	2.197.747	-33.235	2.148.167	-35.124
2.175.199	-26.365	2.197.965	-33.235	2.148.324	-35.125
2.175.350	-26.365	2.198.139	-33.236	2.148.496	-35.126
2.175.473	-26.365	2.198.298	-33.237	2.148.658	-35.126
2.175.645	-26.365	2.198.497	-33.237	2.148.802	-35.127
2.175.815	-26.365	2.198.631	-33.238	2.148.999	-35.128
2.175.959	-26.365	2.198.717	-33.239	2.149.243	-35.128
2.176.138	-26.366	2.198.839	-33.240	2.149.384	-35.129
2.176.273	-26.366	2.198.996	-33.240	2.149.482	-35.129
2.176.530	-26.366	2.199.160	-33.241	2.149.664	-35.130
2.176.987	-26.366	2.199.299	-33.242	2.149.800	-35.130
2.177.247	-26.366	2.199.457	-33.243	2.149.949	-35.131
2.177.319	-26.366	2.199.594	-33.244	2.150.128	-35.132
2.177.414	-26.366	2.199.753	-33.245	2.150.302	-35.132
2.177.540	-26.366	2.199.967	-33.245	2.150.522	-35.132
2.177.643	-26.367	2.200.185	-33.246	2.150.789	-35.133

2.177.693	-26.367	2.200.416	-33.247	2.150.882	-35.133
2.177.780	-26.367	2.200.616	-33.248	2.150.940	-35.134
2.177.871	-26.367	2.200.802	-33.249	2.151.133	-35.134
2.177.980	-26.367	2.201.017	-33.250	2.151.335	-35.135
2.178.237	-26.367	2.201.205	-33.251	2.151.541	-35.135
2.178.425	-26.367	2.201.337	-33.252	2.151.668	-35.135
2.178.541	-26.367	2.201.449	-33.253	2.151.778	-35.136
2.178.765	-26.367	2.201.588	-33.254	2.151.996	-35.136
2.178.943	-26.367	2.201.751	-33.255	2.152.137	-35.137
2.179.108	-26.368	2.201.906	-33.256	2.152.361	-35.137
2.179.321	-26.368	2.202.020	-33.258	2.152.605	-35.137
2.179.500	-26.368	2.202.101	-33.259	2.152.713	-35.138
2.179.592	-26.368	2.202.262	-33.260	2.152.914	-35.138
2.179.724	-26.368	2.202.422	-33.261	2.153.096	-35.138
2.179.913	-26.368	2.202.551	-33.262	2.153.239	-35.138
2.180.049	-26.368	2.202.784	-33.263	2.153.375	-35.139
2.180.224	-26.368	2.202.988	-33.264	2.153.524	-35.139
2.180.396	-26.368	2.203.194	-33.266	2.153.700	-35.139
2.180.539	-26.368	2.203.410	-33.267	2.153.844	-35.139
2.180.690	-26.368	2.203.566	-33.268	2.153.985	-35.140
2.180.866	-26.368	2.203.743	-33.269	2.154.151	-35.140
2.181.093	-26.368	2.203.956	-33.270	2.154.297	-35.140
2.181.344	-26.368	2.204.079	-33.272	2.154.556	-35.140
2.181.496	-26.368	2.204.214	-33.273	2.155.017	-35.141
2.181.579	-26.368	2.204.399	-33.274	2.155.396	-35.141
2.181.718	-26.368	2.204.541	-33.275	2.155.592	-35.141
2.181.940	-26.368	2.204.720	-33.277	2.155.725	-35.141
2.182.116	-26.369	2.204.935	-33.278	2.155.838	-35.141
2.182.282	-26.369	2.205.062	-33.279	2.155.885	-35.142
2.182.461	-26.369	2.205.177	-33.281	2.155.855	-35.142

2.182.629	-26.369	2.205.363	-33.282	2.155.887	-35.142
2.182.764	-26.369	2.205.557	-33.283	2.156.010	-35.142
2.182.863	-26.369	2.205.721	-33.285	2.156.127	-35.142
2.183.028	-26.369	2.205.871	-33.286	2.156.214	-35.143
2.183.165	-26.369	2.206.073	-33.287	2.156.413	-35.143
2.183.304	-26.368	2.206.310	-33.289	2.156.682	-35.143
2.183.501	-26.368	2.206.393	-33.290	2.156.852	-35.143
2.183.711	-26.368	2.206.510	-33.292	2.156.976	-35.144
2.183.920	-26.368	2.206.763	-33.293	2.157.155	-35.144
2.184.106	-26.368	2.206.895	-33.294	2.157.368	-35.144
2.184.258	-26.368	2.207.023	-33.296	2.157.538	-35.144
2.184.413	-26.368	2.207.229	-33.297	2.157.665	-35.145
2.184.530	-26.368	2.207.439	-33.299	2.157.851	-35.145
2.184.644	-26.368	2.207.579	-33.300	2.158.026	-35.145
2.184.796	-26.368	2.207.744	-33.301	2.158.207	-35.145
2.184.991	-26.368	2.207.971	-33.303	2.158.333	-35.146
2.185.215	-26.368	2.208.152	-33.304	2.158.432	-35.146
2.185.421	-26.368	2.208.210	-33.306	2.158.564	-35.146
2.185.564	-26.368	2.208.351	-33.307	2.158.770	-35.147
2.185.695	-26.368	2.208.537	-33.308	2.158.999	-35.147
2.185.932	-26.368	2.208.743	-33.310	2.159.182	-35.148
2.186.167	-26.368	2.209.158	-33.311	2.159.354	-35.148
2.186.292	-26.367	2.209.579	-33.313	2.159.476	-35.148
2.186.415	-26.367	2.209.778	-33.314	2.159.628	-35.149
2.186.574	-26.367	2.209.807	-33.315	2.159.688	-35.149
2.186.741	-26.367	2.209.819	-33.317	2.159.820	-35.150
2.186.915	-26.367	2.209.875	-33.318	2.160.100	-35.150
2.187.094	-26.367	2.209.903	-33.320	2.160.333	-35.151
2.187.267	-26.367	2.209.946	-33.321	2.160.495	-35.151
2.187.453	-26.367	2.210.049	-33.322	2.160.622	-35.152

2.187.657	-26.367	2.210.235	-33.324	2.160.816	-35.152
2.187.838	-26.366	2.210.416	-33.325	2.161.010	-35.153
2.188.019	-26.366	2.210.594	-33.327	2.161.209	-35.154
2.188.139	-26.366	2.210.797	-33.328	2.161.402	-35.154
2.188.272	-26.366	2.210.995	-33.329	2.161.592	-35.155
2.188.425	-26.366	2.211.234	-33.331	2.161.738	-35.156
2.188.539	-26.366	2.211.404	-33.332	2.161.870	-35.156
2.188.681	-26.366	2.211.543	-33.333	2.162.103	-35.157
2.188.823	-26.366	2.211.700	-33.335	2.162.289	-35.158
2.188.990	-26.365	2.211.815	-33.336	2.162.375	-35.159
2.189.113	-26.365	2.211.962	-33.338	2.162.502	-35.159
2.189.180	-26.365	2.212.168	-33.339	2.162.668	-35.160
2.189.303	-26.365	2.212.332	-33.340	2.162.847	-35.161
2.189.458	-26.365	2.212.486	-33.342	2.163.030	-35.162
2.189.646	-26.365	2.212.675	-33.343	2.163.197	-35.163
2.189.854	-26.365	2.212.838	-33.344	2.163.356	-35.164
2.190.107	-26.364	2.212.957	-33.346	2.163.514	-35.165
2.190.416	-26.364	2.213.122	-33.347	2.163.622	-35.165
2.190.641	-26.364	2.213.275	-33.348	2.163.788	-35.166
2.190.768	-26.364	2.213.422	-33.349	2.163.962	-35.167
2.190.880	-26.364	2.213.618	-33.351	2.164.055	-35.168
2.191.064	-26.364	2.213.853	-33.352	2.164.221	-35.169
2.191.272	-26.364	2.213.967	-33.353	2.164.440	-35.171
2.191.441	-26.364	2.214.050	-33.355	2.164.617	-35.172
2.191.521	-26.363	2.214.303	-33.356	2.164.753	-35.173
2.191.583	-26.363	2.214.588	-33.357	2.164.877	-35.174
2.191.700	-26.363	2.214.753	-33.358	2.165.029	-35.175
2.191.857	-26.363	2.214.897	-33.360	2.165.206	-35.176
2.192.052	-26.363	2.215.031	-33.361	2.165.403	-35.177
2.192.211	-26.363	2.215.130	-33.362	2.165.609	-35.178

2.192.368	-26.363	2.215.314	-33.363	2.165.806	-35.180
2.192.558	-26.363	2.215.464	-33.364	2.165.968	-35.181
2.192.758	-26.363	2.215.651	-33.366	2.166.099	-35.182
2.192.977	-26.363	2.215.862	-33.367	2.166.277	-35.183
2.193.204	-26.363	2.216.028	-33.368	2.166.480	-35.185
2.193.389	-26.362	2.216.115	-33.369	2.166.615	-35.186
2.193.557	-26.362	2.216.205	-33.370	2.166.776	-35.187
2.193.723	-26.362	2.216.440	-33.371	2.166.967	-35.188
2.193.885	-26.362	2.216.611	-33.373	2.167.151	-35.190
2.194.070	-26.362	2.216.747	-33.374	2.167.290	-35.191
2.194.200	-26.362	2.216.915	-33.375	2.167.388	-35.192
2.194.368	-26.362	2.217.090	-33.376	2.167.573	-35.194
2.194.556	-26.362	2.217.269	-33.377	2.167.827	-35.195
2.194.742	-26.362	2.217.417	-33.378	2.168.006	-35.196
2.194.908	-26.362	2.217.534	-33.379	2.168.170	-35.198
2.195.058	-26.362	2.217.751	-33.380	2.168.372	-35.199
2.195.228	-26.362	2.218.001	-33.381	2.168.521	-35.200
2.195.383	-26.362	2.218.176	-33.382	2.168.667	-35.202
2.195.551	-26.363	2.218.360	-33.383	2.168.815	-35.203
2.195.741	-26.363	2.218.517	-33.384	2.168.932	-35.204
2.195.905	-26.363	2.218.662	-33.385	2.169.115	-35.206
2.196.066	-26.363	2.218.821	-33.386	2.169.289	-35.207
2.196.259	-26.363	2.218.987	-33.387	2.169.409	-35.208
2.196.389	-26.363	2.219.117	-33.388	2.169.574	-35.210
2.196.595	-26.363	2.219.260	-33.389	2.169.722	-35.211
2.196.783	-26.363	2.219.438	-33.390	2.169.823	-35.212
2.196.848	-26.364	2.219.634	-33.391	2.169.982	-35.214
2.196.969	-26.364	2.219.856	-33.392	2.170.168	-35.215
2.197.160	-26.364	2.219.991	-33.393	2.170.294	-35.216
2.197.395	-26.364	2.220.087	-33.394	2.170.454	-35.218

2.197.618	-26.364	2.220.242	-33.395	2.170.686	-35.219
2.197.768	-26.365	2.220.430	-33.396	2.170.817	-35.220
2.197.861	-26.365	2.220.607	-33.397	2.170.965	-35.222
2.198.024	-26.365	2.220.750	-33.397	2.171.238	-35.223
2.198.210	-26.365	2.220.860	-33.398	2.171.508	-35.224
2.198.310	-26.366	2.220.965	-33.399	2.171.740	-35.226
2.198.421	-26.366	2.221.120	-33.400	2.171.926	-35.227
2.198.716	-26.366	2.221.308	-33.401	2.172.065	-35.228
2.199.174	-26.367	2.221.474	-33.401	2.172.220	-35.229
2.199.475	-26.367	2.221.620	-33.402	2.172.372	-35.231
2.199.586	-26.367	2.221.823	-33.403	2.172.538	-35.232
2.199.689	-26.368	2.222.032	-33.404	2.172.702	-35.233
2.199.728	-26.368	2.222.256	-33.404	2.172.784	-35.234
2.199.760	-26.369	2.222.495	-33.405	2.172.879	-35.236
2.199.819	-26.369	2.222.683	-33.406	2.172.990	-35.237
2.199.926	-26.370	2.222.896	-33.406	2.173.152	-35.238
2.200.087	-26.370	2.223.013	-33.407	2.173.372	-35.239
2.200.242	-26.371	2.223.118	-33.408	2.173.537	-35.240
2.200.453	-26.371	2.223.239	-33.408	2.173.667	-35.242
2.200.594	-26.372	2.223.385	-33.409	2.173.759	-35.243
2.200.768	-26.372	2.223.575	-33.409	2.173.956	-35.244
2.200.981	-26.373	2.223.719	-33.410	2.174.223	-35.245
2.201.104	-26.373	2.223.848	-33.411	2.174.398	-35.246
2.201.256	-26.374	2.224.007	-33.411	2.174.554	-35.247
2.201.462	-26.374	2.224.173	-33.412	2.174.713	-35.248
2.201.668	-26.375	2.224.339	-33.412	2.174.901	-35.249
2.201.815	-26.376	2.224.480	-33.413	2.175.060	-35.250
2.201.971	-26.376	2.224.594	-33.413	2.175.166	-35.251
2.202.137	-26.377	2.224.823	-33.414	2.175.381	-35.252
2.202.258	-26.378	2.225.080	-33.414	2.175.625	-35.254

2.202.428	-26.378	2.225.253	-33.415	2.175.813	-35.255
2.202.652	-26.379	2.225.430	-33.415	2.175.961	-35.255
2.202.840	-26.380	2.225.587	-33.416	2.176.096	-35.256
2.202.905	-26.380	2.225.704	-33.416	2.176.299	-35.257
2.203.035	-26.381	2.225.860	-33.416	2.176.487	-35.258
2.203.259	-26.382	2.226.043	-33.417	2.176.635	-35.259
2.203.514	-26.383	2.226.223	-33.417	2.176.797	-35.260
2.203.727	-26.384	2.226.406	-33.418	2.176.947	-35.261
2.203.839	-26.384	2.226.556	-33.418	2.177.137	-35.262
2.204.032	-26.385	2.226.709	-33.418	2.177.348	-35.263
2.204.215	-26.386	2.226.888	-33.419	2.177.522	-35.264
2.204.395	-26.387	2.227.061	-33.419	2.177.661	-35.265
2.204.563	-26.388	2.227.246	-33.419	2.177.731	-35.266
2.204.688	-26.389	2.227.419	-33.420	2.177.825	-35.266
2.204.816	-26.390	2.227.580	-33.420	2.177.995	-35.267
2.204.964	-26.390	2.227.704	-33.420	2.178.298	-35.268
2.205.091	-26.391	2.227.927	-33.421	2.178.586	-35.269
2.205.231	-26.392	2.228.172	-33.421	2.178.718	-35.270
2.205.426	-26.393	2.228.284	-33.421	2.178.803	-35.270
2.205.613	-26.394	2.228.456	-33.421	2.178.897	-35.271
2.205.784	-26.395	2.228.661	-33.422	2.179.072	-35.272
2.205.963	-26.396	2.228.779	-33.422	2.179.267	-35.273
2.206.111	-26.397	2.228.947	-33.422	2.179.413	-35.274
2.206.261	-26.398	2.229.160	-33.422	2.179.612	-35.274
2.206.386	-26.399	2.229.288	-33.423	2.179.734	-35.275
2.206.541	-26.400	2.229.361	-33.423	2.179.924	-35.276
2.206.707	-26.401	2.229.568	-33.423	2.180.147	-35.277
2.206.852	-26.403	2.229.825	-33.423	2.180.303	-35.277
2.207.066	-26.404	2.229.971	-33.424	2.180.470	-35.278
2.207.260	-26.405	2.230.090	-33.424	2.180.583	-35.279

2.207.370	-26.406	2.230.230	-33.424	2.180.835	-35.279
2.207.506	-26.407	2.230.387	-33.424	2.181.008	-35.280
2.207.724	-26.408	2.230.560	-33.424	2.181.066	-35.281
2.207.935	-26.409	2.230.959	-33.424	2.181.255	-35.282
2.208.109	-26.411	2.231.427	-33.425	2.181.550	-35.282
2.208.263	-26.412	2.231.653	-33.425	2.181.904	-35.283
2.208.445	-26.413	2.231.724	-33.425	2.182.235	-35.284
2.208.571	-26.414	2.231.718	-33.425	2.182.399	-35.284
2.208.705	-26.415	2.231.724	-33.425	2.182.502	-35.285
2.208.920	-26.417	2.231.821	-33.425	2.182.576	-35.286
2.209.090	-26.418	2.231.957	-33.426	2.182.616	-35.286
2.209.207	-26.419	2.232.065	-33.426	2.182.647	-35.287
2.209.352	-26.420	2.232.199	-33.426	2.182.728	-35.288
2.209.529	-26.422	2.232.375	-33.426	2.182.851	-35.288
2.209.704	-26.423	2.232.542	-33.426	2.182.941	-35.289
2.209.912	-26.424	2.232.760	-33.426	2.183.098	-35.290
2.210.166	-26.426	2.232.952	-33.426	2.183.277	-35.291
2.210.322	-26.427	2.233.049	-33.426	2.183.407	-35.291
2.210.432	-26.428	2.233.203	-33.426	2.183.584	-35.292
2.210.571	-26.430	2.233.405	-33.426	2.183.853	-35.293
2.210.730	-26.431	2.233.624	-33.427	2.184.023	-35.293
2.210.831	-26.432	2.233.799	-33.427	2.184.182	-35.294
2.210.948	-26.434	2.233.938	-33.427	2.184.420	-35.295
2.211.120	-26.435	2.234.068	-33.427	2.184.590	-35.296
2.211.241	-26.437	2.234.137	-33.427	2.184.711	-35.296
2.211.375	-26.438	2.234.321	-33.427	2.184.928	-35.297
2.211.575	-26.440	2.234.565	-33.427	2.185.129	-35.298
2.211.767	-26.441	2.234.734	-33.427	2.185.251	-35.299
2.211.964	-26.442	2.234.841	-33.427	2.185.383	-35.300
2.212.157	-26.444	2.234.973	-33.427	2.185.484	-35.301

2.212.287	-26.445	2.235.165	-33.428	2.185.654	-35.301
2.212.489	-26.447	2.235.329	-33.428	2.185.905	-35.302
2.212.740	-26.448	2.235.515	-33.428	2.186.084	-35.303
2.212.953	-26.450	2.235.701	-33.428	2.186.216	-35.304
2.213.139	-26.452	2.235.893	-33.428	2.186.279	-35.305
2.213.327	-26.453	2.236.095	-33.428	2.186.397	-35.306
2.213.495	-26.455	2.236.290	-33.428	2.186.528	-35.307
2.213.624	-26.456	2.236.426	-33.428	2.186.694	-35.308
2.213.754	-26.458	2.236.517	-33.428	2.186.901	-35.309
2.213.858	-26.459	2.236.664	-33.429	2.187.134	-35.310
2.213.969	-26.461	2.236.880	-33.429	2.187.290	-35.311
2.214.133	-26.463	2.237.020	-33.429	2.187.448	-35.312
2.214.277	-26.464	2.237.079	-33.429	2.187.614	-35.313
2.214.384	-26.466	2.237.305	-33.429	2.187.750	-35.314
2.214.557	-26.467	2.237.547	-33.429	2.187.995	-35.315
2.214.737	-26.469	2.237.712	-33.429	2.188.223	-35.317
2.214.926	-26.471	2.237.872	-33.430	2.188.354	-35.318
2.215.145	-26.472	2.238.038	-33.430	2.188.450	-35.319
2.215.300	-26.474	2.238.221	-33.430	2.188.589	-35.320
2.215.463	-26.476	2.238.396	-33.430	2.188.848	-35.321
2.215.663	-26.477	2.238.566	-33.430	2.189.061	-35.323
2.215.858	-26.479	2.238.698	-33.431	2.189.178	-35.324
2.216.037	-26.481	2.238.884	-33.431	2.189.353	-35.325
2.216.167	-26.483	2.239.124	-33.431	2.189.507	-35.326
2.216.272	-26.484	2.239.278	-33.431	2.189.641	-35.328
2.216.481	-26.486	2.239.395	-33.431	2.189.783	-35.329
2.216.741	-26.488	2.239.572	-33.432	2.189.905	-35.330
2.216.850	-26.490	2.239.774	-33.432	2.190.035	-35.332
2.217.009	-26.491	2.239.919	-33.432	2.190.197	-35.333
2.217.233	-26.493	2.240.009	-33.432	2.190.334	-35.335

2.217.411	-26.495	2.240.213	-33.433	2.190.538	-35.336
2.217.596	-26.497	2.240.443	-33.433	2.190.831	-35.338
2.217.740	-26.499	2.240.571	-33.433	2.191.030	-35.339
2.217.896	-26.500	2.240.719	-33.433	2.191.155	-35.341
2.218.102	-26.502	2.240.869	-33.434	2.191.257	-35.342
2.218.266	-26.504	2.241.024	-33.434	2.191.391	-35.344
2.218.484	-26.506	2.241.144	-33.434	2.191.547	-35.346
2.218.705	-26.508	2.241.326	-33.434	2.191.673	-35.347
2.218.819	-26.510	2.241.532	-33.435	2.191.828	-35.349
2.218.969	-26.512	2.241.637	-33.435	2.192.090	-35.351
2.219.079	-26.513	2.241.794	-33.435	2.192.301	-35.352
2.219.162	-26.515	2.242.060	-33.435	2.192.497	-35.354
2.219.341	-26.517	2.242.233	-33.436	2.192.679	-35.356
2.219.556	-26.519	2.242.363	-33.436	2.192.755	-35.357
2.219.772	-26.521	2.242.515	-33.436	2.192.910	-35.359
2.219.979	-26.523	2.242.619	-33.436	2.193.078	-35.361
2.220.116	-26.525	2.242.764	-33.437	2.193.181	-35.363
2.220.211	-26.527	2.242.853	-33.437	2.193.400	-35.364
2.220.347	-26.529	2.242.903	-33.437	2.193.625	-35.366
2.220.520	-26.531	2.243.082	-33.438	2.193.802	-35.368
2.220.697	-26.533	2.243.335	-33.438	2.193.988	-35.370
2.220.889	-26.535	2.243.501	-33.438	2.194.099	-35.372
2.221.288	-26.536	2.243.698	-33.438	2.194.229	-35.374
2.221.729	-26.538	2.243.915	-33.439	2.194.406	-35.375
2.221.909	-26.540	2.244.088	-33.439	2.194.572	-35.377
2.221.954	-26.542	2.244.334	-33.439	2.194.754	-35.379
2.221.985	-26.544	2.244.583	-33.439	2.194.960	-35.381
2.222.047	-26.546	2.244.811	-33.440	2.195.099	-35.383
2.222.088	-26.548	2.244.951	-33.440	2.195.241	-35.385
2.222.128	-26.550	2.245.038	-33.440	2.195.401	-35.387

2.222.242	-26.552	2.245.262	-33.440	2.195.511	-35.389
2.222.379	-26.554	2.245.434	-33.441	2.195.730	-35.390
2.222.560	-26.556	2.245.477	-33.441	2.195.987	-35.392
2.222.800	-26.558	2.245.589	-33.441	2.196.097	-35.394
2.223.024	-26.560	2.245.733	-33.441	2.196.182	-35.396
2.223.224	-26.562	2.245.869	-33.442	2.196.310	-35.398
2.223.385	-26.564	2.246.055	-33.442	2.196.444	-35.400
2.223.537	-26.566	2.246.288	-33.442	2.196.595	-35.402
2.223.671	-26.568	2.246.490	-33.442	2.196.724	-35.404
2.223.806	-26.571	2.246.590	-33.443	2.196.873	-35.406
2.223.914	-26.573	2.246.786	-33.443	2.197.057	-35.408
2.224.109	-26.575	2.247.016	-33.443	2.197.209	-35.410
2.224.355	-26.577	2.247.195	-33.443	2.197.374	-35.411
2.224.507	-26.579	2.247.405	-33.444	2.197.547	-35.413
2.224.697	-26.581	2.247.565	-33.444	2.197.746	-35.415
2.224.858	-26.583	2.247.699	-33.444	2.197.957	-35.417
2.224.973	-26.585	2.247.898	-33.444	2.198.167	-35.419
2.225.107	-26.587	2.248.096	-33.444	2.198.430	-35.421
2.225.293	-26.589	2.248.241	-33.445	2.198.662	-35.423
2.225.488	-26.591	2.248.391	-33.445	2.198.826	-35.425
2.225.665	-26.593	2.248.519	-33.445	2.198.980	-35.427
2.225.880	-26.595	2.248.652	-33.445	2.199.151	-35.428
2.226.068	-26.597	2.248.870	-33.445	2.199.283	-35.430
2.226.250	-26.599	2.249.061	-33.446	2.199.417	-35.432
2.226.422	-26.601	2.249.168	-33.446	2.199.601	-35.434
2.226.568	-26.603	2.249.319	-33.446	2.199.724	-35.436
2.226.707	-26.605	2.249.554	-33.446	2.199.834	-35.437
2.226.824	-26.607	2.249.767	-33.446	2.200.013	-35.439
2.226.985	-26.609	2.249.893	-33.447	2.200.155	-35.441
2.227.197	-26.611	2.250.020	-33.447	2.200.309	-35.443

2.227.372	-26.613	2.250.152	-33.447	2.200.457	-35.445
2.227.496	-26.615	2.250.304	-33.447	2.200.609	-35.446
2.227.628	-26.617	2.250.482	-33.448	2.200.763	-35.448
2.227.787	-26.619	2.250.642	-33.448	2.200.891	-35.450
2.227.943	-26.621	2.250.863	-33.448	2.201.069	-35.452
2.228.127	-26.623	2.251.059	-33.448	2.201.303	-35.453
2.228.270	-26.625	2.251.183	-33.448	2.201.525	-35.455
2.228.403	-26.626	2.251.357	-33.449	2.201.700	-35.457
2.228.551	-26.628	2.251.570	-33.449	2.201.872	-35.458
2.228.701	-26.630	2.251.747	-33.449	2.202.056	-35.460
2.228.938	-26.632	2.251.880	-33.449	2.202.224	-35.462
2.229.081	-26.634	2.252.047	-33.449	2.202.376	-35.463
2.229.191	-26.636	2.252.215	-33.449	2.202.511	-35.465
2.229.334	-26.638	2.252.309	-33.450	2.202.600	-35.466
2.229.547	-26.640	2.252.515	-33.450	2.202.762	-35.468
2.229.774	-26.642	2.252.941	-33.450	2.203.008	-35.470
2.229.932	-26.643	2.253.315	-33.450	2.203.172	-35.471
2.230.117	-26.645	2.253.461	-33.450	2.203.315	-35.473
2.230.275	-26.647	2.253.578	-33.451	2.203.481	-35.474
2.230.461	-26.649	2.253.676	-33.451	2.203.649	-35.476
2.230.627	-26.651	2.253.687	-33.451	2.203.821	-35.477
2.230.810	-26.652	2.253.734	-33.451	2.203.969	-35.479
2.230.943	-26.654	2.253.853	-33.451	2.204.144	-35.480
2.231.028	-26.656	2.253.945	-33.452	2.204.334	-35.482
2.231.238	-26.658	2.254.048	-33.452	2.204.500	-35.483
2.231.463	-26.659	2.254.218	-33.452	2.204.691	-35.485
2.231.629	-26.661	2.254.451	-33.452	2.204.830	-35.486
2.231.785	-26.663	2.254.744	-33.452	2.204.955	-35.487
2.231.982	-26.665	2.254.973	-33.452	2.205.170	-35.489
2.232.157	-26.666	2.255.110	-33.453	2.205.392	-35.490

2.232.276	-26.668	2.255.206	-33.453	2.205.553	-35.492
2.232.439	-26.670	2.255.343	-33.453	2.205.726	-35.493
2.232.571	-26.671	2.255.477	-33.453	2.205.763	-35.494
2.232.746	-26.673	2.255.598	-33.453	2.205.872	-35.496
2.232.917	-26.675	2.255.831	-33.454	2.206.125	-35.497
2.233.060	-26.676	2.256.049	-33.454	2.206.332	-35.499
2.233.172	-26.678	2.256.219	-33.454	2.206.550	-35.500
2.233.329	-26.679	2.256.366	-33.454	2.206.738	-35.501
2.233.521	-26.681	2.256.460	-33.454	2.206.949	-35.503
2.233.629	-26.682	2.256.565	-33.455	2.207.139	-35.504
2.233.734	-26.684	2.256.715	-33.455	2.207.260	-35.505
2.233.913	-26.685	2.256.875	-33.455	2.207.318	-35.506
2.234.118	-26.687	2.257.030	-33.455	2.207.452	-35.508
2.234.366	-26.688	2.257.254	-33.455	2.207.688	-35.509
2.234.531	-26.690	2.257.484	-33.456	2.207.862	-35.510
2.234.725	-26.691	2.257.670	-33.456	2.207.965	-35.512
2.234.975	-26.693	2.257.874	-33.456	2.208.104	-35.513
2.235.116	-26.694	2.258.044	-33.456	2.208.449	-35.514
2.235.300	-26.696	2.258.156	-33.457	2.208.885	-35.515
2.235.535	-26.697	2.258.242	-33.457	2.209.147	-35.517
2.235.679	-26.699	2.258.394	-33.457	2.209.276	-35.518
2.235.804	-26.700	2.258.609	-33.457	2.209.408	-35.519
2.235.918	-26.701	2.258.797	-33.458	2.209.500	-35.520
2.236.051	-26.703	2.258.953	-33.458	2.209.547	-35.522
2.236.247	-26.704	2.259.066	-33.458	2.209.587	-35.523
2.236.375	-26.705	2.259.227	-33.459	2.209.666	-35.524
2.236.485	-26.707	2.259.445	-33.459	2.209.751	-35.525
2.236.626	-26.708	2.259.628	-33.459	2.209.825	-35.526
2.236.805	-26.709	2.259.728	-33.459	2.209.982	-35.528
2.237.004	-26.710	2.259.895	-33.460	2.210.210	-35.529

2.237.157	-26.712	2.260.159	-33.460	2.210.390	-35.530
2.237.325	-26.713	2.260.324	-33.460	2.210.557	-35.531
2.237.520	-26.714	2.260.472	-33.461	2.210.775	-35.533
2.237.728	-26.715	2.260.659	-33.461	2.210.928	-35.534
2.237.917	-26.717	2.260.806	-33.461	2.211.039	-35.535
2.238.060	-26.718	2.260.959	-33.462	2.211.256	-35.537
2.238.257	-26.719	2.261.187	-33.462	2.211.485	-35.538
2.238.483	-26.720	2.261.404	-33.462	2.211.656	-35.539
2.238.609	-26.721	2.261.512	-33.463	2.211.798	-35.541
2.238.717	-26.722	2.261.642	-33.463	2.211.945	-35.542
2.238.949	-26.724	2.261.787	-33.464	2.212.121	-35.543
2.239.142	-26.725	2.261.953	-33.464	2.212.285	-35.545
2.239.220	-26.726	2.262.173	-33.464	2.212.422	-35.546
2.239.321	-26.727	2.262.343	-33.465	2.212.576	-35.547
2.239.545	-26.728	2.262.480	-33.465	2.212.766	-35.549
2.239.791	-26.729	2.262.643	-33.466	2.212.887	-35.550
2.239.932	-26.730	2.262.811	-33.466	2.212.986	-35.552
2.240.087	-26.731	2.262.973	-33.466	2.213.177	-35.553
2.240.240	-26.732	2.263.145	-33.467	2.213.242	-35.555
2.240.418	-26.733	2.263.248	-33.467	2.213.383	-35.556
2.240.650	-26.734	2.263.463	-33.468	2.213.667	-35.557
2.240.842	-26.735	2.263.709	-33.468	2.213.850	-35.559
2.240.984	-26.736	2.263.801	-33.468	2.214.021	-35.560
2.241.167	-26.737	2.263.927	-33.469	2.214.189	-35.562
2.241.326	-26.738	2.264.115	-33.469	2.214.456	-35.564
2.241.494	-26.739	2.264.303	-33.470	2.214.641	-35.565
2.241.671	-26.740	2.264.457	-33.470	2.214.836	-35.567
2.241.880	-26.741	2.264.579	-33.470	2.215.036	-35.568
2.242.048	-26.742	2.264.711	-33.471	2.215.121	-35.570
2.242.143	-26.743	2.264.811	-33.471	2.215.193	-35.572

2.242.267	-26.743	2.264.952	-33.472	2.215.383	-35.573
2.242.475	-26.744	2.265.092	-33.472	2.215.535	-35.575
2.242.733	-26.745	2.265.217	-33.472	2.215.715	-35.577
2.242.915	-26.746	2.265.419	-33.473	2.215.929	-35.578
2.243.020	-26.747	2.265.638	-33.473	2.216.041	-35.580
2.243.111	-26.748	2.265.880	-33.473	2.216.183	-35.582
2.243.454	-26.749	2.266.082	-33.474	2.216.366	-35.584
2.243.927	-26.749	2.266.196	-33.474	2.216.525	-35.586
2.244.135	-26.750	2.266.420	-33.474	2.216.671	-35.588
2.244.211	-26.751	2.266.682	-33.475	2.216.810	-35.589
2.244.306	-26.752	2.266.798	-33.475	2.216.978	-35.591
2.244.315	-26.753	2.266.857	-33.475	2.217.153	-35.593
2.244.348	-26.753	2.267.020	-33.476	2.217.327	-35.595
2.244.453	-26.754	2.267.251	-33.476	2.217.489	-35.597
2.244.569	-26.755	2.267.367	-33.476	2.217.675	-35.599
2.244.706	-26.756	2.267.477	-33.477	2.217.867	-35.601
2.244.867	-26.756	2.267.672	-33.477	2.218.013	-35.603
2.245.049	-26.757	2.267.755	-33.477	2.218.129	-35.605
2.245.208	-26.758	2.267.876	-33.477	2.218.281	-35.607
2.245.354	-26.758	2.268.145	-33.478	2.218.450	-35.609
2.245.548	-26.759	2.268.357	-33.478	2.218.590	-35.611
2.245.743	-26.760	2.268.486	-33.478	2.218.750	-35.613
2.245.876	-26.761	2.268.645	-33.478	2.218.981	-35.616
2.246.069	-26.761	2.268.895	-33.479	2.219.205	-35.618
2.246.314	-26.762	2.269.108	-33.479	2.219.357	-35.620
2.246.458	-26.763	2.269.350	-33.479	2.219.534	-35.622
2.246.550	-26.763	2.269.550	-33.479	2.219.720	-35.624
2.246.734	-26.764	2.269.655	-33.479	2.219.879	-35.626
2.246.942	-26.764	2.269.725	-33.480	2.220.033	-35.629
2.247.063	-26.765	2.269.901	-33.480	2.220.181	-35.631

2.247.206	-26.766	2.270.128	-33.480	2.220.340	-35.633
2.247.376	-26.766	2.270.291	-33.480	2.220.461	-35.635
2.247.534	-26.767	2.270.453	-33.480	2.220.609	-35.638
2.247.724	-26.768	2.270.614	-33.480	2.220.822	-35.640
2.247.883	-26.768	2.270.804	-33.481	2.220.961	-35.642
2.248.017	-26.769	2.270.976	-33.481	2.221.039	-35.644
2.248.210	-26.769	2.271.113	-33.481	2.221.281	-35.647
2.248.416	-26.770	2.271.273	-33.481	2.221.586	-35.649
2.248.640	-26.770	2.271.423	-33.481	2.221.738	-35.651
2.248.868	-26.771	2.271.586	-33.481	2.221.924	-35.654
2.249.019	-26.771	2.271.750	-33.481	2.222.101	-35.656
2.249.153	-26.772	2.271.964	-33.481	2.222.182	-35.658
2.249.315	-26.773	2.272.211	-33.482	2.222.332	-35.661
2.249.464	-26.773	2.272.325	-33.482	2.222.489	-35.663
2.249.599	-26.774	2.272.458	-33.482	2.222.617	-35.665
2.249.762	-26.774	2.272.704	-33.482	2.222.800	-35.668
2.249.951	-26.775	2.272.829	-33.482	2.222.941	-35.670
2.250.126	-26.775	2.272.934	-33.482	2.223.120	-35.672
2.250.305	-26.776	2.273.165	-33.482	2.223.288	-35.674
2.250.477	-26.776	2.273.355	-33.482	2.223.409	-35.677
2.250.556	-26.777	2.273.492	-33.482	2.223.555	-35.679
2.250.690	-26.777	2.273.625	-33.482	2.223.676	-35.681
2.250.869	-26.778	2.273.729	-33.482	2.223.811	-35.684
2.250.996	-26.778	2.273.913	-33.482	2.224.012	-35.686
2.251.172	-26.778	2.274.113	-33.482	2.224.233	-35.688
2.251.335	-26.779	2.274.238	-33.482	2.224.408	-35.690
2.251.453	-26.779	2.274.549	-33.482	2.224.594	-35.693
2.251.604	-26.780	2.275.035	-33.482	2.224.805	-35.695
2.251.803	-26.780	2.275.353	-33.482	2.224.975	-35.697
2.251.960	-26.781	2.275.421	-33.482	2.225.161	-35.699

2.252.143	-26.781	2.275.439	-33.482	2.225.359	-35.701
2.252.339	-26.781	2.275.513	-33.482	2.225.542	-35.704
2.252.455	-26.782	2.275.526	-33.482	2.225.743	-35.706
2.252.666	-26.782	2.275.564	-33.482	2.225.900	-35.708
2.252.883	-26.783	2.275.630	-33.482	2.226.012	-35.710
2.253.069	-26.783	2.275.728	-33.482	2.226.189	-35.712
2.253.228	-26.783	2.275.928	-33.482	2.226.369	-35.714
2.253.328	-26.784	2.276.158	-33.482	2.226.521	-35.717
2.253.456	-26.784	2.276.409	-33.483	2.226.633	-35.719
2.253.597	-26.785	2.276.615	-33.483	2.226.711	-35.721
2.253.799	-26.785	2.276.736	-33.483	2.226.814	-35.723
2.253.997	-26.785	2.276.939	-33.483	2.226.989	-35.725
2.254.196	-26.786	2.277.142	-33.483	2.227.193	-35.727
2.254.379	-26.786	2.277.290	-33.483	2.227.390	-35.729
2.254.503	-26.786	2.277.464	-33.483	2.227.531	-35.731
2.254.652	-26.787	2.277.648	-33.483	2.227.665	-35.733
2.254.764	-26.787	2.277.829	-33.483	2.227.852	-35.735
2.254.970	-26.787	2.277.957	-33.483	2.228.129	-35.737
2.255.271	-26.788	2.278.107	-33.483	2.228.389	-35.739
2.255.414	-26.788	2.278.246	-33.483	2.228.519	-35.740
2.255.466	-26.788	2.278.398	-33.483	2.228.700	-35.742
2.255.638	-26.788	2.278.515	-33.484	2.228.879	-35.744
2.255.822	-26.789	2.278.665	-33.484	2.229.035	-35.746
2.255.893	-26.789	2.278.864	-33.484	2.229.233	-35.748
2.256.068	-26.789	2.279.032	-33.484	2.229.395	-35.750
2.256.281	-26.790	2.279.177	-33.484	2.229.482	-35.751
2.256.451	-26.790	2.279.348	-33.484	2.229.697	-35.753
2.256.602	-26.790	2.279.530	-33.484	2.229.917	-35.755
2.256.778	-26.790	2.279.734	-33.485	2.230.024	-35.756
2.257.034	-26.791	2.279.939	-33.485	2.230.138	-35.758

2.257.235	-26.791	2.280.049	-33.485	2.230.277	-35.760
2.257.435	-26.791	2.280.181	-33.485	2.230.457	-35.761
2.257.601	-26.791	2.280.387	-33.486	2.230.672	-35.763
2.257.784	-26.792	2.280.548	-33.486	2.230.865	-35.765
2.258.037	-26.792	2.280.663	-33.486	2.230.997	-35.766
2.258.183	-26.792	2.280.770	-33.487	2.231.178	-35.768
2.258.270	-26.792	2.280.965	-33.487	2.231.367	-35.769
2.258.427	-26.793	2.281.133	-33.487	2.231.559	-35.771
2.258.587	-26.793	2.281.254	-33.488	2.231.720	-35.772
2.258.712	-26.793	2.281.393	-33.488	2.231.861	-35.774
2.258.821	-26.793	2.281.579	-33.489	2.232.105	-35.775
2.258.931	-26.794	2.281.788	-33.489	2.232.298	-35.777
2.259.057	-26.794	2.281.931	-33.490	2.232.375	-35.778
2.259.184	-26.794	2.282.050	-33.490	2.232.482	-35.779
2.259.382	-26.794	2.282.260	-33.491	2.232.654	-35.781
2.259.632	-26.794	2.282.473	-33.491	2.232.825	-35.782
2.259.823	-26.795	2.282.625	-33.492	2.232.999	-35.783
2.259.979	-26.795	2.282.773	-33.492	2.233.189	-35.785
2.260.179	-26.795	2.282.909	-33.493	2.233.326	-35.786
2.260.380	-26.795	2.283.089	-33.494	2.233.452	-35.787
2.260.524	-26.795	2.283.259	-33.494	2.233.643	-35.789
2.260.674	-26.796	2.283.430	-33.495	2.233.829	-35.790
2.260.896	-26.796	2.283.640	-33.496	2.234.046	-35.791
2.261.073	-26.796	2.283.783	-33.496	2.234.236	-35.792
2.261.189	-26.796	2.283.918	-33.497	2.234.401	-35.793
2.261.358	-26.796	2.284.079	-33.498	2.234.556	-35.795
2.261.563	-26.796	2.284.245	-33.499	2.234.677	-35.796
2.261.725	-26.797	2.284.460	-33.500	2.234.798	-35.797
2.261.839	-26.797	2.284.681	-33.500	2.234.910	-35.798
2.261.991	-26.797	2.284.785	-33.501	2.235.240	-35.799

2.262.202	-26.797	2.284.942	-33.502	2.235.672	-35.800
2.262.431	-26.797	2.285.170	-33.503	2.235.979	-35.801
2.262.612	-26.798	2.285.323	-33.504	2.236.131	-35.802
2.262.737	-26.798	2.285.502	-33.505	2.236.203	-35.803
2.262.925	-26.798	2.285.663	-33.506	2.236.323	-35.804
2.263.087	-26.798	2.285.795	-33.507	2.236.409	-35.805
2.263.203	-26.798	2.285.912	-33.508	2.236.444	-35.806
2.263.380	-26.798	2.286.086	-33.509	2.236.465	-35.807
2.263.588	-26.799	2.286.270	-33.510	2.236.534	-35.808
2.263.797	-26.799	2.286.438	-33.511	2.236.653	-35.809
2.263.932	-26.799	2.286.586	-33.512	2.236.868	-35.810
2.264.133	-26.799	2.286.702	-33.513	2.237.004	-35.811
2.264.316	-26.799	2.286.824	-33.515	2.237.287	-35.812
2.264.436	-26.799	2.286.938	-33.516	2.237.543	-35.813
2.264.588	-26.800	2.287.040	-33.517	2.237.645	-35.814
2.264.791	-26.800	2.287.190	-33.518	2.237.840	-35.815
2.264.995	-26.800	2.287.401	-33.519	2.238.030	-35.816
2.265.103	-26.800	2.287.655	-33.520	2.238.147	-35.817
2.265.231	-26.800	2.287.849	-33.522	2.238.331	-35.818
2.265.406	-26.800	2.288.035	-33.523	2.238.481	-35.819
2.265.728	-26.801	2.288.230	-33.524	2.238.638	-35.820
2.266.129	-26.801	2.288.443	-33.525	2.238.839	-35.821
2.266.402	-26.801	2.288.643	-33.527	2.238.972	-35.822
2.266.536	-26.801	2.288.806	-33.528	2.239.073	-35.823
2.266.617	-26.801	2.288.974	-33.529	2.239.229	-35.824
2.266.730	-26.802	2.289.115	-33.531	2.239.354	-35.825
2.266.738	-26.802	2.289.256	-33.532	2.239.495	-35.825
2.266.727	-26.802	2.289.353	-33.533	2.239.742	-35.826
2.266.786	-26.802	2.289.417	-33.535	2.239.986	-35.827
2.266.939	-26.802	2.289.572	-33.536	2.240.096	-35.828

2.267.204	-26.802	2.289.812	-33.538	2.240.210	-35.829
2.267.390	-26.803	2.290.016	-33.539	2.240.392	-35.830
2.267.502	-26.803	2.290.159	-33.540	2.240.497	-35.831
2.267.661	-26.803	2.290.246	-33.542	2.240.672	-35.832
2.267.903	-26.803	2.290.383	-33.543	2.240.927	-35.833
2.268.148	-26.803	2.290.611	-33.545	2.241.109	-35.834
2.268.248	-26.803	2.290.860	-33.546	2.241.279	-35.835
2.268.366	-26.804	2.291.073	-33.548	2.241.444	-35.836
2.268.577	-26.804	2.291.185	-33.549	2.241.664	-35.837
2.268.761	-26.804	2.291.370	-33.551	2.241.850	-35.838
2.268.914	-26.804	2.291.582	-33.552	2.242.034	-35.839
2.269.052	-26.804	2.291.745	-33.554	2.242.202	-35.840
2.269.180	-26.805	2.291.917	-33.555	2.242.258	-35.841
2.269.327	-26.805	2.292.104	-33.557	2.242.399	-35.842
2.269.493	-26.805	2.292.311	-33.558	2.242.538	-35.843
2.269.612	-26.805	2.292.451	-33.560	2.242.641	-35.844
2.269.733	-26.805	2.292.565	-33.561	2.242.798	-35.845
2.269.917	-26.806	2.292.753	-33.563	2.243.009	-35.846
2.270.147	-26.806	2.292.980	-33.564	2.243.183	-35.847
2.270.320	-26.806	2.293.125	-33.566	2.243.322	-35.848
2.270.509	-26.806	2.293.281	-33.567	2.243.548	-35.849
2.270.695	-26.806	2.293.447	-33.569	2.243.759	-35.850
2.270.864	-26.807	2.293.586	-33.571	2.243.906	-35.851
2.271.078	-26.807	2.293.764	-33.572	2.244.092	-35.852
2.271.261	-26.807	2.293.941	-33.574	2.244.198	-35.853
2.271.391	-26.807	2.294.079	-33.575	2.244.297	-35.854
2.271.537	-26.807	2.294.267	-33.577	2.244.525	-35.856
2.271.720	-26.808	2.294.530	-33.578	2.244.760	-35.857
2.271.873	-26.808	2.294.662	-33.580	2.244.968	-35.858
2.272.020	-26.808	2.294.753	-33.582	2.245.108	-35.859

2.272.213	-26.808	2.294.924	-33.583	2.245.204	-35.860
2.272.392	-26.808	2.295.051	-33.585	2.245.320	-35.861
2.272.529	-26.809	2.295.219	-33.586	2.245.472	-35.862
2.272.740	-26.809	2.295.466	-33.588	2.245.710	-35.863
2.272.919	-26.809	2.295.627	-33.590	2.245.909	-35.865
2.273.057	-26.809	2.295.730	-33.591	2.246.082	-35.866
2.273.203	-26.809	2.295.913	-33.593	2.246.276	-35.867
2.273.375	-26.810	2.296.069	-33.595	2.246.438	-35.868
2.273.564	-26.810	2.296.144	-33.596	2.246.573	-35.869
2.273.705	-26.810	2.296.382	-33.598	2.246.769	-35.870
2.273.848	-26.810	2.296.850	-33.599	2.246.982	-35.872
2.273.981	-26.811	2.297.258	-33.601	2.247.132	-35.873
2.274.124	-26.811	2.297.392	-33.603	2.247.256	-35.874
2.274.315	-26.811	2.297.413	-33.604	2.247.401	-35.875
2.274.540	-26.811	2.297.495	-33.606	2.247.585	-35.876
2.274.731	-26.812	2.297.560	-33.608	2.247.712	-35.877
2.274.906	-26.812	2.297.617	-33.609	2.247.847	-35.878
2.275.053	-26.812	2.297.738	-33.611	2.248.066	-35.880
2.275.190	-26.812	2.297.827	-33.613	2.248.228	-35.881
2.275.344	-26.813	2.297.930	-33.614	2.248.375	-35.882
2.275.483	-26.813	2.298.107	-33.616	2.248.552	-35.883
2.275.663	-26.813	2.298.288	-33.618	2.248.719	-35.884
2.275.840	-26.813	2.298.503	-33.620	2.248.886	-35.885
2.275.992	-26.814	2.298.720	-33.621	2.249.018	-35.886
2.276.158	-26.814	2.298.904	-33.623	2.249.209	-35.887
2.276.265	-26.814	2.299.035	-33.625	2.249.392	-35.888
2.276.429	-26.814	2.299.200	-33.626	2.249.540	-35.889
2.276.694	-26.815	2.299.433	-33.628	2.249.697	-35.890
2.276.863	-26.815	2.299.637	-33.630	2.249.863	-35.891
2.276.993	-26.815	2.299.767	-33.632	2.249.991	-35.892

2.277.150	-26.816	2.299.865	-33.633	2.250.067	-35.893
2.277.280	-26.816	2.300.018	-33.635	2.250.199	-35.894
2.277.469	-26.816	2.300.139	-33.637	2.250.362	-35.895
2.277.675	-26.817	2.300.293	-33.639	2.250.522	-35.896
2.277.757	-26.817	2.300.519	-33.641	2.250.726	-35.897
2.277.823	-26.817	2.300.656	-33.642	2.250.956	-35.898
2.277.971	-26.817	2.300.748	-33.644	2.251.102	-35.899
2.278.129	-26.818	2.300.969	-33.646	2.251.227	-35.900
2.278.293	-26.818	2.301.229	-33.648	2.251.433	-35.901
2.278.490	-26.818	2.301.380	-33.650	2.251.664	-35.902
2.278.687	-26.819	2.301.530	-33.652	2.251.938	-35.902
2.278.864	-26.819	2.301.738	-33.654	2.252.177	-35.903
2.279.025	-26.819	2.301.899	-33.656	2.252.314	-35.904
2.279.153	-26.820	2.302.011	-33.658	2.252.455	-35.905
2.279.361	-26.820	2.302.178	-33.660	2.252.639	-35.906
2.279.587	-26.820	2.302.397	-33.662	2.252.805	-35.906
2.279.805	-26.821	2.302.558	-33.664	2.252.934	-35.907
2.279.986	-26.821	2.302.664	-33.666	2.253.127	-35.908
2.280.155	-26.822	2.302.784	-33.668	2.253.286	-35.908
2.280.354	-26.822	2.302.926	-33.670	2.253.389	-35.909
2.280.551	-26.822	2.303.035	-33.672	2.253.540	-35.910
2.280.685	-26.823	2.303.149	-33.674	2.253.682	-35.910
2.280.742	-26.823	2.303.324	-33.676	2.253.779	-35.911
2.280.889	-26.823	2.303.539	-33.678	2.253.922	-35.911
2.281.075	-26.824	2.303.714	-33.680	2.254.153	-35.912
2.281.254	-26.824	2.303.848	-33.683	2.254.333	-35.913
2.281.402	-26.825	2.304.023	-33.685	2.254.464	-35.913
2.281.534	-26.825	2.304.236	-33.687	2.254.596	-35.914
2.281.664	-26.826	2.304.392	-33.689	2.254.726	-35.914
2.281.783	-26.826	2.304.495	-33.692	2.254.962	-35.914

2.281.978	-26.826	2.304.641	-33.694	2.255.219	-35.915
2.282.199	-26.827	2.304.828	-33.696	2.255.445	-35.915
2.282.386	-26.827	2.305.049	-33.699	2.255.658	-35.916
2.282.570	-26.828	2.305.219	-33.701	2.255.824	-35.916
2.282.742	-26.828	2.305.378	-33.703	2.255.963	-35.916
2.282.885	-26.829	2.305.533	-33.706	2.256.120	-35.917
2.283.078	-26.829	2.305.676	-33.708	2.256.295	-35.917
2.283.268	-26.830	2.305.857	-33.711	2.256.386	-35.917
2.283.389	-26.830	2.306.008	-33.713	2.256.505	-35.918
2.283.566	-26.831	2.306.187	-33.716	2.256.691	-35.918
2.283.729	-26.831	2.306.389	-33.718	2.256.872	-35.918
2.283.887	-26.832	2.306.503	-33.721	2.257.039	-35.918
2.284.086	-26.832	2.306.570	-33.724	2.257.175	-35.918
2.284.272	-26.833	2.306.749	-33.726	2.257.370	-35.919
2.284.464	-26.833	2.306.980	-33.729	2.257.540	-35.919
2.284.641	-26.834	2.307.155	-33.732	2.257.661	-35.919
2.284.769	-26.834	2.307.314	-33.734	2.257.842	-35.919
2.284.919	-26.835	2.307.502	-33.737	2.258.019	-35.919
2.285.129	-26.836	2.307.684	-33.740	2.258.189	-35.919
2.285.280	-26.836	2.307.878	-33.743	2.258.412	-35.919
2.285.468	-26.837	2.308.069	-33.746	2.258.617	-35.919
2.285.683	-26.837	2.308.199	-33.748	2.258.752	-35.920
2.285.694	-26.838	2.308.302	-33.751	2.258.904	-35.920
2.285.806	-26.839	2.308.434	-33.754	2.259.027	-35.920
2.286.089	-26.839	2.308.568	-33.757	2.259.144	-35.920
2.286.258	-26.840	2.308.683	-33.760	2.259.286	-35.920
2.286.414	-26.840	2.308.857	-33.763	2.259.411	-35.920
2.286.656	-26.841	2.308.996	-33.766	2.259.616	-35.920
2.286.891	-26.842	2.309.128	-33.769	2.259.868	-35.920
2.287.036	-26.842	2.309.292	-33.772	2.260.049	-35.920

2.287.144	-26.843	2.309.408	-33.775	2.260.194	-35.920
2.287.225	-26.844	2.309.641	-33.778	2.260.336	-35.920
2.287.302	-26.844	2.309.908	-33.781	2.260.513	-35.920
2.287.471	-26.845	2.310.127	-33.785	2.260.723	-35.920
2.287.697	-26.846	2.310.412	-33.788	2.260.900	-35.919
2.288.066	-26.847	2.310.600	-33.791	2.261.102	-35.919
2.288.485	-26.847	2.310.687	-33.794	2.261.299	-35.919
2.288.727	-26.848	2.310.864	-33.797	2.261.442	-35.919
2.288.801	-26.849	2.311.070	-33.801	2.261.590	-35.919
2.288.814	-26.850	2.311.211	-33.804	2.261.691	-35.919
2.288.866	-26.850	2.311.393	-33.807	2.261.810	-35.919
2.289.016	-26.851	2.311.516	-33.810	2.262.132	-35.919
2.289.138	-26.852	2.311.613	-33.814	2.262.578	-35.919
2.289.175	-26.853	2.311.740	-33.817	2.262.890	-35.919
2.289.285	-26.854	2.311.830	-33.820	2.263.027	-35.919
2.289.437	-26.854	2.311.895	-33.824	2.263.100	-35.919
2.289.599	-26.855	2.312.061	-33.827	2.263.183	-35.919
2.289.784	-26.856	2.312.283	-33.831	2.263.279	-35.919
2.289.995	-26.857	2.312.464	-33.834	2.263.337	-35.918
2.290.203	-26.858	2.312.655	-33.837	2.263.382	-35.918
2.290.383	-26.859	2.312.865	-33.841	2.263.486	-35.918
2.290.580	-26.860	2.313.066	-33.844	2.263.624	-35.918
2.290.773	-26.860	2.313.297	-33.848	2.263.750	-35.918
2.290.919	-26.861	2.313.403	-33.851	2.263.882	-35.918
2.291.064	-26.862	2.313.439	-33.855	2.264.072	-35.918
2.291.174	-26.863	2.313.640	-33.858	2.264.211	-35.918
2.291.306	-26.864	2.313.929	-33.862	2.264.415	-35.918
2.291.465	-26.865	2.314.111	-33.865	2.264.639	-35.918
2.291.687	-26.866	2.314.286	-33.869	2.264.810	-35.918
2.291.909	-26.867	2.314.460	-33.872	2.265.017	-35.918

2.292.007	-26.868	2.314.509	-33.876	2.265.224	-35.918
2.292.081	-26.869	2.314.599	-33.879	2.265.412	-35.918
2.292.245	-26.870	2.314.912	-33.883	2.265.530	-35.918
2.292.433	-26.871	2.315.064	-33.887	2.265.625	-35.919
2.292.608	-26.872	2.315.176	-33.890	2.265.754	-35.919
2.292.793	-26.873	2.315.356	-33.894	2.265.969	-35.919
2.292.944	-26.874	2.315.558	-33.897	2.266.149	-35.919
2.293.178	-26.875	2.315.759	-33.901	2.266.281	-35.919
2.293.427	-26.876	2.315.934	-33.905	2.266.467	-35.919
2.293.521	-26.877	2.316.080	-33.908	2.266.626	-35.920
2.293.676	-26.878	2.316.257	-33.912	2.266.745	-35.920
2.293.866	-26.879	2.316.400	-33.915	2.266.857	-35.920
2.293.960	-26.880	2.316.538	-33.919	2.267.027	-35.920
2.294.166	-26.881	2.316.758	-33.923	2.267.213	-35.921
2.294.342	-26.882	2.316.975	-33.926	2.267.294	-35.921
2.294.503	-26.883	2.317.083	-33.930	2.267.529	-35.921
2.294.668	-26.885	2.317.251	-33.934	2.267.890	-35.922
2.294.790	-26.886	2.317.477	-33.937	2.268.062	-35.922
2.295.031	-26.887	2.317.596	-33.941	2.268.154	-35.923
2.295.226	-26.888	2.317.688	-33.945	2.268.281	-35.923
2.295.313	-26.889	2.317.912	-33.948	2.268.459	-35.923
2.295.491	-26.890	2.318.167	-33.952	2.268.738	-35.924
2.295.707	-26.891	2.318.342	-33.956	2.268.940	-35.925
2.295.869	-26.893	2.318.400	-33.959	2.268.994	-35.925
2.296.008	-26.894	2.318.611	-33.963	2.269.171	-35.926
2.296.178	-26.895	2.319.086	-33.967	2.269.402	-35.926
2.296.306	-26.896	2.319.427	-33.970	2.269.551	-35.927
2.296.384	-26.898	2.319.511	-33.974	2.269.715	-35.928
2.296.590	-26.899	2.319.550	-33.978	2.269.870	-35.928
2.296.833	-26.900	2.319.690	-33.981	2.269.959	-35.929

2.297.047	-26.901	2.319.764	-33.985	2.270.062	-35.930
2.297.190	-26.903	2.319.747	-33.989	2.270.287	-35.930
2.297.338	-26.904	2.319.863	-33.992	2.270.475	-35.931
2.297.489	-26.905	2.320.038	-33.996	2.270.618	-35.932
2.297.610	-26.906	2.320.150	-33.999	2.270.801	-35.933
2.297.813	-26.908	2.320.318	-34.003	2.270.927	-35.934
2.297.941	-26.909	2.320.445	-34.007	2.271.059	-35.935
2.298.046	-26.910	2.320.607	-34.010	2.271.252	-35.936
2.298.214	-26.912	2.320.872	-34.014	2.271.462	-35.937
2.298.402	-26.913	2.321.061	-34.018	2.271.653	-35.937
2.298.569	-26.914	2.321.176	-34.021	2.271.776	-35.938
2.298.691	-26.916	2.321.293	-34.025	2.271.897	-35.939
2.298.885	-26.917	2.321.471	-34.028	2.272.034	-35.940
2.299.146	-26.919	2.321.671	-34.032	2.272.179	-35.942
2.299.301	-26.920	2.321.868	-34.036	2.272.327	-35.943
2.299.402	-26.921	2.321.987	-34.039	2.272.543	-35.944
2.299.594	-26.923	2.322.148	-34.043	2.272.721	-35.945
2.299.790	-26.924	2.322.255	-34.046	2.272.914	-35.946
2.299.921	-26.926	2.322.388	-34.050	2.273.130	-35.947
2.300.042	-26.927	2.322.638	-34.053	2.273.295	-35.948
2.300.206	-26.929	2.322.836	-34.057	2.273.449	-35.950
2.300.329	-26.930	2.322.971	-34.060	2.273.624	-35.951
2.300.482	-26.932	2.323.085	-34.064	2.273.817	-35.952
2.300.607	-26.933	2.323.310	-34.067	2.273.969	-35.953
2.300.715	-26.935	2.323.571	-34.071	2.274.086	-35.954
2.300.896	-26.936	2.323.710	-34.074	2.274.252	-35.956
2.301.026	-26.938	2.323.837	-34.078	2.274.484	-35.957
2.301.194	-26.939	2.324.014	-34.081	2.274.679	-35.958
2.301.485	-26.941	2.324.230	-34.085	2.274.799	-35.960
2.301.686	-26.942	2.324.398	-34.088	2.274.957	-35.961

2.301.870	-26.944	2.324.516	-34.092	2.275.181	-35.962
2.302.141	-26.946	2.324.689	-34.095	2.275.371	-35.964
2.302.392	-26.947	2.324.859	-34.099	2.275.481	-35.965
2.302.547	-26.949	2.324.942	-34.102	2.275.640	-35.966
2.302.626	-26.951	2.325.085	-34.105	2.275.874	-35.968
2.302.771	-26.952	2.325.284	-34.109	2.276.057	-35.969
2.302.968	-26.954	2.325.461	-34.112	2.276.160	-35.970
2.303.102	-26.955	2.325.651	-34.116	2.276.277	-35.972
2.303.154	-26.957	2.325.831	-34.119	2.276.478	-35.973
2.303.313	-26.959	2.325.969	-34.122	2.276.650	-35.974
2.303.548	-26.961	2.326.071	-34.126	2.276.776	-35.976
2.303.705	-26.962	2.326.178	-34.129	2.276.842	-35.977
2.303.839	-26.964	2.326.362	-34.132	2.277.000	-35.979
2.303.970	-26.966	2.326.572	-34.136	2.277.204	-35.980
2.304.164	-26.968	2.326.720	-34.139	2.277.310	-35.981
2.304.359	-26.969	2.326.830	-34.142	2.277.439	-35.983
2.304.525	-26.971	2.327.001	-34.146	2.277.605	-35.984
2.304.724	-26.973	2.327.175	-34.149	2.277.759	-35.985
2.304.856	-26.975	2.327.350	-34.152	2.277.944	-35.987
2.305.009	-26.976	2.327.570	-34.156	2.278.151	-35.988
2.305.164	-26.978	2.327.746	-34.159	2.278.344	-35.989
2.305.291	-26.980	2.327.923	-34.162	2.278.543	-35.991
2.305.427	-26.982	2.328.109	-34.165	2.278.750	-35.992
2.305.586	-26.984	2.328.270	-34.169	2.278.931	-35.994
2.305.815	-26.986	2.328.448	-34.172	2.279.097	-35.995
2.306.043	-26.988	2.328.587	-34.175	2.279.259	-35.996
2.306.180	-26.990	2.328.763	-34.178	2.279.426	-35.997
2.306.335	-26.992	2.329.000	-34.181	2.279.637	-35.999
2.306.521	-26.993	2.329.115	-34.184	2.279.780	-36.000
2.306.691	-26.995	2.329.189	-34.188	2.279.978	-36.001

2.306.837	-26.997	2.329.342	-34.191	2.280.233	-36.003
2.306.974	-26.999	2.329.578	-34.194	2.280.327	-36.004
2.307.163	-27.001	2.329.810	-34.197	2.280.361	-36.005
2.307.325	-27.003	2.329.986	-34.200	2.280.443	-36.007
2.307.493	-27.005	2.330.148	-34.203	2.280.592	-36.008
2.307.645	-27.007	2.330.152	-34.206	2.280.739	-36.009
2.307.798	-27.009	2.330.271	-34.209	2.280.887	-36.010
2.307.970	-27.012	2.330.497	-34.212	2.280.992	-36.011
2.308.151	-27.014	2.330.726	-34.215	2.281.153	-36.013
2.308.335	-27.016	2.330.876	-34.218	2.281.388	-36.014
2.308.521	-27.018	2.331.019	-34.221	2.281.604	-36.015
2.308.660	-27.020	2.331.198	-34.224	2.281.705	-36.016
2.308.732	-27.022	2.331.333	-34.227	2.281.891	-36.017
2.308.886	-27.024	2.331.462	-34.230	2.282.186	-36.019
2.309.164	-27.026	2.331.626	-34.233	2.282.372	-36.020
2.309.399	-27.029	2.331.769	-34.236	2.282.514	-36.021
2.309.534	-27.031	2.332.041	-34.238	2.282.691	-36.022
2.309.613	-27.033	2.332.269	-34.241	2.282.818	-36.023
2.309.713	-27.035	2.332.370	-34.244	2.282.970	-36.024
2.309.884	-27.037	2.332.578	-34.247	2.283.179	-36.025
2.310.092	-27.040	2.332.786	-34.250	2.283.333	-36.026
2.310.430	-27.042	2.332.912	-34.252	2.283.501	-36.027
2.310.898	-27.044	2.333.123	-34.255	2.283.710	-36.028
2.311.187	-27.046	2.333.306	-34.258	2.283.893	-36.029
2.311.232	-27.049	2.333.452	-34.260	2.284.030	-36.030
2.311.303	-27.051	2.333.658	-34.263	2.284.196	-36.031
2.311.422	-27.053	2.333.833	-34.265	2.284.357	-36.032
2.311.461	-27.056	2.333.927	-34.268	2.284.487	-36.033
2.311.570	-27.058	2.334.055	-34.270	2.284.642	-36.034
2.311.662	-27.060	2.334.155	-34.273	2.284.825	-36.035

2.311.751	-27.063	2.334.258	-34.275	2.284.984	-36.036
2.311.855	-27.065	2.334.404	-34.278	2.285.103	-36.037
2.312.012	-27.068	2.334.603	-34.280	2.285.251	-36.038
2.312.231	-27.070	2.334.821	-34.282	2.285.441	-36.039
2.312.379	-27.073	2.334.980	-34.285	2.285.596	-36.040
2.312.535	-27.075	2.335.078	-34.287	2.285.754	-36.041
2.312.780	-27.077	2.335.278	-34.289	2.285.952	-36.041
2.312.988	-27.080	2.335.533	-34.291	2.286.109	-36.042
2.313.159	-27.082	2.335.728	-34.293	2.286.290	-36.043
2.313.289	-27.085	2.335.869	-34.296	2.286.532	-36.044
2.313.447	-27.087	2.336.057	-34.298	2.286.723	-36.045
2.313.624	-27.090	2.336.299	-34.300	2.286.816	-36.046
2.313.761	-27.092	2.336.482	-34.302	2.286.944	-36.047
2.313.923	-27.095	2.336.676	-34.304	2.287.128	-36.047
2.314.086	-27.097	2.336.790	-34.306	2.287.284	-36.048
2.314.173	-27.100	2.336.873	-34.308	2.287.446	-36.049
2.314.297	-27.103	2.337.060	-34.310	2.287.672	-36.050
2.314.475	-27.105	2.337.240	-34.312	2.287.856	-36.051
2.314.650	-27.108	2.337.386	-34.313	2.287.980	-36.052
2.314.825	-27.110	2.337.526	-34.315	2.288.103	-36.052
2.314.964	-27.113	2.337.655	-34.317	2.288.241	-36.053
2.315.098	-27.116	2.337.867	-34.319	2.288.407	-36.054
2.315.349	-27.118	2.338.130	-34.321	2.288.587	-36.055
2.315.575	-27.121	2.338.234	-34.322	2.288.774	-36.056
2.315.775	-27.124	2.338.387	-34.324	2.289.093	-36.057
2.315.996	-27.126	2.338.620	-34.325	2.289.549	-36.057
2.316.174	-27.129	2.338.813	-34.327	2.289.814	-36.058
2.316.272	-27.132	2.339.005	-34.329	2.289.872	-36.059
2.316.341	-27.134	2.339.146	-34.330	2.289.957	-36.060
2.316.476	-27.137	2.339.330	-34.332	2.290.091	-36.061

2.316.677	-27.140	2.339.554	-34.333	2.290.148	-36.062
2.316.857	-27.142	2.339.733	-34.334	2.290.145	-36.062
2.317.036	-27.145	2.339.883	-34.336	2.290.206	-36.063
2.317.211	-27.148	2.340.009	-34.337	2.290.360	-36.064
2.317.357	-27.151	2.340.176	-34.339	2.290.443	-36.065
2.317.488	-27.153	2.340.322	-34.340	2.290.569	-36.066
2.317.594	-27.156	2.340.488	-34.341	2.290.800	-36.067
2.317.728	-27.159	2.340.611	-34.342	2.290.966	-36.068
2.317.990	-27.162	2.340.752	-34.344	2.291.140	-36.068
2.318.201	-27.164	2.340.954	-34.345	2.291.335	-36.069
2.318.356	-27.167	2.341.339	-34.346	2.291.525	-36.070
2.318.510	-27.170	2.341.769	-34.347	2.291.724	-36.071
2.318.652	-27.173	2.341.926	-34.348	2.291.929	-36.072
2.318.734	-27.176	2.341.998	-34.349	2.292.132	-36.073
2.318.880	-27.179	2.342.132	-34.350	2.292.291	-36.074
2.319.086	-27.181	2.342.253	-34.351	2.292.469	-36.075
2.319.276	-27.184	2.342.251	-34.352	2.292.619	-36.076
2.319.507	-27.187	2.342.311	-34.353	2.292.759	-36.077
2.319.699	-27.190	2.342.446	-34.354	2.292.903	-36.078
2.319.849	-27.193	2.342.570	-34.355	2.293.038	-36.079
2.320.008	-27.196	2.342.737	-34.356	2.293.233	-36.081
2.320.125	-27.198	2.342.905	-34.356	2.293.286	-36.082
2.320.280	-27.201	2.343.112	-34.357	2.293.409	-36.083
2.320.471	-27.204	2.343.248	-34.358	2.293.615	-36.084
2.320.623	-27.207	2.343.372	-34.359	2.293.775	-36.085
2.320.819	-27.210	2.343.581	-34.360	2.293.882	-36.086
2.320.990	-27.213	2.343.741	-34.360	2.294.009	-36.088
2.321.133	-27.216	2.343.956	-34.361	2.294.182	-36.089
2.321.299	-27.219	2.344.137	-34.362	2.294.399	-36.090
2.321.454	-27.221	2.344.240	-34.362	2.294.644	-36.092

2.321.657	-27.224	2.344.383	-34.363	2.294.771	-36.093
2.321.805	-27.227	2.344.556	-34.363	2.294.923	-36.094
2.321.935	-27.230	2.344.771	-34.364	2.295.190	-36.096
2.322.072	-27.233	2.344.957	-34.365	2.295.405	-36.097
2.322.283	-27.236	2.345.060	-34.365	2.295.583	-36.098
2.322.489	-27.239	2.345.137	-34.366	2.295.730	-36.100
2.322.627	-27.242	2.345.244	-34.366	2.295.883	-36.101
2.322.730	-27.245	2.345.423	-34.367	2.295.992	-36.103
2.322.800	-27.247	2.345.654	-34.367	2.296.118	-36.104
2.322.885	-27.250	2.345.884	-34.368	2.296.290	-36.106
2.323.100	-27.253	2.346.033	-34.368	2.296.406	-36.108
2.323.302	-27.256	2.346.142	-34.369	2.296.544	-36.109
2.323.434	-27.259	2.346.398	-34.369	2.296.691	-36.111
2.323.579	-27.262	2.346.620	-34.369	2.296.902	-36.113
2.323.788	-27.265	2.346.788	-34.370	2.297.086	-36.114
2.323.994	-27.268	2.347.040	-34.370	2.297.224	-36.116
2.324.247	-27.271	2.347.207	-34.371	2.297.430	-36.118
2.324.501	-27.274	2.347.316	-34.371	2.297.618	-36.120
2.324.655	-27.277	2.347.397	-34.371	2.297.764	-36.122
2.324.867	-27.279	2.347.538	-34.372	2.297.926	-36.123
2.325.071	-27.282	2.347.739	-34.372	2.298.087	-36.125
2.325.229	-27.285	2.347.948	-34.372	2.298.297	-36.127
2.325.336	-27.288	2.348.139	-34.373	2.298.484	-36.129
2.325.461	-27.291	2.348.295	-34.373	2.298.615	-36.131
2.325.654	-27.294	2.348.459	-34.373	2.298.725	-36.133
2.325.768	-27.297	2.348.559	-34.374	2.298.846	-36.135
2.325.847	-27.300	2.348.725	-34.374	2.298.998	-36.137
2.325.974	-27.302	2.348.923	-34.374	2.299.169	-36.140
2.326.120	-27.305	2.349.059	-34.375	2.299.377	-36.142
2.326.283	-27.308	2.349.171	-34.375	2.299.557	-36.144

2.326.518	-27.311	2.349.342	-34.375	2.299.771	-36.146
2.326.677	-27.314	2.349.576	-34.376	2.299.984	-36.148
2.326.857	-27.317	2.349.736	-34.376	2.300.163	-36.151
2.327.108	-27.320	2.349.852	-34.376	2.300.332	-36.153
2.327.284	-27.322	2.350.082	-34.377	2.300.504	-36.155
2.327.385	-27.325	2.350.246	-34.377	2.300.660	-36.158
2.327.542	-27.328	2.350.334	-34.377	2.300.768	-36.160
2.327.739	-27.331	2.350.546	-34.378	2.300.923	-36.162
2.327.936	-27.334	2.350.760	-34.378	2.301.122	-36.165
2.328.183	-27.336	2.350.909	-34.378	2.301.276	-36.167
2.328.313	-27.339	2.351.050	-34.379	2.301.469	-36.170
2.328.422	-27.342	2.351.207	-34.379	2.301.687	-36.172
2.328.624	-27.345	2.351.348	-34.379	2.301.835	-36.175
2.328.725	-27.348	2.351.452	-34.380	2.301.989	-36.177
2.328.855	-27.350	2.351.644	-34.380	2.302.159	-36.180
2.329.081	-27.353	2.351.833	-34.380	2.302.262	-36.182
2.329.262	-27.356	2.352.054	-34.381	2.302.408	-36.185
2.329.377	-27.359	2.352.271	-34.381	2.302.605	-36.188
2.329.529	-27.361	2.352.381	-34.381	2.302.746	-36.190
2.329.720	-27.364	2.352.569	-34.382	2.302.865	-36.193
2.329.897	-27.367	2.352.742	-34.382	2.303.040	-36.196
2.330.076	-27.369	2.352.863	-34.383	2.303.253	-36.199
2.330.257	-27.372	2.352.999	-34.383	2.303.448	-36.201
2.330.439	-27.375	2.353.142	-34.383	2.303.548	-36.204
2.330.627	-27.377	2.353.262	-34.384	2.303.593	-36.207
2.330.780	-27.380	2.353.402	-34.384	2.303.741	-36.210
2.330.896	-27.383	2.353.606	-34.384	2.303.925	-36.212
2.331.030	-27.385	2.353.759	-34.385	2.304.081	-36.215
2.331.178	-27.388	2.353.797	-34.385	2.304.227	-36.218
2.331.361	-27.391	2.353.904	-34.386	2.304.426	-36.221

2.331.564	-27.393	2.354.162	-34.386	2.304.626	-36.224
2.331.765	-27.396	2.354.352	-34.387	2.304.758	-36.227
2.331.987	-27.398	2.354.522	-34.387	2.304.921	-36.230
2.332.152	-27.401	2.354.737	-34.387	2.305.060	-36.232
2.332.251	-27.403	2.354.922	-34.388	2.305.215	-36.235
2.332.381	-27.406	2.355.161	-34.388	2.305.387	-36.238
2.332.531	-27.408	2.355.441	-34.389	2.305.593	-36.241
2.332.555	-27.411	2.355.634	-34.389	2.305.889	-36.244
2.332.807	-27.413	2.355.730	-34.389	2.305.999	-36.247
2.333.333	-27.416	2.355.883	-34.390	2.306.131	-36.250
2.333.754	-27.418	2.356.071	-34.390	2.306.395	-36.253
2.333.989	-27.421	2.356.189	-34.391	2.306.601	-36.256
2.334.088	-27.423	2.356.283	-34.391	2.306.709	-36.259
2.334.137	-27.426	2.356.451	-34.391	2.306.827	-36.262
2.334.121	-27.428	2.356.651	-34.392	2.307.016	-36.265
2.334.126	-27.430	2.356.837	-34.392	2.307.152	-36.269
2.334.222	-27.433	2.356.938	-34.393	2.307.307	-36.272
2.334.352	-27.435	2.357.061	-34.393	2.307.426	-36.275
2.334.507	-27.437	2.357.283	-34.393	2.307.561	-36.278
2.334.702	-27.440	2.357.522	-34.394	2.307.693	-36.281
2.334.888	-27.442	2.357.731	-34.394	2.307.885	-36.284
2.335.043	-27.444	2.357.914	-34.395	2.308.083	-36.287
2.335.217	-27.447	2.358.064	-34.395	2.308.161	-36.290
2.335.430	-27.449	2.358.183	-34.395	2.308.329	-36.294
2.335.645	-27.451	2.358.335	-34.396	2.308.521	-36.297
2.335.811	-27.453	2.358.525	-34.396	2.308.693	-36.300
2.335.997	-27.456	2.358.703	-34.396	2.308.953	-36.303
2.336.189	-27.458	2.358.853	-34.397	2.309.180	-36.306
2.336.370	-27.460	2.359.034	-34.397	2.309.316	-36.310
2.336.482	-27.462	2.359.258	-34.398	2.309.493	-36.313

2.336.518	-27.464	2.359.372	-34.398	2.309.686	-36.316
2.336.637	-27.466	2.359.491	-34.398	2.309.816	-36.319
2.336.835	-27.468	2.359.700	-34.399	2.309.921	-36.323
2.337.021	-27.470	2.359.896	-34.399	2.310.038	-36.326
2.337.186	-27.473	2.360.008	-34.399	2.310.206	-36.329
2.337.296	-27.475	2.360.150	-34.400	2.310.371	-36.332
2.337.478	-27.477	2.360.358	-34.400	2.310.571	-36.336
2.337.697	-27.479	2.360.580	-34.400	2.310.728	-36.339
2.337.925	-27.481	2.360.799	-34.401	2.310.872	-36.342
2.338.116	-27.482	2.360.945	-34.401	2.311.066	-36.346
2.338.250	-27.484	2.361.068	-34.401	2.311.266	-36.349
2.338.384	-27.486	2.361.256	-34.401	2.311.422	-36.352
2.338.595	-27.488	2.361.367	-34.402	2.311.608	-36.356
2.338.831	-27.490	2.361.500	-34.402	2.311.724	-36.359
2.338.967	-27.492	2.361.727	-34.402	2.311.817	-36.362
2.339.129	-27.494	2.361.883	-34.403	2.312.039	-36.366
2.339.250	-27.496	2.361.929	-34.403	2.312.269	-36.369
2.339.382	-27.497	2.362.181	-34.403	2.312.439	-36.373
2.339.565	-27.499	2.362.462	-34.403	2.312.576	-36.376
2.339.706	-27.501	2.362.576	-34.404	2.312.661	-36.379
2.339.827	-27.503	2.362.729	-34.404	2.312.789	-36.383
2.340.007	-27.504	2.362.923	-34.404	2.313.055	-36.386
2.340.208	-27.506	2.363.065	-34.404	2.313.281	-36.390
2.340.296	-27.508	2.363.206	-34.404	2.313.439	-36.393
2.340.492	-27.509	2.363.353	-34.405	2.313.573	-36.397
2.340.593	-27.511	2.363.754	-34.405	2.313.725	-36.400
2.340.694	-27.513	2.364.236	-34.405	2.313.788	-36.404
2.340.966	-27.514	2.364.464	-34.405	2.313.904	-36.407
2.341.189	-27.516	2.364.545	-34.405	2.314.173	-36.411
2.341.353	-27.517	2.364.594	-34.405	2.314.426	-36.414

2.341.530	-27.519	2.364.679	-34.406	2.314.650	-36.418
2.341.700	-27.520	2.364.707	-34.406	2.314.807	-36.421
2.341.832	-27.522	2.364.778	-34.406	2.314.906	-36.425
2.341.982	-27.523	2.364.910	-34.406	2.315.047	-36.429
2.342.143	-27.525	2.364.998	-34.406	2.315.240	-36.432
2.342.387	-27.526	2.365.108	-34.406	2.315.398	-36.436
2.342.594	-27.528	2.365.309	-34.406	2.315.520	-36.439
2.342.737	-27.529	2.365.504	-34.406	2.315.696	-36.443
2.342.948	-27.530	2.365.707	-34.406	2.316.084	-36.446
2.343.123	-27.532	2.365.930	-34.407	2.316.404	-36.450
2.343.245	-27.533	2.366.118	-34.407	2.316.698	-36.454
2.343.360	-27.534	2.366.250	-34.407	2.316.853	-36.457
2.343.454	-27.535	2.366.407	-34.407	2.316.900	-36.461
2.343.593	-27.537	2.366.568	-34.407	2.317.009	-36.465
2.343.761	-27.538	2.366.700	-34.407	2.317.173	-36.468
2.344.025	-27.539	2.366.812	-34.407	2.317.231	-36.472
2.344.200	-27.540	2.366.956	-34.407	2.317.249	-36.476
2.344.350	-27.541	2.367.157	-34.407	2.317.381	-36.479
2.344.503	-27.543	2.367.296	-34.407	2.317.489	-36.483
2.344.695	-27.544	2.367.448	-34.407	2.317.589	-36.487
2.344.881	-27.545	2.367.632	-34.407	2.317.715	-36.490
2.345.017	-27.546	2.367.775	-34.407	2.317.897	-36.494
2.345.156	-27.547	2.367.944	-34.407	2.318.074	-36.498
2.345.371	-27.548	2.368.170	-34.407	2.318.291	-36.502
2.345.535	-27.549	2.368.366	-34.407	2.318.483	-36.505
2.345.621	-27.550	2.368.517	-34.407	2.318.631	-36.509
2.345.770	-27.551	2.368.665	-34.407	2.318.831	-36.513
2.345.938	-27.552	2.368.932	-34.407	2.318.982	-36.517
2.346.066	-27.553	2.369.112	-34.407	2.319.121	-36.520
2.346.182	-27.554	2.369.196	-34.407	2.319.303	-36.524

2.346.326	-27.555	2.369.326	-34.407	2.319.513	-36.528
2.346.509	-27.555	2.369.471	-34.407	2.319.670	-36.532
2.346.686	-27.556	2.369.664	-34.407	2.319.820	-36.535
2.346.937	-27.557	2.369.858	-34.407	2.319.982	-36.539
2.347.188	-27.558	2.369.917	-34.408	2.320.094	-36.543
2.347.385	-27.559	2.370.038	-34.408	2.320.230	-36.547
2.347.576	-27.560	2.370.267	-34.408	2.320.448	-36.551
2.347.755	-27.560	2.370.488	-34.408	2.320.645	-36.555
2.347.905	-27.561	2.370.680	-34.408	2.320.745	-36.559
2.348.083	-27.562	2.370.822	-34.408	2.320.863	-36.562
2.348.228	-27.562	2.370.921	-34.408	2.321.046	-36.566
2.348.414	-27.563	2.371.086	-34.408	2.321.227	-36.570
2.348.569	-27.564	2.371.320	-34.408	2.321.362	-36.574
2.348.609	-27.564	2.371.519	-34.408	2.321.516	-36.578
2.348.692	-27.565	2.371.715	-34.408	2.321.655	-36.582
2.348.884	-27.566	2.371.827	-34.408	2.321.796	-36.586
2.349.100	-27.566	2.371.935	-34.408	2.322.054	-36.590
2.349.296	-27.567	2.372.146	-34.408	2.322.290	-36.594
2.349.442	-27.567	2.372.383	-34.409	2.322.518	-36.597
2.349.637	-27.568	2.372.572	-34.409	2.322.719	-36.601
2.349.829	-27.568	2.372.753	-34.409	2.322.914	-36.605
2.349.986	-27.569	2.372.936	-34.409	2.323.084	-36.609
2.350.188	-27.569	2.373.060	-34.409	2.323.225	-36.613
2.350.397	-27.570	2.373.233	-34.409	2.323.371	-36.617
2.350.624	-27.570	2.373.454	-34.409	2.323.496	-36.621
2.350.802	-27.571	2.373.582	-34.409	2.323.631	-36.625
2.350.945	-27.571	2.373.727	-34.410	2.323.797	-36.629
2.351.089	-27.571	2.373.866	-34.410	2.323.956	-36.633
2.351.198	-27.572	2.373.931	-34.410	2.324.129	-36.637
2.351.353	-27.572	2.374.063	-34.410	2.324.227	-36.641

2.351.550	-27.573	2.374.274	-34.410	2.324.368	-36.645
2.351.738	-27.573	2.374.521	-34.410	2.324.583	-36.649
2.351.935	-27.573	2.374.771	-34.411	2.324.810	-36.652
2.352.177	-27.573	2.374.895	-34.411	2.324.957	-36.656
2.352.336	-27.574	2.375.020	-34.411	2.325.071	-36.660
2.352.435	-27.574	2.375.176	-34.411	2.325.244	-36.664
2.352.533	-27.574	2.375.311	-34.411	2.325.365	-36.668
2.352.666	-27.575	2.375.508	-34.412	2.325.463	-36.672
2.352.878	-27.575	2.375.672	-34.412	2.325.618	-36.676
2.353.084	-27.575	2.375.777	-34.412	2.325.801	-36.680
2.353.216	-27.575	2.375.918	-34.412	2.325.974	-36.684
2.353.353	-27.575	2.376.023	-34.412	2.326.134	-36.688
2.353.586	-27.575	2.376.125	-34.413	2.326.299	-36.692
2.353.741	-27.576	2.376.252	-34.413	2.326.547	-36.695
2.353.870	-27.576	2.376.440	-34.413	2.326.770	-36.699
2.354.035	-27.576	2.376.628	-34.413	2.326.890	-36.703
2.354.182	-27.576	2.376.834	-34.413	2.327.059	-36.707
2.354.332	-27.576	2.377.027	-34.414	2.327.312	-36.711
2.354.536	-27.576	2.377.219	-34.414	2.327.482	-36.715
2.354.771	-27.576	2.377.457	-34.414	2.327.535	-36.718
2.354.914	-27.576	2.377.638	-34.414	2.327.674	-36.722
2.355.085	-27.576	2.377.769	-34.414	2.327.892	-36.726
2.355.242	-27.576	2.377.945	-34.415	2.328.064	-36.730
2.355.409	-27.576	2.378.183	-34.415	2.328.188	-36.734
2.355.621	-27.576	2.378.416	-34.415	2.328.313	-36.737
2.355.726	-27.576	2.378.549	-34.415	2.328.528	-36.741
2.355.925	-27.576	2.378.687	-34.415	2.328.736	-36.745
2.356.320	-27.576	2.378.797	-34.415	2.328.909	-36.748
2.356.700	-27.576	2.378.942	-34.416	2.329.063	-36.752
2.356.904	-27.576	2.379.137	-34.416	2.329.241	-36.756

2.356.991	-27.576	2.379.267	-34.416	2.329.419	-36.759
2.357.079	-27.576	2.379.355	-34.416	2.329.531	-36.763
2.357.094	-27.576	2.379.491	-34.416	2.329.707	-36.766
2.357.101	-27.576	2.379.678	-34.416	2.329.928	-36.770
2.357.234	-27.576	2.379.911	-34.416	2.330.145	-36.773
2.357.363	-27.576	2.380.159	-34.417	2.330.362	-36.777
2.357.509	-27.576	2.380.385	-34.417	2.330.517	-36.780
2.357.738	-27.575	2.380.576	-34.417	2.330.624	-36.784
2.357.869	-27.575	2.380.726	-34.417	2.330.735	-36.787
2.357.995	-27.575	2.380.898	-34.417	2.330.900	-36.790
2.358.216	-27.575	2.381.095	-34.417	2.331.064	-36.794
2.358.394	-27.575	2.381.308	-34.417	2.331.140	-36.797
2.358.595	-27.575	2.381.387	-34.417	2.331.243	-36.800
2.358.826	-27.575	2.381.444	-34.417	2.331.416	-36.804
2.358.947	-27.574	2.381.635	-34.417	2.331.635	-36.807
2.359.086	-27.574	2.381.808	-34.417	2.331.772	-36.810
2.359.271	-27.574	2.381.971	-34.417	2.331.915	-36.813
2.359.413	-27.574	2.382.159	-34.417	2.332.096	-36.816
2.359.536	-27.573	2.382.307	-34.418	2.332.280	-36.819
2.359.753	-27.573	2.382.428	-34.418	2.332.457	-36.823
2.359.968	-27.573	2.382.630	-34.418	2.332.645	-36.826
2.360.090	-27.573	2.382.852	-34.418	2.332.914	-36.829
2.360.179	-27.573	2.383.100	-34.418	2.333.212	-36.832
2.360.276	-27.572	2.383.273	-34.418	2.333.342	-36.834
2.360.445	-27.572	2.383.439	-34.418	2.333.394	-36.837
2.360.694	-27.572	2.383.647	-34.417	2.333.522	-36.840
2.360.891	-27.571	2.383.765	-34.417	2.333.721	-36.843
2.361.060	-27.571	2.383.860	-34.417	2.333.929	-36.846
2.361.247	-27.571	2.384.001	-34.417	2.334.162	-36.849
2.361.405	-27.571	2.384.200	-34.417	2.334.268	-36.851

2.361.550	-27.570	2.384.404	-34.417	2.334.290	-36.854
2.361.780	-27.570	2.384.569	-34.417	2.334.402	-36.857
2.361.965	-27.570	2.384.725	-34.417	2.334.595	-36.859
2.362.076	-27.569	2.384.937	-34.417	2.334.787	-36.862
2.362.213	-27.569	2.385.164	-34.417	2.334.966	-36.865
2.362.376	-27.569	2.385.271	-34.417	2.335.094	-36.867
2.362.555	-27.568	2.385.332	-34.417	2.335.211	-36.870
2.362.720	-27.568	2.385.495	-34.417	2.335.363	-36.872
2.362.836	-27.568	2.385.678	-34.416	2.335.549	-36.874
2.363.000	-27.567	2.385.960	-34.416	2.335.757	-36.877
2.363.186	-27.567	2.386.411	-34.416	2.335.954	-36.879
2.363.348	-27.567	2.386.756	-34.416	2.336.169	-36.881
2.363.465	-27.566	2.386.877	-34.416	2.336.375	-36.884
2.363.624	-27.566	2.386.842	-34.416	2.336.541	-36.886
2.363.813	-27.566	2.386.873	-34.415	2.336.680	-36.888
2.364.023	-27.565	2.387.020	-34.415	2.336.827	-36.890
2.364.215	-27.565	2.387.148	-34.415	2.337.020	-36.892
2.364.333	-27.565	2.387.238	-34.415	2.337.135	-36.895
2.364.456	-27.564	2.387.338	-34.415	2.337.311	-36.897
2.364.594	-27.564	2.387.534	-34.415	2.337.421	-36.899
2.364.774	-27.563	2.387.739	-34.414	2.337.590	-36.901
2.364.966	-27.563	2.387.914	-34.414	2.337.878	-36.903
2.365.128	-27.563	2.388.065	-34.414	2.338.060	-36.905
2.365.285	-27.562	2.388.279	-34.414	2.338.232	-36.907
2.365.453	-27.562	2.388.521	-34.413	2.338.411	-36.908
2.365.680	-27.562	2.388.620	-34.413	2.338.582	-36.910
2.365.836	-27.561	2.388.698	-34.413	2.338.788	-36.912
2.366.055	-27.561	2.388.835	-34.413	2.338.987	-36.914
2.366.227	-27.560	2.389.018	-34.413	2.339.162	-36.916
2.366.378	-27.560	2.389.236	-34.412	2.339.280	-36.917

2.366.561	-27.560	2.389.420	-34.412	2.339.402	-36.919
2.366.709	-27.559	2.389.577	-34.412	2.339.566	-36.921
2.366.821	-27.559	2.389.713	-34.412	2.339.706	-36.922
2.366.967	-27.558	2.389.828	-34.411	2.339.892	-36.924
2.367.197	-27.558	2.389.977	-34.411	2.340.119	-36.926
2.367.393	-27.558	2.390.137	-34.411	2.340.240	-36.927
2.367.538	-27.557	2.390.307	-34.411	2.340.332	-36.929
2.367.672	-27.557	2.390.441	-34.411	2.340.506	-36.930
2.367.829	-27.556	2.390.591	-34.410	2.340.683	-36.932
2.368.051	-27.556	2.390.810	-34.410	2.340.921	-36.933
2.368.271	-27.556	2.390.966	-34.410	2.341.113	-36.935
2.368.348	-27.555	2.391.142	-34.410	2.341.255	-36.936
2.368.404	-27.555	2.391.378	-34.410	2.341.471	-36.937
2.368.488	-27.554	2.391.608	-34.410	2.341.630	-36.939
2.368.642	-27.554	2.391.724	-34.409	2.341.810	-36.940
2.368.814	-27.554	2.391.844	-34.409	2.342.051	-36.942
2.369.008	-27.553	2.391.991	-34.409	2.342.195	-36.943
2.369.198	-27.553	2.392.101	-34.409	2.342.312	-36.944
2.369.371	-27.552	2.392.296	-34.409	2.342.451	-36.945
2.369.565	-27.552	2.392.509	-34.409	2.342.555	-36.947
2.369.776	-27.552	2.392.590	-34.409	2.342.737	-36.948
2.369.993	-27.551	2.392.599	-34.409	2.342.986	-36.949
2.370.193	-27.551	2.392.766	-34.409	2.343.073	-36.950
2.370.423	-27.551	2.393.067	-34.409	2.343.263	-36.951
2.370.609	-27.550	2.393.289	-34.409	2.343.720	-36.953
2.370.723	-27.550	2.393.461	-34.408	2.344.053	-36.954
2.370.873	-27.549	2.393.571	-34.408	2.344.177	-36.955
2.371.004	-27.549	2.393.687	-34.409	2.344.305	-36.956
2.371.203	-27.549	2.393.895	-34.409	2.344.427	-36.957
2.371.413	-27.548	2.394.079	-34.409	2.344.473	-36.958

2.371.579	-27.548	2.394.234	-34.409	2.344.464	-36.959
2.371.693	-27.548	2.394.392	-34.409	2.344.509	-36.960
2.371.774	-27.547	2.394.527	-34.409	2.344.663	-36.961
2.371.879	-27.547	2.394.713	-34.409	2.344.832	-36.962
2.371.984	-27.547	2.394.964	-34.409	2.344.999	-36.963
2.372.197	-27.546	2.395.141	-34.409	2.345.167	-36.964
2.372.318	-27.546	2.395.365	-34.409	2.345.345	-36.965
2.372.486	-27.546	2.395.517	-34.410	2.345.441	-36.966
2.372.730	-27.545	2.395.627	-34.410	2.345.656	-36.967
2.372.923	-27.545	2.395.777	-34.410	2.345.878	-36.968
2.373.118	-27.545	2.395.920	-34.410	2.346.003	-36.969
2.373.357	-27.544	2.396.125	-34.411	2.346.151	-36.970
2.373.566	-27.544	2.396.360	-34.411	2.346.402	-36.971
2.373.705	-27.544	2.396.465	-34.411	2.346.620	-36.972
2.373.935	-27.543	2.396.613	-34.412	2.346.676	-36.973
2.374.153	-27.543	2.396.805	-34.412	2.346.758	-36.974
2.374.288	-27.543	2.396.965	-34.412	2.346.927	-36.975
2.374.422	-27.543	2.397.150	-34.413	2.347.177	-36.976
2.374.548	-27.542	2.397.321	-34.413	2.347.394	-36.977
2.374.666	-27.542	2.397.453	-34.413	2.347.543	-36.978
2.374.861	-27.542	2.397.538	-34.414	2.347.686	-36.979
2.375.047	-27.541	2.397.701	-34.414	2.347.838	-36.980
2.375.210	-27.541	2.397.897	-34.415	2.348.033	-36.981
2.375.396	-27.541	2.398.053	-34.415	2.348.180	-36.982
2.375.589	-27.541	2.398.230	-34.416	2.348.279	-36.983
2.375.739	-27.540	2.398.338	-34.416	2.348.431	-36.984
2.375.919	-27.540	2.398.427	-34.417	2.348.646	-36.985
2.376.096	-27.540	2.398.604	-34.417	2.348.810	-36.986
2.376.180	-27.540	2.398.761	-34.418	2.348.985	-36.987
2.376.294	-27.540	2.398.911	-34.418	2.349.160	-36.987

2.376.456	-27.539	2.399.043	-34.419	2.349.296	-36.988
2.376.646	-27.539	2.399.223	-34.420	2.349.507	-36.989
2.376.866	-27.539	2.399.444	-34.420	2.349.678	-36.990
2.377.030	-27.539	2.399.668	-34.421	2.349.820	-36.991
2.377.141	-27.538	2.399.893	-34.422	2.350.014	-36.992
2.377.328	-27.538	2.400.145	-34.422	2.350.152	-36.993
2.377.576	-27.538	2.400.311	-34.423	2.350.363	-36.994
2.377.759	-27.538	2.400.414	-34.424	2.350.569	-36.995
2.377.921	-27.538	2.400.571	-34.424	2.350.686	-36.996
2.378.100	-27.538	2.400.726	-34.425	2.350.818	-36.998
2.378.233	-27.537	2.400.875	-34.426	2.350.969	-36.999
2.378.393	-27.537	2.401.048	-34.426	2.351.118	-37.000
2.378.550	-27.537	2.401.196	-34.427	2.351.259	-37.001
2.378.676	-27.537	2.401.366	-34.428	2.351.451	-37.002
2.379.012	-27.537	2.401.532	-34.429	2.351.583	-37.003
2.379.408	-27.537	2.401.633	-34.429	2.351.714	-37.004
2.379.702	-27.536	2.401.765	-34.430	2.351.951	-37.005
2.379.849	-27.536	2.401.964	-34.431	2.352.130	-37.006
2.379.868	-27.536	2.402.171	-34.432	2.352.310	-37.007
2.379.896	-27.536	2.402.357	-34.433	2.352.488	-37.008
2.380.004	-27.536	2.402.564	-34.433	2.352.632	-37.010
2.380.076	-27.536	2.402.784	-34.434	2.352.834	-37.011
2.380.213	-27.536	2.403.015	-34.435	2.352.943	-37.012
2.380.316	-27.535	2.403.192	-34.436	2.353.066	-37.013
2.380.452	-27.535	2.403.351	-34.437	2.353.246	-37.014
2.380.611	-27.535	2.403.515	-34.437	2.353.391	-37.015
2.380.790	-27.535	2.403.663	-34.438	2.353.579	-37.017
2.381.003	-27.535	2.403.804	-34.439	2.353.768	-37.018
2.381.151	-27.535	2.403.969	-34.440	2.353.965	-37.019
2.381.332	-27.535	2.404.164	-34.441	2.354.091	-37.020

2.381.526	-27.535	2.404.325	-34.441	2.354.294	-37.021
2.381.745	-27.535	2.404.426	-34.442	2.354.578	-37.023
2.381.908	-27.534	2.404.599	-34.443	2.354.781	-37.024
2.382.016	-27.534	2.404.854	-34.444	2.354.923	-37.025
2.382.166	-27.534	2.405.049	-34.445	2.355.067	-37.026
2.382.276	-27.534	2.405.173	-34.446	2.355.173	-37.028
2.382.466	-27.534	2.405.325	-34.446	2.355.304	-37.029
2.382.665	-27.534	2.405.483	-34.447	2.355.527	-37.030
2.382.827	-27.534	2.405.605	-34.448	2.355.748	-37.031
2.383.004	-27.534	2.405.816	-34.449	2.355.874	-37.033
2.383.095	-27.534	2.406.087	-34.450	2.356.001	-37.034
2.383.237	-27.534	2.406.258	-34.451	2.356.160	-37.035
2.383.387	-27.534	2.406.378	-34.451	2.356.348	-37.036
2.383.515	-27.534	2.406.472	-34.452	2.356.501	-37.038
2.383.763	-27.534	2.406.570	-34.453	2.356.630	-37.039
2.384.014	-27.533	2.406.771	-34.454	2.356.837	-37.040
2.384.177	-27.533	2.406.951	-34.455	2.357.056	-37.041
2.384.352	-27.533	2.407.042	-34.455	2.357.219	-37.043
2.384.529	-27.533	2.407.267	-34.456	2.357.349	-37.044
2.384.744	-27.533	2.407.515	-34.457	2.357.466	-37.045
2.384.899	-27.533	2.407.600	-34.458	2.357.605	-37.047
2.385.004	-27.533	2.407.738	-34.459	2.357.773	-37.048
2.385.126	-27.533	2.407.906	-34.459	2.357.955	-37.049
2.385.264	-27.533	2.408.130	-34.460	2.358.141	-37.050
2.385.468	-27.533	2.408.568	-34.461	2.358.302	-37.052
2.385.647	-27.533	2.408.969	-34.462	2.358.445	-37.053
2.385.755	-27.533	2.409.146	-34.462	2.358.539	-37.054
2.385.901	-27.533	2.409.236	-34.463	2.358.673	-37.055
2.386.096	-27.533	2.409.314	-34.464	2.358.828	-37.057
2.386.241	-27.533	2.409.370	-34.465	2.358.996	-37.058

2.386.422	-27.533	2.409.379	-34.465	2.359.214	-37.059
2.386.606	-27.533	2.409.507	-34.466	2.359.386	-37.060
2.386.725	-27.533	2.409.613	-34.467	2.359.512	-37.061
2.386.864	-27.533	2.409.689	-34.467	2.359.646	-37.063
2.387.081	-27.533	2.409.878	-34.468	2.359.848	-37.064
2.387.291	-27.533	2.410.103	-34.469	2.360.098	-37.065
2.387.458	-27.533	2.410.253	-34.469	2.360.322	-37.066
2.387.607	-27.533	2.410.444	-34.470	2.360.551	-37.068
2.387.720	-27.532	2.410.654	-34.471	2.360.725	-37.069
2.387.916	-27.532	2.410.786	-34.471	2.360.918	-37.070
2.388.142	-27.532	2.410.943	-34.472	2.361.095	-37.071
2.388.270	-27.532	2.411.158	-34.473	2.361.295	-37.072
2.388.384	-27.532	2.411.339	-34.473	2.361.373	-37.073
2.388.613	-27.532	2.411.529	-34.474	2.361.456	-37.074
2.388.795	-27.532	2.411.727	-34.474	2.361.689	-37.076
2.388.965	-27.532	2.411.895	-34.475	2.361.873	-37.077
2.389.165	-27.532	2.412.048	-34.475	2.361.983	-37.078
2.389.263	-27.532	2.412.151	-34.476	2.362.143	-37.079
2.389.384	-27.532	2.412.263	-34.477	2.362.320	-37.080
2.389.619	-27.532	2.412.439	-34.477	2.362.443	-37.081
2.389.807	-27.532	2.412.610	-34.478	2.362.596	-37.082
2.389.966	-27.532	2.412.747	-34.478	2.362.766	-37.083
2.390.134	-27.532	2.412.925	-34.479	2.362.959	-37.084
2.390.300	-27.532	2.413.149	-34.479	2.363.111	-37.086
2.390.462	-27.532	2.413.389	-34.480	2.363.239	-37.087
2.390.600	-27.532	2.413.561	-34.480	2.363.414	-37.088
2.390.838	-27.532	2.413.707	-34.481	2.363.654	-37.089
2.391.048	-27.532	2.413.860	-34.481	2.363.855	-37.090
2.391.105	-27.532	2.414.039	-34.482	2.364.056	-37.091
2.391.209	-27.532	2.414.218	-34.482	2.364.207	-37.092

2.391.402	-27.532	2.414.417	-34.483	2.364.381	-37.093
2.391.529	-27.532	2.414.552	-34.483	2.364.594	-37.094
2.391.624	-27.532	2.414.592	-34.484	2.364.767	-37.095
2.391.767	-27.532	2.414.785	-34.484	2.364.932	-37.096
2.391.918	-27.532	2.414.924	-34.485	2.365.107	-37.097
2.392.087	-27.532	2.415.020	-34.485	2.365.237	-37.098
2.392.312	-27.532	2.415.215	-34.486	2.365.378	-37.099
2.392.484	-27.532	2.415.423	-34.486	2.365.535	-37.099
2.392.643	-27.532	2.415.639	-34.486	2.365.690	-37.100
2.392.934	-27.532	2.415.835	-34.487	2.365.882	-37.101
2.393.177	-27.532	2.415.985	-34.487	2.366.099	-37.102
2.393.315	-27.532	2.416.105	-34.488	2.366.252	-37.103
2.393.484	-27.531	2.416.264	-34.488	2.366.378	-37.104
2.393.653	-27.531	2.416.417	-34.488	2.366.548	-37.105
2.393.848	-27.531	2.416.548	-34.489	2.366.705	-37.106
2.394.007	-27.531	2.416.745	-34.489	2.366.814	-37.107
2.394.110	-27.531	2.416.937	-34.490	2.367.009	-37.108
2.394.263	-27.531	2.417.139	-34.490	2.367.228	-37.108
2.394.386	-27.531	2.417.334	-34.490	2.367.357	-37.109
2.394.465	-27.531	2.417.354	-34.491	2.367.544	-37.110
2.394.623	-27.531	2.417.556	-34.491	2.367.726	-37.111
2.394.794	-27.531	2.417.858	-34.491	2.367.889	-37.112
2.394.979	-27.531	2.418.000	-34.492	2.368.044	-37.113
2.395.161	-27.531	2.418.167	-34.492	2.368.195	-37.114
2.395.294	-27.531	2.418.362	-34.492	2.368.416	-37.115
2.395.472	-27.531	2.418.539	-34.493	2.368.568	-37.115
2.395.739	-27.531	2.418.660	-34.493	2.368.687	-37.116
2.395.952	-27.531	2.418.819	-34.493	2.368.875	-37.117
2.396.105	-27.531	2.418.944	-34.493	2.369.053	-37.118
2.396.265	-27.531	2.419.110	-34.494	2.369.184	-37.119

2.396.498	-27.531	2.419.312	-34.494	2.369.326	-37.120
2.396.705	-27.531	2.419.498	-34.494	2.369.560	-37.121
2.396.814	-27.531	2.419.661	-34.494	2.369.776	-37.121
2.396.918	-27.531	2.419.867	-34.495	2.369.861	-37.122
2.397.092	-27.531	2.420.107	-34.495	2.370.000	-37.123
2.397.325	-27.531	2.420.249	-34.495	2.370.197	-37.124
2.397.527	-27.531	2.420.311	-34.495	2.370.387	-37.125
2.397.683	-27.531	2.420.405	-34.495	2.370.564	-37.126
2.397.853	-27.531	2.420.569	-34.496	2.370.775	-37.126
2.398.026	-27.531	2.420.798	-34.496	2.371.142	-37.127
2.398.167	-27.531	2.420.938	-34.496	2.371.518	-37.128
2.398.372	-27.531	2.421.025	-34.496	2.371.714	-37.129
2.398.604	-27.531	2.421.163	-34.496	2.371.884	-37.130
2.398.758	-27.531	2.421.323	-34.496	2.372.000	-37.131
2.398.877	-27.531	2.421.494	-34.496	2.372.051	-37.131
2.399.026	-27.531	2.421.684	-34.496	2.372.121	-37.132
2.399.177	-27.531	2.421.908	-34.496	2.372.126	-37.133
2.399.314	-27.531	2.422.193	-34.496	2.372.168	-37.134
2.399.518	-27.531	2.422.426	-34.496	2.372.325	-37.135
2.399.686	-27.531	2.422.552	-34.496	2.372.493	-37.136
2.399.802	-27.531	2.422.654	-34.496	2.372.650	-37.137
2.399.977	-27.531	2.422.862	-34.496	2.372.793	-37.137
2.400.203	-27.531	2.423.120	-34.496	2.372.948	-37.138
2.400.385	-27.531	2.423.235	-34.496	2.373.120	-37.139
2.400.515	-27.531	2.423.317	-34.496	2.373.324	-37.140
2.400.677	-27.531	2.423.447	-34.496	2.373.539	-37.141
2.400.884	-27.531	2.423.580	-34.496	2.373.703	-37.142
2.400.965	-27.531	2.423.676	-34.496	2.373.879	-37.143
2.401.070	-27.531	2.423.824	-34.496	2.374.043	-37.144
2.401.288	-27.531	2.424.068	-34.496	2.374.189	-37.145

2.401.407	-27.531	2.424.230	-34.495	2.374.323	-37.146
2.401.657	-27.531	2.424.359	-34.495	2.374.500	-37.147
2.402.094	-27.531	2.424.558	-34.495	2.374.686	-37.148
2.402.417	-27.531	2.424.799	-34.495	2.374.838	-37.149
2.402.509	-27.531	2.425.040	-34.494	2.374.998	-37.150
2.402.612	-27.531	2.425.253	-34.494	2.375.184	-37.151
2.402.749	-27.531	2.425.391	-34.494	2.375.350	-37.152
2.402.794	-27.531	2.425.456	-34.494	2.375.509	-37.153
2.402.850	-27.531	2.425.629	-34.493	2.375.659	-37.154
2.402.928	-27.531	2.425.875	-34.493	2.375.815	-37.155
2.403.015	-27.531	2.426.031	-34.493	2.375.947	-37.156
2.403.236	-27.531	2.426.125	-34.492	2.376.040	-37.157
2.403.456	-27.531	2.426.308	-34.492	2.376.219	-37.158
2.403.588	-27.531	2.426.508	-34.491	2.376.476	-37.159
2.403.755	-27.530	2.426.662	-34.491	2.376.633	-37.160
2.403.951	-27.530	2.426.870	-34.491	2.376.796	-37.162
2.404.146	-27.530	2.427.063	-34.490	2.377.000	-37.163
2.404.328	-27.530	2.427.234	-34.490	2.377.202	-37.164
2.404.516	-27.530	2.427.446	-34.489	2.377.345	-37.165
2.404.682	-27.530	2.427.591	-34.489	2.377.540	-37.166
2.404.867	-27.530	2.427.686	-34.488	2.377.757	-37.167
2.405.017	-27.530	2.427.860	-34.488	2.377.930	-37.169
2.405.170	-27.530	2.428.027	-34.487	2.378.102	-37.170
2.405.349	-27.530	2.428.180	-34.487	2.378.270	-37.171
2.405.481	-27.530	2.428.396	-34.486	2.378.422	-37.172
2.405.627	-27.530	2.428.577	-34.485	2.378.579	-37.174
2.405.736	-27.530	2.428.685	-34.485	2.378.761	-37.175
2.405.873	-27.530	2.428.837	-34.484	2.378.893	-37.176
2.406.046	-27.530	2.428.991	-34.483	2.378.985	-37.177
2.406.182	-27.530	2.429.159	-34.483	2.379.104	-37.179

2.406.377	-27.530	2.429.373	-34.482	2.379.310	-37.180
2.406.633	-27.530	2.429.594	-34.481	2.379.558	-37.181
2.406.852	-27.530	2.429.773	-34.481	2.379.693	-37.183
2.407.022	-27.530	2.429.879	-34.480	2.379.832	-37.184
2.407.146	-27.530	2.429.967	-34.479	2.379.951	-37.185
2.407.319	-27.529	2.430.108	-34.478	2.380.134	-37.187
2.407.555	-27.529	2.430.266	-34.478	2.380.307	-37.188
2.407.715	-27.529	2.430.544	-34.477	2.380.432	-37.189
2.407.798	-27.529	2.431.012	-34.476	2.380.629	-37.191
2.407.937	-27.529	2.431.371	-34.475	2.380.777	-37.192
2.408.148	-27.529	2.431.471	-34.474	2.380.900	-37.193
2.408.291	-27.529	2.431.527	-34.473	2.381.080	-37.195
2.408.392	-27.529	2.431.606	-34.473	2.381.261	-37.196
2.408.613	-27.529	2.431.675	-34.472	2.381.441	-37.198
2.408.820	-27.529	2.431.729	-34.471	2.381.638	-37.199
2.408.947	-27.528	2.431.801	-34.470	2.381.861	-37.200
2.409.128	-27.528	2.431.962	-34.469	2.382.048	-37.202
2.409.227	-27.528	2.432.119	-34.468	2.382.169	-37.203
2.409.354	-27.528	2.432.322	-34.467	2.382.332	-37.205
2.409.628	-27.528	2.432.480	-34.466	2.382.542	-37.206
2.409.843	-27.528	2.432.601	-34.465	2.382.672	-37.207
2.409.948	-27.528	2.432.845	-34.465	2.382.813	-37.209
2.410.047	-27.528	2.433.011	-34.464	2.383.008	-37.210
2.410.228	-27.527	2.433.178	-34.463	2.383.133	-37.212
2.410.432	-27.527	2.433.353	-34.462	2.383.203	-37.213
2.410.611	-27.527	2.433.492	-34.461	2.383.400	-37.214
2.410.797	-27.527	2.433.718	-34.460	2.383.631	-37.216
2.410.943	-27.527	2.433.866	-34.459	2.383.743	-37.217
2.411.115	-27.527	2.434.025	-34.458	2.383.951	-37.218
2.411.321	-27.526	2.434.209	-34.457	2.384.182	-37.220

2.411.460	-27.526	2.434.389	-34.456	2.384.321	-37.221
2.411.573	-27.526	2.434.594	-34.455	2.384.437	-37.222
2.411.776	-27.526	2.434.731	-34.454	2.384.586	-37.224
2.412.013	-27.526	2.434.819	-34.453	2.384.734	-37.225
2.412.101	-27.526	2.434.941	-34.452	2.384.939	-37.226
2.412.240	-27.525	2.435.123	-34.451	2.385.096	-37.227
2.412.466	-27.525	2.435.305	-34.450	2.385.223	-37.229
2.412.643	-27.525	2.435.544	-34.449	2.385.419	-37.230
2.412.831	-27.525	2.435.777	-34.448	2.385.602	-37.231
2.412.984	-27.524	2.435.959	-34.448	2.385.820	-37.232
2.413.131	-27.524	2.436.098	-34.447	2.385.954	-37.233
2.413.333	-27.524	2.436.205	-34.446	2.386.030	-37.234
2.413.512	-27.524	2.436.366	-34.445	2.386.154	-37.235
2.413.661	-27.524	2.436.548	-34.444	2.386.313	-37.236
2.413.871	-27.523	2.436.700	-34.443	2.386.409	-37.238
2.414.052	-27.523	2.436.834	-34.442	2.386.564	-37.239
2.414.135	-27.523	2.436.982	-34.441	2.386.786	-37.240
2.414.242	-27.523	2.437.128	-34.441	2.386.973	-37.241
2.414.377	-27.522	2.437.355	-34.440	2.387.186	-37.241
2.414.514	-27.522	2.437.533	-34.439	2.387.346	-37.242
2.414.617	-27.522	2.437.623	-34.438	2.387.558	-37.243
2.414.762	-27.522	2.437.764	-34.437	2.387.791	-37.244
2.415.005	-27.521	2.437.968	-34.437	2.387.867	-37.245
2.415.239	-27.521	2.438.156	-34.436	2.388.053	-37.246
2.415.434	-27.521	2.438.313	-34.435	2.388.335	-37.247
2.415.602	-27.521	2.438.465	-34.434	2.388.573	-37.247
2.415.798	-27.520	2.438.635	-34.434	2.388.691	-37.248
2.416.024	-27.520	2.438.785	-34.433	2.388.790	-37.249
2.416.228	-27.520	2.438.942	-34.432	2.388.943	-37.250
2.416.411	-27.519	2.439.139	-34.432	2.389.043	-37.250

2.416.518	-27.519	2.439.303	-34.431	2.389.198	-37.251
2.416.641	-27.519	2.439.457	-34.431	2.389.439	-37.252
2.416.857	-27.519	2.439.625	-34.430	2.389.590	-37.252
2.416.991	-27.518	2.439.834	-34.429	2.389.749	-37.253
2.417.103	-27.518	2.440.018	-34.429	2.389.902	-37.253
2.417.219	-27.518	2.440.134	-34.428	2.390.049	-37.254
2.417.345	-27.517	2.440.329	-34.428	2.390.193	-37.254
2.417.490	-27.517	2.440.518	-34.427	2.390.324	-37.254
2.417.666	-27.517	2.440.679	-34.427	2.390.499	-37.255
2.417.852	-27.517	2.440.786	-34.426	2.390.639	-37.255
2.417.988	-27.516	2.440.921	-34.426	2.390.775	-37.255
2.418.181	-27.516	2.441.073	-34.426	2.390.966	-37.256
2.418.392	-27.516	2.441.263	-34.425	2.391.214	-37.256
2.418.606	-27.515	2.441.490	-34.425	2.391.431	-37.256
2.418.817	-27.515	2.441.665	-34.425	2.391.631	-37.256
2.419.034	-27.515	2.441.848	-34.424	2.391.844	-37.256
2.419.215	-27.514	2.442.074	-34.424	2.391.951	-37.257
2.419.348	-27.514	2.442.141	-34.424	2.392.018	-37.257
2.419.529	-27.514	2.442.233	-34.423	2.392.177	-37.257
2.419.684	-27.513	2.442.410	-34.423	2.392.435	-37.257
2.419.819	-27.513	2.442.583	-34.423	2.392.652	-37.257
2.419.926	-27.513	2.442.708	-34.423	2.392.832	-37.257
2.420.064	-27.512	2.442.832	-34.422	2.393.012	-37.257
2.420.316	-27.512	2.442.991	-34.422	2.393.074	-37.256
2.420.555	-27.512	2.443.138	-34.422	2.393.250	-37.256
2.420.724	-27.511	2.443.290	-34.422	2.393.365	-37.256
2.420.855	-27.511	2.443.497	-34.422	2.393.607	-37.256
2.421.021	-27.511	2.443.651	-34.422	2.393.746	-37.256
2.421.207	-27.510	2.443.776	-34.422	2.393.935	-37.256
2.421.423	-27.510	2.443.967	-34.422	2.394.122	-37.255

2.421.577	-27.510	2.444.227	-34.421	2.394.221	-37.255
2.421.702	-27.510	2.444.525	-34.421	2.394.370	-37.255
2.421.918	-27.509	2.444.749	-34.421	2.394.590	-37.254
2.422.069	-27.509	2.444.890	-34.421	2.394.782	-37.254
2.422.213	-27.509	2.445.033	-34.421	2.394.921	-37.254
2.422.433	-27.508	2.445.150	-34.421	2.395.105	-37.253
2.422.607	-27.508	2.445.360	-34.421	2.395.318	-37.253
2.422.719	-27.508	2.445.574	-34.421	2.395.470	-37.252
2.422.881	-27.507	2.445.694	-34.421	2.395.629	-37.252
2.423.057	-27.507	2.445.829	-34.421	2.395.819	-37.251
2.423.181	-27.507	2.445.952	-34.421	2.395.952	-37.251
2.423.400	-27.506	2.446.051	-34.421	2.396.099	-37.250
2.423.640	-27.506	2.446.200	-34.422	2.396.339	-37.250
2.423.747	-27.506	2.446.361	-34.422	2.396.559	-37.249
2.423.839	-27.505	2.446.564	-34.422	2.396.666	-37.248
2.423.991	-27.505	2.446.797	-34.422	2.396.742	-37.248
2.424.133	-27.505	2.446.935	-34.422	2.396.886	-37.247
2.424.330	-27.504	2.447.072	-34.422	2.397.067	-37.247
2.424.753	-27.504	2.447.269	-34.422	2.397.244	-37.246
2.425.222	-27.504	2.447.448	-34.422	2.397.381	-37.245
2.425.408	-27.503	2.447.623	-34.422	2.397.533	-37.245
2.425.470	-27.503	2.447.784	-34.422	2.397.750	-37.244
2.425.542	-27.503	2.447.948	-34.423	2.397.881	-37.243
2.425.568	-27.502	2.448.158	-34.423	2.398.075	-37.242
2.425.638	-27.502	2.448.310	-34.423	2.398.476	-37.242
2.425.744	-27.502	2.448.428	-34.423	2.398.896	-37.241
2.425.836	-27.501	2.448.593	-34.423	2.399.128	-37.240
2.425.938	-27.501	2.448.814	-34.423	2.399.141	-37.239
2.426.113	-27.501	2.448.971	-34.424	2.399.212	-37.238
2.426.329	-27.500	2.449.044	-34.424	2.399.352	-37.238

2.426.494	-27.500	2.449.236	-34.424	2.399.422	-37.237
2.426.638	-27.500	2.449.495	-34.424	2.399.496	-37.236
2.426.803	-27.499	2.449.699	-34.424	2.399.570	-37.235
2.427.054	-27.499	2.449.883	-34.424	2.399.625	-37.234
2.427.278	-27.499	2.450.043	-34.425	2.399.762	-37.234
2.427.451	-27.498	2.450.206	-34.425	2.399.937	-37.233
2.427.540	-27.498	2.450.362	-34.425	2.400.110	-37.232
2.427.695	-27.498	2.450.524	-34.425	2.400.302	-37.231
2.427.894	-27.497	2.450.717	-34.425	2.400.477	-37.230
2.428.056	-27.497	2.450.865	-34.426	2.400.625	-37.230
2.428.210	-27.496	2.451.019	-34.426	2.400.806	-37.229
2.428.382	-27.496	2.451.216	-34.426	2.400.985	-37.228
2.428.537	-27.496	2.451.360	-34.426	2.401.234	-37.227
2.428.687	-27.495	2.451.498	-34.426	2.401.420	-37.227
2.428.813	-27.495	2.451.715	-34.426	2.401.590	-37.226
2.428.920	-27.495	2.451.922	-34.427	2.401.812	-37.225
2.429.083	-27.494	2.452.099	-34.427	2.401.983	-37.224
2.429.316	-27.494	2.452.219	-34.427	2.402.133	-37.224
2.429.492	-27.494	2.452.350	-34.427	2.402.289	-37.223
2.429.707	-27.493	2.452.572	-34.427	2.402.467	-37.222
2.429.921	-27.493	2.452.706	-34.427	2.402.616	-37.222
2.430.128	-27.493	2.452.900	-34.428	2.402.706	-37.221
2.430.300	-27.492	2.453.322	-34.428	2.402.834	-37.221
2.430.443	-27.492	2.453.660	-34.428	2.403.028	-37.220
2.430.612	-27.492	2.453.822	-34.428	2.403.239	-37.219
2.430.761	-27.491	2.453.904	-34.428	2.403.430	-37.219
2.430.851	-27.491	2.453.914	-34.428	2.403.555	-37.218
2.430.996	-27.491	2.453.953	-34.428	2.403.667	-37.218
2.431.207	-27.490	2.454.044	-34.429	2.403.788	-37.217
2.431.391	-27.490	2.454.131	-34.429	2.403.990	-37.217

2.431.550	-27.490	2.454.247	-34.429	2.404.242	-37.217
2.431.684	-27.489	2.454.388	-34.429	2.404.457	-37.216
2.431.839	-27.489	2.454.522	-34.429	2.404.585	-37.216
2.432.009	-27.488	2.454.677	-34.429	2.404.654	-37.215
2.432.148	-27.488	2.454.888	-34.429	2.404.807	-37.215
2.432.275	-27.488	2.455.094	-34.429	2.405.027	-37.215
2.432.455	-27.487	2.455.204	-34.429	2.405.233	-37.214
2.432.641	-27.487	2.455.398	-34.429	2.405.415	-37.214
2.432.815	-27.487	2.455.620	-34.429	2.405.504	-37.214
2.432.943	-27.486	2.455.842	-34.430	2.405.678	-37.214
2.433.100	-27.486	2.456.081	-34.430	2.405.911	-37.214
2.433.313	-27.486	2.456.194	-34.430	2.406.084	-37.214
2.433.459	-27.485	2.456.294	-34.430	2.406.223	-37.214
2.433.662	-27.485	2.456.456	-34.430	2.406.340	-37.213
2.433.887	-27.484	2.456.591	-34.430	2.406.496	-37.213
2.433.982	-27.484	2.456.765	-34.430	2.406.666	-37.213
2.434.133	-27.484	2.456.918	-34.430	2.406.859	-37.213
2.434.370	-27.483	2.457.061	-34.430	2.407.020	-37.214
2.434.540	-27.483	2.457.258	-34.430	2.407.168	-37.214
2.434.684	-27.483	2.457.450	-34.430	2.407.404	-37.214
2.434.828	-27.482	2.457.632	-34.430	2.407.527	-37.214
2.434.993	-27.482	2.457.773	-34.430	2.407.616	-37.214
2.435.114	-27.481	2.458.020	-34.430	2.407.818	-37.214
2.435.273	-27.481	2.458.223	-34.430	2.407.948	-37.215
2.435.510	-27.481	2.458.407	-34.430	2.408.091	-37.215
2.435.727	-27.480	2.458.613	-34.430	2.408.250	-37.215
2.435.928	-27.480	2.458.761	-34.430	2.408.447	-37.216
2.436.055	-27.479	2.458.944	-34.430	2.408.667	-37.216
2.436.203	-27.479	2.459.113	-34.430	2.408.754	-37.216
2.436.386	-27.479	2.459.252	-34.430	2.408.933	-37.217

2.436.529	-27.478	2.459.370	-34.431	2.409.195	-37.217
2.436.713	-27.478	2.459.518	-34.431	2.409.323	-37.218
2.436.852	-27.478	2.459.677	-34.431	2.409.451	-37.218
2.436.971	-27.477	2.459.847	-34.431	2.409.628	-37.219
2.437.141	-27.477	2.460.020	-34.431	2.409.902	-37.219
2.437.260	-27.476	2.460.188	-34.431	2.410.105	-37.220
2.437.421	-27.476	2.460.368	-34.431	2.410.250	-37.220
2.437.576	-27.476	2.460.509	-34.431	2.410.390	-37.221
2.437.711	-27.475	2.460.674	-34.431	2.410.529	-37.222
2.437.915	-27.475	2.460.844	-34.431	2.410.616	-37.222
2.438.122	-27.475	2.460.928	-34.431	2.410.811	-37.223
2.438.313	-27.474	2.461.048	-34.431	2.411.022	-37.224
2.438.510	-27.474	2.461.214	-34.431	2.411.239	-37.224
2.438.758	-27.473	2.461.408	-34.432	2.411.379	-37.225
2.438.976	-27.473	2.461.609	-34.432	2.411.500	-37.226
2.439.173	-27.473	2.461.765	-34.432	2.411.665	-37.227
2.439.404	-27.472	2.461.991	-34.432	2.411.824	-37.227
2.439.530	-27.472	2.462.184	-34.432	2.412.020	-37.228
2.439.644	-27.471	2.462.289	-34.432	2.412.254	-37.229
2.439.803	-27.471	2.462.462	-34.432	2.412.334	-37.230
2.439.897	-27.471	2.462.695	-34.433	2.412.410	-37.231
2.440.002	-27.470	2.462.858	-34.433	2.412.567	-37.232
2.440.191	-27.470	2.463.006	-34.433	2.412.779	-37.232
2.440.323	-27.470	2.463.192	-34.433	2.412.970	-37.233
2.440.400	-27.469	2.463.349	-34.433	2.413.105	-37.234
2.440.529	-27.469	2.463.460	-34.434	2.413.223	-37.235
2.440.775	-27.468	2.463.617	-34.434	2.413.380	-37.236
2.440.945	-27.468	2.463.761	-34.434	2.413.513	-37.237
2.441.078	-27.468	2.463.906	-34.434	2.413.629	-37.238
2.441.276	-27.467	2.464.186	-34.435	2.413.801	-37.239

2.441.478	-27.467	2.464.406	-34.435	2.413.951	-37.240
2.441.700	-27.467	2.464.492	-34.435	2.414.129	-37.241
2.441.861	-27.466	2.464.608	-34.435	2.414.271	-37.242
2.442.023	-27.466	2.464.747	-34.436	2.414.453	-37.243
2.442.154	-27.466	2.464.856	-34.436	2.414.699	-37.244
2.442.262	-27.465	2.464.991	-34.436	2.414.897	-37.245
2.442.426	-27.465	2.465.188	-34.437	2.415.123	-37.246
2.442.666	-27.465	2.465.356	-34.437	2.415.329	-37.247
2.442.908	-27.464	2.465.464	-34.437	2.415.493	-37.248
2.443.104	-27.464	2.465.659	-34.438	2.415.672	-37.249
2.443.259	-27.464	2.465.839	-34.438	2.415.910	-37.249
2.443.407	-27.463	2.465.970	-34.438	2.416.102	-37.250
2.443.555	-27.463	2.466.174	-34.439	2.416.146	-37.251
2.443.671	-27.463	2.466.310	-34.439	2.416.301	-37.252
2.443.781	-27.462	2.466.496	-34.439	2.416.525	-37.253
2.444.016	-27.462	2.466.796	-34.440	2.416.635	-37.254
2.444.296	-27.462	2.467.016	-34.440	2.416.714	-37.255
2.444.462	-27.461	2.467.233	-34.440	2.416.924	-37.256
2.444.606	-27.461	2.467.415	-34.441	2.417.101	-37.257
2.444.771	-27.461	2.467.567	-34.441	2.417.253	-37.258
2.444.949	-27.460	2.467.691	-34.441	2.417.423	-37.259
2.445.109	-27.460	2.467.813	-34.442	2.417.545	-37.260
2.445.273	-27.460	2.468.002	-34.442	2.417.737	-37.261
2.445.445	-27.459	2.468.120	-34.442	2.417.952	-37.262
2.445.568	-27.459	2.468.186	-34.443	2.418.102	-37.263
2.445.742	-27.459	2.468.313	-34.443	2.418.250	-37.264
2.446.005	-27.458	2.468.465	-34.444	2.418.469	-37.265
2.446.240	-27.458	2.468.604	-34.444	2.418.680	-37.266
2.446.339	-27.458	2.468.808	-34.444	2.418.869	-37.267
2.446.445	-27.458	2.469.005	-34.445	2.419.079	-37.268

2.446.604	-27.457	2.469.197	-34.445	2.419.243	-37.269
2.446.741	-27.457	2.469.435	-34.446	2.419.332	-37.269
2.446.937	-27.457	2.469.605	-34.446	2.419.458	-37.270
2.447.112	-27.456	2.469.814	-34.446	2.419.612	-37.271
2.447.439	-27.456	2.470.005	-34.447	2.419.798	-37.272
2.447.910	-27.456	2.470.094	-34.447	2.420.045	-37.273
2.448.192	-27.456	2.470.255	-34.448	2.420.197	-37.274
2.448.254	-27.455	2.470.488	-34.448	2.420.327	-37.275
2.448.273	-27.455	2.470.672	-34.448	2.420.524	-37.276
2.448.337	-27.455	2.470.813	-34.449	2.420.701	-37.276
2.448.378	-27.454	2.470.940	-34.449	2.420.849	-37.277
2.448.441	-27.454	2.471.131	-34.450	2.421.021	-37.278
2.448.549	-27.454	2.471.302	-34.450	2.421.176	-37.279
2.448.676	-27.454	2.471.454	-34.450	2.421.320	-37.280
2.448.864	-27.453	2.471.653	-34.451	2.421.469	-37.281
2.449.100	-27.453	2.471.861	-34.451	2.421.677	-37.281
2.449.285	-27.453	2.472.004	-34.452	2.421.897	-37.282
2.449.433	-27.453	2.472.170	-34.452	2.422.070	-37.283
2.449.581	-27.452	2.472.340	-34.452	2.422.267	-37.284
2.449.702	-27.452	2.472.480	-34.453	2.422.376	-37.285
2.449.903	-27.452	2.472.722	-34.453	2.422.504	-37.285
2.450.128	-27.452	2.472.912	-34.454	2.422.676	-37.286
2.450.282	-27.451	2.472.977	-34.454	2.422.844	-37.287
2.450.406	-27.451	2.473.152	-34.454	2.423.064	-37.288
2.450.547	-27.451	2.473.380	-34.455	2.423.277	-37.289
2.450.723	-27.450	2.473.555	-34.455	2.423.402	-37.289
2.450.914	-27.450	2.473.725	-34.455	2.423.548	-37.290
2.451.039	-27.450	2.473.878	-34.456	2.423.727	-37.291
2.451.214	-27.450	2.474.061	-34.456	2.423.817	-37.292
2.451.427	-27.449	2.474.194	-34.457	2.424.021	-37.292

2.451.559	-27.449	2.474.334	-34.457	2.424.285	-37.293
2.451.619	-27.449	2.474.565	-34.457	2.424.418	-37.294
2.451.765	-27.449	2.474.785	-34.458	2.424.516	-37.295
2.452.023	-27.448	2.474.863	-34.458	2.424.587	-37.295
2.452.254	-27.448	2.474.962	-34.458	2.424.725	-37.296
2.452.478	-27.448	2.475.327	-34.459	2.425.040	-37.297
2.452.673	-27.448	2.475.827	-34.459	2.425.210	-37.298
2.452.811	-27.447	2.476.080	-34.459	2.425.362	-37.298
2.452.984	-27.447	2.476.158	-34.460	2.425.706	-37.299
2.453.147	-27.447	2.476.241	-34.460	2.426.122	-37.300
2.453.288	-27.447	2.476.264	-34.460	2.426.413	-37.301
2.453.439	-27.446	2.476.310	-34.461	2.426.517	-37.302
2.453.611	-27.446	2.476.380	-34.461	2.426.534	-37.302
2.453.811	-27.446	2.476.487	-34.461	2.426.597	-37.303
2.453.964	-27.446	2.476.601	-34.462	2.426.718	-37.304
2.454.099	-27.445	2.476.727	-34.462	2.426.834	-37.305
2.454.236	-27.445	2.476.892	-34.462	2.426.889	-37.306
2.454.363	-27.445	2.477.083	-34.463	2.426.984	-37.306
2.454.518	-27.445	2.477.249	-34.463	2.427.122	-37.307
2.454.677	-27.444	2.477.446	-34.463	2.427.294	-37.308
2.454.863	-27.444	2.477.667	-34.464	2.427.475	-37.309
2.455.081	-27.444	2.477.849	-34.464	2.427.648	-37.310
2.455.235	-27.444	2.478.062	-34.464	2.427.840	-37.311
2.455.392	-27.443	2.478.250	-34.465	2.427.936	-37.312
2.455.585	-27.443	2.478.360	-34.465	2.428.121	-37.312
2.455.698	-27.443	2.478.490	-34.465	2.428.371	-37.313
2.455.817	-27.442	2.478.622	-34.466	2.428.537	-37.314
2.456.030	-27.442	2.478.794	-34.466	2.428.687	-37.315
2.456.192	-27.442	2.478.965	-34.466	2.428.855	-37.316
2.456.326	-27.442	2.479.108	-34.467	2.429.008	-37.317

2.456.490	-27.441	2.479.296	-34.467	2.429.205	-37.318
2.456.729	-27.441	2.479.471	-34.467	2.429.355	-37.319
2.456.922	-27.441	2.479.617	-34.468	2.429.439	-37.320
2.457.085	-27.441	2.479.780	-34.468	2.429.549	-37.321
2.457.302	-27.440	2.479.969	-34.468	2.429.675	-37.322
2.457.396	-27.440	2.480.139	-34.469	2.429.843	-37.323
2.457.497	-27.440	2.480.360	-34.469	2.430.038	-37.324
2.457.712	-27.439	2.480.540	-34.470	2.430.257	-37.326
2.457.896	-27.439	2.480.622	-34.470	2.430.443	-37.327
2.458.053	-27.439	2.480.771	-34.471	2.430.593	-37.328
2.458.177	-27.438	2.481.019	-34.471	2.430.699	-37.329
2.458.387	-27.438	2.481.205	-34.472	2.430.851	-37.330
2.458.631	-27.438	2.481.305	-34.472	2.431.024	-37.331
2.458.785	-27.438	2.481.447	-34.473	2.431.189	-37.333
2.458.920	-27.437	2.481.642	-34.473	2.431.429	-37.334
2.459.050	-27.437	2.481.803	-34.474	2.431.639	-37.335
2.459.232	-27.437	2.482.001	-34.474	2.431.729	-37.337
2.459.428	-27.436	2.482.216	-34.475	2.431.888	-37.338
2.459.589	-27.436	2.482.372	-34.476	2.432.097	-37.339
2.459.697	-27.436	2.482.467	-34.476	2.432.275	-37.341
2.459.825	-27.435	2.482.605	-34.477	2.432.378	-37.342
2.459.955	-27.435	2.482.820	-34.478	2.432.525	-37.343
2.460.096	-27.435	2.483.051	-34.479	2.432.789	-37.345
2.460.266	-27.434	2.483.245	-34.479	2.432.977	-37.346
2.460.421	-27.434	2.483.369	-34.480	2.433.168	-37.348
2.460.565	-27.434	2.483.514	-34.481	2.433.347	-37.349
2.460.701	-27.433	2.483.624	-34.482	2.433.414	-37.351
2.460.898	-27.433	2.483.776	-34.483	2.433.526	-37.352
2.461.137	-27.433	2.484.003	-34.484	2.433.761	-37.354
2.461.366	-27.433	2.484.169	-34.485	2.433.978	-37.355

2.461.602	-27.432	2.484.323	-34.486	2.434.095	-37.357
2.461.855	-27.432	2.484.509	-34.487	2.434.242	-37.359
2.462.013	-27.432	2.484.664	-34.488	2.434.430	-37.360
2.462.110	-27.431	2.484.848	-34.489	2.434.552	-37.362
2.462.273	-27.431	2.485.072	-34.490	2.434.715	-37.364
2.462.460	-27.431	2.485.174	-34.491	2.434.975	-37.365
2.462.578	-27.430	2.485.248	-34.492	2.435.141	-37.367
2.462.744	-27.430	2.485.374	-34.494	2.435.320	-37.369
2.462.890	-27.430	2.485.528	-34.495	2.435.490	-37.370
2.463.015	-27.429	2.485.705	-34.496	2.435.593	-37.372
2.463.217	-27.429	2.485.938	-34.497	2.435.716	-37.374
2.463.345	-27.428	2.486.178	-34.499	2.435.872	-37.376
2.463.456	-27.428	2.486.371	-34.500	2.436.005	-37.378
2.463.652	-27.428	2.486.534	-34.502	2.436.133	-37.380
2.463.790	-27.427	2.486.653	-34.503	2.436.314	-37.381
2.463.929	-27.427	2.486.805	-34.505	2.436.577	-37.383
2.464.177	-27.427	2.486.937	-34.506	2.436.761	-37.385
2.464.365	-27.426	2.487.083	-34.508	2.436.924	-37.387
2.464.531	-27.426	2.487.282	-34.509	2.437.059	-37.389
2.464.755	-27.426	2.487.471	-34.511	2.437.238	-37.391
2.464.895	-27.425	2.487.625	-34.513	2.437.437	-37.393
2.465.025	-27.425	2.487.751	-34.514	2.437.607	-37.395
2.465.253	-27.425	2.487.831	-34.516	2.437.750	-37.397
2.465.434	-27.424	2.487.930	-34.518	2.437.869	-37.399
2.465.580	-27.424	2.488.084	-34.519	2.438.000	-37.401
2.465.737	-27.424	2.488.232	-34.521	2.438.176	-37.403
2.465.878	-27.423	2.488.454	-34.523	2.438.356	-37.405
2.466.012	-27.423	2.488.672	-34.525	2.438.549	-37.406
2.466.241	-27.423	2.488.911	-34.527	2.438.759	-37.408
2.466.449	-27.422	2.489.148	-34.529	2.439.009	-37.410

2.466.573	-27.422	2.489.386	-34.531	2.439.180	-37.412
2.466.725	-27.422	2.489.612	-34.533	2.439.307	-37.414
2.466.915	-27.421	2.489.753	-34.535	2.439.471	-37.416
2.467.110	-27.421	2.489.865	-34.537	2.439.614	-37.418
2.467.290	-27.421	2.489.979	-34.539	2.439.731	-37.420
2.467.448	-27.420	2.490.123	-34.541	2.439.865	-37.422
2.467.569	-27.420	2.490.247	-34.543	2.440.078	-37.424
2.467.686	-27.420	2.490.365	-34.545	2.440.201	-37.426
2.467.925	-27.419	2.490.555	-34.548	2.440.260	-37.428
2.468.122	-27.419	2.490.750	-34.550	2.440.427	-37.430
2.468.313	-27.419	2.490.855	-34.552	2.440.694	-37.432
2.468.479	-27.418	2.491.005	-34.554	2.440.880	-37.434
2.468.599	-27.418	2.491.201	-34.557	2.441.003	-37.436
2.468.794	-27.418	2.491.391	-34.559	2.441.095	-37.438
2.469.017	-27.417	2.491.606	-34.561	2.441.236	-37.440
2.469.178	-27.417	2.491.776	-34.564	2.441.422	-37.442
2.469.357	-27.417	2.491.911	-34.566	2.441.559	-37.444
2.469.512	-27.416	2.492.122	-34.568	2.441.750	-37.446
2.469.617	-27.416	2.492.309	-34.571	2.441.980	-37.448
2.469.697	-27.416	2.492.489	-34.573	2.442.204	-37.449
2.469.930	-27.415	2.492.666	-34.576	2.442.370	-37.451
2.470.397	-27.415	2.492.775	-34.578	2.442.527	-37.453
2.470.840	-27.415	2.492.950	-34.581	2.442.726	-37.455
2.471.031	-27.414	2.493.138	-34.583	2.442.908	-37.457
2.471.071	-27.414	2.493.239	-34.586	2.443.136	-37.459
2.471.051	-27.414	2.493.362	-34.588	2.443.326	-37.460
2.471.079	-27.413	2.493.622	-34.591	2.443.503	-37.462
2.471.189	-27.413	2.493.826	-34.594	2.443.673	-37.464
2.471.339	-27.413	2.493.945	-34.596	2.443.792	-37.466
2.471.461	-27.412	2.494.120	-34.599	2.443.936	-37.467

2.471.581	-27.412	2.494.312	-34.601	2.444.077	-37.469
2.471.706	-27.412	2.494.498	-34.604	2.444.135	-37.471
2.471.843	-27.411	2.494.624	-34.607	2.444.251	-37.472
2.472.029	-27.411	2.494.734	-34.609	2.444.457	-37.474
2.472.242	-27.411	2.494.852	-34.612	2.444.657	-37.476
2.472.458	-27.410	2.494.988	-34.615	2.444.762	-37.477
2.472.637	-27.410	2.495.199	-34.617	2.444.913	-37.479
2.472.782	-27.410	2.495.379	-34.620	2.445.098	-37.480
2.473.004	-27.409	2.495.549	-34.623	2.445.313	-37.482
2.473.179	-27.409	2.495.788	-34.625	2.445.452	-37.484
2.473.248	-27.409	2.495.921	-34.628	2.445.632	-37.485
2.473.345	-27.409	2.496.090	-34.631	2.445.844	-37.486
2.473.503	-27.408	2.496.393	-34.633	2.446.055	-37.488
2.473.694	-27.408	2.496.519	-34.636	2.446.283	-37.489
2.473.873	-27.408	2.496.599	-34.638	2.446.451	-37.491
2.474.036	-27.407	2.496.801	-34.641	2.446.519	-37.492
2.474.198	-27.407	2.496.955	-34.644	2.446.655	-37.493
2.474.417	-27.407	2.497.032	-34.646	2.446.897	-37.495
2.474.554	-27.406	2.497.229	-34.649	2.447.125	-37.496
2.474.653	-27.406	2.497.674	-34.651	2.447.255	-37.497
2.474.832	-27.406	2.498.058	-34.654	2.447.358	-37.499
2.475.002	-27.405	2.498.241	-34.656	2.447.523	-37.500
2.475.211	-27.405	2.498.338	-34.659	2.447.726	-37.501
2.475.426	-27.405	2.498.395	-34.661	2.447.941	-37.502
2.475.567	-27.404	2.498.465	-34.664	2.448.100	-37.503
2.475.748	-27.404	2.498.566	-34.666	2.448.225	-37.504
2.475.970	-27.404	2.498.611	-34.669	2.448.302	-37.506
2.476.151	-27.403	2.498.736	-34.671	2.448.510	-37.507
2.476.246	-27.403	2.498.947	-34.673	2.448.717	-37.508
2.476.360	-27.403	2.499.044	-34.676	2.448.848	-37.509

2.476.507	-27.402	2.499.209	-34.678	2.449.046	-37.510
2.476.680	-27.402	2.499.438	-34.680	2.449.220	-37.511
2.476.888	-27.402	2.499.615	-34.683	2.449.386	-37.512
2.477.063	-27.401	2.499.828	-34.685	2.449.606	-37.513
2.477.195	-27.401	2.500.016	-34.687	2.449.793	-37.513
2.477.321	-27.401	2.500.143	-34.689	2.449.964	-37.514
2.477.527	-27.400	2.500.278	-34.691	2.450.139	-37.515
2.477.760	-27.400	2.500.490	-34.693	2.450.277	-37.516
2.477.883	-27.400	2.500.753	-34.695	2.450.417	-37.517
2.477.988	-27.399	2.500.938	-34.697	2.450.565	-37.518
2.478.225	-27.399	2.501.030	-34.699	2.450.844	-37.518
2.478.409	-27.399	2.501.147	-34.701	2.451.064	-37.519
2.478.557	-27.398	2.501.348	-34.703	2.451.180	-37.520
2.478.714	-27.398	2.501.458	-34.705	2.451.297	-37.521
2.478.853	-27.398	2.501.509	-34.706	2.451.414	-37.521
2.479.021	-27.397	2.501.724	-34.708	2.451.637	-37.522
2.479.218	-27.397	2.501.964	-34.710	2.451.848	-37.523
2.479.354	-27.397	2.502.152	-34.711	2.451.969	-37.523
2.479.516	-27.396	2.502.336	-34.713	2.452.070	-37.524
2.479.737	-27.396	2.502.443	-34.715	2.452.271	-37.524
2.479.904	-27.396	2.502.645	-34.716	2.452.496	-37.525
2.480.051	-27.395	2.502.923	-34.717	2.452.621	-37.525
2.480.214	-27.395	2.503.019	-34.719	2.452.784	-37.526
2.480.401	-27.395	2.503.113	-34.720	2.453.159	-37.526
2.480.558	-27.394	2.503.313	-34.722	2.453.624	-37.527
2.480.701	-27.394	2.503.484	-34.723	2.453.884	-37.527
2.480.865	-27.394	2.503.611	-34.724	2.453.941	-37.528
2.480.987	-27.393	2.503.712	-34.725	2.453.998	-37.528
2.481.136	-27.393	2.503.906	-34.726	2.454.106	-37.528
2.481.360	-27.393	2.504.144	-34.727	2.454.227	-37.529

2.481.529	-27.392	2.504.234	-34.728	2.454.272	-37.529
2.481.695	-27.392	2.504.348	-34.729	2.454.324	-37.529
2.481.870	-27.392	2.504.596	-34.730	2.454.471	-37.530
2.482.036	-27.391	2.504.790	-34.731	2.454.601	-37.530
2.482.150	-27.391	2.504.934	-34.732	2.454.769	-37.530
2.482.227	-27.391	2.505.138	-34.733	2.454.922	-37.530
2.482.378	-27.390	2.505.326	-34.734	2.455.040	-37.531
2.482.599	-27.390	2.505.450	-34.734	2.455.181	-37.531
2.482.755	-27.390	2.505.580	-34.735	2.455.425	-37.531
2.482.905	-27.389	2.505.706	-34.736	2.455.643	-37.531
2.483.055	-27.389	2.505.887	-34.736	2.455.730	-37.531
2.483.124	-27.389	2.506.147	-34.737	2.455.828	-37.531
2.483.255	-27.388	2.506.313	-34.737	2.455.996	-37.532
2.483.493	-27.388	2.506.379	-34.738	2.456.248	-37.532
2.483.676	-27.388	2.506.595	-34.738	2.456.472	-37.532
2.483.888	-27.387	2.506.799	-34.738	2.456.644	-37.532
2.484.166	-27.387	2.507.011	-34.739	2.456.850	-37.532
2.484.362	-27.387	2.507.195	-34.739	2.457.007	-37.532
2.484.530	-27.386	2.507.249	-34.739	2.457.144	-37.532
2.484.686	-27.386	2.507.401	-34.739	2.457.319	-37.532
2.484.807	-27.386	2.507.602	-34.739	2.457.517	-37.532
2.485.009	-27.386	2.507.755	-34.739	2.457.710	-37.532
2.485.195	-27.385	2.507.916	-34.740	2.457.829	-37.532
2.485.280	-27.385	2.508.056	-34.740	2.457.909	-37.532
2.485.462	-27.385	2.508.241	-34.740	2.458.057	-37.533
2.485.607	-27.384	2.508.449	-34.739	2.458.210	-37.533
2.485.732	-27.384	2.508.653	-34.739	2.458.422	-37.533
2.485.889	-27.384	2.508.842	-34.739	2.458.617	-37.533
2.486.023	-27.384	2.508.936	-34.739	2.458.758	-37.533
2.486.178	-27.383	2.509.029	-34.739	2.458.929	-37.533

2.486.364	-27.383	2.509.227	-34.738	2.459.044	-37.533
2.486.581	-27.383	2.509.420	-34.738	2.459.245	-37.533
2.486.727	-27.383	2.509.549	-34.738	2.459.485	-37.533
2.486.960	-27.382	2.509.603	-34.737	2.459.695	-37.533
2.487.173	-27.382	2.509.715	-34.737	2.459.870	-37.533
2.487.280	-27.382	2.509.902	-34.736	2.460.020	-37.533
2.487.446	-27.382	2.510.016	-34.736	2.460.152	-37.533
2.487.634	-27.381	2.510.188	-34.735	2.460.258	-37.533
2.487.778	-27.381	2.510.435	-34.735	2.460.367	-37.533
2.487.948	-27.381	2.510.634	-34.734	2.460.567	-37.532
2.488.098	-27.381	2.510.828	-34.733	2.460.779	-37.532
2.488.290	-27.380	2.511.046	-34.733	2.460.947	-37.532
2.488.490	-27.380	2.511.238	-34.732	2.461.066	-37.532
2.488.656	-27.380	2.511.434	-34.731	2.461.169	-37.532
2.488.794	-27.380	2.511.662	-34.730	2.461.404	-37.532
2.488.935	-27.380	2.511.865	-34.730	2.461.559	-37.532
2.489.132	-27.379	2.512.002	-34.729	2.461.664	-37.532
2.489.286	-27.379	2.512.089	-34.728	2.461.875	-37.532
2.489.444	-27.379	2.512.201	-34.727	2.462.092	-37.532
2.489.632	-27.379	2.512.387	-34.726	2.462.226	-37.533
2.489.856	-27.379	2.512.555	-34.725	2.462.374	-37.533
2.490.049	-27.378	2.512.664	-34.724	2.462.531	-37.533
2.490.134	-27.378	2.512.826	-34.723	2.462.682	-37.533
2.490.269	-27.378	2.513.004	-34.722	2.462.867	-37.533
2.490.497	-27.378	2.513.131	-34.721	2.463.085	-37.533
2.490.694	-27.378	2.513.318	-34.720	2.463.254	-37.533
2.490.815	-27.378	2.513.541	-34.718	2.463.353	-37.533
2.490.932	-27.378	2.513.725	-34.717	2.463.515	-37.533
2.491.062	-27.377	2.513.905	-34.716	2.463.743	-37.533
2.491.225	-27.377	2.514.109	-34.715	2.463.956	-37.533

2.491.463	-27.377	2.514.232	-34.714	2.464.124	-37.533
2.491.640	-27.377	2.514.404	-34.712	2.464.312	-37.533
2.491.832	-27.377	2.514.641	-34.711	2.464.482	-37.533
2.492.047	-27.377	2.514.809	-34.710	2.464.612	-37.533
2.492.141	-27.377	2.514.935	-34.709	2.464.816	-37.533
2.492.219	-27.377	2.515.128	-34.707	2.464.975	-37.534
2.492.464	-27.377	2.515.295	-34.706	2.465.094	-37.534
2.492.840	-27.377	2.515.403	-34.705	2.465.296	-37.534
2.493.248	-27.377	2.515.582	-34.703	2.465.427	-37.534
2.493.521	-27.376	2.515.795	-34.702	2.465.553	-37.534
2.493.596	-27.376	2.515.969	-34.701	2.465.742	-37.534
2.493.664	-27.376	2.516.057	-34.699	2.465.941	-37.534
2.493.694	-27.376	2.516.199	-34.698	2.466.117	-37.534
2.493.716	-27.376	2.516.433	-34.697	2.466.293	-37.534
2.493.788	-27.376	2.516.624	-34.695	2.466.503	-37.534
2.493.947	-27.376	2.516.817	-34.694	2.466.714	-37.535
2.494.180	-27.376	2.516.989	-34.693	2.466.877	-37.535
2.494.305	-27.376	2.517.184	-34.691	2.467.036	-37.535
2.494.419	-27.376	2.517.340	-34.690	2.467.148	-37.535
2.494.646	-27.376	2.517.443	-34.688	2.467.233	-37.535
2.494.861	-27.376	2.517.594	-34.687	2.467.417	-37.535
2.494.969	-27.376	2.517.782	-34.686	2.467.609	-37.535
2.495.078	-27.376	2.517.990	-34.684	2.467.789	-37.535
2.495.262	-27.376	2.518.150	-34.683	2.467.905	-37.536
2.495.466	-27.376	2.518.331	-34.681	2.468.042	-37.536
2.495.683	-27.376	2.518.456	-34.680	2.468.207	-37.536
2.495.880	-27.376	2.518.577	-34.679	2.468.327	-37.536
2.496.033	-27.376	2.518.736	-34.677	2.468.430	-37.536
2.496.201	-27.377	2.518.905	-34.676	2.468.559	-37.536
2.496.321	-27.377	2.519.099	-34.675	2.468.687	-37.536

2.496.411	-27.377	2.519.247	-34.673	2.468.828	-37.536
2.496.521	-27.377	2.519.428	-34.672	2.469.053	-37.537
2.496.662	-27.377	2.519.803	-34.670	2.469.247	-37.537
2.496.865	-27.377	2.520.257	-34.669	2.469.413	-37.537
2.496.998	-27.377	2.520.497	-34.668	2.469.548	-37.537
2.497.085	-27.377	2.520.580	-34.666	2.469.803	-37.537
2.497.278	-27.377	2.520.595	-34.665	2.470.036	-37.537
2.497.534	-27.377	2.520.627	-34.664	2.470.190	-37.537
2.497.795	-27.377	2.520.743	-34.662	2.470.352	-37.537
2.498.008	-27.378	2.520.848	-34.661	2.470.573	-37.538
2.498.159	-27.378	2.520.914	-34.660	2.470.824	-37.538
2.498.310	-27.378	2.521.044	-34.659	2.471.037	-37.538
2.498.490	-27.378	2.521.212	-34.657	2.471.185	-37.538
2.498.622	-27.378	2.521.360	-34.656	2.471.312	-37.538
2.498.732	-27.378	2.521.600	-34.655	2.471.407	-37.538
2.498.885	-27.378	2.521.889	-34.654	2.471.465	-37.538
2.498.971	-27.379	2.522.059	-34.652	2.471.592	-37.539
2.499.117	-27.379	2.522.178	-34.651	2.471.780	-37.539
2.499.350	-27.379	2.522.338	-34.650	2.471.960	-37.539
2.499.536	-27.379	2.522.470	-34.649	2.472.169	-37.539
2.499.697	-27.379	2.522.587	-34.648	2.472.314	-37.539
2.499.850	-27.379	2.522.755	-34.646	2.472.488	-37.539
2.500.051	-27.380	2.522.986	-34.645	2.472.713	-37.540
2.500.255	-27.380	2.523.192	-34.644	2.472.878	-37.540
2.500.387	-27.380	2.523.291	-34.643	2.473.051	-37.540
2.500.536	-27.380	2.523.376	-34.642	2.473.214	-37.540
2.500.757	-27.380	2.523.544	-34.641	2.473.351	-37.540
2.500.871	-27.381	2.523.678	-34.640	2.473.564	-37.541
2.500.931	-27.381	2.523.758	-34.639	2.473.815	-37.541
2.501.144	-27.381	2.523.922	-34.638	2.473.979	-37.541

2.501.373	-27.381	2.524.209	-34.637	2.474.099	-37.541
2.501.552	-27.381	2.524.482	-34.636	2.474.258	-37.542
2.501.745	-27.382	2.524.644	-34.635	2.474.379	-37.542
2.501.974	-27.382	2.524.772	-34.634	2.474.548	-37.542
2.502.186	-27.382	2.524.899	-34.633	2.474.760	-37.542
2.502.330	-27.382	2.525.040	-34.632	2.474.861	-37.543
2.502.414	-27.383	2.525.203	-34.631	2.475.024	-37.543
2.502.478	-27.383	2.525.372	-34.630	2.475.222	-37.543
2.502.663	-27.383	2.525.520	-34.630	2.475.424	-37.543
2.502.885	-27.383	2.525.727	-34.629	2.475.636	-37.544
2.503.028	-27.384	2.525.956	-34.628	2.475.777	-37.544
2.503.179	-27.384	2.526.109	-34.627	2.475.952	-37.544
2.503.347	-27.384	2.526.241	-34.626	2.476.108	-37.545
2.503.553	-27.384	2.526.388	-34.626	2.476.247	-37.545
2.503.773	-27.385	2.526.535	-34.625	2.476.384	-37.545
2.503.967	-27.385	2.526.741	-34.624	2.476.584	-37.546
2.504.108	-27.385	2.526.931	-34.624	2.476.807	-37.546
2.504.267	-27.386	2.527.085	-34.623	2.476.980	-37.547
2.504.444	-27.386	2.527.244	-34.622	2.477.141	-37.547
2.504.576	-27.386	2.527.417	-34.622	2.477.289	-37.547
2.504.733	-27.386	2.527.632	-34.621	2.477.410	-37.548
2.504.902	-27.387	2.527.759	-34.621	2.477.506	-37.548
2.504.987	-27.387	2.527.856	-34.620	2.477.695	-37.549
2.505.078	-27.387	2.527.970	-34.619	2.477.912	-37.549
2.505.203	-27.388	2.528.134	-34.619	2.478.078	-37.550
2.505.381	-27.388	2.528.351	-34.618	2.478.232	-37.550
2.505.580	-27.388	2.528.557	-34.618	2.478.418	-37.551
2.505.716	-27.389	2.528.765	-34.617	2.478.595	-37.552
2.505.893	-27.389	2.528.904	-34.617	2.478.741	-37.552
2.506.102	-27.389	2.529.099	-34.617	2.478.945	-37.553

2.506.290	-27.390	2.529.252	-34.616	2.479.124	-37.553
2.506.496	-27.390	2.529.377	-34.616	2.479.227	-37.554
2.506.753	-27.390	2.529.557	-34.615	2.479.408	-37.555
2.506.946	-27.391	2.529.716	-34.615	2.479.583	-37.555
2.507.115	-27.391	2.529.904	-34.615	2.479.751	-37.556
2.507.294	-27.391	2.530.108	-34.614	2.479.919	-37.557
2.507.417	-27.392	2.530.210	-34.614	2.479.977	-37.557
2.507.587	-27.392	2.530.341	-34.614	2.480.331	-37.558
2.507.762	-27.393	2.530.549	-34.614	2.480.867	-37.559
2.507.853	-27.393	2.530.744	-34.613	2.481.126	-37.560
2.507.959	-27.393	2.530.923	-34.613	2.481.210	-37.560
2.508.167	-27.394	2.531.095	-34.613	2.481.273	-37.561
2.508.315	-27.394	2.531.301	-34.613	2.481.413	-37.562
2.508.467	-27.394	2.531.449	-34.613	2.481.518	-37.563
2.508.643	-27.395	2.531.536	-34.612	2.481.568	-37.564
2.508.790	-27.395	2.531.647	-34.612	2.481.626	-37.565
2.509.014	-27.396	2.531.777	-34.612	2.481.736	-37.566
2.509.193	-27.396	2.531.895	-34.612	2.481.861	-37.567
2.509.341	-27.396	2.532.051	-34.612	2.481.994	-37.568
2.509.514	-27.397	2.532.235	-34.612	2.482.164	-37.569
2.509.663	-27.397	2.532.448	-34.611	2.482.325	-37.570
2.509.894	-27.398	2.532.655	-34.611	2.482.544	-37.571
2.510.020	-27.398	2.532.809	-34.611	2.482.764	-37.572
2.510.173	-27.399	2.532.947	-34.611	2.482.923	-37.573
2.510.379	-27.399	2.533.145	-34.611	2.483.078	-37.574
2.510.502	-27.399	2.533.338	-34.611	2.483.223	-37.575
2.510.656	-27.400	2.533.504	-34.611	2.483.427	-37.576
2.510.842	-27.400	2.533.709	-34.611	2.483.607	-37.578
2.511.012	-27.401	2.533.918	-34.611	2.483.717	-37.579
2.511.176	-27.401	2.534.150	-34.611	2.483.861	-37.580

2.511.366	-27.402	2.534.377	-34.611	2.484.081	-37.582
2.511.563	-27.402	2.534.436	-34.611	2.484.216	-37.583
2.511.747	-27.402	2.534.469	-34.611	2.484.294	-37.584
2.511.886	-27.403	2.534.637	-34.611	2.484.493	-37.586
2.512.000	-27.403	2.534.833	-34.611	2.484.686	-37.587
2.512.175	-27.404	2.534.936	-34.611	2.484.816	-37.589
2.512.385	-27.404	2.535.027	-34.611	2.484.961	-37.590
2.512.569	-27.405	2.535.209	-34.611	2.485.072	-37.592
2.512.724	-27.405	2.535.431	-34.611	2.485.237	-37.593
2.512.863	-27.406	2.535.527	-34.610	2.485.412	-37.595
2.512.999	-27.406	2.535.685	-34.611	2.485.562	-37.596
2.513.145	-27.406	2.536.014	-34.611	2.485.742	-37.598
2.513.372	-27.407	2.536.280	-34.611	2.485.891	-37.600
2.513.555	-27.407	2.536.443	-34.611	2.486.088	-37.601
2.513.720	-27.408	2.536.563	-34.611	2.486.332	-37.603
2.513.904	-27.408	2.536.683	-34.611	2.486.481	-37.605
2.514.050	-27.409	2.536.859	-34.611	2.486.653	-37.606
2.514.209	-27.409	2.537.062	-34.611	2.486.877	-37.608
2.514.357	-27.410	2.537.185	-34.611	2.487.049	-37.610
2.514.493	-27.410	2.537.375	-34.611	2.487.195	-37.612
2.514.633	-27.411	2.537.608	-34.611	2.487.390	-37.614
2.514.720	-27.411	2.537.762	-34.611	2.487.560	-37.616
2.515.035	-27.412	2.537.935	-34.611	2.487.676	-37.618
2.515.548	-27.412	2.538.113	-34.611	2.487.639	-37.620
2.515.853	-27.412	2.538.267	-34.611	2.487.778	-37.622
2.515.978	-27.413	2.538.453	-34.611	2.487.997	-37.624
2.516.124	-27.413	2.538.601	-34.611	2.488.204	-37.626
2.516.194	-27.414	2.538.806	-34.611	2.488.461	-37.628
2.516.227	-27.414	2.539.012	-34.611	2.488.669	-37.630
2.516.267	-27.415	2.539.165	-34.611	2.488.808	-37.632

2.516.319	-27.415	2.539.265	-34.611	2.488.990	-37.634
2.516.479	-27.416	2.539.367	-34.611	2.489.135	-37.637
2.516.665	-27.416	2.539.500	-34.611	2.489.289	-37.639
2.516.848	-27.417	2.539.687	-34.611	2.489.458	-37.641
2.517.039	-27.417	2.539.933	-34.611	2.489.601	-37.643
2.517.262	-27.418	2.540.136	-34.611	2.489.774	-37.646
2.517.464	-27.418	2.540.298	-34.611	2.489.957	-37.648
2.517.599	-27.419	2.540.404	-34.612	2.490.163	-37.650
2.517.784	-27.419	2.540.548	-34.612	2.490.313	-37.652
2.517.977	-27.419	2.540.768	-34.612	2.490.430	-37.655
2.518.118	-27.420	2.540.952	-34.612	2.490.546	-37.657
2.518.292	-27.420	2.541.086	-34.612	2.490.719	-37.660
2.518.440	-27.421	2.541.262	-34.612	2.490.951	-37.662
2.518.559	-27.421	2.541.448	-34.612	2.491.138	-37.664
2.518.714	-27.422	2.541.566	-34.612	2.491.265	-37.667
2.518.873	-27.422	2.541.794	-34.612	2.491.440	-37.669
2.519.050	-27.423	2.542.245	-34.612	2.491.662	-37.672
2.519.173	-27.423	2.542.583	-34.612	2.491.834	-37.674
2.519.256	-27.423	2.542.713	-34.612	2.492.009	-37.677
2.519.386	-27.424	2.542.723	-34.612	2.492.133	-37.679
2.519.583	-27.424	2.542.698	-34.612	2.492.251	-37.682
2.519.818	-27.425	2.542.738	-34.612	2.492.435	-37.684
2.520.042	-27.425	2.542.827	-34.612	2.492.639	-37.687
2.520.224	-27.426	2.542.940	-34.612	2.492.852	-37.689
2.520.424	-27.426	2.543.102	-34.612	2.492.999	-37.692
2.520.624	-27.426	2.543.289	-34.612	2.493.145	-37.694
2.520.741	-27.427	2.543.446	-34.612	2.493.315	-37.697
2.520.851	-27.427	2.543.640	-34.612	2.493.436	-37.700
2.521.021	-27.428	2.543.864	-34.612	2.493.633	-37.702
2.521.223	-27.428	2.544.056	-34.612	2.493.835	-37.705

2.521.368	-27.428	2.544.240	-34.612	2.493.969	-37.707
2.521.538	-27.429	2.544.434	-34.612	2.494.121	-37.710
2.521.691	-27.429	2.544.608	-34.612	2.494.341	-37.712
2.521.827	-27.429	2.544.767	-34.612	2.494.507	-37.715
2.521.996	-27.430	2.544.912	-34.612	2.494.645	-37.717
2.522.166	-27.430	2.545.101	-34.611	2.494.809	-37.720
2.522.348	-27.431	2.545.291	-34.611	2.494.970	-37.722
2.522.525	-27.431	2.545.471	-34.611	2.495.121	-37.725
2.522.677	-27.431	2.545.648	-34.611	2.495.237	-37.727
2.522.853	-27.432	2.545.804	-34.611	2.495.378	-37.730
2.523.020	-27.432	2.545.945	-34.611	2.495.524	-37.732
2.523.147	-27.432	2.546.064	-34.611	2.495.651	-37.735
2.523.282	-27.433	2.546.208	-34.611	2.495.871	-37.737
2.523.404	-27.433	2.546.364	-34.611	2.496.064	-37.740
2.523.573	-27.433	2.546.517	-34.611	2.496.219	-37.742
2.523.808	-27.434	2.546.707	-34.611	2.496.387	-37.744
2.524.001	-27.434	2.546.964	-34.611	2.496.600	-37.747
2.524.174	-27.434	2.547.163	-34.611	2.496.780	-37.749
2.524.348	-27.435	2.547.348	-34.611	2.496.940	-37.752
2.524.554	-27.435	2.547.525	-34.611	2.497.139	-37.754
2.524.818	-27.435	2.547.632	-34.611	2.497.345	-37.756
2.524.987	-27.436	2.547.727	-34.611	2.497.551	-37.759
2.525.107	-27.436	2.547.895	-34.611	2.497.735	-37.761
2.525.285	-27.436	2.548.083	-34.611	2.497.892	-37.763
2.525.452	-27.436	2.548.214	-34.611	2.498.093	-37.765
2.525.560	-27.437	2.548.354	-34.611	2.498.309	-37.768
2.525.726	-27.437	2.548.536	-34.611	2.498.394	-37.770
2.525.941	-27.437	2.548.708	-34.611	2.498.522	-37.772
2.526.117	-27.437	2.548.860	-34.611	2.498.709	-37.774
2.526.243	-27.438	2.548.951	-34.611	2.498.833	-37.777

2.526.393	-27.438	2.549.114	-34.611	2.498.978	-37.779
2.526.588	-27.438	2.549.333	-34.611	2.499.162	-37.781
2.526.747	-27.439	2.549.489	-34.611	2.499.294	-37.783
2.526.865	-27.439	2.549.642	-34.611	2.499.424	-37.785
2.526.998	-27.439	2.549.814	-34.611	2.499.548	-37.787
2.527.173	-27.439	2.549.966	-34.611	2.499.664	-37.789
2.527.336	-27.439	2.550.103	-34.611	2.499.767	-37.791
2.527.473	-27.440	2.550.262	-34.611	2.499.919	-37.793
2.527.607	-27.440	2.550.496	-34.611	2.500.168	-37.795
2.527.740	-27.440	2.550.710	-34.611	2.500.406	-37.797
2.527.883	-27.440	2.550.881	-34.611	2.500.582	-37.799
2.527.999	-27.441	2.551.085	-34.611	2.500.752	-37.801
2.528.167	-27.441	2.551.226	-34.611	2.500.941	-37.803
2.528.362	-27.441	2.551.356	-34.611	2.501.077	-37.804
2.528.530	-27.441	2.551.436	-34.611	2.501.225	-37.806
2.528.716	-27.441	2.551.640	-34.611	2.501.485	-37.808
2.528.940	-27.442	2.551.898	-34.611	2.501.676	-37.810
2.529.184	-27.442	2.552.019	-34.611	2.501.794	-37.812
2.529.400	-27.442	2.552.189	-34.611	2.501.913	-37.813
2.529.621	-27.442	2.552.380	-34.611	2.502.070	-37.815
2.529.778	-27.442	2.552.487	-34.611	2.502.233	-37.817
2.529.926	-27.443	2.552.611	-34.611	2.502.453	-37.818
2.530.076	-27.443	2.552.878	-34.611	2.502.646	-37.820
2.530.247	-27.443	2.553.124	-34.611	2.502.823	-37.822
2.530.468	-27.443	2.553.296	-34.611	2.502.946	-37.823
2.530.587	-27.443	2.553.453	-34.611	2.503.058	-37.825
2.530.641	-27.443	2.553.604	-34.611	2.503.212	-37.826
2.530.753	-27.444	2.553.748	-34.612	2.503.477	-37.828
2.530.921	-27.444	2.553.847	-34.612	2.503.671	-37.830
2.531.109	-27.444	2.553.969	-34.612	2.503.819	-37.831

2.531.308	-27.444	2.554.127	-34.612	2.504.025	-37.832
2.531.478	-27.444	2.554.244	-34.612	2.504.212	-37.834
2.531.641	-27.444	2.554.431	-34.612	2.504.370	-37.835
2.531.841	-27.444	2.554.667	-34.612	2.504.543	-37.837
2.532.069	-27.445	2.554.760	-34.612	2.504.751	-37.838
2.532.247	-27.445	2.554.902	-34.612	2.504.939	-37.840
2.532.443	-27.445	2.555.133	-34.612	2.505.026	-37.841
2.532.616	-27.445	2.555.303	-34.612	2.505.105	-37.842
2.532.768	-27.445	2.555.476	-34.612	2.505.300	-37.844
2.532.957	-27.445	2.555.674	-34.612	2.505.521	-37.845
2.533.112	-27.445	2.555.937	-34.612	2.505.724	-37.846
2.533.250	-27.446	2.556.136	-34.612	2.505.902	-37.847
2.533.354	-27.446	2.556.267	-34.611	2.506.061	-37.849
2.533.521	-27.446	2.556.436	-34.611	2.506.249	-37.850
2.533.747	-27.446	2.556.618	-34.611	2.506.393	-37.851
2.533.927	-27.446	2.556.801	-34.611	2.506.500	-37.852
2.534.118	-27.446	2.556.907	-34.611	2.506.671	-37.853
2.534.289	-27.446	2.556.992	-34.611	2.506.850	-37.855
2.534.453	-27.446	2.557.133	-34.611	2.506.998	-37.856
2.534.633	-27.446	2.557.242	-34.611	2.507.152	-37.857
2.534.781	-27.447	2.557.355	-34.611	2.507.314	-37.858
2.534.967	-27.447	2.557.567	-34.611	2.507.545	-37.859
2.535.140	-27.447	2.557.816	-34.611	2.507.934	-37.860
2.535.272	-27.447	2.558.051	-34.611	2.508.335	-37.861
2.535.407	-27.447	2.558.214	-34.610	2.508.582	-37.862
2.535.561	-27.447	2.558.367	-34.610	2.508.699	-37.863
2.535.725	-27.447	2.558.517	-34.610	2.508.768	-37.864
2.535.901	-27.447	2.558.658	-34.610	2.508.790	-37.865
2.536.111	-27.447	2.558.842	-34.610	2.508.812	-37.866
2.536.277	-27.448	2.559.042	-34.610	2.508.906	-37.867

2.536.463	-27.448	2.559.228	-34.609	2.509.003	-37.868
2.536.682	-27.448	2.559.346	-34.609	2.509.048	-37.869
2.536.805	-27.448	2.559.579	-34.609	2.509.184	-37.870
2.536.949	-27.448	2.559.833	-34.609	2.509.397	-37.871
2.537.101	-27.448	2.559.884	-34.609	2.509.576	-37.872
2.537.173	-27.448	2.559.999	-34.609	2.509.798	-37.873
2.537.279	-27.448	2.560.244	-34.608	2.509.957	-37.874
2.537.495	-27.448	2.560.450	-34.608	2.510.094	-37.875
2.537.954	-27.448	2.560.580	-34.608	2.510.320	-37.875
2.538.424	-27.448	2.560.779	-34.608	2.510.508	-37.876
2.538.558	-27.449	2.560.909	-34.608	2.510.685	-37.877
2.538.629	-27.449	2.561.046	-34.607	2.510.878	-37.878
2.538.673	-27.449	2.561.293	-34.607	2.511.044	-37.879
2.538.753	-27.449	2.561.483	-34.607	2.511.164	-37.880
2.538.867	-27.449	2.561.624	-34.607	2.511.295	-37.881
2.538.958	-27.449	2.561.802	-34.606	2.511.485	-37.881
2.539.070	-27.449	2.562.015	-34.606	2.511.659	-37.882
2.539.176	-27.449	2.562.160	-34.606	2.511.815	-37.883
2.539.337	-27.449	2.562.292	-34.606	2.512.014	-37.884
2.539.536	-27.449	2.562.470	-34.605	2.512.199	-37.885
2.539.763	-27.449	2.562.654	-34.605	2.512.334	-37.885
2.539.913	-27.449	2.562.823	-34.605	2.512.467	-37.886
2.540.045	-27.449	2.563.012	-34.604	2.512.630	-37.887
2.540.245	-27.449	2.563.145	-34.604	2.512.833	-37.888
2.540.450	-27.450	2.563.261	-34.604	2.512.990	-37.889
2.540.664	-27.450	2.563.401	-34.604	2.513.143	-37.889
2.540.865	-27.450	2.563.513	-34.603	2.513.300	-37.890
2.541.054	-27.450	2.563.680	-34.603	2.513.427	-37.891
2.541.188	-27.450	2.564.035	-34.603	2.513.626	-37.892
2.541.274	-27.450	2.564.510	-34.602	2.513.884	-37.892

2.541.429	-27.450	2.564.833	-34.602	2.514.070	-37.893
2.541.606	-27.450	2.564.955	-34.602	2.514.206	-37.894
2.541.748	-27.450	2.564.980	-34.601	2.514.379	-37.895
2.541.882	-27.450	2.565.023	-34.601	2.514.574	-37.895
2.541.996	-27.450	2.565.078	-34.601	2.514.763	-37.896
2.542.123	-27.450	2.565.122	-34.600	2.514.913	-37.897
2.542.268	-27.450	2.565.189	-34.600	2.514.987	-37.898
2.542.494	-27.450	2.565.377	-34.600	2.515.116	-37.898
2.542.760	-27.450	2.565.562	-34.599	2.515.326	-37.899
2.542.984	-27.450	2.565.708	-34.599	2.515.530	-37.900
2.543.142	-27.450	2.565.894	-34.599	2.515.677	-37.901
2.543.269	-27.450	2.566.093	-34.598	2.515.801	-37.901
2.543.435	-27.450	2.566.320	-34.598	2.515.963	-37.902
2.543.569	-27.450	2.566.501	-34.598	2.516.122	-37.903
2.543.716	-27.450	2.566.689	-34.597	2.516.299	-37.904
2.543.846	-27.451	2.566.811	-34.597	2.516.505	-37.904
2.543.954	-27.451	2.566.853	-34.597	2.516.653	-37.905
2.544.145	-27.451	2.566.971	-34.596	2.516.803	-37.906
2.544.334	-27.451	2.567.097	-34.596	2.516.982	-37.907
2.544.510	-27.451	2.567.294	-34.596	2.517.137	-37.907
2.544.684	-27.451	2.567.579	-34.595	2.517.337	-37.908
2.544.850	-27.451	2.567.790	-34.595	2.517.473	-37.909
2.545.045	-27.451	2.567.947	-34.595	2.517.531	-37.909
2.545.226	-27.451	2.568.135	-34.594	2.517.760	-37.910
2.545.353	-27.451	2.568.290	-34.594	2.518.049	-37.911
2.545.531	-27.451	2.568.449	-34.594	2.518.154	-37.911
2.545.736	-27.451	2.568.630	-34.594	2.518.248	-37.912
2.545.819	-27.451	2.568.791	-34.593	2.518.472	-37.913
2.545.959	-27.451	2.568.958	-34.593	2.518.700	-37.914
2.546.105	-27.451	2.569.146	-34.593	2.518.858	-37.914

2.546.165	-27.451	2.569.322	-34.592	2.518.994	-37.915
2.546.386	-27.451	2.569.482	-34.592	2.519.184	-37.915
2.546.669	-27.451	2.569.716	-34.592	2.519.321	-37.916
2.546.881	-27.451	2.569.904	-34.591	2.519.471	-37.917
2.547.021	-27.451	2.569.989	-34.591	2.519.704	-37.917
2.547.153	-27.451	2.570.086	-34.591	2.519.883	-37.918
2.547.382	-27.451	2.570.212	-34.591	2.520.029	-37.919
2.547.561	-27.451	2.570.433	-34.590	2.520.150	-37.919
2.547.726	-27.451	2.570.635	-34.590	2.520.291	-37.920
2.547.860	-27.451	2.570.754	-34.590	2.520.479	-37.920
2.547.982	-27.451	2.570.883	-34.590	2.520.678	-37.921
2.548.175	-27.451	2.571.049	-34.589	2.520.901	-37.921
2.548.369	-27.451	2.571.224	-34.589	2.521.073	-37.922
2.548.531	-27.451	2.571.394	-34.589	2.521.214	-37.922
2.548.719	-27.451	2.571.537	-34.589	2.521.367	-37.923
2.548.896	-27.451	2.571.675	-34.589	2.521.532	-37.923
2.549.001	-27.451	2.571.870	-34.588	2.521.731	-37.924
2.549.156	-27.451	2.572.068	-34.588	2.521.864	-37.924
2.549.423	-27.451	2.572.232	-34.588	2.522.032	-37.925
2.549.590	-27.451	2.572.354	-34.588	2.522.206	-37.925
2.549.727	-27.451	2.572.528	-34.588	2.522.343	-37.926
2.549.922	-27.451	2.572.737	-34.588	2.522.569	-37.926
2.550.063	-27.451	2.572.918	-34.588	2.522.701	-37.926
2.550.146	-27.451	2.573.076	-34.588	2.522.796	-37.927
2.550.251	-27.451	2.573.251	-34.588	2.522.935	-37.927
2.550.421	-27.451	2.573.405	-34.587	2.523.082	-37.927
2.550.516	-27.451	2.573.521	-34.587	2.523.207	-37.928
2.550.595	-27.452	2.573.713	-34.587	2.523.318	-37.928
2.550.836	-27.452	2.573.929	-34.587	2.523.490	-37.928
2.551.123	-27.452	2.574.106	-34.587	2.523.719	-37.929

2.551.296	-27.452	2.574.243	-34.587	2.523.918	-37.929
2.551.443	-27.452	2.574.413	-34.587	2.524.061	-37.929
2.551.661	-27.452	2.574.569	-34.587	2.524.316	-37.929
2.551.870	-27.452	2.574.716	-34.587	2.524.565	-37.929
2.552.064	-27.452	2.574.893	-34.587	2.524.713	-37.930
2.552.250	-27.452	2.575.034	-34.587	2.524.928	-37.930
2.552.437	-27.452	2.575.157	-34.587	2.525.098	-37.930
2.552.585	-27.452	2.575.331	-34.587	2.525.248	-37.930
2.552.715	-27.452	2.575.558	-34.587	2.525.438	-37.930
2.552.863	-27.452	2.575.714	-34.587	2.525.566	-37.930
2.552.989	-27.452	2.575.854	-34.587	2.525.726	-37.930
2.553.112	-27.452	2.576.006	-34.587	2.525.884	-37.930
2.553.233	-27.452	2.576.163	-34.587	2.526.001	-37.931
2.553.387	-27.452	2.576.335	-34.587	2.526.167	-37.931
2.553.549	-27.452	2.576.458	-34.588	2.526.370	-37.931
2.553.737	-27.452	2.576.570	-34.588	2.526.473	-37.931
2.553.900	-27.453	2.576.754	-34.588	2.526.579	-37.931
2.554.005	-27.453	2.576.958	-34.588	2.526.727	-37.931
2.554.233	-27.453	2.577.167	-34.588	2.526.866	-37.931
2.554.530	-27.453	2.577.303	-34.588	2.527.045	-37.931
2.554.727	-27.453	2.577.478	-34.588	2.527.195	-37.931
2.554.883	-27.453	2.577.749	-34.588	2.527.386	-37.931
2.555.056	-27.453	2.577.931	-34.588	2.527.607	-37.931
2.555.207	-27.453	2.578.102	-34.588	2.527.793	-37.931
2.555.386	-27.453	2.578.361	-34.588	2.527.965	-37.931
2.555.585	-27.453	2.578.575	-34.588	2.528.219	-37.931
2.555.717	-27.454	2.578.673	-34.588	2.528.466	-37.931
2.555.838	-27.454	2.578.788	-34.588	2.528.570	-37.931
2.556.064	-27.454	2.578.903	-34.588	2.528.684	-37.930
2.556.271	-27.454	2.579.008	-34.589	2.528.862	-37.930

2.556.394	-27.454	2.579.122	-34.589	2.529.038	-37.930
2.556.587	-27.454	2.579.237	-34.589	2.529.227	-37.930
2.556.732	-27.454	2.579.416	-34.589	2.529.424	-37.930
2.556.812	-27.454	2.579.564	-34.589	2.529.623	-37.930
2.557.032	-27.455	2.579.706	-34.589	2.529.846	-37.930
2.557.260	-27.455	2.579.898	-34.589	2.529.970	-37.930
2.557.405	-27.455	2.580.078	-34.589	2.530.098	-37.930
2.557.607	-27.455	2.580.330	-34.589	2.530.170	-37.930
2.557.796	-27.455	2.580.576	-34.589	2.530.279	-37.930
2.557.897	-27.455	2.580.744	-34.589	2.530.539	-37.930
2.558.012	-27.456	2.580.894	-34.589	2.530.786	-37.930
2.558.236	-27.456	2.581.053	-34.589	2.530.940	-37.930
2.558.391	-27.456	2.581.221	-34.589	2.531.084	-37.930
2.558.522	-27.456	2.581.390	-34.589	2.531.274	-37.930
2.558.703	-27.456	2.581.573	-34.589	2.531.452	-37.930
2.558.889	-27.457	2.581.660	-34.589	2.531.590	-37.930
2.559.066	-27.457	2.581.797	-34.589	2.531.704	-37.930
2.559.237	-27.457	2.582.007	-34.590	2.531.862	-37.930
2.559.399	-27.457	2.582.184	-34.590	2.532.083	-37.930
2.559.550	-27.457	2.582.393	-34.590	2.532.289	-37.930
2.559.712	-27.458	2.582.577	-34.590	2.532.403	-37.930
2.559.859	-27.458	2.582.695	-34.590	2.532.554	-37.930
2.560.067	-27.458	2.582.881	-34.590	2.532.757	-37.930
2.560.421	-27.458	2.583.044	-34.590	2.532.941	-37.930
2.560.820	-27.458	2.583.264	-34.590	2.533.067	-37.930
2.561.087	-27.459	2.583.513	-34.590	2.533.252	-37.930
2.561.191	-27.459	2.583.672	-34.590	2.533.465	-37.930
2.561.226	-27.459	2.583.759	-34.590	2.533.604	-37.930
2.561.255	-27.459	2.583.869	-34.590	2.533.741	-37.930
2.561.263	-27.460	2.584.107	-34.590	2.533.902	-37.931

2.561.321	-27.460	2.584.319	-34.590	2.534.094	-37.931
2.561.457	-27.460	2.584.474	-34.590	2.534.276	-37.931
2.561.609	-27.460	2.584.655	-34.590	2.534.380	-37.931
2.561.811	-27.461	2.584.837	-34.590	2.534.487	-37.931
2.561.963	-27.461	2.584.989	-34.590	2.534.792	-37.931
2.562.148	-27.461	2.585.135	-34.590	2.535.247	-37.932
2.562.419	-27.462	2.585.284	-34.590	2.535.544	-37.932
2.562.603	-27.462	2.585.428	-34.590	2.535.616	-37.932
2.562.725	-27.462	2.585.605	-34.590	2.535.769	-37.932
2.562.861	-27.462	2.585.768	-34.590	2.535.913	-37.933
2.563.016	-27.463	2.585.869	-34.590	2.535.938	-37.933
2.563.208	-27.463	2.586.248	-34.590	2.536.018	-37.933
2.563.370	-27.463	2.586.733	-34.590	2.536.100	-37.934
2.563.510	-27.464	2.586.927	-34.591	2.536.169	-37.934
2.563.748	-27.464	2.587.017	-34.591	2.536.364	-37.935
2.563.936	-27.464	2.587.068	-34.591	2.536.540	-37.935
2.564.116	-27.464	2.587.087	-34.591	2.536.661	-37.936
2.564.279	-27.465	2.587.163	-34.591	2.536.844	-37.936
2.564.417	-27.465	2.587.289	-34.591	2.537.032	-37.937
2.564.566	-27.465	2.587.377	-34.591	2.537.198	-37.937
2.564.677	-27.466	2.587.452	-34.591	2.537.375	-37.938
2.564.843	-27.466	2.587.636	-34.591	2.537.589	-37.938
2.565.064	-27.466	2.587.853	-34.591	2.537.809	-37.939
2.565.276	-27.467	2.588.014	-34.591	2.538.018	-37.939
2.565.483	-27.467	2.588.195	-34.591	2.538.112	-37.940
2.565.626	-27.467	2.588.380	-34.591	2.538.203	-37.941
2.565.771	-27.468	2.588.561	-34.591	2.538.432	-37.942
2.565.962	-27.468	2.588.721	-34.591	2.538.604	-37.942
2.566.060	-27.468	2.588.869	-34.592	2.538.734	-37.943
2.566.122	-27.469	2.588.994	-34.592	2.538.886	-37.944

2.566.230	-27.469	2.589.171	-34.592	2.539.005	-37.945
2.566.426	-27.469	2.589.388	-34.592	2.539.174	-37.946
2.566.634	-27.470	2.589.539	-34.592	2.539.331	-37.946
2.566.855	-27.470	2.589.698	-34.592	2.539.521	-37.947
2.567.046	-27.470	2.589.926	-34.592	2.539.725	-37.948
2.567.182	-27.471	2.590.130	-34.593	2.539.839	-37.949
2.567.354	-27.471	2.590.231	-34.593	2.539.944	-37.950
2.567.506	-27.471	2.590.320	-34.593	2.540.113	-37.951
2.567.650	-27.472	2.590.479	-34.593	2.540.298	-37.952
2.567.839	-27.472	2.590.692	-34.593	2.540.502	-37.953
2.568.018	-27.472	2.590.815	-34.594	2.540.757	-37.955
2.568.214	-27.473	2.590.974	-34.594	2.540.915	-37.956
2.568.381	-27.473	2.591.228	-34.594	2.541.047	-37.957
2.568.446	-27.473	2.591.387	-34.594	2.541.215	-37.958
2.568.568	-27.474	2.591.563	-34.595	2.541.436	-37.959
2.568.756	-27.474	2.591.808	-34.595	2.541.599	-37.961
2.568.979	-27.474	2.591.967	-34.595	2.541.693	-37.962
2.569.178	-27.475	2.592.079	-34.595	2.541.840	-37.963
2.569.356	-27.475	2.592.216	-34.596	2.542.021	-37.965
2.569.548	-27.476	2.592.368	-34.596	2.542.170	-37.966
2.569.760	-27.476	2.592.563	-34.596	2.542.296	-37.967
2.569.832	-27.476	2.592.747	-34.597	2.542.491	-37.969
2.570.006	-27.477	2.592.904	-34.597	2.542.664	-37.970
2.570.259	-27.477	2.593.058	-34.598	2.542.856	-37.972
2.570.408	-27.477	2.593.218	-34.598	2.542.949	-37.973
2.570.606	-27.478	2.593.372	-34.599	2.543.083	-37.975
2.570.718	-27.478	2.593.527	-34.599	2.543.369	-37.976
2.570.844	-27.478	2.593.661	-34.599	2.543.554	-37.978
2.570.997	-27.479	2.593.832	-34.600	2.543.623	-37.980
2.571.202	-27.479	2.593.980	-34.600	2.543.803	-37.981

2.571.385	-27.480	2.594.135	-34.601	2.544.023	-37.983
2.571.540	-27.480	2.594.306	-34.602	2.544.214	-37.984
2.571.739	-27.480	2.594.488	-34.602	2.544.405	-37.986
2.571.906	-27.481	2.594.662	-34.603	2.544.533	-37.988
2.572.036	-27.481	2.594.799	-34.603	2.544.685	-37.990
2.572.207	-27.482	2.594.912	-34.604	2.544.869	-37.991
2.572.353	-27.482	2.595.027	-34.605	2.544.962	-37.993
2.572.469	-27.482	2.595.208	-34.605	2.545.128	-37.995
2.572.571	-27.483	2.595.440	-34.606	2.545.370	-37.997
2.572.709	-27.483	2.595.688	-34.607	2.545.556	-37.998
2.572.878	-27.483	2.595.854	-34.607	2.545.685	-38.000
2.573.022	-27.484	2.595.938	-34.608	2.545.881	-38.002
2.573.152	-27.484	2.596.109	-34.609	2.546.065	-38.004
2.573.275	-27.485	2.596.332	-34.609	2.546.209	-38.006
2.573.398	-27.485	2.596.473	-34.610	2.546.411	-38.008
2.573.579	-27.485	2.596.613	-34.611	2.546.631	-38.009
2.573.871	-27.486	2.596.746	-34.612	2.546.721	-38.011
2.574.125	-27.486	2.596.936	-34.613	2.546.855	-38.013
2.574.309	-27.487	2.597.195	-34.613	2.547.065	-38.015
2.574.476	-27.487	2.597.383	-34.614	2.547.221	-38.017
2.574.682	-27.488	2.597.496	-34.615	2.547.339	-38.019
2.574.859	-27.488	2.597.629	-34.616	2.547.474	-38.021
2.574.995	-27.488	2.597.831	-34.617	2.547.686	-38.023
2.575.148	-27.489	2.597.989	-34.618	2.547.857	-38.024
2.575.295	-27.489	2.598.112	-34.618	2.548.019	-38.026
2.575.464	-27.490	2.598.239	-34.619	2.548.195	-38.028
2.575.580	-27.490	2.598.383	-34.620	2.548.374	-38.030
2.575.667	-27.490	2.598.503	-34.621	2.548.503	-38.032
2.575.865	-27.491	2.598.611	-34.622	2.548.632	-38.034
2.576.078	-27.491	2.598.788	-34.623	2.548.788	-38.036

2.576.259	-27.492	2.598.948	-34.624	2.548.963	-38.037
2.576.411	-27.492	2.599.124	-34.625	2.549.185	-38.039
2.576.588	-27.493	2.599.344	-34.626	2.549.406	-38.041
2.576.784	-27.493	2.599.588	-34.627	2.549.518	-38.043
2.576.960	-27.494	2.599.835	-34.628	2.549.615	-38.045
2.577.147	-27.494	2.600.039	-34.629	2.549.747	-38.046
2.577.328	-27.495	2.600.187	-34.630	2.549.879	-38.048
2.577.530	-27.495	2.600.407	-34.630	2.550.081	-38.050
2.577.675	-27.495	2.600.598	-34.631	2.550.226	-38.052
2.577.769	-27.496	2.600.688	-34.632	2.550.323	-38.054
2.577.925	-27.496	2.600.847	-34.633	2.550.498	-38.055
2.578.145	-27.497	2.601.020	-34.634	2.550.645	-38.057
2.578.352	-27.497	2.601.176	-34.635	2.550.808	-38.059
2.578.513	-27.498	2.601.311	-34.636	2.551.013	-38.060
2.578.651	-27.498	2.601.422	-34.637	2.551.172	-38.062
2.578.795	-27.499	2.601.552	-34.638	2.551.405	-38.064
2.578.972	-27.499	2.601.740	-34.639	2.551.692	-38.066
2.579.171	-27.500	2.601.935	-34.640	2.551.917	-38.067
2.579.425	-27.500	2.602.065	-34.641	2.552.079	-38.069
2.579.590	-27.501	2.602.198	-34.642	2.552.263	-38.071
2.579.691	-27.501	2.602.416	-34.643	2.552.422	-38.072
2.579.836	-27.502	2.602.582	-34.644	2.552.535	-38.074
2.579.966	-27.502	2.602.781	-34.645	2.552.697	-38.075
2.580.162	-27.503	2.603.018	-34.646	2.552.852	-38.077
2.580.352	-27.503	2.603.127	-34.647	2.553.001	-38.078
2.580.501	-27.504	2.603.272	-34.648	2.553.213	-38.080
2.580.614	-27.504	2.603.464	-34.649	2.553.385	-38.082
2.580.800	-27.505	2.603.621	-34.649	2.553.503	-38.083
2.581.038	-27.505	2.603.795	-34.650	2.553.636	-38.085
2.581.183	-27.506	2.603.962	-34.651	2.553.755	-38.086

2.581.324	-27.507	2.604.093	-34.652	2.553.941	-38.088
2.581.501	-27.507	2.604.276	-34.653	2.554.099	-38.089
2.581.712	-27.508	2.604.467	-34.654	2.554.233	-38.090
2.581.895	-27.508	2.604.671	-34.655	2.554.384	-38.092
2.582.055	-27.509	2.604.861	-34.656	2.554.530	-38.093
2.582.221	-27.509	2.604.969	-34.657	2.554.687	-38.095
2.582.299	-27.510	2.605.179	-34.658	2.554.918	-38.096
2.582.478	-27.510	2.605.413	-34.658	2.555.180	-38.097
2.582.890	-27.511	2.605.509	-34.659	2.555.341	-38.099
2.583.307	-27.512	2.605.628	-34.660	2.555.526	-38.100
2.583.529	-27.512	2.605.840	-34.661	2.555.671	-38.101
2.583.557	-27.513	2.606.035	-34.662	2.555.797	-38.103
2.583.562	-27.513	2.606.174	-34.663	2.555.995	-38.104
2.583.674	-27.514	2.606.336	-34.664	2.556.192	-38.105
2.583.781	-27.514	2.606.461	-34.664	2.556.320	-38.107
2.583.867	-27.515	2.606.645	-34.665	2.556.490	-38.108
2.583.997	-27.516	2.606.895	-34.666	2.556.685	-38.109
2.584.154	-27.516	2.607.072	-34.667	2.556.820	-38.110
2.584.309	-27.517	2.607.196	-34.667	2.557.022	-38.112
2.584.423	-27.517	2.607.351	-34.668	2.557.252	-38.113
2.584.577	-27.518	2.607.525	-34.669	2.557.390	-38.114
2.584.836	-27.519	2.607.683	-34.670	2.557.505	-38.115
2.585.043	-27.519	2.607.824	-34.670	2.557.661	-38.116
2.585.161	-27.520	2.608.030	-34.671	2.557.823	-38.118
2.585.327	-27.521	2.608.348	-34.672	2.558.004	-38.119
2.585.475	-27.521	2.608.746	-34.673	2.558.174	-38.120
2.585.616	-27.522	2.609.042	-34.673	2.558.314	-38.121
2.585.822	-27.522	2.609.124	-34.674	2.558.491	-38.122
2.585.989	-27.523	2.609.176	-34.675	2.558.662	-38.123
2.586.121	-27.524	2.609.179	-34.675	2.558.850	-38.124

2.586.320	-27.524	2.609.247	-34.676	2.559.069	-38.126
2.586.469	-27.525	2.609.392	-34.677	2.559.240	-38.127
2.586.574	-27.526	2.609.533	-34.677	2.559.407	-38.128
2.586.721	-27.526	2.609.681	-34.678	2.559.579	-38.129
2.586.840	-27.527	2.609.768	-34.679	2.559.772	-38.130
2.587.025	-27.528	2.609.900	-34.679	2.559.942	-38.131
2.587.234	-27.528	2.610.143	-34.680	2.560.077	-38.132
2.587.466	-27.529	2.610.395	-34.680	2.560.215	-38.133
2.587.636	-27.530	2.610.555	-34.681	2.560.410	-38.135
2.587.781	-27.530	2.610.695	-34.682	2.560.605	-38.136
2.587.975	-27.531	2.610.861	-34.682	2.560.779	-38.137
2.588.220	-27.531	2.611.062	-34.683	2.560.981	-38.138
2.588.394	-27.532	2.611.251	-34.683	2.561.144	-38.139
2.588.546	-27.533	2.611.367	-34.684	2.561.273	-38.140
2.588.670	-27.533	2.611.462	-34.684	2.561.430	-38.141
2.588.806	-27.534	2.611.591	-34.685	2.561.559	-38.142
2.588.936	-27.535	2.611.764	-34.686	2.561.634	-38.143
2.589.078	-27.535	2.611.972	-34.686	2.561.855	-38.144
2.589.247	-27.536	2.612.182	-34.687	2.562.264	-38.146
2.589.406	-27.537	2.612.346	-34.687	2.562.657	-38.147
2.589.525	-27.537	2.612.494	-34.688	2.562.899	-38.148
2.589.727	-27.538	2.612.648	-34.688	2.563.026	-38.149
2.589.987	-27.539	2.612.819	-34.689	2.563.033	-38.150
2.590.164	-27.539	2.613.004	-34.689	2.563.101	-38.151
2.590.292	-27.540	2.613.188	-34.690	2.563.188	-38.152
2.590.509	-27.541	2.613.361	-34.690	2.563.235	-38.153
2.590.685	-27.542	2.613.542	-34.691	2.563.289	-38.155
2.590.750	-27.542	2.613.720	-34.692	2.563.394	-38.156
2.590.899	-27.543	2.613.840	-34.692	2.563.576	-38.157
2.591.074	-27.544	2.614.057	-34.693	2.563.795	-38.158

2.591.248	-27.544	2.614.304	-34.693	2.563.932	-38.159
2.591.389	-27.545	2.614.319	-34.694	2.564.070	-38.161
2.591.520	-27.546	2.614.391	-34.694	2.564.255	-38.162
2.591.704	-27.546	2.614.609	-34.695	2.564.406	-38.163
2.591.906	-27.547	2.614.796	-34.695	2.564.597	-38.164
2.592.112	-27.548	2.615.002	-34.696	2.564.806	-38.166
2.592.304	-27.548	2.615.162	-34.697	2.564.970	-38.167
2.592.466	-27.549	2.615.309	-34.697	2.565.122	-38.168
2.592.630	-27.550	2.615.497	-34.698	2.565.296	-38.170
2.592.756	-27.550	2.615.754	-34.698	2.565.471	-38.171
2.592.889	-27.551	2.615.930	-34.699	2.565.627	-38.172
2.593.091	-27.552	2.615.960	-34.700	2.565.779	-38.174
2.593.273	-27.552	2.616.111	-34.700	2.565.874	-38.175
2.593.351	-27.553	2.616.311	-34.701	2.566.027	-38.176
2.593.493	-27.554	2.616.484	-34.702	2.566.292	-38.178
2.593.773	-27.555	2.616.650	-34.702	2.566.476	-38.179
2.593.950	-27.555	2.616.870	-34.703	2.566.519	-38.181
2.594.080	-27.556	2.617.083	-34.703	2.566.664	-38.182
2.594.258	-27.557	2.617.171	-34.704	2.566.859	-38.184
2.594.436	-27.557	2.617.329	-34.705	2.566.972	-38.185
2.594.635	-27.558	2.617.557	-34.706	2.567.144	-38.187
2.594.799	-27.559	2.617.762	-34.706	2.567.418	-38.188
2.594.911	-27.559	2.617.870	-34.707	2.567.552	-38.190
2.595.024	-27.560	2.617.944	-34.708	2.567.726	-38.192
2.595.146	-27.561	2.618.145	-34.708	2.567.893	-38.193
2.595.244	-27.561	2.618.336	-34.709	2.568.036	-38.195
2.595.389	-27.562	2.618.479	-34.710	2.568.233	-38.197
2.595.603	-27.563	2.618.672	-34.711	2.568.491	-38.198
2.595.834	-27.563	2.618.860	-34.711	2.568.648	-38.200
2.596.036	-27.564	2.619.046	-34.712	2.568.851	-38.202

2.596.170	-27.565	2.619.201	-34.713	2.569.019	-38.203
2.596.357	-27.566	2.619.364	-34.714	2.569.194	-38.205
2.596.632	-27.566	2.619.584	-34.714	2.569.356	-38.207
2.596.873	-27.567	2.619.689	-34.715	2.569.486	-38.209
2.597.090	-27.568	2.619.774	-34.716	2.569.673	-38.211
2.597.224	-27.568	2.619.973	-34.717	2.569.832	-38.212
2.597.312	-27.569	2.620.158	-34.717	2.569.965	-38.214
2.597.476	-27.570	2.620.320	-34.718	2.570.093	-38.216
2.597.615	-27.570	2.620.443	-34.719	2.570.229	-38.218
2.597.740	-27.571	2.620.520	-34.720	2.570.397	-38.220
2.597.853	-27.572	2.620.681	-34.721	2.570.540	-38.222
2.598.015	-27.572	2.620.845	-34.721	2.570.693	-38.224
2.598.232	-27.573	2.620.970	-34.722	2.570.930	-38.226
2.598.405	-27.574	2.621.143	-34.723	2.571.154	-38.228
2.598.506	-27.575	2.621.376	-34.724	2.571.311	-38.229
2.598.597	-27.575	2.621.512	-34.725	2.571.473	-38.231
2.598.784	-27.576	2.621.729	-34.725	2.571.634	-38.233
2.598.997	-27.577	2.622.014	-34.726	2.571.765	-38.235
2.599.225	-27.577	2.622.112	-34.727	2.571.942	-38.237
2.599.417	-27.578	2.622.299	-34.728	2.572.062	-38.239
2.599.564	-27.579	2.622.527	-34.729	2.572.148	-38.241
2.599.709	-27.579	2.622.689	-34.729	2.572.310	-38.243
2.599.908	-27.580	2.622.882	-34.730	2.572.549	-38.245
2.600.061	-27.581	2.623.045	-34.731	2.572.788	-38.247
2.600.209	-27.582	2.623.146	-34.732	2.573.037	-38.249
2.600.385	-27.582	2.623.257	-34.732	2.573.244	-38.251
2.600.565	-27.583	2.623.394	-34.733	2.573.394	-38.253
2.600.758	-27.584	2.623.571	-34.734	2.573.529	-38.255
2.600.962	-27.584	2.623.699	-34.735	2.573.632	-38.258
2.601.080	-27.585	2.623.883	-34.735	2.573.778	-38.260

2.601.224	-27.586	2.624.071	-34.736	2.573.965	-38.262
2.601.324	-27.586	2.624.230	-34.737	2.574.128	-38.264
2.601.484	-27.587	2.624.453	-34.737	2.574.261	-38.266
2.601.777	-27.588	2.624.713	-34.738	2.574.427	-38.268
2.601.992	-27.589	2.624.926	-34.739	2.574.627	-38.269
2.602.141	-27.589	2.625.030	-34.740	2.574.846	-38.271
2.602.303	-27.590	2.625.177	-34.740	2.574.991	-38.273
2.602.477	-27.591	2.625.395	-34.741	2.575.137	-38.275
2.602.635	-27.591	2.625.573	-34.742	2.575.295	-38.277
2.602.772	-27.592	2.625.735	-34.742	2.575.457	-38.279
2.602.968	-27.593	2.625.888	-34.743	2.575.630	-38.281
2.603.166	-27.593	2.626.042	-34.743	2.575.800	-38.283
2.603.273	-27.594	2.626.180	-34.744	2.575.956	-38.285
2.603.457	-27.595	2.626.291	-34.745	2.576.116	-38.287
2.603.661	-27.596	2.626.484	-34.745	2.576.306	-38.288
2.603.792	-27.596	2.626.689	-34.746	2.576.488	-38.290
2.603.959	-27.597	2.626.811	-34.747	2.576.693	-38.292
2.604.193	-27.598	2.626.949	-34.747	2.576.889	-38.294
2.604.341	-27.598	2.627.133	-34.748	2.576.997	-38.296
2.604.478	-27.599	2.627.350	-34.748	2.577.076	-38.297
2.604.703	-27.600	2.627.538	-34.749	2.577.174	-38.299
2.604.824	-27.600	2.627.679	-34.749	2.577.346	-38.301
2.604.958	-27.601	2.627.846	-34.750	2.577.507	-38.302
2.605.288	-27.602	2.627.982	-34.750	2.577.614	-38.304
2.605.703	-27.603	2.628.109	-34.751	2.577.788	-38.306
2.605.984	-27.603	2.628.312	-34.751	2.578.027	-38.307
2.606.043	-27.604	2.628.484	-34.752	2.578.214	-38.309
2.606.057	-27.605	2.628.658	-34.752	2.578.380	-38.310
2.606.127	-27.605	2.628.874	-34.753	2.578.560	-38.312
2.606.187	-27.606	2.629.057	-34.753	2.578.746	-38.313

2.606.273	-27.607	2.629.251	-34.753	2.578.943	-38.315
2.606.363	-27.607	2.629.393	-34.754	2.579.138	-38.316
2.606.469	-27.608	2.629.543	-34.754	2.579.341	-38.317
2.606.668	-27.609	2.629.716	-34.755	2.579.554	-38.319
2.606.840	-27.610	2.629.904	-34.755	2.579.736	-38.320
2.607.029	-27.610	2.629.987	-34.755	2.579.850	-38.321
2.607.227	-27.611	2.630.197	-34.756	2.579.985	-38.322
2.607.375	-27.612	2.630.667	-34.756	2.580.160	-38.324
2.607.597	-27.612	2.631.028	-34.756	2.580.327	-38.325
2.607.831	-27.613	2.631.186	-34.757	2.580.424	-38.326
2.607.976	-27.614	2.631.219	-34.757	2.580.537	-38.327
2.608.148	-27.614	2.631.286	-34.757	2.580.728	-38.328
2.608.311	-27.615	2.631.361	-34.757	2.580.919	-38.329
2.608.457	-27.616	2.631.422	-34.758	2.581.068	-38.330
2.608.639	-27.616	2.631.552	-34.758	2.581.237	-38.331
2.608.755	-27.617	2.631.714	-34.758	2.581.418	-38.332
2.608.883	-27.618	2.631.830	-34.758	2.581.613	-38.333
2.609.064	-27.619	2.631.974	-34.759	2.581.765	-38.334
2.609.229	-27.619	2.632.163	-34.759	2.581.853	-38.335
2.609.377	-27.620	2.632.366	-34.759	2.582.043	-38.335
2.609.547	-27.621	2.632.570	-34.759	2.582.243	-38.336
2.609.747	-27.621	2.632.684	-34.759	2.582.343	-38.337
2.609.933	-27.622	2.632.805	-34.760	2.582.483	-38.338
2.610.090	-27.623	2.633.018	-34.760	2.582.741	-38.338
2.610.276	-27.623	2.633.282	-34.760	2.583.001	-38.339
2.610.494	-27.624	2.633.468	-34.760	2.583.167	-38.340
2.610.710	-27.625	2.633.604	-34.760	2.583.344	-38.340
2.610.854	-27.625	2.633.746	-34.760	2.583.517	-38.341
2.610.970	-27.626	2.633.831	-34.760	2.583.687	-38.341
2.611.143	-27.627	2.633.944	-34.761	2.583.828	-38.342

2.611.248	-27.627	2.634.099	-34.761	2.584.005	-38.342
2.611.415	-27.628	2.634.261	-34.761	2.584.142	-38.342
2.611.642	-27.628	2.634.434	-34.761	2.584.262	-38.343
2.611.775	-27.629	2.634.623	-34.761	2.584.490	-38.343
2.611.935	-27.630	2.634.804	-34.761	2.584.705	-38.343
2.612.100	-27.630	2.634.942	-34.761	2.584.861	-38.344
2.612.299	-27.631	2.635.125	-34.762	2.585.017	-38.344
2.612.496	-27.632	2.635.385	-34.762	2.585.187	-38.344
2.612.603	-27.632	2.635.634	-34.762	2.585.339	-38.344
2.612.747	-27.633	2.635.769	-34.762	2.585.500	-38.345
2.612.900	-27.634	2.635.847	-34.762	2.585.699	-38.345
2.613.049	-27.634	2.635.995	-34.762	2.585.885	-38.345
2.613.224	-27.635	2.636.190	-34.762	2.586.029	-38.345
2.613.381	-27.635	2.636.386	-34.763	2.586.221	-38.345
2.613.528	-27.636	2.636.551	-34.763	2.586.443	-38.345
2.613.712	-27.637	2.636.665	-34.763	2.586.629	-38.345
2.613.889	-27.637	2.636.790	-34.763	2.586.718	-38.345
2.614.063	-27.638	2.636.968	-34.763	2.586.817	-38.345
2.614.277	-27.638	2.637.141	-34.764	2.587.007	-38.345
2.614.510	-27.639	2.637.293	-34.764	2.587.100	-38.345
2.614.674	-27.640	2.637.512	-34.764	2.587.256	-38.345
2.614.840	-27.640	2.637.711	-34.764	2.587.516	-38.345
2.615.038	-27.641	2.637.849	-34.765	2.587.733	-38.345
2.615.211	-27.641	2.637.990	-34.765	2.587.919	-38.345
2.615.338	-27.642	2.638.113	-34.765	2.588.073	-38.345
2.615.471	-27.643	2.638.264	-34.766	2.588.214	-38.345
2.615.605	-27.643	2.638.388	-34.766	2.588.300	-38.345
2.615.707	-27.644	2.638.528	-34.766	2.588.481	-38.345
2.615.895	-27.644	2.638.774	-34.767	2.588.684	-38.345
2.616.076	-27.645	2.638.969	-34.767	2.588.829	-38.345

2.616.273	-27.645	2.639.142	-34.767	2.588.958	-38.345
2.616.425	-27.646	2.639.355	-34.768	2.589.169	-38.345
2.616.569	-27.647	2.639.518	-34.768	2.589.611	-38.345
2.616.828	-27.647	2.639.705	-34.769	2.589.976	-38.345
2.616.997	-27.648	2.639.837	-34.769	2.590.103	-38.344
2.617.052	-27.648	2.639.987	-34.770	2.590.219	-38.344
2.617.181	-27.649	2.640.172	-34.770	2.590.321	-38.344
2.617.336	-27.649	2.640.339	-34.771	2.590.396	-38.344
2.617.542	-27.650	2.640.479	-34.772	2.590.399	-38.344
2.617.787	-27.650	2.640.608	-34.772	2.590.482	-38.344
2.617.921	-27.651	2.640.702	-34.773	2.590.661	-38.344
2.617.996	-27.652	2.640.901	-34.774	2.590.818	-38.344
2.618.076	-27.652	2.641.209	-34.774	2.590.938	-38.344
2.618.289	-27.653	2.641.407	-34.775	2.591.064	-38.344
2.618.560	-27.653	2.641.509	-34.776	2.591.234	-38.344
2.618.745	-27.654	2.641.640	-34.777	2.591.416	-38.344
2.618.932	-27.654	2.641.806	-34.777	2.591.591	-38.344
2.619.178	-27.655	2.641.942	-34.778	2.591.750	-38.344
2.619.431	-27.655	2.642.033	-34.779	2.591.931	-38.344
2.619.605	-27.656	2.642.217	-34.780	2.592.087	-38.344
2.619.757	-27.656	2.642.393	-34.781	2.592.228	-38.345
2.619.906	-27.657	2.642.534	-34.782	2.592.423	-38.345
2.620.041	-27.657	2.642.646	-34.783	2.592.632	-38.345
2.620.183	-27.658	2.642.770	-34.784	2.592.803	-38.345
2.620.324	-27.659	2.642.953	-34.785	2.592.961	-38.345
2.620.455	-27.659	2.643.083	-34.786	2.593.150	-38.346
2.620.585	-27.660	2.643.291	-34.787	2.593.336	-38.346
2.620.721	-27.660	2.643.502	-34.788	2.593.488	-38.346
2.620.872	-27.661	2.643.633	-34.789	2.593.692	-38.347
2.621.007	-27.661	2.643.845	-34.790	2.593.820	-38.347

2.621.172	-27.662	2.644.100	-34.791	2.593.911	-38.347
2.621.379	-27.662	2.644.326	-34.793	2.594.008	-38.348
2.621.530	-27.663	2.644.471	-34.794	2.594.124	-38.348
2.621.732	-27.663	2.644.620	-34.795	2.594.333	-38.349
2.621.935	-27.664	2.644.792	-34.796	2.594.550	-38.349
2.622.113	-27.664	2.644.940	-34.798	2.594.700	-38.350
2.622.303	-27.665	2.645.129	-34.799	2.594.857	-38.351
2.622.431	-27.665	2.645.301	-34.800	2.595.089	-38.351
2.622.639	-27.666	2.645.415	-34.802	2.595.269	-38.352
2.622.888	-27.666	2.645.548	-34.803	2.595.446	-38.353
2.622.986	-27.667	2.645.674	-34.804	2.595.603	-38.354
2.623.091	-27.667	2.645.841	-34.806	2.595.765	-38.354
2.623.308	-27.668	2.646.033	-34.807	2.595.962	-38.355
2.623.510	-27.668	2.646.163	-34.809	2.596.115	-38.356
2.623.723	-27.669	2.646.361	-34.810	2.596.223	-38.357
2.623.865	-27.669	2.646.548	-34.812	2.596.354	-38.358
2.624.008	-27.670	2.646.646	-34.813	2.596.559	-38.359
2.624.161	-27.670	2.646.840	-34.815	2.596.775	-38.360
2.624.293	-27.671	2.647.104	-34.816	2.596.931	-38.361
2.624.492	-27.671	2.647.314	-34.818	2.597.039	-38.363
2.624.691	-27.672	2.647.478	-34.820	2.597.185	-38.364
2.624.858	-27.672	2.647.599	-34.821	2.597.379	-38.365
2.625.001	-27.673	2.647.798	-34.823	2.597.572	-38.366
2.625.215	-27.673	2.647.957	-34.825	2.597.752	-38.368
2.625.414	-27.674	2.648.061	-34.826	2.597.917	-38.369
2.625.511	-27.674	2.648.224	-34.828	2.597.979	-38.370
2.625.685	-27.675	2.648.424	-34.830	2.598.149	-38.372
2.625.913	-27.675	2.648.578	-34.831	2.598.392	-38.373
2.626.049	-27.676	2.648.745	-34.833	2.598.502	-38.375
2.626.158	-27.676	2.648.958	-34.835	2.598.720	-38.377

2.626.336	-27.677	2.649.089	-34.837	2.598.930	-38.378
2.626.542	-27.677	2.649.239	-34.839	2.599.034	-38.380
2.626.769	-27.678	2.649.436	-34.840	2.599.192	-38.382
2.626.942	-27.678	2.649.568	-34.842	2.599.337	-38.383
2.627.094	-27.679	2.649.653	-34.844	2.599.521	-38.385
2.627.250	-27.679	2.649.836	-34.846	2.599.765	-38.387
2.627.371	-27.680	2.650.085	-34.848	2.599.991	-38.389
2.627.637	-27.680	2.650.230	-34.850	2.600.169	-38.391
2.628.130	-27.681	2.650.356	-34.851	2.600.350	-38.393
2.628.442	-27.681	2.650.513	-34.853	2.600.562	-38.395
2.628.497	-27.682	2.650.643	-34.855	2.600.681	-38.397
2.628.574	-27.682	2.650.789	-34.857	2.600.782	-38.399
2.628.611	-27.683	2.651.038	-34.859	2.600.970	-38.401
2.628.608	-27.683	2.651.208	-34.861	2.601.121	-38.403
2.628.680	-27.684	2.651.310	-34.863	2.601.267	-38.405
2.628.795	-27.684	2.651.498	-34.865	2.601.410	-38.407
2.628.972	-27.685	2.651.743	-34.867	2.601.570	-38.409
2.629.176	-27.685	2.651.932	-34.869	2.601.755	-38.411
2.629.294	-27.686	2.652.043	-34.871	2.601.905	-38.414
2.629.467	-27.686	2.652.245	-34.873	2.602.069	-38.416
2.629.656	-27.687	2.652.621	-34.875	2.602.199	-38.418
2.629.872	-27.687	2.652.955	-34.877	2.602.364	-38.420
2.630.107	-27.688	2.653.174	-34.879	2.602.589	-38.423
2.630.245	-27.688	2.653.297	-34.881	2.602.744	-38.425
2.630.396	-27.689	2.653.354	-34.883	2.602.874	-38.427
2.630.523	-27.689	2.653.420	-34.885	2.602.968	-38.430
2.630.681	-27.690	2.653.511	-34.887	2.603.112	-38.432
2.630.883	-27.690	2.653.652	-34.889	2.603.323	-38.434
2.631.083	-27.691	2.653.803	-34.891	2.603.551	-38.437
2.631.255	-27.691	2.653.890	-34.893	2.603.683	-38.439

2.631.358	-27.692	2.654.020	-34.895	2.603.788	-38.441
2.631.463	-27.692	2.654.214	-34.897	2.603.919	-38.444
2.631.613	-27.693	2.654.402	-34.899	2.604.099	-38.446
2.631.725	-27.693	2.654.609	-34.901	2.604.255	-38.449
2.631.940	-27.694	2.654.763	-34.903	2.604.402	-38.451
2.632.174	-27.694	2.654.902	-34.905	2.604.554	-38.454
2.632.301	-27.695	2.655.064	-34.907	2.604.687	-38.456
2.632.525	-27.695	2.655.222	-34.909	2.604.837	-38.459
2.632.805	-27.696	2.655.363	-34.911	2.605.053	-38.461
2.632.971	-27.696	2.655.544	-34.913	2.605.222	-38.463
2.633.038	-27.697	2.655.743	-34.915	2.605.400	-38.466
2.633.211	-27.697	2.655.862	-34.917	2.605.660	-38.468
2.633.435	-27.698	2.656.031	-34.919	2.605.869	-38.471
2.633.550	-27.698	2.656.195	-34.921	2.606.042	-38.473
2.633.672	-27.699	2.656.364	-34.923	2.606.245	-38.476
2.633.854	-27.699	2.656.552	-34.925	2.606.502	-38.478
2.634.027	-27.700	2.656.660	-34.927	2.606.696	-38.480
2.634.179	-27.700	2.656.834	-34.929	2.606.790	-38.483
2.634.315	-27.701	2.657.007	-34.931	2.606.956	-38.485
2.634.470	-27.701	2.657.166	-34.933	2.607.142	-38.488
2.634.612	-27.701	2.657.359	-34.935	2.607.259	-38.490
2.634.789	-27.702	2.657.579	-34.937	2.607.379	-38.492
2.635.042	-27.702	2.657.765	-34.939	2.607.545	-38.495
2.635.259	-27.703	2.657.875	-34.941	2.607.704	-38.497
2.635.395	-27.703	2.658.033	-34.943	2.607.874	-38.499
2.635.529	-27.704	2.658.233	-34.945	2.608.058	-38.502
2.635.714	-27.704	2.658.409	-34.947	2.608.185	-38.504
2.635.869	-27.705	2.658.556	-34.949	2.608.287	-38.506
2.636.028	-27.705	2.658.661	-34.951	2.608.449	-38.508
2.636.198	-27.706	2.658.807	-34.952	2.608.611	-38.511

2.636.371	-27.706	2.658.983	-34.954	2.608.807	-38.513
2.636.564	-27.707	2.659.178	-34.956	2.609.008	-38.515
2.636.721	-27.707	2.659.387	-34.958	2.609.189	-38.517
2.636.877	-27.708	2.659.564	-34.960	2.609.425	-38.519
2.637.044	-27.708	2.659.734	-34.962	2.609.582	-38.522
2.637.235	-27.709	2.659.846	-34.964	2.609.695	-38.524
2.637.379	-27.709	2.659.970	-34.966	2.609.854	-38.526
2.637.545	-27.710	2.660.151	-34.968	2.609.995	-38.528
2.637.736	-27.710	2.660.309	-34.970	2.610.233	-38.530
2.637.846	-27.710	2.660.444	-34.971	2.610.419	-38.532
2.637.972	-27.711	2.660.584	-34.973	2.610.527	-38.534
2.638.170	-27.711	2.660.765	-34.975	2.610.697	-38.536
2.638.330	-27.712	2.660.963	-34.977	2.610.912	-38.538
2.638.533	-27.712	2.661.118	-34.979	2.611.123	-38.540
2.638.733	-27.713	2.661.275	-34.981	2.611.228	-38.542
2.638.856	-27.713	2.661.392	-34.983	2.611.346	-38.544
2.639.015	-27.714	2.661.604	-34.984	2.611.476	-38.546
2.639.212	-27.714	2.661.880	-34.986	2.611.642	-38.547
2.639.447	-27.715	2.662.036	-34.988	2.611.844	-38.549
2.639.609	-27.715	2.662.242	-34.990	2.612.073	-38.551
2.639.696	-27.715	2.662.431	-34.991	2.612.263	-38.553
2.639.781	-27.716	2.662.549	-34.993	2.612.423	-38.555
2.639.926	-27.716	2.662.741	-34.995	2.612.586	-38.556
2.640.053	-27.717	2.662.961	-34.997	2.612.731	-38.558
2.640.146	-27.717	2.663.112	-34.998	2.612.893	-38.560
2.640.291	-27.718	2.663.279	-35.000	2.613.043	-38.561
2.640.421	-27.718	2.663.448	-35.002	2.613.185	-38.563
2.640.591	-27.719	2.663.611	-35.004	2.613.390	-38.565
2.640.881	-27.719	2.663.795	-35.005	2.613.543	-38.566
2.641.143	-27.720	2.663.954	-35.007	2.613.741	-38.568

2.641.349	-27.720	2.664.138	-35.009	2.613.987	-38.570
2.641.519	-27.720	2.664.295	-35.010	2.614.146	-38.571
2.641.685	-27.721	2.664.431	-35.012	2.614.294	-38.573
2.641.902	-27.721	2.664.497	-35.014	2.614.447	-38.574
2.642.075	-27.722	2.664.658	-35.015	2.614.629	-38.576
2.642.210	-27.722	2.664.808	-35.017	2.614.810	-38.577
2.642.318	-27.723	2.664.911	-35.018	2.614.962	-38.578
2.642.487	-27.723	2.665.060	-35.020	2.615.135	-38.580
2.642.704	-27.724	2.665.161	-35.022	2.615.346	-38.581
2.642.917	-27.724	2.665.352	-35.023	2.615.526	-38.583
2.643.065	-27.724	2.665.562	-35.025	2.615.645	-38.584
2.643.163	-27.725	2.665.767	-35.026	2.615.819	-38.585
2.643.239	-27.725	2.665.977	-35.028	2.615.997	-38.586
2.643.356	-27.726	2.666.140	-35.029	2.616.133	-38.588
2.643.522	-27.726	2.666.387	-35.031	2.616.353	-38.589
2.643.766	-27.727	2.666.614	-35.032	2.616.712	-38.590
2.643.919	-27.727	2.666.729	-35.033	2.617.079	-38.591
2.644.063	-27.728	2.666.841	-35.035	2.617.285	-38.593
2.644.241	-27.728	2.667.072	-35.036	2.617.354	-38.594
2.644.456	-27.728	2.667.238	-35.038	2.617.419	-38.595
2.644.629	-27.729	2.667.289	-35.039	2.617.466	-38.596
2.644.825	-27.729	2.667.423	-35.040	2.617.546	-38.597
2.645.025	-27.730	2.667.629	-35.042	2.617.672	-38.598
2.645.216	-27.730	2.667.813	-35.043	2.617.766	-38.599
2.645.349	-27.731	2.667.957	-35.044	2.617.910	-38.600
2.645.475	-27.731	2.668.160	-35.045	2.618.047	-38.601
2.645.659	-27.732	2.668.356	-35.047	2.618.177	-38.602
2.645.814	-27.732	2.668.470	-35.048	2.618.300	-38.603
2.645.957	-27.733	2.668.625	-35.049	2.618.478	-38.604
2.646.161	-27.733	2.668.795	-35.050	2.618.654	-38.605

2.646.369	-27.733	2.668.966	-35.051	2.618.840	-38.606
2.646.523	-27.734	2.669.240	-35.053	2.618.957	-38.607
2.646.638	-27.734	2.669.377	-35.054	2.619.138	-38.608
2.646.787	-27.735	2.669.508	-35.055	2.619.435	-38.609
2.646.993	-27.735	2.669.781	-35.056	2.619.605	-38.610
2.647.209	-27.736	2.669.977	-35.057	2.619.717	-38.611
2.647.405	-27.736	2.670.193	-35.058	2.619.832	-38.612
2.647.574	-27.737	2.670.363	-35.059	2.619.933	-38.613
2.647.690	-27.737	2.670.476	-35.060	2.620.107	-38.614
2.647.837	-27.738	2.670.609	-35.061	2.620.335	-38.615
2.648.018	-27.738	2.670.796	-35.062	2.620.468	-38.616
2.648.193	-27.739	2.671.010	-35.063	2.620.648	-38.617
2.648.379	-27.739	2.671.137	-35.064	2.620.840	-38.618
2.648.502	-27.739	2.671.313	-35.065	2.620.984	-38.619
2.648.683	-27.740	2.671.552	-35.065	2.621.112	-38.620
2.648.987	-27.740	2.671.740	-35.066	2.621.248	-38.620
2.649.169	-27.741	2.671.928	-35.067	2.621.398	-38.621
2.649.237	-27.741	2.672.094	-35.068	2.621.473	-38.622
2.649.402	-27.742	2.672.214	-35.069	2.621.638	-38.623
2.649.548	-27.742	2.672.357	-35.069	2.621.931	-38.624
2.649.677	-27.743	2.672.524	-35.070	2.622.174	-38.625
2.649.872	-27.743	2.672.672	-35.071	2.622.357	-38.626
2.650.262	-27.744	2.672.827	-35.072	2.622.507	-38.627
2.650.710	-27.744	2.672.973	-35.072	2.622.640	-38.628
2.650.916	-27.745	2.673.119	-35.073	2.622.827	-38.629
2.650.974	-27.745	2.673.296	-35.073	2.623.023	-38.630
2.651.047	-27.745	2.673.466	-35.074	2.623.156	-38.631
2.651.166	-27.746	2.673.684	-35.075	2.623.350	-38.632
2.651.209	-27.746	2.673.890	-35.075	2.623.513	-38.632
2.651.234	-27.747	2.674.038	-35.076	2.623.645	-38.633

2.651.339	-27.747	2.674.156	-35.076	2.623.821	-38.634
2.651.472	-27.748	2.674.283	-35.077	2.624.034	-38.635
2.651.620	-27.748	2.674.503	-35.077	2.624.222	-38.636
2.651.786	-27.749	2.674.855	-35.078	2.624.348	-38.637
2.651.974	-27.749	2.675.285	-35.078	2.624.478	-38.638
2.652.166	-27.749	2.675.497	-35.078	2.624.617	-38.639
2.652.347	-27.750	2.675.509	-35.079	2.624.710	-38.640
2.652.561	-27.750	2.675.614	-35.079	2.624.888	-38.641
2.652.722	-27.751	2.675.660	-35.080	2.625.117	-38.642
2.652.879	-27.751	2.675.670	-35.080	2.625.320	-38.643
2.653.045	-27.752	2.675.786	-35.080	2.625.536	-38.645
2.653.179	-27.752	2.675.891	-35.080	2.625.739	-38.646
2.653.350	-27.753	2.676.046	-35.081	2.625.883	-38.647
2.653.467	-27.753	2.676.256	-35.081	2.626.031	-38.648
2.653.623	-27.753	2.676.433	-35.081	2.626.163	-38.649
2.653.824	-27.754	2.676.595	-35.081	2.626.267	-38.650
2.654.006	-27.754	2.676.766	-35.082	2.626.416	-38.651
2.654.161	-27.755	2.676.970	-35.082	2.626.610	-38.652
2.654.265	-27.755	2.677.188	-35.082	2.626.776	-38.653
2.654.352	-27.756	2.677.212	-35.082	2.626.895	-38.654
2.654.519	-27.756	2.677.401	-35.082	2.627.119	-38.655
2.654.781	-27.756	2.677.734	-35.082	2.627.351	-38.657
2.654.994	-27.757	2.677.866	-35.082	2.627.528	-38.658
2.655.186	-27.757	2.677.998	-35.082	2.627.700	-38.659
2.655.389	-27.758	2.678.208	-35.082	2.627.842	-38.660
2.655.587	-27.758	2.678.398	-35.082	2.628.011	-38.661
2.655.800	-27.758	2.678.500	-35.082	2.628.204	-38.662
2.655.932	-27.759	2.678.648	-35.082	2.628.348	-38.663
2.656.071	-27.759	2.678.840	-35.082	2.628.459	-38.664
2.656.248	-27.760	2.678.994	-35.082	2.628.648	-38.666

2.656.307	-27.760	2.679.077	-35.082	2.628.878	-38.667
2.656.414	-27.760	2.679.283	-35.082	2.629.019	-38.668
2.656.664	-27.761	2.679.554	-35.082	2.629.164	-38.669
2.656.878	-27.761	2.679.723	-35.082	2.629.326	-38.670
2.657.068	-27.762	2.679.855	-35.082	2.629.482	-38.671
2.657.274	-27.762	2.680.068	-35.082	2.629.630	-38.672
2.657.495	-27.762	2.680.278	-35.082	2.629.750	-38.673
2.657.643	-27.763	2.680.385	-35.082	2.629.937	-38.674
2.657.737	-27.763	2.680.545	-35.082	2.630.164	-38.676
2.657.834	-27.763	2.680.706	-35.082	2.630.316	-38.677
2.657.976	-27.764	2.680.829	-35.081	2.630.479	-38.678
2.658.161	-27.764	2.680.968	-35.081	2.630.591	-38.679
2.658.332	-27.765	2.681.115	-35.081	2.630.785	-38.680
2.658.550	-27.765	2.681.273	-35.081	2.630.988	-38.681
2.658.742	-27.765	2.681.430	-35.081	2.631.133	-38.682
2.658.936	-27.766	2.681.515	-35.081	2.631.299	-38.683
2.659.070	-27.766	2.681.650	-35.081	2.631.367	-38.684
2.659.199	-27.766	2.681.873	-35.080	2.631.494	-38.685
2.659.421	-27.767	2.682.170	-35.080	2.631.718	-38.686
2.659.594	-27.767	2.682.347	-35.080	2.631.920	-38.687
2.659.772	-27.767	2.682.437	-35.080	2.632.039	-38.688
2.659.933	-27.768	2.682.550	-35.080	2.632.076	-38.689
2.660.043	-27.768	2.682.695	-35.079	2.632.234	-38.690
2.660.198	-27.768	2.682.867	-35.079	2.632.505	-38.691
2.660.385	-27.769	2.683.058	-35.079	2.632.690	-38.692
2.660.547	-27.769	2.683.215	-35.079	2.632.819	-38.693
2.660.744	-27.769	2.683.380	-35.079	2.632.991	-38.694
2.660.910	-27.769	2.683.594	-35.079	2.633.214	-38.695
2.661.068	-27.770	2.683.728	-35.078	2.633.362	-38.696
2.661.260	-27.770	2.683.892	-35.078	2.633.601	-38.697

2.661.414	-27.770	2.684.077	-35.078	2.633.869	-38.698
2.661.563	-27.771	2.684.204	-35.078	2.633.994	-38.699
2.661.697	-27.771	2.684.389	-35.078	2.634.120	-38.700
2.661.846	-27.771	2.684.587	-35.077	2.634.323	-38.701
2.662.032	-27.771	2.684.709	-35.077	2.634.460	-38.702
2.662.181	-27.772	2.684.879	-35.077	2.634.554	-38.703
2.662.303	-27.772	2.685.036	-35.077	2.634.681	-38.704
2.662.502	-27.772	2.685.180	-35.077	2.634.854	-38.705
2.662.643	-27.773	2.685.431	-35.077	2.635.065	-38.706
2.662.766	-27.773	2.685.635	-35.076	2.635.193	-38.706
2.662.917	-27.773	2.685.832	-35.076	2.635.288	-38.707
2.663.044	-27.773	2.686.000	-35.076	2.635.435	-38.708
2.663.226	-27.774	2.686.080	-35.076	2.635.663	-38.709
2.663.438	-27.774	2.686.194	-35.076	2.635.890	-38.710
2.663.661	-27.774	2.686.408	-35.076	2.636.035	-38.711
2.663.878	-27.774	2.686.611	-35.076	2.636.174	-38.712
2.664.064	-27.775	2.686.736	-35.076	2.636.404	-38.713
2.664.178	-27.775	2.686.859	-35.076	2.636.581	-38.713
2.664.341	-27.775	2.686.978	-35.075	2.636.823	-38.714
2.664.601	-27.775	2.687.076	-35.075	2.637.069	-38.715
2.664.803	-27.776	2.687.238	-35.075	2.637.177	-38.716
2.664.942	-27.776	2.687.456	-35.075	2.637.283	-38.717
2.665.118	-27.776	2.687.615	-35.075	2.637.393	-38.718
2.665.270	-27.776	2.687.744	-35.075	2.637.599	-38.718
2.665.378	-27.777	2.687.947	-35.075	2.637.817	-38.719
2.665.494	-27.777	2.688.268	-35.075	2.637.982	-38.720
2.665.660	-27.777	2.688.488	-35.075	2.638.148	-38.721
2.665.856	-27.777	2.688.626	-35.075	2.638.383	-38.722
2.666.011	-27.777	2.688.760	-35.075	2.638.529	-38.722
2.666.165	-27.778	2.688.979	-35.075	2.638.654	-38.723

2.666.349	-27.778	2.689.174	-35.075	2.638.813	-38.724
2.666.556	-27.778	2.689.259	-35.075	2.638.998	-38.725
2.666.808	-27.778	2.689.393	-35.075	2.639.124	-38.725
2.666.987	-27.779	2.689.554	-35.075	2.639.218	-38.726
2.667.100	-27.779	2.689.647	-35.075	2.639.424	-38.727
2.667.272	-27.779	2.689.779	-35.075	2.639.736	-38.728
2.667.429	-27.779	2.689.974	-35.075	2.639.918	-38.728
2.667.617	-27.779	2.690.153	-35.075	2.640.000	-38.729
2.667.821	-27.780	2.690.313	-35.075	2.640.083	-38.730
2.667.972	-27.780	2.690.449	-35.075	2.640.265	-38.731
2.668.130	-27.780	2.690.594	-35.075	2.640.487	-38.731
2.668.267	-27.780	2.690.793	-35.075	2.640.695	-38.732
2.668.448	-27.780	2.691.028	-35.075	2.640.837	-38.733
2.668.593	-27.781	2.691.216	-35.075	2.640.932	-38.733
2.668.670	-27.781	2.691.376	-35.075	2.641.072	-38.734
2.668.868	-27.781	2.691.553	-35.075	2.641.284	-38.735
2.669.149	-27.781	2.691.685	-35.076	2.641.434	-38.735
2.669.342	-27.781	2.691.866	-35.076	2.641.602	-38.736
2.669.510	-27.782	2.692.116	-35.076	2.641.714	-38.737
2.669.709	-27.782	2.692.285	-35.076	2.641.907	-38.737
2.669.876	-27.782	2.692.415	-35.076	2.642.083	-38.738
2.669.978	-27.782	2.692.578	-35.076	2.642.235	-38.739
2.670.143	-27.782	2.692.777	-35.076	2.642.447	-38.739
2.670.367	-27.783	2.692.926	-35.077	2.642.596	-38.740
2.670.504	-27.783	2.693.041	-35.077	2.642.747	-38.740
2.670.659	-27.783	2.693.261	-35.077	2.642.896	-38.741
2.670.840	-27.783	2.693.413	-35.077	2.642.944	-38.742
2.670.964	-27.784	2.693.583	-35.077	2.643.184	-38.742
2.671.122	-27.784	2.693.829	-35.078	2.643.683	-38.743
2.671.309	-27.784	2.693.994	-35.078	2.644.077	-38.743

2.671.447	-27.784	2.694.149	-35.078	2.644.294	-38.744
2.671.587	-27.784	2.694.334	-35.078	2.644.416	-38.744
2.671.747	-27.785	2.694.517	-35.078	2.644.423	-38.745
2.671.943	-27.785	2.694.626	-35.079	2.644.493	-38.746
2.672.112	-27.785	2.694.760	-35.079	2.644.566	-38.746
2.672.188	-27.785	2.694.850	-35.079	2.644.640	-38.747
2.672.496	-27.785	2.695.049	-35.080	2.644.738	-38.747
2.672.954	-27.786	2.695.334	-35.080	2.644.859	-38.748
2.673.273	-27.786	2.695.548	-35.080	2.645.000	-38.748
2.673.438	-27.786	2.695.650	-35.080	2.645.092	-38.749
2.673.518	-27.786	2.695.782	-35.081	2.645.219	-38.749
2.673.597	-27.786	2.695.971	-35.081	2.645.428	-38.750
2.673.638	-27.787	2.696.114	-35.081	2.645.643	-38.750
2.673.690	-27.787	2.696.219	-35.082	2.645.811	-38.751
2.673.788	-27.787	2.696.357	-35.082	2.645.999	-38.751
2.673.943	-27.787	2.696.622	-35.083	2.646.151	-38.752
2.674.111	-27.788	2.697.094	-35.083	2.646.358	-38.752
2.674.315	-27.788	2.697.455	-35.083	2.646.571	-38.753
2.674.500	-27.788	2.697.525	-35.084	2.646.754	-38.753
2.674.649	-27.788	2.697.552	-35.084	2.646.920	-38.754
2.674.814	-27.788	2.697.603	-35.085	2.647.079	-38.754
2.675.009	-27.789	2.697.712	-35.085	2.647.213	-38.754
2.675.229	-27.789	2.697.769	-35.085	2.647.401	-38.755
2.675.367	-27.789	2.697.830	-35.086	2.647.564	-38.755
2.675.527	-27.789	2.697.997	-35.086	2.647.679	-38.756
2.675.696	-27.789	2.698.185	-35.087	2.647.832	-38.756
2.675.855	-27.790	2.698.362	-35.087	2.648.037	-38.757
2.676.064	-27.790	2.698.506	-35.088	2.648.167	-38.757
2.676.156	-27.790	2.698.634	-35.088	2.648.262	-38.758
2.676.241	-27.790	2.698.836	-35.089	2.648.397	-38.758

2.676.361	-27.791	2.699.053	-35.089	2.648.560	-38.759
2.676.487	-27.791	2.699.190	-35.090	2.648.755	-38.759
2.676.725	-27.791	2.699.366	-35.090	2.648.929	-38.759
2.676.949	-27.791	2.699.551	-35.091	2.649.125	-38.760
2.677.119	-27.791	2.699.717	-35.091	2.649.342	-38.760
2.677.288	-27.792	2.699.897	-35.092	2.649.472	-38.761
2.677.462	-27.792	2.700.081	-35.092	2.649.605	-38.761
2.677.590	-27.792	2.700.233	-35.093	2.649.814	-38.761
2.677.738	-27.792	2.700.367	-35.094	2.650.053	-38.762
2.677.993	-27.793	2.700.551	-35.094	2.650.161	-38.762
2.678.206	-27.793	2.700.638	-35.095	2.650.306	-38.763
2.678.286	-27.793	2.700.686	-35.095	2.650.543	-38.763
2.678.403	-27.793	2.700.883	-35.096	2.650.695	-38.763
2.678.567	-27.794	2.701.122	-35.097	2.650.804	-38.764
2.678.744	-27.794	2.701.342	-35.097	2.650.975	-38.764
2.678.897	-27.794	2.701.557	-35.098	2.651.159	-38.764
2.679.038	-27.794	2.701.719	-35.098	2.651.303	-38.765
2.679.233	-27.794	2.701.896	-35.099	2.651.437	-38.765
2.679.405	-27.795	2.702.108	-35.100	2.651.595	-38.765
2.679.557	-27.795	2.702.276	-35.100	2.651.743	-38.765
2.679.752	-27.795	2.702.406	-35.101	2.651.913	-38.766
2.679.901	-27.795	2.702.534	-35.102	2.652.116	-38.766
2.680.017	-27.796	2.702.726	-35.102	2.652.275	-38.766
2.680.223	-27.796	2.702.914	-35.103	2.652.456	-38.766
2.680.428	-27.796	2.703.087	-35.104	2.652.686	-38.767
2.680.540	-27.796	2.703.242	-35.105	2.652.812	-38.767
2.680.656	-27.797	2.703.398	-35.105	2.652.890	-38.767
2.680.881	-27.797	2.703.614	-35.106	2.652.917	-38.767
2.681.071	-27.797	2.703.792	-35.107	2.653.087	-38.767
2.681.271	-27.797	2.703.951	-35.107	2.653.356	-38.767

2.681.499	-27.798	2.704.086	-35.108	2.653.611	-38.767
2.681.647	-27.798	2.704.218	-35.109	2.653.781	-38.767
2.681.788	-27.798	2.704.400	-35.110	2.653.932	-38.768
2.681.946	-27.798	2.704.543	-35.110	2.654.064	-38.768
2.682.159	-27.799	2.704.651	-35.111	2.654.219	-38.768
2.682.321	-27.799	2.704.830	-35.112	2.654.417	-38.768
2.682.453	-27.799	2.705.049	-35.113	2.654.615	-38.767
2.682.556	-27.799	2.705.244	-35.114	2.654.799	-38.767
2.682.697	-27.800	2.705.426	-35.114	2.654.998	-38.767
2.682.906	-27.800	2.705.554	-35.115	2.655.136	-38.767
2.683.091	-27.800	2.705.728	-35.116	2.655.291	-38.767
2.683.246	-27.800	2.705.865	-35.117	2.655.480	-38.767
2.683.399	-27.801	2.706.013	-35.117	2.655.588	-38.767
2.683.614	-27.801	2.706.213	-35.118	2.655.742	-38.766
2.683.795	-27.801	2.706.421	-35.119	2.655.939	-38.766
2.683.944	-27.801	2.706.581	-35.120	2.656.090	-38.766
2.684.092	-27.802	2.706.704	-35.121	2.656.278	-38.766
2.684.211	-27.802	2.706.881	-35.121	2.656.425	-38.765
2.684.371	-27.802	2.707.035	-35.122	2.656.566	-38.765
2.684.568	-27.802	2.707.195	-35.123	2.656.694	-38.764
2.684.691	-27.803	2.707.408	-35.124	2.656.899	-38.764
2.684.851	-27.803	2.707.650	-35.125	2.657.086	-38.763
2.685.023	-27.803	2.707.820	-35.125	2.657.199	-38.763
2.685.139	-27.804	2.707.932	-35.126	2.657.390	-38.762
2.685.274	-27.804	2.708.076	-35.127	2.657.524	-38.762
2.685.465	-27.804	2.708.253	-35.128	2.657.617	-38.761
2.685.661	-27.805	2.708.376	-35.128	2.657.777	-38.761
2.685.793	-27.805	2.708.405	-35.129	2.657.924	-38.760
2.685.939	-27.805	2.708.564	-35.130	2.658.036	-38.759
2.686.161	-27.805	2.708.864	-35.131	2.658.204	-38.758

2.686.436	-27.806	2.709.035	-35.131	2.658.379	-38.758
2.686.640	-27.806	2.709.128	-35.132	2.658.517	-38.757
2.686.758	-27.806	2.709.243	-35.133	2.658.721	-38.756
2.686.932	-27.807	2.709.381	-35.134	2.658.851	-38.755
2.687.164	-27.807	2.709.558	-35.134	2.658.916	-38.754
2.687.332	-27.807	2.709.761	-35.135	2.659.111	-38.753
2.687.455	-27.808	2.709.969	-35.136	2.659.337	-38.752
2.687.629	-27.808	2.710.215	-35.136	2.659.505	-38.751
2.687.736	-27.808	2.710.464	-35.137	2.659.739	-38.750
2.687.828	-27.809	2.710.689	-35.138	2.659.955	-38.749
2.687.991	-27.809	2.710.869	-35.138	2.660.099	-38.748
2.688.141	-27.809	2.711.061	-35.139	2.660.240	-38.747
2.688.188	-27.810	2.711.197	-35.140	2.660.338	-38.746
2.688.355	-27.810	2.711.292	-35.140	2.660.588	-38.745
2.688.621	-27.810	2.711.483	-35.141	2.660.809	-38.744
2.688.810	-27.811	2.711.660	-35.141	2.660.927	-38.742
2.688.963	-27.811	2.711.725	-35.142	2.661.107	-38.741
2.689.136	-27.811	2.711.811	-35.142	2.661.278	-38.740
2.689.417	-27.812	2.711.992	-35.143	2.661.387	-38.739
2.689.691	-27.812	2.712.138	-35.143	2.661.534	-38.737
2.689.830	-27.813	2.712.296	-35.144	2.661.714	-38.736
2.689.923	-27.813	2.712.441	-35.144	2.661.949	-38.735
2.690.096	-27.813	2.712.589	-35.145	2.662.144	-38.733
2.690.244	-27.814	2.712.852	-35.145	2.662.263	-38.732
2.690.421	-27.814	2.713.083	-35.146	2.662.377	-38.730
2.690.616	-27.815	2.713.262	-35.146	2.662.487	-38.729
2.690.760	-27.815	2.713.444	-35.147	2.662.624	-38.728
2.690.906	-27.815	2.713.604	-35.147	2.662.742	-38.726
2.691.129	-27.816	2.713.752	-35.148	2.662.979	-38.725
2.691.354	-27.816	2.713.889	-35.148	2.663.218	-38.723

2.691.492	-27.817	2.714.033	-35.148	2.663.430	-38.721
2.691.617	-27.817	2.714.225	-35.149	2.663.586	-38.720
2.691.762	-27.817	2.714.431	-35.149	2.663.723	-38.718
2.691.875	-27.818	2.714.547	-35.149	2.663.918	-38.717
2.692.008	-27.818	2.714.716	-35.149	2.664.124	-38.715
2.692.243	-27.819	2.714.923	-35.150	2.664.297	-38.714
2.692.469	-27.819	2.715.104	-35.150	2.664.414	-38.712
2.692.586	-27.820	2.715.270	-35.150	2.664.532	-38.710
2.692.711	-27.820	2.715.410	-35.150	2.664.731	-38.709
2.692.954	-27.821	2.715.602	-35.151	2.664.909	-38.707
2.693.177	-27.821	2.715.760	-35.151	2.665.085	-38.705
2.693.336	-27.822	2.715.902	-35.151	2.665.263	-38.704
2.693.453	-27.822	2.716.039	-35.151	2.665.363	-38.702
2.693.572	-27.822	2.716.241	-35.151	2.665.442	-38.700
2.693.709	-27.823	2.716.378	-35.152	2.665.594	-38.699
2.693.940	-27.823	2.716.562	-35.152	2.665.791	-38.697
2.694.071	-27.824	2.716.791	-35.152	2.665.956	-38.695
2.694.210	-27.824	2.716.939	-35.152	2.666.165	-38.694
2.694.391	-27.825	2.717.105	-35.152	2.666.339	-38.692
2.694.499	-27.825	2.717.276	-35.152	2.666.443	-38.690
2.694.687	-27.826	2.717.458	-35.152	2.666.645	-38.689
2.695.100	-27.826	2.717.625	-35.152	2.666.870	-38.687
2.695.490	-27.827	2.717.835	-35.152	2.667.030	-38.685
2.695.674	-27.828	2.718.018	-35.152	2.667.167	-38.684
2.695.808	-27.828	2.718.113	-35.152	2.667.329	-38.682
2.695.847	-27.829	2.718.214	-35.152	2.667.496	-38.681
2.695.797	-27.829	2.718.416	-35.152	2.667.651	-38.679
2.695.908	-27.830	2.718.828	-35.152	2.667.827	-38.677
2.696.064	-27.830	2.719.294	-35.152	2.667.968	-38.676
2.696.165	-27.831	2.719.497	-35.152	2.668.160	-38.674

2.696.354	-27.831	2.719.535	-35.152	2.668.333	-38.673
2.696.533	-27.832	2.719.543	-35.152	2.668.446	-38.671
2.696.678	-27.832	2.719.609	-35.152	2.668.627	-38.670
2.696.844	-27.833	2.719.703	-35.152	2.668.817	-38.668
2.697.023	-27.833	2.719.739	-35.152	2.669.008	-38.667
2.697.194	-27.834	2.719.835	-35.152	2.669.192	-38.665
2.697.364	-27.835	2.720.027	-35.151	2.669.381	-38.664
2.697.554	-27.835	2.720.168	-35.151	2.669.518	-38.662
2.697.708	-27.836	2.720.341	-35.151	2.669.653	-38.661
2.697.890	-27.836	2.720.587	-35.151	2.669.792	-38.660
2.698.112	-27.837	2.720.793	-35.151	2.669.904	-38.658
2.698.282	-27.837	2.720.938	-35.151	2.670.095	-38.657
2.698.409	-27.838	2.721.046	-35.151	2.670.486	-38.656
2.698.526	-27.839	2.721.199	-35.150	2.670.872	-38.655
2.698.699	-27.839	2.721.346	-35.150	2.671.093	-38.653
2.698.907	-27.840	2.721.512	-35.150	2.671.203	-38.652
2.699.063	-27.840	2.721.693	-35.150	2.671.328	-38.651
2.699.182	-27.841	2.721.837	-35.150	2.671.465	-38.650
2.699.351	-27.842	2.722.057	-35.150	2.671.551	-38.649
2.699.521	-27.842	2.722.196	-35.150	2.671.548	-38.648
2.699.706	-27.843	2.722.301	-35.150	2.671.596	-38.647
2.699.902	-27.843	2.722.505	-35.149	2.671.752	-38.646
2.700.056	-27.844	2.722.668	-35.149	2.671.855	-38.645
2.700.269	-27.845	2.722.878	-35.149	2.671.971	-38.644
2.700.443	-27.845	2.723.011	-35.149	2.672.164	-38.643
2.700.621	-27.846	2.723.185	-35.149	2.672.380	-38.642
2.700.800	-27.846	2.723.427	-35.149	2.672.532	-38.641
2.700.910	-27.847	2.723.560	-35.149	2.672.643	-38.641
2.701.058	-27.848	2.723.676	-35.149	2.672.830	-38.640
2.701.215	-27.848	2.723.879	-35.149	2.673.065	-38.639

2.701.380	-27.849	2.724.118	-35.149	2.673.235	-38.639
2.701.486	-27.849	2.724.322	-35.149	2.673.423	-38.638
2.701.604	-27.850	2.724.500	-35.149	2.673.604	-38.638
2.701.786	-27.851	2.724.609	-35.149	2.673.723	-38.637
2.701.970	-27.851	2.724.714	-35.150	2.673.861	-38.637
2.702.142	-27.852	2.724.854	-35.150	2.674.012	-38.637
2.702.325	-27.853	2.725.039	-35.150	2.674.192	-38.636
2.702.535	-27.853	2.725.244	-35.150	2.674.370	-38.636
2.702.733	-27.854	2.725.471	-35.150	2.674.496	-38.636
2.702.888	-27.854	2.725.679	-35.151	2.674.648	-38.636
2.703.001	-27.855	2.725.791	-35.151	2.674.767	-38.636
2.703.148	-27.856	2.725.921	-35.151	2.674.919	-38.636
2.703.268	-27.856	2.726.122	-35.151	2.675.150	-38.636
2.703.376	-27.857	2.726.284	-35.152	2.675.307	-38.636
2.703.580	-27.858	2.726.387	-35.152	2.675.406	-38.636
2.703.875	-27.858	2.726.501	-35.153	2.675.554	-38.636
2.704.109	-27.859	2.726.635	-35.153	2.675.743	-38.636
2.704.268	-27.859	2.726.805	-35.154	2.675.923	-38.637
2.704.438	-27.860	2.727.036	-35.154	2.676.170	-38.637
2.704.591	-27.861	2.727.225	-35.155	2.676.394	-38.637
2.704.749	-27.861	2.727.383	-35.155	2.676.552	-38.638
2.704.887	-27.862	2.727.607	-35.156	2.676.716	-38.638
2.705.014	-27.863	2.727.834	-35.156	2.676.845	-38.639
2.705.215	-27.863	2.727.986	-35.157	2.676.999	-38.639
2.705.403	-27.864	2.728.098	-35.158	2.677.194	-38.640
2.705.548	-27.864	2.728.228	-35.159	2.677.357	-38.641
2.705.779	-27.865	2.728.387	-35.159	2.677.513	-38.641
2.706.028	-27.866	2.728.571	-35.160	2.677.720	-38.642
2.706.162	-27.866	2.728.752	-35.161	2.677.874	-38.643
2.706.230	-27.867	2.728.860	-35.162	2.678.019	-38.644

2.706.371	-27.868	2.729.010	-35.163	2.678.167	-38.645
2.706.578	-27.868	2.729.204	-35.164	2.678.298	-38.646
2.706.728	-27.869	2.729.417	-35.165	2.678.495	-38.647
2.706.848	-27.870	2.729.638	-35.166	2.678.661	-38.648
2.706.960	-27.870	2.729.724	-35.167	2.678.777	-38.649
2.707.144	-27.871	2.729.902	-35.168	2.678.934	-38.651
2.707.343	-27.871	2.730.121	-35.170	2.679.049	-38.652
2.707.415	-27.872	2.730.200	-35.171	2.679.185	-38.653
2.707.556	-27.873	2.730.314	-35.172	2.679.406	-38.655
2.707.803	-27.873	2.730.520	-35.173	2.679.649	-38.656
2.708.016	-27.874	2.730.679	-35.175	2.679.855	-38.657
2.708.149	-27.875	2.730.761	-35.176	2.679.999	-38.659
2.708.300	-27.875	2.730.909	-35.177	2.680.151	-38.661
2.708.463	-27.876	2.731.094	-35.179	2.680.291	-38.662
2.708.670	-27.877	2.731.284	-35.180	2.680.382	-38.664
2.708.937	-27.877	2.731.436	-35.182	2.680.537	-38.665
2.709.120	-27.878	2.731.559	-35.183	2.680.735	-38.667
2.709.250	-27.879	2.731.768	-35.185	2.680.934	-38.669
2.709.416	-27.879	2.731.993	-35.186	2.681.143	-38.671
2.709.619	-27.880	2.732.234	-35.188	2.681.282	-38.673
2.709.792	-27.880	2.732.473	-35.190	2.681.414	-38.674
2.709.901	-27.881	2.732.709	-35.191	2.681.629	-38.676
2.710.034	-27.882	2.732.914	-35.193	2.681.812	-38.678
2.710.204	-27.882	2.733.022	-35.195	2.681.954	-38.680
2.710.345	-27.883	2.733.088	-35.197	2.682.133	-38.682
2.710.450	-27.884	2.733.204	-35.198	2.682.256	-38.684
2.710.540	-27.884	2.733.354	-35.200	2.682.332	-38.686
2.710.688	-27.885	2.733.482	-35.202	2.682.538	-38.688
2.710.883	-27.886	2.733.633	-35.204	2.682.723	-38.690
2.711.097	-27.886	2.733.829	-35.206	2.682.809	-38.693

2.711.275	-27.887	2.733.966	-35.208	2.682.986	-38.695
2.711.494	-27.888	2.734.102	-35.210	2.683.163	-38.697
2.711.744	-27.888	2.734.308	-35.212	2.683.410	-38.699
2.711.916	-27.889	2.734.467	-35.214	2.683.640	-38.701
2.712.098	-27.890	2.734.655	-35.216	2.683.781	-38.704
2.712.260	-27.890	2.734.869	-35.218	2.683.954	-38.706
2.712.429	-27.891	2.735.093	-35.220	2.684.098	-38.708
2.712.585	-27.892	2.735.284	-35.222	2.684.204	-38.710
2.712.693	-27.892	2.735.425	-35.225	2.684.326	-38.713
2.712.871	-27.893	2.735.598	-35.227	2.684.501	-38.715
2.713.044	-27.894	2.735.809	-35.229	2.684.674	-38.717
2.713.210	-27.894	2.735.949	-35.231	2.684.782	-38.720
2.713.402	-27.895	2.736.057	-35.234	2.684.973	-38.722
2.713.637	-27.896	2.736.179	-35.236	2.685.197	-38.725
2.713.828	-27.896	2.736.300	-35.238	2.685.323	-38.727
2.713.893	-27.897	2.736.545	-35.240	2.685.450	-38.729
2.714.005	-27.898	2.736.784	-35.243	2.685.605	-38.732
2.714.176	-27.898	2.736.903	-35.245	2.685.771	-38.734
2.714.393	-27.899	2.737.014	-35.247	2.685.924	-38.737
2.714.649	-27.900	2.737.151	-35.250	2.686.118	-38.739
2.714.786	-27.901	2.737.294	-35.252	2.686.371	-38.741
2.714.877	-27.901	2.737.498	-35.255	2.686.548	-38.744
2.714.985	-27.902	2.737.769	-35.257	2.686.769	-38.746
2.715.137	-27.903	2.737.944	-35.260	2.686.993	-38.749
2.715.353	-27.903	2.738.058	-35.262	2.687.173	-38.751
2.715.548	-27.904	2.738.217	-35.264	2.687.348	-38.754
2.715.750	-27.905	2.738.430	-35.267	2.687.449	-38.756
2.715.919	-27.905	2.738.593	-35.269	2.687.610	-38.759
2.716.009	-27.906	2.738.738	-35.272	2.687.774	-38.761
2.716.226	-27.907	2.738.918	-35.274	2.687.879	-38.764

2.716.458	-27.907	2.739.084	-35.277	2.688.056	-38.766
2.716.653	-27.908	2.739.250	-35.279	2.688.217	-38.768
2.716.777	-27.909	2.739.382	-35.282	2.688.315	-38.771
2.716.887	-27.910	2.739.591	-35.284	2.688.473	-38.773
2.717.189	-27.910	2.739.818	-35.287	2.688.684	-38.776
2.717.632	-27.911	2.739.846	-35.289	2.688.875	-38.778
2.717.924	-27.912	2.739.970	-35.292	2.689.016	-38.781
2.718.049	-27.912	2.740.197	-35.294	2.689.165	-38.783
2.718.083	-27.913	2.740.464	-35.296	2.689.306	-38.786
2.718.105	-27.914	2.740.961	-35.299	2.689.453	-38.788
2.718.231	-27.914	2.741.302	-35.301	2.689.652	-38.791
2.718.307	-27.915	2.741.425	-35.304	2.689.840	-38.793
2.718.307	-27.916	2.741.497	-35.306	2.689.996	-38.795
2.718.426	-27.917	2.741.451	-35.309	2.690.146	-38.798
2.718.665	-27.917	2.741.476	-35.311	2.690.350	-38.800
2.718.825	-27.918	2.741.620	-35.313	2.690.605	-38.803
2.718.986	-27.919	2.741.754	-35.316	2.690.776	-38.805
2.719.192	-27.919	2.741.862	-35.318	2.690.905	-38.808
2.719.391	-27.920	2.741.992	-35.320	2.691.094	-38.810
2.719.601	-27.921	2.742.177	-35.323	2.691.231	-38.812
2.719.757	-27.922	2.742.416	-35.325	2.691.328	-38.815
2.719.922	-27.922	2.742.610	-35.327	2.691.501	-38.817
2.720.057	-27.923	2.742.778	-35.330	2.691.758	-38.819
2.720.237	-27.924	2.742.913	-35.332	2.691.891	-38.822
2.720.428	-27.924	2.743.044	-35.334	2.692.017	-38.824
2.720.562	-27.925	2.743.258	-35.336	2.692.239	-38.827
2.720.708	-27.926	2.743.435	-35.339	2.692.372	-38.829
2.720.914	-27.926	2.743.625	-35.341	2.692.552	-38.831
2.721.105	-27.927	2.743.806	-35.343	2.692.751	-38.834
2.721.228	-27.928	2.743.934	-35.345	2.692.932	-38.836

2.721.365	-27.929	2.744.077	-35.347	2.693.069	-38.838
2.721.508	-27.929	2.744.210	-35.349	2.693.264	-38.841
2.721.736	-27.930	2.744.353	-35.352	2.693.484	-38.843
2.721.942	-27.931	2.744.519	-35.354	2.693.589	-38.845
2.722.033	-27.931	2.744.706	-35.356	2.693.724	-38.848
2.722.220	-27.932	2.744.833	-35.358	2.693.879	-38.850
2.722.476	-27.933	2.744.955	-35.360	2.694.077	-38.852
2.722.686	-27.933	2.745.201	-35.362	2.694.279	-38.855
2.722.787	-27.934	2.745.479	-35.364	2.694.431	-38.857
2.722.859	-27.935	2.745.673	-35.366	2.694.583	-38.859
2.722.968	-27.936	2.745.778	-35.368	2.694.721	-38.862
2.723.148	-27.936	2.745.916	-35.370	2.694.908	-38.864
2.723.376	-27.937	2.746.091	-35.372	2.695.161	-38.866
2.723.551	-27.938	2.746.300	-35.374	2.695.334	-38.868
2.723.677	-27.938	2.746.444	-35.376	2.695.439	-38.871
2.723.827	-27.939	2.746.566	-35.378	2.695.562	-38.873
2.724.057	-27.940	2.746.718	-35.380	2.695.711	-38.875
2.724.215	-27.940	2.746.873	-35.382	2.695.937	-38.878
2.724.322	-27.941	2.747.036	-35.384	2.696.085	-38.880
2.724.510	-27.942	2.747.187	-35.386	2.696.210	-38.882
2.724.720	-27.942	2.747.343	-35.387	2.696.404	-38.884
2.724.850	-27.943	2.747.499	-35.389	2.696.465	-38.887
2.725.056	-27.944	2.747.610	-35.391	2.696.617	-38.889
2.725.222	-27.944	2.747.756	-35.393	2.696.990	-38.891
2.725.287	-27.945	2.748.015	-35.395	2.697.455	-38.893
2.725.465	-27.946	2.748.239	-35.397	2.697.702	-38.896
2.725.638	-27.946	2.748.419	-35.399	2.697.857	-38.898
2.725.833	-27.947	2.748.564	-35.401	2.697.943	-38.900
2.726.039	-27.948	2.748.727	-35.403	2.698.018	-38.902
2.726.147	-27.948	2.748.860	-35.404	2.698.080	-38.904

2.726.310	-27.949	2.748.976	-35.406	2.698.143	-38.907
2.726.494	-27.950	2.749.196	-35.408	2.698.294	-38.909
2.726.667	-27.950	2.749.373	-35.410	2.698.474	-38.911
2.726.887	-27.951	2.749.547	-35.412	2.698.578	-38.913
2.727.057	-27.952	2.749.767	-35.414	2.698.709	-38.916
2.727.174	-27.952	2.750.009	-35.416	2.698.865	-38.918
2.727.289	-27.953	2.750.175	-35.418	2.698.908	-38.920
2.727.458	-27.954	2.750.302	-35.420	2.698.969	-38.922
2.727.701	-27.954	2.750.424	-35.422	2.699.194	-38.924
2.727.889	-27.955	2.750.563	-35.424	2.699.476	-38.927
2.728.069	-27.956	2.750.760	-35.425	2.699.713	-38.929
2.728.244	-27.956	2.750.884	-35.427	2.699.904	-38.931
2.728.416	-27.957	2.751.079	-35.429	2.699.958	-38.933
2.728.615	-27.958	2.751.281	-35.431	2.699.978	-38.936
2.728.709	-27.958	2.751.408	-35.433	2.700.234	-38.938
2.728.889	-27.959	2.751.569	-35.435	2.700.515	-38.940
2.729.066	-27.960	2.751.801	-35.437	2.700.749	-38.942
2.729.178	-27.960	2.752.000	-35.439	2.700.921	-38.944
2.729.366	-27.961	2.752.101	-35.441	2.701.047	-38.947
2.729.517	-27.962	2.752.199	-35.443	2.701.201	-38.949
2.729.644	-27.962	2.752.314	-35.445	2.701.371	-38.951
2.729.738	-27.963	2.752.438	-35.447	2.701.548	-38.953
2.729.877	-27.964	2.752.607	-35.449	2.701.694	-38.956
2.730.095	-27.964	2.752.773	-35.451	2.701.880	-38.958
2.730.269	-27.965	2.752.863	-35.453	2.702.010	-38.960
2.730.399	-27.966	2.753.023	-35.455	2.702.123	-38.963
2.730.558	-27.966	2.753.272	-35.457	2.702.267	-38.965
2.730.764	-27.967	2.753.473	-35.460	2.702.453	-38.967
2.730.996	-27.968	2.753.659	-35.462	2.702.640	-38.969
2.731.244	-27.968	2.753.856	-35.464	2.702.859	-38.972

2.731.433	-27.969	2.754.070	-35.466	2.703.105	-38.974
2.731.624	-27.970	2.754.261	-35.468	2.703.327	-38.976
2.731.815	-27.970	2.754.417	-35.470	2.703.452	-38.978
2.731.936	-27.971	2.754.597	-35.472	2.703.578	-38.981
2.732.104	-27.972	2.754.825	-35.475	2.703.694	-38.983
2.732.294	-27.972	2.754.988	-35.477	2.703.880	-38.985
2.732.372	-27.973	2.755.093	-35.479	2.704.063	-38.988
2.732.437	-27.974	2.755.211	-35.481	2.704.201	-38.990
2.732.567	-27.974	2.755.331	-35.484	2.704.364	-38.992
2.732.726	-27.975	2.755.428	-35.486	2.704.537	-38.994
2.732.870	-27.976	2.755.574	-35.488	2.704.671	-38.997
2.733.004	-27.976	2.755.782	-35.491	2.704.786	-38.999
2.733.228	-27.977	2.755.955	-35.493	2.704.978	-39.001
2.733.475	-27.978	2.756.090	-35.495	2.705.139	-39.004
2.733.721	-27.978	2.756.277	-35.498	2.705.298	-39.006
2.733.890	-27.979	2.756.527	-35.500	2.705.562	-39.008
2.734.057	-27.980	2.756.733	-35.503	2.705.765	-39.010
2.734.243	-27.981	2.756.881	-35.505	2.705.794	-39.013
2.734.373	-27.981	2.757.073	-35.508	2.705.916	-39.015
2.734.548	-27.982	2.757.285	-35.511	2.706.108	-39.017
2.734.705	-27.983	2.757.466	-35.513	2.706.288	-39.020
2.734.854	-27.983	2.757.625	-35.516	2.706.481	-39.022
2.735.045	-27.984	2.757.765	-35.519	2.706.581	-39.024
2.735.231	-27.985	2.757.901	-35.521	2.706.823	-39.026
2.735.417	-27.986	2.758.030	-35.524	2.707.028	-39.029
2.735.587	-27.986	2.758.210	-35.527	2.707.112	-39.031
2.735.725	-27.987	2.758.362	-35.530	2.707.310	-39.033
2.735.872	-27.988	2.758.590	-35.532	2.707.455	-39.035
2.736.046	-27.988	2.758.872	-35.535	2.707.614	-39.038
2.736.190	-27.989	2.759.012	-35.538	2.707.809	-39.040

2.736.382	-27.990	2.759.080	-35.541	2.708.036	-39.042
2.736.628	-27.991	2.759.210	-35.544	2.708.294	-39.044
2.736.773	-27.991	2.759.405	-35.547	2.708.488	-39.047
2.736.935	-27.992	2.759.594	-35.550	2.708.549	-39.049
2.737.120	-27.993	2.759.799	-35.553	2.708.630	-39.051
2.737.282	-27.994	2.760.002	-35.556	2.708.831	-39.053
2.737.466	-27.994	2.760.187	-35.560	2.709.049	-39.055
2.737.589	-27.995	2.760.312	-35.563	2.709.211	-39.057
2.737.682	-27.996	2.760.413	-35.566	2.709.410	-39.059
2.737.874	-27.997	2.760.598	-35.569	2.709.550	-39.061
2.738.139	-27.997	2.760.807	-35.573	2.709.638	-39.063
2.738.338	-27.998	2.760.912	-35.576	2.709.901	-39.065
2.738.466	-27.999	2.761.035	-35.579	2.710.009	-39.067
2.738.609	-28.000	2.761.259	-35.583	2.710.053	-39.069
2.738.730	-28.001	2.761.477	-35.586	2.710.334	-39.071
2.738.878	-28.001	2.761.635	-35.590	2.710.646	-39.073
2.739.060	-28.002	2.761.711	-35.593	2.710.834	-39.075
2.739.207	-28.003	2.761.812	-35.597	2.710.826	-39.077
2.739.551	-28.004	2.761.992	-35.601	2.710.909	-39.079
2.740.046	-28.005	2.762.357	-35.604	2.711.250	-39.081
2.740.303	-28.005	2.762.759	-35.608	2.711.437	-39.083
2.740.331	-28.006	2.763.023	-35.612	2.711.551	-39.084
2.740.372	-28.007	2.763.174	-35.616	2.711.636	-39.086
2.740.453	-28.008	2.763.214	-35.619	2.711.678	-39.088
2.740.482	-28.009	2.763.264	-35.623	2.711.878	-39.090
2.740.534	-28.010	2.763.327	-35.627	2.712.083	-39.091
2.740.666	-28.010	2.763.376	-35.631	2.712.198	-39.093
2.740.775	-28.011	2.763.486	-35.635	2.712.317	-39.095
2.740.955	-28.012	2.763.687	-35.639	2.712.357	-39.096
2.741.137	-28.013	2.763.872	-35.643	2.712.632	-39.098

2.741.360	-28.014	2.764.006	-35.647	2.713.132	-39.100
2.741.588	-28.015	2.764.200	-35.651	2.713.217	-39.101
2.741.707	-28.015	2.764.454	-35.655	2.713.148	-39.103
2.741.880	-28.016	2.764.677	-35.659	2.713.349	-39.104
2.742.106	-28.017	2.764.841	-35.663	2.713.687	-39.106
2.742.278	-28.018	2.764.955	-35.668	2.714.041	-39.107
2.742.394	-28.019	2.765.068	-35.672	2.714.342	-39.109
2.742.527	-28.020	2.765.256	-35.676	2.714.407	-39.110
2.742.639	-28.021	2.765.466	-35.681	2.714.355	-39.111
2.742.806	-28.022	2.765.623	-35.685	2.714.568	-39.113
2.743.022	-28.022	2.765.729	-35.689	2.714.807	-39.114
2.743.116	-28.023	2.765.937	-35.694	2.714.905	-39.115
2.743.283	-28.024	2.766.183	-35.698	2.715.032	-39.116
2.743.514	-28.025	2.766.295	-35.703	2.715.201	-39.118
2.743.630	-28.026	2.766.400	-35.707	2.715.317	-39.119
2.743.738	-28.027	2.766.534	-35.712	2.715.422	-39.120
2.743.957	-28.028	2.766.650	-35.716	2.715.623	-39.121
2.744.178	-28.029	2.766.804	-35.721	2.715.780	-39.122
2.744.391	-28.030	2.766.999	-35.725	2.715.898	-39.124
2.744.577	-28.031	2.767.177	-35.730	2.716.151	-39.125
2.744.750	-28.032	2.767.361	-35.735	2.716.346	-39.126
2.744.989	-28.032	2.767.567	-35.739	2.716.466	-39.127
2.745.168	-28.033	2.767.724	-35.744	2.716.645	-39.128
2.745.302	-28.034	2.767.919	-35.749	2.716.809	-39.129
2.745.432	-28.035	2.768.116	-35.754	2.717.030	-39.130
2.745.562	-28.036	2.768.260	-35.759	2.717.229	-39.131
2.745.724	-28.037	2.768.367	-35.763	2.717.429	-39.132
2.745.897	-28.038	2.768.540	-35.768	2.717.633	-39.132
2.746.013	-28.039	2.768.721	-35.773	2.717.816	-39.133
2.746.136	-28.040	2.768.854	-35.778	2.717.888	-39.134

2.746.357	-28.041	2.769.046	-35.783	2.718.138	-39.135
2.746.510	-28.042	2.769.201	-35.788	2.718.417	-39.136
2.746.668	-28.043	2.769.377	-35.793	2.718.439	-39.137
2.746.855	-28.044	2.769.540	-35.799	2.718.556	-39.137
2.747.032	-28.045	2.769.680	-35.804	2.718.791	-39.138
2.747.191	-28.046	2.769.841	-35.809	2.718.940	-39.139
2.747.321	-28.047	2.769.978	-35.814	2.719.017	-39.140
2.747.470	-28.048	2.770.144	-35.819	2.719.149	-39.140
2.747.563	-28.049	2.770.305	-35.825	2.719.297	-39.141
2.747.686	-28.050	2.770.440	-35.830	2.719.543	-39.142
2.747.918	-28.051	2.770.595	-35.835	2.719.786	-39.142
2.748.173	-28.052	2.770.823	-35.841	2.719.900	-39.143
2.748.344	-28.053	2.770.981	-35.846	2.720.041	-39.144
2.748.452	-28.054	2.771.101	-35.852	2.720.209	-39.144
2.748.648	-28.055	2.771.317	-35.857	2.720.381	-39.145
2.748.892	-28.056	2.771.472	-35.863	2.720.562	-39.145
2.749.055	-28.057	2.771.628	-35.869	2.720.720	-39.146
2.749.153	-28.058	2.771.848	-35.874	2.720.905	-39.147
2.749.323	-28.059	2.772.041	-35.880	2.721.036	-39.147
2.749.481	-28.060	2.772.185	-35.886	2.721.212	-39.148
2.749.597	-28.061	2.772.353	-35.891	2.721.440	-39.148
2.749.846	-28.062	2.772.488	-35.897	2.721.627	-39.149
2.750.049	-28.063	2.772.622	-35.903	2.721.799	-39.149
2.750.177	-28.064	2.772.801	-35.909	2.721.914	-39.150
2.750.388	-28.065	2.772.966	-35.915	2.722.052	-39.150
2.750.576	-28.066	2.773.142	-35.921	2.722.267	-39.151
2.750.692	-28.067	2.773.372	-35.927	2.722.455	-39.151
2.750.836	-28.068	2.773.573	-35.933	2.722.606	-39.152
2.751.011	-28.069	2.773.683	-35.939	2.722.788	-39.152
2.751.176	-28.070	2.773.821	-35.945	2.722.939	-39.153

2.751.328	-28.071	2.773.991	-35.952	2.723.119	-39.153
2.751.477	-28.072	2.774.136	-35.958	2.723.195	-39.153
2.751.614	-28.073	2.774.266	-35.964	2.723.279	-39.154
2.751.769	-28.074	2.774.380	-35.971	2.723.554	-39.154
2.751.913	-28.075	2.774.496	-35.977	2.723.912	-39.155
2.752.017	-28.076	2.774.653	-35.984	2.724.370	-39.155
2.752.142	-28.077	2.774.806	-35.990	2.724.633	-39.156
2.752.328	-28.079	2.774.908	-35.997	2.724.714	-39.156
2.752.549	-28.080	2.775.034	-36.004	2.724.839	-39.156
2.752.766	-28.081	2.775.255	-36.011	2.724.877	-39.157
2.752.997	-28.082	2.775.527	-36.018	2.724.918	-39.157
2.753.159	-28.083	2.775.768	-36.025	2.725.014	-39.158
2.753.322	-28.084	2.775.981	-36.032	2.725.115	-39.158
2.753.578	-28.085	2.776.174	-36.039	2.725.233	-39.159
2.753.781	-28.086	2.776.328	-36.046	2.725.374	-39.159
2.753.915	-28.087	2.776.487	-36.053	2.725.523	-39.159
2.754.042	-28.088	2.776.672	-36.060	2.725.635	-39.160
2.754.171	-28.090	2.776.823	-36.068	2.725.822	-39.160
2.754.358	-28.091	2.776.929	-36.075	2.726.071	-39.161
2.754.533	-28.092	2.777.082	-36.083	2.726.253	-39.161
2.754.673	-28.093	2.777.216	-36.091	2.726.405	-39.161
2.754.818	-28.094	2.777.260	-36.098	2.726.556	-39.162
2.754.930	-28.095	2.777.448	-36.106	2.726.689	-39.162
2.755.023	-28.096	2.777.659	-36.114	2.726.866	-39.163
2.755.197	-28.098	2.777.798	-36.122	2.727.068	-39.163
2.755.397	-28.099	2.777.953	-36.131	2.727.234	-39.164
2.755.577	-28.100	2.778.159	-36.139	2.727.346	-39.164
2.755.793	-28.101	2.778.409	-36.147	2.727.498	-39.164
2.756.036	-28.102	2.778.569	-36.156	2.727.689	-39.165
2.756.206	-28.103	2.778.730	-36.164	2.727.937	-39.165

2.756.320	-28.105	2.778.896	-36.173	2.728.240	-39.166
2.756.459	-28.106	2.779.124	-36.182	2.728.372	-39.166
2.756.614	-28.107	2.779.349	-36.191	2.728.300	-39.167
2.756.798	-28.108	2.779.494	-36.200	2.728.484	-39.167
2.756.982	-28.109	2.779.630	-36.209	2.728.695	-39.168
2.757.178	-28.110	2.779.771	-36.218	2.728.735	-39.168
2.757.354	-28.112	2.779.927	-36.227	2.728.862	-39.169
2.757.531	-28.113	2.780.038	-36.237	2.729.004	-39.169
2.757.733	-28.114	2.780.186	-36.247	2.729.261	-39.170
2.757.881	-28.115	2.780.388	-36.256	2.729.460	-39.170
2.758.012	-28.116	2.780.560	-36.266	2.729.649	-39.171
2.758.142	-28.118	2.780.732	-36.276	2.729.821	-39.171
2.758.315	-28.119	2.780.928	-36.287	2.729.875	-39.172
2.758.518	-28.120	2.781.096	-36.297	2.730.168	-39.172
2.758.681	-28.121	2.781.270	-36.307	2.730.419	-39.173
2.758.904	-28.123	2.781.468	-36.318	2.730.668	-39.173
2.759.142	-28.124	2.781.569	-36.329	2.730.941	-39.174
2.759.279	-28.125	2.781.690	-36.339	2.731.002	-39.175
2.759.410	-28.126	2.781.956	-36.350	2.731.020	-39.175
2.759.579	-28.128	2.782.188	-36.361	2.731.082	-39.176
2.759.695	-28.129	2.782.336	-36.373	2.731.292	-39.176
2.759.898	-28.130	2.782.463	-36.384	2.731.474	-39.177
2.760.112	-28.131	2.782.570	-36.396	2.731.638	-39.178
2.760.269	-28.133	2.782.720	-36.407	2.731.784	-39.178
2.760.457	-28.134	2.782.928	-36.419	2.731.917	-39.179
2.760.645	-28.135	2.783.121	-36.431	2.732.119	-39.179
2.760.836	-28.136	2.783.276	-36.443	2.732.368	-39.180
2.761.003	-28.138	2.783.406	-36.456	2.732.579	-39.181
2.761.155	-28.139	2.783.578	-36.468	2.732.742	-39.181
2.761.250	-28.140	2.783.727	-36.481	2.732.907	-39.182

2.761.339	-28.142	2.783.929	-36.493	2.733.043	-39.183
2.761.535	-28.143	2.784.308	-36.506	2.733.175	-39.183
2.761.909	-28.144	2.784.699	-36.519	2.733.309	-39.184
2.762.272	-28.145	2.784.876	-36.532	2.733.437	-39.185
2.762.503	-28.147	2.784.901	-36.546	2.733.609	-39.185
2.762.608	-28.148	2.784.962	-36.559	2.733.832	-39.186
2.762.657	-28.149	2.785.016	-36.573	2.734.059	-39.187
2.762.690	-28.151	2.785.114	-36.586	2.734.298	-39.187
2.762.773	-28.152	2.785.222	-36.600	2.734.475	-39.188
2.762.914	-28.153	2.785.316	-36.614	2.734.529	-39.189
2.763.011	-28.155	2.785.484	-36.628	2.734.659	-39.189
2.763.142	-28.156	2.785.674	-36.643	2.734.901	-39.190
2.763.276	-28.157	2.785.830	-36.657	2.735.034	-39.191
2.763.394	-28.158	2.786.035	-36.672	2.735.146	-39.192
2.763.568	-28.160	2.786.239	-36.687	2.735.313	-39.192
2.763.788	-28.161	2.786.354	-36.702	2.735.476	-39.193
2.763.962	-28.162	2.786.545	-36.717	2.735.689	-39.194
2.764.150	-28.164	2.786.721	-36.732	2.735.876	-39.194
2.764.388	-28.165	2.786.824	-36.747	2.736.057	-39.195
2.764.626	-28.166	2.786.999	-36.763	2.736.234	-39.196
2.764.793	-28.168	2.787.249	-36.779	2.736.342	-39.196
2.764.880	-28.169	2.787.476	-36.794	2.736.385	-39.197
2.765.017	-28.170	2.787.646	-36.810	2.736.534	-39.197
2.765.179	-28.172	2.787.784	-36.827	2.736.795	-39.198
2.765.325	-28.173	2.787.874	-36.843	2.737.086	-39.199
2.765.424	-28.174	2.788.016	-36.859	2.737.402	-39.199
2.765.558	-28.176	2.788.192	-36.876	2.737.418	-39.200
2.765.820	-28.177	2.788.338	-36.893	2.737.429	-39.201
2.766.002	-28.178	2.788.502	-36.909	2.737.607	-39.201
2.766.118	-28.180	2.788.651	-36.926	2.737.780	-39.202

2.766.295	-28.181	2.788.800	-36.944	2.738.018	-39.202
2.766.537	-28.182	2.788.990	-36.961	2.738.174	-39.203
2.766.761	-28.183	2.789.193	-36.978	2.738.233	-39.203
2.766.981	-28.185	2.789.348	-36.996	2.738.391	-39.204
2.767.149	-28.186	2.789.551	-37.014	2.738.590	-39.204
2.767.276	-28.187	2.789.729	-37.031	2.738.708	-39.205
2.767.480	-28.189	2.789.900	-37.049	2.738.853	-39.205
2.767.625	-28.190	2.790.065	-37.067	2.739.023	-39.206
2.767.758	-28.191	2.790.151	-37.086	2.739.164	-39.206
2.767.904	-28.193	2.790.240	-37.104	2.739.317	-39.207
2.768.033	-28.194	2.790.428	-37.122	2.739.467	-39.207
2.768.170	-28.195	2.790.617	-37.141	2.739.700	-39.208
2.768.348	-28.196	2.790.815	-37.160	2.739.918	-39.208
2.768.510	-28.198	2.791.002	-37.178	2.740.077	-39.209
2.768.668	-28.199	2.791.136	-37.197	2.740.292	-39.209
2.768.831	-28.200	2.791.245	-37.216	2.740.580	-39.209
2.768.976	-28.202	2.791.400	-37.235	2.740.801	-39.210
2.769.160	-28.203	2.791.592	-37.255	2.740.944	-39.210
2.769.308	-28.204	2.791.703	-37.274	2.741.096	-39.211
2.769.490	-28.205	2.791.856	-37.293	2.741.244	-39.211
2.769.673	-28.207	2.792.019	-37.313	2.741.400	-39.211
2.769.825	-28.208	2.792.145	-37.332	2.741.508	-39.212
2.769.980	-28.209	2.792.398	-37.352	2.741.649	-39.212
2.770.135	-28.211	2.792.636	-37.372	2.741.784	-39.212
2.770.302	-28.212	2.792.777	-37.391	2.741.954	-39.213
2.770.525	-28.213	2.792.914	-37.411	2.742.126	-39.213
2.770.684	-28.214	2.793.084	-37.431	2.742.260	-39.213
2.770.838	-28.216	2.793.240	-37.451	2.742.375	-39.214
2.770.995	-28.217	2.793.372	-37.471	2.742.498	-39.214
2.771.116	-28.218	2.793.564	-37.491	2.742.626	-39.214

2.771.318	-28.219	2.793.703	-37.511	2.742.809	-39.215
2.771.527	-28.220	2.793.860	-37.531	2.743.033	-39.215
2.771.741	-28.222	2.794.052	-37.551	2.743.206	-39.215
2.771.882	-28.223	2.794.163	-37.571	2.743.432	-39.215
2.771.971	-28.224	2.794.348	-37.591	2.743.633	-39.216
2.772.113	-28.225	2.794.638	-37.611	2.743.703	-39.216
2.772.308	-28.227	2.794.776	-37.631	2.743.802	-39.216
2.772.505	-28.228	2.794.937	-37.652	2.744.009	-39.217
2.772.719	-28.229	2.795.161	-37.672	2.744.223	-39.217
2.772.928	-28.230	2.795.285	-37.692	2.744.453	-39.217
2.773.119	-28.231	2.795.440	-37.712	2.744.641	-39.217
2.773.239	-28.233	2.795.630	-37.732	2.744.793	-39.218
2.773.293	-28.234	2.795.755	-37.752	2.744.995	-39.218
2.773.450	-28.235	2.795.822	-37.772	2.745.158	-39.218
2.773.629	-28.236	2.795.926	-37.792	2.745.314	-39.219
2.773.727	-28.237	2.796.058	-37.812	2.745.457	-39.219
2.773.821	-28.239	2.796.176	-37.832	2.745.592	-39.219
2.774.005	-28.240	2.796.361	-37.852	2.745.771	-39.220
2.774.190	-28.241	2.796.585	-37.872	2.746.013	-39.220
2.774.384	-28.242	2.796.772	-37.892	2.746.234	-39.220
2.774.530	-28.243	2.796.928	-37.911	2.746.404	-39.221
2.774.613	-28.244	2.797.115	-37.931	2.746.512	-39.221
2.774.792	-28.246	2.797.375	-37.950	2.746.606	-39.221
2.775.016	-28.247	2.797.633	-37.970	2.746.824	-39.222
2.775.242	-28.248	2.797.849	-37.989	2.746.985	-39.222
2.775.464	-28.249	2.797.996	-38.009	2.747.148	-39.222
2.775.735	-28.250	2.798.134	-38.028	2.747.347	-39.223
2.775.974	-28.251	2.798.276	-38.047	2.747.513	-39.223
2.776.132	-28.253	2.798.384	-38.066	2.747.675	-39.223
2.776.307	-28.254	2.798.509	-38.085	2.747.794	-39.224

2.776.445	-28.255	2.798.629	-38.104	2.747.907	-39.224
2.776.591	-28.256	2.798.723	-38.123	2.748.145	-39.225
2.776.736	-28.257	2.798.897	-38.141	2.748.280	-39.225
2.776.864	-28.258	2.799.142	-38.160	2.748.311	-39.225
2.776.999	-28.259	2.799.263	-38.179	2.748.752	-39.226
2.777.173	-28.260	2.799.388	-38.197	2.748.983	-39.226
2.777.321	-28.262	2.799.540	-38.215	2.748.994	-39.227
2.777.382	-28.263	2.799.749	-38.233	2.749.258	-39.227
2.777.487	-28.264	2.799.940	-38.251	2.749.490	-39.228
2.777.672	-28.265	2.800.162	-38.269	2.749.613	-39.228
2.777.896	-28.266	2.800.400	-38.287	2.749.721	-39.229
2.778.083	-28.267	2.800.580	-38.305	2.749.837	-39.229
2.778.244	-28.268	2.800.740	-38.322	2.749.969	-39.230
2.778.420	-28.269	2.800.909	-38.340	2.750.309	-39.230
2.778.640	-28.271	2.801.014	-38.357	2.750.500	-39.231
2.778.878	-28.272	2.801.141	-38.374	2.750.993	-39.231
2.779.042	-28.273	2.801.351	-38.391	2.751.325	-39.232
2.779.172	-28.274	2.801.534	-38.408	2.751.191	-39.233
2.779.330	-28.275	2.801.743	-38.425	2.751.051	-39.233
2.779.492	-28.276	2.801.900	-38.442	2.751.201	-39.234
2.779.641	-28.277	2.802.012	-38.458	2.751.379	-39.234
2.779.825	-28.278	2.802.198	-38.475	2.751.526	-39.235
2.780.056	-28.279	2.802.350	-38.491	2.751.609	-39.236
2.780.240	-28.280	2.802.521	-38.507	2.751.740	-39.236
2.780.384	-28.282	2.802.744	-38.523	2.751.981	-39.237
2.780.547	-28.283	2.802.914	-38.539	2.752.050	-39.238
2.780.703	-28.284	2.803.092	-38.555	2.752.192	-39.238
2.780.833	-28.285	2.803.302	-38.570	2.752.462	-39.239
2.781.039	-28.286	2.803.466	-38.586	2.752.642	-39.240
2.781.259	-28.287	2.803.582	-38.601	2.752.770	-39.240

2.781.383	-28.288	2.803.749	-38.616	2.752.975	-39.241
2.781.526	-28.289	2.803.926	-38.631	2.753.164	-39.242
2.781.639	-28.290	2.804.066	-38.646	2.753.363	-39.243
2.781.833	-28.291	2.804.240	-38.661	2.753.471	-39.244
2.782.097	-28.292	2.804.470	-38.675	2.753.551	-39.245
2.782.265	-28.294	2.804.671	-38.690	2.753.744	-39.245
2.782.409	-28.295	2.804.788	-38.704	2.753.923	-39.246
2.782.554	-28.296	2.804.905	-38.718	2.754.167	-39.247
2.782.751	-28.297	2.805.088	-38.732	2.754.277	-39.248
2.782.935	-28.298	2.805.249	-38.746	2.754.324	-39.249
2.783.043	-28.299	2.805.361	-38.759	2.754.544	-39.250
2.783.181	-28.300	2.805.598	-38.773	2.754.781	-39.251
2.783.394	-28.301	2.806.046	-38.786	2.755.006	-39.252
2.783.615	-28.302	2.806.412	-38.799	2.755.133	-39.253
2.783.717	-28.303	2.806.519	-38.812	2.755.233	-39.254
2.783.904	-28.304	2.806.591	-38.825	2.755.396	-39.255
2.784.344	-28.306	2.806.628	-38.838	2.755.554	-39.257
2.784.695	-28.307	2.806.593	-38.850	2.755.735	-39.258
2.784.815	-28.308	2.806.668	-38.863	2.755.924	-39.259
2.784.886	-28.309	2.806.835	-38.875	2.756.096	-39.260
2.784.981	-28.310	2.807.000	-38.887	2.756.292	-39.261
2.785.038	-28.311	2.807.145	-38.899	2.756.458	-39.263
2.785.129	-28.312	2.807.304	-38.910	2.756.643	-39.264
2.785.242	-28.313	2.807.534	-38.922	2.756.846	-39.265
2.785.327	-28.314	2.807.737	-38.933	2.757.032	-39.266
2.785.476	-28.315	2.807.834	-38.944	2.757.180	-39.268
2.785.639	-28.316	2.807.979	-38.956	2.757.312	-39.269
2.785.775	-28.318	2.808.247	-38.966	2.757.453	-39.270
2.785.948	-28.319	2.808.442	-38.977	2.757.592	-39.272
2.786.134	-28.320	2.808.565	-38.988	2.757.807	-39.273

2.786.356	-28.321	2.808.721	-38.998	2.757.951	-39.275
2.786.585	-28.322	2.808.856	-39.009	2.758.066	-39.276
2.786.743	-28.323	2.808.986	-39.019	2.758.278	-39.277
2.786.902	-28.324	2.809.145	-39.029	2.758.438	-39.279
2.787.065	-28.325	2.809.312	-39.039	2.758.668	-39.280
2.787.253	-28.326	2.809.479	-39.048	2.758.872	-39.282
2.787.388	-28.327	2.809.630	-39.058	2.758.940	-39.283
2.787.492	-28.329	2.809.800	-39.068	2.758.991	-39.285
2.787.661	-28.330	2.809.935	-39.077	2.759.142	-39.286
2.787.835	-28.331	2.810.061	-39.086	2.759.370	-39.288
2.788.013	-28.332	2.810.291	-39.095	2.759.606	-39.290
2.788.156	-28.333	2.810.522	-39.104	2.759.810	-39.291
2.788.304	-28.334	2.810.670	-39.113	2.759.909	-39.293
2.788.466	-28.335	2.810.858	-39.122	2.760.034	-39.294
2.788.676	-28.336	2.811.039	-39.130	2.760.237	-39.296
2.788.865	-28.337	2.811.156	-39.139	2.760.389	-39.298
2.789.045	-28.339	2.811.299	-39.147	2.760.576	-39.299
2.789.222	-28.340	2.811.548	-39.155	2.760.764	-39.301
2.789.331	-28.341	2.811.744	-39.163	2.760.908	-39.303
2.789.530	-28.342	2.811.851	-39.171	2.761.105	-39.304
2.789.778	-28.343	2.811.949	-39.179	2.761.286	-39.306
2.789.882	-28.344	2.812.122	-39.187	2.761.491	-39.308
2.789.995	-28.345	2.812.282	-39.194	2.761.725	-39.310
2.790.157	-28.346	2.812.424	-39.202	2.761.801	-39.311
2.790.313	-28.347	2.812.590	-39.210	2.761.859	-39.313
2.790.494	-28.349	2.812.787	-39.217	2.762.068	-39.315
2.790.620	-28.350	2.813.002	-39.224	2.762.254	-39.316
2.790.822	-28.351	2.813.127	-39.231	2.762.440	-39.318
2.791.004	-28.352	2.813.250	-39.238	2.762.635	-39.320
2.791.155	-28.353	2.813.423	-39.245	2.762.810	-39.321

2.791.358	-28.354	2.813.524	-39.252	2.762.993	-39.323
2.791.451	-28.355	2.813.655	-39.259	2.763.124	-39.325
2.791.625	-28.356	2.813.885	-39.266	2.763.285	-39.326
2.791.859	-28.358	2.814.091	-39.273	2.763.474	-39.328
2.792.057	-28.359	2.814.281	-39.279	2.763.656	-39.330
2.792.238	-28.360	2.814.453	-39.286	2.763.820	-39.332
2.792.354	-28.361	2.814.598	-39.292	2.763.908	-39.333
2.792.448	-28.362	2.814.753	-39.299	2.764.070	-39.335
2.792.614	-28.363	2.814.893	-39.305	2.764.323	-39.337
2.792.817	-28.364	2.815.110	-39.311	2.764.500	-39.338
2.792.968	-28.365	2.815.331	-39.317	2.764.652	-39.340
2.793.134	-28.367	2.815.425	-39.323	2.764.828	-39.341
2.793.304	-28.368	2.815.548	-39.329	2.764.966	-39.343
2.793.470	-28.369	2.815.767	-39.335	2.765.059	-39.345
2.793.647	-28.370	2.815.982	-39.341	2.765.197	-39.346
2.793.838	-28.371	2.816.126	-39.347	2.765.379	-39.348
2.793.991	-28.372	2.816.275	-39.353	2.765.522	-39.350
2.794.110	-28.373	2.816.469	-39.359	2.765.696	-39.351
2.794.301	-28.375	2.816.599	-39.364	2.765.902	-39.353
2.794.494	-28.376	2.816.707	-39.370	2.766.024	-39.354
2.794.688	-28.377	2.816.899	-39.375	2.766.145	-39.356
2.794.906	-28.378	2.817.082	-39.381	2.766.354	-39.357
2.795.039	-28.379	2.817.231	-39.386	2.766.530	-39.359
2.795.162	-28.380	2.817.361	-39.392	2.766.692	-39.360
2.795.367	-28.381	2.817.520	-39.397	2.766.924	-39.362
2.795.529	-28.383	2.817.719	-39.402	2.767.187	-39.363
2.795.682	-28.384	2.817.832	-39.407	2.767.416	-39.365
2.795.809	-28.385	2.817.951	-39.413	2.767.528	-39.366
2.795.928	-28.386	2.818.102	-39.418	2.767.639	-39.368
2.796.136	-28.387	2.818.286	-39.423	2.767.817	-39.369

2.796.281	-28.388	2.818.486	-39.428	2.767.955	-39.371
2.796.360	-28.390	2.818.656	-39.433	2.768.098	-39.372
2.796.484	-28.391	2.818.856	-39.437	2.768.221	-39.373
2.796.656	-28.392	2.819.088	-39.442	2.768.420	-39.375
2.796.831	-28.393	2.819.334	-39.447	2.768.569	-39.376
2.796.996	-28.394	2.819.523	-39.452	2.768.640	-39.377
2.797.061	-28.396	2.819.680	-39.456	2.768.795	-39.379
2.797.281	-28.397	2.819.888	-39.461	2.768.986	-39.380
2.797.595	-28.398	2.820.070	-39.466	2.769.161	-39.381
2.797.835	-28.399	2.820.177	-39.470	2.769.319	-39.383
2.798.047	-28.400	2.820.291	-39.475	2.769.505	-39.384
2.798.199	-28.402	2.820.428	-39.479	2.769.729	-39.385
2.798.330	-28.403	2.820.565	-39.484	2.769.935	-39.387
2.798.485	-28.404	2.820.724	-39.488	2.770.053	-39.388
2.798.651	-28.405	2.820.862	-39.492	2.770.215	-39.389
2.798.799	-28.406	2.820.944	-39.497	2.770.493	-39.390
2.798.961	-28.408	2.821.103	-39.501	2.770.707	-39.391
2.799.066	-28.409	2.821.325	-39.505	2.770.807	-39.393
2.799.176	-28.410	2.821.541	-39.509	2.770.938	-39.394
2.799.409	-28.411	2.821.758	-39.514	2.771.122	-39.395
2.799.634	-28.413	2.821.938	-39.518	2.771.320	-39.396
2.799.763	-28.414	2.822.077	-39.522	2.771.537	-39.397
2.799.875	-28.415	2.822.246	-39.526	2.771.645	-39.398
2.800.039	-28.416	2.822.451	-39.530	2.771.781	-39.399
2.800.182	-28.417	2.822.617	-39.534	2.772.018	-39.401
2.800.381	-28.419	2.822.734	-39.538	2.772.166	-39.402
2.800.657	-28.420	2.822.850	-39.542	2.772.341	-39.403
2.800.832	-28.421	2.823.065	-39.546	2.772.516	-39.404
2.800.956	-28.423	2.823.220	-39.550	2.772.647	-39.405
2.801.125	-28.424	2.823.369	-39.554	2.772.789	-39.406

2.801.289	-28.425	2.823.586	-39.558	2.772.975	-39.407
2.801.451	-28.426	2.823.786	-39.561	2.773.185	-39.408
2.801.628	-28.428	2.823.974	-39.565	2.773.373	-39.409
2.801.801	-28.429	2.824.185	-39.569	2.773.575	-39.410
2.802.005	-28.430	2.824.380	-39.573	2.773.717	-39.411
2.802.188	-28.432	2.824.503	-39.577	2.773.875	-39.412
2.802.348	-28.433	2.824.624	-39.581	2.774.062	-39.413
2.802.498	-28.434	2.824.790	-39.584	2.774.221	-39.414
2.802.632	-28.436	2.824.937	-39.588	2.774.388	-39.415
2.802.751	-28.437	2.825.082	-39.592	2.774.546	-39.416
2.802.888	-28.438	2.825.165	-39.596	2.774.656	-39.417
2.803.088	-28.439	2.825.327	-39.600	2.774.819	-39.418
2.803.286	-28.441	2.825.608	-39.603	2.775.012	-39.419
2.803.452	-28.442	2.825.782	-39.607	2.775.177	-39.420
2.803.643	-28.444	2.825.930	-39.611	2.775.331	-39.421
2.803.827	-28.445	2.826.111	-39.615	2.775.466	-39.422
2.803.979	-28.446	2.826.266	-39.618	2.775.634	-39.422
2.804.157	-28.448	2.826.383	-39.622	2.775.836	-39.423
2.804.319	-28.449	2.826.559	-39.626	2.776.031	-39.424
2.804.479	-28.450	2.826.757	-39.630	2.776.181	-39.425
2.804.617	-28.452	2.826.892	-39.633	2.776.299	-39.426
2.804.757	-28.453	2.827.010	-39.637	2.776.466	-39.427
2.804.902	-28.454	2.827.271	-39.641	2.776.599	-39.428
2.805.056	-28.456	2.827.759	-39.645	2.776.880	-39.429
2.805.262	-28.457	2.828.167	-39.648	2.777.300	-39.430
2.805.480	-28.459	2.828.257	-39.652	2.777.617	-39.431
2.805.639	-28.460	2.828.242	-39.656	2.777.843	-39.432
2.805.760	-28.461	2.828.302	-39.660	2.777.932	-39.433
2.805.950	-28.463	2.828.372	-39.663	2.777.986	-39.434
2.806.375	-28.464	2.828.426	-39.667	2.777.991	-39.435

2.806.888	-28.466	2.828.562	-39.671	2.778.044	-39.436
2.807.082	-28.467	2.828.741	-39.675	2.778.236	-39.437
2.807.108	-28.469	2.828.871	-39.679	2.778.376	-39.438
2.807.216	-28.470	2.829.023	-39.683	2.778.482	-39.439
2.807.245	-28.471	2.829.176	-39.686	2.778.625	-39.440
2.807.249	-28.473	2.829.359	-39.690	2.778.752	-39.441
2.807.357	-28.474	2.829.586	-39.694	2.778.915	-39.442
2.807.469	-28.476	2.829.746	-39.698	2.779.124	-39.443
2.807.619	-28.477	2.829.929	-39.702	2.779.344	-39.445
2.807.834	-28.479	2.830.159	-39.706	2.779.492	-39.446
2.808.000	-28.480	2.830.302	-39.710	2.779.626	-39.447
2.808.167	-28.482	2.830.381	-39.714	2.779.854	-39.448
2.808.384	-28.483	2.830.527	-39.718	2.780.064	-39.449
2.808.568	-28.485	2.830.699	-39.722	2.780.182	-39.450
2.808.694	-28.486	2.830.879	-39.726	2.780.307	-39.452
2.808.857	-28.488	2.831.004	-39.730	2.780.453	-39.453
2.809.075	-28.489	2.831.132	-39.734	2.780.630	-39.454
2.809.243	-28.491	2.831.288	-39.738	2.780.845	-39.456
2.809.388	-28.492	2.831.411	-39.742	2.780.999	-39.457
2.809.504	-28.494	2.831.585	-39.746	2.781.129	-39.458
2.809.613	-28.495	2.831.761	-39.750	2.781.296	-39.460
2.809.793	-28.497	2.831.972	-39.754	2.781.512	-39.461
2.809.953	-28.498	2.832.202	-39.758	2.781.694	-39.462
2.810.104	-28.500	2.832.401	-39.762	2.781.799	-39.464
2.810.251	-28.501	2.832.603	-39.766	2.781.953	-39.465
2.810.368	-28.503	2.832.787	-39.771	2.782.126	-39.467
2.810.516	-28.504	2.832.968	-39.775	2.782.263	-39.468
2.810.740	-28.506	2.833.025	-39.779	2.782.422	-39.470
2.810.960	-28.507	2.833.164	-39.783	2.782.610	-39.472
2.811.166	-28.509	2.833.402	-39.788	2.782.767	-39.473

2.811.306	-28.510	2.833.532	-39.792	2.782.932	-39.475
2.811.487	-28.512	2.833.670	-39.796	2.783.134	-39.476
2.811.718	-28.513	2.833.820	-39.800	2.783.265	-39.478
2.811.794	-28.515	2.834.052	-39.805	2.783.441	-39.480
2.811.920	-28.517	2.834.269	-39.809	2.783.640	-39.482
2.812.120	-28.518	2.834.440	-39.814	2.783.806	-39.483
2.812.279	-28.520	2.834.576	-39.818	2.783.979	-39.485
2.812.476	-28.521	2.834.680	-39.822	2.784.102	-39.487
2.812.632	-28.523	2.834.868	-39.827	2.784.240	-39.489
2.812.773	-28.524	2.835.028	-39.831	2.784.395	-39.491
2.812.928	-28.526	2.835.150	-39.836	2.784.519	-39.493
2.813.116	-28.528	2.835.313	-39.840	2.784.667	-39.494
2.813.311	-28.529	2.835.522	-39.845	2.784.837	-39.496
2.813.493	-28.531	2.835.695	-39.849	2.785.017	-39.498
2.813.680	-28.532	2.835.894	-39.854	2.785.207	-39.500
2.813.821	-28.534	2.836.069	-39.859	2.785.448	-39.502
2.813.958	-28.535	2.836.201	-39.863	2.785.645	-39.504
2.814.129	-28.537	2.836.392	-39.868	2.785.815	-39.507
2.814.302	-28.539	2.836.610	-39.872	2.785.942	-39.509
2.814.402	-28.540	2.836.759	-39.877	2.786.104	-39.511
2.814.561	-28.542	2.836.855	-39.882	2.786.281	-39.513
2.814.807	-28.543	2.837.012	-39.886	2.786.376	-39.515
2.815.038	-28.545	2.837.182	-39.891	2.786.516	-39.517
2.815.218	-28.547	2.837.299	-39.896	2.786.765	-39.519
2.815.396	-28.548	2.837.466	-39.900	2.786.981	-39.522
2.815.573	-28.550	2.837.639	-39.905	2.787.094	-39.524
2.815.671	-28.551	2.837.849	-39.910	2.787.270	-39.526
2.815.804	-28.553	2.838.059	-39.915	2.787.491	-39.528
2.816.026	-28.555	2.838.171	-39.919	2.787.693	-39.531
2.816.259	-28.556	2.838.322	-39.924	2.787.892	-39.533

2.816.418	-28.558	2.838.568	-39.929	2.788.045	-39.535
2.816.530	-28.559	2.838.697	-39.934	2.788.181	-39.538
2.816.669	-28.561	2.838.832	-39.938	2.788.376	-39.540
2.816.783	-28.563	2.839.030	-39.943	2.788.506	-39.542
2.816.924	-28.564	2.839.161	-39.948	2.788.668	-39.545
2.817.065	-28.566	2.839.290	-39.953	2.788.889	-39.547
2.817.276	-28.567	2.839.420	-39.957	2.789.056	-39.550
2.817.509	-28.569	2.839.548	-39.962	2.789.228	-39.552
2.817.737	-28.571	2.839.669	-39.967	2.789.342	-39.554
2.817.899	-28.572	2.839.814	-39.972	2.789.442	-39.557
2.817.968	-28.574	2.839.980	-39.976	2.789.664	-39.559
2.818.112	-28.576	2.840.144	-39.981	2.789.890	-39.561
2.818.221	-28.577	2.840.388	-39.986	2.790.034	-39.564
2.818.341	-28.579	2.840.671	-39.990	2.790.139	-39.566
2.818.538	-28.581	2.840.838	-39.995	2.790.260	-39.569
2.818.692	-28.582	2.841.062	-40.000	2.790.450	-39.571
2.818.806	-28.584	2.841.304	-40.005	2.790.648	-39.573
2.819.026	-28.585	2.841.434	-40.009	2.790.757	-39.576
2.819.230	-28.587	2.841.604	-40.014	2.790.905	-39.578
2.819.335	-28.589	2.841.736	-40.019	2.791.096	-39.580
2.819.515	-28.590	2.841.830	-40.023	2.791.252	-39.583
2.819.742	-28.592	2.841.974	-40.028	2.791.383	-39.585
2.820.006	-28.594	2.842.101	-40.032	2.791.523	-39.587
2.820.236	-28.595	2.842.270	-40.037	2.791.692	-39.590
2.820.422	-28.597	2.842.520	-40.041	2.791.820	-39.592
2.820.606	-28.599	2.842.675	-40.046	2.791.935	-39.594
2.820.668	-28.600	2.842.705	-40.050	2.792.054	-39.597
2.820.793	-28.602	2.842.870	-40.055	2.792.252	-39.599
2.820.956	-28.604	2.843.139	-40.059	2.792.397	-39.601
2.821.078	-28.605	2.843.380	-40.064	2.792.632	-39.603

2.821.241	-28.607	2.843.562	-40.068	2.792.866	-39.605
2.821.416	-28.609	2.843.688	-40.073	2.793.060	-39.608
2.821.566	-28.610	2.843.936	-40.077	2.793.250	-39.610
2.821.707	-28.612	2.844.135	-40.081	2.793.264	-39.612
2.821.852	-28.614	2.844.279	-40.086	2.793.441	-39.614
2.822.018	-28.615	2.844.445	-40.090	2.793.748	-39.616
2.822.216	-28.617	2.844.550	-40.094	2.794.012	-39.618
2.822.440	-28.619	2.844.687	-40.098	2.794.186	-39.620
2.822.682	-28.620	2.844.870	-40.102	2.794.360	-39.622
2.822.890	-28.622	2.845.078	-40.107	2.794.486	-39.624
2.823.033	-28.624	2.845.236	-40.111	2.794.615	-39.626
2.823.188	-28.626	2.845.366	-40.115	2.794.771	-39.628
2.823.359	-28.627	2.845.500	-40.119	2.794.912	-39.630
2.823.456	-28.629	2.845.677	-40.123	2.795.063	-39.632
2.823.557	-28.631	2.845.858	-40.127	2.795.273	-39.634
2.823.797	-28.632	2.846.057	-40.131	2.795.399	-39.635
2.824.069	-28.634	2.846.246	-40.135	2.795.479	-39.637
2.824.223	-28.636	2.846.380	-40.138	2.795.652	-39.639
2.824.326	-28.638	2.846.541	-40.142	2.795.800	-39.641
2.824.438	-28.639	2.846.758	-40.146	2.795.921	-39.642
2.824.620	-28.641	2.846.953	-40.150	2.796.116	-39.644
2.824.807	-28.643	2.847.086	-40.154	2.796.389	-39.646
2.824.992	-28.644	2.847.203	-40.157	2.796.629	-39.647
2.825.150	-28.646	2.847.369	-40.161	2.796.806	-39.649
2.825.313	-28.648	2.847.615	-40.164	2.797.028	-39.650
2.825.569	-28.650	2.847.780	-40.168	2.797.235	-39.652
2.825.760	-28.651	2.847.932	-40.171	2.797.341	-39.653
2.825.885	-28.653	2.848.125	-40.175	2.797.372	-39.655
2.826.006	-28.655	2.848.273	-40.178	2.797.483	-39.656
2.826.191	-28.657	2.848.430	-40.182	2.797.682	-39.657

2.826.429	-28.658	2.848.573	-40.185	2.797.856	-39.659
2.826.546	-28.660	2.848.701	-40.188	2.798.065	-39.660
2.826.602	-28.662	2.848.965	-40.192	2.798.282	-39.661
2.826.768	-28.664	2.849.377	-40.195	2.798.470	-39.662
2.827.026	-28.666	2.849.750	-40.198	2.798.663	-39.664
2.827.246	-28.667	2.849.944	-40.201	2.798.791	-39.665
2.827.386	-28.669	2.850.006	-40.204	2.798.921	-39.666
2.827.525	-28.671	2.850.050	-40.207	2.799.093	-39.667
2.827.722	-28.673	2.850.114	-40.210	2.799.272	-39.668
2.827.881	-28.674	2.850.166	-40.213	2.799.457	-39.669
2.827.982	-28.676	2.850.222	-40.216	2.799.601	-39.670
2.828.157	-28.678	2.850.352	-40.219	2.799.757	-39.671
2.828.611	-28.680	2.850.508	-40.222	2.799.933	-39.672
2.828.968	-28.682	2.850.711	-40.224	2.800.078	-39.673
2.829.106	-28.683	2.850.901	-40.227	2.800.296	-39.674
2.829.211	-28.685	2.851.101	-40.230	2.800.500	-39.675
2.829.290	-28.687	2.851.292	-40.233	2.800.639	-39.676
2.829.420	-28.689	2.851.463	-40.235	2.800.771	-39.676
2.829.478	-28.691	2.851.640	-40.238	2.800.959	-39.677
2.829.514	-28.692	2.851.826	-40.240	2.801.173	-39.678
2.829.615	-28.694	2.851.994	-40.243	2.801.219	-39.679
2.829.815	-28.696	2.852.091	-40.245	2.801.313	-39.680
2.830.030	-28.698	2.852.221	-40.248	2.801.419	-39.680
2.830.184	-28.700	2.852.429	-40.250	2.801.596	-39.681
2.830.291	-28.702	2.852.658	-40.252	2.801.953	-39.682
2.830.478	-28.703	2.852.820	-40.255	2.802.225	-39.682
2.830.713	-28.705	2.852.879	-40.257	2.802.375	-39.683
2.830.941	-28.707	2.852.929	-40.259	2.802.520	-39.684
2.831.187	-28.709	2.853.090	-40.261	2.802.697	-39.684
2.831.313	-28.711	2.853.330	-40.263	2.802.813	-39.685

2.831.353	-28.713	2.853.532	-40.266	2.802.953	-39.685
2.831.469	-28.714	2.853.720	-40.268	2.803.073	-39.686
2.831.671	-28.716	2.853.914	-40.270	2.803.221	-39.686
2.831.786	-28.718	2.854.088	-40.272	2.803.659	-39.687
2.831.862	-28.720	2.854.263	-40.274	2.804.109	-39.687
2.832.047	-28.722	2.854.453	-40.276	2.804.319	-39.688
2.832.254	-28.724	2.854.608	-40.278	2.804.460	-39.688
2.832.419	-28.725	2.854.763	-40.280	2.804.521	-39.689
2.832.614	-28.727	2.854.923	-40.282	2.804.536	-39.689
2.832.788	-28.729	2.855.061	-40.283	2.804.660	-39.690
2.832.931	-28.731	2.855.220	-40.285	2.804.792	-39.690
2.833.117	-28.733	2.855.353	-40.287	2.804.857	-39.691
2.833.323	-28.735	2.855.523	-40.289	2.804.905	-39.691
2.833.506	-28.736	2.855.729	-40.291	2.805.021	-39.692
2.833.644	-28.738	2.855.885	-40.293	2.805.242	-39.692
2.833.770	-28.740	2.856.007	-40.294	2.805.432	-39.693
2.833.932	-28.742	2.856.183	-40.296	2.805.591	-39.693
2.834.157	-28.744	2.856.267	-40.298	2.805.812	-39.694
2.834.344	-28.746	2.856.472	-40.299	2.805.870	-39.694
2.834.440	-28.747	2.856.758	-40.301	2.805.880	-39.694
2.834.565	-28.749	2.856.895	-40.303	2.806.129	-39.695
2.834.742	-28.751	2.857.076	-40.304	2.806.307	-39.695
2.834.883	-28.753	2.857.267	-40.306	2.806.490	-39.696
2.835.038	-28.755	2.857.441	-40.307	2.806.726	-39.696
2.835.186	-28.757	2.857.588	-40.309	2.807.021	-39.697
2.835.399	-28.758	2.857.700	-40.311	2.807.246	-39.698
2.835.599	-28.760	2.857.842	-40.312	2.807.282	-39.698
2.835.761	-28.762	2.858.084	-40.314	2.807.546	-39.699
2.835.865	-28.764	2.858.279	-40.315	2.807.776	-39.699
2.836.025	-28.766	2.858.405	-40.317	2.807.722	-39.700

2.836.234	-28.767	2.858.568	-40.318	2.807.742	-39.700
2.836.416	-28.769	2.858.715	-40.320	2.807.955	-39.701
2.836.592	-28.771	2.858.875	-40.321	2.808.177	-39.702
2.836.770	-28.773	2.859.039	-40.323	2.808.367	-39.702
2.836.924	-28.775	2.859.212	-40.324	2.808.515	-39.703
2.837.146	-28.777	2.859.422	-40.326	2.808.641	-39.704
2.837.361	-28.778	2.859.617	-40.327	2.808.795	-39.704
2.837.496	-28.780	2.859.779	-40.329	2.808.994	-39.705
2.837.629	-28.782	2.859.887	-40.330	2.809.255	-39.706
2.837.859	-28.784	2.860.034	-40.332	2.809.456	-39.707
2.838.072	-28.786	2.860.226	-40.333	2.809.565	-39.708
2.838.163	-28.787	2.860.388	-40.335	2.809.753	-39.708
2.838.287	-28.789	2.860.515	-40.336	2.809.944	-39.709
2.838.477	-28.791	2.860.686	-40.337	2.810.071	-39.710
2.838.626	-28.793	2.860.808	-40.339	2.810.259	-39.711
2.838.799	-28.795	2.860.901	-40.340	2.810.428	-39.712
2.838.979	-28.796	2.861.108	-40.342	2.810.594	-39.713
2.839.127	-28.798	2.861.280	-40.343	2.810.796	-39.714
2.839.323	-28.800	2.861.385	-40.344	2.810.932	-39.715
2.839.494	-28.802	2.861.483	-40.346	2.811.076	-39.716
2.839.626	-28.804	2.861.657	-40.347	2.811.216	-39.717
2.839.814	-28.805	2.861.895	-40.349	2.811.394	-39.718
2.839.995	-28.807	2.862.077	-40.350	2.811.618	-39.719
2.840.132	-28.809	2.862.292	-40.351	2.811.779	-39.720
2.840.269	-28.811	2.862.517	-40.353	2.811.874	-39.721
2.840.415	-28.813	2.862.709	-40.354	2.812.003	-39.722
2.840.594	-28.814	2.862.919	-40.356	2.812.258	-39.723
2.840.729	-28.816	2.863.088	-40.357	2.812.451	-39.724
2.840.827	-28.818	2.863.247	-40.358	2.812.592	-39.725
2.841.002	-28.820	2.863.445	-40.360	2.812.780	-39.727

2.841.187	-28.821	2.863.568	-40.361	2.812.908	-39.728
2.841.328	-28.823	2.863.705	-40.362	2.813.029	-39.729
2.841.498	-28.825	2.863.885	-40.364	2.813.081	-39.730
2.841.643	-28.827	2.864.012	-40.365	2.813.359	-39.732
2.841.853	-28.829	2.864.033	-40.367	2.813.796	-39.733
2.842.145	-28.830	2.864.193	-40.368	2.813.727	-39.734
2.842.357	-28.832	2.864.503	-40.369	2.813.654	-39.736
2.842.527	-28.834	2.864.588	-40.371	2.814.048	-39.737
2.842.664	-28.836	2.864.727	-40.372	2.814.492	-39.738
2.842.791	-28.837	2.865.042	-40.374	2.814.547	-39.740
2.842.955	-28.839	2.865.298	-40.375	2.814.370	-39.741
2.843.117	-28.841	2.865.458	-40.376	2.814.537	-39.743
2.843.271	-28.843	2.865.549	-40.378	2.814.864	-39.744
2.843.426	-28.844	2.865.699	-40.379	2.815.072	-39.746
2.843.544	-28.846	2.865.905	-40.381	2.815.245	-39.747
2.843.719	-28.848	2.866.107	-40.382	2.815.411	-39.749
2.843.876	-28.850	2.866.286	-40.383	2.815.555	-39.750
2.843.969	-28.851	2.866.440	-40.385	2.815.728	-39.752
2.844.181	-28.853	2.866.598	-40.386	2.815.901	-39.753
2.844.406	-28.855	2.866.734	-40.388	2.816.046	-39.755
2.844.546	-28.857	2.866.871	-40.389	2.816.242	-39.756
2.844.775	-28.858	2.867.072	-40.390	2.816.440	-39.758
2.845.060	-28.860	2.867.256	-40.392	2.816.603	-39.759
2.845.263	-28.862	2.867.369	-40.393	2.816.751	-39.761
2.845.411	-28.864	2.867.520	-40.395	2.816.946	-39.762
2.845.519	-28.865	2.867.715	-40.396	2.817.135	-39.764
2.845.576	-28.867	2.867.924	-40.398	2.817.265	-39.765
2.845.754	-28.869	2.868.095	-40.399	2.817.393	-39.767
2.846.010	-28.871	2.868.243	-40.401	2.817.570	-39.769
2.846.185	-28.872	2.868.445	-40.402	2.817.731	-39.770

2.846.422	-28.874	2.868.611	-40.403	2.817.878	-39.772
2.846.631	-28.876	2.868.768	-40.405	2.818.069	-39.773
2.846.696	-28.878	2.868.921	-40.406	2.818.217	-39.775
2.846.791	-28.879	2.869.077	-40.408	2.818.359	-39.776
2.846.939	-28.881	2.869.261	-40.409	2.818.468	-39.778
2.847.133	-28.883	2.869.431	-40.411	2.818.568	-39.780
2.847.321	-28.885	2.869.613	-40.412	2.818.810	-39.781
2.847.494	-28.886	2.869.803	-40.414	2.819.017	-39.783
2.847.723	-28.888	2.869.955	-40.415	2.819.066	-39.784
2.847.856	-28.890	2.870.046	-40.417	2.819.293	-39.786
2.847.978	-28.892	2.870.204	-40.418	2.819.541	-39.787
2.848.157	-28.893	2.870.407	-40.420	2.819.688	-39.789
2.848.348	-28.895	2.870.559	-40.421	2.819.883	-39.790
2.848.532	-28.897	2.870.822	-40.423	2.820.107	-39.792
2.848.661	-28.899	2.871.201	-40.424	2.820.331	-39.794
2.848.853	-28.900	2.871.542	-40.426	2.820.426	-39.795
2.849.024	-28.902	2.871.649	-40.427	2.820.572	-39.797
2.849.179	-28.904	2.871.698	-40.429	2.820.768	-39.798
2.849.387	-28.906	2.871.696	-40.430	2.820.945	-39.799
2.849.609	-28.907	2.871.768	-40.432	2.821.118	-39.801
2.849.752	-28.909	2.871.880	-40.433	2.821.220	-39.802
2.849.768	-28.911	2.871.913	-40.435	2.821.313	-39.804
2.849.873	-28.913	2.872.112	-40.437	2.821.461	-39.805
2.850.103	-28.914	2.872.383	-40.438	2.821.647	-39.807
2.850.338	-28.916	2.872.473	-40.440	2.821.835	-39.808
2.850.708	-28.918	2.872.628	-40.441	2.821.972	-39.809
2.851.090	-28.920	2.872.875	-40.443	2.822.090	-39.811
2.851.291	-28.921	2.873.074	-40.444	2.822.195	-39.812
2.851.422	-28.923	2.873.253	-40.446	2.822.398	-39.814
2.851.516	-28.925	2.873.401	-40.447	2.822.677	-39.815

2.851.563	-28.927	2.873.589	-40.449	2.822.892	-39.816
2.851.591	-28.928	2.873.804	-40.450	2.823.008	-39.818
2.851.692	-28.930	2.873.965	-40.452	2.823.177	-39.819
2.851.808	-28.932	2.874.110	-40.454	2.823.497	-39.820
2.851.880	-28.934	2.874.182	-40.455	2.823.723	-39.821
2.852.014	-28.935	2.874.295	-40.457	2.823.781	-39.823
2.852.199	-28.937	2.874.525	-40.458	2.823.943	-39.824
2.852.402	-28.939	2.874.688	-40.460	2.823.997	-39.825
2.852.635	-28.941	2.874.844	-40.461	2.824.128	-39.826
2.852.853	-28.942	2.875.036	-40.463	2.824.420	-39.828
2.853.058	-28.944	2.875.224	-40.465	2.824.577	-39.829
2.853.232	-28.946	2.875.385	-40.466	2.824.709	-39.830
2.853.343	-28.948	2.875.562	-40.468	2.824.723	-39.831
2.853.499	-28.950	2.875.728	-40.469	2.824.807	-39.832
2.853.709	-28.951	2.875.905	-40.471	2.825.195	-39.834
2.853.854	-28.953	2.876.162	-40.472	2.825.548	-39.835
2.853.965	-28.955	2.876.324	-40.474	2.825.527	-39.836
2.854.124	-28.957	2.876.389	-40.476	2.825.630	-39.837
2.854.290	-28.958	2.876.562	-40.477	2.825.956	-39.838
2.854.505	-28.960	2.876.744	-40.479	2.826.042	-39.839
2.854.720	-28.962	2.876.867	-40.480	2.826.205	-39.840
2.854.793	-28.964	2.877.037	-40.482	2.826.494	-39.841
2.854.911	-28.965	2.877.200	-40.484	2.826.622	-39.842
2.855.164	-28.967	2.877.377	-40.485	2.826.761	-39.843
2.855.364	-28.969	2.877.561	-40.487	2.826.993	-39.845
2.855.540	-28.971	2.877.691	-40.489	2.827.234	-39.846
2.855.704	-28.972	2.877.848	-40.490	2.827.398	-39.847
2.855.862	-28.974	2.878.029	-40.492	2.827.530	-39.848
2.856.061	-28.976	2.878.185	-40.493	2.827.654	-39.849
2.856.169	-28.978	2.878.338	-40.495	2.827.791	-39.850

2.856.318	-28.979	2.878.518	-40.497	2.828.044	-39.851
2.856.546	-28.981	2.878.687	-40.498	2.828.203	-39.852
2.856.671	-28.983	2.878.849	-40.500	2.828.289	-39.853
2.856.808	-28.985	2.878.979	-40.502	2.828.409	-39.854
2.857.018	-28.986	2.879.117	-40.503	2.828.395	-39.855
2.857.144	-28.988	2.879.322	-40.505	2.828.680	-39.856
2.857.256	-28.990	2.879.493	-40.507	2.829.034	-39.857
2.857.440	-28.992	2.879.601	-40.508	2.829.117	-39.858
2.857.574	-28.993	2.879.823	-40.510	2.829.178	-39.859
2.857.676	-28.995	2.880.110	-40.512	2.829.373	-39.860
2.857.848	-28.997	2.880.296	-40.513	2.829.478	-39.861
2.858.062	-28.999	2.880.428	-40.515	2.829.711	-39.862
2.858.334	-29.000	2.880.576	-40.517	2.830.137	-39.862
2.858.546	-29.002	2.880.744	-40.518	2.830.537	-39.863
2.858.629	-29.004	2.880.869	-40.520	2.830.726	-39.864
2.858.731	-29.006	2.880.984	-40.522	2.830.755	-39.865
2.858.916	-29.007	2.881.165	-40.523	2.830.852	-39.866
2.859.154	-29.009	2.881.393	-40.525	2.830.977	-39.867
2.859.349	-29.011	2.881.559	-40.526	2.830.981	-39.868
2.859.517	-29.012	2.881.734	-40.528	2.831.053	-39.869
2.859.718	-29.014	2.881.880	-40.530	2.831.187	-39.870
2.859.839	-29.016	2.882.041	-40.531	2.831.309	-39.871
2.859.908	-29.018	2.882.278	-40.533	2.831.492	-39.872
2.860.099	-29.019	2.882.429	-40.535	2.831.718	-39.873
2.860.338	-29.021	2.882.560	-40.536	2.831.806	-39.874
2.860.457	-29.023	2.882.679	-40.538	2.831.914	-39.875
2.860.576	-29.025	2.882.759	-40.539	2.832.075	-39.876
2.860.794	-29.026	2.882.906	-40.541	2.832.191	-39.877
2.861.028	-29.028	2.883.055	-40.543	2.832.455	-39.878
2.861.181	-29.030	2.883.185	-40.544	2.832.738	-39.879

2.861.325	-29.031	2.883.343	-40.546	2.832.903	-39.880
2.861.494	-29.033	2.883.557	-40.547	2.833.076	-39.881
2.861.671	-29.035	2.883.820	-40.549	2.833.264	-39.882
2.861.823	-29.037	2.884.016	-40.550	2.833.412	-39.883
2.862.008	-29.038	2.884.183	-40.552	2.833.568	-39.884
2.862.191	-29.040	2.884.427	-40.553	2.833.730	-39.885
2.862.275	-29.042	2.884.629	-40.555	2.833.889	-39.886
2.862.393	-29.043	2.884.756	-40.556	2.834.075	-39.887
2.862.556	-29.045	2.884.944	-40.558	2.834.187	-39.888
2.862.741	-29.047	2.885.103	-40.559	2.834.352	-39.890
2.862.885	-29.049	2.885.251	-40.561	2.834.539	-39.891
2.863.020	-29.050	2.885.361	-40.562	2.834.689	-39.892
2.863.157	-29.052	2.885.458	-40.564	2.834.837	-39.893
2.863.289	-29.054	2.885.580	-40.565	2.834.890	-39.894
2.863.452	-29.055	2.885.721	-40.567	2.834.879	-39.895
2.863.656	-29.057	2.885.891	-40.568	2.835.074	-39.896
2.863.896	-29.059	2.886.042	-40.569	2.835.479	-39.898
2.864.135	-29.060	2.886.205	-40.571	2.835.722	-39.899
2.864.391	-29.062	2.886.325	-40.572	2.835.872	-39.900
2.864.543	-29.064	2.886.501	-40.573	2.836.051	-39.901
2.864.682	-29.066	2.886.757	-40.575	2.836.447	-39.903
2.864.850	-29.067	2.886.954	-40.576	2.836.573	-39.904
2.864.966	-29.069	2.887.146	-40.577	2.836.570	-39.905
2.865.118	-29.071	2.887.310	-40.579	2.836.740	-39.906
2.865.296	-29.072	2.887.372	-40.580	2.836.827	-39.908
2.865.464	-29.074	2.887.498	-40.581	2.836.909	-39.909
2.865.577	-29.076	2.887.726	-40.583	2.837.112	-39.910
2.865.717	-29.077	2.887.986	-40.584	2.837.245	-39.912
2.865.914	-29.079	2.888.206	-40.585	2.837.359	-39.913
2.866.036	-29.081	2.888.394	-40.586	2.837.618	-39.915

2.866.163	-29.082	2.888.557	-40.588	2.837.810	-39.916
2.866.357	-29.084	2.888.713	-40.589	2.837.936	-39.917
2.866.573	-29.086	2.888.871	-40.590	2.838.008	-39.919
2.866.795	-29.088	2.889.066	-40.591	2.838.062	-39.920
2.866.956	-29.089	2.889.196	-40.592	2.838.315	-39.922
2.867.101	-29.091	2.889.310	-40.594	2.838.728	-39.923
2.867.304	-29.093	2.889.529	-40.595	2.838.857	-39.925
2.867.444	-29.094	2.889.796	-40.596	2.838.890	-39.926
2.867.632	-29.096	2.890.009	-40.597	2.839.212	-39.928
2.867.838	-29.098	2.890.088	-40.598	2.839.414	-39.929
2.867.990	-29.099	2.890.179	-40.599	2.839.429	-39.931
2.868.190	-29.101	2.890.388	-40.600	2.839.534	-39.933
2.868.392	-29.103	2.890.572	-40.601	2.839.731	-39.934
2.868.511	-29.104	2.890.726	-40.602	2.839.974	-39.936
2.868.636	-29.106	2.890.890	-40.603	2.840.032	-39.938
2.868.806	-29.108	2.891.067	-40.605	2.840.126	-39.939
2.868.922	-29.109	2.891.244	-40.606	2.840.395	-39.941
2.869.099	-29.111	2.891.407	-40.607	2.840.652	-39.943
2.869.337	-29.113	2.891.589	-40.608	2.840.843	-39.944
2.869.532	-29.115	2.891.757	-40.609	2.841.032	-39.946
2.869.747	-29.116	2.891.848	-40.610	2.841.231	-39.948
2.869.948	-29.118	2.891.936	-40.611	2.841.414	-39.949
2.870.063	-29.120	2.892.099	-40.612	2.841.551	-39.951
2.870.201	-29.121	2.892.516	-40.613	2.841.642	-39.953
2.870.343	-29.123	2.893.004	-40.613	2.841.833	-39.955
2.870.498	-29.125	2.893.181	-40.614	2.842.026	-39.956
2.870.652	-29.126	2.893.181	-40.615	2.842.188	-39.958
2.870.808	-29.128	2.893.301	-40.616	2.842.328	-39.960
2.870.988	-29.130	2.893.428	-40.617	2.842.458	-39.962
2.871.125	-29.131	2.893.434	-40.618	2.842.625	-39.964

2.871.317	-29.133	2.893.507	-40.619	2.842.773	-39.965
2.871.519	-29.135	2.893.593	-40.620	2.842.932	-39.967
2.871.642	-29.136	2.893.741	-40.621	2.843.161	-39.969
2.871.822	-29.138	2.893.940	-40.622	2.843.330	-39.971
2.871.997	-29.140	2.894.088	-40.623	2.843.442	-39.973
2.872.122	-29.142	2.894.287	-40.623	2.843.632	-39.974
2.872.254	-29.143	2.894.530	-40.624	2.843.818	-39.976
2.872.527	-29.145	2.894.738	-40.625	2.843.968	-39.978
2.873.026	-29.147	2.894.909	-40.626	2.844.099	-39.980
2.873.358	-29.148	2.895.075	-40.627	2.844.200	-39.982
2.873.408	-29.150	2.895.230	-40.628	2.844.331	-39.984
2.873.506	-29.152	2.895.368	-40.629	2.844.528	-39.986
2.873.661	-29.153	2.895.493	-40.629	2.844.653	-39.988
2.873.692	-29.155	2.895.674	-40.630	2.844.799	-39.990
2.873.685	-29.157	2.895.872	-40.631	2.844.976	-39.991
2.873.756	-29.158	2.896.042	-40.632	2.845.107	-39.993
2.873.865	-29.160	2.896.216	-40.633	2.845.291	-39.995
2.873.980	-29.162	2.896.343	-40.633	2.845.451	-39.997
2.874.205	-29.163	2.896.462	-40.634	2.845.631	-39.999
2.874.492	-29.165	2.896.632	-40.635	2.845.874	-40.001
2.874.705	-29.167	2.896.835	-40.636	2.846.065	-40.003
2.874.796	-29.168	2.897.000	-40.637	2.846.259	-40.005
2.874.944	-29.170	2.897.151	-40.638	2.846.470	-40.007
2.875.204	-29.172	2.897.391	-40.638	2.846.694	-40.009
2.875.331	-29.173	2.897.661	-40.639	2.846.810	-40.011
2.875.486	-29.175	2.897.823	-40.640	2.846.884	-40.012
2.875.672	-29.177	2.897.921	-40.641	2.847.075	-40.014
2.875.830	-29.178	2.898.007	-40.642	2.847.247	-40.016
2.875.999	-29.180	2.898.159	-40.642	2.847.438	-40.018
2.876.075	-29.182	2.898.358	-40.643	2.847.589	-40.020

2.876.231	-29.183	2.898.524	-40.644	2.847.672	-40.022
2.876.505	-29.185	2.898.695	-40.645	2.847.803	-40.024
2.876.647	-29.187	2.898.793	-40.646	2.848.001	-40.026
2.876.740	-29.188	2.898.929	-40.647	2.848.188	-40.028
2.876.961	-29.190	2.899.099	-40.648	2.848.289	-40.030
2.877.138	-29.192	2.899.262	-40.648	2.848.484	-40.031
2.877.283	-29.193	2.899.452	-40.649	2.848.756	-40.033
2.877.523	-29.195	2.899.604	-40.650	2.848.927	-40.035
2.877.737	-29.197	2.899.790	-40.651	2.849.073	-40.037
2.877.946	-29.198	2.900.002	-40.652	2.849.214	-40.039
2.878.161	-29.200	2.900.164	-40.653	2.849.395	-40.041
2.878.257	-29.202	2.900.281	-40.654	2.849.615	-40.043
2.878.358	-29.203	2.900.435	-40.655	2.849.792	-40.045
2.878.535	-29.205	2.900.623	-40.656	2.850.009	-40.047
2.878.708	-29.206	2.900.815	-40.657	2.850.241	-40.049
2.878.820	-29.208	2.900.991	-40.658	2.850.388	-40.050
2.878.900	-29.210	2.901.143	-40.658	2.850.476	-40.052
2.879.063	-29.211	2.901.342	-40.659	2.850.613	-40.054
2.879.270	-29.213	2.901.515	-40.660	2.850.809	-40.056
2.879.443	-29.215	2.901.661	-40.661	2.851.014	-40.058
2.879.619	-29.216	2.901.846	-40.662	2.851.234	-40.060
2.879.785	-29.218	2.902.007	-40.663	2.851.360	-40.062
2.879.955	-29.219	2.902.177	-40.665	2.851.415	-40.063
2.880.165	-29.221	2.902.332	-40.666	2.851.523	-40.065
2.880.327	-29.223	2.902.484	-40.667	2.851.730	-40.067
2.880.466	-29.224	2.902.640	-40.668	2.851.922	-40.069
2.880.588	-29.226	2.902.780	-40.669	2.852.180	-40.071
2.880.735	-29.227	2.902.922	-40.670	2.852.390	-40.073
2.880.881	-29.229	2.903.112	-40.671	2.852.481	-40.074
2.881.069	-29.230	2.903.340	-40.672	2.852.643	-40.076

2.881.297	-29.232	2.903.513	-40.673	2.852.828	-40.078
2.881.434	-29.234	2.903.687	-40.674	2.852.990	-40.080
2.881.598	-29.235	2.903.874	-40.675	2.853.208	-40.082
2.881.761	-29.237	2.904.010	-40.676	2.853.420	-40.084
2.881.964	-29.238	2.904.113	-40.678	2.853.550	-40.085
2.882.167	-29.240	2.904.245	-40.679	2.853.670	-40.087
2.882.314	-29.241	2.904.406	-40.680	2.853.836	-40.089
2.882.424	-29.243	2.904.541	-40.681	2.853.932	-40.091
2.882.588	-29.245	2.904.709	-40.682	2.854.093	-40.093
2.882.760	-29.246	2.904.866	-40.683	2.854.411	-40.094
2.882.863	-29.248	2.905.025	-40.684	2.854.518	-40.096
2.883.013	-29.249	2.905.171	-40.686	2.854.557	-40.098
2.883.214	-29.251	2.905.356	-40.687	2.854.796	-40.100
2.883.414	-29.252	2.905.663	-40.688	2.855.024	-40.101
2.883.611	-29.254	2.905.876	-40.689	2.855.269	-40.103
2.883.786	-29.255	2.906.039	-40.690	2.855.222	-40.105
2.883.955	-29.257	2.906.256	-40.691	2.855.096	-40.107
2.884.106	-29.258	2.906.513	-40.693	2.855.379	-40.108
2.884.247	-29.260	2.906.674	-40.694	2.855.643	-40.110
2.884.450	-29.261	2.906.751	-40.695	2.855.964	-40.112
2.884.586	-29.263	2.906.866	-40.696	2.856.505	-40.114
2.884.695	-29.264	2.906.971	-40.697	2.856.844	-40.115
2.884.843	-29.266	2.907.115	-40.699	2.857.011	-40.117
2.884.938	-29.267	2.907.268	-40.700	2.857.088	-40.119
2.885.085	-29.269	2.907.380	-40.701	2.857.170	-40.120
2.885.319	-29.270	2.907.554	-40.702	2.857.285	-40.122
2.885.558	-29.272	2.907.716	-40.703	2.857.325	-40.124
2.885.744	-29.273	2.907.871	-40.705	2.857.336	-40.125
2.885.941	-29.275	2.908.054	-40.706	2.857.448	-40.127
2.886.169	-29.276	2.908.308	-40.707	2.857.610	-40.128

2.886.335	-29.278	2.908.593	-40.708	2.857.781	-40.130
2.886.538	-29.279	2.908.771	-40.709	2.858.009	-40.132
2.886.739	-29.281	2.908.918	-40.711	2.858.199	-40.133
2.886.877	-29.282	2.909.030	-40.712	2.858.344	-40.135
2.886.996	-29.284	2.909.163	-40.713	2.858.499	-40.137
2.887.112	-29.285	2.909.345	-40.714	2.858.654	-40.138
2.887.281	-29.287	2.909.503	-40.715	2.858.849	-40.140
2.887.452	-29.288	2.909.645	-40.717	2.859.084	-40.141
2.887.614	-29.290	2.909.797	-40.718	2.859.229	-40.143
2.887.742	-29.291	2.909.987	-40.719	2.859.380	-40.145
2.887.877	-29.293	2.910.206	-40.720	2.859.555	-40.146
2.888.011	-29.294	2.910.345	-40.721	2.859.642	-40.148
2.888.130	-29.295	2.910.504	-40.723	2.859.811	-40.149
2.888.286	-29.297	2.910.627	-40.724	2.859.906	-40.151
2.888.446	-29.298	2.910.742	-40.725	2.859.995	-40.152
2.888.723	-29.300	2.911.017	-40.726	2.860.242	-40.154
2.889.028	-29.301	2.911.248	-40.727	2.860.432	-40.155
2.889.178	-29.303	2.911.407	-40.729	2.860.599	-40.157
2.889.326	-29.304	2.911.563	-40.730	2.860.779	-40.158
2.889.449	-29.306	2.911.718	-40.731	2.860.946	-40.160
2.889.583	-29.307	2.911.869	-40.732	2.861.111	-40.161
2.889.749	-29.309	2.912.021	-40.733	2.861.273	-40.163
2.889.958	-29.310	2.912.178	-40.735	2.861.440	-40.164
2.890.177	-29.311	2.912.350	-40.736	2.861.658	-40.166
2.890.307	-29.313	2.912.530	-40.737	2.861.823	-40.167
2.890.419	-29.314	2.912.706	-40.738	2.861.967	-40.169
2.890.609	-29.316	2.912.931	-40.739	2.862.146	-40.170
2.890.721	-29.317	2.913.159	-40.741	2.862.358	-40.171
2.890.880	-29.319	2.913.326	-40.742	2.862.565	-40.173
2.891.179	-29.320	2.913.390	-40.743	2.862.747	-40.174

2.891.382	-29.322	2.913.489	-40.744	2.862.871	-40.176
2.891.537	-29.323	2.913.705	-40.745	2.862.975	-40.177
2.891.675	-29.325	2.913.952	-40.747	2.863.120	-40.178
2.891.841	-29.326	2.914.333	-40.748	2.863.262	-40.180
2.892.048	-29.328	2.914.698	-40.749	2.863.481	-40.181
2.892.238	-29.329	2.914.814	-40.750	2.863.640	-40.183
2.892.395	-29.330	2.914.891	-40.751	2.863.741	-40.184
2.892.502	-29.332	2.914.991	-40.753	2.863.896	-40.185
2.892.662	-29.333	2.915.047	-40.754	2.864.066	-40.187
2.892.910	-29.335	2.915.129	-40.755	2.864.250	-40.188
2.893.058	-29.336	2.915.202	-40.756	2.864.447	-40.189
2.893.138	-29.338	2.915.278	-40.757	2.864.670	-40.190
2.893.239	-29.339	2.915.458	-40.759	2.864.857	-40.192
2.893.455	-29.341	2.915.657	-40.760	2.865.024	-40.193
2.893.720	-29.342	2.915.876	-40.761	2.865.164	-40.194
2.893.907	-29.344	2.916.068	-40.762	2.865.332	-40.196
2.894.005	-29.345	2.916.174	-40.763	2.865.544	-40.197
2.894.118	-29.347	2.916.322	-40.765	2.865.674	-40.198
2.894.301	-29.348	2.916.556	-40.766	2.865.822	-40.200
2.894.449	-29.350	2.916.755	-40.767	2.866.040	-40.201
2.894.857	-29.351	2.916.917	-40.768	2.866.190	-40.202
2.895.309	-29.352	2.917.073	-40.769	2.866.342	-40.203
2.895.443	-29.354	2.917.170	-40.771	2.866.516	-40.204
2.895.450	-29.355	2.917.307	-40.772	2.866.667	-40.206
2.895.483	-29.357	2.917.503	-40.773	2.866.871	-40.207
2.895.567	-29.358	2.917.724	-40.774	2.867.061	-40.208
2.895.674	-29.360	2.917.903	-40.776	2.867.163	-40.209
2.895.838	-29.361	2.917.998	-40.777	2.867.303	-40.210
2.895.928	-29.363	2.918.124	-40.778	2.867.478	-40.212
2.896.010	-29.364	2.918.304	-40.779	2.867.629	-40.213

2.896.176	-29.366	2.918.430	-40.781	2.867.824	-40.214
2.896.389	-29.367	2.918.615	-40.782	2.868.001	-40.215
2.896.584	-29.369	2.918.847	-40.783	2.868.189	-40.216
2.896.674	-29.370	2.919.067	-40.784	2.868.384	-40.217
2.896.859	-29.372	2.919.268	-40.786	2.868.513	-40.218
2.897.124	-29.373	2.919.405	-40.787	2.868.636	-40.219
2.897.347	-29.375	2.919.543	-40.788	2.868.775	-40.220
2.897.518	-29.376	2.919.747	-40.790	2.868.940	-40.221
2.897.582	-29.378	2.919.935	-40.791	2.869.069	-40.222
2.897.756	-29.379	2.920.063	-40.792	2.869.215	-40.223
2.897.973	-29.381	2.920.175	-40.794	2.869.452	-40.224
2.898.078	-29.382	2.920.280	-40.795	2.869.695	-40.225
2.898.219	-29.384	2.920.476	-40.796	2.869.893	-40.226
2.898.410	-29.386	2.920.692	-40.798	2.870.023	-40.227
2.898.546	-29.387	2.920.881	-40.799	2.870.172	-40.228
2.898.684	-29.389	2.921.053	-40.801	2.870.313	-40.229
2.898.952	-29.390	2.921.170	-40.802	2.870.426	-40.230
2.899.132	-29.392	2.921.286	-40.804	2.870.529	-40.231
2.899.272	-29.393	2.921.509	-40.805	2.870.708	-40.232
2.899.499	-29.395	2.921.736	-40.807	2.870.887	-40.233
2.899.648	-29.396	2.921.864	-40.808	2.871.010	-40.234
2.899.807	-29.398	2.921.976	-40.810	2.871.150	-40.235
2.900.009	-29.399	2.922.098	-40.811	2.871.320	-40.235
2.900.191	-29.401	2.922.234	-40.813	2.871.519	-40.236
2.900.345	-29.402	2.922.437	-40.814	2.871.719	-40.237
2.900.509	-29.404	2.922.682	-40.816	2.871.909	-40.238
2.900.617	-29.406	2.922.913	-40.817	2.872.111	-40.238
2.900.751	-29.407	2.923.067	-40.819	2.872.393	-40.239
2.900.956	-29.409	2.923.152	-40.821	2.872.604	-40.240
2.901.126	-29.410	2.923.279	-40.822	2.872.727	-40.241

2.901.217	-29.412	2.923.510	-40.824	2.872.968	-40.241
2.901.364	-29.413	2.923.673	-40.826	2.873.188	-40.242
2.901.591	-29.415	2.923.828	-40.827	2.873.289	-40.243
2.901.804	-29.417	2.923.947	-40.829	2.873.374	-40.243
2.902.008	-29.418	2.924.039	-40.831	2.873.493	-40.244
2.902.104	-29.420	2.924.263	-40.833	2.873.705	-40.245
2.902.199	-29.421	2.924.472	-40.834	2.873.933	-40.245
2.902.382	-29.423	2.924.662	-40.836	2.874.041	-40.246
2.902.525	-29.425	2.924.807	-40.838	2.874.116	-40.246
2.902.734	-29.426	2.924.976	-40.840	2.874.261	-40.247
2.902.900	-29.428	2.925.173	-40.842	2.874.438	-40.248
2.903.023	-29.429	2.925.298	-40.843	2.874.662	-40.248
2.903.260	-29.431	2.925.471	-40.845	2.874.792	-40.249
2.903.492	-29.433	2.925.637	-40.847	2.874.923	-40.249
2.903.656	-29.434	2.925.732	-40.849	2.875.070	-40.250
2.903.796	-29.436	2.925.912	-40.851	2.875.224	-40.250
2.903.979	-29.437	2.926.038	-40.853	2.875.413	-40.251
2.904.102	-29.439	2.926.079	-40.855	2.875.627	-40.251
2.904.255	-29.441	2.926.309	-40.857	2.875.847	-40.252
2.904.424	-29.442	2.926.563	-40.859	2.875.953	-40.252
2.904.568	-29.444	2.926.745	-40.861	2.876.161	-40.253
2.904.746	-29.445	2.926.892	-40.863	2.876.375	-40.253
2.904.915	-29.447	2.927.101	-40.865	2.876.486	-40.254
2.905.063	-29.449	2.927.372	-40.866	2.876.643	-40.254
2.905.240	-29.450	2.927.538	-40.868	2.876.866	-40.255
2.905.417	-29.452	2.927.704	-40.870	2.877.017	-40.255
2.905.569	-29.454	2.927.921	-40.872	2.877.127	-40.256
2.905.768	-29.455	2.928.094	-40.875	2.877.301	-40.256
2.905.941	-29.457	2.928.251	-40.877	2.877.469	-40.256
2.906.080	-29.459	2.928.427	-40.879	2.877.653	-40.257

2.906.264	-29.460	2.928.560	-40.881	2.877.866	-40.257
2.906.360	-29.462	2.928.665	-40.883	2.878.055	-40.258
2.906.480	-29.464	2.928.759	-40.885	2.878.182	-40.258
2.906.711	-29.465	2.928.885	-40.887	2.878.279	-40.259
2.906.873	-29.467	2.929.020	-40.889	2.878.452	-40.259
2.907.046	-29.469	2.929.178	-40.891	2.878.629	-40.260
2.907.173	-29.470	2.929.373	-40.893	2.878.839	-40.260
2.907.271	-29.472	2.929.547	-40.895	2.878.976	-40.261
2.907.437	-29.474	2.929.764	-40.897	2.879.034	-40.261
2.907.661	-29.476	2.930.009	-40.899	2.879.244	-40.262
2.907.874	-29.477	2.930.179	-40.902	2.879.519	-40.262
2.908.074	-29.479	2.930.312	-40.904	2.879.714	-40.263
2.908.327	-29.481	2.930.472	-40.906	2.879.876	-40.263
2.908.556	-29.482	2.930.630	-40.908	2.880.024	-40.264
2.908.737	-29.484	2.930.793	-40.910	2.880.205	-40.264
2.908.846	-29.486	2.930.968	-40.912	2.880.361	-40.265
2.909.002	-29.488	2.931.137	-40.915	2.880.513	-40.265
2.909.128	-29.489	2.931.266	-40.917	2.880.703	-40.266
2.909.294	-29.491	2.931.368	-40.919	2.880.894	-40.266
2.909.435	-29.493	2.931.595	-40.921	2.881.098	-40.267
2.909.564	-29.495	2.931.809	-40.923	2.881.277	-40.268
2.909.710	-29.496	2.931.976	-40.926	2.881.423	-40.268
2.909.872	-29.498	2.932.097	-40.928	2.881.562	-40.269
2.910.030	-29.500	2.932.162	-40.930	2.881.714	-40.269
2.910.122	-29.502	2.932.405	-40.932	2.881.870	-40.270
2.910.273	-29.503	2.932.671	-40.934	2.882.003	-40.271
2.910.446	-29.505	2.932.827	-40.937	2.882.109	-40.271
2.910.621	-29.507	2.933.007	-40.939	2.882.506	-40.272
2.910.825	-29.509	2.933.200	-40.941	2.883.036	-40.273
2.911.058	-29.511	2.933.374	-40.943	2.883.297	-40.273

2.911.263	-29.512	2.933.510	-40.946	2.883.348	-40.274
2.911.430	-29.514	2.933.670	-40.948	2.883.385	-40.275
2.911.629	-29.516	2.933.832	-40.950	2.883.448	-40.275
2.911.775	-29.518	2.934.005	-40.952	2.883.503	-40.276
2.911.884	-29.520	2.934.190	-40.955	2.883.608	-40.277
2.912.037	-29.521	2.934.374	-40.957	2.883.683	-40.278
2.912.196	-29.523	2.934.554	-40.959	2.883.788	-40.278
2.912.361	-29.525	2.934.716	-40.962	2.883.923	-40.279
2.912.536	-29.527	2.934.924	-40.964	2.884.084	-40.280
2.912.738	-29.529	2.935.112	-40.966	2.884.251	-40.281
2.912.944	-29.531	2.935.252	-40.968	2.884.428	-40.281
2.913.130	-29.533	2.935.385	-40.971	2.884.601	-40.282
2.913.275	-29.534	2.935.674	-40.973	2.884.761	-40.283
2.913.391	-29.536	2.936.138	-40.975	2.884.948	-40.284
2.913.575	-29.538	2.936.441	-40.977	2.885.078	-40.285
2.913.820	-29.540	2.936.443	-40.980	2.885.323	-40.286
2.914.055	-29.542	2.936.458	-40.982	2.885.558	-40.287
2.914.169	-29.544	2.936.574	-40.984	2.885.681	-40.288
2.914.290	-29.546	2.936.642	-40.987	2.885.834	-40.289
2.914.468	-29.548	2.936.701	-40.989	2.886.047	-40.289
2.914.612	-29.550	2.936.820	-40.991	2.886.255	-40.290
2.914.794	-29.551	2.936.996	-40.993	2.886.350	-40.291
2.914.980	-29.553	2.937.211	-40.996	2.886.425	-40.293
2.915.190	-29.555	2.937.404	-40.998	2.886.533	-40.294
2.915.382	-29.557	2.937.542	-41.000	2.886.700	-40.295
2.915.493	-29.559	2.937.740	-41.002	2.886.917	-40.296
2.915.606	-29.561	2.937.948	-41.004	2.887.133	-40.297
2.915.811	-29.563	2.938.116	-41.007	2.887.321	-40.298
2.916.064	-29.565	2.938.272	-41.009	2.887.509	-40.299
2.916.190	-29.567	2.938.414	-41.011	2.887.651	-40.300

2.916.292	-29.569	2.938.543	-41.013	2.887.734	-40.301
2.916.512	-29.571	2.938.669	-41.015	2.887.944	-40.303
2.916.887	-29.573	2.938.780	-41.018	2.888.172	-40.304
2.917.260	-29.575	2.938.959	-41.020	2.888.369	-40.305
2.917.437	-29.577	2.939.193	-41.022	2.888.565	-40.306
2.917.532	-29.579	2.939.371	-41.024	2.888.665	-40.308
2.917.635	-29.581	2.939.522	-41.026	2.888.846	-40.309
2.917.701	-29.583	2.939.659	-41.028	2.889.114	-40.310
2.917.682	-29.585	2.939.756	-41.031	2.889.275	-40.311
2.917.762	-29.587	2.939.930	-41.033	2.889.373	-40.313
2.917.914	-29.589	2.940.171	-41.035	2.889.511	-40.314
2.918.033	-29.591	2.940.346	-41.037	2.889.677	-40.316
2.918.267	-29.593	2.940.471	-41.039	2.889.828	-40.317
2.918.442	-29.595	2.940.623	-41.041	2.889.996	-40.318
2.918.582	-29.597	2.940.847	-41.043	2.890.173	-40.320
2.918.778	-29.599	2.941.042	-41.045	2.890.331	-40.321
2.918.936	-29.601	2.941.170	-41.047	2.890.469	-40.323
2.919.110	-29.603	2.941.382	-41.049	2.890.632	-40.324
2.919.283	-29.605	2.941.581	-41.051	2.890.743	-40.326
2.919.442	-29.607	2.941.701	-41.053	2.890.916	-40.327
2.919.645	-29.609	2.941.743	-41.056	2.891.093	-40.329
2.919.883	-29.611	2.941.880	-41.058	2.891.227	-40.330
2.920.025	-29.613	2.942.124	-41.060	2.891.361	-40.332
2.920.121	-29.615	2.942.300	-41.062	2.891.526	-40.333
2.920.242	-29.617	2.942.463	-41.064	2.891.743	-40.335
2.920.352	-29.619	2.942.639	-41.066	2.891.866	-40.337
2.920.552	-29.621	2.942.857	-41.068	2.891.960	-40.338
2.920.750	-29.623	2.943.065	-41.070	2.892.130	-40.340
2.920.872	-29.625	2.943.203	-41.072	2.892.299	-40.341
2.921.042	-29.627	2.943.340	-41.074	2.892.473	-40.343

2.921.255	-29.629	2.943.474	-41.076	2.892.709	-40.345
2.921.450	-29.632	2.943.604	-41.078	2.892.958	-40.346
2.921.646	-29.634	2.943.766	-41.080	2.893.138	-40.348
2.921.864	-29.636	2.943.972	-41.081	2.893.260	-40.350
2.922.014	-29.638	2.944.204	-41.083	2.893.388	-40.351
2.922.155	-29.640	2.944.377	-41.085	2.893.578	-40.353
2.922.341	-29.642	2.944.496	-41.087	2.893.763	-40.355
2.922.480	-29.644	2.944.645	-41.089	2.893.936	-40.356
2.922.625	-29.646	2.944.779	-41.091	2.894.088	-40.358
2.922.821	-29.648	2.944.942	-41.093	2.894.263	-40.360
2.922.978	-29.650	2.945.209	-41.095	2.894.445	-40.361
2.923.116	-29.652	2.945.406	-41.097	2.894.514	-40.363
2.923.289	-29.654	2.945.489	-41.099	2.894.630	-40.365
2.923.452	-29.656	2.945.602	-41.100	2.894.864	-40.366
2.923.598	-29.658	2.945.725	-41.102	2.895.075	-40.368
2.923.756	-29.661	2.945.935	-41.104	2.895.320	-40.370
2.923.914	-29.663	2.946.192	-41.106	2.895.555	-40.371
2.924.084	-29.665	2.946.335	-41.108	2.895.681	-40.373
2.924.277	-29.667	2.946.473	-41.110	2.895.779	-40.375
2.924.482	-29.669	2.946.627	-41.111	2.895.901	-40.376
2.924.629	-29.671	2.946.792	-41.113	2.896.065	-40.378
2.924.705	-29.673	2.946.983	-41.115	2.896.282	-40.380
2.924.873	-29.675	2.947.133	-41.117	2.896.455	-40.381
2.925.118	-29.677	2.947.239	-41.118	2.896.613	-40.383
2.925.270	-29.679	2.947.346	-41.120	2.896.805	-40.384
2.925.443	-29.681	2.947.466	-41.122	2.896.931	-40.386
2.925.631	-29.683	2.947.629	-41.123	2.897.004	-40.387
2.925.782	-29.686	2.947.864	-41.125	2.897.148	-40.389
2.926.003	-29.688	2.948.040	-41.127	2.897.336	-40.391
2.926.181	-29.690	2.948.159	-41.128	2.897.498	-40.392

2.926.259	-29.692	2.948.301	-41.130	2.897.684	-40.394
2.926.387	-29.694	2.948.500	-41.131	2.897.857	-40.395
2.926.573	-29.696	2.948.760	-41.133	2.898.029	-40.397
2.926.828	-29.698	2.948.961	-41.135	2.898.189	-40.398
2.927.001	-29.700	2.949.153	-41.136	2.898.340	-40.399
2.927.068	-29.702	2.949.362	-41.138	2.898.575	-40.401
2.927.224	-29.704	2.949.547	-41.139	2.898.792	-40.402
2.927.406	-29.706	2.949.796	-41.141	2.898.954	-40.404
2.927.581	-29.709	2.949.998	-41.142	2.899.102	-40.405
2.927.737	-29.711	2.950.104	-41.143	2.899.276	-40.407
2.927.892	-29.713	2.950.198	-41.145	2.899.469	-40.408
2.928.091	-29.715	2.950.339	-41.146	2.899.638	-40.409
2.928.286	-29.717	2.950.479	-41.147	2.899.785	-40.411
2.928.526	-29.719	2.950.587	-41.149	2.899.933	-40.412
2.928.680	-29.721	2.950.710	-41.150	2.900.100	-40.413
2.928.744	-29.723	2.950.858	-41.151	2.900.237	-40.415
2.928.835	-29.725	2.951.014	-41.152	2.900.354	-40.416
2.928.972	-29.727	2.951.201	-41.154	2.900.529	-40.417
2.929.158	-29.729	2.951.491	-41.155	2.900.708	-40.419
2.929.265	-29.732	2.951.696	-41.156	2.900.804	-40.420
2.929.438	-29.734	2.951.884	-41.157	2.900.971	-40.421
2.929.613	-29.736	2.952.090	-41.158	2.901.194	-40.422
2.929.785	-29.738	2.952.225	-41.159	2.901.346	-40.424
2.930.081	-29.740	2.952.357	-41.160	2.901.479	-40.425
2.930.363	-29.742	2.952.531	-41.161	2.901.671	-40.426
2.930.577	-29.744	2.952.737	-41.163	2.901.913	-40.427
2.930.719	-29.746	2.952.929	-41.164	2.902.113	-40.428
2.930.862	-29.748	2.953.072	-41.165	2.902.235	-40.430
2.931.017	-29.750	2.953.171	-41.165	2.902.390	-40.431
2.931.138	-29.752	2.953.290	-41.166	2.902.610	-40.432

2.931.295	-29.755	2.953.470	-41.167	2.902.827	-40.433
2.931.437	-29.757	2.953.643	-41.168	2.902.972	-40.434
2.931.609	-29.759	2.953.846	-41.169	2.903.130	-40.435
2.931.744	-29.761	2.954.019	-41.170	2.903.302	-40.436
2.931.837	-29.763	2.954.205	-41.171	2.903.506	-40.438
2.932.000	-29.765	2.954.416	-41.172	2.903.727	-40.439
2.932.180	-29.767	2.954.619	-41.172	2.903.901	-40.440
2.932.350	-29.769	2.954.788	-41.173	2.904.102	-40.441
2.932.509	-29.771	2.954.890	-41.174	2.904.200	-40.442
2.932.679	-29.773	2.955.002	-41.175	2.904.312	-40.443
2.932.907	-29.776	2.955.197	-41.175	2.904.519	-40.444
2.933.134	-29.778	2.955.395	-41.176	2.904.641	-40.445
2.933.336	-29.780	2.955.512	-41.177	2.904.754	-40.446
2.933.456	-29.782	2.955.668	-41.177	2.904.894	-40.447
2.933.578	-29.784	2.955.905	-41.178	2.905.143	-40.448
2.933.724	-29.786	2.956.087	-41.179	2.905.371	-40.449
2.933.954	-29.788	2.956.234	-41.179	2.905.515	-40.450
2.934.169	-29.790	2.956.374	-41.180	2.905.650	-40.451
2.934.291	-29.792	2.956.506	-41.181	2.905.815	-40.452
2.934.435	-29.795	2.956.642	-41.181	2.905.984	-40.454
2.934.606	-29.797	2.956.786	-41.182	2.906.100	-40.455
2.934.800	-29.799	2.957.046	-41.182	2.906.313	-40.456
2.934.915	-29.801	2.957.447	-41.183	2.906.472	-40.457
2.935.064	-29.803	2.957.741	-41.183	2.906.573	-40.458
2.935.285	-29.805	2.957.904	-41.184	2.906.820	-40.459
2.935.458	-29.807	2.958.080	-41.184	2.906.992	-40.460
2.935.616	-29.809	2.958.192	-41.185	2.907.093	-40.461
2.935.784	-29.812	2.958.226	-41.185	2.907.216	-40.462
2.935.923	-29.814	2.958.246	-41.186	2.907.393	-40.463
2.936.054	-29.816	2.958.374	-41.186	2.907.589	-40.464

2.936.248	-29.818	2.958.555	-41.187	2.907.809	-40.465
2.936.437	-29.820	2.958.697	-41.187	2.908.020	-40.466
2.936.609	-29.822	2.958.851	-41.187	2.908.096	-40.467
2.936.819	-29.824	2.959.023	-41.188	2.908.232	-40.468
2.936.982	-29.827	2.959.207	-41.188	2.908.441	-40.469
2.937.133	-29.829	2.959.364	-41.188	2.908.871	-40.471
2.937.341	-29.831	2.959.546	-41.189	2.909.330	-40.472
2.937.442	-29.833	2.959.743	-41.189	2.909.530	-40.473
2.937.547	-29.835	2.959.900	-41.190	2.909.580	-40.474
2.937.824	-29.837	2.960.139	-41.190	2.909.616	-40.475
2.938.018	-29.840	2.960.287	-41.190	2.909.734	-40.476
2.938.107	-29.842	2.960.368	-41.191	2.909.848	-40.477
2.938.236	-29.844	2.960.519	-41.191	2.909.872	-40.478
2.938.546	-29.846	2.960.677	-41.191	2.909.960	-40.480
2.939.041	-29.848	2.960.841	-41.192	2.910.096	-40.481
2.939.410	-29.850	2.961.003	-41.192	2.910.245	-40.482
2.939.475	-29.853	2.961.156	-41.192	2.910.414	-40.483
2.939.460	-29.855	2.961.286	-41.192	2.910.591	-40.484
2.939.489	-29.857	2.961.458	-41.193	2.910.760	-40.485
2.939.521	-29.859	2.961.653	-41.193	2.910.919	-40.487
2.939.634	-29.861	2.961.846	-41.193	2.911.107	-40.488
2.939.771	-29.864	2.962.039	-41.194	2.911.270	-40.489
2.939.913	-29.866	2.962.224	-41.194	2.911.461	-40.490
2.940.092	-29.868	2.962.372	-41.194	2.911.716	-40.492
2.940.238	-29.870	2.962.547	-41.195	2.911.932	-40.493
2.940.396	-29.872	2.962.763	-41.195	2.912.086	-40.494
2.940.628	-29.875	2.962.886	-41.195	2.912.228	-40.496
2.940.819	-29.877	2.963.000	-41.196	2.912.361	-40.497
2.940.974	-29.879	2.963.148	-41.196	2.912.343	-40.498
2.941.136	-29.881	2.963.318	-41.196	2.912.542	-40.500

2.941.306	-29.884	2.963.532	-41.197	2.912.742	-40.501
2.941.429	-29.886	2.963.662	-41.197	2.912.839	-40.502
2.941.584	-29.888	2.963.781	-41.197	2.913.011	-40.504
2.941.788	-29.890	2.963.991	-41.198	2.913.167	-40.505
2.941.968	-29.893	2.964.204	-41.198	2.913.387	-40.507
2.942.155	-29.895	2.964.373	-41.198	2.913.564	-40.508
2.942.303	-29.897	2.964.503	-41.199	2.913.641	-40.510
2.942.491	-29.899	2.964.653	-41.199	2.913.797	-40.511
2.942.655	-29.902	2.964.857	-41.199	2.913.997	-40.513
2.942.781	-29.904	2.964.970	-41.200	2.914.215	-40.515
2.942.928	-29.906	2.965.067	-41.200	2.914.413	-40.516
2.943.099	-29.908	2.965.299	-41.201	2.914.581	-40.518
2.943.272	-29.911	2.965.562	-41.201	2.914.742	-40.519
2.943.428	-29.913	2.965.757	-41.201	2.914.919	-40.521
2.943.637	-29.915	2.965.916	-41.202	2.915.121	-40.523
2.943.874	-29.917	2.966.062	-41.202	2.915.338	-40.524
2.944.041	-29.920	2.966.176	-41.203	2.915.508	-40.526
2.944.203	-29.922	2.966.349	-41.203	2.915.609	-40.528
2.944.338	-29.924	2.966.589	-41.204	2.915.753	-40.529
2.944.438	-29.927	2.966.792	-41.204	2.915.917	-40.531
2.944.568	-29.929	2.966.902	-41.205	2.916.069	-40.533
2.944.688	-29.931	2.967.036	-41.205	2.916.205	-40.535
2.944.854	-29.934	2.967.209	-41.206	2.916.349	-40.537
2.945.003	-29.936	2.967.329	-41.206	2.916.635	-40.538
2.945.157	-29.938	2.967.452	-41.207	2.916.779	-40.540
2.945.374	-29.940	2.967.650	-41.208	2.916.870	-40.542
2.945.559	-29.943	2.967.904	-41.208	2.917.146	-40.544
2.945.783	-29.945	2.968.081	-41.209	2.917.386	-40.546
2.945.985	-29.947	2.968.232	-41.209	2.917.372	-40.548
2.946.094	-29.950	2.968.388	-41.210	2.917.369	-40.550

2.946.277	-29.952	2.968.554	-41.211	2.917.687	-40.551
2.946.476	-29.954	2.968.739	-41.211	2.917.940	-40.553
2.946.598	-29.957	2.968.875	-41.212	2.918.081	-40.555
2.946.750	-29.959	2.968.961	-41.213	2.918.246	-40.557
2.946.925	-29.961	2.969.052	-41.213	2.918.351	-40.559
2.947.080	-29.964	2.969.189	-41.214	2.918.488	-40.561
2.947.275	-29.966	2.969.288	-41.215	2.918.735	-40.563
2.947.485	-29.969	2.969.406	-41.215	2.918.943	-40.565
2.947.672	-29.971	2.969.692	-41.216	2.919.075	-40.567
2.947.849	-29.973	2.969.937	-41.217	2.919.196	-40.569
2.947.998	-29.976	2.970.047	-41.217	2.919.270	-40.571
2.948.163	-29.978	2.970.258	-41.218	2.919.528	-40.573
2.948.336	-29.980	2.970.500	-41.219	2.919.789	-40.575
2.948.477	-29.983	2.970.732	-41.220	2.920.007	-40.577
2.948.590	-29.985	2.970.910	-41.220	2.920.096	-40.579
2.948.752	-29.987	2.971.002	-41.221	2.920.360	-40.581
2.948.968	-29.990	2.971.155	-41.222	2.920.518	-40.583
2.949.099	-29.992	2.971.365	-41.223	2.920.594	-40.585
2.949.250	-29.995	2.971.544	-41.223	2.920.726	-40.587
2.949.453	-29.997	2.971.718	-41.224	2.920.892	-40.589
2.949.626	-29.999	2.971.848	-41.225	2.921.089	-40.591
2.949.749	-30.002	2.971.927	-41.226	2.921.274	-40.593
2.949.911	-30.004	2.972.018	-41.227	2.921.400	-40.595
2.950.107	-30.007	2.972.141	-41.227	2.921.555	-40.597
2.950.276	-30.009	2.972.336	-41.228	2.921.773	-40.599
2.950.381	-30.011	2.972.542	-41.229	2.921.949	-40.601
2.950.529	-30.014	2.972.628	-41.230	2.922.059	-40.603
2.950.753	-30.016	2.972.828	-41.230	2.922.202	-40.604
2.950.901	-30.019	2.973.123	-41.231	2.922.390	-40.606
2.950.999	-30.021	2.973.318	-41.232	2.922.568	-40.608

2.951.104	-30.024	2.973.517	-41.233	2.922.758	-40.610
2.951.313	-30.026	2.973.713	-41.234	2.922.917	-40.612
2.951.483	-30.029	2.973.901	-41.235	2.923.076	-40.614
2.951.647	-30.031	2.974.057	-41.235	2.923.206	-40.616
2.951.852	-30.033	2.974.211	-41.236	2.923.327	-40.617
2.952.077	-30.036	2.974.362	-41.237	2.923.423	-40.619
2.952.329	-30.038	2.974.518	-41.238	2.923.604	-40.621
2.952.487	-30.041	2.974.703	-41.239	2.923.802	-40.623
2.952.639	-30.043	2.974.875	-41.239	2.924.001	-40.625
2.952.825	-30.046	2.975.028	-41.240	2.924.165	-40.626
2.953.008	-30.048	2.975.169	-41.241	2.924.232	-40.628
2.953.131	-30.051	2.975.298	-41.242	2.924.446	-40.630
2.953.264	-30.053	2.975.453	-41.243	2.924.792	-40.631
2.953.430	-30.056	2.975.699	-41.243	2.925.027	-40.633
2.953.576	-30.058	2.975.912	-41.244	2.925.111	-40.635
2.953.721	-30.061	2.976.096	-41.245	2.925.208	-40.636
2.953.832	-30.063	2.976.317	-41.246	2.925.407	-40.638
2.953.990	-30.066	2.976.432	-41.246	2.925.421	-40.639
2.954.157	-30.068	2.976.533	-41.247	2.925.390	-40.641
2.954.342	-30.071	2.976.692	-41.248	2.925.796	-40.643
2.954.510	-30.073	2.976.866	-41.249	2.926.080	-40.644
2.954.702	-30.076	2.977.064	-41.250	2.926.244	-40.646
2.954.941	-30.078	2.977.203	-41.250	2.926.405	-40.647
2.955.157	-30.081	2.977.373	-41.251	2.926.523	-40.648
2.955.350	-30.083	2.977.611	-41.252	2.926.696	-40.650
2.955.505	-30.086	2.977.756	-41.253	2.926.862	-40.651
2.955.659	-30.088	2.977.863	-41.254	2.927.022	-40.653
2.955.841	-30.091	2.977.993	-41.254	2.927.195	-40.654
2.955.999	-30.093	2.978.182	-41.255	2.927.401	-40.656
2.956.165	-30.096	2.978.340	-41.256	2.927.577	-40.657

2.956.374	-30.098	2.978.492	-41.257	2.927.712	-40.658
2.956.479	-30.101	2.978.858	-41.257	2.927.845	-40.660
2.956.555	-30.103	2.979.309	-41.258	2.928.047	-40.661
2.956.747	-30.106	2.979.536	-41.259	2.928.286	-40.662
2.956.982	-30.108	2.979.534	-41.260	2.928.466	-40.663
2.957.191	-30.111	2.979.557	-41.260	2.928.626	-40.665
2.957.300	-30.113	2.979.667	-41.261	2.928.836	-40.666
2.957.412	-30.116	2.979.774	-41.262	2.929.049	-40.667
2.957.600	-30.119	2.979.877	-41.263	2.929.180	-40.668
2.957.788	-30.121	2.980.023	-41.263	2.929.276	-40.670
2.957.926	-30.124	2.980.164	-41.264	2.929.432	-40.671
2.958.123	-30.126	2.980.309	-41.265	2.929.626	-40.672
2.958.341	-30.129	2.980.536	-41.266	2.929.775	-40.673
2.958.481	-30.131	2.980.692	-41.266	2.929.966	-40.674
2.958.654	-30.134	2.980.820	-41.267	2.930.053	-40.675
2.958.835	-30.136	2.981.024	-41.268	2.930.161	-40.676
2.958.988	-30.139	2.981.278	-41.269	2.930.377	-40.678
2.959.140	-30.142	2.981.473	-41.269	2.930.601	-40.679
2.959.348	-30.144	2.981.564	-41.270	2.930.787	-40.680
2.959.518	-30.147	2.981.737	-41.271	2.930.899	-40.681
2.959.597	-30.149	2.981.903	-41.272	2.931.078	-40.682
2.959.745	-30.152	2.982.012	-41.272	2.931.289	-40.683
2.959.966	-30.154	2.982.224	-41.273	2.931.512	-40.684
2.960.104	-30.157	2.982.404	-41.274	2.931.715	-40.685
2.960.222	-30.159	2.982.506	-41.274	2.931.862	-40.686
2.960.637	-30.162	2.982.686	-41.275	2.932.025	-40.687
2.961.086	-30.165	2.982.859	-41.276	2.932.198	-40.688
2.961.179	-30.167	2.982.975	-41.277	2.932.337	-40.689
2.961.220	-30.170	2.983.141	-41.277	2.932.485	-40.690
2.961.387	-30.172	2.983.427	-41.278	2.932.647	-40.691

2.961.505	-30.175	2.983.643	-41.279	2.932.859	-40.693
2.961.581	-30.177	2.983.778	-41.280	2.933.033	-40.694
2.961.661	-30.180	2.983.959	-41.281	2.933.178	-40.695
2.961.766	-30.182	2.984.127	-41.281	2.933.352	-40.696
2.961.911	-30.185	2.984.272	-41.282	2.933.535	-40.697
2.962.066	-30.188	2.984.375	-41.283	2.933.651	-40.698
2.962.245	-30.190	2.984.537	-41.284	2.933.744	-40.699
2.962.409	-30.193	2.984.763	-41.285	2.933.945	-40.700
2.962.607	-30.195	2.984.905	-41.285	2.934.149	-40.701
2.962.823	-30.198	2.985.016	-41.286	2.934.355	-40.702
2.963.005	-30.200	2.985.215	-41.287	2.934.511	-40.703
2.963.188	-30.203	2.985.408	-41.288	2.934.648	-40.705
2.963.322	-30.205	2.985.508	-41.289	2.935.143	-40.706
2.963.466	-30.208	2.985.643	-41.290	2.935.504	-40.707
2.963.625	-30.210	2.985.814	-41.290	2.935.607	-40.708
2.963.786	-30.213	2.985.956	-41.291	2.935.753	-40.709
2.963.941	-30.215	2.986.125	-41.292	2.935.822	-40.710
2.964.095	-30.218	2.986.310	-41.293	2.935.939	-40.712
2.964.268	-30.220	2.986.461	-41.294	2.936.046	-40.713
2.964.428	-30.223	2.986.598	-41.295	2.936.100	-40.714
2.964.609	-30.226	2.986.766	-41.296	2.936.086	-40.715
2.964.797	-30.228	2.986.999	-41.297	2.936.150	-40.716
2.964.963	-30.231	2.987.167	-41.298	2.936.440	-40.718
2.965.148	-30.233	2.987.263	-41.299	2.936.609	-40.719
2.965.302	-30.236	2.987.452	-41.300	2.936.625	-40.720
2.965.426	-30.238	2.987.625	-41.301	2.936.754	-40.722
2.965.639	-30.240	2.987.758	-41.302	2.937.029	-40.723
2.965.833	-30.243	2.987.980	-41.303	2.937.310	-40.724
2.965.999	-30.245	2.988.182	-41.304	2.937.447	-40.726
2.966.174	-30.248	2.988.242	-41.305	2.937.596	-40.727

2.966.317	-30.250	2.988.372	-41.306	2.937.794	-40.729
2.966.494	-30.253	2.988.600	-41.307	2.937.890	-40.730
2.966.647	-30.255	2.988.799	-41.308	2.938.060	-40.732
2.966.783	-30.258	2.988.973	-41.309	2.938.287	-40.733
2.966.911	-30.260	2.989.194	-41.310	2.938.467	-40.735
2.967.083	-30.263	2.989.382	-41.312	2.938.638	-40.736
2.967.270	-30.265	2.989.536	-41.313	2.938.788	-40.738
2.967.426	-30.267	2.989.666	-41.314	2.938.929	-40.740
2.967.570	-30.270	2.989.812	-41.315	2.939.019	-40.741
2.967.737	-30.272	2.989.970	-41.316	2.939.221	-40.743
2.967.899	-30.275	2.990.104	-41.318	2.939.362	-40.745
2.968.030	-30.277	2.990.254	-41.319	2.939.558	-40.746
2.968.224	-30.279	2.990.424	-41.320	2.939.767	-40.748
2.968.391	-30.282	2.990.566	-41.321	2.939.942	-40.750
2.968.515	-30.284	2.990.656	-41.323	2.939.940	-40.752
2.968.670	-30.287	2.990.844	-41.324	2.940.032	-40.754
2.968.851	-30.289	2.991.014	-41.325	2.940.258	-40.756
2.969.091	-30.291	2.991.125	-41.326	2.940.527	-40.758
2.969.301	-30.294	2.991.325	-41.328	2.940.775	-40.760
2.969.439	-30.296	2.991.523	-41.329	2.940.945	-40.762
2.969.608	-30.298	2.991.761	-41.330	2.941.111	-40.764
2.969.742	-30.301	2.991.994	-41.332	2.941.237	-40.766
2.969.919	-30.303	2.992.216	-41.333	2.941.447	-40.768
2.970.143	-30.305	2.992.372	-41.335	2.941.654	-40.770
2.970.273	-30.308	2.992.474	-41.336	2.941.775	-40.772
2.970.425	-30.310	2.992.647	-41.337	2.941.902	-40.774
2.970.659	-30.312	2.992.885	-41.339	2.942.010	-40.777
2.970.854	-30.315	2.993.069	-41.340	2.942.198	-40.779
2.970.981	-30.317	2.993.228	-41.342	2.942.400	-40.781
2.971.136	-30.319	2.993.333	-41.343	2.942.554	-40.783

2.971.282	-30.321	2.993.387	-41.345	2.942.708	-40.786
2.971.404	-30.324	2.993.531	-41.346	2.942.896	-40.788
2.971.584	-30.326	2.993.705	-41.348	2.943.069	-40.791
2.971.810	-30.328	2.993.785	-41.349	2.943.206	-40.793
2.972.019	-30.331	2.993.947	-41.351	2.943.369	-40.795
2.972.178	-30.333	2.994.154	-41.352	2.943.571	-40.798
2.972.294	-30.335	2.994.302	-41.354	2.943.705	-40.800
2.972.433	-30.337	2.994.522	-41.355	2.943.864	-40.803
2.972.617	-30.339	2.994.727	-41.357	2.944.049	-40.805
2.972.704	-30.342	2.994.924	-41.359	2.944.190	-40.808
2.972.740	-30.344	2.995.168	-41.360	2.944.341	-40.811
2.972.906	-30.346	2.995.327	-41.362	2.944.532	-40.813
2.973.036	-30.348	2.995.468	-41.363	2.944.727	-40.816
2.973.210	-30.351	2.995.574	-41.365	2.944.869	-40.819
2.973.517	-30.353	2.995.768	-41.367	2.945.038	-40.821
2.973.742	-30.355	2.996.060	-41.368	2.945.244	-40.824
2.973.921	-30.357	2.996.169	-41.370	2.945.466	-40.827
2.974.160	-30.359	2.996.295	-41.372	2.945.632	-40.829
2.974.375	-30.361	2.996.512	-41.374	2.945.746	-40.832
2.974.546	-30.364	2.996.638	-41.375	2.945.877	-40.835
2.974.677	-30.366	2.996.827	-41.377	2.946.090	-40.837
2.974.818	-30.368	2.996.971	-41.379	2.946.313	-40.840
2.974.996	-30.370	2.997.138	-41.380	2.946.454	-40.843
2.975.147	-30.372	2.997.383	-41.382	2.946.596	-40.846
2.975.301	-30.374	2.997.557	-41.384	2.946.775	-40.848
2.975.428	-30.376	2.997.741	-41.386	2.946.964	-40.851
2.975.540	-30.379	2.997.914	-41.388	2.947.095	-40.854
2.975.699	-30.381	2.998.080	-41.389	2.947.246	-40.857
2.975.879	-30.383	2.998.282	-41.391	2.947.419	-40.860
2.976.036	-30.385	2.998.423	-41.393	2.947.603	-40.862

2.976.101	-30.387	2.998.558	-41.395	2.947.767	-40.865
2.976.270	-30.389	2.998.723	-41.397	2.947.914	-40.868
2.976.559	-30.391	2.998.889	-41.398	2.948.138	-40.871
2.976.751	-30.393	2.999.031	-41.400	2.948.297	-40.873
2.976.971	-30.395	2.999.127	-41.402	2.948.457	-40.876
2.977.162	-30.398	2.999.279	-41.404	2.948.656	-40.879
2.977.346	-30.400	2.999.485	-41.406	2.948.789	-40.881
2.977.492	-30.402	2.999.691	-41.408	2.948.882	-40.884
2.977.628	-30.404	2.999.807	-41.410	2.948.961	-40.887
2.977.836	-30.406	2.999.911	-41.411	2.949.196	-40.890
2.978.009	-30.408	3.000.260	-41.413	2.949.453	-40.892
2.978.150	-30.410	3.000.783	-41.415	2.949.575	-40.895
2.978.329	-30.412	3.001.013	-41.417	2.949.648	-40.898
2.978.540	-30.414	3.000.997	-41.419	2.949.771	-40.900
2.978.652	-30.416	3.001.112	-41.421	2.949.977	-40.903
2.978.777	-30.418	3.001.180	-41.423	2.950.129	-40.905
2.979.046	-30.420	3.001.215	-41.425	2.950.287	-40.908
2.979.244	-30.422	3.001.291	-41.426	2.950.530	-40.910
2.979.366	-30.424	3.001.380	-41.428	2.950.754	-40.913
2.979.541	-30.426	3.001.531	-41.430	2.950.888	-40.916
2.979.687	-30.428	3.001.690	-41.432	2.951.071	-40.918
2.979.875	-30.430	3.001.859	-41.434	2.951.324	-40.920
2.980.089	-30.432	3.002.064	-41.436	2.951.578	-40.923
2.980.216	-30.434	3.002.256	-41.438	2.951.751	-40.925
2.980.310	-30.436	3.002.412	-41.440	2.951.913	-40.928
2.980.464	-30.438	3.002.643	-41.442	2.952.068	-40.930
2.980.696	-30.440	3.002.914	-41.444	2.952.185	-40.932
2.980.854	-30.442	3.003.037	-41.445	2.952.364	-40.935
2.981.017	-30.444	3.003.177	-41.447	2.952.500	-40.937
2.981.215	-30.446	3.003.341	-41.449	2.952.542	-40.939

2.981.333	-30.448	3.003.479	-41.451	2.952.677	-40.941
2.981.469	-30.450	3.003.658	-41.453	2.952.889	-40.944
2.981.661	-30.452	3.003.781	-41.455	2.953.030	-40.946
2.981.841	-30.454	3.004.001	-41.457	2.953.188	-40.948
2.981.978	-30.456	3.004.168	-41.459	2.953.358	-40.950
2.982.144	-30.458	3.004.277	-41.461	2.953.547	-40.952
2.982.487	-30.460	3.004.482	-41.463	2.953.784	-40.954
2.982.932	-30.462	3.004.653	-41.465	2.953.945	-40.956
2.983.179	-30.464	3.004.853	-41.467	2.954.060	-40.958
2.983.253	-30.466	3.005.049	-41.468	2.954.265	-40.960
2.983.298	-30.468	3.005.224	-41.470	2.954.483	-40.962
2.983.311	-30.470	3.005.385	-41.472	2.954.682	-40.964
2.983.320	-30.472	3.005.559	-41.474	2.954.843	-40.966
2.983.403	-30.474	3.005.714	-41.476	2.955.000	-40.967
2.983.539	-30.476	3.005.863	-41.478	2.955.157	-40.969
2.983.706	-30.478	3.006.087	-41.480	2.955.313	-40.971
2.983.869	-30.480	3.006.295	-41.482	2.955.504	-40.973
2.984.035	-30.482	3.006.422	-41.484	2.955.672	-40.974
2.984.245	-30.484	3.006.510	-41.486	2.955.764	-40.976
2.984.443	-30.486	3.006.640	-41.487	2.955.906	-40.978
2.984.640	-30.488	3.006.855	-41.489	2.956.161	-40.979
2.984.864	-30.490	3.007.033	-41.491	2.956.372	-40.981
2.985.045	-30.492	3.007.167	-41.493	2.956.515	-40.982
2.985.190	-30.494	3.007.343	-41.495	2.956.622	-40.984
2.985.363	-30.496	3.007.525	-41.497	2.956.701	-40.985
2.985.525	-30.498	3.007.679	-41.499	2.956.983	-40.987
2.985.671	-30.500	3.007.838	-41.501	2.957.268	-40.988
2.985.834	-30.502	3.007.961	-41.503	2.957.362	-40.990
2.986.038	-30.503	3.008.095	-41.504	2.957.477	-40.991
2.986.217	-30.505	3.008.287	-41.506	2.957.682	-40.992

2.986.393	-30.507	3.008.475	-41.508	2.957.926	-40.994
2.986.520	-30.509	3.008.699	-41.510	2.958.087	-40.995
2.986.592	-30.511	3.008.878	-41.512	2.958.226	-40.996
2.986.744	-30.513	3.008.968	-41.514	2.958.366	-40.998
2.986.956	-30.515	3.009.111	-41.516	2.958.528	-40.999
2.987.171	-30.517	3.009.312	-41.518	2.958.698	-41.000
2.987.339	-30.519	3.009.508	-41.519	2.958.869	-41.001
2.987.498	-30.521	3.009.682	-41.521	2.959.055	-41.002
2.987.700	-30.523	3.009.869	-41.523	2.959.225	-41.003
2.987.867	-30.525	3.010.038	-41.525	2.959.388	-41.005
2.988.005	-30.527	3.010.177	-41.527	2.959.579	-41.006
2.988.170	-30.529	3.010.343	-41.529	2.959.752	-41.007
2.988.361	-30.530	3.010.534	-41.530	2.959.886	-41.008
2.988.470	-30.532	3.010.719	-41.532	2.960.005	-41.009
2.988.564	-30.534	3.010.852	-41.534	2.960.172	-41.010
2.988.750	-30.536	3.010.995	-41.536	2.960.382	-41.011
2.988.929	-30.538	3.011.183	-41.538	2.960.552	-41.012
2.989.073	-30.540	3.011.321	-41.539	2.960.605	-41.013
2.989.268	-30.542	3.011.374	-41.541	2.960.736	-41.014
2.989.442	-30.544	3.011.505	-41.543	2.961.176	-41.015
2.989.609	-30.546	3.011.750	-41.545	2.961.614	-41.015
2.989.792	-30.548	3.011.932	-41.546	2.961.885	-41.016
2.989.927	-30.550	3.012.037	-41.548	2.961.992	-41.017
2.990.047	-30.552	3.012.188	-41.550	2.962.011	-41.018
2.990.140	-30.553	3.012.397	-41.551	2.962.059	-41.019
2.990.302	-30.555	3.012.585	-41.553	2.962.144	-41.020
2.990.541	-30.557	3.012.747	-41.555	2.962.217	-41.021
2.990.753	-30.559	3.012.907	-41.556	2.962.289	-41.021
2.990.928	-30.561	3.013.109	-41.558	2.962.416	-41.022
2.991.093	-30.563	3.013.365	-41.560	2.962.575	-41.023

2.991.259	-30.565	3.013.553	-41.561	2.962.701	-41.024
2.991.468	-30.567	3.013.750	-41.563	2.962.856	-41.025
2.991.664	-30.569	3.013.992	-41.564	2.963.130	-41.025
2.991.719	-30.571	3.014.153	-41.566	2.963.338	-41.026
2.991.867	-30.572	3.014.299	-41.567	2.963.503	-41.027
2.992.129	-30.574	3.014.389	-41.569	2.963.691	-41.028
2.992.260	-30.576	3.014.508	-41.570	2.963.847	-41.028
2.992.422	-30.578	3.014.658	-41.572	2.964.023	-41.029
2.992.628	-30.580	3.014.783	-41.573	2.964.221	-41.030
2.992.765	-30.582	3.014.991	-41.575	2.964.418	-41.031
2.992.899	-30.584	3.015.118	-41.576	2.964.575	-41.031
2.993.123	-30.586	3.015.215	-41.578	2.964.617	-41.032
2.993.327	-30.588	3.015.413	-41.579	2.964.741	-41.033
2.993.495	-30.590	3.015.555	-41.581	2.964.922	-41.034
2.993.644	-30.592	3.015.697	-41.582	2.965.099	-41.034
2.993.755	-30.594	3.015.960	-41.583	2.965.280	-41.035
2.993.954	-30.595	3.016.212	-41.585	2.965.464	-41.036
2.994.161	-30.597	3.016.396	-41.586	2.965.682	-41.037
2.994.291	-30.599	3.016.620	-41.587	2.965.804	-41.038
2.994.380	-30.601	3.016.714	-41.589	2.965.981	-41.038
2.994.489	-30.603	3.016.761	-41.590	2.966.195	-41.039
2.994.681	-30.605	3.016.997	-41.591	2.966.350	-41.040
2.994.882	-30.607	3.017.211	-41.593	2.966.571	-41.041
2.995.042	-30.609	3.017.365	-41.594	2.966.801	-41.042
2.995.151	-30.611	3.017.518	-41.595	2.966.934	-41.042
2.995.302	-30.613	3.017.679	-41.596	2.967.099	-41.043
2.995.549	-30.615	3.017.881	-41.598	2.967.254	-41.044
2.995.768	-30.617	3.018.081	-41.599	2.967.390	-41.045
2.995.981	-30.619	3.018.224	-41.600	2.967.550	-41.046
2.996.145	-30.621	3.018.334	-41.601	2.967.708	-41.047

2.996.299	-30.623	3.018.525	-41.603	2.967.892	-41.047
2.996.490	-30.624	3.018.732	-41.604	2.968.041	-41.048
2.996.669	-30.626	3.018.922	-41.605	2.968.152	-41.049
2.996.870	-30.628	3.019.138	-41.606	2.968.325	-41.050
2.996.943	-30.630	3.019.266	-41.607	2.968.499	-41.051
2.997.065	-30.632	3.019.298	-41.608	2.968.597	-41.052
2.997.270	-30.634	3.019.479	-41.610	2.968.719	-41.053
2.997.339	-30.636	3.019.767	-41.611	2.968.893	-41.054
2.997.431	-30.638	3.019.920	-41.612	2.969.113	-41.055
2.997.585	-30.640	3.019.992	-41.613	2.969.340	-41.056
2.997.805	-30.642	3.020.198	-41.614	2.969.500	-41.057
2.998.047	-30.644	3.020.472	-41.615	2.969.648	-41.058
2.998.235	-30.646	3.020.616	-41.616	2.969.811	-41.059
2.998.365	-30.648	3.020.713	-41.617	2.970.000	-41.060
2.998.508	-30.650	3.020.909	-41.618	2.970.154	-41.061
2.998.741	-30.652	3.021.129	-41.620	2.970.242	-41.062
2.998.984	-30.654	3.021.291	-41.621	2.970.378	-41.063
2.999.131	-30.656	3.021.452	-41.622	2.970.587	-41.064
2.999.268	-30.658	3.021.751	-41.623	2.970.758	-41.065
2.999.467	-30.660	3.022.155	-41.624	2.970.941	-41.066
2.999.631	-30.662	3.022.357	-41.625	2.971.061	-41.067
2.999.783	-30.665	3.022.430	-41.626	2.971.158	-41.069
2.999.981	-30.667	3.022.510	-41.627	2.971.349	-41.070
3.000.175	-30.669	3.022.588	-41.628	2.971.559	-41.071
3.000.341	-30.671	3.022.657	-41.629	2.971.719	-41.072
3.000.515	-30.673	3.022.683	-41.630	2.971.891	-41.073
3.000.675	-30.675	3.022.780	-41.631	2.972.099	-41.074
3.000.844	-30.677	3.022.968	-41.632	2.972.361	-41.076
3.001.003	-30.679	3.023.132	-41.633	2.972.516	-41.077
3.001.138	-30.681	3.023.345	-41.634	2.972.625	-41.078

3.001.306	-30.683	3.023.567	-41.635	2.972.870	-41.079
3.001.548	-30.685	3.023.698	-41.636	2.973.044	-41.081
3.001.757	-30.687	3.023.912	-41.637	2.973.137	-41.082
3.001.909	-30.690	3.024.089	-41.638	2.973.333	-41.083
3.002.062	-30.692	3.024.193	-41.639	2.973.528	-41.084
3.002.191	-30.694	3.024.429	-41.640	2.973.666	-41.086
3.002.350	-30.696	3.024.597	-41.641	2.973.822	-41.087
3.002.550	-30.698	3.024.709	-41.642	2.973.999	-41.088
3.002.773	-30.700	3.024.888	-41.643	2.974.216	-41.090
3.002.942	-30.702	3.025.034	-41.644	2.974.398	-41.091
3.003.073	-30.705	3.025.139	-41.645	2.974.490	-41.092
3.003.232	-30.707	3.025.285	-41.646	2.974.631	-41.093
3.003.359	-30.709	3.025.468	-41.647	2.974.861	-41.095
3.003.493	-30.711	3.025.667	-41.648	2.975.027	-41.096
3.003.626	-30.713	3.025.822	-41.650	2.975.111	-41.097
3.003.748	-30.715	3.025.963	-41.651	2.975.240	-41.099
3.003.934	-30.718	3.026.173	-41.652	2.975.421	-41.100
3.004.364	-30.720	3.026.389	-41.653	2.975.598	-41.101
3.004.801	-30.722	3.026.575	-41.654	2.975.750	-41.102
3.005.002	-30.724	3.026.744	-41.655	2.975.909	-41.104
3.005.088	-30.726	3.026.953	-41.656	2.976.053	-41.105
3.005.117	-30.729	3.027.041	-41.657	2.976.179	-41.106
3.005.155	-30.731	3.027.146	-41.658	2.976.383	-41.108
3.005.242	-30.733	3.027.404	-41.659	2.976.564	-41.109
3.005.343	-30.735	3.027.600	-41.661	2.976.718	-41.110
3.005.428	-30.737	3.027.733	-41.662	2.976.927	-41.111
3.005.558	-30.740	3.027.886	-41.663	2.977.141	-41.113
3.005.762	-30.742	3.028.042	-41.664	2.977.336	-41.114
3.005.919	-30.744	3.028.246	-41.665	2.977.491	-41.115
3.006.093	-30.746	3.028.463	-41.667	2.977.686	-41.116

3.006.314	-30.749	3.028.565	-41.668	2.977.904	-41.117
3.006.549	-30.751	3.028.692	-41.669	2.978.040	-41.119
3.006.750	-30.753	3.028.854	-41.670	2.978.192	-41.120
3.006.859	-30.755	3.029.019	-41.672	2.978.377	-41.121
3.006.967	-30.758	3.029.218	-41.673	2.978.557	-41.122
3.007.187	-30.760	3.029.395	-41.674	2.978.676	-41.123
3.007.304	-30.762	3.029.525	-41.676	2.978.701	-41.125
3.007.411	-30.764	3.029.688	-41.677	2.978.824	-41.126
3.007.650	-30.767	3.029.846	-41.678	2.979.088	-41.127
3.007.823	-30.769	3.030.020	-41.680	2.979.265	-41.128
3.007.997	-30.771	3.030.229	-41.681	2.979.378	-41.129
3.008.168	-30.774	3.030.399	-41.682	2.979.501	-41.130
3.008.329	-30.776	3.030.525	-41.684	2.979.688	-41.132
3.008.495	-30.778	3.030.663	-41.685	2.979.880	-41.133
3.008.667	-30.780	3.030.895	-41.687	2.979.973	-41.134
3.008.838	-30.783	3.031.090	-41.688	2.980.190	-41.135
3.008.997	-30.785	3.031.172	-41.689	2.980.500	-41.136
3.009.190	-30.787	3.031.293	-41.691	2.980.664	-41.137
3.009.409	-30.790	3.031.447	-41.692	2.980.769	-41.138
3.009.615	-30.792	3.031.598	-41.694	2.980.938	-41.139
3.009.760	-30.794	3.031.788	-41.695	2.981.192	-41.140
3.009.877	-30.796	3.032.043	-41.697	2.981.356	-41.141
3.010.013	-30.799	3.032.263	-41.698	2.981.490	-41.143
3.010.143	-30.801	3.032.397	-41.700	2.981.665	-41.144
3.010.251	-30.803	3.032.572	-41.702	2.981.882	-41.145
3.010.411	-30.806	3.032.740	-41.703	2.982.115	-41.146
3.010.656	-30.808	3.032.893	-41.705	2.982.264	-41.147
3.010.837	-30.810	3.033.001	-41.706	2.982.372	-41.148
3.010.989	-30.813	3.033.110	-41.708	2.982.469	-41.149
3.011.172	-30.815	3.033.304	-41.709	2.982.621	-41.150

3.011.279	-30.817	3.033.506	-41.711	2.982.823	-41.151
3.011.404	-30.819	3.033.618	-41.713	2.982.994	-41.152
3.011.588	-30.822	3.033.702	-41.714	2.983.195	-41.153
3.011.765	-30.824	3.033.839	-41.716	2.983.391	-41.154
3.011.939	-30.826	3.034.017	-41.717	2.983.585	-41.155
3.012.101	-30.829	3.034.258	-41.719	2.983.741	-41.156
3.012.300	-30.831	3.034.424	-41.721	2.983.839	-41.157
3.012.466	-30.833	3.034.548	-41.722	2.983.979	-41.158
3.012.585	-30.835	3.034.716	-41.724	2.984.164	-41.159
3.012.751	-30.838	3.034.931	-41.725	2.984.330	-41.160
3.012.957	-30.840	3.035.198	-41.727	2.984.515	-41.161
3.013.141	-30.842	3.035.415	-41.729	2.984.716	-41.162
3.013.323	-30.845	3.035.599	-41.730	2.984.893	-41.163
3.013.503	-30.847	3.035.789	-41.732	2.985.056	-41.164
3.013.716	-30.849	3.035.978	-41.734	2.985.194	-41.165
3.013.857	-30.851	3.036.137	-41.735	2.985.338	-41.166
3.013.958	-30.854	3.036.245	-41.737	2.985.498	-41.168
3.014.128	-30.856	3.036.375	-41.738	2.985.679	-41.169
3.014.304	-30.858	3.036.481	-41.740	2.985.898	-41.170
3.014.492	-30.861	3.036.643	-41.742	2.986.094	-41.171
3.014.662	-30.863	3.036.859	-41.743	2.986.212	-41.172
3.014.848	-30.865	3.036.990	-41.745	2.986.360	-41.173
3.015.005	-30.867	3.037.146	-41.746	2.986.598	-41.174
3.015.128	-30.870	3.037.321	-41.748	2.986.750	-41.175
3.015.301	-30.872	3.037.509	-41.750	2.986.830	-41.176
3.015.536	-30.874	3.037.684	-41.751	2.987.111	-41.177
3.015.717	-30.876	3.037.896	-41.753	2.987.541	-41.178
3.015.837	-30.879	3.038.123	-41.754	2.987.848	-41.179
3.016.018	-30.881	3.038.237	-41.756	2.988.015	-41.180
3.016.123	-30.883	3.038.365	-41.758	2.988.059	-41.181

3.016.209	-30.885	3.038.557	-41.759	2.988.048	-41.182
3.016.367	-30.887	3.038.712	-41.761	2.988.102	-41.183
3.016.557	-30.890	3.038.885	-41.762	2.988.243	-41.184
3.016.732	-30.892	3.039.082	-41.764	2.988.372	-41.185
3.016.889	-30.894	3.039.187	-41.766	2.988.466	-41.186
3.017.051	-30.896	3.039.330	-41.767	2.988.607	-41.188
3.017.206	-30.899	3.039.576	-41.769	2.988.730	-41.189
3.017.430	-30.901	3.039.807	-41.770	2.988.843	-41.190
3.017.689	-30.903	3.039.955	-41.772	2.989.052	-41.191
3.017.874	-30.905	3.040.061	-41.773	2.989.320	-41.192
3.018.059	-30.907	3.040.245	-41.775	2.989.492	-41.193
3.018.251	-30.910	3.040.450	-41.776	2.989.674	-41.194
3.018.426	-30.912	3.040.627	-41.778	2.989.872	-41.195
3.018.557	-30.914	3.040.757	-41.779	2.990.060	-41.196
3.018.683	-30.916	3.040.872	-41.781	2.990.204	-41.197
3.018.862	-30.918	3.041.068	-41.783	2.990.335	-41.199
3.019.033	-30.921	3.041.299	-41.784	2.990.493	-41.200
3.019.168	-30.923	3.041.445	-41.785	2.990.678	-41.201
3.019.333	-30.925	3.041.592	-41.787	2.990.910	-41.202
3.019.449	-30.927	3.041.779	-41.788	2.991.098	-41.203
3.019.565	-30.929	3.041.949	-41.790	2.991.217	-41.204
3.019.716	-30.931	3.042.149	-41.791	2.991.292	-41.206
3.019.864	-30.934	3.042.282	-41.793	2.991.432	-41.207
3.020.085	-30.936	3.042.411	-41.794	2.991.660	-41.208
3.020.343	-30.938	3.042.525	-41.796	2.991.784	-41.209
3.020.529	-30.940	3.042.664	-41.797	2.991.902	-41.211
3.020.677	-30.942	3.042.982	-41.799	2.992.034	-41.212
3.020.819	-30.944	3.043.412	-41.800	2.992.187	-41.213
3.020.942	-30.947	3.043.797	-41.801	2.992.328	-41.215
3.021.089	-30.949	3.044.001	-41.803	2.992.553	-41.216

3.021.302	-30.951	3.044.071	-41.804	2.992.821	-41.217
3.021.599	-30.953	3.044.092	-41.806	2.993.045	-41.219
3.021.786	-30.955	3.044.070	-41.807	2.993.207	-41.220
3.021.855	-30.957	3.044.077	-41.808	2.993.354	-41.221
3.021.963	-30.959	3.044.211	-41.810	2.993.558	-41.223
3.022.131	-30.962	3.044.445	-41.811	2.993.694	-41.224
3.022.335	-30.964	3.044.630	-41.812	2.993.825	-41.226
3.022.546	-30.966	3.044.771	-41.814	2.994.001	-41.227
3.022.744	-30.968	3.044.913	-41.815	2.994.183	-41.229
3.022.816	-30.970	3.045.078	-41.817	2.994.366	-41.230
3.023.000	-30.972	3.045.287	-41.818	2.994.478	-41.232
3.023.246	-30.974	3.045.453	-41.819	2.994.627	-41.233
3.023.344	-30.976	3.045.639	-41.820	2.994.800	-41.235
3.023.524	-30.979	3.045.847	-41.822	2.994.958	-41.236
3.023.773	-30.981	3.045.970	-41.823	2.995.111	-41.238
3.023.983	-30.983	3.046.096	-41.824	2.995.245	-41.239
3.024.121	-30.985	3.046.245	-41.826	2.995.418	-41.241
3.024.223	-30.987	3.046.364	-41.827	2.995.585	-41.243
3.024.352	-30.989	3.046.523	-41.828	2.995.754	-41.244
3.024.543	-30.991	3.046.737	-41.830	2.995.948	-41.246
3.024.720	-30.993	3.046.873	-41.831	2.996.127	-41.248
3.024.872	-30.995	3.046.992	-41.832	2.996.343	-41.249
3.025.074	-30.997	3.047.159	-41.833	2.996.483	-41.251
3.025.242	-31.000	3.047.290	-41.835	2.996.614	-41.253
3.025.396	-31.002	3.047.424	-41.836	2.996.841	-41.255
3.025.587	-31.004	3.047.686	-41.837	2.997.036	-41.257
3.025.659	-31.006	3.047.924	-41.838	2.997.120	-41.258
3.025.768	-31.008	3.048.105	-41.840	2.997.249	-41.260
3.026.223	-31.010	3.048.239	-41.841	2.997.473	-41.262
3.026.755	-31.012	3.048.408	-41.842	2.997.715	-41.264

3.026.880	-31.014	3.048.619	-41.844	2.997.968	-41.266
3.026.884	-31.016	3.048.791	-41.845	2.998.120	-41.268
3.026.898	-31.018	3.048.897	-41.846	2.998.178	-41.269
3.026.953	-31.020	3.049.033	-41.847	2.998.301	-41.271
3.027.068	-31.023	3.049.203	-41.849	2.998.481	-41.273
3.027.192	-31.025	3.049.356	-41.850	2.998.636	-41.275
3.027.311	-31.027	3.049.576	-41.851	2.998.792	-41.277
3.027.458	-31.029	3.049.738	-41.853	2.998.997	-41.279
3.027.625	-31.031	3.049.883	-41.854	2.999.212	-41.281
3.027.845	-31.033	3.050.088	-41.855	2.999.388	-41.283
3.028.083	-31.035	3.050.154	-41.857	2.999.541	-41.285
3.028.286	-31.037	3.050.291	-41.858	2.999.673	-41.287
3.028.477	-31.039	3.050.538	-41.859	2.999.828	-41.289
3.028.590	-31.041	3.050.675	-41.861	2.999.973	-41.290
3.028.698	-31.043	3.050.833	-41.862	3.000.182	-41.292
3.028.904	-31.045	3.050.966	-41.863	3.000.414	-41.294
3.029.098	-31.048	3.051.126	-41.865	3.000.548	-41.296
3.029.252	-31.050	3.051.339	-41.866	3.000.667	-41.298
3.029.402	-31.052	3.051.509	-41.868	3.000.818	-41.300
3.029.544	-31.054	3.051.682	-41.869	3.000.956	-41.302
3.029.678	-31.056	3.051.931	-41.870	3.001.105	-41.304
3.029.807	-31.058	3.052.138	-41.872	3.001.291	-41.306
3.029.945	-31.060	3.052.256	-41.873	3.001.429	-41.308
3.030.086	-31.062	3.052.444	-41.875	3.001.534	-41.310
3.030.253	-31.064	3.052.572	-41.876	3.001.704	-41.312
3.030.440	-31.066	3.052.727	-41.878	3.001.869	-41.313
3.030.648	-31.068	3.052.995	-41.879	3.001.960	-41.315
3.030.869	-31.070	3.053.114	-41.881	3.002.133	-41.317
3.031.057	-31.072	3.053.210	-41.882	3.002.328	-41.319
3.031.281	-31.075	3.053.395	-41.884	3.002.503	-41.321

3.031.523	-31.077	3.053.582	-41.885	3.002.686	-41.323
3.031.624	-31.079	3.053.777	-41.887	3.002.861	-41.325
3.031.740	-31.081	3.053.937	-41.888	3.003.174	-41.326
3.031.893	-31.083	3.054.107	-41.890	3.003.410	-41.328
3.032.015	-31.085	3.054.284	-41.891	3.003.571	-41.330
3.032.185	-31.087	3.054.431	-41.893	3.003.708	-41.332
3.032.386	-31.089	3.054.564	-41.894	3.003.835	-41.334
3.032.534	-31.091	3.054.662	-41.896	3.004.052	-41.335
3.032.639	-31.093	3.054.789	-41.897	3.004.254	-41.337
3.032.874	-31.095	3.054.947	-41.899	3.004.380	-41.339
3.033.070	-31.097	3.055.107	-41.900	3.004.530	-41.341
3.033.207	-31.099	3.055.237	-41.902	3.004.676	-41.342
3.033.362	-31.102	3.055.352	-41.904	3.004.774	-41.344
3.033.517	-31.104	3.055.533	-41.905	3.004.941	-41.346
3.033.732	-31.106	3.055.766	-41.907	3.005.150	-41.347
3.033.898	-31.108	3.055.986	-41.908	3.005.294	-41.349
3.034.045	-31.110	3.056.199	-41.910	3.005.437	-41.351
3.034.171	-31.112	3.056.411	-41.912	3.005.598	-41.352
3.034.254	-31.114	3.056.593	-41.913	3.005.815	-41.354
3.034.428	-31.116	3.056.763	-41.915	3.006.014	-41.355
3.034.660	-31.118	3.056.952	-41.916	3.006.154	-41.357
3.034.859	-31.120	3.057.160	-41.918	3.006.256	-41.359
3.035.013	-31.122	3.057.315	-41.920	3.006.385	-41.360
3.035.175	-31.124	3.057.390	-41.921	3.006.632	-41.362
3.035.332	-31.127	3.057.525	-41.923	3.006.936	-41.363
3.035.482	-31.129	3.057.708	-41.925	3.007.173	-41.365
3.035.667	-31.131	3.057.823	-41.926	3.007.289	-41.366
3.035.833	-31.133	3.057.940	-41.928	3.007.359	-41.368
3.036.014	-31.135	3.058.124	-41.929	3.007.455	-41.369
3.036.190	-31.137	3.058.312	-41.931	3.007.648	-41.371

3.036.329	-31.139	3.058.492	-41.933	3.007.860	-41.372
3.036.448	-31.141	3.058.694	-41.934	3.008.036	-41.374
3.036.617	-31.143	3.058.889	-41.936	3.008.214	-41.375
3.036.844	-31.146	3.059.081	-41.938	3.008.359	-41.377
3.037.051	-31.148	3.059.312	-41.939	3.008.485	-41.378
3.037.214	-31.150	3.059.512	-41.941	3.008.658	-41.380
3.037.322	-31.152	3.059.630	-41.942	3.008.861	-41.381
3.037.459	-31.154	3.059.782	-41.944	3.009.002	-41.382
3.037.626	-31.156	3.059.984	-41.946	3.009.156	-41.384
3.037.823	-31.158	3.060.146	-41.947	3.009.338	-41.385
3.037.994	-31.161	3.060.320	-41.949	3.009.481	-41.387
3.038.052	-31.163	3.060.498	-41.951	3.009.642	-41.388
3.038.070	-31.165	3.060.648	-41.952	3.009.865	-41.389
3.038.269	-31.167	3.060.796	-41.954	3.010.063	-41.391
3.038.549	-31.169	3.060.909	-41.956	3.010.193	-41.392
3.038.766	-31.171	3.061.098	-41.957	3.010.343	-41.393
3.038.972	-31.173	3.061.333	-41.959	3.010.541	-41.395
3.039.157	-31.176	3.061.483	-41.961	3.010.711	-41.396
3.039.335	-31.178	3.061.651	-41.962	3.010.852	-41.397
3.039.566	-31.180	3.061.830	-41.964	3.011.003	-41.399
3.039.760	-31.182	3.061.950	-41.966	3.011.140	-41.400
3.039.938	-31.184	3.062.088	-41.967	3.011.375	-41.401
3.040.125	-31.187	3.062.279	-41.969	3.011.694	-41.402
3.040.276	-31.189	3.062.448	-41.970	3.011.874	-41.404
3.040.443	-31.191	3.062.630	-41.972	3.011.953	-41.405
3.040.605	-31.193	3.062.823	-41.974	3.012.072	-41.406
3.040.742	-31.195	3.062.986	-41.975	3.012.246	-41.408
3.040.872	-31.198	3.063.161	-41.977	3.012.400	-41.409
3.040.999	-31.200	3.063.341	-41.979	3.012.517	-41.410
3.041.108	-31.202	3.063.492	-41.980	3.012.657	-41.411

3.041.237	-31.204	3.063.589	-41.982	3.012.835	-41.413
3.041.400	-31.207	3.063.784	-41.984	3.013.152	-41.414
3.041.615	-31.209	3.063.990	-41.985	3.013.611	-41.415
3.041.794	-31.211	3.064.109	-41.987	3.013.940	-41.416
3.041.976	-31.213	3.064.320	-41.989	3.014.073	-41.418
3.042.149	-31.216	3.064.711	-41.990	3.014.147	-41.419
3.042.323	-31.218	3.065.124	-41.992	3.014.240	-41.420
3.042.517	-31.220	3.065.294	-41.994	3.014.276	-41.421
3.042.661	-31.222	3.065.330	-41.995	3.014.258	-41.423
3.042.835	-31.225	3.065.385	-41.997	3.014.346	-41.424
3.042.994	-31.227	3.065.444	-41.999	3.014.486	-41.425
3.043.152	-31.229	3.065.520	-42.000	3.014.648	-41.426
3.043.401	-31.232	3.065.612	-42.002	3.014.804	-41.428
3.043.575	-31.234	3.065.661	-42.003	3.014.894	-41.429
3.043.705	-31.236	3.065.807	-42.005	3.015.063	-41.430
3.043.882	-31.239	3.066.014	-42.007	3.015.287	-41.431
3.044.041	-31.241	3.066.194	-42.008	3.015.433	-41.433
3.044.197	-31.243	3.066.426	-42.010	3.015.605	-41.434
3.044.378	-31.246	3.066.656	-42.012	3.015.836	-41.435
3.044.541	-31.248	3.066.881	-42.013	3.016.049	-41.437
3.044.676	-31.250	3.067.055	-42.015	3.016.210	-41.438
3.044.843	-31.253	3.067.195	-42.017	3.016.365	-41.439
3.045.045	-31.255	3.067.332	-42.018	3.016.531	-41.441
3.045.295	-31.257	3.067.469	-42.020	3.016.776	-41.442
3.045.477	-31.260	3.067.621	-42.022	3.017.019	-41.443
3.045.641	-31.262	3.067.770	-42.023	3.017.123	-41.445
3.045.807	-31.265	3.067.948	-42.025	3.017.198	-41.446
3.045.934	-31.267	3.068.138	-42.027	3.017.305	-41.447
3.046.082	-31.269	3.068.279	-42.028	3.017.477	-41.449
3.046.187	-31.272	3.068.427	-42.030	3.017.680	-41.450

3.046.375	-31.274	3.068.615	-42.031	3.017.828	-41.452
3.046.571	-31.277	3.068.817	-42.033	3.017.982	-41.453
3.046.690	-31.279	3.069.010	-42.035	3.018.109	-41.455
3.046.853	-31.281	3.069.198	-42.036	3.018.269	-41.456
3.047.043	-31.284	3.069.330	-42.038	3.018.468	-41.458
3.047.216	-31.286	3.069.472	-42.040	3.018.698	-41.459
3.047.386	-31.289	3.069.682	-42.042	3.018.867	-41.461
3.047.525	-31.291	3.069.868	-42.043	3.018.974	-41.462
3.047.841	-31.294	3.070.054	-42.045	3.019.185	-41.464
3.048.352	-31.296	3.070.216	-42.047	3.019.388	-41.466
3.048.633	-31.299	3.070.346	-42.048	3.019.557	-41.467
3.048.712	-31.301	3.070.475	-42.050	3.019.682	-41.469
3.048.770	-31.304	3.070.598	-42.052	3.019.779	-41.470
3.048.797	-31.306	3.070.757	-42.053	3.019.940	-41.472
3.048.872	-31.309	3.070.898	-42.055	3.020.106	-41.474
3.049.010	-31.311	3.071.032	-42.057	3.020.328	-41.475
3.049.103	-31.314	3.071.208	-42.058	3.020.515	-41.477
3.049.193	-31.316	3.071.367	-42.060	3.020.663	-41.479
3.049.364	-31.319	3.071.497	-42.062	3.020.847	-41.481
3.049.543	-31.321	3.071.692	-42.064	3.021.003	-41.482
3.049.648	-31.324	3.071.943	-42.065	3.021.129	-41.484
3.049.790	-31.326	3.072.160	-42.067	3.021.277	-41.486
3.050.007	-31.329	3.072.240	-42.069	3.021.512	-41.488
3.050.183	-31.331	3.072.343	-42.070	3.021.721	-41.490
3.050.370	-31.334	3.072.549	-42.072	3.021.874	-41.492
3.050.544	-31.337	3.072.722	-42.074	3.022.012	-41.494
3.050.668	-31.339	3.072.900	-42.076	3.022.162	-41.496
3.050.841	-31.342	3.073.105	-42.077	3.022.321	-41.497
3.051.047	-31.344	3.073.309	-42.079	3.022.495	-41.499
3.051.239	-31.347	3.073.413	-42.081	3.022.643	-41.501

3.051.372	-31.349	3.073.557	-42.082	3.022.737	-41.503
3.051.488	-31.352	3.073.802	-42.084	3.022.965	-41.505
3.051.689	-31.355	3.073.972	-42.086	3.023.182	-41.508
3.051.819	-31.357	3.074.086	-42.088	3.023.316	-41.510
3.051.895	-31.360	3.074.262	-42.089	3.023.466	-41.512
3.052.065	-31.362	3.074.467	-42.091	3.023.705	-41.514
3.052.339	-31.365	3.074.651	-42.093	3.023.979	-41.516
3.052.542	-31.368	3.074.803	-42.095	3.024.121	-41.518
3.052.747	-31.370	3.074.984	-42.096	3.024.218	-41.520
3.052.885	-31.373	3.075.153	-42.098	3.024.422	-41.522
3.053.002	-31.375	3.075.320	-42.100	3.024.586	-41.524
3.053.175	-31.378	3.075.515	-42.102	3.024.700	-41.526
3.053.361	-31.381	3.075.682	-42.103	3.024.887	-41.528
3.053.540	-31.383	3.075.891	-42.105	3.025.054	-41.531
3.053.730	-31.386	3.076.068	-42.107	3.025.273	-41.533
3.053.846	-31.389	3.076.133	-42.108	3.025.486	-41.535
3.054.016	-31.391	3.076.217	-42.110	3.025.595	-41.537
3.054.194	-31.394	3.076.415	-42.112	3.025.672	-41.539
3.054.268	-31.396	3.076.575	-42.114	3.025.789	-41.541
3.054.409	-31.399	3.076.649	-42.115	3.026.086	-41.543
3.054.580	-31.402	3.076.752	-42.117	3.026.322	-41.545
3.054.823	-31.404	3.076.982	-42.119	3.026.432	-41.548
3.055.074	-31.407	3.077.242	-42.120	3.026.581	-41.550
3.055.258	-31.410	3.077.455	-42.122	3.026.642	-41.552
3.055.442	-31.412	3.077.646	-42.123	3.026.794	-41.554
3.055.643	-31.415	3.077.803	-42.125	3.027.057	-41.556
3.055.761	-31.418	3.077.993	-42.127	3.027.220	-41.558
3.055.844	-31.420	3.078.206	-42.128	3.027.332	-41.560
3.055.995	-31.423	3.078.412	-42.130	3.027.469	-41.562
3.056.235	-31.426	3.078.578	-42.131	3.027.621	-41.564

3.056.427	-31.428	3.078.716	-42.133	3.027.763	-41.566
3.056.483	-31.431	3.078.811	-42.134	3.027.890	-41.568
3.056.700	-31.434	3.078.943	-42.136	3.028.018	-41.570
3.056.921	-31.437	3.079.120	-42.137	3.028.172	-41.572
3.057.043	-31.439	3.079.261	-42.139	3.028.354	-41.574
3.057.235	-31.442	3.079.420	-42.140	3.028.573	-41.576
3.057.449	-31.445	3.079.562	-42.142	3.028.789	-41.578
3.057.570	-31.447	3.079.696	-42.143	3.028.976	-41.580
3.057.691	-31.450	3.079.886	-42.145	3.029.164	-41.582
3.057.881	-31.453	3.080.090	-42.146	3.029.355	-41.584
3.058.031	-31.455	3.080.288	-42.148	3.029.568	-41.586
3.058.185	-31.458	3.080.446	-42.149	3.029.800	-41.588
3.058.365	-31.461	3.080.573	-42.150	3.029.953	-41.590
3.058.553	-31.463	3.080.760	-42.152	3.030.092	-41.591
3.058.781	-31.466	3.080.957	-42.153	3.030.218	-41.593
3.058.947	-31.469	3.081.154	-42.154	3.030.411	-41.595
3.059.052	-31.472	3.081.286	-42.156	3.030.587	-41.597
3.059.204	-31.474	3.081.430	-42.157	3.030.714	-41.598
3.059.382	-31.477	3.081.663	-42.158	3.030.844	-41.600
3.059.525	-31.480	3.081.857	-42.159	3.030.995	-41.602
3.059.642	-31.482	3.082.037	-42.161	3.031.133	-41.604
3.059.785	-31.485	3.082.220	-42.162	3.031.296	-41.605
3.059.854	-31.488	3.082.355	-42.163	3.031.542	-41.607
3.059.970	-31.491	3.082.535	-42.164	3.031.685	-41.608
3.060.222	-31.493	3.082.711	-42.165	3.031.844	-41.610
3.060.437	-31.496	3.082.867	-42.167	3.032.003	-41.612
3.060.624	-31.499	3.083.081	-42.168	3.032.167	-41.613
3.060.798	-31.502	3.083.229	-42.169	3.032.332	-41.615
3.060.926	-31.504	3.083.372	-42.170	3.032.523	-41.616
3.061.075	-31.507	3.083.533	-42.171	3.032.762	-41.618

3.061.328	-31.510	3.083.697	-42.172	3.032.939	-41.619
3.061.609	-31.512	3.083.889	-42.173	3.033.099	-41.621
3.061.765	-31.515	3.084.066	-42.174	3.033.203	-41.622
3.061.938	-31.518	3.084.225	-42.175	3.033.365	-41.623
3.062.104	-31.521	3.084.399	-42.176	3.033.569	-41.625
3.062.250	-31.523	3.084.624	-42.177	3.033.727	-41.626
3.062.430	-31.526	3.084.796	-42.178	3.033.878	-41.627
3.062.554	-31.529	3.084.911	-42.179	3.034.052	-41.629
3.062.654	-31.532	3.085.096	-42.180	3.034.230	-41.630
3.062.780	-31.534	3.085.265	-42.181	3.034.304	-41.631
3.062.980	-31.537	3.085.363	-42.181	3.034.504	-41.632
3.063.152	-31.540	3.085.417	-42.182	3.034.763	-41.634
3.063.290	-31.542	3.085.643	-42.183	3.034.901	-41.635
3.063.456	-31.545	3.086.122	-42.184	3.035.107	-41.636
3.063.640	-31.548	3.086.497	-42.185	3.035.348	-41.637
3.063.901	-31.551	3.086.631	-42.185	3.035.479	-41.638
3.064.149	-31.553	3.086.675	-42.186	3.035.592	-41.639
3.064.251	-31.556	3.086.730	-42.187	3.035.753	-41.640
3.064.337	-31.559	3.086.805	-42.188	3.035.905	-41.641
3.064.561	-31.562	3.086.870	-42.189	3.036.096	-41.642
3.064.817	-31.564	3.086.940	-42.189	3.036.313	-41.644
3.065.010	-31.567	3.087.088	-42.190	3.036.444	-41.645
3.065.182	-31.570	3.087.296	-42.191	3.036.595	-41.646
3.065.301	-31.573	3.087.466	-42.191	3.036.779	-41.647
3.065.396	-31.575	3.087.593	-42.192	3.036.906	-41.648
3.065.607	-31.578	3.087.780	-42.193	3.037.061	-41.648
3.065.807	-31.581	3.087.979	-42.193	3.037.180	-41.649
3.065.939	-31.584	3.088.181	-42.194	3.037.307	-41.650
3.066.072	-31.586	3.088.337	-42.195	3.037.538	-41.651
3.066.253	-31.589	3.088.439	-42.195	3.037.803	-41.652

3.066.423	-31.592	3.088.654	-42.196	3.037.971	-41.653
3.066.566	-31.594	3.088.919	-42.197	3.038.074	-41.654
3.066.751	-31.597	3.088.983	-42.197	3.038.293	-41.655
3.066.928	-31.600	3.089.093	-42.198	3.038.526	-41.656
3.067.081	-31.603	3.089.333	-42.198	3.038.659	-41.657
3.067.245	-31.605	3.089.544	-42.199	3.038.767	-41.657
3.067.448	-31.608	3.089.684	-42.200	3.038.973	-41.658
3.067.612	-31.611	3.089.724	-42.200	3.039.337	-41.659
3.067.702	-31.614	3.089.919	-42.201	3.039.702	-41.660
3.067.896	-31.616	3.090.175	-42.201	3.039.915	-41.661
3.068.080	-31.619	3.090.345	-42.202	3.039.989	-41.661
3.068.264	-31.622	3.090.533	-42.203	3.040.047	-41.662
3.068.431	-31.624	3.090.736	-42.203	3.040.130	-41.663
3.068.580	-31.627	3.090.927	-42.204	3.040.135	-41.664
3.068.784	-31.630	3.091.130	-42.205	3.040.218	-41.664
3.069.008	-31.633	3.091.311	-42.205	3.040.368	-41.665
3.069.102	-31.635	3.091.456	-42.206	3.040.458	-41.666
3.069.160	-31.638	3.091.610	-42.206	3.040.580	-41.666
3.069.312	-31.641	3.091.777	-42.207	3.040.794	-41.667
3.069.745	-31.643	3.091.916	-42.208	3.040.989	-41.668
3.070.226	-31.646	3.092.070	-42.208	3.041.151	-41.669
3.070.446	-31.649	3.092.228	-42.209	3.041.324	-41.669
3.070.505	-31.651	3.092.393	-42.210	3.041.502	-41.670
3.070.536	-31.654	3.092.531	-42.210	3.041.697	-41.671
3.070.628	-31.657	3.092.659	-42.211	3.041.910	-41.672
3.070.703	-31.660	3.092.877	-42.212	3.042.086	-41.673
3.070.747	-31.662	3.093.023	-42.212	3.042.220	-41.673
3.070.855	-31.665	3.093.152	-42.213	3.042.344	-41.674
3.070.996	-31.668	3.093.347	-42.214	3.042.509	-41.675
3.071.158	-31.670	3.093.453	-42.215	3.042.698	-41.676

3.071.378	-31.673	3.093.578	-42.215	3.042.904	-41.677
3.071.596	-31.676	3.093.763	-42.216	3.043.026	-41.678
3.071.806	-31.678	3.093.907	-42.217	3.043.120	-41.678
3.071.985	-31.681	3.094.071	-42.218	3.043.297	-41.679
3.072.146	-31.683	3.094.259	-42.219	3.043.490	-41.680
3.072.314	-31.686	3.094.492	-42.220	3.043.632	-41.681
3.072.505	-31.689	3.094.729	-42.220	3.043.784	-41.682
3.072.661	-31.691	3.094.883	-42.221	3.044.006	-41.683
3.072.783	-31.694	3.095.003	-42.222	3.044.169	-41.684
3.072.954	-31.697	3.095.183	-42.223	3.044.324	-41.685
3.073.094	-31.699	3.095.396	-42.224	3.044.528	-41.686
3.073.185	-31.702	3.095.562	-42.225	3.044.716	-41.687
3.073.409	-31.704	3.095.722	-42.226	3.044.904	-41.688
3.073.625	-31.707	3.095.854	-42.227	3.045.035	-41.690
3.073.712	-31.710	3.095.995	-42.228	3.045.219	-41.691
3.073.825	-31.712	3.096.143	-42.229	3.045.425	-41.692
3.074.044	-31.715	3.096.300	-42.230	3.045.587	-41.693
3.074.269	-31.717	3.096.497	-42.231	3.045.765	-41.694
3.074.445	-31.720	3.096.754	-42.232	3.045.901	-41.695
3.074.655	-31.723	3.096.921	-42.234	3.046.032	-41.697
3.074.847	-31.725	3.097.065	-42.235	3.046.199	-41.698
3.074.970	-31.728	3.097.224	-42.236	3.046.364	-41.699
3.075.092	-31.730	3.097.272	-42.237	3.046.488	-41.701
3.075.226	-31.733	3.097.401	-42.238	3.046.671	-41.702
3.075.385	-31.735	3.097.574	-42.240	3.046.849	-41.704
3.075.635	-31.738	3.097.733	-42.241	3.046.964	-41.705
3.075.829	-31.740	3.097.911	-42.242	3.047.140	-41.706
3.075.941	-31.743	3.098.036	-42.243	3.047.311	-41.708
3.076.116	-31.745	3.098.148	-42.245	3.047.458	-41.709
3.076.252	-31.748	3.098.315	-42.246	3.047.630	-41.711

3.076.407	-31.750	3.098.567	-42.247	3.047.807	-41.713
3.076.584	-31.753	3.098.821	-42.249	3.047.975	-41.714
3.076.734	-31.755	3.098.988	-42.250	3.048.130	-41.716
3.076.920	-31.758	3.099.157	-42.251	3.048.282	-41.718
3.077.112	-31.760	3.099.344	-42.253	3.048.459	-41.719
3.077.235	-31.763	3.099.510	-42.254	3.048.636	-41.721
3.077.325	-31.765	3.099.698	-42.256	3.048.792	-41.723
3.077.466	-31.768	3.099.913	-42.257	3.048.958	-41.725
3.077.689	-31.770	3.100.067	-42.258	3.049.109	-41.727
3.077.914	-31.773	3.100.173	-42.260	3.049.302	-41.729
3.078.089	-31.775	3.100.320	-42.261	3.049.515	-41.730
3.078.282	-31.778	3.100.443	-42.263	3.049.700	-41.732
3.078.503	-31.780	3.100.533	-42.264	3.049.841	-41.734
3.078.654	-31.782	3.100.695	-42.266	3.049.938	-41.736
3.078.804	-31.785	3.100.880	-42.268	3.050.128	-41.738
3.079.005	-31.787	3.101.064	-42.269	3.050.357	-41.740
3.079.135	-31.790	3.101.232	-42.271	3.050.580	-41.742
3.079.262	-31.792	3.101.393	-42.272	3.050.787	-41.745
3.079.442	-31.794	3.101.544	-42.274	3.050.912	-41.747
3.079.659	-31.797	3.101.718	-42.276	3.051.017	-41.749
3.079.793	-31.799	3.101.956	-42.277	3.051.129	-41.751
3.079.894	-31.801	3.102.177	-42.279	3.051.263	-41.753
3.080.104	-31.804	3.102.352	-42.281	3.051.492	-41.755
3.080.313	-31.806	3.102.518	-42.282	3.051.703	-41.757
3.080.533	-31.809	3.102.677	-42.284	3.051.871	-41.760
3.080.657	-31.811	3.102.845	-42.286	3.052.093	-41.762
3.080.757	-31.813	3.102.987	-42.287	3.052.263	-41.764
3.080.992	-31.816	3.103.156	-42.289	3.052.404	-41.766
3.081.167	-31.818	3.103.394	-42.291	3.052.541	-41.769
3.081.250	-31.820	3.103.562	-42.293	3.052.625	-41.771

3.081.411	-31.823	3.103.745	-42.294	3.052.771	-41.773
3.081.577	-31.825	3.103.932	-42.296	3.052.966	-41.776
3.081.665	-31.827	3.104.027	-42.298	3.053.161	-41.778
3.081.779	-31.830	3.104.109	-42.300	3.053.290	-41.780
3.081.950	-31.832	3.104.331	-42.301	3.053.344	-41.783
3.082.142	-31.834	3.104.514	-42.303	3.053.473	-41.785
3.082.312	-31.836	3.104.609	-42.305	3.053.702	-41.787
3.082.491	-31.839	3.104.861	-42.307	3.053.891	-41.790
3.082.701	-31.841	3.105.052	-42.309	3.053.951	-41.792
3.082.913	-31.843	3.105.176	-42.310	3.054.086	-41.794
3.083.103	-31.846	3.105.339	-42.312	3.054.360	-41.797
3.083.298	-31.848	3.105.538	-42.314	3.054.594	-41.799
3.083.497	-31.850	3.105.755	-42.316	3.054.745	-41.802
3.083.680	-31.852	3.105.916	-42.318	3.054.933	-41.804
3.083.860	-31.855	3.106.126	-42.320	3.055.172	-41.806
3.084.030	-31.857	3.106.292	-42.321	3.055.365	-41.809
3.084.151	-31.859	3.106.461	-42.323	3.055.537	-41.811
3.084.275	-31.862	3.106.660	-42.325	3.055.697	-41.814
3.084.438	-31.864	3.106.786	-42.327	3.055.873	-41.816
3.084.540	-31.866	3.106.904	-42.329	3.056.071	-41.819
3.084.622	-31.868	3.107.133	-42.331	3.056.205	-41.821
3.084.774	-31.871	3.107.552	-42.332	3.056.304	-41.823
3.084.984	-31.873	3.107.863	-42.334	3.056.426	-41.826
3.085.202	-31.875	3.107.966	-42.336	3.056.593	-41.828
3.085.406	-31.877	3.108.091	-42.338	3.056.736	-41.831
3.085.589	-31.880	3.108.159	-42.340	3.056.819	-41.833
3.085.794	-31.882	3.108.168	-42.342	3.056.964	-41.836
3.085.988	-31.884	3.108.199	-42.344	3.057.182	-41.838
3.086.136	-31.886	3.108.319	-42.345	3.057.364	-41.840
3.086.303	-31.888	3.108.528	-42.347	3.057.470	-41.843

3.086.476	-31.891	3.108.708	-42.349	3.057.637	-41.845
3.086.646	-31.893	3.108.862	-42.351	3.057.881	-41.848
3.086.853	-31.895	3.109.051	-42.353	3.058.113	-41.850
3.087.079	-31.897	3.109.280	-42.355	3.058.334	-41.852
3.087.191	-31.900	3.109.471	-42.356	3.058.558	-41.855
3.087.361	-31.902	3.109.597	-42.358	3.058.735	-41.857
3.087.521	-31.904	3.109.752	-42.360	3.058.851	-41.859
3.087.628	-31.906	3.109.923	-42.362	3.059.015	-41.862
3.087.814	-31.909	3.110.070	-42.364	3.059.218	-41.864
3.088.004	-31.911	3.110.227	-42.365	3.059.339	-41.866
3.088.231	-31.913	3.110.379	-42.367	3.059.472	-41.869
3.088.403	-31.915	3.110.545	-42.369	3.059.642	-41.871
3.088.571	-31.917	3.110.735	-42.371	3.059.826	-41.873
3.088.719	-31.920	3.110.906	-42.373	3.059.995	-41.875
3.088.820	-31.922	3.111.024	-42.374	3.060.176	-41.878
3.088.994	-31.924	3.111.165	-42.376	3.060.345	-41.880
3.089.189	-31.926	3.111.375	-42.378	3.060.454	-41.882
3.089.330	-31.928	3.111.528	-42.380	3.060.696	-41.884
3.089.493	-31.931	3.111.689	-42.381	3.060.989	-41.887
3.089.681	-31.933	3.111.914	-42.383	3.061.096	-41.889
3.089.804	-31.935	3.112.145	-42.385	3.061.172	-41.891
3.089.952	-31.937	3.112.256	-42.387	3.061.390	-41.893
3.090.176	-31.940	3.112.417	-42.388	3.061.584	-41.895
3.090.386	-31.942	3.112.610	-42.390	3.061.714	-41.897
3.090.555	-31.944	3.112.744	-42.392	3.061.895	-41.899
3.090.708	-31.946	3.112.928	-42.394	3.062.058	-41.901
3.090.837	-31.948	3.113.096	-42.395	3.062.195	-41.904
3.090.927	-31.951	3.113.221	-42.397	3.062.346	-41.906
3.091.156	-31.953	3.113.380	-42.399	3.062.491	-41.908
3.091.617	-31.955	3.113.591	-42.400	3.062.617	-41.910

3.091.999	-31.957	3.113.741	-42.402	3.062.798	-41.912
3.092.200	-31.959	3.113.900	-42.404	3.063.065	-41.914
3.092.270	-31.962	3.114.082	-42.406	3.063.290	-41.916
3.092.271	-31.964	3.114.265	-42.407	3.063.394	-41.918
3.092.312	-31.966	3.114.447	-42.409	3.063.557	-41.919
3.092.397	-31.968	3.114.562	-42.410	3.063.759	-41.921
3.092.534	-31.970	3.114.760	-42.412	3.063.893	-41.923
3.092.639	-31.973	3.114.940	-42.414	3.064.074	-41.925
3.092.773	-31.975	3.114.996	-42.415	3.064.247	-41.927
3.092.951	-31.977	3.115.202	-42.417	3.064.388	-41.929
3.093.119	-31.979	3.115.464	-42.419	3.064.492	-41.931
3.093.297	-31.982	3.115.623	-42.420	3.064.581	-41.933
3.093.513	-31.984	3.115.737	-42.422	3.064.930	-41.934
3.093.708	-31.986	3.115.861	-42.424	3.065.432	-41.936
3.093.886	-31.988	3.116.054	-42.425	3.065.793	-41.938
3.094.081	-31.990	3.116.277	-42.427	3.065.884	-41.940
3.094.229	-31.993	3.116.430	-42.428	3.065.851	-41.941
3.094.351	-31.995	3.116.580	-42.430	3.065.971	-41.943
3.094.522	-31.997	3.116.776	-42.431	3.066.129	-41.945
3.094.688	-31.999	3.116.982	-42.433	3.066.184	-41.946
3.094.830	-32.001	3.117.151	-42.435	3.066.235	-41.948
3.095.027	-32.004	3.117.275	-42.436	3.066.350	-41.950
3.095.211	-32.006	3.117.413	-42.438	3.066.502	-41.951
3.095.330	-32.008	3.117.589	-42.439	3.066.646	-41.953
3.095.443	-32.010	3.117.758	-42.441	3.066.758	-41.954
3.095.623	-32.012	3.117.921	-42.442	3.066.904	-41.956
3.095.848	-32.015	3.118.036	-42.444	3.067.108	-41.958
3.096.057	-32.017	3.118.177	-42.445	3.067.343	-41.959
3.096.201	-32.019	3.118.394	-42.447	3.067.525	-41.961
3.096.349	-32.021	3.118.618	-42.448	3.067.679	-41.962

3.096.531	-32.024	3.118.756	-42.450	3.067.892	-41.964
3.096.661	-32.026	3.118.853	-42.451	3.068.091	-41.965
3.096.823	-32.028	3.118.981	-42.453	3.068.184	-41.967
3.097.014	-32.030	3.119.129	-42.454	3.068.348	-41.968
3.097.199	-32.032	3.119.316	-42.456	3.068.600	-41.970
3.097.332	-32.035	3.119.485	-42.457	3.068.766	-41.971
3.097.482	-32.037	3.119.651	-42.458	3.068.875	-41.973
3.097.684	-32.039	3.119.758	-42.460	3.069.019	-41.974
3.097.863	-32.041	3.119.948	-42.461	3.069.201	-41.976
3.098.033	-32.043	3.120.201	-42.462	3.069.362	-41.977
3.098.157	-32.046	3.120.424	-42.464	3.069.515	-41.978
3.098.325	-32.048	3.120.656	-42.465	3.069.641	-41.980
3.098.466	-32.050	3.120.809	-42.466	3.069.702	-41.981
3.098.618	-32.052	3.120.966	-42.468	3.069.873	-41.983
3.098.835	-32.055	3.121.115	-42.469	3.070.132	-41.984
3.099.044	-32.057	3.121.286	-42.470	3.070.280	-41.986
3.099.149	-32.059	3.121.455	-42.472	3.070.461	-41.987
3.099.240	-32.061	3.121.534	-42.473	3.070.674	-41.988
3.099.411	-32.063	3.121.690	-42.474	3.070.858	-41.990
3.099.576	-32.066	3.121.922	-42.475	3.071.058	-41.991
3.099.763	-32.068	3.122.075	-42.477	3.071.232	-41.993
3.099.908	-32.070	3.122.180	-42.478	3.071.390	-41.994
3.100.124	-32.072	3.122.315	-42.479	3.071.589	-41.996
3.100.341	-32.075	3.122.549	-42.480	3.071.732	-41.997
3.100.425	-32.077	3.122.722	-42.481	3.071.830	-41.998
3.100.627	-32.079	3.122.890	-42.482	3.071.981	-42.000
3.100.844	-32.081	3.123.159	-42.484	3.072.076	-42.001
3.101.021	-32.083	3.123.351	-42.485	3.072.253	-42.003
3.101.223	-32.086	3.123.522	-42.486	3.072.485	-42.004
3.101.328	-32.088	3.123.717	-42.487	3.072.653	-42.005

3.101.436	-32.090	3.123.865	-42.488	3.072.838	-42.007
3.101.628	-32.092	3.123.992	-42.489	3.072.993	-42.008
3.101.809	-32.095	3.124.140	-42.490	3.073.062	-42.010
3.101.963	-32.097	3.124.299	-42.491	3.073.228	-42.011
3.102.163	-32.099	3.124.469	-42.492	3.073.506	-42.013
3.102.365	-32.101	3.124.628	-42.493	3.073.745	-42.014
3.102.516	-32.104	3.124.796	-42.494	3.073.904	-42.016
3.102.671	-32.106	3.124.967	-42.495	3.074.044	-42.017
3.102.872	-32.108	3.125.133	-42.496	3.074.232	-42.019
3.103.011	-32.110	3.125.323	-42.497	3.074.375	-42.020
3.103.103	-32.113	3.125.509	-42.498	3.074.471	-42.022
3.103.222	-32.115	3.125.701	-42.499	3.074.608	-42.023
3.103.369	-32.117	3.125.869	-42.500	3.074.756	-42.025
3.103.565	-32.120	3.126.021	-42.501	3.074.940	-42.027
3.103.697	-32.122	3.126.198	-42.501	3.075.168	-42.028
3.103.828	-32.124	3.126.374	-42.502	3.075.356	-42.030
3.103.979	-32.126	3.126.509	-42.503	3.075.526	-42.031
3.104.128	-32.129	3.126.678	-42.504	3.075.732	-42.033
3.104.362	-32.131	3.126.838	-42.505	3.075.913	-42.034
3.104.587	-32.133	3.126.978	-42.505	3.076.071	-42.036
3.104.781	-32.136	3.127.173	-42.506	3.076.241	-42.038
3.105.014	-32.138	3.127.346	-42.507	3.076.404	-42.039
3.105.220	-32.140	3.127.505	-42.508	3.076.544	-42.041
3.105.395	-32.142	3.127.619	-42.509	3.076.675	-42.043
3.105.570	-32.145	3.127.733	-42.509	3.076.845	-42.044
3.105.742	-32.147	3.127.910	-42.510	3.077.012	-42.046
3.105.867	-32.149	3.128.087	-42.511	3.077.138	-42.048
3.105.966	-32.152	3.128.362	-42.511	3.077.257	-42.049
3.106.096	-32.154	3.128.811	-42.512	3.077.462	-42.051
3.106.208	-32.156	3.129.174	-42.513	3.077.737	-42.053

3.106.340	-32.159	3.129.309	-42.513	3.077.888	-42.054
3.106.520	-32.161	3.129.317	-42.514	3.077.971	-42.056
3.106.696	-32.163	3.129.385	-42.515	3.078.098	-42.058
3.106.931	-32.166	3.129.499	-42.515	3.078.343	-42.059
3.107.159	-32.168	3.129.576	-42.516	3.078.539	-42.061
3.107.328	-32.170	3.129.662	-42.516	3.078.663	-42.063
3.107.513	-32.173	3.129.785	-42.517	3.078.777	-42.064
3.107.654	-32.175	3.129.959	-42.518	3.078.894	-42.066
3.107.845	-32.178	3.130.155	-42.518	3.079.037	-42.068
3.108.042	-32.180	3.130.356	-42.519	3.079.257	-42.070
3.108.152	-32.182	3.130.509	-42.519	3.079.417	-42.071
3.108.307	-32.185	3.130.653	-42.520	3.079.537	-42.073
3.108.481	-32.187	3.130.836	-42.520	3.079.699	-42.075
3.108.656	-32.190	3.131.017	-42.521	3.079.799	-42.076
3.108.836	-32.192	3.131.216	-42.521	3.079.947	-42.078
3.109.044	-32.194	3.131.374	-42.522	3.080.193	-42.080
3.109.250	-32.197	3.131.490	-42.522	3.080.426	-42.082
3.109.428	-32.199	3.131.622	-42.523	3.080.627	-42.083
3.109.638	-32.202	3.131.758	-42.523	3.080.721	-42.085
3.109.785	-32.204	3.131.993	-42.524	3.080.888	-42.087
3.109.906	-32.206	3.132.221	-42.524	3.081.180	-42.089
3.110.050	-32.209	3.132.341	-42.524	3.081.423	-42.090
3.110.186	-32.211	3.132.437	-42.525	3.081.589	-42.092
3.110.370	-32.214	3.132.578	-42.525	3.081.715	-42.094
3.110.609	-32.216	3.132.763	-42.526	3.081.899	-42.096
3.110.793	-32.219	3.132.947	-42.526	3.082.041	-42.097
3.110.847	-32.221	3.133.128	-42.527	3.082.176	-42.099
3.110.946	-32.224	3.133.333	-42.527	3.082.377	-42.101
3.111.187	-32.226	3.133.496	-42.528	3.082.535	-42.103
3.111.450	-32.229	3.133.598	-42.528	3.082.640	-42.104

3.111.606	-32.231	3.133.786	-42.529	3.082.783	-42.106
3.111.732	-32.234	3.133.998	-42.529	3.082.929	-42.108
3.111.917	-32.236	3.134.153	-42.529	3.083.038	-42.110
3.112.050	-32.239	3.134.324	-42.530	3.083.189	-42.111
3.112.202	-32.241	3.134.483	-42.530	3.083.354	-42.113
3.112.417	-32.244	3.134.644	-42.531	3.083.526	-42.115
3.112.563	-32.246	3.134.796	-42.531	3.083.723	-42.117
3.112.690	-32.249	3.134.909	-42.532	3.083.922	-42.118
3.112.943	-32.251	3.135.104	-42.532	3.084.167	-42.120
3.113.419	-32.254	3.135.352	-42.533	3.084.346	-42.122
3.113.748	-32.256	3.135.523	-42.533	3.084.526	-42.124
3.113.804	-32.259	3.135.663	-42.534	3.084.698	-42.125
3.113.875	-32.261	3.135.852	-42.534	3.084.828	-42.127
3.113.976	-32.264	3.136.021	-42.535	3.084.980	-42.129
3.114.064	-32.266	3.136.170	-42.535	3.085.117	-42.131
3.114.095	-32.269	3.136.291	-42.536	3.085.291	-42.133
3.114.193	-32.272	3.136.429	-42.537	3.085.482	-42.134
3.114.339	-32.274	3.136.638	-42.537	3.085.635	-42.136
3.114.453	-32.277	3.136.790	-42.538	3.085.805	-42.138
3.114.635	-32.279	3.136.960	-42.538	3.085.981	-42.140
3.114.846	-32.282	3.137.191	-42.539	3.086.151	-42.142
3.114.995	-32.284	3.137.364	-42.540	3.086.299	-42.143
3.115.151	-32.287	3.137.531	-42.540	3.086.472	-42.145
3.115.397	-32.290	3.137.688	-42.541	3.086.689	-42.147
3.115.639	-32.292	3.137.857	-42.541	3.086.860	-42.149
3.115.782	-32.295	3.138.048	-42.542	3.086.986	-42.151
3.115.876	-32.297	3.138.178	-42.543	3.087.166	-42.152
3.116.008	-32.300	3.138.329	-42.543	3.087.343	-42.154
3.116.208	-32.303	3.138.486	-42.544	3.087.527	-42.156
3.116.454	-32.305	3.138.670	-42.545	3.087.722	-42.158

3.116.591	-32.308	3.138.871	-42.546	3.087.882	-42.160
3.116.668	-32.310	3.139.030	-42.546	3.088.030	-42.162
3.116.838	-32.313	3.139.182	-42.547	3.088.206	-42.163
3.117.055	-32.316	3.139.360	-42.548	3.088.367	-42.165
3.117.238	-32.318	3.139.583	-42.549	3.088.489	-42.167
3.117.384	-32.321	3.139.691	-42.550	3.088.680	-42.169
3.117.564	-32.324	3.139.826	-42.550	3.088.961	-42.171
3.117.762	-32.326	3.140.009	-42.551	3.089.139	-42.173
3.117.971	-32.329	3.140.089	-42.552	3.089.211	-42.175
3.118.148	-32.331	3.140.212	-42.553	3.089.323	-42.176
3.118.289	-32.334	3.140.406	-42.554	3.089.550	-42.178
3.118.474	-32.337	3.140.573	-42.555	3.089.800	-42.180
3.118.640	-32.339	3.140.671	-42.555	3.089.973	-42.182
3.118.767	-32.342	3.140.804	-42.556	3.090.119	-42.184
3.118.909	-32.345	3.140.941	-42.557	3.090.263	-42.186
3.119.082	-32.347	3.141.122	-42.558	3.090.341	-42.188
3.119.266	-32.350	3.141.365	-42.559	3.090.461	-42.190
3.119.377	-32.352	3.141.568	-42.560	3.090.836	-42.192
3.119.471	-32.355	3.141.786	-42.561	3.091.297	-42.194
3.119.692	-32.358	3.141.964	-42.562	3.091.600	-42.195
3.119.868	-32.360	3.142.164	-42.563	3.091.712	-42.197
3.119.958	-32.363	3.142.375	-42.564	3.091.798	-42.199
3.120.132	-32.366	3.142.539	-42.565	3.091.895	-42.201
3.120.338	-32.368	3.142.693	-42.566	3.091.940	-42.203
3.120.479	-32.371	3.142.830	-42.567	3.092.033	-42.205
3.120.706	-32.374	3.142.930	-42.568	3.092.149	-42.207
3.120.902	-32.376	3.143.033	-42.569	3.092.217	-42.209
3.120.948	-32.379	3.143.163	-42.571	3.092.330	-42.211
3.121.087	-32.382	3.143.345	-42.572	3.092.484	-42.213
3.121.293	-32.384	3.143.550	-42.573	3.092.601	-42.215

3.121.293	-32.387	3.143.748	-42.574	3.092.723	-42.216
3.121.580	-32.389	3.143.909	-42.575	3.092.881	-42.218
3.121.775	-32.392	3.144.117	-42.576	3.093.148	-42.220
3.122.032	-32.395	3.144.353	-42.578	3.093.432	-42.222
3.122.245	-32.397	3.144.499	-42.579	3.093.607	-42.224
3.122.388	-32.400	3.144.698	-42.580	3.093.770	-42.226
3.122.531	-32.403	3.144.909	-42.581	3.093.907	-42.228
3.122.704	-32.405	3.145.086	-42.583	3.094.052	-42.230
3.122.863	-32.408	3.145.219	-42.584	3.094.236	-42.232
3.123.066	-32.411	3.145.356	-42.585	3.094.370	-42.234
3.123.266	-32.413	3.145.542	-42.587	3.094.469	-42.236
3.123.409	-32.416	3.145.721	-42.588	3.094.663	-42.238
3.123.481	-32.419	3.145.917	-42.589	3.094.897	-42.240
3.123.638	-32.421	3.146.060	-42.591	3.095.049	-42.242
3.123.882	-32.424	3.146.185	-42.592	3.095.219	-42.244
3.124.034	-32.427	3.146.380	-42.593	3.095.342	-42.246
3.124.185	-32.429	3.146.580	-42.595	3.095.417	-42.248
3.124.362	-32.432	3.146.708	-42.596	3.095.605	-42.250
3.124.525	-32.434	3.146.830	-42.598	3.095.840	-42.252
3.124.713	-32.437	3.146.999	-42.599	3.095.995	-42.254
3.124.890	-32.440	3.147.173	-42.601	3.096.176	-42.256
3.125.024	-32.442	3.147.321	-42.602	3.096.347	-42.259
3.125.107	-32.445	3.147.506	-42.604	3.096.501	-42.261
3.125.187	-32.448	3.147.751	-42.605	3.096.685	-42.263
3.125.399	-32.450	3.147.910	-42.607	3.096.863	-42.265
3.125.652	-32.453	3.148.012	-42.608	3.097.120	-42.267
3.125.838	-32.456	3.148.170	-42.610	3.097.297	-42.269
3.126.013	-32.458	3.148.370	-42.612	3.097.411	-42.271
3.126.199	-32.461	3.148.578	-42.613	3.097.599	-42.273
3.126.403	-32.464	3.148.752	-42.615	3.097.771	-42.275

3.126.620	-32.466	3.148.896	-42.617	3.097.926	-42.277
3.126.839	-32.469	3.149.066	-42.618	3.098.058	-42.280
3.127.043	-32.472	3.149.232	-42.620	3.098.163	-42.282
3.127.224	-32.474	3.149.351	-42.622	3.098.349	-42.284
3.127.296	-32.477	3.149.476	-42.623	3.098.528	-42.286
3.127.387	-32.480	3.149.832	-42.625	3.098.672	-42.288
3.127.568	-32.482	3.150.260	-42.627	3.098.857	-42.290
3.127.675	-32.485	3.150.439	-42.628	3.099.023	-42.293
3.127.816	-32.488	3.150.537	-42.630	3.099.211	-42.295
3.127.997	-32.490	3.150.672	-42.632	3.099.387	-42.297
3.128.110	-32.493	3.150.726	-42.634	3.099.539	-42.299
3.128.244	-32.496	3.150.780	-42.636	3.099.698	-42.302
3.128.406	-32.498	3.150.833	-42.637	3.099.890	-42.304
3.128.582	-32.501	3.150.984	-42.639	3.100.059	-42.306
3.128.864	-32.504	3.151.172	-42.641	3.100.150	-42.308
3.129.085	-32.506	3.151.302	-42.643	3.100.305	-42.311
3.129.232	-32.509	3.151.552	-42.645	3.100.482	-42.313
3.129.352	-32.512	3.151.779	-42.646	3.100.610	-42.315
3.129.472	-32.514	3.151.922	-42.648	3.100.775	-42.317
3.129.671	-32.517	3.152.077	-42.650	3.100.974	-42.320
3.129.933	-32.520	3.152.289	-42.652	3.101.187	-42.322
3.130.128	-32.523	3.152.458	-42.654	3.101.338	-42.324
3.130.249	-32.525	3.152.614	-42.656	3.101.499	-42.327
3.130.424	-32.528	3.152.787	-42.658	3.101.725	-42.329
3.130.576	-32.531	3.152.888	-42.660	3.101.848	-42.331
3.130.724	-32.533	3.153.009	-42.662	3.101.971	-42.333
3.130.948	-32.536	3.153.143	-42.663	3.102.206	-42.336
3.131.085	-32.539	3.153.325	-42.665	3.102.404	-42.338
3.131.192	-32.541	3.153.515	-42.667	3.102.535	-42.340
3.131.407	-32.544	3.153.652	-42.669	3.102.662	-42.342

3.131.614	-32.547	3.153.815	-42.671	3.102.823	-42.345
3.131.820	-32.550	3.154.005	-42.673	3.102.953	-42.347
3.131.981	-32.552	3.154.211	-42.675	3.103.112	-42.349
3.132.083	-32.555	3.154.373	-42.677	3.103.349	-42.351
3.132.239	-32.558	3.154.501	-42.679	3.103.573	-42.353
3.132.441	-32.560	3.154.640	-42.681	3.103.752	-42.356
3.132.683	-32.563	3.154.872	-42.683	3.103.904	-42.358
3.132.886	-32.566	3.155.089	-42.685	3.104.049	-42.360
3.132.993	-32.569	3.155.233	-42.687	3.104.221	-42.362
3.133.112	-32.571	3.155.370	-42.689	3.104.373	-42.364
3.133.300	-32.574	3.155.523	-42.691	3.104.514	-42.366
3.133.538	-32.577	3.155.717	-42.693	3.104.688	-42.369
3.133.730	-32.580	3.155.851	-42.695	3.104.821	-42.371
3.133.860	-32.582	3.155.938	-42.697	3.104.944	-42.373
3.133.995	-32.585	3.156.140	-42.699	3.105.121	-42.375
3.134.153	-32.588	3.156.360	-42.701	3.105.285	-42.377
3.134.257	-32.590	3.156.501	-42.703	3.105.476	-42.379
3.134.445	-32.593	3.156.700	-42.705	3.105.696	-42.381
3.134.854	-32.596	3.156.909	-42.707	3.105.851	-42.383
3.135.213	-32.599	3.157.063	-42.710	3.106.035	-42.385
3.135.399	-32.601	3.157.210	-42.712	3.106.188	-42.387
3.135.472	-32.604	3.157.354	-42.714	3.106.320	-42.389
3.135.556	-32.607	3.157.498	-42.716	3.106.524	-42.391
3.135.643	-32.610	3.157.643	-42.718	3.106.743	-42.393
3.135.690	-32.612	3.157.863	-42.720	3.106.954	-42.395
3.135.750	-32.615	3.158.029	-42.722	3.107.119	-42.397
3.135.867	-32.618	3.158.149	-42.724	3.107.304	-42.398
3.136.042	-32.621	3.158.298	-42.726	3.107.507	-42.400
3.136.217	-32.623	3.158.474	-42.728	3.107.617	-42.402
3.136.361	-32.626	3.158.656	-42.730	3.107.715	-42.404

3.136.541	-32.629	3.158.872	-42.733	3.107.850	-42.406
3.136.781	-32.632	3.159.084	-42.735	3.108.044	-42.407
3.136.936	-32.634	3.159.290	-42.737	3.108.244	-42.409
3.137.081	-32.637	3.159.439	-42.739	3.108.354	-42.411
3.137.229	-32.640	3.159.540	-42.741	3.108.459	-42.412
3.137.383	-32.643	3.159.695	-42.743	3.108.590	-42.414
3.137.589	-32.646	3.159.893	-42.745	3.108.768	-42.416
3.137.769	-32.648	3.160.078	-42.747	3.108.979	-42.417
3.137.928	-32.651	3.160.238	-42.749	3.109.135	-42.419
3.138.092	-32.654	3.160.432	-42.752	3.109.295	-42.421
3.138.206	-32.657	3.160.663	-42.754	3.109.486	-42.422
3.138.383	-32.659	3.160.826	-42.756	3.109.702	-42.424
3.138.609	-32.662	3.160.912	-42.758	3.109.897	-42.425
3.138.717	-32.665	3.161.076	-42.760	3.110.070	-42.427
3.138.831	-32.668	3.161.235	-42.762	3.110.252	-42.428
3.138.990	-32.670	3.161.328	-42.764	3.110.417	-42.430
3.139.219	-32.673	3.161.447	-42.766	3.110.598	-42.431
3.139.445	-32.676	3.161.646	-42.768	3.110.794	-42.432
3.139.606	-32.679	3.161.856	-42.770	3.110.953	-42.434
3.139.825	-32.681	3.161.967	-42.772	3.111.071	-42.435
3.139.987	-32.684	3.162.054	-42.774	3.111.163	-42.436
3.140.155	-32.687	3.162.231	-42.776	3.111.405	-42.438
3.140.353	-32.690	3.162.473	-42.778	3.111.678	-42.439
3.140.493	-32.693	3.162.718	-42.780	3.111.827	-42.440
3.140.612	-32.695	3.162.932	-42.782	3.111.910	-42.442
3.140.761	-32.698	3.163.070	-42.784	3.112.030	-42.443
3.140.971	-32.701	3.163.188	-42.786	3.112.257	-42.444
3.141.138	-32.704	3.163.391	-42.788	3.112.473	-42.445
3.141.286	-32.706	3.163.651	-42.790	3.112.583	-42.447
3.141.454	-32.709	3.163.804	-42.791	3.112.718	-42.448

3.141.584	-32.712	3.163.950	-42.793	3.112.937	-42.449
3.141.723	-32.715	3.164.118	-42.795	3.113.123	-42.450
3.141.927	-32.717	3.164.252	-42.797	3.113.313	-42.451
3.142.140	-32.720	3.164.406	-42.799	3.113.475	-42.452
3.142.249	-32.723	3.164.519	-42.800	3.113.632	-42.454
3.142.340	-32.726	3.164.656	-42.802	3.113.784	-42.455
3.142.491	-32.728	3.164.828	-42.804	3.113.963	-42.456
3.142.669	-32.731	3.164.995	-42.806	3.114.201	-42.457
3.142.827	-32.734	3.165.176	-42.807	3.114.324	-42.458
3.142.958	-32.737	3.165.370	-42.809	3.114.420	-42.459
3.143.172	-32.740	3.165.558	-42.811	3.114.583	-42.460
3.143.383	-32.742	3.165.679	-42.812	3.114.764	-42.462
3.143.562	-32.745	3.165.837	-42.814	3.114.977	-42.463
3.143.767	-32.748	3.166.046	-42.815	3.115.200	-42.464
3.143.984	-32.751	3.166.151	-42.817	3.115.352	-42.465
3.144.168	-32.753	3.166.375	-42.819	3.115.448	-42.466
3.144.236	-32.756	3.166.628	-42.820	3.115.627	-42.467
3.144.348	-32.759	3.166.779	-42.822	3.115.773	-42.468
3.144.558	-32.762	3.166.949	-42.823	3.115.928	-42.469
3.144.734	-32.764	3.167.126	-42.825	3.116.125	-42.470
3.144.880	-32.767	3.167.299	-42.826	3.116.275	-42.471
3.145.049	-32.770	3.167.516	-42.827	3.116.531	-42.472
3.145.287	-32.773	3.167.712	-42.829	3.116.891	-42.473
3.145.500	-32.775	3.167.823	-42.830	3.117.214	-42.474
3.145.623	-32.778	3.167.948	-42.832	3.117.415	-42.475
3.145.779	-32.781	3.168.130	-42.833	3.117.484	-42.477
3.145.913	-32.784	3.168.305	-42.834	3.117.556	-42.478
3.146.013	-32.786	3.168.457	-42.835	3.117.592	-42.479
3.146.183	-32.789	3.168.674	-42.837	3.117.647	-42.480
3.146.350	-32.792	3.168.857	-42.838	3.117.765	-42.481

3.146.498	-32.795	3.168.994	-42.839	3.117.935	-42.482
3.146.591	-32.798	3.169.156	-42.840	3.118.073	-42.483
3.146.726	-32.800	3.169.286	-42.842	3.118.160	-42.484
3.146.949	-32.803	3.169.454	-42.843	3.118.356	-42.485
3.147.101	-32.806	3.169.669	-42.844	3.118.539	-42.486
3.147.224	-32.809	3.169.872	-42.845	3.118.726	-42.487
3.147.423	-32.811	3.170.042	-42.846	3.118.922	-42.488
3.147.665	-32.814	3.170.143	-42.847	3.119.095	-42.490
3.147.926	-32.817	3.170.220	-42.848	3.119.309	-42.491
3.148.117	-32.820	3.170.417	-42.849	3.119.485	-42.492
3.148.275	-32.822	3.170.630	-42.850	3.119.602	-42.493
3.148.463	-32.825	3.170.755	-42.851	3.119.805	-42.494
3.148.637	-32.828	3.171.102	-42.852	3.119.995	-42.496
3.148.799	-32.831	3.171.588	-42.853	3.120.151	-42.497
3.148.937	-32.834	3.171.859	-42.854	3.120.295	-42.498
3.149.046	-32.836	3.171.927	-42.855	3.120.429	-42.499
3.149.211	-32.839	3.171.946	-42.855	3.120.594	-42.500
3.149.409	-32.842	3.171.953	-42.856	3.120.753	-42.502
3.149.515	-32.845	3.171.985	-42.857	3.120.883	-42.503
3.149.597	-32.848	3.172.057	-42.858	3.121.042	-42.504
3.149.778	-32.850	3.172.202	-42.859	3.121.221	-42.506
3.149.963	-32.853	3.172.413	-42.859	3.121.389	-42.507
3.150.093	-32.856	3.172.596	-42.860	3.121.613	-42.508
3.150.271	-32.859	3.172.791	-42.861	3.121.776	-42.510
3.150.504	-32.862	3.172.979	-42.861	3.121.904	-42.511
3.150.714	-32.864	3.173.138	-42.862	3.121.993	-42.513
3.150.908	-32.867	3.173.287	-42.863	3.122.158	-42.514
3.151.118	-32.870	3.173.500	-42.863	3.122.437	-42.515
3.151.299	-32.873	3.173.720	-42.864	3.122.672	-42.517
3.151.418	-32.876	3.173.875	-42.864	3.122.896	-42.518

3.151.560	-32.879	3.174.021	-42.865	3.123.062	-42.520
3.151.697	-32.882	3.174.128	-42.865	3.123.143	-42.521
3.151.866	-32.884	3.174.265	-42.866	3.123.296	-42.523
3.152.101	-32.887	3.174.445	-42.866	3.123.463	-42.525
3.152.242	-32.890	3.174.615	-42.867	3.123.565	-42.526
3.152.383	-32.893	3.174.796	-42.867	3.123.699	-42.528
3.152.552	-32.896	3.174.927	-42.868	3.123.820	-42.529
3.152.701	-32.899	3.175.103	-42.868	3.124.019	-42.531
3.152.860	-32.902	3.175.299	-42.868	3.124.243	-42.533
3.153.041	-32.904	3.175.472	-42.869	3.124.384	-42.534
3.153.264	-32.907	3.175.632	-42.869	3.124.554	-42.536
3.153.441	-32.910	3.175.822	-42.869	3.124.688	-42.538
3.153.578	-32.913	3.176.000	-42.870	3.124.807	-42.540
3.153.770	-32.916	3.176.179	-42.870	3.124.989	-42.542
3.154.001	-32.919	3.176.380	-42.870	3.125.201	-42.543
3.154.128	-32.922	3.176.553	-42.871	3.125.414	-42.545
3.154.243	-32.925	3.176.701	-42.871	3.125.572	-42.547
3.154.399	-32.928	3.176.823	-42.871	3.125.735	-42.549
3.154.521	-32.931	3.176.931	-42.871	3.125.823	-42.551
3.154.738	-32.934	3.177.095	-42.872	3.125.914	-42.553
3.154.981	-32.937	3.177.289	-42.872	3.126.134	-42.555
3.155.161	-32.940	3.177.441	-42.872	3.126.321	-42.557
3.155.332	-32.942	3.177.659	-42.872	3.126.541	-42.559
3.155.480	-32.945	3.177.852	-42.872	3.126.740	-42.561
3.155.645	-32.948	3.177.947	-42.873	3.126.873	-42.563
3.155.800	-32.951	3.178.059	-42.873	3.127.040	-42.565
3.155.960	-32.954	3.178.196	-42.873	3.127.144	-42.567
3.156.246	-32.957	3.178.344	-42.873	3.127.318	-42.569
3.156.703	-32.960	3.178.569	-42.873	3.127.590	-42.571
3.157.007	-32.963	3.178.750	-42.873	3.127.749	-42.574

3.157.112	-32.966	3.178.872	-42.873	3.127.896	-42.576
3.157.231	-32.969	3.179.070	-42.873	3.128.031	-42.578
3.157.290	-32.972	3.179.283	-42.874	3.128.161	-42.580
3.157.271	-32.975	3.179.449	-42.874	3.128.391	-42.582
3.157.315	-32.978	3.179.586	-42.874	3.128.602	-42.585
3.157.462	-32.981	3.179.812	-42.874	3.128.723	-42.587
3.157.643	-32.985	3.180.020	-42.874	3.128.905	-42.589
3.157.756	-32.988	3.180.151	-42.874	3.129.147	-42.591
3.157.888	-32.991	3.180.270	-42.874	3.129.327	-42.594
3.158.120	-32.994	3.180.425	-42.874	3.129.497	-42.596
3.158.283	-32.997	3.180.627	-42.874	3.129.651	-42.598
3.158.403	-33.000	3.180.778	-42.874	3.129.754	-42.600
3.158.575	-33.003	3.180.930	-42.874	3.129.890	-42.603
3.158.748	-33.006	3.181.107	-42.875	3.130.089	-42.605
3.158.930	-33.009	3.181.292	-42.875	3.130.309	-42.607
3.159.106	-33.012	3.181.519	-42.875	3.130.466	-42.609
3.159.250	-33.015	3.181.680	-42.875	3.130.594	-42.612
3.159.435	-33.018	3.181.762	-42.875	3.130.726	-42.614
3.159.612	-33.022	3.181.886	-42.875	3.130.822	-42.616
3.159.736	-33.025	3.182.093	-42.875	3.130.941	-42.619
3.159.861	-33.028	3.182.285	-42.875	3.131.083	-42.621
3.160.038	-33.031	3.182.434	-42.875	3.131.246	-42.623
3.160.179	-33.034	3.182.617	-42.875	3.131.393	-42.626
3.160.298	-33.037	3.182.773	-42.875	3.131.519	-42.628
3.160.486	-33.040	3.182.886	-42.876	3.131.722	-42.630
3.160.617	-33.043	3.182.997	-42.876	3.131.976	-42.632
3.160.796	-33.047	3.183.116	-42.876	3.132.211	-42.635
3.161.024	-33.050	3.183.318	-42.876	3.132.404	-42.637
3.161.230	-33.053	3.183.514	-42.876	3.132.588	-42.639
3.161.408	-33.056	3.183.658	-42.876	3.132.774	-42.641

3.161.584	-33.059	3.183.905	-42.876	3.132.953	-42.644
3.161.750	-33.063	3.184.131	-42.876	3.133.141	-42.646
3.161.917	-33.066	3.184.330	-42.876	3.133.307	-42.648
3.162.115	-33.069	3.184.565	-42.876	3.133.456	-42.650
3.162.321	-33.072	3.184.749	-42.877	3.133.615	-42.652
3.162.471	-33.075	3.184.890	-42.877	3.133.732	-42.655
3.162.574	-33.079	3.185.046	-42.877	3.133.865	-42.657
3.162.693	-33.082	3.185.182	-42.877	3.134.033	-42.659
3.162.865	-33.085	3.185.269	-42.877	3.134.161	-42.661
3.163.055	-33.088	3.185.367	-42.877	3.134.312	-42.663
3.163.188	-33.092	3.185.501	-42.877	3.134.492	-42.665
3.163.358	-33.095	3.185.703	-42.878	3.134.675	-42.667
3.163.573	-33.098	3.185.884	-42.878	3.134.862	-42.669
3.163.779	-33.101	3.185.981	-42.878	3.135.021	-42.671
3.163.918	-33.105	3.186.141	-42.878	3.135.197	-42.673
3.164.005	-33.108	3.186.354	-42.878	3.135.417	-42.675
3.164.198	-33.111	3.186.541	-42.879	3.135.567	-42.677
3.164.417	-33.114	3.186.725	-42.879	3.135.743	-42.679
3.164.581	-33.118	3.186.918	-42.879	3.135.948	-42.681
3.164.771	-33.121	3.187.140	-42.879	3.136.075	-42.683
3.164.948	-33.124	3.187.336	-42.880	3.136.259	-42.685
3.165.089	-33.127	3.187.511	-42.880	3.136.433	-42.687
3.165.276	-33.131	3.187.672	-42.880	3.136.609	-42.689
3.165.432	-33.134	3.187.798	-42.880	3.136.773	-42.691
3.165.577	-33.137	3.187.964	-42.881	3.136.892	-42.693
3.165.804	-33.141	3.188.139	-42.881	3.137.068	-42.694
3.166.011	-33.144	3.188.307	-42.881	3.137.285	-42.696
3.166.145	-33.147	3.188.441	-42.882	3.137.539	-42.698
3.166.263	-33.151	3.188.555	-42.882	3.137.720	-42.700
3.166.425	-33.154	3.188.755	-42.882	3.137.845	-42.702

3.166.620	-33.157	3.188.958	-42.883	3.137.971	-42.703
3.166.779	-33.161	3.189.115	-42.883	3.138.123	-42.705
3.166.907	-33.164	3.189.294	-42.884	3.138.298	-42.707
3.167.086	-33.167	3.189.481	-42.884	3.138.459	-42.708
3.167.257	-33.171	3.189.684	-42.885	3.138.615	-42.710
3.167.419	-33.174	3.189.893	-42.885	3.138.766	-42.712
3.167.594	-33.177	3.190.031	-42.886	3.138.932	-42.713
3.167.722	-33.181	3.190.148	-42.886	3.139.175	-42.715
3.167.843	-33.184	3.190.298	-42.887	3.139.398	-42.716
3.167.951	-33.188	3.190.486	-42.887	3.139.507	-42.718
3.168.073	-33.191	3.190.631	-42.888	3.139.616	-42.720
3.168.268	-33.194	3.190.790	-42.888	3.139.794	-42.721
3.168.459	-33.198	3.190.999	-42.889	3.139.941	-42.723
3.168.608	-33.201	3.191.172	-42.890	3.140.088	-42.724
3.168.795	-33.204	3.191.353	-42.890	3.140.276	-42.726
3.168.979	-33.208	3.191.498	-42.891	3.140.472	-42.727
3.169.143	-33.211	3.191.654	-42.892	3.140.689	-42.728
3.169.388	-33.215	3.191.827	-42.893	3.140.862	-42.730
3.169.555	-33.218	3.192.004	-42.893	3.140.959	-42.731
3.169.745	-33.221	3.192.315	-42.894	3.141.151	-42.733
3.169.901	-33.225	3.192.677	-42.895	3.141.387	-42.734
3.170.042	-33.228	3.192.951	-42.896	3.141.480	-42.736
3.170.317	-33.232	3.193.070	-42.897	3.141.629	-42.737
3.170.483	-33.235	3.193.160	-42.898	3.141.801	-42.738
3.170.598	-33.239	3.193.210	-42.899	3.141.929	-42.740
3.170.742	-33.242	3.193.231	-42.900	3.142.253	-42.741
3.170.856	-33.245	3.193.322	-42.901	3.142.695	-42.742
3.171.017	-33.249	3.193.419	-42.902	3.142.991	-42.743
3.171.201	-33.252	3.193.583	-42.903	3.143.058	-42.745
3.171.361	-33.256	3.193.728	-42.904	3.143.098	-42.746

3.171.494	-33.259	3.193.909	-42.905	3.143.224	-42.747
3.171.664	-33.263	3.194.151	-42.906	3.143.319	-42.748
3.171.870	-33.266	3.194.317	-42.907	3.143.349	-42.750
3.172.061	-33.270	3.194.453	-42.909	3.143.446	-42.751
3.172.285	-33.273	3.194.644	-42.910	3.143.622	-42.752
3.172.435	-33.277	3.194.855	-42.911	3.143.749	-42.753
3.172.539	-33.280	3.194.999	-42.913	3.143.864	-42.754
3.172.720	-33.284	3.195.136	-42.914	3.144.012	-42.756
3.172.936	-33.287	3.195.287	-42.915	3.144.234	-42.757
3.173.107	-33.291	3.195.424	-42.917	3.144.479	-42.758
3.173.275	-33.294	3.195.580	-42.918	3.144.677	-42.759
3.173.409	-33.298	3.195.737	-42.920	3.144.886	-42.760
3.173.535	-33.301	3.195.872	-42.921	3.145.052	-42.761
3.173.723	-33.305	3.196.006	-42.923	3.145.188	-42.762
3.173.918	-33.308	3.196.180	-42.924	3.145.336	-42.763
3.174.109	-33.312	3.196.317	-42.926	3.145.479	-42.765
3.174.301	-33.315	3.196.495	-42.928	3.145.616	-42.766
3.174.467	-33.319	3.196.740	-42.929	3.145.779	-42.767
3.174.633	-33.322	3.196.917	-42.931	3.145.959	-42.768
3.174.774	-33.326	3.197.148	-42.933	3.146.154	-42.769
3.174.915	-33.329	3.197.357	-42.935	3.146.270	-42.770
3.175.143	-33.333	3.197.502	-42.936	3.146.409	-42.771
3.175.325	-33.336	3.197.655	-42.938	3.146.591	-42.772
3.175.489	-33.340	3.197.787	-42.940	3.146.721	-42.774
3.175.634	-33.343	3.197.919	-42.942	3.146.904	-42.775
3.175.757	-33.347	3.198.077	-42.944	3.147.073	-42.776
3.175.939	-33.350	3.198.289	-42.946	3.147.242	-42.777
3.176.109	-33.354	3.198.427	-42.948	3.147.440	-42.778
3.176.307	-33.358	3.198.587	-42.950	3.147.578	-42.779
3.176.521	-33.361	3.198.782	-42.952	3.147.741	-42.780

3.176.683	-33.365	3.198.916	-42.954	3.147.903	-42.781
3.176.820	-33.368	3.199.077	-42.956	3.148.072	-42.783
3.176.982	-33.372	3.199.265	-42.959	3.148.268	-42.784
3.177.196	-33.375	3.199.474	-42.961	3.148.419	-42.785
3.177.337	-33.379	3.199.696	-42.963	3.148.591	-42.786
3.177.368	-33.382	3.199.846	-42.965	3.148.786	-42.787
3.177.633	-33.386	3.199.880	-42.968	3.148.979	-42.789
3.178.125	-33.390	3.200.017	-42.970	3.149.117	-42.790
3.178.495	-33.393	3.200.269	-42.972	3.149.243	-42.791
3.178.604	-33.397	3.200.443	-42.975	3.149.366	-42.792
3.178.640	-33.400	3.200.602	-42.977	3.149.510	-42.793
3.178.770	-33.404	3.200.789	-42.980	3.149.709	-42.795
3.178.778	-33.407	3.200.943	-42.982	3.149.865	-42.796
3.178.802	-33.411	3.201.096	-42.985	3.150.003	-42.797
3.178.987	-33.415	3.201.288	-42.987	3.150.190	-42.799
3.179.111	-33.418	3.201.454	-42.990	3.150.314	-42.800
3.179.247	-33.422	3.201.562	-42.992	3.150.511	-42.801
3.179.460	-33.425	3.201.689	-42.995	3.150.773	-42.803
3.179.669	-33.429	3.201.862	-42.998	3.150.927	-42.804
3.179.847	-33.433	3.202.029	-43.000	3.151.050	-42.806
3.180.018	-33.436	3.202.231	-43.003	3.151.219	-42.807
3.180.186	-33.440	3.202.397	-43.006	3.151.380	-42.808
3.180.330	-33.443	3.202.567	-43.008	3.151.502	-42.810
3.180.515	-33.447	3.202.742	-43.011	3.151.680	-42.811
3.180.717	-33.451	3.202.892	-43.014	3.151.877	-42.813
3.180.825	-33.454	3.203.077	-43.017	3.152.083	-42.814
3.180.956	-33.458	3.203.232	-43.020	3.152.265	-42.816
3.181.125	-33.461	3.203.349	-43.022	3.152.379	-42.818
3.181.255	-33.465	3.203.474	-43.025	3.152.556	-42.819
3.181.400	-33.469	3.203.645	-43.028	3.152.794	-42.821

3.181.580	-33.472	3.203.799	-43.031	3.152.986	-42.822
3.181.669	-33.476	3.203.914	-43.034	3.153.123	-42.824
3.181.812	-33.480	3.204.098	-43.037	3.153.260	-42.826
3.182.021	-33.483	3.204.276	-43.040	3.153.441	-42.827
3.182.202	-33.487	3.204.442	-43.042	3.153.625	-42.829
3.182.485	-33.490	3.204.612	-43.045	3.153.770	-42.831
3.182.715	-33.494	3.204.815	-43.048	3.153.907	-42.833
3.182.897	-33.498	3.205.007	-43.051	3.154.021	-42.834
3.183.033	-33.501	3.205.165	-43.054	3.154.198	-42.836
3.183.163	-33.505	3.205.378	-43.057	3.154.323	-42.838
3.183.312	-33.508	3.205.531	-43.060	3.154.474	-42.840
3.183.423	-33.512	3.205.732	-43.063	3.154.727	-42.841
3.183.600	-33.516	3.205.956	-43.066	3.154.891	-42.843
3.183.770	-33.519	3.206.089	-43.069	3.155.076	-42.845
3.183.943	-33.523	3.206.216	-43.072	3.155.316	-42.847
3.184.160	-33.527	3.206.403	-43.074	3.155.435	-42.849
3.184.305	-33.530	3.206.544	-43.077	3.155.518	-42.851
3.184.461	-33.534	3.206.696	-43.080	3.155.732	-42.853
3.184.635	-33.537	3.206.896	-43.083	3.155.858	-42.854
3.184.839	-33.541	3.207.005	-43.086	3.155.927	-42.856
3.185.060	-33.545	3.207.111	-43.089	3.156.073	-42.858
3.185.155	-33.548	3.207.289	-43.092	3.156.242	-42.860
3.185.241	-33.552	3.207.470	-43.095	3.156.429	-42.862
3.185.334	-33.555	3.207.688	-43.098	3.156.610	-42.864
3.185.508	-33.559	3.207.936	-43.100	3.156.766	-42.866
3.185.744	-33.563	3.208.127	-43.103	3.156.934	-42.868
3.185.870	-33.566	3.208.282	-43.106	3.157.109	-42.870
3.186.037	-33.570	3.208.401	-43.109	3.157.297	-42.872
3.186.257	-33.574	3.208.601	-43.112	3.157.464	-42.874
3.186.508	-33.577	3.208.818	-43.115	3.157.621	-42.876

3.186.729	-33.581	3.208.972	-43.117	3.157.849	-42.878
3.186.867	-33.584	3.209.132	-43.120	3.158.067	-42.880
3.187.022	-33.588	3.209.236	-43.123	3.158.242	-42.882
3.187.177	-33.592	3.209.385	-43.126	3.158.459	-42.884
3.187.268	-33.595	3.209.559	-43.128	3.158.695	-42.886
3.187.422	-33.599	3.209.731	-43.131	3.158.865	-42.888
3.187.632	-33.603	3.209.923	-43.134	3.159.008	-42.891
3.187.813	-33.606	3.210.103	-43.136	3.159.157	-42.893
3.188.005	-33.610	3.210.276	-43.139	3.159.284	-42.895
3.188.201	-33.613	3.210.392	-43.142	3.159.403	-42.897
3.188.334	-33.617	3.210.501	-43.144	3.159.521	-42.899
3.188.488	-33.621	3.210.695	-43.147	3.159.687	-42.901
3.188.690	-33.624	3.210.945	-43.149	3.159.843	-42.903
3.188.843	-33.628	3.211.151	-43.152	3.160.034	-42.905
3.188.998	-33.632	3.211.309	-43.154	3.160.262	-42.907
3.189.196	-33.635	3.211.421	-43.157	3.160.396	-42.909
3.189.299	-33.639	3.211.563	-43.159	3.160.554	-42.911
3.189.349	-33.642	3.211.734	-43.162	3.160.688	-42.913
3.189.533	-33.646	3.211.931	-43.164	3.160.855	-42.915
3.189.723	-33.650	3.212.115	-43.167	3.161.133	-42.918
3.189.760	-33.653	3.212.311	-43.169	3.161.313	-42.920
3.189.940	-33.657	3.212.499	-43.171	3.161.441	-42.922
3.190.237	-33.661	3.212.639	-43.174	3.161.602	-42.924
3.190.424	-33.664	3.212.776	-43.176	3.161.723	-42.926
3.190.576	-33.668	3.212.962	-43.178	3.161.862	-42.928
3.190.717	-33.671	3.213.110	-43.181	3.162.043	-42.930
3.190.941	-33.675	3.213.387	-43.183	3.162.300	-42.932
3.191.147	-33.679	3.213.820	-43.185	3.162.469	-42.935
3.191.299	-33.682	3.214.145	-43.187	3.162.581	-42.937
3.191.488	-33.686	3.214.280	-43.190	3.162.742	-42.939

3.191.656	-33.690	3.214.297	-43.192	3.162.903	-42.941
3.191.824	-33.693	3.214.327	-43.194	3.163.102	-42.943
3.192.000	-33.697	3.214.388	-43.196	3.163.279	-42.945
3.192.126	-33.701	3.214.449	-43.198	3.163.456	-42.947
3.192.200	-33.704	3.214.569	-43.200	3.163.627	-42.949
3.192.397	-33.708	3.214.716	-43.202	3.163.748	-42.951
3.192.594	-33.711	3.214.915	-43.204	3.163.904	-42.954
3.192.675	-33.715	3.215.147	-43.206	3.164.077	-42.956
3.192.831	-33.719	3.215.294	-43.208	3.164.244	-42.958
3.193.004	-33.722	3.215.428	-43.210	3.164.427	-42.960
3.193.225	-33.726	3.215.631	-43.212	3.164.580	-42.962
3.193.434	-33.730	3.215.830	-43.214	3.164.753	-42.964
3.193.589	-33.733	3.215.925	-43.216	3.164.955	-42.966
3.193.781	-33.737	3.216.078	-43.217	3.165.108	-42.968
3.193.943	-33.741	3.216.317	-43.219	3.165.238	-42.970
3.194.099	-33.744	3.216.517	-43.221	3.165.415	-42.972
3.194.297	-33.748	3.216.642	-43.223	3.165.645	-42.975
3.194.487	-33.752	3.216.689	-43.224	3.165.726	-42.977
3.194.678	-33.755	3.216.844	-43.226	3.165.895	-42.979
3.194.850	-33.759	3.217.061	-43.228	3.166.213	-42.981
3.195.014	-33.763	3.217.181	-43.230	3.166.357	-42.983
3.195.179	-33.766	3.217.339	-43.231	3.166.470	-42.985
3.195.323	-33.770	3.217.542	-43.233	3.166.639	-42.987
3.195.530	-33.774	3.217.729	-43.235	3.166.806	-42.989
3.195.703	-33.777	3.217.895	-43.236	3.166.983	-42.991
3.195.826	-33.781	3.218.105	-43.238	3.167.155	-42.994
3.195.961	-33.784	3.218.383	-43.239	3.167.281	-42.996
3.196.125	-33.788	3.218.547	-43.241	3.167.434	-42.998
3.196.350	-33.792	3.218.672	-43.242	3.167.781	-43.000
3.196.555	-33.795	3.218.869	-43.244	3.168.156	-43.002

3.196.676	-33.799	3.219.044	-43.245	3.168.445	-43.004
3.196.757	-33.803	3.219.121	-43.247	3.168.596	-43.006
3.196.904	-33.806	3.219.263	-43.248	3.168.680	-43.008
3.197.122	-33.810	3.219.460	-43.250	3.168.792	-43.010
3.197.408	-33.814	3.219.598	-43.251	3.168.839	-43.012
3.197.547	-33.817	3.219.739	-43.253	3.168.864	-43.014
3.197.606	-33.821	3.219.888	-43.254	3.168.988	-43.016
3.197.765	-33.825	3.220.125	-43.255	3.169.086	-43.019
3.198.026	-33.828	3.220.328	-43.257	3.169.178	-43.021
3.198.214	-33.832	3.220.359	-43.258	3.169.409	-43.023
3.198.308	-33.836	3.220.563	-43.259	3.169.626	-43.025
3.198.412	-33.839	3.220.822	-43.261	3.169.819	-43.027
3.198.543	-33.843	3.220.913	-43.262	3.169.941	-43.029
3.198.644	-33.847	3.221.080	-43.263	3.170.078	-43.031
3.198.862	-33.850	3.221.273	-43.265	3.170.251	-43.033
3.199.125	-33.854	3.221.418	-43.266	3.170.432	-43.035
3.199.521	-33.858	3.221.582	-43.267	3.170.664	-43.037
3.199.887	-33.861	3.221.751	-43.268	3.170.867	-43.039
3.200.029	-33.865	3.221.947	-43.270	3.171.006	-43.041
3.200.083	-33.869	3.222.144	-43.271	3.171.143	-43.044
3.200.097	-33.872	3.222.294	-43.272	3.171.300	-43.046
3.200.179	-33.876	3.222.491	-43.273	3.171.483	-43.048
3.200.276	-33.880	3.222.629	-43.274	3.171.661	-43.050
3.200.417	-33.883	3.222.777	-43.276	3.171.812	-43.052
3.200.519	-33.887	3.222.946	-43.277	3.171.960	-43.054
3.200.616	-33.891	3.223.033	-43.278	3.172.111	-43.056
3.200.748	-33.894	3.223.244	-43.279	3.172.250	-43.059
3.200.968	-33.898	3.223.521	-43.280	3.172.401	-43.061
3.201.212	-33.901	3.223.687	-43.282	3.172.648	-43.063
3.201.427	-33.905	3.223.806	-43.283	3.172.802	-43.065

3.201.609	-33.909	3.224.008	-43.284	3.172.947	-43.067
3.201.761	-33.912	3.224.147	-43.285	3.173.167	-43.069
3.201.906	-33.916	3.224.277	-43.286	3.173.334	-43.072
3.202.076	-33.920	3.224.485	-43.287	3.173.488	-43.074
3.202.263	-33.923	3.224.631	-43.288	3.173.619	-43.076
3.202.422	-33.927	3.224.750	-43.289	3.173.826	-43.078
3.202.546	-33.930	3.224.898	-43.290	3.174.042	-43.081
3.202.690	-33.934	3.225.034	-43.292	3.174.204	-43.083
3.202.935	-33.938	3.225.150	-43.293	3.174.386	-43.085
3.203.087	-33.941	3.225.313	-43.294	3.174.514	-43.087
3.203.207	-33.945	3.225.475	-43.295	3.174.662	-43.090
3.203.345	-33.948	3.225.580	-43.296	3.174.793	-43.092
3.203.492	-33.952	3.225.744	-43.297	3.174.944	-43.094
3.203.706	-33.956	3.226.007	-43.298	3.175.173	-43.097
3.203.914	-33.959	3.226.212	-43.299	3.175.325	-43.099
3.204.073	-33.963	3.226.398	-43.300	3.175.489	-43.101
3.204.221	-33.966	3.226.657	-43.301	3.175.682	-43.104
3.204.391	-33.970	3.226.880	-43.301	3.175.866	-43.106
3.204.586	-33.974	3.226.997	-43.302	3.175.959	-43.108
3.204.763	-33.977	3.227.131	-43.303	3.176.060	-43.111
3.204.919	-33.981	3.227.303	-43.304	3.176.296	-43.113
3.205.106	-33.984	3.227.469	-43.305	3.176.462	-43.116
3.205.245	-33.988	3.227.637	-43.306	3.176.588	-43.118
3.205.372	-33.991	3.227.752	-43.307	3.176.754	-43.121
3.205.544	-33.995	3.227.889	-43.308	3.176.927	-43.123
3.205.689	-33.998	3.228.044	-43.308	3.177.079	-43.126
3.205.818	-34.002	3.228.164	-43.309	3.177.227	-43.128
3.205.970	-34.005	3.228.362	-43.310	3.177.422	-43.131
3.206.138	-34.009	3.228.567	-43.311	3.177.600	-43.133
3.206.400	-34.012	3.228.752	-43.312	3.177.787	-43.136

3.206.607	-34.016	3.228.938	-43.312	3.177.991	-43.139
3.206.675	-34.019	3.229.143	-43.313	3.178.168	-43.141
3.206.815	-34.023	3.229.349	-43.314	3.178.356	-43.144
3.206.956	-34.026	3.229.507	-43.315	3.178.515	-43.146
3.207.072	-34.030	3.229.667	-43.315	3.178.672	-43.149
3.207.256	-34.033	3.229.823	-43.316	3.178.849	-43.152
3.207.459	-34.037	3.230.005	-43.317	3.179.001	-43.154
3.207.661	-34.040	3.230.241	-43.317	3.179.135	-43.157
3.207.820	-34.044	3.230.389	-43.318	3.179.287	-43.160
3.207.955	-34.047	3.230.536	-43.318	3.179.450	-43.162
3.208.123	-34.051	3.230.739	-43.319	3.179.641	-43.165
3.208.358	-34.054	3.230.869	-43.320	3.179.807	-43.168
3.208.520	-34.057	3.230.992	-43.320	3.179.894	-43.171
3.208.677	-34.061	3.231.198	-43.321	3.180.032	-43.173
3.208.868	-34.064	3.231.404	-43.321	3.180.259	-43.176
3.209.005	-34.068	3.231.521	-43.322	3.180.487	-43.179
3.209.189	-34.071	3.231.662	-43.322	3.180.692	-43.181
3.209.335	-34.074	3.231.846	-43.323	3.180.910	-43.184
3.209.501	-34.078	3.232.032	-43.323	3.181.007	-43.187
3.209.732	-34.081	3.232.216	-43.324	3.181.031	-43.190
3.209.958	-34.085	3.232.336	-43.324	3.181.217	-43.192
3.210.171	-34.088	3.232.467	-43.325	3.181.487	-43.195
3.210.346	-34.091	3.232.653	-43.325	3.181.645	-43.198
3.210.518	-34.095	3.232.832	-43.326	3.181.740	-43.201
3.210.652	-34.098	3.233.020	-43.326	3.181.898	-43.203
3.210.737	-34.101	3.233.204	-43.326	3.182.048	-43.206
3.210.906	-34.105	3.233.365	-43.327	3.182.198	-43.209
3.211.096	-34.108	3.233.518	-43.327	3.182.332	-43.212
3.211.198	-34.111	3.233.708	-43.327	3.182.453	-43.214
3.211.266	-34.114	3.233.878	-43.328	3.182.630	-43.217

3.211.358	-34.118	3.234.017	-43.328	3.182.816	-43.220
3.211.573	-34.121	3.234.180	-43.328	3.183.018	-43.223
3.211.769	-34.124	3.234.301	-43.329	3.183.237	-43.225
3.211.874	-34.128	3.234.547	-43.329	3.183.468	-43.228
3.212.032	-34.131	3.234.969	-43.329	3.183.698	-43.231
3.212.285	-34.134	3.235.291	-43.330	3.183.878	-43.233
3.212.523	-34.137	3.235.486	-43.330	3.184.006	-43.236
3.212.697	-34.140	3.235.574	-43.330	3.184.175	-43.239
3.212.903	-34.144	3.235.584	-43.330	3.184.369	-43.242
3.213.038	-34.147	3.235.607	-43.331	3.184.557	-43.244
3.213.199	-34.150	3.235.717	-43.331	3.184.747	-43.247
3.213.414	-34.153	3.235.881	-43.331	3.184.886	-43.250
3.213.555	-34.157	3.236.010	-43.331	3.184.991	-43.252
3.213.680	-34.160	3.236.162	-43.331	3.185.139	-43.255
3.213.831	-34.163	3.236.234	-43.332	3.185.302	-43.257
3.214.006	-34.166	3.236.375	-43.332	3.185.461	-43.260
3.214.225	-34.169	3.236.685	-43.332	3.185.635	-43.263
3.214.377	-34.172	3.236.909	-43.332	3.185.740	-43.265
3.214.456	-34.175	3.237.011	-43.332	3.185.861	-43.268
3.214.583	-34.179	3.237.229	-43.333	3.186.026	-43.270
3.214.745	-34.182	3.237.440	-43.333	3.186.201	-43.273
3.214.951	-34.185	3.237.545	-43.333	3.186.436	-43.275
3.215.155	-34.188	3.237.704	-43.333	3.186.678	-43.278
3.215.357	-34.191	3.237.859	-43.333	3.186.864	-43.281
3.215.599	-34.194	3.238.004	-43.333	3.187.003	-43.283
3.215.771	-34.197	3.238.157	-43.334	3.187.174	-43.286
3.215.923	-34.200	3.238.359	-43.334	3.187.366	-43.288
3.216.126	-34.203	3.238.557	-43.334	3.187.509	-43.291
3.216.230	-34.206	3.238.712	-43.334	3.187.639	-43.293
3.216.275	-34.209	3.238.835	-43.334	3.187.805	-43.295

3.216.459	-34.212	3.239.005	-43.334	3.187.986	-43.298
3.216.721	-34.216	3.239.240	-43.335	3.188.222	-43.300
3.216.909	-34.219	3.239.413	-43.335	3.188.343	-43.303
3.217.083	-34.222	3.239.547	-43.335	3.188.495	-43.305
3.217.275	-34.225	3.239.724	-43.335	3.188.676	-43.308
3.217.422	-34.228	3.239.883	-43.335	3.188.820	-43.310
3.217.614	-34.231	3.240.049	-43.336	3.188.880	-43.312
3.217.791	-34.234	3.240.244	-43.336	3.189.073	-43.315
3.217.877	-34.236	3.240.443	-43.336	3.189.391	-43.317
3.218.067	-34.239	3.240.580	-43.336	3.189.559	-43.319
3.218.273	-34.242	3.240.675	-43.337	3.189.698	-43.322
3.218.437	-34.245	3.240.852	-43.337	3.189.890	-43.324
3.218.640	-34.248	3.241.013	-43.337	3.190.090	-43.326
3.218.803	-34.251	3.241.130	-43.338	3.190.216	-43.329
3.218.969	-34.254	3.241.268	-43.338	3.190.368	-43.331
3.219.124	-34.257	3.241.454	-43.338	3.190.526	-43.333
3.219.286	-34.260	3.241.647	-43.339	3.190.710	-43.335
3.219.464	-34.263	3.241.826	-43.339	3.190.934	-43.338
3.219.662	-34.266	3.241.989	-43.339	3.191.071	-43.340
3.219.857	-34.269	3.242.141	-43.340	3.191.202	-43.342
3.219.978	-34.271	3.242.347	-43.340	3.191.371	-43.344
3.220.112	-34.274	3.242.492	-43.340	3.191.512	-43.347
3.220.240	-34.277	3.242.556	-43.341	3.191.705	-43.349
3.220.328	-34.280	3.242.701	-43.341	3.191.880	-43.351
3.220.482	-34.283	3.242.979	-43.342	3.192.079	-43.353
3.220.776	-34.286	3.243.178	-43.342	3.192.337	-43.356
3.221.203	-34.288	3.243.293	-43.343	3.192.482	-43.358
3.221.556	-34.291	3.243.441	-43.343	3.192.585	-43.360
3.221.733	-34.294	3.243.587	-43.344	3.192.706	-43.362
3.221.830	-34.297	3.243.737	-43.345	3.192.827	-43.364

3.221.852	-34.299	3.243.983	-43.345	3.192.910	-43.367
3.221.906	-34.302	3.244.196	-43.346	3.193.159	-43.369
3.221.993	-34.305	3.244.382	-43.347	3.193.596	-43.371
3.222.069	-34.308	3.244.515	-43.347	3.193.965	-43.373
3.222.184	-34.310	3.244.663	-43.348	3.194.194	-43.375
3.222.350	-34.313	3.244.792	-43.349	3.194.301	-43.377
3.222.545	-34.316	3.244.937	-43.349	3.194.319	-43.379
3.222.709	-34.318	3.245.150	-43.350	3.194.342	-43.382
3.222.882	-34.321	3.245.399	-43.351	3.194.440	-43.384
3.223.055	-34.324	3.245.587	-43.352	3.194.533	-43.386
3.223.253	-34.326	3.245.656	-43.353	3.194.655	-43.388
3.223.423	-34.329	3.245.831	-43.354	3.194.779	-43.390
3.223.517	-34.332	3.246.075	-43.355	3.194.868	-43.392
3.223.654	-34.334	3.246.158	-43.356	3.195.023	-43.394
3.223.867	-34.337	3.246.244	-43.357	3.195.251	-43.396
3.224.001	-34.340	3.246.400	-43.358	3.195.486	-43.398
3.224.135	-34.342	3.246.550	-43.359	3.195.634	-43.400
3.224.374	-34.345	3.246.707	-43.360	3.195.807	-43.402
3.224.536	-34.347	3.246.855	-43.361	3.196.021	-43.405
3.224.702	-34.350	3.247.041	-43.362	3.196.102	-43.407
3.224.837	-34.352	3.247.224	-43.363	3.196.255	-43.409
3.224.964	-34.355	3.247.438	-43.364	3.196.526	-43.411
3.225.143	-34.357	3.247.672	-43.365	3.196.639	-43.413
3.225.385	-34.360	3.247.867	-43.366	3.196.779	-43.415
3.225.659	-34.362	3.248.065	-43.368	3.197.007	-43.417
3.225.874	-34.365	3.248.251	-43.369	3.197.144	-43.419
3.226.051	-34.367	3.248.388	-43.370	3.197.281	-43.422
3.226.202	-34.370	3.248.560	-43.371	3.197.481	-43.424
3.226.386	-34.372	3.248.761	-43.373	3.197.650	-43.426
3.226.535	-34.375	3.248.820	-43.374	3.197.770	-43.428

3.226.656	-34.377	3.248.929	-43.376	3.197.926	-43.430
3.226.834	-34.380	3.249.093	-43.377	3.198.105	-43.432
3.226.957	-34.382	3.249.240	-43.378	3.198.250	-43.435
3.227.086	-34.384	3.249.366	-43.380	3.198.390	-43.437
3.227.272	-34.387	3.249.515	-43.381	3.198.596	-43.439
3.227.415	-34.389	3.249.685	-43.383	3.198.788	-43.441
3.227.520	-34.392	3.249.898	-43.384	3.199.010	-43.444
3.227.715	-34.394	3.250.135	-43.386	3.199.243	-43.446
3.227.860	-34.396	3.250.320	-43.387	3.199.416	-43.448
3.227.997	-34.399	3.250.476	-43.389	3.199.536	-43.450
3.228.235	-34.401	3.250.641	-43.391	3.199.703	-43.453
3.228.419	-34.403	3.250.800	-43.392	3.199.930	-43.455
3.228.611	-34.406	3.250.992	-43.394	3.200.057	-43.457
3.228.741	-34.408	3.251.161	-43.396	3.200.197	-43.460
3.228.799	-34.410	3.251.304	-43.397	3.200.349	-43.462
3.228.952	-34.412	3.251.445	-43.399	3.200.428	-43.464
3.229.203	-34.415	3.251.602	-43.401	3.200.562	-43.467
3.229.395	-34.417	3.251.842	-43.403	3.200.750	-43.469
3.229.523	-34.419	3.252.062	-43.404	3.200.891	-43.471
3.229.770	-34.421	3.252.253	-43.406	3.201.028	-43.474
3.229.958	-34.424	3.252.387	-43.408	3.201.232	-43.476
3.230.107	-34.426	3.252.444	-43.410	3.201.469	-43.479
3.230.287	-34.428	3.252.625	-43.412	3.201.599	-43.481
3.230.440	-34.430	3.252.889	-43.413	3.201.710	-43.484
3.230.610	-34.432	3.253.040	-43.415	3.201.853	-43.486
3.230.760	-34.435	3.253.206	-43.417	3.202.014	-43.489
3.230.919	-34.437	3.253.381	-43.419	3.202.223	-43.491
3.231.109	-34.439	3.253.564	-43.421	3.202.325	-43.494
3.231.292	-34.441	3.253.723	-43.423	3.202.476	-43.497
3.231.445	-34.443	3.253.875	-43.425	3.202.676	-43.499

3.231.568	-34.445	3.254.002	-43.427	3.202.890	-43.502
3.231.741	-34.447	3.254.151	-43.429	3.203.052	-43.505
3.231.875	-34.450	3.254.357	-43.431	3.203.193	-43.507
3.231.981	-34.452	3.254.595	-43.433	3.203.316	-43.510
3.232.224	-34.454	3.254.786	-43.435	3.203.448	-43.513
3.232.466	-34.456	3.254.944	-43.437	3.203.674	-43.515
3.232.639	-34.458	3.255.128	-43.439	3.203.923	-43.518
3.232.812	-34.460	3.255.291	-43.441	3.204.125	-43.521
3.232.912	-34.462	3.255.414	-43.443	3.204.298	-43.524
3.233.004	-34.464	3.255.526	-43.445	3.204.451	-43.526
3.233.143	-34.466	3.255.724	-43.447	3.204.604	-43.529
3.233.254	-34.468	3.256.147	-43.449	3.204.781	-43.532
3.233.425	-34.470	3.256.533	-43.451	3.204.917	-43.535
3.233.634	-34.472	3.256.651	-43.453	3.205.074	-43.538
3.233.840	-34.474	3.256.689	-43.455	3.205.248	-43.541
3.234.008	-34.476	3.256.777	-43.457	3.205.414	-43.543
3.234.230	-34.478	3.256.881	-43.459	3.205.594	-43.546
3.234.481	-34.480	3.256.946	-43.461	3.205.721	-43.549
3.234.684	-34.482	3.257.010	-43.463	3.205.880	-43.552
3.234.911	-34.484	3.257.138	-43.465	3.206.112	-43.555
3.235.046	-34.486	3.257.279	-43.467	3.206.314	-43.558
3.235.159	-34.488	3.257.437	-43.469	3.206.451	-43.561
3.235.331	-34.490	3.257.658	-43.471	3.206.592	-43.563
3.235.446	-34.492	3.257.861	-43.473	3.206.794	-43.566
3.235.573	-34.494	3.258.033	-43.475	3.206.971	-43.569
3.235.778	-34.496	3.258.228	-43.477	3.207.065	-43.572
3.235.909	-34.498	3.258.420	-43.479	3.207.185	-43.575
3.236.031	-34.500	3.258.598	-43.481	3.207.370	-43.578
3.236.181	-34.502	3.258.781	-43.484	3.207.502	-43.581
3.236.343	-34.504	3.258.922	-43.486	3.207.654	-43.584

3.236.544	-34.506	3.259.059	-43.488	3.207.816	-43.586
3.236.740	-34.508	3.259.113	-43.490	3.207.925	-43.589
3.236.942	-34.510	3.259.305	-43.492	3.208.062	-43.592
3.237.099	-34.512	3.259.593	-43.494	3.208.201	-43.595
3.237.222	-34.514	3.259.673	-43.496	3.208.392	-43.598
3.237.401	-34.516	3.259.825	-43.498	3.208.575	-43.601
3.237.589	-34.518	3.260.031	-43.500	3.208.784	-43.604
3.237.737	-34.520	3.260.229	-43.502	3.209.056	-43.607
3.237.908	-34.522	3.260.486	-43.504	3.209.308	-43.609
3.238.130	-34.524	3.260.681	-43.506	3.209.507	-43.612
3.238.300	-34.526	3.260.808	-43.508	3.209.666	-43.615
3.238.470	-34.528	3.261.002	-43.510	3.209.857	-43.618
3.238.608	-34.530	3.261.188	-43.512	3.210.031	-43.621
3.238.737	-34.532	3.261.336	-43.514	3.210.165	-43.623
3.238.875	-34.534	3.261.487	-43.515	3.210.307	-43.626
3.239.063	-34.536	3.261.613	-43.517	3.210.484	-43.629
3.239.218	-34.538	3.261.768	-43.519	3.210.619	-43.632
3.239.410	-34.539	3.261.924	-43.521	3.210.725	-43.634
3.239.604	-34.541	3.262.080	-43.523	3.210.830	-43.637
3.239.785	-34.543	3.262.281	-43.525	3.210.956	-43.640
3.239.991	-34.545	3.262.494	-43.527	3.211.140	-43.643
3.240.146	-34.547	3.262.644	-43.529	3.211.300	-43.645
3.240.330	-34.549	3.262.794	-43.531	3.211.432	-43.648
3.240.504	-34.551	3.262.932	-43.533	3.211.588	-43.651
3.240.663	-34.553	3.263.044	-43.535	3.211.819	-43.653
3.240.891	-34.555	3.263.255	-43.536	3.211.997	-43.656
3.241.089	-34.557	3.263.423	-43.538	3.212.109	-43.658
3.241.187	-34.559	3.263.540	-43.540	3.212.305	-43.661
3.241.365	-34.561	3.263.705	-43.542	3.212.545	-43.664
3.241.559	-34.563	3.263.873	-43.544	3.212.769	-43.666

3.241.692	-34.565	3.264.001	-43.545	3.212.973	-43.669
3.241.906	-34.567	3.264.258	-43.547	3.213.131	-43.671
3.242.065	-34.569	3.264.497	-43.549	3.213.275	-43.674
3.242.113	-34.571	3.264.608	-43.551	3.213.435	-43.676
3.242.203	-34.573	3.264.742	-43.552	3.213.669	-43.679
3.242.525	-34.575	3.264.909	-43.554	3.213.840	-43.681
3.243.001	-34.577	3.265.068	-43.556	3.213.994	-43.684
3.243.351	-34.579	3.265.177	-43.558	3.214.172	-43.686
3.243.481	-34.581	3.265.343	-43.559	3.214.309	-43.689
3.243.540	-34.583	3.265.544	-43.561	3.214.481	-43.691
3.243.557	-34.585	3.265.739	-43.563	3.214.658	-43.694
3.243.542	-34.587	3.265.924	-43.564	3.214.818	-43.696
3.243.609	-34.589	3.266.119	-43.566	3.215.013	-43.698
3.243.777	-34.591	3.266.255	-43.567	3.215.179	-43.701
3.243.940	-34.593	3.266.422	-43.569	3.215.318	-43.703
3.244.085	-34.595	3.266.617	-43.571	3.215.482	-43.705
3.244.252	-34.597	3.266.770	-43.572	3.215.681	-43.708
3.244.439	-34.599	3.266.900	-43.574	3.215.809	-43.710
3.244.637	-34.601	3.267.076	-43.575	3.215.943	-43.712
3.244.830	-34.603	3.267.272	-43.577	3.216.141	-43.715
3.245.023	-34.605	3.267.429	-43.578	3.216.288	-43.717
3.245.169	-34.607	3.267.556	-43.579	3.216.451	-43.719
3.245.334	-34.610	3.267.676	-43.581	3.216.624	-43.721
3.245.493	-34.612	3.267.820	-43.582	3.216.781	-43.724
3.245.645	-34.614	3.267.979	-43.584	3.216.938	-43.726
3.245.847	-34.616	3.268.185	-43.585	3.217.090	-43.728
3.246.003	-34.618	3.268.355	-43.586	3.217.238	-43.730
3.246.055	-34.620	3.268.448	-43.587	3.217.406	-43.733
3.246.165	-34.622	3.268.636	-43.589	3.217.599	-43.735
3.246.387	-34.624	3.268.909	-43.590	3.217.813	-43.737

3.246.575	-34.626	3.269.120	-43.591	3.218.004	-43.739
3.246.732	-34.628	3.269.338	-43.592	3.218.138	-43.741
3.246.920	-34.630	3.269.514	-43.594	3.218.316	-43.743
3.247.108	-34.633	3.269.692	-43.595	3.218.513	-43.746
3.247.299	-34.635	3.269.884	-43.596	3.218.616	-43.748
3.247.455	-34.637	3.269.994	-43.597	3.218.739	-43.750
3.247.648	-34.639	3.270.112	-43.598	3.219.106	-43.752
3.247.832	-34.641	3.270.240	-43.599	3.219.569	-43.754
3.247.986	-34.643	3.270.403	-43.600	3.219.803	-43.756
3.248.154	-34.645	3.270.624	-43.601	3.219.883	-43.758
3.248.279	-34.648	3.270.768	-43.602	3.220.005	-43.760
3.248.449	-34.650	3.270.813	-43.603	3.220.108	-43.762
3.248.625	-34.652	3.270.931	-43.604	3.220.180	-43.764
3.248.742	-34.654	3.271.155	-43.605	3.220.267	-43.766
3.248.889	-34.656	3.271.361	-43.606	3.220.349	-43.768
3.249.088	-34.658	3.271.559	-43.607	3.220.406	-43.770
3.249.265	-34.661	3.271.772	-43.608	3.220.515	-43.772
3.249.411	-34.663	3.271.947	-43.609	3.220.713	-43.774
3.249.597	-34.665	3.272.151	-43.609	3.220.881	-43.776
3.249.854	-34.667	3.272.341	-43.610	3.221.118	-43.778
3.250.064	-34.669	3.272.484	-43.611	3.221.284	-43.780
3.250.172	-34.671	3.272.647	-43.612	3.221.422	-43.782
3.250.258	-34.674	3.272.767	-43.612	3.221.609	-43.784
3.250.421	-34.676	3.272.919	-43.613	3.221.801	-43.786
3.250.602	-34.678	3.273.109	-43.614	3.221.982	-43.788
3.250.710	-34.680	3.273.311	-43.615	3.222.108	-43.790
3.250.888	-34.682	3.273.497	-43.615	3.222.261	-43.792
3.251.087	-34.685	3.273.661	-43.616	3.222.481	-43.793
3.251.263	-34.687	3.273.875	-43.617	3.222.651	-43.795
3.251.466	-34.689	3.274.016	-43.617	3.222.774	-43.797

3.251.671	-34.691	3.274.113	-43.618	3.222.906	-43.799
3.251.768	-34.694	3.274.277	-43.618	3.223.127	-43.801
3.251.902	-34.696	3.274.526	-43.619	3.223.311	-43.803
3.252.068	-34.698	3.274.740	-43.619	3.223.437	-43.805
3.252.260	-34.700	3.274.778	-43.620	3.223.589	-43.807
3.252.423	-34.703	3.274.919	-43.620	3.223.749	-43.809
3.252.565	-34.705	3.275.172	-43.621	3.223.905	-43.811
3.252.742	-34.707	3.275.336	-43.621	3.224.074	-43.813
3.252.896	-34.709	3.275.538	-43.622	3.224.250	-43.815
3.253.094	-34.712	3.275.677	-43.622	3.224.398	-43.817
3.253.279	-34.714	3.275.826	-43.623	3.224.605	-43.819
3.253.449	-34.716	3.276.089	-43.623	3.224.822	-43.821
3.253.618	-34.718	3.276.282	-43.624	3.224.974	-43.823
3.253.799	-34.721	3.276.393	-43.624	3.225.183	-43.825
3.253.951	-34.723	3.276.526	-43.624	3.225.426	-43.827
3.254.078	-34.725	3.276.664	-43.625	3.225.572	-43.829
3.254.209	-34.728	3.276.780	-43.625	3.225.648	-43.831
3.254.322	-34.730	3.277.069	-43.626	3.225.750	-43.833
3.254.464	-34.732	3.277.545	-43.626	3.225.885	-43.835
3.254.615	-34.734	3.277.897	-43.626	3.226.076	-43.837
3.254.772	-34.737	3.278.012	-43.626	3.226.270	-43.839
3.254.926	-34.739	3.278.031	-43.627	3.226.426	-43.841
3.255.086	-34.741	3.278.031	-43.627	3.226.598	-43.843
3.255.328	-34.744	3.278.135	-43.627	3.226.741	-43.845
3.255.551	-34.746	3.278.279	-43.628	3.226.885	-43.847
3.255.751	-34.748	3.278.318	-43.628	3.227.076	-43.849
3.256.002	-34.751	3.278.434	-43.628	3.227.278	-43.851
3.256.156	-34.753	3.278.589	-43.628	3.227.456	-43.853
3.256.299	-34.755	3.278.741	-43.629	3.227.597	-43.855
3.256.503	-34.758	3.278.922	-43.629	3.227.658	-43.857

3.256.628	-34.760	3.279.104	-43.629	3.227.776	-43.859
3.256.806	-34.762	3.279.269	-43.629	3.228.091	-43.861
3.257.072	-34.765	3.279.457	-43.629	3.228.349	-43.864
3.257.193	-34.767	3.279.702	-43.630	3.228.434	-43.866
3.257.227	-34.769	3.279.882	-43.630	3.228.578	-43.868
3.257.325	-34.772	3.280.016	-43.630	3.228.788	-43.870
3.257.549	-34.774	3.280.139	-43.630	3.228.969	-43.872
3.257.719	-34.776	3.280.260	-43.630	3.229.139	-43.874
3.257.849	-34.779	3.280.397	-43.630	3.229.315	-43.877
3.258.029	-34.781	3.280.548	-43.631	3.229.485	-43.879
3.258.213	-34.783	3.280.733	-43.631	3.229.648	-43.881
3.258.376	-34.786	3.280.913	-43.631	3.229.843	-43.883
3.258.557	-34.788	3.281.083	-43.631	3.230.005	-43.886
3.258.789	-34.790	3.281.238	-43.631	3.230.201	-43.888
3.259.003	-34.793	3.281.429	-43.632	3.230.401	-43.890
3.259.131	-34.795	3.281.645	-43.632	3.230.560	-43.892
3.259.263	-34.798	3.281.837	-43.632	3.230.729	-43.894
3.259.456	-34.800	3.282.033	-43.632	3.230.884	-43.897
3.259.637	-34.802	3.282.188	-43.633	3.231.051	-43.899
3.259.812	-34.805	3.282.303	-43.633	3.231.154	-43.901
3.259.991	-34.807	3.282.481	-43.633	3.231.284	-43.903
3.260.132	-34.809	3.282.593	-43.633	3.231.505	-43.906
3.260.259	-34.812	3.282.722	-43.634	3.231.660	-43.908
3.260.412	-34.814	3.282.928	-43.634	3.231.790	-43.910
3.260.620	-34.817	3.283.098	-43.634	3.231.994	-43.912
3.260.813	-34.819	3.283.257	-43.635	3.232.187	-43.914
3.260.968	-34.821	3.283.441	-43.635	3.232.311	-43.917
3.261.143	-34.824	3.283.619	-43.635	3.232.448	-43.919
3.261.340	-34.826	3.283.768	-43.636	3.232.643	-43.921
3.261.519	-34.829	3.283.944	-43.636	3.232.773	-43.923

3.261.674	-34.831	3.284.122	-43.637	3.232.925	-43.925
3.261.804	-34.834	3.284.273	-43.637	3.233.116	-43.927
3.261.924	-34.836	3.284.428	-43.637	3.233.254	-43.929
3.262.109	-34.838	3.284.586	-43.638	3.233.367	-43.932
3.262.279	-34.841	3.284.764	-43.638	3.233.528	-43.934
3.262.489	-34.843	3.284.967	-43.639	3.233.719	-43.936
3.262.719	-34.846	3.285.076	-43.639	3.233.864	-43.938
3.262.890	-34.848	3.285.195	-43.640	3.233.980	-43.940
3.263.078	-34.850	3.285.388	-43.641	3.234.063	-43.942
3.263.224	-34.853	3.285.567	-43.641	3.234.214	-43.944
3.263.358	-34.855	3.285.768	-43.642	3.234.489	-43.946
3.263.515	-34.858	3.285.970	-43.642	3.234.758	-43.948
3.263.661	-34.860	3.286.165	-43.643	3.235.010	-43.950
3.263.821	-34.863	3.286.361	-43.644	3.235.237	-43.952
3.263.995	-34.865	3.286.512	-43.644	3.235.432	-43.954
3.264.298	-34.868	3.286.638	-43.645	3.235.556	-43.956
3.264.753	-34.870	3.286.795	-43.646	3.235.641	-43.958
3.265.029	-34.872	3.286.982	-43.647	3.235.843	-43.960
3.265.121	-34.875	3.287.081	-43.648	3.236.054	-43.962
3.265.191	-34.877	3.287.195	-43.648	3.236.223	-43.963
3.265.238	-34.880	3.287.435	-43.649	3.236.310	-43.965
3.265.278	-34.882	3.287.643	-43.650	3.236.382	-43.967
3.265.378	-34.885	3.287.766	-43.651	3.236.582	-43.969
3.265.482	-34.887	3.287.912	-43.652	3.236.779	-43.971
3.265.603	-34.890	3.288.080	-43.653	3.236.884	-43.972
3.265.780	-34.892	3.288.189	-43.654	3.237.036	-43.974
3.265.945	-34.894	3.288.305	-43.655	3.237.276	-43.976
3.266.158	-34.897	3.288.416	-43.656	3.237.471	-43.977
3.266.351	-34.899	3.288.567	-43.657	3.237.614	-43.979
3.266.454	-34.902	3.288.774	-43.658	3.237.814	-43.981

3.266.664	-34.904	3.288.927	-43.660	3.238.016	-43.982
3.266.934	-34.907	3.289.048	-43.661	3.238.201	-43.984
3.267.090	-34.909	3.289.182	-43.662	3.238.412	-43.986
3.267.224	-34.912	3.289.355	-43.663	3.238.528	-43.987
3.267.393	-34.914	3.289.547	-43.664	3.238.726	-43.989
3.267.546	-34.917	3.289.749	-43.665	3.238.958	-43.990
3.267.676	-34.919	3.290.003	-43.667	3.239.046	-43.992
3.267.843	-34.922	3.290.186	-43.668	3.239.149	-43.993
3.268.011	-34.924	3.290.374	-43.669	3.239.367	-43.995
3.268.128	-34.927	3.290.624	-43.671	3.239.590	-43.996
3.268.297	-34.929	3.290.801	-43.672	3.239.753	-43.998
3.268.504	-34.931	3.290.930	-43.673	3.239.927	-43.999
3.268.619	-34.934	3.291.082	-43.675	3.240.049	-44.000
3.268.745	-34.936	3.291.263	-43.676	3.240.209	-44.002
3.268.921	-34.939	3.291.429	-43.678	3.240.424	-44.003
3.269.132	-34.941	3.291.542	-43.679	3.240.641	-44.004
3.269.373	-34.944	3.291.678	-43.681	3.240.753	-44.006
3.269.576	-34.946	3.291.842	-43.682	3.240.845	-44.007
3.269.729	-34.949	3.291.997	-43.684	3.241.020	-44.008
3.269.840	-34.951	3.292.173	-43.685	3.241.213	-44.010
3.270.023	-34.954	3.292.373	-43.687	3.241.398	-44.011
3.270.191	-34.956	3.292.541	-43.689	3.241.545	-44.012
3.270.302	-34.959	3.292.735	-43.690	3.241.721	-44.013
3.270.455	-34.961	3.292.903	-43.692	3.241.974	-44.015
3.270.681	-34.964	3.293.018	-43.694	3.242.149	-44.016
3.270.862	-34.966	3.293.237	-43.695	3.242.245	-44.017
3.271.011	-34.969	3.293.434	-43.697	3.242.395	-44.018
3.271.234	-34.971	3.293.607	-43.699	3.242.578	-44.019
3.271.373	-34.974	3.293.785	-43.701	3.242.708	-44.020
3.271.516	-34.976	3.293.900	-43.703	3.242.853	-44.021

3.271.679	-34.978	3.294.044	-43.705	3.243.059	-44.022
3.271.799	-34.981	3.294.261	-43.707	3.243.278	-44.024
3.271.987	-34.983	3.294.431	-43.708	3.243.344	-44.025
3.272.138	-34.986	3.294.565	-43.710	3.243.495	-44.026
3.272.275	-34.988	3.294.739	-43.712	3.243.793	-44.027
3.272.485	-34.991	3.294.888	-43.714	3.243.929	-44.028
3.272.726	-34.993	3.295.013	-43.716	3.244.117	-44.029
3.272.918	-34.996	3.295.175	-43.719	3.244.245	-44.030
3.273.069	-34.998	3.295.356	-43.721	3.244.261	-44.031
3.273.257	-35.001	3.295.580	-43.723	3.244.514	-44.032
3.273.448	-35.003	3.295.826	-43.725	3.244.960	-44.033
3.273.555	-35.006	3.296.003	-43.727	3.245.349	-44.034
3.273.701	-35.008	3.296.105	-43.729	3.245.605	-44.035
3.273.882	-35.010	3.296.176	-43.732	3.245.685	-44.036
3.274.048	-35.013	3.296.373	-43.734	3.245.729	-44.037
3.274.193	-35.015	3.296.618	-43.736	3.245.858	-44.037
3.274.298	-35.018	3.296.779	-43.738	3.245.988	-44.038
3.274.489	-35.020	3.296.928	-43.741	3.246.028	-44.039
3.274.675	-35.023	3.297.057	-43.743	3.246.064	-44.040
3.274.846	-35.025	3.297.257	-43.746	3.246.174	-44.041
3.275.011	-35.028	3.297.442	-43.748	3.246.321	-44.042
3.275.188	-35.030	3.297.603	-43.750	3.246.559	-44.043
3.275.388	-35.033	3.297.738	-43.753	3.246.732	-44.044
3.275.529	-35.035	3.297.847	-43.755	3.246.864	-44.044
3.275.667	-35.038	3.297.986	-43.758	3.246.996	-44.045
3.275.833	-35.040	3.298.297	-43.761	3.247.160	-44.046
3.275.952	-35.042	3.298.813	-43.763	3.247.373	-44.047
3.276.043	-35.045	3.299.107	-43.766	3.247.547	-44.048
3.276.205	-35.047	3.299.187	-43.768	3.247.715	-44.049
3.276.401	-35.050	3.299.268	-43.771	3.247.849	-44.050

3.276.519	-35.052	3.299.319	-43.774	3.248.037	-44.050
3.276.674	-35.055	3.299.362	-43.777	3.248.297	-44.051
3.276.904	-35.057	3.299.478	-43.779	3.248.432	-44.052
3.277.102	-35.060	3.299.581	-43.782	3.248.518	-44.053
3.277.268	-35.062	3.299.732	-43.785	3.248.649	-44.054
3.277.455	-35.064	3.299.875	-43.788	3.248.802	-44.055
3.277.672	-35.067	3.299.980	-43.790	3.248.983	-44.056
3.277.868	-35.069	3.300.197	-43.793	3.249.218	-44.057
3.278.073	-35.072	3.300.453	-43.796	3.249.391	-44.058
3.278.276	-35.074	3.300.645	-43.799	3.249.503	-44.059
3.278.492	-35.077	3.300.766	-43.802	3.249.651	-44.060
3.278.557	-35.079	3.300.894	-43.805	3.249.756	-44.061
3.278.696	-35.082	3.301.040	-43.808	3.249.905	-44.062
3.278.926	-35.084	3.301.198	-43.811	3.250.139	-44.063
3.279.013	-35.087	3.301.454	-43.814	3.250.291	-44.064
3.279.142	-35.089	3.301.656	-43.817	3.250.428	-44.065
3.279.274	-35.091	3.301.719	-43.820	3.250.674	-44.066
3.279.398	-35.094	3.301.859	-43.823	3.250.948	-44.067
3.279.518	-35.096	3.302.039	-43.827	3.251.057	-44.068
3.279.735	-35.099	3.302.225	-43.830	3.251.156	-44.069
3.279.980	-35.101	3.302.328	-43.833	3.251.407	-44.070
3.280.172	-35.104	3.302.435	-43.836	3.251.647	-44.071
3.280.371	-35.106	3.302.621	-43.839	3.251.816	-44.073
3.280.583	-35.109	3.302.780	-43.843	3.251.904	-44.074
3.280.791	-35.111	3.303.008	-43.846	3.252.012	-44.075
3.280.945	-35.114	3.303.219	-43.849	3.252.174	-44.076
3.281.100	-35.116	3.303.398	-43.853	3.252.350	-44.077
3.281.235	-35.118	3.303.492	-43.856	3.252.487	-44.079
3.281.386	-35.121	3.303.640	-43.860	3.252.619	-44.080
3.281.581	-35.123	3.303.853	-43.863	3.252.803	-44.081

3.281.747	-35.126	3.304.021	-43.867	3.253.033	-44.083
3.281.918	-35.128	3.304.142	-43.870	3.253.284	-44.084
3.282.115	-35.131	3.304.287	-43.874	3.253.427	-44.085
3.282.253	-35.133	3.304.467	-43.877	3.253.575	-44.087
3.282.357	-35.136	3.304.609	-43.881	3.253.792	-44.088
3.282.525	-35.138	3.304.724	-43.884	3.253.926	-44.090
3.282.713	-35.141	3.304.923	-43.888	3.254.009	-44.091
3.282.852	-35.143	3.305.202	-43.892	3.254.099	-44.093
3.283.007	-35.146	3.305.325	-43.896	3.254.262	-44.094
3.283.228	-35.148	3.305.450	-43.899	3.254.501	-44.096
3.283.416	-35.151	3.305.648	-43.903	3.254.663	-44.097
3.283.551	-35.153	3.305.797	-43.907	3.254.797	-44.099
3.283.688	-35.156	3.305.925	-43.911	3.255.020	-44.101
3.283.878	-35.158	3.306.044	-43.915	3.255.235	-44.102
3.284.091	-35.161	3.306.184	-43.919	3.255.419	-44.104
3.284.250	-35.163	3.306.386	-43.923	3.255.609	-44.106
3.284.319	-35.166	3.306.564	-43.927	3.255.778	-44.107
3.284.494	-35.168	3.306.745	-43.931	3.255.898	-44.109
3.284.732	-35.171	3.306.968	-43.935	3.256.011	-44.111
3.284.901	-35.173	3.307.094	-43.939	3.256.194	-44.113
3.285.085	-35.176	3.307.279	-43.943	3.256.409	-44.114
3.285.244	-35.178	3.307.523	-43.947	3.256.589	-44.116
3.285.408	-35.181	3.307.654	-43.951	3.256.682	-44.118
3.285.563	-35.183	3.307.794	-43.955	3.256.851	-44.120
3.285.768	-35.186	3.307.953	-43.959	3.257.034	-44.121
3.286.025	-35.189	3.308.098	-43.964	3.257.216	-44.123
3.286.385	-35.191	3.308.264	-43.968	3.257.370	-44.125
3.286.671	-35.194	3.308.463	-43.972	3.257.505	-44.127
3.286.733	-35.196	3.308.662	-43.977	3.257.733	-44.129
3.286.838	-35.199	3.308.853	-43.981	3.257.883	-44.131

3.286.942	-35.201	3.309.006	-43.985	3.258.054	-44.133
3.286.936	-35.204	3.309.156	-43.990	3.258.336	-44.134
3.286.996	-35.207	3.309.351	-43.994	3.258.486	-44.136
3.287.141	-35.209	3.309.493	-43.999	3.258.562	-44.138
3.287.285	-35.212	3.309.633	-44.003	3.258.719	-44.140
3.287.444	-35.214	3.309.841	-44.008	3.258.792	-44.142
3.287.625	-35.217	3.309.998	-44.012	3.258.922	-44.144
3.287.863	-35.220	3.310.089	-44.017	3.259.122	-44.146
3.288.107	-35.222	3.310.208	-44.021	3.259.265	-44.148
3.288.260	-35.225	3.310.345	-44.026	3.259.439	-44.150
3.288.414	-35.227	3.310.433	-44.030	3.259.565	-44.152
3.288.557	-35.230	3.310.565	-44.035	3.259.706	-44.154
3.288.731	-35.233	3.310.787	-44.039	3.259.924	-44.156
3.288.919	-35.235	3.311.028	-44.044	3.260.143	-44.158
3.289.024	-35.238	3.311.230	-44.048	3.260.317	-44.159
3.289.172	-35.241	3.311.423	-44.053	3.260.527	-44.161
3.289.370	-35.243	3.311.671	-44.058	3.260.751	-44.163
3.289.563	-35.246	3.311.867	-44.062	3.260.981	-44.165
3.289.699	-35.249	3.312.023	-44.067	3.261.201	-44.167
3.289.807	-35.251	3.312.088	-44.072	3.261.339	-44.169
3.290.010	-35.254	3.312.202	-44.076	3.261.468	-44.171
3.290.143	-35.257	3.312.462	-44.081	3.261.609	-44.173
3.290.230	-35.259	3.312.625	-44.086	3.261.783	-44.175
3.290.465	-35.262	3.312.773	-44.090	3.261.925	-44.177
3.290.697	-35.265	3.312.928	-44.095	3.262.090	-44.179
3.290.851	-35.268	3.313.040	-44.099	3.262.260	-44.181
3.291.032	-35.270	3.313.174	-44.104	3.262.379	-44.182
3.291.275	-35.273	3.313.362	-44.109	3.262.517	-44.184
3.291.447	-35.276	3.313.510	-44.113	3.262.647	-44.186
3.291.563	-35.279	3.313.620	-44.118	3.262.780	-44.188

3.291.614	-35.281	3.313.831	-44.123	3.262.958	-44.190
3.291.779	-35.284	3.314.039	-44.127	3.263.103	-44.192
3.291.965	-35.287	3.314.189	-44.132	3.263.228	-44.194
3.292.097	-35.290	3.314.414	-44.137	3.263.461	-44.195
3.292.177	-35.293	3.314.662	-44.141	3.263.732	-44.197
3.292.361	-35.295	3.314.840	-44.146	3.263.915	-44.199
3.292.565	-35.298	3.314.991	-44.151	3.264.034	-44.201
3.292.741	-35.301	3.315.148	-44.155	3.264.245	-44.203
3.292.943	-35.304	3.315.291	-44.160	3.264.487	-44.205
3.293.134	-35.307	3.315.421	-44.164	3.264.612	-44.206
3.293.302	-35.310	3.315.596	-44.169	3.264.727	-44.208
3.293.409	-35.313	3.315.805	-44.174	3.264.893	-44.210
3.293.605	-35.315	3.316.039	-44.178	3.265.061	-44.212
3.293.860	-35.318	3.316.241	-44.183	3.265.266	-44.214
3.294.002	-35.321	3.316.375	-44.187	3.265.418	-44.215
3.294.041	-35.324	3.316.451	-44.192	3.265.617	-44.217
3.294.185	-35.327	3.316.570	-44.196	3.265.797	-44.219
3.294.460	-35.330	3.316.750	-44.201	3.265.938	-44.221
3.294.644	-35.333	3.316.983	-44.205	3.266.149	-44.222
3.294.768	-35.336	3.317.236	-44.210	3.266.321	-44.224
3.294.933	-35.339	3.317.379	-44.214	3.266.523	-44.226
3.295.112	-35.342	3.317.532	-44.219	3.266.617	-44.227
3.295.259	-35.345	3.317.669	-44.223	3.266.716	-44.229
3.295.480	-35.348	3.317.813	-44.228	3.266.975	-44.231
3.295.732	-35.351	3.318.018	-44.232	3.267.202	-44.232
3.295.869	-35.354	3.318.174	-44.237	3.267.372	-44.234
3.296.000	-35.357	3.318.300	-44.241	3.267.542	-44.236
3.296.127	-35.360	3.318.434	-44.246	3.267.719	-44.237
3.296.289	-35.363	3.318.620	-44.250	3.267.830	-44.239
3.296.505	-35.366	3.318.829	-44.254	3.268.022	-44.241

3.296.618	-35.369	3.319.050	-44.259	3.268.193	-44.242
3.296.740	-35.372	3.319.208	-44.263	3.268.322	-44.244
3.296.960	-35.376	3.319.330	-44.267	3.268.450	-44.246
3.297.180	-35.379	3.319.442	-44.271	3.268.598	-44.247
3.297.350	-35.382	3.319.557	-44.276	3.268.813	-44.249
3.297.469	-35.385	3.319.919	-44.280	3.269.019	-44.251
3.297.592	-35.388	3.320.382	-44.284	3.269.218	-44.252
3.297.763	-35.391	3.320.630	-44.288	3.269.418	-44.254
3.297.885	-35.395	3.320.753	-44.292	3.269.543	-44.255
3.297.937	-35.398	3.320.838	-44.296	3.269.623	-44.257
3.298.055	-35.401	3.320.874	-44.300	3.269.797	-44.259
3.298.250	-35.404	3.320.906	-44.304	3.270.007	-44.260
3.298.477	-35.408	3.320.959	-44.308	3.270.140	-44.262
3.298.651	-35.411	3.321.025	-44.312	3.270.378	-44.263
3.298.839	-35.414	3.321.163	-44.316	3.270.804	-44.265
3.299.109	-35.417	3.321.357	-44.320	3.271.185	-44.266
3.299.352	-35.421	3.321.577	-44.324	3.271.385	-44.268
3.299.525	-35.424	3.321.711	-44.328	3.271.445	-44.270
3.299.677	-35.427	3.321.902	-44.332	3.271.483	-44.271
3.299.858	-35.431	3.322.169	-44.336	3.271.560	-44.273
3.300.018	-35.434	3.322.339	-44.339	3.271.614	-44.274
3.300.151	-35.438	3.322.500	-44.343	3.271.668	-44.276
3.300.291	-35.441	3.322.646	-44.347	3.271.787	-44.277
3.300.455	-35.444	3.322.809	-44.350	3.271.902	-44.279
3.300.659	-35.448	3.322.955	-44.354	3.272.034	-44.280
3.300.778	-35.451	3.323.103	-44.358	3.272.184	-44.282
3.300.818	-35.455	3.323.290	-44.361	3.272.368	-44.283
3.300.894	-35.458	3.323.499	-44.365	3.272.614	-44.285
3.301.089	-35.462	3.323.680	-44.368	3.272.830	-44.287
3.301.318	-35.465	3.323.748	-44.372	3.272.977	-44.288

3.301.465	-35.469	3.323.889	-44.375	3.273.154	-44.290
3.301.584	-35.472	3.324.042	-44.378	3.273.352	-44.291
3.301.806	-35.476	3.324.229	-44.382	3.273.474	-44.293
3.302.088	-35.479	3.324.454	-44.385	3.273.633	-44.294
3.302.333	-35.483	3.324.591	-44.388	3.273.760	-44.296
3.302.516	-35.487	3.324.810	-44.392	3.273.869	-44.298
3.302.637	-35.490	3.325.085	-44.395	3.274.122	-44.299
3.302.756	-35.494	3.325.238	-44.398	3.274.355	-44.301
3.302.886	-35.497	3.325.325	-44.401	3.274.398	-44.303
3.303.018	-35.501	3.325.435	-44.404	3.274.514	-44.304
3.303.232	-35.505	3.325.584	-44.407	3.274.742	-44.306
3.303.464	-35.509	3.325.689	-44.410	3.274.963	-44.308
3.303.637	-35.512	3.325.899	-44.413	3.275.197	-44.309
3.303.822	-35.516	3.326.141	-44.416	3.275.306	-44.311
3.303.970	-35.520	3.326.303	-44.419	3.275.442	-44.313
3.304.089	-35.523	3.326.405	-44.422	3.275.616	-44.315
3.304.265	-35.527	3.326.570	-44.425	3.275.764	-44.316
3.304.432	-35.531	3.326.772	-44.427	3.275.953	-44.318
3.304.653	-35.535	3.326.931	-44.430	3.276.161	-44.320
3.304.829	-35.539	3.327.093	-44.433	3.276.318	-44.322
3.304.944	-35.542	3.327.224	-44.436	3.276.492	-44.324
3.305.141	-35.546	3.327.333	-44.438	3.276.690	-44.326
3.305.291	-35.550	3.327.513	-44.441	3.276.904	-44.327
3.305.440	-35.554	3.327.670	-44.443	3.277.054	-44.329
3.305.612	-35.558	3.327.813	-44.446	3.277.173	-44.331
3.305.746	-35.562	3.328.023	-44.449	3.277.336	-44.333
3.305.858	-35.566	3.328.218	-44.451	3.277.496	-44.335
3.306.021	-35.570	3.328.416	-44.454	3.277.646	-44.337
3.306.252	-35.574	3.328.564	-44.456	3.277.817	-44.339
3.306.465	-35.578	3.328.709	-44.458	3.277.969	-44.341

3.306.588	-35.582	3.328.898	-44.461	3.278.081	-44.343
3.306.682	-35.586	3.329.068	-44.463	3.278.210	-44.345
3.306.866	-35.590	3.329.240	-44.465	3.278.412	-44.347
3.307.086	-35.594	3.329.403	-44.468	3.278.651	-44.349
3.307.249	-35.598	3.329.619	-44.470	3.278.856	-44.352
3.307.289	-35.602	3.329.832	-44.472	3.278.947	-44.354
3.307.585	-35.606	3.329.996	-44.474	3.279.010	-44.356
3.308.073	-35.610	3.330.135	-44.476	3.279.165	-44.358
3.308.363	-35.614	3.330.287	-44.478	3.279.398	-44.360
3.308.537	-35.618	3.330.490	-44.481	3.279.599	-44.363
3.308.625	-35.622	3.330.681	-44.483	3.279.724	-44.365
3.308.674	-35.626	3.330.856	-44.485	3.279.938	-44.367
3.308.684	-35.631	3.330.993	-44.487	3.280.146	-44.369
3.308.694	-35.635	3.331.166	-44.488	3.280.219	-44.372
3.308.824	-35.639	3.331.297	-44.490	3.280.341	-44.374
3.308.976	-35.643	3.331.418	-44.492	3.280.565	-44.376
3.309.107	-35.647	3.331.593	-44.494	3.280.739	-44.379
3.309.227	-35.651	3.331.768	-44.496	3.280.924	-44.381
3.309.414	-35.656	3.331.927	-44.498	3.281.129	-44.384
3.309.579	-35.660	3.332.065	-44.499	3.281.328	-44.386
3.309.803	-35.664	3.332.209	-44.501	3.281.502	-44.388
3.310.016	-35.668	3.332.308	-44.503	3.281.682	-44.391
3.310.166	-35.673	3.332.448	-44.504	3.281.864	-44.393
3.310.327	-35.677	3.332.646	-44.506	3.281.997	-44.396
3.310.500	-35.681	3.332.901	-44.508	3.282.156	-44.398
3.310.735	-35.686	3.333.127	-44.509	3.282.319	-44.401
3.310.894	-35.690	3.333.291	-44.511	3.282.437	-44.403
3.311.008	-35.694	3.333.490	-44.512	3.282.617	-44.406
3.311.138	-35.698	3.333.714	-44.513	3.282.792	-44.408
3.311.344	-35.703	3.333.912	-44.515	3.282.932	-44.411

3.311.505	-35.707	3.334.066	-44.516	3.283.132	-44.413
3.311.595	-35.712	3.334.201	-44.518	3.283.294	-44.416
3.311.761	-35.716	3.334.349	-44.519	3.283.457	-44.418
3.311.974	-35.720	3.334.492	-44.520	3.283.658	-44.421
3.312.174	-35.725	3.334.626	-44.521	3.283.822	-44.423
3.312.343	-35.729	3.334.745	-44.522	3.283.994	-44.426
3.312.455	-35.733	3.334.882	-44.524	3.284.172	-44.428
3.312.603	-35.738	3.335.017	-44.525	3.284.319	-44.431
3.312.813	-35.742	3.335.164	-44.526	3.284.485	-44.433
3.312.983	-35.747	3.335.353	-44.527	3.284.630	-44.436
3.313.236	-35.751	3.335.522	-44.528	3.284.706	-44.438
3.313.442	-35.756	3.335.697	-44.529	3.284.815	-44.441
3.313.466	-35.760	3.335.927	-44.530	3.284.944	-44.443
3.313.573	-35.764	3.336.190	-44.531	3.285.045	-44.446
3.313.752	-35.769	3.336.378	-44.532	3.285.247	-44.448
3.313.878	-35.773	3.336.494	-44.533	3.285.459	-44.451
3.314.073	-35.778	3.336.693	-44.533	3.285.591	-44.453
3.314.211	-35.782	3.336.920	-44.534	3.285.746	-44.455
3.314.344	-35.787	3.337.036	-44.535	3.286.017	-44.458
3.314.532	-35.791	3.337.152	-44.536	3.286.252	-44.460
3.314.703	-35.796	3.337.354	-44.537	3.286.437	-44.463
3.314.891	-35.800	3.337.534	-44.537	3.286.613	-44.465
3.315.002	-35.805	3.337.679	-44.538	3.286.759	-44.467
3.315.182	-35.809	3.337.852	-44.539	3.286.958	-44.470
3.315.406	-35.814	3.338.018	-44.539	3.287.133	-44.472
3.315.500	-35.818	3.338.195	-44.540	3.287.254	-44.474
3.315.670	-35.823	3.338.363	-44.541	3.287.440	-44.477
3.315.909	-35.827	3.338.521	-44.541	3.287.676	-44.479
3.316.078	-35.832	3.338.714	-44.542	3.287.823	-44.481
3.316.237	-35.836	3.338.954	-44.542	3.287.944	-44.484

3.316.389	-35.841	3.339.164	-44.543	3.288.059	-44.486
3.316.534	-35.845	3.339.286	-44.543	3.288.114	-44.488
3.316.740	-35.850	3.339.411	-44.544	3.288.265	-44.490
3.316.882	-35.854	3.339.590	-44.544	3.288.471	-44.492
3.317.043	-35.859	3.339.772	-44.545	3.288.632	-44.495
3.317.258	-35.863	3.339.938	-44.545	3.288.792	-44.497
3.317.383	-35.868	3.340.063	-44.545	3.288.977	-44.499
3.317.523	-35.873	3.340.194	-44.546	3.289.182	-44.501
3.317.712	-35.877	3.340.388	-44.546	3.289.359	-44.503
3.317.892	-35.882	3.340.562	-44.546	3.289.583	-44.505
3.318.091	-35.886	3.340.747	-44.547	3.289.828	-44.507
3.318.264	-35.891	3.340.902	-44.547	3.290.005	-44.509
3.318.423	-35.895	3.340.990	-44.547	3.290.150	-44.511
3.318.623	-35.900	3.341.119	-44.548	3.290.314	-44.513
3.318.828	-35.904	3.341.318	-44.548	3.290.540	-44.515
3.318.951	-35.909	3.341.658	-44.548	3.290.681	-44.517
3.319.045	-35.914	3.342.057	-44.548	3.290.801	-44.519
3.319.247	-35.918	3.342.285	-44.549	3.290.977	-44.521
3.319.471	-35.923	3.342.415	-44.549	3.291.126	-44.523
3.319.583	-35.927	3.342.495	-44.549	3.291.320	-44.525
3.319.648	-35.932	3.342.500	-44.549	3.291.502	-44.526
3.319.787	-35.936	3.342.545	-44.550	3.291.603	-44.528
3.319.948	-35.941	3.342.570	-44.550	3.291.763	-44.530
3.320.117	-35.945	3.342.672	-44.550	3.291.989	-44.532
3.320.253	-35.950	3.342.915	-44.550	3.292.200	-44.533
3.320.444	-35.955	3.343.131	-44.550	3.292.415	-44.535
3.320.666	-35.959	3.343.315	-44.550	3.292.632	-44.537
3.320.816	-35.964	3.343.455	-44.551	3.292.780	-44.538
3.320.993	-35.968	3.343.612	-44.551	3.292.903	-44.540
3.321.216	-35.973	3.343.828	-44.551	3.293.051	-44.542

3.321.443	-35.977	3.344.008	-44.551	3.293.226	-44.543
3.321.656	-35.982	3.344.156	-44.551	3.293.394	-44.545
3.321.824	-35.986	3.344.363	-44.551	3.293.542	-44.546
3.322.004	-35.991	3.344.529	-44.552	3.293.655	-44.548
3.322.145	-35.996	3.344.659	-44.552	3.293.785	-44.549
3.322.249	-36.000	3.344.826	-44.552	3.293.955	-44.551
3.322.393	-36.005	3.344.995	-44.552	3.294.127	-44.552
3.322.500	-36.009	3.345.191	-44.552	3.294.330	-44.554
3.322.607	-36.014	3.345.303	-44.552	3.294.521	-44.555
3.322.830	-36.018	3.345.381	-44.552	3.294.688	-44.557
3.323.058	-36.023	3.345.500	-44.553	3.294.848	-44.558
3.323.163	-36.027	3.345.729	-44.553	3.294.976	-44.559
3.323.312	-36.032	3.346.004	-44.553	3.295.107	-44.561
3.323.484	-36.036	3.346.190	-44.553	3.295.269	-44.562
3.323.649	-36.041	3.346.335	-44.553	3.295.417	-44.564
3.323.815	-36.045	3.346.497	-44.553	3.295.617	-44.565
3.324.053	-36.050	3.346.698	-44.554	3.295.794	-44.566
3.324.324	-36.054	3.346.964	-44.554	3.295.894	-44.568
3.324.464	-36.059	3.347.166	-44.554	3.296.238	-44.569
3.324.565	-36.063	3.347.256	-44.554	3.296.694	-44.570
3.324.698	-36.068	3.347.357	-44.554	3.296.971	-44.572
3.324.893	-36.072	3.347.505	-44.555	3.297.156	-44.573
3.325.078	-36.076	3.347.657	-44.555	3.297.229	-44.574
3.325.245	-36.081	3.347.802	-44.555	3.297.221	-44.575
3.325.403	-36.085	3.347.957	-44.555	3.297.205	-44.577
3.325.569	-36.090	3.348.125	-44.556	3.297.308	-44.578
3.325.740	-36.094	3.348.301	-44.556	3.297.456	-44.579
3.325.891	-36.099	3.348.484	-44.556	3.297.579	-44.580
3.326.054	-36.103	3.348.679	-44.556	3.297.693	-44.581
3.326.307	-36.107	3.348.907	-44.557	3.297.780	-44.583

3.326.519	-36.112	3.349.041	-44.557	3.298.019	-44.584
3.326.615	-36.116	3.349.132	-44.557	3.298.323	-44.585
3.326.806	-36.121	3.349.268	-44.558	3.298.474	-44.586
3.326.978	-36.125	3.349.386	-44.558	3.298.658	-44.588
3.327.104	-36.129	3.349.562	-44.558	3.298.795	-44.589
3.327.261	-36.134	3.349.767	-44.559	3.298.897	-44.590
3.327.383	-36.138	3.349.887	-44.559	3.299.104	-44.591
3.327.570	-36.142	3.350.058	-44.559	3.299.339	-44.593
3.327.789	-36.147	3.350.280	-44.560	3.299.576	-44.594
3.327.994	-36.151	3.350.429	-44.560	3.299.738	-44.595
3.328.152	-36.155	3.350.630	-44.560	3.299.868	-44.597
3.328.312	-36.160	3.350.841	-44.561	3.299.985	-44.598
3.328.463	-36.164	3.350.968	-44.561	3.300.092	-44.599
3.328.680	-36.168	3.351.067	-44.562	3.300.247	-44.601
3.328.934	-36.172	3.351.249	-44.562	3.300.453	-44.602
3.329.035	-36.177	3.351.409	-44.563	3.300.623	-44.604
3.329.115	-36.181	3.351.567	-44.563	3.300.700	-44.605
3.329.221	-36.185	3.351.826	-44.563	3.300.854	-44.607
3.329.443	-36.189	3.352.037	-44.564	3.301.090	-44.608
3.329.877	-36.193	3.352.104	-44.564	3.301.270	-44.610
3.330.253	-36.198	3.352.243	-44.565	3.301.443	-44.611
3.330.399	-36.202	3.352.435	-44.565	3.301.669	-44.613
3.330.475	-36.206	3.352.615	-44.566	3.301.864	-44.615
3.330.544	-36.210	3.352.776	-44.566	3.301.978	-44.616
3.330.616	-36.214	3.352.910	-44.567	3.302.137	-44.618
3.330.670	-36.218	3.353.109	-44.567	3.302.382	-44.620
3.330.789	-36.222	3.353.312	-44.568	3.302.549	-44.621
3.330.937	-36.227	3.353.370	-44.568	3.302.648	-44.623
3.331.069	-36.231	3.353.488	-44.569	3.302.823	-44.625
3.331.221	-36.235	3.353.683	-44.569	3.302.998	-44.627

3.331.432	-36.239	3.353.802	-44.570	3.303.154	-44.629
3.331.606	-36.243	3.354.010	-44.570	3.303.305	-44.631
3.331.700	-36.247	3.354.186	-44.571	3.303.430	-44.633
3.331.880	-36.251	3.354.297	-44.571	3.303.533	-44.635
3.332.115	-36.255	3.354.453	-44.572	3.303.672	-44.637
3.332.286	-36.259	3.354.695	-44.572	3.303.815	-44.639
3.332.379	-36.263	3.354.967	-44.573	3.303.963	-44.641
3.332.567	-36.267	3.355.161	-44.573	3.304.169	-44.643
3.332.791	-36.271	3.355.321	-44.574	3.304.374	-44.646
3.332.924	-36.275	3.355.522	-44.574	3.304.548	-44.648
3.333.059	-36.278	3.355.750	-44.575	3.304.720	-44.650
3.333.264	-36.282	3.355.941	-44.575	3.304.902	-44.653
3.333.432	-36.286	3.356.055	-44.576	3.305.045	-44.655
3.333.575	-36.290	3.356.155	-44.576	3.305.157	-44.658
3.333.688	-36.294	3.356.239	-44.577	3.305.313	-44.660
3.333.804	-36.298	3.356.382	-44.577	3.305.475	-44.663
3.333.981	-36.302	3.356.539	-44.578	3.305.549	-44.666
3.334.185	-36.305	3.356.721	-44.578	3.305.725	-44.668
3.334.425	-36.309	3.356.938	-44.579	3.305.977	-44.671
3.334.648	-36.313	3.357.149	-44.579	3.306.147	-44.674
3.334.786	-36.317	3.357.264	-44.580	3.306.307	-44.677
3.334.940	-36.320	3.357.412	-44.580	3.306.451	-44.680
3.335.121	-36.324	3.357.614	-44.581	3.306.653	-44.683
3.335.299	-36.328	3.357.794	-44.581	3.306.888	-44.686
3.335.482	-36.331	3.358.026	-44.582	3.307.081	-44.689
3.335.642	-36.335	3.358.221	-44.582	3.307.263	-44.692
3.335.795	-36.339	3.358.369	-44.583	3.307.420	-44.695
3.335.927	-36.342	3.358.528	-44.583	3.307.506	-44.698
3.336.101	-36.346	3.358.658	-44.584	3.307.700	-44.701
3.336.274	-36.349	3.358.822	-44.584	3.307.914	-44.705

3.336.440	-36.353	3.358.994	-44.585	3.308.045	-44.708
3.336.603	-36.357	3.359.142	-44.585	3.308.181	-44.711
3.336.754	-36.360	3.359.297	-44.586	3.308.394	-44.715
3.336.917	-36.364	3.359.465	-44.586	3.308.568	-44.718
3.337.124	-36.367	3.359.645	-44.586	3.308.708	-44.722
3.337.318	-36.371	3.359.839	-44.587	3.308.836	-44.725
3.337.415	-36.374	3.360.023	-44.587	3.308.965	-44.729
3.337.541	-36.377	3.360.170	-44.588	3.309.055	-44.732
3.337.711	-36.381	3.360.317	-44.588	3.309.262	-44.736
3.337.802	-36.384	3.360.490	-44.589	3.309.471	-44.740
3.337.961	-36.388	3.360.677	-44.589	3.309.627	-44.744
3.338.192	-36.391	3.360.877	-44.589	3.309.782	-44.747
3.338.373	-36.394	3.361.091	-44.590	3.309.930	-44.751
3.338.543	-36.398	3.361.223	-44.590	3.310.100	-44.755
3.338.708	-36.401	3.361.389	-44.590	3.310.247	-44.759
3.338.916	-36.404	3.361.620	-44.591	3.310.353	-44.763
3.339.145	-36.408	3.361.765	-44.591	3.310.494	-44.767
3.339.294	-36.411	3.361.859	-44.592	3.310.632	-44.771
3.339.432	-36.414	3.362.044	-44.592	3.310.772	-44.775
3.339.595	-36.417	3.362.195	-44.592	3.310.931	-44.779
3.339.756	-36.421	3.362.333	-44.593	3.311.053	-44.783
3.339.887	-36.424	3.362.521	-44.593	3.311.169	-44.787
3.340.003	-36.427	3.362.675	-44.593	3.311.331	-44.791
3.340.204	-36.430	3.362.838	-44.594	3.311.533	-44.795
3.340.410	-36.433	3.363.022	-44.594	3.311.820	-44.800
3.340.613	-36.436	3.363.373	-44.594	3.312.101	-44.804
3.340.780	-36.439	3.363.802	-44.595	3.312.310	-44.808
3.340.946	-36.443	3.364.041	-44.595	3.312.426	-44.813
3.341.096	-36.446	3.364.153	-44.595	3.312.599	-44.817
3.341.244	-36.449	3.364.239	-44.596	3.312.821	-44.821

3.341.398	-36.452	3.364.284	-44.596	3.312.947	-44.826
3.341.491	-36.455	3.364.312	-44.596	3.313.034	-44.830
3.341.620	-36.458	3.364.393	-44.597	3.313.141	-44.834
3.341.812	-36.461	3.364.511	-44.597	3.313.354	-44.839
3.342.014	-36.464	3.364.622	-44.597	3.313.508	-44.843
3.342.130	-36.467	3.364.810	-44.597	3.313.690	-44.848
3.342.231	-36.470	3.364.964	-44.598	3.313.904	-44.852
3.342.444	-36.473	3.365.125	-44.598	3.313.963	-44.857
3.342.657	-36.476	3.365.323	-44.598	3.314.109	-44.862
3.342.756	-36.479	3.365.512	-44.599	3.314.290	-44.866
3.342.968	-36.482	3.365.714	-44.599	3.314.439	-44.871
3.343.279	-36.485	3.365.891	-44.599	3.314.626	-44.875
3.343.511	-36.487	3.365.941	-44.599	3.314.781	-44.880
3.343.714	-36.490	3.366.026	-44.600	3.314.964	-44.884
3.343.926	-36.493	3.366.284	-44.600	3.315.169	-44.889
3.344.039	-36.496	3.366.412	-44.600	3.315.364	-44.894
3.344.132	-36.499	3.366.552	-44.601	3.315.578	-44.898
3.344.295	-36.502	3.366.763	-44.601	3.315.761	-44.903
3.344.467	-36.505	3.366.928	-44.601	3.315.912	-44.908
3.344.586	-36.507	3.367.099	-44.602	3.316.078	-44.912
3.344.716	-36.510	3.367.238	-44.602	3.316.216	-44.917
3.344.895	-36.513	3.367.408	-44.602	3.316.378	-44.922
3.345.049	-36.516	3.367.628	-44.603	3.316.588	-44.926
3.345.179	-36.519	3.367.834	-44.603	3.316.743	-44.931
3.345.296	-36.521	3.368.019	-44.603	3.316.873	-44.936
3.345.461	-36.524	3.368.154	-44.604	3.317.072	-44.940
3.345.648	-36.527	3.368.340	-44.604	3.317.245	-44.945
3.345.822	-36.530	3.368.565	-44.604	3.317.406	-44.950
3.345.999	-36.532	3.368.737	-44.605	3.317.589	-44.954
3.346.201	-36.535	3.368.889	-44.605	3.317.744	-44.959

3.346.411	-36.538	3.369.024	-44.605	3.317.912	-44.964
3.346.613	-36.541	3.369.176	-44.606	3.318.106	-44.968
3.346.781	-36.543	3.369.367	-44.606	3.318.300	-44.973
3.346.925	-36.546	3.369.526	-44.607	3.318.457	-44.977
3.347.088	-36.549	3.369.616	-44.607	3.318.589	-44.982
3.347.278	-36.551	3.369.746	-44.608	3.318.745	-44.987
3.347.423	-36.554	3.369.951	-44.608	3.318.929	-44.991
3.347.576	-36.557	3.370.172	-44.609	3.319.077	-44.996
3.347.787	-36.559	3.370.390	-44.609	3.319.245	-45.000
3.347.950	-36.562	3.370.560	-44.610	3.319.431	-45.005
3.348.101	-36.565	3.370.689	-44.610	3.319.583	-45.009
3.348.247	-36.567	3.370.873	-44.611	3.319.740	-45.014
3.348.402	-36.570	3.371.003	-44.611	3.319.893	-45.018
3.348.558	-36.573	3.371.101	-44.612	3.320.070	-45.023
3.348.719	-36.575	3.371.274	-44.612	3.320.270	-45.027
3.348.922	-36.578	3.371.454	-44.613	3.320.414	-45.032
3.349.125	-36.581	3.371.649	-44.613	3.320.596	-45.036
3.349.280	-36.583	3.371.866	-44.614	3.320.793	-45.041
3.349.456	-36.586	3.372.043	-44.615	3.320.927	-45.045
3.349.613	-36.589	3.372.206	-44.615	3.321.068	-45.050
3.349.792	-36.591	3.372.303	-44.616	3.321.260	-45.054
3.350.005	-36.594	3.372.481	-44.617	3.321.396	-45.058
3.350.164	-36.596	3.372.742	-44.617	3.321.503	-45.063
3.350.323	-36.599	3.372.882	-44.618	3.321.712	-45.067
3.350.471	-36.602	3.373.047	-44.619	3.321.945	-45.071
3.350.578	-36.604	3.373.233	-44.619	3.322.236	-45.076
3.350.739	-36.607	3.373.333	-44.620	3.322.635	-45.080
3.350.981	-36.609	3.373.511	-44.621	3.322.899	-45.084
3.351.187	-36.612	3.373.720	-44.622	3.323.024	-45.088
3.351.313	-36.615	3.373.900	-44.622	3.323.152	-45.092

3.351.456	-36.617	3.374.059	-44.623	3.323.264	-45.096
3.351.794	-36.620	3.374.204	-44.624	3.323.369	-45.100
3.352.249	-36.622	3.374.391	-44.625	3.323.439	-45.105
3.352.487	-36.625	3.374.557	-44.625	3.323.535	-45.109
3.352.538	-36.628	3.374.711	-44.626	3.323.580	-45.113
3.352.614	-36.630	3.374.897	-44.627	3.323.654	-45.117
3.352.682	-36.633	3.375.000	-44.628	3.323.889	-45.121
3.352.711	-36.635	3.375.063	-44.629	3.324.118	-45.125
3.352.805	-36.638	3.375.197	-44.629	3.324.211	-45.129
3.352.968	-36.640	3.375.382	-44.630	3.324.315	-45.132
3.353.081	-36.643	3.375.576	-44.631	3.324.508	-45.136
3.353.178	-36.646	3.375.675	-44.632	3.324.702	-45.140
3.353.347	-36.648	3.375.815	-44.633	3.324.873	-45.144
3.353.517	-36.651	3.376.033	-44.633	3.325.061	-45.148
3.353.694	-36.653	3.376.224	-44.634	3.325.266	-45.152
3.353.922	-36.656	3.376.466	-44.635	3.325.462	-45.155
3.354.136	-36.658	3.376.657	-44.636	3.325.627	-45.159
3.354.341	-36.661	3.376.828	-44.637	3.325.758	-45.163
3.354.526	-36.663	3.377.084	-44.638	3.325.910	-45.167
3.354.669	-36.666	3.377.329	-44.638	3.326.098	-45.170
3.354.785	-36.669	3.377.541	-44.639	3.326.259	-45.174
3.354.916	-36.671	3.377.651	-44.640	3.326.360	-45.178
3.355.064	-36.674	3.377.715	-44.641	3.326.544	-45.182
3.355.188	-36.676	3.377.852	-44.642	3.326.725	-45.185
3.355.365	-36.679	3.377.971	-44.642	3.326.827	-45.189
3.355.562	-36.681	3.378.102	-44.643	3.326.964	-45.192
3.355.695	-36.684	3.378.271	-44.644	3.327.144	-45.196
3.355.840	-36.686	3.378.421	-44.645	3.327.321	-45.200
3.356.017	-36.689	3.378.571	-44.646	3.327.487	-45.203
3.356.216	-36.691	3.378.735	-44.647	3.327.676	-45.207

3.356.419	-36.694	3.378.965	-44.647	3.327.901	-45.210
3.356.585	-36.697	3.379.187	-44.648	3.328.109	-45.214
3.356.743	-36.699	3.379.326	-44.649	3.328.271	-45.217
3.356.913	-36.702	3.379.525	-44.650	3.328.459	-45.221
3.357.137	-36.704	3.379.796	-44.650	3.328.684	-45.224
3.357.336	-36.707	3.380.005	-44.651	3.328.829	-45.228
3.357.448	-36.709	3.380.132	-44.652	3.328.912	-45.231
3.357.592	-36.712	3.380.242	-44.653	3.329.045	-45.234
3.357.771	-36.714	3.380.347	-44.653	3.329.204	-45.238
3.357.917	-36.717	3.380.482	-44.654	3.329.391	-45.241
3.358.033	-36.719	3.380.690	-44.655	3.329.510	-45.244
3.358.178	-36.722	3.380.872	-44.656	3.329.674	-45.248
3.358.365	-36.724	3.381.076	-44.656	3.329.873	-45.251
3.358.590	-36.727	3.381.270	-44.657	3.330.016	-45.254
3.358.779	-36.729	3.381.371	-44.658	3.330.168	-45.258
3.358.943	-36.732	3.381.472	-44.659	3.330.371	-45.261
3.359.138	-36.734	3.381.653	-44.659	3.330.573	-45.264
3.359.266	-36.737	3.381.846	-44.660	3.330.725	-45.267
3.359.377	-36.740	3.382.061	-44.661	3.330.895	-45.271
3.359.563	-36.742	3.382.224	-44.661	3.331.097	-45.274
3.359.716	-36.745	3.382.411	-44.662	3.331.232	-45.277
3.359.854	-36.747	3.382.615	-44.663	3.331.356	-45.280
3.359.991	-36.750	3.382.744	-44.663	3.331.573	-45.283
3.360.161	-36.752	3.382.874	-44.664	3.331.733	-45.287
3.360.435	-36.755	3.383.067	-44.665	3.331.873	-45.290
3.360.657	-36.757	3.383.242	-44.665	3.332.037	-45.293
3.360.815	-36.760	3.383.387	-44.666	3.332.198	-45.296
3.360.966	-36.762	3.383.549	-44.667	3.332.393	-45.299
3.361.064	-36.765	3.383.771	-44.667	3.332.592	-45.302
3.361.190	-36.767	3.383.954	-44.668	3.332.755	-45.305

3.361.393	-36.770	3.384.082	-44.668	3.332.893	-45.308
3.361.591	-36.772	3.384.262	-44.669	3.333.056	-45.311
3.361.696	-36.775	3.384.388	-44.670	3.333.185	-45.315
3.361.841	-36.777	3.384.482	-44.670	3.333.333	-45.318
3.362.065	-36.780	3.384.691	-44.671	3.333.584	-45.321
3.362.257	-36.782	3.385.070	-44.671	3.333.763	-45.324
3.362.419	-36.784	3.385.461	-44.672	3.333.878	-45.326
3.362.601	-36.787	3.385.620	-44.673	3.334.013	-45.329
3.362.755	-36.789	3.385.675	-44.673	3.334.178	-45.332
3.362.878	-36.792	3.385.753	-44.674	3.334.370	-45.335
3.363.083	-36.794	3.385.829	-44.674	3.334.503	-45.338
3.363.210	-36.797	3.385.912	-44.675	3.334.594	-45.341
3.363.369	-36.799	3.385.977	-44.675	3.334.734	-45.344
3.363.569	-36.802	3.386.064	-44.676	3.334.955	-45.347
3.363.663	-36.804	3.386.243	-44.676	3.335.247	-45.350
3.363.781	-36.807	3.386.422	-44.677	3.335.361	-45.352
3.363.896	-36.809	3.386.574	-44.677	3.335.551	-45.355
3.364.041	-36.812	3.386.801	-44.678	3.335.765	-45.358
3.364.225	-36.814	3.387.011	-44.678	3.335.910	-45.361
3.364.367	-36.817	3.387.181	-44.679	3.336.042	-45.363
3.364.568	-36.819	3.387.377	-44.679	3.336.216	-45.366
3.364.793	-36.822	3.387.568	-44.680	3.336.360	-45.369
3.364.940	-36.824	3.387.718	-44.680	3.336.502	-45.371
3.365.128	-36.827	3.387.870	-44.681	3.336.669	-45.374
3.365.417	-36.829	3.388.030	-44.681	3.336.808	-45.376
3.365.641	-36.831	3.388.164	-44.681	3.336.949	-45.379
3.365.818	-36.834	3.388.323	-44.682	3.337.116	-45.381
3.365.966	-36.836	3.388.486	-44.682	3.337.292	-45.384
3.366.086	-36.839	3.388.633	-44.683	3.337.405	-45.386
3.366.271	-36.841	3.388.807	-44.683	3.337.502	-45.389

3.366.405	-36.844	3.388.936	-44.684	3.337.730	-45.391
3.366.539	-36.846	3.389.100	-44.684	3.338.004	-45.394
3.366.683	-36.849	3.389.297	-44.685	3.338.141	-45.396
3.366.768	-36.851	3.389.489	-44.685	3.338.315	-45.398
3.366.909	-36.854	3.389.652	-44.686	3.338.553	-45.401
3.367.101	-36.856	3.389.797	-44.686	3.338.696	-45.403
3.367.296	-36.859	3.390.016	-44.686	3.338.857	-45.405
3.367.534	-36.861	3.390.242	-44.687	3.339.089	-45.408
3.367.730	-36.863	3.390.363	-44.687	3.339.299	-45.410
3.367.849	-36.866	3.390.436	-44.688	3.339.453	-45.412
3.368.060	-36.868	3.390.577	-44.688	3.339.569	-45.414
3.368.258	-36.871	3.390.807	-44.689	3.339.703	-45.416
3.368.438	-36.873	3.390.984	-44.689	3.339.883	-45.418
3.368.586	-36.876	3.391.104	-44.690	3.340.027	-45.420
3.368.719	-36.878	3.391.255	-44.690	3.340.128	-45.422
3.368.887	-36.881	3.391.419	-44.691	3.340.252	-45.424
3.369.049	-36.883	3.391.589	-44.691	3.340.439	-45.426
3.369.200	-36.886	3.391.743	-44.692	3.340.602	-45.428
3.369.353	-36.888	3.391.899	-44.692	3.340.775	-45.430
3.369.514	-36.890	3.392.099	-44.693	3.340.989	-45.432
3.369.713	-36.893	3.392.286	-44.693	3.341.194	-45.434
3.369.944	-36.895	3.392.416	-44.694	3.341.320	-45.436
3.370.074	-36.898	3.392.597	-44.695	3.341.480	-45.437
3.370.212	-36.900	3.392.747	-44.695	3.341.721	-45.439
3.370.382	-36.903	3.392.877	-44.696	3.341.873	-45.441
3.370.558	-36.905	3.392.975	-44.696	3.342.034	-45.443
3.370.779	-36.908	3.393.199	-44.697	3.342.196	-45.444
3.370.934	-36.910	3.393.481	-44.698	3.342.376	-45.446
3.371.109	-36.913	3.393.622	-44.698	3.342.561	-45.448
3.371.292	-36.915	3.393.792	-44.699	3.342.765	-45.449

3.371.468	-36.918	3.393.944	-44.700	3.342.883	-45.451
3.371.609	-36.920	3.394.088	-44.700	3.343.007	-45.452
3.371.714	-36.922	3.394.309	-44.701	3.343.235	-45.454
3.371.902	-36.925	3.394.505	-44.702	3.343.402	-45.455
3.372.065	-36.927	3.394.626	-44.702	3.343.598	-45.456
3.372.216	-36.930	3.394.825	-44.703	3.343.792	-45.458
3.372.351	-36.932	3.394.988	-44.704	3.343.901	-45.459
3.372.552	-36.935	3.395.132	-44.705	3.344.032	-45.461
3.372.820	-36.937	3.395.364	-44.705	3.344.227	-45.462
3.372.959	-36.940	3.395.541	-44.706	3.344.442	-45.463
3.372.980	-36.942	3.395.699	-44.707	3.344.552	-45.464
3.373.175	-36.945	3.395.852	-44.708	3.344.716	-45.466
3.373.405	-36.947	3.395.957	-44.709	3.344.926	-45.467
3.373.690	-36.950	3.396.104	-44.709	3.345.153	-45.468
3.374.200	-36.952	3.396.273	-44.710	3.345.354	-45.469
3.374.518	-36.955	3.396.443	-44.711	3.345.370	-45.470
3.374.591	-36.957	3.396.609	-44.712	3.345.526	-45.472
3.374.648	-36.960	3.396.792	-44.713	3.345.811	-45.473
3.374.745	-36.962	3.396.958	-44.714	3.345.990	-45.474
3.374.812	-36.965	3.397.057	-44.714	3.346.123	-45.475
3.374.846	-36.967	3.397.140	-44.715	3.346.266	-45.476
3.374.915	-36.969	3.397.268	-44.716	3.346.456	-45.477
3.375.056	-36.972	3.397.397	-44.717	3.346.645	-45.478
3.375.211	-36.974	3.397.606	-44.718	3.346.864	-45.479
3.375.385	-36.977	3.397.870	-44.719	3.347.083	-45.480
3.375.536	-36.979	3.398.058	-44.720	3.347.229	-45.481
3.375.683	-36.982	3.398.246	-44.721	3.347.325	-45.482
3.375.917	-36.984	3.398.467	-44.722	3.347.494	-45.483
3.376.137	-36.987	3.398.730	-44.723	3.347.694	-45.484
3.376.328	-36.989	3.398.893	-44.724	3.347.824	-45.485

3.376.553	-36.992	3.398.947	-44.725	3.347.925	-45.486
3.376.716	-36.994	3.399.164	-44.726	3.348.000	-45.486
3.376.809	-36.997	3.399.384	-44.726	3.348.330	-45.487
3.376.994	-36.999	3.399.471	-44.727	3.348.838	-45.488
3.377.151	-37.002	3.399.541	-44.728	3.349.157	-45.489
3.377.227	-37.004	3.399.649	-44.729	3.349.344	-45.490
3.377.357	-37.007	3.399.839	-44.730	3.349.460	-45.491
3.377.568	-37.009	3.400.063	-44.731	3.349.507	-45.491
3.377.777	-37.012	3.400.245	-44.732	3.349.541	-45.492
3.377.899	-37.014	3.400.394	-44.733	3.349.601	-45.493
3.378.036	-37.017	3.400.544	-44.734	3.349.691	-45.494
3.378.261	-37.019	3.400.764	-44.735	3.349.821	-45.495
3.378.464	-37.021	3.400.938	-44.736	3.349.946	-45.495
3.378.666	-37.024	3.401.080	-44.737	3.350.070	-45.496
3.378.849	-37.026	3.401.234	-44.738	3.350.201	-45.497
3.378.985	-37.029	3.401.438	-44.739	3.350.353	-45.498
3.379.124	-37.031	3.401.703	-44.740	3.350.538	-45.498
3.379.305	-37.034	3.401.891	-44.741	3.350.725	-45.499
3.379.456	-37.036	3.402.010	-44.742	3.350.891	-45.500
3.379.619	-37.039	3.402.137	-44.743	3.351.031	-45.500
3.379.792	-37.041	3.402.330	-44.744	3.351.235	-45.501
3.379.923	-37.044	3.402.500	-44.745	3.351.461	-45.502
3.380.076	-37.046	3.402.691	-44.746	3.351.631	-45.503
3.380.184	-37.048	3.402.865	-44.747	3.351.821	-45.503
3.380.359	-37.051	3.403.016	-44.748	3.351.963	-45.504
3.380.589	-37.053	3.403.200	-44.749	3.352.133	-45.505
3.380.739	-37.056	3.403.377	-44.750	3.352.314	-45.506
3.380.870	-37.058	3.403.593	-44.751	3.352.470	-45.507
3.381.036	-37.061	3.403.806	-44.752	3.352.632	-45.507
3.381.230	-37.063	3.403.938	-44.753	3.352.770	-45.508

3.381.454	-37.065	3.404.066	-44.754	3.352.899	-45.509
3.381.591	-37.068	3.404.232	-44.755	3.353.018	-45.510
3.381.676	-37.070	3.404.445	-44.755	3.353.106	-45.511
3.381.857	-37.073	3.404.652	-44.756	3.353.246	-45.512
3.382.112	-37.075	3.404.721	-44.757	3.353.452	-45.512
3.382.269	-37.077	3.404.807	-44.758	3.353.687	-45.513
3.382.437	-37.080	3.405.009	-44.759	3.353.929	-45.514
3.382.592	-37.082	3.405.219	-44.760	3.354.172	-45.515
3.382.758	-37.085	3.405.403	-44.761	3.354.315	-45.516
3.382.939	-37.087	3.405.613	-44.762	3.354.417	-45.517
3.383.121	-37.089	3.405.780	-44.763	3.354.601	-45.518
3.383.336	-37.092	3.405.878	-44.764	3.354.781	-45.519
3.383.506	-37.094	3.405.974	-44.764	3.354.966	-45.520
3.383.589	-37.096	3.406.208	-44.765	3.355.175	-45.521
3.383.716	-37.099	3.406.671	-44.766	3.355.276	-45.522
3.383.918	-37.101	3.407.094	-44.767	3.355.400	-45.523
3.384.056	-37.103	3.407.210	-44.768	3.355.598	-45.524
3.384.207	-37.106	3.407.195	-44.769	3.355.751	-45.525
3.384.457	-37.108	3.407.279	-44.769	3.355.872	-45.527
3.384.673	-37.110	3.407.386	-44.770	3.356.037	-45.528
3.384.818	-37.113	3.407.427	-44.771	3.356.230	-45.529
3.384.951	-37.115	3.407.477	-44.772	3.356.404	-45.530
3.385.110	-37.117	3.407.640	-44.772	3.356.607	-45.531
3.385.284	-37.120	3.407.834	-44.773	3.356.786	-45.533
3.385.392	-37.122	3.407.987	-44.774	3.356.891	-45.534
3.385.562	-37.124	3.408.154	-44.775	3.357.061	-45.535
3.385.685	-37.127	3.408.330	-44.775	3.357.254	-45.537
3.385.758	-37.129	3.408.513	-44.776	3.357.427	-45.538
3.385.924	-37.131	3.408.714	-44.777	3.357.635	-45.539
3.386.122	-37.134	3.408.889	-44.777	3.357.752	-45.541

3.386.284	-37.136	3.408.984	-44.778	3.357.875	-45.542
3.386.432	-37.138	3.409.142	-44.779	3.358.054	-45.544
3.386.628	-37.140	3.409.388	-44.779	3.358.127	-45.545
3.386.772	-37.143	3.409.554	-44.780	3.358.260	-45.547
3.386.938	-37.145	3.409.687	-44.780	3.358.513	-45.549
3.387.185	-37.147	3.409.818	-44.781	3.358.719	-45.550
3.387.438	-37.149	3.410.028	-44.782	3.358.868	-45.552
3.387.666	-37.152	3.410.230	-44.782	3.359.063	-45.553
3.387.834	-37.154	3.410.305	-44.783	3.359.258	-45.555
3.387.972	-37.156	3.410.436	-44.783	3.359.399	-45.557
3.388.120	-37.158	3.410.653	-44.784	3.359.573	-45.559
3.388.298	-37.160	3.410.837	-44.784	3.359.752	-45.560
3.388.453	-37.163	3.411.058	-44.785	3.359.933	-45.562
3.388.564	-37.165	3.411.263	-44.785	3.360.070	-45.564
3.388.748	-37.167	3.411.456	-44.786	3.360.222	-45.566
3.388.820	-37.169	3.411.671	-44.786	3.360.399	-45.567
3.388.894	-37.172	3.411.777	-44.786	3.360.497	-45.569
3.389.121	-37.174	3.411.900	-44.787	3.360.650	-45.571
3.389.337	-37.176	3.412.128	-44.787	3.360.856	-45.573
3.389.543	-37.178	3.412.300	-44.788	3.361.064	-45.575
3.389.724	-37.180	3.412.404	-44.788	3.361.219	-45.577
3.389.890	-37.182	3.412.552	-44.788	3.361.306	-45.579
3.390.081	-37.185	3.412.734	-44.789	3.361.534	-45.581
3.390.306	-37.187	3.412.872	-44.789	3.361.788	-45.583
3.390.454	-37.189	3.412.986	-44.789	3.361.943	-45.585
3.390.602	-37.191	3.413.174	-44.790	3.362.127	-45.587
3.390.789	-37.193	3.413.358	-44.790	3.362.260	-45.589
3.390.959	-37.195	3.413.522	-44.790	3.362.408	-45.591
3.391.147	-37.198	3.413.690	-44.790	3.362.614	-45.592
3.391.288	-37.200	3.413.804	-44.791	3.362.740	-45.594

3.391.383	-37.202	3.413.988	-44.791	3.362.838	-45.596
3.391.560	-37.204	3.414.114	-44.791	3.362.976	-45.598
3.391.754	-37.206	3.414.208	-44.791	3.363.116	-45.600
3.391.904	-37.208	3.414.384	-44.791	3.363.264	-45.602
3.392.109	-37.210	3.414.579	-44.792	3.363.481	-45.605
3.392.339	-37.212	3.414.807	-44.792	3.363.709	-45.607
3.392.513	-37.215	3.414.996	-44.792	3.363.818	-45.609
3.392.650	-37.217	3.415.195	-44.792	3.363.987	-45.611
3.392.794	-37.219	3.415.375	-44.792	3.364.197	-45.613
3.392.948	-37.221	3.415.573	-44.792	3.364.344	-45.615
3.393.098	-37.223	3.415.793	-44.792	3.364.539	-45.617
3.393.280	-37.225	3.415.883	-44.792	3.364.793	-45.619
3.393.475	-37.227	3.416.021	-44.792	3.364.935	-45.621
3.393.553	-37.229	3.416.219	-44.792	3.365.132	-45.623
3.393.708	-37.231	3.416.373	-44.792	3.365.370	-45.625
3.393.896	-37.233	3.416.538	-44.792	3.365.529	-45.627
3.394.034	-37.235	3.416.669	-44.792	3.365.682	-45.629
3.394.205	-37.238	3.416.761	-44.792	3.365.826	-45.630
3.394.427	-37.240	3.416.947	-44.792	3.365.930	-45.632
3.394.649	-37.242	3.417.242	-44.792	3.366.018	-45.634
3.394.817	-37.244	3.417.417	-44.791	3.366.136	-45.636
3.394.912	-37.246	3.417.589	-44.791	3.366.351	-45.638
3.395.063	-37.248	3.417.805	-44.791	3.366.524	-45.640
3.395.219	-37.250	3.417.921	-44.791	3.366.646	-45.642
3.395.321	-37.252	3.418.051	-44.791	3.366.838	-45.644
3.395.554	-37.254	3.418.170	-44.790	3.367.025	-45.646
3.396.029	-37.256	3.418.268	-44.790	3.367.144	-45.648
3.396.357	-37.258	3.418.432	-44.790	3.367.317	-45.650
3.396.479	-37.260	3.418.607	-44.790	3.367.503	-45.652
3.396.584	-37.262	3.418.730	-44.789	3.367.635	-45.654

3.396.650	-37.264	3.418.867	-44.789	3.367.828	-45.655
3.396.718	-37.266	3.419.080	-44.788	3.368.092	-45.657
3.396.781	-37.269	3.419.268	-44.788	3.368.289	-45.659
3.396.886	-37.271	3.419.467	-44.788	3.368.424	-45.661
3.397.018	-37.273	3.419.685	-44.787	3.368.589	-45.663
3.397.159	-37.275	3.419.909	-44.787	3.368.766	-45.665
3.397.368	-37.277	3.420.133	-44.786	3.368.918	-45.666
3.397.574	-37.279	3.420.258	-44.786	3.369.107	-45.668
3.397.697	-37.281	3.420.385	-44.785	3.369.286	-45.670
3.397.868	-37.283	3.420.569	-44.784	3.369.400	-45.672
3.398.062	-37.285	3.420.732	-44.784	3.369.526	-45.673
3.398.244	-37.287	3.420.862	-44.783	3.369.825	-45.675
3.398.441	-37.289	3.421.043	-44.782	3.370.020	-45.677
3.398.637	-37.291	3.421.192	-44.782	3.370.147	-45.679
3.398.795	-37.293	3.421.292	-44.781	3.370.247	-45.680
3.398.897	-37.295	3.421.463	-44.780	3.370.462	-45.682
3.399.077	-37.297	3.421.657	-44.779	3.370.710	-45.683
3.399.261	-37.299	3.421.824	-44.779	3.370.869	-45.685
3.399.366	-37.301	3.421.967	-44.778	3.370.995	-45.687
3.399.523	-37.303	3.422.144	-44.777	3.371.118	-45.688
3.399.688	-37.305	3.422.332	-44.776	3.371.260	-45.690
3.399.819	-37.307	3.422.502	-44.775	3.371.459	-45.691
3.399.995	-37.309	3.422.658	-44.774	3.371.682	-45.693
3.400.188	-37.311	3.422.856	-44.773	3.371.891	-45.694
3.400.365	-37.314	3.423.087	-44.772	3.372.039	-45.696
3.400.559	-37.316	3.423.240	-44.771	3.372.173	-45.697
3.400.736	-37.318	3.423.410	-44.770	3.372.315	-45.699
3.400.912	-37.320	3.423.571	-44.769	3.372.417	-45.700
3.401.057	-37.322	3.423.753	-44.768	3.372.557	-45.702
3.401.226	-37.324	3.423.923	-44.767	3.372.767	-45.703

3.401.440	-37.326	3.424.063	-44.766	3.372.968	-45.705
3.401.614	-37.328	3.424.230	-44.765	3.373.174	-45.706
3.401.737	-37.330	3.424.398	-44.764	3.373.300	-45.707
3.401.888	-37.332	3.424.572	-44.762	3.373.365	-45.709
3.402.058	-37.334	3.424.738	-44.761	3.373.564	-45.710
3.402.133	-37.336	3.424.919	-44.760	3.373.815	-45.711
3.402.304	-37.338	3.425.128	-44.759	3.373.961	-45.713
3.402.560	-37.340	3.425.361	-44.757	3.374.109	-45.714
3.402.731	-37.342	3.425.544	-44.756	3.374.248	-45.715
3.402.841	-37.344	3.425.627	-44.755	3.374.364	-45.717
3.402.958	-37.346	3.425.769	-44.753	3.374.613	-45.718
3.403.153	-37.348	3.425.956	-44.752	3.375.125	-45.719
3.403.355	-37.350	3.426.086	-44.750	3.375.526	-45.720
3.403.493	-37.353	3.426.286	-44.749	3.375.602	-45.721
3.403.625	-37.355	3.426.458	-44.748	3.375.621	-45.723
3.403.786	-37.357	3.426.574	-44.746	3.375.746	-45.724
3.403.969	-37.359	3.426.766	-44.745	3.375.829	-45.725
3.404.156	-37.361	3.426.949	-44.743	3.375.855	-45.726
3.404.342	-37.363	3.427.101	-44.742	3.375.945	-45.727
3.404.552	-37.365	3.427.321	-44.740	3.376.054	-45.728
3.404.706	-37.367	3.427.495	-44.739	3.376.181	-45.729
3.404.846	-37.369	3.427.575	-44.737	3.376.362	-45.730
3.405.039	-37.371	3.427.758	-44.735	3.376.527	-45.731
3.405.249	-37.373	3.428.255	-44.734	3.376.693	-45.732
3.405.383	-37.375	3.428.683	-44.732	3.376.869	-45.733
3.405.508	-37.377	3.428.764	-44.730	3.377.041	-45.734
3.405.726	-37.379	3.428.803	-44.729	3.377.220	-45.735
3.405.881	-37.381	3.428.878	-44.727	3.377.369	-45.736
3.406.054	-37.384	3.428.872	-44.725	3.377.574	-45.737
3.406.234	-37.386	3.428.893	-44.724	3.377.723	-45.738

3.406.422	-37.388	3.429.057	-44.722	3.377.939	-45.739
3.406.602	-37.390	3.429.196	-44.720	3.378.186	-45.740
3.406.743	-37.392	3.429.330	-44.718	3.378.349	-45.741
3.406.874	-37.394	3.429.526	-44.717	3.378.467	-45.742
3.407.016	-37.396	3.429.678	-44.715	3.378.583	-45.743
3.407.206	-37.398	3.429.857	-44.713	3.378.760	-45.744
3.407.352	-37.400	3.430.049	-44.711	3.378.980	-45.744
3.407.532	-37.402	3.430.256	-44.709	3.379.110	-45.745
3.407.701	-37.404	3.430.424	-44.708	3.379.277	-45.746
3.407.776	-37.407	3.430.569	-44.706	3.379.417	-45.747
3.407.870	-37.409	3.430.726	-44.704	3.379.545	-45.748
3.407.975	-37.411	3.430.872	-44.702	3.379.706	-45.749
3.408.121	-37.413	3.431.028	-44.700	3.379.881	-45.749
3.408.301	-37.415	3.431.237	-44.698	3.380.085	-45.750
3.408.515	-37.417	3.431.414	-44.696	3.380.267	-45.751
3.408.716	-37.419	3.431.545	-44.694	3.380.446	-45.752
3.408.907	-37.421	3.431.680	-44.693	3.380.706	-45.753
3.409.169	-37.423	3.431.841	-44.691	3.380.948	-45.753
3.409.406	-37.426	3.432.011	-44.689	3.381.040	-45.754
3.409.543	-37.428	3.432.120	-44.687	3.381.133	-45.755
3.409.681	-37.430	3.432.279	-44.685	3.381.289	-45.756
3.409.876	-37.432	3.432.499	-44.683	3.381.445	-45.757
3.410.028	-37.434	3.432.700	-44.681	3.381.671	-45.757
3.410.164	-37.436	3.432.896	-44.679	3.381.830	-45.758
3.410.247	-37.438	3.433.127	-44.677	3.381.936	-45.759
3.410.400	-37.441	3.433.315	-44.675	3.382.034	-45.759
3.410.609	-37.443	3.433.448	-44.673	3.382.220	-45.760
3.410.735	-37.445	3.433.580	-44.671	3.382.458	-45.761
3.410.845	-37.447	3.433.738	-44.669	3.382.639	-45.762
3.411.014	-37.449	3.433.904	-44.667	3.382.792	-45.762

3.411.214	-37.451	3.434.066	-44.666	3.382.968	-45.763
3.411.379	-37.453	3.434.208	-44.664	3.383.098	-45.764
3.411.537	-37.456	3.434.367	-44.662	3.383.192	-45.764
3.411.772	-37.458	3.434.530	-44.660	3.383.337	-45.765
3.412.025	-37.460	3.434.684	-44.658	3.383.550	-45.766
3.412.229	-37.462	3.434.865	-44.656	3.383.749	-45.766
3.412.358	-37.464	3.435.049	-44.654	3.383.896	-45.767
3.412.507	-37.467	3.435.195	-44.652	3.384.050	-45.768
3.412.610	-37.469	3.435.325	-44.650	3.384.209	-45.768
3.412.716	-37.471	3.435.526	-44.648	3.384.362	-45.769
3.412.935	-37.473	3.435.708	-44.646	3.384.496	-45.770
3.413.139	-37.475	3.435.865	-44.645	3.384.594	-45.770
3.413.387	-37.478	3.436.049	-44.643	3.384.758	-45.771
3.413.579	-37.480	3.436.179	-44.641	3.384.985	-45.772
3.413.694	-37.482	3.436.354	-44.639	3.385.191	-45.772
3.413.833	-37.484	3.436.603	-44.637	3.385.424	-45.773
3.414.024	-37.486	3.436.761	-44.635	3.385.663	-45.774
3.414.215	-37.489	3.436.938	-44.633	3.385.794	-45.774
3.414.406	-37.491	3.437.140	-44.631	3.385.990	-45.775
3.414.505	-37.493	3.437.281	-44.630	3.386.156	-45.775
3.414.626	-37.495	3.437.416	-44.628	3.386.220	-45.776
3.414.861	-37.497	3.437.568	-44.626	3.386.371	-45.776
3.415.063	-37.500	3.437.729	-44.624	3.386.606	-45.777
3.415.227	-37.502	3.437.859	-44.622	3.386.851	-45.777
3.415.349	-37.504	3.438.000	-44.621	3.387.028	-45.778
3.415.513	-37.506	3.438.206	-44.619	3.387.098	-45.778
3.415.663	-37.509	3.438.409	-44.617	3.387.227	-45.779
3.415.827	-37.511	3.438.567	-44.615	3.387.452	-45.779
3.416.083	-37.513	3.438.737	-44.613	3.387.628	-45.780
3.416.248	-37.516	3.438.810	-44.612	3.387.784	-45.780

3.416.331	-37.518	3.439.002	-44.610	3.387.897	-45.781
3.416.456	-37.520	3.439.153	-44.608	3.388.127	-45.781
3.416.716	-37.522	3.439.309	-44.606	3.388.312	-45.781
3.416.891	-37.525	3.439.482	-44.605	3.388.448	-45.782
3.417.021	-37.527	3.439.638	-44.603	3.388.586	-45.782
3.417.185	-37.529	3.439.769	-44.601	3.388.739	-45.782
3.417.394	-37.531	3.439.872	-44.599	3.388.803	-45.783
3.417.740	-37.534	3.440.031	-44.598	3.388.908	-45.783
3.418.071	-37.536	3.440.208	-44.596	3.389.053	-45.783
3.418.302	-37.538	3.440.378	-44.594	3.389.243	-45.784
3.418.412	-37.541	3.440.530	-44.593	3.389.454	-45.784
3.418.459	-37.543	3.440.700	-44.591	3.389.587	-45.784
3.418.576	-37.545	3.440.952	-44.589	3.389.761	-45.784
3.418.633	-37.548	3.441.209	-44.587	3.389.987	-45.784
3.418.667	-37.550	3.441.379	-44.586	3.390.139	-45.785
3.418.807	-37.552	3.441.516	-44.584	3.390.331	-45.785
3.419.001	-37.555	3.441.606	-44.582	3.390.583	-45.785
3.419.160	-37.557	3.441.820	-44.581	3.390.786	-45.785
3.419.327	-37.559	3.442.010	-44.579	3.390.942	-45.785
3.419.435	-37.562	3.442.196	-44.577	3.391.115	-45.785
3.419.575	-37.564	3.442.402	-44.576	3.391.288	-45.785
3.419.841	-37.566	3.442.514	-44.574	3.391.411	-45.785
3.420.047	-37.569	3.442.628	-44.572	3.391.571	-45.785
3.420.184	-37.571	3.442.759	-44.571	3.391.821	-45.785
3.420.313	-37.573	3.442.863	-44.569	3.391.978	-45.785
3.420.433	-37.576	3.443.029	-44.567	3.392.069	-45.785
3.420.537	-37.578	3.443.228	-44.566	3.392.274	-45.785
3.420.766	-37.580	3.443.474	-44.564	3.392.433	-45.785
3.421.004	-37.583	3.443.672	-44.562	3.392.527	-45.784
3.421.183	-37.585	3.443.788	-44.561	3.392.630	-45.784

3.421.350	-37.588	3.444.008	-44.559	3.392.767	-45.784
3.421.473	-37.590	3.444.219	-44.557	3.392.975	-45.784
3.421.653	-37.592	3.444.373	-44.556	3.393.116	-45.784
3.421.852	-37.595	3.444.514	-44.554	3.393.211	-45.783
3.422.000	-37.597	3.444.660	-44.552	3.393.441	-45.783
3.422.182	-37.599	3.444.787	-44.551	3.393.691	-45.783
3.422.336	-37.602	3.444.945	-44.549	3.393.889	-45.782
3.422.458	-37.604	3.445.135	-44.548	3.394.068	-45.782
3.422.723	-37.607	3.445.339	-44.546	3.394.247	-45.781
3.422.962	-37.609	3.445.518	-44.544	3.394.425	-45.781
3.423.103	-37.611	3.445.621	-44.543	3.394.594	-45.781
3.423.302	-37.614	3.445.789	-44.541	3.394.776	-45.780
3.423.484	-37.616	3.446.003	-44.540	3.394.992	-45.780
3.423.499	-37.619	3.446.154	-44.538	3.395.190	-45.779
3.423.591	-37.621	3.446.326	-44.536	3.395.284	-45.779
3.423.756	-37.623	3.446.501	-44.535	3.395.464	-45.778
3.423.958	-37.626	3.446.620	-44.533	3.395.708	-45.778
3.424.168	-37.628	3.446.781	-44.532	3.395.899	-45.777
3.424.315	-37.631	3.446.958	-44.530	3.396.026	-45.777
3.424.438	-37.633	3.447.169	-44.529	3.396.161	-45.776
3.424.599	-37.636	3.447.387	-44.527	3.396.255	-45.775
3.424.750	-37.638	3.447.563	-44.526	3.396.403	-45.775
3.424.944	-37.640	3.447.670	-44.524	3.396.622	-45.774
3.425.141	-37.643	3.447.735	-44.523	3.396.810	-45.773
3.425.273	-37.645	3.447.899	-44.521	3.396.950	-45.773
3.425.439	-37.648	3.448.174	-44.520	3.397.098	-45.772
3.425.653	-37.650	3.448.392	-44.518	3.397.321	-45.771
3.425.815	-37.653	3.448.513	-44.517	3.397.513	-45.771
3.426.024	-37.655	3.448.633	-44.515	3.397.715	-45.770
3.426.196	-37.657	3.448.817	-44.514	3.397.860	-45.769

3.426.314	-37.660	3.449.003	-44.512	3.397.972	-45.769
3.426.479	-37.662	3.449.205	-44.511	3.398.157	-45.768
3.426.613	-37.665	3.449.545	-44.509	3.398.355	-45.767
3.426.810	-37.667	3.449.930	-44.508	3.398.472	-45.766
3.427.028	-37.670	3.450.122	-44.507	3.398.626	-45.766
3.427.202	-37.672	3.450.139	-44.505	3.398.802	-45.765
3.427.379	-37.675	3.450.240	-44.504	3.399.016	-45.764
3.427.493	-37.677	3.450.338	-44.502	3.399.239	-45.763
3.427.590	-37.680	3.450.370	-44.501	3.399.411	-45.763
3.427.791	-37.682	3.450.444	-44.500	3.399.569	-45.762
3.427.989	-37.685	3.450.592	-44.498	3.399.705	-45.761
3.428.168	-37.687	3.450.776	-44.497	3.399.843	-45.760
3.428.326	-37.689	3.450.943	-44.496	3.399.991	-45.760
3.428.402	-37.692	3.451.123	-44.494	3.400.150	-45.759
3.428.580	-37.694	3.451.331	-44.493	3.400.278	-45.758
3.428.828	-37.697	3.451.531	-44.492	3.400.399	-45.757
3.429.013	-37.699	3.451.697	-44.490	3.400.602	-45.756
3.429.189	-37.702	3.451.880	-44.489	3.400.941	-45.756
3.429.355	-37.704	3.452.098	-44.488	3.401.414	-45.755
3.429.503	-37.707	3.452.221	-44.487	3.401.718	-45.754
3.429.627	-37.709	3.452.310	-44.485	3.401.729	-45.753
3.429.796	-37.712	3.452.448	-44.484	3.401.844	-45.752
3.429.991	-37.714	3.452.576	-44.483	3.402.000	-45.751
3.430.085	-37.717	3.452.752	-44.482	3.402.043	-45.751
3.430.186	-37.719	3.452.937	-44.481	3.402.130	-45.750
3.430.374	-37.722	3.453.030	-44.479	3.402.221	-45.749
3.430.558	-37.724	3.453.134	-44.478	3.402.310	-45.748
3.430.753	-37.727	3.453.337	-44.477	3.402.440	-45.747
3.430.999	-37.729	3.453.589	-44.476	3.402.556	-45.746
3.431.210	-37.732	3.453.795	-44.475	3.402.711	-45.746

3.431.360	-37.734	3.453.936	-44.474	3.402.875	-45.745
3.431.505	-37.737	3.454.131	-44.473	3.403.060	-45.744
3.431.662	-37.739	3.454.316	-44.472	3.403.326	-45.743
3.431.870	-37.742	3.454.476	-44.471	3.403.510	-45.742
3.432.039	-37.744	3.454.604	-44.470	3.403.636	-45.742
3.432.177	-37.747	3.454.805	-44.469	3.403.880	-45.741
3.432.300	-37.749	3.455.027	-44.468	3.404.117	-45.740
3.432.441	-37.752	3.455.115	-44.467	3.404.262	-45.739
3.432.599	-37.754	3.455.247	-44.466	3.404.345	-45.738
3.432.722	-37.757	3.455.399	-44.465	3.404.464	-45.738
3.432.821	-37.759	3.455.558	-44.464	3.404.673	-45.737
3.433.022	-37.762	3.455.686	-44.463	3.404.832	-45.736
3.433.271	-37.764	3.455.854	-44.462	3.404.993	-45.736
3.433.485	-37.767	3.456.065	-44.461	3.405.182	-45.735
3.433.672	-37.769	3.456.130	-44.460	3.405.300	-45.734
3.433.869	-37.772	3.456.282	-44.460	3.405.425	-45.733
3.434.017	-37.775	3.456.581	-44.459	3.405.562	-45.733
3.434.136	-37.777	3.456.747	-44.458	3.405.768	-45.732
3.434.265	-37.780	3.456.875	-44.457	3.405.974	-45.731
3.434.420	-37.782	3.457.061	-44.456	3.406.115	-45.731
3.434.633	-37.785	3.457.206	-44.456	3.406.302	-45.730
3.434.850	-37.787	3.457.328	-44.455	3.406.455	-45.730
3.435.016	-37.790	3.457.487	-44.454	3.406.600	-45.729
3.435.175	-37.792	3.457.658	-44.454	3.406.802	-45.728
3.435.359	-37.795	3.457.838	-44.453	3.407.021	-45.728
3.435.465	-37.797	3.458.091	-44.452	3.407.181	-45.727
3.435.612	-37.800	3.458.297	-44.452	3.407.390	-45.727
3.435.826	-37.802	3.458.432	-44.451	3.407.637	-45.726
3.435.972	-37.805	3.458.593	-44.450	3.407.795	-45.726
3.436.136	-37.808	3.458.796	-44.450	3.407.854	-45.725

3.436.308	-37.810	3.458.954	-44.449	3.407.959	-45.725
3.436.383	-37.813	3.459.063	-44.449	3.408.164	-45.724
3.436.603	-37.815	3.459.219	-44.448	3.408.358	-45.724
3.436.888	-37.818	3.459.406	-44.448	3.408.508	-45.723
3.437.061	-37.820	3.459.586	-44.447	3.408.596	-45.723
3.437.242	-37.823	3.459.804	-44.447	3.408.752	-45.722
3.437.423	-37.825	3.459.966	-44.446	3.408.995	-45.722
3.437.554	-37.828	3.460.143	-44.446	3.409.164	-45.721
3.437.661	-37.830	3.460.356	-44.445	3.409.252	-45.721
3.437.813	-37.833	3.460.518	-44.445	3.409.417	-45.721
3.438.013	-37.836	3.460.646	-44.444	3.409.635	-45.720
3.438.168	-37.838	3.460.790	-44.444	3.409.818	-45.720
3.438.257	-37.841	3.460.946	-44.443	3.410.002	-45.720
3.438.405	-37.843	3.461.039	-44.443	3.410.193	-45.719
3.438.644	-37.846	3.461.119	-44.442	3.410.372	-45.719
3.438.900	-37.848	3.461.223	-44.442	3.410.458	-45.719
3.439.073	-37.851	3.461.400	-44.441	3.410.602	-45.718
3.439.154	-37.853	3.461.555	-44.441	3.410.833	-45.718
3.439.424	-37.856	3.461.660	-44.440	3.411.089	-45.718
3.439.868	-37.858	3.461.888	-44.440	3.411.249	-45.718
3.440.179	-37.861	3.462.174	-44.439	3.411.367	-45.718
3.440.339	-37.864	3.462.404	-44.439	3.411.566	-45.717
3.440.378	-37.866	3.462.629	-44.438	3.411.776	-45.717
3.440.389	-37.869	3.462.850	-44.438	3.411.891	-45.717
3.440.432	-37.871	3.463.009	-44.438	3.412.070	-45.717
3.440.475	-37.874	3.463.128	-44.437	3.412.279	-45.717
3.440.588	-37.876	3.463.221	-44.437	3.412.422	-45.716
3.440.764	-37.879	3.463.395	-44.436	3.412.599	-45.716
3.440.895	-37.881	3.463.573	-44.436	3.412.745	-45.716
3.441.084	-37.884	3.463.680	-44.435	3.412.890	-45.716

3.441.285	-37.886	3.463.817	-44.435	3.413.037	-45.716
3.441.376	-37.889	3.463.937	-44.434	3.413.109	-45.715
3.441.530	-37.891	3.463.994	-44.434	3.413.255	-45.715
3.441.759	-37.894	3.464.165	-44.433	3.413.481	-45.715
3.441.896	-37.896	3.464.507	-44.432	3.413.666	-45.715
3.442.061	-37.899	3.464.699	-44.432	3.413.808	-45.714
3.442.271	-37.901	3.464.805	-44.431	3.413.994	-45.714
3.442.426	-37.904	3.464.998	-44.431	3.414.189	-45.714
3.442.571	-37.906	3.465.191	-44.430	3.414.349	-45.714
3.442.729	-37.909	3.465.406	-44.430	3.414.485	-45.713
3.442.877	-37.911	3.465.641	-44.429	3.414.620	-45.713
3.443.034	-37.914	3.465.854	-44.429	3.414.774	-45.713
3.443.243	-37.916	3.466.014	-44.428	3.414.951	-45.713
3.443.430	-37.919	3.466.111	-44.427	3.415.103	-45.712
3.443.553	-37.921	3.466.296	-44.427	3.415.230	-45.712
3.443.669	-37.924	3.466.458	-44.426	3.415.343	-45.712
3.443.820	-37.926	3.466.595	-44.425	3.415.515	-45.711
3.444.125	-37.929	3.466.765	-44.425	3.415.693	-45.711
3.444.373	-37.931	3.466.935	-44.424	3.415.834	-45.711
3.444.456	-37.934	3.467.090	-44.423	3.415.993	-45.710
3.444.626	-37.936	3.467.218	-44.423	3.416.169	-45.710
3.444.790	-37.939	3.467.413	-44.422	3.416.409	-45.709
3.444.919	-37.941	3.467.585	-44.421	3.416.581	-45.709
3.445.119	-37.944	3.467.733	-44.421	3.416.740	-45.708
3.445.266	-37.946	3.467.948	-44.420	3.416.989	-45.708
3.445.385	-37.948	3.468.192	-44.419	3.417.140	-45.708
3.445.544	-37.951	3.468.383	-44.418	3.417.318	-45.707
3.445.757	-37.953	3.468.485	-44.418	3.417.576	-45.706
3.445.952	-37.956	3.468.587	-44.417	3.417.744	-45.706
3.446.046	-37.958	3.468.761	-44.416	3.417.910	-45.705

3.446.161	-37.961	3.468.944	-44.415	3.418.051	-45.705
3.446.335	-37.963	3.469.121	-44.415	3.418.160	-45.704
3.446.617	-37.965	3.469.301	-44.414	3.418.307	-45.704
3.446.885	-37.968	3.469.478	-44.413	3.418.495	-45.703
3.446.964	-37.970	3.469.628	-44.412	3.418.638	-45.702
3.447.054	-37.973	3.469.781	-44.411	3.418.768	-45.702
3.447.192	-37.975	3.469.940	-44.410	3.418.900	-45.701
3.447.352	-37.977	3.470.081	-44.410	3.419.034	-45.700
3.447.552	-37.980	3.470.229	-44.409	3.419.290	-45.699
3.447.755	-37.982	3.470.385	-44.408	3.419.464	-45.699
3.447.930	-37.984	3.470.710	-44.407	3.419.558	-45.698
3.448.087	-37.987	3.471.126	-44.406	3.419.743	-45.697
3.448.214	-37.989	3.471.386	-44.405	3.419.944	-45.696
3.448.369	-37.992	3.471.462	-44.405	3.420.093	-45.696
3.448.586	-37.994	3.471.481	-44.404	3.420.341	-45.695
3.448.723	-37.996	3.471.530	-44.403	3.420.621	-45.694
3.448.862	-37.999	3.471.591	-44.402	3.420.739	-45.693
3.449.102	-38.001	3.471.743	-44.401	3.420.847	-45.692
3.449.283	-38.003	3.471.902	-44.400	3.421.003	-45.691
3.449.427	-38.006	3.471.961	-44.399	3.421.169	-45.690
3.449.604	-38.008	3.472.072	-44.398	3.421.335	-45.689
3.449.746	-38.010	3.472.350	-44.397	3.421.505	-45.688
3.449.848	-38.012	3.472.585	-44.397	3.421.658	-45.687
3.450.034	-38.015	3.472.763	-44.396	3.421.841	-45.686
3.450.292	-38.017	3.472.972	-44.395	3.422.021	-45.685
3.450.414	-38.019	3.473.120	-44.394	3.422.144	-45.684
3.450.472	-38.022	3.473.272	-44.393	3.422.301	-45.683
3.450.627	-38.024	3.473.477	-44.392	3.422.513	-45.682
3.450.833	-38.026	3.473.645	-44.391	3.422.708	-45.681
3.451.014	-38.029	3.473.771	-44.390	3.422.861	-45.680

3.451.154	-38.031	3.473.920	-44.389	3.422.998	-45.678
3.451.273	-38.033	3.474.059	-44.388	3.423.167	-45.677
3.451.483	-38.035	3.474.226	-44.387	3.423.344	-45.676
3.451.714	-38.038	3.474.435	-44.386	3.423.526	-45.675
3.451.841	-38.040	3.474.601	-44.385	3.423.748	-45.674
3.451.924	-38.042	3.474.707	-44.385	3.423.914	-45.672
3.452.011	-38.044	3.474.774	-44.384	3.424.050	-45.671
3.452.195	-38.047	3.474.945	-44.383	3.424.215	-45.670
3.452.478	-38.049	3.475.241	-44.382	3.424.418	-45.669
3.452.777	-38.051	3.475.482	-44.381	3.424.557	-45.667
3.453.018	-38.053	3.475.663	-44.380	3.424.684	-45.666
3.453.188	-38.056	3.475.801	-44.379	3.424.839	-45.665
3.453.318	-38.058	3.475.943	-44.378	3.424.973	-45.664
3.453.484	-38.060	3.476.112	-44.378	3.425.172	-45.662
3.453.717	-38.062	3.476.279	-44.377	3.425.378	-45.661
3.453.878	-38.064	3.476.425	-44.376	3.425.576	-45.660
3.453.972	-38.067	3.476.519	-44.375	3.425.764	-45.658
3.454.097	-38.069	3.476.629	-44.374	3.425.941	-45.657
3.454.262	-38.071	3.476.852	-44.374	3.426.147	-45.656
3.454.399	-38.073	3.477.023	-44.373	3.426.318	-45.655
3.454.570	-38.075	3.477.135	-44.372	3.426.459	-45.653
3.454.734	-38.078	3.477.332	-44.371	3.426.562	-45.652
3.454.832	-38.080	3.477.528	-44.371	3.426.725	-45.650
3.455.000	-38.082	3.477.737	-44.370	3.427.184	-45.649
3.455.173	-38.084	3.477.885	-44.369	3.427.604	-45.648
3.455.353	-38.086	3.478.034	-44.368	3.427.756	-45.646
3.455.569	-38.089	3.478.217	-44.368	3.427.849	-45.645
3.455.812	-38.091	3.478.369	-44.367	3.427.953	-45.644
3.455.996	-38.093	3.478.507	-44.366	3.428.034	-45.642
3.456.154	-38.095	3.478.669	-44.366	3.428.114	-45.641

3.456.364	-38.097	3.478.825	-44.365	3.428.135	-45.639
3.456.539	-38.100	3.478.958	-44.364	3.428.208	-45.638
3.456.693	-38.102	3.479.135	-44.364	3.428.383	-45.637
3.456.819	-38.104	3.479.330	-44.363	3.428.542	-45.635
3.456.992	-38.106	3.479.530	-44.363	3.428.676	-45.634
3.457.214	-38.108	3.479.735	-44.362	3.428.813	-45.633
3.457.361	-38.110	3.479.865	-44.362	3.428.941	-45.631
3.457.469	-38.113	3.479.976	-44.361	3.429.150	-45.630
3.457.586	-38.115	3.480.157	-44.361	3.429.396	-45.628
3.457.737	-38.117	3.480.334	-44.360	3.429.586	-45.627
3.457.939	-38.119	3.480.506	-44.360	3.429.785	-45.626
3.458.128	-38.121	3.480.677	-44.359	3.429.915	-45.625
3.458.297	-38.123	3.480.840	-44.359	3.430.035	-45.623
3.458.497	-38.126	3.481.025	-44.358	3.430.182	-45.622
3.458.647	-38.128	3.481.242	-44.358	3.430.352	-45.621
3.458.755	-38.130	3.481.385	-44.358	3.430.558	-45.620
3.458.959	-38.132	3.481.497	-44.357	3.430.753	-45.619
3.459.150	-38.134	3.481.669	-44.357	3.430.903	-45.617
3.459.265	-38.136	3.481.849	-44.356	3.431.014	-45.616
3.459.402	-38.139	3.482.030	-44.356	3.431.143	-45.615
3.459.626	-38.141	3.482.189	-44.356	3.431.317	-45.614
3.459.837	-38.143	3.482.354	-44.355	3.431.494	-45.613
3.460.013	-38.145	3.482.523	-44.355	3.431.679	-45.612
3.460.126	-38.147	3.482.596	-44.355	3.431.815	-45.611
3.460.262	-38.149	3.482.654	-44.354	3.431.960	-45.610
3.460.479	-38.152	3.482.863	-44.354	3.432.173	-45.609
3.460.645	-38.154	3.483.101	-44.354	3.432.328	-45.608
3.460.813	-38.156	3.483.316	-44.354	3.432.506	-45.608
3.460.923	-38.158	3.483.419	-44.353	3.432.758	-45.607
3.461.147	-38.160	3.483.586	-44.353	3.432.928	-45.606

3.461.596	-38.162	3.483.843	-44.353	3.433.052	-45.605
3.461.935	-38.165	3.484.092	-44.353	3.433.265	-45.605
3.462.041	-38.167	3.484.216	-44.352	3.433.446	-45.604
3.462.126	-38.169	3.484.368	-44.352	3.433.622	-45.603
3.462.268	-38.171	3.484.595	-44.352	3.433.802	-45.603
3.462.352	-38.173	3.484.742	-44.352	3.433.889	-45.602
3.462.364	-38.175	3.484.873	-44.351	3.434.017	-45.601
3.462.424	-38.177	3.484.966	-44.351	3.434.207	-45.601
3.462.567	-38.180	3.485.082	-44.351	3.434.312	-45.600
3.462.680	-38.182	3.485.244	-44.351	3.434.424	-45.600
3.462.798	-38.184	3.485.383	-44.350	3.434.638	-45.599
3.462.994	-38.186	3.485.524	-44.350	3.434.781	-45.599
3.463.200	-38.188	3.485.692	-44.350	3.434.940	-45.599
3.463.445	-38.191	3.485.928	-44.350	3.435.139	-45.598
3.463.634	-38.193	3.486.149	-44.349	3.435.306	-45.598
3.463.745	-38.195	3.486.342	-44.349	3.435.489	-45.598
3.463.897	-38.197	3.486.490	-44.349	3.435.715	-45.598
3.464.113	-38.199	3.486.618	-44.349	3.435.873	-45.598
3.464.297	-38.201	3.486.790	-44.348	3.435.974	-45.598
3.464.494	-38.204	3.487.021	-44.348	3.436.145	-45.598
3.464.655	-38.206	3.487.227	-44.348	3.436.351	-45.598
3.464.738	-38.208	3.487.404	-44.348	3.436.527	-45.598
3.464.890	-38.210	3.487.554	-44.347	3.436.661	-45.598
3.465.110	-38.212	3.487.650	-44.347	3.436.830	-45.598
3.465.237	-38.214	3.487.766	-44.347	3.437.019	-45.598
3.465.359	-38.217	3.487.973	-44.346	3.437.224	-45.598
3.465.562	-38.219	3.488.210	-44.346	3.437.383	-45.598
3.465.730	-38.221	3.488.308	-44.346	3.437.535	-45.599
3.465.937	-38.223	3.488.489	-44.345	3.437.719	-45.599
3.466.169	-38.225	3.488.730	-44.345	3.437.899	-45.599

3.466.328	-38.228	3.488.869	-44.345	3.438.088	-45.600
3.466.490	-38.230	3.489.017	-44.344	3.438.228	-45.600
3.466.679	-38.232	3.489.215	-44.344	3.438.358	-45.600
3.466.888	-38.234	3.489.399	-44.344	3.438.547	-45.601
3.467.021	-38.236	3.489.548	-44.343	3.438.681	-45.601
3.467.097	-38.238	3.489.682	-44.343	3.438.804	-45.602
3.467.247	-38.241	3.489.843	-44.342	3.439.019	-45.602
3.467.434	-38.243	3.490.060	-44.342	3.439.218	-45.603
3.467.630	-38.245	3.490.320	-44.342	3.439.345	-45.604
3.467.805	-38.247	3.490.509	-44.341	3.439.482	-45.604
3.467.943	-38.249	3.490.580	-44.341	3.439.637	-45.605
3.468.008	-38.252	3.490.686	-44.340	3.439.862	-45.605
3.468.170	-38.254	3.490.898	-44.340	3.440.097	-45.606
3.468.403	-38.256	3.491.083	-44.340	3.440.247	-45.607
3.468.546	-38.258	3.491.232	-44.339	3.440.349	-45.607
3.468.742	-38.261	3.491.371	-44.339	3.440.477	-45.608
3.468.959	-38.263	3.491.472	-44.338	3.440.679	-45.609
3.469.064	-38.265	3.491.631	-44.338	3.440.858	-45.610
3.469.167	-38.267	3.492.037	-44.337	3.441.014	-45.610
3.469.355	-38.269	3.492.467	-44.337	3.441.161	-45.611
3.469.514	-38.272	3.492.708	-44.336	3.441.288	-45.612
3.469.677	-38.274	3.492.794	-44.336	3.441.425	-45.613
3.469.890	-38.276	3.492.885	-44.335	3.441.602	-45.614
3.470.089	-38.278	3.492.980	-44.335	3.441.719	-45.615
3.470.247	-38.281	3.493.037	-44.334	3.441.856	-45.615
3.470.453	-38.283	3.493.059	-44.334	3.442.043	-45.616
3.470.645	-38.285	3.493.148	-44.333	3.442.162	-45.617
3.470.780	-38.287	3.493.322	-44.333	3.442.326	-45.618
3.470.883	-38.290	3.493.513	-44.332	3.442.561	-45.619
3.471.049	-38.292	3.493.640	-44.331	3.442.767	-45.620

3.471.226	-38.294	3.493.793	-44.331	3.443.009	-45.621
3.471.382	-38.296	3.493.994	-44.330	3.443.261	-45.622
3.471.582	-38.299	3.494.144	-44.330	3.443.423	-45.623
3.471.682	-38.301	3.494.266	-44.329	3.443.569	-45.624
3.471.844	-38.303	3.494.485	-44.329	3.443.763	-45.625
3.472.072	-38.306	3.494.675	-44.328	3.443.944	-45.625
3.472.225	-38.308	3.494.858	-44.327	3.444.085	-45.626
3.472.437	-38.310	3.495.121	-44.327	3.444.243	-45.627
3.472.623	-38.312	3.495.247	-44.326	3.444.402	-45.628
3.472.763	-38.315	3.495.318	-44.326	3.444.521	-45.629
3.472.943	-38.317	3.495.475	-44.325	3.444.649	-45.630
3.473.102	-38.319	3.495.653	-44.324	3.444.794	-45.631
3.473.215	-38.322	3.495.818	-44.324	3.444.949	-45.632
3.473.327	-38.324	3.495.954	-44.323	3.445.108	-45.633
3.473.409	-38.326	3.496.062	-44.323	3.445.216	-45.634
3.473.517	-38.329	3.496.256	-44.322	3.445.341	-45.635
3.473.652	-38.331	3.496.479	-44.322	3.445.555	-45.636
3.473.773	-38.333	3.496.669	-44.321	3.445.793	-45.637
3.473.967	-38.336	3.496.896	-44.320	3.446.002	-45.637
3.474.174	-38.338	3.497.108	-44.320	3.446.183	-45.638
3.474.438	-38.341	3.497.278	-44.319	3.446.343	-45.639
3.474.710	-38.343	3.497.441	-44.319	3.446.585	-45.640
3.474.915	-38.345	3.497.565	-44.318	3.446.787	-45.641
3.475.125	-38.348	3.497.687	-44.318	3.446.903	-45.642
3.475.288	-38.350	3.497.856	-44.317	3.447.099	-45.642
3.475.418	-38.353	3.498.008	-44.317	3.447.232	-45.643
3.475.576	-38.355	3.498.110	-44.316	3.447.372	-45.644
3.475.783	-38.358	3.498.257	-44.315	3.447.556	-45.645
3.475.930	-38.360	3.498.477	-44.315	3.447.716	-45.645
3.476.028	-38.363	3.498.644	-44.314	3.447.889	-45.646

3.476.125	-38.365	3.498.828	-44.314	3.448.056	-45.647
3.476.284	-38.368	3.499.019	-44.313	3.448.211	-45.648
3.476.444	-38.370	3.499.138	-44.313	3.448.380	-45.648
3.476.603	-38.373	3.499.232	-44.312	3.448.584	-45.649
3.476.781	-38.375	3.499.411	-44.312	3.448.732	-45.650
3.477.000	-38.378	3.499.612	-44.311	3.448.889	-45.650
3.477.195	-38.380	3.499.710	-44.311	3.449.081	-45.651
3.477.408	-38.383	3.499.774	-44.311	3.449.277	-45.651
3.477.639	-38.385	3.499.948	-44.310	3.449.465	-45.652
3.477.787	-38.388	3.500.205	-44.310	3.449.557	-45.652
3.477.917	-38.390	3.500.428	-44.309	3.449.732	-45.653
3.478.083	-38.393	3.500.659	-44.309	3.449.980	-45.653
3.478.224	-38.396	3.500.876	-44.308	3.450.074	-45.654
3.478.387	-38.398	3.500.992	-44.308	3.450.227	-45.654
3.478.576	-38.401	3.501.123	-44.308	3.450.417	-45.655
3.478.742	-38.403	3.501.320	-44.307	3.450.558	-45.655
3.478.889	-38.406	3.501.450	-44.307	3.450.757	-45.656
3.478.994	-38.409	3.501.560	-44.307	3.450.963	-45.656
3.479.161	-38.411	3.501.750	-44.306	3.451.140	-45.656
3.479.346	-38.414	3.501.961	-44.306	3.451.310	-45.657
3.479.521	-38.417	3.502.110	-44.306	3.451.487	-45.657
3.479.742	-38.420	3.502.282	-44.305	3.451.660	-45.657
3.479.911	-38.422	3.502.480	-44.305	3.451.805	-45.658
3.480.052	-38.425	3.502.657	-44.305	3.451.978	-45.658
3.480.212	-38.428	3.502.817	-44.304	3.452.189	-45.658
3.480.383	-38.431	3.503.009	-44.304	3.452.339	-45.658
3.480.536	-38.433	3.503.132	-44.304	3.452.499	-45.659
3.480.678	-38.436	3.503.289	-44.303	3.452.593	-45.659
3.480.780	-38.439	3.503.486	-44.303	3.452.847	-45.659
3.480.938	-38.442	3.503.553	-44.303	3.453.347	-45.659

3.481.169	-38.445	3.503.663	-44.303	3.453.680	-45.659
3.481.393	-38.447	3.503.843	-44.302	3.453.752	-45.659
3.481.566	-38.450	3.503.994	-44.302	3.453.806	-45.659
3.481.712	-38.453	3.504.164	-44.302	3.453.896	-45.659
3.481.898	-38.456	3.504.292	-44.302	3.453.954	-45.659
3.482.063	-38.459	3.504.457	-44.301	3.454.050	-45.659
3.482.221	-38.462	3.504.641	-44.301	3.454.182	-45.659
3.482.397	-38.465	3.504.846	-44.301	3.454.279	-45.659
3.482.511	-38.468	3.505.129	-44.301	3.454.373	-45.659
3.482.628	-38.471	3.505.383	-44.300	3.454.521	-45.659
3.482.881	-38.474	3.505.536	-44.300	3.454.656	-45.659
3.483.268	-38.477	3.505.700	-44.300	3.454.818	-45.659
3.483.627	-38.480	3.505.869	-44.300	3.455.085	-45.658
3.483.839	-38.483	3.506.039	-44.299	3.455.282	-45.658
3.483.925	-38.486	3.506.278	-44.299	3.455.401	-45.658
3.483.972	-38.489	3.506.365	-44.299	3.455.562	-45.658
3.483.951	-38.492	3.506.436	-44.299	3.455.746	-45.658
3.483.991	-38.495	3.506.591	-44.298	3.455.992	-45.658
3.484.190	-38.498	3.506.678	-44.298	3.456.179	-45.657
3.484.345	-38.501	3.506.810	-44.298	3.456.324	-45.657
3.484.449	-38.504	3.506.992	-44.298	3.456.472	-45.657
3.484.595	-38.507	3.507.211	-44.297	3.456.622	-45.657
3.484.815	-38.510	3.507.402	-44.297	3.456.732	-45.656
3.485.013	-38.514	3.507.560	-44.297	3.456.921	-45.656
3.485.176	-38.517	3.507.737	-44.296	3.457.155	-45.656
3.485.364	-38.520	3.507.976	-44.296	3.457.292	-45.656
3.485.512	-38.523	3.508.186	-44.296	3.457.449	-45.655
3.485.711	-38.526	3.508.330	-44.295	3.457.578	-45.655
3.485.907	-38.530	3.508.507	-44.295	3.457.738	-45.655
3.486.064	-38.533	3.508.673	-44.295	3.457.917	-45.655

3.486.184	-38.536	3.508.818	-44.294	3.458.131	-45.654
3.486.360	-38.539	3.508.900	-44.294	3.458.322	-45.654
3.486.516	-38.543	3.509.066	-44.294	3.458.468	-45.654
3.486.642	-38.546	3.509.341	-44.293	3.458.607	-45.653
3.486.834	-38.549	3.509.485	-44.293	3.458.779	-45.653
3.486.987	-38.552	3.509.608	-44.292	3.458.929	-45.653
3.487.184	-38.556	3.509.742	-44.292	3.459.103	-45.653
3.487.340	-38.559	3.509.906	-44.291	3.459.305	-45.652
3.487.491	-38.562	3.510.175	-44.291	3.459.460	-45.652
3.487.708	-38.566	3.510.367	-44.290	3.459.598	-45.652
3.487.856	-38.569	3.510.541	-44.290	3.459.724	-45.651
3.487.977	-38.572	3.510.721	-44.289	3.459.904	-45.651
3.488.163	-38.576	3.510.898	-44.289	3.460.092	-45.651
3.488.387	-38.579	3.511.031	-44.288	3.460.206	-45.650
3.488.589	-38.583	3.511.148	-44.288	3.460.327	-45.650
3.488.688	-38.586	3.511.339	-44.287	3.460.458	-45.650
3.488.808	-38.589	3.511.499	-44.286	3.460.668	-45.650
3.488.984	-38.593	3.511.614	-44.286	3.460.888	-45.649
3.489.122	-38.596	3.511.827	-44.285	3.461.125	-45.649
3.489.243	-38.600	3.512.048	-44.284	3.461.297	-45.649
3.489.373	-38.603	3.512.250	-44.284	3.461.364	-45.648
3.489.526	-38.607	3.512.458	-44.283	3.461.477	-45.648
3.489.724	-38.610	3.512.636	-44.282	3.461.646	-45.648
3.489.894	-38.614	3.512.762	-44.281	3.461.815	-45.648
3.490.094	-38.617	3.512.863	-44.281	3.462.037	-45.647
3.490.298	-38.621	3.513.037	-44.280	3.462.182	-45.647
3.490.411	-38.624	3.513.295	-44.279	3.462.304	-45.647
3.490.569	-38.628	3.513.730	-44.278	3.462.464	-45.647
3.490.753	-38.631	3.514.110	-44.277	3.462.592	-45.647
3.490.913	-38.635	3.514.194	-44.276	3.462.744	-45.646

3.491.098	-38.638	3.514.240	-44.275	3.462.973	-45.646
3.491.209	-38.642	3.514.337	-44.274	3.463.141	-45.646
3.491.339	-38.645	3.514.445	-44.273	3.463.323	-45.646
3.491.555	-38.649	3.514.510	-44.272	3.463.576	-45.646
3.491.721	-38.653	3.514.530	-44.271	3.463.746	-45.645
3.491.942	-38.656	3.514.695	-44.270	3.463.799	-45.645
3.492.130	-38.660	3.514.886	-44.269	3.463.959	-45.645
3.492.278	-38.663	3.515.028	-44.268	3.464.167	-45.645
3.492.473	-38.667	3.515.206	-44.266	3.464.317	-45.645
3.492.639	-38.671	3.515.377	-44.265	3.464.485	-45.644
3.492.755	-38.674	3.515.508	-44.264	3.464.641	-45.644
3.492.872	-38.678	3.515.648	-44.263	3.464.847	-45.644
3.493.041	-38.681	3.515.885	-44.261	3.465.064	-45.644
3.493.208	-38.685	3.516.060	-44.260	3.465.187	-45.644
3.493.383	-38.689	3.516.205	-44.259	3.465.354	-45.643
3.493.640	-38.692	3.516.317	-44.257	3.465.547	-45.643
3.493.904	-38.696	3.516.492	-44.256	3.465.744	-45.643
3.494.052	-38.700	3.516.707	-44.254	3.465.928	-45.643
3.494.146	-38.703	3.516.868	-44.253	3.466.119	-45.642
3.494.250	-38.707	3.516.976	-44.251	3.466.286	-45.642
3.494.409	-38.711	3.517.122	-44.250	3.466.494	-45.642
3.494.608	-38.714	3.517.260	-44.248	3.466.693	-45.641
3.494.745	-38.718	3.517.362	-44.247	3.466.777	-45.641
3.494.876	-38.722	3.517.528	-44.245	3.466.889	-45.641
3.495.092	-38.725	3.517.762	-44.244	3.467.021	-45.640
3.495.190	-38.729	3.518.015	-44.242	3.467.200	-45.640
3.495.288	-38.733	3.518.237	-44.240	3.467.336	-45.640
3.495.437	-38.737	3.518.401	-44.239	3.467.384	-45.639
3.495.541	-38.740	3.518.553	-44.237	3.467.523	-45.639
3.495.724	-38.744	3.518.676	-44.235	3.467.727	-45.638

3.495.939	-38.748	3.518.795	-44.233	3.467.917	-45.638
3.496.133	-38.751	3.518.981	-44.231	3.468.102	-45.637
3.496.372	-38.755	3.519.200	-44.230	3.468.247	-45.637
3.496.581	-38.759	3.519.351	-44.228	3.468.467	-45.636
3.496.805	-38.763	3.519.432	-44.226	3.468.743	-45.636
3.497.028	-38.766	3.519.644	-44.224	3.469.012	-45.635
3.497.198	-38.770	3.519.877	-44.222	3.469.178	-45.635
3.497.348	-38.774	3.519.984	-44.220	3.469.227	-45.634
3.497.387	-38.778	3.520.132	-44.218	3.469.411	-45.633
3.497.502	-38.781	3.520.354	-44.216	3.469.623	-45.633
3.497.722	-38.785	3.520.605	-44.214	3.469.805	-45.632
3.497.900	-38.789	3.520.765	-44.212	3.469.977	-45.631
3.498.094	-38.793	3.520.914	-44.210	3.470.132	-45.631
3.498.208	-38.796	3.521.093	-44.208	3.470.269	-45.630
3.498.300	-38.800	3.521.250	-44.206	3.470.419	-45.629
3.498.485	-38.804	3.521.438	-44.203	3.470.587	-45.628
3.498.669	-38.808	3.521.562	-44.201	3.470.735	-45.627
3.498.878	-38.811	3.521.691	-44.199	3.470.858	-45.626
3.499.077	-38.815	3.521.853	-44.197	3.470.977	-45.625
3.499.232	-38.819	3.522.021	-44.195	3.471.102	-45.624
3.499.421	-38.823	3.522.218	-44.192	3.471.268	-45.623
3.499.630	-38.826	3.522.390	-44.190	3.471.472	-45.622
3.499.829	-38.830	3.522.589	-44.188	3.471.635	-45.621
3.499.955	-38.834	3.522.805	-44.185	3.471.841	-45.620
3.500.081	-38.838	3.522.948	-44.183	3.472.043	-45.619
3.500.288	-38.841	3.523.098	-44.181	3.472.157	-45.618
3.500.506	-38.845	3.523.228	-44.178	3.472.343	-45.616
3.500.677	-38.849	3.523.344	-44.176	3.472.552	-45.615
3.500.854	-38.853	3.523.540	-44.174	3.472.744	-45.614
3.500.996	-38.857	3.523.784	-44.171	3.472.964	-45.613

3.501.169	-38.860	3.523.918	-44.169	3.473.076	-45.611
3.501.386	-38.864	3.524.067	-44.166	3.473.196	-45.610
3.501.526	-38.868	3.524.233	-44.164	3.473.434	-45.608
3.501.674	-38.872	3.524.402	-44.161	3.473.578	-45.607
3.501.856	-38.875	3.524.617	-44.159	3.473.701	-45.605
3.502.065	-38.879	3.524.786	-44.156	3.473.945	-45.604
3.502.271	-38.883	3.524.901	-44.154	3.474.149	-45.602
3.502.399	-38.887	3.524.977	-44.151	3.474.256	-45.601
3.502.507	-38.890	3.525.108	-44.148	3.474.420	-45.599
3.502.679	-38.894	3.525.244	-44.146	3.474.633	-45.597
3.502.885	-38.898	3.525.382	-44.143	3.474.825	-45.596
3.503.015	-38.902	3.525.518	-44.141	3.474.985	-45.594
3.503.123	-38.905	3.525.703	-44.138	3.475.107	-45.592
3.503.278	-38.909	3.525.952	-44.135	3.475.245	-45.590
3.503.466	-38.913	3.526.191	-44.133	3.475.403	-45.588
3.503.633	-38.917	3.526.433	-44.130	3.475.573	-45.586
3.503.802	-38.920	3.526.624	-44.127	3.475.812	-45.585
3.503.983	-38.924	3.526.819	-44.125	3.476.000	-45.583
3.504.135	-38.928	3.527.010	-44.122	3.476.144	-45.581
3.504.333	-38.931	3.527.184	-44.119	3.476.310	-45.579
3.504.500	-38.935	3.527.368	-44.116	3.476.479	-45.577
3.504.742	-38.939	3.527.477	-44.114	3.476.609	-45.574
3.505.143	-38.943	3.527.628	-44.111	3.476.791	-45.572
3.505.506	-38.946	3.527.838	-44.108	3.476.979	-45.570
3.505.665	-38.950	3.528.004	-44.105	3.477.120	-45.568
3.505.721	-38.954	3.528.107	-44.102	3.477.278	-45.566
3.505.754	-38.957	3.528.200	-44.100	3.477.422	-45.564
3.505.742	-38.961	3.528.365	-44.097	3.477.604	-45.561
3.505.836	-38.965	3.528.513	-44.094	3.477.781	-45.559
3.505.981	-38.968	3.528.690	-44.091	3.477.926	-45.557

3.506.055	-38.972	3.528.941	-44.088	3.478.087	-45.555
3.506.176	-38.976	3.529.147	-44.085	3.478.280	-45.552
3.506.364	-38.979	3.529.344	-44.083	3.478.434	-45.550
3.506.573	-38.983	3.529.536	-44.080	3.478.652	-45.547
3.506.783	-38.987	3.529.669	-44.077	3.478.990	-45.545
3.506.983	-38.990	3.529.876	-44.074	3.479.370	-45.542
3.507.188	-38.994	3.530.020	-44.071	3.479.630	-45.540
3.507.368	-38.998	3.530.078	-44.068	3.479.673	-45.537
3.507.545	-39.001	3.530.245	-44.066	3.479.758	-45.535
3.507.690	-39.005	3.530.468	-44.063	3.479.919	-45.532
3.507.867	-39.008	3.530.688	-44.060	3.479.995	-45.530
3.508.060	-39.012	3.530.852	-44.057	3.480.011	-45.527
3.508.217	-39.016	3.530.974	-44.054	3.480.016	-45.524
3.508.372	-39.019	3.531.075	-44.052	3.480.166	-45.522
3.508.503	-39.023	3.531.238	-44.049	3.480.389	-45.519
3.508.661	-39.026	3.531.450	-44.046	3.480.511	-45.516
3.508.828	-39.030	3.531.643	-44.043	3.480.670	-45.514
3.508.936	-39.033	3.531.844	-44.040	3.480.876	-45.511
3.509.074	-39.037	3.532.022	-44.038	3.481.031	-45.508
3.509.352	-39.040	3.532.145	-44.035	3.481.190	-45.505
3.509.584	-39.044	3.532.285	-44.032	3.481.361	-45.503
3.509.743	-39.047	3.532.507	-44.029	3.481.578	-45.500
3.509.913	-39.051	3.532.712	-44.027	3.481.833	-45.497
3.510.071	-39.054	3.532.864	-44.024	3.482.025	-45.494
3.510.280	-39.058	3.533.008	-44.021	3.482.144	-45.491
3.510.469	-39.061	3.533.183	-44.019	3.482.232	-45.489
3.510.606	-39.065	3.533.297	-44.016	3.482.419	-45.486
3.510.750	-39.068	3.533.407	-44.013	3.482.581	-45.483
3.510.914	-39.072	3.533.593	-44.011	3.482.715	-45.480
3.511.031	-39.075	3.533.771	-44.008	3.482.901	-45.478

3.511.129	-39.078	3.533.970	-44.006	3.483.088	-45.475
3.511.261	-39.082	3.534.138	-44.003	3.483.177	-45.472
3.511.461	-39.085	3.534.291	-44.001	3.483.250	-45.469
3.511.624	-39.089	3.534.464	-43.998	3.483.405	-45.467
3.511.812	-39.092	3.534.800	-43.996	3.483.640	-45.464
3.511.980	-39.095	3.535.227	-43.993	3.483.929	-45.461
3.512.175	-39.099	3.535.457	-43.991	3.484.096	-45.459
3.512.329	-39.102	3.535.549	-43.988	3.484.222	-45.456
3.512.529	-39.105	3.535.558	-43.986	3.484.380	-45.453
3.512.691	-39.109	3.535.609	-43.983	3.484.507	-45.450
3.512.773	-39.112	3.535.678	-43.981	3.484.704	-45.448
3.512.881	-39.115	3.535.733	-43.979	3.484.906	-45.445
3.513.101	-39.118	3.535.941	-43.976	3.485.090	-45.443
3.513.336	-39.122	3.536.173	-43.974	3.485.216	-45.440
3.513.441	-39.125	3.536.321	-43.972	3.485.381	-45.437
3.513.593	-39.128	3.536.443	-43.969	3.485.574	-45.435
3.513.840	-39.131	3.536.621	-43.967	3.485.718	-45.432
3.514.070	-39.135	3.536.794	-43.965	3.485.883	-45.430
3.514.200	-39.138	3.537.014	-43.963	3.486.019	-45.427
3.514.352	-39.141	3.537.264	-43.961	3.486.143	-45.424
3.514.492	-39.144	3.537.394	-43.959	3.486.357	-45.422
3.514.605	-39.147	3.537.554	-43.956	3.486.577	-45.420
3.514.801	-39.150	3.537.748	-43.954	3.486.745	-45.417
3.514.973	-39.154	3.537.878	-43.952	3.486.857	-45.415
3.515.133	-39.157	3.538.009	-43.950	3.486.974	-45.412
3.515.371	-39.160	3.538.149	-43.948	3.487.155	-45.410
3.515.558	-39.163	3.538.307	-43.947	3.487.332	-45.408
3.515.699	-39.166	3.538.489	-43.945	3.487.502	-45.405
3.515.931	-39.169	3.538.622	-43.943	3.487.712	-45.403
3.516.107	-39.172	3.538.739	-43.941	3.487.908	-45.401

3.516.226	-39.175	3.538.943	-43.939	3.488.016	-45.399
3.516.369	-39.178	3.539.165	-43.937	3.488.150	-45.397
3.516.523	-39.181	3.539.345	-43.936	3.488.239	-45.394
3.516.694	-39.184	3.539.523	-43.934	3.488.376	-45.392
3.516.808	-39.187	3.539.703	-43.932	3.488.596	-45.390
3.516.915	-39.190	3.539.825	-43.931	3.488.796	-45.388
3.517.137	-39.193	3.540.002	-43.929	3.488.997	-45.386
3.517.274	-39.196	3.540.244	-43.928	3.489.193	-45.384
3.517.368	-39.199	3.540.361	-43.926	3.489.391	-45.382
3.517.521	-39.202	3.540.437	-43.925	3.489.536	-45.380
3.517.722	-39.205	3.540.587	-43.923	3.489.713	-45.378
3.517.982	-39.208	3.540.794	-43.922	3.489.890	-45.376
3.518.224	-39.211	3.541.006	-43.921	3.490.071	-45.374
3.518.427	-39.214	3.541.167	-43.919	3.490.280	-45.373
3.518.575	-39.216	3.541.317	-43.918	3.490.429	-45.371
3.518.757	-39.219	3.541.441	-43.917	3.490.573	-45.369
3.518.961	-39.222	3.541.573	-43.916	3.490.753	-45.367
3.519.089	-39.225	3.541.763	-43.914	3.490.920	-45.365
3.519.229	-39.228	3.541.906	-43.913	3.491.028	-45.364
3.519.417	-39.231	3.542.086	-43.912	3.491.104	-45.362
3.519.568	-39.233	3.542.260	-43.911	3.491.315	-45.360
3.519.670	-39.236	3.542.381	-43.910	3.491.573	-45.359
3.519.772	-39.239	3.542.543	-43.909	3.491.744	-45.357
3.519.890	-39.242	3.542.737	-43.908	3.491.914	-45.355
3.520.027	-39.244	3.542.932	-43.907	3.492.094	-45.354
3.520.237	-39.247	3.543.019	-43.906	3.492.189	-45.352
3.520.466	-39.250	3.543.200	-43.906	3.492.314	-45.350
3.520.614	-39.253	3.543.443	-43.905	3.492.412	-45.349
3.520.808	-39.255	3.543.659	-43.904	3.492.557	-45.347
3.521.006	-39.258	3.543.896	-43.903	3.492.776	-45.346

3.521.163	-39.261	3.544.046	-43.902	3.492.917	-45.344
3.521.310	-39.263	3.544.174	-43.902	3.493.018	-45.343
3.521.465	-39.266	3.544.352	-43.901	3.493.156	-45.341
3.521.687	-39.269	3.544.536	-43.901	3.493.311	-45.340
3.521.862	-39.271	3.544.702	-43.900	3.493.492	-45.338
3.522.012	-39.274	3.544.865	-43.899	3.493.676	-45.337
3.522.195	-39.277	3.545.021	-43.899	3.493.817	-45.335
3.522.402	-39.279	3.545.237	-43.898	3.493.908	-45.334
3.522.583	-39.282	3.545.403	-43.898	3.494.120	-45.333
3.522.738	-39.285	3.545.513	-43.897	3.494.313	-45.331
3.522.921	-39.287	3.545.703	-43.897	3.494.547	-45.330
3.523.073	-39.290	3.545.860	-43.897	3.494.821	-45.328
3.523.219	-39.292	3.545.972	-43.896	3.494.992	-45.327
3.523.333	-39.295	3.546.155	-43.896	3.495.172	-45.326
3.523.478	-39.297	3.546.324	-43.895	3.495.367	-45.324
3.523.739	-39.300	3.546.479	-43.895	3.495.520	-45.323
3.523.943	-39.302	3.546.606	-43.895	3.495.708	-45.322
3.524.118	-39.305	3.546.707	-43.894	3.495.873	-45.320
3.524.273	-39.308	3.546.839	-43.894	3.495.930	-45.319
3.524.409	-39.310	3.546.994	-43.894	3.496.068	-45.318
3.524.602	-39.313	3.547.181	-43.893	3.496.232	-45.316
3.524.760	-39.315	3.547.350	-43.893	3.496.310	-45.315
3.524.919	-39.318	3.547.578	-43.893	3.496.479	-45.314
3.525.118	-39.320	3.547.766	-43.893	3.496.701	-45.313
3.525.306	-39.323	3.547.950	-43.892	3.496.889	-45.311
3.525.462	-39.325	3.548.201	-43.892	3.497.007	-45.310
3.525.630	-39.327	3.548.425	-43.892	3.497.177	-45.309
3.525.826	-39.330	3.548.636	-43.891	3.497.397	-45.308
3.526.024	-39.332	3.548.766	-43.891	3.497.561	-45.306
3.526.162	-39.335	3.548.867	-43.891	3.497.738	-45.305

3.526.328	-39.337	3.548.998	-43.891	3.497.973	-45.304
3.526.573	-39.340	3.549.081	-43.890	3.498.203	-45.303
3.526.898	-39.342	3.549.128	-43.890	3.498.348	-45.301
3.527.250	-39.344	3.549.258	-43.890	3.498.478	-45.300
3.527.426	-39.347	3.549.478	-43.889	3.498.634	-45.299
3.527.477	-39.349	3.549.634	-43.889	3.498.815	-45.298
3.527.514	-39.352	3.549.808	-43.889	3.499.017	-45.296
3.527.594	-39.354	3.549.991	-43.889	3.499.207	-45.295
3.527.690	-39.356	3.550.169	-43.888	3.499.357	-45.294
3.527.730	-39.359	3.550.367	-43.888	3.499.497	-45.293
3.527.849	-39.361	3.550.563	-43.888	3.499.606	-45.291
3.528.034	-39.364	3.550.722	-43.887	3.499.826	-45.290
3.528.228	-39.366	3.550.854	-43.887	3.499.998	-45.289
3.528.443	-39.368	3.551.006	-43.887	3.500.099	-45.288
3.528.619	-39.371	3.551.261	-43.886	3.500.299	-45.287
3.528.753	-39.373	3.551.476	-43.886	3.500.475	-45.285
3.528.919	-39.375	3.551.595	-43.886	3.500.674	-45.284
3.529.157	-39.378	3.551.658	-43.885	3.500.859	-45.283
3.529.371	-39.380	3.551.838	-43.885	3.500.974	-45.282
3.529.489	-39.382	3.552.108	-43.884	3.501.107	-45.280
3.529.619	-39.384	3.552.322	-43.884	3.501.291	-45.279
3.529.781	-39.387	3.552.491	-43.883	3.501.454	-45.278
3.529.897	-39.389	3.552.633	-43.883	3.501.639	-45.277
3.530.082	-39.391	3.552.753	-43.882	3.501.812	-45.276
3.530.280	-39.394	3.552.957	-43.882	3.501.993	-45.274
3.530.368	-39.396	3.553.172	-43.881	3.502.170	-45.273
3.530.471	-39.398	3.553.369	-43.881	3.502.258	-45.272
3.530.630	-39.400	3.553.460	-43.880	3.502.387	-45.271
3.530.833	-39.403	3.553.578	-43.880	3.502.647	-45.269
3.531.017	-39.405	3.553.748	-43.879	3.502.814	-45.268

3.531.206	-39.407	3.553.969	-43.878	3.502.874	-45.267
3.531.422	-39.410	3.554.132	-43.878	3.503.070	-45.266
3.531.667	-39.412	3.554.232	-43.877	3.503.360	-45.265
3.531.852	-39.414	3.554.398	-43.876	3.503.558	-45.264
3.531.982	-39.416	3.554.579	-43.876	3.503.698	-45.262
3.532.236	-39.419	3.554.716	-43.875	3.503.789	-45.261
3.532.401	-39.421	3.554.931	-43.874	3.503.932	-45.260
3.532.440	-39.423	3.555.045	-43.873	3.504.149	-45.259
3.532.617	-39.425	3.555.157	-43.872	3.504.279	-45.258
3.532.800	-39.427	3.555.352	-43.871	3.504.660	-45.257
3.532.925	-39.430	3.555.508	-43.871	3.505.043	-45.255
3.533.080	-39.432	3.555.671	-43.870	3.505.262	-45.254
3.533.260	-39.434	3.556.084	-43.869	3.505.412	-45.253
3.533.430	-39.436	3.556.516	-43.868	3.505.558	-45.252
3.533.562	-39.438	3.556.685	-43.867	3.505.623	-45.251
3.533.807	-39.441	3.556.794	-43.866	3.505.648	-45.250
3.534.024	-39.443	3.556.851	-43.865	3.505.681	-45.249
3.534.168	-39.445	3.556.925	-43.863	3.505.735	-45.248
3.534.333	-39.447	3.557.023	-43.862	3.505.854	-45.246
3.534.510	-39.449	3.557.076	-43.861	3.506.078	-45.245
3.534.675	-39.452	3.557.151	-43.860	3.506.304	-45.244
3.534.803	-39.454	3.557.323	-43.859	3.506.407	-45.243
3.534.980	-39.456	3.557.563	-43.858	3.506.534	-45.242
3.535.144	-39.458	3.557.760	-43.856	3.506.718	-45.241
3.535.241	-39.460	3.557.957	-43.855	3.506.862	-45.240
3.535.403	-39.463	3.558.174	-43.854	3.507.091	-45.239
3.535.630	-39.465	3.558.301	-43.852	3.507.272	-45.238
3.535.758	-39.467	3.558.430	-43.851	3.507.446	-45.237
3.535.881	-39.469	3.558.636	-43.849	3.507.654	-45.236
3.536.068	-39.471	3.558.839	-43.848	3.507.856	-45.235

3.536.282	-39.473	3.559.048	-43.847	3.507.939	-45.234
3.536.490	-39.476	3.559.178	-43.845	3.508.055	-45.233
3.536.644	-39.478	3.559.266	-43.844	3.508.257	-45.232
3.536.781	-39.480	3.559.432	-43.842	3.508.445	-45.231
3.536.953	-39.482	3.559.626	-43.840	3.508.533	-45.231
3.537.144	-39.484	3.559.750	-43.839	3.508.669	-45.230
3.537.289	-39.486	3.559.839	-43.837	3.508.773	-45.229
3.537.462	-39.489	3.559.973	-43.836	3.508.962	-45.228
3.537.664	-39.491	3.560.143	-43.834	3.509.160	-45.227
3.537.810	-39.493	3.560.396	-43.832	3.509.335	-45.227
3.537.958	-39.495	3.560.650	-43.830	3.509.482	-45.226
3.538.112	-39.497	3.560.921	-43.829	3.509.673	-45.225
3.538.248	-39.499	3.561.006	-43.827	3.509.854	-45.224
3.538.387	-39.502	3.561.183	-43.825	3.510.009	-45.224
3.538.517	-39.504	3.561.396	-43.823	3.510.226	-45.223
3.538.600	-39.506	3.561.591	-43.822	3.510.440	-45.222
3.538.766	-39.508	3.561.792	-43.820	3.510.618	-45.222
3.538.914	-39.510	3.561.892	-43.818	3.510.773	-45.221
3.539.048	-39.513	3.561.985	-43.816	3.510.876	-45.220
3.539.263	-39.515	3.562.171	-43.814	3.511.090	-45.220
3.539.433	-39.517	3.562.292	-43.812	3.511.239	-45.219
3.539.572	-39.519	3.562.473	-43.810	3.511.383	-45.219
3.539.821	-39.521	3.562.683	-43.808	3.511.523	-45.218
3.540.121	-39.523	3.562.803	-43.807	3.511.686	-45.218
3.540.345	-39.526	3.562.888	-43.805	3.511.868	-45.217
3.540.494	-39.528	3.563.080	-43.803	3.512.014	-45.217
3.540.628	-39.530	3.563.338	-43.801	3.512.199	-45.216
3.540.854	-39.532	3.563.481	-43.799	3.512.393	-45.216
3.541.089	-39.535	3.563.566	-43.797	3.512.531	-45.215
3.541.183	-39.537	3.563.734	-43.795	3.512.661	-45.215

3.541.241	-39.539	3.563.943	-43.793	3.512.831	-45.215
3.541.418	-39.541	3.564.091	-43.791	3.512.989	-45.214
3.541.564	-39.543	3.564.268	-43.789	3.513.168	-45.214
3.541.705	-39.546	3.564.431	-43.787	3.513.334	-45.213
3.541.859	-39.548	3.564.609	-43.785	3.513.490	-45.213
3.542.022	-39.550	3.564.839	-43.782	3.513.692	-45.213
3.542.224	-39.552	3.565.016	-43.780	3.513.849	-45.212
3.542.430	-39.555	3.565.195	-43.778	3.514.008	-45.212
3.542.570	-39.557	3.565.343	-43.776	3.514.214	-45.212
3.542.758	-39.559	3.565.475	-43.774	3.514.406	-45.212
3.542.961	-39.562	3.565.652	-43.772	3.514.566	-45.211
3.543.116	-39.564	3.565.842	-43.770	3.514.698	-45.211
3.543.318	-39.566	3.566.017	-43.768	3.514.868	-45.211
3.543.463	-39.568	3.566.161	-43.766	3.515.070	-45.211
3.543.596	-39.571	3.566.311	-43.764	3.515.278	-45.210
3.543.806	-39.573	3.566.501	-43.762	3.515.448	-45.210
3.544.019	-39.575	3.566.667	-43.759	3.515.570	-45.210
3.544.175	-39.578	3.566.779	-43.757	3.515.761	-45.210
3.544.330	-39.580	3.566.873	-43.755	3.515.961	-45.209
3.544.494	-39.583	3.567.123	-43.753	3.516.089	-45.209
3.544.652	-39.585	3.567.328	-43.751	3.516.206	-45.209
3.544.836	-39.587	3.567.428	-43.749	3.516.400	-45.209
3.544.978	-39.590	3.567.572	-43.747	3.516.611	-45.208
3.545.123	-39.592	3.567.787	-43.744	3.516.711	-45.208
3.545.281	-39.594	3.567.906	-43.742	3.516.853	-45.208
3.545.421	-39.597	3.568.008	-43.740	3.517.079	-45.208
3.545.606	-39.599	3.568.135	-43.738	3.517.267	-45.207
3.545.862	-39.602	3.568.264	-43.736	3.517.404	-45.207
3.546.032	-39.604	3.568.478	-43.733	3.517.574	-45.207
3.546.118	-39.607	3.568.737	-43.731	3.517.782	-45.206

3.546.259	-39.609	3.568.940	-43.729	3.517.946	-45.206
3.546.444	-39.612	3.569.093	-43.727	3.518.055	-45.206
3.546.620	-39.614	3.569.263	-43.724	3.518.185	-45.205
3.546.768	-39.617	3.569.493	-43.722	3.518.347	-45.205
3.547.001	-39.619	3.569.760	-43.720	3.518.488	-45.205
3.547.180	-39.622	3.569.941	-43.717	3.518.648	-45.204
3.547.361	-39.624	3.570.064	-43.715	3.518.795	-45.204
3.547.581	-39.627	3.570.219	-43.713	3.518.958	-45.203
3.547.690	-39.629	3.570.417	-43.710	3.519.050	-45.203
3.547.787	-39.632	3.570.569	-43.708	3.519.191	-45.202
3.547.865	-39.634	3.570.617	-43.706	3.519.429	-45.202
3.548.053	-39.637	3.570.719	-43.703	3.519.595	-45.201
3.548.504	-39.640	3.570.867	-43.701	3.519.790	-45.201
3.548.956	-39.642	3.570.999	-43.699	3.519.919	-45.200
3.549.147	-39.645	3.571.205	-43.696	3.520.151	-45.200
3.549.237	-39.648	3.571.416	-43.694	3.520.545	-45.199
3.549.326	-39.650	3.571.574	-43.691	3.520.739	-45.198
3.549.359	-39.653	3.571.786	-43.689	3.520.833	-45.198
3.549.398	-39.656	3.572.025	-43.687	3.521.002	-45.197
3.549.537	-39.658	3.572.180	-43.684	3.521.190	-45.196
3.549.688	-39.661	3.572.402	-43.682	3.521.346	-45.195
3.549.781	-39.664	3.572.583	-43.679	3.521.490	-45.194
3.549.917	-39.667	3.572.651	-43.677	3.521.613	-45.194
3.550.076	-39.669	3.572.823	-43.674	3.521.791	-45.193
3.550.258	-39.672	3.572.980	-43.672	3.522.015	-45.192
3.550.425	-39.675	3.573.138	-43.669	3.522.140	-45.191
3.550.623	-39.678	3.573.315	-43.667	3.522.258	-45.190
3.550.859	-39.680	3.573.488	-43.664	3.522.430	-45.189
3.551.028	-39.683	3.573.708	-43.661	3.522.589	-45.188
3.551.172	-39.686	3.573.919	-43.659	3.522.752	-45.187

3.551.343	-39.689	3.574.023	-43.656	3.522.894	-45.186
3.551.519	-39.692	3.574.165	-43.654	3.523.027	-45.184
3.551.671	-39.695	3.574.406	-43.651	3.523.195	-45.183
3.551.809	-39.698	3.574.572	-43.648	3.523.454	-45.182
3.551.978	-39.701	3.574.753	-43.646	3.523.698	-45.181
3.552.145	-39.704	3.574.825	-43.643	3.523.871	-45.179
3.552.274	-39.706	3.574.946	-43.640	3.523.990	-45.178
3.552.453	-39.709	3.575.201	-43.638	3.524.086	-45.177
3.552.629	-39.712	3.575.421	-43.635	3.524.256	-45.175
3.552.809	-39.715	3.575.574	-43.632	3.524.439	-45.174
3.552.977	-39.718	3.575.719	-43.629	3.524.669	-45.172
3.553.148	-39.721	3.575.967	-43.626	3.524.826	-45.171
3.553.362	-39.724	3.576.194	-43.624	3.524.897	-45.169
3.553.532	-39.728	3.576.339	-43.621	3.525.049	-45.168
3.553.705	-39.731	3.576.476	-43.618	3.525.282	-45.166
3.553.886	-39.734	3.576.653	-43.615	3.525.531	-45.164
3.554.048	-39.737	3.576.813	-43.612	3.525.715	-45.163
3.554.198	-39.740	3.576.928	-43.609	3.525.838	-45.161
3.554.345	-39.743	3.577.045	-43.606	3.525.992	-45.159
3.554.497	-39.746	3.577.444	-43.603	3.526.152	-45.157
3.554.656	-39.749	3.577.926	-43.600	3.526.278	-45.156
3.554.774	-39.752	3.578.113	-43.597	3.526.441	-45.154
3.554.895	-39.756	3.578.192	-43.594	3.526.703	-45.152
3.555.100	-39.759	3.578.248	-43.591	3.526.931	-45.150
3.555.302	-39.762	3.578.307	-43.588	3.527.106	-45.148
3.555.468	-39.765	3.578.374	-43.585	3.527.258	-45.146
3.555.569	-39.768	3.578.441	-43.581	3.527.379	-45.144
3.555.714	-39.772	3.578.513	-43.578	3.527.558	-45.142
3.555.924	-39.775	3.578.667	-43.575	3.527.711	-45.140
3.556.149	-39.778	3.578.822	-43.571	3.527.813	-45.138

3.556.307	-39.782	3.578.976	-43.568	3.527.935	-45.135
3.556.407	-39.785	3.579.236	-43.565	3.528.094	-45.133
3.556.555	-39.788	3.579.469	-43.561	3.528.330	-45.131
3.556.751	-39.792	3.579.617	-43.558	3.528.575	-45.129
3.556.967	-39.795	3.579.764	-43.554	3.528.702	-45.126
3.557.155	-39.798	3.579.982	-43.551	3.528.860	-45.124
3.557.334	-39.802	3.580.083	-43.547	3.529.099	-45.122
3.557.493	-39.805	3.580.229	-43.544	3.529.286	-45.119
3.557.687	-39.808	3.580.437	-43.540	3.529.442	-45.117
3.557.879	-39.812	3.580.617	-43.536	3.529.543	-45.115
3.557.986	-39.815	3.580.762	-43.533	3.529.669	-45.112
3.558.120	-39.819	3.580.901	-43.529	3.529.808	-45.110
3.558.277	-39.822	3.581.013	-43.525	3.529.940	-45.107
3.558.428	-39.826	3.581.111	-43.521	3.530.328	-45.105
3.558.593	-39.829	3.581.303	-43.517	3.530.793	-45.102
3.558.745	-39.833	3.581.512	-43.514	3.531.039	-45.100
3.558.923	-39.836	3.581.686	-43.510	3.531.165	-45.097
3.559.070	-39.840	3.581.862	-43.506	3.531.224	-45.094
3.559.203	-39.843	3.582.044	-43.502	3.531.277	-45.092
3.559.398	-39.847	3.582.267	-43.498	3.531.398	-45.089
3.559.641	-39.850	3.582.509	-43.494	3.531.451	-45.087
3.559.818	-39.854	3.582.712	-43.489	3.531.517	-45.084
3.559.964	-39.858	3.582.807	-43.485	3.531.638	-45.081
3.560.065	-39.861	3.582.896	-43.481	3.531.714	-45.078
3.560.108	-39.865	3.583.063	-43.477	3.531.864	-45.076
3.560.219	-39.868	3.583.247	-43.473	3.532.104	-45.073
3.560.408	-39.872	3.583.425	-43.468	3.532.278	-45.070
3.560.620	-39.876	3.583.629	-43.464	3.532.455	-45.067
3.560.768	-39.879	3.583.777	-43.460	3.532.560	-45.065
3.560.866	-39.883	3.583.907	-43.455	3.532.686	-45.062

3.561.086	-39.887	3.584.106	-43.451	3.532.933	-45.059
3.561.389	-39.891	3.584.262	-43.447	3.533.127	-45.056
3.561.537	-39.894	3.584.351	-43.442	3.533.282	-45.053
3.561.674	-39.898	3.584.516	-43.438	3.533.419	-45.051
3.561.820	-39.902	3.584.727	-43.433	3.533.546	-45.048
3.562.003	-39.906	3.584.930	-43.428	3.533.709	-45.045
3.562.254	-39.909	3.585.158	-43.424	3.533.878	-45.042
3.562.444	-39.913	3.585.338	-43.419	3.534.056	-45.039
3.562.599	-39.917	3.585.473	-43.415	3.534.232	-45.036
3.562.758	-39.921	3.585.632	-43.410	3.534.453	-45.034
3.562.849	-39.925	3.585.783	-43.405	3.534.608	-45.031
3.562.947	-39.929	3.585.981	-43.400	3.534.745	-45.028
3.563.175	-39.932	3.586.163	-43.396	3.534.898	-45.025
3.563.372	-39.936	3.586.302	-43.391	3.534.982	-45.022
3.563.542	-39.940	3.586.465	-43.386	3.535.173	-45.020
3.563.678	-39.944	3.586.636	-43.381	3.535.320	-45.017
3.563.799	-39.948	3.586.805	-43.376	3.535.511	-45.014
3.563.976	-39.952	3.586.929	-43.371	3.535.746	-45.011
3.564.200	-39.956	3.587.104	-43.366	3.535.872	-45.009
3.564.463	-39.960	3.587.282	-43.361	3.536.039	-45.006
3.564.655	-39.964	3.587.406	-43.356	3.536.248	-45.003
3.564.803	-39.968	3.587.563	-43.351	3.536.443	-45.000
3.564.923	-39.972	3.587.758	-43.346	3.536.568	-44.997
3.565.099	-39.976	3.587.925	-43.341	3.536.691	-44.995
3.565.316	-39.980	3.588.092	-43.336	3.536.881	-44.992
3.565.435	-39.984	3.588.266	-43.331	3.537.083	-44.989
3.565.558	-39.988	3.588.452	-43.326	3.537.285	-44.987
3.565.688	-39.992	3.588.627	-43.321	3.537.433	-44.984
3.565.844	-39.996	3.588.774	-43.315	3.537.553	-44.981
3.566.075	-40.000	3.588.900	-43.310	3.537.751	-44.978

3.566.293	-40.004	3.589.009	-43.305	3.537.872	-44.976
3.566.440	-40.008	3.589.149	-43.300	3.538.033	-44.973
3.566.588	-40.012	3.589.315	-43.294	3.538.248	-44.970
3.566.788	-40.016	3.589.463	-43.289	3.538.391	-44.968
3.566.947	-40.020	3.589.604	-43.284	3.538.555	-44.965
3.567.090	-40.025	3.589.789	-43.278	3.538.753	-44.963
3.567.289	-40.029	3.590.020	-43.273	3.538.872	-44.960
3.567.511	-40.033	3.590.216	-43.267	3.538.999	-44.957
3.567.668	-40.037	3.590.406	-43.262	3.539.200	-44.955
3.567.817	-40.041	3.590.576	-43.256	3.539.337	-44.952
3.567.957	-40.045	3.590.688	-43.251	3.539.435	-44.950
3.568.091	-40.050	3.590.949	-43.245	3.539.586	-44.947
3.568.225	-40.054	3.591.180	-43.240	3.539.778	-44.945
3.568.360	-40.058	3.591.302	-43.234	3.539.944	-44.942
3.568.557	-40.062	3.591.503	-43.229	3.540.186	-44.940
3.568.784	-40.067	3.591.656	-43.223	3.540.404	-44.937
3.568.972	-40.071	3.591.770	-43.217	3.540.547	-44.935
3.569.135	-40.075	3.591.902	-43.212	3.540.764	-44.933
3.569.346	-40.079	3.592.080	-43.206	3.540.974	-44.930
3.569.532	-40.084	3.592.251	-43.200	3.541.115	-44.928
3.569.610	-40.088	3.592.373	-43.194	3.541.237	-44.925
3.569.706	-40.092	3.592.536	-43.189	3.541.400	-44.923
3.570.123	-40.097	3.592.781	-43.183	3.541.597	-44.921
3.570.555	-40.101	3.593.026	-43.177	3.541.740	-44.918
3.570.649	-40.105	3.593.217	-43.171	3.541.885	-44.916
3.570.700	-40.110	3.593.389	-43.165	3.542.043	-44.914
3.570.849	-40.114	3.593.579	-43.160	3.542.159	-44.911
3.570.927	-40.118	3.593.694	-43.154	3.542.359	-44.909
3.571.000	-40.123	3.593.828	-43.148	3.542.575	-44.907
3.571.169	-40.127	3.594.041	-43.142	3.542.787	-44.904

3.571.302	-40.131	3.594.225	-43.136	3.542.971	-44.902
3.571.479	-40.136	3.594.411	-43.130	3.543.123	-44.899
3.571.656	-40.140	3.594.595	-43.124	3.543.230	-44.897
3.571.791	-40.144	3.594.734	-43.118	3.543.374	-44.895
3.571.954	-40.149	3.594.832	-43.112	3.543.597	-44.893
3.572.141	-40.153	3.594.953	-43.106	3.543.723	-44.890
3.572.314	-40.158	3.595.150	-43.100	3.543.766	-44.888
3.572.502	-40.162	3.595.392	-43.094	3.543.955	-44.886
3.572.711	-40.167	3.595.598	-43.088	3.544.221	-44.883
3.572.871	-40.171	3.595.757	-43.082	3.544.349	-44.881
3.573.065	-40.175	3.595.880	-43.076	3.544.498	-44.879
3.573.224	-40.180	3.595.999	-43.070	3.544.637	-44.876
3.573.293	-40.184	3.596.187	-43.064	3.544.763	-44.874
3.573.409	-40.189	3.596.387	-43.058	3.544.912	-44.872
3.573.602	-40.193	3.596.501	-43.052	3.545.061	-44.869
3.573.771	-40.198	3.596.599	-43.046	3.545.237	-44.867
3.573.947	-40.202	3.596.772	-43.040	3.545.429	-44.865
3.574.109	-40.207	3.596.942	-43.034	3.545.650	-44.863
3.574.306	-40.211	3.597.156	-43.027	3.545.891	-44.860
3.574.497	-40.216	3.597.359	-43.021	3.546.104	-44.858
3.574.651	-40.220	3.597.487	-43.015	3.546.293	-44.856
3.574.822	-40.225	3.597.705	-43.009	3.546.538	-44.853
3.574.963	-40.229	3.597.932	-43.003	3.546.729	-44.851
3.575.146	-40.234	3.598.083	-42.997	3.546.839	-44.849
3.575.353	-40.238	3.598.167	-42.991	3.546.980	-44.846
3.575.537	-40.243	3.598.377	-42.985	3.547.169	-44.844
3.575.722	-40.247	3.598.800	-42.978	3.547.343	-44.842
3.575.872	-40.252	3.599.117	-42.972	3.547.455	-44.839
3.575.949	-40.257	3.599.279	-42.966	3.547.593	-44.837
3.576.093	-40.261	3.599.346	-42.960	3.547.767	-44.835

3.576.289	-40.266	3.599.425	-42.954	3.547.937	-44.832
3.576.407	-40.270	3.599.566	-42.948	3.548.080	-44.830
3.576.546	-40.275	3.599.652	-42.941	3.548.221	-44.828
3.576.689	-40.279	3.599.728	-42.935	3.548.365	-44.825
3.576.790	-40.284	3.599.836	-42.929	3.548.463	-44.823
3.576.935	-40.288	3.599.967	-42.923	3.548.623	-44.821
3.577.188	-40.293	3.600.146	-42.917	3.548.867	-44.818
3.577.413	-40.298	3.600.346	-42.910	3.549.030	-44.816
3.577.540	-40.302	3.600.589	-42.904	3.549.138	-44.813
3.577.673	-40.307	3.600.800	-42.898	3.549.353	-44.811
3.577.870	-40.311	3.600.963	-42.892	3.549.635	-44.809
3.578.019	-40.316	3.601.149	-42.886	3.549.807	-44.806
3.578.206	-40.320	3.601.311	-42.879	3.549.909	-44.804
3.578.424	-40.325	3.601.484	-42.873	3.550.031	-44.802
3.578.597	-40.330	3.601.629	-42.867	3.550.223	-44.799
3.578.824	-40.334	3.601.758	-42.861	3.550.406	-44.797
3.579.014	-40.339	3.601.951	-42.854	3.550.588	-44.795
3.579.113	-40.343	3.602.088	-42.848	3.550.775	-44.792
3.579.286	-40.348	3.602.245	-42.842	3.550.920	-44.790
3.579.457	-40.353	3.602.369	-42.836	3.551.021	-44.787
3.579.586	-40.357	3.602.442	-42.830	3.551.217	-44.785
3.579.742	-40.362	3.602.593	-42.823	3.551.402	-44.783
3.579.922	-40.366	3.602.800	-42.817	3.551.523	-44.780
3.580.096	-40.371	3.603.031	-42.811	3.551.675	-44.778
3.580.211	-40.376	3.603.222	-42.805	3.551.866	-44.776
3.580.363	-40.380	3.603.358	-42.798	3.552.047	-44.773
3.580.599	-40.385	3.603.572	-42.792	3.552.292	-44.771
3.580.822	-40.390	3.603.831	-42.786	3.552.534	-44.768
3.581.001	-40.394	3.603.940	-42.780	3.552.647	-44.766
3.581.148	-40.399	3.604.041	-42.774	3.552.782	-44.764

3.581.329	-40.403	3.604.221	-42.767	3.552.946	-44.761
3.581.534	-40.408	3.604.374	-42.761	3.553.121	-44.759
3.581.656	-40.413	3.604.472	-42.755	3.553.271	-44.757
3.581.732	-40.417	3.604.613	-42.749	3.553.430	-44.754
3.581.862	-40.422	3.604.818	-42.742	3.553.649	-44.752
3.582.004	-40.427	3.605.017	-42.736	3.553.855	-44.750
3.582.105	-40.431	3.605.135	-42.730	3.553.994	-44.747
3.582.238	-40.436	3.605.289	-42.724	3.554.129	-44.745
3.582.428	-40.440	3.605.515	-42.718	3.554.323	-44.743
3.582.582	-40.445	3.605.646	-42.711	3.554.521	-44.740
3.582.751	-40.450	3.605.807	-42.705	3.554.680	-44.738
3.582.940	-40.454	3.605.990	-42.699	3.554.843	-44.736
3.583.214	-40.459	3.606.082	-42.693	3.555.028	-44.734
3.583.481	-40.464	3.606.315	-42.686	3.555.183	-44.731
3.583.644	-40.468	3.606.490	-42.680	3.555.334	-44.729
3.583.807	-40.473	3.606.618	-42.674	3.555.476	-44.727
3.583.958	-40.478	3.606.848	-42.668	3.555.571	-44.725
3.584.048	-40.482	3.607.036	-42.662	3.555.801	-44.722
3.584.255	-40.487	3.607.286	-42.655	3.556.227	-44.720
3.584.489	-40.492	3.607.464	-42.649	3.556.631	-44.718
3.584.592	-40.496	3.607.553	-42.643	3.556.904	-44.716
3.584.698	-40.501	3.607.742	-42.637	3.556.971	-44.714
3.584.848	-40.506	3.607.950	-42.631	3.556.967	-44.712
3.584.995	-40.510	3.608.051	-42.624	3.557.004	-44.709
3.585.161	-40.515	3.608.128	-42.618	3.557.047	-44.707
3.585.338	-40.520	3.608.254	-42.612	3.557.144	-44.705
3.585.476	-40.524	3.608.414	-42.606	3.557.272	-44.703
3.585.643	-40.529	3.608.737	-42.600	3.557.428	-44.701
3.585.870	-40.534	3.608.998	-42.593	3.557.592	-44.699
3.586.065	-40.538	3.609.068	-42.587	3.557.650	-44.697

3.586.228	-40.543	3.609.227	-42.581	3.557.753	-44.695
3.586.448	-40.548	3.609.456	-42.575	3.557.990	-44.692
3.586.632	-40.553	3.609.602	-42.569	3.558.214	-44.690
3.586.795	-40.557	3.609.742	-42.562	3.558.441	-44.688
3.586.954	-40.562	3.609.884	-42.556	3.558.633	-44.686
3.587.152	-40.567	3.610.053	-42.550	3.558.781	-44.684
3.587.350	-40.571	3.610.212	-42.544	3.558.954	-44.683
3.587.528	-40.576	3.610.316	-42.538	3.559.104	-44.681
3.587.680	-40.581	3.610.465	-42.531	3.559.272	-44.679
3.587.821	-40.586	3.610.634	-42.525	3.559.415	-44.677
3.588.000	-40.590	3.610.764	-42.519	3.559.563	-44.675
3.588.150	-40.595	3.610.910	-42.513	3.559.779	-44.673
3.588.266	-40.600	3.611.096	-42.506	3.559.987	-44.671
3.588.446	-40.605	3.611.324	-42.500	3.560.179	-44.670
3.588.681	-40.609	3.611.629	-42.494	3.560.347	-44.668
3.588.842	-40.614	3.611.877	-42.488	3.560.498	-44.666
3.589.008	-40.619	3.612.022	-42.481	3.560.625	-44.665
3.589.160	-40.624	3.612.145	-42.475	3.560.736	-44.663
3.589.330	-40.629	3.612.325	-42.469	3.560.858	-44.661
3.589.498	-40.633	3.612.474	-42.462	3.560.992	-44.660
3.589.623	-40.638	3.612.581	-42.456	3.561.187	-44.658
3.589.807	-40.643	3.612.715	-42.450	3.561.473	-44.657
3.590.058	-40.648	3.612.886	-42.443	3.561.664	-44.655
3.590.238	-40.653	3.613.044	-42.437	3.561.758	-44.653
3.590.346	-40.657	3.613.204	-42.431	3.561.906	-44.652
3.590.495	-40.662	3.613.329	-42.424	3.562.152	-44.651
3.590.682	-40.667	3.613.442	-42.418	3.562.375	-44.649
3.590.898	-40.672	3.613.620	-42.412	3.562.507	-44.648
3.591.036	-40.677	3.613.828	-42.405	3.562.682	-44.646
3.591.158	-40.682	3.614.068	-42.399	3.562.849	-44.645

3.591.224	-40.687	3.614.281	-42.392	3.562.950	-44.644
3.591.550	-40.691	3.614.364	-42.386	3.563.092	-44.643
3.592.068	-40.696	3.614.522	-42.380	3.563.229	-44.641
3.592.282	-40.701	3.614.760	-42.373	3.563.401	-44.640
3.592.321	-40.706	3.614.977	-42.367	3.563.641	-44.639
3.592.408	-40.711	3.615.169	-42.361	3.563.779	-44.638
3.592.529	-40.716	3.615.270	-42.354	3.563.950	-44.637
3.592.635	-40.721	3.615.443	-42.348	3.564.096	-44.636
3.592.708	-40.726	3.615.674	-42.341	3.564.233	-44.635
3.592.720	-40.731	3.615.830	-42.335	3.564.443	-44.634
3.592.838	-40.736	3.615.930	-42.329	3.564.558	-44.633
3.592.987	-40.741	3.616.069	-42.322	3.564.692	-44.632
3.593.159	-40.746	3.616.277	-42.316	3.564.890	-44.631
3.593.395	-40.751	3.616.476	-42.309	3.565.078	-44.630
3.593.582	-40.756	3.616.665	-42.303	3.565.255	-44.629
3.593.759	-40.761	3.616.857	-42.296	3.565.376	-44.628
3.593.941	-40.766	3.617.016	-42.290	3.565.504	-44.628
3.594.140	-40.771	3.617.180	-42.284	3.565.670	-44.627
3.594.317	-40.776	3.617.350	-42.277	3.565.876	-44.626
3.594.366	-40.781	3.617.538	-42.271	3.566.082	-44.625
3.594.443	-40.786	3.617.683	-42.264	3.566.256	-44.625
3.594.691	-40.791	3.617.799	-42.258	3.566.382	-44.624
3.594.944	-40.796	3.617.998	-42.252	3.566.584	-44.624
3.595.152	-40.801	3.618.199	-42.245	3.566.759	-44.623
3.595.292	-40.806	3.618.399	-42.239	3.566.983	-44.622
3.595.419	-40.811	3.618.550	-42.232	3.567.211	-44.622
3.595.555	-40.816	3.618.688	-42.226	3.567.278	-44.621
3.595.686	-40.822	3.618.842	-42.220	3.567.404	-44.621
3.595.921	-40.827	3.618.991	-42.213	3.567.545	-44.620
3.596.188	-40.832	3.619.161	-42.207	3.567.664	-44.620

3.596.427	-40.837	3.619.223	-42.200	3.567.847	-44.619
3.596.571	-40.842	3.619.493	-42.194	3.567.971	-44.619
3.596.675	-40.847	3.620.017	-42.188	3.568.168	-44.619
3.596.866	-40.853	3.620.328	-42.181	3.568.403	-44.618
3.597.041	-40.858	3.620.403	-42.175	3.568.584	-44.618
3.597.175	-40.863	3.620.401	-42.169	3.568.701	-44.617
3.597.318	-40.868	3.620.444	-42.162	3.568.878	-44.617
3.597.480	-40.874	3.620.530	-42.156	3.569.109	-44.617
3.597.659	-40.879	3.620.613	-42.150	3.569.281	-44.616
3.597.859	-40.884	3.620.739	-42.143	3.569.456	-44.616
3.598.002	-40.890	3.620.891	-42.137	3.569.586	-44.615
3.598.112	-40.895	3.621.004	-42.131	3.569.713	-44.615
3.598.250	-40.900	3.621.243	-42.124	3.569.873	-44.615
3.598.421	-40.906	3.621.488	-42.118	3.569.998	-44.614
3.598.601	-40.911	3.621.628	-42.112	3.570.154	-44.614
3.598.760	-40.917	3.621.821	-42.105	3.570.328	-44.614
3.598.948	-40.922	3.622.010	-42.099	3.570.477	-44.613
3.599.138	-40.927	3.622.192	-42.093	3.570.634	-44.613
3.599.301	-40.933	3.622.299	-42.087	3.570.787	-44.613
3.599.450	-40.938	3.622.473	-42.080	3.570.938	-44.612
3.599.539	-40.944	3.622.676	-42.074	3.571.055	-44.612
3.599.648	-40.949	3.622.769	-42.068	3.571.238	-44.611
3.599.887	-40.955	3.622.937	-42.062	3.571.487	-44.611
3.600.105	-40.960	3.623.136	-42.056	3.571.704	-44.611
3.600.258	-40.966	3.623.242	-42.050	3.571.909	-44.610
3.600.404	-40.972	3.623.372	-42.044	3.572.029	-44.610
3.600.565	-40.977	3.623.589	-42.037	3.572.156	-44.609
3.600.746	-40.983	3.623.731	-42.031	3.572.361	-44.609
3.600.953	-40.988	3.623.873	-42.025	3.572.601	-44.608
3.601.118	-40.994	3.624.034	-42.019	3.572.755	-44.608

3.601.252	-41.000	3.624.216	-42.013	3.572.865	-44.607
3.601.440	-41.006	3.624.453	-42.007	3.573.045	-44.607
3.601.604	-41.011	3.624.722	-42.001	3.573.235	-44.606
3.601.718	-41.017	3.624.940	-41.995	3.573.341	-44.606
3.601.830	-41.023	3.625.060	-41.990	3.573.489	-44.605
3.602.048	-41.029	3.625.143	-41.984	3.573.618	-44.605
3.602.289	-41.034	3.625.247	-41.978	3.573.760	-44.604
3.602.445	-41.040	3.625.435	-41.972	3.573.923	-44.603
3.602.626	-41.046	3.625.643	-41.966	3.574.079	-44.603
3.602.838	-41.052	3.625.782	-41.960	3.574.229	-44.602
3.603.004	-41.058	3.625.910	-41.954	3.574.338	-44.601
3.603.138	-41.064	3.626.089	-41.949	3.574.519	-44.601
3.603.275	-41.070	3.626.249	-41.943	3.574.731	-44.600
3.603.414	-41.076	3.626.364	-41.937	3.574.953	-44.599
3.603.542	-41.082	3.626.508	-41.932	3.575.177	-44.598
3.603.629	-41.088	3.626.696	-41.926	3.575.334	-44.598
3.603.756	-41.094	3.626.862	-41.920	3.575.512	-44.597
3.603.997	-41.100	3.627.054	-41.915	3.575.681	-44.596
3.604.205	-41.106	3.627.214	-41.909	3.575.885	-44.595
3.604.377	-41.112	3.627.344	-41.904	3.576.111	-44.594
3.604.561	-41.118	3.627.498	-41.898	3.576.230	-44.593
3.604.758	-41.124	3.627.704	-41.893	3.576.371	-44.592
3.604.991	-41.131	3.627.907	-41.887	3.576.609	-44.591
3.605.244	-41.137	3.628.076	-41.882	3.576.827	-44.590
3.605.455	-41.143	3.628.266	-41.876	3.576.940	-44.589
3.605.551	-41.149	3.628.441	-41.871	3.577.023	-44.588
3.605.701	-41.156	3.628.641	-41.866	3.577.155	-44.587
3.605.852	-41.162	3.628.846	-41.860	3.577.419	-44.586
3.605.960	-41.168	3.628.958	-41.855	3.577.646	-44.584
3.606.102	-41.175	3.629.086	-41.850	3.577.825	-44.583

3.606.252	-41.181	3.629.270	-41.844	3.578.026	-44.582
3.606.402	-41.188	3.629.438	-41.839	3.578.181	-44.580
3.606.537	-41.194	3.629.586	-41.834	3.578.289	-44.579
3.606.726	-41.201	3.629.760	-41.829	3.578.442	-44.578
3.606.899	-41.207	3.629.974	-41.824	3.578.611	-44.576
3.607.041	-41.214	3.630.099	-41.819	3.578.820	-44.575
3.607.227	-41.220	3.630.247	-41.814	3.579.057	-44.573
3.607.456	-41.227	3.630.475	-41.809	3.579.233	-44.572
3.607.641	-41.234	3.630.653	-41.804	3.579.317	-44.570
3.607.827	-41.240	3.630.772	-41.799	3.579.445	-44.569
3.608.029	-41.247	3.630.919	-41.794	3.579.622	-44.567
3.608.142	-41.254	3.631.131	-41.789	3.579.790	-44.565
3.608.277	-41.260	3.631.235	-41.784	3.580.002	-44.563
3.608.470	-41.267	3.631.293	-41.779	3.580.184	-44.562
3.608.658	-41.274	3.631.437	-41.774	3.580.349	-44.560
3.608.872	-41.281	3.631.597	-41.769	3.580.508	-44.558
3.609.001	-41.288	3.631.795	-41.764	3.580.677	-44.556
3.609.129	-41.295	3.632.018	-41.759	3.580.820	-44.554
3.609.337	-41.302	3.632.202	-41.754	3.580.977	-44.552
3.609.440	-41.309	3.632.435	-41.749	3.581.119	-44.550
3.609.583	-41.316	3.632.664	-41.744	3.581.260	-44.548
3.609.751	-41.323	3.632.882	-41.739	3.581.433	-44.546
3.609.935	-41.330	3.633.091	-41.735	3.581.643	-44.544
3.610.104	-41.337	3.633.242	-41.730	3.582.023	-44.541
3.610.247	-41.344	3.633.387	-41.725	3.582.437	-44.539
3.610.459	-41.351	3.633.499	-41.720	3.582.599	-44.537
3.610.681	-41.359	3.633.667	-41.715	3.582.697	-44.534
3.610.869	-41.366	3.633.835	-41.710	3.582.827	-44.532
3.611.001	-41.373	3.633.901	-41.706	3.582.878	-44.530
3.611.154	-41.380	3.633.992	-41.701	3.582.943	-44.527

3.611.344	-41.388	3.634.136	-41.696	3.583.029	-44.524
3.611.538	-41.395	3.634.305	-41.691	3.583.170	-44.522
3.611.740	-41.403	3.634.550	-41.687	3.583.284	-44.519
3.611.902	-41.410	3.634.742	-41.682	3.583.435	-44.516
3.612.045	-41.418	3.634.894	-41.677	3.583.633	-44.514
3.612.180	-41.425	3.635.020	-41.672	3.583.807	-44.511
3.612.361	-41.433	3.635.179	-41.668	3.583.984	-44.508
3.612.511	-41.441	3.635.425	-41.663	3.584.110	-44.505
3.612.625	-41.448	3.635.638	-41.658	3.584.326	-44.502
3.612.799	-41.456	3.635.842	-41.653	3.584.547	-44.499
3.613.171	-41.464	3.636.028	-41.649	3.584.677	-44.496
3.613.576	-41.472	3.636.180	-41.644	3.584.818	-44.493
3.613.763	-41.479	3.636.286	-41.639	3.584.988	-44.490
3.613.847	-41.487	3.636.426	-41.634	3.585.247	-44.487
3.613.915	-41.495	3.636.643	-41.630	3.585.457	-44.484
3.613.984	-41.503	3.636.821	-41.625	3.585.603	-44.481
3.614.027	-41.511	3.636.964	-41.620	3.585.777	-44.478
3.614.062	-41.520	3.637.131	-41.615	3.585.916	-44.475
3.614.169	-41.528	3.637.325	-41.611	3.586.042	-44.471
3.614.366	-41.536	3.637.495	-41.606	3.586.172	-44.468
3.614.511	-41.544	3.637.698	-41.601	3.586.362	-44.465
3.614.641	-41.553	3.637.876	-41.596	3.586.595	-44.461
3.614.872	-41.561	3.637.915	-41.591	3.586.694	-44.458
3.615.063	-41.569	3.638.078	-41.587	3.586.781	-44.455
3.615.220	-41.578	3.638.324	-41.582	3.586.971	-44.451
3.615.426	-41.587	3.638.456	-41.577	3.587.174	-44.448
3.615.621	-41.595	3.638.612	-41.572	3.587.334	-44.444
3.615.771	-41.604	3.638.846	-41.567	3.587.491	-44.441
3.615.930	-41.613	3.639.111	-41.562	3.587.700	-44.437
3.616.091	-41.622	3.639.287	-41.558	3.587.925	-44.433

3.616.237	-41.631	3.639.381	-41.553	3.588.096	-44.430
3.616.386	-41.640	3.639.554	-41.548	3.588.284	-44.426
3.616.559	-41.649	3.639.760	-41.543	3.588.475	-44.422
3.616.685	-41.658	3.639.887	-41.538	3.588.615	-44.419
3.616.815	-41.667	3.640.034	-41.533	3.588.741	-44.415
3.616.951	-41.676	3.640.211	-41.528	3.588.882	-44.411
3.617.137	-41.686	3.640.371	-41.523	3.589.049	-44.408
3.617.357	-41.695	3.640.677	-41.518	3.589.218	-44.404
3.617.546	-41.705	3.641.080	-41.513	3.589.396	-44.400
3.617.766	-41.715	3.641.326	-41.508	3.589.521	-44.396
3.617.910	-41.724	3.641.419	-41.503	3.589.641	-44.392
3.618.019	-41.734	3.641.488	-41.498	3.589.804	-44.388
3.618.239	-41.744	3.641.544	-41.493	3.589.966	-44.385
3.618.472	-41.754	3.641.628	-41.488	3.590.148	-44.381
3.618.586	-41.764	3.641.656	-41.483	3.590.345	-44.377
3.618.727	-41.774	3.641.750	-41.478	3.590.562	-44.373
3.618.948	-41.785	3.641.918	-41.472	3.590.766	-44.369
3.619.135	-41.795	3.642.094	-41.467	3.590.869	-44.365
3.619.272	-41.806	3.642.332	-41.462	3.590.995	-44.361
3.619.377	-41.816	3.642.532	-41.457	3.591.154	-44.357
3.619.529	-41.827	3.642.680	-41.452	3.591.333	-44.353
3.619.663	-41.838	3.642.832	-41.446	3.591.521	-44.349
3.619.839	-41.849	3.643.019	-41.441	3.591.661	-44.345
3.620.072	-41.860	3.643.177	-41.436	3.591.812	-44.341
3.620.262	-41.871	3.643.330	-41.431	3.592.055	-44.337
3.620.447	-41.882	3.643.546	-41.425	3.592.278	-44.333
3.620.612	-41.894	3.643.676	-41.420	3.592.438	-44.329
3.620.699	-41.905	3.643.799	-41.415	3.592.599	-44.325
3.620.826	-41.917	3.643.965	-41.409	3.592.682	-44.320
3.621.043	-41.929	3.644.066	-41.404	3.592.809	-44.316

3.621.208	-41.941	3.644.240	-41.398	3.593.018	-44.312
3.621.404	-41.953	3.644.420	-41.393	3.593.196	-44.308
3.621.606	-41.965	3.644.550	-41.387	3.593.372	-44.304
3.621.738	-41.977	3.644.698	-41.382	3.593.566	-44.299
3.621.895	-41.989	3.644.904	-41.377	3.593.811	-44.295
3.622.052	-42.002	3.645.137	-41.371	3.593.997	-44.291
3.622.213	-42.015	3.645.368	-41.365	3.594.081	-44.287
3.622.351	-42.027	3.645.562	-41.360	3.594.222	-44.282
3.622.470	-42.040	3.645.722	-41.354	3.594.409	-44.278
3.622.676	-42.053	3.645.787	-41.349	3.594.565	-44.273
3.622.892	-42.066	3.645.919	-41.343	3.594.760	-44.269
3.623.062	-42.080	3.646.143	-41.338	3.594.969	-44.265
3.623.208	-42.093	3.646.296	-41.332	3.595.049	-44.260
3.623.318	-42.107	3.646.492	-41.326	3.595.121	-44.256
3.623.539	-42.120	3.646.690	-41.321	3.595.313	-44.251
3.623.811	-42.134	3.646.795	-41.315	3.595.562	-44.247
3.623.929	-42.148	3.646.917	-41.309	3.595.739	-44.242
3.624.031	-42.162	3.647.113	-41.304	3.595.844	-44.238
3.624.218	-42.176	3.647.316	-41.298	3.595.953	-44.233
3.624.386	-42.191	3.647.453	-41.292	3.596.089	-44.229
3.624.501	-42.205	3.647.614	-41.286	3.596.227	-44.224
3.624.583	-42.220	3.647.836	-41.281	3.596.422	-44.219
3.624.695	-42.235	3.648.040	-41.275	3.596.618	-44.215
3.624.872	-42.250	3.648.214	-41.269	3.596.743	-44.210
3.625.035	-42.265	3.648.322	-41.263	3.596.917	-44.205
3.625.190	-42.280	3.648.443	-41.258	3.597.079	-44.201
3.625.327	-42.295	3.648.579	-41.252	3.597.282	-44.196
3.625.504	-42.311	3.648.746	-41.246	3.597.520	-44.191
3.625.639	-42.327	3.648.967	-41.240	3.597.749	-44.187
3.625.838	-42.343	3.649.156	-41.234	3.597.908	-44.182

3.626.209	-42.359	3.649.269	-41.228	3.598.060	-44.177
3.626.440	-42.375	3.649.436	-41.222	3.598.320	-44.172
3.626.566	-42.391	3.649.706	-41.217	3.598.463	-44.167
3.626.714	-42.407	3.649.868	-41.211	3.598.546	-44.162
3.626.877	-42.424	3.649.962	-41.205	3.598.731	-44.158
3.627.025	-42.441	3.650.122	-41.199	3.598.919	-44.153
3.627.231	-42.458	3.650.294	-41.193	3.599.078	-44.148
3.627.381	-42.475	3.650.477	-41.187	3.599.265	-44.143
3.627.466	-42.492	3.650.659	-41.181	3.599.359	-44.138
3.627.643	-42.509	3.650.804	-41.175	3.599.461	-44.133
3.627.748	-42.527	3.650.986	-41.169	3.599.648	-44.128
3.627.867	-42.544	3.651.154	-41.163	3.599.796	-44.123
3.628.083	-42.562	3.651.306	-41.157	3.599.942	-44.118
3.628.304	-42.580	3.651.461	-41.151	3.600.114	-44.113
3.628.495	-42.598	3.651.613	-41.145	3.600.294	-44.108
3.628.708	-42.616	3.651.786	-41.139	3.600.504	-44.103
3.628.934	-42.635	3.651.866	-41.133	3.600.725	-44.098
3.629.073	-42.653	3.652.040	-41.127	3.600.921	-44.093
3.629.238	-42.672	3.652.220	-41.121	3.601.133	-44.087
3.629.396	-42.691	3.652.372	-41.115	3.601.340	-44.082
3.629.540	-42.710	3.652.545	-41.109	3.601.481	-44.077
3.629.720	-42.729	3.652.632	-41.103	3.601.611	-44.072
3.629.909	-42.748	3.652.817	-41.096	3.601.850	-44.067
3.630.110	-42.767	3.653.076	-41.090	3.602.058	-44.062
3.630.244	-42.787	3.653.276	-41.084	3.602.144	-44.056
3.630.320	-42.806	3.653.443	-41.078	3.602.242	-44.051
3.630.432	-42.826	3.653.566	-41.072	3.602.354	-44.046
3.630.625	-42.846	3.653.775	-41.065	3.602.529	-44.041
3.630.870	-42.866	3.654.031	-41.059	3.602.763	-44.035
3.631.054	-42.886	3.654.175	-41.053	3.602.953	-44.030

3.631.208	-42.906	3.654.338	-41.047	3.603.125	-44.025
3.631.376	-42.927	3.654.514	-41.040	3.603.315	-44.020
3.631.509	-42.947	3.654.657	-41.034	3.603.489	-44.014
3.631.680	-42.968	3.654.822	-41.028	3.603.625	-44.009
3.631.882	-42.988	3.654.985	-41.022	3.603.781	-44.004
3.632.022	-43.009	3.655.110	-41.015	3.603.976	-43.998
3.632.204	-43.030	3.655.235	-41.009	3.604.146	-43.993
3.632.387	-43.051	3.655.404	-41.003	3.604.323	-43.988
3.632.528	-43.072	3.655.562	-40.996	3.604.469	-43.982
3.632.679	-43.093	3.655.732	-40.990	3.604.633	-43.977
3.632.881	-43.115	3.656.000	-40.983	3.604.840	-43.972
3.633.060	-43.136	3.656.210	-40.977	3.604.988	-43.966
3.633.174	-43.157	3.656.351	-40.971	3.605.099	-43.961
3.633.336	-43.179	3.656.541	-40.964	3.605.255	-43.955
3.633.532	-43.201	3.656.730	-40.958	3.605.441	-43.950
3.633.703	-43.222	3.656.867	-40.951	3.605.627	-43.945
3.633.822	-43.244	3.657.008	-40.945	3.605.800	-43.939
3.633.973	-43.266	3.657.159	-40.938	3.606.010	-43.934
3.634.259	-43.288	3.657.315	-40.932	3.606.155	-43.928
3.634.695	-43.310	3.657.505	-40.925	3.606.315	-43.923
3.634.987	-43.332	3.657.726	-40.919	3.606.564	-43.917
3.635.110	-43.354	3.657.915	-40.912	3.606.756	-43.912
3.635.273	-43.376	3.658.053	-40.906	3.606.906	-43.907
3.635.349	-43.398	3.658.189	-40.899	3.607.055	-43.901
3.635.323	-43.420	3.658.365	-40.893	3.607.173	-43.896
3.635.410	-43.442	3.658.551	-40.886	3.607.300	-43.890
3.635.555	-43.465	3.658.698	-40.879	3.607.635	-43.885
3.635.627	-43.487	3.658.847	-40.873	3.608.112	-43.879
3.635.782	-43.509	3.658.995	-40.866	3.608.387	-43.874
3.635.990	-43.532	3.659.185	-40.860	3.608.450	-43.868

3.636.115	-43.554	3.659.377	-40.853	3.608.521	-43.863
3.636.292	-43.576	3.659.507	-40.846	3.608.644	-43.858
3.636.528	-43.599	3.659.649	-40.840	3.608.716	-43.852
3.636.733	-43.621	3.659.829	-40.833	3.608.761	-43.847
3.636.896	-43.644	3.660.038	-40.826	3.608.842	-43.841
3.637.019	-43.666	3.660.248	-40.820	3.608.938	-43.836
3.637.182	-43.689	3.660.430	-40.813	3.609.068	-43.830
3.637.325	-43.711	3.660.614	-40.806	3.609.265	-43.825
3.637.473	-43.733	3.660.771	-40.799	3.609.460	-43.819
3.637.646	-43.756	3.660.917	-40.793	3.609.639	-43.814
3.637.838	-43.778	3.661.044	-40.786	3.609.807	-43.808
3.637.977	-43.801	3.661.194	-40.779	3.610.018	-43.803
3.638.076	-43.823	3.661.440	-40.772	3.610.230	-43.798
3.638.237	-43.845	3.661.755	-40.766	3.610.367	-43.792
3.638.416	-43.868	3.662.099	-40.759	3.610.565	-43.787
3.638.568	-43.890	3.662.350	-40.752	3.610.782	-43.781
3.638.774	-43.912	3.662.455	-40.745	3.610.934	-43.776
3.639.003	-43.934	3.662.489	-40.738	3.611.039	-43.771
3.639.200	-43.957	3.662.545	-40.732	3.611.154	-43.765
3.639.349	-43.979	3.662.599	-40.725	3.611.278	-43.760
3.639.483	-44.001	3.662.675	-40.718	3.611.422	-43.755
3.639.659	-44.023	3.662.832	-40.711	3.611.649	-43.749
3.639.787	-44.045	3.663.044	-40.704	3.611.815	-43.744
3.639.970	-44.067	3.663.239	-40.697	3.611.918	-43.739
3.640.211	-44.089	3.663.425	-40.690	3.612.029	-43.734
3.640.378	-44.111	3.663.593	-40.683	3.612.162	-43.729
3.640.493	-44.133	3.663.702	-40.677	3.612.315	-43.723
3.640.638	-44.154	3.663.860	-40.670	3.612.491	-43.718
3.640.780	-44.176	3.664.024	-40.663	3.612.711	-43.713
3.640.935	-44.198	3.664.212	-40.656	3.612.943	-43.708

3.641.140	-44.219	3.664.417	-40.649	3.613.134	-43.703
3.641.267	-44.241	3.664.584	-40.642	3.613.312	-43.698
3.641.339	-44.262	3.664.720	-40.635	3.613.536	-43.693
3.641.497	-44.284	3.664.891	-40.628	3.613.708	-43.688
3.641.743	-44.305	3.665.089	-40.621	3.613.821	-43.683
3.641.882	-44.326	3.665.215	-40.614	3.613.974	-43.678
3.641.961	-44.348	3.665.307	-40.607	3.614.135	-43.673
3.642.112	-44.369	3.665.421	-40.600	3.614.211	-43.668
3.642.228	-44.390	3.665.583	-40.593	3.614.364	-43.663
3.642.319	-44.411	3.665.771	-40.586	3.614.601	-43.658
3.642.532	-44.431	3.665.952	-40.578	3.614.781	-43.653
3.642.785	-44.452	3.666.172	-40.571	3.614.937	-43.649
3.642.892	-44.473	3.666.380	-40.564	3.615.060	-43.644
3.643.024	-44.493	3.666.526	-40.557	3.615.211	-43.639
3.643.228	-44.514	3.666.733	-40.550	3.615.367	-43.634
3.643.352	-44.534	3.666.960	-40.543	3.615.547	-43.630
3.643.501	-44.554	3.667.097	-40.536	3.615.733	-43.625
3.643.702	-44.575	3.667.240	-40.529	3.615.829	-43.620
3.643.820	-44.595	3.667.299	-40.522	3.616.003	-43.616
3.643.977	-44.615	3.667.366	-40.514	3.616.263	-43.611
3.644.189	-44.635	3.667.578	-40.507	3.616.479	-43.607
3.644.315	-44.654	3.667.867	-40.500	3.616.628	-43.602
3.644.533	-44.674	3.668.040	-40.493	3.616.751	-43.598
3.644.756	-44.694	3.668.159	-40.486	3.616.924	-43.593
3.644.861	-44.713	3.668.347	-40.479	3.617.052	-43.589
3.645.005	-44.732	3.668.513	-40.471	3.617.214	-43.585
3.645.255	-44.752	3.668.674	-40.464	3.617.387	-43.580
3.645.457	-44.771	3.668.826	-40.457	3.617.581	-43.576
3.645.526	-44.790	3.669.012	-40.450	3.617.733	-43.572
3.645.645	-44.809	3.669.174	-40.442	3.617.930	-43.567

3.645.775	-44.827	3.669.250	-40.435	3.618.112	-43.563
3.645.876	-44.846	3.669.442	-40.428	3.618.290	-43.559
3.645.970	-44.864	3.669.702	-40.421	3.618.466	-43.555
3.646.122	-44.883	3.669.913	-40.413	3.618.654	-43.550
3.646.396	-44.901	3.670.089	-40.406	3.618.828	-43.546
3.646.593	-44.919	3.670.229	-40.399	3.618.951	-43.542
3.646.709	-44.937	3.670.421	-40.391	3.619.044	-43.538
3.646.891	-44.955	3.670.606	-40.384	3.619.236	-43.534
3.647.104	-44.973	3.670.769	-40.377	3.619.467	-43.530
3.647.375	-44.990	3.670.909	-40.369	3.619.612	-43.526
3.647.690	-45.008	3.671.087	-40.362	3.619.738	-43.522
3.647.818	-45.025	3.671.253	-40.355	3.619.915	-43.518
3.647.917	-45.042	3.671.389	-40.347	3.620.117	-43.514
3.648.113	-45.059	3.671.588	-40.340	3.620.255	-43.510
3.648.279	-45.076	3.671.776	-40.333	3.620.422	-43.506
3.648.377	-45.093	3.671.927	-40.325	3.620.650	-43.502
3.648.489	-45.110	3.672.117	-40.318	3.620.807	-43.498
3.648.666	-45.126	3.672.274	-40.310	3.620.892	-43.494
3.648.837	-45.143	3.672.415	-40.303	3.621.044	-43.490
3.648.959	-45.159	3.672.601	-40.295	3.621.299	-43.486
3.649.120	-45.175	3.672.705	-40.288	3.621.501	-43.482
3.649.341	-45.191	3.672.842	-40.280	3.621.585	-43.478
3.649.522	-45.206	3.673.063	-40.273	3.621.625	-43.474
3.649.706	-45.222	3.673.196	-40.265	3.621.730	-43.471
3.649.958	-45.238	3.673.291	-40.258	3.621.909	-43.467
3.650.147	-45.253	3.673.434	-40.250	3.622.086	-43.463
3.650.266	-45.268	3.673.551	-40.243	3.622.281	-43.459
3.650.390	-45.283	3.673.713	-40.235	3.622.480	-43.455
3.650.549	-45.298	3.673.904	-40.227	3.622.633	-43.451
3.650.735	-45.313	3.674.070	-40.220	3.622.802	-43.447

3.650.957	-45.327	3.674.232	-40.212	3.623.040	-43.444
3.651.172	-45.341	3.674.454	-40.204	3.623.302	-43.440
3.651.307	-45.356	3.674.779	-40.197	3.623.529	-43.436
3.651.512	-45.370	3.674.982	-40.189	3.623.630	-43.432
3.651.721	-45.384	3.675.111	-40.181	3.623.813	-43.428
3.651.871	-45.397	3.675.233	-40.173	3.624.010	-43.424
3.652.036	-45.411	3.675.368	-40.166	3.624.164	-43.421
3.652.141	-45.424	3.675.558	-40.158	3.624.250	-43.417
3.652.299	-45.438	3.675.708	-40.150	3.624.413	-43.413
3.652.523	-45.451	3.675.829	-40.142	3.624.609	-43.409
3.652.658	-45.463	3.675.941	-40.134	3.624.727	-43.405
3.652.864	-45.476	3.676.086	-40.127	3.624.870	-43.402
3.653.047	-45.489	3.676.273	-40.119	3.625.070	-43.398
3.653.154	-45.501	3.676.414	-40.111	3.625.216	-43.394
3.653.271	-45.513	3.676.617	-40.103	3.625.359	-43.390
3.653.423	-45.526	3.676.855	-40.095	3.625.527	-43.386
3.653.633	-45.537	3.677.045	-40.087	3.625.645	-43.382
3.653.790	-45.549	3.677.160	-40.079	3.625.840	-43.379
3.653.984	-45.561	3.677.332	-40.071	3.626.061	-43.375
3.654.196	-45.572	3.677.587	-40.063	3.626.267	-43.371
3.654.342	-45.583	3.677.802	-40.055	3.626.467	-43.367
3.654.521	-45.594	3.677.986	-40.047	3.626.646	-43.363
3.654.656	-45.605	3.678.163	-40.039	3.626.791	-43.359
3.654.821	-45.616	3.678.333	-40.031	3.626.961	-43.356
3.655.013	-45.626	3.678.500	-40.023	3.627.169	-43.352
3.655.079	-45.637	3.678.721	-40.015	3.627.315	-43.348
3.655.284	-45.647	3.678.854	-40.007	3.627.466	-43.344
3.655.753	-45.657	3.678.932	-39.999	3.627.579	-43.340
3.656.158	-45.666	3.679.149	-39.991	3.627.776	-43.336
3.656.318	-45.676	3.679.356	-39.983	3.628.011	-43.332

3.656.339	-45.685	3.679.496	-39.975	3.628.192	-43.329
3.656.407	-45.695	3.679.688	-39.967	3.628.394	-43.325
3.656.490	-45.704	3.679.879	-39.959	3.628.584	-43.321
3.656.577	-45.713	3.680.023	-39.951	3.628.690	-43.317
3.656.689	-45.721	3.680.161	-39.943	3.628.761	-43.313
3.656.816	-45.730	3.680.298	-39.935	3.628.855	-43.309
3.656.957	-45.738	3.680.504	-39.926	3.629.038	-43.305
3.656.958	-45.746	3.680.693	-39.918	3.629.219	-43.301
3.657.151	-45.754	3.680.847	-39.910	3.629.410	-43.298
3.657.473	-45.762	3.680.999	-39.902	3.629.631	-43.294
3.657.697	-45.769	3.681.133	-39.894	3.629.841	-43.290
3.657.954	-45.777	3.681.317	-39.886	3.629.977	-43.286
3.658.164	-45.784	3.681.498	-39.878	3.630.150	-43.282
3.658.264	-45.791	3.681.654	-39.869	3.630.318	-43.278
3.658.355	-45.798	3.681.819	-39.861	3.630.428	-43.274
3.658.539	-45.804	3.682.034	-39.853	3.630.617	-43.270
3.658.717	-45.811	3.682.180	-39.845	3.630.793	-43.266
3.658.858	-45.817	3.682.274	-39.837	3.630.928	-43.263
3.659.028	-45.823	3.682.565	-39.829	3.631.096	-43.259
3.659.279	-45.829	3.683.023	-39.821	3.631.300	-43.255
3.659.403	-45.835	3.683.324	-39.813	3.631.494	-43.251
3.659.493	-45.840	3.683.439	-39.804	3.631.693	-43.247
3.659.648	-45.845	3.683.492	-39.796	3.631.907	-43.243
3.659.765	-45.850	3.683.513	-39.788	3.632.058	-43.239
3.659.946	-45.855	3.683.564	-39.780	3.632.180	-43.235
3.660.168	-45.860	3.683.619	-39.772	3.632.336	-43.232
3.660.404	-45.864	3.683.719	-39.764	3.632.499	-43.228
3.660.612	-45.869	3.683.875	-39.756	3.632.652	-43.224
3.660.757	-45.873	3.684.030	-39.748	3.632.782	-43.220
3.660.939	-45.877	3.684.219	-39.740	3.633.063	-43.216

3.661.086	-45.880	3.684.396	-39.732	3.633.544	-43.212
3.661.185	-45.884	3.684.552	-39.724	3.633.840	-43.208
3.661.353	-45.887	3.684.756	-39.716	3.633.954	-43.204
3.661.483	-45.890	3.684.996	-39.708	3.634.093	-43.200
3.661.604	-45.893	3.685.155	-39.699	3.634.182	-43.197
3.661.839	-45.896	3.685.305	-39.691	3.634.247	-43.193
3.662.086	-45.898	3.685.461	-39.683	3.634.298	-43.189
3.662.211	-45.901	3.685.621	-39.675	3.634.370	-43.185
3.662.361	-45.903	3.685.829	-39.667	3.634.460	-43.181
3.662.521	-45.905	3.686.008	-39.659	3.634.612	-43.177
3.662.612	-45.907	3.686.163	-39.651	3.634.783	-43.173
3.662.776	-45.908	3.686.314	-39.644	3.634.902	-43.169
3.662.976	-45.910	3.686.454	-39.636	3.634.969	-43.165
3.663.120	-45.911	3.686.578	-39.628	3.635.161	-43.161
3.663.283	-45.912	3.686.732	-39.620	3.635.406	-43.157
3.663.420	-45.913	3.686.933	-39.612	3.635.648	-43.154
3.663.549	-45.914	3.687.081	-39.604	3.635.816	-43.150
3.663.789	-45.914	3.687.196	-39.596	3.635.913	-43.146
3.664.030	-45.915	3.687.456	-39.588	3.636.093	-43.142
3.664.196	-45.915	3.687.809	-39.580	3.636.324	-43.138
3.664.342	-45.915	3.687.979	-39.573	3.636.495	-43.134
3.664.526	-45.915	3.688.024	-39.565	3.636.657	-43.130
3.664.680	-45.914	3.688.149	-39.557	3.636.803	-43.127
3.664.810	-45.914	3.688.323	-39.549	3.636.898	-43.123
3.664.958	-45.913	3.688.526	-39.541	3.637.048	-43.119
3.665.164	-45.913	3.688.709	-39.534	3.637.279	-43.115
3.665.352	-45.912	3.688.828	-39.526	3.637.415	-43.111
3.665.511	-45.911	3.688.949	-39.518	3.637.545	-43.108
3.665.729	-45.909	3.689.092	-39.510	3.637.731	-43.104
3.665.891	-45.908	3.689.262	-39.503	3.637.846	-43.100

3.666.061	-45.907	3.689.421	-39.495	3.637.997	-43.096
3.666.227	-45.905	3.689.579	-39.487	3.638.225	-43.092
3.666.317	-45.903	3.689.711	-39.480	3.638.369	-43.089
3.666.437	-45.901	3.689.898	-39.472	3.638.551	-43.085
3.666.664	-45.899	3.690.125	-39.465	3.638.789	-43.081
3.666.914	-45.897	3.690.244	-39.457	3.638.925	-43.078
3.667.061	-45.895	3.690.371	-39.449	3.639.146	-43.074
3.667.189	-45.893	3.690.509	-39.442	3.639.393	-43.070
3.667.328	-45.890	3.690.706	-39.434	3.639.514	-43.067
3.667.459	-45.888	3.690.957	-39.427	3.639.635	-43.063
3.667.608	-45.885	3.691.113	-39.419	3.639.834	-43.059
3.667.735	-45.882	3.691.274	-39.412	3.640.009	-43.056
3.667.876	-45.879	3.691.476	-39.404	3.640.139	-43.052
3.668.076	-45.876	3.691.693	-39.397	3.640.312	-43.049
3.668.280	-45.873	3.691.826	-39.389	3.640.490	-43.045
3.668.497	-45.870	3.691.953	-39.382	3.640.599	-43.042
3.668.705	-45.867	3.692.079	-39.374	3.640.754	-43.038
3.668.864	-45.863	3.692.144	-39.367	3.640.981	-43.034
3.669.037	-45.860	3.692.307	-39.359	3.641.161	-43.031
3.669.226	-45.856	3.692.560	-39.352	3.641.328	-43.027
3.669.415	-45.853	3.692.782	-39.345	3.641.491	-43.024
3.669.601	-45.849	3.692.907	-39.337	3.641.640	-43.021
3.669.745	-45.846	3.692.984	-39.330	3.641.791	-43.017
3.669.872	-45.842	3.693.214	-39.322	3.641.957	-43.014
3.670.006	-45.838	3.693.486	-39.315	3.642.166	-43.010
3.670.137	-45.834	3.693.623	-39.308	3.642.339	-43.007
3.670.284	-45.830	3.693.779	-39.300	3.642.464	-43.003
3.670.446	-45.826	3.693.914	-39.293	3.642.608	-43.000
3.670.591	-45.822	3.694.039	-39.286	3.642.759	-42.997
3.670.771	-45.818	3.694.227	-39.278	3.642.935	-42.993

3.670.942	-45.814	3.694.344	-39.271	3.643.114	-42.990
3.671.109	-45.810	3.694.478	-39.263	3.643.284	-42.987
3.671.351	-45.806	3.694.666	-39.256	3.643.474	-42.983
3.671.599	-45.802	3.694.803	-39.249	3.643.719	-42.980
3.671.711	-45.797	3.694.940	-39.241	3.643.943	-42.977
3.671.826	-45.793	3.695.175	-39.234	3.644.056	-42.973
3.671.998	-45.789	3.695.435	-39.227	3.644.160	-42.970
3.672.128	-45.784	3.695.635	-39.219	3.644.315	-42.967
3.672.258	-45.780	3.695.848	-39.212	3.644.523	-42.963
3.672.480	-45.776	3.696.006	-39.204	3.644.707	-42.960
3.672.669	-45.771	3.696.167	-39.197	3.644.811	-42.957
3.672.874	-45.767	3.696.385	-39.189	3.644.945	-42.953
3.673.045	-45.763	3.696.523	-39.182	3.645.076	-42.950
3.673.168	-45.758	3.696.664	-39.174	3.645.209	-42.947
3.673.354	-45.754	3.696.816	-39.167	3.645.458	-42.943
3.673.547	-45.749	3.696.906	-39.159	3.645.620	-42.940
3.673.717	-45.745	3.697.083	-39.152	3.645.800	-42.937
3.673.836	-45.741	3.697.243	-39.144	3.645.999	-42.933
3.673.979	-45.736	3.697.340	-39.137	3.646.174	-42.930
3.674.176	-45.732	3.697.513	-39.129	3.646.300	-42.926
3.674.321	-45.727	3.697.686	-39.121	3.646.451	-42.923
3.674.501	-45.723	3.697.901	-39.114	3.646.669	-42.919
3.674.725	-45.719	3.698.116	-39.106	3.646.766	-42.916
3.674.894	-45.714	3.698.233	-39.098	3.646.925	-42.912
3.675.029	-45.710	3.698.396	-39.091	3.647.163	-42.909
3.675.244	-45.706	3.698.647	-39.083	3.647.265	-42.905
3.675.375	-45.701	3.698.851	-39.075	3.647.361	-42.902
3.675.472	-45.697	3.698.991	-39.068	3.647.553	-42.898
3.675.733	-45.693	3.699.135	-39.060	3.647.762	-42.894
3.675.982	-45.689	3.699.321	-39.052	3.647.870	-42.891

3.676.129	-45.684	3.699.510	-39.044	3.648.011	-42.887
3.676.266	-45.680	3.699.680	-39.036	3.648.214	-42.883
3.676.371	-45.676	3.699.843	-39.028	3.648.412	-42.880
3.676.426	-45.672	3.700.020	-39.021	3.648.597	-42.876
3.676.610	-45.668	3.700.166	-39.013	3.648.790	-42.872
3.676.992	-45.664	3.700.357	-39.005	3.649.030	-42.868
3.677.415	-45.660	3.700.565	-38.997	3.649.266	-42.865
3.677.687	-45.656	3.700.735	-38.989	3.649.417	-42.861
3.677.771	-45.652	3.700.815	-38.981	3.649.598	-42.857
3.677.802	-45.648	3.700.884	-38.972	3.649.749	-42.853
3.677.827	-45.644	3.701.133	-38.964	3.649.901	-42.849
3.677.860	-45.641	3.701.396	-38.956	3.650.128	-42.845
3.677.969	-45.637	3.701.613	-38.948	3.650.274	-42.841
3.678.112	-45.633	3.701.786	-38.940	3.650.374	-42.837
3.678.282	-45.630	3.701.903	-38.932	3.650.448	-42.833
3.678.452	-45.626	3.702.043	-38.923	3.650.587	-42.829
3.678.625	-45.622	3.702.263	-38.915	3.650.757	-42.824
3.678.813	-45.619	3.702.460	-38.907	3.650.927	-42.820
3.679.012	-45.615	3.702.589	-38.898	3.651.143	-42.816
3.679.171	-45.612	3.702.715	-38.890	3.651.263	-42.812
3.679.366	-45.609	3.702.845	-38.882	3.651.387	-42.807
3.679.514	-45.605	3.703.000	-38.873	3.651.620	-42.803
3.679.579	-45.602	3.703.201	-38.865	3.651.805	-42.799
3.679.812	-45.599	3.703.427	-38.856	3.652.008	-42.794
3.680.016	-45.596	3.703.732	-38.848	3.652.224	-42.790
3.680.050	-45.593	3.704.135	-38.839	3.652.415	-42.785
3.680.219	-45.590	3.704.406	-38.831	3.652.594	-42.781
3.680.422	-45.587	3.704.489	-38.822	3.652.722	-42.776
3.680.576	-45.584	3.704.550	-38.813	3.652.870	-42.772
3.680.717	-45.581	3.704.534	-38.805	3.653.053	-42.767

3.680.880	-45.578	3.704.543	-38.796	3.653.295	-42.763
3.681.057	-45.575	3.704.700	-38.787	3.653.475	-42.758
3.681.248	-45.573	3.704.807	-38.779	3.653.593	-42.753
3.681.516	-45.570	3.704.958	-38.770	3.653.759	-42.748
3.681.732	-45.567	3.705.175	-38.761	3.653.918	-42.744
3.681.895	-45.565	3.705.294	-38.752	3.654.027	-42.739
3.682.051	-45.562	3.705.399	-38.743	3.654.160	-42.734
3.682.220	-45.560	3.705.584	-38.735	3.654.333	-42.729
3.682.383	-45.557	3.705.813	-38.726	3.654.532	-42.724
3.682.556	-45.555	3.706.015	-38.717	3.654.688	-42.719
3.682.662	-45.553	3.706.216	-38.708	3.654.844	-42.715
3.682.820	-45.550	3.706.357	-38.699	3.655.092	-42.710
3.683.067	-45.548	3.706.473	-38.690	3.655.252	-42.705
3.683.279	-45.546	3.706.613	-38.681	3.655.390	-42.700
3.683.414	-45.544	3.706.765	-38.672	3.655.589	-42.694
3.683.526	-45.542	3.706.929	-38.663	3.655.751	-42.689
3.683.649	-45.540	3.707.126	-38.654	3.655.912	-42.684
3.683.797	-45.538	3.707.321	-38.645	3.656.090	-42.679
3.683.990	-45.536	3.707.498	-38.636	3.656.241	-42.674
3.684.158	-45.534	3.707.630	-38.627	3.656.361	-42.669
3.684.270	-45.532	3.707.737	-38.618	3.656.567	-42.664
3.684.410	-45.530	3.707.946	-38.609	3.656.783	-42.658
3.684.610	-45.528	3.708.135	-38.599	3.656.947	-42.653
3.684.756	-45.527	3.708.336	-38.590	3.657.145	-42.648
3.684.843	-45.525	3.708.535	-38.581	3.657.310	-42.643
3.685.006	-45.523	3.708.714	-38.572	3.657.448	-42.637
3.685.258	-45.522	3.708.896	-38.563	3.657.542	-42.632
3.685.498	-45.520	3.709.070	-38.554	3.657.737	-42.627
3.685.671	-45.519	3.709.283	-38.545	3.657.941	-42.621
3.685.783	-45.517	3.709.403	-38.535	3.658.116	-42.616

3.685.949	-45.516	3.709.529	-38.526	3.658.290	-42.610
3.686.181	-45.514	3.709.729	-38.517	3.658.445	-42.605
3.686.358	-45.513	3.709.886	-38.508	3.658.766	-42.600
3.686.456	-45.511	3.710.016	-38.498	3.659.131	-42.594
3.686.570	-45.510	3.710.216	-38.489	3.659.389	-42.589
3.686.722	-45.509	3.710.415	-38.480	3.659.503	-42.583
3.686.927	-45.507	3.710.613	-38.471	3.659.572	-42.578
3.687.123	-45.506	3.710.855	-38.461	3.659.688	-42.572
3.687.258	-45.505	3.710.981	-38.452	3.659.706	-42.567
3.687.458	-45.503	3.711.082	-38.443	3.659.699	-42.561
3.687.686	-45.502	3.711.237	-38.434	3.659.873	-42.555
3.687.863	-45.501	3.711.332	-38.424	3.660.056	-42.550
3.687.976	-45.500	3.711.458	-38.415	3.660.197	-42.544
3.688.051	-45.499	3.711.653	-38.406	3.660.363	-42.539
3.688.177	-45.498	3.711.885	-38.396	3.660.522	-42.533
3.688.430	-45.497	3.712.104	-38.387	3.660.645	-42.528
3.688.600	-45.496	3.712.257	-38.378	3.660.808	-42.522
3.688.745	-45.494	3.712.404	-38.368	3.661.019	-42.516
3.688.900	-45.493	3.712.581	-38.359	3.661.195	-42.511
3.688.962	-45.492	3.712.769	-38.350	3.661.411	-42.505
3.689.096	-45.491	3.712.912	-38.341	3.661.595	-42.500
3.689.252	-45.491	3.713.036	-38.331	3.661.738	-42.494
3.689.461	-45.490	3.713.196	-38.322	3.661.938	-42.489
3.689.695	-45.489	3.713.342	-38.313	3.662.126	-42.483
3.689.930	-45.488	3.713.470	-38.303	3.662.234	-42.478
3.690.201	-45.487	3.713.636	-38.294	3.662.377	-42.472
3.690.411	-45.486	3.713.839	-38.285	3.662.563	-42.467
3.690.552	-45.485	3.714.066	-38.276	3.662.708	-42.461
3.690.746	-45.484	3.714.222	-38.266	3.662.885	-42.456
3.690.961	-45.483	3.714.413	-38.257	3.663.052	-42.450

3.691.076	-45.483	3.714.605	-38.248	3.663.214	-42.445
3.691.183	-45.482	3.714.692	-38.238	3.663.398	-42.440
3.691.293	-45.481	3.714.814	-38.229	3.663.602	-42.434
3.691.440	-45.480	3.714.966	-38.220	3.663.770	-42.429
3.691.596	-45.480	3.715.161	-38.211	3.663.909	-42.424
3.691.722	-45.479	3.715.385	-38.201	3.664.063	-42.418
3.691.823	-45.478	3.715.482	-38.192	3.664.221	-42.413
3.691.933	-45.477	3.715.504	-38.183	3.664.429	-42.408
3.692.108	-45.477	3.715.659	-38.174	3.664.583	-42.403
3.692.350	-45.476	3.715.941	-38.164	3.664.756	-42.397
3.692.574	-45.475	3.716.107	-38.155	3.664.987	-42.392
3.692.758	-45.475	3.716.284	-38.146	3.665.104	-42.387
3.692.954	-45.474	3.716.432	-38.137	3.665.224	-42.382
3.693.105	-45.473	3.716.674	-38.127	3.665.464	-42.377
3.693.268	-45.473	3.716.962	-38.118	3.665.706	-42.372
3.693.477	-45.472	3.717.162	-38.109	3.665.804	-42.367
3.693.623	-45.471	3.717.322	-38.100	3.665.919	-42.362
3.693.748	-45.471	3.717.442	-38.090	3.666.089	-42.357
3.693.952	-45.470	3.717.625	-38.081	3.666.147	-42.352
3.694.149	-45.469	3.717.776	-38.072	3.666.318	-42.347
3.694.276	-45.469	3.717.854	-38.063	3.666.603	-42.342
3.694.427	-45.468	3.717.964	-38.054	3.666.747	-42.337
3.694.601	-45.468	3.718.159	-38.044	3.666.848	-42.333
3.694.796	-45.467	3.718.297	-38.035	3.666.993	-42.328
3.694.998	-45.467	3.718.425	-38.026	3.667.119	-42.323
3.695.176	-45.466	3.718.636	-38.017	3.667.300	-42.318
3.695.323	-45.466	3.718.864	-38.008	3.667.585	-42.314
3.695.486	-45.465	3.719.113	-37.999	3.667.796	-42.309
3.695.636	-45.464	3.719.305	-37.990	3.667.878	-42.305
3.695.786	-45.464	3.719.360	-37.981	3.667.987	-42.300

3.695.921	-45.463	3.719.489	-37.972	3.668.172	-42.296
3.696.102	-45.463	3.719.757	-37.962	3.668.366	-42.291
3.696.321	-45.462	3.719.964	-37.953	3.668.557	-42.287
3.696.445	-45.462	3.720.083	-37.944	3.668.753	-42.282
3.696.607	-45.461	3.720.227	-37.935	3.668.897	-42.278
3.696.816	-45.461	3.720.458	-37.926	3.669.073	-42.274
3.696.982	-45.460	3.720.614	-37.918	3.669.245	-42.270
3.697.141	-45.460	3.720.768	-37.909	3.669.333	-42.265
3.697.289	-45.460	3.720.956	-37.900	3.669.414	-42.261
3.697.451	-45.459	3.721.101	-37.891	3.669.656	-42.257
3.697.661	-45.459	3.721.252	-37.882	3.669.930	-42.253
3.697.809	-45.458	3.721.474	-37.873	3.670.096	-42.248
3.697.969	-45.458	3.721.611	-37.864	3.670.255	-42.244
3.698.334	-45.457	3.721.743	-37.855	3.670.404	-42.240
3.698.767	-45.457	3.721.892	-37.847	3.670.551	-42.236
3.698.945	-45.456	3.722.101	-37.838	3.670.718	-42.232
3.698.997	-45.456	3.722.299	-37.829	3.670.883	-42.228
3.699.169	-45.456	3.722.458	-37.820	3.671.098	-42.224
3.699.225	-45.455	3.722.603	-37.812	3.671.295	-42.220
3.699.234	-45.455	3.722.726	-37.803	3.671.458	-42.216
3.699.341	-45.454	3.722.874	-37.794	3.671.602	-42.212
3.699.456	-45.454	3.723.060	-37.786	3.671.729	-42.208
3.699.608	-45.454	3.723.188	-37.777	3.671.902	-42.204
3.699.765	-45.453	3.723.337	-37.768	3.672.128	-42.200
3.699.924	-45.453	3.723.539	-37.760	3.672.329	-42.196
3.700.135	-45.452	3.723.710	-37.751	3.672.451	-42.192
3.700.296	-45.452	3.723.871	-37.743	3.672.563	-42.188
3.700.469	-45.452	3.723.992	-37.734	3.672.701	-42.184
3.700.715	-45.451	3.724.114	-37.726	3.672.831	-42.180
3.700.891	-45.451	3.724.297	-37.717	3.672.975	-42.176

3.701.068	-45.451	3.724.671	-37.709	3.673.143	-42.172
3.701.225	-45.450	3.725.075	-37.700	3.673.316	-42.168
3.701.324	-45.450	3.725.266	-37.692	3.673.488	-42.164
3.701.461	-45.449	3.725.363	-37.683	3.673.627	-42.160
3.701.574	-45.449	3.725.459	-37.675	3.673.815	-42.156
3.701.721	-45.449	3.725.518	-37.667	3.674.034	-42.152
3.701.903	-45.448	3.725.602	-37.658	3.674.268	-42.148
3.702.051	-45.448	3.725.668	-37.650	3.674.536	-42.144
3.702.240	-45.448	3.725.768	-37.642	3.674.711	-42.140
3.702.431	-45.447	3.725.941	-37.634	3.674.829	-42.136
3.702.575	-45.447	3.726.176	-37.625	3.674.991	-42.132
3.702.747	-45.447	3.726.364	-37.617	3.675.147	-42.128
3.702.946	-45.446	3.726.495	-37.609	3.675.258	-42.124
3.703.143	-45.446	3.726.682	-37.601	3.675.428	-42.120
3.703.304	-45.446	3.726.857	-37.593	3.675.618	-42.116
3.703.434	-45.446	3.727.032	-37.585	3.675.739	-42.112
3.703.578	-45.445	3.727.231	-37.577	3.675.899	-42.107
3.703.752	-45.445	3.727.433	-37.569	3.676.086	-42.103
3.703.965	-45.445	3.727.654	-37.561	3.676.201	-42.099
3.704.102	-45.444	3.727.802	-37.553	3.676.317	-42.095
3.704.151	-45.444	3.727.879	-37.545	3.676.433	-42.091
3.704.362	-45.444	3.727.991	-37.537	3.676.610	-42.086
3.704.651	-45.443	3.728.177	-37.529	3.676.866	-42.082
3.704.818	-45.443	3.728.336	-37.521	3.677.065	-42.078
3.704.970	-45.443	3.728.466	-37.514	3.677.191	-42.073
3.705.132	-45.443	3.728.605	-37.506	3.677.330	-42.069
3.705.288	-45.442	3.728.773	-37.498	3.677.538	-42.064
3.705.468	-45.442	3.729.005	-37.490	3.677.726	-42.060
3.705.613	-45.442	3.729.211	-37.483	3.677.854	-42.056
3.705.696	-45.442	3.729.406	-37.475	3.678.062	-42.051

3.705.851	-45.441	3.729.606	-37.467	3.678.248	-42.046
3.706.055	-45.441	3.729.796	-37.460	3.678.391	-42.042
3.706.194	-45.441	3.729.926	-37.452	3.678.612	-42.037
3.706.385	-45.441	3.730.009	-37.445	3.678.831	-42.033
3.706.613	-45.440	3.730.173	-37.437	3.679.008	-42.028
3.706.750	-45.440	3.730.414	-37.430	3.679.196	-42.023
3.706.906	-45.440	3.730.559	-37.422	3.679.331	-42.018
3.707.095	-45.440	3.730.616	-37.415	3.679.411	-42.014
3.707.304	-45.439	3.730.789	-37.408	3.679.544	-42.009
3.707.517	-45.439	3.730.966	-37.400	3.679.745	-42.004
3.707.643	-45.439	3.731.118	-37.393	3.679.951	-41.999
3.707.769	-45.439	3.731.324	-37.386	3.680.105	-41.994
3.707.896	-45.438	3.731.505	-37.379	3.680.311	-41.989
3.708.059	-45.438	3.731.694	-37.371	3.680.523	-41.984
3.708.261	-45.438	3.731.833	-37.364	3.680.742	-41.979
3.708.468	-45.438	3.731.990	-37.357	3.680.985	-41.974
3.708.684	-45.438	3.732.144	-37.350	3.681.133	-41.968
3.708.797	-45.437	3.732.210	-37.343	3.681.219	-41.963
3.708.945	-45.437	3.732.416	-37.336	3.681.375	-41.958
3.709.193	-45.437	3.732.651	-37.329	3.681.537	-41.953
3.709.377	-45.437	3.732.828	-37.322	3.681.671	-41.947
3.709.469	-45.437	3.733.029	-37.315	3.681.830	-41.942
3.709.547	-45.437	3.733.203	-37.308	3.681.978	-41.936
3.709.756	-45.436	3.733.362	-37.301	3.682.126	-41.931
3.709.955	-45.436	3.733.558	-37.294	3.682.303	-41.925
3.710.014	-45.436	3.733.743	-37.288	3.682.500	-41.920
3.710.151	-45.436	3.733.878	-37.281	3.682.679	-41.914
3.710.310	-45.436	3.734.066	-37.274	3.682.842	-41.909
3.710.451	-45.436	3.734.175	-37.267	3.683.013	-41.903
3.710.600	-45.435	3.734.353	-37.261	3.683.181	-41.897

3.710.786	-45.435	3.734.537	-37.254	3.683.358	-41.892
3.710.997	-45.435	3.734.695	-37.247	3.683.511	-41.886
3.711.216	-45.435	3.734.879	-37.241	3.683.608	-41.880
3.711.465	-45.435	3.735.034	-37.234	3.683.793	-41.874
3.711.685	-45.435	3.735.201	-37.228	3.684.226	-41.868
3.711.859	-45.435	3.735.360	-37.221	3.684.666	-41.862
3.711.924	-45.434	3.735.537	-37.215	3.684.880	-41.856
3.712.059	-45.434	3.735.717	-37.208	3.684.949	-41.850
3.712.332	-45.434	3.735.844	-37.202	3.685.010	-41.844
3.712.495	-45.434	3.735.974	-37.195	3.685.114	-41.838
3.712.593	-45.434	3.736.100	-37.189	3.685.170	-41.832
3.712.665	-45.434	3.736.199	-37.182	3.685.238	-41.825
3.712.751	-45.434	3.736.320	-37.176	3.685.324	-41.819
3.712.958	-45.434	3.736.465	-37.169	3.685.407	-41.813
3.713.210	-45.434	3.736.635	-37.163	3.685.538	-41.807
3.713.412	-45.433	3.736.797	-37.157	3.685.730	-41.800
3.713.515	-45.433	3.736.993	-37.150	3.685.927	-41.794
3.713.636	-45.433	3.737.243	-37.144	3.686.031	-41.787
3.713.868	-45.433	3.737.458	-37.137	3.686.180	-41.781
3.714.049	-45.433	3.737.675	-37.131	3.686.361	-41.774
3.714.266	-45.433	3.737.903	-37.125	3.686.635	-41.768
3.714.516	-45.433	3.738.081	-37.118	3.686.812	-41.761
3.714.710	-45.433	3.738.164	-37.112	3.686.962	-41.755
3.714.855	-45.433	3.738.255	-37.106	3.687.122	-41.748
3.714.898	-45.433	3.738.478	-37.100	3.687.357	-41.741
3.715.056	-45.433	3.738.685	-37.093	3.687.563	-41.735
3.715.314	-45.433	3.738.815	-37.087	3.687.722	-41.728
3.715.511	-45.433	3.738.940	-37.081	3.687.881	-41.722
3.715.661	-45.433	3.739.077	-37.074	3.688.011	-41.715
3.715.800	-45.433	3.739.218	-37.068	3.688.170	-41.708

3.715.930	-45.433	3.739.355	-37.062	3.688.362	-41.701
3.716.050	-45.433	3.739.541	-37.056	3.688.557	-41.695
3.716.271	-45.433	3.739.740	-37.049	3.688.684	-41.688
3.716.492	-45.432	3.739.928	-37.043	3.688.771	-41.681
3.716.672	-45.432	3.740.099	-37.037	3.688.929	-41.674
3.716.821	-45.432	3.740.296	-37.031	3.689.057	-41.668
3.716.956	-45.432	3.740.531	-37.025	3.689.258	-41.661
3.717.124	-45.432	3.740.688	-37.018	3.689.507	-41.654
3.717.315	-45.432	3.740.807	-37.012	3.689.688	-41.647
3.717.484	-45.432	3.740.970	-37.006	3.689.901	-41.641
3.717.578	-45.432	3.741.143	-37.000	3.690.053	-41.634
3.717.708	-45.432	3.741.322	-36.994	3.690.164	-41.627
3.717.892	-45.432	3.741.555	-36.987	3.690.346	-41.620
3.718.106	-45.432	3.741.748	-36.981	3.690.541	-41.613
3.718.268	-45.432	3.741.899	-36.975	3.690.685	-41.606
3.718.445	-45.433	3.742.079	-36.969	3.690.826	-41.600
3.718.637	-45.433	3.742.271	-36.963	3.691.006	-41.593
3.718.814	-45.433	3.742.422	-36.956	3.691.125	-41.586
3.718.961	-45.433	3.742.563	-36.950	3.691.202	-41.579
3.719.044	-45.433	3.742.726	-36.944	3.691.361	-41.572
3.719.230	-45.433	3.742.859	-36.938	3.691.546	-41.566
3.719.662	-45.433	3.743.051	-36.932	3.691.729	-41.559
3.720.104	-45.433	3.743.295	-36.926	3.691.888	-41.552
3.720.302	-45.433	3.743.442	-36.919	3.692.050	-41.545
3.720.329	-45.433	3.743.557	-36.913	3.692.171	-41.539
3.720.403	-45.433	3.743.766	-36.907	3.692.362	-41.532
3.720.486	-45.433	3.743.934	-36.901	3.692.610	-41.525
3.720.506	-45.433	3.744.052	-36.895	3.692.794	-41.519
3.720.562	-45.433	3.744.241	-36.888	3.692.968	-41.512
3.720.679	-45.433	3.744.407	-36.882	3.693.127	-41.505

3.720.880	-45.433	3.744.557	-36.876	3.693.253	-41.499
3.721.075	-45.433	3.744.709	-36.870	3.693.340	-41.492
3.721.220	-45.433	3.744.872	-36.864	3.693.484	-41.486
3.721.387	-45.433	3.745.063	-36.857	3.693.701	-41.479
3.721.584	-45.433	3.745.169	-36.851	3.693.922	-41.473
3.721.761	-45.433	3.745.317	-36.845	3.694.149	-41.466
3.721.985	-45.433	3.745.703	-36.839	3.694.330	-41.460
3.722.189	-45.434	3.746.195	-36.833	3.694.478	-41.453
3.722.300	-45.434	3.746.344	-36.826	3.694.642	-41.447
3.722.449	-45.434	3.746.384	-36.820	3.694.796	-41.440
3.722.614	-45.434	3.746.452	-36.814	3.694.927	-41.434
3.722.747	-45.434	3.746.512	-36.808	3.695.135	-41.428
3.722.911	-45.434	3.746.568	-36.801	3.695.365	-41.421
3.723.148	-45.434	3.746.700	-36.795	3.695.494	-41.415
3.723.349	-45.434	3.746.797	-36.789	3.695.625	-41.409
3.723.468	-45.434	3.746.925	-36.783	3.695.819	-41.402
3.723.544	-45.434	3.747.141	-36.776	3.696.029	-41.396
3.723.694	-45.434	3.747.314	-36.770	3.696.263	-41.390
3.723.902	-45.434	3.747.491	-36.764	3.696.429	-41.384
3.724.121	-45.434	3.747.726	-36.757	3.696.559	-41.377
3.724.316	-45.434	3.747.879	-36.751	3.696.689	-41.371
3.724.435	-45.435	3.747.979	-36.745	3.696.833	-41.365
3.724.572	-45.435	3.748.196	-36.738	3.697.008	-41.359
3.724.796	-45.435	3.748.389	-36.732	3.697.159	-41.353
3.725.023	-45.435	3.748.493	-36.726	3.697.346	-41.346
3.725.132	-45.435	3.748.684	-36.719	3.697.558	-41.340
3.725.204	-45.435	3.748.855	-36.713	3.697.668	-41.334
3.725.376	-45.435	3.749.003	-36.707	3.697.726	-41.328
3.725.581	-45.435	3.749.189	-36.700	3.697.816	-41.322
3.725.739	-45.435	3.749.359	-36.694	3.697.903	-41.316

3.725.899	-45.435	3.749.516	-36.687	3.698.091	-41.310
3.726.143	-45.435	3.749.662	-36.681	3.698.342	-41.304
3.726.306	-45.435	3.749.880	-36.674	3.698.508	-41.298
3.726.383	-45.435	3.750.045	-36.668	3.698.645	-41.292
3.726.559	-45.436	3.750.205	-36.661	3.698.824	-41.286
3.726.712	-45.436	3.750.426	-36.655	3.698.976	-41.280
3.726.888	-45.436	3.750.620	-36.648	3.699.138	-41.274
3.727.052	-45.436	3.750.804	-36.642	3.699.346	-41.268
3.727.189	-45.436	3.750.981	-36.635	3.699.595	-41.262
3.727.397	-45.436	3.751.119	-36.629	3.699.869	-41.256
3.727.509	-45.436	3.751.296	-36.622	3.700.079	-41.250
3.727.621	-45.436	3.751.485	-36.616	3.700.260	-41.244
3.727.846	-45.436	3.751.599	-36.609	3.700.411	-41.239
3.728.070	-45.436	3.751.747	-36.602	3.700.541	-41.233
3.728.239	-45.436	3.751.880	-36.596	3.700.711	-41.227
3.728.456	-45.436	3.752.011	-36.589	3.700.873	-41.221
3.728.701	-45.437	3.752.177	-36.582	3.701.011	-41.215
3.728.873	-45.437	3.752.340	-36.576	3.701.105	-41.209
3.728.970	-45.437	3.752.510	-36.569	3.701.248	-41.204
3.729.078	-45.437	3.752.659	-36.562	3.701.423	-41.198
3.729.276	-45.437	3.752.827	-36.555	3.701.542	-41.192
3.729.501	-45.437	3.753.011	-36.549	3.701.689	-41.186
3.729.684	-45.437	3.753.147	-36.542	3.701.860	-41.181
3.729.855	-45.437	3.753.289	-36.535	3.702.054	-41.175
3.730.010	-45.437	3.753.456	-36.528	3.702.305	-41.169
3.730.139	-45.437	3.753.602	-36.521	3.702.458	-41.164
3.730.269	-45.437	3.753.806	-36.514	3.702.610	-41.158
3.730.417	-45.437	3.754.032	-36.507	3.702.820	-41.152
3.730.620	-45.437	3.754.196	-36.500	3.703.004	-41.147
3.730.844	-45.438	3.754.320	-36.493	3.703.134	-41.141

3.731.025	-45.438	3.754.467	-36.486	3.703.272	-41.135
3.731.141	-45.438	3.754.652	-36.479	3.703.526	-41.130
3.731.238	-45.438	3.754.823	-36.472	3.703.775	-41.124
3.731.342	-45.438	3.755.002	-36.465	3.703.952	-41.119
3.731.472	-45.438	3.755.179	-36.458	3.704.078	-41.113
3.731.617	-45.438	3.755.356	-36.451	3.704.222	-41.107
3.731.819	-45.438	3.755.529	-36.444	3.704.425	-41.102
3.732.032	-45.438	3.755.681	-36.436	3.704.587	-41.096
3.732.198	-45.438	3.755.845	-36.429	3.704.746	-41.091
3.732.388	-45.438	3.756.007	-36.422	3.704.893	-41.085
3.732.654	-45.438	3.756.196	-36.415	3.705.035	-41.080
3.732.890	-45.439	3.756.387	-36.407	3.705.209	-41.074
3.733.019	-45.439	3.756.563	-36.400	3.705.418	-41.069
3.733.159	-45.439	3.756.718	-36.392	3.705.648	-41.063
3.733.271	-45.439	3.756.855	-36.385	3.705.782	-41.058
3.733.419	-45.439	3.757.026	-36.377	3.705.833	-41.052
3.733.640	-45.439	3.757.124	-36.370	3.706.000	-41.047
3.733.795	-45.439	3.757.213	-36.362	3.706.224	-41.042
3.733.839	-45.439	3.757.380	-36.355	3.706.432	-41.036
3.733.944	-45.439	3.757.581	-36.347	3.706.593	-41.031
3.734.128	-45.439	3.757.773	-36.339	3.706.694	-41.025
3.734.313	-45.439	3.757.947	-36.331	3.706.815	-41.020
3.734.431	-45.439	3.758.167	-36.324	3.706.971	-41.014
3.734.608	-45.440	3.758.383	-36.316	3.707.192	-41.009
3.734.879	-45.440	3.758.634	-36.308	3.707.420	-41.004
3.735.114	-45.440	3.758.842	-36.300	3.707.542	-40.998
3.735.287	-45.440	3.759.003	-36.292	3.707.666	-40.993
3.735.406	-45.440	3.759.182	-36.284	3.707.881	-40.988
3.735.500	-45.440	3.759.279	-36.276	3.708.063	-40.982
3.735.743	-45.440	3.759.431	-36.267	3.708.185	-40.977

3.735.986	-45.440	3.759.630	-36.259	3.708.385	-40.972
3.736.159	-45.440	3.759.756	-36.251	3.708.611	-40.966
3.736.346	-45.440	3.759.873	-36.242	3.708.766	-40.961
3.736.519	-45.440	3.760.036	-36.234	3.708.878	-40.956
3.736.696	-45.440	3.760.175	-36.226	3.708.973	-40.950
3.736.875	-45.440	3.760.306	-36.217	3.709.244	-40.945
3.737.036	-45.441	3.760.469	-36.208	3.709.684	-40.940
3.737.209	-45.441	3.760.627	-36.200	3.710.071	-40.934
3.737.359	-45.441	3.760.829	-36.191	3.710.292	-40.929
3.737.509	-45.441	3.761.006	-36.182	3.710.345	-40.924
3.737.669	-45.441	3.761.167	-36.174	3.710.401	-40.918
3.737.789	-45.441	3.761.350	-36.165	3.710.451	-40.913
3.737.948	-45.441	3.761.484	-36.156	3.710.468	-40.908
3.738.089	-45.441	3.761.736	-36.147	3.710.555	-40.903
3.738.261	-45.441	3.761.918	-36.138	3.710.682	-40.897
3.738.416	-45.441	3.762.036	-36.129	3.710.816	-40.892
3.738.591	-45.441	3.762.258	-36.120	3.711.017	-40.887
3.738.840	-45.441	3.762.426	-36.110	3.711.201	-40.881
3.739.048	-45.441	3.762.568	-36.101	3.711.310	-40.876
3.739.178	-45.441	3.762.713	-36.092	3.711.443	-40.871
3.739.301	-45.442	3.762.852	-36.082	3.711.674	-40.865
3.739.462	-45.442	3.763.059	-36.073	3.711.946	-40.860
3.739.657	-45.442	3.763.257	-36.063	3.712.133	-40.855
3.739.789	-45.442	3.763.420	-36.054	3.712.275	-40.849
3.739.893	-45.442	3.763.558	-36.044	3.712.423	-40.844
3.740.122	-45.442	3.763.738	-36.035	3.712.596	-40.839
3.740.334	-45.442	3.763.984	-36.025	3.712.724	-40.833
3.740.390	-45.442	3.764.120	-36.015	3.712.867	-40.828
3.740.562	-45.442	3.764.297	-36.005	3.713.076	-40.823
3.741.073	-45.442	3.764.496	-35.995	3.713.279	-40.818

3.741.447	-45.442	3.764.691	-35.985	3.713.445	-40.812
3.741.508	-45.442	3.764.836	-35.975	3.713.542	-40.807
3.741.584	-45.442	3.764.938	-35.965	3.713.685	-40.802
3.741.646	-45.442	3.765.078	-35.955	3.713.813	-40.797
3.741.726	-45.442	3.765.302	-35.945	3.713.991	-40.791
3.741.831	-45.442	3.765.529	-35.935	3.714.131	-40.786
3.741.949	-45.442	3.765.686	-35.924	3.714.254	-40.781
3.742.095	-45.442	3.765.840	-35.914	3.714.450	-40.776
3.742.177	-45.442	3.765.999	-35.904	3.714.669	-40.771
3.742.294	-45.442	3.766.170	-35.893	3.714.836	-40.765
3.742.534	-45.443	3.766.277	-35.883	3.715.005	-40.760
3.742.817	-45.443	3.766.609	-35.872	3.715.197	-40.755
3.743.004	-45.443	3.767.069	-35.862	3.715.424	-40.750
3.743.141	-45.443	3.767.251	-35.851	3.715.638	-40.745
3.743.322	-45.443	3.767.343	-35.840	3.715.757	-40.739
3.743.409	-45.443	3.767.427	-35.830	3.715.925	-40.734
3.743.575	-45.443	3.767.466	-35.819	3.716.084	-40.729
3.743.810	-45.443	3.767.458	-35.808	3.716.202	-40.724
3.743.941	-45.443	3.767.521	-35.797	3.716.342	-40.719
3.744.115	-45.443	3.767.632	-35.787	3.716.470	-40.714
3.744.294	-45.443	3.767.794	-35.776	3.716.609	-40.708
3.744.449	-45.443	3.767.971	-35.765	3.716.812	-40.703
3.744.615	-45.443	3.768.178	-35.754	3.717.055	-40.698
3.744.796	-45.443	3.768.377	-35.743	3.717.249	-40.693
3.744.942	-45.443	3.768.553	-35.732	3.717.381	-40.688
3.745.143	-45.443	3.768.734	-35.720	3.717.502	-40.683
3.745.388	-45.443	3.768.930	-35.709	3.717.582	-40.677
3.745.526	-45.443	3.769.096	-35.698	3.717.784	-40.672
3.745.656	-45.443	3.769.244	-35.687	3.718.051	-40.667
3.745.822	-45.443	3.769.406	-35.676	3.718.189	-40.662

3.745.971	-45.442	3.769.536	-35.665	3.718.307	-40.657
3.746.201	-45.442	3.769.678	-35.653	3.718.501	-40.652
3.746.360	-45.442	3.769.857	-35.642	3.718.687	-40.647
3.746.437	-45.442	3.769.995	-35.631	3.718.849	-40.641
3.746.602	-45.442	3.770.172	-35.619	3.719.034	-40.636
3.746.790	-45.442	3.770.338	-35.608	3.719.198	-40.631
3.746.962	-45.442	3.770.447	-35.597	3.719.377	-40.626
3.747.087	-45.442	3.770.656	-35.585	3.719.547	-40.621
3.747.246	-45.442	3.770.916	-35.574	3.719.740	-40.616
3.747.404	-45.442	3.771.107	-35.562	3.719.935	-40.610
3.747.534	-45.442	3.771.326	-35.551	3.720.135	-40.605
3.747.755	-45.442	3.771.559	-35.540	3.720.295	-40.600
3.747.968	-45.442	3.771.711	-35.528	3.720.440	-40.595
3.748.107	-45.442	3.771.856	-35.517	3.720.635	-40.590
3.748.253	-45.441	3.771.968	-35.505	3.720.804	-40.584
3.748.450	-45.441	3.772.130	-35.494	3.720.938	-40.579
3.748.618	-45.441	3.772.339	-35.482	3.721.107	-40.574
3.748.772	-45.441	3.772.485	-35.471	3.721.255	-40.569
3.748.941	-45.441	3.772.628	-35.459	3.721.357	-40.563
3.749.111	-45.441	3.772.780	-35.447	3.721.451	-40.558
3.749.277	-45.441	3.772.965	-35.436	3.721.685	-40.553
3.749.362	-45.441	3.773.125	-35.424	3.721.880	-40.547
3.749.511	-45.440	3.773.268	-35.413	3.721.986	-40.542
3.749.732	-45.440	3.773.430	-35.401	3.722.152	-40.536
3.750.005	-45.440	3.773.613	-35.390	3.722.355	-40.531
3.750.165	-45.440	3.773.828	-35.378	3.722.540	-40.525
3.750.242	-45.440	3.774.006	-35.367	3.722.748	-40.520
3.750.374	-45.440	3.774.120	-35.355	3.722.919	-40.514
3.750.519	-45.440	3.774.215	-35.343	3.723.012	-40.509
3.750.710	-45.439	3.774.438	-35.332	3.723.127	-40.503

3.750.905	-45.439	3.774.638	-35.320	3.723.300	-40.497
3.751.097	-45.439	3.774.846	-35.309	3.723.475	-40.492
3.751.270	-45.439	3.775.074	-35.297	3.723.597	-40.486
3.751.476	-45.439	3.775.154	-35.286	3.723.759	-40.480
3.751.638	-45.438	3.775.280	-35.274	3.723.994	-40.475
3.751.765	-45.438	3.775.455	-35.263	3.724.131	-40.469
3.751.971	-45.438	3.775.641	-35.251	3.724.283	-40.463
3.752.133	-45.438	3.775.816	-35.240	3.724.521	-40.457
3.752.227	-45.438	3.775.963	-35.228	3.724.663	-40.451
3.752.379	-45.437	3.776.126	-35.217	3.724.837	-40.446
3.752.520	-45.437	3.776.308	-35.205	3.725.081	-40.440
3.752.655	-45.437	3.776.490	-35.194	3.725.260	-40.434
3.752.849	-45.437	3.776.635	-35.182	3.725.482	-40.428
3.752.964	-45.436	3.776.809	-35.171	3.725.657	-40.422
3.753.123	-45.436	3.776.978	-35.159	3.725.807	-40.416
3.753.341	-45.436	3.777.133	-35.148	3.725.971	-40.410
3.753.533	-45.436	3.777.325	-35.137	3.726.109	-40.403
3.753.763	-45.435	3.777.518	-35.125	3.726.263	-40.397
3.753.955	-45.435	3.777.712	-35.114	3.726.434	-40.391
3.754.150	-45.435	3.777.861	-35.103	3.726.584	-40.385
3.754.385	-45.435	3.777.930	-35.091	3.726.705	-40.379
3.754.586	-45.434	3.778.015	-35.080	3.726.870	-40.372
3.754.742	-45.434	3.778.127	-35.069	3.727.000	-40.366
3.754.862	-45.434	3.778.308	-35.057	3.727.116	-40.360
3.754.991	-45.433	3.778.535	-35.046	3.727.285	-40.353
3.755.107	-45.433	3.778.705	-35.035	3.727.525	-40.347
3.755.208	-45.433	3.778.847	-35.024	3.727.776	-40.341
3.755.349	-45.432	3.779.041	-35.013	3.727.955	-40.334
3.755.509	-45.432	3.779.193	-35.002	3.728.170	-40.328
3.755.681	-45.432	3.779.407	-34.990	3.728.354	-40.321

3.755.826	-45.432	3.779.666	-34.979	3.728.459	-40.315
3.755.959	-45.431	3.779.854	-34.968	3.728.600	-40.308
3.756.188	-45.431	3.780.054	-34.957	3.728.778	-40.301
3.756.412	-45.431	3.780.222	-34.946	3.728.954	-40.295
3.756.597	-45.430	3.780.401	-34.935	3.729.118	-40.288
3.756.756	-45.430	3.780.562	-34.924	3.729.288	-40.281
3.756.862	-45.429	3.780.632	-34.913	3.729.385	-40.275
3.757.080	-45.429	3.780.724	-34.903	3.729.518	-40.268
3.757.308	-45.429	3.780.889	-34.892	3.729.742	-40.261
3.757.422	-45.428	3.780.999	-34.881	3.729.963	-40.255
3.757.625	-45.428	3.781.122	-34.870	3.730.177	-40.248
3.757.799	-45.428	3.781.357	-34.859	3.730.392	-40.241
3.757.968	-45.427	3.781.599	-34.849	3.730.544	-40.234
3.758.177	-45.427	3.781.801	-34.838	3.730.660	-40.227
3.758.334	-45.427	3.781.996	-34.827	3.730.793	-40.220
3.758.454	-45.426	3.782.189	-34.817	3.730.919	-40.213
3.758.586	-45.426	3.782.368	-34.806	3.731.087	-40.206
3.758.782	-45.425	3.782.529	-34.796	3.731.289	-40.200
3.759.030	-45.425	3.782.675	-34.785	3.731.466	-40.193
3.759.095	-45.424	3.782.816	-34.775	3.731.649	-40.186
3.759.285	-45.424	3.782.971	-34.764	3.731.788	-40.179
3.759.562	-45.424	3.783.150	-34.754	3.731.960	-40.172
3.759.662	-45.423	3.783.304	-34.744	3.732.152	-40.165
3.759.848	-45.423	3.783.511	-34.733	3.732.279	-40.158
3.760.025	-45.422	3.783.684	-34.723	3.732.485	-40.150
3.760.184	-45.422	3.783.839	-34.713	3.732.727	-40.143
3.760.388	-45.421	3.784.014	-34.703	3.732.867	-40.136
3.760.559	-45.421	3.784.169	-34.692	3.732.961	-40.129
3.760.657	-45.421	3.784.377	-34.682	3.733.112	-40.122
3.760.852	-45.420	3.784.562	-34.672	3.733.354	-40.115

3.761.065	-45.420	3.784.749	-34.662	3.733.499	-40.108
3.761.199	-45.419	3.784.906	-34.652	3.733.626	-40.101
3.761.425	-45.419	3.785.009	-34.642	3.733.785	-40.094
3.761.620	-45.418	3.785.164	-34.632	3.733.967	-40.087
3.761.736	-45.418	3.785.374	-34.622	3.734.164	-40.080
3.761.826	-45.417	3.785.518	-34.612	3.734.303	-40.073
3.762.097	-45.417	3.785.657	-34.603	3.734.547	-40.066
3.762.546	-45.416	3.785.863	-34.593	3.735.022	-40.058
3.762.802	-45.416	3.786.019	-34.583	3.735.403	-40.051
3.762.937	-45.415	3.786.160	-34.573	3.735.502	-40.044
3.762.995	-45.415	3.786.340	-34.564	3.735.563	-40.037
3.763.011	-45.414	3.786.523	-34.554	3.735.630	-40.030
3.763.067	-45.413	3.786.696	-34.544	3.735.667	-40.023
3.763.142	-45.413	3.786.855	-34.535	3.735.746	-40.016
3.763.282	-45.412	3.787.032	-34.525	3.735.865	-40.009
3.763.405	-45.412	3.787.180	-34.516	3.735.974	-40.002
3.763.546	-45.411	3.787.430	-34.506	3.736.057	-39.994
3.763.761	-45.411	3.787.747	-34.497	3.736.137	-39.987
3.763.938	-45.410	3.788.116	-34.487	3.736.303	-39.980
3.764.172	-45.409	3.788.243	-34.478	3.736.517	-39.973
3.764.360	-45.409	3.788.320	-34.469	3.736.711	-39.966
3.764.480	-45.408	3.788.340	-34.459	3.736.925	-39.959
3.764.605	-45.408	3.788.373	-34.450	3.737.155	-39.952
3.764.805	-45.407	3.788.448	-34.441	3.737.307	-39.945
3.764.928	-45.406	3.788.573	-34.432	3.737.388	-39.938
3.765.028	-45.406	3.788.728	-34.422	3.737.547	-39.931
3.765.226	-45.405	3.788.880	-34.413	3.737.697	-39.924
3.765.479	-45.404	3.789.088	-34.404	3.737.857	-39.917
3.765.693	-45.404	3.789.284	-34.395	3.738.076	-39.910
3.765.888	-45.403	3.789.514	-34.386	3.738.283	-39.903

3.766.006	-45.403	3.789.718	-34.377	3.738.467	-39.896
3.766.083	-45.402	3.789.826	-34.368	3.738.647	-39.890
3.766.219	-45.401	3.789.995	-34.359	3.738.855	-39.883
3.766.418	-45.400	3.790.197	-34.350	3.738.994	-39.876
3.766.673	-45.400	3.790.334	-34.341	3.739.082	-39.869
3.766.878	-45.399	3.790.448	-34.332	3.739.247	-39.862
3.766.953	-45.398	3.790.638	-34.323	3.739.392	-39.856
3.767.084	-45.398	3.790.856	-34.315	3.739.561	-39.849
3.767.358	-45.397	3.791.026	-34.306	3.739.760	-39.842
3.767.579	-45.396	3.791.136	-34.297	3.739.919	-39.836
3.767.755	-45.395	3.791.253	-34.289	3.740.125	-39.829
3.767.904	-45.395	3.791.420	-34.280	3.740.310	-39.822
3.768.006	-45.394	3.791.644	-34.271	3.740.453	-39.816
3.768.070	-45.393	3.791.852	-34.263	3.740.663	-39.809
3.768.226	-45.392	3.792.027	-34.254	3.740.833	-39.803
3.768.472	-45.392	3.792.218	-34.246	3.741.000	-39.796
3.768.659	-45.391	3.792.387	-34.237	3.741.149	-39.790
3.768.804	-45.390	3.792.510	-34.229	3.741.267	-39.783
3.768.955	-45.389	3.792.662	-34.220	3.741.459	-39.777
3.769.160	-45.389	3.792.839	-34.212	3.741.653	-39.771
3.769.359	-45.388	3.792.961	-34.204	3.741.837	-39.764
3.769.558	-45.387	3.793.098	-34.195	3.742.066	-39.758
3.769.716	-45.386	3.793.250	-34.187	3.742.265	-39.752
3.769.826	-45.385	3.793.392	-34.179	3.742.368	-39.745
3.769.955	-45.384	3.793.607	-34.171	3.742.495	-39.739
3.770.089	-45.384	3.793.802	-34.163	3.742.632	-39.733
3.770.278	-45.383	3.793.879	-34.155	3.742.800	-39.727
3.770.527	-45.382	3.794.037	-34.146	3.743.011	-39.721
3.770.695	-45.381	3.794.292	-34.138	3.743.170	-39.715
3.770.833	-45.380	3.794.500	-34.130	3.743.318	-39.709

3.770.967	-45.379	3.794.675	-34.122	3.743.496	-39.703
3.771.140	-45.378	3.794.797	-34.114	3.743.680	-39.696
3.771.362	-45.378	3.794.898	-34.107	3.743.828	-39.691
3.771.503	-45.377	3.795.114	-34.099	3.743.974	-39.685
3.771.548	-45.376	3.795.338	-34.091	3.744.055	-39.679
3.771.712	-45.375	3.795.473	-34.083	3.744.229	-39.673
3.771.996	-45.374	3.795.627	-34.075	3.744.533	-39.667
3.772.105	-45.373	3.795.827	-34.067	3.744.677	-39.661
3.772.271	-45.372	3.795.984	-34.060	3.744.775	-39.655
3.772.489	-45.371	3.796.169	-34.052	3.744.942	-39.649
3.772.651	-45.370	3.796.379	-34.044	3.745.118	-39.643
3.772.818	-45.369	3.796.557	-34.037	3.745.332	-39.638
3.772.982	-45.368	3.796.696	-34.029	3.745.497	-39.632
3.773.138	-45.367	3.796.795	-34.021	3.745.625	-39.626
3.773.336	-45.366	3.796.960	-34.014	3.745.815	-39.620
3.773.533	-45.365	3.797.162	-34.006	3.745.995	-39.615
3.773.670	-45.365	3.797.350	-33.999	3.746.172	-39.609
3.773.815	-45.364	3.797.517	-33.991	3.746.328	-39.603
3.773.883	-45.363	3.797.709	-33.984	3.746.492	-39.597
3.773.966	-45.362	3.797.924	-33.976	3.746.683	-39.592
3.774.128	-45.361	3.798.112	-33.969	3.746.826	-39.586
3.774.301	-45.360	3.798.273	-33.961	3.746.949	-39.580
3.774.529	-45.359	3.798.407	-33.954	3.747.110	-39.575
3.774.619	-45.358	3.798.524	-33.947	3.747.318	-39.569
3.774.808	-45.357	3.798.608	-33.939	3.747.516	-39.563
3.775.164	-45.356	3.798.699	-33.932	3.747.679	-39.557
3.775.378	-45.355	3.798.871	-33.924	3.747.845	-39.552
3.775.512	-45.354	3.799.039	-33.917	3.747.940	-39.546
3.775.692	-45.353	3.799.208	-33.910	3.748.100	-39.540
3.775.952	-45.352	3.799.345	-33.902	3.748.341	-39.535

3.776.104	-45.351	3.799.525	-33.895	3.748.484	-39.529
3.776.208	-45.349	3.799.785	-33.888	3.748.600	-39.523
3.776.367	-45.348	3.799.893	-33.881	3.748.763	-39.517
3.776.519	-45.347	3.800.027	-33.873	3.748.898	-39.512
3.776.628	-45.346	3.800.345	-33.866	3.749.010	-39.506
3.776.773	-45.345	3.800.551	-33.859	3.749.243	-39.500
3.776.939	-45.344	3.800.689	-33.851	3.749.431	-39.494
3.777.092	-45.343	3.800.894	-33.844	3.749.570	-39.488
3.777.261	-45.342	3.801.089	-33.837	3.749.732	-39.483
3.777.416	-45.341	3.801.235	-33.830	3.749.948	-39.477
3.777.612	-45.340	3.801.333	-33.823	3.750.199	-39.471
3.777.813	-45.339	3.801.449	-33.815	3.750.368	-39.465
3.777.987	-45.338	3.801.581	-33.808	3.750.587	-39.459
3.778.195	-45.337	3.801.714	-33.801	3.750.789	-39.453
3.778.380	-45.336	3.801.873	-33.794	3.750.970	-39.447
3.778.546	-45.335	3.802.054	-33.787	3.751.158	-39.441
3.778.720	-45.334	3.802.198	-33.779	3.751.290	-39.435
3.778.847	-45.333	3.802.386	-33.772	3.751.419	-39.429
3.778.945	-45.332	3.802.575	-33.765	3.751.541	-39.423
3.779.110	-45.330	3.802.755	-33.758	3.751.711	-39.417
3.779.337	-45.329	3.802.995	-33.751	3.751.906	-39.411
3.779.534	-45.328	3.803.232	-33.744	3.752.029	-39.404
3.779.738	-45.327	3.803.383	-33.736	3.752.137	-39.398
3.779.890	-45.326	3.803.553	-33.729	3.752.339	-39.392
3.779.995	-45.325	3.803.716	-33.722	3.752.513	-39.386
3.780.132	-45.324	3.803.836	-33.715	3.752.629	-39.380
3.780.327	-45.323	3.803.994	-33.708	3.752.794	-39.373
3.780.500	-45.322	3.804.135	-33.701	3.753.011	-39.367
3.780.670	-45.321	3.804.285	-33.694	3.753.226	-39.361
3.780.851	-45.320	3.804.500	-33.687	3.753.371	-39.354

3.780.971	-45.318	3.804.731	-33.680	3.753.619	-39.348
3.781.156	-45.317	3.804.890	-33.673	3.753.806	-39.341
3.781.375	-45.316	3.805.070	-33.665	3.753.887	-39.335
3.781.541	-45.315	3.805.291	-33.658	3.754.089	-39.328
3.781.671	-45.314	3.805.477	-33.651	3.754.259	-39.322
3.781.819	-45.313	3.805.569	-33.644	3.754.373	-39.315
3.781.931	-45.312	3.805.695	-33.637	3.754.559	-39.309
3.782.141	-45.311	3.805.950	-33.630	3.754.761	-39.302
3.782.362	-45.310	3.806.159	-33.623	3.754.897	-39.296
3.782.535	-45.309	3.806.295	-33.616	3.755.053	-39.289
3.782.628	-45.307	3.806.458	-33.609	3.755.273	-39.282
3.782.795	-45.306	3.806.615	-33.602	3.755.493	-39.276
3.782.964	-45.305	3.806.758	-33.595	3.755.632	-39.269
3.783.107	-45.304	3.806.922	-33.588	3.755.728	-39.262
3.783.554	-45.303	3.807.070	-33.581	3.755.885	-39.255
3.784.061	-45.302	3.807.227	-33.574	3.756.069	-39.249
3.784.230	-45.301	3.807.427	-33.567	3.756.260	-39.242
3.784.262	-45.300	3.807.593	-33.560	3.756.483	-39.235
3.784.350	-45.299	3.807.741	-33.553	3.756.632	-39.228
3.784.362	-45.297	3.807.854	-33.546	3.756.779	-39.221
3.784.323	-45.296	3.807.946	-33.539	3.757.036	-39.214
3.784.420	-45.295	3.808.376	-33.533	3.757.181	-39.207
3.784.622	-45.294	3.808.900	-33.526	3.757.263	-39.200
3.784.790	-45.293	3.809.081	-33.519	3.757.459	-39.193
3.784.996	-45.292	3.809.113	-33.512	3.757.636	-39.186
3.785.186	-45.291	3.809.185	-33.505	3.757.805	-39.179
3.785.352	-45.290	3.809.238	-33.498	3.757.991	-39.172
3.785.530	-45.288	3.809.272	-33.491	3.758.127	-39.165
3.785.724	-45.287	3.809.326	-33.484	3.758.298	-39.158
3.785.924	-45.286	3.809.445	-33.478	3.758.493	-39.151

3.786.102	-45.285	3.809.646	-33.471	3.758.659	-39.144
3.786.246	-45.284	3.809.818	-33.464	3.758.824	-39.137
3.786.360	-45.283	3.809.980	-33.457	3.758.994	-39.130
3.786.514	-45.282	3.810.143	-33.450	3.759.201	-39.123
3.786.631	-45.280	3.810.378	-33.444	3.759.417	-39.115
3.786.799	-45.279	3.810.606	-33.437	3.759.487	-39.108
3.787.029	-45.278	3.810.791	-33.430	3.759.597	-39.101
3.787.164	-45.277	3.811.031	-33.423	3.759.964	-39.094
3.787.260	-45.276	3.811.219	-33.417	3.760.471	-39.087
3.787.423	-45.275	3.811.324	-33.410	3.760.722	-39.080
3.787.581	-45.274	3.811.408	-33.403	3.760.757	-39.072
3.787.726	-45.272	3.811.537	-33.397	3.760.876	-39.065
3.787.958	-45.271	3.811.703	-33.390	3.760.909	-39.058
3.788.214	-45.270	3.811.885	-33.383	3.760.916	-39.051
3.788.376	-45.269	3.812.057	-33.377	3.761.043	-39.043
3.788.565	-45.268	3.812.227	-33.370	3.761.134	-39.036
3.788.775	-45.267	3.812.372	-33.364	3.761.234	-39.029
3.788.858	-45.265	3.812.520	-33.357	3.761.427	-39.022
3.788.934	-45.264	3.812.520	-33.350	3.761.604	-39.014
3.789.102	-45.263	3.812.982	-33.344	3.761.759	-39.007
3.789.274	-45.262	3.813.165	-33.337	3.761.949	-39.000
3.789.462	-45.261	3.813.319	-33.331	3.762.070	-38.992
3.789.667	-45.260	3.813.531	-33.324	3.762.192	-38.985
3.789.836	-45.258	3.813.694	-33.318	3.762.428	-38.978
3.790.014	-45.257	3.813.836	-33.311	3.762.643	-38.971
3.790.179	-45.256	3.814.024	-33.305	3.762.825	-38.963
3.790.341	-45.255	3.814.131	-33.298	3.763.000	-38.956
3.790.451	-45.254	3.814.167	-33.292	3.763.114	-38.949
3.790.616	-45.253	3.814.348	-33.286	3.763.272	-38.942
3.790.863	-45.251	3.814.511	-33.279	3.763.481	-38.934

3.791.024	-45.250	3.814.653	-33.273	3.763.645	-38.927
3.791.183	-45.249	3.814.890	-33.266	3.763.828	-38.920
3.791.321	-45.248	3.815.078	-33.260	3.763.987	-38.913
3.791.495	-45.247	3.815.217	-33.254	3.764.115	-38.906
3.791.685	-45.245	3.815.412	-33.247	3.764.236	-38.899
3.791.842	-45.244	3.815.596	-33.241	3.764.360	-38.892
3.792.000	-45.243	3.815.743	-33.235	3.764.533	-38.885
3.792.209	-45.242	3.815.881	-33.228	3.764.656	-38.878
3.792.422	-45.241	3.816.018	-33.222	3.764.847	-38.870
3.792.484	-45.239	3.816.194	-33.216	3.765.117	-38.863
3.792.628	-45.238	3.816.389	-33.210	3.765.316	-38.856
3.792.882	-45.237	3.816.606	-33.203	3.765.479	-38.850
3.793.118	-45.236	3.816.831	-33.197	3.765.652	-38.843
3.793.272	-45.235	3.816.974	-33.191	3.765.887	-38.836
3.793.344	-45.233	3.817.109	-33.185	3.766.093	-38.829
3.793.497	-45.232	3.817.303	-33.178	3.766.227	-38.822
3.793.683	-45.231	3.817.408	-33.172	3.766.385	-38.815
3.793.876	-45.230	3.817.531	-33.166	3.766.490	-38.808
3.794.088	-45.228	3.817.742	-33.160	3.766.596	-38.801
3.794.193	-45.227	3.817.946	-33.153	3.766.790	-38.795
3.794.266	-45.226	3.818.128	-33.147	3.766.972	-38.788
3.794.431	-45.225	3.818.297	-33.141	3.767.148	-38.781
3.794.655	-45.224	3.818.525	-33.135	3.767.308	-38.774
3.794.810	-45.222	3.818.730	-33.128	3.767.440	-38.768
3.794.917	-45.221	3.818.793	-33.122	3.767.629	-38.761
3.795.007	-45.220	3.818.896	-33.116	3.767.796	-38.754
3.795.130	-45.219	3.819.073	-33.110	3.767.975	-38.748
3.795.321	-45.217	3.819.254	-33.103	3.768.156	-38.741
3.795.479	-45.216	3.819.410	-33.097	3.768.300	-38.735
3.795.605	-45.215	3.819.576	-33.091	3.768.506	-38.728

3.795.797	-45.214	3.819.739	-33.085	3.768.730	-38.722
3.796.033	-45.213	3.819.851	-33.078	3.768.871	-38.715
3.796.272	-45.211	3.820.028	-33.072	3.768.933	-38.709
3.796.465	-45.210	3.820.169	-33.066	3.769.075	-38.702
3.796.672	-45.209	3.820.273	-33.059	3.769.247	-38.696
3.796.896	-45.208	3.820.439	-33.053	3.769.409	-38.690
3.797.039	-45.206	3.820.646	-33.046	3.769.648	-38.684
3.797.174	-45.205	3.820.844	-33.040	3.769.872	-38.677
3.797.325	-45.204	3.821.043	-33.033	3.770.081	-38.671
3.797.505	-45.203	3.821.203	-33.027	3.770.241	-38.665
3.797.690	-45.201	3.821.440	-33.020	3.770.303	-38.659
3.797.777	-45.200	3.821.703	-33.014	3.770.457	-38.653
3.797.849	-45.199	3.821.881	-33.007	3.770.742	-38.647
3.797.993	-45.198	3.821.982	-33.001	3.770.920	-38.640
3.798.149	-45.197	3.822.108	-32.994	3.771.024	-38.634
3.798.276	-45.195	3.822.271	-32.987	3.771.237	-38.628
3.798.529	-45.194	3.822.395	-32.981	3.771.378	-38.622
3.798.777	-45.193	3.822.557	-32.974	3.771.469	-38.616
3.799.001	-45.192	3.822.713	-32.967	3.771.643	-38.610
3.799.208	-45.190	3.822.825	-32.961	3.771.791	-38.604
3.799.341	-45.189	3.822.935	-32.954	3.771.975	-38.598
3.799.492	-45.188	3.823.096	-32.947	3.772.131	-38.592
3.799.667	-45.187	3.823.323	-32.940	3.772.294	-38.586
3.799.803	-45.185	3.823.560	-32.933	3.772.552	-38.580
3.799.995	-45.184	3.823.759	-32.926	3.772.705	-38.574
3.800.230	-45.183	3.823.891	-32.919	3.772.799	-38.569
3.800.401	-45.182	3.824.056	-32.912	3.773.000	-38.563
3.800.477	-45.180	3.824.258	-32.905	3.773.246	-38.557
3.800.696	-45.179	3.824.442	-32.898	3.773.413	-38.551
3.800.934	-45.178	3.824.617	-32.891	3.773.528	-38.545

3.801.057	-45.177	3.824.732	-32.884	3.773.637	-38.539
3.801.216	-45.175	3.824.877	-32.877	3.773.793	-38.533
3.801.401	-45.174	3.825.078	-32.870	3.773.961	-38.528
3.801.571	-45.173	3.825.273	-32.862	3.774.114	-38.522
3.801.734	-45.172	3.825.459	-32.855	3.774.272	-38.516
3.801.892	-45.170	3.825.566	-32.848	3.774.417	-38.510
3.802.048	-45.169	3.825.730	-32.840	3.774.605	-38.504
3.802.193	-45.168	3.825.930	-32.833	3.774.826	-38.498
3.802.301	-45.167	3.826.066	-32.825	3.775.045	-38.493
3.802.453	-45.165	3.826.183	-32.818	3.775.211	-38.487
3.802.629	-45.164	3.826.347	-32.810	3.775.396	-38.481
3.802.823	-45.163	3.826.582	-32.803	3.775.584	-38.475
3.803.027	-45.162	3.826.766	-32.795	3.775.766	-38.469
3.803.224	-45.160	3.826.914	-32.788	3.775.981	-38.464
3.803.396	-45.159	3.827.073	-32.780	3.776.147	-38.458
3.803.528	-45.158	3.827.289	-32.772	3.776.268	-38.452
3.803.665	-45.157	3.827.444	-32.764	3.776.393	-38.446
3.803.828	-45.155	3.827.552	-32.757	3.776.546	-38.440
3.804.008	-45.154	3.827.759	-32.749	3.776.747	-38.435
3.804.153	-45.153	3.828.002	-32.741	3.776.929	-38.429
3.804.277	-45.152	3.828.163	-32.733	3.777.047	-38.423
3.804.645	-45.150	3.828.300	-32.725	3.777.144	-38.417
3.805.101	-45.149	3.828.484	-32.717	3.777.314	-38.411
3.805.307	-45.148	3.828.631	-32.709	3.777.481	-38.405
3.805.383	-45.146	3.828.716	-32.701	3.777.650	-38.399
3.805.424	-45.145	3.828.994	-32.693	3.777.791	-38.394
3.805.515	-45.144	3.829.464	-32.685	3.777.933	-38.388
3.805.599	-45.143	3.829.850	-32.677	3.778.123	-38.382
3.805.639	-45.141	3.829.998	-32.669	3.778.273	-38.376
3.805.735	-45.140	3.830.011	-32.661	3.778.463	-38.370

3.805.934	-45.139	3.830.025	-32.652	3.778.582	-38.364
3.806.008	-45.137	3.830.013	-32.644	3.778.759	-38.358
3.806.216	-45.136	3.830.099	-32.636	3.779.021	-38.352
3.806.563	-45.135	3.830.233	-32.627	3.779.221	-38.346
3.806.716	-45.134	3.830.386	-32.619	3.779.410	-38.340
3.806.874	-45.132	3.830.540	-32.611	3.779.619	-38.334
3.807.079	-45.131	3.830.750	-32.602	3.779.775	-38.329
3.807.238	-45.130	3.830.981	-32.594	3.779.875	-38.323
3.807.401	-45.128	3.831.118	-32.585	3.780.027	-38.317
3.807.506	-45.127	3.831.322	-32.577	3.780.229	-38.311
3.807.619	-45.126	3.831.530	-32.568	3.780.421	-38.305
3.807.818	-45.124	3.831.689	-32.560	3.780.500	-38.299
3.808.022	-45.123	3.831.830	-32.551	3.780.643	-38.293
3.808.146	-45.122	3.831.980	-32.543	3.780.906	-38.287
3.808.304	-45.120	3.832.162	-32.534	3.781.075	-38.281
3.808.507	-45.119	3.832.275	-32.526	3.781.245	-38.275
3.808.739	-45.118	3.832.359	-32.517	3.781.389	-38.269
3.808.884	-45.116	3.832.500	-32.508	3.781.563	-38.263
3.809.034	-45.115	3.832.766	-32.500	3.781.759	-38.257
3.809.223	-45.114	3.832.935	-32.491	3.781.949	-38.251
3.809.312	-45.112	3.833.013	-32.482	3.782.139	-38.244
3.809.514	-45.111	3.833.199	-32.474	3.782.238	-38.238
3.809.760	-45.110	3.833.405	-32.465	3.782.339	-38.232
3.809.924	-45.108	3.833.674	-32.456	3.782.604	-38.226
3.810.074	-45.107	3.833.885	-32.448	3.782.858	-38.220
3.810.242	-45.105	3.833.918	-32.439	3.782.986	-38.214
3.810.371	-45.104	3.834.070	-32.430	3.783.168	-38.208
3.810.482	-45.103	3.834.310	-32.422	3.783.349	-38.202
3.810.609	-45.101	3.834.483	-32.413	3.783.441	-38.196
3.810.719	-45.100	3.834.698	-32.405	3.783.598	-38.190

3.810.844	-45.099	3.834.828	-32.396	3.783.817	-38.184
3.811.069	-45.097	3.834.924	-32.387	3.783.972	-38.178
3.811.337	-45.096	3.835.063	-32.379	3.784.099	-38.171
3.811.513	-45.094	3.835.208	-32.370	3.784.263	-38.165
3.811.658	-45.093	3.835.444	-32.361	3.784.428	-38.159
3.811.808	-45.091	3.835.630	-32.353	3.784.569	-38.153
3.811.953	-45.090	3.835.758	-32.344	3.784.778	-38.147
3.812.113	-45.089	3.835.999	-32.336	3.785.154	-38.141
3.812.285	-45.087	3.836.198	-32.327	3.785.551	-38.135
3.812.426	-45.086	3.836.342	-32.319	3.785.773	-38.129
3.812.581	-45.084	3.836.455	-32.310	3.785.830	-38.123
3.812.734	-45.083	3.836.591	-32.302	3.785.867	-38.117
3.812.917	-45.081	3.836.744	-32.293	3.786.006	-38.111
3.813.131	-45.080	3.836.915	-32.285	3.786.084	-38.104
3.813.337	-45.078	3.837.138	-32.276	3.786.134	-38.098
3.813.507	-45.077	3.837.351	-32.268	3.786.227	-38.092
3.813.626	-45.075	3.837.478	-32.260	3.786.360	-38.086
3.813.773	-45.074	3.837.539	-32.251	3.786.480	-38.080
3.813.926	-45.072	3.837.686	-32.243	3.786.649	-38.074
3.814.144	-45.071	3.837.857	-32.235	3.786.907	-38.068
3.814.384	-45.069	3.838.026	-32.227	3.787.050	-38.062
3.814.580	-45.068	3.838.225	-32.218	3.787.246	-38.055
3.814.711	-45.066	3.838.423	-32.210	3.787.412	-38.049
3.814.841	-45.065	3.838.638	-32.202	3.787.601	-38.043
3.814.995	-45.063	3.838.849	-32.194	3.787.841	-38.037
3.815.134	-45.062	3.839.008	-32.186	3.787.908	-38.031
3.815.345	-45.060	3.839.089	-32.178	3.788.012	-38.025
3.815.558	-45.059	3.839.238	-32.170	3.788.210	-38.019
3.815.730	-45.057	3.839.475	-32.162	3.788.387	-38.013
3.815.896	-45.056	3.839.663	-32.154	3.788.518	-38.007

3.816.050	-45.054	3.839.843	-32.146	3.788.690	-38.001
3.816.129	-45.053	3.839.992	-32.138	3.788.937	-37.995
3.816.246	-45.051	3.840.105	-32.130	3.789.147	-37.988
3.816.411	-45.050	3.840.231	-32.122	3.789.301	-37.982
3.816.548	-45.048	3.840.378	-32.114	3.789.406	-37.976
3.816.707	-45.046	3.840.519	-32.107	3.789.543	-37.970
3.816.891	-45.045	3.840.663	-32.099	3.789.709	-37.964
3.817.108	-45.043	3.840.794	-32.091	3.789.844	-37.958
3.817.315	-45.042	3.840.906	-32.083	3.790.034	-37.952
3.817.453	-45.040	3.841.066	-32.076	3.790.216	-37.946
3.817.641	-45.039	3.841.293	-32.068	3.790.386	-37.940
3.817.885	-45.037	3.841.537	-32.060	3.790.576	-37.934
3.818.063	-45.035	3.841.736	-32.053	3.790.793	-37.929
3.818.210	-45.034	3.841.931	-32.045	3.791.094	-37.923
3.818.420	-45.032	3.842.169	-32.037	3.791.255	-37.917
3.818.629	-45.031	3.842.341	-32.030	3.791.346	-37.911
3.818.723	-45.029	3.842.493	-32.022	3.791.483	-37.905
3.818.806	-45.028	3.842.695	-32.015	3.791.654	-37.899
3.818.936	-45.026	3.842.849	-32.007	3.791.892	-37.893
3.819.068	-45.024	3.842.986	-32.000	3.792.005	-37.887
3.819.247	-45.023	3.843.154	-31.992	3.792.102	-37.881
3.819.406	-45.021	3.843.279	-31.985	3.792.267	-37.875
3.819.561	-45.020	3.843.398	-31.977	3.792.426	-37.869
3.819.760	-45.018	3.843.506	-31.970	3.792.532	-37.863
3.819.919	-45.016	3.843.630	-31.962	3.792.677	-37.858
3.820.057	-45.015	3.843.813	-31.955	3.792.860	-37.852
3.820.276	-45.013	3.844.016	-31.947	3.793.013	-37.846
3.820.526	-45.012	3.844.247	-31.940	3.793.214	-37.840
3.820.735	-45.010	3.844.457	-31.933	3.793.424	-37.834
3.820.874	-45.008	3.844.628	-31.925	3.793.616	-37.828

3.821.024	-45.007	3.844.840	-31.918	3.793.784	-37.823
3.821.219	-45.005	3.845.061	-31.911	3.793.915	-37.817
3.821.346	-45.003	3.845.219	-31.903	3.794.008	-37.811
3.821.466	-45.002	3.845.349	-31.896	3.794.135	-37.805
3.821.633	-45.000	3.845.479	-31.889	3.794.315	-37.800
3.821.750	-44.999	3.845.647	-31.881	3.794.522	-37.794
3.821.943	-44.997	3.845.808	-31.874	3.794.711	-37.788
3.822.209	-44.995	3.845.953	-31.867	3.794.906	-37.783
3.822.431	-44.994	3.846.188	-31.859	3.795.168	-37.777
3.822.597	-44.992	3.846.372	-31.852	3.795.338	-37.771
3.822.709	-44.991	3.846.481	-31.845	3.795.455	-37.765
3.822.889	-44.989	3.846.635	-31.838	3.795.595	-37.760
3.823.065	-44.987	3.846.820	-31.830	3.795.786	-37.754
3.823.235	-44.986	3.847.004	-31.823	3.795.974	-37.748
3.823.405	-44.984	3.847.152	-31.816	3.796.127	-37.743
3.823.544	-44.982	3.847.300	-31.808	3.796.225	-37.737
3.823.712	-44.981	3.847.499	-31.801	3.796.326	-37.731
3.823.932	-44.979	3.847.650	-31.794	3.796.509	-37.726
3.824.066	-44.978	3.847.758	-31.787	3.796.714	-37.720
3.824.201	-44.976	3.847.973	-31.779	3.796.929	-37.714
3.824.450	-44.974	3.848.230	-31.772	3.797.139	-37.708
3.824.633	-44.973	3.848.395	-31.765	3.797.268	-37.703
3.824.760	-44.971	3.848.515	-31.757	3.797.386	-37.697
3.824.933	-44.970	3.848.663	-31.750	3.797.567	-37.691
3.825.121	-44.968	3.848.829	-31.743	3.797.755	-37.685
3.825.266	-44.966	3.849.027	-31.736	3.797.911	-37.680
3.825.410	-44.965	3.849.214	-31.728	3.798.056	-37.674
3.825.614	-44.963	3.849.366	-31.721	3.798.273	-37.668
3.825.948	-44.961	3.849.510	-31.714	3.798.483	-37.662
3.826.293	-44.960	3.849.619	-31.706	3.798.706	-37.657

3.826.473	-44.958	3.849.922	-31.699	3.798.883	-37.651
3.826.570	-44.957	3.850.417	-31.692	3.798.967	-37.645
3.826.697	-44.955	3.850.715	-31.684	3.799.128	-37.639
3.826.743	-44.953	3.850.789	-31.677	3.799.279	-37.633
3.826.703	-44.952	3.850.794	-31.670	3.799.359	-37.627
3.826.779	-44.950	3.850.818	-31.662	3.799.485	-37.621
3.826.943	-44.949	3.850.935	-31.655	3.799.662	-37.616
3.827.091	-44.947	3.851.042	-31.647	3.799.857	-37.610
3.827.271	-44.945	3.851.089	-31.640	3.800.042	-37.604
3.827.473	-44.944	3.851.223	-31.633	3.800.217	-37.598
3.827.654	-44.942	3.851.430	-31.625	3.800.396	-37.592
3.827.921	-44.940	3.851.635	-31.618	3.800.618	-37.586
3.828.143	-44.939	3.851.783	-31.610	3.800.894	-37.580
3.828.300	-44.937	3.851.982	-31.603	3.801.108	-37.574
3.828.454	-44.936	3.852.207	-31.595	3.801.235	-37.568
3.828.593	-44.934	3.852.361	-31.588	3.801.375	-37.561
3.828.777	-44.932	3.852.540	-31.580	3.801.534	-37.555
3.828.894	-44.931	3.852.744	-31.573	3.801.711	-37.549
3.829.024	-44.929	3.852.885	-31.565	3.801.809	-37.543
3.829.187	-44.927	3.853.022	-31.558	3.801.892	-37.537
3.829.371	-44.926	3.853.192	-31.550	3.802.079	-37.531
3.829.511	-44.924	3.853.313	-31.542	3.802.242	-37.524
3.829.572	-44.923	3.853.392	-31.535	3.802.388	-37.518
3.829.789	-44.921	3.853.511	-31.527	3.802.557	-37.512
3.830.034	-44.919	3.853.728	-31.520	3.802.745	-37.506
3.830.216	-44.918	3.853.951	-31.512	3.802.939	-37.499
3.830.389	-44.916	3.854.126	-31.505	3.803.116	-37.493
3.830.522	-44.915	3.854.342	-31.497	3.803.295	-37.487
3.830.721	-44.913	3.854.576	-31.489	3.803.466	-37.480
3.830.956	-44.911	3.854.749	-31.482	3.803.672	-37.474

3.831.125	-44.910	3.854.883	-31.474	3.803.847	-37.467
3.831.234	-44.908	3.855.034	-31.466	3.803.983	-37.461
3.831.411	-44.906	3.855.195	-31.459	3.804.204	-37.455
3.831.578	-44.905	3.855.327	-31.451	3.804.422	-37.448
3.831.693	-44.903	3.855.484	-31.444	3.804.623	-37.442
3.831.773	-44.902	3.855.726	-31.436	3.804.825	-37.435
3.831.960	-44.900	3.855.877	-31.428	3.804.940	-37.429
3.832.193	-44.898	3.855.963	-31.421	3.805.085	-37.422
3.832.343	-44.897	3.856.111	-31.413	3.805.216	-37.416
3.832.513	-44.895	3.856.264	-31.405	3.805.357	-37.409
3.832.679	-44.893	3.856.443	-31.398	3.805.583	-37.403
3.832.897	-44.892	3.856.608	-31.390	3.805.772	-37.396
3.833.088	-44.890	3.856.754	-31.383	3.805.873	-37.390
3.833.181	-44.889	3.856.946	-31.375	3.805.957	-37.383
3.833.326	-44.887	3.857.121	-31.368	3.806.167	-37.376
3.833.474	-44.885	3.857.253	-31.360	3.806.416	-37.370
3.833.651	-44.884	3.857.411	-31.352	3.806.588	-37.363
3.833.818	-44.882	3.857.605	-31.345	3.806.725	-37.357
3.833.985	-44.880	3.857.810	-31.337	3.806.821	-37.350
3.834.185	-44.879	3.857.946	-31.330	3.806.964	-37.343
3.834.333	-44.877	3.858.067	-31.322	3.807.180	-37.337
3.834.492	-44.875	3.858.311	-31.315	3.807.355	-37.330
3.834.673	-44.874	3.858.586	-31.308	3.807.571	-37.323
3.834.876	-44.872	3.858.755	-31.300	3.807.764	-37.317
3.835.002	-44.871	3.858.854	-31.293	3.807.892	-37.310
3.835.128	-44.869	3.858.979	-31.285	3.808.059	-37.304
3.835.327	-44.867	3.859.175	-31.278	3.808.186	-37.297
3.835.490	-44.866	3.859.352	-31.271	3.808.355	-37.290
3.835.624	-44.864	3.859.504	-31.264	3.808.543	-37.284
3.835.797	-44.862	3.859.680	-31.256	3.808.720	-37.277

3.836.050	-44.861	3.859.857	-31.249	3.808.918	-37.270
3.836.243	-44.859	3.860.067	-31.242	3.809.073	-37.264
3.836.391	-44.857	3.860.204	-31.235	3.809.215	-37.257
3.836.573	-44.856	3.860.323	-31.228	3.809.397	-37.251
3.836.707	-44.854	3.860.515	-31.221	3.809.505	-37.244
3.836.841	-44.853	3.860.721	-31.214	3.809.597	-37.237
3.837.047	-44.851	3.860.862	-31.207	3.809.938	-37.231
3.837.202	-44.849	3.860.959	-31.200	3.810.368	-37.224
3.837.275	-44.848	3.861.093	-31.193	3.810.674	-37.218
3.837.336	-44.846	3.861.237	-31.186	3.810.826	-37.211
3.837.507	-44.844	3.861.364	-31.179	3.810.863	-37.204
3.837.759	-44.843	3.861.499	-31.172	3.810.948	-37.198
3.837.935	-44.841	3.861.669	-31.165	3.811.062	-37.191
3.838.036	-44.839	3.861.805	-31.159	3.811.136	-37.185
3.838.186	-44.838	3.861.982	-31.152	3.811.259	-37.178
3.838.403	-44.836	3.862.233	-31.145	3.811.360	-37.172
3.838.629	-44.834	3.862.417	-31.139	3.811.425	-37.165
3.838.882	-44.833	3.862.643	-31.132	3.811.606	-37.158
3.839.113	-44.831	3.862.894	-31.125	3.811.866	-37.152
3.839.309	-44.830	3.863.071	-31.119	3.812.051	-37.145
3.839.445	-44.828	3.863.242	-31.112	3.812.236	-37.139
3.839.586	-44.826	3.863.391	-31.106	3.812.480	-37.132
3.839.749	-44.825	3.863.571	-31.100	3.812.610	-37.126
3.839.946	-44.823	3.863.717	-31.093	3.812.742	-37.119
3.840.103	-44.821	3.863.802	-31.087	3.812.947	-37.113
3.840.170	-44.820	3.863.940	-31.081	3.813.094	-37.106
3.840.240	-44.818	3.864.104	-31.074	3.813.233	-37.100
3.840.338	-44.817	3.864.234	-31.068	3.813.378	-37.093
3.840.531	-44.815	3.864.421	-31.062	3.813.528	-37.087
3.840.813	-44.813	3.864.597	-31.056	3.813.680	-37.081

3.841.018	-44.812	3.864.731	-31.050	3.813.883	-37.074
3.841.112	-44.810	3.864.868	-31.044	3.814.041	-37.068
3.841.260	-44.808	3.864.935	-31.038	3.814.161	-37.061
3.841.477	-44.807	3.865.191	-31.032	3.814.380	-37.055
3.841.671	-44.805	3.865.504	-31.026	3.814.539	-37.049
3.841.845	-44.804	3.865.667	-31.020	3.814.623	-37.043
3.842.023	-44.802	3.865.837	-31.014	3.814.756	-37.036
3.842.192	-44.800	3.866.001	-31.008	3.814.988	-37.030
3.842.372	-44.799	3.866.137	-31.003	3.815.255	-37.024
3.842.592	-44.797	3.866.351	-30.997	3.815.433	-37.018
3.842.753	-44.795	3.866.584	-30.991	3.815.544	-37.011
3.842.863	-44.794	3.866.679	-30.986	3.815.728	-37.005
3.843.005	-44.792	3.866.781	-30.980	3.815.894	-36.999
3.843.135	-44.791	3.866.967	-30.975	3.815.990	-36.993
3.843.297	-44.789	3.867.173	-30.969	3.816.217	-36.987
3.843.506	-44.787	3.867.399	-30.964	3.816.429	-36.981
3.843.690	-44.786	3.867.589	-30.959	3.816.521	-36.975
3.843.882	-44.784	3.867.759	-30.953	3.816.718	-36.969
3.844.048	-44.782	3.867.925	-30.948	3.816.913	-36.963
3.844.232	-44.781	3.868.067	-30.943	3.817.000	-36.956
3.844.471	-44.779	3.868.203	-30.938	3.817.169	-36.951
3.844.630	-44.778	3.868.363	-30.933	3.817.325	-36.945
3.844.733	-44.776	3.868.582	-30.928	3.817.475	-36.939
3.844.955	-44.774	3.868.752	-30.923	3.817.706	-36.933
3.845.173	-44.773	3.868.889	-30.918	3.817.897	-36.927
3.845.249	-44.771	3.869.026	-30.913	3.817.987	-36.921
3.845.349	-44.770	3.869.178	-30.908	3.818.163	-36.915
3.845.569	-44.768	3.869.366	-30.903	3.818.365	-36.909
3.845.758	-44.766	3.869.541	-30.898	3.818.506	-36.903
3.845.901	-44.765	3.869.696	-30.894	3.818.701	-36.898

3.846.086	-44.763	3.869.850	-30.889	3.818.876	-36.892
3.846.255	-44.762	3.870.053	-30.884	3.818.987	-36.886
3.846.369	-44.760	3.870.234	-30.880	3.819.074	-36.880
3.846.416	-44.758	3.870.299	-30.875	3.819.302	-36.875
3.846.635	-44.757	3.870.464	-30.871	3.819.579	-36.869
3.847.138	-44.755	3.870.860	-30.866	3.819.648	-36.863
3.847.460	-44.754	3.871.259	-30.862	3.819.742	-36.858
3.847.599	-44.752	3.871.448	-30.858	3.819.999	-36.852
3.847.705	-44.750	3.871.537	-30.853	3.820.204	-36.846
3.847.800	-44.749	3.871.617	-30.849	3.820.386	-36.841
3.847.890	-44.747	3.871.679	-30.845	3.820.547	-36.835
3.847.986	-44.746	3.871.736	-30.841	3.820.652	-36.829
3.848.042	-44.744	3.871.770	-30.836	3.820.819	-36.824
3.848.138	-44.742	3.871.898	-30.832	3.820.988	-36.818
3.848.324	-44.741	3.872.104	-30.828	3.821.133	-36.813
3.848.478	-44.739	3.872.267	-30.824	3.821.292	-36.807
3.848.630	-44.738	3.872.451	-30.820	3.821.479	-36.801
3.848.835	-44.736	3.872.621	-30.816	3.821.711	-36.796
3.849.001	-44.734	3.872.777	-30.812	3.821.874	-36.790
3.849.171	-44.733	3.873.004	-30.808	3.822.068	-36.784
3.849.356	-44.731	3.873.255	-30.805	3.822.224	-36.779
3.849.572	-44.730	3.873.448	-30.801	3.822.433	-36.773
3.849.720	-44.728	3.873.515	-30.797	3.822.628	-36.767
3.849.764	-44.726	3.873.669	-30.793	3.822.758	-36.762
3.849.937	-44.725	3.873.885	-30.789	3.822.889	-36.756
3.850.123	-44.723	3.874.003	-30.786	3.823.087	-36.750
3.850.269	-44.722	3.874.168	-30.782	3.823.311	-36.745
3.850.439	-44.720	3.874.335	-30.778	3.823.389	-36.739
3.850.553	-44.718	3.874.462	-30.775	3.823.477	-36.733
3.850.773	-44.717	3.874.639	-30.771	3.823.604	-36.727

3.850.971	-44.715	3.874.848	-30.768	3.823.730	-36.721
3.851.118	-44.714	3.875.071	-30.764	3.823.871	-36.716
3.851.310	-44.712	3.875.273	-30.761	3.824.067	-36.710
3.851.528	-44.710	3.875.419	-30.757	3.824.276	-36.704
3.851.736	-44.709	3.875.599	-30.754	3.824.456	-36.698
3.851.875	-44.707	3.875.818	-30.750	3.824.628	-36.692
3.852.068	-44.705	3.875.910	-30.747	3.824.775	-36.686
3.852.265	-44.704	3.876.017	-30.743	3.824.967	-36.680
3.852.390	-44.702	3.876.151	-30.740	3.825.208	-36.674
3.852.477	-44.701	3.876.332	-30.737	3.825.446	-36.668
3.852.704	-44.699	3.876.519	-30.733	3.825.663	-36.662
3.852.915	-44.697	3.876.661	-30.730	3.825.844	-36.656
3.852.939	-44.696	3.876.787	-30.727	3.825.985	-36.650
3.853.041	-44.694	3.876.971	-30.723	3.826.098	-36.644
3.853.315	-44.692	3.877.191	-30.720	3.826.275	-36.637
3.853.593	-44.691	3.877.333	-30.717	3.826.451	-36.631
3.853.734	-44.689	3.877.437	-30.713	3.826.544	-36.625
3.853.867	-44.688	3.877.617	-30.710	3.826.714	-36.619
3.854.044	-44.686	3.877.854	-30.707	3.826.927	-36.612
3.854.191	-44.684	3.878.067	-30.703	3.827.004	-36.606
3.854.345	-44.683	3.878.217	-30.700	3.827.094	-36.599
3.854.512	-44.681	3.878.336	-30.697	3.827.278	-36.593
3.854.633	-44.679	3.878.475	-30.694	3.827.445	-36.586
3.854.789	-44.678	3.878.598	-30.690	3.827.652	-36.580
3.855.038	-44.676	3.878.748	-30.687	3.827.876	-36.573
3.855.244	-44.674	3.878.880	-30.684	3.828.100	-36.567
3.855.423	-44.673	3.879.048	-30.681	3.828.269	-36.560
3.855.652	-44.671	3.879.232	-30.677	3.828.383	-36.553
3.855.807	-44.670	3.879.400	-30.674	3.828.582	-36.546
3.855.889	-44.668	3.879.610	-30.671	3.828.774	-36.540

3.856.060	-44.666	3.879.816	-30.668	3.828.956	-36.533
3.856.235	-44.665	3.879.964	-30.664	3.829.182	-36.526
3.856.349	-44.663	3.880.115	-30.661	3.829.330	-36.519
3.856.484	-44.661	3.880.223	-30.658	3.829.482	-36.512
3.856.667	-44.660	3.880.394	-30.654	3.829.641	-36.505
3.856.824	-44.658	3.880.638	-30.651	3.829.884	-36.498
3.856.978	-44.656	3.880.855	-30.648	3.830.117	-36.491
3.857.144	-44.655	3.881.028	-30.645	3.830.152	-36.484
3.857.328	-44.653	3.881.131	-30.641	3.830.260	-36.477
3.857.549	-44.651	3.881.324	-30.638	3.830.479	-36.470
3.857.755	-44.650	3.881.560	-30.635	3.830.656	-36.463
3.857.935	-44.648	3.881.696	-30.631	3.830.838	-36.456
3.858.058	-44.646	3.881.816	-30.628	3.831.014	-36.448
3.858.185	-44.645	3.881.960	-30.624	3.831.176	-36.441
3.858.354	-44.643	3.882.072	-30.621	3.831.328	-36.434
3.858.472	-44.641	3.882.239	-30.618	3.831.469	-36.427
3.858.568	-44.640	3.882.394	-30.614	3.831.638	-36.419
3.858.735	-44.638	3.882.477	-30.611	3.831.768	-36.412
3.858.882	-44.636	3.882.664	-30.607	3.831.917	-36.404
3.858.983	-44.635	3.882.911	-30.604	3.832.148	-36.397
3.859.128	-44.633	3.883.106	-30.600	3.832.257	-36.389
3.859.392	-44.631	3.883.286	-30.596	3.832.386	-36.382
3.859.620	-44.630	3.883.495	-30.593	3.832.640	-36.374
3.859.798	-44.628	3.883.670	-30.589	3.832.817	-36.366
3.860.045	-44.627	3.883.857	-30.585	3.832.957	-36.359
3.860.262	-44.625	3.884.114	-30.582	3.833.124	-36.351
3.860.421	-44.623	3.884.287	-30.578	3.833.300	-36.343
3.860.591	-44.622	3.884.427	-30.574	3.833.442	-36.336
3.860.815	-44.620	3.884.601	-30.570	3.833.548	-36.328
3.860.930	-44.618	3.884.720	-30.567	3.833.748	-36.320

3.860.978	-44.617	3.884.807	-30.563	3.833.969	-36.312
3.861.143	-44.615	3.884.910	-30.559	3.834.167	-36.305
3.861.319	-44.613	3.885.002	-30.555	3.834.326	-36.297
3.861.456	-44.612	3.885.166	-30.551	3.834.440	-36.289
3.861.609	-44.610	3.885.367	-30.547	3.834.689	-36.281
3.861.770	-44.608	3.885.535	-30.543	3.835.049	-36.273
3.861.949	-44.607	3.885.750	-30.539	3.835.446	-36.265
3.862.195	-44.605	3.885.934	-30.535	3.835.704	-36.257
3.862.397	-44.603	3.886.102	-30.531	3.835.787	-36.249
3.862.542	-44.602	3.886.277	-30.527	3.835.838	-36.241
3.862.691	-44.600	3.886.466	-30.523	3.835.912	-36.233
3.862.875	-44.598	3.886.636	-30.519	3.835.982	-36.225
3.863.062	-44.597	3.886.756	-30.514	3.836.060	-36.217
3.863.251	-44.595	3.886.913	-30.510	3.836.169	-36.209
3.863.398	-44.593	3.887.130	-30.506	3.836.264	-36.200
3.863.492	-44.592	3.887.289	-30.502	3.836.454	-36.192
3.863.687	-44.590	3.887.422	-30.497	3.836.674	-36.184
3.863.929	-44.589	3.887.614	-30.493	3.836.837	-36.176
3.864.057	-44.587	3.887.787	-30.488	3.836.935	-36.167
3.864.190	-44.585	3.887.928	-30.484	3.837.047	-36.159
3.864.403	-44.584	3.888.128	-30.480	3.837.227	-36.151
3.864.616	-44.582	3.888.311	-30.475	3.837.386	-36.143
3.864.749	-44.580	3.888.481	-30.471	3.837.599	-36.134
3.864.865	-44.579	3.888.688	-30.466	3.837.827	-36.126
3.865.022	-44.577	3.888.880	-30.461	3.838.035	-36.118
3.865.180	-44.575	3.889.008	-30.457	3.838.232	-36.109
3.865.368	-44.574	3.889.161	-30.452	3.838.363	-36.101
3.865.553	-44.572	3.889.342	-30.448	3.838.493	-36.093
3.865.714	-44.571	3.889.507	-30.443	3.838.684	-36.084
3.865.891	-44.569	3.889.720	-30.438	3.838.904	-36.076

3.866.058	-44.567	3.889.848	-30.433	3.839.016	-36.068
3.866.198	-44.566	3.889.922	-30.428	3.839.128	-36.060
3.866.436	-44.564	3.890.134	-30.424	3.839.252	-36.051
3.866.654	-44.562	3.890.397	-30.419	3.839.395	-36.043
3.866.797	-44.561	3.890.541	-30.414	3.839.568	-36.035
3.866.949	-44.559	3.890.677	-30.409	3.839.680	-36.026
3.867.109	-44.558	3.890.862	-30.404	3.839.875	-36.018
3.867.263	-44.556	3.891.024	-30.399	3.840.140	-36.010
3.867.386	-44.554	3.891.133	-30.394	3.840.325	-36.001
3.867.528	-44.553	3.891.433	-30.389	3.840.562	-35.993
3.867.661	-44.551	3.891.903	-30.384	3.840.773	-35.985
3.868.011	-44.549	3.892.159	-30.379	3.840.863	-35.977
3.868.477	-44.548	3.892.213	-30.374	3.840.970	-35.968
3.868.672	-44.546	3.892.271	-30.369	3.841.147	-35.960
3.868.766	-44.545	3.892.321	-30.364	3.841.310	-35.952
3.868.867	-44.543	3.892.361	-30.359	3.841.419	-35.944
3.868.893	-44.541	3.892.467	-30.354	3.841.620	-35.935
3.869.002	-44.540	3.892.556	-30.348	3.841.848	-35.927
3.869.128	-44.538	3.892.673	-30.343	3.841.990	-35.919
3.869.200	-44.537	3.892.882	-30.338	3.842.062	-35.911
3.869.339	-44.535	3.893.091	-30.333	3.842.188	-35.903
3.869.474	-44.533	3.893.286	-30.327	3.842.368	-35.895
3.869.615	-44.532	3.893.477	-30.322	3.842.587	-35.886
3.869.861	-44.530	3.893.633	-30.317	3.842.777	-35.878
3.870.082	-44.528	3.893.814	-30.312	3.842.899	-35.870
3.870.270	-44.527	3.893.992	-30.306	3.843.029	-35.862
3.870.470	-44.525	3.894.125	-30.301	3.843.196	-35.854
3.870.571	-44.524	3.894.273	-30.296	3.843.482	-35.846
3.870.700	-44.522	3.894.457	-30.290	3.843.676	-35.838
3.870.883	-44.520	3.894.626	-30.285	3.843.799	-35.830

3.871.028	-44.519	3.894.760	-30.280	3.843.969	-35.822
3.871.131	-44.517	3.894.928	-30.274	3.844.070	-35.814
3.871.313	-44.515	3.895.121	-30.269	3.844.247	-35.807
3.871.519	-44.514	3.895.245	-30.264	3.844.472	-35.799
3.871.736	-44.512	3.895.399	-30.258	3.844.671	-35.791
3.871.904	-44.511	3.895.589	-30.253	3.844.828	-35.783
3.872.032	-44.509	3.895.753	-30.248	3.844.998	-35.775
3.872.178	-44.507	3.895.948	-30.242	3.845.227	-35.768
3.872.314	-44.506	3.896.096	-30.237	3.845.386	-35.760
3.872.502	-44.504	3.896.268	-30.231	3.845.556	-35.752
3.872.712	-44.502	3.896.539	-30.226	3.845.688	-35.745
3.872.921	-44.501	3.896.765	-30.221	3.845.869	-35.737
3.873.107	-44.499	3.896.924	-30.215	3.846.019	-35.729
3.873.261	-44.497	3.897.025	-30.210	3.846.064	-35.722
3.873.407	-44.496	3.897.115	-30.204	3.846.199	-35.714
3.873.503	-44.494	3.897.287	-30.199	3.846.375	-35.707
3.873.660	-44.493	3.897.466	-30.194	3.846.557	-35.699
3.873.857	-44.491	3.897.581	-30.188	3.846.765	-35.691
3.874.044	-44.489	3.897.723	-30.183	3.846.978	-35.684
3.874.178	-44.488	3.897.885	-30.177	3.847.138	-35.676
3.874.312	-44.486	3.898.071	-30.172	3.847.271	-35.669
3.874.507	-44.484	3.898.265	-30.167	3.847.464	-35.662
3.874.616	-44.483	3.898.471	-30.161	3.847.687	-35.654
3.874.800	-44.481	3.898.654	-30.156	3.847.809	-35.647
3.875.002	-44.479	3.898.761	-30.150	3.847.919	-35.639
3.875.105	-44.478	3.898.844	-30.145	3.848.107	-35.632
3.875.296	-44.476	3.898.965	-30.140	3.848.253	-35.624
3.875.497	-44.474	3.899.164	-30.134	3.848.405	-35.617
3.875.688	-44.473	3.899.386	-30.129	3.848.602	-35.610
3.875.836	-44.471	3.899.551	-30.124	3.848.768	-35.602

3.875.902	-44.469	3.899.764	-30.118	3.848.907	-35.595
3.876.089	-44.468	3.899.958	-30.113	3.848.994	-35.588
3.876.339	-44.466	3.900.100	-30.107	3.849.162	-35.581
3.876.458	-44.464	3.900.284	-30.102	3.849.381	-35.573
3.876.653	-44.463	3.900.457	-30.097	3.849.537	-35.566
3.876.898	-44.461	3.900.618	-30.091	3.849.689	-35.559
3.877.070	-44.459	3.900.728	-30.086	3.849.865	-35.552
3.877.250	-44.458	3.900.887	-30.081	3.850.123	-35.545
3.877.379	-44.456	3.901.118	-30.075	3.850.331	-35.537
3.877.487	-44.454	3.901.278	-30.070	3.850.526	-35.530
3.877.650	-44.453	3.901.452	-30.065	3.850.671	-35.523
3.877.838	-44.451	3.901.622	-30.059	3.850.854	-35.516
3.877.975	-44.449	3.901.754	-30.054	3.851.044	-35.509
3.878.154	-44.447	3.901.925	-30.049	3.851.227	-35.502
3.878.387	-44.446	3.902.081	-30.044	3.851.407	-35.495
3.878.620	-44.444	3.902.227	-30.038	3.851.476	-35.488
3.878.755	-44.442	3.902.408	-30.033	3.851.614	-35.481
3.878.926	-44.441	3.902.517	-30.028	3.851.815	-35.473
3.879.089	-44.439	3.902.654	-30.022	3.851.942	-35.466
3.879.201	-44.437	3.902.850	-30.017	3.852.034	-35.459
3.879.381	-44.436	3.903.026	-30.012	3.852.204	-35.452
3.879.505	-44.434	3.903.170	-30.007	3.852.362	-35.445
3.879.588	-44.432	3.903.291	-30.001	3.852.549	-35.438
3.879.761	-44.430	3.903.443	-29.996	3.852.702	-35.431
3.879.959	-44.429	3.903.575	-29.991	3.852.867	-35.424
3.880.074	-44.427	3.903.676	-29.985	3.853.105	-35.417
3.880.217	-44.425	3.903.911	-29.980	3.853.319	-35.411
3.880.406	-44.424	3.904.214	-29.975	3.853.544	-35.404
3.880.599	-44.422	3.904.508	-29.969	3.853.719	-35.397
3.880.822	-44.420	3.904.711	-29.964	3.853.889	-35.390

3.881.017	-44.419	3.904.865	-29.959	3.854.086	-35.383
3.881.205	-44.417	3.905.078	-29.953	3.854.236	-35.376
3.881.393	-44.415	3.905.216	-29.948	3.854.378	-35.369
3.881.549	-44.413	3.905.285	-29.943	3.854.530	-35.363
3.881.701	-44.412	3.905.397	-29.937	3.854.736	-35.356
3.881.809	-44.410	3.905.563	-29.932	3.854.872	-35.349
3.881.927	-44.408	3.905.719	-29.927	3.854.941	-35.342
3.882.068	-44.407	3.905.858	-29.922	3.855.150	-35.336
3.882.274	-44.405	3.905.982	-29.916	3.855.318	-35.329
3.882.433	-44.403	3.906.162	-29.911	3.855.486	-35.322
3.882.539	-44.402	3.906.400	-29.906	3.855.688	-35.315
3.882.708	-44.400	3.906.570	-29.900	3.855.818	-35.309
3.882.899	-44.398	3.906.716	-29.895	3.856.004	-35.302
3.883.124	-44.396	3.906.906	-29.890	3.856.238	-35.295
3.883.239	-44.395	3.907.202	-29.884	3.856.408	-35.289
3.883.405	-44.393	3.907.464	-29.879	3.856.557	-35.282
3.883.609	-44.391	3.907.543	-29.874	3.856.704	-35.276
3.883.785	-44.390	3.907.666	-29.868	3.856.820	-35.269
3.884.021	-44.388	3.907.865	-29.863	3.857.029	-35.262
3.884.211	-44.386	3.907.973	-29.858	3.857.234	-35.256
3.884.299	-44.385	3.908.091	-29.852	3.857.326	-35.249
3.884.464	-44.383	3.908.279	-29.847	3.857.491	-35.243
3.884.670	-44.381	3.908.430	-29.842	3.857.749	-35.236
3.884.810	-44.380	3.908.578	-29.836	3.857.900	-35.230
3.884.993	-44.378	3.908.756	-29.831	3.857.968	-35.223
3.885.182	-44.376	3.908.948	-29.826	3.858.156	-35.217
3.885.350	-44.375	3.909.099	-29.820	3.858.432	-35.211
3.885.538	-44.373	3.909.244	-29.815	3.858.654	-35.204
3.885.728	-44.371	3.909.503	-29.810	3.858.761	-35.198
3.885.894	-44.369	3.909.709	-29.804	3.858.833	-35.191

3.886.107	-44.368	3.909.825	-29.799	3.859.048	-35.185
3.886.320	-44.366	3.909.993	-29.793	3.859.227	-35.179
3.886.459	-44.364	3.910.190	-29.788	3.859.355	-35.172
3.886.573	-44.363	3.910.352	-29.783	3.859.677	-35.166
3.886.725	-44.361	3.910.462	-29.777	3.860.086	-35.160
3.886.909	-44.359	3.910.631	-29.772	3.860.370	-35.154
3.887.030	-44.358	3.910.823	-29.767	3.860.529	-35.147
3.887.162	-44.356	3.910.977	-29.761	3.860.634	-35.141
3.887.348	-44.355	3.911.148	-29.756	3.860.724	-35.135
3.887.527	-44.353	3.911.304	-29.751	3.860.795	-35.129
3.887.751	-44.351	3.911.420	-29.745	3.860.815	-35.122
3.887.946	-44.350	3.911.550	-29.740	3.860.934	-35.116
3.888.036	-44.348	3.911.707	-29.734	3.861.089	-35.110
3.888.182	-44.346	3.912.036	-29.729	3.861.177	-35.104
3.888.372	-44.345	3.912.502	-29.724	3.861.319	-35.098
3.888.477	-44.343	3.912.806	-29.718	3.861.491	-35.092
3.888.654	-44.341	3.912.892	-29.713	3.861.682	-35.086
3.889.001	-44.340	3.912.917	-29.708	3.861.868	-35.079
3.889.410	-44.338	3.912.921	-29.703	3.862.079	-35.073
3.889.630	-44.336	3.912.991	-29.697	3.862.243	-35.067
3.889.662	-44.335	3.913.172	-29.692	3.862.345	-35.061
3.889.710	-44.333	3.913.286	-29.687	3.862.583	-35.055
3.889.779	-44.332	3.913.387	-29.682	3.862.759	-35.049
3.889.815	-44.330	3.913.580	-29.676	3.862.842	-35.043
3.889.875	-44.328	3.913.766	-29.671	3.863.062	-35.037
3.890.060	-44.327	3.913.966	-29.666	3.863.326	-35.031
3.890.235	-44.325	3.914.187	-29.661	3.863.419	-35.025
3.890.363	-44.323	3.914.353	-29.656	3.863.493	-35.019
3.890.571	-44.322	3.914.482	-29.650	3.863.630	-35.013
3.890.819	-44.320	3.914.670	-29.645	3.863.789	-35.007

3.890.983	-44.319	3.914.928	-29.640	3.863.972	-35.001
3.891.109	-44.317	3.915.117	-29.635	3.864.077	-34.995
3.891.277	-44.315	3.915.219	-29.630	3.864.226	-34.989
3.891.458	-44.314	3.915.260	-29.625	3.864.413	-34.983
3.891.628	-44.312	3.915.349	-29.620	3.864.615	-34.977
3.891.810	-44.310	3.915.522	-29.615	3.864.839	-34.972
3.891.956	-44.309	3.915.692	-29.610	3.864.985	-34.966
3.892.062	-44.307	3.915.804	-29.605	3.865.148	-34.960
3.892.269	-44.306	3.915.963	-29.600	3.865.404	-34.954
3.892.448	-44.304	3.916.234	-29.596	3.865.631	-34.948
3.892.583	-44.302	3.916.472	-29.591	3.865.750	-34.942
3.892.729	-44.301	3.916.593	-29.586	3.865.841	-34.937
3.892.882	-44.299	3.916.730	-29.581	3.866.036	-34.931
3.893.076	-44.298	3.916.922	-29.577	3.866.259	-34.925
3.893.257	-44.296	3.917.098	-29.572	3.866.407	-34.919
3.893.445	-44.294	3.917.281	-29.567	3.866.521	-34.913
3.893.615	-44.293	3.917.433	-29.563	3.866.589	-34.908
3.893.797	-44.291	3.917.589	-29.558	3.866.781	-34.902
3.893.990	-44.290	3.917.802	-29.554	3.867.057	-34.896
3.894.174	-44.288	3.917.951	-29.549	3.867.242	-34.890
3.894.388	-44.286	3.918.076	-29.545	3.867.339	-34.885
3.894.505	-44.285	3.918.224	-29.541	3.867.462	-34.879
3.894.597	-44.283	3.918.348	-29.536	3.867.681	-34.873
3.894.709	-44.282	3.918.510	-29.532	3.867.838	-34.867
3.894.902	-44.280	3.918.691	-29.528	3.868.018	-34.862
3.895.104	-44.278	3.918.900	-29.524	3.868.211	-34.856
3.895.281	-44.277	3.919.120	-29.519	3.868.416	-34.850
3.895.465	-44.275	3.919.297	-29.515	3.868.593	-34.845
3.895.598	-44.274	3.919.327	-29.511	3.868.716	-34.839
3.895.804	-44.272	3.919.446	-29.507	3.868.893	-34.833

3.895.920	-44.270	3.919.718	-29.503	3.869.046	-34.828
3.896.082	-44.269	3.919.869	-29.499	3.869.200	-34.822
3.896.266	-44.267	3.920.045	-29.495	3.869.392	-34.816
3.896.436	-44.266	3.920.278	-29.491	3.869.624	-34.811
3.896.568	-44.264	3.920.414	-29.488	3.869.799	-34.805
3.896.707	-44.262	3.920.549	-29.484	3.869.922	-34.799
3.896.881	-44.261	3.920.728	-29.480	3.870.101	-34.794
3.897.076	-44.259	3.920.938	-29.477	3.870.291	-34.788
3.897.250	-44.258	3.921.143	-29.473	3.870.477	-34.782
3.897.440	-44.256	3.921.263	-29.469	3.870.627	-34.777
3.897.589	-44.255	3.921.384	-29.466	3.870.757	-34.771
3.897.742	-44.253	3.921.552	-29.462	3.870.876	-34.765
3.897.928	-44.251	3.921.725	-29.459	3.871.024	-34.760
3.898.067	-44.250	3.921.898	-29.456	3.871.230	-34.754
3.898.260	-44.248	3.922.110	-29.452	3.871.437	-34.748
3.898.495	-44.247	3.922.316	-29.449	3.871.657	-34.742
3.898.659	-44.245	3.922.491	-29.446	3.871.842	-34.737
3.898.763	-44.244	3.922.607	-29.443	3.871.992	-34.731
3.898.951	-44.242	3.922.780	-29.440	3.872.112	-34.725
3.899.153	-44.240	3.922.991	-29.436	3.872.263	-34.719
3.899.290	-44.239	3.923.135	-29.433	3.872.433	-34.713
3.899.424	-44.237	3.923.225	-29.430	3.872.520	-34.708
3.899.620	-44.236	3.923.342	-29.427	3.872.643	-34.702
3.899.789	-44.234	3.923.531	-29.425	3.872.868	-34.696
3.899.904	-44.233	3.923.643	-29.422	3.873.073	-34.690
3.900.060	-44.231	3.923.714	-29.419	3.873.152	-34.684
3.900.223	-44.229	3.923.826	-29.416	3.873.282	-34.678
3.900.388	-44.228	3.924.059	-29.413	3.873.478	-34.672
3.900.540	-44.226	3.924.232	-29.411	3.873.611	-34.666
3.900.672	-44.225	3.924.464	-29.408	3.873.759	-34.660

3.900.802	-44.223	3.924.749	-29.405	3.873.979	-34.654
3.900.938	-44.222	3.924.916	-29.403	3.874.164	-34.648
3.901.111	-44.220	3.925.134	-29.400	3.874.244	-34.642
3.901.331	-44.219	3.925.356	-29.398	3.874.396	-34.636
3.901.563	-44.217	3.925.471	-29.396	3.874.662	-34.630
3.901.801	-44.216	3.925.563	-29.393	3.874.886	-34.624
3.901.982	-44.214	3.925.707	-29.391	3.875.023	-34.618
3.902.193	-44.213	3.925.914	-29.389	3.875.242	-34.612
3.902.405	-44.211	3.926.064	-29.386	3.875.569	-34.606
3.902.553	-44.210	3.926.147	-29.384	3.875.724	-34.600
3.902.683	-44.208	3.926.293	-29.382	3.875.815	-34.593
3.902.811	-44.207	3.926.458	-29.380	3.875.979	-34.587
3.902.965	-44.205	3.926.636	-29.378	3.876.162	-34.581
3.903.116	-44.204	3.926.856	-29.376	3.876.328	-34.575
3.903.212	-44.202	3.926.994	-29.374	3.876.438	-34.568
3.903.362	-44.201	3.927.104	-29.372	3.876.571	-34.562
3.903.519	-44.199	3.927.343	-29.371	3.876.707	-34.556
3.903.640	-44.198	3.927.632	-29.369	3.876.859	-34.550
3.903.822	-44.196	3.927.810	-29.367	3.877.073	-34.543
3.903.951	-44.195	3.928.000	-29.366	3.877.211	-34.537
3.904.143	-44.193	3.928.211	-29.364	3.877.336	-34.530
3.904.432	-44.192	3.928.311	-29.363	3.877.527	-34.524
3.904.657	-44.190	3.928.468	-29.361	3.877.720	-34.518
3.904.797	-44.189	3.928.647	-29.360	3.877.907	-34.511
3.904.913	-44.188	3.928.788	-29.359	3.878.065	-34.505
3.905.043	-44.186	3.928.955	-29.357	3.878.300	-34.498
3.905.206	-44.185	3.929.124	-29.356	3.878.503	-34.492
3.905.411	-44.183	3.929.265	-29.355	3.878.643	-34.485
3.905.623	-44.182	3.929.464	-29.354	3.878.782	-34.479
3.905.798	-44.181	3.929.684	-29.353	3.878.947	-34.472

3.905.924	-44.179	3.929.845	-29.352	3.879.133	-34.466
3.906.065	-44.178	3.929.992	-29.351	3.879.211	-34.459
3.906.252	-44.176	3.930.157	-29.350	3.879.313	-34.453
3.906.443	-44.175	3.930.334	-29.349	3.879.512	-34.446
3.906.578	-44.174	3.930.504	-29.349	3.879.768	-34.440
3.906.769	-44.172	3.930.715	-29.348	3.880.005	-34.433
3.907.015	-44.171	3.930.881	-29.347	3.880.154	-34.427
3.907.169	-44.170	3.930.999	-29.347	3.880.273	-34.420
3.907.350	-44.168	3.931.131	-29.346	3.880.363	-34.414
3.907.540	-44.167	3.931.270	-29.346	3.880.556	-34.407
3.907.635	-44.166	3.931.426	-29.346	3.880.794	-34.401
3.907.753	-44.164	3.931.673	-29.345	3.880.954	-34.394
3.907.914	-44.163	3.931.831	-29.345	3.881.122	-34.387
3.908.120	-44.162	3.931.922	-29.345	3.881.306	-34.381
3.908.362	-44.160	3.932.077	-29.345	3.881.498	-34.374
3.908.540	-44.159	3.932.243	-29.345	3.881.653	-34.368
3.908.630	-44.158	3.932.440	-29.345	3.881.774	-34.361
3.908.766	-44.157	3.932.731	-29.345	3.881.910	-34.355
3.908.994	-44.155	3.933.125	-29.345	3.882.074	-34.348
3.909.157	-44.154	3.933.405	-29.345	3.882.236	-34.342
3.909.326	-44.153	3.933.493	-29.346	3.882.475	-34.335
3.909.461	-44.152	3.933.519	-29.346	3.882.683	-34.328
3.909.656	-44.150	3.933.593	-29.346	3.882.834	-34.322
3.910.049	-44.149	3.933.676	-29.347	3.883.004	-34.315
3.910.419	-44.148	3.933.752	-29.347	3.883.112	-34.309
3.910.634	-44.147	3.933.867	-29.348	3.883.295	-34.302
3.910.672	-44.146	3.934.023	-29.349	3.883.506	-34.296
3.910.708	-44.144	3.934.204	-29.349	3.883.633	-34.289
3.910.766	-44.143	3.934.400	-29.350	3.883.773	-34.283
3.910.881	-44.142	3.934.580	-29.351	3.883.943	-34.276

3.911.053	-44.141	3.934.753	-29.352	3.884.113	-34.270
3.911.116	-44.140	3.934.912	-29.353	3.884.439	-34.263
3.911.176	-44.139	3.935.070	-29.354	3.884.904	-34.257
3.911.339	-44.137	3.935.341	-29.355	3.885.204	-34.250
3.911.579	-44.136	3.935.545	-29.356	3.885.276	-34.244
3.911.776	-44.135	3.935.645	-29.357	3.885.338	-34.238
3.911.888	-44.134	3.935.787	-29.359	3.885.372	-34.231
3.912.094	-44.133	3.935.953	-29.360	3.885.407	-34.225
3.912.301	-44.132	3.936.087	-29.361	3.885.538	-34.218
3.912.482	-44.131	3.936.194	-29.363	3.885.675	-34.212
3.912.661	-44.130	3.936.357	-29.364	3.885.777	-34.205
3.912.829	-44.129	3.936.574	-29.366	3.885.876	-34.199
3.912.950	-44.128	3.936.786	-29.368	3.885.989	-34.193
3.913.048	-44.126	3.936.896	-29.370	3.886.147	-34.186
3.913.271	-44.125	3.936.992	-29.371	3.886.394	-34.180
3.913.409	-44.124	3.937.202	-29.373	3.886.613	-34.174
3.913.546	-44.123	3.937.442	-29.375	3.886.776	-34.167
3.913.703	-44.122	3.937.664	-29.377	3.886.946	-34.161
3.913.940	-44.121	3.937.859	-29.380	3.887.112	-34.155
3.914.128	-44.120	3.938.018	-29.382	3.887.271	-34.148
3.914.238	-44.119	3.938.142	-29.384	3.887.467	-34.142
3.914.464	-44.118	3.938.282	-29.386	3.887.662	-34.136
3.914.677	-44.117	3.938.432	-29.389	3.887.784	-34.130
3.914.868	-44.116	3.938.547	-29.391	3.887.950	-34.123
3.915.067	-44.115	3.938.695	-29.394	3.888.153	-34.117
3.915.263	-44.115	3.938.885	-29.397	3.888.271	-34.111
3.915.429	-44.114	3.939.060	-29.399	3.888.347	-34.105
3.915.576	-44.113	3.939.240	-29.402	3.888.532	-34.099
3.915.697	-44.112	3.939.436	-29.405	3.888.788	-34.092
3.915.826	-44.111	3.939.612	-29.408	3.888.947	-34.086

3.915.982	-44.110	3.939.749	-29.411	3.888.990	-34.080
3.916.166	-44.109	3.939.886	-29.414	3.889.138	-34.074
3.916.304	-44.108	3.940.067	-29.417	3.889.382	-34.068
3.916.419	-44.107	3.940.209	-29.420	3.889.613	-34.062
3.916.628	-44.106	3.940.345	-29.424	3.889.800	-34.056
3.916.830	-44.105	3.940.469	-29.427	3.889.953	-34.050
3.916.992	-44.105	3.940.674	-29.430	3.890.146	-34.044
3.917.117	-44.104	3.940.894	-29.434	3.890.327	-34.038
3.917.232	-44.103	3.941.024	-29.438	3.890.535	-34.032
3.917.404	-44.102	3.941.225	-29.441	3.890.685	-34.026
3.917.582	-44.101	3.941.354	-29.445	3.890.790	-34.020
3.917.787	-44.100	3.941.492	-29.449	3.890.959	-34.014
3.917.980	-44.100	3.941.638	-29.453	3.891.181	-34.008
3.918.156	-44.099	3.941.837	-29.457	3.891.378	-34.002
3.918.305	-44.098	3.942.083	-29.461	3.891.508	-33.997
3.918.430	-44.097	3.942.286	-29.465	3.891.632	-33.991
3.918.589	-44.096	3.942.458	-29.469	3.891.810	-33.985
3.918.781	-44.096	3.942.582	-29.473	3.892.000	-33.979
3.918.907	-44.095	3.942.670	-29.477	3.892.156	-33.973
3.919.037	-44.094	3.942.827	-29.482	3.892.329	-33.967
3.919.227	-44.093	3.943.056	-29.486	3.892.500	-33.961
3.919.377	-44.093	3.943.259	-29.490	3.892.617	-33.956
3.919.547	-44.092	3.943.427	-29.495	3.892.776	-33.950
3.919.731	-44.091	3.943.593	-29.499	3.892.964	-33.944
3.919.870	-44.091	3.943.786	-29.504	3.893.149	-33.938
3.920.082	-44.090	3.943.940	-29.509	3.893.341	-33.932
3.920.282	-44.089	3.944.060	-29.513	3.893.501	-33.926
3.920.418	-44.088	3.944.189	-29.518	3.893.633	-33.921
3.920.559	-44.088	3.944.303	-29.523	3.893.730	-33.915
3.920.670	-44.087	3.944.385	-29.527	3.893.914	-33.909

3.920.871	-44.086	3.944.522	-29.532	3.894.160	-33.903
3.921.078	-44.086	3.944.738	-29.537	3.894.341	-33.897
3.921.252	-44.085	3.944.966	-29.542	3.894.496	-33.892
3.921.384	-44.085	3.945.155	-29.547	3.894.613	-33.886
3.921.494	-44.084	3.945.332	-29.552	3.894.783	-33.880
3.921.657	-44.083	3.945.553	-29.556	3.894.969	-33.874
3.921.754	-44.083	3.945.760	-29.561	3.895.114	-33.868
3.921.859	-44.082	3.945.989	-29.566	3.895.328	-33.863
3.922.040	-44.081	3.946.147	-29.571	3.895.501	-33.857
3.922.286	-44.081	3.946.241	-29.576	3.895.650	-33.851
3.922.553	-44.080	3.946.397	-29.581	3.895.837	-33.845
3.922.766	-44.080	3.946.556	-29.586	3.895.996	-33.839
3.922.935	-44.079	3.946.714	-29.591	3.896.125	-33.833
3.923.018	-44.079	3.946.808	-29.596	3.896.281	-33.827
3.923.204	-44.078	3.946.909	-29.601	3.896.498	-33.821
3.923.492	-44.078	3.947.074	-29.606	3.896.685	-33.815
3.923.705	-44.077	3.947.209	-29.611	3.896.870	-33.809
3.923.817	-44.077	3.947.397	-29.615	3.897.029	-33.803
3.923.868	-44.076	3.947.583	-29.620	3.897.174	-33.797
3.924.006	-44.076	3.947.760	-29.625	3.897.296	-33.791
3.924.171	-44.075	3.947.997	-29.630	3.897.481	-33.785
3.924.281	-44.075	3.948.217	-29.635	3.897.683	-33.779
3.924.457	-44.074	3.948.381	-29.640	3.897.690	-33.773
3.924.662	-44.074	3.948.518	-29.645	3.897.787	-33.767
3.924.800	-44.073	3.948.719	-29.649	3.898.018	-33.761
3.924.980	-44.073	3.948.936	-29.654	3.898.208	-33.754
3.925.180	-44.072	3.949.091	-29.659	3.898.323	-33.748
3.925.372	-44.072	3.949.207	-29.663	3.898.452	-33.742
3.925.620	-44.072	3.949.353	-29.668	3.898.604	-33.736
3.925.818	-44.071	3.949.543	-29.673	3.898.842	-33.729

3.925.914	-44.071	3.949.713	-29.677	3.899.099	-33.723
3.926.097	-44.070	3.949.919	-29.682	3.899.266	-33.717
3.926.329	-44.070	3.950.121	-29.686	3.899.432	-33.710
3.926.429	-44.070	3.950.242	-29.691	3.899.663	-33.704
3.926.552	-44.069	3.950.338	-29.695	3.899.865	-33.698
3.926.776	-44.069	3.950.487	-29.699	3.900.043	-33.691
3.926.982	-44.069	3.950.725	-29.704	3.900.231	-33.685
3.927.108	-44.068	3.950.931	-29.708	3.900.435	-33.678
3.927.238	-44.068	3.951.047	-29.712	3.900.600	-33.672
3.927.417	-44.068	3.951.217	-29.716	3.900.757	-33.665
3.927.550	-44.067	3.951.362	-29.720	3.900.907	-33.659
3.927.712	-44.067	3.951.531	-29.724	3.901.060	-33.652
3.927.921	-44.067	3.951.769	-29.728	3.901.196	-33.646
3.928.171	-44.067	3.951.899	-29.732	3.901.263	-33.639
3.928.312	-44.066	3.952.054	-29.736	3.901.328	-33.633
3.928.374	-44.066	3.952.198	-29.740	3.901.494	-33.626
3.928.524	-44.066	3.952.358	-29.743	3.901.700	-33.619
3.928.701	-44.066	3.952.594	-29.747	3.901.907	-33.613
3.928.850	-44.065	3.952.795	-29.751	3.902.113	-33.606
3.929.032	-44.065	3.952.933	-29.754	3.902.234	-33.599
3.929.234	-44.065	3.953.071	-29.757	3.902.357	-33.593
3.929.375	-44.065	3.953.438	-29.761	3.902.568	-33.586
3.929.562	-44.064	3.953.847	-29.764	3.902.805	-33.579
3.929.765	-44.064	3.954.071	-29.767	3.902.972	-33.573
3.929.960	-44.064	3.954.088	-29.770	3.903.134	-33.566
3.930.125	-44.064	3.954.108	-29.773	3.903.391	-33.559
3.930.223	-44.064	3.954.201	-29.776	3.903.652	-33.552
3.930.378	-44.064	3.954.240	-29.779	3.903.808	-33.546
3.930.804	-44.063	3.954.333	-29.782	3.903.857	-33.539
3.931.234	-44.063	3.954.461	-29.785	3.903.987	-33.532

3.931.328	-44.063	3.954.615	-29.787	3.904.222	-33.525
3.931.389	-44.063	3.954.778	-29.790	3.904.388	-33.519
3.931.584	-44.063	3.954.877	-29.792	3.904.525	-33.512
3.931.650	-44.063	3.955.092	-29.795	3.904.717	-33.505
3.931.654	-44.063	3.955.376	-29.797	3.904.881	-33.498
3.931.815	-44.062	3.955.583	-29.799	3.905.006	-33.492
3.931.946	-44.062	3.955.740	-29.801	3.905.139	-33.485
3.932.018	-44.062	3.955.920	-29.803	3.905.281	-33.478
3.932.203	-44.062	3.956.093	-29.805	3.905.493	-33.471
3.932.441	-44.062	3.956.227	-29.807	3.905.736	-33.464
3.932.670	-44.062	3.956.358	-29.809	3.905.938	-33.458
3.932.829	-44.062	3.956.574	-29.810	3.906.091	-33.451
3.932.957	-44.062	3.956.733	-29.812	3.906.238	-33.444
3.933.157	-44.062	3.956.864	-29.813	3.906.378	-33.437
3.933.322	-44.062	3.957.050	-29.815	3.906.503	-33.431
3.933.493	-44.061	3.957.168	-29.816	3.906.672	-33.424
3.933.737	-44.061	3.957.261	-29.817	3.906.903	-33.417
3.933.860	-44.061	3.957.495	-29.818	3.907.023	-33.411
3.933.914	-44.061	3.957.762	-29.819	3.907.144	-33.404
3.934.059	-44.061	3.957.907	-29.820	3.907.390	-33.397
3.934.237	-44.061	3.958.047	-29.821	3.907.604	-33.391
3.934.337	-44.061	3.958.149	-29.822	3.907.784	-33.384
3.934.431	-44.061	3.958.326	-29.823	3.907.976	-33.377
3.934.638	-44.061	3.958.596	-29.823	3.908.159	-33.371
3.934.839	-44.061	3.958.767	-29.823	3.908.308	-33.364
3.935.032	-44.061	3.958.871	-29.824	3.908.407	-33.358
3.935.296	-44.061	3.959.017	-29.824	3.908.477	-33.351
3.935.529	-44.061	3.959.227	-29.824	3.908.608	-33.345
3.935.688	-44.061	3.959.380	-29.824	3.908.894	-33.338
3.935.807	-44.061	3.959.507	-29.824	3.909.352	-33.332

3.935.977	-44.061	3.959.656	-29.824	3.909.706	-33.325
3.936.159	-44.061	3.959.836	-29.824	3.909.846	-33.319
3.936.362	-44.061	3.959.999	-29.823	3.909.951	-33.313
3.936.535	-44.061	3.960.195	-29.823	3.909.980	-33.306
3.936.568	-44.061	3.960.414	-29.822	3.910.028	-33.300
3.936.703	-44.061	3.960.612	-29.822	3.910.140	-33.294
3.936.910	-44.061	3.960.711	-29.821	3.910.260	-33.287
3.936.989	-44.061	3.960.775	-29.820	3.910.378	-33.281
3.937.180	-44.061	3.960.934	-29.819	3.910.459	-33.275
3.937.435	-44.061	3.961.107	-29.818	3.910.576	-33.269
3.937.578	-44.061	3.961.304	-29.817	3.910.760	-33.263
3.937.704	-44.061	3.961.545	-29.816	3.910.942	-33.256
3.937.820	-44.061	3.961.732	-29.814	3.911.120	-33.250
3.938.002	-44.061	3.961.867	-29.813	3.911.303	-33.244
3.938.153	-44.061	3.962.043	-29.811	3.911.495	-33.238
3.938.320	-44.060	3.962.209	-29.810	3.911.703	-33.232
3.938.489	-44.060	3.962.339	-29.808	3.911.830	-33.227
3.938.535	-44.060	3.962.493	-29.806	3.911.998	-33.221
3.938.734	-44.060	3.962.686	-29.804	3.912.209	-33.215
3.939.057	-44.060	3.962.911	-29.802	3.912.344	-33.209
3.939.211	-44.060	3.963.074	-29.800	3.912.488	-33.203
3.939.379	-44.060	3.963.207	-29.798	3.912.630	-33.198
3.939.525	-44.060	3.963.387	-29.795	3.912.817	-33.192
3.939.662	-44.060	3.963.558	-29.793	3.913.034	-33.186
3.939.873	-44.060	3.963.724	-29.790	3.913.132	-33.181
3.940.061	-44.060	3.963.919	-29.788	3.913.253	-33.175
3.940.219	-44.060	3.964.052	-29.785	3.913.481	-33.170
3.940.321	-44.060	3.964.197	-29.782	3.913.683	-33.165
3.940.512	-44.060	3.964.384	-29.779	3.913.800	-33.159
3.940.773	-44.060	3.964.514	-29.776	3.913.947	-33.154

3.941.004	-44.060	3.964.615	-29.773	3.914.169	-33.149
3.941.198	-44.060	3.964.731	-29.770	3.914.406	-33.143
3.941.310	-44.060	3.964.873	-29.766	3.914.557	-33.138
3.941.445	-44.060	3.965.025	-29.763	3.914.698	-33.133
3.941.592	-44.060	3.965.186	-29.759	3.914.915	-33.128
3.941.693	-44.060	3.965.367	-29.755	3.915.093	-33.123
3.941.817	-44.060	3.965.617	-29.752	3.915.234	-33.118
3.942.027	-44.060	3.965.793	-29.748	3.915.360	-33.113
3.942.146	-44.060	3.966.003	-29.744	3.915.518	-33.108
3.942.184	-44.060	3.966.266	-29.740	3.915.715	-33.103
3.942.334	-44.060	3.966.434	-29.735	3.915.896	-33.098
3.942.534	-44.060	3.966.615	-29.731	3.916.010	-33.093
3.942.738	-44.060	3.966.780	-29.727	3.916.086	-33.088
3.942.889	-44.060	3.966.886	-29.722	3.916.249	-33.084
3.943.047	-44.059	3.967.003	-29.718	3.916.502	-33.079
3.943.224	-44.059	3.967.131	-29.713	3.916.747	-33.074
3.943.461	-44.059	3.967.267	-29.708	3.916.928	-33.070
3.943.703	-44.059	3.967.404	-29.703	3.917.072	-33.065
3.943.880	-44.059	3.967.578	-29.698	3.917.236	-33.060
3.944.037	-44.059	3.967.787	-29.693	3.917.343	-33.056
3.944.222	-44.059	3.967.965	-29.687	3.917.417	-33.051
3.944.425	-44.059	3.968.135	-29.682	3.917.650	-33.047
3.944.561	-44.059	3.968.331	-29.677	3.917.856	-33.042
3.944.608	-44.059	3.968.507	-29.671	3.917.995	-33.038
3.944.735	-44.059	3.968.644	-29.665	3.918.142	-33.033
3.944.926	-44.059	3.968.836	-29.659	3.918.254	-33.029
3.945.107	-44.059	3.969.060	-29.653	3.918.439	-33.025
3.945.271	-44.059	3.969.265	-29.647	3.918.596	-33.020
3.945.421	-44.058	3.969.421	-29.641	3.918.759	-33.016
3.945.562	-44.058	3.969.548	-29.635	3.919.030	-33.012

3.945.717	-44.058	3.969.751	-29.629	3.919.266	-33.007
3.945.903	-44.058	3.969.892	-29.622	3.919.462	-33.003
3.946.071	-44.058	3.970.011	-29.616	3.919.601	-32.999
3.946.288	-44.058	3.970.166	-29.609	3.919.706	-32.994
3.946.483	-44.058	3.970.305	-29.602	3.919.908	-32.990
3.946.701	-44.058	3.970.512	-29.595	3.920.071	-32.986
3.946.917	-44.058	3.970.663	-29.588	3.920.213	-32.981
3.947.079	-44.058	3.970.823	-29.581	3.920.350	-32.977
3.947.213	-44.057	3.971.046	-29.574	3.920.486	-32.973
3.947.354	-44.057	3.971.205	-29.567	3.920.641	-32.968
3.947.589	-44.057	3.971.364	-29.559	3.920.808	-32.964
3.947.809	-44.057	3.971.508	-29.552	3.921.033	-32.960
3.947.964	-44.057	3.971.660	-29.544	3.921.158	-32.955
3.948.092	-44.057	3.971.893	-29.536	3.921.324	-32.951
3.948.201	-44.057	3.972.062	-29.528	3.921.560	-32.947
3.948.366	-44.057	3.972.186	-29.520	3.921.707	-32.942
3.948.573	-44.057	3.972.379	-29.512	3.921.888	-32.938
3.948.779	-44.056	3.972.560	-29.504	3.922.081	-32.934
3.948.979	-44.056	3.972.722	-29.495	3.922.193	-32.929
3.949.149	-44.056	3.972.918	-29.487	3.922.298	-32.925
3.949.265	-44.056	3.973.073	-29.478	3.922.482	-32.920
3.949.424	-44.056	3.973.167	-29.470	3.922.670	-32.916
3.949.606	-44.056	3.973.372	-29.461	3.922.784	-32.911
3.949.753	-44.056	3.973.589	-29.452	3.922.892	-32.906
3.949.881	-44.055	3.973.743	-29.443	3.923.058	-32.902
3.950.023	-44.055	3.974.081	-29.434	3.923.250	-32.897
3.950.229	-44.055	3.974.490	-29.425	3.923.419	-32.893
3.950.423	-44.055	3.974.673	-29.415	3.923.600	-32.888
3.950.585	-44.055	3.974.699	-29.406	3.923.752	-32.883
3.950.768	-44.055	3.974.764	-29.396	3.923.891	-32.879

3.950.949	-44.055	3.974.879	-29.386	3.924.160	-32.874
3.951.068	-44.054	3.974.930	-29.376	3.924.438	-32.869
3.951.205	-44.054	3.974.944	-29.366	3.924.637	-32.864
3.951.465	-44.054	3.975.075	-29.356	3.924.798	-32.859
3.951.892	-44.054	3.975.271	-29.346	3.924.944	-32.854
3.952.191	-44.054	3.975.459	-29.336	3.925.069	-32.849
3.952.285	-44.054	3.975.688	-29.325	3.925.191	-32.844
3.952.401	-44.054	3.975.923	-29.315	3.925.386	-32.839
3.952.469	-44.053	3.976.086	-29.304	3.925.533	-32.834
3.952.433	-44.053	3.976.209	-29.293	3.925.652	-32.829
3.952.463	-44.053	3.976.373	-29.282	3.925.764	-32.824
3.952.617	-44.053	3.976.530	-29.271	3.925.913	-32.819
3.952.809	-44.053	3.976.698	-29.260	3.926.108	-32.813
3.953.001	-44.053	3.976.888	-29.249	3.926.282	-32.808
3.953.199	-44.052	3.977.108	-29.238	3.926.467	-32.803
3.953.341	-44.052	3.977.275	-29.226	3.926.638	-32.797
3.953.470	-44.052	3.977.372	-29.215	3.926.772	-32.792
3.953.670	-44.052	3.977.481	-29.203	3.926.920	-32.786
3.953.889	-44.052	3.977.625	-29.191	3.927.145	-32.781
3.954.081	-44.052	3.977.794	-29.179	3.927.391	-32.775
3.954.117	-44.051	3.977.935	-29.168	3.927.621	-32.770
3.954.248	-44.051	3.978.112	-29.155	3.927.773	-32.764
3.954.475	-44.051	3.978.337	-29.143	3.927.896	-32.759
3.954.644	-44.051	3.978.579	-29.131	3.928.045	-32.753
3.954.855	-44.051	3.978.795	-29.119	3.928.210	-32.747
3.955.027	-44.051	3.978.918	-29.106	3.928.387	-32.741
3.955.172	-44.050	3.979.056	-29.094	3.928.593	-32.735
3.955.328	-44.050	3.979.218	-29.081	3.928.723	-32.730
3.955.508	-44.050	3.979.370	-29.068	3.928.821	-32.724
3.955.674	-44.050	3.979.536	-29.056	3.928.996	-32.718

3.955.811	-44.050	3.979.666	-29.043	3.929.214	-32.712
3.956.030	-44.049	3.979.829	-29.030	3.929.471	-32.706
3.956.257	-44.049	3.980.021	-29.017	3.929.662	-32.700
3.956.465	-44.049	3.980.211	-29.004	3.929.812	-32.693
3.956.650	-44.049	3.980.346	-28.990	3.929.958	-32.687
3.956.790	-44.049	3.980.486	-28.977	3.930.114	-32.681
3.956.925	-44.048	3.980.697	-28.964	3.930.325	-32.675
3.957.007	-44.048	3.980.818	-28.950	3.930.488	-32.669
3.957.159	-44.048	3.980.971	-28.937	3.930.639	-32.662
3.957.348	-44.048	3.981.140	-28.923	3.930.819	-32.656
3.957.487	-44.048	3.981.259	-28.909	3.930.971	-32.650
3.957.600	-44.048	3.981.431	-28.896	3.931.156	-32.643
3.957.796	-44.047	3.981.642	-28.882	3.931.324	-32.637
3.957.993	-44.047	3.981.859	-28.868	3.931.495	-32.630
3.958.127	-44.047	3.982.050	-28.854	3.931.664	-32.624
3.958.336	-44.047	3.982.216	-28.840	3.931.776	-32.617
3.958.517	-44.047	3.982.365	-28.826	3.931.878	-32.611
3.958.655	-44.046	3.982.502	-28.812	3.932.018	-32.604
3.958.800	-44.046	3.982.669	-28.798	3.932.260	-32.597
3.958.926	-44.046	3.982.825	-28.784	3.932.514	-32.591
3.959.092	-44.046	3.983.053	-28.770	3.932.695	-32.584
3.959.297	-44.046	3.983.262	-28.756	3.932.802	-32.577
3.959.504	-44.045	3.983.398	-28.741	3.932.953	-32.571
3.959.675	-44.045	3.983.580	-28.727	3.933.062	-32.564
3.959.805	-44.045	3.983.753	-28.713	3.933.177	-32.557
3.959.948	-44.045	3.983.897	-28.698	3.933.531	-32.550
3.960.126	-44.045	3.984.049	-28.684	3.933.955	-32.544
3.960.332	-44.044	3.984.203	-28.670	3.934.291	-32.537
3.960.495	-44.044	3.984.368	-28.655	3.934.471	-32.530
3.960.587	-44.044	3.984.527	-28.641	3.934.472	-32.523

3.960.802	-44.044	3.984.728	-28.626	3.934.510	-32.516
3.961.010	-44.044	3.984.911	-28.612	3.934.561	-32.509
3.961.083	-44.043	3.985.000	-28.597	3.934.634	-32.502
3.961.289	-44.043	3.985.115	-28.583	3.934.771	-32.495
3.961.494	-44.043	3.985.248	-28.568	3.934.836	-32.488
3.961.664	-44.043	3.985.396	-28.553	3.934.959	-32.481
3.961.877	-44.043	3.985.566	-28.539	3.935.104	-32.474
3.962.000	-44.042	3.985.726	-28.524	3.935.303	-32.467
3.962.151	-44.042	3.985.942	-28.509	3.935.562	-32.460
3.962.386	-44.042	3.986.170	-28.495	3.935.753	-32.453
3.962.502	-44.042	3.986.371	-28.480	3.935.912	-32.446
3.962.679	-44.042	3.986.549	-28.465	3.936.042	-32.439
3.962.894	-44.042	3.986.743	-28.451	3.936.256	-32.432
3.962.939	-44.041	3.986.954	-28.436	3.936.479	-32.425
3.963.044	-44.041	3.987.094	-28.421	3.936.660	-32.418
3.963.201	-44.041	3.987.227	-28.407	3.936.779	-32.411
3.963.342	-44.041	3.987.390	-28.392	3.936.913	-32.403
3.963.506	-44.041	3.987.542	-28.377	3.937.095	-32.396
3.963.666	-44.041	3.987.695	-28.362	3.937.286	-32.389
3.963.867	-44.040	3.987.823	-28.347	3.937.452	-32.382
3.964.074	-44.040	3.987.932	-28.333	3.937.572	-32.375
3.964.305	-44.040	3.988.085	-28.318	3.937.734	-32.368
3.964.522	-44.040	3.988.222	-28.303	3.937.807	-32.361
3.964.718	-44.040	3.988.401	-28.288	3.937.941	-32.354
3.964.883	-44.040	3.988.620	-28.274	3.938.192	-32.347
3.965.022	-44.040	3.988.792	-28.259	3.938.333	-32.340
3.965.172	-44.040	3.989.012	-28.244	3.938.542	-32.333
3.965.320	-44.039	3.989.167	-28.229	3.938.788	-32.326
3.965.468	-44.039	3.989.323	-28.215	3.938.952	-32.319
3.965.569	-44.039	3.989.527	-28.200	3.939.078	-32.312

3.965.683	-44.039	3.989.664	-28.185	3.939.238	-32.305
3.965.789	-44.039	3.989.819	-28.170	3.939.472	-32.299
3.965.923	-44.039	3.989.991	-28.156	3.939.637	-32.292
3.966.154	-44.039	3.990.179	-28.141	3.939.821	-32.285
3.966.335	-44.039	3.990.405	-28.126	3.939.996	-32.278
3.966.488	-44.039	3.990.565	-28.112	3.940.119	-32.271
3.966.664	-44.039	3.990.717	-28.097	3.940.224	-32.264
3.966.844	-44.039	3.990.901	-28.082	3.940.317	-32.257
3.967.080	-44.038	3.991.028	-28.068	3.940.450	-32.251
3.967.289	-44.038	3.991.230	-28.053	3.940.605	-32.244
3.967.440	-44.038	3.991.454	-28.039	3.940.808	-32.237
3.967.652	-44.038	3.991.624	-28.024	3.941.019	-32.230
3.967.881	-44.038	3.991.794	-28.009	3.941.143	-32.224
3.968.011	-44.038	3.991.943	-27.995	3.941.293	-32.217
3.968.080	-44.038	3.992.115	-27.980	3.941.490	-32.210
3.968.175	-44.038	3.992.272	-27.966	3.941.709	-32.204
3.968.320	-44.038	3.992.417	-27.951	3.941.891	-32.197
3.968.539	-44.038	3.992.552	-27.937	3.941.986	-32.191
3.968.759	-44.038	3.992.744	-27.922	3.942.163	-32.184
3.968.936	-44.038	3.992.946	-27.908	3.942.328	-32.178
3.969.084	-44.038	3.993.029	-27.894	3.942.448	-32.171
3.969.225	-44.038	3.993.190	-27.879	3.942.635	-32.165
3.969.443	-44.038	3.993.405	-27.865	3.942.845	-32.158
3.969.669	-44.038	3.993.620	-27.851	3.943.056	-32.152
3.969.830	-44.038	3.993.817	-27.836	3.943.257	-32.146
3.969.999	-44.038	3.993.967	-27.822	3.943.438	-32.139
3.970.150	-44.038	3.994.097	-27.808	3.943.678	-32.133
3.970.314	-44.039	3.994.240	-27.793	3.943.914	-32.127
3.970.511	-44.039	3.994.626	-27.779	3.943.976	-32.120
3.970.690	-44.039	3.995.042	-27.765	3.944.009	-32.114

3.970.854	-44.039	3.995.190	-27.751	3.944.168	-32.108
3.971.047	-44.039	3.995.292	-27.737	3.944.338	-32.102
3.971.230	-44.039	3.995.407	-27.722	3.944.447	-32.096
3.971.389	-44.039	3.995.446	-27.708	3.944.678	-32.090
3.971.557	-44.039	3.995.476	-27.694	3.944.913	-32.083
3.971.691	-44.039	3.995.522	-27.680	3.945.053	-32.077
3.971.781	-44.040	3.995.629	-27.666	3.945.263	-32.071
3.971.960	-44.040	3.995.769	-27.652	3.945.450	-32.065
3.972.267	-44.040	3.995.963	-27.638	3.945.591	-32.059
3.972.706	-44.040	3.996.154	-27.624	3.945.827	-32.053
3.973.011	-44.040	3.996.328	-27.610	3.946.017	-32.047
3.973.070	-44.040	3.996.534	-27.596	3.946.055	-32.041
3.973.145	-44.041	3.996.718	-27.582	3.946.202	-32.035
3.973.257	-44.041	3.996.954	-27.568	3.946.380	-32.029
3.973.272	-44.041	3.997.137	-27.554	3.946.513	-32.023
3.973.251	-44.041	3.997.225	-27.540	3.946.714	-32.017
3.973.376	-44.041	3.997.340	-27.526	3.946.971	-32.011
3.973.550	-44.042	3.997.520	-27.512	3.947.135	-32.005
3.973.706	-44.042	3.997.705	-27.498	3.947.177	-31.999
3.973.925	-44.042	3.997.881	-27.484	3.947.289	-31.994
3.974.186	-44.042	3.998.141	-27.470	3.947.433	-31.988
3.974.363	-44.043	3.998.311	-27.456	3.947.625	-31.982
3.974.496	-44.043	3.998.383	-27.442	3.947.785	-31.976
3.974.652	-44.043	3.998.575	-27.428	3.947.896	-31.970
3.974.836	-44.043	3.998.742	-27.414	3.948.146	-31.965
3.975.039	-44.044	3.998.914	-27.400	3.948.456	-31.959
3.975.139	-44.044	3.999.106	-27.386	3.948.661	-31.953
3.975.211	-44.044	3.999.287	-27.372	3.948.815	-31.947
3.975.316	-44.044	3.999.450	-27.358	3.948.995	-31.942
3.975.493	-44.045	3.999.550	-27.344	3.949.142	-31.936

3.975.753	-44.045	3.999.659	-27.330	3.949.258	-31.930
3.975.916	-44.045	3.999.848	-27.316	3.949.444	-31.925
3.976.031	-44.046	4.000.099	-27.302	3.949.595	-31.919
3.976.210	-44.046	4.000.213	-27.288	3.949.756	-31.913
3.976.422	-44.046	4.000.327	-27.274	3.949.955	-31.908
3.976.608	-44.047	4.000.529	-27.260	3.950.060	-31.902
3.976.812	-44.047	4.000.697	-27.246	3.950.132	-31.896
3.976.968	-44.048	4.000.862	-27.231	3.950.274	-31.891
3.977.126	-44.048	4.001.028	-27.217	3.950.388	-31.885
3.977.325	-44.048	4.001.174	-27.203	3.950.553	-31.880
3.977.491	-44.049	4.001.365	-27.189	3.950.779	-31.874
3.977.643	-44.049	4.001.517	-27.175	3.950.925	-31.869
3.977.758	-44.049	4.001.628	-27.160	3.951.163	-31.863
3.977.946	-44.050	4.001.791	-27.146	3.951.429	-31.858
3.978.123	-44.050	4.001.933	-27.132	3.951.625	-31.853
3.978.221	-44.051	4.002.062	-27.117	3.951.819	-31.847
3.978.398	-44.051	4.002.207	-27.103	3.951.943	-31.842
3.978.598	-44.052	4.002.401	-27.089	3.952.080	-31.837
3.978.721	-44.052	4.002.658	-27.074	3.952.224	-31.831
3.978.889	-44.052	4.002.865	-27.060	3.952.318	-31.826
3.979.128	-44.053	4.003.027	-27.045	3.952.549	-31.821
3.979.324	-44.053	4.003.163	-27.031	3.952.821	-31.816
3.979.476	-44.054	4.003.340	-27.016	3.953.001	-31.811
3.979.597	-44.054	4.003.553	-27.001	3.953.156	-31.806
3.979.731	-44.055	4.003.739	-26.987	3.953.383	-31.800
3.979.904	-44.055	4.003.875	-26.972	3.953.611	-31.795
3.980.092	-44.056	4.004.006	-26.957	3.953.789	-31.790
3.980.231	-44.056	4.004.146	-26.943	3.953.883	-31.785
3.980.367	-44.057	4.004.292	-26.928	3.953.911	-31.781
3.980.518	-44.057	4.004.474	-26.913	3.954.125	-31.776

3.980.759	-44.057	4.004.651	-26.898	3.954.350	-31.771
3.980.966	-44.058	4.004.843	-26.883	3.954.534	-31.766
3.981.080	-44.058	4.005.056	-26.869	3.954.710	-31.761
3.981.274	-44.059	4.005.253	-26.854	3.954.789	-31.756
3.981.419	-44.059	4.005.399	-26.839	3.954.933	-31.752
3.981.564	-44.060	4.005.518	-26.824	3.955.118	-31.747
3.981.732	-44.060	4.005.652	-26.809	3.955.295	-31.743
3.981.884	-44.061	4.005.744	-26.794	3.955.500	-31.738
3.982.036	-44.062	4.005.856	-26.779	3.955.660	-31.733
3.982.188	-44.062	4.006.071	-26.764	3.955.790	-31.729
3.982.375	-44.063	4.006.245	-26.749	3.955.949	-31.725
3.982.564	-44.063	4.006.393	-26.733	3.956.130	-31.720
3.982.726	-44.064	4.006.590	-26.718	3.956.347	-31.716
3.982.903	-44.064	4.006.773	-26.703	3.956.539	-31.712
3.983.077	-44.065	4.007.009	-26.688	3.956.649	-31.708
3.983.207	-44.065	4.007.238	-26.673	3.956.752	-31.703
3.983.376	-44.066	4.007.413	-26.658	3.956.907	-31.699
3.983.510	-44.066	4.007.592	-26.643	3.957.120	-31.695
3.983.607	-44.067	4.007.737	-26.627	3.957.336	-31.692
3.983.795	-44.067	4.007.925	-26.612	3.957.446	-31.688
3.983.966	-44.068	4.008.127	-26.597	3.957.506	-31.684
3.984.031	-44.068	4.008.199	-26.582	3.957.709	-31.680
3.984.129	-44.069	4.008.313	-26.567	3.958.160	-31.676
3.984.313	-44.070	4.008.448	-26.551	3.958.579	-31.673
3.984.558	-44.070	4.008.586	-26.536	3.958.731	-31.669
3.984.810	-44.071	4.008.810	-26.521	3.958.778	-31.666
3.985.006	-44.071	4.008.967	-26.506	3.958.828	-31.662
3.985.197	-44.072	4.009.100	-26.491	3.958.907	-31.659
3.985.404	-44.072	4.009.312	-26.476	3.959.027	-31.655
3.985.595	-44.073	4.009.532	-26.460	3.959.089	-31.652

3.985.742	-44.073	4.009.756	-26.445	3.959.121	-31.649
3.985.924	-44.074	4.009.938	-26.430	3.959.220	-31.646
3.986.111	-44.075	4.010.061	-26.415	3.959.429	-31.643
3.986.256	-44.075	4.010.215	-26.400	3.959.587	-31.640
3.986.382	-44.076	4.010.370	-26.385	3.959.765	-31.637
3.986.448	-44.076	4.010.464	-26.370	3.959.980	-31.634
3.986.588	-44.077	4.010.600	-26.355	3.960.121	-31.631
3.986.769	-44.077	4.010.822	-26.340	3.960.361	-31.628
3.986.931	-44.078	4.011.014	-26.325	3.960.638	-31.626
3.987.126	-44.078	4.011.143	-26.310	3.960.775	-31.623
3.987.355	-44.079	4.011.243	-26.295	3.960.856	-31.620
3.987.597	-44.080	4.011.454	-26.280	3.961.071	-31.618
3.987.777	-44.080	4.011.660	-26.265	3.961.255	-31.616
3.987.917	-44.081	4.011.831	-26.250	3.961.360	-31.613
3.988.082	-44.081	4.012.065	-26.236	3.961.474	-31.611
3.988.244	-44.082	4.012.203	-26.222	3.961.653	-31.609
3.988.427	-44.082	4.012.351	-26.207	3.961.845	-31.607
3.988.623	-44.083	4.012.576	-26.193	3.962.073	-31.605
3.988.767	-44.083	4.012.760	-26.179	3.962.268	-31.603
3.988.861	-44.084	4.012.879	-26.166	3.962.358	-31.601
3.988.958	-44.085	4.013.033	-26.152	3.962.484	-31.599
3.989.113	-44.085	4.013.214	-26.138	3.962.648	-31.598
3.989.308	-44.086	4.013.372	-26.125	3.962.729	-31.596
3.989.496	-44.086	4.013.550	-26.112	3.962.914	-31.594
3.989.655	-44.087	4.013.759	-26.099	3.963.221	-31.593
3.989.823	-44.087	4.013.943	-26.086	3.963.438	-31.592
3.990.099	-44.088	4.014.067	-26.073	3.963.669	-31.590
3.990.305	-44.088	4.014.240	-26.060	3.963.824	-31.589
3.990.370	-44.089	4.014.431	-26.048	3.963.908	-31.588
3.990.505	-44.089	4.014.537	-26.036	3.964.070	-31.587

3.990.689	-44.090	4.014.724	-26.023	3.964.301	-31.586
3.990.901	-44.090	4.015.177	-26.011	3.964.462	-31.585
3.991.079	-44.091	4.015.649	-25.999	3.964.509	-31.584
3.991.192	-44.091	4.015.768	-25.988	3.964.652	-31.583
3.991.310	-44.092	4.015.760	-25.976	3.964.843	-31.582
3.991.487	-44.092	4.015.903	-25.965	3.964.991	-31.582
3.991.718	-44.093	4.015.952	-25.953	3.965.180	-31.581
3.991.915	-44.093	4.015.909	-25.942	3.965.269	-31.580
3.992.050	-44.094	4.015.968	-25.931	3.965.388	-31.580
3.992.180	-44.094	4.016.093	-25.920	3.965.536	-31.579
3.992.383	-44.095	4.016.288	-25.910	3.965.747	-31.579
3.992.522	-44.095	4.016.514	-25.899	3.965.989	-31.579
3.992.658	-44.096	4.016.702	-25.889	3.966.160	-31.579
3.993.040	-44.096	4.016.862	-25.878	3.966.324	-31.578
3.993.470	-44.097	4.017.056	-25.868	3.966.455	-31.578
3.993.676	-44.097	4.017.307	-25.858	3.966.597	-31.578
3.993.709	-44.098	4.017.505	-25.849	3.966.734	-31.578
3.993.745	-44.098	4.017.648	-25.839	3.966.863	-31.578
3.993.896	-44.099	4.017.777	-25.830	3.967.040	-31.578
3.993.984	-44.099	4.017.921	-25.820	3.967.253	-31.578
3.994.052	-44.100	4.018.059	-25.811	3.967.466	-31.579
3.994.143	-44.100	4.018.208	-25.802	3.967.632	-31.579
3.994.185	-44.100	4.018.356	-25.794	3.967.733	-31.579
3.994.335	-44.101	4.018.472	-25.785	3.968.008	-31.580
3.994.590	-44.101	4.018.607	-25.777	3.968.277	-31.580
3.994.790	-44.102	4.018.771	-25.768	3.968.410	-31.580
3.995.013	-44.102	4.018.983	-25.760	3.968.513	-31.581
3.995.269	-44.102	4.019.167	-25.752	3.968.640	-31.581
3.995.410	-44.103	4.019.337	-25.745	3.968.813	-31.582
3.995.511	-44.103	4.019.579	-25.737	3.968.925	-31.582

3.995.717	-44.103	4.019.785	-25.730	3.969.106	-31.583
3.995.845	-44.104	4.019.902	-25.722	3.969.298	-31.583
3.995.945	-44.104			3.969.439	-31.584
3.996.115	-44.104			3.969.619	-31.584
3.996.237	-44.105			3.969.857	-31.585
3.996.418	-44.105			3.970.050	-31.586
3.996.656	-44.105			3.970.202	-31.586
3.996.754	-44.106			3.970.363	-31.587
3.996.927	-44.106			3.970.476	-31.588
3.997.180	-44.106			3.970.627	-31.588
3.997.319	-44.107			3.970.782	-31.589
3.997.449	-44.107			3.970.949	-31.589
3.997.654	-44.107			3.971.143	-31.590
3.997.906	-44.107			3.971.242	-31.591
3.998.033	-44.108			3.971.423	-31.591
3.998.099	-44.108			3.971.626	-31.592
3.998.192	-44.108			3.971.678	-31.593
3.998.402	-44.108			3.971.791	-31.593
3.998.625	-44.109			3.971.979	-31.594
3.998.759	-44.109			3.972.206	-31.594
3.998.951	-44.109			3.972.428	-31.595
3.999.106	-44.109			3.972.623	-31.595
3.999.233	-44.109			3.972.874	-31.596
3.999.438	-44.110			3.973.102	-31.596
3.999.662	-44.110			3.973.246	-31.596
3.999.844	-44.110			3.973.315	-31.597
3.999.969	-44.110			3.973.445	-31.597
4.000.105	-44.110			3.973.633	-31.597
4.000.270	-44.110			3.973.836	-31.597
4.000.444	-44.111			3.974.102	-31.598

4.000.468	-44.111			3.974.276	-31.598
4.000.661	-44.111			3.974.324	-31.598
4.000.974	-44.111			3.974.395	-31.598
4.001.105	-44.111			3.974.543	-31.598
4.001.243	-44.111			3.974.681	-31.598
4.001.375	-44.111			3.974.797	-31.597
4.001.561	-44.111			3.974.987	-31.597
4.001.820	-44.111			3.975.197	-31.597
4.002.027	-44.111			3.975.428	-31.596
4.002.121	-44.111			3.975.630	-31.596
4.002.214	-44.111			3.975.804	-31.596
4.002.366	-44.111			3.975.986	-31.595
4.002.563	-44.111			3.976.138	-31.594
4.002.773	-44.111			3.976.350	-31.594
4.003.044	-44.111			3.976.505	-31.593
4.003.233	-44.111			3.976.650	-31.592
4.003.329	-44.111			3.976.828	-31.591
4.003.510	-44.111			3.976.996	-31.590
4.003.723	-44.111			3.977.164	-31.589
4.003.878	-44.111			3.977.282	-31.588
4.003.994	-44.111			3.977.459	-31.587
4.004.154	-44.111			3.977.615	-31.586
4.004.273	-44.111			3.977.776	-31.584
4.004.308	-44.111			3.977.970	-31.583
4.004.418	-44.111			3.978.141	-31.582
4.004.619	-44.111			3.978.301	-31.580
4.004.756	-44.111			3.978.522	-31.578
4.004.964	-44.111			3.978.723	-31.577
4.005.133	-44.111			3.978.882	-31.575
4.005.309	-44.111			3.978.987	-31.573

4.005.573	-44.111			3.979.103	-31.571
4.005.804	-44.111			3.979.306	-31.569
4.005.967	-44.110			3.979.460	-31.567
4.006.089	-44.110			3.979.639	-31.565
4.006.255	-44.110			3.979.799	-31.563
4.006.396	-44.110			3.979.960	-31.560
4.006.574	-44.110			3.980.128	-31.558
4.006.823	-44.110			3.980.278	-31.555
4.006.991	-44.110			3.980.400	-31.553
4.007.000	-44.110			3.980.565	-31.550
4.007.081	-44.110			3.980.819	-31.547
4.007.301	-44.109			3.981.025	-31.545
4.007.496	-44.109			3.981.167	-31.542
4.007.670	-44.109			3.981.242	-31.539
4.007.825	-44.109			3.981.371	-31.536
4.008.026	-44.109			3.981.550	-31.533
4.008.272	-44.109			3.981.703	-31.529
4.008.456	-44.109			3.981.998	-31.526
4.008.636	-44.109			3.982.412	-31.523
4.008.839	-44.108			3.982.755	-31.520
4.009.001	-44.108			3.982.953	-31.516
4.009.131	-44.108			3.983.059	-31.513
4.009.273	-44.108			3.983.170	-31.509
4.009.402	-44.108			3.983.171	-31.505
4.009.588	-44.108			3.983.132	-31.502
4.009.804	-44.107			3.983.219	-31.498
4.009.991	-44.107			3.983.377	-31.494
4.010.129	-44.107			3.983.524	-31.490
4.010.255	-44.107			3.983.631	-31.486
4.010.443	-44.107			3.983.799	-31.482

4.010.623	-44.106			3.984.012	-31.478
4.010.771	-44.106			3.984.205	-31.473
4.010.928	-44.106			3.984.442	-31.469
4.011.148	-44.106			3.984.688	-31.465
4.011.310	-44.106			3.984.872	-31.461
4.011.462	-44.106			3.985.021	-31.456
4.011.657	-44.105			3.985.208	-31.452
4.011.816	-44.105			3.985.381	-31.447
4.011.949	-44.105			3.985.502	-31.442
4.012.077	-44.105			3.985.617	-31.438
4.012.251	-44.104			3.985.754	-31.433
4.012.422	-44.104			3.985.927	-31.428
4.012.581	-44.104			3.986.112	-31.424
4.012.734	-44.104			3.986.250	-31.419
4.012.886	-44.104			3.986.371	-31.414
4.013.067	-44.103			3.986.520	-31.409
4.013.224	-44.103			3.986.679	-31.404
4.013.510	-44.103			3.986.857	-31.399
4.013.977	-44.103			3.987.102	-31.394
4.014.240	-44.102			3.987.271	-31.389
4.014.240	-44.102			3.987.397	-31.384
4.014.288	-44.102			3.987.574	-31.378
4.014.362	-44.102			3.987.785	-31.373
4.014.381	-44.101			3.988.038	-31.368
4.014.453	-44.101			3.988.194	-31.363
4.014.626	-44.101			3.988.264	-31.357
4.014.780	-44.101			3.988.398	-31.352
4.014.912	-44.101			3.988.565	-31.347
4.015.140	-44.100			3.988.694	-31.341
4.015.305	-44.100			3.988.785	-31.336

4.015.435	-44.100			3.988.972	-31.330
4.015.693	-44.100			3.989.200	-31.325
4.015.895	-44.099			3.989.348	-31.319
4.015.963	-44.099			3.989.539	-31.314
4.016.111	-44.099			3.989.724	-31.308
4.016.353	-44.098			3.989.843	-31.303
4.016.535	-44.098			3.989.959	-31.297
4.016.682	-44.098			3.990.118	-31.291
4.016.874	-44.098			3.990.317	-31.286
4.017.014	-44.097			3.990.458	-31.280
4.017.168	-44.097			3.990.634	-31.275
4.017.375	-44.097			3.990.771	-31.269
4.017.510	-44.097			3.990.921	-31.264
4.017.628	-44.096			3.991.152	-31.258
4.017.799	-44.096			3.991.320	-31.252
4.018.036	-44.096			3.991.497	-31.247
4.018.289	-44.096			3.991.716	-31.241
4.018.442	-44.095			3.991.885	-31.236
4.018.589	-44.095			3.992.059	-31.230
4.018.716	-44.095			3.992.257	-31.224
4.018.802	-44.094			3.992.453	-31.219
4.019.034	-44.094			3.992.589	-31.213
4.019.265	-44.094			3.992.672	-31.207
4.019.417	-44.094			3.992.915	-31.202
4.019.479	-44.093			3.993.149	-31.196
4.019.660	-44.093			3.993.275	-31.191
4.019.854	-44.093			3.993.383	-31.185
4.019.941	-44.092			3.993.504	-31.179
				3.993.673	-31.174
				3.993.857	-31.168

				3.994.092	-31.162
				3.994.312	-31.157
				3.994.482	-31.151
				3.994.574	-31.145
				3.994.663	-31.140
				3.994.843	-31.134
				3.994.974	-31.128
				3.995.076	-31.123
				3.995.262	-31.117
				3.995.386	-31.112
				3.995.523	-31.106
				3.995.747	-31.100
				3.995.903	-31.095
				3.996.047	-31.089
				3.996.282	-31.083
				3.996.477	-31.078
				3.996.516	-31.072
				3.996.708	-31.067
				3.997.021	-31.061
				3.997.204	-31.056
				3.997.343	-31.050
				3.997.492	-31.044
				3.997.697	-31.039
				3.997.914	-31.033
				3.998.012	-31.028
				3.998.034	-31.022
				3.998.185	-31.016
				3.998.484	-31.011
				3.998.625	-31.005
				3.998.725	-31.000

				3.998.922	-30.994
				3.999.086	-30.989
				3.999.258	-30.983
				3.999.395	-30.977
				3.999.588	-30.972
				3.999.819	-30.966
				3.999.995	-30.961
				4.000.143	-30.955
				4.000.317	-30.950
				4.000.538	-30.944
				4.000.697	-30.939
				4.000.813	-30.933
				4.000.992	-30.927
				4.001.169	-30.922
				4.001.335	-30.916
				4.001.491	-30.911
				4.001.673	-30.906
				4.001.886	-30.900
				4.002.025	-30.895
				4.002.112	-30.889
				4.002.253	-30.884
				4.002.442	-30.878
				4.002.615	-30.873
				4.002.834	-30.867
				4.003.033	-30.862
				4.003.163	-30.857
				4.003.316	-30.851
				4.003.488	-30.846
				4.003.629	-30.840
				4.003.774	-30.835

				4.004.026	-30.830
				4.004.200	-30.824
				4.004.250	-30.819
				4.004.424	-30.814
				4.004.662	-30.809
				4.004.837	-30.803
				4.005.002	-30.798
				4.005.193	-30.793
				4.005.360	-30.788
				4.005.451	-30.783
				4.005.638	-30.777
				4.005.822	-30.772
				4.005.974	-30.767
				4.006.361	-30.762
				4.006.669	-30.757
				4.006.824	-30.752
				4.007.016	-30.747
				4.007.145	-30.742
				4.007.218	-30.737
				4.007.298	-30.732
				4.007.341	-30.727
				4.007.424	-30.722
				4.007.552	-30.717
				4.007.661	-30.712
				4.007.867	-30.707
				4.008.052	-30.702
				4.008.253	-30.698
				4.008.470	-30.693
				4.008.674	-30.688
				4.008.878	-30.683

				4.008.996	-30.679
				4.009.117	-30.674
				4.009.238	-30.669
				4.009.395	-30.665
				4.009.561	-30.660
				4.009.691	-30.656
				4.009.804	-30.651
				4.010.018	-30.647
				4.010.245	-30.642
				4.010.378	-30.638
				4.010.468	-30.634
				4.010.620	-30.629
				4.010.819	-30.625
				4.011.010	-30.621
				4.011.237	-30.617
				4.011.371	-30.613
				4.011.527	-30.609
				4.011.772	-30.605
				4.012.011	-30.601
				4.012.188	-30.597
				4.012.318	-30.593
				4.012.448	-30.589
				4.012.572	-30.585
				4.012.661	-30.582
				4.012.751	-30.578
				4.012.955	-30.574
				4.013.109	-30.571
				4.013.273	-30.567
				4.013.470	-30.564
				4.013.644	-30.561

				4.013.875	-30.557
				4.013.996	-30.554
				4.014.115	-30.551
				4.014.309	-30.548
				4.014.472	-30.544
				4.014.666	-30.541
				4.014.821	-30.538
				4.014.927	-30.536
				4.015.096	-30.533
				4.015.241	-30.530
				4.015.479	-30.527
				4.015.768	-30.524
				4.015.942	-30.522
				4.016.091	-30.519
				4.016.292	-30.517
				4.016.458	-30.514
				4.016.470	-30.512
				4.016.592	-30.509
				4.016.815	-30.507
				4.016.997	-30.505
				4.017.130	-30.503
				4.017.269	-30.500
				4.017.455	-30.498
				4.017.639	-30.496
				4.017.796	-30.494
				4.017.903	-30.492
				4.018.085	-30.490
				4.018.352	-30.488
				4.018.583	-30.487
				4.018.741	-30.485

				4.018.839	-30.483
				4.018.931	-30.481
				4.019.089	-30.480
				4.019.297	-30.478
				4.019.360	-30.477
				4.019.395	-30.475
				4.019.537	-30.474
				4.019.702	-30.472
				4.019.866	-30.471
