# Supplementary material for: Data on statistical experimental design to formulate amphotericin B-loaded Eudragit RL100 nanoparticles coated with hyaluronic acid for the treatment of vulvovaginal candidiasis
Source: Data Brief. 2020 Mar 5;29:105311. doi: 10.1016/j.dib.2020.105311 (PMC7082528; doi:10.1016/j.dib.2020.105311)
Supplement: Multimedia component 10 [file mmc10.pdf]

File Name:	**AMP EUD nano HA**	
[Data]		
Temp	**DSC**	
C	mW	
227.813	-16	
227.768	-21	
227.729	-27	
227.785	-33	
227.824	-39	
227.759	-45	
227.715	-52	
227.754	-58	
227.749	-66	
227.652	-73	
227.615	-81	
227.661	-89	
227.691	-97	
227.647	-105	
227.633	-114	
227.701	-123	
227.717	-133	
227.708	-143	
227.663	-153	
227.577	-164	
227.542	-174	
227.577	-186	
227.591	-197	
227.572	-209	
227.617	-222	

227.708	-234	
227.775	-247	
227.806	-261	
227.794	-275	
227.791	-289	
227.747	-304	
227.773	-319	
227.896	-335	
227.852	-351	
227.829	-367	
227.896	-384	
227.868	-401	
227.771	-419	
227.698	-438	
227.670	-456	
227.670	-476	
227.724	-495	
227.759	-516	
227.728	-536	
227.705	-558	
227.782	-580	
227.848	-602	
227.855	-625	
227.966	-648	
228.058	-672	
228.039	-696	
228.060	-721	
228.032	-747	
227.983	-773	
227.995	-799	

228.036	-827	
228.092	-854	
228.163	-883	
228.202	-911	
228.242	-941	
228.267	-971	
228.262	-1.001	
228.309	-1.032	
228.396	-1.064	
228.421	-1.096	
228.403	-1.129	
228.484	-1.162	
228.592	-1.196	
228.570	-1.230	
228.566	-1.265	
228.652	-1.301	
228.680	-1.337	
228.694	-1.374	
228.738	-1.411	
228.755	-1.449	
228.783	-1.487	
228.922	-1.526	
228.993	-1.566	
228.929	-1.606	
228.944	-1.646	
229.067	-1.687	
229.226	-1.729	
229.298	-1.771	
229.275	-1.814	
229.265	-1.857	

229.289	-1.901	
229.352	-1.945	
229.601	-1.990	
229.858	-2.036	
230.005	-2.082	
230.112	-2.128	
230.135	-2.175	
230.082	-2.222	
229.975	-2.270	
229.869	-2.319	
229.839	-2.368	
229.862	-2.417	
229.827	-2.467	
229.939	-2.517	
230.038	-2.568	
230.094	-2.619	
230.191	-2.671	
230.226	-2.723	
230.305	-2.776	
230.410	-2.829	
230.515	-2.882	
230.613	-2.936	
230.569	-2.991	
230.541	-3.045	
230.677	-3.100	
230.882	-3.156	
231.000	-3.212	
231.000	-3.268	
231.026	-3.325	
231.110	-3.382	

231.236	-3.439	
231.215	-3.497	
231.210	-3.555	
231.315	-3.613	
231.392	-3.672	
231.569	-3.731	
231.791	-3.790	
231.896	-3.850	
231.857	-3.910	
231.875	-3.970	
232.066	-4.031	
232.236	-4.091	
232.304	-4.152	
232.376	-4.214	
232.482	-4.275	
232.512	-4.337	
232.481	-4.399	
232.554	-4.461	
232.696	-4.523	
232.724	-4.586	
232.784	-4.649	
232.859	-4.711	
232.971	-4.775	
233.134	-4.838	
233.195	-4.901	
233.328	-4.965	
233.496	-5.028	
233.559	-5.092	
233.615	-5.156	
233.710	-5.220	

233.787	-5.284	
233.871	-5.348	
233.958	-5.412	
234.076	-5.477	
234.156	-5.541	
234.335	-5.605	
234.618	-5.670	
234.757	-5.734	
234.792	-5.799	
234.776	-5.863	
234.867	-5.928	
235.072	-5.992	
235.208	-6.057	
235.291	-6.121	
235.408	-6.186	
235.529	-6.250	
235.648	-6.314	
235.751	-6.379	
235.865	-6.443	
236.042	-6.507	
236.148	-6.571	
236.178	-6.635	
236.302	-6.699	
236.404	-6.763	
236.458	-6.827	
236.530	-6.890	
236.576	-6.954	
236.714	-7.017	
236.831	-7.080	
236.912	-7.143	

237.015	-7.206	
237.055	-7.269	
237.204	-7.332	
237.386	-7.394	
237.565	-7.457	
237.633	-7.519	
237.656	-7.581	
237.785	-7.643	
238.060	-7.704	
238.361	-7.766	
238.512	-7.827	
238.594	-7.888	
238.659	-7.949	
238.787	-8.010	
238.960	-8.070	
239.016	-8.130	
239.093	-8.190	
239.223	-8.250	
239.296	-8.310	
239.384	-8.369	
239.468	-8.428	
239.622	-8.487	
239.867	-8.545	
239.998	-8.604	
239.970	-8.662	
239.991	-8.720	
240.194	-8.777	
240.427	-8.834	
240.613	-8.891	
240.844	-8.948	

240.963	-9.005	
241.035	-9.061	
241.189	-9.117	
241.336	-9.172	
241.434	-9.228	
241.525	-9.283	
241.668	-9.338	
241.861	-9.392	
242.083	-9.446	
242.216	-9.500	
242.316	-9.554	
242.432	-9.607	
242.563	-9.660	
242.670	-9.713	
242.799	-9.766	
242.941	-9.818	
243.111	-9.870	
243.218	-9.921	
243.191	-9.972	
243.228	-10.023	
243.477	-10.074	
243.699	-10.124	
243.892	-10.174	
244.112	-10.224	
244.189	-10.273	
244.310	-10.322	
244.471	-10.371	
244.585	-10.420	
244.695	-10.468	
244.779	-10.516	

244.909	-10.563	
245.154	-10.610	
245.368	-10.657	
245.557	-10.704	
245.653	-10.750	
245.665	-10.796	
245.781	-10.842	
246.008	-10.888	
246.273	-10.933	
246.625	-10.978	
246.887	-11.022	
246.948	-11.066	
247.045	-11.110	
247.164	-11.154	
247.183	-11.197	
247.166	-11.240	
247.271	-11.283	
247.477	-11.325	
247.647	-11.367	
247.701	-11.409	
247.696	-11.451	
247.866	-11.492	
248.176	-11.533	
248.363	-11.573	
248.421	-11.614	
248.568	-11.654	
248.795	-11.693	
248.806	-11.733	
248.927	-11.772	
249.186	-11.811	

249.347	-11.849	
249.548	-11.888	
249.673	-11.926	
249.743	-11.964	
249.823	-12.001	
249.818	-12.038	
250.021	-12.075	
250.236	-12.112	
250.361	-12.148	
250.527	-12.184	
250.644	-12.220	
250.737	-12.256	
250.935	-12.291	
251.231	-12.326	
251.411	-12.361	
251.507	-12.395	
251.600	-12.430	
251.733	-12.464	
251.943	-12.497	
252.157	-12.531	
252.278	-12.564	
252.411	-12.597	
252.544	-12.630	
252.664	-12.662	
252.733	-12.694	
252.817	-12.726	
252.903	-12.758	
253.076	-12.790	
253.312	-12.821	
253.473	-12.852	

253.636	-12.883	
253.827	-12.913	
253.983	-12.944	
254.186	-12.974	
254.356	-13.003	
254.385	-13.033	
254.517	-13.062	
254.676	-13.092	
254.872	-13.121	
255.049	-13.149	
255.177	-13.178	
255.357	-13.206	
255.567	-13.234	
255.711	-13.262	
255.784	-13.290	
255.961	-13.317	
256.213	-13.344	
256.427	-13.371	
256.498	-13.398	
256.632	-13.425	
256.826	-13.451	
256.917	-13.477	
257.032	-13.503	
257.234	-13.529	
257.388	-13.554	
257.519	-13.580	
257.696	-13.605	
257.887	-13.630	
258.043	-13.655	
258.200	-13.679	

258.403	-13.704	
258.477	-13.728	
258.524	-13.752	
258.652	-13.775	
258.762	-13.799	
258.946	-13.823	
259.160	-13.846	
259.265	-13.869	
259.382	-13.892	
259.576	-13.914	
259.758	-13.937	
259.916	-13.959	
260.035	-13.981	
260.180	-14.003	
260.424	-14.025	
260.527	-14.046	
260.737	-14.068	
261.026	-14.089	
261.224	-14.110	
261.402	-14.131	
261.430	-14.151	
261.556	-14.172	
261.742	-14.192	
261.906	-14.212	
262.031	-14.232	
262.101	-14.252	
262.160	-14.272	
262.339	-14.291	
262.540	-14.311	
262.661	-14.330	

262.803	-14.349	
262.936	-14.368	
263.135	-14.386	
263.389	-14.405	
263.535	-14.423	
263.710	-14.442	
263.962	-14.460	
264.153	-14.478	
264.314	-14.495	
264.349	-14.513	
264.440	-14.530	
264.639	-14.548	
264.827	-14.565	
265.037	-14.582	
265.154	-14.599	
265.287	-14.616	
265.441	-14.632	
265.641	-14.649	
265.884	-14.665	
266.024	-14.681	
266.049	-14.697	
266.206	-14.713	
266.474	-14.729	
266.672	-14.745	
266.789	-14.760	
266.877	-14.776	
267.043	-14.791	
267.215	-14.806	
267.386	-14.821	
267.456	-14.836	

267.659	-14.851	
267.876	-14.865	
267.922	-14.880	
268.088	-14.894	
268.351	-14.908	
268.589	-14.922	
268.694	-14.936	
268.855	-14.950	
269.109	-14.964	
269.277	-14.978	
269.373	-14.991	
269.466	-15.005	
269.632	-15.018	
269.737	-15.031	
269.930	-15.044	
270.287	-15.057	
270.651	-15.070	
270.956	-15.083	
271.049	-15.096	
271.182	-15.108	
271.329	-15.121	
271.297	-15.133	
271.318	-15.145	
271.409	-15.157	
271.486	-15.169	
271.586	-15.181	
271.768	-15.193	
271.976	-15.205	
272.120	-15.216	
272.313	-15.228	

272.488	-15.239	
272.647	-15.251	
272.846	-15.262	
273.001	-15.273	
273.149	-15.284	
273.321	-15.295	
273.501	-15.306	
273.601	-15.317	
273.706	-15.328	
273.853	-15.339	
274.051	-15.349	
274.343	-15.360	
274.461	-15.370	
274.518	-15.381	
274.650	-15.391	
274.818	-15.401	
274.967	-15.411	
275.091	-15.421	
275.215	-15.431	
275.388	-15.441	
275.592	-15.451	
275.889	-15.461	
276.126	-15.471	
276.189	-15.480	
276.327	-15.490	
276.476	-15.499	
276.616	-15.509	
276.796	-15.518	
276.947	-15.527	
277.158	-15.537	

277.318	-15.546	
277.416	-15.555	
277.577	-15.564	
277.719	-15.573	
277.895	-15.582	
278.069	-15.591	
278.134	-15.600	
278.298	-15.608	
278.608	-15.617	
278.839	-15.626	
278.860	-15.634	
278.927	-15.643	
279.153	-15.651	
279.277	-15.660	
279.410	-15.668	
279.653	-15.677	
279.846	-15.685	
279.932	-15.693	
280.079	-15.701	
280.354	-15.710	
280.511	-15.718	
280.569	-15.726	
280.712	-15.734	
280.938	-15.742	
281.131	-15.749	
281.252	-15.757	
281.390	-15.765	
281.488	-15.773	
281.607	-15.781	
281.880	-15.788	

282.088	-15.796	
282.227	-15.803	
282.376	-15.811	
282.472	-15.819	
282.631	-15.826	
282.794	-15.833	
282.971	-15.841	
283.156	-15.848	
283.305	-15.855	
283.440	-15.863	
283.659	-15.870	
283.843	-15.877	
283.916	-15.884	
284.025	-15.891	
284.163	-15.898	
284.317	-15.905	
284.447	-15.912	
284.667	-15.919	
284.862	-15.926	
284.946	-15.933	
285.115	-15.940	
285.346	-15.946	
285.464	-15.953	
285.642	-15.960	
285.875	-15.967	
286.017	-15.973	
286.241	-15.980	
286.418	-15.986	
286.549	-15.993	
286.728	-15.999	

286.868	-16.006	
286.941	-16.012	
287.013	-16.019	
287.225	-16.025	
287.458	-16.031	
287.586	-16.038	
287.703	-16.044	
287.902	-16.050	
288.022	-16.056	
288.104	-16.062	
288.281	-16.068	
288.524	-16.074	
288.773	-16.081	
288.897	-16.087	
289.016	-16.093	
289.219	-16.098	
289.447	-16.104	
289.643	-16.110	
289.774	-16.116	
289.902	-16.122	
290.019	-16.128	
290.154	-16.133	
290.287	-16.139	
290.441	-16.145	
290.623	-16.151	
290.831	-16.156	
290.982	-16.162	
291.129	-16.167	
291.290	-16.173	
291.369	-16.179	

291.569	-16.184	
291.789	-16.190	
291.957	-16.195	
292.179	-16.200	
292.319	-16.206	
292.439	-16.211	
292.629	-16.217	
292.773	-16.222	
292.923	-16.227	
293.053	-16.233	
293.183	-16.238	
293.342	-16.243	
293.570	-16.248	
293.809	-16.253	
293.935	-16.259	
294.067	-16.264	
294.268	-16.269	
294.419	-16.274	
294.539	-16.279	
294.635	-16.284	
294.834	-16.289	
294.966	-16.294	
295.120	-16.299	
295.494	-16.304	
295.888	-16.309	
296.089	-16.314	
296.164	-16.319	
296.240	-16.324	
296.307	-16.329	
296.342	-16.334	

296.444	-16.339	
296.608	-16.344	
296.691	-16.348	
296.742	-16.353	
296.887	-16.358	
297.087	-16.363	
297.303	-16.368	
297.485	-16.373	
297.652	-16.377	
297.749	-16.382	
297.941	-16.387	
298.128	-16.392	
298.232	-16.396	
298.485	-16.401	
298.658	-16.406	
298.710	-16.410	
298.901	-16.415	
299.050	-16.420	
299.195	-16.424	
299.309	-16.429	
299.471	-16.434	
299.694	-16.438	
299.835	-16.443	
299.979	-16.448	
300.114	-16.452	
300.246	-16.457	
300.459	-16.462	
300.558	-16.466	
300.738	-16.471	
301.010	-16.476	

301.117	-16.480	
301.256	-16.485	
301.430	-16.489	
301.583	-16.494	
301.713	-16.499	
301.902	-16.503	
302.116	-16.508	
302.283	-16.513	
302.375	-16.517	
302.431	-16.522	
302.590	-16.526	
302.830	-16.531	
303.014	-16.536	
303.112	-16.540	
303.218	-16.545	
303.416	-16.549	
303.593	-16.554	
303.691	-16.559	
303.813	-16.563	
304.005	-16.568	
304.299	-16.573	
304.484	-16.577	
304.454	-16.582	
304.550	-16.586	
304.811	-16.591	
304.961	-16.596	
305.091	-16.600	
305.263	-16.605	
305.417	-16.610	
305.613	-16.614	

305.871	-16.619	
306.070	-16.624	
306.201	-16.628	
306.340	-16.633	
306.556	-16.638	
306.790	-16.642	
306.925	-16.647	
306.984	-16.652	
307.049	-16.656	
307.194	-16.661	
307.372	-16.666	
307.607	-16.670	
307.823	-16.675	
307.984	-16.680	
308.155	-16.684	
308.323	-16.689	
308.462	-16.694	
308.603	-16.698	
308.753	-16.703	
308.892	-16.708	
308.971	-16.712	
309.116	-16.717	
309.186	-16.722	
309.237	-16.726	
309.444	-16.731	
309.652	-16.736	
309.821	-16.740	
310.002	-16.745	
310.132	-16.750	
310.282	-16.754	

310.456	-16.759	
310.632	-16.764	
310.922	-16.768	
311.145	-16.773	
311.289	-16.778	
311.458	-16.782	
311.565	-16.787	
311.686	-16.792	
311.911	-16.796	
312.008	-16.801	
312.129	-16.806	
312.334	-16.810	
312.410	-16.815	
312.531	-16.819	
312.718	-16.824	
312.848	-16.829	
313.011	-16.833	
313.115	-16.838	
313.249	-16.843	
313.450	-16.847	
313.710	-16.852	
314.018	-16.856	
314.200	-16.861	
314.322	-16.865	
314.474	-16.870	
314.653	-16.875	
314.827	-16.879	
314.979	-16.884	
315.100	-16.888	
315.263	-16.893	

315.360	-16.897	
315.454	-16.902	
315.626	-16.906	
315.772	-16.911	
316.023	-16.915	
316.282	-16.920	
316.413	-16.924	
316.518	-16.929	
316.667	-16.933	
316.878	-16.938	
317.074	-16.942	
317.182	-16.946	
317.299	-16.951	
317.442	-16.955	
317.579	-16.960	
317.744	-16.964	
317.913	-16.969	
318.094	-16.973	
318.218	-16.977	
318.370	-16.982	
318.499	-16.986	
318.646	-16.990	
318.851	-16.995	
319.019	-16.999	
319.160	-17.004	
319.318	-17.008	
319.471	-17.012	
319.628	-17.017	
319.841	-17.021	
320.035	-17.025	

320.194	-17.029	
320.276	-17.034	
320.578	-17.038	
321.032	-17.042	
321.407	-17.047	
321.528	-17.051	
321.538	-17.055	
321.679	-17.059	
321.692	-17.064	
321.687	-17.068	
321.802	-17.072	
321.924	-17.076	
321.996	-17.080	
322.111	-17.085	
322.331	-17.089	
322.549	-17.093	
322.756	-17.097	
322.946	-17.101	
323.078	-17.106	
323.235	-17.110	
323.408	-17.114	
323.659	-17.118	
323.844	-17.122	
323.929	-17.126	
324.048	-17.130	
324.143	-17.135	
324.300	-17.139	
324.383	-17.143	
324.458	-17.147	
324.661	-17.151	

324.883	-17.155	
325.109	-17.159	
325.254	-17.163	
325.339	-17.168	
325.474	-17.172	
325.642	-17.176	
325.797	-17.180	
325.947	-17.184	
326.180	-17.188	
326.430	-17.192	
326.659	-17.196	
326.876	-17.200	
327.060	-17.205	
327.192	-17.209	
327.256	-17.213	
327.323	-17.217	
327.514	-17.221	
327.693	-17.225	
327.806	-17.229	
327.985	-17.233	
328.170	-17.237	
328.230	-17.241	
328.443	-17.245	
328.660	-17.250	
328.748	-17.254	
328.905	-17.258	
329.099	-17.262	
329.255	-17.266	
329.373	-17.270	
329.554	-17.274	

329.686	-17.278	
329.854	-17.282	
329.988	-17.286	
330.082	-17.290	
330.265	-17.295	
330.558	-17.299	
330.785	-17.303	
330.867	-17.307	
331.028	-17.311	
331.199	-17.315	
331.334	-17.319	
331.487	-17.323	
331.681	-17.327	
331.853	-17.331	
332.000	-17.335	
332.126	-17.339	
332.327	-17.343	
332.538	-17.348	
332.633	-17.352	
332.723	-17.356	
332.916	-17.360	
333.094	-17.364	
333.224	-17.368	
333.471	-17.372	
333.619	-17.376	
333.633	-17.380	
333.756	-17.384	
333.959	-17.388	
334.082	-17.392	
334.258	-17.396	

334.513	-17.400	
334.639	-17.404	
334.682	-17.408	
334.844	-17.412	
335.066	-17.416	
335.148	-17.420	
335.296	-17.424	
335.494	-17.428	
335.600	-17.432	
335.756	-17.436	
336.040	-17.440	
336.342	-17.444	
336.497	-17.448	
336.635	-17.452	
336.912	-17.455	
337.185	-17.459	
337.307	-17.463	
337.340	-17.467	
337.411	-17.471	
337.641	-17.475	
337.892	-17.479	
337.966	-17.483	
338.036	-17.486	
338.208	-17.490	
338.404	-17.494	
338.584	-17.498	
338.717	-17.502	
338.887	-17.506	
339.088	-17.509	
339.254	-17.513	

339.448	-17.517	
339.641	-17.521	
339.797	-17.524	
340.022	-17.528	
340.248	-17.532	
340.337	-17.536	
340.356	-17.539	
340.474	-17.543	
340.716	-17.547	
340.889	-17.550	
340.960	-17.554	
341.129	-17.558	
341.324	-17.562	
341.436	-17.565	
341.558	-17.569	
341.768	-17.572	
342.058	-17.576	
342.352	-17.580	
342.491	-17.583	
342.504	-17.587	
342.639	-17.590	
342.837	-17.594	
343.009	-17.598	
343.160	-17.601	
343.296	-17.605	
343.438	-17.608	
343.596	-17.612	
343.819	-17.615	
343.947	-17.619	
344.054	-17.622	

344.280	-17.626	
344.467	-17.629	
344.680	-17.633	
344.833	-17.636	
344.927	-17.640	
345.047	-17.643	
345.243	-17.646	
345.443	-17.650	
345.584	-17.653	
345.780	-17.657	
345.840	-17.660	
345.939	-17.664	
346.359	-17.667	
346.764	-17.670	
346.864	-17.674	
347.029	-17.677	
347.250	-17.681	
347.308	-17.684	
347.253	-17.687	
347.245	-17.691	
347.436	-17.694	
347.672	-17.697	
347.815	-17.701	
347.905	-17.704	
348.111	-17.707	
348.278	-17.711	
348.379	-17.714	
348.583	-17.717	
348.779	-17.721	
348.985	-17.724	

349.222	-17.727	
349.301	-17.731	
349.409	-17.734	
349.647	-17.737	
349.814	-17.741	
349.966	-17.744	
350.125	-17.747	
350.217	-17.750	
350.296	-17.754	
350.448	-17.757	
350.645	-17.760	
350.738	-17.764	
350.852	-17.767	
351.003	-17.770	
351.097	-17.773	
351.347	-17.777	
351.620	-17.780	
351.787	-17.783	
351.994	-17.787	
352.255	-17.790	
352.421	-17.793	
352.612	-17.796	
352.716	-17.800	
352.697	-17.803	
352.921	-17.806	
353.166	-17.810	
353.350	-17.813	
353.517	-17.816	
353.678	-17.820	
353.874	-17.823	

354.034	-17.826	
354.131	-17.830	
354.304	-17.833	
354.460	-17.836	
354.553	-17.839	
354.705	-17.843	
354.821	-17.846	
355.088	-17.849	
355.213	-17.853	
355.369	-17.856	
355.669	-17.860	
355.833	-17.863	
355.909	-17.866	
356.049	-17.870	
356.203	-17.873	
356.282	-17.876	
356.471	-17.880	
356.711	-17.883	
356.892	-17.886	
357.077	-17.890	
357.300	-17.893	
357.368	-17.896	
357.479	-17.900	
357.623	-17.903	
357.739	-17.907	
357.949	-17.910	
358.139	-17.913	
358.326	-17.917	
358.496	-17.920	
358.673	-17.923	

358.908	-17.927	
358.997	-17.930	
359.098	-17.934	
359.354	-17.937	
359.483	-17.940	
359.572	-17.944	
359.709	-17.947	
359.934	-17.950	
360.077	-17.954	
360.251	-17.957	
360.489	-17.960	
360.554	-17.964	
360.615	-17.967	
360.805	-17.970	
360.970	-17.974	
361.060	-17.977	
361.172	-17.980	
361.408	-17.984	
361.666	-17.987	
361.844	-17.990	
362.028	-17.994	
362.245	-17.997	
362.400	-18.000	
362.618	-18.003	
362.873	-18.007	
362.988	-18.010	
363.130	-18.013	
363.183	-18.016	
363.249	-18.020	
363.394	-18.023	

363.546	-18.026	
363.794	-18.029	
363.995	-18.033	
364.173	-18.036	
364.378	-18.039	
364.423	-18.042	
364.514	-18.045	
364.696	-18.048	
364.877	-18.052	
365.080	-18.055	
365.183	-18.058	
365.246	-18.061	
365.498	-18.064	
365.792	-18.067	
365.922	-18.070	
366.117	-18.073	
366.340	-18.077	
366.659	-18.080	
366.721	-18.083	
366.707	-18.086	
366.955	-18.089	
367.064	-18.092	
367.191	-18.095	
367.387	-18.098	
367.607	-18.101	
367.859	-18.104	
368.048	-18.107	
368.166	-18.110	
368.272	-18.113	
368.343	-18.116	

368.641	-18.119	
368.817	-18.122	
368.912	-18.125	
369.180	-18.128	
369.298	-18.131	
369.367	-18.134	
369.477	-18.137	
369.571	-18.139	
369.766	-18.142	
370.146	-18.145	
370.344	-18.148	
370.386	-18.151	
370.552	-18.154	
370.786	-18.157	
370.920	-18.160	
370.986	-18.162	
371.087	-18.165	
371.290	-18.168	
371.471	-18.171	
371.675	-18.174	
371.744	-18.176	
372.083	-18.179	
372.607	-18.182	
372.825	-18.185	
373.000	-18.188	
373.053	-18.190	
373.189	-18.193	
373.324	-18.196	
373.250	-18.199	
373.119	-18.201	

373.281	-18.204	
373.569	-18.207	
373.583	-18.210	
373.649	-18.212	
373.884	-18.215	
373.967	-18.218	
374.039	-18.220	
374.363	-18.223	
374.573	-18.226	
374.783	-18.228	
375.250	-18.231	
375.294	-18.234	
375.267	-18.237	
375.431	-18.239	
375.640	-18.242	
375.944	-18.245	
375.970	-18.247	
376.096	-18.250	
376.394	-18.253	
376.655	-18.255	
376.801	-18.258	
376.851	-18.261	
376.921	-18.263	
377.213	-18.266	
377.517	-18.269	
377.577	-18.272	
377.778	-18.274	
377.781	-18.277	
377.834	-18.280	
378.155	-18.282	

378.319	-18.285	
378.418	-18.288	
378.533	-18.290	
378.829	-18.293	
378.877	-18.296	
378.958	-18.299	
379.217	-18.301	
379.337	-18.304	
379.526	-18.307	
379.678	-18.310	
379.796	-18.312	
380.031	-18.315	
380.246	-18.318	
380.346	-18.321	
380.527	-18.323	
380.695	-18.326	
380.770	-18.329	
381.012	-18.332	
381.371	-18.334	
381.374	-18.337	
381.311	-18.340	
381.601	-18.343	
381.918	-18.345	
382.045	-18.348	
382.180	-18.351	
382.316	-18.354	
382.467	-18.357	
382.556	-18.359	
382.667	-18.362	
382.881	-18.365	

383.116	-18.368	
383.426	-18.371	
383.549	-18.373	
383.697	-18.376	
383.926	-18.379	
384.011	-18.382	
384.018	-18.384	
384.026	-18.387	
384.262	-18.390	
384.579	-18.393	
384.789	-18.396	
384.947	-18.398	
385.072	-18.401	
385.227	-18.404	
385.447	-18.407	
385.526	-18.410	
385.652	-18.412	
385.695	-18.415	
385.884	-18.418	
386.315	-18.421	
386.368	-18.424	
386.442	-18.426	
386.608	-18.429	
386.783	-18.432	
387.098	-18.435	
387.348	-18.437	
387.414	-18.440	
387.336	-18.443	
387.373	-18.446	
387.553	-18.448	

387.902	-18.451	
388.197	-18.454	
388.275	-18.457	
388.516	-18.459	
388.743	-18.462	
388.859	-18.465	
389.066	-18.467	
389.214	-18.470	
389.435	-18.473	
389.623	-18.476	
389.620	-18.478	
389.655	-18.481	
389.741	-18.484	
389.900	-18.486	
390.045	-18.489	
390.159	-18.491	
390.333	-18.494	
390.534	-18.497	
390.758	-18.499	
390.988	-18.502	
391.152	-18.505	
391.262	-18.507	
391.457	-18.510	
391.670	-18.512	
391.880	-18.515	
392.023	-18.517	
392.161	-18.520	
392.406	-18.523	
392.603	-18.525	
392.658	-18.528	

392.701	-18.530	
392.854	-18.533	
393.067	-18.535	
393.318	-18.538	
393.504	-18.540	
393.572	-18.543	
393.682	-18.545	
393.915	-18.548	
394.022	-18.550	
394.201	-18.553	
394.600	-18.555	
394.846	-18.558	
394.838	-18.560	
394.906	-18.563	
395.094	-18.565	
395.288	-18.568	
395.577	-18.570	
395.725	-18.572	
395.756	-18.575	
395.801	-18.577	
396.045	-18.580	
396.245	-18.582	
396.317	-18.584	
396.546	-18.587	
396.728	-18.589	
396.903	-18.592	
397.084	-18.594	
397.218	-18.596	
397.373	-18.599	
397.421	-18.601	

397.508	-18.604	
397.831	-18.606	
398.263	-18.608	
398.640	-18.611	
398.855	-18.613	
398.867	-18.616	
398.939	-18.618	
399.116	-18.620	
399.113	-18.623	
398.997	-18.625	
399.054	-18.628	
399.360	-18.630	
399.652	-18.632	
399.712	-18.635	
399.807	-18.637	
400.082	-18.640	
400.270	-18.642	
400.429	-18.644	
400.639	-18.647	
400.834	-18.649	
401.040	-18.652	
401.173	-18.654	
401.243	-18.656	
401.395	-18.659	
401.509	-18.661	
401.704	-18.664	
401.915	-18.666	
402.037	-18.669	
402.189	-18.671	
402.285	-18.674	

402.556	-18.676	
402.699	-18.679	
402.657	-18.681	
402.846	-18.684	
402.958	-18.686	
403.123	-18.689	
403.444	-18.691	
403.726	-18.694	
403.882	-18.696	
404.017	-18.699	
404.073	-18.701	
404.185	-18.704	
404.499	-18.706	
404.618	-18.709	
404.800	-18.711	
404.881	-18.714	
405.038	-18.717	
405.302	-18.719	
405.420	-18.722	
405.465	-18.724	
405.599	-18.727	
405.896	-18.730	
406.060	-18.732	
406.187	-18.735	
406.308	-18.738	
406.484	-18.740	
406.690	-18.743	
406.834	-18.746	
406.946	-18.748	
407.089	-18.751	

407.238	-18.754	
407.403	-18.757	
407.539	-18.759	
407.667	-18.762	
407.717	-18.765	
407.889	-18.768	
408.357	-18.770	
408.646	-18.773	
408.698	-18.776	
408.726	-18.779	
408.795	-18.782	
408.948	-18.784	
409.188	-18.787	
409.420	-18.790	
409.553	-18.793	
409.711	-18.796	
409.899	-18.799	
410.048	-18.801	
410.171	-18.804	
410.301	-18.807	
410.530	-18.810	
410.676	-18.813	
410.853	-18.816	
411.075	-18.819	
411.296	-18.821	
411.458	-18.824	
411.560	-18.827	
411.938	-18.830	
412.048	-18.833	
412.053	-18.836	

412.081	-18.839	
412.165	-18.842	
412.734	-18.844	
412.919	-18.847	
412.400	-18.850	
412.411	-18.853	
412.827	-18.856	
412.995	-18.859	
413.366	-18.862	
413.603	-18.864	
413.558	-18.867	
413.845	-18.870	
414.093	-18.873	
414.226	-18.876	
414.603	-18.879	
414.787	-18.881	
414.909	-18.884	
414.934	-18.887	
415.009	-18.890	
415.311	-18.893	
415.454	-18.895	
415.551	-18.898	
415.711	-18.901	
415.851	-18.904	
416.036	-18.906	
416.241	-18.909	
416.341	-18.912	
416.411	-18.915	
416.600	-18.917	
416.853	-18.920	

417.017	-18.923	
417.179	-18.925	
417.394	-18.928	
417.585	-18.931	
417.781	-18.933	
417.902	-18.936	
418.051	-18.939	
418.177	-18.941	
418.267	-18.944	
418.391	-18.946	
418.518	-18.949	
418.761	-18.952	
418.975	-18.954	
419.082	-18.957	
419.241	-18.959	
419.461	-18.962	
419.660	-18.965	
419.798	-18.967	
419.842	-18.970	
419.978	-18.972	
420.248	-18.975	
420.492	-18.977	
420.638	-18.980	
420.738	-18.982	
420.958	-18.985	
421.174	-18.987	
421.205	-18.990	
421.302	-18.992	
421.512	-18.994	
421.730	-18.997	

421.877	-18.999	
422.044	-19.002	
422.281	-19.004	
422.422	-19.007	
422.586	-19.009	
422.769	-19.011	
422.951	-19.014	
423.023	-19.016	
423.149	-19.018	
423.382	-19.021	
423.546	-19.023	
423.831	-19.026	
424.289	-19.028	
424.648	-19.030	
424.807	-19.033	
424.857	-19.035	
424.856	-19.037	
424.884	-19.040	
424.977	-19.042	
425.075	-19.044	
425.126	-19.047	
425.265	-19.049	
425.387	-19.051	
425.510	-19.053	
425.728	-19.056	
425.814	-19.058	
425.835	-19.060	
426.010	-19.063	
426.296	-19.065	
426.546	-19.067	

426.708	-19.069	
426.907	-19.072	
427.140	-19.074	
427.303	-19.076	
427.508	-19.079	
427.621	-19.081	
427.627	-19.083	
427.842	-19.085	
428.096	-19.088	
428.235	-19.090	
428.318	-19.092	
428.404	-19.094	
428.523	-19.097	
428.678	-19.099	
428.909	-19.101	
429.135	-19.104	
429.285	-19.106	
429.417	-19.108	
429.672	-19.111	
429.940	-19.113	
430.097	-19.115	
430.264	-19.118	
430.372	-19.120	
430.509	-19.122	
430.656	-19.125	
430.723	-19.127	
430.883	-19.129	
430.966	-19.132	
431.128	-19.134	
431.447	-19.137	

431.539	-19.139	
431.540	-19.141	
431.787	-19.144	
432.069	-19.146	
432.192	-19.149	
432.346	-19.151	
432.608	-19.154	
432.742	-19.156	
432.820	-19.159	
433.013	-19.161	
433.166	-19.164	
433.316	-19.166	
433.497	-19.169	
433.580	-19.171	
433.708	-19.174	
433.994	-19.176	
434.276	-19.179	
434.408	-19.181	
434.497	-19.184	
434.608	-19.187	
434.745	-19.189	
435.000	-19.192	
435.223	-19.194	
435.349	-19.197	
435.497	-19.200	
435.708	-19.202	
435.850	-19.205	
435.916	-19.208	
436.064	-19.210	
436.205	-19.213	

436.304	-19.216	
436.532	-19.219	
436.753	-19.221	
436.949	-19.224	
437.139	-19.227	
437.239	-19.229	
437.371	-19.232	
437.589	-19.235	
437.761	-19.238	
437.838	-19.240	
437.924	-19.243	
438.090	-19.246	
438.229	-19.249	
438.369	-19.251	
438.585	-19.254	
438.716	-19.257	
438.833	-19.260	
438.958	-19.262	
439.194	-19.265	
439.471	-19.268	
439.591	-19.271	
439.739	-19.274	
439.919	-19.276	
440.107	-19.279	
440.297	-19.282	
440.480	-19.285	
440.716	-19.287	
440.919	-19.290	
441.049	-19.293	
441.209	-19.296	

441.304	-19.299	
441.372	-19.301	
441.501	-19.304	
441.695	-19.307	
441.857	-19.310	
441.984	-19.313	
442.123	-19.315	
442.295	-19.318	
442.455	-19.321	
442.609	-19.324	
442.852	-19.327	
443.049	-19.329	
443.231	-19.332	
443.405	-19.335	
443.546	-19.338	
443.650	-19.341	
443.766	-19.344	
443.986	-19.346	
444.233	-19.349	
444.386	-19.352	
444.557	-19.355	
444.708	-19.358	
444.787	-19.360	
444.904	-19.363	
445.155	-19.366	
445.420	-19.369	
445.644	-19.372	
445.876	-19.374	
445.947	-19.377	
446.027	-19.380	

446.231	-19.383	
446.472	-19.386	
446.634	-19.388	
446.772	-19.391	
446.933	-19.394	
447.014	-19.397	
447.136	-19.400	
447.282	-19.402	
447.464	-19.405	
447.674	-19.408	
447.854	-19.411	
448.007	-19.414	
448.138	-19.416	
448.270	-19.419	
448.401	-19.422	
448.567	-19.425	
448.794	-19.427	
448.959	-19.430	
449.127	-19.433	
449.301	-19.436	
449.406	-19.439	
449.673	-19.441	
450.073	-19.444	
450.436	-19.447	
450.588	-19.450	
450.652	-19.452	
450.810	-19.455	
450.901	-19.458	
450.879	-19.461	
450.836	-19.464	

450.862	-19.466	
451.060	-19.469	
451.270	-19.472	
451.433	-19.475	
451.733	-19.478	
451.952	-19.480	
452.025	-19.483	
452.224	-19.486	
452.423	-19.489	
452.613	-19.491	
452.731	-19.494	
452.840	-19.497	
453.039	-19.500	
453.142	-19.503	
453.344	-19.505	
453.595	-19.508	
453.735	-19.511	
453.856	-19.514	
453.945	-19.517	
454.054	-19.520	
454.241	-19.522	
454.500	-19.525	
454.688	-19.528	
454.689	-19.531	
454.775	-19.534	
454.935	-19.537	
455.095	-19.540	
455.324	-19.542	
455.547	-19.545	
455.766	-19.548	

455.964	-19.551	
456.101	-19.554	
456.259	-19.557	
456.321	-19.560	
456.456	-19.563	
456.797	-19.566	
456.867	-19.569	
456.921	-19.572	
457.171	-19.575	
457.329	-19.578	
457.496	-19.581	
457.688	-19.584	
457.719	-19.587	
457.823	-19.590	
458.096	-19.593	
458.299	-19.596	
458.610	-19.599	
458.923	-19.602	
459.195	-19.605	
459.176	-19.608	
458.963	-19.611	
459.105	-19.614	
459.297	-19.617	
459.480	-19.620	
459.665	-19.623	
459.853	-19.626	
460.181	-19.629	
460.283	-19.632	
460.337	-19.635	
460.524	-19.638	

460.756	-19.642	
461.079	-19.645	
461.069	-19.648	
461.093	-19.651	
461.400	-19.654	
461.644	-19.657	
461.815	-19.660	
461.964	-19.663	
462.118	-19.667	
462.187	-19.670	
462.272	-19.673	
462.365	-19.676	
462.586	-19.679	
462.758	-19.682	
462.849	-19.685	
463.216	-19.688	
463.446	-19.692	
463.446	-19.695	
463.568	-19.698	
463.750	-19.701	
463.904	-19.704	
464.060	-19.707	
464.197	-19.710	
464.381	-19.713	
464.560	-19.717	
464.608	-19.720	
464.833	-19.723	
465.122	-19.726	
465.299	-19.729	
465.467	-19.732	

465.522	-19.735	
465.589	-19.738	
465.903	-19.741	
466.232	-19.744	
466.395	-19.747	
466.601	-19.750	
466.766	-19.753	
466.862	-19.756	
466.969	-19.759	
467.153	-19.762	
467.280	-19.765	
467.299	-19.768	
467.504	-19.771	
467.695	-19.774	
467.738	-19.777	
467.912	-19.780	
468.214	-19.783	
468.284	-19.786	
468.308	-19.789	
468.617	-19.792	
468.836	-19.795	
468.913	-19.798	
469.179	-19.801	
469.427	-19.803	
469.566	-19.806	
469.762	-19.809	
469.914	-19.812	
470.180	-19.815	
470.318	-19.818	
470.394	-19.820	

470.513	-19.823	
470.612	-19.826	
470.807	-19.829	
470.933	-19.831	
471.157	-19.834	
471.445	-19.837	
471.692	-19.839	
471.828	-19.842	
471.867	-19.845	
471.992	-19.847	
472.188	-19.850	
472.364	-19.853	
472.551	-19.855	
472.730	-19.858	
472.845	-19.861	
472.970	-19.863	
473.193	-19.866	
473.343	-19.868	
473.463	-19.871	
473.634	-19.873	
473.754	-19.876	
473.990	-19.878	
474.202	-19.881	
474.456	-19.883	
474.620	-19.886	
474.617	-19.888	
474.717	-19.891	
474.895	-19.893	
475.083	-19.896	
475.244	-19.898	

475.426	-19.900	
475.596	-19.903	
476.062	-19.905	
476.569	-19.908	
476.766	-19.910	
477.118	-19.912	
477.134	-19.915	
476.899	-19.917	
476.951	-19.919	
477.143	-19.922	
477.239	-19.924	
477.343	-19.926	
477.461	-19.928	
477.512	-19.931	
477.712	-19.933	
477.924	-19.935	
478.191	-19.938	
478.295	-19.940	
478.534	-19.942	
479.060	-19.944	
478.967	-19.946	
478.647	-19.949	
478.815	-19.951	
479.409	-19.953	
479.522	-19.955	
479.723	-19.958	
479.962	-19.960	
479.717	-19.962	
479.831	-19.964	
479.900	-19.966	

479.959	-19.969	
480.338	-19.971	
480.634	-19.973	
480.706	-19.975	
480.839	-19.977	
481.030	-19.980	
481.249	-19.982	
481.348	-19.984	
481.427	-19.986	
481.655	-19.988	
481.932	-19.990	
482.053	-19.993	
482.317	-19.995	
482.532	-19.997	
482.672	-19.999	
482.862	-20.002	
482.982	-20.004	
483.133	-20.006	
483.229	-20.008	
483.353	-20.010	
483.395	-20.013	
483.527	-20.015	
483.808	-20.017	
483.959	-20.019	
484.070	-20.022	
484.306	-20.024	
484.517	-20.026	
484.640	-20.028	
484.679	-20.031	
484.789	-20.033	

485.070	-20.035	
485.112	-20.038	
485.424	-20.040	
485.796	-20.042	
485.738	-20.044	
485.825	-20.047	
485.940	-20.049	
486.048	-20.051	
486.411	-20.054	
486.618	-20.056	
486.730	-20.058	
486.960	-20.061	
487.043	-20.063	
487.216	-20.065	
487.662	-20.068	
487.818	-20.070	
487.805	-20.072	
487.935	-20.075	
488.015	-20.077	
488.203	-20.080	
488.207	-20.082	
488.418	-20.084	
488.733	-20.087	
488.781	-20.089	
488.908	-20.092	
489.185	-20.094	
489.326	-20.096	
489.379	-20.099	
489.560	-20.101	
489.678	-20.104	

489.905	-20.106	
490.071	-20.108	
490.274	-20.111	
490.342	-20.113	
490.367	-20.116	
490.557	-20.118	
490.686	-20.120	
490.836	-20.123	
491.159	-20.125	
491.491	-20.128	
491.675	-20.130	
491.849	-20.132	
492.056	-20.135	
492.209	-20.137	
492.337	-20.140	
492.371	-20.142	
492.495	-20.144	
492.716	-20.147	
492.999	-20.149	
493.232	-20.152	
493.295	-20.154	
493.386	-20.156	
493.546	-20.159	
493.640	-20.161	
493.782	-20.164	
493.969	-20.166	
494.184	-20.168	
494.416	-20.171	
494.550	-20.173	
494.588	-20.175	

494.788	-20.178	
495.054	-20.180	
495.234	-20.182	
495.363	-20.185	
495.516	-20.187	
495.731	-20.189	
495.815	-20.192	
495.938	-20.194	
496.130	-20.196	
496.281	-20.199	
496.459	-20.201	
496.633	-20.203	
496.843	-20.206	
497.040	-20.208	
497.133	-20.210	
497.266	-20.212	
497.461	-20.215	
497.514	-20.217	
497.698	-20.219	
497.895	-20.221	
498.068	-20.224	
498.237	-20.226	
498.399	-20.228	
498.519	-20.230	
498.632	-20.232	
498.799	-20.235	
498.997	-20.237	
499.272	-20.239	
499.455	-20.241	
499.560	-20.243	

499.744	-20.246	
499.909	-20.248	
500.152	-20.250	
500.394	-20.252	
500.413	-20.254	
500.447	-20.256	
500.716	-20.258	
500.967	-20.261	
501.029	-20.263	
501.094	-20.265	
501.255	-20.267	
501.560	-20.269	
501.909	-20.271	
502.314	-20.273	
502.607	-20.275	
502.612	-20.277	
502.695	-20.279	
502.863	-20.281	
502.850	-20.284	
502.856	-20.286	
502.977	-20.288	
503.132	-20.290	
503.231	-20.292	
503.303	-20.294	
503.446	-20.296	
503.627	-20.298	
503.894	-20.300	
504.136	-20.302	
504.234	-20.304	
504.390	-20.306	

504.564	-20.308	
504.744	-20.310	
504.984	-20.312	
505.062	-20.314	
505.090	-20.316	
505.274	-20.318	
505.479	-20.320	
505.723	-20.322	
505.930	-20.324	
506.055	-20.326	
506.160	-20.328	
506.268	-20.330	
506.478	-20.332	
506.712	-20.334	
506.861	-20.336	
506.885	-20.338	
507.007	-20.340	
507.269	-20.342	
507.489	-20.345	
507.694	-20.347	
507.916	-20.349	
508.084	-20.351	
508.234	-20.353	
508.387	-20.355	
508.468	-20.357	
508.622	-20.359	
508.778	-20.361	
509.003	-20.363	
509.127	-20.365	
509.172	-20.367	

509.395	-20.369	
509.604	-20.371	
509.748	-20.374	
509.883	-20.376	
509.972	-20.378	
510.058	-20.380	
510.313	-20.382	
510.599	-20.384	
510.750	-20.386	
510.852	-20.389	
510.953	-20.391	
511.097	-20.393	
511.252	-20.395	
511.419	-20.397	
511.593	-20.399	
511.797	-20.402	
512.037	-20.404	
512.154	-20.406	
512.245	-20.408	
512.523	-20.410	
512.757	-20.413	
512.899	-20.415	
513.006	-20.417	
513.076	-20.419	
513.228	-20.422	
513.481	-20.424	
513.707	-20.426	
513.909	-20.428	
514.033	-20.431	
514.111	-20.433	

514.247	-20.435	
514.387	-20.438	
514.672	-20.440	
514.907	-20.442	
514.965	-20.444	
515.098	-20.447	
515.291	-20.449	
515.452	-20.451	
515.641	-20.454	
515.804	-20.456	
515.886	-20.458	
516.006	-20.461	
516.202	-20.463	
516.366	-20.465	
516.427	-20.468	
516.521	-20.470	
516.752	-20.472	
516.939	-20.475	
517.004	-20.477	
517.201	-20.479	
517.491	-20.482	
517.628	-20.484	
517.752	-20.486	
517.943	-20.489	
518.187	-20.491	
518.423	-20.493	
518.575	-20.495	
518.725	-20.498	
518.852	-20.500	
519.009	-20.502	

519.208	-20.505	
519.323	-20.507	
519.449	-20.509	
519.568	-20.512	
519.649	-20.514	
519.740	-20.516	
519.855	-20.519	
520.088	-20.521	
520.350	-20.523	
520.515	-20.525	
520.541	-20.528	
520.672	-20.530	
521.042	-20.532	
521.314	-20.535	
521.426	-20.537	
521.563	-20.539	
521.749	-20.541	
521.914	-20.544	
522.020	-20.546	
522.212	-20.548	
522.435	-20.550	
522.510	-20.553	
522.603	-20.555	
522.786	-20.557	
522.918	-20.559	
523.063	-20.562	
523.299	-20.564	
523.526	-20.566	
523.727	-20.568	
523.950	-20.570	

524.125	-20.573	
524.213	-20.575	
524.279	-20.577	
524.417	-20.579	
524.618	-20.581	
524.832	-20.584	
525.034	-20.586	
525.166	-20.588	
525.334	-20.590	
525.497	-20.592	
525.695	-20.594	
525.874	-20.596	
525.995	-20.599	
526.173	-20.601	
526.249	-20.603	
526.388	-20.605	
526.631	-20.607	
526.856	-20.609	
527.051	-20.611	
527.189	-20.614	
527.167	-20.616	
527.251	-20.618	
527.701	-20.620	
528.139	-20.622	
528.331	-20.624	
528.441	-20.626	
528.515	-20.628	
528.640	-20.630	
528.699	-20.632	
528.723	-20.635	

528.705	-20.637	
528.786	-20.639	
529.069	-20.641	
529.309	-20.643	
529.432	-20.645	
529.578	-20.647	
529.709	-20.649	
529.847	-20.651	
530.142	-20.653	
530.336	-20.655	
530.495	-20.657	
530.722	-20.660	
530.898	-20.662	
531.118	-20.664	
531.279	-20.666	
531.407	-20.668	
531.459	-20.670	
531.606	-20.672	
531.738	-20.674	
531.827	-20.676	
531.964	-20.678	
532.117	-20.680	
532.319	-20.682	
532.472	-20.684	
532.621	-20.686	
532.791	-20.688	
532.970	-20.691	
533.287	-20.693	
533.563	-20.695	
533.702	-20.697	

533.766	-20.699	
533.860	-20.701	
534.061	-20.703	
534.178	-20.705	
534.324	-20.707	
534.550	-20.710	
534.640	-20.712	
534.697	-20.714	
534.907	-20.716	
535.089	-20.718	
535.288	-20.720	
535.528	-20.722	
535.635	-20.725	
535.795	-20.727	
535.986	-20.729	
536.105	-20.731	
536.212	-20.733	
536.422	-20.735	
536.578	-20.738	
536.783	-20.740	
537.072	-20.742	
537.222	-20.744	
537.335	-20.746	
537.424	-20.749	
537.505	-20.751	
537.647	-20.753	
537.822	-20.755	
538.055	-20.758	
538.308	-20.760	
538.506	-20.762	

538.737	-20.764	
538.977	-20.766	
539.164	-20.769	
539.332	-20.771	
539.411	-20.773	
539.478	-20.775	
539.600	-20.778	
539.547	-20.780	
539.678	-20.782	
539.848	-20.784	
540.131	-20.787	
540.372	-20.789	
540.419	-20.791	
540.702	-20.793	
540.967	-20.796	
541.110	-20.798	
541.272	-20.800	
541.351	-20.802	
541.497	-20.805	
541.593	-20.807	
541.724	-20.809	
541.799	-20.811	
542.060	-20.814	
542.264	-20.816	
542.485	-20.818	
542.705	-20.820	
542.611	-20.823	
542.785	-20.825	
543.076	-20.827	
543.204	-20.829	

543.228	-20.831	
543.345	-20.834	
543.610	-20.836	
543.876	-20.838	
544.174	-20.840	
544.199	-20.842	
544.161	-20.845	
544.458	-20.847	
544.702	-20.849	
545.051	-20.851	
545.092	-20.853	
545.115	-20.855	
545.283	-20.857	
545.513	-20.859	
545.723	-20.862	
545.696	-20.864	
545.985	-20.866	
546.185	-20.868	
546.229	-20.870	
546.245	-20.872	
546.354	-20.874	
546.588	-20.876	
546.931	-20.878	
547.264	-20.880	
547.531	-20.882	
547.861	-20.884	
547.889	-20.886	
547.826	-20.888	
548.053	-20.890	
548.222	-20.892	

548.252	-20.894	
548.315	-20.896	
548.508	-20.898	
548.718	-20.900	
549.074	-20.902	
549.212	-20.904	
549.187	-20.905	
549.334	-20.907	
549.405	-20.909	
549.534	-20.911	
549.676	-20.913	
549.901	-20.915	
550.122	-20.917	
550.410	-20.918	
550.681	-20.920	
550.677	-20.922	
550.807	-20.924	
551.061	-20.926	
550.981	-20.927	
551.218	-20.929	
551.571	-20.931	
551.799	-20.933	
552.247	-20.934	
552.515	-20.936	
552.478	-20.938	
552.349	-20.939	
552.269	-20.941	
552.443	-20.943	
552.681	-20.945	
553.032	-20.946	

552.972	-20.948	
553.357	-20.950	
553.664	-20.951	
554.137	-20.953	
554.502	-20.955	
554.756	-20.956	
554.903	-20.958	
554.801	-20.960	
554.766	-20.961	
555.045	-20.963	
555.199	-20.965	
555.127	-20.966	
555.002	-20.968	
555.103	-20.969	
555.183	-20.971	
555.440	-20.973	
555.851	-20.974	
555.998	-20.976	
555.967	-20.977	
556.235	-20.979	
556.335	-20.981	
556.632	-20.982	
556.702	-20.984	
556.832	-20.985	
556.988	-20.987	
557.261	-20.989	
557.406	-20.990	
557.580	-20.992	
557.855	-20.993	
557.845	-20.995	

557.892	-20.997	
558.075	-20.998	
558.221	-21.000	
558.404	-21.001	
558.552	-21.003	
558.664	-21.005	
558.912	-21.006	
559.221	-21.008	
559.312	-21.009	
559.432	-21.011	
559.558	-21.013	
559.730	-21.014	
560.093	-21.016	
560.386	-21.018	
560.426	-21.019	
560.538	-21.021	
560.650	-21.023	
560.752	-21.024	
560.913	-21.026	
561.128	-21.028	
561.254	-21.029	
561.338	-21.031	
561.543	-21.033	
561.714	-21.034	
561.899	-21.036	
562.110	-21.038	
562.274	-21.040	
562.403	-21.041	
562.588	-21.043	
562.799	-21.045	

562.977	-21.047	
563.093	-21.048	
563.194	-21.050	
563.369	-21.052	
563.501	-21.054	
563.635	-21.055	
563.840	-21.057	
563.985	-21.059	
564.174	-21.061	
564.391	-21.063	
564.562	-21.065	
564.703	-21.066	
564.837	-21.068	
565.007	-21.070	
565.170	-21.072	
565.316	-21.074	
565.522	-21.076	
565.788	-21.078	
565.926	-21.080	
565.998	-21.081	
566.170	-21.083	
566.379	-21.085	
566.507	-21.087	
566.686	-21.089	
566.942	-21.091	
567.073	-21.093	
567.168	-21.095	
567.259	-21.097	
567.407	-21.099	
567.637	-21.101	

567.799	-21.103	
567.892	-21.105	
567.965	-21.107	
568.107	-21.108	
568.342	-21.110	
568.470	-21.112	
568.622	-21.114	
568.806	-21.116	
568.919	-21.118	
569.085	-21.120	
569.313	-21.122	
569.569	-21.124	
569.642	-21.126	
569.757	-21.128	
570.078	-21.130	
570.320	-21.132	
570.450	-21.133	
570.597	-21.135	
570.758	-21.137	
570.912	-21.139	
571.085	-21.141	
571.209	-21.143	
571.345	-21.145	
571.452	-21.147	
571.532	-21.149	
571.662	-21.150	
571.858	-21.152	
572.056	-21.154	
572.247	-21.156	
572.398	-21.158	

572.569	-21.160	
572.789	-21.161	
572.976	-21.163	
573.213	-21.165	
573.428	-21.167	
573.499	-21.169	
573.569	-21.171	
573.745	-21.172	
573.967	-21.174	
574.151	-21.176	
574.321	-21.178	
574.497	-21.179	
574.676	-21.181	
574.801	-21.183	
574.892	-21.185	
575.115	-21.186	
575.268	-21.188	
575.355	-21.190	
575.554	-21.191	
575.799	-21.193	
575.948	-21.195	
576.126	-21.196	
576.281	-21.198	
576.442	-21.200	
576.597	-21.201	
576.737	-21.203	
576.944	-21.205	
577.112	-21.206	
577.283	-21.208	
577.381	-21.210	

577.448	-21.211	
577.650	-21.213	
577.942	-21.214	
578.126	-21.216	
578.294	-21.217	
578.520	-21.219	
578.661	-21.220	
578.787	-21.222	
578.942	-21.223	
579.066	-21.225	
579.116	-21.226	
579.198	-21.228	
579.451	-21.229	
579.866	-21.231	
580.304	-21.232	
580.598	-21.234	
580.679	-21.235	
580.716	-21.237	
580.746	-21.238	
580.834	-21.240	
580.899	-21.241	
580.993	-21.242	
581.094	-21.244	
581.195	-21.245	
581.285	-21.247	
581.453	-21.248	
581.734	-21.249	
581.994	-21.251	
582.100	-21.252	
582.226	-21.254	

582.395	-21.255	
582.553	-21.256	
582.765	-21.258	
582.972	-21.259	
583.062	-21.260	
583.255	-21.262	
583.472	-21.263	
583.566	-21.264	
583.772	-21.266	
583.983	-21.267	
584.073	-21.268	
584.164	-21.270	
584.358	-21.271	
584.509	-21.272	
584.658	-21.274	
584.884	-21.275	
585.072	-21.276	
585.177	-21.278	
585.233	-21.279	
585.336	-21.280	
585.531	-21.281	
585.835	-21.283	
586.107	-21.284	
586.311	-21.285	
586.379	-21.287	
586.467	-21.288	
586.648	-21.290	
586.792	-21.291	
586.952	-21.292	
587.132	-21.294	

587.238	-21.295	
587.359	-21.296	
587.499	-21.298	
587.675	-21.299	
587.947	-21.300	
588.117	-21.302	
588.237	-21.303	
588.406	-21.304	
588.567	-21.306	
588.670	-21.307	
588.728	-21.309	
588.917	-21.310	
589.098	-21.311	
589.191	-21.313	
589.339	-21.314	
589.604	-21.316	
589.754	-21.317	
589.908	-21.318	
590.151	-21.320	
590.394	-21.321	
590.525	-21.323	
590.748	-21.324	
590.989	-21.326	
591.005	-21.327	
591.175	-21.328	
591.391	-21.330	
591.496	-21.331	
591.616	-21.333	
591.786	-21.334	
591.960	-21.336	

592.071	-21.337	
592.354	-21.339	
592.618	-21.340	
592.704	-21.341	
592.906	-21.343	
593.101	-21.344	
593.101	-21.346	
593.235	-21.347	
593.465	-21.349	
593.688	-21.350	
593.773	-21.352	
593.789	-21.353	
593.982	-21.355	
594.128	-21.356	
594.227	-21.358	
594.344	-21.359	
594.489	-21.360	
594.665	-21.362	
594.823	-21.363	
595.083	-21.365	
595.302	-21.366	
595.460	-21.368	
595.690	-21.369	
595.883	-21.371	
595.980	-21.372	
596.115	-21.373	
596.332	-21.375	
596.527	-21.376	
596.722	-21.378	
596.912	-21.379	

597.053	-21.381	
597.179	-21.382	
597.225	-21.383	
597.385	-21.385	
597.629	-21.386	
597.761	-21.388	
597.836	-21.389	
598.036	-21.390	
598.244	-21.392	
598.379	-21.393	
598.592	-21.395	
598.707	-21.396	
598.876	-21.397	
599.188	-21.399	
599.365	-21.400	
599.489	-21.401	
599.677	-21.403	
599.824	-21.404	
600.028	-21.405	
600.288	-21.407	
600.396	-21.408	
600.481	-21.409	
600.602	-21.411	
600.722	-21.412	
600.838	-21.413	
601.064	-21.415	
601.281	-21.416	
601.416	-21.417	
601.526	-21.419	
601.627	-21.420	

601.817	-21.421	
602.045	-21.422	
602.236	-21.424	
602.372	-21.425	
602.577	-21.426	
602.779	-21.427	
602.931	-21.429	
603.048	-21.430	
603.249	-21.431	
603.447	-21.432	
603.578	-21.434	
603.750	-21.435	
603.913	-21.436	
604.049	-21.437	
604.249	-21.439	
604.369	-21.440	
604.480	-21.441	
604.693	-21.442	
604.929	-21.443	
605.055	-21.445	
605.112	-21.446	
605.281	-21.447	
605.524	-21.448	
605.663	-21.450	
606.110	-21.451	
606.541	-21.452	
606.680	-21.453	
606.641	-21.454	
606.720	-21.456	
606.890	-21.457	

606.920	-21.458	
606.891	-21.459	
606.911	-21.461	
607.147	-21.462	
607.266	-21.463	
607.526	-21.464	
607.723	-21.465	
607.980	-21.467	
608.081	-21.468	
608.050	-21.469	
608.407	-21.470	
608.750	-21.472	
608.865	-21.473	
609.099	-21.474	
609.556	-21.475	
609.530	-21.477	
609.388	-21.478	
609.386	-21.479	
609.589	-21.480	
609.733	-21.482	
609.979	-21.483	
610.245	-21.484	
610.189	-21.486	
610.301	-21.487	
610.495	-21.488	
610.783	-21.490	
611.023	-21.491	
611.096	-21.492	
611.194	-21.494	
611.474	-21.495	

611.724	-21.497	
611.741	-21.498	
611.924	-21.499	
612.444	-21.501	
612.712	-21.502	
612.494	-21.504	
612.438	-21.505	
612.600	-21.506	
612.831	-21.508	
613.036	-21.509	
613.112	-21.511	
613.242	-21.512	
613.432	-21.514	
613.687	-21.515	
614.054	-21.517	
613.956	-21.519	
614.166	-21.520	
614.174	-21.522	
614.183	-21.523	
614.511	-21.525	
614.613	-21.526	
614.816	-21.528	
615.235	-21.530	
615.448	-21.531	
615.436	-21.533	
615.862	-21.534	
616.048	-21.536	
616.173	-21.538	
616.278	-21.539	
616.191	-21.541	

616.297	-21.543	
616.589	-21.545	
616.938	-21.546	
617.038	-21.548	
617.113	-21.550	
617.101	-21.551	
617.381	-21.553	
617.565	-21.555	
617.711	-21.557	
617.862	-21.559	
617.846	-21.560	
618.067	-21.562	
618.483	-21.564	
618.416	-21.566	
618.416	-21.568	
618.697	-21.569	
618.987	-21.571	
618.965	-21.573	
619.025	-21.575	
619.231	-21.577	
619.620	-21.579	
619.857	-21.580	
619.744	-21.582	
619.875	-21.584	
620.043	-21.586	
620.301	-21.588	
620.574	-21.590	
620.694	-21.591	
620.702	-21.593	
620.964	-21.595	

621.231	-21.597	
621.450	-21.599	
621.418	-21.601	
621.503	-21.603	
621.776	-21.604	
622.332	-21.606	
622.629	-21.608	
622.519	-21.610	
622.649	-21.612	
622.903	-21.614	
623.063	-21.615	
623.193	-21.617	
623.142	-21.619	
623.242	-21.621	
623.377	-21.623	
623.515	-21.624	
623.831	-21.626	
624.048	-21.628	
624.258	-21.630	
624.249	-21.632	
624.325	-21.633	
624.697	-21.635	
624.771	-21.637	
624.909	-21.639	
625.156	-21.640	
625.402	-21.642	
625.476	-21.644	
625.713	-21.646	
626.195	-21.647	
626.213	-21.649	

626.152	-21.651	
626.279	-21.652	
626.504	-21.654	
626.671	-21.656	
626.462	-21.657	
626.891	-21.659	
627.184	-21.661	
627.994	-21.662	
628.558	-21.664	
628.602	-21.666	
627.970	-21.667	
628.203	-21.669	
628.060	-21.670	
628.019	-21.672	
629.527	-21.673	
629.976	-21.675	
629.307	-21.676	
628.955	-21.678	
629.125	-21.680	
628.872	-21.681	
629.320	-21.682	
629.919	-21.684	
629.928	-21.685	
629.903	-21.687	
629.860	-21.688	
630.118	-21.690	
630.394	-21.691	
630.826	-21.693	
631.082	-21.694	
630.875	-21.695	

631.018	-21.697	
631.515	-21.698	
631.761	-21.700	
632.280	-21.701	
632.433	-21.702	
632.313	-21.704	
632.621	-21.705	
632.724	-21.706	
632.608	-21.708	
632.727	-21.709	
632.945	-21.710	
632.990	-21.711	
633.088	-21.713	
633.090	-21.714	
633.147	-21.715	
633.356	-21.717	
633.356	-21.718	
633.123	-21.719	
633.327	-21.720	
633.868	-21.721	
634.192	-21.723	
634.341	-21.724	
634.618	-21.725	
634.833	-21.726	
634.926	-21.727	
635.137	-21.729	
635.513	-21.730	
635.526	-21.731	
635.303	-21.732	
635.324	-21.733	

635.653	-21.734	
636.019	-21.735	
636.203	-21.737	
636.306	-21.738	
636.462	-21.739	
636.602	-21.740	
636.848	-21.741	
637.299	-21.742	
637.427	-21.743	
637.268	-21.744	
637.554	-21.745	
637.594	-21.747	
637.710	-21.748	
638.666	-21.749	
638.716	-21.750	
638.145	-21.751	
638.934	-21.752	
639.714	-21.753	
639.357	-21.754	
639.357	-21.755	
639.476	-21.756	
639.625	-21.758	
639.843	-21.759	
639.843	-21.760	
639.851	-21.761	
639.978	-21.762	
640.430	-21.763	
640.580	-21.764	
640.544	-21.765	
640.807	-21.766	

640.817	-21.767	
640.894	-21.768	
641.161	-21.770	
641.270	-21.771	
641.338	-21.772	
641.405	-21.773	
641.768	-21.774	
642.018	-21.775	
641.987	-21.776	
642.133	-21.777	
642.517	-21.778	
642.855	-21.779	
642.778	-21.781	
642.766	-21.782	
643.034	-21.783	
643.230	-21.784	
643.282	-21.785	
643.275	-21.786	
643.543	-21.787	
643.710	-21.788	
643.924	-21.789	
644.016	-21.791	
644.044	-21.792	
644.192	-21.793	
644.655	-21.794	
644.881	-21.795	
644.965	-21.796	
644.921	-21.797	
645.061	-21.798	
645.468	-21.800	

645.564	-21.801	
645.828	-21.802	
645.872	-21.803	
645.945	-21.804	
646.325	-21.805	
646.386	-21.806	
646.472	-21.808	
646.696	-21.809	
646.807	-21.810	
646.814	-21.811	
647.327	-21.812	
647.757	-21.813	
648.061	-21.814	
648.288	-21.815	
647.932	-21.817	
648.167	-21.818	
648.621	-21.819	
648.429	-21.820	
648.232	-21.821	
648.251	-21.822	
648.418	-21.823	
648.775	-21.824	
649.217	-21.825	
649.398	-21.827	
649.459	-21.828	
649.777	-21.829	
649.829	-21.830	
650.248	-21.831	
650.378	-21.832	
650.089	-21.833	

650.313	-21.834	
650.843	-21.835	
650.916	-21.836	
650.982	-21.838	
651.329	-21.839	
651.406	-21.840	
651.498	-21.841	
651.506	-21.842	
651.787	-21.843	
651.804	-21.844	
651.903	-21.845	
652.250	-21.846	
652.234	-21.847	
652.550	-21.848	
652.859	-21.849	
653.342	-21.850	
653.512	-21.852	
653.410	-21.853	
653.699	-21.854	
653.821	-21.855	
653.834	-21.856	
654.019	-21.857	
654.303	-21.858	
654.541	-21.859	
654.744	-21.860	
654.759	-21.861	
654.944	-21.862	
655.138	-21.863	
655.241	-21.864	
655.322	-21.865	

655.545	-21.866	
655.874	-21.867	
656.169	-21.868	
656.248	-21.869	
656.166	-21.870	
656.409	-21.871	
656.569	-21.872	
656.703	-21.873	
656.841	-21.874	
656.932	-21.875	
657.092	-21.876	
657.298	-21.877	
657.441	-21.878	
657.638	-21.879	
658.008	-21.880	
658.389	-21.881	
658.599	-21.883	
658.630	-21.884	
658.653	-21.885	
658.715	-21.886	
658.769	-21.887	
659.011	-21.888	
659.283	-21.889	
659.599	-21.890	
659.610	-21.891	
659.492	-21.892	
659.493	-21.893	
659.415	-21.894	
659.801	-21.895	
660.632	-21.896	

660.607	-21.897	
660.560	-21.898	
660.664	-21.899	
660.924	-21.900	
661.186	-21.901	
661.056	-21.902	
661.760	-21.903	
661.941	-21.904	
662.030	-21.905	
662.691	-21.906	
662.404	-21.907	
662.001	-21.908	
662.190	-21.909	
662.646	-21.910	
662.871	-21.912	
663.279	-21.913	
663.083	-21.914	
663.168	-21.915	
663.538	-21.916	
663.597	-21.917	
663.569	-21.918	
663.465	-21.919	
663.838	-21.920	
663.996	-21.921	
664.118	-21.922	
664.142	-21.924	
664.426	-21.925	
664.911	-21.926	
664.716	-21.927	
664.916	-21.928	

665.206	-21.929	
664.566	-21.930	
665.359	-21.932	
665.198	-21.933	
664.883	-21.934	
665.320	-21.935	
665.961	-21.936	
665.916	-21.937	
665.883	-21.939	
666.447	-21.940	
666.559	-21.941	
666.924	-21.942	
667.402	-21.943	
667.417	-21.945	
667.150	-21.946	
667.429	-21.947	
667.831	-21.948	
668.103	-21.950	
668.244	-21.951	
668.316	-21.952	
668.750	-21.953	
668.687	-21.955	
669.025	-21.956	
669.201	-21.957	
668.899	-21.958	
669.211	-21.960	
669.630	-21.961	
669.521	-21.962	
669.389	-21.964	
669.866	-21.965	

669.715	-21.966	
669.567	-21.967	
670.181	-21.969	
670.753	-21.970	
670.562	-21.971	
670.234	-21.973	
670.654	-21.974	
671.209	-21.975	
671.140	-21.977	
671.275	-21.978	
670.752	-21.979	
670.620	-21.980	
671.140	-21.982	
671.189	-21.983	
671.884	-21.984	
672.567	-21.986	
672.689	-21.987	
673.512	-21.988	
673.494	-21.990	
672.476	-21.991	
672.926	-21.992	
672.880	-21.993	
672.850	-21.995	
673.336	-21.996	
673.879	-21.997	
674.792	-21.998	
674.242	-22.000	
674.028	-22.001	
674.613	-22.002	
674.611	-22.003	

675.309	-22.005	
675.331	-22.006	
675.124	-22.007	
675.497	-22.008	
675.821	-22.009	
675.767	-22.011	
676.450	-22.012	
676.051	-22.013	
675.753	-22.014	
676.152	-22.015	
676.353	-22.016	
676.144	-22.018	
675.770	-22.019	
676.338	-22.020	
676.687	-22.021	
676.427	-22.022	
676.938	-22.023	
676.416	-22.024	
676.506	-22.025	
678.270	-22.027	
679.669	-22.028	
678.790	-22.029	
678.182	-22.030	
679.165	-22.031	
678.747	-22.032	
678.575	-22.033	
679.065	-22.034	
678.763	-22.035	
679.081	-22.036	
679.279	-22.037	

679.825	-22.038	
679.923	-22.039	
680.006	-22.040	
680.744	-22.041	
680.350	-22.042	
680.359	-22.043	
680.854	-22.044	
680.890	-22.045	
680.795	-22.046	
681.273	-22.047	
681.505	-22.048	
681.673	-22.048	
681.507	-22.049	
681.982	-22.050	
682.614	-22.051	
682.785	-22.052	
682.899	-22.053	
682.338	-22.054	
682.411	-22.055	
682.940	-22.056	
683.360	-22.056	
683.858	-22.057	
683.888	-22.058	
684.081	-22.059	
684.251	-22.060	
684.473	-22.061	
684.675	-22.061	
684.326	-22.062	
684.434	-22.063	
684.688	-22.064	

684.771	-22.065	
685.171	-22.065	
685.480	-22.066	
685.491	-22.067	
685.381	-22.068	
685.720	-22.069	
685.891	-22.069	
685.493	-22.070	
685.812	-22.071	
686.224	-22.072	
686.571	-22.073	
686.479	-22.073	
686.381	-22.074	
686.618	-22.075	
687.078	-22.076	
687.335	-22.076	
687.206	-22.077	
687.237	-22.078	
687.548	-22.079	
687.670	-22.080	
687.552	-22.080	
687.817	-22.081	
688.008	-22.082	
688.290	-22.083	
688.679	-22.084	
688.806	-22.085	
688.944	-22.085	
689.044	-22.086	
689.240	-22.087	
689.482	-22.088	

689.734	-22.089	
689.827	-22.090	
689.898	-22.091	
689.830	-22.092	
690.077	-22.092	
690.295	-22.093	
690.352	-22.094	
690.458	-22.095	
690.395	-22.096	
690.424	-22.097	
690.900	-22.098	
691.274	-22.099	
691.814	-22.100	
692.081	-22.101	
691.857	-22.102	
691.970	-22.103	
692.314	-22.104	
692.470	-22.105	
692.651	-22.106	
692.681	-22.108	
692.642	-22.109	
692.661	-22.110	
692.995	-22.111	
693.130	-22.112	
693.463	-22.113	
693.561	-22.114	
693.607	-22.116	
693.988	-22.117	
694.091	-22.118	
694.144	-22.119	

694.440	-22.120	
694.716	-22.122	
694.966	-22.123	
695.023	-22.124	
694.902	-22.126	
695.059	-22.127	
695.304	-22.128	
695.320	-22.130	
695.286	-22.131	
695.415	-22.132	
695.907	-22.134	
696.140	-22.135	
695.989	-22.136	
696.328	-22.138	
696.773	-22.139	
696.761	-22.141	
696.847	-22.142	
697.137	-22.144	
697.420	-22.145	
697.604	-22.147	
697.641	-22.148	
697.748	-22.150	
697.898	-22.151	
698.147	-22.153	
698.173	-22.154	
698.191	-22.156	
698.546	-22.157	
698.760	-22.159	
698.828	-22.161	
698.989	-22.162	

699.142	-22.164	
699.436	-22.165	
699.819	-22.167	
699.916	-22.169	
700.045	-22.170	
700.191	-22.172	
700.365	-22.173	
700.429	-22.175	
700.536	-22.177	
700.910	-22.178	
701.003	-22.180	
700.754	-22.182	
701.222	-22.183	
701.430	-22.185	
701.201	-22.187	
701.610	-22.188	
701.674	-22.190	
701.863	-22.192	
702.302	-22.194	
702.665	-22.195	
702.641	-22.197	
702.707	-22.199	
702.778	-22.200	
702.898	-22.202	
703.221	-22.204	
703.388	-22.206	
703.557	-22.207	
703.737	-22.209	
703.820	-22.211	
704.074	-22.213	

704.181	-22.214	
703.928	-22.216	
704.404	-22.218	
705.115	-22.220	
704.380	-22.221	
704.352	-22.223	
705.042	-22.225	
704.950	-22.227	
705.611	-22.228	
706.213	-22.230	
705.723	-22.232	
705.432	-22.234	
706.066	-22.235	
706.472	-22.237	
706.192	-22.239	
706.343	-22.241	
706.770	-22.242	
707.081	-22.244	
707.705	-22.246	
707.710	-22.248	
707.001	-22.249	
707.661	-22.251	
708.603	-22.253	
708.130	-22.255	
708.158	-22.256	
708.758	-22.258	
709.305	-22.260	
708.970	-22.262	
708.618	-22.263	
709.343	-22.265	

709.389	-22.267	
709.175	-22.269	
709.363	-22.270	
710.108	-22.272	
710.806	-22.274	
710.324	-22.276	
710.614	-22.277	
710.455	-22.279	
709.464	-22.281	
709.467	-22.283	
709.961	-22.285	
709.613	-22.286	
709.693	-22.288	
710.881	-22.290	
711.503	-22.291	
711.534	-22.293	
711.516	-22.295	
711.355	-22.297	
712.412	-22.298	
712.861	-22.300	
711.316	-22.302	
710.976	-22.304	
712.874	-22.305	
714.644	-22.307	
714.044	-22.309	
713.772	-22.311	
714.998	-22.312	
715.621	-22.314	
714.580	-22.316	
712.933	-22.318	

711.988	-22.319	
712.081	-22.321	
713.444	-22.323	
714.096	-22.325	
714.871	-22.327	
714.940	-22.328	
713.822	-22.330	
715.306	-22.332	
716.094	-22.334	
714.618	-22.335	
715.304	-22.337	
716.768	-22.339	
715.477	-22.341	
714.258	-22.343	
714.436	-22.344	
714.446	-22.346	
714.574	-22.348	
716.373	-22.350	
718.347	-22.352	
718.119	-22.354	
717.894	-22.355	
717.963	-22.357	
718.002	-22.359	
718.950	-22.361	
718.131	-22.363	
717.505	-22.365	
718.593	-22.367	
718.941	-22.368	
717.893	-22.370	
717.082	-22.372	

718.630	-22.374	
719.832	-22.376	
718.340	-22.378	
716.954	-22.380	
717.215	-22.381	
719.052	-22.383	
720.495	-22.385	
720.174	-22.387	
720.502	-22.389	
720.833	-22.391	
721.223	-22.393	
722.377	-22.395	
722.088	-22.396	
720.146	-22.398	
720.393	-22.400	
722.468	-22.402	
721.791	-22.404	
720.782	-22.406	
721.269	-22.408	
720.570	-22.410	
720.156	-22.411	
720.429	-22.413	
720.609	-22.415	
721.309	-22.417	
722.657	-22.419	
723.910	-22.421	
723.128	-22.423	
723.176	-22.424	
725.203	-22.426	
725.532	-22.428	

723.825	-22.430	
724.129	-22.432	
725.886	-22.434	
724.575	-22.435	
722.467	-22.437	
722.105	-22.439	
723.027	-22.441	
724.844	-22.443	
725.860	-22.444	
724.733	-22.446	
725.339	-22.448	
727.455	-22.449	
726.839	-22.451	
725.360	-22.453	
726.228	-22.455	
726.892	-22.456	
726.642	-22.458	
726.320	-22.460	
727.079	-22.461	
727.064	-22.463	
726.896	-22.465	
726.768	-22.466	
726.642	-22.468	
726.469	-22.469	
725.883	-22.471	
726.850	-22.472	
727.673	-22.474	
727.505	-22.476	
727.748	-22.477	
728.279	-22.479	

728.696	-22.480	
728.498	-22.482	
728.192	-22.483	
728.850	-22.485	
728.902	-22.486	
729.229	-22.488	
729.237	-22.489	
729.648	-22.490	
729.949	-22.492	
729.861	-22.493	
729.812	-22.495	
730.101	-22.496	
730.947	-22.497	
730.180	-22.499	
729.937	-22.500	
731.213	-22.501	
731.117	-22.503	
731.681	-22.504	
732.464	-22.505	
732.140	-22.506	
731.563	-22.508	
731.707	-22.509	
731.826	-22.510	
732.570	-22.511	
733.986	-22.512	
733.216	-22.513	
732.051	-22.515	
732.083	-22.516	
732.638	-22.517	
732.617	-22.518	

732.794	-22.519	
732.980	-22.520	
733.144	-22.521	
733.915	-22.522	
734.257	-22.524	
734.172	-22.525	
734.681	-22.526	
735.079	-22.527	
735.117	-22.528	
734.683	-22.529	
734.529	-22.530	
735.612	-22.531	
736.041	-22.532	
735.555	-22.533	
735.555	-22.534	
735.537	-22.535	
735.791	-22.536	
735.763	-22.537	
735.566	-22.538	
735.924	-22.539	
736.504	-22.540	
736.758	-22.540	
736.775	-22.541	
737.224	-22.542	
737.305	-22.543	
737.053	-22.544	
737.504	-22.545	
738.036	-22.546	
738.399	-22.547	
738.518	-22.548	

738.365	-22.549	
738.570	-22.550	
738.884	-22.550	
738.966	-22.551	
738.390	-22.552	
739.177	-22.553	
740.047	-22.554	
738.541	-22.555	
737.407	-22.556	
737.610	-22.557	
737.771	-22.558	
737.916	-22.558	
738.285	-22.559	
739.688	-22.560	
739.884	-22.561	
740.337	-22.562	
742.179	-22.563	
741.433	-22.564	
741.628	-22.565	
742.279	-22.566	
740.648	-22.567	
740.163	-22.568	
741.185	-22.569	
741.224	-22.570	
741.127	-22.570	
742.354	-22.571	
742.486	-22.572	
742.673	-22.573	
743.245	-22.574	
742.241	-22.575	

742.295	-22.576	
744.409	-22.577	
745.541	-22.578	
745.114	-22.579	
743.913	-22.580	
744.061	-22.581	
745.201	-22.582	
744.708	-22.584	
743.047	-22.585	
743.350	-22.586	
745.808	-22.587	
746.484	-22.588	
745.263	-22.589	
744.414	-22.590	
745.399	-22.591	
744.912	-22.592	
744.038	-22.593	
743.981	-22.594	
744.033	-22.595	
743.905	-22.597	
744.339	-22.598	
746.040	-22.599	
747.094	-22.600	
746.472	-22.601	
745.479	-22.602	
745.881	-22.603	
747.197	-22.605	
747.251	-22.606	
747.132	-22.607	
747.085	-22.608	

747.113	-22.609	
747.957	-22.611	
748.268	-22.612	
747.841	-22.613	
748.013	-22.614	
748.919	-22.616	
748.818	-22.617	
748.549	-22.618	
749.073	-22.619	
748.639	-22.620	
747.564	-22.622	
748.851	-22.623	
751.441	-22.624	
751.813	-22.625	
750.689	-22.627	
750.695	-22.628	
750.582	-22.629	
750.902	-22.630	
750.936	-22.632	
750.482	-22.633	
750.073	-22.634	
750.660	-22.635	
751.922	-22.637	
751.339	-22.638	
751.774	-22.639	
752.381	-22.640	
751.833	-22.641	
751.841	-22.643	
752.374	-22.644	
752.633	-22.645	

752.721	-22.646	
752.662	-22.648	
752.991	-22.649	
753.611	-22.650	
753.867	-22.651	
753.650	-22.652	
753.214	-22.654	
753.494	-22.655	
753.457	-22.656	
754.127	-22.657	
755.228	-22.658	
754.821	-22.660	
754.220	-22.661	
755.162	-22.662	
755.212	-22.663	
754.136	-22.664	
754.725	-22.665	
754.998	-22.667	
755.524	-22.668	
756.494	-22.669	
758.161	-22.670	
758.778	-22.671	
758.312	-22.672	
756.544	-22.673	
755.128	-22.675	
754.883	-22.676	
754.996	-22.677	
755.654	-22.678	
758.111	-22.679	
759.986	-22.680	

759.212	-22.681	
757.622	-22.682	
756.582	-22.683	
756.839	-22.684	
759.053	-22.685	
759.074	-22.687	
757.142	-22.688	
757.672	-22.689	
758.430	-22.690	
759.363	-22.691	
760.101	-22.692	
759.660	-22.693	
759.569	-22.694	
760.415	-22.695	
760.593	-22.696	
760.426	-22.697	
760.315	-22.698	
760.725	-22.699	
760.266	-22.700	
760.674	-22.701	
761.555	-22.702	
761.017	-22.703	
761.117	-22.704	
761.868	-22.705	
762.026	-22.706	
761.770	-22.707	
762.398	-22.708	
763.117	-22.709	
762.996	-22.710	
762.698	-22.711	

762.491	-22.712	
762.345	-22.713	
762.370	-22.714	
763.030	-22.715	
763.809	-22.716	
763.220	-22.717	
762.781	-22.718	
763.586	-22.719	
764.090	-22.720	
763.482	-22.721	
764.240	-22.722	
765.854	-22.723	
764.713	-22.724	
763.806	-22.725	
765.486	-22.726	
765.911	-22.727	
765.749	-22.728	
765.726	-22.729	
765.247	-22.731	
765.751	-22.732	
767.146	-22.733	
768.448	-22.734	
767.673	-22.735	
767.112	-22.736	
767.439	-22.737	
766.868	-22.738	
766.861	-22.739	
767.309	-22.740	
766.527	-22.741	
765.692	-22.742	

765.467	-22.743	
766.684	-22.744	
767.999	-22.746	
767.957	-22.747	
766.404	-22.748	
765.843	-22.749	
766.180	-22.750	
768.129	-22.751	
770.081	-22.752	
769.476	-22.754	
768.532	-22.755	
770.023	-22.756	
771.534	-22.757	
770.782	-22.758	
769.239	-22.760	
770.042	-22.761	
772.022	-22.762	
771.622	-22.763	
769.629	-22.764	
768.440	-22.766	
770.132	-22.767	
771.021	-22.768	
770.975	-22.769	
771.395	-22.771	
771.126	-22.772	
772.318	-22.773	
773.166	-22.774	
773.113	-22.776	
772.701	-22.777	
771.925	-22.778	

772.616	-22.780	
773.299	-22.781	
772.293	-22.782	
771.978	-22.783	
772.578	-22.785	
772.878	-22.786	
773.441	-22.787	
773.492	-22.789	
773.624	-22.790	
773.930	-22.791	
773.802	-22.793	
773.904	-22.794	
774.000	-22.795	
773.930	-22.797	
774.035	-22.798	
774.342	-22.799	
774.529	-22.801	
774.602	-22.802	
774.525	-22.803	
774.955	-22.805	
775.229	-22.806	
775.420	-22.807	
775.734	-22.808	
775.632	-22.810	
775.560	-22.811	
776.570	-22.812	
777.076	-22.814	
776.851	-22.815	
777.101	-22.816	
777.030	-22.817	

776.402	-22.819	
776.534	-22.820	
777.486	-22.821	
778.084	-22.822	
777.859	-22.823	
777.466	-22.825	
778.540	-22.826	
778.186	-22.827	
777.160	-22.828	
777.897	-22.829	
778.933	-22.831	
778.366	-22.832	
777.569	-22.833	
778.428	-22.834	
779.100	-22.835	
779.156	-22.836	
780.395	-22.837	
782.123	-22.838	
781.003	-22.840	
779.689	-22.841	
781.047	-22.842	
782.837	-22.843	
783.080	-22.844	
782.038	-22.845	
780.468	-22.846	
780.563	-22.847	
782.430	-22.848	
781.962	-22.849	
780.283	-22.850	
780.452	-22.851	

782.011	-22.852	
783.687	-22.853	
784.398	-22.853	
784.185	-22.854	
783.970	-22.855	
784.353	-22.856	
784.995	-22.857	
785.174	-22.858	
783.930	-22.859	
783.122	-22.860	
784.313	-22.860	
785.378	-22.861	
784.864	-22.862	
783.864	-22.863	
784.139	-22.864	
785.059	-22.864	
784.742	-22.865	
784.830	-22.866	
784.072	-22.867	
782.746	-22.868	
783.095	-22.868	
783.419	-22.869	
783.859	-22.870	
785.936	-22.870	
786.814	-22.871	
786.288	-22.872	
786.108	-22.873	
786.790	-22.873	
786.848	-22.874	
786.348	-22.875	

788.251	-22.875	
789.146	-22.876	
787.640	-22.877	
785.856	-22.877	
786.109	-22.878	
787.719	-22.879	
787.059	-22.879	
785.704	-22.880	
786.376	-22.881	
786.767	-22.881	
786.686	-22.882	
787.241	-22.882	
787.935	-22.883	
789.846	-22.884	
791.630	-22.884	
790.520	-22.885	
789.503	-22.886	
790.266	-22.886	
789.276	-22.887	
788.472	-22.888	
789.215	-22.888	
789.973	-22.889	
789.970	-22.889	
790.100	-22.890	
791.153	-22.891	
791.749	-22.891	
791.550	-22.892	
790.993	-22.893	
792.177	-22.893	
792.065	-22.894	

790.628	-22.895	
792.170	-22.895	
792.598	-22.896	
791.169	-22.897	
791.058	-22.897	
791.406	-22.898	
792.608	-22.899	
793.916	-22.900	
792.982	-22.900	
792.524	-22.901	
794.410	-22.902	
795.147	-22.903	
793.355	-22.903	
793.008	-22.904	
793.665	-22.905	
794.044	-22.906	
795.327	-22.907	
796.498	-22.907	
796.676	-22.908	
795.238	-22.909	
794.016	-22.910	
795.412	-22.911	
796.833	-22.912	
795.728	-22.912	
794.660	-22.913	
795.288	-22.914	
796.882	-22.915	
798.261	-22.916	
798.916	-22.917	
797.823	-22.918	

795.763	-22.919	
794.730	-22.920	
795.096	-22.921	
796.476	-22.922	
797.053	-22.923	
796.997	-22.924	
797.132	-22.925	
797.663	-22.926	
799.143	-22.927	
800.188	-22.928	
799.100	-22.929	
798.251	-22.930	
799.205	-22.931	
798.420	-22.932	
797.600	-22.933	
798.761	-22.935	
798.345	-22.936	
798.560	-22.937	
800.857	-22.938	
801.559	-22.939	
800.286	-22.940	
799.090	-22.942	
799.864	-22.943	
801.269	-22.944	
801.165	-22.945	
801.083	-22.946	
802.094	-22.948	
802.831	-22.949	
801.479	-22.950	
801.045	-22.951	

801.460	-22.952	
800.869	-22.954	
800.645	-22.955	
801.188	-22.956	
802.189	-22.957	
801.888	-22.959	
801.794	-22.960	
802.346	-22.961	
801.682	-22.963	
800.936	-22.964	
801.037	-22.965	
802.241	-22.966	
804.761	-22.968	
804.652	-22.969	
802.971	-22.970	
801.814	-22.972	
803.048	-22.973	
803.704	-22.974	
803.602	-22.976	
804.220	-22.977	
806.495	-22.978	
807.190	-22.980	
807.076	-22.981	
807.298	-22.983	
807.673	-22.984	
807.801	-22.985	
807.272	-22.987	
805.675	-22.988	
804.335	-22.989	
803.948	-22.991	

804.569	-22.992	
806.546	-22.994	
808.431	-22.995	
808.382	-22.996	
806.918	-22.998	
806.923	-22.999	
808.468	-23.001	
807.654	-23.002	
806.887	-23.003	
807.656	-23.005	
808.534	-23.006	
808.438	-23.008	
807.701	-23.009	
807.971	-23.010	
807.993	-23.012	
808.224	-23.013	
808.620	-23.015	
810.293	-23.016	
810.910	-23.018	
810.204	-23.019	
809.635	-23.020	
810.877	-23.022	
811.393	-23.023	
810.350	-23.025	
810.592	-23.026	
812.259	-23.028	
812.101	-23.029	
811.366	-23.031	
811.702	-23.032	
811.078	-23.034	

809.930	-23.035	
809.492	-23.037	
810.424	-23.038	
812.168	-23.040	
812.121	-23.041	
812.290	-23.043	
813.466	-23.044	
811.973	-23.046	
810.950	-23.047	
812.194	-23.049	
812.398	-23.050	
812.020	-23.052	
813.282	-23.053	
813.314	-23.055	
813.616	-23.056	
813.144	-23.058	
813.499	-23.059	
815.361	-23.061	
816.442	-23.062	
816.526	-23.064	
816.505	-23.065	
815.484	-23.067	
814.265	-23.069	
814.494	-23.070	
816.127	-23.072	
817.756	-23.073	
817.917	-23.075	
817.046	-23.076	
816.149	-23.078	
815.520	-23.080	

815.204	-23.081	
816.265	-23.083	
816.718	-23.084	
816.165	-23.086	
816.721	-23.087	
816.080	-23.089	
814.840	-23.091	
815.120	-23.092	
816.005	-23.094	
817.010	-23.096	
817.314	-23.097	
816.745	-23.099	
817.086	-23.100	
819.250	-23.102	
820.534	-23.104	
820.611	-23.105	
819.527	-23.107	
817.355	-23.109	
816.370	-23.110	
816.490	-23.112	
816.957	-23.114	
818.912	-23.115	
821.221	-23.117	
821.926	-23.119	
821.552	-23.120	
821.036	-23.122	
820.530	-23.124	
820.249	-23.125	
820.453	-23.127	
821.606	-23.129	

822.663	-23.130	
823.130	-23.132	
822.886	-23.134	
821.648	-23.135	
820.138	-23.137	
820.987	-23.139	
823.205	-23.140	
823.988	-23.142	
824.083	-23.144	
823.839	-23.145	
821.883	-23.147	
820.648	-23.149	
822.045	-23.150	
823.528	-23.152	
822.319	-23.154	
821.068	-23.155	
821.376	-23.157	
821.388	-23.159	
821.409	-23.160	
822.428	-23.162	
824.469	-23.163	
825.203	-23.165	
823.926	-23.167	
823.301	-23.168	
824.246	-23.170	
825.469	-23.171	
826.058	-23.173	
826.687	-23.175	
826.991	-23.176	
826.793	-23.178	

827.433	-23.179	
828.305	-23.181	
828.140	-23.182	
826.227	-23.184	
824.983	-23.185	
826.061	-23.187	
826.840	-23.188	
826.441	-23.189	
826.886	-23.191	
827.000	-23.192	
827.505	-23.194	
828.261	-23.195	
827.872	-23.196	
827.892	-23.198	
827.864	-23.199	
827.857	-23.200	
828.239	-23.202	
828.533	-23.203	
828.643	-23.204	
828.736	-23.206	
829.303	-23.207	
829.204	-23.208	
828.621	-23.209	
829.144	-23.211	
829.584	-23.212	
829.779	-23.213	
829.714	-23.214	
830.098	-23.215	
830.392	-23.216	
830.153	-23.217	

830.376	-23.218	
830.448	-23.220	
830.719	-23.221	
831.040	-23.222	
831.145	-23.223	
831.431	-23.224	
831.536	-23.225	
831.320	-23.226	
831.734	-23.227	
832.020	-23.228	
832.551	-23.228	
832.050	-23.229	
832.513	-23.230	
834.893	-23.231	
835.331	-23.232	
835.072	-23.233	
834.761	-23.234	
832.909	-23.234	
831.350	-23.235	
832.785	-23.236	
835.411	-23.236	
836.165	-23.237	
834.397	-23.238	
834.686	-23.239	
835.780	-23.239	
835.042	-23.240	
835.060	-23.240	
835.291	-23.241	
835.234	-23.242	
835.247	-23.242	

834.426	-23.243	
833.988	-23.243	
834.914	-23.244	
836.553	-23.244	
836.975	-23.245	
835.393	-23.245	
835.937	-23.246	
836.709	-23.246	
835.274	-23.247	
835.426	-23.247	
837.685	-23.248	
839.529	-23.248	
838.664	-23.248	
837.120	-23.249	
838.158	-23.249	
838.839	-23.250	
838.363	-23.250	
838.820	-23.250	
839.031	-23.251	
838.976	-23.251	
838.865	-23.251	
838.811	-23.252	
839.385	-23.252	
839.769	-23.252	
839.996	-23.252	
839.802	-23.253	
840.424	-23.253	
841.746	-23.253	
840.797	-23.253	
838.755	-23.254	

838.301	-23.254	
839.472	-23.254	
839.810	-23.254	
839.221	-23.254	
840.591	-23.255	
842.793	-23.255	
843.491	-23.255	
843.820	-23.255	
844.055	-23.255	
843.820	-23.256	
843.877	-23.256	
843.637	-23.256	
843.383	-23.256	
843.559	-23.256	
842.428	-23.257	
841.112	-23.257	
840.933	-23.257	
841.503	-23.257	
843.120	-23.258	
844.177	-23.258	
843.428	-23.258	
843.351	-23.258	
844.130	-23.259	
843.823	-23.259	
843.601	-23.259	
844.376	-23.259	
844.970	-23.260	
844.948	-23.260	
844.999	-23.260	
845.068	-23.261	

845.262	-23.261	
845.659	-23.261	
845.704	-23.262	
845.957	-23.262	
845.909	-23.262	
845.930	-23.263	
846.302	-23.263	
846.120	-23.263	
846.210	-23.264	
846.818	-23.264	
847.034	-23.264	
847.012	-23.265	
847.031	-23.265	
847.222	-23.266	
847.491	-23.266	
847.614	-23.267	
847.894	-23.267	
848.089	-23.268	
847.941	-23.268	
847.892	-23.269	
848.279	-23.269	
848.545	-23.270	
848.755	-23.270	
849.205	-23.271	
849.292	-23.271	
848.980	-23.272	
849.181	-23.273	
849.643	-23.273	
849.874	-23.274	
849.970	-23.275	

849.991	-23.275	
850.155	-23.276	
850.534	-23.277	
850.756	-23.278	
850.752	-23.278	
850.937	-23.279	
851.064	-23.280	
850.938	-23.281	
851.102	-23.281	
851.449	-23.282	
851.532	-23.283	
851.721	-23.284	
851.963	-23.285	
852.046	-23.286	
852.128	-23.287	
852.312	-23.287	
852.521	-23.288	
852.673	-23.289	
852.836	-23.290	
852.943	-23.291	
853.200	-23.292	
853.472	-23.293	
853.667	-23.294	
853.755	-23.295	
853.997	-23.296	
854.125	-23.297	
854.342	-23.298	
854.502	-23.299	
854.598	-23.300	
854.639	-23.301	

854.706	-23.302	
854.907	-23.303	
855.090	-23.304	
855.378	-23.305	
855.650	-23.306	
855.701	-23.307	
855.708	-23.309	
855.801	-23.310	
855.922	-23.311	
856.150	-23.312	
856.335	-23.313	
856.389	-23.314	
856.505	-23.316	
856.737	-23.317	
857.098	-23.318	
857.344	-23.319	
857.519	-23.320	
857.617	-23.322	
857.723	-23.323	
857.885	-23.324	
858.086	-23.325	
858.279	-23.326	
858.456	-23.328	
858.583	-23.329	
858.825	-23.330	
858.968	-23.331	
858.948	-23.333	
859.072	-23.334	
859.288	-23.335	
859.520	-23.336	

859.742	-23.338	
859.938	-23.339	
860.122	-23.340	
860.253	-23.341	
860.325	-23.343	
860.496	-23.344	
860.650	-23.345	
860.801	-23.347	
860.981	-23.348	
861.103	-23.349	
861.276	-23.350	
861.473	-23.352	
861.634	-23.353	
861.854	-23.354	
862.122	-23.356	
862.245	-23.357	
862.309	-23.358	
862.442	-23.359	
862.669	-23.361	
862.857	-23.362	
862.937	-23.363	
863.249	-23.365	
863.721	-23.366	
864.071	-23.367	
864.206	-23.369	
864.327	-23.370	
864.439	-23.371	
864.305	-23.372	
864.239	-23.374	
864.383	-23.375	

864.548	-23.376	
864.669	-23.377	
864.758	-23.379	
864.931	-23.380	
865.127	-23.381	
865.321	-23.382	
865.545	-23.384	
865.753	-23.385	
865.889	-23.386	
866.008	-23.387	
866.173	-23.389	
866.372	-23.390	
866.579	-23.391	
866.720	-23.392	
866.799	-23.394	
866.895	-23.395	
867.067	-23.396	
867.327	-23.397	
867.505	-23.398	
867.604	-23.400	
867.743	-23.401	
867.838	-23.402	
867.989	-23.403	
868.251	-23.404	
868.459	-23.406	
868.674	-23.407	
868.854	-23.408	
869.055	-23.409	
869.252	-23.410	
869.400	-23.412	

869.527	-23.413	
869.595	-23.414	
869.775	-23.415	
869.973	-23.416	
870.108	-23.417	
870.223	-23.419	
870.424	-23.420	
870.608	-23.421	
870.742	-23.422	
870.896	-23.423	
871.059	-23.424	
871.202	-23.426	
871.255	-23.427	
871.451	-23.428	
871.618	-23.429	
871.788	-23.430	
872.055	-23.431	
872.215	-23.433	
872.338	-23.434	
872.517	-23.435	
872.699	-23.436	
872.768	-23.437	
872.948	-23.438	
873.201	-23.439	
873.413	-23.441	
873.474	-23.442	
873.524	-23.443	
873.760	-23.444	
874.006	-23.445	
874.163	-23.446	

874.273	-23.447	
874.351	-23.448	
874.489	-23.450	
874.700	-23.451	
874.905	-23.452	
875.151	-23.453	
875.338	-23.454	
875.532	-23.455	
875.712	-23.456	
875.861	-23.457	
875.978	-23.459	
876.058	-23.460	
876.244	-23.461	
876.444	-23.462	
876.757	-23.463	
877.026	-23.464	
877.032	-23.465	
876.979	-23.466	
877.067	-23.467	
877.297	-23.468	
877.442	-23.469	
877.544	-23.470	
877.747	-23.471	
877.860	-23.472	
878.009	-23.473	
878.261	-23.474	
878.452	-23.475	
878.687	-23.476	
878.883	-23.477	
879.110	-23.478	

879.425	-23.479	
879.635	-23.480	
879.743	-23.481	
879.862	-23.482	
879.911	-23.483	
880.044	-23.484	
880.202	-23.485	
880.260	-23.486	
880.452	-23.487	
880.708	-23.488	
880.850	-23.489	
880.971	-23.490	
881.066	-23.490	
881.220	-23.491	
881.406	-23.492	
881.571	-23.493	
881.767	-23.494	
881.979	-23.495	
882.122	-23.496	
882.266	-23.497	
882.486	-23.497	
882.667	-23.498	
882.840	-23.499	
882.986	-23.500	
883.192	-23.501	
883.365	-23.501	
883.522	-23.502	
883.705	-23.503	
883.839	-23.504	
883.910	-23.505	

884.006	-23.505	
884.229	-23.506	
884.491	-23.507	
884.628	-23.508	
884.765	-23.508	
884.955	-23.509	
885.131	-23.510	
885.295	-23.510	
885.405	-23.511	
885.623	-23.512	
885.781	-23.513	
885.873	-23.513	
885.995	-23.514	
886.199	-23.515	
886.413	-23.515	
886.581	-23.516	
886.768	-23.517	
886.870	-23.517	
887.040	-23.518	
887.266	-23.519	
887.440	-23.519	
887.578	-23.520	
887.710	-23.521	
887.922	-23.521	
888.149	-23.522	
888.294	-23.523	
888.372	-23.523	
888.494	-23.524	
888.708	-23.525	
889.100	-23.525	

889.490	-23.526	
889.654	-23.526	
889.737	-23.527	
889.849	-23.528	
889.918	-23.528	
889.942	-23.529	
889.986	-23.530	
890.101	-23.530	
890.218	-23.531	
890.436	-23.531	
890.642	-23.532	
890.806	-23.533	
891.042	-23.533	
891.077	-23.534	
891.223	-23.535	
891.461	-23.535	
891.671	-23.536	
891.833	-23.536	
892.048	-23.537	
892.279	-23.538	
892.347	-23.538	
892.572	-23.539	
892.708	-23.540	
892.756	-23.540	
892.914	-23.541	
893.066	-23.542	
893.170	-23.542	
893.289	-23.543	
893.434	-23.544	
893.574	-23.544	

893.701	-23.545	
893.888	-23.546	
894.176	-23.546	
894.364	-23.547	
894.540	-23.548	
894.707	-23.549	
894.931	-23.549	
895.157	-23.550	
895.257	-23.551	
895.404	-23.552	
895.626	-23.552	
895.757	-23.553	
895.829	-23.554	
895.972	-23.555	
896.186	-23.556	
896.284	-23.556	
896.373	-23.557	
896.570	-23.558	
896.755	-23.559	
896.930	-23.560	
897.069	-23.561	
897.202	-23.562	
897.364	-23.562	
897.553	-23.563	
897.799	-23.564	
898.015	-23.565	
898.056	-23.566	
898.113	-23.567	
898.416	-23.568	
898.598	-23.569	

898.742	-23.570	
898.932	-23.571	
899.041	-23.572	
899.148	-23.573	
899.370	-23.574	
899.669	-23.575	
899.831	-23.576	
899.905	-23.577	
899.932	-23.578	
900.127	-23.579	
900.331	-23.580	
900.393	-23.581	
900.565	-23.582	
900.796	-23.583	
900.957	-23.585	
901.106	-23.586	
901.364	-23.587	
901.628	-23.588	
901.693	-23.589	
901.797	-23.590	
901.925	-23.591	
902.006	-23.592	
902.188	-23.594	
902.447	-23.595	
902.544	-23.596	
902.604	-23.597	
902.833	-23.598	
903.006	-23.599	
903.104	-23.600	
903.287	-23.601	

903.413	-23.603	
903.494	-23.604	
903.654	-23.605	
903.868	-23.606	
904.137	-23.607	
904.305	-23.608	
904.467	-23.609	
904.641	-23.611	
904.818	-23.612	
905.031	-23.613	
905.278	-23.614	
905.538	-23.615	
905.649	-23.616	
905.712	-23.617	
905.997	-23.618	
906.174	-23.620	
906.181	-23.621	
906.388	-23.622	
906.579	-23.623	
906.710	-23.624	
906.845	-23.625	
906.992	-23.626	
907.152	-23.627	
907.253	-23.628	
907.355	-23.629	
907.554	-23.631	
907.847	-23.632	
907.990	-23.633	
908.121	-23.634	
908.352	-23.635	

908.516	-23.636	
908.632	-23.637	
908.717	-23.638	
908.893	-23.639	
909.128	-23.640	
909.309	-23.641	
909.436	-23.642	
909.602	-23.643	
909.790	-23.644	
909.923	-23.645	
910.050	-23.646	
910.192	-23.646	
910.378	-23.647	
910.605	-23.648	
910.769	-23.649	
910.883	-23.650	
911.100	-23.651	
911.344	-23.652	
911.480	-23.653	
911.590	-23.654	
911.760	-23.655	
911.974	-23.655	
912.133	-23.656	
912.250	-23.657	
912.434	-23.658	
912.652	-23.659	
912.773	-23.660	
912.895	-23.661	
913.056	-23.661	
913.298	-23.662	

913.566	-23.663	
913.708	-23.664	
913.782	-23.665	
913.849	-23.666	
914.029	-23.666	
914.293	-23.667	
914.763	-23.668	
915.096	-23.669	
915.234	-23.670	
915.319	-23.670	
915.313	-23.671	
915.350	-23.672	
915.418	-23.673	
915.538	-23.673	
915.674	-23.674	
915.797	-23.675	
915.883	-23.676	
916.012	-23.676	
916.255	-23.677	
916.450	-23.678	
916.627	-23.679	
916.812	-23.679	
916.944	-23.680	
917.131	-23.681	
917.268	-23.682	
917.420	-23.682	
917.607	-23.683	
917.756	-23.684	
917.853	-23.685	
918.042	-23.685	

918.331	-23.686	
918.487	-23.687	
918.542	-23.688	
918.658	-23.689	
918.766	-23.689	
918.910	-23.690	
919.068	-23.691	
919.204	-23.692	
919.408	-23.693	
919.604	-23.693	
919.787	-23.694	
919.980	-23.695	
920.200	-23.696	
920.442	-23.697	
920.638	-23.697	
920.715	-23.698	
920.844	-23.699	
920.972	-23.700	
921.140	-23.701	
921.308	-23.702	
921.438	-23.703	
921.585	-23.703	
921.710	-23.704	
921.824	-23.705	
922.086	-23.706	
922.361	-23.707	
922.441	-23.708	
922.515	-23.709	
922.645	-23.710	
922.804	-23.711	

922.980	-23.712	
923.143	-23.713	
923.317	-23.714	
923.464	-23.715	
923.618	-23.716	
923.855	-23.717	
924.072	-23.718	
924.236	-23.719	
924.414	-23.720	
924.569	-23.721	
924.688	-23.722	
924.819	-23.723	
925.007	-23.724	
925.159	-23.725	
925.227	-23.726	
925.362	-23.727	
925.585	-23.728	
925.854	-23.729	
925.982	-23.730	
926.089	-23.731	
926.337	-23.733	
926.488	-23.734	
926.578	-23.735	
926.748	-23.736	
926.957	-23.737	
927.177	-23.738	
927.280	-23.739	
927.373	-23.740	
927.598	-23.742	
927.729	-23.743	

927.895	-23.744	
928.064	-23.745	
928.259	-23.746	
928.320	-23.747	
928.374	-23.748	
928.577	-23.749	
928.691	-23.751	
928.764	-23.752	
928.923	-23.753	
929.227	-23.754	
929.509	-23.755	
929.685	-23.756	
929.880	-23.757	
930.130	-23.758	
930.184	-23.759	
930.281	-23.760	
930.428	-23.761	
930.543	-23.763	
930.803	-23.764	
930.981	-23.765	
931.128	-23.766	
931.307	-23.767	
931.465	-23.768	
931.638	-23.769	
931.780	-23.770	
931.948	-23.771	
932.219	-23.772	
932.241	-23.773	
932.263	-23.774	
932.476	-23.775	

932.601	-23.776	
932.739	-23.777	
933.092	-23.778	
933.419	-23.779	
933.504	-23.779	
933.682	-23.780	
933.753	-23.781	
933.880	-23.782	
934.166	-23.783	
934.327	-23.784	
934.541	-23.785	
934.705	-23.786	
934.896	-23.786	
935.154	-23.787	
935.217	-23.788	
935.184	-23.789	
935.343	-23.790	
935.601	-23.790	
935.865	-23.791	
936.048	-23.792	
935.972	-23.793	
936.027	-23.793	
936.336	-23.794	
936.685	-23.795	
937.039	-23.796	
937.141	-23.796	
937.131	-23.797	
937.253	-23.798	
937.399	-23.798	
937.609	-23.799	

937.806	-23.800	
937.936	-23.800	
937.999	-23.801	
938.156	-23.802	
938.257	-23.802	
938.398	-23.803	
938.746	-23.803	
938.892	-23.804	
938.978	-23.804	
939.184	-23.805	
939.237	-23.806	
939.337	-23.806	
939.685	-23.807	
940.122	-23.807	
940.472	-23.808	
940.627	-23.808	
940.664	-23.809	
940.769	-23.809	
940.931	-23.810	
940.987	-23.810	
941.060	-23.811	
941.208	-23.811	
941.313	-23.812	
941.398	-23.812	
941.527	-23.812	
941.696	-23.813	
941.801	-23.813	
942.001	-23.814	
942.291	-23.814	
942.477	-23.815	

942.607	-23.815	
942.794	-23.815	
943.073	-23.816	
943.243	-23.816	
943.300	-23.817	
943.402	-23.817	
943.654	-23.818	
943.811	-23.818	
943.893	-23.818	
944.103	-23.819	
944.215	-23.819	
944.284	-23.820	
944.411	-23.820	
944.553	-23.820	
944.680	-23.821	
944.885	-23.821	
945.230	-23.822	
945.455	-23.822	
945.526	-23.823	
945.660	-23.823	
945.899	-23.824	
946.086	-23.824	
946.191	-23.825	
946.291	-23.825	
946.397	-23.826	
946.582	-23.826	
946.857	-23.827	
947.072	-23.827	
947.171	-23.828	
947.189	-23.828	

947.270	-23.829	
947.476	-23.829	
947.707	-23.830	
948.019	-23.830	
948.184	-23.831	
948.204	-23.831	
948.410	-23.832	
948.623	-23.832	
948.739	-23.833	
948.901	-23.834	
949.009	-23.834	
949.024	-23.835	
949.087	-23.836	
949.311	-23.836	
949.570	-23.837	
949.733	-23.838	
949.898	-23.838	
950.124	-23.839	
950.385	-23.840	
950.544	-23.840	
950.630	-23.841	
950.827	-23.842	
951.066	-23.842	
951.238	-23.843	
951.280	-23.844	
951.419	-23.845	
951.672	-23.845	
951.768	-23.846	
951.918	-23.847	
952.152	-23.848	

952.262	-23.848	
952.401	-23.849	
952.622	-23.850	
952.747	-23.851	
952.848	-23.852	
953.007	-23.852	
953.170	-23.853	
953.288	-23.854	
953.475	-23.855	
953.736	-23.856	
953.848	-23.856	
953.926	-23.857	
954.018	-23.858	
954.215	-23.859	
954.426	-23.860	
954.567	-23.860	
954.764	-23.861	
954.934	-23.862	
955.086	-23.863	
955.311	-23.864	
955.577	-23.865	
955.731	-23.865	
955.867	-23.866	
956.090	-23.867	
956.301	-23.868	
956.507	-23.869	
956.620	-23.870	
956.640	-23.870	
956.805	-23.871	
956.949	-23.872	

957.040	-23.873	
957.191	-23.874	
957.299	-23.875	
957.426	-23.875	
957.600	-23.876	
957.745	-23.877	
957.962	-23.878	
958.221	-23.879	
958.388	-23.880	
958.480	-23.880	
958.683	-23.881	
958.957	-23.882	
959.200	-23.883	
959.354	-23.884	
959.388	-23.884	
959.517	-23.885	
959.717	-23.886	
959.921	-23.887	
960.146	-23.887	
960.309	-23.888	
960.412	-23.889	
960.555	-23.890	
960.759	-23.891	
960.922	-23.891	
961.021	-23.892	
961.190	-23.893	
961.312	-23.894	
961.384	-23.894	
961.571	-23.895	
961.820	-23.896	

962.100	-23.896	
962.281	-23.897	
962.363	-23.898	
962.478	-23.899	
962.580	-23.899	
962.732	-23.900	
962.897	-23.901	
963.058	-23.902	
963.294	-23.902	
963.506	-23.903	
963.644	-23.904	
963.792	-23.904	
963.990	-23.905	
964.161	-23.906	
964.344	-23.906	
964.460	-23.907	
964.561	-23.908	
964.733	-23.909	
964.933	-23.909	
965.266	-23.910	
965.630	-23.911	
965.869	-23.911	
965.984	-23.912	
966.097	-23.913	
966.266	-23.913	
966.339	-23.914	
966.389	-23.915	
966.489	-23.915	
966.590	-23.916	
966.689	-23.917	

966.820	-23.918	
966.990	-23.918	
967.203	-23.919	
967.339	-23.920	
967.493	-23.920	
967.734	-23.921	
967.946	-23.922	
968.102	-23.923	
968.170	-23.923	
968.300	-23.924	
968.438	-23.925	
968.614	-23.926	
968.800	-23.926	
968.984	-23.927	
969.185	-23.928	
969.317	-23.929	
969.470	-23.930	
969.659	-23.930	
969.735	-23.931	
969.864	-23.932	
970.066	-23.933	
970.158	-23.934	
970.328	-23.935	
970.581	-23.935	
970.773	-23.936	
970.932	-23.937	
971.081	-23.938	
971.223	-23.939	
971.396	-23.940	
971.601	-23.941	

971.749	-23.942	
971.862	-23.943	
971.916	-23.944	
972.104	-23.945	
972.409	-23.946	
972.561	-23.947	
972.694	-23.948	
972.871	-23.949	
973.012	-23.950	
973.141	-23.951	
973.318	-23.952	
973.478	-23.953	
973.603	-23.954	
973.742	-23.955	
973.973	-23.956	
974.219	-23.957	
974.355	-23.958	
974.528	-23.959	
974.576	-23.961	
974.555	-23.962	
974.801	-23.963	
975.096	-23.964	
975.260	-23.965	
975.377	-23.967	
975.539	-23.968	
975.705	-23.969	
975.853	-23.970	
976.021	-23.972	
976.171	-23.973	
976.346	-23.974	

976.500	-23.975	
976.650	-23.977	
976.859	-23.978	
977.012	-23.979	
977.145	-23.981	
977.290	-23.982	
977.512	-23.983	
977.729	-23.985	
977.850	-23.986	
978.002	-23.987	
978.093	-23.989	
978.305	-23.990	
978.533	-23.992	
978.634	-23.993	
978.748	-23.994	
978.872	-23.996	
978.978	-23.997	
979.136	-23.998	
979.242	-24.000	
979.333	-24.001	
979.657	-24.003	
979.891	-24.004	
980.032	-24.005	
980.188	-24.007	
980.323	-24.008	
980.567	-24.010	
980.726	-24.011	
980.938	-24.012	
981.145	-24.014	
981.347	-24.015	

981.535	-24.017	
981.758	-24.018	
981.963	-24.019	
981.965	-24.021	
982.013	-24.022	
982.198	-24.024	
982.434	-24.025	
982.538	-24.026	
982.633	-24.028	
982.838	-24.029	
982.992	-24.030	
983.099	-24.032	
983.267	-24.033	
983.456	-24.034	
983.607	-24.036	
983.789	-24.037	
983.977	-24.038	
984.132	-24.039	
984.312	-24.041	
984.453	-24.042	
984.613	-24.043	
984.858	-24.044	
985.062	-24.046	
985.193	-24.047	
985.332	-24.048	
985.518	-24.049	
985.768	-24.050	
985.887	-24.052	
985.934	-24.053	
986.177	-24.054	

986.385	-24.055	
986.485	-24.056	
986.626	-24.057	
986.759	-24.058	
986.945	-24.060	
987.150	-24.061	
987.234	-24.062	
987.374	-24.063	
987.638	-24.064	
987.850	-24.065	
987.964	-24.066	
988.105	-24.067	
988.277	-24.068	
988.352	-24.069	
988.523	-24.070	
988.829	-24.071	
989.070	-24.072	
989.202	-24.073	
989.342	-24.074	
989.479	-24.075	
989.611	-24.076	
989.777	-24.077	
989.866	-24.078	
990.023	-24.079	
990.231	-24.080	
990.497	-24.081	
990.881	-24.081	
991.187	-24.082	
991.304	-24.083	
991.342	-24.084	

991.456	-24.085	
991.622	-24.086	
991.730	-24.087	
991.827	-24.087	
991.883	-24.088	
991.913	-24.089	
992.121	-24.090	
992.354	-24.091	
992.480	-24.091	
992.653	-24.092	
992.896	-24.093	
993.006	-24.094	
993.095	-24.094	
993.254	-24.095	
993.432	-24.096	
993.689	-24.097	
993.866	-24.097	
993.961	-24.098	
994.089	-24.099	
994.212	-24.100	
994.388	-24.100	
994.585	-24.101	
994.680	-24.102	
994.779	-24.103	
995.015	-24.103	
995.227	-24.104	
995.314	-24.105	
995.449	-24.106	
995.607	-24.106	
995.796	-24.107	

996.055	-24.108	
996.271	-24.108	
996.488	-24.109	
996.584	-24.110	
996.746	-24.111	
996.991	-24.111	
997.105	-24.112	
997.252	-24.113	
997.383	-24.114	
997.542	-24.114	
997.729	-24.115	
997.886	-24.116	
998.025	-24.117	
998.109	-24.118	
998.187	-24.118	
998.419	-24.119	
998.650	-24.120	
998.835	-24.121	
999.007	-24.122	
999.122	-24.122	
999.221	-24.123	
999.390	-24.124	
999.615	-24.125	
999.733	-24.126	
999.877	-24.127	
1.000.066	-24.127	
1.000.132	-24.128	
1.000.249	-24.129	
1.000.430	-24.130	
1.000.626	-24.131	

1.000.837	-24.132	
1.001.012	-24.133	
1.001.202	-24.134	
1.001.423	-24.135	
1.001.610	-24.136	
1.001.699	-24.136	
1.001.790	-24.137	
1.001.970	-24.138	
1.002.122	-24.139	
1.002.293	-24.140	
1.002.474	-24.141	
1.002.651	-24.142	
1.002.848	-24.143	
1.002.992	-24.144	
1.003.180	-24.145	
1.003.380	-24.146	
1.003.532	-24.147	
1.003.681	-24.148	
1.003.809	-24.149	
1.003.961	-24.150	
1.004.137	-24.151	
1.004.259	-24.152	
1.004.409	-24.153	
1.004.584	-24.154	
1.004.728	-24.155	
1.004.850	-24.156	
1.004.911	-24.157	
1.005.044	-24.158	
1.005.270	-24.159	
1.005.468	-24.160	

1.005.675	-24.161	
1.005.835	-24.162	
1.005.976	-24.163	
1.006.199	-24.164	
1.006.425	-24.165	
1.006.630	-24.166	
1.006.824	-24.167	
1.006.985	-24.168	
1.007.111	-24.169	
1.007.188	-24.170	
1.007.301	-24.171	
1.007.453	-24.172	
1.007.589	-24.173	
1.007.793	-24.174	
1.007.981	-24.175	
1.008.087	-24.176	
1.008.168	-24.177	
1.008.362	-24.178	
1.008.584	-24.179	
1.008.732	-24.180	
1.008.836	-24.181	
1.009.031	-24.182	
1.009.328	-24.183	
1.009.491	-24.184	
1.009.558	-24.185	
1.009.665	-24.186	
1.009.839	-24.187	
1.010.114	-24.188	
1.010.293	-24.189	
1.010.443	-24.190	

1.010.632	-24.191	
1.010.816	-24.192	
1.010.949	-24.193	
1.011.099	-24.194	
1.011.283	-24.195	
1.011.433	-24.196	
1.011.628	-24.197	
1.011.832	-24.198	
1.011.986	-24.199	
1.012.093	-24.199	
1.012.296	-24.200	
1.012.525	-24.201	
1.012.682	-24.202	
1.012.813	-24.203	
1.012.937	-24.204	
1.013.143	-24.205	
1.013.372	-24.206	
1.013.492	-24.207	
1.013.583	-24.208	
1.013.727	-24.209	
1.013.893	-24.209	
1.014.072	-24.210	
1.014.220	-24.211	
1.014.360	-24.212	
1.014.549	-24.213	
1.014.775	-24.214	
1.014.914	-24.215	
1.014.984	-24.215	
1.015.178	-24.216	
1.015.356	-24.217	

1.015.400	-24.218	
1.015.554	-24.219	
1.015.965	-24.220	
1.016.352	-24.220	
1.016.601	-24.221	
1.016.808	-24.222	
1.016.828	-24.223	
1.016.870	-24.224	
1.017.016	-24.224	
1.017.102	-24.225	
1.017.157	-24.226	
1.017.313	-24.227	
1.017.405	-24.228	
1.017.530	-24.228	
1.017.736	-24.229	
1.017.868	-24.230	
1.018.018	-24.231	
1.018.216	-24.232	
1.018.454	-24.232	
1.018.614	-24.233	
1.018.731	-24.234	
1.018.915	-24.235	
1.019.119	-24.235	
1.019.270	-24.236	
1.019.389	-24.237	
1.019.539	-24.238	
1.019.640	-24.238	
1.019.799	-24.239	
1.019.997	-24.240	
1.020.137	-24.241	

1.020.302	-24.241	
1.020.515	-24.242	
1.020.697	-24.243	
1.020.778	-24.244	
1.020.900	-24.245	
1.021.081	-24.245	
1.021.279	-24.246	
1.021.460	-24.247	
1.021.650	-24.248	
1.021.855	-24.248	
1.022.036	-24.249	
1.022.231	-24.250	
1.022.423	-24.251	
1.022.592	-24.251	
1.022.719	-24.252	
1.022.841	-24.253	
1.022.969	-24.254	
1.023.100	-24.255	
1.023.217	-24.255	
1.023.334	-24.256	
1.023.481	-24.257	
1.023.700	-24.258	
1.023.902	-24.258	
1.024.066	-24.259	
1.024.178	-24.260	
1.024.267	-24.261	
1.024.441	-24.262	
1.024.701	-24.262	
1.024.892	-24.263	
1.024.986	-24.264	

1.025.135	-24.265	
1.025.319	-24.266	
1.025.499	-24.266	
1.025.688	-24.267	
1.025.890	-24.268	
1.026.112	-24.269	
1.026.258	-24.270	
1.026.339	-24.270	
1.026.441	-24.271	
1.026.639	-24.272	
1.026.852	-24.273	
1.026.990	-24.274	
1.027.175	-24.274	
1.027.338	-24.275	
1.027.489	-24.276	
1.027.636	-24.277	
1.027.782	-24.277	
1.027.971	-24.278	
1.028.207	-24.279	
1.028.378	-24.280	
1.028.516	-24.281	
1.028.671	-24.281	
1.028.809	-24.282	
1.028.916	-24.283	
1.029.044	-24.284	
1.029.231	-24.284	
1.029.409	-24.285	
1.029.589	-24.286	
1.029.716	-24.286	
1.029.799	-24.287	

1.029.936	-24.288	
1.030.132	-24.288	
1.030.219	-24.289	
1.030.324	-24.290	
1.030.508	-24.290	
1.030.732	-24.291	
1.030.977	-24.292	
1.031.132	-24.292	
1.031.262	-24.293	
1.031.490	-24.293	
1.031.702	-24.294	
1.031.898	-24.295	
1.032.113	-24.295	
1.032.215	-24.296	
1.032.390	-24.296	
1.032.535	-24.297	
1.032.615	-24.297	
1.032.749	-24.298	
1.032.865	-24.298	
1.033.037	-24.299	
1.033.215	-24.299	
1.033.423	-24.300	
1.033.614	-24.300	
1.033.731	-24.301	
1.033.885	-24.301	
1.034.071	-24.301	
1.034.257	-24.302	
1.034.371	-24.302	
1.034.516	-24.302	
1.034.813	-24.303	

1.034.998	-24.303	
1.035.115	-24.303	
1.035.263	-24.304	
1.035.430	-24.304	
1.035.647	-24.304	
1.035.781	-24.304	
1.035.909	-24.305	
1.036.012	-24.305	
1.036.105	-24.305	
1.036.300	-24.305	
1.036.543	-24.305	
1.036.695	-24.306	
1.036.856	-24.306	
1.037.030	-24.306	
1.037.223	-24.306	
1.037.326	-24.306	
1.037.461	-24.306	
1.037.602	-24.306	
1.037.775	-24.306	
1.037.941	-24.306	
1.038.106	-24.306	
1.038.267	-24.307	
1.038.445	-24.307	
1.038.668	-24.307	
1.038.838	-24.307	
1.038.969	-24.307	
1.039.128	-24.306	
1.039.302	-24.306	
1.039.457	-24.306	
1.039.597	-24.306	

1.039.744	-24.306	
1.039.986	-24.306	
1.040.120	-24.306	
1.040.188	-24.306	
1.040.357	-24.306	
1.040.556	-24.306	
1.040.751	-24.306	
1.040.872	-24.306	
1.041.040	-24.305	
1.041.370	-24.305	
1.041.702	-24.305	
1.041.961	-24.305	
1.042.107	-24.305	
1.042.217	-24.304	
1.042.309	-24.304	
1.042.322	-24.304	
1.042.372	-24.304	
1.042.507	-24.304	
1.042.615	-24.303	
1.042.728	-24.303	
1.042.915	-24.303	
1.043.130	-24.303	
1.043.266	-24.302	
1.043.387	-24.302	
1.043.574	-24.302	
1.043.735	-24.302	
1.043.942	-24.301	
1.044.164	-24.301	
1.044.268	-24.301	
1.044.337	-24.301	

1.044.524	-24.301	
1.044.751	-24.300	
1.044.932	-24.300	
1.045.046	-24.300	
1.045.208	-24.300	
1.045.348	-24.299	
1.045.398	-24.299	
1.045.522	-24.299	
1.045.679	-24.299	
1.045.887	-24.299	
1.046.074	-24.298	
1.046.208	-24.298	
1.046.385	-24.298	
1.046.559	-24.298	
1.046.721	-24.298	
1.046.893	-24.298	
1.047.122	-24.297	
1.047.344	-24.297	
1.047.344	-24.297	
1.047.604	-24.297	
1.047.796	-24.297	
1.047.934	-24.297	
1.048.100	-24.297	
1.048.279	-24.297	
1.048.395	-24.297	
1.048.447	-24.297	
1.048.550	-24.297	
1.048.817	-24.297	
1.049.042	-24.297	
1.049.174	-24.297	

1.049.345	-24.297	
1.049.483	-24.297	
1.049.699	-24.297	
1.049.895	-24.297	
1.050.008	-24.297	
1.050.191	-24.297	
1.050.284	-24.297	
1.050.380	-24.298	
1.050.608	-24.298	
1.050.813	-24.298	
1.050.929	-24.298	
1.051.098	-24.298	
1.051.293	-24.299	
1.051.441	-24.299	
1.051.607	-24.299	
1.051.779	-24.299	
1.051.944	-24.300	
1.052.126	-24.300	
1.052.288	-24.300	
1.052.402	-24.301	
1.052.524	-24.301	
1.052.701	-24.302	
1.052.850	-24.302	
1.052.989	-24.302	
1.053.142	-24.303	
1.053.290	-24.303	
1.053.523	-24.304	
1.053.733	-24.304	
1.053.871	-24.305	
1.053.990	-24.305	

1.054.165	-24.306	
1.054.313	-24.306	
1.054.426	-24.307	
1.054.632	-24.307	
1.054.796	-24.308	
1.054.914	-24.308	
1.055.055	-24.309	
1.055.212	-24.310	
1.055.339	-24.310	
1.055.487	-24.311	
1.055.658	-24.311	
1.055.794	-24.312	
1.055.912	-24.313	
1.056.084	-24.313	
1.056.256	-24.314	
1.056.460	-24.315	
1.056.724	-24.315	
1.056.947	-24.316	
1.057.129	-24.317	
1.057.280	-24.317	
1.057.436	-24.318	
1.057.591	-24.319	
1.057.716	-24.320	
1.057.854	-24.320	
1.058.045	-24.321	
1.058.235	-24.322	
1.058.328	-24.323	
1.058.394	-24.323	
1.058.584	-24.324	
1.058.759	-24.325	

1.058.857	-24.326	
1.058.997	-24.326	
1.059.182	-24.327	
1.059.361	-24.328	
1.059.520	-24.329	
1.059.722	-24.330	
1.059.932	-24.330	
1.060.172	-24.331	
1.060.326	-24.332	
1.060.404	-24.333	
1.060.602	-24.334	
1.060.807	-24.334	
1.060.978	-24.335	
1.061.114	-24.336	
1.061.206	-24.337	
1.061.380	-24.338	
1.061.617	-24.338	
1.061.812	-24.339	
1.061.949	-24.340	
1.062.078	-24.341	
1.062.266	-24.342	
1.062.459	-24.342	
1.062.546	-24.343	
1.062.673	-24.344	
1.062.869	-24.345	
1.063.027	-24.346	
1.063.148	-24.346	
1.063.350	-24.347	
1.063.600	-24.348	
1.063.764	-24.349	

1.063.859	-24.350	
1.064.007	-24.351	
1.064.223	-24.351	
1.064.416	-24.352	
1.064.602	-24.353	
1.064.727	-24.354	
1.064.863	-24.355	
1.065.042	-24.355	
1.065.219	-24.356	
1.065.395	-24.357	
1.065.517	-24.358	
1.065.666	-24.358	
1.065.826	-24.359	
1.065.938	-24.360	
1.066.170	-24.361	
1.066.572	-24.362	
1.066.909	-24.362	
1.067.125	-24.363	
1.067.269	-24.364	
1.067.322	-24.365	
1.067.384	-24.365	
1.067.430	-24.366	
1.067.492	-24.367	
1.067.636	-24.368	
1.067.740	-24.369	
1.067.818	-24.369	
1.067.974	-24.370	
1.068.198	-24.371	
1.068.400	-24.372	
1.068.558	-24.372	

1.068.720	-24.373	
1.068.923	-24.374	
1.069.117	-24.375	
1.069.242	-24.375	
1.069.346	-24.376	
1.069.480	-24.377	
1.069.628	-24.378	
1.069.833	-24.379	
1.069.942	-24.379	
1.070.128	-24.380	
1.070.417	-24.381	
1.070.501	-24.382	
1.070.582	-24.383	
1.070.755	-24.383	
1.070.865	-24.384	
1.070.993	-24.385	
1.071.159	-24.386	
1.071.326	-24.387	
1.071.556	-24.387	
1.071.762	-24.388	
1.071.938	-24.389	
1.072.113	-24.390	
1.072.264	-24.391	
1.072.412	-24.392	
1.072.516	-24.393	
1.072.664	-24.393	
1.072.928	-24.394	
1.073.176	-24.395	
1.073.290	-24.396	
1.073.352	-24.397	

1.073.484	-24.398	
1.073.624	-24.399	
1.073.780	-24.400	
1.073.954	-24.401	
1.074.119	-24.402	
1.074.296	-24.403	
1.074.462	-24.404	
1.074.650	-24.404	
1.074.802	-24.405	
1.074.980	-24.406	
1.075.177	-24.407	
1.075.341	-24.408	
1.075.508	-24.409	
1.075.600	-24.410	
1.075.639	-24.411	
1.075.764	-24.412	
1.076.012	-24.414	
1.076.255	-24.415	
1.076.403	-24.416	
1.076.559	-24.417	
1.076.674	-24.418	
1.076.871	-24.419	
1.077.072	-24.420	
1.077.217	-24.421	
1.077.377	-24.422	
1.077.503	-24.423	
1.077.682	-24.424	
1.077.858	-24.426	
1.078.001	-24.427	
1.078.207	-24.428	

1.078.367	-24.429	
1.078.496	-24.430	
1.078.658	-24.431	
1.078.875	-24.432	
1.079.072	-24.433	
1.079.271	-24.435	
1.079.442	-24.436	
1.079.546	-24.437	
1.079.706	-24.438	
1.079.861	-24.439	
1.079.948	-24.440	
1.080.089	-24.442	
1.080.229	-24.443	
1.080.331	-24.444	
1.080.521	-24.445	
1.080.680	-24.446	
1.080.828	-24.447	
1.081.021	-24.449	
1.081.124	-24.450	
1.081.246	-24.451	
1.081.476	-24.452	
1.081.681	-24.453	
1.081.876	-24.454	
1.082.069	-24.455	
1.082.306	-24.457	
1.082.520	-24.458	
1.082.655	-24.459	
1.082.806	-24.460	
1.082.959	-24.461	
1.083.128	-24.462	

1.083.278	-24.463	
1.083.375	-24.464	
1.083.492	-24.466	
1.083.652	-24.467	
1.083.861	-24.468	
1.084.016	-24.469	
1.084.072	-24.470	
1.084.208	-24.471	
1.084.336	-24.472	
1.084.545	-24.473	
1.084.858	-24.474	
1.084.980	-24.475	
1.085.134	-24.476	
1.085.391	-24.477	
1.085.500	-24.478	
1.085.630	-24.479	
1.085.871	-24.480	
1.086.060	-24.481	
1.086.192	-24.482	
1.086.338	-24.483	
1.086.490	-24.484	
1.086.662	-24.485	
1.086.805	-24.486	
1.086.944	-24.487	
1.087.129	-24.488	
1.087.313	-24.489	
1.087.522	-24.490	
1.087.683	-24.491	
1.087.769	-24.492	
1.087.941	-24.493	

1.088.161	-24.494	
1.088.389	-24.494	
1.088.516	-24.495	
1.088.534	-24.496	
1.088.751	-24.497	
1.088.970	-24.498	
1.089.107	-24.499	
1.089.290	-24.499	
1.089.439	-24.500	
1.089.567	-24.501	
1.089.706	-24.502	
1.089.889	-24.503	
1.090.042	-24.503	
1.090.191	-24.504	
1.090.361	-24.505	
1.090.542	-24.506	
1.090.740	-24.506	
1.090.889	-24.507	
1.090.982	-24.508	
1.091.047	-24.509	
1.091.296	-24.509	
1.091.720	-24.510	
1.092.119	-24.511	
1.092.382	-24.511	
1.092.447	-24.512	
1.092.516	-24.513	
1.092.586	-24.514	
1.092.583	-24.514	
1.092.590	-24.515	
1.092.746	-24.516	

1.092.965	-24.516	
1.093.075	-24.517	
1.093.213	-24.518	
1.093.399	-24.518	
1.093.578	-24.519	
1.093.755	-24.519	
1.093.960	-24.520	
1.094.181	-24.521	
1.094.305	-24.521	
1.094.421	-24.522	
1.094.657	-24.523	
1.094.917	-24.523	
1.095.024	-24.524	
1.095.083	-24.525	
1.095.246	-24.525	
1.095.385	-24.526	
1.095.474	-24.527	
1.095.639	-24.527	
1.095.866	-24.528	
1.096.077	-24.529	
1.096.151	-24.529	
1.096.217	-24.530	
1.096.441	-24.531	
1.096.618	-24.531	
1.096.734	-24.532	
1.096.960	-24.533	
1.097.192	-24.534	
1.097.348	-24.534	
1.097.546	-24.535	
1.097.735	-24.536	

1.097.873	-24.537	
1.098.071	-24.537	
1.098.223	-24.538	
1.098.334	-24.539	
1.098.456	-24.540	
1.098.578	-24.540	
1.098.736	-24.541	
1.098.901	-24.542	
1.099.055	-24.543	
1.099.223	-24.544	
1.099.399	-24.545	
1.099.574	-24.545	
1.099.726	-24.546	
1.099.875	-24.547	
1.099.967	-24.548	
1.100.146	-24.549	
1.100.378	-24.550	
1.100.572	-24.551	
1.100.768	-24.552	
1.100.916	-24.553	
1.101.050	-24.554	
1.101.155	-24.555	
1.101.286	-24.556	
1.101.508	-24.557	
1.101.712	-24.558	
1.101.841	-24.559	
1.101.976	-24.560	
1.102.178	-24.561	
1.102.374	-24.562	
1.102.514	-24.564	

1.102.718	-24.565	
1.102.909	-24.566	
1.103.050	-24.567	
1.103.221	-24.568	
1.103.329	-24.569	
1.103.437	-24.571	
1.103.618	-24.572	
1.103.781	-24.573	
1.103.892	-24.574	
1.104.055	-24.575	
1.104.234	-24.577	
1.104.368	-24.578	
1.104.510	-24.579	
1.104.682	-24.580	
1.104.824	-24.582	
1.105.001	-24.583	
1.105.173	-24.584	
1.105.281	-24.586	
1.105.396	-24.587	
1.105.525	-24.588	
1.105.697	-24.589	
1.105.854	-24.591	
1.105.994	-24.592	
1.106.144	-24.593	
1.106.283	-24.595	
1.106.509	-24.596	
1.106.722	-24.597	
1.106.896	-24.599	
1.107.103	-24.600	
1.107.347	-24.602	

1.107.533	-24.603	
1.107.623	-24.604	
1.107.747	-24.606	
1.107.956	-24.607	
1.108.178	-24.608	
1.108.356	-24.610	
1.108.539	-24.611	
1.108.695	-24.612	
1.108.783	-24.614	
1.108.912	-24.615	
1.109.093	-24.616	
1.109.241	-24.618	
1.109.356	-24.619	
1.109.458	-24.620	
1.109.629	-24.622	
1.109.899	-24.623	
1.110.072	-24.624	
1.110.182	-24.626	
1.110.445	-24.627	
1.110.645	-24.628	
1.110.790	-24.630	
1.111.003	-24.631	
1.111.197	-24.632	
1.111.364	-24.633	
1.111.460	-24.635	
1.111.603	-24.636	
1.111.776	-24.637	
1.111.921	-24.638	
1.112.091	-24.640	
1.112.226	-24.641	

1.112.386	-24.642	
1.112.578	-24.643	
1.112.699	-24.644	
1.112.787	-24.646	
1.112.943	-24.647	
1.113.175	-24.648	
1.113.386	-24.649	
1.113.531	-24.650	
1.113.661	-24.651	
1.113.726	-24.652	
1.113.852	-24.654	
1.114.047	-24.655	
1.114.239	-24.656	
1.114.445	-24.657	
1.114.622	-24.658	
1.114.793	-24.659	
1.114.967	-24.660	
1.115.079	-24.661	
1.115.193	-24.662	
1.115.386	-24.663	
1.115.577	-24.664	
1.115.763	-24.665	
1.115.981	-24.666	
1.116.152	-24.667	
1.116.293	-24.668	
1.116.413	-24.669	
1.116.527	-24.670	
1.116.902	-24.671	
1.117.393	-24.671	
1.117.663	-24.672	

1.117.718	-24.673	
1.117.702	-24.674	
1.117.781	-24.675	
1.117.919	-24.676	
1.117.945	-24.677	
1.118.009	-24.677	
1.118.207	-24.678	
1.118.373	-24.679	
1.118.504	-24.680	
1.118.644	-24.680	
1.118.792	-24.681	
1.118.960	-24.682	
1.119.137	-24.683	
1.119.287	-24.683	
1.119.395	-24.684	
1.119.608	-24.685	
1.119.840	-24.686	
1.119.986	-24.686	
1.120.126	-24.687	
1.120.285	-24.688	
1.120.449	-24.688	
1.120.550	-24.689	
1.120.662	-24.690	
1.120.862	-24.690	
1.121.054	-24.691	
1.121.214	-24.692	
1.121.370	-24.692	
1.121.548	-24.693	
1.121.720	-24.694	
1.121.847	-24.694	

1.122.008	-24.695	
1.122.185	-24.696	
1.122.365	-24.696	
1.122.571	-24.697	
1.122.754	-24.698	
1.122.906	-24.698	
1.123.094	-24.699	
1.123.273	-24.700	
1.123.384	-24.700	
1.123.516	-24.701	
1.123.718	-24.702	
1.123.913	-24.702	
1.124.018	-24.703	
1.124.128	-24.704	
1.124.263	-24.704	
1.124.491	-24.705	
1.124.670	-24.706	
1.124.779	-24.706	
1.124.924	-24.707	
1.125.055	-24.708	
1.125.227	-24.709	
1.125.449	-24.709	
1.125.667	-24.710	
1.125.798	-24.711	
1.125.892	-24.711	
1.126.012	-24.712	
1.126.182	-24.713	
1.126.363	-24.714	
1.126.538	-24.714	
1.126.718	-24.715	

1.126.888	-24.716	
1.127.067	-24.717	
1.127.245	-24.717	
1.127.411	-24.718	
1.127.554	-24.719	
1.127.680	-24.720	
1.127.853	-24.721	
1.128.026	-24.721	
1.128.117	-24.722	
1.128.269	-24.723	
1.128.486	-24.724	
1.128.675	-24.725	
1.128.846	-24.725	
1.128.941	-24.726	
1.129.051	-24.727	
1.129.265	-24.728	
1.129.468	-24.729	
1.129.626	-24.730	
1.129.789	-24.730	
1.129.939	-24.731	
1.129.993	-24.732	
1.130.175	-24.733	
1.130.414	-24.734	
1.130.529	-24.734	
1.130.698	-24.735	
1.130.846	-24.736	
1.130.962	-24.737	
1.131.099	-24.738	
1.131.208	-24.739	
1.131.419	-24.739	

1.131.673	-24.740	
1.131.836	-24.741	
1.131.988	-24.742	
1.132.083	-24.743	
1.132.275	-24.743	
1.132.563	-24.744	
1.132.794	-24.745	
1.132.964	-24.746	
1.133.139	-24.746	
1.133.320	-24.747	
1.133.440	-24.748	
1.133.479	-24.749	
1.133.604	-24.749	
1.133.772	-24.750	
1.133.933	-24.751	
1.134.107	-24.752	
1.134.185	-24.752	
1.134.306	-24.753	
1.134.480	-24.754	
1.134.614	-24.754	
1.134.801	-24.755	
1.135.030	-24.756	
1.135.210	-24.756	
1.135.436	-24.757	
1.135.584	-24.758	
1.135.706	-24.758	
1.135.886	-24.759	
1.136.081	-24.759	
1.136.275	-24.760	
1.136.370	-24.761	

1.136.565	-24.761	
1.136.778	-24.762	
1.136.914	-24.762	
1.137.055	-24.763	
1.137.215	-24.763	
1.137.385	-24.764	
1.137.503	-24.764	
1.137.652	-24.765	
1.137.824	-24.765	
1.138.008	-24.766	
1.138.189	-24.766	
1.138.326	-24.767	
1.138.478	-24.767	
1.138.697	-24.768	
1.138.870	-24.768	
1.138.994	-24.768	
1.139.212	-24.769	
1.139.389	-24.769	
1.139.493	-24.770	
1.139.651	-24.770	
1.139.820	-24.770	
1.139.974	-24.771	
1.140.197	-24.771	
1.140.408	-24.771	
1.140.559	-24.772	
1.140.726	-24.772	
1.140.885	-24.772	
1.141.011	-24.772	
1.141.133	-24.773	
1.141.294	-24.773	

1.141.448	-24.773	
1.141.516	-24.773	
1.141.637	-24.774	
1.141.998	-24.774	
1.142.474	-24.774	
1.142.779	-24.774	
1.142.893	-24.775	
1.142.894	-24.775	
1.142.905	-24.775	
1.142.999	-24.775	
1.143.086	-24.775	
1.143.176	-24.775	
1.143.260	-24.776	
1.143.357	-24.776	
1.143.537	-24.776	
1.143.671	-24.776	
1.143.804	-24.776	
1.143.996	-24.776	
1.144.187	-24.777	
1.144.391	-24.777	
1.144.557	-24.777	
1.144.747	-24.777	
1.144.916	-24.777	
1.145.016	-24.777	
1.145.241	-24.777	
1.145.445	-24.778	
1.145.529	-24.778	
1.145.723	-24.778	
1.145.970	-24.778	
1.146.086	-24.778	

1.146.189	-24.779	
1.146.310	-24.779	
1.146.423	-24.779	
1.146.564	-24.779	
1.146.748	-24.779	
1.146.976	-24.780	
1.147.194	-24.780	
1.147.392	-24.780	
1.147.518	-24.780	
1.147.619	-24.780	
1.147.775	-24.781	
1.148.033	-24.781	
1.148.280	-24.781	
1.148.380	-24.782	
1.148.465	-24.782	
1.148.596	-24.782	
1.148.766	-24.783	
1.148.908	-24.783	
1.149.026	-24.783	
1.149.194	-24.784	
1.149.395	-24.784	
1.149.546	-24.784	
1.149.675	-24.785	
1.149.865	-24.785	
1.150.071	-24.786	
1.150.253	-24.786	
1.150.378	-24.787	
1.150.515	-24.787	
1.150.659	-24.788	
1.150.837	-24.788	

1.151.060	-24.789	
1.151.196	-24.790	
1.151.312	-24.790	
1.151.479	-24.791	
1.151.628	-24.791	
1.151.783	-24.792	
1.151.955	-24.793	
1.152.144	-24.793	
1.152.301	-24.794	
1.152.406	-24.795	
1.152.608	-24.796	
1.152.803	-24.796	
1.152.915	-24.797	
1.153.053	-24.798	
1.153.240	-24.799	
1.153.423	-24.800	
1.153.604	-24.801	
1.153.743	-24.802	
1.153.845	-24.802	
1.154.070	-24.803	
1.154.290	-24.804	
1.154.421	-24.805	
1.154.554	-24.806	
1.154.726	-24.807	
1.154.921	-24.808	
1.155.050	-24.809	
1.155.191	-24.810	
1.155.361	-24.811	
1.155.520	-24.812	
1.155.621	-24.813	

1.155.667	-24.815	
1.155.806	-24.816	
1.156.002	-24.817	
1.156.171	-24.818	
1.156.315	-24.819	
1.156.485	-24.820	
1.156.648	-24.821	
1.156.827	-24.823	
1.156.995	-24.824	
1.157.144	-24.825	
1.157.371	-24.826	
1.157.605	-24.828	
1.157.801	-24.829	
1.157.991	-24.830	
1.158.108	-24.831	
1.158.236	-24.833	
1.158.371	-24.834	
1.158.576	-24.835	
1.158.796	-24.837	
1.158.895	-24.838	
1.158.948	-24.839	
1.159.081	-24.841	
1.159.268	-24.842	
1.159.419	-24.843	
1.159.582	-24.845	
1.159.707	-24.846	
1.159.846	-24.848	
1.159.995	-24.849	
1.160.220	-24.850	
1.160.418	-24.852	

1.160.627	-24.853	
1.160.863	-24.855	
1.161.033	-24.856	
1.161.134	-24.857	
1.161.281	-24.859	
1.161.453	-24.860	
1.161.590	-24.862	
1.161.763	-24.863	
1.161.917	-24.865	
1.162.121	-24.866	
1.162.367	-24.867	
1.162.515	-24.869	
1.162.632	-24.870	
1.162.738	-24.872	
1.162.866	-24.873	
1.163.072	-24.875	
1.163.248	-24.876	
1.163.360	-24.878	
1.163.543	-24.879	
1.163.775	-24.881	
1.163.907	-24.882	
1.164.034	-24.883	
1.164.229	-24.885	
1.164.399	-24.886	
1.164.588	-24.888	
1.164.755	-24.889	
1.164.903	-24.891	
1.165.021	-24.892	
1.165.154	-24.894	
1.165.341	-24.895	

1.165.469	-24.897	
1.165.660	-24.898	
1.165.838	-24.900	
1.165.976	-24.901	
1.166.132	-24.903	
1.166.311	-24.904	
1.166.482	-24.906	
1.166.553	-24.907	
1.166.693	-24.909	
1.167.089	-24.910	
1.167.551	-24.912	
1.167.801	-24.913	
1.167.849	-24.915	
1.167.923	-24.916	
1.168.040	-24.918	
1.168.090	-24.919	
1.168.181	-24.921	
1.168.300	-24.922	
1.168.355	-24.924	
1.168.457	-24.925	
1.168.607	-24.927	
1.168.763	-24.928	
1.168.973	-24.930	
1.169.172	-24.932	
1.169.276	-24.933	
1.169.426	-24.935	
1.169.664	-24.936	
1.169.867	-24.938	
1.170.004	-24.939	
1.170.167	-24.941	

1.170.401	-24.942	
1.170.569	-24.944	
1.170.611	-24.946	
1.170.735	-24.947	
1.170.956	-24.949	
1.171.115	-24.950	
1.171.240	-24.952	
1.171.385	-24.954	
1.171.507	-24.955	
1.171.618	-24.957	
1.171.762	-24.958	
1.171.952	-24.960	
1.172.115	-24.962	
1.172.316	-24.963	
1.172.485	-24.965	
1.172.676	-24.966	
1.172.881	-24.968	
1.173.001	-24.970	
1.173.153	-24.971	
1.173.342	-24.973	
1.173.529	-24.975	
1.173.689	-24.976	
1.173.855	-24.978	
1.173.995	-24.980	
1.174.099	-24.981	
1.174.205	-24.983	
1.174.392	-24.985	
1.174.613	-24.987	
1.174.652	-24.988	
1.174.757	-24.990	

1.174.933	-24.992	
1.175.082	-24.993	
1.175.261	-24.995	
1.175.534	-24.997	
1.175.735	-24.999	
1.175.811	-25.000	
1.175.920	-25.002	
1.176.116	-25.004	
1.176.272	-25.006	
1.176.425	-25.007	
1.176.632	-25.009	
1.176.869	-25.011	
1.177.031	-25.013	
1.177.132	-25.014	
1.177.311	-25.016	
1.177.516	-25.018	
1.177.733	-25.020	
1.177.941	-25.022	
1.178.040	-25.024	
1.178.132	-25.025	
1.178.254	-25.027	
1.178.423	-25.029	
1.178.569	-25.031	
1.178.722	-25.033	
1.178.894	-25.035	
1.179.025	-25.036	
1.179.253	-25.038	
1.179.458	-25.040	
1.179.645	-25.042	
1.179.790	-25.044	

1.179.887	-25.046	
1.179.995	-25.048	
1.180.158	-25.049	
1.180.380	-25.051	
1.180.587	-25.053	
1.180.695	-25.055	
1.180.743	-25.057	
1.180.905	-25.059	
1.181.070	-25.061	
1.181.175	-25.062	
1.181.281	-25.064	
1.181.435	-25.066	
1.181.673	-25.068	
1.181.898	-25.070	
1.182.046	-25.071	
1.182.215	-25.073	
1.182.478	-25.075	
1.182.689	-25.077	
1.182.841	-25.079	
1.183.012	-25.080	
1.183.145	-25.082	
1.183.337	-25.084	
1.183.529	-25.086	
1.183.655	-25.087	
1.183.764	-25.089	
1.183.886	-25.091	
1.184.072	-25.092	
1.184.202	-25.094	
1.184.311	-25.096	
1.184.425	-25.097	

1.184.549	-25.099	
1.184.734	-25.100	
1.184.966	-25.102	
1.185.172	-25.104	
1.185.386	-25.105	
1.185.620	-25.107	
1.185.839	-25.108	
1.185.981	-25.110	
1.186.099	-25.111	
1.186.267	-25.113	
1.186.432	-25.114	
1.186.575	-25.115	
1.186.681	-25.117	
1.186.796	-25.118	
1.186.916	-25.120	
1.187.085	-25.121	
1.187.352	-25.122	
1.187.593	-25.124	
1.187.747	-25.125	
1.187.905	-25.126	
1.188.013	-25.127	
1.188.155	-25.129	
1.188.339	-25.130	
1.188.495	-25.131	
1.188.613	-25.132	
1.188.780	-25.134	
1.188.995	-25.135	
1.189.112	-25.136	
1.189.203	-25.137	
1.189.365	-25.138	

1.189.541	-25.139	
1.189.697	-25.141	
1.189.946	-25.142	
1.190.154	-25.143	
1.190.263	-25.144	
1.190.426	-25.145	
1.190.609	-25.146	
1.190.768	-25.147	
1.190.945	-25.148	
1.191.099	-25.149	
1.191.235	-25.150	
1.191.398	-25.151	
1.191.582	-25.152	
1.191.719	-25.153	
1.191.898	-25.154	
1.192.303	-25.155	
1.192.708	-25.156	
1.192.924	-25.157	
1.192.997	-25.158	
1.193.068	-25.159	
1.193.184	-25.160	
1.193.251	-25.161	
1.193.281	-25.162	
1.193.371	-25.162	
1.193.509	-25.163	
1.193.634	-25.164	
1.193.759	-25.165	
1.193.901	-25.166	
1.194.094	-25.167	
1.194.293	-25.168	

1.194.503	-25.169	
1.194.670	-25.169	
1.194.816	-25.170	
1.194.979	-25.171	
1.195.167	-25.172	
1.195.336	-25.173	
1.195.515	-25.174	
1.195.638	-25.175	
1.195.715	-25.175	
1.195.844	-25.176	
1.195.996	-25.177	
1.196.193	-25.178	
1.196.345	-25.179	
1.196.548	-25.180	
1.196.762	-25.180	
1.196.912	-25.181	
1.197.068	-25.182	
1.197.158	-25.183	
1.197.276	-25.184	
1.197.486	-25.185	
1.197.728	-25.186	
1.197.919	-25.187	
1.198.053	-25.188	
1.198.168	-25.188	
1.198.334	-25.189	
1.198.534	-25.190	
1.198.652	-25.191	
1.198.752	-25.192	
1.198.920	-25.193	
1.199.149	-25.194	

1.199.255	-25.195	
1.199.426	-25.196	
1.199.607	-25.197	
1.199.725	-25.198	
1.199.909	-25.199	
1.200.061	-25.200	
1.200.223	-25.201	
1.200.379	-25.202	
1.200.515	-25.203	
1.200.692	-25.204	
1.200.821	-25.206	
1.200.954	-25.207	
1.201.165	-25.208	
1.201.343	-25.209	
1.201.494	-25.210	
1.201.684	-25.211	
1.201.824	-25.212	
1.201.952	-25.214	
1.202.173	-25.215	
1.202.325	-25.216	
1.202.480	-25.217	
1.202.681	-25.219	
1.202.844	-25.220	
1.203.031	-25.221	
1.203.214	-25.223	
1.203.361	-25.224	
1.203.475	-25.225	
1.203.586	-25.227	
1.203.696	-25.228	
1.203.829	-25.229	

1.204.064	-25.231	
1.204.249	-25.232	
1.204.395	-25.234	
1.204.548	-25.235	
1.204.737	-25.236	
1.204.949	-25.238	
1.205.153	-25.239	
1.205.360	-25.241	
1.205.498	-25.242	
1.205.620	-25.244	
1.205.777	-25.245	
1.205.861	-25.247	
1.205.925	-25.248	
1.206.101	-25.250	
1.206.292	-25.251	
1.206.445	-25.253	
1.206.543	-25.254	
1.206.664	-25.256	
1.206.928	-25.257	
1.207.154	-25.259	
1.207.322	-25.260	
1.207.532	-25.262	
1.207.718	-25.263	
1.207.926	-25.265	
1.208.131	-25.266	
1.208.257	-25.268	
1.208.450	-25.269	
1.208.618	-25.271	
1.208.722	-25.272	
1.208.879	-25.274	

1.209.029	-25.275	
1.209.147	-25.277	
1.209.283	-25.278	
1.209.399	-25.280	
1.209.546	-25.281	
1.209.700	-25.283	
1.209.826	-25.284	
1.209.977	-25.286	
1.210.155	-25.287	
1.210.358	-25.289	
1.210.585	-25.290	
1.210.793	-25.292	
1.210.922	-25.293	
1.211.029	-25.295	
1.211.198	-25.296	
1.211.463	-25.298	
1.211.646	-25.299	
1.211.719	-25.301	
1.211.851	-25.302	
1.212.066	-25.304	
1.212.254	-25.305	
1.212.485	-25.306	
1.212.681	-25.308	
1.212.761	-25.309	
1.212.906	-25.311	
1.213.074	-25.312	
1.213.209	-25.313	
1.213.364	-25.315	
1.213.513	-25.316	
1.213.737	-25.317	

1.213.988	-25.319	
1.214.080	-25.320	
1.214.210	-25.321	
1.214.481	-25.323	
1.214.641	-25.324	
1.214.743	-25.325	
1.214.891	-25.327	
1.215.026	-25.328	
1.215.164	-25.329	
1.215.338	-25.330	
1.215.531	-25.332	
1.215.666	-25.333	
1.215.746	-25.334	
1.215.922	-25.335	
1.216.112	-25.336	
1.216.246	-25.338	
1.216.411	-25.339	
1.216.595	-25.340	
1.216.802	-25.341	
1.216.942	-25.342	
1.217.160	-25.343	
1.217.542	-25.345	
1.217.840	-25.346	
1.218.022	-25.347	
1.218.111	-25.348	
1.218.181	-25.349	
1.218.325	-25.350	
1.218.410	-25.351	
1.218.440	-25.352	
1.218.561	-25.353	

1.218.720	-25.354	
1.218.829	-25.356	
1.218.938	-25.357	
1.219.046	-25.358	
1.219.248	-25.359	
1.219.510	-25.360	
1.219.641	-25.361	
1.219.761	-25.362	
1.219.952	-25.363	
1.220.138	-25.364	
1.220.335	-25.365	
1.220.460	-25.366	
1.220.559	-25.367	
1.220.785	-25.368	
1.220.993	-25.369	
1.221.108	-25.370	
1.221.165	-25.371	
1.221.318	-25.372	
1.221.589	-25.373	
1.221.768	-25.374	
1.221.891	-25.375	
1.221.976	-25.376	
1.222.057	-25.377	
1.222.224	-25.378	
1.222.476	-25.379	
1.222.710	-25.380	
1.222.920	-25.382	
1.223.063	-25.383	
1.223.176	-25.384	
1.223.338	-25.385	

1.223.582	-25.386	
1.223.778	-25.387	
1.223.831	-25.388	
1.223.948	-25.389	
1.224.157	-25.390	
1.224.317	-25.391	
1.224.436	-25.392	
1.224.557	-25.393	
1.224.697	-25.395	
1.224.838	-25.396	
1.225.033	-25.397	
1.225.219	-25.398	
1.225.343	-25.399	
1.225.469	-25.400	
1.225.655	-25.402	
1.225.807	-25.403	
1.225.956	-25.404	
1.226.171	-25.405	
1.226.332	-25.407	
1.226.479	-25.408	
1.226.598	-25.409	
1.226.697	-25.411	
1.226.831	-25.412	
1.227.049	-25.413	
1.227.327	-25.415	
1.227.564	-25.416	
1.227.748	-25.418	
1.227.897	-25.420	
1.228.002	-25.421	
1.228.103	-25.423	

1.228.286	-25.425	
1.228.463	-25.426	
1.228.600	-25.428	
1.228.674	-25.430	
1.228.834	-25.432	
1.229.042	-25.434	
1.229.228	-25.436	
1.229.426	-25.438	
1.229.557	-25.440	
1.229.720	-25.443	
1.229.897	-25.445	
1.230.035	-25.447	
1.230.182	-25.450	
1.230.324	-25.452	
1.230.456	-25.455	
1.230.599	-25.458	
1.230.761	-25.460	
1.230.916	-25.463	
1.231.071	-25.466	
1.231.222	-25.469	
1.231.380	-25.472	
1.231.583	-25.476	
1.231.721	-25.479	
1.231.870	-25.482	
1.232.045	-25.486	
1.232.198	-25.490	
1.232.340	-25.493	
1.232.553	-25.497	
1.232.783	-25.501	
1.232.910	-25.505	

1.233.088	-25.509	
1.233.298	-25.514	
1.233.475	-25.518	
1.233.641	-25.523	
1.233.777	-25.527	
1.233.895	-25.532	
1.234.018	-25.537	
1.234.108	-25.542	
1.234.257	-25.547	
1.234.386	-25.553	
1.234.476	-25.558	
1.234.662	-25.564	
1.234.903	-25.569	
1.235.097	-25.575	
1.235.210	-25.581	
1.235.434	-25.587	
1.235.648	-25.593	
1.235.835	-25.600	
1.236.060	-25.606	
1.236.207	-25.613	
1.236.323	-25.620	
1.236.431	-25.627	
1.236.650	-25.634	
1.236.819	-25.641	
1.236.946	-25.648	
1.237.094	-25.656	
1.237.227	-25.663	
1.237.430	-25.671	
1.237.648	-25.679	
1.237.814	-25.687	

1.237.934	-25.695	
1.238.082	-25.703	
1.238.262	-25.712	
1.238.456	-25.720	
1.238.621	-25.729	
1.238.731	-25.738	
1.238.901	-25.747	
1.239.076	-25.756	
1.239.268	-25.765	
1.239.451	-25.775	
1.239.548	-25.784	
1.239.647	-25.794	
1.239.817	-25.804	
1.239.987	-25.814	
1.240.152	-25.824	
1.240.322	-25.834	
1.240.472	-25.845	
1.240.647	-25.855	
1.240.852	-25.866	
1.240.998	-25.877	
1.241.132	-25.888	
1.241.269	-25.899	
1.241.413	-25.910	
1.241.544	-25.922	
1.241.665	-25.933	
1.241.800	-25.945	
1.241.945	-25.956	
1.242.242	-25.968	
1.242.713	-25.980	
1.243.067	-25.992	

1.243.152	-26.004	
1.243.203	-26.016	
1.243.276	-26.029	
1.243.336	-26.041	
1.243.408	-26.053	
1.243.433	-26.066	
1.243.477	-26.078	
1.243.626	-26.091	
1.243.804	-26.104	
1.243.968	-26.116	
1.244.167	-26.129	
1.244.381	-26.142	
1.244.584	-26.155	
1.244.691	-26.168	
1.244.855	-26.180	
1.245.006	-26.193	
1.245.180	-26.206	
1.245.385	-26.219	
1.245.586	-26.232	
1.245.674	-26.245	
1.245.769	-26.258	
1.245.903	-26.271	
1.246.069	-26.283	
1.246.237	-26.296	
1.246.370	-26.309	
1.246.598	-26.322	
1.246.788	-26.334	
1.246.841	-26.347	
1.246.949	-26.359	
1.247.169	-26.372	

1.247.365	-26.384	
1.247.509	-26.396	
1.247.604	-26.409	
1.247.811	-26.421	
1.248.084	-26.433	
1.248.262	-26.445	
1.248.419	-26.457	
1.248.524	-26.468	
1.248.652	-26.480	
1.248.796	-26.491	
1.248.883	-26.503	
1.248.989	-26.514	
1.249.165	-26.525	
1.249.310	-26.536	
1.249.491	-26.546	
1.249.705	-26.557	
1.249.871	-26.567	
1.250.028	-26.578	
1.250.198	-26.588	
1.250.400	-26.598	
1.250.540	-26.608	
1.250.630	-26.617	
1.250.798	-26.627	
1.251.000	-26.636	
1.251.193	-26.645	
1.251.336	-26.654	
1.251.412	-26.663	
1.251.563	-26.671	
1.251.737	-26.680	
1.251.891	-26.688	

1.252.122	-26.696	
1.252.355	-26.703	
1.252.546	-26.711	
1.252.672	-26.718	
1.252.778	-26.725	
1.252.923	-26.732	
1.253.097	-26.739	
1.253.299	-26.746	
1.253.455	-26.752	
1.253.597	-26.758	
1.253.776	-26.764	
1.253.896	-26.770	
1.254.029	-26.775	
1.254.212	-26.781	
1.254.370	-26.786	
1.254.510	-26.791	
1.254.738	-26.795	
1.254.973	-26.800	
1.255.072	-26.804	
1.255.161	-26.808	
1.255.314	-26.812	
1.255.466	-26.815	
1.255.573	-26.819	
1.255.719	-26.822	
1.255.938	-26.825	
1.256.086	-26.828	
1.256.171	-26.830	
1.256.315	-26.833	
1.256.456	-26.835	
1.256.572	-26.837	

1.256.759	-26.838	
1.257.014	-26.840	
1.257.233	-26.841	
1.257.328	-26.842	
1.257.509	-26.843	
1.257.805	-26.844	
1.257.993	-26.844	
1.258.152	-26.844	
1.258.383	-26.844	
1.258.504	-26.844	
1.258.602	-26.844	
1.258.840	-26.844	
1.258.967	-26.843	
1.259.028	-26.842	
1.259.167	-26.841	
1.259.312	-26.840	
1.259.429	-26.839	
1.259.603	-26.838	
1.259.832	-26.836	
1.260.013	-26.834	
1.260.139	-26.833	
1.260.255	-26.831	
1.260.399	-26.828	
1.260.571	-26.826	
1.260.813	-26.824	
1.261.015	-26.821	
1.261.216	-26.819	
1.261.371	-26.816	
1.261.499	-26.814	
1.261.674	-26.811	

1.261.846	-26.808	
1.262.036	-26.805	
1.262.164	-26.802	
1.262.260	-26.799	
1.262.408	-26.795	
1.262.630	-26.792	
1.262.840	-26.789	
1.262.997	-26.785	
1.263.125	-26.782	
1.263.279	-26.778	
1.263.441	-26.775	
1.263.566	-26.771	
1.263.736	-26.768	
1.263.900	-26.764	
1.264.005	-26.760	
1.264.160	-26.757	
1.264.373	-26.753	
1.264.565	-26.749	
1.264.715	-26.746	
1.264.836	-26.742	
1.265.007	-26.738	
1.265.231	-26.735	
1.265.423	-26.731	
1.265.562	-26.727	
1.265.708	-26.724	
1.265.874	-26.720	
1.266.019	-26.717	
1.266.212	-26.713	
1.266.398	-26.709	
1.266.579	-26.706	

1.266.729	-26.702	
1.266.794	-26.699	
1.266.908	-26.696	
1.267.213	-26.692	
1.267.607	-26.689	
1.267.953	-26.686	
1.268.154	-26.682	
1.268.244	-26.679	
1.268.282	-26.676	
1.268.351	-26.673	
1.268.382	-26.670	
1.268.403	-26.667	
1.268.573	-26.664	
1.268.681	-26.661	
1.268.743	-26.658	
1.268.951	-26.655	
1.269.198	-26.653	
1.269.341	-26.650	
1.269.456	-26.648	
1.269.642	-26.645	
1.269.798	-26.642	
1.269.928	-26.640	
1.270.121	-26.638	
1.270.343	-26.635	
1.270.546	-26.633	
1.270.667	-26.631	
1.270.746	-26.629	
1.270.903	-26.627	
1.271.062	-26.625	
1.271.196	-26.623	

1.271.366	-26.621	
1.271.534	-26.619	
1.271.700	-26.617	
1.271.875	-26.615	
1.271.974	-26.614	
1.272.090	-26.612	
1.272.260	-26.611	
1.272.482	-26.609	
1.272.740	-26.608	
1.272.937	-26.606	
1.273.091	-26.605	
1.273.250	-26.604	
1.273.369	-26.602	
1.273.472	-26.601	
1.273.669	-26.600	
1.273.895	-26.599	
1.274.041	-26.598	
1.274.162	-26.597	
1.274.279	-26.596	
1.274.413	-26.596	
1.274.619	-26.595	
1.274.821	-26.594	
1.274.986	-26.593	
1.275.150	-26.593	
1.275.296	-26.592	
1.275.397	-26.592	
1.275.527	-26.591	
1.275.717	-26.591	
1.275.894	-26.591	
1.276.048	-26.591	

1.276.225	-26.590	
1.276.414	-26.590	
1.276.514	-26.590	
1.276.561	-26.590	
1.276.705	-26.590	
1.276.949	-26.590	
1.277.157	-26.591	
1.277.316	-26.591	
1.277.500	-26.591	
1.277.684	-26.592	
1.277.905	-26.592	
1.278.058	-26.592	
1.278.120	-26.593	
1.278.217	-26.594	
1.278.401	-26.594	
1.278.605	-26.595	
1.278.717	-26.596	
1.278.820	-26.596	
1.278.974	-26.597	
1.279.124	-26.598	
1.279.254	-26.599	
1.279.541	-26.600	
1.279.700	-26.601	
1.279.857	-26.602	
1.280.005	-26.604	
1.280.157	-26.605	
1.280.307	-26.606	
1.280.462	-26.608	
1.280.616	-26.609	
1.280.759	-26.611	

1.280.889	-26.612	
1.281.046	-26.614	
1.281.160	-26.616	
1.281.290	-26.617	
1.281.395	-26.619	
1.281.559	-26.621	
1.281.767	-26.623	
1.281.929	-26.625	
1.282.103	-26.627	
1.282.274	-26.629	
1.282.435	-26.631	
1.282.654	-26.634	
1.282.899	-26.636	
1.283.094	-26.638	
1.283.273	-26.641	
1.283.418	-26.643	
1.283.620	-26.646	
1.283.831	-26.648	
1.283.940	-26.651	
1.284.025	-26.654	
1.284.174	-26.656	
1.284.303	-26.659	
1.284.476	-26.662	
1.284.646	-26.665	
1.284.724	-26.668	
1.284.845	-26.671	
1.285.007	-26.674	
1.285.186	-26.677	
1.285.374	-26.681	
1.285.546	-26.684	

1.285.679	-26.687	
1.285.858	-26.691	
1.286.084	-26.694	
1.286.248	-26.698	
1.286.387	-26.702	
1.286.552	-26.705	
1.286.702	-26.709	
1.286.922	-26.713	
1.287.166	-26.717	
1.287.325	-26.721	
1.287.507	-26.725	
1.287.686	-26.729	
1.287.757	-26.734	
1.287.858	-26.738	
1.288.089	-26.742	
1.288.284	-26.747	
1.288.391	-26.751	
1.288.540	-26.756	
1.288.717	-26.761	
1.288.846	-26.765	
1.289.007	-26.770	
1.289.223	-26.775	
1.289.370	-26.780	
1.289.503	-26.785	
1.289.686	-26.791	
1.289.886	-26.796	
1.290.069	-26.801	
1.290.152	-26.807	
1.290.298	-26.812	
1.290.517	-26.818	

1.290.674	-26.823	
1.290.788	-26.829	
1.290.914	-26.835	
1.291.098	-26.840	
1.291.290	-26.846	
1.291.456	-26.852	
1.291.590	-26.858	
1.291.687	-26.864	
1.291.823	-26.870	
1.292.032	-26.876	
1.292.289	-26.883	
1.292.706	-26.889	
1.293.067	-26.895	
1.293.230	-26.901	
1.293.268	-26.908	
1.293.273	-26.914	
1.293.434	-26.920	
1.293.533	-26.927	
1.293.562	-26.933	
1.293.652	-26.939	
1.293.775	-26.946	
1.293.878	-26.952	
1.294.012	-26.959	
1.294.236	-26.965	
1.294.444	-26.971	
1.294.624	-26.978	
1.294.783	-26.984	
1.294.942	-26.991	
1.295.137	-26.997	
1.295.271	-27.003	

1.295.372	-27.010	
1.295.558	-27.016	
1.295.748	-27.022	
1.295.903	-27.028	
1.296.075	-27.035	
1.296.295	-27.041	
1.296.443	-27.047	
1.296.595	-27.053	
1.296.729	-27.059	
1.296.801	-27.065	
1.296.922	-27.071	
1.297.146	-27.076	
1.297.339	-27.082	
1.297.471	-27.088	
1.297.637	-27.093	
1.297.776	-27.099	
1.297.964	-27.104	
1.298.145	-27.110	
1.298.275	-27.115	
1.298.461	-27.120	
1.298.620	-27.126	
1.298.844	-27.131	
1.299.055	-27.136	
1.299.099	-27.141	
1.299.209	-27.145	
1.299.408	-27.150	
1.299.563	-27.155	
1.299.697	-27.159	
1.299.809	-27.164	
1.299.939	-27.168	

1.300.126	-27.173	
1.300.336	-27.177	
1.300.522	-27.181	
1.300.663	-27.185	
1.300.804	-27.189	
1.300.947	-27.193	
1.301.134	-27.197	
1.301.310	-27.200	
1.301.443	-27.204	
1.301.572	-27.207	
1.301.745	-27.211	
1.301.983	-27.214	
1.302.180	-27.217	
1.302.323	-27.220	
1.302.460	-27.223	
1.302.646	-27.226	
1.302.816	-27.229	
1.302.921	-27.232	
1.303.053	-27.235	
1.303.208	-27.237	
1.303.387	-27.240	
1.303.562	-27.242	
1.303.698	-27.244	
1.303.871	-27.246	
1.304.086	-27.248	
1.304.202	-27.250	
1.304.342	-27.252	
1.304.520	-27.254	
1.304.688	-27.255	
1.304.865	-27.257	

1.305.011	-27.258	
1.305.170	-27.260	
1.305.267	-27.261	
1.305.419	-27.262	
1.305.647	-27.263	
1.305.770	-27.264	
1.305.907	-27.265	
1.306.066	-27.265	
1.306.136	-27.266	
1.306.304	-27.267	
1.306.534	-27.267	
1.306.642	-27.267	
1.306.756	-27.267	
1.306.913	-27.268	
1.307.009	-27.268	
1.307.139	-27.268	
1.307.357	-27.267	
1.307.563	-27.267	
1.307.744	-27.267	
1.307.968	-27.267	
1.308.203	-27.266	
1.308.418	-27.266	
1.308.578	-27.265	
1.308.721	-27.264	
1.308.837	-27.263	
1.308.934	-27.263	
1.309.106	-27.262	
1.309.274	-27.261	
1.309.391	-27.260	
1.309.547	-27.259	

1.309.695	-27.257	
1.309.807	-27.256	
1.309.935	-27.255	
1.310.092	-27.254	
1.310.284	-27.252	
1.310.443	-27.251	
1.310.618	-27.249	
1.310.826	-27.248	
1.311.024	-27.246	
1.311.234	-27.245	
1.311.357	-27.243	
1.311.514	-27.242	
1.311.720	-27.240	
1.311.902	-27.238	
1.312.054	-27.237	
1.312.229	-27.235	
1.312.410	-27.233	
1.312.522	-27.231	
1.312.634	-27.230	
1.312.737	-27.228	
1.312.867	-27.226	
1.313.076	-27.224	
1.313.262	-27.222	
1.313.405	-27.221	
1.313.596	-27.219	
1.313.752	-27.217	
1.313.889	-27.215	
1.314.115	-27.214	
1.314.283	-27.212	
1.314.357	-27.210	

1.314.456	-27.208	
1.314.626	-27.207	
1.314.823	-27.205	
1.315.016	-27.203	
1.315.213	-27.202	
1.315.347	-27.200	
1.315.522	-27.198	
1.315.746	-27.197	
1.315.849	-27.195	
1.316.003	-27.193	
1.316.178	-27.192	
1.316.302	-27.190	
1.316.501	-27.189	
1.316.675	-27.187	
1.316.832	-27.186	
1.316.888	-27.185	
1.317.029	-27.183	
1.317.231	-27.182	
1.317.343	-27.181	
1.317.673	-27.179	
1.318.120	-27.178	
1.318.428	-27.177	
1.318.584	-27.176	
1.318.656	-27.174	
1.318.746	-27.173	
1.318.860	-27.172	
1.318.887	-27.171	
1.318.898	-27.170	
1.319.025	-27.169	
1.319.187	-27.168	

1.319.341	-27.167	
1.319.476	-27.166	
1.319.650	-27.165	
1.319.818	-27.164	
1.319.975	-27.163	
1.320.107	-27.163	
1.320.228	-27.162	
1.320.425	-27.161	
1.320.627	-27.160	
1.320.802	-27.160	
1.320.941	-27.159	
1.321.122	-27.158	
1.321.301	-27.158	
1.321.387	-27.157	
1.321.530	-27.157	
1.321.729	-27.156	
1.321.875	-27.156	
1.322.000	-27.155	
1.322.115	-27.155	
1.322.244	-27.155	
1.322.383	-27.154	
1.322.439	-27.154	
1.322.608	-27.154	
1.322.966	-27.153	
1.323.215	-27.153	
1.323.369	-27.153	
1.323.510	-27.153	
1.323.622	-27.152	
1.323.801	-27.152	
1.324.019	-27.152	

1.324.160	-27.152	
1.324.283	-27.152	
1.324.424	-27.152	
1.324.574	-27.152	
1.324.751	-27.152	
1.324.877	-27.152	
1.324.993	-27.152	
1.325.152	-27.152	
1.325.334	-27.152	
1.325.549	-27.153	
1.325.737	-27.153	
1.325.874	-27.153	
1.326.035	-27.153	
1.326.207	-27.153	
1.326.342	-27.154	
1.326.463	-27.154	
1.326.660	-27.154	
1.326.902	-27.154	
1.327.016	-27.155	
1.327.099	-27.155	
1.327.233	-27.155	
1.327.408	-27.156	
1.327.616	-27.156	
1.327.802	-27.157	
1.327.923	-27.157	
1.328.109	-27.158	
1.328.320	-27.158	
1.328.463	-27.159	
1.328.640	-27.159	
1.328.754	-27.160	

1.328.887	-27.160	
1.329.084	-27.161	
1.329.240	-27.162	
1.329.413	-27.162	
1.329.523	-27.163	
1.329.624	-27.163	
1.329.792	-27.164	
1.329.989	-27.165	
1.330.146	-27.165	
1.330.257	-27.166	
1.330.408	-27.167	
1.330.621	-27.168	
1.330.795	-27.168	
1.330.878	-27.169	
1.331.042	-27.170	
1.331.252	-27.171	
1.331.389	-27.172	
1.331.535	-27.173	
1.331.709	-27.173	
1.331.882	-27.174	
1.331.920	-27.175	
1.331.989	-27.176	
1.332.195	-27.177	
1.332.392	-27.178	
1.332.527	-27.179	
1.332.614	-27.180	
1.332.831	-27.181	
1.333.024	-27.182	
1.333.237	-27.183	
1.333.533	-27.184	

1.333.714	-27.185	
1.333.898	-27.186	
1.334.093	-27.187	
1.334.258	-27.188	
1.334.447	-27.190	
1.334.613	-27.191	
1.334.718	-27.192	
1.334.857	-27.193	
1.334.966	-27.194	
1.335.108	-27.196	
1.335.257	-27.197	
1.335.356	-27.198	
1.335.493	-27.199	
1.335.647	-27.201	
1.335.816	-27.202	
1.335.934	-27.203	
1.336.064	-27.205	
1.336.306	-27.206	
1.336.539	-27.208	
1.336.671	-27.209	
1.336.868	-27.210	
1.337.144	-27.212	
1.337.356	-27.213	
1.337.430	-27.215	
1.337.502	-27.217	
1.337.659	-27.218	
1.337.856	-27.220	
1.338.040	-27.222	
1.338.190	-27.223	
1.338.353	-27.225	

1.338.503	-27.227	
1.338.689	-27.228	
1.338.884	-27.230	
1.339.008	-27.232	
1.339.097	-27.234	
1.339.236	-27.236	
1.339.435	-27.238	
1.339.671	-27.240	
1.339.814	-27.242	
1.339.991	-27.244	
1.340.132	-27.246	
1.340.233	-27.248	
1.340.455	-27.250	
1.340.629	-27.252	
1.340.737	-27.255	
1.340.842	-27.257	
1.341.021	-27.259	
1.341.263	-27.261	
1.341.436	-27.264	
1.341.566	-27.266	
1.341.754	-27.268	
1.341.940	-27.271	
1.342.081	-27.273	
1.342.195	-27.276	
1.342.383	-27.278	
1.342.542	-27.281	
1.342.670	-27.283	
1.342.782	-27.286	
1.343.009	-27.289	
1.343.412	-27.291	

1.343.772	-27.294	
1.344.063	-27.297	
1.344.171	-27.300	
1.344.140	-27.302	
1.344.229	-27.305	
1.344.319	-27.308	
1.344.281	-27.311	
1.344.379	-27.314	
1.344.579	-27.317	
1.344.677	-27.320	
1.344.762	-27.323	
1.344.919	-27.326	
1.345.083	-27.329	
1.345.204	-27.332	
1.345.392	-27.335	
1.345.609	-27.338	
1.345.822	-27.341	
1.346.031	-27.344	
1.346.180	-27.347	
1.346.328	-27.350	
1.346.514	-27.353	
1.346.673	-27.356	
1.346.776	-27.359	
1.346.935	-27.363	
1.347.086	-27.366	
1.347.200	-27.369	
1.347.307	-27.372	
1.347.455	-27.375	
1.347.635	-27.378	
1.347.805	-27.381	

1.347.955	-27.384	
1.348.136	-27.387	
1.348.351	-27.390	
1.348.486	-27.393	
1.348.615	-27.397	
1.348.846	-27.400	
1.349.046	-27.402	
1.349.223	-27.405	
1.349.368	-27.408	
1.349.491	-27.411	
1.349.711	-27.414	
1.349.866	-27.417	
1.349.955	-27.420	
1.350.096	-27.423	
1.350.275	-27.425	
1.350.455	-27.428	
1.350.598	-27.431	
1.350.723	-27.434	
1.350.865	-27.436	
1.351.008	-27.439	
1.351.190	-27.441	
1.351.443	-27.444	
1.351.622	-27.447	
1.351.696	-27.449	
1.351.799	-27.451	
1.351.976	-27.454	
1.352.162	-27.456	
1.352.318	-27.459	
1.352.491	-27.461	
1.352.621	-27.463	

1.352.742	-27.465	
1.352.876	-27.467	
1.353.020	-27.470	
1.353.167	-27.472	
1.353.374	-27.474	
1.353.609	-27.476	
1.353.810	-27.478	
1.353.965	-27.480	
1.354.063	-27.482	
1.354.220	-27.483	
1.354.453	-27.485	
1.354.615	-27.487	
1.354.711	-27.489	
1.354.776	-27.490	
1.354.937	-27.492	
1.355.179	-27.494	
1.355.325	-27.495	
1.355.515	-27.497	
1.355.714	-27.498	
1.355.938	-27.500	
1.356.107	-27.501	
1.356.178	-27.503	
1.356.295	-27.504	
1.356.447	-27.505	
1.356.609	-27.506	
1.356.772	-27.508	
1.356.938	-27.509	
1.357.097	-27.510	
1.357.224	-27.511	
1.357.354	-27.513	

1.357.556	-27.514	
1.357.724	-27.515	
1.357.831	-27.516	
1.357.986	-27.517	
1.358.123	-27.518	
1.358.293	-27.519	
1.358.501	-27.520	
1.358.676	-27.521	
1.358.878	-27.522	
1.359.095	-27.523	
1.359.324	-27.524	
1.359.478	-27.525	
1.359.527	-27.526	
1.359.682	-27.527	
1.359.901	-27.527	
1.360.067	-27.528	
1.360.224	-27.529	
1.360.385	-27.530	
1.360.509	-27.531	
1.360.542	-27.532	
1.360.645	-27.533	
1.360.795	-27.534	
1.360.941	-27.535	
1.361.145	-27.536	
1.361.328	-27.537	
1.361.534	-27.538	
1.361.767	-27.539	
1.361.947	-27.540	
1.362.099	-27.541	
1.362.242	-27.542	

1.362.422	-27.543	
1.362.592	-27.544	
1.362.753	-27.545	
1.362.928	-27.547	
1.363.092	-27.548	
1.363.286	-27.549	
1.363.434	-27.551	
1.363.544	-27.552	
1.363.678	-27.554	
1.363.880	-27.555	
1.364.129	-27.557	
1.364.281	-27.559	
1.364.366	-27.561	
1.364.514	-27.563	
1.364.733	-27.565	
1.364.942	-27.567	
1.365.105	-27.569	
1.365.240	-27.571	
1.365.327	-27.574	
1.365.446	-27.576	
1.365.681	-27.579	
1.365.928	-27.581	
1.366.086	-27.584	
1.366.147	-27.587	
1.366.292	-27.590	
1.366.476	-27.593	
1.366.557	-27.596	
1.366.714	-27.600	
1.366.884	-27.603	
1.367.043	-27.607	

1.367.236	-27.610	
1.367.435	-27.614	
1.367.592	-27.618	
1.367.724	-27.622	
1.367.878	-27.626	
1.368.006	-27.630	
1.368.107	-27.635	
1.368.201	-27.639	
1.368.528	-27.644	
1.369.059	-27.648	
1.369.399	-27.653	
1.369.525	-27.658	
1.369.550	-27.663	
1.369.599	-27.668	
1.369.743	-27.673	
1.369.809	-27.679	
1.369.812	-27.684	
1.369.843	-27.690	
1.369.953	-27.695	
1.370.134	-27.701	
1.370.307	-27.707	
1.370.497	-27.713	
1.370.694	-27.719	
1.370.885	-27.725	
1.371.001	-27.731	
1.371.118	-27.737	
1.371.304	-27.743	
1.371.443	-27.750	
1.371.619	-27.756	
1.371.767	-27.763	

1.371.920	-27.770	
1.372.157	-27.776	
1.372.374	-27.783	
1.372.525	-27.790	
1.372.648	-27.797	
1.372.749	-27.804	
1.372.881	-27.811	
1.373.060	-27.818	
1.373.210	-27.825	
1.373.322	-27.832	
1.373.452	-27.840	
1.373.665	-27.847	
1.373.896	-27.854	
1.374.052	-27.862	
1.374.149	-27.869	
1.374.308	-27.877	
1.374.509	-27.884	
1.374.671	-27.892	
1.374.868	-27.900	
1.375.027	-27.907	
1.375.121	-27.915	
1.375.287	-27.922	
1.375.450	-27.930	
1.375.607	-27.938	
1.375.755	-27.946	
1.375.887	-27.953	
1.376.024	-27.961	
1.376.180	-27.969	
1.376.366	-27.976	
1.376.537	-27.984	

1.376.709	-27.992	
1.376.868	-28.000	
1.377.032	-28.007	
1.377.197	-28.015	
1.377.402	-28.023	
1.377.592	-28.030	
1.377.545	-28.038	
1.377.628	-28.045	
1.377.937	-28.053	
1.378.129	-28.060	
1.378.262	-28.068	
1.378.434	-28.075	
1.378.640	-28.082	
1.378.810	-28.090	
1.379.012	-28.097	
1.379.216	-28.104	
1.379.312	-28.111	
1.379.442	-28.118	
1.379.612	-28.125	
1.379.742	-28.132	
1.379.881	-28.138	
1.380.096	-28.145	
1.380.311	-28.151	
1.380.461	-28.158	
1.380.573	-28.164	
1.380.710	-28.170	
1.380.876	-28.176	
1.381.015	-28.182	
1.381.183	-28.188	
1.381.375	-28.194	

1.381.530	-28.200	
1.381.689	-28.205	
1.381.866	-28.210	
1.382.081	-28.216	
1.382.290	-28.221	
1.382.350	-28.226	
1.382.411	-28.231	
1.382.507	-28.235	
1.382.626	-28.240	
1.382.811	-28.245	
1.382.975	-28.249	
1.383.127	-28.253	
1.383.266	-28.257	
1.383.432	-28.261	
1.383.645	-28.265	
1.383.819	-28.269	
1.383.974	-28.272	
1.384.209	-28.276	
1.384.442	-28.279	
1.384.621	-28.282	
1.384.789	-28.285	
1.384.980	-28.288	
1.385.148	-28.291	
1.385.262	-28.294	
1.385.390	-28.296	
1.385.546	-28.299	
1.385.694	-28.301	
1.385.858	-28.303	
1.385.995	-28.305	
1.386.059	-28.307	

1.386.198	-28.309	
1.386.427	-28.311	
1.386.608	-28.312	
1.386.806	-28.314	
1.386.991	-28.315	
1.387.128	-28.317	
1.387.289	-28.318	
1.387.498	-28.319	
1.387.719	-28.320	
1.387.874	-28.321	
1.387.971	-28.322	
1.388.145	-28.323	
1.388.376	-28.323	
1.388.533	-28.324	
1.388.687	-28.324	
1.388.846	-28.325	
1.388.979	-28.325	
1.389.090	-28.325	
1.389.236	-28.326	
1.389.413	-28.326	
1.389.566	-28.326	
1.389.720	-28.326	
1.389.856	-28.326	
1.390.004	-28.326	
1.390.188	-28.325	
1.390.365	-28.325	
1.390.524	-28.325	
1.390.744	-28.325	
1.390.992	-28.324	
1.391.158	-28.324	

1.391.230	-28.323	
1.391.308	-28.323	
1.391.478	-28.322	
1.391.658	-28.322	
1.391.776	-28.321	
1.391.902	-28.320	
1.392.081	-28.320	
1.392.309	-28.319	
1.392.504	-28.318	
1.392.673	-28.317	
1.392.888	-28.316	
1.392.952	-28.316	
1.393.060	-28.315	
1.393.255	-28.314	
1.393.421	-28.313	
1.393.616	-28.312	
1.393.768	-28.311	
1.394.043	-28.310	
1.394.429	-28.310	
1.394.809	-28.309	
1.394.984	-28.308	
1.395.005	-28.307	
1.395.125	-28.306	
1.395.197	-28.305	
1.395.186	-28.305	
1.395.233	-28.304	
1.395.390	-28.303	
1.395.522	-28.302	
1.395.645	-28.301	
1.395.829	-28.301	

1.396.019	-28.300	
1.396.221	-28.299	
1.396.335	-28.299	
1.396.441	-28.298	
1.396.608	-28.298	
1.396.788	-28.297	
1.397.036	-28.297	
1.397.164	-28.296	
1.397.307	-28.296	
1.397.543	-28.296	
1.397.661	-28.295	
1.397.796	-28.295	
1.397.961	-28.295	
1.398.062	-28.295	
1.398.190	-28.295	
1.398.367	-28.295	
1.398.493	-28.295	
1.398.580	-28.295	
1.398.727	-28.295	
1.398.929	-28.295	
1.399.097	-28.295	
1.399.263	-28.296	
1.399.453	-28.296	
1.399.610	-28.296	
1.399.821	-28.297	
1.400.031	-28.297	
1.400.186	-28.298	
1.400.343	-28.298	
1.400.495	-28.299	
1.400.607	-28.300	

1.400.804	-28.300	
1.401.042	-28.301	
1.401.158	-28.302	
1.401.270	-28.303	
1.401.402	-28.304	
1.401.519	-28.305	
1.401.707	-28.306	
1.401.895	-28.307	
1.402.076	-28.308	
1.402.233	-28.309	
1.402.383	-28.310	
1.402.578	-28.312	
1.402.717	-28.313	
1.402.858	-28.314	
1.403.069	-28.316	
1.403.183	-28.317	
1.403.302	-28.319	
1.403.488	-28.321	
1.403.667	-28.322	
1.403.848	-28.324	
1.403.965	-28.325	
1.404.052	-28.327	
1.404.270	-28.329	
1.404.469	-28.331	
1.404.621	-28.333	
1.404.774	-28.335	
1.404.930	-28.337	
1.405.098	-28.339	
1.405.257	-28.341	
1.405.421	-28.343	

1.405.567	-28.345	
1.405.719	-28.347	
1.405.820	-28.349	
1.405.947	-28.351	
1.406.207	-28.354	
1.406.447	-28.356	
1.406.582	-28.358	
1.406.741	-28.361	
1.406.897	-28.363	
1.407.009	-28.366	
1.407.195	-28.369	
1.407.363	-28.371	
1.407.453	-28.374	
1.407.601	-28.377	
1.407.757	-28.380	
1.407.896	-28.383	
1.407.977	-28.386	
1.408.073	-28.389	
1.408.311	-28.392	
1.408.521	-28.395	
1.408.642	-28.399	
1.408.799	-28.402	
1.408.938	-28.406	
1.409.084	-28.410	
1.409.346	-28.414	
1.409.534	-28.417	
1.409.695	-28.422	
1.409.883	-28.426	
1.410.058	-28.430	
1.410.244	-28.435	

1.410.459	-28.439	
1.410.623	-28.444	
1.410.759	-28.449	
1.410.936	-28.454	
1.411.062	-28.459	
1.411.208	-28.464	
1.411.382	-28.470	
1.411.449	-28.475	
1.411.557	-28.481	
1.411.721	-28.487	
1.411.864	-28.493	
1.412.029	-28.499	
1.412.141	-28.506	
1.412.301	-28.513	
1.412.475	-28.519	
1.412.619	-28.526	
1.412.829	-28.534	
1.413.036	-28.541	
1.413.237	-28.549	
1.413.418	-28.557	
1.413.559	-28.565	
1.413.741	-28.573	
1.413.934	-28.581	
1.414.081	-28.590	
1.414.223	-28.599	
1.414.406	-28.608	
1.414.577	-28.617	
1.414.733	-28.627	
1.414.904	-28.637	
1.415.042	-28.647	

1.415.145	-28.657	
1.415.320	-28.667	
1.415.497	-28.678	
1.415.697	-28.689	
1.415.809	-28.700	
1.415.871	-28.711	
1.416.113	-28.723	
1.416.322	-28.735	
1.416.566	-28.747	
1.416.738	-28.759	
1.416.893	-28.772	
1.417.047	-28.785	
1.417.135	-28.798	
1.417.296	-28.811	
1.417.406	-28.825	
1.417.542	-28.838	
1.417.747	-28.852	
1.417.966	-28.867	
1.418.154	-28.881	
1.418.309	-28.896	
1.418.468	-28.911	
1.418.557	-28.926	
1.418.625	-28.941	
1.418.773	-28.957	
1.418.987	-28.973	
1.419.109	-28.989	
1.419.305	-29.005	
1.419.733	-29.021	
1.420.134	-29.038	
1.420.313	-29.055	

1.420.376	-29.072	
1.420.459	-29.090	
1.420.537	-29.107	
1.420.600	-29.125	
1.420.699	-29.143	
1.420.788	-29.162	
1.420.882	-29.180	
1.421.021	-29.199	
1.421.149	-29.218	
1.421.263	-29.237	
1.421.422	-29.257	
1.421.588	-29.276	
1.421.740	-29.296	
1.421.942	-29.316	
1.422.133	-29.336	
1.422.247	-29.357	
1.422.401	-29.377	
1.422.612	-29.398	
1.422.798	-29.419	
1.422.955	-29.440	
1.423.121	-29.462	
1.423.257	-29.483	
1.423.313	-29.505	
1.423.447	-29.526	
1.423.631	-29.548	
1.423.835	-29.570	
1.423.985	-29.592	
1.424.035	-29.615	
1.424.164	-29.637	
1.424.324	-29.659	

1.424.511	-29.682	
1.424.767	-29.704	
1.424.955	-29.727	
1.425.112	-29.749	
1.425.305	-29.772	
1.425.453	-29.795	
1.425.567	-29.818	
1.425.695	-29.840	
1.425.838	-29.863	
1.425.984	-29.886	
1.426.136	-29.908	
1.426.326	-29.931	
1.426.534	-29.953	
1.426.655	-29.976	
1.426.734	-29.998	
1.426.841	-30.021	
1.426.954	-30.043	
1.427.123	-30.065	
1.427.368	-30.088	
1.427.570	-30.109	
1.427.686	-30.131	
1.427.858	-30.153	
1.427.990	-30.175	
1.428.145	-30.196	
1.428.329	-30.217	
1.428.425	-30.239	
1.428.530	-30.259	
1.428.739	-30.280	
1.428.907	-30.301	
1.429.014	-30.321	

1.429.200	-30.341	
1.429.442	-30.361	
1.429.619	-30.381	
1.429.781	-30.400	
1.429.948	-30.419	
1.430.098	-30.438	
1.430.320	-30.457	
1.430.509	-30.475	
1.430.600	-30.493	
1.430.668	-30.511	
1.430.871	-30.528	
1.431.097	-30.546	
1.431.245	-30.563	
1.431.400	-30.579	
1.431.554	-30.596	
1.431.758	-30.612	
1.431.949	-30.627	
1.432.121	-30.643	
1.432.256	-30.658	
1.432.406	-30.672	
1.432.549	-30.687	
1.432.697	-30.701	
1.432.798	-30.715	
1.432.935	-30.728	
1.433.107	-30.741	
1.433.214	-30.754	
1.433.356	-30.766	
1.433.557	-30.778	
1.433.734	-30.790	
1.433.817	-30.801	

1.433.904	-30.812	
1.433.994	-30.823	
1.434.135	-30.833	
1.434.380	-30.843	
1.434.612	-30.853	
1.434.805	-30.862	
1.434.991	-30.871	
1.435.235	-30.880	
1.435.484	-30.888	
1.435.629	-30.896	
1.435.759	-30.904	
1.435.956	-30.911	
1.436.059	-30.917	
1.436.174	-30.924	
1.436.315	-30.930	
1.436.451	-30.936	
1.436.584	-30.941	
1.436.700	-30.946	
1.436.826	-30.950	
1.437.000	-30.955	
1.437.146	-30.958	
1.437.337	-30.962	
1.437.498	-30.965	
1.437.650	-30.968	
1.437.863	-30.970	
1.438.102	-30.972	
1.438.277	-30.974	
1.438.378	-30.975	
1.438.539	-30.976	
1.438.777	-30.976	

1.438.965	-30.977	
1.439.104	-30.976	
1.439.245	-30.976	
1.439.415	-30.975	
1.439.565	-30.974	
1.439.749	-30.972	
1.439.978	-30.971	
1.440.085	-30.968	
1.440.170	-30.966	
1.440.367	-30.963	
1.440.567	-30.960	
1.440.748	-30.956	
1.440.882	-30.953	
1.441.019	-30.949	
1.441.189	-30.944	
1.441.310	-30.940	
1.441.492	-30.935	
1.441.725	-30.930	
1.441.882	-30.924	
1.441.947	-30.918	
1.442.079	-30.912	
1.442.305	-30.906	
1.442.467	-30.900	
1.442.614	-30.893	
1.442.778	-30.886	
1.442.970	-30.879	
1.443.192	-30.871	
1.443.347	-30.864	
1.443.468	-30.856	
1.443.618	-30.848	

1.443.734	-30.840	
1.443.875	-30.831	
1.444.008	-30.823	
1.444.140	-30.814	
1.444.328	-30.805	
1.444.588	-30.795	
1.444.995	-30.786	
1.445.412	-30.777	
1.445.621	-30.767	
1.445.695	-30.757	
1.445.764	-30.747	
1.445.804	-30.737	
1.445.831	-30.727	
1.445.889	-30.717	
1.445.898	-30.706	
1.446.008	-30.696	
1.446.205	-30.685	
1.446.362	-30.674	
1.446.557	-30.664	
1.446.787	-30.653	
1.446.902	-30.642	
1.446.980	-30.631	
1.447.200	-30.619	
1.447.426	-30.608	
1.447.641	-30.597	
1.447.813	-30.586	
1.447.888	-30.574	
1.447.999	-30.563	
1.448.143	-30.551	
1.448.338	-30.540	

1.448.456	-30.529	
1.448.633	-30.517	
1.448.871	-30.505	
1.449.050	-30.494	
1.449.205	-30.482	
1.449.339	-30.471	
1.449.433	-30.459	
1.449.545	-30.447	
1.449.677	-30.436	
1.449.881	-30.424	
1.450.164	-30.413	
1.450.365	-30.401	
1.450.455	-30.389	
1.450.674	-30.378	
1.450.862	-30.366	
1.451.033	-30.355	
1.451.171	-30.344	
1.451.236	-30.332	
1.451.414	-30.321	
1.451.624	-30.310	
1.451.756	-30.298	
1.451.875	-30.287	
1.452.081	-30.276	
1.452.220	-30.265	
1.452.292	-30.254	
1.452.453	-30.243	
1.452.619	-30.232	
1.452.722	-30.221	
1.452.935	-30.211	
1.453.141	-30.200	

1.453.251	-30.190	
1.453.398	-30.179	
1.453.548	-30.169	
1.453.723	-30.159	
1.453.900	-30.149	
1.454.048	-30.139	
1.454.153	-30.129	
1.454.272	-30.119	
1.454.500	-30.109	
1.454.781	-30.100	
1.455.051	-30.090	
1.455.191	-30.081	
1.455.305	-30.072	
1.455.426	-30.063	
1.455.569	-30.054	
1.455.744	-30.045	
1.455.858	-30.036	
1.455.995	-30.028	
1.456.183	-30.019	
1.456.440	-30.011	
1.456.613	-30.003	
1.456.721	-29.995	
1.456.843	-29.987	
1.456.956	-29.979	
1.457.103	-29.972	
1.457.299	-29.964	
1.457.509	-29.957	
1.457.623	-29.950	
1.457.735	-29.943	
1.457.912	-29.936	

1.458.100	-29.929	
1.458.257	-29.923	
1.458.400	-29.916	
1.458.549	-29.910	
1.458.667	-29.904	
1.458.831	-29.898	
1.459.034	-29.892	
1.459.048	-29.886	
1.459.072	-29.881	
1.459.283	-29.875	
1.459.512	-29.870	
1.459.704	-29.865	
1.459.884	-29.860	
1.460.018	-29.855	
1.460.195	-29.850	
1.460.513	-29.846	
1.460.741	-29.842	
1.460.788	-29.837	
1.460.948	-29.833	
1.461.129	-29.829	
1.461.283	-29.825	
1.461.458	-29.822	
1.461.651	-29.818	
1.461.828	-29.815	
1.461.960	-29.812	
1.462.025	-29.808	
1.462.141	-29.805	
1.462.374	-29.802	
1.462.552	-29.800	
1.462.686	-29.797	

1.462.849	-29.794	
1.462.930	-29.792	
1.463.029	-29.790	
1.463.208	-29.787	
1.463.421	-29.785	
1.463.656	-29.783	
1.463.860	-29.781	
1.464.032	-29.779	
1.464.144	-29.778	
1.464.328	-29.776	
1.464.489	-29.775	
1.464.633	-29.773	
1.464.778	-29.772	
1.464.924	-29.770	
1.465.116	-29.769	
1.465.313	-29.768	
1.465.443	-29.767	
1.465.607	-29.766	
1.465.739	-29.765	
1.465.849	-29.764	
1.466.028	-29.763	
1.466.189	-29.763	
1.466.344	-29.762	
1.466.507	-29.761	
1.466.711	-29.761	
1.466.880	-29.760	
1.466.980	-29.760	
1.467.142	-29.759	
1.467.415	-29.759	
1.467.628	-29.758	

1.467.737	-29.758	
1.467.852	-29.758	
1.467.941	-29.757	
1.468.102	-29.757	
1.468.333	-29.756	
1.468.466	-29.756	
1.468.658	-29.756	
1.468.772	-29.756	
1.468.922	-29.755	
1.469.072	-29.755	
1.469.310	-29.755	
1.469.516	-29.754	
1.469.660	-29.754	
1.469.751	-29.754	
1.469.863	-29.753	
1.470.222	-29.753	
1.470.667	-29.752	
1.470.865	-29.752	
1.470.999	-29.751	
1.471.145	-29.751	
1.471.279	-29.750	
1.471.364	-29.750	
1.471.335	-29.749	
1.471.377	-29.749	
1.471.510	-29.748	
1.471.622	-29.748	
1.471.819	-29.747	
1.471.973	-29.746	
1.472.090	-29.745	
1.472.222	-29.744	

1.472.401	-29.744	
1.472.571	-29.743	
1.472.737	-29.742	
1.472.972	-29.741	
1.473.192	-29.740	
1.473.335	-29.738	
1.473.461	-29.737	
1.473.584	-29.736	
1.473.716	-29.735	
1.473.875	-29.733	
1.474.084	-29.732	
1.474.308	-29.731	
1.474.413	-29.729	
1.474.498	-29.728	
1.474.606	-29.726	
1.474.711	-29.724	
1.474.874	-29.723	
1.475.045	-29.721	
1.475.233	-29.719	
1.475.401	-29.717	
1.475.580	-29.715	
1.475.786	-29.713	
1.475.992	-29.711	
1.476.232	-29.709	
1.476.465	-29.707	
1.476.586	-29.705	
1.476.669	-29.703	
1.476.799	-29.700	
1.476.989	-29.698	
1.477.130	-29.696	

1.477.242	-29.693	
1.477.410	-29.691	
1.477.529	-29.688	
1.477.648	-29.685	
1.477.816	-29.683	
1.477.995	-29.680	
1.478.170	-29.677	
1.478.284	-29.674	
1.478.461	-29.672	
1.478.721	-29.669	
1.478.925	-29.666	
1.479.028	-29.663	
1.479.166	-29.660	
1.479.370	-29.657	
1.479.471	-29.654	
1.479.583	-29.650	
1.479.753	-29.647	
1.479.843	-29.644	
1.480.022	-29.641	
1.480.240	-29.637	
1.480.421	-29.634	
1.480.560	-29.631	
1.480.674	-29.627	
1.480.914	-29.624	
1.481.171	-29.620	
1.481.329	-29.617	
1.481.432	-29.613	
1.481.561	-29.610	
1.481.671	-29.606	
1.481.823	-29.603	

1.482.054	-29.599	
1.482.186	-29.595	
1.482.278	-29.592	
1.482.493	-29.588	
1.482.728	-29.584	
1.482.854	-29.580	
1.483.000	-29.577	
1.483.147	-29.573	
1.483.316	-29.569	
1.483.450	-29.565	
1.483.571	-29.561	
1.483.757	-29.557	
1.483.900	-29.553	
1.483.983	-29.550	
1.484.151	-29.546	
1.484.343	-29.542	
1.484.447	-29.538	
1.484.601	-29.534	
1.484.794	-29.530	
1.484.924	-29.526	
1.485.014	-29.522	
1.485.206	-29.518	
1.485.493	-29.514	
1.485.670	-29.510	
1.485.869	-29.506	
1.486.084	-29.502	
1.486.250	-29.498	
1.486.431	-29.494	
1.486.608	-29.490	
1.486.767	-29.486	

1.486.899	-29.482	
1.487.057	-29.478	
1.487.254	-29.474	
1.487.372	-29.470	
1.487.467	-29.466	
1.487.610	-29.462	
1.487.726	-29.458	
1.487.838	-29.454	
1.488.026	-29.450	
1.488.219	-29.446	
1.488.353	-29.442	
1.488.481	-29.438	
1.488.678	-29.434	
1.488.896	-29.430	
1.489.109	-29.426	
1.489.301	-29.422	
1.489.435	-29.418	
1.489.592	-29.415	
1.489.738	-29.411	
1.489.872	-29.407	
1.490.013	-29.403	
1.490.197	-29.399	
1.490.370	-29.395	
1.490.535	-29.391	
1.490.685	-29.388	
1.490.847	-29.384	
1.491.048	-29.380	
1.491.217	-29.376	
1.491.340	-29.373	
1.491.516	-29.369	

1.491.646	-29.365	
1.491.821	-29.362	
1.492.072	-29.358	
1.492.253	-29.354	
1.492.384	-29.351	
1.492.518	-29.347	
1.492.760	-29.343	
1.492.894	-29.340	
1.492.970	-29.336	
1.493.096	-29.333	
1.493.252	-29.329	
1.493.436	-29.326	
1.493.609	-29.322	
1.493.801	-29.319	
1.493.940	-29.315	
1.494.111	-29.312	
1.494.332	-29.309	
1.494.521	-29.305	
1.494.718	-29.302	
1.494.883	-29.299	
1.494.980	-29.295	
1.495.114	-29.292	
1.495.260	-29.289	
1.495.416	-29.285	
1.495.710	-29.282	
1.496.059	-29.279	
1.496.272	-29.276	
1.496.445	-29.273	
1.496.608	-29.269	
1.496.716	-29.266	

1.496.752	-29.263	
1.496.759	-29.260	
1.496.870	-29.257	
1.496.987	-29.254	
1.497.076	-29.251	
1.497.155	-29.247	
1.497.366	-29.244	
1.497.610	-29.241	
1.497.733	-29.238	
1.497.865	-29.235	
1.498.071	-29.232	
1.498.279	-29.229	
1.498.454	-29.226	
1.498.624	-29.223	
1.498.784	-29.220	
1.499.007	-29.217	
1.499.140	-29.215	
1.499.231	-29.212	
1.499.274	-29.209	
1.499.458	-29.206	
1.499.767	-29.203	
1.499.944	-29.200	
1.500.047	-29.197	
1.500.166	-29.195	
1.500.316	-29.192	
1.500.435	-29.189	
1.500.614	-29.186	
1.500.826	-29.183	
1.500.927	-29.181	
1.501.062	-29.178	

1.501.223	-29.175	
1.501.449	-29.172	
1.501.669	-29.170	
1.501.833	-29.167	
1.501.983	-29.164	
1.502.132	-29.162	
1.502.292	-29.159	
1.502.444	-29.156	
1.502.608	-29.154	
1.502.766	-29.151	
1.502.924	-29.148	
1.503.074	-29.146	
1.503.129	-29.143	
1.503.251	-29.140	
1.503.465	-29.138	
1.503.667	-29.135	
1.503.817	-29.132	
1.504.010	-29.130	
1.504.214	-29.127	
1.504.361	-29.124	
1.504.474	-29.122	
1.504.548	-29.119	
1.504.760	-29.116	
1.504.960	-29.114	
1.505.054	-29.111	
1.505.206	-29.108	
1.505.410	-29.106	
1.505.596	-29.103	
1.505.728	-29.100	
1.505.912	-29.097	

1.506.116	-29.095	
1.506.281	-29.092	
1.506.469	-29.089	
1.506.617	-29.087	
1.506.785	-29.084	
1.506.812	-29.081	
1.506.976	-29.078	
1.507.244	-29.076	
1.507.428	-29.073	
1.507.569	-29.070	
1.507.731	-29.067	
1.507.914	-29.064	
1.507.999	-29.062	
1.508.158	-29.059	
1.508.367	-29.056	
1.508.560	-29.053	
1.508.708	-29.050	
1.508.893	-29.047	
1.508.983	-29.044	
1.509.129	-29.041	
1.509.337	-29.038	
1.509.503	-29.035	
1.509.655	-29.032	
1.509.780	-29.029	
1.509.911	-29.026	
1.510.036	-29.023	
1.510.186	-29.020	
1.510.331	-29.017	
1.510.423	-29.014	
1.510.583	-29.011	

1.510.806	-29.007	
1.511.024	-29.004	
1.511.169	-29.001	
1.511.386	-28.998	
1.511.566	-28.995	
1.511.676	-28.992	
1.511.821	-28.988	
1.512.065	-28.985	
1.512.309	-28.982	
1.512.406	-28.979	
1.512.536	-28.975	
1.512.719	-28.972	
1.512.823	-28.969	
1.512.917	-28.966	
1.513.076	-28.962	
1.513.201	-28.959	
1.513.382	-28.956	
1.513.649	-28.953	
1.513.772	-28.949	
1.513.860	-28.946	
1.514.021	-28.943	
1.514.198	-28.939	
1.514.382	-28.936	
1.514.512	-28.933	
1.514.742	-28.929	
1.514.986	-28.926	
1.515.164	-28.923	
1.515.305	-28.920	
1.515.493	-28.916	
1.515.688	-28.913	

1.515.815	-28.910	
1.515.916	-28.906	
1.516.048	-28.903	
1.516.203	-28.900	
1.516.375	-28.897	
1.516.559	-28.893	
1.516.758	-28.890	
1.516.942	-28.887	
1.517.117	-28.884	
1.517.242	-28.880	
1.517.330	-28.877	
1.517.484	-28.874	
1.517.677	-28.871	
1.517.881	-28.868	
1.518.065	-28.865	
1.518.219	-28.861	
1.518.309	-28.858	
1.518.483	-28.855	
1.518.656	-28.852	
1.518.808	-28.849	
1.518.960	-28.846	
1.519.111	-28.843	
1.519.283	-28.840	
1.519.465	-28.837	
1.519.633	-28.834	
1.519.783	-28.831	
1.519.926	-28.828	
1.520.152	-28.825	
1.520.275	-28.822	
1.520.350	-28.819	

1.520.536	-28.817	
1.520.752	-28.814	
1.520.862	-28.811	
1.521.171	-28.808	
1.521.604	-28.805	
1.521.801	-28.803	
1.521.942	-28.800	
1.522.124	-28.797	
1.522.247	-28.795	
1.522.300	-28.792	
1.522.247	-28.789	
1.522.278	-28.787	
1.522.413	-28.784	
1.522.645	-28.782	
1.522.881	-28.779	
1.522.959	-28.777	
1.523.051	-28.774	
1.523.224	-28.772	
1.523.450	-28.770	
1.523.604	-28.767	
1.523.714	-28.765	
1.523.936	-28.763	
1.524.138	-28.761	
1.524.317	-28.759	
1.524.505	-28.756	
1.524.604	-28.754	
1.524.682	-28.752	
1.524.895	-28.750	
1.525.024	-28.748	
1.525.139	-28.747	

1.525.367	-28.745	
1.525.549	-28.743	
1.525.663	-28.741	
1.525.757	-28.739	
1.525.938	-28.738	
1.526.125	-28.736	
1.526.277	-28.734	
1.526.440	-28.733	
1.526.577	-28.731	
1.526.768	-28.730	
1.526.971	-28.728	
1.527.204	-28.727	
1.527.393	-28.726	
1.527.464	-28.724	
1.527.592	-28.723	
1.527.755	-28.722	
1.527.905	-28.721	
1.528.067	-28.720	
1.528.224	-28.719	
1.528.394	-28.718	
1.528.521	-28.717	
1.528.651	-28.716	
1.528.837	-28.715	
1.528.985	-28.714	
1.529.129	-28.713	
1.529.337	-28.712	
1.529.480	-28.712	
1.529.581	-28.711	
1.529.678	-28.710	
1.529.890	-28.710	

1.530.163	-28.709	
1.530.289	-28.709	
1.530.399	-28.708	
1.530.594	-28.708	
1.530.715	-28.708	
1.530.833	-28.707	
1.531.044	-28.707	
1.531.221	-28.707	
1.531.400	-28.707	
1.531.624	-28.707	
1.531.788	-28.707	
1.531.933	-28.707	
1.532.155	-28.707	
1.532.372	-28.707	
1.532.493	-28.707	
1.532.538	-28.707	
1.532.692	-28.707	
1.532.948	-28.707	
1.533.132	-28.707	
1.533.244	-28.708	
1.533.340	-28.708	
1.533.501	-28.708	
1.533.698	-28.709	
1.533.853	-28.709	
1.534.001	-28.710	
1.534.149	-28.710	
1.534.277	-28.710	
1.534.473	-28.711	
1.534.668	-28.712	
1.534.805	-28.712	

1.534.922	-28.713	
1.535.024	-28.714	
1.535.201	-28.714	
1.535.323	-28.715	
1.535.425	-28.716	
1.535.587	-28.717	
1.535.746	-28.717	
1.535.918	-28.718	
1.536.113	-28.719	
1.536.306	-28.720	
1.536.501	-28.721	
1.536.709	-28.722	
1.536.913	-28.723	
1.537.097	-28.724	
1.537.243	-28.725	
1.537.393	-28.726	
1.537.505	-28.728	
1.537.683	-28.729	
1.537.878	-28.730	
1.538.019	-28.731	
1.538.185	-28.732	
1.538.374	-28.734	
1.538.454	-28.735	
1.538.490	-28.736	
1.538.678	-28.738	
1.538.893	-28.739	
1.539.016	-28.741	
1.539.173	-28.742	
1.539.373	-28.744	
1.539.552	-28.745	

1.539.776	-28.747	
1.539.993	-28.748	
1.540.157	-28.750	
1.540.264	-28.752	
1.540.424	-28.753	
1.540.627	-28.755	
1.540.804	-28.757	
1.540.961	-28.758	
1.541.107	-28.760	
1.541.268	-28.762	
1.541.396	-28.763	
1.541.496	-28.765	
1.541.660	-28.767	
1.541.890	-28.769	
1.542.132	-28.771	
1.542.290	-28.773	
1.542.417	-28.775	
1.542.527	-28.777	
1.542.605	-28.778	
1.542.742	-28.780	
1.542.968	-28.782	
1.543.194	-28.784	
1.543.313	-28.786	
1.543.452	-28.788	
1.543.696	-28.790	
1.543.833	-28.792	
1.543.933	-28.795	
1.544.140	-28.797	
1.544.341	-28.799	
1.544.487	-28.801	

1.544.621	-28.803	
1.544.736	-28.805	
1.544.846	-28.807	
1.545.009	-28.809	
1.545.276	-28.812	
1.545.540	-28.814	
1.545.674	-28.816	
1.545.771	-28.818	
1.545.916	-28.820	
1.546.062	-28.823	
1.546.187	-28.825	
1.546.337	-28.827	
1.546.664	-28.829	
1.547.135	-28.832	
1.547.379	-28.834	
1.547.495	-28.836	
1.547.699	-28.839	
1.547.794	-28.841	
1.547.785	-28.843	
1.547.832	-28.846	
1.547.924	-28.848	
1.548.044	-28.850	
1.548.190	-28.853	
1.548.336	-28.855	
1.548.512	-28.857	
1.548.645	-28.860	
1.548.734	-28.862	
1.548.896	-28.864	
1.549.111	-28.867	
1.549.303	-28.869	

1.549.518	-28.872	
1.549.688	-28.874	
1.549.839	-28.876	
1.550.011	-28.879	
1.550.107	-28.881	
1.550.208	-28.884	
1.550.374	-28.886	
1.550.535	-28.889	
1.550.656	-28.891	
1.550.793	-28.894	
1.551.010	-28.896	
1.551.162	-28.899	
1.551.264	-28.901	
1.551.418	-28.904	
1.551.586	-28.906	
1.551.776	-28.909	
1.551.938	-28.911	
1.552.141	-28.914	
1.552.334	-28.916	
1.552.471	-28.919	
1.552.672	-28.921	
1.552.887	-28.924	
1.553.024	-28.926	
1.553.141	-28.929	
1.553.324	-28.932	
1.553.479	-28.934	
1.553.566	-28.937	
1.553.741	-28.939	
1.553.902	-28.942	
1.554.025	-28.944	

1.554.211	-28.947	
1.554.321	-28.950	
1.554.469	-28.952	
1.554.668	-28.955	
1.554.861	-28.957	
1.555.013	-28.960	
1.555.186	-28.962	
1.555.374	-28.965	
1.555.486	-28.968	
1.555.573	-28.970	
1.555.737	-28.973	
1.555.865	-28.975	
1.556.026	-28.978	
1.556.171	-28.981	
1.556.310	-28.983	
1.556.503	-28.986	
1.556.684	-28.988	
1.556.819	-28.991	
1.557.005	-28.993	
1.557.236	-28.996	
1.557.408	-28.999	
1.557.571	-29.001	
1.557.713	-29.004	
1.557.840	-29.006	
1.557.948	-29.009	
1.558.141	-29.011	
1.558.354	-29.014	
1.558.484	-29.016	
1.558.631	-29.019	
1.558.828	-29.021	

1.558.998	-29.024	
1.559.162	-29.026	
1.559.301	-29.029	
1.559.451	-29.031	
1.559.630	-29.034	
1.559.780	-29.036	
1.559.940	-29.038	
1.560.054	-29.041	
1.560.087	-29.043	
1.560.224	-29.046	
1.560.513	-29.048	
1.560.670	-29.050	
1.560.826	-29.053	
1.560.909	-29.055	
1.561.050	-29.057	
1.561.243	-29.060	
1.561.364	-29.062	
1.561.525	-29.064	
1.561.740	-29.066	
1.561.908	-29.069	
1.562.113	-29.071	
1.562.354	-29.073	
1.562.610	-29.075	
1.562.793	-29.077	
1.562.888	-29.080	
1.563.047	-29.082	
1.563.221	-29.084	
1.563.405	-29.086	
1.563.582	-29.088	
1.563.732	-29.090	

1.563.839	-29.092	
1.563.923	-29.094	
1.564.025	-29.096	
1.564.120	-29.098	
1.564.308	-29.100	
1.564.467	-29.102	
1.564.621	-29.104	
1.564.810	-29.106	
1.564.998	-29.108	
1.565.159	-29.110	
1.565.340	-29.112	
1.565.506	-29.114	
1.565.603	-29.116	
1.565.735	-29.117	
1.565.981	-29.119	
1.566.221	-29.121	
1.566.366	-29.123	
1.566.488	-29.125	
1.566.662	-29.126	
1.566.810	-29.128	
1.566.971	-29.130	
1.567.108	-29.132	
1.567.253	-29.133	
1.567.446	-29.135	
1.567.650	-29.137	
1.567.840	-29.138	
1.567.970	-29.140	
1.568.192	-29.142	
1.568.383	-29.143	
1.568.515	-29.145	

1.568.698	-29.146	
1.568.804	-29.148	
1.568.922	-29.149	
1.569.106	-29.151	
1.569.294	-29.153	
1.569.480	-29.154	
1.569.635	-29.156	
1.569.792	-29.157	
1.569.962	-29.159	
1.570.121	-29.160	
1.570.304	-29.161	
1.570.437	-29.163	
1.570.544	-29.164	
1.570.739	-29.166	
1.570.920	-29.167	
1.571.064	-29.168	
1.571.239	-29.170	
1.571.454	-29.171	
1.571.633	-29.172	
1.571.664	-29.174	
1.571.758	-29.175	
1.572.162	-29.176	
1.572.511	-29.178	
1.572.724	-29.179	
1.572.872	-29.180	
1.572.968	-29.181	
1.573.089	-29.182	
1.573.116	-29.184	
1.573.116	-29.185	
1.573.230	-29.186	

1.573.362	-29.187	
1.573.483	-29.188	
1.573.595	-29.189	
1.573.786	-29.191	
1.573.920	-29.192	
1.574.086	-29.193	
1.574.330	-29.194	
1.574.550	-29.195	
1.574.657	-29.196	
1.574.846	-29.197	
1.575.056	-29.198	
1.575.157	-29.199	
1.575.327	-29.200	
1.575.517	-29.201	
1.575.676	-29.202	
1.575.826	-29.203	
1.575.994	-29.204	
1.576.189	-29.205	
1.576.393	-29.205	
1.576.543	-29.206	
1.576.653	-29.207	
1.576.738	-29.208	
1.576.848	-29.209	
1.577.072	-29.210	
1.577.278	-29.211	
1.577.430	-29.211	
1.577.617	-29.212	
1.577.854	-29.213	
1.578.053	-29.214	
1.578.201	-29.214	

1.578.378	-29.215	
1.578.535	-29.216	
1.578.674	-29.217	
1.578.817	-29.217	
1.578.967	-29.218	
1.579.090	-29.219	
1.579.196	-29.219	
1.579.350	-29.220	
1.579.507	-29.221	
1.579.660	-29.221	
1.579.825	-29.222	
1.580.002	-29.222	
1.580.141	-29.223	
1.580.295	-29.223	
1.580.484	-29.224	
1.580.598	-29.225	
1.580.743	-29.225	
1.580.930	-29.226	
1.581.068	-29.226	
1.581.174	-29.227	
1.581.339	-29.227	
1.581.584	-29.227	
1.581.785	-29.228	
1.581.946	-29.228	
1.582.113	-29.229	
1.582.300	-29.229	
1.582.486	-29.229	
1.582.592	-29.230	
1.582.747	-29.230	
1.582.946	-29.230	

1.583.067	-29.231	
1.583.192	-29.231	
1.583.398	-29.231	
1.583.656	-29.231	
1.583.876	-29.232	
1.583.972	-29.232	
1.584.034	-29.232	
1.584.218	-29.232	
1.584.437	-29.232	
1.584.632	-29.232	
1.584.809	-29.232	
1.584.973	-29.232	
1.585.136	-29.232	
1.585.303	-29.232	
1.585.448	-29.232	
1.585.502	-29.232	
1.585.674	-29.232	
1.585.876	-29.232	
1.585.976	-29.232	
1.586.111	-29.232	
1.586.268	-29.232	
1.586.436	-29.231	
1.586.555	-29.231	
1.586.673	-29.231	
1.586.819	-29.230	
1.586.927	-29.230	
1.587.094	-29.230	
1.587.330	-29.229	
1.587.542	-29.229	
1.587.764	-29.229	

1.587.941	-29.228	
1.588.105	-29.228	
1.588.289	-29.227	
1.588.461	-29.227	
1.588.661	-29.226	
1.588.839	-29.226	
1.588.958	-29.225	
1.589.050	-29.224	
1.589.173	-29.224	
1.589.332	-29.223	
1.589.534	-29.222	
1.589.668	-29.222	
1.589.819	-29.221	
1.590.002	-29.220	
1.590.146	-29.219	
1.590.336	-29.218	
1.590.508	-29.218	
1.590.697	-29.217	
1.590.898	-29.216	
1.591.053	-29.215	
1.591.144	-29.214	
1.591.335	-29.213	
1.591.512	-29.212	
1.591.647	-29.211	
1.591.783	-29.210	
1.591.956	-29.209	
1.592.126	-29.208	
1.592.245	-29.207	
1.592.421	-29.206	
1.592.677	-29.205	

1.592.881	-29.204	
1.593.022	-29.203	
1.593.143	-29.202	
1.593.313	-29.201	
1.593.457	-29.199	
1.593.629	-29.198	
1.593.801	-29.197	
1.593.967	-29.196	
1.594.169	-29.195	
1.594.344	-29.194	
1.594.510	-29.192	
1.594.675	-29.191	
1.594.805	-29.190	
1.594.919	-29.189	
1.595.052	-29.187	
1.595.190	-29.186	
1.595.381	-29.185	
1.595.620	-29.184	
1.595.795	-29.182	
1.595.898	-29.181	
1.596.015	-29.180	
1.596.194	-29.178	
1.596.368	-29.177	
1.596.559	-29.176	
1.596.711	-29.174	
1.596.868	-29.173	
1.597.043	-29.172	
1.597.081	-29.171	
1.597.247	-29.169	
1.597.708	-29.168	

1.598.130	-29.167	
1.598.329	-29.165	
1.598.466	-29.164	
1.598.663	-29.163	
1.598.698	-29.162	
1.598.566	-29.160	
1.598.672	-29.159	
1.598.876	-29.158	
1.598.963	-29.157	
1.599.099	-29.155	
1.599.259	-29.154	
1.599.397	-29.153	
1.599.541	-29.152	
1.599.711	-29.151	
1.599.949	-29.150	
1.600.157	-29.148	
1.600.294	-29.147	
1.600.434	-29.146	
1.600.607	-29.145	
1.600.856	-29.144	
1.601.053	-29.143	
1.601.104	-29.142	
1.601.187	-29.141	
1.601.434	-29.140	
1.601.655	-29.139	
1.601.745	-29.139	
1.601.848	-29.138	
1.602.014	-29.137	
1.602.092	-29.136	
1.602.202	-29.135	

1.602.372	-29.135	
1.602.598	-29.134	
1.602.749	-29.133	
1.602.912	-29.133	
1.603.096	-29.132	
1.603.320	-29.132	
1.603.562	-29.131	
1.603.685	-29.131	
1.603.835	-29.130	
1.604.068	-29.130	
1.604.212	-29.130	
1.604.294	-29.129	
1.604.436	-29.129	
1.604.623	-29.129	
1.604.792	-29.129	
1.604.930	-29.129	
1.605.067	-29.129	
1.605.253	-29.128	
1.605.423	-29.128	
1.605.544	-29.128	
1.605.730	-29.129	
1.605.923	-29.129	
1.606.089	-29.129	
1.606.246	-29.129	
1.606.386	-29.129	
1.606.525	-29.129	
1.606.656	-29.130	
1.606.853	-29.130	
1.606.998	-29.131	
1.607.105	-29.131	

1.607.314	-29.131	
1.607.551	-29.132	
1.607.692	-29.133	
1.607.832	-29.133	
1.608.008	-29.134	
1.608.183	-29.134	
1.608.360	-29.135	
1.608.537	-29.136	
1.608.676	-29.137	
1.608.817	-29.138	
1.609.005	-29.138	
1.609.175	-29.139	
1.609.303	-29.140	
1.609.442	-29.141	
1.609.585	-29.142	
1.609.751	-29.143	
1.609.955	-29.144	
1.610.146	-29.145	
1.610.343	-29.146	
1.610.493	-29.148	
1.610.621	-29.149	
1.610.768	-29.150	
1.610.916	-29.151	
1.611.084	-29.152	
1.611.216	-29.154	
1.611.306	-29.155	
1.611.456	-29.156	
1.611.604	-29.158	
1.611.700	-29.159	
1.611.897	-29.161	

1.612.097	-29.162	
1.612.184	-29.164	
1.612.280	-29.165	
1.612.437	-29.167	
1.612.648	-29.168	
1.612.921	-29.170	
1.613.167	-29.171	
1.613.391	-29.173	
1.613.640	-29.175	
1.613.810	-29.176	
1.613.884	-29.178	
1.614.091	-29.179	
1.614.314	-29.181	
1.614.393	-29.183	
1.614.561	-29.184	
1.614.731	-29.186	
1.614.852	-29.188	
1.614.948	-29.189	
1.615.061	-29.191	
1.615.193	-29.193	
1.615.369	-29.195	
1.615.605	-29.196	
1.615.784	-29.198	
1.615.925	-29.200	
1.616.064	-29.201	
1.616.212	-29.203	
1.616.427	-29.205	
1.616.619	-29.206	
1.616.765	-29.208	
1.616.954	-29.210	

1.617.099	-29.211	
1.617.271	-29.213	
1.617.457	-29.215	
1.617.610	-29.217	
1.617.742	-29.218	
1.617.930	-29.220	
1.618.078	-29.221	
1.618.165	-29.223	
1.618.363	-29.225	
1.618.586	-29.226	
1.618.709	-29.228	
1.618.844	-29.229	
1.618.988	-29.231	
1.619.196	-29.232	
1.619.386	-29.234	
1.619.518	-29.235	
1.619.684	-29.237	
1.619.874	-29.238	
1.620.022	-29.240	
1.620.159	-29.241	
1.620.352	-29.243	
1.620.546	-29.244	
1.620.706	-29.245	
1.620.795	-29.247	
1.620.903	-29.248	
1.621.127	-29.249	
1.621.313	-29.251	
1.621.391	-29.252	
1.621.613	-29.253	
1.621.861	-29.254	

1.622.021	-29.256	
1.622.166	-29.257	
1.622.301	-29.258	
1.622.500	-29.259	
1.622.641	-29.260	
1.622.747	-29.261	
1.623.046	-29.262	
1.623.483	-29.263	
1.623.799	-29.264	
1.624.001	-29.265	
1.624.088	-29.266	
1.624.133	-29.267	
1.624.131	-29.268	
1.624.148	-29.269	
1.624.243	-29.270	
1.624.395	-29.271	
1.624.510	-29.272	
1.624.650	-29.273	
1.624.773	-29.273	
1.624.953	-29.274	
1.625.201	-29.275	
1.625.311	-29.276	
1.625.424	-29.277	
1.625.636	-29.277	
1.625.847	-29.278	
1.625.983	-29.279	
1.626.144	-29.279	
1.626.290	-29.280	
1.626.422	-29.281	
1.626.602	-29.281	

1.626.812	-29.282	
1.626.976	-29.283	
1.627.101	-29.283	
1.627.229	-29.284	
1.627.345	-29.285	
1.627.554	-29.285	
1.627.762	-29.286	
1.627.832	-29.286	
1.627.981	-29.287	
1.628.232	-29.288	
1.628.418	-29.288	
1.628.591	-29.289	
1.628.752	-29.289	
1.628.907	-29.290	
1.629.124	-29.290	
1.629.373	-29.291	
1.629.547	-29.291	
1.629.648	-29.292	
1.629.738	-29.292	
1.629.884	-29.293	
1.630.105	-29.294	
1.630.295	-29.294	
1.630.419	-29.295	
1.630.528	-29.295	
1.630.703	-29.296	
1.630.919	-29.296	
1.631.075	-29.297	
1.631.176	-29.297	
1.631.330	-29.298	
1.631.541	-29.299	

1.631.702	-29.299	
1.631.790	-29.300	
1.631.953	-29.300	
1.632.130	-29.301	
1.632.281	-29.301	
1.632.411	-29.302	
1.632.556	-29.303	
1.632.677	-29.303	
1.632.825	-29.304	
1.633.082	-29.305	
1.633.288	-29.305	
1.633.501	-29.306	
1.633.689	-29.306	
1.633.835	-29.307	
1.634.016	-29.308	
1.634.182	-29.309	
1.634.355	-29.309	
1.634.465	-29.310	
1.634.574	-29.311	
1.634.742	-29.311	
1.634.861	-29.312	
1.634.977	-29.313	
1.635.118	-29.313	
1.635.302	-29.314	
1.635.513	-29.315	
1.635.715	-29.316	
1.635.959	-29.316	
1.636.136	-29.317	
1.636.221	-29.318	
1.636.288	-29.319	

1.636.458	-29.320	
1.636.579	-29.320	
1.636.727	-29.321	
1.636.987	-29.322	
1.637.159	-29.323	
1.637.256	-29.324	
1.637.343	-29.324	
1.637.446	-29.325	
1.637.663	-29.326	
1.637.881	-29.327	
1.637.988	-29.328	
1.638.098	-29.329	
1.638.320	-29.329	
1.638.571	-29.330	
1.638.810	-29.331	
1.638.996	-29.332	
1.639.194	-29.333	
1.639.399	-29.334	
1.639.514	-29.335	
1.639.632	-29.336	
1.639.827	-29.336	
1.640.034	-29.337	
1.640.166	-29.338	
1.640.210	-29.339	
1.640.293	-29.340	
1.640.515	-29.341	
1.640.706	-29.342	
1.640.726	-29.343	
1.640.862	-29.344	
1.641.158	-29.344	

1.641.340	-29.345	
1.641.395	-29.346	
1.641.539	-29.347	
1.641.776	-29.348	
1.641.982	-29.349	
1.642.226	-29.350	
1.642.439	-29.351	
1.642.565	-29.352	
1.642.668	-29.353	
1.642.836	-29.353	
1.642.993	-29.354	
1.643.141	-29.355	
1.643.372	-29.356	
1.643.555	-29.357	
1.643.696	-29.358	
1.643.821	-29.359	
1.643.949	-29.360	
1.644.167	-29.361	
1.644.359	-29.361	
1.644.464	-29.362	
1.644.675	-29.363	
1.644.879	-29.364	
1.644.984	-29.365	
1.645.190	-29.366	
1.645.406	-29.367	
1.645.582	-29.368	
1.645.784	-29.369	
1.645.910	-29.369	
1.645.981	-29.370	
1.646.147	-29.371	

1.646.364	-29.372	
1.646.510	-29.373	
1.646.656	-29.374	
1.646.839	-29.375	
1.646.935	-29.376	
1.647.079	-29.376	
1.647.260	-29.377	
1.647.464	-29.378	
1.647.654	-29.379	
1.647.782	-29.380	
1.647.968	-29.381	
1.648.055	-29.382	
1.648.112	-29.383	
1.648.425	-29.383	
1.648.880	-29.384	
1.649.220	-29.385	
1.649.382	-29.386	
1.649.406	-29.387	
1.649.509	-29.388	
1.649.624	-29.389	
1.649.639	-29.390	
1.649.700	-29.390	
1.649.818	-29.391	
1.649.946	-29.392	
1.650.103	-29.393	
1.650.278	-29.394	
1.650.428	-29.395	
1.650.587	-29.396	
1.650.775	-29.397	
1.650.941	-29.398	

1.651.120	-29.398	
1.651.299	-29.399	
1.651.456	-29.400	
1.651.590	-29.401	
1.651.797	-29.402	
1.651.991	-29.403	
1.652.092	-29.404	
1.652.251	-29.405	
1.652.468	-29.406	
1.652.666	-29.407	
1.652.809	-29.408	
1.652.964	-29.409	
1.653.085	-29.410	
1.653.214	-29.411	
1.653.419	-29.412	
1.653.611	-29.413	
1.653.777	-29.415	
1.653.902	-29.416	
1.654.048	-29.417	
1.654.259	-29.418	
1.654.474	-29.419	
1.654.644	-29.420	
1.654.780	-29.422	
1.654.960	-29.423	
1.655.134	-29.424	
1.655.271	-29.425	
1.655.425	-29.426	
1.655.598	-29.428	
1.655.701	-29.429	
1.655.788	-29.430	

1.655.947	-29.432	
1.656.116	-29.433	
1.656.337	-29.434	
1.656.532	-29.436	
1.656.642	-29.437	
1.656.801	-29.439	
1.656.964	-29.440	
1.657.119	-29.442	
1.657.309	-29.443	
1.657.486	-29.445	
1.657.627	-29.446	
1.657.755	-29.448	
1.657.878	-29.449	
1.657.999	-29.451	
1.658.156	-29.452	
1.658.345	-29.454	
1.658.578	-29.456	
1.658.741	-29.457	
1.658.880	-29.459	
1.659.095	-29.461	
1.659.283	-29.462	
1.659.408	-29.464	
1.659.576	-29.466	
1.659.780	-29.467	
1.659.924	-29.469	
1.660.105	-29.471	
1.660.244	-29.473	
1.660.347	-29.474	
1.660.515	-29.476	
1.660.670	-29.478	

1.660.869	-29.480	
1.661.060	-29.482	
1.661.167	-29.483	
1.661.319	-29.485	
1.661.554	-29.487	
1.661.682	-29.489	
1.661.821	-29.491	
1.662.038	-29.493	
1.662.235	-29.495	
1.662.370	-29.496	
1.662.428	-29.498	
1.662.529	-29.500	
1.662.691	-29.502	
1.662.847	-29.504	
1.663.051	-29.506	
1.663.214	-29.508	
1.663.327	-29.510	
1.663.392	-29.512	
1.663.541	-29.513	
1.663.795	-29.515	
1.664.007	-29.517	
1.664.196	-29.519	
1.664.431	-29.521	
1.664.666	-29.523	
1.664.863	-29.525	
1.664.973	-29.527	
1.665.083	-29.528	
1.665.257	-29.530	
1.665.437	-29.532	
1.665.636	-29.534	

1.665.770	-29.536	
1.665.858	-29.538	
1.665.938	-29.539	
1.666.044	-29.541	
1.666.207	-29.543	
1.666.420	-29.545	
1.666.588	-29.546	
1.666.765	-29.548	
1.666.938	-29.550	
1.667.112	-29.552	
1.667.240	-29.553	
1.667.471	-29.555	
1.667.720	-29.557	
1.667.860	-29.558	
1.668.022	-29.560	
1.668.195	-29.562	
1.668.360	-29.563	
1.668.517	-29.565	
1.668.718	-29.566	
1.668.898	-29.568	
1.669.081	-29.570	
1.669.227	-29.571	
1.669.352	-29.573	
1.669.496	-29.574	
1.669.686	-29.576	
1.669.874	-29.577	
1.669.995	-29.578	
1.670.145	-29.580	
1.670.320	-29.581	
1.670.536	-29.583	

1.670.735	-29.584	
1.670.813	-29.585	
1.670.948	-29.586	
1.671.152	-29.588	
1.671.324	-29.589	
1.671.452	-29.590	
1.671.611	-29.591	
1.671.721	-29.593	
1.671.853	-29.594	
1.672.083	-29.595	
1.672.300	-29.596	
1.672.457	-29.597	
1.672.589	-29.598	
1.672.753	-29.599	
1.672.874	-29.600	
1.673.026	-29.601	
1.673.219	-29.602	
1.673.360	-29.603	
1.673.497	-29.604	
1.673.667	-29.605	
1.673.931	-29.606	
1.674.337	-29.607	
1.674.702	-29.608	
1.674.903	-29.609	
1.675.005	-29.610	
1.675.045	-29.611	
1.675.114	-29.612	
1.675.139	-29.612	
1.675.195	-29.613	
1.675.325	-29.614	

1.675.443	-29.615	
1.675.574	-29.615	
1.675.705	-29.616	
1.675.862	-29.617	
1.676.089	-29.617	
1.676.308	-29.618	
1.676.537	-29.619	
1.676.736	-29.619	
1.676.897	-29.620	
1.677.099	-29.621	
1.677.276	-29.621	
1.677.448	-29.622	
1.677.598	-29.622	
1.677.744	-29.623	
1.677.872	-29.623	
1.677.963	-29.624	
1.678.111	-29.624	
1.678.362	-29.625	
1.678.526	-29.625	
1.678.613	-29.626	
1.678.703	-29.627	
1.678.851	-29.627	
1.679.068	-29.627	
1.679.223	-29.628	
1.679.406	-29.628	
1.679.615	-29.629	
1.679.785	-29.629	
1.679.939	-29.630	
1.680.143	-29.630	
1.680.358	-29.631	

1.680.526	-29.631	
1.680.584	-29.632	
1.680.741	-29.632	
1.680.981	-29.633	
1.681.111	-29.633	
1.681.196	-29.634	
1.681.386	-29.634	
1.681.530	-29.635	
1.681.693	-29.635	
1.681.906	-29.636	
1.682.029	-29.636	
1.682.159	-29.637	
1.682.345	-29.637	
1.682.569	-29.638	
1.682.643	-29.638	
1.682.755	-29.639	
1.682.991	-29.639	
1.683.131	-29.640	
1.683.250	-29.640	
1.683.439	-29.641	
1.683.629	-29.641	
1.683.763	-29.642	
1.683.947	-29.643	
1.684.151	-29.643	
1.684.350	-29.644	
1.684.550	-29.645	
1.684.707	-29.645	
1.684.895	-29.646	
1.685.080	-29.647	
1.685.204	-29.647	

1.685.378	-29.648	
1.685.477	-29.649	
1.685.602	-29.649	
1.685.822	-29.650	
1.685.993	-29.651	
1.686.118	-29.652	
1.686.234	-29.652	
1.686.420	-29.653	
1.686.622	-29.654	
1.686.808	-29.655	
1.686.953	-29.655	
1.687.038	-29.656	
1.687.213	-29.657	
1.687.408	-29.658	
1.687.516	-29.659	
1.687.643	-29.659	
1.687.825	-29.660	
1.688.040	-29.661	
1.688.140	-29.662	
1.688.232	-29.663	
1.688.387	-29.663	
1.688.524	-29.664	
1.688.689	-29.665	
1.688.810	-29.666	
1.688.949	-29.667	
1.689.202	-29.668	
1.689.402	-29.668	
1.689.595	-29.669	
1.689.758	-29.670	
1.689.951	-29.671	

1.690.152	-29.672	
1.690.410	-29.673	
1.690.531	-29.674	
1.690.649	-29.674	
1.690.894	-29.675	
1.691.084	-29.676	
1.691.248	-29.677	
1.691.351	-29.678	
1.691.429	-29.679	
1.691.579	-29.679	
1.691.741	-29.680	
1.691.857	-29.681	
1.692.016	-29.682	
1.692.195	-29.683	
1.692.372	-29.683	
1.692.592	-29.684	
1.692.836	-29.685	
1.693.008	-29.686	
1.693.076	-29.686	
1.693.212	-29.687	
1.693.425	-29.688	
1.693.622	-29.689	
1.693.793	-29.690	
1.693.954	-29.690	
1.694.124	-29.691	
1.694.297	-29.692	
1.694.503	-29.693	
1.694.660	-29.693	
1.694.807	-29.694	
1.694.946	-29.695	

1.695.099	-29.695	
1.695.300	-29.696	
1.695.497	-29.697	
1.695.681	-29.698	
1.695.829	-29.698	
1.695.999	-29.699	
1.696.180	-29.700	
1.696.362	-29.700	
1.696.501	-29.701	
1.696.608	-29.702	
1.696.765	-29.702	
1.696.962	-29.703	
1.697.126	-29.704	
1.697.269	-29.704	
1.697.399	-29.705	
1.697.527	-29.706	
1.697.730	-29.706	
1.697.948	-29.707	
1.698.181	-29.707	
1.698.360	-29.708	
1.698.512	-29.709	
1.698.698	-29.709	
1.698.829	-29.710	
1.698.913	-29.711	
1.699.061	-29.711	
1.699.249	-29.712	
1.699.377	-29.712	
1.699.642	-29.713	
1.700.165	-29.713	
1.700.479	-29.714	

1.700.555	-29.715	
1.700.679	-29.715	
1.700.768	-29.716	
1.700.804	-29.716	
1.700.797	-29.717	
1.700.919	-29.717	
1.701.115	-29.718	
1.701.227	-29.718	
1.701.359	-29.719	
1.701.557	-29.719	
1.701.734	-29.720	
1.701.855	-29.721	
1.702.023	-29.721	
1.702.265	-29.722	
1.702.426	-29.722	
1.702.518	-29.723	
1.702.701	-29.723	
1.702.859	-29.724	
1.703.056	-29.724	
1.703.190	-29.725	
1.703.306	-29.726	
1.703.484	-29.726	
1.703.642	-29.727	
1.703.759	-29.727	
1.703.902	-29.728	
1.704.063	-29.728	
1.704.214	-29.729	
1.704.330	-29.730	
1.704.527	-29.730	
1.704.684	-29.731	

1.704.807	-29.732	
1.705.024	-29.732	
1.705.251	-29.733	
1.705.412	-29.734	
1.705.529	-29.734	
1.705.728	-29.735	
1.705.896	-29.736	
1.706.080	-29.737	
1.706.386	-29.737	
1.706.535	-29.738	
1.706.593	-29.739	
1.706.676	-29.740	
1.706.859	-29.740	
1.707.063	-29.741	
1.707.238	-29.742	
1.707.392	-29.743	
1.707.576	-29.744	
1.707.710	-29.745	
1.707.816	-29.745	
1.707.932	-29.746	
1.708.084	-29.747	
1.708.284	-29.748	
1.708.465	-29.749	
1.708.629	-29.750	
1.708.732	-29.751	
1.708.866	-29.752	
1.709.082	-29.753	
1.709.258	-29.754	
1.709.399	-29.755	
1.709.581	-29.756	

1.709.814	-29.757	
1.709.978	-29.758	
1.710.126	-29.759	
1.710.253	-29.760	
1.710.387	-29.761	
1.710.587	-29.762	
1.710.760	-29.763	
1.710.892	-29.764	
1.711.109	-29.765	
1.711.321	-29.766	
1.711.413	-29.767	
1.711.519	-29.768	
1.711.709	-29.769	
1.711.902	-29.770	
1.712.119	-29.771	
1.712.287	-29.772	
1.712.433	-29.774	
1.712.605	-29.775	
1.712.764	-29.776	
1.712.935	-29.777	
1.713.046	-29.778	
1.713.174	-29.779	
1.713.380	-29.780	
1.713.553	-29.781	
1.713.669	-29.782	
1.713.775	-29.783	
1.713.940	-29.783	
1.714.153	-29.784	
1.714.296	-29.785	
1.714.411	-29.786	

1.714.585	-29.787	
1.714.756	-29.788	
1.714.911	-29.789	
1.715.089	-29.790	
1.715.345	-29.791	
1.715.576	-29.791	
1.715.782	-29.792	
1.715.995	-29.793	
1.716.147	-29.794	
1.716.319	-29.795	
1.716.445	-29.795	
1.716.514	-29.796	
1.716.646	-29.797	
1.716.895	-29.797	
1.717.063	-29.798	
1.717.157	-29.798	
1.717.272	-29.799	
1.717.377	-29.800	
1.717.543	-29.800	
1.717.735	-29.801	
1.717.881	-29.801	
1.718.096	-29.802	
1.718.293	-29.802	
1.718.441	-29.802	
1.718.611	-29.803	
1.718.788	-29.803	
1.719.005	-29.803	
1.719.254	-29.804	
1.719.449	-29.804	
1.719.561	-29.804	

1.719.637	-29.804	
1.719.790	-29.804	
1.720.018	-29.805	
1.720.145	-29.805	
1.720.345	-29.805	
1.720.519	-29.805	
1.720.585	-29.805	
1.720.757	-29.805	
1.720.959	-29.805	
1.721.174	-29.805	
1.721.339	-29.804	
1.721.431	-29.804	
1.721.640	-29.804	
1.721.877	-29.804	
1.722.048	-29.804	
1.722.193	-29.803	
1.722.354	-29.803	
1.722.561	-29.803	
1.722.748	-29.803	
1.722.892	-29.802	
1.723.018	-29.802	
1.723.221	-29.801	
1.723.409	-29.801	
1.723.513	-29.800	
1.723.700	-29.800	
1.723.952	-29.799	
1.724.135	-29.799	
1.724.249	-29.798	
1.724.337	-29.798	
1.724.491	-29.797	

1.724.785	-29.797	
1.724.928	-29.796	
1.724.924	-29.795	
1.725.110	-29.795	
1.725.459	-29.794	
1.725.858	-29.793	
1.726.171	-29.792	
1.726.245	-29.792	
1.726.243	-29.791	
1.726.385	-29.790	
1.726.550	-29.789	
1.726.561	-29.789	
1.726.613	-29.788	
1.726.752	-29.787	
1.726.891	-29.786	
1.727.079	-29.785	
1.727.280	-29.785	
1.727.424	-29.784	
1.727.576	-29.783	
1.727.710	-29.782	
1.727.822	-29.781	
1.728.002	-29.781	
1.728.206	-29.780	
1.728.445	-29.779	
1.728.606	-29.778	
1.728.716	-29.777	
1.728.878	-29.777	
1.729.072	-29.776	
1.729.207	-29.775	
1.729.399	-29.774	

1.729.628	-29.773	
1.729.733	-29.773	
1.729.816	-29.772	
1.729.993	-29.771	
1.730.161	-29.771	
1.730.332	-29.770	
1.730.526	-29.769	
1.730.681	-29.769	
1.730.898	-29.768	
1.731.095	-29.767	
1.731.277	-29.767	
1.731.460	-29.766	
1.731.586	-29.766	
1.731.713	-29.765	
1.731.911	-29.765	
1.732.070	-29.764	
1.732.231	-29.764	
1.732.431	-29.763	
1.732.592	-29.763	
1.732.724	-29.762	
1.732.845	-29.762	
1.732.979	-29.762	
1.733.172	-29.761	
1.733.326	-29.761	
1.733.443	-29.761	
1.733.569	-29.760	
1.733.716	-29.760	
1.733.933	-29.760	
1.734.101	-29.760	
1.734.259	-29.760	

1.734.352	-29.759	
1.734.520	-29.759	
1.734.671	-29.759	
1.734.789	-29.759	
1.734.946	-29.759	
1.735.117	-29.759	
1.735.307	-29.759	
1.735.569	-29.759	
1.735.761	-29.759	
1.735.925	-29.759	
1.736.086	-29.759	
1.736.212	-29.760	
1.736.360	-29.760	
1.736.593	-29.760	
1.736.754	-29.760	
1.736.832	-29.760	
1.736.996	-29.761	
1.737.227	-29.761	
1.737.403	-29.761	
1.737.518	-29.761	
1.737.670	-29.762	
1.737.885	-29.762	
1.738.083	-29.762	
1.738.210	-29.763	
1.738.324	-29.763	
1.738.486	-29.764	
1.738.662	-29.764	
1.738.826	-29.764	
1.738.967	-29.765	
1.739.138	-29.765	

1.739.335	-29.766	
1.739.534	-29.766	
1.739.632	-29.767	
1.739.697	-29.767	
1.739.846	-29.768	
1.740.042	-29.768	
1.740.260	-29.769	
1.740.394	-29.769	
1.740.475	-29.770	
1.740.643	-29.770	
1.740.919	-29.771	
1.741.171	-29.771	
1.741.281	-29.772	
1.741.451	-29.772	
1.741.709	-29.773	
1.741.873	-29.773	
1.742.032	-29.773	
1.742.204	-29.774	
1.742.377	-29.774	
1.742.527	-29.775	
1.742.589	-29.775	
1.742.665	-29.776	
1.742.818	-29.776	
1.742.939	-29.777	
1.743.116	-29.777	
1.743.273	-29.777	
1.743.414	-29.778	
1.743.584	-29.778	
1.743.799	-29.778	
1.743.978	-29.779	

1.744.106	-29.779	
1.744.283	-29.779	
1.744.552	-29.780	
1.744.769	-29.780	
1.744.977	-29.780	
1.745.112	-29.780	
1.745.273	-29.780	
1.745.450	-29.781	
1.745.636	-29.781	
1.745.807	-29.781	
1.745.954	-29.781	
1.746.097	-29.781	
1.746.322	-29.781	
1.746.525	-29.781	
1.746.628	-29.781	
1.746.756	-29.781	
1.746.944	-29.781	
1.747.119	-29.781	
1.747.272	-29.781	
1.747.403	-29.781	
1.747.583	-29.780	
1.747.832	-29.780	
1.747.943	-29.780	
1.748.044	-29.780	
1.748.208	-29.780	
1.748.349	-29.779	
1.748.533	-29.779	
1.748.795	-29.779	
1.748.979	-29.778	
1.749.120	-29.778	

1.749.268	-29.778	
1.749.408	-29.777	
1.749.639	-29.777	
1.749.805	-29.776	
1.749.944	-29.776	
1.750.094	-29.776	
1.750.208	-29.775	
1.750.336	-29.775	
1.750.480	-29.774	
1.750.645	-29.773	
1.750.902	-29.773	
1.751.266	-29.772	
1.751.617	-29.772	
1.751.839	-29.771	
1.751.973	-29.770	
1.752.023	-29.770	
1.752.061	-29.769	
1.752.119	-29.768	
1.752.222	-29.768	
1.752.289	-29.767	
1.752.399	-29.766	
1.752.601	-29.765	
1.752.726	-29.765	
1.752.901	-29.764	
1.753.105	-29.763	
1.753.284	-29.762	
1.753.456	-29.761	
1.753.661	-29.761	
1.753.864	-29.760	
1.754.057	-29.759	

1.754.204	-29.758	
1.754.353	-29.757	
1.754.586	-29.756	
1.754.751	-29.755	
1.754.870	-29.755	
1.754.953	-29.754	
1.755.092	-29.753	
1.755.264	-29.752	
1.755.394	-29.751	
1.755.562	-29.750	
1.755.703	-29.749	
1.755.822	-29.748	
1.755.995	-29.748	
1.756.158	-29.747	
1.756.393	-29.746	
1.756.618	-29.745	
1.756.721	-29.744	
1.756.911	-29.743	
1.757.195	-29.742	
1.757.379	-29.741	
1.757.448	-29.740	
1.757.587	-29.740	
1.757.755	-29.739	
1.757.908	-29.738	
1.758.064	-29.737	
1.758.177	-29.736	
1.758.298	-29.735	
1.758.409	-29.734	
1.758.568	-29.734	
1.758.779	-29.733	

1.758.958	-29.732	
1.759.126	-29.731	
1.759.328	-29.730	
1.759.539	-29.730	
1.759.678	-29.729	
1.759.832	-29.728	
1.759.919	-29.727	
1.760.072	-29.727	
1.760.336	-29.726	
1.760.470	-29.725	
1.760.607	-29.725	
1.760.760	-29.724	
1.760.947	-29.723	
1.761.178	-29.723	
1.761.384	-29.722	
1.761.541	-29.721	
1.761.702	-29.721	
1.761.861	-29.720	
1.762.023	-29.720	
1.762.238	-29.719	
1.762.403	-29.719	
1.762.556	-29.718	
1.762.706	-29.718	
1.762.832	-29.717	
1.762.982	-29.717	
1.763.177	-29.716	
1.763.405	-29.716	
1.763.562	-29.715	
1.763.609	-29.715	
1.763.719	-29.714	

1.763.929	-29.714	
1.764.129	-29.713	
1.764.308	-29.713	
1.764.433	-29.713	
1.764.572	-29.712	
1.764.722	-29.712	
1.764.895	-29.711	
1.765.069	-29.711	
1.765.170	-29.711	
1.765.311	-29.710	
1.765.497	-29.710	
1.765.643	-29.709	
1.765.782	-29.709	
1.765.929	-29.709	
1.766.104	-29.708	
1.766.266	-29.708	
1.766.424	-29.708	
1.766.581	-29.707	
1.766.743	-29.707	
1.766.973	-29.706	
1.767.244	-29.706	
1.767.471	-29.706	
1.767.655	-29.705	
1.767.753	-29.705	
1.767.889	-29.705	
1.768.118	-29.704	
1.768.304	-29.704	
1.768.450	-29.704	
1.768.597	-29.703	
1.768.734	-29.703	

1.768.828	-29.702	
1.768.960	-29.702	
1.769.113	-29.702	
1.769.285	-29.701	
1.769.507	-29.701	
1.769.686	-29.700	
1.769.870	-29.700	
1.770.051	-29.700	
1.770.237	-29.699	
1.770.434	-29.699	
1.770.546	-29.698	
1.770.714	-29.698	
1.770.974	-29.698	
1.771.199	-29.697	
1.771.364	-29.697	
1.771.474	-29.696	
1.771.552	-29.696	
1.771.711	-29.695	
1.771.911	-29.695	
1.772.106	-29.695	
1.772.233	-29.694	
1.772.350	-29.694	
1.772.571	-29.693	
1.772.760	-29.693	
1.772.894	-29.693	
1.772.986	-29.692	
1.773.138	-29.692	
1.773.382	-29.691	
1.773.582	-29.691	
1.773.716	-29.690	

1.773.866	-29.690	
1.774.109	-29.690	
1.774.339	-29.689	
1.774.417	-29.689	
1.774.525	-29.688	
1.774.749	-29.688	
1.774.895	-29.688	
1.775.011	-29.687	
1.775.168	-29.687	
1.775.338	-29.686	
1.775.419	-29.686	
1.775.607	-29.686	
1.775.846	-29.685	
1.776.093	-29.685	
1.776.210	-29.685	
1.776.357	-29.684	
1.776.503	-29.684	
1.776.810	-29.683	
1.777.244	-29.683	
1.777.558	-29.683	
1.777.681	-29.682	
1.777.760	-29.682	
1.777.822	-29.682	
1.777.883	-29.681	
1.777.926	-29.681	
1.778.009	-29.681	
1.778.127	-29.680	
1.778.237	-29.680	
1.778.421	-29.680	
1.778.593	-29.679	

1.778.750	-29.679	
1.778.929	-29.679	
1.779.054	-29.679	
1.779.176	-29.678	
1.779.361	-29.678	
1.779.617	-29.678	
1.779.828	-29.678	
1.779.948	-29.678	
1.780.076	-29.677	
1.780.246	-29.677	
1.780.428	-29.677	
1.780.555	-29.677	
1.780.732	-29.677	
1.780.871	-29.677	
1.780.979	-29.677	
1.781.129	-29.677	
1.781.315	-29.676	
1.781.485	-29.676	
1.781.638	-29.676	
1.781.864	-29.676	
1.782.034	-29.676	
1.782.206	-29.676	
1.782.455	-29.677	
1.782.643	-29.677	
1.782.793	-29.677	
1.782.997	-29.677	
1.783.201	-29.677	
1.783.365	-29.677	
1.783.427	-29.677	
1.783.524	-29.678	

1.783.705	-29.678	
1.783.857	-29.678	
1.783.996	-29.678	
1.784.133	-29.678	
1.784.343	-29.679	
1.784.579	-29.679	
1.784.700	-29.679	
1.784.812	-29.680	
1.784.991	-29.680	
1.785.195	-29.681	
1.785.410	-29.681	
1.785.566	-29.682	
1.785.690	-29.682	
1.785.820	-29.682	
1.785.968	-29.683	
1.786.180	-29.684	
1.786.362	-29.684	
1.786.514	-29.685	
1.786.667	-29.685	
1.786.821	-29.686	
1.787.021	-29.686	
1.787.211	-29.687	
1.787.377	-29.688	
1.787.520	-29.688	
1.787.666	-29.689	
1.787.838	-29.690	
1.788.040	-29.691	
1.788.185	-29.691	
1.788.364	-29.692	
1.788.531	-29.693	

1.788.667	-29.694	
1.788.812	-29.694	
1.788.918	-29.695	
1.789.023	-29.696	
1.789.205	-29.697	
1.789.393	-29.697	
1.789.534	-29.698	
1.789.688	-29.699	
1.789.926	-29.700	
1.790.134	-29.701	
1.790.282	-29.702	
1.790.383	-29.702	
1.790.509	-29.703	
1.790.708	-29.704	
1.790.911	-29.705	
1.791.001	-29.706	
1.791.084	-29.706	
1.791.236	-29.707	
1.791.422	-29.708	
1.791.599	-29.709	
1.791.796	-29.710	
1.791.998	-29.710	
1.792.126	-29.711	
1.792.283	-29.712	
1.792.509	-29.713	
1.792.728	-29.714	
1.792.897	-29.714	
1.793.075	-29.715	
1.793.259	-29.716	
1.793.472	-29.717	

1.793.661	-29.718	
1.793.759	-29.718	
1.793.922	-29.719	
1.794.106	-29.720	
1.794.272	-29.720	
1.794.379	-29.721	
1.794.431	-29.722	
1.794.554	-29.722	
1.794.715	-29.723	
1.794.906	-29.724	
1.795.061	-29.724	
1.795.228	-29.725	
1.795.423	-29.726	
1.795.629	-29.726	
1.795.844	-29.727	
1.796.021	-29.727	
1.796.216	-29.728	
1.796.395	-29.728	
1.796.561	-29.729	
1.796.702	-29.729	
1.796.833	-29.730	
1.797.020	-29.730	
1.797.202	-29.731	
1.797.368	-29.731	
1.797.509	-29.732	
1.797.656	-29.732	
1.797.878	-29.733	
1.798.116	-29.733	
1.798.230	-29.733	
1.798.389	-29.734	

1.798.635	-29.734	
1.798.743	-29.734	
1.798.811	-29.735	
1.799.016	-29.735	
1.799.267	-29.735	
1.799.428	-29.735	
1.799.563	-29.736	
1.799.753	-29.736	
1.799.935	-29.736	
1.800.069	-29.736	
1.800.206	-29.737	
1.800.381	-29.737	
1.800.522	-29.737	
1.800.692	-29.737	
1.800.903	-29.737	
1.801.068	-29.737	
1.801.225	-29.737	
1.801.386	-29.737	
1.801.535	-29.738	
1.801.743	-29.738	
1.801.904	-29.738	
1.802.005	-29.738	
1.802.188	-29.738	
1.802.585	-29.738	
1.803.058	-29.738	
1.803.295	-29.738	
1.803.372	-29.738	
1.803.461	-29.738	
1.803.501	-29.738	
1.803.503	-29.738	

1.803.604	-29.738	
1.803.725	-29.738	
1.803.835	-29.738	
1.803.936	-29.738	
1.804.070	-29.737	
1.804.236	-29.737	
1.804.370	-29.737	
1.804.511	-29.737	
1.804.754	-29.737	
1.805.004	-29.737	
1.805.166	-29.737	
1.805.332	-29.737	
1.805.486	-29.737	
1.805.686	-29.737	
1.805.898	-29.737	
1.805.979	-29.737	
1.806.060	-29.736	
1.806.265	-29.736	
1.806.385	-29.736	
1.806.512	-29.736	
1.806.765	-29.736	
1.806.962	-29.736	
1.807.083	-29.736	
1.807.200	-29.736	
1.807.411	-29.736	
1.807.592	-29.736	
1.807.675	-29.736	
1.807.878	-29.736	
1.808.143	-29.736	
1.808.327	-29.736	

1.808.472	-29.736	
1.808.663	-29.736	
1.808.878	-29.737	
1.809.025	-29.737	
1.809.148	-29.737	
1.809.285	-29.737	
1.809.415	-29.737	
1.809.516	-29.737	
1.809.722	-29.737	
1.809.944	-29.738	
1.810.043	-29.738	
1.810.170	-29.738	
1.810.307	-29.738	
1.810.470	-29.739	
1.810.663	-29.739	
1.810.855	-29.739	
1.811.050	-29.739	
1.811.250	-29.740	
1.811.441	-29.740	
1.811.525	-29.741	
1.811.620	-29.741	
1.811.752	-29.741	
1.811.886	-29.742	
1.812.070	-29.742	
1.812.207	-29.743	
1.812.448	-29.744	
1.812.717	-29.744	
1.812.892	-29.745	
1.813.055	-29.745	
1.813.201	-29.746	

1.813.360	-29.747	
1.813.540	-29.747	
1.813.714	-29.748	
1.813.896	-29.749	
1.814.061	-29.749	
1.814.263	-29.750	
1.814.398	-29.751	
1.814.475	-29.752	
1.814.693	-29.752	
1.814.933	-29.753	
1.815.051	-29.754	
1.815.202	-29.755	
1.815.363	-29.756	
1.815.513	-29.757	
1.815.730	-29.758	
1.815.840	-29.758	
1.815.954	-29.759	
1.816.185	-29.760	
1.816.367	-29.761	
1.816.501	-29.762	
1.816.572	-29.763	
1.816.664	-29.764	
1.816.868	-29.765	
1.817.108	-29.766	
1.817.267	-29.767	
1.817.372	-29.768	
1.817.527	-29.769	
1.817.701	-29.770	
1.817.894	-29.771	
1.818.057	-29.773	

1.818.203	-29.774	
1.818.438	-29.775	
1.818.689	-29.776	
1.818.929	-29.777	
1.819.124	-29.778	
1.819.213	-29.779	
1.819.333	-29.780	
1.819.534	-29.781	
1.819.691	-29.783	
1.819.823	-29.784	
1.819.917	-29.785	
1.820.025	-29.786	
1.820.192	-29.787	
1.820.334	-29.788	
1.820.533	-29.789	
1.820.737	-29.791	
1.820.909	-29.792	
1.821.075	-29.793	
1.821.263	-29.794	
1.821.498	-29.795	
1.821.649	-29.796	
1.821.772	-29.798	
1.821.879	-29.799	
1.822.065	-29.800	
1.822.350	-29.801	
1.822.540	-29.802	
1.822.666	-29.803	
1.822.814	-29.805	
1.822.986	-29.806	
1.823.232	-29.807	

1.823.439	-29.808	
1.823.562	-29.809	
1.823.662	-29.810	
1.823.777	-29.812	
1.823.969	-29.813	
1.824.135	-29.814	
1.824.312	-29.815	
1.824.521	-29.816	
1.824.695	-29.817	
1.824.872	-29.818	
1.824.980	-29.819	
1.825.132	-29.820	
1.825.342	-29.822	
1.825.497	-29.823	
1.825.672	-29.824	
1.825.831	-29.825	
1.825.936	-29.826	
1.826.062	-29.827	
1.826.174	-29.828	
1.826.353	-29.829	
1.826.568	-29.830	
1.826.783	-29.831	
1.826.899	-29.832	
1.827.036	-29.833	
1.827.229	-29.834	
1.827.422	-29.835	
1.827.594	-29.836	
1.827.686	-29.837	
1.827.921	-29.838	
1.828.423	-29.839	

1.828.862	-29.840	
1.828.962	-29.841	
1.828.980	-29.842	
1.829.081	-29.843	
1.829.157	-29.844	
1.829.227	-29.845	
1.829.305	-29.846	
1.829.415	-29.847	
1.829.547	-29.848	
1.829.655	-29.849	
1.829.852	-29.850	
1.829.996	-29.851	
1.830.143	-29.852	
1.830.410	-29.853	
1.830.603	-29.854	
1.830.725	-29.855	
1.830.898	-29.856	
1.831.113	-29.856	
1.831.252	-29.857	
1.831.359	-29.858	
1.831.489	-29.859	
1.831.684	-29.860	
1.831.913	-29.861	
1.832.063	-29.862	
1.832.195	-29.863	
1.832.336	-29.864	
1.832.460	-29.865	
1.832.612	-29.866	
1.832.746	-29.867	
1.832.897	-29.868	

1.833.116	-29.869	
1.833.309	-29.870	
1.833.504	-29.871	
1.833.689	-29.872	
1.833.828	-29.873	
1.834.018	-29.874	
1.834.207	-29.875	
1.834.366	-29.876	
1.834.509	-29.876	
1.834.697	-29.877	
1.834.870	-29.878	
1.834.995	-29.880	
1.835.132	-29.881	
1.835.220	-29.882	
1.835.381	-29.883	
1.835.546	-29.884	
1.835.715	-29.885	
1.835.903	-29.886	
1.836.069	-29.887	
1.836.183	-29.888	
1.836.342	-29.889	
1.836.552	-29.890	
1.836.704	-29.891	
1.836.844	-29.892	
1.837.065	-29.893	
1.837.193	-29.895	
1.837.251	-29.896	
1.837.440	-29.897	
1.837.650	-29.898	
1.837.804	-29.899	

1.838.002	-29.900	
1.838.208	-29.902	
1.838.441	-29.903	
1.838.699	-29.904	
1.838.842	-29.905	
1.838.979	-29.906	
1.839.138	-29.908	
1.839.249	-29.909	
1.839.435	-29.910	
1.839.684	-29.911	
1.839.836	-29.913	
1.839.944	-29.914	
1.840.089	-29.915	
1.840.199	-29.916	
1.840.387	-29.918	
1.840.627	-29.919	
1.840.806	-29.920	
1.840.934	-29.922	
1.841.068	-29.923	
1.841.203	-29.924	
1.841.342	-29.925	
1.841.588	-29.927	
1.841.768	-29.928	
1.841.893	-29.929	
1.842.045	-29.931	
1.842.191	-29.932	
1.842.263	-29.933	
1.842.388	-29.934	
1.842.589	-29.936	
1.842.742	-29.937	

1.842.876	-29.938	
1.843.044	-29.939	
1.843.219	-29.941	
1.843.427	-29.942	
1.843.596	-29.943	
1.843.777	-29.944	
1.844.021	-29.946	
1.844.213	-29.947	
1.844.402	-29.948	
1.844.583	-29.949	
1.844.686	-29.951	
1.844.852	-29.952	
1.845.060	-29.953	
1.845.210	-29.954	
1.845.365	-29.955	
1.845.528	-29.957	
1.845.676	-29.958	
1.845.784	-29.959	
1.845.905	-29.960	
1.846.084	-29.961	
1.846.234	-29.962	
1.846.371	-29.964	
1.846.534	-29.965	
1.846.725	-29.966	
1.847.005	-29.967	
1.847.222	-29.968	
1.847.359	-29.969	
1.847.527	-29.970	
1.847.681	-29.971	
1.847.822	-29.972	

1.847.986	-29.974	
1.848.121	-29.975	
1.848.354	-29.976	
1.848.564	-29.977	
1.848.654	-29.978	
1.848.829	-29.979	
1.849.036	-29.980	
1.849.198	-29.981	
1.849.323	-29.982	
1.849.447	-29.983	
1.849.612	-29.984	
1.849.798	-29.985	
1.850.020	-29.986	
1.850.221	-29.987	
1.850.365	-29.988	
1.850.515	-29.989	
1.850.676	-29.990	
1.850.824	-29.991	
1.850.979	-29.992	
1.851.142	-29.993	
1.851.326	-29.994	
1.851.510	-29.995	
1.851.649	-29.996	
1.851.805	-29.996	
1.852.014	-29.997	
1.852.204	-29.998	
1.852.372	-29.999	
1.852.440	-30.000	
1.852.592	-30.001	
1.852.704	-30.002	

1.852.842	-30.003	
1.853.073	-30.004	
1.853.234	-30.005	
1.853.416	-30.006	
1.853.851	-30.007	
1.854.326	-30.008	
1.854.514	-30.009	
1.854.605	-30.010	
1.854.668	-30.011	
1.854.697	-30.012	
1.854.736	-30.013	
1.854.809	-30.014	
1.854.953	-30.015	
1.855.054	-30.016	
1.855.168	-30.017	
1.855.358	-30.018	
1.855.564	-30.019	
1.855.764	-30.020	
1.855.961	-30.021	
1.856.142	-30.022	
1.856.302	-30.023	
1.856.469	-30.024	
1.856.682	-30.026	
1.856.837	-30.027	
1.856.911	-30.028	
1.857.041	-30.029	
1.857.198	-30.030	
1.857.354	-30.031	
1.857.534	-30.032	
1.857.764	-30.034	

1.857.870	-30.035	
1.857.943	-30.036	
1.858.074	-30.037	
1.858.273	-30.039	
1.858.418	-30.040	
1.858.609	-30.041	
1.858.810	-30.043	
1.859.021	-30.044	
1.859.211	-30.045	
1.859.427	-30.047	
1.859.601	-30.048	
1.859.691	-30.049	
1.859.823	-30.051	
1.859.973	-30.052	
1.860.154	-30.054	
1.860.367	-30.055	
1.860.517	-30.057	
1.860.607	-30.058	
1.860.770	-30.060	
1.861.001	-30.061	
1.861.162	-30.063	
1.861.274	-30.065	
1.861.445	-30.066	
1.861.673	-30.068	
1.861.855	-30.070	
1.861.976	-30.071	
1.862.143	-30.073	
1.862.347	-30.075	
1.862.520	-30.076	
1.862.654	-30.078	

1.862.800	-30.080	
1.862.905	-30.082	
1.863.029	-30.084	
1.863.194	-30.085	
1.863.336	-30.087	
1.863.533	-30.089	
1.863.810	-30.091	
1.863.987	-30.093	
1.864.128	-30.095	
1.864.272	-30.097	
1.864.404	-30.099	
1.864.595	-30.101	
1.864.783	-30.103	
1.864.919	-30.105	
1.865.085	-30.107	
1.865.273	-30.109	
1.865.463	-30.111	
1.865.636	-30.113	
1.865.751	-30.115	
1.865.883	-30.117	
1.866.060	-30.119	
1.866.272	-30.121	
1.866.461	-30.123	
1.866.588	-30.125	
1.866.750	-30.127	
1.866.928	-30.129	
1.867.117	-30.131	
1.867.336	-30.133	
1.867.466	-30.135	
1.867.542	-30.137	

1.867.670	-30.140	
1.867.784	-30.142	
1.867.945	-30.144	
1.868.018	-30.146	
1.868.143	-30.148	
1.868.425	-30.150	
1.868.600	-30.152	
1.868.756	-30.154	
1.868.972	-30.156	
1.869.200	-30.158	
1.869.393	-30.159	
1.869.590	-30.161	
1.869.764	-30.163	
1.869.953	-30.165	
1.870.155	-30.167	
1.870.343	-30.169	
1.870.535	-30.171	
1.870.690	-30.173	
1.870.858	-30.174	
1.870.986	-30.176	
1.871.064	-30.178	
1.871.167	-30.180	
1.871.339	-30.181	
1.871.550	-30.183	
1.871.695	-30.185	
1.871.790	-30.187	
1.871.877	-30.188	
1.872.056	-30.190	
1.872.305	-30.191	
1.872.534	-30.193	

1.872.746	-30.195	
1.872.928	-30.196	
1.873.112	-30.198	
1.873.302	-30.199	
1.873.430	-30.200	
1.873.571	-30.202	
1.873.723	-30.203	
1.873.859	-30.205	
1.874.053	-30.206	
1.874.205	-30.207	
1.874.357	-30.209	
1.874.534	-30.210	
1.874.677	-30.211	
1.874.837	-30.212	
1.875.040	-30.213	
1.875.248	-30.215	
1.875.441	-30.216	
1.875.564	-30.217	
1.875.674	-30.218	
1.875.920	-30.219	
1.876.104	-30.220	
1.876.274	-30.221	
1.876.465	-30.222	
1.876.573	-30.223	
1.876.718	-30.224	
1.876.875	-30.224	
1.877.097	-30.225	
1.877.305	-30.226	
1.877.426	-30.227	
1.877.590	-30.228	

1.877.791	-30.228	
1.877.914	-30.229	
1.878.051	-30.230	
1.878.230	-30.230	
1.878.445	-30.231	
1.878.645	-30.232	
1.878.781	-30.232	
1.878.962	-30.233	
1.879.167	-30.233	
1.879.514	-30.234	
1.879.867	-30.234	
1.880.060	-30.235	
1.880.161	-30.235	
1.880.202	-30.236	
1.880.271	-30.236	
1.880.307	-30.236	
1.880.361	-30.237	
1.880.484	-30.237	
1.880.611	-30.237	
1.880.784	-30.237	
1.880.961	-30.238	
1.881.087	-30.238	
1.881.243	-30.238	
1.881.432	-30.238	
1.881.619	-30.238	
1.881.772	-30.239	
1.881.951	-30.239	
1.882.175	-30.239	
1.882.332	-30.239	
1.882.498	-30.239	

1.882.677	-30.239	
1.882.802	-30.239	
1.882.896	-30.239	
1.883.071	-30.239	
1.883.295	-30.239	
1.883.423	-30.239	
1.883.512	-30.239	
1.883.658	-30.239	
1.883.826	-30.239	
1.883.983	-30.239	
1.884.178	-30.239	
1.884.397	-30.239	
1.884.565	-30.239	
1.884.709	-30.239	
1.884.935	-30.239	
1.885.172	-30.239	
1.885.316	-30.239	
1.885.444	-30.238	
1.885.616	-30.238	
1.885.782	-30.238	
1.885.949	-30.238	
1.886.131	-30.238	
1.886.234	-30.238	
1.886.402	-30.238	
1.886.611	-30.238	
1.886.749	-30.237	
1.886.868	-30.237	
1.887.020	-30.237	
1.887.184	-30.237	
1.887.325	-30.237	

1.887.486	-30.237	
1.887.684	-30.237	
1.887.887	-30.237	
1.888.035	-30.237	
1.888.152	-30.237	
1.888.284	-30.236	
1.888.472	-30.236	
1.888.652	-30.236	
1.888.781	-30.236	
1.888.954	-30.236	
1.889.151	-30.236	
1.889.334	-30.236	
1.889.493	-30.236	
1.889.677	-30.236	
1.889.875	-30.236	
1.890.070	-30.236	
1.890.215	-30.236	
1.890.374	-30.236	
1.890.529	-30.236	
1.890.674	-30.236	
1.890.835	-30.236	
1.890.896	-30.236	
1.890.990	-30.236	
1.891.203	-30.236	
1.891.478	-30.236	
1.891.624	-30.236	
1.891.777	-30.236	
1.891.967	-30.236	
1.892.121	-30.236	
1.892.280	-30.236	

1.892.455	-30.236	
1.892.605	-30.237	
1.892.748	-30.237	
1.892.883	-30.237	
1.893.033	-30.237	
1.893.194	-30.237	
1.893.318	-30.237	
1.893.438	-30.237	
1.893.578	-30.237	
1.893.709	-30.237	
1.893.909	-30.237	
1.894.084	-30.238	
1.894.252	-30.238	
1.894.498	-30.238	
1.894.771	-30.238	
1.894.955	-30.238	
1.895.096	-30.238	
1.895.325	-30.238	
1.895.509	-30.238	
1.895.658	-30.239	
1.895.818	-30.239	
1.895.945	-30.239	
1.896.080	-30.239	
1.896.236	-30.239	
1.896.406	-30.239	
1.896.527	-30.240	
1.896.613	-30.240	
1.896.792	-30.240	
1.896.973	-30.240	
1.897.143	-30.240	

1.897.251	-30.240	
1.897.381	-30.241	
1.897.623	-30.241	
1.897.834	-30.241	
1.898.000	-30.241	
1.898.179	-30.241	
1.898.385	-30.242	
1.898.535	-30.242	
1.898.705	-30.242	
1.898.895	-30.242	
1.899.035	-30.242	
1.899.176	-30.243	
1.899.393	-30.243	
1.899.534	-30.243	
1.899.657	-30.244	
1.899.836	-30.244	
1.900.013	-30.244	
1.900.161	-30.244	
1.900.316	-30.245	
1.900.556	-30.245	
1.900.770	-30.245	
1.900.851	-30.246	
1.900.956	-30.246	
1.901.173	-30.246	
1.901.409	-30.247	
1.901.554	-30.247	
1.901.644	-30.247	
1.901.796	-30.248	
1.902.036	-30.248	
1.902.260	-30.248	

1.902.431	-30.249	
1.902.561	-30.249	
1.902.710	-30.250	
1.902.860	-30.250	
1.902.982	-30.251	
1.903.136	-30.251	
1.903.282	-30.251	
1.903.405	-30.252	
1.903.600	-30.252	
1.903.884	-30.253	
1.904.010	-30.253	
1.904.068	-30.254	
1.904.267	-30.254	
1.904.684	-30.255	
1.905.079	-30.255	
1.905.314	-30.256	
1.905.450	-30.257	
1.905.528	-30.257	
1.905.551	-30.258	
1.905.580	-30.258	
1.905.692	-30.259	
1.905.826	-30.259	
1.905.938	-30.260	
1.906.046	-30.261	
1.906.189	-30.261	
1.906.340	-30.262	
1.906.579	-30.262	
1.906.803	-30.263	
1.906.946	-30.264	
1.907.150	-30.264	

1.907.350	-30.265	
1.907.486	-30.266	
1.907.590	-30.266	
1.907.751	-30.267	
1.907.988	-30.268	
1.908.159	-30.269	
1.908.261	-30.269	
1.908.416	-30.270	
1.908.672	-30.271	
1.908.855	-30.272	
1.908.976	-30.272	
1.909.099	-30.273	
1.909.211	-30.274	
1.909.343	-30.275	
1.909.520	-30.276	
1.909.693	-30.277	
1.909.875	-30.277	
1.910.081	-30.278	
1.910.251	-30.279	
1.910.398	-30.280	
1.910.531	-30.281	
1.910.723	-30.282	
1.910.938	-30.283	
1.911.126	-30.284	
1.911.286	-30.285	
1.911.418	-30.286	
1.911.559	-30.287	
1.911.678	-30.288	
1.911.805	-30.289	
1.912.001	-30.290	

1.912.188	-30.291	
1.912.262	-30.292	
1.912.379	-30.293	
1.912.607	-30.294	
1.912.775	-30.295	
1.912.934	-30.297	
1.913.125	-30.298	
1.913.355	-30.299	
1.913.535	-30.300	
1.913.658	-30.301	
1.913.797	-30.303	
1.913.916	-30.304	
1.914.032	-30.305	
1.914.225	-30.306	
1.914.408	-30.308	
1.914.583	-30.309	
1.914.783	-30.310	
1.914.951	-30.312	
1.915.098	-30.313	
1.915.253	-30.315	
1.915.457	-30.316	
1.915.640	-30.317	
1.915.813	-30.319	
1.915.994	-30.320	
1.916.129	-30.322	
1.916.236	-30.323	
1.916.413	-30.325	
1.916.604	-30.326	
1.916.823	-30.327	
1.917.030	-30.329	

1.917.178	-30.330	
1.917.327	-30.332	
1.917.469	-30.333	
1.917.621	-30.335	
1.917.818	-30.336	
1.918.027	-30.338	
1.918.147	-30.339	
1.918.205	-30.341	
1.918.322	-30.342	
1.918.514	-30.344	
1.918.640	-30.345	
1.918.777	-30.347	
1.918.990	-30.348	
1.919.115	-30.350	
1.919.202	-30.351	
1.919.368	-30.353	
1.919.614	-30.354	
1.919.845	-30.356	
1.920.043	-30.357	
1.920.233	-30.359	
1.920.423	-30.360	
1.920.609	-30.362	
1.920.759	-30.363	
1.920.938	-30.365	
1.921.106	-30.366	
1.921.232	-30.368	
1.921.402	-30.369	
1.921.583	-30.370	
1.921.732	-30.372	
1.921.935	-30.373	

1.922.103	-30.375	
1.922.206	-30.376	
1.922.319	-30.377	
1.922.413	-30.379	
1.922.552	-30.380	
1.922.767	-30.382	
1.923.035	-30.383	
1.923.252	-30.384	
1.923.371	-30.386	
1.923.533	-30.387	
1.923.761	-30.388	
1.923.940	-30.390	
1.924.109	-30.391	
1.924.292	-30.392	
1.924.456	-30.393	
1.924.549	-30.395	
1.924.677	-30.396	
1.924.895	-30.397	
1.925.023	-30.398	
1.925.165	-30.400	
1.925.390	-30.401	
1.925.536	-30.402	
1.925.614	-30.403	
1.925.789	-30.405	
1.926.087	-30.406	
1.926.357	-30.407	
1.926.518	-30.408	
1.926.642	-30.409	
1.926.743	-30.410	
1.926.850	-30.412	

1.927.007	-30.413	
1.927.186	-30.414	
1.927.327	-30.415	
1.927.471	-30.416	
1.927.708	-30.417	
1.927.883	-30.418	
1.927.943	-30.419	
1.928.074	-30.420	
1.928.293	-30.421	
1.928.512	-30.422	
1.928.683	-30.423	
1.928.860	-30.424	
1.929.036	-30.425	
1.929.162	-30.426	
1.929.272	-30.427	
1.929.384	-30.428	
1.929.529	-30.429	
1.929.870	-30.430	
1.930.372	-30.431	
1.930.728	-30.432	
1.930.898	-30.433	
1.930.900	-30.434	
1.931.012	-30.435	
1.931.162	-30.435	
1.931.122	-30.436	
1.931.142	-30.437	
1.931.328	-30.438	
1.931.470	-30.439	
1.931.577	-30.439	
1.931.740	-30.440	

1.931.956	-30.441	
1.932.164	-30.442	
1.932.359	-30.442	
1.932.505	-30.443	
1.932.623	-30.444	
1.932.820	-30.444	
1.932.999	-30.445	
1.933.154	-30.446	
1.933.373	-30.446	
1.933.542	-30.447	
1.933.690	-30.448	
1.933.817	-30.448	
1.933.938	-30.449	
1.934.100	-30.449	
1.934.234	-30.450	
1.934.422	-30.451	
1.934.556	-30.451	
1.934.704	-30.452	
1.934.872	-30.452	
1.934.980	-30.453	
1.935.139	-30.453	
1.935.345	-30.454	
1.935.562	-30.455	
1.935.757	-30.455	
1.935.939	-30.456	
1.936.138	-30.456	
1.936.328	-30.457	
1.936.458	-30.457	
1.936.615	-30.458	
1.936.750	-30.458	

1.936.951	-30.459	
1.937.128	-30.459	
1.937.262	-30.460	
1.937.379	-30.460	
1.937.439	-30.461	
1.937.619	-30.461	
1.937.834	-30.462	
1.938.002	-30.462	
1.938.192	-30.463	
1.938.393	-30.464	
1.938.611	-30.464	
1.938.750	-30.465	
1.938.876	-30.465	
1.938.978	-30.466	
1.939.120	-30.466	
1.939.324	-30.467	
1.939.460	-30.467	
1.939.597	-30.468	
1.939.803	-30.468	
1.939.982	-30.469	
1.940.119	-30.470	
1.940.273	-30.470	
1.940.450	-30.471	
1.940.627	-30.471	
1.940.831	-30.472	
1.941.030	-30.472	
1.941.151	-30.473	
1.941.256	-30.474	
1.941.413	-30.474	
1.941.555	-30.475	

1.941.720	-30.475	
1.941.904	-30.476	
1.942.094	-30.476	
1.942.354	-30.477	
1.942.549	-30.477	
1.942.704	-30.478	
1.942.814	-30.479	
1.942.926	-30.479	
1.943.123	-30.480	
1.943.272	-30.480	
1.943.392	-30.481	
1.943.586	-30.481	
1.943.755	-30.482	
1.943.896	-30.482	
1.944.007	-30.483	
1.944.106	-30.484	
1.944.272	-30.484	
1.944.398	-30.485	
1.944.570	-30.485	
1.944.733	-30.486	
1.944.940	-30.486	
1.945.118	-30.487	
1.945.277	-30.487	
1.945.549	-30.488	
1.945.770	-30.488	
1.945.920	-30.489	
1.946.111	-30.490	
1.946.341	-30.490	
1.946.469	-30.491	
1.946.579	-30.491	

1.946.823	-30.492	
1.946.998	-30.492	
1.947.079	-30.493	
1.947.177	-30.493	
1.947.329	-30.494	
1.947.527	-30.494	
1.947.646	-30.495	
1.947.757	-30.495	
1.947.892	-30.496	
1.948.058	-30.496	
1.948.297	-30.497	
1.948.551	-30.497	
1.948.741	-30.498	
1.948.911	-30.498	
1.949.034	-30.499	
1.949.171	-30.499	
1.949.381	-30.500	
1.949.536	-30.500	
1.949.691	-30.501	
1.949.888	-30.501	
1.950.083	-30.502	
1.950.222	-30.502	
1.950.320	-30.503	
1.950.468	-30.503	
1.950.665	-30.504	
1.950.871	-30.504	
1.951.052	-30.505	
1.951.203	-30.505	
1.951.375	-30.506	
1.951.516	-30.507	

1.951.696	-30.507	
1.951.899	-30.508	
1.952.049	-30.508	
1.952.188	-30.509	
1.952.321	-30.509	
1.952.460	-30.510	
1.952.635	-30.510	
1.952.777	-30.511	
1.952.975	-30.511	
1.953.196	-30.512	
1.953.308	-30.512	
1.953.428	-30.513	
1.953.596	-30.514	
1.953.808	-30.514	
1.954.070	-30.515	
1.954.355	-30.515	
1.954.462	-30.516	
1.954.500	-30.516	
1.954.650	-30.517	
1.954.796	-30.518	
1.954.949	-30.518	
1.955.282	-30.519	
1.955.766	-30.519	
1.956.093	-30.520	
1.956.178	-30.521	
1.956.169	-30.521	
1.956.288	-30.522	
1.956.458	-30.522	
1.956.521	-30.523	
1.956.552	-30.524	

1.956.633	-30.524	
1.956.675	-30.525	
1.956.779	-30.525	
1.957.018	-30.526	
1.957.240	-30.527	
1.957.386	-30.527	
1.957.518	-30.528	
1.957.739	-30.529	
1.957.939	-30.529	
1.958.109	-30.530	
1.958.307	-30.530	
1.958.425	-30.531	
1.958.575	-30.532	
1.958.790	-30.532	
1.958.961	-30.533	
1.959.115	-30.534	
1.959.314	-30.534	
1.959.436	-30.535	
1.959.522	-30.536	
1.959.713	-30.536	
1.959.899	-30.537	
1.960.038	-30.538	
1.960.201	-30.538	
1.960.379	-30.539	
1.960.497	-30.540	
1.960.692	-30.540	
1.960.984	-30.541	
1.961.135	-30.542	
1.961.322	-30.542	
1.961.559	-30.543	

1.961.633	-30.544	
1.961.722	-30.544	
1.961.911	-30.545	
1.962.061	-30.546	
1.962.193	-30.547	
1.962.363	-30.547	
1.962.542	-30.548	
1.962.690	-30.549	
1.962.802	-30.549	
1.963.004	-30.550	
1.963.239	-30.551	
1.963.384	-30.551	
1.963.488	-30.552	
1.963.669	-30.553	
1.963.875	-30.553	
1.963.969	-30.554	
1.964.102	-30.555	
1.964.285	-30.556	
1.964.335	-30.556	
1.964.408	-30.557	
1.964.626	-30.558	
1.964.865	-30.558	
1.965.042	-30.559	
1.965.240	-30.560	
1.965.424	-30.560	
1.965.569	-30.561	
1.965.759	-30.562	
1.965.930	-30.562	
1.966.079	-30.563	
1.966.277	-30.564	

1.966.476	-30.564	
1.966.604	-30.565	
1.966.790	-30.566	
1.966.937	-30.566	
1.967.030	-30.567	
1.967.226	-30.567	
1.967.430	-30.568	
1.967.592	-30.568	
1.967.757	-30.569	
1.967.906	-30.569	
1.968.078	-30.570	
1.968.210	-30.570	
1.968.337	-30.571	
1.968.557	-30.571	
1.968.708	-30.572	
1.968.826	-30.572	
1.968.931	-30.573	
1.969.079	-30.573	
1.969.242	-30.573	
1.969.382	-30.574	
1.969.556	-30.574	
1.969.713	-30.574	
1.969.879	-30.575	
1.970.063	-30.575	
1.970.242	-30.575	
1.970.463	-30.576	
1.970.676	-30.576	
1.970.835	-30.576	
1.971.075	-30.576	
1.971.328	-30.576	

1.971.476	-30.577	
1.971.618	-30.577	
1.971.792	-30.577	
1.971.909	-30.577	
1.972.061	-30.577	
1.972.231	-30.577	
1.972.341	-30.577	
1.972.480	-30.577	
1.972.626	-30.577	
1.972.758	-30.577	
1.972.898	-30.577	
1.973.060	-30.577	
1.973.179	-30.577	
1.973.302	-30.576	
1.973.555	-30.576	
1.973.761	-30.576	
1.973.923	-30.576	
1.974.135	-30.576	
1.974.292	-30.575	
1.974.453	-30.575	
1.974.713	-30.575	
1.974.975	-30.575	
1.975.112	-30.574	
1.975.210	-30.574	
1.975.360	-30.574	
1.975.488	-30.573	
1.975.631	-30.573	
1.975.813	-30.572	
1.975.932	-30.572	
1.976.113	-30.572	

1.976.353	-30.571	
1.976.550	-30.571	
1.976.711	-30.570	
1.976.870	-30.570	
1.977.034	-30.569	
1.977.222	-30.569	
1.977.421	-30.568	
1.977.565	-30.568	
1.977.686	-30.567	
1.977.827	-30.567	
1.978.004	-30.566	
1.978.132	-30.565	
1.978.286	-30.565	
1.978.486	-30.564	
1.978.669	-30.564	
1.978.833	-30.563	
1.978.996	-30.563	
1.979.225	-30.562	
1.979.399	-30.561	
1.979.529	-30.561	
1.979.693	-30.560	
1.979.807	-30.560	
1.979.977	-30.559	
1.980.109	-30.558	
1.980.248	-30.558	
1.980.639	-30.557	
1.981.080	-30.557	
1.981.301	-30.556	
1.981.397	-30.556	
1.981.460	-30.555	

1.981.528	-30.554	
1.981.620	-30.554	
1.981.713	-30.553	
1.981.754	-30.553	
1.981.805	-30.552	
1.981.935	-30.552	
1.982.186	-30.551	
1.982.321	-30.551	
1.982.431	-30.550	
1.982.693	-30.550	
1.982.910	-30.550	
1.983.060	-30.549	
1.983.214	-30.549	
1.983.387	-30.548	
1.983.564	-30.548	
1.983.714	-30.548	
1.983.891	-30.547	
1.984.036	-30.547	
1.984.200	-30.547	
1.984.366	-30.547	
1.984.514	-30.546	
1.984.689	-30.546	
1.984.834	-30.546	
1.984.962	-30.546	
1.985.109	-30.546	
1.985.336	-30.546	
1.985.515	-30.546	
1.985.661	-30.546	
1.985.891	-30.546	
1.986.093	-30.546	

1.986.236	-30.546	
1.986.413	-30.546	
1.986.597	-30.547	
1.986.759	-30.547	
1.986.940	-30.547	
1.987.130	-30.547	
1.987.294	-30.548	
1.987.359	-30.548	
1.987.448	-30.549	
1.987.635	-30.549	
1.987.753	-30.549	
1.987.910	-30.550	
1.988.150	-30.550	
1.988.304	-30.551	
1.988.391	-30.552	
1.988.564	-30.552	
1.988.802	-30.553	
1.988.943	-30.554	
1.989.115	-30.554	
1.989.314	-30.555	
1.989.420	-30.556	
1.989.566	-30.557	
1.989.753	-30.558	
1.989.914	-30.559	
1.990.065	-30.560	
1.990.217	-30.561	
1.990.381	-30.562	
1.990.605	-30.563	
1.990.793	-30.565	
1.990.950	-30.566	

1.991.088	-30.567	
1.991.263	-30.568	
1.991.467	-30.570	
1.991.633	-30.571	
1.991.781	-30.573	
1.991.891	-30.574	
1.992.052	-30.575	
1.992.263	-30.577	
1.992.419	-30.578	
1.992.569	-30.580	
1.992.710	-30.582	
1.992.856	-30.583	
1.993.091	-30.585	
1.993.333	-30.586	
1.993.535	-30.588	
1.993.689	-30.590	
1.993.794	-30.592	
1.993.934	-30.593	
1.994.054	-30.595	
1.994.234	-30.597	
1.994.440	-30.599	
1.994.491	-30.601	
1.994.610	-30.602	
1.994.809	-30.604	
1.994.958	-30.606	
1.995.074	-30.608	
1.995.190	-30.610	
1.995.385	-30.612	
1.995.598	-30.614	
1.995.831	-30.616	

1.996.037	-30.618	
1.996.200	-30.620	
1.996.427	-30.622	
1.996.653	-30.624	
1.996.824	-30.626	
1.996.980	-30.628	
1.997.087	-30.630	
1.997.220	-30.632	
1.997.388	-30.634	
1.997.556	-30.636	
1.997.751	-30.638	
1.997.867	-30.640	
1.997.986	-30.642	
1.998.143	-30.644	
1.998.241	-30.645	
1.998.398	-30.647	
1.998.609	-30.649	
1.998.734	-30.651	
1.998.855	-30.653	
1.999.090	-30.655	
1.999.323	-30.657	
1.999.501	-30.659	
1.999.738	-30.661	
1.999.930	-30.663	
2.000.079	-30.665	
2.000.228	-30.667	
2.000.367	-30.669	
2.000.582	-30.670	
2.000.781	-30.672	
2.000.902	-30.674	

2.001.082	-30.676	
2.001.281	-30.678	
2.001.425	-30.679	
2.001.577	-30.681	
2.001.747	-30.683	
2.001.917	-30.685	
2.002.092	-30.686	
2.002.301	-30.688	
2.002.428	-30.690	
2.002.544	-30.691	
2.002.773	-30.693	
2.002.962	-30.695	
2.003.075	-30.696	
2.003.230	-30.698	
2.003.373	-30.699	
2.003.508	-30.701	
2.003.680	-30.702	
2.003.855	-30.704	
2.004.095	-30.705	
2.004.240	-30.707	
2.004.379	-30.708	
2.004.595	-30.710	
2.004.764	-30.711	
2.004.940	-30.712	
2.005.083	-30.714	
2.005.212	-30.715	
2.005.347	-30.716	
2.005.495	-30.718	
2.005.836	-30.719	
2.006.328	-30.720	

2.006.591	-30.721	
2.006.689	-30.722	
2.006.803	-30.724	
2.006.888	-30.725	
2.006.899	-30.726	
2.006.908	-30.727	
2.007.014	-30.728	
2.007.153	-30.729	
2.007.294	-30.730	
2.007.466	-30.731	
2.007.695	-30.732	
2.007.865	-30.733	
2.008.017	-30.734	
2.008.192	-30.735	
2.008.383	-30.736	
2.008.548	-30.737	
2.008.705	-30.738	
2.008.904	-30.739	
2.009.090	-30.740	
2.009.251	-30.740	
2.009.375	-30.741	
2.009.529	-30.742	
2.009.731	-30.743	
2.009.901	-30.744	
2.010.056	-30.745	
2.010.206	-30.745	
2.010.287	-30.746	
2.010.399	-30.747	
2.010.589	-30.748	
2.010.748	-30.748	

2.010.880	-30.749	
2.011.077	-30.750	
2.011.234	-30.751	
2.011.424	-30.751	
2.011.637	-30.752	
2.011.812	-30.753	
2.011.982	-30.753	
2.012.206	-30.754	
2.012.393	-30.755	
2.012.505	-30.756	
2.012.630	-30.756	
2.012.769	-30.757	
2.012.870	-30.758	
2.013.037	-30.758	
2.013.228	-30.759	
2.013.436	-30.760	
2.013.618	-30.760	
2.013.772	-30.761	
2.013.927	-30.762	
2.014.082	-30.762	
2.014.245	-30.763	
2.014.384	-30.764	
2.014.543	-30.764	
2.014.706	-30.765	
2.014.861	-30.766	
2.015.016	-30.767	
2.015.182	-30.767	
2.015.369	-30.768	
2.015.564	-30.769	
2.015.755	-30.769	

2.015.928	-30.770	
2.016.129	-30.771	
2.016.308	-30.772	
2.016.534	-30.772	
2.016.759	-30.773	
2.016.881	-30.774	
2.017.054	-30.775	
2.017.220	-30.775	
2.017.316	-30.776	
2.017.444	-30.777	
2.017.612	-30.778	
2.017.776	-30.778	
2.017.944	-30.779	
2.018.094	-30.780	
2.018.239	-30.781	
2.018.440	-30.781	
2.018.586	-30.782	
2.018.725	-30.783	
2.018.904	-30.783	
2.019.028	-30.784	
2.019.238	-30.785	
2.019.418	-30.786	
2.019.476	-30.786	
2.019.581	-30.787	
2.019.780	-30.788	
2.020.002	-30.788	
2.020.154	-30.789	
2.020.224	-30.790	
2.020.372	-30.790	
2.020.625	-30.791	

2.020.831	-30.792	
2.020.970	-30.792	
2.021.104	-30.793	
2.021.337	-30.794	
2.021.557	-30.794	
2.021.689	-30.795	
2.021.884	-30.796	
2.021.989	-30.796	
2.022.137	-30.797	
2.022.446	-30.798	
2.022.663	-30.798	
2.022.822	-30.799	
2.022.912	-30.799	
2.023.033	-30.800	
2.023.190	-30.800	
2.023.286	-30.801	
2.023.485	-30.801	
2.023.627	-30.802	
2.023.719	-30.802	
2.023.884	-30.803	
2.024.097	-30.803	
2.024.315	-30.804	
2.024.487	-30.804	
2.024.680	-30.805	
2.024.839	-30.805	
2.025.065	-30.806	
2.025.273	-30.806	
2.025.423	-30.806	
2.025.571	-30.807	
2.025.678	-30.807	

2.025.817	-30.808	
2.026.001	-30.808	
2.026.217	-30.808	
2.026.409	-30.809	
2.026.570	-30.809	
2.026.713	-30.809	
2.026.877	-30.810	
2.027.104	-30.810	
2.027.240	-30.810	
2.027.343	-30.811	
2.027.567	-30.811	
2.027.771	-30.811	
2.027.912	-30.811	
2.028.055	-30.812	
2.028.230	-30.812	
2.028.448	-30.812	
2.028.625	-30.812	
2.028.750	-30.813	
2.028.898	-30.813	
2.029.041	-30.813	
2.029.166	-30.813	
2.029.346	-30.813	
2.029.527	-30.814	
2.029.657	-30.814	
2.029.818	-30.814	
2.030.060	-30.814	
2.030.333	-30.814	
2.030.511	-30.814	
2.030.629	-30.814	
2.030.752	-30.815	

2.030.891	-30.815	
2.031.198	-30.815	
2.031.653	-30.815	
2.031.931	-30.815	
2.031.980	-30.815	
2.032.048	-30.815	
2.032.193	-30.815	
2.032.267	-30.815	
2.032.321	-30.816	
2.032.435	-30.816	
2.032.576	-30.816	
2.032.634	-30.816	
2.032.730	-30.816	
2.032.975	-30.816	
2.033.215	-30.816	
2.033.364	-30.816	
2.033.488	-30.816	
2.033.676	-30.816	
2.033.855	-30.816	
2.034.014	-30.816	
2.034.193	-30.816	
2.034.323	-30.817	
2.034.469	-30.817	
2.034.691	-30.817	
2.034.841	-30.817	
2.034.962	-30.817	
2.035.143	-30.817	
2.035.363	-30.817	
2.035.553	-30.817	
2.035.648	-30.817	

2.035.781	-30.818	
2.035.984	-30.818	
2.036.167	-30.818	
2.036.303	-30.818	
2.036.451	-30.818	
2.036.613	-30.819	
2.036.798	-30.819	
2.037.050	-30.819	
2.037.274	-30.819	
2.037.441	-30.820	
2.037.504	-30.820	
2.037.637	-30.820	
2.037.903	-30.821	
2.038.078	-30.821	
2.038.161	-30.821	
2.038.255	-30.822	
2.038.412	-30.822	
2.038.591	-30.822	
2.038.734	-30.823	
2.038.868	-30.823	
2.039.064	-30.824	
2.039.359	-30.824	
2.039.596	-30.825	
2.039.713	-30.825	
2.039.830	-30.826	
2.039.986	-30.826	
2.040.132	-30.827	
2.040.226	-30.827	
2.040.363	-30.828	
2.040.603	-30.829	

2.040.788	-30.829	
2.040.889	-30.830	
2.041.044	-30.831	
2.041.209	-30.832	
2.041.433	-30.832	
2.041.707	-30.833	
2.041.897	-30.834	
2.042.030	-30.835	
2.042.189	-30.835	
2.042.345	-30.836	
2.042.458	-30.837	
2.042.585	-30.838	
2.042.746	-30.839	
2.042.936	-30.840	
2.043.131	-30.841	
2.043.248	-30.841	
2.043.375	-30.842	
2.043.553	-30.843	
2.043.714	-30.844	
2.043.873	-30.845	
2.044.074	-30.846	
2.044.281	-30.847	
2.044.433	-30.848	
2.044.605	-30.849	
2.044.731	-30.850	
2.044.848	-30.851	
2.044.930	-30.852	
2.045.047	-30.853	
2.045.197	-30.854	
2.045.426	-30.855	

2.045.632	-30.856	
2.045.784	-30.857	
2.045.956	-30.858	
2.046.131	-30.859	
2.046.335	-30.861	
2.046.611	-30.862	
2.046.870	-30.863	
2.047.018	-30.864	
2.047.099	-30.865	
2.047.245	-30.866	
2.047.509	-30.867	
2.047.693	-30.868	
2.047.805	-30.869	
2.047.959	-30.870	
2.048.165	-30.872	
2.048.365	-30.873	
2.048.476	-30.874	
2.048.530	-30.875	
2.048.616	-30.876	
2.048.812	-30.877	
2.048.985	-30.878	
2.049.068	-30.879	
2.049.267	-30.880	
2.049.454	-30.882	
2.049.624	-30.883	
2.049.904	-30.884	
2.050.108	-30.885	
2.050.291	-30.886	
2.050.457	-30.887	
2.050.616	-30.888	

2.050.771	-30.889	
2.050.872	-30.890	
2.051.015	-30.892	
2.051.223	-30.893	
2.051.445	-30.894	
2.051.635	-30.895	
2.051.797	-30.896	
2.051.955	-30.897	
2.052.097	-30.898	
2.052.227	-30.899	
2.052.352	-30.900	
2.052.515	-30.902	
2.052.762	-30.903	
2.052.921	-30.904	
2.053.102	-30.905	
2.053.337	-30.906	
2.053.497	-30.907	
2.053.660	-30.908	
2.053.781	-30.909	
2.053.918	-30.910	
2.054.093	-30.911	
2.054.258	-30.912	
2.054.399	-30.913	
2.054.536	-30.914	
2.054.738	-30.915	
2.054.894	-30.916	
2.055.087	-30.918	
2.055.322	-30.919	
2.055.486	-30.920	
2.055.616	-30.921	

2.055.768	-30.922	
2.055.880	-30.923	
2.055.950	-30.924	
2.056.125	-30.925	
2.056.519	-30.926	
2.056.993	-30.927	
2.057.273	-30.927	
2.057.381	-30.928	
2.057.404	-30.929	
2.057.460	-30.930	
2.057.493	-30.931	
2.057.523	-30.932	
2.057.652	-30.933	
2.057.776	-30.934	
2.057.923	-30.935	
2.058.131	-30.936	
2.058.284	-30.937	
2.058.409	-30.937	
2.058.586	-30.938	
2.058.776	-30.939	
2.058.967	-30.940	
2.059.184	-30.941	
2.059.334	-30.942	
2.059.494	-30.943	
2.059.655	-30.943	
2.059.740	-30.944	
2.059.895	-30.945	
2.060.121	-30.946	
2.060.318	-30.947	
2.060.423	-30.948	

2.060.536	-30.948	
2.060.775	-30.949	
2.060.956	-30.950	
2.061.039	-30.951	
2.061.216	-30.952	
2.061.422	-30.952	
2.061.599	-30.953	
2.061.799	-30.954	
2.061.953	-30.955	
2.062.186	-30.956	
2.062.368	-30.956	
2.062.457	-30.957	
2.062.666	-30.958	
2.062.825	-30.959	
2.062.982	-30.959	
2.063.140	-30.960	
2.063.221	-30.961	
2.063.396	-30.962	
2.063.573	-30.963	
2.063.703	-30.963	
2.063.873	-30.964	
2.064.026	-30.965	
2.064.212	-30.966	
2.064.395	-30.966	
2.064.576	-30.967	
2.064.733	-30.968	
2.064.870	-30.968	
2.065.065	-30.969	
2.065.273	-30.970	
2.065.387	-30.971	

2.065.542	-30.971	
2.065.746	-30.972	
2.065.878	-30.973	
2.066.028	-30.974	
2.066.187	-30.974	
2.066.367	-30.975	
2.066.588	-30.976	
2.066.819	-30.976	
2.066.989	-30.977	
2.067.159	-30.978	
2.067.334	-30.979	
2.067.500	-30.979	
2.067.681	-30.980	
2.067.793	-30.981	
2.067.953	-30.981	
2.068.121	-30.982	
2.068.190	-30.982	
2.068.340	-30.983	
2.068.604	-30.984	
2.068.848	-30.984	
2.069.037	-30.985	
2.069.176	-30.985	
2.069.341	-30.986	
2.069.496	-30.986	
2.069.612	-30.987	
2.069.758	-30.987	
2.069.922	-30.988	
2.070.110	-30.988	
2.070.271	-30.989	
2.070.383	-30.989	

2.070.558	-30.990	
2.070.710	-30.990	
2.070.795	-30.991	
2.070.910	-30.991	
2.071.115	-30.991	
2.071.331	-30.992	
2.071.483	-30.992	
2.071.639	-30.992	
2.071.889	-30.993	
2.072.168	-30.993	
2.072.336	-30.993	
2.072.500	-30.993	
2.072.661	-30.993	
2.072.749	-30.994	
2.072.916	-30.994	
2.073.154	-30.994	
2.073.279	-30.994	
2.073.367	-30.994	
2.073.514	-30.994	
2.073.653	-30.994	
2.073.817	-30.994	
2.073.963	-30.994	
2.074.109	-30.994	
2.074.294	-30.994	
2.074.435	-30.994	
2.074.578	-30.994	
2.074.765	-30.994	
2.075.029	-30.994	
2.075.277	-30.994	
2.075.397	-30.994	

2.075.517	-30.994	
2.075.678	-30.994	
2.075.865	-30.993	
2.076.015	-30.993	
2.076.163	-30.993	
2.076.373	-30.993	
2.076.608	-30.993	
2.076.801	-30.992	
2.076.953	-30.992	
2.077.065	-30.992	
2.077.255	-30.991	
2.077.477	-30.991	
2.077.626	-30.991	
2.077.775	-30.990	
2.077.977	-30.990	
2.078.114	-30.990	
2.078.250	-30.989	
2.078.447	-30.989	
2.078.604	-30.988	
2.078.709	-30.988	
2.078.850	-30.988	
2.079.088	-30.987	
2.079.296	-30.987	
2.079.437	-30.986	
2.079.592	-30.986	
2.079.755	-30.985	
2.079.937	-30.985	
2.080.087	-30.984	
2.080.143	-30.984	
2.080.275	-30.983	

2.080.502	-30.983	
2.080.685	-30.982	
2.080.863	-30.982	
2.081.035	-30.981	
2.081.198	-30.981	
2.081.341	-30.980	
2.081.597	-30.980	
2.082.018	-30.979	
2.082.384	-30.979	
2.082.581	-30.978	
2.082.646	-30.978	
2.082.711	-30.977	
2.082.744	-30.977	
2.082.759	-30.976	
2.082.905	-30.976	
2.083.062	-30.975	
2.083.143	-30.975	
2.083.271	-30.974	
2.083.369	-30.974	
2.083.407	-30.973	
2.083.678	-30.973	
2.084.005	-30.972	
2.084.186	-30.972	
2.084.357	-30.971	
2.084.518	-30.971	
2.084.677	-30.971	
2.084.848	-30.970	
2.085.072	-30.970	
2.085.289	-30.970	
2.085.452	-30.969	

2.085.560	-30.969	
2.085.650	-30.969	
2.085.760	-30.969	
2.085.900	-30.968	
2.086.057	-30.968	
2.086.252	-30.968	
2.086.346	-30.968	
2.086.498	-30.968	
2.086.714	-30.968	
2.086.926	-30.968	
2.087.133	-30.968	
2.087.349	-30.968	
2.087.535	-30.968	
2.087.702	-30.968	
2.087.872	-30.968	
2.088.000	-30.968	
2.088.139	-30.968	
2.088.326	-30.968	
2.088.510	-30.969	
2.088.674	-30.969	
2.088.808	-30.969	
2.088.951	-30.969	
2.089.083	-30.970	
2.089.193	-30.970	
2.089.334	-30.971	
2.089.572	-30.971	
2.089.758	-30.972	
2.089.912	-30.972	
2.090.085	-30.973	
2.090.231	-30.973	

2.090.385	-30.974	
2.090.585	-30.975	
2.090.748	-30.975	
2.090.831	-30.976	
2.090.993	-30.977	
2.091.120	-30.978	
2.091.238	-30.979	
2.091.465	-30.980	
2.091.743	-30.981	
2.092.003	-30.982	
2.092.157	-30.983	
2.092.290	-30.984	
2.092.469	-30.985	
2.092.605	-30.986	
2.092.755	-30.987	
2.092.898	-30.989	
2.093.089	-30.990	
2.093.284	-30.991	
2.093.461	-30.993	
2.093.582	-30.994	
2.093.723	-30.995	
2.093.907	-30.997	
2.094.061	-30.998	
2.094.214	-31.000	
2.094.417	-31.001	
2.094.632	-31.003	
2.094.780	-31.004	
2.094.931	-31.006	
2.095.062	-31.007	
2.095.139	-31.009	

2.095.251	-31.011	
2.095.388	-31.013	
2.095.573	-31.014	
2.095.755	-31.016	
2.095.869	-31.018	
2.095.992	-31.020	
2.096.176	-31.021	
2.096.328	-31.023	
2.096.532	-31.025	
2.096.776	-31.027	
2.096.906	-31.029	
2.097.078	-31.031	
2.097.300	-31.033	
2.097.507	-31.035	
2.097.746	-31.036	
2.097.936	-31.038	
2.098.140	-31.040	
2.098.293	-31.042	
2.098.416	-31.044	
2.098.542	-31.046	
2.098.627	-31.048	
2.098.792	-31.050	
2.098.929	-31.052	
2.099.059	-31.054	
2.099.280	-31.056	
2.099.462	-31.058	
2.099.641	-31.060	
2.099.830	-31.062	
2.099.958	-31.064	
2.100.108	-31.066	

2.100.303	-31.068	
2.100.530	-31.070	
2.100.735	-31.072	
2.100.936	-31.074	
2.101.129	-31.075	
2.101.308	-31.077	
2.101.503	-31.079	
2.101.646	-31.081	
2.101.736	-31.083	
2.101.810	-31.085	
2.102.014	-31.087	
2.102.323	-31.089	
2.102.471	-31.091	
2.102.522	-31.093	
2.102.668	-31.095	
2.102.885	-31.096	
2.103.013	-31.098	
2.103.196	-31.100	
2.103.393	-31.102	
2.103.557	-31.104	
2.103.804	-31.105	
2.103.969	-31.107	
2.104.050	-31.109	
2.104.229	-31.111	
2.104.440	-31.112	
2.104.578	-31.114	
2.104.722	-31.116	
2.104.908	-31.117	
2.105.085	-31.119	
2.105.237	-31.121	

2.105.370	-31.122	
2.105.517	-31.124	
2.105.694	-31.125	
2.105.893	-31.127	
2.106.113	-31.129	
2.106.297	-31.130	
2.106.442	-31.132	
2.106.548	-31.133	
2.106.579	-31.135	
2.106.929	-31.136	
2.107.478	-31.138	
2.107.758	-31.139	
2.107.879	-31.140	
2.107.912	-31.142	
2.107.973	-31.143	
2.108.143	-31.144	
2.108.243	-31.146	
2.108.259	-31.147	
2.108.384	-31.148	
2.108.578	-31.149	
2.108.694	-31.150	
2.108.822	-31.152	
2.109.007	-31.153	
2.109.198	-31.154	
2.109.395	-31.155	
2.109.529	-31.156	
2.109.670	-31.157	
2.109.921	-31.158	
2.110.141	-31.159	
2.110.251	-31.160	

2.110.378	-31.161	
2.110.504	-31.162	
2.110.620	-31.163	
2.110.827	-31.164	
2.111.017	-31.165	
2.111.134	-31.166	
2.111.288	-31.167	
2.111.479	-31.167	
2.111.677	-31.168	
2.111.854	-31.169	
2.112.009	-31.170	
2.112.135	-31.171	
2.112.354	-31.171	
2.112.567	-31.172	
2.112.719	-31.173	
2.112.935	-31.174	
2.113.111	-31.174	
2.113.268	-31.175	
2.113.468	-31.176	
2.113.615	-31.176	
2.113.716	-31.177	
2.113.835	-31.177	
2.113.999	-31.178	
2.114.155	-31.179	
2.114.363	-31.179	
2.114.552	-31.180	
2.114.664	-31.180	
2.114.823	-31.181	
2.115.023	-31.181	
2.115.190	-31.182	

2.115.300	-31.183	
2.115.471	-31.183	
2.115.703	-31.184	
2.115.896	-31.184	
2.116.015	-31.185	
2.116.104	-31.185	
2.116.223	-31.186	
2.116.413	-31.186	
2.116.597	-31.187	
2.116.833	-31.187	
2.117.115	-31.188	
2.117.258	-31.188	
2.117.379	-31.188	
2.117.574	-31.189	
2.117.733	-31.189	
2.117.820	-31.190	
2.117.948	-31.190	
2.118.148	-31.191	
2.118.324	-31.191	
2.118.443	-31.191	
2.118.575	-31.192	
2.118.795	-31.192	
2.118.974	-31.192	
2.119.146	-31.193	
2.119.352	-31.193	
2.119.504	-31.193	
2.119.679	-31.194	
2.119.812	-31.194	
2.119.944	-31.194	
2.120.182	-31.194	

2.120.385	-31.195	
2.120.482	-31.195	
2.120.589	-31.195	
2.120.757	-31.195	
2.120.914	-31.196	
2.121.035	-31.196	
2.121.160	-31.196	
2.121.317	-31.196	
2.121.519	-31.196	
2.121.711	-31.196	
2.121.872	-31.196	
2.122.039	-31.197	
2.122.264	-31.197	
2.122.531	-31.197	
2.122.701	-31.197	
2.122.867	-31.197	
2.123.055	-31.197	
2.123.204	-31.197	
2.123.353	-31.197	
2.123.533	-31.197	
2.123.709	-31.197	
2.123.869	-31.197	
2.123.980	-31.197	
2.124.057	-31.197	
2.124.205	-31.196	
2.124.386	-31.196	
2.124.556	-31.196	
2.124.715	-31.196	
2.124.884	-31.196	
2.125.103	-31.196	

2.125.287	-31.196	
2.125.403	-31.195	
2.125.600	-31.195	
2.125.804	-31.195	
2.125.979	-31.195	
2.126.187	-31.194	
2.126.330	-31.194	
2.126.487	-31.194	
2.126.689	-31.194	
2.126.794	-31.193	
2.126.922	-31.193	
2.127.179	-31.193	
2.127.375	-31.192	
2.127.506	-31.192	
2.127.672	-31.192	
2.127.822	-31.191	
2.127.925	-31.191	
2.128.092	-31.191	
2.128.293	-31.190	
2.128.458	-31.190	
2.128.631	-31.189	
2.128.873	-31.189	
2.129.081	-31.188	
2.129.232	-31.188	
2.129.381	-31.188	
2.129.500	-31.187	
2.129.617	-31.187	
2.129.787	-31.186	
2.130.025	-31.186	
2.130.201	-31.185	

2.130.336	-31.185	
2.130.524	-31.184	
2.130.690	-31.184	
2.130.854	-31.183	
2.131.040	-31.183	
2.131.209	-31.182	
2.131.367	-31.182	
2.131.581	-31.181	
2.131.759	-31.181	
2.131.821	-31.180	
2.132.105	-31.180	
2.132.592	-31.179	
2.132.861	-31.178	
2.132.943	-31.178	
2.133.036	-31.177	
2.133.136	-31.177	
2.133.172	-31.176	
2.133.233	-31.176	
2.133.391	-31.175	
2.133.503	-31.175	
2.133.535	-31.174	
2.133.716	-31.173	
2.133.958	-31.173	
2.134.147	-31.172	
2.134.294	-31.172	
2.134.453	-31.171	
2.134.643	-31.171	
2.134.798	-31.170	
2.134.958	-31.170	
2.135.163	-31.169	

2.135.347	-31.168	
2.135.454	-31.168	
2.135.592	-31.167	
2.135.810	-31.167	
2.136.004	-31.166	
2.136.154	-31.166	
2.136.315	-31.165	
2.136.467	-31.165	
2.136.574	-31.165	
2.136.733	-31.164	
2.136.935	-31.164	
2.137.079	-31.163	
2.137.246	-31.163	
2.137.408	-31.162	
2.137.612	-31.162	
2.137.829	-31.162	
2.138.057	-31.161	
2.138.279	-31.161	
2.138.409	-31.161	
2.138.505	-31.160	
2.138.635	-31.160	
2.138.792	-31.160	
2.139.001	-31.159	
2.139.182	-31.159	
2.139.298	-31.159	
2.139.454	-31.158	
2.139.621	-31.158	
2.139.767	-31.158	
2.139.906	-31.158	
2.140.083	-31.157	

2.140.277	-31.157	
2.140.436	-31.157	
2.140.591	-31.157	
2.140.757	-31.157	
2.140.878	-31.157	
2.140.990	-31.157	
2.141.155	-31.156	
2.141.329	-31.156	
2.141.527	-31.156	
2.141.725	-31.156	
2.141.855	-31.156	
2.142.056	-31.156	
2.142.310	-31.156	
2.142.478	-31.156	
2.142.610	-31.156	
2.142.758	-31.156	
2.142.887	-31.156	
2.143.037	-31.156	
2.143.212	-31.156	
2.143.369	-31.157	
2.143.530	-31.157	
2.143.738	-31.157	
2.143.947	-31.157	
2.144.112	-31.157	
2.144.243	-31.157	
2.144.386	-31.157	
2.144.576	-31.157	
2.144.738	-31.157	
2.144.893	-31.157	
2.144.988	-31.157	

2.145.159	-31.158	
2.145.432	-31.158	
2.145.638	-31.158	
2.145.763	-31.158	
2.145.880	-31.158	
2.146.001	-31.158	
2.146.093	-31.158	
2.146.236	-31.159	
2.146.360	-31.159	
2.146.526	-31.159	
2.146.758	-31.159	
2.146.938	-31.159	
2.147.155	-31.159	
2.147.375	-31.159	
2.147.612	-31.159	
2.147.816	-31.160	
2.147.943	-31.160	
2.148.121	-31.160	
2.148.346	-31.160	
2.148.535	-31.160	
2.148.669	-31.160	
2.148.779	-31.160	
2.148.871	-31.160	
2.149.016	-31.160	
2.149.160	-31.160	
2.149.294	-31.160	
2.149.480	-31.160	
2.149.651	-31.160	
2.149.791	-31.160	
2.149.944	-31.160	

2.150.123	-31.160	
2.150.345	-31.160	
2.150.531	-31.160	
2.150.687	-31.160	
2.150.875	-31.160	
2.151.082	-31.160	
2.151.265	-31.160	
2.151.398	-31.160	
2.151.559	-31.160	
2.151.794	-31.160	
2.152.013	-31.160	
2.152.124	-31.160	
2.152.206	-31.160	
2.152.446	-31.160	
2.152.618	-31.160	
2.152.692	-31.160	
2.152.867	-31.159	
2.153.057	-31.159	
2.153.252	-31.159	
2.153.452	-31.159	
2.153.625	-31.159	
2.153.738	-31.159	
2.153.902	-31.159	
2.154.115	-31.158	
2.154.307	-31.158	
2.154.505	-31.158	
2.154.628	-31.158	
2.154.755	-31.158	
2.154.935	-31.157	
2.155.096	-31.157	

2.155.295	-31.157	
2.155.454	-31.157	
2.155.592	-31.157	
2.155.760	-31.156	
2.155.940	-31.156	
2.156.113	-31.156	
2.156.246	-31.156	
2.156.431	-31.156	
2.156.602	-31.155	
2.156.772	-31.155	
2.156.931	-31.155	
2.157.083	-31.155	
2.157.506	-31.154	
2.157.944	-31.154	
2.158.104	-31.154	
2.158.158	-31.154	
2.158.289	-31.154	
2.158.387	-31.153	
2.158.380	-31.153	
2.158.409	-31.153	
2.158.470	-31.153	
2.158.605	-31.153	
2.158.779	-31.152	
2.158.911	-31.152	
2.159.124	-31.152	
2.159.401	-31.152	
2.159.605	-31.152	
2.159.765	-31.151	
2.159.921	-31.151	
2.160.078	-31.151	

2.160.220	-31.151	
2.160.418	-31.151	
2.160.611	-31.151	
2.160.793	-31.151	
2.160.930	-31.151	
2.161.003	-31.150	
2.161.207	-31.150	
2.161.449	-31.150	
2.161.612	-31.150	
2.161.738	-31.150	
2.161.863	-31.150	
2.161.989	-31.150	
2.162.112	-31.150	
2.162.285	-31.151	
2.162.495	-31.151	
2.162.728	-31.151	
2.162.950	-31.151	
2.163.125	-31.151	
2.163.242	-31.151	
2.163.412	-31.151	
2.163.620	-31.151	
2.163.793	-31.152	
2.163.960	-31.152	
2.164.093	-31.152	
2.164.200	-31.152	
2.164.292	-31.153	
2.164.422	-31.153	
2.164.594	-31.153	
2.164.801	-31.153	
2.164.960	-31.154	

2.165.083	-31.154	
2.165.316	-31.155	
2.165.542	-31.155	
2.165.724	-31.155	
2.165.876	-31.156	
2.166.017	-31.156	
2.166.201	-31.157	
2.166.335	-31.157	
2.166.438	-31.158	
2.166.577	-31.159	
2.166.731	-31.159	
2.166.942	-31.160	
2.167.108	-31.160	
2.167.249	-31.161	
2.167.461	-31.162	
2.167.657	-31.163	
2.167.836	-31.163	
2.167.997	-31.164	
2.168.186	-31.165	
2.168.344	-31.166	
2.168.465	-31.166	
2.168.642	-31.167	
2.168.853	-31.168	
2.169.030	-31.169	
2.169.144	-31.170	
2.169.305	-31.170	
2.169.545	-31.171	
2.169.753	-31.172	
2.169.939	-31.173	
2.170.135	-31.174	

2.170.306	-31.175	
2.170.369	-31.176	
2.170.479	-31.177	
2.170.754	-31.178	
2.170.902	-31.179	
2.171.004	-31.180	
2.171.200	-31.181	
2.171.322	-31.182	
2.171.382	-31.182	
2.171.516	-31.183	
2.171.707	-31.184	
2.171.848	-31.186	
2.172.025	-31.187	
2.172.244	-31.188	
2.172.383	-31.189	
2.172.632	-31.190	
2.172.885	-31.191	
2.173.028	-31.192	
2.173.210	-31.193	
2.173.443	-31.194	
2.173.640	-31.195	
2.173.745	-31.196	
2.173.866	-31.197	
2.173.996	-31.198	
2.174.159	-31.199	
2.174.312	-31.200	
2.174.444	-31.201	
2.174.606	-31.202	
2.174.772	-31.203	
2.174.893	-31.204	

2.175.071	-31.205	
2.175.313	-31.206	
2.175.499	-31.207	
2.175.656	-31.207	
2.175.766	-31.208	
2.175.907	-31.209	
2.176.131	-31.210	
2.176.344	-31.211	
2.176.561	-31.212	
2.176.754	-31.213	
2.176.884	-31.214	
2.177.007	-31.215	
2.177.166	-31.216	
2.177.323	-31.217	
2.177.475	-31.218	
2.177.654	-31.218	
2.177.809	-31.219	
2.178.060	-31.220	
2.178.289	-31.221	
2.178.380	-31.222	
2.178.465	-31.223	
2.178.629	-31.224	
2.178.826	-31.224	
2.179.007	-31.225	
2.179.211	-31.226	
2.179.375	-31.227	
2.179.538	-31.228	
2.179.724	-31.228	
2.179.843	-31.229	
2.180.016	-31.230	

2.180.147	-31.231	
2.180.280	-31.231	
2.180.461	-31.232	
2.180.645	-31.233	
2.180.815	-31.234	
2.180.978	-31.234	
2.181.189	-31.235	
2.181.348	-31.236	
2.181.479	-31.236	
2.181.680	-31.237	
2.181.839	-31.238	
2.181.946	-31.239	
2.182.130	-31.239	
2.182.509	-31.240	
2.182.921	-31.241	
2.183.143	-31.241	
2.183.250	-31.242	
2.183.367	-31.242	
2.183.517	-31.243	
2.183.604	-31.244	
2.183.655	-31.244	
2.183.701	-31.245	
2.183.795	-31.246	
2.183.983	-31.246	
2.184.111	-31.247	
2.184.232	-31.247	
2.184.451	-31.248	
2.184.669	-31.248	
2.184.892	-31.249	
2.185.067	-31.250	

2.185.213	-31.250	
2.185.371	-31.251	
2.185.464	-31.251	
2.185.604	-31.252	
2.185.845	-31.252	
2.186.041	-31.253	
2.186.187	-31.254	
2.186.342	-31.254	
2.186.483	-31.255	
2.186.624	-31.255	
2.186.805	-31.256	
2.186.989	-31.257	
2.187.110	-31.257	
2.187.217	-31.258	
2.187.386	-31.258	
2.187.603	-31.259	
2.187.751	-31.260	
2.187.932	-31.260	
2.188.120	-31.261	
2.188.291	-31.262	
2.188.532	-31.262	
2.188.665	-31.263	
2.188.764	-31.263	
2.188.935	-31.264	
2.189.137	-31.265	
2.189.245	-31.265	
2.189.303	-31.266	
2.189.500	-31.267	
2.189.724	-31.267	
2.189.846	-31.268	

2.189.986	-31.269	
2.190.164	-31.269	
2.190.347	-31.270	
2.190.506	-31.271	
2.190.686	-31.272	
2.190.907	-31.272	
2.191.102	-31.273	
2.191.238	-31.274	
2.191.367	-31.275	
2.191.555	-31.275	
2.191.727	-31.276	
2.191.861	-31.277	
2.192.020	-31.278	
2.192.208	-31.279	
2.192.426	-31.279	
2.192.654	-31.280	
2.192.831	-31.281	
2.192.972	-31.282	
2.193.158	-31.283	
2.193.345	-31.284	
2.193.458	-31.284	
2.193.553	-31.285	
2.193.689	-31.286	
2.193.882	-31.287	
2.194.084	-31.288	
2.194.240	-31.289	
2.194.389	-31.289	
2.194.549	-31.290	
2.194.684	-31.291	
2.194.837	-31.292	

2.195.014	-31.293	
2.195.201	-31.294	
2.195.385	-31.295	
2.195.553	-31.295	
2.195.694	-31.296	
2.195.824	-31.297	
2.195.992	-31.298	
2.196.131	-31.299	
2.196.246	-31.300	
2.196.376	-31.301	
2.196.526	-31.302	
2.196.698	-31.302	
2.196.819	-31.303	
2.196.974	-31.304	
2.197.159	-31.305	
2.197.384	-31.306	
2.197.652	-31.307	
2.197.778	-31.308	
2.197.934	-31.308	
2.198.183	-31.309	
2.198.374	-31.310	
2.198.573	-31.311	
2.198.759	-31.312	
2.198.804	-31.313	
2.198.913	-31.313	
2.199.108	-31.314	
2.199.305	-31.315	
2.199.466	-31.316	
2.199.678	-31.317	
2.199.753	-31.317	

2.199.830	-31.318	
2.200.027	-31.319	
2.200.219	-31.320	
2.200.358	-31.320	
2.200.538	-31.321	
2.200.726	-31.322	
2.200.939	-31.323	
2.201.163	-31.323	
2.201.364	-31.324	
2.201.561	-31.325	
2.201.691	-31.325	
2.201.834	-31.326	
2.202.007	-31.327	
2.202.206	-31.327	
2.202.347	-31.328	
2.202.473	-31.329	
2.202.629	-31.329	
2.202.794	-31.330	
2.203.000	-31.331	
2.203.168	-31.331	
2.203.338	-31.332	
2.203.519	-31.332	
2.203.698	-31.333	
2.203.806	-31.334	
2.203.931	-31.334	
2.204.169	-31.335	
2.204.342	-31.335	
2.204.493	-31.336	
2.204.680	-31.336	
2.204.794	-31.337	

2.204.986	-31.337	
2.205.172	-31.338	
2.205.318	-31.338	
2.205.457	-31.339	
2.205.638	-31.339	
2.205.808	-31.340	
2.205.916	-31.340	
2.206.165	-31.341	
2.206.350	-31.341	
2.206.449	-31.342	
2.206.609	-31.342	
2.206.823	-31.342	
2.206.987	-31.343	
2.207.079	-31.343	
2.207.321	-31.344	
2.207.764	-31.344	
2.208.149	-31.344	
2.208.347	-31.345	
2.208.470	-31.345	
2.208.570	-31.345	
2.208.638	-31.346	
2.208.640	-31.346	
2.208.699	-31.347	
2.208.813	-31.347	
2.208.900	-31.347	
2.209.050	-31.347	
2.209.227	-31.348	
2.209.406	-31.348	
2.209.632	-31.348	
2.209.847	-31.349	

2.210.011	-31.349	
2.210.150	-31.349	
2.210.311	-31.349	
2.210.453	-31.350	
2.210.598	-31.350	
2.210.789	-31.350	
2.210.968	-31.351	
2.211.169	-31.351	
2.211.376	-31.351	
2.211.534	-31.351	
2.211.655	-31.352	
2.211.776	-31.352	
2.211.834	-31.352	
2.211.940	-31.353	
2.212.139	-31.353	
2.212.330	-31.353	
2.212.538	-31.354	
2.212.733	-31.354	
2.212.950	-31.354	
2.213.169	-31.354	
2.213.280	-31.355	
2.213.407	-31.355	
2.213.597	-31.355	
2.213.772	-31.356	
2.213.927	-31.356	
2.214.099	-31.357	
2.214.263	-31.357	
2.214.446	-31.357	
2.214.632	-31.358	
2.214.715	-31.358	

2.214.859	-31.358	
2.214.997	-31.359	
2.215.116	-31.359	
2.215.336	-31.359	
2.215.562	-31.360	
2.215.694	-31.360	
2.215.847	-31.361	
2.216.037	-31.361	
2.216.198	-31.362	
2.216.395	-31.362	
2.216.572	-31.363	
2.216.741	-31.363	
2.216.944	-31.364	
2.217.041	-31.364	
2.217.197	-31.365	
2.217.466	-31.365	
2.217.655	-31.366	
2.217.777	-31.366	
2.217.941	-31.367	
2.218.114	-31.367	
2.218.257	-31.368	
2.218.431	-31.368	
2.218.604	-31.369	
2.218.741	-31.370	
2.218.886	-31.370	
2.219.023	-31.371	
2.219.177	-31.371	
2.219.333	-31.372	
2.219.491	-31.372	
2.219.669	-31.373	

2.219.823	-31.374	
2.219.984	-31.374	
2.220.226	-31.375	
2.220.428	-31.376	
2.220.562	-31.376	
2.220.714	-31.377	
2.220.853	-31.377	
2.221.033	-31.378	
2.221.172	-31.379	
2.221.292	-31.379	
2.221.409	-31.380	
2.221.543	-31.381	
2.221.729	-31.381	
2.221.870	-31.382	
2.222.081	-31.382	
2.222.290	-31.383	
2.222.466	-31.384	
2.222.627	-31.384	
2.222.760	-31.385	
2.222.979	-31.386	
2.223.237	-31.386	
2.223.416	-31.387	
2.223.615	-31.388	
2.223.761	-31.388	
2.223.920	-31.389	
2.224.081	-31.390	
2.224.222	-31.390	
2.224.393	-31.391	
2.224.576	-31.391	
2.224.742	-31.392	

2.224.830	-31.393	
2.224.957	-31.393	
2.225.125	-31.394	
2.225.249	-31.394	
2.225.383	-31.395	
2.225.567	-31.395	
2.225.762	-31.396	
2.225.952	-31.397	
2.226.161	-31.397	
2.226.333	-31.398	
2.226.491	-31.398	
2.226.693	-31.399	
2.226.872	-31.399	
2.227.034	-31.400	
2.227.177	-31.401	
2.227.305	-31.401	
2.227.473	-31.402	
2.227.681	-31.402	
2.227.852	-31.403	
2.227.983	-31.403	
2.228.158	-31.404	
2.228.372	-31.404	
2.228.514	-31.405	
2.228.653	-31.405	
2.228.857	-31.406	
2.228.989	-31.406	
2.229.171	-31.407	
2.229.370	-31.407	
2.229.500	-31.408	
2.229.583	-31.408	

2.229.745	-31.409	
2.229.995	-31.409	
2.230.188	-31.409	
2.230.331	-31.410	
2.230.506	-31.410	
2.230.759	-31.411	
2.230.984	-31.411	
2.231.052	-31.412	
2.231.138	-31.412	
2.231.375	-31.413	
2.231.621	-31.413	
2.231.770	-31.414	
2.231.913	-31.414	
2.232.058	-31.414	
2.232.231	-31.415	
2.232.365	-31.415	
2.232.522	-31.416	
2.232.924	-31.416	
2.233.337	-31.417	
2.233.587	-31.417	
2.233.705	-31.418	
2.233.755	-31.418	
2.233.808	-31.419	
2.233.867	-31.419	
2.233.938	-31.419	
2.234.009	-31.420	
2.234.128	-31.420	
2.234.260	-31.421	
2.234.418	-31.421	
2.234.613	-31.422	

2.234.791	-31.422	
2.234.955	-31.422	
2.235.107	-31.423	
2.235.307	-31.423	
2.235.470	-31.424	
2.235.650	-31.424	
2.235.828	-31.425	
2.235.986	-31.425	
2.236.151	-31.426	
2.236.299	-31.426	
2.236.425	-31.427	
2.236.547	-31.427	
2.236.730	-31.428	
2.236.933	-31.428	
2.237.150	-31.429	
2.237.282	-31.429	
2.237.359	-31.430	
2.237.547	-31.430	
2.237.738	-31.431	
2.237.932	-31.432	
2.238.154	-31.432	
2.238.354	-31.433	
2.238.550	-31.433	
2.238.678	-31.434	
2.238.837	-31.435	
2.239.057	-31.435	
2.239.166	-31.436	
2.239.319	-31.437	
2.239.489	-31.437	
2.239.614	-31.438	

2.239.789	-31.439	
2.239.966	-31.439	
2.240.121	-31.440	
2.240.289	-31.441	
2.240.497	-31.442	
2.240.654	-31.442	
2.240.782	-31.443	
2.240.947	-31.444	
2.241.113	-31.445	
2.241.259	-31.445	
2.241.368	-31.446	
2.241.550	-31.447	
2.241.707	-31.448	
2.241.815	-31.449	
2.241.940	-31.450	
2.242.173	-31.451	
2.242.332	-31.452	
2.242.531	-31.452	
2.242.795	-31.453	
2.242.974	-31.454	
2.243.136	-31.455	
2.243.295	-31.456	
2.243.475	-31.457	
2.243.642	-31.458	
2.243.795	-31.459	
2.243.924	-31.460	
2.244.088	-31.461	
2.244.265	-31.462	
2.244.404	-31.463	
2.244.596	-31.464	

2.244.762	-31.465	
2.244.895	-31.466	
2.245.085	-31.467	
2.245.383	-31.468	
2.245.544	-31.469	
2.245.652	-31.470	
2.245.836	-31.471	
2.245.985	-31.472	
2.246.167	-31.473	
2.246.319	-31.474	
2.246.424	-31.475	
2.246.572	-31.476	
2.246.733	-31.477	
2.246.875	-31.478	
2.247.011	-31.479	
2.247.128	-31.480	
2.247.316	-31.481	
2.247.524	-31.482	
2.247.637	-31.483	
2.247.814	-31.484	
2.248.094	-31.485	
2.248.322	-31.486	
2.248.443	-31.487	
2.248.579	-31.488	
2.248.772	-31.489	
2.248.918	-31.490	
2.249.050	-31.490	
2.249.247	-31.491	
2.249.415	-31.492	
2.249.610	-31.493	

2.249.776	-31.494	
2.249.919	-31.494	
2.250.085	-31.495	
2.250.188	-31.496	
2.250.293	-31.497	
2.250.464	-31.497	
2.250.674	-31.498	
2.250.835	-31.499	
2.251.057	-31.500	
2.251.284	-31.500	
2.251.449	-31.501	
2.251.595	-31.501	
2.251.729	-31.502	
2.251.940	-31.503	
2.252.095	-31.503	
2.252.222	-31.504	
2.252.487	-31.504	
2.252.701	-31.505	
2.252.789	-31.506	
2.252.932	-31.506	
2.253.138	-31.507	
2.253.346	-31.507	
2.253.494	-31.507	
2.253.640	-31.508	
2.253.891	-31.508	
2.254.021	-31.509	
2.254.110	-31.509	
2.254.286	-31.510	
2.254.493	-31.510	
2.254.664	-31.510	

2.254.867	-31.511	
2.255.036	-31.511	
2.255.148	-31.511	
2.255.284	-31.511	
2.255.482	-31.512	
2.255.638	-31.512	
2.255.791	-31.512	
2.255.976	-31.513	
2.256.176	-31.513	
2.256.329	-31.513	
2.256.499	-31.513	
2.256.684	-31.513	
2.256.770	-31.514	
2.256.897	-31.514	
2.257.128	-31.514	
2.257.365	-31.514	
2.257.466	-31.514	
2.257.545	-31.515	
2.257.744	-31.515	
2.258.196	-31.515	
2.258.622	-31.515	
2.258.745	-31.515	
2.258.817	-31.515	
2.258.952	-31.515	
2.259.003	-31.516	
2.259.036	-31.516	
2.259.126	-31.516	
2.259.205	-31.516	
2.259.355	-31.516	
2.259.504	-31.516	

2.259.659	-31.516	
2.259.836	-31.516	
2.260.018	-31.516	
2.260.231	-31.516	
2.260.428	-31.516	
2.260.560	-31.516	
2.260.782	-31.517	
2.261.026	-31.517	
2.261.153	-31.517	
2.261.279	-31.517	
2.261.404	-31.517	
2.261.559	-31.517	
2.261.740	-31.517	
2.262.009	-31.517	
2.262.182	-31.517	
2.262.289	-31.518	
2.262.452	-31.518	
2.262.609	-31.518	
2.262.730	-31.518	
2.262.850	-31.518	
2.263.011	-31.519	
2.263.260	-31.519	
2.263.526	-31.519	
2.263.689	-31.519	
2.263.799	-31.520	
2.263.960	-31.520	
2.264.184	-31.520	
2.264.382	-31.520	
2.264.500	-31.521	
2.264.641	-31.521	

2.264.754	-31.521	
2.264.863	-31.522	
2.265.051	-31.522	
2.265.159	-31.522	
2.265.285	-31.523	
2.265.502	-31.523	
2.265.689	-31.524	
2.265.845	-31.524	
2.265.990	-31.525	
2.266.181	-31.525	
2.266.384	-31.526	
2.266.481	-31.526	
2.266.595	-31.527	
2.266.870	-31.527	
2.267.115	-31.528	
2.267.215	-31.529	
2.267.271	-31.529	
2.267.417	-31.530	
2.267.652	-31.531	
2.267.806	-31.531	
2.267.999	-31.532	
2.268.214	-31.533	
2.268.371	-31.534	
2.268.531	-31.534	
2.268.680	-31.535	
2.268.866	-31.536	
2.269.070	-31.537	
2.269.265	-31.538	
2.269.427	-31.539	
2.269.579	-31.539	

2.269.711	-31.540	
2.269.854	-31.541	
2.270.022	-31.542	
2.270.192	-31.543	
2.270.390	-31.544	
2.270.594	-31.545	
2.270.791	-31.546	
2.270.984	-31.547	
2.271.131	-31.548	
2.271.261	-31.549	
2.271.418	-31.550	
2.271.570	-31.551	
2.271.727	-31.552	
2.271.895	-31.553	
2.271.946	-31.554	
2.272.005	-31.555	
2.272.168	-31.556	
2.272.338	-31.557	
2.272.572	-31.558	
2.272.800	-31.559	
2.272.950	-31.560	
2.273.087	-31.561	
2.273.333	-31.562	
2.273.492	-31.563	
2.273.573	-31.564	
2.273.841	-31.565	
2.274.122	-31.566	
2.274.247	-31.567	
2.274.333	-31.568	
2.274.516	-31.569	

2.274.713	-31.570	
2.274.884	-31.571	
2.275.013	-31.572	
2.275.118	-31.573	
2.275.303	-31.574	
2.275.470	-31.575	
2.275.627	-31.576	
2.275.854	-31.577	
2.275.956	-31.578	
2.276.070	-31.579	
2.276.294	-31.580	
2.276.503	-31.581	
2.276.676	-31.581	
2.276.850	-31.582	
2.277.009	-31.583	
2.277.159	-31.584	
2.277.385	-31.585	
2.277.623	-31.586	
2.277.782	-31.587	
2.277.932	-31.587	
2.278.103	-31.588	
2.278.266	-31.589	
2.278.423	-31.590	
2.278.543	-31.591	
2.278.710	-31.591	
2.278.885	-31.592	
2.279.052	-31.593	
2.279.271	-31.594	
2.279.445	-31.594	
2.279.597	-31.595	

2.279.689	-31.596	
2.279.811	-31.597	
2.280.007	-31.597	
2.280.208	-31.598	
2.280.368	-31.598	
2.280.533	-31.599	
2.280.710	-31.600	
2.280.844	-31.600	
2.280.999	-31.601	
2.281.209	-31.602	
2.281.386	-31.602	
2.281.494	-31.603	
2.281.667	-31.603	
2.281.899	-31.604	
2.282.068	-31.604	
2.282.225	-31.605	
2.282.405	-31.605	
2.282.565	-31.606	
2.282.679	-31.606	
2.282.789	-31.607	
2.282.952	-31.607	
2.283.351	-31.608	
2.283.792	-31.608	
2.284.030	-31.609	
2.284.206	-31.609	
2.284.160	-31.610	
2.284.168	-31.610	
2.284.319	-31.611	
2.284.375	-31.611	
2.284.431	-31.612	

2.284.561	-31.612	
2.284.749	-31.612	
2.284.865	-31.613	
2.284.960	-31.613	
2.285.138	-31.614	
2.285.385	-31.614	
2.285.647	-31.614	
2.285.836	-31.615	
2.285.981	-31.615	
2.286.221	-31.615	
2.286.405	-31.616	
2.286.518	-31.616	
2.286.694	-31.617	
2.286.875	-31.617	
2.287.022	-31.617	
2.287.215	-31.618	
2.287.381	-31.618	
2.287.468	-31.618	
2.287.598	-31.619	
2.287.755	-31.619	
2.287.840	-31.620	
2.287.973	-31.620	
2.288.194	-31.620	
2.288.362	-31.621	
2.288.591	-31.621	
2.288.777	-31.622	
2.288.889	-31.622	
2.289.158	-31.623	
2.289.392	-31.623	
2.289.532	-31.624	

2.289.655	-31.624	
2.289.736	-31.624	
2.289.827	-31.625	
2.289.973	-31.625	
2.290.155	-31.626	
2.290.329	-31.626	
2.290.414	-31.627	
2.290.611	-31.628	
2.290.820	-31.628	
2.290.894	-31.629	
2.291.096	-31.629	
2.291.366	-31.630	
2.291.465	-31.630	
2.291.669	-31.631	
2.291.919	-31.632	
2.292.074	-31.632	
2.292.202	-31.633	
2.292.292	-31.634	
2.292.428	-31.634	
2.292.638	-31.635	
2.292.797	-31.636	
2.292.955	-31.636	
2.293.176	-31.637	
2.293.409	-31.638	
2.293.637	-31.639	
2.293.823	-31.640	
2.293.961	-31.640	
2.294.026	-31.641	
2.294.182	-31.642	
2.294.353	-31.643	

2.294.536	-31.644	
2.294.782	-31.645	
2.294.967	-31.646	
2.295.135	-31.647	
2.295.224	-31.647	
2.295.313	-31.648	
2.295.493	-31.649	
2.295.681	-31.650	
2.295.865	-31.651	
2.296.008	-31.652	
2.296.138	-31.653	
2.296.333	-31.654	
2.296.509	-31.655	
2.296.659	-31.656	
2.296.852	-31.657	
2.297.036	-31.658	
2.297.126	-31.659	
2.297.166	-31.660	
2.297.311	-31.661	
2.297.520	-31.662	
2.297.703	-31.663	
2.297.849	-31.664	
2.298.022	-31.665	
2.298.194	-31.666	
2.298.380	-31.668	
2.298.672	-31.669	
2.298.904	-31.670	
2.299.050	-31.671	
2.299.191	-31.672	
2.299.344	-31.673	

2.299.581	-31.674	
2.299.801	-31.675	
2.299.919	-31.677	
2.300.051	-31.678	
2.300.195	-31.679	
2.300.387	-31.680	
2.300.577	-31.681	
2.300.661	-31.682	
2.300.775	-31.683	
2.300.940	-31.685	
2.301.095	-31.686	
2.301.254	-31.687	
2.301.424	-31.688	
2.301.584	-31.689	
2.301.758	-31.690	
2.301.978	-31.692	
2.302.153	-31.693	
2.302.329	-31.694	
2.302.589	-31.695	
2.302.750	-31.696	
2.302.831	-31.698	
2.302.990	-31.699	
2.303.163	-31.700	
2.303.320	-31.701	
2.303.468	-31.703	
2.303.562	-31.704	
2.303.700	-31.705	
2.303.956	-31.707	
2.304.229	-31.708	
2.304.389	-31.709	

2.304.400	-31.711	
2.304.560	-31.712	
2.304.787	-31.713	
2.304.940	-31.715	
2.305.148	-31.716	
2.305.318	-31.717	
2.305.441	-31.719	
2.305.659	-31.720	
2.305.860	-31.721	
2.305.972	-31.723	
2.306.100	-31.724	
2.306.295	-31.726	
2.306.462	-31.727	
2.306.626	-31.729	
2.306.779	-31.730	
2.306.948	-31.732	
2.307.101	-31.733	
2.307.262	-31.735	
2.307.517	-31.736	
2.307.661	-31.738	
2.307.800	-31.739	
2.308.031	-31.741	
2.308.181	-31.742	
2.308.221	-31.744	
2.308.405	-31.746	
2.308.824	-31.747	
2.309.171	-31.749	
2.309.364	-31.751	
2.309.590	-31.752	
2.309.734	-31.754	

2.309.778	-31.756	
2.309.731	-31.757	
2.309.715	-31.759	
2.309.830	-31.761	
2.309.966	-31.763	
2.310.143	-31.765	
2.310.381	-31.766	
2.310.533	-31.768	
2.310.616	-31.770	
2.310.790	-31.772	
2.310.972	-31.774	
2.311.185	-31.776	
2.311.413	-31.778	
2.311.552	-31.780	
2.311.684	-31.782	
2.311.879	-31.784	
2.312.051	-31.786	
2.312.227	-31.788	
2.312.363	-31.790	
2.312.457	-31.792	
2.312.654	-31.794	
2.312.917	-31.796	
2.313.030	-31.798	
2.313.055	-31.801	
2.313.210	-31.803	
2.313.427	-31.805	
2.313.613	-31.807	
2.313.743	-31.810	
2.313.839	-31.812	
2.314.045	-31.814	

2.314.268	-31.817	
2.314.458	-31.819	
2.314.641	-31.822	
2.314.830	-31.824	
2.315.002	-31.827	
2.315.094	-31.829	
2.315.320	-31.832	
2.315.574	-31.834	
2.315.690	-31.837	
2.315.782	-31.839	
2.315.869	-31.842	
2.315.983	-31.845	
2.316.174	-31.847	
2.316.393	-31.850	
2.316.581	-31.853	
2.316.720	-31.855	
2.316.815	-31.858	
2.316.995	-31.861	
2.317.301	-31.864	
2.317.533	-31.867	
2.317.661	-31.869	
2.317.748	-31.872	
2.317.883	-31.875	
2.318.036	-31.878	
2.318.215	-31.881	
2.318.443	-31.884	
2.318.636	-31.887	
2.318.766	-31.890	
2.318.913	-31.893	
2.319.090	-31.896	

2.319.256	-31.899	
2.319.440	-31.903	
2.319.603	-31.906	
2.319.731	-31.909	
2.319.854	-31.912	
2.320.015	-31.915	
2.320.135	-31.918	
2.320.280	-31.921	
2.320.483	-31.925	
2.320.665	-31.928	
2.320.811	-31.931	
2.320.993	-31.934	
2.321.202	-31.938	
2.321.409	-31.941	
2.321.586	-31.944	
2.321.740	-31.947	
2.321.893	-31.951	
2.322.065	-31.954	
2.322.170	-31.957	
2.322.366	-31.960	
2.322.513	-31.964	
2.322.607	-31.967	
2.322.806	-31.970	
2.322.950	-31.974	
2.323.042	-31.977	
2.323.198	-31.980	
2.323.396	-31.984	
2.323.548	-31.987	
2.323.693	-31.990	
2.323.915	-31.993	

2.324.147	-31.997	
2.324.366	-32.000	
2.324.569	-32.003	
2.324.749	-32.007	
2.324.937	-32.010	
2.325.161	-32.013	
2.325.306	-32.017	
2.325.423	-32.020	
2.325.535	-32.023	
2.325.667	-32.026	
2.325.800	-32.030	
2.325.974	-32.033	
2.326.174	-32.036	
2.326.344	-32.039	
2.326.485	-32.043	
2.326.588	-32.046	
2.326.727	-32.049	
2.326.866	-32.052	
2.327.016	-32.055	
2.327.206	-32.058	
2.327.415	-32.061	
2.327.652	-32.065	
2.327.874	-32.068	
2.328.008	-32.071	
2.328.178	-32.074	
2.328.389	-32.077	
2.328.523	-32.080	
2.328.662	-32.083	
2.328.849	-32.086	
2.329.039	-32.089	

2.329.203	-32.092	
2.329.332	-32.095	
2.329.521	-32.098	
2.329.660	-32.101	
2.329.787	-32.104	
2.329.971	-32.107	
2.330.141	-32.110	
2.330.329	-32.113	
2.330.479	-32.116	
2.330.535	-32.119	
2.330.739	-32.122	
2.331.086	-32.125	
2.331.279	-32.127	
2.331.387	-32.130	
2.331.494	-32.133	
2.331.662	-32.136	
2.331.852	-32.139	
2.332.083	-32.141	
2.332.219	-32.144	
2.332.352	-32.147	
2.332.502	-32.150	
2.332.699	-32.152	
2.332.897	-32.155	
2.333.096	-32.158	
2.333.308	-32.160	
2.333.407	-32.163	
2.333.541	-32.166	
2.333.758	-32.168	
2.333.885	-32.171	
2.333.947	-32.174	

2.334.180	-32.176	
2.334.655	-32.179	
2.335.009	-32.181	
2.335.177	-32.184	
2.335.322	-32.186	
2.335.376	-32.189	
2.335.360	-32.192	
2.335.405	-32.194	
2.335.551	-32.196	
2.335.665	-32.199	
2.335.779	-32.201	
2.335.934	-32.204	
2.336.057	-32.206	
2.336.221	-32.209	
2.336.425	-32.211	
2.336.667	-32.213	
2.336.812	-32.216	
2.336.926	-32.218	
2.337.137	-32.220	
2.337.339	-32.223	
2.337.567	-32.225	
2.337.706	-32.227	
2.337.863	-32.229	
2.338.069	-32.232	
2.338.205	-32.234	
2.338.299	-32.236	
2.338.485	-32.238	
2.338.584	-32.240	
2.338.728	-32.243	
2.338.958	-32.245	

2.339.041	-32.247	
2.339.180	-32.249	
2.339.413	-32.251	
2.339.637	-32.254	
2.339.906	-32.256	
2.340.062	-32.258	
2.339.993	-32.260	
2.340.120	-32.262	
2.340.475	-32.264	
2.340.739	-32.266	
2.340.856	-32.268	
2.340.990	-32.270	
2.341.212	-32.272	
2.341.370	-32.275	
2.341.483	-32.277	
2.341.626	-32.279	
2.341.754	-32.281	
2.341.926	-32.283	
2.342.144	-32.285	
2.342.294	-32.287	
2.342.430	-32.289	
2.342.547	-32.291	
2.342.664	-32.293	
2.342.800	-32.295	
2.342.977	-32.297	
2.343.170	-32.298	
2.343.420	-32.300	
2.343.653	-32.302	
2.343.770	-32.304	
2.343.904	-32.306	

2.344.030	-32.308	
2.344.215	-32.310	
2.344.426	-32.312	
2.344.624	-32.314	
2.344.771	-32.316	
2.344.968	-32.318	
2.345.217	-32.320	
2.345.352	-32.322	
2.345.470	-32.324	
2.345.672	-32.325	
2.345.860	-32.327	
2.345.974	-32.329	
2.346.071	-32.331	
2.346.202	-32.333	
2.346.407	-32.335	
2.346.671	-32.336	
2.346.870	-32.338	
2.347.049	-32.340	
2.347.152	-32.341	
2.347.269	-32.343	
2.347.442	-32.345	
2.347.655	-32.346	
2.347.865	-32.348	
2.348.064	-32.350	
2.348.333	-32.351	
2.348.510	-32.353	
2.348.626	-32.354	
2.348.802	-32.356	
2.348.866	-32.358	
2.348.875	-32.359	

2.348.978	-32.361	
2.349.126	-32.362	
2.349.294	-32.363	
2.349.478	-32.365	
2.349.682	-32.366	
2.349.866	-32.368	
2.350.107	-32.369	
2.350.282	-32.371	
2.350.443	-32.372	
2.350.642	-32.373	
2.350.851	-32.374	
2.351.032	-32.376	
2.351.105	-32.377	
2.351.210	-32.378	
2.351.497	-32.379	
2.351.698	-32.381	
2.351.776	-32.382	
2.351.937	-32.383	
2.352.029	-32.384	
2.352.115	-32.385	
2.352.309	-32.386	
2.352.536	-32.387	
2.352.715	-32.388	
2.352.758	-32.389	
2.352.878	-32.390	
2.353.120	-32.391	
2.353.405	-32.392	
2.353.634	-32.393	
2.353.779	-32.394	
2.353.982	-32.394	

2.354.126	-32.395	
2.354.247	-32.396	
2.354.393	-32.397	
2.354.590	-32.398	
2.354.856	-32.398	
2.354.951	-32.399	
2.355.034	-32.400	
2.355.237	-32.400	
2.355.396	-32.401	
2.355.568	-32.402	
2.355.829	-32.402	
2.356.008	-32.403	
2.356.149	-32.403	
2.356.305	-32.404	
2.356.429	-32.404	
2.356.556	-32.405	
2.356.769	-32.405	
2.357.007	-32.406	
2.357.181	-32.406	
2.357.320	-32.407	
2.357.467	-32.407	
2.357.639	-32.407	
2.357.872	-32.408	
2.358.109	-32.408	
2.358.221	-32.408	
2.358.331	-32.408	
2.358.490	-32.409	
2.358.671	-32.409	
2.358.830	-32.409	
2.358.940	-32.409	

2.359.077	-32.409	
2.359.292	-32.409	
2.359.464	-32.410	
2.359.621	-32.410	
2.359.767	-32.410	
2.359.966	-32.410	
2.360.336	-32.410	
2.360.748	-32.410	
2.360.993	-32.410	
2.361.066	-32.410	
2.361.152	-32.410	
2.361.216	-32.410	
2.361.102	-32.410	
2.361.104	-32.410	
2.361.310	-32.409	
2.361.532	-32.409	
2.361.668	-32.409	
2.361.783	-32.409	
2.361.987	-32.409	
2.362.350	-32.409	
2.362.666	-32.408	
2.362.766	-32.408	
2.362.834	-32.408	
2.362.948	-32.408	
2.363.082	-32.407	
2.363.245	-32.407	
2.363.523	-32.407	
2.363.737	-32.407	
2.363.889	-32.406	
2.363.958	-32.406	

2.363.976	-32.406	
2.364.216	-32.405	
2.364.458	-32.405	
2.364.576	-32.405	
2.364.749	-32.405	
2.364.865	-32.404	
2.364.930	-32.404	
2.365.110	-32.404	
2.365.340	-32.404	
2.365.542	-32.403	
2.365.773	-32.403	
2.365.987	-32.403	
2.366.136	-32.402	
2.366.351	-32.402	
2.366.552	-32.402	
2.366.751	-32.402	
2.366.953	-32.401	
2.367.003	-32.401	
2.367.051	-32.401	
2.367.284	-32.401	
2.367.531	-32.400	
2.367.581	-32.400	
2.367.786	-32.400	
2.368.080	-32.400	
2.368.248	-32.399	
2.368.384	-32.399	
2.368.488	-32.399	
2.368.624	-32.399	
2.368.790	-32.399	
2.368.924	-32.398	

2.369.063	-32.398	
2.369.227	-32.398	
2.369.415	-32.398	
2.369.617	-32.398	
2.369.765	-32.398	
2.369.917	-32.398	
2.370.137	-32.397	
2.370.347	-32.397	
2.370.374	-32.397	
2.370.426	-32.397	
2.370.674	-32.397	
2.370.956	-32.397	
2.371.173	-32.397	
2.371.322	-32.397	
2.371.447	-32.397	
2.371.579	-32.397	
2.371.777	-32.397	
2.371.994	-32.397	
2.372.157	-32.397	
2.372.345	-32.397	
2.372.493	-32.397	
2.372.641	-32.397	
2.372.815	-32.397	
2.372.992	-32.397	
2.373.114	-32.397	
2.373.260	-32.397	
2.373.514	-32.398	
2.373.689	-32.398	
2.373.813	-32.398	
2.374.036	-32.398	

2.374.158	-32.398	
2.374.305	-32.398	
2.374.484	-32.398	
2.374.542	-32.398	
2.374.675	-32.398	
2.374.801	-32.398	
2.374.867	-32.398	
2.375.069	-32.399	
2.375.271	-32.399	
2.375.455	-32.399	
2.375.658	-32.399	
2.375.918	-32.399	
2.376.055	-32.399	
2.376.196	-32.399	
2.376.431	-32.399	
2.376.585	-32.400	
2.376.745	-32.400	
2.376.994	-32.400	
2.377.150	-32.400	
2.377.291	-32.400	
2.377.450	-32.400	
2.377.587	-32.400	
2.377.679	-32.400	
2.377.843	-32.400	
2.378.029	-32.401	
2.378.138	-32.401	
2.378.244	-32.401	
2.378.439	-32.401	
2.378.653	-32.401	
2.378.826	-32.401	

2.379.029	-32.401	
2.379.247	-32.401	
2.379.456	-32.401	
2.379.619	-32.401	
2.379.716	-32.401	
2.379.935	-32.401	
2.380.172	-32.401	
2.380.352	-32.401	
2.380.539	-32.401	
2.380.694	-32.401	
2.380.848	-32.401	
2.380.963	-32.401	
2.381.115	-32.401	
2.381.198	-32.401	
2.381.413	-32.401	
2.381.572	-32.401	
2.381.700	-32.400	
2.381.899	-32.400	
2.382.215	-32.400	
2.382.468	-32.400	
2.382.499	-32.400	
2.382.627	-32.400	
2.382.805	-32.400	
2.382.971	-32.399	
2.383.109	-32.399	
2.383.217	-32.399	
2.383.349	-32.399	
2.383.533	-32.398	
2.383.725	-32.398	
2.383.922	-32.398	

2.384.165	-32.397	
2.384.305	-32.397	
2.384.437	-32.397	
2.384.650	-32.396	
2.384.857	-32.396	
2.385.011	-32.396	
2.385.235	-32.395	
2.385.419	-32.395	
2.385.376	-32.395	
2.385.477	-32.394	
2.385.724	-32.394	
2.385.894	-32.393	
2.386.256	-32.393	
2.386.792	-32.392	
2.387.038	-32.392	
2.387.086	-32.391	
2.387.074	-32.391	
2.386.998	-32.390	
2.387.195	-32.390	
2.387.444	-32.389	
2.387.498	-32.389	
2.387.607	-32.388	
2.387.697	-32.388	
2.387.834	-32.387	
2.387.981	-32.386	
2.388.252	-32.386	
2.388.400	-32.385	
2.388.595	-32.385	
2.388.770	-32.384	
2.388.902	-32.383	

2.389.094	-32.383	
2.389.277	-32.382	
2.389.480	-32.381	
2.389.628	-32.381	
2.389.744	-32.380	
2.389.886	-32.379	
2.390.042	-32.379	
2.390.219	-32.378	
2.390.394	-32.377	
2.390.546	-32.377	
2.390.672	-32.376	
2.390.815	-32.375	
2.390.997	-32.375	
2.391.163	-32.374	
2.391.376	-32.373	
2.391.581	-32.373	
2.391.749	-32.372	
2.391.949	-32.372	
2.392.123	-32.371	
2.392.253	-32.370	
2.392.408	-32.370	
2.392.554	-32.369	
2.392.732	-32.368	
2.392.909	-32.368	
2.393.087	-32.367	
2.393.201	-32.366	
2.393.308	-32.366	
2.393.501	-32.365	
2.393.694	-32.365	
2.393.853	-32.364	

2.394.038	-32.363	
2.394.203	-32.363	
2.394.271	-32.362	
2.394.422	-32.362	
2.394.633	-32.361	
2.394.825	-32.361	
2.395.058	-32.360	
2.395.224	-32.360	
2.395.336	-32.359	
2.395.466	-32.359	
2.395.663	-32.358	
2.395.772	-32.358	
2.395.828	-32.357	
2.396.021	-32.357	
2.396.270	-32.357	
2.396.481	-32.356	
2.396.684	-32.356	
2.396.879	-32.356	
2.397.074	-32.355	
2.397.289	-32.355	
2.397.433	-32.355	
2.397.529	-32.354	
2.397.730	-32.354	
2.397.919	-32.354	
2.398.026	-32.354	
2.398.118	-32.353	
2.398.270	-32.353	
2.398.514	-32.353	
2.398.705	-32.353	
2.398.844	-32.352	

2.399.023	-32.352	
2.399.189	-32.352	
2.399.371	-32.352	
2.399.540	-32.351	
2.399.660	-32.351	
2.399.736	-32.351	
2.399.905	-32.351	
2.400.094	-32.351	
2.400.220	-32.350	
2.400.345	-32.350	
2.400.501	-32.350	
2.400.680	-32.350	
2.400.824	-32.350	
2.400.939	-32.350	
2.401.105	-32.349	
2.401.310	-32.349	
2.401.518	-32.349	
2.401.749	-32.349	
2.402.022	-32.349	
2.402.159	-32.349	
2.402.356	-32.348	
2.402.607	-32.348	
2.402.737	-32.348	
2.402.910	-32.348	
2.403.062	-32.348	
2.403.226	-32.348	
2.403.404	-32.347	
2.403.485	-32.347	
2.403.562	-32.347	
2.403.736	-32.347	

2.403.904	-32.346	
2.404.036	-32.346	
2.404.140	-32.346	
2.404.277	-32.346	
2.404.455	-32.345	
2.404.681	-32.345	
2.404.868	-32.345	
2.405.132	-32.344	
2.405.314	-32.344	
2.405.473	-32.344	
2.405.654	-32.343	
2.405.813	-32.343	
2.405.972	-32.343	
2.406.152	-32.342	
2.406.346	-32.342	
2.406.547	-32.341	
2.406.674	-32.341	
2.406.724	-32.341	
2.406.879	-32.340	
2.407.121	-32.340	
2.407.301	-32.339	
2.407.470	-32.339	
2.407.682	-32.338	
2.407.800	-32.338	
2.407.941	-32.337	
2.408.196	-32.337	
2.408.398	-32.336	
2.408.521	-32.336	
2.408.682	-32.335	
2.408.833	-32.334	

2.409.003	-32.334	
2.409.168	-32.333	
2.409.306	-32.332	
2.409.462	-32.332	
2.409.568	-32.331	
2.409.650	-32.331	
2.409.809	-32.330	
2.410.237	-32.329	
2.410.557	-32.328	
2.410.580	-32.328	
2.410.755	-32.327	
2.411.014	-32.326	
2.411.096	-32.326	
2.411.333	-32.325	
2.411.550	-32.324	
2.411.594	-32.323	
2.411.752	-32.322	
2.412.144	-32.322	
2.412.538	-32.321	
2.412.661	-32.320	
2.412.778	-32.319	
2.412.923	-32.318	
2.413.008	-32.317	
2.413.046	-32.317	
2.413.078	-32.316	
2.413.243	-32.315	
2.413.454	-32.314	
2.413.633	-32.313	
2.413.801	-32.312	
2.413.671	-32.311	

2.413.723	-32.310	
2.414.119	-32.309	
2.414.361	-32.308	
2.414.619	-32.307	
2.414.855	-32.306	
2.414.940	-32.306	
2.415.054	-32.305	
2.415.190	-32.304	
2.415.428	-32.303	
2.415.604	-32.302	
2.415.658	-32.301	
2.415.809	-32.300	
2.415.938	-32.299	
2.416.128	-32.298	
2.416.276	-32.297	
2.416.402	-32.297	
2.416.525	-32.296	
2.416.648	-32.295	
2.416.733	-32.294	
2.416.919	-32.293	
2.417.256	-32.292	
2.417.538	-32.292	
2.417.638	-32.291	
2.417.797	-32.290	
2.418.096	-32.289	
2.418.248	-32.288	
2.418.430	-32.288	
2.418.378	-32.287	
2.418.357	-32.286	
2.418.819	-32.286	

2.419.174	-32.285	
2.419.366	-32.284	
2.419.323	-32.284	
2.419.321	-32.283	
2.419.576	-32.282	
2.419.702	-32.282	
2.419.841	-32.281	
2.420.056	-32.281	
2.420.275	-32.280	
2.420.459	-32.280	
2.420.553	-32.279	
2.420.689	-32.279	
2.420.887	-32.278	
2.421.087	-32.278	
2.421.230	-32.278	
2.421.397	-32.277	
2.421.503	-32.277	
2.421.606	-32.277	
2.421.881	-32.277	
2.422.063	-32.276	
2.422.142	-32.276	
2.422.289	-32.276	
2.422.558	-32.276	
2.422.804	-32.276	
2.422.935	-32.276	
2.423.084	-32.276	
2.423.324	-32.276	
2.423.481	-32.276	
2.423.560	-32.276	
2.423.716	-32.276	

2.423.828	-32.276	
2.423.989	-32.276	
2.424.159	-32.276	
2.424.371	-32.276	
2.424.538	-32.276	
2.424.702	-32.277	
2.424.886	-32.277	
2.425.016	-32.277	
2.425.134	-32.277	
2.425.320	-32.277	
2.425.486	-32.278	
2.425.634	-32.278	
2.425.736	-32.278	
2.425.918	-32.278	
2.426.183	-32.279	
2.426.321	-32.279	
2.426.406	-32.279	
2.426.535	-32.280	
2.426.697	-32.280	
2.426.919	-32.280	
2.427.050	-32.281	
2.427.271	-32.281	
2.427.502	-32.282	
2.427.666	-32.282	
2.427.881	-32.282	
2.428.118	-32.283	
2.428.306	-32.283	
2.428.425	-32.284	
2.428.499	-32.284	
2.428.640	-32.284	

2.428.859	-32.285	
2.429.101	-32.285	
2.429.240	-32.286	
2.429.346	-32.286	
2.429.397	-32.287	
2.429.478	-32.287	
2.429.708	-32.287	
2.430.072	-32.288	
2.430.331	-32.288	
2.430.376	-32.289	
2.430.436	-32.289	
2.430.580	-32.289	
2.430.768	-32.290	
2.430.993	-32.290	
2.431.181	-32.291	
2.431.369	-32.291	
2.431.586	-32.291	
2.431.709	-32.292	
2.431.850	-32.292	
2.432.012	-32.292	
2.432.208	-32.293	
2.432.442	-32.293	
2.432.623	-32.293	
2.432.732	-32.294	
2.432.891	-32.294	
2.433.122	-32.294	
2.433.310	-32.295	
2.433.427	-32.295	
2.433.584	-32.295	
2.433.770	-32.295	

2.433.904	-32.295	
2.434.135	-32.296	
2.434.346	-32.296	
2.434.431	-32.296	
2.434.612	-32.296	
2.434.852	-32.296	
2.434.995	-32.296	
2.435.130	-32.297	
2.435.238	-32.297	
2.435.435	-32.297	
2.435.665	-32.297	
2.435.786	-32.297	
2.435.960	-32.297	
2.436.178	-32.297	
2.436.319	-32.297	
2.436.510	-32.297	
2.436.712	-32.297	
2.436.841	-32.297	
2.436.989	-32.297	
2.437.132	-32.296	
2.437.249	-32.296	
2.437.547	-32.296	
2.437.986	-32.296	
2.438.304	-32.296	
2.438.419	-32.296	
2.438.472	-32.296	
2.438.608	-32.295	
2.438.696	-32.295	
2.438.720	-32.295	
2.438.819	-32.295	

2.438.951	-32.294	
2.439.072	-32.294	
2.439.184	-32.294	
2.439.292	-32.293	
2.439.469	-32.293	
2.439.666	-32.293	
2.439.834	-32.292	
2.440.042	-32.292	
2.440.279	-32.291	
2.440.461	-32.291	
2.440.627	-32.291	
2.440.826	-32.290	
2.441.055	-32.290	
2.441.165	-32.289	
2.441.263	-32.289	
2.441.445	-32.288	
2.441.597	-32.288	
2.441.642	-32.287	
2.441.821	-32.287	
2.442.048	-32.287	
2.442.142	-32.286	
2.442.329	-32.286	
2.442.589	-32.285	
2.442.746	-32.285	
2.442.811	-32.284	
2.443.001	-32.284	
2.443.273	-32.283	
2.443.477	-32.283	
2.443.649	-32.282	
2.443.821	-32.282	

2.444.023	-32.281	
2.444.225	-32.281	
2.444.373	-32.281	
2.444.483	-32.280	
2.444.420	-32.280	
2.444.418	-32.279	
2.444.684	-32.279	
2.445.053	-32.278	
2.445.291	-32.278	
2.445.369	-32.277	
2.445.654	-32.277	
2.446.021	-32.277	
2.446.106	-32.276	
2.445.945	-32.276	
2.445.920	-32.276	
2.446.200	-32.275	
2.446.543	-32.275	
2.446.724	-32.275	
2.446.776	-32.274	
2.446.922	-32.274	
2.447.152	-32.274	
2.447.411	-32.274	
2.447.630	-32.273	
2.447.746	-32.273	
2.447.852	-32.273	
2.448.030	-32.273	
2.448.261	-32.273	
2.448.396	-32.273	
2.448.564	-32.272	
2.448.767	-32.272	

2.448.906	-32.272	
2.449.038	-32.272	
2.449.234	-32.272	
2.449.417	-32.272	
2.449.556	-32.272	
2.449.718	-32.272	
2.449.809	-32.272	
2.450.035	-32.272	
2.450.322	-32.272	
2.450.432	-32.272	
2.450.566	-32.272	
2.450.760	-32.272	
2.450.882	-32.272	
2.450.994	-32.272	
2.451.172	-32.272	
2.451.400	-32.272	
2.451.521	-32.272	
2.451.640	-32.273	
2.451.783	-32.273	
2.451.991	-32.273	
2.452.148	-32.273	
2.452.249	-32.273	
2.452.328	-32.274	
2.452.533	-32.274	
2.452.843	-32.274	
2.453.141	-32.274	
2.453.414	-32.275	
2.453.542	-32.275	
2.453.532	-32.275	
2.453.550	-32.275	

2.453.752	-32.276	
2.454.077	-32.276	
2.454.321	-32.276	
2.454.563	-32.277	
2.454.751	-32.277	
2.454.837	-32.278	
2.454.885	-32.278	
2.455.052	-32.278	
2.455.191	-32.279	
2.455.273	-32.279	
2.455.398	-32.280	
2.455.521	-32.280	
2.455.725	-32.280	
2.456.022	-32.281	
2.456.185	-32.281	
2.456.286	-32.282	
2.456.462	-32.282	
2.456.597	-32.283	
2.456.750	-32.283	
2.457.027	-32.284	
2.457.341	-32.284	
2.457.533	-32.285	
2.457.652	-32.285	
2.457.755	-32.286	
2.457.888	-32.286	
2.458.093	-32.287	
2.458.212	-32.287	
2.458.326	-32.288	
2.458.501	-32.288	
2.458.766	-32.289	

2.458.987	-32.289	
2.459.140	-32.290	
2.459.274	-32.290	
2.459.406	-32.291	
2.459.561	-32.292	
2.459.769	-32.292	
2.459.921	-32.293	
2.460.126	-32.293	
2.460.322	-32.294	
2.460.453	-32.295	
2.460.607	-32.295	
2.460.777	-32.296	
2.460.977	-32.296	
2.461.075	-32.297	
2.461.173	-32.298	
2.461.351	-32.298	
2.461.505	-32.299	
2.461.721	-32.299	
2.461.960	-32.300	
2.462.166	-32.301	
2.462.291	-32.301	
2.462.423	-32.302	
2.462.628	-32.302	
2.462.773	-32.303	
2.462.878	-32.304	
2.463.058	-32.304	
2.463.459	-32.305	
2.463.896	-32.306	
2.464.077	-32.306	
2.464.141	-32.307	

2.464.262	-32.307	
2.464.377	-32.308	
2.464.460	-32.309	
2.464.511	-32.309	
2.464.574	-32.310	
2.464.679	-32.310	
2.464.883	-32.311	
2.465.096	-32.312	
2.465.217	-32.312	
2.465.335	-32.313	
2.465.454	-32.313	
2.465.686	-32.314	
2.465.925	-32.315	
2.466.073	-32.315	
2.466.317	-32.316	
2.466.566	-32.316	
2.466.751	-32.317	
2.466.871	-32.317	
2.466.933	-32.318	
2.467.056	-32.319	
2.467.251	-32.319	
2.467.426	-32.320	
2.467.616	-32.320	
2.467.784	-32.321	
2.467.918	-32.322	
2.468.069	-32.322	
2.468.252	-32.323	
2.468.384	-32.323	
2.468.501	-32.324	
2.468.786	-32.325	

2.469.019	-32.325	
2.469.128	-32.326	
2.469.305	-32.326	
2.469.511	-32.327	
2.469.634	-32.328	
2.469.725	-32.328	
2.469.885	-32.329	
2.470.091	-32.329	
2.470.302	-32.330	
2.470.450	-32.330	
2.470.564	-32.331	
2.470.618	-32.332	
2.470.719	-32.332	
2.470.923	-32.333	
2.471.194	-32.333	
2.471.389	-32.334	
2.471.543	-32.334	
2.471.768	-32.335	
2.471.962	-32.335	
2.472.059	-32.336	
2.472.182	-32.337	
2.472.368	-32.337	
2.472.581	-32.338	
2.472.719	-32.338	
2.472.784	-32.339	
2.472.990	-32.339	
2.473.225	-32.340	
2.473.428	-32.340	
2.473.638	-32.341	
2.473.720	-32.341	

2.474.014	-32.342	
2.474.283	-32.342	
2.474.362	-32.343	
2.474.487	-32.343	
2.474.612	-32.344	
2.474.734	-32.344	
2.474.704	-32.345	
2.474.823	-32.345	
2.475.105	-32.346	
2.475.199	-32.346	
2.475.448	-32.346	
2.475.862	-32.347	
2.476.071	-32.347	
2.476.124	-32.347	
2.476.163	-32.348	
2.476.358	-32.348	
2.476.516	-32.348	
2.476.686	-32.349	
2.476.964	-32.349	
2.477.182	-32.349	
2.477.148	-32.349	
2.477.216	-32.350	
2.477.468	-32.350	
2.477.596	-32.350	
2.477.726	-32.350	
2.477.876	-32.350	
2.478.042	-32.350	
2.478.250	-32.350	
2.478.431	-32.350	
2.478.607	-32.350	

2.478.776	-32.351	
2.479.034	-32.351	
2.479.357	-32.351	
2.479.501	-32.351	
2.479.460	-32.350	
2.479.513	-32.350	
2.479.878	-32.350	
2.480.091	-32.350	
2.480.264	-32.350	
2.480.483	-32.350	
2.480.536	-32.350	
2.480.695	-32.350	
2.480.849	-32.349	
2.480.978	-32.349	
2.481.104	-32.349	
2.481.185	-32.349	
2.481.344	-32.348	
2.481.629	-32.348	
2.481.725	-32.348	
2.481.998	-32.347	
2.482.345	-32.347	
2.482.466	-32.346	
2.482.627	-32.346	
2.482.823	-32.345	
2.482.983	-32.345	
2.483.107	-32.345	
2.483.284	-32.344	
2.483.499	-32.343	
2.483.654	-32.343	
2.483.833	-32.342	

2.484.052	-32.342	
2.484.207	-32.341	
2.484.442	-32.341	
2.484.610	-32.340	
2.484.765	-32.339	
2.484.921	-32.339	
2.484.958	-32.338	
2.485.107	-32.337	
2.485.320	-32.337	
2.485.479	-32.336	
2.485.614	-32.335	
2.485.836	-32.335	
2.486.040	-32.334	
2.486.115	-32.333	
2.486.183	-32.332	
2.486.319	-32.331	
2.486.496	-32.331	
2.486.718	-32.330	
2.486.938	-32.329	
2.486.969	-32.328	
2.487.215	-32.327	
2.487.452	-32.327	
2.487.525	-32.326	
2.487.802	-32.325	
2.487.959	-32.324	
2.488.075	-32.323	
2.488.266	-32.323	
2.488.481	-32.322	
2.488.759	-32.321	
2.488.860	-32.320	

2.489.117	-32.319	
2.489.587	-32.318	
2.489.733	-32.318	
2.489.811	-32.317	
2.489.798	-32.316	
2.489.776	-32.315	
2.490.034	-32.314	
2.490.361	-32.314	
2.490.405	-32.313	
2.490.347	-32.312	
2.490.488	-32.311	
2.490.692	-32.311	
2.490.984	-32.310	
2.491.208	-32.309	
2.491.333	-32.308	
2.491.479	-32.308	
2.491.629	-32.307	
2.491.803	-32.306	
2.491.969	-32.306	
2.492.081	-32.305	
2.492.222	-32.304	
2.492.413	-32.304	
2.492.592	-32.303	
2.492.759	-32.303	
2.492.876	-32.302	
2.493.009	-32.302	
2.493.158	-32.301	
2.493.257	-32.301	
2.493.366	-32.300	
2.493.530	-32.300	

2.493.640	-32.300	
2.493.846	-32.299	
2.494.124	-32.299	
2.494.344	-32.299	
2.494.518	-32.299	
2.494.675	-32.298	
2.494.868	-32.298	
2.495.073	-32.298	
2.495.201	-32.298	
2.495.329	-32.298	
2.495.515	-32.298	
2.495.667	-32.298	
2.495.768	-32.298	
2.495.931	-32.298	
2.496.111	-32.298	
2.496.203	-32.298	
2.496.352	-32.298	
2.496.534	-32.298	
2.496.727	-32.298	
2.496.915	-32.299	
2.497.083	-32.299	
2.497.265	-32.299	
2.497.442	-32.300	
2.497.650	-32.300	
2.497.871	-32.300	
2.498.039	-32.301	
2.498.165	-32.301	
2.498.268	-32.302	
2.498.422	-32.302	
2.498.625	-32.303	

2.498.810	-32.304	
2.498.996	-32.304	
2.499.171	-32.305	
2.499.325	-32.306	
2.499.552	-32.306	
2.499.789	-32.307	
2.499.932	-32.308	
2.500.047	-32.309	
2.500.237	-32.310	
2.500.394	-32.311	
2.500.468	-32.311	
2.500.598	-32.312	
2.500.789	-32.313	
2.500.995	-32.314	
2.501.131	-32.315	
2.501.322	-32.316	
2.501.557	-32.317	
2.501.727	-32.318	
2.501.886	-32.319	
2.502.056	-32.320	
2.502.188	-32.321	
2.502.299	-32.322	
2.502.478	-32.324	
2.502.665	-32.325	
2.502.883	-32.326	
2.503.048	-32.327	
2.503.096	-32.328	
2.503.194	-32.329	
2.503.353	-32.330	
2.503.506	-32.331	

2.503.647	-32.333	
2.503.817	-32.334	
2.504.048	-32.335	
2.504.328	-32.336	
2.504.500	-32.337	
2.504.663	-32.338	
2.504.892	-32.339	
2.505.008	-32.341	
2.505.152	-32.342	
2.505.360	-32.343	
2.505.594	-32.344	
2.505.742	-32.345	
2.505.857	-32.346	
2.505.950	-32.347	
2.506.055	-32.348	
2.506.267	-32.349	
2.506.492	-32.350	
2.506.612	-32.351	
2.506.729	-32.352	
2.506.921	-32.353	
2.507.132	-32.354	
2.507.278	-32.355	
2.507.401	-32.356	
2.507.579	-32.357	
2.507.773	-32.358	
2.507.983	-32.359	
2.508.192	-32.360	
2.508.382	-32.361	
2.508.591	-32.361	
2.508.738	-32.362	

2.508.858	-32.363	
2.509.014	-32.364	
2.509.171	-32.364	
2.509.301	-32.365	
2.509.442	-32.366	
2.509.686	-32.366	
2.509.921	-32.367	
2.510.029	-32.368	
2.510.143	-32.368	
2.510.397	-32.369	
2.510.634	-32.369	
2.510.730	-32.370	
2.510.880	-32.370	
2.511.087	-32.371	
2.511.241	-32.371	
2.511.409	-32.372	
2.511.538	-32.372	
2.511.680	-32.372	
2.511.917	-32.373	
2.512.126	-32.373	
2.512.282	-32.373	
2.512.361	-32.373	
2.512.529	-32.374	
2.512.776	-32.374	
2.512.956	-32.374	
2.513.093	-32.374	
2.513.289	-32.374	
2.513.510	-32.374	
2.513.674	-32.374	
2.513.826	-32.374	

2.513.969	-32.374	
2.514.101	-32.374	
2.514.225	-32.374	
2.514.568	-32.374	
2.515.022	-32.374	
2.515.335	-32.374	
2.515.493	-32.374	
2.515.526	-32.374	
2.515.557	-32.374	
2.515.656	-32.374	
2.515.701	-32.373	
2.515.714	-32.373	
2.515.851	-32.373	
2.515.994	-32.373	
2.516.164	-32.372	
2.516.348	-32.372	
2.516.523	-32.372	
2.516.706	-32.371	
2.516.877	-32.371	
2.517.085	-32.371	
2.517.249	-32.370	
2.517.412	-32.370	
2.517.616	-32.369	
2.517.843	-32.369	
2.518.013	-32.369	
2.518.107	-32.368	
2.518.275	-32.368	
2.518.436	-32.367	
2.518.561	-32.367	
2.518.692	-32.366	

2.518.812	-32.366	
2.518.972	-32.366	
2.519.129	-32.365	
2.519.267	-32.365	
2.519.428	-32.364	
2.519.653	-32.364	
2.519.831	-32.363	
2.520.069	-32.363	
2.520.268	-32.363	
2.520.406	-32.362	
2.520.557	-32.362	
2.520.732	-32.361	
2.520.813	-32.361	
2.520.939	-32.361	
2.521.199	-32.360	
2.521.348	-32.360	
2.521.500	-32.360	
2.521.619	-32.359	
2.521.696	-32.359	
2.521.883	-32.359	
2.522.155	-32.359	
2.522.365	-32.358	
2.522.493	-32.358	
2.522.609	-32.358	
2.522.771	-32.358	
2.522.950	-32.357	
2.523.112	-32.357	
2.523.277	-32.357	
2.523.410	-32.357	
2.523.569	-32.357	

2.523.776	-32.357	
2.523.885	-32.357	
2.524.081	-32.357	
2.524.330	-32.357	
2.524.475	-32.357	
2.524.619	-32.357	
2.524.816	-32.357	
2.524.993	-32.357	
2.525.152	-32.357	
2.525.315	-32.357	
2.525.434	-32.357	
2.525.658	-32.358	
2.525.902	-32.358	
2.526.069	-32.358	
2.526.180	-32.358	
2.526.218	-32.358	
2.526.406	-32.359	
2.526.570	-32.359	
2.526.718	-32.359	
2.526.966	-32.359	
2.527.168	-32.360	
2.527.328	-32.360	
2.527.479	-32.360	
2.527.598	-32.361	
2.527.749	-32.361	
2.527.948	-32.361	
2.528.145	-32.362	
2.528.306	-32.362	
2.528.459	-32.363	
2.528.537	-32.363	

2.528.667	-32.363	
2.528.926	-32.364	
2.529.068	-32.364	
2.529.209	-32.365	
2.529.388	-32.365	
2.529.500	-32.366	
2.529.715	-32.366	
2.530.000	-32.366	
2.530.156	-32.367	
2.530.340	-32.367	
2.530.546	-32.368	
2.530.678	-32.368	
2.530.762	-32.369	
2.530.956	-32.369	
2.531.264	-32.370	
2.531.456	-32.370	
2.531.588	-32.370	
2.531.732	-32.371	
2.531.817	-32.371	
2.531.892	-32.372	
2.532.047	-32.372	
2.532.291	-32.372	
2.532.486	-32.373	
2.532.598	-32.373	
2.532.762	-32.374	
2.532.957	-32.374	
2.533.183	-32.374	
2.533.326	-32.375	
2.533.474	-32.375	
2.533.673	-32.375	

2.533.853	-32.375	
2.533.994	-32.376	
2.534.117	-32.376	
2.534.277	-32.376	
2.534.473	-32.376	
2.534.665	-32.377	
2.534.828	-32.377	
2.535.013	-32.377	
2.535.175	-32.377	
2.535.294	-32.377	
2.535.461	-32.377	
2.535.641	-32.378	
2.535.797	-32.378	
2.535.975	-32.378	
2.536.151	-32.378	
2.536.298	-32.378	
2.536.440	-32.378	
2.536.649	-32.378	
2.536.895	-32.378	
2.537.094	-32.378	
2.537.261	-32.377	
2.537.391	-32.377	
2.537.574	-32.377	
2.537.772	-32.377	
2.537.895	-32.377	
2.538.012	-32.377	
2.538.167	-32.376	
2.538.380	-32.376	
2.538.619	-32.376	
2.538.815	-32.375	

2.538.969	-32.375	
2.539.129	-32.375	
2.539.281	-32.374	
2.539.377	-32.374	
2.539.496	-32.373	
2.539.583	-32.373	
2.539.851	-32.372	
2.540.364	-32.372	
2.540.772	-32.371	
2.540.939	-32.371	
2.540.988	-32.370	
2.541.024	-32.370	
2.541.078	-32.369	
2.541.147	-32.368	
2.541.253	-32.368	
2.541.310	-32.367	
2.541.428	-32.366	
2.541.635	-32.365	
2.541.805	-32.365	
2.541.931	-32.364	
2.542.097	-32.363	
2.542.300	-32.362	
2.542.521	-32.361	
2.542.682	-32.360	
2.542.820	-32.359	
2.543.022	-32.358	
2.543.210	-32.358	
2.543.413	-32.357	
2.543.596	-32.356	
2.543.785	-32.355	

2.543.919	-32.354	
2.544.002	-32.353	
2.544.160	-32.352	
2.544.370	-32.351	
2.544.435	-32.350	
2.544.544	-32.349	
2.544.801	-32.348	
2.544.983	-32.347	
2.545.204	-32.346	
2.545.392	-32.345	
2.545.554	-32.344	
2.545.729	-32.343	
2.545.891	-32.342	
2.546.042	-32.341	
2.546.208	-32.340	
2.546.404	-32.339	
2.546.573	-32.338	
2.546.747	-32.337	
2.546.898	-32.336	
2.547.050	-32.335	
2.547.188	-32.334	
2.547.303	-32.333	
2.547.426	-32.332	
2.547.651	-32.331	
2.547.841	-32.330	
2.547.978	-32.329	
2.548.148	-32.328	
2.548.275	-32.327	
2.548.421	-32.326	
2.548.560	-32.326	

2.548.695	-32.325	
2.548.904	-32.324	
2.549.120	-32.323	
2.549.316	-32.323	
2.549.525	-32.322	
2.549.699	-32.321	
2.549.818	-32.320	
2.549.905	-32.320	
2.550.017	-32.319	
2.550.262	-32.319	
2.550.486	-32.318	
2.550.670	-32.318	
2.550.801	-32.317	
2.550.930	-32.317	
2.551.140	-32.316	
2.551.336	-32.316	
2.551.499	-32.315	
2.551.660	-32.315	
2.551.815	-32.314	
2.551.950	-32.314	
2.552.156	-32.314	
2.552.282	-32.314	
2.552.419	-32.313	
2.552.592	-32.313	
2.552.760	-32.313	
2.553.016	-32.313	
2.553.195	-32.312	
2.553.326	-32.312	
2.553.481	-32.312	
2.553.636	-32.312	

2.553.792	-32.312	
2.553.948	-32.312	
2.554.077	-32.312	
2.554.214	-32.312	
2.554.377	-32.312	
2.554.561	-32.312	
2.554.709	-32.312	
2.554.786	-32.312	
2.554.945	-32.312	
2.555.204	-32.313	
2.555.426	-32.313	
2.555.667	-32.313	
2.555.866	-32.313	
2.556.002	-32.314	
2.556.202	-32.314	
2.556.387	-32.314	
2.556.553	-32.315	
2.556.740	-32.315	
2.556.855	-32.315	
2.557.019	-32.316	
2.557.184	-32.316	
2.557.281	-32.317	
2.557.411	-32.317	
2.557.516	-32.317	
2.557.618	-32.318	
2.557.781	-32.318	
2.557.997	-32.319	
2.558.173	-32.319	
2.558.326	-32.320	
2.558.531	-32.321	

2.558.788	-32.321	
2.558.991	-32.322	
2.559.147	-32.322	
2.559.291	-32.323	
2.559.464	-32.324	
2.559.655	-32.324	
2.559.839	-32.325	
2.559.991	-32.326	
2.560.067	-32.326	
2.560.219	-32.327	
2.560.446	-32.328	
2.560.642	-32.329	
2.560.807	-32.329	
2.560.991	-32.330	
2.561.202	-32.331	
2.561.371	-32.332	
2.561.522	-32.332	
2.561.685	-32.333	
2.561.869	-32.334	
2.562.023	-32.335	
2.562.148	-32.336	
2.562.371	-32.336	
2.562.615	-32.337	
2.562.791	-32.338	
2.562.936	-32.339	
2.563.094	-32.340	
2.563.250	-32.340	
2.563.419	-32.341	
2.563.623	-32.342	
2.563.748	-32.343	

2.563.846	-32.344	
2.564.028	-32.345	
2.564.244	-32.345	
2.564.434	-32.346	
2.564.555	-32.347	
2.564.703	-32.348	
2.564.869	-32.349	
2.564.992	-32.349	
2.565.139	-32.350	
2.565.349	-32.351	
2.565.667	-32.352	
2.566.003	-32.352	
2.566.302	-32.353	
2.566.560	-32.354	
2.566.603	-32.355	
2.566.660	-32.355	
2.566.794	-32.356	
2.566.864	-32.357	
2.566.917	-32.358	
2.567.022	-32.358	
2.567.137	-32.359	
2.567.264	-32.360	
2.567.462	-32.360	
2.567.668	-32.361	
2.567.831	-32.362	
2.568.011	-32.362	
2.568.166	-32.363	
2.568.419	-32.363	
2.568.625	-32.364	
2.568.766	-32.365	

2.568.968	-32.365	
2.569.125	-32.366	
2.569.269	-32.366	
2.569.406	-32.367	
2.569.533	-32.367	
2.569.680	-32.368	
2.569.875	-32.368	
2.570.025	-32.369	
2.570.161	-32.369	
2.570.352	-32.370	
2.570.502	-32.370	
2.570.659	-32.371	
2.570.859	-32.371	
2.571.028	-32.372	
2.571.180	-32.372	
2.571.380	-32.373	
2.571.620	-32.373	
2.571.823	-32.374	
2.571.963	-32.374	
2.572.062	-32.375	
2.572.249	-32.375	
2.572.460	-32.375	
2.572.561	-32.376	
2.572.625	-32.376	
2.572.759	-32.376	
2.572.993	-32.377	
2.573.181	-32.377	
2.573.297	-32.377	
2.573.486	-32.378	
2.573.679	-32.378	

2.573.831	-32.378	
2.573.981	-32.379	
2.574.178	-32.379	
2.574.392	-32.379	
2.574.583	-32.379	
2.574.703	-32.380	
2.574.817	-32.380	
2.574.948	-32.380	
2.575.038	-32.380	
2.575.233	-32.381	
2.575.536	-32.381	
2.575.775	-32.381	
2.575.872	-32.381	
2.575.916	-32.382	
2.576.107	-32.382	
2.576.387	-32.382	
2.576.559	-32.382	
2.576.708	-32.382	
2.576.898	-32.382	
2.577.072	-32.383	
2.577.236	-32.383	
2.577.401	-32.383	
2.577.567	-32.383	
2.577.700	-32.383	
2.577.810	-32.383	
2.577.983	-32.383	
2.578.224	-32.383	
2.578.474	-32.383	
2.578.650	-32.383	
2.578.728	-32.383	

2.578.861	-32.383	
2.579.002	-32.383	
2.579.164	-32.382	
2.579.391	-32.382	
2.579.514	-32.382	
2.579.651	-32.382	
2.579.759	-32.382	
2.579.833	-32.382	
2.579.998	-32.381	
2.580.143	-32.381	
2.580.313	-32.381	
2.580.486	-32.381	
2.580.660	-32.380	
2.580.869	-32.380	
2.581.096	-32.380	
2.581.339	-32.379	
2.581.548	-32.379	
2.581.656	-32.379	
2.581.871	-32.378	
2.582.054	-32.378	
2.582.152	-32.377	
2.582.310	-32.377	
2.582.473	-32.376	
2.582.592	-32.376	
2.582.734	-32.375	
2.582.922	-32.374	
2.583.052	-32.374	
2.583.178	-32.373	
2.583.370	-32.373	
2.583.567	-32.372	

2.583.681	-32.371	
2.583.792	-32.370	
2.584.016	-32.370	
2.584.254	-32.369	
2.584.387	-32.368	
2.584.541	-32.367	
2.584.793	-32.366	
2.584.966	-32.366	
2.585.114	-32.365	
2.585.295	-32.364	
2.585.468	-32.363	
2.585.656	-32.362	
2.585.823	-32.361	
2.586.000	-32.360	
2.586.159	-32.359	
2.586.310	-32.358	
2.586.497	-32.357	
2.586.642	-32.356	
2.586.783	-32.355	
2.586.939	-32.354	
2.587.163	-32.352	
2.587.430	-32.351	
2.587.552	-32.350	
2.587.661	-32.349	
2.587.816	-32.348	
2.588.000	-32.346	
2.588.157	-32.345	
2.588.287	-32.344	
2.588.450	-32.343	
2.588.623	-32.341	

2.588.800	-32.340	
2.588.925	-32.339	
2.589.091	-32.337	
2.589.283	-32.336	
2.589.438	-32.335	
2.589.604	-32.333	
2.589.764	-32.332	
2.589.926	-32.330	
2.590.099	-32.329	
2.590.294	-32.327	
2.590.482	-32.326	
2.590.617	-32.324	
2.590.713	-32.323	
2.591.009	-32.322	
2.591.444	-32.320	
2.591.736	-32.319	
2.591.896	-32.317	
2.592.012	-32.316	
2.592.091	-32.314	
2.592.106	-32.312	
2.592.123	-32.311	
2.592.247	-32.309	
2.592.383	-32.308	
2.592.473	-32.306	
2.592.632	-32.305	
2.592.861	-32.303	
2.593.078	-32.302	
2.593.242	-32.300	
2.593.409	-32.299	
2.593.542	-32.297	

2.593.768	-32.296	
2.594.045	-32.294	
2.594.225	-32.292	
2.594.366	-32.291	
2.594.465	-32.290	
2.594.605	-32.288	
2.594.789	-32.287	
2.594.976	-32.285	
2.595.124	-32.284	
2.595.237	-32.282	
2.595.341	-32.281	
2.595.487	-32.280	
2.595.677	-32.278	
2.595.850	-32.277	
2.595.971	-32.276	
2.596.100	-32.275	
2.596.320	-32.273	
2.596.537	-32.272	
2.596.708	-32.271	
2.596.830	-32.270	
2.596.993	-32.269	
2.597.159	-32.268	
2.597.347	-32.267	
2.597.593	-32.266	
2.597.715	-32.265	
2.597.839	-32.264	
2.598.015	-32.263	
2.598.167	-32.262	
2.598.294	-32.261	
2.598.423	-32.260	

2.598.578	-32.259	
2.598.763	-32.259	
2.598.980	-32.258	
2.599.174	-32.257	
2.599.327	-32.257	
2.599.380	-32.256	
2.599.546	-32.256	
2.599.792	-32.255	
2.599.988	-32.255	
2.600.175	-32.254	
2.600.270	-32.254	
2.600.415	-32.254	
2.600.610	-32.253	
2.600.796	-32.253	
2.600.996	-32.253	
2.601.154	-32.253	
2.601.304	-32.253	
2.601.509	-32.253	
2.601.722	-32.253	
2.601.928	-32.253	
2.602.120	-32.253	
2.602.279	-32.253	
2.602.413	-32.254	
2.602.565	-32.254	
2.602.745	-32.254	
2.602.928	-32.255	
2.603.098	-32.255	
2.603.285	-32.256	
2.603.413	-32.256	
2.603.513	-32.257	

2.603.618	-32.257	
2.603.763	-32.258	
2.603.940	-32.258	
2.604.114	-32.259	
2.604.297	-32.260	
2.604.423	-32.261	
2.604.510	-32.262	
2.604.669	-32.262	
2.604.815	-32.263	
2.604.952	-32.264	
2.605.154	-32.265	
2.605.255	-32.266	
2.605.363	-32.267	
2.605.533	-32.269	
2.605.753	-32.270	
2.605.995	-32.271	
2.606.137	-32.272	
2.606.389	-32.274	
2.606.609	-32.275	
2.606.711	-32.276	
2.606.996	-32.278	
2.607.220	-32.279	
2.607.346	-32.281	
2.607.426	-32.282	
2.607.523	-32.284	
2.607.751	-32.285	
2.607.924	-32.287	
2.608.000	-32.289	
2.608.114	-32.290	
2.608.286	-32.292	

2.608.492	-32.294	
2.608.593	-32.295	
2.608.781	-32.297	
2.608.932	-32.299	
2.609.110	-32.301	
2.609.290	-32.303	
2.609.507	-32.304	
2.609.815	-32.306	
2.610.016	-32.308	
2.610.143	-32.310	
2.610.244	-32.312	
2.610.458	-32.314	
2.610.667	-32.316	
2.610.742	-32.318	
2.610.888	-32.320	
2.611.071	-32.322	
2.611.216	-32.324	
2.611.389	-32.326	
2.611.533	-32.328	
2.611.656	-32.331	
2.611.838	-32.333	
2.612.032	-32.335	
2.612.227	-32.337	
2.612.375	-32.339	
2.612.509	-32.341	
2.612.686	-32.343	
2.612.841	-32.346	
2.613.083	-32.348	
2.613.228	-32.350	
2.613.344	-32.352	

2.613.520	-32.354	
2.613.723	-32.356	
2.613.916	-32.359	
2.614.085	-32.361	
2.614.216	-32.363	
2.614.330	-32.365	
2.614.521	-32.367	
2.614.735	-32.370	
2.614.944	-32.372	
2.615.139	-32.374	
2.615.161	-32.376	
2.615.320	-32.378	
2.615.583	-32.380	
2.615.791	-32.383	
2.615.989	-32.385	
2.616.118	-32.387	
2.616.483	-32.389	
2.616.935	-32.391	
2.617.120	-32.393	
2.617.184	-32.395	
2.617.304	-32.397	
2.617.388	-32.399	
2.617.451	-32.401	
2.617.528	-32.403	
2.617.585	-32.405	
2.617.705	-32.407	
2.617.853	-32.409	
2.617.994	-32.411	
2.618.182	-32.413	
2.618.384	-32.415	

2.618.560	-32.417	
2.618.763	-32.419	
2.618.934	-32.421	
2.619.042	-32.422	
2.619.167	-32.424	
2.619.407	-32.426	
2.619.584	-32.428	
2.619.738	-32.429	
2.619.898	-32.431	
2.620.083	-32.433	
2.620.303	-32.434	
2.620.484	-32.436	
2.620.623	-32.438	
2.620.703	-32.439	
2.620.773	-32.441	
2.620.981	-32.443	
2.621.158	-32.444	
2.621.306	-32.446	
2.621.432	-32.447	
2.621.679	-32.449	
2.621.885	-32.450	
2.622.041	-32.452	
2.622.261	-32.453	
2.622.406	-32.455	
2.622.539	-32.456	
2.622.787	-32.457	
2.622.947	-32.459	
2.623.051	-32.460	
2.623.153	-32.461	
2.623.283	-32.463	

2.623.495	-32.464	
2.623.636	-32.465	
2.623.753	-32.466	
2.623.871	-32.468	
2.624.116	-32.469	
2.624.413	-32.470	
2.624.586	-32.471	
2.624.752	-32.472	
2.624.904	-32.473	
2.625.031	-32.475	
2.625.121	-32.476	
2.625.298	-32.477	
2.625.483	-32.478	
2.625.661	-32.479	
2.625.874	-32.480	
2.626.028	-32.481	
2.626.198	-32.482	
2.626.400	-32.483	
2.626.584	-32.484	
2.626.748	-32.485	
2.626.877	-32.486	
2.627.000	-32.487	
2.627.206	-32.488	
2.627.390	-32.489	
2.627.506	-32.490	
2.627.712	-32.490	
2.627.907	-32.491	
2.628.055	-32.492	
2.628.199	-32.493	
2.628.358	-32.494	

2.628.515	-32.494	
2.628.658	-32.495	
2.628.849	-32.496	
2.629.067	-32.497	
2.629.240	-32.497	
2.629.373	-32.498	
2.629.536	-32.498	
2.629.739	-32.499	
2.629.905	-32.500	
2.630.016	-32.500	
2.630.189	-32.501	
2.630.371	-32.501	
2.630.464	-32.502	
2.630.543	-32.502	
2.630.702	-32.503	
2.630.811	-32.503	
2.630.995	-32.503	
2.631.298	-32.504	
2.631.541	-32.504	
2.631.741	-32.504	
2.631.895	-32.505	
2.632.061	-32.505	
2.632.254	-32.505	
2.632.506	-32.505	
2.632.738	-32.506	
2.632.820	-32.506	
2.632.921	-32.506	
2.633.119	-32.506	
2.633.318	-32.506	
2.633.459	-32.506	

2.633.600	-32.506	
2.633.748	-32.506	
2.633.878	-32.506	
2.634.023	-32.506	
2.634.135	-32.506	
2.634.280	-32.506	
2.634.474	-32.505	
2.634.658	-32.505	
2.634.848	-32.505	
2.635.099	-32.505	
2.635.305	-32.504	
2.635.414	-32.504	
2.635.572	-32.504	
2.635.772	-32.504	
2.635.985	-32.503	
2.636.186	-32.503	
2.636.288	-32.502	
2.636.414	-32.502	
2.636.578	-32.501	
2.636.758	-32.501	
2.636.892	-32.500	
2.637.059	-32.500	
2.637.267	-32.499	
2.637.415	-32.499	
2.637.574	-32.498	
2.637.729	-32.497	
2.637.897	-32.497	
2.638.048	-32.496	
2.638.272	-32.495	
2.638.503	-32.495	

2.638.608	-32.494	
2.638.764	-32.493	
2.638.893	-32.492	
2.639.088	-32.491	
2.639.326	-32.490	
2.639.482	-32.489	
2.639.637	-32.489	
2.639.749	-32.488	
2.639.962	-32.487	
2.640.164	-32.486	
2.640.319	-32.485	
2.640.509	-32.484	
2.640.655	-32.483	
2.640.811	-32.481	
2.641.013	-32.480	
2.641.166	-32.479	
2.641.350	-32.478	
2.641.466	-32.477	
2.641.661	-32.476	
2.642.115	-32.475	
2.642.484	-32.473	
2.642.650	-32.472	
2.642.745	-32.471	
2.642.868	-32.470	
2.642.901	-32.468	
2.642.906	-32.467	
2.642.986	-32.466	
2.643.087	-32.464	
2.643.214	-32.463	
2.643.370	-32.462	

2.643.568	-32.460	
2.643.760	-32.459	
2.643.936	-32.458	
2.644.073	-32.456	
2.644.250	-32.455	
2.644.424	-32.453	
2.644.626	-32.452	
2.644.771	-32.450	
2.644.931	-32.449	
2.645.177	-32.448	
2.645.346	-32.446	
2.645.475	-32.445	
2.645.572	-32.443	
2.645.761	-32.442	
2.645.930	-32.440	
2.646.049	-32.439	
2.646.169	-32.437	
2.646.310	-32.436	
2.646.418	-32.434	
2.646.534	-32.433	
2.646.732	-32.432	
2.647.014	-32.430	
2.647.213	-32.429	
2.647.433	-32.427	
2.647.677	-32.426	
2.647.866	-32.425	
2.647.957	-32.423	
2.648.145	-32.422	
2.648.338	-32.420	
2.648.489	-32.419	

2.648.514	-32.418	
2.648.665	-32.416	
2.648.782	-32.415	
2.648.828	-32.414	
2.649.156	-32.413	
2.649.382	-32.411	
2.649.547	-32.410	
2.649.742	-32.409	
2.649.897	-32.408	
2.650.119	-32.407	
2.650.276	-32.405	
2.650.404	-32.404	
2.650.554	-32.403	
2.650.750	-32.402	
2.650.912	-32.401	
2.651.024	-32.400	
2.651.166	-32.399	
2.651.299	-32.398	
2.651.404	-32.397	
2.651.606	-32.397	
2.651.866	-32.396	
2.652.079	-32.395	
2.652.254	-32.394	
2.652.336	-32.393	
2.652.492	-32.393	
2.652.712	-32.392	
2.652.897	-32.391	
2.653.081	-32.391	
2.653.193	-32.390	
2.653.316	-32.389	

2.653.510	-32.389	
2.653.647	-32.388	
2.653.799	-32.388	
2.653.990	-32.387	
2.654.196	-32.387	
2.654.467	-32.387	
2.654.555	-32.386	
2.654.644	-32.386	
2.654.846	-32.386	
2.655.002	-32.385	
2.655.201	-32.385	
2.655.396	-32.385	
2.655.511	-32.384	
2.655.643	-32.384	
2.655.771	-32.384	
2.655.916	-32.384	
2.656.087	-32.384	
2.656.227	-32.384	
2.656.412	-32.384	
2.656.611	-32.384	
2.656.804	-32.384	
2.656.993	-32.384	
2.657.188	-32.384	
2.657.380	-32.384	
2.657.554	-32.384	
2.657.780	-32.384	
2.658.015	-32.385	
2.658.181	-32.385	
2.658.319	-32.385	
2.658.442	-32.385	

2.658.560	-32.386	
2.658.694	-32.386	
2.658.829	-32.386	
2.658.973	-32.386	
2.659.122	-32.387	
2.659.288	-32.387	
2.659.442	-32.388	
2.659.548	-32.388	
2.659.670	-32.388	
2.659.882	-32.389	
2.660.097	-32.389	
2.660.294	-32.390	
2.660.526	-32.390	
2.660.721	-32.391	
2.660.919	-32.391	
2.661.104	-32.392	
2.661.249	-32.392	
2.661.408	-32.393	
2.661.584	-32.393	
2.661.718	-32.394	
2.661.806	-32.395	
2.661.985	-32.395	
2.662.234	-32.396	
2.662.426	-32.397	
2.662.557	-32.397	
2.662.700	-32.398	
2.662.904	-32.399	
2.663.120	-32.400	
2.663.253	-32.400	
2.663.377	-32.401	

2.663.564	-32.402	
2.663.773	-32.403	
2.663.945	-32.403	
2.664.104	-32.404	
2.664.243	-32.405	
2.664.382	-32.406	
2.664.575	-32.407	
2.664.765	-32.408	
2.664.873	-32.408	
2.664.948	-32.409	
2.665.121	-32.410	
2.665.352	-32.411	
2.665.547	-32.412	
2.665.750	-32.413	
2.665.887	-32.414	
2.665.981	-32.415	
2.666.107	-32.416	
2.666.288	-32.416	
2.666.472	-32.417	
2.666.599	-32.418	
2.666.935	-32.419	
2.667.369	-32.420	
2.667.686	-32.421	
2.667.922	-32.422	
2.667.978	-32.423	
2.667.980	-32.424	
2.668.040	-32.425	
2.668.124	-32.426	
2.668.217	-32.427	
2.668.320	-32.428	

2.668.478	-32.429	
2.668.683	-32.430	
2.668.811	-32.431	
2.668.999	-32.432	
2.669.247	-32.433	
2.669.382	-32.434	
2.669.536	-32.435	
2.669.745	-32.436	
2.669.906	-32.437	
2.670.049	-32.438	
2.670.223	-32.439	
2.670.422	-32.440	
2.670.591	-32.441	
2.670.695	-32.442	
2.670.847	-32.443	
2.671.089	-32.444	
2.671.303	-32.445	
2.671.382	-32.446	
2.671.542	-32.447	
2.671.740	-32.448	
2.671.795	-32.449	
2.671.972	-32.450	
2.672.195	-32.451	
2.672.339	-32.452	
2.672.561	-32.453	
2.672.762	-32.453	
2.672.863	-32.454	
2.673.008	-32.455	
2.673.226	-32.456	
2.673.423	-32.457	

2.673.593	-32.458	
2.673.770	-32.459	
2.673.898	-32.460	
2.674.044	-32.461	
2.674.190	-32.462	
2.674.338	-32.463	
2.674.514	-32.464	
2.674.642	-32.465	
2.674.799	-32.466	
2.675.013	-32.467	
2.675.205	-32.468	
2.675.377	-32.469	
2.675.529	-32.470	
2.675.663	-32.471	
2.675.826	-32.472	
2.676.003	-32.473	
2.676.154	-32.474	
2.676.273	-32.475	
2.676.401	-32.476	
2.676.596	-32.477	
2.676.772	-32.478	
2.676.989	-32.479	
2.677.188	-32.480	
2.677.314	-32.481	
2.677.480	-32.482	
2.677.695	-32.483	
2.677.921	-32.484	
2.678.073	-32.485	
2.678.159	-32.486	
2.678.318	-32.487	

2.678.511	-32.488	
2.678.674	-32.488	
2.678.820	-32.489	
2.678.954	-32.490	
2.679.117	-32.491	
2.679.294	-32.492	
2.679.519	-32.493	
2.679.756	-32.494	
2.679.893	-32.495	
2.679.976	-32.496	
2.680.110	-32.497	
2.680.298	-32.498	
2.680.534	-32.498	
2.680.725	-32.499	
2.680.833	-32.500	
2.680.963	-32.501	
2.681.086	-32.502	
2.681.202	-32.502	
2.681.394	-32.503	
2.681.560	-32.504	
2.681.726	-32.505	
2.681.921	-32.506	
2.682.079	-32.506	
2.682.246	-32.507	
2.682.466	-32.508	
2.682.697	-32.509	
2.682.911	-32.509	
2.683.098	-32.510	
2.683.271	-32.510	
2.683.413	-32.511	

2.683.482	-32.512	
2.683.661	-32.512	
2.683.857	-32.513	
2.683.951	-32.513	
2.684.078	-32.514	
2.684.265	-32.514	
2.684.447	-32.515	
2.684.552	-32.515	
2.684.662	-32.516	
2.684.859	-32.516	
2.685.020	-32.517	
2.685.223	-32.517	
2.685.429	-32.517	
2.685.567	-32.518	
2.685.743	-32.518	
2.685.869	-32.519	
2.685.993	-32.519	
2.686.281	-32.519	
2.686.463	-32.519	
2.686.653	-32.520	
2.686.895	-32.520	
2.687.059	-32.520	
2.687.140	-32.520	
2.687.325	-32.520	
2.687.521	-32.521	
2.687.700	-32.521	
2.687.899	-32.521	
2.688.085	-32.521	
2.688.267	-32.521	
2.688.398	-32.521	

2.688.568	-32.521	
2.688.712	-32.521	
2.688.806	-32.521	
2.689.012	-32.521	
2.689.240	-32.521	
2.689.384	-32.521	
2.689.550	-32.521	
2.689.709	-32.521	
2.689.815	-32.521	
2.689.948	-32.521	
2.690.118	-32.521	
2.690.321	-32.521	
2.690.536	-32.521	
2.690.697	-32.520	
2.690.894	-32.520	
2.691.161	-32.520	
2.691.286	-32.520	
2.691.382	-32.520	
2.691.552	-32.519	
2.691.723	-32.519	
2.691.823	-32.519	
2.691.946	-32.519	
2.692.270	-32.518	
2.692.719	-32.518	
2.693.059	-32.518	
2.693.179	-32.518	
2.693.244	-32.517	
2.693.344	-32.517	
2.693.445	-32.517	
2.693.470	-32.516	

2.693.514	-32.516	
2.693.632	-32.515	
2.693.749	-32.515	
2.693.947	-32.515	
2.694.134	-32.514	
2.694.330	-32.514	
2.694.512	-32.513	
2.694.655	-32.513	
2.694.818	-32.512	
2.694.981	-32.512	
2.695.184	-32.511	
2.695.374	-32.511	
2.695.558	-32.510	
2.695.740	-32.510	
2.695.877	-32.509	
2.696.031	-32.509	
2.696.195	-32.508	
2.696.328	-32.508	
2.696.477	-32.507	
2.696.588	-32.506	
2.696.747	-32.506	
2.696.910	-32.505	
2.697.102	-32.505	
2.697.243	-32.504	
2.697.365	-32.504	
2.697.553	-32.503	
2.697.759	-32.503	
2.697.968	-32.502	
2.698.149	-32.501	
2.698.286	-32.501	

2.698.444	-32.500	
2.698.608	-32.500	
2.698.757	-32.499	
2.698.893	-32.498	
2.699.038	-32.498	
2.699.232	-32.497	
2.699.400	-32.496	
2.699.487	-32.496	
2.699.617	-32.495	
2.699.868	-32.495	
2.700.100	-32.494	
2.700.278	-32.493	
2.700.372	-32.493	
2.700.512	-32.492	
2.700.696	-32.492	
2.700.830	-32.491	
2.701.024	-32.490	
2.701.223	-32.490	
2.701.313	-32.489	
2.701.382	-32.489	
2.701.635	-32.488	
2.701.827	-32.488	
2.702.028	-32.487	
2.702.191	-32.487	
2.702.376	-32.486	
2.702.552	-32.485	
2.702.736	-32.485	
2.702.908	-32.484	
2.703.045	-32.484	
2.703.239	-32.483	

2.703.402	-32.483	
2.703.596	-32.482	
2.703.767	-32.482	
2.703.918	-32.481	
2.704.114	-32.481	
2.704.218	-32.480	
2.704.308	-32.480	
2.704.445	-32.479	
2.704.713	-32.479	
2.704.933	-32.478	
2.705.103	-32.478	
2.705.216	-32.477	
2.705.364	-32.477	
2.705.520	-32.476	
2.705.664	-32.476	
2.705.808	-32.475	
2.705.942	-32.475	
2.706.093	-32.474	
2.706.228	-32.474	
2.706.364	-32.473	
2.706.527	-32.473	
2.706.657	-32.472	
2.706.811	-32.472	
2.707.021	-32.471	
2.707.221	-32.471	
2.707.401	-32.470	
2.707.571	-32.470	
2.707.792	-32.469	
2.708.016	-32.469	
2.708.203	-32.468	

2.708.380	-32.468	
2.708.517	-32.467	
2.708.686	-32.467	
2.708.885	-32.466	
2.709.046	-32.466	
2.709.186	-32.465	
2.709.214	-32.465	
2.709.370	-32.464	
2.709.609	-32.464	
2.709.786	-32.463	
2.709.965	-32.463	
2.710.079	-32.462	
2.710.182	-32.462	
2.710.328	-32.461	
2.710.588	-32.461	
2.710.830	-32.460	
2.710.988	-32.459	
2.711.144	-32.459	
2.711.383	-32.458	
2.711.593	-32.458	
2.711.765	-32.457	
2.711.899	-32.457	
2.712.043	-32.456	
2.712.198	-32.456	
2.712.343	-32.455	
2.712.554	-32.454	
2.712.769	-32.454	
2.712.946	-32.453	
2.713.047	-32.453	
2.713.153	-32.452	

2.713.287	-32.452	
2.713.423	-32.451	
2.713.629	-32.450	
2.713.806	-32.450	
2.713.929	-32.449	
2.714.092	-32.449	
2.714.323	-32.448	
2.714.518	-32.447	
2.714.688	-32.447	
2.714.832	-32.446	
2.714.988	-32.446	
2.715.142	-32.445	
2.715.251	-32.445	
2.715.444	-32.444	
2.715.710	-32.443	
2.715.948	-32.443	
2.716.100	-32.442	
2.716.216	-32.442	
2.716.353	-32.441	
2.716.539	-32.441	
2.716.678	-32.440	
2.716.837	-32.440	
2.716.972	-32.439	
2.717.108	-32.439	
2.717.567	-32.438	
2.718.002	-32.438	
2.718.217	-32.437	
2.718.305	-32.437	
2.718.287	-32.436	
2.718.333	-32.436	

2.718.414	-32.435	
2.718.526	-32.435	
2.718.672	-32.434	
2.718.820	-32.434	
2.718.954	-32.433	
2.719.117	-32.433	
2.719.305	-32.433	
2.719.475	-32.432	
2.719.617	-32.432	
2.719.796	-32.431	
2.719.980	-32.431	
2.720.110	-32.430	
2.720.288	-32.430	
2.720.534	-32.430	
2.720.708	-32.429	
2.720.797	-32.429	
2.720.901	-32.429	
2.721.060	-32.428	
2.721.270	-32.428	
2.721.441	-32.428	
2.721.595	-32.427	
2.721.729	-32.427	
2.721.833	-32.427	
2.721.968	-32.427	
2.722.177	-32.426	
2.722.462	-32.426	
2.722.679	-32.426	
2.722.834	-32.426	
2.722.954	-32.426	
2.723.061	-32.426	

2.723.253	-32.425	
2.723.463	-32.425	
2.723.643	-32.425	
2.723.857	-32.425	
2.724.048	-32.425	
2.724.142	-32.425	
2.724.280	-32.425	
2.724.445	-32.425	
2.724.572	-32.425	
2.724.731	-32.425	
2.724.875	-32.426	
2.725.056	-32.426	
2.725.302	-32.426	
2.725.483	-32.426	
2.725.580	-32.426	
2.725.812	-32.427	
2.726.013	-32.427	
2.726.071	-32.427	
2.726.216	-32.428	
2.726.357	-32.428	
2.726.519	-32.428	
2.726.721	-32.429	
2.726.902	-32.429	
2.727.079	-32.430	
2.727.272	-32.430	
2.727.441	-32.431	
2.727.608	-32.432	
2.727.807	-32.432	
2.727.954	-32.433	
2.728.105	-32.434	

2.728.362	-32.434	
2.728.549	-32.435	
2.728.647	-32.436	
2.728.777	-32.437	
2.728.880	-32.437	
2.728.988	-32.438	
2.729.160	-32.439	
2.729.391	-32.440	
2.729.586	-32.441	
2.729.757	-32.442	
2.729.938	-32.443	
2.730.089	-32.444	
2.730.252	-32.445	
2.730.411	-32.446	
2.730.565	-32.447	
2.730.726	-32.448	
2.730.851	-32.449	
2.730.968	-32.450	
2.731.180	-32.452	
2.731.357	-32.453	
2.731.461	-32.454	
2.731.548	-32.455	
2.731.671	-32.457	
2.731.859	-32.458	
2.732.051	-32.459	
2.732.270	-32.461	
2.732.554	-32.462	
2.732.756	-32.463	
2.732.866	-32.465	
2.733.040	-32.466	

2.733.260	-32.468	
2.733.461	-32.469	
2.733.637	-32.471	
2.733.827	-32.472	
2.733.922	-32.473	
2.734.070	-32.475	
2.734.277	-32.477	
2.734.398	-32.478	
2.734.543	-32.480	
2.734.644	-32.481	
2.734.778	-32.483	
2.735.031	-32.484	
2.735.197	-32.486	
2.735.291	-32.488	
2.735.414	-32.489	
2.735.653	-32.491	
2.735.902	-32.492	
2.736.021	-32.494	
2.736.154	-32.496	
2.736.412	-32.498	
2.736.627	-32.499	
2.736.736	-32.501	
2.736.880	-32.503	
2.737.057	-32.505	
2.737.200	-32.506	
2.737.361	-32.508	
2.737.525	-32.510	
2.737.724	-32.512	
2.737.904	-32.513	
2.738.038	-32.515	

2.738.235	-32.517	
2.738.408	-32.519	
2.738.564	-32.521	
2.738.715	-32.523	
2.738.937	-32.524	
2.739.143	-32.526	
2.739.279	-32.528	
2.739.439	-32.530	
2.739.537	-32.532	
2.739.709	-32.534	
2.739.908	-32.536	
2.740.049	-32.537	
2.740.229	-32.539	
2.740.414	-32.541	
2.740.496	-32.543	
2.740.666	-32.545	
2.740.917	-32.547	
2.741.094	-32.549	
2.741.263	-32.550	
2.741.432	-32.552	
2.741.591	-32.554	
2.741.747	-32.556	
2.741.935	-32.558	
2.742.072	-32.560	
2.742.355	-32.562	
2.742.769	-32.563	
2.743.062	-32.565	
2.743.280	-32.567	
2.743.416	-32.569	
2.743.507	-32.571	

2.743.568	-32.572	
2.743.585	-32.574	
2.743.607	-32.576	
2.743.734	-32.578	
2.743.910	-32.579	
2.744.016	-32.581	
2.744.124	-32.583	
2.744.276	-32.585	
2.744.476	-32.586	
2.744.676	-32.588	
2.744.873	-32.590	
2.745.100	-32.591	
2.745.338	-32.593	
2.745.464	-32.594	
2.745.587	-32.596	
2.745.696	-32.598	
2.745.837	-32.599	
2.746.022	-32.601	
2.746.148	-32.603	
2.746.304	-32.604	
2.746.501	-32.606	
2.746.687	-32.607	
2.746.870	-32.609	
2.747.028	-32.610	
2.747.124	-32.612	
2.747.274	-32.614	
2.747.502	-32.615	
2.747.740	-32.617	
2.747.939	-32.618	
2.748.134	-32.620	

2.748.311	-32.621	
2.748.397	-32.623	
2.748.557	-32.624	
2.748.768	-32.626	
2.748.958	-32.627	
2.749.064	-32.628	
2.749.151	-32.630	
2.749.341	-32.631	
2.749.554	-32.633	
2.749.749	-32.634	
2.749.891	-32.636	
2.750.018	-32.637	
2.750.193	-32.639	
2.750.384	-32.640	
2.750.560	-32.641	
2.750.767	-32.643	
2.750.916	-32.644	
2.751.035	-32.646	
2.751.248	-32.647	
2.751.432	-32.648	
2.751.530	-32.650	
2.751.638	-32.651	
2.751.819	-32.653	
2.751.990	-32.654	
2.752.133	-32.656	
2.752.328	-32.657	
2.752.552	-32.658	
2.752.756	-32.660	
2.752.896	-32.661	
2.753.063	-32.663	

2.753.233	-32.664	
2.753.373	-32.665	
2.753.528	-32.667	
2.753.684	-32.668	
2.753.820	-32.670	
2.753.962	-32.671	
2.754.156	-32.672	
2.754.315	-32.674	
2.754.474	-32.675	
2.754.673	-32.676	
2.754.815	-32.678	
2.754.992	-32.679	
2.755.186	-32.680	
2.755.332	-32.682	
2.755.509	-32.683	
2.755.704	-32.684	
2.755.825	-32.686	
2.755.979	-32.687	
2.756.169	-32.688	
2.756.281	-32.689	
2.756.378	-32.691	
2.756.539	-32.692	
2.756.712	-32.693	
2.756.862	-32.694	
2.757.015	-32.696	
2.757.152	-32.697	
2.757.369	-32.698	
2.757.646	-32.699	
2.757.870	-32.701	
2.758.102	-32.702	

2.758.316	-32.703	
2.758.496	-32.704	
2.758.650	-32.706	
2.758.768	-32.707	
2.758.896	-32.708	
2.759.012	-32.709	
2.759.200	-32.710	
2.759.409	-32.711	
2.759.512	-32.712	
2.759.608	-32.714	
2.759.752	-32.715	
2.759.908	-32.716	
2.760.047	-32.717	
2.760.202	-32.718	
2.760.399	-32.719	
2.760.552	-32.720	
2.760.749	-32.721	
2.760.988	-32.722	
2.761.133	-32.723	
2.761.282	-32.724	
2.761.538	-32.725	
2.761.741	-32.726	
2.761.895	-32.727	
2.762.017	-32.728	
2.762.057	-32.729	
2.762.169	-32.730	
2.762.433	-32.731	
2.762.657	-32.732	
2.762.845	-32.733	
2.762.984	-32.734	

2.763.143	-32.735	
2.763.337	-32.735	
2.763.457	-32.736	
2.763.633	-32.737	
2.763.897	-32.738	
2.764.030	-32.739	
2.764.154	-32.740	
2.764.395	-32.740	
2.764.568	-32.741	
2.764.714	-32.742	
2.764.861	-32.743	
2.765.061	-32.743	
2.765.260	-32.744	
2.765.393	-32.745	
2.765.551	-32.746	
2.765.749	-32.746	
2.765.945	-32.747	
2.766.112	-32.748	
2.766.300	-32.748	
2.766.465	-32.749	
2.766.613	-32.749	
2.766.747	-32.750	
2.766.874	-32.751	
2.767.029	-32.751	
2.767.184	-32.752	
2.767.517	-32.752	
2.767.971	-32.753	
2.768.242	-32.753	
2.768.329	-32.754	
2.768.381	-32.754	

2.768.455	-32.755	
2.768.526	-32.755	
2.768.640	-32.756	
2.768.764	-32.756	
2.768.829	-32.756	
2.768.948	-32.757	
2.769.138	-32.757	
2.769.317	-32.757	
2.769.471	-32.758	
2.769.619	-32.758	
2.769.857	-32.758	
2.770.038	-32.758	
2.770.146	-32.759	
2.770.343	-32.759	
2.770.548	-32.759	
2.770.685	-32.759	
2.770.826	-32.760	
2.770.975	-32.760	
2.771.100	-32.760	
2.771.292	-32.760	
2.771.461	-32.760	
2.771.613	-32.761	
2.771.810	-32.761	
2.771.964	-32.761	
2.772.106	-32.761	
2.772.263	-32.761	
2.772.451	-32.761	
2.772.639	-32.761	
2.772.814	-32.762	
2.772.997	-32.762	

2.773.221	-32.762	
2.773.412	-32.762	
2.773.579	-32.762	
2.773.721	-32.762	
2.773.860	-32.762	
2.774.070	-32.762	
2.774.233	-32.762	
2.774.344	-32.762	
2.774.472	-32.763	
2.774.644	-32.763	
2.774.767	-32.763	
2.774.898	-32.763	
2.775.053	-32.763	
2.775.247	-32.763	
2.775.490	-32.763	
2.775.617	-32.763	
2.775.737	-32.763	
2.775.890	-32.763	
2.776.031	-32.763	
2.776.221	-32.764	
2.776.385	-32.764	
2.776.559	-32.764	
2.776.761	-32.764	
2.776.918	-32.764	
2.777.054	-32.764	
2.777.207	-32.765	
2.777.448	-32.765	
2.777.664	-32.765	
2.777.806	-32.765	
2.777.966	-32.766	

2.778.117	-32.766	
2.778.280	-32.766	
2.778.434	-32.766	
2.778.583	-32.766	
2.778.773	-32.767	
2.778.939	-32.767	
2.779.092	-32.767	
2.779.279	-32.768	
2.779.482	-32.768	
2.779.644	-32.768	
2.779.803	-32.768	
2.780.027	-32.769	
2.780.251	-32.769	
2.780.350	-32.769	
2.780.382	-32.770	
2.780.516	-32.770	
2.780.742	-32.770	
2.780.944	-32.771	
2.781.090	-32.771	
2.781.181	-32.772	
2.781.335	-32.772	
2.781.534	-32.772	
2.781.700	-32.773	
2.781.877	-32.773	
2.782.029	-32.774	
2.782.159	-32.774	
2.782.263	-32.775	
2.782.404	-32.775	
2.782.672	-32.775	
2.782.955	-32.776	

2.783.170	-32.776	
2.783.319	-32.777	
2.783.486	-32.777	
2.783.683	-32.778	
2.783.822	-32.778	
2.783.937	-32.779	
2.784.085	-32.779	
2.784.270	-32.780	
2.784.435	-32.780	
2.784.591	-32.781	
2.784.731	-32.781	
2.784.864	-32.782	
2.785.025	-32.783	
2.785.143	-32.783	
2.785.266	-32.784	
2.785.501	-32.784	
2.785.703	-32.785	
2.785.909	-32.785	
2.786.126	-32.786	
2.786.242	-32.786	
2.786.382	-32.787	
2.786.570	-32.787	
2.786.783	-32.788	
2.786.935	-32.789	
2.787.115	-32.789	
2.787.346	-32.790	
2.787.539	-32.790	
2.787.731	-32.791	
2.787.839	-32.792	
2.787.972	-32.792	

2.788.134	-32.793	
2.788.268	-32.793	
2.788.439	-32.794	
2.788.626	-32.794	
2.788.822	-32.795	
2.789.013	-32.796	
2.789.164	-32.796	
2.789.305	-32.797	
2.789.420	-32.797	
2.789.608	-32.798	
2.789.873	-32.799	
2.790.060	-32.799	
2.790.157	-32.800	
2.790.313	-32.800	
2.790.563	-32.801	
2.790.739	-32.801	
2.790.822	-32.802	
2.791.007	-32.802	
2.791.191	-32.803	
2.791.315	-32.804	
2.791.487	-32.804	
2.791.653	-32.805	
2.791.795	-32.805	
2.791.979	-32.806	
2.792.111	-32.806	
2.792.235	-32.807	
2.792.647	-32.807	
2.793.074	-32.808	
2.793.272	-32.808	
2.793.399	-32.809	

2.793.510	-32.809	
2.793.564	-32.810	
2.793.557	-32.810	
2.793.587	-32.811	
2.793.712	-32.811	
2.793.846	-32.812	
2.793.959	-32.812	
2.794.171	-32.813	
2.794.328	-32.813	
2.794.510	-32.814	
2.794.745	-32.814	
2.794.930	-32.814	
2.795.063	-32.815	
2.795.285	-32.815	
2.795.536	-32.816	
2.795.648	-32.816	
2.795.797	-32.817	
2.795.966	-32.817	
2.796.107	-32.817	
2.796.281	-32.818	
2.796.458	-32.818	
2.796.595	-32.819	
2.796.750	-32.819	
2.796.917	-32.820	
2.797.066	-32.820	
2.797.285	-32.820	
2.797.477	-32.821	
2.797.603	-32.821	
2.797.785	-32.822	
2.797.932	-32.822	

2.798.120	-32.823	
2.798.326	-32.823	
2.798.503	-32.824	
2.798.683	-32.824	
2.798.874	-32.824	
2.799.046	-32.825	
2.799.183	-32.825	
2.799.309	-32.826	
2.799.453	-32.826	
2.799.601	-32.827	
2.799.734	-32.827	
2.799.909	-32.827	
2.800.129	-32.828	
2.800.271	-32.828	
2.800.366	-32.829	
2.800.505	-32.829	
2.800.657	-32.830	
2.800.822	-32.830	
2.800.997	-32.831	
2.801.176	-32.831	
2.801.423	-32.832	
2.801.531	-32.832	
2.801.624	-32.833	
2.801.801	-32.833	
2.801.943	-32.834	
2.802.079	-32.835	
2.802.299	-32.835	
2.802.500	-32.836	
2.802.684	-32.836	
2.802.843	-32.837	

2.802.975	-32.837	
2.803.232	-32.838	
2.803.473	-32.839	
2.803.596	-32.839	
2.803.755	-32.840	
2.803.900	-32.841	
2.804.034	-32.841	
2.804.175	-32.842	
2.804.364	-32.842	
2.804.572	-32.843	
2.804.756	-32.844	
2.804.922	-32.844	
2.805.081	-32.845	
2.805.220	-32.846	
2.805.377	-32.846	
2.805.558	-32.847	
2.805.710	-32.847	
2.805.896	-32.848	
2.806.069	-32.849	
2.806.219	-32.849	
2.806.385	-32.850	
2.806.468	-32.851	
2.806.504	-32.851	
2.806.663	-32.852	
2.806.870	-32.853	
2.807.032	-32.853	
2.807.177	-32.854	
2.807.404	-32.855	
2.807.690	-32.855	
2.807.913	-32.856	

2.808.062	-32.857	
2.808.275	-32.857	
2.808.426	-32.858	
2.808.615	-32.859	
2.808.828	-32.859	
2.809.016	-32.860	
2.809.142	-32.861	
2.809.287	-32.861	
2.809.391	-32.862	
2.809.474	-32.862	
2.809.638	-32.863	
2.809.835	-32.864	
2.809.981	-32.864	
2.810.130	-32.865	
2.810.271	-32.865	
2.810.431	-32.866	
2.810.659	-32.867	
2.810.852	-32.867	
2.811.017	-32.868	
2.811.212	-32.868	
2.811.354	-32.869	
2.811.599	-32.870	
2.811.855	-32.870	
2.811.982	-32.871	
2.812.127	-32.871	
2.812.282	-32.872	
2.812.458	-32.872	
2.812.669	-32.873	
2.812.827	-32.873	
2.812.971	-32.874	

2.813.175	-32.874	
2.813.315	-32.875	
2.813.456	-32.875	
2.813.661	-32.876	
2.813.811	-32.876	
2.814.015	-32.877	
2.814.243	-32.877	
2.814.406	-32.878	
2.814.532	-32.878	
2.814.652	-32.879	
2.814.785	-32.879	
2.814.965	-32.880	
2.815.128	-32.880	
2.815.244	-32.880	
2.815.407	-32.881	
2.815.533	-32.881	
2.815.653	-32.882	
2.815.830	-32.882	
2.816.123	-32.882	
2.816.268	-32.883	
2.816.436	-32.883	
2.816.609	-32.884	
2.816.733	-32.884	
2.816.852	-32.884	
2.817.052	-32.885	
2.817.257	-32.885	
2.817.570	-32.886	
2.817.975	-32.886	
2.818.254	-32.886	
2.818.367	-32.887	

2.818.403	-32.887	
2.818.526	-32.887	
2.818.629	-32.888	
2.818.575	-32.888	
2.818.688	-32.888	
2.818.896	-32.889	
2.818.990	-32.889	
2.819.143	-32.889	
2.819.351	-32.890	
2.819.552	-32.890	
2.819.749	-32.890	
2.819.934	-32.891	
2.820.135	-32.891	
2.820.278	-32.891	
2.820.443	-32.892	
2.820.655	-32.892	
2.820.793	-32.893	
2.820.945	-32.893	
2.821.116	-32.893	
2.821.210	-32.894	
2.821.365	-32.894	
2.821.581	-32.894	
2.821.768	-32.895	
2.821.913	-32.895	
2.822.072	-32.896	
2.822.227	-32.896	
2.822.368	-32.896	
2.822.567	-32.897	
2.822.781	-32.897	
2.822.910	-32.898	

2.823.094	-32.898	
2.823.283	-32.899	
2.823.431	-32.899	
2.823.668	-32.900	
2.823.813	-32.900	
2.823.929	-32.901	
2.824.116	-32.902	
2.824.287	-32.902	
2.824.396	-32.903	
2.824.492	-32.903	
2.824.660	-32.904	
2.824.875	-32.904	
2.825.049	-32.905	
2.825.190	-32.906	
2.825.327	-32.906	
2.825.493	-32.907	
2.825.684	-32.908	
2.825.838	-32.908	
2.825.997	-32.909	
2.826.209	-32.910	
2.826.302	-32.911	
2.826.409	-32.911	
2.826.581	-32.912	
2.826.750	-32.913	
2.826.982	-32.914	
2.827.211	-32.915	
2.827.384	-32.916	
2.827.509	-32.917	
2.827.643	-32.917	
2.827.849	-32.918	

2.828.047	-32.919	
2.828.150	-32.920	
2.828.336	-32.921	
2.828.571	-32.922	
2.828.759	-32.923	
2.828.918	-32.924	
2.829.081	-32.925	
2.829.250	-32.926	
2.829.405	-32.927	
2.829.547	-32.928	
2.829.695	-32.929	
2.829.857	-32.930	
2.829.974	-32.932	
2.830.148	-32.933	
2.830.349	-32.934	
2.830.529	-32.935	
2.830.732	-32.936	
2.830.964	-32.937	
2.831.062	-32.938	
2.831.126	-32.939	
2.831.295	-32.940	
2.831.459	-32.941	
2.831.573	-32.942	
2.831.711	-32.944	
2.831.885	-32.945	
2.832.040	-32.946	
2.832.191	-32.947	
2.832.369	-32.948	
2.832.590	-32.949	
2.832.874	-32.950	

2.833.016	-32.952	
2.833.199	-32.953	
2.833.356	-32.954	
2.833.484	-32.955	
2.833.703	-32.956	
2.833.871	-32.957	
2.833.997	-32.958	
2.834.113	-32.960	
2.834.225	-32.961	
2.834.418	-32.962	
2.834.575	-32.963	
2.834.709	-32.964	
2.834.861	-32.965	
2.835.024	-32.966	
2.835.222	-32.967	
2.835.428	-32.968	
2.835.616	-32.969	
2.835.794	-32.970	
2.835.966	-32.971	
2.836.158	-32.973	
2.836.372	-32.974	
2.836.564	-32.975	
2.836.732	-32.976	
2.836.877	-32.977	
2.836.961	-32.978	
2.837.086	-32.979	
2.837.258	-32.980	
2.837.431	-32.981	
2.837.597	-32.982	
2.837.831	-32.983	

2.838.027	-32.984	
2.838.221	-32.985	
2.838.391	-32.986	
2.838.535	-32.987	
2.838.737	-32.988	
2.838.885	-32.988	
2.839.030	-32.989	
2.839.207	-32.990	
2.839.319	-32.991	
2.839.525	-32.992	
2.839.718	-32.993	
2.839.868	-32.994	
2.840.034	-32.995	
2.840.128	-32.996	
2.840.256	-32.997	
2.840.462	-32.997	
2.840.689	-32.998	
2.840.903	-32.999	
2.841.069	-33.000	
2.841.235	-33.001	
2.841.418	-33.002	
2.841.553	-33.003	
2.841.693	-33.004	
2.841.863	-33.004	
2.841.927	-33.005	
2.842.068	-33.006	
2.842.442	-33.007	
2.842.899	-33.008	
2.843.242	-33.009	
2.843.347	-33.009	

2.843.370	-33.010	
2.843.474	-33.011	
2.843.616	-33.012	
2.843.719	-33.013	
2.843.777	-33.013	
2.843.814	-33.014	
2.843.911	-33.015	
2.844.107	-33.016	
2.844.312	-33.017	
2.844.464	-33.017	
2.844.635	-33.018	
2.844.810	-33.019	
2.844.962	-33.020	
2.845.143	-33.021	
2.845.411	-33.021	
2.845.565	-33.022	
2.845.663	-33.023	
2.845.851	-33.024	
2.846.064	-33.025	
2.846.180	-33.025	
2.846.281	-33.026	
2.846.415	-33.027	
2.846.571	-33.028	
2.846.776	-33.029	
2.846.911	-33.030	
2.847.018	-33.030	
2.847.192	-33.031	
2.847.366	-33.032	
2.847.557	-33.033	
2.847.766	-33.034	

2.847.979	-33.035	
2.848.138	-33.036	
2.848.320	-33.037	
2.848.553	-33.038	
2.848.699	-33.039	
2.848.824	-33.040	
2.848.976	-33.041	
2.849.156	-33.042	
2.849.355	-33.043	
2.849.471	-33.044	
2.849.550	-33.045	
2.849.627	-33.046	
2.849.846	-33.047	
2.850.164	-33.048	
2.850.332	-33.049	
2.850.493	-33.050	
2.850.638	-33.051	
2.850.791	-33.052	
2.850.977	-33.054	
2.851.154	-33.055	
2.851.322	-33.056	
2.851.461	-33.057	
2.851.621	-33.058	
2.851.750	-33.060	
2.851.906	-33.061	
2.852.166	-33.062	
2.852.339	-33.064	
2.852.474	-33.065	
2.852.635	-33.066	
2.852.785	-33.068	

2.853.036	-33.069	
2.853.228	-33.070	
2.853.344	-33.072	
2.853.492	-33.073	
2.853.616	-33.075	
2.853.748	-33.076	
2.853.896	-33.077	
2.854.081	-33.079	
2.854.275	-33.080	
2.854.434	-33.082	
2.854.641	-33.083	
2.854.853	-33.085	
2.854.984	-33.086	
2.855.128	-33.088	
2.855.320	-33.089	
2.855.497	-33.090	
2.855.645	-33.092	
2.855.807	-33.093	
2.856.010	-33.095	
2.856.143	-33.096	
2.856.231	-33.098	
2.856.362	-33.099	
2.856.538	-33.101	
2.856.646	-33.102	
2.856.795	-33.104	
2.856.952	-33.105	
2.857.090	-33.106	
2.857.310	-33.108	
2.857.518	-33.109	
2.857.715	-33.111	

2.857.971	-33.112	
2.858.213	-33.114	
2.858.391	-33.115	
2.858.514	-33.116	
2.858.673	-33.118	
2.858.864	-33.119	
2.859.015	-33.120	
2.859.139	-33.122	
2.859.288	-33.123	
2.859.440	-33.124	
2.859.579	-33.126	
2.859.734	-33.127	
2.859.929	-33.128	
2.860.074	-33.129	
2.860.193	-33.130	
2.860.361	-33.132	
2.860.565	-33.133	
2.860.784	-33.134	
2.860.988	-33.135	
2.861.118	-33.136	
2.861.245	-33.137	
2.861.476	-33.138	
2.861.672	-33.139	
2.861.813	-33.140	
2.861.971	-33.141	
2.862.122	-33.142	
2.862.271	-33.143	
2.862.465	-33.144	
2.862.713	-33.145	
2.862.882	-33.146	

2.862.972	-33.147	
2.863.112	-33.148	
2.863.297	-33.148	
2.863.489	-33.149	
2.863.604	-33.150	
2.863.739	-33.151	
2.863.904	-33.152	
2.864.044	-33.152	
2.864.221	-33.153	
2.864.471	-33.154	
2.864.644	-33.154	
2.864.803	-33.155	
2.865.031	-33.155	
2.865.212	-33.156	
2.865.349	-33.156	
2.865.509	-33.157	
2.865.693	-33.157	
2.865.808	-33.158	
2.865.949	-33.158	
2.866.126	-33.159	
2.866.322	-33.159	
2.866.588	-33.160	
2.866.750	-33.160	
2.866.889	-33.160	
2.867.048	-33.161	
2.867.202	-33.161	
2.867.492	-33.161	
2.867.940	-33.161	
2.868.250	-33.162	
2.868.344	-33.162	

2.868.430	-33.162	
2.868.484	-33.162	
2.868.518	-33.162	
2.868.604	-33.162	
2.868.708	-33.162	
2.868.785	-33.162	
2.868.929	-33.162	
2.869.109	-33.162	
2.869.262	-33.162	
2.869.435	-33.162	
2.869.593	-33.162	
2.869.789	-33.162	
2.869.969	-33.162	
2.870.147	-33.162	
2.870.359	-33.162	
2.870.490	-33.161	
2.870.667	-33.161	
2.870.851	-33.161	
2.870.952	-33.161	
2.871.098	-33.161	
2.871.246	-33.160	
2.871.419	-33.160	
2.871.635	-33.160	
2.871.812	-33.160	
2.872.007	-33.160	
2.872.156	-33.159	
2.872.289	-33.159	
2.872.409	-33.159	
2.872.567	-33.158	
2.872.827	-33.158	

2.873.022	-33.158	
2.873.096	-33.158	
2.873.246	-33.157	
2.873.524	-33.157	
2.873.738	-33.157	
2.873.876	-33.156	
2.874.028	-33.156	
2.874.189	-33.156	
2.874.331	-33.156	
2.874.485	-33.155	
2.874.608	-33.155	
2.874.727	-33.155	
2.874.897	-33.154	
2.875.135	-33.154	
2.875.317	-33.154	
2.875.490	-33.154	
2.875.603	-33.153	
2.875.728	-33.153	
2.875.892	-33.153	
2.876.071	-33.153	
2.876.289	-33.153	
2.876.447	-33.153	
2.876.567	-33.152	
2.876.714	-33.152	
2.876.893	-33.152	
2.877.090	-33.152	
2.877.253	-33.152	
2.877.387	-33.152	
2.877.552	-33.152	
2.877.771	-33.152	

2.877.965	-33.152	
2.878.123	-33.152	
2.878.255	-33.152	
2.878.459	-33.152	
2.878.661	-33.152	
2.878.770	-33.152	
2.878.963	-33.152	
2.879.125	-33.152	
2.879.183	-33.153	
2.879.411	-33.153	
2.879.670	-33.153	
2.879.823	-33.153	
2.879.956	-33.153	
2.880.097	-33.153	
2.880.236	-33.154	
2.880.415	-33.154	
2.880.605	-33.154	
2.880.787	-33.154	
2.880.816	-33.155	
2.880.913	-33.155	
2.881.148	-33.155	
2.881.278	-33.156	
2.881.457	-33.156	
2.881.627	-33.156	
2.881.848	-33.157	
2.882.015	-33.157	
2.882.106	-33.157	
2.882.293	-33.158	
2.882.506	-33.158	
2.882.763	-33.159	

2.882.973	-33.159	
2.883.182	-33.160	
2.883.427	-33.160	
2.883.567	-33.161	
2.883.683	-33.161	
2.883.804	-33.162	
2.883.976	-33.162	
2.884.151	-33.163	
2.884.283	-33.163	
2.884.349	-33.164	
2.884.485	-33.164	
2.884.666	-33.165	
2.884.757	-33.165	
2.884.934	-33.166	
2.885.126	-33.167	
2.885.303	-33.167	
2.885.526	-33.168	
2.885.755	-33.168	
2.885.924	-33.169	
2.886.078	-33.170	
2.886.268	-33.170	
2.886.443	-33.171	
2.886.653	-33.171	
2.886.844	-33.172	
2.886.968	-33.173	
2.887.094	-33.173	
2.887.268	-33.174	
2.887.491	-33.175	
2.887.664	-33.175	
2.887.816	-33.176	

2.887.982	-33.177	
2.888.135	-33.178	
2.888.278	-33.178	
2.888.471	-33.179	
2.888.633	-33.180	
2.888.724	-33.180	
2.888.929	-33.181	
2.889.156	-33.182	
2.889.355	-33.183	
2.889.523	-33.183	
2.889.676	-33.184	
2.889.821	-33.185	
2.889.977	-33.185	
2.890.172	-33.186	
2.890.306	-33.187	
2.890.457	-33.188	
2.890.666	-33.188	
2.890.840	-33.189	
2.891.040	-33.190	
2.891.205	-33.190	
2.891.295	-33.191	
2.891.408	-33.192	
2.891.575	-33.193	
2.891.766	-33.193	
2.891.934	-33.194	
2.892.229	-33.195	
2.892.666	-33.195	
2.892.971	-33.196	
2.893.087	-33.197	
2.893.197	-33.198	

2.893.300	-33.198	
2.893.370	-33.199	
2.893.416	-33.200	
2.893.517	-33.200	
2.893.652	-33.201	
2.893.713	-33.202	
2.893.833	-33.202	
2.894.017	-33.203	
2.894.232	-33.204	
2.894.458	-33.204	
2.894.633	-33.205	
2.894.792	-33.206	
2.894.942	-33.206	
2.895.129	-33.207	
2.895.301	-33.208	
2.895.471	-33.208	
2.895.576	-33.209	
2.895.735	-33.209	
2.895.894	-33.210	
2.895.996	-33.211	
2.896.231	-33.211	
2.896.382	-33.212	
2.896.487	-33.213	
2.896.700	-33.213	
2.896.857	-33.214	
2.897.012	-33.215	
2.897.216	-33.215	
2.897.463	-33.216	
2.897.697	-33.217	
2.897.903	-33.217	

2.898.105	-33.218	
2.898.253	-33.219	
2.898.343	-33.220	
2.898.484	-33.220	
2.898.676	-33.221	
2.898.857	-33.222	
2.899.019	-33.223	
2.899.125	-33.223	
2.899.228	-33.224	
2.899.399	-33.225	
2.899.597	-33.226	
2.899.774	-33.226	
2.899.969	-33.227	
2.900.136	-33.228	
2.900.216	-33.229	
2.900.316	-33.230	
2.900.504	-33.230	
2.900.682	-33.231	
2.900.917	-33.232	
2.901.064	-33.233	
2.901.165	-33.234	
2.901.335	-33.235	
2.901.497	-33.236	
2.901.719	-33.237	
2.901.920	-33.238	
2.902.043	-33.239	
2.902.202	-33.240	
2.902.427	-33.241	
2.902.612	-33.242	
2.902.843	-33.243	

2.903.040	-33.244	
2.903.145	-33.245	
2.903.243	-33.246	
2.903.394	-33.247	
2.903.551	-33.248	
2.903.717	-33.250	
2.903.951	-33.251	
2.904.151	-33.252	
2.904.265	-33.253	
2.904.420	-33.254	
2.904.680	-33.255	
2.904.857	-33.257	
2.904.974	-33.258	
2.905.129	-33.259	
2.905.306	-33.260	
2.905.447	-33.262	
2.905.580	-33.263	
2.905.714	-33.264	
2.905.833	-33.265	
2.906.026	-33.267	
2.906.176	-33.268	
2.906.275	-33.269	
2.906.421	-33.271	
2.906.613	-33.272	
2.906.801	-33.273	
2.906.949	-33.275	
2.907.133	-33.276	
2.907.361	-33.277	
2.907.607	-33.279	
2.907.854	-33.280	

2.908.008	-33.282	
2.908.170	-33.283	
2.908.302	-33.285	
2.908.459	-33.286	
2.908.658	-33.287	
2.908.789	-33.289	
2.908.936	-33.290	
2.909.093	-33.292	
2.909.225	-33.293	
2.909.352	-33.295	
2.909.460	-33.296	
2.909.577	-33.298	
2.909.760	-33.299	
2.909.941	-33.301	
2.910.125	-33.302	
2.910.338	-33.303	
2.910.554	-33.305	
2.910.742	-33.306	
2.910.927	-33.308	
2.911.122	-33.309	
2.911.262	-33.311	
2.911.393	-33.312	
2.911.559	-33.314	
2.911.776	-33.315	
2.911.924	-33.317	
2.912.039	-33.318	
2.912.247	-33.320	
2.912.452	-33.321	
2.912.661	-33.323	
2.912.915	-33.324	

2.913.084	-33.326	
2.913.168	-33.327	
2.913.307	-33.328	
2.913.522	-33.330	
2.913.727	-33.331	
2.913.813	-33.333	
2.913.944	-33.334	
2.914.139	-33.336	
2.914.327	-33.337	
2.914.496	-33.338	
2.914.655	-33.340	
2.914.846	-33.341	
2.914.998	-33.342	
2.915.202	-33.344	
2.915.374	-33.345	
2.915.495	-33.347	
2.915.638	-33.348	
2.915.816	-33.349	
2.915.975	-33.350	
2.916.158	-33.352	
2.916.353	-33.353	
2.916.454	-33.354	
2.916.621	-33.356	
2.916.732	-33.357	
2.917.039	-33.358	
2.917.554	-33.359	
2.917.841	-33.360	
2.917.940	-33.362	
2.918.009	-33.363	
2.918.109	-33.364	

2.918.178	-33.365	
2.918.239	-33.366	
2.918.385	-33.367	
2.918.496	-33.368	
2.918.538	-33.370	
2.918.681	-33.371	
2.918.878	-33.372	
2.919.073	-33.373	
2.919.328	-33.374	
2.919.515	-33.375	
2.919.659	-33.376	
2.919.763	-33.377	
2.919.944	-33.378	
2.920.176	-33.379	
2.920.321	-33.380	
2.920.540	-33.381	
2.920.699	-33.381	
2.920.844	-33.382	
2.920.986	-33.383	
2.921.107	-33.384	
2.921.318	-33.385	
2.921.523	-33.386	
2.921.631	-33.387	
2.921.812	-33.388	
2.922.011	-33.389	
2.922.163	-33.390	
2.922.368	-33.390	
2.922.496	-33.391	
2.922.630	-33.392	
2.922.845	-33.393	

2.923.067	-33.394	
2.923.272	-33.395	
2.923.402	-33.396	
2.923.522	-33.396	
2.923.648	-33.397	
2.923.753	-33.398	
2.923.889	-33.399	
2.924.045	-33.400	
2.924.210	-33.401	
2.924.445	-33.401	
2.924.671	-33.402	
2.924.794	-33.403	
2.924.886	-33.404	
2.925.052	-33.405	
2.925.245	-33.406	
2.925.411	-33.406	
2.925.558	-33.407	
2.925.728	-33.408	
2.925.941	-33.409	
2.926.112	-33.410	
2.926.208	-33.410	
2.926.342	-33.411	
2.926.501	-33.412	
2.926.687	-33.413	
2.926.877	-33.414	
2.927.057	-33.415	
2.927.274	-33.416	
2.927.471	-33.416	
2.927.618	-33.417	
2.927.762	-33.418	

2.927.961	-33.419	
2.928.141	-33.420	
2.928.325	-33.421	
2.928.508	-33.422	
2.928.625	-33.423	
2.928.773	-33.423	
2.928.965	-33.424	
2.929.116	-33.425	
2.929.275	-33.426	
2.929.519	-33.427	
2.929.670	-33.428	
2.929.810	-33.429	
2.929.951	-33.430	
2.930.057	-33.430	
2.930.164	-33.431	
2.930.345	-33.432	
2.930.540	-33.433	
2.930.713	-33.434	
2.930.894	-33.435	
2.930.986	-33.436	
2.931.097	-33.437	
2.931.285	-33.437	
2.931.443	-33.438	
2.931.624	-33.439	
2.931.799	-33.440	
2.931.917	-33.441	
2.932.122	-33.442	
2.932.397	-33.443	
2.932.608	-33.444	
2.932.806	-33.444	

2.932.997	-33.445	
2.933.177	-33.446	
2.933.352	-33.447	
2.933.443	-33.448	
2.933.544	-33.449	
2.933.757	-33.450	
2.933.976	-33.450	
2.934.051	-33.451	
2.934.163	-33.452	
2.934.301	-33.453	
2.934.501	-33.454	
2.934.669	-33.455	
2.934.815	-33.455	
2.935.005	-33.456	
2.935.173	-33.457	
2.935.313	-33.458	
2.935.537	-33.458	
2.935.820	-33.459	
2.935.988	-33.460	
2.936.145	-33.461	
2.936.320	-33.461	
2.936.461	-33.462	
2.936.636	-33.463	
2.936.880	-33.464	
2.937.054	-33.464	
2.937.173	-33.465	
2.937.329	-33.466	
2.937.523	-33.466	
2.937.672	-33.467	
2.937.801	-33.468	

2.937.993	-33.468	
2.938.193	-33.469	
2.938.343	-33.470	
2.938.445	-33.470	
2.938.622	-33.471	
2.938.864	-33.471	
2.939.016	-33.472	
2.939.109	-33.473	
2.939.286	-33.473	
2.939.492	-33.474	
2.939.655	-33.474	
2.939.794	-33.475	
2.939.971	-33.475	
2.940.157	-33.476	
2.940.339	-33.476	
2.940.523	-33.477	
2.940.667	-33.477	
2.940.794	-33.477	
2.940.991	-33.478	
2.941.168	-33.478	
2.941.299	-33.479	
2.941.439	-33.479	
2.941.571	-33.479	
2.941.956	-33.480	
2.942.411	-33.480	
2.942.615	-33.481	
2.942.686	-33.481	
2.942.763	-33.481	
2.942.889	-33.481	
2.942.958	-33.482	

2.943.047	-33.482	
2.943.113	-33.482	
2.943.192	-33.483	
2.943.329	-33.483	
2.943.531	-33.483	
2.943.734	-33.483	
2.943.900	-33.483	
2.944.066	-33.484	
2.944.240	-33.484	
2.944.501	-33.484	
2.944.702	-33.484	
2.944.855	-33.484	
2.945.005	-33.484	
2.945.165	-33.485	
2.945.425	-33.485	
2.945.623	-33.485	
2.945.739	-33.485	
2.945.814	-33.485	
2.945.901	-33.485	
2.946.073	-33.485	
2.946.245	-33.485	
2.946.393	-33.486	
2.946.548	-33.486	
2.946.707	-33.486	
2.946.895	-33.486	
2.947.058	-33.486	
2.947.231	-33.486	
2.947.465	-33.486	
2.947.693	-33.487	
2.947.864	-33.487	

2.948.056	-33.487	
2.948.231	-33.487	
2.948.331	-33.487	
2.948.474	-33.487	
2.948.587	-33.488	
2.948.676	-33.488	
2.948.835	-33.488	
2.949.044	-33.488	
2.949.248	-33.488	
2.949.403	-33.488	
2.949.568	-33.489	
2.949.774	-33.489	
2.949.977	-33.489	
2.950.150	-33.489	
2.950.298	-33.490	
2.950.419	-33.490	
2.950.605	-33.490	
2.950.794	-33.490	
2.950.858	-33.491	
2.950.993	-33.491	
2.951.223	-33.491	
2.951.378	-33.492	
2.951.534	-33.492	
2.951.752	-33.492	
2.952.004	-33.493	
2.952.158	-33.493	
2.952.297	-33.494	
2.952.482	-33.494	
2.952.661	-33.495	
2.952.809	-33.495	

2.952.910	-33.495	
2.953.034	-33.496	
2.953.222	-33.496	
2.953.387	-33.497	
2.953.544	-33.497	
2.953.708	-33.498	
2.953.865	-33.499	
2.954.075	-33.499	
2.954.294	-33.500	
2.954.453	-33.500	
2.954.594	-33.501	
2.954.750	-33.501	
2.954.882	-33.502	
2.955.061	-33.503	
2.955.197	-33.503	
2.955.269	-33.504	
2.955.473	-33.504	
2.955.667	-33.505	
2.955.802	-33.506	
2.955.924	-33.506	
2.956.020	-33.507	
2.956.119	-33.508	
2.956.285	-33.509	
2.956.520	-33.509	
2.956.750	-33.510	
2.957.032	-33.511	
2.957.285	-33.511	
2.957.470	-33.512	
2.957.715	-33.513	
2.957.893	-33.514	

2.957.986	-33.514	
2.958.105	-33.515	
2.958.246	-33.516	
2.958.366	-33.517	
2.958.492	-33.518	
2.958.576	-33.518	
2.958.763	-33.519	
2.958.999	-33.520	
2.959.178	-33.521	
2.959.353	-33.522	
2.959.576	-33.522	
2.959.774	-33.523	
2.959.890	-33.524	
2.960.081	-33.525	
2.960.247	-33.525	
2.960.408	-33.526	
2.960.674	-33.527	
2.960.891	-33.528	
2.961.018	-33.529	
2.961.145	-33.529	
2.961.289	-33.530	
2.961.448	-33.531	
2.961.639	-33.532	
2.961.817	-33.533	
2.962.014	-33.533	
2.962.188	-33.534	
2.962.293	-33.535	
2.962.419	-33.536	
2.962.606	-33.537	
2.962.791	-33.537	

2.962.989	-33.538	
2.963.177	-33.539	
2.963.333	-33.540	
2.963.478	-33.540	
2.963.659	-33.541	
2.963.824	-33.542	
2.963.930	-33.543	
2.964.131	-33.543	
2.964.338	-33.544	
2.964.439	-33.545	
2.964.526	-33.545	
2.964.727	-33.546	
2.964.973	-33.547	
2.965.139	-33.547	
2.965.299	-33.548	
2.965.414	-33.549	
2.965.565	-33.549	
2.965.789	-33.550	
2.965.942	-33.551	
2.966.031	-33.551	
2.966.198	-33.552	
2.966.465	-33.552	
2.966.841	-33.553	
2.967.242	-33.554	
2.967.440	-33.554	
2.967.495	-33.555	
2.967.568	-33.555	
2.967.630	-33.556	
2.967.708	-33.557	
2.967.820	-33.557	

2.967.914	-33.558	
2.968.055	-33.558	
2.968.229	-33.559	
2.968.387	-33.559	
2.968.542	-33.560	
2.968.713	-33.560	
2.968.911	-33.561	
2.969.122	-33.562	
2.969.272	-33.562	
2.969.460	-33.563	
2.969.653	-33.563	
2.969.826	-33.564	
2.970.020	-33.564	
2.970.111	-33.565	
2.970.242	-33.565	
2.970.414	-33.566	
2.970.602	-33.566	
2.970.800	-33.567	
2.970.901	-33.567	
2.971.025	-33.568	
2.971.235	-33.568	
2.971.440	-33.569	
2.971.574	-33.569	
2.971.707	-33.570	
2.971.929	-33.571	
2.972.130	-33.571	
2.972.286	-33.572	
2.972.488	-33.572	
2.972.693	-33.573	
2.972.861	-33.573	

2.972.971	-33.574	
2.973.095	-33.575	
2.973.206	-33.575	
2.973.340	-33.576	
2.973.500	-33.577	
2.973.663	-33.577	
2.973.894	-33.578	
2.974.030	-33.578	
2.974.102	-33.579	
2.974.323	-33.580	
2.974.608	-33.581	
2.974.803	-33.581	
2.974.958	-33.582	
2.975.117	-33.583	
2.975.227	-33.583	
2.975.392	-33.584	
2.975.585	-33.585	
2.975.711	-33.586	
2.975.898	-33.587	
2.976.079	-33.588	
2.976.217	-33.588	
2.976.361	-33.589	
2.976.577	-33.590	
2.976.844	-33.591	
2.977.022	-33.592	
2.977.166	-33.593	
2.977.344	-33.594	
2.977.460	-33.595	
2.977.570	-33.596	
2.977.769	-33.597	

2.977.908	-33.598	
2.978.020	-33.599	
2.978.170	-33.600	
2.978.376	-33.601	
2.978.611	-33.603	
2.978.773	-33.604	
2.978.918	-33.605	
2.979.081	-33.606	
2.979.228	-33.607	
2.979.400	-33.608	
2.979.594	-33.609	
2.979.735	-33.611	
2.979.902	-33.612	
2.980.053	-33.613	
2.980.121	-33.614	
2.980.245	-33.615	
2.980.461	-33.617	
2.980.656	-33.618	
2.980.766	-33.619	
2.980.869	-33.620	
2.981.064	-33.622	
2.981.271	-33.623	
2.981.497	-33.624	
2.981.726	-33.626	
2.981.857	-33.627	
2.982.075	-33.628	
2.982.329	-33.630	
2.982.480	-33.631	
2.982.643	-33.633	
2.982.848	-33.634	

2.983.018	-33.635	
2.983.179	-33.637	
2.983.323	-33.638	
2.983.449	-33.639	
2.983.614	-33.641	
2.983.749	-33.642	
2.983.882	-33.643	
2.984.053	-33.645	
2.984.201	-33.646	
2.984.345	-33.647	
2.984.572	-33.649	
2.984.789	-33.650	
2.984.942	-33.652	
2.985.140	-33.653	
2.985.301	-33.654	
2.985.430	-33.656	
2.985.594	-33.657	
2.985.747	-33.658	
2.985.913	-33.659	
2.986.129	-33.661	
2.986.282	-33.662	
2.986.407	-33.663	
2.986.611	-33.665	
2.986.786	-33.666	
2.986.997	-33.667	
2.987.188	-33.669	
2.987.253	-33.670	
2.987.354	-33.671	
2.987.541	-33.672	
2.987.697	-33.674	

2.987.866	-33.675	
2.988.127	-33.676	
2.988.362	-33.677	
2.988.500	-33.678	
2.988.650	-33.680	
2.988.846	-33.681	
2.989.026	-33.682	
2.989.156	-33.683	
2.989.266	-33.684	
2.989.494	-33.685	
2.989.727	-33.686	
2.989.800	-33.687	
2.989.933	-33.688	
2.990.190	-33.690	
2.990.370	-33.691	
2.990.520	-33.692	
2.990.638	-33.693	
2.990.772	-33.694	
2.990.928	-33.695	
2.991.172	-33.696	
2.991.642	-33.697	
2.992.050	-33.698	
2.992.199	-33.698	
2.992.236	-33.699	
2.992.372	-33.700	
2.992.481	-33.701	
2.992.440	-33.702	
2.992.529	-33.703	
2.992.665	-33.704	
2.992.778	-33.704	

2.992.971	-33.705	
2.993.167	-33.706	
2.993.363	-33.707	
2.993.540	-33.707	
2.993.654	-33.708	
2.993.789	-33.709	
2.993.990	-33.709	
2.994.205	-33.710	
2.994.407	-33.711	
2.994.569	-33.711	
2.994.698	-33.712	
2.994.796	-33.713	
2.994.933	-33.713	
2.995.117	-33.714	
2.995.285	-33.715	
2.995.379	-33.715	
2.995.537	-33.716	
2.995.742	-33.716	
2.995.923	-33.717	
2.996.079	-33.717	
2.996.231	-33.718	
2.996.474	-33.718	
2.996.678	-33.719	
2.996.812	-33.719	
2.996.972	-33.720	
2.997.180	-33.720	
2.997.365	-33.721	
2.997.477	-33.721	
2.997.596	-33.722	
2.997.801	-33.722	

2.997.950	-33.723	
2.998.102	-33.723	
2.998.326	-33.724	
2.998.456	-33.724	
2.998.591	-33.725	
2.998.770	-33.725	
2.998.981	-33.725	
2.999.203	-33.726	
2.999.301	-33.726	
2.999.414	-33.727	
2.999.601	-33.727	
2.999.811	-33.728	
2.999.977	-33.728	
3.000.186	-33.729	
3.000.366	-33.729	
3.000.486	-33.729	
3.000.645	-33.730	
3.000.833	-33.730	
3.001.003	-33.731	
3.001.166	-33.731	
3.001.375	-33.732	
3.001.523	-33.732	
3.001.667	-33.733	
3.001.866	-33.733	
3.002.054	-33.734	
3.002.195	-33.734	
3.002.372	-33.735	
3.002.571	-33.735	
3.002.689	-33.736	
3.002.813	-33.736	

3.002.962	-33.737	
3.003.083	-33.737	
3.003.280	-33.738	
3.003.398	-33.738	
3.003.562	-33.739	
3.003.846	-33.739	
3.004.001	-33.740	
3.004.131	-33.740	
3.004.287	-33.741	
3.004.447	-33.741	
3.004.550	-33.742	
3.004.709	-33.742	
3.004.937	-33.742	
3.005.039	-33.743	
3.005.068	-33.743	
3.005.262	-33.744	
3.005.515	-33.744	
3.005.637	-33.745	
3.005.786	-33.745	
3.005.979	-33.746	
3.006.203	-33.746	
3.006.456	-33.747	
3.006.657	-33.747	
3.006.852	-33.748	
3.007.099	-33.748	
3.007.292	-33.749	
3.007.444	-33.749	
3.007.578	-33.750	
3.007.724	-33.750	
3.007.901	-33.750	

3.008.052	-33.751	
3.008.163	-33.751	
3.008.287	-33.752	
3.008.493	-33.752	
3.008.651	-33.752	
3.008.755	-33.753	
3.008.904	-33.753	
3.009.055	-33.754	
3.009.236	-33.754	
3.009.438	-33.754	
3.009.619	-33.755	
3.009.783	-33.755	
3.009.973	-33.755	
3.010.186	-33.756	
3.010.342	-33.756	
3.010.505	-33.756	
3.010.703	-33.757	
3.010.843	-33.757	
3.010.991	-33.757	
3.011.138	-33.757	
3.011.255	-33.758	
3.011.455	-33.758	
3.011.645	-33.758	
3.011.781	-33.759	
3.011.947	-33.759	
3.012.133	-33.759	
3.012.321	-33.759	
3.012.529	-33.760	
3.012.729	-33.760	
3.012.870	-33.760	

3.012.907	-33.760	
3.013.098	-33.760	
3.013.420	-33.761	
3.013.644	-33.761	
3.013.788	-33.761	
3.013.947	-33.761	
3.014.121	-33.761	
3.014.288	-33.762	
3.014.464	-33.762	
3.014.586	-33.762	
3.014.720	-33.762	
3.014.865	-33.762	
3.015.056	-33.762	
3.015.193	-33.762	
3.015.343	-33.762	
3.015.493	-33.763	
3.015.737	-33.763	
3.016.161	-33.763	
3.016.495	-33.763	
3.016.693	-33.763	
3.016.833	-33.763	
3.016.942	-33.763	
3.016.942	-33.763	
3.016.946	-33.763	
3.017.068	-33.763	
3.017.206	-33.763	
3.017.275	-33.763	
3.017.413	-33.763	
3.017.643	-33.763	
3.017.791	-33.763	

3.017.964	-33.763	
3.018.199	-33.763	
3.018.355	-33.763	
3.018.536	-33.763	
3.018.712	-33.763	
3.018.882	-33.763	
3.019.092	-33.763	
3.019.229	-33.764	
3.019.388	-33.764	
3.019.530	-33.764	
3.019.677	-33.764	
3.019.854	-33.764	
3.019.960	-33.764	
3.020.099	-33.764	
3.020.296	-33.764	
3.020.464	-33.764	
3.020.591	-33.764	
3.020.717	-33.764	
3.020.878	-33.765	
3.021.263	-33.765	
3.021.436	-33.765	
3.021.541	-33.765	
3.021.694	-33.765	
3.021.870	-33.766	
3.022.065	-33.766	
3.022.218	-33.766	
3.022.362	-33.766	
3.022.498	-33.767	
3.022.647	-33.767	
3.022.796	-33.767	

3.022.947	-33.767	
3.023.153	-33.768	
3.023.305	-33.768	
3.023.484	-33.769	
3.023.690	-33.769	
3.023.813	-33.769	
3.024.002	-33.770	
3.024.183	-33.770	
3.024.301	-33.771	
3.024.417	-33.771	
3.024.620	-33.772	
3.024.799	-33.772	
3.024.908	-33.773	
3.025.050	-33.774	
3.025.222	-33.774	
3.025.429	-33.775	
3.025.661	-33.776	
3.025.859	-33.776	
3.026.053	-33.777	
3.026.237	-33.778	
3.026.335	-33.779	
3.026.412	-33.779	
3.026.551	-33.780	
3.026.732	-33.781	
3.027.007	-33.782	
3.027.249	-33.783	
3.027.361	-33.784	
3.027.448	-33.785	
3.027.567	-33.786	
3.027.758	-33.787	

3.027.971	-33.788	
3.028.154	-33.789	
3.028.358	-33.790	
3.028.423	-33.791	
3.028.553	-33.792	
3.028.807	-33.793	
3.028.976	-33.794	
3.029.135	-33.796	
3.029.236	-33.797	
3.029.377	-33.798	
3.029.518	-33.799	
3.029.634	-33.800	
3.029.788	-33.802	
3.030.005	-33.803	
3.030.198	-33.804	
3.030.361	-33.805	
3.030.620	-33.807	
3.030.843	-33.808	
3.031.004	-33.809	
3.031.216	-33.811	
3.031.389	-33.812	
3.031.534	-33.814	
3.031.700	-33.815	
3.031.857	-33.816	
3.031.981	-33.818	
3.032.094	-33.819	
3.032.221	-33.821	
3.032.312	-33.822	
3.032.480	-33.824	
3.032.722	-33.825	

3.032.928	-33.827	
3.033.034	-33.828	
3.033.178	-33.829	
3.033.351	-33.831	
3.033.482	-33.832	
3.033.665	-33.834	
3.033.842	-33.836	
3.034.084	-33.837	
3.034.328	-33.839	
3.034.442	-33.840	
3.034.612	-33.842	
3.034.886	-33.843	
3.035.063	-33.845	
3.035.179	-33.846	
3.035.336	-33.848	
3.035.520	-33.849	
3.035.742	-33.851	
3.035.919	-33.852	
3.036.049	-33.854	
3.036.172	-33.855	
3.036.309	-33.857	
3.036.484	-33.859	
3.036.683	-33.860	
3.036.877	-33.862	
3.037.095	-33.863	
3.037.354	-33.865	
3.037.500	-33.866	
3.037.553	-33.868	
3.037.632	-33.869	
3.037.801	-33.871	

3.038.012	-33.872	
3.038.229	-33.874	
3.038.485	-33.875	
3.038.676	-33.877	
3.038.770	-33.878	
3.038.889	-33.879	
3.039.024	-33.881	
3.039.176	-33.882	
3.039.420	-33.884	
3.039.638	-33.885	
3.039.818	-33.887	
3.039.915	-33.888	
3.039.985	-33.889	
3.040.155	-33.891	
3.040.555	-33.892	
3.041.002	-33.894	
3.041.187	-33.895	
3.041.241	-33.896	
3.041.365	-33.898	
3.041.486	-33.899	
3.041.508	-33.900	
3.041.589	-33.901	
3.041.676	-33.903	
3.041.820	-33.904	
3.041.990	-33.905	
3.042.047	-33.906	
3.042.198	-33.908	
3.042.458	-33.909	
3.042.686	-33.910	
3.042.850	-33.911	

3.043.058	-33.912	
3.043.246	-33.913	
3.043.334	-33.914	
3.043.517	-33.916	
3.043.731	-33.917	
3.043.847	-33.918	
3.043.987	-33.919	
3.044.180	-33.920	
3.044.306	-33.921	
3.044.461	-33.922	
3.044.713	-33.923	
3.044.841	-33.924	
3.044.970	-33.925	
3.045.154	-33.926	
3.045.310	-33.927	
3.045.526	-33.928	
3.045.735	-33.929	
3.045.891	-33.930	
3.046.068	-33.931	
3.046.281	-33.932	
3.046.479	-33.933	
3.046.578	-33.934	
3.046.698	-33.935	
3.046.898	-33.936	
3.047.099	-33.937	
3.047.234	-33.938	
3.047.278	-33.939	
3.047.447	-33.940	
3.047.711	-33.941	
3.047.886	-33.941	

3.048.059	-33.942	
3.048.192	-33.943	
3.048.329	-33.944	
3.048.518	-33.945	
3.048.663	-33.946	
3.048.822	-33.947	
3.048.988	-33.948	
3.049.149	-33.949	
3.049.306	-33.950	
3.049.449	-33.950	
3.049.626	-33.951	
3.049.825	-33.952	
3.050.034	-33.953	
3.050.148	-33.954	
3.050.270	-33.955	
3.050.486	-33.956	
3.050.722	-33.957	
3.050.905	-33.958	
3.051.010	-33.959	
3.051.194	-33.960	
3.051.418	-33.960	
3.051.563	-33.961	
3.051.703	-33.962	
3.051.855	-33.963	
3.052.011	-33.964	
3.052.205	-33.965	
3.052.352	-33.966	
3.052.487	-33.967	
3.052.697	-33.967	
3.052.914	-33.968	

3.053.096	-33.969	
3.053.272	-33.970	
3.053.448	-33.971	
3.053.609	-33.972	
3.053.752	-33.972	
3.053.914	-33.973	
3.054.003	-33.974	
3.054.059	-33.975	
3.054.243	-33.976	
3.054.465	-33.976	
3.054.653	-33.977	
3.054.770	-33.978	
3.054.937	-33.979	
3.055.140	-33.979	
3.055.334	-33.980	
3.055.569	-33.981	
3.055.746	-33.982	
3.055.898	-33.982	
3.056.109	-33.983	
3.056.340	-33.984	
3.056.452	-33.984	
3.056.544	-33.985	
3.056.687	-33.986	
3.056.826	-33.986	
3.057.044	-33.987	
3.057.231	-33.987	
3.057.299	-33.988	
3.057.411	-33.989	
3.057.574	-33.989	
3.057.751	-33.990	

3.057.935	-33.990	
3.058.103	-33.991	
3.058.271	-33.991	
3.058.446	-33.992	
3.058.679	-33.992	
3.058.939	-33.993	
3.059.091	-33.993	
3.059.229	-33.994	
3.059.403	-33.994	
3.059.583	-33.995	
3.059.810	-33.995	
3.059.984	-33.995	
3.060.112	-33.996	
3.060.318	-33.996	
3.060.529	-33.997	
3.060.700	-33.997	
3.060.829	-33.997	
3.060.973	-33.998	
3.061.137	-33.998	
3.061.315	-33.998	
3.061.454	-33.999	
3.061.571	-33.999	
3.061.770	-33.999	
3.061.967	-33.999	
3.062.158	-34.000	
3.062.364	-34.000	
3.062.520	-34.000	
3.062.654	-34.000	
3.062.827	-34.000	
3.062.987	-34.001	

3.063.150	-34.001	
3.063.365	-34.001	
3.063.521	-34.001	
3.063.640	-34.001	
3.063.825	-34.001	
3.063.979	-34.001	
3.064.146	-34.002	
3.064.299	-34.002	
3.064.388	-34.002	
3.064.561	-34.002	
3.064.916	-34.002	
3.065.392	-34.002	
3.065.707	-34.002	
3.065.794	-34.002	
3.065.794	-34.002	
3.065.856	-34.002	
3.065.982	-34.002	
3.066.058	-34.002	
3.066.161	-34.002	
3.066.297	-34.002	
3.066.397	-34.002	
3.066.519	-34.002	
3.066.690	-34.002	
3.066.852	-34.002	
3.067.072	-34.002	
3.067.328	-34.001	
3.067.469	-34.001	
3.067.632	-34.001	
3.067.888	-34.001	
3.068.102	-34.001	

3.068.293	-34.001	
3.068.409	-34.001	
3.068.528	-34.000	
3.068.716	-34.000	
3.068.890	-34.000	
3.069.019	-34.000	
3.069.102	-34.000	
3.069.229	-34.000	
3.069.373	-34.000	
3.069.579	-33.999	
3.069.789	-33.999	
3.069.944	-33.999	
3.070.103	-33.999	
3.070.295	-33.999	
3.070.508	-33.999	
3.070.732	-33.999	
3.070.948	-33.999	
3.071.107	-33.998	
3.071.198	-33.998	
3.071.281	-33.998	
3.071.416	-33.998	
3.071.593	-33.998	
3.071.769	-33.998	
3.071.945	-33.998	
3.072.105	-33.998	
3.072.253	-33.998	
3.072.430	-33.998	
3.072.599	-33.998	
3.072.791	-33.998	
3.072.957	-33.998	

3.073.062	-33.998	
3.073.236	-33.998	
3.073.443	-33.998	
3.073.626	-33.998	
3.073.807	-33.998	
3.073.871	-33.998	
3.073.999	-33.998	
3.074.214	-33.998	
3.074.406	-33.998	
3.074.601	-33.999	
3.074.782	-33.999	
3.074.937	-33.999	
3.075.027	-33.999	
3.075.226	-33.999	
3.075.493	-34.000	
3.075.707	-34.000	
3.075.862	-34.000	
3.075.981	-34.000	
3.076.156	-34.001	
3.076.361	-34.001	
3.076.492	-34.001	
3.076.584	-34.001	
3.076.762	-34.002	
3.076.993	-34.002	
3.077.205	-34.002	
3.077.352	-34.003	
3.077.466	-34.003	
3.077.647	-34.003	
3.077.805	-34.004	
3.077.890	-34.004	

3.078.076	-34.004	
3.078.275	-34.005	
3.078.394	-34.005	
3.078.508	-34.005	
3.078.647	-34.006	
3.078.763	-34.006	
3.078.965	-34.007	
3.079.210	-34.007	
3.079.297	-34.007	
3.079.451	-34.008	
3.079.767	-34.008	
3.080.002	-34.009	
3.080.187	-34.009	
3.080.385	-34.010	
3.080.518	-34.010	
3.080.671	-34.010	
3.080.905	-34.011	
3.081.068	-34.011	
3.081.220	-34.012	
3.081.326	-34.012	
3.081.380	-34.013	
3.081.502	-34.013	
3.081.647	-34.013	
3.081.815	-34.014	
3.081.989	-34.014	
3.082.131	-34.015	
3.082.270	-34.015	
3.082.488	-34.016	
3.082.643	-34.016	
3.082.832	-34.017	

3.083.130	-34.017	
3.083.344	-34.017	
3.083.496	-34.018	
3.083.647	-34.018	
3.083.871	-34.019	
3.084.095	-34.019	
3.084.251	-34.020	
3.084.402	-34.020	
3.084.528	-34.021	
3.084.653	-34.021	
3.084.846	-34.021	
3.085.005	-34.022	
3.085.189	-34.022	
3.085.406	-34.023	
3.085.493	-34.023	
3.085.573	-34.024	
3.085.800	-34.024	
3.086.044	-34.025	
3.086.246	-34.025	
3.086.476	-34.025	
3.086.591	-34.026	
3.086.711	-34.026	
3.086.841	-34.027	
3.086.985	-34.027	
3.087.137	-34.028	
3.087.308	-34.028	
3.087.484	-34.028	
3.087.640	-34.029	
3.087.789	-34.029	
3.087.924	-34.029	

3.088.128	-34.030	
3.088.331	-34.030	
3.088.531	-34.031	
3.088.735	-34.031	
3.088.814	-34.031	
3.088.934	-34.032	
3.089.161	-34.032	
3.089.583	-34.033	
3.089.995	-34.033	
3.090.179	-34.033	
3.090.273	-34.034	
3.090.302	-34.034	
3.090.371	-34.034	
3.090.425	-34.035	
3.090.468	-34.035	
3.090.642	-34.035	
3.090.815	-34.036	
3.090.913	-34.036	
3.091.036	-34.036	
3.091.191	-34.037	
3.091.353	-34.037	
3.091.551	-34.037	
3.091.780	-34.038	
3.091.978	-34.038	
3.092.162	-34.038	
3.092.357	-34.038	
3.092.494	-34.039	
3.092.650	-34.039	
3.092.863	-34.039	
3.093.015	-34.040	

3.093.110	-34.040	
3.093.250	-34.040	
3.093.456	-34.041	
3.093.589	-34.041	
3.093.712	-34.041	
3.093.882	-34.042	
3.094.005	-34.042	
3.094.192	-34.042	
3.094.370	-34.043	
3.094.508	-34.043	
3.094.720	-34.043	
3.094.967	-34.044	
3.095.218	-34.044	
3.095.411	-34.044	
3.095.544	-34.045	
3.095.688	-34.045	
3.095.840	-34.046	
3.095.995	-34.046	
3.096.140	-34.046	
3.096.241	-34.047	
3.096.411	-34.047	
3.096.622	-34.048	
3.096.769	-34.048	
3.096.961	-34.049	
3.097.108	-34.049	
3.097.188	-34.049	
3.097.355	-34.050	
3.097.567	-34.050	
3.097.762	-34.051	
3.097.971	-34.052	

3.098.123	-34.052	
3.098.166	-34.053	
3.098.351	-34.053	
3.098.601	-34.054	
3.098.695	-34.054	
3.098.896	-34.055	
3.099.236	-34.056	
3.099.384	-34.056	
3.099.481	-34.057	
3.099.635	-34.058	
3.099.793	-34.058	
3.100.020	-34.059	
3.100.204	-34.060	
3.100.332	-34.060	
3.100.443	-34.061	
3.100.583	-34.062	
3.100.771	-34.063	
3.100.921	-34.063	
3.101.107	-34.064	
3.101.317	-34.065	
3.101.494	-34.066	
3.101.643	-34.066	
3.101.765	-34.067	
3.101.932	-34.068	
3.102.106	-34.069	
3.102.265	-34.069	
3.102.401	-34.070	
3.102.505	-34.071	
3.102.672	-34.072	
3.102.850	-34.072	

3.103.022	-34.073	
3.103.163	-34.074	
3.103.282	-34.075	
3.103.401	-34.076	
3.103.568	-34.077	
3.103.791	-34.077	
3.104.016	-34.078	
3.104.229	-34.079	
3.104.445	-34.080	
3.104.674	-34.081	
3.104.839	-34.081	
3.105.000	-34.082	
3.105.197	-34.083	
3.105.406	-34.084	
3.105.548	-34.085	
3.105.645	-34.086	
3.105.779	-34.086	
3.105.891	-34.087	
3.106.032	-34.088	
3.106.190	-34.089	
3.106.354	-34.090	
3.106.548	-34.090	
3.106.716	-34.091	
3.106.918	-34.092	
3.107.073	-34.093	
3.107.261	-34.094	
3.107.451	-34.094	
3.107.607	-34.095	
3.107.860	-34.096	
3.108.062	-34.097	

3.108.174	-34.097	
3.108.327	-34.098	
3.108.471	-34.099	
3.108.611	-34.100	
3.108.802	-34.101	
3.108.969	-34.101	
3.109.136	-34.102	
3.109.333	-34.103	
3.109.469	-34.104	
3.109.590	-34.104	
3.109.808	-34.105	
3.110.043	-34.106	
3.110.169	-34.107	
3.110.298	-34.107	
3.110.507	-34.108	
3.110.620	-34.109	
3.110.754	-34.110	
3.110.985	-34.110	
3.111.180	-34.111	
3.111.299	-34.112	
3.111.454	-34.112	
3.111.654	-34.113	
3.111.835	-34.114	
3.111.987	-34.114	
3.112.148	-34.115	
3.112.303	-34.116	
3.112.485	-34.116	
3.112.694	-34.117	
3.112.879	-34.118	
3.113.018	-34.118	

3.113.148	-34.119	
3.113.318	-34.120	
3.113.629	-34.120	
3.113.979	-34.121	
3.114.308	-34.121	
3.114.539	-34.122	
3.114.615	-34.123	
3.114.698	-34.123	
3.114.756	-34.124	
3.114.771	-34.124	
3.114.890	-34.125	
3.115.035	-34.125	
3.115.125	-34.126	
3.115.298	-34.126	
3.115.502	-34.127	
3.115.645	-34.127	
3.115.815	-34.128	
3.116.028	-34.128	
3.116.252	-34.129	
3.116.454	-34.129	
3.116.581	-34.130	
3.116.769	-34.130	
3.116.922	-34.130	
3.117.097	-34.131	
3.117.289	-34.131	
3.117.429	-34.132	
3.117.610	-34.132	
3.117.711	-34.132	
3.117.830	-34.133	
3.117.989	-34.133	

3.118.109	-34.134	
3.118.249	-34.134	
3.118.416	-34.134	
3.118.629	-34.135	
3.118.835	-34.135	
3.118.979	-34.136	
3.119.175	-34.136	
3.119.373	-34.136	
3.119.521	-34.137	
3.119.670	-34.137	
3.119.875	-34.138	
3.120.076	-34.138	
3.120.173	-34.138	
3.120.270	-34.139	
3.120.436	-34.139	
3.120.639	-34.140	
3.120.840	-34.140	
3.121.021	-34.140	
3.121.154	-34.141	
3.121.270	-34.141	
3.121.443	-34.142	
3.121.656	-34.142	
3.121.830	-34.142	
3.121.936	-34.143	
3.122.081	-34.143	
3.122.332	-34.144	
3.122.552	-34.144	
3.122.639	-34.144	
3.122.755	-34.145	
3.122.940	-34.145	

3.123.150	-34.146	
3.123.356	-34.146	
3.123.538	-34.147	
3.123.719	-34.147	
3.123.893	-34.148	
3.123.997	-34.148	
3.124.132	-34.149	
3.124.355	-34.149	
3.124.554	-34.150	
3.124.693	-34.150	
3.124.832	-34.151	
3.125.009	-34.151	
3.125.176	-34.152	
3.125.352	-34.152	
3.125.486	-34.153	
3.125.646	-34.153	
3.125.881	-34.154	
3.126.069	-34.154	
3.126.183	-34.155	
3.126.273	-34.155	
3.126.421	-34.156	
3.126.589	-34.156	
3.126.743	-34.157	
3.126.910	-34.157	
3.127.077	-34.158	
3.127.225	-34.158	
3.127.348	-34.159	
3.127.574	-34.159	
3.127.763	-34.160	
3.127.864	-34.160	

3.128.060	-34.161	
3.128.251	-34.161	
3.128.428	-34.162	
3.128.645	-34.162	
3.128.864	-34.163	
3.129.021	-34.163	
3.129.121	-34.164	
3.129.341	-34.164	
3.129.634	-34.165	
3.129.792	-34.165	
3.129.920	-34.166	
3.130.024	-34.166	
3.130.147	-34.167	
3.130.298	-34.167	
3.130.399	-34.167	
3.130.617	-34.168	
3.130.809	-34.168	
3.130.927	-34.169	
3.131.078	-34.169	
3.131.213	-34.170	
3.131.448	-34.170	
3.131.660	-34.170	
3.131.765	-34.171	
3.132.000	-34.171	
3.132.263	-34.172	
3.132.386	-34.172	
3.132.527	-34.173	
3.132.672	-34.173	
3.132.784	-34.173	
3.132.975	-34.174	

3.133.195	-34.174	
3.133.394	-34.175	
3.133.529	-34.175	
3.133.698	-34.175	
3.133.879	-34.176	
3.134.027	-34.176	
3.134.175	-34.177	
3.134.322	-34.177	
3.134.514	-34.178	
3.134.716	-34.178	
3.134.894	-34.178	
3.135.078	-34.179	
3.135.255	-34.179	
3.135.436	-34.180	
3.135.588	-34.180	
3.135.696	-34.180	
3.135.874	-34.181	
3.136.085	-34.181	
3.136.226	-34.182	
3.136.385	-34.182	
3.136.517	-34.182	
3.136.725	-34.183	
3.136.862	-34.183	
3.136.983	-34.184	
3.137.231	-34.184	
3.137.391	-34.184	
3.137.517	-34.185	
3.137.698	-34.185	
3.138.036	-34.186	
3.138.481	-34.186	

3.138.771	-34.186	
3.138.857	-34.187	
3.138.890	-34.187	
3.138.951	-34.188	
3.139.021	-34.188	
3.139.138	-34.188	
3.139.286	-34.189	
3.139.370	-34.189	
3.139.489	-34.190	
3.139.673	-34.190	
3.139.857	-34.190	
3.140.023	-34.191	
3.140.231	-34.191	
3.140.435	-34.192	
3.140.587	-34.192	
3.140.749	-34.192	
3.140.941	-34.193	
3.141.140	-34.193	
3.141.282	-34.193	
3.141.405	-34.194	
3.141.549	-34.194	
3.141.729	-34.195	
3.141.887	-34.195	
3.142.014	-34.196	
3.142.112	-34.196	
3.142.260	-34.196	
3.142.494	-34.197	
3.142.658	-34.197	
3.142.792	-34.198	
3.142.979	-34.198	

3.143.190	-34.199	
3.143.427	-34.199	
3.143.618	-34.200	
3.143.774	-34.200	
3.143.936	-34.201	
3.144.106	-34.201	
3.144.241	-34.202	
3.144.366	-34.202	
3.144.584	-34.203	
3.144.801	-34.203	
3.144.958	-34.204	
3.145.039	-34.205	
3.145.168	-34.205	
3.145.385	-34.206	
3.145.576	-34.206	
3.145.733	-34.207	
3.145.881	-34.208	
3.145.999	-34.208	
3.146.163	-34.209	
3.146.375	-34.210	
3.146.607	-34.210	
3.146.763	-34.211	
3.146.880	-34.212	
3.147.077	-34.212	
3.147.227	-34.213	
3.147.393	-34.214	
3.147.585	-34.215	
3.147.722	-34.215	
3.147.892	-34.216	
3.148.076	-34.217	

3.148.219	-34.218	
3.148.362	-34.219	
3.148.555	-34.219	
3.148.752	-34.220	
3.148.886	-34.221	
3.149.005	-34.222	
3.149.142	-34.223	
3.149.341	-34.224	
3.149.530	-34.224	
3.149.720	-34.225	
3.149.919	-34.226	
3.150.101	-34.227	
3.150.256	-34.228	
3.150.424	-34.229	
3.150.620	-34.229	
3.150.714	-34.230	
3.150.786	-34.231	
3.150.930	-34.232	
3.151.125	-34.233	
3.151.295	-34.234	
3.151.365	-34.235	
3.151.454	-34.236	
3.151.645	-34.236	
3.151.874	-34.237	
3.152.057	-34.238	
3.152.209	-34.239	
3.152.458	-34.240	
3.152.683	-34.241	
3.152.845	-34.242	
3.153.081	-34.243	

3.153.302	-34.243	
3.153.438	-34.244	
3.153.607	-34.245	
3.153.788	-34.246	
3.153.943	-34.247	
3.154.118	-34.248	
3.154.266	-34.249	
3.154.393	-34.249	
3.154.539	-34.250	
3.154.631	-34.251	
3.154.736	-34.252	
3.154.915	-34.253	
3.155.103	-34.254	
3.155.266	-34.254	
3.155.500	-34.255	
3.155.677	-34.256	
3.155.844	-34.257	
3.156.072	-34.258	
3.156.230	-34.259	
3.156.360	-34.259	
3.156.503	-34.260	
3.156.653	-34.261	
3.156.831	-34.262	
3.157.034	-34.263	
3.157.198	-34.264	
3.157.335	-34.265	
3.157.498	-34.266	
3.157.697	-34.267	
3.157.901	-34.267	
3.158.048	-34.268	

3.158.188	-34.269	
3.158.383	-34.270	
3.158.622	-34.271	
3.158.793	-34.272	
3.158.885	-34.273	
3.159.042	-34.274	
3.159.236	-34.275	
3.159.435	-34.276	
3.159.641	-34.277	
3.159.796	-34.278	
3.159.940	-34.279	
3.160.078	-34.280	
3.160.241	-34.281	
3.160.421	-34.282	
3.160.595	-34.283	
3.160.822	-34.284	
3.160.955	-34.285	
3.161.071	-34.287	
3.161.284	-34.288	
3.161.458	-34.289	
3.161.664	-34.290	
3.161.855	-34.291	
3.161.971	-34.292	
3.162.256	-34.293	
3.162.682	-34.295	
3.162.971	-34.296	
3.163.070	-34.297	
3.163.105	-34.298	
3.163.174	-34.299	
3.163.254	-34.301	

3.163.355	-34.302	
3.163.441	-34.303	
3.163.466	-34.305	
3.163.587	-34.306	
3.163.817	-34.307	
3.164.020	-34.309	
3.164.196	-34.310	
3.164.328	-34.311	
3.164.518	-34.313	
3.164.763	-34.314	
3.164.915	-34.316	
3.165.070	-34.317	
3.165.277	-34.318	
3.165.437	-34.320	
3.165.602	-34.321	
3.165.728	-34.323	
3.165.865	-34.325	
3.166.028	-34.326	
3.166.195	-34.328	
3.166.385	-34.329	
3.166.542	-34.331	
3.166.650	-34.333	
3.166.827	-34.334	
3.167.076	-34.336	
3.167.191	-34.338	
3.167.326	-34.340	
3.167.547	-34.342	
3.167.738	-34.344	
3.167.968	-34.345	
3.168.152	-34.347	

3.168.279	-34.349	
3.168.456	-34.351	
3.168.585	-34.353	
3.168.708	-34.355	
3.168.930	-34.357	
3.169.138	-34.359	
3.169.229	-34.361	
3.169.355	-34.364	
3.169.554	-34.366	
3.169.702	-34.368	
3.169.850	-34.370	
3.170.078	-34.372	
3.170.285	-34.375	
3.170.433	-34.377	
3.170.605	-34.379	
3.170.811	-34.382	
3.170.967	-34.384	
3.171.114	-34.386	
3.171.277	-34.389	
3.171.375	-34.391	
3.171.538	-34.394	
3.171.733	-34.396	
3.171.878	-34.399	
3.172.047	-34.401	
3.172.195	-34.404	
3.172.384	-34.407	
3.172.589	-34.409	
3.172.778	-34.412	
3.172.957	-34.414	
3.173.130	-34.417	

3.173.280	-34.420	
3.173.419	-34.422	
3.173.578	-34.425	
3.173.714	-34.428	
3.173.879	-34.430	
3.174.124	-34.433	
3.174.331	-34.435	
3.174.493	-34.438	
3.174.641	-34.441	
3.174.742	-34.443	
3.174.915	-34.446	
3.175.089	-34.449	
3.175.208	-34.451	
3.175.313	-34.454	
3.175.494	-34.457	
3.175.731	-34.459	
3.175.851	-34.462	
3.175.982	-34.464	
3.176.144	-34.467	
3.176.242	-34.470	
3.176.396	-34.472	
3.176.628	-34.475	
3.176.877	-34.477	
3.177.086	-34.480	
3.177.271	-34.482	
3.177.511	-34.485	
3.177.722	-34.487	
3.177.906	-34.490	
3.178.074	-34.492	
3.178.210	-34.495	

3.178.344	-34.497	
3.178.474	-34.499	
3.178.647	-34.502	
3.178.800	-34.504	
3.178.871	-34.506	
3.179.003	-34.509	
3.179.165	-34.511	
3.179.308	-34.513	
3.179.490	-34.515	
3.179.703	-34.518	
3.179.865	-34.520	
3.180.005	-34.522	
3.180.204	-34.524	
3.180.403	-34.526	
3.180.588	-34.528	
3.180.791	-34.530	
3.180.974	-34.532	
3.181.152	-34.534	
3.181.320	-34.536	
3.181.459	-34.538	
3.181.599	-34.540	
3.181.745	-34.542	
3.181.913	-34.544	
3.182.123	-34.546	
3.182.299	-34.548	
3.182.498	-34.550	
3.182.647	-34.552	
3.182.773	-34.553	
3.182.973	-34.555	
3.183.130	-34.557	

3.183.286	-34.559	
3.183.503	-34.560	
3.183.701	-34.562	
3.183.904	-34.563	
3.184.128	-34.565	
3.184.273	-34.567	
3.184.391	-34.568	
3.184.557	-34.570	
3.184.693	-34.571	
3.184.879	-34.573	
3.185.089	-34.574	
3.185.222	-34.576	
3.185.408	-34.577	
3.185.621	-34.578	
3.185.773	-34.580	
3.185.931	-34.581	
3.186.039	-34.583	
3.186.129	-34.584	
3.186.487	-34.585	
3.186.950	-34.586	
3.187.235	-34.588	
3.187.350	-34.589	
3.187.377	-34.590	
3.187.460	-34.591	
3.187.499	-34.593	
3.187.527	-34.594	
3.187.657	-34.595	
3.187.816	-34.596	
3.187.918	-34.597	
3.188.058	-34.598	

3.188.268	-34.599	
3.188.403	-34.600	
3.188.572	-34.601	
3.188.756	-34.603	
3.188.932	-34.604	
3.189.221	-34.605	
3.189.378	-34.606	
3.189.493	-34.607	
3.189.749	-34.608	
3.189.966	-34.609	
3.190.064	-34.610	
3.190.136	-34.611	
3.190.296	-34.612	
3.190.447	-34.613	
3.190.537	-34.614	
3.190.693	-34.615	
3.190.808	-34.616	
3.190.989	-34.617	
3.191.255	-34.618	
3.191.432	-34.619	
3.191.595	-34.620	
3.191.795	-34.621	
3.192.026	-34.622	
3.192.238	-34.624	
3.192.422	-34.625	
3.192.583	-34.626	
3.192.701	-34.627	
3.192.853	-34.628	
3.193.052	-34.629	
3.193.240	-34.630	

3.193.391	-34.632	
3.193.495	-34.633	
3.193.600	-34.634	
3.193.714	-34.635	
3.193.882	-34.637	
3.194.102	-34.638	
3.194.286	-34.639	
3.194.460	-34.640	
3.194.648	-34.642	
3.194.774	-34.643	
3.194.912	-34.644	
3.195.125	-34.646	
3.195.259	-34.647	
3.195.389	-34.649	
3.195.540	-34.650	
3.195.654	-34.652	
3.195.912	-34.653	
3.196.122	-34.655	
3.196.230	-34.656	
3.196.393	-34.658	
3.196.609	-34.659	
3.196.813	-34.661	
3.196.938	-34.663	
3.197.108	-34.664	
3.197.300	-34.666	
3.197.377	-34.668	
3.197.545	-34.669	
3.197.778	-34.671	
3.197.895	-34.673	
3.198.056	-34.674	

3.198.265	-34.676	
3.198.427	-34.678	
3.198.586	-34.680	
3.198.814	-34.681	
3.199.037	-34.683	
3.199.176	-34.685	
3.199.313	-34.687	
3.199.457	-34.689	
3.199.598	-34.690	
3.199.736	-34.692	
3.199.893	-34.694	
3.200.013	-34.696	
3.200.112	-34.698	
3.200.251	-34.700	
3.200.457	-34.701	
3.200.607	-34.703	
3.200.714	-34.705	
3.200.963	-34.707	
3.201.246	-34.709	
3.201.499	-34.711	
3.201.676	-34.713	
3.201.827	-34.714	
3.202.048	-34.716	
3.202.184	-34.718	
3.202.325	-34.720	
3.202.505	-34.722	
3.202.615	-34.724	
3.202.759	-34.725	
3.202.890	-34.727	
3.203.008	-34.729	

3.203.179	-34.731	
3.203.347	-34.733	
3.203.479	-34.734	
3.203.659	-34.736	
3.203.893	-34.738	
3.204.075	-34.740	
3.204.221	-34.741	
3.204.418	-34.743	
3.204.646	-34.745	
3.204.861	-34.747	
3.205.050	-34.748	
3.205.201	-34.750	
3.205.324	-34.752	
3.205.471	-34.753	
3.205.679	-34.755	
3.205.854	-34.757	
3.205.990	-34.758	
3.206.137	-34.760	
3.206.309	-34.761	
3.206.451	-34.763	
3.206.669	-34.764	
3.206.882	-34.766	
3.206.992	-34.768	
3.207.180	-34.769	
3.207.372	-34.770	
3.207.549	-34.772	
3.207.733	-34.773	
3.207.904	-34.775	
3.208.059	-34.776	
3.208.214	-34.778	

3.208.392	-34.779	
3.208.549	-34.780	
3.208.719	-34.782	
3.208.930	-34.783	
3.209.059	-34.784	
3.209.205	-34.785	
3.209.380	-34.787	
3.209.562	-34.788	
3.209.742	-34.789	
3.209.888	-34.790	
3.210.068	-34.791	
3.210.237	-34.792	
3.210.359	-34.793	
3.210.558	-34.794	
3.210.898	-34.796	
3.211.273	-34.797	
3.211.513	-34.798	
3.211.596	-34.799	
3.211.700	-34.799	
3.211.784	-34.800	
3.211.841	-34.801	
3.211.931	-34.802	
3.212.010	-34.803	
3.212.115	-34.804	
3.212.263	-34.805	
3.212.411	-34.806	
3.212.607	-34.806	
3.212.802	-34.807	
3.212.961	-34.808	
3.213.089	-34.809	

3.213.268	-34.809	
3.213.571	-34.810	
3.213.800	-34.811	
3.213.944	-34.811	
3.214.115	-34.812	
3.214.208	-34.813	
3.214.374	-34.813	
3.214.598	-34.814	
3.214.716	-34.815	
3.214.872	-34.815	
3.215.023	-34.816	
3.215.128	-34.816	
3.215.287	-34.817	
3.215.455	-34.817	
3.215.652	-34.818	
3.215.834	-34.818	
3.216.021	-34.819	
3.216.208	-34.820	
3.216.375	-34.820	
3.216.607	-34.821	
3.216.845	-34.821	
3.216.965	-34.822	
3.217.057	-34.822	
3.217.171	-34.822	
3.217.322	-34.823	
3.217.505	-34.823	
3.217.684	-34.824	
3.217.841	-34.824	
3.217.972	-34.825	
3.218.107	-34.825	

3.218.242	-34.826	
3.218.474	-34.826	
3.218.661	-34.827	
3.218.791	-34.827	
3.218.983	-34.828	
3.219.153	-34.828	
3.219.304	-34.828	
3.219.494	-34.829	
3.219.705	-34.829	
3.219.886	-34.830	
3.219.998	-34.830	
3.220.144	-34.831	
3.220.364	-34.831	
3.220.537	-34.832	
3.220.700	-34.832	
3.220.901	-34.833	
3.221.083	-34.833	
3.221.249	-34.834	
3.221.398	-34.835	
3.221.513	-34.835	
3.221.654	-34.836	
3.221.824	-34.836	
3.222.001	-34.837	
3.222.148	-34.837	
3.222.253	-34.838	
3.222.440	-34.838	
3.222.657	-34.839	
3.222.814	-34.839	
3.223.007	-34.840	
3.223.178	-34.841	

3.223.298	-34.841	
3.223.470	-34.842	
3.223.676	-34.842	
3.223.806	-34.843	
3.223.940	-34.843	
3.224.086	-34.844	
3.224.252	-34.845	
3.224.353	-34.845	
3.224.431	-34.846	
3.224.622	-34.846	
3.224.833	-34.847	
3.225.002	-34.847	
3.225.179	-34.848	
3.225.397	-34.848	
3.225.630	-34.849	
3.225.819	-34.850	
3.226.022	-34.850	
3.226.260	-34.851	
3.226.429	-34.851	
3.226.606	-34.852	
3.226.741	-34.852	
3.226.823	-34.853	
3.226.956	-34.853	
3.227.122	-34.854	
3.227.285	-34.854	
3.227.426	-34.855	
3.227.557	-34.855	
3.227.720	-34.856	
3.227.854	-34.856	
3.227.975	-34.857	

3.228.192	-34.857	
3.228.412	-34.858	
3.228.615	-34.858	
3.228.817	-34.859	
3.228.990	-34.859	
3.229.189	-34.860	
3.229.319	-34.860	
3.229.469	-34.861	
3.229.652	-34.861	
3.229.792	-34.862	
3.229.987	-34.862	
3.230.176	-34.862	
3.230.328	-34.863	
3.230.482	-34.863	
3.230.688	-34.864	
3.230.880	-34.864	
3.231.036	-34.864	
3.231.259	-34.865	
3.231.458	-34.865	
3.231.564	-34.866	
3.231.719	-34.866	
3.231.881	-34.866	
3.232.036	-34.867	
3.232.257	-34.867	
3.232.408	-34.867	
3.232.554	-34.868	
3.232.715	-34.868	
3.232.885	-34.868	
3.233.141	-34.868	
3.233.297	-34.869	

3.233.441	-34.869	
3.233.567	-34.869	
3.233.687	-34.870	
3.233.916	-34.870	
3.234.099	-34.870	
3.234.243	-34.870	
3.234.393	-34.871	
3.234.561	-34.871	
3.234.711	-34.871	
3.234.846	-34.871	
3.235.130	-34.871	
3.235.570	-34.872	
3.235.852	-34.872	
3.235.970	-34.872	
3.236.072	-34.872	
3.236.133	-34.872	
3.236.176	-34.873	
3.236.255	-34.873	
3.236.353	-34.873	
3.236.495	-34.873	
3.236.721	-34.873	
3.236.873	-34.873	
3.236.982	-34.873	
3.237.207	-34.873	
3.237.387	-34.874	
3.237.466	-34.874	
3.237.628	-34.874	
3.237.899	-34.874	
3.238.095	-34.874	
3.238.203	-34.874	

3.238.370	-34.874	
3.238.615	-34.874	
3.238.781	-34.874	
3.238.885	-34.874	
3.239.021	-34.875	
3.239.174	-34.875	
3.239.308	-34.875	
3.239.489	-34.875	
3.239.684	-34.875	
3.239.839	-34.875	
3.239.994	-34.875	
3.240.176	-34.875	
3.240.393	-34.876	
3.240.610	-34.876	
3.240.778	-34.876	
3.240.919	-34.876	
3.241.140	-34.876	
3.241.311	-34.876	
3.241.436	-34.877	
3.241.588	-34.877	
3.241.708	-34.877	
3.241.866	-34.877	
3.242.012	-34.877	
3.242.148	-34.878	
3.242.315	-34.878	
3.242.495	-34.878	
3.242.673	-34.878	
3.242.785	-34.879	
3.242.867	-34.879	
3.243.063	-34.879	

3.243.279	-34.880	
3.243.448	-34.880	
3.243.658	-34.880	
3.243.853	-34.881	
3.243.965	-34.881	
3.244.097	-34.881	
3.244.323	-34.882	
3.244.536	-34.882	
3.244.705	-34.883	
3.244.807	-34.883	
3.244.938	-34.884	
3.245.168	-34.884	
3.245.368	-34.885	
3.245.554	-34.885	
3.245.742	-34.886	
3.245.862	-34.886	
3.246.039	-34.887	
3.246.259	-34.887	
3.246.404	-34.888	
3.246.523	-34.888	
3.246.690	-34.889	
3.246.928	-34.890	
3.247.113	-34.890	
3.247.203	-34.891	
3.247.357	-34.891	
3.247.574	-34.892	
3.247.748	-34.893	
3.247.928	-34.893	
3.248.088	-34.894	
3.248.214	-34.895	

3.248.343	-34.895	
3.248.550	-34.896	
3.248.720	-34.897	
3.248.768	-34.897	
3.248.919	-34.898	
3.249.077	-34.899	
3.249.194	-34.899	
3.249.335	-34.900	
3.249.514	-34.901	
3.249.747	-34.902	
3.249.958	-34.902	
3.250.118	-34.903	
3.250.309	-34.904	
3.250.565	-34.904	
3.250.784	-34.905	
3.250.952	-34.906	
3.251.093	-34.907	
3.251.208	-34.907	
3.251.416	-34.908	
3.251.614	-34.909	
3.251.707	-34.910	
3.251.851	-34.910	
3.252.003	-34.911	
3.252.112	-34.912	
3.252.299	-34.912	
3.252.513	-34.913	
3.252.607	-34.914	
3.252.767	-34.915	
3.253.055	-34.915	
3.253.224	-34.916	

3.253.381	-34.917	
3.253.579	-34.917	
3.253.719	-34.918	
3.253.889	-34.919	
3.254.067	-34.920	
3.254.214	-34.920	
3.254.378	-34.921	
3.254.599	-34.922	
3.254.782	-34.922	
3.254.908	-34.923	
3.255.085	-34.924	
3.255.287	-34.924	
3.255.507	-34.925	
3.255.689	-34.926	
3.255.765	-34.926	
3.255.849	-34.927	
3.256.075	-34.928	
3.256.275	-34.928	
3.256.422	-34.929	
3.256.645	-34.930	
3.256.797	-34.930	
3.256.904	-34.931	
3.257.043	-34.932	
3.257.214	-34.932	
3.257.445	-34.933	
3.257.629	-34.933	
3.257.770	-34.934	
3.257.964	-34.935	
3.258.141	-34.935	
3.258.311	-34.936	

3.258.456	-34.936	
3.258.605	-34.937	
3.258.825	-34.937	
3.259.016	-34.938	
3.259.113	-34.938	
3.259.261	-34.939	
3.259.703	-34.939	
3.260.043	-34.940	
3.260.270	-34.940	
3.260.334	-34.941	
3.260.390	-34.941	
3.260.461	-34.942	
3.260.580	-34.942	
3.260.623	-34.943	
3.260.660	-34.943	
3.260.695	-34.943	
3.260.967	-34.944	
3.261.214	-34.944	
3.261.371	-34.945	
3.261.562	-34.945	
3.261.736	-34.945	
3.261.898	-34.946	
3.262.086	-34.946	
3.262.323	-34.947	
3.262.494	-34.947	
3.262.625	-34.947	
3.262.796	-34.948	
3.262.937	-34.948	
3.263.074	-34.948	
3.263.254	-34.949	

3.263.412	-34.949	
3.263.528	-34.949	
3.263.661	-34.950	
3.263.849	-34.950	
3.264.081	-34.950	
3.264.263	-34.951	
3.264.404	-34.951	
3.264.577	-34.951	
3.264.749	-34.952	
3.264.941	-34.952	
3.265.151	-34.952	
3.265.367	-34.953	
3.265.540	-34.953	
3.265.696	-34.953	
3.265.834	-34.954	
3.265.916	-34.954	
3.266.054	-34.954	
3.266.237	-34.955	
3.266.339	-34.955	
3.266.418	-34.955	
3.266.656	-34.956	
3.266.841	-34.956	
3.267.003	-34.957	
3.267.140	-34.957	
3.267.293	-34.957	
3.267.488	-34.958	
3.267.647	-34.958	
3.267.802	-34.959	
3.267.989	-34.959	
3.268.160	-34.960	

3.268.343	-34.960	
3.268.504	-34.961	
3.268.665	-34.961	
3.268.878	-34.962	
3.269.038	-34.962	
3.269.203	-34.963	
3.269.421	-34.963	
3.269.602	-34.964	
3.269.779	-34.964	
3.269.987	-34.965	
3.270.162	-34.965	
3.270.273	-34.966	
3.270.396	-34.967	
3.270.601	-34.967	
3.270.829	-34.968	
3.270.972	-34.968	
3.271.137	-34.969	
3.271.314	-34.970	
3.271.479	-34.970	
3.271.646	-34.971	
3.271.795	-34.972	
3.271.953	-34.972	
3.272.152	-34.973	
3.272.296	-34.974	
3.272.395	-34.974	
3.272.535	-34.975	
3.272.702	-34.976	
3.272.899	-34.976	
3.273.045	-34.977	
3.273.193	-34.978	

3.273.313	-34.978	
3.273.423	-34.979	
3.273.600	-34.980	
3.273.799	-34.981	
3.274.006	-34.981	
3.274.185	-34.982	
3.274.363	-34.983	
3.274.588	-34.984	
3.274.819	-34.984	
3.275.013	-34.985	
3.275.186	-34.986	
3.275.316	-34.987	
3.275.432	-34.987	
3.275.596	-34.988	
3.275.750	-34.989	
3.275.865	-34.990	
3.276.018	-34.990	
3.276.223	-34.991	
3.276.378	-34.992	
3.276.440	-34.993	
3.276.635	-34.993	
3.276.902	-34.994	
3.277.068	-34.995	
3.277.238	-34.995	
3.277.401	-34.996	
3.277.589	-34.997	
3.277.789	-34.998	
3.277.930	-34.998	
3.278.121	-34.999	
3.278.337	-35.000	

3.278.463	-35.001	
3.278.629	-35.001	
3.278.826	-35.002	
3.279.002	-35.003	
3.279.143	-35.004	
3.279.286	-35.004	
3.279.468	-35.005	
3.279.641	-35.006	
3.279.792	-35.007	
3.279.923	-35.007	
3.280.119	-35.008	
3.280.305	-35.009	
3.280.442	-35.009	
3.280.612	-35.010	
3.280.800	-35.011	
3.280.992	-35.012	
3.281.133	-35.012	
3.281.252	-35.013	
3.281.407	-35.014	
3.281.599	-35.014	
3.281.834	-35.015	
3.282.012	-35.016	
3.282.144	-35.017	
3.282.318	-35.017	
3.282.513	-35.018	
3.282.635	-35.019	
3.282.813	-35.019	
3.282.982	-35.020	
3.283.145	-35.021	
3.283.275	-35.021	

3.283.354	-35.022	
3.283.565	-35.023	
3.284.042	-35.023	
3.284.476	-35.024	
3.284.645	-35.025	
3.284.659	-35.025	
3.284.749	-35.026	
3.284.837	-35.027	
3.284.915	-35.027	
3.285.016	-35.028	
3.285.078	-35.029	
3.285.234	-35.029	
3.285.407	-35.030	
3.285.585	-35.031	
3.285.751	-35.031	
3.285.869	-35.032	
3.286.087	-35.032	
3.286.356	-35.033	
3.286.544	-35.034	
3.286.685	-35.034	
3.286.849	-35.035	
3.287.095	-35.035	
3.287.236	-35.036	
3.287.364	-35.036	
3.287.560	-35.037	
3.287.697	-35.038	
3.287.827	-35.038	
3.287.928	-35.039	
3.288.029	-35.039	
3.288.184	-35.040	

3.288.365	-35.041	
3.288.518	-35.041	
3.288.620	-35.042	
3.288.829	-35.042	
3.289.041	-35.043	
3.289.200	-35.044	
3.289.417	-35.044	
3.289.633	-35.045	
3.289.786	-35.046	
3.289.940	-35.046	
3.290.146	-35.047	
3.290.251	-35.048	
3.290.316	-35.048	
3.290.479	-35.049	
3.290.704	-35.050	
3.290.851	-35.050	
3.290.970	-35.051	
3.291.144	-35.052	
3.291.310	-35.052	
3.291.499	-35.053	
3.291.729	-35.054	
3.291.938	-35.055	
3.292.012	-35.055	
3.292.137	-35.056	
3.292.399	-35.057	
3.292.571	-35.058	
3.292.697	-35.058	
3.292.859	-35.059	
3.293.076	-35.060	
3.293.258	-35.061	

3.293.414	-35.062	
3.293.587	-35.063	
3.293.752	-35.064	
3.293.944	-35.065	
3.294.106	-35.065	
3.294.281	-35.066	
3.294.471	-35.067	
3.294.635	-35.068	
3.294.752	-35.069	
3.294.915	-35.070	
3.295.096	-35.071	
3.295.277	-35.072	
3.295.466	-35.073	
3.295.620	-35.074	
3.295.780	-35.075	
3.295.923	-35.077	
3.296.125	-35.078	
3.296.310	-35.079	
3.296.448	-35.080	
3.296.577	-35.081	
3.296.664	-35.082	
3.296.821	-35.083	
3.297.043	-35.084	
3.297.184	-35.085	
3.297.231	-35.087	
3.297.388	-35.088	
3.297.606	-35.089	
3.297.738	-35.090	
3.297.859	-35.091	
3.298.024	-35.093	

3.298.271	-35.094	
3.298.457	-35.095	
3.298.619	-35.096	
3.298.835	-35.098	
3.299.060	-35.099	
3.299.255	-35.100	
3.299.417	-35.102	
3.299.615	-35.103	
3.299.807	-35.104	
3.299.894	-35.106	
3.300.078	-35.107	
3.300.292	-35.108	
3.300.390	-35.110	
3.300.526	-35.111	
3.300.688	-35.112	
3.300.859	-35.114	
3.300.993	-35.115	
3.301.199	-35.117	
3.301.411	-35.118	
3.301.512	-35.120	
3.301.649	-35.121	
3.301.855	-35.123	
3.302.094	-35.124	
3.302.299	-35.126	
3.302.471	-35.127	
3.302.572	-35.129	
3.302.738	-35.130	
3.302.937	-35.132	
3.303.066	-35.133	
3.303.225	-35.135	

3.303.372	-35.137	
3.303.553	-35.138	
3.303.759	-35.140	
3.303.923	-35.142	
3.304.089	-35.143	
3.304.239	-35.145	
3.304.342	-35.147	
3.304.518	-35.149	
3.304.763	-35.150	
3.304.944	-35.152	
3.305.064	-35.154	
3.305.224	-35.156	
3.305.356	-35.158	
3.305.556	-35.159	
3.305.772	-35.161	
3.305.888	-35.163	
3.306.017	-35.165	
3.306.199	-35.167	
3.306.375	-35.169	
3.306.510	-35.171	
3.306.671	-35.173	
3.306.845	-35.175	
3.307.011	-35.177	
3.307.178	-35.179	
3.307.334	-35.181	
3.307.473	-35.183	
3.307.610	-35.186	
3.307.745	-35.188	
3.307.995	-35.190	
3.308.515	-35.192	

3.308.950	-35.194	
3.308.984	-35.197	
3.309.002	-35.199	
3.309.160	-35.201	
3.309.272	-35.203	
3.309.298	-35.206	
3.309.323	-35.208	
3.309.456	-35.211	
3.309.576	-35.213	
3.309.685	-35.215	
3.309.865	-35.218	
3.310.101	-35.220	
3.310.317	-35.223	
3.310.487	-35.225	
3.310.623	-35.228	
3.310.822	-35.230	
3.310.990	-35.233	
3.311.187	-35.236	
3.311.412	-35.238	
3.311.524	-35.241	
3.311.627	-35.244	
3.311.779	-35.246	
3.311.960	-35.249	
3.312.094	-35.252	
3.312.268	-35.255	
3.312.453	-35.257	
3.312.558	-35.260	
3.312.722	-35.263	
3.312.863	-35.266	
3.312.975	-35.269	

3.313.200	-35.272	
3.313.448	-35.275	
3.313.598	-35.278	
3.313.821	-35.281	
3.313.997	-35.284	
3.314.169	-35.288	
3.314.367	-35.291	
3.314.467	-35.294	
3.314.575	-35.297	
3.314.753	-35.300	
3.314.959	-35.304	
3.315.064	-35.307	
3.315.148	-35.310	
3.315.363	-35.314	
3.315.518	-35.317	
3.315.670	-35.320	
3.315.888	-35.324	
3.316.021	-35.327	
3.316.156	-35.331	
3.316.383	-35.334	
3.316.581	-35.338	
3.316.747	-35.342	
3.316.938	-35.345	
3.317.070	-35.349	
3.317.202	-35.353	
3.317.341	-35.356	
3.317.534	-35.360	
3.317.784	-35.364	
3.317.976	-35.368	
3.318.112	-35.371	

3.318.311	-35.375	
3.318.477	-35.379	
3.318.598	-35.383	
3.318.727	-35.387	
3.318.893	-35.391	
3.319.071	-35.395	
3.319.240	-35.399	
3.319.432	-35.403	
3.319.572	-35.407	
3.319.707	-35.411	
3.319.967	-35.415	
3.320.162	-35.419	
3.320.244	-35.423	
3.320.432	-35.427	
3.320.621	-35.431	
3.320.682	-35.435	
3.320.800	-35.439	
3.321.075	-35.443	
3.321.310	-35.447	
3.321.434	-35.451	
3.321.513	-35.455	
3.321.627	-35.459	
3.321.750	-35.463	
3.321.907	-35.467	
3.322.079	-35.471	
3.322.211	-35.476	
3.322.332	-35.480	
3.322.536	-35.484	
3.322.737	-35.488	
3.322.914	-35.492	

3.323.109	-35.496	
3.323.365	-35.500	
3.323.604	-35.504	
3.323.850	-35.508	
3.324.035	-35.512	
3.324.122	-35.516	
3.324.295	-35.520	
3.324.510	-35.524	
3.324.626	-35.528	
3.324.767	-35.532	
3.324.922	-35.536	
3.325.043	-35.540	
3.325.170	-35.544	
3.325.305	-35.548	
3.325.471	-35.552	
3.325.623	-35.556	
3.325.811	-35.560	
3.326.026	-35.564	
3.326.192	-35.568	
3.326.398	-35.572	
3.326.555	-35.576	
3.326.732	-35.580	
3.326.863	-35.583	
3.327.043	-35.587	
3.327.278	-35.591	
3.327.474	-35.595	
3.327.619	-35.598	
3.327.724	-35.602	
3.327.841	-35.606	
3.328.009	-35.610	

3.328.156	-35.613	
3.328.341	-35.617	
3.328.535	-35.621	
3.328.701	-35.624	
3.328.860	-35.628	
3.328.991	-35.631	
3.329.193	-35.635	
3.329.438	-35.639	
3.329.637	-35.642	
3.329.740	-35.646	
3.329.846	-35.649	
3.330.038	-35.653	
3.330.179	-35.656	
3.330.318	-35.659	
3.330.497	-35.663	
3.330.703	-35.666	
3.330.892	-35.669	
3.331.112	-35.673	
3.331.295	-35.676	
3.331.391	-35.679	
3.331.492	-35.682	
3.331.667	-35.686	
3.331.815	-35.689	
3.331.967	-35.692	
3.332.206	-35.695	
3.332.440	-35.698	
3.332.581	-35.701	
3.332.796	-35.705	
3.333.210	-35.708	
3.333.600	-35.711	

3.333.829	-35.714	
3.333.864	-35.717	
3.333.875	-35.720	
3.333.943	-35.723	
3.334.030	-35.725	
3.334.157	-35.728	
3.334.248	-35.731	
3.334.344	-35.734	
3.334.528	-35.737	
3.334.742	-35.740	
3.334.897	-35.742	
3.335.032	-35.745	
3.335.162	-35.748	
3.335.350	-35.750	
3.335.536	-35.753	
3.335.700	-35.756	
3.335.883	-35.758	
3.336.031	-35.761	
3.336.184	-35.764	
3.336.336	-35.766	
3.336.481	-35.769	
3.336.643	-35.771	
3.336.784	-35.774	
3.336.960	-35.776	
3.337.157	-35.779	
3.337.339	-35.781	
3.337.502	-35.784	
3.337.635	-35.786	
3.337.762	-35.789	
3.337.926	-35.791	

3.338.152	-35.794	
3.338.352	-35.796	
3.338.484	-35.798	
3.338.645	-35.801	
3.338.940	-35.803	
3.339.118	-35.805	
3.339.214	-35.808	
3.339.339	-35.810	
3.339.440	-35.812	
3.339.645	-35.815	
3.339.872	-35.817	
3.340.042	-35.819	
3.340.117	-35.822	
3.340.271	-35.824	
3.340.466	-35.826	
3.340.584	-35.828	
3.340.732	-35.831	
3.340.999	-35.833	
3.341.246	-35.835	
3.341.418	-35.837	
3.341.591	-35.840	
3.341.787	-35.842	
3.341.940	-35.844	
3.342.018	-35.846	
3.342.144	-35.849	
3.342.296	-35.851	
3.342.455	-35.853	
3.342.635	-35.855	
3.342.818	-35.858	
3.342.997	-35.860	

3.343.167	-35.862	
3.343.358	-35.864	
3.343.464	-35.866	
3.343.607	-35.869	
3.343.854	-35.871	
3.344.026	-35.873	
3.344.106	-35.875	
3.344.265	-35.877	
3.344.471	-35.879	
3.344.635	-35.882	
3.344.822	-35.884	
3.345.031	-35.886	
3.345.166	-35.888	
3.345.320	-35.890	
3.345.524	-35.892	
3.345.675	-35.894	
3.345.795	-35.896	
3.345.959	-35.898	
3.346.132	-35.900	
3.346.288	-35.903	
3.346.438	-35.905	
3.346.602	-35.907	
3.346.686	-35.909	
3.346.824	-35.911	
3.346.964	-35.913	
3.347.069	-35.915	
3.347.193	-35.917	
3.347.469	-35.918	
3.347.759	-35.920	
3.347.933	-35.922	

3.348.109	-35.924	
3.348.362	-35.926	
3.348.553	-35.928	
3.348.705	-35.930	
3.348.898	-35.932	
3.349.085	-35.934	
3.349.319	-35.935	
3.349.498	-35.937	
3.349.619	-35.939	
3.349.710	-35.941	
3.349.821	-35.943	
3.350.002	-35.944	
3.350.110	-35.946	
3.350.240	-35.948	
3.350.439	-35.949	
3.350.628	-35.951	
3.350.841	-35.953	
3.351.003	-35.954	
3.351.158	-35.956	
3.351.372	-35.957	
3.351.548	-35.959	
3.351.678	-35.960	
3.351.839	-35.962	
3.352.005	-35.963	
3.352.185	-35.965	
3.352.401	-35.966	
3.352.579	-35.968	
3.352.737	-35.969	
3.352.915	-35.971	
3.353.092	-35.972	

3.353.236	-35.973	
3.353.372	-35.975	
3.353.517	-35.976	
3.353.687	-35.977	
3.353.860	-35.979	
3.354.012	-35.980	
3.354.222	-35.981	
3.354.342	-35.982	
3.354.503	-35.984	
3.354.710	-35.985	
3.354.882	-35.986	
3.355.115	-35.987	
3.355.287	-35.988	
3.355.399	-35.989	
3.355.500	-35.990	
3.355.634	-35.991	
3.355.870	-35.992	
3.356.123	-35.993	
3.356.308	-35.994	
3.356.490	-35.995	
3.356.644	-35.996	
3.356.736	-35.997	
3.356.917	-35.998	
3.357.119	-35.999	
3.357.292	-36.000	
3.357.380	-36.000	
3.357.529	-36.001	
3.357.892	-36.002	
3.358.298	-36.003	
3.358.614	-36.003	

3.358.760	-36.004	
3.358.779	-36.005	
3.358.826	-36.005	
3.358.900	-36.006	
3.359.016	-36.006	
3.359.077	-36.007	
3.359.167	-36.007	
3.359.269	-36.008	
3.359.467	-36.008	
3.359.660	-36.009	
3.359.848	-36.009	
3.360.035	-36.009	
3.360.169	-36.010	
3.360.363	-36.010	
3.360.584	-36.010	
3.360.744	-36.010	
3.360.899	-36.011	
3.361.055	-36.011	
3.361.208	-36.011	
3.361.349	-36.011	
3.361.523	-36.011	
3.361.727	-36.011	
3.361.922	-36.011	
3.362.091	-36.011	
3.362.209	-36.011	
3.362.300	-36.011	
3.362.440	-36.011	
3.362.589	-36.011	
3.362.814	-36.011	
3.363.009	-36.011	

3.363.189	-36.011	
3.363.336	-36.010	
3.363.452	-36.010	
3.363.672	-36.010	
3.363.936	-36.010	
3.364.114	-36.009	
3.364.255	-36.009	
3.364.346	-36.009	
3.364.518	-36.008	
3.364.728	-36.008	
3.364.875	-36.008	
3.365.052	-36.007	
3.365.282	-36.007	
3.365.453	-36.007	
3.365.607	-36.006	
3.365.706	-36.006	
3.365.863	-36.005	
3.366.021	-36.005	
3.366.190	-36.004	
3.366.357	-36.004	
3.366.562	-36.003	
3.366.712	-36.002	
3.366.873	-36.002	
3.367.039	-36.001	
3.367.155	-36.001	
3.367.318	-36.000	
3.367.495	-36.000	
3.367.630	-35.999	
3.367.796	-35.998	
3.367.984	-35.998	

3.368.181	-35.997	
3.368.330	-35.996	
3.368.488	-35.996	
3.368.692	-35.995	
3.368.896	-35.994	
3.369.115	-35.994	
3.369.236	-35.993	
3.369.290	-35.992	
3.369.406	-35.991	
3.369.572	-35.991	
3.369.810	-35.990	
3.370.072	-35.989	
3.370.233	-35.988	
3.370.364	-35.988	
3.370.468	-35.987	
3.370.639	-35.986	
3.370.856	-35.985	
3.371.055	-35.984	
3.371.221	-35.983	
3.371.333	-35.983	
3.371.447	-35.982	
3.371.582	-35.981	
3.371.792	-35.980	
3.371.990	-35.979	
3.372.116	-35.978	
3.372.239	-35.977	
3.372.368	-35.976	
3.372.553	-35.975	
3.372.789	-35.974	
3.373.034	-35.973	

3.373.210	-35.972	
3.373.401	-35.972	
3.373.706	-35.971	
3.373.869	-35.970	
3.373.972	-35.969	
3.374.153	-35.968	
3.374.324	-35.967	
3.374.435	-35.966	
3.374.568	-35.965	
3.374.716	-35.964	
3.374.830	-35.963	
3.374.920	-35.962	
3.375.017	-35.961	
3.375.202	-35.960	
3.375.410	-35.958	
3.375.612	-35.957	
3.375.851	-35.956	
3.376.072	-35.955	
3.376.219	-35.954	
3.376.375	-35.953	
3.376.555	-35.952	
3.376.750	-35.951	
3.376.928	-35.950	
3.377.066	-35.949	
3.377.263	-35.948	
3.377.437	-35.947	
3.377.549	-35.946	
3.377.677	-35.945	
3.377.818	-35.944	
3.378.018	-35.943	

3.378.192	-35.942	
3.378.358	-35.941	
3.378.565	-35.941	
3.378.735	-35.940	
3.378.907	-35.939	
3.379.115	-35.938	
3.379.274	-35.937	
3.379.331	-35.936	
3.379.559	-35.935	
3.379.814	-35.934	
3.379.952	-35.933	
3.380.042	-35.932	
3.380.186	-35.932	
3.380.363	-35.931	
3.380.515	-35.930	
3.380.630	-35.929	
3.380.798	-35.928	
3.381.028	-35.928	
3.381.216	-35.927	
3.381.419	-35.926	
3.381.607	-35.925	
3.381.851	-35.925	
3.382.007	-35.924	
3.382.101	-35.923	
3.382.184	-35.923	
3.382.328	-35.922	
3.382.715	-35.922	
3.383.163	-35.921	
3.383.409	-35.920	
3.383.506	-35.920	

3.383.564	-35.919	
3.383.640	-35.919	
3.383.694	-35.918	
3.383.731	-35.918	
3.383.824	-35.917	
3.383.962	-35.917	
3.384.127	-35.917	
3.384.294	-35.916	
3.384.474	-35.916	
3.384.655	-35.916	
3.384.844	-35.915	
3.384.998	-35.915	
3.385.155	-35.915	
3.385.314	-35.914	
3.385.483	-35.914	
3.385.713	-35.914	
3.385.872	-35.914	
3.386.010	-35.914	
3.386.172	-35.913	
3.386.338	-35.913	
3.386.549	-35.913	
3.386.732	-35.913	
3.386.902	-35.913	
3.387.072	-35.913	
3.387.142	-35.913	
3.387.231	-35.913	
3.387.401	-35.913	
3.387.640	-35.913	
3.387.859	-35.914	
3.388.024	-35.914	

3.388.208	-35.914	
3.388.419	-35.914	
3.388.596	-35.914	
3.388.777	-35.915	
3.388.950	-35.915	
3.389.091	-35.915	
3.389.232	-35.916	
3.389.366	-35.916	
3.389.525	-35.916	
3.389.713	-35.917	
3.389.875	-35.917	
3.389.987	-35.918	
3.390.086	-35.918	
3.390.292	-35.919	
3.390.490	-35.919	
3.390.612	-35.920	
3.390.779	-35.921	
3.391.025	-35.921	
3.391.252	-35.922	
3.391.336	-35.923	
3.391.411	-35.923	
3.391.591	-35.924	
3.391.758	-35.925	
3.391.909	-35.926	
3.392.116	-35.926	
3.392.325	-35.927	
3.392.469	-35.928	
3.392.630	-35.929	
3.392.838	-35.930	
3.393.000	-35.931	

3.393.146	-35.932	
3.393.279	-35.933	
3.393.398	-35.934	
3.393.582	-35.935	
3.393.813	-35.936	
3.393.958	-35.937	
3.394.122	-35.938	
3.394.308	-35.939	
3.394.467	-35.940	
3.394.615	-35.942	
3.394.826	-35.943	
3.395.002	-35.944	
3.395.153	-35.945	
3.395.350	-35.946	
3.395.511	-35.947	
3.395.643	-35.948	
3.395.800	-35.950	
3.395.945	-35.951	
3.396.035	-35.952	
3.396.187	-35.953	
3.396.317	-35.954	
3.396.444	-35.955	
3.396.629	-35.957	
3.396.791	-35.958	
3.396.964	-35.959	
3.397.166	-35.960	
3.397.416	-35.961	
3.397.622	-35.963	
3.397.814	-35.964	
3.397.980	-35.965	

3.398.149	-35.966	
3.398.381	-35.967	
3.398.607	-35.968	
3.398.761	-35.970	
3.398.857	-35.971	
3.398.983	-35.972	
3.399.135	-35.973	
3.399.276	-35.974	
3.399.417	-35.975	
3.399.554	-35.976	
3.399.695	-35.977	
3.399.893	-35.978	
3.400.049	-35.979	
3.400.186	-35.980	
3.400.415	-35.981	
3.400.632	-35.982	
3.400.822	-35.983	
3.401.060	-35.984	
3.401.213	-35.984	
3.401.350	-35.985	
3.401.559	-35.986	
3.401.747	-35.987	
3.401.917	-35.988	
3.402.079	-35.988	
3.402.207	-35.989	
3.402.366	-35.990	
3.402.528	-35.990	
3.402.683	-35.991	
3.402.842	-35.992	
3.403.044	-35.992	

3.403.203	-35.993	
3.403.362	-35.993	
3.403.565	-35.994	
3.403.698	-35.994	
3.403.867	-35.995	
3.404.048	-35.995	
3.404.233	-35.996	
3.404.398	-35.996	
3.404.566	-35.996	
3.404.775	-35.997	
3.404.934	-35.997	
3.405.013	-35.997	
3.405.182	-35.997	
3.405.457	-35.997	
3.405.612	-35.998	
3.405.753	-35.998	
3.405.891	-35.998	
3.406.055	-35.998	
3.406.267	-35.998	
3.406.419	-35.998	
3.406.577	-35.998	
3.406.776	-35.998	
3.406.886	-35.998	
3.406.990	-35.998	
3.407.275	-35.998	
3.407.741	-35.998	
3.408.073	-35.998	
3.408.141	-35.997	
3.408.154	-35.997	
3.408.268	-35.997	

3.408.402	-35.997	
3.408.508	-35.997	
3.408.629	-35.996	
3.408.690	-35.996	
3.408.774	-35.996	
3.408.948	-35.995	
3.409.154	-35.995	
3.409.305	-35.994	
3.409.467	-35.994	
3.409.693	-35.994	
3.409.872	-35.993	
3.410.056	-35.993	
3.410.238	-35.992	
3.410.386	-35.992	
3.410.526	-35.991	
3.410.701	-35.990	
3.410.894	-35.990	
3.411.051	-35.989	
3.411.156	-35.989	
3.411.277	-35.988	
3.411.473	-35.988	
3.411.664	-35.987	
3.411.808	-35.986	
3.411.953	-35.986	
3.412.137	-35.985	
3.412.332	-35.984	
3.412.510	-35.984	
3.412.724	-35.983	
3.412.933	-35.982	
3.413.085	-35.982	

3.413.232	-35.981	
3.413.450	-35.980	
3.413.631	-35.980	
3.413.752	-35.979	
3.413.927	-35.978	
3.414.084	-35.978	
3.414.240	-35.977	
3.414.445	-35.976	
3.414.572	-35.976	
3.414.652	-35.975	
3.414.776	-35.974	
3.414.966	-35.974	
3.415.208	-35.973	
3.415.343	-35.972	
3.415.459	-35.972	
3.415.634	-35.971	
3.415.840	-35.971	
3.416.049	-35.970	
3.416.176	-35.969	
3.416.288	-35.969	
3.416.429	-35.968	
3.416.588	-35.968	
3.416.812	-35.967	
3.417.030	-35.967	
3.417.149	-35.966	
3.417.305	-35.966	
3.417.469	-35.965	
3.417.686	-35.965	
3.417.915	-35.965	
3.418.031	-35.964	

3.418.195	-35.964	
3.418.409	-35.963	
3.418.593	-35.963	
3.418.696	-35.963	
3.418.828	-35.963	
3.418.997	-35.962	
3.419.158	-35.962	
3.419.323	-35.962	
3.419.498	-35.961	
3.419.678	-35.961	
3.419.881	-35.961	
3.420.067	-35.961	
3.420.186	-35.961	
3.420.336	-35.960	
3.420.504	-35.960	
3.420.677	-35.960	
3.420.840	-35.960	
3.420.992	-35.960	
3.421.107	-35.960	
3.421.213	-35.960	
3.421.394	-35.960	
3.421.584	-35.960	
3.421.745	-35.960	
3.421.922	-35.960	
3.422.141	-35.960	
3.422.321	-35.960	
3.422.498	-35.960	
3.422.740	-35.960	
3.422.946	-35.960	
3.423.070	-35.960	

3.423.177	-35.960	
3.423.366	-35.960	
3.423.564	-35.960	
3.423.706	-35.960	
3.423.860	-35.961	
3.423.995	-35.961	
3.424.154	-35.961	
3.424.299	-35.961	
3.424.418	-35.961	
3.424.511	-35.961	
3.424.649	-35.962	
3.424.866	-35.962	
3.425.081	-35.962	
3.425.262	-35.962	
3.425.459	-35.962	
3.425.699	-35.963	
3.425.896	-35.963	
3.426.008	-35.963	
3.426.131	-35.964	
3.426.340	-35.964	
3.426.483	-35.964	
3.426.639	-35.965	
3.426.733	-35.965	
3.426.904	-35.965	
3.427.095	-35.966	
3.427.286	-35.966	
3.427.545	-35.966	
3.427.751	-35.967	
3.427.881	-35.967	
3.428.024	-35.968	

3.428.226	-35.968	
3.428.427	-35.968	
3.428.499	-35.969	
3.428.662	-35.969	
3.428.868	-35.970	
3.429.012	-35.970	
3.429.189	-35.971	
3.429.391	-35.971	
3.429.561	-35.972	
3.429.713	-35.972	
3.429.859	-35.973	
3.430.041	-35.973	
3.430.271	-35.974	
3.430.479	-35.974	
3.430.656	-35.975	
3.430.800	-35.976	
3.430.943	-35.976	
3.431.064	-35.977	
3.431.208	-35.977	
3.431.339	-35.978	
3.431.426	-35.979	
3.431.674	-35.979	
3.432.104	-35.980	
3.432.541	-35.981	
3.432.731	-35.981	
3.432.770	-35.982	
3.432.911	-35.982	
3.432.979	-35.983	
3.432.984	-35.984	
3.433.049	-35.984	

3.433.171	-35.985	
3.433.319	-35.986	
3.433.423	-35.987	
3.433.578	-35.987	
3.433.723	-35.988	
3.433.860	-35.989	
3.434.109	-35.989	
3.434.386	-35.990	
3.434.594	-35.991	
3.434.753	-35.991	
3.434.931	-35.992	
3.435.094	-35.993	
3.435.227	-35.994	
3.435.407	-35.994	
3.435.609	-35.995	
3.435.753	-35.996	
3.435.866	-35.996	
3.435.985	-35.997	
3.436.131	-35.998	
3.436.279	-35.999	
3.436.485	-35.999	
3.436.703	-36.000	
3.436.838	-36.001	
3.436.960	-36.002	
3.437.177	-36.003	
3.437.419	-36.003	
3.437.586	-36.004	
3.437.729	-36.005	
3.437.854	-36.006	
3.438.065	-36.007	

3.438.232	-36.007	
3.438.358	-36.008	
3.438.497	-36.009	
3.438.611	-36.010	
3.438.784	-36.011	
3.439.037	-36.011	
3.439.214	-36.012	
3.439.339	-36.013	
3.439.528	-36.014	
3.439.706	-36.015	
3.439.862	-36.016	
3.440.016	-36.016	
3.440.172	-36.017	
3.440.331	-36.018	
3.440.494	-36.019	
3.440.631	-36.020	
3.440.733	-36.021	
3.440.920	-36.021	
3.441.108	-36.022	
3.441.253	-36.023	
3.441.488	-36.024	
3.441.665	-36.025	
3.441.810	-36.026	
3.441.989	-36.027	
3.442.151	-36.027	
3.442.351	-36.028	
3.442.536	-36.029	
3.442.737	-36.030	
3.442.906	-36.031	
3.443.062	-36.032	

3.443.217	-36.033	
3.443.349	-36.033	
3.443.518	-36.034	
3.443.739	-36.035	
3.443.930	-36.036	
3.444.041	-36.036	
3.444.186	-36.037	
3.444.344	-36.038	
3.444.519	-36.039	
3.444.673	-36.039	
3.444.817	-36.040	
3.444.973	-36.041	
3.445.136	-36.041	
3.445.325	-36.042	
3.445.475	-36.043	
3.445.623	-36.043	
3.445.760	-36.044	
3.445.894	-36.044	
3.446.033	-36.045	
3.446.217	-36.046	
3.446.390	-36.046	
3.446.552	-36.047	
3.446.741	-36.047	
3.446.954	-36.048	
3.447.166	-36.048	
3.447.377	-36.048	
3.447.586	-36.049	
3.447.791	-36.049	
3.447.947	-36.050	
3.448.042	-36.050	

3.448.236	-36.050	
3.448.430	-36.050	
3.448.524	-36.051	
3.448.630	-36.051	
3.448.795	-36.051	
3.448.934	-36.051	
3.449.059	-36.051	
3.449.254	-36.052	
3.449.476	-36.052	
3.449.695	-36.052	
3.449.868	-36.052	
3.450.006	-36.052	
3.450.205	-36.052	
3.450.404	-36.052	
3.450.508	-36.052	
3.450.558	-36.052	
3.450.800	-36.052	
3.451.093	-36.052	
3.451.253	-36.051	
3.451.396	-36.051	
3.451.559	-36.051	
3.451.758	-36.051	
3.451.921	-36.051	
3.452.016	-36.051	
3.452.171	-36.050	
3.452.402	-36.050	
3.452.635	-36.050	
3.452.845	-36.050	
3.452.964	-36.049	
3.453.138	-36.049	

3.453.323	-36.049	
3.453.488	-36.048	
3.453.607	-36.048	
3.453.773	-36.048	
3.453.923	-36.047	
3.454.046	-36.047	
3.454.248	-36.046	
3.454.433	-36.046	
3.454.514	-36.045	
3.454.669	-36.045	
3.454.872	-36.044	
3.455.057	-36.044	
3.455.238	-36.043	
3.455.385	-36.043	
3.455.565	-36.042	
3.455.827	-36.042	
3.455.954	-36.041	
3.456.008	-36.041	
3.456.165	-36.040	
3.456.492	-36.040	
3.456.933	-36.039	
3.457.184	-36.038	
3.457.287	-36.038	
3.457.455	-36.037	
3.457.538	-36.037	
3.457.505	-36.036	
3.457.531	-36.035	
3.457.662	-36.035	
3.457.778	-36.034	
3.457.922	-36.034	

3.458.118	-36.033	
3.458.275	-36.032	
3.458.445	-36.032	
3.458.620	-36.031	
3.458.795	-36.030	
3.458.990	-36.030	
3.459.219	-36.029	
3.459.416	-36.028	
3.459.590	-36.028	
3.459.785	-36.027	
3.459.933	-36.026	
3.460.067	-36.026	
3.460.190	-36.025	
3.460.343	-36.024	
3.460.500	-36.024	
3.460.660	-36.023	
3.460.860	-36.023	
3.460.985	-36.022	
3.461.129	-36.021	
3.461.335	-36.021	
3.461.526	-36.020	
3.461.683	-36.020	
3.461.902	-36.019	
3.462.135	-36.019	
3.462.265	-36.018	
3.462.384	-36.018	
3.462.545	-36.017	
3.462.690	-36.017	
3.462.859	-36.016	
3.463.029	-36.016	

3.463.153	-36.015	
3.463.301	-36.015	
3.463.459	-36.015	
3.463.587	-36.014	
3.463.757	-36.014	
3.463.969	-36.013	
3.464.122	-36.013	
3.464.280	-36.013	
3.464.485	-36.012	
3.464.648	-36.012	
3.464.792	-36.012	
3.464.993	-36.011	
3.465.190	-36.011	
3.465.331	-36.011	
3.465.401	-36.011	
3.465.578	-36.011	
3.465.775	-36.010	
3.465.945	-36.010	
3.466.093	-36.010	
3.466.293	-36.010	
3.466.418	-36.010	
3.466.570	-36.010	
3.466.841	-36.010	
3.467.034	-36.009	
3.467.199	-36.009	
3.467.365	-36.009	
3.467.492	-36.009	
3.467.646	-36.009	
3.467.846	-36.009	
3.468.011	-36.009	

3.468.182	-36.009	
3.468.320	-36.009	
3.468.463	-36.009	
3.468.699	-36.009	
3.468.911	-36.009	
3.469.056	-36.009	
3.469.189	-36.009	
3.469.312	-36.009	
3.469.465	-36.008	
3.469.617	-36.008	
3.469.818	-36.008	
3.470.018	-36.008	
3.470.054	-36.008	
3.470.161	-36.008	
3.470.352	-36.008	
3.470.508	-36.008	
3.470.598	-36.008	
3.470.771	-36.008	
3.471.042	-36.008	
3.471.203	-36.007	
3.471.400	-36.007	
3.471.611	-36.007	
3.471.784	-36.007	
3.471.994	-36.007	
3.472.193	-36.007	
3.472.387	-36.006	
3.472.520	-36.006	
3.472.650	-36.006	
3.472.853	-36.006	
3.472.964	-36.005	

3.473.074	-36.005	
3.473.218	-36.005	
3.473.318	-36.004	
3.473.478	-36.004	
3.473.654	-36.004	
3.473.846	-36.003	
3.474.026	-36.003	
3.474.225	-36.002	
3.474.378	-36.002	
3.474.533	-36.001	
3.474.793	-36.001	
3.475.013	-36.001	
3.475.194	-36.000	
3.475.394	-35.999	
3.475.584	-35.999	
3.475.717	-35.998	
3.475.877	-35.998	
3.476.043	-35.997	
3.476.154	-35.997	
3.476.329	-35.996	
3.476.566	-35.995	
3.476.765	-35.995	
3.476.917	-35.994	
3.477.063	-35.993	
3.477.231	-35.993	
3.477.347	-35.992	
3.477.493	-35.991	
3.477.712	-35.990	
3.477.906	-35.989	
3.478.066	-35.989	

3.478.221	-35.988	
3.478.354	-35.987	
3.478.517	-35.986	
3.478.680	-35.985	
3.478.826	-35.984	
3.479.006	-35.983	
3.479.158	-35.982	
3.479.341	-35.981	
3.479.576	-35.980	
3.479.765	-35.979	
3.479.855	-35.978	
3.480.005	-35.977	
3.480.164	-35.976	
3.480.329	-35.975	
3.480.501	-35.974	
3.480.648	-35.973	
3.480.934	-35.972	
3.481.332	-35.971	
3.481.658	-35.969	
3.481.779	-35.968	
3.481.831	-35.967	
3.481.969	-35.966	
3.482.090	-35.965	
3.482.090	-35.963	
3.482.193	-35.962	
3.482.361	-35.961	
3.482.464	-35.960	
3.482.594	-35.958	
3.482.796	-35.957	
3.483.026	-35.956	

3.483.174	-35.955	
3.483.278	-35.953	
3.483.457	-35.952	
3.483.690	-35.951	
3.483.862	-35.949	
3.484.071	-35.948	
3.484.256	-35.947	
3.484.367	-35.945	
3.484.510	-35.944	
3.484.670	-35.943	
3.484.865	-35.941	
3.485.078	-35.940	
3.485.226	-35.939	
3.485.365	-35.938	
3.485.553	-35.936	
3.485.672	-35.935	
3.485.789	-35.934	
3.485.945	-35.932	
3.486.097	-35.931	
3.486.264	-35.930	
3.486.447	-35.929	
3.486.622	-35.928	
3.486.809	-35.926	
3.486.971	-35.925	
3.487.149	-35.924	
3.487.370	-35.923	
3.487.539	-35.922	
3.487.690	-35.921	
3.487.834	-35.920	
3.487.954	-35.919	

3.488.077	-35.918	
3.488.262	-35.916	
3.488.470	-35.915	
3.488.641	-35.915	
3.488.795	-35.914	
3.488.994	-35.913	
3.489.171	-35.912	
3.489.334	-35.911	
3.489.508	-35.910	
3.489.680	-35.909	
3.489.843	-35.908	
3.490.000	-35.908	
3.490.075	-35.907	
3.490.153	-35.906	
3.490.381	-35.906	
3.490.620	-35.905	
3.490.823	-35.904	
3.491.021	-35.904	
3.491.206	-35.903	
3.491.390	-35.903	
3.491.541	-35.902	
3.491.674	-35.902	
3.491.881	-35.901	
3.492.016	-35.901	
3.492.164	-35.900	
3.492.350	-35.900	
3.492.545	-35.900	
3.492.669	-35.899	
3.492.807	-35.899	
3.492.958	-35.899	

3.493.157	-35.898	
3.493.330	-35.898	
3.493.562	-35.898	
3.493.721	-35.898	
3.493.879	-35.897	
3.494.013	-35.897	
3.494.118	-35.897	
3.494.232	-35.897	
3.494.381	-35.897	
3.494.421	-35.897	
3.494.595	-35.897	
3.494.796	-35.896	
3.494.912	-35.896	
3.495.107	-35.896	
3.495.298	-35.896	
3.495.464	-35.896	
3.495.688	-35.896	
3.495.917	-35.896	
3.496.165	-35.896	
3.496.369	-35.896	
3.496.539	-35.896	
3.496.712	-35.897	
3.496.859	-35.897	
3.497.036	-35.897	
3.497.192	-35.897	
3.497.339	-35.897	
3.497.478	-35.897	
3.497.563	-35.897	
3.497.651	-35.897	
3.497.805	-35.897	

3.497.922	-35.897	
3.498.109	-35.898	
3.498.381	-35.898	
3.498.571	-35.898	
3.498.730	-35.898	
3.498.907	-35.898	
3.499.104	-35.898	
3.499.315	-35.898	
3.499.545	-35.899	
3.499.727	-35.899	
3.499.904	-35.899	
3.500.078	-35.899	
3.500.238	-35.899	
3.500.390	-35.900	
3.500.522	-35.900	
3.500.652	-35.900	
3.500.758	-35.900	
3.500.920	-35.900	
3.501.105	-35.901	
3.501.293	-35.901	
3.501.552	-35.901	
3.501.777	-35.901	
3.501.924	-35.901	
3.502.043	-35.902	
3.502.249	-35.902	
3.502.453	-35.902	
3.502.574	-35.902	
3.502.693	-35.902	
3.502.899	-35.902	
3.503.107	-35.903	

3.503.221	-35.903	
3.503.359	-35.903	
3.503.543	-35.903	
3.503.786	-35.903	
3.503.918	-35.903	
3.503.983	-35.903	
3.504.212	-35.903	
3.504.482	-35.903	
3.504.649	-35.904	
3.504.775	-35.904	
3.504.883	-35.904	
3.504.974	-35.904	
3.505.169	-35.904	
3.505.642	-35.904	
3.506.094	-35.904	
3.506.237	-35.904	
3.506.322	-35.904	
3.506.393	-35.904	
3.506.479	-35.904	
3.506.528	-35.904	
3.506.560	-35.903	
3.506.714	-35.903	
3.506.878	-35.903	
3.507.003	-35.903	
3.507.166	-35.903	
3.507.398	-35.903	
3.507.575	-35.903	
3.507.744	-35.902	
3.507.947	-35.902	
3.508.182	-35.902	

3.508.369	-35.902	
3.508.503	-35.901	
3.508.636	-35.901	
3.508.775	-35.901	
3.508.922	-35.901	
3.509.086	-35.900	
3.509.273	-35.900	
3.509.414	-35.900	
3.509.563	-35.899	
3.509.718	-35.899	
3.509.825	-35.899	
3.510.002	-35.898	
3.510.195	-35.898	
3.510.329	-35.898	
3.510.526	-35.897	
3.510.733	-35.897	
3.510.932	-35.897	
3.511.143	-35.896	
3.511.322	-35.896	
3.511.465	-35.896	
3.511.624	-35.895	
3.511.790	-35.895	
3.511.898	-35.894	
3.512.077	-35.894	
3.512.265	-35.894	
3.512.406	-35.893	
3.512.560	-35.893	
3.512.749	-35.893	
3.512.910	-35.892	
3.513.062	-35.892	

3.513.211	-35.891	
3.513.337	-35.891	
3.513.608	-35.891	
3.513.824	-35.890	
3.513.922	-35.890	
3.514.135	-35.890	
3.514.339	-35.889	
3.514.407	-35.889	
3.514.476	-35.889	
3.514.646	-35.888	
3.514.852	-35.888	
3.515.009	-35.888	
3.515.204	-35.888	
3.515.461	-35.887	
3.515.612	-35.887	
3.515.768	-35.887	
3.515.995	-35.887	
3.516.201	-35.886	
3.516.351	-35.886	
3.516.411	-35.886	
3.516.553	-35.886	
3.516.762	-35.886	
3.516.924	-35.885	
3.517.032	-35.885	
3.517.167	-35.885	
3.517.370	-35.885	
3.517.581	-35.885	
3.517.762	-35.884	
3.517.900	-35.884	
3.518.062	-35.884	

3.518.286	-35.884	
3.518.463	-35.884	
3.518.607	-35.884	
3.518.748	-35.884	
3.518.864	-35.883	
3.519.050	-35.883	
3.519.196	-35.883	
3.519.316	-35.883	
3.519.476	-35.883	
3.519.659	-35.883	
3.519.840	-35.883	
3.519.991	-35.883	
3.520.175	-35.883	
3.520.316	-35.883	
3.520.540	-35.883	
3.520.863	-35.883	
3.521.055	-35.883	
3.521.154	-35.883	
3.521.224	-35.883	
3.521.375	-35.883	
3.521.575	-35.883	
3.521.758	-35.883	
3.521.922	-35.883	
3.522.091	-35.883	
3.522.191	-35.883	
3.522.312	-35.883	
3.522.466	-35.883	
3.522.693	-35.883	
3.522.834	-35.883	
3.522.977	-35.883	

3.523.141	-35.883	
3.523.370	-35.883	
3.523.568	-35.883	
3.523.703	-35.884	
3.523.871	-35.884	
3.524.063	-35.884	
3.524.236	-35.884	
3.524.398	-35.884	
3.524.525	-35.884	
3.524.648	-35.884	
3.524.904	-35.884	
3.525.125	-35.885	
3.525.282	-35.885	
3.525.396	-35.885	
3.525.529	-35.885	
3.525.760	-35.885	
3.525.923	-35.885	
3.526.042	-35.886	
3.526.221	-35.886	
3.526.347	-35.886	
3.526.497	-35.886	
3.526.721	-35.886	
3.526.909	-35.887	
3.527.069	-35.887	
3.527.209	-35.887	
3.527.347	-35.887	
3.527.525	-35.887	
3.527.769	-35.888	
3.527.893	-35.888	
3.528.024	-35.888	

3.528.199	-35.888	
3.528.362	-35.888	
3.528.519	-35.889	
3.528.687	-35.889	
3.528.846	-35.889	
3.528.987	-35.889	
3.529.221	-35.890	
3.529.420	-35.890	
3.529.544	-35.890	
3.529.781	-35.890	
3.530.179	-35.890	
3.530.598	-35.891	
3.530.779	-35.891	
3.530.823	-35.891	
3.530.891	-35.891	
3.530.934	-35.891	
3.530.956	-35.892	
3.531.031	-35.892	
3.531.166	-35.892	
3.531.338	-35.892	
3.531.524	-35.892	
3.531.718	-35.893	
3.531.936	-35.893	
3.532.130	-35.893	
3.532.321	-35.893	
3.532.469	-35.893	
3.532.604	-35.893	
3.532.789	-35.893	
3.532.990	-35.893	
3.533.174	-35.894	

3.533.275	-35.894	
3.533.438	-35.894	
3.533.644	-35.894	
3.533.820	-35.894	
3.533.967	-35.894	
3.534.063	-35.894	
3.534.186	-35.894	
3.534.317	-35.894	
3.534.429	-35.894	
3.534.612	-35.894	
3.534.839	-35.894	
3.535.046	-35.894	
3.535.260	-35.895	
3.535.444	-35.895	
3.535.573	-35.895	
3.535.744	-35.895	
3.535.993	-35.895	
3.536.174	-35.895	
3.536.304	-35.895	
3.536.448	-35.895	
3.536.635	-35.895	
3.536.777	-35.895	
3.536.882	-35.895	
3.537.090	-35.895	
3.537.296	-35.894	
3.537.401	-35.894	
3.537.502	-35.894	
3.537.640	-35.894	
3.537.890	-35.894	
3.538.138	-35.894	

3.538.322	-35.894	
3.538.526	-35.894	
3.538.647	-35.894	
3.538.727	-35.894	
3.538.875	-35.894	
3.539.063	-35.894	
3.539.240	-35.894	
3.539.391	-35.894	
3.539.545	-35.894	
3.539.740	-35.893	
3.539.904	-35.893	
3.540.103	-35.893	
3.540.306	-35.893	
3.540.487	-35.893	
3.540.650	-35.893	
3.540.773	-35.893	
3.540.891	-35.893	
3.541.100	-35.893	
3.541.237	-35.893	
3.541.389	-35.892	
3.541.520	-35.892	
3.541.701	-35.892	
3.541.967	-35.892	
3.542.133	-35.892	
3.542.222	-35.892	
3.542.398	-35.891	
3.542.628	-35.891	
3.542.784	-35.891	
3.542.903	-35.891	
3.543.029	-35.890	

3.543.154	-35.890	
3.543.271	-35.890	
3.543.399	-35.890	
3.543.594	-35.889	
3.543.806	-35.889	
3.543.961	-35.889	
3.544.084	-35.888	
3.544.247	-35.888	
3.544.467	-35.888	
3.544.677	-35.887	
3.544.876	-35.887	
3.545.121	-35.887	
3.545.327	-35.886	
3.545.500	-35.886	
3.545.601	-35.886	
3.545.771	-35.885	
3.546.010	-35.885	
3.546.154	-35.884	
3.546.321	-35.884	
3.546.462	-35.883	
3.546.577	-35.883	
3.546.679	-35.882	
3.546.831	-35.882	
3.547.039	-35.881	
3.547.151	-35.881	
3.547.310	-35.880	
3.547.542	-35.880	
3.547.713	-35.879	
3.547.944	-35.879	
3.548.255	-35.878	

3.548.416	-35.877	
3.548.448	-35.877	
3.548.590	-35.876	
3.548.850	-35.876	
3.549.044	-35.875	
3.549.149	-35.874	
3.549.291	-35.874	
3.549.479	-35.873	
3.549.628	-35.872	
3.549.760	-35.872	
3.549.922	-35.871	
3.550.088	-35.870	
3.550.260	-35.869	
3.550.457	-35.869	
3.550.632	-35.868	
3.550.815	-35.867	
3.551.013	-35.866	
3.551.161	-35.866	
3.551.281	-35.865	
3.551.467	-35.864	
3.551.660	-35.863	
3.551.830	-35.863	
3.552.003	-35.862	
3.552.148	-35.861	
3.552.351	-35.860	
3.552.517	-35.859	
3.552.662	-35.858	
3.552.831	-35.858	
3.553.008	-35.857	
3.553.159	-35.856	

3.553.313	-35.855	
3.553.500	-35.854	
3.553.683	-35.853	
3.553.849	-35.852	
3.553.987	-35.852	
3.554.225	-35.851	
3.554.592	-35.850	
3.554.938	-35.849	
3.555.208	-35.848	
3.555.296	-35.847	
3.555.385	-35.846	
3.555.419	-35.845	
3.555.457	-35.844	
3.555.574	-35.843	
3.555.643	-35.842	
3.555.686	-35.841	
3.555.811	-35.840	
3.556.031	-35.839	
3.556.260	-35.838	
3.556.432	-35.837	
3.556.606	-35.836	
3.556.747	-35.835	
3.556.967	-35.834	
3.557.174	-35.833	
3.557.379	-35.832	
3.557.547	-35.831	
3.557.617	-35.830	
3.557.708	-35.829	
3.557.903	-35.828	
3.558.059	-35.827	

3.558.286	-35.826	
3.558.419	-35.825	
3.558.560	-35.824	
3.558.676	-35.823	
3.558.851	-35.822	
3.559.059	-35.821	
3.559.175	-35.820	
3.559.277	-35.819	
3.559.471	-35.818	
3.559.688	-35.817	
3.559.890	-35.816	
3.560.052	-35.815	
3.560.244	-35.814	
3.560.407	-35.813	
3.560.569	-35.812	
3.560.791	-35.811	
3.560.930	-35.810	
3.561.031	-35.809	
3.561.242	-35.807	
3.561.418	-35.806	
3.561.566	-35.805	
3.561.691	-35.804	
3.561.902	-35.803	
3.562.094	-35.802	
3.562.285	-35.801	
3.562.430	-35.800	
3.562.564	-35.799	
3.562.742	-35.798	
3.562.919	-35.797	
3.563.102	-35.796	

3.563.302	-35.795	
3.563.452	-35.794	
3.563.640	-35.793	
3.563.810	-35.792	
3.563.958	-35.791	
3.564.120	-35.790	
3.564.283	-35.789	
3.564.447	-35.788	
3.564.557	-35.787	
3.564.749	-35.786	
3.564.988	-35.786	
3.565.187	-35.785	
3.565.323	-35.784	
3.565.412	-35.783	
3.565.602	-35.782	
3.565.757	-35.781	
3.565.873	-35.780	
3.566.078	-35.779	
3.566.255	-35.778	
3.566.404	-35.777	
3.566.589	-35.776	
3.566.783	-35.775	
3.566.881	-35.774	
3.566.996	-35.773	
3.567.231	-35.772	
3.567.393	-35.771	
3.567.516	-35.770	
3.567.713	-35.769	
3.567.886	-35.768	
3.568.066	-35.767	

3.568.177	-35.766	
3.568.251	-35.764	
3.568.428	-35.763	
3.568.663	-35.762	
3.568.889	-35.761	
3.569.044	-35.760	
3.569.189	-35.759	
3.569.427	-35.758	
3.569.674	-35.757	
3.569.969	-35.756	
3.570.155	-35.754	
3.570.266	-35.753	
3.570.378	-35.752	
3.570.509	-35.751	
3.570.699	-35.750	
3.570.829	-35.748	
3.570.883	-35.747	
3.571.071	-35.746	
3.571.314	-35.745	
3.571.437	-35.743	
3.571.575	-35.742	
3.571.686	-35.741	
3.571.893	-35.739	
3.572.159	-35.738	
3.572.312	-35.737	
3.572.444	-35.735	
3.572.657	-35.734	
3.572.888	-35.733	
3.573.011	-35.731	
3.573.125	-35.730	

3.573.340	-35.728	
3.573.539	-35.727	
3.573.636	-35.726	
3.573.796	-35.724	
3.573.976	-35.723	
3.574.142	-35.721	
3.574.303	-35.720	
3.574.521	-35.718	
3.574.778	-35.717	
3.574.974	-35.715	
3.575.099	-35.714	
3.575.183	-35.713	
3.575.346	-35.711	
3.575.566	-35.710	
3.575.761	-35.708	
3.575.898	-35.707	
3.576.031	-35.705	
3.576.213	-35.704	
3.576.408	-35.702	
3.576.582	-35.701	
3.576.715	-35.699	
3.576.838	-35.697	
3.577.001	-35.696	
3.577.167	-35.694	
3.577.405	-35.693	
3.577.592	-35.691	
3.577.742	-35.690	
3.577.888	-35.688	
3.578.065	-35.686	
3.578.255	-35.685	

3.578.322	-35.683	
3.578.488	-35.681	
3.578.934	-35.680	
3.579.421	-35.678	
3.579.606	-35.677	
3.579.714	-35.675	
3.579.799	-35.673	
3.579.850	-35.672	
3.579.940	-35.670	
3.580.023	-35.668	
3.580.042	-35.666	
3.580.168	-35.665	
3.580.417	-35.663	
3.580.583	-35.661	
3.580.760	-35.659	
3.580.916	-35.658	
3.581.044	-35.656	
3.581.202	-35.654	
3.581.393	-35.652	
3.581.629	-35.650	
3.581.833	-35.649	
3.581.982	-35.647	
3.582.087	-35.645	
3.582.204	-35.643	
3.582.412	-35.641	
3.582.592	-35.639	
3.582.708	-35.638	
3.582.881	-35.636	
3.583.095	-35.634	
3.583.258	-35.632	

3.583.362	-35.630	
3.583.460	-35.628	
3.583.605	-35.626	
3.583.822	-35.624	
3.584.030	-35.623	
3.584.211	-35.621	
3.584.410	-35.619	
3.584.637	-35.617	
3.584.818	-35.615	
3.584.937	-35.613	
3.585.075	-35.611	
3.585.245	-35.609	
3.585.428	-35.608	
3.585.598	-35.606	
3.585.722	-35.604	
3.585.862	-35.602	
3.586.057	-35.600	
3.586.270	-35.598	
3.586.462	-35.596	
3.586.613	-35.594	
3.586.747	-35.592	
3.586.913	-35.591	
3.587.058	-35.589	
3.587.200	-35.587	
3.587.375	-35.585	
3.587.599	-35.583	
3.587.798	-35.581	
3.587.856	-35.579	
3.588.016	-35.577	
3.588.213	-35.575	

3.588.385	-35.574	
3.588.626	-35.572	
3.588.831	-35.570	
3.588.994	-35.568	
3.589.157	-35.566	
3.589.292	-35.564	
3.589.421	-35.562	
3.589.622	-35.560	
3.589.793	-35.558	
3.589.955	-35.556	
3.590.154	-35.554	
3.590.288	-35.553	
3.590.401	-35.551	
3.590.596	-35.549	
3.590.862	-35.547	
3.591.039	-35.545	
3.591.129	-35.543	
3.591.266	-35.541	
3.591.463	-35.538	
3.591.559	-35.536	
3.591.701	-35.534	
3.591.910	-35.532	
3.592.029	-35.530	
3.592.166	-35.528	
3.592.332	-35.526	
3.592.488	-35.524	
3.592.643	-35.521	
3.592.820	-35.519	
3.592.979	-35.517	
3.593.038	-35.515	

3.593.225	-35.512	
3.593.521	-35.510	
3.593.731	-35.508	
3.593.945	-35.506	
3.594.191	-35.503	
3.594.413	-35.501	
3.594.565	-35.499	
3.594.709	-35.496	
3.594.877	-35.494	
3.595.039	-35.491	
3.595.175	-35.489	
3.595.274	-35.487	
3.595.439	-35.484	
3.595.625	-35.482	
3.595.757	-35.479	
3.595.858	-35.476	
3.595.957	-35.474	
3.596.112	-35.471	
3.596.360	-35.469	
3.596.622	-35.466	
3.596.801	-35.464	
3.596.986	-35.461	
3.597.133	-35.458	
3.597.289	-35.456	
3.597.470	-35.453	
3.597.662	-35.450	
3.597.875	-35.447	
3.597.993	-35.445	
3.598.141	-35.442	
3.598.312	-35.439	

3.598.472	-35.436	
3.598.701	-35.434	
3.598.851	-35.431	
3.598.941	-35.428	
3.599.104	-35.425	
3.599.305	-35.422	
3.599.468	-35.420	
3.599.677	-35.417	
3.599.942	-35.414	
3.600.086	-35.411	
3.600.180	-35.408	
3.600.346	-35.405	
3.600.513	-35.402	
3.600.656	-35.399	
3.600.816	-35.397	
3.601.028	-35.394	
3.601.156	-35.391	
3.601.313	-35.388	
3.601.516	-35.385	
3.601.714	-35.382	
3.601.903	-35.379	
3.602.057	-35.376	
3.602.245	-35.373	
3.602.390	-35.370	
3.602.493	-35.367	
3.602.657	-35.364	
3.602.828	-35.361	
3.603.163	-35.358	
3.603.612	-35.355	
3.603.890	-35.352	

3.604.016	-35.349	
3.604.109	-35.345	
3.604.227	-35.342	
3.604.244	-35.339	
3.604.263	-35.336	
3.604.391	-35.333	
3.604.518	-35.330	
3.604.610	-35.327	
3.604.738	-35.324	
3.604.958	-35.321	
3.605.139	-35.318	
3.605.306	-35.314	
3.605.486	-35.311	
3.605.677	-35.308	
3.605.891	-35.305	
3.606.097	-35.302	
3.606.216	-35.299	
3.606.325	-35.296	
3.606.476	-35.292	
3.606.671	-35.289	
3.606.837	-35.286	
3.606.978	-35.283	
3.607.144	-35.280	
3.607.352	-35.277	
3.607.553	-35.274	
3.607.639	-35.271	
3.607.717	-35.268	
3.607.899	-35.265	
3.608.136	-35.261	
3.608.344	-35.258	

3.608.441	-35.255	
3.608.597	-35.252	
3.608.909	-35.249	
3.609.062	-35.246	
3.609.189	-35.243	
3.609.409	-35.240	
3.609.570	-35.237	
3.609.753	-35.234	
3.609.927	-35.231	
3.610.065	-35.228	
3.610.201	-35.225	
3.610.328	-35.222	
3.610.459	-35.219	
3.610.692	-35.216	
3.610.865	-35.213	
3.611.010	-35.210	
3.611.191	-35.207	
3.611.389	-35.204	
3.611.552	-35.201	
3.611.643	-35.198	
3.611.784	-35.195	
3.611.964	-35.192	
3.612.180	-35.189	
3.612.343	-35.187	
3.612.491	-35.184	
3.612.651	-35.181	
3.612.807	-35.178	
3.612.958	-35.175	
3.613.145	-35.172	
3.613.378	-35.169	

3.613.564	-35.166	
3.613.705	-35.163	
3.613.853	-35.160	
3.614.001	-35.158	
3.614.138	-35.155	
3.614.301	-35.152	
3.614.464	-35.149	
3.614.720	-35.146	
3.614.964	-35.143	
3.615.068	-35.140	
3.615.186	-35.137	
3.615.360	-35.134	
3.615.578	-35.131	
3.615.751	-35.128	
3.615.866	-35.125	
3.616.019	-35.122	
3.616.191	-35.119	
3.616.333	-35.116	
3.616.494	-35.113	
3.616.588	-35.110	
3.616.650	-35.107	
3.616.844	-35.104	
3.617.050	-35.101	
3.617.229	-35.098	
3.617.419	-35.095	
3.617.574	-35.092	
3.617.781	-35.089	
3.618.023	-35.086	
3.618.226	-35.082	
3.618.372	-35.079	

3.618.572	-35.076	
3.618.802	-35.073	
3.618.966	-35.070	
3.619.068	-35.066	
3.619.176	-35.063	
3.619.319	-35.060	
3.619.443	-35.057	
3.619.561	-35.053	
3.619.713	-35.050	
3.619.895	-35.047	
3.620.096	-35.043	
3.620.273	-35.040	
3.620.428	-35.037	
3.620.612	-35.033	
3.620.779	-35.030	
3.620.968	-35.026	
3.621.208	-35.023	
3.621.436	-35.020	
3.621.626	-35.016	
3.621.727	-35.013	
3.621.902	-35.009	
3.622.106	-35.006	
3.622.286	-35.002	
3.622.438	-34.999	
3.622.587	-34.995	
3.622.758	-34.991	
3.622.882	-34.988	
3.623.006	-34.984	
3.623.203	-34.981	
3.623.409	-34.977	

3.623.525	-34.973	
3.623.690	-34.970	
3.623.886	-34.966	
3.624.059	-34.962	
3.624.207	-34.959	
3.624.367	-34.955	
3.624.543	-34.951	
3.624.731	-34.948	
3.624.890	-34.944	
3.625.031	-34.940	
3.625.182	-34.936	
3.625.381	-34.933	
3.625.563	-34.929	
3.625.677	-34.925	
3.625.847	-34.921	
3.626.093	-34.918	
3.626.299	-34.914	
3.626.436	-34.910	
3.626.626	-34.906	
3.626.823	-34.903	
3.626.904	-34.899	
3.627.090	-34.895	
3.627.528	-34.891	
3.627.870	-34.887	
3.628.015	-34.884	
3.628.091	-34.880	
3.628.195	-34.876	
3.628.315	-34.872	
3.628.374	-34.869	
3.628.445	-34.865	

3.628.551	-34.861	
3.628.672	-34.857	
3.628.846	-34.854	
3.629.037	-34.850	
3.629.201	-34.846	
3.629.337	-34.842	
3.629.515	-34.839	
3.629.722	-34.835	
3.629.865	-34.831	
3.630.042	-34.828	
3.630.231	-34.824	
3.630.390	-34.820	
3.630.545	-34.817	
3.630.697	-34.813	
3.630.836	-34.809	
3.631.026	-34.806	
3.631.185	-34.802	
3.631.271	-34.799	
3.631.400	-34.795	
3.631.620	-34.792	
3.631.837	-34.788	
3.631.990	-34.785	
3.632.131	-34.781	
3.632.299	-34.778	
3.632.456	-34.775	
3.632.648	-34.771	
3.632.896	-34.768	
3.633.059	-34.765	
3.633.243	-34.762	
3.633.428	-34.758	

3.633.564	-34.755	
3.633.703	-34.752	
3.633.893	-34.749	
3.634.077	-34.746	
3.634.182	-34.743	
3.634.328	-34.740	
3.634.496	-34.737	
3.634.673	-34.734	
3.634.859	-34.731	
3.634.977	-34.728	
3.635.133	-34.725	
3.635.318	-34.723	
3.635.511	-34.720	
3.635.724	-34.717	
3.635.858	-34.714	
3.635.943	-34.712	
3.636.100	-34.709	
3.636.322	-34.706	
3.636.505	-34.704	
3.636.618	-34.701	
3.636.783	-34.699	
3.637.055	-34.696	
3.637.228	-34.694	
3.637.359	-34.691	
3.637.493	-34.689	
3.637.668	-34.687	
3.637.882	-34.684	
3.637.964	-34.682	
3.638.084	-34.680	
3.638.311	-34.678	

3.638.506	-34.675	
3.638.667	-34.673	
3.638.831	-34.671	
3.639.042	-34.669	
3.639.197	-34.667	
3.639.316	-34.665	
3.639.503	-34.662	
3.639.677	-34.660	
3.639.828	-34.658	
3.639.971	-34.656	
3.640.121	-34.654	
3.640.237	-34.652	
3.640.368	-34.650	
3.640.516	-34.648	
3.640.699	-34.646	
3.640.880	-34.644	
3.641.033	-34.642	
3.641.177	-34.640	
3.641.306	-34.638	
3.641.526	-34.636	
3.641.797	-34.634	
3.642.008	-34.633	
3.642.184	-34.631	
3.642.372	-34.629	
3.642.589	-34.627	
3.642.752	-34.625	
3.642.881	-34.623	
3.643.076	-34.621	
3.643.258	-34.619	
3.643.406	-34.617	

3.643.568	-34.615	
3.643.655	-34.613	
3.643.821	-34.611	
3.643.973	-34.609	
3.644.085	-34.608	
3.644.250	-34.606	
3.644.377	-34.604	
3.644.570	-34.602	
3.644.767	-34.600	
3.644.930	-34.598	
3.645.086	-34.596	
3.645.324	-34.594	
3.645.560	-34.592	
3.645.645	-34.590	
3.645.804	-34.588	
3.645.974	-34.586	
3.646.137	-34.584	
3.646.360	-34.582	
3.646.526	-34.579	
3.646.722	-34.577	
3.646.886	-34.575	
3.647.029	-34.573	
3.647.232	-34.571	
3.647.390	-34.569	
3.647.469	-34.567	
3.647.633	-34.565	
3.647.853	-34.562	
3.648.034	-34.560	
3.648.275	-34.558	
3.648.470	-34.556	

3.648.564	-34.553	
3.648.666	-34.551	
3.648.860	-34.549	
3.649.045	-34.546	
3.649.204	-34.544	
3.649.438	-34.542	
3.649.594	-34.539	
3.649.687	-34.537	
3.649.870	-34.535	
3.650.058	-34.532	
3.650.222	-34.530	
3.650.389	-34.527	
3.650.555	-34.525	
3.650.726	-34.522	
3.650.874	-34.520	
3.650.997	-34.517	
3.651.306	-34.514	
3.651.797	-34.512	
3.652.119	-34.509	
3.652.216	-34.506	
3.652.210	-34.504	
3.652.216	-34.501	
3.652.305	-34.498	
3.652.426	-34.496	
3.652.545	-34.493	
3.652.684	-34.490	
3.652.778	-34.487	
3.652.914	-34.484	
3.653.110	-34.481	
3.653.293	-34.478	

3.653.463	-34.476	
3.653.732	-34.473	
3.653.956	-34.470	
3.654.073	-34.467	
3.654.283	-34.464	
3.654.451	-34.461	
3.654.558	-34.458	
3.654.677	-34.454	
3.654.862	-34.451	
3.655.063	-34.448	
3.655.139	-34.445	
3.655.287	-34.442	
3.655.562	-34.439	
3.655.777	-34.436	
3.655.849	-34.433	
3.655.902	-34.430	
3.656.014	-34.426	
3.656.257	-34.423	
3.656.528	-34.420	
3.656.678	-34.417	
3.656.900	-34.414	
3.657.059	-34.410	
3.657.272	-34.407	
3.657.473	-34.404	
3.657.593	-34.401	
3.657.802	-34.397	
3.657.933	-34.394	
3.658.027	-34.391	
3.658.132	-34.388	
3.658.264	-34.384	

3.658.499	-34.381	
3.658.695	-34.378	
3.658.847	-34.374	
3.659.026	-34.371	
3.659.149	-34.368	
3.659.335	-34.365	
3.659.558	-34.361	
3.659.695	-34.358	
3.659.841	-34.355	
3.659.992	-34.351	
3.660.083	-34.348	
3.660.190	-34.345	
3.660.394	-34.342	
3.660.609	-34.338	
3.660.787	-34.335	
3.661.006	-34.332	
3.661.201	-34.328	
3.661.347	-34.325	
3.661.544	-34.322	
3.661.762	-34.319	
3.661.855	-34.315	
3.661.936	-34.312	
3.662.162	-34.309	
3.662.339	-34.305	
3.662.445	-34.302	
3.662.650	-34.299	
3.662.809	-34.296	
3.662.975	-34.292	
3.663.212	-34.289	
3.663.366	-34.286	

3.663.471	-34.282	
3.663.589	-34.279	
3.663.828	-34.276	
3.663.958	-34.272	
3.664.052	-34.269	
3.664.261	-34.265	
3.664.447	-34.262	
3.664.569	-34.259	
3.664.728	-34.255	
3.664.879	-34.252	
3.665.023	-34.248	
3.665.237	-34.245	
3.665.404	-34.242	
3.665.553	-34.238	
3.665.755	-34.235	
3.665.977	-34.231	
3.666.187	-34.228	
3.666.389	-34.224	
3.666.654	-34.221	
3.666.868	-34.217	
3.666.947	-34.214	
3.667.074	-34.210	
3.667.326	-34.207	
3.667.491	-34.203	
3.667.563	-34.200	
3.667.646	-34.196	
3.667.741	-34.192	
3.667.890	-34.189	
3.668.055	-34.185	
3.668.217	-34.181	

3.668.372	-34.178	
3.668.553	-34.174	
3.668.774	-34.171	
3.668.981	-34.167	
3.669.149	-34.163	
3.669.363	-34.160	
3.669.581	-34.156	
3.669.685	-34.152	
3.669.803	-34.148	
3.669.993	-34.145	
3.670.244	-34.141	
3.670.406	-34.137	
3.670.516	-34.134	
3.670.704	-34.130	
3.670.845	-34.126	
3.670.961	-34.122	
3.671.148	-34.118	
3.671.313	-34.115	
3.671.514	-34.111	
3.671.750	-34.107	
3.671.842	-34.103	
3.672.012	-34.100	
3.672.242	-34.096	
3.672.384	-34.092	
3.672.554	-34.088	
3.672.776	-34.084	
3.672.987	-34.081	
3.673.152	-34.077	
3.673.293	-34.073	
3.673.441	-34.069	

3.673.615	-34.065	
3.673.849	-34.061	
3.674.030	-34.057	
3.674.157	-34.054	
3.674.368	-34.050	
3.674.529	-34.046	
3.674.655	-34.042	
3.674.794	-34.038	
3.674.911	-34.034	
3.675.056	-34.030	
3.675.305	-34.026	
3.675.692	-34.023	
3.676.087	-34.019	
3.676.310	-34.015	
3.676.375	-34.011	
3.676.476	-34.007	
3.676.494	-34.003	
3.676.508	-33.999	
3.676.628	-33.995	
3.676.772	-33.991	
3.676.881	-33.988	
3.676.990	-33.984	
3.677.195	-33.980	
3.677.376	-33.976	
3.677.563	-33.972	
3.677.769	-33.968	
3.677.925	-33.964	
3.678.078	-33.960	
3.678.230	-33.956	
3.678.452	-33.953	

3.678.608	-33.949	
3.678.737	-33.945	
3.678.929	-33.941	
3.679.070	-33.937	
3.679.219	-33.933	
3.679.395	-33.930	
3.679.605	-33.926	
3.679.688	-33.922	
3.679.754	-33.918	
3.679.980	-33.914	
3.680.227	-33.911	
3.680.441	-33.907	
3.680.581	-33.903	
3.680.750	-33.899	
3.680.941	-33.896	
3.681.156	-33.892	
3.681.357	-33.888	
3.681.570	-33.885	
3.681.718	-33.881	
3.681.853	-33.877	
3.682.033	-33.874	
3.682.086	-33.870	
3.682.247	-33.866	
3.682.459	-33.863	
3.682.578	-33.859	
3.682.682	-33.856	
3.682.847	-33.852	
3.683.029	-33.849	
3.683.250	-33.845	
3.683.463	-33.842	

3.683.597	-33.838	
3.683.705	-33.835	
3.683.858	-33.831	
3.684.113	-33.828	
3.684.286	-33.824	
3.684.370	-33.821	
3.684.498	-33.817	
3.684.686	-33.814	
3.684.893	-33.810	
3.685.117	-33.807	
3.685.360	-33.804	
3.685.562	-33.800	
3.685.703	-33.797	
3.685.829	-33.794	
3.685.975	-33.790	
3.686.040	-33.787	
3.686.163	-33.784	
3.686.320	-33.780	
3.686.432	-33.777	
3.686.607	-33.774	
3.686.824	-33.770	
3.687.076	-33.767	
3.687.307	-33.764	
3.687.491	-33.760	
3.687.632	-33.757	
3.687.697	-33.753	
3.687.820	-33.750	
3.688.083	-33.747	
3.688.271	-33.743	
3.688.401	-33.740	

3.688.560	-33.737	
3.688.701	-33.733	
3.688.828	-33.730	
3.688.985	-33.727	
3.689.126	-33.723	
3.689.272	-33.720	
3.689.404	-33.716	
3.689.635	-33.713	
3.689.872	-33.709	
3.690.006	-33.706	
3.690.230	-33.703	
3.690.504	-33.699	
3.690.679	-33.696	
3.690.786	-33.692	
3.690.948	-33.689	
3.691.156	-33.685	
3.691.331	-33.682	
3.691.438	-33.678	
3.691.532	-33.675	
3.691.732	-33.671	
3.691.924	-33.667	
3.692.014	-33.664	
3.692.134	-33.660	
3.692.316	-33.657	
3.692.567	-33.653	
3.692.749	-33.649	
3.692.850	-33.646	
3.693.034	-33.642	
3.693.243	-33.638	
3.693.424	-33.634	

3.693.622	-33.631	
3.693.814	-33.627	
3.693.916	-33.623	
3.694.067	-33.619	
3.694.269	-33.616	
3.694.439	-33.612	
3.694.615	-33.608	
3.694.803	-33.604	
3.694.919	-33.600	
3.695.060	-33.596	
3.695.282	-33.592	
3.695.396	-33.589	
3.695.556	-33.585	
3.695.840	-33.581	
3.696.051	-33.577	
3.696.190	-33.573	
3.696.367	-33.569	
3.696.549	-33.565	
3.696.664	-33.561	
3.696.798	-33.557	
3.696.873	-33.553	
3.697.052	-33.548	
3.697.322	-33.544	
3.697.453	-33.540	
3.697.582	-33.536	
3.697.771	-33.532	
3.697.983	-33.528	
3.698.218	-33.524	
3.698.439	-33.519	
3.698.543	-33.515	

3.698.659	-33.511	
3.698.813	-33.507	
3.698.926	-33.503	
3.699.081	-33.498	
3.699.392	-33.494	
3.699.850	-33.490	
3.700.168	-33.485	
3.700.321	-33.481	
3.700.365	-33.477	
3.700.432	-33.473	
3.700.526	-33.468	
3.700.502	-33.464	
3.700.571	-33.459	
3.700.724	-33.455	
3.700.870	-33.451	
3.701.022	-33.446	
3.701.199	-33.442	
3.701.436	-33.437	
3.701.644	-33.433	
3.701.868	-33.429	
3.702.043	-33.424	
3.702.195	-33.420	
3.702.383	-33.415	
3.702.542	-33.411	
3.702.716	-33.406	
3.702.810	-33.402	
3.702.930	-33.397	
3.703.157	-33.393	
3.703.398	-33.389	
3.703.593	-33.384	

3.703.720	-33.380	
3.703.820	-33.375	
3.703.911	-33.371	
3.704.068	-33.366	
3.704.245	-33.362	
3.704.428	-33.357	
3.704.644	-33.353	
3.704.816	-33.349	
3.704.940	-33.344	
3.705.108	-33.340	
3.705.424	-33.335	
3.705.674	-33.331	
3.705.733	-33.326	
3.705.872	-33.322	
3.706.090	-33.317	
3.706.216	-33.313	
3.706.289	-33.309	
3.706.462	-33.304	
3.706.665	-33.300	
3.706.805	-33.295	
3.706.983	-33.291	
3.707.141	-33.287	
3.707.297	-33.282	
3.707.524	-33.278	
3.707.708	-33.274	
3.707.806	-33.269	
3.707.973	-33.265	
3.708.182	-33.261	
3.708.330	-33.256	
3.708.477	-33.252	

3.708.645	-33.248	
3.708.828	-33.243	
3.709.002	-33.239	
3.709.221	-33.235	
3.709.359	-33.230	
3.709.450	-33.226	
3.709.666	-33.222	
3.709.870	-33.218	
3.709.967	-33.213	
3.710.081	-33.209	
3.710.335	-33.205	
3.710.511	-33.201	
3.710.670	-33.196	
3.710.865	-33.192	
3.710.981	-33.188	
3.711.084	-33.183	
3.711.273	-33.179	
3.711.514	-33.175	
3.711.675	-33.171	
3.711.849	-33.166	
3.712.010	-33.162	
3.712.166	-33.158	
3.712.323	-33.153	
3.712.464	-33.149	
3.712.646	-33.145	
3.712.792	-33.140	
3.712.921	-33.136	
3.713.038	-33.132	
3.713.230	-33.127	
3.713.445	-33.123	

3.713.597	-33.119	
3.713.828	-33.114	
3.713.990	-33.110	
3.714.110	-33.105	
3.714.373	-33.101	
3.714.644	-33.097	
3.714.756	-33.092	
3.714.881	-33.088	
3.715.067	-33.083	
3.715.157	-33.079	
3.715.252	-33.075	
3.715.410	-33.070	
3.715.598	-33.066	
3.715.762	-33.061	
3.715.910	-33.057	
3.716.100	-33.052	
3.716.255	-33.048	
3.716.375	-33.043	
3.716.544	-33.039	
3.716.752	-33.034	
3.716.961	-33.029	
3.717.151	-33.025	
3.717.325	-33.020	
3.717.488	-33.016	
3.717.648	-33.011	
3.717.850	-33.007	
3.717.983	-33.002	
3.718.153	-32.997	
3.718.409	-32.993	
3.718.535	-32.988	

3.718.630	-32.984	
3.718.820	-32.979	
3.719.037	-32.974	
3.719.211	-32.970	
3.719.338	-32.965	
3.719.516	-32.960	
3.719.725	-32.956	
3.719.930	-32.951	
3.720.074	-32.947	
3.720.212	-32.942	
3.720.414	-32.937	
3.720.613	-32.933	
3.720.746	-32.928	
3.720.888	-32.923	
3.721.064	-32.919	
3.721.185	-32.914	
3.721.389	-32.909	
3.721.613	-32.905	
3.721.759	-32.900	
3.721.915	-32.895	
3.722.058	-32.891	
3.722.216	-32.886	
3.722.430	-32.881	
3.722.614	-32.877	
3.722.769	-32.872	
3.722.899	-32.867	
3.723.006	-32.863	
3.723.239	-32.858	
3.723.683	-32.854	
3.724.055	-32.849	

3.724.187	-32.844	
3.724.294	-32.840	
3.724.366	-32.835	
3.724.420	-32.830	
3.724.465	-32.826	
3.724.543	-32.821	
3.724.704	-32.817	
3.724.870	-32.812	
3.725.060	-32.807	
3.725.215	-32.803	
3.725.324	-32.798	
3.725.526	-32.794	
3.725.703	-32.789	
3.725.837	-32.784	
3.726.018	-32.780	
3.726.187	-32.775	
3.726.400	-32.771	
3.726.579	-32.766	
3.726.721	-32.762	
3.726.827	-32.757	
3.726.936	-32.753	
3.727.102	-32.748	
3.727.294	-32.744	
3.727.502	-32.739	
3.727.693	-32.735	
3.727.806	-32.730	
3.727.946	-32.726	
3.728.185	-32.722	
3.728.391	-32.717	
3.728.565	-32.713	

3.728.680	-32.709	
3.728.833	-32.704	
3.729.071	-32.700	
3.729.305	-32.696	
3.729.429	-32.691	
3.729.489	-32.687	
3.729.702	-32.683	
3.729.901	-32.679	
3.730.040	-32.674	
3.730.248	-32.670	
3.730.374	-32.666	
3.730.461	-32.662	
3.730.638	-32.658	
3.730.847	-32.654	
3.731.040	-32.649	
3.731.199	-32.645	
3.731.369	-32.641	
3.731.534	-32.637	
3.731.638	-32.633	
3.731.780	-32.629	
3.731.986	-32.625	
3.732.126	-32.621	
3.732.281	-32.617	
3.732.505	-32.613	
3.732.744	-32.609	
3.732.908	-32.606	
3.733.041	-32.602	
3.733.219	-32.598	
3.733.414	-32.594	
3.733.580	-32.590	

3.733.701	-32.586	
3.733.893	-32.582	
3.734.034	-32.579	
3.734.140	-32.575	
3.734.357	-32.571	
3.734.527	-32.567	
3.734.699	-32.563	
3.734.861	-32.559	
3.735.074	-32.556	
3.735.324	-32.552	
3.735.486	-32.548	
3.735.620	-32.544	
3.735.764	-32.540	
3.735.934	-32.537	
3.736.078	-32.533	
3.736.227	-32.529	
3.736.438	-32.525	
3.736.635	-32.521	
3.736.721	-32.517	
3.736.850	-32.514	
3.737.039	-32.510	
3.737.166	-32.506	
3.737.299	-32.502	
3.737.452	-32.498	
3.737.621	-32.494	
3.737.831	-32.490	
3.738.069	-32.486	
3.738.293	-32.482	
3.738.430	-32.478	
3.738.583	-32.474	

3.738.806	-32.470	
3.739.001	-32.466	
3.739.171	-32.462	
3.739.334	-32.458	
3.739.512	-32.454	
3.739.673	-32.450	
3.739.763	-32.446	
3.739.862	-32.442	
3.740.009	-32.438	
3.740.158	-32.434	
3.740.352	-32.429	
3.740.508	-32.425	
3.740.642	-32.421	
3.740.830	-32.417	
3.741.104	-32.412	
3.741.355	-32.408	
3.741.541	-32.404	
3.741.711	-32.399	
3.741.839	-32.395	
3.741.982	-32.391	
3.742.098	-32.386	
3.742.278	-32.382	
3.742.438	-32.377	
3.742.523	-32.373	
3.742.664	-32.368	
3.742.881	-32.364	
3.743.060	-32.359	
3.743.286	-32.355	
3.743.488	-32.350	
3.743.619	-32.346	

3.743.790	-32.341	
3.743.972	-32.337	
3.744.164	-32.332	
3.744.348	-32.327	
3.744.461	-32.323	
3.744.615	-32.318	
3.744.787	-32.313	
3.744.971	-32.309	
3.745.123	-32.304	
3.745.294	-32.299	
3.745.486	-32.294	
3.745.652	-32.289	
3.745.800	-32.285	
3.745.992	-32.280	
3.746.201	-32.275	
3.746.320	-32.270	
3.746.454	-32.265	
3.746.611	-32.261	
3.746.765	-32.256	
3.746.943	-32.251	
3.747.164	-32.246	
3.747.553	-32.241	
3.747.929	-32.236	
3.748.136	-32.231	
3.748.208	-32.226	
3.748.248	-32.221	
3.748.374	-32.216	
3.748.370	-32.211	
3.748.398	-32.207	
3.748.604	-32.202	

3.748.728	-32.197	
3.748.807	-32.192	
3.749.012	-32.187	
3.749.182	-32.182	
3.749.359	-32.177	
3.749.602	-32.172	
3.749.881	-32.167	
3.750.119	-32.162	
3.750.186	-32.157	
3.750.317	-32.152	
3.750.537	-32.147	
3.750.612	-32.142	
3.750.635	-32.137	
3.750.881	-32.132	
3.751.133	-32.127	
3.751.300	-32.122	
3.751.465	-32.117	
3.751.687	-32.113	
3.751.815	-32.108	
3.751.849	-32.103	
3.752.033	-32.098	
3.752.260	-32.093	
3.752.484	-32.088	
3.752.662	-32.084	
3.752.850	-32.079	
3.753.069	-32.074	
3.753.188	-32.069	
3.753.331	-32.065	
3.753.468	-32.060	
3.753.647	-32.055	

3.753.865	-32.050	
3.754.008	-32.046	
3.754.075	-32.041	
3.754.240	-32.037	
3.754.422	-32.032	
3.754.561	-32.027	
3.754.731	-32.023	
3.754.891	-32.018	
3.755.085	-32.014	
3.755.245	-32.010	
3.755.411	-32.005	
3.755.596	-32.001	
3.755.757	-31.996	
3.755.863	-31.992	
3.756.006	-31.988	
3.756.210	-31.983	
3.756.353	-31.979	
3.756.491	-31.975	
3.756.667	-31.971	
3.756.877	-31.967	
3.757.121	-31.963	
3.757.321	-31.958	
3.757.484	-31.954	
3.757.628	-31.950	
3.757.782	-31.946	
3.757.968	-31.942	
3.758.114	-31.938	
3.758.243	-31.935	
3.758.354	-31.931	
3.758.525	-31.927	

3.758.743	-31.923	
3.758.891	-31.919	
3.759.081	-31.915	
3.759.315	-31.912	
3.759.451	-31.908	
3.759.616	-31.904	
3.759.786	-31.901	
3.759.913	-31.897	
3.760.072	-31.893	
3.760.215	-31.890	
3.760.389	-31.886	
3.760.556	-31.883	
3.760.695	-31.879	
3.760.871	-31.876	
3.761.040	-31.872	
3.761.154	-31.869	
3.761.306	-31.865	
3.761.542	-31.862	
3.761.762	-31.858	
3.761.958	-31.855	
3.762.151	-31.852	
3.762.299	-31.848	
3.762.496	-31.845	
3.762.704	-31.842	
3.762.809	-31.839	
3.762.990	-31.835	
3.763.177	-31.832	
3.763.307	-31.829	
3.763.428	-31.826	
3.763.575	-31.823	

3.763.756	-31.819	
3.763.911	-31.816	
3.764.081	-31.813	
3.764.262	-31.810	
3.764.362	-31.807	
3.764.461	-31.804	
3.764.674	-31.801	
3.764.887	-31.798	
3.765.096	-31.795	
3.765.278	-31.792	
3.765.487	-31.789	
3.765.674	-31.786	
3.765.775	-31.783	
3.765.923	-31.780	
3.766.143	-31.777	
3.766.393	-31.774	
3.766.541	-31.771	
3.766.664	-31.768	
3.766.839	-31.765	
3.767.018	-31.762	
3.767.229	-31.759	
3.767.424	-31.756	
3.767.585	-31.753	
3.767.723	-31.751	
3.767.845	-31.748	
3.768.011	-31.745	
3.768.222	-31.742	
3.768.409	-31.739	
3.768.623	-31.736	
3.768.777	-31.734	

3.768.857	-31.731	
3.768.990	-31.728	
3.769.172	-31.725	
3.769.366	-31.722	
3.769.559	-31.720	
3.769.734	-31.717	
3.769.945	-31.714	
3.770.130	-31.712	
3.770.266	-31.709	
3.770.403	-31.706	
3.770.522	-31.703	
3.770.678	-31.701	
3.770.808	-31.698	
3.771.102	-31.695	
3.771.559	-31.693	
3.771.906	-31.690	
3.772.068	-31.688	
3.772.097	-31.685	
3.772.173	-31.682	
3.772.269	-31.680	
3.772.350	-31.677	
3.772.431	-31.674	
3.772.503	-31.672	
3.772.639	-31.669	
3.772.773	-31.667	
3.772.899	-31.664	
3.773.083	-31.662	
3.773.330	-31.659	
3.773.560	-31.656	
3.773.728	-31.654	

3.773.885	-31.651	
3.774.023	-31.649	
3.774.171	-31.646	
3.774.313	-31.644	
3.774.522	-31.641	
3.774.829	-31.639	
3.775.016	-31.636	
3.775.118	-31.634	
3.775.269	-31.631	
3.775.403	-31.629	
3.775.500	-31.626	
3.775.693	-31.624	
3.775.898	-31.621	
3.776.031	-31.619	
3.776.188	-31.617	
3.776.376	-31.614	
3.776.550	-31.612	
3.776.714	-31.609	
3.776.891	-31.607	
3.777.068	-31.604	
3.777.234	-31.602	
3.777.427	-31.599	
3.777.603	-31.597	
3.777.702	-31.595	
3.777.843	-31.592	
3.778.060	-31.590	
3.778.233	-31.587	
3.778.320	-31.585	
3.778.454	-31.582	
3.778.654	-31.580	

3.778.854	-31.578	
3.779.035	-31.575	
3.779.197	-31.573	
3.779.330	-31.570	
3.779.456	-31.568	
3.779.670	-31.565	
3.779.958	-31.563	
3.780.104	-31.560	
3.780.168	-31.558	
3.780.375	-31.556	
3.780.558	-31.553	
3.780.612	-31.551	
3.780.750	-31.548	
3.780.981	-31.546	
3.781.130	-31.543	
3.781.314	-31.540	
3.781.557	-31.538	
3.781.703	-31.535	
3.781.820	-31.533	
3.781.967	-31.530	
3.782.173	-31.528	
3.782.433	-31.525	
3.782.621	-31.522	
3.782.755	-31.520	
3.782.923	-31.517	
3.783.120	-31.514	
3.783.279	-31.511	
3.783.441	-31.509	
3.783.572	-31.506	
3.783.695	-31.503	

3.783.860	-31.500	
3.784.050	-31.497	
3.784.208	-31.495	
3.784.359	-31.492	
3.784.472	-31.489	
3.784.529	-31.486	
3.784.711	-31.483	
3.784.923	-31.480	
3.785.141	-31.477	
3.785.390	-31.474	
3.785.534	-31.471	
3.785.670	-31.468	
3.785.873	-31.464	
3.786.069	-31.461	
3.786.176	-31.458	
3.786.333	-31.455	
3.786.519	-31.451	
3.786.696	-31.448	
3.786.795	-31.445	
3.786.967	-31.441	
3.787.173	-31.438	
3.787.283	-31.435	
3.787.409	-31.431	
3.787.550	-31.428	
3.787.729	-31.424	
3.787.932	-31.420	
3.788.146	-31.417	
3.788.340	-31.413	
3.788.443	-31.409	
3.788.590	-31.406	

3.788.828	-31.402	
3.789.023	-31.398	
3.789.171	-31.394	
3.789.357	-31.391	
3.789.498	-31.387	
3.789.514	-31.383	
3.789.746	-31.379	
3.790.092	-31.375	
3.790.258	-31.371	
3.790.422	-31.367	
3.790.580	-31.363	
3.790.753	-31.359	
3.790.931	-31.355	
3.791.048	-31.351	
3.791.174	-31.347	
3.791.407	-31.342	
3.791.618	-31.338	
3.791.743	-31.334	
3.791.928	-31.330	
3.792.169	-31.325	
3.792.339	-31.321	
3.792.422	-31.317	
3.792.557	-31.312	
3.792.742	-31.308	
3.792.946	-31.303	
3.793.148	-31.299	
3.793.262	-31.295	
3.793.369	-31.290	
3.793.596	-31.286	
3.793.759	-31.281	

3.793.929	-31.277	
3.794.092	-31.272	
3.794.250	-31.267	
3.794.432	-31.263	
3.794.547	-31.258	
3.794.680	-31.254	
3.795.068	-31.249	
3.795.536	-31.244	
3.795.735	-31.240	
3.795.822	-31.235	
3.795.935	-31.230	
3.796.006	-31.225	
3.796.040	-31.221	
3.796.084	-31.216	
3.796.158	-31.211	
3.796.289	-31.206	
3.796.422	-31.201	
3.796.592	-31.197	
3.796.852	-31.192	
3.797.039	-31.187	
3.797.148	-31.182	
3.797.362	-31.177	
3.797.560	-31.172	
3.797.722	-31.168	
3.797.881	-31.163	
3.798.030	-31.158	
3.798.181	-31.153	
3.798.344	-31.148	
3.798.586	-31.143	
3.798.786	-31.138	

3.798.894	-31.133	
3.799.008	-31.129	
3.799.211	-31.124	
3.799.398	-31.119	
3.799.532	-31.114	
3.799.691	-31.109	
3.799.843	-31.104	
3.800.020	-31.100	
3.800.227	-31.095	
3.800.388	-31.090	
3.800.580	-31.085	
3.800.804	-31.080	
3.801.006	-31.076	
3.801.156	-31.071	
3.801.187	-31.066	
3.801.306	-31.061	
3.801.495	-31.057	
3.801.722	-31.052	
3.801.891	-31.047	
3.801.998	-31.042	
3.802.238	-31.038	
3.802.393	-31.033	
3.802.520	-31.028	
3.802.659	-31.023	
3.802.829	-31.019	
3.803.062	-31.014	
3.803.273	-31.010	
3.803.454	-31.005	
3.803.506	-31.000	
3.803.591	-30.996	

3.803.807	-30.991	
3.803.963	-30.987	
3.804.154	-30.982	
3.804.373	-30.978	
3.804.557	-30.973	
3.804.724	-30.968	
3.804.865	-30.964	
3.805.028	-30.959	
3.805.151	-30.955	
3.805.213	-30.950	
3.805.406	-30.946	
3.805.710	-30.942	
3.805.887	-30.937	
3.806.078	-30.933	
3.806.256	-30.928	
3.806.337	-30.924	
3.806.514	-30.919	
3.806.762	-30.915	
3.806.960	-30.910	
3.807.152	-30.906	
3.807.344	-30.901	
3.807.500	-30.897	
3.807.643	-30.893	
3.807.720	-30.888	
3.807.838	-30.884	
3.808.041	-30.879	
3.808.222	-30.875	
3.808.342	-30.870	
3.808.477	-30.866	
3.808.665	-30.862	

3.808.824	-30.857	
3.808.990	-30.853	
3.809.168	-30.848	
3.809.321	-30.844	
3.809.545	-30.839	
3.809.779	-30.835	
3.809.930	-30.830	
3.810.103	-30.826	
3.810.292	-30.822	
3.810.479	-30.817	
3.810.717	-30.813	
3.810.883	-30.808	
3.811.003	-30.804	
3.811.131	-30.799	
3.811.187	-30.795	
3.811.337	-30.790	
3.811.535	-30.786	
3.811.611	-30.781	
3.811.716	-30.777	
3.811.942	-30.772	
3.812.113	-30.768	
3.812.264	-30.763	
3.812.487	-30.759	
3.812.708	-30.754	
3.812.861	-30.750	
3.813.026	-30.745	
3.813.222	-30.740	
3.813.464	-30.736	
3.813.680	-30.731	
3.813.808	-30.727	

3.813.930	-30.722	
3.814.089	-30.718	
3.814.297	-30.713	
3.814.500	-30.709	
3.814.677	-30.704	
3.814.800	-30.700	
3.814.948	-30.695	
3.815.121	-30.691	
3.815.266	-30.686	
3.815.470	-30.682	
3.815.715	-30.677	
3.815.856	-30.673	
3.815.925	-30.668	
3.816.104	-30.664	
3.816.331	-30.659	
3.816.459	-30.655	
3.816.581	-30.650	
3.816.790	-30.646	
3.817.014	-30.641	
3.817.177	-30.637	
3.817.312	-30.632	
3.817.485	-30.628	
3.817.665	-30.623	
3.817.784	-30.619	
3.817.954	-30.615	
3.818.130	-30.610	
3.818.239	-30.606	
3.818.510	-30.601	
3.818.927	-30.597	
3.819.196	-30.592	

3.819.359	-30.588	
3.819.519	-30.583	
3.819.598	-30.579	
3.819.659	-30.575	
3.819.783	-30.570	
3.819.883	-30.566	
3.819.919	-30.561	
3.820.005	-30.557	
3.820.244	-30.552	
3.820.450	-30.548	
3.820.486	-30.544	
3.820.625	-30.539	
3.820.899	-30.535	
3.821.149	-30.530	
3.821.344	-30.526	
3.821.510	-30.522	
3.821.678	-30.517	
3.821.795	-30.513	
3.821.951	-30.509	
3.822.155	-30.504	
3.822.303	-30.500	
3.822.456	-30.496	
3.822.612	-30.492	
3.822.777	-30.487	
3.822.948	-30.483	
3.823.088	-30.479	
3.823.261	-30.475	
3.823.481	-30.471	
3.823.716	-30.466	
3.823.890	-30.462	

3.824.032	-30.458	
3.824.149	-30.454	
3.824.334	-30.450	
3.824.496	-30.446	
3.824.579	-30.442	
3.824.733	-30.438	
3.824.904	-30.434	
3.825.096	-30.430	
3.825.298	-30.426	
3.825.466	-30.422	
3.825.627	-30.418	
3.825.735	-30.414	
3.825.869	-30.410	
3.826.058	-30.406	
3.826.227	-30.402	
3.826.436	-30.398	
3.826.618	-30.395	
3.826.750	-30.391	
3.826.873	-30.387	
3.827.000	-30.383	
3.827.185	-30.380	
3.827.383	-30.376	
3.827.535	-30.372	
3.827.747	-30.369	
3.827.950	-30.365	
3.828.112	-30.361	
3.828.360	-30.358	
3.828.613	-30.354	
3.828.690	-30.351	
3.828.742	-30.347	

3.828.927	-30.344	
3.829.082	-30.340	
3.829.263	-30.337	
3.829.500	-30.333	
3.829.702	-30.330	
3.829.899	-30.326	
3.830.063	-30.323	
3.830.213	-30.319	
3.830.374	-30.316	
3.830.553	-30.312	
3.830.718	-30.309	
3.830.841	-30.305	
3.830.977	-30.302	
3.831.156	-30.298	
3.831.311	-30.295	
3.831.484	-30.291	
3.831.624	-30.288	
3.831.689	-30.285	
3.831.794	-30.281	
3.832.029	-30.278	
3.832.220	-30.274	
3.832.332	-30.271	
3.832.474	-30.267	
3.832.690	-30.264	
3.832.888	-30.261	
3.833.033	-30.257	
3.833.224	-30.254	
3.833.442	-30.250	
3.833.692	-30.247	
3.833.857	-30.243	

3.833.938	-30.240	
3.834.131	-30.236	
3.834.399	-30.233	
3.834.525	-30.230	
3.834.572	-30.226	
3.834.757	-30.223	
3.834.966	-30.219	
3.835.045	-30.216	
3.835.157	-30.212	
3.835.320	-30.209	
3.835.464	-30.205	
3.835.635	-30.202	
3.835.841	-30.198	
3.836.060	-30.195	
3.836.224	-30.192	
3.836.357	-30.188	
3.836.508	-30.185	
3.836.729	-30.181	
3.836.942	-30.178	
3.837.149	-30.174	
3.837.358	-30.171	
3.837.487	-30.167	
3.837.632	-30.164	
3.837.799	-30.160	
3.838.024	-30.157	
3.838.218	-30.154	
3.838.365	-30.150	
3.838.524	-30.147	
3.838.638	-30.143	
3.838.790	-30.140	

3.838.981	-30.136	
3.839.167	-30.133	
3.839.393	-30.130	
3.839.601	-30.126	
3.839.717	-30.123	
3.839.804	-30.119	
3.839.884	-30.116	
3.840.038	-30.112	
3.840.224	-30.109	
3.840.422	-30.105	
3.840.661	-30.102	
3.840.845	-30.099	
3.841.031	-30.095	
3.841.196	-30.092	
3.841.375	-30.088	
3.841.574	-30.085	
3.841.667	-30.082	
3.841.776	-30.078	
3.841.950	-30.075	
3.842.243	-30.071	
3.842.621	-30.068	
3.842.925	-30.064	
3.843.112	-30.061	
3.843.194	-30.058	
3.843.264	-30.054	
3.843.323	-30.051	
3.843.366	-30.047	
3.843.488	-30.044	
3.843.595	-30.040	
3.843.702	-30.037	

3.843.896	-30.033	
3.844.086	-30.030	
3.844.310	-30.027	
3.844.504	-30.023	
3.844.663	-30.020	
3.844.825	-30.016	
3.845.027	-30.013	
3.845.193	-30.009	
3.845.302	-30.006	
3.845.464	-30.002	
3.845.618	-29.999	
3.845.753	-29.995	
3.845.941	-29.992	
3.846.143	-29.988	
3.846.315	-29.985	
3.846.467	-29.982	
3.846.599	-29.978	
3.846.791	-29.975	
3.847.003	-29.971	
3.847.184	-29.968	
3.847.330	-29.964	
3.847.491	-29.961	
3.847.751	-29.958	
3.847.951	-29.954	
3.848.066	-29.951	
3.848.177	-29.947	
3.848.344	-29.944	
3.848.571	-29.941	
3.848.753	-29.937	
3.848.872	-29.934	

3.848.962	-29.930	
3.849.100	-29.927	
3.849.268	-29.924	
3.849.442	-29.920	
3.849.609	-29.917	
3.849.775	-29.914	
3.849.998	-29.910	
3.850.193	-29.907	
3.850.356	-29.904	
3.850.523	-29.900	
3.850.699	-29.897	
3.850.871	-29.894	
3.850.954	-29.890	
3.851.017	-29.887	
3.851.176	-29.884	
3.851.449	-29.880	
3.851.694	-29.877	
3.851.852	-29.874	
3.852.026	-29.870	
3.852.191	-29.867	
3.852.332	-29.864	
3.852.523	-29.860	
3.852.762	-29.857	
3.852.906	-29.854	
3.853.037	-29.850	
3.853.229	-29.847	
3.853.441	-29.844	
3.853.612	-29.840	
3.853.734	-29.837	
3.853.914	-29.833	

3.854.074	-29.830	
3.854.197	-29.827	
3.854.431	-29.823	
3.854.606	-29.820	
3.854.731	-29.816	
3.854.940	-29.813	
3.855.060	-29.809	
3.855.121	-29.806	
3.855.244	-29.802	
3.855.412	-29.799	
3.855.547	-29.795	
3.855.733	-29.792	
3.855.854	-29.788	
3.856.010	-29.784	
3.856.206	-29.781	
3.856.380	-29.777	
3.856.549	-29.773	
3.856.776	-29.770	
3.857.040	-29.766	
3.857.287	-29.762	
3.857.471	-29.759	
3.857.592	-29.755	
3.857.681	-29.751	
3.857.850	-29.747	
3.858.004	-29.744	
3.858.105	-29.740	
3.858.271	-29.736	
3.858.423	-29.732	
3.858.608	-29.728	
3.858.790	-29.724	

3.858.882	-29.720	
3.858.976	-29.717	
3.859.150	-29.713	
3.859.393	-29.709	
3.859.645	-29.705	
3.859.825	-29.701	
3.859.953	-29.697	
3.860.151	-29.693	
3.860.320	-29.689	
3.860.479	-29.685	
3.860.688	-29.681	
3.860.852	-29.677	
3.861.024	-29.673	
3.861.234	-29.669	
3.861.425	-29.665	
3.861.566	-29.661	
3.861.689	-29.657	
3.861.845	-29.653	
3.861.962	-29.649	
3.862.153	-29.646	
3.862.310	-29.642	
3.862.507	-29.638	
3.862.697	-29.634	
3.862.805	-29.630	
3.862.932	-29.626	
3.863.125	-29.622	
3.863.338	-29.618	
3.863.456	-29.614	
3.863.605	-29.610	
3.863.820	-29.606	

3.863.983	-29.602	
3.864.104	-29.598	
3.864.276	-29.594	
3.864.409	-29.590	
3.864.601	-29.586	
3.864.850	-29.582	
3.865.029	-29.579	
3.865.193	-29.575	
3.865.323	-29.571	
3.865.473	-29.567	
3.865.689	-29.563	
3.866.026	-29.559	
3.866.371	-29.556	
3.866.618	-29.552	
3.866.754	-29.548	
3.866.779	-29.544	
3.866.848	-29.541	
3.866.944	-29.537	
3.867.008	-29.533	
3.867.097	-29.530	
3.867.258	-29.526	
3.867.435	-29.522	
3.867.579	-29.519	
3.867.729	-29.515	
3.867.897	-29.512	
3.868.128	-29.508	
3.868.360	-29.505	
3.868.525	-29.501	
3.868.698	-29.498	
3.868.847	-29.494	

3.868.966	-29.491	
3.869.102	-29.487	
3.869.248	-29.484	
3.869.431	-29.481	
3.869.659	-29.477	
3.869.844	-29.474	
3.870.017	-29.471	
3.870.121	-29.468	
3.870.229	-29.464	
3.870.410	-29.461	
3.870.599	-29.458	
3.870.754	-29.455	
3.870.970	-29.452	
3.871.228	-29.449	
3.871.401	-29.446	
3.871.519	-29.443	
3.871.643	-29.440	
3.871.837	-29.437	
3.871.949	-29.434	
3.872.094	-29.431	
3.872.318	-29.429	
3.872.499	-29.426	
3.872.701	-29.423	
3.872.788	-29.420	
3.872.883	-29.418	
3.873.071	-29.415	
3.873.294	-29.412	
3.873.441	-29.410	
3.873.522	-29.407	
3.873.734	-29.405	

3.873.965	-29.402	
3.874.168	-29.400	
3.874.326	-29.397	
3.874.461	-29.395	
3.874.550	-29.392	
3.874.646	-29.390	
3.874.877	-29.388	
3.875.117	-29.385	
3.875.316	-29.383	
3.875.444	-29.381	
3.875.549	-29.379	
3.875.721	-29.377	
3.875.948	-29.374	
3.876.120	-29.372	
3.876.205	-29.370	
3.876.425	-29.368	
3.876.703	-29.366	
3.876.828	-29.364	
3.876.971	-29.362	
3.877.157	-29.360	
3.877.272	-29.358	
3.877.441	-29.356	
3.877.677	-29.354	
3.877.863	-29.352	
3.878.013	-29.350	
3.878.156	-29.348	
3.878.297	-29.346	
3.878.504	-29.344	
3.878.602	-29.342	
3.878.759	-29.340	

3.879.005	-29.339	
3.879.073	-29.337	
3.879.107	-29.335	
3.879.204	-29.333	
3.879.420	-29.331	
3.879.691	-29.329	
3.879.917	-29.327	
3.880.042	-29.326	
3.880.182	-29.324	
3.880.418	-29.322	
3.880.717	-29.320	
3.880.945	-29.318	
3.881.029	-29.317	
3.881.138	-29.315	
3.881.306	-29.313	
3.881.498	-29.311	
3.881.660	-29.309	
3.881.808	-29.307	
3.881.997	-29.306	
3.882.188	-29.304	
3.882.316	-29.302	
3.882.352	-29.300	
3.882.426	-29.298	
3.882.640	-29.296	
3.882.836	-29.295	
3.883.022	-29.293	
3.883.246	-29.291	
3.883.329	-29.289	
3.883.500	-29.287	
3.883.767	-29.285	

3.884.041	-29.283	
3.884.290	-29.282	
3.884.391	-29.280	
3.884.503	-29.278	
3.884.674	-29.276	
3.884.852	-29.274	
3.885.034	-29.272	
3.885.195	-29.270	
3.885.341	-29.268	
3.885.527	-29.266	
3.885.714	-29.265	
3.885.885	-29.263	
3.886.098	-29.261	
3.886.198	-29.259	
3.886.248	-29.257	
3.886.462	-29.255	
3.886.682	-29.253	
3.886.862	-29.251	
3.887.088	-29.249	
3.887.236	-29.247	
3.887.314	-29.245	
3.887.493	-29.243	
3.887.672	-29.241	
3.887.832	-29.240	
3.888.011	-29.238	
3.888.192	-29.236	
3.888.417	-29.234	
3.888.557	-29.232	
3.888.672	-29.230	
3.888.849	-29.228	

3.888.943	-29.226	
3.889.044	-29.224	
3.889.362	-29.222	
3.889.778	-29.220	
3.890.069	-29.218	
3.890.266	-29.216	
3.890.346	-29.214	
3.890.417	-29.212	
3.890.529	-29.211	
3.890.536	-29.209	
3.890.595	-29.207	
3.890.750	-29.205	
3.890.901	-29.203	
3.891.032	-29.201	
3.891.137	-29.199	
3.891.364	-29.197	
3.891.595	-29.195	
3.891.751	-29.193	
3.891.968	-29.191	
3.892.195	-29.189	
3.892.350	-29.187	
3.892.484	-29.185	
3.892.628	-29.183	
3.892.802	-29.181	
3.892.991	-29.179	
3.893.172	-29.177	
3.893.311	-29.175	
3.893.508	-29.173	
3.893.716	-29.170	
3.893.799	-29.168	

3.893.902	-29.166	
3.894.077	-29.164	
3.894.258	-29.162	
3.894.422	-29.160	
3.894.674	-29.158	
3.894.883	-29.156	
3.895.024	-29.154	
3.895.190	-29.152	
3.895.346	-29.150	
3.895.505	-29.147	
3.895.612	-29.145	
3.895.739	-29.143	
3.895.932	-29.141	
3.896.107	-29.139	
3.896.273	-29.136	
3.896.476	-29.134	
3.896.649	-29.132	
3.896.783	-29.130	
3.896.888	-29.127	
3.897.008	-29.125	
3.897.213	-29.123	
3.897.422	-29.120	
3.897.635	-29.118	
3.897.782	-29.116	
3.897.930	-29.113	
3.898.076	-29.111	
3.898.141	-29.108	
3.898.365	-29.106	
3.898.611	-29.104	
3.898.770	-29.101	

3.899.002	-29.099	
3.899.125	-29.096	
3.899.250	-29.093	
3.899.449	-29.091	
3.899.663	-29.088	
3.899.866	-29.086	
3.899.991	-29.083	
3.900.132	-29.080	
3.900.295	-29.077	
3.900.422	-29.075	
3.900.598	-29.072	
3.900.878	-29.069	
3.901.082	-29.066	
3.901.176	-29.063	
3.901.354	-29.060	
3.901.568	-29.057	
3.901.698	-29.054	
3.901.774	-29.051	
3.901.891	-29.048	
3.902.062	-29.045	
3.902.257	-29.042	
3.902.462	-29.039	
3.902.513	-29.035	
3.902.535	-29.032	
3.902.698	-29.029	
3.902.935	-29.025	
3.903.149	-29.022	
3.903.333	-29.019	
3.903.528	-29.015	
3.903.719	-29.012	

3.903.926	-29.008	
3.904.095	-29.004	
3.904.274	-29.001	
3.904.503	-28.997	
3.904.721	-28.993	
3.904.837	-28.989	
3.904.941	-28.985	
3.905.126	-28.981	
3.905.262	-28.977	
3.905.363	-28.973	
3.905.540	-28.969	
3.905.667	-28.965	
3.905.789	-28.961	
3.905.981	-28.956	
3.906.144	-28.952	
3.906.346	-28.948	
3.906.603	-28.943	
3.906.715	-28.939	
3.906.891	-28.934	
3.907.231	-28.930	
3.907.401	-28.925	
3.907.516	-28.921	
3.907.665	-28.916	
3.907.839	-28.912	
3.907.991	-28.907	
3.908.143	-28.902	
3.908.363	-28.897	
3.908.477	-28.892	
3.908.662	-28.888	
3.908.949	-28.883	

3.909.106	-28.878	
3.909.232	-28.873	
3.909.353	-28.868	
3.909.557	-28.863	
3.909.758	-28.858	
3.909.868	-28.853	
3.910.031	-28.848	
3.910.201	-28.843	
3.910.332	-28.838	
3.910.475	-28.832	
3.910.627	-28.827	
3.910.815	-28.822	
3.911.019	-28.817	
3.911.178	-28.812	
3.911.378	-28.806	
3.911.561	-28.801	
3.911.689	-28.796	
3.911.846	-28.791	
3.912.007	-28.785	
3.912.159	-28.780	
3.912.344	-28.775	
3.912.463	-28.769	
3.912.675	-28.764	
3.913.089	-28.759	
3.913.470	-28.754	
3.913.713	-28.748	
3.913.788	-28.743	
3.913.788	-28.738	
3.913.842	-28.732	
3.913.969	-28.727	

3.914.075	-28.722	
3.914.144	-28.716	
3.914.220	-28.711	
3.914.370	-28.706	
3.914.518	-28.700	
3.914.721	-28.695	
3.915.021	-28.690	
3.915.179	-28.685	
3.915.282	-28.679	
3.915.454	-28.674	
3.915.617	-28.669	
3.915.829	-28.663	
3.916.086	-28.658	
3.916.256	-28.653	
3.916.358	-28.648	
3.916.483	-28.643	
3.916.660	-28.637	
3.916.841	-28.632	
3.916.979	-28.627	
3.917.171	-28.622	
3.917.298	-28.617	
3.917.368	-28.612	
3.917.600	-28.607	
3.917.842	-28.602	
3.917.986	-28.596	
3.918.131	-28.591	
3.918.336	-28.586	
3.918.466	-28.581	
3.918.643	-28.576	
3.918.885	-28.572	

3.919.048	-28.567	
3.919.143	-28.562	
3.919.247	-28.557	
3.919.409	-28.552	
3.919.604	-28.547	
3.919.789	-28.542	
3.919.969	-28.537	
3.920.118	-28.533	
3.920.249	-28.528	
3.920.448	-28.523	
3.920.641	-28.518	
3.920.800	-28.514	
3.920.954	-28.509	
3.921.071	-28.504	
3.921.223	-28.500	
3.921.394	-28.495	
3.921.524	-28.491	
3.921.697	-28.486	
3.921.875	-28.482	
3.922.076	-28.477	
3.922.344	-28.473	
3.922.567	-28.468	
3.922.655	-28.464	
3.922.803	-28.460	
3.923.033	-28.455	
3.923.171	-28.451	
3.923.302	-28.446	
3.923.486	-28.442	
3.923.654	-28.438	
3.923.792	-28.433	

3.923.972	-28.429	
3.924.156	-28.425	
3.924.402	-28.421	
3.924.604	-28.416	
3.924.720	-28.412	
3.924.898	-28.408	
3.925.002	-28.403	
3.925.104	-28.399	
3.925.307	-28.395	
3.925.538	-28.391	
3.925.675	-28.386	
3.925.755	-28.382	
3.925.842	-28.378	
3.925.983	-28.374	
3.926.187	-28.370	
3.926.358	-28.365	
3.926.555	-28.361	
3.926.750	-28.357	
3.926.895	-28.353	
3.927.054	-28.348	
3.927.263	-28.344	
3.927.473	-28.340	
3.927.719	-28.335	
3.927.890	-28.331	
3.927.995	-28.327	
3.928.152	-28.322	
3.928.340	-28.318	
3.928.526	-28.314	
3.928.625	-28.309	
3.928.763	-28.305	

3.928.925	-28.301	
3.929.052	-28.296	
3.929.191	-28.292	
3.929.388	-28.288	
3.929.566	-28.283	
3.929.720	-28.279	
3.929.927	-28.274	
3.930.151	-28.270	
3.930.385	-28.265	
3.930.529	-28.261	
3.930.706	-28.256	
3.930.883	-28.252	
3.931.068	-28.247	
3.931.246	-28.243	
3.931.369	-28.238	
3.931.582	-28.234	
3.931.761	-28.229	
3.931.877	-28.225	
3.932.047	-28.220	
3.932.236	-28.216	
3.932.419	-28.211	
3.932.565	-28.207	
3.932.704	-28.202	
3.932.847	-28.198	
3.933.076	-28.193	
3.933.307	-28.188	
3.933.478	-28.184	
3.933.622	-28.179	
3.933.750	-28.175	
3.933.861	-28.170	

3.933.980	-28.165	
3.934.136	-28.161	
3.934.287	-28.156	
3.934.514	-28.151	
3.934.685	-28.147	
3.934.804	-28.142	
3.934.958	-28.138	
3.935.130	-28.133	
3.935.350	-28.128	
3.935.585	-28.124	
3.935.786	-28.119	
3.935.889	-28.114	
3.936.039	-28.110	
3.936.404	-28.105	
3.936.786	-28.101	
3.937.011	-28.096	
3.937.092	-28.092	
3.937.144	-28.087	
3.937.222	-28.082	
3.937.243	-28.078	
3.937.301	-28.073	
3.937.433	-28.069	
3.937.518	-28.064	
3.937.643	-28.060	
3.937.829	-28.055	
3.938.033	-28.051	
3.938.287	-28.046	
3.938.479	-28.042	
3.938.665	-28.037	
3.938.839	-28.033	

3.939.089	-28.029	
3.939.355	-28.024	
3.939.474	-28.020	
3.939.536	-28.016	
3.939.652	-28.011	
3.939.908	-28.007	
3.940.117	-28.003	
3.940.276	-27.998	
3.940.414	-27.994	
3.940.451	-27.990	
3.940.544	-27.986	
3.940.754	-27.982	
3.941.015	-27.977	
3.941.219	-27.973	
3.941.394	-27.969	
3.941.566	-27.965	
3.941.697	-27.961	
3.941.902	-27.957	
3.942.098	-27.953	
3.942.162	-27.949	
3.942.269	-27.946	
3.942.455	-27.942	
3.942.603	-27.938	
3.942.794	-27.934	
3.943.004	-27.930	
3.943.125	-27.927	
3.943.264	-27.923	
3.943.481	-27.919	
3.943.636	-27.916	
3.943.806	-27.912	

3.944.008	-27.909	
3.944.154	-27.905	
3.944.321	-27.902	
3.944.500	-27.899	
3.944.613	-27.895	
3.944.713	-27.892	
3.944.868	-27.889	
3.945.061	-27.886	
3.945.266	-27.882	
3.945.479	-27.879	
3.945.625	-27.876	
3.945.797	-27.873	
3.946.075	-27.870	
3.946.331	-27.867	
3.946.429	-27.864	
3.946.465	-27.861	
3.946.638	-27.858	
3.946.828	-27.856	
3.946.979	-27.853	
3.947.177	-27.850	
3.947.341	-27.847	
3.947.438	-27.845	
3.947.545	-27.842	
3.947.778	-27.839	
3.948.098	-27.837	
3.948.289	-27.834	
3.948.377	-27.832	
3.948.477	-27.829	
3.948.608	-27.827	
3.948.742	-27.824	

3.948.878	-27.822	
3.948.979	-27.819	
3.949.093	-27.817	
3.949.353	-27.815	
3.949.541	-27.812	
3.949.677	-27.810	
3.949.868	-27.808	
3.950.034	-27.805	
3.950.224	-27.803	
3.950.439	-27.801	
3.950.654	-27.799	
3.950.851	-27.797	
3.951.028	-27.795	
3.951.190	-27.793	
3.951.353	-27.791	
3.951.513	-27.789	
3.951.614	-27.787	
3.951.737	-27.785	
3.951.938	-27.783	
3.952.065	-27.781	
3.952.234	-27.779	
3.952.411	-27.777	
3.952.540	-27.775	
3.952.733	-27.773	
3.952.946	-27.771	
3.953.098	-27.769	
3.953.188	-27.768	
3.953.383	-27.766	
3.953.622	-27.764	
3.953.842	-27.762	

3.954.042	-27.761	
3.954.218	-27.759	
3.954.378	-27.757	
3.954.510	-27.756	
3.954.648	-27.754	
3.954.828	-27.753	
3.955.009	-27.751	
3.955.183	-27.749	
3.955.392	-27.748	
3.955.545	-27.747	
3.955.677	-27.745	
3.955.849	-27.744	
3.956.022	-27.742	
3.956.165	-27.741	
3.956.372	-27.740	
3.956.552	-27.738	
3.956.667	-27.737	
3.956.844	-27.736	
3.957.036	-27.735	
3.957.169	-27.733	
3.957.281	-27.732	
3.957.437	-27.731	
3.957.659	-27.730	
3.957.820	-27.729	
3.957.950	-27.728	
3.958.128	-27.727	
3.958.320	-27.726	
3.958.475	-27.725	
3.958.680	-27.724	
3.958.929	-27.723	

3.959.079	-27.722	
3.959.200	-27.721	
3.959.334	-27.720	
3.959.653	-27.719	
3.960.097	-27.718	
3.960.404	-27.718	
3.960.459	-27.717	
3.960.375	-27.716	
3.960.425	-27.715	
3.960.570	-27.715	
3.960.670	-27.714	
3.960.721	-27.713	
3.960.849	-27.713	
3.960.986	-27.712	
3.961.133	-27.712	
3.961.423	-27.711	
3.961.578	-27.710	
3.961.740	-27.710	
3.961.990	-27.709	
3.962.224	-27.709	
3.962.442	-27.709	
3.962.525	-27.708	
3.962.648	-27.708	
3.962.867	-27.707	
3.963.044	-27.707	
3.963.159	-27.707	
3.963.297	-27.706	
3.963.566	-27.706	
3.963.683	-27.706	
3.963.737	-27.706	

3.963.901	-27.705	
3.964.091	-27.705	
3.964.295	-27.705	
3.964.424	-27.705	
3.964.615	-27.705	
3.964.829	-27.705	
3.965.060	-27.705	
3.965.255	-27.705	
3.965.388	-27.705	
3.965.490	-27.705	
3.965.660	-27.705	
3.965.880	-27.705	
3.965.983	-27.705	
3.966.049	-27.705	
3.966.162	-27.705	
3.966.263	-27.705	
3.966.397	-27.705	
3.966.703	-27.705	
3.966.949	-27.706	
3.967.039	-27.706	
3.967.229	-27.706	
3.967.485	-27.706	
3.967.639	-27.706	
3.967.802	-27.706	
3.967.983	-27.707	
3.968.084	-27.707	
3.968.217	-27.707	
3.968.318	-27.707	
3.968.445	-27.708	
3.968.684	-27.708	

3.968.869	-27.708	
3.969.063	-27.708	
3.969.295	-27.709	
3.969.476	-27.709	
3.969.693	-27.709	
3.969.902	-27.709	
3.970.092	-27.710	
3.970.235	-27.710	
3.970.277	-27.710	
3.970.400	-27.710	
3.970.599	-27.710	
3.970.829	-27.711	
3.971.030	-27.711	
3.971.144	-27.711	
3.971.324	-27.711	
3.971.508	-27.711	
3.971.646	-27.711	
3.971.750	-27.711	
3.971.900	-27.711	
3.972.122	-27.711	
3.972.391	-27.711	
3.972.554	-27.711	
3.972.592	-27.711	
3.972.633	-27.711	
3.972.762	-27.711	
3.972.932	-27.710	
3.973.084	-27.710	
3.973.272	-27.710	
3.973.501	-27.710	
3.973.777	-27.709	

3.973.977	-27.709	
3.974.117	-27.709	
3.974.283	-27.708	
3.974.515	-27.708	
3.974.714	-27.707	
3.974.845	-27.707	
3.974.970	-27.706	
3.975.093	-27.706	
3.975.199	-27.705	
3.975.357	-27.705	
3.975.562	-27.704	
3.975.737	-27.703	
3.975.862	-27.702	
3.976.015	-27.702	
3.976.227	-27.701	
3.976.415	-27.700	
3.976.595	-27.699	
3.976.676	-27.698	
3.976.834	-27.697	
3.977.101	-27.696	
3.977.289	-27.695	
3.977.455	-27.694	
3.977.697	-27.693	
3.977.917	-27.692	
3.978.052	-27.690	
3.978.214	-27.689	
3.978.315	-27.688	
3.978.463	-27.687	
3.978.685	-27.685	
3.978.884	-27.684	

3.979.060	-27.683	
3.979.165	-27.681	
3.979.269	-27.680	
3.979.422	-27.678	
3.979.581	-27.677	
3.979.804	-27.675	
3.980.093	-27.674	
3.980.226	-27.672	
3.980.353	-27.671	
3.980.544	-27.669	
3.980.623	-27.667	
3.980.742	-27.666	
3.980.946	-27.664	
3.981.115	-27.662	
3.981.252	-27.660	
3.981.396	-27.659	
3.981.566	-27.657	
3.981.765	-27.655	
3.981.927	-27.653	
3.982.101	-27.651	
3.982.279	-27.649	
3.982.433	-27.647	
3.982.539	-27.645	
3.982.816	-27.644	
3.983.273	-27.642	
3.983.557	-27.640	
3.983.661	-27.638	
3.983.770	-27.636	
3.983.902	-27.634	
3.983.956	-27.632	

3.984.020	-27.629	
3.984.068	-27.627	
3.984.201	-27.625	
3.984.375	-27.623	
3.984.537	-27.621	
3.984.764	-27.619	
3.984.941	-27.617	
3.985.083	-27.615	
3.985.227	-27.613	
3.985.422	-27.610	
3.985.635	-27.608	
3.985.823	-27.606	
3.985.935	-27.604	
3.986.123	-27.602	
3.986.303	-27.600	
3.986.419	-27.597	
3.986.671	-27.595	
3.986.855	-27.593	
3.986.956	-27.591	
3.987.069	-27.589	
3.987.162	-27.587	
3.987.307	-27.585	
3.987.487	-27.582	
3.987.675	-27.580	
3.987.906	-27.578	
3.988.152	-27.576	
3.988.365	-27.574	
3.988.575	-27.572	
3.988.663	-27.570	
3.988.713	-27.568	

3.988.923	-27.566	
3.989.135	-27.564	
3.989.211	-27.561	
3.989.281	-27.559	
3.989.478	-27.557	
3.989.673	-27.555	
3.989.814	-27.553	
3.989.988	-27.551	
3.990.168	-27.549	
3.990.305	-27.547	
3.990.479	-27.545	
3.990.671	-27.544	
3.990.775	-27.542	
3.990.963	-27.540	
3.991.198	-27.538	
3.991.375	-27.536	
3.991.559	-27.534	
3.991.733	-27.532	
3.991.897	-27.530	
3.992.057	-27.528	
3.992.234	-27.526	
3.992.365	-27.524	
3.992.484	-27.523	
3.992.668	-27.521	
3.992.787	-27.519	
3.992.928	-27.517	
3.993.127	-27.515	
3.993.376	-27.513	
3.993.551	-27.511	
3.993.627	-27.509	

3.993.820	-27.507	
3.994.059	-27.505	
3.994.259	-27.503	
3.994.389	-27.501	
3.994.597	-27.499	
3.994.811	-27.498	
3.994.948	-27.496	
3.995.076	-27.494	
3.995.183	-27.491	
3.995.332	-27.489	
3.995.548	-27.487	
3.995.677	-27.485	
3.995.791	-27.483	
3.995.954	-27.481	
3.996.075	-27.479	
3.996.196	-27.477	
3.996.317	-27.475	
3.996.541	-27.472	
3.996.770	-27.470	
3.996.928	-27.468	
3.997.185	-27.466	
3.997.399	-27.463	
3.997.527	-27.461	
3.997.626	-27.459	
3.997.816	-27.456	
3.997.979	-27.454	
3.998.066	-27.452	
3.998.264	-27.449	
3.998.488	-27.447	
3.998.629	-27.444	

3.998.731	-27.442	
3.998.918	-27.439	
3.999.102	-27.436	
3.999.229	-27.434	
3.999.384	-27.431	
3.999.565	-27.428	
3.999.706	-27.426	
3.999.920	-27.423	
4.000.147	-27.420	
4.000.295	-27.417	
4.000.471	-27.414	
4.000.667	-27.411	
4.000.855	-27.408	
4.000.963	-27.405	
4.001.147	-27.402	
4.001.342	-27.399	
4.001.534	-27.396	
4.001.733	-27.393	
4.001.824	-27.390	
4.001.982	-27.387	
4.002.137	-27.384	
4.002.267	-27.381	
4.002.431	-27.378	
4.002.607	-27.374	
4.002.749	-27.371	
4.002.935	-27.368	
4.003.228	-27.364	
4.003.355	-27.361	
4.003.427	-27.358	
4.003.655	-27.354	

4.003.853	-27.351	
4.004.012	-27.347	
4.004.171	-27.344	
4.004.247	-27.340	
4.004.378	-27.337	
4.004.572	-27.333	
4.004.811	-27.329	
4.005.060	-27.326	
4.005.204	-27.322	
4.005.370	-27.318	
4.005.465	-27.315	
4.005.576	-27.311	
4.005.924	-27.307	
4.006.456	-27.303	
4.006.798	-27.299	
4.006.839	-27.296	
4.006.886	-27.292	
4.006.978	-27.288	
4.007.022	-27.284	
4.007.069	-27.280	
4.007.188	-27.276	
4.007.272	-27.272	
4.007.373	-27.268	
4.007.523	-27.264	
4.007.701	-27.260	
4.007.932	-27.256	
4.008.141	-27.252	
4.008.344	-27.248	
4.008.506	-27.244	
4.008.615	-27.240	

4.008.761	-27.236	
4.008.947	-27.232	
4.009.124	-27.228	
4.009.316	-27.224	
4.009.505	-27.220	
4.009.604	-27.216	
4.009.736	-27.212	
4.009.971	-27.208	
4.010.117	-27.204	
4.010.195	-27.200	
4.010.368	-27.196	
4.010.603	-27.192	
4.010.787	-27.188	
4.010.952	-27.184	
4.011.095	-27.180	
4.011.281	-27.176	
4.011.434	-27.172	
4.011.552	-27.168	
4.011.732	-27.164	
4.011.915	-27.160	
4.012.159	-27.156	
4.012.369	-27.153	
4.012.453	-27.149	
4.012.510	-27.145	
4.012.647	-27.142	
4.012.857	-27.138	
4.013.065	-27.134	
4.013.142	-27.131	
4.013.246	-27.127	
4.013.490	-27.124	

4.013.716	-27.121	
4.013.842	-27.117	
4.013.947	-27.114	
4.014.092	-27.111	
4.014.255	-27.108	
4.014.428	-27.104	
4.014.568	-27.101	
4.014.760	-27.098	
4.015.060	-27.095	
4.015.194	-27.092	
4.015.255	-27.089	
4.015.475	-27.086	
4.015.677	-27.084	
4.015.865	-27.081	
4.016.097	-27.078	
4.016.234	-27.075	
4.016.358	-27.073	
4.016.599	-27.070	
4.016.722	-27.068	
4.016.784	-27.065	
4.016.943	-27.063	
4.017.191	-27.060	
4.017.478	-27.058	
4.017.657	-27.055	
4.017.734	-27.053	
4.017.796	-27.051	
4.017.836	-27.049	
4.018.029	-27.047	
4.018.307	-27.045	
4.018.553	-27.042	

4.018.717	-27.040	
4.018.761	-27.039	
4.018.938	-27.037	
4.019.102	-27.035	
4.019.151	-27.033	
4.019.332	-27.031	
4.019.550	-27.029	
4.019.720	-27.028	
