# Supplementary material for: Data on statistical experimental design to formulate amphotericin B-loaded Eudragit RL100 nanoparticles coated with hyaluronic acid for the treatment of vulvovaginal candidiasis
Source: Data Brief. 2020 Mar 5;29:105311. doi: 10.1016/j.dib.2020.105311 (PMC7082528; doi:10.1016/j.dib.2020.105311)
Supplement: Multimedia component 11 [file mmc11.pdf]

DATATYPE	**IR Spectrum pure EUD**		DATATYPE	**IR Spectrum pure AMP**	
XYUNITS	**Wavenumber;PercentTransmittance**		XYUNITS	**Wavenumber;PercentTransmittance**	
DECIMALSYMBOL	,		DECIMALSYMB(	,	
399,2356	150		399,2356	150	
401,1643	150		401,1643	1,349	
403,0929	-1		403,0929	150	
405,0216	-1		405,0216	150	
406,9503	-1		406,9503	150	
408,879	150		408,879	-1	
410,8076	-1		410,8076	150	
412,7363	150		412,7363	150	
414,665	-1		414,665	-1	
416,5937	117,356		416,5937	-1	
418,5223	150		418,5223	-1	
420,451	150		420,451	-1	
422,3797	150		422,3797	144,4142	
424,3084	64,606		424,3084	46,9205	
426,237	5,3563		426,237	69,5148	
428,1657	44,8701		428,1657	66,8851	
430,0944	83,1307		430,0944	22,5411	
432,0231	-1		432,0231	-1	
433,9517	-1		433,9517	-1	
435,8804	-1		435,8804	-1	
437,8091	-1		437,8091	3,3645	
439,7377	-1		439,7377	-1	
441,6664	40,2163		441,6664	-1	
443,5951	63,258		443,5951	-1	
445,5238	115,1248		445,5238	-1	
447,4524	38,0081		447,4524	-1	
449,3811	118,3475		449,3811	-1	

451,3098	-1		451,3098	-1	
453,2385	150		453,2385	-1	
455,1671	150		455,1671	-1	
457,0958	129,0793		457,0958	-1	
459,0245	150		459,0245	-1	
460,9532	-1		460,9532	-1	
462,8818	112,3757		462,8818	80,9801	
464,8105	-1		464,8105	111,0474	
466,7392	150		466,7392	-1	
468,6679	150		468,6679	-1	
470,5965	100,6183		470,5965	150	
472,5252	40,3882		472,5252	-1	
474,4539	150		474,4539	-1	
476,3826	-1		476,3826	40,3825	
478,3112	143,8142		478,3112	16,7492	
480,2399	-1		480,2399	-1	
482,1686	-1		482,1686	-1	
484,0973	150		484,0973	150	
486,0259	150		486,0259	150	
487,9546	150		487,9546	64,6881	
489,8833	150		489,8833	49,076	
491,812	150		491,812	75,8936	
493,7406	-1		493,7406	-1	
495,6693	1,3466		495,6693	-1	
497,598	26,2912		497,598	150	
499,5267	59,7258		499,5267	122,0966	
501,4553	133,4654		501,4553	20,5121	
503,384	150		503,384	-1	
505,3127	-1		505,3127	-1	
507,2414	-1		507,2414	-1	

509,17	150		509,17	-1	
511,0987	65,6334		511,0987	-1	
513,0274	150		513,0274	-1	
514,956	150		514,956	-1	
516,8847	134,8459		516,8847	150	
518,8134	132,6396		518,8134	144,6719	
520,7421	85,8129		520,7421	46,8078	
522,6707	102,0535		522,6707	20,2421	
524,5994	150		524,5994	42,0553	
526,5281	143,3076		526,5281	84,4339	
528,4568	96,5408		528,4568	119,514	
530,3854	98,2905		530,3854	134,1599	
532,3141	91,8965		532,3141	123,6728	
534,2428	86,6487		534,2428	89,6919	
536,1715	90,4769		536,1715	62,4054	
538,1001	80,6083		538,1001	55,1137	
540,0288	74,5537		540,0288	56,3756	
541,9575	84,9225		541,9575	52,7161	
543,8862	93,5539		543,8862	54,0419	
545,8148	88,2957		545,8148	61,0961	
547,7435	83,4974		547,7435	58,3367	
549,6722	81,8396		549,6722	60,7121	
551,6009	78,7222		551,6009	67,9361	
553,5295	76,7607		553,5295	67,5678	
555,4582	76,0453		555,4582	65,4034	
557,3869	76,5189		557,3869	62,7125	
559,3156	77,577		559,3156	59,0458	
561,2442	71,8488		561,2442	61,7292	
563,1729	64,7044		563,1729	66,7587	
565,1016	67,8321		565,1016	66,806	

567,0303	73,1765		567,0303	67,0863	
568,9589	66,8346		568,9589	67,5327	
570,8876	64,0016		570,8876	68,0241	
572,8163	73,661		572,8163	66,6031	
574,745	78,3166		574,745	64,2793	
576,6736	72,001		576,6736	65,3473	
578,6023	71,927		578,6023	64,9424	
580,531	73,9107		580,531	61,4559	
582,4597	74,8268		582,4597	59,6677	
584,3883	76,1505		584,3883	60,0819	
586,317	73,368		586,317	61,0802	
588,2457	70,1357		588,2457	61,9872	
590,1743	68,5753		590,1743	61,5635	
592,103	68,2937		592,103	61,2567	
594,0317	68,7305		594,0317	62,0108	
595,9604	69,5718		595,9604	62,3935	
597,889	70,1129		597,889	63,0442	
599,8177	68,6561		599,8177	62,1936	
601,7464	67,9678		601,7464	59,934	
603,6751	70,1809		603,6751	59,4271	
605,6037	71,5011		605,6037	59,9003	
607,5324	70,1796		607,5324	60,2694	
609,4611	69,435		609,4611	61,1081	
611,3898	68,8475		611,3898	61,7737	
613,3184	68,6863		613,3184	61,3398	
615,2471	70,233		615,2471	61,3997	
617,1758	71,6567		617,1758	62,1674	
619,1045	71,8634		619,1045	62,384	
621,0331	71,4911		621,0331	61,5974	
622,9618	70,9483		622,9618	61,3106	

624,8905	70,6789		624,8905	62,5441	
626,8192	71,1775		626,8192	63,1999	
628,7478	71,6049		628,7478	63,1153	
630,6765	71,842		630,6765	63,4747	
632,6052	71,9277		632,6052	64,0198	
634,5339	71,8389		634,5339	64,454	
636,4625	71,8242		636,4625	65,0183	
638,3912	71,7107		638,3912	65,8302	
640,3199	71,7572		640,3199	65,9624	
642,2486	72,1441		642,2486	65,1782	
644,1772	72,5331		644,1772	64,8098	
646,1059	72,884		646,1059	65,0139	
648,0346	72,9417		648,0346	65,1885	
649,9633	72,583		649,9633	65,4717	
651,8919	72,3409		651,8919	65,8456	
653,8206	72,33		653,8206	66,1855	
655,7493	72,4355		655,7493	66,3269	
657,678	72,6852		657,678	66,3459	
659,6066	72,8979		659,6066	66,6241	
661,5353	72,9951		661,5353	66,6334	
663,464	73,1925		663,464	66,3762	
665,3926	73,4175		665,3926	66,4368	
667,3213	73,5069		667,3213	66,7735	
669,25	73,3902		669,25	67,0779	
671,1787	73,5596		671,1787	67,1437	
673,1073	74,0388		673,1073	67,2473	
675,036	74,5551		675,036	67,3004	
676,9647	74,9697		676,9647	67,3934	
678,8934	75,2		678,8934	67,5331	
680,822	75,1911		680,822	67,6626	

682,7507	75,3097		682,7507	67,6753	
684,6794	75,4845		684,6794	67,4715	
686,6081	75,6668		686,6081	67,4978	
688,5367	76,0427		688,5367	67,7798	
690,4654	76,203		690,4654	68,1324	
692,3941	76,1536		692,3941	68,5125	
694,3228	76,2136		694,3228	68,7848	
696,2514	76,2746		696,2514	69,2245	
698,1801	76,339		698,1801	69,7217	
700,1088	76,5225		700,1088	70,0502	
702,0375	76,7022		702,0375	70,3977	
703,9661	76,8118		703,9661	70,6346	
705,8948	76,9946		705,8948	70,8789	
707,8235	77,1941		707,8235	71,2149	
709,7522	77,375		709,7522	71,2608	
711,6808	77,5256		711,6808	71,1537	
713,6095	77,5444		713,6095	71,276	
715,5382	77,6143		715,5382	71,4183	
717,4669	77,7758		717,4669	71,4776	
719,3955	77,9035		719,3955	71,6341	
721,3242	78,0782		721,3242	71,9083	
723,2529	78,3078		723,2529	72,2223	
725,1816	78,5821		725,1816	72,3828	
727,1102	78,8884		727,1102	72,4437	
729,0389	79,1238		729,0389	72,5446	
730,9676	79,3345		730,9676	72,5857	
732,8962	79,5531		732,8962	72,6744	
734,8249	79,7131		734,8249	72,792	
736,7536	79,8097		736,7536	72,824	
738,6823	79,7377		738,6823	72,9642	

740,6109	79,4887		740,6109	73,2007	
742,5396	79,2345		742,5396	73,3779	
744,4683	78,8789		744,4683	73,4868	
746,397	78,3235		746,397	73,548	
748,3256	77,8659		748,3256	73,5642	
750,2543	77,6249		750,2543	73,5632	
752,183	77,6502		752,183	73,5621	
754,1117	78,0197		754,1117	73,5294	
756,0403	78,4489		756,0403	73,5072	
757,969	78,7731		757,969	73,6167	
759,8977	79,2403		759,8977	73,9895	
761,8264	79,8204		761,8264	74,4329	
763,755	80,2289		763,755	74,7778	
765,6837	80,5229		765,6837	75,1528	
767,6124	80,699		767,6124	75,4555	
769,5411	80,9222		769,5411	75,5538	
771,4697	81,3246		771,4697	75,4877	
773,3984	81,6054		773,3984	75,4655	
775,3271	81,7931		775,3271	75,5152	
777,2558	81,9666		777,2558	75,4898	
779,1844	81,9704		779,1844	75,4741	
781,1131	81,8792		781,1131	75,4964	
783,0418	81,8593		783,0418	75,4766	
784,9705	81,8735		784,9705	75,393	
786,8991	81,8473		786,8991	75,3402	
788,8278	81,7617		788,8278	75,3671	
790,7565	81,5761		790,7565	75,4712	
792,6852	81,3916		792,6852	75,6634	
794,6138	81,2978		794,6138	75,8204	
796,5425	81,224		796,5425	75,9638	

798,4712	81,0644		798,4712	76,1324	
800,3999	80,8005		800,3999	76,2836	
802,3285	80,6197		802,3285	76,4532	
804,2572	80,4548		804,2572	76,5916	
806,1859	80,1123		806,1859	76,8112	
808,1145	79,7625		808,1145	77,1428	
810,0432	79,5514		810,0432	77,4133	
811,9719	79,5		811,9719	77,7094	
813,9006	79,5182		813,9006	78,0927	
815,8292	79,4882		815,8292	78,4755	
817,7579	79,4583		817,7579	78,8108	
819,6866	79,4537		819,6866	79,057	
821,6153	79,2844		821,6153	79,1477	
823,5439	79,0033		823,5439	79,0288	
825,4726	78,862		825,4726	78,7191	
827,4013	78,7		827,4013	78,3125	
829,33	78,3816		829,33	77,8745	
831,2586	77,9443		831,2586	77,3345	
833,1873	77,4154		833,1873	76,7135	
835,116	76,8154		835,116	76,1492	
837,0447	76,1386		837,0447	75,6173	
838,9733	75,3904		838,9733	75,0943	
840,902	74,745		840,902	74,6905	
842,8307	74,3864		842,8307	74,3799	
844,7594	74,1486		844,7594	74,1579	
846,688	73,9588		846,688	74,0024	
848,6167	73,9397		848,6167	73,845	
850,5454	74,0504		850,5454	73,8799	
852,4741	74,3212		852,4741	74,2665	
854,4027	74,8136		854,4027	74,9839	

856,3314	75,3718		856,3314	75,8686	
858,2601	75,9177		858,2601	76,7943	
860,1888	76,3971		860,1888	77,8598	
862,1174	76,7284		862,1174	78,9403	
864,0461	76,9515		864,0461	79,7744	
865,9748	77,0566		865,9748	80,3706	
867,9035	77,105		867,9035	80,6933	
869,8321	77,1784		869,8321	80,7057	
871,7608	77,223		871,7608	80,6483	
873,6895	77,223		873,6895	80,5786	
875,6182	77,1023		875,6182	80,3834	
877,5468	76,8189		877,5468	80,0486	
879,4755	76,516		879,4755	79,6019	
881,4042	76,2769		881,4042	79,141	
883,3328	76,0839		883,3328	78,6847	
885,2615	75,9447		885,2615	78,3602	
887,1902	75,9198		887,1902	78,3213	
889,1189	76,0199		889,1189	78,4712	
891,0475	76,2383		891,0475	78,7049	
892,9762	76,4849		892,9762	78,9764	
894,9049	76,5997		894,9049	79,1173	
896,8336	76,6506		896,8336	79,1106	
898,7622	76,803		898,7622	79,0452	
900,6909	76,9005		900,6909	78,8111	
902,6196	76,6996		902,6196	78,5605	
904,5483	76,4668		904,5483	78,5664	
906,4769	76,3897		906,4769	78,7085	
908,4056	76,1165		908,4056	78,7228	
910,3343	75,6737		910,3343	78,5051	
912,263	75,3613		912,263	78,2258	

914,1916	75,1284		914,1916	78,2899	
916,1203	74,9192		916,1203	78,7086	
918,049	74,6838		918,049	79,053	
919,9777	74,3557		919,9777	79,0818	
921,9063	73,9334		921,9063	78,9864	
923,835	73,4846		923,835	79,2675	
925,7637	73,049		925,7637	79,9184	
927,6924	72,5178		927,6924	80,4411	
929,621	71,8672		929,621	80,6164	
931,5497	71,2173		931,5497	80,4795	
933,4784	70,5978		933,4784	80,0407	
935,4071	69,979		935,4071	79,3545	
937,3357	69,4112		937,3357	78,5378	
939,2644	68,8949		939,2644	77,5678	
941,1931	68,339		941,1931	76,4249	
943,1218	67,6478		943,1218	75,5903	
945,0504	66,9128		945,0504	75,5457	
946,9791	66,396		946,9791	75,9648	
948,9078	66,1508		948,9078	76,3168	
950,8364	66,0495		950,8364	76,3849	
952,7651	66,1137		952,7651	76,309	
954,6938	66,4243		954,6938	76,3254	
956,6225	66,9314		956,6225	76,4474	
958,5511	67,4521		958,5511	76,4657	
960,4798	67,8707		960,4798	76,081	
962,4085	68,2413		962,4085	75,2356	
964,3372	68,529		964,3372	74,1244	
966,2658	68,6292		966,2658	73,0517	
968,1945	68,5128		968,1945	72,4228	
970,1232	68,1591		970,1232	72,458	

972,0519	67,6279		972,0519	72,8953	
973,9805	66,9094		973,9805	73,4264	
975,9092	65,8327		975,9092	73,7205	
977,8379	64,4725		977,8379	73,4843	
979,7666	63,0586		979,7666	72,8719	
981,6952	61,7005		981,6952	72,1594	
983,6239	60,6011		983,6239	71,5414	
985,5526	59,8703		985,5526	71,0289	
987,4813	59,3705		987,4813	70,5074	
989,4099	59,0046		989,4099	69,9253	
991,3386	58,7111		991,3386	69,0822	
993,2673	58,5207		993,2673	67,7855	
995,196	58,4776		995,196	66,1549	
997,1246	58,43		997,1246	64,4697	
999,0533	58,2908		999,0533	62,8716	
1000,982	58,1444		1000,982	61,2175	
1002,9107	57,915		1002,9107	59,3107	
1004,8393	57,4812		1004,8393	57,2743	
1006,768	56,8125		1006,768	55,5769	
1008,6967	56,0061		1008,6967	54,6505	
1010,6254	55,235		1010,6254	54,7336	
1012,554	54,4872		1012,554	55,8581	
1014,4827	53,7098		1014,4827	57,7701	
1016,4114	52,9428		1016,4114	59,9591	
1018,3401	52,2544		1018,3401	61,9189	
1020,2687	51,5652		1020,2687	63,5555	
1022,1974	50,8848		1022,1974	64,8802	
1024,1261	50,2977		1024,1261	65,64	
1026,0547	49,7748		1026,0547	65,6928	
1027,9834	49,3569		1027,9834	65,0186	

1029,9121	49,1006		1029,9121	63,7855	
1031,8408	49,0478		1031,8408	62,4273	
1033,7694	49,1582		1033,7694	61,2549	
1035,6981	49,4654		1035,6981	60,4	
1037,6268	49,975		1037,6268	59,7967	
1039,5555	50,4101		1039,5555	59,3394	
1041,4841	50,6557		1041,4841	59,2291	
1043,4128	50,763		1043,4128	59,933	
1045,3415	50,7289		1045,3415	61,5887	
1047,2702	50,569		1047,2702	63,7322	
1049,1988	50,3602		1049,1988	65,7801	
1051,1275	50,1466		1051,1275	67,3587	
1053,0562	49,8901		1053,0562	68,433	
1054,9849	49,5993		1054,9849	68,9722	
1056,9135	49,2427		1056,9135	68,7101	
1058,8422	48,9434		1058,8422	67,7961	
1060,7709	48,8283		1060,7709	66,7626	
1062,6996	48,7597		1062,6996	65,8632	
1064,6282	48,7358		1064,6282	65,1843	
1066,5569	48,7715		1066,5569	64,9438	
1068,4856	48,682		1068,4856	65,1714	
1070,4143	48,4065		1070,4143	65,6451	
1072,3429	48,0666		1072,3429	66,3886	
1074,2716	47,6947		1074,2716	67,4787	
1076,2003	47,2576		1076,2003	68,6365	
1078,129	46,7854		1078,129	69,6431	
1080,0576	46,3012		1080,0576	70,5676	
1081,9863	45,7966		1081,9863	71,2695	
1083,915	45,2315		1083,915	71,5447	
1085,8437	44,6422		1085,8437	71,5253	

1087,7723	44,1212		1087,7723	71,4569	
1089,701	43,6388		1089,701	71,6312	
1091,6297	43,1567		1091,6297	72,1243	
1093,5584	42,7462		1093,5584	72,6584	
1095,487	42,4768		1095,487	73,0059	
1097,4157	42,3601		1097,4157	73,204	
1099,3444	42,4316		1099,3444	73,3918	
1101,273	42,6919		1101,273	73,6201	
1103,2017	43,0241		1103,2017	73,8126	
1105,1304	43,3821		1105,1304	73,9018	
1107,0591	43,7857		1107,0591	73,9557	
1108,9877	44,2521		1108,9877	74,0028	
1110,9164	44,824		1110,9164	74,1844	
1112,8451	45,4921		1112,8451	74,674	
1114,7738	46,2412		1114,7738	75,4136	
1116,7024	47,0141		1116,7024	76,2613	
1118,6311	47,788		1118,6311	77,001	
1120,5598	48,5834		1120,5598	77,411	
1122,4885	49,2299		1122,4885	77,4545	
1124,4171	49,763		1124,4171	77,26	
1126,3458	50,374		1126,3458	76,9678	
1128,2745	50,8633		1128,2745	76,7138	
1130,2032	51,1002		1130,2032	76,5416	
1132,1318	51,2296		1132,1318	76,5273	
1134,0605	51,2651		1134,0605	76,7534	
1135,9892	51,2258		1135,9892	77,1497	
1137,9179	51,2002		1137,9179	77,6776	
1139,8465	51,2206		1139,8465	78,309	
1141,7752	51,3727		1141,7752	78,8813	
1143,7039	51,732		1143,7039	79,319	

1145,6326	52,2567		1145,6326	79,6379	
1147,5612	52,9775		1147,5612	79,6912	
1149,4899	54,049		1149,4899	79,5461	
1151,4186	55,4179		1151,4186	79,3566	
1153,3473	56,9306		1153,3473	79,0322	
1155,2759	58,5595		1155,2759	78,6013	
1157,2046	60,2112		1157,2046	78,2515	
1159,1333	61,8465		1159,1333	78,0148	
1161,062	63,4345		1161,062	77,7514	
1162,9906	64,8335		1162,9906	77,4064	
1164,9193	66,0935		1164,9193	76,9583	
1166,848	67,2215		1166,848	76,4209	
1168,7767	68,0476		1168,7767	75,8763	
1170,7053	68,5392		1170,7053	75,3494	
1172,634	68,8193		1172,634	74,9633	
1174,5627	69,0626		1174,5627	74,8147	
1176,4913	69,3908		1176,4913	74,7817	
1178,42	69,8317		1178,42	74,7977	
1180,3487	70,3177		1180,3487	74,8308	
1182,2774	70,8417		1182,2774	74,7979	
1184,206	71,3785		1184,206	74,7359	
1186,1347	71,808		1186,1347	74,7029	
1188,0634	72,1694		1188,0634	74,7852	
1189,9921	72,5663		1189,9921	75,0467	
1191,9207	73,0972		1191,9207	75,4008	
1193,8494	73,798		1193,8494	75,7905	
1195,7781	74,6392		1195,7781	76,2424	
1197,7068	75,5789		1197,7068	76,6844	
1199,6354	76,4456		1199,6354	77,0635	
1201,5641	77,1964		1201,5641	77,4401	

1203,4928	77,9103		1203,4928	77,7569	
1205,4215	78,4799		1205,4215	77,979	
1207,3501	78,857		1207,3501	78,1195	
1209,2788	79,1641		1209,2788	78,2446	
1211,2075	79,3399		1211,2075	78,4508	
1213,1362	79,2529		1213,1362	78,6173	
1215,0648	78,992		1215,0648	78,745	
1216,9935	78,6596		1216,9935	78,9944	
1218,9222	78,2494		1218,9222	79,3166	
1220,8509	77,7817		1220,8509	79,6277	
1222,7795	77,2594		1222,7795	79,8952	
1224,7082	76,6176		1224,7082	80,0662	
1226,6369	75,8744		1226,6369	80,0724	
1228,5656	75,038		1228,5656	79,9108	
1230,4942	74,1272		1230,4942	79,7043	
1232,4229	73,2866		1232,4229	79,4958	
1234,3516	72,5268		1234,3516	79,2829	
1236,2803	71,864		1236,2803	79,138	
1238,2089	71,4007		1238,2089	79,0771	
1240,1376	71,0972		1240,1376	79,0167	
1242,0663	70,9615		1242,0663	78,886	
1243,9949	71,0615		1243,9949	78,7367	
1245,9236	71,3448		1245,9236	78,5627	
1247,8523	71,8099		1247,8523	78,2895	
1249,781	72,4288		1249,781	77,9851	
1251,7096	73,0558		1251,7096	77,657	
1253,6383	73,679		1253,6383	77,2975	
1255,567	74,354		1255,567	77,0887	
1257,4957	75,0671		1257,4957	77,1167	
1259,4243	75,758		1259,4243	77,2519	

1261,353	76,3303		1261,353	77,3732	
1263,2817	76,7912		1263,2817	77,4367	
1265,2104	77,1998		1265,2104	77,4679	
1267,139	77,4563		1267,139	77,4723	
1269,0677	77,5404		1269,0677	77,4253	
1270,9964	77,5734		1270,9964	77,39	
1272,9251	77,5704		1272,9251	77,4636	
1274,8537	77,5702		1274,8537	77,6166	
1276,7824	77,6359		1276,7824	77,7255	
1278,7111	77,736		1278,7111	77,762	
1280,6398	77,9097		1280,6398	77,7476	
1282,5684	78,2184		1282,5684	77,6474	
1284,4971	78,5387		1284,4971	77,4494	
1286,4258	78,8175		1286,4258	77,1748	
1288,3545	79,0891		1288,3545	76,8766	
1290,2831	79,2708		1290,2831	76,6317	
1292,2118	79,4054		1292,2118	76,4452	
1294,1405	79,5347		1294,1405	76,3127	
1296,0692	79,6353		1296,0692	76,2528	
1297,9978	79,7468		1297,9978	76,2131	
1299,9265	79,9142		1299,9265	76,1759	
1301,8552	80,1813		1301,8552	76,1854	
1303,7839	80,5654		1303,7839	76,1814	
1305,7125	81,0602		1305,7125	76,1166	
1307,6412	81,593		1307,6412	76,0861	
1309,5699	82,0958		1309,5699	76,1303	
1311,4986	82,5744		1311,4986	76,2111	
1313,4272	82,9848		1313,4272	76,3283	
1315,3559	83,2765		1315,3559	76,4277	
1317,2846	83,4376		1317,2846	76,4598	

1319,2132	83,5286		1319,2132	76,4707	
1321,1419	83,6307		1321,1419	76,4371	
1323,0706	83,696		1323,0706	76,3326	
1324,9993	83,8416		1324,9993	76,266	
1326,9279	84,11		1326,9279	76,2023	
1328,8566	84,3304		1328,8566	76,0698	
1330,7853	84,4381		1330,7853	75,898	
1332,714	84,4891		1332,714	75,6712	
1334,6426	84,5255		1334,6426	75,4857	
1336,5713	84,5128		1336,5713	75,3977	
1338,5	84,4226		1338,5	75,4371	
1340,4287	84,0858		1340,4287	75,6415	
1342,3573	83,3877		1342,3573	75,746	
1344,286	82,425		1344,286	75,7132	
1346,2147	81,4817		1346,2147	75,5936	
1348,1434	80,9712		1348,1434	75,348	
1350,072	80,9395		1350,072	75,0049	
1352,0007	81,2517		1352,0007	74,5808	
1353,9294	81,7794		1353,9294	74,0474	
1355,8581	82,3595		1355,8581	73,4834	
1357,7867	82,8945		1357,7867	72,9438	
1359,7154	83,2642		1359,7154	72,2875	
1361,6441	83,4604		1361,6441	71,5015	
1363,5728	83,5815		1363,5728	70,6687	
1365,5014	83,5759		1365,5014	69,8273	
1367,4301	83,552		1367,4301	68,9941	
1369,3588	83,5838		1369,3588	68,2247	
1371,2875	83,5931		1371,2875	67,4501	
1373,2161	83,5836		1373,2161	66,5859	
1375,1448	83,5381		1375,1448	65,9072	

1377,0735	83,3916		1377,0735	65,6269	
1379,0022	83,2405		1379,0022	65,512	
1380,9308	83,2203		1380,9308	65,4507	
1382,8595	83,3627		1382,8595	65,3884	
1384,7882	83,5711		1384,7882	65,269	
1386,7169	83,9585		1386,7169	65,1573	
1388,6455	84,5736		1388,6455	65,049	
1390,5742	85,0892		1390,5742	64,7447	
1392,5029	85,4892		1392,5029	64,3168	
1394,4315	85,9109		1394,4315	63,8949	
1396,3602	86,3063		1396,3602	63,5751	
1398,2889	86,6313		1398,2889	63,392	
1400,2176	87,0224		1400,2176	63,3732	
1402,1462	87,2816		1402,1462	63,5052	
1404,0749	87,532		1404,0749	63,9162	
1406,0036	87,858		1406,0036	64,6864	
1407,9323	88,0769		1407,9323	65,5278	
1409,8609	88,192		1409,8609	66,5312	
1411,7896	88,2059		1411,7896	67,6774	
1413,7183	88,1241		1413,7183	68,7461	
1415,647	87,9462		1415,647	69,661	
1417,5756	87,7319		1417,5756	70,5259	
1419,5043	87,3948		1419,5043	71,7318	
1421,433	86,9809		1421,433	72,4508	
1423,3617	86,5361		1423,3617	72,9347	
1425,2903	85,9016		1425,2903	73,5887	
1427,219	85,1629		1427,219	74,0587	
1429,1477	84,191		1429,1477	74,5046	
1431,0764	83,1279		1431,0764	74,8307	
1433,005	82,2986		1433,005	74,8392	

1434,9337	81,6382		1434,9337	74,7423	
1436,8624	81,1101		1436,8624	74,7442	
1438,7911	80,7498		1438,7911	74,6891	
1440,7197	80,3211		1440,7197	74,4148	
1442,6484	79,7469		1442,6484	73,8994	
1444,5771	79,1852		1444,5771	73,3464	
1446,5058	78,7108		1446,5058	73,1225	
1448,4344	78,3614		1448,4344	73,4759	
1450,3631	78,3187		1450,3631	74,15	
1452,2918	78,5721		1452,2918	74,9373	
1454,2205	78,8363		1454,2205	75,5757	
1456,1491	79,196		1456,1491	76,2543	
1458,0778	80,0929		1458,0778	77,5415	
1460,0065	80,5861		1460,0065	78,1634	
1461,9351	80,8611		1461,9351	78,4847	
1463,8638	81,2125		1463,8638	78,9499	
1465,7925	81,7253		1465,7925	79,65	
1467,7212	82,1774		1467,7212	80,2382	
1469,6498	82,5383		1469,6498	80,7877	
1471,5785	83,0977		1471,5785	81,6713	
1473,5072	84,0437		1473,5072	82,9759	
1475,4359	84,6993		1475,4359	83,8064	
1477,3645	85,3847		1477,3645	84,559	
1479,2932	85,9635		1479,2932	85,1376	
1481,2219	86,5335		1481,2219	85,6457	
1483,1506	87,1577		1483,1506	86,0431	
1485,0792	87,6635		1485,0792	86,1825	
1487,0079	88,2951		1487,0079	86,2461	
1488,9366	89,1939		1488,9366	86,2851	
1490,8653	90,3654		1490,8653	86,2849	

1492,7939	91,1136		1492,7939	86,1444	
1494,7226	91,6683		1494,7226	85,931	
1496,6513	92,4134		1496,6513	85,7583	
1498,58	92,981		1498,58	85,6333	
1500,5086	93,2591		1500,5086	85,4004	
1502,4373	93,3676		1502,4373	85,0583	
1504,366	93,3104		1504,366	84,7018	
1506,2947	93,1826		1506,2947	84,3677	
1508,2233	93,3062		1508,2233	83,8497	
1510,152	93,3925		1510,152	83,4595	
1512,0807	93,4471		1512,0807	83,068	
1514,0094	93,4428		1514,0094	82,6003	
1515,938	93,5118		1515,938	82,1638	
1517,8667	93,7373		1517,8667	81,6408	
1519,7954	93,7845		1519,7954	81,1189	
1521,7241	93,8959		1521,7241	80,5006	
1523,6527	94,0988		1523,6527	79,8681	
1525,5814	94,2113		1525,5814	79,1564	
1527,5101	94,266		1527,5101	78,2676	
1529,4388	94,1879		1529,4388	77,3749	
1531,3674	94,066		1531,3674	76,5846	
1533,2961	94,0141		1533,2961	75,6307	
1535,2248	93,9852		1535,2248	74,4869	
1537,1534	93,8707		1537,1534	73,6034	
1539,0821	93,7012		1539,0821	72,6723	
1541,0108	93,693		1541,0108	71,2825	
1542,9395	93,7729		1542,9395	69,9864	
1544,8681	93,8133		1544,8681	68,66	
1546,7968	93,7789		1546,7968	67,513	
1548,7255	93,7375		1548,7255	66,5502	

1550,6542	93,7781		1550,6542	65,5164	
1552,5828	93,8355		1552,5828	64,662	
1554,5115	93,94		1554,5115	63,8849	
1556,4402	93,9552		1556,4402	63,282	
1558,3689	93,861		1558,3689	63,0843	
1560,2975	94,1812		1560,2975	63,3708	
1562,2262	94,2161		1562,2262	63,151	
1564,1549	94,201		1564,1549	63,1821	
1566,0836	94,1857		1566,0836	63,4861	
1568,0122	94,1116		1568,0122	63,7708	
1569,9409	94,13		1569,9409	64,5556	
1571,8696	94,1931		1571,8696	65,2275	
1573,7983	94,1691		1573,7983	65,5381	
1575,7269	94,1143		1575,7269	66,4372	
1577,6556	94,0544		1577,6556	67,8095	
1579,5843	94,0104		1579,5843	68,5091	
1581,513	93,964		1581,513	69,2736	
1583,4416	93,8832		1583,4416	70,1844	
1585,3703	93,7814		1585,3703	71,0782	
1587,299	93,6993		1587,299	71,9678	
1589,2277	93,5816		1589,2277	72,8742	
1591,1563	93,4498		1591,1563	73,7665	
1593,085	93,4119		1593,085	74,565	
1595,0137	93,3726		1595,0137	75,4341	
1596,9424	93,277		1596,9424	76,3008	
1598,871	93,2585		1598,871	77,0319	
1600,7997	93,2468		1600,7997	77,7198	
1602,7284	93,1623		1602,7284	78,4591	
1604,6571	93,0346		1604,6571	79,181	
1606,5857	92,8781		1606,5857	79,8234	

1608,5144	92,7868		1608,5144	80,4781	
1610,4431	92,6896		1610,4431	81,1252	
1612,3717	92,5049		1612,3717	81,6327	
1614,3004	92,3412		1614,3004	82,0003	
1616,2291	92,171		1616,2291	82,5992	
1618,1578	91,9719		1618,1578	83,2931	
1620,0864	91,7731		1620,0864	83,5447	
1622,0151	91,5595		1622,0151	83,858	
1623,9438	91,3796		1623,9438	84,3674	
1625,8725	91,1952		1625,8725	84,6842	
1627,8011	91,0151		1627,8011	85,0466	
1629,7298	90,8367		1629,7298	85,4223	
1631,6585	90,6351		1631,6585	85,5715	
1633,5872	90,4872		1633,5872	85,677	
1635,5158	90,4465		1635,5158	86,0261	
1637,4445	90,4153		1637,4445	86,4246	
1639,3732	90,3396		1639,3732	86,5529	
1641,3019	90,2435		1641,3019	86,6897	
1643,2305	90,1347		1643,2305	86,811	
1645,1592	89,9955		1645,1592	86,8798	
1647,0879	89,9761		1647,0879	87,1184	
1649,0166	90,0685		1649,0166	87,3113	
1650,9452	90,003		1650,9452	87,2434	
1652,8739	90,1873		1652,8739	87,4232	
1654,8026	90,5903		1654,8026	87,8678	
1656,7313	90,5714		1656,7313	87,8394	
1658,6599	90,6949		1658,6599	87,8896	
1660,5886	90,8732		1660,5886	87,9505	
1662,5173	91,1463		1662,5173	88,0882	
1664,446	91,4999		1664,446	88,266	

1666,3746	91,6314		1666,3746	88,2374	
1668,3033	91,7509		1668,3033	88,2577	
1670,232	92,0593		1670,232	88,458	
1672,1607	92,2226		1672,1607	88,4738	
1674,0893	92,3272		1674,0893	88,4414	
1676,018	92,5535		1676,018	88,4493	
1677,9467	92,6065		1677,9467	88,3941	
1679,8754	92,5185		1679,8754	88,2999	
1681,804	92,4211		1681,804	88,1109	
1683,7327	92,4261		1683,7327	87,8091	
1685,6614	92,4708		1685,6614	87,7636	
1687,59	92,4424		1687,59	87,7273	
1689,5187	92,5082		1689,5187	87,6102	
1691,4474	92,5241		1691,4474	87,4809	
1693,3761	92,3899		1693,3761	87,3041	
1695,3047	92,1427		1695,3047	87,2511	
1697,2334	91,8301		1697,2334	87,3009	
1699,1621	91,5493		1699,1621	87,2497	
1701,0908	90,7896		1701,0908	87,5452	
1703,0194	90,0701		1703,0194	87,6349	
1704,9481	89,0994		1704,9481	87,6804	
1706,8768	87,517		1706,8768	87,9784	
1708,8055	85,8228		1708,8055	88,2553	
1710,7341	83,6851		1710,7341	88,5247	
1712,6628	81,2857		1712,6628	88,7593	
1714,5915	78,2289		1714,5915	89,0199	
1716,5202	74,5459		1716,5202	89,353	
1718,4488	69,5768		1718,4488	89,8555	
1720,3775	65,8318		1720,3775	90,2293	
1722,3062	62,9386		1722,3062	90,4889	

1724,2349	60,1008		1724,2349	90,7912	
1726,1635	58,3201		1726,1635	91,0485	
1728,0922	57,4566		1728,0922	91,3059	
1730,0209	57,6331		1730,0209	91,6792	
1731,9496	58,3871		1731,9496	91,9619	
1733,8782	60,789		1733,8782	92,3398	
1735,8069	64,3962		1735,8069	92,7994	
1737,7356	66,6803		1737,7356	93,0114	
1739,6643	70,842		1739,6643	93,4101	
1741,5929	75,4903		1741,5929	93,806	
1743,5216	79,6694		1743,5216	94,0032	
1745,4503	83,6954		1745,4503	94,1606	
1747,379	86,9173		1747,379	94,31	
1749,3076	89,8877		1749,3076	94,3933	
1751,2363	92,2227		1751,2363	94,5356	
1753,165	93,7148		1753,165	94,739	
1755,0936	94,5592		1755,0936	94,809	
1757,0223	95,3677		1757,0223	94,875	
1758,951	95,8778		1758,951	94,9588	
1760,8797	96,2144		1760,8797	95,0111	
1762,8083	96,623		1762,8083	95,0693	
1764,737	96,8081		1764,737	95,0276	
1766,6657	96,8893		1766,6657	94,9608	
1768,5944	97,0423		1768,5944	94,9831	
1770,523	97,2124		1770,523	95,0284	
1772,4517	97,4021		1772,4517	95,0843	
1774,3804	97,4989		1774,3804	95,1209	
1776,3091	97,5348		1776,3091	95,1267	
1778,2377	97,609		1778,2377	95,1035	
1780,1664	97,7317		1780,1664	95,0652	

1782,0951	97,7895		1782,0951	95,0855	
1784,0238	97,8011		1784,0238	95,1396	
1785,9524	97,8653		1785,9524	95,1926	
1787,8811	97,8877		1787,8811	95,1763	
1789,8098	97,889		1789,8098	95,1607	
1791,7385	97,966		1791,7385	95,2572	
1793,6671	98,0392		1793,6671	95,2288	
1795,5958	98,032		1795,5958	95,062	
1797,5245	98,017		1797,5245	95,0044	
1799,4532	98,0098		1799,4532	95,1131	
1801,3818	98,0687		1801,3818	95,2026	
1803,3105	98,1598		1803,3105	95,1661	
1805,2392	98,1797		1805,2392	95,1561	
1807,1679	98,1951		1807,1679	95,1865	
1809,0965	98,226		1809,0965	95,1845	
1811,0252	98,231		1811,0252	95,1674	
1812,9539	98,2472		1812,9539	95,1724	
1814,8826	98,2915		1814,8826	95,2165	
1816,8112	98,3397		1816,8112	95,1853	
1818,7399	98,3149		1818,7399	95,0569	
1820,6686	98,2554		1820,6686	95,0386	
1822,5973	98,1986		1822,5973	95,0696	
1824,5259	98,1588		1824,5259	95,0269	
1826,4546	98,2413		1826,4546	94,937	
1828,3833	98,3119		1828,3833	94,8765	
1830,3119	98,3767		1830,3119	94,9596	
1832,2406	98,4025		1832,2406	95,0735	
1834,1693	98,3533		1834,1693	95,0185	
1836,098	98,3411		1836,098	94,8897	
1838,0266	98,3361		1838,0266	94,8742	

1839,9553	98,3848		1839,9553	94,9189	
1841,884	98,45		1841,884	94,956	
1843,8127	98,384		1843,8127	95,0208	
1845,7413	98,3433		1845,7413	95,0182	
1847,67	98,3912		1847,67	94,9169	
1849,5987	98,3843		1849,5987	94,8006	
1851,5274	98,3662		1851,5274	94,7426	
1853,456	98,3658		1853,456	94,7076	
1855,3847	98,395		1855,3847	94,6927	
1857,3134	98,4573		1857,3134	94,7392	
1859,2421	98,471		1859,2421	94,7659	
1861,1707	98,4961		1861,1707	94,7437	
1863,0994	98,4862		1863,0994	94,7679	
1865,0281	98,3641		1865,0281	94,8361	
1866,9568	98,2718		1866,9568	94,8605	
1868,8854	98,3359		1868,8854	94,8546	
1870,8141	98,3886		1870,8141	94,7479	
1872,7428	98,3471		1872,7428	94,6326	
1874,6715	98,3419		1874,6715	94,6294	
1876,6001	98,3619		1876,6001	94,6097	
1878,5288	98,3371		1878,5288	94,574	
1880,4575	98,2775		1880,4575	94,6005	
1882,3862	98,2123		1882,3862	94,6168	
1884,3148	98,1421		1884,3148	94,6174	
1886,2435	98,1524		1886,2435	94,6537	
1888,1722	98,2781		1888,1722	94,6513	
1890,1009	98,3699		1890,1009	94,5568	
1892,0295	98,3167		1892,0295	94,5353	
1893,9582	98,2404		1893,9582	94,536	
1895,8869	98,1605		1895,8869	94,4035	

1897,8156	97,9947		1897,8156	94,4298	
1899,7442	97,8847		1899,7442	94,6474	
1901,6729	98,0491		1901,6729	94,6966	
1903,6016	98,3185		1903,6016	94,5923	
1905,5302	98,3664		1905,5302	94,4708	
1907,4589	98,2576		1907,4589	94,4153	
1909,3876	98,1184		1909,3876	94,4737	
1911,3163	98,0209		1911,3163	94,4829	
1913,2449	98,0542		1913,2449	94,4847	
1915,1736	98,103		1915,1736	94,5595	
1917,1023	98,0692		1917,1023	94,5653	
1919,031	98,0374		1919,031	94,4195	
1920,9596	97,9894		1920,9596	94,3126	
1922,8883	97,8318		1922,8883	94,3599	
1924,817	97,7484		1924,817	94,3905	
1926,7457	97,85		1926,7457	94,3641	
1928,6743	97,94		1928,6743	94,418	
1930,603	97,8216		1930,603	94,4466	
1932,5317	97,6974		1932,5317	94,3289	
1934,4604	97,7389		1934,4604	94,2551	
1936,389	97,7244		1936,389	94,2797	
1938,3177	97,683		1938,3177	94,2344	
1940,2464	97,7119		1940,2464	94,0715	
1942,1751	97,6767		1942,1751	93,9482	
1944,1037	97,6704		1944,1037	93,9779	
1946,0324	97,7225		1946,0324	94,0558	
1947,9611	97,6438		1947,9611	94,2105	
1949,8898	97,6073		1949,8898	94,3086	
1951,8184	97,5406		1951,8184	94,4341	
1953,7471	97,4975		1953,7471	94,649	

1955,6758	97,7468		1955,6758	94,5704	
1957,6045	97,8536		1957,6045	94,3515	
1959,5331	97,6958		1959,5331	94,1417	
1961,4618	97,523		1961,4618	93,893	
1963,3905	97,4442		1963,3905	93,8893	
1965,3192	97,579		1965,3192	94,0128	
1967,2478	97,6755		1967,2478	94,1173	
1969,1765	97,4314		1969,1765	94,2544	
1971,1052	97,2494		1971,1052	94,3235	
1973,0339	97,2626		1973,0339	94,199	
1974,9625	97,2248		1974,9625	93,943	
1976,8912	97,0375		1976,8912	93,7047	
1978,8199	96,7982		1978,8199	93,5466	
1980,7485	96,9734		1980,7485	93,4654	
1982,6772	97,253		1982,6772	93,4097	
1984,6059	97,2075		1984,6059	93,4777	
1986,5346	97,2091		1986,5346	93,601	
1988,4632	97,3351		1988,4632	93,7615	
1990,3919	97,3411		1990,3919	93,8486	
1992,3206	97,2928		1992,3206	93,7033	
1994,2493	97,2555		1994,2493	93,9057	
1996,1779	97,3043		1996,1779	94,3497	
1998,1066	97,4672		1998,1066	94,3498	
2000,0353	97,5972		2000,0353	94,114	
2001,964	97,7761		2001,964	94,1159	
2003,8926	98,0555		2003,8926	94,1516	
2005,8213	98,0844		2005,8213	93,8917	
2007,75	97,6366		2007,75	93,5682	
2009,6787	97,3085		2009,6787	93,5154	
2011,6073	97,3331		2011,6073	93,7862	

2013,536	97,4		2013,536	94,1773	
2015,4647	97,3298		2015,4647	94,327	
2017,3934	97,3245		2017,3934	94,1393	
2019,322	97,4944		2019,322	93,8079	
2021,2507	97,4506		2021,2507	93,5205	
2023,1794	97,4524		2023,1794	93,4207	
2025,1081	97,5971		2025,1081	93,5671	
2027,0367	97,568		2027,0367	93,702	
2028,9654	97,4161		2028,9654	93,6455	
2030,8941	97,23		2030,8941	93,6747	
2032,8228	97,1565		2032,8228	93,6655	
2034,7514	97,3389		2034,7514	93,4191	
2036,6801	97,5275		2036,6801	93,3027	
2038,6088	97,5975		2038,6088	93,4685	
2040,5375	97,6928		2040,5375	93,633	
2042,4661	97,4676		2042,4661	93,4626	
2044,3948	97,1124		2044,3948	93,3303	
2046,3235	97,2288		2046,3235	93,5806	
2048,2521	97,3484		2048,2521	93,6888	
2050,1808	97,339		2050,1808	93,5284	
2052,1095	97,5232		2052,1095	93,5492	
2054,0382	97,6717		2054,0382	93,6547	
2055,9668	97,7562		2055,9668	93,6101	
2057,8955	97,7148		2057,8955	93,5376	
2059,8242	97,6525		2059,8242	93,5484	
2061,7529	97,692		2061,7529	93,6055	
2063,6815	97,668		2063,6815	93,776	
2065,6102	97,8656		2065,6102	93,9107	
2067,5389	98,0061		2067,5389	93,8798	
2069,4676	97,787		2069,4676	93,8559	

2071,3962	97,6195		2071,3962	93,7636	
2073,3249	97,6616		2073,3249	93,6645	
2075,2536	97,7785		2075,2536	93,6864	
2077,1823	97,8479		2077,1823	93,6458	
2079,1109	97,9107		2079,1109	93,5128	
2081,0396	97,8728		2081,0396	93,4889	
2082,9683	97,7391		2082,9683	93,5641	
2084,897	97,6981		2084,897	93,731	
2086,8256	97,8189		2086,8256	93,8678	
2088,7543	98,0183		2088,7543	93,8918	
2090,683	98,1037		2090,683	93,9454	
2092,6117	97,9829		2092,6117	93,9536	
2094,5403	97,8652		2094,5403	93,8779	
2096,469	97,8873		2096,469	93,7943	
2098,3977	97,8632		2098,3977	93,7489	
2100,3264	97,8387		2100,3264	93,7376	
2102,255	97,8759		2102,255	93,7232	
2104,1837	97,9303		2104,1837	93,7139	
2106,1124	98,0007		2106,1124	93,7524	
2108,0411	97,9637		2108,0411	93,7478	
2109,9697	97,8249		2109,9697	93,6695	
2111,8984	97,7795		2111,8984	93,6521	
2113,8271	97,9326		2113,8271	93,7068	
2115,7558	98,0494		2115,7558	93,7977	
2117,6844	98,043		2117,6844	93,8923	
2119,6131	98,1364		2119,6131	93,8484	
2121,5418	98,1742		2121,5418	93,794	
2123,4704	98,0579		2123,4704	93,8726	
2125,3991	98,0917		2125,3991	93,9387	
2127,3278	98,2401		2127,3278	93,9346	

2129,2565	98,3773			2129,2565	93,857	
2131,1851	98,4503			2131,1851	93,8519	
2133,1138	98,2904			2133,1138	93,8733	
2135,0425	98,1157			2135,0425	93,9318	
2136,9712	98,1011			2136,9712	93,9806	
2138,8998	98,0749			2138,8998	93,7881	
2140,8285	98,0372			2140,8285	93,6897	
2142,7572	98,0552			2142,7572	93,7871	
2144,6859	97,9381			2144,6859	93,9255	
2146,6145	97,7132			2146,6145	94,0354	
2148,5432	97,7762			2148,5432	94,0822	
2150,4719	98,0193			2150,4719	94,1465	
2152,4006	98,0957			2152,4006	94,2062	
2154,3292	98,2439			2154,3292	94,1296	
2156,2579	98,3683			2156,2579	93,8438	
2158,1866	97,8891			2158,1866	93,3244	
2160,1153	97,3507			2160,1153	92,9052	
2162,0439	97,3242			2162,0439	93,062	
2163,9726	97,4472			2163,9726	93,3303	
2165,9013	97,5123			2165,9013	93,1948	
2167,83	97,3567			2167,83	93,0924	
2169,7586	97,3023			2169,7586	93,2773	
2171,6873	97,6743			2171,6873	93,2516	
2173,616	97,834			2173,616	93,2841	
2175,5447	97,6086			2175,5447	93,5154	
2177,4733	97,6553			2177,4733	93,3705	
2179,402	97,8884			2179,402	93,2083	
2181,3307	97,9648			2181,3307	93,1663	
2183,2594	97,9279			2183,2594	93,1502	
2185,188	97,7619			2185,188	93,3049	

2187,1167	97,7183		2187,1167	93,3483	
2189,0454	97,7868		2189,0454	93,3453	
2190,9741	97,7987		2190,9741	93,4407	
2192,9027	97,8346		2192,9027	93,4781	
2194,8314	97,8361		2194,8314	93,3287	
2196,7601	97,7723		2196,7601	93,1864	
2198,6887	97,7795		2198,6887	93,3279	
2200,6174	97,9861		2200,6174	93,4901	
2202,5461	98,048		2202,5461	93,4371	
2204,4748	97,9768		2204,4748	93,2525	
2206,4034	97,9828		2206,4034	93,2015	
2208,3321	97,8857		2208,3321	93,435	
2210,2608	97,9119		2210,2608	93,6116	
2212,1895	98,1124		2212,1895	93,5192	
2214,1181	98,1266		2214,1181	93,3233	
2216,0468	97,8587		2216,0468	93,1596	
2217,9755	97,627		2217,9755	93,1909	
2219,9042	97,842		2219,9042	93,4854	
2221,8328	98,1278		2221,8328	93,6437	
2223,7615	98,093		2223,7615	93,5448	
2225,6902	98,0075		2225,6902	93,5259	
2227,6189	97,9797		2227,6189	93,5232	
2229,5475	97,996		2229,5475	93,4529	
2231,4762	97,8581		2231,4762	93,4	
2233,4049	97,6839		2233,4049	93,3142	
2235,3336	97,8422		2235,3336	93,2734	
2237,2622	98,0779		2237,2622	93,3628	
2239,1909	98,1344		2239,1909	93,4822	
2241,1196	98,0824		2241,1196	93,4565	
2243,0483	98,0953		2243,0483	93,3912	

2244,9769	98,266		2244,9769	93,5466	
2246,9056	98,3364		2246,9056	93,747	
2248,8343	98,1917		2248,8343	93,7268	
2250,763	98,1628		2250,763	93,6172	
2252,6916	98,2223		2252,6916	93,5106	
2254,6203	98,1083		2254,6203	93,315	
2256,549	97,9257		2256,549	93,1528	
2258,4777	97,9618		2258,4777	93,1557	
2260,4063	98,1239		2260,4063	93,2057	
2262,335	98,0165		2262,335	93,2962	
2264,2637	97,9129		2264,2637	93,3953	
2266,1923	98,0669		2266,1923	93,3394	
2268,121	98,1051		2268,121	93,3571	
2270,0497	98,025		2270,0497	93,4662	
2271,9784	98,0459		2271,9784	93,3845	
2273,907	98,1171		2273,907	93,2588	
2275,8357	98,1513		2275,8357	93,2583	
2277,7644	98,1188		2277,7644	93,3017	
2279,6931	98,0509		2279,6931	93,3219	
2281,6217	98,0322		2281,6217	93,3531	
2283,5504	98,038		2283,5504	93,3422	
2285,4791	97,9772		2285,4791	93,2909	
2287,4078	98,0215		2287,4078	93,2712	
2289,3364	98,1072		2289,3364	93,2852	
2291,2651	98,0628		2291,2651	93,2868	
2293,1938	98,0317		2293,1938	93,2838	
2295,1225	98,0352		2295,1225	93,3507	
2297,0511	98,1074		2297,0511	93,3877	
2298,9798	98,1855		2298,9798	93,36	
2300,9085	98,0932		2300,9085	93,3614	

2302,8372	97,9729		2302,8372	93,3805	
2304,7658	98,0348		2304,7658	93,3122	
2306,6945	98,1011		2306,6945	93,1467	
2308,6232	98,0363		2308,6232	93,0443	
2310,5519	98,0542		2310,5519	93,0461	
2312,4805	98,0595		2312,4805	93,0097	
2314,4092	98,0023		2314,4092	92,9539	
2316,3379	98,007		2316,3379	92,8777	
2318,2666	97,8608		2318,2666	92,6605	
2320,1952	97,6856		2320,1952	92,5043	
2322,1239	97,6748		2322,1239	92,4906	
2324,0526	97,6932		2324,0526	92,494	
2325,9813	97,7783		2325,9813	92,5657	
2327,9099	97,8607		2327,9099	92,6616	
2329,8386	97,7826		2329,8386	92,7541	
2331,7673	97,7391		2331,7673	92,7655	
2333,696	97,8553		2333,696	92,6516	
2335,6246	97,8754		2335,6246	92,6011	
2337,5533	97,765		2337,5533	92,6429	
2339,482	97,7354		2339,482	92,5529	
2341,4106	97,7357		2341,4106	92,369	
2343,3393	97,6073		2343,3393	92,502	
2345,268	97,6461		2345,268	92,8968	
2347,1967	97,8712		2347,1967	93,0424	
2349,1253	97,9624		2349,1253	92,8984	
2351,054	97,984		2351,054	92,6788	
2352,9827	97,926		2352,9827	92,5424	
2354,9114	97,6506		2354,9114	92,4835	
2356,84	97,5866		2356,84	92,3414	
2358,7687	97,8212		2358,7687	92,1451	

2360,6974	97,7076		2360,6974	92,0347	
2362,6261	97,528		2362,6261	91,9947	
2364,5547	97,6208		2364,5547	92,0852	
2366,4834	97,7964		2366,4834	92,424	
2368,4121	97,901		2368,4121	92,6839	
2370,3408	97,9146		2370,3408	92,664	
2372,2694	97,9754		2372,2694	92,6345	
2374,1981	98,0148		2374,1981	92,786	
2376,1268	98,0185		2376,1268	92,9822	
2378,0555	98,1453		2378,0555	93,0755	
2379,9841	98,2889		2379,9841	93,1864	
2381,9128	98,3199		2381,9128	93,3217	
2383,8415	98,3575		2383,8415	93,3718	
2385,7702	98,4126		2385,7702	93,3893	
2387,6988	98,3942		2387,6988	93,4053	
2389,6275	98,3645		2389,6275	93,3662	
2391,5562	98,4438		2391,5562	93,3335	
2393,4849	98,5326		2393,4849	93,3899	
2395,4135	98,5099		2395,4135	93,4172	
2397,3422	98,4702		2397,3422	93,3869	
2399,2709	98,527		2399,2709	93,3677	
2401,1996	98,5454		2401,1996	93,3386	
2403,1282	98,4272		2403,1282	93,3166	
2405,0569	98,3821		2405,0569	93,2687	
2406,9856	98,3987		2406,9856	93,217	
2408,9143	98,3874		2408,9143	93,2335	
2410,8429	98,3861		2410,8429	93,2436	
2412,7716	98,4106		2412,7716	93,2511	
2414,7003	98,3723		2414,7003	93,2629	
2416,6289	98,2925		2416,6289	93,2214	

2418,5576	98,3309		2418,5576	93,1723	
2420,4863	98,337		2420,4863	93,1158	
2422,415	98,265		2422,415	93,0921	
2424,3436	98,3084		2424,3436	93,1101	
2426,2723	98,4169		2426,2723	93,0418	
2428,201	98,3855		2428,201	93,0119	
2430,1297	98,3156		2430,1297	93,117	
2432,0583	98,3716		2432,0583	93,0718	
2433,987	98,3863		2433,987	92,9281	
2435,9157	98,302		2435,9157	92,9409	
2437,8444	98,2872		2437,8444	92,946	
2439,773	98,3115		2439,773	92,8882	
2441,7017	98,2865		2441,7017	92,9104	
2443,6304	98,255		2443,6304	92,9613	
2445,5591	98,24		2445,5591	92,923	
2447,4877	98,2719		2447,4877	92,8273	
2449,4164	98,3247		2449,4164	92,7776	
2451,3451	98,3281		2451,3451	92,7828	
2453,2738	98,2697		2453,2738	92,8517	
2455,2024	98,221		2455,2024	92,8645	
2457,1311	98,2209		2457,1311	92,7737	
2459,0598	98,1879		2459,0598	92,691	
2460,9885	98,1762		2460,9885	92,6148	
2462,9171	98,2523		2462,9171	92,5825	
2464,8458	98,3075		2464,8458	92,5886	
2466,7745	98,2346		2466,7745	92,6376	
2468,7032	98,1201		2468,7032	92,6857	
2470,6318	98,1263		2470,6318	92,6572	
2472,5605	98,1933		2472,5605	92,6164	
2474,4892	98,2437		2474,4892	92,6187	

2476,4179	98,2755		2476,4179	92,6738	
2478,3465	98,2981		2478,3465	92,6571	
2480,2752	98,2791		2480,2752	92,5623	
2482,2039	98,1725		2482,2039	92,5238	
2484,1326	97,9963		2484,1326	92,486	
2486,0612	97,8442		2486,0612	92,4792	
2487,9899	97,9263		2487,9899	92,5224	
2489,9186	98,0722		2489,9186	92,4911	
2491,8472	98,0456		2491,8472	92,4155	
2493,7759	98,0444		2493,7759	92,3513	
2495,7046	98,0673		2495,7046	92,315	
2497,6333	98,0674		2497,6333	92,3488	
2499,5619	98,1318		2499,5619	92,4076	
2501,4906	98,163		2501,4906	92,375	
2503,4193	98,1503		2503,4193	92,2912	
2505,348	98,1397		2505,348	92,2733	
2507,2766	98,0967		2507,2766	92,2818	
2509,2053	98,0686		2509,2053	92,2499	
2511,134	98,0633		2511,134	92,206	
2513,0627	98,0455		2513,0627	92,21	
2514,9913	98,0714		2514,9913	92,2207	
2516,92	98,0932		2516,92	92,2692	
2518,8487	97,9557		2518,8487	92,3405	
2520,7774	97,8313		2520,7774	92,228	
2522,706	97,9176		2522,706	92,1229	
2524,6347	98,0492		2524,6347	92,1652	
2526,5634	98,0793		2526,5634	92,1708	
2528,4921	98,0524		2528,4921	92,1502	
2530,4207	98,0895		2530,4207	92,1583	
2532,3494	98,0943		2532,3494	92,1654	

2534,2781	97,9716		2534,2781	92,129	
2536,2068	97,9134		2536,2068	92,0845	
2538,1354	97,984		2538,1354	92,0774	
2540,0641	98,0739		2540,0641	92,1063	
2541,9928	98,0822		2541,9928	92,063	
2543,9215	98,0231		2543,9215	91,958	
2545,8501	97,9912		2545,8501	91,8794	
2547,7788	98,0116		2547,7788	91,7883	
2549,7075	98,0323		2549,7075	91,8106	
2551,6362	97,9781		2551,6362	91,8879	
2553,5648	97,9492		2553,5648	91,8413	
2555,4935	98,0168		2555,4935	91,8186	
2557,4222	98,0687		2557,4222	91,8074	
2559,3508	97,9997		2559,3508	91,7541	
2561,2795	97,8513		2561,2795	91,7197	
2563,2082	97,7886		2563,2082	91,6548	
2565,1369	97,8262		2565,1369	91,6707	
2567,0655	97,8923		2567,0655	91,7823	
2568,9942	97,943		2568,9942	91,7922	
2570,9229	97,947		2570,9229	91,7044	
2572,8516	97,9844		2572,8516	91,6143	
2574,7802	98,0367		2574,7802	91,5828	
2576,7089	97,9845		2576,7089	91,6525	
2578,6376	97,8848		2578,6376	91,6911	
2580,5663	97,8277		2580,5663	91,638	
2582,4949	97,7738		2582,4949	91,6135	
2584,4236	97,7284		2584,4236	91,5991	
2586,3523	97,7531		2586,3523	91,5138	
2588,281	97,8007		2588,281	91,4613	
2590,2096	97,8369		2590,2096	91,4945	

2592,1383	97,8906		2592,1383	91,483	
2594,067	97,8715		2594,067	91,4046	
2595,9957	97,8063		2595,9957	91,3714	
2597,9243	97,8213		2597,9243	91,4219	
2599,853	97,8194		2599,853	91,4082	
2601,7817	97,7708		2601,7817	91,3394	
2603,7104	97,7676		2603,7104	91,307	
2605,639	97,8038		2605,639	91,3049	
2607,5677	97,7899		2607,5677	91,3348	
2609,4964	97,7528		2609,4964	91,2718	
2611,4251	97,7182		2611,4251	91,1623	
2613,3537	97,6784		2613,3537	91,1682	
2615,2824	97,704		2615,2824	91,2275	
2617,2111	97,7115		2617,2111	91,2377	
2619,1398	97,6743		2619,1398	91,2041	
2621,0684	97,6628		2621,0684	91,1443	
2622,9971	97,6194		2622,9971	91,0968	
2624,9258	97,6372		2624,9258	91,0813	
2626,8545	97,7319		2626,8545	91,0456	
2628,7831	97,6877		2628,7831	90,9779	
2630,7118	97,6036		2630,7118	90,9597	
2632,6405	97,6415		2632,6405	91,0313	
2634,5691	97,6989		2634,5691	91,0775	
2636,4978	97,6834		2636,4978	91,0078	
2638,4265	97,6483		2638,4265	90,9316	
2640,3552	97,6459		2640,3552	90,9702	
2642,2838	97,6778		2642,2838	91,0147	
2644,2125	97,6789		2644,2125	90,9205	
2646,1412	97,6449		2646,1412	90,8179	
2648,0699	97,6423		2648,0699	90,8342	

2649,9985	97,6012		2649,9985	90,8612	
2651,9272	97,5872		2651,9272	90,85	
2653,8559	97,6531		2653,8559	90,8612	
2655,7846	97,6632		2655,7846	90,8853	
2657,7132	97,6231		2657,7132	90,8598	
2659,6419	97,6205		2659,6419	90,8392	
2661,5706	97,608		2661,5706	90,8654	
2663,4993	97,5732		2663,4993	90,8288	
2665,4279	97,6156		2665,4279	90,752	
2667,3566	97,6754		2667,3566	90,7503	
2669,2853	97,6319		2669,2853	90,7466	
2671,214	97,5481		2671,214	90,7135	
2673,1426	97,5332		2673,1426	90,7358	
2675,0713	97,5245		2675,0713	90,7413	
2677	97,4917		2677	90,6937	
2678,9287	97,4718		2678,9287	90,6498	
2680,8573	97,4082		2680,8573	90,6246	
2682,786	97,3631		2682,786	90,6003	
2684,7147	97,3655		2684,7147	90,566	
2686,6434	97,3282		2686,6434	90,5422	
2688,572	97,2608		2688,572	90,5049	
2690,5007	97,195		2690,5007	90,4782	
2692,4294	97,1419		2692,4294	90,4752	
2694,3581	97,1136		2694,3581	90,4505	
2696,2867	97,0764		2696,2867	90,4268	
2698,2154	97,0242		2698,2154	90,4021	
2700,1441	96,9829		2700,1441	90,4034	
2702,0728	96,9386		2702,0728	90,4206	
2704,0014	96,8931		2704,0014	90,3944	
2705,9301	96,8561		2705,9301	90,3704	

2707,8588	96,8049		2707,8588	90,3469	
2709,7874	96,7464		2709,7874	90,2861	
2711,7161	96,7393		2711,7161	90,2782	
2713,6448	96,7292		2713,6448	90,3375	
2715,5735	96,6659		2715,5735	90,3034	
2717,5021	96,6255		2717,5021	90,2126	
2719,4308	96,5845		2719,4308	90,2171	
2721,3595	96,5161		2721,3595	90,239	
2723,2882	96,4861		2723,2882	90,2134	
2725,2168	96,4991		2725,2168	90,1748	
2727,1455	96,4928		2727,1455	90,1254	
2729,0742	96,4624		2729,0742	90,0833	
2731,0029	96,4279		2731,0029	90,0677	
2732,9315	96,3648		2732,9315	90,0876	
2734,8602	96,3205		2734,8602	90,0658	
2736,7889	96,3311		2736,7889	90,0031	
2738,7176	96,3115		2738,7176	89,9749	
2740,6462	96,2317		2740,6462	89,9606	
2742,5749	96,1841		2742,5749	89,9358	
2744,5036	96,1888		2744,5036	89,9056	
2746,4323	96,1936		2746,4323	89,8934	
2748,3609	96,1925		2748,3609	89,9072	
2750,2896	96,1806		2750,2896	89,9152	
2752,2183	96,1515		2752,2183	89,8866	
2754,147	96,1138		2754,147	89,8524	
2756,0756	96,0954		2756,0756	89,822	
2758,0043	96,1313		2758,0043	89,7576	
2759,933	96,164		2759,933	89,6863	
2761,8617	96,1067		2761,8617	89,6453	
2763,7903	95,9914		2763,7903	89,6208	

2765,719	95,9098		2765,719	89,59	
2767,6477	95,8973		2767,6477	89,5154	
2769,5764	95,8888		2769,5764	89,4587	
2771,505	95,825		2771,505	89,4559	
2773,4337	95,7127		2773,4337	89,3977	
2775,3624	95,6007		2775,3624	89,3403	
2777,291	95,5659		2777,291	89,3212	
2779,2197	95,576		2779,2197	89,2319	
2781,1484	95,491		2781,1484	89,1357	
2783,0771	95,3349		2783,0771	89,0725	
2785,0057	95,2568		2785,0057	88,9874	
2786,9344	95,2089		2786,9344	88,9276	
2788,8631	95,096		2788,8631	88,8712	
2790,7918	94,9417		2790,7918	88,7649	
2792,7204	94,776		2792,7204	88,6853	
2794,6491	94,6565		2794,6491	88,6245	
2796,5778	94,5635		2796,5778	88,5261	
2798,5065	94,4		2798,5065	88,4313	
2800,4351	94,2467		2800,4351	88,3758	
2802,3638	94,1689		2802,3638	88,3125	
2804,2925	94,0285		2804,2925	88,195	
2806,2212	93,8021		2806,2212	88,0865	
2808,1498	93,6153		2808,1498	87,9485	
2810,0785	93,4616		2810,0785	87,7673	
2812,0072	93,2737		2812,0072	87,6568	
2813,9359	93,0882		2813,9359	87,52	
2815,8645	92,8899		2815,8645	87,341	
2817,7932	92,6893		2817,7932	87,219	
2819,7219	92,4701		2819,7219	87,064	
2821,6506	92,1781		2821,6506	86,8772	

2823,5792	91,901		2823,5792	86,7276	
2825,5079	91,646		2825,5079	86,567	
2827,4366	91,3289		2827,4366	86,3693	
2829,3653	90,9464		2829,3653	86,1497	
2831,2939	90,5563		2831,2939	85,9063	
2833,2226	90,1087		2833,2226	85,5965	
2835,1513	89,5393		2835,1513	85,1849	
2837,08	88,907		2837,08	84,7595	
2839,0086	88,2263		2839,0086	84,3314	
2840,9373	87,4336		2840,9373	83,7969	
2842,866	86,5058		2842,866	83,1507	
2844,7947	85,4807		2844,7947	82,4021	
2846,7233	84,4427		2846,7233	81,5628	
2848,652	83,4198		2848,652	80,6829	
2850,5807	82,4338		2850,5807	79,8356	
2852,5093	81,6122		2852,5093	79,0718	
2854,438	80,9992		2854,438	78,3653	
2856,3667	80,5965		2856,3667	77,6755	
2858,2954	80,3251		2858,2954	77,0723	
2860,224	80,0712		2860,224	76,6699	
2862,1527	79,8795		2862,1527	76,4962	
2864,0814	79,7955		2864,0814	76,5847	
2866,0101	79,7667		2866,0101	76,8431	
2867,9387	79,7387		2867,9387	77,1337	
2869,8674	79,7337		2869,8674	77,5111	
2871,7961	79,8138		2871,7961	77,9872	
2873,7248	80,0077		2873,7248	78,4791	
2875,6534	80,3004		2875,6534	78,8899	
2877,5821	80,5685		2877,5821	79,2071	
2879,5108	80,7313		2879,5108	79,5201	

2881,4395	80,9308		2881,4395	79,749	
2883,3681	81,1947		2883,3681	79,8286	
2885,2968	81,3774		2885,2968	79,8045	
2887,2255	81,4442		2887,2255	79,6999	
2889,1542	81,5078		2889,1542	79,5619	
2891,0828	81,6436		2891,0828	79,3866	
2893,0115	81,7485		2893,0115	79,1117	
2894,9402	81,7596		2894,9402	78,7409	
2896,8689	81,6997		2896,8689	78,368	
2898,7975	81,5669		2898,7975	77,9785	
2900,7262	81,4546		2900,7262	77,5528	
2902,6549	81,3659		2902,6549	77,1566	
2904,5836	81,285		2904,5836	76,811	
2906,5122	81,2525		2906,5122	76,4827	
2908,4409	81,1864		2908,4409	76,139	
2910,3696	81,1231		2910,3696	75,7484	
2912,2983	81,0356		2912,2983	75,3173	
2914,2269	80,8236		2914,2269	74,8897	
2916,1556	80,5437		2916,1556	74,4745	
2918,0843	80,3282		2918,0843	74,1268	
2920,013	80,2465		2920,013	73,8603	
2921,9416	80,2007		2921,9416	73,6603	
2923,8703	80,2041		2923,8703	73,5529	
2925,799	80,3534		2925,799	73,5102	
2927,7276	80,6405		2927,7276	73,4673	
2929,6563	80,9775		2929,6563	73,3937	
2931,585	81,3457		2931,585	73,3293	
2933,5137	81,7791		2933,5137	73,3608	
2935,4423	82,2543		2935,4423	73,4977	
2937,371	82,7631		2937,371	73,7962	

2939,2997	83,2215		2939,2997	74,3012	
2941,2284	83,5629		2941,2284	74,8805	
2943,157	83,8435		2943,157	75,5294	
2945,0857	84,0753		2945,0857	76,2905	
2947,0144	84,2871		2947,0144	77,0851	
2948,9431	84,5597		2948,9431	77,8017	
2950,8717	84,9053		2950,8717	78,4018	
2952,8004	85,3118		2952,8004	78,9618	
2954,7291	85,8141		2954,7291	79,5337	
2956,6578	86,4538		2956,6578	80,09	
2958,5864	87,1675		2958,5864	80,5392	
2960,5151	87,8574		2960,5151	80,8961	
2962,4438	88,5055		2962,4438	81,1924	
2964,3725	89,1367		2964,3725	81,4036	
2966,3011	89,753		2966,3011	81,6214	
2968,2298	90,325		2968,2298	81,9014	
2970,1585	90,8024		2970,1585	82,1251	
2972,0872	91,1629		2972,0872	82,2777	
2974,0158	91,4239		2974,0158	82,49	
2975,9445	91,6425		2975,9445	82,7575	
2977,8732	91,8617		2977,8732	83,1525	
2979,8019	92,0196		2979,8019	83,724	
2981,7305	92,1544		2981,7305	84,3089	
2983,6592	92,3337		2983,6592	84,8152	
2985,5879	92,4938		2985,5879	85,3166	
2987,5166	92,6003		2987,5166	85,8123	
2989,4452	92,7321		2989,4452	86,196	
2991,3739	92,9284		2991,3739	86,5545	
2993,3026	93,1234		2993,3026	86,9169	
2995,2313	93,3812		2995,2313	87,2168	

2997,1599	93,631		2997,1599	87,4508	
2999,0886	93,758		2999,0886	87,5273	
3001,0173	93,9559		3001,0173	87,5326	
3002,9459	94,2709		3002,9459	87,5443	
3004,8746	94,4761		3004,8746	87,4821	
3006,8033	94,5993		3006,8033	87,4227	
3008,732	94,8337		3008,732	87,3644	
3010,6606	95,1185		3010,6606	87,3007	
3012,5893	95,3077		3012,5893	87,3046	
3014,518	95,4001		3014,518	87,331	
3016,4467	95,5365		3016,4467	87,4138	
3018,3753	95,726		3018,3753	87,5741	
3020,304	95,8422		3020,304	87,7011	
3022,2327	95,9273		3022,2327	87,7967	
3024,1614	96,0411		3024,1614	87,9149	
3026,09	96,1064		3026,09	88,0269	
3028,0187	96,1523		3028,0187	88,0888	
3029,9474	96,2654		3029,9474	88,1361	
3031,8761	96,4167		3031,8761	88,1815	
3033,8047	96,5235		3033,8047	88,1435	
3035,7334	96,585		3035,7334	88,0505	
3037,6621	96,632		3037,6621	88,0135	
3039,5908	96,6266		3039,5908	88,0334	
3041,5194	96,6067		3041,5194	88,0057	
3043,4481	96,6736		3043,4481	87,8713	
3045,3768	96,7178		3045,3768	87,7608	
3047,3055	96,6842		3047,3055	87,7603	
3049,2341	96,7148		3049,2341	87,739	
3051,1628	96,7826		3051,1628	87,6685	
3053,0915	96,789		3053,0915	87,6011	

3055,0202	96,7562		3055,0202	87,5382	
3056,9488	96,7881		3056,9488	87,4945	
3058,8775	96,8468		3058,8775	87,3958	
3060,8062	96,8881		3060,8062	87,2585	
3062,7349	96,8948		3062,7349	87,1733	
3064,6635	96,8275		3064,6635	87,1201	
3066,5922	96,7817		3066,5922	87,088	
3068,5209	96,7878		3068,5209	87,0729	
3070,4495	96,809		3070,4495	86,9947	
3072,3782	96,7643		3072,3782	86,8388	
3074,3069	96,7083		3074,3069	86,7168	
3076,2356	96,7752		3076,2356	86,6389	
3078,1642	96,8128		3078,1642	86,5639	
3080,0929	96,7396		3080,0929	86,4943	
3082,0216	96,7149		3082,0216	86,4073	
3083,9503	96,7344		3083,9503	86,3437	
3085,8789	96,6732		3085,8789	86,3467	
3087,8076	96,6003		3087,8076	86,3382	
3089,7363	96,6131		3089,7363	86,2143	
3091,665	96,6539		3091,665	86,0377	
3093,5936	96,6656		3093,5936	85,9233	
3095,5223	96,6372		3095,5223	85,8615	
3097,451	96,5739		3097,451	85,8391	
3099,3797	96,5205		3099,3797	85,8071	
3101,3083	96,5016		3101,3083	85,7288	
3103,237	96,4732		3103,237	85,6596	
3105,1657	96,4722		3105,1657	85,6045	
3107,0944	96,4795		3107,0944	85,5245	
3109,023	96,4122		3109,023	85,435	
3110,9517	96,3039		3110,9517	85,3775	

3112,8804	96,257		3112,8804	85,3343	
3114,8091	96,2927		3114,8091	85,2476	
3116,7377	96,2896		3116,7377	85,1025	
3118,6664	96,2466		3118,6664	84,9863	
3120,5951	96,1991		3120,5951	84,9545	
3122,5238	96,1638		3122,5238	84,8901	
3124,4524	96,1424		3124,4524	84,8178	
3126,3811	96,0747		3126,3811	84,791	
3128,3098	96,0173		3128,3098	84,716	
3130,2385	95,9979		3130,2385	84,6198	
3132,1671	95,953		3132,1671	84,5186	
3134,0958	95,9112		3134,0958	84,4389	
3136,0245	95,9161		3136,0245	84,3977	
3137,9532	95,9119		3137,9532	84,3753	
3139,8818	95,8564		3139,8818	84,3579	
3141,8105	95,7586		3141,8105	84,2463	
3143,7392	95,7093		3143,7392	84,0795	
3145,6678	95,7453		3145,6678	83,9609	
3147,5965	95,7628		3147,5965	83,9117	
3149,5252	95,7103		3149,5252	83,8438	
3151,4539	95,5998		3151,4539	83,6975	
3153,3825	95,5019		3153,3825	83,6407	
3155,3112	95,48		3155,3112	83,5798	
3157,2399	95,4526		3157,2399	83,4334	
3159,1686	95,3632		3159,1686	83,4136	
3161,0972	95,287		3161,0972	83,4252	
3163,0259	95,2091		3163,0259	83,3469	
3164,9546	95,1515		3164,9546	83,2777	
3166,8833	95,1524		3166,8833	83,2287	
3168,8119	95,089		3168,8119	83,1529	

3170,7406	94,9973		3170,7406	83,0705	
3172,6693	94,959		3172,6693	82,9798	
3174,598	94,9144		3174,598	82,8783	
3176,5266	94,8533		3176,5266	82,822	
3178,4553	94,7834		3178,4553	82,7875	
3180,384	94,7164		3180,384	82,7169	
3182,3127	94,6722		3182,3127	82,6301	
3184,2413	94,6427		3184,2413	82,5605	
3186,17	94,5471		3186,17	82,4986	
3188,0987	94,3954		3188,0987	82,4477	
3190,0274	94,3471		3190,0274	82,3896	
3191,956	94,3679		3191,956	82,2827	
3193,8847	94,3388		3193,8847	82,2041	
3195,8134	94,2865		3195,8134	82,1689	
3197,7421	94,2889		3197,7421	82,1067	
3199,6707	94,2926		3199,6707	82,0458	
3201,5994	94,1916		3201,5994	82,002	
3203,5281	94,0363		3203,5281	81,9703	
3205,4568	93,9166		3205,4568	81,916	
3207,3854	93,8096		3207,3854	81,809	
3209,3141	93,6765		3209,3141	81,7227	
3211,2428	93,6271		3211,2428	81,7447	
3213,1715	93,6401		3213,1715	81,7541	
3215,1001	93,5998		3215,1001	81,6038	
3217,0288	93,532		3217,0288	81,4515	
3218,9575	93,479		3218,9575	81,4046	
3220,8861	93,4332		3220,8861	81,3634	
3222,8148	93,3199		3222,8148	81,3276	
3224,7435	93,2053		3224,7435	81,343	
3226,6722	93,147		3226,6722	81,3149	

3228,6008	93,0393		3228,6008	81,2178	
3230,5295	92,952		3230,5295	81,1724	
3232,4582	92,9022		3232,4582	81,1712	
3234,3869	92,8004		3234,3869	81,1072	
3236,3155	92,7345		3236,3155	81,0349	
3238,2442	92,6811		3238,2442	80,9919	
3240,1729	92,5638		3240,1729	80,9532	
3242,1016	92,4885		3242,1016	80,9395	
3244,0302	92,4695		3244,0302	80,9271	
3245,9589	92,4027		3245,9589	80,8525	
3247,8876	92,2863		3247,8876	80,7555	
3249,8163	92,1789		3249,8163	80,7402	
3251,7449	92,1038		3251,7449	80,7106	
3253,6736	92,0338		3253,6736	80,6648	
3255,6023	91,9551		3255,6023	80,6811	
3257,531	91,8212		3257,531	80,686	
3259,4596	91,6416		3259,4596	80,6547	
3261,3883	91,5519		3261,3883	80,5969	
3263,317	91,5509		3263,317	80,5394	
3265,2457	91,5284		3265,2457	80,5054	
3267,1743	91,4332		3267,1743	80,4954	
3269,103	91,3101		3269,103	80,4419	
3271,0317	91,2352		3271,0317	80,3618	
3272,9604	91,1655		3272,9604	80,3989	
3274,889	91,1165		3274,889	80,4689	
3276,8177	91,0864		3276,8177	80,3812	
3278,7464	90,9944		3278,7464	80,2361	
3280,6751	90,9333		3280,6751	80,2363	
3282,6037	90,9343		3282,6037	80,2532	
3284,5324	90,9015		3284,5324	80,21	

3286,4611	90,8291		3286,4611	80,2173	
3288,3898	90,7951		3288,3898	80,2453	
3290,3184	90,7874		3290,3184	80,1915	
3292,2471	90,7657		3292,2471	80,0727	
3294,1758	90,7338		3294,1758	80,0669	
3296,1044	90,707		3296,1044	80,1168	
3298,0331	90,7059		3298,0331	80,1	
3299,9618	90,6716		3299,9618	80,0872	
3301,8905	90,6429		3301,8905	80,077	
3303,8191	90,6807		3303,8191	80,0387	
3305,7478	90,6873		3305,7478	80,0184	
3307,6765	90,6744		3307,6765	80,0509	
3309,6052	90,6618		3309,6052	80,0725	
3311,5338	90,5978		3311,5338	80,0512	
3313,4625	90,5172		3313,4625	80,0383	
3315,3912	90,45		3315,3912	80,0206	
3317,3199	90,4347		3317,3199	80,0434	
3319,2485	90,4207		3319,2485	80,0949	
3321,1772	90,3443		3321,1772	80,0176	
3323,1059	90,2717		3323,1059	79,8814	
3325,0346	90,2343		3325,0346	79,8603	
3326,9632	90,2394		3326,9632	79,8863	
3328,8919	90,2357		3328,8919	79,9053	
3330,8206	90,1389		3330,8206	79,9175	
3332,7493	90,0843		3332,7493	79,891	
3334,6779	90,1059		3334,6779	79,8544	
3336,6066	90,1558		3336,6066	79,8257	
3338,5353	90,2127		3338,5353	79,8128	
3340,464	90,1859		3340,464	79,868	
3342,3926	90,1625		3342,3926	79,9483	

3344,3213	90,2035		3344,3213	79,8778	
3346,25	90,2747		3346,25	79,7862	
3348,1787	90,2628		3348,1787	79,8281	
3350,1073	90,2081		3350,1073	79,8511	
3352,036	90,2773		3352,036	79,837	
3353,9647	90,3738		3353,9647	79,8539	
3355,8934	90,3663		3355,8934	79,8705	
3357,822	90,2846		3357,822	79,8423	
3359,7507	90,3229		3359,7507	79,8253	
3361,6794	90,4778		3361,6794	79,8945	
3363,608	90,567		3363,608	80,0015	
3365,5367	90,6292		3365,5367	80,0909	
3367,4654	90,6745		3367,4654	80,0899	
3369,3941	90,6997		3369,3941	80,0388	
3371,3227	90,7649		3371,3227	80,0836	
3373,2514	90,8279		3373,2514	80,1447	
3375,1801	90,8717		3375,1801	80,1784	
3377,1088	90,902		3377,1088	80,2302	
3379,0374	90,8815		3379,0374	80,2719	
3380,9661	90,8903		3380,9661	80,311	
3382,8948	90,9845		3382,8948	80,3488	
3384,8235	90,9952		3384,8235	80,4445	
3386,7521	90,9654		3386,7521	80,5598	
3388,6808	91,0569		3388,6808	80,5879	
3390,6095	91,1392		3390,6095	80,5913	
3392,5382	91,1305		3392,5382	80,6179	
3394,4668	91,153		3394,4668	80,6256	
3396,3955	91,2427		3396,3955	80,6927	
3398,3242	91,2476		3398,3242	80,8721	
3400,2529	91,173		3400,2529	81,007	

3402,1815	91,1778		3402,1815	81,0678	
3404,1102	91,2789		3404,1102	81,1531	
3406,0389	91,3758		3406,0389	81,1904	
3407,9676	91,4303		3407,9676	81,189	
3409,8962	91,5198		3409,8962	81,2808	
3411,8249	91,5333		3411,8249	81,3978	
3413,7536	91,4723		3413,7536	81,4828	
3415,6823	91,5408		3415,6823	81,5973	
3417,6109	91,5828		3417,6109	81,7046	
3419,5396	91,5574		3419,5396	81,785	
3421,4683	91,6574		3421,4683	81,8662	
3423,397	91,7685		3423,397	81,951	
3425,3256	91,8271		3425,3256	82,0486	
3427,2543	91,9067		3427,2543	82,1563	
3429,183	91,9649		3429,183	82,312	
3431,1117	92,0019		3431,1117	82,452	
3433,0403	92,0174		3433,0403	82,5163	
3434,969	92,0228		3434,969	82,6087	
3436,8977	92,0572		3436,8977	82,7531	
3438,8263	92,0687		3438,8263	82,8222	
3440,755	92,1134		3440,755	82,8296	
3442,6837	92,2126		3442,6837	82,9393	
3444,6124	92,2554		3444,6124	83,108	
3446,541	92,3037		3446,541	83,2784	
3448,4697	92,4022		3448,4697	83,4546	
3450,3984	92,4768		3450,3984	83,55	
3452,3271	92,5214		3452,3271	83,6083	
3454,2557	92,6112		3454,2557	83,7207	
3456,1844	92,6944		3456,1844	83,8286	
3458,1131	92,7159		3458,1131	83,9115	

3460,0418	92,8061		3460,0418	84,0929	
3461,9704	92,9182		3461,9704	84,3148	
3463,8991	92,9293		3463,8991	84,3718	
3465,8278	92,9289		3465,8278	84,4054	
3467,7565	93,0637		3467,7565	84,5691	
3469,6851	93,238		3469,6851	84,7261	
3471,6138	93,3293		3471,6138	84,8323	
3473,5425	93,4212		3473,5425	84,9385	
3475,4712	93,4742		3475,4712	85,0846	
3477,3998	93,4638		3477,3998	85,2278	
3479,3285	93,5077		3479,3285	85,3273	
3481,2572	93,5852		3481,2572	85,4023	
3483,1859	93,6711		3483,1859	85,5057	
3485,1145	93,774		3485,1145	85,6778	
3487,0432	93,86		3487,0432	85,8883	
3488,9719	93,9144		3488,9719	86,106	
3490,9006	93,9038		3490,9006	86,2472	
3492,8292	93,8879		3492,8292	86,3201	
3494,7579	93,9785		3494,7579	86,393	
3496,6866	94,0747		3496,6866	86,4963	
3498,6153	94,097		3498,6153	86,6137	
3500,5439	94,1413		3500,5439	86,7322	
3502,4726	94,2168		3502,4726	86,9048	
3504,4013	94,2801		3504,4013	87,1032	
3506,33	94,3115		3506,33	87,2401	
3508,2586	94,3871		3508,2586	87,4013	
3510,1873	94,5365		3510,1873	87,6579	
3512,116	94,6347		3512,116	87,802	
3514,0446	94,6586		3514,0446	87,8495	
3515,9733	94,6725		3515,9733	88,0311	

3517,902	94,7256		3517,902	88,272	
3519,8307	94,8295		3519,8307	88,4432	
3521,7593	94,8945		3521,7593	88,5982	
3523,688	94,8914		3523,688	88,7075	
3525,6167	95,0028		3525,6167	88,7411	
3527,5454	95,2159		3527,5454	88,8214	
3529,474	95,3057		3529,474	89,0555	
3531,4027	95,3078		3531,4027	89,3014	
3533,3314	95,3247		3533,3314	89,4203	
3535,2601	95,3436		3535,2601	89,5897	
3537,1887	95,3607		3537,1887	89,8262	
3539,1174	95,4083		3539,1174	89,9663	
3541,0461	95,5327		3541,0461	89,9829	
3542,9748	95,6586		3542,9748	90,0051	
3544,9034	95,7855		3544,9034	90,3512	
3546,8321	95,9425		3546,8321	90,7788	
3548,7608	96,0508		3548,7608	90,8572	
3550,6895	96,1598		3550,6895	90,8472	
3552,6181	96,2502		3552,6181	90,9219	
3554,5468	96,234		3554,5468	91,0388	
3556,4755	96,21		3556,4755	91,2323	
3558,4042	96,3108		3558,4042	91,4137	
3560,3328	96,4214		3560,3328	91,5284	
3562,2615	96,4488		3562,2615	91,5969	
3564,1902	96,4574		3564,1902	91,5661	
3566,1189	96,4873		3566,1189	91,6978	
3568,0475	96,6078		3568,0475	92,1045	
3569,9762	96,644		3569,9762	92,3458	
3571,9049	96,614		3571,9049	92,3941	
3573,8336	96,6258		3573,8336	92,4301	

3575,7622	96,7302		3575,7622	92,5633	
3577,6909	96,9056		3577,6909	92,5513	
3579,6196	97,0467		3579,6196	92,5245	
3581,5482	97,1527		3581,5482	92,7515	
3583,4769	97,1724		3583,4769	92,9428	
3585,4056	97,1203		3585,4056	93,0334	
3587,3343	97,2021		3587,3343	93,2385	
3589,2629	97,3326		3589,2629	93,3231	
3591,1916	97,3529		3591,1916	93,2539	
3593,1203	97,3388		3593,1203	93,2967	
3595,049	97,3555		3595,049	93,4557	
3596,9776	97,4306		3596,9776	93,5815	
3598,9063	97,5025		3598,9063	93,7341	
3600,835	97,5758		3600,835	93,9223	
3602,7637	97,688		3602,7637	94,002	
3604,6923	97,8135		3604,6923	93,9987	
3606,621	97,8171		3606,621	94,0361	
3608,5497	97,8685		3608,5497	94,1491	
3610,4784	98,1342		3610,4784	94,2225	
3612,407	98,1596		3612,407	94,2463	
3614,3357	98,0648		3614,3357	94,2266	
3616,2644	98,1484		3616,2644	94,2704	
3618,1931	98,2177		3618,1931	94,5132	
3620,1217	98,1915		3620,1217	94,6495	
3622,0504	98,2033		3622,0504	94,634	
3623,9791	98,343		3623,9791	94,7579	
3625,9078	98,4361		3625,9078	94,8574	
3627,8364	98,4067		3627,8364	94,9353	
3629,7651	98,4462		3629,7651	95,1541	
3631,6938	98,5344		3631,6938	95,1712	

3633,6225	98,6635		3633,6225	95,2462	
3635,5511	98,7433		3635,5511	95,3986	
3637,4798	98,7575		3637,4798	95,4236	
3639,4085	98,8092		3639,4085	95,4775	
3641,3372	98,8663		3641,3372	95,5639	
3643,2658	98,9151		3643,2658	95,5686	
3645,1945	98,8818		3645,1945	95,5771	
3647,1232	98,8845		3647,1232	95,6548	
3649,0519	99,099		3649,0519	95,6814	
3650,9805	99,0857		3650,9805	95,7437	
3652,9092	98,9899		3652,9092	95,7943	
3654,8379	98,946		3654,8379	95,8534	
3656,7665	99,0114		3656,7665	95,9952	
3658,6952	99,1107		3658,6952	95,999	
3660,6239	99,1466		3660,6239	95,9877	
3662,5526	99,2203		3662,5526	96,0862	
3664,4812	99,2546		3664,4812	96,1801	
3666,4099	99,2462		3666,4099	96,1596	
3668,3386	99,2463		3668,3386	96,1365	
3670,2673	99,2431		3670,2673	96,2333	
3672,1959	99,2612		3672,1959	96,1474	
3674,1246	99,3424		3674,1246	96,0636	
3676,0533	99,5373		3676,0533	96,1224	
3677,982	99,4608		3677,982	96,1936	
3679,9106	99,3941		3679,9106	96,3942	
3681,8393	99,4463		3681,8393	96,5187	
3683,768	99,4398		3683,768	96,537	
3685,6967	99,3844		3685,6967	96,5665	
3687,6253	99,3023		3687,6253	96,5413	
3689,554	99,2683		3689,554	96,525	

3691,4827	99,2719		3691,4827	96,4912	
3693,4114	99,225		3693,4114	96,4713	
3695,34	99,2395		3695,34	96,603	
3697,2687	99,3032		3697,2687	96,7154	
3699,1974	99,3153		3699,1974	96,619	
3701,1261	99,3033		3701,1261	96,6072	
3703,0547	99,4176		3703,0547	96,7264	
3704,9834	99,5429		3704,9834	96,7076	
3706,9121	99,5401		3706,9121	96,6841	
3708,8408	99,4745		3708,8408	96,6268	
3710,7694	99,4071		3710,7694	96,5601	
3712,6981	99,366		3712,6981	96,6416	
3714,6268	99,4328		3714,6268	96,6189	
3716,5555	99,5268		3716,5555	96,5989	
3718,4841	99,5712		3718,4841	96,6607	
3720,4128	99,5672		3720,4128	96,6836	
3722,3415	99,5086		3722,3415	96,7507	
3724,2702	99,4961		3724,2702	96,7438	
3726,1988	99,5678		3726,1988	96,6924	
3728,1275	99,4847		3728,1275	96,7044	
3730,0562	99,4474		3730,0562	96,6085	
3731,9848	99,5537		3731,9848	96,5396	
3733,9135	99,5174		3733,9135	96,5429	
3735,8422	99,5364		3735,8422	96,6599	
3737,7709	99,7058		3737,7709	96,7487	
3739,6995	99,762		3739,6995	96,6462	
3741,6282	99,7191		3741,6282	96,5335	
3743,5569	99,6205		3743,5569	96,5463	
3745,4856	99,5076		3745,4856	96,6813	
3747,4142	99,404		3747,4142	96,634	

3749,3429	99,3841		3749,3429	96,5832	
3751,2716	99,533		3751,2716	96,62	
3753,2003	99,6256		3753,2003	96,7079	
3755,1289	99,6036		3755,1289	96,8099	
3757,0576	99,5795		3757,0576	96,8612	
3758,9863	99,6726		3758,9863	96,8507	
3760,915	99,656		3760,915	96,8065	
3762,8436	99,5325		3762,8436	96,7066	
3764,7723	99,5904		3764,7723	96,6842	
3766,701	99,6411		3766,701	96,7269	
3768,6297	99,4367		3768,6297	96,702	
3770,5583	99,3595		3770,5583	96,8292	
3772,487	99,4903		3772,487	96,8845	
3774,4157	99,5409		3774,4157	96,8034	
3776,3444	99,5495		3776,3444	96,7464	
3778,273	99,6087		3778,273	96,7653	
3780,2017	99,6587		3780,2017	96,8586	
3782,1304	99,6421		3782,1304	96,8601	
3784,0591	99,6634		3784,0591	96,8376	
3785,9877	99,653		3785,9877	96,8548	
3787,9164	99,5842		3787,9164	96,8448	
3789,8451	99,6272		3789,8451	96,7879	
3791,7738	99,771		3791,7738	96,7328	
3793,7024	99,803		3793,7024	96,6463	
3795,6311	99,7528		3795,6311	96,5798	
3797,5598	99,7308		3797,5598	96,6252	
3799,4885	99,6054		3799,4885	96,5866	
3801,4171	99,4759		3801,4171	96,5909	
3803,3458	99,6222		3803,3458	96,73	
3805,2745	99,6493		3805,2745	96,7565	

3807,2031	99,5651		3807,2031	96,7899	
3809,1318	99,6387		3809,1318	96,7761	
3811,0605	99,7482		3811,0605	96,7381	
3812,9892	99,7411		3812,9892	96,6716	
3814,9178	99,6334		3814,9178	96,544	
3816,8465	99,5333		3816,8465	96,5956	
3818,7752	99,5192		3818,7752	96,6407	
3820,7039	99,5734		3820,7039	96,6407	
3822,6325	99,6021		3822,6325	96,7553	
3824,5612	99,6452		3824,5612	96,7068	
3826,4899	99,6618		3826,4899	96,6204	
3828,4186	99,5659		3828,4186	96,7136	
3830,3472	99,5351		3830,3472	96,815	
3832,2759	99,5678		3832,2759	96,6901	
3834,2046	99,5723		3834,2046	96,6142	
3836,1333	99,5762		3836,1333	96,6919	
3838,0619	99,6024		3838,0619	96,7896	
3839,9906	99,6424		3839,9906	96,835	
3841,9193	99,6601		3841,9193	96,7444	
3843,848	99,6402		3843,848	96,6785	
3845,7766	99,5402		3845,7766	96,6681	
3847,7053	99,4607		3847,7053	96,7046	
3849,634	99,4971		3849,634	96,6749	
3851,5627	99,5114		3851,5627	96,3921	
3853,4913	99,4234		3853,4913	96,242	
3855,42	99,5848		3855,42	96,5418	
3857,3487	99,7029		3857,3487	96,6738	
3859,2774	99,6022		3859,2774	96,7595	
3861,206	99,4491		3861,206	96,7465	
3863,1347	99,4815		3863,1347	96,6564	

3865,0634	99,6114		3865,0634	96,6149	
3866,9921	99,6608		3866,9921	96,5365	
3868,9207	99,6684		3868,9207	96,5425	
3870,8494	99,6141		3870,8494	96,6751	
3872,7781	99,5949		3872,7781	96,6466	
3874,7067	99,6043		3874,7067	96,6604	
3876,6354	99,6025		3876,6354	96,6734	
3878,5641	99,6761		3878,5641	96,6317	
3880,4928	99,7892		3880,4928	96,6811	
3882,4214	99,7523		3882,4214	96,6698	
3884,3501	99,6943		3884,3501	96,5885	
3886,2788	99,679		3886,2788	96,5815	
3888,2075	99,6677		3888,2075	96,5969	
3890,1361	99,6828		3890,1361	96,6062	
3892,0648	99,7347		3892,0648	96,6164	
3893,9935	99,7345		3893,9935	96,5951	
3895,9222	99,6684		3895,9222	96,5603	
3897,8508	99,6067		3897,8508	96,5434	
3899,7795	99,5765		3899,7795	96,5344	
3901,7082	99,5669		3901,7082	96,4772	
3903,6369	99,5981		3903,6369	96,4773	
3905,5655	99,5613		3905,5655	96,534	
3907,4942	99,5313		3907,4942	96,5948	
3909,4229	99,6047		3909,4229	96,6665	
3911,3516	99,6527		3911,3516	96,6538	
3913,2802	99,6581		3913,2802	96,5431	
3915,2089	99,6679		3915,2089	96,4375	
3917,1376	99,6265		3917,1376	96,504	
3919,0663	99,5544		3919,0663	96,6634	
3920,9949	99,5217		3920,9949	96,6497	

3922,9236	99,5527		3922,9236	96,5955	
3924,8523	99,5403		3924,8523	96,6534	
3926,781	99,5489		3926,781	96,6672	
3928,7096	99,6141		3928,7096	96,5755	
3930,6383	99,5834		3930,6383	96,5079	
3932,567	99,4847		3932,567	96,546	
3934,4957	99,4678		3934,4957	96,6231	
3936,4243	99,562		3936,4243	96,6356	
3938,353	99,663		3938,353	96,5842	
3940,2817	99,6481		3940,2817	96,5528	
3942,2104	99,5168		3942,2104	96,6077	
3944,139	99,4746		3944,139	96,6871	
3946,0677	99,554		3946,0677	96,6546	
3947,9964	99,5555		3947,9964	96,5587	
3949,925	99,5059		3949,925	96,5522	
3951,8537	99,5067		3951,8537	96,6081	
3953,7824	99,5675		3953,7824	96,5454	
3955,7111	99,5961		3955,7111	96,47	
3957,6397	99,5328		3957,6397	96,5516	
3959,5684	99,4835		3959,5684	96,5914	
3961,4971	99,4903		3961,4971	96,5089	
3963,4258	99,5296		3963,4258	96,4951	
3965,3544	99,5472		3965,3544	96,5624	
3967,2831	99,5485		3967,2831	96,5387	
3969,2118	99,5709		3969,2118	96,4652	
3971,1405	99,6214		3971,1405	96,5117	
3973,0691	99,6356		3973,0691	96,593	
3974,9978	99,5424		3974,9978	96,6266	
3976,9265	99,4324		3976,9265	96,602	
3978,8552	99,3979		3978,8552	96,5174	

3980,7838	99,4492		3980,7838	96,514	
3982,7125	99,5029		3982,7125	96,5608	
3984,6412	99,4801		3984,6412	96,5652	
3986,5699	99,4435		3986,5699	96,5808	
3988,4985	99,473		3988,4985	96,5402	
3990,4272	99,5249		3990,4272	96,4629	
3992,3559	99,5024		3992,3559	96,4396	
3994,2846	99,4919		3994,2846	96,4358	
3996,2132	99,5313		3996,2132	96,4761	
3998,1419	99,4886		3998,1419	96,4931	
4000,0706	99,3782		4000,0706	96,4508	
Comment=			Comment=		
