# Supplementary material for: Data on statistical experimental design to formulate amphotericin B-loaded Eudragit RL100 nanoparticles coated with hyaluronic acid for the treatment of vulvovaginal candidiasis
Source: Data Brief. 2020 Mar 5;29:105311. doi: 10.1016/j.dib.2020.105311 (PMC7082528; doi:10.1016/j.dib.2020.105311)
Supplement: Multimedia component 12 [file mmc12.pdf]

DATATYPE	**IR Spectrum pure HA**		**DATATYPE**	**IR Spectrum EUD nanoparticles**	
XYUNITS	**Wavenumber;PercentTransmittance**		**XYUNITS**	**Wavenumber;PercentTransmittance**	
DECIMALSYMB(	,		DECIMALSYMB(	,	
399,2356	121,9053		399,2356	150	
401,1643	14,1819		401,1643	-1	
403,0929	4,2541		403,0929	-1	
405,0216	150		405,0216	150	
406,9503	150		406,9503	150	
408,879	150		408,879	-1	
410,8076	65,6255		410,8076	-1	
412,7363	47,2554		412,7363	-1	
414,665	-1		414,665	41,2252	
416,5937	-1		416,5937	49,2118	
418,5223	-1		418,5223	17,3218	
420,451	71,2557		420,451	150	
422,3797	-1		422,3797	11,4303	
424,3084	-1		424,3084	61,0078	
426,237	97,4652		426,237	93,8056	
428,1657	150		428,1657	86,6158	
430,0944	10,2502		430,0944	40,0524	
432,0231	-1		432,0231	-1	
433,9517	-1		433,9517	-1	
435,8804	-1		435,8804	13,5012	
437,8091	-1		437,8091	-1	
439,7377	2,6906		439,7377	-1	
441,6664	41,4469		441,6664	-1	
443,5951	150		443,5951	15,0508	
445,5238	69,7859		445,5238	-1	
447,4524	90,6854		447,4524	-1	
449,3811	4,2905		449,3811	-1	

451,3098	-1		451,3098	-1	
453,2385	150		453,2385	-1	
455,1671	150		455,1671	-1	
457,0958	-1		457,0958	-1	
459,0245	150		459,0245	-1	
460,9532	-1		460,9532	-1	
462,8818	-1		462,8818	-1	
464,8105	88,012		464,8105	14,4605	
466,7392	-1		466,7392	-1	
468,6679	92,6179		468,6679	-1	
470,5965	7,2349		470,5965	-1	
472,5252	-1		472,5252	150	
474,4539	150		474,4539	150	
476,3826	24,743		476,3826	-1	
478,3112	-1		478,3112	41,1627	
480,2399	-1		480,2399	-1	
482,1686	-1		482,1686	-1	
484,0973	-1		484,0973	-1	
486,0259	-1		486,0259	-1	
487,9546	-1		487,9546	-1	
489,8833	-1		489,8833	-1	
491,812	-1		491,812	17,6988	
493,7406	150		493,7406	101,9511	
495,6693	-1		495,6693	150	
497,598	-1		497,598	-1	
499,5267	-1		499,5267	6,0928	
501,4553	90,3322		501,4553	52,6613	
503,384	57,5967		503,384	38,022	
505,3127	13,7645		505,3127	23,8144	
507,2414	150		507,2414	11,4563	

509,17	-1		509,17	4,662	
511,0987	-1		511,0987	23,6062	
513,0274	-1		513,0274	122,5865	
514,956	150		514,956	-1	
516,8847	103,8944		516,8847	150	
518,8134	115,6497		518,8134	105,7276	
520,7421	-1		520,7421	77,8205	
522,6707	-1		522,6707	61,6118	
524,5994	44,6618		524,5994	59,8486	
526,5281	79,9127		526,5281	81,0383	
528,4568	73,5667		528,4568	119,3354	
530,3854	63,3774		530,3854	116,683	
532,3141	65,6097		532,3141	81,8776	
534,2428	79,209		534,2428	61,0233	
536,1715	76,4263		536,1715	62,1823	
538,1001	57,1544		538,1001	73,9154	
540,0288	55,4273		540,0288	84,9182	
541,9575	67,4541		541,9575	84,2993	
543,8862	73,1293		543,8862	84,7672	
545,8148	71,9797		545,8148	78,9367	
547,7435	71,324		547,7435	64,7296	
549,6722	70,2146		549,6722	69,4615	
551,6009	66,4343		551,6009	83,9028	
553,5295	63,8549		553,5295	80,8226	
555,4582	60,51		555,4582	72,2751	
557,3869	61,4093		557,3869	71,9703	
559,3156	63,0524		559,3156	72,4144	
561,2442	58,4368		561,2442	72,309	
563,1729	58,2838		563,1729	76,2358	
565,1016	61,0938		565,1016	78,4823	

567,0303	60,8409		567,0303	75,467	
568,9589	61,1055		568,9589	73,8566	
570,8876	61,2703		570,8876	74,3033	
572,8163	61,8407		572,8163	73,0136	
574,745	64,6421		574,745	72,975	
576,6736	65,6797		576,6736	73,543	
578,6023	64,2124		578,6023	71,4728	
580,531	62,5308		580,531	70,456	
582,4597	64,0726		582,4597	73,7498	
584,3883	66,6373		584,3883	75,4065	
586,317	65,0184		586,317	73,7227	
588,2457	64,8558		588,2457	75,058	
590,1743	66,044		590,1743	76,566	
592,103	65,1939		592,103	75,3785	
594,0317	64,0962		594,0317	75,594	
595,9604	64,2289		595,9604	76,1781	
597,889	64,0634		597,889	75,957	
599,8177	62,7692		599,8177	76,6326	
601,7464	62,3048		601,7464	76,4346	
603,6751	62,438		603,6751	75,4774	
605,6037	62,655		605,6037	75,0233	
607,5324	63,2768		607,5324	74,2076	
609,4611	63,0224		609,4611	74,668	
611,3898	62,8948		611,3898	75,9125	
613,3184	64,6662		613,3184	75,6063	
615,2471	65,5961		615,2471	75,5337	
617,1758	65,4536		617,1758	76,1452	
619,1045	66,122		619,1045	76,1481	
621,0331	66,6961		621,0331	76,5113	
622,9618	66,8507		622,9618	76,7421	

624,8905	67,0171		624,8905	77,0725	
626,8192	67,4292		626,8192	77,8206	
628,7478	68,113		628,7478	77,5759	
630,6765	68,9592		630,6765	77,0033	
632,6052	69,6356		632,6052	77,0014	
634,5339	69,9429		634,5339	77,2896	
636,4625	70,197		636,4625	77,2881	
638,3912	70,5927		638,3912	77,3244	
640,3199	70,9471		640,3199	77,315	
642,2486	71,1469		642,2486	77,1533	
644,1772	71,3773		644,1772	77,389	
646,1059	71,5474		646,1059	77,4443	
648,0346	71,4576		648,0346	77,1974	
649,9633	71,5538		649,9633	77,1408	
651,8919	71,8944		651,8919	77,1475	
653,8206	72,0963		653,8206	77,3124	
655,7493	72,1905		655,7493	77,5601	
657,678	72,1654		657,678	77,4221	
659,6066	72,3019		659,6066	77,4328	
661,5353	72,7206		661,5353	77,7559	
663,464	72,8252		663,464	77,811	
665,3926	72,5033		665,3926	77,3786	
667,3213	72,2936		667,3213	76,3341	
669,25	72,7679		669,25	76,6105	
671,1787	73,0405		671,1787	77,6918	
673,1073	73,2256		673,1073	78,0217	
675,036	73,2455		675,036	78,2122	
676,9647	73,0726		676,9647	78,6402	
678,8934	73,3264		678,8934	79,0212	
680,822	73,6856		680,822	79,3299	

682,7507	73,7786		682,7507	79,4675	
684,6794	73,8694		684,6794	79,472	
686,6081	73,8594		686,6081	79,6209	
688,5367	73,9343		688,5367	79,6688	
690,4654	74,3715		690,4654	79,7378	
692,3941	74,6081		692,3941	79,8928	
694,3228	74,4234		694,3228	79,8967	
696,2514	74,2579		696,2514	79,9666	
698,1801	74,3711		698,1801	80,148	
700,1088	74,6884		700,1088	80,3523	
702,0375	74,9573		702,0375	80,5105	
703,9661	75,164		703,9661	80,6319	
705,8948	75,4208		705,8948	80,7678	
707,8235	75,653		707,8235	80,8989	
709,7522	75,7913		709,7522	81,0081	
711,6808	75,9646		711,6808	81,0956	
713,6095	76,1745		713,6095	81,0946	
715,5382	76,2953		715,5382	80,888	
717,4669	76,3458		717,4669	80,6998	
719,3955	76,4924		719,3955	80,6051	
721,3242	76,8288		721,3242	80,7006	
723,2529	77,021		723,2529	81,0076	
725,1816	77,0327		725,1816	81,2283	
727,1102	77,08		727,1102	81,3427	
729,0389	77,1619		729,0389	81,3866	
730,9676	77,316		730,9676	81,4626	
732,8962	77,4383		732,8962	81,6131	
734,8249	77,4737		734,8249	81,7268	
736,7536	77,5523		736,7536	81,8512	
738,6823	77,7355		738,6823	81,8933	

740,6109	77,9429		740,6109	81,8046	
742,5396	78,0558		742,5396	81,6852	
744,4683	78,1154		744,4683	81,2411	
746,397	78,212		746,397	80,2925	
748,3256	78,3383		748,3256	79,4788	
750,2543	78,4392		750,2543	79,3059	
752,183	78,5871		752,183	79,477	
754,1117	78,8481		754,1117	79,6926	
756,0403	79,2325		756,0403	79,9872	
757,969	79,6979		757,969	80,4558	
759,8977	80,0939		759,8977	81,0761	
761,8264	80,4082		761,8264	81,5322	
763,755	80,6789		763,755	81,7578	
765,6837	80,9336		765,6837	82,0949	
767,6124	81,1863		767,6124	82,4832	
769,5411	81,4452		769,5411	82,7519	
771,4697	81,6927		771,4697	82,8943	
773,3984	81,8636		773,3984	82,9941	
775,3271	82,0418		775,3271	83,1281	
777,2558	82,2152		777,2558	83,181	
779,1844	82,3522		779,1844	83,2139	
781,1131	82,4316		781,1131	83,2558	
783,0418	82,4083		783,0418	83,2501	
784,9705	82,3984		784,9705	83,1376	
786,8991	82,394		786,8991	82,8977	
788,8278	82,3838		788,8278	82,6929	
790,7565	82,4287		790,7565	82,6277	
792,6852	82,5293		792,6852	82,6734	
794,6138	82,6764		794,6138	82,6747	
796,5425	82,8895		796,5425	82,5441	

798,4712	83,1192		798,4712	82,3264	
800,3999	83,2444		800,3999	82,0704	
802,3285	83,4125		802,3285	81,8489	
804,2572	83,7531		804,2572	81,6552	
806,1859	84,0645		806,1859	81,4436	
808,1145	84,2608		808,1145	81,2771	
810,0432	84,4452		810,0432	81,0993	
811,9719	84,6123		811,9719	80,936	
813,9006	84,8136		813,9006	80,9184	
815,8292	85,108		815,8292	80,8859	
817,7579	85,381		817,7579	80,7217	
819,6866	85,6536		819,6866	80,4839	
821,6153	85,9132		821,6153	80,1734	
823,5439	86,0214		823,5439	79,8391	
825,4726	86,0553		825,4726	79,4747	
827,4013	86,1604		827,4013	79,0507	
829,33	86,3516		829,33	78,6401	
831,2586	86,5217		831,2586	78,2454	
833,1873	86,6911		833,1873	77,7389	
835,116	86,9194		835,116	77,0511	
837,0447	87,0256		837,0447	76,3303	
838,9733	87,0094		838,9733	75,685	
840,902	87,0693		840,902	75,1318	
842,8307	87,2873		842,8307	74,6848	
844,7594	87,4776		844,7594	74,4053	
846,688	87,5701		846,688	74,2764	
848,6167	87,6373		848,6167	74,2492	
850,5454	87,62		850,5454	74,4262	
852,4741	87,657		852,4741	74,8232	
854,4027	87,7993		854,4027	75,3597	

856,3314	87,9105		856,3314	75,9958	
858,2601	87,9362		858,2601	76,6156	
860,1888	87,9498		860,1888	77,1445	
862,1174	88,0382		862,1174	77,6038	
864,0461	88,1313		864,0461	77,8674	
865,9748	88,105		865,9748	77,9743	
867,9035	88,0791		867,9035	78,1546	
869,8321	88,1411		869,8321	78,453	
871,7608	88,1078		871,7608	78,719	
873,6895	87,9965		873,6895	78,7088	
875,6182	87,9375		875,6182	78,549	
877,5468	87,8536		877,5468	78,4201	
879,4755	87,6866		879,4755	78,1674	
881,4042	87,5165		881,4042	77,8756	
883,3328	87,3367		883,3328	77,6426	
885,2615	87,0671		885,2615	77,4889	
887,1902	86,7386		887,1902	77,4906	
889,1189	86,4653		889,1189	77,5951	
891,0475	86,3414		891,0475	77,8083	
892,9762	86,278		892,9762	78,1235	
894,9049	86,1487		894,9049	78,4097	
896,8336	86,1167		896,8336	78,6286	
898,7622	86,2667		898,7622	78,7995	
900,6909	86,437		900,6909	78,8147	
902,6196	86,6236		902,6196	78,6846	
904,5483	86,8543		904,5483	78,4828	
906,4769	87,0178		906,4769	78,1404	
908,4056	87,115		908,4056	77,7004	
910,3343	87,1096		910,3343	77,2896	
912,263	87,0195		912,263	76,9793	

914,1916	86,9684		914,1916	76,7796	
916,1203	86,8549		916,1203	76,5348	
918,049	86,7015		918,049	76,2483	
919,9777	86,5196		919,9777	75,9904	
921,9063	86,257		921,9063	75,6366	
923,835	85,9817		923,835	75,2453	
925,7637	85,6854		925,7637	74,8782	
927,6924	85,3422		927,6924	74,5	
929,621	84,9707		929,621	74,0422	
931,5497	84,4746		931,5497	73,507	
933,4784	83,8738		933,4784	73,0106	
935,4071	83,3201		935,4071	72,5851	
937,3357	82,7179		937,3357	72,1586	
939,2644	82,1643		939,2644	71,624	
941,1931	81,7679		941,1931	70,997	
943,1218	81,4298		943,1218	70,3413	
945,0504	81,173		945,0504	69,7764	
946,9791	81,0638		946,9791	69,4547	
948,9078	81,1876		948,9078	69,4055	
950,8364	81,5248		950,8364	69,5932	
952,7651	81,8948		952,7651	69,937	
954,6938	82,1786		954,6938	70,3597	
956,6225	82,4091		956,6225	70,9596	
958,5511	82,5297		958,5511	71,6986	
960,4798	82,4094		960,4798	72,4044	
962,4085	82,0976		962,4085	73,0592	
964,3372	81,6837		964,3372	73,5525	
966,2658	81,1839		966,2658	73,8497	
968,1945	80,5695		968,1945	74,092	
970,1232	79,829		970,1232	74,2009	

972,0519	79,1174		972,0519	74,0067	
973,9805	78,3692		973,9805	73,6331	
975,9092	77,3911		975,9092	73,1229	
977,8379	76,3757		977,8379	72,353	
979,7666	75,4144		979,7666	71,3999	
981,6952	74,4306		981,6952	70,4081	
983,6239	73,4509		983,6239	69,5491	
985,5526	72,4656		985,5526	68,918	
987,4813	71,5524		987,4813	68,5003	
989,4099	70,7543		989,4099	68,2027	
991,3386	70,0459		991,3386	67,9904	
993,2673	69,3574		993,2673	67,9408	
995,196	68,6538		995,196	67,9945	
997,1246	68,0173		997,1246	68,1468	
999,0533	67,3708		999,0533	68,3587	
1000,982	66,6011		1000,982	68,4921	
1002,9107	65,746		1002,9107	68,4372	
1004,8393	64,9385		1004,8393	68,1025	
1006,768	64,0938		1006,768	67,545	
1008,6967	63,0524		1008,6967	66,8375	
1010,6254	61,9016		1010,6254	66,0268	
1012,554	60,7736		1012,554	65,2151	
1014,4827	59,7656		1014,4827	64,4775	
1016,4114	58,8903		1016,4114	63,8079	
1018,3401	58,1231		1018,3401	63,1824	
1020,2687	57,4784		1020,2687	62,5501	
1022,1974	56,9707		1022,1974	61,8842	
1024,1261	56,6384		1024,1261	61,1926	
1026,0547	56,4483		1026,0547	60,537	
1027,9834	56,3317		1027,9834	59,9575	

1029,9121	56,2767		1029,9121	59,4543	
1031,8408	56,2487		1031,8408	58,988	
1033,7694	56,3198		1033,7694	58,6158	
1035,6981	56,5407		1035,6981	58,5236	
1037,6268	56,8846		1037,6268	58,6361	
1039,5555	57,3366		1039,5555	58,833	
1041,4841	57,7789		1041,4841	58,9967	
1043,4128	58,2818		1043,4128	59,0836	
1045,3415	58,9835		1045,3415	59,1582	
1047,2702	59,7435		1047,2702	59,1103	
1049,1988	60,4735		1049,1988	58,8067	
1051,1275	61,2293		1051,1275	58,2176	
1053,0562	61,9798		1053,0562	57,5	
1054,9849	62,7321		1054,9849	56,6676	
1056,9135	63,4638		1056,9135	55,61	
1058,8422	64,0035		1058,8422	54,5188	
1060,7709	64,4027		1060,7709	53,5919	
1062,6996	64,8277		1062,6996	52,7884	
1064,6282	65,1911		1064,6282	52,0084	
1066,5569	65,404		1066,5569	51,282	
1068,4856	65,5396		1068,4856	50,6356	
1070,4143	65,6476		1070,4143	49,9663	
1072,3429	65,6835		1072,3429	49,2821	
1074,2716	65,7911		1074,2716	48,6507	
1076,2003	66,1035		1076,2003	47,9714	
1078,129	66,4823		1078,129	47,2156	
1080,0576	67,0456		1080,0576	46,4985	
1081,9863	67,9028		1081,9863	45,7484	
1083,915	68,8824		1083,915	44,9152	
1085,8437	69,8937		1085,8437	44,1481	

1087,7723	70,9111		1087,7723	43,474	
1089,701	71,9329		1089,701	42,8115	
1091,6297	72,9429		1091,6297	42,2466	
1093,5584	73,9032		1093,5584	41,8983	
1095,487	74,7405		1095,487	41,689	
1097,4157	75,4505		1097,4157	41,6353	
1099,3444	76,0727		1099,3444	41,8049	
1101,273	76,7201		1101,273	42,0973	
1103,2017	77,449		1103,2017	42,4531	
1105,1304	78,0433		1105,1304	42,8237	
1107,0591	78,4635		1107,0591	43,1785	
1108,9877	78,8757		1108,9877	43,5815	
1110,9164	79,3024		1110,9164	44,0409	
1112,8451	79,6644		1112,8451	44,5697	
1114,7738	79,9989		1114,7738	45,2109	
1116,7024	80,3409		1116,7024	45,9201	
1118,6311	80,626		1118,6311	46,6226	
1120,5598	80,8736		1120,5598	47,2639	
1122,4885	81,1461		1122,4885	47,9235	
1124,4171	81,408		1124,4171	48,6269	
1126,3458	81,6503		1126,3458	49,2249	
1128,2745	81,9225		1128,2745	49,7002	
1130,2032	82,1029		1130,2032	50,0768	
1132,1318	82,132		1132,1318	50,3691	
1134,0605	82,2254		1134,0605	50,6409	
1135,9892	82,3145		1135,9892	50,9063	
1137,9179	82,1652		1137,9179	51,1751	
1139,8465	81,9401		1139,8465	51,5253	
1141,7752	81,748		1141,7752	52,0268	
1143,7039	81,5617		1143,7039	52,6726	

1145,6326	81,4268		1145,6326	53,4852	
1147,5612	81,3588		1147,5612	54,5057	
1149,4899	81,4702		1149,4899	55,8164	
1151,4186	81,8085		1151,4186	57,4087	
1153,3473	82,2599		1153,3473	59,1655	
1155,2759	82,773		1155,2759	61,0254	
1157,2046	83,3227		1157,2046	62,8435	
1159,1333	83,9604		1159,1333	64,5435	
1161,062	84,6774		1161,062	66,1436	
1162,9906	85,3982		1162,9906	67,5818	
1164,9193	86,2381		1164,9193	68,7651	
1166,848	87,1213		1166,848	69,6744	
1168,7767	87,8804		1168,7767	70,2808	
1170,7053	88,5695		1170,7053	70,5391	
1172,634	89,2322		1172,634	70,657	
1174,5627	89,9125		1174,5627	70,803	
1176,4913	90,5642		1176,4913	71,0122	
1178,42	91,0994		1178,42	71,4237	
1180,3487	91,5849		1180,3487	71,9538	
1182,2774	92,0361		1182,2774	72,4425	
1184,206	92,4194		1184,206	72,9016	
1186,1347	92,6358		1186,1347	73,2925	
1188,0634	92,6412		1188,0634	73,67	
1189,9921	92,5583		1189,9921	74,1178	
1191,9207	92,3261		1191,9207	74,6572	
1193,8494	92,0022		1193,8494	75,3405	
1195,7781	91,7798		1195,7781	76,1788	
1197,7068	91,5384		1197,7068	77,1243	
1199,6354	91,2088		1199,6354	78,049	
1201,5641	90,9431		1201,5641	78,8916	

1203,4928	90,8243		1203,4928	79,6276	
1205,4215	90,8018		1205,4215	80,1948	
1207,3501	90,7779		1207,3501	80,6165	
1209,2788	90,7986		1209,2788	80,8986	
1211,2075	90,9889		1211,2075	80,9843	
1213,1362	91,2021		1213,1362	80,9291	
1215,0648	91,3066		1215,0648	80,796	
1216,9935	91,408		1216,9935	80,5086	
1218,9222	91,4353		1218,9222	80,0957	
1220,8509	91,2858		1220,8509	79,6255	
1222,7795	91,0627		1222,7795	79,0629	
1224,7082	90,8661		1224,7082	78,4646	
1226,6369	90,6863		1226,6369	77,8273	
1228,5656	90,524		1228,5656	77,0911	
1230,4942	90,3837		1230,4942	76,2713	
1232,4229	90,2207		1232,4229	75,368	
1234,3516	90,0593		1234,3516	74,4837	
1236,2803	89,9787		1236,2803	73,768	
1238,2089	89,9484		1238,2089	73,2831	
1240,1376	89,9157		1240,1376	72,996	
1242,0663	89,9359		1242,0663	72,7939	
1243,9949	90,0456		1243,9949	72,7321	
1245,9236	90,1529		1245,9236	72,9792	
1247,8523	90,1644		1247,8523	73,4382	
1249,781	90,1159		1249,781	73,9938	
1251,7096	90,0324		1251,7096	74,6569	
1253,6383	89,9651		1253,6383	75,4029	
1255,567	89,8839		1255,567	76,2216	
1257,4957	89,692		1257,4957	77,01	
1259,4243	89,4984		1259,4243	77,7269	

1261,353	89,3655		1261,353	78,373	
1263,2817	89,1907		1263,2817	78,8872	
1265,2104	88,9672		1265,2104	79,2779	
1267,139	88,7229		1267,139	79,5217	
1269,0677	88,4831		1269,0677	79,6353	
1270,9964	88,3035		1270,9964	79,6306	
1272,9251	88,0835		1272,9251	79,512	
1274,8537	87,8069		1274,8537	79,3792	
1276,7824	87,5995		1276,7824	79,2839	
1278,7111	87,4172		1278,7111	79,2702	
1280,6398	87,1692		1280,6398	79,3567	
1282,5684	86,9284		1282,5684	79,466	
1284,4971	86,7562		1284,4971	79,5918	
1286,4258	86,5552		1286,4258	79,7594	
1288,3545	86,2981		1288,3545	79,9125	
1290,2831	86,0916		1290,2831	80,0232	
1292,2118	85,9433		1292,2118	80,1348	
1294,1405	85,7716		1294,1405	80,2524	
1296,0692	85,6511		1296,0692	80,3413	
1297,9978	85,6297		1297,9978	80,4326	
1299,9265	85,5883		1299,9265	80,6301	
1301,8552	85,5062		1301,8552	81,0131	
1303,7839	85,3973		1303,7839	81,533	
1305,7125	85,2956		1305,7125	82,0666	
1307,6412	85,2694		1307,6412	82,6386	
1309,5699	85,215		1309,5699	83,2625	
1311,4986	85,0714		1311,4986	83,8011	
1313,4272	84,9931		1313,4272	84,2244	
1315,3559	85,012		1315,3559	84,5254	
1317,2846	84,9689		1317,2846	84,6637	

1319,2132	84,9032		1319,2132	84,7311	
1321,1419	84,909		1321,1419	84,7503	
1323,0706	84,9213		1323,0706	84,6834	
1324,9993	85,0234		1324,9993	84,7313	
1326,9279	85,1907		1326,9279	84,9374	
1328,8566	85,2998		1328,8566	85,2029	
1330,7853	85,4531		1330,7853	85,4979	
1332,714	85,6342		1332,714	85,6209	
1334,6426	85,7502		1334,6426	85,585	
1336,5713	85,8108		1336,5713	85,5212	
1338,5	85,8076		1338,5	85,324	
1340,4287	85,8698		1340,4287	84,8637	
1342,3573	85,9525		1342,3573	84,0202	
1344,286	85,9122		1344,286	82,7667	
1346,2147	85,826		1346,2147	81,5222	
1348,1434	85,6945		1348,1434	80,856	
1350,072	85,5106		1350,072	80,8348	
1352,0007	85,3521		1352,0007	81,259	
1353,9294	85,1492		1353,9294	81,9134	
1355,8581	84,8527		1355,8581	82,6357	
1357,7867	84,5342		1357,7867	83,2808	
1359,7154	84,1968		1359,7154	83,7096	
1361,6441	83,8085		1361,6441	83,9847	
1363,5728	83,3589		1363,5728	84,3097	
1365,5014	82,8719		1365,5014	84,4721	
1367,4301	82,3118		1367,4301	84,5161	
1369,3588	81,6804		1369,3588	84,6918	
1371,2875	81,1296		1371,2875	84,8454	
1373,2161	80,7506		1373,2161	84,846	
1375,1448	80,6022		1375,1448	84,7415	

1377,0735	80,6375		1377,0735	84,5727	
1379,0022	80,8808		1379,0022	84,4434	
1380,9308	81,2539		1380,9308	84,4287	
1382,8595	81,5741		1382,8595	84,5093	
1384,7882	81,7821		1384,7882	84,635	
1386,7169	81,9678		1386,7169	85,017	
1388,6455	82,1346		1388,6455	85,6792	
1390,5742	82,0249		1390,5742	86,1684	
1392,5029	81,7021		1392,5029	86,531	
1394,4315	81,2871		1394,4315	86,9516	
1396,3602	80,8563		1396,3602	87,3613	
1398,2889	80,4875		1398,2889	87,6575	
1400,2176	80,1863		1400,2176	88,0095	
1402,1462	79,9349		1402,1462	88,2916	
1404,0749	79,7574		1404,0749	88,584	
1406,0036	79,7649		1406,0036	88,9248	
1407,9323	79,8685		1407,9323	89,1133	
1409,8609	80,0868		1409,8609	89,2395	
1411,7896	80,5039		1411,7896	89,3294	
1413,7183	80,9605		1413,7183	89,2925	
1415,647	81,3276		1415,647	89,1496	
1417,5756	81,7393		1417,5756	88,98	
1419,5043	82,5324		1419,5043	88,7844	
1421,433	83,1229		1421,433	88,499	
1423,3617	83,6022		1423,3617	88,0487	
1425,2903	84,271		1425,2903	87,4366	
1427,219	84,7879		1427,219	86,7253	
1429,1477	85,3055		1429,1477	85,7361	
1431,0764	85,9268		1431,0764	84,6549	
1433,005	86,4304		1433,005	83,7557	

1434,9337	86,8176		1434,9337	83,0492	
1436,8624	87,3747		1436,8624	82,4712	
1438,7911	87,8149		1438,7911	82,0371	
1440,7197	88,1048		1440,7197	81,6166	
1442,6484	88,4804		1442,6484	81,0554	
1444,5771	88,7508		1444,5771	80,4516	
1446,5058	88,9129		1446,5058	79,9511	
1448,4344	89,1465		1448,4344	79,6286	
1450,3631	89,374		1450,3631	79,5429	
1452,2918	89,5582		1452,2918	79,6351	
1454,2205	89,6955		1454,2205	79,7427	
1456,1491	89,8542		1456,1491	80,0029	
1458,0778	90,2285		1458,0778	80,8319	
1460,0065	90,4519		1460,0065	81,2832	
1461,9351	90,5322		1461,9351	81,4909	
1463,8638	90,6056		1463,8638	81,7248	
1465,7925	90,8411		1465,7925	82,1069	
1467,7212	91,0185		1467,7212	82,5085	
1469,6498	91,0266		1469,6498	82,8608	
1471,5785	91,044		1471,5785	83,4528	
1473,5072	91,2568		1473,5072	84,4471	
1475,4359	91,4234		1475,4359	85,1191	
1477,3645	91,4989		1477,3645	85,7762	
1479,2932	91,5095		1479,2932	86,3154	
1481,2219	91,5383		1481,2219	86,8494	
1483,1506	91,5506		1483,1506	87,4364	
1485,0792	91,4891		1485,0792	87,9076	
1487,0079	91,4436		1487,0079	88,4308	
1488,9366	91,3873		1488,9366	89,1624	
1490,8653	91,3555		1490,8653	90,3084	

1492,7939	91,34		1492,7939	91,1308	
1494,7226	91,2838		1494,7226	91,6971	
1496,6513	91,2106		1496,6513	92,4657	
1498,58	91,1029		1498,58	93,179	
1500,5086	90,9835		1500,5086	93,5815	
1502,4373	90,8218		1502,4373	93,7636	
1504,366	90,5632		1504,366	93,7622	
1506,2947	90,1944		1506,2947	93,7143	
1508,2233	90,0654		1508,2233	93,96	
1510,152	89,9562		1510,152	94,0898	
1512,0807	89,7215		1512,0807	94,1517	
1514,0094	89,4202		1514,0094	94,1362	
1515,938	89,0141		1515,938	94,1366	
1517,8667	88,5396		1517,8667	94,2251	
1519,7954	88,1957		1519,7954	94,162	
1521,7241	87,7292		1521,7241	94,1757	
1523,6527	87,2777		1523,6527	94,298	
1525,5814	86,8227		1525,5814	94,2392	
1527,5101	86,372		1527,5101	94,1558	
1529,4388	85,9286		1529,4388	94,12	
1531,3674	85,4079		1531,3674	94,0798	
1533,2961	84,866		1533,2961	94,0747	
1535,2248	84,4191		1535,2248	94,0174	
1537,1534	84,0141		1537,1534	93,8691	
1539,0821	83,5066		1539,0821	93,7975	
1541,0108	82,8938		1541,0108	93,8685	
1542,9395	82,4049		1542,9395	93,7969	
1544,8681	82,0005		1544,8681	93,9548	
1546,7968	81,6396		1546,7968	94,115	
1548,7255	81,2696		1548,7255	94,163	

1550,6542	80,9644		1550,6542	94,217	
1552,5828	80,7363		1552,5828	94,1947	
1554,5115	80,4334		1554,5115	94,1579	
1556,4402	80,1105		1556,4402	94,1423	
1558,3689	79,9578		1558,3689	94,2035	
1560,2975	80,2514		1560,2975	94,5641	
1562,2262	80,07		1562,2262	94,5373	
1564,1549	79,9495		1564,1549	94,5197	
1566,0836	79,9821		1566,0836	94,5305	
1568,0122	79,9331		1568,0122	94,4416	
1569,9409	80,0277		1569,9409	94,4168	
1571,8696	80,0971		1571,8696	94,4453	
1573,7983	79,9546		1573,7983	94,3446	
1575,7269	79,8636		1575,7269	94,2372	
1577,6556	79,8719		1577,6556	94,3384	
1579,5843	79,6748		1579,5843	94,4004	
1581,513	79,3665		1581,513	94,3939	
1583,4416	79,0245		1583,4416	94,3978	
1585,3703	78,5755		1585,3703	94,4327	
1587,299	78,0114		1587,299	94,4652	
1589,2277	77,3675		1589,2277	94,4241	
1591,1563	76,676		1591,1563	94,334	
1593,085	75,995		1593,085	94,2704	
1595,0137	75,3292		1595,0137	94,2295	
1596,9424	74,7028		1596,9424	94,1771	
1598,871	74,1127		1598,871	94,1534	
1600,7997	73,6075		1600,7997	94,1107	
1602,7284	73,2989		1602,7284	93,9839	
1604,6571	73,1863		1604,6571	93,8665	
1606,5857	73,1611		1606,5857	93,7391	

1608,5144	73,2486		1608,5144	93,5703	
1610,4431	73,532		1610,4431	93,4421	
1612,3717	73,8722		1612,3717	93,293	
1614,3004	74,1262		1614,3004	93,0682	
1616,2291	74,7038		1616,2291	92,8203	
1618,1578	75,5554		1618,1578	92,6727	
1620,0864	75,8869		1620,0864	92,541	
1622,0151	76,2685		1622,0151	92,3627	
1623,9438	77,0183		1623,9438	92,1813	
1625,8725	77,5366		1625,8725	92,0187	
1627,8011	78,0154		1627,8011	91,8729	
1629,7298	78,6002		1629,7298	91,7063	
1631,6585	79,0045		1631,6585	91,4829	
1633,5872	79,2456		1633,5872	91,2986	
1635,5158	79,6693		1635,5158	91,232	
1637,4445	80,3507		1637,4445	91,2689	
1639,3732	80,6927		1639,3732	91,2397	
1641,3019	80,9983		1641,3019	91,1416	
1643,2305	81,267		1643,2305	91,0192	
1645,1592	81,4784		1645,1592	90,8899	
1647,0879	82,0954		1647,0879	90,9186	
1649,0166	82,6588		1649,0166	91,05	
1650,9452	82,703		1650,9452	91,0215	
1652,8739	83,225		1652,8739	91,1286	
1654,8026	84,3361		1654,8026	91,4866	
1656,7313	84,5313		1656,7313	91,5769	
1658,6599	84,8707		1658,6599	91,737	
1660,5886	85,307		1660,5886	91,8717	
1662,5173	85,9605		1662,5173	92,024	
1664,446	86,7239		1664,446	92,2371	

1666,3746	87,1797		1666,3746	92,2948	
1668,3033	87,6874		1668,3033	92,2797	
1670,232	88,5685		1670,232	92,3509	
1672,1607	89,1908		1672,1607	92,4828	
1674,0893	89,742		1674,0893	92,5622	
1676,018	90,7116		1676,018	92,6291	
1677,9467	91,2677		1677,9467	92,6335	
1679,8754	91,7046		1679,8754	92,5516	
1681,804	92,1127		1681,804	92,3766	
1683,7327	92,5782		1683,7327	92,0762	
1685,6614	93,5426		1685,6614	92,0571	
1687,59	93,9621		1687,59	92,1002	
1689,5187	94,3302		1689,5187	92,0384	
1691,4474	94,6905		1691,4474	91,8932	
1693,3761	94,9049		1693,3761	91,6717	
1695,3047	95,2001		1695,3047	91,4002	
1697,2334	95,5675		1697,2334	91,0594	
1699,1621	95,7469		1699,1621	90,6315	
1701,0908	96,0275		1701,0908	89,761	
1703,0194	96,1503		1703,0194	89,0654	
1704,9481	96,3357		1704,9481	88,1146	
1706,8768	96,5778		1706,8768	86,7546	
1708,8055	96,6561		1708,8055	85,3573	
1710,7341	96,7191		1710,7341	83,492	
1712,6628	96,771		1712,6628	81,3667	
1714,5915	96,7682		1714,5915	78,6769	
1716,5202	96,7598		1716,5202	75,4376	
1718,4488	96,8726		1718,4488	71,2641	
1720,3775	96,9597		1720,3775	68,1558	
1722,3062	96,9945		1722,3062	65,6383	

1724,2349	97,0512		1724,2349	63,1362	
1726,1635	97,086		1726,1635	61,5584	
1728,0922	97,1182		1728,0922	60,7976	
1730,0209	97,2058		1730,0209	60,9268	
1731,9496	97,1896		1731,9496	61,5551	
1733,8782	97,0949		1733,8782	63,8105	
1735,8069	97,1619		1735,8069	67,0448	
1737,7356	97,2163		1737,7356	69,0506	
1739,6643	97,2685		1739,6643	72,8676	
1741,5929	97,3036		1741,5929	77,0416	
1743,5216	97,2919		1743,5216	80,676	
1745,4503	97,262		1745,4503	84,2546	
1747,379	97,2287		1747,379	87,1002	
1749,3076	97,2642		1749,3076	89,4946	
1751,2363	97,4151		1751,2363	91,4061	
1753,165	97,4915		1753,165	92,8127	
1755,0936	97,4678		1755,0936	93,5474	
1757,0223	97,4429		1757,0223	94,1975	
1758,951	97,447		1758,951	94,6774	
1760,8797	97,4469		1760,8797	94,9251	
1762,8083	97,4498		1762,8083	95,2138	
1764,737	97,4384		1764,737	95,4061	
1766,6657	97,3962		1766,6657	95,4895	
1768,5944	97,3633		1768,5944	95,5844	
1770,523	97,3922		1770,523	95,6223	
1772,4517	97,5084		1772,4517	95,711	
1774,3804	97,5122		1774,3804	95,8401	
1776,3091	97,4646		1776,3091	95,9067	
1778,2377	97,4338		1778,2377	95,9684	
1780,1664	97,3978		1780,1664	96,0647	

1782,0951	97,3703		1782,0951	96,1771	
1784,0238	97,3156		1784,0238	96,2489	
1785,9524	97,3407		1785,9524	96,2978	
1787,8811	97,3725		1787,8811	96,2911	
1789,8098	97,3367		1789,8098	96,3031	
1791,7385	97,3075		1791,7385	96,3449	
1793,6671	97,2832		1793,6671	96,328	
1795,5958	97,3326		1795,5958	96,3064	
1797,5245	97,3981		1797,5245	96,3037	
1799,4532	97,3422		1799,4532	96,3542	
1801,3818	97,2844		1801,3818	96,4039	
1803,3105	97,2938		1803,3105	96,3944	
1805,2392	97,2979		1805,2392	96,4303	
1807,1679	97,3147		1807,1679	96,4838	
1809,0965	97,2753		1809,0965	96,4629	
1811,0252	97,2318		1811,0252	96,3824	
1812,9539	97,2813		1812,9539	96,3812	
1814,8826	97,325		1814,8826	96,4953	
1816,8112	97,322		1816,8112	96,5788	
1818,7399	97,3285		1818,7399	96,5443	
1820,6686	97,3394		1820,6686	96,5051	
1822,5973	97,2663		1822,5973	96,5098	
1824,5259	97,1653		1824,5259	96,4897	
1826,4546	97,1264		1826,4546	96,4274	
1828,3833	97,1377		1828,3833	96,3567	
1830,3119	97,2345		1830,3119	96,427	
1832,2406	97,2615		1832,2406	96,579	
1834,1693	97,2314		1834,1693	96,5816	
1836,098	97,2315		1836,098	96,5241	
1838,0266	97,2204		1838,0266	96,5366	

1839,9553	97,1944		1839,9553	96,5673	
1841,884	97,2007		1841,884	96,5561	
1843,8127	97,2477		1843,8127	96,5939	
1845,7413	97,2514		1845,7413	96,6517	
1847,67	97,215		1847,67	96,5783	
1849,5987	97,1897		1849,5987	96,5282	
1851,5274	97,155		1851,5274	96,5781	
1853,456	97,1032		1853,456	96,6156	
1855,3847	97,1409		1855,3847	96,6044	
1857,3134	97,1729		1857,3134	96,582	
1859,2421	97,0904		1859,2421	96,5998	
1861,1707	97,033		1861,1707	96,6628	
1863,0994	97,008		1863,0994	96,6527	
1865,0281	97,0066		1865,0281	96,5547	
1866,9568	96,9882		1866,9568	96,491	
1868,8854	96,9517		1868,8854	96,5497	
1870,8141	96,9411		1870,8141	96,5935	
1872,7428	96,9071		1872,7428	96,5515	
1874,6715	96,8963		1874,6715	96,5422	
1876,6001	96,9328		1876,6001	96,5125	
1878,5288	96,9747		1878,5288	96,5151	
1880,4575	96,9777		1880,4575	96,5289	
1882,3862	96,9433		1882,3862	96,5043	
1884,3148	96,9634		1884,3148	96,641	
1886,2435	97,0526		1886,2435	96,745	
1888,1722	97,0369		1888,1722	96,6094	
1890,1009	96,9493		1890,1009	96,4682	
1892,0295	96,9418		1892,0295	96,493	
1893,9582	97,0061		1893,9582	96,518	
1895,8869	96,9767		1895,8869	96,4445	

1897,8156	96,8544		1897,8156	96,4573	
1899,7442	96,8428		1899,7442	96,5992	
1901,6729	96,8738		1901,6729	96,6274	
1903,6016	96,9262		1903,6016	96,5305	
1905,5302	96,9414		1905,5302	96,4313	
1907,4589	96,888		1907,4589	96,3276	
1909,3876	96,9498		1909,3876	96,2922	
1911,3163	96,9862		1911,3163	96,3782	
1913,2449	96,8833		1913,2449	96,4578	
1915,1736	96,798		1915,1736	96,4635	
1917,1023	96,6449		1917,1023	96,4516	
1919,031	96,6025		1919,031	96,3756	
1920,9596	96,6965		1920,9596	96,2175	
1922,8883	96,6677		1922,8883	96,146	
1924,817	96,623		1924,817	96,1686	
1926,7457	96,5877		1926,7457	96,0979	
1928,6743	96,6883		1928,6743	96,1432	
1930,603	96,8125		1930,603	96,2658	
1932,5317	96,7926		1932,5317	96,219	
1934,4604	96,6782		1934,4604	96,2028	
1936,389	96,5777		1936,389	96,1489	
1938,3177	96,6935		1938,3177	96,1121	
1940,2464	96,7197		1940,2464	96,1751	
1942,1751	96,5468		1942,1751	96,0467	
1944,1037	96,4924		1944,1037	95,8705	
1946,0324	96,579		1946,0324	95,9595	
1947,9611	96,7088		1947,9611	96,1267	
1949,8898	96,6319		1949,8898	96,1304	
1951,8184	96,3914		1951,8184	96,0937	
1953,7471	96,3139		1953,7471	96,2613	

1955,6758	96,3925		1955,6758	96,4672	
1957,6045	96,4384		1957,6045	96,4635	
1959,5331	96,4001		1959,5331	96,2961	
1961,4618	96,3985		1961,4618	96,0897	
1963,3905	96,3785		1963,3905	96,1175	
1965,3192	96,3293		1965,3192	96,3084	
1967,2478	96,3941		1967,2478	96,2472	
1969,1765	96,3968		1969,1765	95,9174	
1971,1052	96,224		1971,1052	95,7025	
1973,0339	96,0492		1973,0339	95,674	
1974,9625	96,0128		1974,9625	95,7793	
1976,8912	95,9733		1976,8912	95,9083	
1978,8199	95,8584		1978,8199	95,8454	
1980,7485	95,917		1980,7485	95,6241	
1982,6772	96,1329		1982,6772	95,5099	
1984,6059	96,1998		1984,6059	95,5514	
1986,5346	96,0978		1986,5346	95,4832	
1988,4632	96,032		1988,4632	95,5841	
1990,3919	95,9791		1990,3919	95,8836	
1992,3206	96,0432		1992,3206	96,0226	
1994,2493	96,1745		1994,2493	96,1691	
1996,1779	96,1236		1996,1779	96,3038	
1998,1066	95,9848		1998,1066	96,2794	
2000,0353	95,9241		2000,0353	96,0769	
2001,964	96,0108		2001,964	96,0814	
2003,8926	96,2674		2003,8926	96,4658	
2005,8213	96,1901		2005,8213	96,5068	
2007,75	95,7326		2007,75	96,064	
2009,6787	95,7621		2009,6787	95,9028	
2011,6073	96,016		2011,6073	96,193	

2013,536	95,9805		2013,536	96,5145	
2015,4647	95,8819		2015,4647	96,715	
2017,3934	95,6934		2017,3934	96,5732	
2019,322	95,5759		2019,322	96,1764	
2021,2507	95,7922		2021,2507	96,1039	
2023,1794	96,058		2023,1794	96,3536	
2025,1081	96,1476		2025,1081	96,4176	
2027,0367	96,1406		2027,0367	96,1376	
2028,9654	96,1657		2028,9654	95,8865	
2030,8941	95,986		2030,8941	95,8281	
2032,8228	95,5577		2032,8228	95,8053	
2034,7514	95,4888		2034,7514	95,8722	
2036,6801	95,7808		2036,6801	95,9028	
2038,6088	95,7594		2038,6088	95,922	
2040,5375	95,6633		2040,5375	96,1424	
2042,4661	95,7965		2042,4661	96,1998	
2044,3948	95,7402		2044,3948	96,1642	
2046,3235	95,6017		2046,3235	96,176	
2048,2521	95,5597		2048,2521	96,0254	
2050,1808	95,6842		2050,1808	95,8755	
2052,1095	95,8161		2052,1095	96,0147	
2054,0382	95,8619		2054,0382	96,2471	
2055,9668	95,9959		2055,9668	96,3081	
2057,8955	95,8845		2057,8955	96,3805	
2059,8242	95,648		2059,8242	96,3425	
2061,7529	95,7156		2061,7529	96,1729	
2063,6815	95,7757		2063,6815	96,1755	
2065,6102	95,7979		2065,6102	96,1915	
2067,5389	96,031		2067,5389	96,229	
2069,4676	96,2229		2069,4676	96,29	

2071,3962	96,2439		2071,3962	96,1805	
2073,3249	96,1561		2073,3249	96,1834	
2075,2536	96,0287		2075,2536	96,2523	
2077,1823	96,0031		2077,1823	96,1744	
2079,1109	96,0626		2079,1109	96,129	
2081,0396	95,975		2081,0396	96,1723	
2082,9683	95,9314		2082,9683	96,1915	
2084,897	96,0917		2084,897	96,2227	
2086,8256	96,1416		2086,8256	96,3532	
2088,7543	96,1148		2088,7543	96,4688	
2090,683	96,117		2090,683	96,4663	
2092,6117	96,0854		2092,6117	96,3668	
2094,5403	95,9333		2094,5403	96,2698	
2096,469	95,8256		2096,469	96,271	
2098,3977	95,7864		2098,3977	96,3677	
2100,3264	95,764		2100,3264	96,4129	
2102,255	95,8786		2102,255	96,332	
2104,1837	95,8241		2104,1837	96,2466	
2106,1124	95,7266		2106,1124	96,3452	
2108,0411	95,8964		2108,0411	96,5045	
2109,9697	95,9455		2109,9697	96,3586	
2111,8984	95,6926		2111,8984	96,1897	
2113,8271	95,5627		2113,8271	96,2394	
2115,7558	95,7455		2115,7558	96,3334	
2117,6844	95,9412		2117,6844	96,4489	
2119,6131	95,9597		2119,6131	96,4733	
2121,5418	95,9804		2121,5418	96,4871	
2123,4704	96,0977		2123,4704	96,5453	
2125,3991	96,1147		2125,3991	96,5331	
2127,3278	96,0194		2127,3278	96,5478	

2129,2565	95,9547		2129,2565	96,622	
2131,1851	96,0641		2131,1851	96,6249	
2133,1138	96,2878		2133,1138	96,4429	
2135,0425	96,3754		2135,0425	96,3039	
2136,9712	96,2532		2136,9712	96,3423	
2138,8998	96,0513		2138,8998	96,2529	
2140,8285	96,0305		2140,8285	96,2208	
2142,7572	96,1094		2142,7572	96,4349	
2144,6859	96,0097		2144,6859	96,579	
2146,6145	95,9147		2146,6145	96,6221	
2148,5432	96,1374		2148,5432	96,6504	
2150,4719	96,4374		2150,4719	96,7285	
2152,4006	96,621		2152,4006	96,7911	
2154,3292	96,4939		2154,3292	96,853	
2156,2579	96,0699		2156,2579	96,9718	
2158,1866	95,6263		2158,1866	96,7705	
2160,1153	95,1028		2160,1153	96,4075	
2162,0439	94,9482		2162,0439	96,3995	
2163,9726	95,2228		2163,9726	96,4854	
2165,9013	95,396		2165,9013	96,2566	
2167,83	95,438		2167,83	95,9509	
2169,7586	95,5279		2169,7586	95,9232	
2171,6873	95,5806		2171,6873	96,1256	
2173,616	95,6747		2173,616	96,5069	
2175,5447	95,6972		2175,5447	96,8162	
2177,4733	95,4907		2177,4733	96,5631	
2179,402	95,4234		2179,402	96,2162	
2181,3307	95,6812		2181,3307	96,3376	
2183,2594	95,8524		2183,2594	96,5217	
2185,188	95,5879		2185,188	96,4544	

2187,1167	95,2459		2187,1167	96,3658	
2189,0454	95,3094		2189,0454	96,4649	
2190,9741	95,4524		2190,9741	96,4813	
2192,9027	95,3873		2192,9027	96,3637	
2194,8314	95,4189		2194,8314	96,3818	
2196,7601	95,5107		2196,7601	96,4237	
2198,6887	95,4858		2198,6887	96,4669	
2200,6174	95,4021		2200,6174	96,6802	
2202,5461	95,5005		2202,5461	96,7145	
2204,4748	95,6327		2204,4748	96,3562	
2206,4034	95,6196		2206,4034	96,1906	
2208,3321	95,7366		2208,3321	96,484	
2210,2608	95,8438		2210,2608	96,6958	
2212,1895	95,7724		2212,1895	96,4404	
2214,1181	95,6686		2214,1181	96,1154	
2216,0468	95,556		2216,0468	96,0657	
2217,9755	95,548		2217,9755	96,1485	
2219,9042	95,7199		2219,9042	96,3667	
2221,8328	95,8023		2221,8328	96,6397	
2223,7615	95,6873		2223,7615	96,7494	
2225,6902	95,6523		2225,6902	96,6621	
2227,6189	95,7097		2227,6189	96,4884	
2229,5475	95,6731		2229,5475	96,4614	
2231,4762	95,6693		2231,4762	96,517	
2233,4049	95,725		2233,4049	96,5204	
2235,3336	95,7109		2235,3336	96,5296	
2237,2622	95,6654		2237,2622	96,4873	
2239,1909	95,6616		2239,1909	96,4785	
2241,1196	95,7366		2241,1196	96,5875	
2243,0483	95,8253		2243,0483	96,5974	

2244,9769	95,8146		2244,9769	96,517	
2246,9056	95,736		2246,9056	96,5712	
2248,8343	95,7467		2248,8343	96,5841	
2250,763	95,8615		2250,763	96,5238	
2252,6916	95,8653		2252,6916	96,5628	
2254,6203	95,7529		2254,6203	96,5224	
2256,549	95,7534		2256,549	96,4153	
2258,4777	95,881		2258,4777	96,416	
2260,4063	95,9668		2260,4063	96,4107	
2262,335	95,9248		2262,335	96,4751	
2264,2637	95,8005		2264,2637	96,5599	
2266,1923	95,6868		2266,1923	96,3716	
2268,121	95,6985		2268,121	96,2074	
2270,0497	95,8055		2270,0497	96,2951	
2271,9784	95,88		2271,9784	96,3462	
2273,907	95,8004		2273,907	96,3241	
2275,8357	95,6372		2275,8357	96,3881	
2277,7644	95,5911		2277,7644	96,3584	
2279,6931	95,626		2279,6931	96,3214	
2281,6217	95,679		2281,6217	96,5155	
2283,5504	95,7451		2283,5504	96,5564	
2285,4791	95,7501		2285,4791	96,4058	
2287,4078	95,738		2287,4078	96,2678	
2289,3364	95,8102		2289,3364	96,1124	
2291,2651	95,8767		2291,2651	96,083	
2293,1938	95,8934		2293,1938	96,1044	
2295,1225	95,8953		2295,1225	96,113	
2297,0511	95,8392		2297,0511	96,1284	
2298,9798	95,8017		2298,9798	95,9725	
2300,9085	95,7725		2300,9085	95,7989	

2302,8372	95,7225		2302,8372	95,7717	
2304,7658	95,7065		2304,7658	95,6103	
2306,6945	95,6375		2306,6945	95,2991	
2308,6232	95,5647		2308,6232	95,057	
2310,5519	95,5398		2310,5519	94,9205	
2312,4805	95,5123		2312,4805	94,818	
2314,4092	95,4672		2314,4092	94,6524	
2316,3379	95,4488		2316,3379	94,3452	
2318,2666	95,4854		2318,2666	93,8772	
2320,1952	95,4048		2320,1952	93,4833	
2322,1239	95,2029		2322,1239	93,2853	
2324,0526	95,2235		2324,0526	93,2009	
2325,9813	95,4511		2325,9813	93,1224	
2327,9099	95,5623		2327,9099	92,9032	
2329,8386	95,5801		2329,8386	92,7439	
2331,7673	95,5607		2331,7673	92,6978	
2333,696	95,4325		2333,696	92,5331	
2335,6246	95,2533		2335,6246	92,4324	
2337,5533	95,1389		2337,5533	92,4151	
2339,482	95,2436		2339,482	92,2	
2341,4106	95,3578		2341,4106	92,001	
2343,3393	95,3418		2343,3393	92,1587	
2345,268	95,3695		2345,268	92,8142	
2347,1967	95,391		2347,1967	93,6174	
2349,1253	95,4561		2349,1253	93,6896	
2351,054	95,5259		2351,054	93,0401	
2352,9827	95,3601		2352,9827	92,2855	
2354,9114	95,0152		2354,9114	91,7052	
2356,84	94,9216		2356,84	91,1078	
2358,7687	94,9984		2358,7687	90,7065	

2360,6974	94,8072		2360,6974	90,7321	
2362,6261	94,7107		2362,6261	90,936	
2364,5547	95,004		2364,5547	91,2451	
2366,4834	95,289		2366,4834	91,8616	
2368,4121	95,3781		2368,4121	92,4856	
2370,3408	95,3882		2370,3408	92,9495	
2372,2694	95,3338		2372,2694	93,4475	
2374,1981	95,373		2374,1981	94,0281	
2376,1268	95,6032		2376,1268	94,6079	
2378,0555	95,7524		2378,0555	95,1519	
2379,9841	95,7461		2379,9841	95,6903	
2381,9128	95,8161		2381,9128	96,1915	
2383,8415	96,0021		2383,8415	96,5265	
2385,7702	96,0755		2385,7702	96,7301	
2387,6988	96,0059		2387,6988	96,8834	
2389,6275	95,9452		2389,6275	96,9129	
2391,5562	95,9473		2391,5562	96,9155	
2393,4849	95,9973		2393,4849	96,9503	
2395,4135	96,0464		2395,4135	96,9509	
2397,3422	96,055		2397,3422	96,9942	
2399,2709	96,0238		2399,2709	96,981	
2401,1996	96,0151		2401,1996	96,8635	
2403,1282	96,0207		2403,1282	96,8386	
2405,0569	95,9238		2405,0569	96,8874	
2406,9856	95,8035		2406,9856	96,8723	
2408,9143	95,8515		2408,9143	96,8892	
2410,8429	95,95		2410,8429	96,9877	
2412,7716	95,9552		2412,7716	97,0161	
2414,7003	95,8855		2414,7003	96,917	
2416,6289	95,7906		2416,6289	96,8005	

2418,5576	95,7504		2418,5576	96,7561	
2420,4863	95,7477		2420,4863	96,7501	
2422,415	95,7654		2422,415	96,8154	
2424,3436	95,7701		2424,3436	96,8664	
2426,2723	95,7089		2426,2723	96,7932	
2428,201	95,6834		2428,201	96,745	
2430,1297	95,7828		2430,1297	96,8225	
2432,0583	95,8172		2432,0583	96,9132	
2433,987	95,722		2433,987	96,9351	
2435,9157	95,6624		2435,9157	96,9061	
2437,8444	95,5871		2437,8444	96,8121	
2439,773	95,5277		2439,773	96,8135	
2441,7017	95,6337		2441,7017	96,9118	
2443,6304	95,7773		2443,6304	96,8583	
2445,5591	95,7913		2445,5591	96,759	
2447,4877	95,7118		2447,4877	96,7612	
2449,4164	95,6636		2449,4164	96,7768	
2451,3451	95,6546		2451,3451	96,7797	
2453,2738	95,6324		2453,2738	96,834	
2455,2024	95,643		2455,2024	96,8732	
2457,1311	95,6197		2457,1311	96,8198	
2459,0598	95,63		2459,0598	96,8002	
2460,9885	95,7098		2460,9885	96,8338	
2462,9171	95,6807		2462,9171	96,8521	
2464,8458	95,6258		2464,8458	96,8303	
2466,7745	95,6368		2466,7745	96,7815	
2468,7032	95,6308		2468,7032	96,7816	
2470,6318	95,6194		2470,6318	96,794	
2472,5605	95,6536		2472,5605	96,7165	
2474,4892	95,6591		2474,4892	96,6641	

2476,4179	95,6373		2476,4179	96,7612	
2478,3465	95,5661		2478,3465	96,7895	
2480,2752	95,425		2480,2752	96,7326	
2482,2039	95,3751		2482,2039	96,7818	
2484,1326	95,4403		2484,1326	96,8107	
2486,0612	95,4651		2486,0612	96,8006	
2487,9899	95,4149		2487,9899	96,8571	
2489,9186	95,4847		2489,9186	96,8598	
2491,8472	95,5751		2491,8472	96,7984	
2493,7759	95,5247		2493,7759	96,7336	
2495,7046	95,5034		2495,7046	96,6129	
2497,6333	95,5009		2497,6333	96,5802	
2499,5619	95,4821		2499,5619	96,7025	
2501,4906	95,5		2501,4906	96,6962	
2503,4193	95,5082		2503,4193	96,591	
2505,348	95,4576		2505,348	96,6371	
2507,2766	95,374		2507,2766	96,7053	
2509,2053	95,3308		2509,2053	96,6792	
2511,134	95,3222		2511,134	96,6407	
2513,0627	95,354		2513,0627	96,6478	
2514,9913	95,3969		2514,9913	96,6608	
2516,92	95,3478		2516,92	96,6959	
2518,8487	95,299		2518,8487	96,758	
2520,7774	95,3102		2520,7774	96,666	
2522,706	95,2946		2522,706	96,56	
2524,6347	95,2643		2524,6347	96,6086	
2526,5634	95,2451		2526,5634	96,6758	
2528,4921	95,2397		2528,4921	96,7022	
2530,4207	95,235		2530,4207	96,6701	
2532,3494	95,233		2532,3494	96,6013	

2534,2781	95,2331		2534,2781	96,5979	
2536,2068	95,2543		2536,2068	96,6382	
2538,1354	95,2851		2538,1354	96,6294	
2540,0641	95,2555		2540,0641	96,6207	
2541,9928	95,2154		2541,9928	96,5992	
2543,9215	95,1758		2543,9215	96,604	
2545,8501	95,1249		2545,8501	96,6638	
2547,7788	95,1189		2547,7788	96,6767	
2549,7075	95,1719		2549,7075	96,6513	
2551,6362	95,1903		2551,6362	96,6514	
2553,5648	95,1113		2553,5648	96,6885	
2555,4935	95,0772		2555,4935	96,6841	
2557,4222	95,1288		2557,4222	96,6051	
2559,3508	95,1425		2559,3508	96,5452	
2561,2795	95,1457		2561,2795	96,563	
2563,2082	95,1554		2563,2082	96,5802	
2565,1369	95,1077		2565,1369	96,5788	
2567,0655	95,039		2567,0655	96,602	
2568,9942	95,0472		2568,9942	96,5909	
2570,9229	95,0911		2570,9229	96,5078	
2572,8516	95,0826		2572,8516	96,448	
2574,7802	95,0925		2574,7802	96,4589	
2576,7089	95,1057		2576,7089	96,5048	
2578,6376	95,0622		2578,6376	96,5658	
2580,5663	95,023		2580,5663	96,6081	
2582,4949	95,0495		2582,4949	96,5968	
2584,4236	95,0563		2584,4236	96,5065	
2586,3523	95,0367		2586,3523	96,3959	
2588,281	95,0254		2588,281	96,3867	
2590,2096	94,9525		2590,2096	96,4606	

2592,1383	94,8882		2592,1383	96,4672	
2594,067	94,836		2594,067	96,4406	
2595,9957	94,8409		2595,9957	96,4431	
2597,9243	94,9559		2597,9243	96,4542	
2599,853	94,9824		2599,853	96,443	
2601,7817	94,9205		2601,7817	96,4116	
2603,7104	94,9274		2603,7104	96,4142	
2605,639	94,9459		2605,639	96,4195	
2607,5677	94,9352		2607,5677	96,4893	
2609,4964	94,9123		2609,4964	96,5002	
2611,4251	94,8676		2611,4251	96,3895	
2613,3537	94,8348		2613,3537	96,3961	
2615,2824	94,7978		2615,2824	96,4399	
2617,2111	94,7778		2617,2111	96,4391	
2619,1398	94,7961		2619,1398	96,452	
2621,0684	94,8151		2621,0684	96,4509	
2622,9971	94,847		2622,9971	96,4293	
2624,9258	94,8649		2624,9258	96,3783	
2626,8545	94,7931		2626,8545	96,3677	
2628,7831	94,662		2628,7831	96,3599	
2630,7118	94,6041		2630,7118	96,3053	
2632,6405	94,5976		2632,6405	96,3337	
2634,5691	94,5968		2634,5691	96,4155	
2636,4978	94,6818		2636,4978	96,4156	
2638,4265	94,7521		2638,4265	96,352	
2640,3552	94,6999		2640,3552	96,3412	
2642,2838	94,6736		2642,2838	96,4119	
2644,2125	94,6846		2644,2125	96,4154	
2646,1412	94,5999		2646,1412	96,3297	
2648,0699	94,5355		2648,0699	96,2987	

2649,9985	94,5791		2649,9985	96,2889	
2651,9272	94,6001		2651,9272	96,273	
2653,8559	94,5707		2653,8559	96,2876	
2655,7846	94,5326		2655,7846	96,2779	
2657,7132	94,5157		2657,7132	96,265	
2659,6419	94,5571		2659,6419	96,2967	
2661,5706	94,6039		2661,5706	96,3229	
2663,4993	94,611		2663,4993	96,2801	
2665,4279	94,5786		2665,4279	96,2392	
2667,3566	94,5394		2667,3566	96,2622	
2669,2853	94,5215		2669,2853	96,2523	
2671,214	94,4857		2671,214	96,235	
2673,1426	94,4528		2673,1426	96,2347	
2675,0713	94,4428		2675,0713	96,1985	
2677	94,3929		2677	96,1739	
2678,9287	94,3466		2678,9287	96,1362	
2680,8573	94,3612		2680,8573	96,0719	
2682,786	94,3388		2682,786	96,0232	
2684,7147	94,2816		2684,7147	95,9924	
2686,6434	94,3124		2686,6434	95,9175	
2688,572	94,3572		2688,572	95,8493	
2690,5007	94,3256		2690,5007	95,8608	
2692,4294	94,3143		2692,4294	95,845	
2694,3581	94,3143		2694,3581	95,7727	
2696,2867	94,2921		2696,2867	95,715	
2698,2154	94,2815		2698,2154	95,6721	
2700,1441	94,2526		2700,1441	95,6045	
2702,0728	94,2154		2702,0728	95,5817	
2704,0014	94,1776		2704,0014	95,5717	
2705,9301	94,1238		2705,9301	95,5293	

2707,8588	94,0907		2707,8588	95,4975	
2709,7874	94,1054		2709,7874	95,4471	
2711,7161	94,1243		2711,7161	95,4122	
2713,6448	94,1221		2713,6448	95,4183	
2715,5735	94,1061		2715,5735	95,4044	
2717,5021	94,0786		2717,5021	95,3556	
2719,4308	94,0336		2719,4308	95,3397	
2721,3595	94,0033		2721,3595	95,3298	
2723,2882	94,0153		2723,2882	95,2878	
2725,2168	94,0223		2725,2168	95,2402	
2727,1455	94,0231		2727,1455	95,1884	
2729,0742	93,9864		2729,0742	95,1625	
2731,0029	93,8998		2731,0029	95,1602	
2732,9315	93,8773		2732,9315	95,1259	
2734,8602	93,9278		2734,8602	95,0215	
2736,7889	93,9298		2736,7889	94,9387	
2738,7176	93,8722		2738,7176	94,9319	
2740,6462	93,8004		2740,6462	94,9208	
2742,5749	93,7724		2742,5749	94,8966	
2744,5036	93,8376		2744,5036	94,9103	
2746,4323	93,8887		2746,4323	94,9277	
2748,3609	93,877		2748,3609	94,919	
2750,2896	93,8467		2750,2896	94,927	
2752,2183	93,7705		2752,2183	94,9467	
2754,147	93,6977		2754,147	94,9376	
2756,0756	93,7101		2756,0756	94,8783	
2758,0043	93,7479		2758,0043	94,8093	
2759,933	93,7375		2759,933	94,7724	
2761,8617	93,703		2761,8617	94,7513	
2763,7903	93,6901		2763,7903	94,6959	

2765,719	93,6959		2765,719	94,6491	
2767,6477	93,6953		2767,6477	94,6224	
2769,5764	93,6252		2769,5764	94,5628	
2771,505	93,5298		2771,505	94,5207	
2773,4337	93,4899		2773,4337	94,4797	
2775,3624	93,4607		2775,3624	94,4189	
2777,291	93,4448		2777,291	94,3532	
2779,2197	93,4049		2779,2197	94,2576	
2781,1484	93,3369		2781,1484	94,143	
2783,0771	93,3139		2783,0771	94,0082	
2785,0057	93,2799		2785,0057	93,8744	
2786,9344	93,2427		2786,9344	93,7761	
2788,8631	93,2525		2788,8631	93,6923	
2790,7918	93,2353		2790,7918	93,5995	
2792,7204	93,1899		2792,7204	93,5278	
2794,6491	93,1627		2794,6491	93,4641	
2796,5778	93,1278		2796,5778	93,3125	
2798,5065	93,0992		2798,5065	93,1033	
2800,4351	93,1116		2800,4351	92,9682	
2802,3638	93,124		2802,3638	92,8718	
2804,2925	93,1019		2804,2925	92,6916	
2806,2212	93,0306		2806,2212	92,4816	
2808,1498	92,9382		2808,1498	92,3023	
2810,0785	92,902		2810,0785	92,1439	
2812,0072	92,905		2812,0072	92,0283	
2813,9359	92,8582		2813,9359	91,854	
2815,8645	92,7748		2815,8645	91,5927	
2817,7932	92,7215		2817,7932	91,3708	
2819,7219	92,6376		2819,7219	91,1691	
2821,6506	92,5682		2821,6506	90,9313	

2823,5792	92,5577		2823,5792	90,6804	
2825,5079	92,5241		2825,5079	90,3802	
2827,4366	92,4695		2827,4366	90,0195	
2829,3653	92,4395		2829,3653	89,6699	
2831,2939	92,4415		2831,2939	89,2833	
2833,2226	92,3789		2833,2226	88,7959	
2835,1513	92,2356		2835,1513	88,2236	
2837,08	92,113		2837,08	87,5584	
2839,0086	92,0959		2839,0086	86,7905	
2840,9373	92,0929		2840,9373	85,9086	
2842,866	91,9923		2842,866	84,9224	
2844,7947	91,8843		2844,7947	83,8283	
2846,7233	91,8089		2846,7233	82,6115	
2848,652	91,7373		2848,652	81,3693	
2850,5807	91,6745		2850,5807	80,2247	
2852,5093	91,6025		2852,5093	79,287	
2854,438	91,5175		2854,438	78,6751	
2856,3667	91,4508		2856,3667	78,2863	
2858,2954	91,3917		2858,2954	77,9964	
2860,224	91,3267		2860,224	77,8572	
2862,1527	91,2711		2862,1527	77,7863	
2864,0814	91,174		2864,0814	77,6986	
2866,0101	91,0396		2866,0101	77,6243	
2867,9387	90,9679		2867,9387	77,5731	
2869,8674	90,9231		2869,8674	77,572	
2871,7961	90,8388		2871,7961	77,6828	
2873,7248	90,7578		2873,7248	77,9451	
2875,6534	90,7479		2875,6534	78,2776	
2877,5821	90,7608		2877,5821	78,5969	
2879,5108	90,6976		2879,5108	78,9068	

2881,4395	90,6277		2881,4395	79,1578	
2883,3681	90,5929		2883,3681	79,3461	
2885,2968	90,5623		2885,2968	79,5484	
2887,2255	90,5911		2887,2255	79,7278	
2889,1542	90,6226		2889,1542	79,8456	
2891,0828	90,6057		2891,0828	79,9761	
2893,0115	90,6323		2893,0115	80,0902	
2894,9402	90,6825		2894,9402	80,0902	
2896,8689	90,6966		2896,8689	80,049	
2898,7975	90,7004		2898,7975	79,9968	
2900,7262	90,7331		2900,7262	79,9053	
2902,6549	90,7616		2902,6549	79,8628	
2904,5836	90,7788		2904,5836	79,834	
2906,5122	90,8283		2906,5122	79,7136	
2908,4409	90,8517		2908,4409	79,5784	
2910,3696	90,834		2910,3696	79,4715	
2912,2983	90,8477		2912,2983	79,3631	
2914,2269	90,9061		2914,2269	79,2169	
2916,1556	90,9689		2916,1556	78,9981	
2918,0843	91,0298		2918,0843	78,8098	
2920,013	91,0729		2920,013	78,6904	
2921,9416	91,0156		2921,9416	78,6206	
2923,8703	90,9431		2923,8703	78,7048	
2925,799	90,9327		2925,799	78,9517	
2927,7276	90,8908		2927,7276	79,2771	
2929,6563	90,8864		2929,6563	79,6965	
2931,585	90,9453		2931,585	80,1999	
2933,5137	90,979		2933,5137	80,7715	
2935,4423	91,0193		2935,4423	81,3266	
2937,371	91,0922		2937,371	81,8214	

2939,2997	91,1771		2939,2997	82,3139	
2941,2284	91,2537		2941,2284	82,7575	
2943,157	91,2973		2943,157	83,1193	
2945,0857	91,2909		2945,0857	83,44	
2947,0144	91,2884		2947,0144	83,7716	
2948,9431	91,338		2948,9431	84,0668	
2950,8717	91,4252		2950,8717	84,3779	
2952,8004	91,5138		2952,8004	84,8248	
2954,7291	91,5536		2954,7291	85,3993	
2956,6578	91,5784		2956,6578	86,0409	
2958,5864	91,5957		2958,5864	86,6819	
2960,5151	91,6046		2960,5151	87,3386	
2962,4438	91,6381		2962,4438	88,0132	
2964,3725	91,678		2964,3725	88,645	
2966,3011	91,6893		2966,3011	89,2209	
2968,2298	91,6397		2968,2298	89,7696	
2970,1585	91,6073		2970,1585	90,2338	
2972,0872	91,6533		2972,0872	90,6007	
2974,0158	91,7316		2974,0158	90,9234	
2975,9445	91,7966		2975,9445	91,1873	
2977,8732	91,7759		2977,8732	91,4201	
2979,8019	91,6658		2979,8019	91,5844	
2981,7305	91,6184		2981,7305	91,707	
2983,6592	91,6662		2983,6592	91,8597	
2985,5879	91,6611		2985,5879	92,0159	
2987,5166	91,565		2987,5166	92,206	
2989,4452	91,4704		2989,4452	92,4009	
2991,3739	91,4226		2991,3739	92,5886	
2993,3026	91,4246		2993,3026	92,7945	
2995,2313	91,4539		2995,2313	92,9834	

2997,1599	91,4229		2997,1599	93,1626	
2999,0886	91,3403		2999,0886	93,2969	
3001,0173	91,2579		3001,0173	93,4222	
3002,9459	91,1556		3002,9459	93,6086	
3004,8746	91,0643		3004,8746	93,8085	
3006,8033	91,0491		3006,8033	94,0111	
3008,732	91,0782		3008,732	94,1709	
3010,6606	91,0388		3010,6606	94,2556	
3012,5893	90,9491		3012,5893	94,3486	
3014,518	90,8754		3014,518	94,4723	
3016,4467	90,8295		3016,4467	94,5958	
3018,3753	90,8202		3018,3753	94,7527	
3020,304	90,785		3020,304	94,8883	
3022,2327	90,695		3022,2327	94,9804	
3024,1614	90,5897		3024,1614	95,0995	
3026,09	90,5445		3026,09	95,1744	
3028,0187	90,5209		3028,0187	95,2	
3029,9474	90,4557		3029,9474	95,2968	
3031,8761	90,4282		3031,8761	95,406	
3033,8047	90,4334		3033,8047	95,3997	
3035,7334	90,4101		3035,7334	95,394	
3037,6621	90,337		3037,6621	95,4748	
3039,5908	90,1944		3039,5908	95,5364	
3041,5194	90,0614		3041,5194	95,5724	
3043,4481	89,9953		3043,4481	95,5742	
3045,3768	89,9333		3045,3768	95,5318	
3047,3055	89,8598		3047,3055	95,5526	
3049,2341	89,7931		3049,2341	95,6198	
3051,1628	89,7194		3051,1628	95,6522	
3053,0915	89,6344		3053,0915	95,6553	

3055,0202	89,5616		3055,0202	95,6206	
3056,9488	89,4701		3056,9488	95,6029	
3058,8775	89,3641		3058,8775	95,6029	
3060,8062	89,293		3060,8062	95,5444	
3062,7349	89,2007		3062,7349	95,4872	
3064,6635	89,1068		3064,6635	95,5023	
3066,5922	89,0639		3066,5922	95,5496	
3068,5209	88,9969		3068,5209	95,5683	
3070,4495	88,9052		3070,4495	95,5166	
3072,3782	88,8679		3072,3782	95,4223	
3074,3069	88,8812		3074,3069	95,3606	
3076,2356	88,8732		3076,2356	95,3439	
3078,1642	88,7312		3078,1642	95,3437	
3080,0929	88,5336		3080,0929	95,3437	
3082,0216	88,454		3082,0216	95,356	
3083,9503	88,4726		3083,9503	95,3558	
3085,8789	88,5206		3085,8789	95,3521	
3087,8076	88,4769		3087,8076	95,3727	
3089,7363	88,3369		3089,7363	95,3611	
3091,665	88,2583		3091,665	95,2993	
3093,5936	88,247		3093,5936	95,2184	
3095,5223	88,2467		3095,5223	95,1089	
3097,451	88,2549		3097,451	95,0767	
3099,3797	88,1949		3099,3797	95,1303	
3101,3083	88,1021		3101,3083	95,1355	
3103,237	88,0554		3103,237	95,1518	
3105,1657	87,9974		3105,1657	95,1731	
3107,0944	87,9692		3107,0944	95,0844	
3109,023	87,9647		3109,023	95,0073	
3110,9517	87,9285		3110,9517	95,0595	

3112,8804	87,8944		3112,8804	95,0806	
3114,8091	87,8629		3114,8091	94,9641	
3116,7377	87,8262		3116,7377	94,8447	
3118,6664	87,8081		3118,6664	94,8373	
3120,5951	87,7827		3120,5951	94,848	
3122,5238	87,739		3122,5238	94,7915	
3124,4524	87,6862		3124,4524	94,7433	
3126,3811	87,5981		3126,3811	94,7331	
3128,3098	87,5681		3128,3098	94,7367	
3130,2385	87,5915		3130,2385	94,7162	
3132,1671	87,5341		3132,1671	94,6584	
3134,0958	87,426		3134,0958	94,6351	
3136,0245	87,3695		3136,0245	94,5989	
3137,9532	87,3794		3137,9532	94,5064	
3139,8818	87,39		3139,8818	94,4446	
3141,8105	87,3337		3141,8105	94,437	
3143,7392	87,2317		3143,7392	94,3884	
3145,6678	87,1086		3145,6678	94,3429	
3147,5965	87,0201		3147,5965	94,3816	
3149,5252	87,0095		3149,5252	94,3479	
3151,4539	86,9834		3151,4539	94,2393	
3153,3825	86,9183		3153,3825	94,1795	
3155,3112	86,8407		3155,3112	94,1516	
3157,2399	86,7526		3157,2399	94,1329	
3159,1686	86,6699		3159,1686	94,1409	
3161,0972	86,5797		3161,0972	94,116	
3163,0259	86,5012		3163,0259	94,0057	
3164,9546	86,4735		3164,9546	93,9324	
3166,8833	86,4401		3166,8833	93,918	
3168,8119	86,3108		3168,8119	93,8987	

3170,7406	86,1742		3170,7406	93,8864	
3172,6693	86,14		3172,6693	93,834	
3174,598	86,099		3174,598	93,7616	
3176,5266	86,0005		3176,5266	93,7449	
3178,4553	85,9211		3178,4553	93,761	
3180,384	85,886		3180,384	93,7175	
3182,3127	85,8332		3182,3127	93,6313	
3184,2413	85,7309		3184,2413	93,5806	
3186,17	85,6498		3186,17	93,4816	
3188,0987	85,5739		3188,0987	93,4063	
3190,0274	85,5231		3190,0274	93,4084	
3191,956	85,5024		3191,956	93,3414	
3193,8847	85,4403		3193,8847	93,2514	
3195,8134	85,39		3195,8134	93,1904	
3197,7421	85,3181		3197,7421	93,1657	
3199,6707	85,1993		3199,6707	93,1679	
3201,5994	85,1735		3201,5994	93,1848	
3203,5281	85,2162		3203,5281	93,19	
3205,4568	85,1775		3205,4568	93,1316	
3207,3854	85,0248		3207,3854	93,0595	
3209,3141	84,8999		3209,3141	92,9692	
3211,2428	84,92		3211,2428	92,9252	
3213,1715	84,9282		3213,1715	92,8738	
3215,1001	84,8825		3215,1001	92,7008	
3217,0288	84,8389		3217,0288	92,6273	
3218,9575	84,7241		3218,9575	92,6844	
3220,8861	84,6108		3220,8861	92,6624	
3222,8148	84,5844		3222,8148	92,5888	
3224,7435	84,6159		3224,7435	92,5903	
3226,6722	84,6485		3226,6722	92,5335	

3228,6008	84,6311		3228,6008	92,3305	
3230,5295	84,569		3230,5295	92,2586	
3232,4582	84,4953		3232,4582	92,3091	
3234,3869	84,4275		3234,3869	92,2737	
3236,3155	84,4123		3236,3155	92,2456	
3238,2442	84,4033		3238,2442	92,2397	
3240,1729	84,3455		3240,1729	92,2099	
3242,1016	84,3061		3242,1016	92,1426	
3244,0302	84,2791		3244,0302	92,0402	
3245,9589	84,2509		3245,9589	91,9658	
3247,8876	84,2295		3247,8876	91,9351	
3249,8163	84,2207		3249,8163	91,9231	
3251,7449	84,1878		3251,7449	91,8549	
3253,6736	84,118		3253,6736	91,7811	
3255,6023	84,1117		3255,6023	91,7896	
3257,531	84,1097		3257,531	91,8036	
3259,4596	84,0819		3259,4596	91,7694	
3261,3883	84,1071		3261,3883	91,6817	
3263,317	84,1047		3263,317	91,6051	
3265,2457	84,1222		3265,2457	91,5985	
3267,1743	84,1874		3267,1743	91,5367	
3269,103	84,1299		3269,103	91,4206	
3271,0317	84,0623		3271,0317	91,3467	
3272,9604	84,0719		3272,9604	91,343	
3274,889	84,0934		3274,889	91,3994	
3276,8177	84,1825		3276,8177	91,3747	
3278,7464	84,1829		3278,7464	91,3354	
3280,6751	84,1021		3280,6751	91,3663	
3282,6037	84,137		3282,6037	91,3224	
3284,5324	84,1426		3284,5324	91,2709	

3286,4611	84,0662		3286,4611	91,3066	
3288,3898	84,1063		3288,3898	91,3407	
3290,3184	84,1831		3290,3184	91,2873	
3292,2471	84,1578		3292,2471	91,2199	
3294,1758	84,1852		3294,1758	91,2454	
3296,1044	84,312		3296,1044	91,2497	
3298,0331	84,3587		3298,0331	91,2244	
3299,9618	84,305		3299,9618	91,2281	
3301,8905	84,3042		3301,8905	91,2272	
3303,8191	84,3546		3303,8191	91,2351	
3305,7478	84,3841		3305,7478	91,1887	
3307,6765	84,4125		3307,6765	91,1276	
3309,6052	84,4365		3309,6052	91,1211	
3311,5338	84,4119		3311,5338	91,1144	
3313,4625	84,3767		3313,4625	91,0991	
3315,3912	84,3956		3315,3912	91,0311	
3317,3199	84,486		3317,3199	91,0065	
3319,2485	84,5668		3319,2485	91,0791	
3321,1772	84,5884		3321,1772	91,0278	
3323,1059	84,6506		3323,1059	90,903	
3325,0346	84,711		3325,0346	90,897	
3326,9632	84,696		3326,9632	90,9352	
3328,8919	84,6969		3328,8919	90,9317	
3330,8206	84,7406		3330,8206	90,9215	
3332,7493	84,7442		3332,7493	90,9279	
3334,6779	84,7264		3334,6779	90,9008	
3336,6066	84,7504		3336,6066	90,8589	
3338,5353	84,7785		3338,5353	90,8783	
3340,464	84,8021		3340,464	90,9603	
3342,3926	84,8293		3342,3926	90,9638	

3344,3213	84,8392		3344,3213	90,8259	
3346,25	84,8613		3346,25	90,7661	
3348,1787	84,9613		3348,1787	90,8414	
3350,1073	85,0509		3350,1073	90,8902	
3352,036	85,0236		3352,036	90,9007	
3353,9647	84,9955		3353,9647	90,933	
3355,8934	85,0242		3355,8934	91,0161	
3357,822	85,0599		3357,822	91,0251	
3359,7507	85,1088		3359,7507	90,9423	
3361,6794	85,1704		3361,6794	90,991	
3363,608	85,2387		3363,608	91,1201	
3365,5367	85,2774		3365,5367	91,1813	
3367,4654	85,2629		3367,4654	91,1183	
3369,3941	85,2606		3369,3941	91,0392	
3371,3227	85,3024		3371,3227	91,0594	
3373,2514	85,401		3373,2514	91,0697	
3375,1801	85,4954		3375,1801	91,0949	
3377,1088	85,5384		3377,1088	91,1679	
3379,0374	85,5736		3379,0374	91,2099	
3380,9661	85,5875		3380,9661	91,1693	
3382,8948	85,6236		3382,8948	91,0701	
3384,8235	85,6882		3384,8235	91,0038	
3386,7521	85,7608		3386,7521	91,0267	
3388,6808	85,8806		3388,6808	91,1001	
3390,6095	85,9571		3390,6095	91,1138	
3392,5382	85,9888		3392,5382	91,0602	
3394,4668	86,0436		3394,4668	91,0154	
3396,3955	86,1063		3396,3955	91,0023	
3398,3242	86,2015		3398,3242	91,0552	
3400,2529	86,2		3400,2529	91,1454	

3402,1815	86,1924		3402,1815	91,2196	
3404,1102	86,3144		3404,1102	91,2764	
3406,0389	86,4088		3406,0389	91,2476	
3407,9676	86,5316		3407,9676	91,1682	
3409,8962	86,7095		3409,8962	91,1793	
3411,8249	86,8049		3411,8249	91,2457	
3413,7536	86,7698		3413,7536	91,2308	
3415,6823	86,7442		3415,6823	91,232	
3417,6109	86,8169		3417,6109	91,3077	
3419,5396	86,9468		3419,5396	91,2853	
3421,4683	87,0735		3421,4683	91,2683	
3423,397	87,093		3423,397	91,3452	
3425,3256	87,0931		3425,3256	91,3651	
3427,2543	87,1645		3427,2543	91,3222	
3429,183	87,2433		3429,183	91,333	
3431,1117	87,3379		3431,1117	91,4364	
3433,0403	87,4587		3433,0403	91,4551	
3434,969	87,5716		3434,969	91,3806	
3436,8977	87,6636		3436,8977	91,3766	
3438,8263	87,7298		3438,8263	91,3724	
3440,755	87,8118		3440,755	91,3908	
3442,6837	87,8324		3442,6837	91,4682	
3444,6124	87,7975		3444,6124	91,4883	
3446,541	87,952		3446,541	91,5246	
3448,4697	88,1754		3448,4697	91,6095	
3450,3984	88,2002		3450,3984	91,6539	
3452,3271	88,2576		3452,3271	91,6853	
3454,2557	88,4404		3454,2557	91,7567	
3456,1844	88,5708		3456,1844	91,7702	
3458,1131	88,6423		3458,1131	91,7239	

3460,0418	88,6847		3460,0418	91,7691	
3461,9704	88,6583		3461,9704	91,8687	
3463,8991	88,6796		3463,8991	91,9457	
3465,8278	88,8228		3465,8278	91,958	
3467,7565	88,9585		3467,7565	91,9265	
3469,6851	89,0648		3469,6851	91,9638	
3471,6138	89,1783		3471,6138	92,0039	
3473,5425	89,3355		3473,5425	92,0003	
3475,4712	89,4555		3475,4712	92,0554	
3477,3998	89,4805		3477,3998	92,149	
3479,3285	89,5383		3479,3285	92,1807	
3481,2572	89,6397		3481,2572	92,1437	
3483,1859	89,7189		3483,1859	92,1805	
3485,1145	89,7513		3485,1145	92,2588	
3487,0432	89,829		3487,0432	92,3198	
3488,9719	89,9606		3488,9719	92,4637	
3490,9006	90,0566		3490,9006	92,5827	
3492,8292	90,0753		3492,8292	92,589	
3494,7579	90,1075		3494,7579	92,5598	
3496,6866	90,2694		3496,6866	92,5624	
3498,6153	90,3742		3498,6153	92,5584	
3500,5439	90,4132		3500,5439	92,5563	
3502,4726	90,5872		3502,4726	92,63	
3504,4013	90,8031		3504,4013	92,7558	
3506,33	90,814		3506,33	92,876	
3508,2586	90,7304		3508,2586	92,9584	
3510,1873	90,7576		3510,1873	93,0241	
3512,116	90,8583		3512,116	93,0498	
3514,0446	91,0263		3514,0446	93,0659	
3515,9733	91,1508		3515,9733	93,1971	

3517,902	91,2188		3517,902	93,3005	
3519,8307	91,3662		3519,8307	93,2654	
3521,7593	91,4067		3521,7593	93,3206	
3523,688	91,3471		3523,688	93,4685	
3525,6167	91,4248		3525,6167	93,4882	
3527,5454	91,6032		3527,5454	93,4267	
3529,474	91,7852		3529,474	93,4962	
3531,4027	91,9994		3531,4027	93,6718	
3533,3314	92,1503		3533,3314	93,7954	
3535,2601	92,1877		3535,2601	93,8777	
3537,1887	92,1728		3537,1887	93,9274	
3539,1174	92,1586		3539,1174	93,9387	
3541,0461	92,2898		3541,0461	93,9092	
3542,9748	92,4978		3542,9748	93,9586	
3544,9034	92,6402		3544,9034	94,1653	
3546,8321	92,7631		3546,8321	94,2951	
3548,7608	92,8292		3548,7608	94,3313	
3550,6895	92,8304		3550,6895	94,4408	
3552,6181	92,9054		3552,6181	94,4871	
3554,5468	93,0228		3554,5468	94,4612	
3556,4755	93,1322		3556,4755	94,573	
3558,4042	93,2465		3558,4042	94,6684	
3560,3328	93,3356		3560,3328	94,6846	
3562,2615	93,4448		3562,2615	94,7265	
3564,1902	93,5229		3564,1902	94,7075	
3566,1189	93,5927		3566,1189	94,6518	
3568,0475	93,6116		3568,0475	94,7971	
3569,9762	93,639		3569,9762	95,0446	
3571,9049	93,7703		3571,9049	95,1334	
3573,8336	93,9125		3573,8336	95,0819	

3575,7622	94,0081		3575,7622	95,0665	
3577,6909	94,0206		3577,6909	95,1137	
3579,6196	94,107		3579,6196	95,2137	
3581,5482	94,2774		3581,5482	95,3823	
3583,4769	94,3789		3583,4769	95,4336	
3585,4056	94,4827		3585,4056	95,3443	
3587,3343	94,6329		3587,3343	95,3773	
3589,2629	94,7022		3589,2629	95,5242	
3591,1916	94,6654		3591,1916	95,5172	
3593,1203	94,6346		3593,1203	95,5332	
3595,049	94,775		3595,049	95,7146	
3596,9776	95,0053		3596,9776	95,7958	
3598,9063	95,0622		3598,9063	95,7873	
3600,835	95,051		3600,835	95,8889	
3602,7637	95,0858		3602,7637	96,0081	
3604,6923	95,1066		3604,6923	96,037	
3606,621	95,1635		3606,621	96,0336	
3608,5497	95,3964		3608,5497	96,2286	
3610,4784	95,6262		3610,4784	96,411	
3612,407	95,6479		3612,407	96,4257	
3614,3357	95,6134		3614,3357	96,4463	
3616,2644	95,6604		3616,2644	96,4318	
3618,1931	95,8358		3618,1931	96,5356	
3620,1217	95,9974		3620,1217	96,6728	
3622,0504	96,0489		3622,0504	96,6756	
3623,9791	96,0628		3623,9791	96,7087	
3625,9078	95,9307		3625,9078	96,6846	
3627,8364	95,897		3627,8364	96,6997	
3629,7651	96,2725		3629,7651	97,0087	
3631,6938	96,4083		3631,6938	97,0736	

3633,6225	96,4559		3633,6225	97,1063	
3635,5511	96,4785		3635,5511	97,2388	
3637,4798	96,475		3637,4798	97,2883	
3639,4085	96,5246		3639,4085	97,3552	
3641,3372	96,5697		3641,3372	97,4396	
3643,2658	96,6334		3643,2658	97,4244	
3645,1945	96,746		3645,1945	97,3293	
3647,1232	96,8977		3647,1232	97,3205	
3649,0519	96,9903		3649,0519	97,4765	
3650,9805	96,9824		3650,9805	97,6096	
3652,9092	96,9718		3652,9092	97,6671	
3654,8379	97,0132		3654,8379	97,6425	
3656,7665	97,1079		3656,7665	97,6929	
3658,6952	97,1658		3658,6952	97,8525	
3660,6239	97,1874		3660,6239	97,9831	
3662,5526	97,1667		3662,5526	98,036	
3664,4812	97,1525		3664,4812	97,9786	
3666,4099	97,0916		3666,4099	97,8648	
3668,3386	97,0241		3668,3386	97,718	
3670,2673	97,2393		3670,2673	97,7066	
3672,1959	97,3033		3672,1959	97,7814	
3674,1246	97,1252		3674,1246	97,7493	
3676,0533	97,1592		3676,0533	97,7548	
3677,982	97,3425		3677,982	97,8839	
3679,9106	97,3905		3679,9106	98,0493	
3681,8393	97,3561		3681,8393	98,0885	
3683,768	97,3335		3683,768	98,0724	
3685,6967	97,3436		3685,6967	98,0709	
3687,6253	97,2806		3687,6253	97,9747	
3689,554	97,198		3689,554	97,887	

3691,4827	97,2432		3691,4827	97,8544	
3693,4114	97,2917		3693,4114	97,8486	
3695,34	97,3271		3695,34	97,9058	
3697,2687	97,4082		3697,2687	97,9287	
3699,1974	97,4091		3699,1974	97,8912	
3701,1261	97,3323		3701,1261	97,9257	
3703,0547	97,3387		3703,0547	98,0014	
3704,9834	97,3668		3704,9834	97,9764	
3706,9121	97,3862		3706,9121	97,9205	
3708,8408	97,3498		3708,8408	97,9312	
3710,7694	97,1506		3710,7694	97,9921	
3712,6981	97,1246		3712,6981	98,037	
3714,6268	97,3169		3714,6268	98,0121	
3716,5555	97,3376		3716,5555	97,9816	
3718,4841	97,2506		3718,4841	98,0326	
3720,4128	97,2364		3720,4128	97,9489	
3722,3415	97,288		3722,3415	97,8	
3724,2702	97,2678		3724,2702	97,8047	
3726,1988	97,2143		3726,1988	97,8303	
3728,1275	97,2485		3728,1275	97,8787	
3730,0562	97,1777		3730,0562	97,9089	
3731,9848	97,0788		3731,9848	97,8628	
3733,9135	97,1648		3733,9135	97,7655	
3735,8422	97,1941		3735,8422	97,9735	
3737,7709	97,188		3737,7709	98,105	
3739,6995	97,2512		3739,6995	98,0133	
3741,6282	97,2579		3741,6282	98,0596	
3743,5569	97,2295		3743,5569	98,2041	
3745,4856	97,2206		3745,4856	98,2292	
3747,4142	97,2159		3747,4142	98,1926	

3749,3429	97,3467		3749,3429	98,1361	
3751,2716	97,5058		3751,2716	98,0907	
3753,2003	97,4868		3753,2003	98,1946	
3755,1289	97,4236		3755,1289	98,3015	
3757,0576	97,3224		3757,0576	98,3757	
3758,9863	97,3075		3758,9863	98,3876	
3760,915	97,4058		3760,915	98,3728	
3762,8436	97,4833		3762,8436	98,3218	
3764,7723	97,511		3764,7723	98,2771	
3766,701	97,3642		3766,701	98,2801	
3768,6297	97,1952		3768,6297	98,2248	
3770,5583	97,2377		3770,5583	98,2714	
3772,487	97,3288		3772,487	98,4075	
3774,4157	97,3611		3774,4157	98,4684	
3776,3444	97,343		3776,3444	98,4769	
3778,273	97,2739		3778,273	98,3939	
3780,2017	97,242		3780,2017	98,2983	
3782,1304	97,2435		3782,1304	98,3332	
3784,0591	97,2686		3784,0591	98,417	
3785,9877	97,3436		3785,9877	98,4413	
3787,9164	97,3864		3787,9164	98,3838	
3789,8451	97,355		3789,8451	98,3529	
3791,7738	97,2651		3791,7738	98,3614	
3793,7024	97,1919		3793,7024	98,326	
3795,6311	97,2867		3795,6311	98,2874	
3797,5598	97,3643		3797,5598	98,3105	
3799,4885	97,2465		3799,4885	98,3044	
3801,4171	97,2809		3801,4171	98,2999	
3803,3458	97,3611		3803,3458	98,3598	
3805,2745	97,293		3805,2745	98,3632	

3807,2031	97,2433		3807,2031	98,3296	
3809,1318	97,2589		3809,1318	98,3128	
3811,0605	97,2661		3811,0605	98,2956	
3812,9892	97,2184		3812,9892	98,2084	
3814,9178	97,18		3814,9178	98,1083	
3816,8465	97,1527		3816,8465	98,2592	
3818,7752	97,0905		3818,7752	98,2973	
3820,7039	97,1935		3820,7039	98,1389	
3822,6325	97,3818		3822,6325	98,2446	
3824,5612	97,3166		3824,5612	98,3454	
3826,4899	97,2245		3826,4899	98,3568	
3828,4186	97,2346		3828,4186	98,386	
3830,3472	97,2505		3830,3472	98,3535	
3832,2759	97,2416		3832,2759	98,2688	
3834,2046	97,2215		3834,2046	98,28	
3836,1333	97,1599		3836,1333	98,2741	
3838,0619	97,0941		3838,0619	98,1566	
3839,9906	97,1504		3839,9906	98,1344	
3841,9193	97,2234		3841,9193	98,2534	
3843,848	97,1977		3843,848	98,3153	
3845,7766	97,1253		3845,7766	98,2701	
3847,7053	97,0692		3847,7053	98,2726	
3849,634	97,0272		3849,634	98,2808	
3851,5627	97,0061		3851,5627	98,104	
3853,4913	97,1378		3853,4913	97,8947	
3855,42	97,1732		3855,42	98,178	
3857,3487	97,1035		3857,3487	98,3698	
3859,2774	97,11		3859,2774	98,4566	
3861,206	97,1605		3861,206	98,4167	
3863,1347	97,1595		3863,1347	98,2119	

3865,0634	97,1437		3865,0634	98,1561	
3866,9921	97,1543		3866,9921	98,2147	
3868,9207	97,151		3868,9207	98,2983	
3870,8494	97,176		3870,8494	98,4356	
3872,7781	97,1094		3872,7781	98,3834	
3874,7067	97,0533		3874,7067	98,3152	
3876,6354	97,0774		3876,6354	98,2823	
3878,5641	97,0532		3878,5641	98,2532	
3880,4928	97,0955		3880,4928	98,3121	
3882,4214	97,1008		3882,4214	98,3545	
3884,3501	97,0565		3884,3501	98,3213	
3886,2788	97,1726		3886,2788	98,36	
3888,2075	97,1686		3888,2075	98,3534	
3890,1361	97,1682		3890,1361	98,2252	
3892,0648	97,3048		3892,0648	98,1735	
3893,9935	97,2514		3893,9935	98,2346	
3895,9222	97,1289		3895,9222	98,2815	
3897,8508	97,0814		3897,8508	98,3256	
3899,7795	97,0902		3899,7795	98,314	
3901,7082	97,0681		3901,7082	98,2093	
3903,6369	97,1069		3903,6369	98,1161	
3905,5655	97,1458		3905,5655	98,1242	
3907,4942	97,08		3907,4942	98,2116	
3909,4229	97,0801		3909,4229	98,3011	
3911,3516	97,1412		3911,3516	98,315	
3913,2802	97,1444		3913,2802	98,2693	
3915,2089	97,0834		3915,2089	98,2061	
3917,1376	97,0273		3917,1376	98,2872	
3919,0663	97,0649		3919,0663	98,4708	
3920,9949	97,1284		3920,9949	98,4071	

3922,9236	97,1129		3922,9236	98,2133	
3924,8523	97,0696		3924,8523	98,1915	
3926,781	97,0777		3926,781	98,3048	
3928,7096	97,0617		3928,7096	98,3556	
3930,6383	97,0187		3930,6383	98,2912	
3932,567	97,0454		3932,567	98,1706	
3934,4957	97,0657		3934,4957	98,162	
3936,4243	97,0469		3936,4243	98,2034	
3938,353	97,0606		3938,353	98,2136	
3940,2817	97,093		3940,2817	98,2382	
3942,2104	97,1196		3942,2104	98,3272	
3944,139	97,1065		3944,139	98,4141	
3946,0677	97,0574		3946,0677	98,3102	
3947,9964	97,0265		3947,9964	98,1141	
3949,925	97,0086		3949,925	98,1265	
3951,8537	96,9972		3951,8537	98,2114	
3953,7824	97,0134		3953,7824	98,1629	
3955,7111	97,0334		3955,7111	98,1286	
3957,6397	97,0257		3957,6397	98,2178	
3959,5684	96,9973		3959,5684	98,3282	
3961,4971	97,0363		3961,4971	98,2819	
3963,4258	97,1212		3963,4258	98,1646	
3965,3544	97,1156		3965,3544	98,1502	
3967,2831	97,0792		3967,2831	98,1763	
3969,2118	97,0888		3969,2118	98,2241	
3971,1405	97,1156		3971,1405	98,2665	
3973,0691	97,0957		3973,0691	98,2266	
3974,9978	97,0941		3974,9978	98,2294	
3976,9265	97,1521		3976,9265	98,3066	
3978,8552	97,1117		3978,8552	98,2579	

3980,7838	97,0467		3980,7838	98,1758	
3982,7125	97,0427		3982,7125	98,1789	
3984,6412	97,0432		3984,6412	98,1593	
3986,5699	97,0148		3986,5699	98,1523	
3988,4985	96,9663		3988,4985	98,1845	
3990,4272	97,021		3990,4272	98,1927	
3992,3559	97,0633		3992,3559	98,1863	
3994,2846	97,0058		3994,2846	98,159	
3996,2132	96,9747		3996,2132	98,1324	
3998,1419	96,964		3998,1419	98,1014	
4000,0706	97,0122		4000,0706	98,1174	
Comment=			Comment=		
