# Supplementary material for: Data on statistical experimental design to formulate amphotericin B-loaded Eudragit RL100 nanoparticles coated with hyaluronic acid for the treatment of vulvovaginal candidiasis
Source: Data Brief. 2020 Mar 5;29:105311. doi: 10.1016/j.dib.2020.105311 (PMC7082528; doi:10.1016/j.dib.2020.105311)
Supplement: Multimedia component 13 [file mmc13.pdf]

DATATYPE	**IR Spectrum AMP EUD nanoparticles**		DATATYPE	**IR Spectrum EDU nanoparticles HA**	
XYUNITS	**Wavenumber;PercentTransmittance**		XYUNITS	**Wavenumber;PercentTransmittance**	
DECIMALSYMB(	,		DECIMALSYMB(	,	
399,2356	7,6176		399,2356	55,5865	
401,1643	-1		401,1643	-1	
403,0929	-1		403,0929	-1	
405,0216	-1		405,0216	150	
406,9503	-1		406,9503	150	
408,879	-1		408,879	150	
410,8076	-1		410,8076	150	
412,7363	73,6623		412,7363	150	
414,665	150		414,665	150	
416,5937	-1		416,5937	-1	
418,5223	-1		418,5223	-1	
420,451	106,0773		420,451	21,492	
422,3797	-1		422,3797	-1	
424,3084	-1		424,3084	-1	
426,237	-1		426,237	-1	
428,1657	-1		428,1657	-1	
430,0944	150		430,0944	150	
432,0231	150		432,0231	150	
433,9517	-1		433,9517	11,033	
435,8804	150		435,8804	-1	
437,8091	55,7598		437,8091	29,809	
439,7377	46,3219		439,7377	150	
441,6664	43,646		441,6664	136,024	
443,5951	100,989		443,5951	-1	
445,5238	84,3508		445,5238	-1	
447,4524	40,9665		447,4524	3,6422	
449,3811	12,792		449,3811	-1	

451,3098	-1		451,3098	84,5536	
453,2385	-1		453,2385	-1	
455,1671	-1		455,1671	-1	
457,0958	15,0992		457,0958	32,674	
459,0245	36,0765		459,0245	-1	
460,9532	13,0939		460,9532	-1	
462,8818	-1		462,8818	-1	
464,8105	150		464,8105	118,1042	
466,7392	150		466,7392	132,204	
468,6679	150		468,6679	52,4359	
470,5965	150		470,5965	26,8567	
472,5252	150		472,5252	-1	
474,4539	98,7503		474,4539	150	
476,3826	-1		476,3826	-1	
478,3112	-1		478,3112	150	
480,2399	-1		480,2399	111,1127	
482,1686	-1		482,1686	140,4486	
484,0973	83,9593		484,0973	150	
486,0259	150		486,0259	-1	
487,9546	150		487,9546	150	
489,8833	-1		489,8833	79,5101	
491,812	-1		491,812	150	
493,7406	3,6277		493,7406	150	
495,6693	10,5236		495,6693	22,9233	
497,598	53,4124		497,598	6,9755	
499,5267	28,7085		499,5267	-1	
501,4553	-1		501,4553	-1	
503,384	-1		503,384	150	
505,3127	-1		505,3127	150	
507,2414	-1		507,2414	-1	

509,17	150		509,17	9,6176	
511,0987	150		511,0987	-1	
513,0274	94,3019		513,0274	119,3288	
514,956	102,718		514,956	150	
516,8847	150		516,8847	132,2956	
518,8134	150		518,8134	82,1776	
520,7421	61,455		520,7421	63,9352	
522,6707	50,2165		522,6707	37,4584	
524,5994	104,5001		524,5994	22,9685	
526,5281	146,3856		526,5281	77,4935	
528,4568	76,2508		528,4568	111,5832	
530,3854	46,4621		530,3854	82,6685	
532,3141	42,6014		532,3141	64,8847	
534,2428	53,0201		534,2428	57,2794	
536,1715	61,0261		536,1715	62,5274	
538,1001	65,7667		538,1001	82,9736	
540,0288	68,6605		540,0288	87,8196	
541,9575	72,1325		541,9575	76,9692	
543,8862	76,3457		543,8862	80,2923	
545,8148	80,2482		545,8148	89,4017	
547,7435	79,0167		547,7435	93,0854	
549,6722	70,9378		549,6722	92,7851	
551,6009	65,6709		551,6009	85,8965	
553,5295	64,2958		553,5295	77,9844	
555,4582	63,4644		555,4582	73,3729	
557,3869	66,7753		557,3869	68,0215	
559,3156	72,604		559,3156	67,3626	
561,2442	75,8717		561,2442	75,4389	
563,1729	78,9428		563,1729	80,7516	
565,1016	79,8164		565,1016	82,5227	

567,0303	78,7781		567,0303	82,6222	
568,9589	80,1586		568,9589	76,3826	
570,8876	80,5824		570,8876	72,0297	
572,8163	76,5172		572,8163	72,5651	
574,745	72,4717		574,745	74,5743	
576,6736	71,3184		576,6736	77,9013	
578,6023	71,6036		578,6023	81,4572	
580,531	70,815		580,531	80,8317	
582,4597	69,9657		582,4597	78,4475	
584,3883	72,8304		584,3883	77,0271	
586,317	75,6209		586,317	76,9716	
588,2457	73,6114		588,2457	77,4965	
590,1743	70,1044		590,1743	77,5096	
592,103	69,4537		592,103	77,8867	
594,0317	72,8765		594,0317	78,5329	
595,9604	74,009		595,9604	79,2421	
597,889	72,1727		597,889	79,1689	
599,8177	73,4143		599,8177	78,3092	
601,7464	74,5617		601,7464	77,666	
603,6751	73,6258		603,6751	77,3716	
605,6037	72,6801		605,6037	78,1903	
607,5324	71,4545		607,5324	78,9406	
609,4611	72,349		609,4611	78,7669	
611,3898	74,0351		611,3898	78,9522	
613,3184	72,9763		613,3184	77,3446	
615,2471	71,8836		615,2471	75,8327	
617,1758	72,6115		617,1758	77,66	
619,1045	73,5218		619,1045	78,9222	
621,0331	74,458		621,0331	78,0039	
622,9618	74,3617		622,9618	77,7072	

624,8905	73,1116		624,8905	78,3927	
626,8192	72,4999		626,8192	79,012	
628,7478	72,8939		628,7478	79,0354	
630,6765	73,4323		630,6765	78,8661	
632,6052	73,6423		632,6052	78,9269	
634,5339	73,3891		634,5339	78,9711	
636,4625	73,0145		636,4625	79,2921	
638,3912	73,5136		638,3912	79,4047	
640,3199	74,1763		640,3199	79,3526	
642,2486	74,5126		642,2486	79,7996	
644,1772	74,6935		644,1772	79,9424	
646,1059	74,4791		646,1059	79,9429	
648,0346	74,6872		648,0346	80,167	
649,9633	75,1903		649,9633	80,3339	
651,8919	75,0291		651,8919	80,5555	
653,8206	74,5703		653,8206	80,3163	
655,7493	74,3774		655,7493	80,0954	
657,678	74,5299		657,678	80,3951	
659,6066	74,6251		659,6066	80,4731	
661,5353	74,5057		661,5353	80,3593	
663,464	74,5213		663,464	80,4186	
665,3926	74,7408		665,3926	80,5124	
667,3213	74,9188		667,3213	80,3073	
669,25	74,7412		669,25	80,3348	
671,1787	74,9657		671,1787	80,68	
673,1073	75,4396		673,1073	80,8136	
675,036	75,6782		675,036	81,1412	
676,9647	75,7144		676,9647	81,6157	
678,8934	75,9907		678,8934	81,751	
680,822	76,4966		680,822	81,704	

682,7507	76,7048		682,7507	81,6686	
684,6794	76,7078		684,6794	81,7672	
686,6081	76,841		686,6081	82,0238	
688,5367	76,8929		688,5367	82,168	
690,4654	76,8961		690,4654	82,1736	
692,3941	77,0192		692,3941	82,2179	
694,3228	77,1989		694,3228	82,3315	
696,2514	77,3069		696,2514	82,394	
698,1801	77,2596		698,1801	82,3825	
700,1088	77,1948		700,1088	82,4637	
702,0375	77,2874		702,0375	82,5854	
703,9661	77,5271		703,9661	82,65	
705,8948	77,7224		705,8948	82,8284	
707,8235	77,7448		707,8235	82,9039	
709,7522	77,8284		709,7522	82,7629	
711,6808	78,0398		711,6808	82,7709	
713,6095	78,1109		713,6095	82,9739	
715,5382	78,2189		715,5382	83,115	
717,4669	78,4607		717,4669	83,0311	
719,3955	78,5502		719,3955	82,8082	
721,3242	78,6919		721,3242	82,6615	
723,2529	79,0033		723,2529	82,6993	
725,1816	79,2486		725,1816	82,9018	
727,1102	79,518		727,1102	83,1667	
729,0389	79,8262		729,0389	83,2793	
730,9676	80,0723		730,9676	83,2067	
732,8962	80,2281		732,8962	83,1577	
734,8249	80,3783		734,8249	83,2145	
736,7536	80,5353		736,7536	83,2746	
738,6823	80,5309		738,6823	83,2202	

740,6109	80,4711		740,6109	82,921	
742,5396	80,4528		742,5396	82,3849	
744,4683	80,2715		744,4683	81,8234	
746,397	79,8996		746,397	81,1439	
748,3256	79,6273		748,3256	80,3181	
750,2543	79,4732		750,2543	79,819	
752,183	79,385		752,183	79,8016	
754,1117	79,5989		754,1117	80,071	
756,0403	80,0918		756,0403	80,5258	
757,969	80,4873		757,969	80,9964	
759,8977	80,7289		759,8977	81,4609	
761,8264	80,9872		761,8264	81,984	
763,755	81,2578		763,755	82,4765	
765,6837	81,5662		765,6837	82,8631	
767,6124	81,8205		767,6124	83,1978	
769,5411	82,0101		769,5411	83,5329	
771,4697	82,1773		771,4697	83,8239	
773,3984	82,2437		773,3984	84,0904	
775,3271	82,2971		775,3271	84,2532	
777,2558	82,3156		777,2558	84,3139	
779,1844	82,2641		779,1844	84,3443	
781,1131	82,1914		781,1131	84,3176	
783,0418	82,1643		783,0418	84,3734	
784,9705	82,2469		784,9705	84,4367	
786,8991	82,2481		786,8991	84,3326	
788,8278	82,1011		788,8278	84,286	
790,7565	81,9804		790,7565	84,3659	
792,6852	81,9157		792,6852	84,3173	
794,6138	81,9053		794,6138	84,2238	
796,5425	81,8625		796,5425	84,2693	

798,4712	81,697		798,4712	84,3289	
800,3999	81,5128		800,3999	84,2242	
802,3285	81,3514		802,3285	84,0103	
804,2572	81,141		804,2572	83,831	
806,1859	80,911		806,1859	83,6855	
808,1145	80,8087		808,1145	83,5213	
810,0432	80,8228		810,0432	83,3644	
811,9719	80,8755		811,9719	83,3146	
813,9006	80,9326		813,9006	83,3292	
815,8292	80,9151		815,8292	83,3375	
817,7579	80,7475		817,7579	83,305	
819,6866	80,5217		819,6866	83,1201	
821,6153	80,3724		821,6153	82,8001	
823,5439	80,1861		823,5439	82,5693	
825,4726	79,8651		825,4726	82,4513	
827,4013	79,4663		827,4013	82,2767	
829,33	78,9789		829,33	82,0593	
831,2586	78,3964		831,2586	81,7576	
833,1873	77,8334		833,1873	81,2104	
835,116	77,2934		835,116	80,4469	
837,0447	76,6871		837,0447	79,5846	
838,9733	76,0503		838,9733	78,6505	
840,902	75,5517		840,902	77,8305	
842,8307	75,2156		842,8307	77,2596	
844,7594	74,9082		844,7594	76,8749	
846,688	74,6809		846,688	76,6067	
848,6167	74,675		848,6167	76,3659	
850,5454	74,8581		850,5454	76,3229	
852,4741	75,1585		852,4741	76,6438	
854,4027	75,5496		854,4027	77,337	

856,3314	75,9737		856,3314	78,2494	
858,2601	76,5338		858,2601	79,1196	
860,1888	77,1419		860,1888	79,9084	
862,1174	77,6021		862,1174	80,5629	
864,0461	78,0398		864,0461	81,0918	
865,9748	78,3735		865,9748	81,5422	
867,9035	78,4794		867,9035	81,8196	
869,8321	78,5475		869,8321	81,9499	
871,7608	78,6091		871,7608	82,09	
873,6895	78,5565		873,6895	82,2527	
875,6182	78,4293		875,6182	82,2319	
877,5468	78,2262		877,5468	82,0623	
879,4755	77,8995		879,4755	81,9421	
881,4042	77,515		881,4042	81,8315	
883,3328	77,1794		883,3328	81,7068	
885,2615	76,9388		885,2615	81,6168	
887,1902	76,8379		887,1902	81,6873	
889,1189	76,9683		889,1189	81,8917	
891,0475	77,2939		891,0475	82,1232	
892,9762	77,6129		892,9762	82,4252	
894,9049	77,8109		894,9049	82,6646	
896,8336	77,9179		896,8336	82,7305	
898,7622	77,9284		898,7622	82,6838	
900,6909	77,8941		900,6909	82,5934	
902,6196	77,76		902,6196	82,4467	
904,5483	77,5253		904,5483	82,1359	
906,4769	77,3644		906,4769	81,751	
908,4056	77,1552		908,4056	81,4279	
910,3343	76,8657		910,3343	81,089	
912,263	76,5849		912,263	80,8265	

914,1916	76,3027		914,1916	80,6471	
916,1203	76,1162		916,1203	80,4405	
918,049	76,0031		918,049	80,309	
919,9777	75,8895		919,9777	80,1951	
921,9063	75,6658		921,9063	79,9683	
923,835	75,2281		923,835	79,5772	
925,7637	74,7543		925,7637	79,1298	
927,6924	74,3319		927,6924	78,7171	
929,621	73,9087		929,621	78,2556	
931,5497	73,4783		931,5497	77,8252	
933,4784	72,9948		933,4784	77,4409	
935,4071	72,4645		935,4071	76,9937	
937,3357	71,9109		937,3357	76,4905	
939,2644	71,3205		939,2644	75,954	
941,1931	70,7047		941,1931	75,3085	
943,1218	70,1233		943,1218	74,5763	
945,0504	69,601		945,0504	73,9588	
946,9791	69,1438		946,9791	73,5183	
948,9078	68,8775		948,9078	73,3064	
950,8364	68,9059		950,8364	73,3648	
952,7651	69,0973		952,7651	73,5609	
954,6938	69,3335		954,6938	73,8818	
956,6225	69,6346		956,6225	74,4377	
958,5511	70,0444		958,5511	75,1357	
960,4798	70,5345		960,4798	75,6605	
962,4085	70,8213		962,4085	76,0472	
964,3372	70,7844		964,3372	76,5265	
966,2658	70,6503		966,2658	76,8708	
968,1945	70,462		968,1945	76,9391	
970,1232	70,13		970,1232	76,964	

972,0519	69,6609		972,0519	76,9034	
973,9805	68,9902		973,9805	76,5439	
975,9092	68,0752		975,9092	75,9209	
977,8379	66,9372		977,8379	75,1484	
979,7666	65,5912		979,7666	74,3729	
981,6952	64,2749		981,6952	73,6944	
983,6239	63,3213		983,6239	73,1552	
985,5526	62,823		985,5526	72,8326	
987,4813	62,5517		987,4813	72,6708	
989,4099	62,2805		989,4099	72,6311	
991,3386	61,9848		991,3386	72,752	
993,2673	61,7219		993,2673	72,9475	
995,196	61,5105		995,196	73,1551	
997,1246	61,2769		997,1246	73,3922	
999,0533	61,0014		999,0533	73,5638	
1000,982	60,6616		1000,982	73,5971	
1002,9107	60,1478		1002,9107	73,4751	
1004,8393	59,3654		1004,8393	73,0712	
1006,768	58,3934		1006,768	72,3169	
1008,6967	57,4696		1008,6967	71,2856	
1010,6254	56,7871		1010,6254	70,1439	
1012,554	56,3853		1012,554	69,0488	
1014,4827	56,1948		1014,4827	68,0352	
1016,4114	56,0702		1016,4114	67,0207	
1018,3401	55,8815		1018,3401	66,0526	
1020,2687	55,6525		1020,2687	65,266	
1022,1974	55,3559		1022,1974	64,5865	
1024,1261	54,8966		1024,1261	63,9898	
1026,0547	54,3595		1026,0547	63,497	
1027,9834	53,8402		1027,9834	63,0527	

1029,9121	53,3143		1029,9121	62,6338	
1031,8408	52,871		1031,8408	62,281	
1033,7694	52,6689		1033,7694	62,1047	
1035,6981	52,6885		1035,6981	62,0757	
1037,6268	52,8598		1037,6268	62,1253	
1039,5555	53,1113		1039,5555	62,2679	
1041,4841	53,3444		1041,4841	62,4312	
1043,4128	53,4383		1043,4128	62,5157	
1045,3415	53,3296		1045,3415	62,4665	
1047,2702	53,0161		1047,2702	62,3253	
1049,1988	52,4613		1049,1988	62,0769	
1051,1275	51,8307		1051,1275	61,6468	
1053,0562	51,3105		1053,0562	61,088	
1054,9849	50,8317		1054,9849	60,4006	
1056,9135	50,3358		1056,9135	59,6409	
1058,8422	49,9391		1058,8422	58,9669	
1060,7709	49,6795		1060,7709	58,3422	
1062,6996	49,544		1062,6996	57,8217	
1064,6282	49,4725		1064,6282	57,4462	
1066,5569	49,4156		1066,5569	57,0354	
1068,4856	49,3895		1068,4856	56,5858	
1070,4143	49,2694		1070,4143	56,0464	
1072,3429	49,0478		1072,3429	55,3195	
1074,2716	48,7831		1074,2716	54,5233	
1076,2003	48,4763		1076,2003	53,7821	
1078,129	48,165		1078,129	53,0149	
1080,0576	47,8109		1080,0576	52,1616	
1081,9863	47,3451		1081,9863	51,3171	
1083,915	46,7875		1083,915	50,5339	
1085,8437	46,1862		1085,8437	49,7932	

1087,7723	45,5362		1087,7723	48,9943	
1089,701	44,9203		1089,701	48,1842	
1091,6297	44,4125		1091,6297	47,488	
1093,5584	43,9272		1093,5584	46,8791	
1095,487	43,5244		1095,487	46,5024	
1097,4157	43,276		1097,4157	46,4386	
1099,3444	43,1761		1099,3444	46,5062	
1101,273	43,269		1101,273	46,6673	
1103,2017	43,4797		1103,2017	46,945	
1105,1304	43,7695		1105,1304	47,2816	
1107,0591	44,1572		1107,0591	47,5638	
1108,9877	44,5783		1108,9877	47,7669	
1110,9164	45,082		1110,9164	47,9512	
1112,8451	45,808		1112,8451	48,1946	
1114,7738	46,7301		1114,7738	48,5989	
1116,7024	47,7416		1116,7024	49,0847	
1118,6311	48,6997		1118,6311	49,4864	
1120,5598	49,4669		1120,5598	49,8084	
1122,4885	50,1362		1122,4885	50,1012	
1124,4171	50,8443		1124,4171	50,2684	
1126,3458	51,5913		1126,3458	50,311	
1128,2745	52,3393		1128,2745	50,3255	
1130,2032	52,889		1130,2032	50,2744	
1132,1318	53,1623		1132,1318	50,0548	
1134,0605	53,4402		1134,0605	49,7214	
1135,9892	53,8274		1135,9892	49,4835	
1137,9179	54,1878		1137,9179	49,3506	
1139,8465	54,4979		1139,8465	49,2767	
1141,7752	54,7721		1141,7752	49,3032	
1143,7039	55,1216		1143,7039	49,4742	

1145,6326	55,7279		1145,6326	49,829	
1147,5612	56,5767		1147,5612	50,3972	
1149,4899	57,5482		1149,4899	51,2728	
1151,4186	58,6923		1151,4186	52,4516	
1153,3473	60,0358		1153,3473	53,7959	
1155,2759	61,4596		1155,2759	55,1772	
1157,2046	62,9174		1157,2046	56,5595	
1159,1333	64,2782		1159,1333	57,951	
1161,062	65,5582		1161,062	59,2749	
1162,9906	66,8895		1162,9906	60,49	
1164,9193	68,1566		1164,9193	61,5525	
1166,848	69,2175		1166,848	62,2797	
1168,7767	69,9758		1168,7767	62,5736	
1170,7053	70,3984		1170,7053	62,5506	
1172,634	70,6025		1172,634	62,3853	
1174,5627	70,7815		1174,5627	62,2674	
1176,4913	71,0539		1176,4913	62,4443	
1178,42	71,4628		1178,42	62,9412	
1180,3487	72,0312		1180,3487	63,6524	
1182,2774	72,6545		1182,2774	64,5211	
1184,206	73,1758		1184,206	65,3519	
1186,1347	73,5897		1186,1347	66,0647	
1188,0634	74,0014		1188,0634	66,8494	
1189,9921	74,4632		1189,9921	67,7402	
1191,9207	74,9859		1191,9207	68,7112	
1193,8494	75,6319		1193,8494	69,9243	
1195,7781	76,4018		1195,7781	71,3194	
1197,7068	77,1751		1197,7068	72,683	
1199,6354	77,9043		1199,6354	73,9948	
1201,5641	78,6083		1201,5641	75,1829	

1203,4928	79,2209		1203,4928	76,1309	
1205,4215	79,6658		1205,4215	76,9072	
1207,3501	80,0152		1207,3501	77,5056	
1209,2788	80,3748		1209,2788	77,8268	
1211,2075	80,6163		1211,2075	77,9212	
1213,1362	80,6351		1213,1362	77,8689	
1215,0648	80,5632		1215,0648	77,6685	
1216,9935	80,4193		1216,9935	77,3431	
1218,9222	80,1597		1218,9222	76,9431	
1220,8509	79,8007		1220,8509	76,4268	
1222,7795	79,3733		1222,7795	75,7972	
1224,7082	78,8807		1224,7082	75,0799	
1226,6369	78,2596		1226,6369	74,287	
1228,5656	77,5532		1228,5656	73,5145	
1230,4942	76,8227		1230,4942	72,7065	
1232,4229	76,0546		1232,4229	71,8631	
1234,3516	75,304		1234,3516	71,1301	
1236,2803	74,7176		1236,2803	70,5483	
1238,2089	74,3421		1238,2089	70,156	
1240,1376	74,045		1240,1376	69,9814	
1242,0663	73,8251		1242,0663	70,0457	
1243,9949	73,8096		1243,9949	70,3402	
1245,9236	73,9949		1245,9236	70,7841	
1247,8523	74,3213		1247,8523	71,3625	
1249,781	74,779		1249,781	72,0707	
1251,7096	75,3869		1251,7096	72,8119	
1253,6383	76,0793		1253,6383	73,5364	
1255,567	76,7654		1255,567	74,2437	
1257,4957	77,409		1257,4957	74,9014	
1259,4243	78,0207		1259,4243	75,5199	

1261,353	78,5911		1261,353	76,1382	
1263,2817	79,0705		1263,2817	76,7058	
1265,2104	79,451		1265,2104	77,1872	
1267,139	79,7139		1267,139	77,6171	
1269,0677	79,8405		1269,0677	77,9369	
1270,9964	79,865		1270,9964	78,1579	
1272,9251	79,8066		1272,9251	78,3786	
1274,8537	79,6944		1274,8537	78,5195	
1276,7824	79,5714		1276,7824	78,6141	
1278,7111	79,4713		1278,7111	78,8292	
1280,6398	79,4844		1280,6398	79,098	
1282,5684	79,6084		1282,5684	79,3429	
1284,4971	79,7657		1284,4971	79,5918	
1286,4258	79,9005		1286,4258	79,7744	
1288,3545	79,9996		1288,3545	79,905	
1290,2831	80,0775		1290,2831	80,0303	
1292,2118	80,1292		1292,2118	80,1218	
1294,1405	80,1637		1294,1405	80,2414	
1296,0692	80,1747		1296,0692	80,413	
1297,9978	80,1798		1297,9978	80,6021	
1299,9265	80,2792		1299,9265	80,8538	
1301,8552	80,5155		1301,8552	81,2464	
1303,7839	80,8735		1303,7839	81,7778	
1305,7125	81,3151		1305,7125	82,4522	
1307,6412	81,7305		1307,6412	83,2855	
1309,5699	82,0649		1309,5699	84,1175	
1311,4986	82,3587		1311,4986	84,8407	
1313,4272	82,6408		1313,4272	85,5155	
1315,3559	82,8605		1315,3559	86,0336	
1317,2846	83,0165		1317,2846	86,344	

1319,2132	83,1704		1319,2132	86,577	
1321,1419	83,3007		1321,1419	86,703	
1323,0706	83,3858		1323,0706	86,7306	
1324,9993	83,5403		1324,9993	86,8472	
1326,9279	83,8145		1326,9279	87,0963	
1328,8566	84,0704		1328,8566	87,3376	
1330,7853	84,1903		1330,7853	87,5191	
1332,714	84,2212		1332,714	87,6789	
1334,6426	84,2486		1334,6426	87,7627	
1336,5713	84,2417		1336,5713	87,7182	
1338,5	84,1038		1338,5	87,5515	
1340,4287	83,7124		1340,4287	87,1803	
1342,3573	83,03		1342,3573	86,4901	
1344,286	82,0897		1344,286	85,5184	
1346,2147	81,1505		1346,2147	84,58	
1348,1434	80,6191		1348,1434	84,0721	
1350,072	80,5775		1350,072	84,0682	
1352,0007	80,8732		1352,0007	84,3996	
1353,9294	81,3554		1353,9294	84,8792	
1355,8581	81,9036		1355,8581	85,3644	
1357,7867	82,3599		1357,7867	85,7512	
1359,7154	82,6692		1359,7154	85,9952	
1361,6441	82,8985		1361,6441	86,0649	
1363,5728	83,0602		1363,5728	85,9517	
1365,5014	83,0916		1365,5014	85,6895	
1367,4301	83,0886		1367,4301	85,455	
1369,3588	83,1024		1369,3588	85,3028	
1371,2875	83,0711		1371,2875	85,103	
1373,2161	83,0077		1373,2161	84,7569	
1375,1448	82,9656		1375,1448	84,2345	

1377,0735	82,9032		1377,0735	83,6827	
1379,0022	82,8947		1379,0022	83,187	
1380,9308	82,9977		1380,9308	82,9071	
1382,8595	83,2041		1382,8595	82,9282	
1384,7882	83,4664		1384,7882	83,182	
1386,7169	83,8203		1386,7169	83,7854	
1388,6455	84,2884		1388,6455	84,6753	
1390,5742	84,6863		1390,5742	85,3782	
1392,5029	85,01		1392,5029	85,9159	
1394,4315	85,3256		1394,4315	86,5171	
1396,3602	85,6098		1396,3602	87,1403	
1398,2889	85,8776		1398,2889	87,6939	
1400,2176	86,1788		1400,2176	88,3627	
1402,1462	86,3662		1402,1462	88,8155	
1404,0749	86,6011		1404,0749	89,1819	
1406,0036	86,9628		1406,0036	89,6724	
1407,9323	87,2365		1407,9323	90,0805	
1409,8609	87,4076		1409,8609	90,3691	
1411,7896	87,5374		1411,7896	90,5579	
1413,7183	87,5926		1413,7183	90,6292	
1415,647	87,4694		1415,647	90,5727	
1417,5756	87,2594		1417,5756	90,4038	
1419,5043	87,1462		1419,5043	90,1023	
1421,433	86,9566		1421,433	89,7619	
1423,3617	86,5778		1423,3617	89,2334	
1425,2903	86,1107		1425,2903	88,3116	
1427,219	85,5695		1427,219	87,3064	
1429,1477	84,7808		1429,1477	86,0941	
1431,0764	83,9456		1431,0764	84,654	
1433,005	83,2692		1433,005	83,4695	

1434,9337	82,706		1434,9337	82,6396	
1436,8624	82,2406		1436,8624	81,9856	
1438,7911	81,9128		1438,7911	81,4661	
1440,7197	81,5688		1440,7197	80,8698	
1442,6484	81,046		1442,6484	80,1155	
1444,5771	80,4608		1444,5771	79,3844	
1446,5058	79,9987		1446,5058	78,8445	
1448,4344	79,7706		1448,4344	78,6985	
1450,3631	79,7493		1450,3631	78,8573	
1452,2918	79,8609		1452,2918	79,1876	
1454,2205	80,0721		1454,2205	79,5872	
1456,1491	80,4717		1456,1491	80,2268	
1458,0778	81,2342		1458,0778	81,3403	
1460,0065	81,6275		1460,0065	81,7824	
1461,9351	81,8816		1461,9351	82,0199	
1463,8638	82,2476		1463,8638	82,3392	
1465,7925	82,8197		1465,7925	82,7686	
1467,7212	83,3197		1467,7212	83,1687	
1469,6498	83,7349		1469,6498	83,54	
1471,5785	84,4956		1471,5785	84,1008	
1473,5072	85,7235		1473,5072	85,0307	
1475,4359	86,3611		1475,4359	85,6507	
1477,3645	86,9498		1477,3645	86,2462	
1479,2932	87,4824		1479,2932	86,8467	
1481,2219	87,9751		1481,2219	87,5382	
1483,1506	88,472		1483,1506	88,2321	
1485,0792	88,8912		1485,0792	88,811	
1487,0079	89,3903		1487,0079	89,5515	
1488,9366	90,0257		1488,9366	90,464	
1490,8653	91,0111		1490,8653	91,6974	

1492,7939	91,703		1492,7939	92,568	
1494,7226	92,1046		1494,7226	93,1501	
1496,6513	92,5531		1496,6513	93,8819	
1498,58	92,9989		1498,58	94,5342	
1500,5086	93,3003		1500,5086	94,9161	
1502,4373	93,392		1502,4373	95,0998	
1504,366	93,3332		1504,366	95,1422	
1506,2947	93,3213		1506,2947	95,1602	
1508,2233	93,4205		1508,2233	95,3044	
1510,152	93,3007		1510,152	95,2996	
1512,0807	93,1369		1512,0807	95,1747	
1514,0094	92,9948		1514,0094	95,009	
1515,938	92,9207		1515,938	94,9219	
1517,8667	92,8877		1517,8667	94,8868	
1519,7954	92,7717		1519,7954	94,7209	
1521,7241	92,5987		1521,7241	94,6152	
1523,6527	92,4577		1523,6527	94,7428	
1525,5814	92,3495		1525,5814	94,7131	
1527,5101	92,3357		1527,5101	94,5526	
1529,4388	92,2752		1529,4388	94,4447	
1531,3674	92,1311		1531,3674	94,3612	
1533,2961	92,043		1533,2961	94,2493	
1535,2248	91,9592		1535,2248	94,0855	
1537,1534	91,7616		1537,1534	93,9322	
1539,0821	91,463		1539,0821	93,8219	
1541,0108	91,3677		1541,0108	93,7596	
1542,9395	91,3394		1542,9395	93,6762	
1544,8681	91,2802		1544,8681	93,6445	
1546,7968	91,2821		1546,7968	93,616	
1548,7255	91,27		1548,7255	93,6057	

1550,6542	91,3088		1550,6542	93,6902	
1552,5828	91,4059		1552,5828	93,7882	
1554,5115	91,5044		1554,5115	93,8815	
1556,4402	91,4898		1556,4402	93,8624	
1558,3689	91,4718		1558,3689	93,7861	
1560,2975	91,854		1560,2975	94,2596	
1562,2262	91,8981		1562,2262	94,3674	
1564,1549	91,9111		1564,1549	94,4305	
1566,0836	91,9545		1566,0836	94,4834	
1568,0122	91,9911		1568,0122	94,4718	
1569,9409	92,1917		1569,9409	94,5658	
1571,8696	92,3362		1571,8696	94,627	
1573,7983	92,2941		1573,7983	94,6043	
1575,7269	92,382		1575,7269	94,6615	
1577,6556	92,6753		1577,6556	94,745	
1579,5843	92,7676		1579,5843	94,7351	
1581,513	92,8572		1581,513	94,7035	
1583,4416	93,0161		1583,4416	94,6899	
1585,3703	93,1028		1585,3703	94,6727	
1587,299	93,1506		1587,299	94,607	
1589,2277	93,2648		1589,2277	94,5462	
1591,1563	93,3485		1591,1563	94,484	
1593,085	93,3121		1593,085	94,3445	
1595,0137	93,2916		1595,0137	94,153	
1596,9424	93,3506		1596,9424	93,9719	
1598,871	93,3564		1598,871	93,7804	
1600,7997	93,3061		1600,7997	93,5974	
1602,7284	93,2435		1602,7284	93,4189	
1604,6571	93,1537		1604,6571	93,2045	
1606,5857	93,0912		1606,5857	92,9884	

1608,5144	93,0647			1608,5144	92,7975		
1610,4431	92,9771			1610,4431	92,6054		
1612,3717	92,8082			1612,3717	92,359		
1614,3004	92,6318			1614,3004	92,0947		
1616,2291	92,4642			1616,2291	91,8026		
1618,1578	92,2634			1618,1578	91,5587		
1620,0864	91,9851			1620,0864	91,388		
1622,0151	91,6256			1622,0151	91,1966		
1623,9438	91,3615			1623,9438	90,8946		
1625,8725	91,2024			1625,8725	90,5429		
1627,8011	90,8789			1627,8011	90,2348		
1629,7298	90,5538			1629,7298	90,0052		
1631,6585	90,3476			1631,6585	89,7532		
1633,5872	90,1647			1633,5872	89,4609		
1635,5158	89,9671			1635,5158	89,3113		
1637,4445	89,7514			1637,4445	89,2454		
1639,3732	89,6149			1639,3732	89,0856		
1641,3019	89,5214			1641,3019	88,9348		
1643,2305	89,4192			1643,2305	88,7848		
1645,1592	89,2511			1645,1592	88,6106		
1647,0879	89,0807			1647,0879	88,4946		
1649,0166	89,0373			1649,0166	88,4543		
1650,9452	88,9308			1650,9452	88,331		
1652,8739	89,0154			1652,8739	88,431		
1654,8026	89,3849			1654,8026	88,8872		
1656,7313	89,4279			1656,7313	88,9304		
1658,6599	89,6157			1658,6599	89,0624		
1660,5886	89,8641			1660,5886	89,2983		
1662,5173	90,1998			1662,5173	89,6752		
1664,446	90,5739			1664,446	90,0371		

1666,3746	90,718		1666,3746	90,2122	
1668,3033	90,8141		1668,3033	90,4452	
1670,232	91,1554		1670,232	90,8794	
1672,1607	91,3735		1672,1607	91,0605	
1674,0893	91,4251		1674,0893	91,085	
1676,018	91,6162		1676,018	91,2945	
1677,9467	91,7271		1677,9467	91,4021	
1679,8754	91,6991		1679,8754	91,3398	
1681,804	91,563		1681,804	91,2359	
1683,7327	91,443		1683,7327	91,1898	
1685,6614	91,581		1685,6614	91,2337	
1687,59	91,5777		1687,59	91,1288	
1689,5187	91,534		1689,5187	90,9812	
1691,4474	91,525		1691,4474	90,8042	
1693,3761	91,4902		1693,3761	90,6248	
1695,3047	91,4534		1695,3047	90,3899	
1697,2334	91,3597		1697,2334	89,9954	
1699,1621	91,1761		1699,1621	89,5847	
1701,0908	90,8131		1701,0908	88,3634	
1703,0194	90,4277		1703,0194	87,2308	
1704,9481	89,7791		1704,9481	85,8025	
1706,8768	88,8342		1706,8768	83,5743	
1708,8055	87,888		1708,8055	81,3037	
1710,7341	86,5285		1710,7341	78,3885	
1712,6628	84,8806		1712,6628	75,0695	
1714,5915	82,7753		1714,5915	71,0242	
1716,5202	80,179		1716,5202	66,287	
1718,4488	76,2575		1718,4488	60,1258	
1720,3775	73,0843		1720,3775	55,7392	
1722,3062	70,4307		1722,3062	52,6461	

1724,2349	67,4951		1724,2349	49,969	
1726,1635	65,309		1726,1635	48,6835	
1728,0922	64,0039		1728,0922	48,4547	
1730,0209	63,5089		1730,0209	49,3559	
1731,9496	63,5433		1731,9496	50,6262	
1733,8782	65,1593		1733,8782	53,779	
1735,8069	68,001		1735,8069	58,2291	
1737,7356	69,8505		1737,7356	60,8903	
1739,6643	73,3895		1739,6643	65,6563	
1741,5929	77,4715		1741,5929	70,8607	
1743,5216	81,2731		1743,5216	75,4852	
1745,4503	84,8973		1745,4503	80,0908	
1747,379	87,7402		1747,379	83,924	
1749,3076	90,4145		1749,3076	87,439	
1751,2363	92,4904		1751,2363	90,3317	
1753,165	93,879		1753,165	92,3412	
1755,0936	94,7049		1755,0936	93,479	
1757,0223	95,4216		1757,0223	94,6143	
1758,951	95,869		1758,951	95,4248	
1760,8797	96,1651		1760,8797	95,9355	
1762,8083	96,519		1762,8083	96,4118	
1764,737	96,7464		1764,737	96,6387	
1766,6657	96,86		1766,6657	96,8468	
1768,5944	96,9427		1768,5944	97,0711	
1770,523	97,0298		1770,523	97,1834	
1772,4517	97,1025		1772,4517	97,318	
1774,3804	97,1628		1774,3804	97,4686	
1776,3091	97,2841		1776,3091	97,5993	
1778,2377	97,3939		1778,2377	97,7079	
1780,1664	97,4349		1780,1664	97,8054	

1782,0951	97,4482		1782,0951	97,8969	
1784,0238	97,4525		1784,0238	97,9185	
1785,9524	97,4976		1785,9524	97,8832	
1787,8811	97,5123		1787,8811	97,8805	
1789,8098	97,4167		1789,8098	97,9253	
1791,7385	97,3581		1791,7385	98,0358	
1793,6671	97,4726		1793,6671	98,171	
1795,5958	97,5559		1795,5958	98,2281	
1797,5245	97,5881		1797,5245	98,2729	
1799,4532	97,5531		1799,4532	98,347	
1801,3818	97,5733		1801,3818	98,4287	
1803,3105	97,6694		1803,3105	98,4306	
1805,2392	97,6563		1805,2392	98,3903	
1807,1679	97,6747		1807,1679	98,4292	
1809,0965	97,8063		1809,0965	98,5033	
1811,0252	97,9064		1811,0252	98,5115	
1812,9539	97,8915		1812,9539	98,5211	
1814,8826	97,8609		1814,8826	98,5342	
1816,8112	97,8372		1816,8112	98,5366	
1818,7399	97,7735		1818,7399	98,5736	
1820,6686	97,7331		1820,6686	98,595	
1822,5973	97,7338		1822,5973	98,553	
1824,5259	97,7872		1824,5259	98,5033	
1826,4546	97,8041		1826,4546	98,5103	
1828,3833	97,7034		1828,3833	98,5701	
1830,3119	97,6424		1830,3119	98,6391	
1832,2406	97,6943		1832,2406	98,6403	
1834,1693	97,7742		1834,1693	98,6413	
1836,098	97,8737		1836,098	98,6608	
1838,0266	97,8782		1838,0266	98,6889	

1839,9553	97,8527		1839,9553	98,6783	
1841,884	97,9328		1841,884	98,6699	
1843,8127	97,9641		1843,8127	98,7061	
1845,7413	97,8841		1845,7413	98,6797	
1847,67	97,9209		1847,67	98,6444	
1849,5987	97,8961		1849,5987	98,6786	
1851,5274	97,7932		1851,5274	98,705	
1853,456	97,8099		1853,456	98,662	
1855,3847	97,8923		1855,3847	98,6973	
1857,3134	97,9421		1857,3134	98,7535	
1859,2421	97,9169		1859,2421	98,7335	
1861,1707	97,8845		1861,1707	98,7175	
1863,0994	97,8682		1863,0994	98,7584	
1865,0281	97,8652		1865,0281	98,791	
1866,9568	97,8305		1866,9568	98,679	
1868,8854	97,686		1868,8854	98,6188	
1870,8141	97,6578		1870,8141	98,6709	
1872,7428	97,7049		1872,7428	98,6449	
1874,6715	97,7113		1874,6715	98,6206	
1876,6001	97,6905		1876,6001	98,5795	
1878,5288	97,6738		1878,5288	98,5353	
1880,4575	97,7231		1880,4575	98,5702	
1882,3862	97,7764		1882,3862	98,6139	
1884,3148	97,7923		1884,3148	98,6245	
1886,2435	97,7963		1886,2435	98,6225	
1888,1722	97,6935		1888,1722	98,5901	
1890,1009	97,5557		1890,1009	98,558	
1892,0295	97,5333		1892,0295	98,5404	
1893,9582	97,5883		1893,9582	98,4403	
1895,8869	97,6926		1895,8869	98,3748	

1897,8156	97,6937		1897,8156	98,4507	
1899,7442	97,6341		1899,7442	98,4046	
1901,6729	97,5526		1901,6729	98,3171	
1903,6016	97,5012		1903,6016	98,3948	
1905,5302	97,5938		1905,5302	98,4192	
1907,4589	97,6808		1907,4589	98,3954	
1909,3876	97,6741		1909,3876	98,3582	
1911,3163	97,6217		1911,3163	98,2428	
1913,2449	97,5888		1913,2449	98,2194	
1915,1736	97,5681		1915,1736	98,2794	
1917,1023	97,5675		1917,1023	98,254	
1919,031	97,5715		1919,031	98,2103	
1920,9596	97,5556		1920,9596	98,2953	
1922,8883	97,5363		1922,8883	98,3368	
1924,817	97,4541		1924,817	98,308	
1926,7457	97,3699		1926,7457	98,3429	
1928,6743	97,3027		1928,6743	98,3575	
1930,603	97,3187		1930,603	98,2816	
1932,5317	97,3631		1932,5317	98,1698	
1934,4604	97,363		1934,4604	98,1227	
1936,389	97,3868		1936,389	98,2074	
1938,3177	97,3297		1938,3177	98,2562	
1940,2464	97,3106		1940,2464	98,1872	
1942,1751	97,337		1942,1751	98,0416	
1944,1037	97,2567		1944,1037	97,931	
1946,0324	97,2329		1946,0324	98,0513	
1947,9611	97,2486		1947,9611	98,0656	
1949,8898	97,2102		1949,8898	97,8147	
1951,8184	97,1281		1951,8184	97,6606	
1953,7471	97,0829		1953,7471	97,6511	

1955,6758	97,1987		1955,6758	97,7437	
1957,6045	97,3014		1957,6045	97,8383	
1959,5331	97,1721		1959,5331	97,7519	
1961,4618	96,9305		1961,4618	97,7458	
1963,3905	96,8563		1963,3905	97,8488	
1965,3192	97,1497		1965,3192	97,8864	
1967,2478	97,2728		1967,2478	97,9361	
1969,1765	96,995		1969,1765	97,6125	
1971,1052	96,9446		1971,1052	97,204	
1973,0339	96,9711		1973,0339	97,3067	
1974,9625	96,8979		1974,9625	97,4413	
1976,8912	96,7195		1976,8912	97,1853	
1978,8199	96,3752		1978,8199	96,9996	
1980,7485	96,3663		1980,7485	97,2869	
1982,6772	96,7761		1982,6772	97,5482	
1984,6059	97,0189		1984,6059	97,6152	
1986,5346	97,0117		1986,5346	97,7295	
1988,4632	97,0813		1988,4632	97,7297	
1990,3919	97,1673		1990,3919	97,622	
1992,3206	97,1413		1992,3206	97,7177	
1994,2493	97,0947		1994,2493	97,8861	
1996,1779	97,1284		1996,1779	97,8557	
1998,1066	97,2005		1998,1066	97,8155	
2000,0353	97,2013		2000,0353	97,8595	
2001,964	97,2157		2001,964	97,9051	
2003,8926	97,3054		2003,8926	97,825	
2005,8213	97,2058		2005,8213	97,7333	
2007,75	97,012		2007,75	97,9324	
2009,6787	97,0347		2009,6787	98,1889	
2011,6073	97,0844		2011,6073	97,9651	

2013,536	97,1386		2013,536	97,6348	
2015,4647	97,1466		2015,4647	97,7009	
2017,3934	96,98		2017,3934	97,8107	
2019,322	96,7886		2019,322	97,7975	
2021,2507	96,7975		2021,2507	97,8358	
2023,1794	97,0762		2023,1794	97,9441	
2025,1081	97,091		2025,1081	98,0436	
2027,0367	96,8925		2027,0367	97,9485	
2028,9654	96,9041		2028,9654	97,7256	
2030,8941	96,9796		2030,8941	97,7663	
2032,8228	97,0234		2032,8228	97,7026	
2034,7514	96,8497		2034,7514	97,5478	
2036,6801	96,6541		2036,6801	97,8741	
2038,6088	96,7552		2038,6088	98,1115	
2040,5375	97,0033		2040,5375	97,9956	
2042,4661	97,1521		2042,4661	97,8797	
2044,3948	97,1076		2044,3948	97,8471	
2046,3235	97,0817		2046,3235	97,9272	
2048,2521	97,1023		2048,2521	97,9089	
2050,1808	97,2085		2050,1808	97,7766	
2052,1095	97,3966		2052,1095	97,8196	
2054,0382	97,511		2054,0382	97,9667	
2055,9668	97,5995		2055,9668	98,0802	
2057,8955	97,548		2057,8955	98,1988	
2059,8242	97,5071		2059,8242	98,409	
2061,7529	97,5722		2061,7529	98,581	
2063,6815	97,4769		2063,6815	98,5042	
2065,6102	97,3628		2065,6102	98,2176	
2067,5389	97,3785		2067,5389	98,0343	
2069,4676	97,3828		2069,4676	98,0776	

2071,3962	97,3248		2071,3962	98,1291	
2073,3249	97,2575		2073,3249	98,1538	
2075,2536	97,2993		2075,2536	98,2284	
2077,1823	97,5114		2077,1823	98,2916	
2079,1109	97,5543		2079,1109	98,1833	
2081,0396	97,3466		2081,0396	98,0523	
2082,9683	97,3789		2082,9683	98,1618	
2084,897	97,5663		2084,897	98,297	
2086,8256	97,4698		2086,8256	98,2924	
2088,7543	97,4603		2088,7543	98,2785	
2090,683	97,6476		2090,683	98,2383	
2092,6117	97,5756		2092,6117	98,1989	
2094,5403	97,4357		2094,5403	98,1362	
2096,469	97,4954		2096,469	98,0647	
2098,3977	97,5711		2098,3977	98,205	
2100,3264	97,6099		2100,3264	98,3836	
2102,255	97,7017		2102,255	98,3408	
2104,1837	97,6987		2104,1837	98,2	
2106,1124	97,7077		2106,1124	98,1973	
2108,0411	97,7313		2108,0411	98,2947	
2109,9697	97,5859		2109,9697	98,3587	
2111,8984	97,4425		2111,8984	98,3152	
2113,8271	97,4315		2113,8271	98,2448	
2115,7558	97,5695		2115,7558	98,3286	
2117,6844	97,6002		2117,6844	98,4014	
2119,6131	97,5518		2119,6131	98,4141	
2121,5418	97,6898		2121,5418	98,4392	
2123,4704	97,8335		2123,4704	98,4262	
2125,3991	97,842		2125,3991	98,3562	
2127,3278	97,789		2127,3278	98,2926	

2129,2565	97,7528		2129,2565	98,401	
2131,1851	97,8188		2131,1851	98,5187	
2133,1138	97,8527		2133,1138	98,5241	
2135,0425	97,7194		2135,0425	98,4924	
2136,9712	97,654		2136,9712	98,3784	
2138,8998	97,6141		2138,8998	98,2186	
2140,8285	97,5948		2140,8285	98,2057	
2142,7572	97,5306		2142,7572	98,3505	
2144,6859	97,3418		2144,6859	98,3881	
2146,6145	97,4196		2146,6145	98,2492	
2148,5432	97,6401		2148,5432	98,0772	
2150,4719	97,7108		2150,4719	98,0786	
2152,4006	97,6182		2152,4006	98,3317	
2154,3292	97,6801		2154,3292	98,4762	
2156,2579	97,9402		2156,2579	98,2067	
2158,1866	97,6199		2158,1866	97,9035	
2160,1153	97,0221		2160,1153	97,6078	
2162,0439	96,9357		2162,0439	97,3322	
2163,9726	97,0583		2163,9726	97,3302	
2165,9013	97,178		2165,9013	97,3411	
2167,83	97,2704		2167,83	97,5787	
2169,7586	97,3423		2169,7586	98,0562	
2171,6873	97,3458		2171,6873	98,2519	
2173,616	97,3826		2173,616	98,1685	
2175,5447	97,5099		2175,5447	97,9434	
2177,4733	97,5598		2177,4733	97,9327	
2179,402	97,5759		2179,402	98,0887	
2181,3307	97,3979		2181,3307	98,006	
2183,2594	97,0435		2183,2594	98,1092	
2185,188	97,006		2185,188	98,2639	

2187,1167	97,381		2187,1167	97,9475	
2189,0454	97,4968		2189,0454	97,9098	
2190,9741	97,3018		2190,9741	98,2604	
2192,9027	97,4682		2192,9027	98,329	
2194,8314	97,6546		2194,8314	98,1894	
2196,7601	97,4072		2196,7601	98,1037	
2198,6887	97,2278		2198,6887	98,3714	
2200,6174	97,3262		2200,6174	98,5824	
2202,5461	97,3782		2202,5461	98,4003	
2204,4748	97,5826		2204,4748	98,3184	
2206,4034	97,7998		2206,4034	98,3869	
2208,3321	97,721		2208,3321	98,3018	
2210,2608	97,6243		2210,2608	98,2654	
2212,1895	97,4445		2212,1895	98,2961	
2214,1181	97,3871		2214,1181	98,187	
2216,0468	97,5715		2216,0468	98,2837	
2217,9755	97,6103		2217,9755	98,4332	
2219,9042	97,6491		2219,9042	98,4602	
2221,8328	97,8627		2221,8328	98,549	
2223,7615	97,9411		2223,7615	98,5459	
2225,6902	97,7521		2225,6902	98,5152	
2227,6189	97,6237		2227,6189	98,5296	
2229,5475	97,5626		2229,5475	98,3938	
2231,4762	97,4576		2231,4762	98,2989	
2233,4049	97,4949		2233,4049	98,417	
2235,3336	97,4952		2235,3336	98,384	
2237,2622	97,3724		2237,2622	98,254	
2239,1909	97,3757		2239,1909	98,3614	
2241,1196	97,5415		2241,1196	98,6223	
2243,0483	97,615		2243,0483	98,7371	

2244,9769	97,6196		2244,9769	98,668	
2246,9056	97,66		2246,9056	98,5672	
2248,8343	97,654		2248,8343	98,4592	
2250,763	97,697		2250,763	98,3907	
2252,6916	97,6485		2252,6916	98,3704	
2254,6203	97,5215		2254,6203	98,4477	
2256,549	97,6653		2256,549	98,5102	
2258,4777	97,8582		2258,4777	98,4523	
2260,4063	97,7142		2260,4063	98,502	
2262,335	97,5853		2262,335	98,6485	
2264,2637	97,6467		2264,2637	98,6145	
2266,1923	97,6063		2266,1923	98,4546	
2268,121	97,5596		2268,121	98,4112	
2270,0497	97,6129		2270,0497	98,4601	
2271,9784	97,6878		2271,9784	98,4259	
2273,907	97,7898		2273,907	98,3742	
2275,8357	97,9031		2275,8357	98,4562	
2277,7644	97,9052		2277,7644	98,5682	
2279,6931	97,7522		2279,6931	98,5857	
2281,6217	97,6238		2281,6217	98,4828	
2283,5504	97,6464		2283,5504	98,4069	
2285,4791	97,6416		2285,4791	98,4534	
2287,4078	97,6162		2287,4078	98,5245	
2289,3364	97,7206		2289,3364	98,593	
2291,2651	97,7909		2291,2651	98,6096	
2293,1938	97,7558		2293,1938	98,572	
2295,1225	97,7617		2295,1225	98,5274	
2297,0511	97,7958		2297,0511	98,5287	
2298,9798	97,6935		2298,9798	98,5618	
2300,9085	97,5332		2300,9085	98,4924	

2302,8372	97,5163		2302,8372	98,4389	
2304,7658	97,6846		2304,7658	98,4414	
2306,6945	97,7581		2306,6945	98,3263	
2308,6232	97,5651		2308,6232	98,1196	
2310,5519	97,4451		2310,5519	97,9643	
2312,4805	97,4653		2312,4805	97,9298	
2314,4092	97,5286		2314,4092	97,8966	
2316,3379	97,6266		2316,3379	97,829	
2318,2666	97,5343		2318,2666	97,7776	
2320,1952	97,265		2320,1952	97,6976	
2322,1239	97,1192		2322,1239	97,5139	
2324,0526	97,1488		2324,0526	97,3101	
2325,9813	97,2443		2325,9813	97,3493	
2327,9099	97,2579		2327,9099	97,4893	
2329,8386	97,1718		2329,8386	97,4181	
2331,7673	97,2136		2331,7673	97,3507	
2333,696	97,2976		2333,696	97,4988	
2335,6246	97,2896		2335,6246	97,5336	
2337,5533	97,2643		2337,5533	97,2589	
2339,482	97,1992		2339,482	97,0699	
2341,4106	97,144		2341,4106	97,1202	
2343,3393	97,1033		2343,3393	97,0869	
2345,268	97,1775		2345,268	97,2728	
2347,1967	97,4909		2347,1967	97,6912	
2349,1253	97,6651		2349,1253	97,8045	
2351,054	97,493		2351,054	97,6151	
2352,9827	97,144		2352,9827	97,3323	
2354,9114	97,0003		2354,9114	97,0967	
2356,84	97,0537		2356,84	96,9072	
2358,7687	97,1184		2358,7687	96,8311	

2360,6974	97,141		2360,6974	96,7386	
2362,6261	97,0977		2362,6261	96,6587	
2364,5547	97,0307		2364,5547	96,759	
2366,4834	97,0449		2366,4834	97,0285	
2368,4121	97,1797		2368,4121	97,3046	
2370,3408	97,3266		2370,3408	97,4314	
2372,2694	97,4285		2372,2694	97,6207	
2374,1981	97,4575		2374,1981	97,9363	
2376,1268	97,5002		2376,1268	98,1424	
2378,0555	97,5782		2378,0555	98,309	
2379,9841	97,6731		2379,9841	98,5513	
2381,9128	97,8581		2381,9128	98,7524	
2383,8415	98,0191		2383,8415	98,8779	
2385,7702	98,0916		2385,7702	98,9434	
2387,6988	98,0798		2387,6988	98,9253	
2389,6275	98,0635		2389,6275	98,8896	
2391,5562	98,1115		2391,5562	98,9338	
2393,4849	98,1434		2393,4849	98,9882	
2395,4135	98,1281		2395,4135	98,9529	
2397,3422	98,0135		2397,3422	98,9271	
2399,2709	97,9193		2399,2709	98,9877	
2401,1996	97,9443		2401,1996	99,0147	
2403,1282	97,9793		2403,1282	98,9361	
2405,0569	97,9438		2405,0569	98,8786	
2406,9856	97,9028		2406,9856	98,9274	
2408,9143	97,9738		2408,9143	98,9824	
2410,8429	98,0149		2410,8429	98,9766	
2412,7716	97,9789		2412,7716	98,9619	
2414,7003	97,9611		2414,7003	98,9609	
2416,6289	97,9497		2416,6289	98,9172	

2418,5576	97,9121		2418,5576	98,8511	
2420,4863	97,8447		2420,4863	98,866	
2422,415	97,861		2422,415	98,9162	
2424,3436	97,9566		2424,3436	98,9623	
2426,2723	97,9622		2426,2723	98,9513	
2428,201	97,8904		2428,201	98,854	
2430,1297	97,8839		2430,1297	98,8158	
2432,0583	97,9018		2432,0583	98,7959	
2433,987	97,8602		2433,987	98,8607	
2435,9157	97,8046		2435,9157	99,0501	
2437,8444	97,8038		2437,8444	99,0779	
2439,773	97,836		2439,773	98,9621	
2441,7017	97,846		2441,7017	98,8361	
2443,6304	97,8688		2443,6304	98,7622	
2445,5591	97,892		2445,5591	98,8237	
2447,4877	97,8567		2447,4877	98,8749	
2449,4164	97,8225		2449,4164	98,8802	
2451,3451	97,8071		2451,3451	98,8664	
2453,2738	97,7885		2453,2738	98,772	
2455,2024	97,8336		2455,2024	98,7317	
2457,1311	97,8315		2457,1311	98,8304	
2459,0598	97,7332		2459,0598	98,9096	
2460,9885	97,7943		2460,9885	98,8931	
2462,9171	97,8973		2462,9171	98,8625	
2464,8458	97,8742		2464,8458	98,8297	
2466,7745	97,8624		2466,7745	98,8214	
2468,7032	97,8289		2468,7032	98,8573	
2470,6318	97,8176		2470,6318	98,9185	
2472,5605	97,8488		2472,5605	98,9643	
2474,4892	97,828		2474,4892	98,9315	

2476,4179	97,7686		2476,4179	98,9011	
2478,3465	97,7148		2478,3465	98,9384	
2480,2752	97,7032		2480,2752	98,9881	
2482,2039	97,6689		2482,2039	98,9784	
2484,1326	97,5955		2484,1326	98,9225	
2486,0612	97,5542		2486,0612	98,8722	
2487,9899	97,5485		2487,9899	98,8103	
2489,9186	97,558		2489,9186	98,7679	
2491,8472	97,5893		2491,8472	98,8175	
2493,7759	97,6554		2493,7759	98,8597	
2495,7046	97,6496		2495,7046	98,7735	
2497,6333	97,586		2497,6333	98,786	
2499,5619	97,6051		2499,5619	98,9064	
2501,4906	97,5931		2501,4906	98,8932	
2503,4193	97,4892		2503,4193	98,8339	
2505,348	97,4402		2505,348	98,8321	
2507,2766	97,435		2507,2766	98,8525	
2509,2053	97,4496		2509,2053	98,8168	
2511,134	97,5072		2511,134	98,7268	
2513,0627	97,525		2513,0627	98,7196	
2514,9913	97,5095		2514,9913	98,783	
2516,92	97,4678		2516,92	98,8367	
2518,8487	97,4401		2518,8487	98,832	
2520,7774	97,5415		2520,7774	98,7209	
2522,706	97,6073		2522,706	98,6637	
2524,6347	97,5501		2524,6347	98,7387	
2526,5634	97,5231		2526,5634	98,7844	
2528,4921	97,5461		2528,4921	98,7058	
2530,4207	97,5442		2530,4207	98,6593	
2532,3494	97,5195		2532,3494	98,6971	

2534,2781	97,5271		2534,2781	98,6863	
2536,2068	97,5278		2536,2068	98,6665	
2538,1354	97,5072		2538,1354	98,6476	
2540,0641	97,5198		2540,0641	98,6358	
2541,9928	97,5612		2541,9928	98,7172	
2543,9215	97,5411		2543,9215	98,8068	
2545,8501	97,5128		2545,8501	98,7739	
2547,7788	97,5266		2547,7788	98,7378	
2549,7075	97,4987		2549,7075	98,7575	
2551,6362	97,497		2551,6362	98,7502	
2553,5648	97,5759		2553,5648	98,7417	
2555,4935	97,6089		2555,4935	98,7288	
2557,4222	97,5209		2557,4222	98,709	
2559,3508	97,4598		2559,3508	98,6815	
2561,2795	97,4826		2561,2795	98,6625	
2563,2082	97,4316		2563,2082	98,6818	
2565,1369	97,3835		2565,1369	98,6768	
2567,0655	97,4492		2567,0655	98,6401	
2568,9942	97,4784		2568,9942	98,6161	
2570,9229	97,4893		2570,9229	98,6141	
2572,8516	97,5028		2572,8516	98,647	
2574,7802	97,4779		2574,7802	98,7001	
2576,7089	97,4637		2576,7089	98,6772	
2578,6376	97,3968		2578,6376	98,5956	
2580,5663	97,3453		2580,5663	98,592	
2582,4949	97,371		2582,4949	98,6523	
2584,4236	97,3476		2584,4236	98,6334	
2586,3523	97,3075		2586,3523	98,5452	
2588,281	97,3023		2588,281	98,5381	
2590,2096	97,2733		2590,2096	98,5803	

2592,1383	97,2564		2592,1383	98,5938	
2594,067	97,2684		2594,067	98,6346	
2595,9957	97,2573		2595,9957	98,6823	
2597,9243	97,2479		2597,9243	98,6806	
2599,853	97,3026		2599,853	98,6915	
2601,7817	97,3554		2601,7817	98,6767	
2603,7104	97,2655		2603,7104	98,6205	
2605,639	97,1207		2605,639	98,6118	
2607,5677	97,1196		2607,5677	98,6223	
2609,4964	97,2131		2609,4964	98,5969	
2611,4251	97,2312		2611,4251	98,5546	
2613,3537	97,1936		2613,3537	98,5591	
2615,2824	97,2175		2615,2824	98,6284	
2617,2111	97,2321		2617,2111	98,6995	
2619,1398	97,2011		2619,1398	98,7046	
2621,0684	97,1964		2621,0684	98,6326	
2622,9971	97,1809		2622,9971	98,5283	
2624,9258	97,1547		2624,9258	98,5367	
2626,8545	97,1539		2626,8545	98,5873	
2628,7831	97,1544		2628,7831	98,5512	
2630,7118	97,1413		2630,7118	98,5908	
2632,6405	97,1681		2632,6405	98,7009	
2634,5691	97,2274		2634,5691	98,6955	
2636,4978	97,2346		2636,4978	98,5744	
2638,4265	97,1842		2638,4265	98,5155	
2640,3552	97,1558		2640,3552	98,538	
2642,2838	97,1331		2642,2838	98,5789	
2644,2125	97,077		2644,2125	98,6367	
2646,1412	97,066		2646,1412	98,639	
2648,0699	97,1075		2648,0699	98,6367	

2649,9985	97,1565		2649,9985	98,6288	
2651,9272	97,1844		2651,9272	98,6023	
2653,8559	97,166		2653,8559	98,611	
2655,7846	97,128		2655,7846	98,6063	
2657,7132	97,085		2657,7132	98,5868	
2659,6419	97,0663		2659,6419	98,5406	
2661,5706	97,0885		2661,5706	98,5149	
2663,4993	97,0968		2663,4993	98,5678	
2665,4279	97,1017		2665,4279	98,5692	
2667,3566	97,0758		2667,3566	98,5201	
2669,2853	97,0104		2669,2853	98,5378	
2671,214	97,0212		2671,214	98,6031	
2673,1426	97,0472		2673,1426	98,6469	
2675,0713	96,9671		2675,0713	98,581	
2677	96,8772		2677	98,5113	
2678,9287	96,8522		2678,9287	98,5245	
2680,8573	96,8281		2680,8573	98,5297	
2682,786	96,7828		2682,786	98,5093	
2684,7147	96,7404		2684,7147	98,4423	
2686,6434	96,7196		2686,6434	98,3859	
2688,572	96,6954		2688,572	98,3768	
2690,5007	96,6641		2690,5007	98,3794	
2692,4294	96,6398		2692,4294	98,356	
2694,3581	96,5711		2694,3581	98,2813	
2696,2867	96,5007		2696,2867	98,2166	
2698,2154	96,448		2698,2154	98,159	
2700,1441	96,3853		2700,1441	98,1255	
2702,0728	96,3601		2702,0728	98,1058	
2704,0014	96,3399		2704,0014	98,0373	
2705,9301	96,297		2705,9301	98,001	

2707,8588	96,2722		2707,8588	98,0138	
2709,7874	96,2467		2709,7874	98,0105	
2711,7161	96,1803		2711,7161	98,0055	
2713,6448	96,1442		2713,6448	97,9787	
2715,5735	96,1647		2715,5735	97,9267	
2717,5021	96,1524		2717,5021	97,8908	
2719,4308	96,1075		2719,4308	97,8751	
2721,3595	96,0408		2721,3595	97,8521	
2723,2882	95,9749		2723,2882	97,8316	
2725,2168	95,9457		2725,2168	97,832	
2727,1455	95,9149		2727,1455	97,7907	
2729,0742	95,8899		2729,0742	97,7348	
2731,0029	95,8811		2731,0029	97,7198	
2732,9315	95,864		2732,9315	97,736	
2734,8602	95,8101		2734,8602	97,7621	
2736,7889	95,7271		2736,7889	97,7462	
2738,7176	95,6989		2738,7176	97,6575	
2740,6462	95,7161		2740,6462	97,5545	
2742,5749	95,7175		2742,5749	97,507	
2744,5036	95,7006		2744,5036	97,52	
2746,4323	95,6954		2746,4323	97,558	
2748,3609	95,6917		2748,3609	97,5767	
2750,2896	95,6264		2750,2896	97,595	
2752,2183	95,5745		2752,2183	97,6044	
2754,147	95,5914		2754,147	97,5548	
2756,0756	95,5873		2756,0756	97,5008	
2758,0043	95,5212		2758,0043	97,5268	
2759,933	95,4694		2759,933	97,5438	
2761,8617	95,4771		2761,8617	97,4863	
2763,7903	95,4483		2763,7903	97,4322	

2765,719	95,3425		2765,719	97,4163	
2767,6477	95,2389		2767,6477	97,3887	
2769,5764	95,223		2769,5764	97,3044	
2771,505	95,2534		2771,505	97,2051	
2773,4337	95,1997		2773,4337	97,1469	
2775,3624	95,1031		2775,3624	97,1101	
2777,291	95,0651		2777,291	97,0676	
2779,2197	95,0252		2779,2197	97,0029	
2781,1484	94,9		2781,1484	96,9064	
2783,0771	94,7435		2783,0771	96,8619	
2785,0057	94,6524		2785,0057	96,8497	
2786,9344	94,6032		2786,9344	96,8012	
2788,8631	94,525		2788,8631	96,7154	
2790,7918	94,4159		2790,7918	96,6032	
2792,7204	94,2647		2792,7204	96,5187	
2794,6491	94,1017		2794,6491	96,4636	
2796,5778	93,9787		2796,5778	96,3774	
2798,5065	93,8366		2798,5065	96,2623	
2800,4351	93,6642		2800,4351	96,1425	
2802,3638	93,5238		2802,3638	95,9991	
2804,2925	93,3911		2804,2925	95,8601	
2806,2212	93,2341		2806,2212	95,7462	
2808,1498	93,0893		2808,1498	95,6709	
2810,0785	92,9131		2810,0785	95,5538	
2812,0072	92,6612		2812,0072	95,3741	
2813,9359	92,4279		2813,9359	95,2184	
2815,8645	92,2006		2815,8645	95,0379	
2817,7932	91,9261		2817,7932	94,8623	
2819,7219	91,699		2819,7219	94,7218	
2821,6506	91,5133		2821,6506	94,5639	

2823,5792	91,2815		2823,5792	94,3788	
2825,5079	90,9683		2825,5079	94,1532	
2827,4366	90,6001		2827,4366	93,8683	
2829,3653	90,2353		2829,3653	93,5267	
2831,2939	89,8154		2831,2939	93,1551	
2833,2226	89,2909		2833,2226	92,792	
2835,1513	88,7166		2835,1513	92,3947	
2837,08	88,0707		2837,08	91,845	
2839,0086	87,2697		2839,0086	91,2042	
2840,9373	86,3476		2840,9373	90,5291	
2842,866	85,3467		2842,866	89,725	
2844,7947	84,2087		2844,7947	88,8075	
2846,7233	82,957		2846,7233	87,8115	
2848,652	81,7449		2848,652	86,8087	
2850,5807	80,6703		2850,5807	85,9727	
2852,5093	79,782		2852,5093	85,2729	
2854,438	79,2011		2854,438	84,6815	
2856,3667	78,873		2856,3667	84,3275	
2858,2954	78,6788		2858,2954	84,1667	
2860,224	78,633		2860,224	84,0831	
2862,1527	78,6791		2862,1527	84,0605	
2864,0814	78,7122		2864,0814	84,0363	
2866,0101	78,679		2866,0101	83,9171	
2867,9387	78,6714		2867,9387	83,7681	
2869,8674	78,7444		2869,8674	83,71	
2871,7961	78,8632		2871,7961	83,7415	
2873,7248	79,0638		2873,7248	83,8913	
2875,6534	79,3333		2875,6534	84,1263	
2877,5821	79,6483		2877,5821	84,3447	
2879,5108	79,9879		2879,5108	84,5599	

2881,4395	80,2504		2881,4395	84,7142	
2883,3681	80,4168		2883,3681	84,8156	
2885,2968	80,5458		2885,2968	84,9382	
2887,2255	80,6962		2887,2255	85,0342	
2889,1542	80,8187		2889,1542	85,1007	
2891,0828	80,7802		2891,0828	85,1527	
2893,0115	80,7208		2893,0115	85,1694	
2894,9402	80,7366		2894,9402	85,1146	
2896,8689	80,6821		2896,8689	84,997	
2898,7975	80,5982		2898,7975	84,8851	
2900,7262	80,4953		2900,7262	84,7367	
2902,6549	80,3357		2902,6549	84,5499	
2904,5836	80,2285		2904,5836	84,4064	
2906,5122	80,159		2906,5122	84,2951	
2908,4409	80,0533		2908,4409	84,1603	
2910,3696	79,8954		2910,3696	83,9565	
2912,2983	79,658		2912,2983	83,7374	
2914,2269	79,3567		2914,2269	83,494	
2916,1556	79,0858		2916,1556	83,1634	
2918,0843	78,88		2918,0843	82,8842	
2920,013	78,7258		2920,013	82,6513	
2921,9416	78,6758		2921,9416	82,4499	
2923,8703	78,7805		2923,8703	82,4267	
2925,799	79,0177		2925,799	82,4838	
2927,7276	79,413		2927,7276	82,6336	
2929,6563	79,9565		2929,6563	82,9857	
2931,585	80,4932		2931,585	83,3615	
2933,5137	80,9816		2933,5137	83,6507	
2935,4423	81,5044		2935,4423	84,0426	
2937,371	82,0818		2937,371	84,5658	

2939,2997	82,6187		2939,2997	84,95	
2941,2284	83,0712		2941,2284	85,1742	
2943,157	83,4972		2943,157	85,378	
2945,0857	83,8645		2945,0857	85,5743	
2947,0144	84,1422		2947,0144	85,7309	
2948,9431	84,4241		2948,9431	85,8455	
2950,8717	84,8245		2950,8717	86,0813	
2952,8004	85,3403		2952,8004	86,4953	
2954,7291	85,892		2954,7291	86,9557	
2956,6578	86,4691		2956,6578	87,4556	
2958,5864	87,1101		2958,5864	88,0602	
2960,5151	87,761		2960,5151	88,7265	
2962,4438	88,3688		2962,4438	89,3691	
2964,3725	88,9849		2964,3725	89,9828	
2966,3011	89,5495		2966,3011	90,5635	
2968,2298	90,0472		2968,2298	91,0607	
2970,1585	90,5282		2970,1585	91,4069	
2972,0872	90,9093		2972,0872	91,5994	
2974,0158	91,212		2974,0158	91,7228	
2975,9445	91,5131		2975,9445	91,7575	
2977,8732	91,7318		2977,8732	91,7599	
2979,8019	91,8855		2979,8019	91,7929	
2981,7305	92,1139		2981,7305	91,8247	
2983,6592	92,3232		2983,6592	91,9883	
2985,5879	92,4562		2985,5879	92,2244	
2987,5166	92,6433		2987,5166	92,3879	
2989,4452	92,8909		2989,4452	92,5712	
2991,3739	93,13		2991,3739	92,8104	
2993,3026	93,335		2993,3026	93,0568	
2995,2313	93,4818		2995,2313	93,3072	

2997,1599	93,5876		2997,1599	93,5733	
2999,0886	93,7402		2999,0886	93,8642	
3001,0173	93,917		3001,0173	94,1697	
3002,9459	94,1054		3002,9459	94,5033	
3004,8746	94,3222		3004,8746	94,8581	
3006,8033	94,4894		3006,8033	95,1762	
3008,732	94,6044		3008,732	95,4797	
3010,6606	94,708		3010,6606	95,7567	
3012,5893	94,8958		3012,5893	95,946	
3014,518	95,0811		3014,518	96,1189	
3016,4467	95,1662		3016,4467	96,2853	
3018,3753	95,274		3018,3753	96,3874	
3020,304	95,3475		3020,304	96,5276	
3022,2327	95,3839		3022,2327	96,7183	
3024,1614	95,4967		3024,1614	96,8323	
3026,09	95,6658		3026,09	96,9072	
3028,0187	95,7728		3028,0187	96,9958	
3029,9474	95,8266		3029,9474	97,0956	
3031,8761	95,9389		3031,8761	97,2344	
3033,8047	95,9847		3033,8047	97,3694	
3035,7334	95,9479		3035,7334	97,4391	
3037,6621	95,9922		3037,6621	97,4272	
3039,5908	96,0875		3039,5908	97,4363	
3041,5194	96,1674		3041,5194	97,5472	
3043,4481	96,232		3043,4481	97,6669	
3045,3768	96,2288		3045,3768	97,6672	
3047,3055	96,1441		3047,3055	97,6587	
3049,2341	96,1307		3049,2341	97,7247	
3051,1628	96,1891		3051,1628	97,7445	
3053,0915	96,1644		3053,0915	97,7147	

3055,0202	96,13		3055,0202	97,6944	
3056,9488	96,1818		3056,9488	97,6886	
3058,8775	96,2008		3058,8775	97,7014	
3060,8062	96,1385		3060,8062	97,7445	
3062,7349	96,0862		3062,7349	97,7708	
3064,6635	96,1022		3064,6635	97,7585	
3066,5922	96,1109		3066,5922	97,7657	
3068,5209	96,0606		3068,5209	97,8066	
3070,4495	96,0078		3070,4495	97,7713	
3072,3782	95,9353		3072,3782	97,7051	
3074,3069	95,9527		3074,3069	97,6944	
3076,2356	96,0293		3076,2356	97,7094	
3078,1642	96,0157		3078,1642	97,7359	
3080,0929	95,9709		3080,0929	97,7355	
3082,0216	95,959		3082,0216	97,6955	
3083,9503	95,9687		3083,9503	97,6865	
3085,8789	95,9012		3085,8789	97,741	
3087,8076	95,7888		3087,8076	97,7127	
3089,7363	95,7523		3089,7363	97,6548	
3091,665	95,7924		3091,665	97,6667	
3093,5936	95,7879		3093,5936	97,6955	
3095,5223	95,7254		3095,5223	97,7576	
3097,451	95,7121		3097,451	97,7421	
3099,3797	95,7195		3099,3797	97,6351	
3101,3083	95,7036		3101,3083	97,5862	
3103,237	95,6682		3103,237	97,5968	
3105,1657	95,6787		3105,1657	97,5994	
3107,0944	95,7147		3107,0944	97,594	
3109,023	95,6677		3109,023	97,6142	
3110,9517	95,6145		3110,9517	97,6038	

3112,8804	95,591		3112,8804	97,5957	
3114,8091	95,5339		3114,8091	97,6331	
3116,7377	95,4334		3116,7377	97,6035	
3118,6664	95,3279		3118,6664	97,5685	
3120,5951	95,3293		3120,5951	97,5842	
3122,5238	95,3489		3122,5238	97,556	
3124,4524	95,3066		3124,4524	97,4912	
3126,3811	95,262		3126,3811	97,4304	
3128,3098	95,1655		3128,3098	97,394	
3130,2385	95,0953		3130,2385	97,3829	
3132,1671	95,1101		3132,1671	97,3689	
3134,0958	95,0481		3134,0958	97,3926	
3136,0245	94,9379		3136,0245	97,3975	
3137,9532	94,9494		3137,9532	97,3343	
3139,8818	94,9802		3139,8818	97,33	
3141,8105	94,8965		3141,8105	97,3568	
3143,7392	94,7874		3143,7392	97,2841	
3145,6678	94,7742		3145,6678	97,2102	
3147,5965	94,7648		3147,5965	97,1999	
3149,5252	94,6405		3149,5252	97,1851	
3151,4539	94,5361		3151,4539	97,2081	
3153,3825	94,5246		3153,3825	97,2069	
3155,3112	94,5438		3155,3112	97,1206	
3157,2399	94,5487		3157,2399	97,0606	
3159,1686	94,4717		3159,1686	97,0375	
3161,0972	94,3552		3161,0972	96,9987	
3163,0259	94,2902		3163,0259	96,9538	
3164,9546	94,2455		3164,9546	96,8983	
3166,8833	94,164		3166,8833	96,831	
3168,8119	94,0813		3168,8119	96,8364	

3170,7406	94,0201		3170,7406	96,8539	
3172,6693	93,979		3172,6693	96,7987	
3174,598	93,9559		3174,598	96,7151	
3176,5266	93,8783		3176,5266	96,6576	
3178,4553	93,7866		3178,4553	96,6665	
3180,384	93,7515		3180,384	96,6577	
3182,3127	93,714		3182,3127	96,6558	
3184,2413	93,6715		3184,2413	96,6345	
3186,17	93,6216		3186,17	96,511	
3188,0987	93,5406		3188,0987	96,4341	
3190,0274	93,46		3190,0274	96,4445	
3191,956	93,396		3191,956	96,434	
3193,8847	93,299		3193,8847	96,3789	
3195,8134	93,1997		3195,8134	96,2436	
3197,7421	93,1813		3197,7421	96,1602	
3199,6707	93,1727		3199,6707	96,196	
3201,5994	93,106		3201,5994	96,1736	
3203,5281	93,0317		3203,5281	96,1297	
3205,4568	92,9756		3205,4568	96,1307	
3207,3854	92,9082		3207,3854	96,144	
3209,3141	92,8496		3209,3141	96,1189	
3211,2428	92,7507		3211,2428	96,0233	
3213,1715	92,5926		3213,1715	95,9393	
3215,1001	92,4845		3215,1001	95,8951	
3217,0288	92,4153		3217,0288	95,8543	
3218,9575	92,3672		3218,9575	95,8438	
3220,8861	92,3091		3220,8861	95,8603	
3222,8148	92,2165		3222,8148	95,8127	
3224,7435	92,1773		3224,7435	95,6971	
3226,6722	92,1163		3226,6722	95,6139	

3228,6008	92,0317		3228,6008	95,5791	
3230,5295	92,0059		3230,5295	95,5465	
3232,4582	91,9213		3232,4582	95,4959	
3234,3869	91,7866		3234,3869	95,4619	
3236,3155	91,6851		3236,3155	95,4732	
3238,2442	91,578		3238,2442	95,4613	
3240,1729	91,4689		3240,1729	95,3904	
3242,1016	91,4088		3242,1016	95,2902	
3244,0302	91,3794		3244,0302	95,1989	
3245,9589	91,3182		3245,9589	95,1929	
3247,8876	91,2167		3247,8876	95,1951	
3249,8163	91,0787		3249,8163	95,1504	
3251,7449	90,9135		3251,7449	95,1631	
3253,6736	90,7908		3253,6736	95,1835	
3255,6023	90,7483		3255,6023	95,1332	
3257,531	90,7		3257,531	95,0195	
3259,4596	90,5634		3259,4596	94,8731	
3261,3883	90,4448		3261,3883	94,7724	
3263,317	90,4146		3263,317	94,7231	
3265,2457	90,3452		3265,2457	94,7104	
3267,1743	90,2123		3267,1743	94,6927	
3269,103	90,0865		3269,103	94,6362	
3271,0317	89,9628		3271,0317	94,5936	
3272,9604	89,8918		3272,9604	94,5448	
3274,889	89,9066		3274,889	94,4815	
3276,8177	89,8568		3276,8177	94,4394	
3278,7464	89,7604		3278,7464	94,4559	
3280,6751	89,7629		3280,6751	94,5267	
3282,6037	89,7765		3282,6037	94,5665	
3284,5324	89,6938		3284,5324	94,5239	

3286,4611	89,6072		3286,4611	94,4369	
3288,3898	89,5814		3288,3898	94,4352	
3290,3184	89,5764		3290,3184	94,4763	
3292,2471	89,5699		3292,2471	94,4707	
3294,1758	89,5528		3294,1758	94,447	
3296,1044	89,5657		3296,1044	94,4181	
3298,0331	89,5393		3298,0331	94,399	
3299,9618	89,4959		3299,9618	94,3602	
3301,8905	89,5653		3301,8905	94,3547	
3303,8191	89,6211		3303,8191	94,3381	
3305,7478	89,5584		3305,7478	94,2839	
3307,6765	89,4681		3307,6765	94,2733	
3309,6052	89,4617		3309,6052	94,2589	
3311,5338	89,4941		3311,5338	94,2791	
3313,4625	89,4943		3313,4625	94,324	
3315,3912	89,4825		3315,3912	94,3242	
3317,3199	89,4313		3317,3199	94,3248	
3319,2485	89,3587		3319,2485	94,3028	
3321,1772	89,2701		3321,1772	94,2819	
3323,1059	89,1985		3323,1059	94,27	
3325,0346	89,1459		3325,0346	94,2195	
3326,9632	89,1004		3326,9632	94,157	
3328,8919	89,121		3328,8919	94,1159	
3330,8206	89,1384		3330,8206	94,1242	
3332,7493	89,1016		3332,7493	94,1781	
3334,6779	89,0437		3334,6779	94,1905	
3336,6066	89,0349		3336,6066	94,1509	
3338,5353	89,1137		3338,5353	94,1681	
3340,464	89,1926		3340,464	94,1806	
3342,3926	89,1955		3342,3926	94,1648	

3344,3213	89,1984		3344,3213	94,1436	
3346,25	89,2741		3346,25	94,1264	
3348,1787	89,3654		3348,1787	94,1285	
3350,1073	89,4077		3350,1073	94,0866	
3352,036	89,4883		3352,036	94,065	
3353,9647	89,5847		3353,9647	94,1696	
3355,8934	89,5962		3355,8934	94,2917	
3357,822	89,6635		3357,822	94,2614	
3359,7507	89,7218		3359,7507	94,1525	
3361,6794	89,7545		3361,6794	94,1037	
3363,608	89,9107		3363,608	94,1004	
3365,5367	89,9907		3365,5367	94,1174	
3367,4654	89,9797		3367,4654	94,1495	
3369,3941	90,0722		3369,3941	94,1157	
3371,3227	90,1582		3371,3227	94,1267	
3373,2514	90,2201		3373,2514	94,2006	
3375,1801	90,2763		3375,1801	94,15	
3377,1088	90,2658		3377,1088	94,1311	
3379,0374	90,3181		3379,0374	94,1554	
3380,9661	90,39		3380,9661	94,0526	
3382,8948	90,4329		3382,8948	93,9854	
3384,8235	90,4786		3384,8235	93,9813	
3386,7521	90,5077		3386,7521	93,9552	
3388,6808	90,5553		3388,6808	93,9419	
3390,6095	90,511		3390,6095	93,9115	
3392,5382	90,4407		3392,5382	93,9082	
3394,4668	90,507		3394,4668	93,9463	
3396,3955	90,5871		3396,3955	93,9963	
3398,3242	90,6295		3398,3242	94,0693	
3400,2529	90,6977		3400,2529	94,1118	

3402,1815	90,7199		3402,1815	94,0753	
3404,1102	90,6947		3404,1102	94,0149	
3406,0389	90,7706		3406,0389	94,0141	
3407,9676	90,94		3407,9676	94,0062	
3409,8962	91,0083		3409,8962	93,9895	
3411,8249	90,9481		3411,8249	94,0071	
3413,7536	90,9601		3413,7536	94,0344	
3415,6823	91,0733		3415,6823	94,0568	
3417,6109	91,1532		3417,6109	94,0173	
3419,5396	91,1975		3419,5396	94,0106	
3421,4683	91,2384		3421,4683	94,0567	
3423,397	91,3211		3423,397	94,0067	
3425,3256	91,4246		3425,3256	93,9563	
3427,2543	91,4914		3427,2543	93,9727	
3429,183	91,5375		3429,183	94	
3431,1117	91,5308		3431,1117	94,026	
3433,0403	91,5317		3433,0403	93,9848	
3434,969	91,5337		3434,969	93,9205	
3436,8977	91,5148		3436,8977	93,941	
3438,8263	91,545		3438,8263	93,9994	
3440,755	91,5923		3440,755	94,0335	
3442,6837	91,6321		3442,6837	94,0092	
3444,6124	91,6552		3444,6124	93,947	
3446,541	91,7517		3446,541	93,949	
3448,4697	91,9318		3448,4697	94,0275	
3450,3984	92,0356		3450,3984	94,0664	
3452,3271	92,0684		3452,3271	94,0995	
3454,2557	92,1669		3454,2557	94,165	
3456,1844	92,3136		3456,1844	94,2397	
3458,1131	92,3818		3458,1131	94,3297	

3460,0418	92,4422		3460,0418	94,4038	
3461,9704	92,5034		3461,9704	94,4548	
3463,8991	92,5531		3463,8991	94,4335	
3465,8278	92,629		3465,8278	94,389	
3467,7565	92,728		3467,7565	94,4294	
3469,6851	92,8542		3469,6851	94,5554	
3471,6138	92,9169		3471,6138	94,6352	
3473,5425	92,9968		3473,5425	94,6042	
3475,4712	93,1242		3475,4712	94,5688	
3477,3998	93,1444		3477,3998	94,5964	
3479,3285	93,1518		3479,3285	94,6469	
3481,2572	93,2697		3481,2572	94,649	
3483,1859	93,3607		3483,1859	94,6182	
3485,1145	93,3043		3485,1145	94,6553	
3487,0432	93,2962		3487,0432	94,7708	
3488,9719	93,4151		3488,9719	94,853	
3490,9006	93,5629		3490,9006	94,8642	
3492,8292	93,649		3492,8292	94,8165	
3494,7579	93,6856		3494,7579	94,7993	
3496,6866	93,7724		3496,6866	94,8196	
3498,6153	93,8157		3498,6153	94,7902	
3500,5439	93,8651		3500,5439	94,8159	
3502,4726	93,9588		3502,4726	94,8871	
3504,4013	94,0074		3504,4013	94,9332	
3506,33	94,1422		3506,33	94,9334	
3508,2586	94,2623		3508,2586	94,9556	
3510,1873	94,2094		3510,1873	95,0795	
3512,116	94,2933		3512,116	95,0967	
3514,0446	94,4904		3514,0446	95,0388	
3515,9733	94,5543		3515,9733	95,0879	

3517,902	94,5742		3517,902	95,1962	
3519,8307	94,6036		3519,8307	95,2901	
3521,7593	94,6704		3521,7593	95,3466	
3523,688	94,8037		3523,688	95,3263	
3525,6167	94,8796		3525,6167	95,2718	
3527,5454	94,9762		3527,5454	95,3789	
3529,474	95,1647		3529,474	95,5056	
3531,4027	95,2371		3531,4027	95,4985	
3533,3314	95,2248		3533,3314	95,4799	
3535,2601	95,3035		3535,2601	95,4704	
3537,1887	95,4977		3537,1887	95,552	
3539,1174	95,6344		3539,1174	95,6511	
3541,0461	95,564		3541,0461	95,7126	
3542,9748	95,5431		3542,9748	95,7608	
3544,9034	95,6985		3544,9034	95,801	
3546,8321	95,7058		3546,8321	95,9248	
3548,7608	95,7283		3548,7608	96,0108	
3550,6895	95,9431		3550,6895	96,0001	
3552,6181	96,149		3552,6181	95,988	
3554,5468	96,1754		3554,5468	96,0169	
3556,4755	96,1589		3556,4755	96,0689	
3558,4042	96,252		3558,4042	96,1906	
3560,3328	96,3496		3560,3328	96,3234	
3562,2615	96,3841		3562,2615	96,3596	
3564,1902	96,4059		3564,1902	96,345	
3566,1189	96,4158		3566,1189	96,3387	
3568,0475	96,5328		3568,0475	96,3881	
3569,9762	96,6979		3569,9762	96,5114	
3571,9049	96,8138		3571,9049	96,6996	
3573,8336	96,8641		3573,8336	96,8006	

3575,7622	96,8298		3575,7622	96,7627	
3577,6909	96,8573		3577,6909	96,7368	
3579,6196	96,9373		3579,6196	96,731	
3581,5482	96,9651		3581,5482	96,7461	
3583,4769	97,0709		3583,4769	96,8268	
3585,4056	97,1788		3585,4056	96,9639	
3587,3343	97,1659		3587,3343	97,1302	
3589,2629	97,283		3589,2629	97,2076	
3591,1916	97,3396		3591,1916	97,2024	
3593,1203	97,289		3593,1203	97,15	
3595,049	97,3148		3595,049	97,1009	
3596,9776	97,388		3596,9776	97,1764	
3598,9063	97,4929		3598,9063	97,2738	
3600,835	97,6133		3600,835	97,3065	
3602,7637	97,6791		3602,7637	97,3511	
3604,6923	97,6753		3604,6923	97,3855	
3606,621	97,6541		3606,621	97,4154	
3608,5497	97,7052		3608,5497	97,5565	
3610,4784	97,8005		3610,4784	97,6756	
3612,407	97,8507		3612,407	97,707	
3614,3357	97,9506		3614,3357	97,7634	
3616,2644	98,0654		3616,2644	97,8184	
3618,1931	98,0763		3618,1931	97,8621	
3620,1217	98,13		3620,1217	97,9501	
3622,0504	98,2361		3622,0504	98,0252	
3623,9791	98,2505		3623,9791	98,0282	
3625,9078	98,1926		3625,9078	98,0068	
3627,8364	98,2391		3627,8364	98,0921	
3629,7651	98,3882		3629,7651	98,2898	
3631,6938	98,4045		3631,6938	98,3609	

3633,6225	98,5269		3633,6225	98,4306	
3635,5511	98,6581		3635,5511	98,5488	
3637,4798	98,6581		3637,4798	98,6345	
3639,4085	98,6407		3639,4085	98,6548	
3641,3372	98,6284		3641,3372	98,7245	
3643,2658	98,5185		3643,2658	98,8201	
3645,1945	98,4251		3645,1945	98,7734	
3647,1232	98,5467		3647,1232	98,6673	
3649,0519	98,7319		3649,0519	98,7976	
3650,9805	98,7603		3650,9805	99,0427	
3652,9092	98,7905		3652,9092	98,9993	
3654,8379	98,8683		3654,8379	98,968	
3656,7665	98,9341		3656,7665	99,1662	
3658,6952	98,8895		3658,6952	99,2404	
3660,6239	98,8708		3660,6239	99,2405	
3662,5526	98,9077		3662,5526	99,3712	
3664,4812	99,007		3664,4812	99,4066	
3666,4099	99,1118		3666,4099	99,3251	
3668,3386	99,1361		3668,3386	99,3252	
3670,2673	99,1512		3670,2673	99,3819	
3672,1959	99,1107		3672,1959	99,3899	
3674,1246	99,0235		3674,1246	99,3477	
3676,0533	99,1195		3676,0533	99,2399	
3677,982	99,2586		3677,982	99,3201	
3679,9106	99,2598		3679,9106	99,4973	
3681,8393	99,1952		3681,8393	99,5733	
3683,768	99,1348		3683,768	99,5082	
3685,6967	99,0731		3685,6967	99,4606	
3687,6253	99,0106		3687,6253	99,4408	
3689,554	99,0556		3689,554	99,3917	

3691,4827	99,1257		3691,4827	99,4675	
3693,4114	99,1407		3693,4114	99,5223	
3695,34	99,1919		3695,34	99,5223	
3697,2687	99,2327		3697,2687	99,548	
3699,1974	99,1647		3699,1974	99,5828	
3701,1261	99,1355		3701,1261	99,6199	
3703,0547	99,2254		3703,0547	99,6097	
3704,9834	99,2348		3704,9834	99,6009	
3706,9121	99,2013		3706,9121	99,6358	
3708,8408	99,1931		3708,8408	99,6587	
3710,7694	99,2496		3710,7694	99,6656	
3712,6981	99,3331		3712,6981	99,6562	
3714,6268	99,2902		3714,6268	99,6003	
3716,5555	99,243		3716,5555	99,5844	
3718,4841	99,3111		3718,4841	99,6446	
3720,4128	99,4017		3720,4128	99,7496	
3722,3415	99,363		3722,3415	99,7077	
3724,2702	99,3089		3724,2702	99,4513	
3726,1988	99,2926		3726,1988	99,3698	
3728,1275	99,2684		3728,1275	99,5267	
3730,0562	99,2655		3730,0562	99,6017	
3731,9848	99,2508		3731,9848	99,5201	
3733,9135	99,1572		3733,9135	99,3996	
3735,8422	99,132		3735,8422	99,5374	
3737,7709	99,321		3737,7709	99,7108	
3739,6995	99,3832		3739,6995	99,7179	
3741,6282	99,2819		3741,6282	99,7055	
3743,5569	99,2386		3743,5569	99,6979	
3745,4856	99,3888		3745,4856	99,7494	
3747,4142	99,357		3747,4142	99,7933	

3749,3429	99,3087		3749,3429	99,7392	
3751,2716	99,4425		3751,2716	99,6601	
3753,2003	99,4684		3753,2003	99,7753	
3755,1289	99,3878		3755,1289	99,8873	
3757,0576	99,3029		3757,0576	99,8709	
3758,9863	99,3495		3758,9863	99,7701	
3760,915	99,4927		3760,915	99,7786	
3762,8436	99,5036		3762,8436	99,8229	
3764,7723	99,3612		3764,7723	99,8157	
3766,701	99,2747		3766,701	99,7828	
3768,6297	99,4081		3768,6297	99,7324	
3770,5583	99,487		3770,5583	99,7486	
3772,487	99,4364		3772,487	99,8148	
3774,4157	99,496		3774,4157	99,8416	
3776,3444	99,566		3776,3444	99,7623	
3778,273	99,563		3778,273	99,6652	
3780,2017	99,5717		3780,2017	99,7302	
3782,1304	99,5399		3782,1304	99,7452	
3784,0591	99,5089		3784,0591	99,7282	
3785,9877	99,4734		3785,9877	99,8148	
3787,9164	99,3965		3787,9164	99,8809	
3789,8451	99,3873		3789,8451	99,8515	
3791,7738	99,4316		3791,7738	99,7612	
3793,7024	99,3783		3793,7024	99,7112	
3795,6311	99,2833		3795,6311	99,7409	
3797,5598	99,4061		3797,5598	99,8447	
3799,4885	99,5211		3799,4885	99,8677	
3801,4171	99,4649		3801,4171	99,7552	
3803,3458	99,482		3803,3458	99,7857	
3805,2745	99,4946		3805,2745	99,8544	

3807,2031	99,4201		3807,2031	99,7838	
3809,1318	99,3593		3809,1318	99,7683	
3811,0605	99,3594		3811,0605	99,8427	
3812,9892	99,3778		3812,9892	99,8683	
3814,9178	99,2963		3814,9178	99,8764	
3816,8465	99,2197		3816,8465	99,9497	
3818,7752	99,3849		3818,7752	99,9835	
3820,7039	99,5922		3820,7039	99,9294	
3822,6325	99,5188		3822,6325	99,8336	
3824,5612	99,3704		3824,5612	99,7924	
3826,4899	99,3311		3826,4899	99,7171	
3828,4186	99,3573		3828,4186	99,6712	
3830,3472	99,3305		3830,3472	99,7335	
3832,2759	99,3891		3832,2759	99,7982	
3834,2046	99,4158		3834,2046	99,7856	
3836,1333	99,2741		3836,1333	99,7118	
3838,0619	99,2586		3838,0619	99,6606	
3839,9906	99,3663		3839,9906	99,7182	
3841,9193	99,3898		3841,9193	99,8016	
3843,848	99,3661		3843,848	99,8832	
3845,7766	99,3748		3845,7766	99,9193	
3847,7053	99,387		3847,7053	99,8904	
3849,634	99,3414		3849,634	99,8594	
3851,5627	99,2677		3851,5627	99,9059	
3853,4913	99,242		3853,4913	99,8967	
3855,42	99,3649		3855,42	99,7318	
3857,3487	99,4198		3857,3487	99,7051	
3859,2774	99,401		3859,2774	99,7412	
3861,206	99,3831		3861,206	99,7067	
3863,1347	99,3303		3863,1347	99,65	

3865,0634	99,2911		3865,0634	99,6742	
3866,9921	99,3576		3866,9921	99,6808	
3868,9207	99,4506		3868,9207	99,7532	
3870,8494	99,4975		3870,8494	99,8858	
3872,7781	99,4141		3872,7781	99,8586	
3874,7067	99,3986		3874,7067	99,8549	
3876,6354	99,429		3876,6354	99,9022	
3878,5641	99,379		3878,5641	99,8974	
3880,4928	99,3542		3880,4928	99,8526	
3882,4214	99,4188		3882,4214	99,7776	
3884,3501	99,4661		3884,3501	99,7294	
3886,2788	99,3991		3886,2788	99,7961	
3888,2075	99,296		3888,2075	99,8251	
3890,1361	99,2479		3890,1361	99,7258	
3892,0648	99,3035		3892,0648	99,6818	
3893,9935	99,3929		3893,9935	99,8028	
3895,9222	99,4004		3895,9222	99,8147	
3897,8508	99,3418		3897,8508	99,7196	
3899,7795	99,2832		3899,7795	99,7275	
3901,7082	99,3175		3901,7082	99,7951	
3903,6369	99,4318		3903,6369	99,7925	
3905,5655	99,5402		3905,5655	99,773	
3907,4942	99,4972		3907,4942	99,7662	
3909,4229	99,3786		3909,4229	99,7952	
3911,3516	99,2917		3911,3516	99,8586	
3913,2802	99,2624		3913,2802	99,8637	
3915,2089	99,2735		3915,2089	99,7254	
3917,1376	99,2066		3917,1376	99,5743	
3919,0663	99,1896		3919,0663	99,5848	
3920,9949	99,3207		3920,9949	99,6723	

3922,9236	99,3876		3922,9236	99,7428	
3924,8523	99,3585		3924,8523	99,7217	
3926,781	99,3529		3926,781	99,7409	
3928,7096	99,3555		3928,7096	99,8567	
3930,6383	99,3205		3930,6383	99,8255	
3932,567	99,3305		3932,567	99,7265	
3934,4957	99,3757		3934,4957	99,7668	
3936,4243	99,3558		3936,4243	99,7635	
3938,353	99,301		3938,353	99,7293	
3940,2817	99,3306		3940,2817	99,7382	
3942,2104	99,4167		3942,2104	99,7101	
3944,139	99,4074		3944,139	99,678	
3946,0677	99,3467		3946,0677	99,6784	
3947,9964	99,3761		3947,9964	99,7085	
3949,925	99,416		3949,925	99,8101	
3951,8537	99,3567		3951,8537	99,8881	
3953,7824	99,3387		3953,7824	99,7962	
3955,7111	99,359		3955,7111	99,7119	
3957,6397	99,309		3957,6397	99,8102	
3959,5684	99,224		3959,5684	99,824	
3961,4971	99,2131		3961,4971	99,743	
3963,4258	99,2604		3963,4258	99,7559	
3965,3544	99,2921		3965,3544	99,7743	
3967,2831	99,3158		3967,2831	99,7323	
3969,2118	99,3415		3969,2118	99,7014	
3971,1405	99,373		3971,1405	99,6872	
3973,0691	99,3402		3973,0691	99,6849	
3974,9978	99,2738		3974,9978	99,6955	
3976,9265	99,2411		3976,9265	99,6346	
3978,8552	99,2261		3978,8552	99,6324	

3980,7838	99,2634		3980,7838	99,7268	
3982,7125	99,2614		3982,7125	99,7584	
3984,6412	99,2083		3984,6412	99,7185	
3986,5699	99,1987		3986,5699	99,6712	
3988,4985	99,2096		3988,4985	99,6791	
3990,4272	99,2508		3990,4272	99,7017	
3992,3559	99,2882		3992,3559	99,6812	
3994,2846	99,2726		3994,2846	99,6997	
3996,2132	99,2579		3996,2132	99,7777	
3998,1419	99,29		3998,1419	99,8144	
4000,0706	99,3067		4000,0706	99,7531	
Comment=			Comment=		
