# Supplementary material for: Data on statistical experimental design to formulate amphotericin B-loaded Eudragit RL100 nanoparticles coated with hyaluronic acid for the treatment of vulvovaginal candidiasis
Source: Data Brief. 2020 Mar 5;29:105311. doi: 10.1016/j.dib.2020.105311 (PMC7082528; doi:10.1016/j.dib.2020.105311)
Supplement: Multimedia component 14 [file mmc14.pdf]

DATATYPE	**IR Spectrum AMP EUD nanoparticles HA**		
XYUNITS	**Wavenumber;PercentTransmittance**		
DECIMALSYMB(	,		
399,2356	150		
401,1643	150		
403,0929	-1		
405,0216	-1		
406,9503	-1		
408,879	7,8181		
410,8076	25,5263		
412,7363	72,2245		
414,665	109,5726		
416,5937	51,2004		
418,5223	10,1541		
420,451	38,4199		
422,3797	52,3183		
424,3084	74,633		
426,237	137,1316		
428,1657	150		
430,0944	150		
432,0231	-1		
433,9517	-1		
435,8804	-1		
437,8091	150		
439,7377	150		
441,6664	150		
443,5951	150		
445,5238	150		
447,4524	66,9937		
449,3811	33,7664		

451,3098	17,312		
453,2385	150		
455,1671	-1		
457,0958	-1		
459,0245	150		
460,9532	-1		
462,8818	18,4512		
464,8105	-1		
466,7392	-1		
468,6679	53,9094		
470,5965	-1		
472,5252	-1		
474,4539	-1		
476,3826	73,3591		
478,3112	-1		
480,2399	-1		
482,1686	-1		
484,0973	-1		
486,0259	-1		
487,9546	-1		
489,8833	-1		
491,812	150		
493,7406	5,5971		
495,6693	-1		
497,598	-1		
499,5267	-1		
501,4553	-1		
503,384	-1		
505,3127	-1		
507,2414	150		

509,17	-1		
511,0987	-1		
513,0274	150		
514,956	101,1093		
516,8847	124,1017		
518,8134	150		
520,7421	45,9013		
522,6707	38,1669		
524,5994	101,871		
526,5281	116,0689		
528,4568	93,0733		
530,3854	83,8328		
532,3141	68,1086		
534,2428	60,6501		
536,1715	68,3261		
538,1001	74,3911		
540,0288	69,9002		
541,9575	65,7899		
543,8862	73,9846		
545,8148	84,2857		
547,7435	86,7127		
549,6722	78,4357		
551,6009	67,5796		
553,5295	64,6717		
555,4582	70,1038		
557,3869	74,3684		
559,3156	74,8242		
561,2442	71,0854		
563,1729	64,9778		
565,1016	63,4285		

567,0303	67,728		
568,9589	74,0082		
570,8876	74,3738		
572,8163	72,6637		
574,745	77,9104		
576,6736	79,0989		
578,6023	68,8644		
580,531	63,0241		
582,4597	64,3261		
584,3883	66,6725		
586,317	70,0701		
588,2457	72,3034		
590,1743	72,1912		
592,103	73,1823		
594,0317	74,6193		
595,9604	73,8569		
597,889	71,9661		
599,8177	70,4343		
601,7464	71,3795		
603,6751	73,5198		
605,6037	72,9006		
607,5324	70,8602		
609,4611	71,4083		
611,3898	73,7704		
613,3184	74,8209		
615,2471	73,9717		
617,1758	72,1681		
619,1045	72,2147		
621,0331	74,0674		
622,9618	74,8967		

624,8905	75,1097		
626,8192	75,6181		
628,7478	75,9046		
630,6765	75,8227		
632,6052	75,4326		
634,5339	75,5149		
636,4625	76,0357		
638,3912	76,152		
640,3199	76,0342		
642,2486	76,2199		
644,1772	76,6513		
646,1059	77,0128		
648,0346	77,2668		
649,9633	77,3376		
651,8919	77,5911		
653,8206	77,9635		
655,7493	77,8354		
657,678	77,4047		
659,6066	77,2858		
661,5353	77,2926		
663,464	77,2945		
665,3926	77,5321		
667,3213	77,777		
669,25	77,9939		
671,1787	78,119		
673,1073	78,1296		
675,036	78,3365		
676,9647	78,6274		
678,8934	78,7686		
680,822	78,9237		

682,7507	79,0407		
684,6794	79,0051		
686,6081	78,9522		
688,5367	78,9826		
690,4654	79,0445		
692,3941	79,1992		
694,3228	79,4367		
696,2514	79,6578		
698,1801	79,8627		
700,1088	80,0014		
702,0375	80,1405		
703,9661	80,2828		
705,8948	80,3586		
707,8235	80,5176		
709,7522	80,7376		
711,6808	80,7979		
713,6095	80,7232		
715,5382	80,7082		
717,4669	80,7794		
719,3955	80,7243		
721,3242	80,6244		
723,2529	80,7146		
725,1816	80,7868		
727,1102	80,8269		
729,0389	80,9226		
730,9676	80,9823		
732,8962	81,0834		
734,8249	81,1294		
736,7536	81,0112		
738,6823	80,8738		

740,6109	80,6913		
742,5396	80,1712		
744,4683	79,2705		
746,397	78,2227		
748,3256	77,3339		
750,2543	76,9047		
752,183	76,9904		
754,1117	77,3558		
756,0403	77,8287		
757,969	78,4284		
759,8977	79,0125		
761,8264	79,5341		
763,755	80,0768		
765,6837	80,6169		
767,6124	81,0808		
769,5411	81,4595		
771,4697	81,7942		
773,3984	82,0634		
775,3271	82,2522		
777,2558	82,28		
779,1844	82,2433		
781,1131	82,2112		
783,0418	82,1253		
784,9705	82,1141		
786,8991	82,1675		
788,8278	82,1898		
790,7565	82,1664		
792,6852	82,0991		
794,6138	82,0208		
796,5425	81,9667		

798,4712	81,9342		
800,3999	81,8745		
802,3285	81,734		
804,2572	81,4849		
806,1859	81,2356		
808,1145	81,1053		
810,0432	81,1602		
811,9719	81,3323		
813,9006	81,4708		
815,8292	81,6007		
817,7579	81,6047		
819,6866	81,3985		
821,6153	81,132		
823,5439	80,8164		
825,4726	80,4628		
827,4013	80,1109		
829,33	79,7431		
831,2586	79,2629		
833,1873	78,5135		
835,116	77,4689		
837,0447	76,2698		
838,9733	75,1496		
840,902	74,1434		
842,8307	73,332		
844,7594	72,8379		
846,688	72,5745		
848,6167	72,5614		
850,5454	72,7247		
852,4741	73,1039		
854,4027	73,8639		

856,3314	74,9026		
858,2601	76,1155		
860,1888	77,3356		
862,1174	78,3242		
864,0461	79,0321		
865,9748	79,5619		
867,9035	79,9232		
869,8321	80,1343		
871,7608	80,2534		
873,6895	80,2395		
875,6182	80,1217		
877,5468	79,9135		
879,4755	79,5737		
881,4042	79,185		
883,3328	78,9105		
885,2615	78,7905		
887,1902	78,8303		
889,1189	79,0815		
891,0475	79,4511		
892,9762	79,8094		
894,9049	80,0605		
896,8336	80,1413		
898,7622	80,0889		
900,6909	79,941		
902,6196	79,6862		
904,5483	79,3134		
906,4769	78,9515		
908,4056	78,6144		
910,3343	78,2439		
912,263	77,9602		

914,1916	77,7428		
916,1203	77,6232		
918,049	77,6163		
919,9777	77,5132		
921,9063	77,2707		
923,835	76,9312		
925,7637	76,5082		
927,6924	76,0679		
929,621	75,6147		
931,5497	75,0692		
933,4784	74,479		
935,4071	73,9488		
937,3357	73,3919		
939,2644	72,7025		
941,1931	71,9401		
943,1218	71,1868		
945,0504	70,4251		
946,9791	69,8024		
948,9078	69,576		
950,8364	69,6553		
952,7651	69,847		
954,6938	70,1214		
956,6225	70,5813		
958,5511	71,19		
960,4798	71,6463		
962,4085	71,9724		
964,3372	72,2932		
966,2658	72,4577		
968,1945	72,4788		
970,1232	72,3931		

972,0519	72,1055		
973,9805	71,6175		
975,9092	70,8787		
977,8379	69,9381		
979,7666	68,9051		
981,6952	67,8926		
983,6239	67,1563		
985,5526	66,7987		
987,4813	66,6861		
989,4099	66,5896		
991,3386	66,5021		
993,2673	66,4399		
995,196	66,2626		
997,1246	66,0337		
999,0533	65,7229		
1000,982	65,1961		
1002,9107	64,3529		
1004,8393	63,1862		
1006,768	61,8405		
1008,6967	60,4513		
1010,6254	59,3473		
1012,554	58,6948		
1014,4827	58,3941		
1016,4114	58,3737		
1018,3401	58,4871		
1020,2687	58,5812		
1022,1974	58,5887		
1024,1261	58,5168		
1026,0547	58,3352		
1027,9834	57,9763		

1029,9121	57,4948		
1031,8408	57,0032		
1033,7694	56,6138		
1035,6981	56,501		
1037,6268	56,6582		
1039,5555	56,9368		
1041,4841	57,2887		
1043,4128	57,6737		
1045,3415	57,9937		
1047,2702	58,1338		
1049,1988	58,1082		
1051,1275	57,9484		
1053,0562	57,5439		
1054,9849	56,8352		
1056,9135	55,9438		
1058,8422	55,0806		
1060,7709	54,2827		
1062,6996	53,4612		
1064,6282	52,6877		
1066,5569	51,9464		
1068,4856	51,2372		
1070,4143	50,7522		
1072,3429	50,4406		
1074,2716	50,1431		
1076,2003	49,8109		
1078,129	49,4553		
1080,0576	49,0635		
1081,9863	48,6384		
1083,915	48,162		
1085,8437	47,5295		

1087,7723	46,8775		
1089,701	46,3413		
1091,6297	45,7467		
1093,5584	45,1432		
1095,487	44,816		
1097,4157	44,7608		
1099,3444	44,8089		
1101,273	44,8972		
1103,2017	45,0955		
1105,1304	45,324		
1107,0591	45,5234		
1108,9877	45,783		
1110,9164	46,0314		
1112,8451	46,438		
1114,7738	47,0576		
1116,7024	47,6018		
1118,6311	48,0767		
1120,5598	48,4808		
1122,4885	48,746		
1124,4171	48,9444		
1126,3458	49,0747		
1128,2745	49,046		
1130,2032	48,9099		
1132,1318	48,7854		
1134,0605	48,6121		
1135,9892	48,4549		
1137,9179	48,3619		
1139,8465	48,3179		
1141,7752	48,4465		
1143,7039	48,6909		

1145,6326	49,0389		
1147,5612	49,6652		
1149,4899	50,598		
1151,4186	51,755		
1153,3473	53,1066		
1155,2759	54,5322		
1157,2046	55,9104		
1159,1333	57,2627		
1161,062	58,5272		
1162,9906	59,6503		
1164,9193	60,6282		
1166,848	61,3131		
1168,7767	61,5928		
1170,7053	61,6035		
1172,634	61,5327		
1174,5627	61,4755		
1176,4913	61,6217		
1178,42	62,1482		
1180,3487	62,9054		
1182,2774	63,7085		
1184,206	64,5289		
1186,1347	65,3026		
1188,0634	66,0841		
1189,9921	66,9692		
1191,9207	67,9793		
1193,8494	69,195		
1195,7781	70,5989		
1197,7068	71,9882		
1199,6354	73,3197		
1201,5641	74,5884		

1203,4928	75,6328		
1205,4215	76,4183		
1207,3501	77,0158		
1209,2788	77,4194		
1211,2075	77,5887		
1213,1362	77,5764		
1215,0648	77,4149		
1216,9935	77,1084		
1218,9222	76,7184		
1220,8509	76,2726		
1222,7795	75,7478		
1224,7082	75,0699		
1226,6369	74,2171		
1228,5656	73,3166		
1230,4942	72,4149		
1232,4229	71,522		
1234,3516	70,7528		
1236,2803	70,1702		
1238,2089	69,7444		
1240,1376	69,5359		
1242,0663	69,6757		
1243,9949	70,0553		
1245,9236	70,5569		
1247,8523	71,2248		
1249,781	71,9951		
1251,7096	72,7549		
1253,6383	73,4949		
1255,567	74,2522		
1257,4957	75,0047		
1259,4243	75,6723		

1261,353	76,2282		
1263,2817	76,7273		
1265,2104	77,1628		
1267,139	77,4418		
1269,0677	77,5887		
1270,9964	77,7037		
1272,9251	77,7619		
1274,8537	77,8247		
1276,7824	78,0023		
1278,7111	78,2195		
1280,6398	78,4491		
1282,5684	78,7442		
1284,4971	79,0539		
1286,4258	79,3238		
1288,3545	79,5668		
1290,2831	79,7424		
1292,2118	79,8104		
1294,1405	79,9116		
1296,0692	80,1762		
1297,9978	80,4952		
1299,9265	80,8248		
1301,8552	81,2634		
1303,7839	81,8216		
1305,7125	82,4486		
1307,6412	83,1181		
1309,5699	83,8002		
1311,4986	84,3722		
1313,4272	84,7845		
1315,3559	85,0782		
1317,2846	85,2766		

1319,2132	85,3609		
1321,1419	85,3162		
1323,0706	85,2776		
1324,9993	85,3632		
1326,9279	85,5624		
1328,8566	85,8045		
1330,7853	86,0007		
1332,714	86,1339		
1334,6426	86,1875		
1336,5713	86,1335		
1338,5	86,0454		
1340,4287	85,8055		
1342,3573	85,1931		
1344,286	84,3123		
1346,2147	83,4386		
1348,1434	82,8863		
1350,072	82,7847		
1352,0007	82,9886		
1353,9294	83,2846		
1355,8581	83,6432		
1357,7867	83,9745		
1359,7154	84,1365		
1361,6441	84,129		
1363,5728	84,0084		
1365,5014	83,7702		
1367,4301	83,5109		
1369,3588	83,299		
1371,2875	83,0528		
1373,2161	82,7271		
1375,1448	82,2898		

1377,0735	81,7775		
1379,0022	81,3217		
1380,9308	81,1286		
1382,8595	81,1978		
1384,7882	81,3884		
1386,7169	81,7957		
1388,6455	82,4224		
1390,5742	82,9583		
1392,5029	83,3768		
1394,4315	83,8003		
1396,3602	84,2114		
1398,2889	84,5446		
1400,2176	84,9739		
1402,1462	85,3382		
1404,0749	85,758		
1406,0036	86,3489		
1407,9323	86,8285		
1409,8609	87,2073		
1411,7896	87,538		
1413,7183	87,7485		
1415,647	87,739		
1417,5756	87,598		
1419,5043	87,4265		
1421,433	87,1482		
1423,3617	86,6486		
1425,2903	85,8488		
1427,219	84,9297		
1429,1477	83,6783		
1431,0764	82,2728		
1433,005	81,2008		

1434,9337	80,4161		
1436,8624	79,8192		
1438,7911	79,3886		
1440,7197	78,9044		
1442,6484	78,2658		
1444,5771	77,6265		
1446,5058	77,1601		
1448,4344	77,0665		
1450,3631	77,3192		
1452,2918	77,7782		
1454,2205	78,2596		
1456,1491	78,9057		
1458,0778	80,1167		
1460,0065	80,6005		
1461,9351	80,7946		
1463,8638	81,1742		
1465,7925	81,7748		
1467,7212	82,1973		
1469,6498	82,5483		
1471,5785	83,0353		
1473,5072	83,78		
1475,4359	84,4253		
1477,3645	85,1198		
1479,2932	85,752		
1481,2219	86,4752		
1483,1506	87,2475		
1485,0792	87,8699		
1487,0079	88,6007		
1488,9366	89,5806		
1490,8653	90,8799		

1492,7939	91,7506		
1494,7226	92,3847		
1496,6513	93,2261		
1498,58	93,9892		
1500,5086	94,4445		
1502,4373	94,6662		
1504,366	94,6897		
1506,2947	94,6279		
1508,2233	94,8048		
1510,152	94,8595		
1512,0807	94,8656		
1514,0094	94,8356		
1515,938	94,7172		
1517,8667	94,608		
1519,7954	94,5052		
1521,7241	94,4635		
1523,6527	94,5382		
1525,5814	94,4675		
1527,5101	94,2985		
1529,4388	94,1341		
1531,3674	93,9808		
1533,2961	93,838		
1535,2248	93,7279		
1537,1534	93,6036		
1539,0821	93,4766		
1541,0108	93,3864		
1542,9395	93,2023		
1544,8681	92,9821		
1546,7968	92,7375		
1548,7255	92,5		

1550,6542	92,307		
1552,5828	92,1899		
1554,5115	92,0539		
1556,4402	91,8908		
1558,3689	91,7101		
1560,2975	91,6575		
1562,2262	91,6305		
1564,1549	91,5948		
1566,0836	91,6127		
1568,0122	91,6561		
1569,9409	91,782		
1571,8696	91,932		
1573,7983	92,036		
1575,7269	92,1808		
1577,6556	92,3988		
1579,5843	92,5617		
1581,513	92,7038		
1583,4416	92,83		
1585,3703	92,9045		
1587,299	93,023		
1589,2277	93,2075		
1591,1563	93,2842		
1593,085	93,2492		
1595,0137	93,2155		
1596,9424	93,1699		
1598,871	93,0676		
1600,7997	92,9149		
1602,7284	92,7559		
1604,6571	92,6312		
1606,5857	92,5224		

1608,5144	92,4341		
1610,4431	92,3339		
1612,3717	92,1544		
1614,3004	91,9652		
1616,2291	91,8314		
1618,1578	91,7626		
1620,0864	91,7005		
1622,0151	91,6236		
1623,9438	91,4887		
1625,8725	91,379		
1627,8011	91,3095		
1629,7298	91,2303		
1631,6585	91,1273		
1633,5872	90,9826		
1635,5158	90,9315		
1637,4445	91,0075		
1639,3732	90,9797		
1641,3019	90,9812		
1643,2305	90,9873		
1645,1592	90,9183		
1647,0879	90,859		
1649,0166	90,8874		
1650,9452	90,7898		
1652,8739	90,735		
1654,8026	91,1393		
1656,7313	91,2851		
1658,6599	91,3882		
1660,5886	91,5112		
1662,5173	91,7938		
1664,446	92,0027		

1666,3746	92,0233		
1668,3033	92,1189		
1670,232	92,3043		
1672,1607	92,32		
1674,0893	92,2462		
1676,018	92,2047		
1677,9467	92,1686		
1679,8754	92,0272		
1681,804	91,8042		
1683,7327	91,4697		
1685,6614	91,1302		
1687,59	90,9316		
1689,5187	90,6402		
1691,4474	90,3306		
1693,3761	90,0721		
1695,3047	89,6329		
1697,2334	88,9903		
1699,1621	88,4659		
1701,0908	87,1316		
1703,0194	85,962		
1704,9481	84,4715		
1706,8768	82,1827		
1708,8055	79,8315		
1710,7341	76,8589		
1712,6628	73,6105		
1714,5915	69,7108		
1716,5202	65,1297		
1718,4488	59,2822		
1720,3775	55,2112		
1722,3062	52,409		

1724,2349	50,0493		
1726,1635	49,0259		
1728,0922	49,0334		
1730,0209	50,1169		
1731,9496	51,5248		
1733,8782	54,8742		
1735,8069	59,3874		
1737,7356	62,0797		
1739,6643	66,9679		
1741,5929	72,3272		
1743,5216	77,1305		
1745,4503	81,7331		
1747,379	85,4444		
1749,3076	88,9179		
1751,2363	91,5706		
1753,165	93,335		
1755,0936	94,3277		
1757,0223	95,2263		
1758,951	95,8468		
1760,8797	96,1834		
1762,8083	96,5438		
1764,737	96,8254		
1766,6657	97,1081		
1768,5944	97,3594		
1770,523	97,433		
1772,4517	97,479		
1774,3804	97,6017		
1776,3091	97,7162		
1778,2377	97,7496		
1780,1664	97,8313		

1782,0951	97,9412		
1784,0238	97,9556		
1785,9524	98,0068		
1787,8811	98,0913		
1789,8098	98,1193		
1791,7385	98,0965		
1793,6671	98,1346		
1795,5958	98,2517		
1797,5245	98,3419		
1799,4532	98,3381		
1801,3818	98,3569		
1803,3105	98,4313		
1805,2392	98,4382		
1807,1679	98,4301		
1809,0965	98,43		
1811,0252	98,4196		
1812,9539	98,5197		
1814,8826	98,6342		
1816,8112	98,6231		
1818,7399	98,5181		
1820,6686	98,488		
1822,5973	98,5777		
1824,5259	98,6458		
1826,4546	98,6469		
1828,3833	98,5711		
1830,3119	98,4975		
1832,2406	98,6138		
1834,1693	98,7199		
1836,098	98,718		
1838,0266	98,729		

1839,9553	98,7775		
1841,884	98,8006		
1843,8127	98,8404		
1845,7413	98,8703		
1847,67	98,8471		
1849,5987	98,8465		
1851,5274	98,8027		
1853,456	98,7667		
1855,3847	98,8591		
1857,3134	98,9099		
1859,2421	98,8359		
1861,1707	98,7388		
1863,0994	98,7013		
1865,0281	98,7328		
1866,9568	98,7273		
1868,8854	98,6434		
1870,8141	98,6351		
1872,7428	98,6961		
1874,6715	98,7527		
1876,6001	98,7641		
1878,5288	98,7524		
1880,4575	98,7206		
1882,3862	98,6442		
1884,3148	98,6327		
1886,2435	98,6971		
1888,1722	98,7392		
1890,1009	98,7796		
1892,0295	98,7909		
1893,9582	98,6775		
1895,8869	98,5499		

1897,8156	98,6259		
1899,7442	98,7479		
1901,6729	98,6589		
1903,6016	98,4639		
1905,5302	98,3713		
1907,4589	98,4147		
1909,3876	98,5179		
1911,3163	98,6058		
1913,2449	98,6412		
1915,1736	98,5561		
1917,1023	98,3627		
1919,031	98,3126		
1920,9596	98,4047		
1922,8883	98,3841		
1924,817	98,3867		
1926,7457	98,4087		
1928,6743	98,3857		
1930,603	98,4065		
1932,5317	98,4632		
1934,4604	98,4744		
1936,389	98,4219		
1938,3177	98,3655		
1940,2464	98,1862		
1942,1751	97,9697		
1944,1037	98,0029		
1946,0324	98,1634		
1947,9611	98,2515		
1949,8898	98,2061		
1951,8184	98,0273		
1953,7471	97,9896		

1955,6758	98,1576		
1957,6045	98,1874		
1959,5331	98,1082		
1961,4618	98,1553		
1963,3905	98,1658		
1965,3192	98,0292		
1967,2478	97,9747		
1969,1765	98,1013		
1971,1052	98,2571		
1973,0339	98,2674		
1974,9625	98,1209		
1976,8912	97,8794		
1978,8199	97,6502		
1980,7485	97,5856		
1982,6772	97,7368		
1984,6059	98,0339		
1986,5346	98,2908		
1988,4632	98,1456		
1990,3919	97,8119		
1992,3206	97,9012		
1994,2493	98,083		
1996,1779	98,165		
1998,1066	98,2522		
2000,0353	98,19		
2001,964	98,1319		
2003,8926	98,2734		
2005,8213	98,3564		
2007,75	98,1449		
2009,6787	97,9274		
2011,6073	97,8632		

2013,536	97,9822		
2015,4647	98,1356		
2017,3934	98,0283		
2019,322	97,7467		
2021,2507	97,5782		
2023,1794	97,6302		
2025,1081	97,6769		
2027,0367	97,6224		
2028,9654	97,7123		
2030,8941	97,822		
2032,8228	97,8641		
2034,7514	98,049		
2036,6801	98,1328		
2038,6088	98,0042		
2040,5375	97,9429		
2042,4661	97,8645		
2044,3948	97,98		
2046,3235	98,3034		
2048,2521	98,264		
2050,1808	98,0011		
2052,1095	98,0744		
2054,0382	98,3131		
2055,9668	98,4297		
2057,8955	98,4634		
2059,8242	98,338		
2061,7529	98,3383		
2063,6815	98,436		
2065,6102	98,2881		
2067,5389	98,1365		
2069,4676	98,2326		

2071,3962	98,2803		
2073,3249	98,1935		
2075,2536	98,2627		
2077,1823	98,2592		
2079,1109	98,3005		
2081,0396	98,5622		
2082,9683	98,6483		
2084,897	98,6379		
2086,8256	98,6938		
2088,7543	98,6576		
2090,683	98,6444		
2092,6117	98,6847		
2094,5403	98,5883		
2096,469	98,469		
2098,3977	98,4438		
2100,3264	98,5618		
2102,255	98,6263		
2104,1837	98,4822		
2106,1124	98,4321		
2108,0411	98,4774		
2109,9697	98,4457		
2111,8984	98,3647		
2113,8271	98,3838		
2115,7558	98,584		
2117,6844	98,6675		
2119,6131	98,6129		
2121,5418	98,7404		
2123,4704	98,8351		
2125,3991	98,6359		
2127,3278	98,4822		

2129,2565	98,5555		
2131,1851	98,6617		
2133,1138	98,714		
2135,0425	98,6027		
2136,9712	98,4188		
2138,8998	98,3469		
2140,8285	98,3543		
2142,7572	98,4056		
2144,6859	98,3968		
2146,6145	98,2858		
2148,5432	98,1979		
2150,4719	98,2396		
2152,4006	98,2985		
2154,3292	98,3371		
2156,2579	98,2186		
2158,1866	97,8307		
2160,1153	97,6992		
2162,0439	97,8406		
2163,9726	97,9389		
2165,9013	97,9585		
2167,83	98,001		
2169,7586	98,2093		
2171,6873	98,1216		
2173,616	97,7708		
2175,5447	97,9383		
2177,4733	98,5038		
2179,402	98,6603		
2181,3307	98,3049		
2183,2594	98,2071		
2185,188	98,5606		

2187,1167	98,8475		
2189,0454	98,789		
2190,9741	98,4226		
2192,9027	98,1446		
2194,8314	98,1537		
2196,7601	98,2199		
2198,6887	98,3241		
2200,6174	98,4734		
2202,5461	98,4528		
2204,4748	98,2676		
2206,4034	98,1798		
2208,3321	98,2139		
2210,2608	98,1314		
2212,1895	98,1443		
2214,1181	98,2748		
2216,0468	98,3285		
2217,9755	98,5488		
2219,9042	98,7212		
2221,8328	98,611		
2223,7615	98,3735		
2225,6902	98,3127		
2227,6189	98,5376		
2229,5475	98,567		
2231,4762	98,4212		
2233,4049	98,4607		
2235,3336	98,5605		
2237,2622	98,5187		
2239,1909	98,5215		
2241,1196	98,5699		
2243,0483	98,4741		

2244,9769	98,4509		
2246,9056	98,5219		
2248,8343	98,5643		
2250,763	98,5835		
2252,6916	98,5414		
2254,6203	98,505		
2256,549	98,5606		
2258,4777	98,5885		
2260,4063	98,5359		
2262,335	98,6008		
2264,2637	98,6767		
2266,1923	98,5752		
2268,121	98,514		
2270,0497	98,5728		
2271,9784	98,6303		
2273,907	98,5969		
2275,8357	98,5722		
2277,7644	98,5782		
2279,6931	98,5216		
2281,6217	98,5335		
2283,5504	98,5357		
2285,4791	98,4671		
2287,4078	98,4405		
2289,3364	98,4294		
2291,2651	98,4524		
2293,1938	98,5568		
2295,1225	98,6647		
2297,0511	98,7219		
2298,9798	98,7085		
2300,9085	98,5598		

2302,8372	98,3841		
2304,7658	98,3601		
2306,6945	98,3907		
2308,6232	98,4033		
2310,5519	98,4409		
2312,4805	98,3986		
2314,4092	98,3083		
2316,3379	98,1675		
2318,2666	97,999		
2320,1952	98,002		
2322,1239	97,9444		
2324,0526	97,7923		
2325,9813	97,8763		
2327,9099	98,0243		
2329,8386	98,0586		
2331,7673	98,0683		
2333,696	97,9844		
2335,6246	97,8445		
2337,5533	97,941		
2339,482	98,075		
2341,4106	97,9146		
2343,3393	97,779		
2345,268	97,8824		
2347,1967	98,0428		
2349,1253	98,0949		
2351,054	97,9952		
2352,9827	97,7559		
2354,9114	97,5526		
2356,84	97,5193		
2358,7687	97,386		

2360,6974	97,2776		
2362,6261	97,5872		
2364,5547	97,7453		
2366,4834	97,7557		
2368,4121	97,8798		
2370,3408	97,9643		
2372,2694	98,1446		
2374,1981	98,189		
2376,1268	98,1792		
2378,0555	98,358		
2379,9841	98,5788		
2381,9128	98,6903		
2383,8415	98,7169		
2385,7702	98,773		
2387,6988	98,807		
2389,6275	98,8318		
2391,5562	98,8739		
2393,4849	98,8971		
2395,4135	98,9165		
2397,3422	98,8793		
2399,2709	98,8247		
2401,1996	98,7888		
2403,1282	98,7935		
2405,0569	98,8399		
2406,9856	98,8108		
2408,9143	98,7705		
2410,8429	98,8001		
2412,7716	98,8121		
2414,7003	98,7792		
2416,6289	98,6977		

2418,5576	98,6608		
2420,4863	98,7832		
2422,415	98,847		
2424,3436	98,7584		
2426,2723	98,7136		
2428,201	98,7796		
2430,1297	98,8366		
2432,0583	98,7212		
2433,987	98,6321		
2435,9157	98,7481		
2437,8444	98,7971		
2439,773	98,7195		
2441,7017	98,7316		
2443,6304	98,7904		
2445,5591	98,8411		
2447,4877	98,9351		
2449,4164	98,8976		
2451,3451	98,6972		
2453,2738	98,5946		
2455,2024	98,6704		
2457,1311	98,6817		
2459,0598	98,5957		
2460,9885	98,6326		
2462,9171	98,6734		
2464,8458	98,645		
2466,7745	98,6545		
2468,7032	98,7102		
2470,6318	98,735		
2472,5605	98,6732		
2474,4892	98,6752		

2476,4179	98,7253		
2478,3465	98,7006		
2480,2752	98,693		
2482,2039	98,682		
2484,1326	98,6287		
2486,0612	98,6351		
2487,9899	98,6058		
2489,9186	98,4943		
2491,8472	98,4893		
2493,7759	98,5583		
2495,7046	98,5794		
2497,6333	98,5937		
2499,5619	98,5939		
2501,4906	98,5525		
2503,4193	98,5154		
2505,348	98,5255		
2507,2766	98,4931		
2509,2053	98,435		
2511,134	98,4342		
2513,0627	98,4188		
2514,9913	98,4188		
2516,92	98,4033		
2518,8487	98,4289		
2520,7774	98,5274		
2522,706	98,5253		
2524,6347	98,4893		
2526,5634	98,4981		
2528,4921	98,4826		
2530,4207	98,4302		
2532,3494	98,3791		

2534,2781	98,362		
2536,2068	98,3567		
2538,1354	98,3953		
2540,0641	98,5128		
2541,9928	98,5613		
2543,9215	98,5154		
2545,8501	98,4845		
2547,7788	98,4272		
2549,7075	98,3122		
2551,6362	98,241		
2553,5648	98,3059		
2555,4935	98,4032		
2557,4222	98,4275		
2559,3508	98,4192		
2561,2795	98,3754		
2563,2082	98,3608		
2565,1369	98,3845		
2567,0655	98,3463		
2568,9942	98,2806		
2570,9229	98,2964		
2572,8516	98,2835		
2574,7802	98,1983		
2576,7089	98,2265		
2578,6376	98,2675		
2580,5663	98,2492		
2582,4949	98,2621		
2584,4236	98,2323		
2586,3523	98,1545		
2588,281	98,1187		
2590,2096	98,0951		

2592,1383	98,116		
2594,067	98,2358		
2595,9957	98,2784		
2597,9243	98,1652		
2599,853	98,0796		
2601,7817	98,109		
2603,7104	98,2168		
2605,639	98,2802		
2607,5677	98,1809		
2609,4964	98,0968		
2611,4251	98,1368		
2613,3537	98,184		
2615,2824	98,1948		
2617,2111	98,1797		
2619,1398	98,1448		
2621,0684	98,102		
2622,9971	98,1053		
2624,9258	98,1291		
2626,8545	98,139		
2628,7831	98,1257		
2630,7118	98,0937		
2632,6405	98,1108		
2634,5691	98,1319		
2636,4978	98,1315		
2638,4265	98,0879		
2640,3552	98,04		
2642,2838	98,0694		
2644,2125	98,0771		
2646,1412	98,0426		
2648,0699	98,0318		

2649,9985	98,0626		
2651,9272	98,0892		
2653,8559	98,083		
2655,7846	98,0781		
2657,7132	98,0876		
2659,6419	98,0956		
2661,5706	98,0765		
2663,4993	98,049		
2665,4279	97,9934		
2667,3566	97,9535		
2669,2853	98,006		
2671,214	98,0295		
2673,1426	97,9779		
2675,0713	97,9187		
2677	97,8865		
2678,9287	97,9061		
2680,8573	97,8804		
2682,786	97,7955		
2684,7147	97,7796		
2686,6434	97,7742		
2688,572	97,6846		
2690,5007	97,6247		
2692,4294	97,619		
2694,3581	97,557		
2696,2867	97,4867		
2698,2154	97,4798		
2700,1441	97,4824		
2702,0728	97,4725		
2704,0014	97,4611		
2705,9301	97,4471		

2707,8588	97,4292		
2709,7874	97,3812		
2711,7161	97,3244		
2713,6448	97,3076		
2715,5735	97,3108		
2717,5021	97,2887		
2719,4308	97,2155		
2721,3595	97,1146		
2723,2882	97,0523		
2725,2168	97,0457		
2727,1455	97,0635		
2729,0742	97,049		
2731,0029	96,993		
2732,9315	96,9467		
2734,8602	96,9285		
2736,7889	96,9584		
2738,7176	96,9686		
2740,6462	96,925		
2742,5749	96,8849		
2744,5036	96,8496		
2746,4323	96,8656		
2748,3609	96,8646		
2750,2896	96,8061		
2752,2183	96,7854		
2754,147	96,7877		
2756,0756	96,7641		
2758,0043	96,7039		
2759,933	96,6607		
2761,8617	96,6452		
2763,7903	96,6203		

2765,719	96,596		
2767,6477	96,5381		
2769,5764	96,4455		
2771,505	96,4113		
2773,4337	96,4151		
2775,3624	96,3487		
2777,291	96,241		
2779,2197	96,1439		
2781,1484	96,1022		
2783,0771	96,0641		
2785,0057	95,9604		
2786,9344	95,8822		
2788,8631	95,7966		
2790,7918	95,6964		
2792,7204	95,6345		
2794,6491	95,5229		
2796,5778	95,3838		
2798,5065	95,301		
2800,4351	95,191		
2802,3638	95,0146		
2804,2925	94,872		
2806,2212	94,741		
2808,1498	94,5514		
2810,0785	94,3792		
2812,0072	94,2303		
2813,9359	94,0813		
2815,8645	93,9271		
2817,7932	93,7164		
2819,7219	93,4793		
2821,6506	93,2594		

2823,5792	93,0519		
2825,5079	92,8295		
2827,4366	92,5912		
2829,3653	92,2986		
2831,2939	91,9114		
2833,2226	91,4412		
2835,1513	90,9414		
2837,08	90,4218		
2839,0086	89,8042		
2840,9373	89,1032		
2842,866	88,3217		
2844,7947	87,4174		
2846,7233	86,4854		
2848,652	85,6063		
2850,5807	84,8154		
2852,5093	84,1528		
2854,438	83,5871		
2856,3667	83,1774		
2858,2954	82,9567		
2860,224	82,8168		
2862,1527	82,6862		
2864,0814	82,5536		
2866,0101	82,4248		
2867,9387	82,3423		
2869,8674	82,3349		
2871,7961	82,3617		
2873,7248	82,4435		
2875,6534	82,6075		
2877,5821	82,8546		
2879,5108	83,1094		

2881,4395	83,2721		
2883,3681	83,4139		
2885,2968	83,5215		
2887,2255	83,5617		
2889,1542	83,5972		
2891,0828	83,6007		
2893,0115	83,5927		
2894,9402	83,5871		
2896,8689	83,5357		
2898,7975	83,4675		
2900,7262	83,3137		
2902,6549	83,046		
2904,5836	82,8231		
2906,5122	82,7068		
2908,4409	82,5789		
2910,3696	82,3802		
2912,2983	82,1803		
2914,2269	81,9603		
2916,1556	81,7444		
2918,0843	81,5249		
2920,013	81,2587		
2921,9416	81,1159		
2923,8703	81,1163		
2925,799	81,1805		
2927,7276	81,3544		
2929,6563	81,5673		
2931,585	81,8183		
2933,5137	82,2034		
2935,4423	82,6286		
2937,371	83,0077		

2939,2997	83,367		
2941,2284	83,6499		
2943,157	83,8389		
2945,0857	84,0235		
2947,0144	84,1837		
2948,9431	84,3457		
2950,8717	84,6412		
2952,8004	85,0496		
2954,7291	85,5029		
2956,6578	86,0571		
2958,5864	86,7388		
2960,5151	87,4244		
2962,4438	88,0474		
2964,3725	88,6693		
2966,3011	89,2363		
2968,2298	89,6649		
2970,1585	89,989		
2972,0872	90,2165		
2974,0158	90,3661		
2975,9445	90,4702		
2977,8732	90,5001		
2979,8019	90,5396		
2981,7305	90,6832		
2983,6592	90,8569		
2985,5879	91,0117		
2987,5166	91,196		
2989,4452	91,4234		
2991,3739	91,6786		
2993,3026	91,9704		
2995,2313	92,2622		

2997,1599	92,514		
2999,0886	92,757		
3001,0173	93,0194		
3002,9459	93,2624		
3004,8746	93,4643		
3006,8033	93,6938		
3008,732	93,9519		
3010,6606	94,1723		
3012,5893	94,4204		
3014,518	94,6623		
3016,4467	94,8483		
3018,3753	95,024		
3020,304	95,2015		
3022,2327	95,428		
3024,1614	95,618		
3026,09	95,7226		
3028,0187	95,8028		
3029,9474	95,8961		
3031,8761	96,0142		
3033,8047	96,1486		
3035,7334	96,2883		
3037,6621	96,3284		
3039,5908	96,3024		
3041,5194	96,3736		
3043,4481	96,4782		
3045,3768	96,5298		
3047,3055	96,5509		
3049,2341	96,5638		
3051,1628	96,6148		
3053,0915	96,6323		

3055,0202	96,6065		
3056,9488	96,6646		
3058,8775	96,769		
3060,8062	96,7644		
3062,7349	96,6568		
3064,6635	96,5869		
3066,5922	96,6254		
3068,5209	96,653		
3070,4495	96,6316		
3072,3782	96,6503		
3074,3069	96,6237		
3076,2356	96,5497		
3078,1642	96,5847		
3080,0929	96,6429		
3082,0216	96,5679		
3083,9503	96,5106		
3085,8789	96,5147		
3087,8076	96,466		
3089,7363	96,4402		
3091,665	96,4367		
3093,5936	96,3926		
3095,5223	96,3968		
3097,451	96,4226		
3099,3797	96,3651		
3101,3083	96,3208		
3103,237	96,3436		
3105,1657	96,3272		
3107,0944	96,2773		
3109,023	96,2556		
3110,9517	96,2685		

3112,8804	96,271		
3114,8091	96,2479		
3116,7377	96,2071		
3118,6664	96,0922		
3120,5951	96,0105		
3122,5238	96,0563		
3124,4524	96,087		
3126,3811	96,0681		
3128,3098	96,0419		
3130,2385	95,9715		
3132,1671	95,9308		
3134,0958	95,9636		
3136,0245	95,938		
3137,9532	95,8419		
3139,8818	95,7684		
3141,8105	95,7318		
3143,7392	95,7452		
3145,6678	95,7409		
3147,5965	95,639		
3149,5252	95,5727		
3151,4539	95,5897		
3153,3825	95,527		
3155,3112	95,4724		
3157,2399	95,5032		
3159,1686	95,4718		
3161,0972	95,4276		
3163,0259	95,3905		
3164,9546	95,27		
3166,8833	95,1381		
3168,8119	95,1237		

3170,7406	95,179		
3172,6693	95,1854		
3174,598	95,1415		
3176,5266	95,0593		
3178,4553	94,9799		
3180,384	94,959		
3182,3127	94,9256		
3184,2413	94,8448		
3186,17	94,7809		
3188,0987	94,7832		
3190,0274	94,7987		
3191,956	94,7485		
3193,8847	94,7208		
3195,8134	94,67		
3197,7421	94,5712		
3199,6707	94,5461		
3201,5994	94,4991		
3203,5281	94,3673		
3205,4568	94,2687		
3207,3854	94,3126		
3209,3141	94,2919		
3211,2428	94,0918		
3213,1715	94,0116		
3215,1001	94,0541		
3217,0288	94,0639		
3218,9575	94,0533		
3220,8861	93,9855		
3222,8148	93,9333		
3224,7435	93,9819		
3226,6722	94,0081		

3228,6008	93,9518		
3230,5295	93,8425		
3232,4582	93,753		
3234,3869	93,7328		
3236,3155	93,6505		
3238,2442	93,5976		
3240,1729	93,6275		
3242,1016	93,5863		
3244,0302	93,4921		
3245,9589	93,3849		
3247,8876	93,3195		
3249,8163	93,2937		
3251,7449	93,2028		
3253,6736	93,1318		
3255,6023	93,1884		
3257,531	93,1695		
3259,4596	93,0653		
3261,3883	92,997		
3263,317	92,9606		
3265,2457	92,9644		
3267,1743	92,9537		
3269,103	92,9353		
3271,0317	92,914		
3272,9604	92,8756		
3274,889	92,8859		
3276,8177	92,8863		
3278,7464	92,7974		
3280,6751	92,6964		
3282,6037	92,6362		
3284,5324	92,6486		

3286,4611	92,6989		
3288,3898	92,7525		
3290,3184	92,7702		
3292,2471	92,7529		
3294,1758	92,7131		
3296,1044	92,6367		
3298,0331	92,6142		
3299,9618	92,6		
3301,8905	92,5561		
3303,8191	92,5119		
3305,7478	92,4776		
3307,6765	92,5286		
3309,6052	92,5066		
3311,5338	92,3755		
3313,4625	92,3576		
3315,3912	92,3712		
3317,3199	92,3226		
3319,2485	92,3017		
3321,1772	92,3204		
3323,1059	92,2799		
3325,0346	92,1789		
3326,9632	92,1075		
3328,8919	92,1314		
3330,8206	92,2066		
3332,7493	92,2126		
3334,6779	92,1674		
3336,6066	92,1209		
3338,5353	92,0962		
3340,464	92,0704		
3342,3926	92,0074		

3344,3213	92,0024		
3346,25	92,0339		
3348,1787	91,9803		
3350,1073	91,9338		
3352,036	91,9545		
3353,9647	91,9886		
3355,8934	92,0014		
3357,822	91,9977		
3359,7507	91,9811		
3361,6794	91,9612		
3363,608	92,0162		
3365,5367	92,096		
3367,4654	92,0874		
3369,3941	92,0402		
3371,3227	92,0065		
3373,2514	91,9473		
3375,1801	91,897		
3377,1088	91,9463		
3379,0374	91,9348		
3380,9661	91,8432		
3382,8948	91,9194		
3384,8235	92,0372		
3386,7521	92,0031		
3388,6808	91,9577		
3390,6095	91,9967		
3392,5382	92,0356		
3394,4668	92,0109		
3396,3955	91,8969		
3398,3242	91,7903		
3400,2529	91,7984		

3402,1815	91,819		
3404,1102	91,8234		
3406,0389	91,9226		
3407,9676	91,9876		
3409,8962	91,9348		
3411,8249	91,9294		
3413,7536	91,9531		
3415,6823	91,929		
3417,6109	91,9137		
3419,5396	91,921		
3421,4683	91,9729		
3423,397	92,0669		
3425,3256	92,0929		
3427,2543	92,0566		
3429,183	92,0682		
3431,1117	92,0824		
3433,0403	92,1011		
3434,969	92,1898		
3436,8977	92,1994		
3438,8263	92,1681		
3440,755	92,1898		
3442,6837	92,1536		
3444,6124	92,1119		
3446,541	92,2182		
3448,4697	92,3802		
3450,3984	92,4195		
3452,3271	92,4786		
3454,2557	92,5663		
3456,1844	92,6019		
3458,1131	92,6834		

3460,0418	92,7861		
3461,9704	92,8323		
3463,8991	92,7862		
3465,8278	92,8086		
3467,7565	92,9708		
3469,6851	93,103		
3471,6138	93,1541		
3473,5425	93,1508		
3475,4712	93,1393		
3477,3998	93,1575		
3479,3285	93,2113		
3481,2572	93,2594		
3483,1859	93,2747		
3485,1145	93,2982		
3487,0432	93,3043		
3488,9719	93,3145		
3490,9006	93,4353		
3492,8292	93,5173		
3494,7579	93,5106		
3496,6866	93,5788		
3498,6153	93,6512		
3500,5439	93,7293		
3502,4726	93,8105		
3504,4013	93,7857		
3506,33	93,8218		
3508,2586	93,9169		
3510,1873	93,929		
3512,116	94,0132		
3514,0446	94,1263		
3515,9733	94,1496		

3517,902	94,209		
3519,8307	94,28		
3521,7593	94,3628		
3523,688	94,4621		
3525,6167	94,4902		
3527,5454	94,4752		
3529,474	94,5127		
3531,4027	94,6114		
3533,3314	94,7067		
3535,2601	94,8352		
3537,1887	94,9634		
3539,1174	95,0574		
3541,0461	95,1386		
3542,9748	95,1908		
3544,9034	95,2141		
3546,8321	95,3292		
3548,7608	95,4649		
3550,6895	95,5257		
3552,6181	95,6217		
3554,5468	95,6244		
3556,4755	95,6024		
3558,4042	95,7519		
3560,3328	95,8811		
3562,2615	95,9372		
3564,1902	96,0297		
3566,1189	96,0266		
3568,0475	96,0203		
3569,9762	96,1633		
3571,9049	96,2997		
3573,8336	96,3722		

3575,7622	96,3659		
3577,6909	96,3645		
3579,6196	96,4864		
3581,5482	96,6382		
3583,4769	96,6618		
3585,4056	96,6938		
3587,3343	96,8751		
3589,2629	96,9856		
3591,1916	97,0472		
3593,1203	97,0293		
3595,049	96,9319		
3596,9776	97,1132		
3598,9063	97,335		
3600,835	97,3077		
3602,7637	97,3097		
3604,6923	97,4365		
3606,621	97,4751		
3608,5497	97,4675		
3610,4784	97,5355		
3612,407	97,5989		
3614,3357	97,7529		
3616,2644	97,8775		
3618,1931	97,9454		
3620,1217	98,0083		
3622,0504	97,9693		
3623,9791	98,0065		
3625,9078	98,105		
3627,8364	98,2304		
3629,7651	98,4458		
3631,6938	98,4345		

3633,6225	98,3565		
3635,5511	98,3571		
3637,4798	98,4876		
3639,4085	98,6903		
3641,3372	98,794		
3643,2658	98,7836		
3645,1945	98,7135		
3647,1232	98,615		
3649,0519	98,6256		
3650,9805	98,8295		
3652,9092	98,9702		
3654,8379	99,0343		
3656,7665	99,0979		
3658,6952	99,1466		
3660,6239	99,1363		
3662,5526	99,092		
3664,4812	99,1753		
3666,4099	99,3609		
3668,3386	99,459		
3670,2673	99,3734		
3672,1959	99,3133		
3674,1246	99,3414		
3676,0533	99,3853		
3677,982	99,4289		
3679,9106	99,5202		
3681,8393	99,5656		
3683,768	99,5443		
3685,6967	99,5269		
3687,6253	99,3909		
3689,554	99,2607		

3691,4827	99,4132		
3693,4114	99,495		
3695,34	99,4736		
3697,2687	99,4754		
3699,1974	99,5163		
3701,1261	99,4779		
3703,0547	99,4525		
3704,9834	99,5656		
3706,9121	99,6147		
3708,8408	99,4971		
3710,7694	99,3734		
3712,6981	99,4678		
3714,6268	99,6655		
3716,5555	99,7772		
3718,4841	99,7507		
3720,4128	99,7377		
3722,3415	99,8037		
3724,2702	99,8261		
3726,1988	99,7828		
3728,1275	99,6299		
3730,0562	99,5088		
3731,9848	99,6463		
3733,9135	99,9799		
3735,8422	100,0184		
3737,7709	99,8539		
3739,6995	99,8236		
3741,6282	99,8319		
3743,5569	99,822		
3745,4856	99,7709		
3747,4142	99,716		

3749,3429	99,6012		
3751,2716	99,4271		
3753,2003	99,498		
3755,1289	99,6436		
3757,0576	99,7139		
3758,9863	99,753		
3760,915	99,8058		
3762,8436	99,8041		
3764,7723	99,7632		
3766,701	99,7659		
3768,6297	99,8606		
3770,5583	99,8837		
3772,487	99,7776		
3774,4157	99,7423		
3776,3444	99,7407		
3778,273	99,7065		
3780,2017	99,767		
3782,1304	99,8591		
3784,0591	99,8707		
3785,9877	99,8256		
3787,9164	99,7868		
3789,8451	99,8579		
3791,7738	99,9209		
3793,7024	99,8612		
3795,6311	99,844		
3797,5598	99,8441		
3799,4885	99,7879		
3801,4171	99,8015		
3803,3458	99,8459		
3805,2745	99,8427		

3807,2031	99,7623		
3809,1318	99,7308		
3811,0605	99,7957		
3812,9892	99,807		
3814,9178	99,6829		
3816,8465	99,5776		
3818,7752	99,6646		
3820,7039	99,7304		
3822,6325	99,7559		
3824,5612	99,8418		
3826,4899	99,9225		
3828,4186	99,9113		
3830,3472	99,8621		
3832,2759	99,8582		
3834,2046	99,8465		
3836,1333	99,7635		
3838,0619	99,6494		
3839,9906	99,6031		
3841,9193	99,6595		
3843,848	99,7725		
3845,7766	99,8697		
3847,7053	99,9124		
3849,634	99,9215		
3851,5627	99,8418		
3853,4913	99,6495		
3855,42	99,7006		
3857,3487	99,7588		
3859,2774	99,7243		
3861,206	99,7069		
3863,1347	99,7214		

3865,0634	99,6678		
3866,9921	99,6582		
3868,9207	99,6946		
3870,8494	99,6697		
3872,7781	99,6231		
3874,7067	99,7144		
3876,6354	99,8211		
3878,5641	99,7961		
3880,4928	99,7326		
3882,4214	99,7438		
3884,3501	99,7477		
3886,2788	99,7619		
3888,2075	99,8168		
3890,1361	99,7946		
3892,0648	99,778		
3893,9935	99,8297		
3895,9222	99,8215		
3897,8508	99,7467		
3899,7795	99,6413		
3901,7082	99,5845		
3903,6369	99,6412		
3905,5655	99,7956		
3907,4942	99,8622		
3909,4229	99,8518		
3911,3516	99,8243		
3913,2802	99,8022		
3915,2089	99,8006		
3917,1376	99,7419		
3919,0663	99,6803		
3920,9949	99,7601		

3922,9236	99,8178		
3924,8523	99,7084		
3926,781	99,6471		
3928,7096	99,7651		
3930,6383	99,848		
3932,567	99,76		
3934,4957	99,6738		
3936,4243	99,6793		
3938,353	99,7239		
3940,2817	99,692		
3942,2104	99,6594		
3944,139	99,7279		
3946,0677	99,7269		
3947,9964	99,6409		
3949,925	99,6193		
3951,8537	99,669		
3953,7824	99,6932		
3955,7111	99,6902		
3957,6397	99,7236		
3959,5684	99,7699		
3961,4971	99,7414		
3963,4258	99,7016		
3965,3544	99,7066		
3967,2831	99,6888		
3969,2118	99,6815		
3971,1405	99,65		
3973,0691	99,5866		
3974,9978	99,6572		
3976,9265	99,7472		
3978,8552	99,725		

3980,7838	99,7016		
3982,7125	99,6872		
3984,6412	99,6618		
3986,5699	99,6763		
3988,4985	99,7015		
3990,4272	99,6641		
3992,3559	99,6554		
3994,2846	99,7333		
3996,2132	99,7714		
3998,1419	99,7529		
4000,0706	99,7167		
Comment=			
