# Supplementary material for: Data on statistical experimental design to formulate amphotericin B-loaded Eudragit RL100 nanoparticles coated with hyaluronic acid for the treatment of vulvovaginal candidiasis
Source: Data Brief. 2020 Mar 5;29:105311. doi: 10.1016/j.dib.2020.105311 (PMC7082528; doi:10.1016/j.dib.2020.105311)
Supplement: Multimedia component 15 [file mmc15.pdf]

**/// Profile Data Ascii Dump (XRD)**	**/// Profile Data Ascii Dump (XRD)**
	//
**Data : I-EUD-Pure**	**Data : II-AMP-Pure**
File Name : I-EUD-Pure.RAW	File Name : II-AMP-Pure.RAW
# Profile Datafile	# Profile Datafile
comment = I-EUD-Pure	comment = II-AMP-Pure
date & time = 07-29-19 09:53:16	date & time = 07-29-19 08:29:25
# Measurement Condition	# Measurement Condition
X-ray tube	X-ray tube
target = Cu	target = Cu
voltage = 40.0 (kV)	voltage = 40.0 (kV)
current = 30.0 (mA)	current = 30.0 (mA)
Slits	Slits
divergence slit = 1.00000 (deg)	divergence slit = 1.00000 (deg)
scatter slit = 1.00000 (deg)	scatter slit = 1.00000 (deg)
receiving slit = 0.30000 (mm)	receiving slit = 0.30000 (mm)
Scanning	Scanning
drive axis = Theta-2Theta	drive axis = Theta-2Theta
scan range = 5.000 - 35.600	scan range = 5.000 - 40.000
scan mode = Continuous Scan	scan mode = Continuous Scan
scan speed = 1.0000 (deg/min)	scan speed = 1.0000 (deg/min)
sampling pitch = 0.0200 (deg)	sampling pitch = 0.0200 (deg)
preset time = 1.20 (sec)	preset time = 1.20 (sec)
# Data [Total No. = 1531]	# Data [Total No. = 1751]

<2Theta>	< I >
5.0000	2104
5.0200	2044
5.0400	2114
5.0600	2120
5.0800	1982
5.1000	2112
5.1200	2084
5.1400	2100
5.1600	2006
5.1800	2056
5.2000	2118
5.2200	2042
5.2400	2112
5.2600	2124
5.2800	2030
5.3000	1936
5.3200	1974
5.3400	2030
5.3600	2038
5.3800	1972
5.4000	1998
5.4200	1982
5.4400	1892
5.4600	2008
5.4800	1992
5.5000	1952
5.5200	1972
5.5400	1930
5.5600	1870

<2Theta>	< I >
5.0000	2082
5.0200	2202
5.0400	2122
5.0600	2156
5.0800	2180
5.1000	2104
5.1200	2184
5.1400	2216
5.1600	2088
5.1800	1970
5.2000	2120
5.2200	2132
5.2400	2112
5.2600	1942
5.2800	1930
5.3000	2020
5.3200	2028
5.3400	1988
5.3600	1916
5.3800	2024
5.4000	2050
5.4200	1968
5.4400	1944
5.4600	1910
5.4800	2024
5.5000	1966
5.5200	2018
5.5400	1800
5.5600	1820

5.5800	1880				5.5800	1914			
5.6000	1922				5.6000	2046			
5.6200	1930				5.6200	1876			
5.6400	1880				5.6400	1848			
5.6600	1956				5.6600	1844			
5.6800	1888				5.6800	1866			
5.7000	1860				5.7000	1932			
5.7200	1990				5.7200	1868			
5.7400	1888				5.7400	1868			
5.7600	1920				5.7600	1822			
5.7800	1902				5.7800	1964			
5.8000	1992				5.8000	1874			
5.8200	1930				5.8200	1826			
5.8400	1904				5.8400	1762			
5.8600	1896				5.8600	1734			
5.8800	1842				5.8800	1858			
5.9000	1878				5.9000	1814			
5.9200	1862				5.9200	1770			
5.9400	1944				5.9400	1740			
5.9600	1874				5.9600	1844			
5.9800	1856				5.9800	1832			
6.0000	1882				6.0000	1792			
6.0200	1964				6.0200	1702			
6.0400	1828				6.0400	1756			
6.0600	1866				6.0600	1852			
6.0800	1864				6.0800	1694			
6.1000	1844				6.1000	1716			
6.1200	1902				6.1200	1692			
6.1400	1814				6.1400	1688			
6.1600	1920				6.1600	1798			

6.1800	1840				6.1800	1672			
6.2000	1806				6.2000	1612			
6.2200	1918				6.2200	1646			
6.2400	1754				6.2400	1704			
6.2600	1860				6.2600	1758			
6.2800	1838				6.2800	1648			
6.3000	1896				6.3000	1716			
6.3200	1804				6.3200	1650			
6.3400	1762				6.3400	1692			
6.3600	1744				6.3600	1734			
6.3800	1856				6.3800	1592			
6.4000	1808				6.4000	1638			
6.4200	1836				6.4200	1690			
6.4400	1748				6.4400	1650			
6.4600	1792				6.4600	1730			
6.4800	1794				6.4800	1592			
6.5000	1806				6.5000	1572			
6.5200	1758				6.5200	1724			
6.5400	1818				6.5400	1600			
6.5600	1790				6.5600	1614			
6.5800	1818				6.5800	1556			
6.6000	1732				6.6000	1616			
6.6200	1708				6.6200	1606			
6.6400	1774				6.6400	1632			
6.6600	1800				6.6600	1658			
6.6800	1824				6.6800	1486			
6.7000	1708				6.7000	1652			
6.7200	1796				6.7200	1682			
6.7400	1778				6.7400	1522			
6.7600	1764				6.7600	1638			

6.7800	1798				6.7800	1568			
6.8000	1806				6.8000	1608			
6.8200	1762				6.8200	1614			
6.8400	1718				6.8400	1524			
6.8600	1756				6.8600	1510			
6.8800	1796				6.8800	1526			
6.9000	1708				6.9000	1628			
6.9200	1772				6.9200	1586			
6.9400	1694				6.9400	1452			
6.9600	1788				6.9600	1524			
6.9800	1814				6.9800	1562			
7.0000	1752				7.0000	1588			
7.0200	1778				7.0200	1574			
7.0400	1776				7.0400	1514			
7.0600	1774				7.0600	1514			
7.0800	1710				7.0800	1564			
7.1000	1688				7.1000	1542			
7.1200	1788				7.1200	1574			
7.1400	1754				7.1400	1470			
7.1600	1798				7.1600	1522			
7.1800	1704				7.1800	1530			
7.2000	1762				7.2000	1608			
7.2200	1836				7.2200	1522			
7.2400	1812				7.2400	1436			
7.2600	1692				7.2600	1544			
7.2800	1818				7.2800	1544			
7.3000	1646				7.3000	1592			
7.3200	1770				7.3200	1522			
7.3400	1776				7.3400	1492			
7.3600	1818				7.3600	1556			

7.3800	1810				7.3800	1542			
7.4000	1846				7.4000	1530			
7.4200	1770				7.4200	1498			
7.4400	1780				7.4400	1462			
7.4600	1808				7.4600	1552			
7.4800	1700				7.4800	1460			
7.5000	1750				7.5000	1518			
7.5200	1778				7.5200	1446			
7.5400	1800				7.5400	1506			
7.5600	1742				7.5600	1604			
7.5800	1774				7.5800	1496			
7.6000	1826				7.6000	1446			
7.6200	1732				7.6200	1456			
7.6400	1758				7.6400	1432			
7.6600	1728				7.6600	1482			
7.6800	1842				7.6800	1482			
7.7000	1728				7.7000	1440			
7.7200	1728				7.7200	1430			
7.7400	1796				7.7400	1498			
7.7600	1774				7.7600	1418			
7.7800	1874				7.7800	1438			
7.8000	1710				7.8000	1404			
7.8200	1878				7.8200	1452			
7.8400	1804				7.8400	1482			
7.8600	1796				7.8600	1482			
7.8800	1838				7.8800	1378			
7.9000	1786				7.9000	1358			
7.9200	1772				7.9200	1422			
7.9400	1748				7.9400	1462			
7.9600	1722				7.9600	1460			

7.9800 1852				7.9800 1438			
8.0000 1688				8.0000 1364			
8.0200 1740				8.0200 1462			
8.0400 1804				8.0400 1412			
8.0600 1824				8.0600 1418			
8.0800 1738				8.0800 1416			
8.1000 1754				8.1000 1420			
8.1200 1740				8.1200 1456			
8.1400 1784				8.1400 1426			
8.1600 1722				8.1600 1372			
8.1800 1838				8.1800 1340			
8.2000 1834				8.2000 1376			
8.2200 1698				8.2200 1424			
8.2400 1752				8.2400 1388			
8.2600 1798				8.2600 1322			
8.2800 1776				8.2800 1366			
8.3000 1796				8.3000 1318			
8.3200 1728				8.3200 1376			
8.3400 1782				8.3400 1374			
8.3600 1788				8.3600 1346			
8.3800 1836				8.3800 1332			
8.4000 1746				8.4000 1396			
8.4200 1838				8.4200 1412			
8.4400 1776				8.4400 1312			
8.4600 1854				8.4600 1248			
8.4800 1742				8.4800 1382			
8.5000 1800				8.5000 1448			
8.5200 1828				8.5200 1380			
8.5400 1808				8.5400 1320			
8.5600 1802				8.5600 1362			

8.5800	1772				8.5800	1310			
8.6000	1792				8.6000	1358			
8.6200	1810				8.6200	1342			
8.6400	1868				8.6400	1322			
8.6600	1830				8.6600	1330			
8.6800	1830				8.6800	1422			
8.7000	1764				8.7000	1380			
8.7200	1800				8.7200	1238			
8.7400	1790				8.7400	1316			
8.7600	1814				8.7600	1344			
8.7800	1768				8.7800	1374			
8.8000	1860				8.8000	1400			
8.8200	1930				8.8200	1296			
8.8400	1770				8.8400	1282			
8.8600	1798				8.8600	1342			
8.8800	1792				8.8800	1362			
8.9000	1862				8.9000	1352			
8.9200	1846				8.9200	1218			
8.9400	1828				8.9400	1330			
8.9600	1818				8.9600	1370			
8.9800	1774				8.9800	1332			
9.0000	1904				9.0000	1288			
9.0200	1814				9.0200	1326			
9.0400	1864				9.0400	1352			
9.0600	1816				9.0600	1330			
9.0800	1930				9.0800	1354			
9.1000	1928				9.1000	1304			
9.1200	1858				9.1200	1240			
9.1400	1806				9.1400	1330			
9.1600	1816				9.1600	1376			

9.1800	1916				9.1800	1334			
9.2000	1894				9.2000	1298			
9.2200	1834				9.2200	1284			
9.2400	1916				9.2400	1328			
9.2600	1874				9.2600	1356			
9.2800	1962				9.2800	1264			
9.3000	1910				9.3000	1306			
9.3200	1972				9.3200	1348			
9.3400	1822				9.3400	1304			
9.3600	1922				9.3600	1270			
9.3800	1946				9.3800	1282			
9.4000	1944				9.4000	1272			
9.4200	1800				9.4200	1362			
9.4400	1902				9.4400	1294			
9.4600	1950				9.4600	1312			
9.4800	1962				9.4800	1202			
9.5000	1980				9.5000	1284			
9.5200	1864				9.5200	1320			
9.5400	1904				9.5400	1350			
9.5600	1990				9.5600	1318			
9.5800	1950				9.5800	1286			
9.6000	1982				9.6000	1284			
9.6200	1970				9.6200	1322			
9.6400	1930				9.6400	1352			
9.6600	1970				9.6600	1258			
9.6800	1970				9.6800	1250			
9.7000	2006				9.7000	1326			
9.7200	1960				9.7200	1342			
9.7400	1958				9.7400	1268			
9.7600	1876				9.7600	1258			

9.7800 1964				9.7800 1286			
9.8000 2040				9.8000 1306			
9.8200 1926				9.8200 1292			
9.8400 2050				9.8400 1310			
9.8600 2028				9.8600 1302			
9.8800 2110				9.8800 1238			
9.9000 1918				9.9000 1402			
9.9200 2048				9.9200 1254			
9.9400 2020				9.9400 1166			
9.9600 2114				9.9600 1272			
9.9800 2024				9.9800 1298			
10.0000 2080				10.0000 1360			
10.0200 2010				10.0200 1290			
10.0400 2116				10.0400 1216			
10.0600 2036				10.0600 1268			
10.0800 1960				10.0800 1266			
10.1000 2026				10.1000 1256			
10.1200 2106				10.1200 1244			
10.1400 2114				10.1400 1214			
10.1600 2080				10.1600 1306			
10.1800 1978				10.1800 1272			
10.2000 2028				10.2000 1256			
10.2200 2138				10.2200 1188			
10.2400 2134				10.2400 1228			
10.2600 2098				10.2600 1288			
10.2800 2042				10.2800 1302			
10.3000 2166				10.3000 1266			
10.3200 2164				10.3200 1228			
10.3400 2046				10.3400 1204			
10.3600 2124				10.3600 1270			

10.3800	2042				10.3800	1322			
10.4000	2178				10.4000	1324			
10.4200	2168				10.4200	1190			
10.4400	2130				10.4400	1226			
10.4600	2154				10.4600	1298			
10.4800	2044				10.4800	1290			
10.5000	2054				10.5000	1240			
10.5200	2130				10.5200	1304			
10.5400	2174				10.5400	1312			
10.5600	2106				10.5600	1314			
10.5800	2188				10.5800	1286			
10.6000	2272				10.6000	1278			
10.6200	2222				10.6200	1248			
10.6400	2120				10.6400	1284			
10.6600	2186				10.6600	1350			
10.6800	2168				10.6800	1260			
10.7000	2256				10.7000	1232			
10.7200	2244				10.7200	1226			
10.7400	2216				10.7400	1286			
10.7600	2150				10.7600	1344			
10.7800	2198				10.7800	1290			
10.8000	2268				10.8000	1256			
10.8200	2266				10.8200	1310			
10.8400	2100				10.8400	1338			
10.8600	2200				10.8600	1270			
10.8800	2330				10.8800	1236			
10.9000	2188				10.9000	1338			
10.9200	2216				10.9200	1302			
10.9400	2134				10.9400	1238			
10.9600	2266				10.9600	1264			

10.9800	2298				10.9800	1264			
11.0000	2300				11.0000	1294			
11.0200	2226				11.0200	1326			
11.0400	2306				11.0400	1352			
11.0600	2336				11.0600	1256			
11.0800	2392				11.0800	1300			
11.1000	2280				11.1000	1312			
11.1200	2328				11.1200	1256			
11.1400	2364				11.1400	1272			
11.1600	2318				11.1600	1234			
11.1800	2250				11.1800	1346			
11.2000	2272				11.2000	1378			
11.2200	2250				11.2200	1290			
11.2400	2348				11.2400	1364			
11.2600	2278				11.2600	1258			
11.2800	2354				11.2800	1282			
11.3000	2274				11.3000	1350			
11.3200	2296				11.3200	1320			
11.3400	2364				11.3400	1300			
11.3600	2318				11.3600	1288			
11.3800	2292				11.3800	1312			
11.4000	2308				11.4000	1338			
11.4200	2378				11.4200	1282			
11.4400	2334				11.4400	1258			
11.4600	2382				11.4600	1294			
11.4800	2398				11.4800	1312			
11.5000	2310				11.5000	1378			
11.5200	2368				11.5200	1292			
11.5400	2410				11.5400	1308			
11.5600	2456				11.5600	1284			

11.5800	2304				11.5800	1306			
11.6000	2392				11.6000	1330			
11.6200	2412				11.6200	1328			
11.6400	2418				11.6400	1316			
11.6600	2442				11.6600	1382			
11.6800	2374				11.6800	1370			
11.7000	2454				11.7000	1338			
11.7200	2444				11.7200	1300			
11.7400	2500				11.7400	1306			
11.7600	2438				11.7600	1326			
11.7800	2408				11.7800	1412			
11.8000	2512				11.8000	1376			
11.8200	2488				11.8200	1320			
11.8400	2482				11.8400	1258			
11.8600	2476				11.8600	1430			
11.8800	2482				11.8800	1340			
11.9000	2556				11.9000	1352			
11.9200	2532				11.9200	1278			
11.9400	2624				11.9400	1434			
11.9600	2440				11.9600	1348			
11.9800	2598				11.9800	1348			
12.0000	2514				12.0000	1314			
12.0200	2460				12.0200	1352			
12.0400	2516				12.0400	1342			
12.0600	2422				12.0600	1440			
12.0800	2494				12.0800	1336			
12.1000	2564				12.1000	1374			
12.1200	2562				12.1200	1336			
12.1400	2542				12.1400	1408			
12.1600	2426				12.1600	1468			

12.1800	2616				12.1800	1326			
12.2000	2622				12.2000	1370			
12.2200	2622				12.2200	1340			
12.2400	2606				12.2400	1336			
12.2600	2618				12.2600	1408			
12.2800	2564				12.2800	1386			
12.3000	2546				12.3000	1324			
12.3200	2506				12.3200	1412			
12.3400	2652				12.3400	1404			
12.3600	2572				12.3600	1388			
12.3800	2570				12.3800	1314			
12.4000	2516				12.4000	1358			
12.4200	2578				12.4200	1384			
12.4400	2598				12.4400	1332			
12.4600	2488				12.4600	1372			
12.4800	2596				12.4800	1440			
12.5000	2618				12.5000	1420			
12.5200	2490				12.5200	1428			
12.5400	2536				12.5400	1390			
12.5600	2580				12.5600	1374			
12.5800	2552				12.5800	1484			
12.6000	2650				12.6000	1446			
12.6200	2638				12.6200	1434			
12.6400	2602				12.6400	1406			
12.6600	2708				12.6600	1402			
12.6800	2588				12.6800	1418			
12.7000	2608				12.7000	1430			
12.7200	2650				12.7200	1392			
12.7400	2640				12.7400	1476			
12.7600	2666				12.7600	1398			

12.7800	2592				12.7800	1406			
12.8000	2578				12.8000	1406			
12.8200	2666				12.8200	1438			
12.8400	2588				12.8400	1422			
12.8600	2758				12.8600	1444			
12.8800	2606				12.8800	1492			
12.9000	2602				12.9000	1492			
12.9200	2646				12.9200	1464			
12.9400	2660				12.9400	1528			
12.9600	2614				12.9600	1392			
12.9800	2592				12.9800	1394			
13.0000	2708				13.0000	1502			
13.0200	2662				13.0200	1412			
13.0400	2602				13.0400	1366			
13.0600	2634				13.0600	1428			
13.0800	2546				13.0800	1494			
13.1000	2662				13.1000	1378			
13.1200	2714				13.1200	1446			
13.1400	2656				13.1400	1448			
13.1600	2646				13.1600	1524			
13.1800	2604				13.1800	1414			
13.2000	2686				13.2000	1404			
13.2200	2718				13.2200	1486			
13.2400	2682				13.2400	1426			
13.2600	2702				13.2600	1480			
13.2800	2576				13.2800	1502			
13.3000	2636				13.3000	1446			
13.3200	2680				13.3200	1384			
13.3400	2728				13.3400	1496			
13.3600	2544				13.3600	1538			

13.3800	2612				13.3800	1452			
13.4000	2614				13.4000	1432			
13.4200	2656				13.4200	1446			
13.4400	2634				13.4400	1446			
13.4600	2648				13.4600	1528			
13.4800	2600				13.4800	1498			
13.5000	2672				13.5000	1490			
13.5200	2694				13.5200	1492			
13.5400	2518				13.5400	1508			
13.5600	2620				13.5600	1514			
13.5800	2686				13.5800	1540			
13.6000	2664				13.6000	1498			
13.6200	2652				13.6200	1486			
13.6400	2614				13.6400	1538			
13.6600	2724				13.6600	1528			
13.6800	2662				13.6800	1382			
13.7000	2560				13.7000	1516			
13.7200	2624				13.7200	1612			
13.7400	2674				13.7400	1542			
13.7600	2714				13.7600	1510			
13.7800	2672				13.7800	1524			
13.8000	2680				13.8000	1454			
13.8200	2744				13.8200	1548			
13.8400	2560				13.8400	1588			
13.8600	2726				13.8600	1502			
13.8800	2730				13.8800	1526			
13.9000	2650				13.9000	1644			
13.9200	2684				13.9200	1638			
13.9400	2626				13.9400	1478			
13.9600	2626				13.9600	1608			

13.9800	2706				13.9800	1514			
14.0000	2620				14.0000	1544			
14.0200	2628				14.0200	1524			
14.0400	2614				14.0400	1516			
14.0600	2564				14.0600	1552			
14.0800	2674				14.0800	1554			
14.1000	2648				14.1000	1634			
14.1200	2636				14.1200	1550			
14.1400	2704				14.1400	1526			
14.1600	2664				14.1600	1460			
14.1800	2694				14.1800	1554			
14.2000	2698				14.2000	1552			
14.2200	2632				14.2200	1536			
14.2400	2590				14.2400	1482			
14.2600	2604				14.2600	1576			
14.2800	2628				14.2800	1514			
14.3000	2558				14.3000	1552			
14.3200	2648				14.3200	1606			
14.3400	2510				14.3400	1502			
14.3600	2622				14.3600	1570			
14.3800	2630				14.3800	1542			
14.4000	2610				14.4000	1584			
14.4200	2646				14.4200	1544			
14.4400	2684				14.4400	1560			
14.4600	2670				14.4600	1514			
14.4800	2670				14.4800	1618			
14.5000	2490				14.5000	1626			
14.5200	2578				14.5200	1616			
14.5400	2662				14.5400	1488			
14.5600	2638				14.5600	1626			

14.5800	2484				14.5800	1580			
14.6000	2628				14.6000	1604			
14.6200	2670				14.6200	1494			
14.6400	2602				14.6400	1602			
14.6600	2594				14.6600	1552			
14.6800	2536				14.6800	1510			
14.7000	2582				14.7000	1546			
14.7200	2566				14.7200	1554			
14.7400	2628				14.7400	1482			
14.7600	2552				14.7600	1600			
14.7800	2610				14.7800	1574			
14.8000	2652				14.8000	1608			
14.8200	2658				14.8200	1594			
14.8400	2684				14.8400	1532			
14.8600	2486				14.8600	1624			
14.8800	2612				14.8800	1516			
14.9000	2708				14.9000	1526			
14.9200	2564				14.9200	1472			
14.9400	2532				14.9400	1562			
14.9600	2614				14.9600	1578			
14.9800	2594				14.9800	1582			
15.0000	2612				15.0000	1464			
15.0200	2524				15.0200	1546			
15.0400	2580				15.0400	1518			
15.0600	2572				15.0600	1532			
15.0800	2690				15.0800	1580			
15.1000	2664				15.1000	1512			
15.1200	2602				15.1200	1524			
15.1400	2632				15.1400	1572			
15.1600	2554				15.1600	1510			

15.1800	2666				15.1800	1474			
15.2000	2632				15.2000	1506			
15.2200	2458				15.2200	1542			
15.2400	2546				15.2400	1502			
15.2600	2576				15.2600	1532			
15.2800	2538				15.2800	1552			
15.3000	2662				15.3000	1536			
15.3200	2494				15.3200	1546			
15.3400	2504				15.3400	1448			
15.3600	2566				15.3600	1524			
15.3800	2540				15.3800	1536			
15.4000	2564				15.4000	1522			
15.4200	2526				15.4200	1546			
15.4400	2572				15.4400	1558			
15.4600	2562				15.4600	1458			
15.4800	2528				15.4800	1566			
15.5000	2554				15.5000	1512			
15.5200	2494				15.5200	1484			
15.5400	2632				15.5400	1524			
15.5600	2604				15.5600	1390			
15.5800	2510				15.5800	1512			
15.6000	2456				15.6000	1596			
15.6200	2630				15.6200	1520			
15.6400	2522				15.6400	1496			
15.6600	2570				15.6600	1504			
15.6800	2486				15.6800	1436			
15.7000	2552				15.7000	1464			
15.7200	2482				15.7200	1508			
15.7400	2564				15.7400	1494			
15.7600	2564				15.7600	1478			

15.7800	2430				15.7800	1522			
15.8000	2510				15.8000	1514			
15.8200	2498				15.8200	1502			
15.8400	2488				15.8400	1446			
15.8600	2526				15.8600	1448			
15.8800	2444				15.8800	1526			
15.9000	2474				15.9000	1540			
15.9200	2510				15.9200	1452			
15.9400	2558				15.9400	1530			
15.9600	2546				15.9600	1458			
15.9800	2420				15.9800	1456			
16.0000	2494				16.0000	1464			
16.0200	2544				16.0200	1390			
16.0400	2448				16.0400	1504			
16.0600	2442				16.0600	1458			
16.0800	2380				16.0800	1456			
16.1000	2508				16.1000	1480			
16.1200	2464				16.1200	1364			
16.1400	2480				16.1400	1508			
16.1600	2506				16.1600	1436			
16.1800	2422				16.1800	1430			
16.2000	2454				16.2000	1432			
16.2200	2544				16.2200	1394			
16.2400	2414				16.2400	1444			
16.2600	2438				16.2600	1438			
16.2800	2396				16.2800	1342			
16.3000	2456				16.3000	1396			
16.3200	2484				16.3200	1426			
16.3400	2378				16.3400	1458			
16.3600	2458				16.3600	1406			

16.3800	2500				16.3800	1448			
16.4000	2430				16.4000	1336			
16.4200	2470				16.4200	1372			
16.4400	2382				16.4400	1368			
16.4600	2492				16.4600	1388			
16.4800	2578				16.4800	1442			
16.5000	2472				16.5000	1416			
16.5200	2404				16.5200	1440			
16.5400	2338				16.5400	1380			
16.5600	2404				16.5600	1480			
16.5800	2470				16.5800	1334			
16.6000	2358				16.6000	1330			
16.6200	2386				16.6200	1342			
16.6400	2432				16.6400	1370			
16.6600	2494				16.6600	1366			
16.6800	2444				16.6800	1330			
16.7000	2382				16.7000	1408			
16.7200	2446				16.7200	1392			
16.7400	2384				16.7400	1414			
16.7600	2356				16.7600	1388			
16.7800	2420				16.7800	1358			
16.8000	2388				16.8000	1424			
16.8200	2322				16.8200	1386			
16.8400	2480				16.8400	1368			
16.8600	2450				16.8600	1374			
16.8800	2466				16.8800	1330			
16.9000	2380				16.9000	1334			
16.9200	2376				16.9200	1370			
16.9400	2396				16.9400	1340			
16.9600	2416				16.9600	1358			

16.9800	2410				16.9800	1366			
17.0000	2426				17.0000	1382			
17.0200	2368				17.0200	1342			
17.0400	2414				17.0400	1362			
17.0600	2372				17.0600	1332			
17.0800	2352				17.0800	1312			
17.1000	2376				17.1000	1372			
17.1200	2434				17.1200	1292			
17.1400	2514				17.1400	1352			
17.1600	2416				17.1600	1368			
17.1800	2434				17.1800	1254			
17.2000	2310				17.2000	1360			
17.2200	2386				17.2200	1362			
17.2400	2324				17.2400	1296			
17.2600	2310				17.2600	1322			
17.2800	2414				17.2800	1280			
17.3000	2290				17.3000	1306			
17.3200	2304				17.3200	1272			
17.3400	2358				17.3400	1266			
17.3600	2352				17.3600	1268			
17.3800	2292				17.3800	1296			
17.4000	2222				17.4000	1302			
17.4200	2328				17.4200	1302			
17.4400	2430				17.4400	1348			
17.4600	2266				17.4600	1314			
17.4800	2376				17.4800	1194			
17.5000	2304				17.5000	1280			
17.5200	2440				17.5200	1330			
17.5400	2444				17.5400	1266			
17.5600	2342				17.5600	1304			

17.5800	2300			17.5800	1332		
17.6000	2354			17.6000	1316		
17.6200	2372			17.6200	1286		
17.6400	2306			17.6400	1308		
17.6600	2344			17.6600	1318		
17.6800	2372			17.6800	1276		
17.7000	2420			17.7000	1258		
17.7200	2412			17.7200	1318		
17.7400	2416			17.7400	1290		
17.7600	2280			17.7600	1308		
17.7800	2312			17.7800	1256		
17.8000	2280			17.8000	1264		
17.8200	2354			17.8200	1268		
17.8400	2376			17.8400	1282		
17.8600	2294			17.8600	1294		
17.8800	2336			17.8800	1330		
17.9000	2428			17.9000	1362		
17.9200	2294			17.9200	1248		
17.9400	2352			17.9400	1232		
17.9600	2298			17.9600	1266		
17.9800	2226			17.9800	1286		
18.0000	2386			18.0000	1250		
18.0200	2266			18.0200	1270		
18.0400	2264			18.0400	1258		
18.0600	2310			18.0600	1206		
18.0800	2246			18.0800	1272		
18.1000	2410			18.1000	1312		
18.1200	2280			18.1200	1306		
18.1400	2292			18.1400	1276		
18.1600	2232			18.1600	1280		

18.1800	2418				18.1800	1210			
18.2000	2384				18.2000	1224			
18.2200	2236				18.2200	1226			
18.2400	2296				18.2400	1286			
18.2600	2266				18.2600	1272			
18.2800	2362				18.2800	1250			
18.3000	2218				18.3000	1250			
18.3200	2276				18.3200	1302			
18.3400	2298				18.3400	1238			
18.3600	2318				18.3600	1180			
18.3800	2332				18.3800	1246			
18.4000	2250				18.4000	1254			
18.4200	2228				18.4200	1240			
18.4400	2254				18.4400	1244			
18.4600	2206				18.4600	1268			
18.4800	2284				18.4800	1272			
18.5000	2356				18.5000	1230			
18.5200	2178				18.5200	1292			
18.5400	2222				18.5400	1278			
18.5600	2254				18.5600	1232			
18.5800	2220				18.5800	1256			
18.6000	2396				18.6000	1216			
18.6200	2194				18.6200	1296			
18.6400	2216				18.6400	1198			
18.6600	2238				18.6600	1220			
18.6800	2140				18.6800	1236			
18.7000	2338				18.7000	1226			
18.7200	2288				18.7200	1266			
18.7400	2232				18.7400	1202			
18.7600	2232				18.7600	1190			

18.7800	2262			18.7800	1238		
18.8000	2218			18.8000	1318		
18.8200	2174			18.8200	1238		
18.8400	2266			18.8400	1252		
18.8600	2320			18.8600	1212		
18.8800	2188			18.8800	1268		
18.9000	2244			18.9000	1224		
18.9200	2214			18.9200	1186		
18.9400	2186			18.9400	1192		
18.9600	2224			18.9600	1276		
18.9800	2184			18.9800	1270		
19.0000	2224			19.0000	1218		
19.0200	2240			19.0200	1234		
19.0400	2248			19.0400	1254		
19.0600	2208			19.0600	1252		
19.0800	2298			19.0800	1244		
19.1000	2162			19.1000	1282		
19.1200	2270			19.1200	1252		
19.1400	2230			19.1400	1276		
19.1600	2194			19.1600	1204		
19.1800	2156			19.1800	1288		
19.2000	2164			19.2000	1272		
19.2200	2170			19.2200	1198		
19.2400	2148			19.2400	1246		
19.2600	2138			19.2600	1196		
19.2800	2174			19.2800	1224		
19.3000	2144			19.3000	1210		
19.3200	2142			19.3200	1258		
19.3400	2138			19.3400	1248		
19.3600	2234			19.3600	1238		

19.3800	2164				19.3800	1218			
19.4000	2234				19.4000	1262			
19.4200	2176				19.4200	1198			
19.4400	2218				19.4400	1234			
19.4600	2158				19.4600	1220			
19.4800	2246				19.4800	1176			
19.5000	2126				19.5000	1232			
19.5200	2128				19.5200	1252			
19.5400	2134				19.5400	1284			
19.5600	2176				19.5600	1166			
19.5800	2192				19.5800	1202			
19.6000	2188				19.6000	1226			
19.6200	2108				19.6200	1254			
19.6400	2130				19.6400	1288			
19.6600	2232				19.6600	1270			
19.6800	2146				19.6800	1212			
19.7000	2088				19.7000	1234			
19.7200	2106				19.7200	1288			
19.7400	2164				19.7400	1174			
19.7600	2212				19.7600	1216			
19.7800	2178				19.7800	1228			
19.8000	2152				19.8000	1292			
19.8200	2092				19.8200	1266			
19.8400	2198				19.8400	1270			
19.8600	2178				19.8600	1318			
19.8800	2154				19.8800	1176			
19.9000	2044				19.9000	1338			
19.9200	2192				19.9200	1300			
19.9400	2128				19.9400	1192			
19.9600	2080				19.9600	1256			

19.9800	2202				19.9800	1244			
20.0000	2058				20.0000	1278			
20.0200	2154				20.0200	1232			
20.0400	2156				20.0400	1206			
20.0600	2202				20.0600	1208			
20.0800	2164				20.0800	1196			
20.1000	2072				20.1000	1212			
20.1200	2170				20.1200	1264			
20.1400	2076				20.1400	1186			
20.1600	2062				20.1600	1248			
20.1800	2088				20.1800	1252			
20.2000	2024				20.2000	1188			
20.2200	2122				20.2200	1258			
20.2400	2142				20.2400	1230			
20.2600	2036				20.2600	1230			
20.2800	2160				20.2800	1236			
20.3000	2058				20.3000	1182			
20.3200	2022				20.3200	1260			
20.3400	2098				20.3400	1270			
20.3600	2050				20.3600	1282			
20.3800	2066				20.3800	1278			
20.4000	2178				20.4000	1232			
20.4200	2078				20.4200	1278			
20.4400	2142				20.4400	1304			
20.4600	2124				20.4600	1306			
20.4800	2006				20.4800	1240			
20.5000	2062				20.5000	1256			
20.5200	2064				20.5200	1266			
20.5400	2048				20.5400	1188			
20.5600	1972				20.5600	1264			

20.5800	2044				20.5800	1302			
20.6000	2040				20.6000	1246			
20.6200	2052				20.6200	1290			
20.6400	2144				20.6400	1202			
20.6600	2014				20.6600	1240			
20.6800	2108				20.6800	1286			
20.7000	2116				20.7000	1206			
20.7200	2058				20.7200	1270			
20.7400	2130				20.7400	1242			
20.7600	1974				20.7600	1264			
20.7800	1954				20.7800	1244			
20.8000	2098				20.8000	1214			
20.8200	2056				20.8200	1288			
20.8400	1998				20.8400	1184			
20.8600	1958				20.8600	1264			
20.8800	1938				20.8800	1298			
20.9000	1964				20.9000	1240			
20.9200	2024				20.9200	1250			
20.9400	2000				20.9400	1330			
20.9600	2062				20.9600	1244			
20.9800	1940				20.9800	1268			
21.0000	1990				21.0000	1240			
21.0200	1962				21.0200	1386			
21.0400	2000				21.0400	1268			
21.0600	1992				21.0600	1298			
21.0800	1996				21.0800	1260			
21.1000	2006				21.1000	1210			
21.1200	1950				21.1200	1296			
21.1400	2074				21.1400	1286			
21.1600	2062				21.1600	1270			

21.1800	1954			21.1800	1280		
21.2000	2010			21.2000	1244		
21.2200	1946			21.2200	1296		
21.2400	1982			21.2400	1212		
21.2600	2038			21.2600	1200		
21.2800	1988			21.2800	1262		
21.3000	1994			21.3000	1272		
21.3200	1878			21.3200	1274		
21.3400	1964			21.3400	1268		
21.3600	2036			21.3600	1184		
21.3800	1974			21.3800	1218		
21.4000	2002			21.4000	1218		
21.4200	1958			21.4200	1224		
21.4400	1968			21.4400	1236		
21.4600	1992			21.4600	1232		
21.4800	1926			21.4800	1320		
21.5000	1888			21.5000	1284		
21.5200	1932			21.5200	1200		
21.5400	2024			21.5400	1168		
21.5600	1904			21.5600	1210		
21.5800	1932			21.5800	1206		
21.6000	2016			21.6000	1286		
21.6200	1956			21.6200	1318		
21.6400	1930			21.6400	1254		
21.6600	1910			21.6600	1176		
21.6800	1936			21.6800	1278		
21.7000	1880			21.7000	1274		
21.7200	1890			21.7200	1266		
21.7400	1858			21.7400	1240		
21.7600	1906			21.7600	1222		

21.7800 1820				21.7800 1216			
21.8000 1892				21.8000 1256			
21.8200 1856				21.8200 1206			
21.8400 1902				21.8400 1264			
21.8600 1946				21.8600 1260			
21.8800 1876				21.8800 1222			
21.9000 1884				21.9000 1230			
21.9200 1892				21.9200 1226			
21.9400 1950				21.9400 1334			
21.9600 1914				21.9600 1242			
21.9800 1824				21.9800 1200			
22.0000 1872				22.0000 1246			
22.0200 1928				22.0200 1162			
22.0400 1772				22.0400 1236			
22.0600 1878				22.0600 1202			
22.0800 1860				22.0800 1194			
22.1000 1866				22.1000 1298			
22.1200 1824				22.1200 1160			
22.1400 1796				22.1400 1270			
22.1600 1862				22.1600 1254			
22.1800 1842				22.1800 1260			
22.2000 1948				22.2000 1182			
22.2200 1820				22.2200 1160			
22.2400 1740				22.2400 1258			
22.2600 1818				22.2600 1312			
22.2800 1812				22.2800 1206			
22.3000 1746				22.3000 1224			
22.3200 1788				22.3200 1184			
22.3400 1792				22.3400 1184			
22.3600 1804				22.3600 1190			

22.3800	1828				22.3800	1228			
22.4000	1822				22.4000	1244			
22.4200	1914				22.4200	1152			
22.4400	1800				22.4400	1274			
22.4600	1842				22.4600	1264			
22.4800	1846				22.4800	1140			
22.5000	1842				22.5000	1156			
22.5200	1894				22.5200	1096			
22.5400	1742				22.5400	1156			
22.5600	1762				22.5600	1156			
22.5800	1840				22.5800	1116			
22.6000	1774				22.6000	1180			
22.6200	1852				22.6200	1118			
22.6400	1676				22.6400	1164			
22.6600	1856				22.6600	1120			
22.6800	1824				22.6800	1142			
22.7000	1776				22.7000	1102			
22.7200	1750				22.7200	1158			
22.7400	1800				22.7400	1116			
22.7600	1672				22.7600	1114			
22.7800	1824				22.7800	1170			
22.8000	1758				22.8000	1148			
22.8200	1764				22.8200	1120			
22.8400	1732				22.8400	1122			
22.8600	1814				22.8600	1104			
22.8800	1714				22.8800	1034			
22.9000	1810				22.9000	1150			
22.9200	1788				22.9200	1134			
22.9400	1736				22.9400	1164			
22.9600	1702				22.9600	1086			

22.9800	1790				22.9800	1088			
23.0000	1740				23.0000	1100			
23.0200	1710				23.0200	1084			
23.0400	1744				23.0400	1034			
23.0600	1770				23.0600	1084			
23.0800	1768				23.0800	1036			
23.1000	1774				23.1000	1108			
23.1200	1782				23.1200	1058			
23.1400	1752				23.1400	1036			
23.1600	1794				23.1600	1082			
23.1800	1812				23.1800	1048			
23.2000	1656				23.2000	1038			
23.2200	1706				23.2200	1056			
23.2400	1836				23.2400	1036			
23.2600	1684				23.2600	1062			
23.2800	1802				23.2800	1050			
23.3000	1746				23.3000	1028			
23.3200	1762				23.3200	1014			
23.3400	1762				23.3400	1080			
23.3600	1686				23.3600	1070			
23.3800	1692				23.3800	1060			
23.4000	1708				23.4000	982			
23.4200	1778				23.4200	1030			
23.4400	1750				23.4400	976			
23.4600	1710				23.4600	1002			
23.4800	1756				23.4800	1012			
23.5000	1708				23.5000	996			
23.5200	1734				23.5200	1024			
23.5400	1706				23.5400	956			
23.5600	1746				23.5600	992			

23.5800	1730				23.5800	964			
23.6000	1720				23.6000	1018			
23.6200	1706				23.6200	1048			
23.6400	1734				23.6400	1014			
23.6600	1720				23.6600	1044			
23.6800	1710				23.6800	964			
23.7000	1762				23.7000	966			
23.7200	1726				23.7200	1026			
23.7400	1738				23.7400	962			
23.7600	1616				23.7600	1002			
23.7800	1710				23.7800	992			
23.8000	1702				23.8000	980			
23.8200	1670				23.8200	984			
23.8400	1744				23.8400	976			
23.8600	1662				23.8600	982			
23.8800	1638				23.8800	990			
23.9000	1744				23.9000	958			
23.9200	1638				23.9200	960			
23.9400	1662				23.9400	992			
23.9600	1660				23.9600	984			
23.9800	1670				23.9800	972			
24.0000	1738				24.0000	940			
24.0200	1652				24.0200	928			
24.0400	1692				24.0400	952			
24.0600	1656				24.0600	938			
24.0800	1686				24.0800	928			
24.1000	1714				24.1000	1004			
24.1200	1576				24.1200	890			
24.1400	1742				24.1400	970			
24.1600	1690				24.1600	914			

24.1800	1580				24.1800	982			
24.2000	1684				24.2000	918			
24.2200	1656				24.2200	926			
24.2400	1640				24.2400	990			
24.2600	1658				24.2600	888			
24.2800	1682				24.2800	924			
24.3000	1688				24.3000	946			
24.3200	1588				24.3200	906			
24.3400	1654				24.3400	910			
24.3600	1600				24.3600	900			
24.3800	1626				24.3800	922			
24.4000	1660				24.4000	910			
24.4200	1642				24.4200	920			
24.4400	1648				24.4400	922			
24.4600	1714				24.4600	894			
24.4800	1616				24.4800	898			
24.5000	1612				24.5000	888			
24.5200	1646				24.5200	908			
24.5400	1638				24.5400	906			
24.5600	1672				24.5600	890			
24.5800	1624				24.5800	874			
24.6000	1636				24.6000	866			
24.6200	1664				24.6200	890			
24.6400	1652				24.6400	878			
24.6600	1636				24.6600	864			
24.6800	1616				24.6800	852			
24.7000	1684				24.7000	878			
24.7200	1646				24.7200	886			
24.7400	1588				24.7400	892			
24.7600	1660				24.7600	860			

24.7800	1636				24.7800	834			
24.8000	1696				24.8000	898			
24.8200	1550				24.8200	874			
24.8400	1620				24.8400	850			
24.8600	1640				24.8600	874			
24.8800	1566				24.8800	906			
24.9000	1630				24.9000	870			
24.9200	1610				24.9200	832			
24.9400	1652				24.9400	906			
24.9600	1586				24.9600	850			
24.9800	1624				24.9800	842			
25.0000	1702				25.0000	874			
25.0200	1642				25.0200	902			
25.0400	1582				25.0400	868			
25.0600	1666				25.0600	820			
25.0800	1610				25.0800	846			
25.1000	1694				25.1000	874			
25.1200	1596				25.1200	836			
25.1400	1572				25.1400	856			
25.1600	1600				25.1600	818			
25.1800	1590				25.1800	810			
25.2000	1640				25.2000	850			
25.2200	1676				25.2200	802			
25.2400	1548				25.2400	882			
25.2600	1664				25.2600	844			
25.2800	1644				25.2800	834			
25.3000	1586				25.3000	814			
25.3200	1622				25.3200	798			
25.3400	1612				25.3400	806			
25.3600	1642				25.3600	796			

25.3800	1658				25.3800	862			
25.4000	1598				25.4000	834			
25.4200	1674				25.4200	768			
25.4400	1546				25.4400	776			
25.4600	1568				25.4600	804			
25.4800	1638				25.4800	830			
25.5000	1584				25.5000	824			
25.5200	1606				25.5200	794			
25.5400	1568				25.5400	788			
25.5600	1618				25.5600	804			
25.5800	1658				25.5800	806			
25.6000	1634				25.6000	796			
25.6200	1634				25.6200	832			
25.6400	1616				25.6400	830			
25.6600	1640				25.6600	810			
25.6800	1652				25.6800	830			
25.7000	1522				25.7000	814			
25.7200	1574				25.7200	784			
25.7400	1648				25.7400	832			
25.7600	1598				25.7600	780			
25.7800	1730				25.7800	798			
25.8000	1504				25.8000	794			
25.8200	1616				25.8200	728			
25.8400	1660				25.8400	802			
25.8600	1634				25.8600	788			
25.8800	1648				25.8800	818			
25.9000	1576				25.9000	842			
25.9200	1588				25.9200	790			
25.9400	1576				25.9400	742			
25.9600	1606				25.9600	786			

25.9800	1654			25.9800	788		
26.0000	1572			26.0000	736		
26.0200	1626			26.0200	806		
26.0400	1626			26.0400	724		
26.0600	1618			26.0600	810		
26.0800	1610			26.0800	784		
26.1000	1580			26.1000	778		
26.1200	1626			26.1200	786		
26.1400	1710			26.1400	730		
26.1600	1584			26.1600	778		
26.1800	1554			26.1800	788		
26.2000	1608			26.2000	798		
26.2200	1626			26.2200	762		
26.2400	1646			26.2400	714		
26.2600	1582			26.2600	762		
26.2800	1574			26.2800	740		
26.3000	1610			26.3000	770		
26.3200	1628			26.3200	760		
26.3400	1674			26.3400	764		
26.3600	1514			26.3600	770		
26.3800	1624			26.3800	730		
26.4000	1636			26.4000	718		
26.4200	1614			26.4200	760		
26.4400	1608			26.4400	774		
26.4600	1512			26.4600	812		
26.4800	1628			26.4800	750		
26.5000	1646			26.5000	746		
26.5200	1612			26.5200	714		
26.5400	1632			26.5400	746		
26.5600	1554			26.5600	764		

26.5800	1648				26.5800	758			
26.6000	1628				26.6000	718			
26.6200	1626				26.6200	740			
26.6400	1696				26.6400	772			
26.6600	1574				26.6600	758			
26.6800	1620				26.6800	756			
26.7000	1570				26.7000	694			
26.7200	1648				26.7200	738			
26.7400	1618				26.7400	732			
26.7600	1576				26.7600	720			
26.7800	1582				26.7800	782			
26.8000	1678				26.8000	716			
26.8200	1552				26.8200	716			
26.8400	1584				26.8400	722			
26.8600	1582				26.8600	760			
26.8800	1626				26.8800	702			
26.9000	1634				26.9000	738			
26.9200	1518				26.9200	712			
26.9400	1660				26.9400	780			
26.9600	1606				26.9600	690			
26.9800	1624				26.9800	774			
27.0000	1638				27.0000	684			
27.0200	1598				27.0200	724			
27.0400	1596				27.0400	772			
27.0600	1702				27.0600	762			
27.0800	1636				27.0800	732			
27.1000	1660				27.1000	690			
27.1200	1560				27.1200	682			
27.1400	1618				27.1400	710			
27.1600	1548				27.1600	744			

27.1800	1606				27.1800	644			
27.2000	1642				27.2000	658			
27.2200	1608				27.2200	688			
27.2400	1636				27.2400	740			
27.2600	1694				27.2600	680			
27.2800	1638				27.2800	674			
27.3000	1598				27.3000	760			
27.3200	1544				27.3200	750			
27.3400	1658				27.3400	732			
27.3600	1696				27.3600	680			
27.3800	1640				27.3800	744			
27.4000	1636				27.4000	714			
27.4200	1620				27.4200	664			
27.4400	1660				27.4400	708			
27.4600	1716				27.4600	684			
27.4800	1586				27.4800	696			
27.5000	1662				27.5000	716			
27.5200	1606				27.5200	684			
27.5400	1628				27.5400	678			
27.5600	1692				27.5600	678			
27.5800	1608				27.5800	660			
27.6000	1574				27.6000	686			
27.6200	1586				27.6200	668			
27.6400	1642				27.6400	704			
27.6600	1716				27.6600	680			
27.6800	1644				27.6800	710			
27.7000	1658				27.7000	670			
27.7200	1746				27.7200	648			
27.7400	1568				27.7400	696			
27.7600	1650				27.7600	704			

27.7800	1622				27.7800	646			
27.8000	1652				27.8000	712			
27.8200	1706				27.8200	672			
27.8400	1660				27.8400	658			
27.8600	1662				27.8600	716			
27.8800	1620				27.8800	684			
27.9000	1632				27.9000	722			
27.9200	1690				27.9200	654			
27.9400	1616				27.9400	700			
27.9600	1668				27.9600	710			
27.9800	1634				27.9800	738			
28.0000	1646				28.0000	670			
28.0200	1660				28.0200	668			
28.0400	1594				28.0400	642			
28.0600	1626				28.0600	624			
28.0800	1642				28.0800	688			
28.1000	1646				28.1000	620			
28.1200	1664				28.1200	672			
28.1400	1572				28.1400	680			
28.1600	1710				28.1600	636			
28.1800	1642				28.1800	668			
28.2000	1626				28.2000	658			
28.2200	1704				28.2200	644			
28.2400	1560				28.2400	690			
28.2600	1610				28.2600	664			
28.2800	1660				28.2800	656			
28.3000	1584				28.3000	662			
28.3200	1634				28.3200	694			
28.3400	1520				28.3400	648			
28.3600	1642				28.3600	676			

28.3800	1668				28.3800	626			
28.4000	1638				28.4000	680			
28.4200	1644				28.4200	624			
28.4400	1662				28.4400	664			
28.4600	1624				28.4600	676			
28.4800	1640				28.4800	604			
28.5000	1650				28.5000	626			
28.5200	1558				28.5200	648			
28.5400	1586				28.5400	642			
28.5600	1658				28.5600	680			
28.5800	1658				28.5800	670			
28.6000	1624				28.6000	686			
28.6200	1610				28.6200	658			
28.6400	1634				28.6400	614			
28.6600	1664				28.6600	678			
28.6800	1686				28.6800	664			
28.7000	1622				28.7000	680			
28.7200	1714				28.7200	664			
28.7400	1678				28.7400	660			
28.7600	1612				28.7600	644			
28.7800	1680				28.7800	618			
28.8000	1576				28.8000	656			
28.8200	1664				28.8200	648			
28.8400	1616				28.8400	634			
28.8600	1624				28.8600	658			
28.8800	1710				28.8800	610			
28.9000	1658				28.9000	622			
28.9200	1642				28.9200	642			
28.9400	1652				28.9400	652			
28.9600	1586				28.9600	690			

28.9800	1684				28.9800	678			
29.0000	1560				29.0000	672			
29.0200	1728				29.0200	616			
29.0400	1690				29.0400	638			
29.0600	1626				29.0600	678			
29.0800	1670				29.0800	652			
29.1000	1632				29.1000	630			
29.1200	1670				29.1200	594			
29.1400	1746				29.1400	620			
29.1600	1580				29.1600	614			
29.1800	1656				29.1800	634			
29.2000	1602				29.2000	612			
29.2200	1718				29.2200	662			
29.2400	1666				29.2400	586			
29.2600	1594				29.2600	596			
29.2800	1686				29.2800	584			
29.3000	1664				29.3000	602			
29.3200	1700				29.3200	648			
29.3400	1744				29.3400	686			
29.3600	1646				29.3600	608			
29.3800	1672				29.3800	612			
29.4000	1626				29.4000	616			
29.4200	1726				29.4200	642			
29.4400	1628				29.4400	562			
29.4600	1598				29.4600	672			
29.4800	1700				29.4800	596			
29.5000	1588				29.5000	594			
29.5200	1604				29.5200	634			
29.5400	1622				29.5400	632			
29.5600	1642				29.5600	610			

29.5800	1682				29.5800	642			
29.6000	1722				29.6000	596			
29.6200	1594				29.6200	638			
29.6400	1726				29.6400	610			
29.6600	1576				29.6600	630			
29.6800	1682				29.6800	628			
29.7000	1666				29.7000	630			
29.7200	1596				29.7200	626			
29.7400	1670				29.7400	576			
29.7600	1674				29.7600	650			
29.7800	1556				29.7800	638			
29.8000	1668				29.8000	562			
29.8200	1610				29.8200	596			
29.8400	1696				29.8400	572			
29.8600	1604				29.8600	632			
29.8800	1564				29.8800	628			
29.9000	1682				29.9000	572			
29.9200	1648				29.9200	602			
29.9400	1652				29.9400	614			
29.9600	1694				29.9600	562			
29.9800	1572				29.9800	578			
30.0000	1656				30.0000	600			
30.0200	1516				30.0200	652			
30.0400	1662				30.0400	606			
30.0600	1686				30.0600	608			
30.0800	1636				30.0800	652			
30.1000	1608				30.1000	672			
30.1200	1552				30.1200	588			
30.1400	1636				30.1400	626			
30.1600	1654				30.1600	610			

30.1800	1570				30.1800	636			
30.2000	1636				30.2000	586			
30.2200	1638				30.2200	624			
30.2400	1672				30.2400	608			
30.2600	1634				30.2600	570			
30.2800	1608				30.2800	624			
30.3000	1656				30.3000	570			
30.3200	1648				30.3200	588			
30.3400	1574				30.3400	572			
30.3600	1746				30.3600	570			
30.3800	1560				30.3800	558			
30.4000	1724				30.4000	610			
30.4200	1686				30.4200	594			
30.4400	1656				30.4400	578			
30.4600	1672				30.4600	574			
30.4800	1552				30.4800	582			
30.5000	1618				30.5000	594			
30.5200	1652				30.5200	596			
30.5400	1678				30.5400	604			
30.5600	1698				30.5600	588			
30.5800	1592				30.5800	588			
30.6000	1612				30.6000	614			
30.6200	1596				30.6200	582			
30.6400	1568				30.6400	618			
30.6600	1612				30.6600	576			
30.6800	1540				30.6800	624			
30.7000	1624				30.7000	632			
30.7200	1584				30.7200	612			
30.7400	1618				30.7400	560			
30.7600	1600				30.7600	564			

30.7800	1576				30.7800	594			
30.8000	1572				30.8000	604			
30.8200	1696				30.8200	634			
30.8400	1630				30.8400	608			
30.8600	1548				30.8600	538			
30.8800	1642				30.8800	574			
30.9000	1600				30.9000	600			
30.9200	1626				30.9200	598			
30.9400	1558				30.9400	580			
30.9600	1602				30.9600	588			
30.9800	1580				30.9800	550			
31.0000	1608				31.0000	554			
31.0200	1692				31.0200	658			
31.0400	1508				31.0400	550			
31.0600	1648				31.0600	574			
31.0800	1594				31.0800	604			
31.1000	1616				31.1000	576			
31.1200	1668				31.1200	566			
31.1400	1504				31.1400	576			
31.1600	1600				31.1600	532			
31.1800	1614				31.1800	600			
31.2000	1570				31.2000	582			
31.2200	1574				31.2200	622			
31.2400	1580				31.2400	608			
31.2600	1644				31.2600	584			
31.2800	1596				31.2800	536			
31.3000	1526				31.3000	582			
31.3200	1608				31.3200	552			
31.3400	1532				31.3400	546			
31.3600	1544				31.3600	586			

31.3800	1708				31.3800	594			
31.4000	1502				31.4000	598			
31.4200	1640				31.4200	574			
31.4400	1580				31.4400	582			
31.4600	1664				31.4600	546			
31.4800	1624				31.4800	584			
31.5000	1576				31.5000	578			
31.5200	1610				31.5200	596			
31.5400	1550				31.5400	528			
31.5600	1542				31.5600	558			
31.5800	1550				31.5800	582			
31.6000	1472				31.6000	560			
31.6200	1594				31.6200	552			
31.6400	1542				31.6400	590			
31.6600	1468				31.6600	548			
31.6800	1644				31.6800	566			
31.7000	1508				31.7000	560			
31.7200	1544				31.7200	588			
31.7400	1612				31.7400	544			
31.7600	1530				31.7600	550			
31.7800	1612				31.7800	546			
31.8000	1552				31.8000	584			
31.8200	1542				31.8200	584			
31.8400	1624				31.8400	578			
31.8600	1462				31.8600	500			
31.8800	1552				31.8800	544			
31.9000	1498				31.9000	550			
31.9200	1550				31.9200	560			
31.9400	1612				31.9400	566			
31.9600	1584				31.9600	578			

31.9800	1628				31.9800	576			
32.0000	1538				32.0000	580			
32.0200	1476				32.0200	602			
32.0400	1526				32.0400	546			
32.0600	1520				32.0600	546			
32.0800	1576				32.0800	554			
32.1000	1480				32.1000	570			
32.1200	1504				32.1200	552			
32.1400	1580				32.1400	570			
32.1600	1510				32.1600	590			
32.1800	1598				32.1800	570			
32.2000	1474				32.2000	564			
32.2200	1594				32.2200	556			
32.2400	1580				32.2400	580			
32.2600	1534				32.2600	546			
32.2800	1558				32.2800	558			
32.3000	1578				32.3000	536			
32.3200	1510				32.3200	548			
32.3400	1530				32.3400	564			
32.3600	1492				32.3600	512			
32.3800	1588				32.3800	566			
32.4000	1602				32.4000	508			
32.4200	1536				32.4200	564			
32.4400	1500				32.4400	538			
32.4600	1484				32.4600	570			
32.4800	1520				32.4800	546			
32.5000	1606				32.5000	494			
32.5200	1504				32.5200	616			
32.5400	1504				32.5400	558			
32.5600	1520				32.5600	594			

32.5800	1548				32.5800	542			
32.6000	1524				32.6000	574			
32.6200	1492				32.6200	592			
32.6400	1524				32.6400	566			
32.6600	1472				32.6600	544			
32.6800	1486				32.6800	536			
32.7000	1508				32.7000	526			
32.7200	1426				32.7200	574			
32.7400	1452				32.7400	566			
32.7600	1480				32.7600	528			
32.7800	1496				32.7800	536			
32.8000	1534				32.8000	582			
32.8200	1436				32.8200	548			
32.8400	1466				32.8400	566			
32.8600	1504				32.8600	542			
32.8800	1516				32.8800	540			
32.9000	1522				32.9000	530			
32.9200	1532				32.9200	542			
32.9400	1526				32.9400	602			
32.9600	1604				32.9600	516			
32.9800	1470				32.9800	548			
33.0000	1542				33.0000	520			
33.0200	1412				33.0200	580			
33.0400	1476				33.0400	526			
33.0600	1456				33.0600	558			
33.0800	1440				33.0800	520			
33.1000	1424				33.1000	598			
33.1200	1438				33.1200	558			
33.1400	1544				33.1400	520			
33.1600	1560				33.1600	546			

33.1800	1430				33.1800	562			
33.2000	1566				33.2000	542			
33.2200	1460				33.2200	556			
33.2400	1460				33.2400	544			
33.2600	1506				33.2600	510			
33.2800	1328				33.2800	522			
33.3000	1504				33.3000	544			
33.3200	1528				33.3200	528			
33.3400	1456				33.3400	554			
33.3600	1532				33.3600	516			
33.3800	1476				33.3800	560			
33.4000	1502				33.4000	562			
33.4200	1474				33.4200	512			
33.4400	1466				33.4400	502			
33.4600	1464				33.4600	540			
33.4800	1368				33.4800	544			
33.5000	1448				33.5000	530			
33.5200	1512				33.5200	582			
33.5400	1458				33.5400	572			
33.5600	1520				33.5600	554			
33.5800	1474				33.5800	492			
33.6000	1470				33.6000	546			
33.6200	1460				33.6200	548			
33.6400	1450				33.6400	510			
33.6600	1422				33.6600	584			
33.6800	1384				33.6800	524			
33.7000	1490				33.7000	534			
33.7200	1424				33.7200	584			
33.7400	1360				33.7400	564			
33.7600	1386				33.7600	494			

33.7800	1392				33.7800	530			
33.8000	1412				33.8000	546			
33.8200	1420				33.8200	536			
33.8400	1340				33.8400	518			
33.8600	1412				33.8600	534			
33.8800	1452				33.8800	554			
33.9000	1446				33.9000	534			
33.9200	1474				33.9200	526			
33.9400	1428				33.9400	516			
33.9600	1478				33.9600	520			
33.9800	1434				33.9800	514			
34.0000	1404				34.0000	598			
34.0200	1460				34.0200	588			
34.0400	1462				34.0400	482			
34.0600	1464				34.0600	522			
34.0800	1430				34.0800	546			
34.1000	1430				34.1000	520			
34.1200	1440				34.1200	528			
34.1400	1364				34.1400	542			
34.1600	1416				34.1600	498			
34.1800	1462				34.1800	530			
34.2000	1372				34.2000	534			
34.2200	1452				34.2200	514			
34.2400	1392				34.2400	516			
34.2600	1414				34.2600	508			
34.2800	1464				34.2800	526			
34.3000	1350				34.3000	502			
34.3200	1468				34.3200	524			
34.3400	1444				34.3400	538			
34.3600	1402				34.3600	532			

34.3800	1424				34.3800	558			
34.4000	1416				34.4000	560			
34.4200	1406				34.4200	526			
34.4400	1360				34.4400	512			
34.4600	1380				34.4600	532			
34.4800	1378				34.4800	522			
34.5000	1386				34.5000	542			
34.5200	1394				34.5200	542			
34.5400	1424				34.5400	546			
34.5600	1378				34.5600	522			
34.5800	1430				34.5800	506			
34.6000	1294				34.6000	568			
34.6200	1354				34.6200	512			
34.6400	1408				34.6400	500			
34.6600	1354				34.6600	540			
34.6800	1426				34.6800	474			
34.7000	1310				34.7000	510			
34.7200	1384				34.7200	502			
34.7400	1426				34.7400	524			
34.7600	1326				34.7600	570			
34.7800	1416				34.7800	532			
34.8000	1370				34.8000	544			
34.8200	1414				34.8200	544			
34.8400	1468				34.8400	504			
34.8600	1426				34.8600	496			
34.8800	1438				34.8800	508			
34.9000	1448				34.9000	504			
34.9200	1426				34.9200	560			
34.9400	1402				34.9400	522			
34.9600	1354				34.9600	576			

34.9800	1378				34.9800	514			
35.0000	1352				35.0000	508			
35.0200	1414				35.0200	556			
35.0400	1410				35.0400	498			
35.0600	1396				35.0600	504			
35.0800	1318				35.0800	512			
35.1000	1300				35.1000	540			
35.1200	1368				35.1200	524			
35.1400	1424				35.1400	538			
35.1600	1300				35.1600	502			
35.1800	1340				35.1800	518			
35.2000	1356				35.2000	548			
35.2200	1340				35.2200	564			
35.2400	1388				35.2400	482			
35.2600	1312				35.2600	528			
35.2800	1386				35.2800	542			
35.3000	1360				35.3000	544			
35.3200	1340				35.3200	578			
35.3400	1402				35.3400	494			
35.3600	1310				35.3600	550			
35.3800	1350				35.3800	522			
35.4000	1456				35.4000	518			
35.4200	1310				35.4200	526			
35.4400	1420				35.4400	540			
35.4600	1298				35.4600	510			
35.4800	1372				35.4800	528			
35.5000	1444				35.5000	544			
35.5200	1322				35.5200	508			
35.5400	1408				35.5400	504			
35.5600	1312				35.5600	520			

35.5800	1344				35.5800	504			
35.6000	1380				35.6000	554			
					35.6200	512			
					35.6400	516			
					35.6600	548			
					35.6800	536			
					35.7000	542			
					35.7200	516			
					35.7400	530			
					35.7600	510			
					35.7800	494			
					35.8000	540			
					35.8200	526			
					35.8400	512			
					35.8600	536			
					35.8800	518			
					35.9000	558			
					35.9200	522			
					35.9400	504			
					35.9600	506			
					35.9800	510			
					36.0000	476			
					36.0200	544			
					36.0400	552			
					36.0600	576			
					36.0800	506			
					36.1000	552			
					36.1200	558			
					36.1400	544			
					36.1600	534			

				36.1800	532			
				36.2000	540			
				36.2200	524			
				36.2400	516			
				36.2600	572			
				36.2800	544			
				36.3000	572			
				36.3200	524			
				36.3400	556			
				36.3600	518			
				36.3800	570			
				36.4000	530			
				36.4200	522			
				36.4400	528			
				36.4600	478			
				36.4800	536			
				36.5000	508			
				36.5200	526			
				36.5400	512			
				36.5600	572			
				36.5800	528			
				36.6000	574			
				36.6200	480			
				36.6400	526			
				36.6600	494			
				36.6800	564			
				36.7000	550			
				36.7200	520			
				36.7400	548			
				36.7600	554			

				36.7800 520			
				36.8000 530			
				36.8200 506			
				36.8400 538			
				36.8600 504			
				36.8800 544			
				36.9000 494			
				36.9200 508			
				36.9400 488			
				36.9600 540			
				36.9800 512			
				37.0000 570			
				37.0200 512			
				37.0400 486			
				37.0600 524			
				37.0800 588			
				37.1000 590			
				37.1200 476			
				37.1400 526			
				37.1600 524			
				37.1800 514			
				37.2000 558			
				37.2200 556			
				37.2400 550			
				37.2600 524			
				37.2800 554			
				37.3000 576			
				37.3200 542			
				37.3400 542			
				37.3600 550			

				37.3800 558			
				37.4000 550			
				37.4200 584			
				37.4400 594			
				37.4600 626			
				37.4800 616			
				37.5000 688			
				37.5200 612			
				37.5400 696			
				37.5600 690			
				37.5800 720			
				37.6000 804			
				37.6200 728			
				37.6400 840			
				37.6600 836			
				37.6800 822			
				37.7000 1048			
				37.7200 978			
				37.7400 1096			
				37.7600 1124			
				37.7800 1040			
				37.8000 1342			
				37.8200 1154			
				37.8400 1374			
				37.8600 1492			
				37.8800 1394			
				37.9000 1734			
				37.9200 1664			
				37.9400 1990			
				37.9600 2206			

				37.9800 2130			
				38.0000 2762			
				38.0200 2500			
				38.0400 3018			
				38.0600 3484			
				38.0800 3038			
				38.1000 3970			
				38.1200 3736			
				38.1400 4044			
				38.1600 4318			
				38.1800 3786			
				38.2000 4596			
				38.2200 4144			
				38.2400 3986			
				38.2600 4306			
				38.2800 3568			
				38.3000 3756			
				38.3200 3306			
				38.3400 3064			
				38.3600 2988			
				38.3800 2436			
				38.4000 2444			
				38.4200 2112			
				38.4400 1804			
				38.4600 1638			
				38.4800 1452			
				38.5000 1292			
				38.5200 1114			
				38.5400 1022			
				38.5600 1002			

				38.5800 904			
				38.6000 814			
				38.6200 798			
				38.6400 714			
				38.6600 716			
				38.6800 674			
				38.7000 668			
				38.7200 670			
				38.7400 614			
				38.7600 572			
				38.7800 624			
				38.8000 612			
				38.8200 612			
				38.8400 544			
				38.8600 562			
				38.8800 532			
				38.9000 556			
				38.9200 554			
				38.9400 554			
				38.9600 538			
				38.9800 528			
				39.0000 498			
				39.0200 570			
				39.0400 514			
				39.0600 536			
				39.0800 528			
				39.1000 554			
				39.1200 532			
				39.1400 540			
				39.1600 482			

				39.1800 516			
				39.2000 500			
				39.2200 562			
				39.2400 548			
				39.2600 486			
				39.2800 492			
				39.3000 482			
				39.3200 530			
				39.3400 564			
				39.3600 492			
				39.3800 502			
				39.4000 538			
				39.4200 516			
				39.4400 506			
				39.4600 480			
				39.4800 490			
				39.5000 508			
				39.5200 452			
				39.5400 530			
				39.5600 536			
				39.5800 464			
				39.6000 536			
				39.6200 498			
				39.6400 526			
				39.6600 468			
				39.6800 504			
				39.7000 500			
				39.7200 522			
				39.7400 486			
				39.7600 526			

				39.7800 484			
				39.8000 530			
				39.8200 470			
				39.8400 500			
				39.8600 548			
				39.8800 496			
				39.9000 558			
				39.9200 546			
				39.9400 514			
				39.9600 506			
				39.9800 488			
				40.0000 540			
