# Supplementary material for: Data on statistical experimental design to formulate amphotericin B-loaded Eudragit RL100 nanoparticles coated with hyaluronic acid for the treatment of vulvovaginal candidiasis
Source: Data Brief. 2020 Mar 5;29:105311. doi: 10.1016/j.dib.2020.105311 (PMC7082528; doi:10.1016/j.dib.2020.105311)
Supplement: Multimedia component 16 [file mmc16.pdf]

**/// Profile Data Ascii Dump (XRD)**				**/// Profile Data Ascii Dump (XRD)**			
///				///			
**Data : III-HA-Pure**				**Data : IV-EUD-Nanoparticles**			
File Name : III-HA-Pure.RAW				File Name : IV-EUD-Nanoparticles.RAW			
# Profile Datafile				# Profile Datafile			
comment = III-HA-Pure				comment = IV-EUD-Nanoparticle			
date & time = 07-29-19 07:58:34				date & time = 07-29-19 10:27:05			
# Measurement Condition				# Measurement Condition			
X-ray tube				X-ray tube			
target = Cu				target = Cu			
voltage = 40.0 (kV)				voltage = 40.0 (kV)			
current = 30.0 (mA)				current = 30.0 (mA)			
Slits				Slits			
divergence slit = 1.00000 (deg)				divergence slit = 1.00000 (deg)			
scatter slit = 1.00000 (deg)				scatter slit = 1.00000 (deg)			
receiving slit = 0.30000 (mm)				receiving slit = 0.30000 (mm)			
Scanning				Scanning			
drive axis = Theta-2Theta				drive axis = Theta-2Theta			
scan range = 5.000 - 39.460				scan range = 5.000 - 40.000			
scan mode = Continuous Scan				scan mode = Continuous Scan			
scan speed = 1.0000 (deg/min)				scan speed = 1.0000 (deg/min)			
sampling pitch = 0.0200 (deg)				sampling pitch = 0.0200 (deg)			
preset time = 1.20 (sec)				preset time = 1.20 (sec)			
# Data [Total No. = 1724]				# Data [Total No. = 1751]			

<2Theta>	< I >	<2Theta>	< I >
5.0000	2118	5.0000	2274
5.0200	2138	5.0200	2370
5.0400	2112	5.0400	2272
5.0600	2146	5.0600	2254
5.0800	2154	5.0800	2226
5.1000	2088	5.1000	2250
5.1200	1932	5.1200	2252
5.1400	2040	5.1400	2188
5.1600	2136	5.1600	2132
5.1800	2134	5.1800	2168
5.2000	2022	5.2000	2246
5.2200	2132	5.2200	2174
5.2400	2106	5.2400	2120
5.2600	2208	5.2600	2104
5.2800	1976	5.2800	2282
5.3000	1986	5.3000	2160
5.3200	2116	5.3200	2078
5.3400	2082	5.3400	2098
5.3600	2050	5.3600	2060
5.3800	2002	5.3800	2216
5.4000	1936	5.4000	2098
5.4200	2110	5.4200	1952
5.4400	2020	5.4400	2000
5.4600	1892	5.4600	2062
5.4800	1912	5.4800	2118
5.5000	1930	5.5000	2036
5.5200	2042	5.5200	1970
5.5400	2000	5.5400	1914
5.5600	1898	5.5600	2062

5.5800	1874				5.5800	2076			
5.6000	1972				5.6000	2080			
5.6200	1914				5.6200	1976			
5.6400	2004				5.6400	2088			
5.6600	1944				5.6600	2050			
5.6800	1882				5.6800	2026			
5.7000	1964				5.7000	1968			
5.7200	1908				5.7200	1900			
5.7400	1918				5.7400	1864			
5.7600	1828				5.7600	2010			
5.7800	1898				5.7800	1942			
5.8000	1872				5.8000	1946			
5.8200	1942				5.8200	1884			
5.8400	1938				5.8400	1890			
5.8600	1864				5.8600	2006			
5.8800	1810				5.8800	1970			
5.9000	1916				5.9000	1968			
5.9200	1890				5.9200	1982			
5.9400	1904				5.9400	1916			
5.9600	1910				5.9600	1876			
5.9800	1890				5.9800	1882			
6.0000	1896				6.0000	1834			
6.0200	1864				6.0200	1868			
6.0400	1730				6.0400	1976			
6.0600	1836				6.0600	1956			
6.0800	1794				6.0800	1832			
6.1000	1846				6.1000	1906			
6.1200	1846				6.1200	1938			
6.1400	1772				6.1400	1914			
6.1600	1758				6.1600	1924			

6.1800	1814				6.1800	1864			
6.2000	1896				6.2000	1806			
6.2200	1834				6.2200	1984			
6.2400	1816				6.2400	1854			
6.2600	1824				6.2600	1950			
6.2800	1922				6.2800	1806			
6.3000	1840				6.3000	1740			
6.3200	1858				6.3200	1942			
6.3400	1750				6.3400	1894			
6.3600	1850				6.3600	1808			
6.3800	1790				6.3800	1752			
6.4000	1770				6.4000	1736			
6.4200	1748				6.4200	1872			
6.4400	1774				6.4400	1864			
6.4600	1816				6.4600	1722			
6.4800	1824				6.4800	1728			
6.5000	1754				6.5000	1822			
6.5200	1766				6.5200	1830			
6.5400	1872				6.5400	1854			
6.5600	1720				6.5600	1768			
6.5800	1748				6.5800	1838			
6.6000	1708				6.6000	1792			
6.6200	1692				6.6200	1886			
6.6400	1774				6.6400	1760			
6.6600	1818				6.6600	1762			
6.6800	1766				6.6800	1732			
6.7000	1786				6.7000	1816			
6.7200	1766				6.7200	1768			
6.7400	1812				6.7400	1704			
6.7600	1820				6.7600	1692			

6.7800	1752				6.7800	1730			
6.8000	1754				6.8000	1738			
6.8200	1748				6.8200	1738			
6.8400	1786				6.8400	1650			
6.8600	1720				6.8600	1692			
6.8800	1716				6.8800	1728			
6.9000	1834				6.9000	1768			
6.9200	1762				6.9200	1670			
6.9400	1810				6.9400	1666			
6.9600	1734				6.9600	1716			
6.9800	1706				6.9800	1760			
7.0000	1754				7.0000	1698			
7.0200	1816				7.0200	1738			
7.0400	1804				7.0400	1702			
7.0600	1758				7.0600	1692			
7.0800	1718				7.0800	1676			
7.1000	1758				7.1000	1624			
7.1200	1764				7.1200	1686			
7.1400	1712				7.1400	1616			
7.1600	1738				7.1600	1796			
7.1800	1702				7.1800	1694			
7.2000	1760				7.2000	1586			
7.2200	1810				7.2200	1576			
7.2400	1846				7.2400	1614			
7.2600	1796				7.2600	1660			
7.2800	1730				7.2800	1706			
7.3000	1768				7.3000	1614			
7.3200	1918				7.3200	1502			
7.3400	1774				7.3400	1746			
7.3600	1724				7.3600	1650			

7.3800	1612				7.3800	1716			
7.4000	1762				7.4000	1632			
7.4200	1816				7.4200	1662			
7.4400	1724				7.4400	1748			
7.4600	1696				7.4600	1644			
7.4800	1718				7.4800	1580			
7.5000	1802				7.5000	1574			
7.5200	1802				7.5200	1612			
7.5400	1690				7.5400	1680			
7.5600	1724				7.5600	1690			
7.5800	1792				7.5800	1688			
7.6000	1774				7.6000	1550			
7.6200	1706				7.6200	1642			
7.6400	1696				7.6400	1646			
7.6600	1766				7.6600	1658			
7.6800	1738				7.6800	1582			
7.7000	1736				7.7000	1568			
7.7200	1804				7.7200	1692			
7.7400	1672				7.7400	1566			
7.7600	1802				7.7600	1560			
7.7800	1746				7.7800	1598			
7.8000	1764				7.8000	1482			
7.8200	1744				7.8200	1622			
7.8400	1702				7.8400	1596			
7.8600	1832				7.8600	1538			
7.8800	1698				7.8800	1552			
7.9000	1700				7.9000	1580			
7.9200	1708				7.9200	1570			
7.9400	1662				7.9400	1586			
7.9600	1688				7.9600	1564			

7.9800	1756				7.9800	1506			
8.0000	1724				8.0000	1590			
8.0200	1724				8.0200	1604			
8.0400	1766				8.0400	1540			
8.0600	1846				8.0600	1518			
8.0800	1708				8.0800	1548			
8.1000	1662				8.1000	1568			
8.1200	1692				8.1200	1610			
8.1400	1768				8.1400	1444			
8.1600	1806				8.1600	1444			
8.1800	1712				8.1800	1546			
8.2000	1648				8.2000	1562			
8.2200	1714				8.2200	1538			
8.2400	1774				8.2400	1544			
8.2600	1718				8.2600	1468			
8.2800	1620				8.2800	1550			
8.3000	1688				8.3000	1500			
8.3200	1740				8.3200	1500			
8.3400	1786				8.3400	1442			
8.3600	1724				8.3600	1490			
8.3800	1708				8.3800	1552			
8.4000	1610				8.4000	1464			
8.4200	1794				8.4200	1502			
8.4400	1704				8.4400	1468			
8.4600	1752				8.4600	1502			
8.4800	1692				8.4800	1534			
8.5000	1736				8.5000	1476			
8.5200	1758				8.5200	1480			
8.5400	1778				8.5400	1378			
8.5600	1646				8.5600	1466			

8.5800	1762				8.5800	1536			
8.6000	1816				8.6000	1472			
8.6200	1758				8.6200	1368			
8.6400	1864				8.6400	1392			
8.6600	1746				8.6600	1488			
8.6800	1682				8.6800	1552			
8.7000	1804				8.7000	1536			
8.7200	1772				8.7200	1414			
8.7400	1786				8.7400	1412			
8.7600	1716				8.7600	1498			
8.7800	1758				8.7800	1496			
8.8000	1752				8.8000	1502			
8.8200	1748				8.8200	1440			
8.8400	1740				8.8400	1466			
8.8600	1772				8.8600	1496			
8.8800	1780				8.8800	1416			
8.9000	1728				8.9000	1416			
8.9200	1680				8.9200	1384			
8.9400	1766				8.9400	1414			
8.9600	1712				8.9600	1534			
8.9800	1850				8.9800	1394			
9.0000	1786				9.0000	1406			
9.0200	1676				9.0200	1460			
9.0400	1726				9.0400	1480			
9.0600	1716				9.0600	1416			
9.0800	1776				9.0800	1368			
9.1000	1764				9.1000	1288			
9.1200	1672				9.1200	1486			
9.1400	1690				9.1400	1356			
9.1600	1754				9.1600	1506			

9.1800	1768				9.1800	1300			
9.2000	1740				9.2000	1360			
9.2200	1732				9.2200	1474			
9.2400	1696				9.2400	1468			
9.2600	1764				9.2600	1370			
9.2800	1798				9.2800	1332			
9.3000	1812				9.3000	1422			
9.3200	1578				9.3200	1408			
9.3400	1706				9.3400	1414			
9.3600	1810				9.3600	1392			
9.3800	1728				9.3800	1296			
9.4000	1784				9.4000	1392			
9.4200	1806				9.4200	1376			
9.4400	1826				9.4400	1420			
9.4600	1798				9.4600	1344			
9.4800	1764				9.4800	1328			
9.5000	1736				9.5000	1504			
9.5200	1714				9.5200	1396			
9.5400	1906				9.5400	1310			
9.5600	1728				9.5600	1394			
9.5800	1822				9.5800	1318			
9.6000	1744				9.6000	1358			
9.6200	1724				9.6200	1454			
9.6400	1750				9.6400	1350			
9.6600	1732				9.6600	1268			
9.6800	1780				9.6800	1360			
9.7000	1726				9.7000	1360			
9.7200	1736				9.7200	1384			
9.7400	1742				9.7400	1318			
9.7600	1776				9.7600	1294			

9.7800	1726				9.7800	1366			
9.8000	1728				9.8000	1364			
9.8200	1842				9.8200	1344			
9.8400	1736				9.8400	1296			
9.8600	1764				9.8600	1308			
9.8800	1664				9.8800	1334			
9.9000	1764				9.9000	1372			
9.9200	1786				9.9200	1300			
9.9400	1786				9.9400	1290			
9.9600	1726				9.9600	1272			
9.9800	1664				9.9800	1384			
10.0000	1812				10.0000	1348			
10.0200	1780				10.0200	1276			
10.0400	1804				10.0400	1246			
10.0600	1760				10.0600	1328			
10.0800	1670				10.0800	1378			
10.1000	1848				10.1000	1348			
10.1200	1760				10.1200	1254			
10.1400	1818				10.1400	1318			
10.1600	1740				10.1600	1272			
10.1800	1736				10.1800	1376			
10.2000	1738				10.2000	1292			
10.2200	1760				10.2200	1270			
10.2400	1724				10.2400	1278			
10.2600	1692				10.2600	1322			
10.2800	1760				10.2800	1400			
10.3000	1882				10.3000	1226			
10.3200	1772				10.3200	1310			
10.3400	1750				10.3400	1336			
10.3600	1744				10.3600	1278			

10.3800	1776				10.3800	1296		
10.4000	1736				10.4000	1210		
10.4200	1718				10.4200	1232		
10.4400	1710				10.4400	1334		
10.4600	1730				10.4600	1302		
10.4800	1780				10.4800	1236		
10.5000	1808				10.5000	1240		
10.5200	1776				10.5200	1282		
10.5400	1694				10.5400	1290		
10.5600	1788				10.5600	1254		
10.5800	1844				10.5800	1264		
10.6000	1706				10.6000	1266		
10.6200	1666				10.6200	1224		
10.6400	1698				10.6400	1328		
10.6600	1858				10.6600	1268		
10.6800	1752				10.6800	1266		
10.7000	1818				10.7000	1258		
10.7200	1724				10.7200	1262		
10.7400	1692				10.7400	1340		
10.7600	1744				10.7600	1274		
10.7800	1762				10.7800	1248		
10.8000	1744				10.8000	1246		
10.8200	1748				10.8200	1292		
10.8400	1816				10.8400	1310		
10.8600	1834				10.8600	1218		
10.8800	1788				10.8800	1222		
10.9000	1716				10.9000	1272		
10.9200	1752				10.9200	1328		
10.9400	1842				10.9400	1312		
10.9600	1812				10.9600	1236		

10.9800	1780				10.9800	1200			
11.0000	1744				11.0000	1260			
11.0200	1720				11.0200	1282			
11.0400	1752				11.0400	1230			
11.0600	1820				11.0600	1174			
11.0800	1768				11.0800	1216			
11.1000	1658				11.1000	1322			
11.1200	1768				11.1200	1280			
11.1400	1752				11.1400	1258			
11.1600	1840				11.1600	1222			
11.1800	1756				11.1800	1280			
11.2000	1664				11.2000	1314			
11.2200	1774				11.2200	1248			
11.2400	1786				11.2400	1248			
11.2600	1828				11.2600	1192			
11.2800	1752				11.2800	1194			
11.3000	1784				11.3000	1332			
11.3200	1768				11.3200	1282			
11.3400	1812				11.3400	1164			
11.3600	1782				11.3600	1174			
11.3800	1830				11.3800	1242			
11.4000	1698				11.4000	1234			
11.4200	1838				11.4200	1196			
11.4400	1792				11.4400	1204			
11.4600	1832				11.4600	1234			
11.4800	1826				11.4800	1180			
11.5000	1718				11.5000	1272			
11.5200	1774				11.5200	1164			
11.5400	1754				11.5400	1186			
11.5600	1738				11.5600	1256			

11.5800	1732				11.5800	1240			
11.6000	1834				11.6000	1248			
11.6200	1786				11.6200	1262			
11.6400	1764				11.6400	1218			
11.6600	1750				11.6600	1258			
11.6800	1788				11.6800	1272			
11.7000	1916				11.7000	1252			
11.7200	1774				11.7200	1170			
11.7400	1802				11.7400	1294			
11.7600	1822				11.7600	1310			
11.7800	1838				11.7800	1196			
11.8000	1824				11.8000	1216			
11.8200	1842				11.8200	1210			
11.8400	1808				11.8400	1182			
11.8600	1680				11.8600	1274			
11.8800	1800				11.8800	1232			
11.9000	1798				11.9000	1186			
11.9200	1822				11.9200	1176			
11.9400	1752				11.9400	1248			
11.9600	1860				11.9600	1246			
11.9800	1900				11.9800	1222			
12.0000	1808				12.0000	1176			
12.0200	1752				12.0200	1216			
12.0400	1834				12.0400	1228			
12.0600	1770				12.0600	1254			
12.0800	1782				12.0800	1170			
12.1000	1844				12.1000	1218			
12.1200	1676				12.1200	1246			
12.1400	1742				12.1400	1214			
12.1600	1850				12.1600	1236			

12.1800	1820				12.1800	1214			
12.2000	1868				12.2000	1218			
12.2200	1720				12.2200	1280			
12.2400	1818				12.2400	1268			
12.2600	1808				12.2600	1132			
12.2800	1838				12.2800	1248			
12.3000	1746				12.3000	1196			
12.3200	1800				12.3200	1258			
12.3400	1848				12.3400	1242			
12.3600	1818				12.3600	1166			
12.3800	1776				12.3800	1156			
12.4000	1800				12.4000	1220			
12.4200	1784				12.4200	1264			
12.4400	1856				12.4400	1194			
12.4600	1786				12.4600	1130			
12.4800	1796				12.4800	1180			
12.5000	1814				12.5000	1174			
12.5200	1814				12.5200	1196			
12.5400	1818				12.5400	1228			
12.5600	1836				12.5600	1188			
12.5800	1748				12.5800	1216			
12.6000	1756				12.6000	1230			
12.6200	1800				12.6200	1274			
12.6400	1882				12.6400	1212			
12.6600	1748				12.6600	1174			
12.6800	1808				12.6800	1202			
12.7000	1752				12.7000	1198			
12.7200	1820				12.7200	1234			
12.7400	1868				12.7400	1124			
12.7600	1834				12.7600	1188			

12.7800	1752				12.7800	1170			
12.8000	1788				12.8000	1168			
12.8200	1820				12.8200	1186			
12.8400	1840				12.8400	1176			
12.8600	1768				12.8600	1186			
12.8800	1750				12.8800	1202			
12.9000	1806				12.9000	1206			
12.9200	1762				12.9200	1174			
12.9400	1828				12.9400	1154			
12.9600	1758				12.9600	1176			
12.9800	1804				12.9800	1254			
13.0000	1818				13.0000	1192			
13.0200	1794				13.0200	1152			
13.0400	1812				13.0400	1190			
13.0600	1792				13.0600	1236			
13.0800	1834				13.0800	1204			
13.1000	1896				13.1000	1182			
13.1200	1882				13.1200	1100			
13.1400	1776				13.1400	1206			
13.1600	1862				13.1600	1186			
13.1800	1724				13.1800	1218			
13.2000	1834				13.2000	1152			
13.2200	1822				13.2200	1180			
13.2400	1780				13.2400	1224			
13.2600	1806				13.2600	1280			
13.2800	1806				13.2800	1224			
13.3000	1844				13.3000	1168			
13.3200	1814				13.3200	1212			
13.3400	1742				13.3400	1214			
13.3600	1768				13.3600	1238			

13.3800	1866				13.3800	1194			
13.4000	1856				13.4000	1162			
13.4200	1800				13.4200	1154			
13.4400	1788				13.4400	1220			
13.4600	1750				13.4600	1164			
13.4800	1738				13.4800	1232			
13.5000	1750				13.5000	1166			
13.5200	1814				13.5200	1144			
13.5400	1848				13.5400	1276			
13.5600	1872				13.5600	1222			
13.5800	1756				13.5800	1178			
13.6000	1750				13.6000	1140			
13.6200	1712				13.6200	1178			
13.6400	1760				13.6400	1288			
13.6600	1838				13.6600	1186			
13.6800	1770				13.6800	1162			
13.7000	1820				13.7000	1200			
13.7200	1826				13.7200	1176			
13.7400	1856				13.7400	1258			
13.7600	1858				13.7600	1122			
13.7800	1724				13.7800	1132			
13.8000	1818				13.8000	1202			
13.8200	1812				13.8200	1246			
13.8400	1808				13.8400	1222			
13.8600	1934				13.8600	1238			
13.8800	1798				13.8800	1176			
13.9000	1824				13.9000	1254			
13.9200	1778				13.9200	1200			
13.9400	1774				13.9400	1180			
13.9600	1804				13.9600	1116			

13.9800 1754				13.9800 1166			
14.0000 1796				14.0000 1208			
14.0200 1784				14.0200 1230			
14.0400 1792				14.0400 1162			
14.0600 1844				14.0600 1168			
14.0800 1698				14.0800 1168			
14.1000 1726				14.1000 1222			
14.1200 1856				14.1200 1218			
14.1400 1822				14.1400 1174			
14.1600 1874				14.1600 1186			
14.1800 1818				14.1800 1198			
14.2000 1888				14.2000 1238			
14.2200 1856				14.2200 1196			
14.2400 1850				14.2400 1188			
14.2600 1834				14.2600 1226			
14.2800 1752				14.2800 1212			
14.3000 1822				14.3000 1270			
14.3200 1902				14.3200 1152			
14.3400 1812				14.3400 1160			
14.3600 1772				14.3600 1152			
14.3800 1760				14.3800 1210			
14.4000 1770				14.4000 1254			
14.4200 1814				14.4200 1244			
14.4400 1794				14.4400 1146			
14.4600 1868				14.4600 1246			
14.4800 1866				14.4800 1256			
14.5000 1772				14.5000 1184			
14.5200 1914				14.5200 1124			
14.5400 1746				14.5400 1200			
14.5600 1762				14.5600 1288			

14.5800	1880				14.5800	1174			
14.6000	1834				14.6000	1192			
14.6200	1812				14.6200	1114			
14.6400	1824				14.6400	1208			
14.6600	1778				14.6600	1218			
14.6800	1864				14.6800	1226			
14.7000	1832				14.7000	1140			
14.7200	1928				14.7200	1122			
14.7400	1792				14.7400	1174			
14.7600	1790				14.7600	1218			
14.7800	1876				14.7800	1178			
14.8000	1818				14.8000	1088			
14.8200	1836				14.8200	1160			
14.8400	1786				14.8400	1178			
14.8600	1836				14.8600	1234			
14.8800	1980				14.8800	1148			
14.9000	1772				14.9000	1166			
14.9200	1812				14.9200	1160			
14.9400	1814				14.9400	1212			
14.9600	1776				14.9600	1208			
14.9800	1796				14.9800	1190			
15.0000	1800				15.0000	1156			
15.0200	1832				15.0200	1218			
15.0400	1888				15.0400	1214			
15.0600	1886				15.0600	1280			
15.0800	1900				15.0800	1150			
15.1000	1894				15.1000	1162			
15.1200	1826				15.1200	1246			
15.1400	1910				15.1400	1178			
15.1600	1874				15.1600	1230			

15.1800	1902			15.1800	1200		
15.2000	1782			15.2000	1144		
15.2200	1836			15.2200	1276		
15.2400	1828			15.2400	1244		
15.2600	1764			15.2600	1186		
15.2800	1842			15.2800	1222		
15.3000	1884			15.3000	1184		
15.3200	1790			15.3200	1224		
15.3400	1784			15.3400	1192		
15.3600	1910			15.3600	1146		
15.3800	1860			15.3800	1152		
15.4000	1796			15.4000	1214		
15.4200	1866			15.4200	1270		
15.4400	1906			15.4400	1230		
15.4600	1882			15.4600	1198		
15.4800	1814			15.4800	1234		
15.5000	1818			15.5000	1196		
15.5200	1880			15.5200	1364		
15.5400	1902			15.5400	1280		
15.5600	1880			15.5600	1176		
15.5800	1898			15.5800	1202		
15.6000	1902			15.6000	1244		
15.6200	1858			15.6200	1264		
15.6400	1928			15.6400	1200		
15.6600	1886			15.6600	1200		
15.6800	1786			15.6800	1208		
15.7000	1914			15.7000	1334		
15.7200	1842			15.7200	1270		
15.7400	1976			15.7400	1208		
15.7600	1890			15.7600	1210		

15.7800	1846				15.7800	1270			
15.8000	1944				15.8000	1256			
15.8200	1914				15.8200	1308			
15.8400	1928				15.8400	1240			
15.8600	1882				15.8600	1206			
15.8800	1878				15.8800	1258			
15.9000	2004				15.9000	1230			
15.9200	1960				15.9200	1218			
15.9400	1938				15.9400	1300			
15.9600	1802				15.9600	1220			
15.9800	1820				15.9800	1262			
16.0000	1902				16.0000	1310			
16.0200	1932				16.0200	1196			
16.0400	2000				16.0400	1224			
16.0600	1926				16.0600	1194			
16.0800	1860				16.0800	1308			
16.1000	1952				16.1000	1238			
16.1200	1940				16.1200	1196			
16.1400	1984				16.1400	1178			
16.1600	1940				16.1600	1246			
16.1800	1886				16.1800	1326			
16.2000	2048				16.2000	1200			
16.2200	1986				16.2200	1190			
16.2400	1920				16.2400	1238			
16.2600	1910				16.2600	1296			
16.2800	1972				16.2800	1278			
16.3000	1936				16.3000	1234			
16.3200	1896				16.3200	1188			
16.3400	1990				16.3400	1340			
16.3600	2020				16.3600	1282			

16.3800	1970				16.3800	1244			
16.4000	2004				16.4000	1264			
16.4200	2030				16.4200	1208			
16.4400	1970				16.4400	1282			
16.4600	2010				16.4600	1264			
16.4800	1994				16.4800	1212			
16.5000	2026				16.5000	1208			
16.5200	1958				16.5200	1168			
16.5400	1888				16.5400	1370			
16.5600	2040				16.5600	1270			
16.5800	1956				16.5800	1300			
16.6000	1906				16.6000	1228			
16.6200	1946				16.6200	1222			
16.6400	1962				16.6400	1384			
16.6600	1978				16.6600	1268			
16.6800	1976				16.6800	1216			
16.7000	1996				16.7000	1270			
16.7200	1996				16.7200	1280			
16.7400	2026				16.7400	1308			
16.7600	2078				16.7600	1256			
16.7800	2106				16.7800	1222			
16.8000	2022				16.8000	1228			
16.8200	2078				16.8200	1208			
16.8400	1956				16.8400	1356			
16.8600	2072				16.8600	1288			
16.8800	1988				16.8800	1294			
16.9000	2016				16.9000	1330			
16.9200	2032				16.9200	1356			
16.9400	2020				16.9400	1302			
16.9600	2054				16.9600	1296			

16.9800	2084				16.9800	1262			
17.0000	2052				17.0000	1342			
17.0200	2126				17.0200	1264			
17.0400	2148				17.0400	1302			
17.0600	2054				17.0600	1340			
17.0800	1996				17.0800	1270			
17.1000	1986				17.1000	1462			
17.1200	2096				17.1200	1296			
17.1400	2078				17.1400	1226			
17.1600	2068				17.1600	1290			
17.1800	2070				17.1800	1226			
17.2000	2044				17.2000	1338			
17.2200	2128				17.2200	1328			
17.2400	2134				17.2400	1238			
17.2600	2162				17.2600	1300			
17.2800	2056				17.2800	1356			
17.3000	2060				17.3000	1412			
17.3200	2178				17.3200	1364			
17.3400	2126				17.3400	1154			
17.3600	2084				17.3600	1408			
17.3800	2134				17.3800	1324			
17.4000	2156				17.4000	1330			
17.4200	2254				17.4200	1306			
17.4400	2164				17.4400	1278			
17.4600	2144				17.4600	1344			
17.4800	2180				17.4800	1364			
17.5000	2126				17.5000	1424			
17.5200	2252				17.5200	1326			
17.5400	2124				17.5400	1296			
17.5600	2128				17.5600	1456			

17.5800	2218				17.5800	1348			
17.6000	2152				17.6000	1330			
17.6200	2226				17.6200	1290			
17.6400	2224				17.6400	1306			
17.6600	2202				17.6600	1434			
17.6800	2230				17.6800	1348			
17.7000	2276				17.7000	1320			
17.7200	2230				17.7200	1336			
17.7400	2158				17.7400	1262			
17.7600	2178				17.7600	1458			
17.7800	2218				17.7800	1328			
17.8000	2120				17.8000	1286			
17.8200	2226				17.8200	1412			
17.8400	2292				17.8400	1330			
17.8600	2270				17.8600	1432			
17.8800	2250				17.8800	1334			
17.9000	2276				17.9000	1274			
17.9200	2260				17.9200	1348			
17.9400	2330				17.9400	1350			
17.9600	2238				17.9600	1358			
17.9800	2346				17.9800	1286			
18.0000	2276				18.0000	1238			
18.0200	2282				18.0200	1432			
18.0400	2388				18.0400	1394			
18.0600	2270				18.0600	1454			
18.0800	2334				18.0800	1284			
18.1000	2256				18.1000	1276			
18.1200	2278				18.1200	1422			
18.1400	2344				18.1400	1286			
18.1600	2352				18.1600	1366			

18.1800	2378				18.1800	1348			
18.2000	2262				18.2000	1310			
18.2200	2330				18.2200	1530			
18.2400	2406				18.2400	1394			
18.2600	2338				18.2600	1324			
18.2800	2424				18.2800	1332			
18.3000	2326				18.3000	1334			
18.3200	2348				18.3200	1402			
18.3400	2354				18.3400	1396			
18.3600	2342				18.3600	1366			
18.3800	2440				18.3800	1402			
18.4000	2246				18.4000	1316			
18.4200	2368				18.4200	1494			
18.4400	2430				18.4400	1384			
18.4600	2350				18.4600	1276			
18.4800	2402				18.4800	1396			
18.5000	2398				18.5000	1394			
18.5200	2510				18.5200	1478			
18.5400	2514				18.5400	1410			
18.5600	2396				18.5600	1368			
18.5800	2400				18.5800	1434			
18.6000	2476				18.6000	1424			
18.6200	2406				18.6200	1460			
18.6400	2556				18.6400	1392			
18.6600	2412				18.6600	1340			
18.6800	2418				18.6800	1442			
18.7000	2464				18.7000	1444			
18.7200	2330				18.7200	1398			
18.7400	2538				18.7400	1394			
18.7600	2468				18.7600	1344			

18.7800	2560				18.7800	1486			
18.8000	2554				18.8000	1428			
18.8200	2470				18.8200	1372			
18.8400	2460				18.8400	1450			
18.8600	2504				18.8600	1416			
18.8800	2574				18.8800	1532			
18.9000	2530				18.9000	1470			
18.9200	2540				18.9200	1358			
18.9400	2620				18.9400	1450			
18.9600	2554				18.9600	1392			
18.9800	2478				18.9800	1502			
19.0000	2600				19.0000	1538			
19.0200	2390				19.0200	1326			
19.0400	2600				19.0400	1438			
19.0600	2604				19.0600	1418			
19.0800	2592				19.0800	1474			
19.1000	2608				19.1000	1464			
19.1200	2550				19.1200	1408			
19.1400	2468				19.1400	1434			
19.1600	2490				19.1600	1460			
19.1800	2588				19.1800	1462			
19.2000	2672				19.2000	1402			
19.2200	2510				19.2200	1398			
19.2400	2616				19.2400	1494			
19.2600	2526				19.2600	1474			
19.2800	2516				19.2800	1444			
19.3000	2582				19.3000	1480			
19.3200	2686				19.3200	1358			
19.3400	2584				19.3400	1602			
19.3600	2652				19.3600	1508			

19.3800	2564				19.3800	1434			
19.4000	2652				19.4000	1438			
19.4200	2646				19.4200	1382			
19.4400	2490				19.4400	1500			
19.4600	2694				19.4600	1502			
19.4800	2650				19.4800	1392			
19.5000	2592				19.5000	1484			
19.5200	2572				19.5200	1382			
19.5400	2704				19.5400	1582			
19.5600	2772				19.5600	1532			
19.5800	2618				19.5800	1380			
19.6000	2726				19.6000	1452			
19.6200	2542				19.6200	1506			
19.6400	2610				19.6400	1546			
19.6600	2638				19.6600	1470			
19.6800	2630				19.6800	1406			
19.7000	2636				19.7000	1482			
19.7200	2644				19.7200	1438			
19.7400	2820				19.7400	1452			
19.7600	2750				19.7600	1398			
19.7800	2578				19.7800	1396			
19.8000	2708				19.8000	1532			
19.8200	2682				19.8200	1538			
19.8400	2504				19.8400	1486			
19.8600	2648				19.8600	1418			
19.8800	2616				19.8800	1390			
19.9000	2616				19.9000	1508			
19.9200	2786				19.9200	1508			
19.9400	2624				19.9400	1514			
19.9600	2726				19.9600	1474			

19.9800	2638			19.9800	1394		
20.0000	2650			20.0000	1554		
20.0200	2754			20.0200	1496		
20.0400	2616			20.0400	1386		
20.0600	2600			20.0600	1424		
20.0800	2658			20.0800	1342		
20.1000	2596			20.1000	1516		
20.1200	2784			20.1200	1450		
20.1400	2588			20.1400	1390		
20.1600	2786			20.1600	1460		
20.1800	2606			20.1800	1424		
20.2000	2606			20.2000	1586		
20.2200	2828			20.2200	1450		
20.2400	2802			20.2400	1270		
20.2600	2726			20.2600	1424		
20.2800	2700			20.2800	1508		
20.3000	2752			20.3000	1510		
20.3200	2730			20.3200	1434		
20.3400	2700			20.3400	1320		
20.3600	2638			20.3600	1460		
20.3800	2708			20.3800	1544		
20.4000	2680			20.4000	1436		
20.4200	2740			20.4200	1352		
20.4400	2644			20.4400	1318		
20.4600	2674			20.4600	1576		
20.4800	2722			20.4800	1494		
20.5000	2638			20.5000	1438		
20.5200	2772			20.5200	1394		
20.5400	2736			20.5400	1410		
20.5600	2654			20.5600	1550		

20.5800	2770				20.5800	1492			
20.6000	2644				20.6000	1400			
20.6200	2742				20.6200	1384			
20.6400	2756				20.6400	1300			
20.6600	2610				20.6600	1474			
20.6800	2786				20.6800	1480			
20.7000	2644				20.7000	1398			
20.7200	2692				20.7200	1358			
20.7400	2688				20.7400	1438			
20.7600	2694				20.7600	1534			
20.7800	2756				20.7800	1512			
20.8000	2636				20.8000	1342			
20.8200	2526				20.8200	1428			
20.8400	2688				20.8400	1498			
20.8600	2708				20.8600	1444			
20.8800	2784				20.8800	1508			
20.9000	2684				20.9000	1324			
20.9200	2648				20.9200	1476			
20.9400	2720				20.9400	1374			
20.9600	2602				20.9600	1460			
20.9800	2780				20.9800	1434			
21.0000	2670				21.0000	1318			
21.0200	2708				21.0200	1524			
21.0400	2652				21.0400	1412			
21.0600	2688				21.0600	1414			
21.0800	2760				21.0800	1394			
21.1000	2676				21.1000	1338			
21.1200	2756				21.1200	1494			
21.1400	2814				21.1400	1476			
21.1600	2668				21.1600	1360			

21.1800	2684				21.1800	1380			
21.2000	2636				21.2000	1346			
21.2200	2628				21.2200	1474			
21.2400	2812				21.2400	1394			
21.2600	2680				21.2600	1318			
21.2800	2710				21.2800	1368			
21.3000	2604				21.3000	1386			
21.3200	2696				21.3200	1444			
21.3400	2766				21.3400	1368			
21.3600	2574				21.3600	1370			
21.3800	2756				21.3800	1418			
21.4000	2664				21.4000	1400			
21.4200	2648				21.4200	1486			
21.4400	2754				21.4400	1420			
21.4600	2598				21.4600	1386			
21.4800	2732				21.4800	1436			
21.5000	2728				21.5000	1342			
21.5200	2554				21.5200	1486			
21.5400	2684				21.5400	1350			
21.5600	2614				21.5600	1306			
21.5800	2678				21.5800	1448			
21.6000	2696				21.6000	1390			
21.6200	2612				21.6200	1370			
21.6400	2706				21.6400	1348			
21.6600	2694				21.6600	1324			
21.6800	2692				21.6800	1430			
21.7000	2768				21.7000	1396			
21.7200	2620				21.7200	1362			
21.7400	2714				21.7400	1332			
21.7600	2694				21.7600	1284			

21.7800	2604				21.7800	1518			
21.8000	2736				21.8000	1410			
21.8200	2684				21.8200	1284			
21.8400	2636				21.8400	1342			
21.8600	2662				21.8600	1344			
21.8800	2690				21.8800	1466			
21.9000	2722				21.9000	1444			
21.9200	2578				21.9200	1312			
21.9400	2678				21.9400	1354			
21.9600	2604				21.9600	1304			
21.9800	2664				21.9800	1388			
22.0000	2698				22.0000	1384			
22.0200	2664				22.0200	1294			
22.0400	2688				22.0400	1388			
22.0600	2776				22.0600	1354			
22.0800	2632				22.0800	1454			
22.1000	2728				22.1000	1364			
22.1200	2578				22.1200	1266			
22.1400	2652				22.1400	1374			
22.1600	2716				22.1600	1328			
22.1800	2626				22.1800	1358			
22.2000	2736				22.2000	1326			
22.2200	2594				22.2200	1290			
22.2400	2674				22.2400	1426			
22.2600	2740				22.2600	1374			
22.2800	2642				22.2800	1312			
22.3000	2694				22.3000	1296			
22.3200	2616				22.3200	1260			
22.3400	2616				22.3400	1392			
22.3600	2734				22.3600	1260			

22.3800	2592				22.3800	1184			
22.4000	2680				22.4000	1300			
22.4200	2608				22.4200	1304			
22.4400	2562				22.4400	1398			
22.4600	2712				22.4600	1380			
22.4800	2616				22.4800	1252			
22.5000	2648				22.5000	1296			
22.5200	2616				22.5200	1320			
22.5400	2622				22.5400	1376			
22.5600	2796				22.5600	1340			
22.5800	2508				22.5800	1210			
22.6000	2542				22.6000	1332			
22.6200	2750				22.6200	1306			
22.6400	2504				22.6400	1352			
22.6600	2768				22.6600	1296			
22.6800	2568				22.6800	1186			
22.7000	2642				22.7000	1300			
22.7200	2656				22.7200	1314			
22.7400	2566				22.7400	1318			
22.7600	2658				22.7600	1236			
22.7800	2588				22.7800	1178			
22.8000	2570				22.8000	1308			
22.8200	2638				22.8200	1336			
22.8400	2610				22.8400	1300			
22.8600	2644				22.8600	1268			
22.8800	2614				22.8800	1322			
22.9000	2576				22.9000	1370			
22.9200	2730				22.9200	1282			
22.9400	2642				22.9400	1238			
22.9600	2618				22.9600	1202			

22.9800	2626				22.9800	1246			
23.0000	2652				23.0000	1312			
23.0200	2704				23.0200	1286			
23.0400	2610				23.0400	1174			
23.0600	2590				23.0600	1266			
23.0800	2582				23.0800	1180			
23.1000	2652				23.1000	1282			
23.1200	2678				23.1200	1226			
23.1400	2600				23.1400	1210			
23.1600	2688				23.1600	1290			
23.1800	2690				23.1800	1178			
23.2000	2538				23.2000	1270			
23.2200	2664				23.2200	1244			
23.2400	2618				23.2400	1164			
23.2600	2660				23.2600	1272			
23.2800	2600				23.2800	1236			
23.3000	2556				23.3000	1294			
23.3200	2594				23.3200	1226			
23.3400	2708				23.3400	1144			
23.3600	2750				23.3600	1304			
23.3800	2684				23.3800	1182			
23.4000	2592				23.4000	1232			
23.4200	2640				23.4200	1238			
23.4400	2622				23.4400	1132			
23.4600	2730				23.4600	1256			
23.4800	2560				23.4800	1230			
23.5000	2608				23.5000	1158			
23.5200	2596				23.5200	1204			
23.5400	2594				23.5400	1152			
23.5600	2644				23.5600	1258			

23.5800	2760				23.5800	1222			
23.6000	2626				23.6000	1164			
23.6200	2590				23.6200	1160			
23.6400	2608				23.6400	1182			
23.6600	2490				23.6600	1296			
23.6800	2640				23.6800	1140			
23.7000	2640				23.7000	1116			
23.7200	2682				23.7200	1202			
23.7400	2698				23.7400	1228			
23.7600	2582				23.7600	1256			
23.7800	2632				23.7800	1214			
23.8000	2658				23.8000	1154			
23.8200	2538				23.8200	1226			
23.8400	2632				23.8400	1250			
23.8600	2532				23.8600	1184			
23.8800	2670				23.8800	1194			
23.9000	2704				23.9000	1112			
23.9200	2558				23.9200	1226			
23.9400	2684				23.9400	1100			
23.9600	2580				23.9600	1222			
23.9800	2618				23.9800	1138			
24.0000	2500				24.0000	1122			
24.0200	2566				24.0200	1228			
24.0400	2678				24.0400	1208			
24.0600	2568				24.0600	1180			
24.0800	2654				24.0800	1150			
24.1000	2584				24.1000	1150			
24.1200	2580				24.1200	1248			
24.1400	2636				24.1400	1216			
24.1600	2628				24.1600	1056			

24.1800	2578				24.1800	1136			
24.2000	2608				24.2000	1162			
24.2200	2640				24.2200	1180			
24.2400	2702				24.2400	1248			
24.2600	2570				24.2600	1142			
24.2800	2644				24.2800	1182			
24.3000	2586				24.3000	1104			
24.3200	2518				24.3200	1158			
24.3400	2642				24.3400	1088			
24.3600	2520				24.3600	1100			
24.3800	2614				24.3800	1166			
24.4000	2678				24.4000	1176			
24.4200	2508				24.4200	1144			
24.4400	2634				24.4400	1084			
24.4600	2464				24.4600	1042			
24.4800	2504				24.4800	1054			
24.5000	2648				24.5000	1122			
24.5200	2492				24.5200	1128			
24.5400	2638				24.5400	1070			
24.5600	2568				24.5600	1060			
24.5800	2562				24.5800	1126			
24.6000	2636				24.6000	1194			
24.6200	2492				24.6200	1074			
24.6400	2648				24.6400	1162			
24.6600	2564				24.6600	1092			
24.6800	2506				24.6800	1110			
24.7000	2586				24.7000	1134			
24.7200	2636				24.7200	1082			
24.7400	2624				24.7400	1094			
24.7600	2482				24.7600	1028			

24.7800	2440				24.7800	1132			
24.8000	2732				24.8000	1076			
24.8200	2482				24.8200	1084			
24.8400	2594				24.8400	1086			
24.8600	2436				24.8600	1080			
24.8800	2394				24.8800	1112			
24.9000	2636				24.9000	1104			
24.9200	2496				24.9200	1010			
24.9400	2554				24.9400	1070			
24.9600	2490				24.9600	1136			
24.9800	2474				24.9800	1130			
25.0000	2616				25.0000	1056			
25.0200	2466				25.0200	1038			
25.0400	2504				25.0400	1014			
25.0600	2586				25.0600	1000			
25.0800	2398				25.0800	1076			
25.1000	2614				25.1000	1092			
25.1200	2520				25.1200	1030			
25.1400	2580				25.1400	1120			
25.1600	2684				25.1600	1116			
25.1800	2530				25.1800	1106			
25.2000	2712				25.2000	1036			
25.2200	2520				25.2200	1030			
25.2400	2496				25.2400	1116			
25.2600	2586				25.2600	1094			
25.2800	2512				25.2800	1054			
25.3000	2642				25.3000	996			
25.3200	2484				25.3200	1078			
25.3400	2528				25.3400	1114			
25.3600	2562				25.3600	1036			

25.3800	2450				25.3800	1004			
25.4000	2514				25.4000	1028			
25.4200	2534				25.4200	980			
25.4400	2454				25.4400	1090			
25.4600	2496				25.4600	1098			
25.4800	2520				25.4800	1062			
25.5000	2440				25.5000	984			
25.5200	2538				25.5200	1066			
25.5400	2590				25.5400	1074			
25.5600	2588				25.5600	1030			
25.5800	2506				25.5800	988			
25.6000	2572				25.6000	1104			
25.6200	2560				25.6200	972			
25.6400	2450				25.6400	1046			
25.6600	2610				25.6600	1038			
25.6800	2448				25.6800	1032			
25.7000	2510				25.7000	1058			
25.7200	2516				25.7200	1072			
25.7400	2526				25.7400	988			
25.7600	2510				25.7600	1062			
25.7800	2498				25.7800	914			
25.8000	2406				25.8000	1004			
25.8200	2578				25.8200	1044			
25.8400	2396				25.8400	964			
25.8600	2568				25.8600	986			
25.8800	2406				25.8800	960			
25.9000	2534				25.9000	1020			
25.9200	2492				25.9200	1036			
25.9400	2402				25.9400	926			
25.9600	2540				25.9600	1024			

25.9800	2466			25.9800	944		
26.0000	2442			26.0000	1048		
26.0200	2534			26.0200	986		
26.0400	2424			26.0400	940		
26.0600	2464			26.0600	998		
26.0800	2440			26.0800	1002		
26.1000	2400			26.1000	984		
26.1200	2488			26.1200	952		
26.1400	2442			26.1400	960		
26.1600	2396			26.1600	984		
26.1800	2462			26.1800	1056		
26.2000	2438			26.2000	996		
26.2200	2514			26.2200	982		
26.2400	2464			26.2400	972		
26.2600	2376			26.2600	1044		
26.2800	2446			26.2800	1006		
26.3000	2370			26.3000	968		
26.3200	2470			26.3200	910		
26.3400	2402			26.3400	930		
26.3600	2440			26.3600	966		
26.3800	2466			26.3800	944		
26.4000	2326			26.4000	928		
26.4200	2444			26.4200	1008		
26.4400	2356			26.4400	946		
26.4600	2340			26.4600	1044		
26.4800	2464			26.4800	946		
26.5000	2414			26.5000	986		
26.5200	2544			26.5200	962		
26.5400	2404			26.5400	942		
26.5600	2388			26.5600	1024		

26.5800	2366			26.5800	920			
26.6000	2282			26.6000	952			
26.6200	2320			26.6200	990			
26.6400	2468			26.6400	980			
26.6600	2308			26.6600	1046			
26.6800	2458			26.6800	960			
26.7000	2244			26.7000	946			
26.7200	2446			26.7200	980			
26.7400	2386			26.7400	904			
26.7600	2336			26.7600	976			
26.7800	2472			26.7800	946			
26.8000	2322			26.8000	898			
26.8200	2486			26.8200	902			
26.8400	2428			26.8400	944			
26.8600	2332			26.8600	982			
26.8800	2432			26.8800	944			
26.9000	2322			26.9000	864			
26.9200	2366			26.9200	956			
26.9400	2432			26.9400	908			
26.9600	2288			26.9600	950			
26.9800	2394			26.9800	918			
27.0000	2332			27.0000	912			
27.0200	2326			27.0200	978			
27.0400	2394			27.0400	914			
27.0600	2344			27.0600	868			
27.0800	2262			27.0800	866			
27.1000	2336			27.1000	856			
27.1200	2384			27.1200	968			
27.1400	2382			27.1400	926			
27.1600	2280			27.1600	898			

27.1800	2302				27.1800	946			
27.2000	2298				27.2000	866			
27.2200	2262				27.2200	890			
27.2400	2356				27.2400	924			
27.2600	2350				27.2600	840			
27.2800	2276				27.2800	948			
27.3000	2324				27.3000	888			
27.3200	2328				27.3200	890			
27.3400	2362				27.3400	922			
27.3600	2306				27.3600	838			
27.3800	2284				27.3800	960			
27.4000	2314				27.4000	944			
27.4200	2232				27.4200	874			
27.4400	2386				27.4400	942			
27.4600	2260				27.4600	854			
27.4800	2338				27.4800	888			
27.5000	2346				27.5000	924			
27.5200	2266				27.5200	870			
27.5400	2360				27.5400	880			
27.5600	2304				27.5600	868			
27.5800	2224				27.5800	954			
27.6000	2330				27.6000	866			
27.6200	2200				27.6200	854			
27.6400	2278				27.6400	870			
27.6600	2244				27.6600	886			
27.6800	2312				27.6800	928			
27.7000	2346				27.7000	884			
27.7200	2300				27.7200	936			
27.7400	2270				27.7400	872			
27.7600	2176				27.7600	886			

27.7800	2308				27.7800	942			
27.8000	2322				27.8000	888			
27.8200	2122				27.8200	880			
27.8400	2286				27.8400	906			
27.8600	2268				27.8600	852			
27.8800	2314				27.8800	886			
27.9000	2182				27.9000	842			
27.9200	2238				27.9200	832			
27.9400	2216				27.9400	872			
27.9600	2310				27.9600	830			
27.9800	2286				27.9800	850			
28.0000	2404				28.0000	878			
28.0200	2216				28.0200	844			
28.0400	2164				28.0400	908			
28.0600	2256				28.0600	888			
28.0800	2206				28.0800	910			
28.1000	2232				28.1000	828			
28.1200	2234				28.1200	848			
28.1400	2192				28.1400	882			
28.1600	2214				28.1600	878			
28.1800	2288				28.1800	848			
28.2000	2242				28.2000	872			
28.2200	2132				28.2200	838			
28.2400	2180				28.2400	870			
28.2600	2192				28.2600	902			
28.2800	2150				28.2800	870			
28.3000	2184				28.3000	808			
28.3200	2156				28.3200	864			
28.3400	2174				28.3400	928			
28.3600	2282				28.3600	882			

28.3800	2138				28.3800	822			
28.4000	2194				28.4000	812			
28.4200	2164				28.4200	834			
28.4400	2198				28.4400	800			
28.4600	2162				28.4600	824			
28.4800	2160				28.4800	808			
28.5000	2204				28.5000	820			
28.5200	2208				28.5200	862			
28.5400	2136				28.5400	800			
28.5600	2208				28.5600	832			
28.5800	2164				28.5800	754			
28.6000	2060				28.6000	878			
28.6200	2194				28.6200	798			
28.6400	2086				28.6400	848			
28.6600	2250				28.6600	818			
28.6800	2182				28.6800	808			
28.7000	2034				28.7000	804			
28.7200	2146				28.7200	792			
28.7400	2132				28.7400	798			
28.7600	2168				28.7600	824			
28.7800	2106				28.7800	802			
28.8000	2126				28.8000	798			
28.8200	2100				28.8200	796			
28.8400	2084				28.8400	836			
28.8600	2102				28.8600	834			
28.8800	2070				28.8800	778			
28.9000	2114				28.9000	832			
28.9200	2132				28.9200	834			
28.9400	2030				28.9400	760			
28.9600	2158				28.9600	810			

28.9800	2128				28.9800	802			
29.0000	2052				29.0000	800			
29.0200	2172				29.0200	842			
29.0400	2038				29.0400	794			
29.0600	2088				29.0600	800			
29.0800	2070				29.0800	826			
29.1000	2106				29.1000	844			
29.1200	2126				29.1200	846			
29.1400	2078				29.1400	786			
29.1600	2120				29.1600	800			
29.1800	2078				29.1800	786			
29.2000	2010				29.2000	844			
29.2200	2152				29.2200	768			
29.2400	2088				29.2400	718			
29.2600	2044				29.2600	860			
29.2800	2072				29.2800	812			
29.3000	2026				29.3000	780			
29.3200	2098				29.3200	764			
29.3400	1998				29.3400	794			
29.3600	1996				29.3600	786			
29.3800	1962				29.3800	848			
29.4000	2036				29.4000	800			
29.4200	1944				29.4200	770			
29.4400	1970				29.4400	672			
29.4600	2016				29.4600	822			
29.4800	2084				29.4800	800			
29.5000	2042				29.5000	762			
29.5200	2092				29.5200	748			
29.5400	2062				29.5400	758			
29.5600	1986				29.5600	808			

29.5800	2006				29.5800	818			
29.6000	2004				29.6000	744			
29.6200	1964				29.6200	736			
29.6400	2006				29.6400	834			
29.6600	2004				29.6600	768			
29.6800	1956				29.6800	776			
29.7000	1996				29.7000	754			
29.7200	2024				29.7200	762			
29.7400	1972				29.7400	762			
29.7600	1994				29.7600	796			
29.7800	2064				29.7800	802			
29.8000	2020				29.8000	798			
29.8200	1986				29.8200	822			
29.8400	2048				29.8400	782			
29.8600	1880				29.8600	776			
29.8800	2018				29.8800	774			
29.9000	1938				29.9000	702			
29.9200	1982				29.9200	816			
29.9400	2084				29.9400	770			
29.9600	1950				29.9600	794			
29.9800	2014				29.9800	758			
30.0000	1932				30.0000	728			
30.0200	1936				30.0200	780			
30.0400	1958				30.0400	754			
30.0600	2000				30.0600	748			
30.0800	1994				30.0800	736			
30.1000	1930				30.1000	712			
30.1200	1926				30.1200	790			
30.1400	1932				30.1400	730			
30.1600	1926				30.1600	748			

30.1800	1922				30.1800	688			
30.2000	1950				30.2000	746			
30.2200	2020				30.2200	786			
30.2400	1942				30.2400	742			
30.2600	1898				30.2600	668			
30.2800	1844				30.2800	768			
30.3000	1996				30.3000	728			
30.3200	1880				30.3200	696			
30.3400	2006				30.3400	726			
30.3600	1898				30.3600	744			
30.3800	1862				30.3800	782			
30.4000	1890				30.4000	734			
30.4200	1916				30.4200	730			
30.4400	1952				30.4400	742			
30.4600	1832				30.4600	682			
30.4800	1780				30.4800	754			
30.5000	1978				30.5000	710			
30.5200	1846				30.5200	724			
30.5400	1808				30.5400	740			
30.5600	1888				30.5600	708			
30.5800	1960				30.5800	750			
30.6000	1900				30.6000	710			
30.6200	1814				30.6200	706			
30.6400	1902				30.6400	704			
30.6600	1820				30.6600	738			
30.6800	1818				30.6800	728			
30.7000	1936				30.7000	732			
30.7200	1854				30.7200	706			
30.7400	1886				30.7400	704			
30.7600	1926				30.7600	762			

30.7800	1886				30.7800	724			
30.8000	2002				30.8000	706			
30.8200	1820				30.8200	708			
30.8400	1884				30.8400	732			
30.8600	1938				30.8600	764			
30.8800	1876				30.8800	776			
30.9000	1950				30.9000	732			
30.9200	1876				30.9200	712			
30.9400	1836				30.9400	710			
30.9600	1954				30.9600	700			
30.9800	1828				30.9800	718			
31.0000	1888				31.0000	726			
31.0200	1834				31.0200	694			
31.0400	1782				31.0400	730			
31.0600	1932				31.0600	736			
31.0800	1900				31.0800	730			
31.1000	1872				31.1000	724			
31.1200	1822				31.1200	712			
31.1400	1844				31.1400	750			
31.1600	1944				31.1600	764			
31.1800	1890				31.1800	700			
31.2000	1832				31.2000	754			
31.2200	1870				31.2200	698			
31.2400	1776				31.2400	750			
31.2600	1818				31.2600	718			
31.2800	1878				31.2800	726			
31.3000	1864				31.3000	726			
31.3200	1786				31.3200	710			
31.3400	1860				31.3400	724			
31.3600	1882				31.3600	734			

31.3800	1806				31.3800	696			
31.4000	1848				31.4000	714			
31.4200	1796				31.4200	720			
31.4400	1832				31.4400	706			
31.4600	1834				31.4600	708			
31.4800	1824				31.4800	642			
31.5000	1788				31.5000	712			
31.5200	1824				31.5200	732			
31.5400	1844				31.5400	716			
31.5600	1798				31.5600	654			
31.5800	1782				31.5800	642			
31.6000	1784				31.6000	710			
31.6200	1882				31.6200	702			
31.6400	1902				31.6400	728			
31.6600	1776				31.6600	670			
31.6800	1806				31.6800	684			
31.7000	1802				31.7000	746			
31.7200	1826				31.7200	718			
31.7400	1808				31.7400	678			
31.7600	1864				31.7600	704			
31.7800	1884				31.7800	682			
31.8000	1842				31.8000	706			
31.8200	1788				31.8200	692			
31.8400	1850				31.8400	710			
31.8600	1888				31.8600	740			
31.8800	1880				31.8800	678			
31.9000	1802				31.9000	726			
31.9200	1858				31.9200	730			
31.9400	1752				31.9400	716			
31.9600	1858				31.9600	700			

31.9800	1852				31.9800	716			
32.0000	1774				32.0000	696			
32.0200	1906				32.0200	682			
32.0400	1770				32.0400	662			
32.0600	1798				32.0600	680			
32.0800	1808				32.0800	728			
32.1000	1824				32.1000	628			
32.1200	1848				32.1200	648			
32.1400	1726				32.1400	614			
32.1600	1880				32.1600	722			
32.1800	1784				32.1800	680			
32.2000	1790				32.2000	688			
32.2200	1822				32.2200	652			
32.2400	1746				32.2400	640			
32.2600	1854				32.2600	672			
32.2800	1848				32.2800	646			
32.3000	1736				32.3000	702			
32.3200	1808				32.3200	684			
32.3400	1800				32.3400	654			
32.3600	1752				32.3600	652			
32.3800	1830				32.3800	718			
32.4000	1824				32.4000	640			
32.4200	1842				32.4200	614			
32.4400	1728				32.4400	622			
32.4600	1800				32.4600	690			
32.4800	1866				32.4800	680			
32.5000	1816				32.5000	660			
32.5200	1798				32.5200	644			
32.5400	1736				32.5400	702			
32.5600	1826				32.5600	702			

32.5800	1852				32.5800	620			
32.6000	1718				32.6000	566			
32.6200	1814				32.6200	668			
32.6400	1762				32.6400	670			
32.6600	1784				32.6600	676			
32.6800	1750				32.6800	620			
32.7000	1744				32.7000	614			
32.7200	1788				32.7200	658			
32.7400	1874				32.7400	666			
32.7600	1758				32.7600	602			
32.7800	1848				32.7800	658			
32.8000	1766				32.8000	658			
32.8200	1748				32.8200	684			
32.8400	1842				32.8400	698			
32.8600	1834				32.8600	664			
32.8800	1724				32.8800	678			
32.9000	1856				32.9000	622			
32.9200	1730				32.9200	692			
32.9400	1780				32.9400	652			
32.9600	1726				32.9600	650			
32.9800	1754				32.9800	694			
33.0000	1758				33.0000	650			
33.0200	1720				33.0200	664			
33.0400	1772				33.0400	634			
33.0600	1708				33.0600	608			
33.0800	1832				33.0800	680			
33.1000	1728				33.1000	702			
33.1200	1700				33.1200	670			
33.1400	1828				33.1400	700			
33.1600	1784				33.1600	596			

33.1800	1772				33.1800	644			
33.2000	1754				33.2000	608			
33.2200	1720				33.2200	672			
33.2400	1714				33.2400	660			
33.2600	1702				33.2600	638			
33.2800	1700				33.2800	628			
33.3000	1742				33.3000	666			
33.3200	1752				33.3200	628			
33.3400	1802				33.3400	622			
33.3600	1660				33.3600	640			
33.3800	1764				33.3800	630			
33.4000	1734				33.4000	618			
33.4200	1654				33.4200	588			
33.4400	1794				33.4400	616			
33.4600	1760				33.4600	642			
33.4800	1680				33.4800	694			
33.5000	1788				33.5000	592			
33.5200	1752				33.5200	618			
33.5400	1750				33.5400	642			
33.5600	1780				33.5600	620			
33.5800	1670				33.5800	646			
33.6000	1800				33.6000	664			
33.6200	1770				33.6200	634			
33.6400	1792				33.6400	648			
33.6600	1676				33.6600	592			
33.6800	1722				33.6800	660			
33.7000	1838				33.7000	620			
33.7200	1676				33.7200	586			
33.7400	1742				33.7400	634			
33.7600	1762				33.7600	672			

33.7800	1674			33.7800	632			
33.8000	1824			33.8000	646			
33.8200	1654			33.8200	584			
33.8400	1792			33.8400	560			
33.8600	1700			33.8600	638			
33.8800	1796			33.8800	660			
33.9000	1758			33.9000	684			
33.9200	1680			33.9200	630			
33.9400	1680			33.9400	630			
33.9600	1732			33.9600	638			
33.9800	1794			33.9800	620			
34.0000	1772			34.0000	580			
34.0200	1728			34.0200	562			
34.0400	1750			34.0400	680			
34.0600	1758			34.0600	654			
34.0800	1682			34.0800	602			
34.1000	1744			34.1000	624			
34.1200	1766			34.1200	600			
34.1400	1678			34.1400	636			
34.1600	1686			34.1600	622			
34.1800	1790			34.1800	616			
34.2000	1636			34.2000	640			
34.2200	1716			34.2200	602			
34.2400	1742			34.2400	662			
34.2600	1732			34.2600	610			
34.2800	1792			34.2800	636			
34.3000	1800			34.3000	662			
34.3200	1772			34.3200	602			
34.3400	1766			34.3400	616			
34.3600	1736			34.3600	632			

34.3800	1744				34.3800	614			
34.4000	1746				34.4000	608			
34.4200	1732				34.4200	616			
34.4400	1688				34.4400	628			
34.4600	1822				34.4600	562			
34.4800	1672				34.4800	568			
34.5000	1732				34.5000	622			
34.5200	1660				34.5200	608			
34.5400	1766				34.5400	578			
34.5600	1772				34.5600	600			
34.5800	1628				34.5800	604			
34.6000	1656				34.6000	642			
34.6200	1702				34.6200	614			
34.6400	1642				34.6400	602			
34.6600	1774				34.6600	614			
34.6800	1660				34.6800	606			
34.7000	1704				34.7000	626			
34.7200	1704				34.7200	630			
34.7400	1642				34.7400	618			
34.7600	1720				34.7600	562			
34.7800	1738				34.7800	618			
34.8000	1628				34.8000	642			
34.8200	1724				34.8200	612			
34.8400	1654				34.8400	554			
34.8600	1680				34.8600	630			
34.8800	1642				34.8800	594			
34.9000	1714				34.9000	612			
34.9200	1704				34.9200	614			
34.9400	1640				34.9400	634			
34.9600	1706				34.9600	616			

34.9800	1606				34.9800	606			
35.0000	1696				35.0000	630			
35.0200	1664				35.0200	596			
35.0400	1644				35.0400	618			
35.0600	1692				35.0600	622			
35.0800	1698				35.0800	632			
35.1000	1680				35.1000	566			
35.1200	1776				35.1200	634			
35.1400	1580				35.1400	520			
35.1600	1706				35.1600	622			
35.1800	1682				35.1800	616			
35.2000	1684				35.2000	600			
35.2200	1724				35.2200	612			
35.2400	1654				35.2400	582			
35.2600	1690				35.2600	618			
35.2800	1664				35.2800	668			
35.3000	1644				35.3000	594			
35.3200	1624				35.3200	594			
35.3400	1636				35.3400	558			
35.3600	1734				35.3600	634			
35.3800	1640				35.3800	600			
35.4000	1764				35.4000	594			
35.4200	1670				35.4200	550			
35.4400	1618				35.4400	586			
35.4600	1660				35.4600	636			
35.4800	1606				35.4800	572			
35.5000	1658				35.5000	554			
35.5200	1684				35.5200	600			
35.5400	1646				35.5400	568			
35.5600	1676				35.5600	560			

35.5800	1640				35.5800	616			
35.6000	1736				35.6000	586			
35.6200	1588				35.6200	614			
35.6400	1688				35.6400	616			
35.6600	1646				35.6600	596			
35.6800	1658				35.6800	626			
35.7000	1672				35.7000	546			
35.7200	1632				35.7200	656			
35.7400	1710				35.7400	606			
35.7600	1630				35.7600	592			
35.7800	1642				35.7800	550			
35.8000	1602				35.8000	574			
35.8200	1700				35.8200	626			
35.8400	1700				35.8400	562			
35.8600	1562				35.8600	544			
35.8800	1704				35.8800	602			
35.9000	1598				35.9000	552			
35.9200	1554				35.9200	592			
35.9400	1592				35.9400	592			
35.9600	1638				35.9600	530			
35.9800	1642				35.9800	580			
36.0000	1636				36.0000	548			
36.0200	1626				36.0200	608			
36.0400	1644				36.0400	592			
36.0600	1666				36.0600	624			
36.0800	1704				36.0800	598			
36.1000	1668				36.1000	556			
36.1200	1678				36.1200	588			
36.1400	1744				36.1400	600			
36.1600	1682				36.1600	556			

36.1800	1632				36.1800	588			
36.2000	1608				36.2000	614			
36.2200	1640				36.2200	618			
36.2400	1610				36.2400	610			
36.2600	1678				36.2600	574			
36.2800	1656				36.2800	614			
36.3000	1688				36.3000	606			
36.3200	1650				36.3200	574			
36.3400	1664				36.3400	608			
36.3600	1614				36.3600	590			
36.3800	1600				36.3800	616			
36.4000	1716				36.4000	602			
36.4200	1622				36.4200	596			
36.4400	1628				36.4400	626			
36.4600	1584				36.4600	600			
36.4800	1634				36.4800	602			
36.5000	1582				36.5000	644			
36.5200	1586				36.5200	608			
36.5400	1650				36.5400	606			
36.5600	1660				36.5600	558			
36.5800	1588				36.5800	636			
36.6000	1634				36.6000	602			
36.6200	1692				36.6200	522			
36.6400	1592				36.6400	572			
36.6600	1578				36.6600	568			
36.6800	1654				36.6800	616			
36.7000	1658				36.7000	610			
36.7200	1556				36.7200	524			
36.7400	1686				36.7400	642			
36.7600	1704				36.7600	618			

36.7800	1670				36.7800	572			
36.8000	1712				36.8000	582			
36.8200	1586				36.8200	584			
36.8400	1660				36.8400	610			
36.8600	1614				36.8600	624			
36.8800	1588				36.8800	604			
36.9000	1634				36.9000	586			
36.9200	1630				36.9200	596			
36.9400	1570				36.9400	618			
36.9600	1662				36.9600	568			
36.9800	1650				36.9800	554			
37.0000	1608				37.0000	566			
37.0200	1578				37.0200	516			
37.0400	1666				37.0400	582			
37.0600	1600				37.0600	580			
37.0800	1654				37.0800	558			
37.1000	1646				37.1000	604			
37.1200	1588				37.1200	580			
37.1400	1590				37.1400	606			
37.1600	1612				37.1600	558			
37.1800	1598				37.1800	534			
37.2000	1630				37.2000	572			
37.2200	1550				37.2200	550			
37.2400	1598				37.2400	566			
37.2600	1710				37.2600	560			
37.2800	1594				37.2800	516			
37.3000	1612				37.3000	596			
37.3200	1666				37.3200	580			
37.3400	1646				37.3400	568			
37.3600	1656				37.3600	522			

37.3800	1646				37.3800	534			
37.4000	1684				37.4000	596			
37.4200	1640				37.4200	562			
37.4400	1654				37.4400	554			
37.4600	1612				37.4600	600			
37.4800	1612				37.4800	554			
37.5000	1678				37.5000	624			
37.5200	1712				37.5200	612			
37.5400	1716				37.5400	578			
37.5600	1752				37.5600	622			
37.5800	1702				37.5800	588			
37.6000	1662				37.6000	684			
37.6200	1680				37.6200	610			
37.6400	1744				37.6400	614			
37.6600	1684				37.6600	662			
37.6800	1814				37.6800	716			
37.7000	1798				37.7000	746			
37.7200	1800				37.7200	710			
37.7400	1874				37.7400	698			
37.7600	1866				37.7600	808			
37.7800	1916				37.7800	790			
37.8000	1920				37.8000	844			
37.8200	1856				37.8200	878			
37.8400	2010				37.8400	830			
37.8600	1892				37.8600	1024			
37.8800	2002				37.8800	1082			
37.9000	2176				37.9000	1164			
37.9200	1996				37.9200	1234			
37.9400	2306				37.9400	1350			
37.9600	2258				37.9600	1460			

37.9800	2292				37.9800	1568			
38.0000	2558				38.0000	1780			
38.0200	2402				38.0200	2026			
38.0400	2770				38.0400	2070			
38.0600	2676				38.0600	2362			
38.0800	2820				38.0800	2506			
38.1000	3062				38.1000	2874			
38.1200	2750				38.1200	2924			
38.1400	3068				38.1400	3190			
38.1600	3080				38.1600	3360			
38.1800	2950				38.1800	3458			
38.2000	3192				38.2000	3676			
38.2200	2764				38.2200	3752			
38.2400	3010				38.2400	3640			
38.2600	2996				38.2600	3620			
38.2800	2702				38.2800	3524			
38.3000	2824				38.3000	3404			
38.3200	2492				38.3200	3414			
38.3400	2550				38.3400	3058			
38.3600	2440				38.3600	2808			
38.3800	2202				38.3800	2728			
38.4000	2196				38.4000	2492			
38.4200	2052				38.4200	2250			
38.4400	2080				38.4400	1998			
38.4600	1882				38.4600	1794			
38.4800	1862				38.4800	1660			
38.5000	1772				38.5000	1430			
38.5200	1760				38.5200	1338			
38.5400	1718				38.5400	1122			
38.5600	1720				38.5600	1068			

38.5800	1632				38.5800	972			
38.6000	1656				38.6000	886			
38.6200	1594				38.6200	824			
38.6400	1632				38.6400	764			
38.6600	1562				38.6600	746			
38.6800	1642				38.6800	736			
38.7000	1538				38.7000	722			
38.7200	1610				38.7200	700			
38.7400	1586				38.7400	628			
38.7600	1576				38.7600	600			
38.7800	1596				38.7800	652			
38.8000	1498				38.8000	580			
38.8200	1524				38.8200	624			
38.8400	1622				38.8400	618			
38.8600	1546				38.8600	614			
38.8800	1560				38.8800	590			
38.9000	1514				38.9000	548			
38.9200	1518				38.9200	558			
38.9400	1618				38.9400	588			
38.9600	1552				38.9600	550			
38.9800	1484				38.9800	570			
39.0000	1552				39.0000	542			
39.0200	1524				39.0200	536			
39.0400	1562				39.0400	560			
39.0600	1492				39.0600	540			
39.0800	1510				39.0800	568			
39.1000	1490				39.1000	530			
39.1200	1528				39.1200	554			
39.1400	1518				39.1400	542			
39.1600	1468				39.1600	536			

39.1800 1562				39.1800 544			
39.2000 1552				39.2000 564			
39.2200 1534				39.2200 518			
39.2400 1558				39.2400 562			
39.2600 1574				39.2600 516			
39.2800 1526				39.2800 546			
39.3000 1466				39.3000 554			
39.3200 1550				39.3200 546			
39.3400 1490				39.3400 542			
39.3600 1520				39.3600 510			
39.3800 1492				39.3800 522			
39.4000 1526				39.4000 558			
39.4200 1508				39.4200 530			
39.4400 1516				39.4400 574			
39.4600 1476				39.4600 520			
				39.4800 580			
				39.5000 560			
				39.5200 530			
				39.5400 584			
				39.5600 516			
				39.5800 572			
				39.6000 502			
				39.6200 514			
				39.6400 586			
				39.6600 556			
				39.6800 516			
				39.7000 566			
				39.7200 542			
				39.7400 568			
				39.7600 578			

				39.7800 546			
				39.8000 576			
				39.8200 548			
				39.8400 606			
				39.8600 552			
				39.8800 608			
				39.9000 592			
				39.9200 590			
				39.9400 628			
				39.9600 564			
				39.9800 562			
				40.0000 564			
