# Supplementary material for: Data on statistical experimental design to formulate amphotericin B-loaded Eudragit RL100 nanoparticles coated with hyaluronic acid for the treatment of vulvovaginal candidiasis
Source: Data Brief. 2020 Mar 5;29:105311. doi: 10.1016/j.dib.2020.105311 (PMC7082528; doi:10.1016/j.dib.2020.105311)
Supplement: Multimedia component 17 [file mmc17.pdf]

**/// Profile Data Ascii Dump (XRD)**	**/// Profile Data Ascii Dump (XRD)**
//	//
**Data : V-AMP-EUD-Nanoparticles**	**Data : VI-EUD-Nano-HA**
File Name : V-AMP-EUD-Nanoparticles.RAW	File Name : VI-EUD-Nano-HA.RAW
# Profile Datafile	# Profile Datafile
comment = V-AMP-EUD-Nanoparticles	comment = VI-EUD-Nano-HA
date & time = 07-29-19 11:04:13	date & time = 07-29-19 12:02:20
# Measurement Condition	# Measurement Condition
X-ray tube	X-ray tube
target = Cu	target = Cu
voltage = 40.0 (kV)	voltage = 40.0 (kV)
current = 30.0 (mA)	current = 30.0 (mA)
Slits	Slits
divergence slit = 1.00000 (deg)	divergence slit = 1.00000 (deg)
scatter slit = 1.00000 (deg)	scatter slit = 1.00000 (deg)
receiving slit = 0.30000 (mm)	receiving slit = 0.30000 (mm)
Scanning	Scanning
drive axis = Theta-2Theta	drive axis = Theta-2Theta
scan range = 5.000 - 40.000	scan range = 5.000 - 33.600
scan mode = Continuous Scan	scan mode = Continuous Scan
scan speed = 1.0000 (deg/min)	scan speed = 1.0000 (deg/min)
sampling pitch = 0.0200 (deg)	sampling pitch = 0.0200 (deg)
preset time = 1.20 (sec)	preset time = 1.20 (sec)
# Data [Total No. = 1751]	# Data [Total No. = 1431]

<2Theta>	< I >
5.0000	2162
5.0200	2178
5.0400	2228
5.0600	2168
5.0800	2092
5.1000	2122
5.1200	2184
5.1400	2116
5.1600	2108
5.1800	2126
5.2000	2094
5.2200	2120
5.2400	2136
5.2600	2054
5.2800	2008
5.3000	2214
5.3200	2162
5.3400	2074
5.3600	2018
5.3800	2032
5.4000	2040
5.4200	2108
5.4400	2064
5.4600	1944
5.4800	2086
5.5000	2070
5.5200	2012
5.5400	2026
5.5600	1940

<2Theta>	< I >
5.0000	2244
5.0200	2142
5.0400	2220
5.0600	2168
5.0800	2094
5.1000	2076
5.1200	2144
5.1400	2212
5.1600	2070
5.1800	2052
5.2000	2034
5.2200	2196
5.2400	2200
5.2600	2036
5.2800	1982
5.3000	1996
5.3200	2004
5.3400	2030
5.3600	1960
5.3800	1998
5.4000	2004
5.4200	2050
5.4400	2024
5.4600	1898
5.4800	1930
5.5000	2046
5.5200	2034
5.5400	1944
5.5600	1790

5.5800	1976				5.5800	1956			
5.6000	2002				5.6000	1956			
5.6200	1914				5.6200	1950			
5.6400	1948				5.6400	1844			
5.6600	1904				5.6600	2008			
5.6800	2066				5.6800	1944			
5.7000	2022				5.7000	2018			
5.7200	1964				5.7200	1838			
5.7400	1856				5.7400	1806			
5.7600	1902				5.7600	1770			
5.7800	1998				5.7800	1956			
5.8000	2018				5.8000	1904			
5.8200	1826				5.8200	1844			
5.8400	1876				5.8400	1804			
5.8600	1898				5.8600	1800			
5.8800	1876				5.8800	1914			
5.9000	1894				5.9000	1904			
5.9200	1848				5.9200	1790			
5.9400	1864				5.9400	1876			
5.9600	1880				5.9600	1886			
5.9800	1990				5.9800	1838			
6.0000	1876				6.0000	1912			
6.0200	1786				6.0200	1692			
6.0400	1762				6.0400	1778			
6.0600	1862				6.0600	1736			
6.0800	1954				6.0800	1806			
6.1000	1794				6.1000	1788			
6.1200	1846				6.1200	1684			
6.1400	1836				6.1400	1772			
6.1600	1868				6.1600	1798			

6.1800	1862				6.1800	1750			
6.2000	1770				6.2000	1796			
6.2200	1740				6.2200	1732			
6.2400	1846				6.2400	1824			
6.2600	1822				6.2600	1718			
6.2800	1844				6.2800	1890			
6.3000	1726				6.3000	1704			
6.3200	1788				6.3200	1698			
6.3400	1778				6.3400	1738			
6.3600	1852				6.3600	1748			
6.3800	1738				6.3800	1644			
6.4000	1684				6.4000	1658			
6.4200	1764				6.4200	1640			
6.4400	1744				6.4400	1720			
6.4600	1870				6.4600	1692			
6.4800	1730				6.4800	1658			
6.5000	1706				6.5000	1664			
6.5200	1818				6.5200	1658			
6.5400	1842				6.5400	1636			
6.5600	1754				6.5600	1784			
6.5800	1642				6.5800	1582			
6.6000	1758				6.6000	1698			
6.6200	1702				6.6200	1664			
6.6400	1776				6.6400	1662			
6.6600	1644				6.6600	1760			
6.6800	1670				6.6800	1636			
6.7000	1694				6.7000	1604			
6.7200	1768				6.7200	1696			
6.7400	1788				6.7400	1620			
6.7600	1700				6.7600	1604			

6.7800 1612				6.7800 1622			
6.8000 1668				6.8000 1604			
6.8200 1702				6.8200 1698			
6.8400 1734				6.8400 1670			
6.8600 1720				6.8600 1648			
6.8800 1654				6.8800 1646			
6.9000 1740				6.9000 1668			
6.9200 1796				6.9200 1646			
6.9400 1658				6.9400 1610			
6.9600 1676				6.9600 1680			
6.9800 1660				6.9800 1590			
7.0000 1726				7.0000 1652			
7.0200 1726				7.0200 1668			
7.0400 1594				7.0400 1582			
7.0600 1614				7.0600 1604			
7.0800 1682				7.0800 1586			
7.1000 1744				7.1000 1624			
7.1200 1766				7.1200 1566			
7.1400 1672				7.1400 1462			
7.1600 1650				7.1600 1580			
7.1800 1636				7.1800 1598			
7.2000 1660				7.2000 1588			
7.2200 1726				7.2200 1610			
7.2400 1596				7.2400 1526			
7.2600 1584				7.2600 1562			
7.2800 1626				7.2800 1526			
7.3000 1662				7.3000 1592			
7.3200 1686				7.3200 1626			
7.3400 1502				7.3400 1528			
7.3600 1638				7.3600 1566			

7.3800	1596				7.3800	1610			
7.4000	1566				7.4000	1536			
7.4200	1560				7.4200	1478			
7.4400	1528				7.4400	1460			
7.4600	1646				7.4600	1588			
7.4800	1564				7.4800	1580			
7.5000	1542				7.5000	1566			
7.5200	1586				7.5200	1492			
7.5400	1510				7.5400	1450			
7.5600	1716				7.5600	1586			
7.5800	1648				7.5800	1536			
7.6000	1590				7.6000	1476			
7.6200	1526				7.6200	1448			
7.6400	1506				7.6400	1466			
7.6600	1660				7.6600	1536			
7.6800	1612				7.6800	1550			
7.7000	1480				7.7000	1366			
7.7200	1468				7.7200	1428			
7.7400	1512				7.7400	1510			
7.7600	1522				7.7600	1482			
7.7800	1510				7.7800	1458			
7.8000	1500				7.8000	1416			
7.8200	1530				7.8200	1428			
7.8400	1588				7.8400	1440			
7.8600	1610				7.8600	1478			
7.8800	1532				7.8800	1450			
7.9000	1456				7.9000	1420			
7.9200	1470				7.9200	1422			
7.9400	1550				7.9400	1374			
7.9600	1510				7.9600	1538			

7.9800	1440				7.9800	1418			
8.0000	1508				8.0000	1350			
8.0200	1500				8.0200	1414			
8.0400	1622				8.0400	1550			
8.0600	1444				8.0600	1354			
8.0800	1508				8.0800	1354			
8.1000	1492				8.1000	1346			
8.1200	1470				8.1200	1420			
8.1400	1480				8.1400	1456			
8.1600	1424				8.1600	1334			
8.1800	1454				8.1800	1386			
8.2000	1488				8.2000	1408			
8.2200	1522				8.2200	1474			
8.2400	1470				8.2400	1386			
8.2600	1412				8.2600	1372			
8.2800	1418				8.2800	1324			
8.3000	1462				8.3000	1310			
8.3200	1494				8.3200	1414			
8.3400	1468				8.3400	1366			
8.3600	1380				8.3600	1350			
8.3800	1392				8.3800	1310			
8.4000	1444				8.4000	1434			
8.4200	1426				8.4200	1432			
8.4400	1364				8.4400	1314			
8.4600	1330				8.4600	1312			
8.4800	1468				8.4800	1368			
8.5000	1534				8.5000	1406			
8.5200	1420				8.5200	1386			
8.5400	1426				8.5400	1342			
8.5600	1356				8.5600	1332			

8.5800 1472				8.5800 1424			
8.6000 1494				8.6000 1438			
8.6200 1350				8.6200 1304			
8.6400 1402				8.6400 1368			
8.6600 1364				8.6600 1352			
8.6800 1456				8.6800 1372			
8.7000 1456				8.7000 1460			
8.7200 1416				8.7200 1330			
8.7400 1434				8.7400 1300			
8.7600 1440				8.7600 1280			
8.7800 1508				8.7800 1382			
8.8000 1424				8.8000 1364			
8.8200 1350				8.8200 1270			
8.8400 1312				8.8400 1262			
8.8600 1466				8.8600 1288			
8.8800 1406				8.8800 1326			
8.9000 1480				8.9000 1280			
8.9200 1364				8.9200 1254			
8.9400 1344				8.9400 1310			
8.9600 1430				8.9600 1342			
8.9800 1454				8.9800 1346			
9.0000 1372				9.0000 1332			
9.0200 1418				9.0200 1224			
9.0400 1354				9.0400 1254			
9.0600 1398				9.0600 1312			
9.0800 1350				9.0800 1290			
9.1000 1294				9.1000 1274			
9.1200 1318				9.1200 1182			
9.1400 1336				9.1400 1302			
9.1600 1480				9.1600 1378			

9.1800	1438				9.1800	1268			
9.2000	1296				9.2000	1190			
9.2200	1340				9.2200	1222			
9.2400	1368				9.2400	1302			
9.2600	1434				9.2600	1306			
9.2800	1372				9.2800	1228			
9.3000	1330				9.3000	1254			
9.3200	1286				9.3200	1228			
9.3400	1404				9.3400	1346			
9.3600	1398				9.3600	1262			
9.3800	1306				9.3800	1200			
9.4000	1254				9.4000	1230			
9.4200	1362				9.4200	1250			
9.4400	1332				9.4400	1232			
9.4600	1360				9.4600	1260			
9.4800	1280				9.4800	1168			
9.5000	1240				9.5000	1214			
9.5200	1222				9.5200	1194			
9.5400	1342				9.5400	1340			
9.5600	1290				9.5600	1212			
9.5800	1324				9.5800	1160			
9.6000	1308				9.6000	1202			
9.6200	1354				9.6200	1190			
9.6400	1376				9.6400	1248			
9.6600	1240				9.6600	1218			
9.6800	1196				9.6800	1152			
9.7000	1314				9.7000	1198			
9.7200	1324				9.7200	1212			
9.7400	1322				9.7400	1198			
9.7600	1282				9.7600	1200			

9.7800	1238				9.7800	1110			
9.8000	1290				9.8000	1216			
9.8200	1346				9.8200	1250			
9.8400	1260				9.8400	1168			
9.8600	1198				9.8600	1156			
9.8800	1224				9.8800	1132			
9.9000	1328				9.9000	1154			
9.9200	1352				9.9200	1222			
9.9400	1206				9.9400	1172			
9.9600	1248				9.9600	1116			
9.9800	1300				9.9800	1136			
10.0000	1316				10.0000	1200			
10.0200	1252				10.0200	1172			
10.0400	1220				10.0400	1142			
10.0600	1196				10.0600	1146			
10.0800	1280				10.0800	1148			
10.1000	1340				10.1000	1234			
10.1200	1278				10.1200	1170			
10.1400	1206				10.1400	1122			
10.1600	1274				10.1600	1082			
10.1800	1280				10.1800	1186			
10.2000	1292				10.2000	1192			
10.2200	1284				10.2200	1116			
10.2400	1200				10.2400	1062			
10.2600	1284				10.2600	1170			
10.2800	1254				10.2800	1142			
10.3000	1302				10.3000	1194			
10.3200	1248				10.3200	1042			
10.3400	1184				10.3400	1114			
10.3600	1272				10.3600	1164			

10.3800	1358				10.3800	1150			
10.4000	1230				10.4000	1132			
10.4200	1252				10.4200	1064			
10.4400	1226				10.4400	1100			
10.4600	1294				10.4600	1122			
10.4800	1238				10.4800	1124			
10.5000	1240				10.5000	1092			
10.5200	1176				10.5200	1058			
10.5400	1294				10.5400	1192			
10.5600	1210				10.5600	1150			
10.5800	1268				10.5800	1146			
10.6000	1108				10.6000	1058			
10.6200	1150				10.6200	1048			
10.6400	1232				10.6400	1166			
10.6600	1238				10.6600	1142			
10.6800	1218				10.6800	1092			
10.7000	1160				10.7000	1082			
10.7200	1142				10.7200	1092			
10.7400	1188				10.7400	1100			
10.7600	1206				10.7600	1188			
10.7800	1156				10.7800	996			
10.8000	1140				10.8000	1044			
10.8200	1254				10.8200	1048			
10.8400	1266				10.8400	1182			
10.8600	1224				10.8600	1138			
10.8800	1156				10.8800	1006			
10.9000	1076				10.9000	1056			
10.9200	1180				10.9200	1070			
10.9400	1272				10.9400	1072			
10.9600	1174				10.9600	1102			

10.9800	1216				10.9800	996			
11.0000	1144				11.0000	1052			
11.0200	1226				11.0200	1018			
11.0400	1242				11.0400	1104			
11.0600	1130				11.0600	1116			
11.0800	1116				11.0800	1000			
11.1000	1154				11.1000	1056			
11.1200	1176				11.1200	1160			
11.1400	1232				11.1400	1104			
11.1600	1170				11.1600	1028			
11.1800	1206				11.1800	1040			
11.2000	1154				11.2000	1024			
11.2200	1310				11.2200	1086			
11.2400	1184				11.2400	1032			
11.2600	1198				11.2600	992			
11.2800	1140				11.2800	1014			
11.3000	1190				11.3000	1032			
11.3200	1234				11.3200	1082			
11.3400	1152				11.3400	992			
11.3600	1128				11.3600	1022			
11.3800	1130				11.3800	1060			
11.4000	1168				11.4000	1074			
11.4200	1132				11.4200	1044			
11.4400	1180				11.4400	1012			
11.4600	1124				11.4600	1064			
11.4800	1132				11.4800	1044			
11.5000	1202				11.5000	1082			
11.5200	1142				11.5200	1094			
11.5400	1134				11.5400	944			
11.5600	1158				11.5600	1054			

11.5800	1198			11.5800	1084		
11.6000	1232			11.6000	1014		
11.6200	1176			11.6200	1040		
11.6400	1082			11.6400	1006		
11.6600	1124			11.6600	1032		
11.6800	1162			11.6800	1048		
11.7000	1102			11.7000	1004		
11.7200	1080			11.7200	966		
11.7400	1168			11.7400	1018		
11.7600	1162			11.7600	1052		
11.7800	1200			11.7800	1038		
11.8000	1182			11.8000	1030		
11.8200	1114			11.8200	994		
11.8400	1096			11.8400	974		
11.8600	1184			11.8600	1104		
11.8800	1172			11.8800	1094		
11.9000	1132			11.9000	1024		
11.9200	1142			11.9200	996		
11.9400	1118			11.9400	942		
11.9600	1136			11.9600	1054		
11.9800	1196			11.9800	1136		
12.0000	1108			12.0000	998		
12.0200	1100			12.0200	1010		
12.0400	1162			12.0400	1000		
12.0600	1130			12.0600	1042		
12.0800	1144			12.0800	1052		
12.1000	1104			12.1000	1000		
12.1200	1170			12.1200	1022		
12.1400	1166			12.1400	992		
12.1600	1106			12.1600	1040		

12.1800	1120				12.1800	1006			
12.2000	1182				12.2000	972			
12.2200	1146				12.2200	948			
12.2400	1172				12.2400	982			
12.2600	1158				12.2600	1014			
12.2800	1130				12.2800	974			
12.3000	1026				12.3000	956			
12.3200	1176				12.3200	976			
12.3400	1134				12.3400	998			
12.3600	1144				12.3600	998			
12.3800	1136				12.3800	946			
12.4000	1110				12.4000	1000			
12.4200	1162				12.4200	1016			
12.4400	1214				12.4400	1014			
12.4600	1108				12.4600	962			
12.4800	1092				12.4800	968			
12.5000	1078				12.5000	972			
12.5200	1246				12.5200	992			
12.5400	1082				12.5400	1048			
12.5600	1108				12.5600	960			
12.5800	1094				12.5800	992			
12.6000	1146				12.6000	986			
12.6200	1194				12.6200	1058			
12.6400	1060				12.6400	1022			
12.6600	1138				12.6600	898			
12.6800	1044				12.6800	916			
12.7000	1114				12.7000	980			
12.7200	1176				12.7200	1032			
12.7400	1078				12.7400	972			
12.7600	1086				12.7600	900			

12.7800	1124				12.7800	936			
12.8000	1140				12.8000	1000			
12.8200	1180				12.8200	928			
12.8400	1088				12.8400	1012			
12.8600	1036				12.8600	904			
12.8800	1068				12.8800	954			
12.9000	1134				12.9000	1000			
12.9200	1152				12.9200	908			
12.9400	1026				12.9400	952			
12.9600	1010				12.9600	922			
12.9800	1148				12.9800	924			
13.0000	1108				13.0000	1008			
13.0200	1196				13.0200	996			
13.0400	1020				13.0400	966			
13.0600	1066				13.0600	944			
13.0800	1190				13.0800	968			
13.1000	1052				13.1000	986			
13.1200	1106				13.1200	942			
13.1400	1138				13.1400	930			
13.1600	1166				13.1600	944			
13.1800	1126				13.1800	974			
13.2000	1132				13.2000	1010			
13.2200	1094				13.2200	884			
13.2400	1064				13.2400	1016			
13.2600	1152				13.2600	956			
13.2800	1136				13.2800	986			
13.3000	1120				13.3000	978			
13.3200	1152				13.3200	874			
13.3400	1092				13.3400	902			
13.3600	1120				13.3600	944			

13.3800	1182				13.3800	930			
13.4000	1114				13.4000	934			
13.4200	1040				13.4200	916			
13.4400	1116				13.4400	884			
13.4600	1152				13.4600	976			
13.4800	1134				13.4800	952			
13.5000	1072				13.5000	960			
13.5200	1028				13.5200	926			
13.5400	1130				13.5400	928			
13.5600	1148				13.5600	964			
13.5800	1076				13.5800	910			
13.6000	1100				13.6000	976			
13.6200	1078				13.6200	846			
13.6400	1132				13.6400	1026			
13.6600	1134				13.6600	1066			
13.6800	1094				13.6800	924			
13.7000	1126				13.7000	926			
13.7200	1104				13.7200	894			
13.7400	1104				13.7400	1004			
13.7600	1116				13.7600	930			
13.7800	1070				13.7800	926			
13.8000	1076				13.8000	886			
13.8200	1078				13.8200	930			
13.8400	1166				13.8400	964			
13.8600	1134				13.8600	950			
13.8800	1022				13.8800	886			
13.9000	1094				13.9000	1014			
13.9200	1098				13.9200	898			
13.9400	1106				13.9400	960			
13.9600	1060				13.9600	986			

13.9800	1094				13.9800	874			
14.0000	1078				14.0000	940			
14.0200	1140				14.0200	948			
14.0400	1162				14.0400	948			
14.0600	1072				14.0600	920			
14.0800	1030				14.0800	916			
14.1000	1112				14.1000	948			
14.1200	1120				14.1200	960			
14.1400	1122				14.1400	938			
14.1600	1030				14.1600	940			
14.1800	1038				14.1800	922			
14.2000	1204				14.2000	988			
14.2200	1140				14.2200	1016			
14.2400	1182				14.2400	946			
14.2600	1130				14.2600	926			
14.2800	1040				14.2800	900			
14.3000	1128				14.3000	878			
14.3200	1136				14.3200	960			
14.3400	1104				14.3400	892			
14.3600	1066				14.3600	846			
14.3800	1046				14.3800	874			
14.4000	1192				14.4000	970			
14.4200	1170				14.4200	910			
14.4400	1104				14.4400	942			
14.4600	1086				14.4600	912			
14.4800	1096				14.4800	910			
14.5000	1132				14.5000	964			
14.5200	1072				14.5200	922			
14.5400	1032				14.5400	838			
14.5600	1110				14.5600	880			

14.5800	1122				14.5800	886			
14.6000	1106				14.6000	962			
14.6200	1046				14.6200	874			
14.6400	1050				14.6400	872			
14.6600	1112				14.6600	884			
14.6800	1096				14.6800	918			
14.7000	1178				14.7000	906			
14.7200	1030				14.7200	898			
14.7400	1052				14.7400	804			
14.7600	1080				14.7600	948			
14.7800	1136				14.7800	962			
14.8000	1114				14.8000	886			
14.8200	1042				14.8200	916			
14.8400	1060				14.8400	820			
14.8600	1144				14.8600	876			
14.8800	1188				14.8800	974			
14.9000	1062				14.9000	858			
14.9200	1066				14.9200	846			
14.9400	1018				14.9400	838			
14.9600	1124				14.9600	870			
14.9800	1128				14.9800	938			
15.0000	1074				15.0000	850			
15.0200	1064				15.0200	888			
15.0400	1106				15.0400	882			
15.0600	1228				15.0600	942			
15.0800	1096				15.0800	888			
15.1000	1054				15.1000	838			
15.1200	1144				15.1200	916			
15.1400	1106				15.1400	872			
15.1600	1154				15.1600	896			

15.1800	1106				15.1800	894			
15.2000	1080				15.2000	872			
15.2200	1174				15.2200	868			
15.2400	1130				15.2400	932			
15.2600	1150				15.2600	900			
15.2800	1074				15.2800	922			
15.3000	1078				15.3000	852			
15.3200	1156				15.3200	908			
15.3400	1140				15.3400	930			
15.3600	1140				15.3600	906			
15.3800	1038				15.3800	888			
15.4000	1048				15.4000	880			
15.4200	1184				15.4200	906			
15.4400	1156				15.4400	934			
15.4600	1086				15.4600	848			
15.4800	1078				15.4800	922			
15.5000	1098				15.5000	876			
15.5200	1212				15.5200	978			
15.5400	1174				15.5400	962			
15.5600	1110				15.5600	842			
15.5800	1092				15.5800	868			
15.6000	1078				15.6000	942			
15.6200	1130				15.6200	930			
15.6400	1180				15.6400	896			
15.6600	1008				15.6600	862			
15.6800	1136				15.6800	904			
15.7000	1184				15.7000	892			
15.7200	1184				15.7200	914			
15.7400	1134				15.7400	914			
15.7600	1098				15.7600	802			

15.7800	1148			15.7800	850		
15.8000	1158			15.8000	838		
15.8200	1106			15.8200	912		
15.8400	1116			15.8400	886		
15.8600	1006			15.8600	868		
15.8800	1120			15.8800	896		
15.9000	1146			15.9000	854		
15.9200	1132			15.9200	914		
15.9400	1142			15.9400	908		
15.9600	1056			15.9600	832		
15.9800	1200			15.9800	950		
16.0000	1242			16.0000	938		
16.0200	1112			16.0200	868		
16.0400	1096			16.0400	878		
16.0600	1134			16.0600	880		
16.0800	1142			16.0800	938		
16.1000	1164			16.1000	948		
16.1200	1082			16.1200	888		
16.1400	1062			16.1400	896		
16.1600	1066			16.1600	884		
16.1800	1198			16.1800	1008		
16.2000	1146			16.2000	956		
16.2200	1072			16.2200	848		
16.2400	1168			16.2400	874		
16.2600	1116			16.2600	864		
16.2800	1214			16.2800	964		
16.3000	1154			16.3000	932		
16.3200	1084			16.3200	866		
16.3400	1158			16.3400	884		
16.3600	1104			16.3600	852		

16.3800	1262				16.3800	862			
16.4000	1120				16.4000	928			
16.4200	1034				16.4200	796			
16.4400	1192				16.4400	908			
16.4600	1200				16.4600	906			
16.4800	1130				16.4800	844			
16.5000	1126				16.5000	856			
16.5200	1090				16.5200	866			
16.5400	1156				16.5400	860			
16.5600	1204				16.5600	952			
16.5800	1158				16.5800	894			
16.6000	1192				16.6000	908			
16.6200	1120				16.6200	870			
16.6400	1208				16.6400	838			
16.6600	1250				16.6600	920			
16.6800	1076				16.6800	850			
16.7000	1150				16.7000	924			
16.7200	1090				16.7200	838			
16.7400	1276				16.7400	932			
16.7600	1236				16.7600	946			
16.7800	1038				16.7800	858			
16.8000	1174				16.8000	908			
16.8200	1108				16.8200	818			
16.8400	1174				16.8400	880			
16.8600	1142				16.8600	898			
16.8800	1136				16.8800	850			
16.9000	1154				16.9000	886			
16.9200	1178				16.9200	884			
16.9400	1194				16.9400	910			
16.9600	1226				16.9600	952			

16.9800	1090				16.9800	822			
17.0000	1204				17.0000	886			
17.0200	1224				17.0200	880			
17.0400	1184				17.0400	916			
17.0600	1140				17.0600	950			
17.0800	1128				17.0800	816			
17.1000	1262				17.1000	920			
17.1200	1252				17.1200	974			
17.1400	1178				17.1400	860			
17.1600	1140				17.1600	916			
17.1800	1062				17.1800	872			
17.2000	1240				17.2000	932			
17.2200	1232				17.2200	946			
17.2400	1192				17.2400	874			
17.2600	1176				17.2600	852			
17.2800	1114				17.2800	896			
17.3000	1238				17.3000	948			
17.3200	1162				17.3200	916			
17.3400	1168				17.3400	842			
17.3600	1146				17.3600	868			
17.3800	1150				17.3800	890			
17.4000	1256				17.4000	908			
17.4200	1236				17.4200	862			
17.4400	1106				17.4400	824			
17.4600	1192				17.4600	950			
17.4800	1192				17.4800	944			
17.5000	1244				17.5000	932			
17.5200	1214				17.5200	894			
17.5400	1110				17.5400	810			
17.5600	1266				17.5600	962			

17.5800	1208			17.5800	910		
17.6000	1242			17.6000	904		
17.6200	1188			17.6200	900		
17.6400	1164			17.6400	894		
17.6600	1252			17.6600	956		
17.6800	1258			17.6800	912		
17.7000	1216			17.7000	920		
17.7200	1248			17.7200	930		
17.7400	1052			17.7400	814		
17.7600	1264			17.7600	926		
17.7800	1264			17.7800	926		
17.8000	1150			17.8000	830		
17.8200	1242			17.8200	896		
17.8400	1172			17.8400	834		
17.8600	1226			17.8600	980		
17.8800	1308			17.8800	948		
17.9000	1098			17.9000	864		
17.9200	1224			17.9200	938		
17.9400	1170			17.9400	818		
17.9600	1386			17.9600	940		
17.9800	1242			17.9800	966		
18.0000	1140			18.0000	860		
18.0200	1226			18.0200	896		
18.0400	1160			18.0400	862		
18.0600	1352			18.0600	928		
18.0800	1286			18.0800	932		
18.1000	1128			18.1000	836		
18.1200	1270			18.1200	936		
18.1400	1306			18.1400	948		
18.1600	1278			18.1600	924		

18.1800	1280					18.1800	920				
18.2000	1116					18.2000	818				
18.2200	1190					18.2200	896				
18.2400	1294					18.2400	960				
18.2600	1214					18.2600	900				
18.2800	1184					18.2800	850				
18.3000	1142					18.3000	816				
18.3200	1278					18.3200	856				
18.3400	1370					18.3400	946				
18.3600	1198					18.3600	896				
18.3800	1288					18.3800	960				
18.4000	1174					18.4000	858				
18.4200	1314					18.4200	952				
18.4400	1284					18.4400	972				
18.4600	1140					18.4600	790				
18.4800	1284					18.4800	930				
18.5000	1154					18.5000	850				
18.5200	1320					18.5200	910				
18.5400	1304					18.5400	926				
18.5600	1184					18.5600	864				
18.5800	1226					18.5800	930				
18.6000	1136					18.6000	920				
18.6200	1376					18.6200	896				
18.6400	1272					18.6400	940				
18.6600	1164					18.6600	866				
18.6800	1272					18.6800	942				
18.7000	1290					18.7000	892				
18.7200	1344					18.7200	990				
18.7400	1198					18.7400	900				
18.7600	1188					18.7600	860				

18.7800	1330				18.7800	890			
18.8000	1322				18.8000	972			
18.8200	1298				18.8200	926			
18.8400	1284				18.8400	916			
18.8600	1116				18.8600	798			
18.8800	1280				18.8800	952			
18.9000	1338				18.9000	952			
18.9200	1282				18.9200	866			
18.9400	1246				18.9400	956			
18.9600	1184				18.9600	890			
18.9800	1380				18.9800	974			
19.0000	1386				19.0000	972			
19.0200	1222				19.0200	834			
19.0400	1378				19.0400	924			
19.0600	1230				19.0600	884			
19.0800	1366				19.0800	964			
19.1000	1266				19.1000	948			
19.1200	1220				19.1200	888			
19.1400	1244				19.1400	922			
19.1600	1262				19.1600	910			
19.1800	1394				19.1800	942			
19.2000	1300				19.2000	928			
19.2200	1128				19.2200	816			
19.2400	1308				19.2400	992			
19.2600	1280				19.2600	908			
19.2800	1316				19.2800	936			
19.3000	1276				19.3000	976			
19.3200	1176				19.3200	818			
19.3400	1322				19.3400	914			
19.3600	1332				19.3600	956			

19.3800	1306			19.3800	922		
19.4000	1310			19.4000	888		
19.4200	1184			19.4200	834		
19.4400	1450			19.4400	940		
19.4600	1268			19.4600	1004		
19.4800	1276			19.4800	854		
19.5000	1300			19.5000	952		
19.5200	1188			19.5200	868		
19.5400	1344			19.5400	968		
19.5600	1404			19.5600	1002		
19.5800	1298			19.5800	900		
19.6000	1424			19.6000	902		
19.6200	1232			19.6200	898		
19.6400	1380			19.6400	942		
19.6600	1346			19.6600	974		
19.6800	1172			19.6800	884		
19.7000	1338			19.7000	966		
19.7200	1268			19.7200	900		
19.7400	1420			19.7400	960		
19.7600	1354			19.7600	1006		
19.7800	1224			19.7800	818		
19.8000	1372			19.8000	964		
19.8200	1298			19.8200	920		
19.8400	1460			19.8400	984		
19.8600	1284			19.8600	932		
19.8800	1250			19.8800	846		
19.9000	1426			19.9000	916		
19.9200	1282			19.9200	938		
19.9400	1372			19.9400	938		
19.9600	1280			19.9600	962		

19.9800	1182				19.9800	850			
20.0000	1394				20.0000	942			
20.0200	1332				20.0200	988			
20.0400	1374				20.0400	956			
20.0600	1274				20.0600	1000			
20.0800	1196				20.0800	872			
20.1000	1396				20.1000	1006			
20.1200	1338				20.1200	974			
20.1400	1290				20.1400	862			
20.1600	1314				20.1600	908			
20.1800	1180				20.1800	832			
20.2000	1494				20.2000	1026			
20.2200	1328				20.2200	998			
20.2400	1204				20.2400	772			
20.2600	1376				20.2600	934			
20.2800	1234				20.2800	898			
20.3000	1314				20.3000	974			
20.3200	1316				20.3200	964			
20.3400	1226				20.3400	828			
20.3600	1296				20.3600	952			
20.3800	1270				20.3800	868			
20.4000	1360				20.4000	946			
20.4200	1388				20.4200	954			
20.4400	1172				20.4400	826			
20.4600	1358				20.4600	980			
20.4800	1252				20.4800	882			
20.5000	1328				20.5000	970			
20.5200	1360				20.5200	960			
20.5400	1238				20.5400	890			
20.5600	1318				20.5600	960			

20.5800	1378				20.5800	944			
20.6000	1322				20.6000	922			
20.6200	1364				20.6200	978			
20.6400	1142				20.6400	850			
20.6600	1378				20.6600	932			
20.6800	1402				20.6800	990			
20.7000	1306				20.7000	834			
20.7200	1364				20.7200	906			
20.7400	1248				20.7400	840			
20.7600	1392				20.7600	938			
20.7800	1364				20.7800	980			
20.8000	1198				20.8000	922			
20.8200	1370				20.8200	904			
20.8400	1216				20.8400	890			
20.8600	1388				20.8600	964			
20.8800	1332				20.8800	920			
20.9000	1150				20.9000	790			
20.9200	1340				20.9200	882			
20.9400	1218				20.9400	866			
20.9600	1350				20.9600	1004			
20.9800	1358				20.9800	938			
21.0000	1224				21.0000	816			
21.0200	1338				21.0200	930			
21.0400	1346				21.0400	932			
21.0600	1352				21.0600	906			
21.0800	1306				21.0800	958			
21.1000	1214				21.1000	858			
21.1200	1370				21.1200	996			
21.1400	1366				21.1400	934			
21.1600	1260				21.1600	894			

21.1800	1324				21.1800	900			
21.2000	1232				21.2000	868			
21.2200	1342				21.2200	982			
21.2400	1316				21.2400	938			
21.2600	1224				21.2600	866			
21.2800	1308				21.2800	936			
21.3000	1172				21.3000	846			
21.3200	1402				21.3200	976			
21.3400	1274				21.3400	958			
21.3600	1230				21.3600	828			
21.3800	1262				21.3800	914			
21.4000	1178				21.4000	894			
21.4200	1376				21.4200	922			
21.4400	1326				21.4400	1020			
21.4600	1164				21.4600	878			
21.4800	1320				21.4800	946			
21.5000	1200				21.5000	876			
21.5200	1386				21.5200	948			
21.5400	1300				21.5400	888			
21.5600	1236				21.5600	816			
21.5800	1326				21.5800	930			
21.6000	1204				21.6000	868			
21.6200	1316				21.6200	934			
21.6400	1306				21.6400	880			
21.6600	1196				21.6600	872			
21.6800	1326				21.6800	972			
21.7000	1248				21.7000	960			
21.7200	1256				21.7200	958			
21.7400	1322				21.7400	924			
21.7600	1110				21.7600	818			

21.7800	1336				21.7800	904			
21.8000	1320				21.8000	902			
21.8200	1166				21.8200	912			
21.8400	1364				21.8400	942			
21.8600	1160				21.8600	848			
21.8800	1340				21.8800	930			
21.9000	1334				21.9000	960			
21.9200	1242				21.9200	826			
21.9400	1246				21.9400	924			
21.9600	1122				21.9600	838			
21.9800	1330				21.9800	922			
22.0000	1340				22.0000	968			
22.0200	1150				22.0200	876			
22.0400	1318				22.0400	930			
22.0600	1178				22.0600	922			
22.0800	1368				22.0800	980			
22.1000	1316				22.1000	910			
22.1200	1056				22.1200	854			
22.1400	1258				22.1400	894			
22.1600	1232				22.1600	856			
22.1800	1294				22.1800	940			
22.2000	1154				22.2000	900			
22.2200	1122				22.2200	870			
22.2400	1280				22.2400	934			
22.2600	1228				22.2600	904			
22.2800	1242				22.2800	892			
22.3000	1202				22.3000	856			
22.3200	1138				22.3200	818			
22.3400	1270				22.3400	936			
22.3600	1266				22.3600	854			

22.3800	1208				22.3800	916			
22.4000	1222				22.4000	840			
22.4200	1138				22.4200	798			
22.4400	1316				22.4400	848			
22.4600	1272				22.4600	894			
22.4800	1182				22.4800	816			
22.5000	1280				22.5000	882			
22.5200	1106				22.5200	834			
22.5400	1306				22.5400	858			
22.5600	1264				22.5600	888			
22.5800	1100				22.5800	836			
22.6000	1256				22.6000	882			
22.6200	1140				22.6200	874			
22.6400	1254				22.6400	914			
22.6600	1246				22.6600	914			
22.6800	1088				22.6800	828			
22.7000	1252				22.7000	888			
22.7200	1128				22.7200	828			
22.7400	1264				22.7400	878			
22.7600	1208				22.7600	890			
22.7800	1010				22.7800	848			
22.8000	1216				22.8000	850			
22.8200	1154				22.8200	910			
22.8400	1230				22.8400	874			
22.8600	1232				22.8600	874			
22.8800	1100				22.8800	798			
22.9000	1226				22.9000	924			
22.9200	1238				22.9200	908			
22.9400	1088				22.9400	878			
22.9600	1156				22.9600	860			

22.9800	1106				22.9800	838			
23.0000	1218				23.0000	856			
23.0200	1192				23.0200	872			
23.0400	1072				23.0400	798			
23.0600	1168				23.0600	942			
23.0800	1058				23.0800	786			
23.1000	1258				23.1000	906			
23.1200	1240				23.1200	880			
23.1400	1072				23.1400	846			
23.1600	1182				23.1600	864			
23.1800	1022				23.1800	824			
23.2000	1270				23.2000	900			
23.2200	1216				23.2200	854			
23.2400	1144				23.2400	824			
23.2600	1206				23.2600	852			
23.2800	1074				23.2800	808			
23.3000	1132				23.3000	916			
23.3200	1126				23.3200	914			
23.3400	1030				23.3400	812			
23.3600	1180				23.3600	852			
23.3800	1162				23.3800	830			
23.4000	1112				23.4000	928			
23.4200	1202				23.4200	846			
23.4400	1030				23.4400	786			
23.4600	1156				23.4600	850			
23.4800	1136				23.4800	876			
23.5000	1066				23.5000	856			
23.5200	1162				23.5200	858			
23.5400	1072				23.5400	768			
23.5600	1128				23.5600	860			

23.5800	1132				23.5800	890			
23.6000	1088				23.6000	822			
23.6200	1116				23.6200	868			
23.6400	996				23.6400	800			
23.6600	1158				23.6600	820			
23.6800	1172				23.6800	882			
23.7000	1084				23.7000	824			
23.7200	1084				23.7200	844			
23.7400	1032				23.7400	856			
23.7600	1166				23.7600	876			
23.7800	1104				23.7800	910			
23.8000	1054				23.8000	820			
23.8200	1126				23.8200	848			
23.8400	1072				23.8400	802			
23.8600	1194				23.8600	908			
23.8800	1136				23.8800	874			
23.9000	1064				23.9000	828			
23.9200	1138				23.9200	834			
23.9400	1128				23.9400	822			
23.9600	1166				23.9600	898			
23.9800	1138				23.9800	900			
24.0000	1014				24.0000	810			
24.0200	1076				24.0200	824			
24.0400	1124				24.0400	856			
24.0600	1076				24.0600	966			
24.0800	1162				24.0800	900			
24.1000	1020				24.1000	806			
24.1200	1182				24.1200	910			
24.1400	1164				24.1400	986			
24.1600	1092				24.1600	780			

24.1800	1124				24.1800	906			
24.2000	992				24.2000	842			
24.2200	1120				24.2200	884			
24.2400	1166				24.2400	828			
24.2600	1026				24.2600	814			
24.2800	1120				24.2800	824			
24.3000	978				24.3000	766			
24.3200	1176				24.3200	862			
24.3400	1082				24.3400	854			
24.3600	954				24.3600	820			
24.3800	1066				24.3800	864			
24.4000	1022				24.4000	802			
24.4200	1116				24.4200	838			
24.4400	1072				24.4400	850			
24.4600	970				24.4600	774			
24.4800	1030				24.4800	842			
24.5000	1084				24.5000	772			
24.5200	1024				24.5200	816			
24.5400	1116				24.5400	826			
24.5600	990				24.5600	748			
24.5800	1022				24.5800	814			
24.6000	1076				24.6000	868			
24.6200	1088				24.6200	812			
24.6400	1060				24.6400	742			
24.6600	988				24.6600	790			
24.6800	1030				24.6800	818			
24.7000	1078				24.7000	824			
24.7200	1008				24.7200	802			
24.7400	1080				24.7400	800			
24.7600	990				24.7600	796			

24.7800	1128				24.7800	810			
24.8000	1080				24.8000	822			
24.8200	1000				24.8200	788			
24.8400	1068				24.8400	810			
24.8600	934				24.8600	784			
24.8800	1038				24.8800	808			
24.9000	1096				24.9000	804			
24.9200	978				24.9200	768			
24.9400	1002				24.9400	804			
24.9600	978				24.9600	772			
24.9800	1104				24.9800	828			
25.0000	1062				25.0000	786			
25.0200	1018				25.0200	742			
25.0400	1010				25.0400	790			
25.0600	1006				25.0600	808			
25.0800	1110				25.0800	780			
25.1000	992				25.1000	774			
25.1200	946				25.1200	734			
25.1400	1016				25.1400	746			
25.1600	986				25.1600	760			
25.1800	1000				25.1800	818			
25.2000	976				25.2000	788			
25.2200	886				25.2200	746			
25.2400	1044				25.2400	832			
25.2600	1038				25.2600	824			
25.2800	1014				25.2800	754			
25.3000	962				25.3000	794			
25.3200	878				25.3200	754			
25.3400	1020				25.3400	816			
25.3600	1056				25.3600	846			

25.3800	954				25.3800	798			
25.4000	1024				25.4000	804			
25.4200	920				25.4200	780			
25.4400	1040				25.4400	822			
25.4600	1026				25.4600	756			
25.4800	970				25.4800	724			
25.5000	970				25.5000	806			
25.5200	914				25.5200	752			
25.5400	1056				25.5400	818			
25.5600	1028				25.5600	784			
25.5800	986				25.5800	766			
25.6000	978				25.6000	764			
25.6200	942				25.6200	764			
25.6400	1020				25.6400	824			
25.6600	1022				25.6600	788			
25.6800	918				25.6800	716			
25.7000	1020				25.7000	772			
25.7200	1032				25.7200	718			
25.7400	966				25.7400	792			
25.7600	992				25.7600	746			
25.7800	900				25.7800	774			
25.8000	1034				25.8000	762			
25.8200	894				25.8200	790			
25.8400	978				25.8400	748			
25.8600	1036				25.8600	776			
25.8800	860				25.8800	720			
25.9000	1010				25.9000	804			
25.9200	924				25.9200	768			
25.9400	950				25.9400	716			
25.9600	982				25.9600	760			

25.9800	942				25.9800	752			
26.0000	1004				26.0000	770			
26.0200	996				26.0200	750			
26.0400	940				26.0400	694			
26.0600	996				26.0600	740			
26.0800	940				26.0800	734			
26.1000	992				26.1000	742			
26.1200	922				26.1200	800			
26.1400	890				26.1400	722			
26.1600	918				26.1600	804			
26.1800	912				26.1800	714			
26.2000	976				26.2000	714			
26.2200	976				26.2200	754			
26.2400	858				26.2400	740			
26.2600	942				26.2600	774			
26.2800	914				26.2800	730			
26.3000	950				26.3000	738			
26.3200	974				26.3200	758			
26.3400	820				26.3400	764			
26.3600	928				26.3600	784			
26.3800	934				26.3800	766			
26.4000	910				26.4000	756			
26.4200	970				26.4200	730			
26.4400	870				26.4400	718			
26.4600	966				26.4600	760			
26.4800	900				26.4800	714			
26.5000	900				26.5000	742			
26.5200	892				26.5200	756			
26.5400	882				26.5400	724			
26.5600	928				26.5600	752			

26.5800	944				26.5800	760			
26.6000	846				26.6000	694			
26.6200	948				26.6200	746			
26.6400	846				26.6400	654			
26.6600	990				26.6600	772			
26.6800	940				26.6800	748			
26.7000	872				26.7000	714			
26.7200	932				26.7200	742			
26.7400	934				26.7400	750			
26.7600	882				26.7600	766			
26.7800	966				26.7800	732			
26.8000	848				26.8000	698			
26.8200	952				26.8200	740			
26.8400	870				26.8400	686			
26.8600	912				26.8600	742			
26.8800	880				26.8800	684			
26.9000	810				26.9000	718			
26.9200	952				26.9200	736			
26.9400	906				26.9400	670			
26.9600	884				26.9600	766			
26.9800	880				26.9800	748			
27.0000	834				27.0000	744			
27.0200	906				27.0200	724			
27.0400	864				27.0400	714			
27.0600	876				27.0600	690			
27.0800	914				27.0800	708			
27.1000	814				27.1000	700			
27.1200	962				27.1200	718			
27.1400	872				27.1400	752			
27.1600	850				27.1600	664			

27.1800	876				27.1800	692			
27.2000	858				27.2000	654			
27.2200	874				27.2200	710			
27.2400	894				27.2400	706			
27.2600	808				27.2600	730			
27.2800	900				27.2800	690			
27.3000	784				27.3000	636			
27.3200	850				27.3200	766			
27.3400	884				27.3400	740			
27.3600	780				27.3600	704			
27.3800	914				27.3800	662			
27.4000	848				27.4000	684			
27.4200	846				27.4200	736			
27.4400	900				27.4400	670			
27.4600	784				27.4600	652			
27.4800	870				27.4800	694			
27.5000	852				27.5000	658			
27.5200	864				27.5200	736			
27.5400	874				27.5400	686			
27.5600	792				27.5600	668			
27.5800	888				27.5800	748			
27.6000	834				27.6000	702			
27.6200	828				27.6200	692			
27.6400	860				27.6400	698			
27.6600	820				27.6600	654			
27.6800	864				27.6800	696			
27.7000	904				27.7000	668			
27.7200	800				27.7200	658			
27.7400	884				27.7400	694			
27.7600	768				27.7600	696			

27.7800	878				27.7800	682			
27.8000	868				27.8000	704			
27.8200	804				27.8200	710			
27.8400	856				27.8400	688			
27.8600	836				27.8600	634			
27.8800	830				27.8800	710			
27.9000	870				27.9000	706			
27.9200	832				27.9200	664			
27.9400	798				27.9400	678			
27.9600	822				27.9600	626			
27.9800	874				27.9800	684			
28.0000	840				28.0000	654			
28.0200	748				28.0200	704			
28.0400	828				28.0400	738			
28.0600	778				28.0600	624			
28.0800	820				28.0800	628			
28.1000	854				28.1000	634			
28.1200	808				28.1200	678			
28.1400	814				28.1400	684			
28.1600	838				28.1600	640			
28.1800	822				28.1800	666			
28.2000	822				28.2000	662			
28.2200	786				28.2200	638			
28.2400	822				28.2400	638			
28.2600	842				28.2600	700			
28.2800	856				28.2800	602			
28.3000	844				28.3000	690			
28.3200	736				28.3200	678			
28.3400	824				28.3400	662			
28.3600	792				28.3600	702			

28.3800	768				28.3800	628			
28.4000	774				28.4000	626			
28.4200	740				28.4200	664			
28.4400	848				28.4400	678			
28.4600	832				28.4600	648			
28.4800	752				28.4800	634			
28.5000	802				28.5000	686			
28.5200	794				28.5200	618			
28.5400	798				28.5400	602			
28.5600	772				28.5600	664			
28.5800	764				28.5800	616			
28.6000	820				28.6000	636			
28.6200	744				28.6200	580			
28.6400	830				28.6400	632			
28.6600	802				28.6600	640			
28.6800	780				28.6800	626			
28.7000	804				28.7000	630			
28.7200	782				28.7200	670			
28.7400	792				28.7400	622			
28.7600	772				28.7600	680			
28.7800	776				28.7800	600			
28.8000	764				28.8000	678			
28.8200	774				28.8200	642			
28.8400	726				28.8400	640			
28.8600	796				28.8600	658			
28.8800	696				28.8800	628			
28.9000	788				28.9000	664			
28.9200	788				28.9200	648			
28.9400	762				28.9400	626			
28.9600	768				28.9600	624			

28.9800	738				28.9800	622			
29.0000	812				29.0000	628			
29.0200	812				29.0200	710			
29.0400	780				29.0400	614			
29.0600	778				29.0600	690			
29.0800	774				29.0800	624			
29.1000	804				29.1000	644			
29.1200	788				29.1200	590			
29.1400	708				29.1400	610			
29.1600	782				29.1600	624			
29.1800	760				29.1800	632			
29.2000	720				29.2000	628			
29.2200	788				29.2200	622			
29.2400	654				29.2400	588			
29.2600	754				29.2600	592			
29.2800	862				29.2800	596			
29.3000	694				29.3000	608			
29.3200	758				29.3200	660			
29.3400	684				29.3400	572			
29.3600	790				29.3600	608			
29.3800	738				29.3800	652			
29.4000	808				29.4000	602			
29.4200	722				29.4200	622			
29.4400	702				29.4400	590			
29.4600	814				29.4600	628			
29.4800	766				29.4800	626			
29.5000	706				29.5000	572			
29.5200	792				29.5200	628			
29.5400	710				29.5400	566			
29.5600	770				29.5600	634			

29.5800	780				29.5800	612			
29.6000	778				29.6000	556			
29.6200	748				29.6200	608			
29.6400	678				29.6400	600			
29.6600	780				29.6600	630			
29.6800	734				29.6800	644			
29.7000	708				29.7000	550			
29.7200	782				29.7200	590			
29.7400	726				29.7400	614			
29.7600	726				29.7600	604			
29.7800	766				29.7800	648			
29.8000	702				29.8000	580			
29.8200	738				29.8200	624			
29.8400	740				29.8400	610			
29.8600	738				29.8600	596			
29.8800	724				29.8800	578			
29.9000	674				29.9000	558			
29.9200	806				29.9200	574			
29.9400	782				29.9400	596			
29.9600	728				29.9600	622			
29.9800	694				29.9800	588			
30.0000	724				30.0000	546			
30.0200	744				30.0200	646			
30.0400	806				30.0400	562			
30.0600	726				30.0600	542			
30.0800	732				30.0800	576			
30.1000	728				30.1000	552			
30.1200	708				30.1200	596			
30.1400	740				30.1400	582			
30.1600	666				30.1600	588			

30.1800	694				30.1800	614			
30.2000	710				30.2000	544			
30.2200	750				30.2200	622			
30.2400	728				30.2400	636			
30.2600	718				30.2600	498			
30.2800	736				30.2800	554			
30.3000	658				30.3000	596			
30.3200	708				30.3200	580			
30.3400	718				30.3400	592			
30.3600	706				30.3600	566			
30.3800	702				30.3800	618			
30.4000	712				30.4000	592			
30.4200	708				30.4200	608			
30.4400	656				30.4400	582			
30.4600	656				30.4600	566			
30.4800	720				30.4800	586			
30.5000	700				30.5000	598			
30.5200	666				30.5200	598			
30.5400	718				30.5400	602			
30.5600	608				30.5600	574			
30.5800	676				30.5800	592			
30.6000	692				30.6000	598			
30.6200	724				30.6200	558			
30.6400	726				30.6400	580			
30.6600	654				30.6600	532			
30.6800	778				30.6800	556			
30.7000	666				30.7000	620			
30.7200	654				30.7200	572			
30.7400	692				30.7400	586			
30.7600	668				30.7600	530			

30.7800	726				30.7800	566			
30.8000	692				30.8000	558			
30.8200	666				30.8200	580			
30.8400	706				30.8400	586			
30.8600	716				30.8600	540			
30.8800	684				30.8800	554			
30.9000	690				30.9000	624			
30.9200	692				30.9200	544			
30.9400	734				30.9400	580			
30.9600	654				30.9600	562			
30.9800	688				30.9800	548			
31.0000	672				31.0000	584			
31.0200	670				31.0200	528			
31.0400	714				31.0400	556			
31.0600	696				31.0600	570			
31.0800	702				31.0800	544			
31.1000	680				31.1000	614			
31.1200	674				31.1200	576			
31.1400	668				31.1400	560			
31.1600	748				31.1600	562			
31.1800	662				31.1800	550			
31.2000	702				31.2000	558			
31.2200	674				31.2200	546			
31.2400	678				31.2400	564			
31.2600	706				31.2600	596			
31.2800	638				31.2800	526			
31.3000	686				31.3000	568			
31.3200	676				31.3200	542			
31.3400	676				31.3400	546			
31.3600	718				31.3600	586			

31.3800	664				31.3800	492			
31.4000	662				31.4000	550			
31.4200	658				31.4200	562			
31.4400	732				31.4400	522			
31.4600	644				31.4600	524			
31.4800	644				31.4800	508			
31.5000	730				31.5000	520			
31.5200	666				31.5200	538			
31.5400	692				31.5400	584			
31.5600	690				31.5600	564			
31.5800	636				31.5800	536			
31.6000	668				31.6000	556			
31.6200	654				31.6200	534			
31.6400	684				31.6400	524			
31.6600	680				31.6600	522			
31.6800	636				31.6800	532			
31.7000	684				31.7000	564			
31.7200	694				31.7200	560			
31.7400	634				31.7400	496			
31.7600	686				31.7600	574			
31.7800	602				31.7800	562			
31.8000	680				31.8000	540			
31.8200	646				31.8200	500			
31.8400	692				31.8400	510			
31.8600	650				31.8600	604			
31.8800	628				31.8800	502			
31.9000	702				31.9000	542			
31.9200	704				31.9200	542			
31.9400	610				31.9400	514			
31.9600	688				31.9600	548			

31.9800	690				31.9800	502			
32.0000	678				32.0000	560			
32.0200	658				32.0200	510			
32.0400	616				32.0400	488			
32.0600	654				32.0600	530			
32.0800	618				32.0800	518			
32.1000	656				32.1000	548			
32.1200	694				32.1200	518			
32.1400	590				32.1400	506			
32.1600	700				32.1600	472			
32.1800	602				32.1800	508			
32.2000	636				32.2000	540			
32.2200	626				32.2200	500			
32.2400	606				32.2400	520			
32.2600	612				32.2600	504			
32.2800	658				32.2800	532			
32.3000	652				32.3000	504			
32.3200	656				32.3200	528			
32.3400	590				32.3400	558			
32.3600	636				32.3600	530			
32.3800	646				32.3800	512			
32.4000	626				32.4000	548			
32.4200	620				32.4200	494			
32.4400	568				32.4400	480			
32.4600	672				32.4600	524			
32.4800	636				32.4800	514			
32.5000	590				32.5000	500			
32.5200	608				32.5200	508			
32.5400	628				32.5400	478			
32.5600	620				32.5600	536			

32.5800	674				32.5800	516			
32.6000	582				32.6000	476			
32.6200	638				32.6200	524			
32.6400	626				32.6400	502			
32.6600	676				32.6600	508			
32.6800	626				32.6800	492			
32.7000	616				32.7000	536			
32.7200	662				32.7200	504			
32.7400	600				32.7400	492			
32.7600	640				32.7600	504			
32.7800	590				32.7800	498			
32.8000	610				32.8000	534			
32.8200	618				32.8200	530			
32.8400	616				32.8400	534			
32.8600	628				32.8600	534			
32.8800	654				32.8800	520			
32.9000	590				32.9000	498			
32.9200	666				32.9200	494			
32.9400	632				32.9400	514			
32.9600	668				32.9600	496			
32.9800	650				32.9800	486			
33.0000	580				33.0000	498			
33.0200	656				33.0200	508			
33.0400	670				33.0400	482			
33.0600	632				33.0600	472			
33.0800	658				33.0800	526			
33.1000	566				33.1000	436			
33.1200	638				33.1200	522			
33.1400	666				33.1400	490			
33.1600	586				33.1600	508			

33.1800 658				33.1800 482			
33.2000 632				33.2000 456			
33.2200 704				33.2200 494			
33.2400 706				33.2400 492			
33.2600 578				33.2600 470			
33.2800 648				33.2800 480			
33.3000 594				33.3000 468			
33.3200 596				33.3200 486			
33.3400 634				33.3400 508			
33.3600 552				33.3600 476			
33.3800 670				33.3800 504			
33.4000 608				33.4000 462			
33.4200 608				33.4200 476			
33.4400 666				33.4400 508			
33.4600 588				33.4600 540			
33.4800 632				33.4800 488			
33.5000 622				33.5000 484			
33.5200 574				33.5200 454			
33.5400 646				33.5400 482			
33.5600 570				33.5600 466			
33.5800 650				33.5800 488			
33.6000 648				33.6000 500			
33.6200 546							
33.6400 614							
33.6600 620							
33.6800 634							
33.7000 654							
33.7200 622							
33.7400 666							
33.7600 564							

33.7800	638							
33.8000	676							
33.8200	496							
33.8400	638							
33.8600	596							
33.8800	648							
33.9000	602							
33.9200	542							
33.9400	606							
33.9600	586							
33.9800	656							
34.0000	580							
34.0200	558							
34.0400	596							
34.0600	612							
34.0800	576							
34.1000	624							
34.1200	550							
34.1400	622							
34.1600	650							
34.1800	528							
34.2000	616							
34.2200	574							
34.2400	616							
34.2600	606							
34.2800	574							
34.3000	620							
34.3200	566							
34.3400	606							
34.3600	600							

34.3800	532							
34.4000	602							
34.4200	586							
34.4400	582							
34.4600	622							
34.4800	550							
34.5000	584							
34.5200	590							
34.5400	600							
34.5600	576							
34.5800	524							
34.6000	586							
34.6200	568							
34.6400	606							
34.6600	596							
34.6800	530							
34.7000	604							
34.7200	574							
34.7400	550							
34.7600	582							
34.7800	560							
34.8000	610							
34.8200	578							
34.8400	580							
34.8600	620							
34.8800	522							
34.9000	622							
34.9200	608							
34.9400	548							
34.9600	558							

34.9800	528						
35.0000	648						
35.0200	576						
35.0400	556						
35.0600	642						
35.0800	562						
35.1000	572						
35.1200	594						
35.1400	512						
35.1600	532						
35.1800	596						
35.2000	536						
35.2200	554						
35.2400	524						
35.2600	588						
35.2800	558						
35.3000	524						
35.3200	568						
35.3400	566						
35.3600	586						
35.3800	566						
35.4000	552						
35.4200	588						
35.4400	526						
35.4600	602						
35.4800	566						
35.5000	506						
35.5200	572						
35.5400	538						
35.5600	578						

35.5800	586						
35.6000	524						
35.6200	614						
35.6400	544						
35.6600	582						
35.6800	550						
35.7000	558						
35.7200	606						
35.7400	576						
35.7600	544						
35.7800	564						
35.8000	548						
35.8200	582						
35.8400	584						
35.8600	562						
35.8800	538						
35.9000	524						
35.9200	640						
35.9400	620						
35.9600	512						
35.9800	582						
36.0000	518						
36.0200	604						
36.0400	612						
36.0600	566						
36.0800	592						
36.1000	582						
36.1200	618						
36.1400	610						
36.1600	562						

36.1800	576							
36.2000	568							
36.2200	576							
36.2400	610							
36.2600	566							
36.2800	600							
36.3000	542							
36.3200	616							
36.3400	620							
36.3600	528							
36.3800	624							
36.4000	574							
36.4200	594							
36.4400	638							
36.4600	506							
36.4800	598							
36.5000	556							
36.5200	538							
36.5400	568							
36.5600	558							
36.5800	586							
36.6000	590							
36.6200	526							
36.6400	598							
36.6600	524							
36.6800	594							
36.7000	550							
36.7200	558							
36.7400	568							
36.7600	548							

36.7800	568							
36.8000	592							
36.8200	532							
36.8400	544							
36.8600	556							
36.8800	588							
36.9000	574							
36.9200	488							
36.9400	534							
36.9600	522							
36.9800	526							
37.0000	626							
37.0200	484							
37.0400	582							
37.0600	552							
37.0800	566							
37.1000	526							
37.1200	496							
37.1400	598							
37.1600	534							
37.1800	506							
37.2000	592							
37.2200	472							
37.2400	526							
37.2600	560							
37.2800	510							
37.3000	568							
37.3200	532							
37.3400	558							
37.3600	600							

37.3800	528						
37.4000	600						
37.4200	562						
37.4400	560						
37.4600	620						
37.4800	528						
37.5000	582						
37.5200	570						
37.5400	610						
37.5600	646						
37.5800	566						
37.6000	680						
37.6200	604						
37.6400	618						
37.6600	686						
37.6800	654						
37.7000	714						
37.7200	796						
37.7400	686						
37.7600	812						
37.7800	782						
37.8000	868						
37.8200	906						
37.8400	908						
37.8600	1116						
37.8800	1052						
37.9000	1212						
37.9200	1254						
37.9400	1410						
37.9600	1538						

37.9800	1666						
38.0000	1850						
38.0200	2072						
38.0400	2236						
38.0600	2506						
38.0800	2724						
38.1000	2954						
38.1200	3182						
38.1400	3476						
38.1600	3674						
38.1800	3830						
38.2000	3886						
38.2200	3950						
38.2400	4114						
38.2600	3970						
38.2800	3848						
38.3000	3836						
38.3200	3528						
38.3400	3246						
38.3600	3124						
38.3800	2772						
38.4000	2562						
38.4200	2250						
38.4400	2090						
38.4600	1868						
38.4800	1670						
38.5000	1420						
38.5200	1414						
38.5400	1272						
38.5600	1028						

38.5800	998							
38.6000	892							
38.6200	916							
38.6400	802							
38.6600	710							
38.6800	684							
38.7000	634							
38.7200	672							
38.7400	610							
38.7600	658							
38.7800	590							
38.8000	556							
38.8200	618							
38.8400	606							
38.8600	572							
38.8800	544							
38.9000	520							
38.9200	560							
38.9400	578							
38.9600	516							
38.9800	564							
39.0000	496							
39.0200	576							
39.0400	556							
39.0600	536							
39.0800	542							
39.1000	454							
39.1200	574							
39.1400	584							
39.1600	492							

39.1800	550							
39.2000	558							
39.2200	532							
39.2400	556							
39.2600	510							
39.2800	564							
39.3000	550							
39.3200	524							
39.3400	540							
39.3600	454							
39.3800	524							
39.4000	512							
39.4200	520							
39.4400	542							
39.4600	492							
39.4800	550							
39.5000	552							
39.5200	474							
39.5400	504							
39.5600	466							
39.5800	560							
39.6000	530							
39.6200	476							
39.6400	552							
39.6600	518							
39.6800	574							
39.7000	550							
39.7200	508							
39.7400	560							
39.7600	506							

39.7800	568						
39.8000	540						
39.8200	486						
39.8400	560						
39.8600	538						
39.8800	544						
39.9000	568						
39.9200	532						
39.9400	598						
39.9600	610						
39.9800	604						
40.0000	550						
