# Supplementary material for: Data on statistical experimental design to formulate amphotericin B-loaded Eudragit RL100 nanoparticles coated with hyaluronic acid for the treatment of vulvovaginal candidiasis
Source: Data Brief. 2020 Mar 5;29:105311. doi: 10.1016/j.dib.2020.105311 (PMC7082528; doi:10.1016/j.dib.2020.105311)
Supplement: Multimedia component 18 [file mmc18.pdf]

/// **Profile Data Ascii Dump (XRD)**

//

Data : VII-AMP-EUD-Nano-HA

File Name : VII-AMP-EUD-Nano-HA.RAW

Profile Datafile

comment = VII-AMP-EUD-Nano-HA

date & time = 07-29-19 12:33:11

Measurement Condition

X-ray tube

target = Cu

voltage = 40.0 (kV)

current = 30.0 (mA)

Slits

divergence slit = 1.00000 (deg)

scatter slit = 1.00000 (deg)

receiving slit = 0.30000 (mm)

Scanning

drive axis = Theta-2Theta

scan range = 5.000 - 35.060

scan mode = Continuous Scan

scan speed = 1.0000 (deg/min)

sampling pitch = 0.0200 (deg)

preset time = 1.20 (sec)

Data [Total No. = 1504]

<2Theta>	< I >
5.0000	2220
5.0200	2040
5.0400	2136
5.0600	2096
5.0800	2084
5.1000	2084
5.1200	2114
5.1400	1994
5.1600	2110
5.1800	2058
5.2000	2090
5.2200	2022
5.2400	1992
5.2600	2062
5.2800	2090
5.3000	1988
5.3200	2074
5.3400	2040
5.3600	2010
5.3800	2068
5.4000	1968
5.4200	1932
5.4400	2096
5.4600	1980
5.4800	1956
5.5000	1898
5.5200	1944
5.5400	2038
5.5600	1952

5.5800	1846			
5.6000	1882			
5.6200	1970			
5.6400	1958			
5.6600	1908			
5.6800	1836			
5.7000	1880			
5.7200	1906			
5.7400	1918			
5.7600	1832			
5.7800	1866			
5.8000	1868			
5.8200	1884			
5.8400	1942			
5.8600	1850			
5.8800	1770			
5.9000	1884			
5.9200	1872			
5.9400	1790			
5.9600	1770			
5.9800	1764			
6.0000	1882			
6.0200	1856			
6.0400	1820			
6.0600	1698			
6.0800	1752			
6.1000	1854			
6.1200	1770			
6.1400	1676			
6.1600	1690			

6.1800	1874			
6.2000	1764			
6.2200	1730			
6.2400	1678			
6.2600	1732			
6.2800	1832			
6.3000	1856			
6.3200	1672			
6.3400	1690			
6.3600	1722			
6.3800	1816			
6.4000	1752			
6.4200	1580			
6.4400	1584			
6.4600	1720			
6.4800	1776			
6.5000	1720			
6.5200	1590			
6.5400	1636			
6.5600	1712			
6.5800	1718			
6.6000	1716			
6.6200	1658			
6.6400	1658			
6.6600	1790			
6.6800	1712			
6.7000	1570			
6.7200	1618			
6.7400	1628			
6.7600	1640			

6.7800	1740			
6.8000	1582			
6.8200	1632			
6.8400	1602			
6.8600	1600			
6.8800	1672			
6.9000	1534			
6.9200	1598			
6.9400	1658			
6.9600	1622			
6.9800	1564			
7.0000	1514			
7.0200	1630			
7.0400	1678			
7.0600	1594			
7.0800	1566			
7.1000	1542			
7.1200	1600			
7.1400	1610			
7.1600	1534			
7.1800	1542			
7.2000	1480			
7.2200	1562			
7.2400	1688			
7.2600	1580			
7.2800	1584			
7.3000	1530			
7.3200	1596			
7.3400	1558			
7.3600	1528			

7.3800	1596			
7.4000	1588			
7.4200	1680			
7.4400	1544			
7.4600	1494			
7.4800	1614			
7.5000	1514			
7.5200	1512			
7.5400	1506			
7.5600	1490			
7.5800	1552			
7.6000	1632			
7.6200	1460			
7.6400	1490			
7.6600	1424			
7.6800	1496			
7.7000	1544			
7.7200	1472			
7.7400	1418			
7.7600	1390			
7.7800	1482			
7.8000	1488			
7.8200	1442			
7.8400	1406			
7.8600	1458			
7.8800	1544			
7.9000	1480			
7.9200	1390			
7.9400	1418			
7.9600	1444			

7.9800	1570			
8.0000	1416			
8.0200	1366			
8.0400	1366			
8.0600	1426			
8.0800	1440			
8.1000	1420			
8.1200	1340			
8.1400	1460			
8.1600	1460			
8.1800	1432			
8.2000	1404			
8.2200	1316			
8.2400	1500			
8.2600	1506			
8.2800	1346			
8.3000	1352			
8.3200	1322			
8.3400	1502			
8.3600	1420			
8.3800	1416			
8.4000	1354			
8.4200	1404			
8.4400	1438			
8.4600	1356			
8.4800	1326			
8.5000	1316			
8.5200	1382			
8.5400	1392			
8.5600	1356			

8.5800	1326			
8.6000	1324			
8.6200	1390			
8.6400	1410			
8.6600	1388			
8.6800	1190			
8.7000	1306			
8.7200	1378			
8.7400	1366			
8.7600	1356			
8.7800	1262			
8.8000	1396			
8.8200	1340			
8.8400	1276			
8.8600	1286			
8.8800	1286			
8.9000	1368			
8.9200	1382			
8.9400	1292			
8.9600	1292			
8.9800	1266			
9.0000	1296			
9.0200	1348			
9.0400	1214			
9.0600	1292			
9.0800	1314			
9.1000	1366			
9.1200	1306			
9.1400	1280			
9.1600	1250			

9.1800	1260			
9.2000	1386			
9.2200	1290			
9.2400	1240			
9.2600	1232			
9.2800	1252			
9.3000	1308			
9.3200	1230			
9.3400	1214			
9.3600	1266			
9.3800	1292			
9.4000	1262			
9.4200	1236			
9.4400	1176			
9.4600	1216			
9.4800	1314			
9.5000	1184			
9.5200	1228			
9.5400	1196			
9.5600	1338			
9.5800	1234			
9.6000	1208			
9.6200	1272			
9.6400	1198			
9.6600	1340			
9.6800	1180			
9.7000	1186			
9.7200	1188			
9.7400	1210			
9.7600	1196			

9.7800	1232			
9.8000	1246			
9.8200	1220			
9.8400	1180			
9.8600	1200			
9.8800	1128			
9.9000	1162			
9.9200	1182			
9.9400	1232			
9.9600	1178			
9.9800	1172			
10.0000	1190			
10.0200	1210			
10.0400	1246			
10.0600	1154			
10.0800	1166			
10.1000	1100			
10.1200	1172			
10.1400	1218			
10.1600	1122			
10.1800	1150			
10.2000	1154			
10.2200	1188			
10.2400	1106			
10.2600	1130			
10.2800	1136			
10.3000	1178			
10.3200	1124			
10.3400	1170			
10.3600	1096			

10.3800	1086			
10.4000	1176			
10.4200	1142			
10.4400	1132			
10.4600	1048			
10.4800	1130			
10.5000	1098			
10.5200	1144			
10.5400	1098			
10.5600	1086			
10.5800	1140			
10.6000	1130			
10.6200	1110			
10.6400	1120			
10.6600	1034			
10.6800	1078			
10.7000	1142			
10.7200	1120			
10.7400	1028			
10.7600	1084			
10.7800	1196			
10.8000	1080			
10.8200	1074			
10.8400	1094			
10.8600	1168			
10.8800	1128			
10.9000	1080			
10.9200	1080			
10.9400	1060			
10.9600	1098			

10.9800	1108			
11.0000	1092			
11.0200	1034			
11.0400	1086			
11.0600	1180			
11.0800	1090			
11.1000	1100			
11.1200	1110			
11.1400	1040			
11.1600	1120			
11.1800	1070			
11.2000	1010			
11.2200	1058			
11.2400	1140			
11.2600	1144			
11.2800	1072			
11.3000	1088			
11.3200	1058			
11.3400	1078			
11.3600	1080			
11.3800	1116			
11.4000	1066			
11.4200	1044			
11.4400	1060			
11.4600	1056			
11.4800	1044			
11.5000	992			
11.5200	1100			
11.5400	1060			
11.5600	1066			

11.5800	1048			
11.6000	1000			
11.6200	1066			
11.6400	1128			
11.6600	1002			
11.6800	1016			
11.7000	1098			
11.7200	1074			
11.7400	1024			
11.7600	988			
11.7800	982			
11.8000	1044			
11.8200	1060			
11.8400	1006			
11.8600	992			
11.8800	1052			
11.9000	1028			
11.9200	1072			
11.9400	1044			
11.9600	998			
11.9800	1076			
12.0000	1116			
12.0200	1082			
12.0400	988			
12.0600	964			
12.0800	1026			
12.1000	1036			
12.1200	984			
12.1400	1018			
12.1600	1034			

12.1800	1058			
12.2000	1038			
12.2200	1100			
12.2400	1030			
12.2600	994			
12.2800	1010			
12.3000	1034			
12.3200	990			
12.3400	1008			
12.3600	1014			
12.3800	1032			
12.4000	972			
12.4200	992			
12.4400	980			
12.4600	1086			
12.4800	1014			
12.5000	958			
12.5200	930			
12.5400	978			
12.5600	1034			
12.5800	970			
12.6000	916			
12.6200	958			
12.6400	1020			
12.6600	1070			
12.6800	950			
12.7000	980			
12.7200	958			
12.7400	1010			
12.7600	1014			

12.7800	958			
12.8000	968			
12.8200	990			
12.8400	1002			
12.8600	1036			
12.8800	930			
12.9000	922			
12.9200	1000			
12.9400	1040			
12.9600	958			
12.9800	984			
13.0000	948			
13.0200	1002			
13.0400	1028			
13.0600	962			
13.0800	950			
13.1000	956			
13.1200	1020			
13.1400	1026			
13.1600	892			
13.1800	972			
13.2000	964			
13.2200	992			
13.2400	954			
13.2600	912			
13.2800	902			
13.3000	966			
13.3200	1004			
13.3400	952			
13.3600	966			

13.3800	928			
13.4000	990			
13.4200	910			
13.4400	934			
13.4600	936			
13.4800	932			
13.5000	956			
13.5200	1038			
13.5400	952			
13.5600	936			
13.5800	1004			
13.6000	950			
13.6200	1002			
13.6400	940			
13.6600	918			
13.6800	988			
13.7000	998			
13.7200	970			
13.7400	904			
13.7600	948			
13.7800	984			
13.8000	926			
13.8200	966			
13.8400	924			
13.8600	996			
13.8800	1000			
13.9000	976			
13.9200	944			
13.9400	980			
13.9600	912			

13.9800	966			
14.0000	882			
14.0200	910			
14.0400	932			
14.0600	940			
14.0800	952			
14.1000	892			
14.1200	908			
14.1400	920			
14.1600	1008			
14.1800	1008			
14.2000	894			
14.2200	968			
14.2400	940			
14.2600	992			
14.2800	914			
14.3000	864			
14.3200	992			
14.3400	982			
14.3600	930			
14.3800	882			
14.4000	922			
14.4200	942			
14.4400	998			
14.4600	986			
14.4800	962			
14.5000	912			
14.5200	958			
14.5400	1004			
14.5600	1008			

14.5800	944			
14.6000	932			
14.6200	980			
14.6400	960			
14.6600	888			
14.6800	930			
14.7000	970			
14.7200	970			
14.7400	944			
14.7600	966			
14.7800	936			
14.8000	978			
14.8200	948			
14.8400	912			
14.8600	954			
14.8800	938			
14.9000	944			
14.9200	918			
14.9400	918			
14.9600	886			
14.9800	900			
15.0000	976			
15.0200	896			
15.0400	930			
15.0600	880			
15.0800	960			
15.1000	906			
15.1200	828			
15.1400	856			
15.1600	812			

15.1800	990			
15.2000	958			
15.2200	840			
15.2400	900			
15.2600	928			
15.2800	944			
15.3000	878			
15.3200	886			
15.3400	870			
15.3600	960			
15.3800	978			
15.4000	910			
15.4200	904			
15.4400	952			
15.4600	920			
15.4800	876			
15.5000	936			
15.5200	852			
15.5400	958			
15.5600	938			
15.5800	916			
15.6000	912			
15.6200	830			
15.6400	954			
15.6600	948			
15.6800	918			
15.7000	908			
15.7200	882			
15.7400	898			
15.7600	942			

15.7800	898			
15.8000	974			
15.8200	896			
15.8400	960			
15.8600	912			
15.8800	866			
15.9000	932			
15.9200	994			
15.9400	922			
15.9600	924			
15.9800	880			
16.0000	942			
16.0200	926			
16.0400	862			
16.0600	874			
16.0800	828			
16.1000	948			
16.1200	974			
16.1400	958			
16.1600	874			
16.1800	912			
16.2000	938			
16.2200	918			
16.2400	928			
16.2600	870			
16.2800	870			
16.3000	896			
16.3200	962			
16.3400	876			
16.3600	920			

16.3800	854			
16.4000	906			
16.4200	910			
16.4400	838			
16.4600	914			
16.4800	920			
16.5000	942			
16.5200	926			
16.5400	856			
16.5600	892			
16.5800	940			
16.6000	884			
16.6200	900			
16.6400	844			
16.6600	848			
16.6800	934			
16.7000	896			
16.7200	892			
16.7400	890			
16.7600	930			
16.7800	914			
16.8000	880			
16.8200	894			
16.8400	872			
16.8600	972			
16.8800	890			
16.9000	884			
16.9200	924			
16.9400	864			
16.9600	928			

16.9800	930			
17.0000	838			
17.0200	872			
17.0400	920			
17.0600	948			
17.0800	898			
17.1000	850			
17.1200	898			
17.1400	884			
17.1600	914			
17.1800	826			
17.2000	842			
17.2200	930			
17.2400	884			
17.2600	910			
17.2800	864			
17.3000	870			
17.3200	904			
17.3400	968			
17.3600	920			
17.3800	862			
17.4000	810			
17.4200	962			
17.4400	924			
17.4600	848			
17.4800	890			
17.5000	900			
17.5200	894			
17.5400	982			
17.5600	858			

17.5800	916			
17.6000	856			
17.6200	922			
17.6400	946			
17.6600	874			
17.6800	892			
17.7000	902			
17.7200	876			
17.7400	916			
17.7600	878			
17.7800	924			
17.8000	902			
17.8200	942			
17.8400	910			
17.8600	876			
17.8800	994			
17.9000	914			
17.9200	864			
17.9400	926			
17.9600	884			
17.9800	920			
18.0000	966			
18.0200	840			
18.0400	920			
18.0600	900			
18.0800	934			
18.1000	902			
18.1200	870			
18.1400	910			
18.1600	890			

18.1800	932			
18.2000	930			
18.2200	852			
18.2400	908			
18.2600	892			
18.2800	942			
18.3000	932			
18.3200	882			
18.3400	908			
18.3600	924			
18.3800	960			
18.4000	924			
18.4200	838			
18.4400	980			
18.4600	920			
18.4800	848			
18.5000	914			
18.5200	890			
18.5400	860			
18.5600	976			
18.5800	856			
18.6000	908			
18.6200	882			
18.6400	950			
18.6600	942			
18.6800	866			
18.7000	960			
18.7200	888			
18.7400	962			
18.7600	956			

18.7800	866			
18.8000	918			
18.8200	928			
18.8400	956			
18.8600	930			
18.8800	870			
18.9000	948			
18.9200	942			
18.9400	954			
18.9600	1004			
18.9800	888			
19.0000	978			
19.0200	952			
19.0400	894			
19.0600	948			
19.0800	856			
19.1000	1028			
19.1200	988			
19.1400	870			
19.1600	888			
19.1800	842			
19.2000	998			
19.2200	946			
19.2400	822			
19.2600	884			
19.2800	902			
19.3000	988			
19.3200	920			
19.3400	846			
19.3600	914			

19.3800	890			
19.4000	1014			
19.4200	940			
19.4400	896			
19.4600	918			
19.4800	912			
19.5000	938			
19.5200	906			
19.5400	934			
19.5600	914			
19.5800	1006			
19.6000	894			
19.6200	918			
19.6400	918			
19.6600	934			
19.6800	994			
19.7000	940			
19.7200	914			
19.7400	918			
19.7600	942			
19.7800	1010			
19.8000	854			
19.8200	910			
19.8400	976			
19.8600	986			
19.8800	886			
19.9000	906			
19.9200	956			
19.9400	970			
19.9600	974			

19.9800	1042			
20.0000	888			
20.0200	924			
20.0400	978			
20.0600	940			
20.0800	946			
20.1000	884			
20.1200	938			
20.1400	964			
20.1600	956			
20.1800	916			
20.2000	884			
20.2200	1012			
20.2400	968			
20.2600	896			
20.2800	896			
20.3000	882			
20.3200	962			
20.3400	952			
20.3600	864			
20.3800	918			
20.4000	892			
20.4200	1004			
20.4400	934			
20.4600	868			
20.4800	958			
20.5000	860			
20.5200	936			
20.5400	944			
20.5600	872			

20.5800	940			
20.6000	920			
20.6200	950			
20.6400	976			
20.6600	920			
20.6800	920			
20.7000	902			
20.7200	936			
20.7400	958			
20.7600	886			
20.7800	944			
20.8000	902			
20.8200	886			
20.8400	916			
20.8600	880			
20.8800	954			
20.9000	980			
20.9200	886			
20.9400	950			
20.9600	900			
20.9800	924			
21.0000	948			
21.0200	890			
21.0400	900			
21.0600	892			
21.0800	966			
21.1000	954			
21.1200	938			
21.1400	958			
21.1600	920			

21.1800	948			
21.2000	852			
21.2200	842			
21.2400	968			
21.2600	976			
21.2800	884			
21.3000	902			
21.3200	854			
21.3400	926			
21.3600	946			
21.3800	906			
21.4000	910			
21.4200	902			
21.4400	960			
21.4600	968			
21.4800	830			
21.5000	920			
21.5200	876			
21.5400	968			
21.5600	948			
21.5800	866			
21.6000	898			
21.6200	944			
21.6400	970			
21.6600	918			
21.6800	884			
21.7000	932			
21.7200	940			
21.7400	910			
21.7600	956			

21.7800	880			
21.8000	898			
21.8200	974			
21.8400	990			
21.8600	914			
21.8800	864			
21.9000	962			
21.9200	956			
21.9400	926			
21.9600	960			
21.9800	882			
22.0000	938			
22.0200	940			
22.0400	864			
22.0600	930			
22.0800	904			
22.1000	916			
22.1200	932			
22.1400	896			
22.1600	854			
22.1800	946			
22.2000	966			
22.2200	920			
22.2400	868			
22.2600	914			
22.2800	858			
22.3000	932			
22.3200	932			
22.3400	906			
22.3600	990			

22.3800	952			
22.4000	922			
22.4200	910			
22.4400	838			
22.4600	950			
22.4800	886			
22.5000	902			
22.5200	904			
22.5400	840			
22.5600	976			
22.5800	926			
22.6000	856			
22.6200	908			
22.6400	906			
22.6600	880			
22.6800	1012			
22.7000	876			
22.7200	920			
22.7400	900			
22.7600	848			
22.7800	878			
22.8000	870			
22.8200	916			
22.8400	882			
22.8600	944			
22.8800	898			
22.9000	804			
22.9200	906			
22.9400	856			
22.9600	882			

22.9800	928			
23.0000	864			
23.0200	906			
23.0400	938			
23.0600	910			
23.0800	934			
23.1000	886			
23.1200	900			
23.1400	884			
23.1600	898			
23.1800	902			
23.2000	816			
23.2200	880			
23.2400	878			
23.2600	870			
23.2800	922			
23.3000	820			
23.3200	940			
23.3400	904			
23.3600	860			
23.3800	858			
23.4000	850			
23.4200	934			
23.4400	926			
23.4600	820			
23.4800	854			
23.5000	852			
23.5200	858			
23.5400	852			
23.5600	858			

23.5800	862			
23.6000	880			
23.6200	822			
23.6400	848			
23.6600	818			
23.6800	896			
23.7000	920			
23.7200	862			
23.7400	902			
23.7600	812			
23.7800	966			
23.8000	912			
23.8200	820			
23.8400	884			
23.8600	908			
23.8800	890			
23.9000	924			
23.9200	828			
23.9400	890			
23.9600	874			
23.9800	880			
24.0000	926			
24.0200	868			
24.0400	910			
24.0600	908			
24.0800	954			
24.1000	890			
24.1200	914			
24.1400	918			
24.1600	864			

24.1800	882			
24.2000	924			
24.2200	826			
24.2400	894			
24.2600	936			
24.2800	858			
24.3000	834			
24.3200	856			
24.3400	878			
24.3600	862			
24.3800	840			
24.4000	866			
24.4200	842			
24.4400	876			
24.4600	880			
24.4800	860			
24.5000	846			
24.5200	814			
24.5400	862			
24.5600	834			
24.5800	838			
24.6000	864			
24.6200	824			
24.6400	816			
24.6600	874			
24.6800	866			
24.7000	872			
24.7200	822			
24.7400	830			
24.7600	866			

24.7800	784			
24.8000	840			
24.8200	858			
24.8400	832			
24.8600	828			
24.8800	776			
24.9000	866			
24.9200	868			
24.9400	792			
24.9600	866			
24.9800	792			
25.0000	806			
25.0200	834			
25.0400	862			
25.0600	846			
25.0800	808			
25.1000	878			
25.1200	814			
25.1400	812			
25.1600	894			
25.1800	832			
25.2000	858			
25.2200	828			
25.2400	772			
25.2600	846			
25.2800	822			
25.3000	854			
25.3200	798			
25.3400	808			
25.3600	838			

25.3800	790			
25.4000	864			
25.4200	836			
25.4400	756			
25.4600	826			
25.4800	834			
25.5000	844			
25.5200	774			
25.5400	806			
25.5600	846			
25.5800	826			
25.6000	778			
25.6200	812			
25.6400	828			
25.6600	824			
25.6800	880			
25.7000	810			
25.7200	822			
25.7400	756			
25.7600	834			
25.7800	810			
25.8000	816			
25.8200	796			
25.8400	790			
25.8600	832			
25.8800	792			
25.9000	774			
25.9200	802			
25.9400	734			
25.9600	764			

25.9800	834			
26.0000	796			
26.0200	786			
26.0400	796			
26.0600	764			
26.0800	774			
26.1000	738			
26.1200	818			
26.1400	786			
26.1600	756			
26.1800	786			
26.2000	758			
26.2200	808			
26.2400	846			
26.2600	760			
26.2800	728			
26.3000	716			
26.3200	844			
26.3400	806			
26.3600	760			
26.3800	752			
26.4000	738			
26.4200	764			
26.4400	806			
26.4600	728			
26.4800	788			
26.5000	722			
26.5200	770			
26.5400	812			
26.5600	800			

26.5800	766			
26.6000	810			
26.6200	766			
26.6400	758			
26.6600	742			
26.6800	742			
26.7000	750			
26.7200	716			
26.7400	746			
26.7600	718			
26.7800	752			
26.8000	776			
26.8200	756			
26.8400	794			
26.8600	758			
26.8800	786			
26.9000	734			
26.9200	724			
26.9400	720			
26.9600	724			
26.9800	742			
27.0000	806			
27.0200	714			
27.0400	712			
27.0600	758			
27.0800	748			
27.1000	736			
27.1200	720			
27.1400	776			
27.1600	780			

27.1800	768			
27.2000	720			
27.2200	724			
27.2400	834			
27.2600	776			
27.2800	732			
27.3000	692			
27.3200	742			
27.3400	770			
27.3600	768			
27.3800	666			
27.4000	694			
27.4200	730			
27.4400	734			
27.4600	720			
27.4800	734			
27.5000	722			
27.5200	708			
27.5400	722			
27.5600	744			
27.5800	716			
27.6000	776			
27.6200	716			
27.6400	676			
27.6600	716			
27.6800	688			
27.7000	684			
27.7200	680			
27.7400	688			
27.7600	742			

27.7800	694			
27.8000	700			
27.8200	646			
27.8400	678			
27.8600	708			
27.8800	724			
27.9000	718			
27.9200	736			
27.9400	728			
27.9600	584			
27.9800	750			
28.0000	700			
28.0200	694			
28.0400	698			
28.0600	702			
28.0800	736			
28.1000	730			
28.1200	668			
28.1400	672			
28.1600	750			
28.1800	696			
28.2000	702			
28.2200	712			
28.2400	678			
28.2600	730			
28.2800	688			
28.3000	692			
28.3200	650			
28.3400	660			
28.3600	668			

28.3800	708			
28.4000	688			
28.4200	696			
28.4400	634			
28.4600	734			
28.4800	688			
28.5000	638			
28.5200	672			
28.5400	622			
28.5600	690			
28.5800	696			
28.6000	658			
28.6200	706			
28.6400	652			
28.6600	682			
28.6800	696			
28.7000	630			
28.7200	768			
28.7400	706			
28.7600	630			
28.7800	670			
28.8000	652			
28.8200	670			
28.8400	638			
28.8600	686			
28.8800	696			
28.9000	688			
28.9200	672			
28.9400	684			
28.9600	700			

28.9800	640			
29.0000	614			
29.0200	724			
29.0400	680			
29.0600	680			
29.0800	628			
29.1000	674			
29.1200	684			
29.1400	650			
29.1600	638			
29.1800	626			
29.2000	666			
29.2200	690			
29.2400	686			
29.2600	664			
29.2800	656			
29.3000	638			
29.3200	674			
29.3400	640			
29.3600	674			
29.3800	656			
29.4000	632			
29.4200	662			
29.4400	658			
29.4600	622			
29.4800	634			
29.5000	640			
29.5200	652			
29.5400	642			
29.5600	636			

29.5800	650			
29.6000	656			
29.6200	684			
29.6400	622			
29.6600	642			
29.6800	646			
29.7000	632			
29.7200	630			
29.7400	680			
29.7600	664			
29.7800	620			
29.8000	634			
29.8200	598			
29.8400	632			
29.8600	634			
29.8800	616			
29.9000	620			
29.9200	544			
29.9400	638			
29.9600	638			
29.9800	628			
30.0000	632			
30.0200	592			
30.0400	636			
30.0600	598			
30.0800	634			
30.1000	586			
30.1200	578			
30.1400	626			
30.1600	584			

30.1800	668			
30.2000	626			
30.2200	562			
30.2400	612			
30.2600	628			
30.2800	622			
30.3000	612			
30.3200	588			
30.3400	582			
30.3600	594			
30.3800	570			
30.4000	614			
30.4200	614			
30.4400	636			
30.4600	616			
30.4800	626			
30.5000	608			
30.5200	604			
30.5400	612			
30.5600	584			
30.5800	612			
30.6000	598			
30.6200	620			
30.6400	600			
30.6600	598			
30.6800	556			
30.7000	660			
30.7200	582			
30.7400	646			
30.7600	588			

30.7800	574			
30.8000	594			
30.8200	592			
30.8400	606			
30.8600	586			
30.8800	576			
30.9000	608			
30.9200	600			
30.9400	568			
30.9600	592			
30.9800	572			
31.0000	614			
31.0200	562			
31.0400	548			
31.0600	606			
31.0800	632			
31.1000	594			
31.1200	548			
31.1400	568			
31.1600	590			
31.1800	592			
31.2000	580			
31.2200	642			
31.2400	562			
31.2600	584			
31.2800	540			
31.3000	576			
31.3200	560			
31.3400	598			
31.3600	590			

31.3800	588			
31.4000	570			
31.4200	592			
31.4400	576			
31.4600	558			
31.4800	582			
31.5000	584			
31.5200	570			
31.5400	562			
31.5600	556			
31.5800	588			
31.6000	524			
31.6200	588			
31.6400	514			
31.6600	574			
31.6800	566			
31.7000	530			
31.7200	570			
31.7400	554			
31.7600	560			
31.7800	530			
31.8000	586			
31.8200	568			
31.8400	566			
31.8600	592			
31.8800	590			
31.9000	520			
31.9200	500			
31.9400	550			
31.9600	534			

31.9800	592			
32.0000	552			
32.0200	598			
32.0400	566			
32.0600	542			
32.0800	576			
32.1000	532			
32.1200	582			
32.1400	538			
32.1600	572			
32.1800	560			
32.2000	544			
32.2200	532			
32.2400	570			
32.2600	508			
32.2800	566			
32.3000	556			
32.3200	550			
32.3400	552			
32.3600	536			
32.3800	500			
32.4000	536			
32.4200	500			
32.4400	566			
32.4600	548			
32.4800	564			
32.5000	558			
32.5200	554			
32.5400	538			
32.5600	562			

32.5800	496			
32.6000	532			
32.6200	480			
32.6400	546			
32.6600	516			
32.6800	568			
32.7000	522			
32.7200	546			
32.7400	544			
32.7600	502			
32.7800	534			
32.8000	570			
32.8200	528			
32.8400	546			
32.8600	526			
32.8800	524			
32.9000	544			
32.9200	542			
32.9400	510			
32.9600	518			
32.9800	508			
33.0000	574			
33.0200	558			
33.0400	528			
33.0600	550			
33.0800	496			
33.1000	576			
33.1200	518			
33.1400	548			
33.1600	554			

33.1800	546			
33.2000	536			
33.2200	520			
33.2400	524			
33.2600	494			
33.2800	520			
33.3000	512			
33.3200	504			
33.3400	550			
33.3600	518			
33.3800	510			
33.4000	516			
33.4200	562			
33.4400	554			
33.4600	508			
33.4800	482			
33.5000	502			
33.5200	502			
33.5400	560			
33.5600	574			
33.5800	506			
33.6000	530			
33.6200	518			
33.6400	522			
33.6600	520			
33.6800	506			
33.7000	550			
33.7200	538			
33.7400	506			
33.7600	492			

33.7800	498			
33.8000	540			
33.8200	558			
33.8400	474			
33.8600	542			
33.8800	480			
33.9000	518			
33.9200	502			
33.9400	482			
33.9600	492			
33.9800	514			
34.0000	466			
34.0200	470			
34.0400	484			
34.0600	476			
34.0800	532			
34.1000	490			
34.1200	480			
34.1400	538			
34.1600	534			
34.1800	490			
34.2000	490			
34.2200	506			
34.2400	516			
34.2600	492			
34.2800	510			
34.3000	486			
34.3200	486			
34.3400	504			
34.3600	484			

34.3800	504			
34.4000	512			
34.4200	484			
34.4400	480			
34.4600	490			
34.4800	496			
34.5000	490			
34.5200	474			
34.5400	496			
34.5600	508			
34.5800	540			
34.6000	494			
34.6200	468			
34.6400	450			
34.6600	500			
34.6800	524			
34.7000	494			
34.7200	502			
34.7400	510			
34.7600	480			
34.7800	506			
34.8000	522			
34.8200	510			
34.8400	518			
34.8600	468			
34.8800	520			
34.9000	476			
34.9200	506			
34.9400	474			
34.9600	446			

34.9800	518			
35.0000	520			
35.0200	530			
35.0400	448			
35.0600	476			
